# Global, regional, and national age-sex-specific mortality for 282 causes of death in 195 countries and territories, 1980–2017: a systematic analysis for the Global Burden of Disease Study 2017

**DOI:** 10.1016/S0140-6736(18)32203-7

**Published:** 2018-11-10

**Authors:** GBD 2017 Causes of Death Collaborators, Gregory A Roth, Degu Abate, Kalkidan Hassen Abate, Solomon M Abay, Cristiana Abbafati, Nooshin Abbasi, Hedayat Abbastabar, Foad Abd-Allah, Jemal Abdela, Ahmed Abdelalim, Ibrahim Abdollahpour, Rizwan Suliankatchi Abdulkader, Haftom Temesgen Abebe, Molla Abebe, Zegeye Abebe, Ayenew Negesse Abejie, Semaw F Abera, Olifan Zewdie Abil, Haftom Niguse Abraha, Aklilu Roba Abrham, Laith Jamal Abu-Raddad, Manfred Mario Kokou Accrombessi, Dilaram Acharya, Abdu A Adamu, Oladimeji M Adebayo, Rufus Adesoji Adedoyin, Victor Adekanmbi, Olatunji O Adetokunboh, Beyene Meressa Adhena, Mina G Adib, Amha Admasie, Ashkan Afshin, Gina Agarwal, Kareha M Agesa, Anurag Agrawal, Sutapa Agrawal, Alireza Ahmadi, Mehdi Ahmadi, Muktar Beshir Ahmed, Sayem Ahmed, Amani Nidhal Aichour, Ibtihel Aichour, Miloud Taki Eddine Aichour, Mohammad Esmaeil Akbari, Rufus Olusola Akinyemi, Nadia Akseer, Ziyad Al-Aly, Ayman Al-Eyadhy, Rajaa M Al-Raddadi, Fares Alahdab, Khurshid Alam, Tahiya Alam, Animut Alebel, Kefyalew Addis Alene, Mehran Alijanzadeh, Reza Alizadeh-Navaei, Syed Mohamed Aljunid, Ala'a Alkerwi, François Alla, Peter Allebeck, Jordi Alonso, Khalid Altirkawi, Nelson Alvis-Guzman, Azmeraw T Amare, Leopold N Aminde, Erfan Amini, Walid Ammar, Yaw Ampem Amoako, Nahla Hamed Anber, Catalina Liliana Andrei, Sofia Androudi, Megbaru Debalkie Animut, Mina Anjomshoa, Hossein Ansari, Mustafa Geleto Ansha, Carl Abelardo T Antonio, Palwasha Anwari, Olatunde Aremu, Johan Ärnlöv, Amit Arora, Monika Arora, Al Artaman, Krishna K Aryal, Hamid Asayesh, Ephrem Tsegay Asfaw, Zerihun Ataro, Suleman Atique, Sachin R Atre, Marcel Ausloos, Euripide F G A Avokpaho, Ashish Awasthi, Beatriz Paulina Ayala Quintanilla, Yohanes Ayele, Rakesh Ayer, Peter S Azzopardi, Arefeh Babazadeh, Umar Bacha, Hamid Badali, Alaa Badawi, Ayele Geleto Bali, Katherine E Ballesteros, Maciej Banach, Kajori Banerjee, Marlena S Bannick, Joseph Adel Mattar Banoub, Miguel A Barboza, Suzanne Lyn Barker-Collo, Till Winfried Bärnighausen, Simon Barquera, Lope H Barrero, Quique Bassat, Sanjay Basu, Bernhard T Baune, Habtamu Wondifraw Baynes, Shahrzad Bazargan-Hejazi, Neeraj Bedi, Ettore Beghi, Masoud Behzadifar, Meysam Behzadifar, Yannick Béjot, Bayu Begashaw Bekele, Abate Bekele Belachew, Ezra Belay, Yihalem Abebe Belay, Michelle L Bell, Aminu K Bello, Derrick A Bennett, Isabela M Bensenor, Adam E Berman, Eduardo Bernabe, Robert S Bernstein, Gregory J Bertolacci, Mircea Beuran, Tina Beyranvand, Ashish Bhalla, Suraj Bhattarai, Soumyadeeep Bhaumik, Zulfiqar A Bhutta, Belete Biadgo, Molly H Biehl, Ali Bijani, Boris Bikbov, Ver Bilano, Nigus Bililign, Muhammad Shahdaat Bin Sayeed, Donal Bisanzio, Tuhin Biswas, Brigette F Blacker, Berrak Bora Basara, Rohan Borschmann, Cristina Bosetti, Kayvan Bozorgmehr, Oliver J Brady, Luisa C Brant, Carol Brayne, Alexandra Brazinova, Nicholas J K Breitborde, Hermann Brenner, Paul Svitil Briant, Gabrielle Britton, Traolach Brugha, Reinhard Busse, Zahid A Butt, Charlton S K H Callender, Ismael R Campos-Nonato, Julio Cesar Campuzano Rincon, Jorge Cano, Mate Car, Rosario Cárdenas, Giulia Carreras, Juan J Carrero, Austin Carter, Félix Carvalho, Carlos A Castañeda-Orjuela, Jacqueline Castillo Rivas, Chris D Castle, Clara Castro, Franz Castro, Ferrán Catalá-López, Ester Cerin, Yazan Chaiah, Jung-Chen Chang, Fiona J Charlson, Pankaj Chaturvedi, Peggy Pei-Chia Chiang, Odgerel Chimed-Ochir, Vesper Hichilombwe Chisumpa, Abdulaal Chitheer, Rajiv Chowdhury, Hanne Christensen, Devasahayam J Christopher, Sheng-Chia Chung, Flavia M Cicuttini, Liliana G Ciobanu, Massimo Cirillo, Aaron J Cohen, Leslie Trumbull Cooper, Paolo Angelo Cortesi, Monica Cortinovis, Ewerton Cousin, Benjamin C Cowie, Michael H Criqui, Elizabeth A Cromwell, Christopher Stephen Crowe, John A Crump, Matthew Cunningham, Alemneh Kabeta Daba, Abel Fekadu Dadi, Lalit Dandona, Rakhi Dandona, Anh Kim Dang, Paul I Dargan, Ahmad Daryani, Siddharth K Das, Rajat Das Gupta, José Das Neves, Tamirat Tesfaye Dasa, Aditya Prasad Dash, Adrian C Davis, Nicole Davis Weaver, Dragos Virgil Davitoiu, Kairat Davletov, Fernando Pio De La Hoz, Jan-Walter De Neve, Meaza Girma Degefa, Louisa Degenhardt, Tizta T Degfie, Selina Deiparine, Gebre Teklemariam Demoz, Balem Betsu Demtsu, Edgar Denova-Gutiérrez, Kebede Deribe, Nikolaos Dervenis, Don C Des Jarlais, Getenet Ayalew Dessie, Subhojit Dey, Samath D Dharmaratne, Daniel Dicker, Mesfin Tadese Dinberu, Eric L Ding, M Ashworth Dirac, Shirin Djalalinia, Klara Dokova, David Teye Doku, Christl A Donnelly, E Ray Dorsey, Pratik P Doshi, Dirk Douwes-Schultz, Kerrie E Doyle, Tim R Driscoll, Manisha Dubey, Eleonora Dubljanin, Eyasu Ejeta Duken, Bruce B Duncan, Andre R Duraes, Hedyeh Ebrahimi, Soheil Ebrahimpour, Dumessa Edessa, David Edvardsson, Anne Elise Eggen, Charbel El Bcheraoui, Maysaa El Sayed Zaki, Ziad El-Khatib, Hajer Elkout, Christian Lycke Ellingsen, Matthias Endres, Aman Yesuf Endries, Benjamin Er, Holly E Erskine, Babak Eshrati, Sharareh Eskandarieh, Reza Esmaeili, Alireza Esteghamati, Mahdi Fakhar, Hamed Fakhim, Mahbobeh Faramarzi, Mohammad Fareed, Farzaneh Farhadi, Carla Sofia E sá Farinha, Andre Faro, Maryam S Farvid, Farshad Farzadfar, Mohammad Hosein Farzaei, Valery L Feigin, Andrea B Feigl, Netsanet Fentahun, Seyed-Mohammad Fereshtehnejad, Eduarda Fernandes, Joao C Fernandes, Alize J Ferrari, Garumma Tolu Feyissa, Irina Filip, Samuel Finegold, Florian Fischer, Christina Fitzmaurice, Nataliya A Foigt, Kyle J Foreman, Carla Fornari, Tahvi D Frank, Takeshi Fukumoto, John E Fuller, Nancy Fullman, Thomas Fürst, João M Furtado, Neal D Futran, Silvano Gallus, Alberto L Garcia-Basteiro, Miguel A Garcia-Gordillo, William M Gardner, Abadi Kahsu Gebre, Tsegaye Tewelde Gebrehiwot, Amanuel Tesfay Gebremedhin, Bereket Gebremichael, Teklu Gebrehiwo Gebremichael, Tilayie Feto Gelano, Johanna M Geleijnse, Ricard Genova-Maleras, Yilma Chisha Dea Geramo, Peter W Gething, Kebede Embaye Gezae, Mohammad Rasoul Ghadami, Reza Ghadimi, Khalil Ghasemi Falavarjani, Maryam Ghasemi-Kasman, Mamata Ghimire, Katherine B Gibney, Paramjit Singh Gill, Tiffany K Gill, Richard F Gillum, Ibrahim Abdelmageed Ginawi, Maurice Giroud, Giorgia Giussani, Shifalika Goenka, Ellen M Goldberg, Srinivas Goli, Hector Gómez-Dantés, Philimon N Gona, Sameer Vali Gopalani, Taren M Gorman, Atsushi Goto, Alessandra C Goulart, Elena V Gnedovskaya, Ayman Grada, Giuseppe Grosso, Harish Chander Gugnani, Andre Luiz Sena Guimaraes, Yuming Guo, Prakash C Gupta, Rahul Gupta, Rajeev Gupta, Tanush Gupta, Reyna Alma Gutiérrez, Bishal Gyawali, Juanita A Haagsma, Nima Hafezi-Nejad, Tekleberhan B Hagos, Tewodros Tesfa Hailegiyorgis, Gessessew Bugssa Hailu, Arvin Haj-Mirzaian, Arya Haj-Mirzaian, Randah R Hamadeh, Samer Hamidi, Alexis J Handal, Graeme J Hankey, Hilda L Harb, Sivadasanpillai Harikrishnan, Josep Maria Haro, Mehedi Hasan, Hadi Hassankhani, Hamid Yimam Hassen, Rasmus Havmoeller, Roderick J Hay, Simon I Hay, Yihua He, Akbar Hedayatizadeh-Omran, Mohamed I Hegazy, Behzad Heibati, Mohsen Heidari, Delia Hendrie, Andualem Henok, Nathaniel J Henry, Claudiu Herteliu, Fatemeh Heydarpour, Pouria Heydarpour, Sousan Heydarpour, Desalegn Tsegaw Hibstu, Hans W Hoek, Michael K Hole, Enayatollah Homaie Rad, Praveen Hoogar, H Dean Hosgood, Seyed Mostafa Hosseini, Mehdi Hosseinzadeh, Mihaela Hostiuc, Sorin Hostiuc, Peter J Hotez, Damian G Hoy, Thomas Hsiao, Guoqing Hu, John J Huang, Abdullatif Husseini, Mohammedaman Mama Hussen, Susan Hutfless, Bulat Idrisov, Olayinka Stephen Ilesanmi, Usman Iqbal, Seyed Sina Naghibi Irvani, Caleb Mackay Salpeter Irvine, Nazrul Islam, Sheikh Mohammed Shariful Islam, Farhad Islami, Kathryn H Jacobsen, Leila Jahangiry, Nader Jahanmehr, Sudhir Kumar Jain, Mihajlo Jakovljevic, Moti Tolera Jalu, Spencer L James, Mehdi Javanbakht, Achala Upendra Jayatilleke, Panniyammakal Jeemon, Kathy J Jenkins, Ravi Prakash Jha, Vivekanand Jha, Catherine O Johnson, Sarah C Johnson, Jost B Jonas, Ankur Joshi, Jacek Jerzy Jozwiak, Suresh Banayya Jungari, Mikk Jürisson, Zubair Kabir, Rajendra Kadel, Amaha Kahsay, Rizwan Kalani, Manoochehr Karami, Behzad Karami Matin, André Karch, Corine Karema, Hamidreza Karimi-Sari, Amir Kasaeian, Dessalegn H Kassa, Getachew Mullu Kassa, Tesfaye Dessale Kassa, Nicholas J Kassebaum, Srinivasa Vittal Katikireddi, Anil Kaul, Zhila Kazemi, Ali Kazemi Karyani, Dhruv Satish Kazi, Adane Teshome Kefale, Peter Njenga Keiyoro, Grant Rodgers Kemp, Andre Pascal Kengne, Andre Keren, Chandrasekharan Nair Kesavachandran, Yousef Saleh Khader, Behzad Khafaei, Morteza Abdullatif Khafaie, Alireza Khajavi, Nauman Khalid, Ibrahim A Khalil, Ejaz Ahmad Khan, Muhammad Shahzeb Khan, Muhammad Ali Khan, Young-Ho Khang, Mona M Khater, Abdullah T Khoja, Ardeshir Khosravi, Mohammad Hossein Khosravi, Jagdish Khubchandani, Aliasghar A Kiadaliri, Getiye D Kibret, Zelalem Teklemariam Kidanemariam, Daniel N Kiirithio, Daniel Kim, Young-Eun Kim, Yun Jin Kim, Ruth W Kimokoti, Yohannes Kinfu, Adnan Kisa, Katarzyna Kissimova-Skarbek, Mika Kivimäki, Ann Kristin Skrindo Knudsen, Jonathan M Kocarnik, Sonali Kochhar, Yoshihiro Kokubo, Tufa Kolola, Jacek A Kopec, Parvaiz A Koul, Ai Koyanagi, Michael A Kravchenko, Kewal Krishan, Barthelemy Kuate Defo, Burcu Kucuk Bicer, G Anil Kumar, Manasi Kumar, Pushpendra Kumar, Michael J Kutz, Igor Kuzin, Hmwe Hmwe Kyu, Deepesh P Lad, Sheetal D Lad, Alessandra Lafranconi, Dharmesh Kumar Lal, Ratilal Lalloo, Tea Lallukka, Jennifer O Lam, Faris Hasan Lami, Van C Lansingh, Sonia Lansky, Heidi J Larson, Arman Latifi, Kathryn Mei-Ming Lau, Jeffrey V Lazarus, Georgy Lebedev, Paul H Lee, James Leigh, Mostafa Leili, Cheru Tesema Leshargie, Shanshan Li, Yichong Li, Juan Liang, Lee-Ling Lim, Stephen S Lim, Miteku Andualem Limenih, Shai Linn, Shiwei Liu, Yang Liu, Rakesh Lodha, Chris Lonsdale, Alan D Lopez, Stefan Lorkowski, Paulo A Lotufo, Rafael Lozano, Raimundas Lunevicius, Stefan Ma, Erlyn Rachelle King Macarayan, Mark T Mackay, Jennifer H MacLachlan, Emilie R Maddison, Fabiana Madotto, Hassan Magdy Abd El Razek, Muhammed Magdy Abd El Razek, Dhaval P Maghavani, Marek Majdan, Reza Majdzadeh, Azeem Majeed, Reza Malekzadeh, Deborah Carvalho Malta, Ana-Laura Manda, Luiz Garcia Mandarano-Filho, Helena Manguerra, Mohammad Ali Mansournia, Chabila Christopher Mapoma, Dadi Marami, Joemer C Maravilla, Wagner Marcenes, Laurie Marczak, Ashley Marks, Guy B Marks, Gabriel Martinez, Francisco Rogerlândio Martins-Melo, Ira Martopullo, Winfried März, Melvin B Marzan, Joseph R Masci, Benjamin Ballard Massenburg, Manu Raj Mathur, Prashant Mathur, Richard Matzopoulos, Pallab K Maulik, Mohsen Mazidi, Colm McAlinden, John J McGrath, Martin McKee, Brian J McMahon, Suresh Mehata, Man Mohan Mehndiratta, Ravi Mehrotra, Kala M Mehta, Varshil Mehta, Tefera C Mekonnen, Addisu Melese, Mulugeta Melku, Peter T N Memiah, Ziad A Memish, Walter Mendoza, Desalegn Tadese Mengistu, Getnet Mengistu, George A Mensah, Seid Tiku Mereta, Atte Meretoja, Tuomo J Meretoja, Tomislav Mestrovic, Haftay Berhane Mezgebe, Bartosz Miazgowski, Tomasz Miazgowski, Anoushka I Millear, Ted R Miller, Molly Katherine Miller-Petrie, G K Mini, Parvaneh Mirabi, Mojde Mirarefin, Andreea Mirica, Erkin M Mirrakhimov, Awoke Temesgen Misganaw, Habtamu Mitiku, Babak Moazen, Karzan Abdulmuhsin Mohammad, Moslem Mohammadi, Noushin Mohammadifard, Mohammed A Mohammed, Shafiu Mohammed, Viswanathan Mohan, Ali H Mokdad, Mariam Molokhia, Lorenzo Monasta, Ghobad Moradi, Maziar Moradi-Lakeh, Mehdi Moradinazar, Paula Moraga, Lidia Morawska, Ilais Moreno Velásquez, Joana Morgado-Da-Costa, Shane Douglas Morrison, Marilita M Moschos, Simin Mouodi, Seyyed Meysam Mousavi, Kindie Fentahun Muchie, Ulrich Otto Mueller, Satinath Mukhopadhyay, Kate Muller, John Everett Mumford, Jonah Musa, Kamarul Imran Musa, Ghulam Mustafa, Saravanan Muthupandian, Jean B Nachega, Gabriele Nagel, Aliya Naheed, Azin Nahvijou, Gurudatta Naik, Sanjeev Nair, Farid Najafi, Luigi Naldi, Hae Sung Nam, Vinay Nangia, Jobert Richie Nansseu, Bruno Ramos Nascimento, Gopalakrishnan Natarajan, Nahid Neamati, Ionut Negoi, Ruxandra Irina Negoi, Subas Neupane, Charles R J Newton, Frida N Ngalesoni, Josephine W Ngunjiri, Anh Quynh Nguyen, Grant Nguyen, Ha Thu Nguyen, Huong Thanh Nguyen, Long Hoang Nguyen, Minh Nguyen, Trang Huyen Nguyen, Emma Nichols, Dina Nur Anggraini Ningrum, Yirga Legesse Nirayo, Molly R Nixon, Nomonde Nolutshungu, Shuhei Nomura, Ole F Norheim, Mehdi Noroozi, Bo Norrving, Jean Jacques Noubiap, Hamid Reza Nouri, Malihe Nourollahpour Shiadeh, Mohammad Reza Nowroozi, Peter S Nyasulu, Christopher M Odell, Richard Ofori-Asenso, Felix Akpojene Ogbo, In-Hwan Oh, Olanrewaju Oladimeji, Andrew T Olagunju, Pedro R Olivares, Helen Elizabeth Olsen, Bolajoko Olubukunola Olusanya, Jacob Olusegun Olusanya, Kanyin L Ong, Sok King Sk Ong, Eyal Oren, Heather M Orpana, Alberto Ortiz, Justin R Ortiz, Stanislav S Otstavnov, Simon Øverland, Mayowa Ojo Owolabi, Raziye Özdemir, Mahesh P A, Rosana Pacella, Smita Pakhale, Abhijit P Pakhare, Amir H Pakpour, Adrian Pana, Songhomitra Panda-Jonas, Jeyaraj Durai Pandian, Andrea Parisi, Eun-Kee Park, Charles D H Parry, Hadi Parsian, Shanti Patel, Sanghamitra Pati, George C Patton, Vishnupriya Rao Paturi, Katherine R Paulson, Alexandre Pereira, David M Pereira, Norberto Perico, Konrad Pesudovs, Max Petzold, Michael R Phillips, Frédéric B Piel, David M Pigott, Julian David Pillay, Meghdad Pirsaheb, Farhad Pishgar, Suzanne Polinder, Maarten J Postma, Akram Pourshams, Hossein Poustchi, Ashwini Pujar, Swayam Prakash, Narayan Prasad, Caroline A Purcell, Mostafa Qorbani, Hedley Quintana, D Alex Quistberg, Kirankumar Waman Rade, Amir Radfar, Anwar Rafay, Alireza Rafiei, Fakher Rahim, Kazem Rahimi, Afarin Rahimi-Movaghar, Mahfuzar Rahman, Mohammad Hifz Ur Rahman, Muhammad Aziz Rahman, Rajesh Kumar Rai, Sasa Rajsic, Usha Ram, Chhabi Lal Ranabhat, Prabhat Ranjan, Puja C Rao, David Laith Rawaf, Salman Rawaf, Christian Razo-García, K Srinath Reddy, Robert C Reiner, Marissa B Reitsma, Giuseppe Remuzzi, Andre M N Renzaho, Serge Resnikoff, Satar Rezaei, Shahab Rezaeian, Mohammad Sadegh Rezai, Seyed Mohammad Riahi, Antonio Luiz P Ribeiro, Maria Jesus Rios-Blancas, Kedir Teji Roba, Nicholas L S Roberts, Stephen R Robinson, Leonardo Roever, Luca Ronfani, Gholamreza Roshandel, Ali Rostami, Dietrich Rothenbacher, Ambuj Roy, Enrico Rubagotti, Perminder S Sachdev, Basema Saddik, Ehsan Sadeghi, Hosein Safari, Mahdi Safdarian, Sare Safi, Saeid Safiri, Rajesh Sagar, Amirhossein Sahebkar, Mohammad Ali Sahraian, Nasir Salam, Joseph S Salama, Payman Salamati, Raphael De Freitas Saldanha, Zikria Saleem, Yahya Salimi, Sundeep Santosh Salvi, Inbal Salz, Evanson Zondani Sambala, Abdallah M Samy, Juan Sanabria, Maria Dolores Sanchez-Niño, Damian Francesco Santomauro, Itamar S Santos, João Vasco Santos, Milena M Santric Milicevic, Bruno Piassi Sao Jose, Abdur Razzaque Sarker, Rodrigo Sarmiento-Suárez, Nizal Sarrafzadegan, Benn Sartorius, Shahabeddin Sarvi, Brijesh Sathian, Maheswar Satpathy, Arundhati R Sawant, Monika Sawhney, Sonia Saxena, Mehdi Sayyah, Elke Schaeffner, Maria Inês Schmidt, Ione J C Schneider, Ben Schöttker, Aletta Elisabeth Schutte, David C Schwebel, Falk Schwendicke, James G Scott, Mario Sekerija, Sadaf G Sepanlou, Edson Serván-Mori, Seyedmojtaba Seyedmousavi, Hosein Shabaninejad, Katya Anne Shackelford, Azadeh Shafieesabet, Mehdi Shahbazi, Amira A Shaheen, Masood Ali Shaikh, Mehran Shams-Beyranvand, Mohammadbagher Shamsi, Morteza Shamsizadeh, Kiomars Sharafi, Mehdi Sharif, Mahdi Sharif-Alhoseini, Rajesh Sharma, Jun She, Aziz Sheikh, Peilin Shi, Mekonnen Sisay Shiferaw, Mika Shigematsu, Rahman Shiri, Reza Shirkoohi, Ivy Shiue, Farhad Shokraneh, Mark G Shrime, Si Si, Soraya Siabani, Tariq J Siddiqi, Inga Dora Sigfusdottir, Rannveig Sigurvinsdottir, Donald H Silberberg, Diego Augusto Santos Silva, João Pedro Silva, Natacha Torres Da Silva, Dayane Gabriele Alves Silveira, Jasvinder A Singh, Narinder Pal Singh, Prashant Kumar Singh, Virendra Singh, Dhirendra Narain Sinha, Karen Sliwa, Mari Smith, Badr Hasan Sobaih, Soheila Sobhani, Eugène Sobngwi, Samir S Soneji, Moslem Soofi, Reed J D Sorensen, Joan B Soriano, Ireneous N Soyiri, Luciano A Sposato, Chandrashekhar T Sreeramareddy, Vinay Srinivasan, Jeffrey D Stanaway, Vladimir I Starodubov, Vasiliki Stathopoulou, Dan J Stein, Caitlyn Steiner, Leo G Stewart, Mark A Stokes, Michelle L Subart, Agus Sudaryanto, Mu'awiyyah Babale Sufiyan, Patrick John Sur, Ipsita Sutradhar, Bryan L Sykes, P N Sylaja, Dillon O Sylte, Cassandra E I Szoeke, Rafael Tabarés-Seisdedos, Takahiro Tabuchi, Santosh Kumar Tadakamadla, Ken Takahashi, Nikhil Tandon, Segen Gebremeskel Tassew, Nuno Taveira, Arash Tehrani-Banihashemi, Tigist Gashaw Tekalign, Merhawi Gebremedhin Tekle, Mohamad-Hani Temsah, Omar Temsah, Abdullah Sulieman Terkawi, Manaye Yihune Teshale, Belay Tessema, Gizachew Assefa Tessema, Kavumpurathu Raman Thankappan, Sathish Thirunavukkarasu, Nihal Thomas, Amanda G Thrift, George D Thurston, Binyam Tilahun, Quyen G To, Ruoyan Tobe-Gai, Marcello Tonelli, Roman Topor-Madry, Anna E Torre, Miguel Tortajada-Girbés, Mathilde Touvier, Marcos Roberto Tovani-Palone, Bach Xuan Tran, Khanh Bao Tran, Suryakant Tripathi, Christopher E Troeger, Thomas Clement Truelsen, Nu Thi Truong, Afewerki Gebremeskel Tsadik, Derrick Tsoi, Lorainne Tudor Car, E Murat Tuzcu, Stefanos Tyrovolas, Kingsley N Ukwaja, Irfan Ullah, Eduardo A Undurraga, Rachel L Updike, Muhammad Shariq Usman, Olalekan A Uthman, Selen Begüm Uzun, Muthiah Vaduganathan, Afsane Vaezi, Gaurang Vaidya, Pascual R Valdez, Elena Varavikova, Tommi Juhani Vasankari, Narayanaswamy Venketasubramanian, Santos Villafaina, Francesco S Violante, Sergey Konstantinovitch Vladimirov, Vasily Vlassov, Stein Emil Vollset, Theo Vos, Gregory R Wagner, Fasil Shiferaw Wagnew, Yasir Waheed, Mitchell Taylor Wallin, Judd L Walson, Yanping Wang, Yuan-Pang Wang, Molla Mesele Wassie, Elisabete Weiderpass, Robert G Weintraub, Fitsum Weldegebreal, Kidu Gidey Weldegwergs, Andrea Werdecker, Adhena Ayaliew Werkneh, T Eoin West, Ronny Westerman, Harvey A Whiteford, Justyna Widecka, Lauren B Wilner, Shadrach Wilson, Andrea Sylvia Winkler, Charles Shey Wiysonge, Charles D A Wolfe, Shouling Wu, Yun-Chun Wu, Grant M A Wyper, Denis Xavier, Gelin Xu, Simon Yadgir, Ali Yadollahpour, Seyed Hossein Yahyazadeh Jabbari, Bereket Yakob, Lijing L Yan, Yuichiro Yano, Mehdi Yaseri, Yasin Jemal Yasin, Gökalp Kadri Yentür, Alex Yeshaneh, Ebrahim M Yimer, Paul Yip, Biruck Desalegn Yirsaw, Engida Yisma, Naohiro Yonemoto, Gerald Yonga, Seok-Jun Yoon, Marcel Yotebieng, Mustafa Z Younis, Mahmoud Yousefifard, Chuanhua Yu, Vesna Zadnik, Zoubida Zaidi, Sojib Bin Zaman, Mohammad Zamani, Zohreh Zare, Ayalew Jejaw Zeleke, Zerihun Menlkalew Zenebe, Anthony Lin Zhang, Kai Zhang, Maigeng Zhou, Sanjay Zodpey, Liesl Joanna Zuhlke, Mohsen Naghavi, Christopher J L Murray

## Abstract

**Background:**

Global development goals increasingly rely on country-specific estimates for benchmarking a nation's progress. To meet this need, the Global Burden of Diseases, Injuries, and Risk Factors Study (GBD) 2016 estimated global, regional, national, and, for selected locations, subnational cause-specific mortality beginning in the year 1980. Here we report an update to that study, making use of newly available data and improved methods. GBD 2017 provides a comprehensive assessment of cause-specific mortality for 282 causes in 195 countries and territories from 1980 to 2017.

**Methods:**

The causes of death database is composed of vital registration (VR), verbal autopsy (VA), registry, survey, police, and surveillance data. GBD 2017 added ten VA studies, 127 country-years of VR data, 502 cancer-registry country-years, and an additional surveillance country-year. Expansions of the GBD cause of death hierarchy resulted in 18 additional causes estimated for GBD 2017. Newly available data led to subnational estimates for five additional countries—Ethiopia, Iran, New Zealand, Norway, and Russia. Deaths assigned International Classification of Diseases (ICD) codes for non-specific, implausible, or intermediate causes of death were reassigned to underlying causes by redistribution algorithms that were incorporated into uncertainty estimation. We used statistical modelling tools developed for GBD, including the Cause of Death Ensemble model (CODEm), to generate cause fractions and cause-specific death rates for each location, year, age, and sex. Instead of using UN estimates as in previous versions, GBD 2017 independently estimated population size and fertility rate for all locations. Years of life lost (YLLs) were then calculated as the sum of each death multiplied by the standard life expectancy at each age. All rates reported here are age-standardised.

**Findings:**

At the broadest grouping of causes of death (Level 1), non-communicable diseases (NCDs) comprised the greatest fraction of deaths, contributing to 73·4% (95% uncertainty interval [UI] 72·5–74·1) of total deaths in 2017, while communicable, maternal, neonatal, and nutritional (CMNN) causes accounted for 18·6% (17·9–19·6), and injuries 8·0% (7·7–8·2). Total numbers of deaths from NCD causes increased from 2007 to 2017 by 22·7% (21·5–23·9), representing an additional 7·61 million (7·20–8·01) deaths estimated in 2017 versus 2007. The death rate from NCDs decreased globally by 7·9% (7·0–8·8). The number of deaths for CMNN causes decreased by 22·2% (20·0–24·0) and the death rate by 31·8% (30·1–33·3). Total deaths from injuries increased by 2·3% (0·5–4·0) between 2007 and 2017, and the death rate from injuries decreased by 13·7% (12·2–15·1) to 57·9 deaths (55·9–59·2) per 100 000 in 2017. Deaths from substance use disorders also increased, rising from 284 000 deaths (268 000–289 000) globally in 2007 to 352 000 (334 000–363 000) in 2017. Between 2007 and 2017, total deaths from conflict and terrorism increased by 118·0% (88·8–148·6). A greater reduction in total deaths and death rates was observed for some CMNN causes among children younger than 5 years than for older adults, such as a 36·4% (32·2–40·6) reduction in deaths from lower respiratory infections for children younger than 5 years compared with a 33·6% (31·2–36·1) increase in adults older than 70 years. Globally, the number of deaths was greater for men than for women at most ages in 2017, except at ages older than 85 years. Trends in global YLLs reflect an epidemiological transition, with decreases in total YLLs from enteric infections, respiratory infections and tuberculosis, and maternal and neonatal disorders between 1990 and 2017; these were generally greater in magnitude at the lowest levels of the Socio-demographic Index (SDI). At the same time, there were large increases in YLLs from neoplasms and cardiovascular diseases. YLL rates decreased across the five leading Level 2 causes in all SDI quintiles. The leading causes of YLLs in 1990—neonatal disorders, lower respiratory infections, and diarrhoeal diseases—were ranked second, fourth, and fifth, in 2017. Meanwhile, estimated YLLs increased for ischaemic heart disease (ranked first in 2017) and stroke (ranked third), even though YLL rates decreased. Population growth contributed to increased total deaths across the 20 leading Level 2 causes of mortality between 2007 and 2017. Decreases in the cause-specific mortality rate reduced the effect of population growth for all but three causes: substance use disorders, neurological disorders, and skin and subcutaneous diseases.

**Interpretation:**

Improvements in global health have been unevenly distributed among populations. Deaths due to injuries, substance use disorders, armed conflict and terrorism, neoplasms, and cardiovascular disease are expanding threats to global health. For causes of death such as lower respiratory and enteric infections, more rapid progress occurred for children than for the oldest adults, and there is continuing disparity in mortality rates by sex across age groups. Reductions in the death rate of some common diseases are themselves slowing or have ceased, primarily for NCDs, and the death rate for selected causes has increased in the past decade.

**Funding:**

Bill & Melinda Gates Foundation.

## Introduction

Systematic recording and analysis of causes of human death remains one of the most resilient successes for public health, beginning with routine and continuous reporting of deaths by physicians starting in the 15th century.^1^ Today, hundreds of thousands of physicians evaluate and select the cause of death for millions of deaths annually, codifying the results according to the International Classification of Diseases (ICD) system.^2^ These efforts form the basis of a global mortality reporting system that is widely relied upon to prioritise health system investments, track progress towards global development goals, and guide scientific research. Although there remains a need for wider adoption and improvement of these systems, continuous reporting of cause-specific mortality in many countries represents a success for global health.^3^

More mortality data are now becoming available because of broader adoption of vital registration systems and increased information-sharing made possible by digital communication. At the same time, efforts to correct, sort, analyse, and report this massive amount of global data are evolving to keep pace with increasing demands for timely assessment of global, regional, and local mortality patterns. In addition to shifts in mortality patterns due to an ongoing epidemiological transition, rapid spikes in mortality due to specific causes are frequently observed and require recurrent updates to global estimates. Examples of mortality spikes include opioid-associated deaths in parts of the USA,^4^ suicide in eastern Europe in the 1990s,^5^ and conflict-associated deaths in the eastern Mediterranean and North Africa region.^6^ Causes of death are now reported digitally in many locations, allowing health authorities to improve the quality and timeliness of mortality reporting.^7^, ^8^ Global development goals increasingly rely on country-specific estimates for benchmarking a nation's progress. Global commitments, such as the UN's Sustainable Development Goals (SDGs),^9^ the Moscow Declaration to End Tuberculosis,^10^ WHO's First Global Conference on Air Pollution and Health^11^ in October, 2018, and the UN High-level Meetings on NCDs^12^ and tuberculosis,^13^ both in September, 2018, will require ongoing tracking of cause-specific mortality, including in locations where mortality surveillance data remain limited.

**Research in context**

**Evidence before this study**

Previously, the Global Burden of Diseases, Injuries, and Risk Factors Study (GBD) 2016 provided estimates for 264 causes of death for 195 countries and territories, by age and sex, from 1980 to 2016. GBD 2016 incorporated newly available data for many locations, expanded and refined the included causes of death, improved modelling techniques, and developed a star rating system for the quality of cause of death data. To better assess mortality among the oldest adults, terminal age categories for age 90–94 years and 95 years and older were added. Other organisations periodically produce estimates of cause-specific mortality, including for a wide list of causes and across multiple age groups (WHO), for selected cancers (the International Agency for Research on Cancer), and for child deaths (the Maternal and Child Epidemiology Estimation [MCEE] group). GBD continues to provide the only peer-reviewed annual estimates of cause-specific mortality available for all locations over time.

**Added value of this study**

GBD 2017 includes estimates for 2017 and also updates the entire series from 1980 produced for GBD 2016. The list of included causes has been expanded and study methods have been improved in multiple ways. First, inclusion of an independent estimation of population and fertility developed for GBD 2017 substantially improved estimates in selected countries. Second, additional data were identified, including 127 country-years of vital registration and ten verbal autopsy studies. Third, new subnational assessments were developed for five countries in 2017: Ethiopia, Iran, New Zealand, Norway, and Russia. Fourth, a new stratum was developed for subnational-level estimation in New Zealand to characterise populations by ethnicity as Māori or non-Māori. Fifth, we revised adjustments made for misclassified deaths due to dementia, Parkinson's disease, and atrial fibrillation. Finally, additional diseases are now estimated, including non-rheumatic calcific aortic and degenerative mitral valve disease; subarachnoid haemorrhage; myelodysplastic, myeloproliferative, and other haemopoietic disorders; diabetes mellitus as type 1 and type 2 (previously combined); poisoning by carbon monoxide; liver cancer due to non-alcoholic steatohepatitis; ectopic pregnancy; and invasive non-typhoidal salmonella.

**Implications of all the available evidence**

Deaths due to communicable, maternal, neonatal, and nutritional causes continue to decline, while deaths from non-communicable diseases increase and injury deaths are stable. Declines in death rates of some non-communicable diseases have slowed or ceased. GBD 2017 has increased its collaboration with governments, leading to additional data for subnational estimation. Engagement with GBD collaborators, policy makers, disease experts, and the public is guiding expansions of the cause list and resulting decreasing burden classified in residual “other” categories. Non-communicable diseases remain the leading causes of death globally, and their burden is rising. GBD 2017 is motivated by the same goals as GBD 2016, including the belief that annual updates, reflecting improvements due to improved data availability, new causes estimated, and better methods to reduce bias and improve transparency in reporting, are contributing to the formulation and tracking of new evidence-based health policy. We intend for GBD 2017 to serve as a global public good, freely available for policy makers and the public seeking to improve human health.

The following study represents an annual update to the Global Burden of Diseases, Injuries, and Risk Factors Study (GBD), an effort to produce consistent and comparable estimates of cause-specific mortality for all locations globally. GBD 2017 includes results by age and sex, for the years 1980 through to 2017, for 195 countries and territories. A cycle of continuous quality improvement has led to substantial changes, including new data sources, new causes of death, and updated methods. For the first time, population estimates have been independently produced by GBD 2017,^14^ and subnational estimates have been produced for Ethiopia, Iran, New Zealand, Norway, and Russia. The purpose of GBD 2017 is to serve as a global public good, freely available for policy makers and the public seeking to improve human health.

## Methods

### Overview

GBD cause of death estimation incorporates methods to adjust for incomplete or missing vital registration (VR) and verbal autopsy (VA) data, general heterogeneity in data completeness and quality, and the redistribution of so-called garbage codes (insufficiently specific or implausible cause of death codes). A general description of these methods is provided in this section, with further detail presented in appendix 1. GBD 2017 complied with the Guidelines for Accurate and Transparent Health Estimates Reporting (GATHER)^15^ statement (appendix 1 section 1.3). Analyses were completed with Python version 2.7.14, Stata version 13.1, and R version 3.3.2. Statistical code used for GBD estimation is publicly available online.

### Geographical units and time periods

The locations included in GBD 2017 have been arranged into a set of hierarchical categories composed of seven super-regions and a further nested set of 21 regions containing 195 countries and territories (appendix 1). Each year, GBD includes subnational analyses for a few new countries and continues to provide subnational estimates for countries that were added in previous cycles. Subnational estimation in GBD 2017 includes five new countries (Ethiopia, Iran, New Zealand, Norway, Russia) and countries previously estimated at subnational levels (GBD 2013: China, Mexico, and the UK [regional level]; GBD 2015: Brazil, India, Japan, Kenya, South Africa, Sweden, and the USA; GBD 2016: Indonesia and the UK [local government authority level]). All analyses are at the first level of administrative organisation within each country except for New Zealand (by Māori ethnicity), Sweden (by Stockholm and non-Stockholm), and the UK (by local government authorities). All subnational estimates for these countries were incorporated into model development and evaluation as part of GBD 2017. To meet data use requirements, in this publication we present all subnational estimates excluding those pending publication (Brazil, India, Japan, Kenya, Mexico, Sweden, the UK, and the USA); because of space constraints these selected subnational results are presented in appendix 2. Subnational estimates for countries with populations larger than 200 million (measured with our most recent year of published estimates) that have not yet been published elsewhere are presented wherever estimates are illustrated with maps but are not included in data tables.

The complete cause-specific estimation results include the years 1980 through to 2017, and are available for exploration by an online data visualisation tool. To better support current health policy assessment, we include a subset of analyses in the current study featuring the most recent interval, 2007–17.

### The GBD cause of death hierarchy

The GBD study attributes each death to a single underlying cause that began the series of events leading to death, in accordance with ICD principles. The GBD study organises causes of death in a hierarchical list containing four levels (appendix 1 section 7). At the highest level (Level 1), all disease burden is divided among three mutually exclusive and collectively exhaustive categories: communicable, maternal, neonatal, and nutritional (CMNN) diseases; non-communicable diseases (NCDs); and injuries. Level 2 distinguishes these Level 1 categories into 21 cause groups, such as cardiovascular diseases; diarrhoeal diseases, lower respiratory infections (LRIs), and other common infectious diseases; or transport injuries. Level 3 disaggregates these causes further; in most cases this disaggregation represents the finest level of detail by cause, such as stroke, ischaemic heart disease, or road injuries. Where data are sufficiently available or specific policy relevance has been sought, selected causes are further disaggregated at Level 4, such as drug-susceptible tuberculosis, multidrug-resistant tuberculosis without extensive drug resistance, and extensively drug-resistant tuberculosis. For GBD 2017, the cause hierarchy was further refined to separately estimate causes with substantial policy interest or high levels of burden. Specific changes included separate estimation of non-rheumatic calcific aortic and degenerative mitral valve diseases, and myelodysplastic, myeloproliferative, and other haemopoietic neoplasms, resulting in a reduction in the estimates of some residual causes. Disaggregation of residual causes also allowed separate estimation of type 1 and type 2 diabetes, chronic kidney disease due to type 1 and type 2 diabetes, poisoning by carbon monoxide, liver cancer due to non-alcoholic steatohepatitis (NASH), subarachnoid haemorrhage, ectopic pregnancy, and invasive non-typhoidal salmonella. Maternal and neonatal disorders, previously estimated as separate cause groupings at Level 2 of the hierarchy, were estimated for GBD 2017 at Level 3 of the hierarchy, and then aggregated up to Level 2 to better capture the epidemiological connections and linked burden between them. The complete hierarchy of causes included in GBD 2017 and their corresponding ICD9 and ICD10 codes are described in appendix 1 (section 7).

### Cause of death data

**Figure 1:**
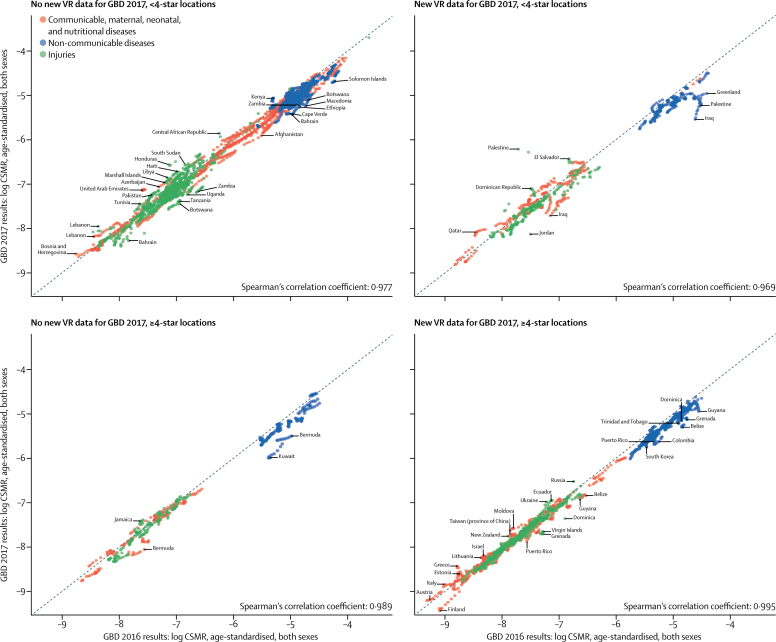
Effect of new VR data on Level 1 cause estimates from GBD 2016 to GBD 2017, based on national locations with varying quality of VR data, 2008–16 The figure shows the degree of consistency between GBD 2016 and GBD 2017 estimates for Level 1 causes at the national level from 2008 to 2016. The diagonal line represents no change from GBD 2016 to GBD 2017. Each point represents one country-year, with colours indicating the Level 1 cause grouping (communicable, maternal, neonatal, and nutritional diseases; non-communicable diseases; and injuries). Panels indicate whether or not any new VR data between 2008 and 2016 were added for that location for GBD 2017, and whether or not a location has 4-star or 5-star VR quality. Points that are outside of the standard 95% prediction interval for a linear regression of 2017 values on 2016 values are annotated (if the same location-cause had multiple points in a time series, only the furthest-most point was annotated). The Spearman's correlation coefficient is noted in the lower right-hand corner of each panel. CSMR=cause-specific mortality rate. GBD=Global Burden of Diseases, Injuries, and Risk Factors Study. VR=vital registration.

The GBD cause of death database consists of VR and VA data; survey and census data for injuries and maternal mortality; surveillance data for maternal mortality and child death; cancer registries; and police records for interpersonal violence and road injuries. Self-harm estimates incorporate VR data and are based on ICD categorisation as described in appendix 1 (section 7). In this iteration of GBD, ten new VA studies and 127 new country-years of VR data were added at the country level. 502 new cancer-registry country-years were added, as was one additional new surveillance country-year. Data sources comprising the GBD cause of death database can be reviewed on the Global Health Data Exchange website. Multiple factors can influence changes between GBD studies in estimates for a given cause-location-year, including the quality of a country's data system (as represented by the GBD star rating system) and the addition of more recent data. Figure 1 shows the relative stability of GBD estimates between study iterations. Variation between GBD 2016 and GBD 2017 estimates was greater in countries with both low star ratings and no new VR data updates occurring between these iterations of the study. Changes to estimates can be seen even in high star rating locations because of changes in modelling strategy or model covariates even when no new VR data were available between cycles.

### Data standardisation and processing

To standardise cause of death data, we used protocols to address the minor proportion of deaths that were assigned to age groups broader than the GBD five-year age groups or were not assigned an age or sex, and to address differences in ICD codes due to national variation or revision, as described in appendix 1 (section 2). Garbage codes, deaths with non-specific codes (eg, unspecified stroke), deaths assigned to ICD codes that could not be underlying causes of death (eg, senility), or deaths assigned to intermediate but not underlying causes of death (eg, heart failure), were redistributed by age, sex, location, and year to the most likely causes of death. Methods used for this redistribution included regression models, redistribution based on fixed proportions, proportional reassignment, and fractional assignment of a death assigned to multiple causes, as developed by Naghavi and colleagues^16^ and detailed in appendix 1 (section 2.7). We excluded all data sources with more than 50% of deaths assigned to major garbage codes (those at Level 1 or Level 2 of the GBD hierarchy) in any location-year to mitigate the potential for bias from these sources. The proportion of VR data assigned to major garbage code categories for each location-year is shown, with supporting detail, in appendix 1 (section 7). New to GBD 2017, the uncertainty around redistribution methods was also estimated. Additional details for this process are provided in appendix 1 (section 2.7). Because mortality due to HIV/AIDS is sometimes coded to other causes of death such as tuberculosis, meningitis, or toxoplasmosis, we also corrected the cause of death assignment to HIV/AIDS for peak epidemic times. Tuberculosis deaths can be misclassified as pneumonia deaths in children in locations with a high tuberculosis burden. Methods to adjust for this potential misclassification are described in detail in appendix 1 (section 3.3).

Mortality rates from dementia and Parkinson's disease reported in VR systems cannot be reconciled with observed trends in prevalence and excess mortality—a disparity that can be attributed to variation in death certification practices for these causes across countries and over time.^17^ For GBD 2017, we sought to address this known bias by using details from multiple cause of death data. For GBD 2017, multiple cause of death data were available to investigators only for the USA, where recent years show improved use of previously under-utilised codes such as dementia. Statistical models of these USA data were used to reclassify deaths from other GBD causes and garbage codes to dementia and Parkinson's disease according to the pattern of intermediate and immediate causes observed in the most recent years. Model results were applied to all countries. A similar reallocation process was used for atrial fibrillation deaths misclassified as deaths due to heart failure or thromboembolic events. A detailed description of these redistribution procedures and the manner in which they were applied to all countries is available in section 2 of appendix 1. This reallocation is illustrated in appendix 1 (section 7).

For the first time in GBD 2017, we separately estimated deaths from diabetes by type. Deaths due to diabetes can be reported in VR and VA data as type 1, type 2, or unspecified. Two data manipulation steps were necessary. First, we assumed all deaths reported in individuals younger than 15 years were type 1 regardless of the original code assignment. Second, we redistributed unspecified diabetes deaths on the basis of a regression in which the true proportions of type 1 and type 2 deaths by age-sex-location-year are a function of the proportion of unspecified deaths, age, the age-standardised prevalence of obesity, and an interaction term for age and obesity prevalence. These methods are described in detail in appendix 1 (section 3.3).

### Data completeness assessment

Completeness of VR data was assessed by location-year, and sources with less than 50% completeness were excluded. We multiplied the estimated all-cause mortality for each age-sex-location-year by the cause fraction for the corresponding age-sex-location-year to adjust all included sources to 100% completeness. VA and VR data availability and completeness are shown for each location-year in appendix 1 (section 7). To further characterise the quality of data available in each country, the GBD study rated each location-year from 1980 to 2017 on a level of 0 to 5 stars according to methods previously described.^18^ Ratings convey an overall measure of the reliability of cause of death estimates for each location-year but do not directly affect the estimation process.

### Cause of death estimation with CODEm

**Figure 2:**
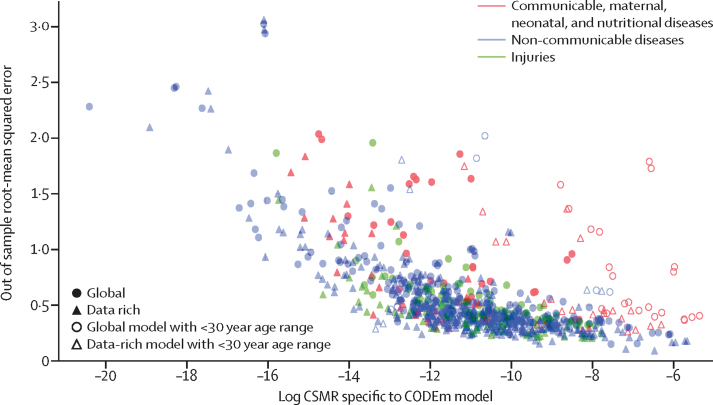
Out-of-sample model performance for CODEm models and age-standardised cause-specific mortality rate by Level 1 causes Model performance was defined by the root-mean squared error of the ensemble model predictions of the log of the age-specific death rates for a cause with 15% of the data held out from the statistical model building. The figure shows the association between the root-mean squared error and the log of the CSMR, aggregated over 1980–2017. Each point represents one CODEm model specific for model-specific age ranges and sex. Circles denote models run with all locations. Triangles denote models run on only data-rich locations. Colours denote the Level 1 cause categories. Open circles and triangles denote models that were run with restricted age groups of less than 30 years. CODEm=Cause of Death Ensemble model. CSMR=cause-specific mortality rate.

The GBD Cause of Death Ensemble model (CODEm) systematically tested and combined results from different statistical models according to their out-of-sample predictive validity. Results are incorporated into a weighted ensemble model as detailed in appendix 1 (section 3.1) and below. For GBD 2017, CODEm was used to estimate 192 causes of death (appendix 1 section 7). To predict the level for each cause of death, we used CODEm to systematically test a large number of functional forms and permutations of covariates.^18^ Each resulting model that met the predetermined requirements for regression coefficient significance and direction was fit on 70% of the data, holding out 30% for cross-validation (appendix 1 section 3.1). Out-of-sample predictive validity of these models was assessed by use of repeated cross-validation tests on the first 15% of the held-out data. Various ensemble models with different weighting parameters were created from the combination of these models, with the highest weights assigned to models with the best out-of-sample prediction error for trends and levels, as detailed in appendix 1 (section 7). Model performance of these ensembles was assessed against the root-mean squared error (RMSE) of the ensemble model predictions of the log of the age-specific death rates for a cause, assessed with the same 15% of the data. The ensemble model performing best was subsequently selected and assessed against the other 15% of the data withheld from the statistical model building. CODEm was run independently by sex for each cause of death. A separate model was run for countries with 4-star or greater VR systems to avert uncertainty inflation from more heterogeneous data. The distribution of RMSE relative to cause-specific mortality rates (CSMRs) at Level 2 of the GBD hierarchy shows that model performance was weakest for causes of death with comparatively low mortality rates (figure 2; appendix 2), while models for more common causes of death such as stroke, chronic obstructive pulmonary disease, and self-harm and interpersonal violence generally had low RMSE.

### Cause of death estimation with alternative estimation strategies

Alternative estimation strategies were used to model a subset of causes of death with unique epidemiology, large changes in reporting over time, or particularly limited data availability, including HIV/AIDS, malaria, chronic kidney disease, cirrhosis, liver cancer, meningitis, dementia, and atrial fibrillation. Alternative strategies included prevalence-based models, incidence and case fatality models, and sub-cause proportion models as described in appendix 1 (section 7). Mortality-incidence ratio models based on registry data were used to estimate mortality from 32 cancers (appendix 1 section 3.3). Negative-binomial models were used for eight causes of death with typically low death counts or causes that typically have no deaths in countries with a high Socio-demographic Index (SDI), including ascariasis, cystic echinococcosis, cysticercosis, diphtheria, iodine deficiency, other intestinal infectious diseases, schistosomiasis, and varicella and herpes zoster virus. Once underlying cause of death estimates and accompanying uncertainty were generated, these models were combined with the cause of death correction procedure (CoDCorrect) to establish estimates consistent with all-cause mortality levels for each age-sex-year location.

### Estimation of fatal discontinuities

Fatal discontinuities are large changes in deaths due to unexpected spikes in injuries or epidemics—defined by GBD as more than one per million or more than 25 deaths—in a specific location-year. We classified fatal discontinuities as conflict and terrorism, major transportation accidents, natural disasters, other forms of disaster such as large fires or the collapse of large buildings, or major outbreaks of infectious diseases. Data on fatal discontinuities came from VR data in the 75 countries with a 4-star or 5-star data quality rating for the interval of 1980–2017. For the remaining 120 countries with a rating of 3 stars or lower, we used alternative databases (appendix 1 section 7). Cholera and meningitis were estimated as fatal discontinuities to reduce the risk of underestimation for small-magnitude outbreaks caused by the smoothing of VR or VA data over time in CODEm. To address lags in reporting and publishing of data, we included news reports and other supplemental data sources when known gaps existed. Further detail about fatal discontinuity estimation is presented in appendix 1 (section 3.3).

### Pathogen counterfactual analysis

Aetiology-specific mortality was estimated for LRIs and diarrhoeal diseases by use of a counterfactual approach that relates the frequency of each aetiology in a population and the association with that aetiology and either LRI or diarrhoea. LRI and diarrhoea were selected as initial candidates for this counterfactual analysis approach given the large disease burden they represent and the broad interest in interventions, mostly vaccine-based, to reduce their burden.^19^ We attributed LRI deaths to four aetiologies: *Haemophilus influenzae* type B pneumonia, *Streptococcus pneumoniae* pneumococcal pneumonia, influenza, and respiratory syncytial virus pneumonia. Diarrhoeal deaths were attributed to 13 aetiologies: adenovirus, Aeromonas spp, *Campylobacter* spp, *Clostridium difficile*, cryptosporidiosis (*Cryptosporidium* spp), amoebiasis (*Entamoeba histolytica*), typical enteropathogenic *Escherichia coli*, enterotoxigenic *E coli*, norovirus, rotavirus, non-typhoidal *Salmonella* spp, shigellosis (*Shigella* spp), and cholera (*Vibrio cholerae*). The mortality attributable to each aetiology is the product of the attributable fraction and the mortality due to LRI or diarrhoea. The current counterfactual analysis is an extension of work begun in GBD 2010, based on the most common pathogens and available data. This method allows for less common aetiologies to be added in the future.

### YLL computation

Years of life lost (YLLs) are a measure of premature death calculated as the sum of each death multiplied by the standard life expectancy at each age. The standard life expectancy was taken from the lowest observed risk of death for each five-year age group in all populations greater than 5 million. In 2017, GBD 2017 included a new demographic assessment of population, fertility, migration, and all-cause mortality.^14^ We used these components to generate single calendar-year and single age-year estimates of the population using transparent and replicable methods.^14^ This independent assessment of the population was subsequently used in the calculation of YLL rates and age-standardised mortality rates. Details of these calculations are available in appendix 1 (section 4.3).

### Decomposition of change in global deaths

Using methods adapted from demographic research from Das Gupta,^20^ we decomposed change in numbers of deaths by cause from 2007 to 2017, using three explanatory components: as change occurring from growth in the total population; as shifts in population structure by age; or as changes in cause-specific mortality rates. We calculated the fraction of change in deaths by cause from each component using counterfactual scenarios, changing the level of one factor from 2007 to 2017, with all other factors held constant. Since the effect depends on the order of entry of the factor, we calculated the average of all combinations of the three factors. Thus, the change in global deaths due to shifts in population age structure could be calculated by comparing the number of deaths in 2007 to the number of deaths in 2017, using the population age structure from 2017 and holding both population size and cause-specific mortality rates at 2007 levels (appendix 1 section 7).

### Uncertainty analysis

Uncertainty in our estimates was attributable to cause-specific model specifications; varied availability of data by age, sex, location, or year; and variability of sample size within data sources. We quantified and propagated uncertainty into final estimates by calculating uncertainty intervals (UIs) for cause-specific estimation components based on 1000 draws from the posterior distribution of cause-specific mortality by age, sex, location, and year.^21^ 95% UIs were calculated with the 2·5th and 97·5th percentiles, and point estimates were calculated from the mean of the draws. Changes over time were considered statistically significant when the uncertainty interval of the percentage change over time did not cross zero.

### Socio-demographic Index and epidemiological transition analysis

The SDI is a value between 0·0 and 1·0 calculated from the geometric mean of three rescaled components: total fertility rate under 25 years (TFRU25), lag-distributed income per capita (LDI), and average educational attainment in the population older than 15 years.^22^ Because the total fertility rate—used in the calculation of SDI for GBD 2016—has a U-shaped association at the highest levels of development, for GBD 2017 we recomputed the SDI using TFRU25 only, an age range for which the association with development is clearest.^14^ We used a generalised additive model with a Loess smoother on SDI to estimate the association between SDI and each age-sex-cause death rate using GBD estimates from all national locations across all years from 1980 to 2017 (appendix 1 section 7). Expected cause-specific death rates were scaled to the expected all-cause death rate to ensure internal consistency. We then computed the number of YLLs and deaths expected for each age-sex-location-year based on SDI alone and compared these estimates to observed rates. Additional details of the development and calculation of SDI for GBD 2017 are described in appendix 1 (section 5).

### Role of the funding source

The funders had no role in study design, data collection, data analysis, data interpretation, or writing of the report. All authors had full access to all the data in the study and had final responsibility for the decision to submit for publication.

## Results

### Global causes of death

Mortality estimates by cause for the years 1990, 2007, and 2017 are available by age and sex through the GBD results tool and for each year in the GBD estimation period 1980–2017 through the online data visualisation tool. All reported rates are age-standardised.

In 2017, at the broadest level of cause of death classification in the GBD cause list (Level 1), CMNN causes accounted for 18·6% (95% UI 17·9–19·6) of total deaths or 10·4 million (10·0–11·0) deaths in 2017, while non-communicable causes (NCDs) accounted for 73·4% (72·5–74·1) or 41·1 million (40·5–41·5) deaths, and injuries accounted for 8·0% (7·7–8·2) of deaths or 4·48 million (4·33–4·59) deaths (table 1). Of the 1·65 billion (1·62–1·67) global YLLs in 2017, 35·1% (34·2–36·2) were from CMNN causes, 53·0% (52·2–53·8) were from NCDs, and the remaining 11·9% (11·5–12·1) were from injuries. Both the number of deaths and death rates from CMNN causes decreased from 2007 to 2017, by 22·2% (20·0–24·0) in terms of total deaths and by 31·8% (30·1–33·3) in terms of mortality rate. Decreases in the number and rate of YLLs from CMNN causes were similar in magnitude (30·4% [28·2–32·4] decrease in YLLs; 35·4% [33·4–37·3] decrease in YLL rate) over the same time period. By contrast, total deaths from NCD causes increased between 2007 and 2017 by 22·7% (21·5–23·9) and total YLLs from NCD causes increased by 13·6% (12·2–14·9), representing an additional 7·61 million (7·20–8·01) deaths and 105 million (94·3–114·0) YLLs estimated in 2017. Rates of both deaths and YLLs from NCD causes decreased over the same time period, by 7·9% (7·0–8·8) to 536·1 deaths (528·4–542·2) per 100 000, with a 9·6% (8·6–10·7) decrease in the YLL rate to 11 100 YLLs (10 900–11 300) per 100 000 in 2017. Total deaths from injuries varied little between 2007 and 2017, with an increase of 2·3% (0·5–4·0) to 4·48 million (4·33–4·59) deaths, while death rates from injury decreased by 13·7% (12·2–15·1) to 57·9 deaths (55·9–59·2) per 100 000 in 2017. Decreases in the number of YLLs (by 6·4% [4·8–7·8] to 195 million [189–200] YLLs in 2017) and YLL rate (by 16·9% [15·3–18·2] to 2550 [2460–2610] YLLs per 100 000 in 2017) for injuries were estimated during the same period.

**Table 1:** Global death and YLL numbers, age-standardised rates per 100 000, and percentage change between 2007 and 2017 for both sexes combined for all GBD causes and Levels 1 through 4 of the cause hierarchy

			**All-age deaths (thousands)**		**Age-standardised death rate (per 100 000)**		**All-age YLLs (thousands)**		**Age-standardised YLL rate (per 100 000)**	
			2017	Percentage change, 2007–17	2017	Percentage change, 2007–17	2017	Percentage change, 2007–17	2017	Percentage change, 2007–17
**All causes**			**55 945·7 (55 356·4 to 56 516·7)**	9·3% (8·2 to 10·2)*	**737·7 (729·9 to 745·4)**	−14·2% (−15·0 to −13·5)*	**1 646 249·6 (1 622 870·6 to 1 673 178·4)**	−9·0% (−10·1 to −7·6)*	**21 926·4 (21 601·1 to 22 314·9)**	−22·2% (−23·2 to −21·0)*
**Communicable, maternal, neonatal, and nutritional diseases**			**10 389·9 (10 004·0 to 10 975·9)**	−22·2% (−24·0 to −20·0)*	**143·8 (138·4 to 151·6)**	−31·8% (−33·3 to −30·1)*	**578 416·6 (558 815·0 to 600 759·1)**	−30·4% (−32·4 to −28·2)*	**8280·6 (8005·4 to 8602·8)**	−35·4% (−37·3 to −33·4)*
	**HIV/AIDS and sexually transmitted infections**		**1073·6 (983·3 to 1182·4)**	−47·7% (−50·0 to −45·1)*	**13·9 (12·6 to 15·5)**	−53·6% (−55·8 to −51·0)*	**60 550·2 (53 533·7 to 69 156·3)**	−47·3% (−50·2 to −44·0)*	**806·4 (703·1 to 936·7)**	−52·1% (−55·2 to −48·6)*
	HIV/AIDS		954·5 (907·3 to 1009·7)	−50·3% (−52·1 to −48·3)*	12·1 (11·5 to 12·9)	−56·5% (−58·0 to −54·7)*	50 497·1 (47 658·0 to 53 595·8)	−51·2% (−52·9 to −49·2)*	655·1 (617·5 to 696·4)	−56·6% (−58·1 to −54·8)*
		HIV/AIDS and drug-susceptible tuberculosis co-infection	194·6 (137·7 to 253·0)	−55·4% (−58·4 to −51·6)*	2·5 (1·8 to 3·2)	−61·1% (−63·7 to −57·7)*	10 664·8 (7613·4 to 13 757·1)	−55·6% (−58·7 to −51·7)*	140·0 (100·2 to 180·0)	−60·5% (−63·1 to −57·0)*
		HIV/AIDS and multidrug-resistant tuberculosis without extensive drug resistance co-infection	22·6 (13·4 to 34·5)	−52·2% (−66·4 to −33·2)*	0·3 (0·2 to 0·4)	−58·1% (−70·5 to −41·5)*	1247·8 (746·6 to 1906·7)	−51·7% (−65·7 to −33·2)*	16·4 (9·8 to 25·1)	−56·8% (−69·3 to −40·4)*
		HIV/AIDS and extensively drug-resistant tuberculosis co-infection	1·2 (0·8 to 1·8)	−8·3% (−26·8 to 14·7)	0·0 (0·0 to 0·0)	−20·3% (−36·4 to −0·2)*	62·7 (38·3 to 92·9)	−10·5% (−28·4 to 11·5)	0·8 (0·5 to 1·2)	−21·0% (−36·7 to −1·4)*
		HIV/AIDS resulting in other diseases	736·0 (659·5 to 817·7)	−48·7% (−51·1 to −45·9)*	9·3 (8·4 to 10·4)	−55·1% (−57·2 to −52·6)*	38 521·8 (34 381·3 to 43 095·5)	−49·8% (−52·3 to −46·9)*	497·9 (444·2 to 558·4)	−55·4% (−57·6 to −52·8)*
	Sexually transmitted infections excluding HIV		119·1 (50·8 to 220·4)	−10·8% (−18·4 to −2·5)*	1·8 (0·7 to 3·3)	−14·4% (−21·5 to −6·6)*	10 053·1 (4057·0 to 18 915·2)	−11·4% (−19·0 to −3·2)*	151·3 (60·6 to 285·3)	−14·4% (−21·8 to −6·6)*
		Syphilis	113·5 (45·2 to 214·5)	−11·3% (−19·1 to −2·8)*	1·7 (0·7 to 3·2)	−14·3% (−21·8 to −6·4)*	9836·1 (3848·5 to 18 676·4)	−11·5% (−19·3 to −3·1)*	148·6 (58·0 to 282·3)	−14·3% (−21·8 to −6·2)*
		Chlamydial infection	1·1 (0·9 to 1·2)	2·5% (−4·5 to 11·3)	0·0 (0·0 to 0·0)	−15·2% (−21·0 to −8·4)*	40·5 (32·6 to 45·0)	−5·5% (−12·2 to 2·5)	0·5 (0·4 to 0·6)	−17·9% (−23·7 to −11·0)*
		Gonococcal infection	3·0 (2·4 to 3·3)	3·7% (−3·4 to 12·5)	0·0 (0·0 to 0·0)	−14·9% (−20·8 to −8·2)*	112·8 (90·2 to 124·9)	−3·8% (−10·7 to 4·3)	1·4 (1·1 to 1·6)	−17·4% (−23·5 to −10·7)*
		Other sexually transmitted infections	1·5 (1·2 to 1·7)	0·2% (−6·4 to 8·3)	0·0 (0·0 to 0·0)	−15·9% (−21·6 to −9·5)*	63·6 (51·0 to 70·7)	−6·2% (−12·7 to 1·1)	0·8 (0·6 to 0·9)	−18·2% (−23·9 to −11·7)*
	**Respiratory infections and tuberculosis**		**3752·3 (3629·4 to 3889·3)**	−8·0% (−10·3 to −5·5)*	**50·5 (48·8 to 52·3)**	−24·5% (−26·4 to −22·6)*	**148 233·5 (141 335·1 to 155 291·4)**	−24·7% (−27·4 to −21·7)*	**2056·0 (1956·3 to 2160·7)**	−32·8% (−35·4 to −30·0)*
	Tuberculosis		1183·7 (1129·8 to 1245·3)	−14·9% (−18·2 to −10·3)*	14·9 (14·3 to 15·7)	−31·4% (−34·1 to −27·6)*	41 876·9 (39 972·4 to 44 120·5)	−21·2% (−24·4 to −17·4)*	533·4 (509·1 to 562·6)	−33·3% (−35·9 to −30·0)*
		Drug-susceptible tuberculosis	1044·1 (951·6 to 1129·2)	−15·5% (−22·3 to −8·6)*	13·2 (12·0 to 14·2)	−31·9% (−37·3 to −26·4)*	36 932·5 (33 846·8 to 39 919·1)	−21·9% (−27·8 to −16·0)*	470·7 (431·3 to 508·4)	−33·8% (−38·7 to −29·0)*
		Multidrug-resistant tuberculosis without extensive drug resistance	126·9 (70·1 to 202·2)	−11·6% (−47·4 to 38·1)	1·6 (0·9 to 2·5)	−28·6% (−57·4 to 11·4)	4505·1 (2582·5 to 6984·6)	−17·6% (−49·4 to 26·5)	57·2 (33·0 to 88·4)	−30·2% (−56·9 to 6·6)
		Extensively drug-resistant tuberculosis	12·6 (8·6 to 18·0)	14·0% (−18·7 to 58·7)	0·2 (0·1 to 0·2)	−7·7% (−34·1 to 28·8)	439·2 (306·2 to 616·5)	5·5% (−23·2 to 44·9)	5·5 (3·8 to 7·7)	−11·1% (−35·2 to 22·1)
	Lower respiratory infections		2558·6 (2442·2 to 2655·4)	−4·3% (−6·9 to −1·5)*	35·4 (33·8 to 36·8)	−21·1% (−23·2 to −18·9)*	105 834·5 (99 746·4 to 111 767·8)	−25·9% (−29·2 to −22·2)*	1515·1 (1424·8 to 1602·2)	−32·6% (−35·7 to −29·2)*
	Upper respiratory infections		9·1 (6·1 to 12·4)	−30·5% (−41·0 to −14·5)*	0·1 (0·1 to 0·2)	−42·1% (−49·6 to −29·9)*	477·3 (247·3 to 730·5)	−33·2% (−44·1 to −12·9)*	6·9 (3·5 to 10·6)	−38·6% (−48·3 to −19·4)*
	Otitis media		0·9 (0·7 to 1·5)	−41·4% (−51·6 to −28·4)*	0·0 (0·0 to 0·0)	−50·4% (−58·8 to −39·9)*	44·8 (31·2 to 72·1)	−49·4% (−59·9 to −35·5)*	0·6 (0·4 to 1·0)	−54·5% (−64·1 to −41·8)*
	**Enteric infections**		**1766·0 (1398·0 to 2386·0)**	−17·2% (−24·6 to −8·2)*	**24·4 (19·5 to 32·4)**	−29·9% (−34·9 to −23·1)*	**84 625·5 (73 770·6 to 100 720·2)**	−30·6% (−36·3 to −23·7)*	**1208·6 (1064·1 to 1424·7)**	−36·6% (−41·8 to −30·7)*
	Diarrhoeal diseases		1569·6 (1176·0 to 2193·0)	−16·6% (−25·3 to −6·7)*	21·6 (16·4 to 29·7)	−30·2% (−36·1 to −22·7)*	70 574·3 (60 421·1 to 86 165·2)	−32·0% (−38·6 to −23·9)*	1009·1 (870·5 to 1211·0)	−38·1% (−43·9 to −31·3)*
	Typhoid and paratyphoid		135·9 (76·9 to 218·9)	−22·3% (−27·3 to −18·1)*	1·9 (1·1 to 3·0)	−27·8% (−32·8 to −23·9)*	9686·1 (5484·9 to 15 746·2)	−23·8% (−29·3 to −19·4)*	136·3 (77·0 to 220·9)	−28·7% (−34·0 to −24·4)*
		Typhoid fever	116·8 (65·4 to 187·7)	−23·7% (−29·0 to −19·3)*	1·6 (0·9 to 2·6)	−29·1% (−34·1 to −25·0)*	8331·7 (4632·5 to 13 419·2)	−25·3% (−31·0 to −20·8)*	117·3 (65·5 to 188·5)	−30·1% (−35·6 to −25·7)*
		Paratyphoid fever	19·1 (8·7 to 37·3)	−12·7% (−20·1 to −4·2)*	0·3 (0·1 to 0·5)	−18·9% (−26·1 to −10·8)*	1354·4 (622·3 to 2620·2)	−13·2% (−21·3 to −3·8)*	19·0 (8·8 to 36·6)	−18·6% (−26·5 to −9·7)*
	Invasive non-typhoidal salmonella		59·1 (33·3 to 98·1)	−17·9% (−25·1 to −8·7)*	0·8 (0·5 to 1·4)	−24·8% (−31·9 to −15·6)*	4260·8 (2382·0 to 7378·6)	−17·2% (−25·7 to −6·8)*	61·6 (34·7 to 107·6)	−22·6% (−30·7 to −12·5)*
	Other intestinal infectious diseases		1·4 (1·0 to 2·2)	−39·7% (−67·1 to 9·7)	0·0 (0·0 to 0·0)	−44·7% (−70·1 to 2·3)	104·4 (67·8 to 170·7)	−43·6% (−71·6 to 11·9)	1·5 (1·0 to 2·5)	−46·9% (−73·7 to 6·3)
	**Neglected tropical diseases and malaria**		**720·1 (530·7 to 938·8)**	−29·0% (−37·3 to −19·3)*	**10·1 (7·5 to 13·2)**	−36·1% (−43·7 to −27·3)*	**48 656·2 (35 574·6 to 64 934·2)**	−33·7% (−42·4 to −23·7)*	**699·9 (508·0 to 933·6)**	−38·6% (−46·7 to −29·2)*
	Malaria		619·8 (440·1 to 839·5)	−30·8% (−39·4 to −20·8)*	8·7 (6·1 to 11·9)	−37·3% (−45·4 to −27·9)*	43 546·6 (29 966·3 to 59 772·4)	−34·5% (−43·8 to −23·6)*	629·4 (432·6 to 858·7)	−39·2% (−48·2 to −28·8)*
	Chagas disease		7·9 (7·5 to 8·6)	3·8% (−1·6 to 12·9)	0·1 (0·1 to 0·1)	−21·1% (−25·2 to −14·3)*	174·9 (166·1 to 193·5)	−4·2% (−9·0 to 4·8)	2·2 (2·0 to 2·4)	−25·1% (−28·9 to −18·1)*
	Leishmaniasis		7·5 (0·0 to 34·5)	−64·8% (−96·8 to −44·5)*	0·1 (0·0 to 0·5)	−67·8% (−97·5 to −50·3)*	509·8 (0·3 to 2440·2)	−63·8% (−92·1 to −39·7)*	7·2 (0·0 to 34·6)	−66·2% (−93·2 to −43·8)*
		Visceral leishmaniasis	7·5 (0·0 to 34·5)	−64·8% (−96·8 to −44·5)*	0·1 (0·0 to 0·5)	−67·8% (−97·5 to −50·3)*	509·8 (0·3 to 2440·2)	−63·8% (−92·1 to −39·7)*	7·2 (0·0 to 34·6)	−66·2% (−93·2 to −43·8)*
	African trypanosomiasis		1·4 (0·3 to 4·9)	−80·7% (−95·6 to −27·8)*	0·0 (0·0 to 0·1)	−82·8% (−96·0 to −34·3)*	77·6 (15·0 to 283·6)	−80·8% (−95·6 to −27·2)*	1·0 (0·2 to 3·8)	−82·3% (−96·0 to −33·6)*
	Schistosomiasis		8·8 (8·0 to 9·8)	−12·3% (−17·6 to −6·4)*	0·1 (0·1 to 0·1)	−28·5% (−32·7 to −23·7)*	342·3 (305·3 to 384·3)	−15·6% (−21·9 to −8·8)*	4·4 (3·9 to 5·0)	−27·4% (−32·9 to −21·4)*
	Cysticercosis		0·7 (0·5 to 1·0)	−15·9% (−42·7 to 23·3)	0·0 (0·0 to 0·0)	−27·3% (−50·5 to 5·3)	39·6 (26·9 to 55·0)	−20·5% (−46·9 to 18·2)	0·5 (0·4 to 0·7)	−28·9% (−52·5 to 4·8)
	Cystic echinococcosis		1·2 (0·9 to 1·5)	−30·0% (−52·1 to −1·3)*	0·0 (0·0 to 0·0)	−41·9% (−59·8 to −19·0)*	52·0 (38·1 to 68·0)	−38·8% (−56·8 to −12·9)*	0·7 (0·5 to 0·9)	−46·4% (−62·0 to −24·1)*
	Dengue		40·5 (17·6 to 49·8)	65·5% (21·7 to 99·7)*	0·5 (0·2 to 0·7)	40·7% (3·6 to 69·7)*	1902·9 (716·6 to 2312·9)	32·0% (−1·8 to 61·2)	26·1 (9·8 to 31·7)	18·2% (−12·0 to 45·0)
	Yellow fever		4·8 (1·0 to 13·8)	−16·6% (−28·7 to −2·0)*	0·1 (0·0 to 0·2)	−23·3% (−34·4 to −9·6)*	313·9 (67·2 to 900·2)	−16·0% (−28·9 to 0·0)	4·3 (0·9 to 12·4)	−21·3% (−33·6 to −5·8)*
	Rabies		11·7 (9·3 to 14·7)	−48·1% (−58·8 to −37·3)*	0·2 (0·1 to 0·2)	−54·8% (−63·8 to −45·0)*	633·7 (504·4 to 836·4)	−51·5% (−61·3 to −38·9)*	8·6 (6·8 to 11·5)	−56·2% (−65·1 to −44·3)*
	Intestinal nematode infections		3·2 (2·5 to 4·1)	−43·1% (−56·1 to −25·0)*	0·0 (0·0 to 0·1)	−47·2% (−59·5 to −30·1)*	257·1 (194·1 to 336·3)	−44·1% (−57·6 to −25·0)*	3·8 (2·9 to 5·0)	−47·6% (−60·4 to −29·6)*
		Ascariasis	3·2 (2·5 to 4·1)	−43·1% (−56·1 to −25·0)*	0·0 (0·0 to 0·1)	−47·2% (−59·5 to −30·1)*	257·1 (194·1 to 336·3)	−44·1% (−57·6 to −25·0)*	3·8 (2·9 to 5·0)	−47·6% (−60·4 to −29·6)*
	Ebola virus disease		0·0 (0·0 to 0·0)	−98·2% (−98·4 to −98·0)*	0·0 (0·0 to 0·0)	−98·4% (−98·6 to −98·2)*	0·5 (0·5 to 0·5)	−98·1% (−98·3 to −97·9)*	0·0 (0·0 to 0·0)	−98·2% (−98·4 to −98·0)*
	Zika virus disease		0·0 (0·0 to 0·1)	..	0·0 (0·0 to 0·0)	..	1·0 (0·2 to 3·4)	..	0·0 (0·0 to 0·0)	..
	Other neglected tropical diseases		12·6 (8·0 to 36·3)	8·1% (−8·1 to 28·2)	0·2 (0·1 to 0·5)	−3·7% (−18·3 to 13·9)	804·3 (442·8 to 2696·6)	3·9% (−16·3 to 29·4)	11·6 (6·3 to 39·6)	−3·5% (−22·2 to 20·7)
	**Other infectious diseases**		**830·5 (732·2 to 947·8)**	−25·9% (−32·4 to −18·8)*	**11·6 (10·1 to 13·3)**	−33·8% (−39·3 to −27·4)*	**53 008·6 (44 786·0 to 63 000·4)**	−33·0% (−39·6 to −25·1)*	**762·8 (640·5 to 911·5)**	−37·9% (−44·0 to −30·5)*
	Meningitis		288·0 (254·3 to 333·2)	−20·1% (−26·0 to −11·0)*	4·0 (3·6 to 4·6)	−27·8% (−33·1 to −19·3)*	19 436·9 (16 935·1 to 22 335·8)	−25·2% (−31·5 to −15·7)*	280·5 (243·6 to 323·2)	−30·2% (−36·3 to −21·4)*
		Pneumococcal meningitis	42·1 (36·6 to 49·4)	−13·4% (−20·6 to −2·3)*	0·6 (0·5 to 0·7)	−22·4% (−28·9 to −12·4)*	2751·8 (2325·8 to 3276·5)	−18·5% (−26·8 to −6·5)*	39·6 (33·4 to 47·0)	−24·2% (−32·1 to −12·8)*
		*H influenzae* type B meningitis	75·7 (66·7 to 92·0)	−33·7% (−39·6 to −26·0)*	1·1 (0·9 to 1·3)	−40·6% (−45·8 to −33·9)*	4907·3 (4232·2 to 5813·6)	−40·4% (−46·1 to −33·0)*	70·5 (60·6 to 83·9)	−44·7% (−50·1 to −37·7)*
		Meningococcal infection	30·0 (25·7 to 35·7)	−31·5% (−37·4 to −22·8)*	0·4 (0·4 to 0·5)	−37·1% (−42·6 to −29·2)*	2180·3 (1819·8 to 2614·5)	−34·9% (−41·4 to −26·4)*	31·9 (26·5 to 38·4)	−38·8% (−45·0 to −30·5)*
		Other meningitis	140·3 (121·4 to 161·8)	−8·9% (−15·4 to 1·4)	2·0 (1·7 to 2·3)	−17·3% (−23·4 to −7·5)*	9597·5 (8195·6 to 11 118·5)	−12·8% (−20·4 to −0·7)*	138·5 (118·3 to 160·5)	−18·4% (−25·7 to −7·4)*
	Encephalitis		92·4 (83·1 to 107·9)	0·0% (−14·2 to 16·2)	1·2 (1·1 to 1·4)	−14·3% (−26·5 to −0·9)*	4588·2 (4059·5 to 5230·7)	−12·1% (−28·1 to 4·5)	64·1 (56·6 to 72·4)	−20·1% (−35·0 to −5·0)*
	Diphtheria		3·6 (2·2 to 6·1)	−23·9% (−55·6 to 36·4)	0·1 (0·0 to 0·1)	−28·6% (−58·8 to 29·2)	298·7 (181·8 to 510·0)	−23·9% (−56·7 to 38·7)	4·4 (2·7 to 7·6)	−28·3% (−59·5 to 31·4)
	Whooping cough		91·8 (45·9 to 163·2)	−23·3% (−54·8 to 35·6)	1·4 (0·7 to 2·4)	−27·1% (−57·1 to 28·8)	7879·2 (3938·1 to 14 010·3)	−23·3% (−54·8 to 35·4)	117·9 (58·9 to 209·6)	−27·1% (−57·0 to 28·8)
	Tetanus		38·1 (25·9 to 48·8)	−54·9% (−65·9 to −39·1)*	0·5 (0·4 to 0·7)	−59·6% (−69·3 to −45·0)*	2447·7 (1734·9 to 3199·0)	−59·3% (−69·9 to −43·5)*	35·1 (25·0 to 46·3)	−62·1% (−72·1 to −47·0)*
	Measles		95·3 (34·5 to 205·2)	−57·0% (−61·9 to −51·9)*	1·4 (0·5 to 3·1)	−59·3% (−64·0 to −54·4)*	8105·1 (2935·7 to 17 469·0)	−56·9% (−61·8 to −51·8)*	120·8 (43·7 to 260·4)	−59·2% (−63·9 to −54·3)*
	Varicella and herpes zoster		15·6 (14·4 to 17·3)	−16·4% (−22·9 to −9·5)*	0·2 (0·2 to 0·2)	−29·2% (−34·7 to −23·4)*	833·0 (742·3 to 938·1)	−22·5% (−31·4 to −13·2)*	12·1 (10·7 to 13·6)	−28·4% (−36·6 to −19·4)*
	Acute hepatitis		126·4 (94·5 to 143·7)	−9·8% (−15·5 to −2·3)*	1·6 (1·2 to 1·9)	−24·5% (−29·2 to −18·4)*	5478·4 (4040·3 to 6330·0)	−21·7% (−27·7 to −14·4)*	72·3 (52·9 to 83·9)	−31·2% (−36·5 to −24·9)*
		Acute hepatitis A	18·6 (13·6 to 23·8)	−33·1% (−41·9 to −22·5)*	0·3 (0·2 to 0·3)	−38·7% (−46·8 to −28·6)*	1286·7 (935·2 to 1633·7)	−36·0% (−45·1 to −24·3)*	18·0 (13·0 to 22·9)	−40·7% (−49·1 to −29·0)*
		Acute hepatitis B	89·6 (66·1 to 102·5)	−0·8% (−8·4 to 8·5)	1·1 (0·8 to 1·3)	−19·6% (−25·4 to −12·4)*	3262·4 (2367·8 to 3819·1)	−12·2% (−19·7 to −2·7)*	41·8 (30·1 to 49·3)	−25·6% (−31·9 to −17·5)*
		Acute hepatitis C	3·5 (1·9 to 6·0)	−23·7% (−35·9 to −9·4)*	0·0 (0·0 to 0·1)	−32·1% (−42·4 to −19·6)*	219·7 (120·1 to 371·3)	−31·0% (−43·3 to −15·3)*	3·2 (1·8 to 5·4)	−35·5% (−47·2 to −20·7)*
		Acute hepatitis E	14·7 (10·4 to 18·5)	−15·8% (−27·2 to −3·1)*	0·2 (0·1 to 0·2)	−25·8% (−35·3 to −15·6)*	709·6 (489·6 to 903·9)	−25·5% (−35·2 to −14·5)*	9·3 (6·4 to 11·8)	−31·9% (−40·6 to −22·0)*
	Other unspecified infectious diseases		79·3 (59·9 to 85·1)	1·6% (−3·1 to 7·9)	1·1 (0·8 to 1·2)	−13·4% (−17·5 to −8·1)*	3941·3 (2831·7 to 4325·8)	−10·2% (−16·2 to −2·4)*	55·6 (39·6 to 61·3)	−17·9% (−23·6 to −10·6)*
	**Maternal and neonatal disorders**		**1977·4 (1890·1 to 2060·6)**	−24·1% (−26·9 to −21·0)*	**29·5 (28·2 to 30·8)**	−26·6% (−29·3 to −23·5)*	**167 684·6 (160 060·7 to 174 918·2)**	−24·2% (−27·1 to −20·9)*	**2518·2 (2403·8 to 2627·1)**	−26·5% (−29·3 to −23·3)*
	Maternal disorders		193·6 (179·9 to 209·6)	−24·0% (−28·4 to −19·5)*	2·5 (2·3 to 2·7)	−30·7% (−34·8 to −26·6)*	10 993·1 (10 198·9 to 11 928·5)	−25·3% (−29·7 to −20·9)*	140·9 (130·8 to 153·0)	−31·5% (−35·5 to −27·5)*
		Maternal haemorrhage	38·5 (33·2 to 45·2)	−52·1% (−59·0 to −44·2)*	0·5 (0·4 to 0·6)	−56·4% (−62·7 to −49·3)*	2173·8 (1859·7 to 2552·5)	−53·0% (−60·1 to −45·0)*	27·8 (23·8 to 32·7)	−57·1% (−63·6 to −49·7)*
		Maternal sepsis and other pregnancy-related infections	21·2 (18·2 to 25·0)	−27·1% (−38·8 to −15·1)*	0·3 (0·2 to 0·3)	−33·5% (−44·2 to −22·6)*	1198·0 (1022·8 to 1420·8)	−28·9% (−41·1 to −16·2)*	15·4 (13·1 to 18·3)	−34·5% (−45·4 to −22·5)*
		Maternal hypertensive disorders	29·4 (25·4 to 34·5)	−5·5% (−20·7 to 11·2)	0·4 (0·3 to 0·4)	−13·0% (−27·3 to 2·6)	1729·6 (1487·6 to 2033·2)	−6·6% (−22·1 to 10·2)	22·3 (19·2 to 26·4)	−13·6% (−28·1 to 2·0)
		Maternal obstructed labour and uterine rupture	13·0 (10·2 to 16·8)	−17·7% (−35·9 to 2·9)	0·2 (0·1 to 0·2)	−25·2% (−41·0 to −6·3)*	720·9 (565·5 to 946·4)	−18·9% (−37·6 to 1·9)	9·2 (7·2 to 12·1)	−25·8% (−42·9 to −6·9)*
		Maternal abortive outcome	17·4 (14·7 to 20·8)	−7·0% (−22·3 to 10·1)	0·2 (0·2 to 0·3)	−15·7% (−29·3 to −0·4)*	963·4 (807·6 to 1161·1)	−8·9% (−24·2 to 8·7)	12·3 (10·3 to 14·9)	−16·8% (−30·7 to −0·5)*
		Ectopic pregnancy	10·2 (7·1 to 15·2)	−11·6% (−41·4 to 27·9)	0·1 (0·1 to 0·2)	−19·2% (−46·2 to 16·8)	590·6 (409·0 to 881·4)	−13·3% (−43·8 to 26·9)	7·6 (5·3 to 11·4)	−20·3% (−48·1 to 17·0)
		Indirect maternal deaths	34·1 (30·0 to 38·7)	−4·1% (−16·7 to 8·5)	0·4 (0·4 to 0·5)	−12·5% (−24·0 to −1·0)*	1934·4 (1694·2 to 2216·7)	−6·1% (−19·2 to 6·8)	24·8 (21·7 to 28·5)	−13·9% (−25·8 to −2·3)*
		Late maternal deaths	3·4 (2·6 to 4·3)	−0·9% (−7·0 to 5·5)	0·0 (0·0 to 0·1)	−9·5% (−14·7 to −4·0)*	194·7 (152·2 to 251·4)	−2·0% (−8·2 to 4·1)	2·5 (2·0 to 3·2)	−10·1% (−15·4 to −4·5)*
		Maternal deaths aggravated by HIV/AIDS	1·6 (1·0 to 2·1)	−23·9% (−31·0 to −16·0)*	0·0 (0·0 to 0·0)	−32·1% (−38·4 to −25·2)*	84·4 (53·0 to 113·8)	−26·7% (−33·6 to −19·2)*	1·1 (0·7 to 1·4)	−34·2% (−40·6 to −27·5)*
		Other maternal disorders	24·8 (20·8 to 29·8)	−8·5% (−24·7 to 11·2)	0·3 (0·3 to 0·4)	−16·5% (−31·2 to 1·5)	1403·1 (1159·5 to 1690·3)	−9·8% (−26·7 to 10·8)	18·0 (14·9 to 21·7)	−17·2% (−32·9 to 1·2)
	Neonatal disorders		1783·8 (1698·5 to 1864·7)	−24·1% (−27·2 to −20·6)*	27·1 (25·8 to 28·3)	−26·2% (−29·1 to −22·7)*	156 691·6 (149 207·2 to 163 802·2)	−24·1% (−27·2 to −20·6)*	2377·2 (2263·7 to 2485·1)	−26·2% (−29·1 to −22·7)*
		Neonatal preterm birth	649·4 (605·4 to 721·3)	−26·2% (−31·3 to −21·5)*	9·9 (9·2 to 10·9)	−28·1% (−33·2 to −23·6)*	57 052·0 (53 182·3 to 63 367·1)	−26·2% (−31·3 to −21·5)*	865·6 (806·9 to 961·5)	−28·1% (−33·2 to −23·6)*
		Neonatal encephalopathy due to birth asphyxia and trauma	533·3 (476·9 to 580·3)	−24·5% (−30·2 to −18·0)*	8·1 (7·2 to 8·8)	−26·5% (−32·0 to −20·2)*	46 845·9 (41 894·1 to 50 985·7)	−24·5% (−30·2 to −18·0)*	710·8 (635·7 to 773·7)	−26·5% (−32·0 to −20·2)*
		Neonatal sepsis and other neonatal infections	203·0 (178·7 to 267·1)	−11·9% (−20·5 to −1·7)*	3·1 (2·7 to 4·1)	−14·4% (−22·7 to −4·4)*	17 830·7 (15 692·9 to 23 459·0)	−11·9% (−20·5 to −1·7)*	270·4 (238·0 to 355·8)	−14·4% (−22·7 to −4·4)*
		Haemolytic disease and other neonatal jaundice	49·1 (42·9 to 55·9)	−37·5% (−45·3 to −28·2)*	0·7 (0·7 to 0·8)	−39·3% (−46·8 to −30·2)*	4309·1 (3771·2 to 4914·0)	−37·5% (−45·3 to −28·2)*	65·4 (57·2 to 74·5)	−39·3% (−46·8 to −30·2)*
		Other neonatal disorders	349·0 (294·9 to 382·3)	−23·6% (−29·8 to −15·5)*	5·3 (4·5 to 5·8)	−25·7% (−31·7 to −17·8)*	30 654·0 (25 899·7 to 33 578·7)	−23·6% (−29·8 to −15·5)*	465·0 (392·9 to 509·4)	−25·7% (−31·7 to −17·8)*
	**Nutritional deficiencies**		**270·0 (249·3 to 295·5)**	−23·9% (−29·2 to −15·7)*	**3·8 (3·5 to 4·2)**	−33·6% (−38·1 to −26·5)*	**15 658·0 (14 051·5 to 17 506·6)**	−34·7% (−40·5 to −26·1)*	**228·7 (204·9 to 255·9)**	−39·4% (−44·8 to −31·4)*
	Protein-energy malnutrition		231·8 (212·4 to 254·2)	−26·1% (−31·7 to −17·9)*	3·3 (3·0 to 3·7)	−34·6% (−39·4 to −27·5)*	14 405·4 (12 873·5 to 16 128·0)	−35·1% (−41·1 to −26·7)*	211·8 (189·0 to 237·3)	−39·4% (−45·0 to −31·6)*
	Other nutritional deficiencies		38·2 (33·7 to 44·6)	−7·2% (−14·6 to 3·1)	0·5 (0·4 to 0·6)	−25·8% (−31·7 to −17·5)*	1252·7 (1087·5 to 1435·2)	−29·2% (−36·9 to −19·7)*	16·9 (14·6 to 19·5)	−38·6% (−45·4 to −30·4)*
**Non-communicable diseases**			**41 071·1 (40 470·9 to 41 548·9)**	22·7% (21·5 to 23·9)*	**536·1 (528·4 to 542·2)**	−7·9% (−8·8 to −7·0)*	**872 601·8 (859 538·6 to 884 787·7)**	13·6% (12·2 to 14·9)*	**11 097·4 (10 928·6 to 11 253·8)**	−9·6% (−10·7 to −8·6)*
	**Neoplasms**		**9556·2 (9395·7 to 9692·3)**	25·4% (23·9 to 27·0)*	**121·2 (119·1 to 122·9)**	−4·4% (−5·6 to −3·3)*	**225 738·1 (221 608·8 to 229 322·4)**	19·6% (17·8 to 21·4)*	**2803·4 (2751·5 to 2848·8)**	−5·6% (−7·0 to −4·1)*
	Lip and oral cavity cancer		193·7 (184·7 to 201·6)	35·6% (29·5 to 40·8)*	2·4 (2·3 to 2·5)	4·0% (−0·6 to 8·0)	5090·6 (4819·5 to 5328·3)	30·5% (23·8 to 36·4)*	62·2 (58·9 to 65·1)	3·0% (−2·3 to 7·6)
	Nasopharynx cancer		69·5 (66·9 to 72·3)	24·4% (20·0 to 28·8)*	0·9 (0·8 to 0·9)	−3·0% (−6·4 to 0·4)	2034·5 (1954·7 to 2117·4)	18·3% (13·9 to 23·1)*	24·8 (23·8 to 25·8)	−5·0% (−8·5 to −1·3)*
	Other pharynx cancer		117·4 (102·1 to 124·5)	40·4% (29·7 to 48·4)*	1·4 (1·3 to 1·5)	7·9% (−0·3 to 14·0)	3204·2 (2766·3 to 3405·1)	36·0% (25·4 to 44·2)*	38·9 (33·5 to 41·3)	6·5% (−1·7 to 12·8)
	Oesophageal cancer		436·0 (425·0 to 447·6)	13·0% (9·9 to 16·3)*	5·5 (5·3 to 5·6)	−14·5% (−16·9 to −12·0)*	9647·5 (9410·7 to 9903·5)	8·9% (5·8 to 12·2)*	118·3 (115·4 to 121·4)	−16·2% (−18·6 to −13·7)*
	Stomach cancer		865·0 (848·3 to 884·7)	9·4% (7·1 to 12·1)*	11·0 (10·8 to 11·2)	−17·1% (−18·8 to −15·1)*	18 782·0 (18 409·7 to 19 207·7)	4·8% (2·4 to 7·4)*	231·6 (227·0 to 236·8)	−18·6% (−20·5 to −16·6)*
	Colon and rectum cancer		896·0 (876·3 to 915·7)	27·8% (24·0 to 31·3)*	11·5 (11·3 to 11·8)	−4·3% (−7·1 to −1·8)*	18 106·7 (17 678·0 to 18 525·0)	23·8% (19·2 to 27·6)*	224·7 (219·4 to 229·9)	−4·5% (−8·0 to −1·7)*
	Liver cancer		819·4 (789·7 to 855·5)	27·0% (23·0 to 32·9)*	10·2 (9·8 to 10·7)	−2·5% (−5·6 to 2·0)	20 536·2 (19 678·7 to 21 551·9)	21·2% (17·0 to 27·4)*	250·7 (240·4 to 263·0)	−4·6% (−8·0 to 0·1)
		Liver cancer due to hepatitis B	325·4 (304·6 to 348·2)	20·3% (15·3 to 28·2)*	4·0 (3·7 to 4·3)	−6·2% (−10·0 to 0·1)	9449·0 (8837·3 to 10 138·6)	14·7% (9·7 to 21·9)*	114·6 (107·3 to 123·0)	−8·4% (−12·2 to −2·6)*
		Liver cancer due to hepatitis C	234·3 (219·4 to 250·6)	30·4% (26·7 to 35·0)*	3·0 (2·8 to 3·2)	−2·1% (−4·9 to 1·4)	4898·4 (4554·0 to 5259·3)	26·9% (23·3 to 31·6)*	60·3 (56·2 to 64·7)	−3·0% (−5·8 to 0·5)
		Liver cancer due to alcohol use	129·3 (114·5 to 147·3)	31·7% (26·8 to 37·3)*	1·6 (1·4 to 1·8)	0·6% (−3·0 to 4·8)	3040·7 (2647·6 to 3549·8)	27·8% (22·4 to 33·9)*	37·2 (32·5 to 43·3)	−0·6% (−4·5 to 3·9)
		Liver cancer due to NASH	66·9 (59·6 to 74·5)	42·3% (38·0 to 47·6)*	0·8 (0·8 to 0·9)	7·6% (4·4 to 11·7)*	1443·8 (1288·9 to 1605·9)	37·3% (32·7 to 42·8)*	17·8 (15·9 to 19·7)	6·3% (2·9 to 10·5)*
		Liver cancer due to other causes	63·5 (57·4 to 70·6)	28·2% (23·6 to 34·3)*	0·8 (0·7 to 0·9)	−0·9% (−4·2 to 3·6)	1704·2 (1528·4 to 1903·8)	21·1% (16·0 to 27·4)*	20·9 (18·8 to 23·3)	−3·5% (−7·2 to 1·4)
	Gallbladder and biliary tract cancer		174·0 (154·2 to 184·9)	25·0% (21·5 to 28·7)*	2·2 (2·0 to 2·4)	−6·7% (−9·4 to −4·0)*	3434·0 (3009·7 to 3660·0)	21·8% (17·8 to 26·3)*	42·6 (37·3 to 45·4)	−6·8% (−9·9 to −3·5)*
	Pancreatic cancer		441·1 (432·8 to 449·0)	39·9% (36·7 to 42·6)*	5·6 (5·5 to 5·7)	4·8% (2·5 to 6·8)*	8988·1 (8806·6 to 9162·9)	35·8% (32·5 to 38·6)*	111·1 (108·9 to 113·2)	4·0% (1·5 to 6·1)*
	Larynx cancer		126·5 (123·4 to 129·9)	21·1% (17·8 to 24·4)*	1·6 (1·5 to 1·6)	−7·7% (−10·1 to −5·2)*	3170·0 (3089·7 to 3260·3)	17·3% (13·9 to 20·9)*	38·5 (37·6 to 39·6)	−9·1% (−11·7 to −6·4)*
	Tracheal, bronchus, and lung cancer		1883·1 (1844·2 to 1922·8)	29·6% (26·5 to 32·5)*	23·7 (23·3 to 24·2)	−2·0% (−4·3 to 0·1)	40 391·6 (39 506·7 to 41 285·6)	24·8% (21·7 to 27·6)*	496·4 (485·5 to 507·2)	−4·1% (−6·5 to −2·0)*
	Malignant skin melanoma		61·7 (47·9 to 70·3)	23·6% (19·0 to 26·9)*	0·8 (0·6 to 0·9)	−5·1% (−8·5 to −2·5)*	1513·2 (1220·7 to 1774·4)	16·1% (12·7 to 20·0)*	18·7 (15·1 to 21·9)	−7·2% (−9·8 to −3·8)*
	Non-melanoma skin cancer		65·1 (63·1 to 66·5)	38·6% (34·9 to 41·2)*	0·8 (0·8 to 0·9)	2·7% (0·0 to 4·5)*	1239·1 (1200·2 to 1266·6)	30·0% (26·2 to 32·7)*	15·5 (15·0 to 15·8)	0·5% (−2·3 to 2·6)
		Non-melanoma skin cancer (squamous-cell carcinoma)	65·1 (63·1 to 66·5)	38·6% (34·9 to 41·2)*	0·8 (0·8 to 0·9)	2·7% (0·0 to 4·5)*	1239·1 (1200·2 to 1266·6)	30·0% (26·2 to 32·7)*	15·5 (15·0 to 15·8)	0·5% (−2·3 to 2·6)
	Breast cancer		611·6 (589·2 to 640·7)	27·0% (21·3 to 31·2)*	7·6 (7·4 to 8·0)	−2·6% (−6·9 to 0·4)	16 400·7 (15 737·0 to 17 320·2)	23·9% (17·3 to 28·7)*	200·2 (192·1 to 211·4)	−1·7% (−6·8 to 2·1)
	Cervical cancer		259·7 (241·1 to 269·2)	18·8% (12·9 to 22·8)*	3·2 (3·0 to 3·3)	−7·2% (−11·7 to −4·0)*	7773·5 (7227·4 to 8087·8)	15·1% (9·4 to 19·1)*	94·6 (88·1 to 98·5)	−7·2% (−11·8 to −3·9)*
	Uterine cancer		85·2 (83·2 to 87·4)	18·8% (15·8 to 22·5)*	1·1 (1·0 to 1·1)	−10·4% (−12·5 to −7·7)*	1930·0 (1879·9 to 1983·0)	14·8% (11·6 to 19·0)*	23·7 (23·1 to 24·3)	−11·2% (−13·7 to −8·0)*
	Ovarian cancer		176·0 (171·4 to 181·2)	30·3% (26·8 to 33·7)*	2·2 (2·1 to 2·3)	−1·0% (−3·6 to 1·6)	4496·9 (4370·7 to 4642·1)	29·1% (24·8 to 33·1)*	54·9 (53·4 to 56·7)	1·1% (−2·2 to 4·2)
	Prostate cancer		415·9 (357·3 to 489·5)	32·5% (29·3 to 38·4)*	5·5 (4·7 to 6·5)	−2·5% (−4·9 to 1·9)	6214·5 (5324·2 to 7293·0)	28·3% (24·9 to 34·5)*	79·3 (68·1 to 93·0)	−3·6% (−6·2 to 1·2)
	Testicular cancer		7·7 (7·4 to 8·0)	6·1% (2·3 to 10·9)*	0·1 (0·1 to 0·1)	−9·4% (−12·6 to −5·2)*	338·7 (323·8 to 357·4)	0·9% (−3·3 to 6·3)	4·3 (4·1 to 4·5)	−10·8% (−14·5 to −6·1)*
	Kidney cancer		138·5 (128·7 to 142·5)	30·1% (26·2 to 34·1)*	1·8 (1·6 to 1·8)	−1·3% (−4·3 to 1·7)	3143·3 (2952·2 to 3234·1)	23·1% (18·5 to 27·3)*	39·4 (37·0 to 40·5)	−3·3% (−6·9 to 0·0)
	Bladder cancer		196·5 (191·5 to 205·8)	27·8% (25·1 to 30·4)*	2·6 (2·5 to 2·7)	−5·4% (−7·3 to −3·4)*	3350·1 (3257·4 to 3511·6)	22·6% (19·9 to 25·3)*	42·2 (41·0 to 44·1)	−6·9% (−8·9 to −4·8)*
	Brain and nervous system cancer		247·1 (213·0 to 265·0)	29·2% (23·2 to 33·4)*	3·1 (2·7 to 3·3)	3·8% (−1·0 to 7·0)	8577·8 (7527·0 to 9359·3)	18·4% (11·9 to 24·6)*	109·8 (96·1 to 120·0)	0·0% (−5·6 to 5·3)
	Thyroid cancer		41·2 (39·9 to 44·1)	28·9% (24·3 to 33·3)*	0·5 (0·5 to 0·6)	−1·2% (−4·5 to 2·0)	1001·2 (963·6 to 1074·0)	22·1% (16·7 to 28·0)*	12·4 (12·0 to 13·4)	−2·3% (−6·6 to 2·4)
	Mesothelioma		29·9 (29·1 to 30·6)	26·9% (20·1 to 32·6)*	0·4 (0·4 to 0·4)	−3·4% (−8·4 to 0·7)	655·7 (635·2 to 677·0)	21·0% (13·8 to 27·3)*	8·1 (7·9 to 8·4)	−5·4% (−10·8 to −0·8)*
	Hodgkin lymphoma		32·6 (27·6 to 38·1)	0·2% (−3·5 to 3·6)	0·4 (0·4 to 0·5)	−16·8% (−19·8 to −14·0)*	1327·6 (1110·1 to 1567·7)	−5·2% (−8·6 to −1·8)*	17·1 (14·3 to 20·2)	−17·1% (−20·1 to −13·9)*
	Non-Hodgkin lymphoma		248·6 (243·5 to 253·1)	29·4% (25·5 to 32·4)*	3·2 (3·1 to 3·2)	0·1% (−2·7 to 2·4)	6828·8 (6611·8 to 7020·0)	22·1% (15·6 to 26·9)*	86·8 (84·0 to 89·5)	0·2% (−5·2 to 4·3)
	Multiple myeloma		107·1 (98·5 to 118·9)	32·7% (28·4 to 36·4)*	1·4 (1·3 to 1·5)	−0·4% (−3·5 to 2·4)	2234·7 (2091·4 to 2493·2)	30·4% (25·6 to 34·4)*	27·7 (25·9 to 30·8)	0·3% (−3·3 to 3·4)
	Leukaemia		347·6 (317·3 to 364·9)	12·8% (9·5 to 15·6)*	4·5 (4·1 to 4·7)	−9·6% (−12·2 to −7·4)*	11 712·0 (10 531·4 to 12 523·3)	2·3% (−3·7 to 6·2)	153·4 (137·9 to 164·5)	−12·0% (−17·3 to −8·5)*
		Acute lymphoid leukaemia	52·2 (46·0 to 56·7)	14·1% (2·6 to 23·2)*	0·7 (0·6 to 0·7)	−1·5% (−11·6 to 6·2)	2661·7 (2341·7 to 2941·1)	5·3% (−8·6 to 15·4)	36·1 (31·7 to 40·0)	−4·7% (−17·6 to 4·7)
		Chronic lymphoid leukaemia	35·2 (33·5 to 36·9)	21·4% (17·7 to 25·0)*	0·5 (0·4 to 0·5)	−10·3% (−13·0 to −7·6)*	634·1 (595·7 to 674·2)	18·3% (14·2 to 22·4)*	8·0 (7·5 to 8·5)	−9·2% (−12·3 to −6·1)*
		Acute myeloid leukaemia	99·9 (91·3 to 104·6)	24·6% (17·1 to 29·8)*	1·3 (1·2 to 1·3)	−1·0% (−6·6 to 3·0)	3192·6 (2868·8 to 3405·6)	16·2% (4·4 to 24·6)*	41·3 (37·0 to 44·1)	−1·4% (−11·3 to 5·8)
		Chronic myeloid leukaemia	24·1 (22·2 to 26·1)	3·3% (0·4 to 6·4)*	0·3 (0·3 to 0·3)	−19·9% (−22·2 to −17·6)*	643·3 (583·4 to 699·1)	−1·7% (−5·2 to 1·5)	8·0 (7·3 to 8·7)	−19·7% (−22·4 to −17·1)*
		Other leukaemia	136·2 (121·0 to 146·8)	4·9% (0·9 to 9·7)*	1·8 (1·6 to 1·9)	−15·6% (−18·7 to −12·1)*	4580·2 (3955·1 to 5013·3)	−8·1% (−14·6 to −1·8)*	60·0 (51·9 to 65·7)	−20·8% (−26·5 to −15·4)*
	Other malignant cancers		359·5 (331·4 to 370·8)	26·8% (23·3 to 29·5)*	4·6 (4·2 to 4·8)	0·1% (−2·6 to 2·2)	11 189·0 (10 386·5 to 11 664·8)	18·4% (12·8 to 22·8)*	144·4 (133·8 to 150·9)	−0·3% (−5·1 to 3·5)
	Other neoplasms		102·9 (80·2 to 122·4)	42·0% (35·6 to 51·7)*	1·3 (1·0 to 1·6)	7·4% (2·1 to 15·8)*	2425·8 (2024·4 to 2932·1)	32·9% (25·9 to 42·7)*	31·1 (25·9 to 37·4)	7·9% (2·0 to 16·5)*
		Myelodysplastic, myeloproliferative, and other haemopoietic neoplasms	98·8 (76·7 to 118·1)	42·6% (36·2 to 52·2)*	1·3 (1·0 to 1·5)	7·1% (1·8 to 15·3)*	2189·1 (1820·8 to 2665·5)	33·9% (26·6 to 43·3)*	27·9 (23·2 to 33·8)	7·2% (1·2 to 15·6)*
		Other benign and in-situ neoplasms	4·1 (3·2 to 4·8)	29·6% (17·2 to 44·5)*	0·1 (0·0 to 0·1)	15·5% (4·1 to 29·2)*	236·8 (186·4 to 277·7)	25·0% (12·7 to 38·6)*	3·2 (2·5 to 3·7)	14·3% (3·0 to 27·0)*
	**Cardiovascular diseases**		**17 790·9 (17 527·1 to 18 042·7)**	21·1% (19·7 to 22·6)*	**233·1 (229·7 to 236·4)**	−10·3% (−11·4 to −9·3)*	**330 172·6 (324 899·3 to 335 159·9)**	14·7% (13·3 to 16·2)*	**4148·0 (4082·0 to 4210·8)**	−11·3% (−12·4 to −10·1)*
	Rheumatic heart disease		285·5 (266·2 to 303·3)	1·3% (−3·9 to 6·0)	3·7 (3·4 to 3·9)	−21·3% (−25·2 to −17·8)*	7492·6 (6926·7 to 8046·7)	−10·2% (−15·4 to −6·2)*	94·5 (87·5 to 101·4)	−25·9% (−30·0 to −22·7)*
	Ischaemic heart disease		8930·4 (8790·7 to 9138·7)	22·3% (20·6 to 23·8)*	116·9 (115·1 to 119·7)	−9·7% (−11·0 to −8·7)*	164 983·4 (162 168·9 to 168 584·2)	17·3% (15·4 to 19·0)*	2065·9 (2030·6 to 2111·7)	−9·8% (−11·2 to −8·5)*
	Stroke		6167·3 (6044·3 to 6327·6)	16·6% (14·7 to 18·6)*	80·5 (78·9 to 82·6)	−13·6% (−15·0 to −12·1)*	113 355·9 (110 957·8 to 116 180·6)	12·1% (9·9 to 14·1)*	1422·2 (1392·0 to 1457·7)	−13·8% (−15·5 to −12·3)*
		Ischaemic stroke	2747·4 (2657·1 to 2857·6)	21·2% (19·0 to 23·3)*	36·6 (35·5 to 38·0)	−11·8% (−13·4 to −10·3)*	40 834·1 (39 133·3 to 43 140·9)	16·9% (14·3 to 19·3)*	521·8 (500·5 to 550·2)	−12·0% (−13·9 to −10·3)*
		Intracerebral haemorrhage	2974·9 (2880·8 to 3072·8)	12·5% (9·6 to 15·1)*	38·2 (37·0 to 39·4)	−15·7% (−17·8 to −13·8)*	61 562·6 (59 598·2 to 63 531·4)	9·3% (6·5 to 11·8)*	764·1 (739·7 to 788·4)	−15·4% (−17·6 to −13·5)*
		Subarachnoid haemorrhage	445·0 (417·2 to 492·3)	18·4% (13·4 to 24·6)*	5·7 (5·3 to 6·3)	−9·4% (−13·1 to −4·9)*	10 959·3 (10 294·3 to 12 264·1)	10·7% (6·8 to 16·5)*	136·4 (128·2 to 152·5)	−11·4% (−14·5 to −7·0)*
	Hypertensive heart disease		925·7 (681·4 to 994·9)	46·6% (26·3 to 59·3)*	12·3 (9·0 to 13·2)	7·5% (−7·3 to 16·3)	15 135·2 (11 349·8 to 16 311·7)	35·7% (19·1 to 47·9)*	191·5 (143·3 to 206·2)	3·8% (−8·8 to 12·9)
	Non-rheumatic valvular heart disease		144·9 (121·8 to 150·4)	31·8% (27·7 to 34·7)*	2·0 (1·6 to 2·0)	−5·3% (−7·9 to −3·2)*	2168·4 (1980·3 to 2322·7)	21·8% (18·6 to 25·0)*	27·9 (25·4 to 29·6)	−6·2% (−8·5 to −3·8)*
		Non-rheumatic calcific aortic valve disease	102·7 (82·7 to 108·0)	40·0% (33·0 to 44·9)*	1·4 (1·1 to 1·5)	−1·0% (−5·6 to 2·2)	1345·1 (1185·5 to 1432·5)	30·4% (25·1 to 35·3)*	17·5 (15·3 to 18·6)	−1·7% (−5·3 to 1·6)
		Non-rheumatic degenerative mitral valve disease	35·7 (30·5 to 42·5)	16·4% (11·0 to 23·4)*	0·5 (0·4 to 0·6)	−14·0% (−18·1 to −8·6)*	683·6 (592·6 to 787·0)	10·3% (4·9 to 16·2)*	8·7 (7·5 to 10·0)	−13·0% (−16·9 to −8·3)*
		Other non-rheumatic valve diseases	6·4 (4·9 to 8·7)	9·7% (−4·1 to 42·2)	0·1 (0·1 to 0·1)	−17·8% (−28·5 to 8·0)	139·7 (105·8 to 187·5)	8·1% (−2·4 to 27·6)	1·8 (1·4 to 2·4)	−12·4% (−21·3 to 4·7)
	Cardiomyopathy and myocarditis		368·5 (341·9 to 386·9)	8·1% (3·8 to 18·2)*	4·8 (4·5 to 5·0)	−16·6% (−19·8 to −9·4)*	9623·3 (8867·5 to 10 208·8)	−5·1% (−9·6 to 5·5)	122·4 (113·0 to 129·7)	−21·5% (−25·1 to −13·0)*
		Myocarditis	46·5 (39·7 to 51·8)	14·4% (5·6 to 29·7)*	0·6 (0·5 to 0·7)	−13·3% (−20·4 to −0·1)*	1259·3 (1100·1 to 1415·5)	−0·3% (−6·9 to 7·6)	16·6 (14·5 to 18·5)	−15·2% (−21·1 to −7·7)*
		Alcoholic cardiomyopathy	88·9 (80·9 to 96·3)	−25·3% (−29·5 to −8·3)*	1·1 (1·0 to 1·2)	−40·5% (−43·7 to −27·6)*	2849·2 (2599·0 to 3073·1)	−30·7% (−34·7 to −12·1)*	34·7 (31·7 to 37·5)	−43·2% (−46·5 to −28·2)*
		Other cardiomyopathy	233·2 (213·7 to 248·3)	28·5% (24·5 to 32·4)*	3·1 (2·8 to 3·3)	−3·6% (−6·7 to −0·7)*	5514·8 (4946·7 to 5992·9)	15·7% (10·9 to 19·9)*	71·1 (64·0 to 77·0)	−5·4% (−9·3 to −2·0)*
	Atrial fibrillation and flutter		287·2 (276·4 to 304·8)	47·8% (45·4 to 50·6)*	4·0 (3·9 to 4·2)	2·6% (0·9 to 4·6)*	3054·5 (2923·0 to 3235·4)	40·5% (37·9 to 43·4)*	40·6 (38·9 to 43·1)	2·2% (0·3 to 4·2)*
	Aortic aneurysm		167·2 (159·8 to 174·1)	23·7% (19·9 to 27·6)*	2·2 (2·1 to 2·3)	−8·5% (−11·2 to −5·8)*	3039·9 (2877·2 to 3186·4)	19·0% (14·5 to 23·6)*	38·2 (36·2 to 40·0)	−8·5% (−11·9 to −5·1)*
	Peripheral vascular disease		70·2 (43·2 to 123·3)	55·7% (31·0 to 74·2)*	1·0 (0·6 to 1·7)	10·5% (−6·8 to 24·1)	916·9 (576·9 to 1540·0)	48·3% (25·0 to 65·6)*	11·8 (7·4 to 20·0)	9·7% (−7·5 to 22·6)
	Endocarditis		83·4 (74·3 to 94·3)	32·2% (25·2 to 36·8)*	1·1 (1·0 to 1·2)	1·0% (−4·0 to 5·0)	2174·5 (2033·2 to 2373·0)	16·9% (8·9 to 22·2)*	28·3 (26·4 to 30·9)	−2·3% (−8·8 to 2·1)
	Other cardiovascular and circulatory diseases		360·7 (338·1 to 392·9)	21·9% (17·9 to 24·8)*	4·7 (4·4 to 5·1)	−7·9% (−10·9 to −5·9)*	8228·0 (7681·4 to 9061·9)	12·6% (9·5 to 15·7)*	104·7 (97·8 to 115·2)	−9·4% (−12·0 to −7·1)*
	**Chronic respiratory diseases**		**3914·2 (3790·6 to 4044·8)**	15·8% (12·7 to 19·3)*	**51·4 (49·7 to 53·1)**	−14·2% (−16·5 to −11·5)*	**68 004·9 (65 869·4 to 70 592·2)**	9·7% (7·0 to 13·2)*	**861·9 (835·4 to 895·0)**	−15·7% (−17·7 to −13·0)*
	Chronic obstructive pulmonary disease		3197·8 (3029·0 to 3358·9)	17·5% (13·3 to 21·1)*	42·2 (40·0 to 44·2)	−13·6% (−16·5 to −11·0)*	50 990·0 (47 678·7 to 54 146·9)	13·2% (8·8 to 16·9)*	647·3 (605·9 to 686·4)	−14·3% (−17·5 to −11·6)*
	Pneumoconiosis		21·6 (20·5 to 22·7)	10·7% (5·1 to 16·6)*	0·3 (0·3 to 0·3)	−16·7% (−20·8 to −12·4)*	426·9 (403·6 to 452·9)	7·9% (1·8 to 14·6)*	5·3 (5·0 to 5·6)	−16·4% (−21·1 to −11·3)*
		Silicosis	11·3 (10·4 to 12·5)	12·0% (1·2 to 22·8)*	0·1 (0·1 to 0·2)	−15·5% (−23·6 to −7·4)*	235·7 (210·3 to 258·2)	11·8% (−0·7 to 23·6)	2·9 (2·6 to 3·2)	−13·4% (−23·1 to −4·3)*
		Asbestosis	3·4 (2·3 to 3·9)	23·3% (15·1 to 33·9)*	0·0 (0·0 to 0·1)	−8·3% (−14·1 to −0·4)*	54·6 (38·6 to 65·6)	15·6% (7·4 to 28·5)*	0·7 (0·5 to 0·8)	−11·4% (−17·5 to −1·3)*
		Coal worker pneumoconiosis	3·2 (2·9 to 4·0)	−2·2% (−12·0 to 11·7)	0·0 (0·0 to 0·1)	−26·6% (−33·8 to −16·7)*	58·9 (52·2 to 76·4)	−6·4% (−16·3 to 8·3)	0·7 (0·7 to 1·0)	−27·9% (−35·4 to −16·9)*
		Other pneumoconiosis	3·6 (3·1 to 4·5)	8·9% (0·0 to 25·4)*	0·0 (0·0 to 0·1)	−17·5% (−24·1 to −5·0)*	77·6 (66·1 to 96·4)	4·2% (−3·8 to 19·5)	1·0 (0·8 to 1·2)	−18·3% (−24·7 to −5·9)*
	Asthma		495·1 (338·2 to 641·2)	−0·7% (−6·2 to 8·1)	6·3 (4·3 to 8·2)	−23·9% (−28·1 to −17·2)*	12 139·9 (8538·5 to 15 576·3)	−7·5% (−11·4 to −1·6)*	152·8 (108·3 to 195·8)	−25·8% (−28·9 to −20·4)*
	Interstitial lung disease and pulmonary sarcoidosis		147·6 (114·9 to 181·3)	49·8% (39·0 to 58·6)*	1·9 (1·5 to 2·4)	11·4% (4·0 to 17·9)*	2716·7 (2156·9 to 3371·3)	43·0% (32·1 to 53·4)*	34·2 (27·1 to 42·4)	10·4% (2·3 to 18·6)*
	Other chronic respiratory diseases		52·1 (45·9 to 59·6)	21·3% (14·1 to 34·2)*	0·7 (0·6 to 0·8)	−3·2% (−8·7 to 6·7)	1731·4 (1504·5 to 1998·9)	10·8% (3·2 to 24·3)*	22·1 (19·3 to 25·5)	−6·3% (−12·6 to 5·3)
	**Digestive diseases**		**2377·7 (2295·1 to 2518·0)**	15·3% (12·1 to 19·7)*	**30·3 (29·2 to 32·1)**	−10·7% (−13·1 to −7·3)*	**65 348·4 (62 343·9 to 69 371·3)**	7·5% (4·2 to 11·9)*	**819·8 (781·7 to 869·7)**	−12·2% (−14·9 to −8·5)*
	Cirrhosis and other chronic liver diseases		1322·9 (1268·2 to 1449·1)	15·0% (8·7 to 21·5)*	16·5 (15·8 to 18·1)	−9·7% (−14·7 to −4·6)*	39 652·4 (37 985·2 to 43 624·9)	8·9% (3·4 to 14·4)*	488·9 (468·0 to 537·5)	−11·3% (−15·8 to −6·9)*
		Cirrhosis and other chronic liver diseases due to hepatitis B	384·0 (349·1 to 441·7)	8·6% (1·1 to 17·3)*	4·8 (4·3 to 5·5)	−14·3% (−20·2 to −7·3)*	11 721·5 (10 648·0 to 13 431·7)	3·4% (−3·3 to 10·7)	144·1 (130·8 to 165·3)	−15·5% (−20·9 to −9·5)*
		Cirrhosis and other chronic liver diseases due to hepatitis C	342·2 (312·6 to 381·1)	17·4% (11·3 to 23·0)*	4·2 (3·9 to 4·7)	−8·4% (−13·0 to −3·9)*	9980·1 (9074·7 to 11 116·9)	12·2% (6·8 to 17·3)*	121·9 (111·0 to 135·8)	−9·6% (−13·9 to −5·5)*
		Cirrhosis and other chronic liver diseases due to alcohol use	332·3 (303·0 to 373·3)	16·9% (11·2 to 23·7)*	4·1 (3·7 to 4·6)	−8·8% (−13·2 to −3·4)*	9785·4 (8919·3 to 10 962·1)	12·3% (7·1 to 18·3)*	119·0 (108·6 to 133·5)	−10·0% (−14·2 to −5·2)*
		Cirrhosis due to NASH	118·0 (108·6 to 128·6)	27·6% (21·2 to 33·3)*	1·5 (1·3 to 1·6)	−1·4% (−6·3 to 3·1)	3285·5 (3011·9 to 3586·8)	22·2% (16·6 to 27·2)*	40·0 (36·6 to 43·6)	−3·0% (−7·4 to 1·0)
		Cirrhosis and other chronic liver diseases due to other causes	146·4 (130·9 to 164·6)	14·2% (8·2 to 20·2)*	1·9 (1·7 to 2·1)	−8·6% (−13·4 to −3·8)*	4880·0 (4392·5 to 5457·1)	2·1% (−4·3 to 10·7)	63·9 (57·5 to 71·4)	−12·0% (−17·5 to −4·5)*
	Upper digestive system diseases		292·1 (279·7 to 312·3)	2·9% (−1·3 to 8·6)	3·8 (3·6 to 4·0)	−21·6% (−24·8 to −17·3)*	6789·9 (6413·1 to 7259·0)	−4·5% (−9·5 to 1·8)	85·2 (80·4 to 91·2)	−23·3% (−27·3 to −18·4)*
		Peptic ulcer disease	240·3 (229·4 to 258·8)	0·6% (−3·6 to 5·6)	3·1 (3·0 to 3·3)	−23·5% (−26·6 to −19·7)*	5513·3 (5202·4 to 5947·8)	−6·8% (−11·4 to −1·5)*	69·1 (65·1 to 74·7)	−25·4% (−29·0 to −21·0)*
		Gastritis and duodenitis	51·8 (43·0 to 56·9)	15·5% (7·5 to 28·9)*	0·7 (0·6 to 0·7)	−11·7% (−17·6 to −2·2)*	1276·6 (1047·1 to 1419·7)	6·8% (−3·1 to 22·4)	16·1 (13·2 to 17·9)	−13·2% (−21·1 to −1·3)*
	Appendicitis		43·9 (40·2 to 47·5)	1·8% (−4·0 to 9·6)	0·6 (0·5 to 0·6)	−17·0% (−21·5 to −10·7)*	1633·2 (1473·2 to 1772·7)	−8·7% (−16·7 to 0·6)	21·4 (19·3 to 23·3)	−20·1% (−27·2 to −12·1)*
	Paralytic ileus and intestinal obstruction		240·5 (198·7 to 261·6)	21·1% (14·4 to 29·0)*	3·2 (2·7 to 3·5)	−5·8% (−11·0 to 0·3)	7245·9 (5866·8 to 7980·6)	6·5% (−3·1 to 15·5)	97·0 (78·9 to 106·8)	−8·7% (−16·8 to −0·8)*
	Inguinal, femoral, and abdominal hernia		44·2 (38·6 to 50·0)	21·7% (16·2 to 28·4)*	0·6 (0·5 to 0·7)	−8·9% (−12·9 to −4·2)*	914·3 (792·8 to 1021·9)	12·1% (4·5 to 20·4)*	11·7 (10·1 to 13·1)	−10·6% (−16·5 to −3·9)*
	Inflammatory bowel disease		38·6 (31·6 to 41·2)	20·4% (11·5 to 27·2)*	0·5 (0·4 to 0·5)	−10·5% (−16·0 to −5·9)*	829·7 (711·4 to 900·7)	10·3% (−2·7 to 19·5)	10·7 (9·1 to 11·7)	−11·3% (−20·7 to −4·5)*
	Vascular intestinal disorders		96·1 (89·0 to 100·8)	22·6% (17·0 to 28·1)*	1·3 (1·2 to 1·3)	−10·2% (−14·2 to −6·2)*	1570·1 (1433·3 to 1667·3)	17·6% (10·7 to 24·8)*	20·0 (18·3 to 21·3)	−10·0% (−15·3 to −5·0)*
	Gallbladder and biliary diseases		110·5 (105·5 to 116·6)	28·8% (25·3 to 33·8)*	1·5 (1·4 to 1·6)	−5·0% (−7·5 to −1·7)*	1983·2 (1863·2 to 2092·0)	18·5% (13·4 to 25·3)*	25·4 (23·8 to 26·8)	−6·9% (−10·9 to −1·8)*
	Pancreatitis		101·6 (89·5 to 108·3)	20·6% (16·4 to 25·7)*	1·3 (1·1 to 1·4)	−5·7% (−9·0 to −1·7)*	2890·0 (2537·1 to 3102·9)	13·8% (8·7 to 19·5)*	35·8 (31·4 to 38·4)	−6·8% (−10·9 to −2·1)*
	Other digestive diseases		87·3 (81·9 to 93·3)	25·4% (18·1 to 32·3)*	1·2 (1·1 to 1·2)	−7·1% (−12·1 to −2·4)*	1839·7 (1663·9 to 2038·5)	16·4% (5·8 to 27·4)*	23·7 (21·5 to 26·3)	−6·5% (−14·9 to 1·8)
	**Neurological disorders**		**3094·2 (3039·6 to 3142·6)**	42·1% (40·2 to 43·9)*	**43·1 (42·3 to 43·7)**	**0·1% (−1·2 to 1·3)**	**38 004·5 (37 134·8 to 39 174·6)**	26·2% (23·9 to 30·2)*	**507·6 (496·1 to 523·4)**	−3·1% (−4·8 to −0·1)*
	Alzheimer's disease and other dementias		2514·6 (2470·5 to 2550·3)	46·2% (43·9 to 48·0)*	35·4 (34·8 to 35·9)	0·6% (−0·9 to 1·8)	23 951·1 (23 523·6 to 24 326·8)	38·6% (35·7 to 40·9)*	323·7 (317·9 to 328·7)	−0·3% (−2·3 to 1·2)
	Parkinson's disease		340·6 (324·4 to 355·1)	38·3% (33·3 to 41·4)*	4·6 (4·4 to 4·8)	0·8% (−2·8 to 3·0)	4361·2 (4182·8 to 4578·7)	33·8% (28·5 to 37·0)*	56·9 (54·5 to 59·8)	0·3% (−3·6 to 2·6)
	Epilepsy		130·2 (117·0 to 150·8)	3·8% (−1·6 to 15·7)	1·7 (1·5 to 2·0)	−10·7% (−15·4 to −0·5)*	6232·1 (5709·8 to 7289·7)	−5·5% (−11·6 to 8·3)	82·6 (75·5 to 96·6)	−14·9% (−20·6 to −2·1)*
	Multiple sclerosis		20·7 (17·7 to 22·2)	22·4% (8·0 to 27·8)*	0·3 (0·2 to 0·3)	−3·9% (−14·5 to 0·4)	628·2 (563·0 to 682·4)	17·1% (4·1 to 24·5)*	7·7 (6·9 to 8·3)	−5·5% (−15·1 to 0·6)
	Motor neuron disease		34·1 (32·8 to 37·1)	32·7% (28·0 to 37·0)*	0·4 (0·4 to 0·5)	1·2% (−2·4 to 4·5)	828·1 (796·7 to 917·1)	27·2% (22·6 to 31·3)*	10·3 (9·9 to 11·4)	0·1% (−3·5 to 3·3)
	Other neurological disorders		53·9 (51·6 to 59·0)	25·4% (17·8 to 32·3)*	0·7 (0·7 to 0·8)	2·0% (−3·9 to 6·8)	2003·8 (1856·8 to 2269·5)	11·4% (3·6 to 21·1)*	26·5 (24·3 to 30·1)	−2·8% (−9·3 to 5·1)
	**Mental disorders**		**0·3 (0·3 to 0·4)**	19·9% (10·0 to 29·2)*	**0·0 (0·0 to 0·0)**	**7·5% (−1·4 to 15·9)**	**17·5 (15·9 to 19·2)**	18·5% (8·8 to 27·5)*	**0·2 (0·2 to 0·2)**	**7·2% (−1·6 to 15·3)**
	Eating disorders		0·3 (0·3 to 0·4)	19·9% (10·0 to 29·2)*	0·0 (0·0 to 0·0)	7·5% (−1·4 to 15·9)	17·5 (15·9 to 19·2)	18·5% (8·8 to 27·5)*	0·2 (0·2 to 0·2)	7·2% (−1·6 to 15·3)
		Anorexia nervosa	0·2 (0·2 to 0·3)	17·6% (7·0 to 27·6)*	0·0 (0·0 to 0·0)	5·5% (−4·1 to 14·4)	12·7 (10·9 to 14·1)	15·9% (5·6 to 25·6)*	0·2 (0·1 to 0·2)	5·0% (−4·4 to 13·7)
		Bulimia nervosa	0·1 (0·1 to 0·1)	26·4% (12·9 to 40·5)*	0·0 (0·0 to 0·0)	13·5% (1·0 to 26·2)*	4·8 (4·0 to 6·7)	25·9% (12·0 to 40·0)*	0·1 (0·1 to 0·1)	13·6% (1·1 to 26·3)*
	**Substance use disorders**		**351·5 (334·1 to 362·7)**	23·8% (20·2 to 27·3)*	**4·3 (4·1 to 4·5)**	**2·0% (−1·0 to 5·0)**	**13 597·6 (12 979·5 to 14 033·3)**	18·8% (15·3 to 22·4)*	**168·0 (160·4 to 173·3)**	**0·8% (−2·2 to 3·9)**
	Alcohol use disorders		184·9 (166·7 to 193·0)	2·7% (−2·2 to 7·7)	2·3 (2·0 to 2·4)	−16·5% (−20·4 to −12·4)*	6750·4 (6113·2 to 7082·7)	−2·1% (−7·2 to 3·3)	82·4 (74·7 to 86·5)	−18·4% (−22·7 to −13·9)*
	Drug use disorders		166·6 (163·4 to 170·3)	60·2% (56·9 to 63·6)*	2·1 (2·0 to 2·1)	34·1% (31·4 to 36·9)*	6847·2 (6704·5 to 7004·4)	50·4% (47·0 to 54·0)*	85·5 (83·7 to 87·5)	30·5% (27·6 to 33·5)*
		Opioid use disorders	109·5 (105·7 to 113·6)	77·0% (68·8 to 88·5)*	1·4 (1·3 to 1·4)	49·4% (42·5 to 59·2)*	4641·2 (4480·6 to 4818·9)	65·0% (57·3 to 75·0)*	58·0 (56·1 to 60·3)	43·9% (37·1 to 52·6)*
		Cocaine use disorders	7·3 (6·6 to 8·1)	42·2% (30·1 to 58·3)*	0·1 (0·1 to 0·1)	19·6% (9·2 to 33·0)*	311·5 (281·5 to 344·1)	35·6% (24·0 to 51·2)*	3·9 (3·5 to 4·3)	16·7% (6·5 to 30·0)*
		Amphetamine use disorders	4·5 (3·3 to 5·0)	27·2% (0·8 to 41·0)*	0·1 (0·0 to 0·1)	8·7% (−14·0 to 20·7)	206·9 (151·6 to 227·8)	21·0% (−3·6 to 34·4)	2·6 (1·9 to 2·8)	5·6% (−15·5 to 17·4)
		Other drug use disorders	45·3 (42·9 to 48·2)	35·2% (22·8 to 46·1)*	0·6 (0·5 to 0·6)	11·3% (1·2 to 19·9)*	1687·6 (1589·4 to 1805·9)	25·9% (14·0 to 37·3)*	21·0 (19·8 to 22·5)	8·2% (−2·0 to 17·8)
	**Diabetes and kidney diseases**		**2611·2 (2557·8 to 2667·2)**	34·2% (32·0 to 36·2)*	**33·6 (32·9 to 34·3)**	**1·3% (−0·3 to 2·7)**	**58 116·9 (56 801·5 to 59 525·7)**	25·1% (23·0 to 27·2)*	**726·4 (710·0 to 744·4)**	**−1·1% (−2·8 to 0·6)**
	Diabetes mellitus		1369·8 (1340·3 to 1401·9)	34·7% (32·2 to 37·3)*	17·5 (17·1 to 17·9)	1·2% (−0·7 to 3·1)	29 300·2 (28 711·5 to 29 950·1)	29·9% (27·2 to 32·4)*	363·1 (355·7 to 371·2)	0·7% (−1·4 to 2·6)
		Type 1 diabetes mellitus	345·5 (319·3 to 371·1)	15·1% (10·5 to 19·0)*	4·3 (4·0 to 4·7)	−11·0% (−14·6 to −7·8)*	9477·3 (8944·6 to 10 079·9)	11·1% (7·2 to 14·3)*	117·3 (110·8 to 124·6)	−10·6% (−13·9 to −7·9)*
		Type 2 diabetes mellitus	1024·3 (985·5 to 1066·8)	43·0% (40·4 to 45·8)*	13·2 (12·7 to 13·7)	5·9% (4·1 to 8·0)*	19 822·9 (19 013·8 to 20 687·8)	41·3% (38·3 to 44·4)*	245·8 (235·8 to 256·5)	7·1% (5·0 to 9·4)*
	Chronic kidney disease		1230·2 (1195·1 to 1258·8)	33·7% (30·5 to 36·1)*	15·9 (15·5 to 16·3)	1·5% (−0·9 to 3·2)	28 508·5 (27 610·2 to 29 314·0)	21·0% (18·2 to 23·5)*	359·4 (348·2 to 369·6)	−2·5% (−4·7 to −0·6)*
		Chronic kidney disease due to type 1 diabetes mellitus	77·3 (62·4 to 95·2)	23·2% (19·0 to 27·4)*	0·9 (0·8 to 1·2)	−1·2% (−4·0 to 1·2)	2622·0 (2121·7 to 3205·5)	17·8% (13·6 to 22·3)*	31·9 (25·9 to 38·9)	−2·9% (−5·6 to −0·3)*
		Chronic kidney disease due to type 2 diabetes mellitus	349·0 (306·8 to 395·9)	40·5% (36·4 to 43·6)*	4·5 (4·0 to 5·1)	4·2% (1·4 to 6·2)*	6671·9 (5825·5 to 7625·9)	35·4% (31·0 to 38·7)*	82·8 (72·4 to 94·5)	2·9% (−0·2 to 5·2)
		Chronic kidney disease due to hypertension	347·4 (304·6 to 391·5)	41·4% (37·4 to 44·2)*	4·6 (4·0 to 5·2)	3·2% (0·4 to 5·2)*	5954·8 (5175·1 to 6741·9)	33·4% (29·3 to 36·5)*	75·2 (65·4 to 84·9)	2·3% (−0·7 to 4·5)
		Chronic kidney disease due to glomerulonephritis	189·7 (165·2 to 217·3)	25·5% (22·1 to 28·8)*	2·4 (2·1 to 2·8)	−1·3% (−3·2 to 0·7)	5554·9 (4929·1 to 6250·8)	12·7% (9·6 to 16·1)*	70·6 (62·8 to 79·4)	−5·5% (−7·5 to −3·3)*
		Chronic kidney disease due to other and unspecified causes	266·8 (232·8 to 304·0)	25·9% (22·4 to 29·4)*	3·4 (3·0 to 3·9)	−1·4% (−3·7 to 0·6)	7704·8 (6794·9 to 8614·8)	10·0% (6·8 to 13·4)*	98·9 (87·4 to 110·0)	−7·7% (−9·9 to −5·4)*
	Acute glomerulonephritis		11·2 (10·5 to 12·1)	14·7% (8·7 to 22·3)*	0·1 (0·1 to 0·2)	−9·5% (−14·5 to −3·5)*	308·2 (282·4 to 336·8)	−5·5% (−10·4 to 2·2)	3·9 (3·6 to 4·3)	−20·9% (−25·1 to −15·1)*
	**Skin and subcutaneous diseases**		**100·3 (65·3 to 131·7)**	42·3% (34·9 to 52·0)*	**1·3 (0·9 to 1·7)**	8·1% (2·7 to 16·5)*	**2517·9 (1703·3 to 3283·8)**	26·1% (18·6 to 35·7)*	**33·1 (22·4 to 43·2)**	**5·0% (−1·2 to 13·8)**
	Bacterial skin diseases		76·0 (48·7 to 95·6)	45·5% (36·8 to 54·9)*	1·0 (0·6 to 1·3)	12·7% (6·0 to 20·7)*	2096·6 (1378·0 to 2691·9)	26·4% (18·0 to 36·9)*	27·6 (18·2 to 35·6)	6·4% (−0·6 to 15·9)
		Cellulitis	18·9 (10·3 to 26·0)	57·0% (45·8 to 67·1)*	0·2 (0·1 to 0·3)	19·6% (9·8 to 28·2)*	480·1 (264·6 to 640·2)	38·3% (30·8 to 50·4)*	6·2 (3·4 to 8·3)	13·7% (7·3 to 23·9)*
		Pyoderma	57·1 (35·8 to 70·8)	42·1% (32·4 to 52·4)*	0·8 (0·5 to 0·9)	10·5% (3·2 to 19·0)*	1616·4 (1051·6 to 2136·7)	23·3% (14·3 to 35·0)*	21·5 (14·1 to 28·8)	4·5% (−3·2 to 15·0)
	Decubitus ulcer		20·3 (13·2 to 30·6)	32·4% (22·9 to 51·0)*	0·3 (0·2 to 0·4)	−5·1% (−12·2 to 9·2)	321·7 (211·2 to 471·5)	26·2% (17·9 to 42·5)*	4·2 (2·7 to 6·1)	−2·3% (−8·8 to 11·5)
	Other skin and subcutaneous diseases		3·9 (2·6 to 7·2)	35·8% (26·6 to 49·6)*	0·1 (0·0 to 0·1)	3·3% (−3·5 to 14·4)	99·6 (69·4 to 165·8)	19·1% (10·8 to 34·1)*	1·3 (0·9 to 2·2)	0·7% (−6·1 to 13·1)
	**Musculoskeletal disorders**		**121·3 (105·6 to 126·2)**	30·9% (25·1 to 35·1)*	**1·6 (1·4 to 1·6)**	**−0·1% (−4·4 to 3·2)**	**2842·7 (2440·7 to 2953·1)**	19·6% (13·7 to 23·2)*	**35·9 (30·8 to 37·3)**	**−2·5% (−7·1 to 0·4)**
	Rheumatoid arthritis		47·3 (39·0 to 51·2)	25·8% (16·2 to 31·9)*	0·6 (0·5 to 0·7)	−5·9% (−12·9 to −1·2)*	866·0 (707·8 to 941·4)	17·9% (8·6 to 23·3)*	10·9 (8·9 to 11·8)	−9·1% (−16·1 to −5·0)*
	Other musculoskeletal disorders		74·0 (66·1 to 78·7)	34·4% (30·2 to 38·8)*	1·0 (0·9 to 1·0)	3·9% (0·9 to 7·5)*	1976·6 (1730·3 to 2089·1)	20·3% (15·6 to 24·0)*	25·0 (21·9 to 26·4)	0·8% (−3·0 to 3·8)
	**Other non-communicable diseases**		**1153·3 (1101·8 to 1208·3)**	**0·8% (−3·9 to 4·0)**	**16·3 (15·5 to 17·1)**	−11·2% (−15·3 to −8·5)*	**68 240·8 (64 835·4 to 72 452·1)**	−10·6% (−15·8 to −6·9)*	**993·0 (941·3 to 1054·3)**	−16·4% (−21·3 to −12·8)*
	Congenital anomalies		584·9 (556·3 to 618·3)	−14·3% (−21·1 to −10·1)*	8·7 (8·2 to 9·2)	−18·2% (−24·7 to −14·1)*	48 860·4 (46 405·7 to 51 687·3)	−15·3% (−22·0 to −11·0)*	729·4 (692·5 to 771·7)	−18·8% (−25·2 to −14·6)*
		Neural tube defects	61·7 (46·7 to 83·7)	−13·1% (−24·5 to −1·0)*	0·9 (0·7 to 1·3)	−16·5% (−27·6 to −4·8)*	5317·5 (4017·1 to 7217·5)	−13·4% (−24·8 to −1·4)*	80·0 (60·4 to 108·6)	−16·7% (−27·7 to −5·0)*
		Congenital heart anomalies	261·2 (216·6 to 308·2)	−17·9% (−24·6 to −9·8)*	3·9 (3·2 to 4·6)	−21·8% (−28·1 to −14·1)*	21 634·4 (17 770·6 to 25 604·8)	−18·9% (−25·5 to −10·8)*	321·7 (263·6 to 381·4)	−22·4% (−28·7 to −14·6)*
		Orofacial clefts	3·8 (1·5 to 8·8)	−40·0% (−54·5 to −22·5)*	0·1 (0·0 to 0·1)	−41·9% (−55·9 to −25·1)*	331·3 (130·1 to 770·5)	−40·0% (−54·5 to −22·7)*	5·0 (2·0 to 11·7)	−41·9% (−56·0 to −25·2)*
		Down syndrome	26·1 (21·3 to 35·1)	3·1% (−7·4 to 17·4)	0·4 (0·3 to 0·5)	−5·2% (−14·2 to 7·0)	1906·1 (1481·7 to 2707·9)	−1·4% (−11·5 to 13·9)	27·7 (21·3 to 39·8)	−7·3% (−16·7 to 7·1)
		Other chromosomal abnormalities	17·9 (12·0 to 26·3)	4·6% (−6·3 to 18·2)	0·3 (0·2 to 0·4)	0·3% (−10·1 to 13·2)	1507·9 (1012·2 to 2233·3)	3·9% (−6·9 to 17·4)	22·6 (15·1 to 33·5)	0·0% (−10·4 to 13·0)
		Congenital musculoskeletal and limb anomalies	11·0 (8·6 to 14·0)	−8·7% (−17·3 to 0·0)	0·2 (0·1 to 0·2)	−12·8% (−20·9 to −4·5)*	912·2 (708·9 to 1172·9)	−9·8% (−18·2 to −1·0)*	13·6 (10·6 to 17·5)	−13·3% (−21·5 to −4·9)*
		Urogenital congenital anomalies	14·1 (10·3 to 16·9)	−2·5% (−11·8 to 9·2)	0·2 (0·1 to 0·2)	−8·5% (−17·1 to 2·1)	1105·8 (781·3 to 1347·8)	−5·5% (−14·6 to 6·3)	16·4 (11·5 to 20·0)	−9·7% (−18·3 to 1·3)
		Digestive congenital anomalies	50·8 (37·7 to 71·8)	−16·2% (−27·1 to −6·4)*	0·8 (0·6 to 1·1)	−19·3% (−29·8 to −9·8)*	4398·7 (3253·9 to 6229·0)	−16·5% (−27·3 to −6·7)*	66·3 (49·0 to 93·9)	−19·4% (−29·9 to −9·9)*
		Other congenital anomalies	138·3 (102·3 to 175·6)	−12·4% (−20·1 to −0·5)*	2·1 (1·5 to 2·6)	−15·9% (−23·3 to −4·5)*	11 746·6 (8613·3 to 14 951·0)	−13·0% (−20·7 to −1·1)*	176·1 (128·8 to 224·2)	−16·3% (−23·7 to −4·8)*
	Urinary diseases and male infertility		271·2 (263·9 to 282·2)	39·6% (34·9 to 43·4)*	3·6 (3·5 to 3·7)	5·7% (2·2 to 8·5)*	6255·2 (6044·8 to 6542·1)	20·8% (15·5 to 24·9)*	81·1 (78·3 to 84·8)	−0·7% (−5·1 to 2·7)
		Urinary tract infections	206·4 (197·9 to 223·2)	48·3% (42·9 to 53·5)*	2·7 (2·6 to 3·0)	10·9% (7·2 to 14·5)*	4522·3 (4285·2 to 5016·3)	31·4% (24·4 to 38·8)*	58·4 (55·2 to 65·0)	7·2% (1·7 to 13·0)*
		Urolithiasis	12·3 (10·5 to 15·7)	30·4% (19·0 to 49·4)*	0·2 (0·1 to 0·2)	−1·2% (−9·7 to 12·9)	255·1 (216·0 to 323·5)	19·6% (9·7 to 36·9)*	3·2 (2·7 to 4·0)	−5·9% (−13·6 to 7·7)
		Other urinary diseases	52·5 (42·3 to 58·0)	15·0% (8·0 to 25·5)*	0·7 (0·6 to 0·8)	−9·9% (−15·3 to −2·2)*	1477·8 (1172·2 to 1660·2)	−3·0% (−9·6 to 6·4)	19·4 (15·4 to 21·9)	−18·2% (−23·3 to −10·6)*
	Gynaecological diseases		8·2 (7·4 to 8·7)	19·1% (5·1 to 30·0)*	0·1 (0·1 to 0·1)	−2·6% (−13·6 to 6·0)	292·9 (272·6 to 318·7)	9·2% (−2·8 to 20·6)	3·7 (3·4 to 4·0)	−6·0% (−15·4 to 3·6)
		Uterine fibroids	2·4 (1·6 to 3·0)	33·3% (6·7 to 54·6)*	0·0 (0·0 to 0·0)	8·1% (−14·9 to 24·7)	74·2 (52·7 to 95·5)	13·0% (−4·2 to 31·6)	0·9 (0·6 to 1·2)	−4·8% (−20·0 to 10·5)
		Polycystic ovarian syndrome	0·0 (0·0 to 0·0)	12·9% (−12·8 to 50·4)	0·0 (0·0 to 0·0)	1·0% (−22·5 to 34·8)	0·7 (0·1 to 1·5)	10·1% (−15·8 to 51·2)	0·0 (0·0 to 0·0)	−0·1% (−24·3 to 37·2)
		Endometriosis	0·2 (0·1 to 0·2)	11·8% (−12·4 to 45·5)	0·0 (0·0 to 0·0)	−3·2% (−23·8 to 25·5)	7·7 (3·2 to 12·0)	10·4% (−12·9 to 41·7)	0·1 (0·0 to 0·1)	−3·2% (−23·2 to 24·3)
		Genital prolapse	0·6 (0·3 to 0·9)	0·6% (−15·4 to 16·1)	0·0 (0·0 to 0·0)	−24·1% (−36·0 to −13·0)*	14·5 (6·8 to 20·1)	−4·4% (−18·4 to 10·4)	0·2 (0·1 to 0·2)	−24·0% (−35·2 to −11·9)*
		Other gynaecological diseases	5·0 (4·1 to 5·6)	16·0% (4·1 to 27·9)*	0·1 (0·1 to 0·1)	−3·6% (−12·2 to 5·9)	195·8 (163·0 to 228·9)	8·9% (−2·9 to 20·6)	2·5 (2·1 to 2·9)	−4·8% (−14·2 to 5·1)
	Haemoglobinopathies and haemolytic anaemias		104·6 (82·0 to 132·2)	5·8% (−1·4 to 13·4)	1·4 (1·1 to 1·8)	−11·3% (−17·6 to −4·8)*	4831·6 (3643·1 to 6268·9)	−1·8% (−13·1 to 9·4)	66·6 (50·0 to 86·2)	−11·1% (−21·6 to −0·5)*
		Thalassaemias	7·2 (6·0 to 8·4)	−23·7% (−32·6 to −12·7)*	0·1 (0·1 to 0·1)	−27·9% (−36·5 to −17·2)*	564·7 (474·8 to 667·6)	−24·6% (−33·9 to −13·2)*	8·2 (6·9 to 9·7)	−28·6% (−37·6 to −17·6)*
		Sickle cell disorders	38·4 (24·0 to 54·8)	3·7% (−11·6 to 17·7)	0·5 (0·3 to 0·8)	−3·1% (−17·6 to 10·3)	2796·4 (1747·3 to 3913·6)	2·1% (−13·7 to 17·3)	39·7 (24·8 to 55·3)	−3·9% (−19·1 to 11·0)
		G6PD deficiency	16·7 (12·1 to 22·5)	11·8% (4·7 to 19·6)*	0·2 (0·2 to 0·3)	−7·1% (−12·1 to −1·0)*	692·6 (522·0 to 896·1)	4·4% (−2·5 to 12·3)	8·8 (6·7 to 11·4)	−9·6% (−15·0 to −3·3)*
		Other haemoglobinopathies and haemolytic anaemias	42·2 (35·1 to 49·2)	13·0% (9·3 to 16·5)*	0·6 (0·5 to 0·6)	−16·1% (−18·7 to −13·4)*	777·8 (634·5 to 917·2)	1·3% (−2·2 to 4·8)	9·9 (8·1 to 11·7)	−19·9% (−22·3 to −17·4)*
	Endocrine, metabolic, blood, and immune disorders		144·5 (115·1 to 152·3)	28·2% (19·7 to 33·3)*	1·9 (1·5 to 2·0)	0·8% (−5·0 to 4·4)	4506·4 (3762·3 to 4919·9)	10·4% (2·7 to 16·9)*	59·7 (50·0 to 65·5)	−5·5% (−11·2 to −0·2)*
	Sudden infant death syndrome		40·0 (18·0 to 77·0)	−17·3% (−28·6 to −1·4)*	0·6 (0·3 to 1·2)	−20·2% (−31·2 to −4·9)*	3494·3 (1570·1 to 6734·0)	−17·3% (−28·6 to −1·4)*	52·7 (23·7 to 101·5)	−20·2% (−31·2 to −4·9)*
**Injuries**			**4484·7 (4332·0 to 4585·6)**	2·3% (0·5 to 4·0)*	**57·9 (55·9 to 59·2)**	−13·7% (−15·1 to −12·2)*	**195 231·1 (188 807·7 to 199 825·5)**	−6·4% (−7·8 to −4·8)*	**2548·3 (2461·9 to 2609·6)**	−16·9% (−18·2 to −15·3)*
	**Transport injuries**		**1335·0 (1289·1 to 1369·5)**	−3·1% (−6·0 to −0·6)*	**17·0 (16·4 to 17·4)**	−17·0% (−19·5 to −14·9)*	**61 937·8 (60 031·2 to 63 736·5)**	−9·6% (−11·8 to −7·3)*	**800·5 (775·9 to 823·3)**	−19·5% (−21·4 to −17·5)*
	Road injuries		1243·1 (1191·9 to 1276·9)	−3·2% (−6·3 to −0·5)*	15·8 (15·2 to 16·3)	−17·1% (−19·7 to −14·9)*	57 638·4 (55 500·8 to 59 369·2)	−9·7% (−12·0 to −7·3)*	745·0 (718·1 to 767·4)	−19·6% (−21·6 to −17·5)*
		Pedestrian road injuries	486·2 (459·7 to 535·0)	−6·4% (−11·7 to −2·1)*	6·2 (5·9 to 6·8)	−21·4% (−25·5 to −17·9)*	20 850·8 (19 596·0 to 23 164·4)	−14·8% (−18·7 to −11·0)*	270·4 (253·9 to 300·8)	−25·1% (−28·3 to −21·9)*
		Cyclist road injuries	68·9 (59·2 to 76·2)	9·1% (1·8 to 16·4)*	0·9 (0·7 to 1·0)	−8·8% (−14·8 to −2·5)*	2853·5 (2471·6 to 3209·0)	1·0% (−5·7 to 8·3)	36·3 (31·5 to 41·0)	−11·8% (−17·8 to −5·3)*
		Motorcyclist road injuries	225·7 (196·1 to 238·6)	−0·6% (−8·9 to 5·2)	2·9 (2·5 to 3·0)	−12·4% (−19·5 to −7·3)*	11 416·3 (9969·6 to 12 098·0)	−5·7% (−12·5 to −0·5)*	146·2 (127·5 to 154·9)	−14·8% (−20·7 to −10·1)*
		Motor vehicle road injuries	451·1 (423·4 to 472·9)	−2·5% (−6·2 to 1·3)	5·8 (5·4 to 6·0)	−15·6% (−18·6 to −12·2)*	22 004·1 (20 639·8 to 23 130·9)	−7·8% (−10·4 to −3·0)*	285·3 (267·6 to 299·7)	−17·2% (−19·6 to −12·8)*
		Other road injuries	11·2 (9·9 to 12·8)	−5·5% (−11·0 to 16·1)	0·1 (0·1 to 0·2)	−19·4% (−24·1 to −1·3)*	513·8 (454·1 to 583·4)	−11·7% (−17·3 to 10·6)	6·7 (5·9 to 7·6)	−21·4% (−26·5 to −1·7)*
	Other transport injuries		91·9 (84·5 to 107·2)	−1·5% (−6·2 to 3·7)	1·2 (1·1 to 1·4)	−15·5% (−19·5 to −10·9)*	4299·4 (3919·6 to 5048·3)	−7·8% (−12·6 to −2·4)*	55·4 (50·5 to 65·0)	−17·9% (−22·2 to −13·2)*
	**Unintentional injuries**		**1804·9 (1695·7 to 1872·0)**	2·9% (0·5 to 6·0)*	**23·8 (22·4 to 24·7)**	−15·3% (−17·3 to −12·8)*	**69 430·5 (64 685·1 to 72 366·8)**	−12·8% (−15·0 to −9·6)*	**928·8 (865·6 to 969·3)**	−23·0% (−25·0 to −20·0)*
	Falls		695·8 (644·9 to 741·7)	27·4% (21·2 to 35·6)*	9·2 (8·5 to 9·8)	−2·8% (−7·4 to 3·4)	16 688·1 (15 101·9 to 17 636·8)	10·1% (4·8 to 17·2)*	216·6 (196·4 to 228·6)	−8·4% (−12·7 to −2·5)*
	Drowning		295·2 (284·5 to 306·2)	−17·2% (−19·8 to −14·1)*	4·0 (3·8 to 4·1)	−27·3% (−29·6 to −24·5)*	16 563·3 (15 784·2 to 17 350·0)	−26·1% (−29·0 to −22·4)*	228·3 (217·2 to 239·7)	−32·8% (−35·5 to −29·3)*
	Fire heat and hot substances		120·6 (101·6 to 129·4)	−7·9% (−10·9 to −1·2)*	1·6 (1·3 to 1·7)	−22·9% (−25·4 to −17·3)*	5286·3 (4308·9 to 5836·4)	−16·5% (−21·0 to −7·3)*	71·0 (57·8 to 78·6)	−25·5% (−29·6 to −17·1)*
	Poisonings		72·4 (52·7 to 79·4)	−6·8% (−16·1 to 2·9)	0·9 (0·7 to 1·0)	−20·8% (−28·4 to −12·5)*	3321·7 (2454·1 to 3669·2)	−14·6% (−22·7 to −3·8)*	44·1 (32·7 to 48·8)	−23·9% (−31·0 to −14·1)*
		Poisoning by carbon monoxide	35·5 (25·7 to 38·8)	−12·5% (−22·4 to −5·0)*	0·5 (0·3 to 0·5)	−26·6% (−34·8 to −20·3)*	1462·4 (1073·0 to 1613·6)	−19·1% (−27·2 to −11·8)*	18·9 (13·8 to 20·9)	−29·0% (−36·2 to −22·4)*
		Poisoning by other means	36·9 (26·8 to 41·0)	−0·5% (−10·1 to 11·9)	0·5 (0·4 to 0·5)	−14·4% (−22·4 to −3·9)*	1859·3 (1385·8 to 2072·9)	−10·7% (−19·6 to 3·3)	25·2 (19·0 to 28·1)	−19·6% (−27·7 to −6·8)*
	Exposure to mechanical forces		136·5 (117·6 to 143·2)	−6·7% (−9·8 to −3·7)*	1·8 (1·5 to 1·8)	−20·3% (−22·9 to −17·8)*	6385·5 (5500·4 to 6710·8)	−13·8% (−16·6 to −10·8)*	84·0 (72·3 to 88·3)	−23·0% (−25·5 to −20·3)*
		Unintentional firearm injuries	22·6 (21·1 to 25·8)	−2·9% (−7·5 to 2·8)	0·3 (0·3 to 0·3)	−16·4% (−20·3 to −11·5)*	1094·5 (1013·5 to 1275·4)	−7·4% (−12·2 to −1·3)*	14·4 (13·3 to 16·9)	−16·5% (−20·9 to −11·1)*
		Other exposure to mechanical forces	113·9 (94·7 to 120·8)	−7·4% (−10·6 to −4·1)*	1·5 (1·2 to 1·6)	−21·0% (−23·7 to −18·3)*	5291·0 (4401·1 to 5626·1)	−15·0% (−18·0 to −11·7)*	69·6 (57·8 to 74·0)	−24·3% (−26·9 to −21·2)*
	Adverse effects of medical treatment		121·6 (103·6 to 137·6)	16·6% (12·0 to 20·9)*	1·6 (1·4 to 1·8)	−6·2% (−10·0 to −2·5)*	4363·9 (3619·9 to 5234·0)	4·0% (−1·2 to 11·0)	58·1 (48·0 to 70·7)	−9·5% (−13·9 to −3·6)*
	Animal contact		81·1 (44·9 to 94·0)	−1·4% (−6·8 to 6·2)	1·1 (0·6 to 1·2)	−16·0% (−20·5 to −9·6)*	3911·9 (2167·6 to 4585·6)	−9·5% (−15·8 to 0·2)	52·4 (29·0 to 61·8)	−19·2% (−25·2 to −10·2)*
		Venomous animal contact	70·9 (37·0 to 83·8)	−1·3% (−7·5 to 6·2)	0·9 (0·5 to 1·1)	−16·0% (−21·0 to −9·7)*	3407·7 (1758·4 to 4087·5)	−9·7% (−16·4 to −0·7)*	45·5 (23·4 to 54·9)	−19·4% (−25·8 to −11·3)*
		Non-venomous animal contact	10·1 (7·1 to 14·4)	−1·6% (−15·3 to 10·2)	0·1 (0·1 to 0·2)	−16·1% (−27·4 to −6·2)*	504·2 (335·8 to 750·1)	−7·9% (−26·1 to 6·5)	6·9 (4·5 to 10·3)	−17·2% (−33·6 to −4·3)*
	Foreign body		124·1 (119·3 to 130·0)	1·7% (−1·9 to 4·8)	1·7 (1·6 to 1·8)	−14·1% (−17·0 to −11·6)*	5907·0 (5566·3 to 6301·2)	−12·4% (−16·4 to −8·3)*	83·3 (78·3 to 88·9)	−20·1% (−23·8 to −16·3)*
		Pulmonary aspiration and foreign body in airway	115·7 (111·4 to 121·3)	1·9% (−1·9 to 5·0)	1·6 (1·5 to 1·7)	−13·9% (−17·0 to −11·4)*	5526·1 (5212·6 to 5910·0)	−12·2% (−16·6 to −8·0)*	78·1 (73·5 to 83·7)	−19·9% (−23·8 to −16·0)*
		Foreign body in other body part	8·4 (7·5 to 10·3)	−0·5% (−6·9 to 7·1)	0·1 (0·1 to 0·1)	−15·8% (−20·8 to −10·0)*	381·0 (326·2 to 474·4)	−14·4% (−21·1 to −6·2)*	5·2 (4·4 to 6·5)	−23·3% (−29·2 to −16·1)*
	Environmental heat and cold exposure		53·3 (36·8 to 59·2)	−13·2% (−22·4 to −8·4)*	0·7 (0·5 to 0·8)	−29·4% (−37·1 to −25·4)*	1845·6 (1246·6 to 2066·2)	−21·4% (−28·8 to −17·5)*	23·7 (15·8 to 26·7)	−32·7% (−39·5 to −29·1)*
	Exposure to forces of nature		9·6 (8·7 to 11·0)	−38·0% (−43·9 to −28·9)*	0·1 (0·1 to 0·1)	−45·8% (−50·8 to −37·9)*	477·6 (438·4 to 544·3)	−45·0% (−49·4 to −37·3)*	6·3 (5·8 to 7·2)	−50·2% (−54·2 to −43·2)*
	Other unintentional injuries		94·7 (91·9 to 98·3)	−14·5% (−16·7 to −12·1)*	1·2 (1·2 to 1·3)	−25·8% (−27·6 to −23·8)*	4679·6 (4519·4 to 4888·2)	−20·7% (−22·9 to −18·1)*	60·9 (58·8 to 63·7)	−28·9% (−30·9 to −26·6)*
	**Self-harm and interpersonal violence**		**1344·8 (1283·1 to 1380·4)**	7·3% (4·6 to 9·7)*	**17·1 (16·3 to 17·5)**	−7·6% (−9·9 to −5·5)*	**63 862·9 (61 029·9 to 65 755·7)**	5·4% (2·8 to 7·7)*	**819·0 (782·2 to 843·4)**	−5·7% (−7·9 to −3·7)*
	Self-harm		793·8 (743·5 to 819·7)	1·1% (−2·6 to 3·7)	10·0 (9·4 to 10·3)	−14·8% (−18·0 to −12·6)*	33 577·2 (31 449·3 to 34 719·1)	−3·4% (−7·0 to −0·9)*	423·6 (396·9 to 438·2)	−15·1% (−18·4 to −12·9)*
		Self-harm by firearm	63·8 (54·6 to 78·6)	6·8% (2·3 to 10·8)*	0·8 (0·7 to 1·0)	−10·3% (−13·9 to −7·2)*	2653·6 (2241·9 to 3288·1)	0·9% (−3·5 to 5·5)	33·5 (28·2 to 41·6)	−11·5% (−15·2 to −7·6)*
		Self-harm by other specified means	730·0 (678·5 to 754·9)	0·6% (−3·2 to 3·4)	9·2 (8·5 to 9·5)	−15·2% (−18·4 to −12·8)*	30 923·6 (28 832·4 to 32 098·2)	−3·7% (−7·5 to −1·1)*	390·1 (363·6 to 405·1)	−15·4% (−18·8 to −13·1)*
	Interpersonal violence		405·3 (365·2 to 431·7)	0·5% (−2·0 to 3·2)	5·2 (4·7 to 5·5)	−11·1% (−13·3 to −8·7)*	21 439·8 (19 275·8 to 22 799·8)	−1·6% (−4·4 to 1·3)	276·8 (248·4 to 294·2)	−10·9% (−13·4 to −8·2)*
		Assault by firearm	174·4 (147·9 to 188·9)	7·5% (4·3 to 10·8)*	2·2 (1·9 to 2·4)	−3·6% (−6·5 to −0·5)*	9541·2 (8106·2 to 10 291·7)	5·4% (2·1 to 9·0)*	122·9 (104·3 to 132·4)	−3·7% (−6·7 to −0·4)*
		Assault by sharp object	91·4 (74·4 to 111·2)	−11·5% (−15·3 to −6·0)*	1·2 (0·9 to 1·4)	−22·3% (−25·6 to −17·6)*	4634·5 (3747·0 to 5648·9)	−13·9% (−17·6 to −8·5)*	59·2 (47·8 to 72·1)	−22·6% (−25·9 to −17·8)*
		Assault by other means	139·5 (123·6 to 164·4)	1·3% (−3·4 to 5·6)	1·8 (1·6 to 2·1)	−11·5% (−15·4 to −7·6)*	7264·1 (6400·8 to 8583·0)	−1·3% (−5·4 to 3·6)	94·7 (83·3 to 111·5)	−11·2% (−14·9 to −6·8)*
	Conflict and terrorism		129·7 (118·1 to 143·2)	118·0% (88·8 to 148·6)*	1·7 (1·6 to 1·9)	98·4% (72·4 to 126·1)*	7966·6 (7244·5 to 8855·9)	113·5% (84·5 to 146·8)*	107·3 (97·6 to 119·1)	97·9% (71·0 to 128·8)*
	Executions and police conflict		16·0 (15·7 to 16·3)	203·9% (186·9 to 220·9)*	0·2 (0·2 to 0·2)	172·4% (156·8 to 187·6)*	879·3 (862·3 to 898·1)	202·1% (184·8 to 219·8)*	11·4 (11·2 to 11·7)	176·4% (160·5 to 192·9)*

Data in parentheses are 95% uncertainty intervals. G6PD=glucose-6-phosphate dehydrogenase. GBD=Global Burden of Diseases, Injuries, and Risk Factors Study. *H influenzae=Haemophilus influenzae*. NASH=non-alcoholic steatohepatitis. YLL=years of life lost.

* Percentage changes that are statistically significant.

#### Communicable, maternal, neonatal, and nutritional diseases

The overall decrease in communicable causes of death included reductions in some of the largest contributors to global mortality, including HIV/AIDS, tuberculosis, diarrhoeal diseases, and malaria (table 1). The peak in HIV/AIDS mortality occurred in 2006 with 1·95 million deaths (95% UI 1·87–2·04) and a rate of 28·8 deaths (27·7–30·1) per 100 000, but between 2007 and 2017, total mortality from HIV/AIDS decreased from 1·92 million (1·84–2·00) deaths to 0·954 million (0·907–1·01) deaths with a commensurate decrease (56·5% [54·7–58·0]) in the mortality rate from 27·9 deaths (26·8–29·1) per 100 000 in 2007 to 12·1 deaths (11·5–12·9) per 100 000 in 2017. Although tuberculosis caused an estimated 1·18 million (1·13–1·25) deaths in 2017, this was nonetheless a decrease of 14·9% (10·3–18·2) from levels in 2007, when tuberculosis caused 1·39 million (1·34–1·46) deaths. Drug-susceptible tuberculosis deaths were the largest component of tuberculosis deaths in 2017 (88·2% [81·4–93·3]) and decreased the most since 2007 (15·5% [8·6–22·3]) in comparison with other tuberculosis sub-causes. All HIV/AIDS and tuberculosis co-infections also decreased, with declines occurring for deaths from HIV/AIDS and drug-resistant tuberculosis co-infection (8·3% [–26·8 to 14·7]), HIV/AIDS and multidrug-resistant tuberculosis co-infection (52·2% [33·2–66·4]), and HIV/AIDS and drug-susceptible tuberculosis co-infection (55·4% [51·6–58·4]). The total number of deaths from diarrhoeal diseases decreased by 16·6% (6·7–25·3) between 2007 and 2017, from 1·88 million (1·53–2·47) deaths in 2007 to 1·57 million (1·18–2·19) deaths in 2017. There was a parallel decrease in the death rate (30·2% [22·7–36·1]) from diarrhoeal diseases, from 31·0 deaths (25·0–40·9) per 100 000 in 2007 to 21·6 deaths (16·4–29·7) per 100 000 in 2017. There were 620 000 deaths (440 000–840 000) from malaria in 2017, a decrease of 30·8% (20·8–39·4) from 2007 when 896 000 deaths (664 000–1 180 000) were estimated. Deaths due to measles decreased by 57·0% (51·9–61·9) from 222 000 deaths (82 300–457 000) in 2007 to 95 300 (34 500–205 000) in 2017. Invasive non-typhoidal salmonella deaths were estimated to have decreased from 71 900 deaths (42 200–116 000) in 2007 to 59 100 deaths (33 300–98 100) in 2017. A notable exception to the estimated improvements for communicable diseases occurred for dengue, where deaths increased by 65·5% (21·7–99·7) from 24 500 (11 500–29 600) in 2007 to 40 500 (17 600–49 800) in 2017, with a similar increase in mortality rate (40·7% [3·6–69·7], from 0·4 deaths [0·2–0·5] per 100 000 in 2007 to 0·5 deaths [0·2–0·7] per 100 000 in 2017).

At Level 2 of the GBD cause hierarchy, there were 1·98 million (95% UI 1·89–2·06) deaths from maternal and neonatal disorders globally in 2017, and 90·2% (89·4–90·9) of these deaths were from neonatal disorders (table 1). Deaths from neonatal disorders decreased by 24·1% (20·6–27·2), from 2·35 million (2·27–2·44) deaths in 2007 to 1·78 million (1·70–1·86) in 2017. A 26·2% (22·7–29·1) decrease in death rates for neonatal disorders was also estimated, from 36·7 deaths (35·3–38·0) per 100 000 in 2007 to 27·1 (25·8–28·3) per 100 000 in 2017. Deaths from maternal disorders decreased by 24·0% (19·5–28·4), from 255 000 deaths (241 000–268 000) in 2007 to 194 000 deaths (180 000–210 000) in 2017. The mortality rate for maternal disorders decreased by 30·7% (26·6–34·8), from 3·6 deaths (3·4–3·8) per 100 000 in 2007 to 2·5 (2·3–2·7) per 100 000 in 2017.

There were 270 000 deaths (95% UI 249 000–295 000) from nutritional deficiencies in 2017, representing 2·60% (2·37–2·86) of all deaths from CMNN causes in that year (table 1). Decreases in death rates from nutritional deficiencies followed a trajectory similar to that of maternal and neonatal disorders, with mortality rates from nutritional deficiencies decreasing by 33·6% (26·5–38·1), from 5·8 deaths (5·4–6·2) per 100 000 in 2007 to 3·8 (3·5–4·2) per 100 000 in 2017.

#### Non-communicable diseases

At Level 2 of the GBD hierarchy, the largest numbers of deaths from NCDs were estimated for cardiovascular diseases (17·8 million [95% UI 17·5–18·0] deaths), followed by neoplasms (9·56 million [9·40–9·69] deaths) and chronic respiratory diseases (3·91 million [3·79–4·04] deaths; table 1). Overall, deaths from NCDs increased globally, from 33·5 million (33·1–33·8) in 2007 to 41·1 million (40·5–41·5) in 2017, while the death rate decreased (from 582·1 deaths [575·1–587·8] per 100 000 in 2007 to 536·1 deaths [528·4–542·2] per 100 000 in 2017). Total deaths from NCDs decreased significantly for only two Level 3 causes: sudden infant death syndrome (17·3% [1·4–28·6]) and congenital anomalies (14·3% [10·1–21·1]). During the past decade the estimated number of deaths from neurological disorders increased by 42·1% (40·2–43·9), from 2·18 million (2·14–2·20) deaths in 2007 to 3·09 million (3·04–3·14) deaths in 2017; although the death rate increased, this change was not significant (0·1% [–1·2 to 1·3], from 43·0 deaths [42·3–43·4] per 100 000 in 2007 to 43·1 deaths [42·3–43·7] per 100 000 in 2017). Among neurological disorders, the greatest increase between 2007 and 2017 occurred for deaths from Alzheimer's disease and other dementias (an increase of 46·2% [43·9–48·0], from 1·72 million [1·70–1·74] deaths in 2007 to 2·51 million [2·47–2·55] in 2017; and from 35·2 deaths [34·7–35·5] per 100 000 in 2007 to 35·4 [34·8–35·9] per 100 000 in 2017).

At a global level, total deaths from cardiovascular diseases increased by 21·1% (95% UI 19·7–22·6) between 2007 and 2017 but death rates decreased from 259·9 deaths (257·1–263·7) per 100 000 in 2007 to 233·1 (229·7–236·4) per 100 000 in 2017 (table 1). In combination, ischaemic heart disease and stroke—at Level 3 of the cause hierarchy—accounted for 84·9% (84·3–86·3) of cardiovascular disease deaths in 2017. Deaths from both these causes increased between 2007 and 2017, from 7·30 million (7·22–7·46) deaths to 8·93 million (8·79–9·14) deaths for ischaemic heart disease, and from 5·29 million (5·22–5·40) deaths to 6·17 million (6·04–6·33) deaths for stroke. The largest decline in mortality rates among cardiovascular diseases during the same decade occurred for rheumatic heart disease, which decreased by 21·3% (17·8–25·2) between 2007 and 2017, from 4·7 deaths (4·4–5·0) per 100 000 to 3·7 deaths (3·4–3·9) per 100 000.

Neoplasms contributed to 23·3% (95% UI 23·0–23·5) of deaths from NCDs in 2017, with tracheal, bronchus, and lung cancer leading to the most deaths (1·88 million [1·84–1·92]), followed by colon and rectum cancer (896 000 [876 000–916 000]; table 1). Newly estimated for GBD 2017, liver cancer due to NASH caused 66 900 deaths (59 600–74 500) in 2017, representing an increase of 42·3% (38·0–47·6) from 2007. Globally, deaths from cancers increased by 25·4% (23·9–27·0) between 2007 and 2017, from 7·62 million (7·51–7·70) deaths in 2007 to 9·56 million (9·40–9·69) deaths in 2017. The largest increases occurred for other neoplasms, which includes myelodysplastic, myeloproliferative, and other haemopoietic neoplasms, benign and in-situ intestinal, cervical, and uterine neoplasms, and other benign and in-situ neoplasms (increase of 42·0% [35·6–51·7] to 103 000 deaths [80 200–122 000]), other pharynx cancer (increase of 40·4% [29·7–48·4] to 117 000 deaths [102 000–124 000]), and pancreatic cancer (increase of 39·9% [36·7–42·6] to 441 000 deaths [433 000–449 000]). Mortality rates for most types of cancer decreased in the decade 2007–17; the largest statistically significant decreases occurred for stomach cancer (decrease of 17·1% [15·1–18·8] to 11·0 deaths [10·8–11·2] per 100 000), Hodgkin lymphoma (decrease of 16·8% [14·0–19·8] to 0·4 deaths [0·4–0·5] per 100 000), and oesophageal cancer (decrease of 14·5% [12·0–16·9] to 5·5 deaths [5·3–5·6] per 100 000).

Several non-communicable causes were separately estimated for the first time in GBD 2017. Among these, diabetes mellitus resulted in 1·37 million (95% UI 1·34–1·40) deaths in 2017, of which 25·2% (23·0–27·3) were from type 1 diabetes (table 1). Total deaths from type 1 diabetes increased from 2007 to 2017 by 15·1% (10·5–19·0) and those from type 2 diabetes by 43·0% (40·4–45·8). During this time period, the mortality rate decreased by 11·0% (7·8–14·6) for type 1 diabetes and increased by 5·9% (4·1–8·0) for type 2 diabetes. Deaths from diabetes-related chronic kidney disease also increased over the past decade, rising from 62 800 deaths (51 100–76 200) in 2007 to 77 300 deaths (62 400–95 200) in 2017 for chronic kidney disease due to type 1 diabetes and from 248 000 deaths (219 000–282 000) in 2007 to 349 000 (307 000–396 000) in 2017 for chronic kidney disease due to type 2 diabetes. Among other newly estimated causes, subarachnoid haemorrhage was estimated to have caused 445 000 deaths (417 000–492 000) in 2017, representing 0·8% (0·7–0·9) of global deaths and 2·5% (2·3–2·8) of cardiovascular disease deaths in 2017; deaths from non-rheumatic valvular heart disease (145 000 [122 000–150 000]) represented 0·3% (0·2–0·3) of global deaths and 0·8% (0·7–0·9) of cardiovascular disease deaths in 2017. Deaths from substance use disorders increased between 2007 and 2017, rising from 284 000 deaths (268 000–289 000) to 352 000 deaths (334 000–363 000) globally; although not statistically significant, the death rate for these disorders increased by 2·0% (–1·0 to 5·0) during this period, rising from 4·3 deaths (4·0 to 4·3) per 100 000 to 4·3 (4·1 to 4·5) per 100 000 (table 1). The greatest number of deaths from drug use disorders were due to opioid use disorders, which resulted in 110 000 deaths (106 000–114 000) globally in 2017, comprising 65·7% (63·8–67·4) of global deaths from drug use disorders.

#### Injuries

At Level 3 of the cause hierarchy, most injury deaths were from road injuries, which caused 1·24 million (95% UI 1·19–1·28) deaths in 2017, representing 27·7% (26·7–28·9) of all injury deaths in that year (table 1). 794 000 deaths (744 000–820 000) in 2017 were from self-harm, followed by 696 000 deaths (645 000–742 000) from falls, 405 000 deaths (365 000–432 000) from interpersonal violence, and 295 000 deaths (285 000–306 000) from drowning. Mortality rates in 2017 were highest among injury causes of death at Level 3 of the GBD hierarchy for road injuries (15·8 deaths [15·2–16·3] per 100 000), self-harm (10·0 deaths [9·4–10·3] per 100 000), and falls (9·2 deaths [8·5–9·8] per 100 000). Overall, from 2007 to 2017, there were 20·1 million (18·7–20·8) deaths from unintentional injuries, 15·1 million (14·8–15·4) deaths from transport injuries, and 14·4 million (13·7–14·7) deaths from self-harm and interpersonal violence. Poisoning by carbon monoxide, estimated for the first time for GBD 2017, caused 35 500 deaths (25 700–38 800) in 2017.

**Figure 3:**
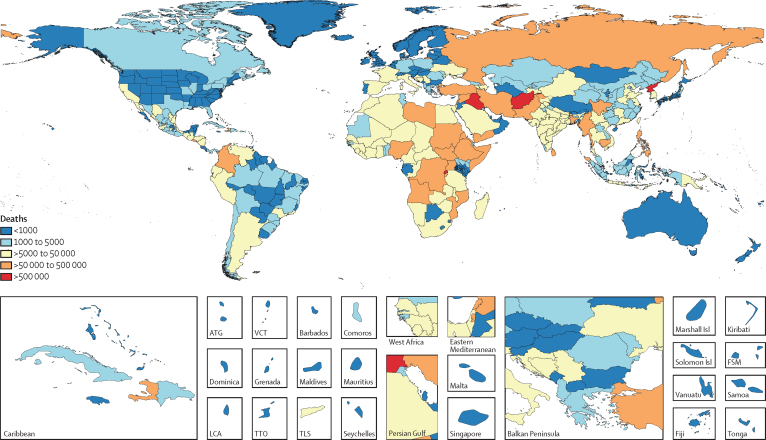
All-age deaths due to fatal discontinuities (violence, disasters, famine, and disease outbreak), for both sexes combined, 1980–2017 We have chosen to show this map in counts to capture the wide range of discontinuity-related deaths ranging from motor vehicle accidents with a smaller number of deaths to natural disasters and conflicts with a larger number of deaths. Deaths are coded to the location of residence for the deceased. Maps by each subtype—violence, disasters, famine, and disease outbreak—are provided in appendix 2. ATG=Antigua and Barbuda. FSM=Federated States of Micronesia. Isl=Islands. LCA=Saint Lucia. TLS=Timor-Leste. TTO=Trinidad and Tobago. VCT=Saint Vincent and the Grenadines.

Since 1980, sudden changes in the expected number of deaths—described as fatal discontinuities in the GBD study—were found in several countries (figure 3). To emphasise the magnitude of these events, we describe total deaths rather than rates in this report. Figure 3 combines total deaths across disparate types of fatal discontinuity; appendix 2 separates these deaths by category. Deaths from conflict and terrorism, despite substantial limitations to their enumeration, were estimated to have increased greatly, rising by 118·0% (95% UI 88·8–148·6) in 2007–17 (table 1). Of deaths related to conflict and terrorism, 16 200 (13 400–19 800) were among people aged 5–14 years and 14 300 (11 700–17 300) occurred for children younger than 5 years; combined, these deaths represented 23·5% (20·5–26·9) of all deaths from conflict and terrorism (table 2).

**Table 2:** Selected causes of global deaths by age groups (<5 years, 5–14 years, 15–49 years, 50–69 years, and ≥70 years) in 2017, with percentage change between 2007 and 2017, for both sexes combined

			**All-age deaths (thousands)**		**Under-5 deaths (thousands)**		**Deaths at age 5–14 years (thousands)**		**Deaths at age 15–49 years (thousands)**		**Deaths at age 50–69 years (thousands)**		**Deaths at age ≥70 years (thousands)**	
			2017	Percentage change, 2007–17	2017	Percentage change, 2007–17	2017	Percentage change, 2007–2017	2017	Percentage change, 2007–17	2017	Percentage change, 2007–17	2017	Percentage change, 2007–17
**All causes**			**55 945·7 (55 356·4 to 56 516·7)**	9·3% (8·2 to 10·2)*	**5391·6 (5195·4 to 5612·9)**	−31·4% (−33·8 to −28·7)*	**731·7 (720·0 to 744·1)**	−21·9% (−23·2 to −20·5)*	**7614·0 (7496·5 to 7741·4)**	−11·2% (−12·3 to −9·9)*	**14 998·6 (14 827·9 to 15 170·8)**	22·7% (21·2 to 24·0)*	**27 209·8 (26 976·2 to 27 441·9)**	25·9% (24·7 to 26·9)*
**Communicable, maternal, neonatal, and nutritional diseases**			**10 389·9 (10 004·0 to 10 975·9)**	−22·2% (−24·0 to −20·0)*	**4366·5 (4193·1 to 4563·3)**	−33·6% (−36·3 to −30·8)*	**352·2 (330·8 to 380·6)**	−29·4% (−31·7 to −27·0)*	**1896·8 (1813·8 to 1992·6)**	−33·2% (−34·8 to −31·5)*	**1463·3 (1377·8 to 1606·9)**	**−1·8% (−4·2 to 1·3)**	**2311·0 (2113·6 to 2616·4)**	18·4% (15·0 to 22·7)*
	**HIV/AIDS and sexually transmitted infection**		**1073·6 s (983·3 to 1182·4)**	−47·7% (−50·0 to −45·1)*	**187·8 (117·0 to 286·3)**	−47·9% (−58·3 to −38·4)*	**46·2 (43·1 to 49·3)**	−12·9% (−17·6 to −7·9)*	**679·6 (633·3 to 727·6)**	−50·1% (−52·1 to −47·9)*	**144·8 (135·6 to 156·1)**	−42·2% (−45·6 to −38·4)*	**15·2 (14·0 to 16·6)**	−46·3% (−50·4 to −41·3)*
	HIV/AIDS		954·5 (907·3 to 1009·7)	−50·3% (−52·1 to −48·3)*	77·5 (69·0 to 86·8)	−67·1% (−70·3 to −63·4)*	44·8 (42·0 to 47·7)	−13·0% (−17·9 to −7·9)*	676·1 (629·7 to 724·5)	−50·2% (−52·3 to −48·0)*	142·6 (133·3 to 153·7)	−42·7% (−46·0 to −38·8)*	13·5 (12·3 to 14·9)	−49·8% (−54·0 to −44·6)*
		HIV/AIDS resulting in other diseases	736·0 (659·5 to 817·7)	−48·7% (−51·1 to −45·9)*	54·6 (46·0 to 64·9)	−64·5% (−68·8 to −59·2)*	30·1 (26·3 to 35·0)	−11·3% (−17·4 to −4·4)*	531·1 (470·1 to 596·1)	−49·8% (−52·6 to −46·9)*	109·5 (97·1 to 124·8)	−35·9% (−40·3 to −30·2)*	10·7 (9·3 to 12·3)	−38·0% (−43·7 to −30·3)*
	**Respiratory infections and tuberculosis**		**3752·3 (3629·4 to 3889·3)**	−8·0% (−10·3 to −5·5)*	**870·5 (803·3 to 941·4)**	−36·7% (−40·6 to −32·4)*	**58·1 (52·1 to 64·1)**	−27·4% (−31·5 to −23·3)*	**543·2 (520·8 to 570·8)**	−18·2% (−20·7 to −15·2)*	**836·8 (805·0 to 868·5)**	7·8% (4·5 to 12·0)*	**1443·7 (1392·6 to 1503·7)**	21·9% (19·4 to 24·7)*
	Tuberculosis		1183·7 (1129·8 to 1245·3)	−14·9% (−18·2 to −10·3)*	57·4 (51·3 to 63·6)	−39·8% (−44·5 to −34·2)*	13·7 (12·2 to 15·5)	−38·4% (−42·7 to −33·8)*	371·7 (353·1 to 392·2)	−23·4% (−26·5 to −19·8)*	429·7 (410·6 to 452·7)	−5·2% (−9·6 to 0·9)	311·2 (294·2 to 330·7)	−6·9% (−11·2 to −0·4)*
		Drug-susceptible tuberculosis	1044·1 (951·6 to 1129·2)	−15·5% (−22·3 to −8·6)*	51·4 (45·3 to 57·6)	−40·5% (−46·0 to −34·1)*	12·2 (10·7 to 13·9)	−39·0% (−44·2 to −33·5)*	326·5 (297·0 to 353·9)	−23·8% (−29·9 to −18·1)*	377·7 (342·6 to 409·5)	−6·0% (−14·3 to 2·4)	276·4 (251·1 to 300·9)	−7·6% (−15·6 to 0·8)
	Lower respiratory infections		2558·6 (2442·2 to 2655·4)	−4·3% (−6·9 to −1·5)*	808·9 (747·3 to 873·6)	−36·4% (−40·6 to −32·2)*	43·9 (38·9 to 48·7)	−23·0% (−27·5 to −18·5)*	170·5 (157·0 to 182·6)	−4·0% (−6·3 to −1·5)*	405·8 (366·8 to 422·6)	26·5% (23·2 to 29·7)*	1129·4 (1078·5 to 1180·4)	33·6% (31·2 to 36·1)*
	**Enteric infections**		**1766·0 (1398·0 to 2386·0)**	−17·2% (−24·6 to −8·2)*	**589·4 (528·3 to 653·6)**	−39·1% (−46·1 to −31·0)*	**111·9 (83·6 to 151·3)**	−27·9% (−32·7 to −21·7)*	**187·1 (135·4 to 278·7)**	−14·4% (−20·0 to −5·5)*	**249·3 (158·0 to 413·5)**	**2·8% (−5·3 to 16·8)**	**628·3 (395·4 to 975·5)**	14·8% (4·3 to 30·7)*
	Diarrhoeal diseases		1569·6 (1176·0 to 2193·0)	−16·6% (−25·3 to −6·7)*	533·8 (477·2 to 593·1)	−40·6% (−47·8 to −32·2)*	44·5 (27·5 to 73·1)	−27·2% (−36·6 to −14·1)*	128·2 (77·2 to 216·2)	−14·1% (−21·6 to −1·9)*	239·1 (145·9 to 404·4)	3·5% (−4·7 to 18·0)	624·0 (390·4 to 972·3)	15·0% (4·5 to 31·1)*
	**Neglected tropical diseases and malaria**		**720·1 (530·7 to 938·8)**	−29·0% (−37·3 to −19·3)*	**375·9 (250·3 to 527·8)**	−39·2% (−50·3 to −27·4)*	**66·1 (49·5 to 87·7)**	−32·3% (−41·8 to −23·5)*	**135·6 (98·0 to 186·4)**	−13·9% (−26·4 to −4·2)*	**91·9 (66·5 to 126·7)**	**−1·3% (−11·2 to 8·0)**	**50·6 (38·5 to 68·2)**	**4·6% (−7·2 to 18·0)**
	Malaria		619·8 (440·1 to 839·5)	−30·8% (−39·4 to −20·8)*	354·3 (226·3 to 508·1)	−39·8% (−51·4 to −27·2)*	54·3 (38·9 to 75·3)	−30·7% (−39·4 to −21·7)*	109·0 (72·2 to 161·0)	−9·5% (−17·1 to −1·1)*	71·2 (46·5 to 107·2)	−3·3% (−12·6 to 6·2)	31·0 (20·8 to 46·4)	−10·8% (−19·4 to −2·0)*
	**Other infectious diseases**		**830·5 (732·2 to 947·8)**	−25·9% (−32·4 to −18·8)*	**414·0 (331·4 to 515·2)**	−38·1% (−45·4 to −28·9)*	**60·6 (51·0 to 74·0)**	−38·8% (−45·2 to −31·7)*	**143·3 (127·1 to 157·6)**	−14·7% (−18·0 to −10·0)*	**110·5 (102·4 to 121·6)**	10·8% (6·3 to 17·4)*	**102·1 (94·1 to 110·9)**	19·6% (14·7 to 24·5)*
	Meningitis		288·0 (254·3 to 333·2)	−20·1% (−26·0 to −11·0)*	153·1 (127·7 to 179·4)	−30·0% (−38·2 to −19·2)*	23·4 (19·1 to 29·9)	−25·2% (−30·6 to −12·8)*	54·7 (46·2 to 69·3)	−9·5% (−13·6 to −1·6)*	31·8 (28·7 to 44·2)	12·3% (6·0 to 20·0)*	25·1 (22·5 to 35·7)	13·8% (6·3 to 21·0)*
	**Maternal and neonatal disorders**		**1977·4 (1890·1 to 2060·6)**	−24·1% (−26·9 to −21·0)*	**1783·8 (1698·5 to 1864·7)**	−24·1% (−27·2 to −20·6)*	**0·8 (0·7 to 0·9)**	−18·5% (−28·5 to −4·9)*	**191·1 (177·5 to 206·9)**	−24·2% (−28·6 to −19·6)*	**1·8 (1·6 to 2·0)**	**2·1% (−7·6 to 14·1)**	**..**	**..**
	Neonatal disorders		1783·8 (1698·5 to 1864·7)	−24·1% (−27·2 to −20·6)*	1783·8 (1698·5 to 1864·7)	−24·1% (−27·2 to −20·6)*	..	..	..	..	..	..	..	..
		Neonatal preterm birth	649·4 (605·4 to 721·3)	−26·2% (−31·3 to −21·5)*	649·4 (605·4 to 721·3)	−26·2% (−31·3 to −21·5)*	..	..	..	..	..	..	..	..
		Neonatal encephalopathy due to birth asphyxia and trauma	533·3 (476·9 to 580·3)	−24·5% (−30·2 to −18·0)*	533·3 (476·9 to 580·3)	−24·5% (−30·2 to −18·0)*	..	..	..	..	..	..	..	..
		Other neonatal disorders	349·0 (294·9 to 382·3)	−23·6% (−29·8 to −15·5)*	349·0 (294·9 to 382·3)	−23·6% (−29·8 to −15·5)*	..	..	..	..	..	..	..	..
	**Nutritional deficiencies**		**270·0 (249·3 to 295·5)**	−23·9% (−29·2 to −15·7)*	**145·1 (128·0 to 163·6)**	−38·7% (−44·8 to −30·2)*	**8·5 (7·3 to 9·9)**	−35·3% (−42·7 to −25·8)*	**16·9 (15·6 to 18·8)**	−12·9% (−17·8 to −4·5)*	**28·3 (26·6 to 31·0)**	8·1% (1·6 to 18·4)*	**71·2 (68·7 to 75·1)**	20·5% (15·6 to 27·9)*
	Protein-energy malnutrition		231·8 (212·4 to 254·2)	−26·1% (−31·7 to −17·9)*	140·3 (123·6 to 158·8)	−38·3% (−44·4 to −29·8)*	7·3 (6·2 to 8·5)	−32·1% (−39·8 to −21·3)*	11·5 (10·4 to 13·1)	−10·6% (−15·9 to −1·1)*	19·2 (17·6 to 21·1)	6·2% (0·7 to 15·5)*	53·6 (49·3 to 56·7)	20·2% (16·3 to 26·0)*
**Non-communicable diseases**			**41071·1 (40470·9 to 41548·9)**	22·7% (21·5 to 23·9)*	**754·6 (707·5 to 804·4)**	−16·9% (−21·7 to −12·6)*	**170·9 (157·6 to 182·2)**	−9·1% (−12·5 to −5·5)*	**3654·7 (3583·2 to 3726·5)**	3·2% (1·7 to 4·7)*	**12516·7 (12332·4 to 12686·5)**	26·7% (25·1 to 28·2)*	**23974·3 (23625·0 to 24257·0)**	26·5% (25·3 to 27·7)*
	**Neoplasms**		**9556·2 (9395·7 to 9692·3)**	25·4% (23·9 to 27·0)*	**49·9 (44·4 to 54·8)**	**−4·8% (−21·5 to 12·6)**	**62·0 (56·7 to 66·8)**	**−2·8% (−9·6 to 3·4)**	**1048·5 (1024·9 to 1072·5)**	5·7% (3·8 to 7·8)*	**3962·3 (3896·5 to 4024·2)**	31·0% (29·2 to 32·9)*	**4433·5 (4351·5 to 4493·0)**	27·2% (25·7 to 28·7)*
	Colon and rectum cancer		896·0 (876·3 to 915·7)	27·8% (24·0 to 31·3)*	..	..	..	..	68·2 (66·1 to 70·0)	11·8% (6·0 to 16·3)*	323·2 (314·8 to 331·3)	32·2% (27·3 to 36·5)*	504·7 (494·2 to 515·3)	27·5% (24·5 to 30·5)*
	Tracheal, bronchus, and lung cancer		1883·1 (1844·2 to 1922·8)	29·6% (26·5 to 32·5)*	..	..	..	..	105·5 (102·5 to 108·9)	−1·0% (−4·1 to 1·8)	861·5 (841·1 to 882·3)	34·9% (31·3 to 38·1)*	916·1 (897·6 to 934·8)	29·5% (26·7 to 32·3)*
	**Cardiovascular diseases**		**17790·9 (17527·1 to 18042·7)**	21·1% (19·7 to 22·6)*	**30·1 (28·2 to 32·3)**	−31·3% (−35·2 to −27·2)*	**15·6 (14·5 to 16·9)**	−18·7% (−22·9 to −15·4)*	**1258·0 (1234·6 to 1284·7)**	**1·6% (−0·2 to 3·4)**	**5152·1 (5068·6 to 5233·7)**	23·6% (21·7 to 25·5)*	**11335·1 (11173·0 to 11494·9)**	22·9% (21·6 to 24·3)*
	Ischaemic heart disease		8930·4 (8790·7 to 9138·7)	22·3% (20·6 to 23·8)*	..	..	..	..	643·8 (628·9 to 661·2)	5·9% (3·7 to 8·3)*	2649·1 (2602·9 to 2699·1)	24·5% (22·3 to 26·6)*	5637·5 (5547·9 to 5786·4)	23·4% (21·8 to 24·8)*
	Stroke		6167·3 (6044·3 to 6327·6)	16·6% (14·7 to 18·6)*	7·5 (6·7 to 8·5)	−40·2% (−45·3 to −35·0)*	5·1 (4·7 to 5·6)	−18·1% (−23·8 to −13·2)*	364·2 (354·9 to 375·0)	0·1% (−2·3 to 2·4)	1836·6 (1795·9 to 1879·2)	21·7% (19·2 to 24·3)*	3953·8 (3875·7 to 4067·2)	16·4% (14·6 to 18·3)*
		Ischaemic stroke	2747·4 (2657·1 to 2857·6)	21·2% (19·0 to 23·3)*	1·0 (0·8 to 1·3)	−36·8% (−44·6 to −29·3)*	0·5 (0·4 to 0·6)	−18·1% (−27·8 to −10·6)*	58·3 (53·4 to 64·9)	2·0% (−1·6 to 5·5)	575·2 (545·6 to 619·7)	27·2% (23·7 to 30·8)*	2112·4 (2052·7 to 2177·3)	20·3% (18·3 to 22·3)*
		Intracerebral haemorrhage	2974·9 (2880·8 to 3072·8)	12·5% (9·6 to 15·1)*	3·3 (2·7 to 4·4)	−44·5% (−49·5 to −40·1)*	2·7 (2·5 to 2·9)	−18·6% (−25·9 to −12·9)*	238·9 (230·5 to 248·1)	−0·4% (−3·3 to 2·4)	1088·7 (1050·2 to 1121·4)	18·7% (15·8 to 21·7)*	1641·3 (1589·9 to 1706·2)	11·1% (8·2 to 13·6)*
	Hypertensive heart disease		925·7 (681·4 to 994·9)	46·6% (26·3 to 59·3)*	..	..	..	..	43·8 (32·4 to 49·2)	10·7% (−0·3 to 21·3)	224·2 (172·2 to 242·8)	39·5% (25·0 to 52·5)*	657·7 (473·8 to 710·6)	52·5% (29·1 to 64·5)*
	**Chronic respiratory diseases**		**3914·2 (3790·6 to 4044·8)**	15·8% (12·7 to 19·3)*	**10·7 (9·3 to 12·4)**	−34·3% (−41·7 to −20·2)*	**6·8 (6·1 to 8·2)**	−24·9% (−29·0 to −18·8)*	**163·0 (156·2 to 175·0)**	−5·9% (−9·1 to −2·0)*	**1004·4 (971·0 to 1040·7)**	15·9% (12·4 to 20·0)*	**2729·3 (2637·9 to 2820·0)**	17·9% (14·6 to 21·4)*
	Chronic obstructive pulmonary disease		3197·8 (3029·0 to 3358·9)	17·5% (13·3 to 21·1)*	1·2 (0·9 to 1·8)	−29·1% (−40·3 to −15·6)*	0·8 (0·7 to 1·0)	−16·6% (−28·1 to −5·2)*	75·8 (67·9 to 90·1)	−2·9% (−8·6 to 1·7)	760·8 (700·2 to 819·6)	17·8% (13·0 to 22·6)*	2359·1 (2256·9 to 2448·6)	18·3% (14·3 to 21·6)*
	**Digestive diseases**		**2377·7 (2295·1 to 2518·0)**	15·3% (12·1 to 19·7)*	**40·2 (34·5 to 45·6)**	**−14·8% (−31·9 to 0·2)**	**20·4 (17·0 to 23·3)**	−14·0% (−23·9 to −3·2)*	**478·2 (454·0 to 510·7)**	**−1·0% (−4·2 to 3·6)**	**884·1 (853·6 to 951·9)**	20·7% (16·6 to 26·4)*	**954·9 (927·8 to 1014·4)**	23·1% (19·2 to 28·9)*
	Cirrhosis and other chronic liver diseases		1322·9 (1268·2 to 1449·1)	15·0% (8·7 to 21·5)*	7·8 (6·3 to 9·6)	−14·9% (−36·9 to 11·1)	8·4 (7·0 to 10·0)	−14·1% (−23·5 to −0·1)*	332·1 (316·4 to 362·3)	−0·4% (−5·2 to 4·7)	592·2 (567·4 to 653·6)	21·0% (14·4 to 28·7)*	382·3 (364·3 to 425·8)	24·0% (15·6 to 33·9)*
	**Neurological disorders**		**3094·2 (3039·6 to 3142·6)**	42·1% (40·2 to 43·9)*	**18·2 (16·1 to 21·4)**	**−18·2% (−27·7 to 6·6)**	**11·4 (10·2 to 13·2)**	−14·1% (−21·2 to −0·7)*	**86·6 (80·8 to 98·1)**	**0·5% (−3·7 to 8·1)**	**244·5 (239·4 to 250·8)**	38·2% (36·0 to 40·6)*	**2733·5 (2683·3 to 2772·6)**	45·5% (43·5 to 47·2)*
	Alzheimer's disease and other dementias		2514·6 (2470·5 to 2550·3)	46·2% (43·9 to 48·0)*	..	..	..	..	2·8 (2·8 to 2·9)	11·2% (7·5 to 14·9)*	138·3 (135·4 to 141·4)	39·6% (36·0 to 42·5)*	2373·5 (2329·6 to 2407·2)	46·7% (44·4 to 48·5)*
	**Diabetes and kidney diseases**		**2611·2 (2557·8 to 2667·2)**	34·2% (32·0 to 36·2)*	**15·0 (13·6 to 16·5)**	−22·3% (−27·6 to −16·3)*	**10·2 (9·3 to 11·1)**	−14·6% (−20·0 to −9·9)*	**278·3 (270·4 to 287·1)**	8·5% (6·2 to 11·0)*	**940·9 (921·7 to 959·9)**	39·4% (36·7 to 42·0)*	**1366·7 (1338·8 to 1395·2)**	38·9% (36·8 to 40·8)*
	Diabetes mellitus		1369·8 (1340·3 to 1401·9)	34·7% (32·2 to 37·3)*	1·7 (1·4 to 2·0)	−11·2% (−19·6 to −2·9)*	1·9 (1·5 to 2·2)	−10·6% (−21·1 to −1·2)*	113·8 (111·0 to 116·8)	13·5% (10·5 to 16·2)*	535·0 (523·9 to 547·0)	38·9% (36·0 to 41·9)*	717·4 (700·6 to 736·2)	36·1% (33·4 to 38·7)*
		Type 2 diabetes mellitus	1024·3 (985·5 to 1066·8)	43·0% (40·4 to 45·8)*	..	..	..	..	49·3 (46·6 to 52·3)	31·1% (26·2 to 35·7)*	380·9 (365·6 to 396·6)	48·0% (44·6 to 51·7)*	594·1 (572·3 to 620·5)	41·0% (38·6 to 43·6)*
	Chronic kidney disease		1230·2 (1195·1 to 1258·8)	33·7% (30·5 to 36·1)*	13·0 (11·7 to 14·4)	−23·0% (−28·9 to −16·7)*	8·0 (7·3 to 8·8)	−15·0% (−20·3 to −10·4)*	162·6 (155·5 to 169·4)	5·7% (3·0 to 8·6)*	402·3 (383·6 to 412·1)	40·3% (35·6 to 43·7)*	644·3 (628·3 to 659·3)	42·2% (38·9 to 44·4)*
	**Other non-communicable diseases**		**1153·3 (1101·8 to 1208·3)**	**0·8% (−3·9 to 4·0)**	**584·4 (544·2 to 628·8)**	−16·6% (−22·5 to −12·2)*	**42·0 (37·4 to 46·4)**	−5·0% (−10·5 to −0·6)*	**130·3 (120·8 to 141·4)**	5·6% (2·6 to 8·3)*	**142·8 (131·2 to 150·3)**	39·3% (36·3 to 42·3)*	**253·7 (236·5 to 262·6)**	46·4% (43·8 to 49·4)*
**Injuries**			**4484·7 (4332·0 to 4585·6)**	2·3% (0·5 to 4·0)*	**270·5 (249·7 to 289·4)**	−26·6% (−31·3 to −18·6)*	**208·7 (194·4 to 221·4)**	−16·5% (−18·7 to −13·7)*	**2062·5 (1998·4 to 2105·8)**	−5·8% (−7·2 to −4·4)*	**1018·6 (975·0 to 1047·8)**	18·9% (14·8 to 21·9)*	**924·5 (889·7 to 956·7)**	28·3% (23·4 to 33·6)*
	**Transport injuries**		**1335·0 (1289·1 to 1369·5)**	−3·1% (−6·0 to −0·6)*	**52·4 (47·0 to 57·7)**	−30·1% (−36·1 to −16·0)*	**66·7 (61·9 to 71·7)**	−19·2% (−22·4 to −15·6)*	**720·2 (699·9 to 740·6)**	−10·3% (−13·3 to −7·5)*	**335·0 (316·0 to 344·8)**	19·9% (12·4 to 24·4)*	**160·7 (153·8 to 165·0)**	16·9% (10·2 to 20·7)*
	Road injuries		1243·1 (1191·9 to 1276·9)	−3·2% (−6·3 to −0·5)*	49·1 (44·0 to 54·2)	−30·0% (−36·1 to −14·9)*	62·4 (57·9 to 67·3)	−19·3% (−22·4 to −15·4)*	669·1 (644·9 to 688·7)	−10·5% (−13·5 to −7·6)*	311·7 (292·2 to 321·5)	20·0% (12·3 to 24·5)*	150·8 (143·8 to 155·1)	16·7% (9·8 to 20·5)*
		Pedestrian road injuries	486·2 (459·7 to 535·0)	−6·4% (−11·7 to −2·1)*	24·0 (21·1 to 28·3)	−36·8% (−42·8 to −26·4)*	31·2 (28·0 to 35·3)	−25·0% (−29·1 to −20·5)*	203·7 (191·6 to 227·0)	−15·2% (−19·8 to −10·5)*	141·8 (133·0 to 154·8)	14·7% (5·4 to 21·5)*	85·5 (80·8 to 91·1)	12·7% (3·6 to 18·2)*
		Motorcyclist road injuries	225·7 (196·1 to 238·6)	−0·6% (−8·9 to 5·2)	3·5 (3·0 to 4·1)	−25·4% (−37·6 to −3·7)*	5·2 (4·4 to 5·9)	−13·4% (−21·3 to −5·2)*	161·5 (142·6 to 171·6)	−8·0% (−15·0 to −2·6)*	45·6 (37·4 to 49·3)	36·8% (17·1 to 48·9)*	9·8 (7·9 to 10·6)	32·5% (12·4 to 44·8)*
		Motor vehicle road injuries	451·1 (423·4 to 472·9)	−2·5% (−6·2 to 1·3)	19·9 (16·5 to 22·9)	−21·5% (−31·6 to 3·0)	21·3 (19·2 to 23·4)	−12·3% (−17·1 to −3·0)*	268·5 (254·4 to 284·6)	−8·9% (−12·5 to −5·5)*	97·6 (88·9 to 103·9)	19·8% (10·1 to 25·2)*	43·7 (40·5 to 46·1)	18·8% (9·9 to 23·4)*
	**Unintentional injuries**		**1804·9 (1695·7 to 1872·0)**	2·9% (0·5 to 6·0)*	**191·5 (175·1 to 206·9)**	−29·2% (−34·2 to −22·4)*	**106·2 (96·6 to 114·9)**	−22·7% (−25·6 to −19·4)*	**486·0 (447·7 to 509·0)**	−12·3% (−14·0 to −10·2)*	**395·8 (363·7 to 416·0)**	19·4% (15·0 to 24·2)*	**625·4 (591·5 to 653·4)**	35·9% (29·8 to 42·9)*
	Falls		695·8 (644·9 to 741·7)	27·4% (21·2 to 35·6)*	20·4 (17·5 to 22·9)	−16·7% (−31·4 to 1·0)	12·9 (11·1 to 14·7)	−7·0% (−18·0 to 4·0)	102·6 (90·7 to 109·2)	−1·0% (−5·6 to 5·7)	155·4 (140·0 to 169·0)	31·1% (22·9 to 42·0)*	404·5 (381·7 to 433·2)	41·6% (33·4 to 52·1)*
	Drowning		295·2 (284·5 to 306·2)	−17·2% (−19·8 to −14·1)*	59·8 (54·3 to 65·9)	−41·8% (−46·7 to −34·9)*	49·7 (45·8 to 53·5)	−26·3% (−29·5 to −22·9)*	99·2 (96·5 to 102·3)	−14·5% (−16·9 to −11·7)*	48·8 (46·6 to 50·2)	19·6% (16·2 to 22·9)*	37·6 (35·1 to 38·7)	28·6% (24·7 to 32·0)*
	Fire, heat, and hot substances		120·6 (101·6 to 129·4)	−7·9% (−10·9 to −1·2)*	17·2 (13·1 to 20·0)	−25·3% (−34·6 to −5·2)*	5·9 (4·7 to 7·0)	−22·4% (−28·2 to −12·2)*	40·8 (32·6 to 45·8)	−16·1% (−19·8 to −9·7)*	25·1 (21·5 to 26·8)	4·0% (−4·5 to 9·6)	31·6 (28·3 to 33·1)	14·4% (7·4 to 19·2)*
	Exposure to mechanical forces		136·5 (117·6 to 143·2)	−6·7% (−9·8 to −3·7)*	13·5 (11·0 to 15·2)	−22·4% (−28·4 to −15·2)*	7·1 (6·2 to 7·8)	−19·9% (−23·6 to −15·9)*	63·0 (54·9 to 66·0)	−15·9% (−19·1 to −12·2)*	33·3 (27·5 to 35·2)	14·0% (9·1 to 18·6)*	19·6 (17·3 to 20·9)	22·8% (17·8 to 28·0)*
	Adverse effects of medical treatment		121·6 (103·6 to 137·6)	16·6% (12·0 to 20·9)*	13·5 (9·8 to 20·0)	−12·1% (−24·2 to 4·9)	3·4 (2·9 to 4·0)	−9·4% (−16·9 to 1·4)	28·6 (23·7 to 31·1)	4·1% (−1·7 to 10·2)	31·6 (26·7 to 34·8)	30·8% (22·7 to 40·5)*	44·5 (39·4 to 49·8)	32·9% (26·4 to 40·6)*
	Foreign body		124·1 (119·3 to 130·0)	1·7% (−1·9 to 4·8)	38·5 (34·9 to 42·0)	−21·8% (−26·8 to −15·7)*	5·1 (4·7 to 5·6)	−4·2% (−9·7 to 0·7)	21·8 (21·0 to 22·8)	−4·5% (−7·4 to −2·6)*	21·3 (20·6 to 21·9)	17·4% (14·4 to 19·5)*	37·4 (36·5 to 38·5)	41·3% (38·3 to 44·1)*
		Pulmonary aspiration and foreign body in airway	115·7 (111·4 to 121·3)	1·9% (−1·9 to 5·0)	37·0 (33·9 to 40·5)	−21·3% (−26·5 to −15·3)*	4·7 (4·3 to 5·1)	−2·2% (−8·2 to 3·2)	19·0 (18·3 to 19·8)	−4·5% (−7·2 to −2·5)*	19·8 (19·1 to 20·3)	17·1% (14·2 to 19·2)*	35·3 (34·3 to 36·2)	41·3% (38·3 to 44·2)*
	**Self-harm and interpersonal violence**		**1344·8 (1283·1 to 1380·4)**	7·3% (4·6 to 9·7)*	**26·6 (24·2 to 28·6)**	15·0% (8·1 to 25·1)*	**35·8 (34·1 to 37·4)**	19·9% (15·8 to 24·3)*	**856·3 (817·6 to 882·2)**	2·9% (0·3 to 5·2)*	**287·7 (273·1 to 295·9)**	17·2% (13·2 to 21·4)*	**138·4 (131·3 to 143·1)**	12·9% (8·6 to 20·6)*
	Self-harm		793·8 (743·5 to 819·7)	1·1% (−2·6 to 3·7)	..	..	8·1 (7·3 to 8·8)	−13·0% (−19·5 to −7·2)*	453·8 (425·2 to 469·5)	−6·1% (−9·8 to −3·3)*	213·1 (199·0 to 219·5)	14·6% (10·4 to 19·0)*	118·8 (111·8 to 123·1)	11·2% (6·8 to 19·5)*
		Self-harm by other specified means	730·0 (678·5 to 754·9)	0·6% (−3·2 to 3·4)	..	..	7·7 (6·9 to 8·5)	−13·2% (−19·9 to −7·2)*	418·6 (389·8 to 434·5)	−6·2% (−10·1 to −3·4)*	195·1 (180·2 to 201·8)	13·8% (9·5 to 18·2)*	108·6 (101·2 to 112·8)	10·1% (5·6 to 18·7)*
	Interpersonal violence		405·3 (365·2 to 431·7)	0·5% (−2·0 to 3·2)	11·8 (9·5 to 13·7)	−21·2% (−29·1 to −7·7)*	10·8 (9·2 to 12·3)	−15·6% (−20·1 to −10·4)*	304·7 (275·0 to 322·3)	−0·5% (−3·1 to 2·4)	62·4 (56·2 to 68·5)	13·0% (8·0 to 17·8)*	15·6 (13·9 to 17·1)	10·1% (4·5 to 14·9)*
		Physical violence by firearm	174·4 (147·9 to 188·9)	7·5% (4·3 to 10·8)*	2·0 (1·3 to 2·5)	−14·0% (−24·6 to 2·6)	2·8 (2·3 to 3·2)	−12·8% (−17·3 to −7·7)*	145·5 (124·4 to 156·8)	5·6% (2·3 to 9·3)*	20·4 (16·8 to 22·8)	27·2% (22·6 to 32·0)*	3·8 (3·0 to 4·4)	24·7% (20·1 to 29·8)*
		Physical violence by other means	139·5 (123·6 to 164·4)	1·3% (−3·4 to 5·6)	8·4 (6·9 to 9·9)	−23·8% (−31·7 to −10·5)*	6·5 (5·5 to 7·7)	−16·4% (−21·9 to −9·7)*	90·9 (79·9 to 107·7)	3·5% (−1·9 to 8·3)	25·9 (23·3 to 30·3)	8·8% (0·3 to 16·3)*	7·9 (7·0 to 9·1)	6·6% (−2·5 to 13·7)
	Conflict and terrorism		129·7 (118·1 to 143·2)	118·0% (88·8 to 148·6)*	14·3 (11·7 to 17·4)	78·7% (33·3 to 136·7)*	16·2 (13·4 to 19·9)	116·3% (64·0 to 187·1)*	85·4 (75·1 to 98·4)	121·3% (80·6 to 165·5)*	10·1 (8·6 to 12·1)	158·6% (108·0 to 220·8)*	3·7 (3·2 to 4·3)	144·1% (103·4 to 193·7)*

Data in parentheses are 95% uncertainty intervals. YLL=years of life lost. GBD=Global Burden of Diseases, Injuries, and Risk Factors Study.

* Statistically significant increases or decreases.

### Age-specific and sex-specific mortality for causes of death

Progress in reducing deaths was not equal between age groups (table 2). Total deaths from lower respiratory infections decreased by 36·4% (95% UI 32·2–40·6) between 2007 and 2017 for children younger than 5 years, while an increase of 33·6% (31·2–36·1) was estimated among older adults (≥70 years). A parallel pattern occurred for deaths from diarrhoeal diseases between 2007 and 2017, which decreased by 40·6% (32·2–47·8) for children younger than 5 years, and increased by 15·0% (4·5–31·1) for adults older than 70 years.

Decreases among aetiologies of infection over the wider time period 1990–2017 included decreases in deaths from pneumococcal pneumonia (71·2% [95% UI 67·1–75·1]), respiratory syncytial virus pneumonia (64·2% [59·4–68·2]), influenza (66·0% [61·6–69·9]), and *H influenzae* type B pneumonia (82·5% [80·0–85·2]) for children younger than 5 years (appendix 2). Among adults older than 70 years, deaths increased from pneumococcal pneumonia (60·4% [39·7–79·9]), influenza (91·1% [82·3–99·6]), and respiratory syncytial virus pneumonia (100·3% [92·4–108·6]) between 1990 and 2017. Diarrhoeal deaths due to *C difficile* increased among adults older than 70 years (779·9% [736·7–831·0]), and for diarrhoeal diseases overall (44·0% [24·7–84·6]); by contrast, diarrhoeal disease deaths declined by 67·9% (61·1–73·1) between 1990 and 2017 for children younger than 5 years.

**Figure 4:**
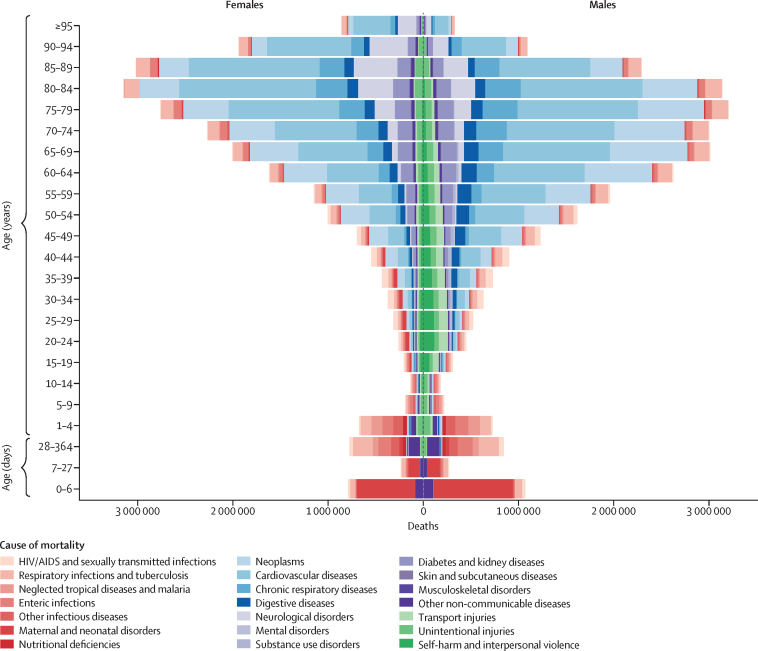
Sex difference in global mortality for 21 Level 2 causes by age, 2017 This figure represents the difference in mortality between females and males, as well as the cause composition of those differences for each GBD age group for the Level 2 causes in GBD 2017. GBD=Global Burden of Diseases, Injuries, and Risk Factors Study.

At a global scale, total deaths were greater for men than for women at most ages in 2017; exceptions included ages 80–84 years (women, 3·30 million [95% UI 3·26–3·35] deaths; men, 3·14 million [3·10–3·18] deaths), 85–89 years (women, 3·02 million [2·99–3·05] deaths; men, 2·29 million [2·27–2·32] deaths), 90–94 years (women, 1·94 million [1·92–1·96] deaths; men, 1·09 million [1·09–1·10] deaths), and 95 years and older (women, 858 000 deaths [852 000–864 000]; men, 329 000 deaths [327 000–331 000]; figure 4). Across causes, the largest female-to-male ratio of deaths occurred for neurological disorders (women ≥85 years, 1·05 million [1·04–1·07] deaths; men ≥85 years, 475 000 deaths [464 000–483 000]) and for cardiovascular diseases (women ≥85 years, 2·65 million [2·61–2·69] deaths; men ≥85 years, 1·56 million [1·53–1·58] deaths). Overall, deaths from injury were also greater for men than for women (3·07 million [2·95–3·14] *vs* 1·42 million [1·36–1·46]) and in each five-year age group up to age 85 years (122 000 deaths [112 000–128 000] for men aged ≥85 years *vs* 173 000 deaths [166 000–181 000] for women aged ≥85 years).

### Patterns in rates of change in global cause-specific mortality rate

**Figure 5:**
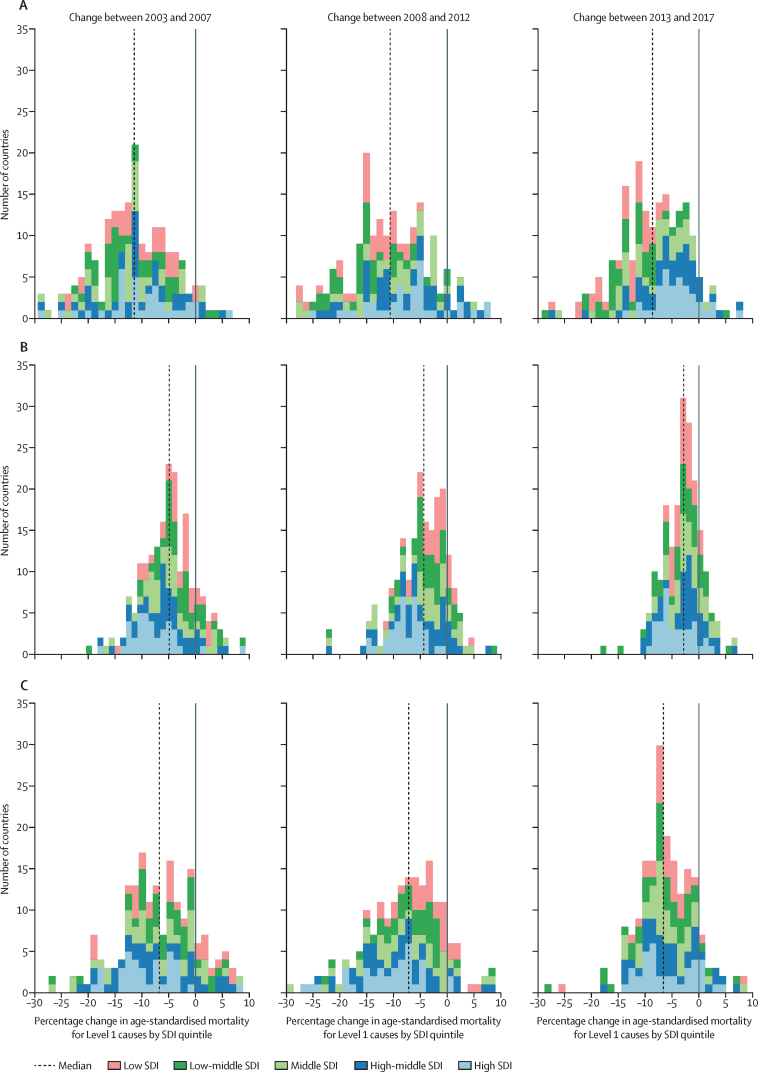
Distribution of percentage change in age-standardised mortality rate for Level 1 causes by SDI quintile (A) Communicable, maternal, neonatal, and nutritional diseases. (B) Non-communicable diseases. (C) Injuries. The figure shows the distribution of the percentage change in the age-standardised mortality rate by Level 1 cause over the three 5-year periods (2003–07, 2008–12, and 2013–17). The colours represent SDI quintiles. The solid line represents no change in the age-standardised mortality rate during the specified 5-year period. The dotted line represents the median over all countries in the percentage change. Countries that were outliers (>30% decrease or a 10% increase in a given time period) were removed from the figure in order to better distinguish the shape of the distribution. For communicable, maternal, neonatal, and nutritional diseases, the following countries were excluded: Finland, Georgia, Lithuania, Rwanda, Serbia, South Africa, Turkey, and Ukraine in 2003–07; Botswana, Croatia, Dominica, Malawi, Namibia, Zambia, and Zimbabwe in 2008–12; and Botswana, Lesotho, South Africa, and Swaziland in 2013–17. For injuries, the following were excluded: Afghanistan, Burundi, Cape Verde, Comoros, Georgia, Iran, Iraq, Jamaica, Liberia, São Tomé and Príncipe, Spain, and Trinidad and Tobago in 2003–07; El Salvador, Honduras, Israel, Libya, Mexico, Myanmar, Palestine, Samoa, South Sudan, Sri Lanka, Syria, and Ukraine in 2008–12; and Afghanistan, Honduras, Iraq, Libya, Puerto Rico, Ukraine, and Yemen in 2013–17. SDI=Socio-demographic Index.

To better understand recent changes across a wide range of causes, we present the distribution of the percentage change in mortality rate at the country level by Level 1 causes and over three time periods (2003–07, 2008–12, and 2013–17; figure 5A, 5B, 5C). At Level 1 of the GBD cause hierarchy, a decrease in the global percentage change in CSMR was evident between time periods, particularly for NCDs, although this varied by SDI quintile. Globally, the percentage change in mortality rate for NCDs was smaller in the most recent period, slowing from a decrease of 7·8% (95% UI 7·5–8·2) over the 2003–07 period to a decrease of 2·1% (1·5–2·7) for 2013–17. For CMNN causes, the largest decrease in percentage change occurred at high SDI quintiles, from a decrease of 8·8% (8·1–9·4) for 2003–07 to a decrease of 3·0% (1·7–4·2) for 2013–17. Increases in the magnitude of the percentage change between 2003–07 and 2013–17 for CMNN causes were estimated at low SDI quintiles (from a decrease of 12·9% [11·6–14·1] for 2003–07 to a decrease of 13·9% [12·1–15·5] for 2013–17), low-middle SDI quintiles (from a decrease of 11·2% [9·8–12·6] for 2003–07 to a decrease of 13·5% [11·4–15·5] for 2013–17), and middle SDI quintiles (from a decrease of 11·8% [10·8–12·8] for 2003–07 to a decrease of 15·6% [14·3–16·7] for 2013–17; figure 5A). For NCDs, the largest decrease in percentage change was for high-middle SDI quintiles, from 11·5% (10·8–12·2) in 2003–07 to 4·5% (3·2–5·7) in 2013–17 (figure 5B). Across injury causes of death, the largest decrease in the magnitude of change occurred at high-middle SDI quintiles (from a decrease of 16·1% [15·2–16·9] for 2003–07 to a decrease of 7·0% [5·5–8·3] for 2013–17), while an increase in magnitude was estimated at low-middle SDI quintiles (from a decrease of 2·1% [0·7–3·6] for 2003–07 to a decrease of 6·3% [4·0–8·6] for 2013–17; figure 5C).

### Epidemiological transitions

**Figure 6:**
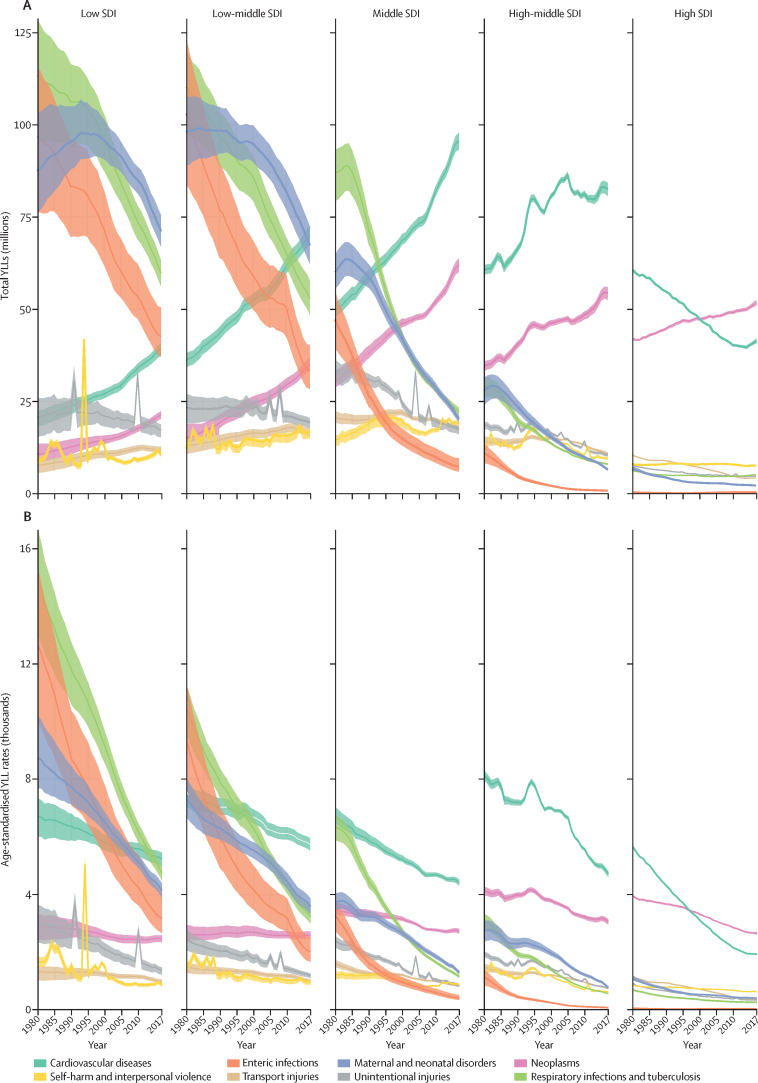
Trends of total YLLs (A) and age-standardised YLL rates (B) for both sexes combined from 1980 to 2017, by top five GBD Level 2 causes in 2017, by SDI quintile Shaded areas show 95% uncertainty intervals. GBD=Global Burden of Diseases, Injuries, and Risk Factors Study. SDI=Socio-demographic Index. YLLs=years of life lost.

The five leading causes of YLLs at Level 2 of the GBD cause hierarchy, together with injuries, by SDI level are shown in figure 6A. The greatest total YLLs for enteric infections in 2017 were at low SDI (42·6 million [95% UI 37·1–50·5] YLLs) and low-middle SDI quintiles (33·4 million [28·4–40·3] YLLs), but with a large decrease from 1990, when YLLs were 83·2 million (69·6–95·8) in low SDI countries and 76·9 million (63·9–89·4) in low-middle SDI countries; changes in respiratory infections and tuberculosis followed a similar pattern. Similarly, YLLs from maternal and neonatal disorders remained high for both low SDI and low-middle SDI countries, despite a large decrease in total YLLs from 87·5 million (76·0–103·0) in 1980 to 71·1 million (66·9–75·5) in 2017 for low SDI countries and from 98·0 million (89·2–108·0) in 1980 to 67·3 million (62·3–72·4) in 2017 for low-middle SDI countries. The impact of premature death due to neoplasms has risen across SDI levels but with the largest increases in low-middle SDI countries (15·1 million [13·6–17·3] YLLs in 1980 *vs* 35·0 million [33·4–36·8] YLLs in 2017) and middle SDI countries (30·6 million [28·9–33·5] YLLs in 1980 *vs* 62·0 million [60·1–63·8] YLLs in 2017). YLLs from cardiovascular diseases increased at all SDI levels with the exception of high SDI countries, where total YLLs fell from 60·6 million (60·2–61·0) in 1980 to 41·4 million (40·8–42·2) in 2017. Total YLLs from injuries (self-harm and interpersonal violence, transport injuries, and unintentional injuries) at certain time periods exceeded those from the global leading causes of death; from 1980 to 2017 this increase occurred most often at lower SDI levels, and even with YLL rates slowly decreasing during this time period. Low SDI countries had a decrease in the rate of YLLs due to self-harm, falling from 604·4 (499·7–712·0) per 100 000 in 1980 to 441·4 (410·9–479·2) per 100 000 in 2017, but still remained higher than in all other SDI quintiles. In general, the precision of estimates was lower at lower SDI levels, represented by the wider 95% UIs across causes, which reflects the availability of data for these locations.

Despite increasing populations and changes in population age-structure, YLL rates decreased across the five leading Level 2 GBD causes of YLLs in all SDI quintiles (figure 6B). Large decreases in YLL rates were estimated at low SDI levels for respiratory infections and tuberculosis (from 14 900 [95% UI 13 000–16 600] YLLs per 100 000 in 1980 to 4750 [4505–4990] YLLs per 100 000 in 2017). Rates for enteric infections also decreased rapidly at low SDI levels (from 12 600 [9990–15 300] YLLs per 100 000 in 1980 to 3180 [2670–4090] YLLs per 100 000 in 2017) and low-middle SDI levels (9310 [7640–11 300] YLLs per 100 000 in 1980 to 2020 [1670–2500] YLLs per 100 000 in 2017). The YLL rate also decreased for cardiovascular diseases and for neoplasms across all SDI levels despite increases in the total number of YLLs. YLL rates for cervical cancer at low SDI levels—a cancer of infectious aetiology—decreased from 317 (242–373) YLLs per 100 000 to 191 (173–211) YLLs per 100 000 (appendix 2). At the same time, cancers such as pancreatic cancer—driven substantially by non-infectious risks—increased at low SDI levels from 38·6 (31·2–50·0) YLLs per 100 000 to 55·9 (51·9–60·0) YLLs per 100 000 (appendix 2).

### Leading causes of global YLLs

**Figure 7:**
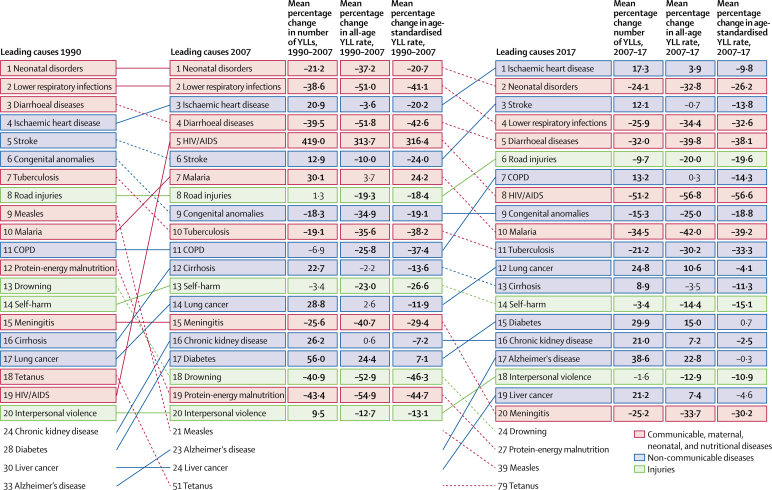
Leading 20 Level 3 causes of global YLLs for 1990, 2007, and 2017 with percentage change in number of YLLs, in all-age and age-standardised rates for both sexes combined Causes are connected by lines between time periods; solid lines are increases and dashed lines are decreases. For the time period 1990–2007 and for 2007–17, three measures of change are shown: percentage change in the number of YLLs, percentage change in the all-age YLL rate, and percentage change in the age-standardised YLL rate. Communicable, maternal, neonatal, and nutritional diseases are shown in red, non-communicable causes in blue, and injuries in green. Statistically significant changes are shown in bold. COPD=chronic obstructive pulmonary disease. YLLs=years of life lost.

Figure 7 shows the ongoing epidemiological shift in leading causes of total YLLs from CMNN diseases to NCDs at Level 3 of the GBD cause hierarchy over the 1990–2007 period and for 2007–17. Globally, the leading causes of YLLs in 1990 were neonatal disorders (ranked first), lower respiratory infections (second), and diarrhoeal diseases (third). Estimated YLLs decreased by 21·2% (95% UI 16·6–25·8) for neonatal disorders, by 38·6% (34·3–42·0) for lower respiratory infections, and by 39·5% (32·6– 45·4) for diarrhoeal diseases, from 1990 to 2007, and by a further 24·1% (20·6–27·2), 25·9% (22·2–29·2), and 32·0% (23·9–38·6), from 2007 to 2017. YLL rates also decreased during the entire 1990–2017 time period for neonatal disorders (from 4059·1 [3802·1–4336·0] YLLs per 100 000 to 2377·2 [2263·7–2485·1] YLLs per 100 000), lower respiratory infections (from 3821·4 [3509·9–4093·3] YLLs per 100 000 to 1515·1 [1424·8–1602·2] YLLs per 100 000), and diarrhoeal diseases (from 2843·6 [2415·0–3280·8] YLLs per 100 000 to 1009·1 [870·5–1211·0] YLLs per 100 000). In 2017, neonatal disorders were ranked second, lower respiratory infections fourth, and diarrhoeal diseases fifth in terms of total YLLs. Estimated YLLs from ischaemic heart disease, ranked first, increased by 20·9% (19·0–22·9) from 1990 to 2007, and by a further 17·3% (15·4–19·0) from 2007 to 2017, while estimated YLLs from stroke, ranked third, increased by 12·9% (10·6–15·2) from 1990 to 2007, and by a further 12·1% (9·9–14·1) from 2007 to 2017. However, decreases in YLL rates were estimated for ischaemic heart disease between 1990 and 2007 (20·2% [19·0–21·4]), and from 2007 to 2017 (9·8% [8·5–11·2]). YLL rates for stroke decreased by 24·0% (22·5–25·4) from 1990 to 2007, and by 13·8% (12·3–15·5) from 2007 to 2017. Other leading NCD causes of YLLs in 2017 included congenital anomalies (ranked ninth), and chronic obstructive pulmonary disease (seventh); other leading CMNN causes included lower respiratory infections (fourth), diarrhoeal diseases (fifth), HIV/AIDS (eighth), and malaria (tenth). The only injury cause of death in the leading ten causes of YLLs in 2017 was road injuries, for which the YLL rate decreased by 18·4% (14·4–22·0) from 1990 to 2007, with a further decrease of 19·6% (17·5–21·6) from 2007 to 2017, but with an increase in relative rank as a source of total YLLs from eighth in 1990 to sixth in 2017.

### YLLs and Socio-demographic Index level

**Figure 8:**
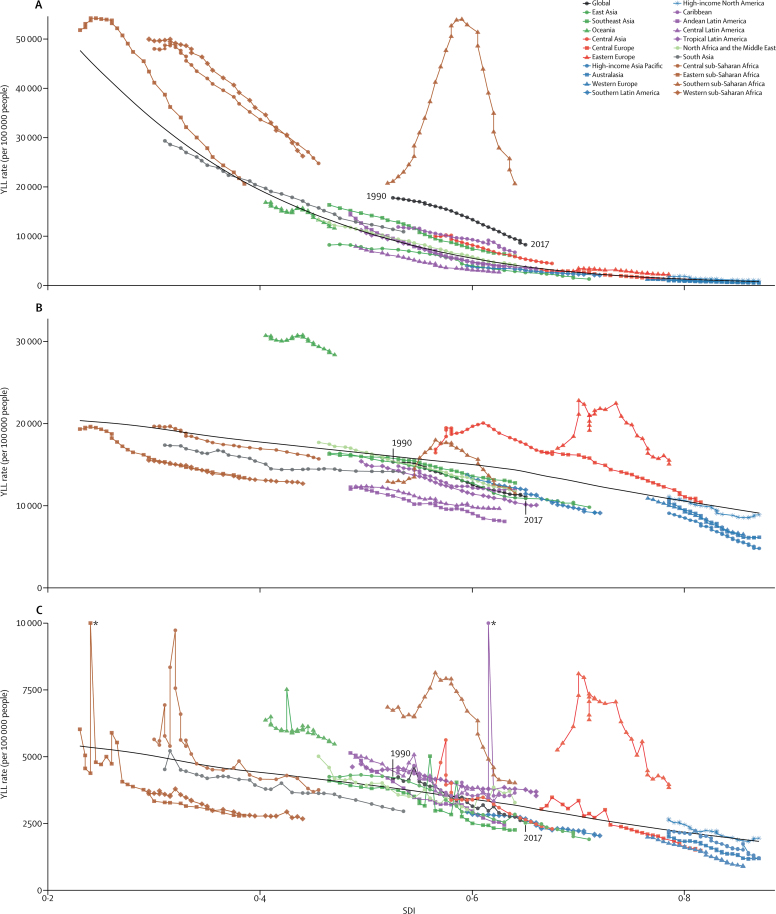
Co-evolution of age-standardised YLLs with SDI globally and for GBD regions for Level 1 causes, for both sexes combined, 1990–2017 (A) Communicable, maternal, neonatal, and nutritional diseases. (B) Non-communicable diseases. (C) Injuries. Coloured lines show global and region values for YLL rates. Each point in a line represents one year starting at 1990 and ending at 2017. In all regions, SDI has increased over time so progress in SDI is associated with points further to the right and later years for a given region. The black lines indicate expected trajectories for each geography expected on the basis of SDI alone. GBD=Global Burden of Diseases, Injuries, and Risk Factors Study. SDI=Socio-demographic Index. YLLs=years of life lost. *Values denoted by asterisks are 18 926·3 for eastern sub-Saharan Africa in 1994 and 35 078·7 for the Caribbean in 2010.

The association between SDI level and YLL rates for each GBD region for each year between 1990 and 2017 is illustrated for CMNN causes, NCDs, and injuries in figure 8. In general, YLL rates decreased as SDI for a given region increased, with some exceptions. Among these, the YLL rate in southern sub-Saharan Africa was distinctly non-linear, increasing across CMNN causes even as SDI level increased before decreasing by a similar amount against a backdrop of rising SDI level. Southern sub-Saharan Africa achieved a higher SDI level than did other regions of sub-Saharan Africa, although not necessarily consistently lower YLL rates for CMNN causes than the other regions of sub-Saharan Africa. Variation in the association between YLL rate for NCDs and SDI in the regions of central Asia and eastern Europe was observed, with the highest YLL rate observed in 2005 for central Asia, and in 1994 for eastern Europe. An end to previous declines in the YLL rate for NCDs at highest SDI (in the most recent years) was observed for high-income North America and Australasia. The impact of fatal discontinuities can be seen in the large spikes in YLL rates estimated in eastern sub-Saharan Africa in 1994, reflecting mortality from the genocide in Rwanda, and in the Caribbean region where the 2010 earthquake in Haiti resulted in a YLL rate 2·5 times greater than the level expected in that year given the SDI level.

### Decomposition of driving factors in epidemiological change for selected causes

**Figure 9:**
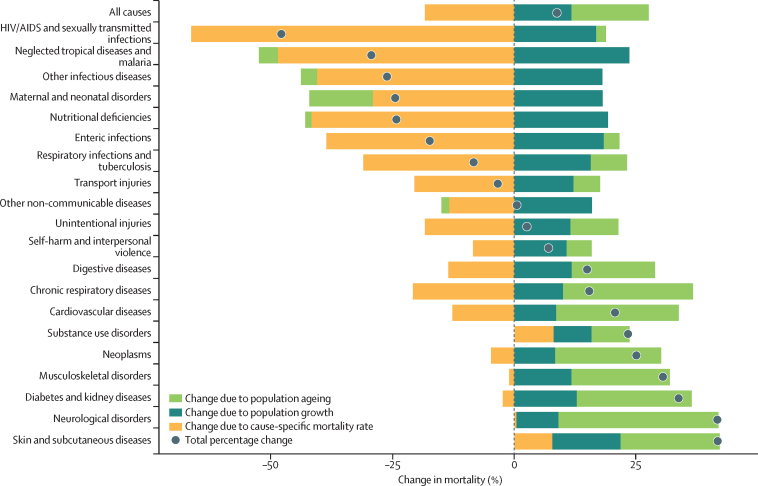
Percentage change in all-age mortality by Level 2 causes at the global level from 2007 to 2017, due to population growth, population ageing, and cause-specific mortality Mental disorders, for which there were 272 deaths globally in 2007 and 327 deaths globally in 2017, are not shown separately but are included in the all-cause category.

Changes in mortality are driven by population growth, population ageing, and changes in CSMR. The relative contributions of these factors to the change in total mortality for the 20 leading Level 2 causes of mortality from 2007 to 2017 are shown in figure 9. Population growth was an important contributor to increased levels of mortality across all causes. Declines in CSMR counterbalanced this effect for all but three causes—substance use disorders, neurological disorders, and skin and subcutaneous diseases. Without these decreases in CSMR, population ageing and growth would have resulted in increased mortality for most causes. Although population ageing led to increases in total deaths for most leading causes, for some causes—particularly neonatal conditions or other causes that primarily affect children—population ageing contributed to reductions for maternal and neonatal disorders (13·0%), neglected tropical diseases and malaria (3·9%), other infectious diseases (3·3%), other NCDs (1·6%), and nutritional deficiencies (1·3%). Changes in CSMR contributed the largest fraction to estimated changes in total deaths for 12 leading causes. CSMR contributed to 66·5% of the decrease in deaths from HIV/AIDS and to 8·1% of the increase in substance use disorders.

## Discussion

### High-level conclusions from the new health estimates of GBD 2017

#### General trends

The results of GBD 2017 show that, globally, CMNN diseases have declined steadily since 1990 in terms of total numbers of deaths and death rates. Global malaria deaths peaked in 2004 and HIV/AIDS-related deaths peaked in 2006, reflecting investments in the delivery of antiretroviral therapies, insecticide-treated bednets, and other interventions. NCDs, including both cardiovascular diseases and cancers, have risen steadily since 1990 in terms of total number of deaths, driven by ageing and population growth, while death rates have decreased, more slowly in the most recent years, as a result of improvements in prevention strategies and health-care interventions. Injury-related death rates have continued to decline since 1990.

#### Diseases of obesity

Our results show that a large number of deaths are known to be caused by high body-mass index, including cardiovascular diseases, neoplasms, dementia, asthma, hepatobiliary diseases, as well as diabetes and kidney diseases.^23^ The prevalence of obesity continues to rise in almost every country in the world, with more than 1 million deaths estimated as being due to type 2 diabetes, half a million deaths due to diabetes-related chronic kidney disease, and 180 000 due to NASH-related liver cancer and cirrhosis in this analysis.^24^ NASH is most often due to chronic insulin resistance secondary to obesity and might be present among 10–35% of the global adult population.^25^ The increasing prevalence of obesity might explain why death rates for cardiovascular disease are no longer declining in Australia, Austria, Brazil, Germany, Netherlands, the UK, and the USA.^26^ There is concern that global rates of ischaemic heart disease and ischaemic stroke might begin to rise for the first time since the 1970s.^27^ Increasing obesity could be the result of increased national wealth leading to complex changes in food systems, food quality, nutrition, technology, and levels of physical activity.^28^ Adult obesity has been identified as a key challenge to global nutrition, in particular for locations where malnutrition and obesity co-occur.^29^ Evidence-based nutrition policies should address this double burden, including in countries where obesity prevalence is low but estimated to be increasing.^24^ At the same time, cost-effective therapies that lower elevated blood pressure, cholesterol, and glucose, and reductions in tobacco smoking will remain important interventions.

#### Lower respiratory and enteric infections

Given the UN General Assembly High-level Meeting on tuberculosis in September, 2018, it is notable that total deaths due to tuberculosis have decreased since 2007, falling most rapidly for children younger than 5 years, but also that the majority of tuberculosis deaths were due to drug-susceptible tuberculosis (88·2% [95% UI 81·4–93·3] of total tuberculosis deaths in 2017). Deaths due to other lower respiratory infections remain a greater concern for children younger than 5 years due to a mortality rate more than ten times higher than that of tuberculosis.

Pneumococcal pneumonia was estimated to be the leading cause of death due to LRI in 2017 for children younger than 5 years, followed by respiratory syncytial virus pneumonia, *H influenzae* type B pneumonia, and influenza (appendix 2). Although the GBD counterfactual methodology for causes of death does smooth over some epidemics, our results show that over the past 27 years, mortality due to influenza and pneumococcal pneumonia has decreased but not at the same rate, reflecting differences in the age patterns and vaccination trends during this time period.^30^ Declines in other vaccine-preventable causes of child mortality, such as measles, suggest that achievements are possible over short periods of time.

Reductions in deaths due to pneumonia between 1990 and 2017 have been far larger for children than for older adults, with death rates due to pneumococcal pneumonia in adults older than 70 years having fallen by less than half as much as those of children, while death rates due to influenza and respiratory syncytial virus pneumonia have changed only minimally (appendix 2).

Similar patterns are seen for deaths due to enteric infections. Deaths related to *C difficile* increased among older adults, with only moderate declines in deaths due to other infectious causes of diarrhoea, while diarrhoeal deaths for children younger than 5 years continued to decline. The epidemic of *C difficile* might reflect increased incidence and pathogenicity of this bacterium, due to changing patterns of antibiotic resistance, comorbidity, and susceptibility among an ageing adult population.^31^ The age pattern for deaths due to multiple infectious diseases reflects investments in interventions that reduce childhood mortality as well as the challenges of delivering more complex health interventions to older adults with comorbid conditions. Evidence-based health policies will need to consider whether large increases in the use of antibiotics^32^ are leading to meaningful reductions in adult infections.^33^

#### The geography of conflict

Conflict-related deaths represent the fastest growing cause of injury-related deaths. Since 2007, conflicts have resulted in 1·14 million deaths, concentrated in the North Africa and Middle East region.^34^ Parts of South Asia, sub-Saharan Africa, and Latin America are also experiencing increasing rates of conflict-related deaths since 2007. Childhood deaths due to conflict were disproportionate. Regions with ongoing conflict are likely to face recurring health emergencies and present a particular challenge for achieving global development targets.^35^ Regional efforts to promote public health in areas with ongoing conflict, such as the recently established Africa Centres for Disease Control and Prevention,^36^ might help to support surveillance and implementation of evidence-based health policies in these often dire situations.

#### Countervailing patterns

The identification of exceptions to any large-scale patterns is an important result of GBD 2017. There are notable exceptions to the overall pattern of increasing total deaths from NCDs. For example, the total number of deaths from various congenital anomalies, including neural tube defects, congenital heart anomalies, and orofacial clefts decreased during the past decade. Some of the decline in neural tube defects and orofacial clefts could be related to improved nutritional status of women and more widespread introduction of folic acid fortification programmes, and some reductions might also be related to improvements in prenatal screening, access to abortion, supportive and interventional care services for infants born with birth defects, and broader reductions in infectious diseases to which such infants are especially susceptible.^37^ Conversely, for selected causes, both the number of deaths and death rate are increasing, including opioid, cocaine, amphetamine, and other drug use disorders, and liver cancers due to hepatitis B and hepatitis C. Inadequate access to treatment for hepatitis C, and restricted implementation of risk-prevention and treatment strategies for addiction and substance abuse might explain, in part, why death rates due to these preventable diseases are increasing.^38^ The total number of fall-related deaths has also risen steadily with no decline in rates, reflecting the way in which population ageing might be having a differential effect on injury-related deaths.

### Public health significance

#### Recent plateaus in mortality rates

The results of GBD 2017 show that declines in death rates of some common diseases are slowing or have ceased, primarily for NCDs. Declines in cardiovascular disease and neoplasms are slowing for many high-income countries. This observation is clearest for estimates at the subnational level, where deaths from these causes are increasing for some states in the USA and local authorities in the UK. Medications that lower blood pressure and blood cholesterol, and therefore the risk of atherosclerotic vascular disease events and deaths, are among the most cost-effective interventions available to health systems but are not being delivered effectively. Increasing evidence of a plateau in the decline, or even increases, in atherosclerotic vascular diseases should drive investment towards innovative systems that can effectively deliver these medications as well as public health measures and behaviour changes that need to accompany them.^39^ Plateaus in mortality are not restricted to high-income countries and can also be seen for leading causes in low-income countries. Decreases in the death rate of malaria have slowed for many regions, perhaps related to a period of slowing in the decline in incident infections observed in many regions between 2011 and 2013. Improved malaria mortality surveillance will be required to understand these most recent patterns.

#### Beyond the leading causes of death

Some common causes of death receive relatively less attention from the global community, because of their position as second or lower-ranked causes within a larger category. These highly ranked but non-leading causes include stomach cancer, asthma, syphilis, chronic kidney disease, congenital heart disease, and rheumatic heart disease. These causes combined resulted in more than 3 million deaths in 2017. Deaths due to these causes are at least partially amenable to primary or secondary prevention strategies, suggesting that improvements in the continuum of care, including in food quality, sanitation, diagnosis and screening, ongoing case management, and increased access to essential medicines via expanded universal health coverage, will have an important role in their reduction.^40^ GBD 2017 added several important but less heralded causes of death, including liver cancer due to NASH, subarachnoid haemorrhage, and non-rheumatic valvular heart diseases. These diseases sometimes do not have the more easily addressed risk exposures associated with the leading causes of death such as LRI, HIV/AIDS, lung cancer, or ischaemic heart disease, or they might be prevalent in locations where medical technologies or effective health-care interventions are less widely available. Diseases ranked lower within a larger cause category also might not benefit from the large-scale, focused advocacy efforts for the leading causes of death yet represent important future targets for research and public health. A rational, disease-burden-based approach to priority setting for health policy, now being adopted in some countries,^41^ might help to accelerate the scale-up of interventions that will have the largest impact on disability and premature death.

#### Emerging diseases and disorders due to antibiotic and opioid use

Although GBD provides estimates starting in the year 1980, changes in the most recent years are of particular importance for governments working to improve responsiveness to emerging threats to human health. Since 2007, rapid increases in death rates have been observed for a small number of diseases and disorders, including dengue, extensively drug-resistant tuberculosis, cellulitis, *C difficile* diarrhoea, and opioid, cocaine, and amphetamine use disorders. Of the infectious causes (other than dengue, the increase of which might reflect changes in the range of its primary vector, *Aedes aegypti*), increased pathogenicity due to antibiotic use or resistance is likely to be a major factor. Rapid increases in opioid-related deaths, particularly in the USA and Canada^42^ but also in other high SDI locations,^43^ have been attributed in part to wider availability of high-potency opioid analgesics^44^ and to international illegal trade of synthetic opioids.^45^

These emerging causes are associated with wider use of pharmaceuticals. The global health armamentarium will need to expand beyond its traditional approach of increasing access to treatment to include antimicrobial stewardship^46^ and programmes to manage the use of synthetic opioids. Policy makers will need particular expertise to balance these initiatives with equally vocal calls to address sepsis^47^ and cancer pain^48^, ^49^ as global health priorities.

#### Epidemiological transition for injuries and cancer

The epidemiological transition is most commonly thought of as a decline in CMNN deaths and a rise in chronic NCDs. GBD 2017 shows that deaths due to injuries and cancers also undergo characteristic transitions. Although specific locations have substantial spikes in injury-related mortality during natural disasters or conflicts, the mortality rates from specific injuries can vary as a function of development along the SDI spectrum. Road injuries, for example, might initially increase early in development when more of the population has exposure to transport-related injuries. As development increases, however, it becomes increasingly important for countries to invest in specific resources that can protect against mortality from these injuries, such as advanced trauma care, emergency medical response, vehicle safety initiatives such as seatbelt laws, and interventions to reduce distractions while driving.^50^

By contrast, other injuries such as falls and self-harm might more reliably decline as SDI increases and access to medical care improves. For example, deaths related to self-harm have declined drastically throughout China, possibly because of improved economic prospects among the poorest individuals and decreased access to lethal pesticides.^51^ There are also exceptions where deaths related to self-harm are not declining, including Australia, Brazil, the Philippines, Turkey, and the USA. Further research is needed to fully examine the underlying factors driving these divergent trends.

The epidemiological transition can also be observed among the different types of cancer. In many countries with lower SDI, cancers of infectious aetiology^52^ or related to poor nutrition are decreasing, whereas cancers typically associated with obesity and alcohol consumption are becoming more common. The concept that future demands on health systems might be, at least in part, predictable is an attractive feature of the theory of epidemiological transition that merits further exploration, such as recent work to produce health forecasts using results of the GBD study and projections of SDI.^53^

#### Continuous quality improvement

GBD 2017 has developed new methods to address the observation that some common causes of death are reported by surveillance systems as having implausible trends across time and varying widely between countries.^54^, ^55^ This concern has been noted for dementia and Parkinson's disease, where there has been a rapid rise in reported deaths attributed to these causes despite stable incidence and case fatality in most epidemiological studies.^56^, ^57^, ^58^, ^59^, ^60^ A new analysis by GBD 2017 uses 35 years of person-level underlying and intermediate cause of death data from the USA—a database of 80·4 million deaths—to better understand how physicians are choosing from a range of alternative diseases when selecting an underlying cause of death that was actually dementia. Countries should consider better use of important contextual data already being collected, such as intermediate and immediate causes of death, to improve the stability and robustness of their mortality surveillance systems. Both New Zealand and Brazil have already made intermediate cause of death data available for this kind of analysis, and other countries should consider this low-cost, high-impact path in the future.

### Changes in health estimates between GBD 2016 and GBD 2017

Each iteration of GBD re-analyses the entire time series by use of newly available data sources from across all estimation years and continually improved methods. New data and modelling approaches effectively improve model validity and decrease uncertainty from various sources with the consequence that estimates for a given cause, location, and year might differ between GBD iterations. The magnitude of these differences between GBD 2017 and GBD 2016 is presented in figure 1. Below we discuss some specific data and methodology changes underlying distinct differences in estimation.

A novel, integrated demographic assessment of population, fertility, and all-cause mortality was completed for GBD 2017. This development affected all causes because of the inclusion of population estimates in age-sex splitting algorithms, but most directly affected maternal and HIV/AIDS-related mortality estimates. The GBD 2017 assessment of the proportion of all-cause deaths due to maternal and neonatal disorders (3·7% [95% UI 3·5–3·8]) was similar to that of GBD 2016 (3·6% [3·4–3·8]), with the difference largely due to addition of new data and expanded subnational estimation. Additionally, GBD 2017 combined maternal and neonatal conditions as a category at Level 2 of the cause hierarchy; as a result, causes within this grouping are now separately reported at Level 3 (rather than Level 2), with neonatal conditions appearing for the first time as the second most common Level 3 source of YLLs.

Access to additional data sources led to several differences in estimation, including the addition of 2778 deaths in children younger than 5 years attributable to the inclusion of additional VA data for Nigeria^61^—a high-population, high-burden location—in GBD 2017. Similarly, introduction of these new data resulted in a decrease in estimated mortality from malaria, so that the GBD 2017 estimate for the year 2016 included 78 200 fewer deaths for Nigeria than were estimated by GBD 2016 for that year.

GBD 2017 also included an additional 502 country-years of cancer registry data and 127 country-years of VR system data compared with GBD 2016; 49·6% of the new cancer registry data came from the newly released Cancer Incidence in Five Continents (CI5 XI) database.^62^

For GBD 2017, major changes to the modelling strategy for dementia and Parkinson's disease included reallocating deaths from causes identified in multiple cause of death data in the USA as the likely alternative cause of death if dementia had not been assigned as the underlying cause. This approach allowed for more accurate identification of deaths to be reassigned to dementia and Parkinson's disease, including a sizeable proportion that had been assigned to garbage code categories as well as common, more specific causes of death in people with dementia or Parkinson's disease.

Changes in data sources and methods have led to improved estimates for several causes. Comparing the most recent decade between GBD 2016 and GBD 2017, 2006 to 2016, the estimated increase in deaths from drug use disorders in GBD 2017 (55·9% [95% UI 53·1–58·8]) was greater than the estimated increase for the same period in GBD 2016 (15·2% [4·8–26·4]), driven by a better fit to most recent years of data in the USA and the use of more appropriate covariates, including sales of prescription opioids by country and the prevalence of injecting drug use. Estimates of HIV/AIDS deaths among children in 2016 were higher in GBD 2017 (84 500 deaths [75 800–94 200]) than those (61 700 deaths [56 000–68 000]) estimated by GBD 2016 for the same year. In countries with high-quality VR data, child incidence was adjusted to produce mortality estimates that better align with recorded HIV/AIDS deaths. Additionally, the paediatric HIV/AIDS mortality estimates were produced with the CD4-count-specific mortality and progression parameters developed by UNAIDS.^63^

### Comparison of GBD 2017 to other estimates

The primary comparison dataset for malaria is the World Malaria Report (WMR)^64^ produced by WHO. As the Malaria Atlas Project produces results for both GBD and the many countries in the WMR, it is not surprising that these results align closely. WHO hepatitis estimates for 2015 present combined mortality results for different stages of viral hepatitis infection (acute hepatitis, cirrhosis, and liver cancer), but the methods and data sources used to generate these estimates are incompletely described;^65^ at least some results were based on additional modelling of GBD 2013 results. Total deaths were estimated at 1·34 million by WHO in 2015, compared with 1·07 million (95% UI 1·02–1·11) for the same year in the present analysis (139 000 deaths [116 000–158 000] for acute hepatitis, 306 000 deaths [281 000–305 000] for hepatitis B cirrhosis, 165 000 deaths [159 000–172 000] for hepatitis B liver cancer, 294 000 deaths [268 000–319 000] for hepatitis C cirrhosis, and 165 000 deaths [159 000–172 000] for hepatitis C liver cancer).

The estimates from the WHO Maternal Child Epidemiology Estimation (MCEE) for cause-specific under-5 mortality in 2016 at the global level differ from those produced by GBD 2017. Notable differences include the absence of estimates for haemoglobinopathies by WHO-MCEE and estimates of deaths from congenital anomalies (303 000 deaths) that are lower than those estimated by GBD (516 000 deaths [95% UI 446 000–595 000]). Lower congenital estimates are primarily due to inclusion of VA studies by WHO-MCEE that were assessed for GBD but found to be unreliable and implausibly low, as described above.^66^ The number of LRI deaths estimated by GBD 2017 for children younger than 5 years (828 000 deaths [774 000–884 000]) was smaller globally than the number estimated by the MCEE group (920 000 deaths in 2015), with the main differences in India and Pakistan. GBD uses the sample registration system by Indian state, whereas the MCEE group uses the Million Deaths Study and the INDEPTH network mortality data,^66^ driving much of the difference between those estimates and those from the present study. Iuliano and colleagues^67^ recently estimated 290 000–650 000 seasonal influenza-associated respiratory deaths globally. Differences in modelling strategy and underlying premise account for much of the difference in that estimate from those of the present study. Chiefly, the estimate of Iuliano and colleagues accounts for any deaths that potentially could be associated with influenza, whereas the GBD 2017 approach estimates only LRI deaths attributable to influenza within the GBD counterfactual framework. The UN Maternal Mortality Estimation Inter-Agency Group (MMEIG) has not updated its estimates since 2015.^68^

The Globocan project, led by the International Agency for Research on Cancer (IARC),^69^ provides estimates of the global and national-level cancer burden for 2012. Whereas Globocan only estimates cancer mortality for a single year, GBD provides mortality estimates for all diseases over time, including for selected subnational locations. This approach allows GBD to account for unknown causes of death by redistributing these to the most likely underlying cause—including cancer. To estimate cancer mortality, six different methods are used in Globocan. In GBD, cancer mortality was estimated with a single ensemble model approach. Despite these differences, estimates at the global level were similar, with Globocan estimating 8·2 million cancer deaths and GBD estimating 8·38 million (95% UI 8·26–8·48) for the year 2012 (appendix 2).

GBD 2017 estimates of deaths from all cardiovascular diseases were generally similar to WHO estimates from recent years. Non-GBD estimates for specific cardiovascular diseases are less common. A recent study estimating global and national deaths due to alcoholic cardiomyopathy used a model based on all-cause mortality and alcohol-attributable fractions.^70^ The investigators estimated 25 997 deaths (95% CI 17 358–49 096) in 2015 compared with GBD 2017 estimates of 90 700 (95% UI 82 800–97 500). Higher GBD estimates are the result of the garbage code redistribution method used by GBD, although the geographical distribution of deaths by country is similar for both studies. In GBD, a substantial number of garbage-coded deaths (eg, heart failure, senility, and atherosclerosis) are redistributed to cardiovascular disease causes, including ischaemic heart disease and alcoholic cardiomyopathy.

In this iteration, we estimated that 136 000 deaths were from drug use disorders globally in 2015; by contrast, the 2017 World Drug Report by the UN Office on Drugs and Crime (UNODC) estimated a total of 191 000 drug-attributable deaths in 2015. Differences might reflect the fact that UNODC data are reported directly by member states and the definition of drug-related deaths differs between countries; some countries include overdose deaths, whereas others can include deaths for which drug use was considered to be a contributing factor.

A direct comparison on a global level is possible for selected injuries and locations. Globally, WHO estimated 1·25 million road traffic deaths in 2013,^71^ a lower figure than that of GBD 2017, which estimated 1·32 million (95% UI 1·29–1·36) road traffic deaths for the year 2013. Although this difference might be partly due to different modelling strategies, the relatively lower estimate was also present in locations with reliable VR data—for example, for the USA, WHO estimated 34 064 deaths for 2013, whereas the GBD 2017 study estimated 41 600 deaths (39 800–43 000). These differences might also be partly due to modelling differences from internal consistency requirements in the GBD framework, as well as differences in ICD mapping to the underlying cause of death. Our estimate of 645 000 deaths (559 000–679 000) globally due to falls was only slightly lower than WHO's estimate for 2015 of 646 000 deaths,^72^ and a similar difference was present for estimates of deaths from self-harm,^73^ with WHO estimating approximately 800 000 deaths annually in recent years and GBD estimating approximately 800 000–820 000 deaths annually in recent years.

### Limitations

Limitations remain in GBD 2017 despite advances in methodology that addressed some of the difficulties of estimating cause-specific mortality at global, regional, national, and subnational scales. Limitations that primarily affect specific causes—such as identification of covariates to address the non-linearity in the association between some injuries and the SDI or accounting for changes in awareness of NASH as an explanation for estimated increased mortality—are described in detail in appendix 1 (section 3). Here, we identify limitations with applicability across many causes. First, time lags in available data, absence of data from specific regions, age groups, or time periods, or unreliability in the data that are available—as is the case for malaria estimation, where a key limitation is the rare and punctuated nature of nationally representative surveys of parasite rate; or diarrhoea mortality estimation, where data are restricted among adults and for the geographical areas with the highest mortality levels—can affect the precision of estimations. Second, the accuracy with which underlying cause of death is assigned is a key limitation for both VR data and VA data sources, which is complicated by multimorbidity at the time of death. GBD 2017 makes substantial efforts to enhance the comparability of results by applying corrections for under-registration and garbage code redistribution algorithms. Levels or estimated time trends might still be affected by systematic problems in selected locations. Third, to separately estimate type 1 and type 2 diabetes we used a regression method to redistribute unspecified diabetes deaths on the basis of the specified type 1 and type 2 deaths. As the proportion of unspecified deaths is high, even in many good-quality VR systems, the type-specific estimates are more uncertain. An additional complication is that many excess deaths in people with diabetes are preferentially coded to macrovascular complications such as stroke and ischaemic heart disease. This approach leaves the more direct consequences of diabetes such as ketoacidosis or hyperosmolar coma as reasons to code a death to diabetes as the underlying cause. These complications can affect type 1 and type 2 diabetes differently. Ascertaining the correct proportions of diabetes deaths that should be assigned to type 1 or type 2 diabetes is difficult. Fourth, the percentage of well certified data is a useful indicator of data completeness; however, quality or accuracy in cause of death certification is not necessarily indicated by a low level of identified garbage coding for a given location. Fifth, some sources of uncertainty will not have been captured by the GBD 2017 estimation process, including among the covariates used in models. Sixth, although some causes use negative binomial modelling approaches to improve estimation with overdispersed data, we have not yet developed a standardised empirical approach for selecting causes to use this method. Seventh, the ICD coding convention does not distinguish between suicide and deaths associated with self-harm, and thus our estimate includes both intentional and unintentional self-harm. Finally, because GBD results are a combination of data and estimation, lags in data reporting mean that estimates for the most recent years rely more on the modelling process, as do estimates for locations with low levels of data completeness. However, for causes with scarce data, the provision of an estimate with an adequate measure of uncertainty is preferable to no information, and identification of these causes is an important step in improving the certification of deaths globally.

### Future directions

Re-estimation of the entire GBD mortality time series from 1980 onwards as part of the GBD annual cycle offers multiple opportunities for strengthening global health estimates. This kind of continuous quality improvement^74^ remains a hallmark of GBD. However, the task of informing national and global policy responses to changing mortality patterns is still reliant on cause of death information that in many cases remains sparse or outdated. The improvements in estimation methods represented in each iteration of GBD do not mitigate the pressing need for investments in data and surveillance on a global scale.

Further work is needed to address the misclassification that occurs in VR data. For example, multiple death codes can be assigned for drug overdoses, and these coding and attribution issues can vary across countries. Additional work is needed to understand the impact of rapid diagnostic testing on the GBD malaria model. Better approaches are needed to make use of location, age, and time patterns when assigning deaths where the disease subtype remains unspecified, such as for diabetes and stroke. More specific subtypes will need to be added for other conditions, such as vascular dementia, breast cancer, and lung cancer. Multiple cause of death data should be put to wider use. Information about intermediate causes of death can be used to improve estimation of disorders that exist as final common pathways to death, including sepsis, heart failure, and acute kidney injury. Multiple cause of death data might also better inform maternal and neonatal death estimates, especially if combined models can borrow strength across related conditions. Associations between inborn and congenital diseases, developmental disorders, and infectious or malnutrition-related deaths are also likely. Additional data about maternal exposures, including tobacco, air pollution, alcohol, and obesity, could lead to cause-specific mortality estimates among newborn babies.

An important goal of the GBD collaboration is the production of estimates for increasingly granular locations, down to areas as small as a 5 × 5 km grid. Estimates of diarrhoea, LRI, and tuberculosis mortality would all have greater impact if produced with a higher degree of geographical precision. Ascertaining the location of injury for causes such as road injuries, falls, drowning, and fires is also an important goal and could improve understanding of where investments in civil infrastructure might most benefit the population. Injury models could also make use of satellite and other open data sources to incorporate more information about the presence and use of improved roads, use of seatbelts, or availability of firearms.

### Conclusion

GBD 2017 reveals both long-term and more recent patterns in global health. The number of deaths due to communicable, maternal, neonatal, and nutritional causes continues to decline, although at varying rates, whereas the number of deaths from NCDs is increasing and those from injuries remains stable. There is evidence that previously observed declines in death rates of some common diseases are now either slowing or have ceased, primarily for NCDs. Mortality estimates are being made with increasing detail as a result of improved methods for correcting biases in the data and the addition of new data sources, new causes, and new subnational locations. The GBD collaboration has expanded to include experts from 140 countries, with formal government engagement leading to the production of subnational estimates. Investments are being made to extend the reach of high-quality mortality surveillance and VR. SDG targets tied to mortality rates will be able to use annual GBD results to benchmark progress and identify best practices in every country.

Correspondence to: Dr Gregory Roth, Institute for Health Metrics and Evaluation, Seattle, WA 98121, USA rothg@uw.edu

**This online publication has been corrected. The corrected version first appeared at thelancet.com on November 9, 2018**

## Data sharing

To download the data used in these analyses, please visit the Global Health Data Exchange at http://ghdx.healthdata.org/gbd–2017.
