# Department of Error

**DOI:** 10.1016/S0140-6736(18)32833-2

**Published:** 2018-11-17

*GBD 2017 Causes of Death Collaborators. Global, regional, and national age-sex-specific mortality for 282 causes of death in 195 countries and territories, 1980–2017: a systematic analysis for the Global Burden of Disease Study 2017.* Lancet *2018; **392:** 1736–88—*The bottom row in figure 7 was cut off. This correction has been made to the online version as of Nov 9, 2018, and has been made to the printed Article.

