# Department of Error

**DOI:** 10.1016/S0140-6736(19)31049-9

**Published:** 2019-06-22

*GBD 2017 Causes of Death Collaborators. Global, regional, and national age-sex-specific mortality for 282 causes of death in 195 countries and territories, 1980–2017: a systematic analysis for the Global Burden of Disease Study 2017.* Lancet *2018; **392:** 1736–88*—In this Global Health Metrics paper, the affiliations have been amended for Yasin Jemal Yasin, Joseph Adel Mattar Banoub, and Abdullatif Husseini; and the declaration of interests statement has been amended for Boris Bikbov. These corrections have been made to the online version as of June 20, 2019.

