# Supplementary material for: Global, regional, and national age-sex-specific mortality for 282 causes of death in 195 countries and territories, 1980–2017: a systematic analysis for the Global Burden of Disease Study 2017
Source: Lancet. 2018 Nov 10;392(10159):1736–88. doi: 10.1016/S0140-6736(18)32203-7 (PMC6227606; doi:10.1016/S0140-6736(18)32203-7)
Supplement: Supplementary appendix 2 [file mmc2.pdf]

THE LANCET

Supplementary appendix 2

This appendix formed part of the original submission and has been peer reviewed. We post it as supplied by the authors.

Supplement to: GBD 2017 Causes of Death Collaborators. Global, regional, and national age-sex-specific mortality for 282 causes of death in 195 countries and territories, 1980–2017: a systematic analysis for the Global Burden of Disease Study 2017. *Lancet* 2018; **392:** 1736–88.

Supplementary Results for “Global, regional, and national age-sex specific mortality for 282 causes of death, 1980–2017: a systematic analysis for the Global Burden of Disease Study 2017”

This supplement provides additional figures and more detailed results for causes of death and years of life lost.

Complete results from the Global Burden of Disease Study (GBD 2017) are now measured in terabytes. These results are available in an interactive data downloading tool in the Global Health Data Exchange (GHDx). The tool contains the complete set of results from all GBD 2017 papers; however, specialised tables from the papers will be available as separate entries in the GHDx, as was made available in GBD 2016. Access to the results and tables through the GHDx are available at http://ghdx.healthdata.org/gbd−2017.

Supplementary Results Figures and Tables:

SR Figure 1 a-j. Leading 20 GBD cause hierarchy Level 3 causes of YLLs by SDI grouping and sex for 1990, 2007, and 2017, with percentage change in number of YLLs, and all-age and age-standardised rates. GBD=Global Burden of Disease. SDI=Socio-demographic Index. YLL=years of life lost. 3

SR Figure 2. Difference between GBD 2017 and WHO 2015 deaths for selected causes, all ages, by sex, 1990, 2005, 2010, and 2015. GBD=Global Burden of Disease. WHO=World Health Organization. 13

SR Figure 3a-b. Difference between GBD 2017 and GBD 2016 change in deaths and percent change in deaths by cause and sex for all ages, 1990, 2005, 2010, and 2016. GBD=Global Burden of Disease..........14

SR Figure 4 a-d. Death counts due to fatal discontinuities by type, 1980 – 2017.......................................16

SR Figure 5. Out-of-sample model performance for CODEm models for GBD 2017 and age-standardised cause-specific mortality rate by level 2 causes – Annotated 20

SR Table 1. Diarrhoeal diseases and lower respiratory infection aetiologies deaths, mortality rates, and annual percentage change from 1990 to 2017...21

SR Table 2. Distribution of percentage change in age-standardised deaths for Level 1 causes by location from 2003 to 2007, 2008 to 2012, and 2013 to 2017, both sexes 22

SR Table 3. Global deaths and YLL numbers, age-standardised rates per 100,000, and percentage change between 2007 and 2017 for females for all GBD causes and Levels 1 through 4 of the cause hierarchy. 95% UIs are in parentheses. GBD=Global Burden of Disease. UI=uncertainty interval. YLL=years of life lost. 40

SR Table 4. Global deaths and YLL numbers, age-standardised rates per 100,000, and percentage change between 2007 and 2017 for males for all GBD causes and Levels 1 through 4 of the cause hierarchy. 95% UIs are in parentheses.
GBD=Global Burden of Disease. UI=uncertainty interval. YLL=years of life lost. 44

SR Table 5. Global deaths by cause and age group for females (with 95% uncertainty intervals), 1990, 1995, 2000, 2005, 2010, and 2017...48

SR Table 6. Global deaths by cause and age group for males (with 95% uncertainty intervals), 1990, 1995, 2000, 2005, 2010, and 2017……………………………………………………………………………………………66

SR Table 7. Global deaths by cause and age group for both sexes combined (with 95% uncertainty intervals), 1990, 1995, 2000, 2005, 2010, and 2017…………………………………………………………………………84

SR Table 8. Global YLLs by cause and age group for females (with 95% uncertainty intervals), 1990, 1995, 2000, 2005, 2010, and 2017. YLL=years of life lost ………………………………………………………………………102

SR Table 9. Global YLLs by cause and age group for males (with 95% uncertainty intervals), 1990, 1995, 2000, 2005, 2010, and 2017. YLL=years of life lost…………………………………………………………………………120

SR Table 10. Global YLLs by cause and age group for both sexes combined (with 95% uncertainty intervals), 1990, 1995, 2000, 2005, 2010, and 2017. YLL=years of life lost...138

SR Table 11 a-e. Deaths due to fatal discontinuities by country, all ages, for both sexes combined, 1980–2017………156

SR Table 12. Comparison of GBD 2017 and GLOBOCAN estimated cancer mortality by cause and sex, 2012. GBD=Global Burden of Disease. ……………………………………………………………………………………174

SR Table 13. Deaths by location, cause, and age group for males (with 95% uncertainty intervals), 2017…….179

SR Table 14. Deaths by location, cause, and age group for females (with 95% uncertainty intervals), 2017………..3310

SR Table 15. Deaths by location, cause, and age group for both sexes combined (with 95% uncertainty intervals), 2017 ……….6445

SR Table 16. Age-standardised YLL rates per 100,000 by SDI and cause for both sexes combined (with 95% confidence intervals), 1980, 2017 ……………………………………………………………………………………..9580

SR Figure 1a: Leading 20 GBD cause hierarchy level 3 causes of High SDI YLLs for males for 1990, 2007 and 2017.

Causes are connected by arrows between time periods. Communicable maternal, neonatal and nutritional disorders are shown in red, non-communicable causes in blue and injuries in green. For the time period 1990 to 2007 and for 2007 to 2017, three measures of change are shown: percent change in the number of YLLs, percent change in the all-age YLL rate and percent change in the age-standardized YLL rate. Numbers in bold highlight statistically significant changes.

Leading causes 1990	Leading causes 2007	Mean % change number of YLLs 1990-2007	Mean % change all-age YLL rate 1990-2007	Mean % change age-standardized YLL rate 1990-2007	Leading causes 2017	Mean % change number of YLLs 2007-2017	Mean % change all-age YLL rate 2007-2017	Mean % change age-standardized YLL rate 2007-2017
1 Ischaemic heart disease	1 Ischaemic heart disease	**-28.2**	**-35.6**	**-48.9**	1 Ischaemic heart disease	**-3.1**	**-8.6**	**-22.1**
2 Lung cancer	2 Lung cancer	0.9	**-9.6**	**-27.3**	2 Lung cancer	1.7	**-4.1**	**-17.8**
3 Stroke	3 Stroke	**-19.9**	**-28.3**	**-43.9**	3 Stroke	**-2.9**	**-8.4**	**-22.5**
4 Road injuries	4 Self-harm	**10.6**	-0.9	**-4.1**	4 Self-harm	**-3.5**	**-9.0**	**-7.7**
5 Self-harm	5 Road injuries	**-38.8**	**-45.2**	**-44.1**	5 COPD	**10.9**	**4.6**	**-14.1**
6 Cirrhosis	6 Cirrhosis	-1.5	**-11.8**	**-26.2**	6 Colorectal cancer	**9.4**	3.2	**-10.9**
7 COPD	7 COPD	0.7	**-9.8**	**-30.7**	7 Cirrhosis	0.2	**-5.5**	**-13.4**
8 Colorectal cancer	8 Colorectal cancer	**19.9**	**7.4**	**-14.2**	8 Alzheimer's disease	**35.9**	**28.1**	-1.4
9 Stomach cancer	9 Lower respiratory infect	**3.7**	**-7.1**	**-28.9**	9 Road injuries	**-25.7**	**-30.0**	**-29.0**
10 Neonatal disorders	10 Alzheimer's disease	**56.1**	**39.8**	**-2.8**	10 Lower respiratory infect	**14.9**	**8.3**	**-10.5**
11 Lower respiratory infect	11 Stomach cancer	**-20.0**	**-28.3**	**-42.5**	11 Drug use disorders	**95.2**	**84.1**	**90.0**
12 Congenital anomalies	12 Liver cancer	**30.0**	**16.5**	**-5.1**	12 Liver cancer	**7.5**	1.3	**-11.2**
13 HIV/AIDS	13 Diabetes	**35.6**	**21.5**	-1.6	13 Prostate cancer	**13.2**	**6.7**	**-13.0**
14 Prostate cancer	14 Prostate cancer	**13.7**	1.9	**-23.1**	14 Stomach cancer	**-6.4**	**-11.8**	**-24.1**
15 Interpersonal violence	15 Pancreatic cancer	**38.4**	**24.0**	-0.3	15 Pancreatic cancer	**19.9**	**13.1**	-2.4
16 Liver cancer	16 Neonatal disorders	**-37.5**	**-44.0**	**-33.5**	16 Diabetes	**3.5**	-2.4	**-14.8**
17 Alzheimer's disease	17 Chronic kidney disease	**31.6**	**17.9**	**-7.3**	17 Chronic kidney disease	**21.0**	**14.1**	**-4.2**
18 Diabetes	18 Drug use disorders	**142.2**	**117.0**	**119.8**	18 Oesophageal cancer	**8.1**	2.0	**-11.3**
19 Leukaemia	19 Leukaemia	1.0	**-9.5**	**-22.6**	19 Falls	**7.5**	1.4	**-11.4**
20 Cardiomyopathy	20 Interpersonal violence	**-22.4**	**-30.5**	**-24.3**	20 Neonatal disorders	**-22.0**	**-26.4**	**-17.8**
21 Pancreatic cancer	21 Oesophageal cancer				21 Leukaemia			
22 Falls	22 Falls				23 Cardiomyopathy			
23 Chronic kidney disease	23 Congenital anomalies				24 Interpersonal violence			
25 Oesophageal cancer	24 Cardiomyopathy				29 Congenital anomalies			
38 Drug use disorders	37 HIV/AIDS				48 HIV/AIDS			

SR Figure 1b: Leading 20 GBD cause hierarchy level 3 causes of High-middle SDI YLLs for males for 1990, 2007 and 2017.

Causes are connected by arrows between time periods. Communicable maternal, neonatal and nutritional disorders are shown in red, non-communicable causes in blue and injuries in green. For the time period 1990 to 2007 and for 2007 to 2017, three measures of change are shown: percent change in the number of YLLs, percent change in the all-age YLL rate and percent change in the age-standardized YLL rate. Numbers in bold highlight statistically significant changes.

Leading causes 1990
1 Ischaemic heart disease
2 Stroke
3 Neonatal disorders
4 Road injuries
5 Lower respiratory infect
6 Congenital anomalies
7 COPD
8 Lung cancer
9 Self-harm
10 Drowning
11 Stomach cancer
12 Interpersonal violence
13 Cirrhosis
14 Liver cancer
15 Tuberculosis
16 Diarrhoeal diseases
17 Falls
18 Oesophageal cancer
19 Leukaemia
20 Nature disaster
21 Chronic kidney disease
22 Alcohol use disorders
23 Colorectal cancer
25 Cardiomyopathy
29 Diabetes
33 Alzheimer's disease

Leading causes 2007	Mean % change number of YLLs 1990-2007	Mean % change all-age YLL rate 1990-2007	Mean % change age-standardized YLL rate 1990-2007
1 Ischaemic heart disease	**36.0**	**17.5**	**-10.7**
2 Stroke	**17.2**	1.3	**-22.8**
3 Road injuries	-0.2	**-13.8**	**-17.0**
4 Lung cancer	**19.6**	3.3	**-16.6**
5 Neonatal disorders	**-53.0**	**-59.4**	**-39.4**
6 Self-harm	**8.5**	-6.3	**-17.4**
7 COPD	**-22.8**	**-33.3**	**-49.3**
8 Lower respiratory infect	**-47.1**	**-54.3**	**-49.7**
9 Cirrhosis	**43.1**	**23.6**	-1.6
10 Congenital anomalies	**-44.1**	**-51.7**	**-28.8**
11 Stomach cancer	**-6.4**	**-19.2**	**-35.9**
12 Liver cancer	**24.5**	7.5	**-14.0**
13 Interpersonal violence	-2.6	**-15.9**	**-20.8**
14 Cardiomyopathy	**176.0**	**138.4**	**88.6**
15 Alcohol use disorders	**66.2**	**43.5**	**18.0**
16 Drowning	**-46.1**	**-53.5**	**-48.3**
17 Colorectal cancer	**44.7**	**25.0**	-0.8
18 Tuberculosis	**-18.4**	**-29.5**	**-42.3**
19 Oesophageal cancer	**18.9**	2.7	**-20.1**
20 Falls	12.0	-3.2	**-10.3**
21 Chronic kidney disease			
22 Diabetes			
23 Alzheimer's disease			
25 Leukaemia			
49 Diarrhoeal diseases			
109 Nature disaster			

Leading causes 2017	Mean % change number of YLLs 2007-2017	Mean % change all-age YLL rate 2007-2017	Mean % change age-standardized YLL rate 2007-2017
1 Ischaemic heart disease	2.9	**-5.4**	**-20.5**
2 Stroke	**6.0**	-2.5	**-19.1**
3 Lung cancer	**23.5**	**13.6**	**-5.4**
4 Road injuries	**-22.0**	**-28.3**	**-27.5**
5 Self-harm	**-17.2**	**-23.8**	**-24.3**
6 COPD	**-4.6**	**-12.3**	**-28.9**
7 Liver cancer	**23.4**	**13.5**	-2.5
8 Cirrhosis	-1.0	**-8.9**	**-19.4**
9 Lower respiratory infect	**-14.1**	**-21.0**	**-32.1**
10 Stomach cancer	2.4	-5.8	**-21.0**
11 Neonatal disorders	**-41.4**	**-46.1**	**-44.4**
12 Colorectal cancer	**28.5**	**18.2**	-1.4
13 Cardiomyopathy	-23.2	**-29.3**	**-34.8**
14 Congenital anomalies	**-39.4**	**-44.3**	**-43.6**
15 Interpersonal violence	**-27.6**	**-33.4**	**-29.6**
16 Oesophageal cancer	**7.5**	-1.1	**-17.9**
17 Alzheimer's disease	**45.7**	**34.0**	2.4
18 Chronic kidney disease	**17.7**	**8.2**	**-7.3**
19 Diabetes	**25.2**	**15.2**	**-3.5**
20 Falls	-3.2	**-11.0**	**-16.6**
22 Alcohol use disorders			
23 Drowning			
26 Leukaemia			
30 Tuberculosis			
60 Diarrhoeal diseases			
87 Nature disaster			

SR Figure 1c: Leading 20 GBD cause hierarchy level 3 causes of Middle SDI YLLs for males for 1990, 2007 and 2017.

Causes are connected by arrows between time periods. Communicable maternal, neonatal and nutritional disorders are shown in red, non-communicable causes in blue and injuries in green. For the time period 1990 to 2007 and for 2007 to 2017, three measures of change are shown: percent change in the number of YLLs, percent change in the all-age YLL rate and percent change in the age-standardized YLL rate. Numbers in bold highlight statistically significant changes.

Leading causes 1990
1 Neonatal disorders
2 Lower respiratory infect
3 Road injuries
4 Ischaemic heart disease
5 Stroke
6 Diarrhoeal diseases
7 Congenital anomalies
8 Drowning
9 Tuberculosis
10 COPD
11 Interpersonal violence
12 Cirrhosis
13 Self-harm
14 Measles
15 Liver cancer
16 Lung cancer
17 Meningitis
18 Stomach cancer
19 Chronic kidney disease
20 Protein-energy malnutrition
21 Falls
26 Diabetes
56 HIV/AIDS

Leading causes 2007	Mean % change number of YLLs 1990-2007	Mean % change all-age YLL rate 1990-2007	Mean % change age-standardized YLL rate 1990-2007
1 Ischaemic heart disease	**54.8**	**26.6**	**-4.9**
2 Stroke	**34.9**	**10.4**	**-14.5**
3 Neonatal disorders	**-44.0**	**-54.2**	**-33.5**
4 Road injuries	3.5	**-15.3**	**-16.0**
5 HIV/AIDS	**2,073.4**	**1,678.1**	**1,616.1**
6 Lower respiratory infect	**-61.5**	**-68.5**	**-58.1**
7 COPD	0.1	**-18.1**	**-38.1**
8 Congenital anomalies	**-36.8**	**-48.3**	**-27.4**
9 Cirrhosis	**22.2**	0.0	**-19.2**
10 Interpersonal violence	**10.7**	**-9.4**	**-11.4**
11 Tuberculosis	**-27.0**	**-40.3**	**-48.2**
12 Lung cancer	**64.0**	**34.2**	4.6
13 Self-harm	-7.2	**-24.1**	**-29.5**
14 Liver cancer	**36.1**	**11.3**	**-11.1**
15 Diarrhoeal diseases	**-61.2**	**-68.3**	**-59.9**
16 Drowning	**-52.9**	**-61.4**	**-54.4**
17 Chronic kidney disease	**44.7**	**18.4**	1.7
18 Diabetes	**85.8**	**52.0**	**17.3**
19 Stomach cancer	**10.6**	**-9.5**	**-30.0**
20 Falls	12.6	-7.9	-12.0
27 Meningitis			
41 Protein-energy malnutrition			
49 Measles			

Leading causes 2017	Mean % change number of YLLs 2007-2017	Mean % change all-age YLL rate 2007-2017	Mean % change age-standardized YLL rate 2007-2017
1 Ischaemic heart disease	**34.6**	**23.4**	0.7
2 Stroke	**25.0**	**14.5**	**-8.4**
3 Road injuries	**-9.8**	**-17.3**	**-17.7**
4 Neonatal disorders	**-37.1**	**-42.3**	**-37.5**
5 COPD	**9.0**	-0.2	**-21.8**
6 Lower respiratory infect	**-27.5**	**-33.5**	**-36.1**
7 Lung cancer	**42.9**	**30.9**	5.3
8 Cirrhosis	**14.0**	4.5	**-9.0**
9 Interpersonal violence	**13.1**	3.6	**6.9**
10 HIV/AIDS	**-41.0**	**-46.0**	**-47.7**
11 Liver cancer	**26.2**	**15.6**	-3.1
12 Chronic kidney disease	**30.8**	**19.9**	2.9
13 Self-harm	3.4	**-5.2**	**-7.8**
14 Diabetes	**45.2**	**33.1**	**8.4**
15 Congenital anomalies	**-30.1**	**-35.9**	**-31.4**
16 Tuberculosis	**-24.2**	**-30.5**	**-38.0**
17 Stomach cancer	**9.9**	0.7	**-18.8**
18 Drowning	**-22.7**	**-29.2**	**-27.0**
19 Falls	**10.9**	1.6	-6.5
20 Diarrhoeal diseases	**-34.8**	**-40.2**	**-41.9**
39 Meningitis			
54 Protein-energy malnutrition			
69 Measles			

SR Figure 1d: Leading 20 GBD cause hierarchy level 3 causes of Low-middle SDI YLLs for males for 1990, 2007 and 2017.

Causes are connected by arrows between time periods. Communicable maternal, neonatal and nutritional disorders are shown in red, non-communicable causes in blue and injuries in green. For the time period 1990 to 2007 and for 2007 to 2017, three measures of change are shown: percent change in the number of YLLs, percent change in the all-age YLL rate and percent change in the age-standardized YLL rate. Numbers in bold highlight statistically significant changes.

Leading causes 1990	Leading causes 2007	Mean % change number of YLLs 1990-2007	Mean % change all-age YLL rate 1990-2007	Mean % change age-standardized YLL rate 1990-2007	Leading causes 2017	Mean % change number of YLLs 2007-2017	Mean % change all-age YLL rate 2007-2017	Mean % change age-standardized YLL rate 2007-2017
1 Neonatal disorders	1 Neonatal disorders	**-12.0**	**-35.7**	**-21.4**	1 Neonatal disorders	**-22.1**	**-33.9**	**-25.0**
2 Lower respiratory infect	2 Lower respiratory infect	**-29.1**	**-48.3**	**-37.1**	2 Ischaemic heart disease	**27.3**	**8.0**	-1.9
3 Diarrhoeal diseases	3 Diarrhoeal diseases	**-31.7**	**-50.2**	**-41.9**	3 Lower respiratory infect	**-25.0**	**-36.4**	**-29.0**
4 Tuberculosis	4 HIV/AIDS	**620.3**	**425.9**	**430.5**	4 Diarrhoeal diseases	**-40.5**	**-49.5**	**-44.3**
5 Ischaemic heart disease	5 Ischaemic heart disease	**51.4**	**10.5**	0.2	5 Stroke	**16.8**	-0.9	**-9.8**
6 Measles	6 Malaria	**62.9**	18.9	**34.2**	6 Road injuries	-0.7	**-15.8**	**-15.8**
7 Congenital anomalies	7 Road injuries	**20.9**	**-11.7**	**-10.0**	7 HIV/AIDS	**-47.3**	**-55.3**	**-56.0**
8 Road injuries	8 Tuberculosis	**-9.8**	**-34.1**	**-36.3**	8 Malaria	**-35.1**	**-44.9**	**-40.7**
9 Malaria	9 Stroke	**28.6**	**-6.2**	**-12.9**	9 Tuberculosis	**-21.8**	**-33.7**	**-36.3**
10 Stroke	10 Congenital anomalies	-3.8	**-29.8**	**-16.1**	10 Congenital anomalies	**-13.5**	**-26.6**	**-17.9**
11 Meningitis	11 Cirrhosis	**34.1**	-2.1	-8.9	11 Cirrhosis	**15.6**	-1.9	**-8.2**
12 Protein-energy malnutrition	12 COPD	4.9	**-23.4**	**-28.8**	12 COPD	**17.1**	-0.7	**-10.9**
13 COPD	13 Meningitis	**-18.6**	**-40.6**	**-31.8**	13 Self-harm	5.1	**-10.8**	**-12.3**
14 Cirrhosis	14 Self-harm	**30.1**	-5.0	-9.7	14 Meningitis	-19.0	**-31.2**	**-25.4**
15 Drowning	15 Interpersonal violence	**51.3**	**10.5**	6.2	15 Interpersonal violence	4.3	**-11.5**	**-12.1**
16 Self-harm	16 Drowning	**-23.5**	**-44.1**	**-37.9**	16 Chronic kidney disease	**17.3**	-0.5	**-5.3**
17 Tetanus	17 Measles	**-67.0**	**-75.9**	**-71.9**	17 Diabetes	**36.0**	**15.4**	**5.3**
18 Asthma	18 Protein-energy malnutrition	**-42.0**	**-57.6**	**-49.9**	18 Drowning	**-20.9**	**-32.9**	**-29.2**
19 Typhoid & paratyph	19 Chronic kidney disease	**39.8**	2.1	0.1	19 Falls	9.9	-6.8	-8.3
20 Interpersonal violence	20 Diabetes	**83.3**	**33.8**	**25.3**	20 Protein-energy malnutrition	**-40.0**	**-49.1**	**-43.9**
21 Chronic kidney disease	21 Falls				23 Asthma			
22 HIV/AIDS	22 Asthma				25 Typhoid & paratyph			
23 Falls	24 Typhoid & paratyph				26 Measles			
30 Diabetes	47 Tetanus				64 Tetanus			

SR Figure 1e: Leading 20 GBD cause hierarchy level 3 causes of Low SDI YLLs for males for 1990, 2007 and 2017.

Causes are connected by arrows between time periods. Communicable maternal, neonatal and nutritional disorders are shown in red, non-communicable causes in blue and injuries in green. For the time period 1990 to 2007 and for 2007 to 2017, three measures of change are shown: percent change in the number of YLLs, percent change in the all-age YLL rate and percent change in the age-standardized YLL rate. Numbers in bold highlight statistically significant changes.

Leading causes 1990	Leading causes 2007	Mean % change number of YLLs 1990-2007	Mean % change all-age YLL rate 1990-2007	Mean % change age-standardized YLL rate 1990-2007	Leading causes 2017	Mean % change number of YLLs 2007-2017	Mean % change all-age YLL rate 2007-2017	Mean % change age-standardized YLL rate 2007-2017
1 Neonatal disorders	1 Neonatal disorders	-4.9	**-35.8**	**-24.2**	1 Neonatal disorders	**-19.1**	**-34.5**	**-22.6**
2 Lower respiratory infect	2 Lower respiratory infect	**-27.9**	**-51.3**	**-41.4**	2 Lower respiratory infect	**-29.2**	**-42.6**	**-31.7**
3 Diarrhoeal diseases	3 Diarrhoeal diseases	**-27.7**	**-51.2**	**-42.3**	3 Diarrhoeal diseases	**-22.3**	**-37.0**	**-29.7**
4 Malaria	4 Malaria	15.7	**-21.9**	-13.1	4 Malaria	**-32.1**	**-45.0**	**-38.4**
5 Measles	5 HIV/AIDS	**138.5**	**61.2**	**56.0**	5 Ischaemic heart disease	**31.4**	**6.4**	-1.3
6 Tuberculosis	6 Tuberculosis	-6.1	**-36.6**	**-34.2**	6 Tuberculosis	**-13.5**	**-29.9**	**-30.4**
7 Protein-energy malnutrition	7 Congenital anomalies	15.6	**-21.9**	-10.7	7 Congenital anomalies	1.7	**-17.6**	-4.3
8 Leishmaniasis	8 Ischaemic heart disease	**53.5**	3.7	1.6	8 Road injuries	3.7	**-16.0**	**-14.9**
9 Congenital anomalies	9 Road injuries	**32.9**	-10.2	-5.8	9 HIV/AIDS	**-54.1**	**-62.8**	**-64.8**
10 Tetanus	10 Protein-energy malnutrition	**-41.9**	**-60.8**	**-56.5**	10 Stroke	**16.2**	**-5.9**	**-13.1**
11 Meningitis	11 Meningitis	-15.1	**-42.7**	**-35.6**	11 COPD	**28.0**	3.7	-5.5
12 HIV/AIDS	12 Stroke	**34.2**	**-9.3**	**-8.8**	12 Meningitis	**-24.6**	**-38.9**	**-31.0**
13 Road injuries	13 Measles	**-66.1**	**-77.1**	**-74.8**	13 Cirrhosis	8.1	-12.4	**-15.6**
14 Ischaemic heart disease	14 COPD	8.2	**-26.9**	**-26.2**	14 Protein-energy malnutrition	**-41.3**	**-52.5**	**-45.9**
15 Drowning	15 Drowning	**-25.1**	**-49.4**	**-44.1**	15 Self-harm	**20.4**	-2.4	-4.8
16 Typhoid & paratyph	16 Cirrhosis	**35.1**	-8.7	-5.7	16 STIs	-10.1	**-27.1**	**-14.4**
17 Stroke	17 Typhoid & paratyph	**-19.0**	**-45.3**	**-43.4**	17 Drowning	**-30.7**	**-43.8**	**-36.9**
18 COPD	18 STIs	-4.4	**-35.4**	**-26.2**	18 Typhoid & paratyph	**-24.3**	**-38.7**	**-35.3**
19 Asthma	19 Whooping cough	6.8	-27.8	-19.8	19 Interpersonal violence	5.1	**-14.9**	**-16.5**
20 STIs	20 Asthma	**-28.1**	**-51.4**	**-47.2**	20 Asthma	-8.7	**-26.0**	**-29.6**
22 Whooping cough	21 Self-harm				22 Whooping cough			
23 Cirrhosis	22 Interpersonal violence				24 Measles			
24 Self-harm	23 Tetanus				47 Tetanus			
25 Interpersonal violence	47 Leishmaniasis				72 Leishmaniasis			

SR Figure 1f: Leading 20 GBD cause hierarchy level 3 causes of High SDI YLLs for females for 1990, 2007 and 2017.

Causes are connected by arrows between time periods. Communicable maternal, neonatal and nutritional disorders are shown in red, non-communicable causes in blue and injuries in green. For the time period 1990 to 2007 and for 2007 to 2017, three measures of change are shown: percent change in the number of YLLs, percent change in the all-age YLL rate and percent change in the age-standardized YLL rate. Numbers in bold highlight statistically significant changes.

Leading causes 1990	Leading causes 2007	Mean % change number of YLLs 1990-2007	Mean % change all-age YLL rate 1990-2007	Mean % change age-standardized YLL rate 1990-2007	Leading causes 2017	Mean % change number of YLLs 2007-2017	Mean % change all-age YLL rate 2007-2017	Mean % change age-standardized YLL rate 2007-2017
1 Ischaemic heart disease	1 Ischaemic heart disease	**-31.9**	**-38.7**	**-50.8**	1 Ischaemic heart disease	**-5.9**	**-11.1**	**-22.9**
2 Stroke	2 Stroke	**-27.2**	**-34.5**	**-47.5**	2 Alzheimer's disease	**24.7**	**17.8**	**-2.7**
3 Breast cancer	3 Alzheimer's disease	**41.8**	**27.6**	**-6.4**	3 Stroke	**-7.6**	**-12.7**	**-24.4**
4 Alzheimer's disease	4 Lung cancer	**40.5**	**26.5**	**7.3**	4 Lung cancer	**14.2**	**7.9**	**-4.2**
5 Lung cancer	5 Breast cancer	-0.5	**-10.5**	**-23.7**	5 Breast cancer	0.8	**-4.7**	**-12.6**
6 Colorectal cancer	6 Colorectal cancer	**4.0**	**-6.4**	**-20.8**	6 COPD	**16.0**	**9.6**	**-3.5**
7 Road injuries	7 COPD	**32.3**	**19.0**	-2.8	7 Colorectal cancer	**3.8**	-1.9	**-12.1**
8 Lower respiratory infect	8 Lower respiratory infect	1.0	**-9.1**	**-30.8**	8 Lower respiratory infect	**10.8**	**4.6**	**-10.5**
9 Neonatal disorders	9 Self-harm	**4.4**	**-6.1**	**-7.1**	9 Pancreatic cancer	**22.2**	**15.4**	2.1
10 COPD	10 Diabetes	-1.5	**-11.3**	**-25.2**	10 Cirrhosis	**5.5**	-0.3	**-8.1**
11 Congenital anomalies	11 Cirrhosis	0.7	**-9.4**	**-22.2**	11 Self-harm	**-5.0**	**-10.3**	**-9.1**
12 Self-harm	12 Road injuries	**-36.3**	**-42.6**	**-42.4**	12 Chronic kidney disease	**19.6**	**12.9**	-1.8
13 Stomach cancer	13 Pancreatic cancer	**34.0**	**20.6**	**2.7**	13 Diabetes	**-6.8**	**-12.0**	**-21.1**
14 Diabetes	14 Chronic kidney disease	**28.8**	**15.9**	**-7.7**	14 Ovarian cancer	**3.6**	-2.2	**-11.1**
15 Cirrhosis	15 Ovarian cancer	**6.1**	**-4.5**	**-18.5**	15 Drug use disorders	**140.4**	**127.1**	**133.9**
16 Ovarian cancer	16 Neonatal disorders	**-35.7**	**-42.2**	**-31.7**	16 Road injuries	**-20.5**	**-24.9**	**-24.5**
17 Pancreatic cancer	17 Stomach cancer	**-28.2**	**-35.4**	**-45.8**	17 Stomach cancer	**-9.3**	**-14.3**	**-23.6**
18 Chronic kidney disease	18 Congenital anomalies	**-39.7**	**-45.7**	**-38.3**	18 Hypertensive heart disease	**18.6**	**12.0**	-3.3
19 Leukaemia	19 Leukaemia	**-6.1**	**-15.5**	**-26.2**	19 Neonatal disorders	**-21.5**	**-25.9**	**-17.3**
20 Cervical cancer	20 Hypertensive heart disease	5.4	-5.1	**-24.7**	20 Leukaemia	**-4.1**	**-9.4**	**-17.0**
23 Hypertensive heart disease	22 Cervical cancer				23 Congenital anomalies			
62 Drug use disorders	30 Drug use disorders				25 Cervical cancer			

SR Figure 1g: Leading 20 GBD cause hierarchy level 3 causes of High-middle SDI YLLs for females for 1990, 2007 and 2017.

Causes are connected by arrows between time periods. Communicable maternal, neonatal and nutritional disorders are shown in red, non-communicable causes in blue and injuries in green. For the time period 1990 to 2007 and for 2007 to 2017, three measures of change are shown: percent change in the number of YLLs, percent change in the all-age YLL rate and percent change in the age-standardized YLL rate. Numbers in bold highlight statistically significant changes.

Leading causes 1990	Leading causes 2007	Mean % change number of YLLs 1990-2007	Mean % change all-age YLL rate 1990-2007	Mean % change age-standardized YLL rate 1990-2007	Leading causes 2017	Mean % change number of YLLs 2007-2017	Mean % change all-age YLL rate 2007-2017	Mean % change age-standardized YLL rate 2007-2017
1 Ischaemic heart disease	1 Ischaemic heart disease	**24.5**	**7.8**	**-15.3**	1 Ischaemic heart disease	0.5	**-6.1**	**-22.4**
2 Stroke	2 Stroke	1.0	**-12.6**	**-30.4**	2 Stroke	**-5.3**	**-11.5**	**-26.5**
3 Neonatal disorders	3 Neonatal disorders	**-53.2**	**-59.4**	**-38.2**	3 Alzheimer's disease	**36.3**	**27.3**	-0.7
4 Lower respiratory infect	4 Congenital anomalies	**-45.2**	**-52.6**	**-28.3**	4 Lung cancer	**39.0**	**29.8**	**8.7**
5 Congenital anomalies	5 COPD	**-38.7**	**-46.9**	**-57.3**	5 Breast cancer	**12.8**	5.3	**-8.9**
6 COPD	6 Road injuries	**-6.1**	**-18.7**	**-17.2**	6 COPD	-9.1	**-15.2**	**-30.5**
7 Road injuries	7 Lower respiratory infect	**-61.0**	**-66.2**	**-60.9**	7 Neonatal disorders	**-41.1**	**-45.0**	**-44.5**
8 Self-harm	8 Breast cancer	**35.2**	**17.1**	**-6.4**	8 Lower respiratory infect	**-13.7**	**-19.4**	**-35.3**
9 Stomach cancer	9 Alzheimer's disease	**45.1**	**25.7**	**-6.6**	9 Road injuries	**-24.8**	**-29.7**	**-30.9**
10 Breast cancer	10 Lung cancer	**46.2**	**26.6**	3.0	10 Diabetes	**17.9**	**10.1**	**-7.9**
11 Diarrhoeal diseases	11 Cirrhosis	**42.9**	**23.7**	2.8	11 Colorectal cancer	**16.8**	**9.1**	**-7.9**
12 Alzheimer's disease	12 Stomach cancer	**-17.1**	**-28.2**	**-41.6**	12 Cirrhosis	-3.6	**-10.0**	**-20.9**
13 Lung cancer	13 Self-harm	**-40.8**	**-48.8**	**-53.8**	13 Congenital anomalies	**-39.6**	**-43.6**	**-44.1**
14 Cirrhosis	14 Colorectal cancer	**23.6**	**7.0**	**-13.0**	14 Stomach cancer	**-5.5**	**-11.7**	**-25.0**
15 Colorectal cancer	15 Diabetes	**26.9**	**9.9**	**-8.7**	15 Chronic kidney disease	**16.6**	**8.9**	**-7.7**
16 Drowning	16 Chronic kidney disease	2.5	**-11.3**	**-23.8**	16 Hypertensive heart disease	**27.4**	**19.0**	-2.3
17 Chronic kidney disease	17 Cardiomyopathy	**99.1**	**72.4**	**44.7**	17 Liver cancer	**16.6**	8.9	-8.1
18 Diabetes	18 Liver cancer	8.0	-6.5	**-23.5**	18 Self-harm	**-25.2**	**-30.2**	**-30.0**
19 Leukaemia	19 Hypertensive heart disease	3.8	-10.1	**-28.4**	19 Cervical cancer	6.2	-0.8	**-12.9**
20 Hypertensive heart disease	20 Cervical cancer	8.8	**-5.8**	**-24.2**	20 Cardiomyopathy	**-21.2**	**-26.4**	**-34.2**
21 Liver cancer	21 Leukaemia				24 Leukaemia			
24 Cervical cancer	29 Drowning				32 Drowning			
33 Cardiomyopathy	37 Diarrhoeal diseases				45 Diarrhoeal diseases			

SR Figure 1h: Leading 20 GBD cause hierarchy level 3 causes of Middle SDI YLLs for females for 1990, 2007 and 2017.

Causes are connected by arrows between time periods. Communicable maternal, neonatal and nutritional disorders are shown in red, non-communicable causes in blue and injuries in green. For the time period 1990 to 2007 and for 2007 to 2017, three measures of change are shown: percent change in the number of YLLs, percent change in the all-age YLL rate and percent change in the age-standardized YLL rate. Numbers in bold highlight statistically significant changes.

Leading causes 1990	Leading causes 2007	Mean % change number of YLLs 1990-2007	Mean % change all-age YLL rate 1990-2007	Mean % change age-standardized YLL rate 1990-2007	Leading causes 2017	Mean % change number of YLLs 2007-2017	Mean % change all-age YLL rate 2007-2017	Mean % change age-standardized YLL rate 2007-2017
1 Lower respiratory infect	1 Stroke	**11.5**	**-9.7**	**-31.7**	1 Ischaemic heart disease	**34.5**	**21.9**	-2.8
2 Neonatal disorders	2 Neonatal disorders	**-47.4**	**-57.4**	**-36.7**	2 Stroke	**18.6**	**7.5**	**-14.4**
3 Diarrhoeal diseases	3 HIV/AIDS	**4,046.0**	**3,259.1**	**3,260.9**	3 Neonatal disorders	**-35.8**	**-41.8**	**-36.4**
4 Stroke	4 Ischaemic heart disease	**40.0**	**13.5**	**-15.7**	4 Lower respiratory infect	**-29.8**	**-36.4**	**-39.4**
5 Ischaemic heart disease	5 Lower respiratory infect	**-64.7**	**-71.4**	**-61.8**	5 COPD	7.0	-3.0	**-24.4**
6 Congenital anomalies	6 Congenital anomalies	**-37.8**	**-49.6**	**-27.5**	6 HIV/AIDS	**-59.0**	**-62.8**	**-63.5**
7 COPD	7 COPD	**-24.0**	**-38.5**	**-54.4**	7 Diabetes	**39.5**	**26.4**	1.8
8 Tuberculosis	8 Diarrhoeal diseases	**-62.2**	**-69.3**	**-60.6**	8 Chronic kidney disease	**24.9**	**13.2**	**-4.0**
9 Self-harm	9 Road injuries	**-11.8**	**-28.6**	**-26.6**	9 Breast cancer	**35.1**	**22.5**	4.1
10 Road injuries	10 Diabetes	**63.1**	**32.2**	1.6	10 Congenital anomalies	**-28.9**	**-35.6**	**-30.5**
11 Drowning	11 Tuberculosis	**-41.1**	**-52.2**	**-56.4**	11 Road injuries	**-13.5**	**-21.6**	**-22.9**
12 Maternal disorders	12 Chronic kidney disease	**29.6**	**5.0**	**-10.7**	12 Alzheimer's disease	**48.5**	**34.6**	0.2
13 Measles	13 Breast cancer	**62.1**	**31.3**	-0.7	13 Lung cancer	**44.4**	**30.9**	5.9
14 Chronic kidney disease	14 Self-harm	**-43.0**	**-53.8**	**-55.8**	14 Diarrhoeal diseases	**-35.8**	**-41.8**	**-45.6**
15 Cirrhosis	15 Cirrhosis	3.9	**-15.8**	**-31.4**	15 Cirrhosis	**7.2**	-2.8	**-16.9**
16 Protein-energy malnutrition	16 Alzheimer's disease	**69.0**	**37.0**	**-10.2**	16 Self-harm	**-10.9**	**-19.2**	**-19.9**
17 Diabetes	17 Lung cancer	**53.8**	**24.6**	**-4.9**	17 Tuberculosis	**-31.9**	**-38.3**	**-43.3**
18 Meningitis	18 Cervical cancer	**28.7**	4.2	**-21.1**	18 Hypertensive heart disease	**47.2**	**33.5**	6.1
19 Rheumatic heart disease	19 Stomach cancer	**-7.7**	**-25.2**	**-42.9**	19 Cervical cancer	**18.2**	7.1	**-8.6**
20 Hypertensive heart disease	20 Maternal disorders	**-47.4**	**-57.4**	**-58.5**	20 Liver cancer	**19.5**	**8.3**	**-11.1**
21 Stomach cancer	21 Liver cancer				21 Stomach cancer			
22 Breast cancer	22 Hypertensive heart disease				25 Drowning			
26 Cervical cancer	23 Drowning				29 Maternal disorders			
28 Liver cancer	29 Rheumatic heart disease				31 Rheumatic heart disease			
30 Lung cancer	30 Meningitis				38 Meningitis			
32 Alzheimer's disease	34 Protein-energy malnutrition				46 Protein-energy malnutrition			
62 HIV/AIDS	40 Measles				63 Measles			

SR Figure 1i: Leading 20 GBD cause hierarchy level 3 causes of Low-middle SDI YLLs for females for 1990, 2007 and 2017.

Causes are connected by arrows between time periods. Communicable maternal, neonatal and nutritional disorders are shown in red, non-communicable causes in blue and injuries in green. For the time period 1990 to 2007 and for 2007 to 2017, three measures of change are shown: percent change in the number of YLLs, percent change in the all-age YLL rate and percent change in the age-standardized YLL rate. Numbers in bold highlight statistically significant changes.

Leading causes 1990	Leading causes 2007	Mean % change number of YLLs 1990-2007	Mean % change all-age YLL rate 1990-2007	Mean % change age-standardized YLL rate 1990-2007	Leading causes 2017	Mean % change number of YLLs 2007-2017	Mean % change all-age YLL rate 2007-2017	Mean % change age-standardized YLL rate 2007-2017
1 Neonatal disorders	1 Neonatal disorders	**-11.3**	**-35.7**	**-20.4**	1 Neonatal disorders	**-22.4**	**-35.1**	**-25.3**
2 Lower respiratory infect	2 Lower respiratory infect	**-31.3**	**-50.2**	**-39.9**	2 Lower respiratory infect	**-27.3**	**-39.3**	**-31.9**
3 Diarrhoeal diseases	3 Diarrhoeal diseases	**-37.3**	**-54.5**	**-45.6**	3 Diarrhoeal diseases	**-35.0**	**-45.7**	**-40.1**
4 Measles	4 HIV/AIDS	**951.7**	**662.4**	**718.8**	4 Ischaemic heart disease	**29.8**	**8.5**	-4.4
5 Tuberculosis	5 Malaria	**52.6**	10.6	**24.9**	5 HIV/AIDS	**-49.6**	**-57.9**	**-58.4**
6 Congenital anomalies	6 Ischaemic heart disease	**30.9**	-5.1	**-16.4**	6 Stroke	**18.5**	-0.9	**-12.0**
7 Malaria	7 Stroke	**21.7**	**-11.8**	**-21.3**	7 Malaria	**-39.6**	**-49.5**	**-45.0**
8 Ischaemic heart disease	8 Congenital anomalies	-4.1	**-30.5**	**-16.3**	8 Congenital anomalies	**-11.9**	**-26.4**	**-16.8**
9 Stroke	9 Tuberculosis	**-22.3**	**-43.7**	**-44.8**	9 Tuberculosis	**-23.4**	**-36.0**	**-37.0**
10 Maternal disorders	10 Maternal disorders	**-17.2**	**-39.9**	**-43.2**	10 COPD	**23.9**	3.5	**-9.6**
11 Meningitis	11 Meningitis	**-17.6**	**-40.3**	**-31.4**	11 Maternal disorders	**-23.6**	**-36.1**	**-36.1**
12 Protein-energy malnutrition	12 COPD	5.8	**-23.3**	**-31.7**	12 Meningitis	**-23.4**	**-36.0**	**-30.1**
13 COPD	13 Measles	**-65.6**	**-75.0**	**-70.4**	13 Road injuries	-3.0	**-18.9**	**-17.2**
14 Road injuries	14 Road injuries	4.9	**-23.9**	**-19.4**	14 Diabetes	**34.5**	**12.4**	0.5
15 Self-harm	15 Self-harm	-4.5	**-30.8**	**-31.9**	15 Cirrhosis	**17.3**	-2.0	-8.5
16 Tetanus	16 Protein-energy malnutrition	**-37.2**	**-54.5**	**-45.7**	16 Chronic kidney disease	**19.3**	-0.3	-5.5
17 Drowning	17 Cirrhosis	**24.2**	-10.0	**-18.0**	17 Breast cancer	**40.2**	**17.1**	5.9
18 Typhoid & paratyph	18 Chronic kidney disease	**23.3**	**-10.6**	**-13.6**	18 Self-harm	**-6.2**	**-21.6**	**-20.9**
19 Asthma	19 Diabetes	**80.0**	**30.5**	**18.3**	19 Protein-energy malnutrition	**-30.5**	**-41.9**	**-35.5**
20 Cirrhosis	20 Breast cancer	**76.3**	**27.8**	12.4	20 Asthma	0.8	**-15.8**	**-22.6**
21 Chronic kidney disease	21 Asthma				23 Measles			
23 HIV/AIDS	22 Drowning				26 Drowning			
24 Diabetes	23 Typhoid & paratyph				27 Typhoid & paratyph			
27 Breast cancer	43 Tetanus				63 Tetanus			

SR Figure 1j: Leading 20 GBD cause hierarchy level 3 causes of Low SDI YLLs for females for 1990, 2007 and 2017.

Causes are connected by arrows between time periods. Communicable maternal, neonatal and nutritional disorders are shown in red, non-communicable causes in blue and injuries in green. For the time period 1990 to 2007 and for 2007 to 2017, three measures of change are shown: percent change in the number of YLLs, percent change in the all-age YLL rate and percent change in the age-standardized YLL rate. Numbers in bold highlight statistically significant changes.

Leading causes 1990
1 Lower respiratory infect
2 Diarrhoeal diseases
3 Neonatal disorders
4 Malaria
5 Measles
6 Tuberculosis
7 Protein-energy malnutrition
8 Maternal disorders
9 Congenital anomalies
10 Leishmaniasis
11 Meningitis
12 Tetanus
13 HIV/AIDS
14 Stroke
15 Ischaemic heart disease
16 Drowning
17 Typhoid & paratyph
18 Whooping cough
19 COPD
20 Asthma
21 Road injuries
22 STIs
23 Self-harm
32 Diabetes

Leading causes 2007	Mean % change number of YLLs 1990-2007	Mean % change all-age YLL rate 1990-2007	Mean % change age-standardized YLL rate 1990-2007
1 Neonatal disorders	-8.9	**-39.2**	**-27.1**
2 Lower respiratory infect	**-29.0**	**-52.6**	**-43.9**
3 Diarrhoeal diseases	**-41.1**	**-60.8**	**-53.0**
4 HIV/AIDS	**314.4**	**176.3**	**211.4**
5 Malaria	**18.3**	**-21.1**	-12.2
6 Tuberculosis	**-18.7**	**-45.8**	**-42.8**
7 Congenital anomalies	**19.5**	**-20.3**	-7.9
8 Maternal disorders	**-13.7**	**-42.4**	**-44.5**
9 Protein-energy malnutrition	**-34.5**	**-56.3**	**-50.9**
10 Meningitis	-13.0	**-42.0**	**-35.8**
11 Ischaemic heart disease	**35.5**	-9.6	**-14.7**
12 Stroke	**24.5**	**-17.0**	**-19.7**
13 Measles	**-65.7**	**-77.1**	**-74.6**
14 Whooping cough	7.1	-28.6	-19.6
15 COPD	9.3	**-27.1**	**-29.0**
16 Road injuries	9.2	**-27.2**	**-23.1**
17 Typhoid & paratyph	**-18.7**	**-45.8**	**-43.5**
18 Drowning	**-27.0**	**-51.3**	**-46.3**
19 Asthma	**-23.0**	**-48.7**	**-45.1**
20 STIs	-4.8	**-36.5**	**-27.9**
21 Self-harm			
23 Tetanus			
25 Diabetes			
55 Leishmaniasis			

Leading causes 2017	Mean % change number of YLLs 2007-2017	Mean % change all-age YLL rate 2007-2017	Mean % change age-standardized YLL rate 2007-2017
1 Neonatal disorders	**-19.5**	**-35.5**	**-23.1**
2 Lower respiratory infect	**-29.3**	**-43.4**	**-32.2**
3 Diarrhoeal diseases	**-29.9**	**-43.8**	**-33.7**
4 Malaria	**-33.4**	**-46.7**	**-39.6**
5 Congenital anomalies	4.1	**-16.7**	-2.4
6 HIV/AIDS	**-62.0**	**-69.6**	**-71.9**
7 Tuberculosis	**-16.3**	**-33.0**	**-31.2**
8 Ischaemic heart disease	**35.2**	**8.3**	-2.7
9 Stroke	**23.0**	-1.5	**-11.0**
10 Maternal disorders	**-24.1**	**-39.2**	**-40.2**
11 Protein-energy malnutrition	**-30.6**	**-44.4**	**-35.9**
12 COPD	**41.9**	13.6	1.5
13 Meningitis	**-27.1**	**-41.7**	**-33.8**
14 Road injuries	1.4	**-18.8**	**-13.5**
15 Whooping cough	-22.4	-37.8	-28.3
16 Asthma	8.8	**-12.9**	**-15.9**
17 Typhoid & paratyph	**-20.5**	**-36.3**	**-32.5**
18 Measles	**-55.8**	**-64.6**	**-59.7**
19 Self-harm	4.4	**-16.4**	**-16.8**
20 Diabetes	**40.2**	**12.3**	3.8
22 STIs			
24 Drowning			
51 Tetanus			
82 Leishmaniasis			

SR Figure 2. Difference between GBD 2017 and WHO 2015 deaths for selected causes, all ages, by sex, 1990, 2005, 2010, and 2015. GBD=Global Burden of Disease. WHO=World Health Organization.

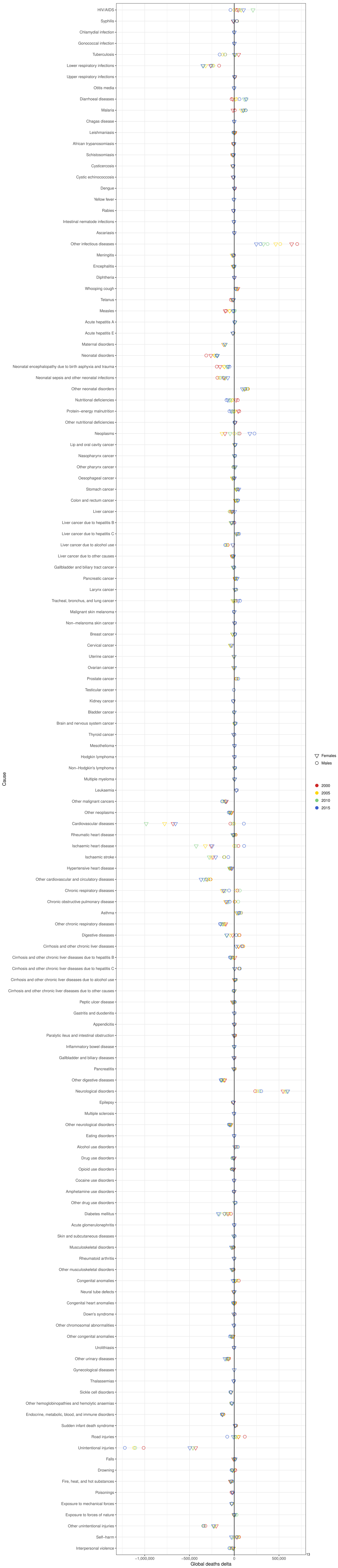

SR Figure 3b. Percentage difference between GBD 2017 and GBD 2016 deaths by cause and sex for all ages, 1990, 2005, 2010, 2016

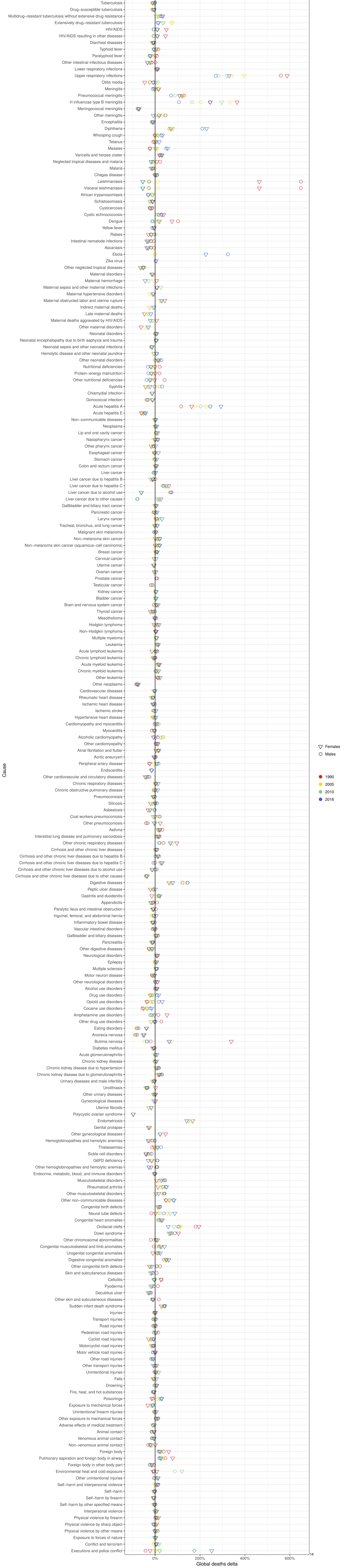

SR Figure 3. Difference between GBD 2017 and GBD 2016 deaths by cause and sex for all ages, 1990, 2005, 2010, and 2016. GBD=Global Burden of Disease.

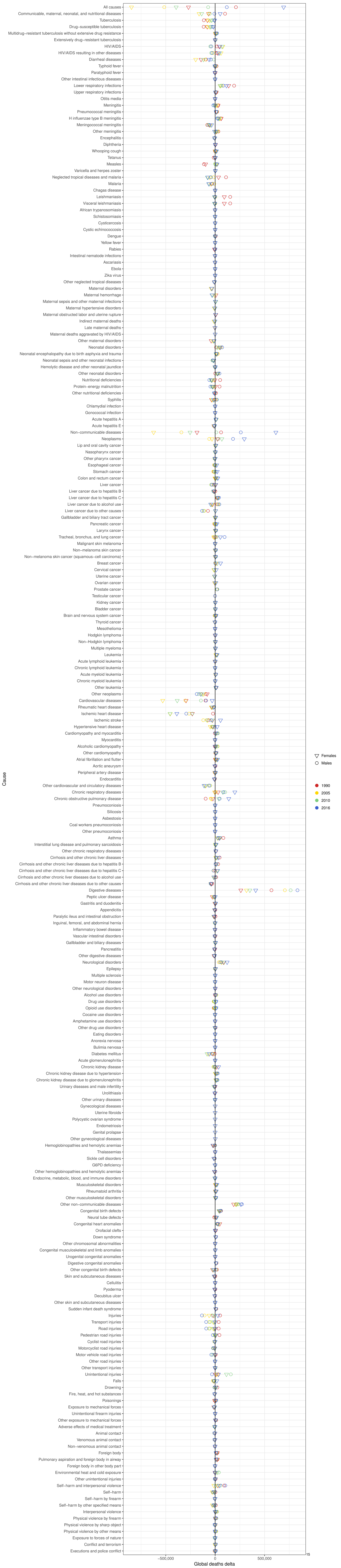

SR Figure 4a. Death counts due to disasters, 1980–2017

Causes included: Exposure to forces of nature, Fire, heat, and hot substances, Environmental heat and cold exposure, Poisonings, Other transport injuries, and Road injuries

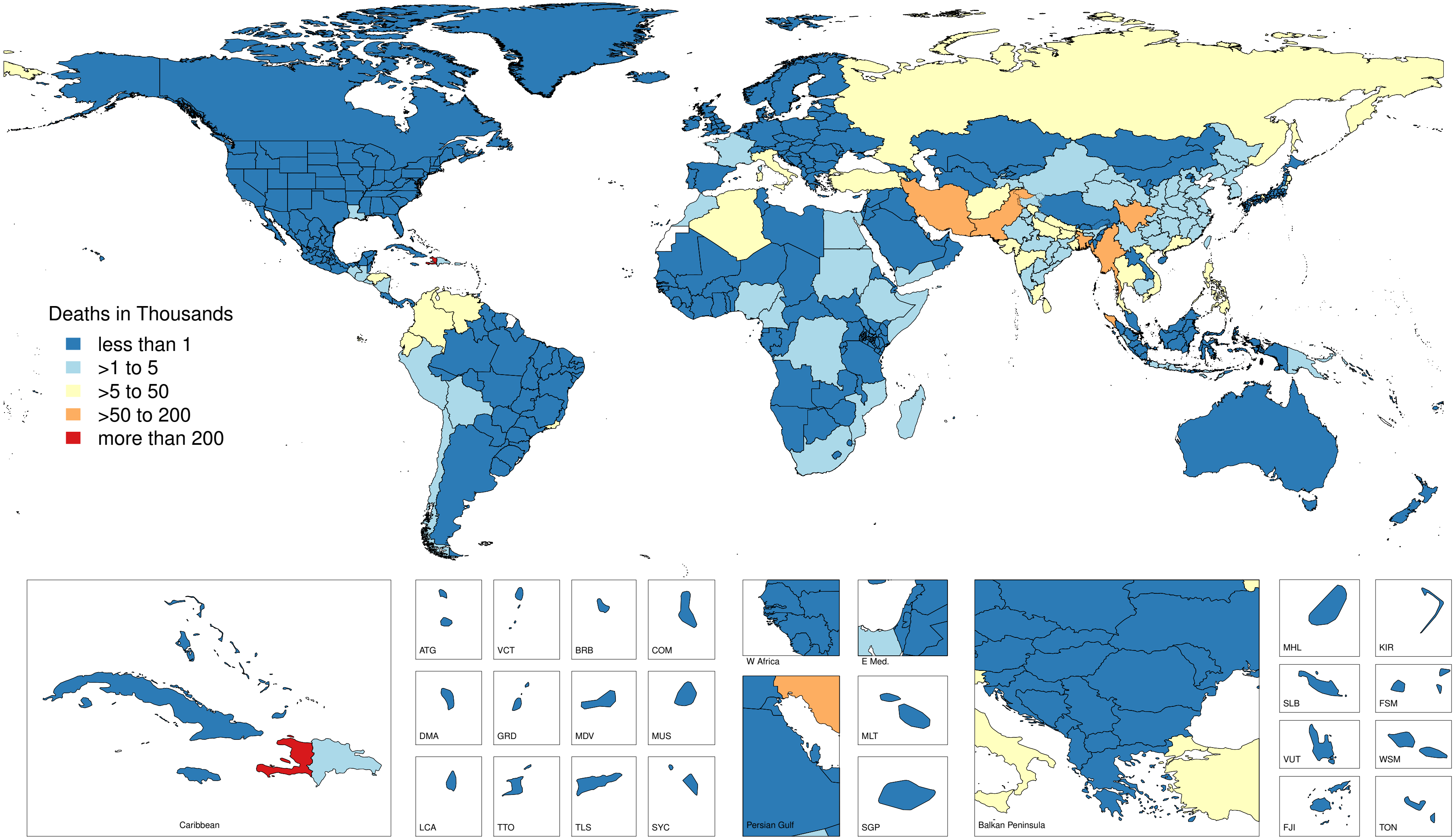

SR Figure 4b. Death counts due to disease outbreak, 1980–2017

Causes included: Malaria, Measles, Meningococcal meningitis, Diarrheal diseases, Dengue Ebola, and Other unspecified infectious diseases

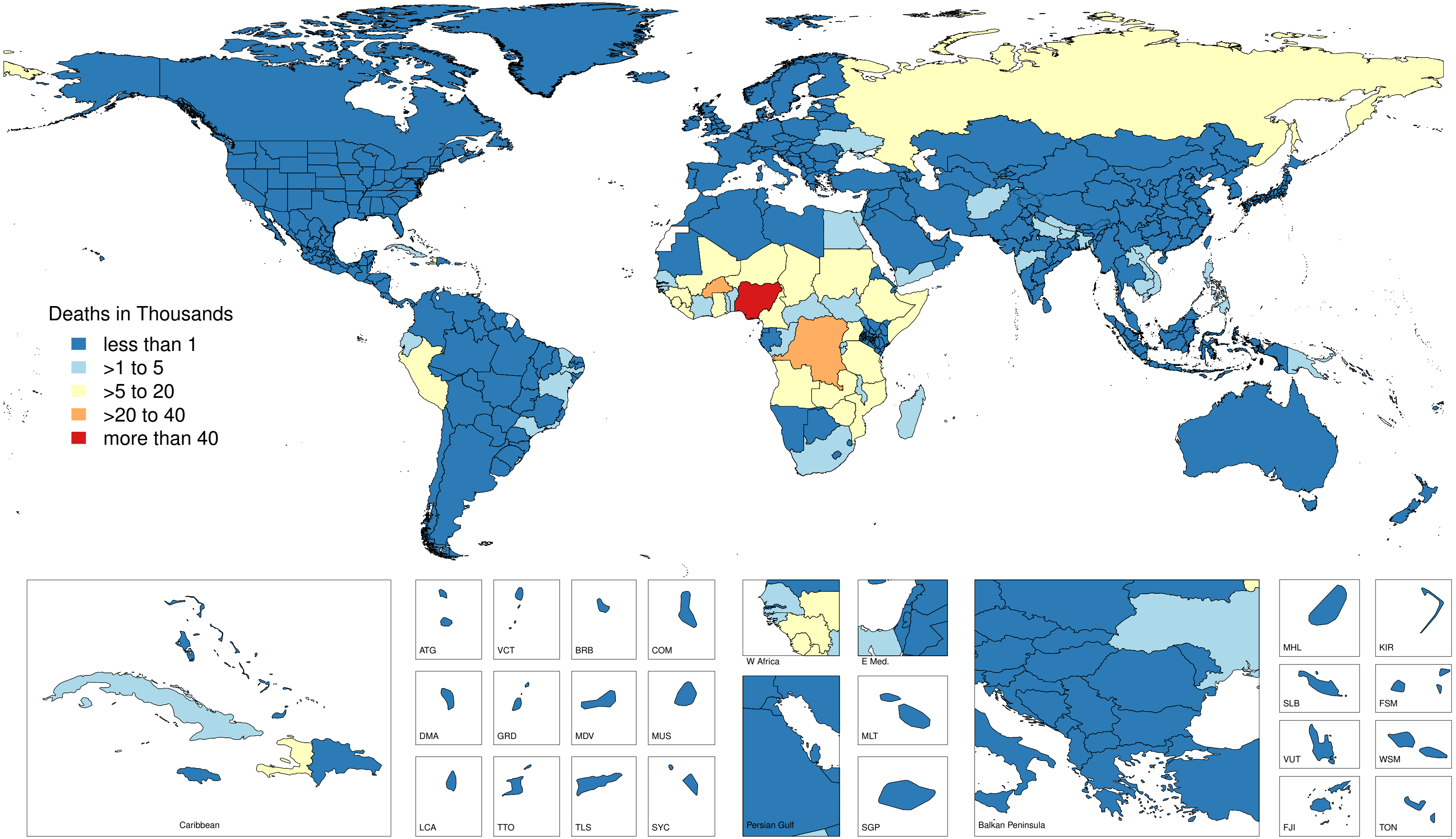

SR Figure 4c. Death counts due to famine, 1980–2017

Cause included: Protein–energy malnutrition

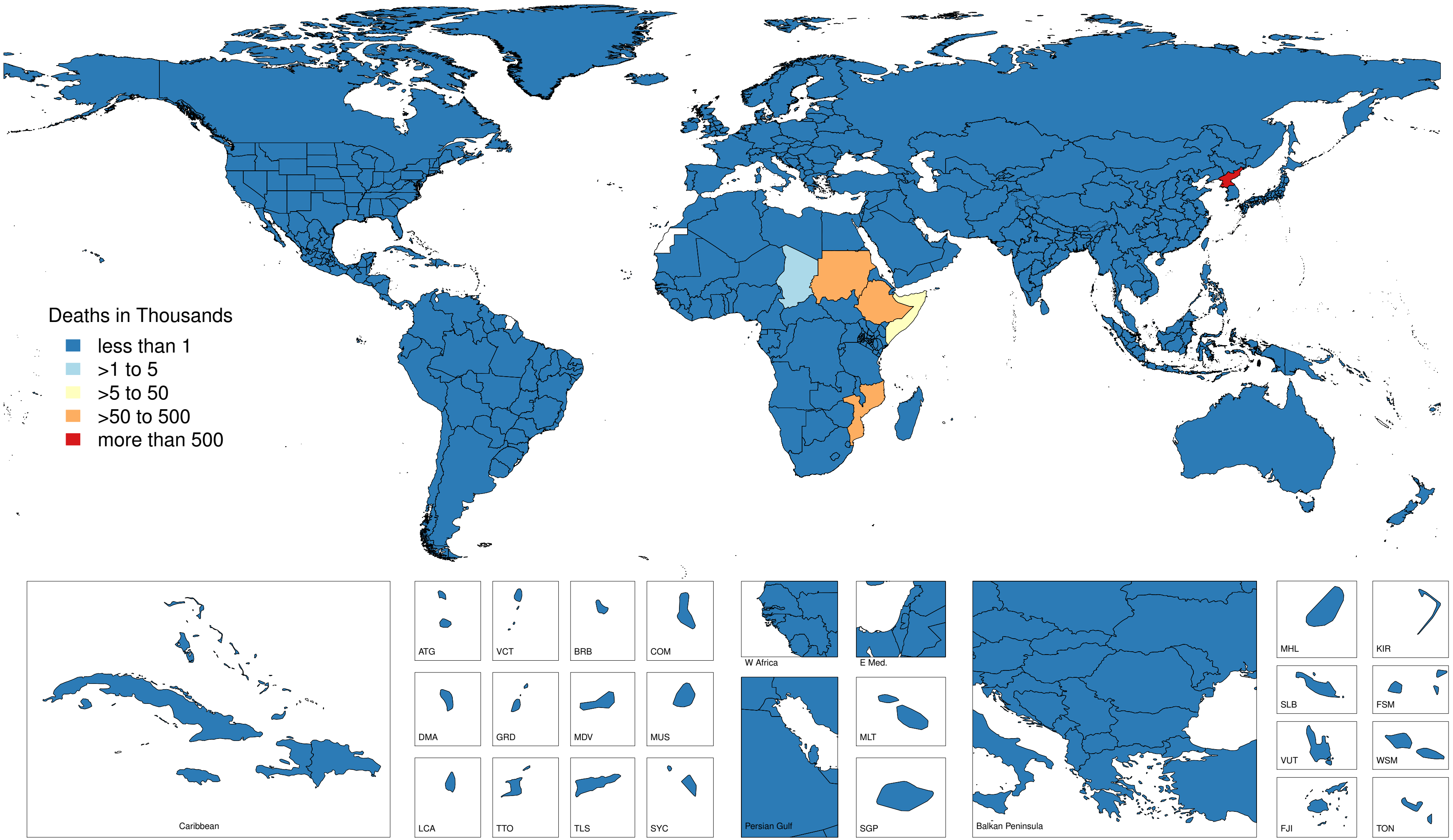

SR Figure 4d. Death counts due to violence, 1980–2017

Causes included: Conflict and terrorism, Interpersonal violence, and Executions and police conflict

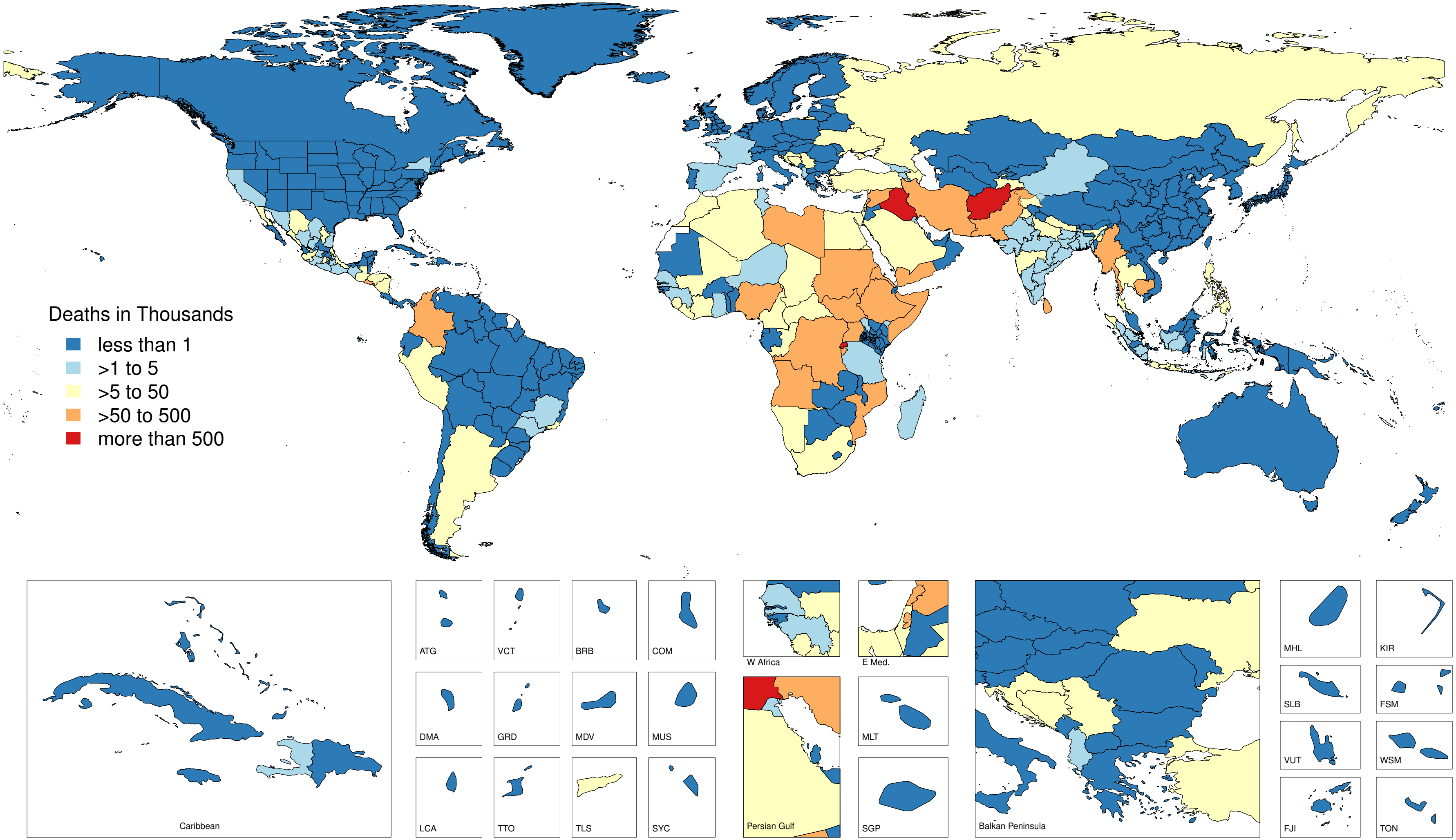

SR Figure 5. Out-of-sample model performance for CODEm models for GBD 2017 and age-standardised cause-specific mortality rate by level 2 causes

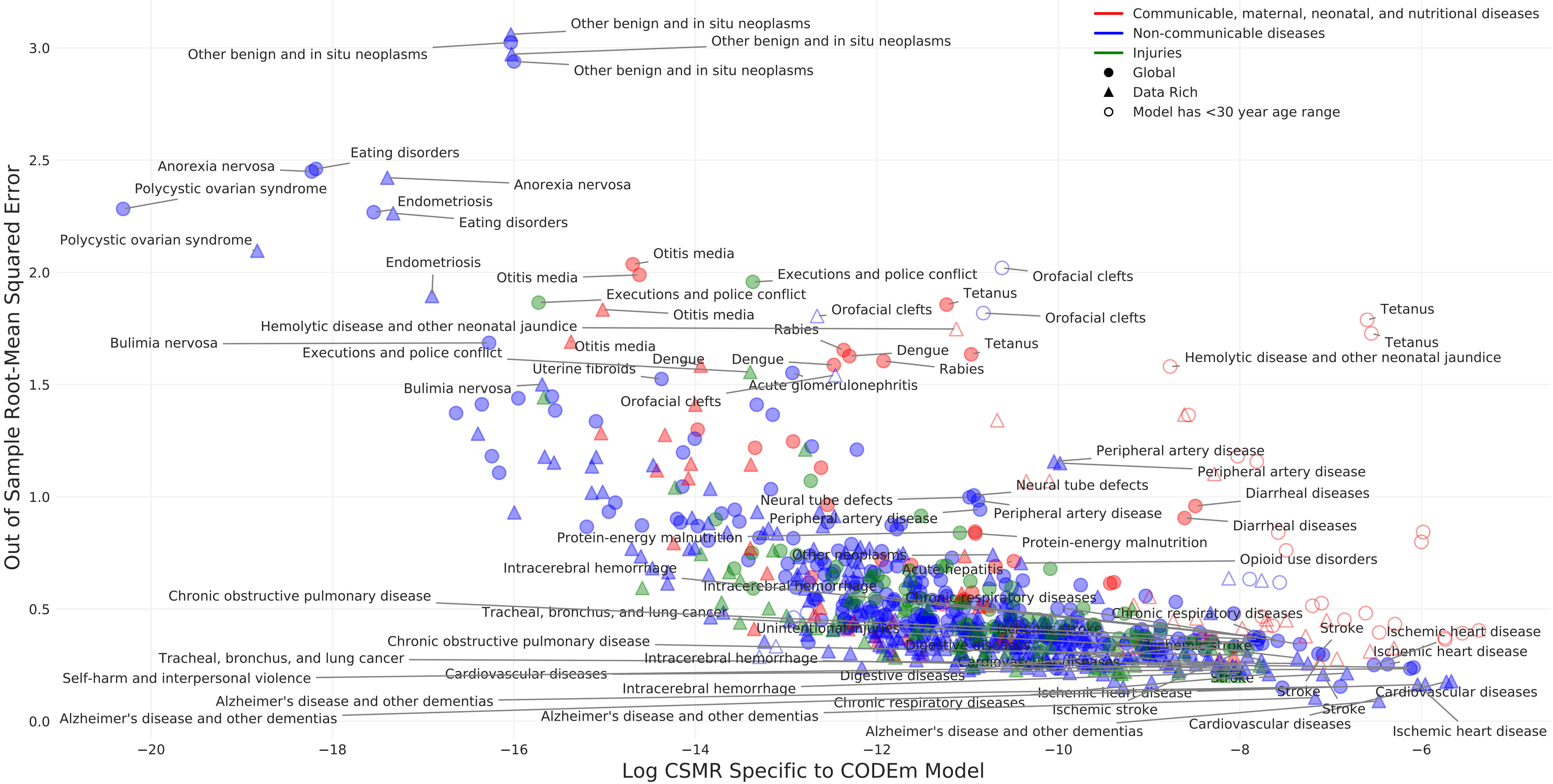

Diarrhoeal Diseases and Lower Respiratory Infection Aetiologies deaths, mortality rate and annual percent change from 1990 to 2017

Aetiology	Deaths among all ages (95% UI)	Deaths per 100,000 all ages (95% UI)	Percent change 1990-2017 all ages (95% UI)	Deaths among children under-5 (95% UI)	Deaths per 100,000 among children under-5 (95% UI)	Percent change 1990-2017 among children under-5 (95% UI)	Deaths among adults 70+ (95% UI)	Deaths per 100,000 among adults 70+ (95% UI)	Percent change 1990-2017 among adults 70+ (95% UI)
Adenovirus	72,187 (47,294-104,229)	0.9 (0.6-1.4)	-51.3 (-62.0 to -34.7)	44,620 (27,391-67,441)	6.6 (4.0-9.9)	-63.8 (-71.6 to -53.1)	16,478 (7,449-29,587)	3.8 (1.7-6.8)	37.3 (8.8 to 85.2)
Aeromonas	11,771 (4,443-24,522)	0.2 (0.1-0.3)	-73.0 (-83.1 to -51.2)	5,584 (2,083-11,160)	0.8 (0.3-1.6)	-83.7 (-90.1 to -72.0)	3,154 (626-9,326)	0.7 (0.1-2.2)	-28.6 (-55.0 to 18.4)
Campylobacter	89,640 (38,743-159,736)	1.2 (0.5-2.1)	-59.2 (-68.0 to -47.0)	67,300 (29,723-117,221)	9.9 (4.4-17.2)	-66.0 (-73.5 to -55.2)	9,806 (1,396-28,586)	2.3 (0.3-6.6)	30.5 (3.3 to 70.5)
Cholera	13,631 (10,884-21,142)	0.2 (0.1-0.3)	-75.3 (-77.6 to -72.6)	4,606 (3,462-6,259)	0.7 (0.5-0.9)	-80.9 (-82.7 to -79.1)	571 (431-896)	0.1 (0.1-0.2)	-68.8 (-72.0 to -66.2)
Clostridium difficile	25,392 (24,137-26,534)	0.3 (0.3-0.3)	679.0 (648.1 to 717.0)	434 (345-540)	0.1 (0.1-0.1)	231.9 (205.3 to 261.7)	21,381 (20,158-22,475)	4.9 (4.7-5.2)	779.9 (736.7 to 831.0)
Cryptosporidium	114,863 (69,289-172,689)	1.5 (0.9-2.3)	-42.8 (-55.1 to -22.7)	92,633 (52,786-143,773)	13.6 (7.8-21.1)	-48.7 (-60.3 to -29.6)	8,495 (3,086-20,492)	2.0 (0.7-4.7)	26.3 (-7.1 to 75.3)
Entamoeba	53,894 (14,373-131,680)	0.7 (0.2-1.7)	-37.8 (-59.2 to -3.2)	15,600 (2,876-43,201)	2.3 (0.4-6.3)	-71.2 (-76.6 to -64.1)	19,749 (2,658-53,078)	4.6 (0.6-12.3)	65.4 (36.1 to 126.3)
Enteropathogenic E coli	38,833 (15,079-77,046)	0.5 (0.2-1.0)	-54.9 (-64.6 to -40.9)	33,646 (12,402-67,393)	4.9 (1.8-9.9)	-58.4 (-67.7 to -44.5)	1,691 (339-5,848)	0.4 (0.1-1.4)	23.2 (-15.2 to 81.5)
Enterotoxigenic E coli	27,817 (9,798-59,255)	0.4 (0.1-0.8)	-56.2 (-67.7 to -34.2)	15,777 (4,286-39,426)	2.3 (0.6-5.8)	-69.0 (-75.7 to -59.7)	4,834 (1,121-14,463)	1.1 (0.3-3.3)	14.0 (-17.0 to 62.5)
Non-typhoidal Salmonella	50,771 (2,825-129,736)	0.7 (0.0-1.7)	-48.3 (-72.1 to -27.9)	25,853 (2,825-60,350)	3.8 (0.4-8.9)	-66.2 (-72.9 to -57.8)	12,355 (0-40,233)	2.9 (0.0-9.3)	39.1 (0 to 42.1)
Norovirus	66,540 (9,078-173,932)	0.9 (0.1-2.3)	-44.8 (-62.3 to -27.3)	22,389 (4,722-55,806)	3.3 (0.7-8.2)	-70.4 (-75.6 to -62.9)	24,332 (1,461-70,844)	5.6 (0.3-16.4)	20.1 (-6.5 to 55.6)
Rotavirus	329,360 (259,772-424,023)	4.3 (3.4-5.5)	-46.1 (-57.1 to -30.9)	185,390 (145,565-224,346)	27.2 (21.4-33.0)	-63.7 (-70.8 to -53.8)	86,608 (50,100-138,291)	20.0 (11.6-32.0)	101.4 (66.3 to 170.4)
Shigella	237,846 (151,304-355,664)	3.1 (2.0-4.7)	-42.7 (-54.2 to -25.3)	95,861 (57,479-155,566)	14.1 (8.4-22.9)	-65.9 (-72.0 to -57.3)	77,511 (42,984-127,489)	17.9 (9.9-29.5)	29.1 (8.9 to 68.2)

Diarrhoeal Diseases and Lower Respiratory Infection Aetiologies deaths, mortality rate and annual percent change from 1990 to 2017

Aetiology	Deaths among all ages (95% UI)	Deaths per 100,000 all ages (95% UI)	Percent change 1990-2017 all ages (95% UI)	Deaths among children under-5 (95% UI)	Deaths per 100,000 among children under-5 (95% UI)	Percent change 1990-2017 among children under-5 (95% UI)	Deaths among adults 70+ (95% UI)	Deaths per 100,000 among adults 70+ (95% UI)	Percent change 1990-2017 among adults 70+ (95% UI)
H influenzae type B pneumonia	69,213 (17,552-127,174)	0.9 (0.2-1.7)	-82.5 (-85.2 to -80.0)	69,213 (17,552-127,174)	10.2 (2.6-18.7)	-82.5 (-85.2 to -80.0)	0 (0-0)	0.0 (0.0-0.0)	--
Influenza	144,505 (110,702-180,125)	1.9 (1.4-2.4)	-0.2 (-13.1 to 14.3)	23,577 (15,360-34,785)	3.5 (2.3-5.1)	-66.0 (-69.9 to -61.6)	70,912 (51,615-92,160)	16.4 (11.9-21.3)	91.1 (82.3 to 99.6)
Pneumococcal pneumonia	1,166,374 (628,827-1,736,413)	15.3 (8.2-22.7)	-39.0 (-53.7 to -19.8)	380,931 (196,142-540,312)	56.0 (28.8-79.4)	-71.2 (-75.1 to -67.1)	456,096 (166,041-866,727)	105.4 (38.4-200.2)	60.4 (39.7 to 79.9)
Respiratory syncytial virus pneumonia	187,614 (139,944-247,475)	2.5 (1.8-3.2)	-45.9 (-52.5 to -37.0)	104,410 (66,220-155,448)	15.3 (9.7-22.8)	-64.2 (-68.2 to -59.4)	51,630 (39,232-67,695)	11.9 (9.1-15.6)	100.3 (92.4 to 108.6)

SR Table 2a: Percent change in age-standardized mortality of Communicable, maternal, neonatal, and nutritional disorders, by SDI and country

Location	2003-2007	2008-2012	2013-2017
Low SDI	-12.9 (-14.1 to -11.6)	-15.5 (-16.7 to -14.0)	-13.9 (-15.5 to -12.1)
Afghanistan	-14.8 (-20.9 to -9.7)	-19.1 (-24.4 to -13.4)	-12.3 (-17.7 to -5.3)
Bangladesh	-23.3 (-28.0 to -18.4)	-20.4 (-25.6 to -14.9)	-13.5 (-19.6 to -6.8)
Benin	-5.1 (-9.7 to -0.9)	-12.5 (-17.0 to -7.8)	-11.3 (-16.0 to -6.2)
Burkina Faso	-12.7 (-16.4 to -8.9)	-13.7 (-17.7 to -9.3)	-13.7 (-18.4 to -8.6)
Burundi	-21.2 (-24.9 to -17.2)	-20.1 (-23.9 to -16.5)	-9.4 (-13.5 to -5.0)
Central African Republic	-5.8 (-10.0 to -1.5)	-14.8 (-18.8 to -10.3)	-11.0 (-15.7 to -5.9)
Chad	-10.6 (-14.1 to -6.4)	-13.6 (-17.6 to -9.1)	-11.7 (-16.2 to -7.1)
Comoros	-13.8 (-18.0 to -9.1)	-11.4 (-16.0 to -6.7)	-8.9 (-13.3 to -4.6)
Cote d'Ivoire	-12.1 (-15.7 to -8.3)	-15.6 (-19.1 to -12.0)	-19.1 (-23.4 to -14.1)
DR Congo	-7.8 (-11.4 to -4.0)	-12.9 (-16.8 to -9.0)	-17.3 (-21.5 to -12.9)
Eritrea	-8.5 (-12.4 to -4.1)	-12.5 (-16.8 to -8.2)	-13.6 (-17.7 to -9.6)
Ethiopia	-24.2 (-26.3 to -22.3)	-26.8 (-28.9 to -24.5)	-18.4 (-21.0 to -15.7)
Guinea	-5.5 (-10.0 to -0.7)	-10.5 (-14.7 to -5.8)	-12.4 (-16.9 to -7.3)
Guinea-Bissau	-6.4 (-11.2 to -1.9)	-11.9 (-16.2 to -7.3)	-14.7 (-18.9 to -10.4)
Haiti	-14.4 (-18.3 to -10.0)	-14.0 (-18.3 to -9.2)	-19.5 (-24.4 to -14.5)
Kiribati	-8.0 (-12.8 to -3.2)	-6.2 (-11.4 to -0.7)	-7.2 (-11.9 to -2.1)
Liberia	-16.9 (-21.4 to -12.2)	-15.1 (-19.3 to -10.7)	-9.6 (-14.3 to -4.6)
Madagascar	-4.1 (-9.3 to 1.2)	-4.7 (-10.6 to 1.3)	-7.7 (-12.7 to -2.3)
Malawi	-16.8 (-20.2 to -12.2)	-37.1 (-40.3 to -32.4)	-22.8 (-26.8 to -18.8)
Mali	-12.6 (-16.4 to -8.5)	-10.0 (-14.6 to -5.2)	-11.4 (-16.5 to -5.3)
Mozambique	0.1 (-3.2 to 3.5)	-7.2 (-10.2 to -4.0)	-26.9 (-30.5 to -23.1)
Nepal	-13.5 (-18.5 to -7.1)	-15.0 (-19.8 to -9.8)	-14.1 (-19.0 to -9.3)
Niger	-12.8 (-18.2 to -7.9)	-9.5 (-14.2 to -4.1)	-12.7 (-17.8 to -7.4)
Papua New Guinea	0.0 (-4.9 to 5.3)	-9.1 (-13.7 to -4.6)	-11.1 (-15.3 to -6.7)
Rwanda	-33.8 (-37.4 to -29.3)	-27.9 (-31.2 to -24.6)	-12.1 (-16.0 to -8.3)
Senegal	-15.4 (-18.7 to -11.7)	-12.5 (-16.3 to -8.4)	-10.3 (-14.0 to -6.2)
Sierra Leone	-5.2 (-9.5 to -0.7)	-15.3 (-19.4 to -11.2)	-13.1 (-17.9 to -7.9)
Solomon Islands	-3.7 (-8.0 to 0.6)	-6.8 (-11.0 to -2.3)	-6.1 (-10.5 to -1.1)
Somalia	-10.3 (-16.1 to -4.2)	-9.8 (-15.1 to -3.9)	-9.8 (-15.8 to -3.8)
South Sudan	-8.1 (-14.5 to -1.6)	-6.9 (-11.7 to -1.6)	-5.8 (-12.2 to 1.1)
Tanzania	-16.3 (-20.6 to -12.3)	-23.6 (-27.6 to -19.6)	-22.6 (-27.5 to -17.5)
The Gambia	-6.8 (-11.4 to -1.8)	-10.8 (-14.7 to -6.5)	-10.2 (-14.4 to -5.9)
Togo	-4.3 (-8.1 to 0.1)	-17.1 (-21.1 to -12.9)	-19.2 (-23.5 to -15.0)
Uganda	-21.3 (-24.0 to -18.5)	-27.0 (-30.2 to -23.7)	-20.6 (-25.0 to -16.8)
Yemen	-20.1 (-25.7 to -14.7)	-15.1 (-21.7 to -7.6)	-8.7 (-15.6 to -1.4)
Low-middle SDI	-11.2 (-12.6 to -9.8)	-16.1 (-17.7 to -14.5)	-13.5 (-15.5 to -11.4)

SR Table 2a: Percent change in age-standardized mortality of Communicable, maternal, neonatal, and nutritional disorders, by SDI and country

Location	2003-2007	2008-2012	2013-2017
Angola	-14.6<br>(-18.8 to -10.1)	-13.5<br>(-17.5 to -9.4)	-13.5<br>(-17.7 to -8.8)
Belize	-19.2<br>(-22.2 to -16.1)	-3.3<br>(-7.2 to 1.0)	-2.4<br>(-6.7 to 2.6)
Bhutan	-20.1<br>(-25.4 to -14.2)	-22.8<br>(-28.6 to -15.8)	-14.1<br>(-19.7 to -7.8)
Bolivia	-14.0<br>(-19.2 to -8.2)	-14.9<br>(-20.5 to -8.1)	-11.0<br>(-17.6 to -3.8)
Cambodia	-22.9<br>(-26.7 to -19.0)	-20.2<br>(-24.2 to -15.8)	-13.4<br>(-17.5 to -9.1)
Cameroon	-6.2<br>(-9.8 to -2.4)	-16.0<br>(-19.6 to -12.3)	-18.6<br>(-22.5 to -14.4)
Cape Verde	-14.9<br>(-19.2 to -10.3)	-14.3<br>(-18.5 to -10.0)	-12.6<br>(-17.3 to -7.5)
Congo (Brazzaville)	-15.9<br>(-19.5 to -12.2)	-16.4<br>(-20.0 to -12.6)	-9.0<br>(-12.9 to -5.1)
Djibouti	-5.3<br>(-11.8 to 1.1)	-15.4<br>(-21.2 to -8.8)	-16.9<br>(-22.2 to -11.1)
Dominican Republic	-2.4<br>(-10.5 to 5.8)	-7.1<br>(-15.1 to 1.9)	-11.2<br>(-21.3 to -0.2)
Egypt	-15.6<br>(-24.2 to -6.3)	-21.1<br>(-28.6 to -12.5)	-11.4<br>(-20.9 to -0.1)
El Salvador	-6.8<br>(-11.9 to -1.6)	-9.9<br>(-15.1 to -4.6)	-4.4<br>(-13.8 to 5.7)
Federated States of Micronesia	-5.5<br>(-12.0 to 5.2)	-0.5<br>(-9.6 to 16.3)	5.6<br>(-7.8 to 30.2)
Ghana	-7.0<br>(-10.4 to -3.6)	-15.5<br>(-18.7 to -11.9)	-16.3<br>(-19.8 to -12.9)
Guatemala	-20.9<br>(-24.0 to -17.4)	-19.6<br>(-23.0 to -15.9)	-3.7<br>(-11.4 to 5.1)
Guyana	-15.1<br>(-18.2 to -11.6)	-1.9<br>(-6.0 to 2.5)	-10.5<br>(-16.0 to -4.2)
Honduras	-14.7<br>(-21.9 to -6.9)	-15.9<br>(-23.1 to -8.0)	-8.8<br>(-16.7 to 0.6)
India	-11.8<br>(-13.7 to -10.0)	-14.4<br>(-16.2 to -12.4)	-11.7<br>(-14.0 to -9.2)
Iraq	-14.7<br>(-22.3 to -7.3)	-22.9<br>(-29.4 to -15.9)	-18.4<br>(-25.3 to -10.2)
Kenya	-14.9<br>(-16.4 to -13.4)	-24.2<br>(-25.7 to -22.7)	-16.5<br>(-18.0 to -14.6)
Kyrgyzstan	-14.2<br>(-19.4 to -8.9)	-25.3<br>(-27.8 to -22.8)	-15.7<br>(-20.3 to -10.4)
Laos	-14.7<br>(-19.8 to -8.0)	-17.9<br>(-23.6 to -11.0)	-17.3<br>(-22.5 to -11.6)
Lesotho	3.6<br>(-0.9 to 9.0)	-21.9<br>(-25.1 to -18.8)	-31.7<br>(-36.5 to -26.1)
Marshall Islands	-8.2<br>(-12.8 to -3.5)	-9.7<br>(-14.7 to -4.1)	-8.7<br>(-13.9 to -2.2)
Mauritania	-16.2<br>(-20.2 to -12.2)	-14.7<br>(-19.2 to -10.2)	-10.5<br>(-15.0 to -5.6)
Morocco	-16.1<br>(-21.4 to -10.6)	-16.0<br>(-21.7 to -9.4)	-16.8<br>(-23.0 to -10.9)
Myanmar	-16.6<br>(-21.0 to -12.3)	-21.5<br>(-25.8 to -17.1)	-21.6<br>(-26.4 to -16.7)
Nicaragua	-18.4<br>(-24.1 to -12.3)	-10.2<br>(-16.5 to -3.3)	-11.0<br>(-17.3 to -4.5)
Nigeria	-11.4<br>(-15.9 to -7.4)	-15.5<br>(-19.4 to -11.2)	-16.2<br>(-20.7 to -11.2)
North Korea	-10.1<br>(-16.9 to -2.8)	-9.0<br>(-15.9 to -1.0)	-8.5<br>(-16.1 to 1.2)
Pakistan	-9.8<br>(-15.2 to -4.2)	-13.2<br>(-19.0 to -6.9)	-13.9<br>(-19.7 to -7.5)
Palestine	-8.5<br>(-13.6 to -2.6)	-15.8<br>(-20.8 to -10.7)	-14.5<br>(-20.1 to -8.7)
Samoa	-3.5<br>(-9.2 to 2.7)	0.3<br>(-6.8 to 7.9)	-2.1<br>(-9.7 to 6.7)
Sao Tome and Principe	-12.6<br>(-16.8 to -8.4)	-10.2<br>(-15.0 to -4.8)	-9.7<br>(-14.3 to -5.5)
Sudan	-17.1<br>(-22.3 to -11.4)	-12.1<br>(-17.6 to -6.3)	-14.6<br>(-20.5 to -8.1)
Swaziland	3.0<br>(-1.4 to 8.6)	-24.1<br>(-27.2 to -20.7)	-50.2<br>(-56.3 to -42.8)
Tajikistan	-15.6<br>(-21.2 to -9.2)	-6.9<br>(-14.0 to 1.2)	-8.7<br>(-16.5 to -0.6)

SR Table 2a: Percent change in age-standardized mortality of Communicable, maternal, neonatal, and nutritional disorders, by SDI and country			
Location	2003-2007	2008-2012	2013-2017
Timor-Leste	-20.3 (-25.1 to -14.3)	-16.4 (-21.7 to -10.3)	-6.7 (-11.7 to -1.2)
Vanuatu	-4.8 (-10.5 to 1.9)	-5.7 (-11.8 to 1.4)	-5.2 (-11.4 to 1.9)
Zambia	-19.0 (-21.9 to -15.4)	-31.7 (-34.7 to -28.2)	-21.9 (-26.3 to -17.6)
Zimbabwe	-4.2 (-9.6 to 1.2)	-43.7 (-49.0 to -36.7)	-28.6 (-33.5 to -23.9)
Middle SDI	-11.8 (-12.8 to -10.8)	-15.4 (-16.4 to -14.3)	-15.6 (-16.7 to -14.3)
Albania	-24.7 (-31.1 to -16.5)	-20.0 (-28.1 to -10.7)	-7.9 (-17.0 to 2.7)
Algeria	-11.2 (-16.4 to -5.6)	-15.5 (-21.3 to -9.0)	-14.4 (-20.1 to -8.1)
Botswana	-20.9 (-26.1 to -12.3)	-36.2 (-40.3 to -32.1)	-35.3 (-42.1 to -28.0)
Brazil	-12.5 (-13.9 to -11.1)	-7.5 (-9.3 to -5.9)	-6.1 (-7.7 to -4.5)
Colombia	-13.0 (-18.3 to -7.3)	-16.1 (-21.9 to -9.8)	-7.1 (-15.5 to 2.4)
Costa Rica	-19.0 (-22.5 to -15.3)	0.4 (-3.1 to 3.9)	-3.8 (-11.5 to 5.0)
Cuba	-12.1 (-14.6 to -9.7)	-5.2 (-7.8 to -2.5)	1.0 (-7.3 to 9.3)
Dominica	-3.1 (-8.5 to 2.8)	14.0 (6.4 to 22.6)	2.7 (-5.4 to 11.2)
Ecuador	-8.7 (-17.5 to 0.3)	-16.7 (-24.6 to -8.2)	-7.4 (-17.3 to 3.9)
Equatorial Guinea	-11.2 (-17.6 to -4.4)	-2.6 (-9.6 to 5.2)	-16.0 (-22.7 to -7.6)
Fiji	-10.1 (-14.9 to -5.1)	-8.7 (-13.5 to -3.5)	-4.3 (-11.3 to 3.0)
Gabon	-11.9 (-16.7 to -7.0)	-18.2 (-22.8 to -13.4)	-26.6 (-31.9 to -21.1)
Grenada	-10.9 (-15.1 to -6.5)	-0.7 (-5.4 to 4.1)	-3.9 (-9.2 to 1.7)
Indonesia	-11.2 (-13.5 to -8.7)	-12.7 (-15.0 to -10.2)	-10.7 (-12.9 to -8.5)
Jamaica	-16.5 (-21.3 to -11.5)	-2.2 (-8.8 to 5.5)	-2.5 (-9.9 to 5.4)
Jordan	-16.1 (-21.5 to -10.4)	-13.3 (-19.4 to -6.7)	-7.2 (-14.2 to 0.8)
Maldives	-28.2 (-32.7 to -23.8)	-26.4 (-30.5 to -21.9)	-13.5 (-18.5 to -8.4)
Mexico	-19.8 (-21.2 to -18.4)	-4.9 (-6.2 to -3.6)	-7.7 (-10.0 to -5.6)
Moldova	-2.6 (-7.2 to 2.4)	-16.3 (-21.4 to -10.4)	-8.5 (-14.9 to -1.5)
Mongolia	-24.4 (-29.2 to -18.8)	-21.2 (-27.4 to -14.7)	-13.9 (-21.2 to -6.2)
Namibia	-6.2 (-10.3 to -1.6)	-35.3 (-39.2 to -31.0)	-20.5 (-25.0 to -15.6)
Panama	1.5 (-4.3 to 7.2)	-2.7 (-8.5 to 3.2)	-11.3 (-18.8 to -3.0)
Paraguay	-11.1 (-17.1 to -4.9)	-6.6 (-13.8 to 0.5)	-14.1 (-22.1 to -5.2)
Peru	-16.6 (-22.0 to -11.2)	-8.0 (-14.3 to -1.2)	-15.4 (-24.3 to -5.2)
Philippines	-4.9 (-9.6 to 0.4)	-3.0 (-8.3 to 2.6)	-6.2 (-14.4 to 2.8)
Saint Lucia	-9.7 (-14.6 to -4.1)	-3.1 (-8.9 to 3.8)	-4.4 (-9.8 to 2.3)
Saint Vincent and the Grenadines	-11.2 (-15.2 to -7.1)	-4.9 (-9.7 to 0.3)	-2.9 (-7.9 to 2.6)
Seychelles	1.0 (-3.5 to 5.6)	2.1 (-2.8 to 6.8)	-3.1 (-7.2 to 1.6)
South Africa	12.8 (10.4 to 15.1)	-27.2 (-29.0 to -25.4)	-34.3 (-37.0 to -31.7)
Sri Lanka	-19.2 (-22.2 to -15.4)	-8.5 (-12.9 to -3.6)	-10.6 (-21.3 to 1.0)
Suriname	-13.0 (-17.6 to -7.9)	-10.1 (-14.8 to -4.9)	-1.7 (-7.7 to 4.3)
Syria	-2.6 (-12.9 to 5.7)	4.9 (-3.2 to 13.9)	-13.7 (-22.3 to -2.9)

SR Table 2a: Percent change in age-standardized mortality of Communicable, maternal, neonatal, and nutritional disorders, by SDI and country

Location	2003-2007	2008-2012	2013-2017
Thailand	-22.3 (-29.0 to -13.0)	-7.1 (-17.5 to 1.2)	-0.8 (-8.1 to 6.4)
Tonga	-6.4 (-11.5 to -1.3)	-8.7 (-15.9 to -1.7)	-4.5 (-10.4 to 1.4)
Trinidad and Tobago	-28.0 (-32.4 to -23.2)	-11.1 (-17.7 to -3.0)	-1.3 (-10.5 to 8.4)
Tunisia	-12.0 (-18.3 to -5.2)	-9.5 (-16.3 to -2.2)	-10.9 (-18.1 to -3.5)
Turkmenistan	-16.7 (-22.8 to -10.2)	-27.9 (-34.5 to -20.9)	-15.6 (-23.1 to -7.3)
Uzbekistan	-10.5 (-17.3 to -3.3)	-15.0 (-21.6 to -7.5)	-12.8 (-20.4 to -3.8)
Venezuela	-20.1 (-22.5 to -17.5)	-2.6 (-5.4 to 0.2)	-4.6 (-14.8 to 8.0)
Vietnam	-12.5 (-17.1 to -7.8)	-12.0 (-16.6 to -7.2)	-11.6 (-16.2 to -7.2)
High-middle SDI	-17.5 (-18.6 to -16.3)	-10.9 (-12.1 to -9.8)	-13.0 (-14.3 to -11.8)
American Samoa	-6.8 (-11.3 to -1.9)	-3.3 (-8.1 to 1.6)	-2.3 (-7.3 to 3.3)
Antigua and Barbuda	-11.8 (-15.5 to -7.7)	-5.3 (-10.0 to -0.8)	-1.6 (-7.0 to 4.4)
Argentina	-2.0 (-4.4 to 0.2)	-0.9 (-3.3 to 1.5)	-5.3 (-12.2 to 2.3)
Armenia	-25.4 (-30.6 to -20.1)	-11.0 (-14.5 to -7.6)	-5.2 (-10.9 to 1.4)
Azerbaijan	-20.1 (-26.4 to -13.6)	-15.2 (-22.9 to -6.5)	-11.0 (-19.5 to -1.2)
Bahrain	-11.3 (-18.7 to -6.4)	-22.8 (-27.2 to -15.0)	-11.8 (-17.2 to -5.9)
Barbados	-3.6 (-7.4 to 0.5)	-7.0 (-11.8 to -1.9)	-0.6 (-5.7 to 5.1)
Belarus	-12.9 (-16.9 to -8.7)	-15.0 (-19.2 to -10.3)	-13.7 (-19.4 to -7.5)
Bermuda	-19.4 (-22.6 to -16.0)	-10.9 (-14.8 to -6.8)	-0.7 (-5.1 to 4.3)
Bosnia and Herzegovina	-11.0 (-17.5 to -5.0)	-10.8 (-16.4 to -4.5)	-1.4 (-9.6 to 7.1)
Bulgaria	-11.7 (-15.8 to -7.4)	-12.5 (-16.2 to -8.6)	-7.9 (-16.9 to 3.1)
Chile	-13.7 (-15.6 to -11.6)	-3.8 (-6.0 to -1.7)	-5.8 (-13.9 to 2.9)
China	-28.5 (-30.4 to -26.2)	-20.6 (-22.6 to -18.7)	-20.8 (-22.8 to -19.0)
Georgia	-30.0 (-35.3 to -24.2)	-11.7 (-18.8 to -3.3)	-7.8 (-14.0 to -1.0)
Greenland	-6.2 (-10.7 to -1.2)	-5.9 (-11.1 to -0.7)	-11.2 (-17.1 to -5.4)
Guam	-2.5 (-7.7 to 2.7)	-0.5 (-5.7 to 5.3)	4.4 (-1.5 to 10.2)
Hungary	-15.2 (-18.8 to -11.7)	-3.9 (-8.6 to 1.2)	-14.3 (-23.9 to -3.2)
Iran	-10.8 (-13.9 to -8.1)	-12.0 (-14.2 to -9.6)	-27.6 (-29.8 to -24.8)
Israel	1.7 (-2.3 to 5.4)	2.6 (-1.2 to 6.6)	-6.8 (-12.1 to -1.4)
Kazakhstan	-16.0 (-20.4 to -11.5)	-24.4 (-28.7 to -19.5)	-13.7 (-18.9 to -7.7)
Kuwait	5.6 (1.2 to 10.1)	2.3 (-2.7 to 8.0)	-5.5 (-13.0 to 2.7)
Lebanon	-10.2 (-16.3 to -2.6)	-15.0 (-21.7 to -7.5)	-3.2 (-10.0 to 3.7)
Libya	-21.2 (-26.2 to -15.5)	-4.6 (-10.4 to 1.7)	1.9 (-4.5 to 8.7)
Macedonia	-20.0 (-25.8 to -13.5)	-14.5 (-20.8 to -7.6)	-7.8 (-18.7 to 4.3)
Malaysia	-1.6 (-11.1 to 2.7)	4.0 (-6.6 to 8.3)	-1.3 (-8.8 to 6.4)
Mauritius	1.7 (-3.6 to 7.6)	5.9 (0.9 to 11.2)	-4.3 (-10.7 to 2.7)
Montenegro	-18.5 (-24.5 to -11.9)	-21.7 (-27.2 to -15.6)	-10.4 (-17.5 to -2.0)
Northern Mariana Islands	-7.1 (-11.9 to -1.8)	-2.8 (-8.9 to 4.9)	-2.0 (-8.3 to 5.1)

SR Table 2a: Percent change in age-standardized mortality of Communicable, maternal, neonatal, and nutritional disorders, by SDI and country			
**Location**	**2003-2007**	**2008-2012**	**2013-2017**
Oman	-9.2 (-14.6 to -3.6)	3.9 (-1.7 to 10.0)	-8.8 (-13.8 to -3.5)
Portugal	-7.7 (-10.1 to -5.0)	-7.8 (-10.6 to -5.1)	-6.7 (-12.0 to -1.6)
Puerto Rico	-11.1 (-14.6 to -7.6)	-15.3 (-18.4 to -12.2)	0.0 (-5.2 to 5.7)
Qatar	-11.1 (-17.0 to -4.9)	-5.0 (-13.9 to 2.4)	-9.2 (-15.2 to -2.5)
Romania	-22.7 (-25.1 to -20.3)	-11.1 (-13.8 to -8.4)	-4.1 (-9.9 to 2.7)
Russian Federation	-12.9 (-14.3 to -11.3)	-9.7 (-11.0 to -8.2)	-10.4 (-11.7 to -9.3)
Saudi Arabia	-13.6 (-19.4 to -7.5)	-12.1 (-20.0 to -4.7)	-15.6 (-22.0 to -6.8)
Serbia	-30.2 (-38.3 to -21.7)	-2.5 (-10.2 to 3.5)	-2.5 (-10.2 to 6.1)
The Bahamas	-19.5 (-21.8 to -16.9)	-5.8 (-8.9 to -2.2)	-2.7 (-6.7 to 2.0)
Turkey	-34.3 (-40.5 to -26.7)	-12.0 (-20.7 to -2.1)	-9.7 (-17.7 to -0.7)
Ukraine	13.8 (11.5 to 16.1)	-20.8 (-23.1 to -18.5)	8.1 (2.5 to 14.0)
United Arab Emirates	-3.5 (-9.3 to 2.7)	-0.1 (-7.1 to 12.8)	-0.5 (-10.2 to 43.4)
Uruguay	-7.6 (-10.7 to -4.2)	-2.1 (-5.1 to 1.1)	-6.2 (-13.8 to 1.4)
Virgin Islands	-11.7 (-15.8 to -7.6)	0.8 (-4.7 to 6.8)	-2.1 (-8.2 to 4.5)
High SDI	-8.8 (-9.4 to -8.1)	-6.8 (-7.4 to -6.2)	-3.0 (-4.2 to -1.7)
Andorra	-4.4 (-11.4 to 3.4)	-4.4 (-10.9 to 2.6)	-5.2 (-11.3 to 0.9)
Australia	-11.3 (-14.1 to -8.2)	-6.1 (-9.4 to -2.9)	0.3 (-9.3 to 10.1)
Austria	-14.2 (-19.2 to -9.3)	-13.8 (-18.3 to -8.9)	-5.6 (-13.9 to 3.3)
Belgium	-9.9 (-13.0 to -7.0)	-6.2 (-9.0 to -3.4)	-8.1 (-13.6 to -2.0)
Brunei	-0.4 (-7.4 to 5.5)	7.5 (-1.1 to 14.7)	-3.1 (-9.2 to 4.1)
Canada	-1.4 (-4.2 to 1.4)	-5.5 (-8.3 to -2.7)	-4.3 (-11.8 to 4.1)
Croatia	-24.5 (-27.3 to -21.3)	-31.5 (-34.9 to -28.2)	-3.2 (-10.3 to 4.0)
Cyprus	-17.7 (-22.5 to -12.7)	-18.8 (-23.5 to -14.4)	-1.2 (-7.1 to 5.5)
Czech Republic	-2.8 (-6.3 to 1.5)	-2.8 (-5.8 to 0.1)	-4.3 (-10.1 to 1.5)
Denmark	5.6 (2.0 to 9.6)	-5.5 (-8.9 to -2.1)	-5.9 (-11.7 to 0.4)
Estonia	-20.4 (-24.1 to -16.5)	-15.4 (-18.8 to -12.0)	-11.3 (-19.0 to -3.4)
Finland	-45.8 (-48.1 to -43.6)	-25.3 (-28.6 to -21.9)	2.6 (-4.7 to 10.4)
France	-15.6 (-18.3 to -12.9)	-8.9 (-11.4 to -6.4)	-7.2 (-13.0 to -0.9)
Germany	-1.2 (-4.6 to 2.3)	-4.5 (-7.4 to -1.6)	-2.7 (-10.6 to 5.3)
Greece	-11.4 (-14.9 to -7.6)	-5.4 (-9.1 to -1.2)	7.4 (-0.4 to 16.5)
Iceland	-10.7 (-14.5 to -7.1)	-9.4 (-12.8 to -5.5)	0.3 (-4.6 to 4.9)
Ireland	-29.4 (-32.0 to -26.8)	-18.0 (-20.9 to -14.9)	-1.0 (-7.4 to 5.6)
Italy	-13.9 (-16.8 to -11.1)	0.0 (-3.0 to 3.5)	-7.1 (-13.7 to 0.6)
Japan	-7.5 (-8.4 to -6.6)	-6.0 (-7.2 to -4.9)	-4.0 (-6.6 to -1.1)
Latvia	-7.9 (-11.7 to -4.0)	-9.4 (-12.5 to -6.2)	-13.5 (-19.9 to -6.5)
Lithuania	11.3 (6.2 to 16.0)	-19.9 (-22.6 to -16.8)	-4.7 (-10.3 to 1.7)
Luxembourg	-10.9 (-14.4 to -7.3)	-6.5 (-9.9 to -2.4)	-3.4 (-10.1 to 5.4)

SR Table 2a: Percent change in age-standardized mortality of Communicable, maternal, neonatal, and nutritional disorders, by SDI and country

Location	2003-2007	2008-2012	2013-2017
Malta	-9.3 (-12.7 to -5.4)	-8.6 (-12.5 to -4.3)	-2.4 (-8.2 to 3.6)
Netherlands	-14.0 (-16.6 to -11.2)	-13.1 (-15.6 to -10.6)	-6.4 (-12.1 to 0.1)
New Zealand	-6.8 (-10.6 to -2.6)	4.9 (0.6 to 9.2)	-10.5 (-15.2 to -5.5)
Norway	-7.3 (-8.9 to -5.9)	-5.3 (-7.0 to -3.8)	-6.8 (-9.2 to -4.3)
Poland	-3.8 (-6.3 to -1.3)	-7.6 (-10.0 to -5.0)	-8.6 (-14.4 to -2.1)
Singapore	-8.2 (-12.2 to -4.2)	-4.9 (-8.0 to -1.7)	-6.1 (-11.9 to 0.4)
Slovakia	-5.9 (-10.2 to -1.9)	-10.7 (-14.6 to -6.8)	-7.8 (-14.9 to 0.0)
Slovenia	-23.1 (-26.1 to -19.9)	-14.8 (-18.2 to -11.1)	-11.1 (-16.5 to -5.3)
South Korea	-13.2 (-15.4 to -11.1)	7.8 (4.7 to 11.2)	-6.8 (-12.6 to -1.1)
Spain	-11.5 (-14.2 to -8.7)	-13.5 (-15.9 to -11.1)	-2.6 (-8.4 to 3.5)
Sweden	-7.3 (-10.6 to -4.1)	1.9 (-1.4 to 5.7)	-5.6 (-10.2 to -0.7)
Switzerland	-13.2 (-17.8 to -8.1)	-5.4 (-9.1 to -1.3)	-6.2 (-13.8 to 3.0)
Taiwan (Province of China)	0.1 (-2.5 to 3.0)	2.3 (-0.6 to 5.3)	2.1 (-3.0 to 7.6)
USA	-8.5 (-9.4 to -7.5)	-8.2 (-9.0 to -7.4)	-1.9 (-3.5 to -0.2)
United Kingdom	-13.6 (-14.4 to -12.7)	-9.7 (-10.8 to -8.7)	0.8 (-0.4 to 2.1)

SR Table 2: Percent change in age-standardized mortality of Injuries, by SDI and country			
**Location**	**2003-2007**	**2008-2012**	**2013-2017**
Low SDI	-5.8<br>(-7.4 to -4.2)	-4.1<br>(-5.6 to -2.6)	-4.9<br>(-7.5 to -2.2)
Afghanistan	14.2<br>(7.5 to 23.2)	0.3<br>(-4.1 to 5.0)	-30.3<br>(-35.1 to -25.9)
Bangladesh	1.3<br>(-5.2 to 8.6)	-15.4<br>(-20.4 to -10.0)	-12.7<br>(-18.3 to -6.9)
Benin	-10.9<br>(-15.5 to -5.8)	-0.7<br>(-5.9 to 5.1)	-8.5<br>(-13.5 to -3.5)
Burkina Faso	-4.9<br>(-8.9 to -0.6)	2.0<br>(-2.6 to 7.1)	-1.2<br>(-6.3 to 4.6)
Burundi	-68.6<br>(-72.1 to -64.8)	-7.3<br>(-12.7 to -2.0)	-1.9<br>(-6.6 to 3.0)
Central African Republic	7.7<br>(0.1 to 15.1)	-5.0<br>(-10.9 to 1.3)	-25.1<br>(-29.5 to -20.6)
Chad	6.4<br>(0.1 to 12.6)	-12.5<br>(-17.6 to -7.4)	-2.5<br>(-7.3 to 2.5)
Comoros	15.7<br>(9.6 to 22.1)	4.9<br>(-0.3 to 10.2)	-11.4<br>(-15.6 to -6.8)
Cote d'Ivoire	-8.7<br>(-13.5 to -3.7)	-3.6<br>(-9.0 to 2.0)	-6.5<br>(-11.7 to -0.5)
DR Congo	-12.8<br>(-17.1 to -8.3)	-5.1<br>(-10.1 to 0.5)	-5.2<br>(-10.1 to 0.3)
Eritrea	-9.0<br>(-14.2 to -3.4)	-10.2<br>(-15.3 to -5.2)	-9.8<br>(-14.6 to -4.3)
Ethiopia	-11.0<br>(-14.4 to -7.2)	-12.1<br>(-15.1 to -8.8)	-7.2<br>(-10.2 to -4.0)
Guinea	2.9<br>(-2.8 to 8.6)	-0.3<br>(-5.1 to 5.2)	-6.8<br>(-11.9 to -1.5)
Guinea-Bissau	-3.7<br>(-8.9 to 1.6)	-5.7<br>(-10.3 to -0.4)	-7.0<br>(-11.9 to -1.9)
Haiti	-4.0<br>(-9.6 to 1.6)	-10.5<br>(-15.5 to -5.3)	-6.0<br>(-11.2 to -0.4)
Kiribati	-1.4<br>(-6.9 to 3.6)	-1.9<br>(-7.2 to 3.6)	-3.8<br>(-9.3 to 1.6)
Liberia	-63.5<br>(-67.1 to -59.1)	0.8<br>(-4.3 to 6.4)	-7.2<br>(-12.1 to -2.1)
Madagascar	-10.6<br>(-15.9 to -5.0)	-1.6<br>(-7.4 to 4.0)	-3.9<br>(-9.3 to 1.6)
Malawi	-9.2<br>(-14.6 to -3.3)	-3.1<br>(-9.0 to 2.8)	-6.2<br>(-11.0 to -1.4)
Mali	-18.9<br>(-23.9 to -13.7)	-0.7<br>(-5.6 to 4.8)	-3.9<br>(-9.0 to 1.8)
Mozambique	4.3<br>(-1.9 to 10.2)	-3.8<br>(-9.6 to 2.1)	-6.4<br>(-11.9 to -0.9)
Nepal	-10.4<br>(-18.8 to -5.0)	-0.4<br>(-6.7 to 5.5)	-4.0<br>(-8.8 to 1.7)
Niger	-7.1<br>(-15.6 to 0.7)	-5.0<br>(-10.7 to 1.2)	0.4<br>(-5.6 to 8.4)
Papua New Guinea	1.9<br>(-3.5 to 7.4)	-0.3<br>(-5.4 to 5.3)	-5.4<br>(-10.6 to -0.2)
Rwanda	-18.2<br>(-24.2 to -10.7)	-8.4<br>(-13.1 to -3.5)	-7.0<br>(-11.4 to -2.0)
Senegal	-3.5<br>(-8.3 to 1.9)	-3.5<br>(-8.3 to 1.5)	-1.4<br>(-6.5 to 3.2)
Sierra Leone	2.1<br>(-3.8 to 8.2)	-2.5<br>(-7.7 to 3.4)	-3.7<br>(-8.4 to 1.0)
Solomon Islands	7.7<br>(1.9 to 13.5)	-3.9<br>(-9.1 to 1.7)	-5.2<br>(-10.4 to 0.1)
Somalia	1.1<br>(-5.1 to 7.5)	5.9<br>(0.7 to 12.4)	8.2<br>(1.4 to 16.3)
South Sudan	-5.0<br>(-12.9 to 3.7)	13.5<br>(6.1 to 22.7)	-0.5<br>(-5.8 to 4.9)
Tanzania	-3.9<br>(-9.2 to 1.8)	-1.0<br>(-6.3 to 4.2)	-2.7<br>(-7.9 to 2.4)
The Gambia	3.2<br>(-2.3 to 9.2)	-2.6<br>(-7.6 to 2.5)	-3.3<br>(-8.0 to 1.7)
Togo	0.2<br>(-7.3 to 6.6)	-5.5<br>(-10.7 to 0.1)	-7.8<br>(-12.9 to -2.7)
Uganda	-18.4<br>(-23.6 to -13.9)	-9.0<br>(-14.3 to -3.5)	-9.6<br>(-14.5 to -4.6)
Yemen	-8.1<br>(-14.4 to -0.9)	2.8<br>(-4.1 to 11.3)	62.1<br>(44.4 to 81.7)

SR Table 2: Percent change in age-standardized mortality of Injuries, by SDI and country			
Location	2003-2007	2008-2012	2013-2017
Low-middle SDI	-2.1 (-3.6 to -0.7)	-14.4 (-15.7 to -13.2)	-6.3 (-8.6 to -4.0)
Angola	-9.2 (-15.0 to -2.6)	-13.7 (-18.8 to -8.7)	-12.5 (-17.7 to -7.1)
Belize	-6.4 (-9.0 to -3.9)	-4.1 (-6.6 to -1.6)	-0.8 (-5.2 to 3.4)
Bhutan	-9.8 (-14.9 to -5.1)	-10.2 (-15.5 to -5.0)	-6.9 (-12.5 to -1.7)
Bolivia	-11.7 (-17.1 to -6.4)	-9.4 (-16.6 to -3.0)	-9.1 (-16.0 to -2.9)
Cambodia	-9.2 (-13.3 to -5.1)	-5.8 (-10.2 to -1.5)	-7.4 (-11.7 to -2.6)
Cameroon	-3.9 (-9.6 to 1.6)	-6.6 (-12.1 to -0.9)	-6.7 (-12.4 to -1.3)
Cape Verde	14.4 (8.4 to 20.7)	-1.5 (-6.5 to 4.0)	-4.7 (-10.5 to 1.3)
Congo (Brazzaville)	-7.7 (-13.8 to -0.9)	-1.5 (-6.6 to 4.2)	-4.7 (-10.6 to 1.1)
Djibouti	-7.6 (-14.8 to 0.2)	-12.6 (-19.2 to -5.1)	-6.5 (-13.5 to 1.3)
Dominican Republic	3.5 (-8.7 to 16.7)	8.9 (-3.4 to 22.8)	0.7 (-15.0 to 17.1)
Egypt	-7.7 (-10.7 to -4.4)	-2.2 (-6.4 to 1.1)	-6.6 (-15.2 to 3.1)
El Salvador	4.4 (-3.4 to 12.3)	24.6 (17.6 to 32.8)	-28.0 (-39.2 to -15.0)
Federated States of Micronesia	-2.7 (-9.1 to 3.6)	-2.8 (-12.0 to 3.8)	-2.1 (-14.7 to 5.8)
Ghana	0.9 (-4.7 to 6.3)	-1.7 (-6.2 to 3.4)	-3.1 (-8.0 to 1.6)
Guatemala	-1.3 (-2.9 to 0.3)	-8.6 (-10.1 to -7.1)	2.0 (-8.3 to 13.6)
Guyana	-1.0 (-3.5 to 1.7)	2.6 (-0.1 to 5.4)	-11.3 (-20.5 to -1.3)
Honduras	-5.2 (-16.6 to 7.5)	55.9 (39.3 to 76.6)	-40.4 (-47.2 to -33.5)
India	-3.1 (-4.8 to -1.6)	-2.5 (-4.4 to -0.2)	-6.8 (-10.3 to -3.1)
Iraq	82.3 (68.4 to 100.5)	-23.3 (-26.5 to -19.6)	24.8 (20.9 to 28.9)
Kenya	-0.6 (-3.2 to 2.2)	-8.8 (-10.8 to -6.8)	-8.2 (-9.9 to -6.3)
Kyrgyzstan	-6.1 (-10.4 to -1.9)	-16.2 (-17.8 to -14.7)	-17.1 (-20.2 to -13.4)
Laos	-10.5 (-17.3 to -5.0)	-12.4 (-18.2 to -7.4)	-11.8 (-16.8 to -7.0)
Lesotho	5.9 (-0.8 to 13.4)	-4.2 (-10.7 to 2.0)	-9.9 (-16.3 to -3.8)
Marshall Islands	-1.0 (-5.5 to 3.7)	-4.6 (-9.5 to 0.5)	-5.2 (-9.9 to 0.2)
Mauritania	-5.0 (-9.5 to -0.2)	-5.7 (-10.4 to -1.1)	-6.1 (-10.9 to -1.6)
Morocco	-9.7 (-14.8 to -4.2)	-6.7 (-12.6 to -0.4)	-7.1 (-13.3 to -0.2)
Myanmar	-7.5 (-12.7 to -2.0)	-80.4 (-82.2 to -78.6)	7.4 (2.4 to 13.2)
Nicaragua	-8.4 (-16.5 to 0.7)	-11.1 (-17.4 to -4.0)	-14.0 (-23.3 to -3.5)
Nigeria	-6.8 (-15.4 to 1.8)	-1.7 (-9.0 to 6.6)	-2.6 (-9.6 to 5.0)
North Korea	3.8 (-2.7 to 10.3)	-2.2 (-7.7 to 4.4)	-3.2 (-9.7 to 3.4)
Pakistan	-1.3 (-7.6 to 5.0)	-2.6 (-9.1 to 4.1)	-10.3 (-16.9 to -2.8)
Palestine	-21.5 (-22.9 to -20.5)	-70.8 (-72.6 to -69.8)	-4.3 (-9.0 to 0.7)
Samoa	-4.1 (-9.6 to 1.4)	11.7 (5.6 to 18.3)	-2.0 (-8.8 to 4.0)
Sao Tome and Principe	19.9 (10.6 to 27.4)	-21.0 (-26.6 to -15.4)	-5.5 (-10.3 to -0.7)
Sudan	-26.7 (-31.9 to -20.5)	-10.4 (-15.1 to -5.0)	-16.2 (-21.0 to -11.1)

SR Table 2: Percent change in age-standardized mortality of Injuries, by SDI and country			
**Location**	**2003-2007**	**2008-2012**	**2013-2017**
Swaziland	-1.6<br>(-8.7 to 4.7)	-8.0<br>(-15.1 to -1.0)	-18.1<br>(-24.9 to -10.5)
Tajikistan	-2.6<br>(-8.8 to 4.0)	-3.2<br>(-9.1 to 3.3)	-2.2<br>(-9.0 to 5.4)
Timor-Leste	-12.0<br>(-17.3 to -6.8)	-5.8<br>(-12.2 to 0.3)	-0.3<br>(-6.4 to 7.3)
Vanuatu	-0.6<br>(-7.3 to 6.5)	-3.3<br>(-9.4 to 4.3)	-1.6<br>(-8.5 to 6.3)
Zambia	-9.5<br>(-14.5 to -3.6)	-6.5<br>(-11.9 to -1.2)	-5.8<br>(-10.9 to -0.9)
Zimbabwe	-1.8<br>(-8.1 to 4.2)	-14.5<br>(-19.9 to -8.2)	-9.6<br>(-14.4 to -4.5)
Middle SDI	-7.2<br>(-8.5 to -6.1)	-8.4<br>(-9.3 to -7.5)	-6.3<br>(-8.2 to -4.6)
Albania	-23.3<br>(-28.8 to -15.7)	-8.0<br>(-17.5 to 2.2)	-3.3<br>(-13.3 to 9.1)
Algeria	-22.8<br>(-26.3 to -19.1)	-11.6<br>(-15.7 to -7.3)	-10.4<br>(-14.7 to -6.2)
Botswana	-9.7<br>(-19.2 to 2.8)	-5.5<br>(-13.1 to 2.9)	-7.8<br>(-14.0 to -2.1)
Brazil	-6.5<br>(-7.3 to -5.7)	0.2<br>(-0.7 to 1.0)	-5.2<br>(-6.3 to -4.3)
Colombia	-26.7<br>(-27.5 to -25.7)	-12.0<br>(-13.6 to -10.6)	-8.8<br>(-19.4 to 2.3)
Costa Rica	-10.3<br>(-12.5 to -7.8)	-0.2<br>(-2.5 to 2.1)	8.4<br>(1.6 to 16.2)
Cuba	-11.0<br>(-12.4 to -9.5)	-0.3<br>(-1.9 to 1.3)	2.4<br>(-6.9 to 13.3)
Dominica	6.4<br>(3.5 to 9.3)	7.2<br>(3.7 to 10.9)	-0.7<br>(-5.2 to 4.3)
Ecuador	8.5<br>(1.4 to 16.6)	-9.6<br>(-15.8 to -2.4)	-10.8<br>(-19.4 to 0.4)
Equatorial Guinea	-11.7<br>(-20.4 to -3.5)	-15.6<br>(-23.9 to -6.6)	-1.9<br>(-11.0 to 8.7)
Fiji	-0.2<br>(-3.3 to 2.9)	-6.5<br>(-9.6 to -3.1)	-3.2<br>(-11.8 to 5.8)
Gabon	-1.0<br>(-7.8 to 6.3)	-12.5<br>(-18.1 to -6.8)	-9.4<br>(-14.8 to -3.7)
Grenada	1.8<br>(-1.0 to 4.7)	-3.2<br>(-6.0 to -0.5)	-0.4<br>(-4.4 to 3.6)
Indonesia	-9.1<br>(-11.4 to -6.8)	-14.3<br>(-16.5 to -11.6)	-7.4<br>(-9.4 to -5.2)
Jamaica	46.3<br>(41.1 to 51.4)	9.1<br>(3.9 to 14.2)	-7.5<br>(-18.1 to 4.2)
Jordan	-16.4<br>(-20.4 to -11.7)	-14.8<br>(-19.0 to -10.6)	-4.4<br>(-10.1 to 1.4)
Maldives	-12.6<br>(-15.7 to -9.1)	-19.9<br>(-23.2 to -14.6)	-6.9<br>(-11.2 to -2.2)
Mexico	-5.9<br>(-6.7 to -5.1)	11.8<br>(11.1 to 12.4)	13.9<br>(12.2 to 15.6)
Moldova	-1.8<br>(-4.1 to 0.4)	-16.6<br>(-18.4 to -14.8)	-6.9<br>(-10.1 to -4.0)
Mongolia	-6.0<br>(-10.0 to -1.6)	-10.4<br>(-14.9 to -5.8)	-6.6<br>(-13.9 to 0.6)
Namibia	-18.2<br>(-24.3 to -10.8)	-18.6<br>(-25.5 to -11.6)	-9.2<br>(-15.7 to -2.3)
Panama	-0.1<br>(-2.7 to 2.7)	-2.5<br>(-5.1 to -0.1)	-13.0<br>(-19.0 to -6.8)
Paraguay	-0.5<br>(-9.2 to 9.0)	-3.4<br>(-11.8 to 5.9)	-7.4<br>(-22.9 to 10.9)
Peru	-9.3<br>(-16.3 to -2.0)	1.8<br>(-6.8 to 11.2)	-13.5<br>(-25.9 to 0.7)
Philippines	-7.0<br>(-13.9 to 0.7)	-1.4<br>(-9.3 to 7.3)	-11.2<br>(-22.6 to 1.4)
Saint Lucia	-4.8<br>(-7.4 to -2.3)	0.0<br>(-2.7 to 3.0)	-5.1<br>(-9.4 to -0.3)
Saint Vincent and the Grenadines	2.4<br>(-0.9 to 5.5)	0.8<br>(-2.5 to 4.1)	-13.8<br>(-17.7 to -9.9)
Seychelles	-10.1<br>(-12.9 to -7.2)	-4.0<br>(-7.0 to -0.9)	0.1<br>(-4.4 to 4.8)
South Africa	-16.5<br>(-18.8 to -13.6)	-30.0<br>(-32.6 to -24.8)	-5.0<br>(-8.4 to -1.3)

SR Table 2: Percent change in age-standardized mortality of Injuries, by SDI and country			
**Location**	**2003-2007**	**2008-2012**	**2013-2017**
Sri Lanka	1.9 (-0.1 to 4.2)	-44.1 (-46.7 to -41.0)	-6.4 (-20.2 to 9.1)
Suriname	0.7 (-4.6 to 5.9)	-10.9 (-16.2 to -5.5)	-1.9 (-9.2 to 5.7)
Syria	-4.6 (-10.5 to 1.8)	975.4 (885.3 to 1093.4)	-8.8 (-9.6 to -8.0)
Thailand	-18.1 (-23.8 to -12.4)	-10.6 (-16.0 to -4.4)	-1.2 (-11.6 to 9.7)
Tonga	-0.4 (-5.3 to 4.1)	-0.5 (-5.8 to 4.7)	-3.4 (-8.0 to 1.7)
Trinidad and Tobago	15.8 (12.2 to 19.4)	-21.2 (-27.1 to -15.4)	-5.3 (-21.9 to 12.8)
Tunisia	-11.8 (-18.6 to -4.9)	-9.1 (-16.3 to -1.5)	-8.5 (-16.1 to -0.0)
Turkmenistan	-4.7 (-8.0 to -1.8)	-22.6 (-25.2 to -19.9)	-6.5 (-13.4 to 0.5)
Uzbekistan	-8.1 (-13.0 to -2.8)	-8.4 (-13.3 to -3.3)	-6.7 (-15.5 to 3.4)
Venezuela	-2.6 (-3.7 to -1.5)	-5.1 (-6.1 to -4.2)	-2.4 (-17.4 to 14.1)
Vietnam	-1.5 (-6.3 to 4.1)	-5.9 (-10.2 to -1.5)	-4.7 (-9.3 to -0.1)
High-middle SDI	-16.1 (-16.9 to -15.2)	-13.8 (-14.8 to -12.8)	-7.0 (-8.3 to -5.5)
American Samoa	-14.9 (-17.3 to -12.3)	-7.1 (-10.6 to -3.7)	-9.2 (-12.7 to -4.9)
Antigua and Barbuda	1.5 (-2.0 to 5.1)	-5.3 (-8.6 to -1.7)	-1.8 (-6.0 to 3.2)
Argentina	-4.5 (-5.7 to -3.3)	-2.0 (-3.1 to -0.8)	-7.5 (-16.8 to 3.3)
Armenia	-5.7 (-8.3 to -3.1)	-5.2 (-7.2 to -3.2)	-5.6 (-9.0 to -2.3)
Azerbaijan	-3.0 (-8.1 to 2.8)	-5.1 (-10.0 to 0.0)	-6.7 (-13.6 to 1.4)
Bahrain	-16.8 (-20.2 to -13.1)	-15.8 (-19.5 to -11.6)	-7.1 (-12.7 to -1.0)
Barbados	-2.9 (-5.4 to -0.3)	-6.8 (-9.8 to -3.7)	-1.8 (-7.6 to 4.6)
Belarus	-14.6 (-15.9 to -13.2)	-21.2 (-22.8 to -19.7)	-17.3 (-21.9 to -12.5)
Bermuda	-19.5 (-22.3 to -16.8)	-8.0 (-12.0 to -4.2)	-7.3 (-12.8 to -1.7)
Bosnia and Herzegovina	-0.2 (-3.2 to 3.6)	-8.5 (-11.1 to -5.8)	0.1 (-6.0 to 6.9)
Bulgaria	-7.9 (-9.6 to -6.3)	-14.8 (-16.3 to -13.0)	-0.9 (-6.7 to 4.7)
Chile	-6.8 (-8.1 to -5.3)	-8.7 (-10.0 to -7.4)	-3.7 (-14.0 to 7.7)
China	-8.6 (-11.6 to -6.4)	-17.3 (-18.9 to -15.8)	-9.8 (-11.9 to -7.8)
Georgia	11.4 (7.5 to 15.1)	-13.9 (-15.5 to -12.2)	2.9 (-0.8 to 6.5)
Greenland	-10.3 (-12.7 to -7.7)	-9.5 (-12.1 to -6.6)	-11.3 (-15.3 to -7.4)
Guam	6.6 (3.5 to 9.7)	-2.9 (-6.4 to 0.8)	-0.2 (-5.2 to 5.0)
Hungary	-14.2 (-15.6 to -12.9)	-15.3 (-16.7 to -13.9)	-8.8 (-13.4 to -3.8)
Iran	-37.8 (-39.6 to -36.5)	-13.0 (-14.8 to -11.2)	-13.6 (-15.0 to -10.7)
Israel	-21.1 (-22.4 to -19.6)	-33.4 (-35.0 to -31.7)	-4.8 (-10.0 to 1.0)
Kazakhstan	-1.7 (-3.7 to 0.2)	-17.4 (-19.1 to -15.7)	-14.0 (-17.9 to -9.7)
Kuwait	-0.7 (-4.3 to 2.1)	-23.7 (-25.9 to -20.7)	-8.2 (-14.3 to -2.1)
Lebanon	-0.5 (-7.0 to 6.1)	-11.9 (-18.0 to -5.9)	-4.2 (-9.0 to 1.4)
Libya	-9.1 (-14.8 to -2.9)	28.1 (18.8 to 36.5)	33.5 (25.6 to 46.3)
Macedonia	-13.9 (-17.2 to -11.0)	-10.0 (-12.7 to -7.3)	3.6 (-3.1 to 10.5)

SR Table 2: Percent change in age-standardized mortality of Injuries, by SDI and country			
Location	2003-2007	2008-2012	2013-2017
Malaysia	-8.0<br>(-10.0 to -5.7)	-7.0<br>(-8.9 to -4.7)	2.9<br>(-5.8 to 12.9)
Mauritius	-0.9<br>(-3.5 to 1.7)	-4.3<br>(-7.0 to -1.7)	-5.1<br>(-11.5 to 1.4)
Montenegro	-10.5<br>(-15.4 to -5.6)	-11.1<br>(-16.1 to -5.8)	-9.3<br>(-16.8 to -0.6)
Northern Mariana Islands	4.9<br>(-0.1 to 9.7)	-2.2<br>(-7.3 to 2.6)	-0.1<br>(-4.9 to 4.8)
Oman	-9.9<br>(-16.1 to -3.4)	-3.4<br>(-10.9 to 4.4)	-13.1<br>(-19.5 to -5.2)
Portugal	-19.2<br>(-20.4 to -17.7)	-14.9<br>(-16.5 to -13.2)	-8.7<br>(-14.3 to -3.3)
Puerto Rico	4.6<br>(2.5 to 6.8)	0.3<br>(-1.8 to 2.3)	98.8<br>(91.8 to 105.6)
Qatar	-6.4<br>(-13.1 to 1.3)	-15.6<br>(-22.2 to -8.3)	-9.8<br>(-16.9 to -1.5)
Romania	-12.0<br>(-13.3 to -10.4)	-14.1<br>(-15.4 to -12.8)	-3.7<br>(-8.5 to 1.3)
Russian Federation	-19.1<br>(-19.7 to -18.5)	-21.5<br>(-22.4 to -20.2)	-12.4<br>(-13.2 to -11.6)
Saudi Arabia	-2.3<br>(-10.3 to 4.5)	-4.9<br>(-13.3 to 2.1)	0.3<br>(-5.2 to 6.9)
Serbia	-11.7<br>(-13.8 to -8.9)	-13.0<br>(-14.7 to -11.1)	-6.1<br>(-11.3 to -1.2)
The Bahamas	7.5<br>(4.3 to 11.3)	9.5<br>(5.7 to 13.1)	-6.1<br>(-12.2 to 0.3)
Turkey	-8.8<br>(-14.3 to -3.3)	2.7<br>(-3.0 to 9.0)	5.2<br>(-2.7 to 13.5)
Ukraine	-9.8<br>(-12.0 to -8.4)	-32.3<br>(-33.8 to -29.6)	25.5<br>(18.6 to 32.6)
United Arab Emirates	1.1<br>(-6.3 to 9.3)	-3.1<br>(-10.3 to 4.1)	-4.9<br>(-12.2 to 2.6)
Uruguay	2.7<br>(0.4 to 4.7)	7.3<br>(5.0 to 9.5)	-6.5<br>(-16.5 to 3.5)
Virgin Islands	6.2<br>(2.6 to 10.7)	2.1<br>(-2.3 to 7.2)	7.5<br>(2.6 to 13.0)
High SDI	-8.3<br>(-8.6 to -8.0)	-8.7<br>(-9.1 to -8.3)	-1.8<br>(-3.1 to -0.6)
Andorra	-4.3<br>(-9.1 to 0.5)	-4.6<br>(-9.3 to 0.5)	-4.3<br>(-9.1 to 0.9)
Australia	-4.5<br>(-6.1 to -3.0)	-6.7<br>(-8.4 to -5.0)	2.9<br>(-6.8 to 13.5)
Austria	-10.8<br>(-12.5 to -9.0)	-7.3<br>(-9.1 to -5.3)	-7.9<br>(-13.3 to -2.5)
Belgium	-8.2<br>(-9.6 to -6.7)	-4.8<br>(-6.4 to -3.1)	-9.6<br>(-14.4 to -4.6)
Brunei	-4.9<br>(-8.2 to -1.5)	0.8<br>(-3.1 to 4.9)	-1.4<br>(-6.1 to 3.5)
Canada	-3.5<br>(-4.9 to -2.2)	-6.3<br>(-7.7 to -4.8)	-0.6<br>(-5.5 to 4.9)
Croatia	-4.0<br>(-5.8 to -2.1)	-14.6<br>(-16.5 to -12.8)	-7.8<br>(-12.5 to -3.1)
Cyprus	-16.9<br>(-20.9 to -12.7)	-18.2<br>(-22.3 to -14.0)	-4.3<br>(-10.9 to 2.7)
Czech Republic	-17.7<br>(-19.0 to -16.3)	-11.3<br>(-12.7 to -9.9)	-8.9<br>(-14.0 to -3.3)
Denmark	-12.3<br>(-14.0 to -10.4)	-19.3<br>(-21.3 to -17.3)	-1.1<br>(-6.4 to 4.7)
Estonia	-18.4<br>(-20.8 to -16.2)	-26.6<br>(-28.7 to -24.4)	-9.4<br>(-20.5 to 2.7)
Finland	-6.7<br>(-8.5 to -4.7)	-14.9<br>(-16.7 to -13.1)	-7.2<br>(-12.6 to -1.1)
France	-16.8<br>(-18.0 to -15.5)	-8.6<br>(-9.8 to -7.3)	-8.9<br>(-13.6 to -3.9)
Germany	-14.0<br>(-15.3 to -12.7)	-4.9<br>(-6.3 to -3.5)	0.9<br>(-8.7 to 11.2)
Greece	-0.8<br>(-2.8 to 1.3)	-9.9<br>(-11.6 to -8.2)	-0.4<br>(-5.8 to 5.2)
Iceland	-9.8<br>(-12.3 to -7.2)	-3.9<br>(-6.6 to -1.0)	0.9<br>(-3.1 to 5.4)
Ireland	-10.6<br>(-12.6 to -8.6)	-14.0<br>(-16.0 to -12.0)	-11.5<br>(-17.3 to -5.4)

SR Table 2: Percent change in age-standardized mortality of Injuries, by SDI and country			
**Location**	**2003-2007**	**2008-2012**	**2013-2017**
Italy	-13.8<br>(-14.9 to -12.5)	-11.5<br>(-12.7 to -10.2)	-11.3<br>(-16.4 to -6.3)
Japan	-6.3<br>(-6.7 to -5.8)	-7.7<br>(-8.2 to -7.0)	-6.4<br>(-8.8 to -4.0)
Latvia	-9.8<br>(-12.6 to -7.5)	-24.9<br>(-26.6 to -23.1)	-8.6<br>(-18.9 to 2.0)
Lithuania	8.6<br>(4.6 to 11.7)	-24.2<br>(-25.9 to -22.3)	-12.7<br>(-18.2 to -7.1)
Luxembourg	-14.2<br>(-16.6 to -11.7)	-7.3<br>(-10.3 to -4.0)	-3.6<br>(-10.9 to 3.9)
Malta	-2.7<br>(-5.2 to -0.0)	-12.2<br>(-15.1 to -9.4)	1.3<br>(-3.2 to 5.9)
Netherlands	-12.4<br>(-14.2 to -10.3)	0.8<br>(-1.2 to 2.9)	-0.6<br>(-5.8 to 5.2)
New Zealand	-7.0<br>(-8.7 to -5.2)	-9.1<br>(-10.9 to -7.3)	-0.4<br>(-5.2 to 4.8)
Norway	-10.2<br>(-11.1 to -9.1)	-7.9<br>(-8.9 to -6.8)	-10.0<br>(-12.0 to -7.6)
Poland	-2.9<br>(-4.4 to -1.5)	-13.8<br>(-15.0 to -12.5)	-9.6<br>(-14.3 to -4.2)
Singapore	-10.7<br>(-13.2 to -8.2)	-15.4<br>(-17.4 to -13.3)	-11.6<br>(-17.4 to -5.6)
Slovakia	-6.7<br>(-8.6 to -4.6)	-8.6<br>(-12.6 to -5.7)	-8.4<br>(-13.5 to -2.8)
Slovenia	-11.9<br>(-14.0 to -9.6)	-16.9<br>(-19.1 to -14.6)	-8.6<br>(-14.6 to -2.6)
South Korea	-11.8<br>(-13.0 to -10.6)	-9.5<br>(-12.3 to -6.8)	-14.1<br>(-20.7 to -7.4)
Spain	-32.0<br>(-32.8 to -30.9)	-18.7<br>(-19.9 to -17.4)	-5.6<br>(-10.6 to -0.9)
Sweden	-4.6<br>(-6.1 to -2.9)	-6.9<br>(-8.6 to -5.2)	-5.5<br>(-10.1 to -1.0)
Switzerland	-11.6<br>(-13.4 to -9.7)	-7.6<br>(-9.6 to -5.5)	-12.5<br>(-18.0 to -6.8)
Taiwan (Province of China)	-10.6<br>(-11.9 to -9.4)	-11.6<br>(-12.8 to -10.3)	-1.3<br>(-6.0 to 3.8)
USA	-2.5<br>(-3.0 to -1.9)	-6.3<br>(-6.7 to -5.9)	5.4<br>(2.6 to 8.1)
United Kingdom	-6.1<br>(-6.6 to -5.6)	-12.6<br>(-13.9 to -11.2)	0.8<br>(-0.7 to 2.2)

SR Table 2b: Percent change in age-standardized mortality of Non-communicable diseases, by SDI and country			
**Location**	**2003-2007**	**2008-2012**	**2013-2017**
Low SDI	-3.0 (-4.2 to -1.7)	-3.1 (-4.5 to -1.7)	-0.6 (-2.5 to 1.4)
Afghanistan	-4.8 (-8.1 to -1.3)	-6.8 (-9.7 to -3.7)	-3.7 (-7.0 to -0.4)
Bangladesh	3.2 (-0.2 to 6.6)	-11.0 (-14.4 to -7.4)	-3.8 (-8.8 to 1.4)
Benin	-3.8 (-7.9 to 0.9)	0.0 (-4.2 to 4.4)	-4.4 (-8.1 to -0.3)
Burkina Faso	-1.9 (-5.5 to 1.7)	0.7 (-3.1 to 4.6)	-2.0 (-5.6 to 2.1)
Burundi	-9.3 (-13.7 to -4.8)	-5.0 (-9.1 to -0.7)	-3.6 (-7.5 to 0.2)
Central African Republic	-2.3 (-6.1 to 1.7)	-2.1 (-6.3 to 1.8)	-3.0 (-6.9 to 1.2)
Chad	-2.2 (-6.4 to 2.1)	-0.1 (-4.1 to 4.4)	-2.0 (-5.8 to 2.1)
Comoros	-6.1 (-10.1 to -2.1)	-2.6 (-6.6 to 2.0)	-1.8 (-6.1 to 2.6)
Cote d'Ivoire	-2.3 (-6.7 to 2.6)	-4.0 (-8.7 to 0.4)	-5.6 (-9.9 to -1.0)
DR Congo	-3.6 (-7.4 to 0.3)	-0.9 (-5.0 to 3.5)	-3.4 (-7.3 to 0.6)
Eritrea	-4.8 (-8.7 to -0.7)	-5.0 (-9.1 to -1.2)	-4.8 (-9.0 to -0.5)
Ethiopia	-10.0 (-12.4 to -7.4)	-10.0 (-12.5 to -7.3)	-4.7 (-7.2 to -2.1)
Guinea	2.2 (-1.7 to 6.1)	1.7 (-2.6 to 5.9)	-2.1 (-6.3 to 2.2)
Guinea-Bissau	-1.3 (-5.4 to 3.0)	-4.5 (-8.9 to -0.4)	-4.7 (-8.8 to -0.7)
Haiti	-2.6 (-7.4 to 2.0)	-1.2 (-5.2 to 3.2)	-3.1 (-7.2 to 1.6)
Kiribati	-0.6 (-3.9 to 2.5)	-1.2 (-4.5 to 2.2)	-2.4 (-5.8 to 0.9)
Liberia	-2.3 (-6.9 to 2.4)	1.7 (-2.3 to 6.4)	-2.9 (-7.0 to 1.6)
Madagascar	-8.8 (-13.3 to -4.1)	-2.0 (-6.8 to 2.7)	-2.9 (-7.8 to 2.1)
Malawi	-10.1 (-15.1 to -5.1)	-5.4 (-10.4 to -0.1)	-3.8 (-7.7 to 0.5)
Mali	-4.1 (-7.5 to -0.7)	-2.1 (-6.1 to 1.8)	-0.2 (-4.4 to 4.4)
Mozambique	1.9 (-2.7 to 6.7)	-1.3 (-6.1 to 3.7)	-4.5 (-9.4 to 0.4)
Nepal	2.0 (-2.5 to 7.0)	4.7 (0.2 to 9.3)	-0.4 (-5.0 to 3.7)
Niger	-3.6 (-8.0 to 2.4)	-2.1 (-6.6 to 2.1)	-0.6 (-5.2 to 4.3)
Papua New Guinea	0.1 (-2.8 to 3.1)	-2.5 (-5.5 to 0.3)	-3.2 (-5.9 to -0.1)
Rwanda	-14.3 (-18.7 to -9.9)	-8.2 (-12.2 to -4.1)	-3.0 (-7.0 to 1.0)
Senegal	-2.3 (-5.9 to 1.4)	0.4 (-3.9 to 4.8)	0.1 (-4.0 to 4.3)
Sierra Leone	-0.1 (-4.4 to 4.2)	-1.9 (-6.1 to 2.5)	-4.7 (-8.4 to -1.1)
Solomon Islands	-0.9 (-4.4 to 2.7)	-2.3 (-5.7 to 1.1)	-1.5 (-4.8 to 1.8)
Somalia	-4.5 (-10.0 to 0.7)	-2.2 (-7.1 to 3.7)	-3.1 (-8.2 to 3.1)
South Sudan	-2.3 (-8.6 to 4.6)	-3.5 (-9.1 to 3.9)	-1.6 (-7.3 to 4.9)
Tanzania	-4.0 (-8.5 to 0.7)	-1.5 (-6.3 to 3.2)	-1.1 (-5.1 to 3.0)
The Gambia	1.3 (-3.1 to 6.1)	-0.9 (-5.0 to 3.9)	-2.2 (-6.4 to 2.1)
Togo	-3.4 (-8.2 to 1.5)	-2.7 (-7.2 to 2.3)	-4.6 (-8.9 to -0.0)
Uganda	-8.1 (-12.5 to -3.6)	-7.0 (-11.6 to -2.4)	-5.9 (-10.3 to -1.9)
Yemen	-5.6 (-11.2 to 0.4)	-4.2 (-9.1 to 1.9)	0.5 (-4.8 to 6.8)

SR Table 2b: Percent change in age-standardized mortality of Non-communicable diseases, by SDI and country			
Location	2003-2007	2008-2012	2013-2017
Low-middle SDI	-4.6 (-5.6 to -3.5)	-1.7 (-2.9 to -0.6)	-1.8 (-3.3 to -0.3)
Angola	-7.2 (-11.1 to -2.9)	-6.3 (-10.5 to -1.9)	-6.4 (-10.7 to -1.6)
Belize	-10.4 (-11.8 to -9.0)	-4.7 (-6.1 to -3.0)	-0.1 (-2.9 to 2.8)
Bhutan	-5.6 (-9.5 to -1.7)	-5.1 (-10.0 to -0.6)	-4.7 (-9.5 to 0.6)
Bolivia	-4.1 (-8.8 to 0.9)	-4.0 (-8.9 to 1.2)	-2.9 (-8.7 to 2.7)
Cambodia	-6.8 (-10.0 to -3.2)	-4.9 (-8.3 to -1.5)	-2.6 (-6.1 to 1.4)
Cameroon	-2.2 (-7.0 to 2.6)	-4.9 (-9.9 to 0.2)	-6.8 (-11.7 to -2.2)
Cape Verde	-1.6 (-6.0 to 1.9)	0.0 (-2.9 to 3.3)	-0.7 (-5.3 to 3.5)
Congo (Brazzaville)	-5.2 (-9.5 to -1.1)	-5.3 (-9.8 to -1.3)	-2.0 (-6.5 to 2.0)
Djibouti	-5.2 (-12.4 to 2.5)	-3.6 (-10.1 to 4.9)	-1.8 (-8.5 to 6.0)
Dominican Republic	4.4 (-5.5 to 16.1)	1.8 (-6.8 to 11.6)	5.4 (-7.5 to 18.2)
Egypt	-3.1 (-3.9 to -2.5)	1.2 (0.7 to 1.8)	0.4 (-7.1 to 8.8)
El Salvador	9.0 (3.8 to 14.1)	-3.7 (-8.2 to 1.1)	1.3 (-11.4 to 15.5)
Federated States of Micronesia	-1.0 (-4.0 to 2.4)	-1.8 (-5.3 to 1.7)	-0.9 (-5.2 to 2.9)
Ghana	1.5 (-2.9 to 6.3)	-1.9 (-6.4 to 2.8)	-2.9 (-7.0 to 1.2)
Guatemala	-4.8 (-5.7 to -4.0)	-0.7 (-1.6 to 0.2)	2.7 (-4.8 to 11.6)
Guyana	-5.3 (-6.8 to -3.7)	9.3 (7.4 to 11.3)	-6.3 (-14.6 to 1.9)
Honduras	-2.3 (-12.4 to 8.3)	-1.6 (-11.4 to 9.1)	-1.8 (-11.0 to 9.0)
India	-4.9 (-6.1 to -3.7)	0.7 (-0.5 to 2.2)	0.7 (-1.0 to 2.6)
Iraq	-19.7 (-22.6 to -16.3)	-22.5 (-25.8 to -18.8)	-18.1 (-22.1 to -13.3)
Kenya	-2.4 (-4.2 to -0.5)	-3.0 (-4.7 to -1.3)	-4.3 (-5.7 to -2.9)
Kyrgyzstan	1.5 (-1.9 to 5.1)	-10.2 (-10.9 to -9.4)	-10.8 (-12.8 to -8.8)
Laos	-6.7 (-10.4 to -2.8)	-8.0 (-12.0 to -4.2)	-5.8 (-9.6 to -2.0)
Lesotho	4.0 (-3.2 to 10.2)	-1.7 (-8.8 to 4.6)	-8.3 (-13.6 to -2.8)
Marshall Islands	-1.9 (-4.8 to 0.9)	-4.5 (-7.7 to -1.3)	-4.4 (-7.4 to -0.9)
Mauritania	-4.9 (-8.6 to -0.8)	-2.7 (-7.0 to 1.8)	-2.5 (-6.8 to 1.5)
Morocco	-1.7 (-5.9 to 3.3)	-5.1 (-9.5 to -0.2)	-4.2 (-9.7 to 2.1)
Myanmar	-7.7 (-11.4 to -3.8)	-9.7 (-13.1 to -6.0)	-5.7 (-9.1 to -2.1)
Nicaragua	-6.2 (-13.1 to 0.8)	-5.3 (-10.1 to 0.1)	-7.9 (-15.6 to 0.7)
Nigeria	-7.1 (-15.0 to 0.9)	-2.6 (-10.0 to 6.9)	0.0 (-7.7 to 8.8)
North Korea	-4.9 (-8.1 to -1.3)	-3.2 (-7.0 to 0.3)	-3.1 (-8.0 to 1.7)
Pakistan	-0.0 (-5.5 to 5.4)	-0.7 (-6.8 to 5.2)	-1.2 (-7.0 to 5.4)
Palestine	-2.8 (-5.8 to 0.1)	-9.2 (-11.5 to -6.7)	-7.6 (-11.9 to -3.5)
Samoa	0.2 (-3.8 to 4.5)	2.1 (-2.7 to 6.7)	-1.6 (-6.4 to 2.5)
Sao Tome and Principe	-3.5 (-7.0 to 0.2)	1.7 (-2.7 to 6.0)	-0.7 (-4.8 to 3.4)
Sudan	-4.4 (-8.1 to -0.9)	-4.8 (-9.0 to -0.7)	-3.8 (-8.7 to 0.7)

SR Table 2b: Percent change in age-standardized mortality of Non-communicable diseases, by SDI and country			
**Location**	**2003-2007**	**2008-2012**	**2013-2017**
Swaziland	-1.6 (-9.7 to 4.7)	-6.1 (-13.0 to 0.8)	-14.8 (-20.8 to -8.1)
Tajikistan	3.2 (-1.5 to 8.5)	-1.3 (-6.2 to 3.8)	1.7 (-5.2 to 9.0)
Timor-Leste	-4.0 (-7.7 to -0.3)	-0.5 (-4.7 to 3.9)	1.8 (-2.8 to 6.7)
Vanuatu	-0.5 (-4.9 to 4.4)	-1.4 (-5.6 to 4.0)	-0.6 (-4.8 to 4.1)
Zambia	-10.5 (-14.5 to -6.4)	-4.6 (-9.6 to 0.3)	-3.2 (-7.3 to 0.9)
Zimbabwe	1.2 (-3.8 to 5.7)	-10.4 (-15.3 to -5.7)	-8.3 (-12.8 to -3.9)
Middle SDI	-8.0 (-8.8 to -7.2)	-2.4 (-3.4 to -1.4)	-1.5 (-2.9 to -0.2)
Albania	-7.6 (-11.9 to -2.6)	-5.3 (-13.6 to 2.8)	-2.6 (-11.1 to 6.9)
Algeria	-4.7 (-7.6 to -1.7)	-5.4 (-8.6 to -2.2)	-5.7 (-9.1 to -2.1)
Botswana	0.1 (-8.3 to 10.5)	0.1 (-6.9 to 8.5)	-5.3 (-12.0 to 1.2)
Brazil	-7.8 (-8.4 to -7.4)	-4.5 (-5.0 to -4.1)	-1.0 (-1.7 to -0.4)
Colombia	-9.4 (-10.0 to -8.9)	-12.0 (-12.6 to -11.5)	-2.1 (-10.1 to 7.0)
Costa Rica	-15.7 (-17.0 to -14.4)	2.5 (1.0 to 4.0)	6.5 (1.3 to 11.9)
Cuba	-3.2 (-4.1 to -2.3)	-4.3 (-5.3 to -3.5)	1.6 (-6.8 to 10.7)
Dominica	-2.0 (-4.1 to 0.2)	4.9 (2.4 to 7.5)	1.9 (-1.8 to 5.5)
Ecuador	3.8 (-1.2 to 9.4)	-3.0 (-7.8 to 2.6)	-4.1 (-11.3 to 4.6)
Equatorial Guinea	-8.7 (-16.3 to -1.2)	-5.9 (-13.4 to 2.4)	-2.0 (-9.8 to 7.0)
Fiji	-4.4 (-5.8 to -2.9)	-4.1 (-5.7 to -2.3)	-3.0 (-11.3 to 5.4)
Gabon	-4.6 (-9.6 to 0.3)	-7.3 (-11.7 to -3.1)	-4.2 (-8.0 to 0.0)
Grenada	1.3 (-0.9 to 3.5)	1.8 (-0.9 to 4.5)	-0.7 (-4.4 to 3.1)
Indonesia	-0.6 (-2.5 to 1.1)	-1.8 (-3.7 to 0.1)	-2.0 (-4.1 to 0.1)
Jamaica	-6.9 (-8.7 to -5.1)	0.4 (-2.7 to 3.8)	3.2 (-5.4 to 12.5)
Jordan	-8.8 (-11.9 to -5.1)	-8.6 (-12.0 to -4.9)	-3.4 (-8.6 to 2.4)
Maldives	-12.6 (-14.1 to -11.1)	-22.2 (-23.8 to -20.5)	-4.5 (-8.0 to -0.7)
Mexico	-5.8 (-6.1 to -5.6)	-1.2 (-1.4 to -1.0)	-0.2 (-1.5 to 0.9)
Moldova	-3.1 (-3.9 to -2.2)	-11.1 (-11.9 to -10.2)	-5.0 (-7.3 to -2.9)
Mongolia	-8.2 (-10.7 to -5.5)	-10.0 (-12.8 to -7.3)	-6.1 (-11.1 to -0.4)
Namibia	-16.5 (-21.5 to -10.7)	-14.0 (-20.5 to -7.2)	-3.5 (-9.3 to 2.1)
Panama	-5.0 (-6.7 to -3.3)	-1.3 (-2.9 to 0.3)	-3.4 (-8.2 to 1.2)
Paraguay	2.1 (-4.7 to 9.4)	2.0 (-4.9 to 9.2)	-6.3 (-18.7 to 8.4)
Peru	-11.7 (-18.0 to -5.0)	-1.6 (-8.6 to 6.2)	-8.4 (-19.2 to 3.9)
Philippines	3.6 (-2.7 to 11.0)	2.2 (-3.9 to 9.3)	-1.0 (-12.8 to 11.1)
Saint Lucia	-6.9 (-8.6 to -5.3)	-1.2 (-3.2 to 0.8)	1.7 (-1.7 to 5.3)
Saint Vincent and the Grenadines	-4.3 (-6.0 to -2.5)	3.4 (1.3 to 5.3)	0.1 (-3.2 to 3.6)
Seychelles	-4.1 (-5.9 to -2.1)	-2.5 (-4.4 to -0.4)	-1.2 (-4.2 to 2.3)
South Africa	-4.8 (-5.6 to -3.8)	-13.1 (-13.9 to -12.2)	-9.7 (-11.0 to -8.5)

SR Table 2b: Percent change in age-standardized mortality of Non-communicable diseases, by SDI and country			
**Location**	**2003-2007**	**2008-2012**	**2013-2017**
Sri Lanka	-4.4 (-5.1 to -3.7)	-7.9 (-11.7 to -4.6)	-8.1 (-19.9 to 2.8)
Suriname	-4.9 (-8.6 to -1.4)	-4.8 (-8.8 to -0.5)	3.7 (-2.0 to 9.6)
Syria	-7.2 (-12.3 to -1.8)	-3.1 (-9.5 to 3.2)	-0.4 (-6.6 to 6.0)
Thailand	-8.7 (-13.2 to -4.0)	-9.6 (-13.8 to -5.1)	0.4 (-7.9 to 8.6)
Tonga	-1.5 (-5.5 to 1.9)	-1.2 (-5.9 to 3.7)	-2.6 (-7.1 to 2.2)
Trinidad and Tobago	-10.8 (-12.4 to -9.1)	-13.9 (-18.1 to -9.1)	2.5 (-11.4 to 18.1)
Tunisia	-5.9 (-12.1 to 0.3)	-3.6 (-10.2 to 3.5)	-2.5 (-9.6 to 5.5)
Turkmenistan	5.1 (4.2 to 6.0)	-14.5 (-15.4 to -13.8)	-3.0 (-8.2 to 2.7)
Uzbekistan	3.6 (-0.5 to 8.3)	-2.5 (-6.6 to 1.7)	-4.7 (-12.1 to 3.3)
Venezuela	-8.0 (-8.6 to -7.4)	-1.9 (-2.6 to -1.3)	1.6 (-9.0 to 14.5)
Vietnam	-4.3 (-8.2 to -0.5)	-3.9 (-7.8 to 0.4)	-1.3 (-5.4 to 2.9)
High-middle SDI	-11.5 (-12.2 to -10.8)	-6.7 (-7.6 to -5.8)	-4.5 (-5.7 to -3.2)
American Samoa	1.2 (-2.0 to 4.8)	-0.9 (-5.0 to 3.1)	-1.5 (-6.4 to 3.4)
Antigua and Barbuda	-6.7 (-9.2 to -4.2)	-2.6 (-5.6 to 0.4)	-2.2 (-6.1 to 2.0)
Argentina	-6.6 (-7.1 to -6.0)	-2.7 (-3.4 to -2.1)	-2.6 (-10.5 to 6.4)
Armenia	-5.8 (-6.9 to -4.8)	-5.9 (-7.1 to -4.7)	-2.8 (-5.4 to -0.0)
Azerbaijan	-5.7 (-9.1 to -2.2)	-4.7 (-8.0 to -1.1)	-1.5 (-7.3 to 4.8)
Bahrain	-18.0 (-20.0 to -15.7)	-14.0 (-16.8 to -11.3)	-6.7 (-11.1 to -2.1)
Barbados	-4.5 (-6.3 to -2.5)	-4.5 (-6.7 to -2.2)	0.8 (-3.9 to 5.4)
Belarus	-9.0 (-9.7 to -8.3)	-8.1 (-8.9 to -7.4)	-6.9 (-10.9 to -2.8)
Bermuda	-10.2 (-13.0 to -7.4)	-6.5 (-9.7 to -3.1)	-2.8 (-6.3 to 0.8)
Bosnia and Herzegovina	4.6 (3.1 to 6.2)	-0.4 (-1.9 to 1.1)	-0.5 (-5.0 to 4.4)
Bulgaria	-4.5 (-5.4 to -3.7)	-7.1 (-8.0 to -6.1)	-1.7 (-6.1 to 2.3)
Chile	-7.2 (-8.0 to -6.4)	-2.5 (-3.4 to -1.7)	-2.6 (-10.8 to 6.4)
China	-12.6 (-13.7 to -11.5)	-1.6 (-3.0 to -0.2)	-3.0 (-4.7 to -1.3)
Georgia	-7.7 (-10.0 to -5.3)	1.5 (0.4 to 2.6)	6.8 (4.1 to 9.5)
Greenland	-7.6 (-9.6 to -5.7)	-7.9 (-10.1 to -5.6)	-3.4 (-7.7 to 0.2)
Guam	0.5 (-1.7 to 2.9)	0.4 (-2.4 to 3.2)	2.4 (-1.5 to 6.6)
Hungary	-5.1 (-5.8 to -4.3)	-6.3 (-7.2 to -5.5)	-9.4 (-13.2 to -5.3)
Iran	-1.0 (-1.7 to -0.3)	-1.2 (-1.7 to -0.6)	-1.7 (-2.4 to -1.2)
Israel	-9.3 (-10.5 to -8.0)	-9.4 (-10.7 to -8.0)	-6.2 (-10.8 to -1.4)
Kazakhstan	-4.0 (-4.4 to -3.5)	-7.2 (-7.7 to -6.8)	-9.4 (-12.5 to -6.0)
Kuwait	-0.1 (-1.5 to 1.3)	-21.7 (-23.0 to -20.5)	-2.2 (-7.1 to 3.1)
Lebanon	-1.9 (-5.7 to 2.2)	-3.1 (-6.2 to 1.2)	-0.8 (-4.1 to 2.4)
Libya	-5.5 (-9.6 to -1.3)	7.4 (2.1 to 12.8)	0.2 (-5.0 to 4.9)
Macedonia	-6.6 (-8.1 to -4.9)	-10.5 (-12.0 to -8.9)	0.9 (-3.4 to 5.7)

SR Table 2b: Percent change in age-standardized mortality of Non-communicable diseases, by SDI and country			
Location	2003-2007	2008-2012	2013-2017
Malaysia	-11.8 (-12.7 to -9.8)	-8.8 (-10.0 to -6.3)	3.4 (-3.4 to 11.0)
Mauritius	-6.5 (-8.3 to -4.6)	-5.8 (-7.6 to -3.9)	-0.6 (-5.6 to 4.8)
Montenegro	-2.8 (-6.4 to 0.9)	-4.3 (-7.8 to -0.6)	0.3 (-5.6 to 7.0)
Northern Mariana Islands	-3.7 (-8.1 to 0.6)	0.4 (-6.3 to 8.9)	-1.5 (-7.7 to 4.9)
Oman	-5.7 (-10.7 to -0.6)	2.6 (-2.7 to 8.4)	-9.8 (-14.7 to -4.4)
Portugal	-15.3 (-16.2 to -14.4)	-9.3 (-10.4 to -8.3)	-4.1 (-8.4 to 0.2)
Puerto Rico	-5.9 (-7.3 to -4.4)	-6.8 (-8.3 to -5.4)	-0.8 (-5.6 to 4.1)
Qatar	-12.3 (-16.6 to -7.8)	-13.8 (-18.3 to -9.0)	-9.2 (-14.0 to -4.1)
Romania	-11.0 (-11.5 to -10.5)	-7.5 (-8.1 to -6.9)	-2.8 (-6.8 to 0.8)
Russian Federation	-13.0 (-13.2 to -12.9)	-14.9 (-15.2 to -14.7)	-7.4 (-8.1 to -6.8)
Saudi Arabia	5.1 (-0.4 to 11.4)	-0.7 (-11.3 to 8.0)	-9.8 (-16.2 to 0.2)
Serbia	-2.5 (-3.9 to -1.4)	-0.5 (-1.6 to 0.4)	-4.3 (-8.4 to -0.2)
The Bahamas	-1.4 (-3.3 to 0.5)	-0.1 (-2.6 to 2.5)	-0.2 (-5.7 to 4.8)
Turkey	-6.1 (-10.4 to -1.1)	-0.6 (-5.2 to 4.4)	-8.3 (-14.9 to -1.2)
Ukraine	0.7 (0.2 to 1.1)	-15.2 (-15.6 to -14.9)	3.4 (-0.5 to 7.6)
United Arab Emirates	-5.0 (-9.9 to 0.2)	-0.1 (-5.2 to 4.9)	-1.9 (-8.0 to 4.2)
Uruguay	-4.1 (-5.5 to -2.7)	-2.2 (-3.8 to -0.7)	-2.2 (-10.8 to 6.6)
Virgin Islands	-1.2 (-6.5 to 4.0)	1.7 (-3.6 to 7.0)	-0.1 (-6.1 to 4.7)
High SDI	-8.7 (-8.9 to -8.6)	-6.1 (-6.2 to -5.9)	-2.4 (-3.6 to -1.3)
Andorra	-3.5 (-8.5 to 0.7)	-1.6 (-6.4 to 3.6)	-1.8 (-6.1 to 2.5)
Australia	-8.1 (-8.9 to -7.2)	-9.1 (-10.0 to -8.3)	2.4 (-6.1 to 11.4)
Austria	-9.9 (-11.0 to -8.7)	-4.8 (-6.0 to -3.4)	-4.8 (-9.1 to -0.2)
Belgium	-9.5 (-10.5 to -8.5)	-5.3 (-6.3 to -4.2)	-7.9 (-12.2 to -3.6)
Brunei	-5.6 (-8.3 to -2.9)	-0.4 (-3.9 to 3.2)	-2.6 (-7.4 to 2.2)
Canada	-8.4 (-9.0 to -7.8)	-8.1 (-8.8 to -7.5)	-0.5 (-4.8 to 4.2)
Croatia	-4.2 (-5.6 to -2.8)	-10.9 (-12.3 to -9.5)	-5.7 (-10.3 to -1.0)
Cyprus	-12.3 (-15.8 to -8.8)	-14.3 (-17.8 to -10.8)	-5.6 (-10.6 to -0.3)
Czech Republic	-12.9 (-13.8 to -12.1)	-8.1 (-9.1 to -7.2)	-6.6 (-10.9 to -1.9)
Denmark	-7.7 (-9.0 to -6.3)	-10.5 (-11.9 to -9.0)	-3.5 (-8.1 to 1.4)
Estonia	-6.1 (-7.9 to -4.4)	-13.1 (-14.9 to -11.1)	-8.2 (-17.5 to 1.0)
Finland	-6.0 (-7.3 to -4.6)	-6.1 (-7.5 to -4.7)	-3.6 (-8.2 to 1.4)
France	-10.6 (-11.0 to -10.1)	-6.0 (-6.6 to -5.6)	-6.8 (-10.8 to -2.4)
Germany	-10.6 (-11.0 to -10.2)	-4.4 (-4.9 to -3.9)	-0.6 (-9.1 to 8.0)
Greece	-4.4 (-5.5 to -3.3)	-5.8 (-6.8 to -4.6)	-3.0 (-7.5 to 1.4)
Iceland	-7.8 (-9.6 to -5.9)	-5.7 (-7.6 to -3.7)	-1.3 (-4.4 to 1.9)
Ireland	-11.0 (-12.6 to -9.5)	-8.6 (-10.0 to -7.1)	-6.6 (-11.2 to -1.7)

SR Table 2b: Percent change in age-standardized mortality of Non-communicable diseases, by SDI and country			
**Location**	**2003-2007**	**2008-2012**	**2013-2017**
Italy	-10.6 (-11.1 to -10.1)	-5.6 (-6.2 to -5.1)	-6.4 (-10.7 to -2.2)
Japan	-5.5 (-5.7 to -5.2)	-4.2 (-4.4 to -3.9)	-5.2 (-7.0 to -3.4)
Latvia	1.5 (0.1 to 3.0)	-10.1 (-11.6 to -8.8)	-7.1 (-15.2 to 1.5)
Lithuania	8.6 (7.1 to 10.0)	-11.7 (-12.8 to -10.5)	-6.0 (-10.5 to -1.4)
Luxembourg	-11.1 (-12.9 to -9.1)	-5.2 (-7.7 to -2.7)	-2.2 (-7.5 to 3.9)
Malta	-5.0 (-7.1 to -3.1)	-9.0 (-11.0 to -7.0)	1.4 (-2.1 to 5.1)
Netherlands	-13.3 (-14.1 to -12.5)	-6.3 (-7.2 to -5.3)	-1.6 (-6.0 to 3.5)
New Zealand	-9.4 (-10.8 to -8.1)	-7.8 (-9.3 to -6.4)	-2.6 (-6.7 to 2.0)
Norway	-8.8 (-9.4 to -8.2)	-6.8 (-7.5 to -6.2)	-7.6 (-9.0 to -6.2)
Poland	-5.1 (-5.6 to -4.7)	-8.5 (-9.0 to -8.1)	-6.5 (-10.2 to -2.4)
Singapore	-12.3 (-14.2 to -10.4)	-14.9 (-16.6 to -13.0)	-9.0 (-13.3 to -4.0)
Slovakia	-2.6 (-3.7 to -1.4)	-9.1 (-10.2 to -7.9)	-6.4 (-10.7 to -1.8)
Slovenia	-12.7 (-14.5 to -10.8)	-9.0 (-11.0 to -7.1)	-8.2 (-13.5 to -2.3)
South Korea	-17.5 (-17.9 to -17.1)	-10.8 (-13.0 to -8.6)	-7.0 (-12.5 to -1.8)
Spain	-9.2 (-9.8 to -8.7)	-8.4 (-8.9 to -7.7)	-4.2 (-8.2 to -0.2)
Sweden	-5.4 (-6.5 to -4.3)	-7.7 (-8.7 to -6.5)	-5.2 (-9.1 to -1.2)
Switzerland	-9.1 (-10.4 to -7.8)	-6.4 (-7.7 to -4.9)	-8.5 (-13.0 to -4.1)
Taiwan (Province of China)	-7.4 (-8.1 to -6.8)	-5.3 (-6.0 to -4.5)	0.2 (-3.8 to 4.4)
USA	-7.5 (-7.7 to -7.4)	-4.4 (-4.6 to -4.2)	1.8 (-0.0 to 3.6)
United Kingdom	-10.4 (-10.6 to -10.2)	-8.7 (-8.9 to -8.4)	-0.9 (-1.7 to 0.0)

SR Table 3: Global deaths and YLL numbers, age-standardised rates per 100,000, and percent change between 2007 and 2017 for females for all GBD causes and Levels 1 through 4 of the cause hierarchy. 95% UIs are in parentheses. UI=uncertainty interval. YLL=year of life lost.								
Cause	All Age Deaths (thousands)		Age-standardised Death Rate (per 100,000)		All Age YLLs (thousands)		Age-standardised YLL Rate (per 100,000)	
	2017	Percent Change 2007-2017	2017	Percent Change 2007-2017	2017	Percent Change 2007-2017	2017	Percent Change 2007-2017
**All causes**	25,558.2 (25,224.1 to 25,885.0)	**9.5 (8.1 to 10.8)**	619.7 (611.7 to 627.9)	**-15.0 (-16.1 to -14.0)**	698,329.1 (687,437.6 to 710,664.2)	**-10.9 (-12.3 to -9.5)**	18,463.0 (18,162.7 to 18,819.2)	**-24.1 (-25.4 to -22.9)**
**Communicable, maternal, neonatal, and nutritional diseases**	4,912.8 (4,654.6 to 5,327.8)	**-22.9 (-25.0 to -20.0)**	133.4 (127.0 to 142.8)	**-33.0 (-34.6 to -30.8)**	269,005.6 (259,578.0 to 279,982.0)	**-31.8 (-33.7 to -29.6)**	7,865.2 (7,594.4 to 8,183.0)	**-36.8 (-38.6 to -34.7)**
**HIV/AIDS and sexually transmitted infections**	494.8 (454.6 to 542.5)	**-53.1 (-55.4 to -50.4)**	13.0 (11.8 to 14.4)	**-58.2 (-60.4 to -55.7)**	28,032.6 (25,120.6 to 31,630.3)	**-52.2 (-54.9 to -49.1)**	756.9 (668.9 to 865.5)	**-56.5 (-59.3 to -53.3)**
HIV/AIDS	446.1 (416.9 to 478.6)	**-55.5 (-57.5 to -53.3)**	11.5 (10.7 to 12.3)	**-60.9 (-62.6 to -59.0)**	24,086.2 (22,451.2 to 25,847.5)	**-55.6 (-57.6 to -53.5)**	634.7 (591.5 to 682.1)	**-60.3 (-62.0 to -58.4)**
HIV/AIDS - Drug-susceptible Tuberculosis	91.2 (64.4 to 118.8)	**-58.8 (-61.8 to -55.1)**	2.4 (1.7 to 3.1)	**-63.9 (-66.5 to -60.6)**	5,066.2 (3,601.6 to 6,528.8)	**-58.5 (-61.5 to -54.8)**	135.3 (96.4 to 173.7)	**-62.9 (-65.6 to -59.5)**
HIV/AIDS - Multidrug-resistant Tuberculosis without extensive drug resistance	10.4 (6.2 to 16.0)	**-55.3 (-69.0 to -37.6)**	0.3 (0.2 to 0.4)	**-60.6 (-72.7 to -44.8)**	582.0 (342.9 to 895.4)	**-54.4 (-68.2 to -36.4)**	15.6 (9.2 to 24.0)	**-59.1 (-71.5 to -42.6)**
HIV/AIDS - Extensively drug-resistant Tuberculosis	0.5 (0.3 to 0.7)	-12.0 (-32.5 to 13.2)	0.0 (0.0 to 0.0)	**-23.3 (-41.0 to -1.8)**	23.7 (14.7 to 35.1)	-13.8 (-33.3 to 10.1)	0.6 (0.4 to 0.9)	**-23.5 (-40.6 to -1.9)**
HIV/AIDS resulting in other diseases	344.1 (304.5 to 384.9)	**-54.5 (-57.2 to -51.7)**	8.8 (7.8 to 9.9)	**-60.0 (-62.3 to -57.6)**	18,414.3 (16,204.0 to 20,713.2)	**-54.8 (-57.5 to -52.0)**	483.3 (425.3 to 544.4)	**-59.6 (-61.9 to -57.0)**
Sexually transmitted infections excluding HIV	48.7 (22.5 to 86.9)	**-8.9 (-16.0 to -1.0)**	1.5 (0.7 to 2.7)	**-13.5 (-20.3 to -5.8)**	3,946.4 (1,664.5 to 7,278.5)	**-10.2 (-17.8 to -2.0)**	122.2 (50.7 to 226.5)	**-13.6 (-21.1 to -5.6)**
Syphilis	43.2 (17.1 to 81.1)	**-10.2 (-18.3 to -1.6)**	1.3 (0.5 to 2.5)	**-13.4 (-21.2 to -5.1)**	3,734.5 (1,447.7 to 7,047.4)	**-10.4 (-18.6 to -1.8)**	116.9 (45.2 to 220.8)	**-13.4 (-21.3 to -5.0)**
Chlamydial infection	1.1 (0.9 to 1.2)	2.5 (-4.5 to 11.3)	0.0 (0.0 to 0.0)	**-15.0 (-21.0 to -8.1)**	40.5 (32.6 to 45.0)	-5.5 (-12.2 to 2.5)	1.0 (0.8 to 1.1)	**-17.9 (-23.7 to -10.9)**
Gonococcal infection	3.0 (2.4 to 3.2)	3.9 (-3.7 to 12.9)	0.1 (0.1 to 0.1)	**-14.7 (-21.0 to -7.7)**	110.0 (87.9 to 122.3)	-3.7 (-10.9 to 4.7)	2.7 (2.2 to 3.0)	**-17.3 (-23.7 to -10.3)**
Other sexually transmitted infections	1.5 (1.2 to 1.6)	0.3 (-6.9 to 8.9)	0.0 (0.0 to 0.0)	**-15.8 (-22.0 to -8.6)**	61.3 (49.2 to 68.4)	-6.0 (-12.8 to 1.9)	1.5 (1.2 to 1.7)	**-18.1 (-24.2 to -11.3)**
**Respiratory infections and tuberculosis**	1,658.8 (1,580.9 to 1,731.5)	**-7.1 (-10.2 to -3.4)**	42.4 (40.4 to 44.3)	**-24.6 (-27.2 to -21.7)**	65,632.0 (61,788.0 to 69,147.4)	**-25.9 (-29.4 to -22.1)**	1,858.4 (1,746.8 to 1,963.7)	**-33.7 (-37.0 to -30.3)**
Tuberculosis	437.2 (410.8 to 474.6)	**-13.7 (-18.6 to -3.1)**	10.7 (10.1 to 11.6)	**-30.1 (-34.0 to -21.6)**	15,776.3 (14,908.0 to 16,893.9)	**-22.3 (-26.4 to -14.4)**	406.1 (382.9 to 434.4)	**-33.6 (-37.1 to -26.8)**
Drug-susceptible tuberculosis	386.6 (348.3 to 427.6)	**-14.8 (-23.1 to -2.6)**	9.5 (8.6 to 10.5)	**-30.9 (-37.6 to -21.1)**	13,952.8 (12,549.0 to 15,245.7)	**-23.3 (-30.1 to -14.4)**	359.3 (323.2 to 392.5)	**-34.4 (-40.2 to -26.9)**
Multidrug-resistant tuberculosis without extensive drug resistance	46.6 (24.6 to 77.4)	-6.7 (-47.1 to 47.4)	1.1 (0.6 to 1.9)	-24.3 (-56.8 to 19.5)	1,684.0 (938.6 to 2,695.6)	-15.5 (-49.9 to 32.6)	43.2 (24.4 to 68.4)	-27.8 (-56.7 to 12.8)
Extensively drug-resistant tuberculosis	4.0 (2.6 to 6.0)	27.0 (-15.2 to 86.6)	0.1 (0.1 to 0.1)	2.8 (-31.4 to 50.6)	139.5 (89.9 to 208.5)	15.2 (-21.2 to 68.4)	3.5 (2.3 to 5.3)	-2.1 (-32.7 to 42.7)
Lower respiratory infections	1,216.8 (1,147.6 to 1,273.7)	**-4.3 (-7.7 to -1.0)**	31.6 (29.9 to 33.0)	**-22.4 (-25.2 to -19.6)**	49,611.2 (46,332.5 to 52,631.5)	**-26.9 (-30.9 to -22.8)**	1,445.2 (1,347.3 to 1,538.5)	**-33.7 (-37.4 to -29.8)**
Upper respiratory infections	4.4 (2.6 to 7.8)	-30.2 (-44.2 to 10.6)	0.1 (0.1 to 0.2)	**-41.3 (-52.5 to -14.8)**	222.6 (88.9 to 488.8)	-30.5 (-46.0 to 1.8)	6.6 (2.5 to 14.7)	**-35.8 (-50.1 to -4.6)**
Otitis media	0.5 (0.3 to 0.7)	**-39.1 (-53.8 to -16.7)**	0.0 (0.0 to 0.0)	**-48.9 (-61.7 to -29.6)**	21.9 (15.2 to 39.1)	**-50.1 (-65.1 to -26.7)**	0.6 (0.4 to 1.1)	**-55.1 (-69.0 to -34.1)**
**Enteric infections**	922.4 (637.3 to 1,374.5)	**-13.8 (-23.8 to -0.7)**	24.1 (17.3 to 34.7)	**-28.7 (-35.4 to -18.6)**	40,593.3 (33,579.5 to 51,780.3)	**-30.9 (-37.4 to -22.0)**	1,170.6 (989.8 to 1,455.4)	**-37.6 (-43.3 to -29.7)**
Diarrheal diseases	831.6 (542.9 to 1,264.4)	-13.1 (-24.2 to 1.6)	21.5 (14.7 to 32.0)	**-28.9 (-36.4 to -17.5)**	34,108.5 (26,976.1 to 44,896.4)	**-32.5 (-40.3 to -21.9)**	981.2 (802.0 to 1,250.5)	**-39.4 (-46.0 to -30.0)**
Typhoid and paratyphoid	61.9 (34.8 to 101.3)	**-20.3 (-25.7 to -15.6)**	1.8 (1.0 to 2.9)	**-26.1 (-31.3 to -21.8)**	4,402.2 (2,469.5 to 7,253.9)	**-21.9 (-27.7 to -16.9)**	127.4 (71.3 to 210.1)	**-27.0 (-32.6 to -22.2)**
Typhoid fever	53.0 (29.4 to 86.6)	**-21.9 (-28.1 to -16.8)**	1.5 (0.8 to 2.5)	**-27.7 (-33.7 to -22.9)**	3,773.0 (2,087.0 to 6,169.5)	**-23.7 (-30.4 to -18.6)**	109.2 (60.6 to 179.2)	**-28.7 (-35.0 to -23.9)**
Paratyphoid fever	8.9 (4.0 to 17.5)	-9.0 (-19.0 to 1.5)	0.3 (0.1 to 0.5)	**-15.4 (-25.1 to -5.3)**	629.2 (282.5 to 1,218.3)	-9.3 (-20.3 to 2.6)	18.2 (8.2 to 35.0)	**-15.1 (-25.6 to -3.7)**
Invasive Non-typhoidal Salmonella (iNTS)	28.1 (16.0 to 46.6)	**-18.9 (-26.8 to -9.6)**	0.8 (0.5 to 1.4)	**-25.7 (-33.3 to -16.7)**	2,023.1 (1,146.5 to 3,499.5)	**-18.5 (-27.7 to -8.1)**	60.3 (34.0 to 104.8)	**-23.8 (-32.6 to -13.9)**
Other intestinal infectious diseases	0.8 (0.5 to 1.5)	-40.1 (-74.3 to 37.6)	0.0 (0.0 to 0.0)	-45.3 (-77.2 to 30.0)	59.4 (33.1 to 119.7)	-44.0 (-77.9 to 39.5)	1.8 (1.0 to 3.7)	-47.3 (-79.4 to 33.2)
**Neglected tropical diseases and malaria**	332.4 (245.2 to 434.4)	**-29.5 (-38.2 to -19.8)**	9.4 (7.0 to 12.4)	**-37.1 (-45.1 to -27.9)**	22,328.4 (16,338.0 to 29,702.0)	**-34.9 (-44.1 to -24.4)**	661.9 (478.6 to 885.5)	**-39.8 (-48.6 to -30.1)**
Malaria	288.9 (205.2 to 387.1)	**-31.8 (-41.0 to -21.7)**	8.3 (5.9 to 11.2)	**-38.5 (-47.2 to -29.5)**	20,207.6 (14,018.4 to 27,554.9)	**-35.9 (-45.9 to -25.4)**	602.3 (412.6 to 818.4)	**-40.6 (-49.9 to -30.7)**
Chagas disease	3.3 (3.1 to 3.7)	5.7 (-0.8 to 14.2)	0.1 (0.1 to 0.1)	**-20.1 (-25.0 to -13.6)**	67.4 (63.4 to 74.4)	-3.1 (-9.1 to 5.1)	1.6 (1.5 to 1.8)	**-24.8 (-29.5 to -18.4)**
Leishmaniasis	2.7 (0.0 to 12.2)	**-64.6 (-96.8 to -41.4)**	0.1 (0.0 to 0.4)	**-67.4 (-97.3 to -46.9)**	184.0 (0.1 to 877.2)	**-63.8 (-90.4 to -36.2)**	5.4 (0.0 to 25.7)	**-66.1 (-91.2 to -41.1)**
Visceral leishmaniasis	2.7 (0.0 to 12.2)	**-64.6 (-96.8 to -41.4)**	0.1 (0.0 to 0.4)	**-67.4 (-97.3 to -46.9)**	184.0 (0.1 to 877.2)	**-63.8 (-90.4 to -36.2)**	5.4 (0.0 to 25.7)	**-66.1 (-91.2 to -41.1)**
African trypanosomiasis	0.6 (0.1 to 2.4)	**-80.9 (-95.9 to -29.8)**	0.0 (0.0 to 0.1)	**-82.9 (-96.4 to -37.0)**	36.8 (6.9 to 138.3)	**-80.8 (-95.8 to -30.3)**	1.0 (0.2 to 3.7)	**-82.4 (-96.2 to -33.4)**
Schistosomiasis	4.0 (3.6 to 4.5)	**-12.8 (-20.6 to -4.4)**	0.1 (0.1 to 0.1)	**-28.9 (-35.5 to -22.1)**	151.5 (133.2 to 171.8)	**-17.2 (-26.6 to -7.0)**	3.9 (3.5 to 4.5)	**-28.8 (-37.4 to -19.5)**
Cysticercosis	0.4 (0.2 to 0.5)	-17.5 (-53.2 to 39.6)	0.0 (0.0 to 0.0)	-29.0 (-59.5 to 20.3)	19.3 (11.7 to 28.4)	-22.6 (-56.4 to 31.7)	0.5 (0.3 to 0.8)	-30.9 (-61.2 to 16.9)
Cystic echinococcosis	0.6 (0.4 to 0.8)	-28.9 (-58.2 to 15.0)	0.0 (0.0 to 0.0)	**-41.6 (-65.3 to -6.0)**	24.8 (15.5 to 35.0)	**-39.2 (-63.2 to -1.6)**	0.7 (0.4 to 1.0)	**-47.1 (-68.2 to -14.4)**
Dengue	19.9 (5.3 to 25.2)	**75.4 (11.8 to 122.8)**	0.5 (0.1 to 0.6)	45.6 (-6.4 to 86.5)	880.2 (218.5 to 1,118.5)	36.0 (-13.5 to 81.6)	24.4 (6.1 to 31.2)	20.4 (-23.5 to 61.7)
Yellow fever	1.4 (0.3 to 4.1)	-14.4 (-30.9 to 5.4)	0.0 (0.0 to 0.1)	**-21.2 (-36.4 to -2.4)**	92.3 (18.7 to 267.0)	-13.6 (-31.2 to 8.6)	2.6 (0.5 to 7.6)	-19.1 (-35.6 to 2.3)
Rabies	3.7 (1.9 to 5.3)	**-52.3 (-69.9 to -33.6)**	0.1 (0.1 to 0.1)	**-59.1 (-73.9 to -42.5)**	193.0 (98.0 to 286.7)	**-54.8 (-70.4 to -34.7)**	5.3 (2.7 to 8.1)	**-59.5 (-73.7 to -40.8)**
Intestinal nematode infections	1.6 (1.1 to 2.3)	**-43.7 (-60.7 to -14.9)**	0.0 (0.0 to 0.1)	**-47.8 (-63.7 to -20.6)**	128.2 (88.6 to 186.1)	**-44.7 (-62.2 to -14.7)**	3.9 (2.7 to 5.7)	**-48.2 (-64.7 to -20.0)**
Ascariasis	1.6 (1.1 to 2.3)	**-43.7 (-60.7 to -14.9)**	0.0 (0.0 to 0.1)	**-47.8 (-63.7 to -20.6)**	128.2 (88.6 to 186.1)	**-44.7 (-62.2 to -14.7)**	3.9 (2.7 to 5.7)	**-48.2 (-64.7 to -20.0)**
Ebola	0.0 (0.0 to 0.0)	**-98.8 (-99.0 to -98.7)**	0.0 (0.0 to 0.0)	**-98.9 (-99.1 to -98.7)**	0.2 (0.2 to 0.2)	**-98.6 (-98.9 to -98.4)**	0.0 (0.0 to 0.0)	**-98.7 (-98.9 to -98.5)**
Zika virus	0.0 (0.0 to 0.0)	-	0.0 (0.0 to 0.0)	-	0.6 (0.1 to 2.1)	-	0.0 (0.0 to 0.1)	-
Other neglected tropical diseases	5.3 (2.9 to 16.6)	13.3 (-3.6 to 36.8)	0.1 (0.1 to 0.5)	1.0 (-14.4 to 21.3)	342.4 (176.8 to 1,237.0)	7.2 (-13.1 to 34.2)	10.2 (5.2 to 37.7)	-0.4 (-19.8 to 25.4)
**Other infectious diseases**	394.3 (340.2 to 457.7)	**-26.5 (-33.5 to -19.0)**	11.1 (9.5 to 13.0)	**-34.8 (-40.8 to -28.0)**	25,381.1 (21,259.6 to 30,471.8)	**-33.9 (-40.6 to -25.7)**	753.1 (627.9 to 907.3)	**-38.7 (-44.9 to -30.9)**
Meningitis	133.9 (108.5 to 165.3)	**-21.2 (-28.0 to -12.4)**	3.8 (3.2 to 4.6)	**-29.1 (-35.4 to -20.6)**	8,964.8 (7,423.0 to 10,657.5)	**-26.5 (-33.8 to -16.0)**	266.2 (219.9 to 314.4)	**-31.6 (-38.5 to -21.5)**
Pneumococcal meningitis	18.0 (14.7 to 22.2)	**-14.2 (-21.8 to -5.0)**	0.5 (0.4 to 0.6)	**-23.6 (-30.4 to -14.9)**	1,144.0 (928.2 to 1,378.5)	**-19.9 (-28.5 to -8.3)**	33.7 (27.2 to 40.6)	**-25.7 (-33.8 to -14.4)**
H influenzae type B meningitis	37.4 (31.8 to 47.8)	**-34.7 (-41.6 to -25.7)**	1.1 (0.9 to 1.3)	**-41.9 (-47.9 to -34.2)**	2,416.8 (2,031.4 to 2,968.9)	**-41.5 (-48.0 to -32.8)**	71.5 (59.7 to 87.5)	**-45.8 (-51.9 to -37.5)**
Meningococcal meningitis	13.2 (10.8 to 16.1)	**-32.1 (-38.9 to -22.6)**	0.4 (0.3 to 0.5)	**-37.9 (-44.3 to -29.0)**	954.2 (770.3 to 1,146.8)	**-35.8 (-42.9 to -25.7)**	28.8 (23.0 to 34.6)	**-39.7 (-46.5 to -30.0)**
Other meningitis	65.3 (53.8 to 79.7)	-9.7 (-17.8 to 1.3)	1.9 (1.5 to 2.2)	**-18.2 (-25.7 to -7.5)**	4,449.9 (3,635.8 to 5,264.4)	**-13.7 (-22.8 to -0.8)**	132.3 (107.6 to 156.8)	**-19.3 (-27.9 to -6.8)**
Encephalitis	46.0 (41.1 to 53.5)	4.9 (-9.8 to 22.4)	1.2 (1.1 to 1.4)	-11.2 (-24.3 to 3.2)	2,223.8 (1,960.8 to 2,529.9)	-10.1 (-26.9 to 8.3)	63.2 (55.5 to 71.9)	**-18.7 (-34.5 to -2.0)**
Diphtheria	1.7 (0.9 to 3.4)	-26.0 (-64.6 to 68.3)	0.1 (0.0 to 0.1)	-30.5 (-67.2 to 59.3)	143.3 (74.1 to 290.5)	-26.1 (-65.7 to 71.5)	4.4 (2.3 to 8.9)	-30.4 (-67.9 to 62.6)
Whooping cough	51.1 (25.6 to 89.7)	-22.6 (-54.5 to 35.7)	1.6 (0.8 to 2.8)	-26.5 (-56.8 to 28.8)	4,383.3 (2,198.3 to 7,699.6)	-22.6 (-54.5 to 35.8)	135.7 (68.1 to 238.4)	-26.5 (-56.8 to 28.9)
Tetanus	15.8 (10.9 to 21.6)	**-56.6 (-68.6 to -37.2)**	0.4 (0.3 to 0.6)	**-61.0 (-71.7 to -43.9)**	1,051.6 (751.9 to 1,404.5)	**-60.9 (-72.2 to -42.0)**	31.3 (22.5 to 42.2)	**-63.4 (-74.1 to -45.6)**
Measles	47.7 (17.3 to 101.9)	**-57.4 (-62.8 to -51.8)**	1.5 (0.5 to 3.1)	**-59.7 (-64.8 to -54.4)**	4,050.8 (1,472.2 to 8,658.3)	**-57.3 (-62.7 to -51.6)**	124.9 (45.4 to 266.9)	**-59.6 (-64.8 to -54.3)**
Varicella and herpes zoster	8.3 (7.4 to 9.7)	**-14.1 (-23.2 to -4.5)**	0.2 (0.2 to 0.3)	**-27.5 (-35.6 to -19.1)**	422.8 (367.5 to 491.8)	**-20.9 (-32.1 to -7.4)**	12.5 (10.8 to 14.6)	**-26.9 (-37.6 to -14.1)**
Acute hepatitis	56.1 (39.9 to 68.3)	**-10.2 (-19.7 to -0.6)**	1.4 (1.0 to 1.8)	**-24.7 (-32.7 to -16.6)**	2,521.2 (1,713.9 to 3,146.3)	**-22.9 (-31.3 to -13.9)**	68.2 (46.0 to 85.8)	**-31.9 (-39.4 to -23.5)**
Acute hepatitis A	9.8 (6.7 to 13.0)	**-33.7 (-45.6 to -21.0)**	0.3 (0.2 to 0.4)	**-38.8 (-50.1 to -26.6)**	698.4 (464.1 to 943.9)	**-36.1 (-48.3 to -22.5)**	20.3 (13.4 to 27.6)	**-40.6 (-52.0 to -27.4)**
Acute hepatitis B	37.4 (27.1 to 45.7)	1.1 (-10.5 to 14.3)	0.9 (0.7 to 1.1)	**-18.3 (-27.6 to -7.9)**	1,362.4 (937.3 to 1,701.3)	-11.9 (-22.6 to 0.4)	35.2 (24.1 to 43.8)	**-25.2 (-34.4 to -14.6)**
Acute hepatitis C	1.8 (1.0 to 3.2)	**-23.8 (-38.0 to -4.7)**	0.1 (0.0 to 0.1)	**-32.3 (-44.8 to -15.1)**	117.7 (62.1 to 202.2)	**-30.6 (-45.5 to -10.2)**	3.5 (1.9 to 6.1)	**-35.1 (-49.1 to -15.5)**
Acute hepatitis E	7.1 (4.9 to 9.2)	-14.7 (-30.5 to 3.7)	0.2 (0.1 to 0.2)	**-25.2 (-38.7 to -10.2)**	342.7 (233.2 to 445.9)	**-25.5 (-39.8 to -10.1)**	9.1 (6.2 to 11.8)	**-31.7 (-44.9 to -18.0)**
Other unspecified infectious diseases	33.7 (24.3 to 37.9)	-1.4 (-7.5 to 5.3)	0.9 (0.6 to 1.0)	**-17.1 (-22.3 to -11.1)**	1,619.4 (1,108.4 to 1,893.4)	**-14.5 (-21.3 to -8.1)**	46.7 (31.6 to 55.1)	**-22.2 (-28.5 to -16.3)**
**Maternal and neonatal disorders**	963.0 (920.3 to 1,006.5)	**-24.1 (-26.9 to -21.1)**	29.2 (27.9 to 30.5)	**-27.0 (-29.8 to -24.1)**	78,579.1 (75,091.7 to 82,156.1)	**-24.3 (-27.2 to -21.2)**	2,411.0 (2,303.3 to 2,521.4)	**-26.9 (-29.8 to -23.8)**
Maternal disorders	193.6 (179.9 to 209.6)	**-24.0 (-28.4 to -19.5)**	5.0 (4.6 to 5.4)	**-30.5 (-34.6 to -26.4)**	10,993.1 (10,198.9 to 11,928.5)	**-25.3 (-29.7 to -20.9)**	284.8 (264.3 to 309.2)	**-31.3 (-35.3 to -27.2)**
Maternal hemorrhage	38.5 (33.2 to 45.2)	**-52.1 (-59.0 to -44.2)**	1.0 (0.9 to 1.2)	**-56.3 (-62.6 to -49.1)**	2,173.8 (1,859.7 to 2,552.5)	**-53.0 (-60.1 to -45.0)**	56.2 (48.1 to 65.9)	**-56.9 (-63.4 to -49.5)**
Maternal sepsis and other maternal infections	21.2 (18.2 to 25.0)	**-27.1 (-38.8 to -15.1)**	0.5 (0.5 to 0.6)	**-33.4 (-44.1 to -22.4)**	1,198.0 (1,022.8 to 1,420.8)	**-28.9 (-41.1 to -16.2)**	31.0 (26.4 to 36.9)	**-34.4 (-45.3 to -22.4)**
Maternal hypertensive disorders	29.4 (25.4 to 34.5)	-5.5 (-20.7 to 11.2)	0.8 (0.7 to 0.9)	-12.7 (-27.0 to 2.9)	1,729.6 (1,487.6 to 2,033.2)	-6.6 (-22.1 to 10.2)	45.2 (38.9 to 53.4)	-13.3 (-27.9 to 2.3)
Maternal obstructed labor and uterine rupture	13.0 (10.2 to 16.8)	-17.7 (-35.9 to 2.9)	0.3 (0.3 to 0.4)	**-25.1 (-40.8 to -6.2)**	720.9 (565.5 to 946.4)	-18.9 (-37.6 to 1.9)	18.6 (14.5 to 24.5)	**-25.6 (-42.8 to -6.7)**
Maternal abortion and miscarriage	17.4 (14.7 to 20.8)	-7.0 (-22.3 to 10.1)	0.4 (0.4 to 0.5)	**-15.6 (-29.2 to -0.3)**	963.4 (807.6 to 1,161.1)	-8.9 (-24.2 to 8.7)	24.8 (20.8 to 30.1)	**-16.6 (-30.5 to -0.3)**
Ectopic pregnancy	10.2 (7.1 to 15.2)	-11.6 (-41.4 to 27.9)	0.3 (0.2 to 0.4)	-18.9 (-46.1 to 17.0)	590.6 (409.0 to 881.4)	-13.3 (-43.8 to 26.9)	15.3 (10.6 to 23.0)	-20.1 (-48.0 to 17.3)
Indirect maternal deaths	34.1 (30.0 to 38.7)	-4.1 (-16.7 to 8.5)	0.9 (0.8 to 1.0)	**-12.3 (-23.9 to -0.7)**	1,934.4 (1,694.2 to 2,216.7)	-6.1 (-19.2 to 6.8)	50.1 (43.9 to 57.5)	**-13.6 (-25.5 to -2.0)**
Late maternal deaths	3.4 (2.6 to 4.3)	-0.9 (-7.0 to 5.5)	0.1 (0.1 to 0.1)	**-9.3 (-14.5 to -3.7)**	194.7 (152.2 to 251.4)	-2.0 (-8.2 to 4.1)	5.1 (4.0 to 6.5)	**-9.8 (-15.1 to -4.3)**
Maternal deaths aggravated by HIV/AIDS	1.6 (1.0 to 2.1)	**-23.9 (-31.0 to -16.0)**	0.0 (0.0 to 0.1)	**-31.9 (-38.3 to -25.0)**	84.4 (53.0 to 113.8)	**-26.7 (-33.6 to -19.2)**	2.2 (1.4 to 2.9)	**-34.1 (-40.4 to -27.3)**
Other maternal disorders	24.8 (20.8 to 29.8)	-8.5 (-24.7 to 11.2)	0.6 (0.5 to 0.8)	-16.4 (-31.0 to 1.6)	1,403.1 (1,159.5 to 1,690.3)	-9.8 (-26.7 to 10.8)	36.3 (30.1 to 43.8)	-17.0 (-32.6 to 1.4)
Neonatal disorders	769.4 (732.4 to 807.1)	**-24.1 (-27.5 to -20.4)**	24.2 (23.0 to 25.4)	**-26.2 (-29.5 to -22.7)**	67,586.1 (64,338.3 to 70,892.5)	**-24.1 (-27.5 to -20.4)**	2,126.2 (2,024.1 to 2,230.2)	**-26.2 (-29.5 to -22.7)**
Neonatal preterm birth	279.1 (254.3 to 320.6)	**-26.6 (-32.5 to -20.9)**	8.8 (8.0 to 10.1)	**-28.6 (-34.4 to -23.1)**	24,519.7 (22,343.5 to 28,164.3)	**-26.6 (-32.5 to -20.9)**	771.5 (703.0 to 886.2)	**-28.6 (-34.4 to -23.1)**
Neonatal encephalopathy due to birth asphyxia and trauma	222.6 (194.1 to 246.2)	**-24.7 (-32.0 to -16.8)**	7.0 (6.1 to 7.7)	**-26.8 (-33.9 to -19.2)**	19,557.6 (17,054.6 to 21,629.0)	**-24.7 (-32.0 to -16.8)**	615.4 (536.6 to 680.6)	**-26.8 (-33.9 to -19.1)**
Neonatal sepsis and other neonatal infections	90.7 (67.4 to 129.1)	-7.9 (-20.4 to 3.5)	2.9 (2.1 to 4.1)	-10.5 (-22.8 to 0.5)	7,961.6 (5,920.5 to 11,337.5)	-7.9 (-20.5 to 3.5)	250.4 (186.2 to 356.5)	-10.5 (-22.8 to 0.5)
Hemolytic disease and other neonatal jaundice	21.3 (17.8 to 25.3)	**-38.2 (-48.5 to -23.3)**	0.7 (0.6 to 0.8)	**-40.0 (-50.0 to -25.5)**	1,874.2 (1,560.8 to 2,223.5)	**-38.2 (-48.5 to -23.3)**	58.9 (49.1 to 69.9)	**-40.0 (-50.0 to -25.5)**
Other neonatal disorders	155.7 (132.2 to 175.7)	**-24.1 (-32.6 to -14.2)**	4.9 (4.2 to 5.5)	**-26.3 (-34.6 to -16.6)**	13,673.0 (11,608.2 to 15,435.7)	**-24.1 (-32.6 to -14.2)**	430.1 (365.2 to 485.6)	**-26.3 (-34.6 to -16.6)**
**Nutritional deficiencies**	147.0 (135.8 to 157.2)	**-18.0 (-23.3 to -12.2)**	4.1 (3.7 to 4.4)	**-29.5 (-34.2 to -24.2)**	8,459.1 (7,588.2 to 9,261.2)	**-29.9 (-36.0 to -23.5)**	253.2 (226.6 to 277.8)	**-35.1 (-40.9 to -29.1)**
Protein-energy malnutrition	122.8 (111.3 to 134.1)	**-20.9 (-26.7 to -15.0)**	3.5 (3.1 to 3.8)	**-30.8 (-36.1 to -25.4)**	7,656.8 (6,773.8 to 8,461.6)	**-30.5 (-37.0 to -23.9)**	231.6 (204.5 to 256.4)	**-35.2 (-41.3 to -28.9)**
Other nutritional deficiencies	24.1 (20.6 to 29.4)	1.0 (-8.1 to 11.7)	0.6 (0.5 to 0.7)	**-20.2 (-27.6 to -11.8)**	802.3 (668.5 to 939.9)	**-23.7 (-33.2 to -14.5)**	21.6 (17.9 to 25.5)	**-34.5 (-43.0 to -26.3)**

SR Table 3: Global deaths and YLL numbers, age-standardised rates per 100,000, and percent change between 2007 and 2017 for females for all GBD causes and Levels 1 through 4 of the cause hierarchy. 95% UIs are in parentheses. UI=uncertainty interval. YLL=year of life lost.

Cause	All Age Deaths (thousands)		Age-standardised Death Rate (per 100,000)		All Age YLLs (thousands)		Age-standardised YLL Rate (per 100,000)	
**Non-communicable diseases**	19,228.2 (18,804.5 to 19,569.5)	**23.0 (21.3 to 24.8)**	450.9 (441.1 to 459.0)	**-7.8 (-9.2 to -6.5)**	373,244.6 (364,778.7 to 380,747.8)	**13.7 (11.7 to 15.4)**	9,105.0 (8,896.0 to 9,293.5)	**-9.6 (-11.2 to -8.3)**
**Neoplasms**	4,114.0 (4,015.7 to 4,200.6)	**25.0 (22.6 to 27.3)**	96.9 (94.5 to 98.9)	**-4.2 (-6.1 to -2.5)**	98,155.9 (95,600.6 to 100,440.2)	**19.6 (16.6 to 22.3)**	2,359.5 (2,297.4 to 2,414.0)	**-5.0 (-7.4 to -2.8)**
Lip and oral cavity cancer	68.6 (65.2 to 72.3)	**42.7 (36.1 to 49.6)**	1.6 (1.5 to 1.7)	**9.1 (4.0 to 14.3)**	1,703.0 (1,599.0 to 1,814.3)	**38.1 (30.2 to 46.9)**	40.7 (38.1 to 43.4)	**9.3 (3.0 to 16.3)**
Nasopharynx cancer	18.6 (17.8 to 19.4)	**24.8 (19.0 to 31.8)**	0.4 (0.4 to 0.5)	-2.2 (-6.8 to 3.2)	551.3 (527.4 to 576.8)	**17.0 (11.3 to 23.6)**	13.3 (12.7 to 13.9)	**-5.5 (-10.1 to -0.2)**
Other pharynx cancer	33.5 (31.2 to 35.8)	**44.6 (34.0 to 53.6)**	0.8 (0.7 to 0.8)	**11.3 (3.2 to 18.2)**	911.5 (845.7 to 972.0)	**40.1 (30.0 to 49.3)**	21.7 (20.1 to 23.1)	**10.4 (2.4 to 17.8)**
Esophageal cancer	125.2 (120.1 to 130.4)	**6.9 (1.4 to 11.9)**	2.9 (2.8 to 3.1)	**-19.1 (-23.2 to -15.2)**	2,547.6 (2,444.6 to 2,650.8)	3.2 (-1.9 to 8.0)	60.0 (57.6 to 62.4)	**-20.5 (-24.4 to -16.8)**
Stomach cancer	318.5 (309.8 to 327.9)	**8.6 (5.0 to 12.1)**	7.5 (7.3 to 7.7)	**-17.4 (-20.2 to -14.9)**	6,766.5 (6,575.5 to 6,963.9)	**4.2 (0.5 to 7.7)**	160.3 (155.8 to 164.9)	**-18.3 (-21.2 to -15.7)**
Colon and rectum cancer	413.5 (401.1 to 423.4)	**24.7 (19.9 to 28.7)**	9.6 (9.4 to 9.9)	**-6.3 (-10.0 to -3.3)**	7,941.7 (7,675.6 to 8,141.0)	**21.0 (15.2 to 25.4)**	187.3 (181.0 to 192.0)	**-6.4 (-10.8 to -3.0)**
Liver cancer	247.3 (238.5 to 257.3)	**23.9 (18.4 to 30.5)**	5.8 (5.6 to 6.0)	**-5.4 (-9.6 to -0.5)**	5,468.6 (5,263.9 to 5,685.4)	**17.5 (11.9 to 24.9)**	129.5 (124.6 to 134.6)	**-8.2 (-12.6 to -2.5)**
Liver cancer due to hepatitis B	73.1 (67.7 to 78.8)	**16.2 (9.3 to 24.3)**	1.7 (1.6 to 1.9)	**-9.8 (-15.2 to -3.4)**	1,950.7 (1,816.5 to 2,090.7)	**9.1 (2.4 to 17.2)**	46.5 (43.4 to 49.9)	**-13.2 (-18.4 to -6.8)**
Liver cancer due to hepatitis C	94.1 (88.9 to 99.2)	**26.6 (22.1 to 32.3)**	2.2 (2.1 to 2.3)	**-4.8 (-8.3 to -0.5)**	1,774.3 (1,669.5 to 1,881.4)	**22.9 (17.9 to 29.4)**	41.6 (39.1 to 44.1)	**-6.1 (-10.0 to -1.2)**
Liver cancer due to alcohol use	27.6 (24.4 to 31.6)	**27.0 (22.0 to 32.8)**	0.6 (0.6 to 0.7)	-3.0 (-6.7 to 1.4)	581.8 (508.1 to 680.3)	**23.5 (18.3 to 30.0)**	13.7 (12.0 to 16.0)	-4.3 (-8.2 to 0.6)
Liver cancer due to NASH	25.7 (22.9 to 28.7)	**36.5 (30.6 to 43.4)**	0.6 (0.5 to 0.7)	3.3 (-1.2 to 8.4)	499.2 (446.6 to 554.0)	**31.5 (25.2 to 39.1)**	11.8 (10.5 to 13.0)	1.6 (-3.2 to 7.2)
Liver cancer due to other causes	26.8 (24.3 to 29.8)	**23.3 (17.0 to 31.3)**	0.6 (0.6 to 0.7)	-5.1 (-9.9 to 1.0)	662.5 (600.8 to 735.1)	**15.9 (9.5 to 24.3)**	15.9 (14.4 to 17.6)	**-8.2 (-13.1 to -1.6)**
Gallbladder and biliary tract cancer	102.4 (88.8 to 109.6)	**23.8 (19.2 to 28.5)**	2.4 (2.1 to 2.6)	**-7.1 (-10.6 to -3.6)**	1,993.9 (1,680.9 to 2,131.3)	**21.9 (16.6 to 27.2)**	46.9 (39.5 to 50.1)	**-6.5 (-10.7 to -2.5)**
Pancreatic cancer	215.0 (210.6 to 219.7)	**40.9 (38.1 to 43.9)**	5.0 (4.9 to 5.1)	**5.8 (3.7 to 8.1)**	4,048.8 (3,962.5 to 4,147.2)	**38.6 (35.7 to 41.3)**	95.1 (93.0 to 97.4)	**6.0 (3.8 to 8.1)**
Larynx cancer	20.9 (20.0 to 21.7)	**26.2 (21.2 to 31.3)**	0.5 (0.5 to 0.5)	-3.2 (-7.1 to 0.7)	529.0 (506.2 to 551.2)	**22.0 (16.7 to 27.6)**	12.6 (12.0 to 13.1)	-4.3 (-8.5 to 0.1)
Tracheal, bronchus, and lung cancer	596.3 (578.6 to 614.2)	**36.4 (31.9 to 40.6)**	13.9 (13.5 to 14.4)	3.3 (0.0 to 6.6)	12,396.7 (12,004.7 to 12,790.2)	**31.5 (27.4 to 35.7)**	291.5 (282.3 to 300.8)	1.3 (-1.9 to 4.5)
Malignant skin melanoma	28.7 (22.0 to 36.5)	**22.6 (16.3 to 28.8)**	0.7 (0.5 to 0.9)	**-6.0 (-10.7 to -0.9)**	679.5 (538.8 to 880.9)	**16.2 (9.8 to 24.6)**	16.3 (12.9 to 21.1)	-7.0 (-12.0 to 0.1)
Non-melanoma skin cancer	21.8 (21.2 to 22.3)	**44.7 (38.6 to 49.6)**	0.5 (0.5 to 0.5)	**6.3 (1.9 to 9.8)**	364.8 (354.2 to 375.6)	**34.9 (27.5 to 41.0)**	8.6 (8.4 to 8.9)	4.4 (-1.2 to 9.0)
Non-melanoma skin cancer (squamous-cell carcinoma)	21.8 (21.2 to 22.3)	**44.7 (38.6 to 49.6)**	0.5 (0.5 to 0.5)	**6.3 (1.9 to 9.8)**	364.8 (354.2 to 375.6)	**34.9 (27.5 to 41.0)**	8.6 (8.4 to 8.9)	4.4 (-1.2 to 9.0)
Breast cancer	600.7 (578.7 to 629.9)	**26.8 (20.9 to 31.0)**	14.1 (13.6 to 14.8)	-2.2 (-6.7 to 1.0)	16,132.2 (15,470.6 to 17,055.7)	**23.7 (17.0 to 28.5)**	384.1 (368.2 to 406.3)	-1.8 (-7.1 to 2.1)
Cervical cancer	259.7 (241.1 to 269.2)	**18.8 (12.9 to 22.8)**	6.1 (5.7 to 6.4)	**-6.9 (-11.5 to -3.7)**	7,773.5 (7,227.4 to 8,087.8)	**15.1 (9.4 to 19.1)**	186.0 (173.1 to 193.6)	**-7.3 (-11.9 to -4.1)**
Uterine cancer	85.2 (83.2 to 87.4)	**18.8 (15.8 to 22.5)**	2.0 (1.9 to 2.0)	**-9.8 (-12.1 to -7.0)**	1,930.0 (1,879.9 to 1,983.0)	**14.8 (11.6 to 19.0)**	45.5 (44.3 to 46.8)	**-11.2 (-13.7 to -7.9)**
Ovarian cancer	176.0 (171.4 to 181.2)	**30.3 (26.8 to 33.7)**	4.1 (4.0 to 4.3)	-0.1 (-2.8 to 2.5)	4,496.9 (4,370.7 to 4,642.1)	**29.1 (24.8 to 33.1)**	106.7 (103.7 to 110.2)	1.3 (-2.1 to 4.5)
Prostate cancer	-	-	-	-	-	-	-	-
Testicular cancer	-	-	-	-	-	-	-	-
Kidney cancer	48.9 (42.9 to 50.6)	**26.2 (21.2 to 31.3)**	1.2 (1.0 to 1.2)	-4.1 (-7.9 to 0.1)	1,062.2 (979.2 to 1,099.1)	**19.2 (11.8 to 25.7)**	25.8 (23.8 to 26.8)	**-5.7 (-11.6 to -0.3)**
Bladder cancer	51.8 (50.3 to 53.3)	**24.5 (18.7 to 29.6)**	1.2 (1.2 to 1.2)	**-7.8 (-12.1 to -4.0)**	830.6 (802.5 to 856.7)	**20.0 (13.7 to 25.6)**	19.5 (18.9 to 20.1)	**-8.1 (-13.0 to -3.8)**
Brain and nervous system cancer	106.9 (76.1 to 119.1)	**23.3 (7.3 to 38.4)**	2.6 (1.9 to 2.9)	-1.3 (-13.8 to 11.0)	3,641.3 (2,636.3 to 4,128.3)	11.8 (-4.2 to 30.8)	93.0 (67.2 to 105.6)	-5.7 (-19.4 to 10.6)
Thyroid cancer	24.1 (23.1 to 26.8)	**23.9 (18.2 to 29.2)**	0.6 (0.5 to 0.6)	**-4.5 (-8.9 to -0.4)**	574.0 (543.2 to 642.9)	**17.6 (10.5 to 25.2)**	13.8 (13.1 to 15.5)	-5.5 (-11.4 to 0.8)
Mesothelioma	8.2 (7.9 to 8.6)	**22.3 (13.7 to 29.7)**	0.2 (0.2 to 0.2)	**-5.9 (-12.3 to -0.6)**	189.4 (177.7 to 204.6)	**16.6 (7.0 to 25.5)**	4.5 (4.2 to 4.9)	**-7.0 (-14.2 to -0.6)**
Hodgkin lymphoma	11.8 (9.6 to 14.1)	2.4 (-2.7 to 7.3)	0.3 (0.2 to 0.3)	**-14.0 (-18.1 to -9.7)**	487.1 (383.2 to 583.1)	-2.5 (-8.6 to 4.0)	12.6 (9.8 to 15.1)	**-13.6 (-19.0 to -7.5)**
Non-Hodgkin lymphoma	104.4 (101.6 to 107.3)	**27.0 (21.7 to 31.1)**	2.5 (2.4 to 2.6)	-1.7 (-5.8 to 1.5)	2,682.3 (2,599.6 to 2,776.4)	**21.1 (14.0 to 27.4)**	66.5 (64.2 to 69.2)	-1.0 (-6.9 to 4.5)
Multiple myeloma	52.0 (48.9 to 58.4)	**29.7 (24.9 to 34.8)**	1.2 (1.1 to 1.4)	-2.0 (-5.6 to 1.8)	1,043.7 (996.1 to 1,183.7)	**28.9 (23.6 to 34.2)**	24.6 (23.5 to 27.9)	-0.7 (-4.8 to 3.3)
Leukemia	150.3 (131.4 to 160.5)	**10.3 (4.4 to 14.0)**	3.7 (3.2 to 4.0)	**-11.5 (-16.4 to -8.4)**	5,033.9 (4,315.7 to 5,494.0)	0.2 (-8.9 to 5.6)	131.2 (112.5 to 144.1)	**-13.7 (-21.9 to -8.8)**
Acute lymphoid leukemia	21.6 (17.6 to 24.3)	15.5 (-2.8 to 24.3)	0.6 (0.5 to 0.6)	-0.3 (-16.5 to 7.4)	1,106.9 (907.7 to 1,286.5)	7.4 (-14.0 to 17.7)	30.6 (25.1 to 35.7)	-2.6 (-22.7 to 7.0)
Chronic lymphoid leukemia	14.4 (13.4 to 15.2)	**19.9 (15.9 to 24.2)**	0.3 (0.3 to 0.4)	**-11.1 (-14.0 to -7.8)**	243.5 (219.5 to 263.9)	**18.3 (14.0 to 22.3)**	5.8 (5.2 to 6.3)	**-8.6 (-11.9 to -5.4)**
Acute myeloid leukemia	42.5 (37.9 to 46.2)	**19.5 (7.9 to 25.5)**	1.0 (0.9 to 1.1)	**-4.7 (-13.8 to 0.0)**	1,339.6 (1,168.1 to 1,509.2)	10.8 (-6.2 to 19.9)	34.2 (29.7 to 38.7)	-6.0 (-20.7 to 2.0)
Chronic myeloid leukemia	11.1 (9.7 to 12.7)	3.0 (-2.1 to 7.9)	0.3 (0.2 to 0.3)	**-19.7 (-23.5 to -15.8)**	302.7 (254.5 to 352.9)	-1.5 (-7.5 to 3.6)	7.4 (6.2 to 8.6)	**-19.0 (-24.0 to -14.8)**
Other leukemia	60.7 (50.8 to 65.4)	2.5 (-4.9 to 7.5)	1.5 (1.2 to 1.6)	**-17.7 (-23.6 to -13.6)**	2,041.3 (1,659.4 to 2,257.5)	**-10.1 (-20.3 to -2.8)**	53.3 (43.2 to 59.3)	**-22.6 (-31.7 to -16.1)**
Other malignant neoplasms	172.8 (156.1 to 182.2)	**26.8 (23.8 to 29.7)**	4.2 (3.8 to 4.4)	0.0 (-2.4 to 2.4)	5,214.6 (4,748.5 to 5,556.1)	**19.0 (13.5 to 24.1)**	133.1 (121.4 to 142.3)	0.1 (-4.9 to 4.8)
Other neoplasms	50.9 (36.4 to 65.0)	**38.5 (29.2 to 53.3)**	1.2 (0.9 to 1.5)	5.5 (-2.0 to 18.1)	1,161.1 (903.3 to 1,515.3)	**30.2 (20.1 to 43.6)**	28.8 (22.4 to 37.6)	6.0 (-2.4 to 17.8)
Myelodysplastic, myeloproliferative, and other hematopoietic neoplasms	48.7 (34.8 to 62.5)	**39.1 (30.0 to 53.8)**	1.1 (0.8 to 1.5)	5.2 (-2.2 to 17.6)	1,035.1 (799.4 to 1,360.8)	**31.3 (22.1 to 44.8)**	25.3 (19.7 to 33.4)	5.4 (-2.5 to 16.9)
Other benign and in situ neoplasms	2.3 (1.6 to 2.7)	**27.3 (11.6 to 47.2)**	0.1 (0.0 to 0.1)	12.5 (-1.8 to 29.9)	126.0 (89.7 to 149.8)	**21.5 (5.6 to 39.3)**	3.4 (2.4 to 4.1)	10.6 (-4.1 to 26.8)
**Cardiovascular diseases**	8,444.6 (8,266.5 to 8,616.0)	**19.9 (18.0 to 21.9)**	196.1 (191.9 to 200.1)	**-11.4 (-12.8 to -9.9)**	136,669.0 (133,485.3 to 139,685.6)	**13.3 (11.2 to 15.5)**	3,230.4 (3,155.3 to 3,301.7)	**-12.6 (-14.2 to -10.9)**
Rheumatic heart disease	166.7 (151.4 to 181.9)	4.3 (-1.9 to 11.6)	4.0 (3.6 to 4.3)	**-19.3 (-24.2 to -13.5)**	4,217.3 (3,779.1 to 4,679.2)	**-7.1 (-13.3 to -0.7)**	103.2 (92.5 to 114.4)	**-24.0 (-29.1 to -18.8)**
Ischemic heart disease	4,026.4 (3,934.1 to 4,144.5)	**21.3 (19.1 to 23.4)**	93.3 (91.2 to 96.1)	**-10.8 (-12.4 to -9.2)**	62,707.4 (61,018.9 to 64,428.8)	**16.7 (14.2 to 19.2)**	1,474.2 (1,434.5 to 1,515.1)	**-10.6 (-12.5 to -8.7)**
Stroke	2,993.1 (2,913.1 to 3,092.1)	**14.2 (11.5 to 16.9)**	69.6 (67.7 to 71.9)	**-15.1 (-17.2 to -13.2)**	49,595.1 (48,199.8 to 51,207.5)	**9.1 (6.3 to 12.0)**	1,169.1 (1,136.3 to 1,207.2)	**-16.0 (-18.2 to -13.8)**
Ischemic stroke	1,416.6 (1,359.1 to 1,476.0)	**16.7 (13.9 to 19.6)**	32.8 (31.4 to 34.2)	**-14.5 (-16.6 to -12.4)**	18,917.7 (17,842.1 to 20,031.0)	**11.4 (8.2 to 14.7)**	442.7 (417.2 to 469.0)	**-15.6 (-18.0 to -13.1)**
Intracerebral hemorrhage	1,353.4 (1,295.9 to 1,412.1)	**11.5 (7.8 to 15.3)**	31.6 (30.2 to 33.0)	**-16.3 (-19.1 to -13.4)**	25,543.1 (24,322.5 to 26,715.9)	**7.6 (3.9 to 11.4)**	603.1 (574.3 to 630.8)	**-16.8 (-19.6 to -13.8)**
Subarachnoid hemorrhage	223.1 (210.0 to 252.3)	**16.0 (11.8 to 20.5)**	5.2 (4.9 to 5.9)	**-11.4 (-14.6 to -8.0)**	5,134.2 (4,791.2 to 5,900.9)	**8.7 (4.4 to 12.9)**	123.3 (115.1 to 142.0)	**-13.5 (-17.0 to -10.2)**
Hypertensive heart disease	523.9 (372.2 to 572.2)	**43.3 (24.7 to 58.3)**	12.1 (8.6 to 13.2)	5.1 (-8.1 to 15.6)	7,767.6 (5,781.6 to 8,542.6)	**31.8 (17.9 to 47.2)**	182.2 (135.7 to 200.6)	0.6 (-9.8 to 11.9)
Non-rheumatic valvular heart disease	81.3 (63.9 to 86.3)	**31.2 (23.6 to 35.4)**	1.9 (1.5 to 2.0)	**-6.0 (-10.2 to -2.6)**	1,052.5 (924.1 to 1,148.2)	**22.0 (17.2 to 26.7)**	24.9 (22.0 to 27.2)	**-6.3 (-9.4 to -2.3)**
Non-rheumatic calcific aortic valve disease	57.4 (41.6 to 62.0)	**38.4 (28.6 to 44.0)**	1.3 (1.0 to 1.4)	-2.3 (-7.9 to 1.3)	628.1 (516.1 to 703.8)	**29.8 (22.2 to 36.0)**	14.7 (12.1 to 16.5)	-2.7 (-7.2 to 1.5)
Non-rheumatic degenerative mitral valve disease	20.5 (16.7 to 27.0)	**17.8 (11.0 to 25.3)**	0.5 (0.4 to 0.6)	**-12.8 (-17.5 to -7.4)**	355.3 (296.1 to 450.7)	**12.5 (6.0 to 19.1)**	8.5 (7.1 to 10.8)	**-11.0 (-15.5 to -5.8)**
Other non-rheumatic valve diseases	3.5 (2.4 to 4.9)	9.8 (-4.6 to 47.0)	0.1 (0.1 to 0.1)	-17.2 (-28.2 to 11.2)	69.1 (47.6 to 100.4)	9.2 (-2.0 to 32.1)	1.7 (1.2 to 2.4)	-11.4 (-20.7 to 7.4)
Cardiomyopathy and myocarditis	155.6 (149.2 to 163.3)	**10.0 (7.0 to 14.3)**	3.7 (3.5 to 3.9)	**-17.1 (-19.3 to -14.0)**	3,411.0 (3,203.5 to 3,660.7)	**-6.0 (-8.8 to -1.8)**	85.3 (79.9 to 91.7)	**-22.7 (-25.1 to -19.5)**
Myocarditis	24.6 (20.1 to 27.1)	**10.0 (0.3 to 29.3)**	0.6 (0.5 to 0.6)	**-17.4 (-24.8 to -2.5)**	572.4 (488.9 to 643.4)	-6.4 (-13.3 to 3.9)	14.8 (12.7 to 16.7)	**-20.8 (-26.7 to -12.0)**
Alcoholic cardiomyopathy	20.3 (18.7 to 24.2)	**-35.5 (-38.5 to -29.9)**	0.5 (0.4 to 0.6)	**-49.3 (-51.6 to -45.2)**	614.1 (566.1 to 703.0)	**-39.1 (-41.9 to -33.4)**	14.8 (13.6 to 16.9)	**-50.5 (-52.8 to -45.9)**
Other cardiomyopathy	110.7 (103.5 to 117.3)	**26.4 (21.8 to 30.3)**	2.6 (2.4 to 2.8)	**-5.9 (-9.7 to -3.0)**	2,224.5 (1,979.3 to 2,424.6)	**10.8 (6.4 to 17.0)**	55.7 (49.4 to 60.8)	**-9.9 (-13.4 to -5.4)**
Atrial fibrillation and flutter	177.3 (170.9 to 183.8)	**45.9 (42.9 to 49.1)**	4.1 (3.9 to 4.2)	**2.6 (0.5 to 4.9)**	1,748.5 (1,675.0 to 1,826.4)	**39.4 (36.5 to 42.5)**	40.5 (38.8 to 42.4)	**2.4 (0.2 to 4.7)**
Aortic aneurysm	60.9 (58.7 to 65.3)	**26.8 (22.2 to 30.7)**	1.4 (1.4 to 1.5)	**-6.6 (-10.0 to -3.8)**	948.6 (907.1 to 1,049.6)	**21.6 (16.2 to 25.9)**	22.3 (21.4 to 24.7)	**-6.6 (-10.6 to -3.2)**
Peripheral artery disease	36.4 (16.4 to 84.6)	**55.8 (25.6 to 79.9)**	0.8 (0.4 to 1.9)	10.2 (-11.1 to 27.4)	395.3 (183.8 to 889.5)	**49.4 (19.2 to 73.6)**	9.2 (4.3 to 20.6)	10.4 (-12.0 to 28.2)
Endocarditis	43.8 (34.5 to 53.5)	**28.8 (22.5 to 34.8)**	1.0 (0.8 to 1.3)	-2.1 (-7.0 to 3.1)	1,038.7 (886.5 to 1,192.9)	**13.3 (5.3 to 20.4)**	26.6 (22.8 to 30.4)	-5.2 (-11.7 to 0.7)
Other cardiovascular and circulatory diseases	179.1 (165.4 to 199.1)	**22.9 (18.5 to 27.8)**	4.2 (3.9 to 4.7)	**-7.2 (-10.6 to -3.6)**	3,786.9 (3,407.4 to 4,407.2)	**14.3 (10.4 to 20.4)**	92.9 (83.4 to 109.0)	**-8.1 (-11.2 to -3.4)**
**Chronic respiratory diseases**	1,749.8 (1,626.3 to 1,877.8)	**19.1 (11.4 to 26.2)**	40.8 (37.9 to 43.8)	**-11.3 (-17.0 to -5.9)**	29,086.6 (27,197.0 to 31,655.3)	**12.7 (5.7 to 20.6)**	690.2 (645.4 to 751.7)	**-12.9 (-18.3 to -6.7)**
Chronic obstructive pulmonary disease	1,408.0 (1,279.9 to 1,516.6)	**19.8 (10.4 to 27.0)**	32.7 (29.7 to 35.3)	**-11.4 (-18.2 to -6.1)**	21,130.0 (18,882.3 to 23,092.0)	**15.6 (5.7 to 23.7)**	495.9 (443.2 to 542.2)	**-12.1 (-19.4 to -5.9)**
Pneumoconiosis	2.3 (1.9 to 2.7)	**16.4 (1.4 to 31.0)**	0.1 (0.0 to 0.1)	**-12.2 (-23.4 to -1.4)**	43.2 (35.7 to 50.1)	8.7 (-4.4 to 21.3)	1.0 (0.8 to 1.2)	**-14.6 (-25.1 to -4.7)**
Silicosis	0.6 (0.4 to 0.8)	**25.5 (10.3 to 42.1)**	0.0 (0.0 to 0.0)	-5.3 (-16.8 to 7.4)	11.1 (6.7 to 14.8)	**16.4 (2.5 to 32.3)**	0.3 (0.2 to 0.3)	-8.8 (-19.8 to 3.8)
Asbestosis	0.5 (0.3 to 0.6)	14.1 (-3.6 to 37.1)	0.0 (0.0 to 0.0)	-14.6 (-27.6 to 3.3)	8.2 (5.0 to 11.0)	7.7 (-7.7 to 30.2)	0.2 (0.1 to 0.3)	-16.0 (-28.0 to 2.0)
Coal workers pneumoconiosis	0.3 (0.1 to 0.4)	**15.4 (0.2 to 38.9)**	0.0 (0.0 to 0.0)	-13.2 (-24.6 to 4.4)	4.8 (2.6 to 6.7)	5.2 (-8.5 to 28.2)	0.1 (0.1 to 0.2)	-17.6 (-28.4 to 0.2)
Other pneumoconiosis	1.0 (0.8 to 1.3)	12.6 (-2.4 to 27.8)	0.0 (0.0 to 0.0)	**-14.7 (-26.1 to -3.3)**	19.1 (14.3 to 24.7)	5.9 (-7.4 to 19.3)	0.5 (0.3 to 0.6)	**-16.3 (-26.9 to -5.7)**
Asthma	253.6 (173.2 to 330.3)	**9.4 (1.9 to 24.0)**	6.0 (4.1 to 7.8)	**-15.9 (-21.7 to -4.6)**	6,074.0 (4,302.3 to 8,084.8)	0.4 (-5.7 to 11.1)	148.2 (106.0 to 197.1)	**-19.4 (-24.3 to -10.3)**
Interstitial lung disease and pulmonary sarcoidosis	67.7 (45.3 to 90.2)	**49.1 (32.8 to 65.6)**	1.6 (1.1 to 2.1)	11.6 (-0.2 to 24.2)	1,213.1 (842.3 to 1,665.5)	**42.7 (24.6 to 60.8)**	28.8 (20.0 to 39.6)	10.8 (-3.1 to 25.1)
Other chronic respiratory diseases	18.1 (14.7 to 21.3)	**22.1 (10.5 to 40.0)**	0.4 (0.4 to 0.5)	-2.0 (-10.9 to 12.9)	626.4 (508.8 to 743.3)	8.2 (-3.3 to 28.8)	16.2 (13.3 to 19.4)	-7.4 (-17.2 to 11.3)
**Digestive diseases**	945.9 (902.0 to 997.2)	**16.8 (13.3 to 21.2)**	22.4 (21.3 to 23.6)	**-10.5 (-13.2 to -7.2)**	22,838.7 (21,519.9 to 24,242.4)	**7.4 (3.8 to 12.0)**	563.2 (529.4 to 599.2)	**-12.6 (-15.7 to -8.6)**
Cirrhosis and other chronic liver diseases	440.2 (415.5 to 518.4)	**15.9 (8.4 to 24.7)**	10.5 (9.9 to 12.3)	**-9.9 (-15.7 to -3.0)**	11,937.6 (11,229.9 to 14,028.0)	**8.3 (1.6 to 14.7)**	290.7 (273.4 to 342.3)	**-12.2 (-17.7 to -7.0)**
Cirrhosis and other chronic liver diseases due to hepatitis B	105.8 (93.9 to 129.6)	8.8 (-0.6 to 21.1)	2.5 (2.2 to 3.1)	**-15.1 (-22.5 to -5.6)**	2,860.1 (2,548.7 to 3,427.7)	2.6 (-6.1 to 12.3)	69.0 (61.4 to 82.7)	**-16.9 (-23.8 to -9.2)**
Cirrhosis and other chronic liver diseases due to hepatitis C	117.6 (106.7 to 134.8)	**18.2 (10.5 to 26.3)**	2.8 (2.5 to 3.2)	**-8.6 (-14.6 to -2.4)**	3,075.0 (2,794.5 to 3,524.2)	**12.0 (4.8 to 19.1)**	73.6 (67.0 to 84.3)	**-10.5 (-16.2 to -5.0)**
Cirrhosis and other chronic liver diseases due to alcohol use	90.9 (81.6 to 109.7)	**17.5 (10.8 to 28.3)**	2.1 (1.9 to 2.6)	**-9.4 (-14.6 to -1.1)**	2,387.9 (2,143.6 to 2,849.8)	**12.1 (5.8 to 21.7)**	56.8 (51.0 to 67.8)	**-11.3 (-16.3 to -3.8)**
Cirrhosis due to NASH	49.9 (45.6 to 55.9)	**26.8 (19.4 to 33.7)**	1.2 (1.1 to 1.3)	-2.8 (-8.4 to 2.4)	1,265.1 (1,150.8 to 1,406.1)	**20.7 (14.1 to 26.8)**	30.1 (27.3 to 33.4)	**-5.0 (-10.3 to -0.4)**
Cirrhosis and other chronic liver diseases due to other causes	76.0 (67.0 to 89.6)	**14.7 (7.7 to 20.9)**	1.9 (1.6 to 2.2)	**-8.8 (-14.3 to -3.7)**	2,349.6 (2,070.9 to 2,806.2)	1.5 (-6.0 to 11.1)	61.2 (53.8 to 73.4)	**-12.8 (-19.4 to -4.6)**
Upper digestive system diseases	129.4 (114.8 to 139.7)	**7.5 (2.4 to 13.7)**	3.0 (2.7 to 3.3)	**-18.7 (-22.5 to -14.0)**	2,664.6 (2,250.0 to 2,889.2)	-1.0 (-6.4 to 6.0)	64.5 (54.4 to 69.9)	**-20.8 (-25.2 to -15.3)**
Peptic ulcer disease	107.1 (94.7 to 120.0)	**5.5 (0.4 to 11.6)**	2.5 (2.2 to 2.8)	**-20.2 (-24.0 to -15.7)**	2,198.0 (1,858.8 to 2,479.0)	-2.3 (-7.8 to 4.9)	53.1 (44.9 to 59.8)	**-22.0 (-26.2 to -16.2)**
Gastritis and duodenitis	22.3 (13.4 to 26.1)	**18.1 (7.7 to 37.9)**	0.5 (0.3 to 0.6)	-10.5 (-18.1 to 3.3)	466.6 (266.9 to 546.0)	5.7 (-5.0 to 25.8)	11.3 (6.5 to 13.4)	-14.9 (-23.6 to 0.1)

SR Table 3: Global deaths and YLL numbers, age-standardised rates per 100,000, and percent change between 2007 and 2017 for females for all GBD causes and Levels 1 through 4 of the cause hierarchy. 95% UIs are in parentheses. UI=uncertainty interval. YLL=year of life lost.

Cause	All Age Deaths (thousands)		Age-standardised Death Rate (per 100,000)		All Age YLLs (thousands)		Age-standardised YLL Rate (per 100,000)	
Appendicitis	22.3 (19.1 to 24.8)	3.0 (-4.2 to 12.1)	0.6 (0.5 to 0.6)	**-15.8 (-21.8 to -8.3)**	832.1 (703.0 to 932.1)	-8.2 (-16.8 to 1.8)	21.9 (18.5 to 24.6)	**-19.3 (-27.2 to -10.1)**
Paralytic ileus and intestinal obstruction	115.6 (88.4 to 130.0)	**22.5 (15.7 to 31.8)**	2.8 (2.1 to 3.2)	-5.3 (-10.9 to 2.4)	3,192.3 (2,325.7 to 3,624.3)	7.3 (-3.4 to 18.6)	84.4 (61.7 to 95.6)	-8.3 (-17.3 to 1.5)
Inguinal, femoral, and abdominal hernia	18.4 (13.5 to 23.0)	**22.9 (13.4 to 33.2)**	0.4 (0.3 to 0.5)	**-8.7 (-15.5 to -1.1)**	330.2 (229.1 to 388.9)	**17.1 (8.1 to 28.4)**	8.0 (5.5 to 9.3)	-7.9 (-14.8 to 1.1)
Inflammatory bowel disease	20.3 (15.2 to 22.4)	**17.9 (9.0 to 24.4)**	0.5 (0.4 to 0.5)	**-12.4 (-17.8 to -7.8)**	387.4 (297.6 to 443.4)	8.7 (-4.5 to 18.7)	9.5 (7.3 to 11.0)	**-12.9 (-22.5 to -5.4)**
Vascular intestinal disorders	54.6 (48.9 to 59.3)	**22.7 (16.8 to 28.8)**	1.3 (1.1 to 1.4)	**-9.8 (-14.2 to -5.2)**	777.0 (671.6 to 853.1)	**18.5 (11.8 to 24.5)**	18.3 (15.8 to 20.0)	**-9.5 (-14.6 to -4.9)**
Gallbladder and biliary diseases	62.7 (56.4 to 66.6)	**25.8 (21.3 to 31.8)**	1.5 (1.3 to 1.5)	**-7.2 (-10.5 to -2.7)**	1,053.0 (916.0 to 1,124.0)	**15.2 (9.7 to 23.9)**	25.4 (22.0 to 27.2)	**-9.4 (-13.8 to -2.4)**
Pancreatitis	38.3 (29.5 to 42.0)	**20.7 (14.3 to 27.7)**	0.9 (0.7 to 1.0)	**-7.6 (-12.3 to -2.4)**	884.0 (681.5 to 985.5)	**13.8 (5.4 to 21.7)**	21.3 (16.4 to 23.8)	**-8.5 (-15.2 to -2.3)**
Other digestive diseases	44.1 (41.8 to 47.5)	**23.2 (18.0 to 31.3)**	1.0 (1.0 to 1.1)	**-9.1 (-12.8 to -3.1)**	780.4 (698.0 to 849.2)	**13.2 (4.8 to 25.0)**	19.2 (17.0 to 21.1)	**-9.7 (-16.8 to -0.1)**
**Neurological disorders**	1,901.8 (1,861.8 to 1,932.2)	**41.0 (38.3 to 43.0)**	43.7 (42.7 to 44.4)	-0.2 (-2.0 to 1.3)	20,727.0 (20,203.3 to 21,294.4)	**27.1 (24.3 to 30.5)**	492.9 (480.3 to 507.9)	**-3.3 (-5.3 to -0.5)**
Alzheimer's disease and other dementias	1,648.7 (1,618.0 to 1,675.5)	**43.7 (40.5 to 46.0)**	37.6 (36.9 to 38.2)	0.3 (-1.9 to 1.9)	14,788.8 (14,490.5 to 15,058.2)	**36.4 (32.2 to 39.1)**	342.0 (335.1 to 348.3)	-0.6 (-3.6 to 1.3)
Parkinson's disease	147.2 (138.6 to 151.9)	**34.5 (29.2 to 38.3)**	3.4 (3.2 to 3.5)	-0.7 (-4.6 to 2.0)	1,808.6 (1,708.4 to 1,873.7)	**30.3 (24.8 to 34.1)**	42.2 (39.8 to 43.7)	-1.2 (-5.3 to 1.7)
Epilepsy	54.8 (48.2 to 62.2)	5.5 (-0.6 to 17.0)	1.4 (1.2 to 1.6)	-9.9 (-15.3 to 0.2)	2,569.3 (2,282.5 to 2,979.5)	-5.8 (-12.5 to 9.1)	69.2 (61.4 to 80.3)	**-15.3 (-21.5 to -1.4)**
Multiple sclerosis	12.2 (10.1 to 13.6)	**22.1 (8.4 to 28.7)**	0.3 (0.2 to 0.3)	-4.3 (-14.4 to 1.1)	366.2 (321.1 to 418.0)	**16.7 (4.7 to 25.7)**	8.8 (7.7 to 10.0)	-6.1 (-14.9 to 1.4)
Motor neuron disease	15.0 (14.0 to 16.2)	**29.9 (22.2 to 35.5)**	0.4 (0.3 to 0.4)	-0.6 (-6.5 to 3.6)	348.4 (324.9 to 387.2)	**26.2 (19.7 to 31.6)**	8.4 (7.8 to 9.4)	-1.2 (-6.4 to 3.0)
Other neurological disorders	23.9 (22.5 to 28.5)	**25.3 (14.0 to 33.7)**	0.6 (0.5 to 0.7)	0.7 (-7.8 to 6.6)	845.7 (739.3 to 1,101.8)	11.6 (-1.8 to 24.0)	22.3 (19.1 to 29.7)	-3.8 (-14.4 to 5.9)
**Mental disorders**	0.3 (0.3 to 0.3)	**16.2 (5.6 to 26.2)**	0.0 (0.0 to 0.0)	4.3 (-5.2 to 13.3)	15.2 (13.6 to 16.9)	**15.1 (4.5 to 24.9)**	0.4 (0.3 to 0.4)	4.3 (-5.3 to 13.2)
Eating disorders	0.3 (0.3 to 0.3)	**16.2 (5.6 to 26.2)**	0.0 (0.0 to 0.0)	4.3 (-5.2 to 13.3)	15.2 (13.6 to 16.9)	**15.1 (4.5 to 24.9)**	0.4 (0.3 to 0.4)	4.3 (-5.3 to 13.2)
Anorexia nervosa	0.2 (0.2 to 0.2)	**13.3 (2.3 to 24.3)**	0.0 (0.0 to 0.0)	1.7 (-8.1 to 11.4)	11.2 (9.5 to 12.5)	**12.0 (1.0 to 22.6)**	0.3 (0.2 to 0.3)	1.6 (-8.4 to 11.1)
Bulimia nervosa	0.1 (0.1 to 0.1)	**25.2 (9.3 to 40.4)**	0.0 (0.0 to 0.0)	12.6 (-1.9 to 26.1)	4.0 (3.2 to 5.9)	**24.9 (8.9 to 40.0)**	0.1 (0.1 to 0.2)	12.9 (-1.5 to 26.3)
**Substance use disorders**	82.2 (79.3 to 87.8)	**40.2 (35.4 to 51.4)**	2.0 (1.9 to 2.1)	**13.6 (9.7 to 23.1)**	2,942.8 (2,834.8 to 3,214.9)	**33.8 (29.4 to 46.0)**	72.3 (69.7 to 79.0)	**12.8 (9.1 to 23.3)**
Alcohol use disorders	29.2 (26.8 to 34.0)	-0.3 (-8.0 to 16.8)	0.7 (0.6 to 0.8)	**-20.3 (-26.5 to -5.8)**	981.8 (905.2 to 1,221.8)	-6.9 (-14.1 to 14.3)	23.8 (21.9 to 29.7)	**-23.3 (-29.2 to -5.3)**
Drug use disorders	53.0 (51.6 to 54.5)	**80.6 (75.9 to 85.6)**	1.3 (1.2 to 1.3)	**47.8 (43.9 to 51.9)**	1,961.0 (1,903.3 to 2,023.2)	**71.5 (66.4 to 76.7)**	48.5 (47.1 to 50.0)	**46.7 (42.5 to 51.3)**
Opioid use disorders	32.0 (29.2 to 34.4)	**103.3 (79.9 to 131.2)**	0.8 (0.7 to 0.8)	**67.5 (48.3 to 90.5)**	1,225.9 (1,111.8 to 1,325.0)	**90.8 (67.0 to 118.3)**	30.4 (27.6 to 32.9)	**64.0 (43.6 to 87.6)**
Cocaine use disorders	1.8 (1.5 to 2.3)	**72.4 (32.3 to 121.7)**	0.0 (0.0 to 0.1)	**44.4 (11.0 to 85.7)**	78.0 (62.9 to 96.8)	**67.0 (27.4 to 116.4)**	1.9 (1.6 to 2.4)	**43.6 (9.7 to 85.6)**
Amphetamine use disorders	1.3 (1.0 to 1.5)	**54.4 (13.7 to 111.6)**	0.0 (0.0 to 0.0)	31.7 (-3.1 to 80.9)	58.6 (46.9 to 68.6)	**46.1 (5.8 to 100.8)**	1.5 (1.2 to 1.7)	28.1 (-6.7 to 76.2)
Other drug use disorders	17.9 (16.2 to 20.2)	**52.8 (30.7 to 76.4)**	0.4 (0.4 to 0.5)	**23.0 (5.2 to 42.0)**	598.5 (525.4 to 688.9)	**44.4 (20.3 to 71.5)**	14.7 (12.9 to 16.9)	**22.4 (1.9 to 45.1)**
**Diabetes and kidney diseases**	1,294.9 (1,261.1 to 1,328.1)	**33.9 (31.1 to 36.5)**	30.4 (29.6 to 31.2)	1.2 (-0.9 to 3.3)	27,428.2 (26,683.0 to 28,189.9)	**24.7 (21.6 to 27.5)**	657.9 (640.0 to 676.2)	-1.7 (-4.1 to 0.5)
Diabetes mellitus	711.1 (688.9 to 734.1)	**33.8 (30.1 to 37.5)**	16.6 (16.1 to 17.2)	0.9 (-1.8 to 3.7)	14,527.3 (14,081.7 to 14,980.5)	**29.0 (25.1 to 32.7)**	343.9 (333.3 to 354.5)	0.0 (-3.0 to 2.8)
Diabetes mellitus type 1	178.6 (159.2 to 198.9)	**12.4 (6.6 to 19.0)**	4.2 (3.8 to 4.7)	**-13.0 (-17.5 to -7.7)**	4,731.5 (4,227.9 to 5,206.3)	**8.7 (3.1 to 13.9)**	114.2 (102.2 to 125.6)	**-12.8 (-17.4 to -8.5)**
Diabetes mellitus type 2	532.5 (504.2 to 558.4)	**43.0 (39.0 to 47.5)**	12.4 (11.7 to 13.0)	**6.8 (3.8 to 10.1)**	9,795.8 (9,218.3 to 10,322.2)	**41.8 (37.3 to 47.2)**	229.7 (216.1 to 242.1)	**7.8 (4.6 to 11.9)**
Chronic kidney disease	579.0 (565.5 to 593.5)	**34.2 (31.2 to 37.0)**	13.6 (13.3 to 14.0)	1.7 (-0.6 to 3.8)	12,771.6 (12,407.2 to 13,163.5)	**20.5 (17.4 to 23.5)**	310.7 (301.8 to 320.5)	**-3.3 (-5.8 to -0.9)**
Chronic kidney disease due to diabetes mellitus type 1	35.7 (28.8 to 44.1)	**23.2 (18.9 to 27.5)**	0.8 (0.7 to 1.0)	-1.8 (-4.8 to 1.2)	1,178.9 (955.1 to 1,438.3)	**17.3 (12.5 to 22.2)**	28.4 (23.1 to 34.6)	**-3.8 (-7.0 to -0.7)**
Chronic kidney disease due to diabetes mellitus type 2	171.2 (151.4 to 192.5)	**41.2 (37.4 to 44.9)**	4.0 (3.5 to 4.5)	**4.9 (2.4 to 7.5)**	3,063.0 (2,683.2 to 3,484.8)	**35.4 (31.6 to 39.1)**	71.8 (62.9 to 81.7)	**2.9 (0.3 to 5.7)**
Chronic kidney disease due to hypertension	163.1 (142.8 to 183.8)	**41.5 (37.7 to 45.0)**	3.8 (3.3 to 4.3)	**3.8 (1.2 to 6.1)**	2,603.8 (2,270.7 to 2,966.6)	**33.3 (29.5 to 36.8)**	61.3 (53.5 to 69.8)	2.3 (-0.5 to 4.9)
Chronic kidney disease due to glomerulonephritis	89.5 (78.0 to 102.7)	**25.5 (21.8 to 29.2)**	2.1 (1.9 to 2.4)	-1.7 (-4.2 to 0.8)	2,522.8 (2,227.0 to 2,834.4)	**11.2 (7.4 to 15.2)**	63.1 (55.8 to 70.7)	**-7.1 (-9.8 to -4.4)**
Chronic kidney disease due to other and unspecified causes	119.6 (104.1 to 136.4)	**26.2 (22.3 to 30.5)**	2.9 (2.5 to 3.3)	-1.5 (-4.2 to 1.2)	3,403.2 (3,006.3 to 3,798.6)	**9.3 (5.3 to 13.6)**	86.1 (76.4 to 95.5)	**-8.6 (-11.8 to -5.6)**
Acute glomerulonephritis	4.8 (4.5 to 5.3)	**14.9 (8.1 to 23.5)**	0.1 (0.1 to 0.1)	**-10.0 (-15.4 to -3.3)**	129.3 (115.9 to 143.6)	-7.7 (-13.2 to 0.5)	3.3 (2.9 to 3.6)	**-23.1 (-27.6 to -16.4)**
**Skin and subcutaneous diseases**	52.7 (29.3 to 76.9)	**45.0 (38.2 to 54.2)**	1.3 (0.7 to 1.8)	**10.1 (4.6 to 18.3)**	1,249.2 (687.2 to 1,855.7)	**27.6 (18.9 to 42.1)**	32.3 (17.9 to 47.4)	6.4 (-1.5 to 19.1)
Bacterial skin diseases	38.4 (19.6 to 54.6)	**49.6 (39.7 to 59.0)**	0.9 (0.5 to 1.3)	**15.3 (8.0 to 23.9)**	1,024.7 (543.8 to 1,457.1)	**27.9 (18.0 to 45.0)**	26.9 (14.1 to 38.5)	7.8 (-1.1 to 22.5)
Cellulitis	9.3 (4.1 to 14.2)	**54.1 (29.3 to 69.5)**	0.2 (0.1 to 0.3)	16.9 (-1.7 to 29.9)	220.0 (99.2 to 334.5)	**35.2 (16.8 to 58.5)**	5.5 (2.5 to 8.3)	10.6 (-4.3 to 30.3)
Pyoderma	29.1 (14.9 to 41.9)	**48.3 (40.0 to 57.7)**	0.7 (0.4 to 1.0)	**14.8 (8.5 to 23.4)**	804.6 (415.4 to 1,172.3)	**26.1 (16.2 to 42.8)**	21.3 (11.1 to 31.4)	7.1 (-2.1 to 22.0)
Decubitus ulcer	11.9 (6.7 to 19.4)	**33.1 (23.6 to 46.8)**	0.3 (0.2 to 0.4)	-3.7 (-10.7 to 7.1)	168.9 (95.4 to 266.5)	**27.6 (18.3 to 39.1)**	4.0 (2.3 to 6.4)	-0.8 (-8.3 to 9.5)
Other skin and subcutaneous diseases	2.4 (1.3 to 5.4)	**38.3 (26.6 to 52.8)**	0.1 (0.0 to 0.1)	5.2 (-3.4 to 17.3)	55.6 (32.2 to 115.7)	**21.9 (8.5 to 39.8)**	1.4 (0.8 to 2.9)	2.7 (-8.4 to 19.5)
**Musculoskeletal disorders**	80.3 (67.9 to 85.7)	**31.6 (22.4 to 37.6)**	1.9 (1.6 to 2.0)	1.4 (-5.5 to 6.0)	1,904.2 (1,607.5 to 2,019.3)	**19.8 (11.1 to 24.8)**	46.5 (39.2 to 49.4)	-1.6 (-8.6 to 2.5)
Rheumatoid arthritis	32.3 (26.0 to 36.2)	**29.3 (15.3 to 37.6)**	0.8 (0.6 to 0.8)	-2.6 (-13.1 to 3.7)	580.4 (454.3 to 652.0)	**20.9 (7.6 to 28.7)**	13.7 (10.7 to 15.4)	**-6.9 (-17.0 to -0.9)**
Other musculoskeletal disorders	48.0 (41.0 to 53.1)	**33.3 (26.6 to 39.7)**	1.1 (1.0 to 1.3)	4.3 (-0.8 to 9.2)	1,323.8 (1,131.2 to 1,442.0)	**19.4 (12.2 to 24.5)**	32.8 (28.0 to 35.7)	0.7 (-5.2 to 5.0)
**Other non-communicable diseases**	561.7 (535.6 to 590.6)	3.1 (-1.5 to 6.3)	15.5 (14.7 to 16.4)	**-10.4 (-14.3 to -7.6)**	32,227.8 (30,221.3 to 34,565.2)	**-9.3 (-14.1 to -5.5)**	959.4 (896.6 to 1,032.7)	**-15.5 (-20.1 to -11.8)**
Congenital birth defects	275.8 (260.5 to 292.3)	**-12.9 (-17.9 to -8.4)**	8.5 (8.0 to 9.0)	**-17.0 (-21.9 to -12.7)**	23,037.8 (21,736.4 to 24,462.3)	**-13.8 (-19.1 to -9.3)**	711.5 (670.9 to 755.8)	**-17.5 (-22.6 to -13.1)**
Neural tube defects	30.4 (22.2 to 42.8)	-11.8 (-25.0 to 3.4)	0.9 (0.7 to 1.3)	**-15.4 (-28.2 to -0.7)**	2,616.1 (1,905.3 to 3,697.3)	-12.1 (-25.2 to 3.0)	81.5 (59.3 to 115.1)	**-15.6 (-28.4 to -0.9)**
Congenital heart anomalies	125.4 (97.7 to 147.7)	**-17.1 (-22.8 to -9.3)**	3.8 (3.0 to 4.5)	**-21.1 (-26.7 to -13.9)**	10,401.2 (8,002.0 to 12,305.0)	**-18.1 (-23.9 to -10.7)**	320.1 (245.4 to 379.0)	**-21.7 (-27.3 to -14.7)**
Orofacial clefts	1.7 (0.7 to 5.2)	**-42.0 (-57.3 to -23.3)**	0.1 (0.0 to 0.2)	**-44.0 (-58.8 to -26.1)**	144.5 (63.8 to 455.4)	**-42.0 (-57.4 to -23.4)**	4.5 (2.0 to 14.3)	**-44.0 (-58.8 to -26.2)**
Down syndrome	11.6 (9.0 to 17.8)	5.9 (-7.5 to 21.1)	0.3 (0.3 to 0.5)	-3.2 (-14.4 to 10.6)	832.8 (614.7 to 1,365.0)	1.2 (-11.3 to 17.8)	24.8 (18.1 to 41.2)	-5.2 (-16.9 to 10.1)
Other chromosomal abnormalities	10.3 (5.7 to 19.3)	2.9 (-10.5 to 19.6)	0.3 (0.2 to 0.6)	-1.3 (-14.1 to 14.8)	872.8 (485.0 to 1,662.3)	2.3 (-11.0 to 19.3)	27.1 (15.0 to 51.8)	-1.6 (-14.4 to 14.7)
Congenital musculoskeletal and limb anomalies	5.5 (2.9 to 8.4)	-9.5 (-19.0 to 2.7)	0.2 (0.1 to 0.3)	**-13.8 (-22.9 to -2.1)**	457.4 (237.5 to 700.4)	-10.8 (-20.3 to 1.7)	14.1 (7.3 to 21.6)	**-14.5 (-23.7 to -2.5)**
Urogenital congenital anomalies	6.0 (4.3 to 7.5)	-2.4 (-13.3 to 10.6)	0.2 (0.1 to 0.2)	-8.8 (-18.7 to 3.4)	470.3 (326.1 to 594.2)	-5.4 (-16.2 to 8.0)	14.4 (9.9 to 18.3)	-9.8 (-20.3 to 3.0)
Digestive congenital anomalies	21.4 (12.6 to 33.7)	**-12.1 (-20.7 to -0.4)**	0.7 (0.4 to 1.1)	**-15.5 (-23.8 to -4.0)**	1,854.4 (1,086.2 to 2,924.0)	**-12.4 (-20.9 to -0.7)**	57.9 (33.9 to 91.3)	**-15.6 (-23.8 to -4.1)**
Other congenital birth defects	63.5 (45.1 to 84.4)	-9.7 (-19.8 to 2.3)	2.0 (1.4 to 2.6)	**-13.5 (-23.2 to -1.9)**	5,388.3 (3,795.1 to 7,187.1)	-10.4 (-20.3 to 1.7)	167.1 (117.4 to 223.2)	**-13.9 (-23.5 to -2.1)**
Urinary diseases and male infertility	136.5 (131.6 to 142.3)	**45.8 (39.5 to 49.6)**	3.2 (3.1 to 3.4)	**10.1 (5.4 to 13.0)**	3,008.9 (2,876.6 to 3,148.1)	**25.9 (18.5 to 29.9)**	75.2 (71.6 to 78.8)	3.0 (-3.3 to 6.4)
Urinary tract infections	116.6 (111.3 to 125.7)	**52.4 (46.1 to 57.9)**	2.7 (2.6 to 3.0)	**14.3 (9.6 to 18.4)**	2,440.7 (2,292.0 to 2,690.0)	**37.6 (29.1 to 45.8)**	60.4 (56.4 to 66.9)	**11.8 (4.8 to 18.5)**
Urolithiasis	5.6 (4.7 to 7.6)	**39.6 (27.3 to 62.5)**	0.1 (0.1 to 0.2)	5.5 (-3.9 to 22.4)	112.2 (92.4 to 149.5)	**26.3 (16.2 to 47.3)**	2.7 (2.2 to 3.6)	-0.4 (-8.4 to 15.7)
Other urinary diseases	14.4 (8.6 to 17.4)	9.4 (-9.2 to 24.0)	0.4 (0.2 to 0.4)	**-13.5 (-27.2 to -2.5)**	456.0 (280.7 to 577.3)	**-13.3 (-26.0 to -2.8)**	12.1 (7.5 to 15.5)	**-25.7 (-35.8 to -17.1)**
Gynecological diseases	8.2 (7.4 to 8.7)	**19.1 (5.1 to 30.0)**	0.2 (0.2 to 0.2)	-2.7 (-13.6 to 6.1)	292.9 (272.6 to 318.7)	9.2 (-2.8 to 20.6)	7.3 (6.8 to 7.9)	-6.0 (-15.3 to 3.6)
Uterine fibroids	2.4 (1.6 to 3.0)	**33.3 (6.7 to 54.6)**	0.1 (0.0 to 0.1)	6.5 (-15.3 to 22.7)	74.2 (52.7 to 95.5)	13.0 (-4.2 to 31.6)	1.8 (1.3 to 2.3)	-5.5 (-20.2 to 9.8)
Polycystic ovarian syndrome	0.0 (0.0 to 0.0)	12.9 (-12.8 to 50.4)	0.0 (0.0 to 0.0)	1.0 (-22.4 to 35.0)	0.7 (0.1 to 1.5)	10.1 (-15.8 to 51.2)	0.0 (0.0 to 0.0)	0.0 (-24.1 to 37.7)
Endometriosis	0.2 (0.1 to 0.2)	11.8 (-12.4 to 45.5)	0.0 (0.0 to 0.0)	-3.4 (-23.9 to 25.4)	7.7 (3.2 to 12.0)	10.4 (-12.9 to 41.7)	0.2 (0.1 to 0.3)	-3.2 (-23.3 to 24.2)
Genital prolapse	0.6 (0.3 to 0.9)	0.6 (-15.4 to 16.1)	0.0 (0.0 to 0.0)	**-23.4 (-35.4 to -12.1)**	14.5 (6.8 to 20.1)	-4.4 (-18.4 to 10.4)	0.3 (0.2 to 0.5)	**-23.7 (-34.9 to -11.4)**
Other gynecological diseases	5.0 (4.1 to 5.6)	**16.0 (4.1 to 27.9)**	0.1 (0.1 to 0.1)	-3.4 (-12.0 to 6.3)	195.8 (163.0 to 228.9)	8.9 (-2.9 to 20.6)	4.9 (4.1 to 5.8)	-4.8 (-14.2 to 5.2)
Hemoglobinopathies and hemolytic anemias	50.3 (43.4 to 62.3)	2.4 (-4.1 to 11.7)	1.3 (1.1 to 1.6)	**-14.5 (-20.4 to -6.6)**	2,248.1 (1,840.9 to 2,968.7)	-6.3 (-16.6 to 8.2)	62.9 (50.8 to 84.1)	**-15.3 (-25.1 to -1.8)**
Thalassemias	3.4 (2.7 to 4.3)	**-27.0 (-35.5 to -12.6)**	0.1 (0.1 to 0.1)	**-31.4 (-39.6 to -17.5)**	266.0 (212.8 to 330.0)	**-28.2 (-37.0 to -13.4)**	7.9 (6.3 to 9.8)	**-32.3 (-40.7 to -17.9)**
Sickle cell disorders	18.4 (13.0 to 26.2)	-1.4 (-15.1 to 19.5)	0.5 (0.4 to 0.8)	-8.4 (-21.6 to 11.5)	1,338.6 (928.8 to 1,940.5)	-3.3 (-18.2 to 19.2)	39.2 (27.0 to 57.2)	-9.3 (-23.7 to 12.9)
G6PD deficiency	5.2 (4.1 to 6.6)	**6.4 (1.3 to 13.6)**	0.1 (0.1 to 0.2)	**-10.6 (-14.7 to -4.9)**	223.8 (178.1 to 279.7)	-0.6 (-6.0 to 8.2)	5.7 (4.6 to 7.2)	**-13.3 (-18.1 to -5.5)**
Other hemoglobinopathies and hemolytic anemias	23.3 (19.4 to 27.3)	**11.5 (6.8 to 15.7)**	0.5 (0.5 to 0.6)	**-16.9 (-20.3 to -13.7)**	419.8 (348.2 to 495.9)	-0.2 (-4.7 to 4.1)	10.1 (8.3 to 11.9)	**-20.9 (-24.2 to -17.4)**
Endocrine, metabolic, blood, and immune disorders	74.3 (55.3 to 81.2)	**26.2 (19.5 to 30.8)**	1.8 (1.4 to 2.0)	-1.5 (-5.7 to 1.6)	2,186.9 (1,767.5 to 2,547.3)	**8.8 (2.3 to 15.7)**	57.3 (46.7 to 67.3)	**-7.3 (-12.4 to -1.1)**
Sudden infant death syndrome	16.6 (6.5 to 36.9)	-16.1 (-30.7 to 6.4)	0.5 (0.2 to 1.2)	-19.2 (-33.2 to 2.5)	1,453.2 (571.6 to 3,230.6)	-16.1 (-30.7 to 6.4)	45.4 (17.8 to 100.9)	-19.2 (-33.2 to 2.5)
**Injuries**	1,417.2 (1,355.3 to 1,457.7)	**4.8 (2.2 to 7.4)**	35.5 (33.9 to 36.5)	**-13.6 (-15.6 to -11.5)**	56,078.9 (53,094.6 to 58,080.6)	**-7.7 (-9.7 to -5.6)**	1,492.9 (1,413.0 to 1,547.6)	**-18.3 (-20.1 to -16.4)**
**Transport injuries**	332.7 (314.3 to 344.6)	-2.1 (-5.3 to 0.8)	8.4 (7.9 to 8.7)	**-17.5 (-20.1 to -15.2)**	14,759.8 (13,966.6 to 15,350.2)	**-10.9 (-13.9 to -8.1)**	389.9 (369.1 to 405.7)	**-21.2 (-23.9 to -18.5)**
Road injuries	315.7 (298.2 to 327.2)	-2.1 (-5.3 to 0.8)	8.0 (7.5 to 8.3)	**-17.5 (-20.1 to -15.1)**	13,995.9 (13,242.0 to 14,574.9)	**-10.9 (-13.8 to -7.9)**	369.7 (349.8 to 385.1)	**-21.1 (-23.7 to -18.3)**
Pedestrian road injuries	144.4 (133.9 to 157.2)	**-4.8 (-8.9 to -0.7)**	3.6 (3.3 to 3.9)	**-21.2 (-24.5 to -17.6)**	5,942.4 (5,441.5 to 6,559.1)	**-15.3 (-19.4 to -10.5)**	157.0 (143.6 to 173.8)	**-25.9 (-29.6 to -21.7)**
Cyclist road injuries	14.4 (12.6 to 16.6)	7.5 (-0.6 to 16.2)	0.4 (0.3 to 0.4)	**-10.6 (-17.3 to -3.4)**	588.2 (516.2 to 680.8)	-2.2 (-9.7 to 6.1)	15.1 (13.2 to 17.5)	**-14.9 (-21.6 to -7.3)**
Motorcyclist road injuries	32.4 (26.4 to 36.8)	-3.6 (-14.6 to 10.9)	0.8 (0.7 to 0.9)	**-16.8 (-26.0 to -4.7)**	1,571.8 (1,308.0 to 1,785.8)	-10.5 (-18.8 to 1.4)	41.0 (34.2 to 46.7)	**-20.0 (-27.1 to -9.8)**
Motor vehicle road injuries	121.4 (109.9 to 129.2)	0.6 (-2.6 to 4.1)	3.1 (2.8 to 3.3)	**-13.9 (-16.6 to -10.8)**	5,742.4 (5,183.1 to 6,143.6)	**-7.0 (-10.7 to -1.1)**	152.5 (137.5 to 163.3)	**-16.7 (-20.1 to -10.9)**
Other road injuries	3.1 (2.5 to 3.8)	5.0 (-3.2 to 23.8)	0.1 (0.1 to 0.1)	-9.5 (-16.7 to 6.2)	151.1 (123.7 to 188.5)	-1.3 (-10.9 to 19.2)	4.1 (3.3 to 5.1)	-11.2 (-19.9 to 7.1)
Other transport injuries	17.0 (15.8 to 19.2)	-1.7 (-6.4 to 5.6)	0.4 (0.4 to 0.5)	**-17.1 (-21.1 to -11.2)**	763.9 (702.8 to 856.3)	**-12.0 (-17.0 to -5.4)**	20.2 (18.5 to 22.7)	**-22.0 (-27.0 to -16.1)**
**Unintentional injuries**	715.9 (669.6 to 746.7)	**9.8 (5.5 to 15.0)**	17.8 (16.6 to 18.5)	**-12.5 (-15.6 to -8.8)**	24,015.4 (21,847.6 to 25,417.8)	**-10.6 (-13.5 to -7.0)**	650.7 (590.4 to 690.6)	**-21.8 (-24.4 to -18.4)**
Falls	316.3 (297.4 to 346.6)	**33.1 (24.8 to 43.4)**	7.4 (7.0 to 8.1)	-0.9 (-7.0 to 6.7)	5,831.7 (5,500.4 to 6,313.1)	**15.3 (8.3 to 22.5)**	146.2 (138.0 to 157.3)	**-6.4 (-12.4 to -0.7)**

SR Table 3: Global deaths and YLL numbers, age-standardised rates per 100,000, and percent change between 2007 and 2017 for females for all GBD causes and Levels 1 through 4 of the cause hierarchy. 95% UIs are in parentheses. UI=uncertainty interval. YLL=year of life lost.

Cause	All Age Deaths (thousands)		Age-standardised Death Rate (per 100,000)		All Age YLLs (thousands)		Age-standardised YLL Rate (per 100,000)	
Drowning	92.2<br>(83.2 to 97.0)	**-16.8<br>(-20.8 to -11.7)**	2.5<br>(2.3 to 2.6)	**-28.3<br>(-32.0 to -23.4)**	5,048.8<br>(4,610.0 to 5,445.2)	**-28.2<br>(-32.8 to -20.7)**	144.4<br>(132.0 to 156.6)	**-34.9<br>(-39.3 to -27.7)**
Fire, heat, and hot substances	60.6<br>(47.5 to 66.6)	**-6.3<br>(-10.0 to -0.7)**	1.5<br>(1.2 to 1.7)	**-21.5<br>(-24.6 to -16.8)**	2,671.2<br>(1,991.4 to 2,997.0)	**-16.3<br>(-21.2 to -5.9)**	72.8<br>(54.2 to 81.8)	**-24.9<br>(-29.5 to -15.0)**
Poisonings	26.6<br>(17.9 to 29.2)	-3.2<br>(-12.4 to 7.5)	0.7<br>(0.5 to 0.7)	**-18.1<br>(-25.7 to -9.0)**	1,220.6<br>(841.5 to 1,340.2)	**-13.4<br>(-21.2 to -1.9)**	33.3<br>(23.2 to 36.6)	**-22.4<br>(-29.2 to -11.9)**
Poisoning by carbon monoxide	11.8<br>(7.5 to 13.1)	-5.9<br>(-18.6 to 1.8)	0.3<br>(0.2 to 0.3)	**-21.9<br>(-32.3 to -15.4)**	460.7<br>(295.7 to 517.3)	**-15.3<br>(-24.4 to -7.4)**	12.1<br>(7.7 to 13.6)	**-25.3<br>(-33.3 to -18.1)**
Poisoning by other means	14.9<br>(10.0 to 16.4)	-0.9<br>(-10.6 to 11.8)	0.4<br>(0.3 to 0.4)	**-14.9<br>(-22.7 to -3.9)**	759.9<br>(533.2 to 841.7)	-12.2<br>(-20.8 to 1.7)	21.2<br>(15.0 to 23.5)	**-20.5<br>(-28.2 to -7.9)**
Exposure to mechanical forces	34.5<br>(26.1 to 39.3)	**7.7<br>(2.6 to 17.0)**	0.9<br>(0.7 to 1.0)	**-10.1<br>(-14.4 to -2.4)**	1,508.0<br>(1,129.8 to 1,708.9)	-2.8<br>(-8.8 to 5.1)	41.1<br>(30.8 to 46.6)	**-13.2<br>(-19.0 to -6.1)**
Unintentional firearm injuries	5.5<br>(4.6 to 6.6)	**10.7<br>(2.2 to 21.4)**	0.1<br>(0.1 to 0.2)	**-9.2<br>(-16.1 to -0.2)**	212.1<br>(177.5 to 262.0)	1.8<br>(-7.9 to 16.9)	5.6<br>(4.7 to 7.0)	-9.6<br>(-18.4 to 4.1)
Other exposure to mechanical forces	29.0<br>(20.8 to 33.7)	**7.1<br>(1.9 to 17.7)**	0.7<br>(0.5 to 0.9)	**-10.3<br>(-14.8 to -1.7)**	1,295.9<br>(924.5 to 1,496.9)	-3.5<br>(-9.5 to 4.9)	35.5<br>(25.4 to 40.9)	**-13.7<br>(-19.3 to -6.1)**
Adverse effects of medical treatment	59.9<br>(48.9 to 67.8)	**16.7<br>(12.1 to 20.2)**	1.5<br>(1.2 to 1.7)	**-6.8<br>(-10.5 to -3.9)**	2,033.6<br>(1,576.4 to 2,463.1)	2.5<br>(-2.7 to 9.4)	53.9<br>(41.5 to 66.2)	**-11.3<br>(-15.9 to -5.4)**
Animal contact	36.6<br>(14.4 to 47.1)	2.3<br>(-7.8 to 12.7)	1.0<br>(0.4 to 1.2)	**-12.5<br>(-20.9 to -4.0)**	1,823.8<br>(702.8 to 2,282.1)	-4.7<br>(-12.6 to 5.7)	50.1<br>(19.3 to 62.6)	**-14.5<br>(-21.5 to -5.0)**
Venomous animal contact	32.8<br>(11.9 to 42.9)	2.7<br>(-8.2 to 15.9)	0.9<br>(0.3 to 1.1)	**-12.2<br>(-21.2 to -2.2)**	1,623.6<br>(586.7 to 2,078.3)	-4.0<br>(-12.7 to 6.1)	44.4<br>(16.1 to 57.1)	**-14.0<br>(-21.5 to -5.2)**
Non-venomous animal contact	3.8<br>(1.8 to 8.2)	-1.3<br>(-26.1 to 31.3)	0.1<br>(0.0 to 0.2)	-15.2<br>(-36.2 to 11.5)	200.1<br>(86.0 to 473.2)	-9.6<br>(-36.4 to 30.0)	5.7<br>(2.4 to 13.5)	-17.9<br>(-42.3 to 17.8)
Foreign body	50.1<br>(47.6 to 52.0)	4.9<br>(-1.7 to 9.0)	1.3<br>(1.2 to 1.4)	**-13.1<br>(-18.5 to -9.6)**	2,284.0<br>(2,108.9 to 2,416.1)	**-12.6<br>(-19.7 to -7.0)**	66.2<br>(60.9 to 70.3)	**-20.2<br>(-26.9 to -15.0)**
Pulmonary aspiration and foreign body in airway	46.3<br>(44.1 to 48.2)	4.9<br>(-1.8 to 9.3)	1.2<br>(1.2 to 1.3)	**-13.1<br>(-18.6 to -9.4)**	2,127.0<br>(1,967.0 to 2,257.3)	**-12.7<br>(-20.0 to -7.0)**	62.0<br>(57.1 to 66.0)	**-20.2<br>(-26.9 to -14.8)**
Foreign body in other body part	3.8<br>(3.4 to 4.9)	4.7<br>(-5.1 to 16.9)	0.1<br>(0.1 to 0.1)	**-13.3<br>(-20.6 to -3.4)**	157.0<br>(135.1 to 215.5)	-11.3<br>(-21.6 to 1.9)	4.3<br>(3.6 to 5.9)	**-21.1<br>(-30.6 to -8.9)**
Environmental heat and cold exposure	16.4<br>(10.0 to 18.5)	-3.4<br>(-17.5 to 2.9)	0.4<br>(0.2 to 0.5)	**-23.6<br>(-35.3 to -18.5)**	497.0<br>(287.1 to 571.2)	**-16.2<br>(-28.5 to -9.7)**	12.9<br>(7.3 to 14.9)	**-28.5<br>(-39.7 to -22.3)**
Exposure to forces of nature	3.8<br>(3.3 to 4.7)	**-34.2<br>(-43.0 to -17.6)**	0.1<br>(0.1 to 0.1)	**-43.7<br>(-50.7 to -30.3)**	180.5<br>(162.1 to 212.3)	**-43.9<br>(-49.8 to -33.5)**	4.9<br>(4.4 to 5.7)	**-49.6<br>(-54.7 to -40.7)**
Other unintentional injuries	19.0<br>(18.2 to 19.9)	**-13.0<br>(-17.0 to -8.3)**	0.5<br>(0.5 to 0.5)	**-25.8<br>(-29.3 to -21.9)**	916.2<br>(869.0 to 972.8)	**-23.6<br>(-28.0 to -18.5)**	24.8<br>(23.5 to 26.5)	**-31.6<br>(-35.6 to -26.9)**
**Self-harm and interpersonal violence**	368.6<br>(352.6 to 381.8)	2.1<br>(-1.3 to 4.7)	9.3<br>(8.9 to 9.6)	**-11.9<br>(-14.8 to -9.7)**	17,303.6<br>(16,574.4 to 17,975.0)	-0.2<br>(-3.4 to 2.7)	452.3<br>(432.9 to 470.2)	**-9.9<br>(-12.7 to -7.2)**
Self-harm	253.6<br>(242.6 to 262.3)	**-3.5<br>(-7.8 to -0.6)**	6.3<br>(6.0 to 6.5)	**-18.1<br>(-21.8 to -15.6)**	10,839.2<br>(10,321.4 to 11,245.2)	**-8.3<br>(-13.0 to -5.3)**	275.9<br>(262.3 to 286.5)	**-18.4<br>(-22.6 to -15.7)**
Self-harm by firearm	9.3<br>(8.0 to 10.6)	0.3<br>(-7.0 to 9.1)	0.2<br>(0.2 to 0.3)	**-14.7<br>(-20.5 to -7.5)**	403.0<br>(332.0 to 473.9)	-6.3<br>(-14.2 to 3.1)	10.2<br>(8.4 to 12.1)	**-16.7<br>(-23.1 to -8.5)**
Self-harm by other specified means	244.3<br>(233.8 to 252.7)	**-3.7<br>(-8.1 to -0.7)**	6.1<br>(5.8 to 6.3)	**-18.2<br>(-22.0 to -15.7)**	10,436.2<br>(9,943.4 to 10,843.1)	**-8.4<br>(-13.2 to -5.3)**	265.6<br>(252.7 to 276.2)	**-18.5<br>(-22.8 to -15.8)**
Interpersonal violence	73.5<br>(61.2 to 85.0)	**-7.7<br>(-10.4 to -2.3)**	1.9<br>(1.6 to 2.2)	**-19.1<br>(-21.6 to -14.3)**	3,809.7<br>(3,145.7 to 4,405.3)	**-11.0<br>(-14.3 to -3.9)**	101.2<br>(83.6 to 117.2)	**-19.6<br>(-22.7 to -12.8)**
Physical violence by firearm	18.1<br>(15.0 to 21.3)	-1.7<br>(-6.2 to 3.8)	0.5<br>(0.4 to 0.5)	**-13.3<br>(-17.2 to -8.5)**	944.4<br>(782.4 to 1,117.2)	-5.0<br>(-9.2 to 0.8)	24.8<br>(20.5 to 29.4)	**-13.8<br>(-17.5 to -8.5)**
Physical violence by sharp object	17.8<br>(14.4 to 20.7)	**-12.1<br>(-14.9 to -8.0)**	0.4<br>(0.4 to 0.5)	**-23.7<br>(-26.3 to -20.0)**	860.1<br>(689.6 to 1,003.3)	**-15.0<br>(-18.4 to -9.5)**	22.3<br>(17.8 to 26.0)	**-23.9<br>(-27.0 to -18.8)**
Physical violence by other means	37.6<br>(30.3 to 45.6)	**-8.2<br>(-12.1 to -0.9)**	1.0<br>(0.8 to 1.2)	**-19.5<br>(-23.1 to -12.8)**	2,005.2<br>(1,592.7 to 2,442.5)	**-11.9<br>(-16.5 to -2.0)**	54.1<br>(42.8 to 65.9)	**-20.1<br>(-24.4 to -10.9)**
Conflict and terrorism	38.5<br>(34.5 to 43.1)	**114.0<br>(80.5 to 153.5)**	1.1<br>(1.0 to 1.2)	**94.7<br>(63.8 to 131.5)**	2,496.8<br>(2,211.1 to 2,849.4)	**108.5<br>(71.8 to 152.3)**	71.0<br>(62.4 to 81.0)	**93.6<br>(58.4 to 134.8)**
Executions and police conflict	3.0<br>(2.9 to 3.1)	**382.6<br>(344.4 to 411.1)**	0.1<br>(0.1 to 0.1)	**320.6<br>(287.2 to 347.1)**	158.0<br>(153.4 to 164.4)	**358.1<br>(315.5 to 388.3)**	4.2<br>(4.1 to 4.4)	**312.4<br>(271.5 to 340.8)**

SR Table 4: Global deaths and YLL numbers, age-standardised rates per 100,000, and percent change between 2007 and 2017 for males for all GBD causes and Levels 1 through 4 of the cause hierarchy. 95% UIs are in parentheses. UI=uncertainty interval. YLL=year of life lost.

Cause	All Age Deaths (thousands)		Age-standardised Death Rate (per 100,000)		All Age YLLs (thousands)		Age-standardised YLL Rate (per 100,000)	
	2017	Percent Change 2007-2017	2017	Percent Change 2007-2017	2017	Percent Change 2007-2017	2017	Percent Change 2007-2017
**All causes**	30,387.6 (29,985.7 to 30,775.5)	**9.1 (7.7 to 10.4)**	873.5 (862.6 to 884.1)	**-13.8 (-14.9 to -12.8)**	947,920.4 (933,279.9 to 963,909.5)	**-7.5 (-8.8 to -6.1)**	25,564.9 (25,164.8 to 26,013.5)	**-20.7 (-21.8 to -19.5)**
**Communicable, maternal, neonatal, and nutritional diseases**	5,477.1 (5,230.2 to 5,873.7)	**-21.7 (-23.6 to -19.5)**	155.9 (148.7 to 167.5)	**-31.0 (-32.6 to -29.2)**	309,410.9 (298,515.0 to 322,899.1)	**-29.2 (-31.3 to -26.9)**	8,695.4 (8,384.1 to 9,069.6)	**-34.2 (-36.2 to -32.1)**
**HIV/AIDS and sexually transmitted infections**	578.8 (525.4 to 643.0)	**-42.0 (-44.4 to -39.2)**	14.9 (13.4 to 16.7)	**-48.6 (-51.1 to -45.8)**	32,517.6 (28,374.1 to 37,836.1)	**-42.1 (-45.2 to -38.4)**	854.1 (733.9 to 1,005.1)	**-47.5 (-50.9 to -43.6)**
HIV/AIDS	508.4 (483.0 to 539.6)	**-44.6 (-46.5 to -42.3)**	12.8 (12.2 to 13.6)	**-51.6 (-53.2 to -49.6)**	26,410.9 (25,030.7 to 28,030.0)	**-46.3 (-48.1 to -44.1)**	675.8 (640.7 to 717.8)	**-52.4 (-53.9 to -50.4)**
HIV/AIDS - Drug-susceptible Tuberculosis	103.4 (73.6 to 133.4)	**-51.9 (-55.0 to -48.1)**	2.6 (1.9 to 3.4)	**-58.1 (-60.8 to -54.8)**	5,598.6 (4,014.1 to 7,203.1)	**-52.6 (-55.7 to -48.8)**	144.7 (104.0 to 186.1)	**-57.8 (-60.5 to -54.5)**
HIV/AIDS - Multidrug-resistant Tuberculosis without extensive drug resistance	12.2 (7.3 to 18.6)	**-49.2 (-63.4 to -29.4)**	0.3 (0.2 to 0.5)	**-55.6 (-68.0 to -38.5)**	665.9 (396.9 to 1,017.2)	**-49.1 (-63.0 to -29.7)**	17.2 (10.3 to 26.3)	**-54.6 (-67.1 to -37.3)**
HIV/AIDS - Extensively drug-resistant Tuberculosis	0.8 (0.5 to 1.2)	-6.0 (-23.5 to 16.3)	0.0 (0.0 to 0.0)	-18.4 (-33.6 to 1.0)	39.0 (23.5 to 57.8)	-8.4 (-25.3 to 13.0)	1.0 (0.6 to 1.5)	**-19.3 (-34.1 to -0.6)**
HIV/AIDS resulting in other diseases	391.9 (348.2 to 435.4)	**-42.2 (-45.0 to -38.7)**	9.9 (8.8 to 10.9)	**-49.5 (-51.9 to -46.5)**	20,107.5 (17,868.2 to 22,431.1)	**-44.2 (-46.9 to -40.7)**	512.9 (455.4 to 571.7)	**-50.5 (-52.9 to -47.6)**
Sexually transmitted infections excluding HIV	70.4 (27.9 to 132.6)	**-12.0 (-19.8 to -3.0)**	2.1 (0.8 to 3.9)	**-14.9 (-22.5 to -6.3)**	6,106.7 (2,387.0 to 11,555.3)	**-12.2 (-20.0 to -3.1)**	178.3 (69.6 to 337.5)	**-14.9 (-22.5 to -6.2)**
Syphilis	70.3 (27.7 to 132.5)	**-12.0 (-19.8 to -3.0)**	2.1 (0.8 to 3.9)	**-14.9 (-22.5 to -6.3)**	6,101.6 (2,382.4 to 11,550.1)	**-12.2 (-20.0 to -3.1)**	178.2 (69.5 to 337.4)	**-14.9 (-22.5 to -6.2)**
Chlamydial infection	0.0 (0.0 to 0.0)	-	0.0 (0.0 to 0.0)	-	0.0 (0.0 to 0.0)	-	0.0 (0.0 to 0.0)	-
Gonococcal infection	0.1 (0.0 to 0.1)	-5.4 (-24.7 to 17.3)	0.0 (0.0 to 0.0)	**-20.6 (-37.2 to -0.8)**	2.8 (2.0 to 4.0)	-9.8 (-25.7 to 6.9)	0.1 (0.1 to 0.1)	**-20.9 (-35.1 to -5.5)**
Other sexually transmitted infections	0.1 (0.0 to 0.1)	-2.1 (-24.6 to 24.5)	0.0 (0.0 to 0.0)	-19.5 (-39.2 to 3.9)	2.3 (1.6 to 3.3)	-10.2 (-26.4 to 8.7)	0.1 (0.0 to 0.1)	**-21.4 (-35.6 to -4.6)**
**Respiratory infections and tuberculosis**	2,093.5 (1,998.9 to 2,193.2)	**-8.7 (-11.7 to -5.8)**	60.3 (57.6 to 63.3)	**-24.7 (-27.0 to -22.6)**	82,601.4 (78,649.0 to 87,047.3)	**-23.7 (-27.1 to -20.1)**	2,270.8 (2,158.1 to 2,395.5)	**-32.1 (-35.0 to -28.8)**
Tuberculosis	746.5 (701.0 to 794.9)	**-15.5 (-20.4 to -11.3)**	19.6 (18.4 to 20.9)	**-32.5 (-36.3 to -29.1)**	26,100.6 (24,595.6 to 27,876.0)	**-20.6 (-24.9 to -16.5)**	666.3 (627.6 to 712.1)	**-33.2 (-36.9 to -29.9)**
Drug-susceptible tuberculosis	657.6 (593.2 to 717.2)	**-15.9 (-22.9 to -9.5)**	17.3 (15.6 to 18.9)	**-32.9 (-38.4 to -27.7)**	22,979.7 (20,783.2 to 25,046.8)	**-21.0 (-27.4 to -14.9)**	587.0 (531.6 to 639.3)	**-33.6 (-39.0 to -28.5)**
Multidrug-resistant tuberculosis without extensive drug resistance	80.3 (45.5 to 127.6)	-14.1 (-48.8 to 34.8)	2.1 (1.2 to 3.3)	-31.2 (-59.1 to 7.8)	2,821.2 (1,655.2 to 4,383.0)	-18.8 (-49.6 to 23.9)	71.8 (42.2 to 111.3)	-31.7 (-57.6 to 3.8)
Extensively drug-resistant tuberculosis	8.6 (6.1 to 11.9)	8.7 (-20.5 to 48.4)	0.2 (0.2 to 0.3)	-12.0 (-35.7 to 20.8)	299.7 (215.7 to 408.8)	1.5 (-24.3 to 37.7)	7.5 (5.4 to 10.3)	-14.8 (-36.4 to 15.6)
Lower respiratory infections	1,341.8 (1,244.2 to 1,413.6)	**-4.3 (-7.5 to -0.7)**	40.5 (37.8 to 42.7)	**-20.2 (-22.6 to -17.6)**	56,223.3 (52,410.7 to 60,109.3)	**-25.0 (-29.3 to -20.6)**	1,596.7 (1,489.1 to 1,708.7)	**-31.6 (-35.4 to -27.6)**
Upper respiratory infections	4.7 (3.1 to 6.2)	**-30.8 (-41.0 to -0.3)**	0.1 (0.1 to 0.2)	**-42.5 (-49.7 to -20.3)**	254.6 (130.4 to 385.5)	**-35.4 (-44.8 to -12.3)**	7.2 (3.7 to 11.0)	**-40.8 (-48.9 to -21.6)**
Otitis media	0.5 (0.3 to 0.9)	**-43.4 (-54.4 to -27.3)**	0.0 (0.0 to 0.0)	**-52.1 (-61.1 to -39.2)**	22.9 (13.2 to 46.1)	**-48.7 (-60.6 to -31.1)**	0.6 (0.4 to 1.2)	**-54.1 (-64.7 to -38.6)**
**Enteric infections**	843.6 (596.7 to 1,288.3)	**-20.5 (-29.6 to -7.2)**	24.5 (17.3 to 37.3)	**-32.0 (-38.5 to -20.9)**	44,032.2 (36,202.5 to 56,115.6)	**-30.2 (-39.1 to -19.5)**	1,243.0 (1,028.3 to 1,560.0)	**-35.8 (-43.8 to -25.9)**
Diarrheal diseases	738.0 (492.9 to 1,187.4)	**-20.3 (-30.8 to -4.8)**	21.6 (14.4 to 34.8)	**-32.5 (-40.1 to -20.2)**	36,465.8 (29,144.2 to 48,750.1)	**-31.5 (-41.7 to -18.8)**	1,034.0 (834.8 to 1,368.0)	**-37.2 (-46.2 to -25.4)**
Typhoid and paratyphoid	74.0 (42.1 to 119.2)	**-23.9 (-28.7 to -19.5)**	2.0 (1.1 to 3.2)	**-29.2 (-33.9 to -25.1)**	5,283.9 (3,017.0 to 8,471.6)	**-25.4 (-30.4 to -20.7)**	144.8 (82.7 to 232.6)	**-30.0 (-35.0 to -25.5)**
Typhoid fever	63.8 (35.6 to 102.7)	**-25.1 (-30.2 to -20.6)**	1.7 (1.0 to 2.8)	**-30.3 (-35.3 to -26.0)**	4,558.7 (2,535.7 to 7,273.8)	**-26.6 (-32.3 to -21.8)**	125.0 (69.8 to 200.8)	**-31.2 (-36.6 to -26.5)**
Paratyphoid fever	10.2 (4.7 to 19.9)	**-15.8 (-24.6 to -5.9)**	0.3 (0.1 to 0.5)	**-21.8 (-30.1 to -12.3)**	725.2 (329.1 to 1,399.2)	**-16.3 (-25.7 to -5.6)**	19.8 (9.0 to 38.2)	**-21.5 (-30.4 to -11.5)**
Invasive Non-typhoidal Salmonella (iNTS)	31.0 (17.4 to 51.9)	**-16.9 (-25.6 to -5.5)**	0.9 (0.5 to 1.4)	**-24.1 (-32.4 to -13.4)**	2,237.7 (1,234.8 to 3,852.1)	**-16.0 (-25.9 to -3.1)**	63.0 (34.9 to 108.7)	**-21.4 (-31.0 to -9.1)**
Other intestinal infectious diseases	0.6 (0.4 to 1.1)	-39.1 (-70.6 to 35.8)	0.0 (0.0 to 0.0)	-44.0 (-73.0 to 22.4)	44.9 (26.1 to 87.1)	-43.1 (-75.4 to 38.9)	1.3 (0.7 to 2.5)	-46.4 (-77.0 to 31.9)
**Neglected tropical diseases and malaria**	387.6 (287.6 to 514.3)	**-28.6 (-37.2 to -19.3)**	10.7 (7.9 to 14.1)	**-35.4 (-43.2 to -27.1)**	26,327.7 (19,182.7 to 35,402.6)	**-32.7 (-41.7 to -22.6)**	736.6 (533.5 to 991.2)	**-37.5 (-46.2 to -28.1)**
Malaria	330.9 (233.0 to 456.4)	**-29.9 (-38.7 to -19.7)**	9.1 (6.4 to 12.6)	**-36.3 (-44.5 to -27.1)**	23,339.0 (16,305.7 to 32,218.1)	**-33.2 (-43.0 to -22.3)**	655.6 (457.2 to 906.8)	**-37.9 (-47.2 to -27.6)**
Chagas disease	4.5 (4.2 to 5.1)	2.4 (-4.4 to 13.3)	0.1 (0.1 to 0.1)	**-22.0 (-27.2 to -13.9)**	107.5 (100.3 to 120.4)	-4.9 (-11.3 to 5.3)	2.8 (2.6 to 3.1)	**-25.3 (-30.4 to -17.5)**
Leishmaniasis	4.9 (0.0 to 22.5)	**-64.9 (-96.8 to -45.3)**	0.1 (0.0 to 0.6)	**-68.0 (-97.6 to -52.2)**	325.8 (0.2 to 1,572.8)	**-63.9 (-93.3 to -40.3)**	8.9 (0.0 to 43.3)	**-66.2 (-94.5 to -45.4)**
Visceral leishmaniasis	4.9 (0.0 to 22.5)	**-64.9 (-96.8 to -45.3)**	0.1 (0.0 to 0.6)	**-68.0 (-97.6 to -52.2)**	325.8 (0.2 to 1,572.8)	**-63.9 (-93.3 to -40.3)**	8.9 (0.0 to 43.3)	**-66.2 (-94.5 to -45.4)**
African trypanosomiasis	0.7 (0.1 to 2.8)	**-80.6 (-95.9 to -22.7)**	0.0 (0.0 to 0.1)	**-82.7 (-96.4 to -31.0)**	40.8 (7.3 to 158.1)	**-80.7 (-96.0 to -22.4)**	1.1 (0.2 to 4.1)	**-82.3 (-96.3 to -30.8)**
Schistosomiasis	4.8 (4.3 to 5.4)	**-11.8 (-18.4 to -4.1)**	0.1 (0.1 to 0.1)	**-28.4 (-33.6 to -22.1)**	190.8 (167.9 to 216.5)	**-14.4 (-22.4 to -5.9)**	4.9 (4.3 to 5.6)	**-26.4 (-33.5 to -18.7)**
Cysticercosis	0.4 (0.2 to 0.6)	-14.3 (-53.8 to 57.1)	0.0 (0.0 to 0.0)	-25.8 (-59.0 to 31.7)	20.3 (11.8 to 29.4)	-18.3 (-56.1 to 51.7)	0.5 (0.3 to 0.8)	-27.0 (-59.8 to 35.2)
Cystic echinococcosis	0.6 (0.4 to 0.8)	-31.1 (-59.9 to 15.3)	0.0 (0.0 to 0.0)	**-42.3 (-65.9 to -5.5)**	27.2 (17.1 to 38.4)	-38.5 (-63.5 to 1.6)	0.7 (0.5 to 1.0)	**-45.8 (-67.8 to -11.0)**
Dengue	20.6 (5.9 to 26.3)	**57.0 (5.7 to 102.8)**	0.6 (0.2 to 0.7)	35.7 (-8.2 to 74.4)	1,022.6 (295.2 to 1,282.0)	28.8 (-12.6 to 68.8)	27.7 (8.1 to 34.5)	16.3 (-20.9 to 52.8)
Yellow fever	3.4 (0.7 to 9.8)	-17.5 (-32.3 to 1.4)	0.1 (0.0 to 0.3)	**-24.3 (-38.1 to -6.6)**	221.6 (45.9 to 631.5)	-16.9 (-33.2 to 4.4)	6.0 (1.2 to 17.0)	**-22.3 (-37.8 to -1.8)**
Rabies	7.9 (6.6 to 10.1)	**-45.9 (-56.6 to -31.5)**	0.2 (0.2 to 0.3)	**-52.4 (-61.6 to -39.1)**	440.7 (355.7 to 598.4)	**-49.9 (-60.1 to -33.9)**	11.7 (9.4 to 16.1)	**-54.5 (-63.8 to -39.4)**
Intestinal nematode infections	1.6 (1.2 to 2.2)	**-42.5 (-58.7 to -18.3)**	0.0 (0.0 to 0.1)	**-46.5 (-61.7 to -24.0)**	128.9 (93.8 to 177.9)	**-43.4 (-60.4 to -18.0)**	3.7 (2.7 to 5.1)	**-47.0 (-62.9 to -22.8)**
Ascariasis	1.6 (1.2 to 2.2)	**-42.5 (-58.7 to -18.3)**	0.0 (0.0 to 0.1)	**-46.5 (-61.7 to -24.0)**	128.9 (93.8 to 177.9)	**-43.4 (-60.4 to -18.0)**	3.7 (2.7 to 5.1)	**-47.0 (-62.9 to -22.8)**
Ebola	0.0 (0.0 to 0.0)	**-97.6 (-97.9 to -97.3)**	0.0 (0.0 to 0.0)	**-97.9 (-98.1 to -97.6)**	0.3 (0.3 to 0.4)	**-97.5 (-97.8 to -97.2)**	0.0 (0.0 to 0.0)	**-97.7 (-98.0 to -97.4)**
Zika virus	0.0 (0.0 to 0.0)	-	0.0 (0.0 to 0.0)	-	0.4 (0.1 to 1.3)	-	0.0 (0.0 to 0.0)	-
Other neglected tropical diseases	7.3 (4.4 to 21.0)	4.6 (-15.5 to 28.1)	0.2 (0.1 to 0.6)	-7.2 (-25.5 to 12.8)	461.9 (241.8 to 1,579.1)	1.6 (-23.9 to 31.6)	13.0 (6.7 to 45.1)	-5.8 (-30.1 to 22.1)
**Other infectious diseases**	436.2 (383.5 to 498.5)	**-25.3 (-31.6 to -18.0)**	12.1 (10.6 to 13.9)	**-33.0 (-38.5 to -26.7)**	27,627.5 (23,436.6 to 32,732.6)	**-32.2 (-38.9 to -24.2)**	773.8 (655.3 to 920.6)	**-37.1 (-43.2 to -29.5)**
Meningitis	154.1 (135.7 to 183.7)	**-19.1 (-26.4 to -8.2)**	4.3 (3.8 to 5.1)	**-26.7 (-33.3 to -17.0)**	10,472.1 (9,030.6 to 12,190.3)	**-24.0 (-31.8 to -12.4)**	294.4 (253.4 to 342.8)	**-29.0 (-36.3 to -18.1)**
Pneumococcal meningitis	24.0 (20.4 to 29.1)	-12.8 (-22.0 to 0.7)	0.7 (0.6 to 0.8)	**-21.5 (-29.6 to -8.7)**	1,607.9 (1,324.1 to 1,941.5)	**-17.6 (-28.0 to -2.2)**	45.2 (37.0 to 54.8)	**-23.2 (-33.2 to -8.6)**
H influenzae type B meningitis	38.3 (32.8 to 48.8)	**-32.8 (-39.4 to -23.6)**	1.1 (0.9 to 1.4)	**-39.4 (-45.1 to -31.4)**	2,490.5 (2,075.4 to 3,095.7)	**-39.4 (-46.2 to -30.4)**	69.7 (57.8 to 86.9)	**-43.6 (-49.9 to -35.2)**
Meningococcal meningitis	16.7 (14.0 to 20.1)	**-31.0 (-38.6 to -19.7)**	0.5 (0.4 to 0.6)	**-36.3 (-43.4 to -26.2)**	1,226.1 (1,002.5 to 1,499.7)	**-34.3 (-42.4 to -22.3)**	34.8 (28.4 to 42.8)	**-38.1 (-45.8 to -26.8)**
Other meningitis	75.0 (65.1 to 88.2)	-8.2 (-16.5 to 4.4)	2.1 (1.8 to 2.4)	**-16.6 (-24.2 to -5.1)**	5,147.6 (4,391.5 to 6,024.0)	-12.0 (-21.3 to 2.1)	144.6 (122.8 to 169.4)	**-17.7 (-26.6 to -4.0)**
Encephalitis	46.4 (41.3 to 56.0)	-4.5 (-19.0 to 14.7)	1.3 (1.1 to 1.5)	**-17.7 (-30.0 to -2.1)**	2,364.4 (2,067.6 to 2,777.0)	-13.9 (-30.3 to 4.4)	65.0 (56.7 to 76.0)	**-21.5 (-36.5 to -4.9)**
Diphtheria	1.9 (1.0 to 3.9)	-22.0 (-62.7 to 71.1)	0.1 (0.0 to 0.1)	-26.6 (-65.3 to 62.3)	155.4 (80.2 to 330.5)	-21.8 (-63.8 to 75.5)	4.4 (2.3 to 9.5)	-26.3 (-66.2 to 66.6)
Whooping cough	40.7 (20.5 to 73.5)	-24.2 (-55.1 to 34.3)	1.2 (0.6 to 2.1)	-27.9 (-57.3 to 27.9)	3,495.8 (1,758.1 to 6,309.7)	-24.2 (-55.1 to 34.3)	101.2 (50.9 to 182.6)	-27.8 (-57.2 to 28.0)
Tetanus	22.3 (14.4 to 30.4)	**-53.6 (-65.7 to -34.9)**	0.6 (0.4 to 0.8)	**-58.5 (-69.1 to -41.2)**	1,396.1 (954.8 to 1,928.1)	**-58.1 (-70.2 to -37.9)**	38.9 (26.6 to 54.3)	**-61.0 (-72.2 to -42.0)**
Measles	47.6 (17.0 to 103.7)	**-56.6 (-62.0 to -50.7)**	1.4 (0.5 to 3.0)	**-58.9 (-64.0 to -53.3)**	4,054.3 (1,448.0 to 8,821.6)	**-56.5 (-61.9 to -50.7)**	117.0 (41.8 to 254.4)	**-58.8 (-63.9 to -53.2)**
Varicella and herpes zoster	7.3 (6.6 to 8.2)	**-19.0 (-27.2 to -9.3)**	0.2 (0.2 to 0.2)	**-31.5 (-38.2 to -23.8)**	410.3 (358.1 to 473.0)	**-24.1 (-34.3 to -10.4)**	11.7 (10.2 to 13.5)	**-29.9 (-39.2 to -17.1)**
Acute hepatitis	70.3 (44.8 to 83.8)	-9.5 (-16.8 to 2.6)	1.8 (1.2 to 2.2)	**-24.3 (-30.4 to -13.9)**	2,957.2 (1,851.9 to 3,653.5)	**-20.7 (-27.9 to -5.5)**	76.9 (48.0 to 95.9)	**-30.7 (-37.0 to -16.6)**
Acute hepatitis A	8.9 (5.7 to 12.1)	**-32.5 (-43.9 to -12.8)**	0.2 (0.1 to 0.3)	**-38.5 (-49.0 to -19.4)**	588.3 (365.9 to 819.5)	**-35.9 (-47.2 to -12.9)**	15.9 (9.9 to 22.3)	**-40.8 (-51.4 to -18.6)**
Acute hepatitis B	52.2 (33.7 to 61.5)	-2.1 (-10.6 to 11.0)	1.4 (0.9 to 1.6)	**-20.5 (-27.4 to -10.5)**	1,900.0 (1,220.1 to 2,277.3)	-12.5 (-21.7 to 1.2)	48.5 (31.0 to 58.2)	**-25.8 (-33.7 to -14.1)**
Acute hepatitis C	1.6 (0.8 to 2.9)	-23.7 (-38.6 to 1.7)	0.0 (0.0 to 0.1)	**-31.9 (-44.5 to -9.5)**	102.0 (51.2 to 183.1)	**-31.4 (-46.0 to -1.9)**	2.9 (1.4 to 5.2)	**-35.9 (-49.4 to -7.0)**
Acute hepatitis E	7.6 (4.4 to 9.9)	**-16.8 (-30.0 to -2.0)**	0.2 (0.1 to 0.3)	**-26.6 (-37.9 to -13.9)**	366.9 (207.3 to 489.4)	**-25.4 (-37.3 to -10.8)**	9.5 (5.4 to 12.7)	**-32.1 (-42.4 to -19.2)**
Other unspecified infectious diseases	45.6 (34.0 to 50.0)	4.0 (-2.1 to 15.9)	1.3 (0.9 to 1.4)	**-10.8 (-16.0 to -1.0)**	2,321.9 (1,658.5 to 2,598.0)	-6.8 (-15.5 to 6.9)	64.3 (45.5 to 72.1)	**-14.7 (-22.7 to -2.0)**
**Maternal and neonatal disorders**	1,014.4 (962.3 to 1,065.5)	**-24.2 (-27.4 to -20.5)**	29.7 (28.2 to 31.2)	**-26.1 (-29.3 to -22.5)**	89,105.5 (84,535.9 to 93,597.0)	**-24.2 (-27.4 to -20.5)**	2,611.0 (2,477.3 to 2,742.6)	**-26.1 (-29.3 to -22.5)**
Maternal disorders	-	-	-	-	-	-	-	-
Maternal hemorrhage	-	-	-	-	-	-	-	-
Maternal sepsis and other maternal infections	-	-	-	-	-	-	-	-
Maternal hypertensive disorders	-	-	-	-	-	-	-	-
Maternal obstructed labor and uterine rupture	-	-	-	-	-	-	-	-
Maternal abortion and miscarriage	-	-	-	-	-	-	-	-
Ectopic pregnancy	-	-	-	-	-	-	-	-
Indirect maternal deaths	-	-	-	-	-	-	-	-
Late maternal deaths	-	-	-	-	-	-	-	-
Maternal deaths aggravated by HIV/AIDS	-	-	-	-	-	-	-	-
Other maternal disorders	-	-	-	-	-	-	-	-
Neonatal disorders	1,014.4 (962.3 to 1,065.5)	**-24.2 (-27.4 to -20.5)**	29.7 (28.2 to 31.2)	**-26.1 (-29.3 to -22.5)**	89,105.5 (84,535.9 to 93,597.0)	**-24.2 (-27.4 to -20.5)**	2,611.0 (2,477.3 to 2,742.6)	**-26.1 (-29.3 to -22.5)**
Neonatal preterm birth	370.3 (340.6 to 421.1)	**-25.8 (-31.5 to -20.2)**	10.9 (10.0 to 12.3)	**-27.7 (-33.3 to -22.3)**	32,532.4 (29,920.1 to 36,994.9)	**-25.8 (-31.5 to -20.2)**	953.3 (876.7 to 1,084.1)	**-27.7 (-33.3 to -22.3)**
Neonatal encephalopathy due to birth asphyxia and trauma	310.6 (272.5 to 344.1)	**-24.4 (-31.7 to -16.0)**	9.1 (8.0 to 10.1)	**-26.3 (-33.4 to -18.2)**	27,288.3 (23,941.4 to 30,230.3)	**-24.4 (-31.7 to -16.0)**	799.7 (701.6 to 885.9)	**-26.3 (-33.4 to -18.2)**
Neonatal sepsis and other neonatal infections	112.4 (97.4 to 138.3)	**-15.0 (-25.6 to -2.1)**	3.3 (2.9 to 4.1)	**-17.2 (-27.6 to -4.7)**	9,869.0 (8,556.2 to 12,149.6)	**-15.0 (-25.6 to -2.1)**	289.2 (250.7 to 356.0)	**-17.2 (-27.6 to -4.7)**
Hemolytic disease and other neonatal jaundice	27.7 (23.4 to 32.2)	**-37.0 (-46.5 to -25.6)**	0.8 (0.7 to 0.9)	**-38.7 (-47.9 to -27.6)**	2,434.8 (2,059.1 to 2,831.2)	**-37.0 (-46.5 to -25.6)**	71.3 (60.3 to 83.0)	**-38.7 (-47.9 to -27.6)**
Other neonatal disorders	193.3 (150.9 to 218.0)	**-23.2 (-31.3 to -12.9)**	5.7 (4.4 to 6.4)	**-25.2 (-33.1 to -15.2)**	16,981.0 (13,253.3 to 19,149.8)	**-23.2 (-31.3 to -12.9)**	497.5 (388.3 to 561.1)	**-25.2 (-33.1 to -15.2)**
**Nutritional deficiencies**	123.0 (111.6 to 143.6)	**-29.9 (-36.5 to -15.4)**	3.7 (3.3 to 4.2)	**-37.8 (-43.3 to -25.6)**	7,198.9 (6,304.4 to 8,560.2)	**-39.5 (-46.6 to -24.6)**	206.1 (180.4 to 245.2)	**-43.7 (-50.3 to -29.8)**
Protein-energy malnutrition	108.9 (98.4 to 126.2)	**-31.1 (-37.9 to -17.0)**	3.2 (2.9 to 3.7)	**-38.2 (-44.0 to -26.1)**	6,748.5 (5,885.1 to 8,030.8)	**-39.7 (-47.0 to -24.9)**	193.7 (168.7 to 230.7)	**-43.6 (-50.3 to -29.9)**
Other nutritional deficiencies	14.1 (11.9 to 18.5)	-18.4 (-27.0 to 6.3)	0.4 (0.4 to 0.6)	**-34.0 (-40.6 to -14.6)**	450.4 (391.2 to 581.9)	**-37.3 (-45.2 to -16.3)**	12.4 (10.8 to 16.0)	**-45.0 (-51.9 to -27.2)**
**Non-communicable diseases**	21,842.9 (21,430.4 to 22,207.9)	**22.5 (20.7 to 24.2)**	636.7 (624.9 to 647.0)	**-8.3 (-9.5 to -7.0)**	499,357.2 (489,396.7 to 508,589.1)	**13.6 (11.7 to 15.3)**	13,273.8 (13,008.0 to 13,517.2)	**-9.7 (-11.1 to -8.4)**
**Neoplasms**	5,442.2 (5,325.0 to 5,553.7)	**25.8 (23.4 to 28.2)**	151.5 (148.2 to 154.6)	**-5.0 (-6.7 to -3.3)**	127,582.2 (124,839.1 to 130,571.8)	**19.6 (17.3 to 22.0)**	3,314.8 (3,242.6 to 3,390.5)	**-6.1 (-7.9 to -4.3)**

SR Table 4: Global deaths and YLL numbers, age-standardised rates per 100,000, and percent change between 2007 and 2017 for males for all GBD causes and Levels 1 through 4 of the cause hierarchy. 95% UIs are in parentheses. UI=uncertainty interval. YLL=year of life lost.

Cause	All Age Deaths (thousands)		Age-standardised Death Rate (per 100,000)		All Age YLLs (thousands)		Age-standardised YLL Rate (per 100,000)	
Lip and oral cavity cancer	125.1 (117.0 to 131.2)	**31.9 (23.5 to 38.5)**	3.3 (3.1 to 3.5)	1.3 (-5.0 to 6.2)	3,387.6 (3,148.5 to 3,575.3)	**27.0 (18.0 to 34.0)**	85.0 (79.1 to 89.6)	0.2 (-6.8 to 5.6)
Nasopharynx cancer	51.0 (48.4 to 53.6)	**24.2 (18.8 to 30.4)**	1.3 (1.3 to 1.4)	-3.7 (-7.8 to 1.0)	1,483.2 (1,405.4 to 1,563.8)	**18.9 (13.2 to 25.3)**	37.0 (35.0 to 38.9)	-4.8 (-9.3 to 0.3)
Other pharynx cancer	83.9 (69.8 to 91.3)	**38.8 (24.5 to 51.5)**	2.2 (1.8 to 2.4)	6.5 (-4.6 to 16.2)	2,292.7 (1,905.2 to 2,495.9)	**34.4 (20.5 to 47.0)**	57.1 (47.4 to 62.2)	5.3 (-5.7 to 15.1)
Esophageal cancer	310.7 (300.2 to 320.6)	**15.6 (11.5 to 19.7)**	8.4 (8.1 to 8.7)	**-12.8 (-15.9 to -9.7)**	7,099.8 (6,865.0 to 7,324.6)	**11.1 (7.1 to 15.0)**	181.9 (175.8 to 187.6)	**-14.5 (-17.6 to -11.5)**
Stomach cancer	546.4 (530.9 to 564.0)	**9.9 (6.8 to 13.4)**	15.2 (14.8 to 15.7)	**-17.2 (-19.5 to -14.6)**	12,015.5 (11,671.6 to 12,393.9)	**5.1 (2.0 to 8.7)**	311.5 (302.8 to 321.2)	**-18.9 (-21.2 to -16.1)**
Colon and rectum cancer	482.5 (464.7 to 497.8)	**30.6 (24.9 to 35.0)**	13.8 (13.3 to 14.2)	-2.8 (-6.9 to 0.4)	10,165.0 (9,773.3 to 10,504.8)	**26.2 (20.2 to 30.8)**	267.0 (256.9 to 275.6)	-3.1 (-7.6 to 0.3)
Liver cancer	572.1 (543.4 to 610.0)	**28.4 (22.3 to 37.6)**	15.1 (14.4 to 16.1)	-1.4 (-6.1 to 5.6)	15,067.6 (14,197.5 to 16,112.2)	**22.7 (16.7 to 31.6)**	378.6 (357.3 to 404.3)	-3.2 (-7.8 to 3.9)
Liver cancer due to hepatitis B	252.3 (234.3 to 275.2)	**21.5 (15.0 to 32.9)**	6.4 (6.0 to 7.0)	-5.2 (-10.3 to 3.9)	7,498.3 (6,950.7 to 8,184.9)	**16.2 (10.0 to 26.4)**	185.3 (171.9 to 202.1)	-6.8 (-11.8 to 1.2)
Liver cancer due to hepatitis C	140.2 (129.0 to 152.0)	**33.1 (27.0 to 41.1)**	3.9 (3.6 to 4.2)	-0.5 (-5.1 to 5.6)	3,124.1 (2,853.5 to 3,403.9)	**29.3 (23.4 to 36.7)**	80.5 (73.8 to 87.5)	-1.1 (-5.5 to 4.5)
Liver cancer due to alcohol use	101.7 (89.6 to 116.5)	**33.0 (26.6 to 40.4)**	2.7 (2.4 to 3.1)	1.1 (-3.7 to 6.4)	2,458.9 (2,131.6 to 2,872.5)	**28.9 (21.9 to 36.6)**	62.5 (54.5 to 72.6)	0.3 (-4.7 to 5.9)
Liver cancer due to NASH	41.1 (36.4 to 46.0)	**46.2 (38.8 to 55.9)**	1.1 (1.0 to 1.3)	**10.0 (4.5 to 17.8)**	944.6 (830.6 to 1,060.7)	**40.5 (33.2 to 49.5)**	24.3 (21.4 to 27.3)	**8.9 (3.5 to 16.0)**
Liver cancer due to other causes	36.7 (32.5 to 41.5)	**32.0 (24.7 to 41.7)**	1.0 (0.9 to 1.1)	2.4 (-3.1 to 9.9)	1,041.6 (920.1 to 1,182.7)	**24.6 (17.3 to 33.8)**	26.0 (23.0 to 29.4)	-0.1 (-5.5 to 7.0)
Gallbladder and biliary tract cancer	71.6 (60.2 to 79.0)	**26.6 (21.0 to 32.2)**	2.1 (1.8 to 2.3)	**-6.0 (-10.0 to -1.9)**	1,440.1 (1,194.9 to 1,600.8)	**21.7 (15.6 to 27.8)**	38.1 (31.7 to 42.3)	**-7.0 (-11.5 to -2.6)**
Pancreatic cancer	226.1 (219.1 to 233.0)	**38.9 (33.5 to 43.2)**	6.3 (6.1 to 6.5)	**4.1 (0.1 to 7.3)**	4,939.3 (4,783.8 to 5,091.5)	**33.6 (28.1 to 38.1)**	128.1 (124.1 to 132.0)	2.6 (-1.5 to 5.9)
Larynx cancer	105.6 (102.7 to 108.8)	**20.1 (16.6 to 23.8)**	2.8 (2.7 to 2.9)	**-8.9 (-11.5 to -6.3)**	2,641.0 (2,565.4 to 2,721.2)	**16.4 (12.7 to 20.4)**	66.6 (64.7 to 68.6)	**-9.9 (-12.8 to -6.9)**
Tracheal, bronchus, and lung cancer	1,286.8 (1,250.0 to 1,322.4)	**26.7 (22.5 to 30.6)**	35.4 (34.4 to 36.3)	**-4.8 (-7.9 to -2.0)**	27,994.9 (27,160.0 to 28,794.6)	**22.1 (17.8 to 25.8)**	724.4 (703.1 to 744.8)	**-6.5 (-9.7 to -3.7)**
Malignant skin melanoma	33.0 (20.4 to 39.2)	**24.5 (14.5 to 31.4)**	0.9 (0.6 to 1.1)	-4.2 (-11.9 to 1.0)	833.6 (558.7 to 1,029.6)	**16.0 (8.4 to 21.6)**	21.4 (14.2 to 26.3)	**-7.2 (-12.9 to -2.7)**
Non-melanoma skin cancer	43.3 (41.4 to 44.6)	**35.7 (32.2 to 38.4)**	1.3 (1.2 to 1.3)	0.0 (-2.5 to 2.0)	874.3 (836.7 to 899.2)	**28.0 (24.7 to 30.7)**	23.3 (22.3 to 23.9)	-1.4 (-3.9 to 0.6)
Non-melanoma skin cancer (squamous-cell carcinoma)	43.3 (41.4 to 44.6)	**35.7 (32.2 to 38.4)**	1.3 (1.2 to 1.3)	0.0 (-2.5 to 2.0)	874.3 (836.7 to 899.2)	**28.0 (24.7 to 30.7)**	23.3 (22.3 to 23.9)	-1.4 (-3.9 to 0.6)
Breast cancer	10.9 (10.5 to 11.4)	**41.7 (34.4 to 48.2)**	0.3 (0.3 to 0.3)	**6.1 (1.0 to 10.8)**	268.5 (257.3 to 280.4)	**38.3 (30.7 to 45.4)**	6.8 (6.6 to 7.1)	**7.4 (1.7 to 12.7)**
Cervical cancer	-	**-**	-	**-**	-	**-**	-	**-**
Uterine cancer	-	**-**	-	**-**	-	**-**	-	**-**
Ovarian cancer	-	**-**	-	**-**	-	**-**	-	**-**
Prostate cancer	415.9 (357.3 to 489.5)	**32.5 (29.3 to 38.4)**	13.1 (11.2 to 15.3)	**-5.3 (-7.5 to -1.1)**	6,214.5 (5,324.2 to 7,293.0)	**28.3 (24.9 to 34.5)**	177.6 (152.7 to 207.9)	**-5.4 (-7.9 to -0.7)**
Testicular cancer	7.7 (7.4 to 8.0)	**6.1 (2.3 to 10.9)**	0.2 (0.2 to 0.2)	**-9.5 (-12.6 to -5.4)**	338.7 (323.8 to 357.4)	0.9 (-3.3 to 6.3)	8.6 (8.2 to 9.1)	**-11.0 (-14.6 to -6.3)**
Kidney cancer	89.6 (84.6 to 92.5)	**32.3 (27.6 to 36.8)**	2.5 (2.4 to 2.6)	0.0 (-3.5 to 3.3)	2,081.1 (1,935.3 to 2,152.2)	**25.3 (20.3 to 29.5)**	54.3 (50.6 to 56.2)	-1.9 (-5.8 to 1.4)
Bladder cancer	144.7 (139.6 to 154.0)	**29.0 (25.9 to 32.3)**	4.4 (4.2 to 4.7)	**-5.6 (-7.8 to -3.2)**	2,519.5 (2,425.4 to 2,681.3)	**23.5 (20.5 to 26.6)**	69.1 (66.5 to 73.4)	**-7.1 (-9.2 to -4.7)**
Brain and nervous system cancer	140.3 (118.0 to 157.7)	**34.1 (25.9 to 45.3)**	3.7 (3.1 to 4.1)	**7.8 (1.3 to 17.0)**	4,936.5 (4,133.4 to 5,645.5)	**23.8 (16.4 to 33.5)**	127.0 (106.2 to 145.0)	4.7 (-1.6 to 13.0)
Thyroid cancer	17.2 (16.4 to 17.8)	**36.6 (28.6 to 42.5)**	0.5 (0.5 to 0.5)	4.5 (-1.4 to 8.8)	427.2 (407.3 to 443.0)	**28.9 (20.2 to 35.1)**	11.0 (10.5 to 11.4)	2.6 (-4.1 to 7.4)
Mesothelioma	21.7 (21.0 to 22.5)	**28.7 (21.8 to 34.5)**	0.6 (0.6 to 0.6)	-3.2 (-8.2 to 1.1)	466.3 (450.4 to 481.2)	**22.9 (15.8 to 28.9)**	12.2 (11.8 to 12.6)	**-4.9 (-10.2 to -0.4)**
Hodgkin lymphoma	20.7 (16.8 to 25.8)	-1.1 (-5.2 to 2.6)	0.5 (0.4 to 0.7)	**-18.4 (-21.7 to -15.4)**	840.5 (681.8 to 1,058.4)	**-6.8 (-10.4 to -3.0)**	21.7 (17.6 to 27.3)	**-18.9 (-22.1 to -15.6)**
Non-Hodgkin lymphoma	144.3 (139.8 to 147.9)	**31.1 (23.4 to 35.3)**	4.0 (3.9 to 4.1)	1.3 (-4.2 to 4.3)	4,146.5 (3,973.0 to 4,298.1)	**22.7 (12.2 to 28.3)**	108.1 (103.5 to 112.2)	0.9 (-7.6 to 5.5)
Multiple myeloma	55.2 (46.4 to 64.0)	**35.6 (27.7 to 41.4)**	1.6 (1.3 to 1.8)	0.9 (-4.5 to 4.9)	1,191.0 (1,038.6 to 1,394.0)	**31.8 (23.0 to 38.0)**	31.1 (27.0 to 36.3)	1.2 (-5.2 to 5.9)
Leukemia	197.3 (178.4 to 209.1)	**14.8 (11.9 to 18.0)**	5.5 (5.0 to 5.8)	**-8.5 (-10.7 to -6.0)**	6,678.1 (5,882.9 to 7,155.6)	3.9 (-0.1 to 8.6)	177.0 (155.8 to 189.7)	**-10.7 (-14.2 to -6.8)**
Acute lymphoid leukemia	30.6 (26.6 to 33.8)	**13.2 (4.3 to 25.7)**	0.8 (0.7 to 0.9)	-2.3 (-9.9 to 8.2)	1,554.8 (1,332.4 to 1,718.9)	3.9 (-6.4 to 18.7)	41.5 (35.6 to 46.1)	-6.0 (-15.4 to 7.5)
Chronic lymphoid leukemia	20.8 (19.6 to 22.1)	**22.5 (17.6 to 28.6)**	0.6 (0.6 to 0.7)	**-10.3 (-13.7 to -5.8)**	390.6 (360.0 to 421.9)	**18.4 (12.8 to 24.4)**	10.6 (9.8 to 11.4)	**-9.9 (-14.0 to -5.3)**
Acute myeloid leukemia	57.4 (50.9 to 61.0)	**28.7 (22.7 to 34.2)**	1.6 (1.4 to 1.7)	1.7 (-2.8 to 5.8)	1,853.1 (1,571.2 to 2,000.4)	**20.5 (11.1 to 29.1)**	48.8 (41.4 to 52.7)	1.9 (-5.8 to 9.1)
Chronic myeloid leukemia	12.9 (11.6 to 14.1)	3.5 (-0.5 to 7.7)	0.4 (0.3 to 0.4)	**-20.4 (-23.4 to -17.3)**	340.6 (302.9 to 375.7)	-2.0 (-6.3 to 2.9)	8.8 (7.9 to 9.7)	**-20.4 (-23.8 to -16.5)**
Other leukemia	75.6 (65.4 to 84.3)	**6.9 (1.8 to 13.1)**	2.1 (1.8 to 2.3)	**-14.2 (-18.0 to -9.8)**	2,538.9 (2,135.7 to 2,865.2)	-6.4 (-11.8 to 1.0)	67.3 (56.8 to 75.8)	**-19.3 (-23.8 to -12.9)**
Other malignant neoplasms	186.8 (166.9 to 194.4)	**26.9 (21.2 to 31.4)**	5.1 (4.6 to 5.3)	0.1 (-4.5 to 3.5)	5,974.4 (5,345.2 to 6,278.9)	**17.9 (11.5 to 23.8)**	156.4 (140.0 to 164.7)	-0.7 (-6.0 to 4.2)
Other neoplasms	52.0 (35.7 to 67.9)	**45.6 (33.5 to 60.0)**	1.5 (1.0 to 2.0)	8.9 (-0.6 to 20.7)	1,264.7 (936.1 to 1,660.8)	**35.6 (23.3 to 50.9)**	33.8 (24.7 to 44.2)	9.6 (-0.6 to 22.2)
Myelodysplastic, myeloproliferative, and other hematopoietic neoplasms	50.1 (34.2 to 65.9)	**46.1 (33.8 to 60.9)**	1.5 (1.0 to 1.9)	8.6 (-0.9 to 20.5)	1,154.0 (846.9 to 1,518.3)	**36.2 (23.5 to 52.3)**	30.8 (22.5 to 40.5)	8.8 (-1.6 to 21.9)
Other benign and in situ neoplasms	1.9 (1.3 to 2.5)	**32.4 (21.7 to 51.1)**	0.0 (0.0 to 0.1)	**19.1 (9.5 to 36.6)**	110.7 (77.4 to 147.5)	**29.1 (18.4 to 45.4)**	2.9 (2.0 to 3.9)	**18.7 (8.9 to 33.9)**
**Cardiovascular diseases**	9,346.3 (9,173.3 to 9,526.5)	**22.2 (20.3 to 24.2)**	275.5 (270.5 to 280.6)	**-9.6 (-10.9 to -8.2)**	193,503.6 (189,476.5 to 197,539.2)	**15.8 (13.7 to 17.8)**	5,137.5 (5,036.2 to 5,241.9)	**-10.4 (-11.9 to -8.8)**
Rheumatic heart disease	118.8 (111.8 to 132.1)	-2.8 (-8.8 to 4.0)	3.4 (3.2 to 3.7)	**-24.0 (-28.1 to -18.9)**	3,275.3 (3,062.6 to 3,666.0)	**-13.8 (-19.5 to -7.7)**	85.6 (80.2 to 95.6)	**-28.2 (-32.8 to -23.4)**
Ischemic heart disease	4,904.0 (4,806.6 to 5,023.3)	**23.1 (21.1 to 25.2)**	144.4 (141.5 to 147.9)	**-9.0 (-10.5 to -7.6)**	102,276.0 (100,080.3 to 104,704.7)	**17.7 (15.5 to 19.9)**	2,702.9 (2,645.8 to 2,766.8)	**-9.3 (-10.9 to -7.6)**
Stroke	3,174.2 (3,085.6 to 3,262.5)	**19.0 (16.2 to 21.7)**	93.0 (90.4 to 95.6)	**-12.4 (-14.4 to -10.5)**	63,760.8 (61,883.7 to 65,556.5)	**14.4 (11.5 to 17.3)**	1,698.4 (1,649.2 to 1,745.3)	**-12.2 (-14.4 to -10.1)**
Ischemic stroke	1,330.8 (1,275.6 to 1,400.2)	**26.3 (23.0 to 29.5)**	41.1 (39.5 to 43.1)	**-9.1 (-11.3 to -6.8)**	21,916.4 (20,865.4 to 23,369.0)	**22.0 (18.4 to 25.5)**	610.4 (582.4 to 648.9)	**-8.9 (-11.4 to -6.4)**
Intracerebral hemorrhage	1,621.5 (1,563.7 to 1,696.7)	**13.4 (10.0 to 17.1)**	45.7 (44.1 to 47.9)	**-15.8 (-18.3 to -13.1)**	36,019.4 (34,660.1 to 37,601.9)	**10.6 (7.3 to 14.2)**	938.0 (903.3 to 979.5)	**-14.7 (-17.2 to -11.9)**
Subarachnoid hemorrhage	221.9 (194.2 to 254.0)	**21.0 (12.4 to 33.7)**	6.1 (5.4 to 7.0)	-7.3 (-13.7 to 1.8)	5,825.0 (5,176.3 to 6,729.2)	**12.5 (6.3 to 24.6)**	150.0 (133.4 to 172.9)	**-9.4 (-14.7 to -0.2)**
Hypertensive heart disease	401.7 (271.6 to 434.5)	**51.1 (25.4 to 63.6)**	12.3 (8.2 to 13.2)	10.9 (-8.0 to 19.4)	7,367.6 (5,052.3 to 8,043.5)	**39.9 (18.2 to 52.7)**	200.3 (136.6 to 218.2)	7.5 (-9.6 to 16.8)
Non-rheumatic valvular heart disease	63.5 (52.8 to 67.7)	**32.5 (27.7 to 37.2)**	2.1 (1.6 to 2.2)	**-3.9 (-7.2 to -0.4)**	1,115.9 (1,001.0 to 1,233.4)	**21.6 (16.9 to 26.2)**	31.1 (27.2 to 33.9)	**-5.8 (-9.3 to -2.2)**
Non-rheumatic calcific aortic valve disease	45.3 (35.6 to 48.6)	**42.0 (33.9 to 47.6)**	1.5 (1.1 to 1.6)	1.2 (-4.3 to 5.0)	717.0 (607.3 to 787.2)	**31.0 (24.8 to 36.2)**	20.4 (17.2 to 22.2)	-0.6 (-5.1 to 3.2)
Non-rheumatic degenerative mitral valve disease	15.3 (12.4 to 18.5)	**14.5 (8.6 to 25.8)**	0.5 (0.4 to 0.6)	**-15.1 (-19.1 to -6.6)**	328.3 (268.6 to 387.3)	**8.0 (1.8 to 16.6)**	8.8 (7.2 to 10.5)	**-14.7 (-19.5 to -7.6)**
Other non-rheumatic valve diseases	3.0 (1.9 to 4.3)	9.6 (-5.7 to 45.6)	0.1 (0.1 to 0.1)	-18.4 (-30.4 to 9.9)	70.6 (45.5 to 102.3)	7.0 (-4.5 to 29.6)	1.9 (1.2 to 2.7)	-13.5 (-23.6 to 7.0)
Cardiomyopathy and myocarditis	212.9 (187.4 to 228.8)	**6.7 (0.7 to 23.9)**	6.0 (5.2 to 6.4)	**-15.9 (-20.3 to -2.8)**	6,212.3 (5,563.8 to 6,683.3)	-4.6 (-10.8 to 11.9)	160.5 (143.8 to 172.3)	**-20.6 (-25.6 to -7.0)**
Myocarditis	21.9 (17.8 to 26.6)	**19.8 (9.5 to 35.7)**	0.7 (0.5 to 0.8)	-7.9 (-16.9 to 6.4)	686.9 (565.3 to 836.1)	5.4 (-3.6 to 17.6)	18.3 (15.2 to 22.1)	-9.9 (-17.8 to 0.8)
Alcoholic cardiomyopathy	68.6 (57.5 to 75.2)	-21.6 (-27.0 to 4.4)	1.7 (1.5 to 1.9)	**-36.6 (-41.0 to -17.0)**	2,235.0 (1,902.1 to 2,440.8)	**-28.0 (-33.2 to -0.9)**	55.2 (47.0 to 60.4)	**-40.5 (-44.8 to -18.5)**
Other cardiomyopathy	122.4 (107.5 to 133.5)	**30.5 (24.6 to 38.2)**	3.6 (3.1 to 3.9)	-1.7 (-6.3 to 5.5)	3,290.3 (2,895.1 to 3,613.7)	**19.3 (12.0 to 25.7)**	86.9 (77.0 to 95.1)	-2.3 (-8.0 to 2.9)
Atrial fibrillation and flutter	110.0 (98.2 to 125.0)	**51.0 (45.0 to 55.4)**	3.9 (3.5 to 4.4)	3.1 (-0.7 to 6.0)	1,306.0 (1,175.2 to 1,473.9)	**42.1 (35.5 to 46.5)**	40.4 (36.3 to 45.6)	2.2 (-2.3 to 5.3)
Aortic aneurysm	106.3 (100.0 to 113.8)	**22.0 (17.5 to 27.0)**	3.1 (3.0 to 3.4)	**-10.5 (-13.6 to -7.0)**	2,091.2 (1,946.0 to 2,249.4)	**17.9 (12.9 to 23.6)**	56.0 (52.3 to 60.2)	**-9.9 (-13.6 to -5.7)**
Peripheral artery disease	33.8 (19.6 to 61.8)	**55.6 (27.1 to 72.6)**	1.1 (0.6 to 2.0)	11.1 (-8.7 to 23.8)	521.6 (302.1 to 945.9)	**47.5 (19.4 to 64.0)**	14.7 (8.5 to 26.7)	9.4 (-11.1 to 21.7)
Endocarditis	39.6 (37.3 to 41.9)	**36.2 (27.4 to 41.8)**	1.1 (1.1 to 1.2)	5.2 (-1.3 to 9.3)	1,135.8 (1,066.9 to 1,207.3)	**20.5 (10.7 to 26.2)**	30.2 (28.3 to 32.0)	0.9 (-6.8 to 5.4)
Other cardiovascular and circulatory diseases	181.5 (166.2 to 203.5)	**20.9 (16.1 to 25.7)**	5.2 (4.8 to 5.8)	**-8.7 (-12.5 to -5.0)**	4,441.1 (4,070.7 to 4,986.7)	**11.2 (7.8 to 14.9)**	117.4 (107.8 to 131.3)	**-10.4 (-13.3 to -7.6)**
**Chronic respiratory diseases**	2,164.4 (2,083.0 to 2,227.1)	**13.2 (10.4 to 16.2)**	65.4 (62.9 to 67.3)	**-17.2 (-19.2 to -15.1)**	38,918.3 (37,520.1 to 40,092.1)	**7.5 (4.6 to 10.6)**	1,067.3 (1,028.5 to 1,099.0)	**-18.1 (-20.3 to -15.8)**
Chronic obstructive pulmonary disease	1,789.8 (1,672.7 to 1,888.4)	**15.8 (11.9 to 19.5)**	54.8 (51.5 to 57.6)	**-16.2 (-18.7 to -13.7)**	29,860.1 (27,547.1 to 32,029.0)	**11.6 (7.5 to 15.6)**	829.4 (767.9 to 886.7)	**-16.4 (-19.3 to -13.6)**
Pneumoconiosis	19.2 (18.2 to 20.4)	**10.0 (3.8 to 16.6)**	0.6 (0.5 to 0.6)	**-18.8 (-23.1 to -14.2)**	383.7 (361.7 to 408.9)	**7.8 (1.0 to 15.3)**	10.2 (9.6 to 10.8)	**-17.6 (-22.6 to -12.0)**
Silicosis	10.7 (9.8 to 11.8)	11.3 (-0.3 to 22.4)	0.3 (0.3 to 0.3)	**-17.9 (-26.0 to -10.0)**	224.6 (199.1 to 246.8)	11.6 (-1.9 to 23.6)	5.9 (5.3 to 6.5)	**-14.8 (-24.6 to -5.9)**
Asbestosis	2.9 (1.9 to 3.4)	**24.9 (15.9 to 35.9)**	0.1 (0.1 to 0.1)	**-8.7 (-15.0 to -1.2)**	46.5 (31.6 to 56.8)	**17.1 (8.1 to 30.2)**	1.3 (0.9 to 1.6)	**-11.4 (-17.9 to -2.1)**
Coal workers pneumoconiosis	3.0 (2.7 to 3.8)	-3.5 (-14.0 to 11.4)	0.1 (0.1 to 0.1)	**-29.0 (-36.4 to -18.8)**	54.1 (47.1 to 71.2)	-7.4 (-17.7 to 9.4)	1.5 (1.3 to 1.9)	**-29.5 (-37.1 to -17.7)**
Other pneumoconiosis	2.7 (2.2 to 3.5)	7.5 (-2.8 to 27.5)	0.1 (0.1 to 0.1)	**-19.3 (-26.9 to -4.3)**	58.5 (48.8 to 79.5)	3.7 (-6.3 to 21.4)	1.5 (1.3 to 2.1)	**-19.3 (-27.0 to -5.1)**
Asthma	241.4 (142.8 to 339.5)	-9.5 (-16.9 to 1.7)	6.7 (4.0 to 9.4)	**-31.4 (-37.3 to -22.6)**	6,065.9 (3,688.9 to 8,265.2)	**-14.3 (-20.0 to -6.2)**	158.5 (97.0 to 215.8)	**-31.6 (-36.5 to -24.6)**
Interstitial lung disease and pulmonary sarcoidosis	80.0 (61.4 to 104.6)	**50.4 (31.5 to 61.8)**	2.4 (1.8 to 3.1)	10.4 (-2.5 to 18.2)	1,503.7 (1,179.8 to 2,034.4)	**43.3 (25.1 to 55.3)**	40.8 (31.9 to 54.7)	9.7 (-3.7 to 18.5)
Other chronic respiratory diseases	34.0 (29.3 to 40.8)	**20.9 (12.6 to 33.5)**	0.9 (0.8 to 1.1)	-4.9 (-11.0 to 4.4)	1,105.0 (931.4 to 1,327.5)	**12.3 (4.5 to 24.9)**	28.5 (24.1 to 34.0)	-5.8 (-12.1 to 4.3)
**Digestive diseases**	1,431.8 (1,368.4 to 1,556.9)	**14.4 (10.6 to 21.3)**	38.8 (37.0 to 42.2)	**-11.0 (-13.9 to -5.8)**	42,509.7 (40,340.2 to 46,293.8)	**7.5 (3.9 to 15.1)**	1,084.0 (1,028.1 to 1,178.8)	**-12.0 (-14.9 to -5.7)**
Cirrhosis and other chronic liver diseases	882.7 (838.3 to 966.5)	**14.6 (8.2 to 21.6)**	22.9 (21.8 to 25.1)	**-9.8 (-14.8 to -4.3)**	27,714.8 (26,303.9 to 30,317.6)	**9.2 (3.5 to 15.8)**	692.6 (656.8 to 757.9)	**-10.8 (-15.5 to -5.4)**
Cirrhosis and other chronic liver diseases due to hepatitis B	278.2 (251.8 to 320.5)	**8.6 (1.0 to 17.3)**	7.2 (6.5 to 8.3)	**-14.2 (-20.3 to -7.2)**	8,861.4 (8,031.9 to 10,128.0)	3.7 (-3.3 to 11.3)	221.3 (200.7 to 253.3)	**-14.9 (-20.8 to -8.9)**
Cirrhosis and other chronic liver diseases due to hepatitis C	224.7 (202.9 to 250.5)	**16.9 (10.9 to 23.2)**	5.8 (5.3 to 6.5)	**-8.3 (-13.0 to -3.5)**	6,905.1 (6,208.9 to 7,716.4)	**12.3 (6.6 to 18.6)**	171.5 (154.1 to 191.4)	**-9.1 (-13.5 to -4.1)**
Cirrhosis and other chronic liver diseases due to alcohol use	241.4 (219.5 to 267.8)	**16.6 (10.8 to 23.2)**	6.2 (5.7 to 6.9)	**-8.5 (-13.2 to -3.3)**	7,397.4 (6,710.8 to 8,205.9)	**12.4 (6.9 to 18.5)**	183.1 (166.2 to 202.9)	**-9.4 (-13.8 to -4.5)**
Cirrhosis due to NASH	68.1 (62.0 to 74.9)	**28.2 (21.5 to 35.0)**	1.8 (1.6 to 1.9)	-0.4 (-5.6 to 5.0)	2,020.5 (1,833.8 to 2,234.8)	**23.2 (17.2 to 29.2)**	50.1 (45.4 to 55.3)	-1.5 (-6.3 to 3.4)
Cirrhosis and other chronic liver diseases due to other causes	70.4 (62.1 to 80.4)	**13.7 (7.0 to 22.4)**	1.9 (1.7 to 2.2)	**-8.3 (-13.6 to -1.4)**	2,530.3 (2,251.7 to 2,866.5)	2.8 (-4.7 to 13.4)	66.6 (59.4 to 75.2)	**-11.3 (-17.7 to -1.8)**
Upper digestive system diseases	162.7 (152.4 to 184.3)	-0.4 (-6.3 to 7.1)	4.6 (4.3 to 5.2)	**-24.1 (-28.3 to -18.5)**	4,125.3 (3,803.1 to 4,665.8)	-6.7 (-13.5 to 1.2)	107.0 (98.9 to 120.9)	**-25.0 (-30.2 to -18.8)**
Peptic ulcer disease	133.2 (125.1 to 151.2)	-3.1 (-8.6 to 3.7)	3.8 (3.5 to 4.3)	**-26.1 (-30.1 to -20.8)**	3,315.3 (3,071.6 to 3,737.1)	**-9.6 (-15.7 to -2.8)**	86.0 (79.8 to 97.0)	**-27.5 (-32.3 to -22.1)**
Gastritis and duodenitis	29.5 (26.6 to 33.8)	**13.7 (3.2 to 27.5)**	0.8 (0.8 to 0.9)	**-13.2 (-20.7 to -3.5)**	810.0 (713.0 to 934.7)	7.4 (-4.8 to 24.2)	21.0 (18.6 to 24.2)	-12.5 (-22.1 to 0.1)
Appendicitis	21.7 (19.5 to 24.7)	0.6 (-7.7 to 11.5)	0.6 (0.5 to 0.7)	**-18.5 (-24.9 to -10.0)**	801.0 (714.4 to 915.0)	-9.2 (-19.2 to 3.1)	21.0 (18.7 to 24.0)	**-21.0 (-29.7 to -10.5)**
Paralytic ileus and intestinal obstruction	124.9 (98.6 to 148.8)	**19.9 (11.5 to 29.6)**	3.6 (2.9 to 4.3)	-6.4 (-12.4 to 0.7)	4,053.7 (3,208.6 to 4,672.0)	5.9 (-5.0 to 17.8)	110.0 (87.8 to 126.6)	-9.0 (-18.1 to 1.0)

SR Table 4: Global deaths and YLL numbers, age-standardised rates per 100,000, and percent change between 2007 and 2017 for males for all GBD causes and Levels 1 through 4 of the cause hierarchy. 95% UIs are in parentheses. UI=uncertainty interval. YLL=year of life lost.

Cause	All Age Deaths (thousands)		Age-standardised Death Rate (per 100,000)		All Age YLLs (thousands)		Age-standardised YLL Rate (per 100,000)	
Inguinal, femoral, and abdominal hernia	25.8 (23.3 to 30.1)	**20.9 (13.4 to 31.5)**	0.8 (0.7 to 0.9)	**-9.8 (-15.2 to -2.7)**	584.1 (522.8 to 681.1)	**9.4 (0.4 to 21.5)**	15.7 (14.1 to 18.3)	**-12.0 (-18.8 to -2.6)**
Inflammatory bowel disease	18.3 (15.3 to 19.7)	**23.3 (12.3 to 33.1)**	0.5 (0.4 to 0.6)	**-7.7 (-14.6 to -0.8)**	442.2 (387.1 to 474.9)	11.7 (-2.4 to 23.6)	11.8 (10.3 to 12.7)	**-9.6 (-20.1 to -0.8)**
Vascular intestinal disorders	41.5 (38.5 to 44.3)	**22.4 (16.2 to 32.3)**	1.2 (1.2 to 1.3)	**-10.3 (-14.5 to -3.7)**	793.0 (728.6 to 852.6)	**16.7 (9.2 to 28.2)**	21.6 (19.8 to 23.2)	**-10.3 (-15.8 to -2.3)**
Gallbladder and biliary diseases	47.8 (45.6 to 53.1)	**33.0 (27.5 to 40.8)**	1.5 (1.4 to 1.6)	-2.1 (-5.8 to 3.1)	930.2 (872.0 to 1,031.1)	**22.5 (15.5 to 31.8)**	25.5 (23.9 to 28.1)	-3.9 (-9.0 to 3.0)
Pancreatitis	63.4 (55.7 to 69.7)	**20.5 (13.4 to 27.9)**	1.7 (1.5 to 1.9)	-4.4 (-9.8 to 1.4)	2,006.0 (1,748.4 to 2,220.5)	**13.8 (5.8 to 21.6)**	50.5 (43.9 to 55.8)	-5.9 (-12.3 to 0.3)
Other digestive diseases	43.1 (38.8 to 49.5)	**27.8 (16.6 to 41.7)**	1.3 (1.2 to 1.5)	-4.8 (-12.2 to 4.3)	1,059.3 (921.3 to 1,255.2)	**19.0 (4.7 to 36.6)**	28.3 (24.7 to 33.4)	-4.0 (-14.4 to 9.0)
**Neurological disorders**	1,192.4 (1,155.4 to 1,229.9)	**44.0 (40.9 to 46.9)**	41.3 (40.1 to 42.4)	1.0 (-1.0 to 2.8)	17,277.6 (16,556.9 to 18,293.3)	**25.1 (21.5 to 31.1)**	517.3 (496.8 to 544.9)	-2.7 (-5.3 to 1.3)
Alzheimer's disease and other dementias	866.0 (842.8 to 886.8)	**51.2 (46.9 to 53.9)**	31.5 (30.7 to 32.2)	2.1 (-0.5 to 3.7)	9,162.3 (8,891.3 to 9,421.5)	**42.4 (37.5 to 45.5)**	295.8 (287.4 to 303.7)	1.0 (-2.2 to 3.0)
Parkinson's disease	193.4 (178.2 to 206.0)	**41.4 (35.2 to 45.2)**	6.3 (5.8 to 6.7)	0.3 (-3.9 to 2.9)	2,552.6 (2,391.5 to 2,746.9)	**36.3 (30.0 to 40.2)**	76.0 (71.1 to 81.7)	0.1 (-4.4 to 2.9)
Epilepsy	75.4 (65.0 to 90.3)	2.6 (-3.4 to 16.2)	2.0 (1.7 to 2.4)	-11.4 (-16.6 to 0.1)	3,662.8 (3,233.0 to 4,458.4)	-5.3 (-12.0 to 10.4)	96.0 (84.9 to 116.8)	**-14.7 (-20.9 to -0.4)**
Multiple sclerosis	8.5 (7.1 to 9.2)	**22.7 (8.2 to 27.5)**	0.2 (0.2 to 0.2)	-3.4 (-14.4 to 0.3)	262.0 (225.7 to 291.3)	**17.7 (4.1 to 23.2)**	6.5 (5.6 to 7.2)	**-4.7 (-15.4 to -0.1)**
Motor neuron disease	19.1 (18.1 to 20.7)	**35.0 (27.8 to 39.7)**	0.5 (0.5 to 0.6)	2.6 (-2.7 to 6.2)	479.7 (454.3 to 527.7)	**28.0 (20.7 to 32.2)**	12.4 (11.7 to 13.6)	1.1 (-4.3 to 4.5)
Other neurological disorders	30.0 (28.7 to 31.9)	**25.4 (16.6 to 33.2)**	0.8 (0.8 to 0.9)	3.1 (-3.8 to 8.6)	1,158.1 (1,103.9 to 1,232.9)	**11.2 (2.4 to 21.1)**	30.7 (29.1 to 32.8)	-2.0 (-9.5 to 6.2)
**Mental disorders**	0.0 (0.0 to 0.1)	**50.6 (28.7 to 72.3)**	0.0 (0.0 to 0.0)	**35.0 (15.0 to 55.1)**	2.4 (2.0 to 2.7)	**46.3 (24.3 to 68.7)**	0.1 (0.0 to 0.1)	**32.3 (12.2 to 52.3)**
Eating disorders	0.0 (0.0 to 0.1)	**50.6 (28.7 to 72.3)**	0.0 (0.0 to 0.0)	**35.0 (15.0 to 55.1)**	2.4 (2.0 to 2.7)	**46.3 (24.3 to 68.7)**	0.1 (0.0 to 0.1)	**32.3 (12.2 to 52.3)**
Anorexia nervosa	0.0 (0.0 to 0.0)	**62.2 (33.4 to 88.0)**	0.0 (0.0 to 0.0)	**45.4 (19.6 to 68.6)**	1.5 (1.2 to 1.8)	**56.1 (27.6 to 81.8)**	0.0 (0.0 to 0.0)	**41.5 (16.5 to 64.6)**
Bulimia nervosa	0.0 (0.0 to 0.0)	**32.4 (11.0 to 58.9)**	0.0 (0.0 to 0.0)	18.7 (-0.3 to 41.9)	0.8 (0.7 to 1.1)	**31.1 (9.9 to 58.0)**	0.0 (0.0 to 0.0)	18.3 (-0.9 to 42.5)
**Substance use disorders**	269.3 (250.7 to 278.2)	**19.5 (15.5 to 22.9)**	6.8 (6.3 to 7.0)	-0.9 (-4.2 to 1.9)	10,654.8 (9,909.1 to 11,014.1)	**15.2 (11.3 to 18.5)**	264.2 (245.8 to 273.1)	-1.9 (-5.3 to 0.9)
Alcohol use disorders	155.7 (136.6 to 162.5)	3.3 (-2.2 to 7.8)	3.9 (3.4 to 4.1)	**-15.4 (-19.9 to -11.8)**	5,768.6 (5,022.4 to 6,031.7)	-1.2 (-7.1 to 3.1)	141.8 (123.4 to 148.2)	**-17.3 (-22.3 to -13.7)**
Drug use disorders	113.6 (110.9 to 116.5)	**52.1 (48.2 to 56.0)**	2.9 (2.8 to 3.0)	**28.6 (25.4 to 31.9)**	4,886.2 (4,765.1 to 5,021.8)	**43.3 (39.3 to 47.4)**	122.4 (119.4 to 125.8)	**25.0 (21.5 to 28.5)**
Opioid use disorders	77.5 (74.7 to 80.3)	**68.1 (61.6 to 76.4)**	2.0 (1.9 to 2.0)	**43.2 (37.6 to 50.4)**	3,415.3 (3,292.4 to 3,535.6)	**57.4 (51.3 to 65.1)**	85.6 (82.5 to 88.6)	**37.9 (32.6 to 44.7)**
Cocaine use disorders	5.5 (4.9 to 6.0)	**34.3 (21.7 to 46.5)**	0.1 (0.1 to 0.1)	**13.4 (2.5 to 23.6)**	233.5 (208.4 to 255.6)	**27.6 (15.6 to 38.7)**	5.8 (5.2 to 6.3)	9.9 (-0.4 to 19.6)
Amphetamine use disorders	3.2 (2.2 to 3.6)	18.9 (-4.0 to 31.6)	0.1 (0.1 to 0.1)	1.8 (-17.7 to 12.9)	148.3 (101.0 to 163.7)	13.3 (-6.9 to 25.2)	3.7 (2.5 to 4.1)	-1.2 (-18.5 to 9.2)
Other drug use disorders	27.4 (25.9 to 29.4)	**25.8 (17.2 to 32.0)**	0.7 (0.7 to 0.8)	4.8 (-2.3 to 10.1)	1,089.1 (1,026.5 to 1,175.9)	**17.7 (9.8 to 24.1)**	27.4 (25.8 to 29.5)	1.8 (-5.1 to 7.3)
**Diabetes and kidney diseases**	1,316.3 (1,272.3 to 1,355.2)	**34.4 (31.5 to 36.9)**	37.6 (36.4 to 38.7)	0.7 (-1.3 to 2.4)	30,688.7 (29,661.2 to 31,645.8)	**25.6 (22.9 to 28.2)**	803.2 (776.2 to 828.0)	-0.7 (-2.8 to 1.3)
Diabetes mellitus	658.8 (642.9 to 675.5)	**35.7 (32.8 to 38.8)**	18.6 (18.1 to 19.0)	1.0 (-1.1 to 3.3)	14,772.9 (14,422.2 to 15,174.4)	**30.8 (27.8 to 33.8)**	384.4 (375.3 to 394.8)	1.2 (-1.0 to 3.5)
Diabetes mellitus type 1	167.0 (145.0 to 190.8)	**18.1 (12.6 to 23.9)**	4.5 (3.9 to 5.1)	**-8.8 (-13.3 to -4.3)**	4,745.8 (4,271.5 to 5,283.8)	**13.6 (9.0 to 18.6)**	120.5 (108.4 to 134.3)	**-8.4 (-12.3 to -4.2)**
Diabetes mellitus type 2	491.8 (462.1 to 519.0)	**43.0 (39.4 to 47.2)**	14.1 (13.3 to 14.9)	**4.6 (2.1 to 7.6)**	10,027.2 (9,361.3 to 10,654.3)	**40.8 (36.9 to 45.4)**	263.9 (246.8 to 280.2)	**6.2 (3.4 to 9.5)**
Chronic kidney disease	651.2 (613.4 to 672.8)	**33.3 (28.4 to 36.6)**	18.9 (17.9 to 19.5)	0.5 (-2.9 to 2.8)	15,736.8 (14,899.2 to 16,327.2)	**21.5 (17.7 to 24.8)**	414.1 (391.9 to 430.3)	-2.2 (-5.2 to 0.4)
Chronic kidney disease due to diabetes mellitus type 1	41.7 (33.0 to 51.5)	**23.3 (17.4 to 28.4)**	1.0 (0.8 to 1.3)	-0.8 (-4.9 to 2.5)	1,443.1 (1,145.3 to 1,785.4)	**18.2 (12.7 to 23.5)**	35.5 (28.3 to 43.8)	-2.1 (-6.0 to 1.4)
Chronic kidney disease due to diabetes mellitus type 2	177.8 (152.9 to 203.9)	**39.9 (33.7 to 43.9)**	5.1 (4.4 to 5.9)	3.0 (-1.4 to 5.7)	3,609.0 (3,078.8 to 4,165.5)	**35.3 (29.0 to 39.7)**	95.1 (81.6 to 109.5)	2.6 (-2.1 to 5.6)
Chronic kidney disease due to hypertension	184.3 (160.2 to 207.2)	**41.3 (35.3 to 45.2)**	5.7 (5.0 to 6.4)	1.6 (-2.3 to 4.1)	3,351.0 (2,857.7 to 3,801.0)	**33.5 (27.7 to 37.7)**	91.6 (78.7 to 103.3)	1.7 (-2.5 to 4.6)
Chronic kidney disease due to glomerulonephritis	100.3 (87.1 to 115.4)	**25.5 (21.3 to 29.3)**	2.8 (2.4 to 3.2)	-1.7 (-4.3 to 0.7)	3,032.1 (2,665.7 to 3,451.6)	**13.9 (10.1 to 17.6)**	78.8 (69.8 to 89.3)	**-4.3 (-6.9 to -1.7)**
Chronic kidney disease due to other and unspecified causes	147.2 (128.1 to 168.1)	**25.6 (21.3 to 29.4)**	4.2 (3.6 to 4.8)	-2.1 (-5.1 to 0.3)	4,301.6 (3,762.2 to 4,862.8)	**10.6 (6.7 to 14.5)**	113.1 (99.5 to 127.1)	**-7.2 (-9.9 to -4.5)**
Acute glomerulonephritis	6.4 (5.9 to 7.1)	**14.5 (5.6 to 25.0)**	0.2 (0.2 to 0.2)	**-9.8 (-17.0 to -1.8)**	178.9 (163.9 to 200.3)	-3.9 (-10.5 to 7.0)	4.7 (4.3 to 5.2)	**-19.3 (-24.9 to -10.5)**
**Skin and subcutaneous diseases**	47.6 (28.1 to 69.5)	**39.3 (27.7 to 58.4)**	1.4 (0.8 to 2.1)	5.8 (-2.7 to 20.8)	1,268.7 (752.7 to 1,830.0)	**24.6 (13.8 to 40.7)**	34.1 (20.2 to 49.1)	3.5 (-5.4 to 17.7)
Bacterial skin diseases	37.6 (21.0 to 52.3)	**41.6 (28.7 to 61.4)**	1.1 (0.6 to 1.5)	9.8 (-0.1 to 25.3)	1,071.9 (619.2 to 1,512.7)	**25.0 (13.2 to 42.3)**	28.7 (16.6 to 40.4)	4.9 (-4.8 to 20.0)
Cellulitis	9.6 (4.4 to 15.2)	**59.9 (40.6 to 80.8)**	0.3 (0.1 to 0.4)	**22.8 (6.2 to 41.1)**	260.1 (124.9 to 400.6)	**40.9 (26.5 to 59.7)**	6.9 (3.3 to 10.7)	**16.6 (4.2 to 33.1)**
Pyoderma	28.0 (15.5 to 39.3)	**36.2 (23.4 to 56.4)**	0.8 (0.4 to 1.1)	6.0 (-3.4 to 21.9)	811.8 (453.6 to 1,151.4)	**20.6 (9.0 to 38.3)**	21.8 (12.2 to 31.1)	1.8 (-8.0 to 17.4)
Decubitus ulcer	8.4 (4.6 to 15.1)	**31.3 (19.0 to 58.3)**	0.3 (0.1 to 0.5)	-7.0 (-16.0 to 12.8)	152.8 (84.8 to 270.2)	**24.7 (13.0 to 47.3)**	4.3 (2.4 to 7.6)	-3.9 (-13.0 to 14.4)
Other skin and subcutaneous diseases	1.6 (0.9 to 3.0)	**32.2 (19.7 to 52.0)**	0.0 (0.0 to 0.1)	0.7 (-8.8 to 16.7)	44.0 (26.5 to 75.9)	**15.8 (5.7 to 33.6)**	1.2 (0.7 to 2.1)	-1.8 (-9.9 to 11.8)
**Musculoskeletal disorders**	41.0 (34.7 to 42.7)	**29.4 (25.3 to 33.2)**	1.2 (1.0 to 1.3)	-2.7 (-6.0 to 0.2)	938.4 (797.8 to 982.1)	**19.2 (15.8 to 22.4)**	24.9 (21.3 to 26.1)	**-4.3 (-7.0 to -1.7)**
Rheumatoid arthritis	15.0 (11.8 to 16.4)	**18.9 (13.7 to 24.0)**	0.4 (0.3 to 0.5)	**-12.1 (-16.0 to -8.2)**	285.7 (222.4 to 314.4)	**12.3 (7.7 to 17.6)**	7.7 (6.0 to 8.5)	**-13.7 (-17.3 to -9.8)**
Other musculoskeletal disorders	26.0 (22.1 to 28.0)	**36.4 (30.9 to 41.6)**	0.8 (0.7 to 0.8)	3.6 (-0.7 to 7.8)	652.8 (554.4 to 696.3)	**22.4 (18.3 to 26.1)**	17.2 (14.7 to 18.4)	0.7 (-2.8 to 3.9)
**Other non-communicable diseases**	591.5 (557.8 to 631.3)	-1.2 (-8.5 to 3.2)	17.2 (16.2 to 18.3)	**-12.0 (-18.0 to -8.4)**	36,012.9 (33,809.4 to 39,113.1)	**-11.8 (-19.6 to -6.8)**	1,026.4 (962.9 to 1,116.8)	**-17.1 (-24.4 to -12.4)**
Congenital birth defects	309.1 (291.8 to 334.5)	**-15.6 (-25.2 to -9.9)**	8.9 (8.4 to 9.6)	**-19.3 (-28.5 to -13.8)**	25,822.5 (24,299.9 to 28,010.8)	**-16.6 (-26.2 to -10.9)**	746.1 (701.9 to 809.9)	**-19.9 (-29.0 to -14.3)**
Neural tube defects	31.3 (22.8 to 45.2)	**-14.3 (-30.1 to -0.5)**	0.9 (0.7 to 1.3)	**-17.5 (-32.7 to -4.2)**	2,701.3 (1,953.3 to 3,904.7)	**-14.6 (-30.3 to -0.8)**	78.6 (56.8 to 113.6)	**-17.7 (-32.9 to -4.4)**
Congenital heart anomalies	135.8 (111.9 to 163.4)	**-18.6 (-27.7 to -9.1)**	3.9 (3.2 to 4.7)	**-22.3 (-30.9 to -13.2)**	11,233.3 (9,161.7 to 13,634.0)	**-19.6 (-28.5 to -10.0)**	323.4 (263.0 to 393.2)	**-22.9 (-31.4 to -13.7)**
Orofacial clefts	2.1 (0.6 to 5.8)	**-38.3 (-58.3 to -13.1)**	0.1 (0.0 to 0.2)	**-40.1 (-59.6 to -15.6)**	186.8 (53.7 to 505.2)	**-38.3 (-58.3 to -13.2)**	5.5 (1.6 to 14.8)	**-40.1 (-59.6 to -15.7)**
Down syndrome	14.5 (11.5 to 20.9)	1.0 (-10.1 to 17.5)	0.4 (0.3 to 0.6)	-6.6 (-16.4 to 8.1)	1,073.3 (798.3 to 1,613.9)	-3.4 (-14.5 to 14.5)	30.4 (22.3 to 46.0)	-8.8 (-19.3 to 7.8)
Other chromosomal abnormalities	7.6 (4.8 to 12.6)	7.1 (-7.3 to 23.6)	0.2 (0.1 to 0.4)	2.6 (-11.1 to 18.3)	635.1 (393.6 to 1,067.2)	6.1 (-8.3 to 23.0)	18.4 (11.4 to 30.9)	2.2 (-11.7 to 18.3)
Congenital musculoskeletal and limb anomalies	5.5 (4.1 to 7.5)	-7.9 (-19.2 to 5.0)	0.2 (0.1 to 0.2)	-11.7 (-22.6 to 0.5)	454.8 (335.8 to 628.9)	-8.8 (-20.1 to 4.2)	13.1 (9.6 to 18.2)	-12.1 (-23.1 to 0.3)
Urogenital congenital anomalies	8.1 (5.4 to 10.2)	-2.6 (-13.7 to 11.7)	0.2 (0.2 to 0.3)	-8.3 (-18.5 to 4.6)	635.5 (411.1 to 819.5)	-5.5 (-16.4 to 8.8)	18.3 (11.7 to 23.7)	-9.5 (-19.8 to 3.9)
Digestive congenital anomalies	29.4 (20.8 to 40.3)	**-19.0 (-32.8 to -6.9)**	0.9 (0.6 to 1.2)	**-21.8 (-35.2 to -10.1)**	2,544.3 (1,802.0 to 3,499.7)	**-19.2 (-33.0 to -7.2)**	74.2 (52.5 to 102.0)	**-21.9 (-35.3 to -10.2)**
Other congenital birth defects	74.7 (53.2 to 95.9)	**-14.6 (-23.3 to -1.7)**	2.2 (1.5 to 2.8)	**-17.9 (-26.2 to -5.4)**	6,358.2 (4,504.0 to 8,192.0)	**-15.1 (-23.8 to -2.2)**	184.4 (130.5 to 237.7)	**-18.2 (-26.5 to -5.7)**
Urinary diseases and male infertility	134.6 (130.1 to 140.9)	**33.9 (29.0 to 38.8)**	4.1 (3.9 to 4.3)	1.1 (-2.4 to 4.6)	3,246.3 (3,118.8 to 3,419.1)	**16.3 (10.9 to 21.9)**	88.1 (84.6 to 92.8)	-4.2 (-8.7 to 0.4)
Urinary tract infections	89.8 (84.2 to 99.6)	**43.4 (37.6 to 49.6)**	2.8 (2.6 to 3.0)	**7.0 (3.2 to 11.0)**	2,081.6 (1,929.8 to 2,363.7)	**24.8 (17.8 to 32.1)**	56.8 (52.8 to 64.4)	2.3 (-3.0 to 8.1)
Urolithiasis	6.7 (5.6 to 8.7)	**23.6 (11.2 to 42.1)**	0.2 (0.2 to 0.3)	-7.1 (-16.1 to 6.1)	142.9 (119.6 to 186.6)	**14.7 (3.0 to 32.2)**	3.8 (3.2 to 4.9)	-10.1 (-19.2 to 3.4)
Other urinary diseases	38.1 (32.6 to 44.2)	**17.3 (8.6 to 27.9)**	1.1 (1.0 to 1.3)	**-9.9 (-16.4 to -2.3)**	1,021.8 (849.4 to 1,185.1)	2.3 (-6.2 to 12.9)	27.6 (23.0 to 32.0)	**-14.6 (-21.3 to -6.4)**
Gynecological diseases	-	**-**	-	**-**	-	**-**	-	**-**
Uterine fibroids	-	**-**	-	**-**	-	**-**	-	**-**
Polycystic ovarian syndrome	-	**-**	-	**-**	-	**-**	-	**-**
Endometriosis	-	**-**	-	**-**	-	**-**	-	**-**
Genital prolapse	-	**-**	-	**-**	-	**-**	-	**-**
Other gynecological diseases	-	**-**	-	**-**	-	**-**	-	**-**
Hemoglobinopathies and hemolytic anemias	54.3 (35.9 to 77.2)	9.2 (-0.7 to 18.3)	1.5 (1.0 to 2.2)	**-8.6 (-16.5 to -1.3)**	2,583.4 (1,654.7 to 3,865.7)	2.6 (-13.0 to 14.8)	70.4 (45.3 to 105.3)	-7.1 (-21.4 to 4.1)
Thalassemias	3.7 (2.9 to 4.5)	**-20.3 (-32.8 to -5.5)**	0.1 (0.1 to 0.1)	**-24.5 (-36.6 to -10.1)**	298.8 (229.6 to 358.4)	**-21.0 (-34.1 to -5.6)**	8.4 (6.5 to 10.1)	**-25.0 (-37.5 to -10.1)**
Sickle cell disorders	20.1 (10.2 to 34.7)	8.9 (-12.3 to 23.6)	0.5 (0.3 to 0.9)	2.2 (-17.9 to 16.4)	1,457.8 (752.3 to 2,511.4)	7.7 (-14.2 to 23.7)	40.2 (20.8 to 69.3)	1.7 (-19.2 to 17.1)
G6PD deficiency	11.6 (7.4 to 16.4)	**14.4 (5.0 to 23.7)**	0.3 (0.2 to 0.4)	-5.3 (-11.8 to 2.1)	468.8 (314.2 to 638.8)	6.9 (-2.2 to 16.6)	11.9 (8.0 to 16.2)	**-7.7 (-14.5 to -0.2)**
Other hemoglobinopathies and hemolytic anemias	18.9 (14.1 to 23.2)	**14.8 (10.4 to 20.3)**	0.6 (0.4 to 0.7)	**-15.2 (-18.3 to -11.1)**	358.1 (265.4 to 443.2)	3.1 (-1.7 to 8.1)	9.8 (7.3 to 12.1)	**-18.9 (-22.2 to -15.1)**
Endocrine, metabolic, blood, and immune disorders	70.2 (52.4 to 75.7)	**30.3 (19.4 to 36.2)**	2.0 (1.5 to 2.2)	3.5 (-4.8 to 7.8)	2,319.6 (1,800.0 to 2,598.4)	**11.8 (2.4 to 19.1)**	62.4 (48.1 to 69.9)	-3.7 (-11.0 to 2.0)
Sudden infant death syndrome	23.3 (9.3 to 44.3)	-18.1 (-32.9 to 2.1)	0.7 (0.3 to 1.3)	**-20.9 (-35.2 to -1.4)**	2,041.1 (816.2 to 3,872.5)	-18.1 (-32.9 to 2.1)	59.4 (23.8 to 112.8)	**-20.9 (-35.2 to -1.4)**
**Injuries**	3,067.5 (2,950.4 to 3,143.1)	1.2 (-0.8 to 3.2)	80.9 (77.7 to 83.0)	**-13.8 (-15.6 to -12.1)**	139,152.3 (134,848.5 to 142,501.5)	**-5.9 (-7.5 to -4.0)**	3,595.7 (3,483.2 to 3,685.9)	**-16.4 (-17.8 to -14.6)**
**Transport injuries**	1,002.3 (962.9 to 1,031.8)	**-3.4 (-7.1 to -0.3)**	25.8 (24.8 to 26.5)	**-16.9 (-20.1 to -14.3)**	47,177.9 (45,591.1 to 48,684.6)	**-9.1 (-11.9 to -6.4)**	1,207.4 (1,167.7 to 1,247.0)	**-19.0 (-21.4 to -16.5)**
Road injuries	927.4 (887.1 to 956.9)	**-3.6 (-7.5 to -0.2)**	23.9 (22.8 to 24.6)	**-17.1 (-20.5 to -14.2)**	43,642.5 (41,981.0 to 45,157.4)	**-9.3 (-12.1 to -6.4)**	1,117.1 (1,074.4 to 1,156.7)	**-19.1 (-21.6 to -16.6)**
Pedestrian road injuries	341.8 (315.2 to 382.7)	**-7.0 (-13.5 to -1.4)**	8.9 (8.2 to 9.9)	**-21.5 (-26.9 to -17.0)**	14,908.3 (13,725.5 to 16,857.5)	**-14.6 (-19.6 to -9.7)**	383.6 (352.8 to 433.5)	**-24.7 (-29.0 to -20.5)**
Cyclist road injuries	54.5 (43.9 to 62.4)	**9.5 (1.3 to 17.8)**	1.4 (1.1 to 1.6)	**-8.3 (-15.1 to -1.3)**	2,265.2 (1,840.1 to 2,628.2)	1.9 (-5.9 to 10.3)	57.6 (46.9 to 67.0)	**-10.9 (-17.7 to -3.6)**
Motorcyclist road injuries	193.3 (165.8 to 206.4)	-0.1 (-9.1 to 6.6)	4.9 (4.2 to 5.2)	**-11.6 (-19.4 to -5.8)**	9,844.4 (8,523.6 to 10,542.2)	-4.9 (-12.2 to 0.7)	249.9 (216.2 to 267.6)	**-13.9 (-20.4 to -9.0)**
Motor vehicle road injuries	329.7 (306.3 to 355.7)	-3.6 (-8.4 to 0.7)	8.5 (7.9 to 9.1)	**-16.3 (-20.4 to -12.4)**	16,261.7 (15,163.5 to 17,525.6)	**-8.0 (-11.6 to -3.1)**	416.7 (388.0 to 448.8)	**-17.5 (-20.5 to -12.9)**
Other road injuries	8.1 (7.0 to 9.6)	-9.0 (-15.2 to 13.1)	0.2 (0.2 to 0.3)	**-22.7 (-27.9 to -4.3)**	362.7 (311.5 to 432.5)	-15.4 (-20.8 to 6.9)	9.3 (8.0 to 11.1)	**-25.0 (-29.9 to -5.2)**
Other transport injuries	74.9 (67.8 to 89.2)	-1.4 (-7.0 to 5.1)	1.9 (1.7 to 2.3)	**-15.2 (-19.9 to -9.7)**	3,535.5 (3,167.6 to 4,263.3)	**-6.8 (-12.4 to -0.3)**	90.3 (80.8 to 108.8)	**-16.9 (-22.0 to -11.2)**
**Unintentional injuries**	1,089.0 (1,002.6 to 1,134.7)	-1.2 (-3.9 to 2.2)	30.1 (27.8 to 31.4)	**-16.9 (-19.2 to -14.2)**	45,415.1 (42,612.9 to 47,207.7)	**-13.9 (-16.4 to -10.3)**	1,204.5 (1,130.3 to 1,252.3)	**-23.5 (-25.8 to -20.4)**
Falls	379.5 (319.6 to 408.9)	**22.9 (16.6 to 31.0)**	11.1 (9.4 to 12.0)	-4.1 (-9.1 to 2.2)	10,856.3 (9,198.2 to 11,620.1)	7.4 (-0.5 to 16.3)	287.6 (243.6 to 307.6)	**-9.3 (-15.7 to -2.0)**
Drowning	203.0 (196.0 to 210.6)	**-17.3 (-20.1 to -14.2)**	5.4 (5.2 to 5.6)	**-26.7 (-29.1 to -24.0)**	11,514.4 (11,035.9 to 12,031.5)	**-25.2 (-28.1 to -21.9)**	309.6 (296.4 to 324.1)	**-31.7 (-34.5 to -28.6)**
Fire, heat, and hot substances	60.0 (49.7 to 65.8)	-9.5 (-13.1 to 3.0)	1.6 (1.4 to 1.8)	**-24.4 (-27.2 to -14.5)**	2,615.1 (2,106.0 to 2,941.6)	**-16.7 (-22.1 to -0.3)**	69.6 (56.0 to 78.5)	**-26.2 (-30.9 to -11.9)**
Poisonings	45.7 (32.4 to 51.1)	-8.7 (-18.3 to 2.4)	1.2 (0.9 to 1.3)	**-22.2 (-30.2 to -12.8)**	2,101.1 (1,504.9 to 2,361.8)	**-15.3 (-23.9 to -2.5)**	54.9 (39.4 to 61.7)	**-24.8 (-32.3 to -13.1)**
Poisoning by carbon monoxide	23.7 (17.2 to 26.2)	**-15.4 (-25.1 to -7.1)**	0.6 (0.4 to 0.7)	**-28.6 (-36.6 to -21.6)**	1,001.6 (731.6 to 1,109.2)	**-20.8 (-28.9 to -12.2)**	25.7 (18.7 to 28.5)	**-30.5 (-37.6 to -22.6)**

SR Table 4: Global deaths and YLL numbers, age-standardised rates per 100,000, and percent change between 2007 and 2017 for males for all GBD causes and Levels 1 through 4 of the cause hierarchy. 95% UIs are in parentheses. UI=uncertainty interval. YLL=year of life lost.

Cause	All Age Deaths (thousands)		Age-standardised Death Rate (per 100,000)		All Age YLLs (thousands)		Age-standardised YLL Rate (per 100,000)	
Poisoning by other means	22.0<br>(14.8 to 25.1)	-0.2<br>(-11.1 to 16.5)	0.6<br>(0.4 to 0.7)	-14.2<br>(-23.1 to 0.1)	1,099.5<br>(763.8 to 1,265.9)	-9.6<br>(-19.8 to 10.2)	29.2<br>(20.4 to 33.7)	**-18.9<br>(-28.0 to -0.9)**
Exposure to mechanical forces	102.0<br>(88.8 to 106.2)	**-10.7<br>(-14.1 to -7.1)**	2.6<br>(2.3 to 2.8)	**-23.2<br>(-26.1 to -20.0)**	4,877.5<br>(4,249.5 to 5,090.9)	**-16.7<br>(-19.7 to -13.2)**	126.2<br>(109.9 to 131.9)	**-25.7<br>(-28.4 to -22.5)**
Unintentional firearm injuries	17.1<br>(15.8 to 19.8)	-6.6<br>(-11.0 to 1.1)	0.4<br>(0.4 to 0.5)	**-18.7<br>(-22.6 to -12.1)**	882.4<br>(805.2 to 1,040.9)	**-9.4<br>(-14.3 to -0.9)**	23.0<br>(20.9 to 27.3)	**-18.1<br>(-22.6 to -10.3)**
Other exposure to mechanical forces	84.9<br>(71.7 to 88.8)	**-11.5<br>(-15.0 to -7.7)**	2.2<br>(1.9 to 2.3)	**-24.0<br>(-26.9 to -20.7)**	3,995.1<br>(3,368.3 to 4,194.9)	**-18.2<br>(-21.3 to -14.6)**	103.3<br>(87.0 to 108.5)	**-27.1<br>(-29.9 to -24.0)**
Adverse effects of medical treatment	61.7<br>(51.7 to 74.3)	**16.5<br>(10.5 to 22.6)**	1.7<br>(1.5 to 2.1)	**-5.9<br>(-10.8 to -0.6)**	2,330.3<br>(1,913.5 to 2,938.7)	5.3<br>(-1.0 to 12.8)	62.4<br>(51.3 to 79.6)	**-8.0<br>(-13.2 to -1.9)**
Animal contact	44.5<br>(23.7 to 52.2)	-4.2<br>(-11.3 to 11.3)	1.2<br>(0.6 to 1.4)	**-18.6<br>(-24.5 to -5.6)**	2,088.2<br>(1,110.5 to 2,487.6)	-13.3<br>(-21.3 to 3.7)	55.0<br>(29.3 to 65.7)	**-22.8<br>(-30.1 to -7.8)**
Venomous animal contact	38.1<br>(19.1 to 44.9)	-4.6<br>(-12.5 to 11.8)	1.0<br>(0.5 to 1.2)	**-18.8<br>(-25.3 to -4.8)**	1,784.1<br>(899.9 to 2,149.3)	-14.3<br>(-22.7 to 3.1)	46.9<br>(23.7 to 56.8)	**-23.7<br>(-31.3 to -8.1)**
Non-venomous animal contact	6.4<br>(4.2 to 7.7)	-1.8<br>(-8.7 to 10.4)	0.2<br>(0.1 to 0.2)	**-17.1<br>(-22.7 to -7.1)**	304.0<br>(193.5 to 360.2)	-6.8<br>(-17.2 to 6.5)	8.1<br>(5.1 to 9.6)	**-16.8<br>(-25.8 to -4.9)**
Foreign body	74.0<br>(69.8 to 78.3)	-0.3<br>(-4.1 to 3.1)	2.1<br>(2.0 to 2.2)	**-14.7<br>(-17.7 to -12.0)**	3,623.0<br>(3,370.4 to 3,925.2)	**-12.2<br>(-16.7 to -7.6)**	100.2<br>(93.0 to 108.9)	**-20.0<br>(-24.0 to -15.7)**
Pulmonary aspiration and foreign body in airway	69.4<br>(66.1 to 73.8)	0.0<br>(-4.0 to 3.5)	2.0<br>(1.9 to 2.1)	**-14.5<br>(-17.6 to -11.8)**	3,399.1<br>(3,186.4 to 3,694.8)	**-11.9<br>(-16.6 to -7.2)**	94.2<br>(88.0 to 102.6)	**-19.6<br>(-23.8 to -15.3)**
Foreign body in other body part	4.6<br>(3.6 to 6.0)	-4.5<br>(-13.8 to 4.2)	0.1<br>(0.1 to 0.2)	**-17.7<br>(-24.6 to -11.0)**	223.9<br>(160.8 to 298.8)	**-16.4<br>(-26.3 to -5.6)**	6.0<br>(4.3 to 8.2)	**-24.7<br>(-33.4 to -15.2)**
Environmental heat and cold exposure	37.0<br>(24.9 to 41.4)	**-16.9<br>(-25.3 to -12.1)**	1.0<br>(0.7 to 1.1)	**-31.4<br>(-38.3 to -27.3)**	1,348.6<br>(888.2 to 1,525.2)	**-23.2<br>(-30.4 to -19.2)**	34.6<br>(22.5 to 39.3)	**-34.1<br>(-40.8 to -30.5)**
Exposure to forces of nature	5.8<br>(5.1 to 7.0)	**-40.3<br>(-47.4 to -28.2)**	0.2<br>(0.1 to 0.2)	**-47.2<br>(-53.4 to -36.4)**	297.2<br>(266.0 to 357.4)	**-45.6<br>(-51.6 to -34.5)**	7.8<br>(7.0 to 9.3)	**-50.6<br>(-55.9 to -40.6)**
Other unintentional injuries	75.7<br>(73.2 to 79.1)	**-14.9<br>(-17.4 to -12.1)**	1.9<br>(1.9 to 2.0)	**-25.7<br>(-27.9 to -23.3)**	3,763.4<br>(3,632.9 to 3,942.9)	**-20.0<br>(-22.5 to -17.0)**	96.5<br>(93.0 to 101.1)	**-28.2<br>(-30.5 to -25.6)**
**Self-harm and interpersonal violence**	976.3<br>(916.8 to 1,006.6)	**9.4<br>(5.5 to 12.6)**	25.0<br>(23.5 to 25.8)	**-6.0<br>(-9.4 to -3.0)**	46,559.3<br>(43,729.4 to 48,044.0)	**7.7<br>(4.1 to 10.7)**	1,183.7<br>(1,111.3 to 1,221.7)	**-4.1<br>(-7.3 to -1.4)**
Self-harm	540.2<br>(491.3 to 563.2)	3.4<br>(-2.2 to 7.3)	13.9<br>(12.6 to 14.5)	**-13.3<br>(-18.0 to -9.7)**	22,738.0<br>(20,718.6 to 23,784.1)	-0.8<br>(-6.3 to 3.6)	572.8<br>(521.6 to 599.3)	**-13.5<br>(-18.3 to -9.7)**
Self-harm by firearm	54.5<br>(45.2 to 69.3)	**8.0<br>(2.0 to 13.1)**	1.4<br>(1.2 to 1.8)	**-9.9<br>(-14.4 to -6.2)**	2,250.7<br>(1,832.2 to 2,880.3)	2.3<br>(-3.7 to 7.8)	56.9<br>(46.4 to 72.8)	**-10.6<br>(-15.5 to -6.0)**
Self-harm by other specified means	485.7<br>(434.0 to 508.6)	2.9<br>(-2.8 to 7.2)	12.5<br>(11.2 to 13.1)	**-13.6<br>(-18.5 to -9.7)**	20,487.4<br>(18,416.8 to 21,516.5)	-1.2<br>(-6.9 to 3.7)	515.9<br>(463.0 to 541.7)	**-13.8<br>(-18.8 to -9.6)**
Interpersonal violence	331.9<br>(293.4 to 356.6)	2.5<br>(-0.3 to 5.7)	8.4<br>(7.4 to 9.0)	**-9.2<br>(-11.7 to -6.3)**	17,630.1<br>(15,578.4 to 19,002.8)	0.7<br>(-2.2 to 4.1)	449.4<br>(396.4 to 484.6)	**-8.9<br>(-11.5 to -5.9)**
Physical violence by firearm	156.3<br>(131.0 to 171.3)	**8.6<br>(5.1 to 12.6)**	4.0<br>(3.3 to 4.3)	-2.5<br>(-5.7 to 1.1)	8,596.8<br>(7,235.2 to 9,404.5)	**6.7<br>(3.2 to 10.7)**	219.1<br>(184.3 to 239.8)	-2.7<br>(-5.8 to 1.0)
Physical violence by sharp object	73.6<br>(56.3 to 92.5)	**-11.4<br>(-15.9 to -5.2)**	1.9<br>(1.4 to 2.3)	**-22.0<br>(-25.9 to -16.6)**	3,774.4<br>(2,883.4 to 4,789.5)	**-13.7<br>(-17.9 to -7.5)**	95.6<br>(72.9 to 121.1)	**-22.4<br>(-26.2 to -17.0)**
Physical violence by other means	101.9<br>(88.5 to 125.2)	5.2<br>(-1.1 to 11.2)	2.6<br>(2.2 to 3.2)	**-8.1<br>(-13.5 to -3.1)**	5,258.9<br>(4,531.3 to 6,454.3)	3.5<br>(-2.5 to 10.1)	134.7<br>(116.1 to 165.4)	**-7.2<br>(-12.3 to -1.3)**
Conflict and terrorism	91.3<br>(81.0 to 104.2)	**119.8<br>(81.5 to 160.9)**	2.4<br>(2.1 to 2.7)	**99.5<br>(65.9 to 137.0)**	5,469.8<br>(4,805.0 to 6,317.5)	**115.8<br>(76.8 to 160.8)**	143.1<br>(125.6 to 165.0)	**99.5<br>(63.8 to 141.1)**
Executions and police conflict	13.0<br>(12.7 to 13.3)	**179.9<br>(161.9 to 197.8)**	0.3<br>(0.3 to 0.3)	**150.6<br>(134.7 to 166.6)**	721.3<br>(705.9 to 739.5)	**181.1<br>(162.8 to 199.0)**	18.5<br>(18.1 to 18.9)	**156.5<br>(139.9 to 172.7)**

SR Table 5: Global deaths by cause and age group for females (with 95% confidence intervals), 1990

Cause	Early Neonatal	Late Neonatal	Post Neonatal	1 to 4	5 to 9	10 to 14	15 to 19	20 to 24	25 to 29	30 to 34	35 to 39
All causes	1,300,716 (1,264,650-1,336,58	568,922 (548,046-592,333	1,833,432 (1,790,072-1,880,37	1,830,477 (1,777,576-1,890,09	384,236 (380,097-388,404	200,665 (198,343-203,090	293,751 (288,962-298,706	374,237 (367,470-381,128	379,633 (372,621-386,841	379,225 (372,913-385,921	409,196 (402,065-416,486
Communicable, maternal, neonatal, and nutritional disease	1,194,354 (1,155,125-1,231,91	506,184 (483,864-529,237	1,533,233 (1,474,066-1,588,80	1,470,775 (1,401,406-1,552,99	237,950 (221,786-255,139	98,627 (91,518-108,238	123,934 (116,706-134,336	169,781 (162,800-178,45	169,106 (162,000-178,212	152,096 (145,634-160,653	139,000 (131,960-147,873
HIV/AIDS and sexually transmitted infections	-	8,019 (2,992-14,996	31,031 (23,222-41,841	15,438 (13,747-17,184	1,417 (951-2,035)	265 (214-340)	2,875 (2,375-3,536)	12,480 (10,540-14,820)	19,500 (16,372-23,281)	16,971 (14,423-19,839)	13,257 (11,058-15,882)
HIV/AIDS	-	-	18,770 (16,932-20,832)	14,211 (12,879-15,719)	810 (575-1,031)	191 (154-261)	2,518 (2,015-3,160)	11,897 (9,950-14,286)	18,702 (15,521-22,489)	16,273 (13,755-19,073)	12,611 (10,438-15,183)
HIV/AIDS - Drug-susceptible Tuberculosis	-	-	7,470 (6,033-8,573)	5,784 (4,816-6,574)	283 (187-373)	42 (25-64)	905 (712-1,143)	4,016 (2,996-5,028)	5,715 (4,157-7,366)	5,082 (3,670-6,508)	4,083 (3,015-5,182)
HIV/AIDS - Multidrug-resistant Tuberculosis without extensive drug resistance	-	-	46 (25-84)	41 (23-70)	2 (1-3)	0 (0-1)	7 (4-13)	28 (15-54)	41 (22-78)	38 (20-71)	31 (17-57)
HIV/AIDS - Extensively drug-resistant Tuberculosis	-	-	0 (0-0)	0 (0-0)	0 (0-0)	0 (0-0)	0 (0-0)	0 (0-0)	0 (0-0)	0 (0-0)	0 (0-0)
HIV/AIDS resulting in other diseases	-	-	11,254 (9,825-13,190)	8,386 (7,420-9,710)	525 (385-709)	149 (122-209)	1,606 (1,237-2,162)	7,853 (6,057-10,384)	12,946 (9,830-17,122)	11,153 (8,700-14,179)	8,497 (6,562-11,148)
Sexually transmitted infections excluding HIV	26,396 (10,099-48,664	8,019 (2,992-14,996	12,261 (4,612-23,136	1,227 (449-2,326)	608 (217-1,166)	74 (39-95)	357 (222-444)	583 (336-702)	798 (429-973)	697 (405-829)	647 (396-753)
Syphilis	26,396 (10,099-48,664	8,019 (2,992-14,996	12,261 (4,612-23,136	1,227 (449-2,326)	608 (217-1,166)	27 (14-35)	34 (21-43)	54 (31-65)	69 (37-84)	58 (34-69)	46 (28-54)
Chlamydial infection	-	-	-	-	-	13 (7-17)	55 (34-69)	148 (85-178)	147 (79-179)	80 (46-95)	106 (65-123)
Gonococcal infection	-	-	-	-	-	22 (12-28)	139 (86-173)	226 (130-272)	391 (210-476)	349 (203-415)	297 (182-346)
Other sexually transmitted infections	-	-	-	-	-	12 (6-15)	129 (80-160)	155 (89-186)	192 (103-234)	210 (122-250)	197 (121-230)
Respiratory infections and tuberculosis	-	131,568 (112,851-149,108	601,622 (551,719-649,528	390,676 (330,494-452,528	46,517 (38,000-54,364	20,022 (17,153-22,792	27,494 (24,511-30,316	36,733 (33,187-39,891	41,778 (37,699-45,173	41,036 (37,277-44,603	43,789 (39,586-47,454
Tuberculosis	-	-	40,855 (36,826-44,717	45,431 (37,268-53,629	12,201 (9,797-14,693	8,519 (7,084-10,020	18,644 (16,330-20,995	27,505 (24,566-30,382	32,030 (28,480-35,222	32,079 (28,743-35,287	34,120 (30,344-37,492
Drug-susceptible tuberculosis	-	-	40,337 (36,383-44,118	45,068 (36,933-53,233	12,113 (9,717-14,635	8,443 (6,997-9,950	18,497 (16,170-20,876	27,243 (24,317-30,100	31,735 (28,186-34,919	31,775 (28,431-34,904	33,799 (30,025-37,149
Multidrug-resistant tuberculosis without extensive drug resistance	-	-	517 (236-1,067)	362 (192-670)	88 (42-170)	76 (37-151)	147 (73-287)	262 (129-505)	295 (153-543)	304 (154-557)	321 (162-607)
Extensively drug-resistant tuberculosis	-	-	0 (0-0)	0 (0-0)	0 (0-0)	0 (0-0)	0 (0-0)	0 (0-0)	0 (0-0)	0 (0-0)	0 (0-0)
Lower respiratory infections	108,387 (95,189-126,576	131,317 (112,698-148,752	557,253 (511,039-602,804	342,402 (290,069-398,043	33,748 (26,578-39,447	11,268 (9,343-12,669	8,709 (7,516-9,547	9,077 (7,971-9,867	9,579 (8,193-10,378	8,813 (7,679-9,515	9,522 (8,337-10,340
Upper respiratory infections	94 (33-141)	205 (78-310)	3,226 (1,087-4,816)	2,546 (718-4,988)	411 (118-671)	127 (42-216)	88 (33-151)	123 (39-193)	134 (45-203)	108 (40-162)	106 (40-168)
Otitis media	72 (5-428)	46 (18-141)	288 (155-535)	297 (98-741)	157 (77-315)	108 (59-206)	53 (33-80)	28 (17-47)	34 (19-68)	35 (22-59)	41 (27-65)
Enteric infections	20,997 (16,731-26,019	52,947 (41,073-62,690	408,793 (354,712-464,894	409,642 (330,145-493,236	73,009 (47,695-98,595	33,187 (23,021-46,286	23,237 (14,841-34,720	17,675 (9,880-27,674	14,541 (8,192-23,619	13,513 (7,301-22,214	12,513 (6,823-20,997
Diarrheal diseases	20,997 (16,731-26,019	52,769 (40,869-62,570	404,610 (350,472-460,474	383,337 (305,190-466,819	35,757 (13,591-61,761	12,765 (5,416-23,227	12,426 (5,164-22,928	11,994 (4,850-21,970	10,269 (4,063-19,340	10,194 (4,070-19,072	9,802 (4,187-18,334
Typhoid and paratyphoid	-	0 (0-0)	261 (141-464)	18,293 (8,135-34,800	34,904 (19,796-58,265	19,325 (11,124-30,957	10,024 (4,842-17,938	5,013 (2,554-8,747	3,637 (1,925-6,113	2,729 (1,472-4,510	2,111 (1,124-3,418
Typhoid fever	-	-	240 (128-429)	16,260 (7,199-31,278	30,706 (16,733-51,492	16,948 (9,655-27,522	8,768 (4,259-15,912	4,406 (2,219-7,785	3,160 (1,643-5,441	2,343 (1,244-3,953	1,786 (932-2,979)
Paratyphoid fever	-	-	21 (11-40)	2,033 (745-4,524)	4,198 (1,805-8,237	2,377 (1,017-4,813	1,257 (494-2,658)	607 (242-1,355)	477 (204-1,020)	386 (164-818)	325 (145-649)
Invasive Non-typhoidal Salmonella (iNTS)	-	178 (96-303)	2,547 (1,407-4,402	7,176 (3,547-13,594	2,235 (1,086-4,143	1,048 (467-1,993)	744 (307-1,399)	626 (257-1,191)	610 (238-1,253)	560 (230-1,065)	572 (249-1,102)
Other intestinal infectious diseases	-	-	1,374 (669-3,098)	835 (342-2,221)	113 (36-313)	48 (11-128)	43 (10-124)	43 (11-117)	24 (4-64)	31 (6-80)	28 (5-67)
Neglected tropical diseases and malaria	202 (94-586)	10,074 (6,207-15,472	85,856 (54,100-129,711	188,145 (114,053-306,995	39,734 (22,312-78,723	17,955 (10,263-34,905	13,329 (7,056-29,888	13,597 (7,381-28,903	11,989 (5,937-26,160	11,128 (5,571-23,491	11,401 (5,744-23,278
Malaria	-	9,884 (6,059-15,340	70,591 (45,040-104,011	139,386 (88,853-203,794	22,570 (13,250-32,717	10,035 (5,996-14,192	5,837 (3,917-8,184	6,393 (4,269-9,115	4,959 (3,222-7,116	4,496 (2,967-6,507	4,599 (3,078-6,590
Chagas disease	-	-	3 (2-3)	2 (1-2)	2 (1-2)	4 (3-4)	9 (8-10)	22 (20-25)	46 (41-51)	82 (74-89)	126 (116-136)
Leishmaniasis	-	-	11,941 (13-45,040)	36,297 (29-152,808	10,231 (6-53,200)	4,785 (4-22,963)	5,033 (3-22,319)	4,776 (3-21,039)	4,884 (5-19,493)	4,474 (5-17,014)	4,643 (5-16,533)
Visceral leishmaniasis	-	-	11,941 (13-45,040)	36,297 (29-152,808	10,231 (6-53,200)	4,785 (4-22,963)	5,033 (3-22,319)	4,776 (3-21,039)	4,884 (5-19,493)	4,474 (5-17,014)	4,643 (5-16,533)
African trypanosomiasis	-	-	-	406 (83-1,390)	599 (146-1,503)	636 (174-1,519)	831 (202-2,023)	892 (231-2,227)	804 (217-1,843)	671 (188-1,730)	598 (164-1,532)
Schistosomiasis	-	-	0 (0-0)	557 (354-827)	162 (108-230)	198 (138-269)	202 (149-267)	223 (167-289)	250 (192-314)	253 (198-318)	304 (238-375)
Cysticercosis	0 (0-0)	0 (0-0)	0 (0-0)	131 (56-229)	53 (15-114)	33 (5-80)	27 (3-65)	44 (11-94)	50 (11-109)	50 (11-110)	42 (6-96)
Cystic echinococcosis	-	-	-	377 (233-546)	147 (71-243)	68 (21-139)	38 (5-91)	45 (9-95)	59 (17-120)	57 (14-120)	66 (18-129)
Dengue	-	-	1,438 (196-4,384)	2,074 (341-6,232)	930 (146-2,107)	359 (88-781)	245 (55-522)	253 (59-569)	197 (53-435)	183 (42-403)	146 (38-308)
Yellow fever	-	2 (0-7)	39 (8-123)	352 (69-1,060)	459 (85-1,412)	417 (85-1,241)	378 (71-1,157)	321 (65-930)	257 (49-794)	193 (39-590)	151 (31-459)
Rabies	-	-	498 (222-940)	2,462 (1,029-5,037	3,673 (1,300-6,166	1,217 (386-2,090)	613 (251-989)	525 (206-878)	388 (180-707)	580 (295-933)	639 (310-988)
Intestinal nematode infections	-	-	377 (284-500)	4,187 (3,017-5,578	649 (481-857)	99 (74-128)	29 (21-38)	23 (16-30)	17 (12-22)	21 (15-27)	19 (14-25)
Ascariasis	-	-	377 (284-500)	4,187 (3,017-5,578	649 (481-857)	99 (74-128)	29 (21-38)	23 (16-30)	17 (12-22)	21 (15-27)	19 (14-25)
Ebola	0 (0-0)	0 (0-0)	0 (0-0)	0 (0-0)	0 (0-0)	0 (0-0)	0 (0-0)	0 (0-0)	0 (0-0)	0 (0-0)	0 (0-0)
Zika virus	0 (0-0)	0 (0-0)	0 (0-0)	0 (0-0)	0 (0-0)	0 (0-0)	0 (0-0)	0 (0-0)	0 (0-0)	0 (0-0)	0 (0-0)
Other neglected tropical diseases	202 (94-586)	187 (95-565)	971 (402-3,430)	1,914 (485-8,998)	260 (115-705)	103 (58-260)	88 (50-239)	79 (43-202)	80 (41-198)	68 (34-168)	68 (36-158)
Other infectious diseases	40,013 (32,555-51,773	66,082 (54,098-78,602	241,601 (177,766-347,483	358,876 (234,717-546,717	66,781 (47,310-97,875	23,774 (18,194-32,421	17,280 (14,062-21,630	14,973 (12,366-17,681	11,793 (9,761-13,764	10,570 (8,972-12,205	9,785 (8,271-11,263
Meningitis	4,827 (3,712-6,029)	10,373 (7,630-12,342	61,904 (50,477-71,063	63,961 (47,446-77,675	13,914 (9,173-18,780	7,230 (5,148-9,379)	5,853 (4,078-7,725)	4,799 (3,381-6,530)	4,118 (2,932-5,681)	3,441 (2,501-4,699)	3,353 (2,446-4,659)
Pneumococcal meningitis	646 (496-850)	1,318 (960-1,661)	7,283 (5,703-9,103)	6,648 (4,627-8,817)	1,112 (703-1,596)	622 (431-872)	627 (423-863)	619 (447-885)	574 (396-842)	479 (334-691)	502 (351-716)
H influenzae type B meningitis	1,406 (1,062-1,747)	3,153 (2,353-3,838)	21,124 (16,464-25,333	24,806 (18,561-30,787	6,078 (3,944-8,114)	2,962 (2,032-3,778)	2,226 (1,526-2,869)	1,635 (1,127-2,163)	1,284 (879-1,686)	1,049 (751-1,418)	983 (695-1,358)
Meningococcal meningitis	690 (528-884)	1,421 (1,049-1,731)	8,068 (6,442-9,702)	7,489 (5,352-9,624)	1,346 (907-1,850)	618 (431-828)	471 (337-629)	364 (261-488)	328 (227-462)	287 (213-411)	272 (196-383)
Other meningitis	2,085 (1,589-2,655)	4,481 (3,296-5,481)	25,430 (20,145-30,591	25,018 (18,026-31,408	5,378 (3,486-7,431)	3,028 (2,163-3,999)	2,530 (1,786-3,451)	2,180 (1,557-3,069)	1,933 (1,343-2,726)	1,627 (1,184-2,263)	1,595 (1,164-2,244)
Encephalitis	351 (256-432)	728 (485-1,059)	7,702 (4,757-11,788	9,592 (5,687-18,368	4,729 (3,257-7,225)	2,262 (1,727-3,082)	1,401 (1,168-1,781)	1,313 (1,069-1,721)	1,004 (811-1,320)	987 (832-1,293)	851 (703-1,109)
Diphtheria	-	-	1,482 (947-2,296)	5,448 (3,194-8,649)	1,012 (549-1,880)	247 (149-410)	85 (51-139)	46 (28-76)	39 (25-59)	37 (24-56)	40 (26-62)
Whooping cough	-	-	44,629 (20,218-83,301	42,151 (18,698-79,395	4,671 (1,891-10,124	767 (317-1,578)	294 (125-589)	233 (99-476)	136 (58-275)	90 (38-185)	74 (30-154)
Tetanus	31,526 (23,810-43,929	52,144 (41,146-64,425	14,890 (10,373-24,315	10,374 (3,904-15,369	2,854 (1,519-4,130)	1,651 (954-2,383)	2,274 (1,233-3,403)	2,239 (1,215-3,289)	1,813 (1,006-2,663)	1,904 (1,101-2,772)	1,623 (952-2,398)
Measles	-	-	93,556 (34,294-203,596	209,022 (78,063-428,223	31,586 (11,694-65,872	7,677 (2,731-16,818	3,017 (1,020-6,892)	1,547 (525-3,589)	764 (262-1,774)	434 (148-1,011)	267 (92-618)
Varicella and herpes zoster	109 (85-141)	324 (242-420)	2,382 (1,883-2,998)	2,598 (1,976-3,423)	1,056 (779-1,385)	304 (235-395)	157 (122-198)	165 (131-209)	210 (169-263)	248 (200-307)	216 (176-266)
Acute hepatitis	-	-	7,638 (5,316-8,989)	9,743 (6,754-12,611	4,922 (3,436-6,268)	2,736 (1,905-3,355)	3,266 (2,218-4,034)	3,790 (2,722-4,507)	2,970 (2,176-3,524)	2,690 (1,956-3,131)	2,733 (2,095-3,198)
Acute hepatitis A	-	-	1,273 (565-2,076)	6,192 (3,949-8,452)	3,321 (2,192-4,458)	1,429 (898-1,954)	1,291 (812-1,780)	1,226 (719-1,760)	680 (381-1,018)	474 (239-805)	405 (188-712)
Acute hepatitis B	-	-	4,528 (2,725-6,053)	2,535 (1,239-4,215)	745 (350-1,296)	458 (205-811)	745 (383-1,282)	1,231 (606-2,049)	1,423 (811-2,056)	1,635 (971-2,221)	1,884 (1,247-2,491)
Acute hepatitis C	-	-	1,824 (930-3,029)	802 (333-1,527)	102 (43-213)	28 (11-65)	21 (7-47)	19 (6-42)	15 (4-38)	16 (4-40)	23 (6-66)
Acute hepatitis E	-	-	13 (5-25)	215 (96-412)	754 (370-1,217)	821 (457-1,221)	1,209 (754-1,698)	1,315 (793-1,869)	852 (440-1,306)	565 (267-1,018)	421 (180-765)
Other unspecified infectious diseases	3,200 (1,527-4,181)	2,513 (1,401-3,051)	7,419 (4,246-9,092)	5,989 (3,098-9,226)	2,037 (1,355-2,677)	900 (616-1,145)	934 (691-1,159)	841 (627-1,002)	740 (545-891)	738 (524-890)	627 (477-767)
Maternal and neonatal disorders	998,194 (951,018-1,041,354	237,494 (210,924-275,997	60,927 (49,916-79,482)	5,063 (3,552-8,006)	-	1,191 (969-1,381)	38,122 (33,906-41,704)	72,500 (66,543-77,604)	67,633 (62,230-72,428)	57,110 (52,616-61,450)	46,641 (42,912-50,252)
Maternal disorders	-	-	-	-	-	1,191 (969-1,381)	38,122 (33,906-41,704)	72,500 (66,543-77,604)	67,633 (62,230-72,428)	57,110 (52,616-61,450)	46,641 (42,912-50,252)
Maternal hemorrhage	-	-	-	-	-	415 (326-512)	11,351 (7,088-18,246)	27,281 (17,863-41,146)	27,747 (20,059-38,661)	25,282 (19,244-32,670)	20,914 (16,622-26,176)
Maternal sepsis and other maternal infections	-	-	-	-	-	109 (74-156)	6,016 (3,194-11,218)	9,051 (5,018-17,472)	7,246 (4,325-13,698)	5,470 (3,437-9,353)	4,448 (2,925-7,058)
Maternal hypertensive disorders	-	-	-	-	-	121 (88-162)	5,772 (3,635-10,367)	9,070 (5,713-15,290)	7,946 (5,312-13,204)	5,703 (3,815-9,330)	4,239 (2,920-7,048)
Maternal obstructed labor and uterine rupture	-	-	-	-	-	123 (84-181)	2,355 (1,094-5,821)	4,158 (1,812-10,997)	4,086 (1,952-9,866)	3,703 (1,944-8,221)	3,238 (1,938-6,436)
Maternal abortion and miscarriage	-	-	-	-	-	125 (91-168)	3,671 (2,145-6,500)	4,872 (2,915-8,817)	4,368 (2,778-7,593)	4,087 (2,589-7,075)	3,696 (2,432-5,889)
Ectopic pregnancy	-	-	-	-	-	46 (27-86)	1,943 (586-5,561)	3,136 (1,059-9,709)	2,772 (1,073-7,877)	2,321 (934-6,476)	1,599 (667-4,357)
Indirect maternal deaths	-	-	-	-	-	131 (97-175)	3,959 (2,183-7,598)	8,317 (4,696-16,510)	7,543 (4,668-13,184)	5,878 (3,790-10,478)	4,715 (3,192-7,778)
Late maternal deaths	-	-	-	-	-	13 (9-19)	415 (299-577)	778 (581-1,085)	751 (554-1,025)	636 (457-895)	485 (359-671)
Maternal deaths aggravated by HIV/AIDS	-	-	-	-	-	0 (0-0)	52 (27-80)	151 (86-216)	164 (97-227)	137 (81-192)	97 (57-138)
Other maternal disorders	-	-	-	-	-	108 (79-146)	2,589 (1,298-5,458)	5,684 (2,733-12,055)	5,011 (2,731-10,024)	3,891 (2,349-7,045)	3,209 (2,066-5,514)
Neonatal disorders	998,194 (951,018-1,041,354	237,494 (210,924-275,997	60,927 (49,916-79,482)	5,063 (3,552-8,006)	-	-	-	-	-	-	-
Neonatal preterm birth	444,909 (400,987-486,786)	88,186 (74,080-108,357)	27,569 (21,852-36,667)	1,538 (1,065-2,469)	-	-	-	-	-	-	-
Neonatal encephalopathy due to birth asphyxia and trauma	296,206 (260,781-338,174)	38,799 (31,790-48,842)	10,673 (7,946-15,936)	1,790 (1,172-3,266)	-	-	-	-	-	-	-
Neonatal sepsis and other neonatal infections	51,554 (29,561-82,561)	40,636 (25,865-63,138)	4,793 (2,785-8,523)	200 (74-433)	-	-	-	-	-	-	-
Hemolytic disease and other neonatal jaundice	27,888 (23,069-33,726)	22,666 (17,160-28,537)	4,843 (3,299-6,984)	343 (203-606)	-	-	-	-	-	-	-
Other neonatal disorders	177,636 (147,625-206,727)	47,206 (37,329-67,625)	13,049 (9,855-19,498)	1,190 (815-2,130)	-	-	-	-	-	-	-
Nutritional deficiencies	-	-	103,403 (90,631-117,429)	102,936 (83,022-125,180)	10,492 (8,246-12,809)	2,234 (1,868-2,604)	1,597 (1,381-1,821)	1,823 (1,620-2,033)	1,872 (1,688-2,073)	1,768 (1,598-1,926)	1,613 (1,482-1,750)
Protein-energy malnutrition	-	-	98,873 (86,338-112,523)	96,313 (77,821-117,920)	8,777 (6,784-10,841)	1,623 (1,314-1,945)	982 (832-1,153)	1,032 (880-1,214)	1,082 (919-1,300)	1,100 (940-1,266)	1,038 (879-1,171)
Other nutritional deficiencies	-	-	4,529 (3,230-6,102)	6,623 (4,423-9,402)	1,715 (1,238-2,410)	611 (455-815)	615 (500-755)	791 (665-985)	790 (657-955)	668 (555-818)	575 (485-735)
Non-communicable diseases	97,937 (87,619-112,581)	54,695 (48,461-62,050)	228,461 (202,046-270,018)	171,601 (138,239-205,403)	63,279 (54,441-73,568)	48,116 (42,948-52,844)	74,023 (69,074-78,380)	94,412 (89,595-98,780)	115,469 (110,282-120,285)	147,564 (141,999-153,035)	201,354 (194,817-207,879)
Neoplasms	871 (672-989)	673 (490-864)	7,881 (5,498-10,502)	23,915 (15,993-37,358)	19,227 (15,252-25,346)	14,369 (12,317-16,994)	18,012 (16,358-19,831)	22,379 (20,706-24,466)	31,819 (29,589-34,137)	48,687 (45,578-52,089)	74,395 (70,282-79,158)
Lip and oral cavity cancer	-	-	-	-	-	-	248 (209-296)	360 (315-415)	438 (384-505)	628 (568-703)	859 (780-956)
Nasopharynx cancer	-	-	-	-	159 (126-190)	160 (135-182)	279 (252-305)	358 (321-391)	485 (431-530)	627 (555-693)	924 (834-1,017)
Other pharynx cancer	-	-	-	-	-	-	178 (141-221)	201 (155-248)	275 (211-337)	347 (272-429)	517 (410-636)
Esophageal cancer	-	-	-	-	-	-	165 (149-182)	326 (302-350)	468 (425-506)	758 (673-823)	1,196 (1,085-1,308)
Stomach cancer	-	-	-	-	-	-	609 (560-669)	1,629 (1,513-1,776)	3,192 (2,983-3,461)	5,674 (5,338-6,133)	8,605 (8,085-9,264)
Colon and rectum cancer	-	-	-	-	-	-	363 (317-430)	891 (775-1,084)	1,531 (1,353-1,832)	2,798 (2,465-3,342)	4,154 (3,728-4,840)
Liver cancer	-	-	-	-	603 (438-743)	411 (334-474)	566 (474-636)	1,120 (923-1,265)	1,690 (1,395-1,909)	2,769 (2,267-3,119)	4,252 (3,595-4,742)
Liver cancer due to hepatitis B	-	-	-	-	350 (253-433)	280 (226-325)	387 (320-438)	791 (655-903)	1,146 (946-1,308)	1,800 (1,471-2,070)	2,644 (2,204-3,018)
Liver cancer due to hepatitis C	-	-	-	-	12 (8-16)	10 (7-13)	13 (10-16)	43 (34-54)	112 (81-146)	251 (193-307)	485 (374-610)
Liver cancer due to alcohol use	-	-	-	-	-	-	2 (1-3)	15 (11-21)	50 (35-68)	134 (103-174)	303 (222-407)
Liver cancer due to NASH	-	-	-	-	-	-	44 (33-55)	62 (47-78)	84 (61-108)	126 (93-163)	182 (139-230)
Liver cancer due to other causes	-	-	-	-	242 (172-309)	121 (94-147)	122 (95-148)	208 (163-253)	299 (226-376)	459 (344-577)	637 (486-801)
Gallbladder and biliary tract cancer	-	-	-	-	-	-	55 (43-63)	107 (81-123)	181 (133-207)	379 (285-433)	675 (548-756)
Pancreatic cancer	-	-	-	-	-	-	70 (65-75)	134 (125-144)	255 (236-280)	455 (424-497)	843 (799-906)
Larynx cancer	-	-	-	-	-	-	82 (73-93)	150 (129-176)	206 (184-235)	248 (223-274)	417 (385-453)
Tracheal, bronchus, and lung cancer	-	-	-	-	-	-	337 (308-368)	670 (602-742)	1,319 (1,216-1,451)	2,558 (2,370-2,815)	4,467 (4,189-4,792)
Malignant skin melanoma	-	-	-	-	-	-	109 (79-156)	208 (149-290)	363 (259-470)	535 (380-684)	752 (533-930)
Non-melanoma skin cancer	-	-	-	-	-	-	56 (49-66)	75 (64-92)	101 (86-122)	123 (105-149)	178 (154-214)
Non-melanoma skin cancer (squamous-cell carcinoma)	-	-	-	-	-	-	56 (49-66)	75 (64-92)	101 (86-122)	123 (105-149)	178 (154-214)
Breast cancer	-	-	-	-	-	-	243 (155-309)	756 (524-972)	2,664 (2,112-3,300)	7,825 (6,580-9,410)	15,975 (13,753-18,776)
Cervical cancer	-	-	-	-	-	-	316 (271-351)	1,306 (1,110-1,466)	3,500 (2,981-4,005)	7,184 (6,225-8,119)	11,752 (10,366-13,539)
Uterine cancer	-	-	-	-	-	-	91 (82-100)	243 (213-268)	463 (406-511)	768 (669-848)	1,222 (1,089-1,339)
Ovarian cancer	-	-	-	-	-	-	468 (420-541)	684 (617-798)	929 (835-1,088)	1,436 (1,298-1,674)	2,325 (2,122-2,655)
Prostate cancer	-	-	-	-	-	-	-	-	-	-	-
Testicular cancer	-	-	-	-	-	-	-	-	-	-	-
Kidney cancer	25 (20-32)	16 (12-22)	225 (163-308)	946 (577-1,390)	412 (332-521)	161 (138-195)	102 (86-121)	129 (107-151)	180 (150-209)	229 (190-267)	361 (290-417)
Bladder cancer	-	-	-	-	-	-	50 (44-56)	71 (63-82)	131 (117-148)	179 (161-204)	300 (273-339)
Brain and nervous system cancer	113 (60-184)	117 (51-192)	1,546 (559-2,721)	5,454 (1,976-10,678)	5,019 (2,483-9,074)	2,811 (1,688-4,528)	2,048 (1,250-3,291)	2,052 (1,185-3,193)	2,368 (1,408-3,664)	2,830 (1,693-4,094)	3,175 (2,031-4,571)
Thyroid cancer	-	-	-	-	-	101 (74-125)	247 (184-308)	288 (222-351)	487 (360-597)	320 (248-387)	375 (300-449)

Cause	40 to 44	45 to 49	50 to 54	54 to 59	60 to 64	65 to 69	70 to 74	75 to 79	80 to 84	85 to 89	90 to 94	95 plus
All causes	437,063 (430,318-443,716	501,708 (494,702-508,938	681,500 (672,736-690,644	868,997 (858,536-880,421	1,229,813 (1,216,448-1,243,89	1,517,013 (1,501,099-1,532,95	1,749,053 (1,731,388-1,766,87	2,101,334 (2,083,526-2,117,92	2,097,829 (2,084,512-2,110,37	1,525,206 (1,518,444-1,531,48	695,695 (693,558-697,851	222,007 (221,601-222,374
Communicable, maternal, neonatal, and nutritional disease	111,431 (104,970-119,573	97,749 (90,917-107,221	106,132 (97,174-119,602	117,323 (107,315-134,790	149,567 (134,003-175,567	165,704 (146,563-200,13	192,198 (167,854-230,867	188,289 (162,338-225,664	189,522 (162,896-225,599	129,368 (113,226-148,60	54,013 (48,326-60,930	18,029 (16,705-19,690
HIV/AIDS and sexually transmitted infections	9,031 (7,628-10,467)	7,115 (6,065-8,357)	5,054 (4,320-5,920)	3,813 (3,241-4,509)	2,780 (2,341-3,272)	2,093 (1,739-2,446)	1,032 (814-1,259)	689 (557-828)	321 (232-373)	166 (126-192)	72 (56-82)	19 (17-22)
HIV/AIDS	8,338 (6,999-9,713)	6,541 (5,494-7,771)	4,584 (3,886-5,416)	3,285 (2,740-3,944)	2,335 (1,909-2,824)	1,704 (1,371-2,058)	732 (534-960)	373 (272-498)	62 (50-76)	20 (17-24)	7 (6-9)	2 (2-2)
HIV/AIDS - Drug-susceptible Tuberculosis	2,859 (2,149-3,555)	2,351 (1,796-2,912)	1,711 (1,326-2,072)	1,208 (945-1,493)	841 (667-1,054)	604 (467-764)	271 (195-361)	136 (98-180)	19 (14-24)	5 (4-7)	2 (2-2)	0 (0-0)
HIV/AIDS - Multidrug-resistant Tuberculosis without extensive drug resistance	21 (12-39)	17 (9-32)	13 (7-24)	10 (5-18)	7 (4-13)	5 (3-9)	2 (1-4)	1 (1-2)	0 (0-0)	0 (0-0)	0 (0-0)	0 (0-0)
HIV/AIDS - Extensively drug-resistant Tuberculosis	0 (0-0)	0 (0-0)	0 (0-0)	0 (0-0)	0 (0-0)	0 (0-0)	0 (0-0)	0 (0-0)	0 (0-0)	0 (0-0)	0 (0-0)	0 (0-0)
HIV/AIDS resulting in other diseases	5,457 (4,312-7,020)	4,173 (3,314-5,367)	2,860 (2,315-3,595)	2,067 (1,658-2,648)	1,486 (1,172-1,886)	1,094 (870-1,390)	459 (326-642)	236 (167-329)	43 (34-54)	15 (13-18)	5 (5-6)	1 (1-2)
Sexually transmitted infections excluding HIV	693 (424-803)	574 (364-658)	469 (292-555)	528 (313-641)	445 (257-537)	389 (252-462)	300 (211-345)	316 (212-375)	259 (173-310)	146 (106-172)	64 (48-75)	18 (15-20)
Syphilis	46 (28-54)	37 (24-43)	67 (42-79)	95 (56-115)	79 (46-95)	80 (52-95)	74 (52-85)	68 (46-81)	54 (36-65)	27 (19-31)	11 (8-13)	4 (3-4)
Chlamydial infection	132 (81-153)	101 (64-116)	76 (47-90)	86 (51-104)	74 (43-89)	40 (26-47)	41 (29-47)	44 (29-52)	49 (33-59)	29 (21-34)	12 (9-15)	3 (3-4)
Gonococcal infection	334 (204-386)	277 (176-317)	219 (136-259)	228 (135-276)	181 (105-218)	206 (134-245)	137 (96-158)	160 (108-191)	122 (81-146)	71 (51-83)	32 (24-37)	8 (7-9)
Other sexually transmitted infections	181 (111-210)	159 (101-182)	108 (67-127)	119 (71-144)	111 (64-134)	64 (41-76)	48 (34-55)	44 (29-52)	34 (23-41)	20 (14-23)	9 (7-10)	3 (2-3)
Respiratory infections and tuberculosis	41,148 (37,479-44,568	43,249 (39,417-47,048	56,167 (51,240-61,175	64,110 (58,448-70,085	80,400 (73,372-88,805	88,969 (81,699-98,373	104,113 (95,552-114,328	104,810 (98,038-114,084	106,510 (100,055-115,232	79,591 (76,012-85,315	35,793 (34,531-38,377	12,841 (12,522-13,431
Tuberculosis	31,331 (27,931-34,477	32,716 (29,157-36,089	40,679 (35,982-45,099	43,927 (38,344-49,010	49,073 (42,662-55,883	48,152 (41,598-55,177	49,898 (43,033-56,808	39,311 (33,723-45,006	29,031 (24,813-34,053	12,985 (11,096-14,983	3,356 (2,841-3,883	747 (648-865)
Drug-susceptible tuberculosis	31,023 (27,639-34,168	32,397 (28,905-35,863	40,316 (35,737-44,582	43,500 (37,976-48,566	48,531 (42,259-55,105	47,553 (41,063-54,645	49,237 (42,477-56,179	38,746 (33,217-44,438	28,633 (24,335-33,581	12,805 (10,954-14,829	3,314 (2,802-3,827	738 (639-855)
Multidrug-resistant tuberculosis without extensive drug resistance	308 (151-593)	319 (159-609)	363 (187-688)	427 (214-813)	542 (261-1,047)	599 (287-1,174)	661 (313-1,331)	565 (274-1,136)	398 (197-759)	181 (96-327)	42 (25-69)	9 (6-14)
Extensively drug-resistant tuberculosis	0 (0-0)	0 (0-0)	0 (0-0)	0 (0-0)	0 (0-0)	0 (0-0)	0 (0-0)	0 (0-0)	0 (0-0)	0 (0-0)	0 (0-0)	0 (0-0)
Lower respiratory infections	9,662 (8,491-10,457	10,373 (9,205-11,153	15,313 (13,678-16,686	19,946 (17,779-21,631	31,008 (27,179-33,634	40,386 (35,797-43,817	53,466 (47,549-58,336	64,526 (59,125-69,006	75,987 (71,238-81,226	65,353 (62,738-69,789	31,957 (30,900-34,376	11,917 (11,622-12,577
Upper respiratory infections	114 (37-182)	136 (44-219)	137 (50-221)	179 (67-277)	261 (94-405)	353 (114-540)	691 (202-1,036)	932 (277-1,385)	1,462 (427-2,221)	1,233 (366-1,876)	472 (155-683)	173 (63-239)
Otitis media	42 (28-66)	23 (17-32)	37 (26-52)	58 (39-78)	58 (40-81)	77 (53-108)	58 (41-76)	40 (29-52)	30 (22-39)	20 (16-26)	9 (7-11)	3 (3-4)
Enteric infections	11,962 (6,051-20,021	13,397 (6,794-23,063	17,643 (8,541-30,867	23,386 (11,321-43,022	38,004 (18,043-65,724	45,814 (22,343-83,241	58,412 (27,967-98,363	60,655 (30,991-102,381	66,157 (32,874-106,450	38,981 (20,214-60,419	13,814 (7,515-21,239	3,491 (1,979-5,317)
Diarrheal diseases	9,846 (4,181-18,042	11,654 (5,139-21,688	16,138 (7,130-29,427	22,019 (9,895-41,793	36,773 (17,085-64,739	44,771 (21,466-82,471	57,637 (27,217-97,596	60,117 (30,355-101,981	65,804 (32,655-106,052	38,800 (20,066-60,222	13,756 (7,446-21,209	3,474 (1,956-5,302)
Typhoid and paratyphoid	1,587 (851-2,634)	1,240 (675-2,095)	967 (505-1,665)	807 (392-1,495)	663 (332-1,197)	517 (300-839)	370 (225-585)	238 (149-375)	146 (94-230)	68 (45-113)	22 (13-38)	7 (3-12)
Typhoid fever	1,327 (687-2,266)	1,026 (546-1,754)	801 (411-1,385)	675 (326-1,270)	559 (277-1,014)	439 (251-708)	316 (189-495)	203 (123-325)	127 (82-205)	60 (38-105)	19 (11-36)	5 (3-11)
Paratyphoid fever	260 (118-524)	214 (90-430)	167 (66-343)	132 (53-275)	104 (43-212)	78 (35-153)	54 (25-104)	35 (18-64)	19 (9-34)	9 (4-14)	3 (1-5)	1 (0-2)
Invasive Non-typhoidal Salmonella (iNTS)	510 (238-975)	485 (226-925)	522 (234-990)	547 (251-974)	552 (254-970)	507 (239-871)	392 (186-685)	289 (136-527)	190 (78-361)	95 (38-176)	32 (8-69)	9 (0-25)
Other intestinal infectious diseases	20 (3-50)	19 (3-51)	16 (2-40)	14 (2-33)	16 (2-40)	20 (2-48)	14 (2-33)	11 (1-29)	17 (1-44)	17 (1-48)	3 (0-11)	2 (0-5)
Neglected tropical diseases and malaria	10,443 (5,467-20,355	10,295 (5,421-19,113	11,880 (6,781-20,238	11,709 (6,895-19,635	12,570 (7,765-20,113	11,425 (7,056-18,023	10,758 (6,985-16,126	5,708 (3,864-8,500)	2,733 (1,882-4,172)	1,041 (752-1,569)	300 (221-433)	83 (58-110)
Malaria	4,073 (2,713-5,936)	4,096 (2,724-5,908)	5,339 (3,522-7,812)	5,851 (3,897-8,629)	6,742 (4,458-9,997)	6,432 (4,215-9,658)	6,661 (4,293-10,096)	2,892 (1,930-4,224)	1,161 (800-1,631)	277 (179-371)	78 (42-105)	35 (10-51)
Chagas disease	174 (161-188)	235 (219-253)	308 (289-330)	367 (340-398)	406 (378-436)	400 (370-438)	397 (367-429)	346 (317-376)	242 (220-265)	156 (138-175)	62 (52-74)	11 (9-12)
Leishmaniasis	4,023 (5-13,998)	3,990 (7-12,647)	3,833 (7-12,057)	3,441 (6-10,825)	3,353 (7-10,284)	2,761 (6-8,451)	2,014 (4-6,041)	1,157 (8-3,623)	557 (4-1,808)	199 (9-647)	50 (3-157)	7 (0-23)
Visceral leishmaniasis	4,023 (5-13,998)	3,990 (7-12,647)	3,833 (7-12,057)	3,441 (6-10,825)	3,353 (7-10,284)	2,761 (6-8,451)	2,014 (4-6,041)	1,157 (8-3,623)	557 (4-1,808)	199 (9-647)	50 (3-157)	7 (0-23)
African trypanosomiasis	463 (114-1,254)	480 (118-1,390)	433 (105-1,221)	422 (100-1,231)	355 (76-1,182)	293 (62-920)	187 (37-601)	122 (25-448)	52 (11-170)	19 (4-62)	4 (1-16)	1 (0-3)
Schistosomiasis	316 (251-395)	355 (279-442)	464 (362-584)	526 (420-647)	592 (464-735)	478 (377-591)	644 (515-796)	507 (403-634)	283 (226-357)	122 (91-163)	28 (20-39)	5 (3-8)
Cysticercosis	35 (3-88)	38 (7-89)	31 (2-79)	30 (3-78)	24 (1-65)	23 (2-62)	5 (0-17)	12 (0-37)	8 (0-24)	4 (0-13)	1 (0-4)	0 (0-1)
Cystic echinococcosis	60 (14-131)	60 (14-132)	70 (16-152)	74 (16-155)	76 (15-165)	70 (12-159)	62 (10-142)	50 (5-126)	37 (3-101)	24 (2-73)	8 (0-27)	3 (0-9)
Dengue	157 (36-317)	137 (31-271)	174 (36-351)	186 (39-367)	168 (41-327)	144 (42-281)	254 (93-519)	158 (65-331)	192 (90-482)	113 (55-302)	33 (15-88)	7 (3-22)
Yellow fever	128 (25-380)	123 (22-372)	109 (22-341)	89 (17-276)	67 (13-206)	53 (10-163)	33 (6-108)	18 (4-55)	7 (1-21)	3 (0-9)	1 (0-2)	0 (0-1)
Rabies	925 (417-1,596)	701 (421-1,174)	1,021 (590-1,876)	633 (396-1,156)	658 (386-1,147)	641 (376-1,212)	374 (222-752)	320 (187-634)	102 (58-211)	71 (38-131)	16 (8-31)	6 (3-12)
Intestinal nematode infections	13 (10-18)	14 (10-18)	15 (11-20)	14 (10-19)	31 (24-40)	15 (11-20)	26 (19-33)	21 (15-28)	15 (10-20)	6 (4-9)	3 (2-5)	1 (1-2)
Ascariasis	13 (10-18)	14 (10-18)	15 (11-20)	14 (10-19)	31 (24-40)	15 (11-20)	26 (19-33)	21 (15-28)	15 (10-20)	6 (4-9)	3 (2-5)	1 (1-2)
Ebola	0 (0-0)	0 (0-0)	0 (0-0)	0 (0-0)	0 (0-0)	0 (0-0)	0 (0-0)	0 (0-0)	0 (0-0)	0 (0-0)	0 (0-0)	0 (0-0)
Zika virus	0 (0-0)	0 (0-0)	0 (0-0)	0 (0-0)	0 (0-0)	0 (0-0)	0 (0-0)	0 (0-0)	0 (0-0)	0 (0-0)	0 (0-0)	0 (0-0)
Other neglected tropical diseases	74 (37-152)	67 (35-148)	83 (44-185)	77 (44-158)	98 (53-213)	114 (65-252)	102 (60-222)	105 (66-230)	77 (47-165)	45 (28-89)	15 (9-31)	8 (5-15)
Other infectious diseases	9,449 (7,912-10,814	9,291 (7,811-10,636	11,265 (9,421-12,948	11,291 (9,598-12,829	11,865 (10,159-13,597	12,508 (10,726-14,567	12,295 (10,416-14,551	10,201 (8,775-11,647	8,146 (6,855-9,285)	4,580 (3,868-5,139)	1,564 (1,337-1,755)	511 (425-595)
Meningitis	2,893 (2,098-3,878)	3,041 (2,216-4,232)	3,291 (2,371-4,540)	3,357 (2,505-4,531)	3,479 (2,557-4,866)	3,584 (2,609-4,852)	3,906 (2,819-5,465)	2,853 (2,029-3,775)	2,290 (1,685-2,864)	1,281 (919-1,658)	383 (283-482)	121 (89-150)
Pneumococcal meningitis	444 (311-636)	503 (356-727)	537 (387-753)	527 (380-747)	553 (399-774)	600 (422-813)	699 (490-996)	511 (371-696)	399 (286-529)	215 (156-277)	63 (48-80)	21 (15-28)
H influenzae type B meningitis	944 (661-1,270)	981 (675-1,323)	1,092 (766-1,503)	1,157 (816-1,565)	1,196 (838-1,675)	1,197 (830-1,689)	1,268 (882-1,816)	984 (663-1,324)	810 (553-1,067)	456 (313-597)	146 (101-185)	44 (30-56)
Meningococcal meningitis	226 (164-306)	235 (166-323)	261 (183-362)	266 (191-373)	277 (193-390)	290 (205-407)	315 (223-445)	233 (163-327)	185 (131-255)	93 (67-126)	26 (19-34)	8 (5-12)
Other meningitis	1,279 (936-1,764)	1,322 (965-1,857)	1,401 (1,004-1,933)	1,407 (1,038-1,944)	1,452 (1,070-2,037)	1,496 (1,075-2,019)	1,624 (1,170-2,322)	1,125 (823-1,538)	897 (645-1,148)	517 (376-687)	148 (112-195)	49 (36-64)
Encephalitis	924 (753-1,170)	894 (779-1,105)	1,035 (905-1,284)	1,231 (1,069-1,528)	1,648 (1,422-2,039)	1,648 (1,417-2,046)	1,862 (1,537-2,314)	1,650 (1,357-2,025)	1,463 (1,143-1,760)	662 (540-781)	170 (145-192)	46 (40-52)
Diphtheria	47 (33-68)	72 (49-107)	62 (44-90)	39 (27-57)	-	-	-	-	-	-	-	-
Whooping cough	59 (24-126)	52 (22-107)	56 (23-117)	58 (25-118)	-	-	-	-	-	-	-	-
Tetanus	1,733 (932-2,750)	1,930 (995-2,926)	2,634 (1,371-3,904)	2,153 (1,224-3,225)	1,866 (1,119-2,803)	2,206 (1,353-3,785)	1,734 (1,090-2,838)	1,418 (843-2,472)	677 (397-1,138)	360 (208-592)	92 (53-151)	21 (12-36)
Measles	162 (57-372)	101 (36-232)	78 (27-182)	59 (21-138)	-	-	-	-	-	-	-	-
Varicella and herpes zoster	130 (105-162)	124 (102-152)	127 (104-159)	151 (122-184)	182 (146-225)	340 (275-418)	321 (262-397)	462 (374-570)	710 (569-907)	731 (576-947)	391 (300-501)	181 (135-261)
Acute hepatitis	2,722 (2,104-3,209)	2,301 (1,796-2,777)	2,903 (2,278-3,557)	3,113 (2,574-3,889)	3,195 (2,626-3,919)	3,006 (2,561-3,651)	2,764 (2,315-3,376)	2,223 (1,847-2,642)	1,533 (1,249-1,872)	625 (519-793)	161 (135-203)	30 (24-40)
Acute hepatitis A	309 (139-565)	183 (85-351)	128 (61-226)	84 (42-141)	67 (34-114)	53 (25-89)	25 (10-48)	12 (4-26)	3 (1-8)	0 (0-1)	0 (0-0)	0 (0-0)
Acute hepatitis B	2,092 (1,411-2,696)	1,920 (1,377-2,450)	2,604 (2,032-3,231)	2,889 (2,349-3,612)	2,972 (2,411-3,631)	2,765 (2,280-3,419)	2,512 (2,013-3,098)	1,951 (1,498-2,366)	1,269 (932-1,610)	481 (339-635)	110 (72-149)	18 (10-27)
Acute hepatitis C	23 (6-57)	23 (6-57)	29 (8-72)	32 (10-79)	44 (11-111)	54 (15-147)	62 (18-166)	59 (17-152)	54 (14-152)	30 (8-76)	10 (3-28)	2 (0-6)
Acute hepatitis E	298 (107-577)	175 (68-357)	143 (59-281)	107 (42-212)	113 (44-242)	134 (51-271)	165 (69-333)	201 (79-401)	208 (86-384)	114 (51-203)	41 (21-67)	10 (5-16)
Other unspecified infectious diseases	779 (566-992)	776 (560-959)	1,079 (782-1,322)	1,130 (831-1,395)	1,495 (1,101-1,867)	1,725 (1,264-2,192)	1,707 (1,253-2,262)	1,596 (1,183-2,023)	1,472 (1,016-1,981)	921 (605-1,225)	366 (235-485)	112 (75-142)
Maternal and neonatal disorders	27,503 (25,309-29,831)	12,741 (11,683-13,832)	1,954 (1,763-2,147)	-	-	-	-	-	-	-	-	-
Maternal disorders	27,503 (25,309-29,831)	12,741 (11,683-13,832)	1,954 (1,763-2,147)	-	-	-	-	-	-	-	-	-
Maternal hemorrhage	10,843 (8,572-13,678)	3,660 (2,722-4,682)	604 (478-777)	-	-	-	-	-	-	-	-	-
Maternal sepsis and other maternal infections	3,136 (2,272-4,501)	2,639 (2,040-3,337)	283 (216-389)	-	-	-	-	-	-	-	-	-
Maternal hypertensive disorders	2,335 (1,635-3,523)	645 (464-903)	90 (65-136)	-	-	-	-	-	-	-	-	-
Maternal obstructed labor and uterine rupture	2,177 (1,436-3,774)	1,476 (1,090-2,032)	136 (90-221)	-	-	-	-	-	-	-	-	-
Maternal abortion and miscarriage	2,828 (1,968-4,119)	2,153 (1,671-2,726)	214 (165-288)	-	-	-	-	-	-	-	-	-
Ectopic pregnancy	975 (396-2,514)	445 (192-1,045)	180 (83-327)	-	-	-	-	-	-	-	-	-
Indirect maternal deaths	2,832 (2,021-4,296)	675 (508-972)	208 (161-275)	-	-	-	-	-	-	-	-	-
Late maternal deaths	252 (189-345)	101 (85-121)	15 (12-18)	-	-	-	-	-	-	-	-	-
Maternal deaths aggravated by HIV/AIDS	54 (30-77)	42 (25-61)	5 (3-7)	-	-	-	-	-	-	-	-	-
Other maternal disorders	2,071 (1,339-3,291)	906 (681-1,247)	218 (167-303)	-	-	-	-	-	-	-	-	-
Neonatal disorders	-	-	-	-	-	-	-	-	-	-	-	-
Neonatal preterm birth	-	-	-	-	-	-	-	-	-	-	-	-
Neonatal encephalopathy due to birth asphyxia and trauma	-	-	-	-	-	-	-	-	-	-	-	-
Neonatal sepsis and other neonatal infections	-	-	-	-	-	-	-	-	-	-	-	-
Hemolytic disease and other neonatal jaundice	-	-	-	-	-	-	-	-	-	-	-	-
Other neonatal disorders	-	-	-	-	-	-	-	-	-	-	-	-
Nutritional deficiencies	1,895 (1,717-2,088)	1,661 (1,524-1,804)	2,170 (2,005-2,354)	3,014 (2,745-3,309)	3,948 (3,630-4,349)	4,895 (4,457-5,414)	5,587 (5,134-6,137)	6,226 (5,745-6,900)	5,654 (5,224-6,248)	5,009 (4,680-5,541)	2,470 (2,329-2,668)	1,084 (1,034-1,156)
Protein-energy malnutrition	1,172 (1,001-1,341)	1,084 (934-1,222)	1,349 (1,144-1,528)	1,992 (1,741-2,310)	2,704 (2,410-3,028)	3,415 (3,037-3,834)	4,221 (3,776-4,641)	4,616 (4,035-5,220)	4,294 (3,698-4,882)	3,914 (3,194-4,443)	1,937 (1,489-2,212)	850 (604-976)
Other nutritional deficiencies	723 (617-872)	577 (487-729)	821 (703-1,027)	1,022 (875-1,250)	1,244 (1,055-1,540)	1,480 (1,234-1,869)	1,367 (1,123-1,811)	1,610 (1,278-2,232)	1,360 (1,027-2,123)	1,095 (743-1,911)	533 (342-992)	234 (123-485)
Non-communicable diseases	268,007 (260,782-274,998)	354,268 (345,594-362,260)	523,601 (511,436-533,498)	702,396 (685,234-715,438)	1,025,726 (999,452-1,044,30	1,296,143 (1,264,243-1,317,54	1,501,859 (1,465,935-1,528,54	1,853,356 (1,817,472-1,881,86	1,852,621 (1,816,250-1,879,27	1,355,069 (1,335,839-1,372,23	623,867 (617,144-629,547)	198,688 (197,113-200,064)
Neoplasms	102,579 (97,910-108,479)	131,720 (126,592-138,371)	183,700 (177,752-193,184)	225,753 (219,601-237,246)	290,378 (283,623-305,756)	321,861 (314,708-338,942)	301,268 (294,256-318,606)	297,925 (292,522-311,96	230,984 (227,031-240,440)	127,569 (125,646-131,425)	45,294 (44,725-46,230)	10,779 (10,653-10,975)
Lip and oral cavity cancer	1,403 (1,273-1,541)	1,878 (1,730-2,044)	2,658 (2,442-2,901)	3,179 (2,974-3,419)	4,160 (3,872-4,495)	3,730 (3,476-3,997)	3,333 (3,068-3,604)	3,232 (3,042-3,426)	2,833 (2,662-3,030)	1,706 (1,626-1,787)	652 (623-684)	162 (156-168)
Nasopharynx cancer	1,196 (1,078-1,317)	1,385 (1,271-1,504)	1,750 (1,604-1,905)	2,005 (1,853-2,175)	1,901 (1,730-2,101)	1,674 (1,517-1,857)	1,378 (1,249-1,549)	986 (890-1,120)	644 (582-730)	290 (265-323)	76 (71-84)	15 (15-16)
Other pharynx cancer	722 (596-877)	1,234 (1,068-1,444)	1,761 (1,504-2,070)	2,047 (1,797-2,351)	2,541 (2,270-2,892)	2,266 (2,046-2,561)	1,960 (1,790-2,187)	1,120 (1,023-1,256)	832 (733-915)	403 (361-433)	119 (110-126)	27 (25-28)
Esophageal cancer	2,174 (2,032-2,335)	3,517 (3,286-3,756)	6,134 (5,772-6,506)	9,371 (8,816-9,999)	13,851 (12,978-14,873)	16,750 (15,620-18,025)	17,824 (16,632-19,333)	14,849 (13,908-16,198)	10,627 (9,925-11,581)	5,296 (4,958-5,752)	1,529 (1,442-1,643)	282 (269-299)
Stomach cancer	11,290 (10,699-12,130)	13,293 (12,601-14,169)	18,715 (17,828-19,857)	24,137 (23,114-25,539)	34,499 (33,070-36,598)	40,993 (39,336-43,597)	41,248 (39,487-44,190)	42,162 (40,774-44,561)	33,165 (32,177-35,118)	17,908 (17,431-18,765)	5,819 (5,685-6,027)	1,277 (1,245-1,313)
Colon and rectum cancer	6,003 (5,505-6,836)	8,542 (7,869-9,663)	13,709 (12,767-15,281)	18,096 (17,047-19,825)	25,965 (24,750-27,920)	31,463 (30,148-33,590)	32,250 (30,797-34,501)	39,214 (37,904-41,255)	35,225 (34,276-36,616)	21,789 (21,216-22,453)	8,811 (8,598-9,033)	2,177 (2,126-2,231)
Liver cancer	5,962 (5,127-6,599)	7,819 (6,794-8,565)	11,000 (9,648-11,951)	14,665 (13,024-15,952)	18,824 (16,855-20,411)	21,373 (19,200-23,280)	20,058 (18,138-22,103)	17,695 (16,309-19,262)	11,950 (11,240-12,885)	5,878 (5,605-6,203)	1,562 (1,494-1,628)	304 (291-319)
Liver cancer due to hepatitis B	3,491 (2,941-3,967)	4,192 (3,551-4,753)	5,141 (4,370-5,861)	5,824 (4,866-6,734)	6,448 (5,416-7,471)	6,879 (5,830-7,890)	5,898 (4,956-6,989)	4,071 (3,465-4,776)	2,094 (1,793-2,467)	802 (697-920)	162 (138-187)	24 (19-31)
Liver cancer due to hepatitis C	867 (704-1,047)	1,487 (1,177-1,816)	2,775 (2,285-3,265)	4,701 (4,053-5,380)	6,880 (5,941-7,785)	7,945 (7,071-8,854)	7,827 (7,000-8,699)	7,778 (7,004-8,554)	5,899 (5,391-6,434)	3,209 (2,980-3,424)	919 (853-984)	188 (172-205)
Liver cancer due to alcohol use	493 (372-641)	667 (480-942)	988 (709-1,386)	1,285 (944-1,754)	1,878 (1,419-2,474)	2,633 (2,152-3,262)	2,552 (2,098-3,134)	2,250 (1,938-2,626)	1,459 (1,235-1,717)	686 (585-797)	174 (144-205)	32 (24-39)
Liver cancer due to NASH	263 (204-331)	390 (300-494)	641 (507-811)	989 (770-1,244)	1,425 (1,136-1,754)	1,729 (1,370-2,132)	1,823 (1,456-2,237)	1,883 (1,521-2,313)	1,397 (1,140-1,722)	696 (592-818)	191 (157-231)	38 (30-49)
Liver cancer due to other causes	848 (665-1,068)	1,083 (853-1,330)	1,456 (1,160-1,819)	1,866 (1,505-2,245)	2,194 (1,756-2,677)	2,188 (1,753-2,687)	1,958 (1,535-2,443)	1,713 (1,410-2,077)	1,102 (895-1,348)	485 (408-573)	116 (96-140)	21 (16-27)
Gallbladder and biliary tract cancer	1,113 (955-1,228)	1,997 (1,803-2,264)	3,429 (3,145-3,903)	5,028 (4,722-5,861)	7,744 (7,312-9,062)	9,323 (8,891-10,827)	9,371 (8,912-10,890)	10,683 (10,250-11,967)	8,861 (8,483-9,890)	4,931 (4,739-5,398)	1,747 (1,684-1,845)	394 (380-410)
Pancreatic cancer	1,432 (1,378-1,514)	2,442 (2,370-2,553)	4,386 (4,279-4,531)	6,710 (6,565-6,902)	10,940 (10,710-11,189)	13,804 (13,567-14,088)	14,126 (13,848-14,424)	15,930 (15,642-16,201)	13,026 (12,755-13,291)	7,483 (7,313-7,644)	2,785 (2,731-2,838)	609 (593-625)
Larynx cancer	610 (557-671)	901 (833-972)	1,409 (1,303-1,518)	1,731 (1,620-1,853)	2,163 (2,030-2,333)	2,213 (2,074-2,373)	1,869 (1,750-2,010)	1,387 (1,311-1,482)	977 (919-1,043)	449 (429-474)	119 (114-123)	24 (23-25)
Tracheal, bronchus, and lung cancer	7,718 (7,375-8,123)	11,597 (11,177-12,076)	18,107 (17,577-18,658)	25,870 (25,172-26,634)	37,341 (36,366-38,421)	44,265 (43,146-45,468)	41,348 (40,154-42,723)	36,782 (35,825-37,849)	24,301 (23,478-25,025)	11,677 (11,280-12,014)	3,643 (3,533-3,736)	811 (786-835)
Malignant skin melanoma	926 (653-1,165)	942 (661-1,159)	1,196 (887-1,568)	1,353 (1,032-1,802)	1,660 (1,255-2,276)	1,853 (1,410-2,472)	1,673 (1,269-2,243)	1,961 (1,467-2,717)	1,720 (1,292-2,316)	1,050 (786-1,393)	412 (293-538)	120 (85-159)
Non-melanoma skin cancer	228 (200-270)	297 (265-346)	408 (369-467)	505 (461-577)	707 (648-798)	838 (774-932)	933 (865-1,038)	1,144 (1,091-1,226)	1,350 (1,293-1,418)	1,238 (1,189-1,288)	677 (655-701)	281 (272-291)
Non-melanoma skin cancer (squamous-cell carcinoma)	228 (200-270)	297 (265-346)	408 (369-467)	505 (461-577)	707 (648-798)	838 (774-932)	933 (865-1,038)	1,144 (1,091-1,226)	1,350 (1,293-1,418)	1,238 (1,189-1,288)	677 (655-701)	281 (272-291)
Breast cancer	23,466 (21,350-26,860)	29,484 (27,170-33,308)	37,678 (35,128-42,286)	39,504 (37,202-43,722)	40,968 (39,006-44,796)	39,309 (37,704-42,900)	31,473 (30,142-34,412)	30,258 (29,331-32,366)	24,091 (23,463-25,685)	14,165 (13,834-14,870)	5,524 (5,376-5,758)	1,526 (1,484-1,572)
Cervical cancer	15,898 (14,234-18,422)	18,415 (16,618-20,392)	21,457 (19,322-24,519)	21,884 (20,123-24,414)	22,062 (20,205-24,437)	20,037 (18,382-21,890)	16,515 (15,141-18,331)	13,227 (12,446-14,571)	8,972 (8,554-9,593)	4,308 (4,135-4,616)	1,390 (1,342-1,448)	351 (340-365)
Uterine cancer	1,762 (1,602-1,908)	2,778 (2,562-2,974)	4,293 (3,993-4,546)	5,787 (5,429-6,110)	8,285 (7,855-8,721)	8,975 (8,576-9,414)	7,769 (7,423-8,131)	7,322 (7,114-7,569)	5,297 (5,149-5,458)	2,504 (2,445-2,570)	868 (846-891)	203 (197-211)
Ovarian cancer	3,940 (3,656-4,395)	6,043 (5,646-6,689)	9,329 (8,799-10,132)	10,747 (10,231-11,548)	13,061 (12,493-13,870)	13,353 (12,855-14,042)	10,803 (10,407-11,315)	10,153 (9,856-10,459)	7,125 (6,929-7,325)	3,646 (3,537-3,758)	1,249 (1,215-1,284)	255 (245-264)
Prostate cancer	-	-	-	-	-	-	-	-	-	-	-	-
Testicular cancer	-	-	-	-	-	-	-	-	-	-	-	-
Kidney cancer	573 (442-647)	885 (669-990)	1,526 (1,130-1,685)	2,165 (1,642-2,361)	3,167 (2,432-3,410)	3,754 (2,948-4,010)	3,487 (2,809-3,730)	3,809 (3,002-4,023)	2,900 (2,402-3,039)	1,465 (1,283-1,533)	539 (424-567)	117 (88-125)
Bladder cancer	488 (450-547)	666 (620-736)	1,089 (1,015-1,197)	1,552 (1,456-1,681)	2,517 (2,375-2,695)	3,611 (3,417-3,839)	4,325 (4,070-4,636)	5,820 (5,557-6,086)	5,719 (5,452-5,986)	3,760 (3,608-3,891)	1,504 (1,458-1,547)	384 (372-396)
Brain and nervous system cancer	3,327 (2,283-4,614)	3,772 (2,604-5,291)	4,783 (3,563-6,409)	5,630 (4,360-7,649)	6,384 (5,079-8,640)	6,267 (5,102-8,625)	4,788 (3,941-6,732)	3,863 (3,330-5,346)	2,199 (1,953-3,002)	942 (850-1,262)	255 (232-335)	49 (45-63)
Thyroid cancer	470 (399-550)	538 (473-626)	846 (746-991)	1,109 (1,008-1,296)	1,580 (1,453-1,852)	1,815 (1,678-2,156)	1,804 (1,671-2,164)	1,877 (1,782-2,144)	1,547 (1,474-1,724)	804 (772-886)	246 (236-265)	53 (50-56)

SR Table 5: Global deaths by cause and age group for females (with 95% confidence intervals), 1990

Cause	Early Neonatal	Late Neonatal	Post Neonatal
Mesothelioma	-	-	-
Hodgkin lymphoma	3 (1-3)	2 (1-4)	73 (23-115)
Non-Hodgkin lymphoma	32 (27-37)	34 (27-43)	598 (459-740)
Multiple myeloma	-	-	-
Leukemia	186 (125-236)	188 (117-271)	3,280 (1,865-5,244)
Acute lymphoid leukemia	33 (21-52)	26 (15-53)	475 (257-1,038)
Chronic lymphoid leukemia	-	-	-
Acute myeloid leukemia	22 (14-39)	22 (13-49)	347 (173-806)
Chronic myeloid leukemia	-	-	-
Other leukemia	132 (84-174)	140 (83-211)	2,457 (1,303-3,867)
Other malignant neoplasms	488 (357-562)	302 (201-386)	1,931 (1,148-2,551)
Other neoplasms	24 (17-31)	15 (10-19)	229 (146-288)
Myelodysplastic, myeloproliferative, and other hematopoietic neoplasm	23 (16-30)	12 (8-15)	202 (134-261)
Other benign and in situ neoplasms	1 (1-2)	3 (2-5)	27 (12-48)
Cardiovascular diseases	1,920 (1,763-2,129)	1,866 (1,605-2,122)	15,950 (14,470-17,454)
Rheumatic heart disease	-	-	-
Ischemic heart disease	-	-	-
Stroke	793 (689-950)	777 (606-1,039)	6,055 (4,693-8,220)
Ischemic stroke	130 (104-166)	125 (89-184)	662 (481-932)
Intracerebral hemorrhage	319 (277-391)	303 (239-417)	2,628 (2,063-3,625)
Subarachnoid hemorrhage	344 (251-400)	349 (241-425)	2,765 (1,894-3,537)
Hypertensive heart disease	-	-	-
Non-rheumatic valvular heart disease	-	-	-
Non-rheumatic calcific aortic valve disease	-	-	-
Non-rheumatic degenerative mitral valve disease	-	-	-
Other non-rheumatic valve diseases	-	-	-
Cardiomyopathy and myocarditis	678 (548-799)	626 (461-772)	5,203 (3,710-6,315)
Myocarditis	85 (60-108)	115 (76-146)	1,615 (1,088-2,016)
Alcoholic cardiomyopathy	-	-	-
Other cardiomyopathy	593 (468-702)	511 (360-630)	3,587 (2,449-4,411)
Atrial fibrillation and flutter	-	-	-
Aortic aneurysm	-	-	-
Peripheral artery disease	-	-	-
Endocarditis	96 (78-125)	134 (97-192)	1,792 (1,372-2,368)
Other cardiovascular and circulatory diseases	353 (280-505)	329 (250-483)	2,899 (1,935-4,848)
Chronic respiratory diseases	0 (0-0)	0 (0-0)	0 (0-0)
Chronic obstructive pulmonary disease	-	-	0 (0-0)
Pneumoconiosis	-	-	-
Silicosis	-	-	-
Asbestosis	-	-	-
Coal workers pneumoconiosis	-	-	-
Other pneumoconiosis	-	-	-
Asthma	-	-	-
Interstitial lung disease and pulmonary sarcoidosis	-	-	-
Other chronic respiratory diseases	0 (0-0)	0 (0-0)	0 (0-0)
Digestive diseases	1,322 (1,091-1,478)	1,577 (1,319-2,019)	12,530 (9,875-18,714)
Cirrhosis and other chronic liver diseases	-	-	-
Cirrhosis and other chronic liver diseases due to hepatitis B	-	-	-
Cirrhosis and other chronic liver diseases due to hepatitis C	-	-	-
Cirrhosis and other chronic liver diseases due to alcohol use	-	-	-
Cirrhosis due to NASH	-	-	-
Cirrhosis and other chronic liver diseases due to other causes	-	-	-
Upper digestive system diseases	-	-	-
Peptic ulcer disease	-	-	-
Gastritis and duodenitis	-	-	-
Appendicitis	-	-	-
Paralytic ileus and intestinal obstruction	1,322 (1,091-1,478)	1,577 (1,319-2,019)	12,530 (9,875-18,714)
Inguinal, femoral, and abdominal hernia	-	-	-
Inflammatory bowel disease	-	-	-
Vascular intestinal disorders	-	-	-
Gallbladder and biliary diseases	-	-	-
Pancreatitis	-	-	-
Other digestive diseases	-	-	-
Neurological disorders	-	-	6,370 (3,888-8,137)
Alzheimer's disease and other dementias	-	-	-
Parkinson's disease	-	-	-
Epilepsy	-	-	4,334 (2,614-5,385)
Multiple sclerosis	-	-	-
Motor neuron disease	9 (2-16)	17 (5-27)	335 (233-461)
Other neurological disorders	-	-	1,701 (779-3,196)
Mental disorders	-	-	-
Eating disorders	-	-	-
Anorexia nervosa	-	-	-
Bulimia nervosa	-	-	-
Substance use disorders	0 (0-0)	0 (0-0)	3 (0-9)
Alcohol use disorders	-	-	-
Drug use disorders	0 (0-0)	0 (0-0)	3 (0-9)
Opioid use disorders	0 (0-0)	0 (0-0)	3 (0-9)
Cocaine use disorders	-	-	-
Amphetamine use disorders	-	-	-
Other drug use disorders	-	-	-
Diabetes and kidney diseases	9 (6-11)	29 (21-39)	5,990 (5,390-6,578)
Diabetes mellitus	9 (6-11)	29 (21-39)	657 (463-838)
Diabetes mellitus type 1	9 (6-11)	29 (21-39)	657 (463-838)
Diabetes mellitus type 2	-	-	-
Chronic kidney disease	-	-	5,071 (4,547-5,607)
Chronic kidney disease due to diabetes mellitus type 1	-	-	21 (12-35)
Chronic kidney disease due to diabetes mellitus type 2	-	-	14 (7-25)
Chronic kidney disease due to hypertension	-	-	24 (14-37)
Chronic kidney disease due to glomerulonephritis	-	-	1,250 (880-1,703)
Chronic kidney disease due to other and unspecified causes	-	-	3,760 (3,199-4,288)
Acute glomerulonephritis	-	-	262 (183-320)
Skin and subcutaneous diseases	-	-	2,478 (728-3,616)
Bacterial skin diseases	-	-	2,439 (702-3,569)
Cellulitis	-	-	237 (25-575)
Pyoderma	-	-	2,202 (655-3,225)
Decubitus ulcer	-	-	-
Other skin and subcutaneous diseases	-	-	38 (10-85)
Musculoskeletal disorders	-	-	-
Rheumatoid arthritis	-	-	-
Other musculoskeletal disorders	-	-	-
Other non-communicable diseases	93,805 (83,583-107,814)	50,533 (44,358-57,075)	177,260 (153,741-218,703)
Congenital birth defects	92,119 (81,959-105,963)	46,286 (39,881-53,599)	142,161 (126,817-158,045)
Neural tube defects	15,056 (11,511-19,253)	5,551 (3,588-8,278)	16,066 (9,245-28,819)
Congenital heart anomalies	31,776 (23,478-40,083)	21,570 (15,042-26,538)	78,506 (52,756-101,289)
Orofacial clefts	2,539 (920-6,403)	1,376 (467-3,371)	2,468 (748-6,791)
Down syndrome	1,693 (850-3,052)	872 (354-1,609)	3,999 (1,466-7,684)
Other chromosomal abnormalities	2,484 (1,023-6,671)	1,594 (646-4,177)	3,396 (1,487-8,681)
Congenital musculoskeletal and limb anomalies	2,245 (1,066-3,610)	820 (273-1,563)	2,093 (672-4,542)
Urogenital congenital anomalies	2,223 (1,463-3,009)	851 (501-1,250)	1,373 (642-2,407)
Digestive congenital anomalies	8,380 (4,450-13,795)	4,623 (2,062-7,821)	10,912 (4,010-20,677)
Other congenital birth defects	25,722 (16,625-37,827)	9,029 (4,509-13,889)	23,348 (10,429-40,461)
Urinary diseases and male infertility	256 (225-296)	248 (204-301)	2,838 (2,538-3,246)
Urinary tract infections	43 (30-65)	58 (33-85)	1,672 (1,211-2,163)
Urolithiasis	-	-	-

Cause	1 to 4	5 to 9	10 to 14	15 to 19	20 to 24	25 to 29	30 to 34	35 to 39	40 to 44	45 to 49
Mesothelioma	-	-	-	45 (23-73)	76 (36-129)	110 (57-175)	123 (68-189)	192 (117-290)	300 (201-431)	228 (163-312)
Hodgkin lymphoma	273 (93-504)	350 (167-503)	474 (268-629)	945 (584-1,175)	1,124 (728-1,369)	1,162 (763-1,403)	944 (630-1,126)	759 (520-885)	602 (415-695)	499 (353-565)
Non-Hodgkin lymphoma	2,090 (1,290-2,834)	1,178 (935-1,421)	878 (763-998)	988 (886-1,096)	1,272 (1,122-1,432)	1,383 (1,219-1,560)	1,433 (1,281-1,599)	1,684 (1,532-1,862)	1,941 (1,804-2,120)	2,193 (2,058-2,362)
Multiple myeloma	-	-	-	121 (67-153)	147 (78-187)	144 (87-183)	155 (107-194)	278 (214-332)	460 (387-535)	759 (666-883)
Leukemia	8,990 (5,124-17,200)	8,180 (5,536-12,342)	6,190 (4,715-8,047)	5,819 (4,486-6,987)	5,059 (3,747-6,320)	4,815 (3,356-5,933)	4,324 (3,069-5,316)	4,552 (3,406-5,474)	4,393 (3,463-5,075)	4,614 (3,759-5,227)
Acute lymphoid leukemia	1,816 (1,044-4,723)	2,358 (1,575-4,484)	1,682 (1,315-2,539)	1,238 (990-1,791)	809 (636-1,216)	746 (554-1,174)	494 (373-772)	531 (409-787)	458 (371-637)	406 (344-544)
Chronic lymphoid leukemia	-	-	-	79 (53-118)	91 (53-143)	102 (57-167)	94 (54-150)	100 (70-142)	110 (87-140)	132 (109-161)
Acute myeloid leukemia	1,423 (613-4,113)	931 (549-2,041)	927 (660-1,515)	1,015 (774-1,512)	923 (704-1,410)	924 (708-1,433)	887 (696-1,310)	953 (771-1,349)	995 (833-1,338)	1,131 (965-1,463)
Chronic myeloid leukemia	[illegible]	[illegible]	[illegible]	[illegible]	[illegible]	[illegible]	[illegible]	[illegible]	[illegible]	[illegible]
Other leukemia	[illegible]	[illegible]	[illegible]	[illegible]	[illegible]	[illegible]	[illegible]	[illegible]	[illegible]	[illegible]
Other malignant neoplasms	[illegible]	[illegible]	[illegible]	[illegible]	[illegible]	[illegible]	[illegible]	[illegible]	[illegible]	[illegible]
Other neoplasms	[illegible]	[illegible]	[illegible]	[illegible]	[illegible]	[illegible]	[illegible]	[illegible]	[illegible]	[illegible]
Myelodysplastic, myeloproliferative, and other hematopoietic neoplasm	[illegible]	[illegible]	[illegible]	[illegible]	[illegible]	[illegible]	[illegible]	[illegible]	[illegible]	[illegible]
Other benign and in situ neoplasms	[illegible]	[illegible]	[illegible]	[illegible]	[illegible]	[illegible]	[illegible]	[illegible]	[illegible]	[illegible]
Cardiovascular diseases	[illegible]	[illegible]	[illegible]	[illegible]	[illegible]	[illegible]	[illegible]	[illegible]	[illegible]	[illegible]
Rheumatic heart disease	[illegible]	[illegible]	[illegible]	[illegible]	[illegible]	[illegible]	[illegible]	[illegible]	[illegible]	[illegible]
Ischemic heart disease	[illegible]	[illegible]	[illegible]	[illegible]	[illegible]	[illegible]	[illegible]	[illegible]	[illegible]	[illegible]
Stroke	[illegible]	[illegible]	[illegible]	[illegible]	[illegible]	[illegible]	[illegible]	[illegible]	[illegible]	[illegible]
Ischemic stroke	[illegible]	[illegible]	[illegible]	[illegible]	[illegible]	[illegible]	[illegible]	[illegible]	[illegible]	[illegible]
Intracerebral hemorrhage	[illegible]	[illegible]	[illegible]	[illegible]	[illegible]	[illegible]	[illegible]	[illegible]	[illegible]	[illegible]
Subarachnoid hemorrhage	[illegible]	[illegible]	[illegible]	[illegible]	[illegible]	[illegible]	[illegible]	[illegible]	[illegible]	[illegible]
Hypertensive heart disease	[illegible]	[illegible]	[illegible]	[illegible]	[illegible]	[illegible]	[illegible]	[illegible]	[illegible]	[illegible]
Non-rheumatic valvular heart disease	[illegible]	[illegible]	[illegible]	[illegible]	[illegible]	[illegible]	[illegible]	[illegible]	[illegible]	[illegible]
Non-rheumatic calcific aortic valve disease	[illegible]	[illegible]	[illegible]	[illegible]	[illegible]	[illegible]	[illegible]	[illegible]	[illegible]	[illegible]
Non-rheumatic degenerative mitral valve disease	[illegible]	[illegible]	[illegible]	[illegible]	[illegible]	[illegible]	[illegible]	[illegible]	[illegible]	[illegible]
Other non-rheumatic valve diseases	[illegible]	[illegible]	[illegible]	[illegible]	[illegible]	[illegible]	[illegible]	[illegible]	[illegible]	[illegible]
Cardiomyopathy and myocarditis	[illegible]	[illegible]	[illegible]	[illegible]	[illegible]	[illegible]	[illegible]	[illegible]	[illegible]	[illegible]
Myocarditis	[illegible]	[illegible]	[illegible]	[illegible]	[illegible]	[illegible]	[illegible]	[illegible]	[illegible]	[illegible]
Alcoholic cardiomyopathy	[illegible]	[illegible]	[illegible]	[illegible]	[illegible]	[illegible]	[illegible]	[illegible]	[illegible]	[illegible]
Other cardiomyopathy	[illegible]	[illegible]	[illegible]	[illegible]	[illegible]	[illegible]	[illegible]	[illegible]	[illegible]	[illegible]
Atrial fibrillation and flutter	[illegible]	[illegible]	[illegible]	[illegible]	[illegible]	[illegible]	[illegible]	[illegible]	[illegible]	[illegible]
Aortic aneurysm	[illegible]	[illegible]	[illegible]	[illegible]	[illegible]	[illegible]	[illegible]	[illegible]	[illegible]	[illegible]
Peripheral artery disease	[illegible]	[illegible]	[illegible]	[illegible]	[illegible]	[illegible]	[illegible]	[illegible]	[illegible]	[illegible]
Endocarditis	[illegible]	[illegible]	[illegible]	[illegible]	[illegible]	[illegible]	[illegible]	[illegible]	[illegible]	[illegible]
Other cardiovascular and circulatory diseases	[illegible]	[illegible]	[illegible]	[illegible]	[illegible]	[illegible]	[illegible]	[illegible]	[illegible]	[illegible]
Chronic respiratory diseases	[illegible]	[illegible]	[illegible]	[illegible]	[illegible]	[illegible]	[illegible]	[illegible]	[illegible]	[illegible]
Chronic obstructive pulmonary disease	[illegible]	[illegible]	[illegible]	[illegible]	[illegible]	[illegible]	[illegible]	[illegible]	[illegible]	[illegible]
Pneumoconiosis	[illegible]	[illegible]	[illegible]	[illegible]	[illegible]	[illegible]	[illegible]	[illegible]	[illegible]	[illegible]
Silicosis	[illegible]	[illegible]	[illegible]	[illegible]	[illegible]	[illegible]	[illegible]	[illegible]	[illegible]	[illegible]
Asbestosis	[illegible]	[illegible]	[illegible]	[illegible]	[illegible]	[illegible]	[illegible]	[illegible]	[illegible]	[illegible]
Coal workers pneumoconiosis	[illegible]	[illegible]	[illegible]	[illegible]	[illegible]	[illegible]	[illegible]	[illegible]	[illegible]	[illegible]
Other pneumoconiosis	[illegible]	[illegible]	[illegible]	[illegible]	[illegible]	[illegible]	[illegible]	[illegible]	[illegible]	[illegible]
Asthma	[illegible]	[illegible]	[illegible]	[illegible]	[illegible]	[illegible]	[illegible]	[illegible]	[illegible]	[illegible]
Interstitial lung disease and pulmonary sarcoidosis	[illegible]	[illegible]	[illegible]	[illegible]	[illegible]	[illegible]	[illegible]	[illegible]	[illegible]	[illegible]
Other chronic respiratory diseases	[illegible]	[illegible]	[illegible]	[illegible]	[illegible]	[illegible]	[illegible]	[illegible]	[illegible]	[illegible]
Digestive diseases	[illegible]	[illegible]	[illegible]	[illegible]	[illegible]	[illegible]	[illegible]	[illegible]	[illegible]	[illegible]
Cirrhosis and other chronic liver diseases	[illegible]	[illegible]	[illegible]	[illegible]	[illegible]	[illegible]	[illegible]	[illegible]	[illegible]	[illegible]
Cirrhosis and other chronic liver diseases due to hepatitis B	[illegible]	[illegible]	[illegible]	[illegible]	[illegible]	[illegible]	[illegible]	[illegible]	[illegible]	[illegible]
Cirrhosis and other chronic liver diseases due to hepatitis C	[illegible]	[illegible]	[illegible]	[illegible]	[illegible]	[illegible]	[illegible]	[illegible]	[illegible]	[illegible]
Cirrhosis and other chronic liver diseases due to alcohol use	[illegible]	[illegible]	[illegible]	[illegible]	[illegible]	[illegible]	[illegible]	[illegible]	[illegible]	[illegible]
Cirrhosis due to NASH	[illegible]	[illegible]	[illegible]	[illegible]	[illegible]	[illegible]	[illegible]	[illegible]	[illegible]	[illegible]
Cirrhosis and other chronic liver diseases due to other causes	[illegible]	[illegible]	[illegible]	[illegible]	[illegible]	[illegible]	[illegible]	[illegible]	[illegible]	[illegible]
Upper digestive system diseases	[illegible]	[illegible]	[illegible]	[illegible]	[illegible]	[illegible]	[illegible]	[illegible]	[illegible]	[illegible]
Peptic ulcer disease	[illegible]	[illegible]	[illegible]	[illegible]	[illegible]	[illegible]	[illegible]	[illegible]	[illegible]	[illegible]
Gastritis and duodenitis	[illegible]	[illegible]	[illegible]	[illegible]	[illegible]	[illegible]	[illegible]	[illegible]	[illegible]	[illegible]
Appendicitis	[illegible]	[illegible]	[illegible]	[illegible]	[illegible]	[illegible]	[illegible]	[illegible]	[illegible]	[illegible]
Paralytic ileus and intestinal obstruction	[illegible]	[illegible]	[illegible]	[illegible]	[illegible]	[illegible]	[illegible]	[illegible]	[illegible]	[illegible]
Inguinal, femoral, and abdominal hernia	[illegible]	[illegible]	[illegible]	[illegible]	[illegible]	[illegible]	[illegible]	[illegible]	[illegible]	[illegible]
Inflammatory bowel disease	[illegible]	[illegible]	[illegible]	[illegible]	[illegible]	[illegible]	[illegible]	[illegible]	[illegible]	[illegible]
Vascular intestinal disorders	[illegible]	[illegible]	[illegible]	[illegible]	[illegible]	[illegible]	[illegible]	[illegible]	[illegible]	[illegible]
Gallbladder and biliary diseases	[illegible]	[illegible]	[illegible]	[illegible]	[illegible]	[illegible]	[illegible]	[illegible]	[illegible]	[illegible]
Pancreatitis	[illegible]	[illegible]	[illegible]	[illegible]	[illegible]	[illegible]	[illegible]	[illegible]	[illegible]	[illegible]
Other digestive diseases	[illegible]	[illegible]	[illegible]	[illegible]	[illegible]	[illegible]	[illegible]	[illegible]	[illegible]	[illegible]
Neurological disorders	[illegible]	[illegible]	[illegible]	[illegible]	[illegible]	[illegible]	[illegible]	[illegible]	[illegible]	[illegible]
Alzheimer's disease and other dementias	[illegible]	[illegible]	[illegible]	[illegible]	[illegible]	[illegible]	[illegible]	[illegible]	[illegible]	[illegible]
Parkinson's disease	[illegible]	[illegible]	[illegible]	[illegible]	[illegible]	[illegible]	[illegible]	[illegible]	[illegible]	[illegible]
Epilepsy	[illegible]	[illegible]	[illegible]	[illegible]	[illegible]	[illegible]	[illegible]	[illegible]	[illegible]	[illegible]
Multiple sclerosis	[illegible]	[illegible]	[illegible]	[illegible]	[illegible]	[illegible]	[illegible]	[illegible]	[illegible]	[illegible]
Motor neuron disease	[illegible]	[illegible]	[illegible]	[illegible]	[illegible]	[illegible]	[illegible]	[illegible]	[illegible]	[illegible]
Other neurological disorders	[illegible]	[illegible]	[illegible]	[illegible]	[illegible]	[illegible]	[illegible]	[illegible]	[illegible]	[illegible]
Mental disorders	[illegible]	[illegible]	[illegible]	[illegible]	[illegible]	[illegible]	[illegible]	[illegible]	[illegible]	[illegible]
Eating disorders	[illegible]	[illegible]	[illegible]	[illegible]	[illegible]	[illegible]	[illegible]	[illegible]	[illegible]	[illegible]
Anorexia nervosa	[illegible]	[illegible]	[illegible]	[illegible]	[illegible]	[illegible]	[illegible]	[illegible]	[illegible]	[illegible]
Bulimia nervosa	[illegible]	[illegible]	[illegible]	[illegible]	[illegible]	[illegible]	[illegible]	[illegible]	[illegible]	[illegible]
Substance use disorders	[illegible]	[illegible]	[illegible]	[illegible]	[illegible]	[illegible]	[illegible]	[illegible]	[illegible]	[illegible]
Alcohol use disorders	[illegible]	[illegible]	[illegible]	[illegible]	[illegible]	[illegible]	[illegible]	[illegible]	[illegible]	[illegible]
Drug use disorders	[illegible]	[illegible]	[illegible]	[illegible]	[illegible]	[illegible]	[illegible]	[illegible]	[illegible]	[illegible]
Opioid use disorders	[illegible]	[illegible]	[illegible]	[illegible]	[illegible]	[illegible]	[illegible]	[illegible]	[illegible]	[illegible]
Cocaine use disorders	[illegible]	[illegible]	[illegible]	[illegible]	[illegible]	[illegible]	[illegible]	[illegible]	[illegible]	[illegible]
Amphetamine use disorders	[illegible]	[illegible]	[illegible]	[illegible]	[illegible]	[illegible]	[illegible]	[illegible]	[illegible]	[illegible]
Other drug use disorders	[illegible]	[illegible]	[illegible]	[illegible]	[illegible]	[illegible]	[illegible]	[illegible]	[illegible]	[illegible]
Diabetes and kidney diseases	[illegible]	[illegible]	[illegible]	[illegible]	[illegible]	[illegible]	[illegible]	[illegible]	[illegible]	[illegible]
Diabetes mellitus	[illegible]	[illegible]	[illegible]	[illegible]	[illegible]	[illegible]	[illegible]	[illegible]	[illegible]	[illegible]
Diabetes mellitus type 1	[illegible]	[illegible]	[illegible]	[illegible]	[illegible]	[illegible]	[illegible]	[illegible]	[illegible]	[illegible]
Diabetes mellitus type 2	[illegible]	[illegible]	[illegible]	[illegible]	[illegible]	[illegible]	[illegible]	[illegible]	[illegible]	[illegible]
Chronic kidney disease	[illegible]	[illegible]	[illegible]	[illegible]	[illegible]	[illegible]	[illegible]	[illegible]	[illegible]	[illegible]
Chronic kidney disease due to diabetes mellitus type 1	[illegible]	[illegible]	[illegible]	[illegible]	[illegible]	[illegible]	[illegible]	[illegible]	[illegible]	[illegible]
Chronic kidney disease due to diabetes mellitus type 2	[illegible]	[illegible]	[illegible]	[illegible]	[illegible]	[illegible]	[illegible]	[illegible]	[illegible]	[illegible]
Chronic kidney disease due to hypertension	[illegible]	[illegible]	[illegible]	[illegible]	[illegible]	[illegible]	[illegible]	[illegible]	[illegible]	[illegible]
Chronic kidney disease due to glomerulonephritis	[illegible]	[illegible]	[illegible]	[illegible]	[illegible]	[illegible]	[illegible]	[illegible]	[illegible]	[illegible]
Chronic kidney disease due to other and unspecified causes	[illegible]	[illegible]	[illegible]	[illegible]	[illegible]	[illegible]	[illegible]	[illegible]	[illegible]	[illegible]
Acute glomerulonephritis	[illegible]	[illegible]	[illegible]	[illegible]	[illegible]	[illegible]	[illegible]	[illegible]	[illegible]	[illegible]
Skin and subcutaneous diseases	[illegible]	[illegible]	[illegible]	[illegible]	[illegible]	[illegible]	[illegible]	[illegible]	[illegible]	[illegible]
Bacterial skin diseases	[illegible]	[illegible]	[illegible]	[illegible]	[illegible]	[illegible]	[illegible]	[illegible]	[illegible]	[illegible]
Cellulitis	[illegible]	[illegible]	[illegible]	[illegible]	[illegible]	[illegible]	[illegible]	[illegible]	[illegible]	[illegible]
Pyoderma	[illegible]	[illegible]	[illegible]	[illegible]	[illegible]	[illegible]	[illegible]	[illegible]	[illegible]	[illegible]
Decubitus ulcer	[illegible]	[illegible]	[illegible]	[illegible]	[illegible]	[illegible]	[illegible]	[illegible]	[illegible]	[illegible]
Other skin and subcutaneous diseases	[illegible]	[illegible]	[illegible]	[illegible]	[illegible]	[illegible]	[illegible]	[illegible]	[illegible]	[illegible]
Musculoskeletal disorders	[illegible]	[illegible]	[illegible]	[illegible]	[illegible]	[illegible]	[illegible]	[illegible]	[illegible]	[illegible]
Rheumatoid arthritis	[illegible]	[illegible]	[illegible]	[illegible]	[illegible]	[illegible]	[illegible]	[illegible]	[illegible]	[illegible]
Other musculoskeletal disorders	[illegible]	[illegible]	[illegible]	[illegible]	[illegible]	[illegible]	[illegible]	[illegible]	[illegible]	[illegible]
Other non-communicable diseases	[illegible]	[illegible]	[illegible]	[illegible]	[illegible]	[illegible]	[illegible]	[illegible]	[illegible]	[illegible]
Congenital birth defects	[illegible]	[illegible]	[illegible]	[illegible]	[illegible]	[illegible]	[illegible]	[illegible]	[illegible]	[illegible]
Neural tube defects	[illegible]	[illegible]	[illegible]	[illegible]	[illegible]	[illegible]	[illegible]	[illegible]	[illegible]	[illegible]
Congenital heart anomalies	[illegible]	[illegible]	[illegible]	[illegible]	[illegible]	[illegible]	[illegible]	[illegible]	[illegible]	[illegible]
Orofacial clefts	[illegible]	[illegible]	[illegible]	[illegible]	[illegible]	[illegible]	[illegible]	[illegible]	[illegible]	[illegible]
Down syndrome	[illegible]	[illegible]	[illegible]	[illegible]	[illegible]	[illegible]	[illegible]	[illegible]	[illegible]	[illegible]
Other chromosomal abnormalities	[illegible]	[illegible]	[illegible]	[illegible]	[illegible]	[illegible]	[illegible]	[illegible]	[illegible]	[illegible]
Congenital musculoskeletal and limb anomalies	[illegible]	[illegible]	[illegible]	[illegible]	[illegible]	[illegible]	[illegible]	[illegible]	[illegible]	[illegible]
Urogenital congenital anomalies	[illegible]	[illegible]	[illegible]	[illegible]	[illegible]	[illegible]	[illegible]	[illegible]	[illegible]	[illegible]
Digestive congenital anomalies	[illegible]	[illegible]	[illegible]	[illegible]	[illegible]	[illegible]	[illegible]	[illegible]	[illegible]	[illegible]
Other congenital birth defects	[illegible]	[illegible]	[illegible]	[illegible]	[illegible]	[illegible]	[illegible]	[illegible]	[illegible]	[illegible]
Urinary diseases and male infertility	[illegible]	[illegible]	[illegible]	[illegible]	[illegible]	[illegible]	[illegible]	[illegible]	[illegible]	[illegible]
Urinary tract infections	[illegible]	[illegible]	[illegible]	[illegible]	[illegible]	[illegible]	[illegible]	[illegible]	[illegible]	[illegible]
Urolithiasis	[illegible]	[illegible]	[illegible]	[illegible]	[illegible]	[illegible]	[illegible]	[illegible]	[illegible]	[illegible]

Cause	50 to 54	54 to 59	60 to 64	65 to 69	70 to 74	75 to 79	80 to 84	85 to 89	90 to 94	95 plus
Mesothelioma	337 (262-442)	484 (380-628)	600 (476-763)	678 (541-849)	644 (529-798)	720 (605-866)	521 (436-624)	258 (223-300)	87 (78-101)	19 (17-21)
Hodgkin lymphoma	588 (411-666)	649 (448-739)	790 (559-895)	917 (651-1,034)	825 (593-941)	797 (568-900)	549 (394-624)	233 (175-264)	56 (40-63)	8 (6-9)
Non-Hodgkin lymphoma	3,043 (2,887-3,250)	3,987 (3,803-4,220)	5,735 (5,482-6,016)	6,992 (6,731-7,276)	6,955 (6,689-7,205)	6,883 (6,650-7,087)	5,514 (5,329-5,669)	2,994 (2,897-3,075)	966 (939-990)	198 (192-204)
Multiple myeloma	1,358 (1,211-1,542)	2,134 (1,997-2,394)	3,266 (2,963-3,704)	4,041 (3,828-4,672)	3,962 (3,759-4,664)	4,261 (4,074-5,140)	3,298 (3,150-4,075)	1,719 (1,633-2,204)	563 (534-727)	92 (86-117)
Leukemia	5,509 (4,660-6,368)	6,523 (5,778-7,417)	8,168 (7,410-9,390)	8,900 (8,236-10,216)	8,588 (7,990-9,868)	9,188 (8,735-10,368)	7,582 (7,298-8,360)	4,527 (4,385-4,887)	1,753 (1,703-1,858)	442 (428-458)
Acute lymphoid leukemia	463 (407-626)	551 (489-734)	625 (558-857)	570 (513-741)	488 (440-637)	452 (417-551)	323 (295-401)	164 (151-193)	51 (47-58)	11 (10-12)
Chronic lymphoid leukemia	251 (222-287)	408 (374-457)	768 (710-843)	1,002 (942-1,106)	1,106 (1,052-1,178)	1,581 (1,493-1,677)	1,480 (1,399-1,575)	1,034 (969-1,093)	448 (425-471)	127 (121-134)
Acute myeloid leukemia	1,295 (1,145-1,591)	1,519 (1,362-1,839)	1,974 (1,782-2,381)	2,173 (1,975-2,632)	2,061 (1,887-2,470)	2,156 (1,993-2,524)	1,606 (1,491-1,982)	813 (764-1,030)	273 (256-355)	62 (58-78)
Chronic myeloid leukemia	[illegible]	[illegible]	[illegible]	[illegible]	[illegible]	[illegible]	[illegible]	[illegible]	[illegible]	[illegible]
Other leukemia	[illegible]	[illegible]	[illegible]	[illegible]	[illegible]	[illegible]	[illegible]	[illegible]	[illegible]	[illegible]
Other malignant neoplasms	[illegible]	[illegible]	[illegible]	[illegible]	[illegible]	[illegible]	[illegible]	[illegible]	[illegible]	[illegible]
Other neoplasms	[illegible]	[illegible]	[illegible]	[illegible]	[illegible]	[illegible]	[illegible]	[illegible]	[illegible]	[illegible]
Myelodysplastic, myeloproliferative, and other hematopoietic neoplasm	[illegible]	[illegible]	[illegible]	[illegible]	[illegible]	[illegible]	[illegible]	[illegible]	[illegible]	[illegible]
Other benign and in situ neoplasms	[illegible]	[illegible]	[illegible]	[illegible]	[illegible]	[illegible]	[illegible]	[illegible]	[illegible]	[illegible]
Cardiovascular diseases	[illegible]	[illegible]	[illegible]	[illegible]	[illegible]	[illegible]	[illegible]	[illegible]	[illegible]	[illegible]
Rheumatic heart disease	[illegible]	[illegible]	[illegible]	[illegible]	[illegible]	[illegible]	[illegible]	[illegible]	[illegible]	[illegible]
Ischemic heart disease	[illegible]	[illegible]	[illegible]	[illegible]	[illegible]	[illegible]	[illegible]	[illegible]	[illegible]	[illegible]
Stroke	[illegible]	[illegible]	[illegible]	[illegible]	[illegible]	[illegible]	[illegible]	[illegible]	[illegible]	[illegible]
Ischemic stroke	[illegible]	[illegible]	[illegible]	[illegible]	[illegible]	[illegible]	[illegible]	[illegible]	[illegible]	[illegible]
Intracerebral hemorrhage	[illegible]	[illegible]	[illegible]	[illegible]	[illegible]	[illegible]	[illegible]	[illegible]	[illegible]	[illegible]
Subarachnoid hemorrhage	[illegible]	[illegible]	[illegible]	[illegible]	[illegible]	[illegible]	[illegible]	[illegible]	[illegible]	[illegible]
Hypertensive heart disease	[illegible]	[illegible]	[illegible]	[illegible]	[illegible]	[illegible]	[illegible]	[illegible]	[illegible]	[illegible]
Non-rheumatic valvular heart disease	[illegible]	[illegible]	[illegible]	[illegible]	[illegible]	[illegible]	[illegible]	[illegible]	[illegible]	[illegible]
Non-rheumatic calcific aortic valve disease	[illegible]	[illegible]	[illegible]	[illegible]	[illegible]	[illegible]	[illegible]	[illegible]	[illegible]	[illegible]
Non-rheumatic degenerative mitral valve disease	[illegible]	[illegible]	[illegible]	[illegible]	[illegible]	[illegible]	[illegible]	[illegible]	[illegible]	[illegible]
Other non-rheumatic valve diseases	[illegible]	[illegible]	[illegible]	[illegible]	[illegible]	[illegible]	[illegible]	[illegible]	[illegible]	[illegible]
Cardiomyopathy and myocarditis	[illegible]	[illegible]	[illegible]	[illegible]	[illegible]	[illegible]	[illegible]	[illegible]	[illegible]	[illegible]
Myocarditis	[illegible]	[illegible]	[illegible]	[illegible]	[illegible]	[illegible]	[illegible]	[illegible]	[illegible]	[illegible]
Alcoholic cardiomyopathy	[illegible]	[illegible]	[illegible]	[illegible]	[illegible]	[illegible]	[illegible]	[illegible]	[illegible]	[illegible]
Other cardiomyopathy	[illegible]	[illegible]	[illegible]	[illegible]	[illegible]	[illegible]	[illegible]	[illegible]	[illegible]	[illegible]
Atrial fibrillation and flutter	[illegible]	[illegible]	[illegible]	[illegible]	[illegible]	[illegible]	[illegible]	[illegible]	[illegible]	[illegible]
Aortic aneurysm	[illegible]	[illegible]	[illegible]	[illegible]	[illegible]	[illegible]	[illegible]	[illegible]	[illegible]	[illegible]
Peripheral artery disease	[illegible]	[illegible]	[illegible]	[illegible]	[illegible]	[illegible]	[illegible]	[illegible]	[illegible]	[illegible]
Endocarditis	[illegible]	[illegible]	[illegible]	[illegible]	[illegible]	[illegible]	[illegible]	[illegible]	[illegible]	[illegible]
Other cardiovascular and circulatory diseases	[illegible]	[illegible]	[illegible]	[illegible]	[illegible]	[illegible]	[illegible]	[illegible]	[illegible]	[illegible]
Chronic respiratory diseases	[illegible]	[illegible]	[illegible]	[illegible]	[illegible]	[illegible]	[illegible]	[illegible]	[illegible]	[illegible]
Chronic obstructive pulmonary disease	[illegible]	[illegible]	[illegible]	[illegible]	[illegible]	[illegible]	[illegible]	[illegible]	[illegible]	[illegible]
Pneumoconiosis	[illegible]	[illegible]	[illegible]	[illegible]	[illegible]	[illegible]	[illegible]	[illegible]	[illegible]	[illegible]
Silicosis	[illegible]	[illegible]	[illegible]	[illegible]	[illegible]	[illegible]	[illegible]	[illegible]	[illegible]	[illegible]
Asbestosis	[illegible]	[illegible]	[illegible]	[illegible]	[illegible]	[illegible]	[illegible]	[illegible]	[illegible]	[illegible]
Coal workers pneumoconiosis	[illegible]	[illegible]	[illegible]	[illegible]	[illegible]	[illegible]	[illegible]	[illegible]	[illegible]	[illegible]
Other pneumoconiosis	[illegible]	[illegible]	[illegible]	[illegible]	[illegible]	[illegible]	[illegible]	[illegible]	[illegible]	[illegible]
Asthma	[illegible]	[illegible]	[illegible]	[illegible]	[illegible]	[illegible]	[illegible]	[illegible]	[illegible]	[illegible]
Interstitial lung disease and pulmonary sarcoidosis	[illegible]	[illegible]	[illegible]	[illegible]	[illegible]	[illegible]	[illegible]	[illegible]	[illegible]	[illegible]
Other chronic respiratory diseases	[illegible]	[illegible]	[illegible]	[illegible]	[illegible]	[illegible]	[illegible]	[illegible]	[illegible]	[illegible]
Digestive diseases	[illegible]	[illegible]	[illegible]	[illegible]	[illegible]	[illegible]	[illegible]	[illegible]	[illegible]	[illegible]
Cirrhosis and other chronic liver diseases	[illegible]	[illegible]	[illegible]	[illegible]	[illegible]	[illegible]	[illegible]	[illegible]	[illegible]	[illegible]
Cirrhosis and other chronic liver diseases due to hepatitis B	[illegible]	[illegible]	[illegible]	[illegible]	[illegible]	[illegible]	[illegible]	[illegible]	[illegible]	[illegible]
Cirrhosis and other chronic liver diseases due to hepatitis C	[illegible]	[illegible]	[illegible]	[illegible]	[illegible]	[illegible]	[illegible]	[illegible]	[illegible]	[illegible]
Cirrhosis and other chronic liver diseases due to alcohol use	[illegible]	[illegible]	[illegible]	[illegible]	[illegible]	[illegible]	[illegible]	[illegible]	[illegible]	[illegible]
Cirrhosis due to NASH	[illegible]	[illegible]	[illegible]	[illegible]	[illegible]	[illegible]	[illegible]	[illegible]	[illegible]	[illegible]
Cirrhosis and other chronic liver diseases due to other causes	[illegible]	[illegible]	[illegible]	[illegible]	[illegible]	[illegible]	[illegible]	[illegible]	[illegible]	[illegible]
Upper digestive system diseases	[illegible]	[illegible]	[illegible]	[illegible]	[illegible]	[illegible]	[illegible]	[illegible]	[illegible]	[illegible]
Peptic ulcer disease	[illegible]	[illegible]	[illegible]	[illegible]	[illegible]	[illegible]	[illegible]	[illegible]	[illegible]	[illegible]
Gastritis and duodenitis	[illegible]	[illegible]	[illegible]	[illegible]	[illegible]	[illegible]	[illegible]	[illegible]	[illegible]	[illegible]
Appendicitis	[illegible]	[illegible]	[illegible]	[illegible]	[illegible]	[illegible]	[illegible]	[illegible]	[illegible]	[illegible]
Paralytic ileus and intestinal obstruction	[illegible]	[illegible]	[illegible]	[illegible]	[illegible]	[illegible]	[illegible]	[illegible]	[illegible]	[illegible]
Inguinal, femoral, and abdominal hernia	[illegible]	[illegible]	[illegible]	[illegible]	[illegible]	[illegible]	[illegible]	[illegible]	[illegible]	[illegible]
Inflammatory bowel disease	[illegible]	[illegible]	[illegible]	[illegible]	[illegible]	[illegible]	[illegible]	[illegible]	[illegible]	[illegible]
Vascular intestinal disorders	[illegible]	[illegible]	[illegible]	[illegible]	[illegible]	[illegible]	[illegible]	[illegible]	[illegible]	[illegible]
Gallbladder and biliary diseases	[illegible]	[illegible]	[illegible]	[illegible]	[illegible]	[illegible]	[illegible]	[illegible]	[illegible]	[illegible]
Pancreatitis	[illegible]	[illegible]	[illegible]	[illegible]	[illegible]	[illegible]	[illegible]	[illegible]	[illegible]	[illegible]
Other digestive diseases	[illegible]	[illegible]	[illegible]	[illegible]	[illegible]	[illegible]	[illegible]	[illegible]	[illegible]	[illegible]
Neurological disorders	[illegible]	[illegible]	[illegible]	[illegible]	[illegible]	[illegible]	[illegible]	[illegible]	[illegible]	[illegible]
Alzheimer's disease and other dementias	[illegible]	[illegible]	[illegible]	[illegible]	[illegible]	[illegible]	[illegible]	[illegible]	[illegible]	[illegible]
Parkinson's disease	[illegible]	[illegible]	[illegible]	[illegible]	[illegible]	[illegible]	[illegible]	[illegible]	[illegible]	[illegible]
Epilepsy	[illegible]	[illegible]	[illegible]	[illegible]	[illegible]	[illegible]	[illegible]	[illegible]	[illegible]	[illegible]
Multiple sclerosis	[illegible]	[illegible]	[illegible]	[illegible]	[illegible]	[illegible]	[illegible]	[illegible]	[illegible]	[illegible]
Motor neuron disease	[illegible]	[illegible]	[illegible]	[illegible]	[illegible]	[illegible]	[illegible]	[illegible]	[illegible]	[illegible]
Other neurological disorders	[illegible]	[illegible]	[illegible]	[illegible]	[illegible]	[illegible]	[illegible]	[illegible]	[illegible]	[illegible]
Mental disorders	[illegible]	[illegible]	[illegible]	[illegible]	[illegible]	[illegible]	[illegible]	[illegible]	[illegible]	[illegible]
Eating disorders	[illegible]	[illegible]	[illegible]	[illegible]	[illegible]	[illegible]	[illegible]	[illegible]	[illegible]	[illegible]
Anorexia nervosa	[illegible]	[illegible]	[illegible]	[illegible]	[illegible]	[illegible]	[illegible]	[illegible]	[illegible]	[illegible]
Bulimia nervosa	[illegible]	[illegible]	[illegible]	[illegible]	[illegible]	[illegible]	[illegible]	[illegible]	[illegible]	[illegible]
Substance use disorders	[illegible]	[illegible]	[illegible]	[illegible]	[illegible]	[illegible]	[illegible]	[illegible]	[illegible]	[illegible]
Alcohol use disorders	[illegible]	[illegible]	[illegible]	[illegible]	[illegible]	[illegible]	[illegible]	[illegible]	[illegible]	[illegible]
Drug use disorders	[illegible]	[illegible]	[illegible]	[illegible]	[illegible]	[illegible]	[illegible]	[illegible]	[illegible]	[illegible]
Opioid use disorders	[illegible]	[illegible]	[illegible]	[illegible]	[illegible]	[illegible]	[illegible]	[illegible]	[illegible]	[illegible]
Cocaine use disorders	[illegible]	[illegible]	[illegible]	[illegible]	[illegible]	[illegible]	[illegible]	[illegible]	[illegible]	[illegible]
Amphetamine use disorders	[illegible]	[illegible]	[illegible]	[illegible]	[illegible]	[illegible]	[illegible]	[illegible]	[illegible]	[illegible]
Other drug use disorders	[illegible]	[illegible]	[illegible]	[illegible]	[illegible]	[illegible]	[illegible]	[illegible]	[illegible]	[illegible]
Diabetes and kidney diseases	[illegible]	[illegible]	[illegible]	[illegible]	[illegible]	[illegible]	[illegible]	[illegible]	[illegible]	[illegible]
Diabetes mellitus	[illegible]	[illegible]	[illegible]	[illegible]	[illegible]	[illegible]	[illegible]	[illegible]	[illegible]	[illegible]
Diabetes mellitus type 1	[illegible]	[illegible]	[illegible]	[illegible]	[illegible]	[illegible]	[illegible]	[illegible]	[illegible]	[illegible]
Diabetes mellitus type 2	[illegible]	[illegible]	[illegible]	[illegible]	[illegible]	[illegible]	[illegible]	[illegible]	[illegible]	[illegible]
Chronic kidney disease	[illegible]	[illegible]	[illegible]	[illegible]	[illegible]	[illegible]	[illegible]	[illegible]	[illegible]	[illegible]
Chronic kidney disease due to diabetes mellitus type 1	[illegible]	[illegible]	[illegible]	[illegible]	[illegible]	[illegible]	[illegible]	[illegible]	[illegible]	[illegible]
Chronic kidney disease due to diabetes mellitus type 2	[illegible]	[illegible]	[illegible]	[illegible]	[illegible]	[illegible]	[illegible]	[illegible]	[illegible]	[illegible]
Chronic kidney disease due to hypertension	[illegible]	[illegible]	[illegible]	[illegible]	[illegible]	[illegible]	[illegible]	[illegible]	[illegible]	[illegible]
Chronic kidney disease due to glomerulonephritis	[illegible]	[illegible]	[illegible]	[illegible]	[illegible]	[illegible]	[illegible]	[illegible]	[illegible]	[illegible]
Chronic kidney disease due to other and unspecified causes	[illegible]	[illegible]	[illegible]	[illegible]	[illegible]	[illegible]	[illegible]	[illegible]	[illegible]	[illegible]
Acute glomerulonephritis	[illegible]	[illegible]	[illegible]	[illegible]	[illegible]	[illegible]	[illegible]	[illegible]	[illegible]	[illegible]
Skin and subcutaneous diseases	[illegible]	[illegible]	[illegible]	[illegible]	[illegible]	[illegible]	[illegible]	[illegible]	[illegible]	[illegible]
Bacterial skin diseases	[illegible]	[illegible]	[illegible]	[illegible]	[illegible]	[illegible]	[illegible]	[illegible]	[illegible]	[illegible]
Cellulitis	[illegible]	[illegible]	[illegible]	[illegible]	[illegible]	[illegible]	[illegible]	[illegible]	[illegible]	[illegible]
Pyoderma	[illegible]	[illegible]	[illegible]	[illegible]	[illegible]	[illegible]	[illegible]	[illegible]	[illegible]	[illegible]
Decubitus ulcer	[illegible]	[illegible]	[illegible]	[illegible]	[illegible]	[illegible]	[illegible]	[illegible]	[illegible]	[illegible]
Other skin and subcutaneous diseases	[illegible]	[illegible]	[illegible]	[illegible]	[illegible]	[illegible]	[illegible]	[illegible]	[illegible]	[illegible]
Musculoskeletal disorders	[illegible]	[illegible]	[illegible]	[illegible]	[illegible]	[illegible]	[illegible]	[illegible]	[illegible]	[illegible]
Rheumatoid arthritis	[illegible]	[illegible]	[illegible]	[illegible]	[illegible]	[illegible]	[illegible]	[illegible]	[illegible]	[illegible]
Other musculoskeletal disorders	[illegible]	[illegible]	[illegible]	[illegible]	[illegible]	[illegible]	[illegible]	[illegible]	[illegible]	[illegible]
Other non-communicable diseases	[illegible]	[illegible]	[illegible]	[illegible]	[illegible]	[illegible]	[illegible]	[illegible]	[illegible]	[illegible]
Congenital birth defects	[illegible]	[illegible]	[illegible]	[illegible]	[illegible]	[illegible]	[illegible]	[illegible]	[illegible]	[illegible]
Neural tube defects	[illegible]	[illegible]	[illegible]	[illegible]	[illegible]	[illegible]	[illegible]	[illegible]	[illegible]	[illegible]
Congenital heart anomalies	[illegible]	[illegible]	[illegible]	[illegible]	[illegible]	[illegible]	[illegible]	[illegible]	[illegible]	[illegible]
Orofacial clefts	[illegible]	[illegible]	[illegible]	[illegible]	[illegible]	[illegible]	[illegible]	[illegible]	[illegible]	[illegible]
Down syndrome	[illegible]	[illegible]	[illegible]	[illegible]	[illegible]	[illegible]	[illegible]	[illegible]	[illegible]	[illegible]
Other chromosomal abnormalities	[illegible]	[illegible]	[illegible]	[illegible]	[illegible]	[illegible]	[illegible]	[illegible]	[illegible]	[illegible]
Congenital musculoskeletal and limb anomalies	[illegible]	[illegible]	[illegible]	[illegible]	[illegible]	[illegible]	[illegible]	[illegible]	[illegible]	[illegible]
Urogenital congenital anomalies	[illegible]	[illegible]	[illegible]	[illegible]	[illegible]	[illegible]	[illegible]	[illegible]	[illegible]	[illegible]
Digestive congenital anomalies	[illegible]	[illegible]	[illegible]	[illegible]	[illegible]	[illegible]	[illegible]	[illegible]	[illegible]	[illegible]
Other congenital birth defects	[illegible]	[illegible]	[illegible]	[illegible]	[illegible]	[illegible]	[illegible]	[illegible]	[illegible]	[illegible]
Urinary diseases and male infertility	[illegible]	[illegible]	[illegible]	[illegible]	[illegible]	[illegible]	[illegible]	[illegible]	[illegible]	[illegible]
Urinary tract infections	[illegible]	[illegible]	[illegible]	[illegible]	[illegible]	[illegible]	[illegible]	[illegible]	[illegible]	[illegible]
Urolithiasis	[illegible]	[illegible]	[illegible]	[illegible]	[illegible]	[illegible]	[illegible]	[illegible]	[illegible]	[illegible]

SR Table 5: Global deaths by cause and age group for females (with 95% confidence intervals), 1990

Cause	Early Neonatal	Late Neonatal	Post Neonatal	1 to 4	5 to 9	10 to 14
Other urinary diseases	213 (170-251)	190 (136-235)	1,166 (622-1,643)	2,061 (1,171-2,866	543 (360-750)	348 (261-457)
Gynecological diseases	-	-	-	-	-	-
Uterine fibroids	-	-	-	-	-	-
Polycystic ovarian syndrome	-	-	-	-	-	-
Endometriosis	-	-	-	-	-	-
Genital prolapse	-	-	-	-	-	-
Other gynecological diseases	-	-	-	-	-	-
Hemoglobinopathies and hemolytic anemias	495 (390-677)	383 (280-539)	3,861 (2,113-6,419	11,027 (4,214-17,880	2,934 (1,870-4,068	2,037 (1,617-2,789
Thalassemias	235 (168-326)	168 (114-239)	1,810 (994-2,937)	2,931 (1,299-4,347	1,265 (829-1,669)	655 (482-823)
Sickle cell disorders	226 (178-327)	204 (148-294)	1,962 (1,024-3,378	7,825 (2,673-13,193	1,434 (846-2,144)	1,042 (740-1,544)
G6PD deficiency	4 (3-6)	3 (2-5)	38 (18-72)	149 (58-272)	113 (73-176)	157 (105-235)
Other hemoglobinopathies and hemolytic anemias	29 (22-40)	7 (5-9)	51 (29-84)	121 (58-195)	122 (89-163)	183 (146-245)
Endocrine, metabolic, blood, and immune disorders	936 (571-1,114)	914 (498-1,178	5,279 (2,565-7,455	3,223 (1,443-5,035	1,097 (728-1,413)	766 (588-960)
Sudden infant death syndrome	-	2,702 (913-6,102	23,121 (7,660-56,777	-	-	-
Injuries	8,425 (7,098-9,503	8,043 (6,701-9,273	71,738 (62,646-79,532	188,100 (145,673-214,649	83,007 (73,388-91,758	53,921 (48,827-58,299
Transport injuries	684 (591-796)	1,164 (835-1,420)	10,565 (8,206-11,972	39,198 (25,866-46,115	24,687 (21,351-27,629	14,047 (12,583-15,370
Road injuries	601 (519-702)	1,071 (733-1,314)	9,771 (7,307-11,136	36,723 (23,031-43,291	23,680 (20,489-26,519	13,187 (11,781-14,463
Pedestrian road injuries	332 (251-409)	600 (388-879)	4,572 (3,558-6,147	23,100 (16,481-31,062	15,344 (13,153-17,502	7,216 (6,353-8,063
Cyclist road injuries	-	-	-	617 (292-1,004)	609 (450-826)	603 (497-747)
Motorcyclist road injuries	68 (45-101)	52 (34-78)	1,075 (640-1,643)	1,891 (980-3,584)	1,292 (890-1,935)	1,222 (875-1,734)
Motor vehicle road injuries	193 (150-236)	411 (223-594)	4,008 (2,435-5,083	10,863 (4,692-14,814	6,265 (4,997-7,387	4,043 (3,427-4,564
Other road injuries	8 (5-12)	8 (4-11)	117 (53-190)	252 (86-456)	170 (96-233)	104 (71-136)
Other transport injuries	83 (57-127)	94 (54-193)	794 (585-1,281)	2,475 (1,683-4,510	1,007 (775-1,305)	860 (711-998)
Unintentional injuries	5,696 (4,749-6,587	6,022 (4,992-7,047	56,164 (49,088-63,029	135,484 (105,682-157,209	50,496 (43,325-57,143	27,683 (23,977-30,589
Falls	839 (713-1,050)	401 (306-575)	5,283 (4,003-7,048	8,209 (5,442-12,330	3,291 (2,542-4,165	1,860 (1,556-2,196
Drowning	271 (241-331)	255 (216-286)	7,502 (5,902-8,454	77,688 (58,608-90,293	26,705 (22,616-30,464	12,065 (10,324-13,341
Fire, heat, and hot substances	213 (140-271)	419 (195-560)	5,310 (2,839-6,528	15,211 (6,986-20,008	4,014 (2,715-4,814	2,492 (1,708-3,038
Poisonings	123 (95-147)	159 (110-184)	2,261 (1,396-2,833	5,771 (3,265-7,978	1,575 (1,199-1,925	1,380 (1,124-1,734
Poisoning by carbon monoxide	29 (19-38)	41 (28-50)	540 (333-670)	1,344 (961-1,721)	713 (591-926)	551 (462-765)
Poisoning by other means	93 (74-111)	118 (79-142)	1,721 (1,028-2,291	4,427 (2,246-6,596	862 (587-1,089)	829 (645-1,026)
Exposure to mechanical forces	365 (228-459)	459 (244-609)	2,634 (1,707-3,195	3,414 (2,316-4,210	2,406 (1,706-2,954	1,153 (887-1,319)
Unintentional firearm injuries	35 (21-45)	26 (14-37)	265 (126-361)	376 (215-514)	303 (205-379)	232 (178-268)
Other exposure to mechanical forces	330 (202-419)	433 (227-576)	2,369 (1,528-2,882	3,038 (2,007-3,772	2,103 (1,457-2,618	921 (691-1,072)
Adverse effects of medical treatment	876 (535-1,079)	760 (408-1,040)	5,395 (2,712-7,899	3,792 (1,436-6,922	1,317 (761-1,753)	838 (551-1,014)
Animal contact	131 (42-188)	151 (50-225)	1,854 (624-2,771)	4,692 (1,536-9,234	4,399 (1,508-7,466	2,713 (957-3,893)
Venomous animal contact	87 (21-143)	82 (23-135)	1,417 (482-2,152)	3,482 (1,251-6,933	3,796 (1,283-6,434	2,462 (833-3,530)
Non-venomous animal contact	44 (15-114)	70 (20-166)	438 (86-1,548)	1,211 (136-5,632)	604 (117-2,726)	250 (74-877)
Foreign body	2,467 (2,167-3,062	3,133 (2,646-3,925	22,497 (18,407-26,453	8,305 (5,583-10,329	1,814 (1,608-2,127	1,022 (942-1,161)
Pulmonary aspiration and foreign body in airway	2,360 (2,079-2,936	3,003 (2,538-3,776	20,712 (16,628-24,315	7,312 (4,816-9,313	1,552 (1,363-1,840	875 (793-1,004)
Foreign body in other body part	107 (39-161)	130 (28-186)	1,785 (314-2,891)	993 (265-2,004)	262 (141-383)	147 (93-192)
Environmental heat and cold exposure	172 (66-221)	123 (42-175)	1,511 (479-2,109)	869 (292-1,464)	413 (198-574)	167 (110-191)
Exposure to forces of nature	15 (8-28)	56 (29-115)	457 (237-863)	2,534 (1,242-4,818	2,546 (1,181-4,816	2,335 (1,148-4,732
Other unintentional injuries	225 (191-268)	105 (84-126)	1,460 (1,238-1,693	4,998 (3,949-6,099	2,014 (1,669-2,453	1,658 (1,395-1,953
Self-harm and interpersonal violence	2,046 (1,655-2,280	857 (687-936)	5,008 (3,605-5,678	13,418 (11,075-15,294	7,824 (6,629-8,946	12,191 (11,201-13,346
Self-harm	-	-	-	-	-	6,227 (5,531-7,065
Self-harm by firearm	-	-	-	-	-	210 (123-313)
Self-harm by other specified means	-	-	-	-	-	6,017 (5,352-6,868
Interpersonal violence	1,299 (908-1,533)	452 (282-531)	3,671 (2,273-4,337	6,329 (3,976-8,213	3,999 (2,804-5,121	2,889 (2,323-3,555
Physical violence by firearm	65 (39-97)	34 (17-48)	355 (203-526)	566 (327-935)	423 (313-592)	499 (416-612)
Physical violence by sharp object	64 (46-79)	47 (26-64)	315 (194-409)	677 (354-958)	486 (336-638)	498 (377-631)
Physical violence by other means	1,171 (793-1,388)	371 (225-432)	3,001 (1,809-3,566	5,085 (2,916-6,679	3,090 (1,977-3,941	1,892 (1,412-2,353
Conflict and terrorism	747 (729-770)	406 (396-418)	1,320 (1,290-1,358	7,057 (6,881-7,277	3,796 (3,702-3,916	3,049 (2,969-3,158
Executions and police conflict	-	-	17 (8-25)	32 (21-43)	29 (26-33)	26 (24-29)

Cause	15 to 19	20 to 24	25 to 29	30 to 34	35 to 39	40 to 44
Other urinary diseases	326 (195-482)	419 (256-629)	354 (233-531)	394 (270-561)	377 (237-555)	380 (259-563)
Gynecological diseases	468 (359-607)	498 (369-635)	480 (363-608)	514 (387-623)	557 (439-639)	564 (454-631)
Uterine fibroids	78 (49-104)	101 (60-155)	123 (75-185)	125 (79-187)	145 (97-199)	170 (115-214)
Polycystic ovarian syndrome	1 (0-4)	2 (1-6)	2 (0-4)	1 (0-3)	1 (0-2)	1 (0-2)
Endometriosis	9 (4-14)	11 (5-17)	11 (5-18)	16 (8-26)	15 (7-25)	18 (8-30)
Genital prolapse	18 (10-29)	13 (7-20)	15 (6-24)	16 (7-25)	13 (5-22)	24 (7-43)
Other gynecological diseases	362 (267-515)	371 (259-536)	330 (229-482)	357 (255-467)	383 (284-516)	350 (262-459)
Hemoglobinopathies and hemolytic anemias	2,672 (2,010-3,425	2,511 (1,825-3,180	1,988 (1,471-2,482	1,876 (1,320-2,317	1,337 (984-1,692	1,296 (972-1,631)
Thalassemias	540 (375-691)	422 (269-536)	268 (176-347)	149 (93-197)	65 (46-83)	36 (29-47)
Sickle cell disorders	1,614 (1,183-2,166	1,426 (988-1,969)	1,009 (718-1,393	791 (556-1,096)	426 (309-678)	351 (239-592)
G6PD deficiency	249 (183-349)	295 (215-396)	336 (245-443)	404 (284-520)	324 (228-417)	321 (234-425)
Other hemoglobinopathies and hemolytic anemias	270 (213-362)	369 (289-462)	374 (290-455)	531 (385-638)	522 (394-631)	588 (464-726)
Endocrine, metabolic, blood, and immune disorders	885 (692-1,094)	1,122 (834-1,347	1,198 (872-1,467	1,281 (935-1,594)	1,336 (1,012-1,661	1,276 (1,022-1,656
Sudden infant death syndrome	-	-	-	-	-	-
Injuries	95,795 (90,311-100,288	110,044 (105,189-114,226	95,057 (90,819-98,532	79,565 (75,782-82,559	68,842 (65,582-71,746	57,626 (54,610-60,184
Transport injuries	24,551 (22,894-26,075	26,150 (24,554-27,785	22,800 (21,419-24,149	19,884 (18,812-21,049	18,284 (17,254-19,377	16,532 (15,657-17,601
Road injuries	22,880 (21,349-24,242	24,730 (23,129-26,322	21,498 (20,201-22,843	18,812 (17,723-19,871	17,349 (16,382-18,382	15,610 (14,769-16,581
Pedestrian road injuries	8,297 (7,465-9,226	9,002 (8,081-10,068	7,524 (6,761-8,648	7,481 (6,728-8,587	7,280 (6,491-8,263	6,850 (6,171-7,665
Cyclist road injuries	754 (598-967)	608 (461-826)	550 (391-776)	594 (424-843)	544 (406-769)	540 (419-748)
Motorcyclist road injuries	3,426 (2,654-4,497	4,018 (3,002-5,193	3,298 (2,571-4,372	2,873 (2,215-3,717	2,394 (1,821-3,099	2,182 (1,656-2,869
Motor vehicle road injuries	10,209 (9,124-11,031	10,909 (9,846-11,940	9,973 (8,910-10,970	7,697 (6,874-8,488	6,966 (6,261-7,647	5,910 (5,311-6,481
Other road injuries	195 (130-253)	193 (122-245)	153 (102-198)	168 (110-217)	165 (112-212)	127 (90-163)
Other transport injuries	1,671 (1,433-1,951	1,420 (1,182-1,654	1,302 (1,090-1,492	1,072 (895-1,227	935 (791-1,074	922 (790-1,041)
Unintentional injuries	24,721 (21,433-27,021	23,962 (20,581-26,236	20,871 (17,984-22,768	19,577 (17,059-21,372	17,928 (15,719-19,488	16,253 (14,104-17,633
Falls	2,224 (1,849-2,699	2,181 (1,830-2,647	1,873 (1,615-2,239	2,150 (1,856-2,553	2,448 (2,143-2,880	2,364 (2,110-2,695
Drowning	7,436 (6,478-8,077	5,488 (4,887-5,924	4,269 (3,783-4,590	3,679 (3,355-3,943	3,131 (2,807-3,347	2,605 (2,361-2,820
Fire, heat, and hot substances	4,589 (2,622-5,734	5,463 (3,040-6,870	4,380 (2,625-5,396	3,962 (2,503-4,758	3,014 (2,056-3,591	2,226 (1,625-2,587
Poisonings	1,539 (1,265-2,034	1,779 (1,453-2,408	1,497 (1,223-2,102	1,218 (992-1,618)	1,162 (965-1,491)	1,049 (895-1,329)
Poisoning by carbon monoxide	785 (659-1,081)	893 (748-1,259)	733 (611-1,086	601 (505-836)	560 (478-784)	529 (458-679)
Poisoning by other means	754 (601-972)	886 (691-1,231)	764 (611-1,049	617 (479-826)	602 (481-777)	521 (436-669)
Exposure to mechanical forces	1,473 (1,078-1,705	1,372 (1,026-1,554	1,405 (1,058-1,596	1,402 (1,047-1,603	1,310 (998-1,479	1,346 (1,035-1,524
Unintentional firearm injuries	296 (236-343)	305 (241-348)	312 (223-368)	338 (252-402)	249 (190-291)	237 (180-283)
Other exposure to mechanical forces	1,177 (827-1,392)	1,067 (765-1,228)	1,093 (814-1,258	1,065 (757-1,233)	1,061 (783-1,214	1,109 (824-1,268)
Adverse effects of medical treatment	1,069 (743-1,272)	1,788 (1,200-2,141	1,812 (1,159-2,221	1,509 (1,000-1,811	1,576 (1,054-1,877	1,656 (1,159-1,916
Animal contact	1,821 (594-2,705)	1,532 (460-2,260)	1,355 (448-1,906	1,546 (500-2,190)	1,325 (437-1,853	1,755 (599-2,406)
Venomous animal contact	1,663 (509-2,421)	1,349 (387-1,979)	1,196 (371-1,709	1,422 (441-2,044)	1,213 (377-1,715	1,646 (535-2,295)
Non-venomous animal contact	158 (48-623)	183 (59-674)	160 (50-482)	124 (39-371)	112 (39-312)	108 (38-298)
Foreign body	669 (632-747)	715 (679-827)	757 (712-852)	722 (682-807)	769 (736-848)	757 (730-820)
Pulmonary aspiration and foreign body in airway	476 (431-541)	486 (441-568)	533 (485-610)	552 (510-617)	591 (549-657)	612 (572-664)
Foreign body in other body part	193 (145-224)	229 (166-273)	224 (162-266)	170 (121-204)	177 (131-211)	146 (114-176)
Environmental heat and cold exposure	351 (214-418)	341 (210-403)	369 (260-416)	532 (391-602)	640 (490-718)	735 (556-825)
Exposure to forces of nature	1,628 (826-3,203)	1,421 (719-2,745	1,227 (633-2,255	1,019 (534-1,904)	831 (442-1,572	715 (382-1,310)
Other unintentional injuries	1,922 (1,646-2,206	1,883 (1,526-2,215	1,926 (1,593-2,264	1,837 (1,584-2,125	1,723 (1,511-1,977	1,045 (941-1,156)
Self-harm and interpersonal violence	46,522 (43,611-50,001	59,932 (56,757-63,463	51,386 (48,961-54,603	40,104 (38,069-42,765	32,630 (30,898-34,871	24,841 (23,615-26,519
Self-harm	36,640 (33,937-39,992	48,401 (45,765-51,768	41,163 (39,086-44,181	30,661 (29,160-33,212	24,921 (23,742-26,959	18,973 (18,067-20,430
Self-harm by firearm	1,208 (642-1,889)	1,433 (815-2,114)	1,085 (726-1,490)	949 (725-1,214)	792 (645-968)	685 (596-800)
Self-harm by other specified means	35,432 (32,800-39,012	46,967 (44,146-50,878	40,078 (38,011-43,416	29,712 (28,173-32,418	24,129 (22,969-26,280	18,288 (17,403-19,742
Interpersonal violence	6,300 (5,053-7,457	8,625 (6,946-10,232	8,335 (6,731-9,515	7,809 (6,156-8,785	6,424 (5,149-7,186	4,907 (3,977-5,451
Physical violence by firearm	1,641 (1,333-1,947	2,136 (1,692-2,502	2,101 (1,657-2,447	1,814 (1,417-2,112	1,515 (1,175-1,797	1,086 (847-1,266)
Physical violence by sharp object	1,298 (1,013-1,633	2,243 (1,748-2,820	2,305 (1,829-2,788	2,335 (1,798-2,776	1,930 (1,554-2,224	1,600 (1,247-1,858
Physical violence by other means	3,360 (2,397-4,167	4,247 (3,149-5,334	3,929 (2,925-4,803	3,660 (2,668-4,273	2,979 (2,275-3,437	2,221 (1,758-2,595
Conflict and terrorism	3,553 (3,466-3,665	2,874 (2,801-2,964	1,854 (1,810-1,907	1,597 (1,558-1,646	1,253 (1,219-1,292	931 (906-960)
Executions and police conflict	30 (28-34)	32 (27-38)	34 (24-42)	37 (22-46)	31 (21-39)	29 (18-37)

Cause	45 to 49	50 to 54	54 to 59	60 to 64	65 to 69	70 to 74
Other urinary diseases	476 (313-654)	569 (372-776)	740 (472-977)	986 (666-1,263)	942 (606-1,205	943 (606-1,215)
Gynecological diseases	636 (521-741)	499 (409-583)	336 (280-390)	276 (240-320)	321 (274-379)	201 (177-231)
Uterine fibroids	200 (153-259)	178 (141-255)	79 (64-119)	70 (56-94)	70 (55-103)	45 (35-71)
Polycystic ovarian syndrome	1 (0-2)	1 (0-2)	-	-	-	-
Endometriosis	23 (9-41)	16 (8-29)	-	-	-	-
Genital prolapse	30 (7-54)	61 (17-102)	49 (17-86)	53 (26-86)	87 (41-135)	66 (37-94)
Other gynecological diseases	382 (292-479)	243 (180-327)	208 (163-266)	153 (121-206)	164 (123-229)	89 (67-122)
Hemoglobinopathies and hemolytic anemias	1,110 (843-1,413	1,299 (1,002-1,631	1,462 (1,136-1,909	1,689 (1,329-2,164	2,053 (1,627-2,539	2,320 (1,817-2,890
Thalassemias	23 (18-30)	13 (10-16)	7 (6-10)	4 (3-5)	2 (2-3)	1 (1-1)
Sickle cell disorders	253 (169-467)	192 (125-380)	130 (77-255)	46 (29-82)	21 (14-32)	7 (4-11)
G6PD deficiency	241 (175-317)	226 (161-306)	202 (138-304)	156 (102-240)	128 (84-196)	79 (51-121)
Other hemoglobinopathies and hemolytic anemias	594 (460-732)	868 (685-1,061)	1,122 (881-1,409	1,483 (1,166-1,850	1,902 (1,505-2,330	2,234 (1,748-2,784
Endocrine, metabolic, blood, and immune disorders	1,524 (1,214-1,890	1,955 (1,570-2,364	2,009 (1,621-2,534	2,874 (2,316-3,562	3,321 (2,606-4,107	3,099 (2,488-3,918
Sudden infant death syndrome	-	-	-	-	-	-
Injuries	49,691 (47,203-51,921	51,767 (49,243-53,905	49,279 (46,990-51,315	54,520 (51,759-56,934	55,167 (52,229-57,915	54,997 (51,771-57,830
Transport injuries	14,147 (13,471-15,000	15,152 (14,451-15,912	14,190 (13,518-14,973	14,677 (14,059-15,301	13,183 (12,628-13,812	11,962 (11,461-12,548
Road injuries	13,297 (12,676-14,070	14,298 (13,634-15,034	13,577 (12,933-14,301	13,862 (13,297-14,503	12,512 (11,989-13,128	11,446 (10,936-11,999
Pedestrian road injuries	6,139 (5,590-6,813	6,877 (6,385-7,478	6,969 (6,508-7,452	8,049 (7,547-8,557	7,285 (6,855-7,794	6,995 (6,628-7,516
Cyclist road injuries	549 (436-734)	532 (438-669)	528 (444-649)	512 (434-635)	529 (457-626)	436 (380-505)
Motorcyclist road injuries	1,708 (1,345-2,262	1,759 (1,386-2,346	1,444 (1,139-1,958	1,016 (795-1,411)	785 (612-1,108	565 (429-770)
Motor vehicle road injuries	4,747 (4,309-5,223	4,984 (4,466-5,430	4,499 (4,054-4,917	4,242 (3,874-4,615	3,870 (3,430-4,171	3,419 (3,112-3,698
Other road injuries	153 (111-197)	146 (104-198)	137 (100-190)	44 (32-56)	42 (31-55)	31 (23-41)
Other transport injuries	850 (734-962)	854 (734-972)	613 (535-681)	816 (723-901)	672 (598-747)	517 (461-569)
Unintentional injuries	15,646 (13,786-16,895	17,341 (15,338-18,714	18,884 (17,124-20,310	24,149 (21,863-26,150	27,108 (24,609-29,518	29,481 (26,824-32,020
Falls	2,928 (2,616-3,347	3,768 (3,341-4,367	5,507 (4,868-6,385	8,866 (7,836-10,296	11,450 (10,100-13,343	14,496 (12,840-16,626
Drowning	2,368 (2,166-2,601	2,546 (2,355-2,767	2,416 (2,241-2,648	2,746 (2,441-3,029	2,914 (2,634-3,215	2,724 (2,506-3,033
Fire, heat, and hot substances	2,132 (1,566-2,482	2,049 (1,565-2,350	1,956 (1,525-2,240	2,208 (1,780-2,491	2,642 (2,138-2,961	2,683 (2,170-2,992
Poisonings	861 (747-1,132	1,029 (919-1,296)	931 (826-1,215	971 (872-1,217)	984 (874-1,276	913 (807-1,226)
Poisoning by carbon monoxide	457 (400-622)	587 (534-755)	527 (474-686)	591 (537-761)	579 (512-772)	512 (448-735)
Poisoning by other means	404 (342-548)	442 (379-580)	404 (354-569)	380 (329-501)	405 (351-543)	401 (354-539)
Exposure to mechanical forces	1,117 (892-1,237	1,002 (812-1,121)	1,268 (973-1,433	1,267 (971-1,419)	1,139 (922-1,272	1,051 (857-1,173)
Unintentional firearm injuries	223 (176-261)	201 (164-238)	198 (162-247)	195 (155-248)	197 (156-254)	185 (151-239)
Other exposure to mechanical forces	893 (697-1,003	801 (634-904)	1,070 (776-1,225	1,072 (788-1,221)	942 (752-1,058	866 (681-976)
Adverse effects of medical treatment	1,728 (1,257-1,985	1,546 (1,166-1,765	1,925 (1,503-2,169	2,879 (2,260-3,290	3,007 (2,426-3,441	3,020 (2,462-3,397
Animal contact	1,499 (524-2,053	1,839 (676-2,506)	1,283 (505-1,701	1,489 (543-2,010)	1,455 (510-1,961	1,309 (472-1,751)
Venomous animal contact	1,347 (454-1,876	1,670 (575-2,306)	1,112 (400-1,506	1,371 (482-1,876)	1,333 (438-1,824	1,189 (420-1,620)
Non-venomous animal contact	152 (58-368)	169 (69-424)	171 (72-408)	119 (44-285)	122 (50-293)	119 (49-306)
Foreign body	735 (708-789)	954 (916-1,022)	1,003 (966-1,060	1,115 (1,069-1,173	1,191 (1,143-1,248	1,347 (1,281-1,413
Pulmonary aspiration and foreign body in airway	629 (594-674)	834 (791-895)	883 (840-934)	1,006 (958-1,065)	1,082 (1,029-1,138	1,216 (1,148-1,282
Foreign body in other body part	106 (78-133)	120 (95-151)	119 (90-155)	109 (81-140)	109 (75-139)	131 (89-164)
Environmental heat and cold exposure	758 (582-860)	1,036 (893-1,137)	1,022 (812-1,136	1,243 (926-1,396)	1,001 (738-1,120	1,049 (698-1,206)
Exposure to forces of nature	582 (321-1,035	606 (324-1,082)	659 (335-1,187	637 (337-1,181)	550 (294-968)	453 (252-815)
Other unintentional injuries	941 (852-1,036	966 (867-1,069)	915 (830-1,015	726 (661-808)	774 (702-895)	435 (403-486)
Self-harm and interpersonal violence	19,898 (18,924-21,318	19,275 (18,463-20,434	16,205 (15,526-17,194	15,694 (15,079-16,658	14,875 (14,210-15,733	13,553 (12,902-14,489
Self-harm	15,486 (14,810-16,719	15,181 (14,538-16,221	13,014 (12,488-13,878	12,743 (12,202-13,580	12,404 (11,849-13,221	11,552 (10,936-12,407
Self-harm by firearm	542 (483-626)	491 (434-582)	442 (392-534)	388 (345-475)	376 (328-471)	313 (265-417)
Self-harm by other specified means	14,944 (14,274-16,196	14,690 (14,060-15,732	12,572 (12,042-13,430	12,355 (11,813-13,161	12,027 (11,464-12,838	11,239 (10,602-12,102
Interpersonal violence	3,619 (2,954-4,031	3,365 (2,791-3,753	2,581 (2,126-2,876	2,420 (2,039-2,701	1,996 (1,703-2,300	1,662 (1,396-1,958
Physical violence by firearm	778 (610-903)	676 (498-804)	529 (386-625)	475 (360-565)	348 (271-427)	289 (219-366)
Physical violence by sharp object	1,117 (884-1,307	1,007 (817-1,168)	723 (585-828)	676 (563-797)	476 (410-552)	458 (377-546)
Physical violence by other means	1,724 (1,344-2,011	1,683 (1,348-1,969	1,330 (1,089-1,538	1,269 (1,057-1,467	1,172 (954-1,354	916 (753-1,090)
Conflict and terrorism	768 (748-789)	706 (687-729)	592 (577-609)	513 (499-529)	460 (447-476)	325 (316-336)
Executions and police conflict	26 (15-38)	22 (16-30)	17 (13-24)	18 (14-23)	14 (11-20)	13 (10-18)

Cause	75 to 79	80 to 84	85 to 89	90 to 94	95 plus
Other urinary diseases	953 (619-1,212)	620 (399-801)	434 (283-566)	220 (144-286)	110 (65-137)
Gynecological diseases	177 (161-195)	286 (239-341)	185 (157-225)	69 (60-82)	23 (20-27)
Uterine fibroids	36 (29-51)	72 (49-114)	52 (33-80)	23 (16-33)	8 (5-11)
Polycystic ovarian syndrome	-	-	-	-	-
Endometriosis	-	-	-	-	-
Genital prolapse	57 (31-76)	76 (43-120)	45 (28-69)	17 (10-23)	6 (4-8)
Other gynecological diseases	84 (69-113)	138 (107-181)	88 (70-114)	30 (25-39)	9 (8-12)
Hemoglobinopathies and hemolytic anemias	2,471 (1,902-3,019	2,589 (1,881-3,102	2,161 (1,453-2,606	1,098 (719-1,327)	372 (248-440)
Thalassemias	0 (0-0)	0 (0-0)	0 (0-0)	0 (0-0)	0 (0-0)
Sickle cell disorders	3 (2-4)	1 (0-1)	0 (0-1)	0 (0-0)	0 (0-0)
G6PD deficiency	50 (32-77)	25 (16-38)	10 (7-15)	3 (2-4)	1 (0-1)
Other hemoglobinopathies and hemolytic anemias	2,418 (1,861-2,947	2,563 (1,861-3,065	2,150 (1,446-2,592	1,095 (717-1,324)	372 (248-439)
Endocrine, metabolic, blood, and immune disorders	3,469 (2,573-4,252	3,198 (2,361-4,069	2,467 (1,827-3,078	1,176 (878-1,540)	402 (312-561)
Sudden infant death syndrome	-	-	-	-	-
Injuries	59,689 (56,342-62,399	55,686 (52,287-58,054	40,769 (38,761-42,431	17,815 (17,004-18,520	5,289 (5,074-5,473
Transport injuries	10,475 (10,127-10,914	7,098 (6,861-7,429	3,494 (3,368-3,688	1,145 (1,106-1,206	313 (301-328)
Road injuries	9,985 (9,653-10,412	6,760 (6,536-7,095	3,333 (3,214-3,522	1,094 (1,057-1,158	297 (285-311)
Pedestrian road injuries	6,411 (6,144-6,784	4,459 (4,276-4,717	2,084 (1,989-2,219	610 (583-650)	151 (142-161)
Cyclist road injuries	283 (245-324)	120 (104-139)	52 (44-61)	17 (14-21)	0 (0-0)
Motorcyclist road injuries	304 (229-432)	157 (116-233)	70 (50-110)	21 (16-31)	7 (5-9)
Motor vehicle road injuries	2,910 (2,660-3,115	1,980 (1,831-2,106	1,101 (1,023-1,186	437 (412-469)	137 (128-146)
Other road injuries	77 (57-106)	43 (33-61)	25 (19-37)	9 (7-13)	3 (2-4)
Other transport injuries	490 (445-534)	339 (310-370)	161 (145-178)	50 (45-56)	16 (15-18)
Unintentional injuries	37,047 (34,029-39,569	40,305 (37,225-42,527	33,214 (31,227-34,758	15,600 (14,798-16,321	4,757 (4,542-4,947
Falls	21,108 (19,129-23,197	25,256 (22,931-27,045	21,992 (20,260-23,288	10,711 (10,025-11,338	3,223 (3,049-3,390
Drowning	2,658 (2,434-2,975	2,061 (1,873-2,338	1,012 (934-1,147	263 (246-298)	47 (44-54)
Fire, heat, and hot substances	3,298 (2,791-3,608	3,370 (2,940-3,673	2,585 (2,316-2,790	1,107 (1,018-1,183	363 (342-383)
Poisonings	862 (785-1,124)	697 (641-884)	382 (355-464)	114 (107-132)	27 (25-29)
Poisoning by carbon monoxide	565 (511-765)	438 (395-588)	240 (220-308)	64 (59-79)	12 (11-14)
Poisoning by other means	297 (264-400)	259 (231-324)	142 (124-167)	51 (43-58)	15 (13-17)
Exposure to mechanical forces	1,062 (885-1,182)	1,108 (991-1,254)	863 (793-996)	406 (374-457)	133 (124-147)
Unintentional firearm injuries	265 (212-333)	246 (207-328)	188 (161-263)	86 (73-120)	28 (24-38)
Other exposure to mechanical forces	797 (637-901)	862 (743-977)	674 (619-756)	320 (295-350)	105 (97-114)
Adverse effects of medical treatment	3,384 (2,887-3,711	3,412 (2,908-3,755	2,970 (2,533-3,265	1,414 (1,229-1,530	444 (388-478)
Animal contact	977 (347-1,342)	589 (246-785)	273 (131-356)	81 (43-103)	16 (11-21)
Venomous animal contact	879 (286-1,236	507 (197-699)	219 (93-298)	62 (28-82)	12 (7-16)
Non-venomous animal contact	98 (47-217)	82 (42-162)	54 (29-95)	20 (12-32)	5 (3-7)
Foreign body	1,724 (1,638-1,792	1,940 (1,850-2,015	1,749 (1,663-1,832	889 (842-937)	318 (304-333)
Pulmonary aspiration and foreign body in airway	1,579 (1,496-1,648	1,783 (1,695-1,863	1,633 (1,546-1,721	839 (793-886)	303 (289-318)
Foreign body in other body part	145 (102-176)	157 (107-185)	116 (79-143)	50 (35-59)	16 (12-20)
Environmental heat and cold exposure	1,206 (853-1,347	1,247 (907-1,379)	969 (771-1,068	436 (370-479)	132 (120-143)
Exposure to forces of nature	269 (167-470)	170 (110-285)	119 (67-217)	50 (28-88)	9 (6-16)
Other unintentional injuries	500 (463-565)	455 (422-518)	299 (276-336)	129 (119-142)	44 (42-48)
Self-harm and interpersonal violence	12,167 (11,631-12,867	8,283 (7,906-8,838	4,061 (3,908-4,286	1,071 (1,040-1,114	219 (213-225)
Self-harm	10,357 (9,826-11,048	7,568 (7,190-8,120	3,704 (3,552-3,934	987 (956-1,030)	183 (177-189)
Self-harm by firearm	292 (245-381)	227 (192-289)	134 (117-151)	54 (49-60)	19 (17-21)
Self-harm by other specified means	10,066 (9,542-10,764	7,340 (6,967-7,918	3,571 (3,421-3,801	933 (902-975)	165 (159-171)
Interpersonal violence	1,587 (1,400-1,795	567 (534-609)	282 (268-302)	70 (66-75)	17 (16-19)
Physical violence by firearm	236 (176-288)	54 (48-63)	27 (24-32)	7 (6-8)	2 (2-2)
Physical violence by sharp object	452 (387-529)	138 (126-162)	60 (54-71)	14 (13-16)	3 (3-4)
Physical violence by other means	899 (783-1,028	374 (340-411)	195 (180-211)	49 (46-53)	12 (11-13)
Conflict and terrorism	212 (205-220)	133 (128-139)	65 (62-69)	11 (10-11)	17 (16-18)
Executions and police conflict	10 (8-15)	15 (12-20)	9 (7-12)	3 (2-4)	1 (0-1)

SR Table 5: Global deaths by cause and age group for females (with 95% confidence intervals), 1995

Cause	Early Neonatal	Late Neonatal	Post Neonatal	1 to 4	5 to 9	10 to 14	15 to 19
All causes	1,239,721 (1,205,689-1,272,34	490,192 (473,670-510,554	1,656,540 (1,615,567-1,695,66	1,663,450 (1,615,238-1,714,441	370,134 (366,424-374,072	200,049 (197,948-202,267	280,073 (276,061-284,274
Communicable, maternal, neonatal, and nutritional disease	1,136,206 (1,101,103-1,168,88	434,844 (416,020-455,076	1,385,308 (1,330,515-1,432,80	1,348,023 (1,289,522-1,421,68	221,811 (208,630-236,486	95,990 (89,637-104,228	117,512 (111,723-125,221
HIV/AIDS and sexually transmitted infections	-	8,067 (3,084-15,238	57,771 (49,407-69,094	34,471 (32,075-37,048	4,696 (4,130-5,340	893 (669-1,105	5,387 (4,543-6,555
HIV/AIDS	-	-	45,152 (42,281-48,148	33,189 (30,981-35,522	4,078 (3,723-4,471	820 (597-1,030	5,068 (4,241-6,219
HIV/AIDS - Drug-susceptible Tuberculosis	-	-	16,646 (12,537-19,022	12,655 (10,056-14,129	1,449 (1,103-1,679	262 (167-354)	1,682 (1,299-2,085
HIV/AIDS - Multidrug-resistant Tuberculosis without extensive drug resistance	-	-	697 (412-1,140)	539 (325-865)	58 (33-96)	10 (6-20)	71 (43-116)
HIV/AIDS - Extensively drug-resistant Tuberculosis	-	-	2 (2-3)	3 (2-4)	0 (0-1)	0 (0-0)	0 (0-1)
HIV/AIDS resulting in other diseases	-	-	27,807 (24,787-32,239	19,992 (18,070-23,055	2,571 (2,253-3,013	547 (410-717)	3,314 (2,640-4,372
Sexually transmitted infections excluding HIV	27,249 (10,563-51,125	8,067 (3,084-15,238	12,619 (4,796-24,009	1,282 (477-2,516	617 (225-1,191)	73 (42-93)	319 (213-380)
Syphilis	27,249 (10,563-51,125	8,067 (3,084-15,238	12,619 (4,796-24,009	1,282 (477-2,516	617 (225-1,191)	27 (16-34)	31 (21-37)
Chlamydial infection	-	-	-	-	-	13 (7-16)	49 (33-59)
Gonococcal infection	-	-	-	-	-	22 (13-28)	124 (83-148)
Other sexually transmitted infections	-	-	-	-	-	12 (7-15)	115 (77-137)
Respiratory infections and tuberculosis	-	111,247 (97,219-124,487	517,653 (473,995-557,042	335,486 (281,925-387,889	44,680 (36,565-51,470	19,878 (17,386-22,258	25,884 (23,687-28,184
Tuberculosis	-	-	36,557 (32,867-40,268	41,082 (33,633-47,952	11,350 (9,317-13,425	8,463 (7,226-9,742	17,262 (15,486-19,114
Drug-susceptible tuberculosis	-	-	34,574 (30,826-38,151	39,356 (32,197-46,335	10,828 (8,854-12,887	8,059 (6,878-9,338	16,481 (14,749-18,413
Multidrug-resistant tuberculosis without extensive drug resistance	-	-	1,950 (1,316-2,978	1,707 (1,053-2,956	515 (291-1,017)	397 (237-733)	769 (452-1,464)
Extensively drug-resistant tuberculosis	-	-	32 (22-46)	19 (12-31)	8 (5-13)	6 (4-10)	12 (8-19)
Lower respiratory infections	101,598 (89,533-115,649	111,051 (97,055-124,264	478,289 (437,491-515,220	291,858 (245,889-338,313	32,751 (25,778-37,805	11,182 (9,330-12,459	8,500 (7,358-9,256
Upper respiratory infections	75 (26-109)	158 (59-234)	2,586 (811-3,821)	2,263 (571-4,516	435 (120-649)	128 (41-208)	79 (30-131)
Otitis media	53 (4-341)	37 (13-128)	221 (97-467)	283 (89-719)	145 (69-320)	106 (53-197)	44 (27-70)
Enteric infections	18,598 (15,254-22,548	42,194 (34,071-48,833	346,251 (302,833-386,743	358,831 (287,099-433,438	65,744 (43,884-89,481	31,703 (22,584-44,892	21,986 (14,290-32,630
Diarrheal diseases	18,598 (15,254-22,548	41,980 (33,869-48,696	341,892 (299,131-382,291	333,218 (264,343-404,295	31,819 (12,424-56,254	12,426 (5,422-22,558	11,744 (4,893-21,391
Typhoid and paratyphoid	-	0 (0-0)	209 (116-381)	16,560 (7,334-32,084	31,262 (17,535-51,706	17,977 (10,392-28,800	9,365 (4,600-16,798
Typhoid fever	-	-	190 (102-351)	14,595 (6,511-28,647	27,405 (15,011-45,621	15,733 (8,952-25,291	8,189 (4,018-14,970
Paratyphoid fever	-	-	19 (9-38)	1,965 (709-4,336	3,858 (1,640-7,706	2,244 (974-4,390)	1,175 (467-2,516)
[illegible]	[illegible]	[illegible]	[illegible]	[illegible]	[illegible]	[illegible]	[illegible]

Cause	20 to 24	25 to 29	30 to 34	35 to 39	40 to 44	45 to 49	50 to 54
All causes	391,504 (384,396-399,254	446,129 (436,820-456,525	460,649 (452,262-468,740	450,866 (442,666-459,245	529,227 (522,060-536,640	584,513 (577,491-591,383	680,911 (673,015-688,636
Communicable, maternal, neonatal, and nutritional disease	181,032 (173,917-189,906	210,406 (201,262-221,109	198,353 (190,602-207,618	170,502 (162,915-178,655	143,602 (137,531-151,226	114,048 (108,073-123,310	113,304 (106,144-126,015
HIV/AIDS and sexually transmitted infections	28,911 (25,167-33,758	62,108 (54,734-70,609	62,590 (56,962-68,962	50,085 (44,636-55,868	37,178 (34,109-40,404	25,705 (23,265-28,321	17,453 (15,937-19,069
HIV/AIDS	28,343 (24,638-33,208	61,284 (53,934-69,733	61,861 (56,270-68,285	49,448 (44,071-55,195	36,447 (33,383-39,716	25,107 (22,664-27,727	17,035 (15,513-18,614
HIV/AIDS - Drug-susceptible Tuberculosis	7,876 (5,515-10,087	13,804 (9,383-18,447	14,101 (9,600-18,954	12,241 (8,296-16,186	10,026 (6,798-13,041	7,507 (5,260-9,588	5,674 (4,081-6,808
HIV/AIDS - Multidrug-resistant Tuberculosis without extensive drug resistance	335 (191-549)	599 (344-982)	622 (358-1,018)	533 (304-863)	434 (249-694)	328 (188-525)	244 (142-397)
HIV/AIDS - Extensively drug-resistant Tuberculosis	2 (1-3)	3 (2-5)	4 (2-6)	4 (2-5)	3 (2-4)	2 (1-3)	1 (1-2)
HIV/AIDS resulting in other diseases	20,131 (16,074-25,208	46,877 (38,224-56,691	47,134 (39,795-54,748	36,670 (30,365-43,747	25,984 (21,860-30,479	17,270 (14,327-20,593	11,116 (9,513-13,099
Sexually transmitted infections excluding HIV	568 (347-679)	824 (466-1,000	729 (432-853)	636 (421-723)	732 (485-828)	598 (405-677)	418 (285-476)
Syphilis	53 (32-63)	71 (40-86)	61 (36-71)	46 (30-52)	49 (32-55)	39 (26-44)	60 (41-68)
Chlamydial infection	144 (88-173)	152 (86-184)	83 (49-97)	104 (69-118)	139 (92-158)	105 (71-119)	68 (46-77)
Gonococcal infection	220 (135-263)	403 (228-489)	365 (216-427)	293 (194-332)	352 (233-399)	288 (195-327)	195 (133-222)
Other sexually transmitted infections	151 (92-180)	198 (112-240)	220 (130-257)	194 (128-220)	192 (127-217)	166 (112-188)	96 (65-109)
Respiratory infections and tuberculosis	36,533 (33,838-39,309	42,504 (39,279-45,381	42,454 (39,297-45,283	42,563 (39,297-45,721	43,851 (40,554-46,906	43,534 (40,193-46,733	54,274 (49,756-58,537
Tuberculosis	27,037 (24,619-29,515	32,169 (29,226-34,766	32,628 (29,826-35,257	32,472 (29,344-35,373	32,444 (29,280-35,305	31,659 (28,426-34,624	38,389 (34,158-42,263
Drug-susceptible tuberculosis	25,646 (23,227-28,225	30,496 (27,612-33,096	30,905 (28,142-33,584	30,915 (27,835-33,751	30,747 (27,504-33,597	29,990 (26,698-33,065	36,617 (32,550-40,310
Multidrug-resistant tuberculosis without extensive drug resistance	1,367 (857-2,343	1,644 (1,063-2,820	1,692 (1,082-2,901	1,529 (965-2,698	1,665 (1,058-2,860	1,639 (1,047-2,792	1,744 (1,067-3,225
Extensively drug-resistant tuberculosis	24 (16-35)	29 (20-43)	31 (21-45)	28 (19-40)	32 (22-45)	31 (21-44)	29 (19-44)
Lower respiratory infections	9,356 (8,253-10,180	10,160 (8,736-10,979	9,675 (8,352-10,388	9,962 (8,834-10,830	11,242 (9,971-12,092	11,706 (10,448-12,445	15,742 (14,035-16,961
Upper respiratory infections	115 (37-165)	144 (48-198)	120 (43-167)	99 (39-152)	129 (43-189)	149 (51-214)	117 (45-182)
Otitis media	25 (14-45)	31 (17-66)	31 (19-54)	30 (20-51)	36 (24-59)	19 (14-27)	26 (18-36)
Enteric infections	17,925 (10,178-28,343	15,095 (8,708-24,145	14,037 (7,846-22,677	12,317 (6,892-21,038	12,446 (6,335-20,985	13,133 (6,791-23,000	16,690 (8,213-29,913
Diarrheal diseases	12,055 (4,983-21,919	10,700 (4,285-20,168	10,693 (4,454-19,762	9,776 (4,318-18,290	10,361 (4,452-18,767	11,506 (5,257-21,295	15,290 (6,889-28,802
Typhoid and paratyphoid	5,123 (2,603-8,964	3,711 (1,974-6,348	2,716 (1,431-4,585	1,923 (1,004-3,210	1,503 (786-2,568)	1,107 (599-1,881	844 (436-1,456)
Typhoid fever	4,491 (2,236-7,933	3,212 (1,648-5,642	2,324 (1,214-4,001	1,626 (843-2,784	1,251 (629-2,173)	909 (479-1,545	692 (352-1,223)
Paratyphoid fever	632 (260-1,359)	500 (212-1,046	392 (167-804)	297 (132-593)	252 (114-505)	198 (83-387)	152 (60-308)
[illegible]	[illegible]	[illegible]	[illegible]	[illegible]	[illegible]	[illegible]	[illegible]

Cause	54 to 59	60 to 64	65 to 69	70 to 74	75 to 79	80 to 84	85 to 89	90 to 94	95 plus
All causes	919,453 (909,851-929,025	1,276,013 (1,264,439-1,287,98	1,640,040 (1,626,371-1,653,88	1,988,522 (1,972,404-2,004,69	2,039,792 (2,024,229-2,055,08	2,264,753 (2,252,026-2,277,24	1,791,941 (1,784,545-1,799,29	915,996 (913,279-918,791	297,378 (296,680-298,11
Communicable, maternal, neonatal, and nutritional disease	123,756 (114,017-141,021	155,851 (141,327-182,858	173,928 (154,489-211,226	200,524 (177,072-240,393	194,058 (169,536-234,118	198,175 (172,255-235,256	152,949 (135,323-176,937	72,109 (65,578-80,556	25,556 (23,896-27,796
HIV/AIDS and sexually transmitted infections	11,980 (10,678-13,498	8,415 (7,428-9,426	5,989 (5,226-6,815	3,140 (2,601-3,723	1,598 (1,330-1,897	471 (389-528)	227 (193-249)	104 (87-116)	27 (24-29)
HIV/AIDS	11,485 (10,191-12,956	8,017 (7,022-9,025	5,599 (4,838-6,418	2,835 (2,290-3,409	1,326 (1,056-1,623	229 (184-275)	81 (64-97)	30 (24-36)	6 (5-7)
HIV/AIDS - Drug-susceptible Tuberculosis	3,910 (2,955-4,716	2,771 (2,155-3,327	1,936 (1,509-2,355	1,015 (786-1,264)	474 (357-606)	71 (57-88)	22 (17-28)	8 (6-10)	1 (1-1)
HIV/AIDS - Multidrug-resistant Tuberculosis without extensive drug resistance	172 (103-279)	123 (74-199)	86 (52-140)	43 (25-72)	20 (12-33)	5 (3-7)	2 (1-2)	1 (0-1)	0 (0-0)
HIV/AIDS - Extensively drug-resistant Tuberculosis	1 (1-2)	1 (1-1)	1 (0-1)	0 (0-0)	0 (0-0)	0 (0-0)	0 (0-0)	0 (0-0)	0 (0-0)
HIV/AIDS resulting in other diseases	7,402 (6,279-8,931	5,123 (4,330-6,233	3,577 (2,989-4,403	1,776 (1,401-2,249	831 (644-1,052)	153 (119-189)	57 (44-70)	21 (17-26)	5 (4-5)
Sexually transmitted infections excluding HIV	495 (328-574)	398 (261-458)	390 (270-441)	305 (233-341)	272 (206-308)	242 (179-279)	147 (117-163)	74 (59-84)	21 (18-23)
Syphilis	89 (59-103)	71 (46-81)	80 (55-91)	75 (58-84)	59 (44-66)	51 (38-59)	27 (21-30)	13 (10-15)	4 (4-5)
Chlamydial infection	81 (53-93)	66 (43-76)	40 (28-45)	41 (32-46)	38 (28-43)	46 (34-53)	29 (23-32)	14 (11-16)	4 (3-4)
Gonococcal infection	214 (141-248)	162 (106-186)	206 (143-233)	139 (106-156)	138 (105-157)	114 (84-131)	71 (57-79)	37 (29-42)	10 (9-11)
Other sexually transmitted infections	112 (74-129)	99 (65-114)	64 (44-72)	49 (37-55)	38 (28-43)	32 (23-37)	20 (16-22)	10 (8-12)	3 (3-3)
Respiratory infections and tuberculosis	63,636 (58,715-69,210	81,208 (74,639-89,028	91,286 (84,257-100,111	108,242 (99,722-118,632	106,690 (99,637-115,394	113,891 (107,498-121,890	93,696 (89,539-99,331	47,664 (46,058-50,472	17,884 (17,469-18,629
Tuberculosis	42,138 (37,021-46,977	47,830 (41,770-53,727	47,208 (41,222-53,546	48,713 (42,582-55,693	39,059 (33,906-44,582	28,841 (25,093-33,445	14,330 (12,487-16,550	3,965 (3,448-4,534	889 (781-1,023)
Drug-susceptible tuberculosis	40,048 (34,960-44,657	45,281 (39,204-51,299	44,548 (38,464-50,827	45,884 (39,451-52,425	36,722 (31,570-42,031	27,142 (23,498-31,475	13,493 (11,627-15,719	3,747 (3,256-4,288	844 (736-973)
Multidrug-resistant tuberculosis without extensive drug resistance	2,054 (1,277-3,593	2,503 (1,608-4,244	2,610 (1,692-4,314	2,775 (1,839-4,399	2,292 (1,527-3,513	1,666 (1,107-2,577	819 (562-1,226)	214 (150-307)	44 (32-63)
Extensively drug-resistant tuberculosis	36 (25-54)	46 (32-69)	51 (35-72)	54 (37-77)	46 (32-64)	34 (24-48)	17 (12-24)	5 (3-6)	1 (1-1)
Lower respiratory infections	21,282 (19,038-22,862	33,078 (29,011-35,600	43,671 (38,881-47,488	58,813 (52,850-63,970	66,737 (61,131-71,916	83,566 (78,072-88,462	78,023 (74,650-82,750	43,110 (41,848-45,886	16,793 (16,430-17,516
Upper respiratory infections	167 (69-244)	247 (91-364)	333 (114-476)	664 (196-925)	860 (252-1,188	1,458 (409-2,002)	1,326 (392-1,816	581 (205-773)	199 (83-258)
Otitis media	48 (32-71)	53 (36-82)	73 (50-107)	52 (38-71)	35 (25-47)	26 (19-34)	18 (14-23)	8 (6-10)	3 (3-4)
Enteric infections	23,058 (11,648-42,575	38,093 (18,629-67,200	47,320 (24,002-87,556	58,846 (29,421-101,171	61,664 (32,678-106,717	64,453 (32,754-106,308	44,587 (24,090-71,019	16,751 (9,177-26,101	4,289 (2,506-6,635
Diarrheal diseases	21,730 (9,979-41,484	36,856 (17,435-66,427	46,265 (22,890-86,686	58,051 (28,639-100,247	61,117 (32,051-106,145	64,090 (32,481-105,945	44,396 (23,917-70,782	16,690 (9,106-26,042	4,272 (2,489-6,624
Typhoid and paratyphoid	724 (346-1,357	626 (307-1,123)	492 (281-804)	339 (204-541)	214 (132-336)	123 (76-199)	62 (41-104)	20 (13-35)	5 (3-9)
Typhoid fever	598 (284-1,128	520 (253-950)	413 (233-685)	285 (170-458)	181 (108-290)	106 (65-178)	53 (34-97)	18 (11-33)	4 (2-8)
Paratyphoid fever	126 (50-261)	106 (42-217)	79 (34-152)	53 (25-101)	33 (16-60)	17 (9-30)	8 (5-15)	3 (1-4)	1 (0-2)
[illegible]	[illegible]	[illegible]	[illegible]	[illegible]	[illegible]	[illegible]	[illegible]	[illegible]	[illegible]

SR Table 5: Global deaths by cause and age group for females (with 95% confidence intervals), 1995

Cause	Early Neonatal	Late Neonatal	Post Neonatal	1 to 4	5 to 9	10 to 14	15 to 19	20 to 24	25 to 29	30 to 34	35 to 39	40 to 44	45 to 49	50 to 54	54 to 59	60 to 64	65 to 69	70 to 74	75 to 79	80 to 84	85 to 89	90 to 94	95 plus
Mesothelioma	-	-	-	-	-	-	45 (25-69)	80 (40-128)	124 (69-183)	139 (83-204)	208 (138-298)	356 (254-491)	267 (201-347)	337 (268-428)	519 (422-644)	619 (507-758)	741 (618-885)	796 (677-939)	684 (612-779)	587 (520-661)	318 (283-354)	119 (111-131)	27 (25-29)
Hodgkin lymphoma	2 (1-3)	2 (1-3)	54 (19-81)	240 (89-426)	344 (168-490)	500 (290-655)	932 (593-1,159)	1,162 (766-1,441)	1,224 (799-1,513)	1,005 (681-1,213)	776 (550-901)	671 (484-777)	539 (392-610)	536 (385-603)	640 (465-737)	750 (539-855)	902 (673-1,025)	886 (668-1,005)	715 (530-824)	564 (415-655)	261 (197-300)	68 (50-78)	10 (7-11)
Non-Hodgkin lymphoma	31 (28-36)	31 (25-37)	540 (423-651)	2,010 (1,319-2,658)	1,329 (1,082-1,571)	1,003 (884-1,126)	1,032 (923-1,143)	1,442 (1,253-1,621)	1,616 (1,416-1,812)	1,740 (1,547-1,920)	1,906 (1,746-2,078)	2,421 (2,240-2,628)	2,719 (2,552-2,909)	3,329 (3,163-3,538)	4,504 (4,309-4,747)	6,342 (6,059-6,661)	8,040 (7,795-8,374)	9,140 (8,867-9,450)	7,593 (7,406-7,800)	7,105 (6,947-7,273)	4,196 (4,103-4,294)	1,482 (1,449-1,516)	312 (305-319)
Multiple myeloma	-	-	-	-	-	-	120 (69-149)	163 (83-215)	179 (100-229)	198 (129-246)	311 (241-365)	575 (472-657)	939 (807-1,061)	1,435 (1,261-1,612)	2,381 (2,195-2,642)	3,507 (3,119-3,929)	4,593 (4,314-5,105)	5,064 (4,712-5,716)	4,468 (4,159-5,060)	3,956 (3,741-4,615)	2,274 (2,132-2,748)	813 (778-978)	137 (125-165)
Leukemia	169 (118-215)	161 (107-223)	2,759 (1,618-4,187)	8,161 (4,878-14,305)	8,642 (5,724-12,728)	6,530 (4,953-8,207)	5,489 (4,224-6,569)	5,148 (3,719-6,536)	5,442 (3,679-6,822)	4,963 (3,462-6,186)	4,619 (3,522-5,407)	5,109 (4,034-5,896)	5,325 (4,359-6,044)	5,404 (4,599-6,120)	6,816 (6,082-7,682)	8,334 (7,490-9,575)	9,688 (8,916-10,998)	10,478 (9,773-11,732)	8,974 (8,443-9,950)	8,503 (8,224-9,325)	5,537 (5,382-5,899)	2,334 (2,266-2,442)	607 (590-628)
Acute lymphoid leukemia	32 (22-51)	25 (16-49)	440 (256-902)	1,776 (1,117-4,217)	2,417 (1,603-4,467)	1,819 (1,433-2,691)	1,237 (984-1,748)	851 (654-1,270)	845 (601-1,332)	572 (421-878)	584 (455-811)	558 (447-742)	489 (415-650)	464 (415-613)	601 (537-785)	651 (583-904)	657 (597-838)	604 (550-762)	449 (415-570)	364 (335-451)	193 (179-228)	67 (62-74)	15 (14-16)
Chronic lymphoid leukemia	-	-	-	-	-	-	75 (49-106)	99 (53-159)	135 (68-219)	123 (67-196)	103 (75-134)	136 (108-167)	165 (139-193)	242 (217-273)	452 (416-518)	774 (715-856)	1,106 (1,039-1,213)	1,426 (1,350-1,538)	1,484 (1,413-1,562)	1,692 (1,615-1,783)	1,310 (1,236-1,366)	622 (591-648)	185 (175-193)
Acute myeloid leukemia	21 (14-37)	21 (13-43)	320 (169-700)	1,362 (658-3,625)	942 (561-2,001)	1,008 (734-1,582)	1,029 (802-1,510)	988 (753-1,509)	1,023 (771-1,597)	1,004 (786-1,483)	1,057 (881-1,431)	1,176 (1,003-1,556)	1,349 (1,173-1,705)	1,343 (1,196-1,630)	1,675 (1,514-1,972)	2,085 (1,887-2,488)	2,461 (2,271-2,865)	2,656 (2,480-2,993)	2,239 (2,117-2,515)	1,890 (1,795-2,211)	1,035 (982-1,219)	369 (350-453)	84 (80-105)
Chronic myeloid leukemia	-	-	-	-	-	-	261 (194-339)	372 (262-518)	494 (344-646)	546 (398-704)	568 (448-704)	643 (533-778)	666 (568-786)	774 (654-928)	926 (819-1,120)	1,085 (968-1,297)	1,240 (1,127-1,437)	1,293 (1,185-1,453)	1,087 (1,012-1,195)	1,153 (1,084-1,243)	746 (697-797)	321 (299-342)	85 (79-91)
Other leukemia	116 (77-155)	115 (73-170)	2,000 (1,089-3,092)	5,023 (2,750-9,584)	5,283 (3,263-7,892)	3,704 (2,527-4,784)	2,888 (2,004-3,643)	2,837 (1,773-3,857)	2,946 (1,731-4,092)	2,719 (1,679-3,691)	2,307 (1,541-2,872)	2,596 (1,847-3,166)	2,658 (1,955-3,216)	2,581 (2,005-2,974)	3,162 (2,603-3,648)	3,739 (3,117-4,385)	4,224 (3,623-4,904)	4,500 (3,989-5,199)	3,716 (3,330-4,301)	3,404 (3,194-3,840)	2,254 (2,143-2,484)	955 (911-1,034)	238 (227-250)
Other malignant neoplasms	481 (358-551)	280 (196-346)	1,763 (1,090-2,362)	5,359 (3,220-8,431)	2,975 (2,228-3,798)	3,112 (2,673-3,570)	3,172 (2,813-3,559)	2,826 (2,402-3,250)	2,922 (2,396-3,394)	2,987 (2,427-3,398)	3,251 (2,796-3,559)	4,287 (3,648-4,647)	5,012 (4,381-5,363)	6,069 (5,425-6,454)	8,185 (7,455-8,892)	10,040 (9,210-10,865)	11,706 (10,605-12,750)	11,806 (10,826-12,949)	9,730 (8,974-10,676)	8,884 (7,969-9,711)	5,492 (4,662-5,943)	2,291 (1,940-2,438)	594 (510-634)
Other neoplasms	25 (18-31)	15 (10-18)	226 (140-295)	683 (378-960)	600 (400-752)	393 (274-480)	309 (212-382)	369 (252-436)	453 (312-547)	546 (379-652)	634 (447-773)	748 (503-925)	908 (603-1,109)	1,064 (686-1,344)	1,408 (899-1,788)	1,856 (1,194-2,309)	2,396 (1,559-3,139)	2,962 (1,888-4,035)	2,899 (1,853-4,000)	3,032 (1,948-4,243)	2,115 (1,366-3,095)	949 (610-1,497)	276 (182-446)
Myelodysplastic, myeloproliferative, and other hematopoietic neoplasm	24 (17-30)	12 (8-15)	198 (126-265)	547 (318-826)	405 (287-544)	259 (184-327)	203 (145-258)	257 (184-325)	338 (248-435)	415 (299-518)	518 (372-646)	659 (455-827)	836 (561-1,031)	1,007 (651-1,280)	1,358 (869-1,739)	1,817 (1,174-2,275)	2,369 (1,540-3,117)	2,942 (1,879-4,019)	2,888 (1,847-3,989)	3,023 (1,945-4,236)	2,111 (1,364-3,092)	947 (609-1,495)	275 (182-445)
Other benign and in situ neoplasms	1 (1-2)	3 (2-5)	27 (13-48)	136 (59-287)	195 (106-279)	135 (88-179)	106 (66-145)	112 (66-155)	114 (64-160)	130 (76-184)	116 (68-166)	89 (51-130)	71 (40-105)	56 (29-86)	49 (24-83)	39 (17-69)	27 (12-46)	20 (8-37)	11 (4-20)	9 (3-18)	5 (2-8)	2 (1-3)	0 (0-1)
Cardiovascular diseases	1,743 (1,611-1,895)	1,536 (1,360-1,695)	13,421 (12,332-14,516)	12,262 (10,473-13,998)	7,039 (6,295-7,856)	6,999 (6,391-7,561)	16,818 (15,895-17,757)	26,606 (25,360-27,836)	37,561 (35,934-39,212)	50,631 (48,589-52,957)	64,209 (62,099-66,516)	104,180 (101,172-107,593)	144,965 (140,989-149,768)	191,311 (186,316-196,615)	298,824 (290,942-306,250)	454,899 (443,627-469,727)	652,413 (637,686-672,202)	846,197 (828,560-873,275)	914,027 (897,699-948,459)	1,101,401 (1,085,805-1,136,15[illegible])	902,426 (891,842-923,116)	465,306 (461,461-472,754)	154,993 (153,896-156,630)
Rheumatic heart disease	-	-	-	3,566 (2,684-4,540)	2,519 (2,112-2,939)	2,780 (2,437-3,086)	3,749 (3,323-4,244)	5,229 (4,564-5,839)	6,741 (5,947-7,537)	7,524 (6,632-8,416)	7,291 (6,389-8,156)	10,033 (8,915-11,136)	10,526 (9,495-11,544)	10,739 (9,725-11,930)	13,651 (12,498-14,984)	16,748 (15,419-18,248)	18,270 (17,005-20,230)	19,659 (18,342-21,850)	17,249 (16,089-19,374)	15,230 (14,285-17,033)	9,955 (9,377-11,007)	4,272 (4,048-4,642)	1,195 (1,144-1,277)
Ischemic heart disease	-	-	-	-	-	-	5,224 (4,800-5,663)	8,735 (8,139-9,651)	12,859 (12,017-14,008)	19,127 (17,930-20,715)	24,557 (23,327-26,172)	40,096 (38,276-42,286)	57,322 (54,961-60,169)	78,645 (75,405-82,383)	128,851 (124,361-133,561)	200,529 (193,942-207,363)	296,496 (288,587-304,856)	383,307 (374,177-393,998)	418,672 (410,641-431,449)	533,748 (525,560-547,797)	454,501 (448,371-463,915)	246,847 (243,800-251,630)	88,031 (86,979-89,285)
Stroke	657 (568-763)	584 (472-729)	4,659 (3,679-6,224)	3,081 (2,414-4,169)	2,414 (2,124-2,898)	2,317 (2,075-2,637)	4,483 (4,177-4,854)	7,632 (7,127-8,287)	11,208 (10,512-12,171)	15,968 (15,064-17,175)	22,555 (21,523-23,833)	40,092 (38,633-42,143)	59,360 (57,206-61,962)	79,354 (76,664-82,576)	122,080 (118,254-126,988)	189,686 (183,890-198,009)	270,043 (262,271-282,671)	350,658 (341,897-370,960)	372,036 (362,201-395,295)	420,432 (411,376-442,400)	320,577 (314,191-334,009)	147,451 (144,885-152,243)	43,436 (42,699-44,539)
Ischemic stroke	122 (96-151)	104 (75-145)	549 (386-766)	373 (273-515)	265 (220-326)	225 (188-269)	768 (664-884)	1,283 (1,108-1,461)	1,822 (1,624-2,031)	2,496 (2,223-2,759)	3,336 (3,021-3,664)	5,947 (5,441-6,483)	9,818 (9,123-10,704)	15,006 (13,724-16,491)	28,174 (26,037-30,648)	61,861 (57,942-66,516)	100,872 (95,613-106,972)	155,835 (149,237-164,084)	175,567 (168,546-185,027)	255,393 (248,392-266,129)	205,895 (201,061-213,120)	97,325 (95,374-100,049)	29,838 (29,224-30,550)
Intracerebral hemorrhage	261 (227-312)	222 (179-290)	2,020 (1,598-2,780)	1,205 (882-1,787)	1,157 (992-1,462)	1,204 (1,045-1,408)	2,526 (2,276-2,813)	4,278 (3,871-4,777)	6,467 (5,924-7,208)	9,162 (8,414-10,122)	13,110 (12,150-14,259)	23,699 (22,195-25,620)	37,341 (35,160-40,215)	49,970 (47,098-53,648)	73,184 (68,691-78,638)	104,177 (96,848-113,260)	138,476 (129,883-150,684)	162,078 (152,609-180,583)	164,229 (155,442-183,976)	139,917 (134,031-157,416)	98,567 (95,030-108,161)	43,611 (42,171-46,687)	11,901 (11,556-12,409)
Subarachnoid hemorrhage	274 (211-313)	258 (191-301)	2,090 (1,537-2,654)	1,504 (1,197-1,864)	992 (809-1,135)	888 (758-1,005)	1,188 (1,045-1,318)	2,071 (1,798-2,303)	2,919 (2,498-3,189)	4,310 (3,789-4,699)	6,110 (5,518-6,535)	10,446 (8,895-11,170)	12,201 (10,192-13,030)	14,378 (11,897-15,403)	20,723 (17,103-22,288)	23,648 (17,766-25,705)	30,695 (23,258-33,264)	32,745 (22,910-35,833)	32,239 (22,666-35,672)	25,122 (16,922-28,226)	16,114 (11,686-17,795)	6,515 (5,141-6,935)	1,698 (1,451-1,829)
Hypertensive heart disease	-	-	-	-	-	-	448 (278-595)	793 (505-1,049)	1,263 (816-1,645)	1,992 (1,324-2,525)	2,720 (1,860-3,395)	4,744 (3,388-5,748)	7,269 (5,310-8,616)	10,305 (7,435-12,238)	15,735 (11,767-18,501)	23,325 (17,661-27,002)	33,600 (26,245-37,874)	46,590 (35,841-51,754)	51,553 (40,857-57,071)	54,388 (44,622-60,567)	42,145 (33,819-46,728)	21,654 (17,096-25,182)	6,798 (5,248-7,903)
Non-rheumatic valvular heart disease	-	-	-	-	-	-	327 (233-446)	386 (260-547)	457 (320-634)	387 (289-511)	455 (359-571)	541 (446-667)	655 (554-793)	858 (739-1,014)	1,299 (1,145-1,476)	1,854 (1,585-2,100)	2,876 (2,457-3,223)	4,337 (3,581-5,150)	6,007 (4,950-6,871)	8,656 (7,030-9,803)	8,156 (6,681-9,767)	4,923 (3,859-5,884)	1,755 (1,467-2,172)
Non-rheumatic calcific aortic valve disease	-	-	-	-	-	-	91 (54-181)	101 (55-230)	126 (72-265)	128 (82-234)	160 (111-271)	186 (135-287)	234 (174-358)	338 (260-479)	527 (415-719)	865 (711-1,112)	1,478 (1,230-1,856)	2,478 (2,005-3,094)	3,576 (2,787-4,324)	5,723 (4,437-6,633)	5,804 (4,566-6,921)	3,663 (2,825-4,577)	1,354 (1,055-1,635)
Non-rheumatic degenerative mitral valve disease	-	-	-	-	-	-	184 (116-301)	227 (137-379)	258 (162-421)	213 (142-326)	248 (174-352)	304 (221-406)	360 (273-474)	452 (337-580)	677 (526-847)	889 (653-1,107)	1,267 (901-1,535)	1,710 (1,249-2,165)	2,108 (1,588-2,620)	2,567 (1,942-3,354)	2,088 (1,496-2,841)	1,142 (841-1,567)	369 (272-531)
Other non-rheumatic valve diseases	-	-	-	-	-	-	51 (33-80)	59 (38-93)	72 (44-117)	47 (29-74)	46 (27-72)	51 (32-76)	60 (37-94)	68 (39-101)	95 (54-135)	100 (57-148)	131 (77-182)	149 (86-206)	323 (188-446)	366 (214-493)	264 (155-351)	118 (72-159)	32 (20-44)
Cardiomyopathy and myocarditis	669 (537-775)	549 (409-659)	4,583 (3,379-5,518)	2,810 (1,660-3,663)	1,141 (829-1,453)	951 (776-1,157)	1,145 (882-1,427)	1,616 (1,226-2,016)	2,114 (1,711-2,581)	2,368 (2,034-2,793)	3,002 (2,705-3,432)	3,818 (3,500-4,295)	4,074 (3,767-4,493)	4,258 (3,915-4,775)	6,470 (6,019-7,127)	6,699 (6,217-7,387)	8,029 (7,549-8,640)	9,566 (9,096-10,159)	11,040 (10,468-11,689)	17,357 (16,515-18,215)	19,690 (18,588-20,888)	11,094 (10,529-11,774)	3,945 (3,742-4,155)
Myocarditis	80 (57-102)	97 (67-120)	1,346 (920-1,659)	982 (607-1,296)	488 (366-630)	314 (240-393)	423 (320-553)	639 (479-849)	680 (505-880)	539 (412-694)	512 (382-652)	589 (450-736)	410 (310-504)	398 (300-492)	513 (375-627)	501 (352-613)	645 (470-764)	818 (567-971)	1,004 (695-1,185)	1,630 (1,087-2,221)	2,040 (1,345-3,012)	1,158 (770-1,799)	418 (301-687)
Alcoholic cardiomyopathy	-	-	-	-	-	-	135 (72-190)	237 (142-332)	441 (307-561)	722 (595-825)	1,182 (1,049-1,296)	1,618 (1,454-1,769)	1,854 (1,644-2,079)	1,707 (1,517-1,921)	2,815 (2,539-3,136)	2,124 (1,887-2,430)	2,249 (2,009-2,676)	1,859 (1,621-2,509)	1,679 (1,466-2,435)	1,985 (1,653-3,366)	1,649 (1,303-3,224)	942 (752-1,814)	308 (256-576)
Other cardiomyopathy	590 (459-690)	452 (328-549)	3,237 (2,273-3,949)	1,828 (1,050-2,399)	653 (452-851)	637 (500-780)	587 (443-759)	740 (544-948)	993 (746-1,285)	1,106 (873-1,385)	1,308 (1,057-1,604)	1,611 (1,339-1,961)	1,810 (1,525-2,173)	2,153 (1,820-2,571)	3,142 (2,699-3,711)	4,073 (3,545-4,723)	5,134 (4,579-5,691)	6,890 (6,045-7,451)	8,357 (7,331-8,997)	13,742 (11,956-14,714)	16,001 (13,333-17,261)	8,994 (7,492-9,736)	3,219 (2,652-3,459)
Atrial fibrillation and flutter	-	-	-	-	-	-	-	-	-	30 (28-32)	32 (30-35)	162 (146-190)	266 (242-305)	439 (409-471)	996 (935-1,053)	1,975 (1,851-2,134)	3,626 (3,430-3,852)	6,991 (6,659-7,436)	10,385 (9,949-10,729)	19,528 (18,741-20,171)	20,350 (19,563-20,888)	14,817 (14,132-15,213)	4,880 (4,658-5,006)
Aortic aneurysm	-	-	-	-	-	-	91 (70-129)	173 (121-248)	243 (175-343)	330 (244-454)	416 (344-543)	584 (502-731)	821 (721-1,002)	1,069 (959-1,297)	1,796 (1,642-2,130)	2,486 (2,285-2,916)	4,001 (3,750-4,506)	5,604 (5,299-6,140)	6,444 (6,146-6,925)	7,378 (7,063-7,695)	5,814 (5,585-6,016)	2,739 (2,655-2,820)	790 (769-811)
Peripheral artery disease	-	-	-	-	-	-	-	-	-	-	-	61 (34-106)	87 (42-174)	130 (58-268)	272 (110-552)	473 (199-978)	898 (352-1,961)	1,337 (509-3,002)	1,740 (640-3,685)	2,946 (1,053-6,519)	2,906 (1,047-6,086)	1,969 (705-4,398)	877 (315-1,955)
Endocarditis	98 (78-131)	121 (91-165)	1,668 (1,274-2,190)	1,292 (851-1,856)	311 (252-400)	326 (267-416)	411 (340-516)	627 (517-819)	811 (677-980)	691 (581-876)	744 (621-965)	848 (706-1,092)	690 (567-893)	800 (651-1,055)	985 (785-1,272)	1,454 (1,152-1,855)	1,903 (1,471-2,420)	2,403 (1,774-3,099)	2,687 (1,912-3,505)	3,206 (2,153-4,408)	2,920 (1,896-3,889)	1,713 (1,039-2,329)	620 (393-884)
Other cardiovascular and circulatory diseases	318 (250-477)	282 (218-419)	2,511 (1,725-4,241)	1,512 (864-3,065)	654 (454-1,070)	624 (473-910)	941 (698-1,345)	1,415 (1,036-2,059)	1,866 (1,399-2,725)	2,213 (1,735-2,988)	2,436 (1,961-3,093)	3,201 (2,627-3,937)	3,896 (3,250-4,646)	4,715 (3,940-5,591)	6,688 (5,682-7,857)	9,671 (8,232-11,211)	12,672 (11,009-14,423)	15,745 (13,951-17,758)	16,215 (14,448-17,974)	18,531 (16,831-20,050)	15,413 (14,132-16,525)	7,826 (7,307-8,304)	2,668 (2,516-2,822)
Chronic respiratory diseases	0 (0-0)	0 (0-0)	0 (0-0)	16,696 (12,014-20,101)	4,473 (3,688-5,225)	3,065 (2,610-3,535)	4,720 (4,097-5,198)	6,464 (5,511-7,022)	8,757 (7,364-9,433)	10,846 (9,114-11,624)	12,986 (10,678-13,878)	19,610 (15,723-20,860)	29,520 (22,739-31,329)	44,129 (32,541-47,324)	71,402 (52,452-76,369)	128,587 (94,681-137,339)	177,839 (132,634-190,089)	246,564 (186,391-262,217)	263,105 (203,196-279,857)	255,863 (200,622-272,612)	174,414 (143,138-184,611)	69,626 (59,367-73,026)	16,619 (14,734-17,415)
Chronic obstructive pulmonary disease	-	-	0 (0-0)	2,053 (1,053-3,981)	678 (380-1,136)	380 (233-587)	1,357 (914-1,942)	2,287 (1,526-3,085)	3,704 (2,640-4,668)	5,013 (3,637-6,186)	5,420 (3,810-7,032)	9,806 (6,841-12,377)	16,276 (10,778-20,676)	26,654 (17,027-34,209)	46,941 (30,328-58,461)	96,904 (64,627-114,429)	142,339 (98,724-164,285)	208,647 (150,525-233,487)	226,849 (168,237-252,204)	224,622 (171,923-247,511)	154,811 (124,384-167,062)	62,290 (52,130-66,445)	14,710 (12,923-15,571)
Pneumoconiosis	-	-	-	-	-	-	20 (10-27)	29 (13-39)	42 (20-57)	52 (25-69)	40 (22-52)	61 (35-77)	62 (35-79)	72 (43-96)	105 (60-144)	149 (90-195)	187 (120-241)	257 (172-331)	262 (177-352)	234 (170-322)	142 (112-194)	56 (44-77)	16 (13-21)
Silicosis	-	-	-	-	-	-	4 (2-7)	5 (2-8)	10 (4-17)	13 (6-23)	9 (5-13)	17 (9-26)	21 (11-30)	16 (8-24)	23 (12-35)	38 (22-54)	41 (22-60)	71 (42-98)	77 (47-107)	70 (44-100)	41 (28-57)	15 (10-22)	4 (3-5)
Asbestosis	-	-	-	-	-	-	2 (1-3)	4 (2-8)	6 (2-11)	6 (2-11)	6 (3-11)	7 (3-12)	8 (3-14)	12 (6-22)	19 (10-35)	25 (12-41)	30 (16-49)	47 (24-72)	44 (23-69)	38 (22-59)	25 (14-37)	10 (6-15)	3 (2-4)
Coal workers pneumoconiosis	-	-	-	-	-	-	3 (1-7)	3 (1-6)	6 (1-15)	7 (2-16)	4 (1-8)	9 (3-18)	7 (2-15)	10 (3-17)	13 (4-22)	17 (6-30)	28 (11-47)	31 (11-54)	33 (14-51)	27 (11-39)	18 (8-26)	7 (4-10)	2 (1-3)
Other pneumoconiosis	-	-	-	-	-	-	11 (5-17)	17 (6-27)	20 (7-34)	25 (10-41)	21 (10-32)	28 (13-42)	26 (12-39)	33 (15-50)	50 (24-75)	70 (34-103)	88 (48-125)	108 (61-154)	108 (64-155)	99 (64-143)	58 (39-85)	23 (17-34)	7 (5-10)
Asthma	-	-	-	12,233 (8,046-15,886)	3,103 (2,360-3,923)	2,296 (1,852-2,778)	2,893 (2,217-3,545)	3,583 (2,677-4,360)	4,279 (3,097-5,216)	4,947 (3,560-6,021)	6,401 (4,582-7,704)	8,293 (5,695-10,157)	11,562 (7,708-14,496)	15,065 (9,198-19,695)	20,900 (12,356-27,712)	28,370 (15,705-39,083)	31,021 (17,405-43,151)	32,457 (18,700-44,732)	30,735 (18,302-42,227)	25,657 (16,518-34,679)	15,503 (10,449-20,651)	5,600 (4,043-7,177)	1,404 (1,121-1,699)
Interstitial lung disease and pulmonary sarcoidosis	-	-	-	270 (108-1,082)	118 (59-360)	66 (40-162)	96 (63-207)	136 (86-320)	208 (142-466)	294 (196-591)	445 (290-836)	579 (385-1,006)	828 (509-1,517)	1,187 (717-2,272)	1,766 (1,043-3,257)	2,594 (1,601-5,005)	3,583 (2,249-6,485)	4,421 (2,982-7,283)	4,418 (2,952-6,780)	4,391 (2,950-5,899)	3,085 (2,058-3,960)	1,234 (872-1,520)	351 (263-461)
Other chronic respiratory diseases	0 (0-0)	0 (0-0)	0 (0-0)	2,138 (881-4,248)	575 (341-947)	323 (212-480)	354 (237-524)	429 (294-627)	524 (349-739)	540 (353-754)	679 (426-980)	872 (535-1,262)	791 (495-1,163)	1,151 (702-1,632)	1,689 (1,073-2,300)	570 (369-741)	710 (503-891)	783 (588-956)	841 (640-1,060)	958 (769-1,185)	873 (746-1,026)	446 (391-515)	138 (125-157)
Digestive diseases	1,236 (1,033-1,365)	1,365 (1,183-1,711)	10,889 (8,691-16,116)	14,481 (10,514-22,184)	7,690 (6,171-10,001)	6,109 (5,203-7,277)	9,533 (8,442-10,950)	12,296 (10,916-14,206)	14,061 (12,637-16,117)	16,378 (15,018-18,499)	19,956 (18,362-21,504)	26,657 (24,381-28,399)	31,479 (29,052-33,289)	37,757 (34,915-40,010)	50,346 (46,542-52,943)	62,798 (57,030-65,423)	73,515 (67,081-76,763)	78,943 (72,642-82,659)	74,871 (68,972-78,140)	75,417 (69,849-78,379)	56,872 (53,213-58,699)	27,761 (26,449-28,542)	8,650 (8,253-8,872)
Cirrhosis and other chronic liver diseases	-	-	-	6,216 (3,982-12,132)	3,162 (2,343-5,115)	2,746 (2,262-3,729)	4,222 (3,659-5,175)	5,799 (5,029-6,862)	7,102 (6,233-8,190)	9,620 (8,626-10,999)	12,396 (11,347-13,539)	17,712 (16,289-19,163)	20,796 (19,243-22,247)	24,692 (22,397-26,391)	31,775 (29,183-34,033)	36,476 (32,877-39,094)	38,982 (35,322-41,621)	37,373 (33,699-40,342)	29,842 (26,238-32,242)	24,090 (20,965-26,061)	14,484 (12,574-15,551)	5,814 (5,196-6,176)	1,716 (1,530-1,819)
Cirrhosis and other chronic liver diseases due to hepatitis B	-	-	-	121 (69-251)	158 (112-252)	254 (192-353)	555 (413-755)	1,875 (1,478-2,381)	3,022 (2,457-3,714)	3,875 (3,289-4,632)	4,095 (3,444-4,945)	5,474 (4,614-6,370)	5,776 (4,775-6,751)	6,313 (5,094-7,532)	8,044 (6,601-9,818)	9,606 (7,754-11,559)	10,635 (8,687-12,797)	10,626 (8,574-12,728)	8,676 (6,947-10,345)	6,556 (5,028-7,964)	3,531 (2,780-4,227)	1,203 (930-1,489)	294 (208-397)
Cirrhosis and other chronic liver diseases due to hepatitis C	-	-	-	46 (25-100)	66 (45-112)	115 (84-164)	235 (168-337)	976 (731-1,277)	1,586 (1,186-2,099)	2,481 (2,008-3,051)	3,579 (2,858-4,376)	5,190 (4,287-6,218)	6,156 (5,107-7,312)	7,380 (5,941-8,848)	8,916 (7,517-10,304)	10,071 (8,302-12,102)	10,596 (9,045-12,342)	9,800 (8,317-11,340)	7,407 (6,039-8,967)	5,876 (4,667-7,209)	3,622 (2,966-4,248)	1,496 (1,204-1,825)	452 (344-574)
Cirrhosis and other chronic liver diseases due to alcohol use	-	-	-	-	-	-	20 (9-37)	333 (247-441)	659 (498-843)	1,421 (1,199-1,731)	2,621 (2,157-3,141)	4,125 (3,521-4,781)	5,128 (4,374-6,037)	6,117 (5,186-7,164)	8,043 (6,726-9,446)	8,597 (6,997-10,273)	8,516 (7,083-9,986)	7,503 (6,108-9,078)	5,565 (4,683-6,550)	4,411 (3,648-5,347)	2,579 (2,165-3,030)	1,011 (805-1,249)	288 (206-389)
Cirrhosis due to NASH	-	-	-	-	-	-	70 (52-92)	196 (150-250)	306 (227-396)	553 (448-680)	922 (718-1,158)	1,511 (1,250-1,814)	2,117 (1,740-2,534)	2,818 (2,321-3,337)	3,643 (3,150-4,197)	4,082 (3,460-4,750)	4,099 (3,532-4,718)	3,583 (3,056-4,162)	2,548 (2,103-3,032)	1,981 (1,567-2,412)	1,279 (1,037-1,513)	549 (431-689)	166 (120-225)
Cirrhosis and other chronic liver diseases due to other causes	-	-	-	6,050 (3,870-11,836)	2,938 (2,163-4,798)	2,376 (1,943-3,274)	3,343 (2,854-4,120)	2,418 (1,882-3,098)	1,529 (1,127-1,977)	1,291 (951-1,689)	1,180 (887-1,540)	1,413 (1,048-1,833)	1,619 (1,230-2,090)	2,064 (1,531-2,698)	3,129 (2,225-4,315)	4,120 (2,972-5,474)	5,136 (3,782-6,634)	5,862 (4,562-7,407)	5,646 (4,351-7,136)	5,265 (4,048-6,672)	3,474 (2,839-4,165)	1,554 (1,218-1,890)	515 (372-654)
Upper digestive system diseases	-	-	-	1,126 (776-1,665)	969 (742-1,312)	770 (626-963)	1,241 (1,034-1,569)	2,563 (2,146-3,187)	2,551 (2,167-3,091)	2,634 (2,228-3,152)	2,860 (2,463-3,300)	3,312 (2,780-3,787)	4,101 (3,528-4,577)	5,562 (4,747-6,373)	6,809 (6,035-7,717)	10,356 (8,949-11,504)	13,167 (11,528-14,551)	14,604 (12,934-16,067)	15,337 (13,642-16,700)	16,072 (14,487-17,178)	12,839 (11,862-13,636)	6,172 (5,774-6,496)	1,908 (1,777-2,018)
Peptic ulcer disease	-	-	-	909 (588-1,470)	692 (504-1,080)	612 (489-812)	1,078 (886-1,420)	2,179 (1,781-2,884)	2,101 (1,734-2,755)	2,177 (1,809-2,804)	2,412 (2,063-2,970)	2,871 (2,430-3,426)	3,567 (3,093-4,174)	4,890 (4,197-5,834)	6,001 (5,296-7,033)	9,135 (7,968-10,531)	11,581 (10,414-13,223)	12,882 (11,287-14,655)	13,491 (12,165-15,096)	14,177 (12,935-15,325)	11,384 (10,508-12,290)	5,447 (5,074-5,833)	1,678 (1,559-1,788)
Gastritis and duodenitis	-	-	-	217 (97-351)	277 (136-434)	158 (82-223)	164 (89-243)	384 (167-563)	450 (209-644)	458 (211-633)	448 (206-611)	441 (212-582)	534 (258-704)	673 (312-900)	808 (426-1,077)	1,221 (602-1,572)	1,586 (804-2,005)	1,722 (877-2,143)	1,846 (969-2,280)	1,895 (1,128-2,315)	1,454 (949-1,726)	725 (542-838)	230 (186-254)
Appendicitis	-	-	-	1,836 (862-3,010)	1,147 (827-1,679)	937 (750-1,205)	1,760 (1,427-2,343)	1,248 (1,018-1,658)	1,281 (1,032-1,706)	731 (605-931)	770 (656-937)	647 (528-776)	757 (646-888)	650 (556-771)	1,191 (1,010-1,433)	1,273 (1,038-1,453)	1,285 (1,091-1,449)	1,278 (1,040-1,479)	1,309 (1,087-1,530)	1,122 (940-1,322)	892 (742-1,082)	410 (348-493)	112 (94-134)
Paralytic ileus and intestinal obstruction	1,236 (1,033-1,365)	1,365 (1,183-1,711)	10,889 (8,691-16,116)	1,540 (1,027-2,146)	1,223 (604-1,734)	925 (533-1,167)	1,475 (870-1,861)	1,275 (730-1,623)	1,270 (765-1,584)	1,031 (637-1,253)	1,235 (755-1,484)	1,585 (983-1,867)	1,691 (1,102-1,970)	1,979 (1,335-2,318)	3,143 (2,178-3,668)	4,031 (2,822-4,715)	5,054 (3,734-5,881)	5,899 (4,561-6,975)	6,782 (5,463-8,158)	8,069 (6,500-10,016)	7,084 (5,521-8,739)	4,083 (3,195-4,958)	1,357 (1,021-1,742)
Inguinal, femoral, and abdominal hernia	-	-	-	190 (80-367)	130 (69-207)	82 (43-110)	84 (46-113)	75 (41-100)	69 (42-92)	120 (71-152)	168 (93-211)	156 (103-195)	282 (174-349)	437 (275-523)	780 (416-937)	1,121 (579-1,352)	1,497 (806-1,795)	1,767 (1,037-2,041)	1,797 (1,042-2,071)	2,376 (1,394-2,744)	1,839 (1,106-2,108)	950 (585-1,146)	291 (174-366)
Inflammatory bowel disease	-	-	-	763 (309-1,699)	152 (77-299)	82 (54-137)	118 (78-198)	190 (119-331)	229 (151-390)	232 (166-362)	266 (209-369)	302 (246-401)	364 (300-476)	382 (323-490)	545 (467-707)	724 (618-884)	996 (877-1,200)	1,386 (1,227-1,679)	1,604 (1,403-1,809)	2,018 (1,655-2,279)	1,740 (1,392-1,943)	943 (720-1,053)	324 (248-363)
Vascular intestinal disorders	-	-	-	86 (33-147)	24 (11-33)	17 (11-21)	31 (20-39)	68 (40-89)	85 (53-111)	117 (77-144)	170 (127-200)	279 (221-326)	418 (340-485)	555 (468-623)	974 (837-1,088)	1,694 (1,444-1,876)	3,040 (2,693-3,337)	4,608 (4,128-5,065)	5,527 (4,908-6,046)	7,009 (6,407-7,584)	5,665 (4,735-6,144)	2,737 (2,289-2,932)	742 (613-803)
Gallbladder and biliary diseases	-	-	-	624 (414-901)	244 (186-315)	159 (127-191)	184 (153-213)	335 (270-391)	476 (395-554)	622 (531-715)	715 (583-802)	909 (732-1,006)	1,102 (943-1,226)	1,454 (1,203-1,588)	2,119 (1,764-2,325)	3,211 (2,671-3,493)	4,407 (3,641-4,829)	5,543 (4,767-6,011)	6,092 (5,280-6,639)	7,008 (6,226-7,596)	5,594 (5,215-6,145)	3,127 (2,912-3,420)	1,034 (950-1,120)
Pancreatitis	-	-	-	61 (34-146)	85 (56-176)	93 (69-149)	212 (162-336)	365 (281-600)	523 (392-828)	734 (561-1,089)	876 (702-1,151)	1,158 (911-1,447)	1,290 (991-1,687)	1,248 (981-1,566)	1,858 (1,479-2,358)	2,125 (1,680-2,602)	2,726 (2,108-3,276)	3,076 (2,312-3,597)	2,722 (2,028-3,136)	2,789 (2,148-3,204)	1,996 (1,528-2,228)	896 (670-1,008)	259 (200-291)
Other digestive diseases	-	-	-	2,038 (1,602-2,698)	554 (461-685)	296 (249-352)	205 (166-250)	380 (318-463)	473 (389-575)	535 (450-639)	500 (396-588)	599 (476-680)	678 (589-782)	797 (675-887)	1,152 (982-1,282)	1,787 (1,524-1,976)	2,361 (2,095-2,559)	3,408 (3,052-3,670)	3,860 (3,550-4,143)	4,865 (4,553-5,171)	4,739 (4,452-5,039)	2,628 (2,476-2,797)	909 (857-969)
Neurological disorders	-	-	5,933 (3,904-7,514)	7,662 (3,900-10,452)	4,122 (2,930-5,137)	4,014 (3,086-4,701)	5,394 (4,230-6,055)	5,447 (4,342-6,058)	5,289 (4,257-5,855)	5,366 (4,197-5,908)	4,491 (3,655-4,860)	5,116 (4,379-5,507)	5,236 (4,812-5,597)	7,016 (6,658-7,488)	10,328 (9,914-11,109)	19,707 (18,869-21,382)	37,668 (36,304-40,554)	71,685 (69,147-76,837)	125,796 (122,814-132,575)	210,262 (206,707-218,214)	227,151 (223,934-233,798)	160,641 (159,007-163,252)	56,892 (56,340-57,533)
Alzheimer's disease and other dementias	-	-	-	-	-	-	-	-	-	-	-	285 (261-332)	837 (773-961)	2,179 (2,040-2,479)	5,041 (4,744-5,653)	12,798 (12,041-14,336)	27,367 (26,174-29,822)	55,645 (53,467-60,051)	105,257 (102,659-110,360)	186,118 (182,985-191,581)	210,610 (207,541-216,509)	154,359 (152,793-156,785)	55,556 (55,003-56,204)
Parkinson's disease	-	-	-	-	-	-	-	7 (5-9)	10 (7-12)	19 (13-22)	21 (16-24)	200 (164-235)	407 (351-475)	689 (622-805)	1,150 (1,042-1,325)	2,394 (2,184-2,742)	5,164 (4,785-5,970)	10,956 (10,221-12,599)	16,751 (15,854-18,972)	20,969 (20,002-23,218)	14,469 (13,855-15,985)	5,524 (5,319-6,155)	1,124 (1,085-1,225)
Epilepsy	-	-	4,112 (2,701-5,015)	5,831 (3,008-7,979)	3,384 (2,445-4,109)	3,294 (2,426-3,808)	4,798 (3,681-5,339)	4,877 (3,789-5,422)	4,604 (3,552-5,092)	4,440 (3,319-4,948)	3,304 (2,578-3,609)	2,970 (2,381-3,277)	1,929 (1,598-2,102)	2,024 (1,603-2,232)	1,601 (1,337-1,741)	1,634 (1,280-1,778)	1,813 (1,438-1,975)	1,715 (1,402-1,881)	1,188 (971-1,396)	1,077 (885-1,198)	784 (625-972)	279 (234-331)	76 (67-86)
Multiple sclerosis	-	-	-	-	-	-	-	102 (64-169)	202 (121-307)	363 (235-513)	574 (411-752)	836 (604-1,055)	934 (702-1,133)	886 (726-1,087)	937 (793-1,151)	854 (731-1,083)	801 (688-1,002)	689 (565-854)	442 (371-535)	322 (261-393)	172 (129-191)	50 (40-57)	10 (8-12)
Motor neuron disease	9 (2-15)	16 (5-26)	286 (192-405)	146 (75-215)	23 (18-27)	23 (19-26)	17 (15-20)	17 (14-21)	27 (21-32)	46 (38-54)	80 (71-90)	170 (153-198)	305 (281-358)	458 (435-550)	699 (665-785)	975 (929-1,066)	1,345 (1,288-1,490)	1,398 (1,347-1,442)	1,047 (1,005-1,081)	698 (665-727)	320 (303-331)	79 (74-82)	15 (14-15)
Other neurological disorders	-	-	1,535 (740-2,848)	1,685 (616-3,592)	715 (391-1,373)	697 (439-1,168)	579 (361-968)	444 (297-732)	446 (308-694)	497 (363-727)	512 (429-721)	655 (566-895)	825 (711-1,121)	780 (722-1,054)	900 (843-1,152)	1,051 (988-1,337)	1,178 (1,116-1,430)	1,281 (1,210-1,466)	1,110 (1,042-1,247)	1,078 (922-1,165)	796 (592-851)	349 (264-370)	111 (93-117)
Mental disorders	-	-	-	-	0 (0-0)	3 (3-5)	18 (15-23)	29 (25-38)	32 (28-42)	34 (29-40)	30 (27-35)	28 (25-32)	26 (24-30)	-	-	-	-	-	-	-	-	-	-
Eating disorders	-	-	-	-	0 (0-0)	3 (3-5)	18 (15-23)	29 (25-38)	32 (28-42)	34 (29-40)	30 (27-35)	28 (25-32)	26 (24-30)	-	-	-	-	-	-	-	-	-	-
Anorexia nervosa	-	-	-	-	0 (0-0)	3 (3-4)	16 (14-21)	21 (16-26)	21 (15-28)	24 (17-30)	23 (17-27)	21 (16-25)	22 (18-26)	-	-	-	-	-	-	-	-	-	-
Bulimia nervosa	-	-	-	-	0 (0-0)	0 (0-0)	1 (1-2)	8 (6-15)	11 (8-21)	9 (6-17)	7 (4-14)	7 (4-13)	4 (2-10)	-	-	-	-	-	-	-	-	-	-
Substance use disorders	0 (0-0)	0 (0-0)	0 (0-0)	0 (0-0)	0 (0-0)	0 (0-0)	1,719 (1,594-1,809)	2,978 (2,770-3,158)	4,313 (4,036-4,558)	4,992 (4,560-5,220)	5,158 (4,435-5,419)	5,708 (4,719-6,011)	5,413 (4,568-5,736)	4,508 (3,801-4,770)	5,334 (4,131-5,590)	4,109 (3,541-4,303)	3,771 (3,290-3,965)	2,546 (2,328-2,720)	2,185 (2,025-2,346)	1,584 (1,430-1,686)	1,105 (964-1,180)	443 (391-466)	121 (107-127)
Alcohol use disorders	-	-	-	-	-	-	332 (208-374)	529 (345-595)	940 (734-1,055)	1,660 (1,242-1,829)	2,697 (1,947-2,937)	3,521 (2,550-3,783)	3,745 (2,881-4,071)	3,261 (2,542-3,521)	4,041 (2,832-4,295)	2,710 (2,056-2,871)	2,283 (1,730-2,438)	1,160 (857-1,235)	888 (655-1,014)	569 (326-618)	403 (207-444)	150 (85-163)	41 (25-44)
Drug use disorders	0 (0-0)	0 (0-0)	0 (0-0)	0 (0-0)	0 (0-0)	0 (0-0)	1,387 (1,311-1,463)	2,449 (2,302-2,613)	3,372 (3,159-3,614)	3,331 (3,158-3,514)	2,461 (2,359-2,568)	2,186 (2,094-2,287)	1,667 (1,596-1,755)	1,247 (1,195-1,315)	1,294 (1,237-1,364)	1,399 (1,331-1,494)	1,488 (1,416-1,607)	1,386 (1,317-1,522)	1,297 (1,224-1,439)	1,015 (961-1,118)	703 (661-771)	294 (279-314)	81 (77-84)
Opioid use disorders	0 (0-0)	0 (0-0)	0 (0-0)	0 (0-0)	0 (0-0)	0 (0-0)	703 (555-870)	1,227 (900-1,597)	1,666 (1,273-2,180)	1,687 (1,311-2,124)	1,267 (1,066-1,443)	1,128 (949-1,275)	806 (665-925)	590 (483-675)	628 (522-723)	661 (521-796)	742 (602-898)	565 (449-743)	530 (415-712)	390 (300-518)	288 (220-362)	122 (97-143)	30 (25-34)
Cocaine use disorders	-	-	-	-	-	-	39 (25-56)	62 (37-99)	92 (54-137)	103 (78-132)	96 (83-112)	85 (73-101)	64 (55-76)	32 (28-38)	32 (27-38)	29 (24-36)	27 (23-33)	10 (8-12)	8 (6-9)	5 (4-6)	4 (3-5)	2 (2-2)	1 (0-1)
Amphetamine use disorders	-	-	-	-	-	-	95 (30-228)	225 (51-596)	294 (75-712)	165 (48-399)	103 (41-234)	91 (37-205)	74 (30-170)	26 (13-58)	27 (14-55)	27 (12-62)	23 (11-51)	5 (3-11)	5 (3-9)	2 (1-4)	2 (1-3)	1 (0-1)	0 (0-0)
Other drug use disorders	-	-	-	-	-	-	550 (352-728)	935 (499-1,355)	1,320 (794-1,828)	1,377 (948-1,804)	995 (783-1,221)	882 (722-1,082)	724 (598-887)	599 (517-716)	608 (529-722)	682 (584-828)	696 (585-845)	806 (711-916)	755 (668-864)	618 (564-698)	409 (369-469)	169 (157-193)	50 (47-55)
Diabetes and kidney diseases	8 (6-10)	26 (18-34)	5,275 (4,766-5,759)	8,127 (6,858-9,281)	3,540 (3,107-3,993)	3,944 (3,518-4,386)	5,824 (5,487-6,144)	9,504 (9,026-9,980)	11,894 (11,345-12,445)	14,026 (13,400-14,780)	15,928 (15,156-16,826)	22,480 (21,521-23,612)	27,886 (26,671-29,221)	38,102 (36,355-39,790)	52,976 (50,972-55,041)	74,801 (71,772-77,506)	88,376 (85,273-91,538)	92,525 (89,565-96,104)	85,730 (82,889-89,227)	77,109 (74,919-79,968)	54,074 (52,739-55,695)	25,842 (25,275-26,424)	8,203 (8,042-8,356)
Diabetes mellitus	8 (6-10)	26 (18-34)	571 (390-721)	557 (373-771)	519 (380-674)	862 (624-1,120)	1,575 (1,476-1,683)	2,356 (2,223-2,498)	3,342 (3,163-3,573)	3,974 (3,773-4,255)	5,465 (5,130-5,926)	9,155 (8,686-9,788)	13,142 (12,421-14,053)	20,389 (19,377-21,596)	30,338 (29,017-31,851)	46,563 (44,545-48,847)	56,424 (54,154-58,884)	58,413 (56,294-60,531)	51,570 (49,776-53,574)	44,295 (42,921-45,890)	27,595 (26,767-28,465)	12,302 (11,982-12,619)	3,634 (3,537-3,725)
Diabetes mellitus type 1	8 (6-10)	26 (18-34)	571 (390-721)	557 (373-771)	519 (380-674)	862 (624-1,120)	1,398 (1,278-1,504)	2,043 (1,870-2,195)	2,932 (2,723-3,138)	3,202 (2,941-3,456)	4,313 (3,994-4,711)	7,038 (6,462-7,630)	7,184 (6,267-8,188)	10,586 (9,202-12,148)	15,402 (13,404-17,641)	17,712 (14,941-21,186)	20,755 (17,144-24,365)	17,759 (14,161-21,130)	15,137 (11,927-18,110)	9,417 (7,151-11,494)	5,505 (3,918-6,577)	2,318 (1,645-2,673)	647 (453-740)
Diabetes mellitus type 2	-	-	-	-	-	-	177 (110-299)	313 (191-522)	411 (257-680)	772 (533-1,144)	1,152 (831-1,607)	2,116 (1,550-2,872)	5,958 (4,808-7,219)	9,803 (8,056-11,704)	14,935 (12,744-17,251)	28,850 (25,181-32,279)	35,668 (31,898-39,314)	40,654 (37,360-44,151)	36,432 (33,690-39,929)	34,877 (32,948-37,557)	22,090 (20,933-23,832)	9,984 (9,548-10,749)	2,987 (2,881-3,194)
Chronic kidney disease	-	-	4,507 (4,029-4,986)	7,257 (6,090-8,410)	2,765 (2,413-3,117)	2,923 (2,639-3,197)	4,102 (3,836-4,367)	6,891 (6,501-7,301)	8,228 (7,798-8,684)	9,729 (9,229-10,313)	10,205 (9,696-10,751)	13,015 (12,383-13,669)	14,429 (13,748-15,121)	17,482 (16,609-18,395)	22,327 (21,292-23,332)	27,833 (26,496-29,057)	31,490 (30,140-32,974)	33,615 (32,229-35,232)	33,729 (32,346-35,428)	32,471 (31,424-33,863)	26,271 (25,526-27,190)	13,458 (13,109-13,832)	4,543 (4,438-4,649)
Chronic kidney disease due to diabetes mellitus type 1	-	-	19 (10-30)	34 (17-62)	20 (10-36)	34 (18-57)	90 (48-146)	355 (190-578)	901 (553-1,390)	1,820 (1,210-2,560)	2,327 (1,559-3,234)	3,118 (2,054-4,289)	3,166 (2,139-4,387)	2,906 (1,772-4,240)	2,565 (1,460-3,991)	1,899 (927-3,250)	1,447 (748-2,616)	1,165 (552-2,060)	951 (468-1,735)	834 (415-1,431)	438 (229-758)	92 (44-167)	6 (3-13)
Chronic kidney disease due to diabetes mellitus type 2	-	-	13 (6-22)	21 (10-40)	11 (5-21)	16 (7-31)	38 (17-70)	113 (49-214)	227 (100-436)	430 (202-772)	657 (337-1,112)	1,306 (729-2,146)	2,324 (1,435-3,350)	4,072 (2,753-5,693)	6,428 (4,506-8,325)	9,451 (6,771-12,009)	11,131 (8,290-13,922)	11,777 (8,730-14,580)	11,409 (8,131-14,719)	10,263 (7,206-13,466)	7,601 (5,282-9,812)	3,492 (2,492-4,561)	1,077 (680-1,561)
Chronic kidney disease due to hypertension	-	-	22 (12-33)	52 (28-83)	33 (18-54)	60 (34-95)	147 (88-223)	390 (248-560)	632 (430-892)	908 (625-1,254)	1,117 (777-1,514)	1,630 (1,164-2,159)	2,092 (1,481-2,796)	3,002 (2,194-3,977)	4,405 (3,339-5,763)	6,187 (4,565-7,979)	7,965 (6,150-10,040)	9,688 (7,624-12,163)	10,982 (8,672-13,618)	11,670 (9,236-14,267)	10,878 (8,933-12,977)	6,237 (5,205-7,260)	2,260 (1,841-2,671)
Chronic kidney disease due to glomerulonephritis	-	-	1,125 (795-1,536)	2,262 (1,553-3,176)	903 (602-1,268)	1,044 (699-1,430)	1,647 (1,166-2,190)	2,774 (1,966-3,687)	2,973 (2,085-3,900)	3,009 (2,139-4,073)	2,803 (1,899-3,868)	3,272 (2,173-4,516)	3,318 (2,202-4,768)	3,493 (2,290-4,997)	3,957 (2,644-5,719)	4,081 (2,624-5,985)	4,490 (2,887-6,481)	4,715 (2,970-6,800)	4,545 (2,964-6,591)	4,360 (2,852-6,442)	3,259 (2,161-4,808)	1,594 (1,073-2,326)	535 (297-853)
Chronic kidney disease due to other and unspecified causes	-	-	3,329 (2,826-3,783)	4,889 (3,815-5,947)	1,798 (1,375-2,196)	1,769 (1,402-2,170)	2,180 (1,642-2,722)	3,259 (2,326-4,190)	3,495 (2,540-4,523)	3,562 (2,532-4,600)	3,301 (2,261-4,377)	3,689 (2,469-4,989)	3,529 (2,370-5,007)	4,009 (2,623-5,721)	4,972 (3,254-7,017)	6,214 (3,862-9,002)	6,457 (4,134-9,104)	6,271 (4,058-8,925)	5,841 (3,779-8,801)	5,344 (3,549-7,677)	4,096 (2,800-5,675)	2,043 (1,399-2,863)	666 (427-1,032)
Acute glomerulonephritis	-	-	196 (128-241)	314 (180-409)	256 (209-305)	159 (132-184)	147 (126-167)	257 (222-293)	323 (278-371)	323 (285-364)	258 (229-292)	310 (278-349)	314 (282-351)	231 (205-261)	311 (278-348)	405 (360-458)	463 (406-528)	497 (435-584)	431 (374-509)	344 (300-404)	208 (181-243)	82 (71-98)	26 (21-32)
Skin and subcutaneous diseases	-	-	2,522 (772-3,664)	1,498 (347-2,523)	358 (110-532)	280 (108-376)	331 (137-421)	329 (147-413)	323 (147-400)	419 (202-533)	465 (231-615)	605 (307-800)	692 (345-909)	939 (468-1,249)	1,200 (637-1,662)	1,467 (754-2,015)	1,892 (983-2,666)	2,268 (1,243-3,256)	2,629 (1,508-3,962)	3,047 (1,610-4,724)	2,847 (1,402-4,217)	1,704 (816-2,474)	730 (358-1,162)
Bacterial skin diseases	-	-	2,486 (746-3,620)	1,418 (309-2,422)	308 (77-473)	223 (71-314)	251 (86-335)	254 (98-333)	243 (98-313)	332 (149-418)	380 (176-487)	496 (239-654)	566 (271-728)	794 (377-1,027)	945 (490-1,286)	1,199 (594-1,588)	1,418 (728-1,987)	1,562 (849-2,252)	1,726 (958-2,667)	1,648 (917-2,522)	1,348 (702-2,080)	774 (412-1,199)	309 (166-531)
Cellulitis	-	-	232 (27-546)	276 (35-610)	100 (20-170)	77 (20-118)	76 (23-111)	74 (27-102)	74 (25-101)	96 (41-135)	92 (43-147)	116 (52-191)	126 (55-206)	180 (75-303)	235 (108-414)	281 (129-493)	348 (155-607)	340 (159-641)	337 (150-653)	384 (176-770)	295 (132-587)	190 (86-398)	83 (36-175)
Pyoderma	-	-	2,254 (698-3,298)	1,142 (270-1,952)	208 (53-318)	146 (48-207)	175 (61-233)	180 (69-235)	169 (68-218)	236 (96-301)	288 (124-368)	380 (162-497)	441 (195-576)	614 (273-788)	710 (345-970)	918 (417-1,206)	1,070 (517-1,484)	1,222 (637-1,722)	1,389 (750-2,118)	1,264 (683-1,871)	1,052 (526-1,617)	584 (303-874)	227 (114-375)
Decubitus ulcer	-	-	-	39 (14-109)	24 (13-53)	27 (15-45)	45 (25-77)	31 (16-49)	44 (23-72)	47 (25-79)	49 (28-82)	67 (36-102)	88 (44-130)	106 (57-168)	214 (112-336)	213 (109-327)	399 (200-584)	619 (315-935)	794 (397-1,175)	1,250 (581-1,869)	1,351 (606-1,892)	837 (361-1,152)	387 (165-565)
Other skin and subcutaneous diseases	-	-	37 (10-82)	42 (16-108)	27 (13-55)	30 (16-51)	36 (19-56)	44 (23-71)	36 (18-55)	40 (20-64)	37 (19-65)	42 (19-80)	38 (17-71)	39 (18-84)	41 (17-89)	56 (24-114)	74 (31-152)	87 (39-192)	109 (46-222)	149 (59-316)	149 (58-319)	92 (32-206)	34 (11-72)
Musculoskeletal disorders	-	-	-	-	407 (271-543)	632 (516-799)	1,166 (989-1,509)	1,506 (1,271-2,151)	1,616 (1,377-2,404)	1,768 (1,521-2,421)	1,557 (1,403-1,943)	1,888 (1,629-2,390)	1,939 (1,783-2,285)	1,812 (1,648-2,096)	2,375 (2,171-2,728)	3,597 (3,213-4,119)	4,820 (4,204-5,460)	5,387 (4,822-6,052)	5,067 (4,552-5,749)	4,766 (4,322-5,314)	3,910 (3,612-4,278)	1,846 (1,737-1,969)	633 (604-665)
Rheumatoid arthritis	-	-	-	-	83 (43-138)	56 (36-78)	72 (45-108)	105 (70-158)	140 (98-203)	208 (144-285)	205 (152-265)	347 (253-453)	554 (412-683)	651 (508-797)	1,143 (941-1,363)	2,030 (1,650-2,417)	2,925 (2,403-3,433)	3,327 (2,801-3,917)	3,103 (2,648-3,681)	2,618 (2,239-3,056)	1,761 (1,516-2,034)	656 (572-758)	157 (138-178)
Other musculoskeletal disorders	-	-	-	-	323 (201-433)	576 (470-724)	1,093 (917-1,416)	1,401 (1,170-2,021)	1,476 (1,244-2,216)	1,560 (1,325-2,178)	1,352 (1,208-1,678)	1,542 (1,325-1,969)	1,385 (1,231-1,660)	1,160 (1,035-1,382)	1,232 (1,097-1,483)	1,567 (1,327-2,004)	1,895 (1,585-2,472)	2,060 (1,747-2,600)	1,963 (1,676-2,419)	2,148 (1,882-2,526)	2,150 (1,920-2,410)	1,190 (1,068-1,291)	476 (448-503)
Other non-communicable diseases	91,838 (82,920-103,183)	44,936 (40,400-51,581)	163,326 (141,520-201,078)	73,710 (60,346-88,325)	17,234 (15,316-19,513)	10,288 (9,406-11,528)	9,257 (8,606-10,190)	10,229 (9,512-11,160)	9,369 (8,644-10,169)	8,484 (7,722-9,110)	7,167 (6,662-7,759)	7,926 (7,348-8,517)	7,214 (6,645-7,800)	7,655 (7,048-8,273)	9,037 (8,393-9,801)	10,902 (10,069-11,827)	13,333 (12,205-14,352)	13,298 (12,181-14,419)	12,920 (11,782-14,003)	13,552 (12,282-14,676)	11,571 (10,403-12,224)	6,508 (5,855-6,880)	2,652 (2,409-2,814)
Congenital birth defects	90,216 (81,384-101,757)	41,090 (36,481-47,405)	131,457 (118,861-145,371)	57,204 (48,278-68,482)	12,005 (10,693-13,685)	6,626 (6,040-7,456)	4,006 (3,713-4,525)	4,101 (3,824-4,571)	3,748 (3,481-4,108)	2,477 (2,264-2,769)	1,929 (1,747-2,221)	2,034 (1,843-2,346)	1,007 (877-1,174)	792 (690-960)	817 (721-1,024)	760 (666-959)	743 (654-920)	-	-	-	-	-	-
Neural tube defects	14,714 (11,079-18,316)	4,685 (3,208-6,798)	14,158 (8,103-25,032)	7,544 (3,255-17,719)	630 (433-939)	351 (266-453)	179 (138-229)	147 (109-200)	98 (75-128)	91 (71-117)	62 (51-75)	50 (42-60)	35 (29-41)	25 (21-29)	24 (21-29)	19 (17-23)	15 (13-19)	-	-	-	-	-	-
Congenital heart anomalies	31,137 (22,781-39,178)	19,226 (13,742-23,615)	72,787 (48,156-92,885)	31,935 (17,229-43,538)	7,894 (5,900-9,423)	4,485 (3,773-5,160)	2,594 (2,201-2,972)	2,656 (2,243-3,010)	2,598 (2,286-2,929)	1,512 (1,314-1,721)	1,243 (1,085-1,438)	1,408 (1,234-1,650)	557 (482-671)	377 (320-477)	382 (330-495)	362 (316-470)	389 (335-500)	-	-	-	-	-	-
Orofacial clefts	1,900 (752-4,786)	931 (340-2,541)	1,722 (595-4,898)	379 (101-1,519)	-	-	-	-	-	-	-	-	-	-	-	-	-	-	-	-	-	-	-
Down syndrome	1,656 (887-2,840)	790 (364-1,369)	3,690 (1,543-6,784)	2,063 (655-4,978)	254 (123-456)	185 (116-261)	217 (144-296)	331 (202-494)	377 (231-541)	302 (207-415)	192 (147-240)	221 (175-276)	181 (144-216)	195 (163-247)	193 (160-260)	142 (116-208)	71 (57-115)	-	-	-	-	-	-
Other chromosomal abnormalities	2,496 (1,133-6,214)	1,499 (678-3,733)	3,269 (1,518-8,135)	746 (280-2,470)	160 (76-365)	86 (48-162)	69 (40-137)	66 (34-147)	54 (30-104)	42 (24-78)	31 (19-55)	28 (16-43)	18 (11-30)	16 (10-29)	17 (10-30)	15 (9-28)	13 (9-25)	-	-	-	-	-	-
Congenital musculoskeletal and limb anomalies	2,179 (1,061-3,525)	724 (276-1,270)	1,913 (627-3,963)	803 (205-2,115)	245 (91-461)	162 (81-229)	120 (63-163)	113 (50-168)	76 (38-110)	66 (35-94)	50 (32-66)	35 (24-47)	28 (18-38)	22 (13-28)	24 (15-33)	26 (17-34)	26 (14-35)	-	-	-	-	-	-
Urogenital congenital anomalies	2,223 (1,461-2,941)	813 (504-1,153)	1,345 (643-2,304)	459 (162-945)	174 (97-286)	130 (86-182)	60 (44-83)	70 (48-95)	53 (36-74)	51 (35-65)	54 (39-68)	60 (41-74)	49 (36-60)	50 (38-62)	71 (53-88)	91 (72-116)	121 (96-149)	-	-	-	-	-	-
Digestive congenital anomalies	8,321 (4,369-13,521)	4,119 (2,030-6,774)	10,333 (4,081-19,202)	2,580 (762-5,792)	224 (121-438)	90 (61-138)	52 (34-78)	49 (31-85)	27 (19-39)	26 (17-39)	20 (15-26)	18 (13-24)	17 (12-22)	18 (14-24)	21 (15-27)	24 (18-32)	32 (23-43)	-	-	-	-	-	-
Other congenital birth defects	25,590 (16,721-36,620)	8,304 (4,467-12,374)	22,241 (10,274-37,444)	10,694 (4,068-22,116)	2,424 (1,330-3,927)	1,136 (741-1,666)	714 (499-1,040)	668 (424-981)	465 (303-685)	387 (276-555)	276 (209-380)	212 (160-289)	120 (97-152)	89 (73-115)	86 (71-112)	80 (67-103)	75 (62-100)	-	-	-	-	-	-
Urinary diseases and male infertility	265 (234-303)	228 (194-267)	2,697 (2,408-3,044)	3,174 (2,618-3,834)	1,087 (932-1,266)	736 (646-825)	1,258 (1,153-1,380)	1,804 (1,682-1,975)	1,615 (1,520-1,741)	1,884 (1,770-2,040)	1,836 (1,742-1,971)	2,255 (2,145-2,422)	2,489 (2,367-2,676)	3,055 (2,900-3,277)	4,240 (4,031-4,531)	5,094 (4,861-5,419)	6,480 (6,192-6,847)	6,821 (6,533-7,179)	6,911 (6,598-7,250)	7,010 (6,775-7,313)	6,079 (5,897-6,286)	3,533 (3,439-3,633)	1,577 (1,540-1,614)
Urinary tract infections	45 (33-73)	56 (36-82)	1,603 (1,176-2,065)	1,177 (555-1,915)	476 (299-673)	348 (251-441)	910 (746-1,073)	1,281 (1,043-1,516)	1,092 (871-1,270)	1,258 (1,016-1,458)	1,326 (1,118-1,505)	1,616 (1,347-1,813)	1,709 (1,459-1,936)	2,253 (1,981-2,519)	3,082 (2,728-3,446)	3,600 (3,213-4,111)	4,867 (4,403-5,453)	5,187 (4,747-5,752)	5,376 (5,026-6,036)	5,858 (5,573-6,296)	5,258 (5,039-5,581)	3,100 (2,998-3,253)	1,408 (1,368-1,467)
Urolithiasis	-	-	-	-	57 (24-110)	32 (16-55)	30 (14-54)	76 (33-137)	120 (56-208)	169 (87-273)	125 (79-187)	180 (120-259)	240 (170-333)	231 (184-309)	374 (300-495)	441 (361-576)	605 (488-795)	623 (501-787)	540 (434-674)	481 (381-593)	289 (222-370)	139 (113-175)	40 (32-50)

SR Table 5: Global deaths by cause and age group for females (with 95% confidence intervals), 1995

Cause	Early Neonatal	Late Neonatal	Post Neonatal	1 to 4	5 to 9	10 to 14	15 to 19
Other urinary diseases	219 (172-260)	172 (125-210)	1,094 (581-1,547)	1,997 (1,097-2,817)	554 (359-759)	356 (272-458)	318 (184-448)
Gynecological diseases	-	-	-	-	-	-	434 (347-566)
Uterine fibroids	-	-	-	-	-	-	70 (43-95)
Polycystic ovarian syndrome	-	-	-	-	-	-	1 (0-4)
Endometriosis	-	-	-	-	-	-	9 (4-13)
Genital prolapse	-	-	-	-	-	-	15 (8-24)
Other gynecological diseases	-	-	-	-	-	-	339 (258-490)
Hemoglobinopathies and hemolytic anemias	465 (377-638)	340 (264-482)	3,493 (2,006-5,876)	10,386 (4,184-17,181)	2,999 (1,978-4,242)	2,122 (1,700-2,889)	2,700 (2,072-3,481)
Thalassemias	205 (149-282)	140 (102-202)	1,473 (842-2,397)	2,305 (1,201-3,359)	1,325 (885-1,770)	662 (496-822)	492 (339-622)
Sickle cell disorders	231 (181-340)	191 (145-275)	1,942 (1,057-3,387)	7,850 (2,817-13,386)	1,458 (888-2,179)	1,118 (839-1,664)	1,684 (1,257-2,252)
G6PD deficiency	4 (3-6)	3 (2-5)	36 (19-67)	141 (58-261)	116 (77-178)	168 (116-248)	250 (183-347)
Other hemoglobinopathies and hemolytic anemias	26 (19-35)	6 (4-8)	42 (25-69)	90 (47-143)	101 (74-139)	173 (141-228)	273 (215-361)
Endocrine, metabolic, blood, and immune disorders	892 (564-1,076)	831 (483-1,047)	4,663 (2,481-6,418)	2,946 (1,475-4,522)	1,143 (763-1,504)	805 (631-1,030)	859 (672-1,070)
Sudden infant death syndrome	-	2,447 (838-5,507)	21,017 (6,818-51,990)	-	-	-	-
Injuries	7,840 (6,577-8,690)	6,864 (5,784-7,752)	62,983 (55,515-69,076)	158,716 (126,294-180,236)	82,855 (74,566-90,242)	53,233 (48,505-56,814)	90,108 (85,440-93,862)
Transport injuries	716 (604-834)	927 (722-1,085)	9,574 (7,514-10,745)	34,313 (23,322-40,105)	26,420 (23,514-29,152)	15,164 (13,893-16,253)	24,915 (23,629-26,180)
Road injuries	626 (516-743)	859 (647-1,010)	8,889 (6,821-10,000)	32,126 (20,999-37,620)	25,341 (22,504-27,933)	14,231 (12,991-15,288)	23,229 (22,031-24,488)
Pedestrian road injuries	351 (255-449)	458 (322-628)	4,047 (3,240-5,281)	19,736 (14,485-25,736)	16,555 (14,565-18,677)	7,771 (7,028-8,469)	8,354 (7,606-9,332)
Cyclist road injuries	-	-	-	578 (290-902)	751 (502-991)	712 (579-857)	800 (637-975)
Motorcyclist road injuries	66 (43-98)	43 (29-64)	956 (631-1,415)	1,637 (904-3,029)	1,262 (866-1,950)	1,195 (912-1,643)	3,215 (2,595-4,113)
Motor vehicle road injuries	200 (148-258)	351 (214-476)	3,775 (2,431-4,705)	9,932 (4,763-13,262)	6,591 (5,255-7,741)	4,441 (3,821-4,921)	10,649 (9,621-11,474)
Other road injuries	8 (5-12)	7 (4-10)	111 (52-181)	243 (86-428)	182 (105-248)	112 (79-147)	211 (147-274)
Other transport injuries	90 (58-157)	68 (43-127)	685 (511-1,118)	2,187 (1,544-3,863)	1,079 (836-1,411)	933 (782-1,077)	1,686 (1,462-1,958)
Unintentional injuries	5,469 (4,599-6,178)	5,319 (4,444-6,062)	49,429 (43,569-54,924)	114,659 (91,769-132,555)	49,875 (43,254-55,275)	26,459 (22,932-28,869)	22,356 (19,256-24,242)
Falls	841 (725-1,028)	374 (299-503)	5,034 (4,063-6,315)	7,502 (5,239-10,656)	3,463 (2,778-4,262)	1,909 (1,662-2,182)	2,183 (1,897-2,504)
Drowning	249 (224-290)	210 (177-232)	6,294 (5,120-7,035)	64,658 (50,534-74,200)	28,211 (23,882-31,605)	12,416 (10,490-13,498)	6,781 (6,040-7,250)
Fire, heat, and hot substances	194 (127-238)	350 (173-451)	4,506 (2,439-5,520)	13,049 (6,093-17,200)	3,811 (2,521-4,537)	2,443 (1,704-2,896)	4,318 (2,606-5,287)
Poisonings	107 (81-122)	137 (93-157)	2,050 (1,262-2,554)	5,205 (2,756-7,093)	1,665 (1,197-2,095)	1,428 (1,183-1,753)	1,450 (1,263-1,823)
Poisoning by carbon monoxide	26 (16-33)	34 (22-40)	461 (263-574)	1,093 (719-1,388)	767 (605-1,015)	560 (479-757)	739 (662-955)
Poisoning by other means	81 (62-92)	103 (69-122)	1,589 (950-2,074)	4,113 (2,001-5,944)	898 (603-1,134)	869 (690-1,054)	711 (585-892)
Exposure to mechanical forces	376 (223-475)	462 (231-608)	2,506 (1,449-3,141)	3,085 (1,985-3,853)	2,308 (1,633-2,802)	1,122 (845-1,302)	1,428 (1,027-1,661)
Unintentional firearm injuries	32 (19-41)	23 (13-31)	222 (110-293)	282 (162-396)	250 (169-314)	200 (152-233)	285 (229-328)
Other exposure to mechanical forces	344 (200-440)	439 (216-582)	2,283 (1,313-2,887)	2,804 (1,758-3,520)	2,058 (1,419-2,518)	922 (678-1,085)	1,143 (779-1,355)
Adverse effects of medical treatment	830 (510-1,020)	669 (377-894)	4,840 (2,467-7,128)	3,558 (1,404-6,464)	1,325 (759-1,764)	865 (573-1,036)	1,057 (737-1,238)
Animal contact	126 (40-180)	136 (45-197)	1,828 (618-2,694)	4,586 (1,518-8,695)	4,413 (1,538-6,917)	2,845 (993-3,945)	1,784 (606-2,509)
Venomous animal contact	84 (21-141)	77 (22-127)	1,430 (483-2,126)	3,450 (1,209-6,559)	3,816 (1,309-5,847)	2,591 (866-3,623)	1,634 (531-2,309)
Non-venomous animal contact	41 (13-109)	59 (18-140)	398 (80-1,371)	1,136 (133-5,311)	596 (121-2,537)	254 (78-809)	150 (51-503)
Foreign body	2,383 (2,129-2,792)	2,777 (2,404-3,277)	19,742 (16,263-22,519)	7,218 (4,977-8,675)	1,814 (1,591-2,070)	1,034 (962-1,142)	689 (657-755)
Pulmonary aspiration and foreign body in airway	2,292 (2,034-2,701)	2,681 (2,327-3,168)	18,416 (14,986-20,946)	6,439 (4,376-7,921)	1,552 (1,357-1,785)	886 (810-988)	497 (454-555)
Foreign body in other body part	91 (38-138)	96 (27-137)	1,327 (270-2,084)	779 (240-1,487)	262 (135-395)	147 (94-195)	192 (149-227)
Environmental heat and cold exposure	153 (59-198)	102 (36-142)	1,239 (399-1,744)	806 (269-1,360)	391 (190-537)	170 (112-191)	394 (261-453)
Exposure to forces of nature	4 (3-4)	15 (13-16)	121 (109-136)	625 (564-696)	618 (561-689)	638 (576-710)	522 (468-592)
Other unintentional injuries	207 (179-239)	88 (73-103)	1,268 (1,091-1,457)	4,366 (3,512-5,303)	1,855 (1,582-2,191)	1,591 (1,385-1,818)	1,750 (1,558-1,949)
Self-harm and interpersonal violence	1,655 (1,261-1,829)	618 (471-682)	3,980 (2,763-4,461)	9,743 (7,615-11,211)	6,560 (5,105-7,574)	11,610 (10,614-12,607)	42,836 (40,389-45,527)
Self-harm	-	-	-	-	-	6,508 (5,858-7,261)	33,607 (31,530-36,087)
Self-harm by firearm	-	-	-	-	-	217 (135-311)	1,156 (649-1,782)
Self-harm by other specified means	-	-	-	-	-	6,291 (5,653-7,058)	32,451 (30,386-35,165)
Interpersonal violence	1,250 (856-1,422)	397 (250-461)	3,270 (2,051-3,748)	5,918 (3,792-7,383)	4,333 (2,878-5,344)	3,238 (2,585-3,841)	7,021 (5,600-7,994)
Physical violence by firearm	71 (44-103)	32 (16-46)	346 (205-498)	601 (368-956)	466 (345-638)	601 (507-717)	1,989 (1,640-2,288)
Physical violence by sharp object	64 (45-76)	43 (25-58)	287 (179-358)	640 (349-909)	570 (368-738)	555 (417-684)	1,423 (1,114-1,687)
Physical violence by other means	1,114 (740-1,274)	322 (200-375)	2,636 (1,569-3,060)	4,677 (2,745-5,867)	3,297 (2,048-4,123)	2,082 (1,541-2,553)	3,610 (2,592-4,304)
Conflict and terrorism	406 (348-486)	221 (189-266)	699 (598-838)	3,800 (3,261-4,613)	2,196 (1,864-2,676)	1,835 (1,578-2,168)	2,178 (1,915-2,570)
Executions and police conflict	-	-	12 (6-15)	26 (20-30)	31 (28-35)	28 (26-31)	29 (27-34)

Cause	20 to 24	25 to 29	30 to 34	35 to 39	40 to 44	45 to 49	50 to 54	54 to 59
Other urinary diseases	446 (262-633)	403 (249-565)	456 (299-606)	386 (235-530)	458 (301-629)	540 (344-701)	570 (348-752)	784 (491-1,018)
Gynecological diseases	492 (380-635)	496 (384-638)	545 (432-669)	570 (464-644)	633 (528-709)	683 (583-772)	483 (409-567)	344 (297-390)
Uterine fibroids	94 (53-153)	125 (74-192)	134 (84-201)	148 (94-203)	196 (134-251)	223 (173-280)	163 (126-228)	80 (65-114)
Polycystic ovarian syndrome	3 (1-7)	2 (0-5)	1 (0-3)	1 (0-2)	1 (0-2)	1 (0-2)	1 (0-2)	-
Endometriosis	11 (6-17)	11 (5-18)	16 (8-25)	15 (7-25)	20 (9-33)	26 (11-45)	16 (7-29)	-
Genital prolapse	13 (6-19)	15 (6-23)	16 (6-25)	13 (4-22)	26 (7-45)	30 (7-53)	60 (17-98)	49 (17-86)
Other gynecological diseases	372 (268-546)	343 (249-503)	379 (287-496)	393 (305-512)	390 (299-497)	403 (314-491)	243 (183-319)	215 (174-271)
Hemoglobinopathies and hemolytic anemias	2,653 (1,920-3,460)	2,172 (1,613-2,775)	2,095 (1,511-2,590)	1,396 (1,031-1,757)	1,482 (1,102-1,853)	1,213 (923-1,510)	1,267 (972-1,587)	1,467 (1,153-1,912)
Thalassemias	429 (283-535)	287 (192-370)	167 (109-218)	67 (48-85)	44 (35-57)	28 (22-37)	14 (12-18)	8 (7-11)
Sickle cell disorders	1,582 (1,134-2,227)	1,148 (830-1,599)	904 (650-1,260)	483 (350-747)	421 (287-691)	288 (195-503)	216 (143-404)	154 (94-286)
G6PD deficiency	327 (242-436)	369 (267-484)	455 (319-581)	365 (267-463)	374 (275-484)	277 (204-358)	253 (183-336)	234 (161-349)
Other hemoglobinopathies and hemolytic anemias	315 (246-397)	368 (285-459)	569 (419-688)	481 (368-575)	643 (514-789)	620 (479-757)	784 (623-947)	1,070 (852-1,333)
Endocrine, metabolic, blood, and immune disorders	1,178 (861-1,408)	1,338 (938-1,616)	1,483 (1,075-1,822)	1,437 (1,104-1,765)	1,523 (1,193-1,942)	1,822 (1,416-2,237)	2,058 (1,639-2,478)	2,169 (1,736-2,702)
Sudden infant death syndrome	-	-	-	-	-	-	-	-
Injuries	111,421 (106,647-114,949)	106,268 (101,679-109,759)	92,588 (88,445-95,819)	70,343 (67,085-72,569)	68,998 (65,787-71,362)	59,535 (56,737-61,646)	51,062 (48,656-52,851)	54,000 (51,582-55,817)
Transport injuries	28,044 (26,109-29,448)	26,094 (24,263-27,434)	23,787 (22,333-24,928)	19,682 (18,800-20,450)	20,317 (19,516-21,212)	16,999 (16,291-17,747)	15,169 (14,545-15,782)	15,268 (14,660-15,924)
Road injuries	26,531 (24,622-27,851)	24,605 (22,743-25,881)	22,535 (21,026-23,579)	18,735 (17,898-19,465)	19,236 (18,440-20,096)	16,012 (15,336-16,724)	14,315 (13,772-14,893)	14,622 (14,054-15,246)
Pedestrian road injuries	9,973 (9,014-11,266)	9,160 (8,249-10,777)	9,386 (8,463-10,840)	7,709 (7,102-8,465)	8,543 (7,873-9,340)	7,431 (6,881-8,203)	6,638 (6,178-7,175)	7,388 (6,963-7,910)
Cyclist road injuries	750 (539-996)	801 (513-1,136)	901 (591-1,257)	624 (481-805)	770 (607-988)	770 (618-981)	570 (499-667)	588 (518-678)
Motorcyclist road injuries	4,094 (3,191-5,272)	3,777 (2,910-5,094)	3,409 (2,611-4,445)	2,398 (1,950-2,957)	2,560 (2,003-3,233)	1,904 (1,554-2,443)	1,739 (1,413-2,218)	1,478 (1,193-2,001)
Motor vehicle road injuries	11,498 (10,213-12,578)	10,694 (9,365-11,868)	8,648 (7,639-9,651)	7,818 (7,054-8,456)	7,206 (6,520-7,899)	5,729 (5,188-6,229)	5,221 (4,614-5,660)	5,017 (4,456-5,442)
Other road injuries	216 (144-275)	173 (113-223)	192 (125-245)	186 (131-236)	157 (114-200)	177 (132-225)	148 (107-200)	151 (113-202)
Other transport injuries	1,512 (1,272-1,783)	1,490 (1,258-1,753)	1,251 (1,071-1,481)	947 (831-1,063)	1,080 (947-1,210)	987 (873-1,102)	853 (746-964)	646 (582-704)
Unintentional injuries	23,384 (20,180-25,390)	22,064 (19,187-23,930)	21,384 (18,829-23,134)	18,237 (16,131-19,538)	19,021 (16,803-20,344)	18,147 (16,256-19,272)	17,059 (15,063-18,299)	20,765 (18,958-21,968)
Falls	2,289 (1,955-2,699)	2,148 (1,878-2,497)	2,493 (2,194-2,876)	2,541 (2,310-2,855)	2,807 (2,581-3,092)	3,376 (3,097-3,712)	3,791 (3,449-4,270)	5,887 (5,356-6,564)
Drowning	5,632 (4,968-6,008)	4,936 (4,281-5,283)	4,417 (3,947-4,716)	3,148 (2,886-3,312)	3,134 (2,887-3,319)	2,785 (2,576-2,945)	2,422 (2,261-2,583)	2,543 (2,379-2,716)
Fire, heat, and hot substances	5,334 (3,093-6,531)	4,524 (2,754-5,448)	4,149 (2,706-4,899)	3,041 (2,172-3,512)	2,522 (1,932-2,844)	2,396 (1,830-2,710)	2,025 (1,567-2,273)	2,201 (1,788-2,432)
Poisonings	1,763 (1,478-2,426)	1,675 (1,411-2,411)	1,426 (1,217-1,967)	1,245 (1,102-1,492)	1,354 (1,228-1,672)	1,126 (1,037-1,438)	968 (894-1,190)	1,143 (1,073-1,381)
Poisoning by carbon monoxide	868 (751-1,216)	821 (705-1,224)	713 (619-993)	643 (586-826)	742 (686-914)	666 (620-845)	539 (501-675)	719 (679-857)
Poisoning by other means	895 (705-1,283)	854 (683-1,247)	713 (567-1,013)	603 (492-731)	613 (529-782)	460 (408-626)	429 (380-547)	424 (379-564)
Exposure to mechanical forces	1,382 (1,004-1,567)	1,561 (1,150-1,772)	1,609 (1,189-1,820)	1,407 (1,055-1,585)	1,617 (1,250-1,819)	1,308 (1,037-1,440)	986 (790-1,101)	1,372 (1,041-1,540)
Unintentional firearm injuries	298 (233-347)	317 (218-375)	360 (264-421)	272 (210-310)	274 (208-316)	255 (204-291)	196 (158-229)	214 (176-263)
Other exposure to mechanical forces	1,084 (756-1,252)	1,244 (901-1,420)	1,248 (892-1,425)	1,136 (822-1,295)	1,343 (1,010-1,527)	1,053 (822-1,174)	791 (620-889)	1,158 (842-1,315)
Adverse effects of medical treatment	1,886 (1,269-2,259)	2,013 (1,271-2,430)	1,697 (1,116-2,021)	1,650 (1,137-1,913)	1,875 (1,342-2,137)	1,900 (1,400-2,150)	1,549 (1,161-1,779)	1,986 (1,566-2,225)
Animal contact	1,555 (474-2,246)	1,455 (483-2,028)	1,697 (562-2,358)	1,353 (459-1,866)	1,924 (687-2,582)	1,564 (573-2,087)	1,854 (687-2,506)	1,349 (538-1,753)
Venomous animal contact	1,368 (400-1,990)	1,278 (404-1,800)	1,556 (499-2,169)	1,241 (401-1,715)	1,799 (614-2,449)	1,394 (479-1,905)	1,686 (589-2,308)	1,170 (439-1,564)
Non-venomous animal contact	187 (62-663)	177 (55-514)	142 (45-408)	112 (43-299)	125 (49-320)	170 (70-389)	168 (69-402)	180 (79-399)
Foreign body	773 (730-870)	839 (781-922)	840 (790-913)	893 (858-964)	988 (955-1,052)	989 (955-1,041)	1,017 (978-1,080)	1,227 (1,184-1,281)
Pulmonary aspiration and foreign body in airway	535 (484-612)	598 (544-675)	646 (595-712)	701 (654-761)	810 (759-861)	862 (819-904)	900 (856-956)	1,096 (1,048-1,146)
Foreign body in other body part	238 (176-286)	241 (173-288)	194 (138-236)	193 (149-235)	179 (141-219)	127 (94-156)	116 (93-151)	131 (103-171)
Environmental heat and cold exposure	404 (275-465)	451 (327-502)	718 (553-785)	929 (784-991)	1,233 (1,025-1,328)	1,299 (1,091-1,399)	1,093 (947-1,171)	1,629 (1,391-1,750)
Exposure to forces of nature	541 (472-627)	475 (424-536)	428 (383-488)	374 (340-415)	400 (352-472)	401 (338-497)	443 (352-566)	490 (397-624)
Other unintentional injuries	1,826 (1,551-2,110)	1,987 (1,730-2,275)	1,909 (1,693-2,158)	1,655 (1,497-1,847)	1,166 (1,078-1,267)	1,003 (925-1,086)	912 (828-1,006)	935 (854-1,027)
Self-harm and interpersonal violence	59,992 (57,026-62,918)	58,110 (55,253-61,297)	47,417 (44,968-50,045)	32,425 (30,731-33,920)	29,660 (28,261-30,891)	24,389 (23,175-25,475)	18,834 (18,081-19,575)	17,967 (17,320-18,584)
Self-harm	48,487 (45,964-51,640)	47,430 (45,349-50,496)	36,798 (35,233-39,389)	23,583 (22,670-24,898)	22,109 (21,286-23,282)	18,635 (17,905-19,681)	14,589 (14,132-15,224)	13,749 (13,288-14,351)
Self-harm by firearm	1,462 (846-2,136)	1,095 (729-1,504)	971 (751-1,243)	854 (726-1,009)	772 (681-898)	621 (564-714)	519 (466-619)	443 (395-541)
Self-harm by other specified means	47,025 (44,455-50,594)	46,335 (44,093-49,796)	35,827 (34,221-38,544)	22,729 (21,795-24,103)	21,337 (20,526-22,514)	18,014 (17,293-19,089)	14,070 (13,590-14,702)	13,305 (12,871-13,908)
Interpersonal violence	9,638 (7,555-11,100)	9,420 (7,443-10,571)	9,429 (7,308-10,372)	7,878 (6,524-8,540)	6,798 (5,686-7,367)	5,120 (4,324-5,502)	3,674 (3,053-4,075)	3,628 (3,135-3,899)
Physical violence by firearm	2,437 (1,909-2,797)	2,320 (1,790-2,677)	2,135 (1,657-2,437)	1,896 (1,472-2,169)	1,423 (1,119-1,614)	1,007 (806-1,127)	779 (591-914)	638 (476-739)
Physical violence by sharp object	2,483 (1,923-3,047)	2,655 (2,027-3,195)	2,897 (2,231-3,404)	2,392 (1,985-2,678)	2,285 (1,872-2,618)	1,643 (1,376-1,834)	1,095 (905-1,248)	1,050 (913-1,165)
Physical violence by other means	4,718 (3,422-5,784)	4,445 (3,266-5,347)	4,396 (3,236-5,069)	3,590 (2,877-4,012)	3,090 (2,562-3,462)	2,470 (2,039-2,747)	1,800 (1,464-2,092)	1,941 (1,674-2,121)
Conflict and terrorism	1,835 (1,634-2,143)	1,224 (1,096-1,414)	1,147 (1,027-1,326)	933 (832-1,078)	719 (653-812)	605 (547-690)	551 (504-615)	571 (525-637)
Executions and police conflict	32 (29-35)	36 (27-43)	43 (26-52)	31 (24-35)	34 (22-42)	29 (18-43)	21 (16-30)	19 (14-27)

Cause	60 to 64	65 to 69	70 to 74	75 to 79	80 to 84	85 to 89	90 to 94	95 plus
Other urinary diseases	1,053 (691-1,330)	1,007 (626-1,297)	1,011 (637-1,280)	996 (599-1,279)	671 (420-860)	532 (343-683)	293 (187-367)	129 (88-156)
Gynecological diseases	277 (244-323)	340 (295-395)	214 (191-241)	159 (145-180)	287 (240-336)	205 (171-241)	84 (73-98)	28 (25-33)
Uterine fibroids	67 (54-91)	72 (56-104)	48 (37-72)	33 (26-48)	72 (50-111)	57 (37-85)	29 (21-41)	10 (7-14)
Polycystic ovarian syndrome	-	-	-	-	-	-	-	-
Endometriosis	-	-	-	-	-	-	-	-
Genital prolapse	51 (24-84)	89 (42-138)	69 (38-96)	49 (28-65)	74 (42-114)	46 (28-70)	18 (12-24)	6 (4-8)
Other gynecological diseases	159 (127-209)	178 (137-243)	97 (75-130)	78 (64-106)	142 (109-182)	102 (81-131)	37 (31-48)	12 (10-16)
Hemoglobinopathies and hemolytic anemias	1,720 (1,355-2,160)	2,149 (1,689-2,654)	2,549 (1,982-3,149)	2,391 (1,870-2,973)	2,731 (2,009-3,255)	2,413 (1,639-2,882)	1,362 (886-1,630)	494 (325-581)
Thalassemias	4 (4-6)	2 (2-3)	1 (1-1)	0 (0-0)	0 (0-0)	0 (0-0)	0 (0-0)	0 (0-0)
Sickle cell disorders	58 (37-102)	27 (18-41)	9 (6-14)	3 (2-5)	1 (1-2)	0 (0-1)	0 (0-0)	0 (0-0)
G6PD deficiency	192 (127-295)	162 (105-248)	101 (66-160)	61 (39-95)	31 (20-48)	13 (8-19)	4 (2-5)	1 (0-1)
Other hemoglobinopathies and hemolytic anemias	1,466 (1,165-1,792)	1,958 (1,548-2,404)	2,438 (1,900-3,003)	2,326 (1,821-2,878)	2,698 (1,986-3,210)	2,400 (1,629-2,865)	1,358 (884-1,626)	493 (325-580)
Endocrine, metabolic, blood, and immune disorders	3,050 (2,423-3,709)	3,621 (2,813-4,377)	3,714 (2,934-4,630)	3,459 (2,681-4,355)	3,524 (2,682-4,583)	2,874 (2,167-3,660)	1,530 (1,169-2,018)	553 (428-769)
Sudden infant death syndrome	-	-	-	-	-	-	-	-
Injuries	58,715 (55,499-60,895)	61,716 (58,536-64,044)	64,202 (60,838-66,949)	60,342 (56,823-62,827)	60,112 (56,707-62,257)	46,853 (44,558-48,494)	22,496 (21,594-23,227)	7,067 (6,808-7,286)
Transport injuries	15,953 (15,240-16,697)	14,693 (14,152-15,300)	14,035 (13,470-14,626)	10,603 (10,217-11,060)	7,874 (7,599-8,215)	4,235 (4,082-4,435)	1,513 (1,464-1,575)	420 (404-438)
Road injuries	15,072 (14,409-15,746)	13,950 (13,448-14,533)	13,444 (12,898-14,024)	10,110 (9,738-10,562)	7,502 (7,241-7,853)	4,036 (3,892-4,233)	1,445 (1,398-1,507)	399 (382-416)
Pedestrian road injuries	8,658 (8,204-9,215)	8,089 (7,716-8,631)	8,159 (7,791-8,682)	6,305 (6,037-6,737)	4,846 (4,669-5,170)	2,480 (2,374-2,639)	793 (758-840)	197 (187-211)
Cyclist road injuries	600 (524-707)	613 (550-694)	573 (518-635)	323 (287-360)	148 (133-165)	68 (59-78)	24 (21-29)	0 (0-0)
Motorcyclist road injuries	1,116 (899-1,494)	876 (716-1,174)	635 (504-818)	333 (262-445)	172 (134-237)	85 (65-121)	27 (21-38)	8 (7-11)
Motor vehicle road injuries	4,650 (4,206-5,039)	4,327 (3,879-4,648)	4,041 (3,686-4,370)	3,066 (2,805-3,276)	2,288 (2,138-2,435)	1,373 (1,289-1,477)	589 (561-625)	189 (178-200)
Other road injuries	47 (35-58)	45 (35-58)	36 (27-46)	84 (62-114)	48 (38-66)	30 (23-42)	11 (9-16)	4 (3-5)
Other transport injuries	881 (798-974)	743 (671-816)	591 (546-637)	493 (455-532)	371 (344-402)	199 (182-218)	68 (63-75)	21 (20-23)
Unintentional injuries	25,794 (23,289-27,522)	30,335 (27,569-32,314)	33,972 (31,095-36,048)	37,320 (33,984-39,521)	42,696 (39,505-44,655)	37,673 (35,399-39,217)	19,473 (18,546-20,169)	6,332 (6,080-6,548)
Falls	9,627 (8,704-10,678)	12,793 (11,542-14,310)	16,763 (15,162-18,505)	21,218 (19,253-22,985)	26,325 (24,060-27,953)	24,956 (23,148-26,232)	13,291 (12,481-13,903)	4,257 (4,053-4,451)
Drowning	2,889 (2,597-3,112)	3,133 (2,906-3,383)	3,037 (2,839-3,283)	2,853 (2,642-3,118)	2,359 (2,192-2,592)	1,260 (1,189-1,384)	387 (367-420)	69 (65-75)
Fire, heat, and hot substances	2,300 (1,884-2,541)	2,936 (2,421-3,210)	2,988 (2,512-3,244)	3,144 (2,646-3,414)	3,519 (3,108-3,778)	2,803 (2,542-2,983)	1,354 (1,254-1,431)	476 (451-499)
Poisonings	1,003 (927-1,228)	1,146 (1,063-1,429)	1,065 (980-1,383)	790 (724-1,066)	752 (701-952)	436 (409-522)	147 (138-169)	33 (32-36)
Poisoning by carbon monoxide	606 (565-761)	718 (668-902)	624 (573-842)	494 (446-697)	470 (432-627)	274 (254-348)	83 (78-103)	14 (13-17)
Poisoning by other means	397 (350-512)	428 (374-553)	441 (397-576)	297 (265-407)	283 (250-347)	162 (142-189)	63 (55-71)	19 (17-21)
Exposure to mechanical forces	1,348 (1,026-1,515)	1,265 (1,020-1,398)	1,192 (971-1,319)	1,069 (869-1,190)	1,181 (1,041-1,330)	944 (873-1,092)	485 (451-546)	167 (157-183)
Unintentional firearm injuries	196 (156-248)	215 (170-273)	207 (172-261)	272 (216-344)	273 (235-355)	222 (195-299)	112 (98-149)	37 (33-48)
Other exposure to mechanical forces	1,152 (841-1,316)	1,050 (824-1,170)	985 (777-1,101)	797 (619-900)	908 (775-1,017)	721 (656-805)	373 (345-414)	131 (122-141)
Adverse effects of medical treatment	3,030 (2,366-3,442)	3,246 (2,609-3,750)	3,432 (2,835-3,861)	3,322 (2,786-3,681)	3,593 (3,085-3,939)	3,251 (2,833-3,549)	1,727 (1,561-1,863)	581 (530-623)
Animal contact	1,641 (597-2,176)	1,630 (563-2,175)	1,441 (525-1,887)	1,072 (375-1,441)	620 (266-809)	327 (160-413)	103 (57-127)	21 (14-26)
Venomous animal contact	1,517 (522-2,048)	1,498 (490-2,028)	1,308 (457-1,758)	972 (315-1,332)	529 (211-714)	263 (115-347)	78 (38-100)	15 (9-19)
Non-venomous animal contact	124 (46-291)	133 (58-298)	133 (59-322)	101 (46-219)	90 (48-171)	64 (34-108)	25 (15-40)	6 (4-9)
Foreign body	1,224 (1,179-1,282)	1,353 (1,302-1,403)	1,634 (1,571-1,694)	1,707 (1,632-1,773)	2,124 (2,045-2,198)	2,079 (1,993-2,175)	1,191 (1,133-1,254)	477 (456-499)
Pulmonary aspiration and foreign body in airway	1,111 (1,060-1,164)	1,231 (1,176-1,283)	1,490 (1,422-1,552)	1,564 (1,488-1,633)	1,963 (1,886-2,043)	1,946 (1,859-2,040)	1,127 (1,070-1,188)	457 (437-480)
Foreign body in other body part	113 (86-148)	121 (87-155)	144 (103-182)	143 (101-178)	161 (111-191)	133 (92-161)	65 (45-75)	20 (15-25)
Environmental heat and cold exposure	1,467 (1,135-1,622)	1,387 (1,105-1,519)	1,343 (973-1,510)	1,171 (801-1,319)	1,309 (972-1,435)	1,059 (844-1,147)	547 (466-590)	178 (162-191)
Exposure to forces of nature	535 (420-693)	614 (474-824)	594 (435-805)	505 (376-702)	459 (335-621)	235 (171-330)	85 (62-119)	17 (13-22)
Other unintentional injuries	730 (675-806)	832 (758-942)	484 (452-530)	470 (434-530)	456 (425-510)	325 (302-361)	156 (146-169)	57 (53-61)
Self-harm and interpersonal violence	16,968 (16,304-17,696)	16,689 (16,097-17,472)	16,195 (15,547-17,151)	12,419 (11,916-13,180)	9,541 (9,145-10,239)	4,944 (4,782-5,223)	1,510 (1,471-1,571)	315 (309-323)
Self-harm	13,528 (13,004-14,188)	13,459 (12,961-14,137)	13,595 (13,001-14,507)	10,475 (9,985-11,210)	8,595 (8,195-9,290)	4,467 (4,307-4,749)	1,385 (1,346-1,446)	261 (255-269)
Self-harm by firearm	367 (324-462)	371 (325-477)	340 (292-453)	297 (250-411)	256 (221-332)	158 (140-182)	71 (65-78)	25 (23-27)
Self-harm by other specified means	13,161 (12,638-13,836)	13,088 (12,593-13,798)	13,256 (12,656-14,161)	10,178 (9,693-10,924)	8,339 (7,946-9,066)	4,309 (4,154-4,594)	1,314 (1,276-1,374)	236 (230-244)
Interpersonal violence	2,883 (2,446-3,142)	2,682 (2,336-2,977)	2,221 (1,920-2,511)	1,698 (1,476-1,908)	739 (698-788)	370 (352-391)	102 (97-109)	24 (22-25)
Physical violence by firearm	546 (405-640)	417 (325-507)	359 (281-442)	264 (196-321)	63 (55-71)	34 (31-39)	9 (8-10)	3 (3-3)
Physical violence by sharp object	825 (698-939)	689 (613-779)	628 (533-715)	493 (419-576)	187 (172-213)	82 (74-94)	21 (19-25)	5 (4-5)
Physical violence by other means	1,512 (1,269-1,702)	1,576 (1,332-1,748)	1,234 (1,046-1,411)	941 (811-1,068)	489 (450-522)	254 (238-267)	72 (67-76)	16 (15-17)
Conflict and terrorism	541 (499-605)	533 (494-587)	366 (337-403)	237 (219-263)	197 (184-214)	102 (96-109)	20 (19-21)	30 (28-31)
Executions and police conflict	16 (13-22)	15 (11-21)	13 (10-17)	9 (7-14)	10 (8-13)	6 (4-7)	2 (2-3)	1 (0-1)

SR Table 5: Global deaths by cause and age group for females (with 95% confidence intervals), 2000

Cause	Early Neonatal	Late Neonatal	Post Neonatal	1 to 4
All causes	1,164,607 (1,136,048-1,195,22	420,796 (404,008-440,604	1,475,712 (1,437,807-1,515,47	1,462,851 (1,418,510-1,508,34
Communicable, maternal, neonatal, and nutritional disease	1,062,473 (1,033,274-1,092,73	370,702 (353,648-389,494	1,226,127 (1,176,857-1,270,44	1,187,817 (1,135,099-1,245,531
HIV/AIDS and sexually transmitted infections	-	7,880 (3,039-15,099	85,069 (75,947-96,764	50,300 (46,879-53,795
HIV/AIDS	-	-	72,086 (68,202-76,111	48,995 (45,594-52,222
HIV/AIDS - Drug-susceptible Tuberculosis	-	-	24,752 (18,323-28,676	17,506 (13,538-20,014
HIV/AIDS - Multidrug-resistant Tuberculosis without extensive drug resistance	-	-	2,161 (1,300-3,327)	1,510 (909-2,411)
HIV/AIDS - Extensively drug-resistant Tuberculosis	-	-	18 (11-26)	13 (8-18)
HIV/AIDS resulting in other diseases	-	-	45,155 (40,245-52,271	29,966 (26,501-34,832
Sexually transmitted infections excluding HIV	27,067 (10,581-51,478	7,880 (3,039-15,099	12,983 (4,892-24,921	1,305 (482-2,559
Syphilis	27,067 (10,581-51,478	7,880 (3,039-15,099	12,983 (4,892-24,921	1,305 (482-2,559
Chlamydial infection	-	-	-	-
Gonococcal infection	-	-	-	-
Other sexually transmitted infections	-	-	-	-
Respiratory infections and tuberculosis	-	91,600 (82,500-100,797	431,991 (394,527-465,575	274,865 (228,921-316,690
Tuberculosis	-	-	31,870 (28,267-35,302	35,070 (28,497-41,209
Drug-susceptible tuberculosis	-	-	29,227 (25,722-32,719	32,445 (26,177-38,286
Multidrug-resistant tuberculosis without extensive drug resistance	-	-	2,565 (1,734-3,717	2,572 (1,641-4,000
Extensively drug-resistant tuberculosis	-	-	79 (56-108)	53 (35-79)
Lower respiratory infections	91,429 (82,657-102,275	91,463 (82,401-100,667	398,051 (364,615-430,180	237,785 (197,620-274,148
Upper respiratory infections	51 (19-77)	110 (46-168)	1,897 (608-3,005)	1,788 (432-3,677
Otitis media	33 (3-235)	27 (8-96)	173 (74-375)	222 (75-500)
Enteric infections	15,289 (13,058-17,805	32,337 (26,968-37,351	279,366 (247,869-310,395	293,746 (234,619-351,08[illegible]
Diarrheal diseases	15,289 (13,058-17,805	32,078 (26,799-37,058	274,483 (243,695-305,796	267,715 (212,541-323,98[illegible]
Typhoid and paratyphoid	-	0 (0-0)	174 (89-330)	15,092 (6,841-28,456
Typhoid fever	-	-	158 (81-296)	13,319 (5,999-25,423
Paratyphoid fever	-	-	16 (6-33)	1,773 (649-3,814)
Invasive Non-typhoidal Salmonella (iNTS)	-	259 (145-438)	3,753 (2,114-6,416	10,376 (5,151-18,694
Other intestinal infectious diseases	-	-	956 (428-2,276)	562 (211-1,391)
Neglected tropical diseases and malaria	213 (102-668)	11,660 (7,483-17,695	93,703 (64,748-131,874	184,766 (126,516-258,97[illegible]
Malaria	-	11,487 (7,165-17,588	89,762 (60,295-127,495	171,313 (115,211-241,97[illegible]
Chagas disease	-	-	1 (1-2)	1 (1-1)
Leishmaniasis	-	-	999 (1-4,554)	3,813 (1-20,608)
Visceral leishmaniasis	-	-	999 (1-4,554)	3,813 (1-20,608)
African trypanosomiasis	-	-	-	445 (92-1,623)
Schistosomiasis	-	-	0 (0-0)	463 (290-740)
Cysticercosis	0 (0-0)	0 (0-0)	0 (0-0)	107 (46-191)
Cystic echinococcosis	-	-	-	234 (135-355)
Dengue	-	-	1,450 (275-3,079)	1,800 (408-3,541)
Yellow fever	-	1 (0-4)	23 (5-73)	215 (40-668)
Rabies	-	-	299 (159-534)	1,613 (765-2,825)
Intestinal nematode infections	-	-	274 (209-365)	3,028 (2,160-4,253
Ascariasis	-	-	274 (209-365)	3,028 (2,160-4,253
Ebola	1 (1-2)	1 (1-2)	20 (15-26)	58 (44-75)
Zika virus	0 (0-0)	0 (0-0)	0 (0-0)	0 (0-0)
Other neglected tropical diseases	212 (101-667)	170 (87-535)	874 (395-3,370)	1,675 (512-8,561)
Other infectious diseases	25,362 (21,118-31,555	32,670 (26,949-39,495	195,200 (142,354-274,566	293,335 (194,653-442,46[illegible]
Meningitis	5,111 (4,083-6,283	8,540 (6,868-10,247	54,592 (45,034-63,447	54,721 (40,802-66,527
Pneumococcal meningitis	704 (533-894)	1,103 (843-1,387	6,414 (5,035-7,925	5,826 (4,119-7,585
H influenzae type B meningitis	1,411 (1,091-1,761	2,477 (1,984-3,031	18,037 (14,251-21,427	20,662 (15,188-25,984
Meningococcal meningitis	784 (624-970)	1,326 (1,071-1,611	7,890 (6,503-9,471	6,932 (5,215-8,846
Other meningitis	2,212 (1,709-2,770	3,634 (2,843-4,424	22,252 (17,636-26,775	21,300 (15,657-26,743
Encephalitis	331 (262-385)	656 (504-825)	7,534 (4,997-10,285	9,098 (6,193-14,404
Diphtheria	-	-	1,145 (681-1,856)	4,172 (2,103-7,821
Whooping cough	-	-	37,786 (17,494-70,758	37,914 (16,796-71,803
Tetanus	16,777 (12,568-22,934	20,928 (15,901-27,436	7,173 (4,798-11,971	5,257 (2,632-8,563
Measles	-	-	71,749 (25,718-155,279	165,847 (63,622-333,11[illegible]
Varicella and herpes zoster	93 (71-122)	264 (206-337)	2,081 (1,627-2,640	2,344 (1,773-3,061
Acute hepatitis	-	-	6,420 (4,221-7,668	8,373 (5,763-10,625
Acute hepatitis A	-	-	1,196 (547-1,977)	5,421 (3,428-7,228
Acute hepatitis B	-	-	3,817 (2,267-5,104	2,158 (1,158-3,430
Acute hepatitis C	-	-	1,396 (674-2,410)	615 (266-1,183)
Acute hepatitis E	-	-	11 (5-21)	180 (78-360)
Other unspecified infectious diseases	3,050 (1,348-3,815	2,282 (1,170-2,776	6,720 (3,531-8,191	5,610 (2,698-8,163
Maternal and neonatal disorders	903,029 (867,981-935,018	194,556 (181,115-211,907	51,282 (43,895-61,423	4,440 (3,534-5,706
Maternal disorders	-	-	-	-
Maternal hemorrhage	-	-	-	-
Maternal sepsis and other maternal infections	-	-	-	-
Maternal hypertensive disorders	-	-	-	-
Maternal obstructed labor and uterine rupture	-	-	-	-
Maternal abortion and miscarriage	-	-	-	-
Ectopic pregnancy	-	-	-	-
Indirect maternal deaths	-	-	-	-
Late maternal deaths	-	-	-	-
Maternal deaths aggravated by HIV/AIDS	-	-	-	-
Other maternal disorders	-	-	-	-
Neonatal disorders	903,029 (867,981-935,018	194,556 (181,115-211,907	51,282 (43,895-61,423	4,440 (3,534-5,706
Neonatal preterm birth	373,991 (344,194-419,278	64,807 (57,023-77,552	19,716 (16,505-24,332	1,278 (978-1,757)
Neonatal encephalopathy due to birth asphyxia and trauma	281,951 (250,007-312,249	31,685 (25,894-37,140	9,692 (7,733-12,059	1,587 (1,254-2,204
Neonatal sepsis and other neonatal infections	55,079 (32,847-87,250	40,424 (28,247-58,897	5,731 (3,425-8,646	217 (83-417)
Hemolytic disease and other neonatal jaundice	22,691 (18,210-27,173	15,361 (11,786-19,412	3,628 (2,337-5,338	245 (138-412)
Other neonatal disorders	169,316 (144,137-191,733	42,279 (34,512-51,439	12,516 (9,660-16,516	1,114 (832-1,674)
Nutritional deficiencies	-	-	89,515 (80,703-99,153	86,366 (72,435-100,75[illegible]
Protein-energy malnutrition	-	-	86,148 (77,445-95,736	81,543 (68,146-95,490
Other nutritional deficiencies	-	-	3,367 (2,458-4,638	4,824 (3,382-6,544
Non-communicable diseases	94,644 (86,807-106,693	44,187 (40,754-50,099	193,660 (172,646-224,752	138,225 (115,261-161,35[illegible]
Neoplasms	797 (645-908)	535 (440-636)	5,855 (4,542-7,459	20,173 (14,806-27,942
Lip and oral cavity cancer	-	-	-	-
Nasopharynx cancer	-	-	-	-
Other pharynx cancer	-	-	-	-
Esophageal cancer	-	-	-	-
Stomach cancer	-	-	-	-
Colon and rectum cancer	-	-	-	-
Liver cancer	-	-	-	-
Liver cancer due to hepatitis B	-	-	-	-
Liver cancer due to hepatitis C	-	-	-	-
Liver cancer due to alcohol use	-	-	-	-
Liver cancer due to NASH	-	-	-	-
Liver cancer due to other causes	-	-	-	-
Gallbladder and biliary tract cancer	-	-	-	-
Pancreatic cancer	-	-	-	-
Larynx cancer	-	-	-	-
Tracheal, bronchus, and lung cancer	-	-	-	-
Malignant skin melanoma	-	-	-	-
Non-melanoma skin cancer	-	-	-	-
Non-melanoma skin cancer (squamous-cell carcinoma)	-	-	-	-
Breast cancer	-	-	-	-
Cervical cancer	-	-	-	-
Uterine cancer	-	-	-	-
Ovarian cancer	-	-	-	-
Prostate cancer	-	-	-	-
Testicular cancer	-	-	-	-
Kidney cancer	22 (19-28)	12 (10-16)	171 (126-228)	792 (523-1,143)
Bladder cancer	-	-	-	-
Brain and nervous system cancer	107 (64-151)	89 (53-130)	1,174 (569-1,764)	4,383 (2,119-7,063
Thyroid cancer	-	-	-	-

Cause	5 to 9	10 to 14	15 to 19	20 to 24	25 to 29	30 to 34	35 to 39	40 to 44	45 to 49	50 to 54	54 to 59	60 to 64	65 to 69	70 to 74	75 to 79	80 to 84	85 to 89	90 to 94	95 plus
All causes	318,395 (314,676-322,462	204,528 (202,434-206,677	287,758 (283,285-292,234	386,338 (378,140-395,079	478,415 (464,865-493,472	527,510 (516,808-540,030	532,449 (520,832-544,959	563,667 (555,741-572,266	681,195 (672,563-689,989	763,101 (755,009-771,081	881,738 (872,380-890,705	1,277,396 (1,266,200-1,288,67	1,644,052 (1,630,792-1,658,09	2,128,789 (2,112,095-2,145,73	2,327,973 (2,310,797-2,345,41	2,203,185 (2,189,245-2,217,50	1,977,300 (1,968,926-1,986,04	1,108,165 (1,104,735-1,111,83	400,573 (399,654-401,631
Communicable, maternal, neonatal, and nutritional disease	196,375 (185,502-208,927	96,910 (90,938-105,141	121,388 (115,541-129,660	190,028 (181,980-199,676	255,059 (241,946-269,579	262,750 (252,139-274,359	231,319 (220,603-243,055	185,063 (177,744-193,588	153,005 (146,101-163,051	129,664 (122,771-141,203	129,982 (120,832-146,603	160,784 (147,467-186,929	187,950 (168,323-228,788	217,358 (191,991-262,270	209,468 (182,663-252,863	207,659 (179,753-249,921	163,531 (145,726-189,765	86,748 (78,877-97,936	34,137 (32,261-36,901
HIV/AIDS and sexually transmitted infections	10,315 (9,527-11,170	3,845 (3,492-4,252	8,464 (7,137-10,013	42,452 (36,937-49,851	107,619 (96,077-120,671	124,505 (115,437-134,363	106,908 (97,567-117,177	82,306 (77,253-87,053	59,239 (54,759-63,609	35,931 (33,637-38,113	22,129 (20,264-24,094	15,291 (13,752-16,950	10,856 (9,757-12,054	6,043 (5,302-6,815	3,084 (2,630-3,552	663 (577-743)	304 (265-337)	151 (130-168)	41 (36-44)
HIV/AIDS	9,685 (9,031-10,336	3,773 (3,421-4,175	8,158 (6,848-9,707	41,908 (36,377-49,239	106,818 (95,167-119,882	123,788 (114,742-133,633	106,260 (96,875-116,574	81,605 (76,580-86,299	58,617 (54,124-63,068	35,537 (33,266-37,717	21,686 (19,807-23,627	14,931 (13,383-16,569	10,480 (9,372-11,682	5,735 (4,995-6,504	2,813 (2,352-3,278	435 (362-512)	139 (112-167)	59 (48-71)	10 (9-13)
HIV/AIDS - Drug-susceptible Tuberculosis	3,355 (2,577-3,891	1,271 (980-1,494)	2,647 (2,046-3,285	11,394 (7,914-14,900	22,236 (13,998-30,940	24,977 (15,483-35,706	23,239 (14,573-32,319	20,235 (12,853-26,744	16,325 (10,908-20,865	11,122 (8,083-13,434	7,186 (5,553-8,533	5,066 (3,964-5,948	3,629 (2,929-4,274	2,026 (1,624-2,445	996 (777-1,197)	146 (116-177)	43 (33-54)	18 (14-23)	3 (2-4)
HIV/AIDS - Multidrug-resistant Tuberculosis without extensive drug resistance	271 (162-416)	104 (59-168)	237 (143-379)	954 (553-1,508)	1,807 (990-2,914)	2,016 (1,092-3,337)	1,853 (1,019-2,994)	1,594 (877-2,531)	1,296 (749-1,967)	891 (530-1,315)	573 (352-860)	404 (247-600)	287 (178-427)	160 (102-240)	80 (50-121)	13 (8-20)	4 (3-6)	2 (1-3)	0 (0-0)
HIV/AIDS - Extensively drug-resistant Tuberculosis	2 (2-3)	1 (1-2)	3 (2-5)	11 (7-18)	23 (14-35)	27 (17-42)	26 (16-38)	21 (13-32)	17 (11-25)	11 (7-16)	6 (4-9)	5 (3-7)	3 (2-5)	2 (1-2)	1 (1-1)	0 (0-0)	0 (0-0)	0 (0-0)	0 (0-0)
HIV/AIDS resulting in other diseases	6,057 (5,381-7,013	2,397 (2,088-2,825	5,271 (4,263-6,694	29,549 (23,854-37,199	82,753 (67,774-98,100	96,768 (81,793-110,337	81,142 (67,655-94,119	59,755 (51,163-68,430	40,979 (34,946-47,735	23,513 (20,529-26,962	13,921 (12,020-16,221	9,456 (8,159-11,145	6,560 (5,647-7,772	3,547 (3,006-4,249	1,736 (1,410-2,125	276 (224-338)	92 (73-115)	39 (32-48)	7 (6-9)
Sexually transmitted infections excluding HIV	630 (229-1,222)	72 (44-90)	305 (218-356)	543 (353-637)	801 (480-958)	717 (461-828)	648 (442-728)	701 (485-788)	622 (446-701)	394 (297-437)	443 (305-500)	360 (258-406)	376 (275-421)	308 (246-343)	272 (224-301)	227 (177-259)	165 (135-183)	92 (74-104)	30 (26-33)
Syphilis	630 (229-1,222)	26 (16-33)	29 [illegible]	50 [illegible]	69 [illegible]	60 [illegible]	46 [illegible]	47 [illegible]	40 [illegible]	56 [illegible]	80 [illegible]	64 [illegible]	77 [illegible]	76 [illegible]	59 [illegible]	48 [illegible]	30 [illegible]	16 [illegible]	7 [illegible]
Chlamydial infection	-	12 (8-16)	[illegible]	[illegible]	[illegible]	[illegible]	[illegible]	[illegible]	[illegible]	[illegible]	[illegible]	[illegible]	[illegible]	[illegible]	[illegible]	[illegible]	[illegible]	[illegible]	[illegible]
Gonococcal infection	-	21 (13-27)	[illegible]	[illegible]	[illegible]	[illegible]	[illegible]	[illegible]	[illegible]	[illegible]	[illegible]	[illegible]	[illegible]	[illegible]	[illegible]	[illegible]	[illegible]	[illegible]	[illegible]
Other sexually transmitted infections	-	12 (7-15)	[illegible]	[illegible]	[illegible]	[illegible]	[illegible]	[illegible]	[illegible]	[illegible]	[illegible]	[illegible]	[illegible]	[illegible]	[illegible]	[illegible]	[illegible]	[illegible]	[illegible]
Respiratory infections and tuberculosis	36,403 (30,206-41,718	19,855 (17,566-22,133	[illegible]	[illegible]	[illegible]	[illegible]	[illegible]	[illegible]	[illegible]	[illegible]	[illegible]	[illegible]	[illegible]	[illegible]	[illegible]	[illegible]	[illegible]	[illegible]	[illegible]
Tuberculosis	9,494 (7,927-11,256	8,463 (7,331-9,607	[illegible]	[illegible]	[illegible]	[illegible]	[illegible]	[illegible]	[illegible]	[illegible]	[illegible]	[illegible]	[illegible]	[illegible]	[illegible]	[illegible]	[illegible]	[illegible]	[illegible]
Drug-susceptible tuberculosis	8,711 (7,179-10,328	7,723 (6,670-8,857	[illegible]	[illegible]	[illegible]	[illegible]	[illegible]	[illegible]	[illegible]	[illegible]	[illegible]	[illegible]	[illegible]	[illegible]	[illegible]	[illegible]	[illegible]	[illegible]	[illegible]
Multidrug-resistant tuberculosis without extensive drug resistance	761 (434-1,301)	718 (436-1,171)	[illegible]	[illegible]	[illegible]	[illegible]	[illegible]	[illegible]	[illegible]	[illegible]	[illegible]	[illegible]	[illegible]	[illegible]	[illegible]	[illegible]	[illegible]	[illegible]	[illegible]
Extensively drug-resistant tuberculosis	21 (13-33)	22 (15-34)	[illegible]	[illegible]	[illegible]	[illegible]	[illegible]	[illegible]	[illegible]	[illegible]	[illegible]	[illegible]	[illegible]	[illegible]	[illegible]	[illegible]	[illegible]	[illegible]	[illegible]
Lower respiratory infections	26,495 (21,222-30,654	11,184 (9,347-12,516	[illegible]	[illegible]	[illegible]	[illegible]	[illegible]	[illegible]	[illegible]	[illegible]	[illegible]	[illegible]	[illegible]	[illegible]	[illegible]	[illegible]	[illegible]	[illegible]	[illegible]
Upper respiratory infections	300 (88-510)	113 (39-190)	[illegible]	[illegible]	[illegible]	[illegible]	[illegible]	[illegible]	[illegible]	[illegible]	[illegible]	[illegible]	[illegible]	[illegible]	[illegible]	[illegible]	[illegible]	[illegible]	[illegible]
Otitis media	[illegible]	[illegible]	[illegible]	[illegible]	[illegible]	[illegible]	[illegible]	[illegible]	[illegible]	[illegible]	[illegible]	[illegible]	[illegible]	[illegible]	[illegible]	[illegible]	[illegible]	[illegible]	[illegible]
Enteric infections	[illegible]	[illegible]	[illegible]	[illegible]	[illegible]	[illegible]	[illegible]	[illegible]	[illegible]	[illegible]	[illegible]	[illegible]	[illegible]	[illegible]	[illegible]	[illegible]	[illegible]	[illegible]	[illegible]
Diarrheal diseases	[illegible]	[illegible]	[illegible]	[illegible]	[illegible]	[illegible]	[illegible]	[illegible]	[illegible]	[illegible]	[illegible]	[illegible]	[illegible]	[illegible]	[illegible]	[illegible]	[illegible]	[illegible]	[illegible]
Typhoid and paratyphoid	[illegible]	[illegible]	[illegible]	[illegible]	[illegible]	[illegible]	[illegible]	[illegible]	[illegible]	[illegible]	[illegible]	[illegible]	[illegible]	[illegible]	[illegible]	[illegible]	[illegible]	[illegible]	[illegible]
Typhoid fever	[illegible]	[illegible]	[illegible]	[illegible]	[illegible]	[illegible]	[illegible]	[illegible]	[illegible]	[illegible]	[illegible]	[illegible]	[illegible]	[illegible]	[illegible]	[illegible]	[illegible]	[illegible]	[illegible]
Paratyphoid fever	[illegible]	[illegible]	[illegible]	[illegible]	[illegible]	[illegible]	[illegible]	[illegible]	[illegible]	[illegible]	[illegible]	[illegible]	[illegible]	[illegible]	[illegible]	[illegible]	[illegible]	[illegible]	[illegible]
Invasive Non-typhoidal Salmonella (iNTS)	[illegible]	[illegible]	[illegible]	[illegible]	[illegible]	[illegible]	[illegible]	[illegible]	[illegible]	[illegible]	[illegible]	[illegible]	[illegible]	[illegible]	[illegible]	[illegible]	[illegible]	[illegible]	[illegible]
Other intestinal infectious diseases	[illegible]	[illegible]	[illegible]	[illegible]	[illegible]	[illegible]	[illegible]	[illegible]	[illegible]	[illegible]	[illegible]	[illegible]	[illegible]	[illegible]	[illegible]	[illegible]	[illegible]	[illegible]	[illegible]
Neglected tropical diseases and malaria	[illegible]	[illegible]	[illegible]	[illegible]	[illegible]	[illegible]	[illegible]	[illegible]	[illegible]	[illegible]	[illegible]	[illegible]	[illegible]	[illegible]	[illegible]	[illegible]	[illegible]	[illegible]	[illegible]
Malaria	[illegible]	[illegible]	[illegible]	[illegible]	[illegible]	[illegible]	[illegible]	[illegible]	[illegible]	[illegible]	[illegible]	[illegible]	[illegible]	[illegible]	[illegible]	[illegible]	[illegible]	[illegible]	[illegible]
Chagas disease	[illegible]	[illegible]	[illegible]	[illegible]	[illegible]	[illegible]	[illegible]	[illegible]	[illegible]	[illegible]	[illegible]	[illegible]	[illegible]	[illegible]	[illegible]	[illegible]	[illegible]	[illegible]	[illegible]
Leishmaniasis	[illegible]	[illegible]	[illegible]	[illegible]	[illegible]	[illegible]	[illegible]	[illegible]	[illegible]	[illegible]	[illegible]	[illegible]	[illegible]	[illegible]	[illegible]	[illegible]	[illegible]	[illegible]	[illegible]
Visceral leishmaniasis	[illegible]	[illegible]	[illegible]	[illegible]	[illegible]	[illegible]	[illegible]	[illegible]	[illegible]	[illegible]	[illegible]	[illegible]	[illegible]	[illegible]	[illegible]	[illegible]	[illegible]	[illegible]	[illegible]
African trypanosomiasis	[illegible]	[illegible]	[illegible]	[illegible]	[illegible]	[illegible]	[illegible]	[illegible]	[illegible]	[illegible]	[illegible]	[illegible]	[illegible]	[illegible]	[illegible]	[illegible]	[illegible]	[illegible]	[illegible]
Schistosomiasis	[illegible]	[illegible]	[illegible]	[illegible]	[illegible]	[illegible]	[illegible]	[illegible]	[illegible]	[illegible]	[illegible]	[illegible]	[illegible]	[illegible]	[illegible]	[illegible]	[illegible]	[illegible]	[illegible]
Cysticercosis	[illegible]	[illegible]	[illegible]	[illegible]	[illegible]	[illegible]	[illegible]	[illegible]	[illegible]	[illegible]	[illegible]	[illegible]	[illegible]	[illegible]	[illegible]	[illegible]	[illegible]	[illegible]	[illegible]
Cystic echinococcosis	[illegible]	[illegible]	[illegible]	[illegible]	[illegible]	[illegible]	[illegible]	[illegible]	[illegible]	[illegible]	[illegible]	[illegible]	[illegible]	[illegible]	[illegible]	[illegible]	[illegible]	[illegible]	[illegible]
Dengue	[illegible]	[illegible]	[illegible]	[illegible]	[illegible]	[illegible]	[illegible]	[illegible]	[illegible]	[illegible]	[illegible]	[illegible]	[illegible]	[illegible]	[illegible]	[illegible]	[illegible]	[illegible]	[illegible]
Yellow fever	[illegible]	[illegible]	[illegible]	[illegible]	[illegible]	[illegible]	[illegible]	[illegible]	[illegible]	[illegible]	[illegible]	[illegible]	[illegible]	[illegible]	[illegible]	[illegible]	[illegible]	[illegible]	[illegible]
Rabies	[illegible]	[illegible]	[illegible]	[illegible]	[illegible]	[illegible]	[illegible]	[illegible]	[illegible]	[illegible]	[illegible]	[illegible]	[illegible]	[illegible]	[illegible]	[illegible]	[illegible]	[illegible]	[illegible]
Intestinal nematode infections	[illegible]	[illegible]	[illegible]	[illegible]	[illegible]	[illegible]	[illegible]	[illegible]	[illegible]	[illegible]	[illegible]	[illegible]	[illegible]	[illegible]	[illegible]	[illegible]	[illegible]	[illegible]	[illegible]
Ascariasis	[illegible]	[illegible]	[illegible]	[illegible]	[illegible]	[illegible]	[illegible]	[illegible]	[illegible]	[illegible]	[illegible]	[illegible]	[illegible]	[illegible]	[illegible]	[illegible]	[illegible]	[illegible]	[illegible]
Ebola	[illegible]	[illegible]	[illegible]	[illegible]	[illegible]	[illegible]	[illegible]	[illegible]	[illegible]	[illegible]	[illegible]	[illegible]	[illegible]	[illegible]	[illegible]	[illegible]	[illegible]	[illegible]	[illegible]
Zika virus	[illegible]	[illegible]	[illegible]	[illegible]	[illegible]	[illegible]	[illegible]	[illegible]	[illegible]	[illegible]	[illegible]	[illegible]	[illegible]	[illegible]	[illegible]	[illegible]	[illegible]	[illegible]	[illegible]
Other neglected tropical diseases	[illegible]	[illegible]	[illegible]	[illegible]	[illegible]	[illegible]	[illegible]	[illegible]	[illegible]	[illegible]	[illegible]	[illegible]	[illegible]	[illegible]	[illegible]	[illegible]	[illegible]	[illegible]	[illegible]
Other infectious diseases	[illegible]	[illegible]	[illegible]	[illegible]	[illegible]	[illegible]	[illegible]	[illegible]	[illegible]	[illegible]	[illegible]	[illegible]	[illegible]	[illegible]	[illegible]	[illegible]	[illegible]	[illegible]	[illegible]
Meningitis	[illegible]	[illegible]	[illegible]	[illegible]	[illegible]	[illegible]	[illegible]	[illegible]	[illegible]	[illegible]	[illegible]	[illegible]	[illegible]	[illegible]	[illegible]	[illegible]	[illegible]	[illegible]	[illegible]
Pneumococcal meningitis	[illegible]	[illegible]	[illegible]	[illegible]	[illegible]	[illegible]	[illegible]	[illegible]	[illegible]	[illegible]	[illegible]	[illegible]	[illegible]	[illegible]	[illegible]	[illegible]	[illegible]	[illegible]	[illegible]
H influenzae type B meningitis	[illegible]	[illegible]	[illegible]	[illegible]	[illegible]	[illegible]	[illegible]	[illegible]	[illegible]	[illegible]	[illegible]	[illegible]	[illegible]	[illegible]	[illegible]	[illegible]	[illegible]	[illegible]	[illegible]
Meningococcal meningitis	[illegible]	[illegible]	[illegible]	[illegible]	[illegible]	[illegible]	[illegible]	[illegible]	[illegible]	[illegible]	[illegible]	[illegible]	[illegible]	[illegible]	[illegible]	[illegible]	[illegible]	[illegible]	[illegible]
Other meningitis	[illegible]	[illegible]	[illegible]	[illegible]	[illegible]	[illegible]	[illegible]	[illegible]	[illegible]	[illegible]	[illegible]	[illegible]	[illegible]	[illegible]	[illegible]	[illegible]	[illegible]	[illegible]	[illegible]
Encephalitis	[illegible]	[illegible]	[illegible]	[illegible]	[illegible]	[illegible]	[illegible]	[illegible]	[illegible]	[illegible]	[illegible]	[illegible]	[illegible]	[illegible]	[illegible]	[illegible]	[illegible]	[illegible]	[illegible]
Diphtheria	[illegible]	[illegible]	[illegible]	[illegible]	[illegible]	[illegible]	[illegible]	[illegible]	[illegible]	[illegible]	[illegible]	[illegible]	[illegible]	[illegible]	[illegible]	[illegible]	[illegible]	[illegible]	[illegible]
Whooping cough	[illegible]	[illegible]	[illegible]	[illegible]	[illegible]	[illegible]	[illegible]	[illegible]	[illegible]	[illegible]	[illegible]	[illegible]	[illegible]	[illegible]	[illegible]	[illegible]	[illegible]	[illegible]	[illegible]
Tetanus	[illegible]	[illegible]	[illegible]	[illegible]	[illegible]	[illegible]	[illegible]	[illegible]	[illegible]	[illegible]	[illegible]	[illegible]	[illegible]	[illegible]	[illegible]	[illegible]	[illegible]	[illegible]	[illegible]
Measles	[illegible]	[illegible]	[illegible]	[illegible]	[illegible]	[illegible]	[illegible]	[illegible]	[illegible]	[illegible]	[illegible]	[illegible]	[illegible]	[illegible]	[illegible]	[illegible]	[illegible]	[illegible]	[illegible]
Varicella and herpes zoster	[illegible]	[illegible]	[illegible]	[illegible]	[illegible]	[illegible]	[illegible]	[illegible]	[illegible]	[illegible]	[illegible]	[illegible]	[illegible]	[illegible]	[illegible]	[illegible]	[illegible]	[illegible]	[illegible]
Acute hepatitis	[illegible]	[illegible]	[illegible]	[illegible]	[illegible]	[illegible]	[illegible]	[illegible]	[illegible]	[illegible]	[illegible]	[illegible]	[illegible]	[illegible]	[illegible]	[illegible]	[illegible]	[illegible]	[illegible]
Acute hepatitis A	[illegible]	[illegible]	[illegible]	[illegible]	[illegible]	[illegible]	[illegible]	[illegible]	[illegible]	[illegible]	[illegible]	[illegible]	[illegible]	[illegible]	[illegible]	[illegible]	[illegible]	[illegible]	[illegible]
Acute hepatitis B	[illegible]	[illegible]	[illegible]	[illegible]	[illegible]	[illegible]	[illegible]	[illegible]	[illegible]	[illegible]	[illegible]	[illegible]	[illegible]	[illegible]	[illegible]	[illegible]	[illegible]	[illegible]	[illegible]
Acute hepatitis C	[illegible]	[illegible]	[illegible]	[illegible]	[illegible]	[illegible]	[illegible]	[illegible]	[illegible]	[illegible]	[illegible]	[illegible]	[illegible]	[illegible]	[illegible]	[illegible]	[illegible]	[illegible]	[illegible]
Acute hepatitis E	[illegible]	[illegible]	[illegible]	[illegible]	[illegible]	[illegible]	[illegible]	[illegible]	[illegible]	[illegible]	[illegible]	[illegible]	[illegible]	[illegible]	[illegible]	[illegible]	[illegible]	[illegible]	[illegible]
Other unspecified infectious diseases	[illegible]	[illegible]	[illegible]	[illegible]	[illegible]	[illegible]	[illegible]	[illegible]	[illegible]	[illegible]	[illegible]	[illegible]	[illegible]	[illegible]	[illegible]	[illegible]	[illegible]	[illegible]	[illegible]
Maternal and neonatal disorders	[illegible]	[illegible]	[illegible]	[illegible]	[illegible]	[illegible]	[illegible]	[illegible]	[illegible]	[illegible]	[illegible]	[illegible]	[illegible]	[illegible]	[illegible]	[illegible]	[illegible]	[illegible]	[illegible]
Maternal disorders	[illegible]	[illegible]	[illegible]	[illegible]	[illegible]	[illegible]	[illegible]	[illegible]	[illegible]	[illegible]	[illegible]	[illegible]	[illegible]	[illegible]	[illegible]	[illegible]	[illegible]	[illegible]	[illegible]
Maternal hemorrhage	[illegible]	[illegible]	[illegible]	[illegible]	[illegible]	[illegible]	[illegible]	[illegible]	[illegible]	[illegible]	[illegible]	[illegible]	[illegible]	[illegible]	[illegible]	[illegible]	[illegible]	[illegible]	[illegible]
Maternal sepsis and other maternal infections	[illegible]	[illegible]	[illegible]	[illegible]	[illegible]	[illegible]	[illegible]	[illegible]	[illegible]	[illegible]	[illegible]	[illegible]	[illegible]	[illegible]	[illegible]	[illegible]	[illegible]	[illegible]	[illegible]
Maternal hypertensive disorders	[illegible]	[illegible]	[illegible]	[illegible]	[illegible]	[illegible]	[illegible]	[illegible]	[illegible]	[illegible]	[illegible]	[illegible]	[illegible]	[illegible]	[illegible]	[illegible]	[illegible]	[illegible]	[illegible]
Maternal obstructed labor and uterine rupture	[illegible]	[illegible]	[illegible]	[illegible]	[illegible]	[illegible]	[illegible]	[illegible]	[illegible]	[illegible]	[illegible]	[illegible]	[illegible]	[illegible]	[illegible]	[illegible]	[illegible]	[illegible]	[illegible]
Maternal abortion and miscarriage	[illegible]	[illegible]	[illegible]	[illegible]	[illegible]	[illegible]	[illegible]	[illegible]	[illegible]	[illegible]	[illegible]	[illegible]	[illegible]	[illegible]	[illegible]	[illegible]	[illegible]	[illegible]	[illegible]
Ectopic pregnancy	[illegible]	[illegible]	[illegible]	[illegible]	[illegible]	[illegible]	[illegible]	[illegible]	[illegible]	[illegible]	[illegible]	[illegible]	[illegible]	[illegible]	[illegible]	[illegible]	[illegible]	[illegible]	[illegible]
Indirect maternal deaths	[illegible]	[illegible]	[illegible]	[illegible]	[illegible]	[illegible]	[illegible]	[illegible]	[illegible]	[illegible]	[illegible]	[illegible]	[illegible]	[illegible]	[illegible]	[illegible]	[illegible]	[illegible]	[illegible]
Late maternal deaths	[illegible]	[illegible]	[illegible]	[illegible]	[illegible]	[illegible]	[illegible]	[illegible]	[illegible]	[illegible]	[illegible]	[illegible]	[illegible]	[illegible]	[illegible]	[illegible]	[illegible]	[illegible]	[illegible]
Maternal deaths aggravated by HIV/AIDS	[illegible]	[illegible]	[illegible]	[illegible]	[illegible]	[illegible]	[illegible]	[illegible]	[illegible]	[illegible]	[illegible]	[illegible]	[illegible]	[illegible]	[illegible]	[illegible]	[illegible]	[illegible]	[illegible]
Other maternal disorders	[illegible]	[illegible]	[illegible]	[illegible]	[illegible]	[illegible]	[illegible]	[illegible]	[illegible]	[illegible]	[illegible]	[illegible]	[illegible]	[illegible]	[illegible]	[illegible]	[illegible]	[illegible]	[illegible]
Neonatal disorders	[illegible]	[illegible]	[illegible]	[illegible]	[illegible]	[illegible]	[illegible]	[illegible]	[illegible]	[illegible]	[illegible]	[illegible]	[illegible]	[illegible]	[illegible]	[illegible]	[illegible]	[illegible]	[illegible]
Neonatal preterm birth	[illegible]	[illegible]	[illegible]	[illegible]	[illegible]	[illegible]	[illegible]	[illegible]	[illegible]	[illegible]	[illegible]	[illegible]	[illegible]	[illegible]	[illegible]	[illegible]	[illegible]	[illegible]	[illegible]
Neonatal encephalopathy due to birth asphyxia and trauma	[illegible]	[illegible]	[illegible]	[illegible]	[illegible]	[illegible]	[illegible]	[illegible]	[illegible]	[illegible]	[illegible]	[illegible]	[illegible]	[illegible]	[illegible]	[illegible]	[illegible]	[illegible]	[illegible]
Neonatal sepsis and other neonatal infections	[illegible]	[illegible]	[illegible]	[illegible]	[illegible]	[illegible]	[illegible]	[illegible]	[illegible]	[illegible]	[illegible]	[illegible]	[illegible]	[illegible]	[illegible]	[illegible]	[illegible]	[illegible]	[illegible]
Hemolytic disease and other neonatal jaundice	[illegible]	[illegible]	[illegible]	[illegible]	[illegible]	[illegible]	[illegible]	[illegible]	[illegible]	[illegible]	[illegible]	[illegible]	[illegible]	[illegible]	[illegible]	[illegible]	[illegible]	[illegible]	[illegible]
Other neonatal disorders	[illegible]	[illegible]	[illegible]	[illegible]	[illegible]	[illegible]	[illegible]	[illegible]	[illegible]	[illegible]	[illegible]	[illegible]	[illegible]	[illegible]	[illegible]	[illegible]	[illegible]	[illegible]	[illegible]
Nutritional deficiencies	[illegible]	[illegible]	[illegible]	[illegible]	[illegible]	[illegible]	[illegible]	[illegible]	[illegible]	[illegible]	[illegible]	[illegible]	[illegible]	[illegible]	[illegible]	[illegible]	[illegible]	[illegible]	[illegible]
Protein-energy malnutrition	[illegible]	[illegible]	[illegible]	[illegible]	[illegible]	[illegible]	[illegible]	[illegible]	[illegible]	[illegible]	[illegible]	[illegible]	[illegible]	[illegible]	[illegible]	[illegible]	[illegible]	[illegible]	[illegible]
Other nutritional deficiencies	[illegible]	[illegible]	[illegible]	[illegible]	[illegible]	[illegible]	[illegible]	[illegible]	[illegible]	[illegible]	[illegible]	[illegible]	[illegible]	[illegible]	[illegible]	[illegible]	[illegible]	[illegible]	[illegible]
Non-communicable diseases	[illegible]	[illegible]	[illegible]	[illegible]	[illegible]	[illegible]	[illegible]	[illegible]	[illegible]	[illegible]	[illegible]	[illegible]	[illegible]	[illegible]	[illegible]	[illegible]	[illegible]	[illegible]	[illegible]
Neoplasms	[illegible]	[illegible]	[illegible]	[illegible]	[illegible]	[illegible]	[illegible]	[illegible]	[illegible]	[illegible]	[illegible]	[illegible]	[illegible]	[illegible]	[illegible]	[illegible]	[illegible]	[illegible]	[illegible]
Lip and oral cavity cancer	[illegible]	[illegible]	[illegible]	[illegible]	[illegible]	[illegible]	[illegible]	[illegible]	[illegible]	[illegible]	[illegible]	[illegible]	[illegible]	[illegible]	[illegible]	[illegible]	[illegible]	[illegible]	[illegible]
Nasopharynx cancer	[illegible]	[illegible]	[illegible]	[illegible]	[illegible]	[illegible]	[illegible]	[illegible]	[illegible]	[illegible]	[illegible]	[illegible]	[illegible]	[illegible]	[illegible]	[illegible]	[illegible]	[illegible]	[illegible]
Other pharynx cancer	[illegible]	[illegible]	[illegible]	[illegible]	[illegible]	[illegible]	[illegible]	[illegible]	[illegible]	[illegible]	[illegible]	[illegible]	[illegible]	[illegible]	[illegible]	[illegible]	[illegible]	[illegible]	[illegible]
Esophageal cancer	[illegible]	[illegible]	[illegible]	[illegible]	[illegible]	[illegible]	[illegible]	[illegible]	[illegible]	[illegible]	[illegible]	[illegible]	[illegible]	[illegible]	[illegible]	[illegible]	[illegible]	[illegible]	[illegible]
Stomach cancer	[illegible]	[illegible]	[illegible]	[illegible]	[illegible]	[illegible]	[illegible]	[illegible]	[illegible]	[illegible]	[illegible]	[illegible]	[illegible]	[illegible]	[illegible]	[illegible]	[illegible]	[illegible]	[illegible]
Colon and rectum cancer	[illegible]	[illegible]	[illegible]	[illegible]	[illegible]	[illegible]	[illegible]	[illegible]	[illegible]	[illegible]	[illegible]	[illegible]	[illegible]	[illegible]	[illegible]	[illegible]	[illegible]	[illegible]	[illegible]
Liver cancer	[illegible]	[illegible]	[illegible]	[illegible]	[illegible]	[illegible]	[illegible]	[illegible]	[illegible]	[illegible]	[illegible]	[illegible]	[illegible]	[illegible]	[illegible]	[illegible]	[illegible]	[illegible]	[illegible]
Liver cancer due to hepatitis B	[illegible]	[illegible]	[illegible]	[illegible]	[illegible]	[illegible]	[illegible]	[illegible]	[illegible]	[illegible]	[illegible]	[illegible]	[illegible]	[illegible]	[illegible]	[illegible]	[illegible]	[illegible]	[illegible]
Liver cancer due to hepatitis C	[illegible]	[illegible]	[illegible]	[illegible]	[illegible]	[illegible]	[illegible]	[illegible]	[illegible]	[illegible]	[illegible]	[illegible]	[illegible]	[illegible]	[illegible]	[illegible]	[illegible]	[illegible]	[illegible]
Liver cancer due to alcohol use	[illegible]	[illegible]	[illegible]	[illegible]	[illegible]	[illegible]	[illegible]	[illegible]	[illegible]	[illegible]	[illegible]	[illegible]	[illegible]	[illegible]	[illegible]	[illegible]	[illegible]	[illegible]	[illegible]
Liver cancer due to NASH	[illegible]	[illegible]	[illegible]	[illegible]	[illegible]	[illegible]	[illegible]	[illegible]	[illegible]	[illegible]	[illegible]	[illegible]	[illegible]	[illegible]	[illegible]	[illegible]	[illegible]	[illegible]	[illegible]
Liver cancer due to other causes	[illegible]	[illegible]	[illegible]	[illegible]	[illegible]	[illegible]	[illegible]	[illegible]	[illegible]	[illegible]	[illegible]	[illegible]	[illegible]	[illegible]	[illegible]	[illegible]	[illegible]	[illegible]	[illegible]
Gallbladder and biliary tract cancer	[illegible]	[illegible]	[illegible]	[illegible]	[illegible]	[illegible]	[illegible]	[illegible]	[illegible]	[illegible]	[illegible]	[illegible]	[illegible]	[illegible]	[illegible]	[illegible]	[illegible]	[illegible]	[illegible]
Pancreatic cancer	[illegible]	[illegible]	[illegible]	[illegible]	[illegible]	[illegible]	[illegible]	[illegible]	[illegible]	[illegible]	[illegible]	[illegible]	[illegible]	[illegible]	[illegible]	[illegible]	[illegible]	[illegible]	[illegible]
Larynx cancer	[illegible]	[illegible]	[illegible]	[illegible]	[illegible]	[illegible]	[illegible]	[illegible]	[illegible]	[illegible]	[illegible]	[illegible]	[illegible]	[illegible]	[illegible]	[illegible]	[illegible]	[illegible]	[illegible]
Tracheal, bronchus, and lung cancer	[illegible]	[illegible]	[illegible]	[illegible]	[illegible]	[illegible]	[illegible]	[illegible]	[illegible]	[illegible]	[illegible]	[illegible]	[illegible]	[illegible]	[illegible]	[illegible]	[illegible]	[illegible]	[illegible]
Malignant skin melanoma	[illegible]	[illegible]	[illegible]	[illegible]	[illegible]	[illegible]	[illegible]	[illegible]	[illegible]	[illegible]	[illegible]	[illegible]	[illegible]	[illegible]	[illegible]	[illegible]	[illegible]	[illegible]	[illegible]
Non-melanoma skin cancer	[illegible]	[illegible]	[illegible]	[illegible]	[illegible]	[illegible]	[illegible]	[illegible]	[illegible]	[illegible]	[illegible]	[illegible]	[illegible]	[illegible]	[illegible]	[illegible]	[illegible]	[illegible]	[illegible]
Non-melanoma skin cancer (squamous-cell carcinoma)	[illegible]	[illegible]	[illegible]	[illegible]	[illegible]	[illegible]	[illegible]	[illegible]	[illegible]	[illegible]	[illegible]	[illegible]	[illegible]	[illegible]	[illegible]	[illegible]	[illegible]	[illegible]	[illegible]
Breast cancer	[illegible]	[illegible]	[illegible]	[illegible]	[illegible]	[illegible]	[illegible]	[illegible]	[illegible]	[illegible]	[illegible]	[illegible]	[illegible]	[illegible]	[illegible]	[illegible]	[illegible]	[illegible]	[illegible]
Cervical cancer	[illegible]	[illegible]	[illegible]	[illegible]	[illegible]	[illegible]	[illegible]	[illegible]	[illegible]	[illegible]	[illegible]	[illegible]	[illegible]	[illegible]	[illegible]	[illegible]	[illegible]	[illegible]	[illegible]
Uterine cancer	[illegible]	[illegible]	[illegible]	[illegible]	[illegible]	[illegible]	[illegible]	[illegible]	[illegible]	[illegible]	[illegible]	[illegible]	[illegible]	[illegible]	[illegible]	[illegible]	[illegible]	[illegible]	[illegible]
Ovarian cancer	[illegible]	[illegible]	[illegible]	[illegible]	[illegible]	[illegible]	[illegible]	[illegible]	[illegible]	[illegible]	[illegible]	[illegible]	[illegible]	[illegible]	[illegible]	[illegible]	[illegible]	[illegible]	[illegible]
Prostate cancer	[illegible]	[illegible]	[illegible]	[illegible]	[illegible]	[illegible]	[illegible]	[illegible]	[illegible]	[illegible]	[illegible]	[illegible]	[illegible]	[illegible]	[illegible]	[illegible]	[illegible]	[illegible]	[illegible]
Testicular cancer	[illegible]	[illegible]	[illegible]	[illegible]	[illegible]	[illegible]	[illegible]	[illegible]	[illegible]	[illegible]	[illegible]	[illegible]	[illegible]	[illegible]	[illegible]	[illegible]	[illegible]	[illegible]	[illegible]
Kidney cancer	[illegible]	[illegible]	[illegible]	[illegible]	[illegible]	[illegible]	[illegible]	[illegible]	[illegible]	[illegible]	[illegible]	[illegible]	[illegible]	[illegible]	[illegible]	[illegible]	[illegible]	[illegible]	[illegible]
Bladder cancer	[illegible]	[illegible]	[illegible]	[illegible]	[illegible]	[illegible]	[illegible]	[illegible]	[illegible]	[illegible]	[illegible]	[illegible]	[illegible]	[illegible]	[illegible]	[illegible]	[illegible]	[illegible]	[illegible]
Brain and nervous system cancer	[illegible]	[illegible]	[illegible]	[illegible]	[illegible]	[illegible]	[illegible]	[illegible]	[illegible]	[illegible]	[illegible]	[illegible]	[illegible]	[illegible]	[illegible]	[illegible]	[illegible]	[illegible]	[illegible]
Thyroid cancer	[illegible]	[illegible]	[illegible]	[illegible]	[illegible]	[illegible]	[illegible]	[illegible]	[illegible]	[illegible]	[illegible]	[illegible]	[illegible]	[illegible]	[illegible]	[illegible]	[illegible]	[illegible]	[illegible]

SR Table 5: Global deaths by cause and age group for females (with 95% confidence intervals), 2000

Cause	Early Neonatal	Late Neonatal	Post Neonatal	1 to 4	5 to 9	10 to 14	15 to 19	20 to 24	25 to 29	30 to 34	35 to 39	40 to 44	45 to 49	50 to 54	54 to 59	60 to 64	65 to 69	70 to 74	75 to 79	80 to 84	85 to 89	90 to 94	95 plus
Mesothelioma	-	-	-	-	-	-	46 (28-66)	75 (42-113)	122 (75-171)	143 (91-201)	217 (152-296)	355 (273-465)	284 (230-350)	366 (310-438)	470 (410-553)	612 (545-706)	709 (632-806)	830 (749-930)	840 (779-914)	564 (531-603)	380 (359-405)	154 (147-163)	37 (36-39)
Hodgkin lymphoma	2 (1-2)	1 (1-2)	33 (16-46)	194 (83-379)	281 (156-387)	484 (297-620)	934 (625-1,158)	1,128 (783-1,382)	1,203 (834-1,471)	968 (675-1,184)	763 (540-913)	630 (472-753)	557 (425-654)	556 (414-639)	565 (422-682)	707 (534-846)	832 (645-990)	861 (671-1,012)	764 (594-898)	517 (394-613)	271 (211-317)	77 (58-89)	13 (9-14)
Non-Hodgkin lymphoma	29 (26-34)	27 (22-32)	445 (348-539)	1,831 (1,247-2,412)	1,155 (954-1,355)	1,032 (893-1,169)	1,050 (949-1,157)	1,437 (1,267-1,610)	1,657 (1,454-1,856)	1,785 (1,565-1,984)	2,046 (1,869-2,236)	2,464 (2,300-2,656)	3,051 (2,866-3,268)	3,800 (3,618-4,026)	4,654 (4,500-4,869)	6,727 (6,493-7,038)	8,400 (8,153-8,738)	9,984 (9,705-10,330)	9,619 (9,397-9,872)	7,584 (7,394-7,753)	5,303 (5,200-5,412)	2,024 (1,979-2,067)	463 (452-474)
Multiple myeloma	-	-	-	-	-	-	113 (78-139)	137 (87-175)	162 (110-203)	204 (146-251)	351 (281-406)	600 (522-675)	1,087 (980-1,229)	1,698 (1,539-1,890)	2,411 (2,272-2,660)	3,780 (3,453-4,211)	4,860 (4,502-5,362)	5,714 (5,140-6,281)	5,778 (5,146-6,317)	4,177 (3,757-4,585)	2,820 (2,382-3,151)	1,093 (1,001-1,248)	204 (176-231)
Leukemia	152 (109-192)	138 (100-181)	2,257 (1,464-3,219)	7,297 (4,646-11,817)	7,181 (5,091-10,047)	6,678 (5,050-8,430)	5,590 (4,415-6,483)	4,742 (3,585-5,766)	4,980 (3,595-6,171)	5,040 (3,550-6,327)	5,111 (3,790-6,024)	5,067 (4,043-5,761)	6,030 (4,857-6,866)	6,241 (5,189-6,966)	6,737 (5,934-7,489)	8,741 (7,810-9,674)	10,055 (9,041-11,024)	11,557 (10,492-12,559)	11,213 (10,486-12,057)	8,595 (8,096-9,196)	6,569 (6,300-6,893)	2,932 (2,839-3,040)	814 (792-841)
Acute lymphoid leukemia	32 (22-49)	24 (16-43)	422 (283-781)	1,770 (1,209-3,765)	2,329 (1,665-3,990)	1,924 (1,499-2,810)	1,366 (1,097-1,816)	886 (705-1,225)	895 (661-1,312)	618 (452-923)	676 (507-916)	613 (490-768)	588 (489-757)	571 (491-726)	624 (546-788)	733 (666-922)	713 (641-880)	708 (647-862)	565 (526-677)	373 (345-461)	225 (211-261)	81 (76-90)	20 (18-21)
Chronic lymphoid leukemia	-	-	-	-	-	-	69 (51-85)	77 (49-103)	106 (64-149)	122 (69-179)	116 (80-147)	133 (109-154)	189 (157-221)	286 (252-320)	407 (372-452)	803 (735-884)	1,051 (984-1,142)	1,521 (1,452-1,630)	1,884 (1,801-1,980)	1,615 (1,561-1,682)	1,551 (1,457-1,612)	807 (775-836)	251 (239-261)
Acute myeloid leukemia	21 (14-35)	20 (13-37)	300 (180-608)	1,302 (706-3,243)	872 (557-1,728)	1,048 (765-1,621)	1,113 (880-1,560)	1,038 (822-1,464)	1,072 (838-1,574)	1,081 (848-1,568)	1,188 (974-1,579)	1,285 (1,095-1,615)	1,578 (1,375-1,935)	1,602 (1,422-1,892)	1,764 (1,581-2,024)	2,352 (2,141-2,662)	2,735 (2,495-3,070)	3,107 (2,849-3,374)	3,030 (2,854-3,280)	2,124 (2,012-2,292)	1,365 (1,303-1,472)	504 (483-563)	122 (116-144)
Chronic myeloid leukemia	-	-	-	-	-	-	[illegible]	[illegible]	[illegible]	[illegible]	[illegible]	[illegible]	[illegible]	[illegible]	[illegible]	[illegible]	[illegible]	[illegible]	[illegible]	[illegible]	[illegible]	[illegible]	[illegible]
Other leukemia	99 (69-132)	93 (66-130)	1,535 (922-2,365)	4,225 (2,438-7,653)	3,980 (2,612-5,889)	3,705 (2,503-4,933)	[illegible]	[illegible]	[illegible]	[illegible]	[illegible]	[illegible]	[illegible]	[illegible]	[illegible]	[illegible]	[illegible]	[illegible]	[illegible]	[illegible]	[illegible]	[illegible]	[illegible]
Other malignant neoplasms	459 (356-533)	253 (193-314)	1,558 (1,055-2,129)	5,013 (3,202-7,812)	2,598 (2,099-3,281)	3,226 (2,823-3,766)	[illegible]	[illegible]	[illegible]	[illegible]	[illegible]	[illegible]	[illegible]	[illegible]	[illegible]	[illegible]	[illegible]	[illegible]	[illegible]	[illegible]	[illegible]	[illegible]	[illegible]
Other neoplasms	26 (19-32)	15 (10-18)	216 (139-282)	663 (379-940)	545 (379-704)	433 (314-542)	[illegible]	[illegible]	[illegible]	[illegible]	[illegible]	[illegible]	[illegible]	[illegible]	[illegible]	[illegible]	[illegible]	[illegible]	[illegible]	[illegible]	[illegible]	[illegible]	[illegible]
Myelodysplastic, myeloproliferative, and other hematopoietic neoplasm	25 (18-31)	11 (8-15)	188 (124-256)	524 (314-794)	358 (266-486)	283 (211-367)	[illegible]	[illegible]	[illegible]	[illegible]	[illegible]	[illegible]	[illegible]	[illegible]	[illegible]	[illegible]	[illegible]	[illegible]	[illegible]	[illegible]	[illegible]	[illegible]	[illegible]
Other benign and in situ neoplasms	1 (1-2)	3 (2-5)	29 (15-47)	139 (65-274)	186 (111-248)	149 (102-188)	[illegible]	[illegible]	[illegible]	[illegible]	[illegible]	[illegible]	[illegible]	[illegible]	[illegible]	[illegible]	[illegible]	[illegible]	[illegible]	[illegible]	[illegible]	[illegible]	[illegible]
Cardiovascular diseases	1,603 (1,475-1,746)	1,276 (1,156-1,389)	11,471 (10,630-12,382)	10,171 (8,761-11,404)	5,574 (5,009-6,127)	7,055 (6,495-7,625)	[illegible]	[illegible]	[illegible]	[illegible]	[illegible]	[illegible]	[illegible]	[illegible]	[illegible]	[illegible]	[illegible]	[illegible]	[illegible]	[illegible]	[illegible]	[illegible]	[illegible]
Rheumatic heart disease	-	-	-	2,812 (2,154-3,507)	1,951 (1,644-2,250)	2,776 (2,463-3,084)	[illegible]	[illegible]	[illegible]	[illegible]	[illegible]	[illegible]	[illegible]	[illegible]	[illegible]	[illegible]	[illegible]	[illegible]	[illegible]	[illegible]	[illegible]	[illegible]	[illegible]
Ischemic heart disease	-	-	-	-	-	-	[illegible]	[illegible]	[illegible]	[illegible]	[illegible]	[illegible]	[illegible]	[illegible]	[illegible]	[illegible]	[illegible]	[illegible]	[illegible]	[illegible]	[illegible]	[illegible]	[illegible]
Stroke	551 (473-643)	441 (368-530)	3,494 (2,865-4,706)	2,462 (1,961-3,222)	1,788 (1,588-2,090)	2,261 (2,050-2,565)	[illegible]	[illegible]	[illegible]	[illegible]	[illegible]	[illegible]	[illegible]	[illegible]	[illegible]	[illegible]	[illegible]	[illegible]	[illegible]	[illegible]	[illegible]	[illegible]	[illegible]
Ischemic stroke	114 (84-142)	86 (62-109)	461 (321-631)	[illegible]	[illegible]	[illegible]	[illegible]	[illegible]	[illegible]	[illegible]	[illegible]	[illegible]	[illegible]	[illegible]	[illegible]	[illegible]	[illegible]	[illegible]	[illegible]	[illegible]	[illegible]	[illegible]	[illegible]
Intracerebral hemorrhage	237 (202-282)	177 (144-220)	1,625 (1,294-2,239)	[illegible]	[illegible]	[illegible]	[illegible]	[illegible]	[illegible]	[illegible]	[illegible]	[illegible]	[illegible]	[illegible]	[illegible]	[illegible]	[illegible]	[illegible]	[illegible]	[illegible]	[illegible]	[illegible]	[illegible]
Subarachnoid hemorrhage	199 (169-227)	178 (146-209)	1,407 (1,139-1,741)	[illegible]	[illegible]	[illegible]	[illegible]	[illegible]	[illegible]	[illegible]	[illegible]	[illegible]	[illegible]	[illegible]	[illegible]	[illegible]	[illegible]	[illegible]	[illegible]	[illegible]	[illegible]	[illegible]	[illegible]
Hypertensive heart disease	-	-	-	[illegible]	[illegible]	[illegible]	[illegible]	[illegible]	[illegible]	[illegible]	[illegible]	[illegible]	[illegible]	[illegible]	[illegible]	[illegible]	[illegible]	[illegible]	[illegible]	[illegible]	[illegible]	[illegible]	[illegible]
Non-rheumatic valvular heart disease	-	-	-	[illegible]	[illegible]	[illegible]	[illegible]	[illegible]	[illegible]	[illegible]	[illegible]	[illegible]	[illegible]	[illegible]	[illegible]	[illegible]	[illegible]	[illegible]	[illegible]	[illegible]	[illegible]	[illegible]	[illegible]
Non-rheumatic calcific aortic valve disease	-	-	-	[illegible]	[illegible]	[illegible]	[illegible]	[illegible]	[illegible]	[illegible]	[illegible]	[illegible]	[illegible]	[illegible]	[illegible]	[illegible]	[illegible]	[illegible]	[illegible]	[illegible]	[illegible]	[illegible]	[illegible]
Non-rheumatic degenerative mitral valve disease	-	-	-	[illegible]	[illegible]	[illegible]	[illegible]	[illegible]	[illegible]	[illegible]	[illegible]	[illegible]	[illegible]	[illegible]	[illegible]	[illegible]	[illegible]	[illegible]	[illegible]	[illegible]	[illegible]	[illegible]	[illegible]
Other non-rheumatic valve diseases	-	-	-	[illegible]	[illegible]	[illegible]	[illegible]	[illegible]	[illegible]	[illegible]	[illegible]	[illegible]	[illegible]	[illegible]	[illegible]	[illegible]	[illegible]	[illegible]	[illegible]	[illegible]	[illegible]	[illegible]	[illegible]
Cardiomyopathy and myocarditis	658 (526-760)	479 (375-559)	4,301 (3,322-5,070)	[illegible]	[illegible]	[illegible]	[illegible]	[illegible]	[illegible]	[illegible]	[illegible]	[illegible]	[illegible]	[illegible]	[illegible]	[illegible]	[illegible]	[illegible]	[illegible]	[illegible]	[illegible]	[illegible]	[illegible]
Myocarditis	76 (56-99)	84 (60-103)	1,206 (856-1,467)	[illegible]	[illegible]	[illegible]	[illegible]	[illegible]	[illegible]	[illegible]	[illegible]	[illegible]	[illegible]	[illegible]	[illegible]	[illegible]	[illegible]	[illegible]	[illegible]	[illegible]	[illegible]	[illegible]	[illegible]
Alcoholic cardiomyopathy	-	-	-	[illegible]	[illegible]	[illegible]	[illegible]	[illegible]	[illegible]	[illegible]	[illegible]	[illegible]	[illegible]	[illegible]	[illegible]	[illegible]	[illegible]	[illegible]	[illegible]	[illegible]	[illegible]	[illegible]	[illegible]
Other cardiomyopathy	583 (453-678)	395 (301-465)	3,095 (2,298-3,698)	[illegible]	[illegible]	[illegible]	[illegible]	[illegible]	[illegible]	[illegible]	[illegible]	[illegible]	[illegible]	[illegible]	[illegible]	[illegible]	[illegible]	[illegible]	[illegible]	[illegible]	[illegible]	[illegible]	[illegible]
Atrial fibrillation and flutter	-	-	-	[illegible]	[illegible]	[illegible]	[illegible]	[illegible]	[illegible]	[illegible]	[illegible]	[illegible]	[illegible]	[illegible]	[illegible]	[illegible]	[illegible]	[illegible]	[illegible]	[illegible]	[illegible]	[illegible]	[illegible]
Aortic aneurysm	-	-	-	[illegible]	[illegible]	[illegible]	[illegible]	[illegible]	[illegible]	[illegible]	[illegible]	[illegible]	[illegible]	[illegible]	[illegible]	[illegible]	[illegible]	[illegible]	[illegible]	[illegible]	[illegible]	[illegible]	[illegible]
Peripheral artery disease	-	-	-	[illegible]	[illegible]	[illegible]	[illegible]	[illegible]	[illegible]	[illegible]	[illegible]	[illegible]	[illegible]	[illegible]	[illegible]	[illegible]	[illegible]	[illegible]	[illegible]	[illegible]	[illegible]	[illegible]	[illegible]
Endocarditis	100 (77-134)	110 (82-148)	1,486 (1,145-1,952)	[illegible]	[illegible]	[illegible]	[illegible]	[illegible]	[illegible]	[illegible]	[illegible]	[illegible]	[illegible]	[illegible]	[illegible]	[illegible]	[illegible]	[illegible]	[illegible]	[illegible]	[illegible]	[illegible]	[illegible]
Other cardiovascular and circulatory diseases	294 (233-448)	247 (196-360)	2,189 (1,569-3,563)	[illegible]	[illegible]	[illegible]	[illegible]	[illegible]	[illegible]	[illegible]	[illegible]	[illegible]	[illegible]	[illegible]	[illegible]	[illegible]	[illegible]	[illegible]	[illegible]	[illegible]	[illegible]	[illegible]	[illegible]
Chronic respiratory diseases	0 (0-0)	0 (0-0)	0 (0-0)	[illegible]	[illegible]	[illegible]	[illegible]	[illegible]	[illegible]	[illegible]	[illegible]	[illegible]	[illegible]	[illegible]	[illegible]	[illegible]	[illegible]	[illegible]	[illegible]	[illegible]	[illegible]	[illegible]	[illegible]
Chronic obstructive pulmonary disease	-	-	0 (0-0)	[illegible]	[illegible]	[illegible]	[illegible]	[illegible]	[illegible]	[illegible]	[illegible]	[illegible]	[illegible]	[illegible]	[illegible]	[illegible]	[illegible]	[illegible]	[illegible]	[illegible]	[illegible]	[illegible]	[illegible]
Pneumoconiosis	-	-	-	[illegible]	[illegible]	[illegible]	[illegible]	[illegible]	[illegible]	[illegible]	[illegible]	[illegible]	[illegible]	[illegible]	[illegible]	[illegible]	[illegible]	[illegible]	[illegible]	[illegible]	[illegible]	[illegible]	[illegible]
Silicosis	-	-	-	[illegible]	[illegible]	[illegible]	[illegible]	[illegible]	[illegible]	[illegible]	[illegible]	[illegible]	[illegible]	[illegible]	[illegible]	[illegible]	[illegible]	[illegible]	[illegible]	[illegible]	[illegible]	[illegible]	[illegible]
Asbestosis	-	-	-	[illegible]	[illegible]	[illegible]	[illegible]	[illegible]	[illegible]	[illegible]	[illegible]	[illegible]	[illegible]	[illegible]	[illegible]	[illegible]	[illegible]	[illegible]	[illegible]	[illegible]	[illegible]	[illegible]	[illegible]
Coal workers pneumoconiosis	-	-	-	[illegible]	[illegible]	[illegible]	[illegible]	[illegible]	[illegible]	[illegible]	[illegible]	[illegible]	[illegible]	[illegible]	[illegible]	[illegible]	[illegible]	[illegible]	[illegible]	[illegible]	[illegible]	[illegible]	[illegible]
Other pneumoconiosis	-	-	-	[illegible]	[illegible]	[illegible]	[illegible]	[illegible]	[illegible]	[illegible]	[illegible]	[illegible]	[illegible]	[illegible]	[illegible]	[illegible]	[illegible]	[illegible]	[illegible]	[illegible]	[illegible]	[illegible]	[illegible]
Asthma	-	-	-	[illegible]	[illegible]	[illegible]	[illegible]	[illegible]	[illegible]	[illegible]	[illegible]	[illegible]	[illegible]	[illegible]	[illegible]	[illegible]	[illegible]	[illegible]	[illegible]	[illegible]	[illegible]	[illegible]	[illegible]
Interstitial lung disease and pulmonary sarcoidosis	-	-	-	[illegible]	[illegible]	[illegible]	[illegible]	[illegible]	[illegible]	[illegible]	[illegible]	[illegible]	[illegible]	[illegible]	[illegible]	[illegible]	[illegible]	[illegible]	[illegible]	[illegible]	[illegible]	[illegible]	[illegible]
Other chronic respiratory diseases	0 (0-0)	0 (0-0)	0 (0-0)	[illegible]	[illegible]	[illegible]	[illegible]	[illegible]	[illegible]	[illegible]	[illegible]	[illegible]	[illegible]	[illegible]	[illegible]	[illegible]	[illegible]	[illegible]	[illegible]	[illegible]	[illegible]	[illegible]	[illegible]
Digestive diseases	1,156 (954-1,274)	1,171 (1,033-1,423)	9,745 (8,117-13,562)	[illegible]	[illegible]	[illegible]	[illegible]	[illegible]	[illegible]	[illegible]	[illegible]	[illegible]	[illegible]	[illegible]	[illegible]	[illegible]	[illegible]	[illegible]	[illegible]	[illegible]	[illegible]	[illegible]	[illegible]
Cirrhosis and other chronic liver diseases	-	-	-	[illegible]	[illegible]	[illegible]	[illegible]	[illegible]	[illegible]	[illegible]	[illegible]	[illegible]	[illegible]	[illegible]	[illegible]	[illegible]	[illegible]	[illegible]	[illegible]	[illegible]	[illegible]	[illegible]	[illegible]
Cirrhosis and other chronic liver diseases due to hepatitis B	-	-	-	[illegible]	[illegible]	[illegible]	[illegible]	[illegible]	[illegible]	[illegible]	[illegible]	[illegible]	[illegible]	[illegible]	[illegible]	[illegible]	[illegible]	[illegible]	[illegible]	[illegible]	[illegible]	[illegible]	[illegible]
Cirrhosis and other chronic liver diseases due to hepatitis C	-	-	-	[illegible]	[illegible]	[illegible]	[illegible]	[illegible]	[illegible]	[illegible]	[illegible]	[illegible]	[illegible]	[illegible]	[illegible]	[illegible]	[illegible]	[illegible]	[illegible]	[illegible]	[illegible]	[illegible]	[illegible]
Cirrhosis and other chronic liver diseases due to alcohol use	-	-	-	[illegible]	[illegible]	[illegible]	[illegible]	[illegible]	[illegible]	[illegible]	[illegible]	[illegible]	[illegible]	[illegible]	[illegible]	[illegible]	[illegible]	[illegible]	[illegible]	[illegible]	[illegible]	[illegible]	[illegible]
Cirrhosis due to NASH	-	-	-	[illegible]	[illegible]	[illegible]	[illegible]	[illegible]	[illegible]	[illegible]	[illegible]	[illegible]	[illegible]	[illegible]	[illegible]	[illegible]	[illegible]	[illegible]	[illegible]	[illegible]	[illegible]	[illegible]	[illegible]
Cirrhosis and other chronic liver diseases due to other causes	-	-	-	[illegible]	[illegible]	[illegible]	[illegible]	[illegible]	[illegible]	[illegible]	[illegible]	[illegible]	[illegible]	[illegible]	[illegible]	[illegible]	[illegible]	[illegible]	[illegible]	[illegible]	[illegible]	[illegible]	[illegible]
Upper digestive system diseases	-	-	-	[illegible]	[illegible]	[illegible]	[illegible]	[illegible]	[illegible]	[illegible]	[illegible]	[illegible]	[illegible]	[illegible]	[illegible]	[illegible]	[illegible]	[illegible]	[illegible]	[illegible]	[illegible]	[illegible]	[illegible]
Peptic ulcer disease	-	-	-	[illegible]	[illegible]	[illegible]	[illegible]	[illegible]	[illegible]	[illegible]	[illegible]	[illegible]	[illegible]	[illegible]	[illegible]	[illegible]	[illegible]	[illegible]	[illegible]	[illegible]	[illegible]	[illegible]	[illegible]
Gastritis and duodenitis	-	-	-	[illegible]	[illegible]	[illegible]	[illegible]	[illegible]	[illegible]	[illegible]	[illegible]	[illegible]	[illegible]	[illegible]	[illegible]	[illegible]	[illegible]	[illegible]	[illegible]	[illegible]	[illegible]	[illegible]	[illegible]
Appendicitis	-	-	-	[illegible]	[illegible]	[illegible]	[illegible]	[illegible]	[illegible]	[illegible]	[illegible]	[illegible]	[illegible]	[illegible]	[illegible]	[illegible]	[illegible]	[illegible]	[illegible]	[illegible]	[illegible]	[illegible]	[illegible]
Paralytic ileus and intestinal obstruction	1,156 (954-1,274)	1,171 (1,033-1,423)	9,745 (8,117-13,562)	[illegible]	[illegible]	[illegible]	[illegible]	[illegible]	[illegible]	[illegible]	[illegible]	[illegible]	[illegible]	[illegible]	[illegible]	[illegible]	[illegible]	[illegible]	[illegible]	[illegible]	[illegible]	[illegible]	[illegible]
Inguinal, femoral, and abdominal hernia	-	-	-	[illegible]	[illegible]	[illegible]	[illegible]	[illegible]	[illegible]	[illegible]	[illegible]	[illegible]	[illegible]	[illegible]	[illegible]	[illegible]	[illegible]	[illegible]	[illegible]	[illegible]	[illegible]	[illegible]	[illegible]
Inflammatory bowel disease	-	-	-	[illegible]	[illegible]	[illegible]	[illegible]	[illegible]	[illegible]	[illegible]	[illegible]	[illegible]	[illegible]	[illegible]	[illegible]	[illegible]	[illegible]	[illegible]	[illegible]	[illegible]	[illegible]	[illegible]	[illegible]
Vascular intestinal disorders	-	-	-	[illegible]	[illegible]	[illegible]	[illegible]	[illegible]	[illegible]	[illegible]	[illegible]	[illegible]	[illegible]	[illegible]	[illegible]	[illegible]	[illegible]	[illegible]	[illegible]	[illegible]	[illegible]	[illegible]	[illegible]
Gallbladder and biliary diseases	-	-	-	[illegible]	[illegible]	[illegible]	[illegible]	[illegible]	[illegible]	[illegible]	[illegible]	[illegible]	[illegible]	[illegible]	[illegible]	[illegible]	[illegible]	[illegible]	[illegible]	[illegible]	[illegible]	[illegible]	[illegible]
Pancreatitis	-	-	-	[illegible]	[illegible]	[illegible]	[illegible]	[illegible]	[illegible]	[illegible]	[illegible]	[illegible]	[illegible]	[illegible]	[illegible]	[illegible]	[illegible]	[illegible]	[illegible]	[illegible]	[illegible]	[illegible]	[illegible]
Other digestive diseases	-	-	-	[illegible]	[illegible]	[illegible]	[illegible]	[illegible]	[illegible]	[illegible]	[illegible]	[illegible]	[illegible]	[illegible]	[illegible]	[illegible]	[illegible]	[illegible]	[illegible]	[illegible]	[illegible]	[illegible]	[illegible]
Neurological disorders	-	-	5,479 (3,912-6,947)	[illegible]	[illegible]	[illegible]	[illegible]	[illegible]	[illegible]	[illegible]	[illegible]	[illegible]	[illegible]	[illegible]	[illegible]	[illegible]	[illegible]	[illegible]	[illegible]	[illegible]	[illegible]	[illegible]	[illegible]
Alzheimer's disease and other dementias	-	-	-	[illegible]	[illegible]	[illegible]	[illegible]	[illegible]	[illegible]	[illegible]	[illegible]	[illegible]	[illegible]	[illegible]	[illegible]	[illegible]	[illegible]	[illegible]	[illegible]	[illegible]	[illegible]	[illegible]	[illegible]
Parkinson's disease	-	-	-	[illegible]	[illegible]	[illegible]	[illegible]	[illegible]	[illegible]	[illegible]	[illegible]	[illegible]	[illegible]	[illegible]	[illegible]	[illegible]	[illegible]	[illegible]	[illegible]	[illegible]	[illegible]	[illegible]	[illegible]
Epilepsy	-	-	3,864 (2,780-4,578)	[illegible]	[illegible]	[illegible]	[illegible]	[illegible]	[illegible]	[illegible]	[illegible]	[illegible]	[illegible]	[illegible]	[illegible]	[illegible]	[illegible]	[illegible]	[illegible]	[illegible]	[illegible]	[illegible]	[illegible]
Multiple sclerosis	-	-	-	[illegible]	[illegible]	[illegible]	[illegible]	[illegible]	[illegible]	[illegible]	[illegible]	[illegible]	[illegible]	[illegible]	[illegible]	[illegible]	[illegible]	[illegible]	[illegible]	[illegible]	[illegible]	[illegible]	[illegible]
Motor neuron disease	9 (2-14)	14 (5-21)	237 (166-328)	[illegible]	[illegible]	[illegible]	[illegible]	[illegible]	[illegible]	[illegible]	[illegible]	[illegible]	[illegible]	[illegible]	[illegible]	[illegible]	[illegible]	[illegible]	[illegible]	[illegible]	[illegible]	[illegible]	[illegible]
Other neurological disorders	-	-	1,377 (735-2,556)	[illegible]	[illegible]	[illegible]	[illegible]	[illegible]	[illegible]	[illegible]	[illegible]	[illegible]	[illegible]	[illegible]	[illegible]	[illegible]	[illegible]	[illegible]	[illegible]	[illegible]	[illegible]	[illegible]	[illegible]
Mental disorders	-	-	-	[illegible]	[illegible]	[illegible]	[illegible]	[illegible]	[illegible]	[illegible]	[illegible]	[illegible]	[illegible]	[illegible]	[illegible]	[illegible]	[illegible]	[illegible]	[illegible]	[illegible]	[illegible]	[illegible]	[illegible]
Eating disorders	-	-	-	[illegible]	[illegible]	[illegible]	[illegible]	[illegible]	[illegible]	[illegible]	[illegible]	[illegible]	[illegible]	[illegible]	[illegible]	[illegible]	[illegible]	[illegible]	[illegible]	[illegible]	[illegible]	[illegible]	[illegible]
Anorexia nervosa	-	-	-	[illegible]	[illegible]	[illegible]	[illegible]	[illegible]	[illegible]	[illegible]	[illegible]	[illegible]	[illegible]	[illegible]	[illegible]	[illegible]	[illegible]	[illegible]	[illegible]	[illegible]	[illegible]	[illegible]	[illegible]
Bulimia nervosa	-	-	-	[illegible]	[illegible]	[illegible]	[illegible]	[illegible]	[illegible]	[illegible]	[illegible]	[illegible]	[illegible]	[illegible]	[illegible]	[illegible]	[illegible]	[illegible]	[illegible]	[illegible]	[illegible]	[illegible]	[illegible]
Substance use disorders	0 (0-0)	0 (0-0)	0 (0-0)	[illegible]	[illegible]	[illegible]	[illegible]	[illegible]	[illegible]	[illegible]	[illegible]	[illegible]	[illegible]	[illegible]	[illegible]	[illegible]	[illegible]	[illegible]	[illegible]	[illegible]	[illegible]	[illegible]	[illegible]
Alcohol use disorders	-	-	-	[illegible]	[illegible]	[illegible]	[illegible]	[illegible]	[illegible]	[illegible]	[illegible]	[illegible]	[illegible]	[illegible]	[illegible]	[illegible]	[illegible]	[illegible]	[illegible]	[illegible]	[illegible]	[illegible]	[illegible]
Drug use disorders	0 (0-0)	0 (0-0)	0 (0-0)	[illegible]	[illegible]	[illegible]	[illegible]	[illegible]	[illegible]	[illegible]	[illegible]	[illegible]	[illegible]	[illegible]	[illegible]	[illegible]	[illegible]	[illegible]	[illegible]	[illegible]	[illegible]	[illegible]	[illegible]
Opioid use disorders	0 (0-0)	0 (0-0)	0 (0-0)	[illegible]	[illegible]	[illegible]	[illegible]	[illegible]	[illegible]	[illegible]	[illegible]	[illegible]	[illegible]	[illegible]	[illegible]	[illegible]	[illegible]	[illegible]	[illegible]	[illegible]	[illegible]	[illegible]	[illegible]
Cocaine use disorders	-	-	-	[illegible]	[illegible]	[illegible]	[illegible]	[illegible]	[illegible]	[illegible]	[illegible]	[illegible]	[illegible]	[illegible]	[illegible]	[illegible]	[illegible]	[illegible]	[illegible]	[illegible]	[illegible]	[illegible]	[illegible]
Amphetamine use disorders	-	-	-	[illegible]	[illegible]	[illegible]	[illegible]	[illegible]	[illegible]	[illegible]	[illegible]	[illegible]	[illegible]	[illegible]	[illegible]	[illegible]	[illegible]	[illegible]	[illegible]	[illegible]	[illegible]	[illegible]	[illegible]
Other drug use disorders	-	-	-	[illegible]	[illegible]	[illegible]	[illegible]	[illegible]	[illegible]	[illegible]	[illegible]	[illegible]	[illegible]	[illegible]	[illegible]	[illegible]	[illegible]	[illegible]	[illegible]	[illegible]	[illegible]	[illegible]	[illegible]
Diabetes and kidney diseases	7 (5-9)	23 (17-30)	4,599 (4,166-5,060)	[illegible]	[illegible]	[illegible]	[illegible]	[illegible]	[illegible]	[illegible]	[illegible]	[illegible]	[illegible]	[illegible]	[illegible]	[illegible]	[illegible]	[illegible]	[illegible]	[illegible]	[illegible]	[illegible]	[illegible]
Diabetes mellitus	7 (5-9)	23 (17-30)	496 (343-612)	[illegible]	[illegible]	[illegible]	[illegible]	[illegible]	[illegible]	[illegible]	[illegible]	[illegible]	[illegible]	[illegible]	[illegible]	[illegible]	[illegible]	[illegible]	[illegible]	[illegible]	[illegible]	[illegible]	[illegible]
Diabetes mellitus type 1	7 (5-9)	23 (17-30)	496 (343-612)	[illegible]	[illegible]	[illegible]	[illegible]	[illegible]	[illegible]	[illegible]	[illegible]	[illegible]	[illegible]	[illegible]	[illegible]	[illegible]	[illegible]	[illegible]	[illegible]	[illegible]	[illegible]	[illegible]	[illegible]
Diabetes mellitus type 2	-	-	-	[illegible]	[illegible]	[illegible]	[illegible]	[illegible]	[illegible]	[illegible]	[illegible]	[illegible]	[illegible]	[illegible]	[illegible]	[illegible]	[illegible]	[illegible]	[illegible]	[illegible]	[illegible]	[illegible]	[illegible]
Chronic kidney disease	-	-	3,951 (3,565-4,351)	[illegible]	[illegible]	[illegible]	[illegible]	[illegible]	[illegible]	[illegible]	[illegible]	[illegible]	[illegible]	[illegible]	[illegible]	[illegible]	[illegible]	[illegible]	[illegible]	[illegible]	[illegible]	[illegible]	[illegible]
Chronic kidney disease due to diabetes mellitus type 1	-	-	16 (9-26)	[illegible]	[illegible]	[illegible]	[illegible]	[illegible]	[illegible]	[illegible]	[illegible]	[illegible]	[illegible]	[illegible]	[illegible]	[illegible]	[illegible]	[illegible]	[illegible]	[illegible]	[illegible]	[illegible]	[illegible]
Chronic kidney disease due to diabetes mellitus type 2	-	-	11 (6-19)	[illegible]	[illegible]	[illegible]	[illegible]	[illegible]	[illegible]	[illegible]	[illegible]	[illegible]	[illegible]	[illegible]	[illegible]	[illegible]	[illegible]	[illegible]	[illegible]	[illegible]	[illegible]	[illegible]	[illegible]
Chronic kidney disease due to hypertension	-	-	19 (11-30)	[illegible]	[illegible]	[illegible]	[illegible]	[illegible]	[illegible]	[illegible]	[illegible]	[illegible]	[illegible]	[illegible]	[illegible]	[illegible]	[illegible]	[illegible]	[illegible]	[illegible]	[illegible]	[illegible]	[illegible]
Chronic kidney disease due to glomerulonephritis	-	-	1,001 (704-1,360)	[illegible]	[illegible]	[illegible]	[illegible]	[illegible]	[illegible]	[illegible]	[illegible]	[illegible]	[illegible]	[illegible]	[illegible]	[illegible]	[illegible]	[illegible]	[illegible]	[illegible]	[illegible]	[illegible]	[illegible]
Chronic kidney disease due to other and unspecified causes	-	-	2,904 (2,494-3,308)	[illegible]	[illegible]	[illegible]	[illegible]	[illegible]	[illegible]	[illegible]	[illegible]	[illegible]	[illegible]	[illegible]	[illegible]	[illegible]	[illegible]	[illegible]	[illegible]	[illegible]	[illegible]	[illegible]	[illegible]
Acute glomerulonephritis	-	-	152 (92-189)	[illegible]	[illegible]	[illegible]	[illegible]	[illegible]	[illegible]	[illegible]	[illegible]	[illegible]	[illegible]	[illegible]	[illegible]	[illegible]	[illegible]	[illegible]	[illegible]	[illegible]	[illegible]	[illegible]	[illegible]
Skin and subcutaneous diseases	-	-	2,491 (832-3,508)	[illegible]	[illegible]	[illegible]	[illegible]	[illegible]	[illegible]	[illegible]	[illegible]	[illegible]	[illegible]	[illegible]	[illegible]	[illegible]	[illegible]	[illegible]	[illegible]	[illegible]	[illegible]	[illegible]	[illegible]
Bacterial skin diseases	-	-	2,461 (818-3,456)	[illegible]	[illegible]	[illegible]	[illegible]	[illegible]	[illegible]	[illegible]	[illegible]	[illegible]	[illegible]	[illegible]	[illegible]	[illegible]	[illegible]	[illegible]	[illegible]	[illegible]	[illegible]	[illegible]	[illegible]
Cellulitis	-	-	209 (31-457)	[illegible]	[illegible]	[illegible]	[illegible]	[illegible]	[illegible]	[illegible]	[illegible]	[illegible]	[illegible]	[illegible]	[illegible]	[illegible]	[illegible]	[illegible]	[illegible]	[illegible]	[illegible]	[illegible]	[illegible]
Pyoderma	-	-	2,252 (783-3,151)	[illegible]	[illegible]	[illegible]	[illegible]	[illegible]	[illegible]	[illegible]	[illegible]	[illegible]	[illegible]	[illegible]	[illegible]	[illegible]	[illegible]	[illegible]	[illegible]	[illegible]	[illegible]	[illegible]	[illegible]
Decubitus ulcer	-	-	-	[illegible]	[illegible]	[illegible]	[illegible]	[illegible]	[illegible]	[illegible]	[illegible]	[illegible]	[illegible]	[illegible]	[illegible]	[illegible]	[illegible]	[illegible]	[illegible]	[illegible]	[illegible]	[illegible]	[illegible]
Other skin and subcutaneous diseases	-	-	31 (10-62)	[illegible]	[illegible]	[illegible]	[illegible]	[illegible]	[illegible]	[illegible]	[illegible]	[illegible]	[illegible]	[illegible]	[illegible]	[illegible]	[illegible]	[illegible]	[illegible]	[illegible]	[illegible]	[illegible]	[illegible]
Musculoskeletal disorders	-	-	-	[illegible]	[illegible]	[illegible]	[illegible]	[illegible]	[illegible]	[illegible]	[illegible]	[illegible]	[illegible]	[illegible]	[illegible]	[illegible]	[illegible]	[illegible]	[illegible]	[illegible]	[illegible]	[illegible]	[illegible]
Rheumatoid arthritis	-	-	-	[illegible]	[illegible]	[illegible]	[illegible]	[illegible]	[illegible]	[illegible]	[illegible]	[illegible]	[illegible]	[illegible]	[illegible]	[illegible]	[illegible]	[illegible]	[illegible]	[illegible]	[illegible]	[illegible]	[illegible]
Other musculoskeletal disorders	-	-	-	[illegible]	[illegible]	[illegible]	[illegible]	[illegible]	[illegible]	[illegible]	[illegible]	[illegible]	[illegible]	[illegible]	[illegible]	[illegible]	[illegible]	[illegible]	[illegible]	[illegible]	[illegible]	[illegible]	[illegible]
Other non-communicable diseases	91,072 (83,386-103,225)	41,167 (37,756-47,114)	154,021 (134,331-185,550)	[illegible]	[illegible]	[illegible]	[illegible]	[illegible]	[illegible]	[illegible]	[illegible]	[illegible]	[illegible]	[illegible]	[illegible]	[illegible]	[illegible]	[illegible]	[illegible]	[illegible]	[illegible]	[illegible]	[illegible]
Congenital birth defects	89,568 (81,924-101,725)	37,783 (34,604-41,725)	124,736 (113,539-136,064)	[illegible]	[illegible]	[illegible]	[illegible]	[illegible]	[illegible]	[illegible]	[illegible]	[illegible]	[illegible]	[illegible]	[illegible]	[illegible]	[illegible]	[illegible]	[illegible]	[illegible]	[illegible]	[illegible]	[illegible]
Neural tube defects	14,015 (10,713-17,365)	3,936 (2,845-5,344)	12,204 (7,148-21,466)	[illegible]	[illegible]	[illegible]	[illegible]	[illegible]	[illegible]	[illegible]	[illegible]	[illegible]	[illegible]	[illegible]	[illegible]	[illegible]	[illegible]	[illegible]	[illegible]	[illegible]	[illegible]	[illegible]	[illegible]
Congenital heart anomalies	30,815 (22,125-38,154)	17,545 (13,133-21,293)	69,352 (45,893-85,951)	[illegible]	[illegible]	[illegible]	[illegible]	[illegible]	[illegible]	[illegible]	[illegible]	[illegible]	[illegible]	[illegible]	[illegible]	[illegible]	[illegible]	[illegible]	[illegible]	[illegible]	[illegible]	[illegible]	[illegible]
Orofacial clefts	1,513 (718-3,883)	674 (304-1,759)	1,382 (578-4,067)	[illegible]	[illegible]	[illegible]	[illegible]	[illegible]	[illegible]	[illegible]	[illegible]	[illegible]	[illegible]	[illegible]	[illegible]	[illegible]	[illegible]	[illegible]	[illegible]	[illegible]	[illegible]	[illegible]	[illegible]
Down syndrome	1,739 (996-2,946)	778 (390-1,302)	3,639 (1,719-6,591)	[illegible]	[illegible]	[illegible]	[illegible]	[illegible]	[illegible]	[illegible]	[illegible]	[illegible]	[illegible]	[illegible]	[illegible]	[illegible]	[illegible]	[illegible]	[illegible]	[illegible]	[illegible]	[illegible]	[illegible]
Other chromosomal abnormalities	2,699 (1,273-6,528)	1,517 (745-3,454)	3,460 (1,638-7,975)	[illegible]	[illegible]	[illegible]	[illegible]	[illegible]	[illegible]	[illegible]	[illegible]	[illegible]	[illegible]	[illegible]	[illegible]	[illegible]	[illegible]	[illegible]	[illegible]	[illegible]	[illegible]	[illegible]	[illegible]
Congenital musculoskeletal and limb anomalies	2,159 (1,078-3,330)	665 (283-1,064)	1,778 (658-3,421)	[illegible]	[illegible]	[illegible]	[illegible]	[illegible]	[illegible]	[illegible]	[illegible]	[illegible]	[illegible]	[illegible]	[illegible]	[illegible]	[illegible]	[illegible]	[illegible]	[illegible]	[illegible]	[illegible]	[illegible]
Urogenital congenital anomalies	2,304 (1,533-3,032)	806 (531-1,103)	1,303 (673-2,160)	[illegible]	[illegible]	[illegible]	[illegible]	[illegible]	[illegible]	[illegible]	[illegible]	[illegible]	[illegible]	[illegible]	[illegible]	[illegible]	[illegible]	[illegible]	[illegible]	[illegible]	[illegible]	[illegible]	[illegible]
Digestive congenital anomalies	8,455 (4,509-13,376)	3,920 (1,994-6,142)	10,039 (4,388-17,861)	[illegible]	[illegible]	[illegible]	[illegible]	[illegible]	[illegible]	[illegible]	[illegible]	[illegible]	[illegible]	[illegible]	[illegible]	[illegible]	[illegible]	[illegible]	[illegible]	[illegible]	[illegible]	[illegible]	[illegible]
Other congenital birth defects	25,870 (17,315-36,272)	7,943 (4,611-11,594)	21,579 (10,781-35,431)	[illegible]	[illegible]	[illegible]	[illegible]	[illegible]	[illegible]	[illegible]	[illegible]	[illegible]	[illegible]	[illegible]	[illegible]	[illegible]	[illegible]	[illegible]	[illegible]	[illegible]	[illegible]	[illegible]	[illegible]
Urinary diseases and male infertility	258 (233-286)	198 (176-224)	2,491 (2,249-2,772)	[illegible]	[illegible]	[illegible]	[illegible]	[illegible]	[illegible]	[illegible]	[illegible]	[illegible]	[illegible]	[illegible]	[illegible]	[illegible]	[illegible]	[illegible]	[illegible]	[illegible]	[illegible]	[illegible]	[illegible]
Urinary tract infections	48 (34-77)	56 (38-80)	1,539 (1,143-1,961)	[illegible]	[illegible]	[illegible]	[illegible]	[illegible]	[illegible]	[illegible]	[illegible]	[illegible]	[illegible]	[illegible]	[illegible]	[illegible]	[illegible]	[illegible]	[illegible]	[illegible]	[illegible]	[illegible]	[illegible]
Urolithiasis	-	-	-	[illegible]	[illegible]	[illegible]	[illegible]	[illegible]	[illegible]	[illegible]	[illegible]	[illegible]	[illegible]	[illegible]	[illegible]	[illegible]	[illegible]	[illegible]	[illegible]	[illegible]	[illegible]	[illegible]	[illegible]

SR Table 5: Global deaths by cause and age group for females (with 95% confidence intervals), 2000

Cause	Early Neonatal	Late Neonatal	Post Neonatal	1 to 4	5 to 9	10 to 14	15 to 19	20 to 24
Other urinary diseases	210 (166-241)	141 (110-167)	952 (533-1,359)	1,696 (941-2,446)	477 (308-660)	383 (287-500)	321 (179-462)	437 (256-622)
Gynecological diseases	-	-	-	-	-	-	411 (346-554)	458 (373-611)
Uterine fibroids	-	-	-	-	-	-	59 (38-85)	74 (44-122)
Polycystic ovarian syndrome	-	-	-	-	-	-	1 (0-3)	2 (1-6)
Endometriosis	-	-	-	-	-	-	9 (4-14)	11 (5-16)
Genital prolapse	-	-	-	-	-	-	13 (7-21)	11 (5-17)
Other gynecological diseases	-	-	-	-	-	-	330 (263-482)	360 (275-541)
Hemoglobinopathies and hemolytic anemias	428 (347-588)	293 (243-419)	3,079 (1,934-5,103)	9,550 (4,192-15,515)	2,513 (1,817-3,512)	2,080 (1,660-2,858)	2,784 (2,155-3,550)	2,595 (1,922-3,366)
Thalassemias	172 (130-235)	109 (86-164)	1,136 (724-1,811)	1,794 (1,053-2,577)	961 (717-1,226)	621 (478-772)	447 (305-602)	354 (230-445)
Sickle cell disorders	234 (183-341)	178 (142-251)	1,886 (1,098-3,261)	7,569 (2,926-12,746)	1,385 (894-2,098)	1,152 (882-1,700)	1,816 (1,351-2,415)	1,642 (1,205-2,272)
G6PD deficiency	4 (3-6)	3 (2-4)	31 (17-57)	127 (59-235)	102 (72-153)	169 (118-246)	262 (187-367)	320 (237-427)
Other hemoglobinopathies and hemolytic anemias	19 (14-26)	4 (3-6)	27 (18-44)	60 (35-95)	65 (51-87)	139 (114-182)	259 (191-372)	278 (208-375)
Endocrine, metabolic, blood, and immune disorders	818 (548-1,026)	712 (468-891)	4,085 (2,484-5,727)	2,659 (1,493-3,992)	957 (716-1,274)	803 (659-1,067)	866 (714-1,114)	1,119 (892-1,388)
Sudden infant death syndrome	-	2,182 (752-4,837)	19,629 (6,291-48,759)	-	-	-	-	-
Injuries	7,490 (6,449-8,154)	5,907 (5,080-6,503)	55,925 (49,833-61,530)	136,809 (112,169-152,922)	67,451 (60,255-73,540)	55,357 (50,738-58,855)	92,031 (87,041-95,682)	101,981 (97,161-105,309)
Transport injuries	662 (554-767)	678 (599-747)	8,374 (6,887-9,300)	29,083 (20,528-33,602)	21,579 (19,350-23,624)	15,479 (14,267-16,594)	24,906 (23,476-26,121)	27,571 (25,839-28,912)
Road injuries	582 (493-686)	633 (560-699)	7,796 (6,243-8,672)	27,242 (18,653-31,642)	20,668 (18,488-22,679)	14,433 (13,263-15,484)	23,022 (21,820-24,193)	26,069 (24,270-27,351)
Pedestrian road injuries	317 (227-406)	308 (238-384)	3,379 (2,793-4,260)	16,077 (12,203-20,474)	12,839 (11,411-14,468)	7,839 (7,106-8,622)	7,953 (7,197-8,878)	9,157 (8,312-10,375)
Cyclist road injuries	-	-	-	537 (307-802)	633 (486-763)	829 (651-971)	887 (715-1,054)	786 (596-951)
Motorcyclist road injuries	65 (40-95)	37 (27-51)	862 (603-1,234)	1,441 (838-2,522)	1,030 (763-1,436)	1,154 (911-1,507)	3,091 (2,552-3,870)	3,919 (3,026-4,937)
Motor vehicle road injuries	192 (141-248)	282 (213-346)	3,451 (2,414-4,205)	8,958 (4,774-11,856)	5,989 (4,963-6,845)	4,492 (3,898-4,954)	10,867 (9,924-11,660)	11,974 (10,636-13,007)
Other road injuries	8 (5-12)	6 (4-8)	104 (53-160)	229 (91-392)	176 (111-232)	118 (87-154)	225 (164-294)	233 (162-292)
Other transport injuries	79 (49-142)	45 (32-66)	577 (443-890)	1,841 (1,321-3,212)	912 (739-1,127)	1,045 (881-1,210)	1,883 (1,630-2,222)	1,502 (1,292-1,752)
Unintentional injuries	4,832 (4,117-5,361)	4,418 (3,738-4,955)	43,329 (38,267-48,356)	94,208 (76,010-107,641)	37,868 (32,368-42,424)	26,354 (22,456-28,803)	22,265 (18,911-24,300)	22,077 (18,795-24,123)
Falls	809 (716-937)	342 (289-414)	4,800 (3,979-5,779)	6,587 (4,892-8,857)	2,847 (2,359-3,380)	2,000 (1,738-2,289)	2,289 (1,996-2,615)	2,217 (1,929-2,590)
Drowning	227 (201-259)	168 (149-184)	5,209 (4,297-5,844)	51,198 (39,354-58,518)	19,960 (17,036-22,419)	12,277 (10,232-13,273)	6,356 (5,665-6,819)	4,779 (4,241-5,110)
Fire, heat, and hot substances	171 (111-208)	284 (153-354)	3,786 (2,160-4,629)	10,802 (5,319-14,177)	2,965 (2,065-3,528)	2,388 (1,639-2,812)	4,526 (2,627-5,605)	5,436 (3,084-6,694)
Poisonings	97 (70-109)	122 (79-139)	1,893 (1,173-2,288)	4,623 (2,440-6,134)	1,286 (921-1,471)	1,522 (1,177-1,742)	1,457 (1,210-1,641)	1,621 (1,344-1,895)
Poisoning by carbon monoxide	25 (14-30)	33 (18-39)	433 (224-536)	967 (560-1,216)	565 (414-653)	616 (477-733)	767 (644-853)	813 (664-931)
Poisoning by other means	73 (53-81)	89 (59-104)	1,460 (907-1,865)	3,656 (1,843-5,140)	721 (485-878)	907 (676-1,075)	689 (545-814)	808 (637-1,025)
Exposure to mechanical forces	387 (220-491)	466 (228-620)	2,593 (1,346-3,331)	2,862 (1,832-3,579)	2,023 (1,416-2,471)	1,129 (847-1,304)	1,504 (1,059-1,771)	1,334 (962-1,525)
Unintentional firearm injuries	28 (17-36)	20 (12-26)	188 (108-253)	230 (145-342)	214 (156-273)	176 (136-212)	269 (215-314)	277 (219-321)
Other exposure to mechanical forces	359 (200-460)	446 (215-599)	2,405 (1,213-3,127)	2,632 (1,636-3,309)	1,809 (1,228-2,229)	953 (689-1,117)	1,235 (825-1,482)	1,056 (725-1,231)
Adverse effects of medical treatment	754 (481-912)	573 (348-740)	4,365 (2,400-6,286)	3,170 (1,339-5,641)	1,119 (677-1,456)	872 (582-1,042)	1,098 (784-1,287)	1,958 (1,355-2,316)
Animal contact	112 (38-152)	114 (41-157)	1,774 (593-2,498)	4,294 (1,465-7,629)	4,086 (1,481-6,015)	3,099 (1,059-4,363)	1,983 (662-2,898)	1,651 (510-2,473)
Venomous animal contact	75 (20-121)	69 (20-111)	1,433 (461-2,091)	3,312 (1,154-5,780)	3,623 (1,272-5,195)	2,846 (925-3,959)	1,828 (578-2,634)	1,469 (429-2,185)
Non-venomous animal contact	36 (13-90)	46 (16-104)	341 (83-1,165)	982 (135-4,354)	463 (115-1,878)	254 (80-872)	155 (54-559)	182 (64-673)
Foreign body	1,952 (1,790-2,243)	2,182 (1,964-2,477)	16,716 (13,776-18,850)	5,960 (4,067-7,089)	1,424 (1,263-1,607)	997 (923-1,091)	676 (646-728)	751 (713-821)
Pulmonary aspiration and foreign body in airway	1,897 (1,719-2,188)	2,129 (1,908-2,421)	15,991 (13,115-18,106)	5,429 (3,699-6,635)	1,237 (1,095-1,412)	862 (786-957)	489 (444-542)	525 (473-586)
Foreign body in other body part	55 (28-93)	53 (23-82)	725 (257-1,289)	531 (209-1,084)	188 (115-285)	135 (93-194)	187 (152-233)	227 (184-279)
Environmental heat and cold exposure	129 (49-166)	83 (29-112)	1,009 (338-1,396)	705 (231-1,176)	326 (146-445)	152 (97-176)	359 (215-420)	360 (235-422)
Exposure to forces of nature	3 (2-3)	8 (7-10)	73 (64-86)	319 (283-365)	308 (274-352)	302 (272-337)	248 (225-274)	210 (190-237)
Other unintentional injuries	191 (167-218)	75 (65-86)	1,113 (965-1,259)	3,690 (3,034-4,443)	1,523 (1,309-1,772)	1,616 (1,428-1,838)	1,770 (1,590-1,987)	1,758 (1,558-1,992)
Self-harm and interpersonal violence	1,997 (1,674-2,174)	811 (701-869)	4,222 (3,430-4,713)	13,518 (11,823-14,748)	8,003 (7,021-8,696)	13,524 (12,484-14,556)	44,860 (42,365-47,340)	52,333 (49,540-54,937)
Self-harm	-	-	-	-	-	6,395 (5,700-7,272)	32,836 (30,770-35,208)	38,683 (36,642-41,245)
Self-harm by firearm	-	-	-	-	-	206 (126-295)	1,174 (641-1,777)	1,381 (768-2,023)
Self-harm by other specified means	-	-	-	-	-	6,189 (5,513-7,075)	31,662 (29,339-34,205)	37,302 (35,091-40,286)
Interpersonal violence	1,113 (789-1,290)	330 (220-389)	2,650 (1,859-3,142)	5,204 (3,508-6,434)	3,453 (2,466-4,142)	3,374 (2,684-3,968)	7,523 (5,947-8,506)	10,069 (7,857-11,403)
Physical violence by firearm	71 (45-102)	29 (15-41)	308 (195-445)	566 (357-899)	443 (332-595)	602 (504-714)	2,095 (1,717-2,438)	2,633 (2,048-2,995)
Physical violence by sharp object	60 (44-72)	39 (24-51)	233 (159-290)	584 (335-837)	410 (291-511)	574 (437-713)	1,502 (1,162-1,743)	2,487 (1,986-3,003)
Physical violence by other means	982 (681-1,116)	263 (173-311)	2,108 (1,427-2,534)	4,054 (2,562-5,082)	2,600 (1,711-3,225)	2,198 (1,619-2,694)	3,926 (2,856-4,675)	4,949 (3,609-5,924)
Conflict and terrorism	883 (821-970)	481 (449-529)	1,562 (1,462-1,700)	8,275 (7,696-9,172)	4,514 (4,151-5,017)	3,718 (3,428-4,137)	4,455 (4,140-4,946)	3,537 (3,299-3,881)
Executions and police conflict	-	-	10 (7-18)	39 (36-41)	36 (33-41)	36 (34-40)	46 (44-53)	44 (43-49)

Cause	25 to 29	30 to 34	35 to 39	40 to 44	45 to 49	50 to 54	54 to 59
Other urinary diseases	419 (263-596)	498 (327-679)	427 (264-590)	443 (285-596)	585 (369-753)	569 (348-743)	735 (443-942)
Gynecological diseases	487 (390-638)	562 (454-678)	605 (509-669)	659 (553-728)	773 (667-872)	511 (448-585)	336 (292-379)
Uterine fibroids	107 (69-168)	128 (83-199)	153 (94-215)	200 (131-261)	255 (200-321)	181 (143-244)	73 (59-101)
Polycystic ovarian syndrome	2 (0-5)	1 (0-3)	1 (0-2)	1 (0-2)	1 (0-2)	1 (0-2)	-
Endometriosis	11 (5-18)	16 (8-27)	15 (7-26)	22 (9-37)	32 (12-55)	18 (8-31)	-
Genital prolapse	13 (5-20)	14 (5-22)	12 (4-20)	23 (7-40)	30 (7-52)	58 (17-94)	45 (15-81)
Other gynecological diseases	353 (264-522)	402 (311-519)	424 (334-548)	412 (317-514)	455 (349-550)	254 (188-324)	218 (181-269)
Hemoglobinopathies and hemolytic anemias	2,133 (1,616-2,738)	2,147 (1,592-2,671)	1,499 (1,150-1,890)	1,498 (1,162-1,880)	1,362 (1,045-1,703)	1,370 (1,063-1,706)	1,425 (1,130-1,831)
Thalassemias	256 (175-325)	176 (117-228)	75 (55-95)	44 (35-54)	37 (29-46)	21 (17-25)	10 (9-13)
Sickle cell disorders	1,207 (872-1,724)	1,002 (737-1,437)	534 (382-820)	456 (309-735)	336 (223-590)	238 (158-429)	172 (106-324)
G6PD deficiency	368 (266-480)	517 (363-656)	421 (308-529)	425 (313-536)	355 (265-455)	313 (229-412)	280 (194-411)
Other hemoglobinopathies and hemolytic anemias	302 (233-378)	453 (335-545)	468 (359-559)	573 (457-692)	633 (507-770)	798 (631-954)	962 (776-1,190)
Endocrine, metabolic, blood, and immune disorders	1,410 (1,031-1,743)	1,626 (1,200-2,044)	1,683 (1,299-2,063)	1,690 (1,325-2,115)	2,200 (1,683-2,606)	2,495 (1,945-2,945)	2,320 (1,830-2,822)
Sudden infant death syndrome	-	-	-	-	-	-	-
Injuries	98,549 (93,683-101,827)	92,194 (87,450-95,417)	75,583 (71,655-78,002)	65,608 (62,331-67,656)	65,953 (62,745-68,038)	56,767 (54,178-58,460)	49,400 (47,345-50,937)
Transport injuries	26,997 (24,626-28,350)	26,236 (23,921-27,579)	22,574 (21,356-23,442)	20,761 (19,962-21,509)	20,268 (19,131-21,108)	17,659 (16,952-18,225)	15,036 (14,479-15,634)
Road injuries	25,488 (23,074-26,768)	24,896 (22,621-26,082)	21,493 (20,286-22,325)	19,678 (18,906-20,411)	19,105 (17,954-19,884)	16,657 (16,013-17,197)	14,371 (13,828-14,950)
Pedestrian road injuries	9,048 (8,092-10,618)	10,355 (9,271-12,210)	8,936 (8,244-9,884)	8,408 (7,838-9,198)	8,898 (8,217-9,933)	7,841 (7,310-8,554)	7,027 (6,577-7,550)
Cyclist road injuries	882 (609-1,124)	1,200 (823-1,531)	934 (746-1,105)	873 (721-1,002)	1,121 (889-1,336)	848 (718-961)	659 (587-726)
Motorcyclist road injuries	3,899 (2,941-5,181)	3,867 (3,028-4,968)	2,792 (2,315-3,349)	2,513 (2,055-3,001)	2,189 (1,819-2,695)	1,893 (1,590-2,305)	1,450 (1,197-1,873)
Motor vehicle road injuries	11,472 (9,878-12,683)	9,260 (7,873-10,357)	8,622 (7,673-9,343)	7,713 (6,997-8,365)	6,688 (5,971-7,289)	5,909 (5,305-6,375)	5,085 (4,565-5,476)
Other road injuries	187 (129-233)	213 (144-270)	209 (153-265)	170 (131-212)	210 (159-259)	165 (122-211)	150 (112-198)
Other transport injuries	1,508 (1,313-1,819)	1,340 (1,170-1,609)	1,081 (975-1,207)	1,084 (979-1,199)	1,163 (1,045-1,311)	1,002 (903-1,114)	665 (611-719)
Unintentional injuries	21,600 (18,570-23,676)	21,751 (18,864-23,705)	19,179 (16,850-20,591)	18,062 (15,871-19,231)	19,769 (17,730-20,908)	18,220 (16,281-19,286)	18,937 (17,258-20,058)
Falls	2,137 (1,869-2,464)	2,560 (2,249-2,938)	2,764 (2,509-3,073)	2,708 (2,521-2,919)	3,780 (3,480-4,129)	4,169 (3,863-4,537)	5,856 (5,347-6,463)
Drowning	4,355 (3,796-4,661)	4,357 (3,812-4,665)	3,383 (3,044-3,558)	2,852 (2,637-2,983)	3,075 (2,839-3,249)	2,676 (2,511-2,816)	2,321 (2,168-2,453)
Fire, heat, and hot substances	4,716 (2,770-5,735)	4,366 (2,759-5,206)	3,194 (2,242-3,699)	2,435 (1,876-2,709)	2,593 (1,979-2,910)	2,138 (1,699-2,352)	1,953 (1,561-2,138)
Poisonings	1,620 (1,349-1,954)	1,483 (1,231-1,807)	1,353 (1,129-1,503)	1,303 (1,145-1,458)	1,284 (1,177-1,547)	1,129 (1,021-1,311)	931 (864-1,087)
Poisoning by carbon monoxide	811 (690-975)	747 (624-896)	689 (600-808)	728 (648-805)	780 (716-918)	660 (606-759)	548 (503-629)
Poisoning by other means	809 (620-1,026)	737 (555-977)	664 (508-789)	575 (463-671)	504 (435-651)	469 (399-593)	383 (325-481)
Exposure to mechanical forces	1,562 (1,138-1,787)	1,635 (1,192-1,869)	1,520 (1,099-1,730)	1,597 (1,213-1,801)	1,477 (1,164-1,635)	1,106 (877-1,233)	1,338 (995-1,518)
Unintentional firearm injuries	310 (219-365)	347 (253-407)	268 (205-309)	272 (208-313)	272 (217-313)	207 (169-244)	193 (159-239)
Other exposure to mechanical forces	1,252 (885-1,442)	1,289 (891-1,488)	1,252 (887-1,437)	1,325 (978-1,506)	1,205 (919-1,344)	899 (695-1,015)	1,145 (818-1,323)
Adverse effects of medical treatment	2,159 (1,394-2,580)	1,823 (1,195-2,186)	1,837 (1,260-2,130)	1,949 (1,419-2,202)	2,183 (1,635-2,473)	1,669 (1,268-1,875)	2,009 (1,585-2,260)
Animal contact	1,576 (511-2,309)	1,884 (611-2,716)	1,512 (496-2,083)	1,910 (705-2,493)	1,727 (648-2,256)	1,863 (716-2,434)	1,333 (527-1,703)
Venomous animal contact	1,403 (437-2,035)	1,746 (558-2,513)	1,396 (437-1,924)	1,796 (628-2,386)	1,544 (541-2,038)	1,688 (607-2,231)	1,166 (429-1,538)
Non-venomous animal contact	173 (58-548)	138 (48-413)	116 (47-317)	114 (50-280)	183 (81-403)	175 (79-401)	167 (76-363)
Foreign body	826 (771-901)	818 (756-885)	895 (859-951)	988 (957-1,035)	1,107 (1,074-1,150)	1,189 (1,151-1,236)	1,124 (1,089-1,159)
Pulmonary aspiration and foreign body in airway	593 (532-660)	637 (584-697)	699 (651-754)	808 (761-852)	966 (923-1,010)	1,059 (1,014-1,105)	1,004 (960-1,046)
Foreign body in other body part	233 (185-294)	180 (139-232)	196 (158-248)	180 (152-229)	141 (111-179)	130 (110-170)	120 (98-163)
Environmental heat and cold exposure	394 (296-437)	584 (430-653)	781 (635-845)	1,061 (897-1,142)	1,309 (1,116-1,399)	1,241 (1,099-1,316)	1,102 (884-1,200)
Exposure to forces of nature	206 (188-231)	187 (171-208)	173 (158-191)	155 (143-171)	136 (123-152)	120 (110-131)	118 (109-128)
Other unintentional injuries	2,049 (1,824-2,323)	2,053 (1,851-2,289)	1,766 (1,623-1,930)	1,103 (1,034-1,178)	1,099 (1,024-1,177)	921 (851-998)	852 (782-935)
Self-harm and interpersonal violence	49,953 (46,877-52,718)	44,206 (41,513-46,714)	33,830 (31,768-35,296)	26,785 (25,471-27,776)	25,916 (24,614-26,983)	20,888 (19,993-21,587)	15,426 (14,867-15,919)
Self-harm	37,826 (36,010-40,478)	32,733 (31,174-35,136)	24,098 (23,176-25,357)	18,803 (18,219-19,532)	19,172 (18,421-20,111)	15,776 (15,282-16,342)	11,665 (11,312-12,091)
Self-harm by firearm	1,070 (681-1,507)	942 (691-1,225)	879 (731-1,068)	827 (737-1,002)	697 (631-852)	569 (514-704)	453 (405-586)
Self-harm by other specified means	36,756 (34,908-39,691)	31,791 (30,170-34,434)	23,220 (22,263-24,525)	17,976 (17,412-18,671)	18,475 (17,725-19,439)	15,207 (14,699-15,760)	11,213 (10,875-11,641)
Interpersonal violence	9,895 (7,704-11,026)	9,532 (7,181-10,569)	8,170 (6,525-8,886)	6,826 (5,661-7,317)	5,756 (4,848-6,154)	4,291 (3,605-4,717)	3,078 (2,559-3,338)
Physical violence by firearm	2,501 (1,821-2,940)	2,263 (1,674-2,603)	2,067 (1,588-2,331)	1,601 (1,267-1,787)	1,200 (974-1,330)	902 (701-1,052)	663 (496-766)
Physical violence by sharp object	2,714 (2,050-3,199)	2,877 (2,175-3,439)	2,463 (1,982-2,810)	2,226 (1,801-2,527)	1,835 (1,516-2,060)	1,281 (1,070-1,465)	856 (698-954)
Physical violence by other means	4,680 (3,367-5,539)	4,393 (3,120-5,161)	3,640 (2,784-4,081)	2,999 (2,445-3,323)	2,721 (2,199-2,996)	2,108 (1,732-2,401)	1,559 (1,279-1,739)
Conflict and terrorism	2,189 (2,050-2,378)	1,897 (1,771-2,083)	1,522 (1,414-1,673)	1,119 (1,037-1,232)	953 (885-1,041)	794 (739-870)	661 (617-722)
Executions and police conflict	43 (41-45)	44 (38-52)	40 (36-47)	37 (30-47)	35 (27-53)	27 (22-38)	22 (19-30)

Cause	60 to 64	65 to 69	70 to 74	75 to 79	80 to 84	85 to 89	90 to 94	95 plus
Other urinary diseases	1,046 (662-1,329)	1,046 (618-1,342)	1,094 (659-1,368)	1,091 (652-1,387)	736 (455-932)	597 (372-745)	372 (229-470)	177 (123-213)
Gynecological diseases	284 (252-320)	367 (320-416)	238 (214-263)	182 (167-199)	310 (260-356)	227 (194-261)	102 (89-117)	38 (34-44)
Uterine fibroids	68 (55-89)	75 (58-107)	54 (43-77)	38 (31-51)	78 (57-115)	64 (45-93)	36 (26-49)	15 (10-19)
Polycystic ovarian syndrome	-	-	-	-	-	-	-	-
Endometriosis	-	-	-	-	-	-	-	-
Genital prolapse	51 (24-86)	91 (39-145)	75 (39-104)	55 (29-72)	78 (41-121)	51 (31-76)	21 (13-28)	8 (5-10)
Other gynecological diseases	166 (134-208)	201 (154-264)	110 (83-145)	89 (73-121)	154 (113-199)	111 (89-140)	45 (37-58)	16 (14-21)
Hemoglobinopathies and hemolytic anemias	1,699 (1,351-2,148)	2,186 (1,698-2,716)	2,672 (2,097-3,320)	2,675 (2,134-3,315)	2,636 (2,033-3,207)	2,567 (1,829-3,010)	1,609 (1,074-1,895)	644 (429-753)
Thalassemias	5 (4-7)	3 (2-4)	1 (1-2)	1 (0-1)	0 (0-0)	0 (0-0)	0 (0-0)	0 (0-0)
Sickle cell disorders	68 (44-116)	32 (21-50)	12 (8-18)	4 (2-6)	1 (1-2)	0 (0-1)	0 (0-0)	0 (0-0)
G6PD deficiency	242 (159-369)	218 (142-338)	148 (97-232)	85 (55-131)	42 (27-67)	18 (11-26)	6 (3-8)	1 (1-2)
Other hemoglobinopathies and hemolytic anemias	1,384 (1,090-1,699)	1,933 (1,508-2,384)	2,511 (1,983-3,080)	2,585 (2,059-3,188)	2,593 (2,003-3,147)	2,549 (1,815-2,988)	1,604 (1,069-1,889)	643 (429-751)
Endocrine, metabolic, blood, and immune disorders	3,273 (2,573-3,926)	3,793 (2,984-4,613)	4,083 (3,214-5,025)	4,239 (3,148-5,159)	3,789 (2,836-4,781)	3,551 (2,586-4,382)	2,061 (1,488-2,554)	858 (593-1,068)
Sudden infant death syndrome	-	-	-	-	-	-	-	-
Injuries	58,560 (55,425-60,393)	62,823 (59,185-65,067)	69,167 (65,462-71,996)	68,732 (65,016-71,316)	59,907 (56,609-62,224)	51,192 (49,030-52,809)	27,148 (26,176-27,844)	9,479 (9,203-9,713)
Transport injuries	16,578 (15,967-17,319)	15,725 (15,138-16,345)	15,910 (15,297-16,506)	12,657 (12,219-13,167)	8,349 (8,060-8,740)	5,086 (4,911-5,320)	1,977 (1,915-2,048)	591 (569-616)
Road injuries	15,621 (15,047-16,358)	14,871 (14,330-15,484)	15,199 (14,601-15,778)	12,031 (11,609-12,532)	7,934 (7,663-8,306)	4,840 (4,676-5,062)	1,885 (1,826-1,957)	561 (540-585)
Pedestrian road injuries	8,846 (8,387-9,411)	8,531 (8,136-9,109)	9,301 (8,816-9,973)	7,513 (7,166-7,952)	5,042 (4,820-5,419)	2,920 (2,794-3,111)	1,016 (973-1,079)	274 (260-291)
Cyclist road injuries	664 (591-732)	726 (649-805)	686 (610-765)	427 (383-471)	177 (159-196)	93 (80-107)	35 (29-42)	0 (0-0)
Motorcyclist road injuries	1,153 (959-1,482)	992 (821-1,234)	745 (600-909)	391 (318-482)	205 (162-261)	104 (80-138)	36 (29-47)	12 (10-14)
Motor vehicle road injuries	4,908 (4,471-5,290)	4,572 (4,101-4,891)	4,424 (3,992-4,762)	3,597 (3,300-3,840)	2,455 (2,272-2,609)	1,684 (1,590-1,789)	781 (741-825)	270 (256-285)
Other road injuries	51 (40-62)	50 (40-63)	43 (34-54)	103 (80-133)	56 (45-74)	39 (32-52)	16 (14-21)	6 (5-8)
Other transport injuries	957 (883-1,040)	854 (780-932)	712 (658-764)	626 (581-669)	415 (384-451)	246 (227-266)	92 (85-99)	30 (28-32)
Unintentional injuries	26,285 (23,702-27,807)	31,806 (28,650-33,765)	37,474 (34,321-39,768)	43,232 (40,003-45,480)	42,914 (39,635-44,900)	40,870 (38,782-42,317)	23,414 (22,447-24,128)	8,468 (8,206-8,696)
Falls	10,082 (9,257-10,847)	14,343 (13,096-15,914)	19,357 (17,735-21,166)	25,462 (23,458-27,420)	27,283 (25,130-29,025)	27,702 (26,042-29,003)	16,495 (15,694-17,111)	5,833 (5,612-6,046)
Drowning	2,823 (2,601-3,008)	3,218 (2,942-3,420)	3,337 (3,141-3,534)	3,359 (3,115-3,580)	2,719 (2,468-2,942)	1,577 (1,471-1,682)	524 (495-554)	109 (104-114)
Fire, heat, and hot substances	2,343 (1,959-2,535)	2,873 (2,342-3,115)	3,128 (2,648-3,369)	3,386 (2,903-3,627)	3,239 (2,830-3,492)	2,824 (2,566-3,002)	1,495 (1,394-1,574)	603 (575-626)
Poisonings	1,062 (991-1,191)	1,096 (1,004-1,277)	1,196 (1,109-1,402)	960 (896-1,139)	752 (695-896)	509 (475-571)	182 (170-199)	46 (43-49)
Poisoning by carbon monoxide	693 (644-786)	679 (620-787)	746 (691-882)	639 (594-767)	461 (422-574)	326 (303-381)	107 (99-122)	21 (20-24)
Poisoning by other means	369 (311-445)	417 (346-499)	450 (396-556)	321 (281-400)	291 (249-350)	183 (158-207)	75 (66-83)	25 (23-26)
Exposure to mechanical forces	1,425 (1,065-1,607)	1,381 (1,079-1,529)	1,338 (1,052-1,481)	1,250 (1,006-1,377)	1,210 (1,017-1,331)	1,037 (949-1,169)	581 (545-645)	222 (210-243)
Unintentional firearm injuries	199 (160-255)	216 (170-276)	223 (186-277)	305 (244-378)	285 (235-363)	265 (232-334)	147 (131-184)	52 (47-65)
Other exposure to mechanical forces	1,227 (886-1,406)	1,165 (890-1,306)	1,115 (855-1,249)	945 (727-1,065)	925 (754-1,029)	772 (688-853)	435 (399-477)	169 (159-183)
Adverse effects of medical treatment	3,185 (2,463-3,661)	3,522 (2,759-4,073)	3,721 (3,025-4,230)	3,721 (3,153-4,096)	3,299 (2,811-3,679)	3,107 (2,757-3,478)	1,761 (1,607-1,930)	645 (601-696)
Animal contact	1,691 (622-2,210)	1,868 (642-2,469)	1,643 (598-2,138)	1,219 (426-1,618)	700 (298-897)	362 (183-446)	133 (75-159)	30 (21-36)
Venomous animal contact	1,576 (547-2,093)	1,740 (557-2,322)	1,505 (526-1,991)	1,109 (357-1,505)	605 (238-799)	286 (128-363)	99 (49-123)	21 (13-25)
Non-venomous animal contact	115 (50-252)	127 (59-296)	138 (67-327)	111 (58-232)	95 (50-179)	75 (43-124)	34 (22-52)	9 (7-13)
Foreign body	1,283 (1,241-1,331)	1,368 (1,318-1,416)	1,752 (1,691-1,815)	2,046 (1,965-2,116)	2,097 (2,021-2,168)	2,412 (2,326-2,507)	1,507 (1,446-1,575)	718 (691-747)
Pulmonary aspiration and foreign body in airway	1,163 (1,114-1,210)	1,245 (1,189-1,297)	1,591 (1,522-1,653)	1,880 (1,801-1,952)	1,923 (1,846-2,006)	2,256 (2,172-2,354)	1,422 (1,361-1,492)	688 (660-717)
Foreign body in other body part	120 (102-161)	123 (93-161)	161 (122-206)	166 (124-208)	174 (127-213)	156 (113-189)	85 (59-97)	30 (23-36)
Environmental heat and cold exposure	1,551 (1,207-1,720)	1,166 (870-1,296)	1,389 (979-1,572)	1,235 (843-1,389)	1,111 (756-1,242)	966 (745-1,057)	548 (459-591)	192 (172-206)
Exposure to forces of nature	122 (112-134)	107 (99-117)	101 (93-111)	76 (70-84)	53 (49-58)	31 (29-34)	13 (11-15)	2 (2-3)
Other unintentional injuries	718 (668-789)	864 (794-974)	511 (478-554)	519 (486-576)	450 (419-505)	343 (322-375)	175 (165-188)	67 (63-72)
Self-harm and interpersonal violence	15,698 (15,123-16,209)	15,292 (14,744-15,868)	15,783 (15,229-16,374)	12,843 (12,380-13,326)	8,645 (8,317-9,105)	5,236 (5,074-5,477)	1,757 (1,716-1,806)	420 (411-429)
Self-harm	11,797 (11,426-12,206)	12,056 (11,631-12,576)	12,677 (12,191-13,195)	10,410 (10,008-10,856)	7,775 (7,447-8,236)	4,723 (4,562-4,965)	1,622 (1,581-1,674)	361 (352-370)
Self-harm by firearm	362 (317-488)	338 (286-492)	315 (268-468)	304 (255-453)	234 (199-336)	158 (142-187)	77 (71-83)	30 (27-32)
Self-harm by other specified means	11,435 (11,071-11,841)	11,719 (11,291-12,260)	12,362 (11,872-12,899)	10,106 (9,724-10,569)	7,541 (7,214-8,010)	4,565 (4,404-4,803)	1,545 (1,504-1,598)	331 (323-340)
Interpersonal violence	3,234 (2,773-3,497)	2,625 (2,226-2,925)	2,650 (2,290-2,944)	2,122 (1,841-2,324)	661 (610-706)	405 (383-424)	117 (110-125)	31 (30-33)
Physical violence by firearm	606 (462-714)	450 (346-553)	428 (332-525)	327 (248-391)	65 (56-74)	37 (33-41)	10 (9-11)	4 (3-4)
Physical violence by sharp object	918 (776-1,031)	656 (567-742)	748 (635-836)	616 (521-702)	170 (155-192)	94 (86-106)	26 (24-29)	6 (6-7)
Physical violence by other means	1,709 (1,447-1,890)	1,519 (1,252-1,698)	1,474 (1,243-1,647)	1,178 (1,011-1,297)	426 (385-459)	274 (258-287)	81 (76-86)	21 (20-22)
Conflict and terrorism	648 (605-706)	593 (552-651)	441 (411-480)	300 (280-326)	200 (188-217)	104 (96-113)	16 (15-18)	28 (25-30)
Executions and police conflict	20 (17-26)	18 (14-23)	15 (13-20)	11 (9-15)	8 (7-10)	4 (4-6)	2 (2-3)	1 (0-1)

SR Table 5: Global deaths by cause and age group for females (with 95% confidence intervals), 2005

Cause	Early Neonatal	Late Neonatal	Post Neonatal	1 to 4	5 to 9	10 to 14	15 to 19
All causes	1,069,988 (1,043,621-1,098,02	366,892 (351,840-381,934	1,245,986 (1,213,933-1,278,06	1,209,826 (1,168,741-1,251,72	274,697 (271,395-278,158	177,682 (175,647-179,793	276,876 (272,852-281,344
Communicable, maternal, neonatal, and nutritional disease	970,262 (944,444-997,204	320,751 (306,987-334,639	1,021,036 (982,230-1,057,83	972,838 (929,863-1,018,19	171,926 (163,005-182,633	88,508 (83,327-95,662	115,118 (109,658-122,803
HIV/AIDS and sexually transmitted infections	-	7,606 (2,932-14,332	89,774 (80,272-101,926	49,647 (46,119-52,978	13,547 (12,812-14,374	7,567 (7,074-8,125	13,552 (11,757-15,463
HIV/AIDS	-	-	76,559 (72,638-80,312	48,322 (45,045-51,392	12,899 (12,338-13,502	7,507 (7,012-8,064	13,288 (11,527-15,195
HIV/AIDS - Drug-susceptible Tuberculosis	-	-	24,316 (17,962-28,984	16,176 (12,491-18,876	4,212 (3,135-4,951	2,368 (1,732-2,849	4,111 (3,072-5,078
HIV/AIDS - Multidrug-resistant Tuberculosis without extensive drug resistance	-	-	2,513 (1,515-3,663)	1,644 (1,009-2,425)	405 (248-582)	220 (135-317)	401 (246-590)
HIV/AIDS - Extensively drug-resistant Tuberculosis	-	-	34 (21-48)	21 (13-30)	6 (4-8)	3 (2-5)	7 (5-10)
HIV/AIDS resulting in other diseases	-	-	49,696 (43,566-57,727	30,480 (26,702-35,474	8,276 (7,366-9,508	4,915 (4,266-5,712	8,768 (7,185-10,709
Sexually transmitted infections excluding HIV	26,300 (10,327-49,295	7,606 (2,932-14,332	13,215 (5,098-25,158	1,326 (502-2,574	648 (238-1,248)	60 (42-74)	265 (206-301)
Syphilis	26,300 (10,327-49,295	7,606 (2,932-14,332	13,215 (5,098-25,158	1,326 (502-2,574	648 (238-1,248)	22 (16-27)	26 (20-29)
Chlamydial infection	-	-	-	-	-	10 (7-13)	41 (32-47)
Gonococcal infection	-	-	-	-	-	18 (13-22)	103 (80-117)
Other sexually transmitted infections	-	-	-	-	-	10 (7-12)	95 (74-109)
Respiratory infections and tuberculosis	-	76,921 (69,995-84,171	349,374 (320,150-376,133	217,559 (185,849-246,413	29,756 (24,838-33,877	16,422 (14,544-18,345	23,631 (21,912-25,593
Tuberculosis	-	-	25,800 (23,231-28,358	27,346 (22,914-31,891	7,477 (6,331-8,679	6,826 (5,974-7,763	15,563 (14,127-17,045
Drug-susceptible tuberculosis	-	-	23,335 (20,886-25,817	24,880 (20,774-28,959	6,715 (5,606-7,875	6,108 (5,298-6,982	13,913 (12,572-15,368
Multidrug-resistant tuberculosis without extensive drug resistance	-	-	2,367 (1,802-3,031	2,398 (1,715-3,235	733 (525-1,027)	690 (507-964)	1,579 (1,208-2,136
Extensively drug-resistant tuberculosis	-	-	99 (77-126)	68 (51-90)	28 (21-40)	28 (21-39)	71 (55-93)
Lower respiratory infections	81,272 (73,159-90,559	76,826 (69,904-84,110	322,042 (295,537-346,279	188,588 (160,960-213,847	21,973 (17,736-25,165	9,450 (8,028-10,624	7,983 (7,193-8,812
Upper respiratory infections	34 (15-57)	76 (34-128)	1,405 (460-2,528)	1,477 (344-2,895	230 (66-463)	83 (28-171)	59 (25-106)
Otitis media	23 (2-183)	19 (7-53)	127 (55-276)	148 (56-326)	77 (36-154)	63 (32-115)	26 (16-41)
Enteric infections	12,901 (11,137-14,974	26,315 (22,891-30,035	226,546 (202,512-250,567	230,911 (191,843-273,456	51,490 (37,487-70,377	27,848 (20,301-39,295	21,597 (14,535-32,045
Diarrheal diseases	12,901 (11,137-14,974	25,971 (22,552-29,734	220,547 (197,231-244,261	203,266 (167,165-244,756	23,448 (11,256-43,049	11,089 (5,440-20,300	11,325 (5,306-20,349
Typhoid and paratyphoid	-	0 (0-0)	156 (77-301)	13,771 (6,373-25,965	24,283 (13,853-39,324	14,961 (8,607-24,058	8,975 (4,413-15,983
Typhoid fever	-	-	142 (69-271)	12,197 (5,590-23,048	21,366 (11,937-35,534	13,108 (7,443-20,969	7,830 (3,858-14,130
Paratyphoid fever	-	-	14 (6-30)	1,574 (580-3,390	2,917 (1,254-5,718	1,853 (803-3,669	1,144 (454-2,462)
Invasive Non-typhoidal Salmonella (iNTS)	-	344 (190-599)	5,100 (2,809-8,847	13,445 (7,039-23,107	3,696 (1,909-6,245	1,766 (804-3,249	1,267 (520-2,379)
Other intestinal infectious diseases	-	-	743 (327-1,994)	428 (171-1,104	63 (17-182)	33 (7-92)	31 (6-81)
Neglected tropical diseases and malaria	216 (104-691)	12,194 (7,803-17,811	98,447 (69,534-136,343	198,820 (141,575-268,755	30,811 (21,811-41,671	15,327 (10,959-20,297	10,536 (7,803-14,321
Malaria	-	12,030 (7,686-17,716	95,069 (65,310-132,962	188,261 (132,374-256,622	25,247 (17,117-34,503	12,494 (8,476-17,055	8,161 (5,688-11,470
Chagas disease	-	-	1 (1-1)	1 (1-1)	1 (1-1)	2 (2-2)	4 (3-4)
Leishmaniasis	-	-	613 (0-2,524)	2,574 (1-13,126)	1,368 (0-9,897)	644 (0-4,494)	517 (0-3,132)
Visceral leishmaniasis	-	-	613 (0-2,524)	2,574 (1-13,126)	1,368 (0-9,897)	644 (0-4,494)	517 (0-3,132)
African trypanosomiasis	-	-	-	257 (52-806)	456 (121-1,289)	518 (145-1,430)	584 (166-1,464)

SR Table 5: Global deaths by cause and age group for females (with 95% confidence intervals), 2005

Cause	Early Neonatal	Late Neonatal	Post Neonatal	1 to 4	5 to 9	10 to 14	15 to 19	20 to 24	25 to 29	30 to 34	35 to 39
Mesothelioma	-	-	-	-	-	-	46 (31-63)	69 (45-98)	96 (68-131)	121 (87-165)	206 (151-278)
Hodgkin lymphoma	1 (1-2)	1 (1-1)	20 (13-25)	157 (78-292)	245 (154-337)	433 (285-571)	886 (614-1,146)	1,066 (783-1,346)	1,111 (812-1,380)	885 (650-1,088)	711 (525-888)
Non-Hodgkin lymphoma	26 (23-30)	23 (19-27)	346 (273-419)	1,640 (1,194-2,120)	1,029 (857-1,215)	870 (774-979)	1,065 (967-1,175)	1,412 (1,290-1,558)	1,534 (1,406-1,679)	1,611 (1,491-1,759)	2,053 (1,889-2,247)
Multiple myeloma	-	-	-	-	-	-	103 (89-125)	113 (97-134)	126 (114-145)	165 (150-188)	340 (309-397)
Leukemia	141 (102-174)	123 (92-155)	1,922 (1,401-2,538)	6,519 (4,693-9,654)	6,163 (4,804-7,967)	5,485 (4,560-6,438)	5,629 (4,619-6,384)	4,487 (3,671-5,061)	4,101 (3,412-4,602)	4,246 (3,426-4,794)	5,017 (3,923-5,749)
Acute lymphoid leukemia	32 (23-46)	24 (17-39)	419 (311-664)	1,790 (1,335-3,166)	2,319 (1,789-3,451)	1,897 (1,563-2,466)	1,482 (1,220-1,851)	978 (823-1,221)	910 (746-1,165)	638 (533-800)	740 (574-938)
Chronic lymphoid leukemia	-	-	-	-	-	-	71 (55-84)	64 (48-76)	64 (53-76)	79 (62-96)	112 (86-136)
Acute myeloid leukemia	20 (14-32)	19 (13-32)	283 (195-511)	1,247 (781-2,645)	826 (591-1,444)	1,000 (780-1,376)	1,172 (964-1,506)	1,094 (921-1,364)	1,059 (896-1,320)	1,085 (910-1,360)	1,231 (1,025-1,565)
Chronic myeloid leukemia	-	-	-	-	-	-	242 (190-305)	317 (241-415)	391 (298-507)	454 (352-574)	505 (394-626)
Other leukemia	89 (62-114)	79 (58-104)	1,220 (821-1,761)	3,482 (2,246-5,819)	3,018 (2,237-4,167)	2,588 (1,961-3,078)	2,662 (1,924-3,169)	2,034 (1,456-2,351)	1,677 (1,277-1,942)	1,989 (1,467-2,354)	2,428 (1,715-2,968)
Other malignant neoplasms	451 (357-525)	240 (197-299)	1,417 (1,079-1,899)	4,816 (3,394-7,086)	2,455 (2,077-2,979)	3,048 (2,694-3,447)	3,575 (3,239-4,025)	2,951 (2,678-3,290)	2,807 (2,580-3,126)	2,851 (2,634-3,204)	3,536 (3,247-4,008)
Other neoplasms	26 (19-34)	15 (10-19)	204 (140-255)	647 (399-887)	513 (369-657)	392 (290-503)	374 (270-476)	396 (289-493)	451 (330-570)	610 (451-773)	817 (613-1,032)
Myelodysplastic, myeloproliferative, and other hematopoietic neoplasm	25 (19-32)	11 (9-15)	173 (123-225)	500 (321-739)	327 (250-441)	246 (182-322)	238 (179-314)	266 (200-345)	323 (246-422)	452 (341-588)	664 (506-856)
Other benign and in situ neoplasms	1 (1-2)	3 (2-5)	31 (17-46)	148 (74-262)	186 (118-238)	146 (103-187)	135 (93-172)	131 (86-171)	129 (84-169)	158 (104-209)	154 (102-202)
Cardiovascular diseases	1,506 (1,381-1,638)	1,088 (998-1,183)	9,941 (9,236-10,691)	8,573 (7,611-9,495)	4,543 (4,109-4,953)	5,768 (5,290-6,202)	16,315 (15,493-17,140)	22,412 (21,462-23,429)	29,354 (28,267-30,351)	43,403 (41,958-44,845)	67,623 (65,446-69,810)
Rheumatic heart disease	-	-	-	2,166 (1,703-2,690)	1,511 (1,281-1,743)	2,166 (1,912-2,399)	3,404 (3,062-3,820)	3,922 (3,420-4,433)	4,356 (3,751-4,953)	5,083 (4,423-5,728)	6,418 (5,596-7,198)
Ischemic heart disease	-	-	-	-	-	-	5,269 (4,886-5,668)	7,630 (7,128-8,186)	10,281 (9,661-10,981)	17,028 (16,128-18,049)	26,751 (25,396-28,268)
Stroke	480 (413-560)	343 (297-397)	2,700 (2,284-3,503)	2,001 (1,638-2,556)	1,390 (1,238-1,557)	1,794 (1,619-2,001)	4,074 (3,814-4,370)	5,923 (5,562-6,325)	7,919 (7,538-8,425)	12,773 (12,184-13,443)	22,913 (21,893-24,134)
Ischemic stroke	110 (79-139)	72 (54-88)	399 (280-533)	268 (198-342)	176 (145-211)	191 (158-232)	751 (635-880)	1,104 (927-1,293)	1,446 (1,251-1,662)	2,232 (1,943-2,546)	3,592 (3,194-3,984)
Intracerebral hemorrhage	216 (180-262)	148 (122-175)	1,322 (1,054-1,791)	889 (634-1,225)	718 (613-840)	968 (843-1,112)	2,342 (2,109-2,604)	3,431 (3,115-3,750)	4,575 (4,206-5,018)	7,445 (6,862-8,085)	13,898 (12,934-15,037)
Subarachnoid hemorrhage	154 (136-179)	123 (106-147)	978 (839-1,181)	844 (712-1,012)	496 (432-565)	635 (549-731)	981 (873-1,128)	1,388 (1,225-1,651)	1,898 (1,688-2,178)	3,096 (2,768-3,529)	5,423 (4,929-6,036)
Hypertensive heart disease	-	-	-	-	-	-	418 (256-547)	591 (371-790)	969 (611-1,263)	1,635 (1,081-2,070)	2,771 (1,886-3,459)
Non-rheumatic valvular heart disease	-	-	-	-	-	-	362 (275-471)	395 (297-517)	446 (347-568)	397 (319-489)	512 (423-622)
Non-rheumatic calcific aortic valve disease	-	-	-	-	-	-	102 (67-180)	102 (66-183)	122 (82-206)	130 (94-209)	188 (140-297)
Non-rheumatic degenerative mitral valve disease	-	-	-	-	-	-	200 (134-306)	227 (153-348)	247 (175-364)	212 (152-302)	265 (197-363)
Other non-rheumatic valve diseases	-	-	-	-	-	-	60 (41-92)	65 (45-101)	77 (53-116)	55 (36-82)	58 (35-89)
Cardiomyopathy and myocarditis	620 (497-711)	406 (332-465)	3,879 (3,122-4,487)	2,120 (1,461-2,602)	826 (657-1,010)	877 (749-1,032)	1,267 (1,044-1,546)	1,680 (1,400-2,014)	2,425 (2,138-2,812)	3,065 (2,821-3,437)	4,160 (3,867-4,600)
Myocarditis	70 (53-93)	72 (53-90)	1,080 (813-1,289)	696 (497-863)	305 (237-381)	264 (200-325)	466 (359-585)	561 (433-701)	579 (435-713)	530 (415-644)	639 (487-773)
Alcoholic cardiomyopathy	-	-	-	-	-	-	125 (78-169)	295 (237-349)	765 (685-837)	1,322 (1,244-1,401)	1,962 (1,866-2,058)
Other cardiomyopathy	549 (429-634)	334 (268-386)	2,799 (2,154-3,295)	1,424 (961-1,777)	521 (398-645)	614 (504-726)	676 (527-833)	824 (641-1,017)	1,080 (840-1,331)	1,213 (985-1,465)	1,560 (1,283-1,850)
Atrial fibrillation and flutter	-	-	-	-	-	-	-	-	-	35 (33-38)	42 (39-46)
Aortic aneurysm	-	-	-	-	-	-	90 (73-122)	149 (119-201)	202 (169-262)	288 (238-373)	423 (360-530)
Peripheral artery disease	-	-	-	-	-	-	-	-	-	-	-
Endocarditis	101 (78-137)	100 (76-131)	1,306 (1,013-1,701)	1,037 (697-1,416)	254 (209-323)	317 (268-390)	422 (363-544)	640 (551-832)	833 (719-1,026)	744 (647-911)	856 (742-1,049)
Other cardiovascular and circulatory diseases	306 (246-438)	239 (195-327)	2,057 (1,555-3,100)	1,249 (815-2,353)	561 (434-806)	613 (496-805)	1,009 (785-1,362)	1,482 (1,171-1,956)	1,924 (1,543-2,540)	2,354 (1,935-2,982)	2,777 (2,334-3,395)
Chronic respiratory diseases	0 (0-0)	0 (0-0)	0 (0-0)	9,243 (6,774-12,802)	2,527 (2,122-3,562)	2,241 (1,946-3,019)	3,987 (3,638-4,990)	5,086 (4,701-6,048)	6,363 (5,896-7,200)	8,175 (7,412-9,054)	11,821 (10,663-13,280)
Chronic obstructive pulmonary disease	-	-	0 (0-0)	1,116 (699-1,818)	298 (204-474)	222 (160-339)	1,092 (825-1,496)	1,506 (1,134-2,051)	2,040 (1,568-2,695)	2,961 (2,307-3,824)	4,514 (3,497-5,712)
Pneumoconiosis	-	-	-	-	-	-	15 (11-19)	15 (11-19)	20 (13-25)	30 (19-35)	37 (26-44)
Silicosis	-	-	-	-	-	-	3 (2-4)	3 (1-4)	4 (2-6)	7 (4-10)	8 (5-11)
Asbestosis	-	-	-	-	-	-	2 (1-3)	3 (1-4)	4 (2-5)	5 (2-7)	6 (3-10)
Coal workers pneumoconiosis	-	-	-	-	-	-	2 (1-4)	2 (1-3)	3 (1-4)	4 (1-6)	4 (1-6)
Other pneumoconiosis	-	-	-	-	-	-	8 (5-11)	8 (5-12)	10 (6-14)	14 (9-20)	19 (11-26)
Asthma	-	-	-	6,458 (4,427-9,767)	1,727 (1,321-2,681)	1,642 (1,325-2,412)	2,410 (1,855-3,377)	2,997 (2,312-3,954)	3,570 (2,706-4,654)	4,323 (3,229-5,482)	6,021 (4,411-7,805)
Interstitial lung disease and pulmonary sarcoidosis	-	-	-	189 (98-541)	84 (50-193)	63 (43-114)	112 (77-205)	154 (106-289)	232 (164-395)	327 (224-569)	514 (353-865)
Other chronic respiratory diseases	0 (0-0)	0 (0-0)	0 (0-0)	1,481 (733-2,548)	418 (287-594)	314 (217-422)	358 (258-482)	414 (298-559)	501 (350-656)	534 (361-712)	735 (494-988)
Digestive diseases	1,073 (883-1,188)	1,044 (934-1,221)	8,829 (7,652-11,273)	11,008 (8,669-15,360)	6,085 (5,130-7,248)	5,705 (4,961-6,511)	10,136 (9,025-11,281)	12,698 (10,991-13,874)	14,152 (12,563-15,312)	16,181 (14,747-17,542)	21,262 (19,762-22,865)
Cirrhosis and other chronic liver diseases	-	-	-	4,699 (3,431-8,386)	2,469 (1,931-3,827)	2,486 (2,109-3,417)	4,384 (3,826-5,502)	6,182 (5,403-6,998)	7,499 (6,617-8,393)	9,951 (9,151-11,155)	13,476 (12,502-15,139)
Cirrhosis and other chronic liver diseases due to hepatitis B	-	-	-	88 (57-162)	111 (80-178)	207 (158-293)	533 (397-736)	1,827 (1,419-2,282)	2,906 (2,326-3,608)	3,742 (3,185-4,403)	4,395 (3,690-5,382)
Cirrhosis and other chronic liver diseases due to hepatitis C	-	-	-	34 (21-65)	52 (35-84)	100 (74-140)	232 (166-333)	1,033 (767-1,332)	1,688 (1,263-2,163)	2,572 (2,124-3,082)	3,843 (3,065-4,741)
Cirrhosis and other chronic liver diseases due to alcohol use	-	-	-	-	-	-	20 (9-36)	337 (245-459)	716 (546-927)	1,521 (1,300-1,804)	2,866 (2,365-3,485)
Cirrhosis due to NASH	-	-	-	-	-	-	72 (54-98)	225 (173-287)	373 (279-476)	653 (538-787)	1,070 (835-1,348)
Cirrhosis and other chronic liver diseases due to other causes	-	-	-	4,578 (3,347-8,202)	2,306 (1,808-3,597)	2,178 (1,838-3,011)	3,527 (3,029-4,428)	2,760 (2,171-3,422)	1,817 (1,359-2,340)	1,463 (1,094-1,917)	1,303 (978-1,670)
Upper digestive system diseases	-	-	-	868 (637-1,230)	724 (568-907)	664 (518-797)	1,124 (939-1,410)	2,262 (1,889-2,633)	2,007 (1,674-2,335)	2,121 (1,756-2,421)	2,666 (2,204-3,038)
Peptic ulcer disease	-	-	-	694 (476-1,121)	512 (393-714)	517 (409-651)	956 (786-1,248)	1,861 (1,561-2,354)	1,559 (1,291-1,992)	1,638 (1,352-2,078)	2,150 (1,779-2,664)
Gastritis and duodenitis	-	-	-	174 (85-252)	212 (114-296)	147 (78-200)	168 (94-245)	401 (178-538)	447 (212-590)	483 (228-635)	516 (243-687)
Appendicitis	-	-	-	1,527 (774-2,265)	938 (733-1,240)	908 (725-1,136)	1,882 (1,535-2,412)	1,268 (1,034-1,525)	1,336 (1,130-1,647)	713 (605-855)	820 (689-983)
Paralytic ileus and intestinal obstruction	1,073 (883-1,188)	1,044 (934-1,221)	8,829 (7,652-11,273)	1,562 (996-2,075)	1,231 (576-1,652)	1,072 (585-1,342)	1,922 (1,090-2,376)	1,686 (939-2,085)	1,630 (948-1,979)	1,262 (743-1,514)	1,525 (917-1,837)
Inguinal, femoral, and abdominal hernia	-	-	-	110 (62-180)	94 (58-131)	73 (40-93)	87 (51-117)	80 (46-100)	75 (46-96)	133 (74-174)	186 (100-239)
Inflammatory bowel disease	-	-	-	538 (262-1,082)	93 (55-156)	66 (48-94)	120 (86-184)	184 (134-276)	220 (164-317)	227 (176-305)	290 (233-369)
Vascular intestinal disorders	-	-	-	73 (28-110)	17 (9-21)	15 (11-18)	34 (22-42)	73 (46-89)	92 (60-112)	119 (82-140)	183 (137-215)
Gallbladder and biliary diseases	-	-	-	528 (338-774)	194 (138-256)	143 (106-172)	175 (144-202)	310 (237-366)	402 (327-464)	524 (458-589)	711 (594-809)
Pancreatitis	-	-	-	48 (31-90)	72 (51-115)	96 (66-126)	240 (183-328)	405 (312-522)	555 (415-682)	747 (566-908)	956 (715-1,148)
Other digestive diseases	-	-	-	1,054 (774-1,550)	253 (205-318)	183 (156-214)	168 (132-202)	247 (201-295)	336 (266-412)	384 (331-458)	449 (375-528)
Neurological disorders	-	-	4,890 (3,748-6,020)	6,033 (3,675-7,898)	2,930 (2,369-3,541)	3,373 (2,891-3,900)	5,422 (4,572-6,015)	4,740 (4,136-5,237)	4,174 (3,665-4,578)	4,528 (3,891-5,065)	4,720 (4,058-5,146)
Alzheimer's disease and other dementias	-	-	-	-	-	-	-	-	-	-	-
Parkinson's disease	-	-	-	-	-	-	-	6 (5-7)	6 (6-8)	13 (11-15)	21 (18-25)
Epilepsy	-	-	3,457 (2,658-4,049)	4,587 (2,861-5,920)	2,387 (1,947-2,864)	2,700 (2,245-3,129)	4,764 (3,874-5,344)	4,201 (3,565-4,744)	3,562 (3,075-4,003)	3,692 (3,053-4,226)	3,452 (2,810-3,921)
Multiple sclerosis	-	-	-	-	-	-	-	100 (78-153)	177 (141-254)	316 (261-427)	554 (468-720)
Motor neuron disease	9 (2-14)	11 (6-16)	199 (153-246)	121 (64-170)	18 (15-20)	20 (18-24)	19 (17-23)	18 (16-20)	25 (22-28)	45 (41-56)	95 (88-108)
Other neurological disorders	-	-	1,234 (745-2,252)	1,325 (576-2,734)	525 (339-976)	653 (464-1,022)	639 (432-968)	415 (338-644)	404 (342-601)	462 (405-637)	597 (534-798)
Mental disorders	-	-	-	-	0 (0-0)	3 (3-4)	19 (16-23)	31 (28-39)	34 (31-41)	39 (35-45)	40 (37-46)
Eating disorders	-	-	-	-	0 (0-0)	3 (3-4)	19 (16-23)	31 (28-39)	34 (31-41)	39 (35-45)	40 (37-46)
Anorexia nervosa	-	-	-	-	0 (0-0)	3 (3-3)	17 (15-21)	22 (16-26)	23 (15-27)	29 (21-34)	31 (24-37)
Bulimia nervosa	-	-	-	-	0 (0-0)	0 (0-0)	2 (1-3)	9 (7-18)	12 (9-21)	10 (7-18)	9 (6-17)
Substance use disorders	0 (0-0)	0 (0-0)	0 (0-0)	0 (0-0)	0 (0-0)	0 (0-0)	1,569 (1,497-1,648)	2,261 (2,176-2,342)	3,058 (2,914-3,171)	4,075 (3,800-4,253)	5,262 (4,966-5,625)
Alcohol use disorders	-	-	-	-	-	-	254 (217-280)	445 (356-481)	992 (843-1,069)	1,654 (1,394-1,792)	2,545 (2,237-2,897)
Drug use disorders	0 (0-0)	0 (0-0)	0 (0-0)	0 (0-0)	0 (0-0)	0 (0-0)	1,316 (1,254-1,386)	1,816 (1,759-1,882)	2,065 (2,005-2,129)	2,421 (2,355-2,493)	2,718 (2,632-2,809)
Opioid use disorders	0 (0-0)	0 (0-0)	0 (0-0)	0 (0-0)	0 (0-0)	0 (0-0)	769 (655-902)	1,123 (1,023-1,209)	1,271 (1,174-1,352)	1,391 (1,268-1,499)	1,496 (1,353-1,625)
Cocaine use disorders	-	-	-	-	-	-	34 (26-43)	52 (46-60)	64 (56-71)	109 (98-122)	132 (120-147)
Amphetamine use disorders	-	-	-	-	-	-	65 (36-118)	92 (62-137)	99 (75-138)	86 (62-125)	99 (69-151)
Other drug use disorders	-	-	-	-	-	-	447 (336-568)	549 (468-642)	632 (566-714)	835 (755-942)	991 (884-1,133)
Diabetes and kidney diseases	7 (5-8)	20 (15-27)	4,080 (3,742-4,449)	6,100 (5,265-6,886)	2,629 (2,340-2,920)	3,534 (3,154-3,891)	6,058 (5,746-6,414)	8,520 (8,120-8,986)	10,427 (9,971-10,903)	12,819 (12,320-13,422)	18,023 (17,342-18,765)
Diabetes mellitus	7 (5-8)	20 (15-27)	413 (300-522)	459 (319-600)	429 (311-545)	856 (607-1,080)	1,847 (1,732-1,960)	2,409 (2,280-2,547)	3,377 (3,201-3,587)	4,130 (3,928-4,347)	6,649 (6,324-7,047)
Diabetes mellitus type 1	7 (5-8)	20 (15-27)	413 (300-522)	459 (319-600)	429 (311-545)	856 (607-1,080)	1,590 (1,431-1,725)	2,031 (1,853-2,197)	2,893 (2,656-3,127)	3,102 (2,835-3,373)	4,846 (4,457-5,250)
Diabetes mellitus type 2	-	-	-	-	-	-	257 (172-400)	378 (269-553)	485 (347-707)	1,028 (789-1,364)	1,804 (1,423-2,327)
Chronic kidney disease	-	-	3,544 (3,232-3,861)	5,476 (4,732-6,202)	2,090 (1,858-2,318)	2,592 (2,351-2,830)	4,113 (3,872-4,409)	6,006 (5,651-6,414)	6,945 (6,554-7,372)	8,544 (8,113-9,019)	11,168 (10,708-11,655)
Chronic kidney disease due to diabetes mellitus type 1	-	-	14 (8-23)	25 (13-45)	14 (7-25)	28 (15-48)	89 (47-145)	295 (158-483)	689 (418-1,056)	1,490 (1,003-2,079)	2,553 (1,724-3,519)
Chronic kidney disease due to diabetes mellitus type 2	-	-	9 (5-17)	15 (7-30)	8 (4-15)	14 (6-26)	38 (17-70)	94 (40-180)	172 (78-328)	351 (164-634)	731 (378-1,219)
Chronic kidney disease due to hypertension	-	-	17 (10-26)	39 (21-62)	24 (13-40)	52 (29-85)	151 (90-229)	366 (235-533)	585 (400-829)	858 (603-1,178)	1,263 (889-1,694)
Chronic kidney disease due to glomerulonephritis	-	-	907 (648-1,236)	1,766 (1,205-2,425)	707 (479-995)	926 (631-1,253)	1,615 (1,158-2,125)	2,423 (1,701-3,224)	2,564 (1,812-3,386)	2,716 (1,911-3,594)	2,990 (1,989-4,146)
Chronic kidney disease due to other and unspecified causes	-	-	2,597 (2,231-2,954)	3,631 (2,913-4,323)	1,338 (1,035-1,621)	1,572 (1,244-1,918)	2,221 (1,660-2,766)	2,829 (2,009-3,621)	2,934 (2,127-3,798)	3,130 (2,232-4,038)	3,631 (2,521-4,804)
Acute glomerulonephritis	-	-	123 (72-156)	166 (88-220)	110 (86-133)	85 (70-103)	98 (88-110)	104 (93-116)	104 (92-118)	145 (129-161)	206 (183-228)
Skin and subcutaneous diseases	-	-	2,416 (914-3,361)	1,317 (429-2,139)	306 (113-429)	297 (125-401)	383 (170-493)	339 (175-455)	330 (176-449)	435 (243-630)	570 (298-787)
Bacterial skin diseases	-	-	2,389 (904-3,323)	1,238 (383-2,018)	258 (82-378)	234 (83-332)	287 (111-377)	255 (119-328)	245 (122-329)	341 (180-479)	461 (231-612)
Cellulitis	-	-	182 (35-408)	200 (42-383)	83 (22-132)	80 (22-123)	90 (28-129)	76 (32-103)	71 (31-98)	96 (50-153)	110 (53-173)
Pyoderma	-	-	2,206 (856-3,074)	1,038 (329-1,717)	175 (58-256)	154 (56-220)	197 (76-264)	179 (81-233)	173 (84-244)	245 (119-342)	352 (165-468)
Decubitus ulcer	-	-	-	39 (16-87)	23 (13-43)	31 (18-48)	55 (31-93)	37 (20-57)	46 (28-78)	50 (28-83)	62 (35-106)
Other skin and subcutaneous diseases	-	-	27 (11-48)	41 (18-88)	25 (14-48)	32 (18-54)	41 (24-72)	48 (27-82)	39 (21-64)	44 (24-79)	47 (24-86)
Musculoskeletal disorders	-	-	-	-	319 (245-413)	583 (489-675)	1,444 (1,119-1,663)	1,646 (1,379-1,854)	1,699 (1,473-1,923)	1,897 (1,599-2,188)	2,046 (1,702-2,295)
Rheumatoid arthritis	-	-	-	-	59 (30-89)	44 (28-58)	66 (44-93)	76 (53-103)	99 (73-129)	159 (116-212)	227 (177-290)
Other musculoskeletal disorders	-	-	-	-	261 (191-336)	539 (447-621)	1,378 (1,063-1,581)	1,570 (1,318-1,769)	1,601 (1,376-1,811)	1,738 (1,452-2,015)	1,819 (1,511-2,042)
Other non-communicable diseases	90,385 (83,251-99,927)	38,670 (35,618-43,024)	141,787 (124,741-168,143)	62,300 (53,792-73,091)	12,649 (11,181-14,196)	9,162 (8,339-10,136)	9,770 (9,052-10,752)	9,419 (8,769-10,423)	8,262 (7,715-9,067)	8,058 (7,400-8,756)	8,140 (7,507-8,750)
Congenital birth defects	89,013 (81,850-98,581)	35,591 (33,158-38,930)	114,857 (105,163-126,101)	49,007 (43,328-55,860)	8,723 (7,753-9,760)	5,984 (5,479-6,672)	4,462 (4,172-4,858)	3,679 (3,454-4,090)	3,098 (2,906-3,420)	2,242 (2,077-2,482)	2,230 (2,061-2,479)
Neural tube defects	12,871 (9,910-15,831)	3,161 (2,368-4,217)	10,870 (6,472-19,180)	7,151 (3,335-15,879)	381 (277-548)	251 (206-303)	161 (135-191)	122 (101-147)	84 (70-104)	80 (66-98)	64 (54-77)
Congenital heart anomalies	29,909 (21,329-37,165)	16,393 (12,455-19,665)	62,744 (42,492-76,179)	25,942 (14,185-34,321)	5,385 (4,178-6,391)	3,923 (3,325-4,383)	2,818 (2,413-3,120)	2,162 (1,869-2,420)	2,005 (1,770-2,249)	1,250 (1,089-1,398)	1,374 (1,218-1,543)
Orofacial clefts	1,298 (613-3,673)	533 (248-1,488)	1,089 (467-3,329)	255 (81-1,056)	-	-	-	-	-	-	-
Down syndrome	1,684 (1,093-2,929)	724 (419-1,253)	3,273 (1,896-6,084)	1,817 (782-4,252)	225 (135-377)	182 (141-253)	258 (209-345)	309 (246-463)	269 (225-343)	275 (229-353)	248 (197-307)
Other chromosomal abnormalities	2,989 (1,380-6,806)	1,604 (812-3,589)	3,653 (1,729-7,827)	781 (308-2,254)	163 (82-326)	101 (57-167)	97 (55-165)	87 (48-165)	71 (40-116)	55 (31-87)	45 (27-65)
Congenital musculoskeletal and limb anomalies	2,252 (1,071-3,509)	656 (293-1,057)	1,700 (712-3,148)	689 (205-1,612)	192 (90-307)	140 (83-184)	133 (76-173)	113 (64-152)	70 (45-92)	66 (39-87)	59 (38-78)
Urogenital congenital anomalies	2,524 (1,652-3,235)	841 (570-1,132)	1,314 (729-2,112)	368 (182-721)	151 (101-218)	141 (101-191)	75 (56-95)	82 (63-102)	58 (45-71)	57 (44-69)	69 (53-87)
Digestive congenital anomalies	8,682 (4,696-13,637)	3,820 (1,979-5,939)	9,481 (4,625-16,360)	2,365 (797-5,059)	194 (119-338)	93 (67-138)	62 (41-92)	56 (37-90)	30 (21-41)	28 (20-40)	25 (18-32)
Other congenital birth defects	26,803 (18,284-36,091)	7,860 (4,841-11,250)	20,734 (11,568-32,553)	9,638 (4,207-18,076)	2,032 (1,290-2,948)	1,152 (839-1,597)	859 (646-1,163)	747 (548-1,003)	511 (376-695)	431 (336-560)	346 (277-438)
Urinary diseases and male infertility	236 (214-260)	172 (155-190)	2,132 (1,928-2,329)	2,339 (2,017-2,669)	818 (704-934)	668 (586-753)	1,347 (1,240-1,459)	1,777 (1,659-1,911)	1,490 (1,402-1,581)	1,780 (1,682-1,885)	2,082 (1,985-2,196)
Urinary tract infections	47 (34-75)	55 (38-75)	1,361 (1,021-1,719)	986 (505-1,595)	399 (278-560)	342 (265-429)	1,058 (917-1,207)	1,389 (1,214-1,594)	1,122 (985-1,274)	1,320 (1,164-1,473)	1,605 (1,413-1,775)
Urolithiasis	-	-	-	-	19 (10-34)	17 (10-26)	17 (11-25)	34 (23-49)	40 (30-55)	74 (55-103)	99 (71-135)

Cause	40 to 44	45 to 49	50 to 54	54 to 59	60 to 64	65 to 69	70 to 74	75 to 79	80 to 84	85 to 89	90 to 94	95 plus
Mesothelioma	353 (279-451)	269 (235-316)	394 (346-455)	525 (469-597)	567 (518-636)	736 (683-809)	823 (770-890)	883 (838-940)	730 (699-771)	389 (372-406)	191 (185-199)	52 (50-54)
Hodgkin lymphoma	609 (474-757)	514 (420-634)	569 (445-694)	577 (449-714)	618 (488-782)	791 (643-976)	797 (639-983)	769 (605-928)	591 (455-702)	263 (207-308)	85 (64-100)	15 (11-17)
Non-Hodgkin lymphoma	2,556 (2,382-2,761)	2,983 (2,851-3,164)	4,177 (3,994-4,427)	5,201 (4,999-5,473)	6,677 (6,437-7,022)	8,628 (8,376-8,982)	10,012 (9,727-10,367)	10,120 (9,896-10,364)	9,413 (9,208-9,633)	5,529 (5,418-5,634)	2,465 (2,417-2,515)	649 (634-664)
Multiple myeloma	629 (582-707)	1,105 (1,042-1,248)	1,932 (1,823-2,162)	2,842 (2,708-3,166)	3,834 (3,613-4,325)	5,213 (4,919-5,774)	5,978 (5,407-6,589)	6,330 (5,628-7,002)	5,360 (4,804-5,904)	2,881 (2,449-3,199)	1,303 (1,172-1,514)	284 (241-317)
Leukemia	5,311 (4,354-5,920)	5,723 (4,863-6,262)	7,008 (5,831-7,702)	7,710 (6,698-8,426)	8,545 (7,532-9,412)	10,545 (9,392-11,390)	11,873 (10,511-12,770)	12,380 (11,283-13,187)	11,032 (10,208-11,617)	6,747 (6,329-7,046)	3,459 (3,336-3,579)	1,068 (1,037-1,100)
Acute lymphoid leukemia	702 (565-842)	644 (551-778)	699 (589-852)	759 (635-915)	750 (661-909)	792 (694-935)	772 (685-923)	679 (619-788)	493 (451-577)	249 (231-287)	101 (94-110)	25 (24-27)
Chronic lymphoid leukemia	142 (118-163)	182 (164-207)	334 (294-377)	487 (448-545)	759 (683-841)	1,115 (1,043-1,206)	1,456 (1,373-1,577)	1,991 (1,878-2,095)	2,068 (1,991-2,172)	1,479 (1,421-1,542)	929 (886-964)	328 (314-341)
Acute myeloid leukemia	1,396 (1,216-1,694)	1,699 (1,517-1,957)	1,851 (1,652-2,139)	2,090 (1,883-2,338)	2,470 (2,227-2,728)	3,091 (2,797-3,364)	3,449 (3,090-3,720)	3,570 (3,238-3,786)	2,971 (2,606-3,132)	1,583 (1,441-1,648)	663 (634-695)	178 (170-190)
Chronic myeloid leukemia	561 (462-678)	583 (489-686)	753 (629-893)	776 (682-927)	864 (768-1,052)	1,061 (948-1,248)	1,114 (997-1,284)	1,139 (1,058-1,270)	1,089 (1,016-1,176)	670 (634-716)	364 (344-385)	107 (100-114)
Other leukemia	2,510 (1,897-2,919)	2,615 (2,010-2,952)	3,371 (2,568-3,870)	3,597 (2,872-4,103)	3,703 (3,025-4,142)	4,485 (3,706-4,912)	5,083 (4,259-5,592)	5,001 (4,351-5,417)	4,412 (3,938-4,721)	2,766 (2,552-2,966)	1,402 (1,325-1,473)	429 (409-448)
Other malignant neoplasms	4,577 (4,222-5,107)	5,757 (5,363-6,336)	8,172 (7,558-8,957)	9,446 (8,683-10,353)	10,281 (9,648-11,398)	12,825 (11,830-14,127)	13,489 (12,560-14,836)	13,516 (12,278-14,747)	11,213 (9,861-12,125)	6,481 (5,745-7,044)	3,268 (2,852-3,518)	1,026 (900-1,126)
Other neoplasms	953 (693-1,228)	1,191 (843-1,523)	1,639 (1,174-2,112)	1,879 (1,330-2,492)	2,200 (1,526-2,857)	2,940 (2,056-3,954)	3,822 (2,581-5,145)	4,691 (3,003-6,166)	4,873 (2,905-6,081)	3,501 (1,922-4,347)	2,091 (1,054-2,623)	756 (378-949)
Myelodysplastic, myeloproliferative, and other hematopoietic neoplasm	838 (613-1,084)	1,093 (775-1,416)	1,550 (1,115-2,012)	1,815 (1,280-2,413)	2,154 (1,506-2,809)	2,906 (2,032-3,915)	3,795 (2,568-5,123)	4,672 (2,994-6,151)	4,858 (2,900-6,073)	3,494 (1,918-4,340)	2,088 (1,052-2,620)	755 (378-948)
Other benign and in situ neoplasms	116 (77-150)	98 (62-126)	89 (52-119)	64 (35-91)	46 (23-68)	34 (18-50)	27 (12-42)	20 (8-38)	15 (5-30)	7 (3-12)	3 (1-7)	1 (0-2)
Cardiovascular diseases	106,096 (103,262-108,669)	149,530 (145,989-152,720)	232,976 (227,387-237,599)	305,720 (298,218-312,165)	412,500 (402,567-422,075)	644,811 (628,469-657,355)	860,093 (841,367-876,805)	1,138,389 (1,118,161-1,158,37	1,210,612 (1,192,045-1,230,77	934,769 (924,466-952,028)	611,563 (605,692-617,327)	247,293 (245,442-249,344)
Rheumatic heart disease	7,775 (6,837-8,666)	7,872 (6,988-8,786)	10,201 (9,307-11,103)	11,147 (10,202-12,166)	12,576 (11,436-13,818)	15,736 (14,440-17,194)	18,046 (16,696-19,716)	17,725 (16,731-19,245)	16,378 (15,536-17,614)	11,532 (10,984-12,266)	6,403 (6,170-6,736)	2,298 (2,226-2,392)
Ischemic heart disease	42,539 (40,720-44,299)	62,891 (60,783-65,284)	96,137 (93,106-99,341)	133,510 (129,596-137,541)	188,676 (183,544-194,212)	299,636 (291,538-306,877)	393,900 (384,856-403,117)	537,118 (526,253-547,647)	575,483 (565,365-587,682)	451,796 (444,253-463,793)	314,378 (310,102-321,550)	134,481 (132,434-138,002)
Stroke	39,034 (37,546-40,759)	56,613 (54,810-58,374)	96,616 (93,429-99,872)	123,718 (120,135-127,484)	165,826 (161,111-170,959)	262,953 (255,251-269,959)	356,931 (347,695-366,360)	460,088 (450,524-470,435)	467,229 (458,432-478,230)	340,410 (333,785-349,643)	192,704 (189,529-197,582)	69,126 (67,998-70,920)
Ischemic stroke	6,189 (5,594-6,788)	9,920 (9,047-10,884)	18,627 (17,285-20,173)	28,201 (26,186-30,421)	54,200 (50,280-59,425)	97,861 (92,565-104,462)	158,106 (151,193-165,665)	222,344 (215,765-229,612)	268,238 (261,979-275,700)	201,345 (196,312-207,823)	120,440 (118,028-123,413)	45,659 (44,815-46,928)
Intracerebral hemorrhage	24,003 (22,576-25,503)	36,877 (35,031-38,769)	64,019 (61,005-67,211)	78,370 (74,428-82,401)	96,108 (90,048-101,604)	142,511 (134,861-149,978)	176,135 (167,749-184,367)	210,332 (202,396-218,678)	178,516 (172,983-184,967)	124,525 (120,762-129,732)	64,163 (62,498-66,303)	20,644 (20,134-21,312)
Subarachnoid hemorrhage	8,842 (8,100-9,946)	9,815 (9,011-11,309)	13,970 (12,867-16,054)	17,147 (15,728-19,718)	15,518 (14,205-17,864)	22,580 (20,970-25,390)	22,690 (21,039-25,630)	27,412 (24,887-30,202)	20,475 (18,512-22,090)	14,540 (13,250-15,606)	8,101 (7,189-8,485)	2,823 (2,569-2,966)
Hypertensive heart disease	4,613 (3,297-5,538)	7,243 (5,307-8,557)	11,564 (8,615-13,555)	15,401 (11,610-18,025)	20,271 (15,231-23,730)	31,043 (24,000-35,510)	43,049 (34,806-49,081)	54,151 (45,314-60,968)	60,083 (48,944-67,200)	48,574 (38,183-53,710)	33,235 (22,922-35,909)	13,677 (9,049-14,774)
Non-rheumatic valvular heart disease	615 (526-742)	762 (674-896)	1,093 (969-1,281)	1,443 (1,305-1,670)	1,842 (1,672-2,164)	2,889 (2,600-3,401)	4,436 (3,938-5,500)	7,766 (6,403-8,815)	11,292 (8,796-12,219)	11,097 (8,334-11,808)	8,708 (5,842-9,256)	3,768 (2,615-4,005)
Non-rheumatic calcific aortic valve disease	224 (178-325)	294 (239-409)	459 (379-630)	637 (536-865)	925 (793-1,203)	1,578 (1,375-2,010)	2,640 (2,250-3,391)	4,840 (3,844-5,756)	7,787 (5,735-8,562)	8,255 (5,848-8,921)	6,863 (4,485-7,418)	3,065 (1,969-3,302)
Non-rheumatic degenerative mitral valve disease	326 (252-421)	392 (307-492)	535 (419-669)	690 (555-846)	809 (628-983)	1,162 (879-1,411)	1,609 (1,282-1,993)	2,445 (1,968-3,054)	2,953 (2,244-3,835)	2,423 (1,718-3,272)	1,635 (1,161-2,294)	641 (451-927)
Other non-rheumatic valve diseases	65 (41-97)	76 (50-115)	99 (60-144)	116 (69-163)	108 (63-155)	148 (88-206)	187 (107-257)	481 (278-632)	552 (311-698)	419 (240-529)	210 (122-268)	62 (40-85)
Cardiomyopathy and myocarditis	5,852 (5,557-6,296)	7,093 (6,795-7,548)	8,148 (7,829-8,595)	8,581 (8,214-9,127)	7,000 (6,526-7,662)	8,467 (7,988-9,097)	9,492 (8,958-10,183)	13,316 (12,693-14,065)	17,547 (16,873-18,230)	15,905 (15,243-16,609)	13,089 (12,396-13,829)	6,159 (5,865-6,500)
Myocarditis	714 (547-850)	521 (380-615)	643 (469-748)	711 (513-828)	604 (412-711)	857 (607-991)	1,147 (809-1,316)	1,683 (1,164-1,928)	2,711 (1,759-3,148)	2,970 (1,908-3,512)	2,838 (1,451-3,552)	1,575 (717-2,040)
Alcoholic cardiomyopathy	3,201 (3,071-3,332)	4,273 (4,096-4,478)	4,721 (4,526-4,957)	4,368 (4,132-4,646)	2,422 (2,279-2,579)	2,667 (2,493-2,867)	1,618 (1,474-1,848)	1,902 (1,735-2,292)	1,651 (1,455-2,277)	1,040 (890-1,466)	725 (602-1,118)	297 (250-450)
Other cardiomyopathy	1,937 (1,659-2,232)	2,300 (2,005-2,627)	2,783 (2,465-3,137)	3,502 (3,109-3,957)	3,973 (3,542-4,535)	4,943 (4,522-5,469)	6,727 (6,225-7,290)	9,730 (9,172-10,320)	13,185 (12,490-14,072)	11,895 (11,176-12,922)	9,526 (8,624-11,089)	4,288 (3,808-5,220)
Atrial fibrillation and flutter	200 (184-223)	337 (314-360)	632 (596-671)	1,226 (1,156-1,301)	2,173 (2,058-2,268)	4,168 (3,968-4,333)	8,289 (7,953-8,581)	14,641 (14,135-15,082)	25,276 (24,525-26,006)	25,484 (24,464-26,386)	22,521 (21,923-23,010)	8,868 (8,657-9,092)
Aortic aneurysm	615 (550-738)	922 (849-1,071)	1,379 (1,276-1,583)	1,920 (1,775-2,210)	2,435 (2,278-2,808)	3,902 (3,695-4,390)	5,529 (5,285-6,099)	7,618 (7,366-8,132)	8,657 (8,376-9,009)	6,828 (6,637-7,016)	4,188 (4,088-4,315)	1,562 (1,524-1,613)
Peripheral artery disease	86 (49-167)	137 (68-307)	238 (111-541)	402 (182-884)	630 (291-1,419)	1,163 (505-2,704)	1,749 (746-3,935)	3,036 (1,248-6,797)	4,317 (1,772-10,010)	4,184 (1,729-9,093)	3,739 (1,468-9,068)	1,948 (744-4,889)
Endocarditis	1,008 (869-1,262)	878 (719-1,076)	1,066 (864-1,304)	1,181 (945-1,492)	1,570 (1,264-1,957)	2,105 (1,687-2,681)	2,707 (2,082-3,447)	3,575 (2,539-4,653)	4,203 (2,871-5,605)	3,722 (2,444-4,916)	2,838 (1,667-3,730)	1,287 (768-1,758)
Other cardiovascular and circulatory diseases	3,757 (3,210-4,467)	4,783 (4,194-5,542)	5,905 (5,157-6,835)	7,190 (6,241-8,333)	9,501 (8,176-11,058)	12,751 (11,128-14,563)	15,965 (14,113-17,967)	19,356 (17,481-21,274)	20,149 (18,373-21,885)	15,238 (13,823-16,448)	9,760 (9,117-10,396)	4,119 (3,887-4,373)
Chronic respiratory diseases	16,074 (14,617-17,724)	24,274 (21,734-25,667)	39,644 (34,335-41,768)	59,628 (50,612-62,571)	98,821 (85,042-104,411)	148,654 (128,678-156,384)	216,945 (191,542-228,856)	255,610 (227,925-266,959)	266,392 (235,502-277,452)	192,553 (174,138-200,299)	94,862 (89,244-99,031)	31,078 (29,888-32,435)
Chronic obstructive pulmonary disease	7,079 (5,622-8,794)	12,094 (9,391-15,244)	24,553 (19,413-29,106)	39,887 (30,967-46,608)	72,760 (57,446-84,854)	117,320 (95,372-133,066)	179,346 (152,083-197,195)	216,897 (188,047-234,321)	232,295 (205,414-246,691)	171,132 (154,498-180,951)	85,512 (80,332-89,506)	28,022 (26,918-29,200)
Pneumoconiosis	50 (34-61)	53 (36-68)	84 (61-102)	106 (70-133)	135 (94-176)	190 (134-240)	285 (216-352)	343 (256-418)	319 (239-392)	205 (158-251)	105 (81-123)	38 (32-43)
Silicosis	13 (7-19)	16 (9-21)	21 (11-27)	24 (14-34)	32 (19-46)	40 (22-55)	73 (45-105)	90 (59-127)	84 (57-125)	52 (35-78)	26 (17-39)	8 (5-13)
Asbestosis	7 (3-11)	9 (4-14)	16 (8-24)	20 (11-32)	24 (13-39)	33 (18-49)	56 (31-80)	70 (36-95)	70 (33-95)	47 (21-63)	25 (10-32)	9 (4-11)
Coal workers pneumoconiosis	7 (2-10)	6 (3-9)	11 (5-18)	13 (5-21)	15 (6-24)	27 (12-42)	33 (14-49)	40 (19-58)	34 (16-47)	24 (11-32)	13 (7-17)	5 (3-7)
Other pneumoconiosis	24 (14-34)	23 (14-31)	36 (22-50)	49 (28-68)	63 (37-87)	91 (57-122)	122 (81-164)	142 (98-190)	131 (93-174)	82 (60-108)	42 (31-56)	15 (12-20)
Asthma	7,321 (5,201-9,329)	10,245 (6,964-13,253)	12,240 (7,955-16,145)	15,982 (9,889-21,261)	22,368 (12,692-31,119)	26,128 (14,930-36,814)	30,567 (17,858-43,589)	30,531 (18,688-42,681)	26,061 (17,017-35,661)	15,695 (10,857-21,051)	6,423 (4,827-8,235)	1,980 (1,611-2,386)
Interstitial lung disease and pulmonary sarcoidosis	712 (491-1,083)	1,051 (693-1,622)	1,532 (1,020-2,325)	2,093 (1,347-3,196)	3,017 (1,925-4,937)	4,338 (2,781-6,854)	5,963 (3,838-8,978)	6,919 (4,362-9,783)	6,747 (4,176-8,811)	4,814 (2,906-6,042)	2,414 (1,464-2,804)	873 (536-985)
Other chronic respiratory diseases	913 (615-1,285)	830 (556-1,145)	1,235 (850-1,679)	1,560 (1,052-2,072)	541 (366-709)	677 (482-868)	784 (581-1,006)	921 (683-1,185)	970 (740-1,241)	706 (571-902)	407 (345-494)	166 (146-192)
Digestive diseases	28,310 (26,490-30,172)	35,569 (33,493-37,484)	46,772 (44,372-49,401)	53,698 (50,476-56,554)	62,333 (55,521-65,638)	76,945 (69,333-80,420)	85,725 (77,490-89,661)	92,092 (85,290-95,685)	89,127 (82,274-92,175)	65,430 (60,414-67,524)	38,740 (37,112-39,654)	15,036 (14,539-15,342)
Cirrhosis and other chronic liver diseases	19,411 (18,214-21,759)	24,311 (23,064-26,681)	31,764 (30,155-35,062)	34,780 (32,851-38,628)	37,005 (33,592-39,721)	41,119 (38,199-44,348)	41,099 (37,543-45,184)	37,031 (33,892-40,091)	28,920 (26,016-31,334)	17,098 (15,063-18,554)	7,937 (7,339-8,571)	2,840 (2,671-3,068)
Cirrhosis and other chronic liver diseases due to hepatitis B	5,635 (4,787-6,732)	6,061 (5,028-7,158)	7,631 (6,234-9,170)	8,394 (6,885-10,194)	9,314 (7,565-11,403)	10,806 (8,950-13,141)	11,387 (9,226-13,807)	10,091 (8,152-12,003)	7,708 (6,121-9,351)	4,329 (3,505-5,158)	1,665 (1,322-2,046)	488 (363-636)
Cirrhosis and other chronic liver diseases due to hepatitis C	5,682 (4,701-6,796)	7,167 (5,929-8,549)	9,284 (7,656-11,179)	9,715 (8,277-11,269)	10,208 (8,358-12,164)	11,160 (9,615-12,957)	10,880 (9,198-12,546)	9,328 (7,727-11,303)	7,207 (5,824-8,734)	4,386 (3,644-5,147)	2,092 (1,719-2,521)	792 (620-990)
Cirrhosis and other chronic liver diseases due to alcohol use	4,625 (3,969-5,373)	6,264 (5,350-7,384)	8,202 (6,993-9,506)	8,846 (7,426-10,503)	8,651 (6,978-10,499)	8,952 (7,407-10,675)	8,126 (6,576-9,890)	7,106 (5,994-8,373)	5,185 (4,295-6,274)	2,837 (2,407-3,387)	1,330 (1,069-1,657)	462 (330-624)
Cirrhosis due to NASH	1,858 (1,544-2,203)	2,824 (2,356-3,364)	3,931 (3,290-4,627)	4,336 (3,798-4,962)	4,555 (3,879-5,243)	4,754 (4,135-5,414)	4,364 (3,746-5,024)	3,555 (2,987-4,182)	2,650 (2,170-3,174)	1,652 (1,366-1,938)	798 (631-1,004)	287 (209-391)
Cirrhosis and other chronic liver diseases due to other causes	1,611 (1,198-2,093)	1,995 (1,525-2,532)	2,715 (2,045-3,525)	3,489 (2,517-4,687)	4,277 (3,095-5,643)	5,447 (4,049-7,091)	6,342 (4,925-7,948)	6,951 (5,385-8,878)	6,170 (4,737-7,778)	3,894 (3,187-4,669)	2,053 (1,633-2,519)	810 (596-1,037)
Upper digestive system diseases	2,840 (2,261-3,167)	3,519 (2,900-3,898)	5,325 (4,313-5,878)	6,099 (5,132-6,788)	8,822 (7,071-9,700)	12,686 (10,321-13,791)	15,030 (12,246-16,282)	16,675 (14,133-17,861)	17,068 (14,957-18,078)	12,961 (11,761-13,731)	7,232 (6,798-7,711)	2,647 (2,496-2,836)
Peptic ulcer disease	2,392 (1,914-2,795)	2,992 (2,524-3,495)	4,529 (3,791-5,284)	5,193 (4,414-6,155)	7,567 (6,184-8,682)	10,833 (9,131-12,244)	12,808 (10,466-14,519)	14,180 (12,465-15,982)	14,407 (12,829-15,833)	10,887 (9,875-11,934)	6,068 (5,654-6,675)	2,245 (2,111-2,469)
Gastritis and duodenitis	448 (224-575)	527 (265-661)	796 (385-1,016)	906 (470-1,144)	1,255 (605-1,573)	1,852 (887-2,273)	2,222 (1,092-2,738)	2,494 (1,312-2,980)	2,661 (1,482-3,157)	2,075 (1,202-2,381)	1,164 (804-1,338)	401 (299-463)
Appendicitis	669 (527-765)	834 (690-957)	727 (596-818)	1,243 (998-1,439)	1,333 (1,002-1,549)	1,437 (1,116-1,644)	1,398 (1,089-1,629)	1,551 (1,246-1,787)	1,311 (1,073-1,534)	1,033 (837-1,263)	546 (464-652)	172 (149-200)
Paralytic ileus and intestinal obstruction	1,883 (1,137-2,210)	2,183 (1,366-2,556)	2,802 (1,733-3,299)	3,811 (2,474-4,396)	4,972 (3,083-5,845)	6,491 (4,274-7,519)	7,499 (5,331-8,561)	9,358 (7,337-10,788)	10,572 (8,357-12,513)	9,049 (7,033-10,789)	6,283 (4,786-7,428)	2,586 (1,851-3,136)
Inguinal, femoral, and abdominal hernia	167 (114-213)	319 (206-403)	484 (333-605)	673 (450-808)	929 (609-1,105)	1,322 (895-1,591)	1,706 (1,191-2,073)	1,989 (1,415-2,554)	2,467 (1,642-3,087)	1,879 (1,304-2,423)	1,192 (836-1,612)	465 (306-651)
Inflammatory bowel disease	335 (276-402)	443 (366-510)	534 (433-611)	659 (542-765)	807 (614-907)	1,110 (886-1,228)	1,593 (1,231-1,741)	2,160 (1,659-2,344)	2,688 (1,916-2,934)	2,306 (1,601-2,544)	1,663 (1,073-1,872)	705 (448-812)
Vascular intestinal disorders	323 (262-363)	531 (452-591)	775 (656-861)	1,164 (1,007-1,292)	1,827 (1,508-2,011)	3,369 (2,922-3,636)	5,281 (4,574-5,704)	7,948 (7,098-8,598)	8,768 (7,956-9,502)	6,675 (5,766-7,179)	4,095 (3,432-4,576)	1,379 (1,117-1,546)
Gallbladder and biliary diseases	842 (718-932)	1,065 (944-1,163)	1,641 (1,448-1,804)	1,955 (1,744-2,242)	2,683 (2,287-2,940)	3,966 (3,429-4,288)	5,148 (4,506-5,577)	6,958 (6,131-7,551)	7,990 (7,159-8,608)	6,615 (6,006-7,108)	4,708 (4,338-5,163)	2,094 (1,926-2,301)
Pancreatitis	1,286 (990-1,508)	1,695 (1,233-2,006)	1,801 (1,336-2,094)	2,110 (1,580-2,455)	2,239 (1,672-2,560)	3,101 (2,288-3,472)	3,634 (2,593-4,087)	3,838 (2,741-4,283)	3,532 (2,607-3,856)	2,452 (1,836-2,691)	1,367 (995-1,503)	485 (365-538)
Other digestive diseases	555 (454-635)	669 (595-749)	920 (816-1,018)	1,205 (1,080-1,324)	1,715 (1,469-1,884)	2,344 (2,070-2,537)	3,336 (2,951-3,589)	4,585 (4,282-4,890)	5,813 (5,474-6,158)	5,361 (5,020-5,701)	3,716 (3,521-4,004)	1,662 (1,578-1,833)
Neurological disorders	5,228 (4,762-5,720)	5,902 (5,519-6,225)	9,243 (8,857-9,812)	12,324 (11,928-13,151)	20,982 (20,371-22,335)	42,304 (41,130-44,915)	84,162 (82,020-88,638)	170,702 (167,376-177,835)	264,094 (259,933-272,175)	275,974 (269,701-279,464)	239,054 (236,664-242,254)	104,634 (103,671-105,627)
Alzheimer's disease and other dementias	272 (253-310)	921 (877-1,020)	2,874 (2,708-3,230)	5,857 (5,515-6,593)	13,363 (12,816-14,642)	30,528 (29,487-33,022)	64,987 (63,136-69,216)	141,660 (138,632-148,512)	231,982 (228,260-239,015)	254,855 (249,113-258,100)	229,230 (227,036-232,197)	102,102 (101,155-103,063)
Parkinson's disease	193 (173-220)	413 (383-464)	916 (839-1,068)	1,336 (1,213-1,507)	2,449 (2,262-2,742)	5,796 (5,421-6,400)	13,042 (11,941-14,164)	23,484 (21,817-24,996)	27,508 (25,799-28,943)	18,475 (17,394-19,572)	8,628 (8,210-9,277)	2,137 (2,054-2,206)
Epilepsy	2,932 (2,471-3,391)	2,044 (1,732-2,225)	2,372 (1,925-2,751)	1,723 (1,460-1,837)	1,710 (1,337-1,851)	2,111 (1,629-2,310)	2,192 (1,723-2,418)	1,732 (1,397-1,982)	1,640 (1,332-1,775)	1,128 (906-1,348)	531 (446-599)	173 (151-189)
Multiple sclerosis	851 (720-1,027)	1,089 (907-1,266)	1,252 (1,034-1,418)	1,238 (993-1,397)	1,044 (808-1,178)	928 (730-1,051)	779 (584-864)	644 (475-711)	455 (323-503)	227 (158-250)	82 (60-89)	20 (15-22)
Motor neuron disease	212 (195-256)	391 (368-451)	679 (644-822)	982 (942-1,092)	1,203 (1,148-1,315)	1,596 (1,540-1,720)	1,735 (1,684-1,792)	1,717 (1,661-1,774)	1,202 (1,159-1,242)	495 (474-512)	153 (145-160)	36 (34-37)
Other neurological disorders	768 (716-968)	1,043 (969-1,308)	1,151 (1,088-1,385)	1,189 (1,147-1,404)	1,213 (1,155-1,457)	1,346 (1,289-1,596)	1,427 (1,366-1,623)	1,465 (1,403-1,621)	1,308 (1,209-1,407)	794 (754-847)	429 (404-459)	166 (159-184)
Mental disorders	38 (35-42)	35 (32-38)	-	-	-	-	-	-	-	-	-	-
Eating disorders	38 (35-42)	35 (32-38)	-	-	-	-	-	-	-	-	-	-
Anorexia nervosa	29 (21-34)	29 (23-33)	-	-	-	-	-	-	-	-	-	-
Bulimia nervosa	9 (5-17)	6 (3-12)	-	-	-	-	-	-	-	-	-	-
Substance use disorders	6,535 (5,842-6,744)	7,365 (6,295-7,648)	6,928 (5,912-7,166)	5,722 (4,668-6,004)	3,890 (3,418-4,051)	4,012 (3,451-4,178)	2,615 (2,376-2,692)	2,679 (2,426-2,794)	1,965 (1,737-2,033)	1,366 (1,192-1,421)	737 (643-764)	277 (239-288)
Alcohol use disorders	3,722 (3,036-3,919)	4,825 (3,720-5,069)	4,721 (3,721-4,938)	3,976 (2,880-4,318)	2,504 (2,050-2,639)	2,470 (1,866-2,601)	1,228 (947-1,280)	1,172 (879-1,278)	661 (417-704)	451 (260-482)	234 (132-252)	89 (50-95)
Drug use disorders	2,812 (2,728-2,898)	2,540 (2,445-2,632)	2,207 (2,136-2,280)	1,746 (1,693-1,803)	1,386 (1,332-1,443)	1,542 (1,484-1,608)	1,387 (1,340-1,442)	1,507 (1,459-1,563)	1,303 (1,263-1,346)	915 (881-958)	504 (487-521)	189 (182-195)
Opioid use disorders	1,566 (1,454-1,658)	1,350 (1,247-1,436)	1,155 (1,060-1,239)	928 (848-996)	724 (633-799)	847 (745-945)	621 (556-706)	671 (614-765)	545 (507-623)	390 (362-441)	205 (193-218)	69 (65-72)
Cocaine use disorders	165 (151-182)	165 (150-182)	106 (97-117)	66 (60-73)	40 (36-47)	36 (31-43)	12 (10-13)	11 (10-13)	8 (7-9)	6 (5-6)	4 (4-5)	2 (2-2)
Amphetamine use disorders	97 (76-131)	92 (73-124)	55 (43-78)	41 (31-62)	31 (20-56)	29 (17-53)	7 (5-12)	8 (6-12)	4 (3-4)	3 (2-4)	2 (1-3)	1 (0-2)
Other drug use disorders	984 (908-1,091)	933 (865-1,030)	891 (821-989)	711 (646-798)	590 (525-692)	630 (538-749)	747 (670-824)	816 (742-879)	747 (688-786)	516 (483-546)	292 (277-307)	116 (111-122)
Diabetes and kidney diseases	25,683 (24,811-26,560)	33,772 (32,629-34,837)	51,984 (50,257-53,616)	64,499 (62,572-66,334)	85,181 (82,639-87,565)	107,214 (103,975-109,915)	119,385 (115,889-122,321)	126,334 (122,781-129,446)	111,965 (109,154-114,895)	77,332 (75,838-79,435)	44,511 (43,661-45,345)	16,739 (16,475-17,005)
Diabetes mellitus	11,449 (10,949-12,009)	17,229 (16,516-18,006)	28,693 (27,495-29,898)	37,091 (35,769-38,432)	52,928 (50,940-54,953)	67,539 (65,093-69,857)	74,096 (71,600-76,331)	76,670 (74,268-78,819)	64,987 (63,037-67,009)	40,198 (39,125-41,512)	21,034 (20,543-21,553)	6,887 (6,733-7,047)
Diabetes mellitus type 1	8,067 (7,429-8,714)	8,182 (7,227-9,222)	13,103 (11,595-14,877)	16,272 (14,503-18,244)	16,766 (14,446-20,143)	20,967 (18,210-24,360)	18,865 (16,140-21,894)	18,796 (15,851-21,843)	11,728 (9,319-13,765)	6,780 (5,189-7,984)	3,284 (2,324-3,801)	1,035 (719-1,169)
Diabetes mellitus type 2	3,381 (2,749-4,182)	9,047 (7,897-10,274)	15,590 (13,772-17,404)	20,819 (18,704-23,008)	36,161 (32,510-39,057)	46,572 (42,645-50,075)	55,232 (51,678-58,394)	57,874 (54,450-61,409)	53,259 (50,868-56,339)	33,418 (31,850-35,626)	17,750 (17,124-18,912)	5,852 (5,652-6,186)
Chronic kidney disease	14,014 (13,463-14,558)	16,349 (15,748-16,944)	23,039 (22,212-23,910)	27,131 (26,196-28,026)	31,973 (30,996-33,072)	39,312 (37,953-40,627)	44,848 (43,473-46,206)	49,233 (47,659-50,746)	46,594 (45,452-47,939)	36,883 (36,082-37,894)	23,365 (22,836-23,853)	9,812 (9,620-10,005)
Chronic kidney disease due to diabetes mellitus type 1	3,404 (2,253-4,672)	3,597 (2,414-4,963)	3,908 (2,377-5,660)	3,150 (1,835-4,940)	2,209 (1,074-3,767)	1,849 (947-3,324)	1,606 (775-2,809)	1,441 (703-2,588)	1,213 (612-2,073)	624 (330-1,050)	182 (89-327)	16 (7-31)
Chronic kidney disease due to diabetes mellitus type 2	1,453 (823-2,393)	2,706 (1,691-3,888)	5,572 (3,855-7,568)	8,201 (5,805-10,519)	11,338 (8,286-14,221)	14,443 (10,954-17,852)	16,331 (12,339-19,988)	17,255 (12,557-21,677)	15,453 (10,982-19,850)	11,359 (8,030-14,511)	6,424 (4,651-8,280)	2,511 (1,620-3,593)
Chronic kidney disease due to hypertension	1,781 (1,296-2,365)	2,363 (1,685-3,104)	3,841 (2,821-5,016)	5,255 (3,968-6,902)	6,951 (5,271-8,955)	9,714 (7,519-12,224)	12,623 (9,930-15,765)	15,406 (12,225-19,197)	16,523 (12,996-20,220)	15,237 (12,499-18,077)	10,654 (8,880-12,373)	4,810 (3,857-5,749)
Chronic kidney disease due to glomerulonephritis	3,451 (2,252-4,808)	3,735 (2,490-5,333)	4,631 (3,065-6,616)	4,631 (3,091-6,740)	4,564 (2,948-6,790)	5,457 (3,543-7,870)	6,121 (3,886-8,738)	6,609 (4,320-9,457)	5,943 (3,804-8,758)	4,173 (2,773-6,082)	2,549 (1,713-3,695)	1,031 (565-1,701)
Chronic kidney disease due to other and unspecified causes	3,926 (2,660-5,262)	3,947 (2,653-5,608)	5,088 (3,336-7,219)	5,867 (3,855-8,218)	6,911 (4,226-10,007)	7,849 (5,059-11,171)	8,167 (5,373-11,544)	8,523 (5,491-12,600)	7,461 (4,964-10,666)	5,490 (3,739-7,614)	3,555 (2,427-4,976)	1,444 (913-2,242)
Acute glomerulonephritis	220 (199-241)	194 (176-215)	252 (229-277)	278 (250-306)	280 (253-310)	363 (326-403)	440 (397-487)	431 (388-477)	384 (345-429)	251 (227-280)	112 (101-124)	40 (36-48)
Skin and subcutaneous diseases	754 (412-1,081)	936 (482-1,292)	1,348 (709-1,816)	1,531 (797-2,136)	1,829 (898-2,480)	2,509 (1,273-3,509)	3,150 (1,641-4,335)	3,873 (2,172-5,797)	4,381 (2,352-6,807)	3,921 (1,964-5,964)	2,758 (1,355-4,271)	1,457 (681-2,357)
Bacterial skin diseases	612 (309-854)	764 (378-1,030)	1,131 (574-1,480)	1,212 (605-1,654)	1,513 (710-1,973)	1,943 (942-2,610)	2,267 (1,132-3,104)	2,607 (1,417-3,855)	2,516 (1,318-3,740)	2,091 (1,051-3,105)	1,407 (697-2,174)	689 (333-1,071)
Cellulitis	155 (73-248)	187 (79-294)	302 (134-459)	328 (143-525)	373 (159-594)	485 (208-773)	508 (223-812)	586 (236-943)	648 (263-1,127)	517 (210-917)	375 (150-719)	197 (75-369)
Pyoderma	457 (221-654)	577 (276-791)	829 (399-1,121)	884 (433-1,272)	1,139 (520-1,489)	1,458 (661-1,989)	1,759 (848-2,381)	2,021 (1,093-3,000)	1,868 (972-2,722)	1,573 (780-2,400)	1,032 (503-1,506)	492 (234-758)
Decubitus ulcer	86 (47-142)	116 (61-188)	155 (84-257)	262 (140-435)	249 (127-408)	474 (241-715)	769 (405-1,194)	1,105 (564-1,772)	1,657 (833-2,536)	1,632 (786-2,495)	1,201 (560-1,829)	700 (314-1,091)
Other skin and subcutaneous diseases	56 (27-113)	55 (26-104)	63 (29-136)	57 (25-125)	67 (28-146)	92 (40-198)	114 (53-252)	161 (71-358)	207 (89-465)	198 (83-489)	150 (56-377)	68 (23-177)
Musculoskeletal disorders	2,371 (1,957-2,662)	2,440 (2,190-2,680)	2,638 (2,289-2,866)	2,925 (2,616-3,193)	3,898 (3,302-4,284)	5,574 (4,633-6,096)	6,619 (5,569-7,225)	7,045 (6,061-7,662)	6,735 (5,976-7,234)	4,935 (4,401-5,264)	2,897 (2,693-3,058)	1,277 (1,223-1,331)
Rheumatoid arthritis	359 (275-455)	596 (454-707)	884 (707-1,001)	1,279 (1,046-1,438)	2,060 (1,636-2,355)	3,289 (2,609-3,742)	3,982 (3,250-4,454)	4,152 (3,476-4,631)	3,541 (3,017-3,921)	2,164 (1,848-2,464)	956 (834-1,116)	287 (255-351)
Other musculoskeletal disorders	2,012 (1,638-2,278)	1,843 (1,616-2,045)	1,753 (1,522-1,945)	1,646 (1,442-1,867)	1,838 (1,565-2,232)	2,285 (1,876-2,905)	2,636 (2,169-3,317)	2,892 (2,450-3,440)	3,194 (2,697-3,689)	2,771 (2,397-3,057)	1,941 (1,694-2,086)	990 (893-1,037)
Other non-communicable diseases	8,848 (8,152-9,449)	8,398 (7,643-9,040)	9,817 (8,803-10,501)	10,212 (9,204-10,911)	11,503 (10,408-12,336)	14,824 (13,449-15,826)	15,615 (14,227-16,791)	17,815 (16,191-18,868)	18,918 (16,996-19,966)	16,245 (14,643-17,017)	11,366 (10,141-11,873)	5,832 (5,168-6,097)
Congenital birth defects	2,320 (2,115-2,543)	1,185 (1,016-1,356)	1,140 (958-1,295)	1,083 (892-1,225)	871 (725-1,000)	855 (744-991)	-	-	-	-	-	-
Neural tube defects	50 (43-58)	33 (28-39)	28 (24-33)	25 (21-28)	17 (15-21)	16 (14-19)	-	-	-	-	-	-
Congenital heart anomalies	1,557 (1,394-1,751)	614 (515-708)	486 (408-584)	429 (361-512)	354 (300-433)	401 (341-481)	-	-	-	-	-	-
Orofacial clefts	-	-	-	-	-	-	-	-	-	-	-	-
Down syndrome	277 (237-339)	248 (200-295)	360 (279-408)	362 (253-409)	234 (167-271)	117 (84-146)	-	-	-	-	-	-
Other chromosomal abnormalities	41 (23-56)	28 (16-39)	24 (14-36)	23 (13-35)	18 (10-28)	16 (10-26)	-	-	-	-	-	-
Congenital musculoskeletal and limb anomalies	40 (27-52)	30 (22-42)	26 (17-35)	26 (17-37)	23 (17-33)	21 (15-34)	-	-	-	-	-	-
Urogenital congenital anomalies	74 (53-92)	61 (48-79)	70 (53-83)	88 (69-105)	107 (85-132)	157 (128-185)	-	-	-	-	-	-
Digestive congenital anomalies	22 (16-29)	22 (16-28)	26 (19-32)	26 (19-32)	27 (20-33)	37 (27-47)	-	-	-	-	-	-
Other congenital birth defects	259 (211-328)	149 (123-183)	119 (96-143)	104 (84-126)	90 (74-106)	90 (74-112)	-	-	-	-	-	-
Urinary diseases and male infertility	2,415 (2,309-2,533)	2,774 (2,664-2,905)	3,650 (3,509-3,794)	4,358 (4,180-4,555)	5,191 (4,951-5,434)	7,283 (6,952-7,633)	8,213 (7,871-8,559)	9,926 (9,560-10,306)	10,478 (10,150-10,796)	9,344 (9,113-9,618)	6,709 (6,559-6,847)	3,657 (3,588-3,733)
Urinary tract infections	1,891 (1,705-2,060)	2,107 (1,935-2,323)	2,876 (2,666-3,149)	3,376 (3,113-3,727)	3,942 (3,627-4,441)	5,809 (5,385-6,440)	6,650 (6,223-7,295)	8,216 (7,786-8,916)	9,163 (8,804-9,673)	8,426 (8,166-8,814)	6,138 (5,971-6,341)	3,358 (3,273-3,461)
Urolithiasis	126 (94-168)	167 (131-211)	237 (186-294)	299 (231-381)	298 (236-376)	473 (375-585)	484 (390-618)	571 (483-727)	509 (426-668)	317 (267-434)	195 (169-272)	78 (67-111)

SR Table 5: Global deaths by cause and age group for females (with 95% confidence intervals), 2005

Cause	Early Neonatal	Late Neonatal	Post Neonatal	1 to 4	5 to 9	10 to 14	15 to 19
Other urinary diseases	188 (149-214)	117 (91-140)	771 (418-1,130)	1,353 (712-1,942)	400 (253-544)	309 (233-400)	273 (156-387)
Gynecological diseases	-	-	-	-	-	-	386 (340-560)
Uterine fibroids	-	-	-	-	-	-	47 (34-67)
Polycystic ovarian syndrome	-	-	-	-	-	-	1 (0-3)
Endometriosis	-	-	-	-	-	-	8 (4-13)
Genital prolapse	-	-	-	-	-	-	11 (6-18)
Other gynecological diseases	-	-	-	-	-	-	318 (267-468)
Hemoglobinopathies and hemolytic anemias	394 (316-536)	272 (223-381)	2,759 (1,905-4,374	8,528 (3,979-14,004	2,235 (1,735-3,037	1,778 (1,425-2,498	2,676 (2,125-3,496
Thalassemias	146 (111-192)	105 (81-143)	963 (714-1,429)	1,442 (1,001-1,923	750 (550-955)	450 (333-591)	428 (283-597)
Sickle cell disorders	230 (175-343)	162 (128-232)	1,751 (1,068-2,915	6,929 (2,785-11,934	1,347 (949-2,018)	1,088 (800-1,656)	1,750 (1,362-2,403
G6PD deficiency	4 (2-5)	2 (2-4)	28 (17-50)	117 (57-207)	94 (67-139)	146 (101-210)	270 (190-383)
Other hemoglobinopathies and hemolytic anemias	14 (11-20)	3 (3-5)	18 (13-28)	41 (25-63)	44 (35-56)	95 (75-123)	228 (162-336)
Endocrine, metabolic, blood, and immune disorders	742 (533-986)	625 (455-830)	3,674 (2,502-5,107	2,426 (1,522-3,481	872 (705-1,133)	732 (629-953)	899 (749-1,149)
Sudden infant death syndrome	-	2,009 (717-4,172)	18,364 (6,108-43,872	-	-	-	-
Injuries	5,978 (5,151-6,485	4,819 (4,210-5,267	47,959 (43,181-52,239	114,130 (96,244-126,688	55,666 (49,488-60,557	44,875 (40,893-47,659	88,119 (83,377-91,175
Transport injuries	464 (417-508)	561 (504-609)	7,523 (6,156-8,240	25,433 (18,466-28,902	17,764 (16,048-19,567	12,443 (11,426-13,447	24,872 (23,568-26,164
Road injuries	419 (381-463)	525 (470-570)	7,047 (5,693-7,763	23,933 (16,947-27,329	17,031 (15,362-18,774	11,593 (10,668-12,565	23,133 (21,972-24,349
Pedestrian road injuries	202 (162-243)	241 (196-300)	2,920 (2,443-3,531	13,611 (10,599-16,727	10,075 (8,952-11,374	5,848 (5,261-6,513	8,051 (7,313-9,043
Cyclist road injuries	-	-	-	502 (324-708)	511 (435-601)	634 (541-751)	1,019 (822-1,194)
Motorcyclist road injuries	48 (33-67)	34 (25-45)	816 (588-1,141)	1,338 (838-2,138	900 (700-1,107)	999 (788-1,233)	3,208 (2,696-3,800
Motor vehicle road injuries	162 (128-190)	244 (190-290)	3,213 (2,338-3,792	8,261 (4,905-10,494	5,368 (4,551-6,086	4,001 (3,505-4,424	10,619 (9,700-11,443
Other road injuries	8 (5-10)	6 (4-8)	98 (56-147)	220 (100-367)	176 (116-234)	112 (84-153)	236 (181-308)
Other transport injuries	44 (32-68)	35 (26-49)	476 (377-685)	1,500 (1,121-2,493	733 (609-855)	850 (731-970)	1,740 (1,526-2,024
Unintentional injuries	4,344 (3,733-4,768	3,855 (3,325-4,276	37,892 (33,546-41,758	81,809 (69,871-91,905	33,782 (28,867-37,365	23,851 (20,624-25,924	24,047 (20,879-25,895
Falls	751 (677-844)	314 (274-359)	4,359 (3,707-5,015	5,826 (4,645-7,263	2,481 (2,177-2,797	1,732 (1,545-1,903	2,379 (2,125-2,655
Drowning	207 (184-235)	140 (125-153)	4,336 (3,694-4,881	40,477 (33,058-45,910	14,639 (12,519-16,455	8,227 (6,969-8,999	5,952 (5,335-6,393
Fire, heat, and hot substances	156 (99-186)	246 (134-313)	3,189 (1,893-3,902	9,102 (4,899-11,837	2,411 (1,762-2,833	1,961 (1,371-2,296	4,180 (2,533-5,043
Poisonings	88 (61-97)	105 (70-120)	1,678 (1,097-2,006	3,925 (2,272-5,067	1,009 (720-1,154)	1,176 (871-1,353)	1,611 (1,158-1,744
Poisoning by carbon monoxide	21 (12-26)	26 (14-30)	338 (184-425)	731 (439-921)	402 (268-479)	427 (291-505)	822 (579-904)
Poisoning by other means	67 (47-75)	79 (54-93)	1,341 (883-1,678)	3,193 (1,807-4,335	608 (424-716)	749 (559-871)	789 (559-879)
Exposure to mechanical forces	359 (209-454)	419 (227-547)	2,367 (1,317-3,000	2,676 (1,808-3,226	1,893 (1,315-2,265	1,054 (789-1,205)	1,562 (1,089-1,823
Unintentional firearm injuries	24 (16-33)	18 (11-24)	164 (108-231)	206 (141-312)	199 (148-255)	158 (121-191)	258 (202-301)
Other exposure to mechanical forces	335 (190-430)	401 (213-529)	2,203 (1,186-2,817	2,470 (1,628-3,008	1,695 (1,133-2,053	896 (642-1,037	1,304 (874-1,548)
Adverse effects of medical treatment	673 (444-836)	485 (316-624)	3,795 (2,273-5,443	2,759 (1,297-4,884	962 (642-1,219)	759 (545-896)	1,075 (799-1,245)
Animal contact	98 (35-130)	99 (35-134)	1,635 (555-2,233)	3,986 (1,486-6,317	3,764 (1,374-5,271	2,912 (995-3,886)	1,940 (663-2,724)
Venomous animal contact	65 (17-101)	61 (17-96)	1,333 (427-1,931)	3,147 (1,118-4,716	3,377 (1,189-4,736	2,699 (875-3,659)	1,796 (582-2,492)
Non-venomous animal contact	33 (12-77)	38 (15-88)	302 (83-1,012)	839 (149-3,386)	386 (118-1,475)	213 (80-666)	144 (57-458)
Foreign body	1,696 (1,551-1,888	1,811 (1,646-2,032	13,913 (11,720-15,543	5,237 (3,603-6,196	1,191 (1,085-1,323	849 (794-913)	689 (659-729)
Pulmonary aspiration and foreign body in airway	1,658 (1,509-1,855	1,782 (1,616-2,007	13,541 (11,436-15,234	4,861 (3,363-5,831	1,046 (940-1,183)	741 (682-810)	505 (456-548)
Foreign body in other body part	37 (23-69)	30 (19-54)	372 (227-773)	377 (197-781)	145 (100-219)	108 (82-156)	185 (156-232)
Environmental heat and cold exposure	110 (41-140)	69 (24-93)	841 (290-1,151)	604 (215-971)	275 (129-359)	122 (77-143)	317 (185-376)
Exposure to forces of nature	27 (22-32)	100 (84-119)	814 (679-972)	4,130 (3,503-4,874	3,862 (3,257-4,577	3,712 (3,140-4,402	2,701 (2,289-3,171
Other unintentional injuries	181 (158-208)	66 (58-75)	964 (852-1,089)	3,087 (2,606-3,626	1,294 (1,139-1,473	1,346 (1,203-1,511	1,642 (1,494-1,800
Self-harm and interpersonal violence	1,171 (926-1,350)	403 (326-473)	2,544 (2,064-3,027	6,888 (5,579-7,855	4,119 (3,441-4,830	8,580 (7,860-9,334	39,199 (36,988-41,085
Self-harm	-	-	-	-	-	4,604 (4,131-5,076	30,692 (28,956-32,435
Self-harm by firearm	-	-	-	-	-	171 (107-242)	1,017 (563-1,515)
Self-harm by other specified means	-	-	-	-	-	4,433 (3,978-4,899	29,675 (27,808-31,546
Interpersonal violence	923 (677-1,102)	268 (191-337)	2,084 (1,605-2,577	4,397 (3,091-5,366	2,696 (2,015-3,406	2,744 (2,221-3,254	7,010 (5,454-7,985
Physical violence by firearm	65 (40-92)	25 (13-36)	264 (173-377)	528 (329-789)	413 (305-543)	573 (477-678)	1,994 (1,621-2,343
Physical violence by sharp object	56 (41-69)	35 (22-48)	187 (137-243)	538 (322-765)	279 (213-342)	438 (342-529)	1,460 (1,116-1,698
Physical violence by other means	802 (571-966)	207 (145-261)	1,633 (1,224-2,071	3,331 (2,186-4,099	2,005 (1,443-2,550	1,733 (1,318-2,156	3,556 (2,507-4,362
Conflict and terrorism	247 (196-322)	135 (106-179)	442 (350-578)	2,444 (1,936-3,217	1,383 (1,074-1,816	1,194 (942-1,584)	1,441 (1,146-1,883
Executions and police conflict	-	-	17 (8-46)	47 (44-53)	39 (36-45)	38 (36-43)	56 (53-64)

Cause	20 to 24	25 to 29	30 to 34	35 to 39	40 to 44	45 to 49	50 to 54	54 to 59
Other urinary diseases	354 (205-492)	328 (205-446)	386 (261-514)	378 (234-517)	398 (256-535)	500 (322-643)	537 (324-682)	683 (414-854)
Gynecological diseases	423 (366-586)	441 (381-579)	513 (447-635)	603 (528-667)	690 (607-759)	794 (699-892)	572 (512-639)	358 (327-390)
Uterine fibroids	58 (38-98)	90 (61-141)	116 (78-180)	147 (90-215)	206 (131-278)	271 (211-339)	219 (180-282)	90 (76-120)
Polycystic ovarian syndrome	2 (1-6)	2 (0-5)	1 (0-4)	1 (0-3)	1 (0-3)	1 (0-3)	1 (0-2)	-
Endometriosis	11 (6-17)	11 (5-17)	17 (8-27)	16 (7-26)	24 (9-39)	37 (13-61)	20 (9-36)	-
Genital prolapse	11 (6-16)	11 (4-17)	12 (5-18)	10 (4-17)	20 (7-34)	27 (7-47)	56 (17-89)	41 (15-72)
Other gynecological diseases	341 (270-525)	327 (253-491)	367 (290-482)	428 (339-549)	438 (340-536)	458 (356-554)	276 (197-344)	226 (184-265)
Hemoglobinopathies and hemolytic anemias	2,413 (1,878-3,228	1,865 (1,466-2,487	1,900 (1,480-2,487	1,430 (1,118-1,817	1,520 (1,209-1,901	1,275 (1,008-1,650	1,482 (1,141-1,886	1,502 (1,219-1,937
Thalassemias	298 (183-410)	178 (114-242)	130 (88-171)	67 (49-85)	42 (34-53)	25 (20-31)	21 (18-27)	10 (9-13)
Sickle cell disorders	1,548 (1,161-2,217	1,104 (804-1,612	916 (673-1,361)	521 (372-807)	458 (308-769)	334 (218-613)	275 (177-518)	187 (117-351)
G6PD deficiency	325 (230-446)	347 (251-461)	494 (335-624)	434 (316-533)	460 (335-567)	357 (266-453)	385 (279-499)	319 (225-464)
Other hemoglobinopathies and hemolytic anemias	241 (168-364)	236 (171-323)	360 (253-466)	408 (308-488)	559 (436-669)	558 (442-671)	801 (631-949)	987 (800-1,230
Endocrine, metabolic, blood, and immune disorders	1,126 (923-1,414)	1,368 (1,026-1,665	1,623 (1,218-1,945	1,796 (1,355-2,129	1,903 (1,453-2,298	2,370 (1,795-2,736	2,973 (2,285-3,400	2,911 (2,137-3,395
Sudden infant death syndrome	-	-	-	-	-	-	-	-
Injuries	91,409 (87,280-94,239	80,413 (76,403-82,978	73,585 (69,876-75,839	73,638 (69,840-76,015	68,831 (65,327-70,867	61,690 (58,821-63,288	66,280 (63,162-68,119	59,329 (56,514-60,970
Transport injuries	25,881 (24,578-27,133	22,596 (21,487-23,639	21,333 (20,407-22,091	22,745 (21,455-23,684	22,344 (21,511-23,051	18,807 (18,086-19,432	21,090 (20,244-21,747	18,171 (17,431-18,764
Road injuries	24,576 (23,354-25,755	21,391 (20,320-22,442	20,307 (19,414-21,069	21,738 (20,440-22,606	21,247 (20,481-21,941	17,752 (17,095-18,339	20,024 (19,180-20,678	17,437 (16,745-18,002
Pedestrian road injuries	8,022 (7,261-9,072	6,707 (6,014-7,582	7,515 (6,886-8,308	9,046 (8,361-9,891	9,100 (8,521-9,851	7,628 (7,110-8,232	9,547 (8,853-10,366	8,785 (8,192-9,522
Cyclist road injuries	720 (614-817)	611 (522-691)	847 (724-954)	1,068 (857-1,188	1,105 (898-1,223)	970 (805-1,070	1,231 (1,005-1,370	1,022 (850-1,133
Motorcyclist road injuries	3,643 (3,055-4,334	3,207 (2,656-3,855	3,115 (2,644-3,639	2,850 (2,451-3,303	2,780 (2,301-3,160	2,065 (1,773-2,414	2,216 (1,911-2,573	1,703 (1,459-1,993
Motor vehicle road injuries	11,954 (10,918-12,780	10,689 (9,668-11,564	8,627 (7,721-9,290	8,556 (7,595-9,266	8,076 (7,313-8,672	6,868 (6,213-7,321	6,830 (6,146-7,307	5,749 (5,150-6,153
Other road injuries	237 (177-306)	177 (132-223)	203 (155-254)	218 (166-274)	186 (149-230)	221 (178-276)	201 (156-253)	178 (137-225)
Other transport injuries	1,306 (1,167-1,450	1,205 (1,079-1,341	1,026 (926-1,124)	1,006 (919-1,113	1,097 (1,000-1,203	1,055 (964-1,145	1,065 (978-1,160)	734 (678-800)
Unintentional injuries	22,540 (19,395-24,227	20,670 (17,696-22,234	20,596 (17,854-22,015	20,789 (18,139-22,119	20,612 (18,034-21,913	20,642 (18,399-21,743	22,194 (19,876-23,297	22,843 (20,785-23,950
Falls	2,197 (2,031-2,369	1,955 (1,836-2,064	2,389 (2,265-2,508	3,030 (2,839-3,228	3,126 (2,973-3,276	3,853 (3,667-4,038	5,055 (4,823-5,302	6,796 (6,456-7,173
Drowning	3,971 (3,651-4,245	3,188 (2,860-3,390	3,137 (2,896-3,310	3,239 (2,787-3,413	2,928 (2,595-3,060	2,587 (2,422-2,705	2,984 (2,706-3,121	2,681 (2,428-2,802
Fire, heat, and hot substances	4,964 (3,044-5,916	4,200 (2,684-4,999	3,807 (2,648-4,360	3,035 (2,174-3,468	2,400 (1,857-2,649	2,517 (1,975-2,797	2,304 (1,869-2,510	2,090 (1,717-2,264
Poisonings	1,618 (1,205-1,739	1,419 (1,099-1,535	1,370 (1,019-1,486	1,542 (1,071-1,698	1,549 (1,151-1,666	1,285 (1,053-1,370	1,508 (1,143-1,620	1,331 (1,015-1,431
Poisoning by carbon monoxide	801 (574-876)	723 (541-800)	703 (523-770)	745 (540-817)	821 (617-885)	790 (656-840)	848 (661-908)	766 (602-823)
Poisoning by other means	818 (600-907)	696 (523-764)	667 (470-739)	797 (506-911)	729 (502-802)	495 (376-548)	660 (457-728)	564 (388-629)
Exposure to mechanical forces	1,373 (982-1,564)	1,520 (1,098-1,739	1,596 (1,140-1,804	1,644 (1,143-1,899	1,796 (1,294-2,054	1,540 (1,152-1,730	1,372 (1,013-1,568	1,535 (1,093-1,765
Unintentional firearm injuries	263 (208-305)	301 (223-349)	337 (259-387)	252 (194-286)	265 (208-308)	276 (225-321)	226 (186-267)	205 (170-251)
Other exposure to mechanical forces	1,110 (760-1,285)	1,220 (853-1,411	1,260 (854-1,448)	1,393 (932-1,632	1,531 (1,065-1,778	1,264 (913-1,445	1,146 (811-1,333)	1,330 (890-1,558
Adverse effects of medical treatment	1,928 (1,417-2,293	2,013 (1,426-2,366	1,657 (1,200-1,914	1,838 (1,318-2,111	2,024 (1,502-2,279	2,208 (1,697-2,488	1,801 (1,418-2,014	2,143 (1,735-2,405
Animal contact	1,624 (532-2,209)	1,493 (514-2,015	1,759 (611-2,308)	1,546 (515-2,034	1,962 (727-2,516)	1,680 (643-2,124	1,970 (770-2,528)	1,366 (555-1,723
Venomous animal contact	1,459 (442-2,003	1,345 (433-1,829	1,643 (532-2,185)	1,435 (449-1,910	1,846 (654-2,411)	1,507 (529-1,953	1,782 (657-2,347)	1,188 (437-1,547
Non-venomous animal contact	165 (68-521)	148 (61-414)	115 (50-284)	110 (48-279)	115 (54-255)	173 (86-347)	188 (96-382)	177 (83-352)
Foreign body	757 (725-804)	803 (770-850)	795 (762-841)	920 (886-970)	1,068 (1,037-1,110	1,181 (1,147-1,218	1,401 (1,362-1,449	1,439 (1,399-1,481
Pulmonary aspiration and foreign body in airway	536 (482-588)	587 (525-636)	627 (578-671)	726 (669-777)	874 (817-916)	1,041 (994-1,079	1,254 (1,198-1,302	1,304 (1,245-1,349
Foreign body in other body part	221 (189-279)	217 (186-276)	168 (145-217)	194 (166-252)	193 (168-249)	140 (119-182)	147 (128-193)	135 (117-186)
Environmental heat and cold exposure	323 (215-367)	383 (306-415)	590 (471-638)	767 (628-821)	1,159 (982-1,235)	1,507 (1,305-1,605	1,614 (1,460-1,696	1,481 (1,258-1,578
Exposure to forces of nature	2,292 (1,940-2,709	2,047 (1,739-2,398	1,793 (1,532-2,089	1,491 (1,273-1,740	1,391 (1,183-1,625	1,250 (1,065-1,454	1,143 (971-1,326)	1,053 (911-1,238
Other unintentional injuries	1,492 (1,359-1,631	1,650 (1,509-1,802	1,704 (1,566-1,855	1,736 (1,618-1,871	1,209 (1,142-1,279	1,035 (977-1,101)	1,044 (981-1,106)	927 (870-991)
Self-harm and interpersonal violence	42,988 (41,034-45,070	37,147 (35,262-38,945	31,656 (29,957-32,919	30,104 (28,449-31,211	25,875 (24,739-26,639	22,241 (21,419-22,850	22,996 (22,143-23,612	18,316 (17,616-18,802
Self-harm	32,345 (30,918-33,841	27,469 (26,347-28,786	22,585 (21,791-23,444	22,187 (21,364-23,017	18,954 (18,439-19,501	16,434 (16,008-16,868	18,085 (17,599-18,569	14,614 (14,184-15,044
Self-harm by firearm	1,222 (735-1,734)	954 (646-1,289)	825 (637-1,028)	779 (662-943)	827 (747-1,003)	761 (703-917)	635 (579-791)	512 (467-656)
Self-harm by other specified means	31,124 (29,630-32,664	26,515 (25,370-27,872	21,760 (20,967-22,686	21,408 (20,612-22,262	18,128 (17,629-18,695	15,673 (15,260-16,091	17,450 (16,973-17,962	14,102 (13,673-14,536
Interpersonal violence	9,422 (7,465-10,724	8,896 (7,101-9,971	8,372 (6,705-9,574	7,347 (5,946-8,281	6,481 (5,445-7,107	5,420 (4,679-5,914	4,569 (3,883-5,084	3,425 (2,901-3,710
Physical violence by firearm	2,563 (2,046-2,978	2,333 (1,831-2,698	2,043 (1,609-2,355	1,851 (1,477-2,125	1,540 (1,278-1,723	1,212 (1,024-1,347	960 (769-1,141)	697 (546-813)
Physical violence by sharp object	2,381 (1,877-2,775	2,447 (1,958-2,812	2,522 (2,025-2,952	2,269 (1,834-2,594	2,137 (1,731-2,428	1,706 (1,434-1,901	1,388 (1,158-1,583	973 (808-1,073
Physical violence by other means	4,478 (3,222-5,401	4,116 (3,084-4,925	3,807 (2,879-4,511	3,227 (2,457-3,709	2,804 (2,297-3,194	2,502 (2,088-2,792	2,221 (1,852-2,567	1,755 (1,466-1,939
Conflict and terrorism	1,167 (946-1,492)	735 (610-937)	651 (545-821)	521 (438-636)	394 (330-484)	347 (289-427)	309 (260-375)	250 (212-301)
Executions and police conflict	54 (52-60)	48 (46-52)	49 (45-57)	49 (42-64)	46 (37-61)	39 (31-61)	33 (28-46)	27 (23-37)

Cause	60 to 64	65 to 69	70 to 74	75 to 79	80 to 84	85 to 89	90 to 94	95 plus
Other urinary diseases	951 (574-1,191)	1,001 (576-1,272	1,079 (619-1,355)	1,139 (649-1,428	805 (488-997)	601 (386-753)	376 (238-464)	220 (153-268)
Gynecological diseases	291 (263-316)	400 (348-448)	270 (235-291)	220 (198-234)	366 (310-404)	252 (213-282)	118 (104-130)	48 (43-54)
Uterine fibroids	72 (59-91)	91 (72-119)	69 (58-90)	54 (44-66)	91 (70-123)	67 (49-92)	39 (29-51)	17 (13-22)
Polycystic ovarian syndrome	-	-	-	-	-	-	-	-
Endometriosis	-	-	-	-	-	-	-	-
Genital prolapse	47 (22-77)	91 (39-140)	75 (37-105)	58 (30-76)	87 (46-128)	55 (32-79)	23 (15-30)	10 (6-12)
Other gynecological diseases	172 (141-208)	219 (168-277)	125 (95-159)	108 (90-135)	188 (138-234)	130 (100-158)	55 (45-66)	21 (19-26)
Hemoglobinopathies and hemolytic anemias	1,605 (1,285-2,055	2,126 (1,665-2,716	2,641 (2,085-3,313	2,762 (2,233-3,492	3,012 (2,377-3,690	2,487 (1,885-2,940	1,774 (1,241-2,043	798 (548-912)
Thalassemias	5 (4-6)	3 (2-4)	2 (1-2)	1 (1-1)	0 (0-1)	0 (0-0)	0 (0-0)	0 (0-0)
Sickle cell disorders	77 (50-131)	38 (26-59)	15 (10-23)	5 (3-8)	2 (1-3)	0 (0-1)	0 (0-0)	0 (0-0)
G6PD deficiency	276 (184-435)	262 (171-412)	183 (119-294)	109 (71-173)	55 (35-89)	21 (14-32)	7 (4-10)	1 (1-2)
Other hemoglobinopathies and hemolytic anemias	1,248 (983-1,550)	1,823 (1,448-2,257	2,442 (1,935-3,052	2,646 (2,140-3,334	2,955 (2,336-3,609	2,465 (1,869-2,910	1,766 (1,235-2,031	796 (547-910)
Endocrine, metabolic, blood, and immune disorders	3,545 (2,698-4,113	4,161 (3,155-4,887	4,490 (3,386-5,286	4,908 (3,491-5,637	5,063 (3,455-5,868	4,161 (2,863-4,790	2,766 (1,819-3,132	1,330 (806-1,480)
Sudden infant death syndrome	-	-	-	-	-	-	-	-
Injuries	58,748 (55,016-60,622	68,179 (63,446-70,427	74,575 (69,655-77,167	79,898 (75,116-82,386	77,066 (72,493-79,444	57,705 (54,717-59,350	33,888 (32,582-34,700	13,068 (12,716-13,360
Transport injuries	16,818 (16,094-17,401	16,723 (16,044-17,286	16,594 (15,895-17,197	14,207 (13,738-14,689	10,232 (9,877-10,621	5,613 (5,409-5,841	2,390 (2,318-2,469	772 (745-801)
Road injuries	15,897 (15,174-16,458	15,850 (15,203-16,401	15,889 (15,216-16,482	13,532 (13,068-13,996	9,745 (9,416-10,110	5,359 (5,163-5,580	2,285 (2,216-2,363	734 (707-762)
Pedestrian road injuries	8,902 (8,366-9,520	9,146 (8,664-9,707	9,773 (9,203-10,386	8,653 (8,240-9,152	6,328 (6,044-6,711	3,293 (3,131-3,503	1,245 (1,189-1,315	362 (345-383)
Cyclist road injuries	780 (651-862)	836 (715-927)	781 (668-875)	514 (453-574)	248 (220-274)	123 (105-144)	48 (40-57)	0 (0-0)
Motorcyclist road injuries	1,222 (1,028-1,489	1,074 (922-1,251)	846 (699-983)	486 (390-561)	263 (206-302)	137 (104-163)	48 (39-56)	17 (14-19)
Motor vehicle road injuries	4,934 (4,439-5,313	4,735 (4,286-5,051	4,438 (4,015-4,764	3,753 (3,453-4,039	2,832 (2,609-3,002	1,759 (1,641-1,862	922 (875-969)	344 (327-363)
Other road injuries	58 (45-70)	58 (46-72)	51 (41-64)	126 (99-159)	74 (61-94)	47 (39-61)	23 (19-28)	9 (8-11)
Other transport injuries	922 (843-1,005)	873 (797-948)	705 (653-758)	676 (625-722)	487 (448-525)	254 (234-275)	105 (97-113)	39 (37-41)
Unintentional injuries	26,965 (24,113-28,444	35,362 (31,646-37,163	42,246 (38,211-44,279	51,429 (47,421-53,623	56,332 (52,136-58,447	46,658 (43,848-48,137	29,443 (28,203-30,245	11,770 (11,436-12,061
Falls	10,628 (9,933-11,245	15,969 (14,629-17,020	22,682 (20,688-24,164	30,834 (28,589-32,491	36,788 (34,056-38,554	32,748 (30,784-34,204	21,337 (20,440-22,050	8,311 (8,031-8,588
Drowning	2,728 (2,365-2,876	3,326 (2,954-3,502	3,463 (3,222-3,627	3,873 (3,355-4,072	3,431 (2,834-3,620	1,895 (1,654-1,997	694 (617-728)	161 (146-167)
Fire, heat, and hot substances	2,060 (1,686-2,237	2,903 (2,368-3,137	2,986 (2,485-3,232	3,566 (3,029-3,810	3,673 (3,187-3,895	2,702 (2,380-2,842	1,608 (1,484-1,673	709 (673-733)
Poisonings	1,024 (793-1,097)	1,329 (1,027-1,416	1,341 (1,041-1,427	1,293 (999-1,386)	1,095 (821-1,177)	611 (465-657)	239 (195-253)	63 (55-66)
Poisoning by carbon monoxide	600 (468-641)	828 (651-883)	808 (614-869)	871 (668-940)	685 (507-742)	377 (277-408)	142 (111-152)	30 (25-32)
Poisoning by other means	425 (304-470)	501 (356-549)	532 (393-577)	422 (309-468)	410 (295-453)	234 (173-260)	97 (79-106)	32 (29-34)
Exposure to mechanical forces	1,428 (1,018-1,638	1,514 (1,114-1,696	1,485 (1,106-1,676	1,443 (1,098-1,621	1,510 (1,207-1,658	1,108 (922-1,199)	688 (615-733)	293 (274-310)
Unintentional firearm injuries	180 (145-237)	218 (176-280)	222 (182-282)	326 (263-403)	335 (268-406)	289 (239-350)	184 (157-219)	74 (65-88)
Other exposure to mechanical forces	1,248 (855-1,459)	1,296 (929-1,476	1,263 (909-1,452)	1,117 (799-1,285)	1,175 (898-1,313)	819 (654-903)	504 (441-540)	219 (201-231)
Adverse effects of medical treatment	3,193 (2,453-3,687	3,704 (2,878-4,240	3,948 (3,153-4,514	4,086 (3,455-4,522	3,927 (3,412-4,367	2,997 (2,642-3,402	1,895 (1,733-2,110	763 (718-831)
Animal contact	1,679 (619-2,172)	1,975 (660-2,575	1,829 (654-2,357)	1,403 (486-1,826	802 (347-1,008)	418 (212-503)	160 (97-185)	44 (32-50)
Venomous animal contact	1,570 (547-2,056)	1,846 (570-2,443	1,686 (570-2,223)	1,281 (403-1,703)	689 (274-894)	333 (150-415)	116 (61-141)	30 (20-35)
Non-venomous animal contact	109 (49-236)	128 (63-273)	143 (72-327)	122 (65-246)	112 (64-205)	86 (50-135)	44 (29-64)	14 (10-19)
Foreign body	1,260 (1,218-1,306	1,484 (1,432-1,529	1,826 (1,761-1,884	2,261 (2,183-2,333	2,737 (2,655-2,821	2,563 (2,488-2,641	1,886 (1,821-1,952	1,078 (1,041-1,113
Pulmonary aspiration and foreign body in airway	1,150 (1,097-1,200	1,353 (1,291-1,404	1,665 (1,583-1,730	2,074 (1,985-2,150	2,534 (2,436-2,629	2,391 (2,306-2,478	1,783 (1,720-1,852	1,035 (999-1,072)
Foreign body in other body part	110 (94-153)	131 (108-172)	161 (134-214)	187 (150-238)	203 (163-264)	172 (131-229)	104 (77-130)	42 (33-52)
Environmental heat and cold exposure	1,263 (918-1,438)	1,350 (1,046-1,488	1,360 (900-1,574)	1,410 (965-1,595)	1,328 (934-1,482)	938 (689-1,032	605 (497-648)	234 (208-248)
Exposure to forces of nature	1,002 (856-1,166)	895 (766-1,040)	805 (691-939)	675 (577-784)	511 (432-606)	301 (254-364)	138 (111-179)	40 (31-54)
Other unintentional injuries	700 (654-766)	913 (851-994)	522 (492-561)	585 (548-638)	531 (498-580)	376 (353-410)	193 (183-206)	75 (72-79)
Self-harm and interpersonal violence	14,965 (14,413-15,440	16,093 (15,483-16,612	15,736 (15,169-16,227	14,262 (13,771-14,712	10,502 (10,194-10,848	5,435 (5,276-5,621	2,054 (2,006-2,102	525 (515-537)
Self-harm	12,142 (11,755-12,509	13,170 (12,711-13,597	13,168 (12,739-13,574	11,825 (11,442-12,209	9,675 (9,380-10,006	5,049 (4,889-5,233	1,925 (1,878-1,972	472 (461-482)
Self-harm by firearm	378 (334-526)	330 (280-506)	295 (248-471)	290 (246-464)	249 (215-354)	156 (141-187)	85 (79-91)	36 (33-38)
Self-harm by other specified means	11,765 (11,380-12,124	12,839 (12,403-13,254	12,873 (12,436-13,295	11,535 (11,153-11,904	9,426 (9,138-9,746	4,894 (4,735-5,081	1,840 (1,793-1,888	436 (426-446)
Interpersonal violence	2,586 (2,156-2,878	2,685 (2,293-2,999	2,391 (2,014-2,702	2,299 (1,986-2,529	728 (673-781)	333 (309-358)	117 (109-124)	33 (30-35)
Physical violence by firearm	561 (433-664)	452 (355-555)	421 (322-522)	358 (272-433)	71 (62-82)	37 (32-41)	11 (10-12)	5 (4-5)
Physical violence by sharp object	712 (582-811)	671 (588-755)	671 (546-761)	661 (555-748)	193 (173-213)	83 (74-94)	27 (24-30)	7 (6-8)
Physical violence by other means	1,313 (1,080-1,511	1,562 (1,300-1,741	1,300 (1,069-1,485	1,280 (1,104-1,415	464 (423-502)	213 (198-230)	78 (73-82)	21 (20-22)
Conflict and terrorism	216 (184-262)	220 (190-264)	162 (142-192)	126 (112-145)	90 (82-102)	48 (44-54)	10 (9-11)	21 (19-23)
Executions and police conflict	20 (18-26)	19 (15-25)	15 (13-19)	12 (10-16)	9 (8-11)	4 (4-5)	2 (2-3)	1 (1-1)

SR Table 5: Global deaths by cause and age group for females (with 95% confidence intervals), 2010

Cause	Early Neonatal	Late Neonatal	Post Neonatal	1 to 4	5 to 9	10 to 14	15 to 19	20 to 24	25 to 29	30 to 34	35 to 39	40 to 44	45 to 49	50 to 54	54 to 59	60 to 64	65 to 69	70 to 74	75 to 79	80 to 84	85 to 89	90 to 94	95 plus
All causes	975,710 (947,075-1,006,85	310,266 (296,091-324,818	1,036,051 (1,006,507-1,067,16	981,244 (944,617-1,023,92	246,933 (243,572-250,646	173,802 (171,527-176,068	255,952 (251,233-260,882	340,034 (332,078-348,818	405,919 (392,188-420,264	467,007 (454,465-480,209	528,077 (514,015-542,357	607,638 (597,552-617,442	700,562 (690,163-711,042	836,710 (826,350-847,027	1,069,802 (1,057,511-1,081,95	1,346,953 (1,333,325-1,360,17	1,568,581 (1,553,155-1,583,67	2,148,505 (2,129,833-2,166,93	2,559,218 (2,538,166-2,580,31	2,932,277 (2,911,554-2,951,67	2,476,571 (2,463,115-2,489,41	1,369,395 (1,363,614-1,375,17	651,223 (649,700-652,71
Communicable, maternal, neonatal, and nutritional disease	879,698 (851,749-909,044	268,091 (254,487-281,392	829,532 (796,052-861,318	764,632 (727,187-803,886	144,552 (136,957-154,101	81,466 (76,513-88,634	104,020 (98,452-111,661	152,470 (145,213-161,396	213,757 (200,561-228,683	249,062 (236,890-262,339	245,581 (232,725-258,985	211,347 (203,572-220,110	168,691 (160,897-179,784	143,171 (136,038-153,897	135,073 (125,860-151,754	151,131 (136,995-176,920	170,766 (151,270-203,955	222,308 (192,370-269,286	233,028 (200,084-286,482	244,176 (209,854-305,600	194,066 (170,977-230,823	103,829 (93,451-118,921	51,281 (48,523-55,381
HIV/AIDS and sexually transmitted infections	-	6,814 (2,604-12,785	64,993 (56,266-76,516	32,509 (30,032-35,165	14,602 (13,809-15,534	10,458 (9,860-11,059	18,739 (16,799-20,928	35,600 (30,694-41,903	102,146 (89,625-116,595	143,041 (132,134-154,821	141,907 (130,577-154,156	120,498 (115,030-125,763	86,302 (80,117-92,952	53,708 (50,532-56,822	29,321 (27,063-31,559	16,756 (15,204-18,331	10,603 (9,536-11,652	6,350 (5,682-7,088	3,504 (3,085-3,956	764 (687-854)	382 (343-424)	184 (168-201)	63 (59-66)
HIV/AIDS	-	-	52,266 (49,165-55,503	31,226 (29,062-33,636	13,947 (13,255-14,600	10,400 (9,800-11,004	18,515 (16,555-20,721	35,201 (30,291-41,499	101,596 (89,058-116,046	142,510 (131,606-154,300	141,391 (130,016-153,597	119,906 (114,462-125,264	85,762 (79,615-92,428	53,333 (50,151-56,442	28,871 (26,614-31,096	16,437 (14,890-17,986	10,269 (9,198-11,302	6,049 (5,373-6,789	3,223 (2,814-3,676	509 (432-596)	190 (157-228)	81 (67-95)	15 (13-17)
HIV/AIDS - Drug-susceptible Tuberculosis	-	-	15,993 (11,725-19,149	9,755 (7,486-11,464	4,212 (2,941-5,152	3,015 (2,128-3,741	5,241 (3,704-6,632	9,046 (6,365-11,575	19,634 (12,909-27,113	23,104 (14,953-33,403	23,330 (15,165-33,275	20,761 (13,682-29,206	17,632 (11,742-24,097	13,888 (9,508-18,156	8,578 (6,376-10,220	5,094 (3,956-6,009	3,284 (2,644-3,860	2,004 (1,618-2,379	1,063 (854-1,284	162 (128-199)	59 (45-74)	25 (19-31)	4 (3-5)
HIV/AIDS - Multidrug-resistant Tuberculosis without extensive drug resistance	-	-	1,672 (1,039-2,378)	994 (633-1,365)	413 (246-587)	296 (179-424)	520 (318-769)	991 (584-1,490)	2,124 (1,168-3,395)	2,483 (1,335-4,089)	2,461 (1,337-4,068)	2,173 (1,213-3,412)	1,821 (1,042-2,837)	1,443 (840-2,231)	906 (559-1,321)	529 (339-767)	332 (215-481)	203 (130-296)	108 (70-161)	17 (11-27)	6 (4-10)	3 (2-4)	0 (0-1)
HIV/AIDS - Extensively drug-resistant Tuberculosis	-	-	31 (19-43)	18 (12-26)	8 (5-12)	6 (4-8)	10 (6-15)	27 (16-39)	66 (39-101)	84 (49-130)	81 (47-123)	70 (41-105)	55 (33-82)	40 (24-58)	25 (15-35)	14 (9-20)	8 (5-11)	4 (3-6)	2 (2-3)	0 (0-1)	0 (0-0)	0 (0-0)	0 (0-0)
HIV/AIDS resulting in other diseases	-	-	34,571 (30,065-39,904	20,460 (17,837-23,722	9,313 (8,123-10,810	7,083 (6,109-8,208	12,743 (10,583-15,186	25,138 (20,043-31,950	79,771 (65,083-94,209	116,839 (101,712-130,962	115,519 (100,019-130,520	96,902 (86,695-106,303	66,254 (57,297-74,975	37,962 (32,628-43,623	19,362 (17,001-22,524	10,801 (9,418-12,451	6,645 (5,810-7,684	3,839 (3,276-4,469	2,050 (1,717-2,418	329 (278-392)	125 (102-155)	53 (44-64)	10 (9-12)
Sexually transmitted infections excluding HIV	23,696 (9,034-44,545	6,814 (2,604-12,785	12,726 (4,811-24,142	1,283 (480-2,510	655 (247-1,270)	58 (43-71)	224 (185-255)	398 (307-452)	550 (397-631)	531 (396-598)	516 (392-574)	593 (451-662)	541 (423-605)	376 (298-409)	450 (336-495)	319 (251-355)	334 (251-371)	300 (242-331)	281 (243-310)	255 (222-280)	191 (166-206)	103 (93-111)	48 (44-50)
Syphilis	23,696 (9,034-44,545	6,814 (2,604-12,785	12,726 (4,811-24,142	1,283 (480-2,510	655 (247-1,270)	21 (16-26)	22 (18-25)	37 (28-42)	47 (34-54)	44 (33-50)	37 (28-41)	40 (30-44)	35 (28-39)	54 (43-58)	81 (61-89)	57 (45-63)	69 (52-76)	74 (60-82)	61 (52-67)	54 (47-59)	35 (30-38)	18 (16-19)	10 (10-11)
Chlamydial infection	-	-	-	-	-	10 (8-12)	35 (29-39)	101 (78-115)	101 (73-116)	61 (45-68)	84 (64-94)	113 (86-126)	95 (74-106)	61 (48-66)	73 (55-81)	53 (42-59)	34 (26-38)	41 (33-45)	39 (34-43)	48 (42-53)	37 (33-40)	20 (18-21)	9 (8-9)
Gonococcal infection	-	-	-	-	-	17 (13-21)	87 (72-99)	155 (119-175)	269 (194-309)	266 (198-300)	237 (180-264)	285 (217-318)	261 (204-292)	175 (139-190)	194 (145-214)	130 (102-144)	177 (133-196)	137 (110-151)	143 (124-158)	120 (104-132)	93 (81-100)	51 (46-55)	22 (21-23)
Other sexually transmitted infections	-	-	-	-	-	9 (7-11)	81 (67-92)	106 (81-120)	132 (95-152)	160 (120-180)	157 (120-175)	155 (118-173)	150 (117-168)	86 (68-94)	101 (76-111)	80 (63-89)	55 (41-61)	48 (39-53)	39 (34-43)	33 (29-37)	26 (23-28)	14 (13-15)	7 (6-7)
Respiratory infections and tuberculosis	-	62,113 (56,391-67,744	286,242 (263,253-307,844	173,178 (150,889-195,125	24,605 (20,905-27,729	14,579 (12,943-16,313	19,370 (18,102-21,010	27,869 (26,156-29,954	31,438 (29,482-33,422	32,738 (30,983-34,778	36,582 (34,495-38,749	36,777 (34,833-38,870	40,183 (37,868-42,403	51,250 (48,350-54,155	60,655 (57,048-64,407	74,149 (69,050-78,417	86,579 (79,335-91,626	113,517 (104,254-120,907	123,040 (114,275-131,000	133,373 (125,523-146,107	117,932 (112,985-127,270	69,491 (67,073-74,681	38,150 (37,286-39,756
Tuberculosis	-	-	20,641 (18,771-22,482	21,003 (18,043-24,273	5,667 (4,877-6,567	5,600 (4,934-6,345	12,144 (11,161-13,348	19,574 (18,163-21,163	22,573 (21,109-24,141	23,969 (22,527-25,586	26,340 (24,654-28,138	25,364 (23,816-26,994	26,884 (25,230-28,690	31,399 (29,401-33,571	34,555 (32,040-37,446	36,474 (33,887-39,325	36,810 (34,274-39,516	42,148 (38,937-45,915	36,626 (33,920-40,074	28,286 (25,902-31,268	14,934 (13,757-16,612	4,755 (4,378-5,260	1,317 (1,218-1,443
Drug-susceptible tuberculosis	-	-	18,577 (16,644-20,273	19,042 (16,120-22,056	5,070 (4,307-5,936	4,993 (4,331-5,686	10,826 (9,771-11,932	17,422 (15,910-19,018	20,103 (18,379-21,645	21,286 (19,528-23,004	23,435 (21,636-25,334	22,521 (20,758-24,359	23,956 (22,048-25,854	28,063 (25,761-30,331	30,801 (28,144-33,596	32,607 (29,709-35,414	32,930 (30,098-35,768	37,659 (34,197-41,309	32,745 (29,849-36,047	25,267 (22,879-28,238	13,424 (12,201-14,982	4,299 (3,941-4,766	1,202 (1,107-1,315
Multidrug-resistant tuberculosis without extensive drug resistance	-	-	1,963 (1,430-2,670	1,895 (1,363-2,585	569 (377-827)	577 (382-835)	1,247 (825-1,805)	2,009 (1,379-2,880	2,289 (1,575-3,279	2,480 (1,660-3,589	2,680 (1,779-3,825	2,616 (1,738-3,806	2,694 (1,850-3,834	3,088 (2,132-4,423	3,497 (2,345-5,042	3,621 (2,337-5,333	3,641 (2,388-5,358	4,203 (2,740-6,158	3,624 (2,370-5,289	2,804 (1,877-4,102	1,396 (983-1,935	420 (308-561)	105 (81-135)
Extensively drug-resistant tuberculosis	-	-	101 (76-137)	66 (48-91)	28 (19-40)	30 (21-43)	71 (51-101)	143 (109-187)	182 (142-234)	203 (156-265)	225 (174-290)	228 (176-294)	234 (183-302)	249 (194-321)	257 (197-344)	246 (180-340)	239 (174-329)	286 (209-386)	258 (189-359)	216 (162-288)	115 (89-151)	36 (28-47)	10 (8-12)
Lower respiratory infections	72,944 (64,979-82,109	62,046 (56,315-67,647	264,398 (243,798-284,721	150,816 (131,169-170,569	18,685 (15,657-21,252	8,858 (7,644-9,980	7,161 (6,512-8,006	8,235 (7,513-9,216	8,791 (7,912-9,609	8,708 (7,769-9,519	10,176 (9,087-11,122	11,340 (10,312-12,334	13,226 (11,871-14,328	19,771 (17,763-21,054	25,983 (23,413-27,703	37,531 (32,882-39,795	49,604 (42,753-52,796	71,123 (63,623-76,041	86,104 (78,699-92,281	104,620 (98,596-115,710	102,523 (97,735-111,145	64,464 (62,187-69,422	36,689 (35,891-38,174
Upper respiratory infections	24 (12-44)	56 (28-107)	1,109 (373-2,398)	1,260 (285-2,523)	196 (55-447)	73 (25-170)	46 (19-93)	50 (25-89)	61 (32-100)	51 (27-82)	52 (26-90)	56 (32-92)	65 (39-107)	67 (40-115)	90 (58-152)	112 (67-194)	119 (74-201)	208 (139-329)	279 (192-454)	441 (328-787)	456 (356-821)	264 (214-456)	140 (114-274)
Otitis media	17 (2-140)	11 (5-27)	94 (44-185)	99 (42-215)	57 (28-120)	48 (26-90)	19 (12-31)	10 (7-18)	13 (7-26)	11 (6-21)	15 (8-30)	16 (9-33)	9 (6-16)	13 (8-21)	27 (17-48)	33 (20-59)	46 (31-77)	38 (26-61)	30 (22-45)	26 (20-37)	18 (14-24)	8 (7-10)	4 (4-5)
Enteric infections	11,594 (9,959-13,625	22,026 (19,265-25,197	193,299 (171,844-216,967	188,819 (158,311-224,877	45,385 (33,669-62,976	25,643 (18,831-36,076	19,600 (13,467-29,635	16,989 (10,558-26,743	13,657 (8,475-21,717	12,601 (7,719-20,875	11,786 (6,885-20,341	11,407 (6,238-19,938	12,404 (6,989-23,221	15,476 (8,134-28,593	21,617 (11,276-40,951	35,084 (18,584-65,394	46,749 (23,504-85,674	71,954 (35,979-125,395	81,608 (43,152-141,835	89,180 (48,606-152,508	60,808 (33,889-99,381	26,644 (15,038-42,560	8,694 (5,508-13,112
Diarrheal diseases	11,594 (9,959-13,625	21,745 (19,019-24,932	188,128 (167,038-210,293	163,514 (136,081-197,395	20,704 (11,288-36,740	10,311 (5,440-19,316	9,975 (4,910-18,583	10,606 (5,139-19,783	9,218 (4,333-17,584	9,378 (4,351-17,707	9,224 (4,352-17,543	9,347 (4,386-17,986	10,680 (5,216-21,240	13,980 (6,929-27,198	20,214 (9,615-39,447	33,846 (17,321-64,015	45,660 (22,371-84,935	71,068 (35,314-124,781	80,973 (42,555-140,978	88,748 (48,230-152,136	60,563 (33,660-99,140	26,556 (14,921-42,468	8,663 (5,490-13,077
Typhoid and paratyphoid	-	0 (0-0)	152 (73-300)	12,550 (5,856-23,551	20,801 (11,919-33,612	13,619 (7,692-21,825	8,494 (4,224-15,085	5,390 (2,686-9,482	3,545 (1,842-6,087	2,449 (1,296-4,140	1,819 (941-3,038	1,360 (700-2,326	1,045 (554-1,785	776 (402-1,345	644 (306-1,210	531 (257-959)	448 (251-733)	353 (208-566)	229 (136-365)	123 (72-199)	58 (35-95)	20 (12-34)	6 (4-11)
Typhoid fever	-	-	138 (66-268)	11,164 (5,084-21,118	18,375 (10,459-30,039	11,941 (6,707-19,156	7,399 (3,657-13,235	4,673 (2,283-8,331	3,052 (1,556-5,293	2,089 (1,078-3,617	1,532 (785-2,657	1,134 (573-1,967	861 (452-1,485	639 (326-1,111	532 (252-1,011	440 (208-806)	374 (204-623)	296 (173-480)	193 (113-313)	105 (61-173)	50 (29-84)	17 (10-31)	5 (3-11)
Paratyphoid fever	-	-	14 (6-30)	1,386 (525-2,993	2,426 (1,050-4,894	1,678 (736-3,338	1,095 (420-2,361)	717 (290-1,603	493 (206-1,056	361 (151-758)	287 (123-573)	226 (100-453)	183 (76-373)	137 (54-288)	112 (43-247)	91 (36-191)	74 (33-147)	57 (26-111)	35 (17-65)	18 (8-33)	8 (4-14)	3 (1-5)	1 (0-1)
Invasive Non-typhoidal Salmonella (iNTS)	-	281 (161-501)	4,471 (2,531-7,700	12,450 (6,383-22,071	3,835 (1,947-6,785	1,686 (761-3,153	1,107 (454-2,109)	968 (397-1,846	880 (338-1,854	755 (326-1,454)	726 (320-1,416	686 (319-1,330	666 (316-1,282	709 (326-1,405	749 (346-1,349	696 (306-1,225	627 (291-1,095	522 (243-930)	397 (184-728)	292 (120-557)	166 (64-313)	63 (16-138)	21 (1-64)
Other intestinal infectious diseases	-	-	548 (239-1,382)	305 (112-855)	46 (11-132)	27 (5-80)	24 (5-73)	25 (5-73)	14 (2-39)	19 (3-48)	18 (3-48)	14 (2-38)	13 (2-36)	11 (1-29)	10 (1-27)	11 (1-31)	14 (2-38)	11 (1-29)	10 (1-24)	17 (1-46)	21 (2-54)	5 (0-16)	3 (0-12)
Neglected tropical diseases and malaria	214 (101-712)	9,528 (5,979-14,212	78,728 (54,082-108,785	166,450 (119,330-222,530	25,270 (18,296-33,432	12,748 (9,289-16,917	8,578 (6,221-11,541	9,283 (6,730-12,823	7,650 (5,343-10,646	7,500 (5,433-10,457	7,807 (5,660-10,933	7,849 (5,701-10,887	8,213 (5,884-11,544	9,708 (6,798-13,805	10,575 (7,517-14,859	10,956 (7,708-15,440	10,709 (7,464-15,380	11,834 (8,211-17,123	6,291 (4,737-8,745	3,640 (2,928-5,007	1,597 (1,282-2,352	642 (508-931)	254 (178-332)
Malaria	-	9,373 (5,799-14,088	75,776 (51,283-106,241	158,178 (109,092-216,068	21,138 (14,785-28,615	10,520 (7,327-14,259	6,883 (4,714-9,794	7,649 (5,185-11,114	6,131 (4,061-9,175	5,886 (3,912-8,753	6,222 (4,177-9,300	6,124 (3,962-9,225	6,569 (4,292-9,862	7,833 (5,035-11,843	8,603 (5,560-12,888	8,966 (5,774-13,591	8,878 (5,619-13,524	9,570 (6,050-14,687	4,384 (2,931-6,595	1,893 (1,321-2,780	536 (382-707)	224 (131-286)	106 (41-142)
Chagas disease	-	-	1 (1-1)	1 (0-1)	1 (1-1)	2 (1-2)	3 (3-4)	7 (6-7)	13 (12-15)	26 (24-29)	48 (43-53)	78 (72-85)	120 (112-128)	186 (173-199)	271 (253-290)	312 (292-331)	376 (351-402)	434 (411-458)	447 (418-475)	405 (380-433)	270 (251-291)	140 (127-156)	48 (42-59)
Leishmaniasis	-	-	374 (0-1,479)	1,566 (1-7,769)	751 (0-5,342)	400 (0-2,689)	294 (0-1,751)	268 (0-1,442)	289 (0-1,398)	276 (0-1,265)	251 (0-1,061)	205 (0-818)	168 (0-581)	147 (3-478)	139 (7-431)	126 (4-378)	98 (5-280)	85 (8-232)	59 (7-157)	39 (7-95)	17 (3-42)	3 (0-10)	1 (0-2)
Visceral leishmaniasis	-	-	374 (0-1,479)	1,566 (1-7,769)	751 (0-5,342)	400 (0-2,689)	294 (0-1,751)	268 (0-1,442)	289 (0-1,398)	276 (0-1,265)	251 (0-1,061)	205 (0-818)	168 (0-581)	147 (3-478)	139 (7-431)	126 (4-378)	98 (5-280)	85 (8-232)	59 (7-157)	39 (7-95)	17 (3-42)	3 (0-10)	1 (0-2)
African trypanosomiasis	-	-	-	119 (21-414)	205 (46-693)	242 (59-728)	245 (56-820)	238 (54-743)	215 (45-660)	199 (42-666)	167 (36-577)	143 (31-485)	134 (30-439)	122 (25-477)	115 (18-457)	69 (14-262)	56 (9-235)	37 (7-157)	31 (5-124)	14 (3-55)	6 (1-20)	1 (0-5)	0 (0-1)
Schistosomiasis	-	-	0 (0-0)	301 (198-446)	115 (80-157)	144 (108-193)	136 (105-180)	141 (110-179)	154 (118-197)	162 (126-209)	196 (152-249)	223 (176-280)	262 (207-330)	327 (255-416)	375 (293-472)	374 (300-473)	293 (231-376)	402 (316-497)	357 (282-450)	237 (191-296)	120 (93-150)	37 (26-48)	8 (5-12)
Cysticercosis	0 (0-0)	0 (0-0)	0 (0-0)	76 (30-147)	30 (6-68)	20 (3-53)	17 (2-44)	22 (3-59)	29 (5-73)	30 (6-70)	28 (5-71)	27 (4-70)	29 (5-74)	26 (3-67)	23 (3-58)	18 (2-52)	16 (1-48)	4 (0-13)	10 (0-32)	7 (0-23)	4 (0-13)	1 (0-5)	0 (0-2)
Cystic echinococcosis	-	-	-	119 (58-195)	52 (15-106)	32 (4-74)	16 (0-50)	19 (0-57)	29 (2-74)	30 (2-77)	38 (4-88)	36 (4-92)	36 (4-91)	41 (4-97)	45 (5-112)	46 (5-114)	45 (5-117)	48 (5-121)	39 (3-99)	33 (2-96)	23 (1-68)	9 (0-29)	4 (0-13)
Dengue	-	-	1,441 (264-2,310)	1,955 (406-2,800)	1,388 (224-1,928)	683 (143-936)	471 (95-656)	491 (104-660)	411 (99-561)	438 (87-602)	380 (89-497)	456 (93-599)	403 (87-523)	425 (84-596)	522 (89-720)	561 (92-763)	444 (99-591)	864 (285-1,552	597 (215-1,339	806 (334-1,957	486 (205-1,119	178 (74-427)	60 (27-156)
Yellow fever	-	1 (0-3)	17 (3-54)	166 (33-498)	254 (47-791)	225 (42-663)	188 (35-576)	155 (30-470)	125 (23-378)	95 (17-289)	79 (16-249)	66 (13-201)	58 (12-178)	53 (10-165)	43 (8-139)	30 (6-91)	23 (4-67)	14 (2-43)	8 (2-25)	4 (1-12)	1 (0-5)	0 (0-1)	0 (0-0)
Rabies	-	-	118 (67-196)	707 (355-1,191	837 (381-1,466)	315 (144-512)	200 (102-338)	182 (85-310)	145 (74-242)	256 (122-389)	290 (155-436)	374 (183-547)	328 (178-471)	430 (251-625)	324 (175-476)	307 (144-437)	318 (179-498)	199 (126-335)	176 (117-305)	61 (39-96)	48 (28-79)	12 (7-18)	6 (4-9)
Intestinal nematode infections	-	-	154 (110-210)	1,756 (1,211-2,523	253 (189-334)	49 (36-64)	14 (9-20)	11 (7-15)	8 (5-11)	11 (7-15)	11 (7-15)	8 (5-11)	8 (6-12)	9 (6-13)	8 (6-12)	17 (12-24)	9 (6-12)	16 (11-21)	14 (10-19)	10 (7-14)	5 (3-7)	3 (2-5)	1 (1-2)
Ascariasis	-	-	154 (110-210)	1,756 (1,211-2,523	253 (189-334)	49 (36-64)	14 (9-20)	11 (7-15)	8 (5-11)	11 (7-15)	11 (7-15)	8 (5-11)	8 (6-12)	9 (6-13)	8 (6-12)	17 (12-24)	9 (6-12)	16 (11-21)	14 (10-19)	10 (7-14)	5 (3-7)	3 (2-5)	1 (1-2)
Ebola	0 (0-0)	0 (0-0)	0 (0-0)	0 (0-0)	0 (0-0)	0 (0-0)	0 (0-0)	0 (0-0)	0 (0-0)	0 (0-0)	0 (0-0)	0 (0-0)	0 (0-0)	0 (0-0)	0 (0-0)	0 (0-0)	0 (0-0)	0 (0-0)	0 (0-0)	0 (0-0)	0 (0-0)	0 (0-0)	0 (0-0)
Zika virus	[illegible]	[illegible]	[illegible]	[illegible]	[illegible]	[illegible]	[illegible]	[illegible]	[illegible]	[illegible]	[illegible]	[illegible]	[illegible]	[illegible]	[illegible]	[illegible]	[illegible]	[illegible]	[illegible]	[illegible]	[illegible]	[illegible]	[illegible]
Other neglected tropical diseases	[illegible]	[illegible]	[illegible]	[illegible]	[illegible]	[illegible]	[illegible]	[illegible]	[illegible]	[illegible]	[illegible]	[illegible]	[illegible]	[illegible]	[illegible]	[illegible]	[illegible]	[illegible]	[illegible]	[illegible]	[illegible]	[illegible]	[illegible]
Other infectious diseases	[illegible]	[illegible]	[illegible]	[illegible]	[illegible]	[illegible]	[illegible]	[illegible]	[illegible]	[illegible]	[illegible]	[illegible]	[illegible]	[illegible]	[illegible]	[illegible]	[illegible]	[illegible]	[illegible]	[illegible]	[illegible]	[illegible]	[illegible]
Meningitis	[illegible]	[illegible]	[illegible]	[illegible]	[illegible]	[illegible]	[illegible]	[illegible]	[illegible]	[illegible]	[illegible]	[illegible]	[illegible]	[illegible]	[illegible]	[illegible]	[illegible]	[illegible]	[illegible]	[illegible]	[illegible]	[illegible]	[illegible]
Pneumococcal meningitis	[illegible]	[illegible]	[illegible]	[illegible]	[illegible]	[illegible]	[illegible]	[illegible]	[illegible]	[illegible]	[illegible]	[illegible]	[illegible]	[illegible]	[illegible]	[illegible]	[illegible]	[illegible]	[illegible]	[illegible]	[illegible]	[illegible]	[illegible]
H influenzae type B meningitis	[illegible]	[illegible]	[illegible]	[illegible]	[illegible]	[illegible]	[illegible]	[illegible]	[illegible]	[illegible]	[illegible]	[illegible]	[illegible]	[illegible]	[illegible]	[illegible]	[illegible]	[illegible]	[illegible]	[illegible]	[illegible]	[illegible]	[illegible]
Meningococcal meningitis	[illegible]	[illegible]	[illegible]	[illegible]	[illegible]	[illegible]	[illegible]	[illegible]	[illegible]	[illegible]	[illegible]	[illegible]	[illegible]	[illegible]	[illegible]	[illegible]	[illegible]	[illegible]	[illegible]	[illegible]	[illegible]	[illegible]	[illegible]
Other meningitis	[illegible]	[illegible]	[illegible]	[illegible]	[illegible]	[illegible]	[illegible]	[illegible]	[illegible]	[illegible]	[illegible]	[illegible]	[illegible]	[illegible]	[illegible]	[illegible]	[illegible]	[illegible]	[illegible]	[illegible]	[illegible]	[illegible]	[illegible]
Encephalitis	[illegible]	[illegible]	[illegible]	[illegible]	[illegible]	[illegible]	[illegible]	[illegible]	[illegible]	[illegible]	[illegible]	[illegible]	[illegible]	[illegible]	[illegible]	[illegible]	[illegible]	[illegible]	[illegible]	[illegible]	[illegible]	[illegible]	[illegible]
Diphtheria	[illegible]	[illegible]	[illegible]	[illegible]	[illegible]	[illegible]	[illegible]	[illegible]	[illegible]	[illegible]	[illegible]	[illegible]	[illegible]	[illegible]	[illegible]	[illegible]	[illegible]	[illegible]	[illegible]	[illegible]	[illegible]	[illegible]	[illegible]
Whooping cough	[illegible]	[illegible]	[illegible]	[illegible]	[illegible]	[illegible]	[illegible]	[illegible]	[illegible]	[illegible]	[illegible]	[illegible]	[illegible]	[illegible]	[illegible]	[illegible]	[illegible]	[illegible]	[illegible]	[illegible]	[illegible]	[illegible]	[illegible]
Tetanus	[illegible]	[illegible]	[illegible]	[illegible]	[illegible]	[illegible]	[illegible]	[illegible]	[illegible]	[illegible]	[illegible]	[illegible]	[illegible]	[illegible]	[illegible]	[illegible]	[illegible]	[illegible]	[illegible]	[illegible]	[illegible]	[illegible]	[illegible]
Measles	[illegible]	[illegible]	[illegible]	[illegible]	[illegible]	[illegible]	[illegible]	[illegible]	[illegible]	[illegible]	[illegible]	[illegible]	[illegible]	[illegible]	[illegible]	[illegible]	[illegible]	[illegible]	[illegible]	[illegible]	[illegible]	[illegible]	[illegible]
Varicella and herpes zoster	[illegible]	[illegible]	[illegible]	[illegible]	[illegible]	[illegible]	[illegible]	[illegible]	[illegible]	[illegible]	[illegible]	[illegible]	[illegible]	[illegible]	[illegible]	[illegible]	[illegible]	[illegible]	[illegible]	[illegible]	[illegible]	[illegible]	[illegible]
Acute hepatitis	[illegible]	[illegible]	[illegible]	[illegible]	[illegible]	[illegible]	[illegible]	[illegible]	[illegible]	[illegible]	[illegible]	[illegible]	[illegible]	[illegible]	[illegible]	[illegible]	[illegible]	[illegible]	[illegible]	[illegible]	[illegible]	[illegible]	[illegible]
Acute hepatitis A	[illegible]	[illegible]	[illegible]	[illegible]	[illegible]	[illegible]	[illegible]	[illegible]	[illegible]	[illegible]	[illegible]	[illegible]	[illegible]	[illegible]	[illegible]	[illegible]	[illegible]	[illegible]	[illegible]	[illegible]	[illegible]	[illegible]	[illegible]
Acute hepatitis B	[illegible]	[illegible]	[illegible]	[illegible]	[illegible]	[illegible]	[illegible]	[illegible]	[illegible]	[illegible]	[illegible]	[illegible]	[illegible]	[illegible]	[illegible]	[illegible]	[illegible]	[illegible]	[illegible]	[illegible]	[illegible]	[illegible]	[illegible]
Acute hepatitis C	[illegible]	[illegible]	[illegible]	[illegible]	[illegible]	[illegible]	[illegible]	[illegible]	[illegible]	[illegible]	[illegible]	[illegible]	[illegible]	[illegible]	[illegible]	[illegible]	[illegible]	[illegible]	[illegible]	[illegible]	[illegible]	[illegible]	[illegible]
Acute hepatitis E	[illegible]	[illegible]	[illegible]	[illegible]	[illegible]	[illegible]	[illegible]	[illegible]	[illegible]	[illegible]	[illegible]	[illegible]	[illegible]	[illegible]	[illegible]	[illegible]	[illegible]	[illegible]	[illegible]	[illegible]	[illegible]	[illegible]	[illegible]
Other unspecified infectious diseases	[illegible]	[illegible]	[illegible]	[illegible]	[illegible]	[illegible]	[illegible]	[illegible]	[illegible]	[illegible]	[illegible]	[illegible]	[illegible]	[illegible]	[illegible]	[illegible]	[illegible]	[illegible]	[illegible]	[illegible]	[illegible]	[illegible]	[illegible]
Maternal and neonatal disorders	[illegible]	[illegible]	[illegible]	[illegible]	[illegible]	[illegible]	[illegible]	[illegible]	[illegible]	[illegible]	[illegible]	[illegible]	[illegible]	[illegible]	[illegible]	[illegible]	[illegible]	[illegible]	[illegible]	[illegible]	[illegible]	[illegible]	[illegible]
Maternal disorders	[illegible]	[illegible]	[illegible]	[illegible]	[illegible]	[illegible]	[illegible]	[illegible]	[illegible]	[illegible]	[illegible]	[illegible]	[illegible]	[illegible]	[illegible]	[illegible]	[illegible]	[illegible]	[illegible]	[illegible]	[illegible]	[illegible]	[illegible]
Maternal hemorrhage	[illegible]	[illegible]	[illegible]	[illegible]	[illegible]	[illegible]	[illegible]	[illegible]	[illegible]	[illegible]	[illegible]	[illegible]	[illegible]	[illegible]	[illegible]	[illegible]	[illegible]	[illegible]	[illegible]	[illegible]	[illegible]	[illegible]	[illegible]
Maternal sepsis and other maternal infections	[illegible]	[illegible]	[illegible]	[illegible]	[illegible]	[illegible]	[illegible]	[illegible]	[illegible]	[illegible]	[illegible]	[illegible]	[illegible]	[illegible]	[illegible]	[illegible]	[illegible]	[illegible]	[illegible]	[illegible]	[illegible]	[illegible]	[illegible]
Maternal hypertensive disorders	[illegible]	[illegible]	[illegible]	[illegible]	[illegible]	[illegible]	[illegible]	[illegible]	[illegible]	[illegible]	[illegible]	[illegible]	[illegible]	[illegible]	[illegible]	[illegible]	[illegible]	[illegible]	[illegible]	[illegible]	[illegible]	[illegible]	[illegible]
Maternal obstructed labor and uterine rupture	[illegible]	[illegible]	[illegible]	[illegible]	[illegible]	[illegible]	[illegible]	[illegible]	[illegible]	[illegible]	[illegible]	[illegible]	[illegible]	[illegible]	[illegible]	[illegible]	[illegible]	[illegible]	[illegible]	[illegible]	[illegible]	[illegible]	[illegible]
Maternal abortion and miscarriage	[illegible]	[illegible]	[illegible]	[illegible]	[illegible]	[illegible]	[illegible]	[illegible]	[illegible]	[illegible]	[illegible]	[illegible]	[illegible]	[illegible]	[illegible]	[illegible]	[illegible]	[illegible]	[illegible]	[illegible]	[illegible]	[illegible]	[illegible]
Ectopic pregnancy	[illegible]	[illegible]	[illegible]	[illegible]	[illegible]	[illegible]	[illegible]	[illegible]	[illegible]	[illegible]	[illegible]	[illegible]	[illegible]	[illegible]	[illegible]	[illegible]	[illegible]	[illegible]	[illegible]	[illegible]	[illegible]	[illegible]	[illegible]
Indirect maternal deaths	[illegible]	[illegible]	[illegible]	[illegible]	[illegible]	[illegible]	[illegible]	[illegible]	[illegible]	[illegible]	[illegible]	[illegible]	[illegible]	[illegible]	[illegible]	[illegible]	[illegible]	[illegible]	[illegible]	[illegible]	[illegible]	[illegible]	[illegible]
Late maternal deaths	[illegible]	[illegible]	[illegible]	[illegible]	[illegible]	[illegible]	[illegible]	[illegible]	[illegible]	[illegible]	[illegible]	[illegible]	[illegible]	[illegible]	[illegible]	[illegible]	[illegible]	[illegible]	[illegible]	[illegible]	[illegible]	[illegible]	[illegible]
Maternal deaths aggravated by HIV/AIDS	[illegible]	[illegible]	[illegible]	[illegible]	[illegible]	[illegible]	[illegible]	[illegible]	[illegible]	[illegible]	[illegible]	[illegible]	[illegible]	[illegible]	[illegible]	[illegible]	[illegible]	[illegible]	[illegible]	[illegible]	[illegible]	[illegible]	[illegible]
Other maternal disorders	[illegible]	[illegible]	[illegible]	[illegible]	[illegible]	[illegible]	[illegible]	[illegible]	[illegible]	[illegible]	[illegible]	[illegible]	[illegible]	[illegible]	[illegible]	[illegible]	[illegible]	[illegible]	[illegible]	[illegible]	[illegible]	[illegible]	[illegible]
Neonatal disorders	[illegible]	[illegible]	[illegible]	[illegible]	[illegible]	[illegible]	[illegible]	[illegible]	[illegible]	[illegible]	[illegible]	[illegible]	[illegible]	[illegible]	[illegible]	[illegible]	[illegible]	[illegible]	[illegible]	[illegible]	[illegible]	[illegible]	[illegible]
Neonatal preterm birth	[illegible]	[illegible]	[illegible]	[illegible]	[illegible]	[illegible]	[illegible]	[illegible]	[illegible]	[illegible]	[illegible]	[illegible]	[illegible]	[illegible]	[illegible]	[illegible]	[illegible]	[illegible]	[illegible]	[illegible]	[illegible]	[illegible]	[illegible]
Neonatal encephalopathy due to birth asphyxia and trauma	[illegible]	[illegible]	[illegible]	[illegible]	[illegible]	[illegible]	[illegible]	[illegible]	[illegible]	[illegible]	[illegible]	[illegible]	[illegible]	[illegible]	[illegible]	[illegible]	[illegible]	[illegible]	[illegible]	[illegible]	[illegible]	[illegible]	[illegible]
Neonatal sepsis and other neonatal infections	[illegible]	[illegible]	[illegible]	[illegible]	[illegible]	[illegible]	[illegible]	[illegible]	[illegible]	[illegible]	[illegible]	[illegible]	[illegible]	[illegible]	[illegible]	[illegible]	[illegible]	[illegible]	[illegible]	[illegible]	[illegible]	[illegible]	[illegible]
Hemolytic disease and other neonatal jaundice	[illegible]	[illegible]	[illegible]	[illegible]	[illegible]	[illegible]	[illegible]	[illegible]	[illegible]	[illegible]	[illegible]	[illegible]	[illegible]	[illegible]	[illegible]	[illegible]	[illegible]	[illegible]	[illegible]	[illegible]	[illegible]	[illegible]	[illegible]
Other neonatal disorders	[illegible]	[illegible]	[illegible]	[illegible]	[illegible]	[illegible]	[illegible]	[illegible]	[illegible]	[illegible]	[illegible]	[illegible]	[illegible]	[illegible]	[illegible]	[illegible]	[illegible]	[illegible]	[illegible]	[illegible]	[illegible]	[illegible]	[illegible]
Nutritional deficiencies	[illegible]	[illegible]	[illegible]	[illegible]	[illegible]	[illegible]	[illegible]	[illegible]	[illegible]	[illegible]	[illegible]	[illegible]	[illegible]	[illegible]	[illegible]	[illegible]	[illegible]	[illegible]	[illegible]	[illegible]	[illegible]	[illegible]	[illegible]
Protein-energy malnutrition	[illegible]	[illegible]	[illegible]	[illegible]	[illegible]	[illegible]	[illegible]	[illegible]	[illegible]	[illegible]	[illegible]	[illegible]	[illegible]	[illegible]	[illegible]	[illegible]	[illegible]	[illegible]	[illegible]	[illegible]	[illegible]	[illegible]	[illegible]
Other nutritional deficiencies	[illegible]	[illegible]	[illegible]	[illegible]	[illegible]	[illegible]	[illegible]	[illegible]	[illegible]	[illegible]	[illegible]	[illegible]	[illegible]	[illegible]	[illegible]	[illegible]	[illegible]	[illegible]	[illegible]	[illegible]	[illegible]	[illegible]	[illegible]
Non-communicable diseases	[illegible]	[illegible]	[illegible]	[illegible]	[illegible]	[illegible]	[illegible]	[illegible]	[illegible]	[illegible]	[illegible]	[illegible]	[illegible]	[illegible]	[illegible]	[illegible]	[illegible]	[illegible]	[illegible]	[illegible]	[illegible]	[illegible]	[illegible]
Neoplasms	[illegible]	[illegible]	[illegible]	[illegible]	[illegible]	[illegible]	[illegible]	[illegible]	[illegible]	[illegible]	[illegible]	[illegible]	[illegible]	[illegible]	[illegible]	[illegible]	[illegible]	[illegible]	[illegible]	[illegible]	[illegible]	[illegible]	[illegible]
Lip and oral cavity cancer	[illegible]	[illegible]	[illegible]	[illegible]	[illegible]	[illegible]	[illegible]	[illegible]	[illegible]	[illegible]	[illegible]	[illegible]	[illegible]	[illegible]	[illegible]	[illegible]	[illegible]	[illegible]	[illegible]	[illegible]	[illegible]	[illegible]	[illegible]
Nasopharynx cancer	[illegible]	[illegible]	[illegible]	[illegible]	[illegible]	[illegible]	[illegible]	[illegible]	[illegible]	[illegible]	[illegible]	[illegible]	[illegible]	[illegible]	[illegible]	[illegible]	[illegible]	[illegible]	[illegible]	[illegible]	[illegible]	[illegible]	[illegible]
Other pharynx cancer	[illegible]	[illegible]	[illegible]	[illegible]	[illegible]	[illegible]	[illegible]	[illegible]	[illegible]	[illegible]	[illegible]	[illegible]	[illegible]	[illegible]	[illegible]	[illegible]	[illegible]	[illegible]	[illegible]	[illegible]	[illegible]	[illegible]	[illegible]
Esophageal cancer	[illegible]	[illegible]	[illegible]	[illegible]	[illegible]	[illegible]	[illegible]	[illegible]	[illegible]	[illegible]	[illegible]	[illegible]	[illegible]	[illegible]	[illegible]	[illegible]	[illegible]	[illegible]	[illegible]	[illegible]	[illegible]	[illegible]	[illegible]
Stomach cancer	[illegible]	[illegible]	[illegible]	[illegible]	[illegible]	[illegible]	[illegible]	[illegible]	[illegible]	[illegible]	[illegible]	[illegible]	[illegible]	[illegible]	[illegible]	[illegible]	[illegible]	[illegible]	[illegible]	[illegible]	[illegible]	[illegible]	[illegible]
Colon and rectum cancer	[illegible]	[illegible]	[illegible]	[illegible]	[illegible]	[illegible]	[illegible]	[illegible]	[illegible]	[illegible]	[illegible]	[illegible]	[illegible]	[illegible]	[illegible]	[illegible]	[illegible]	[illegible]	[illegible]	[illegible]	[illegible]	[illegible]	[illegible]
Liver cancer	[illegible]	[illegible]	[illegible]	[illegible]	[illegible]	[illegible]	[illegible]	[illegible]	[illegible]	[illegible]	[illegible]	[illegible]	[illegible]	[illegible]	[illegible]	[illegible]	[illegible]	[illegible]	[illegible]	[illegible]	[illegible]	[illegible]	[illegible]
Liver cancer due to hepatitis B	[illegible]	[illegible]	[illegible]	[illegible]	[illegible]	[illegible]	[illegible]	[illegible]	[illegible]	[illegible]	[illegible]	[illegible]	[illegible]	[illegible]	[illegible]	[illegible]	[illegible]	[illegible]	[illegible]	[illegible]	[illegible]	[illegible]	[illegible]
Liver cancer due to hepatitis C	[illegible]	[illegible]	[illegible]	[illegible]	[illegible]	[illegible]	[illegible]	[illegible]	[illegible]	[illegible]	[illegible]	[illegible]	[illegible]	[illegible]	[illegible]	[illegible]	[illegible]	[illegible]	[illegible]	[illegible]	[illegible]	[illegible]	[illegible]
Liver cancer due to alcohol use	[illegible]	[illegible]	[illegible]	[illegible]	[illegible]	[illegible]	[illegible]	[illegible]	[illegible]	[illegible]	[illegible]	[illegible]	[illegible]	[illegible]	[illegible]	[illegible]	[illegible]	[illegible]	[illegible]	[illegible]	[illegible]	[illegible]	[illegible]
Liver cancer due to NASH	[illegible]	[illegible]	[illegible]	[illegible]	[illegible]	[illegible]	[illegible]	[illegible]	[illegible]	[illegible]	[illegible]	[illegible]	[illegible]	[illegible]	[illegible]	[illegible]	[illegible]	[illegible]	[illegible]	[illegible]	[illegible]	[illegible]	[illegible]
Liver cancer due to other causes	[illegible]	[illegible]	[illegible]	[illegible]	[illegible]	[illegible]	[illegible]	[illegible]	[illegible]	[illegible]	[illegible]	[illegible]	[illegible]	[illegible]	[illegible]	[illegible]	[illegible]	[illegible]	[illegible]	[illegible]	[illegible]	[illegible]	[illegible]
Gallbladder and biliary tract cancer	[illegible]	[illegible]	[illegible]	[illegible]	[illegible]	[illegible]	[illegible]	[illegible]	[illegible]	[illegible]	[illegible]	[illegible]	[illegible]	[illegible]	[illegible]	[illegible]	[illegible]	[illegible]	[illegible]	[illegible]	[illegible]	[illegible]	[illegible]
Pancreatic cancer	[illegible]	[illegible]	[illegible]	[illegible]	[illegible]	[illegible]	[illegible]	[illegible]	[illegible]	[illegible]	[illegible]	[illegible]	[illegible]	[illegible]	[illegible]	[illegible]	[illegible]	[illegible]	[illegible]	[illegible]	[illegible]	[illegible]	[illegible]
Larynx cancer	[illegible]	[illegible]	[illegible]	[illegible]	[illegible]	[illegible]	[illegible]	[illegible]	[illegible]	[illegible]	[illegible]	[illegible]	[illegible]	[illegible]	[illegible]	[illegible]	[illegible]	[illegible]	[illegible]	[illegible]	[illegible]	[illegible]	[illegible]
Tracheal, bronchus, and lung cancer	[illegible]	[illegible]	[illegible]	[illegible]	[illegible]	[illegible]	[illegible]	[illegible]	[illegible]	[illegible]	[illegible]	[illegible]	[illegible]	[illegible]	[illegible]	[illegible]	[illegible]	[illegible]	[illegible]	[illegible]	[illegible]	[illegible]	[illegible]
Malignant skin melanoma	[illegible]	[illegible]	[illegible]	[illegible]	[illegible]	[illegible]	[illegible]	[illegible]	[illegible]	[illegible]	[illegible]	[illegible]	[illegible]	[illegible]	[illegible]	[illegible]	[illegible]	[illegible]	[illegible]	[illegible]	[illegible]	[illegible]	[illegible]
Non-melanoma skin cancer	[illegible]	[illegible]	[illegible]	[illegible]	[illegible]	[illegible]	[illegible]	[illegible]	[illegible]	[illegible]	[illegible]	[illegible]	[illegible]	[illegible]	[illegible]	[illegible]	[illegible]	[illegible]	[illegible]	[illegible]	[illegible]	[illegible]	[illegible]
Non-melanoma skin cancer (squamous-cell carcinoma)	[illegible]	[illegible]	[illegible]	[illegible]	[illegible]	[illegible]	[illegible]	[illegible]	[illegible]	[illegible]	[illegible]	[illegible]	[illegible]	[illegible]	[illegible]	[illegible]	[illegible]	[illegible]	[illegible]	[illegible]	[illegible]	[illegible]	[illegible]
Breast cancer	[illegible]	[illegible]	[illegible]	[illegible]	[illegible]	[illegible]	[illegible]	[illegible]	[illegible]	[illegible]	[illegible]	[illegible]	[illegible]	[illegible]	[illegible]	[illegible]	[illegible]	[illegible]	[illegible]	[illegible]	[illegible]	[illegible]	[illegible]
Cervical cancer	[illegible]	[illegible]	[illegible]	[illegible]	[illegible]	[illegible]	[illegible]	[illegible]	[illegible]	[illegible]	[illegible]	[illegible]	[illegible]	[illegible]	[illegible]	[illegible]	[illegible]	[illegible]	[illegible]	[illegible]	[illegible]	[illegible]	[illegible]
Uterine cancer	[illegible]	[illegible]	[illegible]	[illegible]	[illegible]	[illegible]	[illegible]	[illegible]	[illegible]	[illegible]	[illegible]	[illegible]	[illegible]	[illegible]	[illegible]	[illegible]	[illegible]	[illegible]	[illegible]	[illegible]	[illegible]	[illegible]	[illegible]
Ovarian cancer	[illegible]	[illegible]	[illegible]	[illegible]	[illegible]	[illegible]	[illegible]	[illegible]	[illegible]	[illegible]	[illegible]	[illegible]	[illegible]	[illegible]	[illegible]	[illegible]	[illegible]	[illegible]	[illegible]	[illegible]	[illegible]	[illegible]	[illegible]
Prostate cancer	[illegible]	[illegible]	[illegible]	[illegible]	[illegible]	[illegible]	[illegible]	[illegible]	[illegible]	[illegible]	[illegible]	[illegible]	[illegible]	[illegible]	[illegible]	[illegible]	[illegible]	[illegible]	[illegible]	[illegible]	[illegible]	[illegible]	[illegible]
Testicular cancer	[illegible]	[illegible]	[illegible]	[illegible]	[illegible]	[illegible]	[illegible]	[illegible]	[illegible]	[illegible]	[illegible]	[illegible]	[illegible]	[illegible]	[illegible]	[illegible]	[illegible]	[illegible]	[illegible]	[illegible]	[illegible]	[illegible]	[illegible]
Kidney cancer	[illegible]	[illegible]	[illegible]	[illegible]	[illegible]	[illegible]	[illegible]	[illegible]	[illegible]	[illegible]	[illegible]	[illegible]	[illegible]	[illegible]	[illegible]	[illegible]	[illegible]	[illegible]	[illegible]	[illegible]	[illegible]	[illegible]	[illegible]
Bladder cancer	[illegible]	[illegible]	[illegible]	[illegible]	[illegible]	[illegible]	[illegible]	[illegible]	[illegible]	[illegible]	[illegible]	[illegible]	[illegible]	[illegible]	[illegible]	[illegible]	[illegible]	[illegible]	[illegible]	[illegible]	[illegible]	[illegible]	[illegible]
Brain and nervous system cancer	[illegible]	[illegible]	[illegible]	[illegible]	[illegible]	[illegible]	[illegible]	[illegible]	[illegible]	[illegible]	[illegible]	[illegible]	[illegible]	[illegible]	[illegible]	[illegible]	[illegible]	[illegible]	[illegible]	[illegible]	[illegible]	[illegible]	[illegible]
Thyroid cancer	[illegible]	[illegible]	[illegible]	[illegible]	[illegible]	[illegible]	[illegible]	[illegible]	[illegible]	[illegible]	[illegible]	[illegible]	[illegible]	[illegible]	[illegible]	[illegible]	[illegible]	[illegible]	[illegible]	[illegible]	[illegible]	[illegible]	[illegible]

SR Table 5: Global deaths by cause and age group for females (with 95% confidence intervals), 2010

Cause	Early Neonatal	Late Neonatal	Post Neonatal	1 to 4	5 to 9	10 to 14	15 to 19
Mesothelioma	-	-	-	-	-	-	44 (32-58)
Hodgkin lymphoma	1 (1-2)	1 (0-1)	16 (13-20)	145 (81-250)	249 (163-349)	435 (286-598)	839 (593-1,123)
Non-Hodgkin lymphoma	25 (22-28)	21 (18-24)	320 (270-370)	1,593 (1,278-1,939	1,094 (929-1,303)	915 (808-1,042)	1,064 (954-1,205)
Multiple myeloma	-	-	-	-	-	-	99 (88-113)
Leukemia	131 (96-156)	111 (87-133)	1,688 (1,364-2,081	5,888 (4,691-7,861	5,993 (4,971-7,193	5,299 (4,473-6,046	5,017 (4,148-5,750
Acute lymphoid leukemia	33 (23-43)	24 (18-35)	450 (353-625)	1,850 (1,459-2,722	2,530 (2,006-3,278	2,022 (1,648-2,440	1,531 (1,243-1,808
Chronic lymphoid leukemia	-	-	-	-	-	-	65 (51-79)
Acute myeloid leukemia	20 (14-30)	19 (14-28)	277 (211-434)	1,207 (857-2,099	863 (649-1,230)	1,046 (826-1,302	1,176 (977-1,450)
Chronic myeloid leukemia	-	-	-	-	-	-	216 (173-275)
Other leukemia	78 (56-96)	68 (51-84)	961 (723-1,284)	2,831 (2,057-4,232	2,599 (2,017-3,311	2,231 (1,748-2,662	2,029 (1,601-2,368
Other malignant neoplasms	463 (361-541)	236 (199-280)	1,426 (1,161-1,803	4,976 (3,686-6,661	2,701 (2,293-3,193	3,342 (2,894-3,818	3,678 (3,153-4,217
Other neoplasms	27 (20-36)	15 (11-19)	202 (150-262)	659 (432-901)	542 (392-708)	416 (313-534)	378 (277-485)
Myelodysplastic, myeloproliferative, and other hematopoietic neoplasm	26 (19-34)	11 (9-16)	168 (130-227)	498 (340-724)	339 (263-458)	262 (200-344)	238 (183-317)
Other benign and in situ neoplasms	1 (1-2)	3 (2-4)	34 (20-49)	161 (87-265)	203 (132-257)	154 (110-197)	139 (98-180)
Cardiovascular diseases	1,358 (1,247-1,477	919 (847-996)	8,683 (8,053-9,321	7,388 (6,663-8,098	4,090 (3,706-4,471	5,522 (5,055-5,972	14,581 (13,789-15,490
Rheumatic heart disease	-	-	-	1,705 (1,360-2,061	1,218 (1,046-1,376	1,940 (1,711-2,150	2,814 (2,541-3,098
Ischemic heart disease	-	-	-	-	-	-	4,814 (4,476-5,186
Stroke	379 (327-440)	259 (232-291)	2,067 (1,794-2,592	1,623 (1,356-1,968	1,246 (1,120-1,385	1,717 (1,548-1,907	3,558 (3,305-3,833
Ischemic stroke	98 (70-125)	58 (46-68)	333 (239-436)	221 (169-271)	157 (130-187)	183 (148-227)	668 (558-797)
Intracerebral hemorrhage	157 (130-189)	106 (91-123)	962 (785-1,226)	723 (516-948)	651 (561-747)	933 (817-1,056)	2,036 (1,831-2,235
Subarachnoid hemorrhage	123 (109-145)	96 (83-114)	772 (673-949)	679 (590-797)	439 (383-501)	601 (524-716)	854 (759-1,003)
Hypertensive heart disease	-	-	-	-	-	-	395 (255-509)
Non-rheumatic valvular heart disease	-	-	-	-	-	-	349 (271-447)
Non-rheumatic calcific aortic valve disease	-	-	-	-	-	-	99 (68-166)
Non-rheumatic degenerative mitral valve disease	-	-	-	-	-	-	192 (133-286)
Other non-rheumatic valve diseases	-	-	-	-	-	-	58 (40-90)
Cardiomyopathy and myocarditis	578 (473-655)	345 (299-384)	3,441 (2,874-3,944	1,884 (1,409-2,260	781 (654-916)	885 (776-1,016)	1,151 (976-1,377)
Myocarditis	63 (50-88)	60 (46-78)	923 (730-1,112)	603 (462-730)	291 (236-356)	269 (211-331)	414 (326-510)
Alcoholic cardiomyopathy	-	-	-	-	-	-	94 (61-124)
Other cardiomyopathy	515 (408-590)	286 (242-321)	2,518 (2,005-2,922	1,281 (907-1,586)	490 (395-584)	615 (520-715)	643 (506-781)
Atrial fibrillation and flutter	-	-	-	-	-	-	-
Aortic aneurysm	-	-	-	-	-	-	84 (69-110)
Peripheral artery disease	-	-	-	-	-	-	-
Endocarditis	98 (76-129)	89 (69-117)	1,204 (937-1,523)	968 (657-1,302)	257 (216-308)	333 (287-402)	415 (364-504)
Other cardiovascular and circulatory diseases	303 (249-411)	225 (188-291)	1,970 (1,570-2,776	1,209 (842-2,104	588 (483-789)	647 (536-813)	1,001 (805-1,290)
Chronic respiratory diseases	0 (0-0)	0 (0-0)	0 (0-0)	7,762 (6,268-10,592	2,172 (1,843-3,039	2,082 (1,809-2,845	3,528 (3,206-4,413
Chronic obstructive pulmonary disease	-	-	0 (0-0)	977 (658-1,528	265 (192-405)	211 (159-304)	973 (748-1,320)
Pneumoconiosis	-	-	-	-	-	-	12 (9-15)
Silicosis	-	-	-	-	-	-	2 (1-3)
Asbestosis	-	-	-	-	-	-	1 (1-2)
Coal workers pneumoconiosis	-	-	-	-	-	-	1 (1-2)
Other pneumoconiosis	-	-	-	-	-	-	7 (5-9)
Asthma	-	-	-	5,237 (3,922-7,884	1,405 (1,100-2,199	1,470 (1,193-2,220	2,076 (1,632-2,964
Interstitial lung disease and pulmonary sarcoidosis	-	-	-	186 (100-439)	88 (55-171)	67 (46-114)	119 (80-200)
Other chronic respiratory diseases	0 (0-0)	0 (0-0)	0 (0-0)	1,362 (734-2,171)	414 (310-550)	334 (250-429)	348 (271-448)
Digestive diseases	984 (790-1,096)	914 (816-1,030)	8,076 (7,280-9,431	10,028 (8,432-12,686	5,725 (4,739-6,707	5,636 (4,776-6,436	9,512 (8,367-10,733
Cirrhosis and other chronic liver diseases	-	-	-	4,157 (3,249-6,848	2,246 (1,786-3,458	2,388 (2,006-3,269	4,021 (3,452-5,136
Cirrhosis and other chronic liver diseases due to hepatitis B	-	-	-	76 (52-133)	98 (71-154)	191 (142-270)	474 (343-660)
Cirrhosis and other chronic liver diseases due to hepatitis C	-	-	-	30 (20-54)	49 (34-76)	96 (70-135)	217 (153-321)
Cirrhosis and other chronic liver diseases due to alcohol use	-	-	-	-	-	-	18 (8-32)
Cirrhosis due to NASH	-	-	-	-	-	-	68 (51-93)
Cirrhosis and other chronic liver diseases due to other causes	-	-	-	4,051 (3,172-6,672	2,099 (1,658-3,222	2,101 (1,769-2,902	3,245 (2,724-4,148
Upper digestive system diseases	-	-	-	776 (576-1,155)	646 (474-796)	623 (472-744)	937 (766-1,138)
Peptic ulcer disease	-	-	-	625 (450-1,054)	463 (343-608)	484 (367-591)	795 (633-990)
Gastritis and duodenitis	-	-	-	151 (71-222)	183 (96-243)	139 (75-185)	142 (87-209)
Appendicitis	-	-	-	1,420 (778-1,904)	833 (661-1,028)	876 (676-1,050)	1,671 (1,363-2,065
Paralytic ileus and intestinal obstruction	984 (790-1,096)	914 (816-1,030	8,076 (7,280-9,431	1,565 (986-2,059)	1,284 (611-1,749)	1,166 (631-1,473)	2,075 (1,170-2,620
Inguinal, femoral, and abdominal hernia	-	-	-	101 (56-146)	95 (52-129)	78 (42-100)	91 (48-120)
Inflammatory bowel disease	-	-	-	454 (236-860)	80 (54-127)	61 (46-86)	113 (82-161)
Vascular intestinal disorders	-	-	-	69 (25-98)	15 (9-19)	14 (10-17)	33 (22-40)
Gallbladder and biliary diseases	-	-	-	495 (317-733)	204 (136-274)	150 (106-183)	168 (134-192)
Pancreatitis	-	-	-	46 (30-76)	73 (51-105)	102 (66-130)	242 (176-299)
Other digestive diseases	-	-	-	944 (697-1,448)	250 (197-314)	177 (151-204)	162 (130-192)
Neurological disorders	-	-	4,398 (3,605-5,329	5,293 (3,535-6,846	2,726 (2,348-3,243	3,236 (2,831-3,785	4,962 (4,337-5,744
Alzheimer's disease and other dementias	-	-	-	-	-	-	-
Parkinson's disease	-	-	-	-	-	-	-
Epilepsy	-	-	3,055 (2,388-3,528	3,955 (2,758-5,065	2,190 (1,841-2,670	2,522 (2,152-3,020	4,312 (3,661-5,177
Multiple sclerosis	-	-	-	-	-	-	-
Motor neuron disease	7 (2-12)	11 (6-15)	185 (150-217)	108 (65-133)	19 (15-21)	21 (18-24)	18 (16-21)
Other neurological disorders	-	-	1,159 (774-1,991)	1,230 (595-2,390)	517 (366-829)	693 (507-1,010)	631 (448-895)
Mental disorders	-	-	-	-	0 (0-0)	3 (3-4)	18 (16-22)
Eating disorders	-	-	-	-	0 (0-0)	3 (3-4)	18 (16-22)
Anorexia nervosa	-	-	-	-	0 (0-0)	3 (3-4)	16 (14-20)
Bulimia nervosa	-	-	-	-	0 (0-0)	0 (0-0)	2 (1-3)
Substance use disorders	0 (0-0)	0 (0-0)	0 (0-0)	0 (0-0)	2 (0-8)	3 (0-10)	1,280 (1,220-1,360
Alcohol use disorders	-	-	-	-	-	-	194 (180-238)
Drug use disorders	0 (0-0)	0 (0-0)	0 (0-0)	0 (0-0)	2 (0-8)	3 (0-10)	1,086 (1,033-1,149
Opioid use disorders	0 (0-0)	0 (0-0)	0 (0-0)	0 (0-0)	2 (0-8)	3 (0-10)	651 (579-736)
Cocaine use disorders	-	-	-	-	-	-	29 (22-34)
Amphetamine use disorders	-	-	-	-	-	-	50 (34-78)
Other drug use disorders	-	-	-	-	-	-	356 (284-431)
Diabetes and kidney diseases	6 (4-8)	18 (13-23)	3,601 (3,314-3,901	5,326 (4,684-5,952	2,462 (2,176-2,724	3,450 (3,071-3,778	5,544 (5,235-5,913
Diabetes mellitus	6 (4-8)	18 (13-23)	357 (261-446)	414 (291-525)	419 (288-531)	838 (585-1,057)	1,656 (1,552-1,777
Diabetes mellitus type 1	6 (4-8)	18 (13-23)	357 (261-446)	414 (291-525)	419 (288-531)	838 (585-1,057)	1,419 (1,294-1,553
Diabetes mellitus type 2	-	-	-	-	-	-	237 (169-342)
Chronic kidney disease	-	-	3,143 (2,877-3,413	4,786 (4,218-5,376	1,956 (1,744-2,176	2,539 (2,302-2,777	3,818 (3,565-4,112
Chronic kidney disease due to diabetes mellitus type 1	-	-	12 (7-20)	21 (11-38)	13 (6-24)	28 (15-47)	81 (43-134)
Chronic kidney disease due to diabetes mellitus type 2	-	-	8 (4-15)	13 (6-26)	7 (3-14)	13 (6-25)	35 (15-64)
Chronic kidney disease due to hypertension	-	-	16 (9-23)	35 (20-56)	23 (12-37)	51 (29-84)	141 (84-217)
Chronic kidney disease due to glomerulonephritis	-	-	820 (589-1,099)	1,573 (1,100-2,159	675 (460-946)	916 (620-1,250)	1,506 (1,068-1,984
Chronic kidney disease due to other and unspecified causes	-	-	2,287 (1,964-2,614	3,143 (2,540-3,708	1,238 (964-1,504)	1,531 (1,214-1,876	2,055 (1,533-2,563
Acute glomerulonephritis	-	-	100 (60-128)	126 (66-171)	87 (67-108)	73 (59-88)	71 (62-81)
Skin and subcutaneous diseases	-	-	2,268 (1,028-3,514	1,196 (481-2,161)	285 (123-399)	298 (135-404)	369 (179-530)
Bacterial skin diseases	-	-	2,243 (1,012-3,485	1,117 (435-2,039)	234 (90-334)	232 (91-322)	271 (118-368)
Cellulitis	-	-	167 (43-349)	171 (49-321)	75 (23-116)	79 (25-117)	84 (29-119)
Pyoderma	-	-	2,076 (940-3,218)	945 (375-1,757)	159 (62-231)	154 (62-220)	187 (81-267)
Decubitus ulcer	-	-	-	39 (18-80)	25 (15-45)	33 (19-55)	58 (33-102)
Other skin and subcutaneous diseases	-	-	24 (12-46)	40 (20-85)	26 (16-51)	33 (20-58)	41 (25-77)
Musculoskeletal disorders	-	-	-	-	318 (252-399)	581 (485-664)	1,328 (1,030-1,510
Rheumatoid arthritis	-	-	-	-	53 (29-75)	40 (27-51)	54 (37-73)
Other musculoskeletal disorders	-	-	-	-	265 (206-333)	541 (450-617)	1,274 (990-1,447)
Other non-communicable diseases	87,513 (80,318-95,008)	35,576 (32,795-38,868)	130,643 (115,457-152,978	57,250 (49,740-65,405	12,367 (10,989-13,836	9,095 (8,232-10,182	9,100 (8,344-10,116
Congenital birth defects	86,237 (78,837-93,647	32,817 (30,624-35,163	105,663 (97,309-114,985	45,111 (40,037-51,465	8,492 (7,595-9,446	5,980 (5,462-6,547	4,079 (3,821-4,410
Neural tube defects	11,918 (9,347-14,718	2,677 (2,103-3,448	10,257 (6,432-17,036	7,243 (3,707-14,872	373 (279-504)	242 (200-290)	153 (131-182)
Congenital heart anomalies	28,708 (20,473-35,056	15,007 (12,065-17,725	56,622 (40,509-67,604	22,981 (13,451-30,153	5,196 (4,266-6,066	3,913 (3,361-4,356	2,486 (2,151-2,724
Orofacial clefts	1,022 (462-3,089)	391 (171-1,170	847 (343-2,786)	205 (68-753)	-	-	-
Down syndrome	1,636 (1,147-2,863	692 (462-1,180	3,141 (2,034-5,666	1,755 (841-3,973)	226 (153-371)	185 (149-263)	239 (203-325)
Other chromosomal abnormalities	3,172 (1,521-6,983	1,621 (881-3,333	3,733 (1,864-7,506	789 (325-2,097)	178 (92-330)	110 (63-175)	103 (60-164)
Congenital musculoskeletal and limb anomalies	2,247 (1,093-3,524	607 (296-917)	1,586 (740-2,741)	634 (211-1,386)	185 (96-268)	138 (86-179)	124 (76-163)
Urogenital congenital anomalies	2,506 (1,621-3,157	808 (570-1,062	1,264 (753-1,930)	347 (179-654)	151 (109-208)	144 (105-192)	76 (58-96)
Digestive congenital anomalies	[illegible]	[illegible]	[illegible]	[illegible]	[illegible]	[illegible]	[illegible]
Other congenital birth defects	[illegible]	[illegible]	[illegible]	[illegible]	[illegible]	[illegible]	[illegible]
Urinary diseases and male infertility	[illegible]	[illegible]	[illegible]	[illegible]	[illegible]	[illegible]	[illegible]
Urinary tract infections	[illegible]	[illegible]	[illegible]	[illegible]	[illegible]	[illegible]	[illegible]
Urolithiasis	[illegible]	[illegible]	[illegible]	[illegible]	[illegible]	[illegible]	[illegible]

Cause	20 to 24	25 to 29	30 to 34	35 to 39	40 to 44	45 to 49	50 to 54	54 to 59
Mesothelioma	71 (50-93)	97 (74-123)	117 (90-149)	199 (153-256)	358 (292-444)	301 (269-343)	418 (384-467)	595 (556-652)
Hodgkin lymphoma	1,038 (766-1,344	1,082 (798-1,362	858 (628-1,084)	679 (506-857)	583 (451-735)	504 (401-634)	537 (431-668)	612 (474-781)
Non-Hodgkin lymphoma	1,550 (1,411-1,734	1,683 (1,548-1,847	1,704 (1,585-1,839	2,128 (1,992-2,293	2,711 (2,541-2,907	3,316 (3,163-3,497	4,346 (4,178-4,537	5,918 (5,717-6,155
Multiple myeloma	132 (115-153)	143 (130-159)	176 (162-195)	363 (333-411)	678 (634-755)	1,232 (1,161-1,403	2,091 (1,992-2,410	3,307 (3,162-3,802
Leukemia	4,694 (3,889-5,241	4,255 (3,532-4,752	4,039 (3,252-4,542	4,620 (3,749-5,203	5,299 (4,400-5,869	6,116 (5,139-6,718	6,851 (5,813-7,507	8,601 (7,450-9,408
Acute lymphoid leukemia	1,142 (914-1,344)	1,040 (817-1,248	701 (545-816)	801 (620-957)	804 (620-931)	782 (613-882)	791 (643-885)	987 (791-1,113)
Chronic lymphoid leukemia	85 (64-102)	78 (63-93)	80 (63-98)	112 (90-135)	163 (134-189)	214 (184-248)	344 (302-392)	567 (511-637)
Acute myeloid leukemia	1,198 (1,007-1,457	1,160 (982-1,380	1,148 (962-1,361)	1,285 (1,069-1,546	1,471 (1,263-1,739	1,861 (1,627-2,100	2,012 (1,782-2,246	2,457 (2,156-2,667
Chronic myeloid leukemia	307 (236-393)	376 (292-476)	439 (335-539)	475 (368-583)	531 (430-635)	550 (446-643)	728 (599-853)	796 (678-944)
Other leukemia	1,962 (1,458-2,293	1,602 (1,229-1,847	1,672 (1,285-1,956	1,947 (1,487-2,328	2,330 (1,829-2,712	2,708 (2,081-3,079	2,975 (2,343-3,306	3,795 (3,079-4,213
Other malignant neoplasms	3,321 (2,876-3,757	3,120 (2,772-3,453	3,019 (2,735-3,325	3,669 (3,360-4,039	4,844 (4,467-5,315	6,523 (5,933-7,048	9,155 (7,944-9,761	12,139 (10,317-12,884
Other neoplasms	445 (333-567)	500 (375-642)	631 (476-819)	838 (649-1,101	1,024 (782-1,364)	1,378 (1,026-1,838	1,782 (1,284-2,383	2,262 (1,650-3,097
Myelodysplastic, myeloproliferative, and other hematopoietic neoplasm	297 (227-393)	357 (278-469)	462 (357-619)	677 (529-898)	899 (689-1,204)	1,269 (944-1,702	1,693 (1,221-2,278	2,191 (1,603-3,018
Other benign and in situ neoplasms	149 (101-189)	143 (96-185)	169 (117-215)	161 (114-204)	124 (87-158)	109 (74-140)	89 (59-112)	71 (46-92)
Cardiovascular diseases	22,559 (21,620-23,647	28,715 (27,684-29,795	41,978 (40,556-43,404	63,720 (61,510-65,612	105,658 (102,426-108,407	157,331 (152,962-160,801	219,062 (213,858-223,469	322,371 (315,204-328,804
Rheumatic heart disease	3,653 (3,250-4,041	3,891 (3,372-4,384	4,691 (4,022-5,395	5,707 (4,898-6,532	7,638 (6,542-8,698	7,852 (6,898-8,823	8,506 (7,436-9,644	10,763 (9,555-11,906
Ischemic heart disease	8,015 (7,555-8,573	10,558 (9,967-11,252	17,289 (16,378-18,352	26,124 (24,740-27,544	44,410 (42,398-46,302	68,108 (65,594-70,679	95,556 (92,489-98,853	145,577 (141,439-149,646
Stroke	5,806 (5,486-6,180	7,533 (7,155-7,942	11,676 (11,122-12,302	20,744 (19,871-21,741	37,687 (36,310-39,152	59,280 (57,366-61,182	85,198 (82,599-87,696	124,305 (120,791-127,545
Ischemic stroke	1,085 (899-1,289)	1,396 (1,196-1,618	2,077 (1,785-2,401	3,325 (2,904-3,784	6,160 (5,425-6,933	10,387 (9,341-11,511	16,744 (15,032-18,621	28,366 (25,823-31,226
Intracerebral hemorrhage	3,373 (3,073-3,686	4,328 (3,991-4,688	6,742 (6,213-7,273	12,431 (11,561-13,328	23,071 (21,624-24,465	38,939 (36,958-40,924	55,519 (52,672-58,186	78,728 (74,725-82,226
Subarachnoid hemorrhage	1,348 (1,196-1,633	1,810 (1,624-2,072	2,857 (2,574-3,273	4,988 (4,537-5,671	8,456 (7,725-9,705	9,954 (9,140-11,529	12,936 (11,854-15,195	17,211 (15,814-20,713
Hypertensive heart disease	604 (400-779)	947 (634-1,205	1,546 (1,056-1,933	2,623 (1,821-3,242	4,624 (3,376-5,512	7,718 (5,798-9,036	11,683 (8,842-13,523	17,439 (13,440-20,076
Non-rheumatic valvular heart disease	411 (315-528)	456 (363-566)	396 (324-482)	513 (427-619)	637 (547-760)	827 (731-982)	1,141 (1,015-1,345	1,648 (1,505-1,914
Non-rheumatic calcific aortic valve disease	111 (75-194)	129 (93-206)	134 (100-204)	193 (147-290)	237 (189-336)	327 (270-456)	496 (418-672)	737 (632-999)
Non-rheumatic degenerative mitral valve disease	230 (158-346)	243 (176-353)	206 (151-290)	259 (195-348)	331 (257-428)	414 (331-523)	546 (432-682)	770 (630-945)
Other non-rheumatic valve diseases	71 (49-108)	83 (58-125)	56 (37-85)	60 (36-90)	69 (43-104)	86 (55-131)	100 (61-145)	141 (85-198)
Cardiomyopathy and myocarditis	1,714 (1,449-2,033	2,332 (2,066-2,667	2,938 (2,700-3,284	3,897 (3,597-4,320	4,858 (4,512-5,325	6,083 (5,731-6,568	7,278 (6,898-7,779	8,949 (8,515-9,589
Myocarditis	602 (471-737)	590 (460-713)	525 (417-640)	611 (470-735)	737 (576-876)	582 (440-679)	597 (449-695)	775 (579-900)
Alcoholic cardiomyopathy	234 (184-283)	600 (527-662)	1,127 (1,054-1,203	1,638 (1,542-1,737	2,099 (2,002-2,214	2,986 (2,846-3,172	3,666 (3,508-3,901	4,107 (3,941-4,435
Other cardiomyopathy	879 (684-1,059	1,142 (905-1,371	1,285 (1,052-1,517	1,648 (1,352-1,935	2,022 (1,697-2,346	2,515 (2,172-2,884	3,016 (2,630-3,411	4,067 (3,563-4,601
Atrial fibrillation and flutter	-	-	35 (32-37)	42 (39-46)	216 (198-233)	384 (358-409)	667 (628-698)	1,477 (1,397-1,538
Aortic aneurysm	150 (123-197)	202 (173-255)	283 (239-359)	416 (364-515)	627 (569-753)	973 (900-1,136	1,407 (1,325-1,611	2,145 (2,022-2,450
Peripheral artery disease	-	-	-	-	87 (49-179)	155 (78-368)	285 (133-673)	514 (235-1,168
Endocarditis	659 (577-812)	825 (716-1,004	742 (648-893)	860 (752-1,029	1,030 (910-1,247)	947 (792-1,168	1,170 (960-1,423)	1,377 (1,137-1,749
Other cardiovascular and circulatory diseases	1,546 (1,248-1,967	1,972 (1,628-2,492	2,382 (2,000-2,966	2,794 (2,398-3,329	3,845 (3,320-4,529	5,004 (4,415-5,750	6,170 (5,429-7,128	8,176 (7,190-9,379
Chronic respiratory diseases	4,785 (4,427-5,898	5,821 (5,432-6,782	7,300 (6,812-8,491	10,660 (9,850-12,398	15,318 (14,376-17,407	23,670 (22,287-26,424	36,505 (33,257-39,402	59,353 (53,772-63,624
Chronic obstructive pulmonary disease	1,484 (1,169-1,930	1,914 (1,524-2,445	2,592 (2,082-3,287	3,830 (3,004-4,868	6,542 (5,308-8,052	11,903 (9,865-14,132	21,505 (17,923-25,329	38,719 (32,257-45,067
Pneumoconiosis	15 (11-18)	19 (13-23)	24 (16-28)	30 (21-36)	48 (34-58)	56 (41-66)	74 (56-90)	110 (79-141)
Silicosis	3 (1-4)	4 (2-5)	5 (3-7)	6 (3-9)	13 (7-17)	18 (10-24)	17 (9-23)	26 (14-36)
Asbestosis	3 (1-4)	3 (2-4)	4 (2-5)	5 (3-8)	6 (3-9)	9 (5-13)	14 (8-22)	21 (12-33)
Coal workers pneumoconiosis	1 (1-2)	2 (1-3)	3 (1-4)	3 (1-4)	5 (2-8)	6 (3-9)	9 (4-13)	12 (6-19)
Other pneumoconiosis	8 (6-12)	10 (6-13)	12 (8-17)	16 (10-22)	24 (15-33)	23 (15-32)	33 (22-46)	51 (33-69)
Asthma	2,685 (2,086-3,735	3,136 (2,439-4,258	3,817 (2,905-5,084	5,513 (4,046-7,398	7,006 (5,039-9,289	9,691 (6,717-12,680	11,947 (7,802-16,072	16,223 (10,536-22,277
Interstitial lung disease and pulmonary sarcoidosis	174 (125-291)	253 (185-415)	346 (241-571)	546 (384-897)	760 (542-1,145)	1,140 (792-1,666)	1,716 (1,204-2,545	2,541 (1,719-3,758
Other chronic respiratory diseases	427 (335-551)	499 (371-630)	521 (375-666)	741 (522-970)	963 (690-1,309)	881 (640-1,126)	1,264 (910-1,645)	1,760 (1,266-2,290
Digestive diseases	12,597 (10,943-14,115	13,783 (12,306-15,164	16,090 (14,498-17,391	20,709 (19,119-22,209	27,392 (25,418-29,237	35,891 (34,114-38,226	46,557 (44,396-49,367	60,022 (56,651-63,225
Cirrhosis and other chronic liver diseases	6,190 (5,347-7,257	7,449 (6,571-8,472	9,944 (9,101-11,097	13,162 (12,296-14,875	18,564 (17,494-21,011	24,482 (23,356-27,634	31,563 (29,996-35,549	38,950 (37,119-44,106
Cirrhosis and other chronic liver diseases due to hepatitis B	1,803 (1,404-2,275	2,833 (2,233-3,521	3,622 (3,053-4,336	4,140 (3,434-5,086	5,397 (4,575-6,501	6,107 (5,040-7,328	7,024 (5,682-8,583	8,956 (7,317-11,154
Cirrhosis and other chronic liver diseases due to hepatitis C	1,054 (772-1,373)	1,713 (1,280-2,211	2,597 (2,137-3,096	3,792 (3,051-4,702	5,464 (4,533-6,555	7,292 (6,057-8,743	9,343 (7,723-11,175	10,835 (9,286-12,577
Cirrhosis and other chronic liver diseases due to alcohol use	336 (245-466)	710 (547-916)	1,554 (1,319-1,851	2,843 (2,351-3,448	4,346 (3,736-5,081	6,145 (5,228-7,329	8,168 (6,906-9,610	10,062 (8,414-11,986
Cirrhosis due to NASH	228 (174-294)	380 (285-491)	682 (562-810)	1,096 (858-1,363	1,825 (1,521-2,165	2,931 (2,442-3,497	4,216 (3,548-4,977	5,105 (4,489-5,819
Cirrhosis and other chronic liver diseases due to other causes	2,769 (2,159-3,439	1,812 (1,362-2,359	1,489 (1,110-1,952	1,292 (972-1,643	1,532 (1,138-1,993	2,007 (1,535-2,550	2,812 (2,120-3,608	3,993 (2,887-5,319
Upper digestive system diseases	2,020 (1,670-2,326	1,688 (1,412-1,954	1,940 (1,569-2,199	2,392 (1,917-2,716	2,659 (2,033-2,970	3,429 (2,822-3,855	4,808 (3,898-5,340	6,049 (5,020-6,636
Peptic ulcer disease	1,647 (1,380-2,036	1,324 (1,112-1,667	1,530 (1,256-1,885	1,932 (1,591-2,351	2,242 (1,736-2,605	2,881 (2,388-3,400	4,098 (3,432-4,814	5,122 (4,274-5,907
Gastritis and duodenitis	372 (168-476)	365 (178-466)	409 (188-536)	460 (223-585)	418 (221-529)	549 (288-683)	710 (366-897)	927 (517-1,174
Appendicitis	1,219 (998-1,492	1,279 (1,063-1,579	714 (606-864)	786 (660-951)	676 (531-773)	803 (682-943)	721 (608-838)	1,411 (1,132-1,635
Paralytic ileus and intestinal obstruction	1,815 (1,024-2,258	1,669 (1,017-2,047	1,395 (789-1,715)	1,635 (967-1,996	2,074 (1,225-2,423	2,280 (1,473-2,652	3,219 (1,861-3,870	4,776 (2,952-5,597
Inguinal, femoral, and abdominal hernia	86 (47-111)	81 (46-110)	153 (80-204)	204 (105-269)	171 (116-215)	331 (206-412)	497 (344-638)	752 (524-922)
Inflammatory bowel disease	189 (140-267)	222 (169-307)	225 (176-290)	275 (225-338)	333 (263-389)	458 (369-526)	556 (438-627)	772 (599-860)
Vascular intestinal disorders	75 (49-91)	94 (65-115)	121 (85-140)	188 (138-217)	323 (253-359)	558 (461-616)	821 (692-898)	1,357 (1,129-1,480
Gallbladder and biliary diseases	322 (246-379)	404 (324-465)	507 (435-563)	678 (563-767)	803 (680-893)	1,097 (975-1,208)	1,578 (1,395-1,726	2,096 (1,854-2,372
Pancreatitis	443 (331-543)	584 (432-699)	757 (569-904)	972 (718-1,151	1,250 (957-1,453)	1,762 (1,285-2,079	1,833 (1,375-2,092	2,497 (1,854-2,845
Other digestive diseases	238 (195-278)	312 (249-375)	334 (286-388)	416 (350-478)	537 (445-609)	693 (623-767)	960 (861-1,049)	1,362 (1,221-1,478
Neurological disorders	4,823 (4,298-5,505	4,190 (3,765-4,672	4,396 (3,844-5,014	4,443 (4,014-5,065	5,173 (4,840-5,820	6,331 (6,081-6,772	9,478 (9,083-10,089	14,257 (13,857-14,891
Alzheimer's disease and other dementias	-	-	-	-	270 (253-298)	1,003 (957-1,098)	2,860 (2,736-3,114	6,819 (6,551-7,349
Parkinson's disease	6 (5-7)	6 (6-7)	12 (10-13)	20 (17-23)	199 (180-224)	462 (426-514)	885 (832-1,031)	1,514 (1,413-1,694
Epilepsy	4,239 (3,697-4,966	3,539 (3,104-4,044	3,559 (3,012-4,174	3,192 (2,728-3,812	2,865 (2,516-3,470	2,155 (1,861-2,416	2,441 (2,017-2,935	1,990 (1,689-2,145
Multiple sclerosis	109 (89-157)	185 (154-251)	313 (267-411)	536 (466-682)	807 (720-981)	1,068 (956-1,305	1,270 (1,085-1,512	1,386 (1,112-1,541
Motor neuron disease	20 (18-24)	29 (25-32)	49 (43-58)	100 (92-116)	227 (208-273)	450 (422-536)	771 (733-925)	1,147 (1,096-1,311
Other neurological disorders	449 (378-659)	431 (377-600)	463 (420-628)	595 (551-783)	805 (761-1,005)	1,192 (1,113-1,455	1,251 (1,171-1,481	1,400 (1,339-1,629
Mental disorders	32 (28-40)	38 (34-45)	40 (36-45)	41 (38-47)	42 (38-47)	41 (38-46)	-	-
Eating disorders	32 (28-40)	38 (34-45)	40 (36-45)	41 (38-47)	42 (38-47)	41 (38-46)	-	-
Anorexia nervosa	22 (16-26)	25 (17-30)	29 (22-34)	32 (25-38)	32 (25-37)	34 (27-39)	-	-
Bulimia nervosa	10 (8-18)	13 (10-23)	11 (7-18)	9 (6-17)	10 (6-17)	7 (3-13)	-	-
Substance use disorders	2,328 (2,256-2,475	3,277 (3,175-3,494	4,095 (3,974-4,464	5,066 (4,888-5,704	5,806 (5,593-6,431	7,042 (6,736-7,778	6,873 (6,551-7,550	6,083 (5,726-6,873
Alcohol use disorders	373 (347-461)	836 (785-1,036	1,400 (1,315-1,728	2,132 (2,005-2,763	2,729 (2,576-3,329	3,745 (3,457-4,514	3,829 (3,568-4,507	3,644 (3,258-4,400
Drug use disorders	1,955 (1,891-2,035	2,442 (2,368-2,522	2,694 (2,620-2,777	2,934 (2,844-3,028	3,077 (2,984-3,171	3,297 (3,176-3,430	3,044 (2,928-3,163	2,439 (2,352-2,530
Opioid use disorders	1,210 (1,120-1,304	1,514 (1,422-1,606	1,565 (1,450-1,671	1,662 (1,518-1,788	1,758 (1,627-1,863	1,839 (1,709-1,954	1,676 (1,541-1,799	1,343 (1,233-1,437
Cocaine use disorders	57 (50-64)	73 (66-81)	129 (119-143)	141 (129-156)	161 (147-178)	195 (178-215)	152 (139-168)	98 (89-108)
Amphetamine use disorders	101 (77-133)	117 (96-145)	93 (74-120)	103 (80-140)	107 (89-136)	117 (97-147)	80 (64-103)	59 (48-81)
Other drug use disorders	587 (515-665)	737 (677-814)	907 (835-1,008)	1,029 (937-1,162	1,051 (967-1,167)	1,146 (1,058-1,260	1,137 (1,053-1,257	938 (853-1,035
Diabetes and kidney diseases	8,486 (8,064-8,941	10,209 (9,675-10,774	12,273 (11,740-12,843	17,104 (16,468-17,782	26,176 (25,215-27,042	37,410 (36,116-38,542	54,371 (52,601-55,922	73,254 (71,374-75,027
Diabetes mellitus	2,336 (2,218-2,472	3,264 (3,082-3,486	3,927 (3,752-4,119	6,404 (6,108-6,706	11,771 (11,269-12,268	18,904 (18,119-19,652	29,985 (28,865-31,124	41,419 (40,181-42,674
Diabetes mellitus type 1	1,936 (1,774-2,096	2,752 (2,533-2,983	2,855 (2,569-3,120	4,498 (4,112-4,905	7,965 (7,289-8,643	8,394 (7,416-9,516	12,566 (10,970-14,461	16,859 (14,983-18,962
Diabetes mellitus type 2	399 (298-543)	512 (392-694)	1,073 (866-1,368)	1,906 (1,561-2,372	3,807 (3,184-4,594	10,510 (9,351-11,830	17,419 (15,467-19,243	24,560 (22,290-26,819
Chronic kidney disease	6,038 (5,677-6,441	6,841 (6,420-7,321	8,230 (7,798-8,702	10,538 (10,060-11,061	14,195 (13,596-14,806	18,278 (17,530-18,905	24,187 (23,277-25,126	31,549 (30,561-32,506
Chronic kidney disease due to diabetes mellitus type 1	296 (159-487)	682 (409-1,030	1,386 (926-1,939)	2,329 (1,556-3,200	3,411 (2,272-4,690	4,032 (2,679-5,559	4,066 (2,475-5,955	3,647 (2,115-5,690
Chronic kidney disease due to diabetes mellitus type 2	95 (40-181)	171 (77-323)	327 (153-590)	667 (343-1,121	1,462 (830-2,409)	3,052 (1,902-4,383	5,846 (4,037-7,975	9,529 (6,742-12,102
Chronic kidney disease due to hypertension	370 (234-533)	582 (395-807)	840 (581-1,158)	1,213 (858-1,627	1,827 (1,332-2,422	2,665 (1,894-3,493	4,060 (2,988-5,248	6,148 (4,663-8,017
Chronic kidney disease due to glomerulonephritis	2,408 (1,684-3,197	2,490 (1,748-3,289	2,589 (1,806-3,463	2,829 (1,909-3,899	3,437 (2,233-4,748	4,081 (2,665-5,858	4,837 (3,202-6,921	5,406 (3,578-7,843
Chronic kidney disease due to other and unspecified causes	2,869 (2,042-3,660	2,916 (2,125-3,781	3,089 (2,224-3,991	3,500 (2,415-4,600	4,058 (2,761-5,470	4,449 (2,981-6,256	5,378 (3,523-7,649	6,819 (4,508-9,598
Acute glomerulonephritis	112 (99-125)	105 (93-117)	115 (103-127)	162 (144-179)	209 (188-230)	228 (206-250)	199 (181-219)	286 (261-314)
Skin and subcutaneous diseases	352 (189-495)	341 (191-494)	448 (254-667)	592 (326-856)	809 (449-1,200)	1,025 (559-1,521	1,504 (822-2,167)	1,918 (1,017-2,777
Bacterial skin diseases	263 (129-364)	255 (132-362)	355 (192-509)	485 (251-661)	665 (341-956)	837 (441-1,209	1,267 (674-1,774)	1,539 (782-2,164
Cellulitis	79 (34-113)	75 (34-107)	103 (54-163)	119 (57-193)	167 (78-276)	211 (90-329)	360 (163-572)	431 (188-668)
Pyoderma	184 (87-258)	179 (92-258)	253 (128-374)	366 (180-505)	498 (247-734)	626 (316-941)	907 (466-1,309)	1,107 (551-1,626
Decubitus ulcer	41 (24-65)	47 (29-81)	50 (30-82)	61 (36-105)	88 (48-148)	128 (68-215)	169 (93-289)	312 (161-525)
Other skin and subcutaneous diseases	48 (29-88)	38 (23-71)	43 (25-87)	46 (26-94)	57 (30-129)	60 (31-127)	67 (33-155)	67 (31-160)
Musculoskeletal disorders	1,801 (1,457-1,978	1,838 (1,501-2,025	1,912 (1,560-2,131	2,002 (1,635-2,183	2,478 (1,981-2,753	2,747 (2,346-2,992	2,672 (2,293-2,861	3,313 (2,885-3,572
Rheumatoid arthritis	68 (49-92)	85 (64-111)	130 (95-170)	183 (141-229)	334 (253-415)	619 (480-716)	805 (632-898)	1,381 (1,113-1,546
Other musculoskeletal disorders	1,733 (1,395-1,903	1,753 (1,423-1,935	1,782 (1,458-1,989	1,819 (1,484-1,988	2,143 (1,721-2,385	2,128 (1,792-2,316	1,866 (1,586-2,041	1,932 (1,673-2,135
Other non-communicable diseases	9,854 (9,155-10,845	8,507 (7,942-9,373	7,935 (7,332-8,693	7,992 (7,404-8,606	9,178 (8,441-9,770	9,172 (8,290-9,819	10,453 (9,344-11,172	11,924 (10,724-12,710
Congenital birth defects	4,037 (3,809-4,330	3,386 (3,195-3,642	2,320 (2,159-2,519	2,262 (2,090-2,469	2,567 (2,382-2,768	1,326 (1,151-1,475	1,194 (1,000-1,353	1,235 (1,011-1,355
Neural tube defects	130 (110-154)	95 (80-114)	84 (70-100)	67 (57-81)	55 (48-64)	37 (32-44)	31 (27-36)	29 (26-34)
Congenital heart anomalies	2,320 (1,997-2,547	2,162 (1,923-2,363	1,244 (1,077-1,359	1,353 (1,200-1,493	1,711 (1,535-1,881	667 (567-745)	486 (405-573)	469 (392-539)
Orofacial clefts	-	-	-	-	-	-	-	-
Down syndrome	358 (285-533)	301 (255-396)	284 (241-373)	253 (215-320)	306 (263-384)	293 (237-341)	380 (298-430)	426 (296-479)
Other chromosomal abnormalities	105 (58-186)	87 (51-136)	65 (37-99)	50 (30-71)	47 (27-64)	32 (17-42)	28 (15-38)	27 (14-35)
Congenital musculoskeletal and limb anomalies	129 (74-174)	84 (52-107)	77 (45-102)	66 (41-85)	45 (30-57)	35 (24-47)	29 (19-38)	29 (20-40)
Urogenital congenital anomalies	93 (69-114)	64 (49-76)	61 (47-73)	73 (55-89)	83 (59-103)	70 (54-87)	76 (58-89)	105 (83-125)
Digestive congenital anomalies	[illegible]	[illegible]	[illegible]	[illegible]	[illegible]	[illegible]	[illegible]	[illegible]
Other congenital birth defects	[illegible]	[illegible]	[illegible]	[illegible]	[illegible]	[illegible]	[illegible]	[illegible]
Urinary diseases and male infertility	[illegible]	[illegible]	[illegible]	[illegible]	[illegible]	[illegible]	[illegible]	[illegible]
Urinary tract infections	[illegible]	[illegible]	[illegible]	[illegible]	[illegible]	[illegible]	[illegible]	[illegible]
Urolithiasis	[illegible]	[illegible]	[illegible]	[illegible]	[illegible]	[illegible]	[illegible]	[illegible]

Cause	60 to 64	65 to 69	70 to 74	75 to 79	80 to 84	85 to 89	90 to 94	95 plus
Mesothelioma	691 (647-750)	762 (721-812)	954 (910-1,004)	991 (953-1,031)	872 (843-901)	563 (545-582)	214 (207-221)	75 (73-77)
Hodgkin lymphoma	656 (521-831)	715 (576-903)	797 (639-1,001)	761 (604-919)	633 (484-746)	327 (248-377)	89 (68-104)	19 (14-21)
Non-Hodgkin lymphoma	7,825 (7,558-8,116	8,783 (8,506-9,082	10,749 (10,456-11,066	10,688 (10,418-10,937	10,471 (10,258-10,695	7,367 (7,229-7,506	2,837 (2,781-2,895	925 (905-945)
Multiple myeloma	4,577 (4,343-5,320	5,323 (5,081-6,183	6,513 (6,042-7,455	6,760 (6,109-7,432	6,046 (5,331-6,624	3,886 (3,295-4,217	1,440 (1,266-1,622	394 (317-420)
Leukemia	9,846 (8,591-10,837	10,292 (9,002-11,009	12,653 (11,041-13,476	13,038 (11,493-13,803	12,376 (11,082-12,968	8,887 (8,203-9,236	3,760 (3,566-3,888	1,438 (1,391-1,477
Acute lymphoid leukemia	991 (813-1,116)	865 (705-945)	925 (776-1,021)	793 (671-877)	637 (541-702)	348 (308-382)	122 (106-130)	35 (32-37)
Chronic lymphoid leukemia	900 (804-1,000)	1,038 (957-1,127	1,563 (1,459-1,687	1,975 (1,836-2,096	2,221 (2,116-2,333	1,936 (1,851-2,029	950 (905-987)	428 (408-446)
Acute myeloid leukemia	2,979 (2,628-3,240	3,278 (2,891-3,539	3,947 (3,404-4,216	4,078 (3,396-4,278	3,556 (2,896-3,735	2,286 (1,856-2,386	805 (716-837)	264 (248-276)
Chronic myeloid leukemia	888 (787-1,069)	916 (783-1,083	1,100 (973-1,273)	1,095 (985-1,237	1,115 (1,022-1,217	838 (782-901)	345 (326-365)	135 (128-143)
Other leukemia	4,088 (3,319-4,542	4,195 (3,425-4,573	5,117 (4,262-5,587	5,096 (4,327-5,536	4,847 (4,302-5,176	3,479 (3,215-3,714	1,538 (1,438-1,613	577 (545-599)
Other malignant neoplasms	13,443 (11,557-14,433	13,533 (11,794-14,506	16,591 (13,875-17,758	15,620 (13,217-16,542	14,627 (11,677-15,580	9,194 (7,488-9,781	3,843 (3,294-4,071	1,442 (1,218-1,551
Other neoplasms	2,763 (2,025-3,703	3,156 (2,285-4,350	4,286 (3,015-5,885	5,188 (3,535-7,009	5,660 (3,536-7,232	4,851 (2,719-5,990	2,363 (1,203-2,951	1,070 (534-1,352
Myelodysplastic, myeloproliferative, and other hematopoietic neoplasm	2,713 (1,982-3,641	3,122 (2,259-4,317	4,258 (3,001-5,848	5,163 (3,525-6,990	5,642 (3,524-7,218	4,841 (2,715-5,981	2,360 (1,201-2,946	1,069 (534-1,351
Other benign and in situ neoplasms	51 (31-68)	33 (20-45)	28 (15-40)	24 (10-51)	17 (7-34)	10 (3-18)	3 (1-6)	1 (0-2)
Cardiovascular diseases	451,416 (440,221-461,158	572,127 (557,145-585,266	867,991 (847,813-886,695	1,097,732 (1,076,969-1,119,298	1,339,817 (1,317,007-1,360,40	1,153,263 (1,136,759-1,168,09	627,098 (620,274-635,370	304,016 (301,503-306,586
Rheumatic heart disease	12,815 (11,300-14,234	13,850 (12,393-15,135	16,804 (15,275-18,141	17,165 (15,882-18,363	16,777 (15,802-17,718	13,398 (12,792-14,031	6,856 (6,619-7,159	2,996 (2,914-3,119
Ischemic heart disease	210,285 (204,488-216,234	266,899 (259,244-274,290	412,437 (402,885-423,934	518,027 (507,721-532,997	647,953 (635,943-664,923	568,393 (559,221-583,062	318,334 (312,969-329,255	161,213 (158,090-167,546
Stroke	176,169 (171,123-181,137	226,774 (220,164-233,444	341,234 (332,230-350,920	429,378 (419,146-440,935	496,047 (486,095-508,681	394,948 (387,018-404,807	190,918 (187,335-197,435	82,415 (80,874-85,624
Ischemic stroke	58,271 (53,315-64,034	81,887 (75,780-89,033	154,046 (146,008-162,624	203,868 (195,391-212,940	287,057 (279,645-295,551	237,279 (231,804-243,812	116,985 (114,128-121,194	54,128 (52,998-56,346
Intracerebral hemorrhage	101,530 (95,233-107,134	125,460 (117,864-132,675	165,823 (157,259-174,007	200,394 (191,687-209,115	187,623 (181,156-194,293	140,878 (136,528-145,880	65,782 (63,889-68,293	24,770 (24,102-25,770
Subarachnoid hemorrhage	16,368 (15,075-19,066	19,428 (17,904-22,870	21,365 (20,040-24,288	25,116 (23,580-28,312	21,366 (19,871-23,637	16,791 (15,830-18,057	8,151 (7,425-8,672	3,516 (3,309-3,760
Hypertensive heart disease	23,071 (17,907-26,311	30,633 (23,857-34,848	46,304 (36,671-52,080	61,178 (48,033-67,947	75,629 (57,832-83,248	67,805 (46,657-73,875	40,519 (26,288-44,097	20,466 (11,663-22,304
Non-rheumatic valvular heart disease	2,119 (1,909-2,531	2,896 (2,652-3,485	4,645 (4,186-5,758	8,266 (6,829-9,242	12,754 (9,937-13,458	15,612 (10,852-16,659	10,411 (6,653-11,086	5,669 (3,566-6,084
Non-rheumatic calcific aortic valve disease	1,097 (954-1,444)	1,616 (1,419-2,104	2,820 (2,431-3,662	5,181 (4,105-6,056	8,842 (6,334-9,633	11,938 (7,761-12,972	8,297 (5,006-8,991	4,695 (2,717-5,117
Non-rheumatic degenerative mitral valve disease	893 (713-1,091)	1,126 (872-1,389	1,617 (1,306-2,037	2,483 (2,032-3,169	3,171 (2,486-4,215	3,100 (2,164-4,300	1,833 (1,274-2,635	884 (605-1,347
Other non-rheumatic valve diseases	128 (73-184)	155 (91-212)	209 (115-271)	602 (323-755)	740 (396-903)	575 (308-713)	281 (155-343)	90 (55-118)
Cardiomyopathy and myocarditis	8,049 (7,592-8,711	7,334 (6,809-7,999	9,851 (9,289-10,590	12,996 (12,312-13,779	19,379 (18,666-20,109	20,083 (19,257-21,054	12,242 (11,746-12,774	7,312 (6,954-7,809
Myocarditis	669 (478-777)	788 (590-907)	1,097 (819-1,241)	1,632 (1,251-1,826	2,853 (2,102-3,212	3,556 (2,526-4,072	2,555 (1,686-2,947	1,911 (983-2,395
Alcoholic cardiomyopathy	2,797 (2,628-3,059	1,709 (1,571-1,898	1,836 (1,683-2,148	1,665 (1,503-2,148	1,829 (1,590-2,826	1,119 (937-1,775)	571 (478-917)	291 (245-485)
Other cardiomyopathy	4,583 (4,092-5,175	4,836 (4,373-5,367	6,918 (6,333-7,547	9,699 (8,927-10,403	14,697 (13,621-15,418	15,408 (14,467-16,659	9,116 (8,525-9,988	5,110 (4,585-6,220
Atrial fibrillation and flutter	2,657 (2,520-2,772	4,245 (4,034-4,429	9,190 (8,790-9,535	15,902 (15,200-16,568	29,762 (28,723-30,770	34,170 (33,115-35,151	25,675 (24,838-26,399	11,955 (11,655-12,432
Aortic aneurysm	2,838 (2,695-3,222	3,781 (3,595-4,285	5,647 (5,414-6,250	7,575 (7,314-8,159	9,248 (8,943-9,617	8,508 (8,281-8,755	4,614 (4,495-4,727	2,187 (2,132-2,271
Peripheral artery disease	869 (409-1,985)	1,200 (545-2,859	2,167 (938-5,174)	3,219 (1,398-7,539	5,363 (2,264-12,480	6,099 (2,543-13,705	4,430 (1,797-10,802	2,873 (1,139-7,457
Endocarditis	1,839 (1,492-2,317	2,178 (1,793-2,797	2,923 (2,330-3,758	3,849 (2,858-4,976	4,797 (3,414-6,300	5,051 (3,259-6,497	3,233 (1,967-4,225	1,820 (1,067-2,486
Other cardiovascular and circulatory diseases	10,706 (9,396-12,285	12,337 (10,860-13,966	16,789 (14,881-18,863	20,177 (18,110-22,183	22,108 (19,885-23,935	19,195 (17,730-20,568	9,865 (9,152-10,459	5,112 (4,834-5,497
Chronic respiratory diseases	100,145 (91,270-106,400	135,745 (122,782-143,146	209,599 (189,743-223,115	256,985 (235,003-270,855	284,298 (260,973-299,756	216,959 (202,161-226,117	104,843 (99,747-112,705	39,355 (38,147-41,518
Chronic obstructive pulmonary disease	73,568 (62,389-84,296	106,102 (91,609-117,882	170,886 (149,072-187,917	214,940 (191,535-232,851	245,589 (224,793-263,413	191,509 (177,836-201,013	93,996 (89,448-101,668	35,471 (34,385-37,574
Pneumoconiosis	144 (109-182)	171 (127-212)	277 (222-340)	344 (266-426)	363 (286-457)	261 (204-300)	117 (97-135)	51 (42-57)
Silicosis	36 (21-50)	37 (20-52)	70 (42-98)	94 (60-133)	96 (64-142)	67 (44-109)	29 (19-44)	11 (7-18)
Asbestosis	26 (16-39)	30 (17-44)	55 (33-76)	69 (38-92)	79 (38-106)	67 (27-86)	27 (12-34)	12 (5-16)
Coal workers pneumoconiosis	15 (7-23)	22 (12-33)	29 (14-42)	37 (20-53)	36 (19-49)	27 (15-37)	14 (8-18)	7 (4-9)
Other pneumoconiosis	66 (43-89)	81 (54-110)	124 (87-162)	145 (103-194)	152 (114-199)	100 (76-133)	48 (38-64)	20 (15-28)
Asthma	22,066 (13,214-31,420	23,971 (14,034-34,003	30,540 (18,021-44,113	32,153 (19,787-45,188	28,307 (18,917-38,577	17,324 (12,226-23,048	7,022 (5,276-9,089	2,273 (1,891-2,696
Interstitial lung disease and pulmonary sarcoidosis	3,733 (2,439-5,742	4,792 (3,122-7,364	6,995 (4,609-10,352	8,421 (5,220-11,899	8,778 (5,327-11,651	6,887 (3,936-8,612	3,182 (1,816-3,796	1,323 (765-1,493)
Other chronic respiratory diseases	635 (466-794)	711 (532-858)	901 (696-1,107)	1,127 (872-1,374)	1,261 (996-1,625)	978 (798-1,171)	526 (438-615)	238 (206-265)
Digestive diseases	67,633 (62,506-71,152	73,285 (67,416-76,849	88,385 (81,944-92,644	95,220 (88,654-100,122	98,869 (92,727-102,879	79,573 (75,742-82,573	41,735 (40,132-42,933	19,330 (18,879-19,826
Cirrhosis and other chronic liver diseases	40,418 (37,937-45,313	39,620 (37,036-43,997	43,235 (40,123-49,328	39,474 (36,663-44,464	33,089 (30,292-37,292	21,087 (19,412-23,429	8,863 (8,299-9,815	3,601 (3,410-4,035
Cirrhosis and other chronic liver diseases due to hepatitis B	9,711 (7,884-11,935	10,125 (8,340-12,358	11,546 (9,355-14,201	10,648 (8,760-12,951	8,655 (6,973-10,745	5,054 (4,239-6,016	1,895 (1,542-2,335	595 (446-773)
Cirrhosis and other chronic liver diseases due to hepatitis C	11,059 (9,224-13,087	10,823 (9,303-12,457	11,459 (9,821-13,282	10,038 (8,421-12,104	8,418 (6,903-10,125	5,539 (4,698-6,458	2,399 (2,002-2,880	1,038 (821-1,289)
Cirrhosis and other chronic liver diseases due to alcohol use	9,696 (7,881-11,784	8,520 (6,988-10,364	8,672 (7,056-10,740	7,507 (6,301-8,986	5,911 (4,926-7,260	3,562 (3,018-4,308	1,424 (1,151-1,774	575 (411-782)
Cirrhosis due to NASH	5,186 (4,468-5,958	4,814 (4,229-5,499	4,821 (4,216-5,517	3,933 (3,328-4,622	3,183 (2,645-3,777	2,125 (1,794-2,495	937 (755-1,167)	374 (270-506)
Cirrhosis and other chronic liver diseases due to other causes	4,767 (3,473-6,250	5,337 (3,971-6,950	6,737 (5,240-8,533	7,348 (5,756-9,270	6,921 (5,364-8,719	4,807 (3,975-5,779	2,208 (1,769-2,694	1,019 (756-1,305)
Upper digestive system diseases	8,539 (6,991-9,314	11,194 (9,283-12,289	14,053 (11,656-15,312	16,174 (13,806-17,422	17,395 (15,464-18,524	14,019 (12,975-15,169	7,108 (6,705-7,688	3,000 (2,862-3,280
Peptic ulcer disease	7,266 (6,024-8,228	9,452 (7,984-10,808	11,879 (9,824-13,666	13,601 (11,719-15,275	14,528 (12,959-16,047	11,774 (10,810-13,054	5,863 (5,434-6,539	2,516 (2,386-2,810
Gastritis and duodenitis	1,273 (677-1,571)	1,742 (882-2,137	2,174 (1,140-2,644	2,573 (1,429-3,133	2,867 (1,759-3,514	2,246 (1,510-2,842	1,245 (923-1,592)	483 (381-592)
Appendicitis	1,388 (1,078-1,593	1,397 (1,118-1,581	1,432 (1,149-1,658	1,668 (1,382-1,912	1,441 (1,208-1,669	1,177 (996-1,384)	594 (518-690)	207 (184-234)
Paralytic ileus and intestinal obstruction	5,762 (3,577-6,829	6,662 (4,337-7,714	8,110 (5,733-9,280	10,271 (8,028-11,838	12,181 (9,554-14,334	11,447 (8,764-13,636	7,054 (5,477-8,357	3,436 (2,449-4,148
Inguinal, femoral, and abdominal hernia	1,000 (709-1,242)	1,215 (870-1,549	1,786 (1,301-2,249	2,047 (1,492-2,664	2,762 (1,920-3,530	2,337 (1,659-3,123	1,321 (941-1,731)	610 (403-857)
Inflammatory bowel disease	944 (697-1,037)	1,103 (838-1,210	1,653 (1,227-1,816	2,160 (1,609-2,354	2,811 (1,942-3,090	2,844 (1,900-3,182	1,744 (1,096-1,967	945 (569-1,094)
Vascular intestinal disorders	2,157 (1,789-2,379	3,221 (2,702-3,502	5,733 (4,967-6,198	7,933 (7,045-8,643	9,921 (9,036-10,854	8,471 (7,648-9,404	4,250 (3,825-4,647	1,792 (1,557-2,014
Gallbladder and biliary diseases	2,873 (2,542-3,115	3,643 (3,200-3,946	5,026 (4,478-5,482	6,884 (6,082-7,475	8,964 (8,031-9,613	8,471 (7,667-9,205	5,344 (4,856-5,810	2,958 (2,643-3,247
Pancreatitis	2,629 (1,965-2,966	2,911 (2,121-3,271	3,909 (2,853-4,394	3,978 (2,912-4,425	4,107 (3,004-4,467	3,106 (2,351-3,456	1,499 (1,120-1,647	635 (486-706)
Other digestive diseases	1,922 (1,708-2,073	2,319 (2,105-2,490	3,448 (3,110-3,680	4,633 (4,359-4,971	6,199 (5,887-6,599	6,614 (6,253-7,127	3,957 (3,741-4,254	2,147 (2,037-2,401
Neurological disorders	25,109 (24,481-26,236	43,199 (42,185-45,038	91,628 (89,626-95,196	185,252 (181,821-190,746	311,081 (304,859-317,146	360,162 (355,383-365,942	273,015 (267,633-276,105	143,197 (141,853-144,292
Alzheimer's disease and other dementias	15,925 (15,431-16,936	31,019 (30,212-32,715	70,600 (68,996-73,907	153,555 (150,833-158,730	273,231 (268,538-278,764	332,132 (327,780-337,772	261,719 (256,512-264,781	139,758 (138,531-140,833
Parkinson's disease	2,891 (2,683-3,231	5,764 (5,369-6,258	14,201 (12,871-15,156	25,486 (23,308-26,873	32,433 (30,258-34,006	24,273 (22,658-25,162	9,907 (9,171-10,456	2,877 (2,748-2,943
Epilepsy	1,924 (1,577-2,096	2,083 (1,685-2,266	2,400 (1,947-2,656	2,017 (1,640-2,336	1,982 (1,641-2,163	1,599 (1,293-1,854	621 (517-713)	257 (232-277)
Multiple sclerosis	1,293 (968-1,432)	1,036 (751-1,150	886 (619-978)	682 (469-752)	516 (344-566)	308 (206-342)	96 (67-105)	28 (20-30)
Motor neuron disease	1,547 (1,478-1,674	1,808 (1,731-1,953	1,931 (1,874-1,994	1,878 (1,817-1,935	1,357 (1,307-1,406	707 (680-730)	175 (167-183)	51 (48-53)
Other neurological disorders	1,529 (1,412-1,788	1,488 (1,386-1,734	1,609 (1,516-1,813	1,633 (1,497-1,800	1,561 (1,366-1,666	1,142 (988-1,203)	497 (473-524)	226 (216-248)
Mental disorders	-	-	-	-	-	-	-	-
Eating disorders	-	-	-	-	-	-	-	-
Anorexia nervosa	-	-	-	-	-	-	-	-
Bulimia nervosa	-	-	-	-	-	-	-	-
Substance use disorders	4,588 (4,258-4,841	3,532 (3,266-3,679	2,956 (2,738-3,050	2,997 (2,698-3,109	2,544 (2,237-2,627	1,992 (1,693-2,072	915 (788-948)	421 (361-437)
Alcohol use disorders	2,662 (2,326-2,929	1,862 (1,555-1,950	1,328 (1,093-1,372	1,247 (936-1,337	810 (498-860)	648 (331-701)	286 (162-308)	128 (69-138)
Drug use disorders	1,926 (1,862-1,992	1,670 (1,605-1,739	1,627 (1,571-1,683	1,751 (1,689-1,815	1,734 (1,684-1,787	1,344 (1,295-1,394	629 (611-650)	293 (283-303)
Opioid use disorders	1,058 (956-1,143)	949 (844-1,044	781 (717-869)	842 (780-928)	808 (767-879)	603 (571-647)	278 (264-292)	112 (107-118)
Cocaine use disorders	59 (54-67)	39 (34-46)	13 (11-15)	13 (10-14)	9 (8-10)	8 (7-9)	5 (4-5)	3 (3-4)
Amphetamine use disorders	42 (30-66)	31 (20-55)	9 (7-14)	9 (7-14)	5 (3-5)	5 (3-6)	3 (1-3)	3 (1-3)
Other drug use disorders	766 (687-878)	651 (564-772)	824 (747-901)	887 (812-947)	912 (843-955)	727 (684-769)	344 (327-361)	175 (167-184)
Diabetes and kidney diseases	97,314 (94,575-100,105	109,709 (106,598-112,486	128,070 (124,707-131,517	140,547 (136,837-144,532	133,543 (129,885-137,570	103,664 (101,390-106,149	52,514 (51,422-53,793	23,886 (23,457-24,307
Diabetes mellitus	58,971 (56,822-61,026	67,370 (65,102-69,462	77,222 (74,908-79,670	82,854 (80,279-85,622	75,486 (72,828-77,985	51,442 (50,004-53,038	23,301 (22,714-23,997	9,163 (8,961-9,373
Diabetes mellitus type 1	17,322 (14,961-20,609	19,298 (16,683-22,687	18,210 (15,874-21,022	19,042 (16,593-22,019	12,958 (10,707-15,250	7,948 (6,228-9,186	3,334 (2,566-3,810	1,249 (878-1,400
Diabetes mellitus type 2	41,649 (38,152-44,763	48,072 (44,475-51,376	59,012 (55,516-62,290	63,812 (59,998-67,530	62,528 (59,360-66,408	43,494 (41,685-46,159	19,967 (19,130-21,144	7,913 (7,657-8,354
Chronic kidney disease	38,020 (36,896-39,202	41,983 (40,661-43,318	50,400 (48,983-51,834	57,210 (55,537-59,001	57,600 (56,139-59,320	51,894 (50,672-53,157	29,066 (28,393-29,814	14,668 (14,359-15,036
Chronic kidney disease due to diabetes mellitus type 1	2,620 (1,288-4,479	1,946 (992-3,459	1,800 (869-3,124)	1,651 (806-2,961	1,456 (721-2,491)	926 (494-1,564	223 (111-394)	25 (11-49)
Chronic kidney disease due to diabetes mellitus type 2	13,477 (9,920-16,959	15,590 (11,857-19,213	18,348 (13,819-22,391	20,173 (14,729-25,312	19,344 (13,845-24,828	16,115 (11,533-20,483	8,315 (6,054-10,682	3,838 (2,495-5,448
Chronic kidney disease due to hypertension	8,273 (6,286-10,621	10,501 (8,131-13,190	14,170 (11,240-17,703	18,013 (14,364-22,432	20,376 (16,120-24,970	21,008 (17,371-24,928	13,189 (10,987-15,335	7,043 (5,665-8,458
Chronic kidney disease due to glomerulonephritis	5,449 (3,524-8,071	5,651 (3,637-8,160	6,936 (4,395-9,892	7,567 (4,909-10,836	7,295 (4,727-10,904	5,916 (3,925-8,658	3,016 (2,025-4,387	1,505 (824-2,455)
Chronic kidney disease due to other and unspecified causes	8,200 (5,013-11,859	8,296 (5,306-11,783	9,146 (6,022-12,935	9,806 (6,337-14,475	9,128 (6,071-13,161	7,930 (5,414-10,967	4,322 (2,948-6,090	2,257 (1,439-3,479
Acute glomerulonephritis	323 (295-353)	355 (324-390)	448 (409-491)	483 (443-533)	457 (419-502)	328 (302-356)	147 (136-160)	56 (51-62)
Skin and subcutaneous diseases	2,257 (1,100-3,149	2,717 (1,406-3,871	3,667 (1,906-5,129	4,469 (2,506-6,764	5,209 (2,857-8,230	5,051 (2,634-8,150	3,157 (1,659-5,128	1,929 (934-3,311)
Bacterial skin diseases	1,873 (876-2,522)	2,110 (1,021-2,864	2,712 (1,341-3,677	3,157 (1,670-4,733	3,194 (1,664-4,792	2,876 (1,443-4,351	1,782 (878-2,850)	1,008 (474-1,605)
Cellulitis	511 (216-788)	535 (225-823)	650 (274-1,035)	697 (274-1,097	835 (331-1,432)	729 (293-1,301)	485 (186-924)	295 (104-547)
Pyoderma	1,362 (644-1,828)	1,574 (722-2,212	2,062 (984-2,827)	2,459 (1,298-3,695	2,359 (1,232-3,477	2,146 (1,072-3,290	1,297 (630-1,990)	713 (340-1,129)
Decubitus ulcer	302 (156-502)	510 (266-775)	826 (447-1,328)	1,132 (611-1,844	1,768 (977-2,899)	1,905 (996-3,194)	1,204 (645-2,013)	826 (407-1,436
Other skin and subcutaneous diseases	82 (36-190)	98 (45-229)	129 (62-306)	180 (84-424)	247 (111-581)	270 (117-715)	170 (70-462)	95 (34-268)
Musculoskeletal disorders	4,611 (3,825-4,972	5,821 (4,771-6,383	7,240 (5,940-7,787	7,887 (6,543-8,567	7,768 (6,753-8,436	6,311 (5,703-6,734	3,246 (2,973-3,441	1,636 (1,562-1,703
Rheumatoid arthritis	2,328 (1,818-2,644	3,297 (2,543-3,736	4,254 (3,375-4,772	4,668 (3,689-5,215	4,148 (3,402-4,668	2,701 (2,319-3,060	1,118 (971-1,300)	371 (331-439)
Other musculoskeletal disorders	2,284 (1,948-2,675	2,524 (2,049-3,153	2,986 (2,431-3,713	3,220 (2,649-3,903	3,620 (3,069-4,203	3,610 (3,260-3,962	2,127 (1,887-2,288	1,266 (1,168-1,323
Other non-communicable diseases	13,894 (12,456-14,749	15,731 (14,117-16,762	17,799 (16,102-19,040	20,528 (18,565-21,630	22,898 (20,522-24,041	22,158 (19,620-23,185	13,738 (12,255-14,341	8,235 (7,193-8,645
Congenital birth defects	1,064 (862-1,172)	920 (789-1,035)	-	-	-	-	-	-
Neural tube defects	21 (18-25)	17 (15-21)	-	-	-	-	-	-
Congenital heart anomalies	411 (346-491)	412 (346-482)	-	-	-	-	-	-
Orofacial clefts	-	-	-	-	-	-	-	-
Down syndrome	312 (210-351)	139 (95-165)	-	-	-	-	-	-
Other chromosomal abnormalities	23 (11-32)	18 (10-26)	-	-	-	-	-	-
Congenital musculoskeletal and limb anomalies	27 (20-39)	23 (16-36)	-	-	-	-	-	-
Urogenital congenital anomalies	128 (101-153)	171 (139-199)	-	-	-	-	-	-
Digestive congenital anomalies	[illegible]	[illegible]	[illegible]	[illegible]	[illegible]	[illegible]	[illegible]	[illegible]
Other congenital birth defects	[illegible]	[illegible]	[illegible]	[illegible]	[illegible]	[illegible]	[illegible]	[illegible]
Urinary diseases and male infertility	[illegible]	[illegible]	[illegible]	[illegible]	[illegible]	[illegible]	[illegible]	[illegible]
Urinary tract infections	[illegible]	[illegible]	[illegible]	[illegible]	[illegible]	[illegible]	[illegible]	[illegible]
Urolithiasis	[illegible]	[illegible]	[illegible]	[illegible]	[illegible]	[illegible]	[illegible]	[illegible]

SR Table 5: Global deaths by cause and age group for females (with 95% confidence intervals), 2010

Cause	Early Neonatal	Late Neonatal	Post Neonatal	1 to 4	5 to 9	10 to 14	15 to 19
Other urinary diseases	180 (142-205)	102 (80-124)	660 (342-986)	1,144 (585-1,682)	348 (217-473)	274 (209-359)	227 (133-313)
Gynecological diseases	-	-	-	-	-	-	373 (332-502)
Uterine fibroids	-	-	-	-	-	-	35 (27-51)
Polycystic ovarian syndrome	-	-	-	-	-	-	1 (0-3)
Endometriosis	-	-	-	-	-	-	8 (4-14)
Genital prolapse	-	-	-	-	-	-	10 (5-16)
Other gynecological diseases	-	-	-	-	-	-	319 (276-432)
Hemoglobinopathies and hemolytic anemias	365 (276-502)	237 (191-326)	2,526 (1,833-3,832)	7,766 (3,990-12,413)	2,206 (1,751-2,972)	1,711 (1,347-2,428)	2,498 (1,965-3,400)
Thalassemias	127 (90-156)	86 (64-116)	815 (616-1,145)	1,174 (890-1,523)	697 (502-932)	394 (297-527)	369 (243-524)
Sickle cell disorders	224 (170-340)	147 (115-209)	1,670 (1,068-2,651)	6,450 (2,885-10,626)	1,383 (992-2,077)	1,106 (797-1,684)	1,688 (1,295-2,388)
G6PD deficiency	4 (2-5)	2 (2-3)	27 (17-46)	111 (58-194)	93 (66-136)	142 (98-209)	260 (178-387)
Other hemoglobinopathies and hemolytic anemias	10 (7-14)	2 (1-2)	14 (10-22)	32 (20-48)	33 (25-42)	69 (53-94)	182 (123-304)
Endocrine, metabolic, blood, and immune disorders	684 (517-939)	561 (448-767)	3,489 (2,634-4,756)	2,260 (1,575-3,057)	888 (734-1,119)	744 (642-964)	857 (729-1,099)
Sudden infant death syndrome	-	1,803 (709-3,653)	16,954 (6,063-39,060)	-	-	-	-
Injuries	5,380 (4,664-5,848)	4,275 (3,812-4,611)	44,171 (40,080-47,777)	105,030 (93,215-114,677)	57,097 (51,645-61,376)	48,664 (45,092-51,469)	84,147 (79,660-87,118)
Transport injuries	336 (308-364)	419 (386-456)	6,620 (5,670-7,317)	22,594 (17,370-25,578)	16,640 (14,994-18,390)	11,655 (10,627-12,687)	21,109 (19,943-22,357)
Road injuries	309 (281-337)	393 (361-427)	6,223 (5,245-6,879)	21,334 (16,164-24,186)	15,987 (14,406-17,734)	10,879 (9,948-11,845)	19,646 (18,575-20,830)
Pedestrian road injuries	134 (112-158)	164 (136-199)	2,466 (2,089-2,981)	11,776 (9,371-14,511)	9,410 (8,293-10,702)	5,439 (4,824-6,249)	6,714 (6,010-7,796)
Cyclist road injuries	-	-	-	453 (328-615)	502 (426-607)	617 (511-758)	773 (640-952)
Motorcyclist road injuries	33 (25-42)	28 (22-37)	764 (563-1,026)	1,208 (862-1,739)	846 (670-999)	981 (784-1,159)	2,939 (2,494-3,412)
Motor vehicle road injuries	136 (112-157)	197 (164-227)	2,901 (2,260-3,363)	7,690 (5,025-9,774)	5,057 (4,265-5,712)	3,731 (3,277-4,163)	8,992 (8,235-9,795)
Other road injuries	7 (5-9)	5 (3-7)	92 (57-132)	206 (105-334)	172 (116-230)	111 (83-155)	228 (178-309)
Other transport injuries	26 (20-36)	26 (21-33)	397 (319-538)	1,261 (946-1,937)	653 (554-766)	776 (671-891)	1,463 (1,273-1,696)
Unintentional injuries	4,002 (3,454-4,402)	3,496 (3,073-3,810)	35,253 (31,497-38,504)	75,927 (67,509-83,261)	36,476 (32,088-39,559)	28,534 (25,618-30,465)	26,769 (23,922-28,364)
Falls	710 (634-795)	287 (254-323)	4,203 (3,646-4,743)	5,116 (4,272-6,026)	2,377 (2,070-2,660)	1,766 (1,565-1,951)	2,211 (2,002-2,432)
Drowning	186 (164-209)	116 (105-128)	3,643 (3,140-4,077)	31,630 (26,977-35,212)	12,566 (10,640-14,119)	7,387 (6,368-8,060)	4,791 (4,251-5,207)
Fire, heat, and hot substances	143 (91-173)	205 (125-255)	2,831 (1,776-3,432)	8,022 (4,781-10,201)	2,178 (1,578-2,516)	1,781 (1,246-2,079)	3,719 (2,285-4,487)
Poisonings	80 (55-88)	94 (64-107)	1,573 (1,038-1,831)	3,523 (2,276-4,373)	920 (649-1,037)	1,091 (774-1,232)	1,298 (903-1,404)
Poisoning by carbon monoxide	18 (11-22)	21 (12-25)	286 (157-363)	619 (373-757)	374 (227-432)	384 (234-448)	605 (396-669)
Poisoning by other means	61 (43-69)	73 (50-83)	1,287 (858-1,545)	2,904 (1,848-3,728)	546 (400-638)	707 (532-822)	693 (494-766)
Exposure to mechanical forces	343 (202-433)	383 (227-489)	2,216 (1,290-2,777)	2,493 (1,735-2,963)	1,827 (1,268-2,168)	983 (727-1,127)	1,420 (1,003-1,632)
Unintentional firearm injuries	22 (15-32)	16 (11-23)	158 (113-229)	198 (144-314)	189 (143-263)	148 (115-182)	247 (190-290)
Other exposure to mechanical forces	321 (184-411)	367 (211-472)	2,058 (1,158-2,610)	2,296 (1,538-2,771)	1,638 (1,087-1,977)	835 (595-975)	1,173 (804-1,369)
Adverse effects of medical treatment	614 (415-770)	425 (295-541)	3,453 (2,220-4,735)	2,513 (1,306-4,312)	938 (655-1,182)	735 (553-877)	1,011 (772-1,196)
Animal contact	88 (32-117)	87 (33-118)	1,551 (519-2,091)	3,937 (1,472-5,704)	3,425 (1,277-4,669)	2,769 (974-3,730)	1,802 (646-2,429)
Venomous animal contact	59 (16-89)	54 (16-86)	1,275 (389-1,825)	3,166 (1,124-4,583)	3,079 (1,101-4,284)	2,570 (860-3,527)	1,674 (568-2,268)
Non-venomous animal contact	29 (12-68)	33 (14-73)	276 (87-871)	771 (161-2,844)	346 (117-1,134)	199 (78-551)	128 (54-352)
Foreign body	1,498 (1,365-1,636)	1,504 (1,390-1,638)	11,910 (10,384-13,125)	4,756 (3,432-5,533)	1,151 (1,050-1,262)	817 (764-872)	642 (610-678)
Pulmonary aspiration and foreign body in airway	1,467 (1,326-1,607)	1,482 (1,365-1,617)	11,636 (10,206-12,835)	4,458 (3,228-5,270)	1,028 (933-1,135)	719 (662-777)	474 (430-512)
Foreign body in other body part	31 (20-56)	22 (15-41)	274 (172-530)	298 (172-590)	123 (93-183)	98 (77-143)	168 (144-212)
Environmental heat and cold exposure	110 (46-140)	65 (23-87)	856 (313-1,151)	609 (264-922)	297 (137-391)	136 (90-158)	342 (214-405)
Exposure to forces of nature	72 (32-149)	275 (118-537)	2,222 (960-4,354)	10,919 (4,631-22,320)	9,708 (4,215-19,384)	9,906 (4,484-19,388)	8,186 (3,491-15,990)
Other unintentional injuries	157 (138-178)	53 (47-61)	794 (706-890)	2,410 (2,063-2,790)	1,087 (971-1,220)	1,163 (1,049-1,297)	1,347 (1,234-1,483)
Self-harm and interpersonal violence	1,043 (838-1,227)	360 (302-428)	2,298 (1,879-2,702)	6,509 (5,364-7,333)	3,982 (3,423-4,555)	8,474 (7,763-9,204)	36,268 (34,167-38,181)
Self-harm	-	-	-	-	-	4,506 (4,040-4,994)	28,174 (26,416-29,797)
Self-harm by firearm	-	-	-	-	-	162 (105-228)	912 (526-1,332)
Self-harm by other specified means	-	-	-	-	-	4,344 (3,882-4,805)	27,262 (25,493-28,866)
Interpersonal violence	791 (586-975)	222 (165-291)	1,807 (1,416-2,240)	3,878 (2,732-4,698)	2,377 (1,815-2,947)	2,568 (2,085-3,033)	6,326 (5,069-7,231)
Physical violence by firearm	57 (34-84)	22 (12-32)	251 (161-342)	498 (316-707)	383 (275-494)	553 (458-650)	1,934 (1,604-2,245)
Physical violence by sharp object	50 (36-63)	31 (20-42)	165 (122-215)	503 (321-720)	239 (184-292)	405 (313-489)	1,241 (948-1,435)
Physical violence by other means	684 (494-841)	169 (124-224)	1,392 (1,066-1,746)	2,876 (1,996-3,504)	1,755 (1,286-2,257)	1,611 (1,232-1,997)	3,152 (2,304-3,895)
Conflict and terrorism	251 (225-295)	137 (123-159)	453 (407-525)	2,577 (2,300-3,010)	1,560 (1,388-1,812)	1,359 (1,205-1,585)	1,708 (1,487-2,008)
Executions and police conflict	-	-	38 (9-110)	54 (51-60)	45 (41-51)	42 (39-46)	61 (57-69)

Cause	20 to 24	25 to 29	30 to 34	35 to 39	40 to 44	45 to 49	50 to 54	54 to 59
Other urinary diseases	315 (183-442)	285 (169-391)	323 (207-423)	321 (188-435)	351 (217-454)	485 (304-605)	519 (293-656)	725 (434-895)
Gynecological diseases	419 (373-551)	435 (388-550)	503 (455-595)	589 (531-650)	699 (624-771)	851 (753-946)	601 (546-658)	420 (386-449)
Uterine fibroids	51 (35-82)	83 (57-129)	108 (75-161)	138 (76-202)	199 (116-270)	300 (214-379)	234 (193-291)	120 (98-147)
Polycystic ovarian syndrome	2 (1-7)	2 (0-5)	1 (0-4)	1 (0-3)	1 (0-3)	1 (0-3)	1 (0-2)	-
Endometriosis	12 (5-20)	12 (5-19)	17 (7-27)	16 (7-28)	25 (8-43)	40 (12-65)	22 (9-38)	-
Genital prolapse	10 (6-16)	10 (4-16)	10 (5-16)	9 (4-15)	18 (6-31)	24 (6-42)	52 (16-83)	41 (16-69)
Other gynecological diseases	343 (284-488)	328 (262-466)	366 (300-454)	425 (349-542)	456 (358-551)	486 (377-576)	291 (211-349)	259 (211-296)
Hemoglobinopathies and hemolytic anemias	2,365 (1,858-3,207)	1,824 (1,469-2,475)	1,829 (1,402-2,440)	1,341 (1,056-1,762)	1,508 (1,198-1,931)	1,341 (1,057-1,740)	1,452 (1,124-1,873)	1,649 (1,314-2,163)
Thalassemias	277 (168-387)	167 (104-236)	110 (73-152)	57 (42-72)	44 (34-54)	33 (27-40)	20 (16-24)	13 (11-16)
Sickle cell disorders	1,532 (1,173-2,186)	1,079 (799-1,593)	913 (668-1,387)	522 (368-819)	485 (328-807)	375 (244-688)	304 (195-578)	237 (148-454)
G6PD deficiency	329 (228-471)	361 (254-486)	484 (324-628)	412 (298-514)	476 (342-586)	399 (299-498)	420 (307-539)	400 (281-578)
Other hemoglobinopathies and hemolytic anemias	227 (151-370)	217 (152-316)	322 (224-446)	350 (261-429)	503 (390-607)	534 (426-644)	708 (551-843)	999 (799-1,219)
Endocrine, metabolic, blood, and immune disorders	1,150 (974-1,470)	1,319 (1,054-1,655)	1,471 (1,189-1,776)	1,725 (1,348-2,065)	1,923 (1,460-2,279)	2,647 (1,955-2,962)	3,255 (2,431-3,637)	3,482 (2,514-3,925)
Sudden infant death syndrome	-	-	-	-	-	-	-	-
Injuries	97,253 (92,701-100,137)	84,269 (80,212-86,840)	73,205 (69,391-75,484)	70,238 (66,612-72,423)	70,323 (66,401-72,553)	68,580 (65,080-70,613)	65,751 (62,421-67,528)	68,564 (65,373-70,258)
Transport injuries	25,887 (24,081-27,174)	22,392 (21,361-23,404)	19,688 (18,824-20,434)	20,387 (19,198-21,222)	22,228 (21,337-23,014)	20,993 (20,280-21,709)	20,047 (19,416-20,745)	20,378 (19,758-21,003)
Road injuries	24,613 (22,865-25,867)	21,259 (20,249-22,263)	18,739 (17,907-19,484)	19,480 (18,300-20,283)	21,149 (20,258-21,880)	19,887 (19,223-20,535)	18,999 (18,383-19,667)	19,584 (19,008-20,185)
Pedestrian road injuries	8,539 (7,688-9,703)	7,056 (6,343-8,052)	6,976 (6,389-7,801)	8,146 (7,556-8,892)	9,333 (8,741-10,118)	8,831 (8,269-9,552)	8,476 (7,855-9,357)	9,760 (9,182-10,594)
Cyclist road injuries	802 (664-927)	665 (576-768)	758 (651-865)	777 (665-842)	1,099 (882-1,198)	1,183 (961-1,285)	1,006 (856-1,111)	1,164 (923-1,271)
Motorcyclist road injuries	3,765 (3,078-4,487)	3,268 (2,668-3,869)	3,070 (2,519-3,599)	2,689 (2,131-3,071)	2,945 (2,336-3,332)	2,527 (2,068-2,806)	2,352 (1,892-2,662)	2,021 (1,653-2,257)
Motor vehicle road injuries	11,261 (10,206-12,149)	10,091 (9,218-10,866)	7,744 (7,038-8,341)	7,659 (6,913-8,258)	7,587 (6,828-8,206)	7,109 (6,434-7,625)	6,957 (6,262-7,444)	6,435 (5,789-6,875)
Other road injuries	245 (186-323)	179 (137-230)	192 (151-246)	210 (167-264)	184 (146-228)	238 (193-301)	207 (161-265)	205 (156-261)
Other transport injuries	1,275 (1,141-1,438)	1,132 (1,027-1,261)	949 (867-1,043)	906 (827-1,000)	1,079 (986-1,195)	1,106 (1,018-1,205)	1,048 (962-1,147)	793 (740-845)
Unintentional injuries	28,187 (25,122-29,898)	25,955 (23,009-27,497)	24,785 (21,949-26,278)	23,810 (21,177-25,077)	24,428 (21,617-25,777)	25,357 (22,830-26,555)	25,987 (23,527-27,128)	29,788 (27,431-30,995)
Falls	2,377 (2,161-2,628)	2,058 (1,942-2,187)	2,367 (2,233-2,521)	2,988 (2,810-3,178)	3,359 (3,185-3,549)	4,190 (3,999-4,359)	5,008 (4,756-5,233)	7,775 (7,381-8,198)
Drowning	3,789 (3,374-4,073)	2,971 (2,694-3,162)	2,803 (2,560-2,963)	2,821 (2,423-2,970)	2,736 (2,412-2,866)	2,708 (2,499-2,838)	2,625 (2,434-2,764)	2,855 (2,575-2,987)
Fire, heat, and hot substances	4,837 (2,984-5,803)	4,045 (2,592-4,785)	3,821 (2,512-4,507)	2,839 (2,020-3,270)	2,202 (1,669-2,456)	2,372 (1,816-2,651)	2,160 (1,729-2,368)	2,164 (1,740-2,352)
Poisonings	1,705 (1,157-1,880)	1,472 (1,035-1,586)	1,254 (843-1,361)	1,417 (880-1,551)	1,560 (961-1,709)	1,422 (950-1,539)	1,329 (924-1,431)	1,488 (1,001-1,615)
Poisoning by carbon monoxide	782 (506-868)	705 (472-772)	600 (411-654)	634 (426-695)	705 (448-779)	722 (499-785)	666 (486-717)	743 (533-804)
Poisoning by other means	923 (608-1,044)	767 (520-836)	655 (403-724)	784 (449-879)	855 (488-955)	701 (430-772)	663 (425-731)	746 (440-824)
Exposure to mechanical forces	1,406 (988-1,600)	1,545 (1,089-1,788)	1,537 (1,084-1,757)	1,566 (1,057-1,820)	1,878 (1,278-2,182)	1,659 (1,169-1,905)	1,342 (951-1,550)	1,778 (1,230-2,051)
Unintentional firearm injuries	262 (205-300)	295 (227-347)	337 (260-401)	245 (193-278)	250 (198-303)	271 (220-325)	221 (184-268)	221 (185-283)
Other exposure to mechanical forces	1,144 (765-1,327)	1,250 (841-1,475)	1,200 (792-1,401)	1,321 (848-1,563)	1,628 (1,061-1,924)	1,388 (925-1,628)	1,121 (756-1,325)	1,557 (1,017-1,830)
Adverse effects of medical treatment	2,003 (1,491-2,385)	2,035 (1,481-2,402)	1,637 (1,223-1,920)	1,811 (1,331-2,098)	2,104 (1,569-2,383)	2,296 (1,737-2,630)	1,835 (1,447-2,099)	2,443 (1,980-2,752)
Animal contact	1,620 (548-2,236)	1,513 (550-1,994)	1,806 (632-2,369)	1,537 (520-2,027)	2,049 (774-2,639)	1,737 (691-2,177)	2,045 (814-2,600)	1,514 (634-1,878)
Venomous animal contact	1,463 (464-2,021)	1,377 (461-1,844)	1,699 (564-2,246)	1,435 (465-1,912)	1,937 (701-2,511)	1,564 (569-2,010)	1,864 (706-2,417)	1,324 (500-1,693)
Non-venomous animal contact	157 (67-463)	136 (64-340)	107 (49-254)	101 (45-232)	112 (54-249)	173 (88-330)	181 (96-348)	190 (93-370)
Foreign body	758 (726-792)	785 (754-818)	763 (731-796)	875 (842-912)	970 (937-1,001)	1,111 (1,078-1,144)	1,327 (1,289-1,365)	1,532 (1,487-1,579)
Pulmonary aspiration and foreign body in airway	544 (489-583)	582 (528-624)	602 (553-642)	691 (635-733)	793 (735-831)	973 (924-1,010)	1,182 (1,129-1,223)	1,383 (1,325-1,435)
Foreign body in other body part	214 (186-271)	203 (176-261)	161 (139-208)	183 (159-241)	177 (153-232)	138 (115-180)	145 (126-193)	149 (130-209)
Environmental heat and cold exposure	469 (354-519)	616 (539-646)	926 (787-985)	1,231 (1,089-1,285)	1,715 (1,516-1,797)	2,649 (2,441-2,731)	3,464 (3,306-3,531)	3,412 (3,127-3,531)
Exposure to forces of nature	7,844 (3,539-14,874)	7,413 (3,196-15,378)	6,311 (2,864-12,528)	5,209 (2,328-10,724)	4,692 (2,146-9,349)	4,130 (1,808-8,226)	3,881 (1,771-7,939)	3,826 (1,738-7,391)
Other unintentional injuries	1,379 (1,272-1,501)	1,501 (1,389-1,634)	1,560 (1,439-1,698)	1,518 (1,419-1,635)	1,162 (1,101-1,226)	1,083 (1,028-1,135)	972 (919-1,032)	1,001 (942-1,060)
Self-harm and interpersonal violence	43,178 (41,203-45,480)	35,923 (34,093-37,492)	28,733 (27,303-29,994)	26,041 (24,832-26,976)	23,667 (22,620-24,522)	22,231 (21,366-22,861)	19,716 (19,022-20,271)	18,399 (17,833-18,889)
Self-harm	32,622 (31,191-34,202)	26,613 (25,501-27,718)	20,420 (19,622-21,229)	18,975 (18,321-19,600)	17,650 (17,122-18,172)	16,884 (16,395-17,354)	15,199 (14,848-15,558)	14,651 (14,274-15,022)
Self-harm by firearm	1,154 (698-1,621)	903 (638-1,189)	773 (616-952)	724 (622-853)	744 (673-890)	754 (701-867)	691 (639-789)	566 (527-654)
Self-harm by other specified means	31,468 (30,038-33,086)	25,710 (24,557-26,833)	19,647 (18,855-20,500)	18,251 (17,606-18,904)	16,906 (16,403-17,448)	16,130 (15,658-16,591)	14,508 (14,160-14,871)	14,085 (13,716-14,453)
Interpersonal violence	8,838 (7,030-10,150)	8,155 (6,586-9,318)	7,355 (6,069-8,493)	6,293 (5,238-7,121)	5,450 (4,533-6,206)	4,828 (4,088-5,420)	4,027 (3,415-4,543)	3,301 (2,807-3,717)
Physical violence by firearm	2,432 (2,005-2,847)	2,237 (1,859-2,635)	1,924 (1,604-2,248)	1,696 (1,406-2,001)	1,402 (1,172-1,603)	1,183 (996-1,330)	980 (795-1,163)	746 (588-895)
Physical violence by sharp object	2,148 (1,669-2,499)	2,114 (1,704-2,431)	2,041 (1,667-2,341)	1,840 (1,520-2,140)	1,730 (1,362-2,024)	1,494 (1,225-1,699)	1,189 (984-1,379)	919 (766-1,039)
Physical violence by other means	4,259 (3,103-5,245)	3,804 (2,983-4,617)	3,390 (2,668-4,058)	2,756 (2,172-3,307)	2,319 (1,879-2,743)	2,151 (1,750-2,472)	1,859 (1,548-2,231)	1,636 (1,351-1,901)
Conflict and terrorism	1,653 (1,369-2,052)	1,097 (906-1,358)	900 (769-1,083)	716 (615-855)	515 (445-608)	475 (400-587)	455 (374-571)	415 (335-536)
Executions and police conflict	65 (62-71)	58 (55-62)	57 (50-69)	57 (47-77)	51 (42-67)	44 (35-61)	35 (31-45)	32 (28-39)

Cause	60 to 64	65 to 69	70 to 74	75 to 79	80 to 84	85 to 89	90 to 94	95 plus
Other urinary diseases	1,004 (599-1,227)	987 (526-1,247)	1,184 (646-1,465)	1,355 (709-1,697)	1,055 (572-1,318)	838 (483-1,040)	488 (289-605)	258 (186-325)
Gynecological diseases	339 (304-366)	414 (342-448)	307 (249-330)	259 (208-275)	466 (380-518)	328 (269-363)	151 (127-167)	68 (58-76)
Uterine fibroids	99 (74-120)	110 (79-140)	98 (68-118)	77 (47-94)	148 (113-185)	105 (76-134)	62 (42-77)	29 (19-36)
Polycystic ovarian syndrome	-	-	-	-	-	-	-	-
Endometriosis	-	-	-	-	-	-	-	-
Genital prolapse	44 (21-71)	80 (33-122)	71 (35-96)	54 (27-71)	87 (49-132)	59 (36-82)	23 (15-29)	11 (7-13)
Other gynecological diseases	196 (160-229)	224 (171-279)	138 (104-170)	128 (104-154)	231 (168-284)	164 (129-195)	66 (54-78)	28 (25-34)
Hemoglobinopathies and hemolytic anemias	1,755 (1,372-2,239)	2,070 (1,610-2,680)	2,696 (2,105-3,418)	2,878 (2,332-3,618)	3,284 (2,624-4,048)	3,029 (2,329-3,607)	1,852 (1,347-2,151)	1,027 (697-1,167)
Thalassemias	7 (5-8)	3 (3-5)	2 (1-2)	1 (1-1)	0 (0-1)	0 (0-0)	0 (0-0)	0 (0-0)
Sickle cell disorders	94 (61-164)	45 (30-71)	18 (12-27)	7 (4-10)	2 (1-3)	1 (0-1)	0 (0-0)	0 (0-0)
G6PD deficiency	339 (230-522)	301 (199-470)	216 (141-348)	143 (93-234)	74 (47-119)	31 (20-45)	9 (6-12)	2 (1-3)
Other hemoglobinopathies and hemolytic anemias	1,315 (1,033-1,614)	1,720 (1,358-2,169)	2,460 (1,924-3,063)	2,727 (2,223-3,413)	3,208 (2,567-3,936)	2,998 (2,306-3,565)	1,844 (1,340-2,142)	1,025 (695-1,165)
Endocrine, metabolic, blood, and immune disorders	4,365 (3,238-4,913)	4,627 (3,353-5,196)	5,112 (3,721-5,742)	5,542 (3,796-6,075)	6,132 (4,024-6,738)	5,857 (3,630-6,490)	3,503 (2,149-3,885)	2,000 (1,101-2,275)
Sudden infant death syndrome	-	-	-	-	-	-	-	-
Injuries	69,708 (65,670-71,666)	68,320 (63,921-70,516)	80,291 (75,335-82,773)	87,532 (82,349-90,340)	88,964 (84,085-91,516)	74,335 (71,090-76,558)	38,819 (37,650-39,780)	17,690 (17,314-18,101)
Transport injuries	19,464 (18,684-20,160)	16,345 (15,771-16,953)	16,785 (16,131-17,443)	14,376 (13,886-14,884)	11,153 (10,774-11,595)	6,706 (6,480-6,937)	2,631 (2,551-2,711)	987 (951-1,021)
Road injuries	18,438 (17,719-19,098)	15,493 (14,957-16,066)	16,020 (15,395-16,652)	13,661 (13,194-14,130)	10,577 (10,221-10,988)	6,397 (6,187-6,611)	2,507 (2,429-2,583)	937 (902-972)
Pedestrian road injuries	10,331 (9,674-11,115)	8,723 (8,264-9,401)	9,670 (9,118-10,388)	8,512 (8,111-9,092)	6,871 (6,552-7,305)	3,904 (3,734-4,152)	1,371 (1,313-1,455)	463 (441-488)
Cyclist road injuries	974 (740-1,059)	862 (701-959)	812 (693-914)	578 (511-649)	289 (254-321)	159 (137-183)	60 (50-72)	0 (0-0)
Motorcyclist road injuries	1,557 (1,223-1,780)	1,160 (930-1,312)	926 (730-1,053)	579 (435-653)	319 (236-357)	166 (123-187)	61 (47-68)	22 (18-25)
Motor vehicle road injuries	5,510 (4,931-5,901)	4,689 (4,202-5,000)	4,556 (4,109-4,888)	3,853 (3,532-4,150)	3,013 (2,744-3,201)	2,107 (1,965-2,226)	990 (936-1,035)	439 (416-463)
Other road injuries	67 (50-80)	59 (45-74)	55 (42-68)	140 (111-175)	85 (71-107)	60 (51-75)	25 (22-32)	13 (12-16)
Other transport injuries	1,025 (937-1,122)	851 (773-932)	766 (713-827)	714 (663-770)	575 (531-622)	310 (287-334)	124 (116-134)	49 (47-52)
Unintentional injuries	33,920 (30,856-35,373)	37,705 (34,091-39,470)	48,450 (44,329-50,412)	59,190 (55,097-61,537)	66,762 (62,435-68,983)	61,232 (58,364-63,304)	33,973 (32,846-34,911)	16,017 (15,637-16,405)
Falls	11,966 (11,317-12,744)	16,283 (15,192-17,375)	24,209 (22,465-25,903)	34,123 (32,036-36,499)	41,822 (39,087-44,382)	42,260 (40,176-44,217)	24,787 (23,940-25,747)	11,395 (11,043-11,777)
Drowning	3,085 (2,591-3,236)	3,252 (2,781-3,420)	3,496 (3,208-3,658)	4,243 (3,621-4,452)	3,945 (3,341-4,125)	2,365 (2,057-2,465)	846 (748-879)	216 (195-224)
Fire, heat, and hot substances	2,187 (1,769-2,384)	2,574 (2,061-2,815)	3,025 (2,509-3,262)	3,447 (2,892-3,694)	3,911 (3,383-4,145)	3,150 (2,775-3,326)	1,616 (1,476-1,692)	842 (803-870)
Poisonings	1,293 (831-1,410)	1,245 (799-1,363)	1,458 (1,004-1,569)	1,446 (909-1,585)	1,375 (899-1,501)	825 (567-895)	282 (203-302)	85 (71-89)
Poisoning by carbon monoxide	682 (459-746)	647 (415-712)	819 (567-886)	882 (556-972)	824 (533-907)	490 (324-538)	158 (108-171)	39 (30-42)
Poisoning by other means	611 (352-679)	599 (353-668)	639 (410-701)	564 (335-628)	551 (341-607)	335 (225-366)	124 (91-135)	46 (40-48)
Exposure to mechanical forces	1,633 (1,140-1,876)	1,501 (1,059-1,711)	1,584 (1,153-1,805)	1,601 (1,169-1,827)	1,787 (1,355-1,998)	1,435 (1,158-1,570)	749 (632-804)	384 (351-404)
Unintentional firearm injuries	192 (156-262)	201 (164-264)	227 (191-292)	342 (279-439)	380 (300-464)	362 (297-428)	214 (177-249)	104 (87-118)
Other exposure to mechanical forces	1,441 (963-1,690)	1,300 (887-1,508)	1,357 (944-1,572)	1,259 (859-1,479)	1,406 (1,014-1,608)	1,072 (829-1,193)	535 (433-582)	280 (251-298)
Adverse effects of medical treatment	3,670 (2,852-4,157)	3,733 (2,898-4,275)	4,309 (3,446-4,899)	4,593 (3,786-5,083)	4,522 (3,894-5,068)	3,871 (3,437-4,336)	1,968 (1,796-2,202)	952 (901-1,053)
Animal contact	1,720 (673-2,206)	1,983 (697-2,594)	1,910 (713-2,421)	1,540 (562-1,969)	911 (413-1,129)	495 (262-593)	193 (118-221)	60 (47-69)
Venomous animal contact	1,606 (600-2,087)	1,862 (595-2,466)	1,762 (634-2,261)	1,411 (469-1,832)	785 (329-1,002)	388 (183-481)	142 (77-172)	40 (28-46)
Non-venomous animal contact	114 (56-234)	121 (60-256)	147 (74-319)	129 (67-253)	126 (73-232)	106 (66-161)	51 (35-77)	20 (16-27)
Foreign body	1,446 (1,404-1,491)	1,430 (1,384-1,471)	1,942 (1,880-2,000)	2,450 (2,372-2,527)	3,117 (3,025-3,210)	3,474 (3,376-3,582)	2,089 (2,034-2,152)	1,521 (1,471-1,571)
Pulmonary aspiration and foreign body in airway	1,325 (1,271-1,374)	1,313 (1,254-1,357)	1,772 (1,694-1,838)	2,256 (2,149-2,341)	2,878 (2,760-2,975)	3,256 (3,156-3,360)	1,962 (1,904-2,032)	1,462 (1,412-1,512)
Foreign body in other body part	121 (105-167)	117 (96-158)	170 (144-224)	194 (161-262)	239 (194-329)	218 (161-296)	127 (91-168)	60 (43-76)
Environmental heat and cold exposure	2,930 (2,495-3,155)	1,885 (1,598-2,013)	3,244 (2,733-3,485)	3,064 (2,491-3,319)	3,476 (2,950-3,694)	2,335 (2,014-2,467)	1,046 (903-1,096)	430 (394-448)
Exposure to forces of nature	3,229 (1,542-6,330)	2,922 (1,289-6,220)	2,729 (1,285-5,197)	2,064 (1,002-3,970)	1,295 (633-2,446)	569 (293-1,075)	167 (96-293)	34 (20-58)
Other unintentional injuries	760 (719-817)	897 (836-963)	544 (516-579)	618 (582-667)	602 (568-646)	455 (430-482)	230 (220-242)	98 (94-102)
Self-harm and interpersonal violence	16,324 (15,773-16,796)	14,270 (13,752-14,723)	15,057 (14,490-15,520)	13,966 (13,489-14,444)	11,049 (10,718-11,381)	6,396 (6,217-6,568)	2,215 (2,158-2,269)	687 (672-700)
Self-harm	13,261 (12,898-13,612)	11,785 (11,428-12,156)	12,415 (12,003-12,809)	11,679 (11,311-12,084)	10,174 (9,857-10,505)	5,961 (5,783-6,129)	2,090 (2,033-2,143)	624 (610-638)
Self-harm by firearm	424 (388-508)	335 (298-449)	294 (252-427)	285 (243-439)	263 (231-374)	188 (173-220)	95 (89-102)	48 (45-50)
Self-harm by other specified means	12,838 (12,490-13,182)	11,450 (11,107-11,815)	12,122 (11,713-12,512)	11,394 (11,033-11,802)	9,911 (9,609-10,239)	5,773 (5,597-5,939)	1,995 (1,941-2,047)	577 (564-589)
Interpersonal violence	2,660 (2,242-3,041)	2,143 (1,766-2,478)	2,374 (1,975-2,706)	2,077 (1,744-2,360)	705 (651-781)	358 (333-391)	109 (101-118)	37 (35-39)
Physical violence by firearm	599 (470-712)	440 (345-549)	437 (331-551)	375 (285-470)	74 (64-87)	42 (37-48)	13 (11-14)	6 (5-7)
Physical violence by sharp object	715 (593-822)	486 (406-562)	658 (528-746)	597 (480-689)	191 (170-213)	92 (81-103)	26 (23-30)	8 (7-9)
Physical violence by other means	1,346 (1,115-1,610)	1,217 (967-1,420)	1,279 (1,037-1,481)	1,105 (919-1,290)	440 (403-494)	224 (208-246)	70 (65-76)	23 (22-25)
Conflict and terrorism	379 (303-494)	323 (261-415)	252 (206-325)	199 (159-260)	158 (123-209)	72 (59-90)	13 (11-17)	24 (21-29)
Executions and police conflict	24 (21-29)	19 (15-23)	16 (14-19)	12 (10-15)	10 (9-12)	6 (5-7)	3 (2-3)	1 (1-1)

SR Table 5: Global deaths by cause and age group for females (with 95% confidence intervals), 2017

Cause	Early Neonatal	Late Neonatal	Post Neonatal	1 to 4	5 to 9	10 to 14	15 to 19	20 to 24	25 to 29	30 to 34	35 to 39
All causes	789,766 (761,124-820,738)	235,008 (226,349-244,213)	776,067 (745,764-811,135)	674,526 (644,903-708,292)	191,068 (187,548-194,984)	137,564 (134,890-140,536)	205,371 (199,965-211,025)	260,025 (251,991-268,567)	314,999 (304,357-325,671)	373,371 (362,816-384,290)	434,294 (423,153-446,274)
Communicable, maternal, neonatal, and nutritional disease	704,919 (676,254-734,318)	199,843 (191,908-208,541)	596,448 (565,527-628,188)	502,205 (472,868-535,115)	107,991 (101,780-116,445)	63,400 (59,232-69,409)	82,597 (77,560-89,628)	111,277 (104,866-118,922)	137,532 (128,572-147,824)	156,659 (147,674-166,240)	159,571 (150,481-169,468)
HIV/AIDS and sexually transmitted infections	-	6,158 (2,354-11,729)	36,828 (28,206-48,789)	15,401 (13,404-17,951)	10,638 (9,706-11,639)	10,326 (9,600-11,078)	17,178 (15,419-19,218)	24,509 (21,267-29,204)	48,429 (41,184-57,217)	66,172 (58,778-74,172)	66,808 (59,852-74,326)
HIV/AIDS	-	-	24,035 (21,445-26,760)	14,122 (12,402-16,410)	10,044 (9,228-10,863)	10,271 (9,539-11,015)	17,003 (15,237-19,041)	24,196 (20,969-28,869)	47,954 (40,772-56,723)	65,680 (58,289-73,675)	66,309 (59,328-73,809)
HIV/AIDS - Drug-susceptible Tuberculosis	-	-	6,446 (4,812-7,965)	3,569 (2,756-4,360)	2,953 (2,098-3,624)	2,877 (1,996-3,605)	4,494 (3,269-5,744)	5,782 (4,124-7,481)	9,348 (6,398-12,642)	11,580 (7,870-15,801)	11,260 (7,784-15,027)
HIV/AIDS - Multidrug-resistant Tuberculosis without extensive drug resistance	-	-	821 (473-1,298)	428 (252-675)	341 (195-523)	331 (189-510)	520 (308-801)	681 (399-1,045)	1,065 (609-1,670)	1,300 (738-2,027)	1,259 (732-1,972)
HIV/AIDS - Extensively drug-resistant Tuberculosis	-	-	21 (13-33)	12 (7-19)	8 (5-12)	8 (5-12)	14 (8-20)	22 (14-34)	47 (29-71)	69 (42-104)	68 (42-102)
HIV/AIDS resulting in other diseases	-	-	16,746 (14,136-19,795)	10,113 (8,424-12,550)	6,741 (5,799-7,973)	7,055 (6,029-8,248)	11,975 (10,046-14,297)	17,710 (14,444-22,565)	37,495 (29,693-46,573)	52,731 (44,331-61,775)	53,722 (45,965-62,054)
Sexually transmitted infections excluding HIV	21,514 (8,425-39,969)	6,158 (2,354-11,729)	12,793 (4,892-24,418)	1,279 (469-2,479)	594 (214-1,181)	56 (42-69)	175 (148-201)	313 (250-362)	474 (358-549)	492 (383-563)	499 (389-570)
Syphilis	21,514 (8,425-39,969)	6,158 (2,354-11,729)	12,793 (4,892-24,418)	1,279 (469-2,479)	594 (214-1,181)	20 (15-25)	17 (14-19)	29 (23-34)	41 (31-47)	41 (32-47)	36 (28-41)
Chlamydial infection	-	-	-	-	-	10 (7-12)	27 (23-31)	80 (64-92)	87 (66-101)	56 (44-64)	82 (64-93)
Gonococcal infection	-	-	-	-	-	17 (12-20)	68 (57-78)	121 (97-140)	232 (175-269)	246 (192-282)	229 (179-262)
Other sexually transmitted infections	-	-	-	-	-	9 (7-11)	63 (53-72)	83 (66-96)	114 (86-132)	148 (115-170)	152 (119-174)
Respiratory infections and tuberculosis	-	42,655 (38,058-47,372)	210,242 (191,319-228,826)	116,268 (99,852-134,011)	19,089 (16,402-21,785)	10,819 (9,618-12,153)	14,329 (13,204-15,757)	19,908 (18,402-21,746)	24,638 (23,069-26,586)	27,812 (26,010-29,801)	32,648 (30,520-34,987)
Tuberculosis	-	-	15,035 (13,602-16,491)	13,919 (11,809-16,299)	3,875 (3,314-4,547)	3,665 (3,166-4,235)	8,302 (7,559-9,097)	13,243 (11,988-14,542)	16,826 (15,545-18,176)	19,295 (17,787-20,939)	22,327 (20,585-24,237)
Drug-susceptible tuberculosis	-	-	13,401 (11,896-14,849)	12,516 (10,554-14,745)	3,426 (2,887-4,024)	3,246 (2,765-3,794)	7,357 (6,496-8,208)	11,710 (10,328-13,041)	14,859 (13,208-16,473)	16,966 (15,210-18,722)	19,664 (17,438-21,861)
Multidrug-resistant tuberculosis without extensive drug resistance	-	-	1,541 (953-2,309)	1,347 (836-2,064)	422 (248-661)	393 (236-599)	882 (515-1,395)	1,415 (831-2,260)	1,798 (1,039-2,880)	2,124 (1,170-3,447)	2,423 (1,337-3,975)
Extensively drug-resistant tuberculosis	-	-	93 (60-138)	56 (37-80)	27 (17-42)	26 (16-39)	63 (39-97)	118 (78-174)	169 (116-245)	205 (139-298)	240 (163-346)
Lower respiratory infections	54,872 (47,297-63,507)	42,610 (38,016-47,326)	194,292 (177,010-211,553)	101,329 (87,215-117,421)	15,031 (12,678-17,370)	7,072 (6,150-8,039)	5,982 (5,403-6,807)	6,625 (6,025-7,506)	7,759 (7,077-8,607)	8,470 (7,600-9,287)	10,267 (9,116-11,163)
Upper respiratory infections	16 (7-35)	39 (19-81)	862 (293-2,223)	974 (232-2,235)	155 (48-407)	60 (20-159)	35 (14-83)	34 (16-71)	44 (24-78)	40 (24-66)	43 (24-79)
Otitis media	10 (1-78)	6 (3-15)	54 (28-110)	46 (23-93)	27 (15-52)	22 (13-40)	10 (7-17)	6 (4-10)	8 (5-14)	8 (5-14)	11 (7-22)
Enteric infections	7,730 (6,436-9,474)	14,112 (12,039-16,714)	134,484 (116,867-154,764)	114,631 (93,844-137,817)	34,867 (25,788-48,952)	18,879 (13,649-26,973)	15,784 (10,678-23,861)	14,020 (9,050-21,371)	11,829 (7,464-18,977)	11,228 (6,762-18,696)	10,843 (6,310-18,832)
Diarrheal diseases	7,730 (6,436-9,474)	13,928 (11,871-16,531)	130,569 (113,628-150,915)	93,011 (74,294-112,207)	15,492 (7,849-28,363)	7,020 (3,498-13,596)	7,475 (3,542-13,958)	8,125 (3,787-15,255)	7,780 (3,584-14,943)	8,295 (3,758-16,052)	8,555 (3,883-16,575)
Typhoid and paratyphoid	-	0 (0-0)	143 (69-273)	10,199 (4,746-19,039)	15,879 (9,083-25,566)	10,360 (5,809-16,736)	7,390 (3,667-13,130)	5,096 (2,466-9,006)	3,321 (1,704-5,675)	2,290 (1,180-3,898)	1,679 (871-2,880)
Typhoid fever	-	-	127 (61-243)	8,815 (3,958-16,228)	13,637 (7,661-22,061)	8,892 (4,939-14,658)	6,299 (3,152-11,180)	4,332 (2,090-7,677)	2,821 (1,432-4,933)	1,941 (986-3,380)	1,420 (724-2,476)
Paratyphoid fever	-	-	16 (6-34)	1,384 (518-2,912)	2,242 (979-4,383)	1,468 (636-2,969)	1,091 (423-2,315)	764 (302-1,659)	500 (207-1,044)	349 (146-737)	259 (112-523)
Invasive Non-typhoidal Salmonella (iNTS)	-	184 (101-313)	3,409 (1,928-5,764)	11,231 (5,469-20,563)	3,466 (1,685-6,491)	1,481 (652-2,840)	903 (359-1,775)	783 (309-1,536)	717 (271-1,490)	628 (266-1,223)	594 (262-1,176)
Other intestinal infectious diseases	-	-	363 (149-1,024)	190 (70-522)	30 (7-90)	18 (3-50)	17 (2-47)	17 (3-48)	11 (1-31)	15 (2-47)	15 (2-42)
Neglected tropical diseases and malaria	201 (93-715)	6,473 (3,653-10,104)	57,766 (37,417-83,489)	112,918 (76,271-157,069)	19,723 (14,196-26,415)	10,669 (7,755-14,311)	7,817 (5,667-10,688)	8,205 (5,908-11,489)	6,641 (4,705-9,341)	6,794 (4,818-9,491)	7,483 (5,375-10,464)
Malaria	-	6,328 (3,527-10,009)	55,085 (34,623-80,517)	106,323 (69,730-149,937)	16,646 (11,610-23,388)	8,993 (6,298-12,622)	6,563 (4,463-9,354)	6,977 (4,702-10,174)	5,459 (3,611-8,087)	5,480 (3,611-8,106)	6,123 (4,070-9,075)
Chagas disease	-	-	1 (0-1)	0 (0-1)	1 (0-1)	1 (1-2)	3 (2-3)	5 (5-6)	9 (8-11)	19 (17-22)	44 (39-51)
Leishmaniasis	-	-	191 (0-727)	883 (0-4,119)	329 (0-2,141)	186 (0-1,138)	133 (0-704)	109 (0-547)	116 (0-535)	117 (0-506)	109 (0-435)
Visceral leishmaniasis	-	-	191 (0-727)	883 (0-4,119)	329 (0-2,141)	186 (0-1,138)	133 (0-704)	109 (0-547)	116 (0-535)	117 (0-506)	109 (0-435)
African trypanosomiasis	-	-	-	34 (3-178)	58 (6-287)	70 (8-333)	58 (7-250)	65 (6-334)	54 (5-230)	53 (5-247)	46 (5-206)
Schistosomiasis	-	-	0 (0-0)	234 (146-372)	93 (64-129)	129 (92-177)	118 (87-157)	116 (87-149)	127 (97-163)	142 (111-178)	181 (139-231)
Cysticercosis	0 (0-0)	0 (0-0)	0 (0-0)	55 (19-110)	24 (5-55)	17 (2-47)	13 (1-38)	17 (2-44)	24 (4-58)	26 (4-64)	26 (4-66)
Cystic echinococcosis	-	-	-	70 (26-128)	31 (3-73)	20 (0-49)	10 (0-35)	12 (0-36)	19 (0-53)	22 (0-63)	28 (2-76)
Dengue	-	-	1,430 (302-2,130)	1,871 (512-2,796)	1,439 (353-2,130)	718 (201-1,036)	533 (123-819)	577 (132-828)	545 (125-817)	597 (110-866)	556 (116-816)
Yellow fever	-	1 (0-2)	16 (3-50)	161 (30-466)	233 (44-707)	205 (42-606)	159 (32-485)	132 (24-415)	102 (19-298)	83 (16-262)	67 (13-213)
Rabies	-	-	61 (32-107)	502 (229-1,095)	454 (188-894)	177 (77-343)	115 (53-256)	95 (44-196)	81 (40-144)	150 (76-264)	191 (88-328)
Intestinal nematode infections	-	-	102 (72-143)	1,160 (727-1,832)	160 (114-222)	32 (23-44)	9 (6-13)	7 (4-11)	6 (4-9)	8 (5-11)	8 (5-12)
Ascariasis	-	-	102 (72-143)	1,160 (727-1,832)	160 (114-222)	32 (23-44)	9 (6-13)	7 (4-11)	6 (4-9)	8 (5-11)	8 (5-12)
Ebola	0 (0-0)	0 (0-0)	0 (0-0)	1 (1-1)	0 (0-0)	0 (0-0)	0 (0-0)	0 (0-0)	0 (0-0)	0 (0-0)	0 (0-0)
Zika virus	0 (0-0)	0 (0-0)	0 (0-1)	0 (0-3)	1 (0-3)	1 (0-4)	1 (0-6)	1 (0-7)	1 (0-8)	1 (0-7)	1 (0-6)
Other neglected tropical diseases	201 (93-715)	145 (74-458)	880 (416-3,098)	1,623 (569-7,816)	255 (139-784)	121 (68-370)	100 (61-261)	91 (52-244)	98 (53-223)	95 (48-214)	102 (52-218)
Other infectious diseases	8,752 (6,796-10,440)	9,856 (7,667-12,038)	84,994 (68,102-105,349)	99,827 (74,868-132,629)	20,234 (16,299-25,608)	10,615 (8,894-12,697)	9,149 (7,414-10,807)	8,793 (7,290-10,466)	7,803 (6,626-9,199)	7,924 (6,813-9,322)	8,019 (6,924-9,440)
Meningitis	3,720 (2,839-4,523)	4,686 (3,708-5,830)	33,051 (26,173-39,443)	29,026 (21,670-36,958)	6,700 (4,813-9,740)	4,838 (3,561-6,768)	4,267 (2,953-5,735)	3,586 (2,511-5,089)	3,292 (2,316-4,596)	3,107 (2,184-4,348)	3,197 (2,325-4,553)
Pneumococcal meningitis	524 (395-666)	620 (481-793)	4,183 (3,195-5,195)	3,444 (2,375-4,629)	628 (413-950)	492 (335-723)	527 (333-747)	519 (348-780)	494 (328-722)	454 (305-653)	503 (351-736)
H influenzae type B meningitis	753 (545-941)	1,102 (853-1,384)	8,544 (6,654-10,710)	7,569 (5,585-10,172)	2,333 (1,689-3,493)	1,518 (1,135-2,176)	1,266 (878-1,721)	984 (715-1,416)	843 (616-1,246)	791 (595-1,188)	753 (578-1,169)
Meningococcal meningitis	490 (366-608)	593 (454-756)	4,049 (3,131-5,004)	3,341 (2,439-4,521)	622 (420-936)	384 (264-566)	305 (198-444)	241 (161-351)	235 (154-344)	233 (160-347)	241 (166-346)
Other meningitis	1,953 (1,485-2,423)	2,370 (1,848-2,950)	16,275 (12,687-19,488)	14,672 (10,666-18,853)	3,117 (2,216-4,575)	2,444 (1,724-3,422)	2,169 (1,489-2,909)	1,842 (1,268-2,587)	1,720 (1,173-2,430)	1,629 (1,109-2,295)	1,700 (1,201-2,383)
Encephalitis	247 (212-281)	467 (373-555)	5,525 (4,791-6,443)	5,520 (4,501-6,846)	2,863 (2,222-3,612)	1,653 (1,332-2,049)	1,142 (951-1,350)	1,204 (1,031-1,403)	1,093 (970-1,258)	1,184 (1,069-1,397)	974 (880-1,208)
Diphtheria	-	-	289 (134-591)	1,131 (392-2,873)	149 (74-294)	49 (26-89)	15 (9-25)	9 (5-14)	8 (5-13)	8 (5-14)	9 (6-16)
Whooping cough	-	-	23,098 (11,500-40,999)	24,690 (12,582-43,160)	2,325 (1,151-4,224)	416 (205-771)	143 (71-271)	119 (60-220)	70 (35-129)	48 (24-89)	37 (18-70)
Tetanus	2,918 (2,022-4,170)	3,313 (2,061-5,000)	1,394 (836-2,373)	938 (374-1,922)	371 (181-657)	322 (157-560)	466 (199-808)	401 (175-763)	340 (188-576)	432 (225-756)	378 (193-659)
Measles	-	-	11,869 (4,256-26,228)	29,521 (10,753-61,791)	4,290 (1,579-9,555)	1,105 (403-2,473)	382 (135-843)	207 (71-454)	112 (38-246)	69 (24-153)	43 (15-95)
Varicella and herpes zoster	48 (36-64)	133 (99-179)	1,338 (1,016-1,764)	1,613 (1,206-2,146)	605 (434-878)	192 (141-262)	93 (70-128)	103 (75-148)	137 (103-189)	169 (132-217)	144 (110-187)
Acute hepatitis	-	-	4,027 (2,205-5,367)	3,565 (2,314-5,387)	2,030 (1,316-2,916)	1,549 (1,018-2,011)	2,123 (1,404-2,758)	2,655 (1,921-3,315)	2,243 (1,691-2,785)	2,344 (1,665-2,832)	2,714 (1,911-3,288)
Acute hepatitis A	-	-	821 (370-1,428)	2,326 (1,419-3,622)	1,435 (911-2,097)	876 (552-1,226)	915 (572-1,295)	900 (554-1,310)	564 (314-868)	458 (221-745)	435 (199-789)
Acute hepatitis B	-	-	2,349 (1,149-3,406)	893 (463-1,584)	268 (119-478)	224 (100-402)	444 (219-758)	799 (397-1,356)	973 (554-1,496)	1,319 (778-1,882)	1,789 (1,054-2,441)
Acute hepatitis C	-	-	848 (394-1,516)	267 (114-499)	44 (17-94)	16 (6-37)	14 (5-32)	13 (4-29)	11 (3-26)	13 (4-30)	20 (5-54)
Acute hepatitis E	-	-	8 (3-15)	79 (31-154)	283 (137-489)	432 (245-681)	750 (436-1,088)	942 (552-1,296)	695 (384-1,024)	555 (250-960)	469 (187-855)
Other unspecified infectious diseases	1,819 (1,010-2,301)	1,257 (824-1,463)	4,404 (2,603-5,456)	3,823 (2,209-5,026)	901 (617-1,083)	492 (383-562)	518 (436-593)	510 (427-594)	508 (417-595)	562 (443-663)	523 (423-610)
Maternal and neonatal disorders	611,824 (580,005-643,748)	120,588 (112,105-129,420)	33,796 (26,923-40,437)	3,201 (2,257-3,962)	-	783 (672-906)	17,468 (15,528-19,677)	34,769 (31,567-38,322)	36,938 (34,027-40,116)	35,451 (32,830-38,547)	32,546 (30,008-35,538)
Maternal disorders	-	-	-	-	-	783 (672-906)	17,468 (15,528-19,677)	34,769 (31,567-38,322)	36,938 (34,027-40,116)	35,451 (32,830-38,547)	32,546 (30,008-35,538)
Maternal hemorrhage	-	-	-	-	-	145 (97-209)	2,696 (1,913-4,096)	6,275 (4,322-9,413)	7,344 (5,466-10,674)	8,024 (5,927-11,215)	7,587 (5,904-9,900)
Maternal sepsis and other maternal infections	-	-	-	-	-	65 (47-87)	2,417 (1,751-3,410)	3,732 (2,559-5,998)	3,667 (2,541-5,612)	3,234 (2,346-4,904)	3,138 (2,379-4,282)
Maternal hypertensive disorders	-	-	-	-	-	94 (75-119)	3,486 (2,574-4,810)	6,580 (4,851-9,271)	6,477 (4,687-9,011)	5,290 (3,969-7,130)	4,392 (3,364-5,886)
Maternal obstructed labor and uterine rupture	-	-	-	-	-	95 (66-141)	1,092 (675-1,915)	1,937 (1,082-4,319)	2,102 (1,217-3,941)	2,365 (1,438-4,217)	2,391 (1,541-3,788)
Maternal abortion and miscarriage	-	-	-	-	-	69 (50-93)	1,727 (1,149-2,687)	2,607 (1,614-4,284)	2,688 (1,740-4,313)	2,806 (1,798-4,366)	2,753 (1,906-4,078)
Ectopic pregnancy	-	-	-	-	-	38 (27-54)	940 (447-2,011)	2,017 (907-4,869)	2,176 (1,078-5,027)	2,248 (1,189-4,638)	1,500 (792-3,111)
Indirect maternal deaths	-	-	-	-	-	165 (132-207)	2,873 (2,261-3,873)	6,221 (4,621-8,774)	6,629 (5,119-9,410)	6,034 (4,666-8,141)	5,847 (4,697-7,642)
Late maternal deaths	-	-	-	-	-	13 (9-19)	309 (211-448)	647 (452-935)	741 (528-1,028)	691 (457-1,008)	534 (376-748)
Maternal deaths aggravated by HIV/AIDS	-	-	-	-	-	3 (1-4)	58 (32-88)	151 (88-216)	247 (151-335)	320 (208-432)	339 (215-454)
Other maternal disorders	-	-	-	-	-	95 (77-116)	1,869 (1,187-2,920)	4,602 (2,756-7,614)	4,868 (3,085-7,613)	4,439 (3,035-6,585)	4,064 (2,938-5,788)
Neonatal disorders	611,824 (580,005-643,748)	120,588 (112,105-129,420)	33,796 (26,923-40,437)	3,201 (2,257-3,962)	-	-	-	-	-	-	-
Neonatal preterm birth	232,739 (210,553-272,109)	34,552 (29,801-39,985)	10,996 (8,576-13,196)	830 (509-1,075)	-	-	-	-	-	-	-
Neonatal encephalopathy due to birth asphyxia and trauma	196,491 (171,510-218,190)	18,539 (14,047-22,595)	6,499 (4,305-8,509)	1,099 (741-1,407)	-	-	-	-	-	-	-
Neonatal sepsis and other neonatal infections	51,054 (34,587-84,067)	33,816 (26,640-45,868)	5,437 (3,792-7,184)	345 (113-488)	-	-	-	-	-	-	-
Hemolytic disease and other neonatal jaundice	12,712 (10,494-15,280)	6,899 (5,197-9,125)	1,609 (964-2,719)	122 (75-198)	-	-	-	-	-	-	-
Other neonatal disorders	118,829 (101,043-135,463)	26,783 (22,217-31,221)	9,256 (6,793-11,742)	805 (580-1,012)	-	-	-	-	-	-	-
Nutritional deficiencies	-	-	38,338 (33,639-42,892)	39,960 (33,652-46,480)	3,440 (2,776-3,994)	1,309 (1,104-1,521)	872 (737-1,019)	1,073 (934-1,221)	1,255 (1,108-1,404)	1,277 (1,135-1,431)	1,225 (1,100-1,395)
Protein-energy malnutrition	-	-	37,077 (32,498-41,771)	38,251 (32,196-44,510)	2,870 (2,314-3,363)	1,034 (850-1,228)	577 (475-718)	626 (521-772)	693 (571-850)	743 (624-901)	771 (652-948)
Other nutritional deficiencies	-	-	1,260 (851-1,728)	1,709 (1,131-2,313)	571 (403-760)	275 (202-354)	295 (238-357)	447 (381-525)	561 (458-669)	534 (446-628)	454 (384-530)
Non-communicable diseases	80,054 (73,249-87,187)	31,775 (29,059-34,986)	143,629 (129,741-163,314)	96,290 (85,659-107,312)	40,651 (36,428-44,386)	38,935 (35,781-41,434)	59,518 (55,857-62,731)	77,309 (73,142-80,993)	105,810 (101,729-109,927)	149,993 (144,807-154,855)	213,139 (206,643-219,868)
Neoplasms	709 (588-793)	415 (369-459)	4,390 (3,887-4,898)	16,336 (14,262-18,500)	14,174 (12,323-15,848)	12,860 (11,465-14,073)	16,020 (14,424-17,310)	20,065 (18,320-21,422)	32,183 (30,188-33,836)	52,092 (49,824-54,025)	80,754 (77,892-83,641)
Lip and oral cavity cancer	-	-	-	-	-	-	386 (286-529)	670 (497-919)	814 (633-1,049)	1,162 (959-1,419)	1,566 (1,351-1,878)
Nasopharynx cancer	-	-	-	-	74 (62-87)	97 (85-112)	147 (135-162)	170 (158-184)	272 (255-293)	406 (378-436)	586 (550-630)
Other pharynx cancer	-	-	-	-	-	-	168 (134-208)	226 (183-285)	347 (293-411)	465 (406-522)	768 (676-862)
Esophageal cancer	-	-	-	-	-	-	99 (87-115)	207 (183-246)	351 (319-398)	644 (589-718)	1,007 (935-1,103)
Stomach cancer	-	-	-	-	-	-	322 (299-348)	831 (766-898)	1,913 (1,812-2,035)	3,634 (3,468-3,825)	5,633 (5,364-5,938)
Colon and rectum cancer	-	-	-	-	-	-	268 (238-302)	723 (634-826)	1,397 (1,293-1,507)	2,925 (2,749-3,089)	4,458 (4,270-4,652)
Liver cancer	-	-	-	-	414 (352-480)	300 (266-336)	416 (375-466)	636 (580-722)	1,217 (1,136-1,322)	2,065 (1,939-2,199)	3,002 (2,850-3,169)
Liver cancer due to hepatitis B	-	-	-	-	217 (183-254)	190 (164-216)	254 (223-288)	393 (353-447)	753 (673-836)	1,219 (1,102-1,342)	1,624 (1,454-1,795)
Liver cancer due to hepatitis C	-	-	-	-	11 (8-15)	9 (7-12)	12 (9-17)	37 (28-50)	102 (75-137)	232 (187-280)	440 (343-547)
Liver cancer due to alcohol use	-	-	-	-	-	-	2 (1-2)	15 (12-19)	51 (39-68)	140 (115-172)	316 (248-399)
Liver cancer due to NASH	-	-	-	-	-	-	47 (37-58)	57 (47-70)	85 (67-106)	129 (102-159)	180 (144-220)
Liver cancer due to other causes	-	-	-	-	187 (155-223)	101 (85-120)	102 (83-124)	134 (112-160)	225 (184-274)	345 (285-418)	443 (362-540)
Gallbladder and biliary tract cancer	-	-	-	-	-	-	54 (46-66)	111 (90-136)	197 (158-242)	437 (342-500)	772 (606-873)
Pancreatic cancer	-	-	-	-	-	-	74 (68-83)	129 (117-142)	301 (284-320)	606 (577-637)	1,209 (1,158-1,266)
Larynx cancer	-	-	-	-	-	-	59 (51-69)	114 (90-151)	178 (153-211)	237 (210-272)	415 (378-454)
Tracheal, bronchus, and lung cancer	-	-	-	-	-	-	276 (254-301)	563 (512-626)	1,316 (1,214-1,439)	2,657 (2,498-2,835)	4,423 (4,211-4,688)
Malignant skin melanoma	-	-	-	-	-	-	113 (88-158)	229 (180-315)	411 (326-549)	613 (494-818)	890 (731-1,188)
Non-melanoma skin cancer	-	-	-	-	-	-	49 (45-52)	61 (56-66)	100 (93-107)	139 (131-147)	215 (203-229)
Non-melanoma skin cancer (squamous-cell carcinoma)	-	-	-	-	-	-	49 (45-52)	61 (56-66)	100 (93-107)	139 (131-147)	215 (203-229)
Breast cancer	-	-	-	-	-	-	376 (261-467)	1,110 (918-1,326)	3,890 (3,401-4,396)	10,672 (9,851-11,678)	21,252 (19,978-23,249)
Cervical cancer	-	-	-	-	-	-	207 (187-227)	963 (858-1,076)	3,157 (2,815-3,436)	7,177 (6,468-7,700)	12,369 (11,439-13,155)
Uterine cancer	-	-	-	-	-	-	56 (51-64)	132 (118-155)	328 (301-370)	633 (591-692)	1,048 (980-1,124)
Ovarian cancer	-	-	-	-	-	-	563 (484-696)	854 (741-1,034)	1,295 (1,172-1,509)	2,037 (1,896-2,222)	3,391 (3,179-3,628)
Prostate cancer	-	-	-	-	-	-	-	-	-	-	-
Testicular cancer	-	-	-	-	-	-	-	-	-	-	-
Kidney cancer	18 (17-20)	9 (8-10)	134 (120-150)	656 (572-748)	323 (292-357)	165 (150-179)	104 (94-111)	141 (127-151)	230 (214-245)	317 (297-335)	489 (453-519)
Bladder cancer	[illegible]	[illegible]	[illegible]	[illegible]	[illegible]	[illegible]	43 (37-53)	60 (51-73)	105 (93-121)	163 (148-181)	292 (266-320)
Brain and nervous system cancer	85 (55-99)	57 (38-69)	786 (534-964)	2,906 (2,094-3,587)	3,396 (2,465-4,294)	2,451 (1,757-3,002)	1,866 (1,259-2,299)	1,815 (1,225-2,217)	2,265 (1,745-3,080)	3,291 (2,298-3,782)	[illegible]
Thyroid cancer	[illegible]	[illegible]	[illegible]	[illegible]	[illegible]	[illegible]	[illegible]	[illegible]	[illegible]	[illegible]	[illegible]

Cause	40 to 44	45 to 49	50 to 54	54 to 59	60 to 64	65 to 69	70 to 74	75 to 79	80 to 84	85 to 89	90 to 94	95 plus
All causes	546,078 (534,758-558,220)	697,314 (683,870-712,045)	1,003,195 (985,141-1,021,363)	1,150,787 (1,132,263-1,169,475)	1,619,782 (1,593,028-1,646,529)	2,004,611 (1,971,040-2,036,984)	2,268,859 (2,231,983-2,305,679)	2,758,911 (2,717,369-2,801,084)	3,301,942 (3,257,189-3,345,284)	3,018,320 (2,985,550-3,051,468)	1,938,267 (1,921,050-1,956,038)	858,050 (852,356-863,824)
Communicable, maternal, neonatal, and nutritional disease	148,023 (141,300-156,233)	127,077 (119,738-137,009)	134,886 (125,967-149,594)	129,337 (119,150-148,888)	157,607 (140,996-186,714)	184,367 (161,957-221,236)	231,122 (195,971-279,307)	237,272 (201,403-292,493)	292,384 (245,048-365,533)	241,638 (207,603-289,735)	139,080 (124,561-157,574)	67,541 (63,366-73,408)
HIV/AIDS and sexually transmitted infections	59,323 (55,252-63,158)	45,022 (40,757-50,448)	30,274 (27,869-32,774)	14,739 (13,140-16,437)	9,286 (8,210-10,691)	6,173 (5,358-7,672)	3,106 (2,662-3,726)	1,674 (1,460-1,923)	603 (551-659)	376 (340-406)	195 (181-208)	75 (71-80)
HIV/AIDS	58,708 (54,568-62,547)	44,463 (40,205-49,935)	29,761 (27,349-32,236)	14,212 (12,656-15,935)	8,919 (7,851-10,345)	5,775 (4,984-7,283)	2,783 (2,352-3,411)	1,379 (1,178-1,620)	310 (269-361)	142 (122-167)	56 (48-65)	13 (12-15)
HIV/AIDS - Drug-susceptible Tuberculosis	10,306 (7,246-13,642)	8,725 (6,064-11,675)	6,636 (4,625-8,645)	3,176 (2,268-4,144)	1,923 (1,390-2,498)	1,144 (830-1,496)	600 (439-779)	321 (234-413)	54 (38-71)	23 (16-31)	9 (6-12)	2 (1-3)
HIV/AIDS - Multidrug-resistant Tuberculosis without extensive drug resistance	1,147 (678-1,783)	953 (557-1,471)	725 (422-1,119)	352 (202-564)	210 (122-329)	121 (71-187)	62 (36-97)	33 (19-54)	6 (3-9)	2 (1-4)	1 (1-2)	0 (0-0)
HIV/AIDS - Extensively drug-resistant Tuberculosis	59 (36-89)	47 (29-71)	33 (20-50)	19 (12-28)	12 (8-19)	8 (5-13)	3 (2-5)	1 (1-2)	0 (0-1)	0 (0-0)	0 (0-0)	0 (0-0)
HIV/AIDS resulting in other diseases	47,196 (41,836-52,138)	34,738 (29,522-40,647)	22,366 (19,370-25,718)	10,665 (9,120-12,542)	6,774 (5,714-8,238)	4,503 (3,698-6,052)	2,118 (1,701-2,753)	1,024 (831-1,251)	250 (211-297)	117 (98-140)	46 (38-54)	11 (10-13)
Sexually transmitted infections excluding HIV	615 (467-706)	559 (440-638)	513 (373-583)	526 (385-603)	366 (298-409)	398 (302-451)	323 (257-360)	296 (257-323)	293 (256-318)	234 (200-251)	139 (127-147)	62 (58-65)
Syphilis	41 (31-47)	36 (29-41)	73 (53-83)	95 (69-109)	65 (53-72)	82 (62-93)	80 (64-89)	64 (55-69)	62 (54-67)	43 (37-46)	24 (22-26)	13 (12-14)
Chlamydial infection	117 (89-134)	98 (77-112)	83 (60-95)	86 (63-98)	61 (50-68)	41 (31-46)	44 (35-49)	41 (36-45)	55 (48-60)	46 (39-49)	27 (25-28)	11 (10-12)
Gonococcal infection	296 (225-340)	270 (212-307)	239 (174-272)	227 (166-260)	149 (121-166)	210 (159-239)	147 (117-164)	150 (131-164)	138 (121-149)	113 (97-122)	69 (63-73)	29 (27-30)
Other sexually transmitted infections	161 (122-185)	155 (122-177)	118 (86-134)	119 (87-136)	92 (74-102)	65 (49-74)	52 (41-58)	41 (36-45)	38 (34-42)	32 (27-34)	19 (17-20)	9 (8-9)
Respiratory infections and tuberculosis	36,198 (33,684-38,860)	39,746 (37,124-42,542)	59,587 (55,222-63,956)	65,967 (61,511-70,379)	83,484 (77,221-88,964)	98,177 (89,544-104,648)	120,843 (110,919-128,974)	130,500 (121,090-139,098)	158,332 (148,378-173,040)	145,849 (138,917-157,376)	95,985 (92,383-102,945)	50,843 (49,391-53,132)
Tuberculosis	23,978 (22,009-26,092)	25,416 (23,544-27,597)	35,355 (32,349-39,062)	35,344 (32,350-38,826)	36,997 (33,960-40,594)	36,107 (33,342-39,981)	39,236 (35,999-44,172)	33,470 (30,536-37,699)	30,769 (27,691-34,988)	16,793 (15,258-19,087)	5,611 (5,164-6,365)	1,633 (1,516-1,859)
Drug-susceptible tuberculosis	21,038 (18,624-23,530)	22,449 (19,998-24,939)	31,129 (27,280-35,112)	31,130 (27,357-35,111)	32,709 (28,797-36,699)	31,902 (28,283-36,086)	34,702 (30,351-39,775)	29,693 (26,006-34,030)	27,171 (23,684-31,469)	14,954 (13,339-17,254)	5,052 (4,578-5,772)	1,490 (1,366-1,691)
Multidrug-resistant tuberculosis without extensive drug resistance	2,678 (1,349-4,697)	2,713 (1,436-4,489)	3,894 (1,911-6,918)	3,882 (1,942-6,610)	3,948 (2,017-6,782)	3,875 (1,933-6,708)	4,201 (2,038-7,251)	3,468 (1,766-5,870)	3,299 (1,627-5,728)	1,667 (917-2,684)	500 (302-756)	128 (85-183)
Extensively drug-resistant tuberculosis	263 (170-399)	254 (167-377)	332 (201-525)	332 (205-515)	340 (208-528)	330 (197-515)	333 (188-542)	308 (190-473)	298 (180-462)	172 (114-252)	59 (41-81)	15 (11-21)
Lower respiratory infections	12,159 (10,987-13,202)	14,267 (12,731-15,594)	24,153 (21,251-26,024)	30,523 (27,390-32,753)	46,349 (40,513-49,753)	61,908 (53,915-66,423)	81,398 (71,728-87,655)	96,767 (88,422-103,303)	127,144 (118,945-140,150)	128,636 (122,427-138,879)	90,109 (86,694-96,616)	49,062 (47,639-51,071)
Upper respiratory infections	47 (27-78)	56 (36-92)	65 (42-110)	77 (51-133)	109 (75-186)	121 (88-191)	176 (126-277)	238 (172-401)	394 (289-761)	402 (291-851)	256 (193-557)	143 (107-343)
Otitis media	14 (8-28)	8 (6-14)	13 (10-22)	24 (16-43)	28 (18-51)	40 (26-70)	32 (22-52)	25 (18-40)	25 (19-38)	19 (15-26)	9 (8-11)	4 (4-6)
Enteric infections	11,437 (6,123-20,415)	12,507 (6,895-23,730)	19,038 (9,682-35,839)	22,959 (11,122-44,601)	36,749 (18,315-70,008)	50,771 (23,629-92,545)	75,093 (37,218-132,401)	78,562 (40,221-141,403)	107,534 (55,874-189,744)	75,931 (39,016-130,198)	32,552 (17,298-52,439)	10,843 (6,480-16,501)
Diarrheal diseases	9,602 (4,403-18,441)	10,981 (5,572-22,146)	17,679 (8,426-34,488)	21,716 (10,096-42,946)	35,576 (16,984-68,710)	49,741 (22,671-91,654)	74,306 (36,399-131,660)	77,965 (39,768-140,736)	107,100 (55,572-189,410)	75,681 (38,725-129,961)	32,454 (17,199-52,347)	10,807 (6,432-16,489)
Typhoid and paratyphoid	1,276 (655-2,216)	999 (530-1,728)	773 (396-1,361)	622 (291-1,163)	548 (267-979)	475 (264-784)	344 (201-556)	238 (142-376)	146 (86-228)	69 (42-110)	24 (14-39)	7 (4-14)
Typhoid fever	1,077 (534-1,879)	842 (444-1,444)	655 (332-1,146)	533 (253-1,030)	471 (224-866)	411 (225-684)	299 (174-494)	208 (121-335)	128 (74-208)	61 (36-101)	21 (13-37)	6 (3-12)
Paratyphoid fever	199 (86-413)	158 (66-319)	119 (48-249)	90 (34-197)	77 (31-163)	64 (28-128)	44 (19-88)	30 (14-57)	17 (8-33)	8 (4-15)	3 (1-5)	1 (0-2)
Invasive Non-typhoidal Salmonella (iNTS)	546 (257-1,072)	514 (246-975)	574 (264-1,132)	611 (280-1,093)	614 (278-1,104)	541 (253-949)	433 (191-773)	350 (157-651)	271 (106-534)	158 (58-304)	68 (16-152)	24 (1-75)
Other intestinal infectious diseases	12 (1-34)	13 (1-32)	12 (1-32)	10 (1-26)	11 (1-29)	15 (1-38)	10 (1-27)	9 (0-25)	17 (1-45)	23 (2-61)	7 (0-20)	4 (0-13)
Neglected tropical diseases and malaria	7,775 (5,505-10,944)	8,157 (5,758-11,378)	10,387 (7,317-14,723)	11,315 (7,934-16,054)	12,090 (8,467-17,034)	10,843 (7,506-15,585)	11,942 (8,461-17,119)	6,671 (4,971-9,304)	4,823 (3,587-6,824)	2,438 (1,644-4,093)	923 (644-1,510)	378 (250-548)
Malaria	6,114 (3,939-9,327)	6,495 (4,161-9,733)	8,136 (5,275-12,265)	9,128 (5,870-13,736)	9,781 (6,240-14,745)	8,795 (5,635-13,317)	9,012 (5,724-13,613)	4,398 (2,966-6,523)	2,073 (1,509-2,959)	612 (425-793)	264 (162-347)	142 (58-186)
Chagas disease	70 (62-81)	106 (95-121)	169 (154-194)	268 (240-304)	329 (301-369)	408 (371-454)	455 (416-510)	466 (426-517)	438 (398-486)	325 (296-359)	153 (139-170)	58 (52-66)
Leishmaniasis	91 (0-345)	79 (0-264)	73 (0-239)	59 (0-185)	55 (0-165)	46 (0-132)	35 (0-101)	22 (0-65)	13 (0-39)	6 (0-18)	2 (0-6)	1 (0-2)
Visceral leishmaniasis	91 (0-345)	79 (0-264)	73 (0-239)	59 (0-185)	55 (0-165)	46 (0-132)	35 (0-101)	22 (0-65)	13 (0-39)	6 (0-18)	2 (0-6)	1 (0-2)
African trypanosomiasis	41 (4-171)	40 (4-184)	36 (3-161)	29 (3-145)	23 (2-116)	17 (1-83)	11 (1-60)	7 (1-37)	4 (0-22)	2 (0-9)	1 (0-3)	0 (0-0)
Schistosomiasis	209 (163-261)	246 (190-315)	315 (241-408)	358 (281-467)	378 (298-477)	283 (224-353)	360 (281-457)	334 (258-420)	232 (182-288)	122 (96-153)	39 (29-51)	9 (6-14)
Cysticercosis	26 (4-66)	26 (4-66)	26 (3-72)	23 (3-64)	19 (2-54)	16 (1-46)	4 (0-12)	10 (0-31)	7 (0-24)	4 (0-14)	2 (0-6)	1 (0-2)
Cystic echinococcosis	31 (2-78)	32 (3-81)	41 (4-103)	41 (4-107)	44 (5-107)	42 (4-105)	41 (4-110)	35 (3-96)	32 (2-89)	25 (1-70)	11 (0-34)	4 (0-13)
Dengue	760 (133-1,171)	728 (127-1,115)	1,041 (163-1,759)	997 (161-1,588)	1,045 (158-1,639)	788 (157-1,150)	1,676 (449-2,875)	1,078 (319-2,041)	1,789 (571-3,730)	1,169 (401-2,637)	387 (138-964)	127 (46-338)
Yellow fever	55 (11-186)	49 (9-149)	45 (8-149)	36 (7-109)	26 (5-83)	18 (3-54)	12 (2-36)	7 (1-20)	3 (1-10)	1 (0-3)	0 (0-1)	0 (0-0)
Rabies	252 (116-436)	239 (117-378)	346 (176-495)	239 (125-421)	212 (95-316)	227 (118-346)	136 (76-214)	113 (66-173)	57 (26-80)	60 (24-89)	18 (6-27)	9 (3-14)
Intestinal nematode infections	7 (4-10)	7 (5-11)	9 (6-12)	8 (5-11)	17 (12-22)	8 (5-11)	14 (9-18)	12 (8-17)	11 (7-15)	5 (4-8)	4 (2-6)	1 (1-2)
Ascariasis	7 (4-10)	7 (5-11)	9 (6-12)	8 (5-11)	17 (12-22)	8 (5-11)	14 (9-18)	12 (8-17)	11 (7-15)	5 (4-8)	4 (2-6)	1 (1-2)
Ebola	0 (0-0)	0 (0-0)	0 (0-0)	0 (0-0)	0 (0-0)	0 (0-0)	0 (0-0)	0 (0-0)	0 (0-0)	0 (0-0)	0 (0-0)	0 (0-0)
Zika virus	1 (0-7)	1 (0-5)	1 (0-4)	1 (0-4)	0 (0-2)	0 (0-2)	0 (0-1)	0 (0-1)	0 (0-0)	0 (0-0)	0 (0-0)	0 (0-0)
Other neglected tropical diseases	119 (55-237)	110 (51-240)	150 (74-311)	127 (66-247)	159 (77-317)	197 (93-357)	187 (90-336)	190 (102-372)	164 (89-309)	108 (60-209)	43 (26-78)	27 (18-54)
Other infectious diseases	8,729 (7,576-10,223)	8,738 (7,718-10,217)	11,467 (10,031-13,572)	11,135 (10,018-13,037)	11,867 (10,770-13,956)	13,140 (11,772-15,322)	14,343 (12,756-17,109)	12,436 (11,184-14,405)	13,038 (11,507-14,736)	8,600 (7,530-9,533)	3,451 (2,984-3,783)	1,405 (1,151-1,606)
Meningitis	3,061 (2,293-4,406)	3,348 (2,518-4,845)	3,860 (2,926-5,742)	3,566 (2,769-5,421)	3,461 (2,748-5,503)	3,580 (2,911-5,729)	4,155 (3,277-6,655)	3,163 (2,608-4,971)	3,113 (2,507-4,665)	2,121 (1,662-3,129)	755 (604-1,071)	297 (236-387)
Pneumococcal meningitis	484 (333-703)	569 (398-842)	627 (454-918)	572 (418-854)	555 (412-845)	589 (433-893)	722 (520-1,136)	534 (392-805)	493 (365-722)	322 (244-450)	116 (91-158)	49 (36-66)
H influenzae type B meningitis	887 (666-1,383)	937 (710-1,431)	1,206 (909-1,893)	1,104 (850-1,762)	1,095 (850-1,864)	1,134 (891-1,926)	1,277 (975-2,248)	1,047 (814-1,805)	1,088 (850-1,784)	756 (586-1,183)	288 (227-433)	102 (79-143)
Meningococcal meningitis	214 (149-310)	234 (163-343)	278 (195-409)	261 (186-393)	255 (181-398)	264 (192-420)	307 (217-477)	237 (171-390)	233 (169-363)	144 (108-214)	47 (34-67)	19 (13-27)
Other meningitis	1,476 (1,049-2,076)	1,608 (1,173-2,263)	1,750 (1,264-2,528)	1,629 (1,232-2,429)	1,557 (1,184-2,367)	1,593 (1,228-2,447)	1,849 (1,404-2,899)	1,345 (1,065-2,096)	1,299 (1,009-1,900)	899 (672-1,303)	304 (235-419)	127 (94-161)
Encephalitis	1,244 (1,118-1,550)	1,271 (1,143-1,685)	1,624 (1,441-2,340)	1,795 (1,585-2,442)	2,315 (2,074-3,168)	2,564 (2,274-3,253)	3,437 (2,987-4,111)	3,123 (2,699-3,672)	3,687 (3,142-4,271)	2,141 (1,830-2,447)	670 (586-734)	219 (188-238)
Diphtheria	13 (8-21)	21 (13-33)	18 (12-28)	11 (7-18)	-	-	-	-	-	-	-	-
Whooping cough	33 (16-63)	30 (14-55)	34 (16-65)	32 (16-61)	-	-	-	-	-	-	-	-
Tetanus	453 (220-831)	469 (216-763)	696 (340-1,118)	514 (273-861)	430 (232-679)	554 (299-957)	489 (256-782)	439 (225-696)	250 (122-376)	192 (72-287)	58 (24-84)	17 (7-25)
Measles	30 (11-65)	18 (7-39)	16 (6-34)	11 (4-23)	-	-	-	-	-	-	-	-
Varicella and herpes zoster	97 (76-124)	93 (76-116)	103 (82-131)	108 (87-139)	128 (103-162)	239 (191-303)	240 (190-301)	364 (294-452)	649 (517-809)	782 (622-1,026)	538 (379-770)	360 (205-563)
Acute hepatitis	3,127 (2,166-3,801)	2,686 (1,933-3,281)	3,840 (2,788-4,717)	3,672 (2,753-4,597)	3,730 (2,769-4,575)	4,059 (2,859-4,991)	3,817 (2,783-4,696)	3,150 (2,306-3,868)	2,874 (1,999-3,485)	1,398 (1,027-1,702)	430 (340-502)	102 (85-120)
Acute hepatitis A	393 (179-725)	237 (101-494)	177 (84-319)	100 (51-173)	63 (29-114)	39 (19-68)	18 (8-35)	9 (3-19)	3 (1-8)	1 (0-2)	0 (0-0)	0 (0-0)
Acute hepatitis B	2,303 (1,437-3,015)	2,175 (1,458-2,770)	3,393 (2,411-4,236)	3,371 (2,546-4,215)	3,458 (2,572-4,248)	3,734 (2,648-4,611)	3,448 (2,556-4,271)	2,735 (2,001-3,422)	2,334 (1,556-2,979)	1,058 (714-1,352)	290 (189-383)	59 (32-85)
Acute hepatitis C	24 (6-60)	24 (6-63)	34 (11-83)	38 (10-96)	50 (12-127)	71 (18-186)	82 (23-223)	83 (23-214)	97 (28-273)	62 (17-163)	25 (6-66)	8 (2-19)
Acute hepatitis E	406 (155-807)	250 (96-537)	235 (94-499)	164 (60-327)	160 (61-336)	216 (82-453)	269 (106-544)	323 (115-658)	439 (183-878)	277 (113-535)	115 (52-191)	35 (16-59)
Other unspecified infectious diseases	671 (565-774)	802 (632-923)	1,276 (1,010-1,472)	1,425 (1,062-1,578)	1,803 (1,375-2,012)	2,143 (1,651-2,379)	2,205 (1,729-2,417)	2,198 (1,604-2,404)	2,466 (1,810-2,736)	1,968 (1,416-2,195)	1,000 (719-1,095)	411 (299-448)
Maternal and neonatal disorders	22,733 (20,765-24,978)	11,201 (10,043-12,512)	1,750 (1,577-1,968)	-	-	-	-	-	-	-	-	-
Maternal disorders	22,733 (20,765-24,978)	11,201 (10,043-12,512)	1,750 (1,577-1,968)	-	-	-	-	-	-	-	-	-
Maternal hemorrhage	4,579 (3,341-6,141)	1,577 (1,199-2,034)	315 (228-440)	-	-	-	-	-	-	-	-	-
Maternal sepsis and other maternal infections	2,865 (2,246-3,663)	1,929 (1,553-2,374)	183 (141-236)	-	-	-	-	-	-	-	-	-
Maternal hypertensive disorders	2,313 (1,782-3,030)	657 (524-826)	85 (69-108)	-	-	-	-	-	-	-	-	-
Maternal obstructed labor and uterine rupture	1,564 (1,029-2,392)	1,299 (886-1,878)	131 (91-193)	-	-	-	-	-	-	-	-	-
Maternal abortion and miscarriage	2,445 (1,704-3,475)	2,131 (1,715-2,654)	216 (162-283)	-	-	-	-	-	-	-	-	-
Ectopic pregnancy	880 (463-1,775)	304 (180-564)	98 (61-169)	-	-	-	-	-	-	-	-	-
Indirect maternal deaths	4,294 (3,481-5,406)	1,636 (1,335-1,986)	393 (327-480)	-	-	-	-	-	-	-	-	-
Late maternal deaths	290 (196-415)	123 (97-155)	23 (17-30)	-	-	-	-	-	-	-	-	-
Maternal deaths aggravated by HIV/AIDS	274 (173-369)	169 (107-231)	20 (13-28)	-	-	-	-	-	-	-	-	-
Other maternal disorders	3,230 (2,569-4,209)	1,375 (1,093-1,708)	286 (238-346)	-	-	-	-	-	-	-	-	-
Neonatal disorders	-	-	-	-	-	-	-	-	-	-	-	-
Neonatal preterm birth	-	-	-	-	-	-	-	-	-	-	-	-
Neonatal encephalopathy due to birth asphyxia and trauma	-	-	-	-	-	-	-	-	-	-	-	-
Neonatal sepsis and other neonatal infections	-	-	-	-	-	-	-	-	-	-	-	-
Hemolytic disease and other neonatal jaundice	-	-	-	-	-	-	-	-	-	-	-	-
Other neonatal disorders	-	-	-	-	-	-	-	-	-	-	-	-
Nutritional deficiencies	1,828 (1,637-2,038)	1,704 (1,515-1,957)	2,381 (2,150-2,683)	3,221 (2,905-3,553)	4,131 (3,770-4,496)	5,263 (4,786-5,679)	5,796 (5,358-6,193)	7,428 (6,920-7,892)	8,053 (7,502-8,579)	8,443 (7,929-8,875)	5,973 (5,672-6,281)	3,996 (3,802-4,208)
Protein-energy malnutrition	1,047 (898-1,271)	1,076 (909-1,327)	1,321 (1,116-1,626)	1,999 (1,699-2,357)	2,597 (2,268-3,018)	3,264 (2,887-3,692)	3,823 (3,432-4,159)	5,083 (4,532-5,509)	5,753 (5,144-6,235)	6,328 (5,438-6,838)	4,667 (3,754-5,115)	3,243 (2,412-3,589)
Other nutritional deficiencies	781 (642-919)	629 (528-731)	1,060 (883-1,233)	1,222 (1,011-1,464)	1,533 (1,251-1,854)	1,999 (1,636-2,387)	1,973 (1,659-2,321)	2,345 (1,948-2,780)	2,300 (1,864-2,910)	2,115 (1,664-3,028)	1,306 (968-2,238)	753 (468-1,600)
Non-communicable diseases	335,299 (326,650-344,104)	504,058 (492,020-515,719)	793,467 (774,965-810,722)	955,381 (934,574-974,641)	1,383,000 (1,349,898-1,411,602)	1,736,220 (1,691,768-1,772,586)	1,954,143 (1,896,327-1,998,996)	2,428,983 (2,366,584-2,480,762)	2,906,674 (2,826,367-2,963,142)	2,684,726 (2,631,412-2,727,639)	1,742,882 (1,718,589-1,763,180)	766,267 (758,746-772,640)
Neoplasms	130,891 (126,958-135,430)	201,177 (195,923-207,149)	304,226 (295,904-312,639)	342,840 (335,082-351,600)	451,998 (440,213-462,411)	506,511 (493,733-518,080)	479,365 (466,764-491,078)	476,518 (463,987-487,001)	444,523 (432,015-453,993)	315,052 (307,266-320,845)	156,035 (152,441-158,722)	54,870 (53,678-55,761)
Lip and oral cavity cancer	2,796 (2,440-3,268)	3,704 (3,380-4,103)	5,881 (5,365-6,572)	6,529 (6,027-7,218)	8,328 (7,609-9,174)	7,509 (6,957-8,140)	7,070 (6,547-7,635)	6,741 (6,323-7,167)	6,876 (6,438-7,336)	5,068 (4,784-5,346)	2,583 (2,468-2,700)	935 (899-969)
Nasopharynx cancer	1,014 (950-1,084)	1,592 (1,493-1,697)	2,358 (2,205-2,511)	2,278 (2,161-2,403)	2,372 (2,221-2,535)	2,179 (2,035-2,336)	1,737 (1,624-1,847)	1,277 (1,197-1,359)	1,091 (1,022-1,169)	597 (565-633)	240 (228-253)	70 (68-73)
Other pharynx cancer	1,379 (1,190-1,556)	2,323 (2,103-2,558)	3,806 (3,443-4,186)	4,072 (3,721-4,423)	4,849 (4,445-5,268)	4,383 (4,019-4,748)	4,252 (3,879-4,650)	2,390 (2,138-2,637)	2,230 (1,946-2,523)	1,260 (1,126-1,387)	448 (416-481)	130 (124-137)
Esophageal cancer	2,075 (1,933-2,235)	3,917 (3,695-4,141)	7,100 (6,675-7,501)	8,758 (8,317-9,183)	13,515 (12,847-14,213)	17,677 (16,720-18,618)	18,668 (17,571-19,814)	18,111 (17,108-19,204)	16,763 (15,885-17,754)	10,755 (10,264-11,236)	4,411 (4,218-4,595)	1,167 (1,119-1,210)
Stomach cancer	9,212 (8,780-9,602)	12,814 (12,346-13,287)	19,382 (18,681-20,091)	21,100 (20,376-21,884)	31,953 (30,770-33,134)	39,595 (38,006-41,146)	38,404 (36,880-39,940)	42,081 (40,616-43,703)	41,528 (40,134-42,939)	29,831 (28,932-30,762)	14,982 (14,600-15,393)	5,333 (5,186-5,500)
Colon and rectum cancer	7,681 (7,379-7,968)	13,657 (13,180-14,128)	22,145 (21,409-22,849)	26,910 (26,002-27,750)	37,902 (36,330-39,114)	45,869 (44,127-47,375)	47,509 (45,536-49,098)	56,131 (54,186-57,924)	60,348 (58,244-62,366)	48,438 (47,114-49,882)	26,923 (26,209-27,646)	10,265 (9,978-10,571)
Liver cancer	5,346 (5,087-5,634)	10,158 (9,667-10,692)	16,849 (16,063-17,732)	18,381 (17,626-19,149)	30,170 (28,828-31,723)	36,107 (34,400-38,133)	32,974 (31,424-34,735)	32,615 (31,274-34,072)	29,127 (27,939-30,537)	18,057 (17,412-18,737)	7,341 (6,970-7,650)	2,132 (1,973-2,243)
Liver cancer due to hepatitis B	2,784 (2,510-3,081)	5,080 (4,571-5,625)	7,484 (6,635-8,416)	6,425 (5,666-7,262)	10,038 (8,805-11,394)	11,501 (10,250-12,954)	9,130 (7,939-10,379)	7,211 (6,403-8,103)	5,224 (4,540-5,969)	2,590 (2,317-2,914)	813 (706-924)	175 (139-215)
Liver cancer due to hepatitis C	930 (775-1,101)	2,068 (1,710-2,468)	4,317 (3,661-4,976)	6,250 (5,527-7,012)	10,767 (9,561-12,051)	12,949 (11,864-14,097)	12,704 (11,630-13,749)	14,086 (12,852-15,285)	13,932 (12,816-15,060)	9,617 (8,931-10,217)	4,292 (3,957-4,604)	1,355 (1,213-1,481)
Liver cancer due to alcohol use	582 (463-734)	981 (723-1,373)	1,603 (1,172-2,250)	1,858 (1,376-2,492)	3,086 (2,346-4,143)	4,435 (3,624-5,406)	4,369 (3,627-5,322)	4,080 (3,528-4,766)	3,272 (2,768-3,860)	1,882 (1,615-2,183)	705 (594-830)	182 (141-224)
Liver cancer due to NASH	313 (255-386)	629 (503-781)	1,192 (956-1,465)	1,564 (1,238-1,918)	2,720 (2,216-3,285)	3,458 (2,794-4,182)	3,591 (2,946-4,387)	4,111 (3,404-4,946)	3,972 (3,270-4,751)	2,442 (2,092-2,834)	975 (803-1,168)	277 (215-347)
Liver cancer due to other causes	737 (605-913)	1,401 (1,149-1,702)	2,253 (1,824-2,757)	2,284 (1,873-2,695)	3,558 (2,930-4,270)	3,764 (3,112-4,487)	3,180 (2,567-3,827)	3,128 (2,621-3,714)	2,729 (2,226-3,265)	1,526 (1,293-1,756)	556 (461-660)	143 (109-181)
Gallbladder and biliary tract cancer	1,448 (1,157-1,626)	2,904 (2,339-3,161)	5,562 (4,397-6,074)	7,339 (5,987-7,988)	11,243 (9,160-12,264)	13,106 (10,932-14,203)	13,502 (11,582-14,443)	13,976 (12,436-15,184)	13,838 (12,356-15,209)	10,414 (9,642-11,143)	5,355 (5,074-5,633)	2,166 (2,065-2,250)
Pancreatic cancer	2,544 (2,446-2,663)	5,120 (4,954-5,342)	9,766 (9,438-10,172)	13,950 (13,587-14,366)	22,716 (22,145-23,423)	29,009 (28,243-29,810)	30,543 (29,720-31,345)	31,914 (30,976-32,908)	30,103 (29,271-31,042)	22,353 (21,711-23,003)	11,054 (10,746-11,356)	3,595 (3,485-3,708)
Larynx cancer	724 (656-801)	1,126 (1,055-1,203)	2,033 (1,887-2,194)	2,244 (2,103-2,383)	2,898 (2,726-3,079)	3,009 (2,839-3,208)	2,620 (2,451-2,781)	2,023 (1,919-2,125)	1,771 (1,677-1,872)	965 (923-1,005)	350 (335-363)	92 (89-96)
Tracheal, bronchus, and lung cancer	9,371 (8,903-9,924)	19,705 (18,873-20,643)	36,186 (34,789-37,845)	45,617 (43,981-47,585)	73,166 (70,521-75,755)	90,290 (87,118-93,365)	86,058 (83,161-89,102)	82,525 (79,589-85,434)	72,380 (69,898-74,988)	45,911 (44,258-47,447)	19,617 (18,861-20,209)	6,225 (6,030-6,412)
Malignant skin melanoma	1,182 (969-1,593)	1,451 (1,170-1,905)	1,930 (1,514-2,526)	2,290 (1,832-3,129)	2,685 (2,058-3,441)	2,979 (2,280-3,909)	2,841 (2,200-3,525)	3,200 (2,354-4,034)	3,206 (2,288-3,911)	2,636 (1,833-3,147)	1,463 (938-1,740)	594 (361-722)
Non-melanoma skin cancer	346 (328-364)	596 (564-629)	891 (839-940)	966 (923-1,008)	1,588 (1,507-1,674)	1,960 (1,861-2,059)	2,057 (1,961-2,159)	2,309 (2,213-2,401)	3,038 (2,922-3,154)	3,327 (3,219-3,438)	2,555 (2,487-2,627)	1,554 (1,513-1,601)
Non-melanoma skin cancer (squamous-cell carcinoma)	346 (328-364)	596 (564-629)	891 (839-940)	966 (923-1,008)	1,588 (1,507-1,674)	1,960 (1,861-2,059)	2,057 (1,961-2,159)	2,309 (2,213-2,401)	3,038 (2,922-3,154)	3,327 (3,219-3,438)	2,555 (2,487-2,627)	1,554 (1,513-1,601)
Breast cancer	34,869 (33,029-37,892)	49,671 (47,338-53,935)	67,372 (64,218-71,502)	69,637 (66,429-73,544)	70,295 (67,065-73,948)	65,618 (62,789-68,384)	55,235 (52,543-57,944)	50,038 (48,098-51,896)	43,931 (42,177-45,874)	32,134 (31,151-33,426)	17,705 (17,084-18,255)	6,921 (6,681-7,185)
Cervical cancer	19,963 (18,561-21,096)	26,498 (23,698-27,998)	33,462 (30,190-35,349)	30,422 (28,784-31,903)	30,310 (28,120-31,749)	26,970 (25,338-28,195)	22,075 (20,759-23,195)	17,817 (16,615-18,616)	14,747 (13,886-15,322)	8,793 (8,354-9,147)	3,598 (3,442-3,728)	1,143 (1,100-1,187)
Uterine cancer	1,916 (1,819-2,020)	3,649 (3,472-3,830)	6,104 (5,813-6,393)	8,003 (7,712-8,305)	11,951 (11,520-12,438)	13,057 (12,652-13,572)	11,132 (10,770-11,531)	10,081 (9,767-10,432)	8,445 (8,178-8,761)	5,362 (5,203-5,544)	2,535 (2,454-2,625)	807 (777-843)
Ovarian cancer	6,328 (5,964-6,752)	11,265 (10,769-11,834)	18,051 (17,293-18,877)	20,110 (19,400-20,920)	23,476 (22,665-24,324)	23,776 (23,024-24,594)	20,393 (19,780-21,138)	17,131 (16,601-17,751)	13,441 (12,993-13,926)	8,704 (8,407-9,025)	3,998 (3,840-4,179)	1,170 (1,105-1,233)
Prostate cancer	-	-	-	-	-	-	-	-	-	-	-	-
Testicular cancer	-	-	-	-	-	-	-	-	-	-	-	-
Kidney cancer	816 (764-863)	1,440 (1,360-1,533)	2,486 (2,343-2,700)	3,428 (3,252-3,644)	4,967 (4,630-5,252)	6,024 (5,541-6,325)	5,940 (5,264-6,197)	6,638 (5,543-6,974)	6,397 (4,963-6,746)	4,882 (3,457-5,191)	2,491 (1,656-2,683)	812 (509-882)
Bladder cancer	[illegible]	[illegible]	[illegible]	[illegible]	[illegible]	[illegible]	[illegible]	[illegible]	[illegible]	[illegible]	[illegible]	[illegible]
Brain and nervous system cancer	[illegible]	[illegible]	[illegible]	[illegible]	[illegible]	[illegible]	[illegible]	[illegible]	[illegible]	[illegible]	[illegible]	[illegible]
Thyroid cancer	[illegible]	[illegible]	[illegible]	[illegible]	[illegible]	[illegible]	[illegible]	[illegible]	[illegible]	[illegible]	[illegible]	[illegible]

SR Table 5: Global deaths by cause and age group for females (with 95% confidence intervals), 2017

Cause	Early Neonatal	Late Neonatal	Post Neonatal	1 to 4	5 to 9	10 to 14	15 to 19	20 to 24	25 to 29	30 to 34	35 to 39
Mesothelioma	-	-	-	-	-	-	40 (31-50)	60 (46-75)	94 (77-112)	119 (99-139)	196 (163-231)
Hodgkin lymphoma	1 (1-1)	1 (0-1)	15 (12-19)	150 (86-287)	227 (145-333)	403 (260-560)	758 (527-1,036)	921 (671-1,216)	1,009 (746-1,308)	845 (621-1,103)	673 (504-855)
Non-Hodgkin lymphoma	22 (20-24)	18 (16-20)	298 (268-341)	1,575 (1,287-2,013)	1,093 (894-1,380)	920 (804-1,080)	1,035 (916-1,232)	1,455 (1,284-1,756)	1,793 (1,643-2,018)	1,928 (1,805-2,083)	2,289 (2,151-2,454)
Multiple myeloma	-	-	-	-	-	-	100 (87-113)	126 (109-143)	171 (152-188)	217 (197-237)	410 (377-451)
Leukemia	113 (82-126)	94 (74-109)	1,481 (1,188-1,738)	5,142 (4,292-6,182)	5,341 (4,396-6,319)	4,697 (3,930-5,389)	4,248 (3,613-4,986)	3,833 (3,175-4,493)	4,265 (3,481-4,762)	4,204 (3,390-4,733)	4,366 (3,621-4,894)
Acute lymphoid leukemia	31 (22-36)	23 (17-28)	446 (339-556)	1,791 (1,409-2,385)	2,391 (1,879-2,949)	1,873 (1,518-2,250)	1,389 (1,132-1,714)	1,033 (842-1,283)	1,110 (846-1,281)	799 (604-897)	825 (670-945)
Chronic lymphoid leukemia	-	-	-	-	-	-	55 (45-69)	62 (51-79)	89 (72-109)	99 (78-121)	101 (84-123)
Acute myeloid leukemia	18 (13-23)	17 (13-21)	257 (203-333)	1,093 (864-1,440)	794 (609-1,015)	961 (764-1,159)	1,065 (879-1,312)	1,098 (910-1,383)	1,169 (994-1,363)	1,226 (1,029-1,426)	1,337 (1,131-1,598)
Chronic myeloid leukemia	-	-	-	-	-	-	169 (134-224)	240 (189-316)	328 (254-411)	415 (323-499)	453 (353-546)
Other leukemia	63 (45-72)	55 (42-64)	778 (588-929)	2,258 (1,711-2,714)	2,156 (1,701-2,658)	1,863 (1,473-2,214)	1,570 (1,268-1,888)	1,399 (1,042-1,634)	1,569 (1,189-1,757)	1,666 (1,283-1,908)	1,650 (1,288-1,927)
Other malignant neoplasms	444 (350-514)	221 (193-259)	1,473 (1,215-1,799)	5,243 (4,144-6,565)	2,737 (2,266-3,267)	3,298 (2,799-3,881)	3,544 (3,007-4,153)	3,152 (2,692-3,687)	3,290 (2,926-3,709)	3,372 (3,045-3,754)	3,932 (3,632-4,385)
Other neoplasms	27 (20-36)	15 (11-20)	202 (153-274)	664 (454-928)	568 (413-767)	433 (330-571)	380 (288-498)	436 (330-564)	563 (442-755)	750 (589-1,005)	938 (741-1,254)
Myelodysplastic, myeloproliferative, and other hematopoietic neoplasm	26 (19-34)	11 (9-16)	164 (128-229)	488 (347-723)	348 (265-483)	272 (213-364)	234 (182-314)	284 (220-382)	399 (322-543)	544 (437-750)	748 (595-1,015)
Other benign and in situ neoplasms	2 (1-2)	3 (2-4)	38 (24-52)	176 (106-261)	220 (147-289)	161 (117-207)	146 (104-190)	152 (107-189)	164 (117-214)	207 (149-262)	190 (140-242)
Cardiovascular diseases	1,075 (974-1,181)	686 (629-751)	6,637 (6,155-7,153)	5,451 (4,880-6,030)	3,265 (2,954-3,601)	4,481 (4,067-4,894)	11,821 (10,973-12,687)	17,684 (16,690-18,926)	25,982 (24,875-27,282)	40,730 (39,062-42,432)	61,142 (58,621-63,560)
Rheumatic heart disease	-	-	-	1,039 (842-1,249)	845 (730-950)	1,289 (1,135-1,426)	1,840 (1,665-2,016)	2,366 (2,135-2,604)	3,010 (2,667-3,345)	3,899 (3,404-4,454)	4,819 (4,179-5,594)
Ischemic heart disease	-	-	-	-	-	-	3,966 (3,641-4,311)	6,426 (5,969-7,003)	9,807 (9,248-10,517)	17,329 (16,303-18,382)	25,889 (24,377-27,309)
Stroke	274 (237-319)	175 (156-195)	1,432 (1,243-1,686)	1,122 (950-1,338)	977 (866-1,091)	1,460 (1,299-1,648)	2,952 (2,704-3,245)	4,511 (4,194-4,885)	6,829 (6,468-7,232)	11,153 (10,578-11,781)	19,114 (18,169-20,148)
Ischemic stroke	80 (57-104)	42 (33-50)	258 (185-331)	146 (116-179)	124 (101-148)	154 (125-190)	567 (465-676)	856 (686-1,038)	1,245 (1,063-1,455)	1,988 (1,693-2,302)	3,108 (2,659-3,588)
Intracerebral hemorrhage	107 (87-130)	67 (59-77)	646 (527-785)	506 (360-666)	508 (445-577)	800 (699-906)	1,667 (1,499-1,867)	2,570 (2,346-2,836)	3,909 (3,632-4,218)	6,397 (5,930-6,833)	11,224 (10,379-11,994)
Subarachnoid hemorrhage	88 (76-103)	66 (56-79)	528 (456-629)	470 (405-559)	345 (297-411)	507 (436-616)	719 (636-858)	1,085 (960-1,306)	1,674 (1,496-1,948)	2,768 (2,494-3,228)	4,783 (4,344-5,591)
Hypertensive heart disease	-	-	-	-	-	-	355 (240-448)	507 (347-653)	896 (643-1,120)	1,592 (1,167-1,962)	2,678 (1,964-3,276)
Non-rheumatic valvular heart disease	-	-	-	-	-	-	334 (259-422)	374 (289-484)	448 (360-553)	427 (351-520)	560 (472-662)
Non-rheumatic calcific aortic valve disease	-	-	-	-	-	-	96 (68-154)	104 (73-168)	134 (101-201)	151 (118-218)	219 (175-309)
Non-rheumatic degenerative mitral valve disease	-	-	-	-	-	-	183 (130-272)	206 (145-309)	233 (172-335)	213 (160-301)	273 (210-370)
Other non-rheumatic valve diseases	-	-	-	-	-	-	55 (39-87)	63 (45-98)	81 (56-123)	62 (41-94)	68 (41-105)
Cardiomyopathy and myocarditis	455 (372-517)	255 (228-280)	2,499 (2,165-2,819)	1,365 (1,091-1,593)	666 (578-737)	794 (711-881)	967 (837-1,124)	1,337 (1,135-1,562)	2,043 (1,816-2,327)	2,682 (2,450-2,971)	3,655 (3,367-4,023)
Myocarditis	49 (39-69)	43 (35-57)	625 (500-781)	414 (325-526)	245 (206-298)	242 (196-297)	334 (267-414)	461 (366-566)	574 (459-693)	537 (439-639)	593 (466-724)
Alcoholic cardiomyopathy	-	-	-	-	-	-	67 (46-88)	124 (90-159)	337 (280-391)	725 (646-827)	1,182 (1,071-1,355)
Other cardiomyopathy	406 (324-468)	212 (185-236)	1,875 (1,556-2,147)	950 (715-1,142)	421 (351-479)	552 (478-626)	566 (456-666)	753 (596-894)	1,132 (918-1,327)	1,420 (1,174-1,619)	1,879 (1,534-2,164)
Atrial fibrillation and flutter	-	-	-	-	-	-	-	-	-	39 (36-42)	47 (43-51)
Aortic aneurysm	-	-	-	-	-	-	76 (65-97)	128 (108-164)	199 (174-244)	302 (262-368)	440 (392-530)
Peripheral artery disease	-	-	-	-	-	-	-	-	-	-	-
Endocarditis	87 (67-113)	76 (60-95)	1,082 (838-1,338)	877 (597-1,145)	242 (201-282)	332 (282-387)	404 (353-478)	634 (554-737)	833 (730-972)	809 (710-946)	972 (848-1,142)
Other cardiovascular and circulatory diseases	258 (211-353)	180 (153-231)	1,623 (1,322-2,262)	1,049 (757-1,805)	536 (443-714)	606 (519-771)	926 (759-1,229)	1,400 (1,153-1,802)	1,917 (1,613-2,446)	2,498 (2,127-3,109)	2,969 (2,568-3,556)
Chronic respiratory diseases	0 (0-0)	0 (0-0)	0 (0-0)	5,869 (4,937-7,482)	1,741 (1,464-2,383)	1,677 (1,448-2,232)	2,888 (2,588-3,547)	3,835 (3,461-4,744)	5,188 (4,768-6,167)	6,864 (6,301-8,069)	10,134 (9,235-11,833)
Chronic obstructive pulmonary disease	-	-	0 (0-0)	793 (559-1,248)	236 (182-341)	193 (156-252)	854 (681-1,090)	1,229 (986-1,596)	1,780 (1,462-2,201)	2,530 (2,097-3,082)	3,593 (2,919-4,430)
Pneumoconiosis	-	-	-	-	-	-	9 (6-12)	10 (7-13)	18 (12-22)	23 (17-28)	26 (18-32)
Silicosis	-	-	-	-	-	-	2 (1-2)	2 (1-3)	4 (2-5)	5 (3-7)	6 (3-7)
Asbestosis	-	-	-	-	-	-	1 (1-2)	2 (1-3)	3 (2-4)	4 (2-5)	5 (3-8)
Coal workers pneumoconiosis	-	-	-	-	-	-	1 (0-1)	1 (0-1)	2 (1-3)	2 (1-4)	2 (1-4)
Other pneumoconiosis	-	-	-	-	-	-	5 (4-7)	6 (4-8)	9 (6-12)	12 (8-16)	14 (9-19)
Asthma	-	-	-	3,696 (2,949-5,079)	1,047 (836-1,603)	1,097 (904-1,634)	1,610 (1,286-2,172)	2,065 (1,632-2,838)	2,631 (2,111-3,529)	3,380 (2,625-4,452)	5,134 (3,897-6,798)
Interstitial lung disease and pulmonary sarcoidosis	-	-	-	179 (101-341)	85 (56-142)	62 (46-95)	112 (80-170)	167 (123-249)	271 (206-396)	394 (300-576)	618 (451-905)
Other chronic respiratory diseases	0 (0-0)	0 (0-0)	0 (0-0)	1,200 (699-1,699)	373 (291-488)	325 (236-430)	302 (243-373)	365 (299-458)	488 (388-594)	537 (427-668)	763 (578-957)
Digestive diseases	845 (637-979)	755 (626-872)	7,080 (5,978-8,073)	9,064 (7,456-10,816)	4,839 (3,829-5,887)	4,726 (3,937-5,554)	8,312 (7,147-9,708)	11,127 (9,454-12,805)	13,175 (11,432-14,936)	16,223 (14,376-17,854)	21,021 (19,270-22,942)
Cirrhosis and other chronic liver diseases	-	-	-	3,621 (2,794-5,107)	1,891 (1,454-2,817)	2,038 (1,690-2,690)	3,688 (3,051-4,591)	5,668 (4,760-6,804)	7,109 (6,113-8,287)	10,063 (9,052-11,358)	13,355 (12,299-15,141)
Cirrhosis and other chronic liver diseases due to hepatitis B	-	-	-	62 (43-96)	79 (56-124)	155 (114-219)	407 (290-573)	1,550 (1,144-2,006)	2,605 (1,983-3,290)	3,513 (2,900-4,289)	3,961 (3,192-4,952)
Cirrhosis and other chronic liver diseases due to hepatitis C	-	-	-	24 (16-36)	42 (29-64)	85 (61-118)	207 (142-305)	1,011 (714-1,383)	1,692 (1,234-2,190)	2,683 (2,211-3,227)	3,912 (3,093-4,797)
Cirrhosis and other chronic liver diseases due to alcohol use	-	-	-	-	-	-	15 (7-28)	295 (206-415)	672 (506-883)	1,583 (1,329-1,879)	2,920 (2,422-3,564)
Cirrhosis due to NASH	-	-	-	-	-	-	67 (49-92)	221 (165-289)	375 (274-494)	720 (588-853)	1,189 (933-1,473)
Cirrhosis and other chronic liver diseases due to other causes	-	-	-	3,535 (2,731-4,979)	1,770 (1,363-2,616)	1,797 (1,476-2,383)	2,991 (2,433-3,827)	2,590 (1,950-3,354)	1,765 (1,306-2,370)	1,564 (1,157-2,099)	1,373 (1,030-1,763)
Upper digestive system diseases	-	-	-	627 (429-935)	516 (358-661)	472 (349-583)	662 (530-808)	1,560 (1,226-1,840)	1,466 (1,160-1,718)	1,765 (1,373-2,017)	2,211 (1,717-2,510)
Peptic ulcer disease	-	-	-	519 (362-851)	380 (271-490)	377 (278-470)	559 (441-697)	1,243 (997-1,475)	1,144 (924-1,370)	1,377 (1,106-1,645)	1,760 (1,405-2,111)
Gastritis and duodenitis	-	-	-	109 (47-165)	136 (72-183)	96 (51-129)	102 (64-145)	317 (128-426)	322 (152-422)	388 (178-512)	451 (210-584)
Appendicitis	-	-	-	1,251 (755-1,726)	664 (497-820)	658 (500-790)	1,310 (1,043-1,633)	987 (782-1,238)	1,154 (951-1,435)	684 (560-823)	757 (630-912)
Paralytic ileus and intestinal obstruction	845 (637-979)	755 (626-872)	7,080 (5,978-8,073)	1,678 (958-2,184)	1,085 (549-1,474)	1,024 (578-1,324)	1,888 (1,114-2,507)	1,662 (957-2,206)	1,735 (1,032-2,195)	1,497 (854-1,877)	1,783 (1,030-2,208)
Inguinal, femoral, and abdominal hernia	-	-	-	93 (43-121)	81 (40-113)	67 (33-90)	83 (36-113)	81 (37-112)	89 (40-124)	183 (81-273)	256 (111-369)
Inflammatory bowel disease	-	-	-	361 (204-620)	70 (50-100)	56 (42-71)	106 (78-139)	174 (128-226)	228 (175-294)	239 (186-293)	286 (222-337)
Vascular intestinal disorders	-	-	-	61 (22-91)	13 (8-17)	13 (9-15)	30 (20-38)	69 (46-85)	93 (64-114)	128 (91-152)	203 (149-238)
Gallbladder and biliary diseases	-	-	-	467 (279-719)	199 (125-283)	134 (90-171)	160 (118-188)	311 (229-382)	411 (321-484)	528 (435-596)	719 (568-828)
Pancreatitis	-	-	-	43 (26-61)	65 (45-91)	87 (55-111)	219 (155-278)	390 (280-474)	573 (424-677)	789 (593-917)	1,002 (722-1,167)
Other digestive diseases	-	-	-	862 (571-1,279)	254 (185-329)	177 (144-207)	166 (129-203)	224 (175-271)	317 (242-389)	349 (292-405)	448 (365-522)
Neurological disorders	-	-	3,816 (3,335-4,438)	4,358 (3,467-5,485)	2,379 (2,052-2,860)	2,802 (2,462-3,336)	4,371 (3,802-5,385)	4,076 (3,565-5,012)	4,152 (3,759-4,786)	4,625 (4,154-5,404)	4,559 (4,156-5,335)
Alzheimer's disease and other dementias	-	-	-	-	-	-	-	-	-	-	-
Parkinson's disease	-	-	-	-	-	-	-	5 (4-6)	6 (6-7)	12 (11-14)	20 (17-22)
Epilepsy	-	-	2,596 (2,082-3,058)	3,196 (2,667-4,083)	1,865 (1,549-2,386)	2,029 (1,718-2,524)	3,690 (3,134-4,764)	3,548 (3,027-4,491)	3,472 (3,080-4,153)	3,693 (3,206-4,435)	3,223 (2,787-3,988)
Multiple sclerosis	-	-	-	-	-	-	-	96 (80-131)	184 (155-242)	341 (292-433)	569 (494-726)
Motor neuron disease	5 (2-8)	9 (5-12)	164 (137-198)	91 (63-109)	19 (15-21)	21 (18-24)	16 (14-18)	17 (16-21)	32 (29-37)	58 (51-68)	110 (100-126)
Other neurological disorders	-	-	1,056 (783-1,665)	1,071 (600-1,883)	495 (377-699)	752 (551-981)	665 (464-850)	408 (354-550)	457 (407-586)	522 (475-649)	637 (595-781)
Mental disorders	-	-	-	-	0 (0-0)	4 (3-4)	18 (15-22)	33 (28-39)	42 (36-50)	46 (39-53)	44 (39-51)
Eating disorders	-	-	-	-	0 (0-0)	4 (3-4)	18 (15-22)	33 (28-39)	42 (36-50)	46 (39-53)	44 (39-51)
Anorexia nervosa	-	-	-	-	0 (0-0)	4 (3-4)	16 (14-20)	22 (15-26)	27 (18-33)	32 (25-39)	33 (25-40)
Bulimia nervosa	-	-	-	-	0 (0-0)	0 (0-0)	2 (1-3)	11 (8-19)	15 (12-25)	14 (9-23)	11 (7-20)
Substance use disorders	0 (0-0)	0 (0-0)	0 (0-0)	0 (0-0)	0 (0-0)	0 (0-0)	1,302 (1,234-1,388)	2,563 (2,467-2,717)	4,312 (4,145-4,703)	5,819 (5,594-6,517)	6,876 (6,575-7,960)
Alcohol use disorders	-	-	-	-	-	-	167 (151-217)	285 (260-398)	779 (703-1,106)	1,405 (1,275-1,993)	2,203 (2,006-3,172)
Drug use disorders	0 (0-0)	0 (0-0)	0 (0-0)	0 (0-0)	0 (0-0)	0 (0-0)	1,135 (1,073-1,200)	2,278 (2,192-2,370)	3,533 (3,410-3,677)	4,413 (4,253-4,610)	4,673 (4,479-4,894)
Opioid use disorders	0 (0-0)	0 (0-0)	0 (0-0)	0 (0-0)	0 (0-0)	0 (0-0)	743 (674-815)	1,547 (1,409-1,669)	2,357 (2,139-2,556)	2,784 (2,485-3,040)	2,935 (2,595-3,228)
Cocaine use disorders	-	-	-	-	-	-	29 (23-33)	65 (54-80)	106 (86-133)	220 (176-281)	232 (184-299)
Amphetamine use disorders	-	-	-	-	-	-	46 (35-59)	99 (77-119)	157 (119-195)	138 (106-170)	141 (107-170)
Other drug use disorders	-	-	-	-	-	-	317 (261-374)	568 (484-656)	913 (775-1,081)	1,272 (1,096-1,508)	1,365 (1,169-1,636)
Diabetes and kidney diseases	5 (4-7)	15 (11-21)	3,017 (2,743-3,321)	4,392 (3,821-5,050)	2,109 (1,867-2,371)	2,998 (2,674-3,316)	4,945 (4,587-5,323)	7,328 (6,791-7,928)	9,899 (9,281-10,589)	12,561 (11,945-13,273)	17,433 (16,662-18,300)
Diabetes mellitus	5 (4-7)	15 (11-21)	304 (223-380)	394 (277-527)	365 (256-456)	753 (527-930)	1,556 (1,421-1,708)	2,118 (1,959-2,292)	3,269 (3,031-3,533)	4,119 (3,913-4,383)	6,834 (6,520-7,186)
Diabetes mellitus type 1	5 (4-7)	15 (11-21)	304 (223-380)	394 (277-527)	365 (256-456)	753 (527-930)	1,316 (1,170-1,457)	1,725 (1,557-1,895)	2,692 (2,433-2,957)	2,857 (2,551-3,134)	4,583 (4,123-5,007)
Diabetes mellitus type 2	-	-	-	-	-	-	240 (184-328)	393 (309-514)	576 (460-749)	1,262 (1,071-1,529)	2,251 (1,912-2,676)
Chronic kidney disease	-	-	2,642 (2,403-2,926)	3,917 (3,402-4,508)	1,677 (1,483-1,898)	2,186 (1,968-2,431)	3,343 (3,079-3,641)	5,154 (4,726-5,638)	6,537 (6,075-7,113)	8,338 (7,840-8,934)	10,493 (9,897-11,155)
Chronic kidney disease due to diabetes mellitus type 1	-	-	11 (6-17)	18 (9-32)	12 (6-21)	25 (14-42)	73 (38-121)	255 (135-415)	669 (398-1,007)	1,401 (927-1,979)	2,239 (1,476-3,103)
Chronic kidney disease due to diabetes mellitus type 2	-	-	7 (4-12)	11 (5-21)	6 (3-12)	12 (5-22)	31 (14-57)	81 (35-155)	168 (75-323)	331 (151-595)	640 (327-1,072)
Chronic kidney disease due to hypertension	-	-	13 (7-20)	29 (16-46)	19 (11-32)	44 (24-71)	124 (74-193)	320 (201-460)	560 (374-783)	866 (605-1,198)	1,244 (890-1,675)
Chronic kidney disease due to glomerulonephritis	-	-	712 (512-969)	1,348 (935-1,884)	597 (412-833)	818 (565-1,119)	1,355 (973-1,796)	2,086 (1,461-2,772)	2,384 (1,686-3,156)	2,633 (1,845-3,551)	2,877 (1,932-3,978)
Chronic kidney disease due to other and unspecified causes	-	-	1,899 (1,625-2,171)	2,512 (1,989-3,024)	1,042 (802-1,285)	1,287 (1,005-1,594)	1,759 (1,313-2,199)	2,411 (1,723-3,133)	2,757 (1,995-3,579)	3,108 (2,226-4,016)	3,493 (2,427-4,634)
Acute glomerulonephritis	-	-	72 (44-94)	81 (45-114)	66 (49-84)	59 (46-74)	46 (38-57)	57 (47-66)	93 (80-107)	104 (91-120)	106 (91-121)
Skin and subcutaneous diseases	-	-	2,460 (1,192-3,793)	1,184 (561-2,160)	259 (124-367)	264 (133-390)	366 (190-564)	351 (195-531)	365 (203-553)	506 (294-781)	693 (388-1,009)
Bacterial skin diseases	-	-	2,436 (1,172-3,758)	1,099 (506-2,047)	207 (90-290)	197 (90-286)	264 (124-391)	261 (132-376)	273 (145-400)	405 (220-598)	574 (305-795)
Cellulitis	-	-	179 (56-352)	161 (58-297)	66 (23-100)	65 (24-93)	79 (29-112)	77 (33-112)	77 (35-117)	120 (63-190)	147 (70-225)
Pyoderma	-	-	2,257 (1,099-3,454)	938 (437-1,759)	140 (61-202)	132 (62-200)	185 (88-284)	184 (91-271)	195 (102-294)	285 (151-438)	428 (217-615)
Decubitus ulcer	-	-	-	42 (21-77)	25 (16-47)	34 (20-58)	63 (36-115)	42 (24-69)	51 (32-92)	53 (33-92)	66 (41-121)
Other skin and subcutaneous diseases	-	-	25 (12-52)	43 (23-85)	27 (17-52)	33 (20-57)	40 (24-76)	48 (29-92)	41 (24-76)	49 (29-100)	52 (30-113)
Musculoskeletal disorders	-	-	-	-	300 (240-362)	560 (466-628)	1,184 (938-1,336)	1,481 (1,231-1,608)	1,878 (1,544-2,022)	2,042 (1,656-2,248)	1,998 (1,675-2,130)
Rheumatoid arthritis	-	-	-	-	45 (28-62)	35 (25-45)	43 (31-58)	46 (35-63)	71 (57-92)	114 (88-146)	150 (117-187)
Other musculoskeletal disorders	-	-	-	-	254 (197-306)	524 (437-586)	1,141 (900-1,270)	1,435 (1,196-1,558)	1,807 (1,487-1,951)	1,928 (1,559-2,109)	1,848 (1,546-1,971)
Other non-communicable diseases	77,414 (70,857-84,343)	29,895 (27,179-33,042)	116,228 (103,000-136,098)	49,636 (43,169-57,092)	11,587 (10,261-13,234)	8,564 (7,683-9,663)	8,289 (7,491-9,374)	8,765 (8,023-9,807)	8,634 (8,003-9,581)	8,484 (7,916-9,308)	8,485 (7,898-9,175)
Congenital birth defects	76,308 (69,629-83,211)	27,611 (25,485-30,037)	93,822 (86,588-101,372)	38,495 (33,976-43,652)	8,027 (7,164-9,006)	5,655 (5,158-6,200)	3,656 (3,414-3,961)	3,462 (3,224-3,771)	3,569 (3,331-3,876)	2,608 (2,434-2,834)	2,444 (2,257-2,670)
Neural tube defects	10,196 (8,039-12,650)	2,134 (1,735-2,647)	9,993 (6,750-15,249)	6,670 (3,752-12,613)	347 (266-432)	240 (198-285)	148 (126-175)	123 (104-146)	96 (81-115)	96 (82-113)	76 (65-89)
Congenital heart anomalies	24,398 (17,975-29,825)	12,281 (10,426-14,572)	48,042 (35,547-57,418)	18,564 (11,696-24,568)	4,782 (4,071-5,628)	3,598 (3,199-4,005)	2,105 (1,827-2,316)	1,830 (1,573-2,025)	2,234 (2,000-2,479)	1,336 (1,185-1,468)	1,389 (1,228-1,539)
Orofacial clefts	687 (292-2,298)	243 (98-777)	586 (217-1,982)	135 (49-439)	-	-	-	-	-	-	-
Down syndrome	1,551 (1,148-2,620)	652 (469-1,091)	3,173 (2,125-5,584)	1,731 (892-3,728)	245 (180-381)	204 (166-273)	242 (206-322)	335 (278-492)	353 (297-467)	355 (306-455)	294 (255-369)
Other chromosomal abnormalities	3,190 (1,610-6,520)	1,577 (938-2,960)	3,768 (1,951-7,167)	749 (337-1,848)	190 (106-329)	120 (72-179)	110 (65-171)	110 (63-185)	100 (58-150)	82 (48-119)	61 (36-84)
Congenital musculoskeletal and limb anomalies	2,059 (1,082-3,163)	511 (271-728)	1,425 (718-2,278)	533 (206-1,040)	173 (97-235)	130 (80-166)	116 (69-149)	117 (67-160)	83 (51-107)	87 (50-115)	77 (48-100)
Urogenital congenital anomalies	2,320 (1,478-2,932)	723 (537-931)	1,203 (735-1,761)	317 (181-538)	149 (109-199)	141 (103-188)	73 (55-92)	89 (67-111)	69 (54-83)	69 (54-84)	80 (61-100)
Digestive congenital anomalies	7,616 (4,548-12,186)	3,092 (1,969-4,390)	8,051 (4,630-12,651)	1,955 (772-3,735)	182 (128-273)	93 (69-124)	57 (41-79)	59 (40-89)	35 (25-45)	36 (25-49)	30 (21-38)
Other congenital birth defects	24,291 (17,294-31,973)	6,398 (4,600-8,613)	17,580 (11,452-25,185)	7,840 (4,399-12,997)	1,959 (1,474-2,623)	1,129 (895-1,490)	804 (648-1,057)	800 (621-1,032)	600 (475-793)	548 (446-689)	437 (359-543)
Urinary diseases and male infertility	205 (183-229)	132 (118-148)	1,827 (1,640-2,036)	1,736 (1,493-1,982)	704 (594-823)	551 (481-623)	1,208 (1,091-1,327)	1,786 (1,613-1,977)	1,612 (1,489-1,748)	1,999 (1,860-2,142)	2,343 (2,216-2,485)
Urinary tract infections	49 (35-79)	55 (43-74)	1,343 (1,100-1,632)	927 (564-1,418)	423 (328-581)	349 (295-426)	1,015 (905-1,165)	1,518 (1,353-1,744)	1,333 (1,206-1,498)	1,642 (1,493-1,824)	1,962 (1,815-2,149)
Urolithiasis	-	-	-	-	12 (8-20)	13 (9-19)	12 (8-18)	27 (19-41)	40 (30-55)	74 (56-101)	88 (67-119)

Cause	40 to 44	45 to 49	50 to 54	54 to 59	60 to 64	65 to 69	70 to 74	75 to 79	80 to 84	85 to 89	90 to 94	95 plus
Mesothelioma	363 (312-429)	317 (288-355)	496 (457-552)	639 (608-686)	787 (746-844)	983 (942-1,026)	992 (951-1,038)	1,088 (1,042-1,136)	940 (901-984)	650 (617-685)	313 (299-326)	99 (95-103)
Hodgkin lymphoma	592 (457-759)	520 (403-657)	620 (478-777)	654 (502-830)	765 (603-964)	868 (681-1,084)	819 (651-1,044)	804 (639-975)	678 (527-813)	371 (288-425)	120 (90-137)	25 (17-28)
Non-Hodgkin lymphoma	2,965 (2,797-3,153)	3,797 (3,603-4,008)	5,666 (5,387-5,935)	6,870 (6,600-7,163)	10,059 (9,679-10,483)	12,028 (11,605-12,458)	12,429 (11,996-12,870)	12,197 (11,750-12,655)	11,824 (11,376-12,264)	8,725 (8,385-9,053)	4,137 (3,970-4,302)	1,260 (1,216-1,313)
Multiple myeloma	755 (702-837)	1,417 (1,324-1,610)	2,543 (2,382-2,948)	3,882 (3,679-4,542)	5,831 (5,486-6,836)	7,211 (6,825-8,516)	7,351 (6,851-8,557)	7,808 (7,075-8,669)	6,891 (6,115-7,614)	4,599 (4,018-5,121)	2,123 (1,858-2,357)	519 (412-554)
Leukemia	5,046 (4,318-5,633)	6,488 (5,379-7,211)	8,367 (6,871-9,163)	9,227 (8,110-10,237)	11,922 (10,153-13,046)	13,395 (11,358-14,090)	13,538 (11,677-14,509)	14,042 (12,331-14,889)	13,234 (11,787-14,001)	10,094 (9,248-10,627)	5,331 (5,029-5,557)	1,844 (1,767-1,920)
Acute lymphoid leukemia	835 (658-950)	901 (666-988)	1,035 (772-1,138)	1,138 (916-1,295)	1,328 (1,009-1,449)	1,248 (923-1,359)	1,047 (815-1,140)	929 (753-1,005)	763 (610-832)	453 (379-496)	182 (152-197)	47 (43-51)
Chronic lymphoid leukemia	157 (134-187)	249 (205-289)	440 (358-504)	610 (544-685)	1,122 (974-1,260)	1,392 (1,249-1,510)	1,539 (1,401-1,671)	2,136 (1,960-2,284)	2,269 (2,100-2,443)	2,146 (1,975-2,294)	1,378 (1,282-1,467)	543 (511-577)
Acute myeloid leukemia	1,543 (1,344-1,810)	2,023 (1,745-2,298)	2,412 (2,072-2,772)	2,859 (2,474-3,150)	3,567 (3,118-3,934)	4,217 (3,689-4,577)	4,428 (3,818-4,738)	4,496 (3,866-4,796)	3,870 (3,305-4,132)	2,602 (2,195-2,775)	1,111 (987-1,187)	334 (315-353)
Chronic myeloid leukemia	532 (429-630)	556 (435-651)	844 (671-996)	854 (716-1,012)	969 (840-1,163)	1,046 (901-1,240)	1,056 (927-1,255)	1,081 (971-1,263)	1,112 (988-1,259)	857 (784-950)	463 (423-504)	163 (148-180)
Other leukemia	1,979 (1,631-2,285)	2,760 (2,121-3,174)	3,635 (2,791-4,061)	3,767 (3,028-4,261)	4,936 (3,887-5,438)	5,492 (4,354-5,901)	5,468 (4,427-5,974)	5,399 (4,566-5,903)	5,219 (4,536-5,609)	4,037 (3,676-4,365)	2,197 (2,061-2,335)	757 (713-802)
Other malignant neoplasms	5,208 (4,806-5,739)	7,223 (6,614-7,987)	11,125 (10,111-12,066)	13,349 (12,067-14,423)	16,875 (15,224-18,098)	18,409 (16,378-19,720)	17,174 (15,589-18,404)	17,438 (15,419-18,492)	16,259 (13,857-17,242)	11,362 (9,587-12,151)	5,686 (4,785-6,047)	1,957 (1,613-2,112)
Other neoplasms	1,165 (909-1,549)	1,665 (1,283-2,266)	2,419 (1,880-3,279)	2,818 (2,140-3,932)	3,768 (2,924-5,132)	4,651 (3,495-6,392)	5,355 (3,935-7,423)	6,325 (4,418-8,546)	6,812 (4,529-8,695)	5,997 (3,551-7,415)	3,548 (1,866-4,526)	1,439 (736-1,827)
Myelodysplastic, myeloproliferative, and other hematopoietic neoplasm	1,018 (797-1,362)	1,527 (1,188-2,111)	2,286 (1,768-3,120)	2,721 (2,073-3,824)	3,678 (2,864-5,051)	4,583 (3,442-6,326)	5,308 (3,913-7,389)	6,290 (4,397-8,526)	6,784 (4,497-8,647)	5,982 (3,530-7,404)	3,542 (1,860-4,520)	1,437 (734-1,825)
Other benign and in situ neoplasms	147 (111-189)	138 (97-180)	134 (87-162)	98 (61-124)	90 (46-125)	68 (31-100)	47 (20-72)	35 (13-67)	27 (9-49)	15 (6-25)	6 (2-12)	2 (1-3)
Cardiovascular diseases	105,751 (101,775-109,197)	167,040 (161,308-171,831)	273,933 (265,161-281,849)	345,743 (335,548-354,507)	541,067 (526,024-555,268)	727,470 (707,169-747,064)	856,077 (832,528-880,239)	1,161,029 (1,132,877-1,189,85	1,437,564 (1,406,012-1,470,14	1,373,924 (1,349,145-1,399,96	885,560 (871,972-897,249)	390,500 (385,829-395,17
Rheumatic heart disease	7,307 (6,147-8,583)	7,744 (6,682-9,070)	11,425 (9,659-13,735)	10,528 (9,088-12,371)	13,873 (12,128-15,812)	16,007 (14,334-17,818)	16,563 (14,776-18,374)	16,903 (15,488-18,120)	18,751 (17,294-19,915)	15,662 (14,657-16,471)	9,031 (8,582-9,448)	3,809 (3,645-4,021)
Ischemic heart disease	46,796 (44,433-49,165)	75,012 (71,780-78,056)	122,422 (117,183-127,422)	162,852 (156,770-168,276)	253,545 (244,965-261,588)	338,655 (327,686-349,152)	396,077 (383,873-410,213)	550,445 (535,259-569,576)	687,691 (670,240-710,871)	674,400 (660,071-697,345)	450,447 (440,901-466,247)	204,654 (199,755-212,319)
Stroke	35,102 (33,619-36,630)	61,239 (58,800-63,478)	105,208 (101,264-109,324)	126,156 (121,740-130,524)	208,691 (201,458-216,914)	287,009 (277,081-298,261)	334,837 (322,910-347,962)	441,612 (427,911-458,982)	518,356 (503,124-536,979)	459,989 (448,143-477,103)	260,890 (254,906-270,831)	104,014 (101,458-108,379)
Ischemic stroke	5,903 (5,086-6,796)	10,956 (9,738-12,319)	20,713 (18,346-23,276)	28,553 (25,386-32,126)	69,126 (62,891-76,264)	105,453 (97,908-113,474)	146,276 (136,938-155,795)	211,135 (201,530-220,889)	297,865 (287,108-309,364)	279,279 (271,316-289,562)	163,902 (159,626-170,328)	68,881 (67,155-71,744)
Intracerebral hemorrhage	20,934 (19,512-22,263)	39,898 (37,658-42,008)	68,664 (64,775-72,639)	78,786 (74,112-83,019)	119,492 (112,021-126,474)	156,120 (147,364-164,595)	166,183 (157,140-175,379)	202,801 (193,575-213,698)	196,481 (188,178-206,132)	159,965 (153,589-167,705)	85,166 (82,194-89,083)	30,517 (29,480-31,851)
Subarachnoid hemorrhage	8,264 (7,497-9,637)	10,386 (9,475-12,135)	15,830 (14,375-18,724)	18,816 (17,027-22,611)	20,073 (18,339-23,065)	25,436 (23,445-29,365)	22,378 (20,585-25,805)	27,676 (25,698-30,731)	24,010 (21,758-26,445)	20,745 (19,224-22,283)	11,822 (10,819-12,642)	4,617 (4,337-5,021)
Hypertensive heart disease	4,784 (3,530-5,774)	8,580 (6,347-10,062)	14,817 (11,295-17,185)	19,532 (15,385-22,675)	29,051 (22,048-33,067)	40,817 (30,735-45,557)	52,187 (40,096-58,461)	71,025 (52,810-79,230)	95,602 (68,374-107,152)	91,200 (59,718-101,261)	61,830 (37,053-67,923)	28,476 (16,183-31,347)
Non-rheumatic valvular heart disease	706 (604-831)	921 (807-1,078)	1,436 (1,251-1,655)	1,941 (1,745-2,204)	2,663 (2,394-3,078)	3,804 (3,443-4,484)	5,245 (4,763-6,489)	8,777 (7,516-10,171)	13,610 (10,735-14,639)	17,574 (12,409-18,984)	14,780 (9,186-15,966)	7,733 (4,593-8,426)
Non-rheumatic calcific aortic valve disease	268 (218-356)	376 (315-512)	632 (532-835)	907 (779-1,180)	1,431 (1,256-1,824)	2,221 (1,973-2,801)	3,266 (2,866-4,207)	5,740 (4,577-6,779)	9,696 (7,231-10,686)	13,629 (9,017-14,962)	12,049 (7,005-13,276)	6,479 (3,603-7,169)
Non-rheumatic degenerative mitral valve disease	359 (280-470)	448 (354-572)	677 (525-859)	886 (709-1,093)	1,073 (864-1,330)	1,386 (1,091-1,743)	1,763 (1,431-2,222)	2,529 (2,036-3,206)	3,270 (2,628-4,301)	3,388 (2,496-4,754)	2,423 (1,703-3,728)	1,146 (750-1,834)
Other non-rheumatic valve diseases	79 (51-123)	97 (64-156)	126 (81-199)	147 (96-225)	160 (96-245)	197 (123-295)	215 (127-307)	508 (320-762)	644 (415-943)	557 (350-788)	308 (197-439)	108 (72-156)
Cardiomyopathy and myocarditis	4,671 (4,284-5,158)	5,313 (4,913-5,831)	6,642 (6,134-7,316)	8,546 (7,969-9,251)	9,282 (8,671-10,071)	9,561 (8,963-10,254)	10,004 (9,334-10,781)	14,111 (13,318-14,962)	20,110 (19,059-21,116)	23,644 (22,519-24,918)	17,901 (16,979-18,962)	9,102 (8,585-9,884)
Myocarditis	734 (588-881)	635 (493-742)	775 (589-917)	779 (584-931)	810 (603-931)	1,045 (804-1,227)	1,163 (890-1,334)	1,731 (1,399-2,004)	2,941 (2,397-3,593)	4,043 (3,238-4,971)	3,568 (2,501-4,193)	2,214 (1,364-2,664)
Alcoholic cardiomyopathy	1,557 (1,435-1,755)	1,818 (1,669-2,045)	2,092 (1,924-2,368)	2,789 (2,584-3,170)	2,492 (2,306-2,838)	1,897 (1,726-2,223)	1,164 (1,042-1,391)	1,425 (1,260-1,954)	1,090 (930-1,586)	793 (650-1,189)	522 (438-827)	229 (193-364)
Other cardiomyopathy	2,380 (1,946-2,756)	2,860 (2,408-3,272)	3,776 (3,194-4,349)	4,978 (4,254-5,597)	5,981 (5,301-6,669)	6,620 (5,991-7,219)	7,677 (7,036-8,352)	10,954 (10,045-11,751)	16,079 (14,886-17,064)	18,808 (17,574-20,098)	13,810 (12,844-15,302)	6,660 (6,009-7,873)
Atrial fibrillation and flutter	238 (216-257)	454 (416-487)	895 (824-946)	1,766 (1,654-1,858)	3,556 (3,337-3,736)	5,970 (5,641-6,272)	10,629 (10,067-11,251)	18,813 (17,809-19,831)	35,756 (34,043-37,568)	44,111 (42,393-45,916)	38,414 (37,225-40,085)	16,582 (16,044-17,607)
Aortic aneurysm	666 (608-796)	1,064 (988-1,238)	1,715 (1,602-1,996)	2,501 (2,353-2,873)	3,514 (3,323-3,970)	5,076 (4,807-5,685)	6,307 (5,957-6,999)	8,543 (8,117-9,184)	10,481 (9,955-11,027)	10,329 (9,933-10,698)	6,579 (6,376-6,778)	2,984 (2,884-3,107)
Peripheral artery disease	102 (57-207)	176 (91-414)	350 (175-807)	671 (305-1,542)	1,244 (586-2,748)	1,881 (857-4,472)	2,477 (1,095-5,811)	4,178 (1,842-9,724)	6,296 (2,794-14,298)	7,841 (3,351-17,901)	7,002 (2,956-17,595)	4,167 (1,694-10,509)
Endocarditis	1,192 (1,057-1,425)	1,078 (927-1,320)	1,490 (1,281-1,779)	1,725 (1,457-2,196)	2,436 (2,065-3,019)	2,960 (2,498-3,783)	3,446 (2,801-4,438)	4,375 (3,363-5,756)	5,516 (4,078-7,285)	6,057 (4,074-7,742)	4,706 (2,833-6,109)	2,490 (1,457-3,315)
Other cardiovascular and circulatory diseases	4,187 (3,621-4,955)	5,459 (4,823-6,406)	7,532 (6,611-8,813)	9,525 (8,416-10,960)	13,209 (11,744-15,178)	15,730 (14,274-17,841)	18,305 (16,424-20,626)	22,248 (20,350-24,375)	25,397 (22,936-27,883)	23,116 (21,432-25,084)	13,982 (13,247-15,336)	6,489 (6,109-7,170)
Chronic respiratory diseases	15,643 (14,312-18,216)	25,567 (23,403-29,267)	49,474 (44,726-54,442)	67,548 (61,182-73,715)	116,157 (106,282-125,601)	164,738 (151,026-179,114)	231,966 (208,963-251,751)	267,181 (244,319-287,030)	329,025 (298,397-351,646)	259,122 (240,383-276,335)	134,946 (127,023-144,871)	50,205 (47,690-52,603)
Chronic obstructive pulmonary disease	6,437 (5,297-7,973)	12,899 (10,795-15,291)	29,469 (24,578-34,729)	43,575 (36,553-50,194)	86,256 (74,845-96,992)	130,539 (115,845-144,275)	189,135 (164,937-208,539)	223,890 (200,461-242,270)	281,938 (253,776-303,774)	227,148 (209,739-242,777)	120,484 (113,786-130,734)	45,034 (42,908-47,563)
Pneumoconiosis	42 (29-52)	60 (44-71)	95 (76-114)	116 (81-141)	170 (136-204)	213 (175-259)	278 (235-334)	350 (270-421)	398 (317-478)	303 (242-349)	162 (134-189)	67 (54-76)
Silicosis	10 (5-14)	20 (11-26)	24 (12-31)	26 (14-35)	46 (26-62)	50 (27-70)	73 (45-108)	100 (60-141)	114 (73-160)	82 (54-126)	41 (27-65)	15 (10-24)
Asbestosis	6 (3-9)	10 (5-14)	18 (10-27)	23 (14-35)	31 (19-44)	38 (24-53)	57 (36-79)	68 (41-92)	82 (46-109)	72 (34-92)	39 (17-51)	15 (7-21)
Coal workers pneumoconiosis	4 (2-6)	6 (3-9)	12 (6-17)	13 (6-19)	18 (9-27)	28 (15-42)	31 (16-46)	39 (21-57)	42 (22-58)	34 (19-47)	19 (11-26)	9 (5-12)
Other pneumoconiosis	22 (14-29)	24 (17-34)	41 (29-57)	55 (36-73)	75 (54-101)	97 (73-129)	118 (86-156)	144 (105-190)	160 (122-209)	115 (92-153)	63 (52-86)	26 (19-36)
Asthma	7,198 (5,172-9,584)	10,225 (7,227-13,585)	15,648 (10,368-21,018)	18,399 (12,247-24,825)	24,053 (15,359-32,714)	26,342 (16,832-36,737)	32,391 (20,306-45,809)	31,636 (20,817-42,616)	33,908 (22,912-45,867)	21,375 (15,368-28,125)	8,894 (6,947-10,955)	2,910 (2,460-3,380)
Interstitial lung disease and pulmonary sarcoidosis	908 (665-1,294)	1,414 (1,037-1,952)	2,530 (1,821-3,676)	3,368 (2,402-4,814)	4,901 (3,385-7,202)	6,723 (4,682-9,779)	9,096 (6,037-13,079)	10,028 (6,461-13,483)	11,243 (7,125-14,496)	9,032 (5,460-11,127)	4,654 (2,660-5,471)	1,874 (1,075-2,162)
Other chronic respiratory diseases	1,058 (805-1,431)	969 (753-1,238)	1,732 (1,271-2,262)	2,090 (1,597-2,666)	777 (618-926)	921 (752-1,065)	1,066 (859-1,268)	1,277 (1,035-1,488)	1,538 (1,228-1,866)	1,264 (1,049-1,464)	751 (645-847)	320 (280-356)
Digestive diseases	28,286 (26,078-30,711)	37,175 (35,188-40,124)	53,260 (50,371-58,754)	65,805 (62,607-70,473)	80,706 (76,243-86,489)	91,365 (86,148-98,422)	94,441 (88,352-101,206)	104,296 (97,633-112,232)	113,010 (106,698-119,710)	96,943 (93,165-102,288)	58,167 (56,367-61,006)	25,311 (24,597-26,451)
Cirrhosis and other chronic liver diseases	18,924 (17,548-21,734)	24,851 (23,164-28,684)	34,421 (31,928-42,269)	41,959 (39,456-48,991)	48,622 (45,635-56,965)	49,673 (46,504-58,468)	46,635 (43,658-55,636)	44,103 (41,068-51,881)	39,242 (36,552-46,676)	26,746 (25,009-31,434)	12,640 (11,786-14,966)	4,946 (4,596-5,803)
Cirrhosis and other chronic liver diseases due to hepatitis B	5,242 (4,275-6,474)	6,087 (4,973-7,585)	7,729 (6,113-10,158)	8,894 (7,107-11,446)	11,068 (8,784-14,230)	12,054 (9,836-15,707)	11,807 (9,483-15,153)	11,183 (9,146-14,253)	9,888 (8,004-12,817)	6,185 (5,279-7,731)	2,527 (2,044-3,264)	808 (589-1,077)
Cirrhosis and other chronic liver diseases due to hepatitis C	5,606 (4,589-6,792)	7,478 (6,153-9,097)	10,211 (8,373-12,660)	11,806 (10,066-14,008)	13,389 (11,191-15,970)	13,545 (11,506-16,059)	12,501 (10,632-14,715)	11,305 (9,472-13,731)	10,020 (8,253-12,309)	7,106 (6,018-8,410)	3,499 (2,890-4,229)	1,457 (1,153-1,858)
Cirrhosis and other chronic liver diseases due to alcohol use	4,501 (3,823-5,370)	6,181 (5,218-7,548)	8,795 (7,322-10,952)	10,877 (8,935-13,264)	11,741 (9,460-14,604)	10,991 (9,057-13,938)	9,310 (7,456-12,195)	8,489 (7,143-10,505)	7,101 (5,835-9,115)	4,572 (3,851-5,784)	2,074 (1,646-2,691)	773 (547-1,074)
Cirrhosis due to NASH	1,950 (1,610-2,331)	3,051 (2,527-3,691)	4,638 (3,860-5,607)	5,895 (5,160-6,798)	6,602 (5,673-7,606)	6,290 (5,499-7,243)	5,512 (4,790-6,347)	4,679 (3,943-5,464)	3,962 (3,294-4,744)	2,842 (2,428-3,391)	1,393 (1,122-1,758)	533 (388-731)
Cirrhosis and other chronic liver diseases due to other causes	1,624 (1,192-2,113)	2,054 (1,544-2,630)	3,049 (2,264-3,984)	4,488 (3,214-5,969)	5,823 (4,266-7,618)	6,793 (5,080-8,861)	7,505 (5,850-9,643)	8,446 (6,550-10,754)	8,270 (6,406-10,615)	6,041 (5,050-7,396)	3,148 (2,510-3,950)	1,375 (993-1,787)
Upper digestive system diseases	2,595 (1,917-2,924)	3,435 (2,795-3,855)	6,001 (4,764-6,826)	6,240 (5,016-6,923)	9,420 (7,615-10,394)	12,916 (10,711-14,200)	14,024 (11,536-15,519)	16,479 (14,168-17,986)	19,349 (17,276-20,858)	16,645 (15,479-18,059)	9,286 (8,824-10,382)	3,775 (3,579-4,225)
Peptic ulcer disease	2,184 (1,636-2,528)	2,848 (2,294-3,373)	5,029 (4,008-5,975)	5,283 (4,248-6,080)	7,932 (6,454-8,985)	10,730 (8,987-12,242)	11,659 (9,532-13,395)	13,674 (11,809-15,390)	15,897 (14,160-17,913)	13,763 (12,678-15,551)	7,637 (7,193-8,779)	3,146 (2,971-3,566)
Gastritis and duodenitis	410 (225-517)	587 (306-710)	972 (509-1,220)	957 (553-1,247)	1,488 (801-1,811)	2,185 (1,165-2,704)	2,365 (1,280-2,848)	2,805 (1,530-3,355)	3,453 (2,089-4,373)	2,882 (1,916-3,769)	1,649 (1,252-2,147)	629 (517-804)
Appendicitis	717 (548-832)	861 (713-1,015)	893 (742-1,074)	1,655 (1,321-1,982)	1,607 (1,255-1,846)	1,651 (1,319-1,904)	1,549 (1,233-1,747)	1,768 (1,487-2,013)	1,659 (1,431-1,862)	1,423 (1,242-1,613)	759 (688-849)	270 (247-300)
Paralytic ileus and intestinal obstruction	2,450 (1,356-3,019)	2,748 (1,692-3,344)	4,624 (2,479-5,802)	5,937 (3,562-7,094)	7,000 (4,316-8,420)	8,529 (5,635-10,134)	9,371 (6,493-10,907)	11,481 (8,991-13,577)	14,208 (11,233-16,717)	13,987 (10,994-16,550)	9,779 (7,600-11,456)	4,428 (3,199-5,307)
Inguinal, femoral, and abdominal hernia	192 (125-239)	393 (228-543)	637 (428-803)	871 (595-1,069)	1,168 (808-1,460)	1,466 (1,072-1,944)	1,950 (1,363-2,465)	2,216 (1,635-2,895)	3,095 (2,195-4,059)	2,820 (2,000-3,733)	1,849 (1,317-2,440)	788 (516-1,097)
Inflammatory bowel disease	350 (255-407)	485 (378-553)	661 (510-746)	896 (667-998)	1,145 (826-1,272)	1,429 (1,052-1,581)	1,799 (1,305-2,006)	2,355 (1,716-2,641)	3,003 (2,063-3,390)	3,134 (2,081-3,558)	2,354 (1,459-2,729)	1,191 (726-1,384)
Vascular intestinal disorders	351 (265-402)	594 (468-673)	937 (758-1,044)	1,545 (1,252-1,715)	2,614 (2,106-2,952)	4,206 (3,500-4,663)	5,843 (4,889-6,473)	8,846 (7,756-9,703)	10,678 (9,387-11,873)	10,089 (9,078-11,124)	6,036 (5,446-6,703)	2,238 (1,977-2,550)
Gallbladder and biliary diseases	839 (678-922)	1,189 (1,012-1,310)	1,896 (1,619-2,078)	2,339 (2,041-2,591)	3,521 (3,066-3,844)	4,660 (4,101-5,054)	5,334 (4,677-5,866)	7,441 (6,582-8,063)	10,233 (9,008-10,983)	10,468 (9,156-11,316)	7,810 (6,927-8,667)	4,089 (3,592-4,547)
Pancreatitis	1,293 (946-1,492)	1,882 (1,341-2,256)	2,098 (1,551-2,442)	2,787 (2,048-3,181)	3,203 (2,377-3,623)	3,829 (2,804-4,284)	4,068 (2,974-4,683)	4,420 (3,185-4,956)	4,664 (3,451-5,180)	3,877 (2,988-4,383)	2,146 (1,625-2,387)	831 (642-931)
Other digestive diseases	574 (461-684)	737 (651-812)	1,092 (967-1,191)	1,576 (1,391-1,724)	2,406 (2,123-2,640)	3,005 (2,753-3,230)	3,868 (3,497-4,206)	5,188 (4,826-5,603)	6,877 (6,463-7,428)	7,753 (7,281-8,440)	5,507 (5,170-6,119)	2,755 (2,567-3,118)
Neurological disorders	5,439 (5,050-6,210)	6,959 (6,648-7,522)	12,124 (11,570-13,080)	16,314 (15,798-16,940)	32,048 (31,133-33,281)	58,974 (57,294-61,192)	103,882 (101,115-106,684)	211,777 (205,900-216,863)	365,602 (353,599-374,116)	458,715 (446,829-466,868)	399,340 (393,071-404,540)	195,457 (193,252-198,121)
Alzheimer's disease and other dementias	265 (252-283)	1,080 (1,029-1,159)	3,681 (3,491-3,976)	7,557 (7,314-7,891)	20,419 (19,725-21,366)	42,409 (41,072-44,122)	79,715 (77,685-82,003)	175,525 (170,798-179,407)	320,962 (310,962-328,531)	423,469 (412,797-431,276)	382,821 (376,706-387,887)	190,764 (188,581-193,381)
Parkinson's disease	198 (178-217)	518 (471-578)	1,185 (1,087-1,349)	1,680 (1,568-1,854)	3,708 (3,384-4,124)	7,901 (7,299-8,432)	15,913 (14,145-16,794)	29,015 (26,274-30,127)	38,188 (35,336-39,848)	30,566 (28,350-31,803)	14,382 (13,224-15,049)	3,913 (3,686-4,011)
Epilepsy	3,026 (2,671-3,763)	2,385 (2,039-2,728)	3,313 (2,729-3,949)	2,456 (2,045-2,701)	2,479 (1,997-2,738)	2,701 (2,205-2,983)	2,863 (2,344-3,189)	2,329 (1,877-2,697)	2,536 (2,087-2,791)	2,088 (1,691-2,371)	979 (830-1,077)	360 (322-391)
Multiple sclerosis	848 (746-1,062)	1,114 (995-1,379)	1,436 (1,290-1,742)	1,593 (1,303-1,785)	1,560 (1,154-1,730)	1,403 (969-1,557)	1,099 (712-1,231)	812 (531-912)	587 (384-656)	381 (253-427)	140 (96-155)	40 (27-44)
Motor neuron disease	240 (218-295)	496 (452-612)	934 (857-1,197)	1,373 (1,280-1,582)	1,911 (1,773-2,122)	2,467 (2,275-2,711)	2,322 (2,159-2,464)	2,118 (1,942-2,259)	1,469 (1,333-1,577)	780 (710-831)	250 (227-265)	67 (61-71)
Other neurological disorders	861 (798-1,044)	1,367 (1,260-1,601)	1,575 (1,465-1,854)	1,655 (1,535-1,879)	1,971 (1,818-2,233)	2,094 (1,932-2,340)	1,970 (1,801-2,163)	1,977 (1,754-2,130)	1,860 (1,582-2,013)	1,430 (1,192-1,539)	767 (694-814)	314 (296-332)
Mental disorders	47 (41-52)	49 (43-55)	-	-	-	-	-	-	-	-	-	-
Eating disorders	47 (41-52)	49 (43-55)	-	-	-	-	-	-	-	-	-	-
Anorexia nervosa	35 (27-41)	41 (33-47)	-	-	-	-	-	-	-	-	-	-
Bulimia nervosa	11 (7-19)	8 (4-15)	-	-	-	-	-	-	-	-	-	-
Substance use disorders	7,253 (6,928-8,295)	8,419 (8,011-9,565)	8,960 (8,523-9,927)	8,088 (7,631-9,096)	6,608 (6,192-7,088)	5,585 (5,241-5,898)	3,656 (3,387-3,841)	4,058 (3,666-4,261)	3,459 (3,065-3,615)	2,927 (2,480-3,084)	1,647 (1,429-1,733)	690 (601-725)
Alcohol use disorders	2,747 (2,505-3,693)	3,439 (3,143-4,552)	3,610 (3,291-4,493)	3,622 (3,275-4,578)	3,051 (2,685-3,548)	2,493 (2,129-2,731)	1,285 (1,049-1,398)	1,553 (1,155-1,710)	1,010 (621-1,128)	894 (453-1,016)	457 (245-516)	189 (107-213)
Drug use disorders	4,506 (4,320-4,708)	4,980 (4,738-5,235)	5,349 (5,102-5,621)	4,466 (4,228-4,698)	3,556 (3,412-3,709)	3,092 (2,980-3,221)	2,371 (2,281-2,471)	2,505 (2,406-2,602)	2,449 (2,361-2,537)	2,034 (1,944-2,125)	1,190 (1,142-1,247)	501 (481-521)
Opioid use disorders	2,841 (2,510-3,126)	3,066 (2,670-3,417)	3,238 (2,818-3,607)	2,729 (2,364-3,048)	2,153 (1,964-2,327)	1,937 (1,816-2,077)	1,248 (1,153-1,354)	1,352 (1,261-1,449)	1,271 (1,185-1,354)	1,022 (951-1,094)	560 (519-599)	216 (194-235)
Cocaine use disorders	226 (178-290)	265 (203-346)	237 (180-311)	191 (146-252)	114 (92-143)	73 (61-89)	18 (15-20)	17 (14-19)	11 (10-13)	10 (9-11)	8 (6-9)	5 (4-6)
Amphetamine use disorders	138 (108-164)	159 (121-191)	128 (87-160)	106 (75-133)	74 (54-98)	54 (37-77)	13 (9-18)	14 (9-18)	6 (4-7)	7 (4-8)	5 (1-6)	4 (1-5)
Other drug use disorders	1,301 (1,112-1,557)	1,490 (1,269-1,788)	1,746 (1,496-2,104)	1,440 (1,225-1,743)	1,215 (1,069-1,374)	1,028 (918-1,132)	1,092 (994-1,179)	1,123 (1,034-1,200)	1,161 (1,068-1,235)	995 (912-1,070)	617 (570-671)	276 (255-303)
Diabetes and kidney diseases	28,173 (27,084-29,275)	42,966 (41,329-44,568)	72,122 (69,535-74,672)	87,994 (85,244-90,790)	126,869 (122,750-131,090)	147,758 (143,364-152,524)	149,330 (144,442-154,534)	163,906 (158,652-169,485)	167,358 (161,706-173,204)	133,692 (129,960-137,660)	77,044 (75,175-78,811)	32,941 (32,214-33,624)
Diabetes mellitus	13,242 (12,638-13,871)	22,099 (21,133-23,105)	39,549 (37,905-41,180)	50,444 (48,707-52,339)	77,353 (74,172-80,644)	90,619 (87,312-94,235)	90,003 (86,310-93,737)	97,191 (93,300-101,377)	96,872 (92,693-101,243)	67,402 (64,692-70,195)	34,124 (32,904-35,209)	12,421 (12,020-12,838)
Diabetes mellitus type 1	8,503 (7,659-9,275)	8,929 (7,706-10,078)	15,205 (13,036-17,268)	18,442 (16,311-20,604)	20,224 (17,506-23,293)	23,156 (20,172-26,425)	18,901 (16,482-21,324)	19,791 (17,185-22,627)	14,990 (12,533-17,470)	9,431 (7,838-10,841)	4,461 (3,498-5,047)	1,530 (1,109-1,710)
Diabetes mellitus type 2	4,739 (4,037-5,593)	13,170 (11,934-14,505)	24,344 (21,986-26,643)	32,002 (29,485-34,416)	57,129 (53,412-60,722)	67,463 (63,326-71,608)	71,102 (66,996-75,409)	77,400 (72,761-82,412)	81,882 (77,163-87,067)	57,971 (55,138-61,369)	29,663 (28,432-31,224)	10,891 (10,489-11,396)
Chronic kidney disease	14,791 (14,056-15,507)	20,642 (19,671-21,564)	32,294 (30,930-33,727)	37,329 (35,866-38,889)	49,113 (47,451-50,856)	56,622 (54,764-58,585)	58,812 (56,889-60,881)	66,163 (64,025-68,298)	69,920 (67,901-72,228)	65,867 (64,028-67,800)	42,711 (41,549-43,873)	20,438 (19,900-20,955)
Chronic kidney disease due to diabetes mellitus type 1	3,420 (2,246-4,761)	4,540 (2,982-6,281)	5,487 (3,306-8,038)	4,330 (2,475-6,784)	3,394 (1,680-5,813)	2,642 (1,333-4,680)	2,082 (999-3,639)	1,893 (918-3,446)	1,702 (860-2,892)	1,109 (591-1,903)	337 (164-605)	34 (15-68)
Chronic kidney disease due to diabetes mellitus type 2	1,462 (825-2,429)	3,430 (2,145-4,922)	7,856 (5,389-10,873)	11,369 (7,952-14,482)	17,487 (12,689-22,019)	21,079 (16,120-25,893)	21,703 (16,275-26,593)	23,428 (17,000-29,561)	23,736 (16,902-30,376)	20,705 (14,810-26,248)	12,195 (8,812-15,702)	5,438 (3,533-7,633)
Chronic kidney disease due to hypertension	1,952 (1,415-2,560)	3,048 (2,190-4,009)	5,460 (4,009-7,185)	7,283 (5,566-9,435)	10,774 (8,248-13,884)	14,240 (11,065-17,731)	16,713 (13,332-21,016)	20,848 (16,583-25,994)	24,677 (19,435-30,436)	26,347 (21,656-31,559)	18,922 (15,628-22,144)	9,602 (7,675-11,612)
Chronic kidney disease due to glomerulonephritis	3,616 (2,354-5,002)	4,512 (2,893-6,512)	6,140 (3,994-8,886)	6,264 (4,133-9,164)	6,931 (4,479-10,256)	7,551 (4,866-10,928)	7,779 (4,889-11,239)	8,746 (5,620-12,596)	8,787 (5,605-13,100)	7,578 (5,005-11,180)	4,614 (3,093-6,694)	2,143 (1,184-3,461)
Chronic kidney disease due to other and unspecified causes	4,341 (2,965-5,832)	5,111 (3,443-7,184)	7,352 (4,881-10,391)	8,083 (5,359-11,399)	10,527 (6,370-15,144)	11,110 (7,154-15,752)	10,534 (6,748-15,041)	11,248 (7,282-16,577)	11,018 (7,385-15,803)	10,128 (6,950-13,962)	6,642 (4,566-9,266)	3,221 (2,023-5,025)
Acute glomerulonephritis	140 (123-158)	226 (197-254)	279 (248-318)	221 (200-249)	403 (356-454)	516 (460-578)	515 (456-579)	551 (494-626)	565 (507-636)	424 (383-472)	210 (192-230)	82 (74-91)
Skin and subcutaneous diseases	987 (535-1,461)	1,289 (689-1,960)	2,169 (1,155-3,116)	2,613 (1,358-3,733)	3,190 (1,591-4,515)	3,986 (2,064-5,688)	4,798 (2,464-6,810)	5,754 (3,135-8,665)	6,941 (3,713-10,424)	6,928 (3,604-11,081)	4,884 (2,536-7,809)	2,730 (1,332-4,636)
Bacterial skin diseases	832 (419-1,202)	1,076 (552-1,571)	1,871 (945-2,664)	2,151 (1,068-3,008)	2,677 (1,274-3,742)	3,151 (1,514-4,381)	3,638 (1,779-4,880)	4,202 (2,157-6,191)	4,494 (2,252-6,514)	4,225 (2,117-6,310)	2,869 (1,366-4,340)	1,510 (715-2,336)
Cellulitis	216 (95-329)	264 (113-403)	508 (216-814)	602 (261-902)	762 (320-1,207)	862 (354-1,322)	859 (364-1,325)	946 (367-1,481)	1,132 (447-1,940)	1,011 (401-1,731)	750 (292-1,417)	425 (156-763)
Pyoderma	617 (308-952)	811 (404-1,261)	1,363 (668-1,916)	1,549 (766-2,263)	1,915 (918-2,707)	2,290 (1,068-3,231)	2,779 (1,305-3,893)	3,256 (1,710-4,838)	3,362 (1,683-4,829)	3,214 (1,558-4,882)	2,118 (988-3,060)	1,084 (504-1,651)
Decubitus ulcer	90 (51-156)	141 (77-238)	212 (115-365)	379 (197-635)	400 (206-664)	693 (360-1,085)	997 (536-1,613)	1,327 (719-2,233)	2,136 (1,141-3,491)	2,352 (1,261-4,020)	1,753 (957-3,020)	1,092 (562-1,958)
Other skin and subcutaneous diseases	64 (36-150)	72 (36-151)	86 (43-196)	83 (38-202)	113 (51-257)	142 (67-338)	163 (79-386)	224 (105-526)	312 (147-723)	351 (160-913)	263 (108-724)	129 (48-369)
Musculoskeletal disorders	2,589 (2,062-2,850)	3,096 (2,608-3,389)	3,535 (2,929-3,802)	3,833 (3,315-4,162)	6,009 (4,942-6,543)	8,099 (6,632-8,943)	8,997 (7,371-9,791)	9,331 (7,704-10,181)	10,121 (8,441-11,018)	8,329 (7,224-8,969)	4,771 (4,285-5,083)	2,132 (2,012-2,265)
Rheumatoid arthritis	296 (217-365)	623 (490-724)	1,008 (745-1,156)	1,445 (1,160-1,637)	2,828 (2,192-3,256)	4,293 (3,238-4,982)	4,979 (3,871-5,771)	5,294 (4,174-5,999)	5,363 (4,112-6,099)	3,547 (2,937-4,069)	1,596 (1,347-1,797)	494 (430-573)
Other musculoskeletal disorders	2,294 (1,826-2,547)	2,473 (2,067-2,700)	2,527 (2,090-2,787)	2,388 (2,087-2,634)	3,181 (2,660-3,617)	3,806 (3,099-4,485)	4,018 (3,238-4,797)	4,037 (3,339-4,715)	4,759 (3,944-5,556)	4,782 (4,153-5,286)	3,175 (2,826-3,469)	1,638 (1,522-1,751)
Other non-communicable diseases	10,241 (9,507-10,948)	10,321 (9,458-11,020)	13,664 (12,457-14,468)	14,602 (13,235-15,517)	18,351 (16,629-19,447)	21,735 (19,727-22,889)	21,631 (19,675-22,879)	25,133 (22,917-26,503)	29,071 (26,283-30,489)	29,092 (26,120-30,517)	20,489 (18,185-21,511)	11,431 (9,989-12,061)
Congenital birth defects	3,063 (2,799-3,278)	1,475 (1,313-1,613)	1,533 (1,322-1,686)	1,432 (1,171-1,558)	1,358 (1,105-1,469)	1,285 (1,113-1,426)	-	-	-	-	-	-
Neural tube defects	67 (58-77)	42 (37-49)	39 (34-45)	35 (31-41)	27 (24-33)	24 (22-30)	-	-	-	-	-	-
Congenital heart anomalies	2,008 (1,795-2,180)	706 (615-790)	592 (496-678)	502 (421-572)	500 (426-575)	561 (480-642)	-	-	-	-	-	-
Orofacial clefts	-	-	-	-	-	-	-	-	-	-	-	-
Down syndrome	371 (320-474)	353 (284-410)	531 (420-604)	531 (360-600)	424 (284-480)	216 (146-247)	-	-	-	-	-	-
Other chromosomal abnormalities	58 (33-80)	35 (19-43)	31 (16-40)	31 (16-39)	26 (12-33)	25 (13-33)	-	-	-	-	-	-
Congenital musculoskeletal and limb anomalies	54 (35-70)	39 (27-51)	35 (23-44)	34 (23-45)	34 (25-47)	31 (21-46)	-	-	-	-	-	-
Urogenital congenital anomalies	97 (69-122)	80 (61-98)	97 (72-115)	123 (96-148)	167 (132-197)	233 (186-271)	-	-	-	-	-	-
Digestive congenital anomalies	30 (21-38)	28 (21-34)	35 (26-43)	35 (25-42)	42 (31-50)	56 (42-69)	-	-	-	-	-	-
Other congenital birth defects	379 (304-479)	192 (161-231)	173 (140-204)	143 (118-169)	139 (114-159)	138 (111-168)	-	-	-	-	-	-
Urinary diseases and male infertility	2,968 (2,806-3,147)	3,650 (3,474-3,844)	5,754 (5,466-6,029)	6,960 (6,629-7,330)	8,982 (8,520-9,463)	11,219 (10,641-11,840)	12,304 (11,628-13,037)	15,329 (14,568-16,209)	17,456 (16,652-18,466)	17,764 (17,125-18,516)	12,715 (12,297-13,226)	7,344 (7,115-7,593)
Urinary tract infections	2,501 (2,319-2,719)	2,960 (2,762-3,245)	4,877 (4,558-5,277)	5,826 (5,447-6,346)	7,305 (6,793-8,059)	9,458 (8,844-10,331)	10,459 (9,748-11,583)	13,028 (12,204-14,319)	15,288 (14,478-16,639)	15,957 (15,248-16,985)	11,524 (11,070-12,163)	6,766 (6,523-7,035)
Urolithiasis	127 (100-179)	194 (157-266)	270 (222-360)	341 (277-464)	470 (392-630)	610 (500-826)	558 (449-796)	757 (619-1,096)	777 (633-1,141)	626 (508-971)	406 (331-626)	196 (160-302)

SR Table 5: Global deaths by cause and age group for females (with 95% confidence intervals), 2017

Cause	Early Neonatal	Late Neonatal	Post Neonatal	1 to 4	5 to 9	10 to 14	15 to 19	20 to 24
Other urinary diseases	155 (118-184)	77 (60-94)	484 (246-741)	809 (382-1,238)	269 (164-370)	189 (141-249)	181 (102-247)	241 (133-342)
Gynecological diseases	-	-	-	-	-	-	354 (306-427)	381 (333-469)
Uterine fibroids	-	-	-	-	-	-	30 (23-44)	40 (27-68)
Polycystic ovarian syndrome	-	-	-	-	-	-	1 (0-3)	2 (0-6)
Endometriosis	-	-	-	-	-	-	9 (4-16)	11 (5-20)
Genital prolapse	-	-	-	-	-	-	9 (5-14)	9 (5-15)
Other gynecological diseases	-	-	-	-	-	-	305 (261-369)	319 (263-414)
Hemoglobinopathies and hemolytic anemias	299 (225-429)	184 (143-259)	2,216 (1,621-3,333)	7,400 (4,289-11,543)	2,005 (1,555-2,799)	1,630 (1,223-2,359)	2,272 (1,740-3,164)	2,126 (1,625-2,961)
Thalassemias	92 (65-117)	61 (47-83)	615 (466-864)	826 (645-1,067)	561 (408-795)	332 (256-443)	293 (201-427)	220 (132-328)
Sickle cell disorders	197 (147-311)	120 (89-184)	1,569 (1,049-2,396)	6,459 (3,490-10,360)	1,341 (946-2,000)	1,118 (768-1,716)	1,609 (1,191-2,364)	1,456 (1,084-2,091)
G6PD deficiency	3 (2-4)	2 (1-3)	23 (15-37)	92 (54-157)	78 (54-116)	124 (84-185)	233 (152-365)	284 (189-450)
Other hemoglobinopathies and hemolytic anemias	7 (5-10)	1 (1-2)	10 (8-15)	22 (15-34)	25 (20-34)	58 (44-80)	136 (89-238)	167 (105-295)
Endocrine, metabolic, blood, and immune disorders	604 (479-802)	481 (405-658)	3,233 (2,597-4,281)	2,005 (1,592-2,562)	850 (709-1,106)	727 (626-942)	800 (673-1,033)	1,009 (838-1,334)
Sudden infant death syndrome	-	1,487 (644-2,983)	15,130 (5,836-34,471)	-	-	-	-	-
Injuries	4,792 (4,191-5,237)	3,391 (3,082-3,667)	35,990 (32,415-38,736)	76,031 (68,384-83,249)	42,425 (37,817-46,043)	35,228 (32,196-37,568)	63,257 (59,422-66,274)	71,440 (67,573-74,591)
Transport injuries	236 (214-258)	301 (272-334)	5,261 (4,743-5,846)	16,935 (14,593-19,277)	13,886 (12,434-15,506)	10,041 (9,131-10,945)	17,105 (15,962-18,394)	19,092 (17,901-20,343)
Road injuries	220 (199-241)	282 (255-314)	4,952 (4,448-5,500)	16,033 (13,810-18,271)	13,354 (11,934-14,908)	9,436 (8,575-10,284)	15,931 (14,837-17,132)	18,121 (16,982-19,322)
Pedestrian road injuries	84 (71-100)	106 (86-134)	1,806 (1,525-2,246)	8,023 (6,799-9,769)	7,314 (6,238-8,487)	4,390 (3,813-5,093)	5,022 (4,339-6,147)	5,474 (4,713-6,643)
Cyclist road injuries	-	-	-	385 (305-502)	455 (379-575)	568 (456-729)	622 (487-827)	533 (444-668)
Motorcyclist road injuries	21 (17-26)	20 (16-26)	601 (452-773)	936 (741-1,222)	708 (583-846)	825 (677-979)	2,320 (1,972-2,676)	2,765 (2,242-3,219)
Motor vehicle road injuries	109 (89-127)	152 (127-177)	2,463 (2,063-2,839)	6,502 (4,996-7,975)	4,722 (4,094-5,418)	3,543 (3,089-3,986)	7,757 (7,048-8,479)	9,124 (8,273-9,908)
Other road injuries	5 (4-7)	4 (3-6)	83 (55-116)	187 (111-294)	155 (112-212)	110 (82-156)	211 (163-297)	226 (168-316)
Other transport injuries	17 (14-21)	19 (16-23)	309 (257-383)	902 (701-1,209)	532 (449-628)	606 (514-699)	1,174 (1,008-1,401)	971 (856-1,105)
Unintentional injuries	3,318 (2,850-3,704)	2,585 (2,304-2,831)	28,030 (24,828-30,642)	49,167 (43,075-54,879)	22,189 (18,606-24,878)	15,466 (13,140-17,232)	14,875 (12,399-16,665)	15,778 (13,225-17,487)
Falls	654 (573-768)	250 (206-296)	3,794 (3,141-4,495)	4,287 (3,499-5,196)	2,259 (1,878-2,624)	1,618 (1,355-1,869)	1,957 (1,645-2,252)	1,966 (1,708-2,220)
Drowning	154 (132-184)	89 (79-105)	2,815 (2,434-3,269)	21,118 (18,281-24,319)	9,490 (8,094-10,788)	5,956 (5,182-6,614)	3,596 (3,199-3,965)	2,784 (2,535-3,019)
Fire, heat, and hot substances	115 (75-139)	153 (102-189)	2,325 (1,585-2,789)	6,268 (4,386-7,697)	1,775 (1,342-2,104)	1,336 (985-1,650)	2,772 (1,836-3,624)	3,668 (2,445-4,642)
Poisonings	63 (45-75)	73 (51-85)	1,384 (915-1,610)	2,875 (1,992-3,363)	776 (549-892)	916 (643-1,061)	942 (670-1,052)	1,069 (796-1,196)
Poisoning by carbon monoxide	14 (9-18)	16 (9-21)	244 (127-331)	507 (306-660)	331 (192-392)	354 (197-421)	430 (280-487)	478 (310-541)
Poisoning by other means	48 (35-58)	57 (41-66)	1,140 (766-1,332)	2,368 (1,653-2,857)	444 (338-529)	562 (434-674)	512 (385-576)	591 (448-676)
Exposure to mechanical forces	311 (191-388)	339 (220-419)	1,977 (1,264-2,441)	2,383 (1,750-2,848)	1,595 (1,141-1,891)	831 (630-958)	1,147 (831-1,322)	1,104 (806-1,261)
Unintentional firearm injuries	20 (14-30)	15 (10-23)	160 (113-242)	211 (159-340)	181 (135-262)	137 (108-179)	226 (174-277)	239 (192-282)
Other exposure to mechanical forces	291 (174-367)	323 (208-404)	1,817 (1,127-2,268)	2,173 (1,546-2,619)	1,414 (981-1,700)	693 (513-813)	921 (639-1,088)	864 (592-1,007)
Adverse effects of medical treatment	530 (370-657)	350 (262-436)	3,088 (2,107-4,104)	2,215 (1,320-3,716)	824 (606-1,044)	636 (497-783)	889 (683-1,064)	1,756 (1,315-2,132)
Animal contact	73 (27-98)	70 (29-95)	1,362 (480-1,873)	3,456 (1,434-4,671)	3,073 (1,171-4,308)	2,197 (822-3,076)	1,613 (569-2,252)	1,467 (517-1,967)
Venomous animal contact	47 (12-74)	43 (12-68)	1,109 (321-1,638)	2,787 (1,049-4,080)	2,764 (975-3,942)	2,021 (702-2,906)	1,501 (502-2,156)	1,335 (434-1,849)
Non-venomous animal contact	25 (11-53)	27 (13-51)	254 (91-663)	669 (188-1,789)	309 (113-748)	175 (75-379)	112 (49-257)	132 (60-303)
Foreign body	1,220 (1,121-1,329)	1,173 (1,074-1,291)	9,954 (8,948-10,897)	4,184 (3,136-4,974)	1,121 (1,017-1,242)	784 (729-845)	589 (556-626)	673 (632-714)
Pulmonary aspiration and foreign body in airway	1,196 (1,092-1,308)	1,156 (1,059-1,269)	9,738 (8,773-10,643)	3,955 (2,966-4,738)	1,012 (914-1,128)	702 (650-752)	447 (407-486)	486 (439-524)
Foreign body in other body part	24 (17-42)	17 (13-30)	216 (151-398)	229 (153-426)	108 (83-164)	83 (66-121)	142 (122-183)	187 (162-242)
Environmental heat and cold exposure	77 (26-105)	46 (15-64)	680 (210-968)	441 (193-635)	218 (98-290)	106 (63-131)	218 (108-284)	190 (100-231)
Exposure to forces of nature	2 (1-2)	6 (5-8)	49 (42-68)	262 (223-368)	256 (211-376)	270 (221-440)	221 (177-368)	207 (165-355)
Other unintentional injuries	119 (105-136)	38 (33-44)	603 (534-687)	1,677 (1,434-1,997)	803 (713-915)	816 (731-924)	932 (847-1,045)	894 (801-1,005)
Self-harm and interpersonal violence	1,238 (1,085-1,396)	504 (461-568)	2,698 (2,311-3,069)	9,929 (8,921-10,780)	6,351 (5,895-6,831)	9,721 (9,170-10,280)	31,276 (29,357-33,146)	36,570 (34,622-38,724)
Self-harm	-	-	-	-	-	3,558 (3,137-3,910)	21,128 (19,313-22,852)	25,322 (23,620-26,990)
Self-harm by firearm	-	-	-	-	-	135 (98-172)	704 (447-941)	928 (595-1,235)
Self-harm by other specified means	-	-	-	-	-	3,423 (3,023-3,769)	20,424 (18,621-22,051)	24,395 (22,792-25,968)
Interpersonal violence	635 (482-790)	175 (132-238)	1,562 (1,202-1,964)	3,323 (2,317-4,169)	2,044 (1,587-2,525)	2,195 (1,779-2,585)	5,417 (4,462-6,268)	7,407 (6,033-8,553)
Physical violence by firearm	49 (28-71)	18 (10-27)	222 (140-292)	445 (278-613)	332 (235-434)	480 (400-566)	1,709 (1,429-1,977)	2,232 (1,869-2,601)
Physical violence by sharp object	41 (29-53)	24 (17-34)	145 (105-189)	488 (321-704)	215 (166-267)	351 (270-431)	1,021 (793-1,246)	1,609 (1,253-1,910)
Physical violence by other means	545 (411-694)	132 (100-186)	1,195 (923-1,559)	2,390 (1,671-3,020)	1,497 (1,099-1,920)	1,364 (1,046-1,696)	2,688 (2,100-3,360)	3,565 (2,758-4,380)
Conflict and terrorism	599 (435-825)	329 (241-462)	1,087 (797-1,520)	6,396 (4,567-9,145)	4,176 (2,921-6,066)	3,850 (2,621-5,659)	4,488 (3,158-6,647)	3,577 (2,511-5,189)
Executions and police conflict	-	-	49 (16-113)	210 (206-217)	130 (126-138)	119 (116-123)	243 (239-252)	264 (258-274)

Cause	25 to 29	30 to 34	35 to 39	40 to 44	45 to 49	50 to 54	54 to 59	60 to 64
Other urinary diseases	239 (140-325)	283 (176-369)	293 (164-396)	340 (199-438)	496 (286-623)	607 (329-774)	792 (459-990)	1,207 (679-1,489)
Gynecological diseases	427 (382-504)	504 (459-563)	599 (540-663)	732 (647-820)	913 (801-1,013)	787 (697-860)	475 (427-522)	411 (354-451)
Uterine fibroids	76 (54-118)	117 (78-170)	140 (74-199)	191 (111-259)	317 (218-405)	293 (241-368)	128 (97-157)	132 (89-163)
Polycystic ovarian syndrome	2 (0-5)	1 (0-4)	1 (0-3)	2 (0-4)	1 (0-3)	2 (0-3)	-	-
Endometriosis	11 (5-19)	17 (7-28)	17 (7-28)	27 (9-46)	41 (11-65)	26 (10-47)	-	-
Genital prolapse	9 (4-13)	9 (5-15)	8 (4-15)	17 (6-30)	22 (6-41)	57 (18-89)	41 (17-67)	45 (21-65)
Other gynecological diseases	329 (273-423)	360 (299-444)	432 (358-525)	496 (395-572)	531 (417-614)	409 (313-483)	306 (248-353)	234 (192-271)
Hemoglobinopathies and hemolytic anemias	1,739 (1,390-2,422)	1,816 (1,410-2,511)	1,290 (1,042-1,750)	1,487 (1,202-1,972)	1,375 (1,116-1,819)	1,705 (1,339-2,205)	1,729 (1,383-2,294)	2,045 (1,619-2,652)
Thalassemias	151 (92-225)	107 (68-155)	48 (35-62)	37 (30-48)	39 (32-49)	31 (25-38)	13 (11-17)	8 (7-10)
Sickle cell disorders	1,065 (773-1,593)	966 (696-1,445)	567 (397-896)	555 (381-915)	448 (293-804)	385 (244-702)	295 (181-560)	120 (75-213)
G6PD deficiency	318 (222-450)	465 (319-621)	384 (284-488)	467 (351-585)	429 (326-549)	523 (382-671)	437 (316-639)	390 (269-587)
Other hemoglobinopathies and hemolytic anemias	205 (143-301)	279 (193-406)	291 (218-370)	428 (341-536)	459 (370-565)	767 (604-923)	984 (796-1,236)	1,526 (1,200-1,894)
Endocrine, metabolic, blood, and immune disorders	1,287 (1,056-1,663)	1,556 (1,269-1,964)	1,810 (1,433-2,209)	1,990 (1,528-2,361)	2,909 (2,199-3,289)	3,884 (2,996-4,425)	4,006 (2,899-4,538)	5,555 (4,144-6,217)
Sudden infant death syndrome	-	-	-	-	-	-	-	-
Injuries	71,657 (67,740-74,522)	66,719 (62,823-69,250)	61,583 (58,064-64,184)	62,756 (59,059-65,436)	66,179 (62,772-68,783)	74,842 (69,915-78,743)	66,068 (63,055-68,545)	79,175 (75,274-82,391)
Transport injuries	20,267 (19,272-21,418)	19,264 (18,271-20,154)	18,344 (16,813-19,325)	19,932 (18,001-20,953)	21,576 (20,000-22,600)	25,200 (22,268-26,535)	20,579 (19,049-21,534)	23,910 (22,148-25,082)
Road injuries	19,252 (18,301-20,383)	18,356 (17,405-19,237)	17,510 (16,020-18,457)	18,872 (16,947-19,827)	20,404 (18,876-21,396)	23,812 (20,940-25,076)	19,771 (18,246-20,675)	22,666 (20,969-23,780)
Pedestrian road injuries	6,177 (5,509-7,159)	6,492 (5,863-7,385)	6,612 (5,922-7,433)	7,496 (6,780-8,401)	8,618 (7,895-9,471)	10,523 (9,382-11,846)	9,074 (8,245-10,109)	12,290 (11,285-13,406)
Cyclist road injuries	718 (610-860)	824 (699-976)	595 (523-669)	847 (709-965)	1,304 (1,109-1,473)	1,457 (1,181-1,654)	1,046 (906-1,181)	1,315 (1,002-1,485)
Motorcyclist road injuries	2,828 (2,300-3,333)	2,873 (2,263-3,355)	2,282 (1,810-2,647)	2,588 (2,015-3,057)	2,652 (2,018-3,003)	3,104 (2,294-3,789)	2,094 (1,669-2,413)	1,954 (1,396-2,277)
Motor vehicle road injuries	9,351 (8,535-10,119)	7,971 (7,250-8,540)	7,806 (6,866-8,474)	7,759 (6,620-8,463)	7,571 (6,588-8,256)	8,485 (7,044-9,411)	7,332 (6,440-7,950)	7,023 (6,175-7,614)
Other road injuries	179 (137-235)	197 (157-257)	215 (167-277)	182 (142-228)	257 (206-336)	243 (183-299)	226 (174-285)	84 (62-99)
Other transport injuries	1,015 (912-1,132)	908 (835-1,000)	834 (757-947)	1,060 (947-1,280)	1,172 (1,062-1,384)	1,388 (1,220-1,829)	807 (742-939)	1,244 (1,112-1,456)
Unintentional injuries	16,984 (14,242-18,549)	17,788 (15,027-19,344)	16,968 (14,681-18,354)	18,549 (16,102-20,413)	20,943 (18,460-22,612)	24,942 (22,518-28,106)	26,395 (24,554-28,309)	36,025 (33,306-38,354)
Falls	2,095 (1,911-2,278)	2,481 (2,279-2,676)	2,929 (2,693-3,217)	3,400 (3,028-3,865)	4,752 (4,362-5,268)	6,738 (5,810-8,012)	9,154 (8,059-10,602)	15,356 (13,824-17,452)
Drowning	2,820 (2,487-3,026)	2,727 (2,488-2,899)	2,514 (2,228-2,683)	2,447 (2,192-2,596)	2,855 (2,544-3,020)	3,645 (3,066-3,920)	2,918 (2,598-3,107)	3,851 (3,154-4,096)
Fire, heat, and hot substances	3,433 (2,262-4,174)	3,549 (2,376-4,236)	2,716 (1,916-3,193)	2,233 (1,625-2,590)	2,407 (1,719-2,805)	2,413 (1,736-2,848)	2,292 (1,760-2,592)	2,477 (1,963-2,761)
Poisonings	1,362 (925-1,521)	1,220 (800-1,363)	1,095 (742-1,206)	1,237 (812-1,369)	1,415 (866-1,601)	1,564 (955-1,763)	1,280 (900-1,413)	1,582 (953-1,804)
Poisoning by carbon monoxide	644 (391-738)	586 (375-656)	523 (370-576)	573 (388-635)	655 (416-741)	669 (428-757)	631 (457-690)	800 (484-913)
Poisoning by other means	718 (482-806)	634 (399-718)	572 (357-642)	664 (413-743)	759 (435-866)	895 (514-1,029)	649 (414-731)	783 (427-903)
Exposure to mechanical forces	1,481 (1,036-1,744)	1,601 (1,145-1,852)	1,471 (1,026-1,716)	1,826 (1,296-2,118)	1,794 (1,254-2,086)	1,680 (1,143-1,989)	1,962 (1,391-2,285)	1,995 (1,371-2,344)
Unintentional firearm injuries	287 (228-347)	353 (281-435)	258 (210-302)	264 (212-330)	284 (231-347)	236 (195-296)	239 (199-313)	222 (178-304)
Other exposure to mechanical forces	1,194 (783-1,439)	1,248 (824-1,483)	1,213 (805-1,452)	1,561 (1,061-1,844)	1,510 (989-1,795)	1,444 (933-1,746)	1,723 (1,157-2,052)	1,773 (1,165-2,132)
Adverse effects of medical treatment	1,949 (1,440-2,320)	1,681 (1,295-2,015)	1,868 (1,386-2,232)	2,348 (1,715-2,757)	2,617 (1,953-3,075)	2,459 (1,876-2,952)	2,969 (2,326-3,380)	4,520 (3,528-5,136)
Animal contact	1,452 (542-1,914)	1,808 (642-2,421)	1,517 (534-2,093)	2,179 (825-3,220)	1,886 (767-2,661)	2,515 (939-4,282)	1,718 (696-2,482)	2,012 (784-2,873)
Venomous animal contact	1,324 (461-1,788)	1,701 (574-2,286)	1,419 (473-1,976)	2,065 (739-3,088)	1,698 (628-2,445)	2,291 (798-3,896)	1,507 (549-2,257)	1,875 (680-2,700)
Non-venomous animal contact	128 (62-278)	107 (51-224)	98 (47-224)	115 (53-280)	188 (91-376)	225 (111-557)	211 (102-446)	138 (70-289)
Foreign body	789 (744-833)	827 (772-879)	923 (876-973)	1,017 (967-1,065)	1,143 (1,082-1,195)	1,501 (1,423-1,568)	1,696 (1,631-1,764)	1,800 (1,731-1,875)
Pulmonary aspiration and foreign body in airway	589 (538-635)	655 (606-703)	732 (677-779)	828 (773-875)	999 (942-1,050)	1,334 (1,256-1,400)	1,528 (1,460-1,598)	1,648 (1,580-1,725)
Foreign body in other body part	200 (170-260)	172 (145-225)	192 (162-253)	190 (160-250)	144 (118-192)	167 (143-227)	167 (146-237)	152 (133-208)
Environmental heat and cold exposure	226 (148-258)	403 (264-464)	496 (354-556)	681 (453-791)	817 (556-937)	925 (653-1,061)	1,166 (797-1,334)	1,362 (803-1,647)
Exposure to forces of nature	212 (167-359)	204 (160-333)	191 (145-356)	191 (143-361)	187 (136-363)	197 (136-391)	202 (134-460)	215 (139-474)
Other unintentional injuries	1,164 (1,069-1,286)	1,287 (1,187-1,415)	1,248 (1,156-1,352)	990 (932-1,059)	1,069 (1,008-1,141)	1,304 (1,198-1,421)	1,037 (957-1,123)	855 (808-911)
Self-harm and interpersonal violence	34,407 (32,541-36,200)	29,668 (28,255-31,174)	26,270 (25,006-27,338)	24,275 (23,124-25,326)	23,660 (22,579-24,527)	24,700 (23,564-25,723)	19,095 (18,343-19,783)	19,240 (18,406-20,000)
Self-harm	24,455 (22,944-25,811)	19,935 (18,967-20,888)	17,868 (16,990-18,699)	17,123 (16,321-17,884)	17,343 (16,604-18,004)	19,084 (18,280-19,876)	14,498 (13,938-15,051)	15,224 (14,543-15,862)
Self-harm by firearm	820 (598-1,035)	770 (629-916)	748 (654-869)	754 (666-916)	727 (661-852)	752 (684-897)	677 (622-778)	515 (472-611)
Self-harm by other specified means	23,635 (22,197-24,952)	19,165 (18,216-20,140)	17,120 (16,293-17,936)	16,370 (15,576-17,111)	16,616 (15,915-17,277)	18,332 (17,554-19,080)	13,821 (13,296-14,376)	14,709 (14,049-15,352)
Interpersonal violence	7,521 (6,176-8,823)	7,307 (6,163-8,636)	6,261 (5,312-7,168)	5,491 (4,616-6,255)	4,839 (4,024-5,442)	4,299 (3,521-4,988)	3,456 (2,952-3,930)	3,060 (2,586-3,557)
Physical violence by firearm	2,128 (1,788-2,551)	1,933 (1,627-2,282)	1,765 (1,498-2,092)	1,415 (1,186-1,658)	1,185 (987-1,351)	1,051 (833-1,322)	848 (679-1,025)	706 (559-849)
Physical violence by sharp object	1,854 (1,479-2,172)	1,983 (1,609-2,351)	1,729 (1,444-2,025)	1,665 (1,323-1,991)	1,453 (1,169-1,674)	1,238 (965-1,498)	932 (765-1,060)	814 (664-953)
Physical violence by other means	3,540 (2,806-4,378)	3,390 (2,736-4,144)	2,767 (2,284-3,362)	2,411 (1,991-2,864)	2,202 (1,763-2,579)	2,009 (1,591-2,449)	1,676 (1,378-1,981)	1,539 (1,275-1,867)
Conflict and terrorism	2,183 (1,554-3,094)	2,180 (1,532-3,161)	1,899 (1,307-2,785)	1,436 (1,003-2,155)	1,275 (859-1,887)	1,133 (757-1,714)	940 (641-1,423)	810 (550-1,210)
Executions and police conflict	247 (241-256)	246 (237-259)	242 (235-254)	224 (217-234)	203 (196-214)	184 (180-192)	200 (197-205)	146 (143-151)

Cause	65 to 69	70 to 74	75 to 79	80 to 84	85 to 89	90 to 94	95 plus
Other urinary diseases	1,151 (590-1,460)	1,287 (652-1,609)	1,544 (721-1,953)	1,392 (648-1,808)	1,181 (604-1,517)	785 (418-1,009)	381 (249-476)
Gynecological diseases	508 (403-553)	342 (260-376)	291 (221-319)	623 (489-710)	500 (360-585)	220 (166-249)	91 (74-103)
Uterine fibroids	151 (100-189)	117 (73-141)	91 (47-110)	226 (150-288)	213 (116-281)	100 (54-127)	41 (22-52)
Polycystic ovarian syndrome	-	-	-	-	-	-	-
Endometriosis	-	-	-	-	-	-	-
Genital prolapse	83 (35-118)	68 (33-89)	54 (27-68)	96 (54-137)	66 (40-87)	27 (19-33)	13 (8-15)
Other gynecological diseases	274 (208-332)	156 (122-193)	146 (118-175)	301 (214-369)	222 (174-266)	93 (77-114)	37 (33-45)
Hemoglobinopathies and hemolytic anemias	2,464 (1,959-3,107)	2,800 (2,233-3,533)	2,976 (2,430-3,705)	3,585 (2,914-4,386)	3,479 (2,737-4,146)	2,421 (1,786-2,853)	1,246 (858-1,418)
Thalassemias	5 (4-6)	2 (2-3)	1 (1-2)	0 (0-1)	0 (0-0)	0 (0-0)	0 (0-0)
Sickle cell disorders	59 (39-95)	23 (15-35)	8 (5-13)	2 (1-4)	1 (0-1)	0 (0-0)	0 (0-0)
G6PD deficiency	361 (244-554)	248 (164-384)	161 (105-260)	88 (57-140)	37 (25-56)	11 (7-15)	2 (1-3)
Other hemoglobinopathies and hemolytic anemias	2,039 (1,629-2,509)	2,527 (2,015-3,154)	2,806 (2,284-3,466)	3,494 (2,846-4,284)	3,441 (2,713-4,102)	2,410 (1,775-2,840)	1,244 (856-1,416)
Endocrine, metabolic, blood, and immune disorders	6,259 (4,504-6,930)	6,184 (4,432-6,892)	6,537 (4,541-7,212)	7,406 (4,858-8,254)	7,350 (4,592-8,244)	5,133 (3,028-5,872)	2,750 (1,510-3,186)
Sudden infant death syndrome	-	-	-	-	-	-	-
Injuries	84,024 (79,457-87,508)	83,593 (79,157-88,940)	92,656 (88,337-96,596)	102,883 (97,740-109,478)	91,956 (87,183-97,641)	56,305 (54,237-58,641)	24,241 (23,559-25,023)
Transport injuries	21,502 (20,106-22,496)	18,161 (16,916-19,013)	15,216 (14,575-15,870)	12,737 (11,976-13,339)	8,158 (7,665-8,523)	3,718 (3,533-3,863)	1,300 (1,238-1,355)
Road injuries	20,424 (19,114-21,394)	17,337 (16,116-18,153)	14,449 (13,852-15,044)	12,010 (11,277-12,573)	7,739 (7,281-8,077)	3,542 (3,363-3,679)	1,232 (1,174-1,285)
Pedestrian road injuries	11,361 (10,562-12,312)	10,113 (9,301-11,046)	8,551 (8,045-9,197)	7,652 (7,127-8,223)	4,675 (4,354-5,043)	1,928 (1,818-2,055)	615 (578-654)
Cyclist road injuries	1,362 (1,042-1,572)	1,007 (811-1,190)	691 (589-811)	368 (317-429)	223 (182-269)	91 (74-113)	1 (1-1)
Motorcyclist road injuries	1,505 (1,118-1,745)	1,006 (771-1,184)	623 (468-717)	375 (286-432)	211 (158-244)	82 (63-93)	30 (23-33)
Motor vehicle road injuries	6,120 (5,418-6,554)	5,151 (4,674-5,572)	4,427 (4,030-4,755)	3,513 (3,200-3,755)	2,555 (2,355-2,723)	1,405 (1,307-1,481)	571 (531-605)
Other road injuries	75 (56-90)	61 (46-72)	157 (125-200)	102 (83-125)	75 (63-93)	35 (30-43)	17 (15-20)
Other transport injuries	1,078 (965-1,247)	824 (738-977)	767 (701-849)	727 (645-832)	419 (380-485)	176 (161-197)	68 (64-75)
Unintentional injuries	44,238 (40,914-47,196)	50,388 (47,166-54,915)	63,623 (59,979-67,113)	79,191 (74,809-85,150)	76,837 (72,776-82,223)	49,614 (47,645-51,881)	22,013 (21,340-22,771)
Falls	21,621 (19,515-24,627)	28,729 (25,393-33,990)	40,104 (37,280-44,091)	53,699 (49,428-60,348)	55,308 (51,463-60,873)	37,061 (35,126-39,450)	16,058 (15,394-16,811)
Drowning	4,434 (3,619-4,721)	4,019 (3,592-4,279)	4,667 (3,897-4,963)	4,702 (4,068-5,000)	3,074 (2,651-3,248)	1,205 (1,065-1,266)	309 (276-324)
Fire, heat, and hot substances	3,033 (2,414-3,370)	2,993 (2,413-3,325)	3,604 (3,023-3,918)	4,230 (3,560-4,649)	3,624 (3,143-3,919)	2,183 (1,971-2,312)	1,037 (975-1,080)
Poisonings	1,778 (1,008-2,030)	1,506 (921-1,709)	1,545 (917-1,757)	1,471 (920-1,647)	986 (654-1,097)	395 (285-431)	112 (91-120)
Poisoning by carbon monoxide	911 (508-1,054)	767 (443-882)	915 (543-1,053)	846 (505-961)	583 (368-658)	223 (153-247)	51 (37-57)
Poisoning by other means	867 (463-1,010)	739 (441-839)	630 (356-722)	625 (384-703)	403 (268-449)	173 (128-190)	61 (51-64)
Exposure to mechanical forces	1,952 (1,341-2,304)	1,770 (1,252-2,065)	1,765 (1,260-2,077)	2,114 (1,559-2,418)	1,784 (1,401-2,004)	1,089 (911-1,197)	496 (442-532)
Unintentional firearm injuries	237 (196-310)	233 (190-301)	370 (299-476)	434 (334-534)	450 (353-532)	297 (238-339)	142 (114-158)
Other exposure to mechanical forces	1,715 (1,133-2,063)	1,537 (1,043-1,831)	1,395 (922-1,690)	1,680 (1,181-1,970)	1,334 (996-1,524)	792 (645-881)	354 (308-384)
Adverse effects of medical treatment	4,765 (3,724-5,465)	4,984 (3,916-5,722)	5,120 (4,215-5,686)	5,424 (4,524-6,173)	4,799 (4,200-5,345)	2,864 (2,616-3,210)	1,241 (1,166-1,384)
Animal contact	2,278 (819-3,266)	2,049 (775-2,969)	1,679 (637-2,213)	1,181 (528-1,630)	657 (334-863)	264 (163-323)	83 (64-98)
Venomous animal contact	2,128 (710-3,088)	1,888 (645-2,745)	1,536 (535-2,064)	1,029 (422-1,458)	522 (238-714)	193 (105-254)	56 (40-69)
Non-venomous animal contact	150 (75-321)	162 (83-381)	143 (78-275)	153 (85-309)	135 (81-227)	71 (50-109)	27 (21-37)
Foreign body	1,985 (1,900-2,064)	2,295 (2,206-2,387)	2,871 (2,756-2,979)	3,730 (3,584-3,897)	4,405 (4,215-4,580)	3,264 (3,129-3,418)	2,148 (2,066-2,231)
Pulmonary aspiration and foreign body in airway	1,829 (1,747-1,905)	2,112 (2,025-2,204)	2,650 (2,535-2,770)	3,439 (3,288-3,600)	4,111 (3,936-4,299)	3,074 (2,942-3,241)	2,059 (1,980-2,140)
Foreign body in other body part	156 (126-215)	183 (156-245)	220 (176-295)	292 (225-403)	294 (207-403)	189 (127-254)	89 (60-113)
Environmental heat and cold exposure	1,079 (675-1,281)	1,301 (636-1,663)	1,453 (792-1,751)	1,736 (959-2,076)	1,461 (952-1,665)	902 (674-997)	367 (306-403)
Exposure to forces of nature	207 (124-532)	199 (112-528)	176 (98-467)	152 (81-452)	100 (50-303)	51 (21-160)	22 (7-75)
Other unintentional injuries	1,105 (1,015-1,201)	542 (513-573)	641 (601-681)	752 (691-810)	639 (585-694)	336 (315-359)	139 (131-149)
Self-harm and interpersonal violence	18,284 (17,439-19,058)	15,044 (14,277-15,761)	13,818 (13,133-14,413)	10,956 (10,446-11,445)	6,961 (6,679-7,191)	2,974 (2,882-3,058)	928 (903-949)
Self-harm	14,794 (14,069-15,470)	12,177 (11,549-12,776)	11,154 (10,571-11,662)	9,942 (9,455-10,430)	6,367 (6,103-6,592)	2,784 (2,695-2,868)	840 (816-861)
Self-harm by firearm	417 (377-531)	331 (290-476)	307 (262-470)	292 (252-439)	222 (200-263)	129 (120-139)	65 (60-69)
Self-harm by other specified means	14,376 (13,649-15,058)	11,846 (11,229-12,425)	10,848 (10,263-11,369)	9,649 (9,180-10,142)	6,145 (5,885-6,369)	2,655 (2,574-2,738)	775 (754-796)
Interpersonal violence	2,661 (2,220-3,031)	2,295 (1,863-2,706)	2,234 (1,880-2,574)	695 (627-792)	415 (382-460)	155 (143-169)	48 (44-52)
Physical violence by firearm	543 (431-691)	486 (370-612)	422 (320-534)	79 (69-96)	47 (41-55)	17 (15-18)	8 (7-9)
Physical violence by sharp object	601 (498-706)	620 (471-723)	634 (505-741)	183 (158-209)	107 (93-121)	37 (32-42)	10 (9-12)
Physical violence by other means	1,517 (1,239-1,763)	1,190 (938-1,446)	1,178 (989-1,415)	433 (391-507)	260 (241-293)	101 (93-111)	30 (27-32)
Conflict and terrorism	718 (487-1,034)	500 (340-767)	381 (257-575)	284 (193-427)	162 (109-241)	27 (19-39)	38 (27-54)
Executions and police conflict	112 (109-116)	71 (70-74)	48 (46-50)	36 (34-37)	17 (16-19)	8 (8-9)	2 (2-3)

SR Table 6: Global deaths by cause and age group for males (with 95% confidence intervals), 1990

Cause	Early Neonatal	Late Neonatal	Post Neonatal	1 to 4	5 to 9	10 to 14
All causes	1,736,328 (1,695,419-1,775,925)	694,176 (668,937-720,200)	2,021,200 (1,974,966-2,068,089)	1,785,332 (1,737,104-1,835,003)	469,706 (464,539-474,695)	266,063 (263,545-268,536)
Communicable, maternal, neonatal, and nutritional diseases	1,589,599 (1,534,003-1,634,275)	609,720 (576,548-639,077)	1,664,668 (1,599,718-1,733,027)	1,370,134 (1,299,464-1,450,611)	237,583 (217,611-261,884)	104,273 (93,613-119,026)
HIV/AIDS and sexually transmitted infections	-	15,340 (5,560-29,106)	44,115 (28,730-65,673)	16,362 (14,127-18,899)	1,981 (1,325-2,868)	351 (289-455)
HIV/AIDS	-	-	20,205 (18,015-22,399)	14,265 (12,700-16,024)	1,141 (806-1,477)	292 (238-395)
HIV/AIDS - Drug-susceptible Tuberculosis	-	-	8,003 (6,529-9,253)	5,785 (4,833-6,669)	408 (267-538)	67 (41-100)
HIV/AIDS - Multidrug-resistant Tuberculosis without extensive drug resistance	-	-	49 (27-88)	42 (24-71)	2 (1-4)	1 (0-1)
HIV/AIDS - Extensively drug-resistant Tuberculosis	-	-	0 (0-0)	0 (0-0)	0 (0-0)	0 (0-0)
HIV/AIDS resulting in other diseases	-	-	12,153 (10,554-14,230)	8,437 (7,366-9,887)	731 (524-1,006)	224 (183-312)
Sexually transmitted infections excluding HIV	39,882 (14,942-74,880)	15,340 (5,560-29,106)	23,910 (9,057-45,582)	2,098 (757-4,079)	841 (298-1,630)	59 (38-93)
Syphilis	39,882 (14,942-74,880)	15,340 (5,560-29,106)	23,910 (9,057-45,582)	2,098 (757-4,079)	841 (298-1,630)	51 (32-79)
Chlamydial infection	-	-	-	-	-	0 (0-0)
Gonococcal infection	-	-	-	-	-	5 (3-7)
Other sexually transmitted infections	-	-	-	-	-	4 (3-7)
Respiratory infections and tuberculosis	-	165,027 (133,997-189,249)	640,215 (577,868-694,487)	342,337 (283,799-394,195)	42,289 (33,396-48,102)	18,816 (15,864-21,021)
Tuberculosis	-	-	43,372 (38,743-47,780)	42,750 (34,373-49,928)	9,144 (7,586-10,578)	6,695 (5,617-7,750)
Drug-susceptible tuberculosis	-	-	42,810 (38,171-47,003)	42,419 (34,192-49,593)	9,034 (7,467-10,470)	6,618 (5,548-7,675)
Multidrug-resistant tuberculosis without extensive drug resistance	-	-	562 (259-1,152)	331 (183-579)	110 (52-224)	77 (37-151)
Extensively drug-resistant tuberculosis	-	-	0 (0-0)	0 (0-0)	0 (0-0)	0 (0-0)
Lower respiratory infections	145,074 (126,267-167,816)	164,743 (133,824-188,831)	592,433 (534,748-641,901)	295,929 (244,235-340,740)	32,566 (24,512-37,273)	11,827 (9,454-13,237)
Upper respiratory infections	135 (55-189)	238 (95-373)	4,147 (1,453-6,635)	3,462 (946-6,931)	466 (146-708)	206 (72-291)
Otitis media	35 (7-121)	46 (22-103)	263 (166-464)	195 (70-438)	113 (56-221)	89 (50-174)
Enteric infections	23,708 (18,619-31,138)	48,282 (37,867-62,795)	424,672 (339,919-500,363)	340,091 (248,309-429,459)	70,530 (46,356-99,782)	34,491 (23,541-47,932)
Diarrheal diseases	23,708 (18,619-31,138)	48,079 (37,704-62,598)	420,518 (335,924-496,151)	307,933 (220,848-392,704)	26,554 (11,221-48,015)	10,131 (4,311-19,311)
Typhoid and paratyphoid	-	0 (0-0)	294 (199-447)	23,797 (10,796-44,655)	41,258 (23,200-66,763)	23,202 (13,465-36,287)
Typhoid fever	-	-	268 (184-400)	21,132 (9,627-40,278)	36,310 (20,104-58,777)	20,392 (11,675-32,426)
Paratyphoid fever	-	-	27 (13-48)	2,665 (994-5,757)	4,948 (2,080-9,704)	2,809 (1,240-5,594)
Invasive Non-typhoidal Salmonella (iNTS)	-	203 (110-348)	2,833 (1,552-4,949)	7,792 (3,947-14,797)	2,634 (1,276-4,886)	1,119 (498-2,158)
Other intestinal infectious diseases	-	-	1,027 (488-2,440)	569 (235-1,565)	85 (29-197)	39 (9-94)
Neglected tropical diseases and malaria	276 (140-859)	14,140 (8,845-21,653)	100,826 (61,406-154,432)	207,449 (121,866-351,147)	47,537 (26,592-93,856)	24,584 (14,097-49,350)
Malaria	-	13,823 (8,434-21,320)	81,675 (51,769-120,183)	144,001 (90,351-216,856)	24,375 (13,941-36,476)	11,313 (6,415-16,760)
Chagas disease	-	-	4 (3-4)	2 (1-2)	3 (3-4)	7 (6-9)
Leishmaniasis	-	-	15,277 (17-56,655)	47,779 (43-189,366)	13,867 (10-65,780)	7,877 (6-35,173)
Visceral leishmaniasis	-	-	15,277 (17-56,655)	47,779 (43-189,366)	13,867 (10-65,780)	7,877 (6-35,173)
African trypanosomiasis	-	-	-	431 (96-1,413)	673 (168-1,742)	670 (175-1,606)
Schistosomiasis	-	-	0 (0-0)	531 (350-770)	199 (132-275)	232 (156-318)
Cysticercosis	0 (0-0)	0 (0-0)	0 (0-0)	102 (41-188)	48 (11-108)	33 (5-76)
Cystic echinococcosis	-	-	-	373 (223-560)	158 (75-262)	81 (29-150)
Dengue	-	-	1,403 (210-3,381)	3,689 (848-8,238)	950 (343-1,933)	319 (93-670)
Yellow fever	-	6 (1-19)	94 (19-277)	854 (159-2,661)	1,385 (273-4,050)	1,119 (242-3,179)
Rabies	-	-	567 (323-877)	3,015 (1,545-4,765)	4,734 (3,185-6,998)	2,647 (1,775-3,851)
Intestinal nematode infections	-	-	411 (314-530)	4,019 (2,952-5,424)	710 (532-915)	118 (91-150)
Ascariasis	-	-	411 (314-530)	4,019 (2,952-5,424)	710 (532-915)	118 (91-150)
Ebola	0 (0-0)	0 (0-0)	0 (0-0)	0 (0-0)	0 (0-0)	0 (0-0)
Zika virus	0 (0-0)	0 (0-0)	0 (0-0)	0 (0-0)	0 (0-0)	0 (0-0)
Other neglected tropical diseases	276 (140-859)	311 (143-955)	1,396 (710-4,522)	2,651 (800-10,995)	434 (248-1,086)	169 (112-380)
Other infectious diseases	50,701 (41,109-62,475)	86,974 (69,826-104,096)	267,337 (197,564-377,593)	351,727 (229,554-537,275)	64,601 (45,363-94,414)	23,427 (17,681-31,819)
Meningitis	7,808 (5,660-9,836)	14,180 (10,558-16,834)	78,125 (65,300-89,933)	70,060 (52,430-84,510)	15,294 (9,879-19,175)	7,050 (4,567-8,606)
Pneumococcal meningitis	1,163 (825-1,565)	2,052 (1,442-2,645)	10,672 (8,100-13,535)	9,903 (6,906-13,266)	1,384 (883-1,903)	623 (402-866)
H influenzae type B meningitis	2,314 (1,556-3,079)	4,208 (3,068-5,369)	24,854 (19,548-30,653)	23,399 (16,896-30,019)	6,135 (3,859-7,835)	2,727 (1,681-3,416)
Meningococcal meningitis	1,175 (836-1,531)	2,111 (1,544-2,637)	10,846 (8,534-13,369)	9,332 (6,754-12,362)	1,628 (1,072-2,219)	667 (450-882)
Other meningitis	3,157 (2,306-4,057)	5,808 (4,339-7,181)	31,752 (25,653-37,756)	27,426 (20,084-35,324)	6,146 (3,908-7,831)	3,032 (1,951-3,856)
Encephalitis	500 (353-633)	931 (548-1,462)	9,269 (6,093-14,182)	8,907 (5,690-16,612)	4,614 (3,477-6,506)	2,598 (2,057-3,506)
Diphtheria	-	-	1,605 (1,037-2,371)	5,274 (3,194-8,722)	931 (572-1,451)	257 (152-403)
Whooping cough	-	-	40,099 (18,124-75,016)	32,124 (14,067-60,329)	3,369 (1,342-6,983)	620 (256-1,238)
Tetanus	38,335 (30,794-48,412)	67,752 (51,718-83,439)	14,737 (10,955-21,203)	9,476 (2,598-14,802)	3,530 (1,749-5,096)	1,821 (999-2,560)
Measles	-	-	103,439 (37,492-220,940)	207,174 (78,588-417,166)	29,391 (11,285-62,745)	6,875 (2,428-15,544)
Varicella and herpes zoster	118 (88-154)	359 (258-484)	2,514 (2,033-3,127)	2,619 (1,966-3,393)	1,033 (767-1,336)	304 (231-391)
Acute hepatitis	-	-	8,657 (4,059-10,807)	10,204 (4,782-14,897)	4,179 (2,143-5,438)	2,577 (1,395-3,376)
Acute hepatitis A	-	-	1,170 (486-2,154)	5,893 (2,913-8,549)	2,614 (1,357-3,635)	1,236 (647-1,771)
Acute hepatitis B	-	-	5,576 (2,502-7,704)	3,270 (1,280-5,965)	820 (338-1,445)	516 (228-919)
Acute hepatitis C	-	-	1,898 (826-3,380)	822 (299-1,678)	80 (29-169)	23 (7-51)
Acute hepatitis E	-	-	13 (4-26)	219 (84-437)	665 (292-1,094)	802 (359-1,253)
Other unspecified infectious diseases	3,940 (1,845-5,186)	3,753 (1,729-4,772)	8,893 (5,093-12,115)	5,889 (3,555-9,461)	2,260 (1,457-2,966)	1,325 (805-1,757)
Maternal and neonatal disorders	1,329,789 (1,256,277-1,394,814)	279,956 (237,663-343,353)	68,989 (55,138-93,997)	5,664 (3,930-9,568)	-	-
Maternal disorders	-	-	-	-	-	-
Maternal hemorrhage	-	-	-	-	-	-
Maternal sepsis and other maternal infections	-	-	-	-	-	-
Maternal hypertensive disorders	-	-	-	-	-	-
Maternal obstructed labor and uterine rupture	-	-	-	-	-	-
Maternal abortion and miscarriage	-	-	-	-	-	-
Ectopic pregnancy	-	-	-	-	-	-
Indirect maternal deaths	-	-	-	-	-	-
Late maternal deaths	-	-	-	-	-	-
Maternal deaths aggravated by HIV/AIDS	-	-	-	-	-	-
Other maternal disorders	-	-	-	-	-	-
Neonatal disorders	1,329,789 (1,256,277-1,394,814)	279,956 (237,663-343,353)	68,989 (55,138-93,997)	5,664 (3,930-9,568)	-	-
Neonatal preterm birth	589,535 (532,832-655,583)	98,398 (81,624-131,848)	32,597 (25,811-44,163)	1,702 (1,173-2,829)	-	-
Neonatal encephalopathy due to birth asphyxia and trauma	403,730 (352,486-455,375)	47,963 (38,487-62,611)	12,121 (9,002-19,493)	2,279 (1,490-4,400)	-	-
Neonatal sepsis and other neonatal infections	74,666 (56,185-96,279)	53,219 (38,165-83,223)	5,985 (4,188-10,313)	229 (121-479)	-	-
Hemolytic disease and other neonatal jaundice	39,943 (30,956-52,049)	30,788 (20,875-40,627)	4,689 (3,283-6,542)	300 (169-502)	-	-
Other neonatal disorders	221,916 (174,236-263,893)	49,588 (35,964-76,605)	13,597 (9,830-21,495)	1,155 (758-2,092)	-	-
Nutritional deficiencies	-	-	118,514 (91,768-136,851)	106,503 (79,782-129,476)	10,645 (7,147-13,555)	2,605 (1,855-3,192)
Protein-energy malnutrition	-	-	113,576 (88,232-130,744)	101,403 (76,043-122,751)	8,709 (6,034-11,317)	2,073 (1,533-2,524)
Other nutritional deficiencies	-	-	4,938 (3,204-6,740)	5,101 (2,681-7,200)	1,936 (1,061-2,796)	532 (312-722)
Non-communicable diseases	135,184 (116,674-178,724)	72,986 (60,182-96,724)	270,638 (231,911-323,812)	165,377 (136,653-195,922)	75,471 (66,544-84,711)	54,380 (48,345-58,968)
Neoplasms	978 (743-1,108)	788 (573-1,024)	8,158 (5,968-9,837)	26,009 (19,617-36,876)	25,024 (20,961-29,196)	18,340 (15,857-20,019)
Lip and oral cavity cancer	-	-	-	-	-	-
Nasopharynx cancer	-	-	-	-	303 (237-360)	315 (267-360)
Other pharynx cancer	-	-	-	-	-	-
Esophageal cancer	-	-	-	-	-	-
Stomach cancer	-	-	-	-	-	-
Colon and rectum cancer	-	-	-	-	-	-
Liver cancer	-	-	-	-	722 (562-857)	655 (542-836)
Liver cancer due to hepatitis B	-	-	-	-	539 (422-640)	534 (437-682)
Liver cancer due to hepatitis C	-	-	-	-	11 (8-15)	11 (8-14)
Liver cancer due to alcohol use	-	-	-	-	-	-
Liver cancer due to NASH	-	-	-	-	-	-
Liver cancer due to other causes	-	-	-	-	172 (132-215)	109 (83-140)
Gallbladder and biliary tract cancer	-	-	-	-	-	-
Pancreatic cancer	-	-	-	-	-	-
Larynx cancer	-	-	-	-	-	-
Tracheal, bronchus, and lung cancer	-	-	-	-	-	-
Malignant skin melanoma	-	-	-	-	-	-
Non-melanoma skin cancer	-	-	-	-	-	-
Non-melanoma skin cancer (squamous-cell carcinoma)	-	-	-	-	-	-
Breast cancer	-	-	-	-	-	-
Cervical cancer	-	-	-	-	-	-
Uterine cancer	-	-	-	-	-	-
Ovarian cancer	-	-	-	-	-	-
Prostate cancer	-	-	-	-	-	-
Testicular cancer	-	-	-	-	-	-
Kidney cancer	26 (19-33)	17 (12-25)	226 (150-301)	884 (568-1,358)	386 (305-475)	123 (105-140)
Bladder cancer	-	-	-	-	-	-
Brain and nervous system cancer	97 (65-120)	93 (58-113)	1,224 (733-1,491)	4,162 (2,566-5,526)	4,810 (3,448-5,568)	3,057 (2,372-3,567)
Thyroid cancer	-	-	-	-	-	70 (54-88)
Mesothelioma	-	-	-	-	-	-
Hodgkin lymphoma	19 (10-23)	8 (3-10)	151 (66-192)	879 (342-1,298)	1,485 (782-1,954)	1,111 (701-1,373)
Non-Hodgkin lymphoma	50 (43-64)	50 (39-75)	569 (451-792)	2,959 (1,962-4,734)	2,609 (2,097-3,422)	1,684 (1,438-2,071)

Cause	15 to 19	20 to 24	25 to 29	30 to 34	35 to 39	40 to 44	45 to 49	50 to 54	54 to 59	60 to 64	65 to 69	70 to 74	75 to 79	80 to 84	85 to 89	90 to 94	95 plus
All causes	380,282 (375,501-385,383)	509,654 (503,507-516,497)	536,316 (528,497-544,624)	569,982 (561,747-578,782)	646,176 (636,619-656,148)	732,778 (722,442-743,679)	848,559 (836,968-861,219)	1,145,312 (1,131,589-1,160,413)	1,427,857 (1,411,726-1,445,129)	1,922,589 (1,903,546-1,943,022)	2,041,966 (2,023,127-2,061,592)	2,058,149 (2,039,758-2,077,255)	2,041,492 (2,027,079-2,056,523)	1,677,552 (1,668,442-1,687,020)	959,127 (955,195-963,169)	344,113 (342,990-345,268)	81,900 (81,710-82,088)
Communicable, maternal, neonatal, and nutritional diseases	90,966 (79,238-109,938)	103,773 (92,609-121,931)	124,133 (112,629-140,850)	138,022 (126,986-152,942)	146,845 (135,284-162,156)	150,452 (139,071-164,934)	159,691 (147,753-175,832)	175,106 (161,059-193,414)	191,437 (176,532-214,385)	226,994 (205,851-258,544)	225,835 (202,940-263,984)	231,717 (205,399-274,876)	205,081 (180,804-245,312)	175,845 (151,504-211,579)	106,966 (92,790-127,506)	39,000 (33,777-46,724)	9,995 (8,739-11,665)
HIV/AIDS and sexually transmitted infections	1,548 (1,308-1,881)	11,340 (9,586-13,553)	30,488 (25,430-36,497)	36,054 (30,729-42,093)	32,514 (27,363-38,678)	25,571 (21,950-29,368)	18,500 (15,433-22,125)	11,527 (9,724-13,467)	8,249 (6,970-9,771)	6,020 (5,022-7,286)	4,417 (3,559-5,414)	2,737 (2,126-3,436)	1,842 (1,421-2,300)	371 (257-486)	167 (92-240)	52 (31-75)	14 (8-20)
HIV/AIDS	1,459 (1,233-1,782)	11,254 (9,504-13,488)	30,341 (25,307-36,381)	35,882 (30,591-42,014)	32,351 (27,207-38,475)	25,376 (21,742-29,136)	18,364 (15,327-21,993)	11,370 (9,584-13,303)	8,027 (6,767-9,524)	5,783 (4,800-6,984)	4,183 (3,358-5,203)	2,517 (1,930-3,175)	1,549 (1,186-1,965)	204 (158-258)	51 (40-67)	18 (14-22)	2 (2-3)
HIV/AIDS - Drug-susceptible Tuberculosis	483 (381-599)	3,422 (2,521-4,355)	6,823 (4,843-9,038)	7,412 (5,245-9,842)	6,865 (4,798-9,046)	5,796 (4,130-7,662)	4,796 (3,432-6,280)	3,392 (2,447-4,433)	2,566 (1,912-3,271)	1,939 (1,485-2,436)	1,463 (1,110-1,838)	970 (727-1,251)	599 (454-766)	73 (55-94)	16 (12-22)	6 (4-8)	1 (0-1)
HIV/AIDS - Multidrug-resistant Tuberculosis without extensive drug resistance	4 (2-7)	26 (14-50)	59 (32-102)	71 (40-123)	69 (39-117)	58 (33-97)	44 (25-74)	30 (17-50)	23 (13-39)	17 (10-30)	12 (7-21)	7 (3-13)	5 (3-9)	1 (0-2)	0 (0-0)	0 (0-0)	0 (0-0)
HIV/AIDS - Extensively drug-resistant Tuberculosis	0 (0-0)	0 (0-0)	0 (0-0)	0 (0-0)	0 (0-0)	0 (0-0)	0 (0-0)	0 (0-0)	0 (0-0)	0 (0-0)	0 (0-0)	0 (0-0)	0 (0-0)	0 (0-0)	0 (0-0)	0 (0-0)	0 (0-0)
HIV/AIDS resulting in other diseases	[illegible]	[illegible]	[illegible]	[illegible]	[illegible]	[illegible]	[illegible]	[illegible]	[illegible]	[illegible]	[illegible]	[illegible]	[illegible]	[illegible]	[illegible]	[illegible]	[illegible]
Sexually transmitted infections excluding HIV	[illegible]	[illegible]	[illegible]	[illegible]	[illegible]	[illegible]	[illegible]	[illegible]	[illegible]	[illegible]	[illegible]	[illegible]	[illegible]	[illegible]	[illegible]	[illegible]	[illegible]
Syphilis	[illegible]	[illegible]	[illegible]	[illegible]	[illegible]	[illegible]	[illegible]	[illegible]	[illegible]	[illegible]	[illegible]	[illegible]	[illegible]	[illegible]	[illegible]	[illegible]	[illegible]
Chlamydial infection	[illegible]	[illegible]	[illegible]	[illegible]	[illegible]	[illegible]	[illegible]	[illegible]	[illegible]	[illegible]	[illegible]	[illegible]	[illegible]	[illegible]	[illegible]	[illegible]	[illegible]
Gonococcal infection	[illegible]	[illegible]	[illegible]	[illegible]	[illegible]	[illegible]	[illegible]	[illegible]	[illegible]	[illegible]	[illegible]	[illegible]	[illegible]	[illegible]	[illegible]	[illegible]	[illegible]
Other sexually transmitted infections	[illegible]	[illegible]	[illegible]	[illegible]	[illegible]	[illegible]	[illegible]	[illegible]	[illegible]	[illegible]	[illegible]	[illegible]	[illegible]	[illegible]	[illegible]	[illegible]	[illegible]
Respiratory infections and tuberculosis	[illegible]	[illegible]	[illegible]	[illegible]	[illegible]	[illegible]	[illegible]	[illegible]	[illegible]	[illegible]	[illegible]	[illegible]	[illegible]	[illegible]	[illegible]	[illegible]	[illegible]
Tuberculosis	[illegible]	[illegible]	[illegible]	[illegible]	[illegible]	[illegible]	[illegible]	[illegible]	[illegible]	[illegible]	[illegible]	[illegible]	[illegible]	[illegible]	[illegible]	[illegible]	[illegible]
Drug-susceptible tuberculosis	[illegible]	[illegible]	[illegible]	[illegible]	[illegible]	[illegible]	[illegible]	[illegible]	[illegible]	[illegible]	[illegible]	[illegible]	[illegible]	[illegible]	[illegible]	[illegible]	[illegible]
Multidrug-resistant tuberculosis without extensive drug resistance	[illegible]	[illegible]	[illegible]	[illegible]	[illegible]	[illegible]	[illegible]	[illegible]	[illegible]	[illegible]	[illegible]	[illegible]	[illegible]	[illegible]	[illegible]	[illegible]	[illegible]
Extensively drug-resistant tuberculosis	[illegible]	[illegible]	[illegible]	[illegible]	[illegible]	[illegible]	[illegible]	[illegible]	[illegible]	[illegible]	[illegible]	[illegible]	[illegible]	[illegible]	[illegible]	[illegible]	[illegible]
Lower respiratory infections	[illegible]	[illegible]	[illegible]	[illegible]	[illegible]	[illegible]	[illegible]	[illegible]	[illegible]	[illegible]	[illegible]	[illegible]	[illegible]	[illegible]	[illegible]	[illegible]	[illegible]
Upper respiratory infections	[illegible]	[illegible]	[illegible]	[illegible]	[illegible]	[illegible]	[illegible]	[illegible]	[illegible]	[illegible]	[illegible]	[illegible]	[illegible]	[illegible]	[illegible]	[illegible]	[illegible]
Otitis media	[illegible]	[illegible]	[illegible]	[illegible]	[illegible]	[illegible]	[illegible]	[illegible]	[illegible]	[illegible]	[illegible]	[illegible]	[illegible]	[illegible]	[illegible]	[illegible]	[illegible]
Enteric infections	[illegible]	[illegible]	[illegible]	[illegible]	[illegible]	[illegible]	[illegible]	[illegible]	[illegible]	[illegible]	[illegible]	[illegible]	[illegible]	[illegible]	[illegible]	[illegible]	[illegible]
Diarrheal diseases	[illegible]	[illegible]	[illegible]	[illegible]	[illegible]	[illegible]	[illegible]	[illegible]	[illegible]	[illegible]	[illegible]	[illegible]	[illegible]	[illegible]	[illegible]	[illegible]	[illegible]
Typhoid and paratyphoid	[illegible]	[illegible]	[illegible]	[illegible]	[illegible]	[illegible]	[illegible]	[illegible]	[illegible]	[illegible]	[illegible]	[illegible]	[illegible]	[illegible]	[illegible]	[illegible]	[illegible]
Typhoid fever	[illegible]	[illegible]	[illegible]	[illegible]	[illegible]	[illegible]	[illegible]	[illegible]	[illegible]	[illegible]	[illegible]	[illegible]	[illegible]	[illegible]	[illegible]	[illegible]	[illegible]
Paratyphoid fever	[illegible]	[illegible]	[illegible]	[illegible]	[illegible]	[illegible]	[illegible]	[illegible]	[illegible]	[illegible]	[illegible]	[illegible]	[illegible]	[illegible]	[illegible]	[illegible]	[illegible]
Invasive Non-typhoidal Salmonella (iNTS)	[illegible]	[illegible]	[illegible]	[illegible]	[illegible]	[illegible]	[illegible]	[illegible]	[illegible]	[illegible]	[illegible]	[illegible]	[illegible]	[illegible]	[illegible]	[illegible]	[illegible]
Other intestinal infectious diseases	[illegible]	[illegible]	[illegible]	[illegible]	[illegible]	[illegible]	[illegible]	[illegible]	[illegible]	[illegible]	[illegible]	[illegible]	[illegible]	[illegible]	[illegible]	[illegible]	[illegible]
Neglected tropical diseases and malaria	[illegible]	[illegible]	[illegible]	[illegible]	[illegible]	[illegible]	[illegible]	[illegible]	[illegible]	[illegible]	[illegible]	[illegible]	[illegible]	[illegible]	[illegible]	[illegible]	[illegible]
Malaria	[illegible]	[illegible]	[illegible]	[illegible]	[illegible]	[illegible]	[illegible]	[illegible]	[illegible]	[illegible]	[illegible]	[illegible]	[illegible]	[illegible]	[illegible]	[illegible]	[illegible]
Chagas disease	[illegible]	[illegible]	[illegible]	[illegible]	[illegible]	[illegible]	[illegible]	[illegible]	[illegible]	[illegible]	[illegible]	[illegible]	[illegible]	[illegible]	[illegible]	[illegible]	[illegible]
Leishmaniasis	[illegible]	[illegible]	[illegible]	[illegible]	[illegible]	[illegible]	[illegible]	[illegible]	[illegible]	[illegible]	[illegible]	[illegible]	[illegible]	[illegible]	[illegible]	[illegible]	[illegible]
Visceral leishmaniasis	[illegible]	[illegible]	[illegible]	[illegible]	[illegible]	[illegible]	[illegible]	[illegible]	[illegible]	[illegible]	[illegible]	[illegible]	[illegible]	[illegible]	[illegible]	[illegible]	[illegible]
African trypanosomiasis	[illegible]	[illegible]	[illegible]	[illegible]	[illegible]	[illegible]	[illegible]	[illegible]	[illegible]	[illegible]	[illegible]	[illegible]	[illegible]	[illegible]	[illegible]	[illegible]	[illegible]
Schistosomiasis	[illegible]	[illegible]	[illegible]	[illegible]	[illegible]	[illegible]	[illegible]	[illegible]	[illegible]	[illegible]	[illegible]	[illegible]	[illegible]	[illegible]	[illegible]	[illegible]	[illegible]
Cysticercosis	[illegible]	[illegible]	[illegible]	[illegible]	[illegible]	[illegible]	[illegible]	[illegible]	[illegible]	[illegible]	[illegible]	[illegible]	[illegible]	[illegible]	[illegible]	[illegible]	[illegible]
Cystic echinococcosis	[illegible]	[illegible]	[illegible]	[illegible]	[illegible]	[illegible]	[illegible]	[illegible]	[illegible]	[illegible]	[illegible]	[illegible]	[illegible]	[illegible]	[illegible]	[illegible]	[illegible]
Dengue	[illegible]	[illegible]	[illegible]	[illegible]	[illegible]	[illegible]	[illegible]	[illegible]	[illegible]	[illegible]	[illegible]	[illegible]	[illegible]	[illegible]	[illegible]	[illegible]	[illegible]
Yellow fever	[illegible]	[illegible]	[illegible]	[illegible]	[illegible]	[illegible]	[illegible]	[illegible]	[illegible]	[illegible]	[illegible]	[illegible]	[illegible]	[illegible]	[illegible]	[illegible]	[illegible]
Rabies	[illegible]	[illegible]	[illegible]	[illegible]	[illegible]	[illegible]	[illegible]	[illegible]	[illegible]	[illegible]	[illegible]	[illegible]	[illegible]	[illegible]	[illegible]	[illegible]	[illegible]
Intestinal nematode infections	[illegible]	[illegible]	[illegible]	[illegible]	[illegible]	[illegible]	[illegible]	[illegible]	[illegible]	[illegible]	[illegible]	[illegible]	[illegible]	[illegible]	[illegible]	[illegible]	[illegible]
Ascariasis	[illegible]	[illegible]	[illegible]	[illegible]	[illegible]	[illegible]	[illegible]	[illegible]	[illegible]	[illegible]	[illegible]	[illegible]	[illegible]	[illegible]	[illegible]	[illegible]	[illegible]
Ebola	[illegible]	[illegible]	[illegible]	[illegible]	[illegible]	[illegible]	[illegible]	[illegible]	[illegible]	[illegible]	[illegible]	[illegible]	[illegible]	[illegible]	[illegible]	[illegible]	[illegible]
Zika virus	[illegible]	[illegible]	[illegible]	[illegible]	[illegible]	[illegible]	[illegible]	[illegible]	[illegible]	[illegible]	[illegible]	[illegible]	[illegible]	[illegible]	[illegible]	[illegible]	[illegible]
Other neglected tropical diseases	[illegible]	[illegible]	[illegible]	[illegible]	[illegible]	[illegible]	[illegible]	[illegible]	[illegible]	[illegible]	[illegible]	[illegible]	[illegible]	[illegible]	[illegible]	[illegible]	[illegible]
Other infectious diseases	[illegible]	[illegible]	[illegible]	[illegible]	[illegible]	[illegible]	[illegible]	[illegible]	[illegible]	[illegible]	[illegible]	[illegible]	[illegible]	[illegible]	[illegible]	[illegible]	[illegible]
Meningitis	[illegible]	[illegible]	[illegible]	[illegible]	[illegible]	[illegible]	[illegible]	[illegible]	[illegible]	[illegible]	[illegible]	[illegible]	[illegible]	[illegible]	[illegible]	[illegible]	[illegible]
Pneumococcal meningitis	[illegible]	[illegible]	[illegible]	[illegible]	[illegible]	[illegible]	[illegible]	[illegible]	[illegible]	[illegible]	[illegible]	[illegible]	[illegible]	[illegible]	[illegible]	[illegible]	[illegible]
H influenzae type B meningitis	[illegible]	[illegible]	[illegible]	[illegible]	[illegible]	[illegible]	[illegible]	[illegible]	[illegible]	[illegible]	[illegible]	[illegible]	[illegible]	[illegible]	[illegible]	[illegible]	[illegible]
Meningococcal meningitis	[illegible]	[illegible]	[illegible]	[illegible]	[illegible]	[illegible]	[illegible]	[illegible]	[illegible]	[illegible]	[illegible]	[illegible]	[illegible]	[illegible]	[illegible]	[illegible]	[illegible]
Other meningitis	[illegible]	[illegible]	[illegible]	[illegible]	[illegible]	[illegible]	[illegible]	[illegible]	[illegible]	[illegible]	[illegible]	[illegible]	[illegible]	[illegible]	[illegible]	[illegible]	[illegible]
Encephalitis	[illegible]	[illegible]	[illegible]	[illegible]	[illegible]	[illegible]	[illegible]	[illegible]	[illegible]	[illegible]	[illegible]	[illegible]	[illegible]	[illegible]	[illegible]	[illegible]	[illegible]
Diphtheria	[illegible]	[illegible]	[illegible]	[illegible]	[illegible]	[illegible]	[illegible]	[illegible]	[illegible]	[illegible]	[illegible]	[illegible]	[illegible]	[illegible]	[illegible]	[illegible]	[illegible]
Whooping cough	[illegible]	[illegible]	[illegible]	[illegible]	[illegible]	[illegible]	[illegible]	[illegible]	[illegible]	[illegible]	[illegible]	[illegible]	[illegible]	[illegible]	[illegible]	[illegible]	[illegible]
Tetanus	[illegible]	[illegible]	[illegible]	[illegible]	[illegible]	[illegible]	[illegible]	[illegible]	[illegible]	[illegible]	[illegible]	[illegible]	[illegible]	[illegible]	[illegible]	[illegible]	[illegible]
Measles	[illegible]	[illegible]	[illegible]	[illegible]	[illegible]	[illegible]	[illegible]	[illegible]	[illegible]	[illegible]	[illegible]	[illegible]	[illegible]	[illegible]	[illegible]	[illegible]	[illegible]
Varicella and herpes zoster	[illegible]	[illegible]	[illegible]	[illegible]	[illegible]	[illegible]	[illegible]	[illegible]	[illegible]	[illegible]	[illegible]	[illegible]	[illegible]	[illegible]	[illegible]	[illegible]	[illegible]
Acute hepatitis	[illegible]	[illegible]	[illegible]	[illegible]	[illegible]	[illegible]	[illegible]	[illegible]	[illegible]	[illegible]	[illegible]	[illegible]	[illegible]	[illegible]	[illegible]	[illegible]	[illegible]
Acute hepatitis A	[illegible]	[illegible]	[illegible]	[illegible]	[illegible]	[illegible]	[illegible]	[illegible]	[illegible]	[illegible]	[illegible]	[illegible]	[illegible]	[illegible]	[illegible]	[illegible]	[illegible]
Acute hepatitis B	[illegible]	[illegible]	[illegible]	[illegible]	[illegible]	[illegible]	[illegible]	[illegible]	[illegible]	[illegible]	[illegible]	[illegible]	[illegible]	[illegible]	[illegible]	[illegible]	[illegible]
Acute hepatitis C	[illegible]	[illegible]	[illegible]	[illegible]	[illegible]	[illegible]	[illegible]	[illegible]	[illegible]	[illegible]	[illegible]	[illegible]	[illegible]	[illegible]	[illegible]	[illegible]	[illegible]
Acute hepatitis E	[illegible]	[illegible]	[illegible]	[illegible]	[illegible]	[illegible]	[illegible]	[illegible]	[illegible]	[illegible]	[illegible]	[illegible]	[illegible]	[illegible]	[illegible]	[illegible]	[illegible]
Other unspecified infectious diseases	[illegible]	[illegible]	[illegible]	[illegible]	[illegible]	[illegible]	[illegible]	[illegible]	[illegible]	[illegible]	[illegible]	[illegible]	[illegible]	[illegible]	[illegible]	[illegible]	[illegible]
Maternal and neonatal disorders	[illegible]	[illegible]	[illegible]	[illegible]	[illegible]	[illegible]	[illegible]	[illegible]	[illegible]	[illegible]	[illegible]	[illegible]	[illegible]	[illegible]	[illegible]	[illegible]	[illegible]
Maternal disorders	[illegible]	[illegible]	[illegible]	[illegible]	[illegible]	[illegible]	[illegible]	[illegible]	[illegible]	[illegible]	[illegible]	[illegible]	[illegible]	[illegible]	[illegible]	[illegible]	[illegible]
Maternal hemorrhage	[illegible]	[illegible]	[illegible]	[illegible]	[illegible]	[illegible]	[illegible]	[illegible]	[illegible]	[illegible]	[illegible]	[illegible]	[illegible]	[illegible]	[illegible]	[illegible]	[illegible]
Maternal sepsis and other maternal infections	[illegible]	[illegible]	[illegible]	[illegible]	[illegible]	[illegible]	[illegible]	[illegible]	[illegible]	[illegible]	[illegible]	[illegible]	[illegible]	[illegible]	[illegible]	[illegible]	[illegible]
Maternal hypertensive disorders	[illegible]	[illegible]	[illegible]	[illegible]	[illegible]	[illegible]	[illegible]	[illegible]	[illegible]	[illegible]	[illegible]	[illegible]	[illegible]	[illegible]	[illegible]	[illegible]	[illegible]
Maternal obstructed labor and uterine rupture	[illegible]	[illegible]	[illegible]	[illegible]	[illegible]	[illegible]	[illegible]	[illegible]	[illegible]	[illegible]	[illegible]	[illegible]	[illegible]	[illegible]	[illegible]	[illegible]	[illegible]
Maternal abortion and miscarriage	[illegible]	[illegible]	[illegible]	[illegible]	[illegible]	[illegible]	[illegible]	[illegible]	[illegible]	[illegible]	[illegible]	[illegible]	[illegible]	[illegible]	[illegible]	[illegible]	[illegible]
Ectopic pregnancy	[illegible]	[illegible]	[illegible]	[illegible]	[illegible]	[illegible]	[illegible]	[illegible]	[illegible]	[illegible]	[illegible]	[illegible]	[illegible]	[illegible]	[illegible]	[illegible]	[illegible]
Indirect maternal deaths	[illegible]	[illegible]	[illegible]	[illegible]	[illegible]	[illegible]	[illegible]	[illegible]	[illegible]	[illegible]	[illegible]	[illegible]	[illegible]	[illegible]	[illegible]	[illegible]	[illegible]
Late maternal deaths	[illegible]	[illegible]	[illegible]	[illegible]	[illegible]	[illegible]	[illegible]	[illegible]	[illegible]	[illegible]	[illegible]	[illegible]	[illegible]	[illegible]	[illegible]	[illegible]	[illegible]
Maternal deaths aggravated by HIV/AIDS	[illegible]	[illegible]	[illegible]	[illegible]	[illegible]	[illegible]	[illegible]	[illegible]	[illegible]	[illegible]	[illegible]	[illegible]	[illegible]	[illegible]	[illegible]	[illegible]	[illegible]
Other maternal disorders	[illegible]	[illegible]	[illegible]	[illegible]	[illegible]	[illegible]	[illegible]	[illegible]	[illegible]	[illegible]	[illegible]	[illegible]	[illegible]	[illegible]	[illegible]	[illegible]	[illegible]
Neonatal disorders	[illegible]	[illegible]	[illegible]	[illegible]	[illegible]	[illegible]	[illegible]	[illegible]	[illegible]	[illegible]	[illegible]	[illegible]	[illegible]	[illegible]	[illegible]	[illegible]	[illegible]
Neonatal preterm birth	[illegible]	[illegible]	[illegible]	[illegible]	[illegible]	[illegible]	[illegible]	[illegible]	[illegible]	[illegible]	[illegible]	[illegible]	[illegible]	[illegible]	[illegible]	[illegible]	[illegible]
Neonatal encephalopathy due to birth asphyxia and trauma	[illegible]	[illegible]	[illegible]	[illegible]	[illegible]	[illegible]	[illegible]	[illegible]	[illegible]	[illegible]	[illegible]	[illegible]	[illegible]	[illegible]	[illegible]	[illegible]	[illegible]
Neonatal sepsis and other neonatal infections	[illegible]	[illegible]	[illegible]	[illegible]	[illegible]	[illegible]	[illegible]	[illegible]	[illegible]	[illegible]	[illegible]	[illegible]	[illegible]	[illegible]	[illegible]	[illegible]	[illegible]
Hemolytic disease and other neonatal jaundice	[illegible]	[illegible]	[illegible]	[illegible]	[illegible]	[illegible]	[illegible]	[illegible]	[illegible]	[illegible]	[illegible]	[illegible]	[illegible]	[illegible]	[illegible]	[illegible]	[illegible]
Other neonatal disorders	[illegible]	[illegible]	[illegible]	[illegible]	[illegible]	[illegible]	[illegible]	[illegible]	[illegible]	[illegible]	[illegible]	[illegible]	[illegible]	[illegible]	[illegible]	[illegible]	[illegible]
Nutritional deficiencies	[illegible]	[illegible]	[illegible]	[illegible]	[illegible]	[illegible]	[illegible]	[illegible]	[illegible]	[illegible]	[illegible]	[illegible]	[illegible]	[illegible]	[illegible]	[illegible]	[illegible]
Protein-energy malnutrition	[illegible]	[illegible]	[illegible]	[illegible]	[illegible]	[illegible]	[illegible]	[illegible]	[illegible]	[illegible]	[illegible]	[illegible]	[illegible]	[illegible]	[illegible]	[illegible]	[illegible]
Other nutritional deficiencies	[illegible]	[illegible]	[illegible]	[illegible]	[illegible]	[illegible]	[illegible]	[illegible]	[illegible]	[illegible]	[illegible]	[illegible]	[illegible]	[illegible]	[illegible]	[illegible]	[illegible]
Non-communicable diseases	[illegible]	[illegible]	[illegible]	[illegible]	[illegible]	[illegible]	[illegible]	[illegible]	[illegible]	[illegible]	[illegible]	[illegible]	[illegible]	[illegible]	[illegible]	[illegible]	[illegible]
Neoplasms	[illegible]	[illegible]	[illegible]	[illegible]	[illegible]	[illegible]	[illegible]	[illegible]	[illegible]	[illegible]	[illegible]	[illegible]	[illegible]	[illegible]	[illegible]	[illegible]	[illegible]
Lip and oral cavity cancer	[illegible]	[illegible]	[illegible]	[illegible]	[illegible]	[illegible]	[illegible]	[illegible]	[illegible]	[illegible]	[illegible]	[illegible]	[illegible]	[illegible]	[illegible]	[illegible]	[illegible]
Nasopharynx cancer	[illegible]	[illegible]	[illegible]	[illegible]	[illegible]	[illegible]	[illegible]	[illegible]	[illegible]	[illegible]	[illegible]	[illegible]	[illegible]	[illegible]	[illegible]	[illegible]	[illegible]
Other pharynx cancer	[illegible]	[illegible]	[illegible]	[illegible]	[illegible]	[illegible]	[illegible]	[illegible]	[illegible]	[illegible]	[illegible]	[illegible]	[illegible]	[illegible]	[illegible]	[illegible]	[illegible]
Esophageal cancer	[illegible]	[illegible]	[illegible]	[illegible]	[illegible]	[illegible]	[illegible]	[illegible]	[illegible]	[illegible]	[illegible]	[illegible]	[illegible]	[illegible]	[illegible]	[illegible]	[illegible]
Stomach cancer	[illegible]	[illegible]	[illegible]	[illegible]	[illegible]	[illegible]	[illegible]	[illegible]	[illegible]	[illegible]	[illegible]	[illegible]	[illegible]	[illegible]	[illegible]	[illegible]	[illegible]
Colon and rectum cancer	[illegible]	[illegible]	[illegible]	[illegible]	[illegible]	[illegible]	[illegible]	[illegible]	[illegible]	[illegible]	[illegible]	[illegible]	[illegible]	[illegible]	[illegible]	[illegible]	[illegible]
Liver cancer	[illegible]	[illegible]	[illegible]	[illegible]	[illegible]	[illegible]	[illegible]	[illegible]	[illegible]	[illegible]	[illegible]	[illegible]	[illegible]	[illegible]	[illegible]	[illegible]	[illegible]
Liver cancer due to hepatitis B	[illegible]	[illegible]	[illegible]	[illegible]	[illegible]	[illegible]	[illegible]	[illegible]	[illegible]	[illegible]	[illegible]	[illegible]	[illegible]	[illegible]	[illegible]	[illegible]	[illegible]
Liver cancer due to hepatitis C	[illegible]	[illegible]	[illegible]	[illegible]	[illegible]	[illegible]	[illegible]	[illegible]	[illegible]	[illegible]	[illegible]	[illegible]	[illegible]	[illegible]	[illegible]	[illegible]	[illegible]
Liver cancer due to alcohol use	[illegible]	[illegible]	[illegible]	[illegible]	[illegible]	[illegible]	[illegible]	[illegible]	[illegible]	[illegible]	[illegible]	[illegible]	[illegible]	[illegible]	[illegible]	[illegible]	[illegible]
Liver cancer due to NASH	[illegible]	[illegible]	[illegible]	[illegible]	[illegible]	[illegible]	[illegible]	[illegible]	[illegible]	[illegible]	[illegible]	[illegible]	[illegible]	[illegible]	[illegible]	[illegible]	[illegible]
Liver cancer due to other causes	[illegible]	[illegible]	[illegible]	[illegible]	[illegible]	[illegible]	[illegible]	[illegible]	[illegible]	[illegible]	[illegible]	[illegible]	[illegible]	[illegible]	[illegible]	[illegible]	[illegible]
Gallbladder and biliary tract cancer	[illegible]	[illegible]	[illegible]	[illegible]	[illegible]	[illegible]	[illegible]	[illegible]	[illegible]	[illegible]	[illegible]	[illegible]	[illegible]	[illegible]	[illegible]	[illegible]	[illegible]
Pancreatic cancer	[illegible]	[illegible]	[illegible]	[illegible]	[illegible]	[illegible]	[illegible]	[illegible]	[illegible]	[illegible]	[illegible]	[illegible]	[illegible]	[illegible]	[illegible]	[illegible]	[illegible]
Larynx cancer	[illegible]	[illegible]	[illegible]	[illegible]	[illegible]	[illegible]	[illegible]	[illegible]	[illegible]	[illegible]	[illegible]	[illegible]	[illegible]	[illegible]	[illegible]	[illegible]	[illegible]
Tracheal, bronchus, and lung cancer	[illegible]	[illegible]	[illegible]	[illegible]	[illegible]	[illegible]	[illegible]	[illegible]	[illegible]	[illegible]	[illegible]	[illegible]	[illegible]	[illegible]	[illegible]	[illegible]	[illegible]
Malignant skin melanoma	[illegible]	[illegible]	[illegible]	[illegible]	[illegible]	[illegible]	[illegible]	[illegible]	[illegible]	[illegible]	[illegible]	[illegible]	[illegible]	[illegible]	[illegible]	[illegible]	[illegible]
Non-melanoma skin cancer	[illegible]	[illegible]	[illegible]	[illegible]	[illegible]	[illegible]	[illegible]	[illegible]	[illegible]	[illegible]	[illegible]	[illegible]	[illegible]	[illegible]	[illegible]	[illegible]	[illegible]
Non-melanoma skin cancer (squamous-cell carcinoma)	[illegible]	[illegible]	[illegible]	[illegible]	[illegible]	[illegible]	[illegible]	[illegible]	[illegible]	[illegible]	[illegible]	[illegible]	[illegible]	[illegible]	[illegible]	[illegible]	[illegible]
Breast cancer	[illegible]	[illegible]	[illegible]	[illegible]	[illegible]	[illegible]	[illegible]	[illegible]	[illegible]	[illegible]	[illegible]	[illegible]	[illegible]	[illegible]	[illegible]	[illegible]	[illegible]
Cervical cancer	[illegible]	[illegible]	[illegible]	[illegible]	[illegible]	[illegible]	[illegible]	[illegible]	[illegible]	[illegible]	[illegible]	[illegible]	[illegible]	[illegible]	[illegible]	[illegible]	[illegible]
Uterine cancer	[illegible]	[illegible]	[illegible]	[illegible]	[illegible]	[illegible]	[illegible]	[illegible]	[illegible]	[illegible]	[illegible]	[illegible]	[illegible]	[illegible]	[illegible]	[illegible]	[illegible]
Ovarian cancer	[illegible]	[illegible]	[illegible]	[illegible]	[illegible]	[illegible]	[illegible]	[illegible]	[illegible]	[illegible]	[illegible]	[illegible]	[illegible]	[illegible]	[illegible]	[illegible]	[illegible]
Prostate cancer	[illegible]	[illegible]	[illegible]	[illegible]	[illegible]	[illegible]	[illegible]	[illegible]	[illegible]	[illegible]	[illegible]	[illegible]	[illegible]	[illegible]	[illegible]	[illegible]	[illegible]
Testicular cancer	[illegible]	[illegible]	[illegible]	[illegible]	[illegible]	[illegible]	[illegible]	[illegible]	[illegible]	[illegible]	[illegible]	[illegible]	[illegible]	[illegible]	[illegible]	[illegible]	[illegible]
Kidney cancer	[illegible]	[illegible]	[illegible]	[illegible]	[illegible]	[illegible]	[illegible]	[illegible]	[illegible]	[illegible]	[illegible]	[illegible]	[illegible]	[illegible]	[illegible]	[illegible]	[illegible]
Bladder cancer	[illegible]	[illegible]	[illegible]	[illegible]	[illegible]	[illegible]	[illegible]	[illegible]	[illegible]	[illegible]	[illegible]	[illegible]	[illegible]	[illegible]	[illegible]	[illegible]	[illegible]
Brain and nervous system cancer	[illegible]	[illegible]	[illegible]	[illegible]	[illegible]	[illegible]	[illegible]	[illegible]	[illegible]	[illegible]	[illegible]	[illegible]	[illegible]	[illegible]	[illegible]	[illegible]	[illegible]
Thyroid cancer	[illegible]	[illegible]	[illegible]	[illegible]	[illegible]	[illegible]	[illegible]	[illegible]	[illegible]	[illegible]	[illegible]	[illegible]	[illegible]	[illegible]	[illegible]	[illegible]	[illegible]
Mesothelioma	[illegible]	[illegible]	[illegible]	[illegible]	[illegible]	[illegible]	[illegible]	[illegible]	[illegible]	[illegible]	[illegible]	[illegible]	[illegible]	[illegible]	[illegible]	[illegible]	[illegible]
Hodgkin lymphoma	[illegible]	[illegible]	[illegible]	[illegible]	[illegible]	[illegible]	[illegible]	[illegible]	[illegible]	[illegible]	[illegible]	[illegible]	[illegible]	[illegible]	[illegible]	[illegible]	[illegible]
Non-Hodgkin lymphoma	[illegible]	[illegible]	[illegible]	[illegible]	[illegible]	[illegible]	[illegible]	[illegible]	[illegible]	[illegible]	[illegible]	[illegible]	[illegible]	[illegible]	[illegible]	[illegible]	[illegible]

SR Table 6: Global deaths by cause and age group for males (with 95% confidence intervals), 1990

Cause	Early Neonatal	Late Neonatal	Post Neonatal	1 to 4	5 to 9	10 to 14	15 to 19
Multiple myeloma	-	-	-	-	-	-	112 (67-149)
Leukemia	245 (163-284)	263 (163-345)	3,770 (2,267-4,757)	10,380 (6,641-15,509)	10,954 (7,979-13,862)	7,755 (6,052-8,767)	7,243 (6,031-8,171)
Acute lymphoid leukemia	44 (30-59)	46 (25-80)	712 (385-1,140)	3,040 (1,511-5,418)	3,865 (2,540-5,443)	2,397 (1,820-3,062)	1,935 (1,632-2,341)
Chronic lymphoid leukemia	-	-	-	-	-	-	110 (81-146)
Acute myeloid leukemia	40 (28-54)	41 (26-71)	377 (238-704)	1,443 (810-3,228)	1,035 (689-1,660)	925 (712-1,235)	1,054 (880-1,253)
Chronic myeloid leukemia	-	-	-	-	-	-	219 (181-257)
Other leukemia	161 (92-192)	176 (93-226)	2,680 (1,352-3,345)	5,898 (2,728-9,121)	6,053 (3,307-7,869)	4,433 (2,894-5,296)	3,925 (2,943-4,537)
Other malignant neoplasms	511 (367-594)	334 (214-474)	1,947 (1,263-2,642)	6,194 (3,829-10,490)	3,278 (2,518-4,121)	3,228 (2,717-3,712)	4,215 (3,733-4,685)
Other neoplasms	30 (18-41)	23 (13-31)	270 (144-394)	550 (264-891)	479 (266-703)	342 (223-477)	320 (215-416)
Myelodysplastic, myeloproliferative, and other hematopoietic neoplasms	30 (17-40)	21 (12-29)	244 (125-362)	469 (206-788)	355 (181-551)	242 (149-339)	228 (146-295)
Other benign and in situ neoplasms	0 (0-1)	2 (1-3)	26 (17-39)	82 (48-133)	124 (75-179)	100 (65-146)	92 (60-124)
Cardiovascular diseases	2,972 (2,666-3,371)	2,689 (2,262-3,057)	19,058 (17,316-20,681)	14,142 (11,823-15,967)	7,615 (6,849-8,375)	6,692 (6,004-7,243)	19,003 (17,454-20,441)
Rheumatic heart disease	-	-	-	4,590 (3,642-5,803)	2,604 (2,240-2,990)	2,778 (2,415-3,114)	4,227 (3,598-5,105)
Ischemic heart disease	-	-	-	-	-	-	5,449 (4,944-5,915)
Stroke	1,389 (1,142-1,692)	1,243 (895-1,617)	8,467 (6,490-10,676)	4,818 (3,632-6,063)	3,143 (2,738-3,597)	2,407 (2,156-2,621)	5,764 (5,299-6,178)
Ischemic stroke	178 (128-257)	210 (120-370)	685 (424-1,154)	573 (319-1,060)	275 (218-367)	199 (169-243)	623 (546-716)
Intracerebral hemorrhage	653 (507-868)	530 (350-831)	4,362 (3,044-6,690)	1,955 (1,250-3,149)	1,595 (1,307-2,068)	1,190 (1,037-1,437)	3,487 (3,099-3,981)
Subarachnoid hemorrhage	559 (387-691)	503 (292-695)	3,420 (2,040-4,721)	2,290 (1,319-3,158)	1,274 (857-1,506)	1,018 (774-1,145)	1,654 (1,280-1,960)
Hypertensive heart disease	-	-	-	-	-	-	448 (299-519)
Non-rheumatic valvular heart disease	-	-	-	-	-	-	352 (276-436)
Non-rheumatic calcific aortic valve disease	-	-	-	-	-	-	106 (79-137)
Non-rheumatic degenerative mitral valve disease	-	-	-	-	-	-	170 (128-223)
Other non-rheumatic valve diseases	-	-	-	-	-	-	76 (45-102)
Cardiomyopathy and myocarditis	827 (676-999)	680 (532-915)	3,927 (3,006-5,251)	1,875 (1,333-2,764)	807 (664-1,002)	710 (645-808)	1,406 (1,229-1,583)
Myocarditis	116 (56-167)	156 (78-233)	1,115 (608-1,685)	588 (334-930)	294 (191-398)	228 (171-285)	472 (357-560)
Alcoholic cardiomyopathy	-	-	-	-	-	-	141 (105-182)
Other cardiomyopathy	712 (595-854)	524 (421-691)	2,812 (2,186-3,781)	1,286 (868-1,983)	513 (404-663)	482 (418-553)	793 (667-899)
Atrial fibrillation and flutter	-	-	-	-	-	-	-
Aortic aneurysm	-	-	-	-	-	-	182 (158-223)
Peripheral artery disease	-	-	-	-	-	-	-
Endocarditis	167 (128-230)	157 (115-231)	2,032 (1,501-2,841)	887 (474-1,334)	230 (189-284)	217 (184-258)	288 (248-333)
Other cardiovascular and circulatory diseases	588 (481-734)	609 (441-775)	4,633 (3,083-6,043)	1,972 (1,033-2,924)	831 (590-1,157)	580 (485-746)	886 (751-1,070)
Chronic respiratory diseases	0 (0-0)	0 (0-0)	0 (0-0)	19,463 (16,300-22,747)	5,052 (4,266-5,762)	3,708 (3,212-4,193)	5,841 (5,231-6,427)
Chronic obstructive pulmonary disease	-	-	0 (0-0)	2,022 (1,247-4,854)	732 (566-1,331)	409 (333-725)	1,884 (1,518-2,724)
Pneumoconiosis	-	-	-	-	-	-	32 (23-42)
Silicosis	-	-	-	-	-	-	11 (7-18)
Asbestosis	-	-	-	-	-	-	3 (1-5)
Coal workers pneumoconiosis	-	-	-	-	-	-	4 (2-7)
Other pneumoconiosis	-	-	-	-	-	-	13 (8-20)
Asthma	-	-	-	15,550 (12,359-19,006)	3,703 (3,013-4,396)	2,935 (2,461-3,429)	3,294 (2,206-3,999)
Interstitial lung disease and pulmonary sarcoidosis	-	-	-	279 (79-817)	86 (39-208)	54 (34-105)	75 (50-140)
Other chronic respiratory diseases	0 (0-0)	0 (0-0)	0 (0-0)	1,611 (697-2,853)	531 (331-794)	311 (215-452)	555 (373-778)
Digestive diseases	1,764 (1,521-2,085)	2,371 (1,919-3,178)	16,609 (12,994-24,297)	17,648 (12,725-27,021)	9,641 (7,707-12,485)	6,224 (5,114-7,370)	10,295 (8,569-11,601)
Cirrhosis and other chronic liver diseases	-	-	-	6,853 (4,283-11,527)	3,526 (2,705-4,736)	2,745 (2,176-3,342)	4,448 (3,604-5,207)
Cirrhosis and other chronic liver diseases due to hepatitis B	-	-	-	305 (176-553)	430 (319-605)	540 (412-703)	1,174 (870-1,503)
Cirrhosis and other chronic liver diseases due to hepatitis C	-	-	-	92 (49-177)	118 (79-177)	164 (116-220)	358 (254-473)
Cirrhosis and other chronic liver diseases due to alcohol use	-	-	-	-	-	-	37 (17-66)
Cirrhosis due to NASH	-	-	-	-	-	-	76 (56-98)
Cirrhosis and other chronic liver diseases due to other causes	-	-	-	6,456 (4,043-10,830)	2,979 (2,271-3,977)	2,041 (1,608-2,512)	2,803 (2,223-3,397)
Upper digestive system diseases	-	-	-	1,691 (1,118-2,465)	1,146 (876-1,513)	815 (641-1,012)	1,797 (1,479-2,137)
Peptic ulcer disease	-	-	-	1,211 (752-2,022)	925 (699-1,216)	688 (545-862)	1,502 (1,227-1,780)
Gastritis and duodenitis	-	-	-	480 (206-809)	220 (119-342)	126 (76-188)	295 (197-395)
Appendicitis	-	-	-	1,738 (842-3,048)	1,464 (1,052-2,125)	1,000 (803-1,287)	1,231 (993-1,497)
Paralytic ileus and intestinal obstruction	1,764 (1,521-2,085)	2,371 (1,919-3,178)	16,609 (12,994-24,297)	1,933 (1,261-2,667)	1,822 (1,084-2,383)	780 (491-981)	1,558 (1,041-1,941)
Inguinal, femoral, and abdominal hernia	-	-	-	906 (529-1,568)	341 (247-503)	173 (129-222)	288 (217-367)
Inflammatory bowel disease	-	-	-	869 (402-1,922)	186 (108-350)	95 (64-145)	106 (81-126)
Vascular intestinal disorders	-	-	-	440 (133-669)	83 (41-110)	34 (23-40)	47 (38-53)
Gallbladder and biliary diseases	-	-	-	426 (272-602)	186 (145-241)	139 (113-170)	189 (156-217)
Pancreatitis	-	-	-	111 (59-213)	101 (67-160)	84 (66-120)	241 (197-299)
Other digestive diseases	-	-	-	2,680 (1,739-4,594)	788 (591-1,167)	359 (280-491)	391 (301-560)
Neurological disorders	-	-	6,363 (3,983-8,453)	9,686 (4,680-14,108)	4,692 (3,370-6,040)	4,761 (3,775-5,596)	8,100 (6,811-9,116)
Alzheimer's disease and other dementias	-	-	-	-	-	-	-
Parkinson's disease	-	-	-	-	-	-	-
Epilepsy	-	-	4,082 (2,430-5,394)	7,889 (3,678-11,740)	3,956 (2,767-5,149)	3,798 (2,843-4,482)	6,220 (4,933-7,099)
Multiple sclerosis	-	-	-	-	-	-	-
Motor neuron disease	4 (3-7)	14 (8-25)	439 (319-865)	100 (65-221)	28 (23-48)	38 (32-68)	77 (67-117)
Other neurological disorders	-	-	1,842 (956-2,605)	1,696 (678-2,711)	707 (420-952)	924 (684-1,150)	1,804 (1,630-2,093)
Mental disorders	-	-	-	-	0 (0-0)	0 (0-1)	2 (2-3)
Eating disorders	-	-	-	-	0 (0-0)	0 (0-1)	2 (2-3)
Anorexia nervosa	-	-	-	-	0 (0-0)	0 (0-1)	2 (1-2)
Bulimia nervosa	-	-	-	-	0 (0-0)	0 (0-0)	1 (0-1)
Substance use disorders	0 (0-0)	0 (0-0)	2 (0-8)	0 (0-0)	0 (0-0)	0 (0-0)	3,119 (2,892-3,392)
Alcohol use disorders	-	-	-	-	-	-	940 (825-1,059)
Drug use disorders	0 (0-0)	0 (0-0)	2 (0-8)	0 (0-0)	0 (0-0)	0 (0-0)	2,179 (1,998-2,407)
Opioid use disorders	0 (0-0)	0 (0-0)	2 (0-8)	0 (0-0)	0 (0-0)	0 (0-0)	1,282 (1,046-1,613)
Cocaine use disorders	-	-	-	-	-	-	47 (30-77)
Amphetamine use disorders	-	-	-	-	-	-	135 (66-246)
Other drug use disorders	-	-	-	-	-	-	716 (550-979)
Diabetes and kidney diseases	30 (21-39)	103 (71-127)	7,209 (6,263-8,022)	7,350 (5,960-8,542)	4,465 (3,649-5,090)	3,367 (2,843-3,778)	6,249 (5,459-7,011)
Diabetes mellitus	30 (21-39)	103 (71-127)	854 (617-1,076)	397 (304-505)	463 (359-572)	459 (338-567)	889 (805-965)
Diabetes mellitus type 1	30 (21-39)	103 (71-127)	854 (617-1,076)	397 (304-505)	463 (359-572)	459 (338-567)	812 (732-887)
Diabetes mellitus type 2	-	-	-	-	-	-	77 (61-93)
Chronic kidney disease	-	-	6,068 (5,211-6,863)	6,576 (5,180-7,627)	3,664 (2,893-4,222)	2,692 (2,212-3,048)	5,109 (4,394-5,802)
Chronic kidney disease due to diabetes mellitus type 1	-	-	23 (12-38)	28 (14-51)	23 (11-43)	28 (15-47)	91 (47-150)
Chronic kidney disease due to diabetes mellitus type 2	-	-	18 (9-31)	20 (10-38)	15 (7-29)	15 (7-28)	43 (19-80)
Chronic kidney disease due to hypertension	-	-	31 (17-48)	50 (27-82)	46 (25-77)	59 (33-94)	198 (118-305)
Chronic kidney disease due to glomerulonephritis	-	-	1,353 (941-1,896)	1,880 (1,253-2,654)	1,087 (701-1,564)	893 (573-1,242)	1,968 (1,346-2,670)
Chronic kidney disease due to other and unspecified causes	-	-	4,645 (3,848-5,370)	4,598 (3,509-5,658)	2,493 (1,834-3,072)	1,697 (1,320-2,092)	2,808 (2,100-3,564)
Acute glomerulonephritis	-	-	286 (186-383)	377 (225-566)	339 (264-426)	216 (173-267)	251 (204-316)
Skin and subcutaneous diseases	-	-	1,795 (700-2,382)	946 (317-1,660)	296 (117-400)	265 (120-352)	407 (189-569)
Bacterial skin diseases	-	-	1,730 (647-2,297)	855 (265-1,501)	241 (81-343)	210 (85-291)	342 (152-488)
Cellulitis	-	-	154 (36-289)	147 (37-291)	75 (22-114)	73 (27-107)	82 (27-128)
Pyoderma	-	-	1,576 (594-2,073)	708 (215-1,263)	166 (58-236)	137 (56-188)	260 (119-369)
Decubitus ulcer	-	-	-	28 (14-74)	25 (14-48)	28 (16-51)	36 (18-59)
Other skin and subcutaneous diseases	-	-	66 (32-210)	63 (30-211)	31 (18-66)	27 (17-50)	28 (16-49)
Musculoskeletal disorders	-	-	-	-	367 (231-447)	420 (311-476)	489 (404-540)
Rheumatoid arthritis	-	-	-	-	66 (37-126)	65 (45-98)	59 (45-77)
Other musculoskeletal disorders	-	-	-	-	301 (192-381)	356 (261-413)	430 (354-478)
Other non-communicable diseases	129,436 (111,334-172,796)	67,021 (54,294-90,807)	211,443 (176,207-265,564)	70,133 (56,935-98,071)	18,317 (15,952-22,273)	10,602 (9,209-12,795)	9,150 (7,713-11,188)
Congenital birth defects	127,076 (109,336-170,126)	60,309 (49,080-84,358)	162,765 (143,385-213,329)	57,130 (45,963-87,052)	12,857 (11,180-16,650)	6,442 (5,740-8,152)	4,389 (4,011-5,423)
Neural tube defects	18,230 (12,563-30,063)	6,582 (3,855-11,969)	14,953 (8,332-27,493)	7,795 (3,606-20,363)	815 (541-1,289)	406 (299-539)	189 (154-234)
Congenital heart anomalies	44,467 (28,818-63,370)	27,781 (16,729-37,942)	84,183 (53,747-111,463)	26,429 (13,557-39,825)	7,853 (5,427-10,833)	4,175 (3,351-5,564)	2,841 (2,531-3,577)
Orofacial clefts	3,212 (1,270-7,908)	1,450 (459-4,025)	2,638 (816-7,121)	509 (104-1,989)	-	-	-
Down syndrome	2,640 (1,133-4,614)	1,480 (507-2,738)	6,581 (2,215-10,910)	3,239 (874-5,832)	470 (221-796)	206 (131-295)	276 (170-436)
Other chromosomal abnormalities	1,804 (982-4,073)	850 (380-2,066)	1,731 (834-3,907)	438 (186-1,153)	85 (49-168)	51 (31-91)	56 (35-106)
Congenital musculoskeletal and limb anomalies	2,331 (1,356-3,770)	692 (353-1,087)	1,746 (808-2,875)	575 (230-1,117)	222 (121-360)	132 (89-185)	150 (104-197)
Urogenital congenital anomalies	3,335 (2,138-5,254)	1,120 (607-1,666)	1,897 (1,022-2,925)	530 (229-1,018)	174 (108-286)	113 (81-159)	53 (43-73)
Digestive congenital anomalies	14,598 (9,099-26,413)	8,280 (4,959-17,226)	17,954 (9,983-37,074)	4,186 (1,633-12,862)	352 (201-633)	115 (78-172)	48 (39-61)
Other congenital birth defects	36,457 (22,098-49,455)	12,074 (5,300-19,158)	31,082 (12,947-48,213)	13,428 (4,840-23,304)	2,886 (1,686-4,355)	1,244 (860-1,663)	779 (655-981)
Urinary diseases and male infertility	652 (528-797)	731 (533-907)	4,605 (3,991-5,198)	2,708 (2,139-3,229)	1,138 (920-1,361)	630 (537-716)	966 (822-1,094)
Urinary tract infections	118 (70-166)	208 (106-312)	3,017 (2,236-3,746)	1,098 (635-1,585)	572 (391-789)	364 (286-445)	628 (522-754)
Urolithiasis	-	-	-	-	59 (23-92)	41 (17-61)	36 (18-50)
Other urinary diseases	534 (399-662)	523 (311-697)	1,588 (773-2,407)	1,610 (930-2,295)	507 (327-716)	226 (152-302)	302 (193-419)
Gynecological diseases	-	-	-	-	-	-	-
Uterine fibroids	-	-	-	-	-	-	-
Polycystic ovarian syndrome	-	-	-	-	-	-	-

Cause	20 to 24	25 to 29	30 to 34	35 to 39	40 to 44	45 to 49	50 to 54	54 to 59
Multiple myeloma	131 (81-170)	145 (94-184)	239 (183-293)	393 (305-472)	706 (560-859)	941 (777-1,139)	1,659 (1,375-1,985)	2,530 (2,066-3,078)
Leukemia	6,302 (4,991-7,008)	5,308 (4,399-5,854)	4,879 (3,980-5,372)	5,051 (4,206-5,513)	5,202 (4,325-5,705)	5,293 (4,535-5,795)	6,698 (5,822-7,228)	8,464 (7,482-9,284)
Acute lymphoid leukemia	1,350 (1,139-1,630)	954 (789-1,178)	797 (655-978)	776 (653-936)	688 (586-818)	638 (556-759)	782 (684-909)	891 (778-1,025)
Chronic lymphoid leukemia	116 (83-163)	95 (71-131)	96 (72-131)	106 (84-135)	160 (129-197)	248 (212-284)	500 (444-550)	823 (744-889)
Acute myeloid leukemia	974 (814-1,139)	944 (804-1,106)	972 (832-1,123)	1,046 (895-1,178)	1,147 (982-1,288)	1,295 (1,125-1,459)	1,657 (1,460-1,815)	2,107 (1,863-2,315)
Chronic myeloid leukemia	334 (276-387)	490 (414-560)	569 (489-638)	643 (559-715)	669 (600-739)	705 (632-784)	875 (795-972)	1,054 (964-1,164)
Other leukemia	3,528 (2,456-4,100)	2,824 (2,052-3,264)	2,445 (1,774-2,831)	2,480 (1,857-2,810)	2,538 (1,894-2,887)	2,406 (1,889-2,706)	2,883 (2,323-3,196)	3,589 (3,003-4,114)
Other malignant neoplasms	3,493 (3,168-3,856)	2,855 (2,604-3,098)	2,859 (2,580-3,095)	3,620 (3,258-3,885)	4,590 (4,074-4,910)	5,496 (4,912-5,893)	8,056 (7,095-8,626)	10,279 (8,886-11,083)
Other neoplasms	311 (210-402)	357 (240-473)	452 (304-598)	536 (356-670)	650 (426-846)	722 (466-944)	1,005 (642-1,377)	1,336 (845-1,774)
Myelodysplastic, myeloproliferative, and other hematopoietic neoplasms	229 (149-305)	281 (184-375)	374 (243-501)	468 (307-598)	596 (384-776)	685 (442-893)	976 (620-1,341)	1,315 (832-1,731)
Other benign and in situ neoplasms	82 (53-111)	76 (46-104)	78 (46-110)	68 (39-95)	54 (30-76)	38 (21-54)	29 (16-42)	21 (11-30)
Cardiovascular diseases	30,753 (28,741-33,033)	42,080 (39,715-44,759)	64,285 (61,102-67,733)	101,739 (97,622-106,183)	154,736 (149,047-160,362)	216,157 (209,211-224,223)	345,752 (337,088-355,490)	468,918 (458,486-480,528)
Rheumatic heart disease	4,999 (4,292-6,196)	4,454 (3,862-5,522)	4,858 (4,192-6,062)	5,950 (5,169-7,460)	6,981 (6,090-8,727)	7,374 (6,467-9,217)	9,589 (8,464-11,855)	11,089 (9,931-13,491)
Ischemic heart disease	11,231 (10,396-12,069)	18,004 (16,816-19,209)	31,849 (29,991-33,727)	54,053 (51,494-56,767)	84,331 (80,902-88,055)	122,073 (117,504-127,182)	193,859 (188,129-200,285)	260,387 (253,383-268,426)
Stroke	9,430 (8,709-10,090)	12,521 (11,651-13,339)	18,386 (17,336-19,576)	28,696 (27,116-30,331)	45,662 (43,300-47,931)	64,714 (61,700-67,926)	109,242 (104,929-113,735)	152,855 (148,011-158,247)
Ischemic stroke	1,037 (921-1,184)	1,413 (1,261-1,596)	2,184 (1,948-2,464)	3,495 (3,146-3,933)	6,115 (5,491-6,842)	11,065 (9,982-12,335)	22,663 (20,622-25,014)	36,136 (33,032-39,693)
Intracerebral hemorrhage	5,776 (5,168-6,463)	7,730 (6,999-8,761)	11,902 (10,873-13,132)	18,808 (17,217-20,746)	29,924 (27,376-32,818)	43,559 (40,260-47,624)	71,148 (66,056-77,050)	96,320 (89,593-104,127)
Subarachnoid hemorrhage	2,617 (2,028-3,099)	3,378 (2,691-3,920)	4,300 (3,411-5,052)	6,393 (5,119-7,582)	9,622 (7,646-11,572)	10,090 (7,655-12,381)	15,431 (11,238-19,273)	20,398 (14,178-25,902)
Hypertensive heart disease	765 (528-871)	1,155 (810-1,306)	1,745 (1,305-1,980)	3,022 (2,288-3,420)	4,852 (3,638-5,505)	6,792 (4,981-7,756)	10,964 (8,163-12,453)	15,503 (11,393-17,354)
Non-rheumatic valvular heart disease	417 (333-512)	463 (377-561)	476 (392-569)	658 (554-783)	835 (718-991)	886 (768-1,026)	1,322 (1,168-1,515)	1,896 (1,689-2,151)
Non-rheumatic calcific aortic valve disease	131 (99-166)	175 (135-220)	200 (156-242)	300 (243-361)	405 (322-477)	458 (372-531)	701 (589-809)	1,027 (857-1,159)
Non-rheumatic degenerative mitral valve disease	210 (159-271)	213 (161-276)	228 (174-295)	291 (225-369)	350 (279-440)	392 (324-480)	561 (465-690)	792 (655-962)
Other non-rheumatic valve diseases	76 (43-105)	75 (45-107)	48 (28-71)	66 (37-90)	80 (44-115)	35 (20-50)	59 (32-83)	77 (41-106)
Cardiomyopathy and myocarditis	1,806 (1,568-2,037)	2,559 (2,236-2,955)	3,415 (3,010-3,928)	4,758 (4,159-5,544)	5,958 (5,156-6,807)	6,704 (5,744-7,667)	9,322 (8,006-10,715)	10,825 (9,335-12,282)
Myocarditis	536 (387-639)	595 (441-710)	460 (343-555)	560 (419-682)	669 (471-844)	533 (375-684)	543 (375-710)	698 (470-923)
Alcoholic cardiomyopathy	287 (217-348)	704 (537-859)	1,396 (1,096-1,742)	2,297 (1,864-2,839)	2,872 (2,334-3,472)	3,426 (2,788-4,034)	4,971 (4,097-6,127)	4,990 (4,170-6,010)
Other cardiomyopathy	983 (822-1,129)	1,259 (1,054-1,492)	1,560 (1,339-1,793)	1,901 (1,628-2,230)	2,417 (2,024-2,851)	2,745 (2,253-3,288)	3,808 (3,092-4,524)	5,137 (3,953-6,114)
Atrial fibrillation and flutter	-	-	29 (24-31)	43 (36-46)	183 (167-210)	279 (253-322)	551 (499-651)	1,082 (993-1,279)
Aortic aneurysm	273 (236-333)	454 (383-550)	619 (531-745)	954 (845-1,140)	1,287 (1,166-1,509)	1,739 (1,585-2,046)	2,778 (2,558-3,206)	4,466 (4,124-5,090)
Peripheral artery disease	-	-	-	-	73 (44-105)	123 (70-186)	303 (152-479)	554 (279-925)
Endocarditis	434 (377-496)	598 (503-728)	637 (548-762)	725 (629-867)	791 (696-960)	762 (671-926)	930 (820-1,154)	1,067 (952-1,299)
Other cardiovascular and circulatory diseases	1,397 (1,193-1,702)	1,872 (1,635-2,239)	2,270 (2,013-2,708)	2,880 (2,528-3,488)	3,784 (3,303-4,688)	4,713 (4,106-5,930)	6,892 (6,033-8,581)	9,194 (8,101-11,335)
Chronic respiratory diseases	7,973 (7,326-8,621)	9,564 (8,821-10,466)	11,928 (11,098-12,886)	17,279 (16,084-18,547)	25,862 (24,348-27,528)	39,067 (36,926-41,383)	72,086 (69,044-75,583)	116,244 (111,305-121,262)
Chronic obstructive pulmonary disease	2,853 (2,400-4,068)	3,575 (3,035-4,980)	5,220 (4,542-7,016)	7,789 (6,567-10,731)	13,587 (11,587-17,901)	22,422 (19,017-29,073)	45,959 (39,648-56,791)	79,301 (69,030-95,447)
Pneumoconiosis	52 (37-66)	127 (95-160)	250 (189-314)	493 (369-612)	621 (460-782)	671 (495-842)	985 (726-1,253)	1,376 (1,067-1,732)
Silicosis	23 (16-34)	68 (47-100)	173 (126-230)	357 (260-469)	437 (304-591)	441 (294-602)	629 (453-851)	843 (631-1,127)
Asbestosis	4 (1-6)	6 (2-12)	10 (3-19)	13 (5-23)	18 (7-35)	21 (10-38)	48 (25-79)	88 (47-139)
Coal workers pneumoconiosis	8 (4-13)	26 (11-41)	29 (13-51)	61 (24-107)	100 (42-185)	119 (54-227)	169 (87-295)	264 (163-411)
Other pneumoconiosis	17 (9-26)	27 (15-46)	38 (22-66)	63 (32-108)	67 (35-106)	90 (47-143)	139 (77-210)	181 (101-275)
Asthma	4,224 (2,519-5,006)	5,031 (3,053-5,972)	5,335 (2,897-6,419)	7,568 (3,872-9,217)	9,690 (4,585-12,270)	13,353 (5,514-17,575)	20,962 (8,377-28,664)	29,239 (11,394-40,721)
Interstitial lung disease and pulmonary sarcoidosis	117 (79-221)	171 (117-302)	241 (168-447)	386 (263-734)	552 (370-1,058)	804 (537-1,603)	1,467 (948-2,791)	2,378 (1,507-4,560)
Other chronic respiratory diseases	727 (476-1,064)	660 (439-968)	881 (595-1,289)	1,043 (692-1,517)	1,413 (946-2,026)	1,818 (1,235-2,646)	2,712 (1,960-3,668)	3,951 (2,881-5,246)
Digestive diseases	15,594 (13,151-17,548)	25,033 (21,773-27,393)	36,551 (32,545-39,606)	54,095 (48,524-58,282)	69,835 (61,989-75,142)	79,894 (72,447-85,664)	94,527 (85,110-100,747)	103,547 (93,414-110,634)
Cirrhosis and other chronic liver diseases	8,153 (6,704-9,273)	14,699 (12,444-16,149)	24,254 (21,532-26,335)	37,528 (33,382-40,385)	49,690 (44,197-53,208)	56,456 (50,792-60,259)	65,576 (58,975-70,032)	69,071 (62,002-73,866)
Cirrhosis and other chronic liver diseases due to hepatitis B	3,828 (3,040-4,559)	7,836 (6,344-9,103)	11,679 (10,062-13,260)	15,608 (13,119-17,967)	18,726 (15,485-21,463)	18,959 (15,982-22,030)	20,535 (17,056-24,501)	21,427 (17,238-25,628)
Cirrhosis and other chronic liver diseases due to hepatitis C	1,397 (1,047-1,775)	2,937 (2,208-3,743)	5,532 (4,539-6,561)	9,302 (7,478-11,547)	12,842 (10,502-15,437)	15,097 (12,458-18,104)	17,658 (14,322-21,101)	18,055 (15,224-21,326)
Cirrhosis and other chronic liver diseases due to alcohol use	688 (517-888)	1,841 (1,432-2,348)	4,557 (3,916-5,276)	9,283 (7,827-10,953)	13,628 (11,667-15,691)	16,617 (14,141-19,348)	20,010 (16,831-23,214)	21,245 (17,806-24,850)
Cirrhosis due to NASH	210 (164-262)	438 (333-559)	910 (757-1,062)	1,687 (1,330-2,070)	2,633 (2,187-3,099)	3,644 (2,974-4,332)	4,654 (3,825-5,612)	4,910 (4,173-5,690)
Cirrhosis and other chronic liver diseases due to other causes	2,029 (1,530-2,601)	1,648 (1,197-2,201)	1,575 (1,134-2,076)	1,648 (1,221-2,150)	1,862 (1,381-2,455)	2,140 (1,576-2,835)	2,719 (1,971-3,630)	3,433 (2,319-4,906)
Upper digestive system diseases	2,903 (2,438-3,351)	4,091 (3,432-4,694)	5,344 (4,676-6,078)	7,381 (6,481-8,432)	9,647 (8,454-10,793)	11,911 (10,646-13,321)	15,481 (13,837-17,036)	17,986 (15,936-19,631)
Peptic ulcer disease	2,505 (2,118-2,881)	3,479 (2,979-3,977)	4,479 (3,904-5,048)	6,362 (5,605-7,263)	8,513 (7,497-9,551)	10,544 (9,461-11,836)	13,805 (12,452-15,275)	15,990 (14,168-17,520)
Gastritis and duodenitis	398 (256-527)	612 (395-784)	865 (619-1,088)	1,019 (766-1,249)	1,133 (893-1,363)	1,368 (1,085-1,645)	1,676 (1,322-2,032)	1,995 (1,580-2,444)
Appendicitis	1,035 (849-1,224)	1,156 (939-1,395)	744 (626-877)	978 (816-1,210)	1,176 (948-1,456)	1,004 (835-1,216)	1,079 (914-1,274)	1,208 (1,039-1,414)
Paralytic ileus and intestinal obstruction	1,540 (982-1,910)	1,890 (1,240-2,317)	1,984 (1,254-2,425)	2,584 (1,703-3,150)	2,941 (1,991-3,567)	3,352 (2,127-4,043)	3,672 (2,612-4,417)	4,800 (3,386-5,832)
Inguinal, femoral, and abdominal hernia	309 (241-390)	384 (307-466)	394 (297-488)	530 (429-668)	598 (491-766)	696 (567-894)	1,008 (854-1,238)	1,330 (1,142-1,619)
Inflammatory bowel disease	206 (158-246)	280 (223-334)	319 (261-379)	357 (297-414)	427 (362-487)	510 (435-575)	618 (535-688)	762 (661-875)
Vascular intestinal disorders	95 (74-111)	144 (114-165)	149 (124-166)	263 (221-297)	416 (359-466)	621 (536-689)	1,061 (954-1,171)	1,625 (1,464-1,769)
Gallbladder and biliary diseases	296 (248-336)	349 (297-389)	483 (414-539)	682 (595-761)	866 (746-954)	994 (880-1,089)	1,430 (1,254-1,557)	1,878 (1,655-2,043)
Pancreatitis	665 (543-866)	1,482 (1,246-1,838)	2,181 (1,911-2,609)	2,909 (2,583-3,428)	3,086 (2,696-3,600)	3,136 (2,698-3,636)	3,111 (2,804-3,577)	3,145 (2,737-3,613)
Other digestive diseases	390 (316-516)	558 (435-764)	699 (561-934)	882 (706-1,194)	987 (817-1,291)	1,214 (992-1,627)	1,492 (1,236-1,958)	1,742 (1,484-2,236)
Neurological disorders	7,622 (6,377-8,390)	6,397 (5,556-7,143)	5,610 (4,885-6,236)	5,652 (4,976-6,384)	5,805 (5,157-6,299)	6,164 (5,591-6,658)	7,526 (7,127-7,972)	10,725 (10,231-11,248)
Alzheimer's disease and other dementias	-	-	-	-	330 (308-356)	693 (653-747)	1,898 (1,796-2,052)	4,089 (3,898-4,393)
Parkinson's disease	8 (6-9)	11 (9-12)	25 (20-28)	27 (23-31)	270 (245-318)	511 (462-612)	944 (858-1,130)	1,462 (1,342-1,772)
Epilepsy	6,211 (4,908-6,988)	5,488 (4,631-6,197)	4,749 (4,012-5,321)	4,554 (3,878-5,324)	3,829 (3,242-4,339)	3,260 (2,641-3,754)	2,572 (2,173-2,875)	2,508 (2,111-2,775)
Multiple sclerosis	50 (41-60)	118 (86-144)	226 (170-270)	372 (285-446)	509 (400-601)	565 (455-664)	637 (530-743)	659 (558-788)
Motor neuron disease	64 (56-105)	50 (45-76)	82 (74-116)	156 (143-217)	254 (234-330)	403 (366-562)	574 (530-719)	908 (836-1,162)
Other neurological disorders	1,288 (1,150-1,516)	729 (642-856)	528 (469-608)	544 (501-613)	614 (569-696)	732 (669-835)	900 (823-1,031)	1,099 (1,007-1,256)
Mental disorders	4 (3-5)	3 (3-4)	3 (3-4)	3 (3-3)	3 (3-4)	3 (3-4)	-	-
Eating disorders	4 (3-5)	3 (3-4)	3 (3-4)	3 (3-3)	3 (3-4)	3 (3-4)	-	-
Anorexia nervosa	2 (2-3)	1 (1-2)	1 (1-2)	2 (1-2)	2 (2-3)	2 (2-3)	-	-
Bulimia nervosa	2 (2-3)	2 (2-3)	2 (2-2)	1 (1-2)	1 (1-2)	1 (1-1)	-	-
Substance use disorders	7,668 (7,175-8,319)	11,842 (11,213-12,561)	15,384 (14,695-16,290)	16,509 (15,822-17,482)	16,348 (15,584-17,350)	14,974 (14,064-16,067)	15,339 (14,560-16,092)	12,881 (12,102-13,663)
Alcohol use disorders	2,296 (2,023-2,535)	4,437 (3,960-4,870)	7,909 (7,169-8,600)	10,342 (9,708-11,160)	11,557 (10,817-12,399)	12,015 (11,135-13,081)	12,656 (11,911-13,429)	10,619 (9,845-11,416)
Drug use disorders	5,372 (4,983-5,868)	7,405 (6,907-8,068)	7,475 (7,045-8,038)	6,167 (5,817-6,591)	4,790 (4,505-5,134)	2,959 (2,775-3,187)	2,682 (2,546-2,859)	2,262 (2,133-2,407)
Opioid use disorders	3,141 (2,563-4,008)	4,524 (3,813-5,430)	4,505 (3,803-5,479)	3,650 (3,103-4,313)	2,717 (2,236-3,275)	1,533 (1,204-1,873)	1,351 (1,060-1,632)	1,118 (853-1,350)
Cocaine use disorders	128 (75-261)	185 (125-305)	291 (214-455)	256 (186-403)	190 (134-323)	129 (84-233)	136 (96-226)	105 (70-192)
Amphetamine use disorders	318 (109-631)	398 (178-774)	327 (154-580)	295 (144-520)	239 (103-423)	166 (71-304)	97 (56-164)	78 (40-143)
Other drug use disorders	1,785 (1,315-2,547)	2,298 (1,751-3,214)	2,352 (1,852-3,204)	1,966 (1,591-2,635)	1,644 (1,326-2,228)	1,131 (909-1,557)	1,098 (907-1,438)	961 (767-1,283)
Diabetes and kidney diseases	9,302 (8,386-10,238)	10,458 (9,583-11,352)	13,268 (12,367-14,373)	16,669 (15,579-17,985)	22,134 (20,831-23,765)	27,345 (25,833-29,389)	39,457 (37,469-42,101)	51,533 (49,032-55,410)
Diabetes mellitus	1,663 (1,511-1,801)	2,229 (2,102-2,365)	3,767 (3,551-3,970)	5,153 (4,907-5,403)	8,251 (7,819-8,672)	12,187 (11,562-12,807)	19,170 (18,189-20,111)	26,908 (25,642-28,119)
Diabetes mellitus type 1	1,502 (1,348-1,642)	1,982 (1,850-2,120)	3,059 (2,836-3,274)	4,110 (3,843-4,392)	6,470 (5,956-6,942)	5,899 (5,066-6,906)	8,928 (7,648-10,546)	12,321 (10,553-14,733)
Diabetes mellitus type 2	162 (125-199)	247 (210-292)	708 (610-818)	1,043 (891-1,190)	1,781 (1,454-2,067)	6,288 (5,320-7,020)	10,242 (8,654-11,239)	14,588 (12,369-16,168)
Chronic kidney disease	7,301 (6,420-8,140)	7,861 (7,071-8,668)	9,114 (8,282-10,076)	11,089 (10,130-12,200)	13,469 (12,426-14,916)	14,697 (13,532-16,319)	19,770 (18,458-22,079)	24,008 (22,321-27,520)
Chronic kidney disease due to diabetes mellitus type 1	316 (167-518)	751 (449-1,157)	1,525 (976-2,234)	2,388 (1,544-3,403)	3,088 (1,990-4,378)	3,101 (1,986-4,534)	3,114 (1,830-4,567)	2,569 (1,397-4,071)
Chronic kidney disease due to diabetes mellitus type 2	111 (47-212)	206 (90-389)	395 (181-718)	748 (388-1,292)	1,429 (796-2,331)	2,495 (1,524-3,632)	4,623 (3,144-6,619)	6,861 (4,711-9,086)
Chronic kidney disease due to hypertension	455 (281-665)	668 (444-950)	946 (644-1,334)	1,346 (928-1,860)	1,881 (1,360-2,510)	2,331 (1,648-3,111)	3,657 (2,690-4,856)	5,125 (3,867-6,636)
Chronic kidney disease due to glomerulonephritis	2,839 (1,908-3,851)	2,833 (1,952-3,768)	2,764 (1,916-3,799)	2,923 (1,969-4,133)	3,216 (2,066-4,532)	3,110 (2,024-4,525)	3,808 (2,518-5,416)	3,888 (2,570-5,746)
Chronic kidney disease due to other and unspecified causes	3,581 (2,548-4,635)	3,403 (2,425-4,382)	3,485 (2,420-4,576)	3,685 (2,525-4,999)	3,855 (2,559-5,301)	3,661 (2,422-5,250)	4,569 (2,956-6,471)	5,564 (3,635-7,978)
Acute glomerulonephritis	338 (277-415)	367 (304-439)	387 (323-459)	428 (356-506)	414 (342-491)	460 (385-568)	516 (431-623)	616 (510-765)
Skin and subcutaneous diseases	399 (172-523)	379 (186-479)	630 (309-817)	636 (326-821)	702 (361-947)	885 (468-1,230)	1,038 (537-1,511)	1,354 (697-1,865)
Bacterial skin diseases	328 (138-437)	304 (143-392)	532 (237-697)	526 (250-675)	573 (291-758)	750 (387-1,035)	874 (431-1,214)	1,142 (573-1,524)
Cellulitis	69 (29-93)	64 (29-94)	98 (47-149)	108 (49-182)	115 (53-206)	138 (62-240)	172 (72-332)	203 (86-359)
Pyoderma	258 (105-352)	240 (109-321)	434 (180-581)	417 (184-548)	457 (213-596)	612 (291-850)	701 (329-955)	939 (440-1,251)
Decubitus ulcer	43 (21-74)	51 (25-85)	72 (39-131)	85 (43-150)	99 (50-184)	104 (52-189)	132 (66-242)	173 (83-316)
Other skin and subcutaneous diseases	28 (15-47)	23 (13-39)	26 (14-46)	26 (14-45)	30 (16-55)	30 (16-56)	32 (16-60)	39 (19-78)
Musculoskeletal disorders	560 (443-629)	605 (483-674)	622 (504-696)	739 (596-830)	827 (686-901)	914 (745-994)	946 (806-1,006)	1,386 (1,159-1,485)
Rheumatoid arthritis	65 (48-88)	69 (50-90)	80 (60-104)	113 (87-137)	168 (132-199)	223 (174-260)	364 (299-404)	643 (535-717)
Other musculoskeletal disorders	495 (384-562)	536 (423-601)	542 (437-616)	626 (505-714)	658 (542-734)	690 (554-767)	583 (488-641)	743 (599-835)
Other non-communicable diseases	8,683 (7,399-10,797)	7,227 (6,290-8,562)	6,216 (5,442-7,182)	6,485 (5,697-7,542)	6,867 (6,111-7,878)	6,125 (5,443-6,874)	7,617 (6,679-8,610)	8,533 (7,563-9,604)
Congenital birth defects	3,808 (3,496-4,732)	3,066 (2,824-3,715)	2,097 (1,939-2,510)	2,161 (1,973-2,643)	2,044 (1,868-2,535)	802 (730-980)	775 (707-988)	774 (706-1,031)
Neural tube defects	163 (135-198)	120 (98-145)	77 (64-93)	58 (50-70)	57 (46-71)	36 (30-44)	28 (23-35)	28 (23-34)
Congenital heart anomalies	2,472 (2,212-3,147)	2,161 (1,947-2,681)	1,406 (1,261-1,705)	1,512 (1,348-1,899)	1,358 (1,206-1,719)	412 (363-536)	369 (327-479)	360 (317-495)
Orofacial clefts	-	-	-	-	-	-	-	-
Down syndrome	235 (155-358)	220 (141-333)	210 (133-311)	197 (139-280)	225 (156-327)	159 (114-216)	168 (128-234)	158 (121-252)
Other chromosomal abnormalities	55 (34-102)	46 (30-83)	29 (21-52)	35 (22-59)	23 (15-43)	17 (12-33)	18 (13-36)	18 (13-32)
Congenital musculoskeletal and limb anomalies	97 (67-132)	56 (41-74)	41 (30-55)	38 (28-50)	31 (22-43)	18 (15-24)	19 (15-24)	20 (14-26)
Urogenital congenital anomalies	61 (51-81)	49 (41-64)	45 (34-57)	56 (43-75)	70 (55-94)	42 (33-53)	55 (42-71)	74 (57-100)
Digestive congenital anomalies	38 (31-48)	35 (27-44)	22 (18-27)	29 (23-36)	27 (21-35)	17 (14-22)	20 (17-25)	26 (21-33)
Other congenital birth defects	687 (574-862)	379 (315-459)	267 (223-339)	235 (191-295)	251 (206-309)	101 (85-131)	97 (81-123)	90 (78-116)
Urinary diseases and male infertility	1,297 (1,149-1,434)	1,194 (1,086-1,293)	1,436 (1,323-1,547)	1,661 (1,533-1,796)	2,163 (1,993-2,324)	2,648 (2,450-2,867)	3,468 (3,213-3,742)	3,933 (3,677-4,206)
Urinary tract infections	824 (694-982)	754 (639-888)	811 (681-966)	898 (742-1,070)	1,167 (949-1,386)	1,318 (1,093-1,564)	1,820 (1,527-2,108)	2,092 (1,782-2,439)
Urolithiasis	62 (30-87)	87 (43-123)	106 (55-144)	143 (79-186)	196 (116-250)	242 (150-299)	362 (237-433)	423 (291-515)
Other urinary diseases	411 (283-560)	353 (262-460)	519 (398-654)	620 (479-797)	800 (593-1,016)	1,087 (841-1,369)	1,287 (960-1,651)	1,419 (1,077-1,801)
Gynecological diseases	-	-	-	-	-	-	-	-
Uterine fibroids	-	-	-	-	-	-	-	-
Polycystic ovarian syndrome	-	-	-	-	-	-	-	-

Cause	60 to 64	65 to 69	70 to 74	75 to 79	80 to 84	85 to 89	90 to 94	95 plus
Multiple myeloma	3,502 (2,916-4,278)	3,954 (3,358-5,027)	3,694 (3,097-4,659)	3,508 (2,957-4,554)	2,483 (2,127-3,293)	1,185 (976-1,548)	283 (235-371)	53 (44-73)
Leukemia	10,825 (9,852-11,786)	11,486 (10,634-12,563)	10,528 (9,754-11,448)	10,182 (9,574-11,012)	7,360 (6,947-7,924)	3,632 (3,427-3,911)	1,095 (1,045-1,163)	202 (193-211)
Acute lymphoid leukemia	983 (867-1,132)	943 (844-1,075)	758 (675-880)	451 (411-505)	281 (258-318)	121 (110-134)	33 (31-36)	5 (5-6)
Chronic lymphoid leukemia	1,377 (1,272-1,477)	1,665 (1,543-1,772)	1,707 (1,568-1,819)	1,911 (1,795-2,024)	1,555 (1,464-1,644)	858 (812-905)	281 (268-295)	60 (57-63)
Acute myeloid leukemia	2,641 (2,365-2,888)	2,751 (2,520-2,989)	2,505 (2,286-2,711)	2,173 (2,003-2,317)	1,410 (1,304-1,517)	610 (558-653)	160 (147-171)	25 (23-27)
Chronic myeloid leukemia	1,248 (1,144-1,372)	1,354 (1,257-1,498)	1,195 (1,110-1,307)	1,439 (1,329-1,560)	1,075 (997-1,169)	497 (463-535)	152 (141-163)	27 (25-29)
Other leukemia	4,576 (4,035-5,148)	4,773 (4,224-5,518)	4,364 (3,917-5,024)	4,208 (3,855-4,815)	3,038 (2,789-3,442)	1,546 (1,417-1,730)	469 (437-518)	85 (79-91)
Other malignant neoplasms	12,488 (11,274-13,559)	12,370 (11,065-13,796)	10,378 (9,473-11,653)	9,050 (8,288-10,103)	6,055 (5,501-6,789)	2,888 (2,542-3,180)	844 (744-918)	162 (144-177)
Other neoplasms	1,776 (1,136-2,496)	2,056 (1,344-3,017)	2,004 (1,384-3,047)	2,244 (1,503-3,648)	1,768 (1,227-2,954)	980 (704-1,746)	317 (225-572)	71 (51-129)
Myelodysplastic, myeloproliferative, and other hematopoietic neoplasms	1,760 (1,125-2,481)	2,046 (1,336-3,006)	1,998 (1,381-3,040)	2,239 (1,501-3,645)	1,766 (1,225-2,952)	980 (704-1,746)	316 (225-572)	71 (51-129)
Other benign and in situ neoplasms	15 (8-22)	11 (5-16)	6 (3-9)	4 (2-7)	2 (1-4)	1 (0-1)	0 (0-0)	0 (0-0)
Cardiovascular diseases	684,842 (670,478-700,031)	763,600 (747,972-780,209)	796,508 (779,710-813,774)	850,490 (834,815-866,631)	723,710 (711,219-738,503)	427,317 (419,503-437,003)	158,110 (155,203-161,609)	38,912 (38,269-39,747)
Rheumatic heart disease	13,973 (12,550-17,076)	14,320 (13,051-17,083)	14,038 (12,839-16,414)	11,865 (10,984-13,645)	9,570 (8,871-10,859)	4,900 (4,582-5,536)	1,685 (1,580-1,867)	399 (376-430)
Ischemic heart disease	369,874 (360,879-380,341)	398,878 (388,531-411,133)	398,330 (388,368-412,086)	433,893 (424,560-447,344)	374,390 (366,680-386,792)	228,247 (223,153-235,815)	89,387 (87,578-92,207)	23,317 (22,902-23,946)
Stroke	237,604 (230,161-245,673)	276,256 (268,183-286,105)	302,781 (293,128-314,257)	315,679 (306,949-326,035)	259,902 (252,443-268,953)	144,363 (140,118-150,380)	47,727 (46,191-49,746)	10,471 (10,144-10,904)
Ischemic stroke	77,828 (71,823-84,279)	97,830 (90,775-105,223)	127,488 (119,248-136,151)	148,929 (141,253-157,104)	147,312 (141,186-154,228)	86,383 (83,062-90,418)	29,769 (28,663-31,102)	6,818 (6,596-7,099)
Intracerebral hemorrhage	136,665 (126,417-148,109)	153,572 (142,244-166,545)	151,902 (140,681-165,831)	145,984 (136,088-157,684)	98,441 (91,882-106,744)	51,075 (47,554-55,451)	15,963 (14,918-17,156)	3,273 (3,065-3,499)
Subarachnoid hemorrhage	23,111 (14,852-29,867)	24,854 (15,909-32,449)	23,391 (13,277-30,990)	20,766 (12,590-27,072)	14,149 (8,432-18,567)	6,905 (4,396-9,004)	1,995 (1,424-2,573)	380 (296-468)
Hypertensive heart disease	23,750 (16,943-26,463)	28,949 (20,043-32,007)	34,898 (23,577-38,491)	34,165 (24,253-37,171)	29,555 (21,592-32,093)	16,666 (12,357-17,993)	5,936 (4,538-6,405)	1,386 (1,078-1,482)
Non-rheumatic valvular heart disease	2,695 (2,368-3,009)	3,512 (3,064-3,893)	3,939 (3,418-4,316)	4,869 (4,337-5,404)	4,606 (4,113-5,158)	2,912 (2,691-3,425)	1,170 (1,091-1,430)	277 (257-363)
Non-rheumatic calcific aortic valve disease	1,537 (1,273-1,719)	2,100 (1,729-2,332)	2,375 (1,992-2,648)	3,127 (2,692-3,572)	3,054 (2,646-3,496)	2,008 (1,814-2,375)	834 (766-1,014)	198 (181-264)
Non-rheumatic degenerative mitral valve disease	1,048 (858-1,257)	1,264 (1,013-1,501)	1,387 (1,115-1,643)	1,549 (1,214-1,912)	1,364 (1,026-1,641)	802 (602-982)	301 (223-364)	71 (50-86)
Other non-rheumatic valve diseases	110 (58-151)	148 (77-206)	177 (90-241)	193 (103-266)	188 (103-266)	101 (60-140)	34 (21-49)	8 (5-11)
Cardiomyopathy and myocarditis	12,023 (10,437-13,481)	11,069 (8,576-12,273)	9,573 (6,710-10,685)	11,187 (7,284-12,387)	10,592 (5,959-11,909)	7,949 (3,891-9,173)	3,009 (1,509-3,438)	759 (378-864)
Myocarditis	614 (417-801)	613 (420-798)	613 (429-771)	721 (495-910)	729 (499-959)	602 (412-956)	270 (190-444)	77 (52-115)
Alcoholic cardiomyopathy	4,900 (4,141-6,315)	3,506 (2,931-4,197)	2,313 (1,712-2,983)	2,260 (1,645-3,382)	1,547 (996-2,646)	822 (468-1,514)	280 (162-509)	68 (39-117)
Other cardiomyopathy	6,509 (4,898-7,639)	6,950 (4,832-8,056)	6,647 (4,414-7,697)	8,206 (4,874-9,426)	8,316 (4,277-9,637)	6,524 (2,872-7,752)	2,460 (1,090-2,899)	613 (270-720)
Atrial fibrillation and flutter	2,050 (1,885-2,485)	3,054 (2,803-3,658)	4,616 (4,158-5,514)	7,091 (6,474-8,741)	9,700 (8,556-11,701)	7,737 (6,865-9,361)	4,130 (3,669-4,958)	1,150 (1,028-1,378)
Aortic aneurysm	7,544 (7,016-8,539)	10,255 (9,606-11,343)	11,075 (10,423-12,053)	12,089 (11,463-12,884)	9,141 (8,678-9,731)	4,720 (4,490-4,997)	1,498 (1,424-1,581)	281 (268-296)
Peripheral artery disease	1,060 (538-1,785)	1,276 (670-2,134)	1,364 (697-2,328)	1,884 (945-3,142)	1,678 (844-2,803)	1,074 (520-1,789)	477 (231-780)	164 (79-290)
Endocarditis	1,361 (1,221-1,623)	1,555 (1,396-1,847)	1,487 (1,351-1,749)	1,574 (1,452-1,815)	1,275 (1,180-1,461)	743 (697-833)	285 (267-316)	71 (68-79)
Other cardiovascular and circulatory diseases	12,910 (11,507-15,666)	14,477 (12,919-17,286)	14,406 (12,775-17,315)	16,195 (14,554-19,000)	13,302 (11,908-15,625)	8,008 (7,240-9,264)	2,807 (2,551-3,204)	638 (585-723)
Chronic respiratory diseases	207,698 (200,014-216,111)	258,370 (247,490-268,028)	311,111 (298,150-323,215)	302,145 (289,047-312,961)	249,990 (238,934-259,731)	128,417 (122,747-133,481)	38,408 (36,485-40,028)	7,647 (7,294-7,997)
Chronic obstructive pulmonary disease	156,752 (138,979-180,277)	204,023 (183,264-227,820)	257,840 (236,595-280,898)	254,564 (233,705-273,569)	214,280 (198,049-227,547)	111,701 (103,887-118,336)	33,449 (31,347-35,302)	6,650 (6,272-7,022)
Pneumoconiosis	2,197 (1,753-2,694)	2,831 (2,308-3,449)	2,972 (2,426-3,628)	2,929 (2,510-3,538)	2,017 (1,740-2,484)	888 (769-1,096)	226 (200-266)	41 (37-47)
Silicosis	1,303 (988-1,697)	1,684 (1,311-2,133)	1,714 (1,310-2,195)	1,666 (1,341-2,064)	1,104 (892-1,378)	461 (369-575)	105 (88-128)	18 (16-22)
Asbestosis	138 (76-223)	174 (101-263)	204 (128-309)	203 (130-286)	148 (94-206)	78 (52-110)	26 (18-37)	5 (3-6)
Coal workers pneumoconiosis	490 (313-729)	717 (499-1,033)	794 (530-1,163)	823 (617-1,128)	601 (456-828)	274 (212-367)	70 (58-88)	13 (11-15)
Other pneumoconiosis	266 (149-412)	257 (144-394)	260 (143-407)	237 (133-364)	164 (96-250)	75 (46-114)	25 (16-37)	5 (4-7)
Asthma	43,130 (16,237-62,347)	45,320 (17,547-66,387)	43,725 (17,446-65,812)	37,828 (16,977-60,105)	28,330 (13,773-45,295)	13,008 (6,833-20,831)	3,771 (2,136-6,121)	737 (469-1,130)
Interstitial lung disease and pulmonary sarcoidosis	3,788 (2,345-7,007)	4,360 (2,937-7,852)	4,637 (3,239-7,978)	5,073 (3,664-7,694)	3,749 (2,732-5,376)	1,840 (1,393-2,552)	573 (465-783)	118 (98-165)
Other chronic respiratory diseases	1,831 (1,350-2,433)	1,835 (1,396-2,415)	1,937 (1,538-2,535)	1,750 (1,455-2,182)	1,613 (1,348-2,035)	980 (834-1,217)	389 (332-492)	101 (87-123)
Digestive diseases	116,183 (103,683-123,231)	105,725 (93,857-112,074)	88,908 (75,939-94,436)	78,809 (69,916-83,301)	57,839 (51,376-61,064)	31,426 (28,164-33,147)	10,811 (9,738-11,426)	2,447 (2,231-2,577)
Cirrhosis and other chronic liver diseases	72,588 (64,468-77,548)	60,215 (53,097-64,896)	43,818 (37,175-47,802)	34,245 (29,213-37,388)	21,277 (17,918-23,545)	9,694 (8,127-10,725)	2,981 (2,429-3,327)	651 (535-725)
Cirrhosis and other chronic liver diseases due to hepatitis B	23,007 (18,136-27,463)	19,934 (15,943-23,825)	15,291 (11,632-18,366)	11,681 (8,928-14,074)	7,098 (5,199-8,732)	3,029 (2,253-3,688)	868 (617-1,115)	172 (114-235)
Cirrhosis and other chronic liver diseases due to hepatitis C	18,402 (15,179-22,085)	15,241 (12,708-17,879)	11,027 (8,912-13,060)	8,351 (6,686-10,263)	5,151 (4,067-6,447)	2,506 (1,988-3,021)	818 (633-1,015)	184 (136-241)
Cirrhosis and other chronic liver diseases due to alcohol use	21,785 (17,937-25,762)	16,720 (13,927-19,569)	11,059 (8,923-13,160)	8,725 (7,275-10,151)	5,287 (4,347-6,245)	2,299 (1,936-2,642)	671 (547-812)	146 (109-189)
Cirrhosis due to NASH	5,064 (4,202-5,958)	4,018 (3,359-4,661)	2,758 (2,280-3,239)	1,930 (1,557-2,363)	1,164 (917-1,438)	577 (460-697)	191 (142-242)	45 (32-62)
Cirrhosis and other chronic liver diseases due to other causes	4,330 (2,972-6,025)	4,302 (3,013-5,894)	3,682 (2,728-4,858)	3,558 (2,590-4,845)	2,577 (1,881-3,469)	1,284 (992-1,625)	433 (309-572)	103 (67-147)
Upper digestive system diseases	22,104 (19,441-24,029)	22,123 (18,997-23,948)	21,377 (17,855-23,335)	19,584 (17,249-21,210)	14,677 (12,924-15,789)	8,265 (7,408-8,886)	2,779 (2,515-2,978)	642 (585-691)
Peptic ulcer disease	19,910 (17,708-21,709)	19,967 (17,294-21,739)	19,218 (16,170-21,102)	17,611 (15,602-19,177)	13,207 (11,697-14,315)	7,404 (6,690-8,019)	2,480 (2,248-2,677)	566 (516-611)
Gastritis and duodenitis	2,194 (1,724-2,735)	2,156 (1,693-2,691)	2,159 (1,639-2,714)	1,973 (1,543-2,443)	1,470 (1,160-1,860)	861 (696-1,068)	299 (248-367)	77 (66-90)
Appendicitis	1,451 (1,241-1,689)	1,307 (1,122-1,496)	1,400 (1,158-1,663)	1,239 (1,036-1,487)	1,000 (823-1,206)	559 (461-681)	212 (174-271)	44 (36-56)
Paralytic ileus and intestinal obstruction	6,091 (4,446-7,371)	6,799 (5,097-8,341)	6,494 (4,926-7,899)	6,407 (5,183-7,814)	5,710 (4,677-7,044)	3,992 (3,303-4,761)	1,473 (1,192-1,781)	316 (271-395)
Inguinal, femoral, and abdominal hernia	1,785 (1,516-2,142)	2,049 (1,757-2,373)	2,109 (1,728-2,438)	2,431 (2,032-2,851)	2,388 (2,034-2,759)	1,500 (1,316-1,716)	591 (525-678)	141 (125-159)
Inflammatory bowel disease	931 (808-1,052)	1,026 (880-1,171)	1,058 (913-1,189)	1,166 (989-1,329)	1,078 (868-1,237)	660 (521-755)	244 (191-275)	58 (47-65)
Vascular intestinal disorders	2,502 (2,283-2,679)	3,135 (2,848-3,345)	3,386 (3,058-3,633)	3,930 (3,668-4,178)	3,320 (3,072-3,517)	1,851 (1,733-1,957)	652 (606-692)	138 (128-146)
Gallbladder and biliary diseases	2,859 (2,571-3,087)	3,472 (3,071-3,710)	3,800 (3,349-4,068)	4,400 (3,957-4,693)	3,958 (3,586-4,217)	2,329 (2,129-2,467)	919 (842-977)	219 (202-232)
Pancreatitis	3,177 (2,816-3,616)	2,824 (2,451-3,161)	2,337 (2,004-2,649)	2,313 (1,968-2,632)	1,600 (1,377-1,834)	838 (720-962)	258 (221-297)	52 (45-58)
Other digestive diseases	2,696 (2,206-3,743)	2,776 (2,350-3,564)	3,129 (2,640-4,059)	3,094 (2,697-3,761)	2,833 (2,439-3,428)	1,737 (1,550-1,987)	701 (628-795)	185 (167-208)
Neurological disorders	19,053 (18,436-19,929)	30,433 (29,376-31,665)	48,376 (46,544-50,895)	83,896 (80,888-86,871)	102,368 (98,522-105,333)	83,225 (80,337-85,405)	41,424 (40,133-42,435)	11,278 (10,920-11,510)
Alzheimer's disease and other dementias	10,410 (9,943-11,143)	19,019 (18,403-20,158)	32,659 (31,623-34,567)	60,837 (58,901-63,235)	80,266 (77,562-82,614)	70,994 (68,617-73,025)	38,010 (36,920-38,942)	10,706 (10,370-10,943)
Parkinson's disease	3,139 (2,885-3,844)	6,085 (5,597-7,583)	11,608 (10,664-14,060)	19,435 (18,037-23,075)	19,776 (18,362-23,745)	11,080 (10,251-13,128)	3,111 (2,890-3,715)	511 (480-602)
Epilepsy	2,459 (1,986-2,836)	2,214 (1,740-2,833)	1,623 (1,321-1,965)	1,385 (1,183-1,552)	916 (745-1,037)	493 (413-564)	136 (114-157)	30 (26-35)
Multiple sclerosis	646 (555-772)	519 (449-621)	342 (293-425)	259 (221-316)	140 (118-166)	57 (46-63)	13 (10-15)	2 (2-2)
Motor neuron disease	1,194 (1,117-1,448)	1,356 (1,279-1,572)	1,057 (1,010-1,168)	878 (839-931)	470 (447-493)	169 (161-178)	31 (30-33)	5 (4-5)
Other neurological disorders	1,205 (1,119-1,348)	1,240 (1,161-1,364)	1,087 (1,025-1,189)	1,101 (1,042-1,195)	800 (755-856)	433 (406-462)	122 (116-130)	24 (23-26)
Mental disorders	-	-	-	-	-	-	-	-
Eating disorders	-	-	-	-	-	-	-	-
Anorexia nervosa	-	-	-	-	-	-	-	-
Bulimia nervosa	-	-	-	-	-	-	-	-
Substance use disorders	10,695 (10,094-11,372)	6,648 (6,297-7,038)	4,158 (3,921-4,392)	3,105 (2,908-3,293)	1,624 (1,539-1,712)	824 (784-871)	254 (239-272)	56 (53-59)
Alcohol use disorders	8,667 (8,008-9,308)	5,030 (4,678-5,389)	2,771 (2,547-2,987)	1,968 (1,776-2,142)	878 (810-940)	407 (375-437)	104 (94-114)	24 (22-26)
Drug use disorders	2,028 (1,920-2,160)	1,618 (1,519-1,735)	1,387 (1,299-1,492)	1,137 (1,072-1,222)	746 (697-809)	417 (386-455)	149 (139-166)	32 (30-35)
Opioid use disorders	1,035 (781-1,263)	819 (589-1,002)	548 (357-711)	473 (329-593)	308 (215-393)	190 (132-239)	70 (50-87)	15 (12-19)
Cocaine use disorders	88 (60-150)	64 (39-123)	25 (16-47)	21 (14-37)	4 (3-6)	2 (2-4)	1 (1-2)	0 (0-0)
Amphetamine use disorders	63 (36-102)	46 (22-89)	12 (6-19)	10 (5-16)	3 (2-5)	1 (1-2)	1 (0-1)	0 (0-0)
Other drug use disorders	843 (673-1,136)	689 (536-962)	803 (663-1,001)	634 (524-777)	431 (356-519)	224 (183-276)	78 (63-96)	16 (13-19)
Diabetes and kidney diseases	68,572 (65,460-72,984)	72,686 (69,657-77,011)	70,716 (67,653-75,276)	69,066 (66,090-72,351)	52,733 (50,509-55,365)	30,492 (29,088-31,923)	10,915 (10,436-11,398)	2,660 (2,539-2,764)
Diabetes mellitus	36,979 (35,284-38,524)	39,425 (37,733-40,970)	37,285 (35,735-38,822)	34,452 (33,034-35,734)	24,364 (23,241-25,292)	12,364 (11,746-12,895)	4,049 (3,861-4,228)	876 (837-911)
Diabetes mellitus type 1	12,771 (10,728-15,841)	13,207 (11,225-16,257)	10,511 (8,746-13,294)	9,203 (7,747-11,687)	5,048 (4,209-6,675)	2,532 (2,067-3,341)	783 (637-1,035)	156 (129-201)
Diabetes mellitus type 2	24,208 (21,208-26,093)	26,218 (23,306-28,069)	26,773 (24,074-28,562)	25,248 (22,911-26,830)	19,316 (17,733-20,400)	9,832 (9,015-10,431)	3,266 (3,015-3,463)	719 (673-757)
Chronic kidney disease	30,968 (28,976-35,004)	32,608 (30,518-36,614)	32,786 (30,850-36,850)	34,088 (32,196-36,621)	27,992 (26,618-30,087)	17,928 (16,942-19,186)	6,791 (6,494-7,167)	1,758 (1,680-1,837)
Chronic kidney disease due to diabetes mellitus type 1	1,943 (970-3,354)	1,326 (672-2,392)	948 (466-1,691)	740 (343-1,375)	497 (245-883)	207 (103-377)	35 (16-64)	2 (1-4)
Chronic kidney disease due to diabetes mellitus type 2	10,064 (7,122-13,335)	10,762 (7,768-14,178)	10,272 (7,420-13,374)	9,559 (6,503-12,759)	7,131 (4,792-9,886)	3,982 (2,610-5,401)	1,329 (924-1,797)	320 (192-488)
Chronic kidney disease due to hypertension	7,426 (5,566-9,614)	9,045 (6,996-11,409)	10,408 (8,247-13,062)	11,924 (9,457-14,809)	10,740 (8,520-13,131)	7,887 (6,482-9,314)	3,327 (2,808-3,840)	919 (752-1,081)
Chronic kidney disease due to glomerulonephritis	4,348 (2,786-6,439)	4,406 (2,817-6,457)	4,497 (2,812-6,450)	4,834 (3,109-6,997)	3,845 (2,445-5,689)	2,248 (1,491-3,316)	799 (530-1,191)	201 (113-330)
Chronic kidney disease due to other and unspecified causes	7,187 (4,377-10,415)	7,069 (4,532-10,031)	6,662 (4,371-9,307)	7,031 (4,608-10,128)	5,779 (3,935-8,080)	3,605 (2,500-4,955)	1,302 (879-1,791)	316 (203-476)
Acute glomerulonephritis	625 (519-751)	654 (542-778)	646 (529-795)	526 (436-637)	377 (309-456)	200 (163-248)	75 (58-98)	27 (18-38)
Skin and subcutaneous diseases	1,758 (906-2,476)	1,798 (937-2,549)	2,021 (1,026-2,814)	2,324 (1,162-3,253)	1,931 (1,008-2,798)	1,344 (707-1,876)	553 (282-838)	149 (78-242)
Bacterial skin diseases	1,464 (727-2,069)	1,373 (676-1,914)	1,455 (698-2,061)	1,580 (760-2,299)	1,143 (593-1,739)	724 (382-1,109)	293 (160-481)	74 (40-130)
Cellulitis	265 (107-480)	214 (82-405)	231 (99-436)	234 (88-458)	193 (76-383)	116 (42-230)	50 (19-106)	14 (5-31)
Pyoderma	1,200 (566-1,669)	1,158 (549-1,626)	1,224 (572-1,688)	1,346 (620-1,915)	950 (491-1,472)	607 (310-913)	243 (130-391)	60 (34-107)
Decubitus ulcer	242 (108-463)	362 (162-628)	499 (233-832)	657 (274-1,159)	698 (296-1,129)	551 (218-825)	232 (86-365)	68 (28-108)
Other skin and subcutaneous diseases	52 (27-108)	63 (31-119)	67 (33-127)	87 (42-174)	90 (41-176)	69 (30-139)	28 (11-62)	7 (3-17)
Musculoskeletal disorders	2,377 (1,937-2,556)	2,628 (2,219-2,817)	2,844 (2,423-3,053)	2,887 (2,422-3,130)	2,264 (1,947-2,454)	1,691 (1,514-1,836)	590 (522-644)	136 (122-145)
Rheumatoid arthritis	1,199 (948-1,352)	1,396 (1,165-1,557)	1,576 (1,332-1,747)	1,563 (1,308-1,745)	1,066 (901-1,209)	708 (608-808)	202 (166-233)	41 (35-47)
Other musculoskeletal disorders	1,177 (911-1,349)	1,232 (992-1,403)	1,267 (1,008-1,446)	1,324 (1,039-1,538)	1,197 (983-1,364)	983 (838-1,101)	389 (330-435)	94 (83-103)
Other non-communicable diseases	11,301 (10,121-12,470)	12,680 (11,353-14,093)	12,536 (11,001-13,863)	12,677 (11,155-13,822)	11,661 (10,352-12,423)	7,442 (6,504-7,859)	3,032 (2,710-3,186)	842 (770-879)
Congenital birth defects	759 (691-997)	622 (561-800)	-	-	-	-	-	-
Neural tube defects	24 (20-32)	20 (16-24)	-	-	-	-	-	-
Congenital heart anomalies	342 (304-458)	284 (252-379)	-	-	-	-	-	-
Orofacial clefts	-	-	-	-	-	-	-	-
Down syndrome	136 (104-229)	68 (49-113)	-	-	-	-	-	-
Other chromosomal abnormalities	15 (10-30)	11 (8-20)	-	-	-	-	-	-
Congenital musculoskeletal and limb anomalies	26 (18-35)	30 (17-41)	-	-	-	-	-	-
Urogenital congenital anomalies	100 (75-136)	109 (83-146)	-	-	-	-	-	-
Digestive congenital anomalies	28 (24-37)	32 (26-41)	-	-	-	-	-	-
Other congenital birth defects	87 (77-117)	69 (58-91)	-	-	-	-	-	-
Urinary diseases and male infertility	6,016 (5,620-6,417)	7,170 (6,680-7,641)	7,613 (7,093-8,105)	7,614 (7,218-7,979)	7,397 (6,977-7,752)	4,560 (4,327-4,749)	1,930 (1,830-2,008)	563 (542-583)
Urinary tract infections	3,297 (2,783-3,746)	3,906 (3,378-4,471)	3,946 (3,398-4,617)	4,638 (4,168-5,167)	4,383 (3,955-4,923)	3,065 (2,819-3,380)	1,330 (1,231-1,457)	406 (378-449)
Urolithiasis	677 (464-824)	787 (514-957)	774 (512-933)	793 (571-961)	589 (407-727)	304 (197-367)	110 (75-133)	21 (15-25)
Other urinary diseases	2,043 (1,542-2,611)	2,476 (1,844-3,146)	2,893 (2,210-3,613)	2,183 (1,657-2,730)	2,425 (1,863-2,977)	1,191 (882-1,479)	491 (369-604)	136 (98-164)
Gynecological diseases	-	-	-	-	-	-	-	-
Uterine fibroids	-	-	-	-	-	-	-	-
Polycystic ovarian syndrome	-	-	-	-	-	-	-	-

SR Table 6: Global deaths by cause and age group for males (with 95% confidence intervals), 1990

Cause	Early Neonatal	Late Neonatal	Post Neonatal	1 to 4	5 to 9	10 to 14	15 to 19
Endometriosis	-	-	-	-	-	-	-
Genital prolapse	-	-	-	-	-	-	-
Other gynecological diseases	-	-	-	-	-	-	-
Hemoglobinopathies and hemolytic anemias	731 (451-1,050)	644 (415-885)	4,070 (2,290-5,636)	7,411 (3,528-12,089)	3,102 (1,996-4,167)	2,657 (1,780-3,682)	3,012 (1,783-4,409)
Thalassemias	306 (208-384)	294 (157-493)	1,716 (1,036-2,742)	2,112 (1,171-3,711)	1,129 (746-1,664)	657 (472-836)	432 (303-537)
Sickle cell disorders	372 (170-668)	329 (175-504)	2,212 (1,042-3,517)	4,983 (2,131-8,123)	1,656 (950-2,651)	1,475 (825-2,405)	1,809 (847-3,112)
G6PD deficiency	12 (6-21)	11 (6-17)	87 (45-140)	234 (115-410)	224 (147-325)	348 (238-474)	479 (338-635)
Other hemoglobinopathies and hemolytic anemias	42 (28-58)	10 (6-14)	55 (36-83)	83 (50-135)	93 (70-127)	177 (140-224)	293 (216-380)
Endocrine, metabolic, blood, and immune disorders	977 (600-1,184)	1,058 (581-1,456)	5,461 (3,476-8,163)	2,885 (1,573-4,440)	1,220 (863-1,695)	873 (686-1,132)	783 (635-969)
Sudden infant death syndrome	-	4,280 (1,478-8,427)	34,542 (13,733-71,835)	-	-	-	-
Injuries	11,544 (9,702-13,199)	11,470 (9,149-13,842)	85,894 (72,869-97,401)	249,821 (201,173-286,532)	156,652 (140,471-169,774)	107,410 (98,155-114,744)	205,028 (192,543-214,047)
Transport injuries	1,940 (1,686-2,448)	2,353 (1,844-2,871)	13,625 (10,101-15,843)	51,708 (33,536-62,993)	47,117 (40,963-52,767)	30,791 (27,288-33,991)	79,864 (73,674-85,805)
Road injuries	1,605 (1,373-2,094)	2,039 (1,530-2,533)	12,558 (9,192-14,588)	48,730 (31,561-59,534)	44,835 (39,109-50,268)	28,777 (25,489-31,817)	74,684 (68,917-80,120)
Pedestrian road injuries	890 (708-1,313)	1,063 (788-1,515)	6,024 (4,565-7,279)	31,731 (22,493-39,490)	29,337 (25,278-33,878)	15,783 (13,570-18,149)	21,024 (17,418-25,356)
Cyclist road injuries	-	-	-	990 (449-1,704)	1,859 (1,294-2,620)	1,857 (1,508-2,431)	2,634 (2,170-3,182)
Motorcyclist road injuries	87 (67-137)	139 (76-223)	946 (636-1,270)	2,215 (1,107-3,347)	2,140 (1,575-2,869)	2,767 (2,236-3,582)	19,152 (16,835-23,223)
Motor vehicle road injuries	601 (427-798)	808 (465-1,196)	5,368 (3,340-6,840)	13,341 (6,252-19,048)	11,095 (8,188-14,449)	8,033 (6,387-9,649)	31,309 (27,817-35,164)
Other road injuries	27 (13-47)	30 (10-56)	220 (94-374)	452 (145-914)	405 (207-623)	338 (217-475)	566 (380-896)
Other transport injuries	335 (232-495)	314 (185-492)	1,068 (626-1,414)	2,978 (1,420-4,060)	2,282 (1,682-2,790)	2,014 (1,659-2,354)	5,180 (4,514-5,863)
Unintentional injuries	8,092 (6,495-9,402)	8,545 (6,551-10,394)	67,719 (57,461-77,980)	186,426 (155,209-216,055)	101,150 (90,141-109,987)	61,251 (55,739-65,522)	59,695 (55,338-63,138)
Falls	1,033 (641-1,392)	562 (264-939)	6,142 (3,518-8,894)	11,467 (6,085-18,822)	6,898 (4,376-10,234)	5,074 (3,794-6,643)	5,982 (5,023-7,425)
Drowning	482 (428-539)	416 (338-476)	9,796 (8,707-10,974)	112,677 (93,873-129,138)	63,737 (57,353-69,904)	33,484 (30,372-36,429)	24,473 (22,249-26,576)
Fire, heat, and hot substances	359 (212-448)	366 (178-504)	5,266 (2,535-6,918)	12,981 (5,768-18,102)	3,912 (2,262-4,793)	1,910 (1,265-2,253)	2,442 (1,747-2,967)
Poisonings	289 (156-366)	308 (148-432)	3,046 (1,423-4,290)	7,159 (3,269-10,635)	2,518 (1,417-3,576)	1,887 (1,190-2,634)	2,356 (1,844-3,070)
Poisoning by carbon monoxide	67 (36-85)	76 (37-105)	735 (361-1,015)	1,710 (1,014-2,326)	1,180 (677-1,755)	845 (555-1,229)	1,348 (1,077-1,865)
Poisoning by other means	221 (121-283)	231 (113-328)	2,311 (1,058-3,315)	5,449 (2,260-8,219)	1,338 (732-1,974)	1,042 (636-1,453)	1,008 (724-1,295)
Exposure to mechanical forces	932 (803-1,075)	1,309 (939-1,558)	5,255 (4,121-6,115)	10,381 (6,483-12,511)	5,638 (4,870-6,571)	4,101 (3,635-4,608)	7,380 (6,594-8,513)
Unintentional firearm injuries	120 (83-161)	131 (83-233)	936 (645-1,362)	1,489 (854-2,965)	950 (733-1,147)	1,002 (853-1,145)	2,131 (1,842-2,433)
Other exposure to mechanical forces	811 (691-938)	1,178 (840-1,410)	4,318 (3,347-5,103)	8,892 (5,491-10,848)	4,688 (3,975-5,602)	3,099 (2,695-3,558)	5,249 (4,602-6,232)
Adverse effects of medical treatment	875 (529-1,097)	676 (378-959)	5,265 (2,876-7,558)	3,939 (1,694-7,413)	1,550 (950-1,980)	1,055 (722-1,252)	1,294 (895-1,559)
Animal contact	342 (107-469)	433 (105-684)	2,063 (723-2,808)	6,061 (1,934-9,777)	5,610 (2,060-7,994)	3,767 (1,606-4,969)	2,694 (1,245-3,487)
Venomous animal contact	300 (88-419)	383 (86-616)	1,660 (544-2,461)	5,122 (1,501-8,970)	4,867 (1,699-7,389)	3,421 (1,427-4,620)	2,312 (1,018-3,093)
Non-venomous animal contact	42 (19-57)	50 (20-81)	403 (152-632)	938 (362-1,915)	743 (316-1,115)	346 (169-446)	382 (209-517)
Foreign body	3,123 (2,255-3,772)	4,031 (2,798-4,967)	26,578 (21,670-31,574)	11,654 (8,157-14,024)	3,135 (2,830-3,642)	2,144 (1,964-2,347)	1,361 (1,274-1,529)
Pulmonary aspiration and foreign body in airway	3,014 (2,200-3,658)	3,893 (2,709-4,809)	24,167 (19,595-30,131)	9,425 (6,823-12,232)	2,618 (2,321-3,157)	1,913 (1,756-2,135)	1,214 (1,133-1,408)
Foreign body in other body part	110 (42-154)	138 (40-234)	2,411 (617-3,520)	2,229 (708-3,723)	517 (268-663)	232 (146-282)	147 (92-178)
Environmental heat and cold exposure	316 (102-448)	235 (66-427)	2,280 (697-3,460)	1,290 (456-2,465)	660 (326-869)	499 (296-621)	1,152 (784-1,373)
Exposure to forces of nature	28 (14-52)	61 (31-114)	511 (259-946)	2,533 (1,275-4,885)	2,949 (1,459-5,539)	3,504 (1,781-6,683)	3,594 (1,932-6,852)
Other unintentional injuries	313 (257-370)	146 (112-172)	1,517 (1,269-1,690)	6,284 (4,882-7,381)	4,545 (3,768-5,264)	3,825 (3,288-4,317)	6,967 (6,128-7,731)
Self-harm and interpersonal violence	1,512 (1,173-1,683)	572 (447-637)	4,550 (3,357-5,151)	11,687 (9,622-13,064)	8,384 (6,955-9,227)	15,367 (13,121-16,755)	65,470 (58,703-69,515)
Self-harm	-	-	-	-	-	6,510 (4,710-7,632)	28,644 (23,379-31,805)
Self-harm by firearm	-	-	-	-	-	337 (236-524)	3,660 (2,725-4,823)
Self-harm by other specified means	-	-	-	-	-	6,173 (4,356-7,299)	24,985 (20,245-28,303)
Interpersonal violence	1,133 (795-1,305)	371 (246-436)	3,897 (2,708-4,498)	6,340 (4,273-7,711)	4,798 (3,364-5,644)	5,406 (4,253-6,313)	27,263 (23,528-30,274)
Physical violence by firearm	130 (72-190)	48 (27-70)	507 (328-783)	815 (515-1,411)	607 (468-833)	1,371 (1,139-1,669)	12,355 (10,756-13,641)
Physical violence by sharp object	98 (56-137)	36 (18-61)	409 (195-686)	927 (407-1,886)	920 (424-1,466)	1,023 (605-1,361)	6,928 (4,946-8,574)
Physical violence by other means	905 (627-1,060)	287 (184-342)	2,982 (2,031-3,586)	4,598 (3,021-5,965)	3,271 (2,296-4,109)	3,012 (2,263-3,793)	7,981 (6,500-10,322)
Conflict and terrorism	378 (369-389)	201 (196-207)	615 (600-636)	5,303 (5,176-5,464)	3,527 (3,445-3,644)	3,398 (3,315-3,501)	9,245 (9,007-9,517)
Executions and police conflict	-	-	37 (19-54)	44 (25-59)	58 (37-73)	54 (39-65)	317 (186-407)

Cause	20 to 24	25 to 29	30 to 34	35 to 39	40 to 44	45 to 49	50 to 54	54 to 59
Endometriosis	-	-	-	-	-	-	-	-
Genital prolapse	-	-	-	-	-	-	-	-
Other gynecological diseases	-	-	-	-	-	-	-	-
Hemoglobinopathies and hemolytic anemias	2,719 (1,512-4,109)	1,922 (1,154-2,804)	1,457 (914-2,096)	1,384 (837-2,015)	1,322 (836-1,925)	1,249 (755-1,835)	1,612 (951-2,389)	1,843 (1,142-2,670)
Thalassemias	307 (191-399)	148 (92-195)	61 (41-82)	29 (22-38)	16 (12-21)	10 (8-12)	6 (5-7)	3 (2-3)
Sickle cell disorders	1,497 (618-2,777)	928 (379-1,747)	578 (227-1,135)	547 (185-1,082)	399 (134-823)	237 (75-534)	151 (55-354)	59 (27-125)
G6PD deficiency	541 (385-694)	508 (371-627)	517 (372-628)	491 (354-629)	493 (342-683)	573 (351-846)	726 (381-1,179)	671 (336-1,159)
Other hemoglobinopathies and hemolytic anemias	374 (283-467)	337 (260-402)	302 (230-366)	317 (240-382)	414 (314-507)	428 (310-549)	730 (490-988)	1,110 (754-1,506)
Endocrine, metabolic, blood, and immune disorders	859 (676-1,069)	1,045 (799-1,235)	1,226 (961-1,472)	1,280 (1,003-1,556)	1,338 (1,074-1,632)	1,427 (1,198-1,797)	1,761 (1,440-2,204)	1,983 (1,631-2,472)
Sudden infant death syndrome	-	-	-	-	-	-	-	-
Injuries	293,111 (280,693-302,884)	268,867 (259,184-276,975)	234,226 (225,513-241,725)	211,446 (203,677-218,890)	172,660 (165,649-179,089)	145,270 (138,457-151,022)	139,816 (132,914-146,070)	121,497 (114,330-127,121)
Transport injuries	111,814 (104,327-119,287)	99,956 (94,449-105,925)	82,759 (78,191-87,695)	74,699 (70,478-79,496)	59,747 (56,541-63,761)	48,348 (45,782-51,904)	45,553 (43,294-49,466)	39,031 (37,040-42,479)
Road injuries	105,318 (98,133-112,413)	93,488 (88,036-99,296)	77,590 (73,076-82,402)	69,525 (65,531-74,062)	55,360 (52,215-59,135)	44,509 (41,985-47,702)	41,903 (39,685-45,211)	36,032 (34,112-39,114)
Pedestrian road injuries	28,986 (23,852-34,149)	27,678 (23,378-32,338)	26,224 (22,144-29,887)	25,078 (20,424-28,939)	20,828 (17,224-23,973)	18,352 (15,411-21,097)	18,693 (15,854-21,714)	16,536 (14,211-19,247)
Cyclist road injuries	2,389 (1,972-2,924)	2,216 (1,866-2,695)	2,242 (1,817-2,721)	2,163 (1,748-2,607)	2,226 (1,739-2,689)	2,026 (1,649-2,425)	2,134 (1,783-2,503)	1,776 (1,507-2,100)
Motorcyclist road injuries	26,424 (23,389-32,052)	21,982 (19,603-26,644)	15,930 (14,168-19,300)	13,622 (11,786-16,788)	10,408 (9,104-12,761)	7,302 (6,258-9,079)	5,724 (4,954-7,322)	4,467 (3,872-5,755)
Motor vehicle road injuries	46,690 (42,272-51,881)	40,861 (37,548-45,420)	32,637 (29,877-36,714)	28,142 (25,371-31,867)	21,448 (19,605-24,468)	16,343 (14,640-19,053)	14,850 (13,255-17,478)	12,844 (11,499-15,341)
Other road injuries	830 (551-1,239)	751 (496-1,110)	558 (385-813)	520 (355-756)	450 (308-641)	484 (330-679)	502 (336-710)	409 (285-579)
Other transport injuries	6,496 (5,843-7,180)	6,468 (5,816-7,078)	5,168 (4,686-5,627)	5,174 (4,674-5,683)	4,387 (4,011-4,935)	3,840 (3,489-4,274)	3,650 (3,305-4,160)	2,999 (2,709-3,447)
Unintentional injuries	69,385 (65,332-72,702)	62,652 (59,550-65,357)	59,128 (56,441-61,764)	58,320 (55,680-60,755)	51,045 (48,728-53,179)	48,127 (45,451-50,255)	49,074 (46,110-51,349)	45,394 (42,063-47,409)
Falls	9,200 (7,514-11,065)	9,897 (8,214-11,969)	10,411 (8,854-12,352)	11,328 (9,651-13,280)	11,544 (9,721-13,727)	11,433 (10,021-13,593)	12,455 (10,884-14,755)	13,131 (11,266-15,177)
Drowning	21,910 (19,976-23,697)	15,865 (14,662-16,983)	13,466 (12,542-14,298)	11,788 (10,993-12,509)	9,722 (9,093-10,264)	7,860 (7,376-8,293)	7,879 (7,436-8,270)	6,189 (5,860-6,497)
Fire, heat, and hot substances	3,737 (2,728-4,487)	3,931 (2,924-4,635)	3,922 (3,058-4,488)	3,836 (3,057-4,428)	3,248 (2,682-3,704)	2,997 (2,481-3,474)	3,262 (2,786-3,746)	2,969 (2,519-3,423)
Poisonings	3,452 (2,586-4,671)	3,503 (2,833-4,534)	3,319 (2,730-4,172)	3,441 (2,942-4,048)	3,307 (2,820-3,953)	2,768 (2,410-3,268)	3,183 (2,904-3,643)	2,571 (2,303-3,053)
Poisoning by carbon monoxide	2,016 (1,510-2,764)	2,025 (1,647-2,744)	2,093 (1,779-2,633)	2,249 (1,987-2,658)	2,181 (1,929-2,562)	1,788 (1,614-2,082)	2,160 (2,017-2,422)	1,655 (1,541-1,930)
Poisoning by other means	1,436 (1,032-1,953)	1,478 (1,087-1,873)	1,226 (887-1,578)	1,192 (917-1,460)	1,126 (865-1,370)	980 (756-1,198)	1,023 (811-1,252)	917 (744-1,144)
Exposure to mechanical forces	10,510 (9,423-12,319)	9,936 (9,023-11,526)	9,172 (8,414-10,420)	9,135 (8,379-10,497)	6,909 (6,379-8,136)	6,781 (6,220-7,726)	6,425 (5,948-7,202)	5,133 (4,740-5,855)
Unintentional firearm injuries	3,014 (2,456-3,490)	2,658 (2,179-3,081)	1,984 (1,627-2,276)	1,699 (1,385-1,959)	1,269 (1,025-1,478)	1,284 (1,014-1,502)	1,155 (896-1,368)	807 (673-946)
Other exposure to mechanical forces	7,497 (6,553-9,324)	7,278 (6,493-9,033)	7,188 (6,521-8,542)	7,436 (6,736-8,960)	5,640 (5,167-6,898)	5,497 (5,009-6,329)	5,270 (4,834-5,918)	4,326 (3,987-4,925)
Adverse effects of medical treatment	1,957 (1,296-2,352)	1,786 (1,214-2,114)	1,770 (1,243-2,088)	1,891 (1,317-2,214)	1,931 (1,344-2,252)	1,893 (1,304-2,206)	1,920 (1,352-2,206)	2,329 (1,632-2,691)
Animal contact	2,710 (1,201-3,411)	2,577 (1,165-3,196)	2,409 (1,109-2,997)	2,328 (1,074-2,844)	2,338 (1,025-2,888)	3,108 (1,348-3,941)	2,267 (1,066-2,782)	2,695 (1,216-3,352)
Venomous animal contact	2,265 (963-2,925)	2,087 (912-2,658)	2,077 (951-2,687)	1,996 (917-2,547)	2,029 (879-2,605)	2,755 (1,153-3,600)	1,897 (862-2,404)	2,324 (1,023-2,967)
Non-venomous animal contact	446 (219-595)	489 (249-639)	332 (150-445)	332 (157-460)	309 (134-423)	353 (183-481)	369 (195-497)	371 (191-511)
Foreign body	2,024 (1,904-2,295)	2,105 (2,007-2,369)	2,193 (2,104-2,411)	2,309 (2,221-2,498)	2,254 (2,172-2,441)	2,071 (1,995-2,263)	2,456 (2,361-2,579)	2,296 (2,211-2,388)
Pulmonary aspiration and foreign body in airway	1,813 (1,695-2,077)	1,878 (1,781-2,148)	1,887 (1,806-2,096)	1,987 (1,911-2,177)	1,954 (1,879-2,124)	1,892 (1,817-2,063)	2,264 (2,172-2,390)	2,135 (2,058-2,235)
Foreign body in other body part	212 (133-260)	227 (154-267)	306 (245-358)	322 (247-366)	300 (231-347)	179 (132-207)	192 (143-230)	161 (117-188)
Environmental heat and cold exposure	1,780 (1,176-2,083)	2,175 (1,589-2,499)	2,897 (2,196-3,299)	3,335 (2,550-3,807)	3,321 (2,530-3,836)	3,140 (2,398-3,687)	4,176 (3,365-4,860)	3,611 (2,749-4,147)
Exposure to forces of nature	3,335 (1,759-6,540)	2,868 (1,460-5,381)	2,226 (1,137-4,148)	1,790 (940-3,330)	1,428 (801-2,672)	1,147 (641-2,101)	1,178 (618-2,091)	1,142 (591-2,070)
Other unintentional injuries	8,770 (7,856-9,545)	8,010 (7,342-8,597)	7,343 (6,757-7,849)	7,140 (6,618-7,661)	5,042 (4,699-5,338)	4,930 (4,522-5,262)	3,875 (3,591-4,116)	3,328 (3,104-3,537)
Self-harm and interpersonal violence	111,911 (100,745-117,661)	106,260 (95,583-111,812)	92,339 (82,372-97,129)	78,426 (69,751-82,628)	61,868 (55,094-65,312)	48,795 (42,441-51,824)	45,188 (39,905-47,996)	37,072 (32,087-39,372)
Self-harm	50,982 (42,476-55,685)	52,507 (43,386-56,934)	48,249 (40,334-52,036)	44,664 (37,246-48,159)	37,874 (31,435-40,832)	32,055 (26,684-34,777)	31,563 (26,713-33,955)	27,122 (22,876-29,327)
Self-harm by firearm	6,129 (4,638-8,037)	5,549 (4,258-7,155)	4,912 (3,808-6,545)	4,282 (3,356-5,599)	3,709 (2,948-4,826)	3,196 (2,497-4,115)	3,034 (2,374-3,882)	2,778 (2,203-3,518)
Self-harm by other specified means	44,853 (36,396-49,681)	46,959 (38,241-51,532)	43,337 (35,720-47,241)	40,381 (32,557-44,034)	34,165 (27,957-37,249)	28,860 (23,429-31,586)	28,530 (23,923-30,953)	24,344 (20,095-26,566)
Interpersonal violence	44,846 (39,018-49,202)	42,458 (37,546-45,439)	36,215 (31,888-39,002)	28,394 (24,867-30,902)	20,635 (18,006-22,331)	14,596 (12,621-16,009)	12,146 (10,440-13,182)	8,783 (7,533-9,973)
Physical violence by firearm	20,533 (17,601-22,709)	18,689 (16,013-20,430)	14,754 (12,340-16,023)	11,323 (9,138-12,388)	7,765 (6,260-8,706)	5,071 (4,107-5,696)	3,823 (3,015-4,336)	2,711 (2,144-3,192)
Physical violence by sharp object	11,982 (8,809-14,372)	12,280 (9,405-14,924)	10,650 (8,208-12,958)	8,162 (6,375-10,159)	5,863 (4,460-7,100)	4,108 (3,102-5,134)	3,351 (2,536-4,163)	2,229 (1,733-2,723)
Physical violence by other means	12,330 (10,017-15,510)	11,489 (9,648-14,471)	10,811 (8,923-13,427)	8,909 (7,579-11,041)	7,007 (5,971-8,658)	5,418 (4,541-6,604)	4,973 (4,158-5,926)	3,843 (3,125-4,573)
Conflict and terrorism	14,907 (14,538-15,382)	9,900 (9,655-10,192)	6,784 (6,611-6,981)	4,535 (4,418-4,681)	2,885 (2,809-2,975)	1,856 (1,810-1,918)	1,301 (1,266-1,343)	1,069 (1,040-1,101)
Executions and police conflict	1,176 (564-1,513)	1,394 (803-1,776)	1,091 (528-1,354)	833 (424-1,038)	475 (256-588)	288 (148-359)	178 (112-218)	98 (62-125)

Cause	60 to 64	65 to 69	70 to 74	75 to 79	80 to 84	85 to 89	90 to 94	95 plus
Endometriosis	-	-	-	-	-	-	-	-
Genital prolapse	-	-	-	-	-	-	-	-
Other gynecological diseases	-	-	-	-	-	-	-	-
Hemoglobinopathies and hemolytic anemias	2,143 (1,339-3,007)	2,340 (1,469-3,362)	2,404 (1,481-3,418)	2,507 (1,568-3,327)	2,102 (1,353-2,585)	1,405 (895-1,671)	563 (360-656)	137 (90-157)
Thalassemias	1 (1-1)	0 (0-0)	0 (0-0)	0 (0-0)	0 (0-0)	0 (0-0)	0 (0-0)	0 (0-0)
Sickle cell disorders	27 (17-45)	13 (8-19)	5 (3-7)	1 (1-2)	1 (0-1)	0 (0-0)	0 (0-0)	0 (0-0)
G6PD deficiency	581 (279-1,004)	424 (199-762)	269 (122-486)	146 (68-265)	59 (29-98)	23 (12-37)	5 (3-8)	1 (0-1)
Other hemoglobinopathies and hemolytic anemias	1,534 (1,029-2,065)	1,903 (1,235-2,632)	2,130 (1,355-2,951)	2,359 (1,501-3,108)	2,043 (1,315-2,494)	1,382 (882-1,639)	558 (357-648)	136 (89-156)
Endocrine, metabolic, blood, and immune disorders	2,382 (2,046-2,991)	2,549 (2,180-3,176)	2,518 (2,076-3,111)	2,557 (1,901-3,045)	2,161 (1,540-2,514)	1,476 (1,025-1,668)	538 (360-617)	142 (104-164)
Sudden infant death syndrome	-	-	-	-	-	-	-	-
Injuries	110,862 (102,476-116,092)	90,950 (82,800-96,193)	74,330 (66,657-78,815)	65,282 (58,905-69,335)	49,236 (44,082-52,490)	28,263 (25,225-30,039)	9,814 (8,900-10,425)	2,210 (2,040-2,328)
Transport injuries	33,606 (31,796-36,780)	27,234 (25,717-30,116)	20,938 (19,745-23,175)	16,276 (15,392-17,892)	10,199 (9,669-11,189)	4,577 (4,331-4,987)	1,268 (1,201-1,374)	259 (246-275)
Road injuries	31,351 (29,585-34,131)	25,534 (24,070-28,087)	19,733 (18,579-21,767)	15,343 (14,482-16,825)	9,650 (9,158-10,538)	4,342 (4,106-4,716)	1,199 (1,136-1,296)	241 (229-255)
Pedestrian road injuries	16,175 (14,251-18,425)	13,357 (11,793-15,260)	10,585 (9,465-12,156)	8,850 (8,059-10,106)	5,600 (5,114-6,381)	2,608 (2,382-2,983)	688 (636-778)	136 (127-152)
Cyclist road injuries	1,834 (1,514-2,199)	1,554 (1,323-1,834)	1,217 (1,035-1,451)	928 (821-1,057)	566 (511-644)	202 (181-231)	45 (39-53)	0 (0-0)
Motorcyclist road injuries	2,616 (2,201-3,444)	2,004 (1,701-2,644)	1,369 (1,169-1,788)	758 (642-1,022)	471 (394-632)	154 (126-213)	35 (28-48)	8 (6-9)
Motor vehicle road injuries	10,437 (9,302-12,487)	8,327 (7,392-10,079)	6,321 (5,619-7,718)	4,620 (4,135-5,633)	2,881 (2,598-3,464)	1,317 (1,184-1,598)	419 (381-497)	93 (84-104)
Other road injuries	290 (202-409)	291 (196-424)	239 (162-357)	188 (130-278)	133 (91-207)	60 (42-96)	13 (9-22)	4 (3-6)
Other transport injuries	2,255 (2,057-2,597)	1,700 (1,549-2,008)	1,205 (1,092-1,423)	933 (833-1,127)	549 (488-657)	235 (208-286)	69 (61-84)	18 (16-21)
Unintentional injuries	45,245 (41,105-47,640)	39,257 (34,645-41,755)	34,285 (29,593-36,723)	33,460 (29,077-35,825)	28,893 (25,117-31,239)	18,876 (16,354-20,415)	7,315 (6,512-7,878)	1,719 (1,560-1,831)
Falls	14,713 (12,373-17,245)	14,891 (12,281-17,234)	14,775 (11,725-16,946)	16,835 (13,838-18,962)	16,207 (13,343-18,179)	11,432 (9,555-12,766)	4,689 (4,056-5,193)	1,089 (966-1,198)
Drowning	5,903 (5,592-6,203)	4,380 (4,153-4,624)	3,591 (3,381-3,802)	2,495 (2,359-2,640)	1,850 (1,732-1,989)	878 (821-947)	223 (209-240)	44 (42-47)
Fire, heat, and hot substances	3,142 (2,605-3,580)	2,763 (2,247-3,194)	2,613 (2,059-3,066)	2,502 (2,106-2,865)	2,350 (1,960-2,696)	1,557 (1,344-1,752)	586 (520-648)	146 (133-158)
Poisonings	2,432 (2,111-2,907)	1,559 (1,383-2,009)	1,216 (1,067-1,619)	925 (824-1,173)	638 (563-814)	291 (259-364)	77 (68-91)	14 (12-16)
Poisoning by carbon monoxide	1,498 (1,358-1,799)	857 (758-1,129)	666 (567-888)	563 (495-712)	381 (335-489)	173 (154-218)	44 (39-53)	7 (6-8)
Poisoning by other means	935 (730-1,137)	701 (577-932)	550 (458-741)	363 (302-468)	257 (219-327)	118 (97-146)	33 (28-38)	8 (7-8)
Exposure to mechanical forces	3,970 (3,700-4,442)	3,056 (2,832-3,462)	2,285 (2,106-2,650)	1,750 (1,622-2,043)	1,071 (1,002-1,238)	635 (596-723)	253 (240-287)	71 (67-79)
Unintentional firearm injuries	658 (551-794)	534 (443-643)	441 (355-523)	377 (314-452)	257 (212-307)	141 (119-167)	52 (44-63)	13 (11-16)
Other exposure to mechanical forces	3,312 (3,069-3,687)	2,522 (2,329-2,861)	1,844 (1,678-2,129)	1,373 (1,255-1,607)	814 (746-938)	495 (459-562)	201 (188-225)	58 (55-64)
Adverse effects of medical treatment	3,175 (2,224-3,700)	3,480 (2,400-4,062)	2,934 (2,075-3,421)	2,943 (2,068-3,406)	2,343 (1,626-2,725)	1,488 (986-1,728)	541 (363-634)	124 (89-145)
Animal contact	2,468 (1,047-3,072)	2,193 (928-2,812)	1,608 (718-2,096)	1,255 (572-1,663)	686 (346-912)	270 (148-361)	69 (43-91)	15 (10-19)
Venomous animal contact	2,095 (855-2,681)	1,849 (763-2,353)	1,335 (578-1,710)	1,021 (457-1,309)	552 (268-708)	209 (112-270)	51 (31-66)	10 (7-13)
Non-venomous animal contact	373 (182-542)	344 (162-520)	273 (129-433)	234 (115-378)	135 (68-218)	61 (35-96)	19 (11-29)	5 (3-7)
Foreign body	2,324 (2,224-2,421)	2,039 (1,931-2,141)	1,830 (1,714-1,933)	1,989 (1,920-2,090)	1,861 (1,788-1,955)	1,268 (1,214-1,335)	515 (494-540)	135 (131-140)
Pulmonary aspiration and foreign body in airway	2,142 (2,048-2,230)	1,911 (1,832-2,008)	1,695 (1,604-1,793)	1,842 (1,771-1,947)	1,745 (1,675-1,845)	1,200 (1,146-1,269)	491 (471-517)	128 (124-133)
Foreign body in other body part	182 (129-222)	127 (86-155)	135 (84-169)	147 (96-176)	116 (77-142)	69 (47-84)	24 (16-29)	7 (5-8)
Environmental heat and cold exposure	3,303 (2,471-3,833)	2,332 (1,502-2,759)	1,738 (1,066-2,078)	1,730 (1,054-2,101)	1,217 (744-1,456)	715 (480-838)	243 (175-281)	53 (41-63)
Exposure to forces of nature	1,054 (543-2,053)	817 (416-1,574)	635 (326-1,153)	277 (163-489)	140 (91-223)	97 (54-174)	32 (16-63)	5 (2-8)
Other unintentional injuries	2,760 (2,560-2,935)	1,747 (1,628-1,858)	1,061 (983-1,131)	759 (715-799)	529 (490-568)	245 (228-261)	87 (81-93)	23 (22-25)
Self-harm and interpersonal violence	32,010 (27,199-34,153)	24,459 (20,301-26,255)	19,107 (15,513-20,535)	15,546 (13,104-16,562)	10,144 (8,450-10,839)	4,811 (4,185-5,137)	1,231 (1,100-1,292)	233 (214-243)
Self-harm	24,420 (19,887-26,389)	19,417 (15,584-21,049)	15,814 (12,479-17,201)	13,381 (11,067-14,351)	9,381 (7,724-10,071)	4,464 (3,839-4,787)	1,130 (999-1,190)	177 (161-186)
Self-harm by firearm	2,696 (2,176-3,435)	2,465 (2,034-3,078)	2,142 (1,855-2,646)	2,055 (1,786-2,617)	1,493 (1,281-1,993)	743 (613-1,028)	208 (174-286)	32 (27-43)
Self-harm by other specified means	21,724 (17,302-23,792)	16,952 (13,165-18,646)	13,672 (10,157-15,146)	11,326 (8,909-12,442)	7,888 (6,090-8,707)	3,720 (2,986-4,107)	922 (761-1,003)	145 (124-155)
Interpersonal violence	6,691 (5,712-7,359)	4,384 (3,657-4,956)	2,810 (2,375-3,248)	1,851 (1,595-2,107)	562 (499-631)	247 (226-279)	62 (58-71)	37 (33-40)
Physical violence by firearm	1,779 (1,415-2,077)	1,133 (900-1,353)	718 (544-871)	428 (333-528)	110 (86-132)	45 (36-54)	13 (11-16)	10 (7-11)
Physical violence by sharp object	1,882 (1,422-2,335)	1,183 (843-1,439)	730 (526-894)	475 (367-579)	134 (114-158)	58 (51-70)	16 (14-19)	10 (9-12)
Physical violence by other means	3,031 (2,565-3,576)	2,069 (1,685-2,495)	1,362 (1,145-1,708)	948 (801-1,104)	318 (275-369)	143 (128-167)	33 (31-39)	17 (15-20)
Conflict and terrorism	830 (807-857)	614 (597-635)	458 (444-473)	299 (288-313)	190 (183-199)	96 (92-101)	37 (35-39)	17 (16-18)
Executions and police conflict	69 (47-84)	43 (25-53)	25 (14-31)	15 (11-19)	11 (8-13)	5 (4-7)	2 (1-2)	1 (1-1)

SR Table 6: Global deaths by cause and age group for males (with 95% confidence intervals), 1995

Cause	Early Neonatal	Late Neonatal	Post Neonatal	1 to 4	5 to 9	10 to 14	15 to 19	20 to 24	25 to 29	30 to 34	35 to 39	40 to 44	45 to 49	50 to 54	54 to 59	60 to 64	65 to 69	70 to 74	75 to 79	80 to 84	85 to 89	90 to 94	95 plus
All causes	1,662,202 (1,623,070-1,701,818)	582,691 (564,112-602,982)	1,820,198 (1,781,355-1,862,041)	1,643,905 (1,598,146-1,690,164)	464,404 (459,416-469,405)	269,592 (267,413-271,923)	367,289 (363,282-371,981)	534,566 (528,589-541,387)	637,852 (628,797-646,806)	715,704 (706,626-724,918)	759,457 (749,122-769,890)	943,006 (931,678-954,059)	1,027,695 (1,016,418-1,038,799)	1,141,109 (1,128,698-1,153,429)	1,525,808 (1,511,685-1,540,176)	2,010,030 (1,992,793-2,027,921)	2,367,264 (2,348,793-2,386,221)	2,440,405 (2,421,188-2,459,319)	2,046,982 (2,032,640-2,061,409)	1,836,576 (1,827,783-1,845,972)	1,152,143 (1,147,716-1,156,651)	451,825 (450,350-453,265)	104,956 (104,688-105,222)
Communicable, maternal, neonatal, and nutritional diseases	1,519,972 (1,467,816-1,562,930)	510,950 (485,863-532,037)	1,497,930 (1,436,808-1,561,198)	1,277,560 (1,218,216-1,346,694)	223,477 (206,192-242,543)	102,280 (93,262-113,436)	85,516 (76,328-98,532)	110,013 (101,300-122,975)	156,673 (146,507-169,629)	193,295 (183,225-205,616)	196,031 (185,299-209,978)	202,610 (193,004-214,548)	191,383 (182,296-205,431)	184,151 (173,532-200,977)	199,077 (186,913-220,358)	232,357 (213,556-262,273)	245,032 (222,474-286,110)	250,262 (223,329-295,718)	212,887 (188,762-255,382)	185,956 (163,499-222,207)	124,531 (109,309-149,867)	49,973 (44,126-59,349)	12,894 (11,488-14,960)
HIV/AIDS and sexually transmitted infections	-	15,056 (5,651-28,012)	73,106 (57,203-95,237)	34,442 (31,734-37,600)	6,217 (5,440-7,138)	1,176 (894-1,443)	2,559 (2,222-2,967)	19,040 (16,693-21,924)	58,538 (52,294-65,832)	83,528 (77,180-90,492)	78,127 (70,941-86,282)	66,301 (61,481-71,462)	46,123 (42,390-50,445)	27,492 (25,218-29,757)	17,784 (16,132-19,488)	12,780 (11,390-14,235)	9,098 (8,022-10,201)	5,563 (4,804-6,361)	3,451 (2,949-3,954)	711 (571-852)	277 (187-361)	104 (68-137)	22 (12-31)
HIV/AIDS	-	-	48,689 (45,360-52,240)	32,277 (29,973-34,785)	5,345 (4,868-5,884)	1,118 (836-1,382)	2,486 (2,158-2,893)	18,955 (16,604-21,837)	58,379 (52,131-65,611)	83,315 (76,954-90,355)	77,937 (70,717-86,056)	66,058 (61,175-71,177)	45,932 (42,135-50,212)	27,315 (25,062-29,579)	17,500 (15,870-19,232)	12,513 (11,126-13,982)	8,813 (7,714-9,933)	5,324 (4,556-6,108)	3,167 (2,690-3,637)	543 (458-635)	156 (129-195)	55 (47-65)	7 (6-9)
HIV/AIDS - Drug-susceptible Tuberculosis	-	-	18,009 (13,819-20,509)	12,319 (9,777-13,906)	1,878 (1,433-2,179)	350 (228-462)	757 (593-930)	5,462 (3,986-6,772)	12,253 (8,403-16,398)	14,986 (10,153-20,221)	14,444 (9,843-19,516)	13,321 (9,131-17,810)	10,494 (7,173-14,032)	7,498 (5,181-9,583)	5,227 (3,780-6,454)	3,970 (2,967-4,843)	2,905 (2,244-3,522)	1,908 (1,461-2,314)	1,158 (912-1,388)	181 (145-218)	47 (37-59)	17 (14-21)	2 (1-2)
HIV/AIDS - Multidrug-resistant Tuberculosis without extensive drug resistance	-	-	759 (452-1,217)	529 (323-845)	77 (44-128)	14 (7-26)	36 (23-56)	243 (148-385)	567 (335-896)	729 (444-1,142)	723 (439-1,128)	682 (414-1,070)	526 (317-832)	349 (204-552)	253 (153-391)	187 (114-289)	135 (83-214)	85 (51-137)	51 (31-82)	13 (8-19)	4 (3-6)	1 (1-2)	0 (0-0)
HIV/AIDS - Extensively drug-resistant Tuberculosis	-	-	3 (2-4)	3 (2-4)	1 (0-1)	0 (0-0)	1 (0-1)	2 (1-3)	6 (4-9)	9 (6-14)	11 (7-16)	10 (6-15)	7 (4-11)	4 (2-5)	3 (2-5)	2 (1-3)	1 (1-2)	1 (0-1)	0 (0-0)	0 (0-0)	0 (0-0)	0 (0-0)	0 (0-0)
HIV/AIDS resulting in other diseases	-	-	29,918 (26,722-34,432)	19,426 (17,390-22,517)	3,390 (2,968-3,934)	755 (577-969)	1,691 (1,442-2,089)	13,249 (10,941-16,368)	45,553 (37,905-53,711)	67,591 (58,994-75,377)	62,759 (53,972-71,782)	52,046 (45,364-58,221)	34,905 (29,587-40,241)	19,465 (16,763-22,561)	12,018 (10,367-14,171)	8,355 (7,126-9,824)	5,771 (4,896-6,921)	3,332 (2,768-4,044)	1,958 (1,629-2,368)	349 (281-439)	106 (82-140)	37 (30-46)	5 (5-7)
Sexually transmitted infections excluding HIV	40,913 (15,716-76,446)	15,056 (5,651-28,012)	24,417 (9,251-46,458)	2,165 (812-4,263)	872 (321-1,679)	58 (41-81)	74 (42-107)	85 (46-125)	158 (70-237)	213 (92-322)	190 (111-279)	243 (112-391)	191 (91-292)	177 (106-278)	284 (153-429)	267 (131-441)	285 (154-457)	239 (118-374)	284 (112-467)	168 (63-269)	120 (41-194)	48 (15-80)	15 (6-24)
Syphilis	40,913 (15,716-76,446)	15,056 (5,651-28,012)	24,417 (9,251-46,458)	2,165 (812-4,263)	872 (321-1,679)	49 (35-69)	61 (34-88)	69 (37-102)	133 (59-199)	164 (71-248)	157 (92-231)	208 (96-336)	168 (80-257)	159 (95-249)	257 (139-389)	252 (123-416)	267 (145-428)	222 (109-347)	259 (102-425)	152 (57-244)	105 (36-170)	40 (13-67)	11 (4-18)
Chlamydial infection	-	-	-	-	-	0 (0-0)	0 (0-0)	0 (0-0)	0 (0-0)	0 (0-0)	0 (0-0)	0 (0-0)	0 (0-0)	0 (0-0)	0 (0-0)	0 (0-0)	0 (0-0)	0 (0-0)	0 (0-0)	0 (0-0)	0 (0-0)	0 (0-0)	0 (0-0)
Gonococcal infection	-	-	-	-	-	4 (3-6)	9 (5-12)	10 (5-14)	16 (7-24)	40 (18-61)	9 (5-12)	20 (9-32)	12 (6-18)	8 (5-13)	12 (7-19)	7 (4-13)	9 (5-14)	9 (4-14)	11 (4-18)	7 (3-12)	6 (2-10)	3 (1-4)	1 (0-1)

SR Table 6: Global deaths by cause and age group for males (with 95% confidence intervals), 1995

Cause	Early Neonatal	Late Neonatal	Post Neonatal	1 to 4	5 to 9	10 to 14	15 to 19	20 to 24	25 to 29	30 to 34	35 to 39	40 to 44
Multiple myeloma	-	-	-	-	-	-	102 (68-124)	138 (86-175)	184 (110-239)	297 (214-367)	447 (346-523)	872 (685-1,071)
Leukemia	227 (155-261)	228 (150-282)	3,365 (2,019-4,209)	9,905 (6,582-14,145)	12,127 (8,748-15,081)	8,524 (6,598-9,561)	6,895 (5,797-7,638)	6,405 (5,046-6,933)	6,153 (4,919-7,009)	5,850 (4,668-6,460)	5,321 (4,496-5,739)	6,243 (5,220-6,693)
Acute lymphoid leukemia	42 (30-57)	41 (25-67)	663 (391-1,021)	2,953 (1,604-5,137)	4,056 (2,783-5,643)	2,634 (2,065-3,347)	1,979 (1,708-2,340)	1,428 (1,206-1,710)	1,087 (856-1,391)	946 (752-1,172)	870 (740-1,024)	840 (716-1,004)
Chronic lymphoid leukemia	-	-	-	-	-	-	98 (73-125)	118 (81-168)	114 (74-169)	122 (83-169)	112 (91-140)	199 (168-242)
Acute myeloid leukemia	40 (29-55)	40 (28-64)	376 (250-665)	1,519 (921-3,108)	1,149 (806-1,787)	1,056 (836-1,388)	1,114 (932-1,307)	1,042 (872-1,240)	1,036 (891-1,284)	1,119 (965-1,343)	1,181 (1,025-1,353)	1,362 (1,200-1,557)
Chronic myeloid leukemia	-	-	-	-	-	-	214 (176-249)	339 (277-395)	534 (439-629)	626 (526-716)	670 (585-746)	740 (659-817)
Other leukemia	145 (85-170)	146 (83-178)	2,325 (1,158-2,876)	5,433 (2,740-7,871)	6,923 (3,829-8,836)	4,834 (3,113-5,661)	3,490 (2,653-3,988)	3,478 (2,375-3,974)	3,383 (2,245-3,981)	3,038 (2,044-3,484)	2,487 (1,880-2,769)	3,102 (2,314-3,436)
Other malignant neoplasms	495 (361-577)	301 (209-401)	1,742 (1,184-2,332)	6,090 (3,905-9,850)	3,541 (2,741-4,322)	3,560 (3,054-4,056)	4,254 (3,839-4,702)	3,603 (3,339-3,974)	3,140 (2,805-3,455)	3,201 (2,898-3,543)	3,832 (3,525-4,188)	5,381 (4,875-5,801)
Other neoplasms	32 (19-42)	23 (14-31)	277 (155-401)	559 (303-860)	561 (334-805)	397 (268-522)	340 (234-426)	361 (242-458)	442 (296-564)	571 (385-721)	643 (436-801)	856 (575-1,086)
Myelodysplastic, myeloproliferative, and other hematopoietic neoplasms	31 (19-42)	21 (13-28)	250 (134-368)	474 (236-757)	420 (225-628)	280 (182-382)	238 (159-305)	265 (173-343)	349 (223-452)	473 (305-614)	559 (375-701)	782 (526-998)
Other benign and in situ neoplasms	1 (0-1)	2 (1-3)	27 (18-38)	85 (52-124)	141 (92-184)	117 (80-163)	103 (69-134)	96 (62-125)	93 (56-120)	98 (58-131)	84 (49-115)	74 (42-99)
Cardiovascular diseases	2,756 (2,485-3,071)	2,176 (1,912-2,414)	16,473 (15,123-17,719)	12,476 (10,653-13,945)	7,915 (7,242-8,593)	6,979 (6,463-7,467)	17,727 (16,572-18,898)	31,947 (30,445-33,731)	50,956 (48,962-53,633)	79,059 (76,332-82,580)	115,478 (112,330-119,147)	196,508 (191,914-201,694)
Rheumatic heart disease	-	-	-	3,860 (3,083-4,874)	2,519 (2,242-2,840)	2,761 (2,490-3,034)	3,771 (3,306-4,451)	4,678 (4,136-5,625)	4,940 (4,411-5,859)	5,412 (4,859-6,520)	5,776 (5,144-6,965)	7,483 (6,761-8,935)
Ischemic heart disease	-	-	-	-	-	-	5,148 (4,758-5,532)	12,075 (11,430-12,768)	22,174 (21,217-23,340)	39,435 (37,943-41,088)	62,308 (60,297-64,304)	106,457 (103,795-109,516)
Stroke	1,198 (985-1,425)	917 (700-1,146)	6,772 (5,290-8,454)	4,064 (3,108-5,032)	3,246 (2,878-3,658)	2,470 (2,260-2,662)	5,128 (4,793-5,449)	9,553 (9,091-10,080)	15,259 (14,415-16,253)	22,643 (21,506-23,902)	31,192 (29,908-32,696)	58,704 (56,703-61,225)
Ischemic stroke	148 (107-211)	149 (92-243)	529 (339-864)	473 (268-866)	269 (220-351)	200 (172-243)	570 (510-646)	1,049 (949-1,179)	1,664 (1,505-1,863)	2,672 (2,422-2,986)	3,922 (3,622-4,290)	8,088 (7,511-8,761)
Intracerebral hemorrhage	579 (434-775)	399 (274-590)	3,497 (2,451-5,332)	1,569 (1,016-2,555)	1,648 (1,368-2,052)	1,221 (1,068-1,458)	3,097 (2,773-3,486)	5,867 (5,447-6,463)	9,584 (8,862-10,737)	14,825 (13,810-16,252)	20,452 (19,192-22,139)	38,660 (36,437-41,698)
Subarachnoid hemorrhage	470 (330-570)	369 (241-476)	2,746 (1,648-3,710)	2,022 (1,223-2,744)	1,328 (866-1,544)	1,049 (818-1,173)	1,461 (1,161-1,720)	2,638 (2,110-3,024)	4,011 (3,134-4,484)	5,146 (4,055-5,822)	6,818 (5,617-7,938)	11,956 (9,291-13,832)
Hypertensive heart disease	-	-	-	-	-	-	375 (267-428)	732 (521-828)	1,363 (978-1,542)	2,067 (1,560-2,327)	3,156 (2,423-3,541)	5,843 (4,432-6,502)
Non-rheumatic valvular heart disease	-	-	-	-	-	-	359 (292-434)	443 (363-532)	532 (431-649)	555 (460-666)	731 (625-852)	993 (857-1,168)
Non-rheumatic calcific aortic valve disease	-	-	-	-	-	-	113 (84-141)	142 (107-179)	201 (159-257)	233 (188-286)	344 (282-407)	496 (407-593)
Non-rheumatic degenerative mitral valve disease	-	-	-	-	-	-	168 (130-216)	219 (169-278)	245 (185-316)	265 (203-339)	314 (248-391)	401 (321-502)
Other non-rheumatic valve diseases	-	-	-	-	-	-	78 (46-106)	82 (46-111)	86 (50-124)	57 (33-85)	73 (41-99)	95 (51-138)
Cardiomyopathy and myocarditis	850 (717-992)	626 (518-779)	3,714 (2,928-4,752)	1,863 (1,374-2,591)	994 (841-1,164)	850 (779-934)	1,508 (1,342-1,662)	2,138 (1,945-2,354)	3,210 (2,973-3,536)	4,652 (4,215-5,034)	6,896 (6,161-7,534)	9,364 (7,978-10,497)
Myocarditis	117 (58-167)	144 (82-199)	1,014 (600-1,472)	579 (364-838)	390 (263-524)	280 (218-352)	479 (366-565)	590 (436-693)	747 (577-880)	596 (449-704)	657 (502-791)	857 (618-1,041)
Alcoholic cardiomyopathy	-	-	-	-	-	-	163 (121-206)	414 (334-485)	971 (815-1,113)	2,135 (1,795-2,418)	3,870 (3,187-4,426)	5,334 (4,195-6,146)
Other cardiomyopathy	733 (619-848)	482 (389-593)	2,699 (2,123-3,513)	1,284 (885-1,871)	604 (480-799)	570 (498-654)	866 (744-968)	1,135 (965-1,285)	1,493 (1,262-1,720)	1,922 (1,651-2,177)	2,369 (2,025-2,746)	3,173 (2,638-3,698)
Atrial fibrillation and flutter	-	-	-	-	-	-	-	-	-	33 (28-35)	48 (41-52)	219 (199-251)
Aortic aneurysm	-	-	-	-	-	-	189 (167-225)	298 (259-353)	545 (440-681)	753 (627-931)	1,110 (990-1,297)	1,656 (1,493-1,918)
Peripheral artery disease	-	-	-	-	-	-	-	-	-	-	-	104 (62-148)
Endocarditis	179 (143-238)	147 (112-208)	1,981 (1,499-2,686)	888 (506-1,295)	297 (257-351)	270 (238-312)	329 (291-378)	530 (472-600)	784 (686-934)	849 (749-995)	927 (809-1,084)	1,073 (952-1,249)
Other cardiovascular and circulatory diseases	529 (429-684)	486 (370-633)	4,006 (2,730-5,260)	1,801 (972-2,698)	858 (616-1,196)	628 (530-809)	919 (783-1,109)	1,499 (1,284-1,819)	2,150 (1,889-2,576)	2,659 (2,348-3,183)	3,334 (2,924-4,010)	4,611 (4,044-5,608)
Chronic respiratory diseases	0 (0-0)	0 (0-0)	0 (0-0)	17,010 (14,065-19,690)	4,676 (4,088-5,234)	3,725 (3,321-4,145)	5,431 (4,993-5,874)	8,050 (7,535-8,646)	10,872 (10,262-11,612)	13,816 (13,115-14,672)	17,461 (16,556-18,512)	29,102 (27,850-30,601)
Chronic obstructive pulmonary disease	-	-	0 (0-0)	1,673 (1,024-3,978)	729 (581-1,240)	414 (347-704)	1,675 (1,350-2,389)	2,932 (2,559-4,125)	4,413 (3,949-5,903)	6,450 (5,832-8,455)	7,816 (6,635-10,735)	15,950 (14,080-20,153)
Pneumoconiosis	-	-	-	-	-	-	25 (18-31)	52 (42-63)	166 (135-202)	323 (263-391)	438 (358-520)	744 (602-885)
Silicosis	-	-	-	-	-	-	8 (5-12)	22 (16-31)	90 (63-131)	226 (175-288)	307 (238-383)	523 (408-660)
Asbestosis	-	-	-	-	-	-	3 (1-4)	4 (2-7)	8 (3-16)	13 (4-26)	14 (6-24)	24 (10-41)
Coal workers pneumoconiosis	-	-	-	-	-	-	3 (2-5)	8 (5-12)	33 (18-51)	35 (20-57)	53 (27-87)	113 (60-181)
Other pneumoconiosis	-	-	-	-	-	-	11 (7-15)	18 (12-25)	34 (22-58)	49 (31-84)	64 (42-95)	84 (53-124)
Asthma	-	-	-	13,499 (10,838-16,388)	3,274 (2,697-3,808)	2,897 (2,488-3,310)	3,064 (2,076-3,670)	4,127 (2,510-4,900)	5,307 (3,417-6,197)	5,705 (3,326-6,808)	7,532 (3,960-9,234)	10,023 (5,009-12,639)
Interstitial lung disease and pulmonary sarcoidosis	-	-	-	273 (87-752)	94 (45-216)	62 (40-117)	81 (55-140)	135 (92-239)	202 (136-349)	291 (204-514)	458 (315-797)	689 (466-1,236)
Other chronic respiratory diseases	0 (0-0)	0 (0-0)	0 (0-0)	1,564 (739-2,669)	579 (390-812)	352 (255-499)	587 (408-819)	804 (550-1,141)	783 (549-1,099)	1,046 (745-1,475)	1,216 (849-1,731)	1,696 (1,193-2,389)
Digestive diseases	1,642 (1,448-1,986)	2,003 (1,695-2,537)	14,316 (11,494-20,857)	15,854 (11,730-23,434)	9,604 (8,032-12,004)	6,518 (5,580-7,563)	10,227 (8,696-11,465)	16,204 (13,944-17,787)	28,227 (25,420-30,832)	42,595 (39,170-46,129)	59,940 (55,051-64,213)	84,465 (77,205-90,393)
Cirrhosis and other chronic liver diseases	-	-	-	6,301 (4,175-10,153)	3,575 (2,869-4,654)	2,941 (2,471-3,468)	4,576 (3,745-5,260)	8,659 (7,278-9,630)	16,852 (14,874-18,527)	28,596 (26,131-30,781)	41,959 (38,300-44,902)	61,156 (55,591-65,340)
Cirrhosis and other chronic liver diseases due to hepatitis B	-	-	-	277 (170-481)	456 (351-626)	577 (452-737)	1,153 (854-1,475)	4,028 (3,270-4,744)	9,177 (7,527-10,651)	14,046 (12,312-15,829)	17,072 (14,426-19,790)	23,037 (19,508-26,163)
Cirrhosis and other chronic liver diseases due to hepatitis C	-	-	-	82 (46-151)	122 (87-181)	175 (129-233)	368 (267-487)	1,468 (1,129-1,840)	3,241 (2,475-4,129)	6,322 (5,225-7,457)	10,332 (8,210-12,731)	15,612 (12,974-18,657)
Cirrhosis and other chronic liver diseases due to alcohol use	-	-	-	-	-	-	36 (16-67)	731 (558-928)	2,081 (1,625-2,590)	5,333 (4,642-6,124)	10,711 (8,985-12,664)	16,893 (14,651-19,347)
Cirrhosis due to NASH	-	-	-	-	-	-	80 (58-104)	232 (184-288)	519 (410-647)	1,106 (932-1,287)	1,965 (1,561-2,405)	3,374 (2,804-3,951)
Cirrhosis and other chronic liver diseases due to other causes	-	-	-	5,942 (3,954-9,524)	2,997 (2,395-3,909)	2,189 (1,808-2,617)	2,938 (2,339-3,491)	2,199 (1,687-2,789)	1,836 (1,338-2,416)	1,791 (1,293-2,380)	1,879 (1,385-2,462)	2,240 (1,642-2,972)
Upper digestive system diseases	-	-	-	1,470 (975-2,127)	1,069 (854-1,385)	790 (651-960)	1,635 (1,373-1,904)	2,823 (2,440-3,191)	4,375 (3,885-4,994)	5,921 (5,371-6,718)	7,489 (6,827-8,423)	10,597 (9,620-11,733)
Peptic ulcer disease	-	-	-	1,032 (638-1,731)	856 (675-1,105)	662 (545-809)	1,341 (1,127-1,570)	2,397 (2,064-2,705)	3,669 (3,289-4,180)	4,902 (4,471-5,512)	6,400 (5,817-7,201)	9,299 (8,512-10,267)
Gastritis and duodenitis	-	-	-	438 (209-719)	212 (124-310)	129 (83-177)	294 (204-371)	426 (283-542)	706 (473-889)	1,019 (733-1,266)	1,089 (846-1,294)	1,298 (1,053-1,544)
Appendicitis	-	-	-	1,529 (770-2,555)	1,345 (1,019-1,896)	1,010 (829-1,267)	1,176 (969-1,412)	1,032 (854-1,236)	1,266 (1,060-1,534)	818 (703-974)	1,028 (859-1,247)	1,286 (1,073-1,557)
Paralytic ileus and intestinal obstruction	1,642 (1,448-1,986)	2,003 (1,695-2,537)	14,316 (11,494-20,857)	1,918 (1,269-2,580)	1,889 (1,180-2,380)	871 (559-1,051)	1,610 (1,083-1,956)	1,647 (1,038-2,021)	2,102 (1,410-2,493)	2,335 (1,459-2,834)	3,007 (1,958-3,635)	3,467 (2,299-4,127)
Inguinal, femoral, and abdominal hernia	-	-	-	726 (440-1,178)	307 (232-429)	168 (125-207)	278 (215-348)	322 (254-395)	422 (348-519)	446 (343-541)	588 (480-713)	678 (572-848)
Inflammatory bowel disease	-	-	-	788 (361-1,621)	202 (120-366)	98 (67-148)	100 (80-117)	203 (162-249)	299 (239-386)	357 (297-440)	382 (329-449)	499 (437-578)
Vascular intestinal disorders	-	-	-	430 (143-645)	85 (45-109)	35 (25-41)	48 (40-54)	102 (83-117)	158 (129-183)	163 (137-190)	292 (252-345)	504 (442-579)
Gallbladder and biliary diseases	-	-	-	376 (238-526)	183 (151-230)	147 (120-177)	164 (137-189)	280 (236-310)	381 (333-429)	540 (483-605)	677 (616-735)	985 (884-1,082)
Pancreatitis	-	-	-	97 (53-184)	99 (69-156)	89 (72-128)	250 (212-309)	747 (636-981)	1,729 (1,476-2,264)	2,602 (2,297-3,222)	3,570 (3,232-4,203)	4,115 (3,677-4,844)
Other digestive diseases	-	-	-	2,218 (1,477-3,729)	849 (672-1,207)	370 (305-496)	390 (296-590)	389 (332-492)	642 (533-817)	817 (695-1,030)	947 (792-1,218)	1,177 (1,037-1,461)
Neurological disorders	-	-	5,824 (3,814-7,578)	9,252 (4,881-13,398)	4,813 (3,660-6,172)	5,128 (4,238-5,905)	8,087 (6,788-9,100)	8,057 (6,758-8,823)	7,561 (6,430-8,381)	6,799 (5,845-7,539)	6,328 (5,583-7,193)	7,134 (6,410-7,677)
Alzheimer's disease and other dementias	-	-	-	-	-	-	-	-	-	-	-	409 (381-438)
Parkinson's disease	-	-	-	-	-	-	-	8 (7-9)	13 (10-14)	30 (24-33)	29 (25-33)	329 (303-372)
Epilepsy	-	-	3,756 (2,366-4,854)	7,534 (3,802-11,069)	4,028 (3,024-5,191)	4,086 (3,152-4,679)	6,253 (4,997-7,215)	6,573 (5,196-7,385)	6,501 (5,385-7,255)	5,779 (4,811-6,455)	5,077 (4,300-5,952)	4,692 (3,992-5,260)
Multiple sclerosis	-	-	-	-	-	-	-	54 (43-65)	134 (92-166)	256 (191-311)	416 (316-498)	623 (478-737)
Motor neuron disease	4 (2-6)	11 (7-18)	378 (262-670)	91 (58-171)	32 (24-50)	43 (35-70)	73 (66-99)	68 (59-101)	59 (50-86)	98 (87-134)	191 (178-249)	318 (298-401)
Other neurological disorders	-	-	1,690 (923-2,313)	1,628 (663-2,527)	753 (453-1,014)	1,000 (759-1,241)	1,761 (1,635-2,088)	1,353 (1,210-1,570)	855 (723-1,034)	636 (548-735)	615 (578-699)	763 (714-858)
Mental disorders	-	-	-	-	0 (0-1)	1 (0-1)	3 (2-3)	5 (4-6)	4 (3-5)	4 (3-4)	4 (3-4)	4 (4-5)
Eating disorders	-	-	-	-	0 (0-1)	1 (0-1)	3 (2-3)	5 (4-6)	4 (3-5)	4 (3-4)	4 (3-4)	4 (4-5)
Anorexia nervosa	-	-	-	-	0 (0-0)	0 (0-1)	2 (2-2)	2 (2-3)	2 (1-2)	[illegible] (1-2)	2 (2-3)	3 (2-3)
Bulimia nervosa	-	-	-	-	0 (0-0)	0 (0-0)	1 (1-1)	2 (2-3)	2 (2-3)	2 (2-3)	2 (1-2)	2 (1-2)
Substance use disorders	0 (0-0)	0 (0-0)	0 (0-0)	2 (0-8)	5 (1-13)	5 (1-12)	3,677 (3,491-3,907)	10,399 (9,928-11,001)	16,985 (16,163-18,041)	22,571 (21,752-23,691)	24,558 (23,706-25,440)	27,918 (26,780-29,082)
Alcohol use disorders	-	-	-	-	-	-	1,117 (996-1,215)	3,077 (2,742-3,310)	5,772 (5,296-6,266)	11,015 (10,161-11,816)	15,984 (15,106-16,760)	19,868 (18,776-20,956)
Drug use disorders	0 (0-0)	0 (0-0)	0 (0-0)	2 (0-8)	5 (1-13)	5 (1-12)	2,559 (2,411-2,754)	7,323 (6,886-7,862)	11,213 (10,524-12,091)	11,556 (11,037-12,305)	8,574 (8,226-9,013)	8,050 (7,672-8,473)
Opioid use disorders	0 (0-0)	0 (0-0)	0 (0-0)	2 (0-8)	5 (1-13)	5 (1-12)	1,661 (1,471-1,959)	4,575 (3,884-5,657)	7,053 (5,901-8,687)	7,274 (6,249-8,729)	5,489 (4,966-6,112)	4,949 (4,370-5,665)
Cocaine use disorders	-	-	-	-	-	-	58 (43-81)	179 (118-306)	258 (167-415)	427 (308-636)	384 (308-517)	347 (272-495)
Amphetamine use disorders	-	-	-	-	-	-	122 (65-192)	365 (143-676)	561 (245-1,076)	457 (229-784)	341 (204-523)	341 (180-544)
Other drug use disorders	-	-	-	-	-	-	718 (579-922)	2,203 (1,557-3,015)	3,341 (2,303-4,659)	3,398 (2,523-4,611)	2,359 (1,970-2,993)	2,413 (1,986-3,134)
Diabetes and kidney diseases	28 (20-36)	90 (63-111)	6,393 (5,575-7,113)	6,396 (5,248-7,446)	4,522 (3,741-5,062)	3,515 (2,996-3,883)	6,301 (5,694-6,984)	10,143 (9,363-11,026)	12,986 (12,024-14,015)	16,667 (15,514-17,865)	18,714 (17,771-19,924)	27,511 (26,335-29,088)
Diabetes mellitus	28 (20-36)	90 (63-111)	797 (573-986)	363 (281-443)	484 (376-585)	498 (360-603)	956 (886-1,025)	1,915 (1,798-2,025)	2,749 (2,622-2,886)	4,734 (4,523-4,954)	6,115 (5,885-6,333)	10,697 (10,249-11,100)
Diabetes mellitus type 1	28 (20-36)	90 (63-111)	797 (573-986)	363 (281-443)	484 (376-585)	498 (360-603)	868 (803-934)	1,710 (1,592-1,820)	2,389 (2,268-2,522)	3,749 (3,539-3,970)	4,767 (4,512-5,022)	8,167 (7,671-8,662)
Diabetes mellitus type 2	-	-	-	-	-	-	89 (73-106)	205 (172-251)	361 (298-486)	985 (853-1,200)	1,347 (1,196-1,518)	2,529 (2,221-2,855)
Chronic kidney disease	-	-	5,377 (4,661-6,040)	5,745 (4,627-6,738)	3,716 (2,995-4,181)	2,826 (2,398-3,129)	5,165 (4,576-5,779)	7,943 (7,238-8,720)	9,846 (8,811-10,777)	11,506 (10,218-12,528)	12,230 (11,308-13,256)	16,363 (15,371-17,730)
Chronic kidney disease due to diabetes mellitus type 1	-	-	20 (11-33)	24 (12-43)	24 (11-42)	29 (15-49)	91 (47-148)	345 (182-573)	969 (572-1,507)	1,973 (1,266-2,856)	2,646 (1,718-3,745)	3,794 (2,468-5,330)
Chronic kidney disease due to diabetes mellitus type 2	-	-	15 (8-26)	17 (8-32)	15 (7-29)	16 (7-29)	43 (19-78)	121 (51-231)	267 (113-506)	514 (235-935)	823 (427-1,408)	1,747 (992-2,830)
Chronic kidney disease due to hypertension	-	-	27 (16-43)	44 (24-70)	47 (25-78)	62 (35-99)	201 (120-309)	489 (310-708)	818 (548-1,172)	1,162 (789-1,638)	1,456 (1,009-2,000)	2,218 (1,587-2,944)
Chronic kidney disease due to glomerulonephritis	-	-	1,205 (850-1,672)	1,676 (1,125-2,392)	1,103 (722-1,591)	942 (617-1,305)	2,054 (1,436-2,742)	3,136 (2,150-4,199)	3,502 (2,436-4,693)	3,436 (2,381-4,709)	3,288 (2,206-4,611)	3,991 (2,609-5,582)
Chronic kidney disease due to other and unspecified causes	-	-	4,110 (3,416-4,768)	3,984 (3,118-4,854)	2,527 (1,881-3,073)	1,777 (1,381-2,174)	2,777 (2,084-3,452)	3,853 (2,766-4,979)	4,290 (3,033-5,547)	4,421 (3,058-5,749)	4,016 (2,757-5,390)	4,613 (3,033-6,335)
Acute glomerulonephritis	-	-	219 (139-301)	288 (171-437)	322 (263-394)	192 (162-231)	179 (152-211)	286 (248-333)	392 (336-472)	427 (377-492)	370 (327-414)	452 (404-507)
Skin and subcutaneous diseases	-	-	1,736 (710-2,318)	998 (338-1,707)	322 (134-421)	304 (142-398)	461 (224-622)	448 (206-571)	462 (221-573)	797 (372-1,010)	759 (389-956)	897 (466-1,173)
Bacterial skin diseases	-	-	1,679 (659-2,241)	910 (293-1,580)	260 (93-357)	241 (102-324)	396 (179-549)	372 (162-483)	372 (167-471)	674 (294-869)	640 (312-809)	735 (376-934)
Cellulitis	-	-	154 (37-291)	161 (41-323)	82 (25-123)	86 (32-124)	93 (32-143)	81 (35-106)	78 (34-108)	126 (57-177)	144 (64-227)	170 (74-276)
Pyoderma	-	-	1,525 (606-2,028)	749 (239-1,298)	178 (64-244)	155 (65-209)	303 (145-437)	290 (121-385)	294 (128-380)	549 (228-725)	496 (225-641)	565 (260-733)
Decubitus ulcer	-	-	-	29 (14-76)	28 (16-56)	32 (18-58)	35 (18-58)	47 (23-80)	63 (31-113)	92 (49-165)	91 (46-162)	126 (66-226)
Other skin and subcutaneous diseases	-	-	57 (28-176)	58 (29-182)	34 (20-75)	31 (19-58)	29 (17-52)	30 (17-52)	27 (15-46)	30 (17-52)	29 (15-49)	36 (19-64)
Musculoskeletal disorders	-	-	-	-	376 (240-449)	440 (331-491)	479 (400-525)	602 (456-667)	744 (551-832)	767 (577-836)	849 (674-940)	1,006 (836-1,077)
Rheumatoid arthritis	-	-	-	-	62 (35-121)	60 (42-93)	53 (43-69)	62 (46-89)	76 (52-118)	91 (62-134)	110 (86-138)	184 (147-225)
Other musculoskeletal disorders	-	-	-	-	314 (204-387)	380 (286-431)	426 (354-471)	540 (401-599)	668 (489-756)	677 (508-743)	739 (581-830)	822 (672-892)
Other non-communicable diseases	125,880 (110,343-166,812)	57,313 (48,762-75,651)	195,330 (164,617-242,493)	66,120 (54,560-90,829)	20,339 (18,228-23,384)	11,929 (10,658-13,845)	9,360 (7,880-11,226)	9,561 (8,224-11,352)	8,721 (7,740-9,873)	7,766 (6,834-8,664)	7,491 (6,605-8,485)	8,545 (7,614-9,581)
Congenital birth defects	123,573 (108,551-164,213)	51,447 (43,883-69,169)	151,039 (135,141-193,987)	53,595 (44,139-80,009)	14,551 (13,075-17,325)	7,389 (6,772-8,675)	4,343 (4,005-5,092)	4,213 (3,932-4,923)	3,845 (3,589-4,429)	2,698 (2,522-3,070)	2,458 (2,243-2,875)	2,536 (2,345-2,985)
Neural tube defects	18,284 (12,766-29,724)	5,559 (3,586-9,813)	13,543 (8,065-24,569)	7,142 (3,249-19,008)	1,023 (707-1,416)	495 (372-618)	184 (154-218)	193 (159-229)	180 (141-219)	111 (92-132)	70 (60-84)	80 (64-100)
Congenital heart anomalies	42,682 (28,110-59,649)	23,961 (15,798-32,215)	78,470 (51,885-101,879)	24,781 (13,780-36,470)	8,986 (6,545-11,231)	4,766 (3,921-5,837)	2,771 (2,501-3,340)	2,716 (2,437-3,241)	2,672 (2,408-3,169)	1,778 (1,593-2,095)	1,707 (1,540-2,039)	1,665 (1,494-2,010)
Orofacial clefts	2,758 (1,001-7,122)	1,023 (340-2,752)	2,045 (680-6,069)	410 (81-1,553)	-	-	-	-	-	-	-	-
Down syndrome	2,489 (1,187-4,225)	1,219 (518-2,092)	6,042 (2,365-9,846)	3,069 (995-5,323)	508 (259-810)	231 (153-314)	257 (166-403)	255 (165-397)	295 (169-500)	297 (170-476)	218 (151-295)	296 (193-422)
Other chromosomal abnormalities	1,882 (1,076-3,972)	802 (417-1,722)	1,717 (896-3,663)	438 (194-1,073)	95 (58-171)	61 (38-100)	64 (44-119)	67 (45-114)	59 (40-98)	38 (28-64)	47 (30-72)	31 (20-55)
Congenital musculoskeletal and limb anomalies	2,277 (1,376-3,648)	614 (353-937)	1,643 (831-2,640)	554 (231-1,030)	232 (136-362)	150 (107-208)	153 (109-197)	105 (77-138)	65 (49-85)	51 (38-69)	43 (32-56)	37 (27-51)
Urogenital congenital anomalies	3,266 (2,148-4,998)	1,004 (578-1,478)	1,808 (956-2,822)	530 (211-993)	192 (114-314)	131 (93-182)	57 (47-83)	71 (59-95)	64 (49-85)	61 (45-77)	69 (53-87)	89 (70-116)
Digestive congenital anomalies	14,199 (8,945-25,156)	6,933 (4,379-13,085)	16,575 (9,617-33,022)	3,925 (1,601-12,091)	372 (222-609)	129 (89-176)	49 (41-63)	42 (34-51)	43 (33-54)	27 (21-35)	33 (27-42)	35 (27-45)
Other congenital birth defects	35,736 (21,959-47,752)	10,332 (5,111-15,327)	29,196 (12,854-44,301)	12,746 (4,793-21,432)	3,142 (1,861-4,654)	1,426 (994-1,864)	808 (691-1,001)	765 (631-919)	466 (369-569)	334 (270-418)	272 (226-337)	303 (256-366)
Urinary diseases and male infertility	677 (552-819)	616 (486-740)	4,384 (3,871-4,902)	2,627 (2,123-3,097)	1,132 (952-1,306)	679 (600-754)	1,019 (902-1,123)	1,475 (1,355-1,596)	1,478 (1,374-1,592)	1,845 (1,722-1,967)	1,979 (1,857-2,107)	2,705 (2,551-2,862)
Urinary tract infections	126 (75-179)	184 (103-262)	2,877 (2,160-3,569)	1,026 (599-1,492)	594 (425-776)	406 (333-487)	665 (564-788)	948 (823-1,115)	941 (789-1,083)	1,067 (901-1,261)	1,106 (953-1,295)	1,539 (1,301-1,767)
Urolithiasis	-	-	-	-	55 (24-90)	37 (18-56)	27 (15-38)	58 (30-86)	109 (55-164)	130 (70-191)	138 (86-181)	226 (145-290)
Other urinary diseases	551 (411-674)	433 (277-557)	1,507 (743-2,300)	1,602 (964-2,257)	483 (328-663)	236 (165-306)	327 (223-434)	469 (344-610)	429 (327-556)	647 (508-794)	736 (579-912)	940 (726-1,179)
Gynecological diseases	-	-	-	-	-	-	-	-	-	-	-	-
Uterine fibroids	-	-	-	-	-	-	-	-	-	-	-	-
Polycystic ovarian syndrome	-	-	-	-	-	-	-	-	-	-	-	-

Cause	45 to 49	50 to 54	54 to 59	60 to 64	65 to 69	70 to 74	75 to 79	80 to 84	85 to 89	90 to 94	95 plus
Multiple myeloma	1,172 (962-1,401)	1,771 (1,459-2,130)	2,810 (2,276-3,402)	3,853 (3,169-4,640)	4,730 (3,896-5,797)	4,828 (3,932-5,974)	3,788 (3,067-4,695)	3,052 (2,494-3,800)	1,583 (1,228-1,951)	411 (322-503)	75 (61-98)
Leukemia	6,234 (5,409-6,751)	6,621 (5,871-7,207)	8,892 (8,079-9,803)	11,344 (10,491-12,356)	13,415 (12,642-14,670)	13,299 (12,620-14,561)	10,332 (9,795-11,352)	8,460 (8,101-9,130)	4,552 (4,401-4,868)	1,483 (1,437-1,570)	267 (258-276)
Acute lymphoid leukemia	769 (680-906)	792 (705-935)	975 (889-1,122)	1,037 (939-1,203)	1,139 (1,042-1,319)	959 (879-1,111)	451 (415-513)	321 (297-366)	153 (143-171)	45 (42-48)	7 (7-7)
Chronic lymphoid leukemia	305 (271-342)	493 (450-536)	912 (846-981)	1,420 (1,329-1,520)	1,976 (1,863-2,110)	2,228 (2,113-2,357)	1,858 (1,774-1,952)	1,817 (1,739-1,903)	1,111 (1,063-1,162)	395 (379-411)	82 (79-86)
Acute myeloid leukemia	1,556 (1,391-1,768)	1,736 (1,562-1,930)	2,314 (2,100-2,594)	2,875 (2,644-3,188)	3,338 (3,128-3,668)	3,321 (3,118-3,584)	2,360 (2,209-2,528)	1,733 (1,637-1,866)	795 (749-852)	220 (207-234)	34 (32-35)
Chronic myeloid leukemia	784 (706-863)	832 (758-920)	1,065 (983-1,172)	1,243 (1,147-1,357)	1,523 (1,416-1,668)	1,461 (1,376-1,582)	1,340 (1,251-1,461)	1,176 (1,106-1,270)	618 (585-659)	201 (191-214)	33 (31-35)
Other leukemia	2,820 (2,218-3,134)	2,769 (2,250-3,120)	3,627 (3,073-4,137)	4,768 (4,185-5,407)	5,439 (4,915-6,206)	5,331 (4,898-6,108)	4,323 (3,974-4,969)	3,414 (3,193-3,862)	1,874 (1,768-2,079)	623 (592-686)	111 (105-119)
Other malignant neoplasms	6,377 (5,727-6,827)	7,689 (6,977-8,339)	10,577 (9,474-11,516)	12,822 (11,750-13,872)	14,204 (12,978-15,808)	12,533 (11,575-13,793)	9,067 (8,372-10,273)	6,825 (6,164-7,604)	3,472 (3,059-3,821)	1,118 (985-1,213)	206 (185-224)
Other neoplasms	963 (646-1,221)	1,126 (745-1,466)	1,559 (1,022-2,005)	2,046 (1,382-2,794)	2,609 (1,754-3,590)	2,843 (1,950-4,080)	2,477 (1,637-3,651)	2,287 (1,527-3,533)	1,384 (945-2,199)	483 (340-801)	105 (74-174)
Myelodysplastic, myeloproliferative, and other hematopoietic neoplasms	912 (612-1,160)	1,093 (713-1,429)	1,533 (1,004-1,976)	2,028 (1,369-2,773)	2,594 (1,741-3,574)	2,834 (1,944-4,073)	2,473 (1,633-3,647)	2,284 (1,526-3,531)	1,383 (944-2,198)	483 (340-801)	105 (74-174)
Other benign and in situ neoplasms	51 (28-72)	32 (18-45)	26 (14-35)	18 (9-26)	15 (6-22)	9 (4-15)	5 (2-7)	3 (1-5)	1 (0-2)	0 (0-0)	0 (0-0)
Cardiovascular diseases	264,359 (258,692-270,742)	341,399 (334,545-349,215)	511,417 (502,078-520,365)	716,947 (703,863-729,118)	900,734 (883,956-915,939)	949,442 (930,683-964,387)	817,178 (802,067-832,644)	777,965 (764,254-792,440)	501,604 (491,903-511,261)	201,759 (198,061-205,592)	48,542 (47,680-49,467)
Rheumatic heart disease	7,463 (6,742-8,871)	8,110 (7,289-9,829)	10,315 (9,416-12,177)	13,082 (11,960-15,538)	14,472 (13,335-16,890)	14,390 (13,376-16,551)	10,879 (10,138-12,382)	9,489 (8,918-10,537)	5,587 (5,285-6,145)	2,127 (2,023-2,303)	493 (470-525)
Ischemic heart disease	150,150 (146,526-154,161)	193,566 (188,977-198,652)	288,459 (282,602-294,589)	382,844 (374,991-390,998)	472,521 (462,801-482,245)	475,717 (465,741-486,531)	406,023 (397,817-417,385)	397,911 (390,271-409,135)	264,912 (259,296-272,981)	112,120 (109,979-115,482)	28,734 (28,240-29,496)
Stroke	78,269 (75,802-81,370)	106,141 (102,667-110,000)	163,501 (159,182-168,666)	254,802 (248,198-261,999)	330,293 (321,962-339,823)	362,693 (353,585-373,510)	312,439 (304,115-323,005)	283,045 (276,454-291,336)	171,531 (167,383-177,262)	61,812 (60,288-63,895)	13,188 (12,846-13,663)
Ischemic stroke	13,909 (12,965-14,966)	21,517 (19,882-23,409)	40,050 (37,530-42,797)	82,188 (76,911-87,731)	120,498 (114,054-127,323)	152,777 (145,581-161,101)	141,356 (134,385-148,972)	156,974 (151,540-163,379)	100,548 (97,233-104,739)	37,669 (36,540-39,025)	8,514 (8,277-8,807)
Intracerebral hemorrhage	52,243 (49,377-56,310)	69,604 (65,168-75,123)	102,136 (96,396-109,790)	147,535 (138,395-158,988)	180,610 (170,245-194,169)	181,430 (170,551-195,836)	148,632 (139,462-161,390)	109,458 (103,201-118,379)	62,213 (58,728-66,982)	21,362 (20,278-22,764)	4,175 (3,957-4,426)
Subarachnoid hemorrhage	12,117 (9,089-14,421)	15,021 (11,021-18,572)	21,316 (15,166-26,165)	25,079 (15,762-31,487)	29,184 (18,636-36,790)	28,486 (15,920-36,042)	22,451 (12,857-29,092)	16,613 (9,596-21,290)	8,771 (5,443-10,990)	2,780 (1,962-3,426)	499 (393-596)
Hypertensive heart disease	7,783 (5,933-8,674)	10,473 (7,963-11,685)	15,342 (11,779-16,899)	24,153 (17,896-26,677)	30,514 (22,557-33,518)	38,944 (27,678-42,577)	34,004 (25,060-36,706)	31,445 (23,763-33,881)	19,653 (14,956-21,188)	7,875 (6,130-8,467)	1,762 (1,396-1,894)
Non-rheumatic valvular heart disease	1,036 (916-1,181)	1,352 (1,193-1,547)	1,950 (1,753-2,219)	2,755 (2,446-3,068)	3,860 (3,384-4,286)	4,836 (4,170-5,246)	4,862 (4,136-5,282)	5,469 (4,497-5,955)	3,898 (3,417-4,323)	1,739 (1,532-1,915)	410 (358-456)
Non-rheumatic calcific aortic valve disease	560 (462-641)	744 (629-854)	1,090 (927-1,244)	1,602 (1,342-1,776)	2,378 (1,976-2,633)	3,066 (2,580-3,409)	3,176 (2,672-3,468)	3,807 (3,091-4,176)	2,828 (2,395-3,195)	1,308 (1,126-1,430)	315 (271-350)
Non-rheumatic degenerative mitral valve disease	434 (354-529)	549 (449-673)	782 (653-941)	1,034 (838-1,247)	1,315 (1,047-1,598)	1,559 (1,245-1,851)	1,471 (1,156-1,811)	1,439 (1,118-1,783)	937 (731-1,167)	383 (296-486)	85 (65-106)
Other non-rheumatic valve diseases	42 (24-59)	59 (32-83)	78 (43-106)	118 (62-166)	167 (89-229)	212 (109-287)	214 (111-299)	223 (120-312)	132 (78-177)	48 (30-69)	10 (7-14)
Cardiomyopathy and myocarditis	10,243 (8,517-11,283)	9,889 (8,301-10,862)	13,862 (11,490-15,181)	13,236 (11,133-14,359)	13,779 (10,863-14,838)	12,374 (8,756-13,481)	10,635 (7,272-11,609)	11,396 (6,753-12,532)	9,064 (4,750-10,126)	3,840 (2,082-4,280)	953 (511-1,059)
Myocarditis	666 (488-820)	570 (403-714)	769 (533-984)	703 (493-874)	756 (542-940)	796 (581-963)	718 (518-890)	824 (572-1,058)	740 (527-1,134)	368 (272-589)	99 (69-143)
Alcoholic cardiomyopathy	6,012 (4,805-7,004)	5,155 (4,086-5,897)	7,315 (6,184-8,452)	5,515 (4,812-6,377)	4,959 (4,137-5,954)	3,159 (2,209-4,270)	2,131 (1,459-3,057)	1,780 (1,069-3,014)	1,066 (519-1,955)	391 (214-720)	95 (51-162)
Other cardiomyopathy	3,564 (2,954-4,141)	4,164 (3,387-4,796)	5,778 (4,538-6,636)	7,017 (5,430-8,025)	8,064 (5,841-9,120)	8,420 (5,602-9,527)	7,785 (5,125-8,801)	8,792 (4,957-9,955)	7,259 (3,537-8,403)	3,081 (1,523-3,554)	759 (370-864)
Atrial fibrillation and flutter	335 (302-381)	574 (516-666)	1,160 (1,056-1,371)	2,155 (1,996-2,568)	3,545 (3,285-4,227)	5,723 (5,224-6,810)	7,146 (6,532-8,546)	11,002 (9,754-13,109)	9,510 (8,415-11,406)	5,565 (4,986-6,641)	1,495 (1,318-1,753)
Aortic aneurysm	2,241 (2,065-2,511)	2,924 (2,730-3,261)	5,043 (4,743-5,540)	7,894 (7,361-8,797)	11,775 (11,097-12,793)	13,612 (12,984-14,603)	12,278 (11,624-13,043)	10,558 (10,124-11,069)	5,958 (5,727-6,253)	2,040 (1,961-2,133)	369 (356-384)
Peripheral artery disease	184 (103-281)	324 (175-498)	765 (399-1,221)	1,225 (648-2,022)	1,903 (979-3,134)	2,025 (1,048-3,283)	1,942 (1,014-3,126)	2,148 (1,074-3,473)	1,517 (722-2,476)	721 (355-1,164)	242 (118-413)
Endocarditis	1,005 (897-1,186)	1,054 (936-1,277)	1,243 (1,122-1,487)	1,570 (1,427-1,840)	1,922 (1,755-2,241)	1,988 (1,843-2,263)	1,802 (1,682-2,046)	1,661 (1,559-1,840)	1,061 (1,005-1,152)	443 (420-475)	111 (105-118)
Other cardiovascular and circulatory diseases	5,651 (4,957-6,861)	6,991 (6,121-8,643)	9,777 (8,595-11,839)	13,230 (11,766-15,914)	16,150 (14,473-19,049)	17,140 (15,396-19,958)	15,170 (13,399-17,970)	13,841 (12,430-16,115)	8,912 (8,043-10,186)	3,477 (3,164-3,912)	785 (722-878)
Chronic respiratory diseases	40,922 (39,171-42,985)	65,640 (62,926-68,317)	111,937 (108,103-115,819)	207,536 (200,069-214,195)	284,149 (272,834-292,868)	354,550 (340,420-365,243)	310,378 (297,737-319,663)	271,848 (260,871-280,854)	158,124 (150,927-163,628)	52,711 (50,290-54,514)	10,029 (9,580-10,419)
Chronic obstructive pulmonary disease	24,184 (20,836-30,100)	41,791 (35,697-51,807)	76,822 (67,031-90,995)	159,249 (143,225-181,775)	226,631 (204,827-251,164)	297,712 (277,124-322,712)	263,321 (243,357-282,221)	235,581 (219,844-248,981)	139,154 (131,119-146,476)	46,619 (44,062-48,748)	8,797 (8,336-9,224)
Pneumoconiosis	709 (567-852)	828 (650-1,039)	1,250 (1,032-1,547)	2,041 (1,691-2,469)	2,891 (2,451-3,422)	3,479 (3,037-4,041)	2,719 (2,369-3,311)	2,196 (1,971-2,629)	1,119 (1,005-1,330)	301 (273-350)	54 (49-61)
Silicosis	458 (344-596)	499 (368-654)	694 (521-909)	1,171 (913-1,502)	1,699 (1,377-2,103)	1,999 (1,640-2,455)	1,528 (1,253-1,894)	1,204 (1,017-1,458)	583 (490-702)	140 (120-168)	23 (20-27)
Asbestosis	27 (14-45)	49 (27-80)	105 (54-160)	152 (83-243)	215 (119-311)	266 (167-382)	234 (150-319)	197 (121-261)	115 (73-152)	39 (28-54)	7 (5-9)
Coal workers pneumoconiosis	118 (61-194)	140 (82-230)	246 (169-358)	414 (266-613)	650 (456-906)	878 (648-1,193)	686 (508-950)	591 (462-797)	316 (252-407)	85 (73-106)	16 (14-19)
Other pneumoconiosis	106 (65-156)	140 (88-197)	206 (128-295)	305 (194-444)	326 (217-472)	336 (223-486)	272 (174-403)	205 (138-290)	105 (72-148)	36 (26-51)	8 (6-10)
Asthma	12,969 (5,795-17,044)	19,059 (7,915-26,314)	27,082 (11,377-38,659)	40,556 (16,396-59,490)	47,071 (19,605-70,200)	45,338 (19,465-69,306)	37,306 (16,952-59,766)	27,884 (14,203-44,175)	14,225 (7,655-22,540)	4,455 (2,612-7,113)	874 (557-1,355)
Interstitial lung disease and pulmonary sarcoidosis	974 (659-1,739)	1,475 (987-2,733)	2,600 (1,673-4,674)	3,919 (2,538-7,050)	5,496 (3,625-9,287)	5,869 (4,177-9,511)	5,391 (3,944-8,100)	4,459 (3,233-6,185)	2,454 (1,817-3,258)	824 (652-1,076)	172 (137-225)
Other chronic respiratory diseases	2,085 (1,509-2,869)	2,487 (1,774-3,437)	4,182 (3,166-5,483)	1,771 (1,306-2,355)	2,060 (1,602-2,660)	2,152 (1,727-2,787)	1,641 (1,358-2,057)	1,727 (1,485-2,159)	1,172 (1,019-1,424)	512 (445-628)	132 (116-155)
Digestive diseases	91,951 (85,486-97,249)	95,185 (86,782-101,003)	109,413 (100,394-115,349)	121,268 (110,148-128,257)	119,132 (108,000-124,825)	102,138 (90,238-107,236)	79,110 (70,510-83,741)	62,488 (56,192-65,600)	36,822 (33,632-38,571)	13,822 (12,769-14,469)	3,068 (2,847-3,205)
Cirrhosis and other chronic liver diseases	66,156 (61,003-70,297)	68,013 (61,295-72,239)	74,681 (67,845-79,557)	78,017 (70,577-82,880)	69,545 (62,370-73,928)	52,425 (45,855-56,005)	35,553 (30,573-38,642)	23,802 (20,360-25,879)	11,635 (9,987-12,635)	3,815 (3,255-4,148)	828 (709-899)
Cirrhosis and other chronic liver diseases due to hepatitis B	22,098 (18,819-25,600)	21,108 (17,423-25,171)	22,907 (18,635-27,145)	25,006 (20,063-29,868)	23,159 (19,025-27,419)	18,123 (14,448-21,490)	12,834 (10,022-15,298)	8,221 (6,247-9,865)	3,727 (2,904-4,391)	1,109 (835-1,370)	220 (154-292)
Cirrhosis and other chronic liver diseases due to hepatitis C	17,513 (14,513-20,986)	18,203 (14,850-21,750)	19,135 (16,233-22,463)	19,511 (16,099-23,379)	17,393 (14,657-20,168)	12,969 (10,783-15,240)	8,556 (6,897-10,417)	5,731 (4,617-7,092)	2,940 (2,391-3,485)	1,023 (827-1,236)	236 (180-298)
Cirrhosis and other chronic liver diseases due to alcohol use	19,557 (16,796-22,495)	20,807 (17,704-24,137)	23,365 (19,735-27,233)	23,324 (19,243-27,535)	19,387 (16,307-22,563)	13,605 (11,123-16,047)	8,588 (7,168-9,982)	5,744 (4,782-6,788)	2,759 (2,359-3,171)	884 (730-1,064)	187 (140-239)
Cirrhosis due to NASH	4,501 (3,718-5,343)	5,050 (4,194-6,027)	5,557 (4,802-6,382)	5,629 (4,709-6,530)	4,813 (4,120-5,518)	3,415 (2,894-3,973)	2,102 (1,708-2,499)	1,365 (1,102-1,655)	722 (594-856)	256 (201-316)	59 (44-79)
Cirrhosis and other chronic liver diseases due to other causes	2,487 (1,846-3,295)	2,845 (2,099-3,754)	3,717 (2,504-5,319)	4,547 (3,130-6,274)	4,793 (3,376-6,580)	4,312 (3,225-5,704)	3,472 (2,537-4,636)	2,741 (2,039-3,696)	1,487 (1,158-1,880)	543 (400-711)	126 (83-176)
Upper digestive system diseases	12,486 (11,492-14,004)	13,946 (12,601-15,343)	17,348 (15,803-18,771)	21,268 (18,969-23,106)	23,122 (20,313-24,973)	22,176 (19,211-23,684)	18,640 (16,606-20,092)	14,947 (13,391-15,957)	9,201 (8,356-9,842)	3,434 (3,158-3,652)	762 (702-812)
Peptic ulcer disease	10,999 (10,144-12,362)	12,337 (11,235-13,636)	15,400 (14,105-16,744)	19,045 (17,150-20,770)	20,846 (18,486-22,548)	19,860 (17,296-21,299)	16,583 (14,851-17,977)	13,330 (11,986-14,367)	8,149 (7,437-8,775)	3,039 (2,796-3,239)	667 (615-712)
Gastritis and duodenitis	1,486 (1,203-1,769)	1,609 (1,307-1,931)	1,948 (1,561-2,325)	2,223 (1,796-2,764)	2,276 (1,851-2,766)	2,316 (1,865-2,861)	2,057 (1,666-2,494)	1,617 (1,323-1,986)	1,051 (877-1,263)	395 (335-469)	95 (82-110)
Appendicitis	1,021 (877-1,219)	1,013 (871-1,196)	1,177 (1,021-1,385)	1,391 (1,213-1,635)	1,387 (1,216-1,587)	1,498 (1,262-1,750)	1,214 (1,004-1,485)	1,019 (853-1,231)	622 (519-755)	253 (212-317)	54 (44-68)
Paralytic ileus and intestinal obstruction	3,702 (2,366-4,387)	3,701 (2,643-4,481)	5,041 (3,554-6,002)	6,330 (4,685-7,813)	7,653 (5,773-9,429)	7,433 (5,749-8,992)	6,638 (5,419-8,267)	6,332 (5,239-7,926)	4,712 (3,892-5,646)	1,931 (1,580-2,339)	414 (353-516)
Inguinal, femoral, and abdominal hernia	746 (635-924)	981 (838-1,205)	1,348 (1,168-1,621)	1,808 (1,539-2,168)	2,244 (1,947-2,591)	2,383 (1,980-2,722)	2,388 (1,912-2,817)	2,407 (2,028-2,805)	1,638 (1,444-1,860)	698 (627-797)	162 (145-184)
Inflammatory bowel disease	576 (505-665)	577 (510-660)	796 (700-935)	939 (827-1,072)	1,181 (1,030-1,338)	1,263 (1,115-1,408)	1,215 (1,007-1,391)	1,231 (977-1,413)	826 (659-931)	337 (264-376)	76 (60-84)
Vascular intestinal disorders	737 (654-850)	987 (891-1,107)	1,772 (1,615-1,982)	2,502 (2,277-2,752)	3,763 (3,440-4,124)	4,178 (3,857-4,506)	3,727 (3,488-3,956)	3,663 (3,433-3,878)	2,290 (2,168-2,460)	887 (832-938)	183 (171-192)
Gallbladder and biliary diseases	1,070 (987-1,170)	1,287 (1,173-1,405)	1,784 (1,629-1,905)	2,715 (2,487-2,934)	3,721 (3,363-3,977)	4,223 (3,831-4,489)	4,105 (3,711-4,417)	4,057 (3,745-4,296)	2,691 (2,499-2,854)	1,167 (1,087-1,236)	278 (260-293)
Pancreatitis	4,049 (3,579-4,703)	3,145 (2,854-3,682)	3,670 (3,270-4,239)	3,400 (3,066-3,958)	3,416 (3,047-3,838)	2,947 (2,606-3,289)	2,381 (2,003-2,742)	1,823 (1,597-2,049)	1,058 (931-1,182)	353 (310-394)	70 (62-77)
Other digestive diseases	1,410 (1,189-1,804)	1,535 (1,271-2,052)	1,798 (1,576-2,234)	2,896 (2,423-3,909)	3,102 (2,726-3,890)	3,612 (3,172-4,484)	3,250 (2,868-3,920)	3,206 (2,859-3,737)	2,151 (1,972-2,377)	945 (874-1,034)	242 (225-265)
Neurological disorders	7,329 (6,697-7,889)	7,676 (7,241-8,085)	11,304 (10,868-11,824)	20,378 (19,780-21,371)	35,427 (34,473-37,006)	59,475 (57,777-62,450)	87,536 (84,269-90,236)	117,087 (113,167-120,242)	102,875 (100,340-105,654)	56,125 (54,694-57,459)	14,770 (14,412-15,103)
Alzheimer's disease and other dementias	819 (781-870)	1,877 (1,809-1,988)	4,197 (4,054-4,427)	11,092 (10,606-11,800)	22,094 (21,391-23,250)	39,783 (38,483-41,859)	63,261 (61,195-65,278)	91,483 (88,790-93,614)	87,450 (85,363-89,783)	51,394 (50,179-52,554)	14,000 (13,698-14,307)
Parkinson's disease	598 (555-707)	954 (879-1,146)	1,546 (1,442-1,846)	3,397 (3,177-4,123)	7,171 (6,722-8,798)	14,526 (13,634-17,463)	20,390 (19,026-23,990)	22,876 (21,455-27,114)	13,968 (13,030-16,438)	4,308 (3,946-5,078)	687 (650-788)
Epilepsy	3,789 (3,076-4,370)	2,597 (2,157-2,911)	2,645 (2,210-2,919)	2,637 (2,129-3,001)	2,563 (2,013-3,251)	1,915 (1,552-2,287)	1,508 (1,274-1,681)	1,026 (818-1,139)	611 (507-682)	187 (156-211)	39 (33-45)
Multiple sclerosis	701 (564-810)	651 (533-742)	711 (597-821)	670 (579-796)	595 (505-693)	440 (364-515)	264 (221-306)	164 (133-185)	72 (56-81)	18 (14-19)	3 (2-3)
Motor neuron disease	520 (486-673)	647 (609-775)	1,018 (957-1,247)	1,302 (1,236-1,550)	1,586 (1,516-1,812)	1,423 (1,375-1,544)	965 (931-1,009)	591 (568-613)	232 (221-242)	47 (45-49)	7 (6-7)
Other neurological disorders	902 (853-1,015)	950 (892-1,088)	1,186 (1,113-1,338)	1,281 (1,221-1,408)	1,418 (1,355-1,541)	1,387 (1,335-1,495)	1,149 (1,097-1,242)	947 (905-1,003)	542 (518-570)	171 (164-180)	34 (33-36)
Mental disorders	5 (4-5)	-	-	-	-	-	-	-	-	-	-
Eating disorders	5 (4-5)	-	-	-	-	-	-	-	-	-	-
Anorexia nervosa	3 (3-3)	-	-	-	-	-	-	-	-	-	-
Bulimia nervosa	2 (1-2)	-	-	-	-	-	-	-	-	-	-
Substance use disorders	24,689 (23,358-25,816)	18,389 (17,616-19,229)	19,637 (18,576-20,552)	14,096 (13,522-14,782)	9,961 (9,520-10,380)	5,743 (5,489-5,982)	3,488 (3,263-3,673)	2,027 (1,949-2,118)	1,139 (1,091-1,194)	403 (384-425)	85 (81-88)
Alcohol use disorders	19,783 (18,509-20,923)	15,176 (14,454-15,946)	16,475 (15,384-17,371)	11,481 (10,899-12,134)	7,628 (7,190-8,026)	3,843 (3,564-4,073)	2,114 (1,901-2,274)	1,033 (963-1,093)	517 (474-553)	148 (134-159)	33 (30-35)
Drug use disorders	4,906 (4,670-5,160)	3,212 (3,085-3,364)	3,162 (3,029-3,324)	2,615 (2,501-2,731)	2,333 (2,237-2,449)	1,900 (1,806-2,001)	1,374 (1,309-1,451)	995 (943-1,057)	622 (586-663)	255 (240-273)	52 (49-55)
Opioid use disorders	2,849 (2,488-3,208)	1,724 (1,466-1,961)	1,751 (1,490-1,971)	1,435 (1,169-1,679)	1,292 (1,042-1,503)	814 (599-1,013)	600 (445-727)	434 (323-528)	299 (223-359)	126 (95-150)	26 (21-30)
Cocaine use disorders	237 (184-341)	175 (138-254)	167 (131-250)	113 (84-173)	102 (73-166)	36 (25-61)	27 (18-45)	5 (4-8)	3 (3-5)	2 (1-3)	0 (0-1)
Amphetamine use disorders	226 (127-361)	104 (67-157)	103 (65-158)	71 (45-109)	63 (37-105)	15 (9-23)	11 (7-18)	4 (2-6)	2 (1-3)	1 (1-1)	0 (0-0)
Other drug use disorders	1,593 (1,341-2,040)	1,209 (1,028-1,501)	1,140 (949-1,443)	996 (803-1,287)	876 (699-1,173)	1,034 (857-1,263)	736 (623-885)	552 (468-654)	318 (269-386)	126 (107-153)	25 (22-30)
Diabetes and kidney diseases	32,461 (30,950-34,176)	41,964 (40,239-44,420)	56,865 (54,478-60,582)	76,984 (73,981-80,949)	87,532 (84,188-92,358)	87,749 (84,337-92,466)	75,961 (72,673-79,569)	61,486 (59,047-64,190)	38,791 (37,087-40,375)	15,210 (14,581-15,769)	3,633 (3,483-3,756)
Diabetes mellitus	15,049 (14,444-15,638)	21,386 (20,493-22,250)	30,733 (29,488-31,945)	42,572 (40,724-44,143)	48,622 (46,674-50,460)	47,931 (46,107-49,525)	39,244 (37,700-40,736)	29,633 (28,334-30,740)	16,633 (15,874-17,245)	5,957 (5,699-6,193)	1,257 (1,201-1,305)
Diabetes mellitus type 1	6,958 (6,101-8,039)	9,408 (8,189-11,067)	13,323 (11,575-15,834)	13,767 (11,657-16,856)	15,255 (13,188-18,516)	12,424 (10,599-15,324)	9,801 (8,299-12,525)	5,591 (4,697-7,292)	3,028 (2,510-3,964)	1,038 (861-1,347)	206 (172-261)
Diabetes mellitus type 2	8,091 (7,101-8,898)	11,979 (10,369-13,059)	17,410 (15,114-19,082)	28,805 (25,738-30,826)	33,367 (30,327-35,609)	35,508 (32,730-37,414)	29,443 (26,819-31,234)	24,042 (22,351-25,295)	13,606 (12,744-14,301)	4,919 (4,605-5,177)	1,051 (993-1,107)
Chronic kidney disease	16,937 (15,758-18,432)	20,174 (19,089-22,466)	25,586 (24,118-29,158)	33,853 (31,886-37,817)	38,287 (36,125-42,514)	39,202 (37,161-43,690)	36,244 (34,110-39,122)	31,498 (30,047-33,740)	21,952 (20,758-23,253)	9,167 (8,781-9,579)	2,345 (2,242-2,442)
Chronic kidney disease due to diabetes mellitus type 1	3,582 (2,322-5,141)	3,198 (1,899-4,711)	2,754 (1,523-4,346)	2,143 (1,072-3,671)	1,586 (802-2,833)	1,155 (563-2,061)	788 (370-1,473)	563 (277-1,003)	256 (131-462)	47 (22-85)	2 (1-5)
Chronic kidney disease due to diabetes mellitus type 2	2,858 (1,769-4,114)	4,805 (3,265-6,882)	7,320 (5,086-9,628)	11,208 (8,025-14,570)	12,887 (9,459-16,639)	12,514 (9,165-16,004)	10,550 (7,230-14,025)	8,307 (5,619-11,391)	5,066 (3,315-6,816)	1,876 (1,306-2,536)	442 (270-675)
Chronic kidney disease due to hypertension	2,622 (1,866-3,477)	3,737 (2,778-4,926)	5,371 (4,075-6,947)	8,088 (6,091-10,452)	10,440 (8,104-13,181)	12,282 (9,680-15,488)	12,895 (10,299-15,958)	12,172 (9,679-14,767)	9,603 (7,920-11,314)	4,457 (3,745-5,133)	1,230 (1,010-1,441)
Chronic kidney disease due to glomerulonephritis	3,722 (2,473-5,369)	3,835 (2,509-5,489)	4,293 (2,882-6,263)	4,676 (2,989-6,917)	5,188 (3,351-7,588)	5,343 (3,333-7,658)	4,915 (3,184-7,138)	4,219 (2,685-6,285)	2,710 (1,796-3,969)	1,060 (702-1,552)	261 (145-434)
Chronic kidney disease due to other and unspecified causes	4,153 (2,747-5,910)	4,600 (2,982-6,558)	5,848 (3,821-8,319)	7,736 (4,750-11,260)	8,187 (5,242-11,589)	7,908 (5,204-11,011)	7,096 (4,636-10,302)	6,236 (4,289-8,741)	4,318 (2,973-5,906)	1,727 (1,175-2,364)	410 (264-620)
Acute glomerulonephritis	474 (417-558)	404 (347-485)	547 (475-647)	560 (498-627)	623 (553-703)	615 (539-700)	473 (401-551)	356 (308-415)	205 (175-245)	86 (69-109)	31 (22-44)
Skin and subcutaneous diseases	1,091 (595-1,502)	1,124 (594-1,619)	1,527 (819-2,050)	1,956 (1,022-2,718)	2,174 (1,178-3,021)	2,466 (1,342-3,416)	2,604 (1,360-3,567)	2,287 (1,258-3,307)	1,708 (934-2,357)	789 (415-1,149)	206 (111-315)
Bacterial skin diseases	932 (499-1,250)	954 (486-1,313)	1,306 (671-1,690)	1,649 (815-2,218)	1,699 (892-2,322)	1,788 (904-2,515)	1,828 (906-2,588)	1,372 (736-2,056)	946 (500-1,390)	419 (224-668)	104 (57-174)
Cellulitis	204 (94-330)	199 (85-352)	274 (116-448)	321 (131-507)	302 (123-547)	315 (142-585)	282 (107-525)	254 (102-491)	164 (62-330)	76 (31-161)	21 (7-45)
Pyoderma	727 (353-998)	754 (363-1,015)	1,032 (489-1,357)	1,328 (633-1,834)	1,397 (689-1,956)	1,473 (713-2,052)	1,545 (724-2,174)	1,118 (602-1,705)	782 (400-1,138)	343 (181-531)	83 (46-139)
Decubitus ulcer	124 (64-226)	138 (73-255)	181 (93-342)	253 (123-487)	404 (191-690)	599 (289-1,005)	690 (325-1,197)	816 (362-1,332)	681 (279-992)	332 (124-511)	92 (37-140)
Other skin and subcutaneous diseases	36 (19-65)	32 (17-61)	40 (20-81)	54 (29-108)	71 (36-133)	79 (41-152)	86 (43-169)	99 (48-202)	81 (38-164)	38 (16-83)	10 (4-21)
Musculoskeletal disorders	1,080 (896-1,151)	956 (819-1,009)	1,445 (1,223-1,533)	2,484 (2,045-2,668)	2,954 (2,537-3,125)	3,270 (2,833-3,480)	3,050 (2,566-3,295)	2,482 (2,163-2,648)	1,897 (1,692-2,037)	745 (663-806)	169 (153-179)
Rheumatoid arthritis	231 (183-275)	333 (277-375)	629 (538-702)	1,185 (933-1,344)	1,483 (1,251-1,655)	1,699 (1,468-1,888)	1,579 (1,308-1,767)	1,109 (949-1,267)	760 (654-863)	246 (207-287)	48 (41-55)
Other musculoskeletal disorders	848 (689-920)	623 (531-674)	816 (662-905)	1,299 (1,016-1,471)	1,470 (1,195-1,649)	1,571 (1,279-1,759)	1,470 (1,161-1,689)	1,373 (1,154-1,532)	1,137 (979-1,263)	499 (423-553)	121 (107-131)
Other non-communicable diseases	7,420 (6,650-8,150)	8,017 (7,034-8,943)	9,410 (8,412-10,395)	12,129 (10,913-13,285)	14,880 (13,516-16,234)	14,933 (13,370-16,180)	12,881 (11,519-14,038)	12,909 (11,680-13,791)	8,948 (7,990-9,389)	3,983 (3,616-4,164)	1,084 (1,004-1,133)
Congenital birth defects	1,031 (926-1,239)	830 (752-1,038)	860 (789-1,115)	825 (758-1,052)	718 (651-912)	-	-	-	-	-	-
Neural tube defects	47 (39-59)	30 (25-38)	31 (26-38)	27 (22-34)	23 (20-29)	-	-	-	-	-	-
Congenital heart anomalies	521 (455-664)	386 (340-490)	389 (346-521)	352 (313-459)	316 (278-413)	-	-	-	-	-	-
Orofacial clefts	-	-	-	-	-	-	-	-	-	-	-
Down syndrome	211 (147-277)	187 (146-255)	183 (144-275)	165 (130-248)	83 (61-132)	-	-	-	-	-	-
Other chromosomal abnormalities	23 (18-43)	20 (15-40)	20 (15-37)	16 (11-32)	13 (9-24)	-	-	-	-	-	-
Congenital musculoskeletal and limb anomalies	23 (19-30)	21 (16-26)	21 (15-27)	28 (19-36)	34 (19-44)	-	-	-	-	-	-
Urogenital congenital anomalies	53 (43-66)	60 (47-76)	84 (66-109)	112 (87-148)	131 (103-171)	-	-	-	-	-	-
Digestive congenital anomalies	23 (19-31)	21 (18-27)	30 (25-39)	30 (26-40)	38 (32-49)	-	-	-	-	-	-
Other congenital birth defects	129 (104-162)	105 (88-132)	102 (86-128)	94 (82-123)	80 (67-104)	-	-	-	-	-	-
Urinary diseases and male infertility	3,153 (2,965-3,354)	3,550 (3,337-3,794)	4,394 (4,151-4,635)	6,389 (6,024-6,755)	8,600 (8,105-9,075)	9,033 (8,444-9,508)	7,737 (7,317-8,078)	8,159 (7,679-8,529)	5,550 (5,286-5,771)	2,542 (2,413-2,647)	728 (699-755)
Urinary tract infections	1,660 (1,425-1,903)	1,902 (1,611-2,196)	2,469 (2,156-2,804)	3,597 (3,095-4,119)	4,927 (4,326-5,544)	4,905 (4,278-5,663)	4,730 (4,212-5,292)	4,920 (4,456-5,500)	3,797 (3,501-4,146)	1,766 (1,646-1,922)	541 (505-586)
Urolithiasis	266 (185-327)	305 (211-370)	418 (311-511)	632 (440-769)	859 (611-1,046)	839 (569-1,015)	683 (466-831)	560 (394-690)	325 (222-392)	130 (91-156)	23 (17-28)
Other urinary diseases	1,227 (979-1,521)	1,343 (1,033-1,697)	1,507 (1,177-1,892)	2,161 (1,678-2,751)	2,815 (2,146-3,531)	3,289 (2,564-4,058)	2,324 (1,777-2,926)	2,678 (2,113-3,229)	1,428 (1,088-1,764)	646 (496-778)	164 (124-204)
Gynecological diseases	-	-	-	-	-	-	-	-	-	-	-
Uterine fibroids	-	-	-	-	-	-	-	-	-	-	-
Polycystic ovarian syndrome	-	-	-	-	-	-	-	-	-	-	-

SR Table 6: Global deaths by cause and age group for males (with 95% confidence intervals), 1995

Cause	Early Neonatal	Late Neonatal	Post Neonatal	1 to 4	5 to 9	10 to 14	15 to 19	20 to 24	25 to 29	30 to 34	35 to 39	40 to 44	45 to 49	50 to 54	54 to 59	60 to 64	65 to 69	70 to 74	75 to 79	80 to 84	85 to 89	90 to 94	95 plus
Endometriosis	-	-	-	-	-	-	-	-	-	-	-	-	-	-	-	-	-	-	-	-	-	-	-
Genital prolapse	-	-	-	-	-	-	-	-	-	-	-	-	-	-	-	-	-	-	-	-	-	-	-
Other gynecological diseases	-	-	-	-	-	-	-	-	-	-	-	-	-	-	-	-	-	-	-	-	-	-	-
Hemoglobinopathies and hemolytic anemias	707 (422-1,031)	576 (360-752)	3,831 (2,272-5,280)	7,187 (3,722-11,248)	3,315 (2,230-4,400)	2,903 (1,987-3,939)	3,201 (1,811-4,734)	2,934 (1,616-4,427)	2,164 (1,329-3,084)	1,698 (1,064-2,395)	1,521 (886-2,210)	1,569 (985-2,285)	1,384 (854-1,961)	1,614 (938-2,375)	1,868 (1,161-2,662)	2,228 (1,394-3,128)	2,539 (1,632-3,505)	2,705 (1,726-3,689)	2,490 (1,592-3,370)	2,289 (1,477-2,871)	1,626 (1,056-1,918)	709 (455-805)	165 (113-186)
Thalassemias	276 (193-340)	249 (145-372)	1,484 (969-2,277)	1,884 (1,158-3,088)	1,256 (847-1,817)	693 (491-865)	412 (287-511)	311 (186-405)	180 (111-243)	77 (51-105)	31 (23-40)	21 (16-28)	13 (10-16)	7 (5-8)	3 (3-4)	1 (1-2)	0 (0-1)	0 (0-0)	0 (0-0)	0 (0-0)	0 (0-0)	0 (0-0)	0 (0-0)
Sickle cell disorders	386 (178-689)	310 (169-489)	2,226 (1,056-3,502)	5,017 (2,293-8,056)	1,738 (1,017-2,781)	1,655 (964-2,651)	2,036 (930-3,462)	1,716 (722-3,123)	1,087 (457-1,998)	688 (269-1,355)	634 (217-1,245)	498 (164-1,027)	267 (93-582)	165 (65-380)	69 (34-138)	33 (21-53)	16 (11-24)	6 (4-10)	2 (1-3)	1 (0-1)	0 (0-1)	0 (0-0)	0 (0-0)
G6PD deficiency	12 (7-21)	10 (6-16)	81 (43-128)	223 (114-383)	239 (163-338)	382 (265-512)	498 (343-670)	562 (403-718)	588 (444-709)	628 (452-749)	548 (389-701)	625 (438-842)	683 (429-980)	785 (406-1,247)	783 (405-1,329)	704 (342-1,188)	532 (257-925)	344 (164-602)	177 (80-324)	74 (35-132)	27 (15-43)	6 (4-10)	1 (0-1)
Other hemoglobinopathies and hemolytic anemias	33 (23-46)	7 (5-10)	39 (26-58)	64 (41-100)	82 (63-106)	173 (136-218)	256 (184-337)	344 (266-433)	309 (244-375)	306 (234-375)	308 (231-372)	425 (321-514)	421 (311-527)	657 (437-889)	1,013 (698-1,359)	1,490 (1,003-1,981)	1,990 (1,346-2,691)	2,354 (1,557-3,154)	2,312 (1,498-3,069)	2,214 (1,436-2,751)	1,599 (1,037-1,886)	702 (450-797)	164 (112-185)
Endocrine, metabolic, blood, and immune disorders	923 (565-1,101)	937 (559-1,203)	4,977 (3,333-7,531)	2,710 (1,531-4,143)	1,341 (935-1,855)	958 (750-1,233)	798 (644-961)	939 (721-1,112)	1,234 (889-1,411)	1,525 (1,131-1,753)	1,532 (1,178-1,775)	1,735 (1,353-2,020)	1,852 (1,526-2,241)	2,022 (1,590-2,452)	2,288 (1,820-2,742)	2,686 (2,239-3,265)	3,024 (2,522-3,712)	3,195 (2,615-3,872)	2,654 (2,084-3,260)	2,461 (1,815-2,906)	1,772 (1,324-2,023)	733 (514-840)	190 (146-222)
Sudden infant death syndrome	-	3,738 (1,251-7,036)	31,099 (10,834-65,794)	-	-	-	-	-	-	-	-	-	-	-	-	-	-	-	-	-	-	-	-
Injuries	10,979 (9,118-12,358)	9,445 (7,717-10,930)	74,831 (64,735-83,982)	212,954 (174,711-244,556)	160,377 (147,377-171,493)	108,346 (100,890-114,511)	198,955 (189,843-205,752)	303,991 (295,000-311,358)	308,084 (298,848-314,588)	277,828 (267,985-283,706)	240,180 (232,555-245,403)	222,373 (213,617-227,563)	181,008 (173,252-185,636)	142,141 (135,021-147,029)	139,952 (132,619-144,262)	121,441 (112,389-125,919)	110,349 (101,489-115,163)	89,302 (80,713-93,325)	67,756 (60,596-71,884)	54,862 (49,327-57,828)	34,363 (30,771-36,275)	13,043 (11,897-13,734)	2,927 (2,712-3,061)
Transport injuries	2,052 (1,741-2,426)	1,931 (1,568-2,291)	11,863 (9,297-13,595)	45,856 (30,584-54,928)	48,893 (43,298-53,812)	32,463 (29,748-35,168)	78,618 (74,076-83,313)	116,773 (111,526-123,107)	116,950 (111,941-123,460)	98,797 (94,757-103,368)	81,803 (78,896-85,675)	74,154 (71,678-77,229)	57,183 (55,185-59,589)	45,433 (43,603-48,205)	41,615 (40,022-44,076)	35,887 (34,483-37,867)	31,706 (30,309-33,836)	24,795 (23,757-26,215)	17,126 (16,236-18,598)	11,385 (10,850-12,190)	5,505 (5,242-5,829)	1,645 (1,574-1,725)	329 (313-344)
Road injuries	1,717 (1,443-2,103)	1,694 (1,340-2,031)	10,965 (8,492-12,561)	43,144 (28,753-51,875)	46,427 (41,297-51,274)	30,233 (27,687-32,830)	73,287 (69,058-77,598)	109,863 (104,915-115,941)	109,420 (104,835-115,449)	92,722 (88,844-97,172)	76,081 (73,322-79,817)	68,819 (66,417-71,827)	52,698 (50,711-55,011)	41,711 (39,954-44,180)	38,378 (36,875-40,542)	33,450 (32,109-35,302)	29,669 (28,355-31,590)	23,342 (22,367-24,681)	16,116 (15,265-17,459)	10,775 (10,264-11,502)	5,224 (4,980-5,520)	1,556 (1,490-1,627)	307 (292-322)
Pedestrian road injuries	961 (737-1,300)	872 (652-1,236)	5,073 (4,060-6,122)	27,551 (19,954-34,234)	30,497 (26,933-34,038)	16,412 (14,589-18,236)	20,368 (17,819-24,400)	30,694 (27,320-35,912)	34,617 (31,178-39,232)	32,787 (29,538-36,893)	27,289 (23,830-31,227)	26,459 (23,267-29,665)	21,883 (19,405-24,781)	18,115 (16,057-20,993)	17,531 (15,659-19,960)	17,127 (15,560-19,019)	15,550 (14,130-17,423)	12,457 (11,443-13,941)	9,272 (8,525-10,415)	6,160 (5,709-6,871)	3,094 (2,879-3,388)	885 (831-968)	171 (159-186)
Cyclist road injuries	-	-	-	957 (468-1,534)	2,096 (1,426-2,821)	2,118 (1,695-2,666)	2,690 (2,272-3,196)	2,720 (2,238-3,216)	2,975 (2,339-3,644)	3,059 (2,383-3,713)	2,584 (2,070-2,971)	3,059 (2,350-3,633)	2,633 (2,089-3,042)	2,381 (1,947-2,724)	2,054 (1,771-2,345)	2,204 (1,791-2,540)	1,951 (1,682-2,223)	1,552 (1,334-1,774)	929 (795-1,086)	638 (578-717)	260 (234-292)	62 (55-73)	0 (0-1)
Motorcyclist road injuries	96 (73-144)	123 (72-187)	852 (606-1,107)	2,014 (1,032-3,006)	2,249 (1,626-2,989)	2,904 (2,382-3,611)	18,518 (16,438-22,009)	28,222 (25,458-33,165)	27,604 (24,264-32,224)	20,348 (17,759-23,329)	15,159 (13,351-17,964)	13,211 (11,563-15,453)	8,650 (7,546-10,319)	5,890 (5,188-7,363)	4,760 (4,185-5,939)	2,873 (2,462-3,646)	2,335 (1,998-2,921)	1,594 (1,367-1,963)	809 (672-1,053)	556 (471-709)	202 (167-264)	48 (39-61)	10 (8-12)
Motor vehicle road injuries	632 (426-866)	674 (405-969)	4,850 (3,253-6,052)	12,229 (6,231-16,978)	11,194 (8,637-14,240)	8,472 (7,084-9,993)	31,171 (28,145-34,385)	47,393 (42,912-52,332)	43,402 (39,142-49,134)	35,906 (32,570-40,468)	30,505 (27,478-33,769)	25,572 (23,350-28,656)	19,004 (17,130-21,493)	14,840 (13,325-16,833)	13,609 (12,273-15,390)	10,946 (9,833-12,530)	9,502 (8,563-10,984)	7,460 (6,699-8,545)	4,915 (4,396-5,805)	3,279 (2,980-3,778)	1,598 (1,454-1,829)	545 (497-609)	121 (110-129)
Other road injuries	28 (12-49)	24 (10-43)	189 (92-311)	393 (141-737)	390 (207-601)	327 (219-459)	540 (384-817)	835 (579-1,211)	822 (543-1,189)	622 (428-853)	544 (396-740)	519 (375-694)	527 (385-720)	485 (351-667)	424 (323-571)	301 (229-405)	330 (248-463)	280 (205-390)	191 (143-280)	142 (102-214)	70 (52-105)	16 (12-26)	5 (4-7)
Other transport injuries	335 (216-485)	237 (147-369)	898 (582-1,187)	2,712 (1,400-3,763)	2,466 (1,869-2,965)	2,229 (1,895-2,555)	5,331 (4,745-6,044)	6,910 (6,369-7,593)	7,530 (6,848-8,174)	6,076 (5,590-6,489)	5,722 (5,284-6,247)	5,335 (4,968-5,748)	4,485 (4,168-4,891)	3,722 (3,435-4,221)	3,237 (2,982-3,685)	2,437 (2,256-2,725)	2,037 (1,887-2,308)	1,453 (1,341-1,655)	1,010 (914-1,212)	610 (553-726)	281 (252-332)	89 (79-106)	22 (20-27)
Unintentional injuries	7,624 (6,043-8,654)	7,074 (5,567-8,260)	59,132 (50,997-67,261)	158,323 (133,889-182,469)	104,267 (95,889-111,719)	60,647 (56,385-64,229)	53,358 (50,551-55,760)	68,822 (65,701-71,643)	69,263 (65,665-72,346)	68,262 (65,067-70,965)	65,286 (62,550-67,205)	65,472 (62,384-67,671)	59,341 (56,477-61,344)	48,781 (45,669-50,607)	52,575 (49,008-54,502)	49,196 (44,868-51,445)	47,433 (42,216-50,064)	40,778 (35,637-43,321)	34,927 (30,199-37,425)	32,081 (28,071-34,288)	22,925 (20,023-24,581)	9,694 (8,684-10,310)	2,288 (2,092-2,415)
Falls	1,003 (638-1,305)	484 (251-735)	5,783 (3,394-8,076)	10,573 (5,672-16,607)	7,410 (4,647-10,497)	5,444 (4,131-7,019)	5,770 (4,966-6,900)	9,580 (7,745-11,379)	11,674 (9,086-14,213)	12,527 (10,110-14,834)	12,673 (10,893-14,354)	14,714 (12,627-17,112)	13,908 (12,412-16,107)	12,852 (11,210-14,936)	14,707 (12,747-16,535)	16,253 (13,759-18,722)	17,893 (14,879-20,230)	17,964 (14,562-20,267)	17,753 (14,362-19,873)	17,919 (14,859-19,775)	14,025 (11,728-15,435)	6,224 (5,428-6,759)	1,441 (1,282-1,557)
Drowning	433 (387-478)	314 (267-350)	8,190 (7,345-9,057)	94,373 (80,339-106,664)	69,279 (63,844-74,520)	34,943 (32,696-37,522)	21,841 (20,363-23,373)	22,306 (21,249-23,628)	18,383 (17,571-19,341)	16,341 (15,745-17,019)	13,618 (13,149-14,146)	12,660 (12,238-13,098)	9,807 (9,474-10,129)	7,773 (7,466-8,080)	7,322 (7,082-7,584)	6,527 (6,308-6,763)	5,430 (5,245-5,635)	4,320 (4,151-4,490)	2,675 (2,556-2,791)	2,143 (2,038-2,254)	1,127 (1,070-1,188)	318 (303-334)	61 (58-64)
Fire, heat, and hot substances	341 (204-418)	322 (162-426)	4,444 (2,236-5,751)	11,031 (5,050-15,071)	3,778 (2,215-4,597)	1,920 (1,292-2,228)	2,373 (1,747-2,870)	3,784 (2,828-4,395)	4,254 (3,102-4,864)	4,513 (3,502-5,041)	4,569 (3,818-5,117)	4,511 (3,853-4,946)	4,114 (3,568-4,560)	3,445 (2,984-3,830)	3,875 (3,435-4,285)	3,578 (3,043-3,976)	3,474 (2,960-3,901)	3,042 (2,480-3,452)	2,473 (2,066-2,811)	2,507 (2,117-2,813)	1,744 (1,522-1,923)	735 (654-795)	185 (169-197)
Poisonings	258 (139-317)	262 (139-344)	2,682 (1,333-3,623)	6,075 (2,811-8,941)	2,601 (1,477-3,767)	1,903 (1,211-2,638)	2,153 (1,738-2,704)	3,469 (2,654-4,595)	3,938 (2,980-5,370)	3,878 (3,208-5,070)	4,172 (3,678-4,708)	4,705 (4,130-5,477)	3,940 (3,522-4,490)	3,009 (2,728-3,408)	3,400 (3,116-3,856)	2,665 (2,366-3,144)	2,111 (1,921-2,558)	1,438 (1,275-1,875)	883 (795-1,147)	680 (610-875)	334 (304-425)	96 (86-115)	18 (16-20)
Poisoning by carbon monoxide	60 (32-75)	65 (32-85)	619 (311-831)	1,370 (774-1,822)	1,285 (723-1,967)	864 (574-1,254)	1,243 (1,041-1,588)	2,054 (1,588-2,790)	2,312 (1,812-3,327)	2,495 (2,123-3,251)	2,878 (2,582-3,256)	3,321 (2,968-3,834)	2,800 (2,517-3,153)	2,016 (1,846-2,252)	2,420 (2,228-2,698)	1,691 (1,553-2,003)	1,309 (1,201-1,579)	823 (724-1,084)	526 (468-698)	403 (362-526)	200 (181-259)	55 (49-68)	8 (7-10)
Poisoning by other means	197 (108-245)	197 (106-260)	2,064 (1,019-2,843)	4,705 (2,040-6,985)	1,316 (748-1,920)	1,040 (661-1,412)	910 (660-1,143)	1,415 (1,023-1,883)	1,626 (1,176-2,098)	1,382 (1,009-1,811)	1,294 (1,009-1,493)	1,384 (1,087-1,651)	1,140 (919-1,361)	993 (788-1,173)	980 (819-1,178)	974 (765-1,151)	802 (666-1,013)	615 (521-811)	357 (301-473)	277 (241-354)	134 (111-168)	41 (35-48)	9 (8-10)
Exposure to mechanical forces	909 (756-1,034)	1,138 (851-1,315)	4,733 (3,848-5,284)	8,987 (5,742-10,662)	5,401 (4,754-6,400)	3,977 (3,599-4,427)	6,932 (6,318-7,911)	10,667 (9,950-12,024)	11,217 (10,538-13,063)	10,585 (10,032-12,000)	9,733 (9,188-10,624)	8,318 (7,911-9,560)	7,738 (7,295-8,477)	6,209 (5,805-6,875)	5,346 (5,064-5,955)	4,062 (3,849-4,433)	3,465 (3,258-3,845)	2,592 (2,419-2,916)	1,775 (1,643-2,042)	1,148 (1,081-1,302)	723 (687-807)	316 (301-347)	87 (83-95)
Unintentional firearm injuries	109 (80-155)	106 (73-188)	746 (540-1,175)	1,134 (633-2,422)	785 (602-999)	856 (747-1,009)	2,003 (1,762-2,292)	2,923 (2,460-3,313)	2,754 (2,165-3,120)	2,097 (1,642-2,347)	1,751 (1,480-1,983)	1,436 (1,162-1,610)	1,408 (1,122-1,612)	1,091 (848-1,274)	809 (697-938)	648 (542-746)	580 (492-686)	462 (383-533)	360 (298-424)	259 (217-304)	147 (125-171)	63 (54-74)	14 (12-17)
Other exposure to mechanical forces	800 (660-918)	1,032 (767-1,206)	3,987 (3,231-4,486)	7,853 (5,034-9,406)	4,617 (4,006-5,541)	3,121 (2,785-3,524)	4,928 (4,432-5,718)	7,745 (7,060-8,954)	8,463 (7,842-10,633)	8,488 (7,933-9,933)	7,982 (7,489-8,934)	6,882 (6,469-8,177)	6,329 (5,931-7,047)	5,117 (4,762-5,707)	4,538 (4,271-5,066)	3,415 (3,222-3,718)	2,884 (2,695-3,191)	2,131 (1,983-2,393)	1,415 (1,304-1,634)	889 (828-1,012)	576 (544-645)	252 (240-276)	73 (69-80)
Adverse effects of medical treatment	813 (511-1,000)	576 (348-775)	4,861 (2,732-6,987)	3,859 (1,715-7,258)	1,610 (1,012-2,004)	1,119 (788-1,318)	1,352 (946-1,626)	2,097 (1,435-2,493)	2,026 (1,407-2,366)	2,063 (1,446-2,418)	2,131 (1,488-2,499)	2,301 (1,616-2,668)	2,188 (1,523-2,532)	1,898 (1,350-2,186)	2,402 (1,732-2,768)	3,270 (2,386-3,773)	3,870 (2,802-4,472)	3,447 (2,543-3,981)	3,026 (2,171-3,479)	2,586 (1,837-2,982)	1,721 (1,204-1,983)	706 (497-815)	160 (118-188)
Animal contact	318 (103-422)	360 (100-525)	1,948 (691-2,600)	5,800 (1,890-9,195)	5,485 (2,009-7,292)	3,948 (1,812-5,021)	2,544 (1,225-3,176)	2,672 (1,196-3,259)	2,788 (1,261-3,340)	2,674 (1,218-3,220)	2,348 (1,124-2,832)	2,579 (1,181-3,092)	3,141 (1,412-3,812)	2,222 (1,047-2,685)	2,689 (1,233-3,233)	2,639 (1,136-3,217)	2,508 (1,048-3,154)	1,843 (821-2,335)	1,399 (633-1,796)	750 (384-953)	341 (183-433)	93 (56-117)	20 (13-25)
Venomous animal contact	279 (84-378)	318 (83-467)	1,594 (535-2,313)	4,906 (1,471-8,263)	4,696 (1,662-6,657)	3,587 (1,609-4,679)	2,168 (995-2,759)	2,216 (954-2,791)	2,212 (964-2,761)	2,262 (1,043-2,874)	2,009 (948-2,453)	2,198 (998-2,696)	2,731 (1,179-3,398)	1,854 (845-2,269)	2,299 (1,037-2,839)	2,234 (907-2,789)	2,112 (870-2,653)	1,527 (652-1,895)	1,144 (514-1,439)	597 (297-742)	266 (140-337)	68 (40-85)	14 (9-17)
Non-venomous animal contact	38 (18-52)	42 (18-64)	354 (139-545)	894 (337-1,773)	788 (318-1,185)	361 (179-460)	376 (213-502)	455 (222-585)	575 (274-730)	412 (172-540)	339 (171-450)	382 (171-504)	410 (218-544)	368 (197-502)	390 (207-535)	406 (195-575)	395 (189-577)	316 (156-471)	255 (123-404)	152 (79-232)	75 (43-111)	25 (14-36)	7 (4-9)
Foreign body	2,966 (2,073-3,474)	3,285 (2,347-3,871)	23,095 (18,739-27,434)	10,151 (7,238-12,160)	3,104 (2,809-3,644)	2,095 (1,944-2,317)	1,374 (1,306-1,530)	2,237 (2,125-2,529)	2,372 (2,274-2,739)	2,605 (2,510-2,879)	2,953 (2,858-3,162)	3,280 (3,178-3,504)	3,090 (2,987-3,285)	2,670 (2,577-2,801)	3,196 (3,075-3,320)	2,690 (2,573-2,788)	2,634 (2,476-2,735)	2,304 (2,154-2,398)	2,102 (2,005-2,200)	2,225 (2,151-2,309)	1,662 (1,598-1,730)	749 (719-782)	206 (200-213)
Pulmonary aspiration and foreign body in airway	2,868 (2,022-3,368)	3,188 (2,305-3,768)	21,312 (17,180-26,249)	8,354 (6,088-10,714)	2,576 (2,282-3,167)	1,863 (1,730-2,113)	1,235 (1,167-1,417)	2,007 (1,898-2,294)	2,100 (2,001-2,474)	2,258 (2,168-2,537)	2,603 (2,514-2,777)	2,912 (2,818-3,104)	2,868 (2,772-3,041)	2,488 (2,398-2,600)	3,010 (2,897-3,130)	2,493 (2,384-2,586)	2,481 (2,361-2,572)	2,145 (2,013-2,247)	1,947 (1,865-2,045)	2,097 (2,027-2,199)	1,579 (1,521-1,659)	716 (689-750)	197 (192-204)
Foreign body in other body part	98 (40-142)	97 (31-153)	1,783 (495-2,620)	1,796 (580-3,059)	528 (262-692)	232 (142-280)	139 (90-166)	230 (149-274)	273 (172-319)	347 (267-406)	350 (277-412)	367 (283-429)	222 (167-259)	182 (135-223)	186 (143-220)	198 (140-238)	153 (104-187)	158 (99-191)	155 (99-186)	128 (86-153)	83 (55-99)	33 (22-38)	8 (6-10)
Environmental heat and cold exposure	284 (91-406)	197 (58-329)	1,923 (586-2,908)	1,266 (425-2,359)	675 (325-865)	535 (310-661)	1,374 (1,027-1,576)	2,117 (1,526-2,384)	2,561 (1,837-2,856)	3,783 (2,918-4,188)	4,778 (3,969-5,223)	5,681 (4,758-6,193)	5,319 (4,496-5,821)	4,434 (3,658-4,866)	5,555 (4,670-6,114)	4,057 (3,208-4,507)	3,476 (2,568-3,911)	2,178 (1,418-2,543)	1,813 (1,065-2,191)	1,302 (834-1,524)	841 (585-961)	319 (232-364)	71 (57-80)
Exposure to forces of nature	7 (6-8)	15 (14-17)	144 (130-164)	638 (578-713)	723 (656-803)	914 (837-1,017)	990 (912-1,097)	1,079 (981-1,200)	965 (883-1,064)	826 (759-905)	740 (679-810)	695 (634-770)	603 (544-679)	570 (497-665)	577 (493-689)	573 (484-690)	503 (419-609)	396 (320-501)	285 (228-368)	268 (202-357)	129 (95-176)	30 (22-39)	9 (7-12)
Other unintentional injuries	293 (250-340)	120 (97-138)	1,329 (1,135-1,471)	5,570 (4,450-6,453)	4,200 (3,639-4,736)	3,849 (3,422-4,236)	6,656 (6,061-7,258)	8,812 (8,229-9,440)	9,086 (8,518-9,696)	8,468 (8,010-9,041)	7,570 (7,181-8,022)	6,028 (5,748-6,317)	5,493 (5,181-5,791)	3,698 (3,474-3,897)	3,503 (3,320-3,682)	2,882 (2,715-3,046)	2,069 (1,949-2,182)	1,254 (1,179-1,319)	744 (699-783)	553 (513-588)	278 (259-294)	109 (102-116)	30 (28-31)
Self-harm and interpersonal violence	1,303 (972-1,441)	441 (332-494)	3,837 (2,803-4,361)	8,775 (6,830-10,067)	7,217 (5,553-8,051)	15,236 (12,878-16,475)	66,979 (61,121-70,000)	118,396 (107,552-122,857)	121,871 (106,612-127,326)	110,768 (97,361-115,680)	93,091 (84,291-96,620)	82,747 (74,145-86,157)	64,484 (57,689-67,293)	47,927 (42,859-50,006)	45,762 (40,912-47,716)	36,358 (30,996-38,263)	31,210 (26,547-32,833)	23,729 (19,344-25,110)	15,703 (12,841-16,664)	11,396 (9,458-12,008)	5,934 (5,120-6,217)	1,705 (1,510-1,774)	310 (284-320)
Self-harm	-	-	-	-	-	7,116 (5,229-8,123)	28,823 (24,922-31,082)	54,959 (46,961-58,382)	63,107 (51,548-67,791)	59,750 (49,531-63,711)	52,606 (45,614-55,419)	50,789 (43,296-53,704)	42,324 (36,524-44,825)	32,977 (28,642-34,609)	32,833 (28,727-34,554)	27,435 (22,571-29,178)	24,460 (20,277-25,890)	19,701 (15,420-20,964)	13,334 (10,678-14,201)	10,484 (8,549-11,076)	5,488 (4,672-5,765)	1,562 (1,362-1,630)	238 (215-247)
Self-harm by firearm	-	-	-	-	-	366 (270-555)	3,773 (2,849-4,899)	6,269 (4,880-8,294)	5,705 (4,411-7,583)	5,282 (4,112-7,245)	4,827 (3,841-6,369)	4,487 (3,598-6,027)	3,984 (3,077-5,239)	3,217 (2,501-4,116)	3,018 (2,346-3,915)	2,652 (2,116-3,431)	2,633 (2,125-3,372)	2,447 (2,058-3,093)	1,904 (1,635-2,355)	1,613 (1,400-2,127)	902 (760-1,239)	282 (238-386)	46 (37-60)
Self-harm by other specified means	-	-	-	-	-	6,749 (4,891-7,751)	25,050 (21,454-27,384)	48,690 (41,160-52,456)	57,401 (45,669-62,224)	54,468 (44,415-58,676)	47,780 (40,606-50,792)	46,302 (39,156-49,332)	38,341 (32,693-40,890)	29,759 (25,543-31,457)	29,815 (25,684-31,574)	24,783 (19,952-26,595)	21,827 (17,786-23,321)	17,254 (12,981-18,622)	11,430 (8,710-12,350)	8,870 (6,860-9,595)	4,586 (3,674-4,963)	1,280 (1,038-1,377)	193 (163-205)
Interpersonal violence	1,098 (766-1,236)	333 (224-386)	3,484 (2,451-4,016)	5,910 (3,965-7,197)	5,177 (3,513-6,006)	6,100 (4,752-6,893)	32,371 (28,068-34,662)	52,903 (45,652-56,235)	50,254 (42,871-52,717)	44,576 (38,103-46,934)	36,171 (31,622-38,039)	29,030 (25,446-30,723)	20,383 (17,812-21,736)	13,778 (11,837-14,726)	11,858 (10,380-12,783)	8,030 (6,831-8,573)	6,059 (5,143-6,584)	3,562 (2,973-3,923)	2,061 (1,714-2,309)	663 (586-725)	316 (286-351)	89 (82-98)	50 (44-53)
Physical violence by firearm	145 (84-201)	46 (27-67)	486 (317-715)	838 (529-1,447)	647 (489-872)	1,637 (1,324-1,928)	15,643 (13,342-16,808)	24,342 (20,543-26,557)	21,466 (17,806-23,470)	17,483 (14,112-18,934)	13,770 (11,159-14,935)	9,794 (7,796-10,838)	6,260 (5,061-6,981)	4,336 (3,417-4,869)	3,105 (2,432-3,594)	1,975 (1,544-2,299)	1,345 (1,058-1,566)	870 (651-1,051)	497 (377-606)	129 (101-153)	62 (49-72)	21 (17-24)	13 (10-15)
Physical violence by sharp object	94 (56-136)	32 (16-51)	349 (175-594)	825 (379-1,688)	1,030 (476-1,696)	1,126 (664-1,515)	7,661 (5,584-9,323)	13,883 (10,234-16,535)	14,708 (10,838-17,545)	13,447 (10,082-15,841)	10,639 (8,406-12,638)	8,725 (6,860-10,052)	6,026 (4,801-7,044)	3,822 (2,905-4,592)	3,156 (2,561-3,615)	2,284 (1,762-2,724)	1,657 (1,233-1,943)	926 (670-1,095)	529 (391-627)	165 (142-190)	78 (67-89)	23 (20-27)	14 (11-16)
Physical violence by other means	859 (587-978)	256 (168-300)	2,649 (1,833-3,169)	4,246 (2,755-5,398)	3,501 (2,425-4,438)	3,337 (2,452-4,136)	9,066 (7,551-11,323)	14,678 (12,269-18,257)	14,080 (11,571-17,857)	13,646 (11,287-16,762)	11,761 (10,209-14,078)	10,512 (9,173-12,394)	8,097 (7,077-9,464)	5,620 (4,778-6,556)	5,598 (4,867-6,350)	3,771 (3,241-4,313)	3,057 (2,610-3,545)	1,765 (1,481-2,126)	1,036 (861-1,200)	370 (320-419)	177 (156-204)	45 (41-52)	23 (21-26)
Conflict and terrorism	205 (175-247)	108 (91-132)	324 (277-392)	2,828 (2,433-3,407)	1,979 (1,701-2,380)	1,968 (1,710-2,342)	5,481 (4,892-6,354)	9,267 (8,357-10,704)	6,565 (5,881-7,517)	4,893 (4,401-5,569)	3,467 (3,103-4,017)	2,310 (2,081-2,635)	1,422 (1,302-1,593)	991 (915-1,104)	968 (901-1,067)	818 (758-896)	639 (594-705)	436 (406-477)	291 (272-319)	237 (223-255)	123 (117-131)	52 (50-55)	20 (19-21)
Executions and police conflict	-	-	29 (15-37)	37 (25-44)	62 (41-73)	53 (45-61)	305 (240-359)	1,267 (703-1,515)	1,944 (1,056-2,364)	1,549 (745-1,819)	848 (505-998)	619 (376-733)	354 (209-440)	181 (132-223)	103 (79-134)	75 (56-94)	51 (35-62)	30 (19-36)	16 (12-21)	12 (8-15)	6 (4-8)	3 (2-3)	1 (1-2)

SR Table 6: Global deaths by cause and age group for males (with 95% confidence intervals), 2000

Cause	Early Neonatal	Late Neonatal	Post Neonatal	1 to 4	5 to 9	10 to 14	15 to 19	20 to 24	25 to 29	30 to 34	35 to 39	40 to 44	45 to 49	50 to 54	54 to 59	60 to 64	65 to 69	70 to 74	75 to 79	80 to 84	85 to 89	90 to 94	95 plus
All causes	1,579,056 (1,545,191-1,617,380)	497,350 (480,587-515,158)	1,616,374 (1,584,217-1,653,172)	1,464,121 (1,423,725-1,506,334)	384,908 (381,105-389,138)	279,208 (276,822-281,596)	390,665 (386,535-395,028)	532,871 (526,503-539,513)	653,968 (643,681-664,700)	766,842 (755,544-777,691)	858,811 (846,300-871,694)	963,725 (952,983-974,625)	1,196,184 (1,183,392-1,209,227)	1,293,170 (1,279,944-1,306,001)	1,431,540 (1,418,277-1,444,108)	1,997,530 (1,981,647-2,013,302)	2,392,950 (2,374,141-2,411,011)	2,746,680 (2,726,071-2,766,813)	2,433,489 (2,416,775-2,450,093)	1,878,391 (1,867,482-1,888,881)	1,280,016 (1,274,920-1,285,249)	547,102 (545,263-548,981)	140,559 (140,225-140,895)
Communicable, maternal, neonatal, and nutritional diseases	1,439,773 (1,396,611-1,479,102)	433,871 (414,317-452,739)	1,321,623 (1,270,672-1,372,114)	1,146,734 (1,096,734-1,204,847)	196,496 (183,732-211,496)	104,647 (96,239-114,887)	88,829 (80,692-100,585)	115,131 (107,153-125,886)	179,540 (168,308-192,614)	229,463 (218,613-242,622)	245,691 (233,865-260,958)	241,569 (232,329-254,406)	237,401 (227,401-252,706)	205,705 (195,781-223,184)	199,962 (188,673-220,509)	226,566 (209,927-255,950)	250,645 (227,972-294,094)	272,763 (243,457-321,675)	233,605 (207,750-282,829)	193,706 (170,135-234,331)	131,221 (116,424-157,008)	57,879 (51,506-68,541)	16,410 (14,849-18,946)
HIV/AIDS and sexually transmitted infections	-	14,620 (5,537-27,774)	102,531 (85,976-125,329)	49,152 (45,664-52,949)	12,715 (11,745-13,862)	4,748 (4,335-5,210)	4,504 (3,796-5,288)	24,556 (21,557-28,266)	79,609 (70,815-89,635)	117,044 (108,561-125,894)	119,551 (109,873-130,168)	104,684 (98,551-110,917)	78,807 (73,395-84,960)	44,135 (41,426-47,045)	25,123 (23,001-27,270)	17,287 (15,710-19,009)	12,464 (11,185-13,769)	8,128 (7,228-9,088)	4,959 (4,399-5,544)	868 (761-1,008)	296 (231-366)	120 (95-142)	25 (17-33)
HIV/AIDS	-	-	77,696 (73,293-82,322)	46,981 (43,639-50,155)	11,870 (11,131-12,659)	4,705 (4,289-5,164)	4,456 (3,753-5,241)	24,498 (21,479-28,201)	79,509 (70,711-89,519)	116,915 (108,407-125,778)	119,402 (109,698-129,961)	104,536 (98,408-110,797)	78,674 (73,263-84,800)	43,996 (41,289-46,910)	24,957 (22,837-27,094)	17,095 (15,515-18,810)	12,275 (10,994-13,573)	7,949 (7,051-8,894)	4,774 (4,246-5,349)	770 (678-888)	206 (176-247)	84 (74-97)	11 (10-13)
HIV/AIDS - Drug-susceptible Tuberculosis	-	-	26,445 (19,911-30,729)	16,879 (13,220-19,313)	4,130 (3,213-4,767)	1,604 (1,255-1,871)	1,401 (1,081-1,735)	7,120 (5,203-8,876)	18,421 (12,158-24,843)	23,846 (15,319-33,695)	24,530 (15,934-34,246)	23,376 (14,957-32,280)	19,912 (12,967-26,151)	12,781 (8,808-15,618)	7,784 (5,884-9,289)	5,489 (4,289-6,554)	4,065 (3,259-4,817)	2,736 (2,170-3,218)	1,669 (1,325-1,975)	253 (205-299)	62 (49-75)	26 (21-32)	3 (2-4)
HIV/AIDS - Multidrug-resistant Tuberculosis without extensive drug resistance	-	-	2,306 (1,413-3,560)	1,447 (871-2,252)	330 (199-505)	130 (75-208)	134 (82-214)	644 (390-1,022)	1,628 (939-2,649)	2,122 (1,219-3,475)	2,195 (1,263-3,557)	2,090 (1,196-3,335)	1,774 (1,034-2,749)	1,121 (682-1,677)	670 (420-988)	473 (297-702)	344 (221-517)	231 (147-345)	141 (89-212)	25 (17-36)	7 (4-10)	3 (2-4)	0 (0-0)
HIV/AIDS - Extensively drug-resistant Tuberculosis	-	-	20 (13-29)	13 (8-19)	3 (2-4)	2 (1-3)	3 (2-4)	12 (8-19)	36 (23-53)	53 (34-80)	61 (39-90)	58 (36-86)	45 (28-65)	23 (15-33)	12 (8-17)	9 (6-13)	5 (4-7)	3 (2-5)	2 (1-3)	1 (0-1)	0 (0-0)	0 (0-0)	0 (0-0)
HIV/AIDS resulting in other diseases	-	-	48,926 (43,386-56,553)	28,642 (25,501-33,132)	7,406 (6,591-8,535)	2,969 (2,605-3,460)	2,919 (2,387-3,563)	16,721 (13,994-20,549)	59,424 (48,147-70,960)	90,893 (77,681-103,355)	92,616 (78,167-106,792)	79,012 (67,245-89,546)	56,943 (48,802-66,182)	30,071 (26,434-34,733)	16,491 (14,502-19,202)	11,125 (9,667-13,051)	7,861 (6,752-9,274)	4,979 (4,236-5,995)	2,962 (2,547-3,547)	492 (417-600)	137 (112-177)	55 (46-68)	8 (7-9)
Sexually transmitted infections excluding HIV	40,851 (15,822-77,422)	14,620 (5,537-27,774)	24,835 (9,292-48,542)	2,171 (806-4,282)	845 (309-1,639)	44 (36-65)	48 (40-62)	58 (39-85)	100 (59-143)	130 (74-189)	149 (88-213)	148 (86-240)	133 (81-199)	139 (92-214)	166 (104-263)	192 (109-301)	189 (116-315)	179 (107-282)	185 (102-288)	98 (49-153)	90 (35-145)	36 (14-55)	14 (6-21)
Syphilis	40,851 (15,822-77,422)	14,620 (5,537-27,774)	24,835 (9,292-48,542)	2,171 (806-4,282)	845 (309-1,639)	37 (31-55)	39 (33-51)	47 (32-69)	84 (49-121)	100 (57-146)	124 (73-176)	127 (74-206)	117 (71-176)	125 (82-192)	151 (95-239)	181 (103-284)	177 (109-295)	166 (99-261)	168 (93-262)	89 (44-138)	79 (31-126)	30 (12-46)	11 (5-17)
Chlamydial infection	-	-	-	-	-	0 (0-0)	0 (0-0)	0 (0-0)	0 (0-0)	0 (0-0)	0 (0-0)	0 (0-0)	0 (0-0)	0 (0-0)	0 (0-0)	0 (0-0)	0 (0-0)	0 (0-0)	0 (0-0)	0 (0-0)	0 (0-0)	0 (0-0)	0 (0-0)
Gonococcal infection	-	-	-	-	-	3 (3-5)	6 (5-7)	7 (4-10)	10 (6-15)	25 (14-36)	7 (4-9)	12 (7-19)	8 (5-13)	7 (4-10)	7 (5-11)	6 (3-9)	6 (4-10)	7 (4-10)	7 (4-11)	4 (2-7)	5 (2-7)	2 (1-3)	1 (0-1)
Other sexually transmitted infections	-	-	-	-	-	3 (3-5)	3 (2-4)	4 (3-6)	6 (3-8)	5 (3-8)	19 (11-27)	9 (5-15)	7 (4-11)	8 (5-12)	8 (5-13)	6 (3-9)	6 (4-10)	6 (4-10)	9 (5-15)	5 (2-7)	7 (3-11)	4 (2-6)	3 (1-4)
Respiratory infections and tuberculosis	-	105,621 (94,422-116,608)	454,241 (415,139-490,766)	245,337 (203,354-282,519)	31,730 (25,956-35,673)	18,879 (16,520-20,809)	24,170 (22,140-26,155)	39,504 (36,986-42,569)	54,913 (51,724-58,510)	68,617 (65,062-73,461)	80,108 (76,077-85,239)	91,017 (86,784-96,623)	108,002 (103,090-114,755)	111,678 (106,499-118,341)	119,240 (113,613-125,947)	141,171 (134,267-149,669)	156,303 (147,615-165,880)	170,301 (160,630-181,811)	148,628 (139,219-157,438)	126,309 (117,992-133,795)	88,142 (82,765-92,886)	39,136 (37,251-41,105)	11,213 (10,768-11,784)
Tuberculosis	-	-	32,538 (29,187-35,876)	32,743 (26,891-37,991)	6,758 (5,799-7,658)	6,756 (5,903-7,542)	14,827 (13,453-16,240)	29,749 (27,452-32,401)	43,206 (40,263-46,579)	54,825 (51,641-58,987)	62,821 (59,274-67,631)	70,304 (66,370-75,558)	81,482 (76,819-86,924)	81,476 (76,656-87,210)	85,252 (80,028-91,094)	90,179 (84,778-96,470)	93,421 (86,904-101,008)	89,176 (83,086-96,714)	62,846 (58,470-67,765)	41,924 (38,545-45,574)	19,707 (18,085-21,484)	5,956 (5,465-6,508)	1,038 (946-1,136)
Drug-susceptible tuberculosis	-	-	29,768 (26,460-32,843)	30,311 (24,974-35,162)	6,166 (5,219-7,061)	6,149 (5,337-6,936)	13,560 (12,209-15,000)	27,048 (24,612-29,629)	39,094 (36,018-42,562)	49,555 (45,937-53,618)	56,672 (52,410-61,362)	63,165 (58,387-68,438)	73,057 (67,711-79,019)	73,388 (67,976-79,508)	77,367 (71,153-83,730)	81,337 (75,000-88,253)	84,515 (77,301-91,873)	80,691 (73,756-88,406)	56,964 (52,258-62,052)	38,168 (34,721-41,857)	17,966 (16,315-19,737)	5,449 (4,960-6,010)	952 (865-1,048)
Multidrug-resistant tuberculosis without extensive drug resistance	-	-	2,682 (1,906-3,778)	2,382 (1,591-3,610)	571 (393-822)	586 (381-874)	1,225 (800-1,877)	2,574 (1,801-3,731)	3,909 (2,716-5,579)	5,018 (3,468-7,190)	5,827 (4,040-8,516)	6,730 (4,715-9,676)	7,940 (5,656-11,426)	7,659 (5,420-10,953)	7,535 (5,119-11,312)	8,407 (5,819-12,436)	8,520 (5,764-12,729)	8,118 (5,559-11,813)	5,636 (3,903-8,087)	3,602 (2,478-5,237)	1,667 (1,166-2,366)	485 (344-688)	82 (58-116)
Extensively drug-resistant tuberculosis	-	-	89 (63-122)	51 (35-73)	21 (14-28)	21 (14-29)	41 (29-58)	126 (96-162)	203 (157-256)	251 (194-318)	321 (253-405)	409 (321-509)	484 (381-601)	429 (336-536)	349 (264-460)	435 (334-561)	386 (285-515)	367 (270-490)	246 (177-333)	154 (110-215)	74 (53-102)	22 (16-30)	4 (3-5)
Lower respiratory infections	127,735 (112,930-146,636)	105,463 (94,251-116,461)	419,189 (383,799-453,313)	210,055 (174,656-244,279)	24,578 (19,132-27,779)	11,841 (9,618-13,154)	9,192 (7,727-10,122)	9,620 (8,313-10,725)	11,537 (9,902-12,635)	13,587 (11,972-15,184)	17,055 (14,945-18,524)	20,475 (18,458-22,213)	26,110 (23,240-28,229)	29,813 (26,446-32,295)	33,724 (29,572-36,687)	50,635 (44,024-54,529)	62,366 (54,233-66,713)	80,489 (71,263-87,593)	85,061 (76,306-92,679)	83,496 (75,292-89,367)	67,799 (62,786-72,379)	32,875 (31,138-34,731)	10,103 (9,684-10,659)
Upper respiratory infections	74 (31-100)	133 (58-198)	2,379 (843-3,946)	2,398 (563-4,829)	314 (104-467)	191 (68-265)	99 (41-154)	98 (47-142)	145 (71-198)	184 (85-245)	198 (93-271)	205 (102-301)	243 (118-341)	225 (114-323)	224 (106-329)	310 (139-451)	446 (194-616)	578 (220-775)	684 (257-922)	860 (281-1,178)	623 (225-844)	301 (124-399)	71 (35-92)
Otitis media	19 (4-75)	25 (12-58)	135 (49-323)	142 (42-369)	80 (38-171)	91 (41-199)	52 (28-105)	37 (19-78)	26 (14-52)	22 (11-46)	35 (19-76)	32 (18-65)	167 (76-418)	164 (81-400)	41 (24-74)	47 (30-88)	69 (43-134)	58 (38-108)	37 (25-66)	29 (20-49)	14 (10-23)	4 (3-6)	1 (1-2)
Enteric infections	19,179 (16,089-23,494)	34,367 (28,623-41,713)	292,393 (246,172-339,544)	277,606 (206,469-341,533)	57,381 (40,299-80,137)	33,556 (23,442-46,989)	24,248 (16,082-37,324)	18,306 (11,312-30,173)	15,793 (9,411-26,090)	14,549 (8,594-24,852)	15,664 (8,554-27,386)	15,581 (8,289-29,072)	18,299 (9,059-35,374)	19,358 (9,162-38,556)	24,588 (11,618-50,974)	33,841 (15,639-67,971)	47,692 (21,344-93,855)	59,235 (26,664-118,003)	54,308 (25,454-106,277)	48,567 (21,904-94,827)	31,441 (14,759-60,395)	13,640 (6,422-25,807)	3,498 (1,800-6,379)
Diarrheal diseases	19,179 (16,089-23,494)	34,074 (28,411-41,392)	287,353 (241,664-334,118)	246,629 (180,185-305,578)	21,937 (9,599-40,497)	10,886 (4,650-22,153)	10,806 (4,699-22,263)	10,648 (4,397-21,286)	10,140 (4,190-20,504)	10,184 (4,361-21,045)	12,313 (5,359-24,170)	13,001 (5,511-26,733)	16,162 (6,976-33,722)	17,678 (7,436-36,919)	23,078 (10,029-49,730)	32,409 (14,310-66,636)	46,444 (20,167-92,894)	58,310 (25,735-116,908)	53,726 (24,753-105,938)	48,210 (21,507-94,585)	31,280 (14,594-60,261)	13,594 (6,372-25,771)	3,487 (1,790-6,362)
Typhoid and paratyphoid	-	0 (0-0)	192 (102-342)	19,349 (8,927-36,307)	31,844 (18,396-50,398)	21,001 (11,943-33,511)	12,213 (6,103-21,844)	6,567 (3,394-11,512)	4,748 (2,467-8,074)	3,537 (1,870-6,001)	2,531 (1,304-4,261)	1,802 (936-3,017)	1,405 (746-2,371)	978 (501-1,679)	770 (373-1,420)	668 (330-1,210)	566 (323-930)	402 (240-644)	223 (136-343)	113 (69-171)	50 (32-74)	16 (11-24)	4 (3-6)
Typhoid fever	-	-	173 (92-302)	17,111 (7,844-31,975)	27,953 (15,822-44,810)	18,309 (10,230-29,590)	10,632 (5,185-18,790)	5,718 (2,854-10,115)	4,084 (2,089-7,044)	3,010 (1,522-5,142)	2,138 (1,074-3,626)	1,512 (760-2,571)	1,165 (617-1,960)	811 (413-1,400)	641 (309-1,195)	558 (271-1,026)	477 (264-793)	341 (201-546)	190 (114-297)	97 (59-150)	43 (27-64)	14 (10-21)	4 (3-5)
Paratyphoid fever	-	-	19 (8-40)	2,238 (833-4,971)	3,891 (1,655-7,553)	2,692 (1,176-5,385)	1,582 (607-3,308)	849 (338-1,841)	664 (278-1,410)	527 (227-1,108)	394 (172-788)	290 (123-576)	240 (100-500)	167 (66-351)	129 (50-273)	110 (45-227)	89 (40-174)	61 (28-116)	33 (16-62)	16 (8-29)	7 (3-12)	2 (1-4)	1 (0-1)
Invasive Non-typhoidal Salmonella (iNTS)	-	293 (161-516)	4,150 (2,316-7,545)	11,247 (5,847-20,548)	3,542 (1,700-6,240)	1,633 (722-3,095)	1,197 (503-2,250)	1,058 (444-2,024)	884 (349-1,752)	798 (331-1,555)	794 (340-1,526)	759 (367-1,447)	711 (339-1,341)	688 (317-1,363)	728 (336-1,300)	750 (341-1,346)	665 (311-1,148)	512 (246-914)	352 (168-633)	235 (102-437)	102 (42-183)	29 (8-59)	5 (0-15)
Other intestinal infectious diseases	-	-	698 (313-1,705)	381 (147-1,143)	57 (16-150)	35 (8-99)	31 (6-88)	33 (6-89)	21 (3-53)	30 (5-70)	26 (4-65)	19 (2-47)	20 (2-51)	14 (1-37)	11 (1-30)	13 (1-34)	16 (2-41)	11 (1-29)	7 (0-18)	9 (0-24)	9 (0-25)	2 (0-5)	1 (0-2)
Neglected tropical diseases and malaria	292 (129-1,034)	16,663 (10,565-25,066)	104,401 (70,980-147,579)	196,655 (135,519-275,916)	37,205 (26,339-53,902)	21,942 (15,795-32,025)	16,150 (11,247-24,872)	16,036 (11,322-23,339)	13,569 (9,380-20,320)	12,224 (8,508-18,120)	12,455 (8,700-17,724)	12,098 (8,645-16,817)	11,355 (8,288-15,461)	11,466 (8,264-15,540)	11,066 (7,979-14,905)	12,453 (9,185-17,067)	11,375 (8,182-15,792)	11,797 (8,336-16,606)	5,565 (4,157-7,595)	2,720 (2,106-3,744)	1,018 (810-1,427)	301 (234-447)	74 (55-129)
Malaria	-	16,382 (10,198-24,698)	99,744 (64,997-142,852)	179,157 (118,869-256,411)	27,263 (17,995-38,701)	15,138 (9,997-21,205)	10,263 (6,998-14,423)	10,274 (6,880-14,817)	8,776 (5,909-12,817)	7,791 (5,190-11,294)	8,156 (5,465-12,016)	8,054 (5,332-11,805)	7,099 (4,624-10,561)	8,063 (5,309-11,836)	7,578 (4,932-11,136)	9,114 (5,990-13,583)	8,416 (5,456-12,773)	9,003 (5,712-13,790)	3,388 (2,150-5,268)	1,512 (1,028-2,292)	403 (282-616)	93 (50-155)	19 (11-49)
Chagas disease	-	-	2 (2-3)	1 (1-1)	2 (2-2)	4 (3-4)	10 (8-12)	25 (22-28)	45 (41-51)	96 (87-105)	158 (145-172)	244 (225-263)	352 (325-379)	453 (423-484)	506 (463-554)	659 (607-712)	673 (627-724)	678 (618-742)	397 (361-435)	275 (255-295)	167 (156-178)	58 (53-63)	12 (11-14)
Leishmaniasis	-	-	1,278 (1-5,918)	5,508 (2-30,626)	2,621 (0-18,925)	1,720 (0-11,778)	1,835 (1-10,794)	1,493 (1-7,995)	1,555 (1-7,790)	1,390 (1-6,722)	1,218 (5-5,418)	975 (7-4,076)	867 (8-3,234)	711 (12-2,628)	605 (14-2,274)	581 (15-2,077)	497 (18-1,725)	361 (13-1,236)	211 (5-769)	109 (1-392)	43 (1-152)	11 (0-42)	2 (0-8)
Visceral leishmaniasis	-	-	1,278 (1-5,918)	5,508 (2-30,626)	2,621 (0-18,925)	1,720 (0-11,778)	1,835 (1-10,794)	1,493 (1-7,995)	1,555 (1-7,790)	1,390 (1-6,722)	1,218 (5-5,418)	975 (7-4,076)	867 (8-3,234)	711 (12-2,628)	605 (14-2,274)	581 (15-2,077)	497 (18-1,725)	361 (13-1,236)	211 (5-769)	109 (1-392)	43 (1-152)	11 (0-42)	2 (0-8)
African trypanosomiasis	-	-	-	455 (86-1,530)	759 (176-2,249)	823 (205-2,093)	1,130 (298-2,985)	1,331 (316-3,565)	1,066 (289-3,044)	944 (243-2,479)	859 (240-2,329)	784 (169-2,389)	776 (169-2,763)	446 (102-1,455)	409 (89-1,245)	299 (59-1,047)	227 (49-734)	149 (31-537)	92 (19-335)	40 (8-130)	14 (3-45)	3 (1-10)	0 (0-2)
Schistosomiasis	-	-	0 (0-0)	431 (278-660)	168 (120-235)	217 (160-291)	244 (186-312)	283 (219-360)	270 (214-337)	283 (224-349)	331 (265-411)	379 (304-469)	441 (349-542)	478 (384-589)	524 (414-654)	588 (474-736)	445 (349-549)	566 (448-700)	414 (329-510)	230 (181-285)	91 (70-118)	21 (15-29)	4 (2-5)
Cysticercosis	0 (0-0)	0 (0-0)	0 (0-0)	82 (34-144)	37 (8-87)	27 (3-68)	29 (3-78)	42 (5-103)	56 (11-127)	54 (8-125)	50 (7-122)	47 (7-119)	41 (4-107)	33 (3-89)	28 (2-71)	23 (1-67)	19 (1-53)	4 (0-14)	9 (0-29)	5 (0-17)	2 (0-8)	1 (0-3)	0 (0-1)
Cystic echinococcosis	-	-	-	229 (133-342)	98 (44-171)	63 (18-127)	31 (1-86)	36 (2-93)	49 (6-124)	49 (6-123)	59 (7-144)	56 (6-139)	61 (7-150)	61 (8-148)	64 (9-155)	70 (10-169)	67 (10-161)	62 (9-148)	39 (3-93)	24 (1-64)	15 (0-43)	5 (0-16)	1 (0-5)
Dengue	-	-	1,402 (277-2,588)	2,688 (856-4,555)	979 (367-1,526)	489 (163-803)	536 (187-840)	547 (201-908)	483 (169-786)	304 (105-525)	358 (98-681)	232 (90-456)	280 (85-489)	260 (83-471)	283 (84-473)	267 (91-466)	291 (109-534)	297 (147-593)	285 (128-627)	315 (128-683)	185 (90-505)	69 (33-189)	22 (10-66)
Yellow fever	-	3 (1-10)	54 (11-179)	510 (95-1,569)	780 (155-2,393)	683 (144-2,027)	691 (141-1,938)	621 (130-1,816)	428 (81-1,219)	338 (70-1,048)	291 (58-875)	252 (49-757)	204 (41-607)	155 (29-468)	134 (26-427)	98 (20-308)	69 (14-213)	42 (8-132)	251 (107-382)	8 (1-23)	2 (0-7)	9 (1-28)	2 (0-8)
Rabies	-	-	394 (267-579)	2,321 (1,409-3,637)	3,619 (2,586-5,244)	2,489 (1,809-3,485)	1,213 (865-2,016)	1,217 (885-1,853)	647 (499-909)	773 (553-1,134)	746 (550-1,056)	859 (659-1,185)	1,006 (748-1,524)	601 (468-809)	718 (563-959)	513 (400-765)	443 (342-587)	389 (297-515)	293 (220-399)	83 (61-113)	32 (24-44)	7 (5-9)	2 (2-3)
Intestinal nematode infections	-	-	295 (219-393)	2,932 (2,078-4,035)	493 (377-653)	106 (81-134)	30 (22-39)	25 (19-33)	19 (14-25)	25 (19-33)	24 (17-31)	17 (12-23)	19 (13-24)	17 (12-22)	15 (11-20)	33 (25-43)	17 (12-22)	27 (20-35)	19 (14-26)	12 (8-16)	5 (3-7)	3 (2-4)	1 (0-1)
Ascariasis	-	-	295 (219-393)	2,932 (2,078-4,035)	493 (377-653)	106 (81-134)	30 (22-39)	25 (19-33)	19 (14-25)	25 (19-33)	24 (17-31)	17 (12-23)	19 (13-24)	17 (12-22)	15 (11-20)	33 (25-43)	17 (12-22)	27 (20-35)	19 (14-26)	12 (8-16)	5 (3-7)	3 (2-4)	1 (0-1)
Ebola	1 (1-2)	2 (1-2)	22 (17-29)	54 (41-71)	21 (16-28)	12 (9-15)	8 (6-11)	7 (5-9)	6 (4-8)	6 (4-7)	6 (4-8)	5 (4-7)	5 (4-7)	5 (4-6)	5 (4-7)	5 (4-7)	5 (4-6)	3 (2-4)	2 (2-3)	1 (1-2)	1 (0-1)	0 (0-0)	0 (0-0)
Zika virus	0 (0-0)	0 (0-0)	0 (0-0)	0 (0-0)	0 (0-0)	0 (0-0)	0 (0-0)	0 (0-0)	0 (0-0)	0 (0-0)	0 (0-0)	0 (0-0)	0 (0-0)	0 (0-0)	0 (0-0)	0 (0-0)	0 (0-0)	0 (0-0)	0 (0-0)	0 (0-0)	0 (0-0)	0 (0-0)	0 (0-0)
Other neglected tropical diseases	291 (127-1,033)	275 (120-980)	1,208 (569-4,264)	2,288 (633-10,254)	365 (205-1,084)	171 (107-438)	131 (91-275)	135 (88-305)	168 (105-357)	170 (104-313)	200 (120-381)	193 (124-369)	205 (124-400)	184 (122-382)	196 (128-410)	204 (140-404)	207 (135-430)	215 (151-460)	164 (116-369)	106 (76-236)	58 (40-130)	21 (15-50)	7 (5-17)
Other infectious diseases	32,836 (26,897-39,666)	38,867 (31,709-46,741)	210,107 (155,187-294,214)	282,276 (186,812-432,342)	49,612 (35,405-72,351)	22,914 (18,368-30,015)	17,930 (14,933-21,275)	14,999 (12,612-16,820)	13,952 (11,462-15,495)	15,142 (12,740-16,389)	15,562 (12,886-16,933)	15,540 (12,828-17,057)	17,836 (14,308-19,621)	15,505 (12,635-17,105)	15,217 (12,629-16,858)	15,685 (12,908-17,130)	15,807 (13,262-17,643)	14,293 (12,289-15,768)	11,545 (9,693-12,612)	7,818 (6,599-8,567)	4,442 (3,813-4,886)	1,542 (1,309-1,701)	416 (352-466)
Meningitis	8,272 (6,013-10,318)	11,265 (8,920-13,472)	64,580 (54,610-74,709)	59,699 (45,966-72,683)	12,026 (8,567-15,482)	7,315 (5,313-9,274)	6,308 (4,853-8,428)	5,414 (4,188-7,126)	4,680 (3,474-6,014)	4,907 (3,840-6,522)	4,889 (3,844-6,508)	4,289 (3,421-5,861)	4,746 (3,794-6,291)	4,132 (3,392-5,600)	3,995 (3,218-5,489)	4,280 (3,475-5,878)	3,689 (2,987-5,128)	3,600 (2,866-4,987)	2,928 (2,374-4,122)	1,875 (1,481-2,470)	1,164 (898-1,529)	335 (262-419)	83 (64-100)
Pneumococcal meningitis	1,277 (885-1,716)	1,690 (1,227-2,169)	8,891 (6,813-11,518)	8,843 (6,143-11,991)	1,172 (765-1,591)	648 (454-930)	645 (458-929)	629 (454-869)	542 (397-761)	627 (468-858)	712 (527-957)	677 (499-943)	806 (590-1,129)	731 (560-986)	685 (519-944)	746 (552-1,047)	607 (459-847)	631 (469-883)	512 (383-728)	317 (235-432)	194 (144-256)	50 (37-64)	12 (8-16)
H influenzae type B meningitis	2,250 (1,502-3,037)	3,017 (2,260-4,013)	19,396 (15,257-24,153)	18,855 (13,457-25,132)	4,546 (3,091-5,986)	2,778 (1,925-3,608)	2,146 (1,644-2,883)	1,631 (1,197-2,128)	1,543 (1,117-1,973)	1,521 (1,179-2,006)	1,445 (1,121-1,957)	1,266 (1,000-1,770)	1,434 (1,151-1,907)	1,200 (965-1,661)	1,194 (928-1,712)	1,288 (1,032-1,825)	1,243 (993-1,769)	1,171 (921-1,660)	966 (762-1,388)	638 (487-892)	395 (293-515)	130 (96-161)	32 (23-39)
Meningococcal meningitis	1,326 (963-1,721)	1,887 (1,455-2,332)	10,018 (8,177-12,123)	8,663 (6,433-11,241)	1,459 (1,073-1,921)	754 (571-979)	623 (488-805)	496 (374-640)	410 (305-536)	439 (337-587)	435 (337-586)	377 (293-504)	406 (313-542)	379 (295-515)	366 (280-490)	394 (294-554)	324 (245-451)	315 (238-424)	261 (198-365)	166 (125-228)	96 (73-130)	25 (19-32)	7 (5-9)
Other meningitis	3,420 (2,486-4,424)	4,671 (3,653-5,767)	26,275 (21,413-31,766)	23,339 (17,621-29,973)	4,849 (3,384-6,210)	3,135 (2,225-4,022)	2,894 (2,188-3,898)	2,659 (1,979-3,475)	2,185 (1,587-2,817)	2,321 (1,754-3,101)	2,299 (1,740-3,066)	1,969 (1,525-2,637)	2,100 (1,674-2,820)	1,822 (1,485-2,520)	1,750 (1,392-2,409)	1,851 (1,444-2,517)	1,515 (1,199-2,107)	1,484 (1,154-2,062)	1,189 (940-1,672)	755 (588-1,007)	479 (368-629)	131 (101-168)	33 (25-41)
Encephalitis	430 (356-515)	724 (553-930)	8,347 (6,234-11,296)	8,192 (5,919-12,457)	4,010 (3,296-4,957)	3,253 (2,666-4,090)	2,141 (1,891-2,395)	1,756 (1,579-1,962)	1,589 (1,448-1,786)	1,827 (1,632-2,061)	1,893 (1,711-2,123)	1,679 (1,541-1,836)	1,611 (1,478-1,793)	1,419 (1,307-1,559)	1,643 (1,498-1,832)	1,997 (1,819-2,183)	2,450 (2,163-2,767)	2,616 (2,268-2,950)	2,359 (1,968-2,698)	1,716 (1,389-1,986)	781 (641-891)	232 (197-262)	50 (43-57)
Diphtheria	-	-	1,158 (682-1,837)	3,939 (2,134-7,454)	646 (387-1,060)	238 (143-377)	77 (47-126)	46 (28-72)	40 (26-63)	41 (26-64)	45 (29-71)	55 (37-82)	93 (63-140)	65 (44-96)	41 (27-62)	-	-	-	-	-	-	-	-
Whooping cough	-	-	33,760 (15,626-63,268)	28,588 (12,843-53,906)	2,786 (1,232-5,714)	613 (270-1,244)	276 (122-564)	180 (82-357)	108 (47-221)	88 (38-184)	60 (26-124)	79 (35-163)	64 (28-131)	45 (20-91)	62 (27-125)	-	-	-	-	-	-	-	-
Tetanus	20,287 (15,697-26,756)	23,241 (17,605-30,510)	7,143 (4,987-10,997)	4,529 (1,749-7,777)	1,941 (1,109-2,940)	1,243 (765-1,862)	2,004 (1,085-3,150)	1,772 (852-2,681)	1,879 (795-2,945)	1,723 (1,027-2,401)	1,916 (1,058-2,586)	2,290 (1,083-3,230)	3,153 (1,372-4,510)	2,496 (1,150-3,582)	2,760 (1,121-4,196)	2,117 (943-3,062)	2,100 (1,097-3,350)	1,486 (843-2,109)	978 (513-1,577)	529 (290-903)	244 (132-432)	77 (44-135)	21 (11-33)
Measles	-	-	78,693 (27,845-168,650)	162,438 (61,586-324,826)	22,414 (8,447-47,299)	5,936 (2,156-13,261)	2,147 (727-4,977)	952 (319-2,239)	397 (133-920)	242 (83-559)	155 (53-356)	122 (42-279)	95 (33-217)	69 (24-158)	78 (27-178)	-	-	-	-	-	-	-	-
Varicella and herpes zoster	101 (78-136)	284 (217-374)	2,191 (1,718-2,811)	2,359 (1,767-3,086)	901 (685-1,174)	314 (242-405)	166 (132-213)	176 (141-224)	227 (184-287)	287 (235-348)	246 (204-306)	150 (124-184)	145 (118-177)	126 (102-155)	145 (117-177)	176 (145-214)	339 (278-414)	315 (263-382)	410 (339-501)	548 (446-671)	526 (426-651)	276 (222-357)	112 (88-150)
Acute hepatitis	-	-	6,880 (3,513-8,504)	7,326 (3,891-9,577)	3,147 (1,751-3,980)	2,747 (1,553-3,379)	3,139 (1,852-3,716)	3,456 (2,220-4,013)	3,911 (2,405-4,454)	4,746 (2,855-5,386)	4,842 (3,062-5,474)	5,645 (3,703-6,472)	6,494 (4,315-7,335)	5,766 (3,911-6,457)	5,097 (3,427-5,719)	5,231 (3,380-5,915)	5,156 (3,393-5,818)	4,082 (2,797-4,570)	3,065 (2,084-3,475)	1,765 (1,173-2,014)	822 (570-929)	227 (166-255)	42 (31-48)
Acute hepatitis A	-	-	1,050 (420-1,817)	4,369 (2,165-6,231)	2,017 (1,130-2,717)	1,323 (748-1,824)	1,086 (656-1,507)	935 (539-1,341)	805 (419-1,247)	768 (341-1,296)	728 (326-1,284)	790 (404-1,390)	825 (401-1,441)	572 (296-889)	302 (158-471)	193 (91-318)	121 (58-199)	59 (23-105)	26 (8-52)	5 (1-12)	1 (0-3)	0 (0-0)	0 (0-0)
Acute hepatitis B	-	-	4,409 (2,028-5,942)	2,268 (970-3,780)	594 (252-1,029)	514 (238-901)	741 (389-1,238)	1,173 (629-1,870)	1,951 (1,081-2,806)	3,045 (1,714-4,148)	3,430 (1,992-4,432)	4,316 (2,654-5,349)	5,189 (3,311-6,284)	4,866 (3,140-5,687)	4,556 (2,959-5,217)	4,791 (3,049-5,522)	4,742 (3,085-5,414)	3,708 (2,555-4,262)	2,696 (1,766-3,167)	1,476 (949-1,792)	631 (416-788)	155 (97-202)	25 (14-35)
Acute hepatitis C	-	-	1,410 (617-2,512)	530 (211-1,032)	55 (20-118)	21 (7-45)	13 (4-28)	11 (3-24)	12 (4-31)	18 (5-46)	27 (7-73)	35 (10-81)	52 (14-131)	54 (17-128)	52 (16-118)	69 (19-174)	83 (22-219)	77 (23-189)	70 (19-183)	50 (14-135)	33 (11-82)	12 (3-29)	3 (1-7)
Acute hepatitis E	-	-	11 (4-20)	159 (64-319)	480 (229-792)	888 (420-1,325)	1,298 (678-1,813)	1,337 (717-1,899)	1,142 (558-1,781)	914 (353-1,602)	657 (251-1,254)	504 (196-997)	428 (167-923)	273 (108-566)	187 (71-362)	178 (75-360)	210 (77-427)	237 (97-474)	274 (107-555)	234 (94-450)	156 (70-282)	61 (29-101)	14 (7-24)
Other unspecified infectious diseases	3,745 (1,735-4,770)	3,352 (1,515-4,264)	7,353 (4,252-9,580)	5,207 (3,313-7,554)	1,740 (1,220-2,244)	1,254 (905-1,591)	1,671 (1,174-2,147)	1,246 (869-1,554)	1,121 (823-1,347)	1,279 (947-1,521)	1,515 (1,138-1,803)	1,231 (941-1,450)	1,436 (1,141-1,677)	1,387 (1,096-1,586)	1,395 (1,102-1,604)	1,884 (1,421-2,180)	2,073 (1,602-2,366)	2,195 (1,658-2,576)	1,803 (1,322-2,022)	1,385 (1,006-1,587)	907 (636-1,060)	395 (276-461)	108 (76-127)
Maternal and neonatal disorders	1,218,787 (1,165,616-1,267,987)	223,733 (205,294-251,452)	57,206 (49,067-69,372)	5,237 (4,194-7,034)	-	-	-	-	-	-	-	-	-	-	-	-	-	-	-	-	-	-	-
Maternal disorders	-	-	-	-	-	-	-	-	-	-	-	-	-	-	-	-	-	-	-	-	-	-	-
Maternal hemorrhage	-	-	-	-	-	-	-	-	-	-	-	-	-	-	-	-	-	-	-	-	-	-	-
Maternal sepsis and other maternal infections	-	-	-	-	-	-	-	-	-	-	-	-	-	-	-	-	-	-	-	-	-	-	-
Maternal hypertensive disorders	-	-	-	-	-	-	-	-	-	-	-	-	-	-	-	-	-	-	-	-	-	-	-
Maternal obstructed labor and uterine rupture	-	-	-	-	-	-	-	-	-	-	-	-	-	-	-	-	-	-	-	-	-	-	-
Maternal abortion and miscarriage	-	-	-	-	-	-	-	-	-	-	-	-	-	-	-	-	-	-	-	-	-	-	-
Ectopic pregnancy	-	-	-	-	-	-	-	-	-	-	-	-	-	-	-	-	-	-	-	-	-	-	-
Indirect maternal deaths	-	-	-	-	-	-	-	-	-	-	-	-	-	-	-	-	-	-	-	-	-	-	-
Late maternal deaths	-	-	-	-	-	-	-	-	-	-	-	-	-	-	-	-	-	-	-	-	-	-	-
Maternal deaths aggravated by HIV/AIDS	-	-	-	-	-	-	-	-	-	-	-	-	-	-	-	-	-	-	-	-	-	-	-
Other maternal disorders	-	-	-	-	-	-	-	-	-	-	-	-	-	-	-	-	-	-	-	-	-	-	-
Neonatal disorders	1,218,787 (1,165,616-1,267,987)	223,733 (205,294-251,452)	57,206 (49,067-69,372)	5,237 (4,194-7,034)	-	-	-	-	-	-	-	-	-	-	-	-	-	-	-	-	-	-	-
Neonatal preterm birth	500,818 (462,515-563,431)	70,306 (62,426-84,992)	23,503 (19,931-29,554)	1,478 (1,143-1,978)	-	-	-	-	-	-	-	-	-	-	-	-	-	-	-	-	-	-	-
Neonatal encephalopathy due to birth asphyxia and trauma	390,426 (348,714-428,338)	39,164 (32,098-45,132)	10,675 (8,746-13,673)	2,149 (1,692-3,101)	-	-	-	-	-	-	-	-	-	-	-	-	-	-	-	-	-	-	-
Neonatal sepsis and other neonatal infections	78,106 (63,164-99,890)	51,604 (40,753-70,676)	6,515 (4,986-9,758)	238 (139-470)	-	-	-	-	-	-	-	-	-	-	-	-	-	-	-	-	-	-	-
Hemolytic disease and other neonatal jaundice	33,459 (27,267-41,797)	19,130 (14,523-24,495)	3,528 (2,364-4,967)	230 (140-372)	-	-	-	-	-	-	-	-	-	-	-	-	-	-	-	-	-	-	-
Other neonatal disorders	215,978 (169,028-248,236)	43,529 (33,848-55,775)	12,985 (9,753-17,631)	1,141 (794-1,681)	-	-	-	-	-	-	-	-	-	-	-	-	-	-	-	-	-	-	-
Nutritional deficiencies	-	-	100,744 (84,982-112,610)	90,471 (71,314-107,989)	7,854 (6,327-9,217)	2,609 (2,178-2,960)	1,827 (1,548-2,032)	1,728 (1,479-1,907)	1,704 (1,512-1,837)	1,888 (1,736-1,990)	2,350 (2,135-2,488)	2,649 (2,415-2,797)	3,102 (2,764-3,304)	3,564 (3,208-3,778)	4,728 (4,171-5,075)	6,129 (5,487-6,512)	7,004 (6,152-7,530)	9,009 (7,901-9,793)	8,600 (7,578-9,275)	7,424 (6,675-7,991)	5,882 (5,394-6,331)	3,140 (2,833-3,492)	1,184 (1,066-1,329)
Protein-energy malnutrition	-	-	97,470 (82,428-108,974)	87,280 (68,660-104,375)	6,643 (5,413-7,788)	2,104 (1,813-2,363)	1,386 (1,193-1,532)	1,350 (1,172-1,482)	1,388 (1,255-1,485)	1,628 (1,496-1,718)	1,882 (1,742-1,988)	2,170 (1,988-2,287)	2,461 (2,217-2,614)	2,919 (2,657-3,093)	3,741 (3,402-3,992)	4,972 (4,546-5,256)	5,530 (4,991-5,885)	7,095 (6,367-7,656)	6,867 (6,180-7,403)	6,234 (5,630-6,726)	5,016 (4,535-5,437)	2,663 (2,264-3,023)	1,028 (889-1,177)
Other nutritional deficiencies	-	-	3,274 (2,264-4,200)	3,191 (1,777-4,379)	1,211 (804-1,610)	505 (347-655)	442 (318-570)	378 (279-507)	316 (237-419)	260 (201-349)	468 (352-603)	480 (362-633)	641 (478-827)	645 (476-861)	987 (692-1,289)	1,157 (825-1,500)	1,474 (1,028-1,865)	1,914 (1,356-2,414)	1,733 (1,232-2,218)	1,190 (868-1,653)	866 (647-1,308)	477 (355-789)	156 (112-270)
Non-communicable diseases	129,100 (115,561-160,793)	55,595 (49,540-68,110)	229,668 (199,907-268,214)	136,265 (115,569-157,972)	65,850 (59,716-71,498)	61,244 (56,832-65,270)	88,159 (82,731-92,456)	115,074 (110,136-119,178)	169,528 (163,913-176,185)	251,502 (244,961-259,550)	351,672 (343,497-359,278)	501,126 (491,564-510,088)	749,552 (736,826-760,725)	923,447 (908,794-935,943)	1,102,441 (1,082,863-1,117,107)	1,644,579 (1,617,846-1,665,166)	2,027,863 (1,987,757-2,054,323)	2,370,075 (2,320,671-2,401,496)	2,118,152 (2,076,234-2,145,767)	1,627,203 (1,589,499-1,652,019)	1,110,534 (1,086,382-1,125,918)	473,247 (463,406-479,647)	120,167 (117,783-121,661)
Neoplasms	899 (707-1,000)	630 (504-739)	6,392 (4,988-7,572)	23,671 (18,942-30,831)	23,186 (20,292-25,992)	21,311 (18,825-23,508)	22,600 (20,503-23,824)	23,228 (21,754-24,120)	34,003 (32,014-35,429)	56,311 (53,781-58,432)	80,089 (77,323-82,986)	121,884 (117,709-124,702)	212,991 (208,058-217,707)	273,921 (268,317-278,533)	328,947 (322,609-334,174)	482,411 (474,571-488,760)	570,075 (560,169-578,031)	603,231 (592,011-610,699)	475,836 (464,014-483,078)	304,416 (293,302-309,600)	167,884 (159,224-170,752)	55,087 (51,342-56,161)	10,730 (9,787-10,967)
Lip and oral cavity cancer	-	-	-	-	-	-	314 (281-350)	398 (366-435)	680 (607-756)	1,264 (1,159-1,376)	2,382 (2,208-2,562)	4,341 (4,094-4,622)	7,159 (6,813-7,550)	9,105 (8,727-9,560)	10,249 (9,757-10,836)	11,986 (11,450-12,527)	10,764 (10,291-11,289)	9,197 (8,783-9,641)	6,004 (5,724-6,287)	3,748 (3,562-3,917)	1,973 (1,877-2,045)	710 (673-737)	138 (132-143)
Nasopharynx cancer	-	-	-	-	210 (176-243)	299 (258-337)	349 (323-375)	402 (372-435)	685 (623-744)	1,367 (1,217-1,505)	1,845 (1,660-2,024)	2,825 (2,637-3,033)	4,163 (3,890-4,441)	4,425 (4,182-4,662)	4,732 (4,469-5,016)	4,905 (4,663-5,142)	4,729 (4,501-4,939)	4,056 (3,880-4,234)	2,447 (2,330-2,574)	1,580 (1,492-1,684)	755 (717-798)	243 (233-256)	42 (40-44)
Other pharynx cancer	-	-	-	-	-	-	126 (84-147)	150 (92-173)	223 (134-261)	521 (347-588)	1,097 (821-1,236)	2,451 (2,014-2,677)	4,772 (3,960-5,197)	6,489 (5,573-6,974)	7,311 (6,174-8,005)	8,037 (6,925-8,731)	7,205 (6,084-7,913)	5,631 (4,719-6,145)	3,205 (2,685-3,494)	1,868 (1,491-2,057)	761 (628-830)	206 (172-222)	30 (26-32)
Esophageal cancer	-	-	-	-	-	-	165 (153-182)	267 (252-285)	522 (489-564)	1,373 (1,291-1,480)	3,017 (2,868-3,187)	7,158 (6,826-7,517)	17,263 (16,530-18,086)	22,729 (21,954-23,637)	25,799 (24,895-26,772)	37,399 (36,221-38,651)	42,945 (41,649-44,362)	42,835 (41,338-44,236)	30,496 (29,392-31,504)	17,211 (16,588-17,911)	7,456 (7,209-7,726)	1,929 (1,866-1,993)	301 (290-312)
Stomach cancer	-	-	-	-	-	-	521 (488-558)	1,043 (991-1,100)	2,491 (2,350-2,671)	5,791 (5,481-6,212)	9,013 (8,644-9,438)	14,309 (13,773-14,959)	28,132 (27,135-29,232)	34,593 (33,599-35,778)	41,046 (39,796-42,499)	65,930 (64,328-67,813)	79,250 (77,515-81,195)	84,621 (82,710-86,617)	62,769 (61,450-64,304)	38,642 (37,720-39,689)	19,907 (19,479-20,391)	5,991 (5,862-6,138)	1,109 (1,084-1,136)
Colon and rectum cancer	-	-	-	-	-	-	554 (424-619)	1,014 (837-1,104)	2,009 (1,745-2,170)	3,891 (3,530-4,156)	5,232 (4,825-5,541)	7,444 (7,116-7,810)	13,062 (12,323-13,625)	17,632 (17,070-18,293)	23,476 (22,805-24,311)	36,910 (35,977-38,015)	46,245 (45,124-47,602)	53,179 (51,919-54,535)	45,516 (44,587-46,624)	31,334 (30,635-32,151)	19,215 (18,795-19,683)	6,633 (6,486-6,792)	1,411 (1,377-1,449)
Liver cancer	-	-	-	-	662 (559-766)	791 (676-895)	835 (764-916)	1,556 (1,389-1,686)	4,784 (4,136-5,267)	12,053 (10,221-13,511)	18,130 (15,922-20,049)	25,086 (22,363-27,129)	39,585 (35,541-42,474)	41,167 (38,968-43,514)	41,426 (38,815-45,315)	52,424 (50,097-55,548)	56,237 (53,909-58,960)	51,132 (49,049-53,274)	34,799 (33,431-36,487)	19,122 (18,219-20,198)	8,372 (8,034-8,789)	2,193 (2,098-2,285)	349 (335-364)
Liver cancer due to hepatitis B	-	-	-	-	492 (410-569)	644 (545-733)	664 (603-728)	1,233 (1,086-1,346)	3,714 (3,164-4,149)	9,008 (7,484-10,214)	12,768 (10,808-14,444)	16,736 (14,778-18,452)	25,808 (22,636-28,392)	24,222 (21,592-26,285)	21,263 (18,691-24,082)	24,003 (21,372-26,800)	23,280 (21,053-25,633)	18,865 (16,648-20,990)	11,020 (9,913-12,175)	5,240 (4,634-5,898)	1,882 (1,683-2,083)	416 (361-471)	54 (43-65)
Liver cancer due to hepatitis C	-	-	-	-	10 (7-13)	14 (10-17)	13 (10-16)	42 (35-51)	205 (157-255)	708 (567-850)	1,343 (1,053-1,660)	2,347 (1,924-2,775)	4,549 (3,617-5,441)	6,797 (5,797-7,818)	9,399 (8,047-10,895)	13,785 (11,909-15,718)	15,326 (13,834-16,970)	14,791 (13,369-16,285)	11,220 (10,033-12,461)	7,045 (6,352-7,777)	3,558 (3,267-3,834)	1,036 (948-1,123)	184 (165-201)
Liver cancer due to alcohol use	-	-	-	-	-	-	4 (2-6)	40 (31-52)	196 (138-270)	747 (546-970)	1,825 (1,240-2,454)	3,137 (2,348-4,038)	4,840 (3,516-6,703)	5,569 (4,148-7,587)	5,961 (4,469-7,686)	8,515 (6,694-10,725)	11,284 (9,584-13,207)	11,358 (9,769-13,312)	7,711 (6,798-8,798)	3,891 (3,319-4,473)	1,663 (1,452-1,881)	407 (345-474)	58 (46-71)
Liver cancer due to NASH	-	-	-	-	-	-	52 (41-65)	76 (61-92)	184 (138-233)	422 (298-530)	614 (481-771)	871 (693-1,078)	1,462 (1,111-1,834)	1,771 (1,387-2,191)	2,159 (1,672-2,729)	3,052 (2,441-3,752)	3,466 (2,777-4,261)	3,612 (2,931-4,451)	3,028 (2,474-3,702)	1,906 (1,535-2,320)	847 (715-987)	229 (189-276)	37 (29-47)
Liver cancer due to other causes	-	-	-	-	159 (127-192)	133 (104-160)	101 (81-122)	165 (134-196)	485 (361-600)	1,168 (862-1,464)	1,580 (1,191-2,002)	1,994 (1,520-2,509)	2,926 (2,263-3,671)	2,809 (2,200-3,534)	2,645 (2,044-3,230)	3,070 (2,447-3,774)	2,881 (2,301-3,499)	2,505 (1,993-3,108)	1,820 (1,499-2,198)	1,040 (846-1,273)	422 (352-499)	105 (85-126)	16 (12-20)
Gallbladder and biliary tract cancer	-	-	-	-	-	-	46 (39-58)	68 (56-88)	134 (107-178)	352 (274-470)	589 (475-747)	989 (835-1,215)	1,748 (1,455-2,119)	2,747 (2,365-3,262)	3,672 (3,175-4,382)	5,615 (4,922-6,550)	7,158 (6,354-8,385)	8,189 (7,304-9,485)	7,269 (6,582-8,258)	5,012 (4,472-5,625)	3,059 (2,596-3,337)	1,058 (908-1,135)	194 (165-206)
Pancreatic cancer	-	-	-	-	-	-	90 (83-98)	162 (151-176)	368 (336-393)	999 (910-1,074)	1,923 (1,777-2,056)	3,432 (3,325-3,558)	6,343 (6,060-6,644)	9,395 (9,140-9,700)	12,255 (11,971-12,634)	17,549 (17,140-18,056)	20,717 (20,233-21,375)	21,715 (21,208-22,358)	17,280 (16,921-17,689)	10,655 (10,435-10,866)	5,897 (5,787-6,005)	1,818 (1,779-1,855)	323 (315-331)
Larynx cancer	-	-	-	-	-	-	86 (80-92)	126 (118-133)	195 (186-205)	508 (476-538)	1,276 (1,193-1,348)	3,074 (2,928-3,234)	6,380 (6,059-6,678)	9,324 (8,965-9,694)	10,858 (10,405-11,310)	14,017 (13,523-14,508)	13,984 (13,399-14,520)	12,212 (11,682-12,633)	7,450 (7,172-7,731)	4,404 (4,202-4,648)	1,983 (1,910-2,072)	568 (549-594)	96 (93-100)
Tracheal, bronchus, and lung cancer	-	-	-	-	-	-	609 (568-660)	1,067 (993-1,150)	2,548 (2,395-2,737)	5,772 (5,352-6,218)	10,183 (9,469-10,704)	19,606 (18,895-20,360)	43,131 (41,446-44,590)	63,975 (62,497-65,690)	82,139 (80,217-84,131)	131,014 (128,809-133,764)	157,531 (154,856-160,649)	164,084 (161,423-166,853)	118,736 (116,795-121,018)	64,589 (63,404-65,878)	30,094 (29,504-30,745)	8,103 (7,951-8,262)	1,317 (1,290-1,346)
Malignant skin melanoma	-	-	-	-	-	-	134 (102-178)	226 (173-304)	386 (283-499)	696 (508-925)	1,033 (743-1,358)	1,463 (1,045-1,922)	1,731 (1,204-2,300)	2,072 (1,394-2,675)	2,176 (1,497-2,814)	2,320 (1,522-3,063)	2,475 (1,650-3,210)	2,622 (1,710-3,342)	2,266 (1,440-2,957)	1,538 (996-1,955)	923 (615-1,200)	332 (219-434)	69 (48-94)
Non-melanoma skin cancer	-	-	-	-	-	-	104 (97-111)	144 (136-152)	223 (209-240)	396 (373-425)	495 (471-527)	762 (725-802)	1,032 (995-1,074)	1,422 (1,349-1,489)	1,741 (1,641-1,825)	3,069 (2,896-3,208)	3,669 (3,501-3,817)	4,050 (3,866-4,205)	3,478 (3,312-3,611)	2,736 (2,561-2,856)	1,988 (1,890-2,061)	980 (930-1,017)	296 (282-306)
Non-melanoma skin cancer (squamous-cell carcinoma)	-	-	-	-	-	-	104 (97-111)	144 (136-152)	223 (209-240)	396 (373-425)	495 (471-527)	762 (725-802)	1,032 (995-1,074)	1,422 (1,349-1,489)	1,741 (1,641-1,825)	3,069 (2,896-3,208)	3,669 (3,501-3,817)	4,050 (3,866-4,205)	3,478 (3,312-3,611)	2,736 (2,561-2,856)	1,988 (1,890-2,061)	980 (930-1,017)	296 (282-306)
Breast cancer	-	-	-	-	-	-	33 (31-34)	31 (30-32)	38 (35-40)	68 (60-76)	164 (146-182)	239 (218-258)	411 (375-449)	493 (462-525)	601 (570-638)	790 (754-834)	829 (797-871)	902 (866-942)	633 (612-657)	435 (417-452)	303 (291-314)	107 (103-112)	24 (23-25)
Cervical cancer	-	-	-	-	-	-	-	-	-	-	-	-	-	-	-	-	-	-	-	-	-	-	-
Uterine cancer	-	-	-	-	-	-	-	-	-	-	-	-	-	-	-	-	-	-	-	-	-	-	-
Ovarian cancer	-	-	-	-	-	-	-	-	-	-	-	-	-	-	-	-	-	-	-	-	-	-	-
Prostate cancer	-	-	-	-	-	-	75 (59-83)	113 (88-127)	169 (135-195)	211 (165-236)	268 (212-298)	516 (420-582)	1,451 (1,166-1,672)	3,586 (2,880-4,111)	8,082 (6,481-9,011)	18,451 (14,716-20,962)	31,973 (25,591-36,752)	47,859 (39,350-54,324)	56,191 (44,657-64,615)	49,434 (38,337-54,683)	33,755 (24,872-36,607)	13,548 (10,005-14,444)	2,945 (2,023-3,133)
Testicular cancer	-	-	-	-	-	-	384 (358-414)	849 (794-914)	1,168 (1,083-1,252)	1,129 (1,037-1,203)	884 (825-939)	630 (598-670)	469 (441-493)	310 (294-327)	231 (220-243)	280 (265-297)	264 (251-277)	280 (266-296)	226 (216-236)	171 (163-179)	104 (100-108)	36 (35-38)	11 (10-11)
Kidney cancer	26 (20-31)	15 (12-19)	184 (136-236)	840 (577-1,198)	358 (302-421)	140 (123-161)	107 (99-117)	141 (131-152)	241 (221-267)	375 (344-413)	726 (684-780)	1,423 (1,368-1,501)	2,830 (2,720-2,980)	4,323 (4,179-4,504)	5,356 (5,172-5,544)	7,193 (6,975-7,455)	7,986 (7,756-8,234)	8,411 (8,175-8,677)	6,780 (6,614-7,022)	4,212 (4,103-4,386)	2,483 (2,424-2,581)	806 (782-834)	141 (136-146)
Bladder cancer	-	-	-	-	-	-	55 (48-61)	90 (72-100)	143 (115-158)	326 (268-359)	562 (473-616)	1,124 (958-1,190)	2,152 (1,935-2,286)	3,589 (3,283-3,712)	5,363 (4,910-5,571)	9,961 (9,256-10,299)	13,920 (13,045-14,362)	18,142 (17,372-18,733)	17,527 (16,560-18,075)	13,678 (12,879-14,109)	9,126 (8,757-9,363)	3,413 (3,286-3,515)	712 (689-732)
Brain and nervous system cancer	95 (65-109)	79 (55-90)	1,041 (658-1,253)	3,860 (2,555-4,998)	4,826 (3,530-5,548)	4,000 (2,946-4,523)	2,328 (1,848-2,697)	2,097 (1,691-2,373)	3,123 (2,476-3,482)	4,247 (3,285-4,655)	4,830 (3,837-5,297)	5,761 (4,746-6,580)	6,951 (5,709-7,497)	7,415 (6,079-8,333)	7,878 (6,405-8,953)	9,183 (7,606-10,296)	9,118 (7,552-10,185)	7,768 (6,532-8,560)	5,390 (4,242-5,946)	2,696 (2,065-3,061)	1,134 (846-1,288)	335 (254-385)	45 (33-50)
Thyroid cancer	-	-	-	-	-	87 (72-104)	101 (90-114)	142 (128-157)	276 (251-306)	220 (199-243)	276 (253-308)	380 (358-407)	604 (565-654)	798 (757-846)	943 (898-1,001)	1,121 (1,069-1,191)	1,241 (1,185-1,321)	1,263 (1,213-1,336)	1,195 (1,153-1,239)	777 (749-803)	410 (396-424)	125 (121-129)	23 (22-24)
Mesothelioma	-	-	-	-	-	-	43 (37-52)	55 (48-66)	100 (81-122)	137 (111-168)	212 (183-253)	336 (308-383)	611 (554-699)	941 (885-1,044)	1,406 (1,333-1,536)	1,683 (1,589-1,819)	2,065 (1,925-2,230)	2,288 (2,146-2,451)	2,129 (2,019-2,253)	1,269 (1,221-1,326)	683 (655-716)	196 (189-205)	33 (32-34)
Hodgkin lymphoma	14 (8-16)	4 (2-5)	87 (47-109)	698 (334-1,023)	1,198 (771-1,507)	1,134 (768-1,409)	1,179 (846-1,538)	1,208 (926-1,565)	1,424 (1,105-1,757)	1,424 (1,082-1,765)	1,405 (1,077-1,763)	1,296 (998-1,674)	1,397 (1,077-1,712)	1,392 (1,087-1,731)	1,536 (1,213-1,990)	1,741 (1,348-2,181)	1,901 (1,469-2,366)	1,835 (1,438-2,347)	926 (692-1,156)	555 (416-677)	269 (204-327)	49 (38-61)	7 (6-9)
Non-Hodgkin lymphoma	46 (41-56)	39 (33-51)	454 (367-606)	2,802 (2,001-4,055)	2,523 (2,145-3,072)	1,939 (1,702-2,261)	1,854 (1,710-2,034)	2,043 (1,901-2,229)	2,269 (2,135-2,468)	2,640 (2,489-2,883)	3,194 (3,019-3,487)	3,893 (3,706-4,130)	5,008 (4,777-5,408)	6,218 (5,956-6,622)	7,322 (6,973-7,691)	9,521 (9,122-9,996)	11,153 (10,746-11,690)	11,806 (11,472-12,241)	10,112 (9,827-10,391)	6,757 (6,562-6,920)	3,845 (3,749-3,927)	1,143 (1,111-1,170)	195 (190-200)

SR Table 6: Global deaths by cause and age group for males (with 95% confidence intervals), 2000

Cause	Early Neonatal	Late Neonatal	Post Neonatal	1 to 4	5 to 9	10 to 14	15 to 19	20 to 24	25 to 29	30 to 34	35 to 39	40 to 44	45 to 49	50 to 54	54 to 59	60 to 64	65 to 69	70 to 74	75 to 79	80 to 84	85 to 89	90 to 94	95 plus
Multiple myeloma	-	-	-	-	-	-	105 (75-127)	119 (85-143)	166 (111-210)	316 (238-386)	507 (396-591)	917 (752-1,101)	1,362 (1,130-1,593)	2,104 (1,801-2,533)	2,909 (2,403-3,507)	4,110 (3,435-4,842)	5,066 (4,178-6,041)	5,709 (4,559-6,702)	4,975 (3,924-5,841)	3,305 (2,608-3,872)	1,952 (1,461-2,306)	551 (416-647)	109 (83-135)
Leukemia	209 (148-241)	197 (139-236)	2,804 (1,816-3,490)	9,135 (6,421-12,481)	9,890 (7,543-11,889)	8,759 (6,779-10,140)	7,451 (6,068-8,182)	5,819 (4,628-6,297)	6,035 (4,864-6,575)	6,279 (4,934-6,932)	6,028 (4,853-6,549)	6,128 (5,153-6,628)	7,104 (6,037-7,610)	7,739 (6,756-8,250)	8,896 (7,890-9,587)	11,983 (10,853-12,827)	14,254 (13,187-15,274)	15,743 (14,741-16,856)	13,459 (12,714-14,253)	9,054 (8,521-9,515)	5,540 (5,302-5,807)	1,898 (1,819-1,971)	363 (350-375)
Acute lymphoid leukemia	41 (30-56)	38 (25-57)	606 (391-908)	2,835 (1,724-4,554)	3,722 (2,766-4,948)	2,833 (2,258-3,548)	2,183 (1,873-2,543)	1,443 (1,245-1,675)	1,154 (964-1,400)	1,045 (855-1,251)	1,006 (855-1,171)	898 (777-1,035)	905 (795-1,071)	958 (851-1,088)	994 (882-1,121)	1,148 (1,020-1,297)	1,229 (1,111-1,407)	1,187 (1,087-1,332)	578 (531-642)	347 (316-388)	183 (169-200)	55 (52-59)	10 (9-10)
Chronic lymphoid leukemia	-	-	-	-	-	-	103 (77-127)	96 (71-125)	112 (83-154)	138 (98-187)	126 (98-163)	196 (164-226)	337 (300-377)	562 (510-601)	846 (777-900)	1,509 (1,413-1,589)	1,979 (1,853-2,090)	2,580 (2,450-2,722)	2,405 (2,288-2,506)	1,826 (1,742-1,903)	1,319 (1,273-1,363)	512 (492-533)	111 (106-116)
Acute myeloid leukemia	42 (30-56)	40 (29-58)	384 (273-621)	1,627 (1,058-3,095)	1,106 (833-1,584)	1,159 (926-1,512)	1,254 (1,037-1,456)	1,109 (906-1,268)	1,146 (973-1,346)	1,253 (1,041-1,479)	1,347 (1,132-1,518)	1,527 (1,305-1,710)	1,817 (1,587-2,026)	2,118 (1,877-2,294)	2,528 (2,240-2,778)	3,274 (2,926-3,511)	3,815 (3,516-4,114)	4,224 (3,918-4,525)	3,344 (3,139-3,532)	2,081 (1,953-2,206)	1,093 (1,029-1,149)	313 (293-331)	51 (48-53)
Chronic myeloid leukemia	-	-	-	-	-	-	215 (174-249)	309 (255-354)	511 (423-583)	612 (503-695)	685 (583-762)	694 (610-774)	799 (700-876)	859 (774-946)	940 (854-1,040)	1,162 (1,055-1,271)	1,417 (1,305-1,559)	1,545 (1,430-1,666)	1,592 (1,487-1,716)	1,071 (991-1,154)	663 (629-700)	226 (214-240)	37 (35-39)
Other leukemia	126 (80-148)	119 (74-143)	1,814 (992-2,260)	4,674 (2,563-6,484)	5,063 (3,093-6,209)	4,767 (3,101-5,702)	3,696 (2,652-4,170)	2,862 (2,024-3,218)	3,111 (2,149-3,513)	3,230 (2,164-3,687)	2,864 (1,943-3,269)	2,812 (2,132-3,105)	3,246 (2,514-3,557)	3,243 (2,609-3,533)	3,587 (2,969-4,009)	4,891 (4,198-5,420)	5,814 (5,142-6,540)	6,206 (5,602-6,955)	5,539 (5,070-6,138)	3,729 (3,406-4,074)	2,283 (2,120-2,475)	792 (744-854)	154 (145-162)
Other malignant neoplasms	476 (360-553)	274 (204-346)	1,545 (1,134-2,070)	5,776 (3,950-9,070)	3,002 (2,454-3,596)	3,716 (3,207-4,257)	4,563 (4,127-5,007)	3,528 (3,278-3,907)	3,124 (2,849-3,395)	3,305 (2,976-3,692)	4,068 (3,747-4,530)	5,357 (4,935-5,844)	6,970 (6,423-7,494)	8,503 (7,866-9,226)	9,860 (9,075-10,745)	12,907 (12,052-14,016)	14,383 (13,447-15,696)	14,047 (13,252-15,349)	10,836 (10,201-11,975)	6,820 (6,377-7,562)	3,814 (3,562-4,206)	1,319 (1,220-1,426)	268 (253-300)
Other neoplasms	34 (21-44)	23 (15-31)	278 (167-391)	559 (332-834)	517 (330-713)	445 (312-596)	388 (276-478)	369 (261-477)	480 (333-604)	650 (451-826)	748 (528-950)	945 (671-1,191)	1,172 (806-1,507)	1,434 (988-1,853)	1,682 (1,154-2,159)	2,312 (1,580-3,041)	3,015 (2,048-3,912)	3,653 (2,435-4,816)	3,744 (2,239-4,905)	2,816 (1,652-3,626)	2,082 (1,179-2,772)	798 (463-1,089)	181 (103-253)
Myelodysplastic, myeloproliferative, and other hematopoietic neoplasms	33 (20-43)	21 (13-28)	248 (142-356)	468 (253-727)	381 (227-552)	313 (208-433)	267 (185-335)	266 (182-346)	373 (254-477)	536 (365-693)	650 (454-838)	861 (605-1,093)	1,109 (772-1,436)	1,393 (957-1,800)	1,655 (1,135-2,123)	2,291 (1,568-3,019)	2,998 (2,036-3,888)	3,641 (2,428-4,806)	3,737 (2,233-4,898)	2,813 (1,649-3,624)	2,081 (1,178-2,771)	797 (463-1,089)	181 (103-253)
Other benign and in situ neoplasms	1 (0-1)	2 (1-3)	30 (20-40)	91 (56-126)	136 (91-176)	132 (89-174)	121 (82-152)	104 (69-135)	107 (66-137)	114 (66-151)	98 (57-129)	84 (49-114)	64 (36-90)	41 (24-59)	27 (15-37)	22 (11-30)	16 (7-23)	12 (5-19)	7 (3-11)	3 (1-5)	1 (1-3)	0 (0-1)	0 (0-0)
Cardiovascular diseases	2,533 (2,301-2,791)	1,808 (1,626-1,978)	14,250 (13,219-15,273)	10,552 (9,275-11,653)	5,974 (5,519-6,427)	7,061 (6,598-7,526)	18,593 (17,588-19,668)	30,016 (28,785-31,430)	49,991 (48,035-52,381)	82,115 (79,485-85,345)	125,643 (122,137-129,070)	193,888 (189,595-199,161)	303,213 (297,009-309,569)	387,376 (380,305-395,476)	466,406 (457,807-476,416)	721,533 (709,026-734,315)	894,137 (877,816-911,118)	1,076,941 (1,056,280-1,093,865)	968,202 (951,079-984,969)	757,540 (742,314-773,302)	545,121 (534,983-555,580)	239,044 (234,855-243,749)	63,730 (62,684-64,889)
Rheumatic heart disease	-	-	-	3,114 (2,539-3,888)	1,793 (1,598-1,991)	2,702 (2,471-2,943)	3,863 (3,460-4,519)	4,169 (3,698-4,980)	4,548 (4,090-5,366)	5,150 (4,640-6,144)	5,843 (5,250-6,924)	6,373 (5,703-7,629)	7,528 (6,871-8,784)	8,067 (7,304-9,500)	8,799 (7,982-10,409)	11,626 (10,689-13,561)	13,843 (12,765-16,094)	14,743 (13,761-16,817)	11,841 (11,166-13,193)	9,326 (8,796-10,297)	6,026 (5,736-6,498)	2,566 (2,448-2,741)	660 (634-696)
Ischemic heart disease	-	-	-	-	-	-	5,540 (5,176-5,907)	11,770 (11,193-12,452)	22,726 (21,786-23,854)	41,723 (40,247-43,475)	67,245 (65,078-69,461)	105,598 (102,612-109,287)	168,767 (164,932-173,391)	217,106 (212,506-222,844)	261,346 (255,579-268,173)	386,990 (379,385-395,999)	456,152 (447,233-466,260)	538,847 (527,716-549,970)	474,982 (465,401-486,311)	371,743 (363,845-382,646)	280,008 (274,634-288,760)	129,892 (127,390-134,006)	36,961 (36,328-38,054)
Stroke	1,024 (839-1,210)	696 (558-847)	5,311 (4,273-6,607)	3,302 (2,623-4,064)	2,342 (2,086-2,614)	2,489 (2,275-2,702)	5,248 (4,964-5,546)	8,392 (7,998-8,808)	13,749 (13,119-14,579)	23,219 (22,111-24,537)	34,980 (33,575-36,307)	56,190 (54,314-58,486)	92,988 (90,261-95,828)	122,374 (119,051-126,195)	150,057 (145,617-155,357)	256,559 (250,851-263,442)	341,906 (333,684-351,343)	421,119 (410,999-431,395)	378,846 (370,349-388,704)	289,245 (282,143-297,734)	191,980 (187,559-197,829)	74,695 (72,912-77,137)	17,617 (17,215-18,204)
Ischemic stroke	114 (80-165)	102 (67-158)	404 (274-644)	381 (221-693)	201 (165-252)	204 (174-248)	565 (503-647)	946 (849-1,069)	1,525 (1,377-1,715)	2,665 (2,414-2,969)	4,204 (3,867-4,618)	7,431 (6,889-8,101)	16,159 (15,197-17,270)	24,636 (23,047-26,490)	33,798 (31,464-36,552)	82,328 (77,481-87,516)	118,508 (112,383-125,119)	178,520 (171,439-186,395)	168,707 (161,832-175,984)	151,338 (145,727-157,338)	108,562 (105,074-112,653)	44,177 (42,708-45,805)	11,068 (10,767-11,457)
Intracerebral hemorrhage	533 (391-707)	327 (229-472)	2,872 (2,076-4,351)	1,262 (817-2,034)	1,230 (1,018-1,516)	1,280 (1,095-1,520)	3,241 (2,955-3,582)	5,200 (4,791-5,621)	8,742 (8,136-9,688)	15,642 (14,624-17,114)	23,779 (22,491-25,339)	38,027 (35,613-40,625)	63,848 (61,102-67,377)	81,682 (77,270-86,673)	97,585 (91,344-104,477)	151,588 (143,497-161,225)	195,508 (185,730-207,885)	214,700 (204,260-227,249)	186,484 (178,138-197,851)	122,034 (116,084-129,617)	73,893 (70,587-78,588)	27,090 (25,959-28,636)	5,825 (5,577-6,152)
Subarachnoid hemorrhage	377 (260-456)	268 (178-333)	2,035 (1,283-2,723)	1,659 (1,050-2,202)	912 (668-1,065)	1,005 (791-1,132)	1,442 (1,145-1,708)	2,246 (1,764-2,632)	3,483 (2,876-3,966)	4,911 (3,982-5,620)	6,997 (5,722-8,016)	10,732 (8,842-12,611)	12,981 (10,102-14,935)	16,056 (12,279-19,223)	18,674 (14,161-23,139)	22,643 (15,859-28,041)	27,890 (19,338-34,124)	27,899 (18,184-33,986)	23,655 (15,522-29,063)	15,873 (10,071-19,870)	9,526 (6,408-11,571)	3,428 (2,539-4,096)	724 (569-842)
Hypertensive heart disease	-	-	-	-	-	-	363 (264-412)	598 (437-683)	1,155 (835-1,303)	2,017 (1,528-2,283)	3,361 (2,586-3,787)	5,577 (4,358-6,231)	8,865 (6,778-9,905)	11,740 (9,078-13,025)	14,355 (11,279-16,001)	22,494 (17,448-24,848)	29,747 (23,066-32,761)	38,890 (30,255-42,309)	37,259 (29,143-40,099)	31,895 (25,593-34,319)	21,934 (17,233-23,622)	9,725 (7,553-10,461)	2,463 (1,912-2,634)
Non-rheumatic valvular heart disease	-	-	-	-	-	-	384 (313-459)	436 (365-515)	538 (457-636)	575 (485-684)	795 (680-927)	1,042 (919-1,202)	1,174 (1,036-1,332)	1,525 (1,365-1,757)	1,928 (1,746-2,188)	2,714 (2,434-2,993)	3,847 (3,422-4,325)	5,204 (4,557-5,642)	5,936 (5,176-6,461)	5,583 (4,664-5,963)	4,821 (4,010-5,142)	2,340 (1,966-2,492)	616 (497-662)
Non-rheumatic calcific aortic valve disease	-	-	-	-	-	-	123 (92-153)	144 (109-176)	204 (159-251)	241 (198-290)	375 (307-447)	530 (433-628)	638 (538-738)	866 (745-1,011)	1,092 (949-1,278)	1,607 (1,378-1,792)	2,373 (2,026-2,703)	3,310 (2,864-3,743)	3,977 (3,433-4,349)	3,879 (3,155-4,214)	3,617 (2,910-3,882)	1,820 (1,510-1,936)	490 (381-526)
Non-rheumatic degenerative mitral valve disease	-	-	-	-	-	-	178 (139-224)	211 (168-259)	243 (187-300)	273 (211-341)	341 (270-421)	412 (330-503)	486 (392-587)	591 (480-711)	760 (625-919)	983 (782-1,168)	1,288 (1,012-1,567)	1,646 (1,297-1,956)	1,691 (1,313-2,048)	1,436 (1,096-1,762)	1,034 (806-1,274)	454 (345-582)	111 (84-141)
Other non-rheumatic valve diseases	-	-	-	-	-	-	83 (49-112)	81 (47-112)	90 (54-129)	61 (36-93)	79 (45-111)	101 (56-150)	49 (28-69)	68 (38-98)	77 (42-107)	124 (65-178)	186 (100-259)	249 (129-337)	268 (139-364)	269 (141-369)	170 (100-229)	65 (41-93)	15 (10-21)
Cardiomyopathy and myocarditis	857 (716-984)	576 (495-679)	3,809 (3,133-4,630)	1,805 (1,412-2,382)	887 (767-1,021)	947 (862-1,038)	1,677 (1,514-1,843)	2,303 (2,029-2,516)	3,645 (3,240-3,949)	4,853 (4,277-5,232)	7,446 (6,388-8,044)	10,875 (8,981-11,749)	12,966 (10,584-14,005)	12,792 (10,447-13,866)	12,485 (10,164-13,702)	14,685 (12,363-15,868)	13,681 (11,040-14,838)	13,776 (10,397-14,880)	13,062 (9,325-14,094)	10,436 (6,983-11,398)	9,503 (5,450-10,484)	4,556 (2,647-5,038)	1,291 (738-1,415)
Myocarditis	112 (58-154)	132 (80-173)	1,030 (645-1,430)	568 (385-769)	327 (238-418)	317 (248-397)	545 (417-638)	579 (430-684)	799 (606-945)	681 (495-803)	762 (578-913)	939 (663-1,194)	811 (592-982)	686 (477-872)	792 (521-1,055)	785 (515-1,013)	878 (600-1,094)	1,013 (702-1,247)	1,022 (706-1,257)	924 (632-1,120)	990 (663-1,239)	598 (396-758)	181 (112-218)
Alcoholic cardiomyopathy	-	-	-	-	-	-	157 (125-195)	497 (373-579)	1,191 (943-1,320)	2,107 (1,717-2,300)	4,051 (3,244-4,427)	6,385 (4,754-6,934)	7,965 (6,069-8,671)	7,328 (5,320-7,991)	6,034 (4,811-6,637)	7,094 (5,620-7,762)	5,048 (3,921-5,577)	4,058 (2,811-4,695)	2,782 (1,851-3,640)	1,600 (968-2,240)	1,081 (563-1,710)	418 (241-695)	120 (68-190)
Other cardiomyopathy	745 (631-847)	444 (369-524)	2,779 (2,251-3,500)	1,237 (908-1,716)	560 (461-719)	630 (555-731)	975 (845-1,082)	1,227 (1,024-1,374)	1,654 (1,381-1,855)	2,066 (1,764-2,314)	2,633 (2,244-3,005)	3,551 (2,961-4,094)	4,190 (3,570-4,789)	4,778 (4,073-5,475)	5,659 (4,613-6,479)	6,805 (5,759-7,701)	7,755 (6,265-8,671)	8,706 (6,524-9,648)	9,259 (6,536-10,179)	7,913 (5,224-8,758)	7,432 (4,079-8,336)	3,539 (1,925-3,980)	991 (536-1,100)
Atrial fibrillation and flutter	-	-	-	-	-	-	-	-	-	36 (30-39)	54 (45-58)	237 (212-266)	390 (354-440)	683 (624-799)	1,206 (1,098-1,401)	2,300 (2,128-2,756)	3,752 (3,485-4,398)	6,647 (6,041-7,877)	8,986 (8,281-10,824)	11,602 (10,294-13,599)	11,340 (10,097-13,473)	7,180 (6,387-8,492)	2,109 (1,835-2,451)
Aortic aneurysm	-	-	-	-	-	-	192 (172-227)	286 (254-332)	533 (455-641)	770 (648-944)	1,198 (1,054-1,398)	1,694 (1,573-1,910)	2,540 (2,342-2,858)	3,409 (3,203-3,747)	4,751 (4,449-5,262)	7,849 (7,323-8,650)	11,182 (10,467-12,273)	14,130 (13,502-15,253)	14,243 (13,609-15,099)	10,472 (10,009-11,080)	6,893 (6,656-7,217)	2,557 (2,462-2,667)	501 (483-520)
Peripheral artery disease	-	-	-	-	-	-	-	-	-	-	-	124 (74-181)	245 (138-365)	456 (252-703)	720 (393-1,157)	1,522 (800-2,522)	2,013 (1,083-3,356)	2,772 (1,432-4,506)	2,758 (1,458-4,571)	2,291 (1,185-3,776)	2,007 (991-3,299)	1,040 (533-1,706)	376 (190-665)
Endocarditis	169 (135-228)	127 (98-180)	1,721 (1,319-2,305)	779 (462-1,093)	232 (204-272)	284 (255-331)	356 (321-408)	534 (481-608)	824 (738-976)	939 (846-1,097)	1,068 (958-1,245)	1,202 (1,077-1,411)	1,253 (1,143-1,437)	1,324 (1,203-1,555)	1,356 (1,240-1,625)	1,729 (1,591-2,013)	2,139 (1,973-2,469)	2,371 (2,213-2,680)	2,402 (2,266-2,678)	1,934 (1,827-2,130)	1,408 (1,343-1,513)	634 (606-678)	172 (165-183)
Other cardiovascular and circulatory diseases	483 (395-627)	409 (317-527)	3,409 (2,414-4,407)	1,552 (892-2,261)	720 (551-966)	638 (552-804)	970 (817-1,174)	1,529 (1,297-1,873)	2,274 (1,983-2,742)	2,833 (2,509-3,380)	3,654 (3,229-4,400)	4,975 (4,341-6,121)	6,498 (5,773-7,800)	7,899 (6,941-9,632)	9,402 (8,223-11,598)	13,064 (11,536-15,933)	15,875 (14,136-18,975)	18,440 (16,577-21,621)	17,886 (15,944-20,872)	13,012 (11,539-15,288)	9,200 (8,292-10,505)	3,860 (3,513-4,362)	964 (886-1,077)
Chronic respiratory diseases	0 (0-0)	0 (0-0)	0 (0-0)	12,597 (10,557-14,529)	3,435 (3,026-3,851)	3,557 (3,180-3,948)	5,360 (4,966-5,750)	7,273 (6,814-7,747)	10,250 (9,699-10,941)	13,646 (13,032-14,402)	18,825 (17,938-19,808)	25,729 (24,668-26,965)	43,093 (41,399-44,847)	65,122 (62,830-67,413)	98,462 (94,722-102,120)	181,234 (175,228-187,080)	272,011 (261,516-280,076)	367,030 (352,244-377,886)	348,090 (334,980-357,413)	284,103 (272,930-293,042)	173,132 (166,099-178,502)	64,708 (61,898-66,800)	13,929 (13,393-14,363)
Chronic obstructive pulmonary disease	-	-	0 (0-0)	1,156 (724-2,573)	458 (362-807)	372 (303-648)	1,629 (1,314-2,370)	2,401 (2,017-3,472)	3,854 (3,376-5,515)	6,144 (5,507-8,185)	8,455 (7,346-11,543)	13,156 (11,145-16,908)	25,693 (22,736-31,771)	42,130 (36,141-51,023)	66,973 (57,452-79,779)	139,387 (124,269-157,456)	217,920 (197,646-241,711)	307,306 (284,872-332,802)	297,541 (277,909-317,574)	247,524 (231,200-261,360)	153,461 (145,401-160,216)	57,758 (54,967-60,179)	12,417 (11,869-12,894)
Pneumoconiosis	-	-	-	-	-	-	23 (19-28)	38 (31-45)	128 (109-151)	308 (261-361)	481 (419-549)	523 (446-604)	767 (664-875)	848 (720-1,003)	998 (838-1,219)	1,723 (1,463-2,053)	2,634 (2,289-3,044)	3,485 (3,088-3,947)	3,219 (2,914-3,663)	2,166 (1,962-2,518)	1,307 (1,201-1,476)	388 (358-434)	73 (68-80)
Silicosis	-	-	-	-	-	-	7 (5-10)	14 (11-19)	64 (47-83)	210 (164-263)	337 (276-404)	345 (276-420)	490 (400-605)	514 (405-644)	555 (420-726)	920 (736-1,173)	1,514 (1,256-1,859)	1,967 (1,659-2,370)	1,821 (1,567-2,180)	1,155 (991-1,402)	670 (579-810)	178 (155-211)	29 (25-34)
Asbestosis	-	-	-	-	-	-	3 (1-4)	4 (2-6)	8 (3-13)	14 (5-27)	17 (7-26)	23 (11-37)	33 (17-52)	57 (32-88)	93 (53-141)	155 (91-237)	235 (135-331)	345 (210-467)	356 (216-442)	268 (158-329)	183 (103-221)	63 (41-79)	12 (7-14)
Coal workers pneumoconiosis	-	-	-	-	-	-	3 (2-5)	5 (3-8)	23 (15-33)	31 (20-48)	56 (34-76)	77 (48-119)	124 (75-180)	130 (80-195)	171 (107-257)	331 (217-472)	523 (356-736)	747 (552-1,004)	685 (531-904)	499 (384-669)	320 (259-402)	97 (84-117)	20 (17-23)
Other pneumoconiosis	-	-	-	-	-	-	11 (8-14)	15 (11-19)	33 (24-48)	52 (36-84)	72 (52-104)	77 (55-104)	120 (85-166)	148 (101-204)	179 (122-253)	318 (219-436)	362 (264-497)	426 (305-578)	357 (246-487)	243 (172-327)	134 (98-176)	50 (37-67)	12 (9-15)
Asthma	-	-	-	9,846 (8,034-12,045)	2,396 (1,996-2,795)	2,742 (2,362-3,144)	2,975 (2,003-3,594)	3,843 (2,377-4,624)	5,180 (3,307-6,187)	5,718 (3,475-6,846)	8,026 (4,462-9,840)	9,566 (4,856-12,191)	13,342 (6,421-17,637)	17,985 (7,960-25,339)	24,616 (10,258-36,107)	34,604 (14,600-51,341)	43,978 (18,772-66,805)	46,816 (20,627-71,906)	38,509 (18,157-61,089)	27,700 (13,844-44,164)	13,904 (7,606-22,043)	4,774 (2,789-7,548)	997 (637-1,543)
Interstitial lung disease and pulmonary sarcoidosis	-	-	-	235 (91-604)	78 (41-164)	67 (44-119)	92 (63-155)	145 (101-247)	226 (157-376)	327 (231-564)	528 (373-922)	757 (538-1,278)	1,152 (816-2,006)	1,700 (1,218-2,877)	2,560 (1,794-4,605)	3,881 (2,770-6,577)	5,587 (4,016-9,358)	7,166 (5,202-11,306)	7,046 (5,230-10,120)	5,145 (3,658-7,181)	3,239 (2,318-4,130)	1,187 (883-1,468)	268 (199-325)
Other chronic respiratory diseases	0 (0-0)	0 (0-0)	0 (0-0)	1,360 (696-2,182)	502 (353-691)	376 (281-513)	640 (456-887)	847 (587-1,187)	862 (619-1,206)	1,149 (841-1,566)	1,335 (940-1,864)	1,728 (1,218-2,419)	2,138 (1,537-2,966)	2,458 (1,761-3,475)	3,315 (2,389-4,687)	1,639 (1,220-2,220)	1,891 (1,458-2,500)	2,255 (1,823-2,910)	1,775 (1,484-2,195)	1,569 (1,321-1,975)	1,221 (1,079-1,472)	600 (527-718)	174 (156-204)
Digestive diseases	1,549 (1,361-1,866)	1,738 (1,494-2,138)	12,831 (10,652-17,702)	13,500 (10,181-19,676)	8,039 (6,736-9,722)	6,570 (5,717-7,798)	10,785 (9,415-12,130)	16,176 (14,158-17,776)	28,237 (25,637-31,347)	42,673 (39,879-47,719)	62,997 (59,056-69,391)	83,859 (76,446-89,322)	101,463 (96,053-109,599)	104,190 (96,013-110,950)	104,993 (96,225-111,161)	120,684 (109,481-127,890)	120,522 (110,000-126,860)	113,100 (100,957-119,169)	91,008 (83,447-96,106)	63,609 (57,263-66,869)	40,508 (37,165-42,660)	16,380 (15,181-17,114)	4,042 (3,775-4,193)
Cirrhosis and other chronic liver diseases	-	-	-	5,584 (3,947-8,361)	3,191 (2,629-3,970)	3,030 (2,569-3,632)	4,970 (4,153-5,628)	8,948 (7,632-9,969)	17,055 (15,261-19,085)	29,043 (26,845-32,194)	44,724 (41,815-49,162)	61,738 (55,783-65,998)	74,430 (70,268-79,881)	76,163 (69,598-81,438)	73,570 (66,959-78,893)	79,882 (71,809-85,280)	72,308 (65,324-77,151)	59,241 (52,436-63,337)	41,854 (37,195-45,131)	24,749 (21,431-26,855)	13,022 (11,222-14,169)	4,459 (3,903-4,831)	1,056 (929-1,137)
Cirrhosis and other chronic liver diseases due to hepatitis B	-	-	-	244 (155-405)	367 (281-491)	574 (453-749)	1,216 (906-1,545)	3,995 (3,256-4,736)	9,056 (7,596-10,546)	14,230 (12,569-16,168)	18,386 (15,780-21,307)	22,517 (19,074-25,831)	24,618 (20,825-28,853)	23,245 (19,300-27,544)	22,575 (18,288-27,155)	25,338 (20,456-30,226)	24,369 (20,212-28,853)	20,526 (16,702-24,303)	14,861 (12,091-17,427)	8,921 (7,008-10,561)	4,304 (3,402-5,077)	1,331 (1,036-1,614)	282 (203-368)
Cirrhosis and other chronic liver diseases due to hepatitis C	-	-	-	72 (43-126)	104 (74-150)	175 (128-234)	390 (284-524)	1,503 (1,165-1,879)	3,268 (2,508-4,156)	6,350 (5,255-7,531)	10,852 (8,644-13,460)	15,837 (12,973-18,871)	19,677 (16,184-23,672)	20,533 (16,804-24,649)	18,956 (16,077-22,269)	19,982 (16,430-23,979)	18,050 (15,256-21,021)	14,646 (12,249-17,229)	10,019 (8,206-12,095)	5,893 (4,751-7,234)	3,273 (2,674-3,879)	1,170 (951-1,413)	295 (227-376)
Cirrhosis and other chronic liver diseases due to alcohol use	-	-	-	-	-	-	40 (18-70)	776 (588-1,000)	2,181 (1,696-2,727)	5,432 (4,708-6,360)	11,341 (9,493-13,501)	17,476 (14,950-19,994)	22,102 (19,036-25,665)	23,227 (19,740-26,983)	22,620 (18,987-26,558)	23,816 (19,439-28,266)	19,774 (16,575-23,014)	15,259 (12,422-18,109)	10,306 (8,733-11,937)	5,685 (4,717-6,690)	2,974 (2,536-3,441)	1,017 (839-1,225)	239 (180-306)
Cirrhosis due to NASH	-	-	-	-	-	-	87 (66-115)	247 (197-310)	548 (431-689)	1,154 (970-1,342)	2,140 (1,694-2,604)	3,522 (2,942-4,146)	5,201 (4,330-6,150)	5,913 (4,910-7,042)	5,682 (4,876-6,547)	6,021 (5,033-6,996)	5,171 (4,442-5,945)	4,004 (3,416-4,631)	2,564 (2,133-3,037)	1,491 (1,226-1,803)	836 (687-987)	305 (240-380)	78 (57-103)
Cirrhosis and other chronic liver diseases due to other causes	-	-	-	5,268 (3,728-7,913)	2,719 (2,230-3,350)	2,281 (1,925-2,774)	3,238 (2,603-3,820)	2,426 (1,863-3,068)	2,001 (1,467-2,612)	1,876 (1,353-2,494)	2,005 (1,487-2,611)	2,387 (1,742-3,164)	2,831 (2,091-3,713)	3,246 (2,389-4,270)	3,737 (2,543-5,265)	4,725 (3,261-6,519)	4,944 (3,494-6,678)	4,806 (3,582-6,274)	4,103 (3,056-5,458)	2,760 (2,087-3,670)	1,635 (1,281-2,062)	636 (468-822)	163 (109-228)
Upper digestive system diseases	-	-	-	1,287 (867-1,892)	830 (678-1,036)	741 (617-924)	1,575 (1,356-1,861)	2,564 (2,242-2,939)	3,990 (3,560-4,653)	5,418 (4,955-6,262)	7,301 (6,679-8,461)	8,880 (8,089-9,982)	12,066 (11,198-13,751)	13,177 (12,083-14,530)	14,554 (13,212-16,005)	18,279 (16,426-19,666)	21,182 (18,913-22,948)	22,602 (19,674-24,248)	19,458 (17,520-20,988)	14,206 (12,666-15,274)	9,151 (8,283-9,840)	3,680 (3,393-3,915)	913 (847-969)
Peptic ulcer disease	-	-	-	862 (529-1,551)	658 (526-832)	613 (506-750)	1,265 (1,085-1,490)	2,127 (1,859-2,429)	3,247 (2,889-3,757)	4,329 (3,949-4,974)	6,082 (5,546-7,053)	7,643 (6,989-8,564)	10,437 (9,676-11,782)	11,480 (10,558-12,589)	12,684 (11,508-14,047)	16,155 (14,500-17,409)	18,779 (16,781-20,329)	19,997 (17,467-21,508)	17,082 (15,373-18,456)	12,387 (11,003-13,340)	7,936 (7,190-8,571)	3,190 (2,948-3,407)	786 (731-837)
Gastritis and duodenitis	-	-	-	426 (211-676)	172 (104-241)	129 (86-176)	309 (218-398)	437 (290-558)	743 (524-915)	1,089 (792-1,343)	1,219 (925-1,473)	1,237 (992-1,468)	1,629 (1,307-1,939)	1,696 (1,404-1,999)	1,870 (1,537-2,203)	2,125 (1,773-2,455)	2,403 (2,022-2,799)	2,604 (2,203-3,043)	2,375 (2,043-2,774)	1,819 (1,564-2,120)	1,215 (1,045-1,416)	490 (431-563)	126 (112-143)
Appendicitis	-	-	-	1,241 (605-2,048)	1,124 (873-1,513)	1,041 (862-1,342)	1,252 (1,048-1,538)	1,010 (840-1,232)	1,318 (1,111-1,675)	838 (719-1,008)	1,097 (938-1,363)	1,294 (1,084-1,559)	1,098 (942-1,314)	1,053 (919-1,256)	1,155 (992-1,369)	1,365 (1,184-1,604)	1,413 (1,232-1,633)	1,670 (1,404-1,966)	1,393 (1,182-1,675)	1,093 (915-1,321)	680 (564-836)	295 (248-367)	69 (57-85)
Paralytic ileus and intestinal obstruction	1,549 (1,361-1,866)	1,738 (1,494-2,138)	12,831 (10,652-17,702)	1,807 (1,238-2,369)	1,691 (1,095-2,112)	898 (604-1,084)	1,715 (1,207-2,083)	1,691 (1,103-2,058)	2,181 (1,461-2,593)	2,430 (1,580-2,923)	3,171 (2,125-3,787)	3,682 (2,520-4,397)	4,068 (2,667-4,817)	4,075 (2,917-4,927)	5,149 (3,626-6,296)	6,677 (4,974-8,243)	8,209 (6,226-10,175)	8,612 (6,653-10,350)	7,910 (6,495-9,969)	6,828 (5,579-8,384)	5,527 (4,604-6,601)	2,444 (1,992-2,935)	588 (490-716)
Inguinal, femoral, and abdominal hernia	-	-	-	574 (344-925)	244 (187-331)	164 (126-205)	303 (244-387)	320 (262-402)	419 (353-526)	438 (353-534)	600 (511-757)	659 (564-817)	784 (675-986)	988 (847-1,188)	1,238 (1,072-1,523)	1,676 (1,445-2,002)	2,163 (1,871-2,555)	2,536 (2,112-2,911)	2,676 (2,210-3,100)	2,387 (2,032-2,774)	1,644 (1,456-1,879)	783 (707-893)	202 (182-228)
Inflammatory bowel disease	-	-	-	667 (333-1,322)	151 (98-256)	95 (68-146)	103 (84-120)	188 (153-218)	300 (249-365)	371 (314-458)	409 (356-484)	537 (468-597)	658 (587-747)	673 (595-740)	789 (689-885)	1,007 (863-1,115)	1,250 (1,088-1,380)	1,485 (1,278-1,613)	1,520 (1,239-1,694)	1,361 (1,053-1,546)	1,027 (787-1,145)	464 (344-512)	114 (84-125)
Vascular intestinal disorders	-	-	-	387 (130-579)	75 (42-96)	35 (25-42)	48 (41-55)	102 (84-118)	164 (136-190)	164 (140-190)	306 (264-360)	529 (474-588)	848 (764-950)	1,164 (1,071-1,270)	1,634 (1,502-1,772)	2,626 (2,448-2,837)	3,732 (3,434-4,035)	4,950 (4,586-5,316)	4,682 (4,408-4,959)	3,663 (3,364-3,863)	2,650 (2,476-2,805)	1,142 (1,058-1,204)	264 (245-278)
Gallbladder and biliary diseases	-	-	-	348 (209-491)	143 (115-180)	160 (128-197)	173 (149-202)	260 (229-293)	369 (328-412)	541 (483-621)	727 (661-817)	937 (849-1,030)	1,156 (1,070-1,306)	1,381 (1,278-1,527)	1,606 (1,464-1,737)	2,505 (2,341-2,726)	3,539 (3,245-3,839)	4,574 (4,180-4,919)	4,782 (4,408-5,179)	4,066 (3,747-4,349)	3,102 (2,910-3,294)	1,462 (1,372-1,547)	396 (373-417)
Pancreatitis	-	-	-	81 (45-154)	83 (61-125)	93 (74-137)	265 (226-336)	769 (666-1,012)	1,863 (1,615-2,374)	2,653 (2,373-3,323)	3,703 (3,350-4,475)	4,467 (3,985-5,115)	4,849 (4,327-5,497)	3,819 (3,476-4,406)	3,545 (3,110-4,136)	3,760 (3,389-4,258)	3,631 (3,262-4,087)	3,572 (3,186-3,991)	3,053 (2,651-3,441)	1,934 (1,695-2,161)	1,255 (1,116-1,396)	455 (401-500)	101 (90-110)
Other digestive diseases	-	-	-	1,523 (876-2,881)	507 (372-782)	311 (244-446)	382 (288-618)	325 (271-460)	578 (479-770)	777 (655-1,022)	959 (821-1,256)	1,135 (992-1,477)	1,508 (1,328-1,923)	1,697 (1,459-2,275)	1,754 (1,539-2,310)	2,907 (2,406-4,131)	3,094 (2,738-4,044)	3,858 (3,416-4,972)	3,679 (3,354-4,435)	3,322 (2,981-3,979)	2,449 (2,257-2,719)	1,197 (1,105-1,311)	340 (315-370)
Neurological disorders	-	-	5,270 (3,743-6,714)	8,351 (4,761-11,872)	3,963 (3,170-4,941)	5,438 (4,482-6,259)	8,753 (7,272-9,896)	7,677 (6,428-8,549)	7,389 (6,358-8,279)	7,043 (6,099-7,771)	6,986 (6,099-7,839)	7,239 (6,500-7,932)	8,486 (7,771-9,053)	8,997 (8,517-9,404)	11,532 (11,029-11,946)	21,188 (20,468-21,908)	38,300 (37,204-39,548)	69,547 (67,635-72,439)	109,224 (105,731-112,055)	124,787 (119,743-127,919)	120,232 (116,490-122,333)	70,620 (68,668-71,850)	20,409 (19,910-20,715)
Alzheimer's disease and other dementias	-	-	-	-	-	-	-	-	-	-	-	398 (382-417)	955 (900-1,017)	2,187 (2,107-2,300)	4,195 (4,063-4,366)	11,457 (11,166-11,923)	23,808 (23,174-24,861)	46,263 (45,033-48,227)	78,062 (76,086-80,323)	96,863 (93,489-98,974)	101,685 (98,949-103,371)	64,481 (62,759-65,555)	19,315 (18,851-19,594)
Parkinson's disease	-	-	-	-	-	-	-	7 (6-8)	13 (10-14)	32 (26-35)	33 (27-37)	325 (301-372)	702 (647-805)	1,123 (1,049-1,316)	1,576 (1,464-1,866)	3,580 (3,354-4,225)	7,853 (7,381-9,394)	17,289 (16,274-20,143)	26,201 (24,466-29,557)	24,950 (23,271-27,881)	16,811 (15,060-19,081)	5,597 (5,051-6,422)	979 (919-1,099)
Epilepsy	-	-	3,386 (2,348-4,307)	6,784 (3,820-9,791)	3,303 (2,646-4,150)	4,363 (3,378-4,979)	6,834 (5,405-7,907)	6,261 (5,007-7,156)	6,334 (5,343-7,230)	5,973 (4,988-6,658)	5,624 (4,723-6,527)	4,710 (4,026-5,433)	4,337 (3,533-4,910)	2,930 (2,438-3,268)	2,685 (2,230-2,955)	2,673 (2,145-3,122)	2,801 (2,174-3,515)	2,254 (1,812-2,673)	1,829 (1,523-2,007)	1,166 (911-1,291)	738 (599-819)	244 (198-271)	56 (46-62)
Multiple sclerosis	-	-	-	-	-	-	-	53 (44-61)	129 (97-153)	249 (199-296)	410 (331-488)	627 (515-708)	786 (638-893)	781 (624-871)	726 (607-815)	710 (600-809)	616 (521-697)	497 (406-559)	341 (275-375)	177 (139-192)	87 (66-95)	23 (17-25)	4 (3-4)
Motor neuron disease	4 (3-5)	10 (6-13)	324 (211-522)	89 (51-141)	25 (20-34)	44 (35-67)	76 (68-98)	63 (56-83)	58 (50-75)	99 (88-129)	212 (198-271)	358 (337-423)	616 (582-770)	803 (763-922)	1,096 (1,035-1,297)	1,414 (1,347-1,621)	1,738 (1,670-1,953)	1,726 (1,673-1,858)	1,395 (1,353-1,444)	712 (690-733)	315 (304-327)	67 (65-70)	9 (9-10)
Other neurological disorders	-	-	1,560 (931-2,081)	1,478 (621-2,250)	635 (397-851)	1,032 (791-1,263)	1,843 (1,729-2,153)	1,293 (1,218-1,433)	856 (781-997)	690 (614-771)	706 (659-782)	820 (780-915)	1,090 (1,043-1,195)	1,173 (1,119-1,318)	1,255 (1,183-1,421)	1,353 (1,291-1,497)	1,483 (1,421-1,624)	1,518 (1,459-1,641)	1,397 (1,340-1,502)	919 (881-983)	596 (571-627)	209 (200-219)	47 (45-49)
Mental disorders	-	-	-	-	0 (0-0)	1 (0-1)	3 (2-3)	4 (3-5)	4 (3-5)	4 (3-4)	4 (4-5)	5 (4-5)	5 (4-5)	-	-	-	-	-	-	-	-	-	-
Eating disorders	-	-	-	-	0 (0-0)	1 (0-1)	3 (2-3)	4 (3-5)	4 (3-5)	4 (3-4)	4 (4-5)	5 (4-5)	5 (4-5)	-	-	-	-	-	-	-	-	-	-
Anorexia nervosa	-	-	-	-	0 (0-0)	1 (0-1)	2 (1-3)	2 (2-3)	2 (1-2)	2 (1-2)	3 (2-3)	3 (3-4)	3 (3-4)	-	-	-	-	-	-	-	-	-	-
Bulimia nervosa	-	-	-	-	0 (0-0)	0 (0-0)	1 (1-1)	2 (1-2)	2 (2-3)	2 (2-2)	1 (1-2)	2 (1-2)	1 (1-2)	-	-	-	-	-	-	-	-	-	-
Substance use disorders	0 (0-0)	0 (0-0)	0 (0-0)	7 (2-16)	16 (7-29)	17 (8-29)	3,852 (3,677-4,035)	10,191 (9,790-10,551)	16,600 (15,871-17,366)	21,741 (20,881-22,710)	25,034 (23,974-25,892)	28,467 (27,232-29,284)	29,164 (27,551-30,340)	21,872 (20,835-22,591)	16,417 (15,434-16,977)	15,715 (14,771-16,261)	10,375 (9,901-10,701)	7,172 (6,763-7,411)	4,463 (4,192-4,652)	2,267 (2,159-2,363)	1,374 (1,313-1,433)	532 (509-555)	128 (123-133)
Alcohol use disorders	-	-	-	-	-	-	1,054 (897-1,134)	3,070 (2,667-3,253)	6,121 (5,475-6,553)	10,103 (8,982-10,842)	14,941 (13,603-15,672)	20,035 (18,638-20,802)	22,730 (21,023-23,843)	17,675 (16,553-18,340)	13,453 (12,395-13,997)	12,935 (11,960-13,482)	7,879 (7,340-8,200)	4,937 (4,508-5,170)	2,762 (2,462-2,935)	1,102 (995-1,158)	580 (524-616)	186 (166-199)	46 (42-48)
Drug use disorders	0 (0-0)	0 (0-0)	0 (0-0)	7 (2-16)	16 (7-29)	17 (8-29)	2,798 (2,668-2,952)	7,121 (6,849-7,448)	10,480 (9,951-11,080)	11,638 (11,145-12,253)	10,094 (9,726-10,508)	8,433 (8,136-8,822)	6,435 (6,187-6,702)	4,197 (4,054-4,351)	2,964 (2,842-3,109)	2,780 (2,678-2,896)	2,496 (2,391-2,611)	2,235 (2,144-2,340)	1,702 (1,631-1,779)	1,165 (1,106-1,227)	794 (751-838)	347 (329-366)	82 (78-87)
Opioid use disorders	0 (0-0)	0 (0-0)	0 (0-0)	7 (2-16)	16 (7-29)	17 (8-29)	1,886 (1,714-2,092)	4,669 (4,260-5,194)	6,746 (6,054-7,670)	7,373 (6,571-8,450)	6,429 (5,864-7,148)	5,250 (4,825-5,725)	3,803 (3,451-4,135)	2,332 (2,092-2,564)	1,638 (1,416-1,850)	1,634 (1,440-1,812)	1,460 (1,239-1,666)	1,037 (852-1,238)	807 (666-945)	541 (433-643)	401 (325-466)	177 (145-202)	42 (35-47)
Cocaine use disorders	-	-	-	-	-	-	67 (54-87)	206 (167-266)	288 (223-376)	446 (343-594)	459 (378-580)	432 (375-529)	358 (301-457)	282 (245-352)	173 (140-242)	144 (118-196)	113 (84-175)	55 (42-81)	39 (29-60)	7 (5-10)	5 (4-7)	3 (2-4)	1 (1-1)
Amphetamine use disorders	-	-	-	-	-	-	113 (69-160)	278 (164-407)	470 (264-701)	450 (261-671)	394 (255-551)	334 (227-438)	274 (188-385)	144 (112-187)	101 (69-151)	85 (63-116)	67 (43-104)	21 (15-30)	15 (10-23)	4 (3-6)	3 (2-4)	1 (1-2)	1 (0-1)
Other drug use disorders	-	-	-	-	-	-	732 (606-898)	1,968 (1,627-2,404)	2,976 (2,306-3,651)	3,369 (2,611-4,214)	2,812 (2,334-3,418)	2,416 (2,143-2,836)	2,000 (1,740-2,404)	1,438 (1,276-1,691)	1,052 (895-1,296)	917 (777-1,117)	855 (698-1,110)	1,123 (959-1,314)	840 (732-977)	613 (526-701)	385 (337-448)	165 (144-192)	39 (35-45)
Diabetes and kidney diseases	27 (19-33)	81 (58-98)	5,696 (5,015-6,352)	5,582 (4,631-6,422)	3,735 (3,143-4,175)	3,670 (3,183-4,009)	7,004 (6,360-7,653)	9,978 (9,311-10,801)	12,943 (11,997-13,842)	17,987 (16,638-19,258)	21,774 (20,589-22,972)	28,974 (27,845-30,478)	39,933 (38,283-41,679)	50,331 (48,639-52,675)	63,070 (60,577-66,430)	84,708 (81,738-88,219)	101,382 (97,127-106,053)	109,037 (104,827-113,718)	98,776 (94,960-102,864)	71,419 (68,583-74,396)	47,945 (46,086-49,858)	19,990 (19,248-20,665)	5,184 (4,994-5,349)
Diabetes mellitus	27 (19-33)	81 (58-98)	749 (542-915)	338 (265-404)	446 (345-540)	526 (387-622)	1,139 (1,068-1,214)	2,050 (1,940-2,156)	2,881 (2,747-3,024)	5,234 (5,004-5,481)	7,062 (6,805-7,310)	11,837 (11,414-12,296)	18,850 (18,141-19,531)	25,802 (24,875-26,730)	34,248 (32,949-35,500)	47,333 (45,674-48,938)	56,375 (53,983-58,459)	60,162 (57,974-62,161)	52,542 (50,486-54,326)	35,694 (34,113-37,065)	21,465 (20,484-22,282)	8,050 (7,711-8,361)	1,806 (1,723-1,874)
Diabetes mellitus type 1	27 (19-33)	81 (58-98)	749 (542-915)	338 (265-404)	446 (345-540)	526 (387-622)	1,024 (956-1,095)	1,815 (1,714-1,925)	2,479 (2,362-2,609)	4,033 (3,800-4,280)	5,323 (5,046-5,602)	8,777 (8,231-9,291)	8,268 (7,336-9,463)	10,780 (9,521-12,584)	14,028 (12,072-16,714)	14,345 (12,258-17,798)	16,607 (14,276-20,067)	14,688 (12,493-18,087)	12,275 (10,377-15,404)	6,407 (5,274-8,319)	3,693 (3,007-4,784)	1,319 (1,069-1,696)	282 (235-355)
Diabetes mellitus type 2	-	-	-	-	-	-	115 (94-143)	235 (197-280)	402 (357-485)	1,202 (1,065-1,416)	1,739 (1,583-1,960)	3,060 (2,647-3,454)	10,582 (9,492-11,623)	15,023 (13,219-16,188)	20,220 (17,685-22,215)	32,987 (29,660-35,430)	39,768 (36,368-42,508)	45,474 (42,091-48,124)	40,267 (37,347-42,510)	29,287 (27,302-30,761)	17,772 (16,673-18,851)	6,732 (6,337-7,090)	1,524 (1,442-1,603)
Chronic kidney disease	-	-	4,776 (4,176-5,343)	5,035 (4,110-5,859)	3,082 (2,564-3,463)	2,972 (2,556-3,271)	5,697 (5,119-6,302)	7,730 (7,148-8,446)	9,747 (8,772-10,513)	12,361 (10,935-13,425)	14,358 (13,097-15,383)	16,810 (15,857-18,148)	20,647 (19,013-22,000)	24,142 (23,071-26,230)	28,394 (26,939-31,800)	36,873 (34,911-40,124)	44,403 (41,827-47,995)	48,242 (45,594-52,480)	45,711 (43,398-48,491)	35,343 (33,607-37,454)	26,255 (24,923-27,672)	11,843 (11,389-12,321)	3,342 (3,211-3,461)
Chronic kidney disease due to diabetes mellitus type 1	-	-	18 (10-29)	21 (10-38)	19 (9-35)	30 (16-51)	99 (51-162)	323 (172-531)	923 (548-1,433)	2,100 (1,338-3,041)	3,126 (2,028-4,489)	3,896 (2,520-5,480)	4,411 (2,857-6,305)	3,832 (2,289-5,590)	3,085 (1,717-4,869)	2,339 (1,165-3,995)	1,862 (931-3,329)	1,458 (713-2,578)	1,020 (482-1,913)	615 (303-1,088)	311 (158-551)	64 (31-115)	4 (2-8)
Chronic kidney disease due to diabetes mellitus type 2	-	-	14 (7-24)	15 (7-28)	12 (6-24)	16 (7-30)	47 (21-86)	113 (48-217)	255 (110-482)	551 (256-996)	985 (508-1,686)	1,804 (1,019-2,949)	3,560 (2,210-5,067)	5,802 (3,980-8,195)	8,422 (5,912-10,930)	12,320 (8,786-15,889)	15,385 (11,439-19,794)	15,907 (11,863-20,111)	13,732 (9,491-18,057)	9,755 (6,671-13,388)	6,213 (4,082-8,374)	2,483 (1,741-3,359)	648 (398-985)
Chronic kidney disease due to hypertension	-	-	24 (13-37)	38 (21-61)	38 (21-62)	64 (36-101)	221 (132-337)	481 (309-692)	822 (556-1,161)	1,271 (859-1,786)	1,730 (1,193-2,368)	2,320 (1,668-3,058)	3,199 (2,261-4,253)	4,441 (3,311-5,898)	5,985 (4,549-7,737)	8,735 (6,598-11,250)	11,973 (9,327-15,067)	14,688 (11,623-18,281)	15,844 (12,628-19,600)	13,683 (10,858-16,627)	11,454 (9,494-13,530)	5,686 (4,776-6,550)	1,715 (1,405-2,014)
Chronic kidney disease due to glomerulonephritis	-	-	1,077 (751-1,488)	1,496 (1,012-2,139)	936 (612-1,346)	982 (646-1,354)	2,235 (1,565-2,997)	3,087 (2,140-4,126)	3,500 (2,450-4,697)	3,642 (2,511-5,039)	3,722 (2,485-5,267)	4,049 (2,597-5,651)	4,445 (2,914-6,451)	4,600 (3,043-6,562)	4,489 (2,970-6,664)	5,110 (3,269-7,542)	5,789 (3,722-8,510)	6,484 (4,084-9,345)	6,112 (3,933-8,948)	4,527 (2,896-6,767)	3,156 (2,115-4,650)	1,352 (895-1,981)	376 (206-619)
Chronic kidney disease due to other and unspecified causes	-	-	3,644 (3,030-4,202)	3,465 (2,703-4,213)	2,076 (1,563-2,517)	1,879 (1,462-2,283)	3,096 (2,354-3,828)	3,726 (2,692-4,803)	4,247 (3,009-5,458)	4,798 (3,330-6,221)	4,795 (3,289-6,393)	4,741 (3,118-6,519)	5,031 (3,284-7,185)	5,467 (3,549-7,787)	6,414 (4,214-9,071)	8,369 (5,167-12,123)	9,396 (6,023-13,338)	9,705 (6,413-13,641)	9,004 (5,936-13,080)	6,763 (4,623-9,512)	5,120 (3,541-6,992)	2,258 (1,544-3,100)	599 (386-900)
Acute glomerulonephritis	-	-	171 (106-242)	209 (127-314)	207 (170-248)	172 (141-205)	168 (144-193)	198 (175-225)	314 (271-363)	392 (335-453)	354 (309-406)	327 (288-365)	437 (391-486)	386 (339-438)	428 (366-505)	502 (444-568)	604 (533-675)	633 (562-713)	523 (459-598)	383 (336-440)	225 (196-258)	96 (81-118)	36 (26-49)
Skin and subcutaneous diseases	-	-	1,668 (751-2,181)	974 (366-1,570)	278 (128-358)	342 (160-444)	529 (255-709)	466 (222-594)	478 (238-592)	890 (419-1,098)	874 (449-1,074)	999 (538-1,320)	1,335 (725-1,806)	1,360 (749-1,903)	1,659 (883-2,179)	2,160 (1,162-2,939)	2,468 (1,371-3,313)	3,024 (1,634-4,055)	3,283 (1,730-4,346)	2,634 (1,476-3,655)	2,087 (1,154-2,909)	1,034 (551-1,482)	303 (159-454)
Bacterial skin diseases	-	-	1,621 (715-2,129)	894 (322-1,439)	223 (92-300)	273 (118-362)	456 (206-617)	389 (177-494)	388 (186-483)	752 (341-953)	729 (355-889)	835 (438-1,068)	1,143 (614-1,507)	1,153 (594-1,566)	1,422 (736-1,805)	1,832 (910-2,423)	1,957 (1,038-2,591)	2,257 (1,155-3,061)	2,317 (1,164-3,163)	1,646 (877-2,386)	1,195 (619-1,692)	574 (302-877)	158 (84-251)
Cellulitis	-	-	145 (39-254)	165 (43-315)	71 (24-104)	100 (38-145)	107 (37-160)	87 (37-114)	86 (37-120)	139 (62-190)	167 (72-251)	208 (90-330)	279 (123-433)	287 (119-472)	308 (132-495)	410 (167-620)	366 (153-632)	448 (193-770)	404 (148-701)	335 (131-610)	239 (84-457)	117 (44-225)	35 (12-70)
Pyoderma	-	-	1,476 (643-1,930)	729 (261-1,179)	152 (64-205)	172 (72-230)	349 (167-492)	302 (132-388)	301 (139-388)	613 (256-796)	562 (259-709)	626 (295-819)	864 (430-1,184)	866 (431-1,160)	1,114 (528-1,453)	1,422 (711-1,973)	1,591 (776-2,171)	1,809 (873-2,443)	1,913 (926-2,580)	1,310 (707-1,978)	956 (490-1,368)	457 (240-693)	123 (65-198)
Decubitus ulcer	-	-	-	29 (15-72)	26 (15-47)	35 (19-65)	40 (20-64)	46 (23-75)	61 (29-98)	104 (52-180)	113 (56-194)	126 (65-223)	151 (79-270)	168 (88-312)	195 (100-367)	269 (133-500)	436 (211-725)	675 (339-1,145)	858 (398-1,461)	883 (414-1,452)	794 (334-1,179)	411 (158-648)	130 (52-192)
Other skin and subcutaneous diseases	-	-	47 (23-129)	50 (26-139)	30 (18-57)	35 (21-64)	33 (19-58)	31 (18-53)	29 (16-49)	35 (19-59)	33 (18-55)	39 (21-67)	42 (22-76)	39 (20-74)	43 (20-83)	59 (30-115)	76 (39-137)	92 (46-175)	108 (53-220)	105 (50-203)	98 (46-196)	49 (21-105)	15 (5-31)
Musculoskeletal disorders	-	-	-	-	304 (205-364)	438 (335-492)	500 (413-546)	552 (436-600)	730 (549-793)	812 (602-874)	945 (733-1,020)	1,054 (877-1,142)	1,235 (1,034-1,308)	1,079 (942-1,136)	1,426 (1,208-1,515)	2,449 (2,016-2,629)	3,142 (2,618-3,335)	3,810 (3,216-4,043)	3,681 (3,095-3,928)	2,720 (2,332-2,895)	1,974 (1,740-2,115)	852 (745-917)	217 (196-230)
Rheumatoid arthritis	-	-	-	-	45 (28-81)	55 (39-84)	54 (42-69)	55 (43-74)	72 (51-101)	93 (62-138)	125 (90-166)	178 (140-210)	264 (201-314)	369 (306-410)	609 (508-681)	1,148 (894-1,306)	1,581 (1,275-1,764)	1,979 (1,637-2,210)	1,890 (1,562-2,093)	1,228 (1,021-1,379)	792 (671-901)	274 (227-316)	62 (52-71)
Other musculoskeletal disorders	-	-	-	-	259 (178-317)	383 (292-435)	447 (370-491)	497 (386-540)	658 (483-717)	718 (534-781)	819 (636-893)	875 (726-958)	971 (806-1,042)	709 (609-760)	817 (677-905)	1,301 (1,029-1,463)	1,561 (1,272-1,752)	1,831 (1,464-2,055)	1,791 (1,438-2,024)	1,492 (1,244-1,662)	1,182 (1,024-1,310)	577 (500-641)	155 (137-167)
Other non-communicable diseases	124,090 (110,730-155,869)	51,328 (45,445-64,006)	183,560 (156,323-222,912)	61,032 (51,252-82,058)	16,919 (15,112-19,341)	12,840 (11,492-14,573)	10,180 (8,611-12,098)	9,514 (8,100-11,480)	8,903 (7,848-10,138)	8,281 (7,334-9,161)	8,500 (7,565-9,440)	9,028 (8,050-10,044)	8,634 (7,759-9,443)	9,200 (8,181-10,076)	9,527 (8,478-10,501)	12,498 (11,280-13,588)	15,450 (14,082-16,794)	17,184 (15,654-18,431)	15,588 (14,207-16,767)	13,708 (12,682-14,636)	10,277 (9,465-10,833)	5,000 (4,638-5,239)	1,496 (1,407-1,572)
Congenital birth defects	121,880 (108,773-153,692)	46,163 (41,188-58,626)	142,676 (129,875-177,197)	49,340 (41,424-71,526)	11,837 (10,626-13,943)	8,154 (7,504-9,199)	4,864 (4,558-5,484)	4,153 (3,896-4,749)	3,970 (3,706-4,449)	2,990 (2,795-3,297)	2,997 (2,775-3,362)	2,774 (2,557-3,147)	1,262 (1,111-1,465)	1,052 (914-1,239)	941 (832-1,143)	909 (814-1,110)	776 (701-946)	-	-	-	-	-	-
Neural tube defects	17,701 (12,753-28,144)	4,773 (3,204-8,028)	11,468 (6,947-20,666)	6,545 (3,041-17,463)	685 (490-987)	457 (343-565)	180 (152-218)	151 (129-184)	136 (113-162)	105 (88-126)	76 (66-89)	75 (62-95)	53 (45-64)	35 (29-43)	31 (26-38)	27 (23-35)	24 (21-30)	-	-	-	-	-	-
Congenital heart anomalies	41,541 (28,261-55,840)	21,471 (15,279-27,551)	75,120 (50,635-95,412)	22,571 (13,110-32,843)	7,208 (5,458-8,947)	5,276 (4,261-6,235)	3,097 (2,825-3,612)	2,645 (2,392-3,125)	2,780 (2,534-3,207)	1,965 (1,767-2,231)	2,064 (1,862-2,371)	1,828 (1,648-2,124)	611 (540-758)	459 (393-571)	389 (350-499)	362 (325-453)	320 (282-407)	-	-	-	-	-	-
Orofacial clefts	2,278 (885-5,733)	750 (290-1,930)	1,601 (636-4,430)	310 (71-1,182)	-	-	-	-	-	-	-	-	-	-	-	-	-	-	-	-	-	-	-
Down syndrome	2,505 (1,277-4,011)	1,108 (561-1,798)	5,758 (2,647-9,359)	2,881 (1,063-4,929)	439 (254-659)	266 (176-359)	302 (202-459)	258 (181-370)	304 (184-494)	351 (199-572)	292 (198-400)	305 (214-415)	297 (203-383)	284 (217-344)	250 (191-311)	218 (171-283)	114 (89-151)	-	-	-	-	-	-
Other chromosomal abnormalities	2,159 (1,202-4,270)	852 (465-1,691)	1,906 (999-3,809)	463 (218-1,054)	99 (58-167)	76 (45-117)	82 (54-131)	84 (53-129)	74 (47-113)	49 (32-69)	60 (37-86)	42 (25-62)	32 (23-49)	28 (18-46)	23 (15-37)	18 (12-33)	15 (9-24)	-	-	-	-	-	-
Congenital musculoskeletal and limb anomalies	2,303 (1,440-3,698)	583 (353-858)	1,592 (854-2,517)	534 (237-977)	205 (126-305)	161 (113-225)	168 (124-211)	109 (80-139)	70 (53-87)	56 (44-75)	50 (38-63)	40 (29-52)	25 (21-33)	22 (18-28)	18 (14-23)	22 (18-28)	22 (17-29)	-	-	-	-	-	-
Urogenital congenital anomalies	3,442 (2,159-5,227)	977 (599-1,388)	1,708 (936-2,678)	473 (203-862)	167 (103-260)	146 (101-208)	64 (54-89)	76 (63-103)	68 (54-87)	67 (50-87)	86 (63-110)	105 (82-139)	66 (55-83)	74 (58-91)	93 (73-113)	126 (99-159)	152 (123-186)	-	-	-	-	-	-
Digestive congenital anomalies	14,167 (9,167-23,160)	6,141 (4,045-10,359)	15,501 (9,727-28,988)	3,617 (1,584-10,349)	317 (208-494)	145 (104-193)	56 (48-68)	45 (38-55)	46 (36-55)	29 (24-37)	41 (33-52)	39 (32-50)	28 (23-36)	27 (22-33)	30 (25-37)	33 (27-41)	41 (34-50)	-	-	-	-	-	-
Other congenital birth defects	35,785 (22,515-46,755)	9,508 (5,250-13,216)	28,021 (13,191-42,182)	11,947 (4,784-19,487)	2,717 (1,664-3,904)	1,628 (1,113-2,196)	915 (788-1,111)	786 (671-946)	492 (402-598)	367 (296-459)	329 (266-412)	340 (290-410)	151 (124-191)	124 (103-152)	107 (89-129)	102 (87-129)	87 (72-107)	-	-	-	-	-	-
Urinary diseases and male infertility	674 (572-797)	514 (431-603)	4,151 (3,685-4,644)	2,402 (1,964-2,821)	986 (837-1,117)	733 (657-808)	1,140 (1,034-1,237)	1,537 (1,414-1,656)	1,582 (1,477-1,704)	1,991 (1,860-2,110)	2,225 (2,095-2,355)	2,777 (2,620-2,930)	3,591 (3,389-3,795)	3,896 (3,697-4,103)	4,210 (3,983-4,418)	6,432 (6,101-6,745)	8,776 (8,291-9,226)	10,603 (9,957-11,093)	9,419 (8,944-9,801)	8,966 (8,428-9,386)	6,531 (6,256-6,783)	3,283 (3,140-3,412)	1,016 (983-1,053)
Urinary tract infections	128 (74-189)	163 (100-226)	2,778 (2,115-3,447)	929 (573-1,322)	531 (398-680)	454 (370-538)	777 (663-913)	1,027 (897-1,195)	1,040 (885-1,183)	1,173 (995-1,368)	1,268 (1,078-1,469)	1,621 (1,392-1,858)	1,971 (1,694-2,247)	2,187 (1,887-2,489)	2,382 (2,072-2,739)	3,803 (3,301-4,300)	5,127 (4,466-5,802)	6,027 (5,253-6,816)	5,987 (5,349-6,628)	5,522 (4,975-6,105)	4,633 (4,276-4,977)	2,360 (2,195-2,524)	779 (714-828)
Urolithiasis	-	-	-	-	34 (18-52)	31 (19-47)	24 (17-35)	38 (25-56)	77 (51-115)	109 (66-164)	139 (93-193)	180 (131-235)	277 (210-349)	322 (246-404)	326 (250-408)	552 (414-690)	730 (524-916)	842 (627-1,016)	739 (535-906)	505 (369-631)	324 (243-397)	144 (109-176)	29 (23-36)
Other urinary diseases	546 (420-660)	351 (247-439)	1,373 (716-2,088)	1,473 (925-2,059)	421 (300-571)	247 (175-320)	339 (228-447)	472 (338-613)	465 (352-596)	708 (549-867)	819 (640-1,008)	976 (764-1,219)	1,343 (1,072-1,650)	1,387 (1,085-1,731)	1,502 (1,174-1,869)	2,077 (1,628-2,623)	2,919 (2,265-3,678)	3,734 (2,955-4,585)	2,693 (2,123-3,351)	2,939 (2,387-3,542)	1,573 (1,272-1,954)	779 (622-939)	208 (171-271)
Gynecological diseases	-	-	-	-	-	-	-	-	-	-	-	-	-	-	-	-	-	-	-	-	-	-	-
Uterine fibroids	-	-	-	-	-	-	-	-	-	-	-	-	-	-	-	-	-	-	-	-	-	-	-
Polycystic ovarian syndrome	-	-	-	-	-	-	-	-	-	-	-	-	-	-	-	-	-	-	-	-	-	-	-

SR Table 6: Global deaths by cause and age group for males (with 95% confidence intervals), 2000

Cause	Early Neonatal	Late Neonatal	Post Neonatal	1 to 4	5 to 9	10 to 14
Endometriosis	-	-	-	-	-	-
Genital prolapse	-	-	-	-	-	-
Other gynecological diseases	-	-	-	-	-	-
Hemoglobinopathies and hemolytic anemias	674 (391-1,035)	514 (318-678)	3,635 (2,264-4,973)	6,785 (3,718-10,039)	2,977 (2,044-4,103)	2,964 (2,012-4,096)
Thalassemias	237 (168-289)	206 (128-277)	1,275 (882-1,842)	1,626 (1,059-2,440)	1,001 (735-1,305)	683 (491-840)
Sickle cell disorders	400 (180-738)	294 (163-468)	2,255 (1,114-3,602)	4,906 (2,248-7,469)	1,711 (995-2,792)	1,743 (1,006-2,809)
G6PD deficiency	12 (6-22)	9 (5-14)	78 (42-122)	211 (113-340)	211 (150-290)	387 (271-517)
Other hemoglobinopathies and hemolytic anemias	25 (18-36)	5 (3-7)	28 (19-40)	42 (28-62)	54 (43-67)	151 (118-193)
Endocrine, metabolic, blood, and immune disorders	862 (549-1,022)	827 (523-1,053)	4,496 (3,127-6,798)	2,505 (1,494-3,701)	1,120 (832-1,516)	989 (774-1,276)
Sudden infant death syndrome	-	3,309 (1,115-6,095)	28,601 (9,595-59,002)	-	-	-
Injuries	10,183 (8,682-11,209)	7,884 (6,695-8,749)	65,083 (57,642-72,465)	181,122 (151,739-205,252)	122,561 (113,226-130,637)	113,316 (106,484-118,764)
Transport injuries	1,814 (1,556-2,112)	1,583 (1,353-1,810)	10,171 (8,278-11,506)	39,241 (27,199-46,532)	39,435 (35,701-42,785)	33,350 (30,706-35,802)
Road injuries	1,532 (1,304-1,835)	1,391 (1,185-1,600)	9,387 (7,571-10,624)	36,816 (25,618-43,659)	37,289 (33,819-40,572)	30,789 (28,341-33,120)
Pedestrian road injuries	835 (644-1,159)	697 (532-970)	4,169 (3,512-5,055)	22,789 (16,931-28,604)	23,378 (21,081-25,597)	16,577 (14,998-18,212)
Cyclist road injuries	-	-	-	898 (504-1,336)	1,856 (1,396-2,274)	2,439 (1,895-2,906)
Motorcyclist road injuries	100 (73-139)	117 (75-156)	781 (568-962)	1,846 (1,056-2,617)	1,924 (1,565-2,332)	3,033 (2,523-3,596)
Motor vehicle road injuries	572 (392-758)	558 (359-757)	4,269 (3,093-5,291)	10,945 (6,292-14,846)	9,793 (8,128-11,623)	8,406 (7,278-9,673)
Other road injuries	25 (12-40)	20 (10-31)	168 (91-255)	338 (145-598)	339 (215-472)	335 (239-452)
Other transport injuries	282 (190-403)	192 (128-292)	784 (541-1,058)	2,425 (1,368-3,222)	2,147 (1,776-2,486)	2,561 (2,228-2,905)
Unintentional injuries	6,908 (5,642-7,682)	5,771 (4,714-6,459)	51,195 (44,737-57,861)	130,366 (111,072-149,106)	74,834 (68,508-80,283)	62,217 (58,188-65,763)
Falls	969 (657-1,217)	418 (245-568)	5,288 (3,214-7,004)	9,299 (5,373-13,788)	6,004 (4,182-7,744)	5,648 (4,475-7,326)
Drowning	383 (347-419)	245 (214-270)	6,813 (6,168-7,559)	75,695 (64,002-84,832)	47,017 (43,224-50,448)	36,364 (34,181-38,642)
Fire, heat, and hot substances	321 (192-396)	284 (148-369)	3,715 (2,021-4,724)	9,045 (4,426-12,261)	2,902 (1,851-3,480)	1,795 (1,220-2,070)
Poisonings	243 (131-292)	234 (133-291)	2,392 (1,264-3,064)	5,103 (2,601-7,005)	1,927 (1,175-2,470)	1,955 (1,280-2,518)
Poisoning by carbon monoxide	57 (30-70)	59 (29-75)	541 (267-706)	1,150 (663-1,487)	897 (538-1,173)	873 (595-1,158)
Poisoning by other means	186 (101-225)	174 (102-219)	1,851 (1,004-2,427)	3,954 (1,933-5,631)	1,030 (639-1,359)	1,082 (691-1,399)
Exposure to mechanical forces	900 (710-1,015)	1,016 (755-1,146)	4,594 (3,703-5,095)	7,876 (5,296-9,336)	4,360 (3,861-4,892)	3,887 (3,535-4,226)
Unintentional firearm injuries	100 (75-151)	89 (64-155)	630 (451-1,035)	952 (535-2,071)	550 (464-761)	700 (620-863)
Other exposure to mechanical forces	800 (612-910)	927 (670-1,053)	3,964 (3,026-4,433)	6,925 (4,735-8,234)	3,810 (3,349-4,272)	3,187 (2,862-3,489)
Adverse effects of medical treatment	729 (483-887)	483 (320-619)	4,491 (2,686-6,437)	3,581 (1,680-6,849)	1,346 (898-1,681)	1,133 (807-1,323)
Animal contact	276 (97-354)	287 (93-378)	1,709 (650-2,189)	4,885 (1,735-7,035)	4,504 (1,823-5,719)	4,413 (1,947-5,536)
Venomous animal contact	242 (78-318)	250 (74-338)	1,429 (505-1,923)	4,073 (1,303-6,381)	3,932 (1,529-5,209)	4,067 (1,738-5,201)
Non-venomous animal contact	34 (17-46)	37 (17-53)	280 (128-426)	812 (327-1,593)	571 (274-807)	346 (178-440)
Foreign body	2,568 (1,883-2,935)	2,530 (1,946-2,875)	19,370 (15,680-22,776)	8,574 (6,057-10,443)	2,429 (2,186-2,834)	1,950 (1,814-2,188)
Pulmonary aspiration and foreign body in airway	2,489 (1,846-2,862)	2,470 (1,918-2,813)	18,317 (14,822-21,920)	7,300 (5,345-9,373)	2,078 (1,861-2,480)	1,731 (1,601-1,993)
Foreign body in other body part	79 (34-122)	60 (25-94)	1,053 (386-1,606)	1,274 (464-2,326)	351 (202-475)	219 (137-275)
Environmental heat and cold exposure	243 (78-332)	159 (51-235)	1,558 (490-2,306)	1,139 (377-2,028)	501 (244-638)	497 (261-618)
Exposure to forces of nature	6 (5-7)	9 (8-10)	74 (65-85)	334 (297-380)	361 (327-407)	469 (426-522)
Other unintentional injuries	271 (235-312)	106 (90-121)	1,192 (1,042-1,325)	4,833 (3,934-5,574)	3,483 (3,078-3,871)	4,107 (3,713-4,488)
Self-harm and interpersonal violence	1,460 (1,165-1,582)	530 (444-574)	3,717 (2,983-4,212)	11,515 (9,935-12,643)	8,292 (7,164-8,814)	17,749 (15,548-19,041)
Self-harm	-	-	-	-	-	7,328 (5,776-8,340)
Self-harm by firearm	-	-	-	-	-	336 (241-561)
Self-harm by other specified means	-	-	-	-	-	6,992 (5,372-7,973)
Interpersonal violence	1,013 (717-1,135)	288 (203-333)	2,968 (2,234-3,459)	5,263 (3,680-6,386)	4,105 (2,980-4,627)	6,332 (4,804-7,169)
Physical violence by firearm	147 (86-199)	44 (27-64)	449 (296-628)	824 (516-1,386)	630 (477-826)	1,623 (1,296-1,926)
Physical violence by sharp object	90 (53-125)	28 (15-43)	283 (159-459)	723 (339-1,408)	705 (364-1,077)	1,141 (665-1,557)
Physical violence by other means	776 (546-889)	216 (149-254)	2,236 (1,672-2,698)	3,716 (2,499-4,649)	2,770 (1,982-3,379)	3,568 (2,632-4,410)
Conflict and terrorism	448 (416-489)	241 (224-265)	729 (676-798)	6,208 (5,790-6,768)	4,140 (3,835-4,562)	4,033 (3,730-4,462)
Executions and police conflict	-	-	20 (13-35)	44 (39-49)	47 (43-52)	57 (53-62)

Cause	15 to 19	20 to 24	25 to 29	30 to 34	35 to 39	40 to 44	45 to 49	50 to 54
Endometriosis	-	-	-	-	-	-	-	-
Genital prolapse	-	-	-	-	-	-	-	-
Other gynecological diseases	-	-	-	-	-	-	-	-
Hemoglobinopathies and hemolytic anemias	3,357 (1,901-5,018)	2,939 (1,579-4,527)	2,175 (1,293-3,195)	1,746 (1,095-2,501)	1,609 (957-2,345)	1,593 (957-2,352)	1,549 (966-2,228)	1,723 (1,044-2,431)
Thalassemias	398 (274-498)	260 (156-333)	153 (101-199)	75 (52-102)	34 (25-45)	21 (16-26)	14 (11-18)	8 (7-10)
Sickle cell disorders	2,210 (1,049-3,806)	1,841 (802-3,345)	1,180 (509-2,162)	742 (295-1,460)	679 (240-1,356)	533 (181-1,103)	313 (100-711)	174 (72-387)
G6PD deficiency	528 (364-702)	552 (386-718)	593 (443-720)	663 (484-788)	620 (454-770)	679 (467-918)	789 (502-1,124)	880 (489-1,365)
Other hemoglobinopathies and hemolytic anemias	220 (156-295)	287 (214-365)	249 (195-305)	266 (201-328)	276 (209-332)	361 (264-447)	433 (326-543)	661 (453-875)
Endocrine, metabolic, blood, and immune disorders	819 (668-1,022)	885 (718-1,080)	1,176 (915-1,401)	1,554 (1,176-1,873)	1,668 (1,310-2,020)	1,884 (1,507-2,233)	2,232 (1,843-2,707)	2,530 (1,978-3,016)
Sudden infant death syndrome	-	-	-	-	-	-	-	-
Injuries	213,677 (206,440-219,969)	302,666 (295,354-309,004)	304,900 (296,303-311,149)	285,877 (276,914-292,379)	261,447 (252,650-266,751)	221,030 (213,869-225,531)	209,231 (200,961-213,527)	164,018 (157,366-168,068)
Transport injuries	81,984 (78,037-86,215)	113,549 (109,351-118,748)	116,138 (109,487-121,380)	107,231 (101,196-111,583)	95,300 (91,962-98,824)	75,036 (72,609-77,835)	69,249 (67,079-71,402)	53,030 (51,234-55,434)
Road injuries	75,705 (72,143-79,655)	106,099 (102,129-111,156)	107,858 (101,359-113,017)	100,347 (94,412-104,622)	88,385 (85,220-91,717)	69,183 (66,794-71,835)	63,612 (61,442-65,793)	48,415 (46,751-50,624)
Pedestrian road injuries	21,042 (18,758-24,880)	28,421 (25,890-33,303)	33,181 (30,615-37,378)	35,451 (32,895-39,654)	31,896 (29,219-36,292)	25,086 (22,794-28,677)	26,269 (24,276-29,181)	20,860 (19,096-23,696)
Cyclist road injuries	3,149 (2,545-3,716)	2,865 (2,280-3,299)	3,207 (2,491-3,774)	3,747 (2,694-4,379)	3,429 (2,476-3,944)	3,354 (2,478-3,795)	3,641 (2,733-4,170)	3,160 (2,378-3,603)
Motorcyclist road injuries	19,918 (17,836-23,044)	28,538 (25,813-32,043)	28,513 (24,857-31,933)	24,015 (20,460-27,120)	19,545 (16,545-21,587)	13,936 (12,187-15,559)	11,171 (9,863-12,409)	7,191 (6,391-8,418)
Motor vehicle road injuries	31,046 (28,397-34,603)	45,474 (41,912-50,349)	42,163 (38,208-47,417)	36,472 (32,810-41,394)	32,903 (29,784-36,399)	26,267 (24,400-29,137)	21,915 (19,683-24,452)	16,662 (15,041-18,451)
Other road injuries	550 (413-784)	801 (603-1,090)	795 (569-1,076)	662 (484-870)	612 (465-797)	539 (418-686)	617 (476-800)	542 (406-725)
Other transport injuries	6,279 (5,661-7,144)	7,449 (6,912-8,426)	8,280 (7,690-8,976)	6,884 (6,373-7,366)	6,915 (6,480-7,435)	5,853 (5,513-6,555)	5,636 (5,304-6,071)	4,615 (4,304-5,231)
Unintentional injuries	53,994 (51,434-56,167)	62,047 (59,365-64,162)	64,655 (61,861-67,027)	66,406 (63,246-68,952)	67,192 (63,901-69,142)	62,351 (59,412-64,135)	66,139 (62,805-67,989)	54,418 (51,433-56,180)
Falls	5,926 (5,163-7,094)	8,691 (7,287-10,146)	10,711 (8,725-12,865)	12,370 (10,118-14,995)	13,575 (11,533-15,525)	14,399 (12,558-16,412)	16,110 (14,257-18,238)	14,636 (12,975-16,616)
Drowning	21,915 (20,769-23,205)	19,246 (18,414-20,252)	16,428 (15,772-17,177)	15,392 (14,813-16,050)	13,703 (13,244-14,202)	11,855 (11,453-12,258)	10,940 (10,626-11,278)	8,654 (8,396-8,916)
Fire, heat, and hot substances	2,331 (1,670-2,797)	3,468 (2,617-4,035)	3,933 (2,970-4,511)	4,113 (3,193-4,655)	4,513 (3,743-5,063)	4,525 (3,972-4,965)	4,768 (4,176-5,220)	4,067 (3,570-4,457)
Poisonings	2,186 (1,701-2,629)	3,242 (2,551-3,888)	3,755 (2,923-4,599)	3,767 (2,996-4,671)	4,033 (3,420-4,469)	4,387 (3,839-4,801)	4,334 (3,819-4,798)	3,440 (3,083-3,813)
Poisoning by carbon monoxide	1,278 (1,039-1,556)	1,976 (1,618-2,361)	2,257 (1,819-2,842)	2,439 (2,019-3,023)	2,750 (2,463-3,065)	3,144 (2,870-3,422)	3,141 (2,901-3,455)	2,427 (2,288-2,642)
Poisoning by other means	909 (636-1,116)	1,265 (916-1,567)	1,498 (1,060-1,839)	1,328 (921-1,673)	1,282 (939-1,452)	1,243 (931-1,421)	1,193 (898-1,376)	1,013 (776-1,170)
Exposure to mechanical forces	7,037 (6,487-7,867)	9,818 (9,152-10,846)	10,800 (10,216-11,647)	10,943 (10,381-11,545)	10,690 (10,198-11,175)	7,835 (7,457-8,415)	8,653 (8,259-9,138)	6,804 (6,392-7,379)
Unintentional firearm injuries	1,822 (1,605-2,102)	2,627 (2,294-2,992)	2,547 (2,157-2,953)	1,869 (1,553-2,183)	1,651 (1,396-1,928)	1,308 (1,120-1,475)	1,411 (1,179-1,608)	1,088 (879-1,248)
Other exposure to mechanical forces	5,215 (4,764-5,878)	7,191 (6,635-8,058)	8,253 (7,738-8,985)	9,074 (8,510-9,708)	9,040 (8,540-9,497)	6,527 (6,189-7,079)	7,243 (6,851-7,730)	5,715 (5,388-6,239)
Adverse effects of medical treatment	1,437 (1,032-1,720)	2,131 (1,467-2,552)	2,050 (1,449-2,422)	2,142 (1,540-2,520)	2,308 (1,665-2,693)	2,437 (1,739-2,836)	2,499 (1,802-2,874)	2,090 (1,575-2,425)
Animal contact	2,793 (1,335-3,418)	2,549 (1,177-3,071)	2,727 (1,270-3,255)	2,693 (1,269-3,213)	2,540 (1,217-2,976)	2,447 (1,199-2,918)	3,330 (1,583-3,940)	2,217 (1,100-2,597)
Venomous animal contact	2,421 (1,103-3,044)	2,172 (959-2,681)	2,241 (1,003-2,778)	2,325 (1,084-2,874)	2,200 (1,031-2,642)	2,130 (1,014-2,569)	2,891 (1,319-3,491)	1,821 (862-2,176)
Non-venomous animal contact	372 (221-487)	377 (221-479)	487 (271-591)	367 (177-463)	341 (183-428)	317 (170-409)	439 (256-556)	396 (225-522)
Foreign body	1,329 (1,267-1,468)	2,204 (2,081-2,443)	2,328 (2,229-2,608)	2,415 (2,329-2,684)	2,800 (2,704-3,016)	3,207 (3,123-3,426)	3,398 (3,287-3,614)	3,176 (3,083-3,323)
Pulmonary aspiration and foreign body in airway	1,193 (1,132-1,361)	1,995 (1,888-2,229)	2,076 (1,990-2,374)	2,075 (1,992-2,352)	2,432 (2,343-2,624)	2,850 (2,768-3,051)	3,141 (3,046-3,350)	2,961 (2,874-3,100)
Foreign body in other body part	136 (87-163)	209 (140-249)	252 (166-300)	340 (260-408)	368 (286-440)	357 (288-432)	257 (195-301)	215 (160-256)
Environmental heat and cold exposure	1,133 (749-1,368)	1,684 (1,173-1,936)	2,116 (1,521-2,394)	2,847 (2,028-3,219)	3,851 (2,969-4,268)	4,912 (4,128-5,357)	5,505 (4,612-6,020)	5,020 (4,187-5,449)
Exposure to forces of nature	512 (468-565)	470 (433-520)	440 (406-483)	397 (367-433)	353 (324-384)	306 (283-334)	282 (262-305)	229 (213-249)
Other unintentional injuries	7,395 (6,802-8,015)	8,545 (8,092-9,084)	9,367 (8,829-10,092)	9,327 (8,828-9,941)	8,825 (8,395-9,329)	6,043 (5,792-6,319)	6,320 (5,999-6,640)	4,086 (3,899-4,263)
Self-harm and interpersonal violence	77,698 (71,883-80,382)	127,070 (119,036-130,857)	124,108 (113,466-128,353)	112,240 (100,510-116,726)	98,955 (89,774-102,456)	83,643 (76,883-85,974)	73,843 (66,623-76,510)	56,570 (51,756-58,292)
Self-harm	30,196 (26,777-32,287)	51,691 (46,544-54,760)	58,920 (51,403-62,912)	57,401 (48,912-61,035)	54,474 (47,642-57,230)	49,040 (43,476-50,927)	47,523 (41,603-49,790)	38,287 (33,980-39,701)
Self-harm by firearm	3,602 (2,709-4,961)	5,881 (4,532-7,942)	5,408 (4,132-7,353)	4,758 (3,610-6,614)	4,575 (3,577-6,063)	4,467 (3,492-5,847)	4,254 (3,343-5,548)	3,527 (2,726-4,433)
Self-harm by other specified means	26,595 (23,310-28,673)	45,810 (40,494-49,036)	53,512 (46,086-57,637)	52,643 (44,690-56,416)	49,899 (42,619-52,893)	44,573 (39,218-46,659)	43,269 (37,168-45,623)	34,759 (30,452-36,261)
Interpersonal violence	35,240 (30,406-37,643)	55,832 (48,912-59,238)	51,917 (44,815-54,279)	45,318 (38,463-47,853)	38,012 (32,727-40,443)	30,452 (26,692-32,054)	23,622 (20,681-25,111)	16,554 (14,458-17,631)
Physical violence by firearm	17,187 (14,563-18,455)	27,194 (23,073-29,351)	23,292 (18,903-25,413)	18,761 (14,804-20,445)	15,212 (12,006-16,780)	11,168 (8,870-12,308)	7,470 (5,939-8,249)	5,126 (3,991-5,708)
Physical violence by sharp object	8,164 (5,911-9,990)	13,547 (10,109-16,359)	14,297 (10,547-17,490)	13,059 (9,752-16,053)	10,934 (8,526-13,378)	8,903 (7,135-10,357)	6,996 (5,594-8,313)	4,696 (3,720-5,660)
Physical violence by other means	9,889 (8,190-12,448)	15,091 (12,793-18,614)	14,328 (11,958-17,746)	13,498 (11,089-16,976)	11,866 (10,167-14,502)	10,382 (9,168-12,421)	9,156 (8,003-10,624)	6,733 (5,821-7,788)
Conflict and terrorism	11,858 (11,105-12,912)	18,545 (17,344-20,149)	11,905 (11,190-12,868)	8,172 (7,650-8,806)	5,578 (5,212-6,130)	3,582 (3,321-3,958)	2,336 (2,176-2,549)	1,527 (1,429-1,652)
Executions and police conflict	404 (355-461)	1,001 (842-1,129)	1,366 (1,123-1,578)	1,350 (912-1,579)	891 (708-1,039)	568 (423-685)	361 (285-455)	202 (168-251)

Cause	54 to 59	60 to 64	65 to 69	70 to 74	75 to 79	80 to 84	85 to 89	90 to 94	95 plus
Endometriosis	-	-	-	-	-	-	-	-	-
Genital prolapse	-	-	-	-	-	-	-	-	-
Other gynecological diseases	-	-	-	-	-	-	-	-	-
Hemoglobinopathies and hemolytic anemias	1,821 (1,137-2,598)	2,205 (1,382-3,101)	2,575 (1,667-3,557)	2,839 (1,904-3,803)	2,742 (1,882-3,599)	2,145 (1,509-2,740)	1,686 (1,188-2,008)	812 (548-922)	207 (148-234)
Thalassemias	4 (3-5)	2 (1-2)	1 (0-1)	0 (0-0)	0 (0-0)	0 (0-0)	0 (0-0)	0 (0-0)	0 (0-0)
Sickle cell disorders	74 (38-143)	35 (23-55)	18 (12-25)	8 (5-11)	2 (1-3)	1 (0-1)	0 (0-0)	0 (0-0)	0 (0-0)
G6PD deficiency	830 (442-1,373)	759 (378-1,277)	605 (302-1,034)	419 (210-729)	224 (109-405)	85 (41-153)	33 (18-55)	8 (5-12)	1 (1-2)
Other hemoglobinopathies and hemolytic anemias	913 (631-1,230)	1,410 (950-1,871)	1,952 (1,351-2,611)	2,412 (1,672-3,192)	2,516 (1,752-3,278)	2,059 (1,463-2,598)	1,653 (1,162-1,966)	804 (544-911)	206 (147-232)
Endocrine, metabolic, blood, and immune disorders	2,556 (2,001-3,009)	2,951 (2,421-3,500)	3,323 (2,742-3,993)	3,742 (3,085-4,549)	3,427 (2,675-4,142)	2,597 (2,057-3,191)	2,060 (1,650-2,397)	905 (686-1,087)	272 (219-329)
Sudden infant death syndrome	-	-	-	-	-	-	-	-	-
Injuries	129,137 (122,178-132,671)	126,384 (118,509-130,250)	114,442 (105,788-118,224)	103,841 (95,455-107,609)	81,732 (74,427-85,210)	57,482 (51,900-60,468)	38,260 (35,027-40,018)	15,977 (14,746-16,668)	3,982 (3,734-4,121)
Transport injuries	41,155 (39,598-43,138)	37,386 (36,067-39,132)	34,229 (32,935-35,730)	29,019 (27,912-30,294)	21,025 (20,165-22,180)	12,581 (11,973-13,423)	6,395 (6,119-6,762)	2,028 (1,938-2,118)	439 (422-456)
Road injuries	37,545 (36,100-39,379)	34,537 (33,296-36,067)	31,738 (30,516-33,162)	27,052 (26,003-28,237)	19,631 (18,828-20,658)	11,817 (11,246-12,594)	6,035 (5,778-6,355)	1,908 (1,825-1,991)	407 (391-423)
Pedestrian road injuries	16,410 (15,010-18,933)	17,425 (16,204-19,419)	16,581 (15,466-18,434)	14,497 (13,616-16,043)	11,421 (10,721-12,621)	6,809 (6,352-7,576)	3,526 (3,303-3,843)	1,072 (1,008-1,167)	226 (214-242)
Cyclist road injuries	2,302 (1,841-2,627)	2,560 (2,041-2,892)	2,411 (1,954-2,705)	2,008 (1,637-2,256)	1,205 (990-1,359)	660 (569-761)	302 (266-345)	83 (73-97)	1 (1-1)
Motorcyclist road injuries	5,108 (4,529-6,052)	3,143 (2,723-3,800)	2,691 (2,328-3,171)	1,920 (1,636-2,262)	978 (797-1,173)	658 (551-813)	250 (206-311)	64 (53-80)	14 (12-17)
Motor vehicle road injuries	13,303 (11,889-14,806)	11,089 (10,041-12,409)	9,694 (8,779-10,808)	8,291 (7,413-9,215)	5,791 (5,205-6,539)	3,527 (3,192-4,014)	1,877 (1,713-2,094)	669 (614-728)	160 (147-169)
Other road injuries	423 (330-538)	320 (262-403)	362 (284-470)	336 (259-444)	237 (184-316)	162 (121-231)	79 (62-115)	20 (15-29)	7 (5-10)
Other transport injuries	3,610 (3,355-4,152)	2,849 (2,683-3,243)	2,492 (2,327-2,807)	1,967 (1,842-2,223)	1,395 (1,288-1,617)	764 (692-903)	360 (327-423)	121 (109-141)	32 (29-38)
Unintentional injuries	45,982 (42,603-47,645)	50,790 (46,610-53,022)	48,630 (43,412-51,057)	47,467 (41,674-50,111)	42,059 (36,770-44,487)	33,819 (29,703-35,931)	25,511 (22,738-27,040)	11,956 (10,902-12,580)	3,131 (2,911-3,262)
Falls	14,142 (12,300-15,723)	16,946 (14,460-19,121)	19,252 (16,307-21,481)	21,474 (17,608-23,797)	22,208 (18,429-24,263)	19,594 (16,003-21,293)	16,032 (13,525-17,304)	7,933 (6,996-8,490)	2,021 (1,824-2,142)
Drowning	6,375 (6,170-6,592)	6,803 (6,614-7,005)	5,595 (5,421-5,781)	5,168 (4,994-5,335)	3,321 (3,199-3,441)	2,399 (2,281-2,507)	1,331 (1,266-1,393)	407 (390-424)	86 (83-89)
Fire, heat, and hot substances	3,278 (2,868-3,656)	3,997 (3,494-4,389)	3,426 (2,929-3,843)	3,435 (2,897-3,863)	2,734 (2,341-3,061)	2,358 (1,997-2,668)	1,756 (1,551-1,925)	797 (722-852)	228 (210-240)
Poisonings	2,479 (2,235-2,827)	2,929 (2,630-3,314)	2,088 (1,841-2,478)	1,755 (1,559-2,147)	1,066 (922-1,322)	699 (588-876)	377 (333-469)	114 (101-136)	23 (21-26)
Poisoning by carbon monoxide	1,693 (1,585-1,917)	2,018 (1,885-2,255)	1,318 (1,214-1,563)	1,102 (990-1,320)	670 (589-829)	415 (357-522)	230 (205-288)	69 (62-83)	12 (11-14)
Poisoning by other means	786 (623-944)	912 (699-1,078)	770 (620-964)	653 (539-829)	396 (322-507)	284 (234-362)	147 (120-183)	46 (39-53)	11 (10-12)
Exposure to mechanical forces	4,890 (4,613-5,418)	4,014 (3,826-4,388)	3,536 (3,348-3,861)	2,956 (2,775-3,253)	2,111 (1,974-2,350)	1,223 (1,156-1,369)	803 (767-882)	372 (355-404)	111 (107-118)
Unintentional firearm injuries	699 (614-825)	636 (547-727)	565 (488-665)	506 (435-588)	400 (341-472)	268 (228-314)	160 (139-184)	73 (63-86)	18 (16-22)
Other exposure to mechanical forces	4,191 (3,937-4,685)	3,379 (3,209-3,689)	2,971 (2,777-3,259)	2,450 (2,275-2,694)	1,711 (1,592-1,904)	955 (895-1,071)	643 (611-705)	299 (285-323)	93 (89-100)
Adverse effects of medical treatment	2,372 (1,801-2,733)	3,263 (2,457-3,821)	4,046 (3,059-4,692)	3,788 (2,975-4,441)	3,512 (2,758-4,091)	2,536 (2,006-2,997)	1,747 (1,424-2,085)	768 (632-939)	199 (166-248)
Animal contact	2,612 (1,214-3,077)	2,519 (1,112-2,996)	2,647 (1,130-3,196)	2,115 (957-2,520)	1,622 (733-1,964)	863 (432-1,060)	377 (204-458)	117 (69-141)	28 (18-32)
Venomous animal contact	2,243 (1,005-2,731)	2,117 (889-2,553)	2,242 (910-2,737)	1,764 (760-2,145)	1,330 (588-1,623)	697 (341-852)	293 (154-362)	87 (49-106)	19 (11-23)
Non-venomous animal contact	369 (199-506)	402 (199-561)	405 (198-571)	352 (182-499)	292 (152-425)	166 (88-246)	84 (49-120)	30 (17-41)	9 (6-12)
Foreign body	2,617 (2,540-2,725)	2,953 (2,841-3,062)	2,615 (2,481-2,716)	2,631 (2,442-2,728)	2,535 (2,409-2,626)	2,326 (2,224-2,415)	1,961 (1,895-2,033)	972 (939-1,011)	313 (304-324)
Pulmonary aspiration and foreign body in airway	2,450 (2,377-2,559)	2,753 (2,655-2,847)	2,458 (2,354-2,556)	2,455 (2,294-2,562)	2,352 (2,256-2,472)	2,186 (2,112-2,284)	1,867 (1,806-1,951)	931 (898-972)	301 (293-312)
Foreign body in other body part	167 (126-197)	200 (150-247)	157 (109-190)	177 (115-213)	183 (122-218)	140 (93-168)	94 (63-111)	41 (28-48)	12 (9-14)
Environmental heat and cold exposure	3,849 (2,971-4,327)	4,352 (3,522-4,838)	3,197 (2,231-3,672)	2,577 (1,717-2,985)	1,984 (1,165-2,418)	1,215 (721-1,445)	809 (551-947)	347 (247-394)	84 (65-94)
Exposure to forces of nature	167 (156-182)	173 (160-187)	133 (124-145)	114 (107-124)	80 (74-86)	45 (42-48)	21 (19-22)	6 (6-7)	1 (1-1)
Other unintentional injuries	3,202 (3,032-3,372)	2,841 (2,700-2,976)	2,095 (1,973-2,209)	1,455 (1,366-1,525)	885 (839-932)	563 (524-598)	298 (281-316)	123 (115-129)	37 (35-39)
Self-harm and interpersonal violence	42,000 (37,610-43,465)	38,207 (33,998-39,539)	31,583 (27,210-32,887)	27,355 (23,480-28,441)	18,648 (15,673-19,478)	11,082 (9,155-11,709)	6,354 (5,546-6,648)	1,993 (1,784-2,071)	412 (379-424)
Self-harm	29,891 (26,195-31,179)	28,066 (24,212-29,249)	24,555 (20,745-25,703)	22,225 (18,404-23,215)	15,693 (12,905-16,465)	10,140 (8,246-10,753)	5,887 (5,062-6,175)	1,845 (1,634-1,921)	321 (291-331)
Self-harm by firearm	2,802 (2,171-3,523)	2,514 (1,978-3,188)	2,334 (1,845-2,911)	2,368 (1,940-2,994)	2,055 (1,731-2,564)	1,407 (1,194-1,744)	913 (785-1,206)	307 (262-418)	55 (46-73)
Self-harm by other specified means	27,089 (23,421-28,436)	25,552 (21,789-26,801)	22,221 (18,509-23,440)	19,856 (16,047-20,955)	13,638 (10,767-14,508)	8,733 (6,798-9,402)	4,975 (4,083-5,332)	1,538 (1,283-1,638)	266 (229-280)
Interpersonal violence	10,781 (9,324-11,704)	9,012 (7,864-9,642)	6,149 (5,183-6,682)	4,467 (3,770-4,815)	2,526 (2,116-2,740)	656 (569-726)	333 (304-371)	100 (92-111)	69 (63-73)
Physical violence by firearm	3,367 (2,621-3,831)	2,172 (1,711-2,503)	1,459 (1,124-1,684)	1,043 (778-1,242)	623 (469-742)	145 (112-175)	71 (55-82)	25 (20-29)	17 (12-19)
Physical violence by sharp object	2,773 (2,208-3,284)	2,579 (2,042-3,034)	1,699 (1,252-2,014)	1,174 (879-1,351)	657 (496-776)	159 (134-181)	84 (73-96)	25 (22-29)	18 (15-20)
Physical violence by other means	4,640 (3,937-5,464)	4,261 (3,749-4,844)	2,992 (2,539-3,534)	2,250 (1,918-2,666)	1,246 (1,055-1,459)	352 (296-411)	178 (159-209)	50 (45-59)	35 (32-39)
Conflict and terrorism	1,224 (1,155-1,333)	1,051 (991-1,139)	826 (781-894)	632 (597-678)	410 (387-440)	273 (259-290)	127 (120-135)	45 (43-48)	19 (18-21)
Executions and police conflict	105 (85-136)	78 (64-97)	52 (43-63)	32 (24-39)	18 (15-21)	13 (10-15)	7 (6-9)	4 (3-5)	2 (2-3)

SR Table 6: Global deaths by cause and age group for males (with 95% confidence intervals), 2005

Cause	Early Neonatal	Late Neonatal	Post Neonatal	1 to 4	5 to 9	10 to 14	15 to 19	20 to 24	25 to 29	30 to 34
All causes	1,473,433 (1,440,580-1,508,778)	419,746 (406,033-434,043)	1,358,799 (1,324,489-1,394,700)	1,238,313 (1,203,717-1,273,550)	328,678 (324,961-332,460)	236,831 (234,538-239,109)	389,657 (384,976-394,586)	513,840 (507,218-521,017)	582,140 (571,251-593,340)	710,160 (698,826-721,356)
Communicable, maternal, neonatal, and nutritional diseases	1,338,289 (1,301,927-1,372,979)	364,551 (350,206-378,895)	1,094,455 (1,050,416-1,137,027)	961,625 (922,734-1,005,156)	178,347 (166,881-191,804)	96,371 (88,705-105,669)	89,759 (81,599-101,412)	110,953 (102,758-121,331)	176,077 (163,965-189,897)	239,229 (227,413-252,801)
HIV/AIDS and sexually transmitted infections	-	13,840 (5,339-25,833)	108,025 (91,673-130,975)	48,503 (44,880-52,157)	16,959 (16,054-18,029)	9,450 (8,900-10,074)	9,674 (8,451-11,033)	23,688 (20,767-27,269)	85,827 (76,524-96,060)	136,032 (125,874-146,410)
HIV/AIDS	-	-	82,760 (78,334-87,200)	46,304 (43,198-49,223)	16,119 (15,382-16,859)	9,417 (8,858-10,041)	9,635 (8,414-10,998)	23,643 (20,726-27,227)	85,761 (76,487-95,987)	135,953 (125,785-146,335)
HIV/AIDS - Drug-susceptible Tuberculosis	-	-	25,967 (19,377-30,927)	15,621 (12,186-18,069)	5,296 (3,993-6,181)	3,031 (2,266-3,575)	3,045 (2,279-3,742)	6,386 (4,841-7,969)	18,437 (12,245-25,004)	26,609 (16,914-37,059)
HIV/AIDS - Multidrug-resistant Tuberculosis without extensive drug resistance	-	-	2,671 (1,628-3,936)	1,567 (976-2,302)	496 (311-702)	273 (171-392)	285 (180-417)	764 (468-1,140)	2,123 (1,202-3,277)	3,039 (1,672-4,850)
HIV/AIDS - Extensively drug-resistant Tuberculosis	-	-	38 (24-54)	21 (14-29)	7 (5-10)	4 (3-6)	7 (4-9)	25 (16-36)	78 (49-118)	117 (72-175)
HIV/AIDS resulting in other diseases	-	-	54,084 (47,578-62,671)	29,095 (25,726-33,547)	10,319 (9,203-11,878)	6,109 (5,368-7,033)	6,298 (5,310-7,597)	16,468 (13,765-20,129)	65,122 (53,878-77,371)	106,188 (91,727-120,973)
Sexually transmitted infections excluding HIV	39,917 (15,508-75,359)	13,840 (5,339-25,833)	25,265 (9,588-48,244)	2,199 (814-4,333)	841 (311-1,615)	33 (29-48)	39 (34-46)	45 (32-60)	66 (41-93)	79 (52-118)
Syphilis	39,917 (15,508-75,359)	13,840 (5,339-25,833)	25,265 (9,588-48,244)	2,199 (814-4,333)	841 (311-1,615)	28 (24-41)	32 (28-38)	36 (26-49)	55 (35-78)	61 (40-91)
Chlamydial infection	-	-	-	-	-	0 (0-0)	0 (0-0)	0 (0-0)	0 (0-0)	0 (0-0)
Gonococcal infection	-	-	-	-	-	3 (2-4)	5 (4-5)	5 (4-7)	7 (4-9)	15 (10-22)
Other sexually transmitted infections	-	-	-	-	-	2 (2-3)	2 (2-3)	3 (2-4)	4 (2-5)	3 (2-5)
Respiratory infections and tuberculosis	-	82,407 (75,116-89,957)	364,468 (334,749-391,803)	197,469 (168,102-225,170)	26,289 (21,943-29,578)	15,373 (13,477-17,090)	22,637 (20,900-24,485)	37,136 (34,806-40,108)	48,316 (45,785-51,605)	62,318 (59,370-66,402)
Tuberculosis	-	-	25,869 (23,547-28,244)	25,484 (21,544-29,376)	5,600 (4,831-6,403)	5,426 (4,761-6,131)	13,650 (12,418-14,943)	27,520 (25,489-29,906)	37,350 (34,931-40,310)	48,654 (46,027-51,989)
Drug-susceptible tuberculosis	-	-	23,370 (21,165-25,741)	23,239 (19,500-26,860)	5,058 (4,325-5,773)	4,878 (4,237-5,512)	12,248 (11,074-13,482)	24,552 (22,529-26,786)	33,023 (30,717-35,950)	42,944 (40,408-46,134)
Multidrug-resistant tuberculosis without extensive drug resistance	-	-	2,390 (1,839-3,053)	2,181 (1,567-2,943)	520 (390-675)	524 (402-683)	1,337 (1,040-1,682)	2,765 (2,195-3,461)	3,978 (3,178-4,877)	5,264 (4,223-6,464)
Extensively drug-resistant tuberculosis	-	-	108 (84-136)	65 (50-86)	23 (18-30)	23 (18-30)	65 (52-83)	202 (162-250)	349 (278-423)	447 (364-539)
Lower respiratory infections	114,105 (101,491-129,594)	82,298 (75,028-89,869)	336,763 (309,887-361,900)	170,004 (145,177-195,764)	20,449 (16,333-23,163)	9,792 (8,061-11,053)	8,876 (7,623-9,922)	9,519 (8,197-10,571)	10,871 (9,481-11,902)	13,544 (12,111-15,046)
Upper respiratory infections	47 (25-62)	92 (47-135)	1,734 (673-2,865)	1,892 (471-3,856)	190 (78-287)	105 (47-149)	75 (37-114)	68 (37-103)	76 (45-109)	102 (62-144)
Otitis media	14 (3-58)	16 (8-38)	103 (38-255)	88 (27-239)	49 (23-104)	50 (25-108)	36 (18-81)	29 (14-64)	19 (10-41)	17 (9-37)
Enteric infections	16,444 (14,115-19,646)	28,245 (24,102-33,414)	237,710 (205,612-273,429)	241,603 (188,772-291,904)	54,544 (38,910-75,700)	30,836 (21,742-43,530)	23,560 (15,678-36,139)	18,404 (11,540-30,396)	14,970 (9,099-25,054)	13,813 (8,357-23,959)
Diarrheal diseases	16,444 (14,115-19,646)	27,859 (23,712-32,976)	231,570 (200,032-266,907)	209,616 (160,161-256,457)	20,525 (9,398-38,367)	9,953 (4,463-20,083)	10,216 (4,563-21,630)	10,376 (4,388-20,770)	9,354 (3,949-19,038)	9,453 (4,232-19,955)
Typhoid and paratyphoid	-	0 (0-0)	180 (90-320)	17,811 (8,308-32,781)	29,662 (17,218-47,016)	18,934 (10,798-30,290)	11,904 (5,862-20,940)	6,685 (3,418-11,554)	4,481 (2,324-7,672)	3,300 (1,691-5,589)
Typhoid fever	-	-	163 (82-292)	15,826 (7,385-29,485)	26,115 (14,898-41,559)	16,580 (9,208-26,772)	10,374 (5,058-18,249)	5,832 (2,875-10,303)	3,882 (1,995-6,685)	2,827 (1,429-4,899)
Paratyphoid fever	-	-	17 (7-36)	1,985 (756-4,336)	3,547 (1,510-6,813)	2,354 (1,043-4,718)	1,530 (602-3,195)	852 (342-1,838)	600 (251-1,245)	473 (203-983)
Invasive Non-typhoidal Salmonella (iNTS)	-	386 (217-646)	5,424 (3,093-9,372)	13,886 (7,457-23,901)	4,313 (2,131-7,376)	1,922 (863-3,485)	1,412 (579-2,631)	1,314 (555-2,512)	1,118 (437-2,321)	1,036 (437-1,985)
Other intestinal infectious diseases	-	-	536 (222-1,309)	291 (111-862)	44 (12-122)	27 (6-74)	28 (5-75)	29 (5-73)	16 (2-44)	24 (4-61)
Neglected tropical diseases and malaria	288 (121-1,047)	17,487 (11,203-26,074)	108,901 (75,270-151,902)	208,126 (150,263-282,384)	36,756 (26,748-50,499)	20,837 (15,157-28,952)	16,437 (11,704-23,631)	16,668 (11,763-22,876)	14,058 (9,959-19,714)	12,815 (9,180-17,821)
Malaria	-	17,234 (10,985-25,820)	104,928 (71,198-147,828)	194,189 (134,478-267,684)	28,626 (19,781-39,723)	15,556 (10,697-21,701)	11,629 (7,910-16,294)	12,071 (8,076-17,393)	10,496 (6,959-15,366)	9,480 (6,314-13,838)
Chagas disease	-	-	1 (1-2)	1 (1-1)	1 (1-2)	3 (3-3)	7 (6-8)	18 (16-20)	34 (31-39)	72 (66-78)
Leishmaniasis	-	-	786 (1-3,293)	3,720 (1-19,562)	2,008 (0-14,389)	1,312 (0-8,908)	1,411 (0-8,283)	1,100 (0-5,816)	976 (0-4,588)	922 (0-4,211)
Visceral leishmaniasis	-	-	786 (1-3,293)	3,720 (1-19,562)	2,008 (0-14,389)	1,312 (0-8,908)	1,411 (0-8,283)	1,100 (0-5,816)	976 (0-4,588)	922 (0-4,211)
African trypanosomiasis	-	-	-	278 (51-926)	434 (110-1,204)	484 (129-1,251)	688 (184-1,866)	745 (198-1,889)	622 (167-1,747)	547 (148-1,542)
Schistosomiasis	-	-	0 (0-0)	335 (226-503)	138 (98-193)	179 (130-245)	212 (162-275)	252 (193-322)	239 (189-302)	244 (190-300)
Cysticercosis	0 (0-0)	0 (0-0)	0 (0-0)	68 (27-126)	29 (5-65)	20 (2-54)	23 (2-65)	35 (4-87)	41 (5-96)	43 (6-107)
Cystic echinococcosis	-	-	-	171 (92-265)	78 (28-137)	49 (11-106)	25 (0-69)	30 (1-83)	40 (3-101)	43 (3-112)
Dengue	-	-	1,522 (269-2,504)	2,860 (1,035-4,273)	1,179 (419-1,659)	568 (172-824)	667 (198-1,008)	660 (224-960)	550 (183-775)	354 (106-524)
Yellow fever	-	2 (0-8)	44 (8-138)	437 (87-1,348)	668 (135-1,946)	578 (111-1,710)	549 (104-1,547)	501 (97-1,541)	348 (70-1,019)	278 (55-824)
Rabies	-	-	297 (208-426)	1,854 (1,254-3,087)	2,881 (2,074-4,164)	1,850 (1,367-2,669)	1,069 (793-1,813)	1,101 (823-1,721)	535 (422-782)	652 (505-926)
Intestinal nematode infections	-	-	216 (161-285)	2,199 (1,585-3,024)	366 (272-482)	76 (58-97)	24 (17-32)	19 (14-26)	14 (10-19)	18 (13-24)
Ascariasis	-	-	216 (161-285)	2,199 (1,585-3,024)	366 (272-482)	76 (58-97)	24 (17-32)	19 (14-26)	14 (10-19)	18 (13-24)
Ebola	0 (0-0)	0 (0-0)	0 (0-0)	0 (0-0)	0 (0-0)	0 (0-0)	0 (0-0)	0 (0-0)	4 (3-5)	2 (2-3)
Zika virus	0 (0-0)	0 (0-0)	0 (0-0)	0 (0-0)	0 (0-0)	0 (0-0)	0 (0-0)	0 (0-0)	0 (0-0)	0 (0-0)
Other neglected tropical diseases	288 (121-1,047)	250 (109-883)	1,105 (540-3,964)	2,015 (589-9,638)	347 (195-1,046)	163 (102-421)	133 (93-284)	137 (91-305)	158 (101-340)	161 (101-297)
Other infectious diseases	25,766 (21,281-31,077)	25,759 (21,223-30,404)	158,688 (122,334-211,709)	195,131 (135,852-286,958)	38,421 (28,655-53,763)	18,219 (14,878-22,953)	16,142 (13,324-18,589)	13,824 (11,542-15,402)	11,837 (9,757-13,058)	13,147 (11,101-14,273)
Meningitis	7,831 (5,631-9,734)	9,342 (7,536-11,070)	55,433 (47,103-64,361)	49,794 (39,615-60,591)	10,608 (7,759-13,372)	6,142 (4,702-7,992)	5,951 (4,656-7,984)	5,157 (4,035-6,823)	4,137 (3,231-5,437)	4,504 (3,585-6,005)
Pneumococcal meningitis	1,215 (856-1,617)	1,426 (1,060-1,826)	7,690 (5,905-9,789)	7,465 (5,327-10,108)	1,065 (724-1,454)	572 (396-832)	619 (437-897)	617 (445-860)	512 (369-718)	614 (460-823)
H influenzae type B meningitis	2,135 (1,379-2,891)	2,493 (1,876-3,326)	16,647 (12,982-20,950)	15,382 (11,146-20,296)	3,919 (2,748-5,195)	2,227 (1,683-3,035)	1,997 (1,568-2,773)	1,500 (1,159-2,017)	1,266 (985-1,694)	1,282 (1,037-1,773)
Meningococcal meningitis	1,191 (846-1,568)	1,464 (1,134-1,818)	7,923 (6,409-9,800)	6,839 (5,020-8,995)	1,179 (843-1,572)	584 (431-782)	522 (390-693)	432 (318-580)	340 (249-458)	381 (285-519)
Other meningitis	3,291 (2,371-4,192)	3,959 (3,148-4,810)	23,174 (18,992-27,868)	20,108 (15,379-25,228)	4,445 (3,109-5,591)	2,759 (2,046-3,561)	2,813 (2,157-3,754)	2,609 (1,973-3,420)	2,019 (1,530-2,589)	2,227 (1,668-2,983)
Encephalitis	386 (339-461)	614 (528-721)	7,614 (6,218-9,430)	7,334 (5,793-10,045)	3,518 (2,912-4,220)	2,899 (2,469-3,398)	2,094 (1,860-2,348)	1,688 (1,529-1,890)	1,382 (1,283-1,533)	1,671 (1,520-1,834)
Diphtheria	-	-	577 (340-956)	1,835 (935-3,596)	337 (197-565)	120 (75-197)	43 (27-69)	25 (15-38)	20 (13-32)	22 (14-34)
Whooping cough	-	-	28,660 (13,531-53,938)	24,586 (11,658-46,540)	2,332 (1,055-4,705)	493 (223-1,016)	230 (104-480)	151 (70-307)	79 (36-162)	67 (30-135)
Tetanus	14,167 (10,671-18,628)	12,628 (9,357-16,887)	5,211 (3,396-8,205)	2,598 (1,163-4,645)	1,425 (787-2,235)	779 (481-1,212)	1,337 (737-2,203)	1,196 (628-1,875)	1,285 (571-2,031)	1,179 (666-1,682)
Measles	-	-	46,634 (16,330-99,765)	95,664 (35,851-191,463)	14,670 (5,502-31,156)	3,801 (1,399-8,363)	1,359 (469-3,100)	601 (203-1,382)	241 (81-552)	147 (50-339)
Varicella and herpes zoster	85 (64-112)	230 (175-294)	1,908 (1,508-2,417)	2,111 (1,626-2,730)	800 (608-1,059)	269 (209-345)	145 (112-187)	155 (122-197)	186 (151-229)	238 (190-304)
Acute hepatitis	-	-	6,143 (2,988-7,654)	6,507 (3,165-8,690)	3,104 (1,577-3,960)	2,558 (1,357-3,183)	3,356 (1,796-4,073)	3,629 (2,189-4,265)	3,491 (2,079-4,084)	4,159 (2,439-4,804)
Acute hepatitis A	-	-	891 (328-1,573)	3,846 (1,869-5,454)	2,020 (1,029-2,739)	1,263 (676-1,728)	1,171 (641-1,681)	989 (533-1,448)	718 (368-1,105)	663 (298-1,150)
Acute hepatitis B	-	-	4,032 (1,764-5,565)	2,078 (874-3,444)	570 (230-990)	470 (204-848)	789 (386-1,344)	1,210 (616-1,999)	1,702 (981-2,522)	2,621 (1,464-3,581)
Acute hepatitis C	-	-	1,210 (515-2,183)	442 (185-863)	51 (18-109)	19 (6-41)	13 (4-29)	11 (3-26)	11 (3-28)	15 (4-40)
Acute hepatitis E	-	-	10 (3-20)	141 (54-283)	463 (189-791)	806 (343-1,254)	1,382 (654-1,992)	1,420 (710-2,046)	1,061 (479-1,673)	860 (325-1,622)
Other unspecified infectious diseases	3,297 (1,695-4,117)	2,945 (1,447-3,643)	6,508 (4,022-8,212)	4,702 (3,166-6,378)	1,627 (1,119-2,191)	1,157 (830-1,538)	1,629 (1,168-2,126)	1,221 (863-1,552)	1,017 (754-1,243)	1,160 (861-1,390)
Maternal and neonatal disorders	1,141,709 (1,091,414-1,188,336)	196,814 (183,204-213,108)	52,929 (45,224-60,336)	5,036 (4,163-6,026)	-	-	-	-	-	-
Maternal disorders	-	-	-	-	-	-	-	-	-	-
Maternal hemorrhage	-	-	-	-	-	-	-	-	-	-
Maternal sepsis and other maternal infections	-	-	-	-	-	-	-	-	-	-
Maternal hypertensive disorders	-	-	-	-	-	-	-	-	-	-
Maternal obstructed labor and uterine rupture	-	-	-	-	-	-	-	-	-	-
Maternal abortion and miscarriage	-	-	-	-	-	-	-	-	-	-
Ectopic pregnancy	-	-	-	-	-	-	-	-	-	-
Indirect maternal deaths	-	-	-	-	-	-	-	-	-	-
Late maternal deaths	-	-	-	-	-	-	-	-	-	-
Maternal deaths aggravated by HIV/AIDS	-	-	-	-	-	-	-	-	-	-
Other maternal disorders	-	-	-	-	-	-	-	-	-	-
Neonatal disorders	1,141,709 (1,091,414-1,188,336)	196,814 (183,204-213,108)	52,929 (45,224-60,336)	5,036 (4,163-6,026)	-	-	-	-	-	-
Neonatal preterm birth	444,844 (413,543-516,242)	57,420 (51,133-67,842)	19,642 (16,774-23,561)	1,338 (1,034-1,669)	-	-	-	-	-	-
Neonatal encephalopathy due to birth asphyxia and trauma	381,793 (332,572-413,954)	34,750 (27,186-39,859)	10,406 (8,096-12,329)	2,125 (1,720-2,579)	-	-	-	-	-	-
Neonatal sepsis and other neonatal infections	78,792 (65,080-100,345)	50,252 (41,723-65,115)	7,145 (5,821-9,603)	240 (149-454)	-	-	-	-	-	-
Hemolytic disease and other neonatal jaundice	29,754 (24,764-36,860)	14,740 (11,528-18,702)	2,939 (1,919-4,326)	180 (111-282)	-	-	-	-	-	-
Other neonatal disorders	206,526 (160,351-236,274)	39,651 (31,592-46,954)	12,796 (9,441-16,183)	1,153 (784-1,534)	-	-	-	-	-	-
Nutritional deficiencies	-	-	63,734 (56,117-71,466)	65,758 (53,958-78,521)	5,378 (4,498-6,190)	1,656 (1,437-1,890)	1,309 (1,152-1,448)	1,234 (1,102-1,364)	1,070 (968-1,159)	1,104 (1,014-1,181)
Protein-energy malnutrition	-	-	61,510 (54,109-69,012)	63,639 (51,815-76,064)	4,517 (3,753-5,288)	1,303 (1,115-1,494)	939 (813-1,055)	931 (822-1,039)	834 (743-909)	893 (811-963)
Other nutritional deficiencies	-	-	2,224 (1,822-2,714)	2,119 (1,341-2,758)	861 (663-1,086)	353 (286-434)	369 (311-445)	303 (259-389)	236 (203-301)	212 (181-275)
Non-communicable diseases	126,781 (114,524-152,819)	49,102 (44,953-57,173)	206,860 (183,212-237,325)	122,703 (108,112-140,193)	55,483 (49,946-60,578)	50,711 (46,674-54,221)	87,569 (82,554-91,487)	112,317 (107,696-116,319)	144,806 (140,219-149,068)	218,732 (213,331-224,090)
Neoplasms	861 (695-955)	565 (475-637)	5,532 (4,743-6,385)	22,110 (18,539-27,478)	19,840 (17,831-21,930)	17,824 (16,271-19,233)	22,832 (21,000-23,974)	22,557 (21,181-23,476)	26,294 (24,795-27,238)	42,843 (41,013-44,173)
Lip and oral cavity cancer	-	-	-	-	-	-	329 (295-365)	426 (392-466)	659 (608-718)	1,250 (1,163-1,348)
Nasopharynx cancer	-	-	-	-	141 (120-162)	217 (195-241)	298 (277-318)	316 (296-336)	404 (381-429)	814 (763-863)
Other pharynx cancer	-	-	-	-	-	-	135 (90-157)	160 (99-182)	209 (130-237)	512 (344-573)
Esophageal cancer	-	-	-	-	-	-	175 (161-191)	250 (235-267)	366 (348-387)	945 (903-993)
Stomach cancer	-	-	-	-	-	-	516 (486-552)	910 (867-963)	1,636 (1,567-1,724)	3,768 (3,615-3,946)
Colon and rectum cancer	-	-	-	-	-	-	578 (445-639)	1,011 (820-1,089)	1,620 (1,387-1,737)	3,202 (2,930-3,411)
Liver cancer	-	-	-	-	466 (400-546)	561 (491-711)	826 (750-974)	1,339 (1,208-1,663)	2,653 (2,406-3,198)	7,319 (6,788-8,185)
Liver cancer due to hepatitis B	-	-	-	-	340 (291-408)	451 (394-579)	653 (585-771)	1,040 (933-1,312)	1,964 (1,745-2,388)	5,298 (4,841-5,980)
Liver cancer due to hepatitis C	-	-	-	-	8 (6-11)	11 (8-14)	13 (11-17)	40 (32-50)	133 (101-175)	460 (381-554)
Liver cancer due to alcohol use	-	-	-	-	-	-	4 (2-6)	41 (31-55)	164 (121-227)	590 (461-749)
Liver cancer due to NASH	-	-	-	-	-	-	55 (43-69)	73 (58-97)	123 (93-162)	281 (220-354)
Liver cancer due to other causes	-	-	-	-	118 (97-141)	99 (80-125)	101 (81-125)	145 (117-185)	269 (209-346)	690 (553-857)
Gallbladder and biliary tract cancer	-	-	-	-	-	-	47 (39-57)	67 (55-81)	109 (89-133)	290 (238-370)
Pancreatic cancer	-	-	-	-	-	-	92 (86-99)	158 (150-168)	292 (280-305)	818 (785-855)
Larynx cancer	-	-	-	-	-	-	81 (75-88)	115 (108-123)	142 (135-149)	409 (384-434)
Tracheal, bronchus, and lung cancer	-	-	-	-	-	-	617 (573-667)	927 (870-1,000)	1,571 (1,484-1,671)	3,689 (3,539-3,880)
Malignant skin melanoma	-	-	-	-	-	-	135 (104-178)	230 (180-304)	366 (276-479)	652 (494-871)
Non-melanoma skin cancer	-	-	-	-	-	-	105 (99-113)	142 (134-150)	182 (172-192)	350 (331-371)
Non-melanoma skin cancer (squamous-cell carcinoma)	-	-	-	-	-	-	105 (99-113)	142 (134-150)	182 (172-192)	350 (331-371)
Breast cancer	-	-	-	-	-	-	34 (33-36)	32 (31-34)	35 (33-37)	64 (59-68)
Cervical cancer	-	-	-	-	-	-	-	-	-	-
Uterine cancer	-	-	-	-	-	-	-	-	-	-
Ovarian cancer	-	-	-	-	-	-	-	-	-	-
Prostate cancer	-	-	-	-	-	-	75 (62-87)	116 (96-135)	155 (131-186)	202 (166-233)
Testicular cancer	-	-	-	-	-	-	370 (342-400)	832 (774-898)	1,064 (994-1,142)	1,008 (944-1,072)
Kidney cancer	26 (20-31)	15 (11-17)	179 (143-219)	837 (623-1,120)	329 (286-374)	130 (118-140)	118 (109-127)	152 (138-160)	230 (214-242)	377 (347-398)
Bladder cancer	-	-	-	-	-	-	54 (49-60)	85 (73-96)	114 (96-127)	261 (226-285)
Brain and nervous system cancer	90 (64-102)	69 (52-80)	908 (657-1,089)	3,623 (2,629-4,689)	4,047 (3,362-4,802)	3,327 (2,752-3,967)	2,485 (2,002-2,861)	2,161 (1,754-2,484)	2,687 (2,200-3,120)	3,777 (3,088-4,305)
Thyroid cancer	-	-	-	-	-	79 (68-92)	107 (96-120)	152 (138-166)	262 (237-287)	211 (196-229)
Mesothelioma	-	-	-	-	-	-	46 (41-54)	57 (52-65)	85 (77-97)	119 (109-136)
Hodgkin lymphoma	11 (7-14)	2 (2-3)	62 (41-84)	571 (321-810)	1,036 (713-1,375)	972 (702-1,262)	1,100 (818-1,467)	1,137 (902-1,510)	1,261 (1,022-1,612)	1,241 (996-1,628)
Non-Hodgkin lymphoma	42 (37-51)	33 (29-41)	387 (318-506)	2,604 (1,960-3,537)	2,284 (1,957-2,711)	1,720 (1,535-1,956)	1,877 (1,736-2,064)	2,092 (1,942-2,256)	2,044 (1,912-2,183)	2,441 (2,302-2,616)

Cause	35 to 39	40 to 44	45 to 49	50 to 54	54 to 59	60 to 64	65 to 69	70 to 74	75 to 79	80 to 84	85 to 89	90 to 94	95 plus
All causes	867,312 (854,110-881,172)	1,034,761 (1,024,458-1,046,049)	1,206,824 (1,195,097-1,219,822)	1,505,490 (1,492,142-1,519,923)	1,654,872 (1,641,370-1,669,662)	1,897,260 (1,882,658-1,913,106)	2,417,699 (2,401,447-2,435,411)	2,772,875 (2,754,515-2,793,795)	2,814,874 (2,797,825-2,833,157)	2,283,443 (2,271,815-2,295,648)	1,367,038 (1,360,853-1,373,653)	643,094 (640,875-645,442)	185,265 (184,834-185,727)
Communicable, maternal, neonatal, and nutritional diseases	267,611 (253,706-283,381)	270,297 (260,740-282,881)	256,315 (245,448-271,896)	229,174 (219,437-246,742)	201,993 (191,395-223,556)	212,639 (196,559-240,587)	237,861 (216,454-279,273)	266,043 (236,758-314,908)	250,688 (221,851-306,054)	212,437 (187,355-255,187)	137,202 (121,077-167,560)	63,557 (57,106-74,632)	20,343 (18,596-23,313)
HIV/AIDS and sexually transmitted infections	149,220 (137,253-160,515)	138,109 (131,719-144,371)	105,153 (98,865-112,596)	62,494 (58,956-65,786)	32,130 (29,694-34,360)	19,868 (18,246-21,599)	13,592 (12,351-14,941)	9,010 (8,116-9,897)	5,615 (5,080-6,217)	904 (818-1,008)	278 (242-319)	119 (106-135)	24 (19-27)
HIV/AIDS	149,114 (137,187-160,410)	138,018 (131,594-144,295)	105,079 (98,791-112,527)	62,401 (58,857-65,716)	32,035 (29,590-34,242)	19,780 (18,151-21,517)	13,481 (12,212-14,806)	8,914 (8,023-9,796)	5,508 (4,986-6,110)	849 (764-948)	230 (201-263)	97 (87-110)	14 (13-16)
HIV/AIDS - Drug-susceptible Tuberculosis	28,602 (17,962-40,205)	27,961 (17,547-38,917)	23,946 (15,653-32,631)	16,669 (11,763-20,903)	9,180 (6,994-11,021)	5,996 (4,753-7,139)	4,178 (3,327-4,941)	2,873 (2,294-3,367)	1,780 (1,417-2,086)	260 (213-305)	65 (52-79)	28 (23-33)	3 (3-4)
HIV/AIDS - Multidrug-resistant Tuberculosis without extensive drug resistance	3,223 (1,746-5,151)	3,132 (1,712-4,935)	2,688 (1,517-4,131)	1,855 (1,113-2,782)	1,019 (626-1,498)	645 (400-952)	440 (277-647)	300 (193-442)	189 (122-286)	29 (19-43)	8 (5-11)	3 (2-5)	0 (0-1)
HIV/AIDS - Extensively drug-resistant Tuberculosis	123 (75-182)	122 (75-181)	96 (60-143)	55 (36-80)	29 (19-41)	15 (10-22)	10 (7-14)	6 (4-9)	4 (3-6)	1 (1-1)	0 (0-0)	0 (0-0)	0 (0-0)
HIV/AIDS resulting in other diseases	117,166 (100,680-133,735)	106,803 (92,570-119,139)	78,350 (67,769-89,679)	43,821 (38,473-49,944)	21,807 (19,106-24,988)	13,124 (11,481-15,150)	8,852 (7,716-10,282)	5,735 (4,939-6,772)	3,535 (3,053-4,167)	558 (484-659)	157 (132-191)	66 (57-78)	10 (9-12)
Sexually transmitted infections excluding HIV	106 (62-154)	91 (57-152)	73 (48-116)	93 (61-152)	95 (60-158)	88 (51-156)	112 (66-193)	96 (58-171)	107 (71-164)	56 (37-86)	48 (28-69)	22 (12-32)	10 (5-13)
Syphilis	88 (52-128)	78 (49-131)	65 (43-103)	83 (54-136)	86 (54-144)	83 (48-147)	105 (62-181)	89 (53-158)	97 (65-149)	50 (34-78)	42 (24-60)	18 (10-27)	7 (4-10)
Chlamydial infection	0 (0-0)	0 (0-0)	0 (0-0)	0 (0-0)	0 (0-0)	0 (0-0)	0 (0-0)	0 (0-0)	0 (0-0)	0 (0-0)	0 (0-0)	0 (0-0)	0 (0-0)
Gonococcal infection	5 (3-7)	7 (5-12)	5 (3-7)	4 (3-7)	4 (3-7)	3 (1-4)	3 (2-6)	4 (2-6)	4 (3-6)	2 (2-4)	2 (1-3)	1 (1-2)	0 (0-1)
Other sexually transmitted infections	14 (8-20)	6 (3-9)	4 (3-6)	5 (3-9)	5 (3-8)	3 (1-4)	4 (2-6)	3 (2-6)	5 (4-8)	3 (2-4)	4 (2-5)	3 (1-4)	2 (1-2)
Respiratory infections and tuberculosis	74,506 (70,891-79,084)	87,702 (84,019-92,356)	103,192 (98,693-109,432)	116,260 (111,403-122,824)	116,166 (111,105-122,629)	129,172 (122,715-137,220)	146,218 (138,151-154,307)	163,372 (153,461-175,519)	158,511 (148,550-169,281)	138,800 (129,992-149,269)	91,294 (85,615-97,635)	44,445 (42,456-47,093)	14,552 (14,094-15,378)
Tuberculosis	57,142 (53,819-60,967)	65,791 (62,509-69,767)	75,017 (70,981-79,827)	80,701 (76,403-85,851)	77,059 (72,847-81,842)	78,500 (73,925-83,990)	81,676 (76,110-87,514)	80,174 (75,045-86,034)	62,346 (58,126-66,809)	42,317 (39,102-45,569)	19,681 (18,098-21,374)	5,918 (5,438-6,436)	1,133 (1,036-1,228)
Drug-susceptible tuberculosis	50,292 (47,059-54,098)	57,381 (53,876-61,380)	65,258 (61,177-70,109)	70,401 (65,813-75,589)	67,876 (63,401-72,607)	69,725 (65,108-75,139)	72,464 (67,500-78,069)	71,449 (66,365-77,221)	55,553 (51,601-59,740)	37,916 (34,817-41,082)	17,688 (16,091-19,293)	5,344 (4,888-5,840)	1,025 (939-1,116)
Multidrug-resistant tuberculosis without extensive drug resistance	6,301 (5,044-7,677)	7,647 (6,182-9,332)	8,832 (7,185-10,793)	9,363 (7,575-11,363)	8,464 (6,819-10,300)	8,221 (6,540-10,112)	8,610 (6,910-10,552)	8,205 (6,480-10,159)	6,386 (5,133-7,876)	4,145 (3,274-5,166)	1,879 (1,474-2,385)	540 (423-685)	101 (78-131)
Extensively drug-resistant tuberculosis	550 (444-661)	763 (609-927)	927 (729-1,132)	937 (734-1,148)	720 (577-884)	554 (444-687)	602 (481-741)	520 (412-648)	407 (324-504)	255 (200-329)	115 (89-149)	34 (27-45)	7 (5-9)
Lower respiratory infections	17,196 (15,251-18,768)	21,732 (19,776-23,778)	27,905 (25,290-30,295)	35,254 (31,618-38,369)	38,885 (34,662-42,461)	50,435 (43,609-54,537)	64,214 (56,268-68,564)	82,808 (73,758-91,370)	95,695 (86,662-105,641)	95,911 (87,968-104,784)	71,190 (65,952-77,494)	38,306 (36,564-40,834)	13,360 (12,902-14,165)
Upper respiratory infections	143 (80-201)	155 (94-222)	166 (101-243)	191 (116-264)	192 (117-254)	199 (113-287)	274 (163-367)	340 (195-437)	434 (243-550)	545 (281-648)	409 (215-498)	217 (126-260)	58 (36-70)
Otitis media	25 (13-55)	24 (13-48)	104 (49-250)	114 (58-254)	31 (19-59)	38 (23-78)	54 (33-103)	50 (32-100)	36 (24-70)	28 (18-53)	14 (9-26)	4 (3-7)	1 (1-2)
Enteric infections	15,127 (8,570-26,542)	15,137 (8,228-28,698)	17,809 (9,215-35,388)	19,879 (9,663-39,548)	23,762 (11,630-49,023)	32,857 (15,942-65,536)	46,493 (21,537-90,656)	59,768 (27,443-117,499)	60,814 (29,232-122,350)	54,510 (25,554-106,255)	35,120 (17,016-68,330)	14,465 (7,141-27,303)	4,158 (2,212-7,492)
Diarrheal diseases	11,680 (5,222-22,821)	12,435 (5,357-25,376)	15,551 (6,875-33,025)	17,960 (7,816-37,237)	22,093 (9,917-47,587)	31,288 (14,107-64,555)	45,122 (20,186-89,860)	58,728 (26,249-116,744)	60,109 (28,378-121,956)	54,080 (24,964-105,958)	34,931 (16,836-68,149)	14,410 (7,056-27,248)	4,146 (2,203-7,485)
Typhoid and paratyphoid	2,386 (1,232-4,002)	1,709 (882-2,854)	1,320 (706-2,213)	986 (511-1,686)	731 (351-1,351)	632 (312-1,147)	538 (304-889)	396 (238-637)	242 (147-373)	117 (70-177)	50 (31-76)	15 (10-23)	4 (3-6)
Typhoid fever	2,025 (1,021-3,443)	1,440 (720-2,456)	1,104 (584-1,866)	823 (418-1,401)	613 (297-1,142)	532 (257-964)	456 (254-754)	338 (199-541)	206 (123-323)	101 (61-154)	43 (27-65)	14 (9-20)	4 (3-5)
Paratyphoid fever	360 (157-726)	269 (116-540)	216 (91-447)	163 (63-341)	118 (45-250)	101 (40-207)	83 (37-163)	58 (27-111)	35 (17-65)	16 (8-30)	7 (3-12)	2 (1-3)	0 (0-1)
Invasive Non-typhoidal Salmonella (iNTS)	1,038 (443-2,039)	975 (449-1,854)	921 (442-1,780)	920 (424-1,794)	928 (425-1,635)	925 (425-1,649)	818 (386-1,403)	634 (303-1,131)	456 (208-829)	303 (127-580)	130 (52-236)	37 (11-78)	8 (0-20)
Other intestinal infectious diseases	23 (4-58)	18 (2-43)	17 (2-43)	14 (2-37)	11 (1-30)	11 (1-29)	15 (2-39)	10 (1-26)	8 (1-20)	10 (1-27)	9 (1-25)	2 (0-6)	1 (0-3)
Neglected tropical diseases and malaria	13,088 (9,333-18,078)	13,008 (9,217-17,945)	12,043 (8,584-16,284)	12,670 (9,079-17,467)	12,290 (8,897-17,189)	12,761 (9,173-17,819)	12,340 (8,768-17,281)	13,474 (9,351-19,061)	6,225 (4,575-8,736)	3,169 (2,498-4,316)	1,157 (937-1,557)	328 (259-474)	133 (108-196)
Malaria	9,646 (6,352-14,120)	9,670 (6,401-14,163)	8,597 (5,623-12,769)	9,550 (6,212-14,155)	9,265 (5,882-13,764)	9,823 (6,396-14,703)	9,670 (6,165-14,774)	10,894 (6,949-16,536)	4,256 (2,728-6,575)	1,834 (1,233-2,866)	513 (367-776)	109 (63-191)	26 (15-66)
Chagas disease	128 (118-139)	189 (175-205)	277 (259-295)	389 (363-418)	459 (423-502)	608 (561-657)	649 (603-697)	667 (611-737)	430 (393-471)	297 (275-320)	177 (166-192)	67 (62-73)	16 (14-18)
Leishmaniasis	878 (0-3,777)	726 (0-2,989)	624 (2-2,207)	530 (5-1,814)	399 (3-1,332)	395 (2-1,248)	353 (9-1,080)	255 (11-757)	150 (5-460)	70 (0-221)	28 (0-90)	8 (0-26)	2 (0-5)
Visceral leishmaniasis	878 (0-3,777)	726 (0-2,989)	624 (2-2,207)	530 (5-1,814)	399 (3-1,332)	395 (2-1,248)	353 (9-1,080)	255 (11-757)	150 (5-460)	70 (0-221)	28 (0-90)	8 (0-26)	2 (0-5)
African trypanosomiasis	481 (123-1,415)	446 (118-1,394)	384 (83-1,150)	333 (67-1,139)	206 (45-612)	165 (37-520)	113 (25-398)	79 (18-239)	47 (11-162)	28 (8-91)	9 (2-29)	2 (0-8)	3 (0-10)
Schistosomiasis	291 (232-352)	339 (270-418)	378 (298-472)	454 (364-565)	486 (390-596)	511 (409-636)	384 (307-476)	500 (399-622)	384 (309-480)	208 (165-261)	88 (67-113)	22 (16-31)	4 (2-6)
Cysticercosis	39 (5-100)	38 (5-97)	34 (4-88)	30 (3-81)	24 (2-68)	19 (1-55)	16 (1-48)	4 (0-12)	8 (0-27)	5 (0-17)	2 (0-7)	1 (0-2)	0 (0-1)
Cystic echinococcosis	50 (5-126)	49 (3-123)	52 (4-126)	56 (6-144)	55 (6-142)	56 (7-137)	57 (6-143)	53 (7-129)	39 (3-104)	25 (1-71)	14 (0-41)	5 (0-17)	1 (0-5)
Dengue	435 (115-613)	284 (106-398)	356 (106-504)	337 (105-487)	359 (91-517)	338 (104-506)	364 (124-558)	352 (171-574)	364 (170-645)	397 (152-618)	215 (97-387)	77 (38-135)	26 (15-45)
Yellow fever	230 (45-666)	196 (39-620)	167 (31-542)	130 (25-390)	103 (19-317)	82 (15-247)	54 (10-163)	34 (7-107)	18 (3-58)	74 (21-151)	2 (0-7)	0 (0-1)	42 (29-48)
Rabies	680 (518-963)	847 (684-1,125)	942 (721-1,451)	625 (510-815)	700 (566-943)	527 (429-774)	448 (363-565)	381 (301-482)	316 (247-418)	94 (72-125)	35 (28-48)	8 (6-10)	3 (2-4)
Intestinal nematode infections	18 (13-23)	13 (9-18)	14 (10-19)	14 (10-19)	12 (8-16)	25 (18-32)	12 (8-17)	20 (14-26)	16 (11-21)	9 (6-13)	4 (2-6)	2 (1-3)	1 (0-1)
Ascariasis	18 (13-23)	13 (9-18)	14 (10-19)	14 (10-19)	12 (8-16)	25 (18-32)	12 (8-17)	20 (14-26)	16 (11-21)	9 (6-13)	4 (2-6)	2 (1-3)	1 (0-1)
Ebola	4 (3-5)	4 (3-5)	2 (2-3)	0 (0-0)	0 (0-0)	0 (0-0)	0 (0-0)	0 (0-0)	0 (0-0)	0 (0-0)	0 (0-0)	0 (0-0)	0 (0-0)
Zika virus	0 (0-0)	0 (0-0)	0 (0-0)	0 (0-0)	0 (0-0)	0 (0-0)	0 (0-0)	0 (0-0)	0 (0-0)	0 (0-0)	0 (0-0)	0 (0-0)	0 (0-0)
Other neglected tropical diseases	208 (123-374)	208 (128-380)	217 (127-401)	221 (132-414)	222 (138-433)	213 (140-409)	219 (139-429)	235 (158-470)	196 (132-409)	128 (91-261)	69 (49-146)	26 (19-57)	10 (7-22)
Other infectious diseases	14,266 (11,863-15,507)	14,667 (12,025-16,025)	16,002 (12,902-17,502)	15,446 (12,833-16,968)	14,569 (12,133-15,927)	14,374 (12,106-15,831)	14,531 (12,291-16,124)	13,905 (11,906-15,268)	12,477 (10,713-13,722)	8,706 (7,516-9,570)	4,655 (4,060-5,119)	1,603 (1,393-1,764)	475 (411-524)
Meningitis	4,686 (3,751-6,305)	4,216 (3,397-5,835)	4,494 (3,735-6,202)	4,186 (3,602-5,762)	3,790 (3,204-5,289)	3,905 (3,299-5,646)	3,323 (2,876-4,951)	3,350 (2,831-5,006)	3,019 (2,560-4,588)	1,975 (1,660-2,774)	1,213 (988-1,689)	345 (291-463)	93 (78-123)
Pneumococcal meningitis	701 (523-953)	671 (493-942)	785 (572-1,100)	750 (569-1,022)	666 (504-927)	703 (520-1,011)	557 (418-806)	598 (448-871)	529 (397-784)	338 (249-476)	200 (153-271)	51 (39-68)	14 (10-19)
H influenzae type B meningitis	1,311 (1,048-1,879)	1,214 (980-1,769)	1,298 (1,058-1,853)	1,170 (943-1,735)	1,080 (873-1,652)	1,134 (915-1,779)	1,092 (877-1,735)	1,069 (847-1,704)	1,001 (797-1,622)	669 (527-1,026)	409 (325-596)	131 (104-179)	35 (27-48)
Meningococcal meningitis	389 (295-535)	341 (259-466)	356 (270-492)	350 (274-503)	329 (249-459)	340 (250-498)	275 (206-406)	279 (212-408)	252 (188-382)	165 (124-239)	97 (74-137)	25 (19-34)	7 (5-10)
Other meningitis	2,284 (1,717-3,008)	1,991 (1,546-2,650)	2,056 (1,659-2,781)	1,908 (1,569-2,662)	1,715 (1,403-2,380)	1,728 (1,361-2,450)	1,400 (1,124-2,054)	1,404 (1,125-2,068)	1,237 (996-1,849)	804 (642-1,124)	507 (395-697)	138 (108-184)	38 (30-49)
Encephalitis	1,812 (1,673-1,998)	1,632 (1,508-1,803)	1,547 (1,431-1,780)	1,480 (1,374-1,631)	1,619 (1,490-1,815)	1,881 (1,729-2,076)	2,405 (2,166-2,700)	2,650 (2,347-2,990)	2,746 (2,367-3,122)	2,099 (1,734-2,459)	906 (754-1,045)	253 (217-285)	64 (56-73)
Diphtheria	25 (16-40)	30 (21-44)	48 (33-70)	38 (26-54)	22 (15-34)	-	-	-	-	-	-	-	-
Whooping cough	49 (22-101)	65 (29-130)	51 (23-104)	40 (18-81)	51 (23-105)	-	-	-	-	-	-	-	-
Tetanus	1,395 (730-1,966)	1,690 (808-2,376)	2,378 (969-3,328)	1,953 (906-2,710)	2,061 (846-3,038)	1,511 (709-2,097)	1,516 (815-2,457)	1,170 (639-1,658)	834 (433-1,259)	472 (259-770)	222 (111-391)	69 (38-116)	19 (9-28)
Measles	98 (34-224)	77 (27-176)	60 (21-137)	45 (16-104)	49 (17-111)	-	-	-	-	-	-	-	-
Varicella and herpes zoster	218 (175-269)	134 (110-167)	129 (106-156)	120 (98-146)	131 (107-158)	155 (128-194)	298 (240-366)	285 (235-349)	401 (329-495)	530 (433-658)	496 (404-616)	269 (216-345)	119 (96-156)
Acute hepatitis	4,518 (2,737-5,183)	5,589 (3,414-6,463)	5,818 (3,658-6,790)	6,072 (3,876-6,901)	5,325 (3,385-6,059)	5,009 (3,124-5,746)	4,884 (3,067-5,590)	4,202 (2,677-4,778)	3,462 (2,118-3,951)	2,058 (1,271-2,364)	865 (574-989)	236 (171-266)	49 (36-55)
Acute hepatitis A	646 (276-1,161)	672 (316-1,208)	577 (279-1,068)	467 (252-775)	265 (148-414)	138 (69-231)	80 (42-139)	39 (17-71)	18 (6-39)	4 (1-11)	1 (0-2)	0 (0-0)	0 (0-0)
Acute hepatitis B	3,212 (1,912-4,215)	4,369 (2,592-5,466)	4,802 (3,069-5,921)	5,275 (3,198-6,272)	4,817 (2,949-5,561)	4,638 (2,856-5,391)	4,534 (2,825-5,263)	3,856 (2,400-4,453)	3,082 (1,873-3,633)	1,733 (1,037-2,088)	678 (414-846)	164 (103-214)	30 (17-41)
Acute hepatitis C	24 (6-63)	32 (9-78)	43 (12-105)	51 (15-119)	52 (15-135)	62 (16-144)	75 (19-197)	75 (24-191)	76 (23-192)	58 (17-142)	33 (10-87)	12 (3-29)	3 (1-8)
Acute hepatitis E	636 (234-1,178)	517 (171-1,064)	396 (144-852)	279 (103-583)	191 (73-395)	170 (63-364)	195 (68-429)	231 (90-474)	286 (108-588)	263 (104-513)	153 (66-284)	60 (28-103)	16 (7-27)
Other unspecified infectious diseases	1,464 (1,119-1,730)	1,234 (978-1,444)	1,476 (1,176-1,723)	1,513 (1,232-1,755)	1,521 (1,253-1,766)	1,913 (1,460-2,209)	2,104 (1,621-2,381)	2,248 (1,736-2,637)	2,013 (1,513-2,261)	1,572 (1,170-1,779)	954 (700-1,097)	432 (314-498)	131 (96-150)
Maternal and neonatal disorders	-	-	-	-	-	-	-	-	-	-	-	-	-
Maternal disorders	-	-	-	-	-	-	-	-	-	-	-	-	-
Maternal hemorrhage	-	-	-	-	-	-	-	-	-	-	-	-	-
Maternal sepsis and other maternal infections	-	-	-	-	-	-	-	-	-	-	-	-	-
Maternal hypertensive disorders	-	-	-	-	-	-	-	-	-	-	-	-	-
Maternal obstructed labor and uterine rupture	-	-	-	-	-	-	-	-	-	-	-	-	-
Maternal abortion and miscarriage	-	-	-	-	-	-	-	-	-	-	-	-	-
Ectopic pregnancy	-	-	-	-	-	-	-	-	-	-	-	-	-
Indirect maternal deaths	-	-	-	-	-	-	-	-	-	-	-	-	-
Late maternal deaths	-	-	-	-	-	-	-	-	-	-	-	-	-
Maternal deaths aggravated by HIV/AIDS	-	-	-	-	-	-	-	-	-	-	-	-	-
Other maternal disorders	-	-	-	-	-	-	-	-	-	-	-	-	-
Neonatal disorders	-	-	-	-	-	-	-	-	-	-	-	-	-
Neonatal preterm birth	-	-	-	-	-	-	-	-	-	-	-	-	-
Neonatal encephalopathy due to birth asphyxia and trauma	-	-	-	-	-	-	-	-	-	-	-	-	-
Neonatal sepsis and other neonatal infections	-	-	-	-	-	-	-	-	-	-	-	-	-
Hemolytic disease and other neonatal jaundice	-	-	-	-	-	-	-	-	-	-	-	-	-
Other neonatal disorders	-	-	-	-	-	-	-	-	-	-	-	-	-
Nutritional deficiencies	1,405 (1,301-1,498)	1,674 (1,558-1,780)	2,116 (1,943-2,259)	2,425 (2,214-2,599)	3,076 (2,830-3,281)	3,607 (3,306-3,860)	4,687 (4,329-4,997)	6,514 (6,004-6,966)	7,047 (6,523-7,532)	6,348 (5,894-6,739)	4,698 (4,410-5,015)	2,597 (2,464-2,742)	1,002 (957-1,050)
Protein-energy malnutrition	1,026 (924-1,111)	1,264 (1,139-1,374)	1,571 (1,393-1,713)	1,841 (1,648-2,010)	2,322 (2,095-2,515)	2,683 (2,423-2,912)	3,533 (3,204-3,808)	4,922 (4,446-5,347)	5,384 (4,913-5,835)	5,076 (4,586-5,466)	3,811 (3,461-4,117)	2,095 (1,776-2,263)	815 (698-881)
Other nutritional deficiencies	379 (331-469)	409 (355-521)	545 (468-690)	584 (499-757)	753 (652-938)	924 (793-1,152)	1,154 (995-1,403)	1,593 (1,366-1,922)	1,664 (1,421-2,063)	1,272 (1,066-1,739)	887 (735-1,255)	502 (398-815)	187 (140-314)
Non-communicable diseases	342,299 (334,412-349,734)	525,957 (516,315-535,665)	736,617 (724,506-747,629)	1,078,112 (1,062,779-1,091,658)	1,294,606 (1,273,472-1,309,918)	1,559,937 (1,532,493-1,579,665)	2,055,446 (2,015,129-2,080,847)	2,393,563 (2,343,629-2,427,216)	2,462,887 (2,412,397-2,495,173)	1,996,231 (1,952,198-2,024,732)	1,185,836 (1,158,607-1,202,846)	559,675 (548,512-566,300)	159,324 (156,513-161,085)
Neoplasms	75,438 (73,023-77,290)	124,427 (121,526-127,085)	190,264 (186,226-193,606)	318,901 (313,309-323,603)	403,233 (396,387-408,753)	467,178 (458,972-473,277)	586,498 (576,894-593,967)	635,444 (624,518-643,847)	572,388 (559,900-580,460)	398,908 (386,911-404,985)	185,192 (176,842-188,590)	68,017 (63,852-69,402)	15,130 (13,837-15,445)
Lip and oral cavity cancer	2,539 (2,377-2,712)	4,633 (4,366-4,938)	7,625 (7,274-8,041)	10,709 (10,292-11,153)	11,899 (11,379-12,410)	12,231 (11,660-12,803)	11,808 (11,266-12,270)	10,134 (9,659-10,539)	7,498 (7,143-7,779)	4,816 (4,590-5,017)	2,223 (2,108-2,317)	864 (821-898)	193 (184-201)
Nasopharynx cancer	1,673 (1,582-1,773)	2,800 (2,653-2,951)	3,564 (3,392-3,731)	5,045 (4,835-5,261)	5,450 (5,231-5,669)	4,721 (4,519-4,903)	4,720 (4,525-4,905)	4,220 (4,056-4,375)	2,764 (2,645-2,873)	1,770 (1,703-1,835)	739 (707-770)	268 (258-277)	53 (52-56)
Other pharynx cancer	1,100 (848-1,222)	2,527 (2,106-2,769)	4,932 (4,153-5,275)	7,505 (6,402-7,943)	8,310 (7,214-8,841)	8,459 (7,248-9,095)	7,998 (6,779-8,522)	6,281 (5,217-6,791)	4,120 (3,403-4,435)	2,485 (2,007-2,690)	901 (704-977)	257 (215-275)	43 (38-46)
Esophageal cancer	2,606 (2,494-2,738)	6,778 (6,532-7,063)	13,041 (12,669-13,476)	25,764 (25,067-26,617)	32,418 (31,591-33,311)	36,113 (35,171-37,210)	41,974 (40,936-43,148)	44,564 (43,362-45,814)	35,498 (34,498-36,485)	22,003 (21,400-22,637)	8,961 (8,710-9,230)	2,470 (2,399-2,544)	435 (420-451)
Stomach cancer	7,699 (7,406-8,040)	13,961 (13,538-14,549)	21,554 (20,952-22,300)	38,615 (37,535-39,774)	49,374 (48,262-50,783)	58,632 (57,316-60,066)	76,361 (74,804-77,978)	84,923 (83,140-86,776)	73,414 (72,088-74,859)	47,898 (46,920-48,933)	21,350 (20,887-21,842)	7,180 (7,046-7,340)	1,524 (1,493-1,559)
Colon and rectum cancer	5,109 (4,865-5,353)	8,016 (7,700-8,350)	12,952 (12,548-13,386)	21,033 (20,385-21,716)	29,413 (28,585-30,285)	37,113 (36,217-38,171)	49,682 (48,638-50,903)	56,437 (55,199-57,799)	55,461 (54,381-56,610)	42,243 (41,300-43,182)	20,565 (20,113-21,035)	7,992 (7,824-8,178)	1,976 (1,932-2,022)
Liver cancer	16,639 (15,655-18,027)	25,538 (24,146-27,663)	32,193 (30,459-34,754)	50,339 (47,962-52,890)	53,435 (50,784-56,428)	51,854 (49,693-55,731)	55,896 (53,727-59,330)	56,515 (54,406-59,463)	43,728 (42,145-45,935)	25,565 (24,608-26,887)	10,101 (9,721-10,705)	2,872 (2,759-3,013)	527 (507-549)
Liver cancer due to hepatitis B	11,590 (10,524-12,684)	16,960 (15,568-18,536)	20,035 (18,097-21,936)	29,661 (26,767-32,096)	27,903 (25,192-30,602)	23,678 (21,209-26,612)	23,155 (20,845-25,508)	21,257 (18,946-23,588)	13,835 (12,497-15,157)	6,817 (6,060-7,599)	2,403 (2,145-2,659)	550 (481-618)	82 (66-97)
Liver cancer due to hepatitis C	1,273 (1,028-1,541)	2,430 (2,038-2,854)	3,992 (3,214-4,885)	8,165 (6,947-9,417)	11,811 (10,159-13,479)	13,534 (11,718-15,519)	14,810 (13,336-16,551)	16,072 (14,548-17,741)	14,247 (12,854-15,742)	9,477 (8,563-10,394)	4,234 (3,900-4,566)	1,365 (1,248-1,472)	279 (253-305)
Liver cancer due to alcohol use	1,753 (1,234-2,331)	3,225 (2,442-4,160)	4,558 (3,373-6,227)	6,805 (5,057-9,377)	7,423 (5,530-9,671)	8,443 (6,586-10,689)	11,444 (9,715-13,389)	12,286 (10,570-14,454)	9,460 (8,365-10,726)	5,314 (4,587-6,064)	1,875 (1,631-2,136)	514 (436-595)	86 (68-104)
Liver cancer due to NASH	580 (457-722)	904 (719-1,129)	1,274 (996-1,589)	2,220 (1,757-2,745)	2,800 (2,198-3,506)	3,128 (2,498-3,824)	3,591 (2,863-4,421)	4,075 (3,291-5,038)	3,868 (3,148-4,702)	2,576 (2,083-3,140)	1,056 (883-1,239)	304 (249-368)	57 (43-72)
Liver cancer due to other causes	1,441 (1,125-1,776)	2,019 (1,582-2,519)	2,334 (1,861-2,890)	3,488 (2,791-4,368)	3,498 (2,723-4,223)	3,071 (2,454-3,749)	2,896 (2,333-3,520)	2,825 (2,275-3,446)	2,319 (1,897-2,780)	1,382 (1,119-1,686)	533 (445-630)	139 (113-167)	24 (18-30)
Gallbladder and biliary tract cancer	581 (482-704)	1,040 (858-1,238)	1,762 (1,453-2,017)	3,152 (2,612-3,618)	4,238 (3,536-4,875)	5,687 (4,850-6,419)	7,553 (6,478-8,527)	8,908 (7,608-9,884)	8,899 (7,805-9,715)	6,566 (5,630-7,138)	3,358 (2,844-3,637)	1,335 (1,116-1,429)	286 (234-301)
Pancreatic cancer	1,873 (1,800-1,953)	3,698 (3,557-3,835)	6,554 (6,375-6,753)	11,358 (11,008-11,725)	15,733 (15,299-16,190)	18,595 (18,204-19,079)	23,474 (22,987-24,099)	24,488 (23,948-25,130)	21,872 (21,423-22,395)	14,727 (14,425-15,044)	6,669 (6,558-6,784)	2,353 (2,307-2,398)	478 (467-490)
Larynx cancer	1,136 (1,061-1,207)	2,861 (2,716-3,018)	5,766 (5,508-6,041)	9,808 (9,483-10,197)	11,608 (11,185-12,039)	12,524 (12,053-13,025)	13,655 (13,087-14,197)	11,817 (11,351-12,270)	8,477 (8,169-8,769)	5,281 (5,076-5,465)	2,061 (1,986-2,138)	639 (619-660)	124 (120-129)
Tracheal, bronchus, and lung cancer	8,828 (8,478-9,254)	19,467 (18,920-20,089)	37,910 (37,009-38,885)	74,381 (72,884-76,040)	101,821 (100,003-103,856)	123,473 (121,376-125,810)	161,217 (158,880-164,228)	172,176 (169,610-175,106)	146,433 (144,265-148,872)	88,676 (87,307-90,217)	35,189 (34,641-35,825)	10,502 (10,331-10,682)	1,935 (1,896-1,975)
Malignant skin melanoma	1,005 (755-1,302)	1,507 (1,106-1,977)	1,828 (1,305-2,450)	2,361 (1,627-3,167)	2,705 (1,788-3,441)	2,453 (1,534-3,086)	2,785 (1,705-3,416)	2,802 (1,691-3,314)	2,748 (1,618-3,263)	2,136 (1,244-2,529)	1,077 (653-1,268)	442 (266-540)	104 (65-129)
Non-melanoma skin cancer	504 (483-526)	805 (773-842)	1,069 (1,024-1,118)	1,671 (1,587-1,746)	2,060 (1,971-2,144)	3,146 (2,975-3,288)	3,999 (3,800-4,157)	4,420 (4,217-4,589)	4,261 (4,077-4,411)	3,637 (3,486-3,761)	2,242 (2,145-2,317)	1,236 (1,191-1,277)	422 (409-434)
Non-melanoma skin cancer (squamous-cell carcinoma)	504 (483-526)	805 (773-842)	1,069 (1,024-1,118)	1,671 (1,587-1,746)	2,060 (1,971-2,144)	3,146 (2,975-3,288)	3,999 (3,800-4,157)	4,420 (4,217-4,589)	4,261 (4,077-4,411)	3,637 (3,486-3,761)	2,242 (2,145-2,317)	1,236 (1,191-1,277)	422 (409-434)
Breast cancer	167 (155-179)	258 (241-276)	449 (424-478)	613 (582-645)	740 (710-773)	865 (832-904)	954 (926-987)	1,024 (987-1,063)	744 (720-768)	581 (561-604)	316 (305-329)	128 (123-133)	32 (31-34)
Cervical cancer	-	-	-	-	-	-	-	-	-	-	-	-	-
Uterine cancer	-	-	-	-	-	-	-	-	-	-	-	-	-
Ovarian cancer	-	-	-	-	-	-	-	-	-	-	-	-	-
Prostate cancer	282 (230-323)	550 (454-637)	1,547 (1,266-1,818)	4,160 (3,363-4,800)	9,605 (7,661-10,657)	18,891 (15,112-21,509)	34,259 (27,379-39,118)	50,023 (41,572-57,262)	63,909 (52,394-75,972)	61,071 (48,793-69,451)	35,117 (27,387-39,922)	16,069 (12,255-17,686)	4,008 (2,772-4,266)
Testicular cancer	824 (781-875)	632 (600-672)	466 (447-488)	349 (335-365)	261 (251-273)	245 (234-256)	258 (247-269)	265 (254-276)	255 (246-265)	209 (201-219)	107 (102-111)	43 (41-44)	14 (14-15)
Kidney cancer	781 (728-821)	1,555 (1,465-1,621)	3,116 (2,977-3,224)	5,214 (4,986-5,387)	6,976 (6,658-7,194)	7,335 (7,035-7,547)	8,959 (8,571-9,198)	9,173 (8,739-9,410)	8,727 (8,411-8,925)	6,248 (6,035-6,417)	2,856 (2,784-2,929)	1,100 (1,071-1,137)	224 (217-232)
Bladder cancer	525 (469-578)	1,114 (1,009-1,196)	2,136 (1,966-2,259)	4,028 (3,805-4,228)	6,275 (5,969-6,618)	9,305 (8,844-9,819)	14,150 (13,620-14,761)	17,900 (17,431-18,908)	20,100 (19,431-21,309)	17,550 (17,057-18,505)	9,459 (9,209-9,942)	4,114 (4,013-4,262)	1,002 (975-1,037)
Brain and nervous system cancer	4,975 (4,083-5,695)	6,270 (5,287-7,243)	7,078 (6,052-8,045)	8,932 (7,663-10,076)	9,869 (8,381-10,958)	9,534 (8,097-10,751)	9,951 (8,328-11,193)	8,787 (7,348-9,584)	6,760 (5,354-7,340)	3,721 (2,819-4,077)	1,352 (1,020-1,493)	442 (327-499)	66 (48-73)
Thyroid cancer	294 (275-317)	420 (396-449)	649 (619-683)	987 (942-1,039)	1,203 (1,149-1,266)	1,176 (1,122-1,234)	1,365 (1,301-1,426)	1,407 (1,357-1,471)	1,577 (1,526-1,637)	1,069 (1,033-1,107)	477 (461-493)	164 (159-170)	33 (32-35)
Mesothelioma	212 (194-240)	366 (341-408)	634 (596-700)	1,039 (984-1,130)	1,615 (1,554-1,723)	1,726 (1,659-1,835)	2,254 (2,171-2,378)	2,461 (2,381-2,590)	2,503 (2,430-2,607)	1,785 (1,734-1,847)	788 (766-813)	263 (254-272)	49 (47-50)
Hodgkin lymphoma	1,285 (1,027-1,683)	1,235 (978-1,609)	1,297 (1,051-1,669)	1,437 (1,161-1,850)	1,589 (1,278-2,100)	1,542 (1,245-1,994)	1,792 (1,430-2,283)	1,748 (1,406-2,266)	993 (755-1,255)	631 (483-754)	273 (206-327)	55 (43-68)	9 (7-11)
Non-Hodgkin lymphoma	3,260 (3,097-3,492)	4,184 (4,002-4,443)	5,201 (4,990-5,462)	7,204 (6,973-7,574)	8,658 (8,380-9,026)	9,811 (9,402-10,250)	11,832 (11,380-12,252)	12,644 (12,279-13,043)	11,723 (11,423-12,029)	8,730 (8,528-8,938)	4,260 (4,163-4,347)	1,464 (1,433-1,495)	282 (274-290)

SR Table 6: Global deaths by cause and age group for males (with 95% confidence intervals), 2005

Cause	Early Neonatal	Late Neonatal	Post Neonatal
Multiple myeloma	-	-	-
Leukemia	189 (140-218)	168 (126-194)	2,286 (1,708-2,750)
Acute lymphoid leukemia	38 (28-52)	33 (24-47)	552 (396-787)
Chronic lymphoid leukemia	-	-	-
Acute myeloid leukemia	42 (31-56)	39 (29-52)	390 (297-556)
Chronic myeloid leukemia	-	-	-
Other leukemia	109 (75-128)	95 (66-112)	1,344 (900-1,615)
Other malignant neoplasms	468 (361-543)	256 (201-306)	1,441 (1,159-1,851)
Other neoplasms	35 (22-46)	23 (15-29)	269 (180-356)
Myelodysplastic, myeloproliferative, and other hematopoietic neoplasms	34 (22-45)	20 (14-26)	235 (154-317)
Other benign and in situ neoplasms	1 (0-1)	3 (1-4)	34 (23-45)
Cardiovascular diseases	2,344 (2,135-2,579)	1,509 (1,391-1,631)	12,046 (11,310-12,834)
Rheumatic heart disease	-	-	-
Ischemic heart disease	-	-	-
Stroke	898 (745-1,062)	534 (457-627)	4,032 (3,400-4,934)
Ischemic stroke	99 (70-146)	75 (54-107)	326 (244-490)
Intracerebral hemorrhage	486 (348-643)	262 (196-353)	2,251 (1,681-3,264)
Subarachnoid hemorrhage	313 (210-394)	197 (132-241)	1,455 (998-1,861)
Hypertensive heart disease	-	-	-
Non-rheumatic valvular heart disease	-	-	-
Non-rheumatic calcific aortic valve disease	-	-	-
Non-rheumatic degenerative mitral valve disease	-	-	-
Other non-rheumatic valve diseases	-	-	-
Cardiomyopathy and myocarditis	812 (694-926)	500 (438-559)	3,661 (3,130-4,192)
Myocarditis	99 (54-137)	113 (74-146)	1,012 (681-1,312)
Alcoholic cardiomyopathy	-	-	-
Other cardiomyopathy	713 (606-814)	387 (338-442)	2,650 (2,224-3,194)
Atrial fibrillation and flutter	-	-	-
Aortic aneurysm	-	-	-
Peripheral artery disease	-	-	-
Endocarditis	149 (120-202)	108 (83-149)	1,409 (1,096-1,882)
Other cardiovascular and circulatory diseases	485 (411-595)	368 (302-450)	2,944 (2,248-3,635)
Chronic respiratory diseases	0 (0-0)	0 (0-0)	0 (0-0)
Chronic obstructive pulmonary disease	-	-	0 (0-0)
Pneumoconiosis	-	-	-
Silicosis	-	-	-
Asbestosis	-	-	-
Coal workers pneumoconiosis	-	-	-
Other pneumoconiosis	-	-	-
Asthma	-	-	-
Interstitial lung disease and pulmonary sarcoidosis	-	-	-
Other chronic respiratory diseases	0 (0-0)	0 (0-0)	0 (0-0)
Digestive diseases	1,462 (1,290-1,742)	1,514 (1,338-1,762)	11,807 (10,331-14,678)
Cirrhosis and other chronic liver diseases	-	-	-
Cirrhosis and other chronic liver diseases due to hepatitis B	-	-	-
Cirrhosis and other chronic liver diseases due to hepatitis C	-	-	-
Cirrhosis and other chronic liver diseases due to alcohol use	-	-	-
Cirrhosis due to NASH	-	-	-
Cirrhosis and other chronic liver diseases due to other causes	-	-	-
Upper digestive system diseases	-	-	-
Peptic ulcer disease	-	-	-
Gastritis and duodenitis	-	-	-
Appendicitis	-	-	-
Paralytic ileus and intestinal obstruction	1,462 (1,290-1,742)	1,514 (1,338-1,762)	11,807 (10,331-14,678)
Inguinal, femoral, and abdominal hernia	-	-	-
Inflammatory bowel disease	-	-	-
Vascular intestinal disorders	-	-	-
Gallbladder and biliary diseases	-	-	-
Pancreatitis	-	-	-
Other digestive diseases	-	-	-
Neurological disorders	-	-	4,661 (3,702-5,764)
Alzheimer's disease and other dementias	-	-	-
Parkinson's disease	-	-	-
Epilepsy	-	-	2,950 (2,290-3,649)
Multiple sclerosis	-	-	-
Motor neuron disease	4 (3-4)	10 (7-11)	289 (206-395)
Other neurological disorders	-	-	1,423 (931-1,862)
Mental disorders	-	-	-
Eating disorders	-	-	-
Anorexia nervosa	-	-	-
Bulimia nervosa	-	-	-
Substance use disorders	0 (0-0)	0 (0-0)	0 (0-0)
Alcohol use disorders	-	-	-
Drug use disorders	0 (0-0)	0 (0-0)	0 (0-0)
Opioid use disorders	0 (0-0)	0 (0-0)	0 (0-0)
Cocaine use disorders	-	-	-
Amphetamine use disorders	-	-	-
Other drug use disorders	-	-	-
Diabetes and kidney diseases	25 (18-29)	70 (52-84)	5,073 (4,512-5,578)
Diabetes mellitus	25 (18-29)	70 (52-84)	688 (509-827)
Diabetes mellitus type 1	25 (18-29)	70 (52-84)	688 (509-827)
Diabetes mellitus type 2	-	-	-
Chronic kidney disease	-	-	4,249 (3,753-4,711)
Chronic kidney disease due to diabetes mellitus type 1	-	-	15 (9-25)
Chronic kidney disease due to diabetes mellitus type 2	-	-	12 (6-21)
Chronic kidney disease due to hypertension	-	-	21 (12-33)
Chronic kidney disease due to glomerulonephritis	-	-	964 (687-1,329)
Chronic kidney disease due to other and unspecified causes	-	-	3,237 (2,732-3,737)
Acute glomerulonephritis	-	-	135 (83-191)
Skin and subcutaneous diseases	-	-	1,578 (775-2,134)
Bacterial skin diseases	-	-	1,540 (751-2,069)
Cellulitis	-	-	118 (42-202)
Pyoderma	-	-	1,422 (694-1,931)
Decubitus ulcer	-	-	-
Other skin and subcutaneous diseases	-	-	38 (18-79)
Musculoskeletal disorders	-	-	-
Rheumatoid arthritis	-	-	-
Other musculoskeletal disorders	-	-	-
Other non-communicable diseases	122,085 (110,096-147,413)	45,434 (41,449-53,628)	166,164 (143,570-197,275)
Congenital birth defects	120,200 (108,508-145,616)	40,986 (37,643-48,841)	128,453 (118,281-153,740)
Neural tube defects	16,462 (11,834-24,965)	3,816 (2,656-6,197)	9,394 (5,660-16,814)
Congenital heart anomalies	40,044 (27,855-52,508)	19,048 (14,506-23,544)	67,870 (48,167-82,906)
Orofacial clefts	1,956 (689-4,973)	567 (221-1,379)	1,125 (454-2,983)
Down syndrome	2,380 (1,387-3,857)	966 (614-1,499)	4,969 (2,903-8,168)
Other chromosomal abnormalities	2,504 (1,378-4,964)	923 (534-1,729)	2,122 (1,127-4,137)
Congenital musculoskeletal and limb anomalies	2,423 (1,535-3,794)	566 (369-792)	1,545 (912-2,345)
Urogenital congenital anomalies	3,785 (2,241-5,621)	980 (598-1,355)	1,692 (933-2,581)
Digestive congenital anomalies	14,145 (9,095-21,577)	5,433 (3,827-8,228)	13,931 (9,178-23,624)
Other congenital birth defects	36,500 (23,426-47,428)	8,689 (5,294-11,573)	25,806 (13,667-37,631)
Urinary diseases and male infertility	489 (425-563)	363 (318-411)	3,633 (3,219-4,041)
Urinary tract infections	95 (56-145)	122 (83-164)	2,443 (1,861-3,053)
Urolithiasis	-	-	-
Other urinary diseases	394 (299-464)	241 (176-288)	1,190 (628-1,781)
Gynecological diseases	-	-	-
Uterine fibroids	-	-	-
Polycystic ovarian syndrome	-	-	-

Cause	1 to 4	5 to 9	10 to 14	15 to 19	20 to 24	25 to 29	30 to 34	35 to 39	40 to 44	45 to 49	50 to 54	54 to 59	60 to 64	65 to 69	70 to 74	75 to 79	80 to 84	85 to 89	90 to 94	95 plus
Multiple myeloma	-	-	-	101 (84-122)	106 (91-135)	123 (104-150)	280 (240-332)	502 (422-597)	986 (837-1,206)	1,434 (1,241-1,742)	2,449 (2,147-2,975)	3,513 (3,008-4,210)	4,342 (3,702-5,216)	5,506 (4,606-6,532)	6,170 (4,913-7,115)	5,849 (4,613-6,752)	4,395 (3,411-5,094)	2,104 (1,601-2,445)	703 (522-820)	154 (116-189)
Leukemia	8,289 (6,410-10,820)	8,271 (6,869-9,559)	6,968 (5,872-7,821)	7,331 (6,115-8,044)	5,481 (4,540-5,952)	4,689 (3,945-5,152)	5,075 (4,246-5,500)	5,958 (4,927-6,381)	6,382 (5,463-6,898)	6,931 (6,029-7,563)	8,938 (7,803-9,598)	10,538 (9,448-11,411)	12,095 (10,866-12,869)	15,298 (14,043-16,264)	16,854 (15,539-17,873)	16,160 (15,191-17,072)	12,147 (11,564-12,739)	6,020 (5,742-6,281)	2,345 (2,282-2,450)	503 (490-519)
Acute lymphoid leukemia	2,720 (1,834-4,042)	3,547 (2,817-4,524)	2,618 (2,222-3,141)	2,235 (1,927-2,639)	1,490 (1,289-1,693)	1,069 (916-1,219)	985 (852-1,114)	1,076 (912-1,236)	997 (868-1,147)	981 (863-1,125)	1,169 (1,030-1,322)	1,225 (1,094-1,386)	1,186 (1,035-1,319)	1,402 (1,256-1,569)	1,309 (1,171-1,442)	732 (671-820)	459 (421-512)	205 (190-224)	67 (63-72)	13 (12-14)
Chronic lymphoid leukemia	-	-	-	105 (81-127)	85 (66-102)	75 (61-87)	105 (82-120)	131 (108-158)	204 (177-234)	345 (311-383)	642 (590-694)	998 (937-1,071)	1,451 (1,350-1,522)	2,117 (1,985-2,223)	2,636 (2,465-2,762)	2,820 (2,689-2,946)	2,450 (2,362-2,554)	1,350 (1,303-1,395)	624 (603-646)	157 (151-163)
Acute myeloid leukemia	1,691 (1,187-2,831)	1,088 (857-1,442)	1,122 (929-1,362)	1,303 (1,087-1,531)	1,185 (969-1,344)	1,135 (966-1,292)	1,273 (1,073-1,426)	1,443 (1,217-1,626)	1,661 (1,435-1,867)	2,009 (1,744-2,229)	2,487 (2,188-2,715)	3,074 (2,742-3,390)	3,576 (3,207-3,813)	4,411 (4,024-4,705)	4,883 (4,470-5,162)	4,351 (4,073-4,564)	3,023 (2,884-3,178)	1,347 (1,277-1,414)	433 (414-455)	77 (74-81)
Chronic myeloid leukemia	-	-	-	191 (157-222)	273 (228-318)	423 (362-490)	492 (417-548)	589 (502-657)	625 (551-704)	699 (614-782)	826 (734-921)	910 (826-1,014)	963 (867-1,075)	1,304 (1,188-1,451)	1,392 (1,269-1,513)	1,646 (1,520-1,779)	1,280 (1,197-1,375)	605 (571-641)	248 (236-263)	47 (44-50)
Other leukemia	3,878 (2,468-5,244)	3,636 (2,661-4,352)	3,228 (2,503-3,683)	3,497 (2,630-3,870)	2,448 (1,839-2,698)	1,987 (1,504-2,197)	2,221 (1,667-2,432)	2,720 (2,017-2,971)	2,895 (2,256-3,150)	2,897 (2,327-3,228)	3,815 (3,075-4,158)	4,332 (3,618-4,751)	4,918 (4,116-5,373)	6,063 (5,297-6,728)	6,634 (5,861-7,313)	6,611 (6,022-7,263)	4,935 (4,521-5,344)	2,513 (2,330-2,723)	973 (926-1,047)	209 (200-221)
Other malignant neoplasms	5,612 (4,195-8,041)	[illegible]	[illegible]	[illegible]	[illegible]	[illegible]	[illegible]	[illegible]	[illegible]	[illegible]	[illegible]	[illegible]	[illegible]	[illegible]	[illegible]	[illegible]	[illegible]	[illegible]	[illegible]	[illegible]
Other neoplasms	574 (363-825)	[illegible]	[illegible]	[illegible]	[illegible]	[illegible]	[illegible]	[illegible]	[illegible]	[illegible]	[illegible]	[illegible]	[illegible]	[illegible]	[illegible]	[illegible]	[illegible]	[illegible]	[illegible]	[illegible]
Myelodysplastic, myeloproliferative, and other hematopoietic neoplasms	470 (275-700)	[illegible]	[illegible]	[illegible]	[illegible]	[illegible]	[illegible]	[illegible]	[illegible]	[illegible]	[illegible]	[illegible]	[illegible]	[illegible]	[illegible]	[illegible]	[illegible]	[illegible]	[illegible]	[illegible]
Other benign and in situ neoplasms	105 (64-138)	[illegible]	[illegible]	[illegible]	[illegible]	[illegible]	[illegible]	[illegible]	[illegible]	[illegible]	[illegible]	[illegible]	[illegible]	[illegible]	[illegible]	[illegible]	[illegible]	[illegible]	[illegible]	[illegible]
Cardiovascular diseases	9,065 (8,145-9,941)	[illegible]	[illegible]	[illegible]	[illegible]	[illegible]	[illegible]	[illegible]	[illegible]	[illegible]	[illegible]	[illegible]	[illegible]	[illegible]	[illegible]	[illegible]	[illegible]	[illegible]	[illegible]	[illegible]
Rheumatic heart disease	2,443 (2,042-2,995)	[illegible]	[illegible]	[illegible]	[illegible]	[illegible]	[illegible]	[illegible]	[illegible]	[illegible]	[illegible]	[illegible]	[illegible]	[illegible]	[illegible]	[illegible]	[illegible]	[illegible]	[illegible]	[illegible]
Ischemic heart disease	-	[illegible]	[illegible]	[illegible]	[illegible]	[illegible]	[illegible]	[illegible]	[illegible]	[illegible]	[illegible]	[illegible]	[illegible]	[illegible]	[illegible]	[illegible]	[illegible]	[illegible]	[illegible]	[illegible]
Stroke	2,753 (2,250-3,340)	[illegible]	[illegible]	[illegible]	[illegible]	[illegible]	[illegible]	[illegible]	[illegible]	[illegible]	[illegible]	[illegible]	[illegible]	[illegible]	[illegible]	[illegible]	[illegible]	[illegible]	[illegible]	[illegible]
Ischemic stroke	341 (212-596)	[illegible]	[illegible]	[illegible]	[illegible]	[illegible]	[illegible]	[illegible]	[illegible]	[illegible]	[illegible]	[illegible]	[illegible]	[illegible]	[illegible]	[illegible]	[illegible]	[illegible]	[illegible]	[illegible]
Intracerebral hemorrhage	1,071 (721-1,707)	[illegible]	[illegible]	[illegible]	[illegible]	[illegible]	[illegible]	[illegible]	[illegible]	[illegible]	[illegible]	[illegible]	[illegible]	[illegible]	[illegible]	[illegible]	[illegible]	[illegible]	[illegible]	[illegible]
Subarachnoid hemorrhage	1,341 (876-1,741)	[illegible]	[illegible]	[illegible]	[illegible]	[illegible]	[illegible]	[illegible]	[illegible]	[illegible]	[illegible]	[illegible]	[illegible]	[illegible]	[illegible]	[illegible]	[illegible]	[illegible]	[illegible]	[illegible]
Hypertensive heart disease	[illegible]	[illegible]	[illegible]	[illegible]	[illegible]	[illegible]	[illegible]	[illegible]	[illegible]	[illegible]	[illegible]	[illegible]	[illegible]	[illegible]	[illegible]	[illegible]	[illegible]	[illegible]	[illegible]	[illegible]
Non-rheumatic valvular heart disease	[illegible]	[illegible]	[illegible]	[illegible]	[illegible]	[illegible]	[illegible]	[illegible]	[illegible]	[illegible]	[illegible]	[illegible]	[illegible]	[illegible]	[illegible]	[illegible]	[illegible]	[illegible]	[illegible]	[illegible]
Non-rheumatic calcific aortic valve disease	[illegible]	[illegible]	[illegible]	[illegible]	[illegible]	[illegible]	[illegible]	[illegible]	[illegible]	[illegible]	[illegible]	[illegible]	[illegible]	[illegible]	[illegible]	[illegible]	[illegible]	[illegible]	[illegible]	[illegible]
Non-rheumatic degenerative mitral valve disease	[illegible]	[illegible]	[illegible]	[illegible]	[illegible]	[illegible]	[illegible]	[illegible]	[illegible]	[illegible]	[illegible]	[illegible]	[illegible]	[illegible]	[illegible]	[illegible]	[illegible]	[illegible]	[illegible]	[illegible]
Other non-rheumatic valve diseases	[illegible]	[illegible]	[illegible]	[illegible]	[illegible]	[illegible]	[illegible]	[illegible]	[illegible]	[illegible]	[illegible]	[illegible]	[illegible]	[illegible]	[illegible]	[illegible]	[illegible]	[illegible]	[illegible]	[illegible]
Cardiomyopathy and myocarditis	[illegible]	[illegible]	[illegible]	[illegible]	[illegible]	[illegible]	[illegible]	[illegible]	[illegible]	[illegible]	[illegible]	[illegible]	[illegible]	[illegible]	[illegible]	[illegible]	[illegible]	[illegible]	[illegible]	[illegible]
Myocarditis	[illegible]	[illegible]	[illegible]	[illegible]	[illegible]	[illegible]	[illegible]	[illegible]	[illegible]	[illegible]	[illegible]	[illegible]	[illegible]	[illegible]	[illegible]	[illegible]	[illegible]	[illegible]	[illegible]	[illegible]
Alcoholic cardiomyopathy	[illegible]	[illegible]	[illegible]	[illegible]	[illegible]	[illegible]	[illegible]	[illegible]	[illegible]	[illegible]	[illegible]	[illegible]	[illegible]	[illegible]	[illegible]	[illegible]	[illegible]	[illegible]	[illegible]	[illegible]
Other cardiomyopathy	[illegible]	[illegible]	[illegible]	[illegible]	[illegible]	[illegible]	[illegible]	[illegible]	[illegible]	[illegible]	[illegible]	[illegible]	[illegible]	[illegible]	[illegible]	[illegible]	[illegible]	[illegible]	[illegible]	[illegible]
Atrial fibrillation and flutter	[illegible]	[illegible]	[illegible]	[illegible]	[illegible]	[illegible]	[illegible]	[illegible]	[illegible]	[illegible]	[illegible]	[illegible]	[illegible]	[illegible]	[illegible]	[illegible]	[illegible]	[illegible]	[illegible]	[illegible]
Aortic aneurysm	[illegible]	[illegible]	[illegible]	[illegible]	[illegible]	[illegible]	[illegible]	[illegible]	[illegible]	[illegible]	[illegible]	[illegible]	[illegible]	[illegible]	[illegible]	[illegible]	[illegible]	[illegible]	[illegible]	[illegible]
Peripheral artery disease	[illegible]	[illegible]	[illegible]	[illegible]	[illegible]	[illegible]	[illegible]	[illegible]	[illegible]	[illegible]	[illegible]	[illegible]	[illegible]	[illegible]	[illegible]	[illegible]	[illegible]	[illegible]	[illegible]	[illegible]
Endocarditis	[illegible]	[illegible]	[illegible]	[illegible]	[illegible]	[illegible]	[illegible]	[illegible]	[illegible]	[illegible]	[illegible]	[illegible]	[illegible]	[illegible]	[illegible]	[illegible]	[illegible]	[illegible]	[illegible]	[illegible]
Other cardiovascular and circulatory diseases	[illegible]	[illegible]	[illegible]	[illegible]	[illegible]	[illegible]	[illegible]	[illegible]	[illegible]	[illegible]	[illegible]	[illegible]	[illegible]	[illegible]	[illegible]	[illegible]	[illegible]	[illegible]	[illegible]	[illegible]
Chronic respiratory diseases	[illegible]	[illegible]	[illegible]	[illegible]	[illegible]	[illegible]	[illegible]	[illegible]	[illegible]	[illegible]	[illegible]	[illegible]	[illegible]	[illegible]	[illegible]	[illegible]	[illegible]	[illegible]	[illegible]	[illegible]
Chronic obstructive pulmonary disease	[illegible]	[illegible]	[illegible]	[illegible]	[illegible]	[illegible]	[illegible]	[illegible]	[illegible]	[illegible]	[illegible]	[illegible]	[illegible]	[illegible]	[illegible]	[illegible]	[illegible]	[illegible]	[illegible]	[illegible]
Pneumoconiosis	[illegible]	[illegible]	[illegible]	[illegible]	[illegible]	[illegible]	[illegible]	[illegible]	[illegible]	[illegible]	[illegible]	[illegible]	[illegible]	[illegible]	[illegible]	[illegible]	[illegible]	[illegible]	[illegible]	[illegible]
Silicosis	[illegible]	[illegible]	[illegible]	[illegible]	[illegible]	[illegible]	[illegible]	[illegible]	[illegible]	[illegible]	[illegible]	[illegible]	[illegible]	[illegible]	[illegible]	[illegible]	[illegible]	[illegible]	[illegible]	[illegible]
Asbestosis	[illegible]	[illegible]	[illegible]	[illegible]	[illegible]	[illegible]	[illegible]	[illegible]	[illegible]	[illegible]	[illegible]	[illegible]	[illegible]	[illegible]	[illegible]	[illegible]	[illegible]	[illegible]	[illegible]	[illegible]
Coal workers pneumoconiosis	[illegible]	[illegible]	[illegible]	[illegible]	[illegible]	[illegible]	[illegible]	[illegible]	[illegible]	[illegible]	[illegible]	[illegible]	[illegible]	[illegible]	[illegible]	[illegible]	[illegible]	[illegible]	[illegible]	[illegible]
Other pneumoconiosis	[illegible]	[illegible]	[illegible]	[illegible]	[illegible]	[illegible]	[illegible]	[illegible]	[illegible]	[illegible]	[illegible]	[illegible]	[illegible]	[illegible]	[illegible]	[illegible]	[illegible]	[illegible]	[illegible]	[illegible]
Asthma	[illegible]	[illegible]	[illegible]	[illegible]	[illegible]	[illegible]	[illegible]	[illegible]	[illegible]	[illegible]	[illegible]	[illegible]	[illegible]	[illegible]	[illegible]	[illegible]	[illegible]	[illegible]	[illegible]	[illegible]
Interstitial lung disease and pulmonary sarcoidosis	[illegible]	[illegible]	[illegible]	[illegible]	[illegible]	[illegible]	[illegible]	[illegible]	[illegible]	[illegible]	[illegible]	[illegible]	[illegible]	[illegible]	[illegible]	[illegible]	[illegible]	[illegible]	[illegible]	[illegible]
Other chronic respiratory diseases	[illegible]	[illegible]	[illegible]	[illegible]	[illegible]	[illegible]	[illegible]	[illegible]	[illegible]	[illegible]	[illegible]	[illegible]	[illegible]	[illegible]	[illegible]	[illegible]	[illegible]	[illegible]	[illegible]	[illegible]
Digestive diseases	[illegible]	[illegible]	[illegible]	[illegible]	[illegible]	[illegible]	[illegible]	[illegible]	[illegible]	[illegible]	[illegible]	[illegible]	[illegible]	[illegible]	[illegible]	[illegible]	[illegible]	[illegible]	[illegible]	[illegible]
Cirrhosis and other chronic liver diseases	[illegible]	[illegible]	[illegible]	[illegible]	[illegible]	[illegible]	[illegible]	[illegible]	[illegible]	[illegible]	[illegible]	[illegible]	[illegible]	[illegible]	[illegible]	[illegible]	[illegible]	[illegible]	[illegible]	[illegible]
Cirrhosis and other chronic liver diseases due to hepatitis B	[illegible]	[illegible]	[illegible]	[illegible]	[illegible]	[illegible]	[illegible]	[illegible]	[illegible]	[illegible]	[illegible]	[illegible]	[illegible]	[illegible]	[illegible]	[illegible]	[illegible]	[illegible]	[illegible]	[illegible]
Cirrhosis and other chronic liver diseases due to hepatitis C	[illegible]	[illegible]	[illegible]	[illegible]	[illegible]	[illegible]	[illegible]	[illegible]	[illegible]	[illegible]	[illegible]	[illegible]	[illegible]	[illegible]	[illegible]	[illegible]	[illegible]	[illegible]	[illegible]	[illegible]
Cirrhosis and other chronic liver diseases due to alcohol use	[illegible]	[illegible]	[illegible]	[illegible]	[illegible]	[illegible]	[illegible]	[illegible]	[illegible]	[illegible]	[illegible]	[illegible]	[illegible]	[illegible]	[illegible]	[illegible]	[illegible]	[illegible]	[illegible]	[illegible]
Cirrhosis due to NASH	[illegible]	[illegible]	[illegible]	[illegible]	[illegible]	[illegible]	[illegible]	[illegible]	[illegible]	[illegible]	[illegible]	[illegible]	[illegible]	[illegible]	[illegible]	[illegible]	[illegible]	[illegible]	[illegible]	[illegible]
Cirrhosis and other chronic liver diseases due to other causes	[illegible]	[illegible]	[illegible]	[illegible]	[illegible]	[illegible]	[illegible]	[illegible]	[illegible]	[illegible]	[illegible]	[illegible]	[illegible]	[illegible]	[illegible]	[illegible]	[illegible]	[illegible]	[illegible]	[illegible]
Upper digestive system diseases	[illegible]	[illegible]	[illegible]	[illegible]	[illegible]	[illegible]	[illegible]	[illegible]	[illegible]	[illegible]	[illegible]	[illegible]	[illegible]	[illegible]	[illegible]	[illegible]	[illegible]	[illegible]	[illegible]	[illegible]
Peptic ulcer disease	[illegible]	[illegible]	[illegible]	[illegible]	[illegible]	[illegible]	[illegible]	[illegible]	[illegible]	[illegible]	[illegible]	[illegible]	[illegible]	[illegible]	[illegible]	[illegible]	[illegible]	[illegible]	[illegible]	[illegible]
Gastritis and duodenitis	[illegible]	[illegible]	[illegible]	[illegible]	[illegible]	[illegible]	[illegible]	[illegible]	[illegible]	[illegible]	[illegible]	[illegible]	[illegible]	[illegible]	[illegible]	[illegible]	[illegible]	[illegible]	[illegible]	[illegible]
Appendicitis	[illegible]	[illegible]	[illegible]	[illegible]	[illegible]	[illegible]	[illegible]	[illegible]	[illegible]	[illegible]	[illegible]	[illegible]	[illegible]	[illegible]	[illegible]	[illegible]	[illegible]	[illegible]	[illegible]	[illegible]
Paralytic ileus and intestinal obstruction	[illegible]	[illegible]	[illegible]	[illegible]	[illegible]	[illegible]	[illegible]	[illegible]	[illegible]	[illegible]	[illegible]	[illegible]	[illegible]	[illegible]	[illegible]	[illegible]	[illegible]	[illegible]	[illegible]	[illegible]
Inguinal, femoral, and abdominal hernia	[illegible]	[illegible]	[illegible]	[illegible]	[illegible]	[illegible]	[illegible]	[illegible]	[illegible]	[illegible]	[illegible]	[illegible]	[illegible]	[illegible]	[illegible]	[illegible]	[illegible]	[illegible]	[illegible]	[illegible]
Inflammatory bowel disease	[illegible]	[illegible]	[illegible]	[illegible]	[illegible]	[illegible]	[illegible]	[illegible]	[illegible]	[illegible]	[illegible]	[illegible]	[illegible]	[illegible]	[illegible]	[illegible]	[illegible]	[illegible]	[illegible]	[illegible]
Vascular intestinal disorders	[illegible]	[illegible]	[illegible]	[illegible]	[illegible]	[illegible]	[illegible]	[illegible]	[illegible]	[illegible]	[illegible]	[illegible]	[illegible]	[illegible]	[illegible]	[illegible]	[illegible]	[illegible]	[illegible]	[illegible]
Gallbladder and biliary diseases	[illegible]	[illegible]	[illegible]	[illegible]	[illegible]	[illegible]	[illegible]	[illegible]	[illegible]	[illegible]	[illegible]	[illegible]	[illegible]	[illegible]	[illegible]	[illegible]	[illegible]	[illegible]	[illegible]	[illegible]
Pancreatitis	[illegible]	[illegible]	[illegible]	[illegible]	[illegible]	[illegible]	[illegible]	[illegible]	[illegible]	[illegible]	[illegible]	[illegible]	[illegible]	[illegible]	[illegible]	[illegible]	[illegible]	[illegible]	[illegible]	[illegible]
Other digestive diseases	[illegible]	[illegible]	[illegible]	[illegible]	[illegible]	[illegible]	[illegible]	[illegible]	[illegible]	[illegible]	[illegible]	[illegible]	[illegible]	[illegible]	[illegible]	[illegible]	[illegible]	[illegible]	[illegible]	[illegible]
Neurological disorders	[illegible]	[illegible]	[illegible]	[illegible]	[illegible]	[illegible]	[illegible]	[illegible]	[illegible]	[illegible]	[illegible]	[illegible]	[illegible]	[illegible]	[illegible]	[illegible]	[illegible]	[illegible]	[illegible]	[illegible]
Alzheimer's disease and other dementias	[illegible]	[illegible]	[illegible]	[illegible]	[illegible]	[illegible]	[illegible]	[illegible]	[illegible]	[illegible]	[illegible]	[illegible]	[illegible]	[illegible]	[illegible]	[illegible]	[illegible]	[illegible]	[illegible]	[illegible]
Parkinson's disease	[illegible]	[illegible]	[illegible]	[illegible]	[illegible]	[illegible]	[illegible]	[illegible]	[illegible]	[illegible]	[illegible]	[illegible]	[illegible]	[illegible]	[illegible]	[illegible]	[illegible]	[illegible]	[illegible]	[illegible]
Epilepsy	[illegible]	[illegible]	[illegible]	[illegible]	[illegible]	[illegible]	[illegible]	[illegible]	[illegible]	[illegible]	[illegible]	[illegible]	[illegible]	[illegible]	[illegible]	[illegible]	[illegible]	[illegible]	[illegible]	[illegible]
Multiple sclerosis	[illegible]	[illegible]	[illegible]	[illegible]	[illegible]	[illegible]	[illegible]	[illegible]	[illegible]	[illegible]	[illegible]	[illegible]	[illegible]	[illegible]	[illegible]	[illegible]	[illegible]	[illegible]	[illegible]	[illegible]
Motor neuron disease	[illegible]	[illegible]	[illegible]	[illegible]	[illegible]	[illegible]	[illegible]	[illegible]	[illegible]	[illegible]	[illegible]	[illegible]	[illegible]	[illegible]	[illegible]	[illegible]	[illegible]	[illegible]	[illegible]	[illegible]
Other neurological disorders	[illegible]	[illegible]	[illegible]	[illegible]	[illegible]	[illegible]	[illegible]	[illegible]	[illegible]	[illegible]	[illegible]	[illegible]	[illegible]	[illegible]	[illegible]	[illegible]	[illegible]	[illegible]	[illegible]	[illegible]
Mental disorders	[illegible]	[illegible]	[illegible]	[illegible]	[illegible]	[illegible]	[illegible]	[illegible]	[illegible]	[illegible]	[illegible]	[illegible]	[illegible]	[illegible]	[illegible]	[illegible]	[illegible]	[illegible]	[illegible]	[illegible]
Eating disorders	[illegible]	[illegible]	[illegible]	[illegible]	[illegible]	[illegible]	[illegible]	[illegible]	[illegible]	[illegible]	[illegible]	[illegible]	[illegible]	[illegible]	[illegible]	[illegible]	[illegible]	[illegible]	[illegible]	[illegible]
Anorexia nervosa	[illegible]	[illegible]	[illegible]	[illegible]	[illegible]	[illegible]	[illegible]	[illegible]	[illegible]	[illegible]	[illegible]	[illegible]	[illegible]	[illegible]	[illegible]	[illegible]	[illegible]	[illegible]	[illegible]	[illegible]
Bulimia nervosa	[illegible]	[illegible]	[illegible]	[illegible]	[illegible]	[illegible]	[illegible]	[illegible]	[illegible]	[illegible]	[illegible]	[illegible]	[illegible]	[illegible]	[illegible]	[illegible]	[illegible]	[illegible]	[illegible]	[illegible]
Substance use disorders	[illegible]	[illegible]	[illegible]	[illegible]	[illegible]	[illegible]	[illegible]	[illegible]	[illegible]	[illegible]	[illegible]	[illegible]	[illegible]	[illegible]	[illegible]	[illegible]	[illegible]	[illegible]	[illegible]	[illegible]
Alcohol use disorders	[illegible]	[illegible]	[illegible]	[illegible]	[illegible]	[illegible]	[illegible]	[illegible]	[illegible]	[illegible]	[illegible]	[illegible]	[illegible]	[illegible]	[illegible]	[illegible]	[illegible]	[illegible]	[illegible]	[illegible]
Drug use disorders	[illegible]	[illegible]	[illegible]	[illegible]	[illegible]	[illegible]	[illegible]	[illegible]	[illegible]	[illegible]	[illegible]	[illegible]	[illegible]	[illegible]	[illegible]	[illegible]	[illegible]	[illegible]	[illegible]	[illegible]
Opioid use disorders	[illegible]	[illegible]	[illegible]	[illegible]	[illegible]	[illegible]	[illegible]	[illegible]	[illegible]	[illegible]	[illegible]	[illegible]	[illegible]	[illegible]	[illegible]	[illegible]	[illegible]	[illegible]	[illegible]	[illegible]
Cocaine use disorders	[illegible]	[illegible]	[illegible]	[illegible]	[illegible]	[illegible]	[illegible]	[illegible]	[illegible]	[illegible]	[illegible]	[illegible]	[illegible]	[illegible]	[illegible]	[illegible]	[illegible]	[illegible]	[illegible]	[illegible]
Amphetamine use disorders	[illegible]	[illegible]	[illegible]	[illegible]	[illegible]	[illegible]	[illegible]	[illegible]	[illegible]	[illegible]	[illegible]	[illegible]	[illegible]	[illegible]	[illegible]	[illegible]	[illegible]	[illegible]	[illegible]	[illegible]
Other drug use disorders	[illegible]	[illegible]	[illegible]	[illegible]	[illegible]	[illegible]	[illegible]	[illegible]	[illegible]	[illegible]	[illegible]	[illegible]	[illegible]	[illegible]	[illegible]	[illegible]	[illegible]	[illegible]	[illegible]	[illegible]
Diabetes and kidney diseases	[illegible]	[illegible]	[illegible]	[illegible]	[illegible]	[illegible]	[illegible]	[illegible]	[illegible]	[illegible]	[illegible]	[illegible]	[illegible]	[illegible]	[illegible]	[illegible]	[illegible]	[illegible]	[illegible]	[illegible]
Diabetes mellitus	[illegible]	[illegible]	[illegible]	[illegible]	[illegible]	[illegible]	[illegible]	[illegible]	[illegible]	[illegible]	[illegible]	[illegible]	[illegible]	[illegible]	[illegible]	[illegible]	[illegible]	[illegible]	[illegible]	[illegible]
Diabetes mellitus type 1	[illegible]	[illegible]	[illegible]	[illegible]	[illegible]	[illegible]	[illegible]	[illegible]	[illegible]	[illegible]	[illegible]	[illegible]	[illegible]	[illegible]	[illegible]	[illegible]	[illegible]	[illegible]	[illegible]	[illegible]
Diabetes mellitus type 2	[illegible]	[illegible]	[illegible]	[illegible]	[illegible]	[illegible]	[illegible]	[illegible]	[illegible]	[illegible]	[illegible]	[illegible]	[illegible]	[illegible]	[illegible]	[illegible]	[illegible]	[illegible]	[illegible]	[illegible]
Chronic kidney disease	[illegible]	[illegible]	[illegible]	[illegible]	[illegible]	[illegible]	[illegible]	[illegible]	[illegible]	[illegible]	[illegible]	[illegible]	[illegible]	[illegible]	[illegible]	[illegible]	[illegible]	[illegible]	[illegible]	[illegible]
Chronic kidney disease due to diabetes mellitus type 1	[illegible]	[illegible]	[illegible]	[illegible]	[illegible]	[illegible]	[illegible]	[illegible]	[illegible]	[illegible]	[illegible]	[illegible]	[illegible]	[illegible]	[illegible]	[illegible]	[illegible]	[illegible]	[illegible]	[illegible]
Chronic kidney disease due to diabetes mellitus type 2	[illegible]	[illegible]	[illegible]	[illegible]	[illegible]	[illegible]	[illegible]	[illegible]	[illegible]	[illegible]	[illegible]	[illegible]	[illegible]	[illegible]	[illegible]	[illegible]	[illegible]	[illegible]	[illegible]	[illegible]
Chronic kidney disease due to hypertension	[illegible]	[illegible]	[illegible]	[illegible]	[illegible]	[illegible]	[illegible]	[illegible]	[illegible]	[illegible]	[illegible]	[illegible]	[illegible]	[illegible]	[illegible]	[illegible]	[illegible]	[illegible]	[illegible]	[illegible]
Chronic kidney disease due to glomerulonephritis	[illegible]	[illegible]	[illegible]	[illegible]	[illegible]	[illegible]	[illegible]	[illegible]	[illegible]	[illegible]	[illegible]	[illegible]	[illegible]	[illegible]	[illegible]	[illegible]	[illegible]	[illegible]	[illegible]	[illegible]
Chronic kidney disease due to other and unspecified causes	[illegible]	[illegible]	[illegible]	[illegible]	[illegible]	[illegible]	[illegible]	[illegible]	[illegible]	[illegible]	[illegible]	[illegible]	[illegible]	[illegible]	[illegible]	[illegible]	[illegible]	[illegible]	[illegible]	[illegible]
Acute glomerulonephritis	[illegible]	[illegible]	[illegible]	[illegible]	[illegible]	[illegible]	[illegible]	[illegible]	[illegible]	[illegible]	[illegible]	[illegible]	[illegible]	[illegible]	[illegible]	[illegible]	[illegible]	[illegible]	[illegible]	[illegible]
Skin and subcutaneous diseases	[illegible]	[illegible]	[illegible]	[illegible]	[illegible]	[illegible]	[illegible]	[illegible]	[illegible]	[illegible]	[illegible]	[illegible]	[illegible]	[illegible]	[illegible]	[illegible]	[illegible]	[illegible]	[illegible]	[illegible]
Bacterial skin diseases	[illegible]	[illegible]	[illegible]	[illegible]	[illegible]	[illegible]	[illegible]	[illegible]	[illegible]	[illegible]	[illegible]	[illegible]	[illegible]	[illegible]	[illegible]	[illegible]	[illegible]	[illegible]	[illegible]	[illegible]
Cellulitis	[illegible]	[illegible]	[illegible]	[illegible]	[illegible]	[illegible]	[illegible]	[illegible]	[illegible]	[illegible]	[illegible]	[illegible]	[illegible]	[illegible]	[illegible]	[illegible]	[illegible]	[illegible]	[illegible]	[illegible]
Pyoderma	[illegible]	[illegible]	[illegible]	[illegible]	[illegible]	[illegible]	[illegible]	[illegible]	[illegible]	[illegible]	[illegible]	[illegible]	[illegible]	[illegible]	[illegible]	[illegible]	[illegible]	[illegible]	[illegible]	[illegible]
Decubitus ulcer	[illegible]	[illegible]	[illegible]	[illegible]	[illegible]	[illegible]	[illegible]	[illegible]	[illegible]	[illegible]	[illegible]	[illegible]	[illegible]	[illegible]	[illegible]	[illegible]	[illegible]	[illegible]	[illegible]	[illegible]
Other skin and subcutaneous diseases	[illegible]	[illegible]	[illegible]	[illegible]	[illegible]	[illegible]	[illegible]	[illegible]	[illegible]	[illegible]	[illegible]	[illegible]	[illegible]	[illegible]	[illegible]	[illegible]	[illegible]	[illegible]	[illegible]	[illegible]
Musculoskeletal disorders	[illegible]	[illegible]	[illegible]	[illegible]	[illegible]	[illegible]	[illegible]	[illegible]	[illegible]	[illegible]	[illegible]	[illegible]	[illegible]	[illegible]	[illegible]	[illegible]	[illegible]	[illegible]	[illegible]	[illegible]
Rheumatoid arthritis	[illegible]	[illegible]	[illegible]	[illegible]	[illegible]	[illegible]	[illegible]	[illegible]	[illegible]	[illegible]	[illegible]	[illegible]	[illegible]	[illegible]	[illegible]	[illegible]	[illegible]	[illegible]	[illegible]	[illegible]
Other musculoskeletal disorders	[illegible]	[illegible]	[illegible]	[illegible]	[illegible]	[illegible]	[illegible]	[illegible]	[illegible]	[illegible]	[illegible]	[illegible]	[illegible]	[illegible]	[illegible]	[illegible]	[illegible]	[illegible]	[illegible]	[illegible]
Other non-communicable diseases	[illegible]	[illegible]	[illegible]	[illegible]	[illegible]	[illegible]	[illegible]	[illegible]	[illegible]	[illegible]	[illegible]	[illegible]	[illegible]	[illegible]	[illegible]	[illegible]	[illegible]	[illegible]	[illegible]	[illegible]
Congenital birth defects	[illegible]	[illegible]	[illegible]	[illegible]	[illegible]	[illegible]	[illegible]	[illegible]	[illegible]	[illegible]	[illegible]	[illegible]	[illegible]	[illegible]	[illegible]	[illegible]	[illegible]	[illegible]	[illegible]	[illegible]
Neural tube defects	[illegible]	[illegible]	[illegible]	[illegible]	[illegible]	[illegible]	[illegible]	[illegible]	[illegible]	[illegible]	[illegible]	[illegible]	[illegible]	[illegible]	[illegible]	[illegible]	[illegible]	[illegible]	[illegible]	[illegible]
Congenital heart anomalies	[illegible]	[illegible]	[illegible]	[illegible]	[illegible]	[illegible]	[illegible]	[illegible]	[illegible]	[illegible]	[illegible]	[illegible]	[illegible]	[illegible]	[illegible]	[illegible]	[illegible]	[illegible]	[illegible]	[illegible]
Orofacial clefts	[illegible]	[illegible]	[illegible]	[illegible]	[illegible]	[illegible]	[illegible]	[illegible]	[illegible]	[illegible]	[illegible]	[illegible]	[illegible]	[illegible]	[illegible]	[illegible]	[illegible]	[illegible]	[illegible]	[illegible]
Down syndrome	[illegible]	[illegible]	[illegible]	[illegible]	[illegible]	[illegible]	[illegible]	[illegible]	[illegible]	[illegible]	[illegible]	[illegible]	[illegible]	[illegible]	[illegible]	[illegible]	[illegible]	[illegible]	[illegible]	[illegible]
Other chromosomal abnormalities	[illegible]	[illegible]	[illegible]	[illegible]	[illegible]	[illegible]	[illegible]	[illegible]	[illegible]	[illegible]	[illegible]	[illegible]	[illegible]	[illegible]	[illegible]	[illegible]	[illegible]	[illegible]	[illegible]	[illegible]
Congenital musculoskeletal and limb anomalies	[illegible]	[illegible]	[illegible]	[illegible]	[illegible]	[illegible]	[illegible]	[illegible]	[illegible]	[illegible]	[illegible]	[illegible]	[illegible]	[illegible]	[illegible]	[illegible]	[illegible]	[illegible]	[illegible]	[illegible]
Urogenital congenital anomalies	[illegible]	[illegible]	[illegible]	[illegible]	[illegible]	[illegible]	[illegible]	[illegible]	[illegible]	[illegible]	[illegible]	[illegible]	[illegible]	[illegible]	[illegible]	[illegible]	[illegible]	[illegible]	[illegible]	[illegible]
Digestive congenital anomalies	[illegible]	[illegible]	[illegible]	[illegible]	[illegible]	[illegible]	[illegible]	[illegible]	[illegible]	[illegible]	[illegible]	[illegible]	[illegible]	[illegible]	[illegible]	[illegible]	[illegible]	[illegible]	[illegible]	[illegible]
Other congenital birth defects	[illegible]	[illegible]	[illegible]	[illegible]	[illegible]	[illegible]	[illegible]	[illegible]	[illegible]	[illegible]	[illegible]	[illegible]	[illegible]	[illegible]	[illegible]	[illegible]	[illegible]	[illegible]	[illegible]	[illegible]
Urinary diseases and male infertility	[illegible]	[illegible]	[illegible]	[illegible]	[illegible]	[illegible]	[illegible]	[illegible]	[illegible]	[illegible]	[illegible]	[illegible]	[illegible]	[illegible]	[illegible]	[illegible]	[illegible]	[illegible]	[illegible]	[illegible]
Urinary tract infections	[illegible]	[illegible]	[illegible]	[illegible]	[illegible]	[illegible]	[illegible]	[illegible]	[illegible]	[illegible]	[illegible]	[illegible]	[illegible]	[illegible]	[illegible]	[illegible]	[illegible]	[illegible]	[illegible]	[illegible]
Urolithiasis	[illegible]	[illegible]	[illegible]	[illegible]	[illegible]	[illegible]	[illegible]	[illegible]	[illegible]	[illegible]	[illegible]	[illegible]	[illegible]	[illegible]	[illegible]	[illegible]	[illegible]	[illegible]	[illegible]	[illegible]
Other urinary diseases	[illegible]	[illegible]	[illegible]	[illegible]	[illegible]	[illegible]	[illegible]	[illegible]	[illegible]	[illegible]	[illegible]	[illegible]	[illegible]	[illegible]	[illegible]	[illegible]	[illegible]	[illegible]	[illegible]	[illegible]
Gynecological diseases	[illegible]	[illegible]	[illegible]	[illegible]	[illegible]	[illegible]	[illegible]	[illegible]	[illegible]	[illegible]	[illegible]	[illegible]	[illegible]	[illegible]	[illegible]	[illegible]	[illegible]	[illegible]	[illegible]	[illegible]
Uterine fibroids	[illegible]	[illegible]	[illegible]	[illegible]	[illegible]	[illegible]	[illegible]	[illegible]	[illegible]	[illegible]	[illegible]	[illegible]	[illegible]	[illegible]	[illegible]	[illegible]	[illegible]	[illegible]	[illegible]	[illegible]
Polycystic ovarian syndrome	[illegible]	[illegible]	[illegible]	[illegible]	[illegible]	[illegible]	[illegible]	[illegible]	[illegible]	[illegible]	[illegible]	[illegible]	[illegible]	[illegible]	[illegible]	[illegible]	[illegible]	[illegible]	[illegible]	[illegible]

SR Table 6: Global deaths by cause and age group for males (with 95% confidence intervals), 2005

Cause	Early Neonatal	Late Neonatal	Post Neonatal	1 to 4	5 to 9	10 to 14	15 to 19	20 to 24	25 to 29	30 to 34
Endometriosis	-	-	-	-	-	-	-	-	-	-
Genital prolapse	-	-	-	-	-	-	-	-	-	-
Other gynecological diseases	-	-	-	-	-	-	-	-	-	-
Hemoglobinopathies and hemolytic anemias	626 (357-1,018)	443 (283-628)	3,329 (2,063-4,912)	6,041 (3,320-8,482)	2,690 (1,805-3,925)	2,666 (1,786-3,901)	3,220 (1,920-4,960)	2,857 (1,572-4,519)	2,004 (1,144-3,169)	1,599 (967-2,447)
Thalassemias	201 (145-245)	168 (110-213)	1,085 (793-1,483)	1,358 (929-1,851)	807 (585-1,063)	559 (395-720)	362 (254-441)	223 (140-289)	100 (67-129)	54 (39-69)
Sickle cell disorders	394 (177-779)	264 (142-451)	2,152 (1,046-3,782)	4,461 (2,008-6,811)	1,652 (962-2,808)	1,656 (936-2,775)	2,126 (1,043-3,770)	1,802 (799-3,384)	1,148 (472-2,222)	730 (271-1,489)
G6PD deficiency	11 (6-21)	8 (5-13)	73 (41-115)	194 (105-300)	194 (136-270)	355 (247-494)	539 (377-723)	600 (402-788)	555 (386-699)	606 (428-743)
Other hemoglobinopathies and hemolytic anemias	19 (13-28)	3 (2-4)	20 (14-27)	28 (19-39)	37 (29-47)	96 (70-129)	193 (136-261)	232 (161-307)	201 (145-256)	208 (148-268)
Endocrine, metabolic, blood, and immune disorders	770 (527-934)	722 (506-921)	4,288 (3,121-5,962)	2,463 (1,518-3,486)	1,000 (782-1,283)	888 (733-1,126)	841 (692-1,056)	908 (772-1,118)	1,055 (895-1,302)	1,479 (1,198-1,767)
Sudden infant death syndrome	-	2,919 (1,058-5,333)	26,461 (9,278-51,280)	-	-	-	-	-	-	-
Injuries	8,362 (7,306-9,116)	6,093 (5,375-6,588)	57,485 (52,249-62,686)	153,984 (134,016-171,082)	94,848 (86,671-101,595)	89,749 (83,920-94,703)	212,329 (205,291-218,756)	290,570 (283,587-296,824)	261,257 (254,166-266,155)	252,199 (244,402-256,944)
Transport injuries	1,260 (1,133-1,408)	1,033 (929-1,148)	8,591 (7,349-9,529)	34,129 (24,583-40,186)	31,298 (28,500-34,008)	27,358 (25,276-29,478)	86,043 (82,067-90,789)	115,902 (111,626-120,708)	98,784 (95,673-103,001)	92,437 (89,526-96,252)
Road injuries	1,068 (955-1,213)	903 (807-1,006)	7,930 (6,736-8,842)	32,031 (23,261-37,725)	29,432 (26,812-31,971)	25,021 (23,152-27,060)	79,263 (75,680-83,652)	108,079 (103,980-112,756)	91,219 (88,382-95,133)	86,222 (83,444-89,895)
Pedestrian road injuries	537 (434-702)	434 (348-571)	3,377 (2,902-4,109)	19,205 (14,553-23,909)	17,136 (15,550-19,082)	12,640 (11,297-14,306)	22,065 (20,003-25,952)	27,465 (25,153-32,336)	24,486 (22,390-28,415)	27,117 (25,244-31,098)
Cyclist road injuries	-	-	-	845 (545-1,190)	1,658 (1,319-2,081)	2,040 (1,688-2,426)	3,697 (2,899-4,344)	3,077 (2,417-3,549)	2,730 (2,152-3,172)	3,302 (2,524-3,808)
Motorcyclist road injuries	79 (58-101)	82 (58-105)	722 (543-867)	1,749 (1,094-2,354)	1,714 (1,416-2,024)	2,636 (2,167-3,060)	22,590 (20,102-25,176)	30,934 (27,552-34,014)	24,522 (21,924-26,921)	21,022 (18,411-22,884)
Motor vehicle road injuries	435 (319-543)	373 (272-465)	3,690 (2,896-4,435)	9,934 (6,422-13,127)	8,601 (7,437-9,765)	7,380 (6,480-8,325)	30,318 (27,955-34,261)	45,758 (42,508-50,388)	38,742 (35,769-42,948)	34,172 (31,831-38,024)
Other road injuries	16 (9-23)	13 (8-19)	142 (89-198)	299 (157-497)	323 (233-429)	324 (252-420)	594 (469-777)	846 (657-1,091)	738 (595-961)	609 (492-765)
Other transport injuries	193 (136-269)	130 (92-194)	660 (474-838)	2,097 (1,183-2,793)	1,867 (1,555-2,252)	2,337 (2,012-2,767)	6,779 (6,044-7,865)	7,823 (7,193-9,203)	7,565 (6,960-8,843)	6,215 (5,804-6,986)
Unintentional injuries	6,107 (5,211-6,712)	4,752 (4,112-5,177)	46,140 (41,236-50,855)	113,507 (99,426-126,787)	59,100 (53,446-63,629)	50,220 (46,727-53,173)	59,123 (56,053-61,253)	64,435 (60,950-66,446)	58,959 (55,824-60,872)	63,356 (59,261-65,293)
Falls	914 (621-1,116)	361 (228-457)	4,767 (3,067-5,898)	8,332 (5,288-11,342)	5,320 (3,952-6,453)	5,038 (3,988-5,942)	6,313 (5,184-7,032)	8,828 (7,274-9,709)	9,373 (7,869-10,219)	11,647 (9,579-12,563)
Drowning	342 (312-375)	192 (174-209)	5,657 (5,100-6,233)	61,441 (54,144-68,125)	30,936 (28,010-33,595)	22,752 (21,090-24,335)	20,883 (19,914-21,974)	17,038 (16,317-17,977)	12,483 (12,079-13,034)	12,275 (11,887-12,719)
Fire, heat, and hot substances	296 (183-362)	246 (141-309)	3,203 (1,890-3,951)	7,724 (4,136-10,160)	2,366 (1,637-2,835)	1,433 (1,021-1,684)	2,191 (1,560-2,580)	3,293 (2,513-3,842)	3,506 (2,768-4,076)	3,782 (3,108-4,266)
Poisonings	234 (127-275)	204 (125-242)	2,102 (1,259-2,561)	4,325 (2,537-5,550)	1,508 (976-1,746)	1,507 (1,041-1,723)	2,275 (1,602-2,524)	3,199 (2,411-3,502)	3,314 (2,695-3,605)	3,515 (2,726-3,808)
Poisoning by carbon monoxide	52 (27-64)	51 (27-63)	437 (233-550)	942 (586-1,211)	622 (409-715)	577 (402-654)	1,296 (929-1,432)	1,929 (1,503-2,098)	2,006 (1,702-2,176)	2,318 (1,904-2,496)
Poisoning by other means	182 (99-218)	153 (97-182)	1,665 (1,027-2,045)	3,383 (1,941-4,517)	886 (571-1,062)	930 (612-1,100)	979 (657-1,123)	1,269 (902-1,466)	1,309 (968-1,481)	1,198 (830-1,364)
Exposure to mechanical forces	862 (639-966)	862 (645-964)	4,059 (3,062-4,493)	6,923 (4,843-8,100)	3,632 (3,228-4,034)	3,237 (2,896-3,562)	7,354 (6,613-7,998)	9,716 (8,590-10,627)	9,215 (8,036-9,961)	9,921 (8,222-10,599)
Unintentional firearm injuries	93 (70-140)	78 (57-132)	586 (423-912)	898 (505-1,838)	437 (367-623)	562 (495-715)	1,629 (1,432-1,882)	2,324 (2,053-2,672)	2,273 (1,966-2,656)	1,626 (1,431-1,964)
Other exposure to mechanical forces	769 (550-863)	784 (576-886)	3,473 (2,493-3,881)	6,024 (4,314-7,079)	3,196 (2,786-3,559)	2,675 (2,353-2,975)	5,726 (4,935-6,279)	7,392 (6,439-8,248)	6,942 (5,919-7,566)	8,295 (6,801-8,942)
Adverse effects of medical treatment	646 (446-837)	401 (291-543)	3,950 (2,565-5,802)	3,197 (1,662-6,285)	1,160 (839-1,481)	995 (754-1,178)	1,400 (1,070-1,633)	2,102 (1,529-2,475)	1,837 (1,375-2,129)	1,968 (1,493-2,291)
Animal contact	238 (86-291)	228 (79-292)	1,498 (586-1,894)	4,233 (1,728-5,636)	4,090 (1,787-5,188)	3,964 (1,815-4,894)	2,750 (1,324-3,286)	2,479 (1,191-2,944)	2,328 (1,202-2,721)	2,359 (1,225-2,782)
Venomous animal contact	208 (69-259)	197 (63-256)	1,269 (447-1,659)	3,487 (1,249-4,985)	3,641 (1,514-4,701)	3,685 (1,635-4,594)	2,403 (1,091-2,959)	2,144 (960-2,591)	1,954 (932-2,323)	2,106 (1,050-2,514)
Non-venomous animal contact	30 (16-40)	31 (16-42)	228 (119-336)	746 (327-1,387)	449 (248-626)	280 (159-347)	347 (216-443)	335 (221-425)	374 (248-463)	253 (159-319)
Foreign body	2,060 (1,687-2,347)	1,923 (1,627-2,164)	17,587 (14,892-19,815)	7,752 (5,593-9,180)	1,961 (1,769-2,290)	1,604 (1,492-1,801)	1,311 (1,248-1,450)	2,176 (2,055-2,396)	2,229 (2,141-2,407)	2,395 (2,318-2,582)
Pulmonary aspiration and foreign body in airway	2,004 (1,650-2,294)	1,889 (1,600-2,135)	16,998 (14,533-19,171)	6,841 (5,176-8,412)	1,732 (1,555-1,982)	1,457 (1,352-1,642)	1,190 (1,129-1,342)	1,990 (1,877-2,208)	2,030 (1,951-2,205)	2,102 (2,017-2,280)
Foreign body in other body part	56 (27-85)	34 (20-54)	589 (323-1,049)	911 (365-1,830)	229 (159-324)	147 (109-197)	121 (85-146)	186 (129-224)	199 (145-241)	293 (241-367)
Environmental heat and cold exposure	201 (65-276)	125 (42-175)	1,292 (430-1,823)	1,009 (355-1,722)	415 (204-533)	396 (203-514)	955 (601-1,176)	1,454 (995-1,705)	1,825 (1,393-2,078)	2,680 (2,081-3,014)
Exposure to forces of nature	52 (43-62)	113 (95-134)	933 (781-1,099)	4,284 (3,556-5,118)	4,604 (3,898-5,522)	5,768 (4,883-6,815)	6,025 (5,082-7,131)	5,567 (4,697-6,542)	4,758 (3,973-5,604)	3,892 (3,282-4,570)
Other unintentional injuries	263 (227-304)	98 (84-110)	1,092 (970-1,206)	4,287 (3,679-4,926)	3,108 (2,751-3,467)	3,526 (3,177-3,869)	7,667 (7,089-8,294)	8,583 (8,114-9,170)	8,089 (7,694-8,550)	8,922 (8,518-9,440)
Self-harm and interpersonal violence	995 (739-1,105)	307 (240-347)	2,754 (2,213-3,182)	6,349 (5,032-7,201)	4,450 (3,709-4,927)	12,171 (10,723-13,153)	67,163 (61,619-69,833)	110,233 (103,559-113,773)	103,515 (96,426-106,515)	96,406 (88,876-99,333)
Self-harm	-	-	-	-	-	5,590 (4,764-6,210)	29,771 (26,968-31,925)	50,232 (46,325-53,107)	50,891 (46,615-53,825)	50,857 (46,132-52,898)
Self-harm by firearm	-	-	-	-	-	280 (200-474)	3,304 (2,476-4,659)	5,667 (4,360-7,739)	4,924 (3,764-6,640)	4,309 (3,306-5,874)
Self-harm by other specified means	-	-	-	-	-	5,310 (4,452-5,937)	26,467 (23,624-28,408)	44,565 (40,168-47,416)	45,967 (41,754-48,645)	46,549 (42,022-48,869)
Interpersonal violence	871 (615-980)	241 (173-280)	2,522 (1,986-2,964)	4,479 (3,164-5,335)	3,152 (2,414-3,629)	5,254 (4,071-5,891)	33,313 (28,198-35,579)	53,065 (46,235-56,503)	47,711 (41,487-49,727)	41,890 (36,133-44,482)
Physical violence by firearm	139 (77-187)	41 (24-57)	421 (262-554)	811 (489-1,243)	602 (434-757)	1,632 (1,289-1,920)	17,465 (14,784-18,943)	27,980 (23,920-30,476)	23,977 (20,146-26,023)	18,635 (15,171-20,570)
Physical violence by sharp object	83 (47-114)	25 (13-38)	232 (138-356)	622 (296-1,186)	448 (261-601)	840 (526-1,047)	7,983 (5,779-9,739)	13,090 (9,778-15,490)	12,695 (9,660-15,188)	11,938 (8,934-14,220)
Physical violence by other means	649 (462-761)	174 (127-209)	1,868 (1,470-2,309)	3,045 (2,159-3,827)	2,102 (1,577-2,529)	2,782 (2,077-3,457)	7,866 (6,187-10,578)	11,996 (9,724-15,309)	11,040 (9,084-13,676)	11,317 (9,302-14,260)
Conflict and terrorism	124 (98-163)	67 (52-87)	206 (163-264)	1,821 (1,437-2,347)	1,254 (995-1,643)	1,269 (1,024-1,639)	3,628 (2,883-4,756)	6,044 (4,918-7,676)	4,091 (3,374-5,006)	2,895 (2,401-3,600)
Executions and police conflict	-	-	27 (13-69)	49 (46-56)	43 (40-46)	58 (54-63)	450 (405-509)	892 (806-981)	822 (727-949)	764 (663-915)

Cause	35 to 39	40 to 44	45 to 49	50 to 54	54 to 59	60 to 64	65 to 69	70 to 74	75 to 79	80 to 84	85 to 89	90 to 94	95 plus
Endometriosis	-	-	-	-	-	-	-	-	-	-	-	-	-
Genital prolapse	-	-	-	-	-	-	-	-	-	-	-	-	-
Other gynecological diseases	-	-	-	-	-	-	-	-	-	-	-	-	-
Hemoglobinopathies and hemolytic anemias	1,565 (930-2,362)	1,608 (981-2,447)	1,537 (919-2,274)	1,914 (1,161-2,813)	1,929 (1,277-2,703)	2,109 (1,350-2,996)	2,509 (1,636-3,487)	2,839 (1,910-3,911)	2,881 (2,039-3,833)	2,392 (1,752-3,032)	1,575 (1,166-1,945)	865 (610-999)	253 (180-282)
Thalassemias	34 (26-43)	22 (17-28)	14 (11-17)	10 (9-13)	5 (4-7)	2 (2-3)	1 (1-1)	0 (0-0)	0 (0-0)	0 (0-0)	0 (0-0)	0 (0-0)	0 (0-0)
Sickle cell disorders	682 (234-1,387)	552 (179-1,201)	332 (103-773)	211 (78-507)	83 (43-160)	40 (27-63)	21 (15-30)	9 (6-13)	3 (2-4)	1 (0-1)	0 (0-0)	0 (0-0)	0 (0-0)
G6PD deficiency	619 (442-786)	696 (492-949)	820 (511-1,198)	1,064 (612-1,653)	962 (556-1,541)	851 (447-1,422)	737 (378-1,245)	523 (266-917)	306 (156-537)	119 (61-208)	42 (22-72)	11 (6-17)	2 (1-3)
Other hemoglobinopathies and hemolytic anemias	230 (167-284)	337 (246-418)	370 (269-481)	629 (439-834)	879 (644-1,154)	1,215 (831-1,636)	1,750 (1,232-2,369)	2,307 (1,619-3,116)	2,572 (1,867-3,361)	2,273 (1,681-2,837)	1,533 (1,139-1,885)	854 (602-986)	252 (179-280)
Endocrine, metabolic, blood, and immune disorders	1,738 (1,381-2,061)	2,128 (1,676-2,458)	2,589 (1,992-2,982)	3,211 (2,387-3,753)	3,401 (2,510-3,822)	3,473 (2,654-3,951)	3,851 (2,953-4,417)	4,241 (3,338-4,962)	4,226 (3,153-4,912)	3,593 (2,671-4,154)	2,457 (1,893-2,809)	1,223 (894-1,413)	424 (313-489)
Sudden infant death syndrome	-	-	-	-	-	-	-	-	-	-	-	-	-
Injuries	257,403 (248,633-262,247)	238,507 (228,946-243,236)	213,892 (205,982-217,956)	198,204 (189,795-202,078)	158,273 (149,795-161,800)	124,685 (116,006-128,707)	124,392 (114,443-128,257)	113,269 (102,735-117,406)	101,299 (91,073-105,625)	74,775 (66,310-78,126)	44,000 (39,165-46,163)	19,862 (18,122-20,652)	5,598 (5,261-5,774)
Transport injuries	97,927 (95,147-101,852)	85,959 (83,548-88,877)	71,156 (69,369-73,401)	66,826 (65,213-68,782)	51,842 (50,493-53,362)	39,981 (38,754-41,350)	37,517 (36,333-38,658)	32,394 (31,306-33,376)	25,605 (24,627-26,469)	15,941 (15,262-16,596)	7,420 (7,102-7,757)	2,484 (2,384-2,589)	574 (552-594)
Road injuries	90,952 (88,083-94,605)	79,687 (77,542-82,285)	65,382 (63,645-67,504)	61,388 (59,797-63,393)	47,628 (46,341-49,134)	37,047 (35,916-38,320)	34,823 (33,719-36,006)	30,189 (29,168-31,131)	23,865 (22,974-24,701)	14,973 (14,362-15,587)	6,997 (6,701-7,309)	2,333 (2,242-2,430)	531 (510-550)
Pedestrian road injuries	31,773 (29,500-36,664)	28,705 (26,889-32,978)	25,272 (23,617-28,474)	26,324 (24,673-29,289)	21,168 (19,851-23,585)	18,230 (17,190-20,167)	18,030 (17,033-19,668)	16,267 (15,434-17,785)	14,150 (13,418-15,315)	8,828 (8,365-9,628)	4,206 (3,957-4,588)	1,334 (1,262-1,447)	296 (281-317)
Cyclist road injuries	3,777 (2,740-4,283)	4,227 (3,087-4,840)	3,925 (3,029-4,518)	4,491 (3,200-5,127)	3,493 (2,550-3,936)	3,183 (2,398-3,672)	3,018 (2,365-3,417)	2,612 (2,016-2,949)	1,668 (1,331-1,879)	906 (744-1,028)	358 (292-416)	110 (92-129)	1 (1-1)
Motorcyclist road injuries	21,321 (17,530-23,142)	17,454 (14,574-19,119)	12,276 (10,840-13,472)	9,980 (8,692-10,961)	6,974 (6,098-7,754)	3,914 (3,355-4,479)	3,299 (2,792-3,698)	2,397 (1,961-2,711)	1,329 (1,029-1,498)	897 (722-1,044)	336 (269-404)	89 (68-107)	23 (18-26)
Motor vehicle road injuries	33,422 (30,319-37,245)	28,671 (26,428-32,051)	23,234 (21,073-25,867)	19,916 (17,977-21,475)	15,478 (14,004-16,891)	11,370 (10,314-12,476)	10,063 (9,054-10,966)	8,522 (7,647-9,351)	6,420 (5,776-6,998)	4,129 (3,752-4,484)	1,999 (1,793-2,178)	776 (711-830)	201 (185-213)
Other road injuries	658 (503-808)	631 (489-773)	675 (526-844)	677 (513-867)	516 (406-647)	350 (285-426)	414 (331-515)	391 (309-498)	298 (235-382)	213 (161-282)	99 (80-135)	25 (20-36)	10 (8-13)
Other transport injuries	6,975 (6,478-7,855)	6,272 (5,872-7,187)	5,774 (5,391-6,648)	5,438 (5,049-6,242)	4,214 (3,906-4,867)	2,934 (2,738-3,380)	2,694 (2,504-3,097)	2,205 (2,054-2,521)	1,740 (1,603-2,028)	968 (885-1,121)	423 (378-499)	151 (136-177)	43 (39-50)
Unintentional injuries	71,052 (65,776-73,320)	70,984 (65,799-73,367)	71,292 (66,189-73,454)	68,840 (63,689-70,897)	58,099 (53,253-60,160)	50,430 (45,170-52,749)	54,323 (47,929-57,099)	53,025 (45,553-55,927)	53,120 (45,539-56,261)	44,901 (38,724-47,531)	29,998 (25,564-31,837)	15,126 (13,474-15,870)	4,518 (4,182-4,686)
Falls	14,579 (11,531-15,671)	16,630 (13,430-17,795)	17,286 (14,605-18,409)	18,488 (15,586-19,576)	17,679 (14,761-18,812)	17,857 (14,608-19,172)	21,550 (17,750-23,134)	24,784 (19,402-26,821)	28,545 (22,646-30,792)	26,792 (21,393-28,926)	19,445 (15,397-21,025)	10,263 (8,775-10,943)	2,990 (2,674-3,141)
Drowning	12,326 (11,946-12,805)	11,703 (11,382-12,082)	10,506 (10,236-10,809)	9,813 (9,578-10,084)	7,424 (7,245-7,627)	6,201 (6,037-6,380)	5,951 (5,780-6,130)	5,575 (5,409-5,737)	4,209 (4,077-4,341)	3,018 (2,889-3,132)	1,556 (1,492-1,617)	506 (490-523)	117 (113-120)
Fire, heat, and hot substances	4,161 (3,479-4,684)	4,512 (3,914-4,941)	4,943 (4,375-5,373)	4,781 (4,254-5,198)	3,910 (3,497-4,282)	3,350 (2,855-3,733)	3,643 (3,147-4,054)	3,365 (2,802-3,773)	3,125 (2,652-3,474)	2,744 (2,304-3,045)	1,746 (1,487-1,923)	875 (783-936)	277 (257-292)
Poisonings	3,898 (3,070-4,168)	4,550 (3,725-4,873)	4,517 (3,773-4,835)	4,332 (3,596-4,591)	3,321 (2,772-3,523)	2,489 (1,986-2,681)	2,476 (1,995-2,662)	1,984 (1,552-2,139)	1,530 (1,148-1,657)	1,001 (709-1,092)	471 (343-515)	148 (116-160)	32 (26-34)
Poisoning by carbon monoxide	2,597 (2,150-2,765)	3,173 (2,735-3,372)	3,282 (2,915-3,479)	3,044 (2,682-3,207)	2,302 (2,027-2,431)	1,565 (1,325-1,677)	1,604 (1,356-1,717)	1,217 (960-1,311)	993 (752-1,079)	607 (425-667)	283 (203-311)	89 (69-97)	17 (14-18)
Poisoning by other means	1,301 (903-1,440)	1,377 (957-1,550)	1,235 (859-1,396)	1,288 (893-1,428)	1,019 (731-1,131)	924 (642-1,046)	871 (635-974)	767 (562-864)	537 (387-600)	394 (281-435)	188 (137-210)	58 (46-64)	15 (13-16)
Exposure to mechanical forces	11,702 (9,706-12,414)	9,739 (7,986-10,307)	9,079 (7,985-9,779)	8,661 (7,426-9,170)	6,240 (5,566-6,642)	4,162 (3,767-4,491)	3,795 (3,390-4,135)	3,200 (2,877-3,476)	2,563 (2,373-2,783)	1,586 (1,483-1,697)	906 (858-965)	445 (421-475)	144 (138-150)
Unintentional firearm injuries	1,459 (1,272-1,751)	1,225 (1,076-1,425)	1,293 (1,139-1,514)	1,061 (934-1,231)	718 (650-857)	580 (510-671)	559 (494-656)	489 (429-577)	433 (382-517)	298 (264-363)	160 (143-195)	79 (70-97)	21 (19-26)
Other exposure to mechanical forces	10,243 (8,218-10,948)	8,513 (6,800-9,059)	7,786 (6,649-8,470)	7,600 (6,363-8,102)	5,522 (4,801-5,914)	3,582 (3,148-3,866)	3,236 (2,826-3,547)	2,711 (2,348-2,946)	2,130 (1,917-2,310)	1,289 (1,163-1,381)	746 (682-799)	366 (341-391)	123 (117-129)
Adverse effects of medical treatment	2,262 (1,701-2,631)	2,496 (1,865-2,871)	2,606 (1,919-2,993)	2,333 (1,852-2,732)	2,608 (2,053-3,005)	3,304 (2,589-3,886)	4,214 (3,257-4,946)	4,001 (3,239-4,789)	4,040 (3,264-4,760)	3,114 (2,596-3,704)	1,837 (1,560-2,237)	871 (750-1,121)	255 (226-336)
Animal contact	2,353 (1,188-2,724)	2,429 (1,240-2,838)	3,122 (1,598-3,623)	2,344 (1,232-2,723)	2,548 (1,271-2,952)	2,480 (1,114-2,938)	2,603 (1,170-3,099)	2,182 (1,012-2,584)	1,856 (887-2,185)	1,015 (514-1,202)	439 (232-519)	132 (80-152)	35 (22-40)
Venomous animal contact	2,062 (1,001-2,416)	2,136 (1,030-2,511)	2,729 (1,315-3,213)	1,915 (954-2,254)	2,152 (1,027-2,540)	2,115 (897-2,520)	2,223 (942-2,685)	1,837 (810-2,227)	1,536 (683-1,847)	831 (400-998)	350 (177-423)	99 (56-118)	24 (15-29)
Non-venomous animal contact	291 (186-359)	293 (180-370)	394 (260-500)	429 (270-557)	396 (235-529)	365 (196-508)	379 (206-529)	345 (192-476)	320 (181-445)	184 (107-254)	90 (54-124)	33 (21-44)	11 (7-14)
Foreign body	2,716 (2,620-2,885)	3,316 (3,228-3,513)	3,664 (3,542-3,848)	3,893 (3,781-4,047)	3,434 (3,354-3,580)	2,675 (2,599-2,788)	2,953 (2,838-3,076)	2,773 (2,584-2,881)	3,004 (2,858-3,104)	2,957 (2,828-3,056)	2,173 (2,104-2,239)	1,257 (1,222-1,301)	480 (468-496)
Pulmonary aspiration and foreign body in airway	2,392 (2,286-2,531)	2,971 (2,843-3,107)	3,422 (3,310-3,585)	3,660 (3,543-3,796)	3,246 (3,165-3,388)	2,509 (2,428-2,597)	2,808 (2,677-2,913)	2,615 (2,436-2,720)	2,816 (2,669-2,925)	2,813 (2,697-2,912)	2,082 (2,017-2,149)	1,214 (1,176-1,252)	466 (454-480)
Foreign body in other body part	324 (264-413)	345 (286-442)	242 (199-301)	233 (185-296)	187 (151-236)	166 (134-226)	145 (117-195)	158 (123-223)	188 (152-262)	145 (120-209)	90 (75-129)	43 (36-59)	14 (12-20)
Environmental heat and cold exposure	3,471 (2,731-3,813)	4,943 (4,230-5,307)	6,088 (5,384-6,537)	6,399 (5,582-6,818)	5,026 (4,207-5,471)	3,428 (2,682-3,819)	3,577 (2,644-4,076)	2,506 (1,667-2,930)	2,343 (1,439-2,789)	1,380 (867-1,621)	791 (527-916)	374 (282-421)	107 (88-119)
Exposure to forces of nature	3,402 (2,891-4,007)	2,933 (2,487-3,459)	2,590 (2,217-3,016)	2,220 (1,888-2,588)	1,812 (1,545-2,132)	1,634 (1,397-1,890)	1,275 (1,086-1,479)	1,094 (933-1,287)	813 (696-940)	593 (507-701)	309 (262-364)	115 (96-137)	34 (28-40)
Other unintentional injuries	10,182 (9,721-10,735)	7,735 (7,424-8,098)	6,893 (6,598-7,207)	5,574 (5,347-5,813)	4,097 (3,919-4,274)	2,849 (2,708-2,986)	2,287 (2,168-2,400)	1,562 (1,469-1,637)	1,091 (1,037-1,140)	699 (656-735)	326 (306-342)	140 (132-146)	47 (44-49)
Self-harm and interpersonal violence	88,423 (81,592-91,088)	81,564 (74,593-83,789)	71,443 (65,997-73,441)	62,537 (57,475-64,291)	48,332 (43,478-49,716)	34,275 (29,850-35,504)	32,552 (28,002-33,684)	27,850 (23,280-28,961)	22,573 (18,498-23,537)	13,933 (11,084-14,647)	6,581 (5,530-6,908)	2,253 (2,045-2,322)	506 (474-520)
Self-harm	50,666 (45,545-52,602)	49,970 (44,489-51,685)	46,846 (42,409-48,405)	43,413 (38,956-44,760)	35,610 (31,463-36,837)	26,206 (22,155-27,216)	25,759 (21,772-26,761)	23,241 (18,607-24,261)	19,418 (15,512-20,317)	13,051 (10,254-13,761)	6,198 (5,143-6,505)	2,115 (1,901-2,184)	416 (388-430)
Self-harm by firearm	3,992 (3,099-5,295)	4,230 (3,285-5,519)	4,385 (3,481-5,694)	3,948 (3,084-4,978)	3,248 (2,543-4,074)	2,481 (1,965-3,046)	2,297 (1,780-2,857)	2,164 (1,752-2,685)	2,037 (1,663-2,524)	1,600 (1,354-1,999)	840 (715-1,051)	335 (285-448)	67 (55-90)
Self-harm by other specified means	46,675 (41,129-48,657)	45,740 (40,458-47,616)	42,461 (38,098-44,202)	39,465 (35,059-40,917)	32,363 (28,181-33,745)	23,725 (19,772-24,787)	23,462 (19,450-24,540)	21,078 (16,430-22,189)	17,380 (13,450-18,353)	11,451 (8,580-12,227)	5,358 (4,292-5,696)	1,780 (1,525-1,869)	349 (310-366)
Interpersonal violence	35,097 (30,136-37,685)	29,792 (25,933-31,467)	23,439 (20,722-24,999)	18,323 (16,004-19,499)	12,157 (10,612-13,152)	7,660 (6,528-8,270)	6,469 (5,515-6,996)	4,364 (3,654-4,746)	2,976 (2,516-3,235)	752 (646-831)	318 (282-356)	103 (95-116)	72 (65-77)
Physical violence by firearm	14,937 (11,956-16,788)	11,488 (9,327-12,919)	7,896 (6,464-8,839)	5,703 (4,484-6,432)	3,691 (2,955-4,281)	2,202 (1,756-2,589)	1,520 (1,180-1,762)	1,079 (801-1,297)	715 (535-843)	170 (127-208)	77 (60-89)	28 (22-32)	22 (16-25)
Physical violence by sharp object	10,247 (7,794-12,347)	8,880 (6,996-10,246)	7,040 (5,679-8,193)	5,364 (4,276-6,371)	3,265 (2,642-3,815)	2,204 (1,664-2,622)	1,806 (1,348-2,109)	1,156 (853-1,329)	773 (593-909)	188 (158-213)	78 (66-91)	27 (23-31)	20 (17-23)
Physical violence by other means	9,914 (8,133-12,502)	9,423 (8,076-11,296)	8,503 (7,426-9,759)	7,256 (6,205-8,314)	5,201 (4,419-6,060)	3,255 (2,789-3,838)	3,143 (2,688-3,678)	2,129 (1,805-2,581)	1,488 (1,257-1,726)	394 (328-468)	163 (141-198)	49 (44-58)	30 (27-36)
Conflict and terrorism	1,976 (1,634-2,431)	1,300 (1,097-1,608)	861 (705-1,087)	592 (495-737)	449 (382-549)	339 (293-409)	275 (239-325)	214 (187-254)	161 (143-186)	118 (106-134)	58 (52-66)	30 (28-34)	15 (14-17)
Executions and police conflict	683 (591-819)	502 (424-611)	298 (251-379)	210 (179-268)	115 (98-149)	70 (61-88)	49 (42-60)	30 (26-36)	19 (17-22)	13 (11-14)	7 (5-9)	4 (3-4)	3 (2-3)

SR Table 6: Global deaths by cause and age group for males (with 95% confidence intervals), 2010

Cause	Early Neonatal	Late Neonatal	Post Neonatal	1 to 4	5 to 9	10 to 14	15 to 19	20 to 24	25 to 29	30 to 34	35 to 39	40 to 44
All causes	1,328,256 (1,290,697-1,365,484)	356,678 (343,307-369,297)	1,134,856 (1,100,006-1,173,022)	1,021,728 (976,166-1,066,459)	297,746 (293,601-301,745)	227,314 (224,831-229,943)	376,411 (371,402-382,187)	527,499 (521,063-535,315)	584,441 (575,063-594,578)	682,424 (672,044-693,252)	825,006 (812,538-838,302)	1,005,262 (992,686-1,018,203)
Communicable, maternal, neonatal, and nutritional diseases	1,200,883 (1,161,953-1,238,660)	307,447 (294,768-319,469)	896,770 (855,468-933,984)	775,643 (733,122-821,630)	154,534 (144,617-166,691)	87,669 (80,797-96,992)	87,848 (80,293-97,858)	101,511 (93,957-111,606)	151,162 (140,824-164,054)	206,555 (196,072-219,311)	236,228 (224,264-250,290)	240,974 (232,000-253,874)
HIV/AIDS and sexually transmitted infections	-	12,357 (4,697-23,157)	79,630 (63,601-100,924)	32,047 (29,190-35,231)	18,104 (17,124-19,187)	12,719 (12,094-13,343)	16,453 (14,777-18,276)	20,030 (17,770-22,893)	65,856 (57,913-74,502)	110,853 (102,304-119,914)	125,361 (115,338-135,578)	117,791 (112,484-123,416)
HIV/AIDS	-	-	55,933 (52,159-59,701)	29,936 (27,591-32,512)	17,274 (16,464-18,097)	12,688 (12,063-13,309)	16,417 (14,738-18,241)	19,988 (17,732-22,844)	65,798 (57,843-74,442)	110,789 (102,237-119,878)	125,279 (115,248-135,492)	117,716 (112,379-123,352)
HIV/AIDS - Drug-susceptible Tuberculosis	-	-	17,006 (12,596-20,293)	9,409 (7,268-11,007)	5,265 (3,724-6,373)	3,778 (2,723-4,598)	4,884 (3,596-6,014)	5,390 (4,082-6,658)	14,652 (10,163-19,431)	20,963 (13,770-28,807)	23,745 (15,517-32,901)	23,128 (15,168-31,563)
HIV/AIDS - Multidrug-resistant Tuberculosis without extensive drug resistance	-	-	1,772 (1,103-2,483)	948 (613-1,301)	507 (307-712)	361 (226-518)	472 (297-691)	620 (397-887)	1,756 (1,025-2,625)	2,516 (1,411-3,925)	2,760 (1,512-4,304)	2,637 (1,466-4,052)
HIV/AIDS - Extensively drug-resistant Tuberculosis	-	-	35 (22-49)	18 (12-26)	10 (6-14)	7 (4-10)	10 (7-15)	27 (17-39)	92 (55-139)	147 (88-226)	146 (88-221)	126 (75-189)
HIV/AIDS resulting in other diseases	-	-	37,119 (32,535-43,132)	19,561 (16,995-22,681)	11,492 (10,068-13,284)	8,541 (7,493-9,868)	11,051 (9,403-13,048)	13,951 (11,706-16,894)	49,298 (40,593-59,331)	87,163 (75,232-99,392)	98,627 (84,726-112,208)	91,825 (80,374-102,226)
Sexually transmitted infections excluding HIV	35,675 (13,961-67,165)	12,357 (4,697-23,157)	23,697 (8,971-45,335)	2,112 (773-4,106)	830 (310-1,593)	32 (27-50)	36 (32-41)	42 (32-53)	58 (40-78)	64 (44-97)	82 (49-124)	75 (47-126)
Syphilis	35,675 (13,961-67,165)	12,357 (4,697-23,157)	23,697 (8,971-45,335)	2,112 (773-4,106)	830 (310-1,593)	27 (23-42)	30 (27-34)	34 (26-43)	48 (33-66)	49 (34-75)	68 (41-102)	65 (41-108)
Chlamydial infection	-	-	-	-	-	0 (0-0)	0 (0-0)	0 (0-0)	0 (0-0)	0 (0-0)	0 (0-0)	0 (0-0)
Gonococcal infection	-	-	-	-	-	2 (2-4)	4 (4-5)	5 (4-6)	6 (4-8)	12 (8-18)	4 (2-5)	6 (4-10)
Other sexually transmitted infections	-	-	-	-	-	2 (2-3)	2 (2-3)	3 (2-4)	3 (2-5)	3 (2-4)	11 (6-16)	5 (3-8)
Respiratory infections and tuberculosis	-	67,193 (61,409-73,111)	291,309 (268,150-315,732)	157,406 (136,531-181,115)	22,797 (18,876-25,799)	13,697 (12,057-15,212)	20,001 (18,499-21,579)	34,351 (32,272-36,737)	45,333 (42,896-48,100)	56,821 (54,057-59,906)	69,472 (65,995-72,814)	80,317 (76,534-84,205)
Tuberculosis	-	-	20,055 (18,307-21,983)	19,340 (16,664-22,337)	4,507 (3,900-5,110)	4,397 (3,900-4,925)	11,418 (10,412-12,372)	24,431 (22,790-26,227)	33,946 (31,893-36,304)	43,244 (40,963-45,589)	52,390 (49,531-55,189)	59,592 (56,562-62,905)
Drug-susceptible tuberculosis	-	-	18,043 (16,254-19,904)	17,574 (15,039-20,318)	4,061 (3,479-4,644)	3,950 (3,463-4,435)	10,236 (9,288-11,192)	21,797 (20,164-23,683)	30,026 (27,759-32,395)	38,081 (35,351-40,846)	46,038 (42,645-49,361)	52,303 (48,537-56,002)
Multidrug-resistant tuberculosis without extensive drug resistance	-	-	1,904 (1,401-2,567)	1,704 (1,261-2,264)	423 (298-575)	423 (295-598)	1,118 (776-1,552)	2,433 (1,731-3,356)	3,568 (2,553-4,914)	4,679 (3,301-6,510)	5,751 (4,004-8,023)	6,593 (4,526-9,324)
Extensively drug-resistant tuberculosis	-	-	107 (82-143)	62 (48-82)	23 (17-31)	23 (17-32)	63 (47-87)	201 (161-256)	352 (277-442)	485 (381-609)	601 (471-761)	697 (550-873)
Lower respiratory infections	99,757 (88,656-112,239)	67,111 (61,333-73,043)	269,836 (248,084-292,809)	136,468 (117,845-157,478)	18,103 (14,545-20,655)	9,181 (7,661-10,382)	8,501 (7,194-9,455)	9,844 (8,412-10,966)	11,310 (9,818-12,331)	13,491 (12,046-15,018)	16,958 (14,950-18,695)	20,588 (18,371-22,487)
Upper respiratory infections	32 (21-43)	70 (40-100)	1,340 (586-2,172)	1,536 (420-3,035)	148 (65-220)	83 (37-118)	54 (27-87)	54 (32-80)	61 (39-87)	73 (47-104)	105 (63-149)	118 (76-170)
Otitis media	11 (3-47)	12 (5-29)	78 (29-200)	62 (19-167)	38 (18-86)	37 (18-78)	28 (15-60)	21 (9-49)	15 (7-32)	13 (6-27)	19 (9-40)	19 (9-41)
Enteric infections	14,164 (12,301-16,421)	24,747 (21,452-28,585)	225,099 (194,484-254,180)	220,218 (175,574-268,793)	49,348 (35,668-68,078)	27,601 (19,409-39,274)	21,770 (14,522-33,402)	17,600 (10,900-28,581)	14,423 (8,871-24,159)	13,375 (7,998-24,127)	14,427 (8,150-26,182)	15,037 (8,110-28,720)
Diarrheal diseases	14,164 (12,301-16,421)	24,440 (21,160-28,315)	219,876 (188,960-248,398)	191,562 (146,489-235,489)	19,847 (9,517-37,702)	9,082 (4,316-18,616)	9,525 (4,287-19,498)	9,728 (4,121-19,741)	9,029 (3,874-18,777)	9,387 (4,301-20,512)	11,225 (5,016-22,515)	12,517 (5,543-26,584)
Typhoid and paratyphoid	-	0 (0-0)	172 (87-311)	15,551 (7,295-28,446)	25,275 (14,582-39,869)	16,775 (9,550-26,786)	11,070 (5,366-19,336)	6,767 (3,388-11,759)	4,409 (2,291-7,422)	3,049 (1,581-5,120)	2,271 (1,171-3,821)	1,654 (845-2,822)
Typhoid fever	-	-	156 (79-280)	13,865 (6,519-25,573)	22,292 (12,806-35,717)	14,725 (8,205-23,729)	9,664 (4,701-17,088)	5,895 (2,911-10,413)	3,810 (1,924-6,586)	2,608 (1,307-4,442)	1,919 (978-3,337)	1,383 (687-2,393)
Paratyphoid fever	-	-	16 (6-34)	1,687 (655-3,563)	2,983 (1,274-5,790)	2,050 (888-4,034)	1,406 (545-2,924)	872 (353-1,886)	599 (242-1,252)	441 (182-909)	352 (152-714)	271 (118-553)
Invasive Non-typhoidal Salmonella (iNTS)	-	307 (172-540)	4,663 (2,606-8,316)	12,894 (6,674-23,334)	4,192 (2,056-7,462)	1,722 (765-3,274)	1,153 (465-2,185)	1,079 (439-2,071)	970 (380-1,998)	918 (384-1,748)	910 (402-1,753)	850 (405-1,616)
Other intestinal infectious diseases	-	-	388 (154-1,016)	211 (76-592)	34 (8-95)	22 (4-64)	23 (4-64)	26 (5-65)	15 (2-44)	21 (3-53)	21 (3-52)	16 (3-41)
Neglected tropical diseases and malaria	274 (115-1,024)	13,374 (8,536-20,016)	84,910 (58,451-120,183)	172,720 (122,519-239,211)	31,029 (22,688-41,766)	17,592 (12,832-24,138)	14,168 (10,228-19,459)	15,245 (11,027-20,723)	12,980 (9,192-17,889)	11,783 (8,403-16,376)	12,132 (8,725-16,508)	12,057 (8,596-16,404)
Malaria	-	13,145 (8,350-19,876)	81,565 (55,055-116,988)	162,036 (111,245-228,360)	24,874 (16,956-34,892)	13,423 (9,389-18,985)	10,548 (7,178-14,746)	11,577 (7,660-16,647)	10,072 (6,735-14,735)	9,091 (6,030-13,230)	9,302 (6,178-13,736)	9,244 (6,085-13,453)
Chagas disease	-	-	1 (1-2)	0 (0-1)	1 (1-1)	3 (2-3)	5 (4-6)	13 (11-14)	23 (21-26)	55 (50-60)	105 (96-113)	159 (148-171)
Leishmaniasis	-	-	463 (0-1,884)	2,331 (1-11,680)	1,292 (0-8,973)	910 (0-6,181)	791 (0-4,610)	641 (0-3,365)	635 (0-2,981)	634 (0-2,846)	519 (2-2,214)	463 (4-1,878)
Visceral leishmaniasis	-	-	463 (0-1,884)	2,331 (1-11,680)	1,292 (0-8,973)	910 (0-6,181)	791 (0-4,610)	641 (0-3,365)	635 (0-2,981)	634 (0-2,846)	519 (2-2,214)	463 (4-1,878)
African trypanosomiasis	-	-	-	115 (19-445)	202 (45-697)	227 (49-774)	281 (60-954)	320 (70-1,075)	268 (58-967)	247 (44-854)	207 (48-667)	184 (39-653)
Schistosomiasis	-	-	0 (0-0)	271 (183-399)	127 (88-175)	166 (123-224)	196 (149-248)	234 (179-294)	225 (175-286)	233 (183-300)	272 (213-339)	317 (254-403)
Cysticercosis	0 (0-0)	0 (0-0)	0 (0-0)	57 (21-107)	25 (5-57)	18 (2-48)	21 (2-62)	32 (3-83)	38 (6-91)	38 (6-92)	35 (5-87)	36 (6-92)
Cystic echinococcosis	-	-	-	121 (60-202)	56 (16-113)	38 (7-84)	20 (0-59)	24 (0-69)	34 (1-90)	36 (1-99)	44 (3-118)	43 (2-112)
Dengue	-	-	1,473 (228-2,093)	2,577 (946-3,491)	1,364 (446-1,846)	682 (176-922)	834 (213-1,141)	916 (233-1,226)	779 (225-1,028)	533 (117-719)	698 (131-961)	525 (125-712)
Yellow fever	-	2 (0-6)	40 (8-123)	387 (76-1,137)	632 (130-1,911)	536 (108-1,567)	484 (88-1,510)	423 (85-1,255)	302 (60-917)	239 (51-745)	200 (39-618)	165 (31-494)
Rabies	-	-	176 (128-248)	1,325 (901-2,232)	1,819 (1,282-2,889)	1,359 (981-2,038)	831 (617-1,374)	902 (704-1,336)	412 (330-562)	486 (384-646)	520 (410-696)	685 (565-852)
Intestinal nematode infections	-	-	152 (112-201)	1,630 (1,145-2,292)	268 (199-352)	60 (45-78)	19 (14-26)	16 (11-22)	12 (8-16)	15 (11-20)	15 (10-20)	11 (8-16)
Ascariasis	-	-	152 (112-201)	1,630 (1,145-2,292)	268 (199-352)	60 (45-78)	19 (14-26)	16 (11-22)	12 (8-16)	15 (11-20)	15 (10-20)	11 (8-16)
Ebola	0 (0-0)	0 (0-0)	0 (0-0)	0 (0-0)	0 (0-0)	0 (0-0)	0 (0-0)	0 (0-0)	0 (0-0)	0 (0-0)	0 (0-0)	0 (0-0)
Zika virus	0 (0-0)	0 (0-0)	0 (0-0)	0 (0-0)	0 (0-0)	0 (0-0)	0 (0-0)	0 (0-0)	0 (0-0)	0 (0-0)	0 (0-0)	0 (0-0)
Other neglected tropical diseases	274 (115-1,024)	228 (102-805)	1,040 (526-3,616)	1,869 (573-8,970)	369 (209-1,092)	169 (103-447)	138 (95-284)	148 (98-324)	180 (112-362)	173 (106-308)	217 (122-380)	225 (134-411)
Other infectious diseases	17,822 (14,633-21,080)	18,152 (14,933-21,234)	115,984 (93,081-150,581)	134,421 (98,429-187,062)	28,654 (22,180-38,194)	14,519 (12,058-17,468)	14,217 (11,753-16,107)	13,084 (10,950-14,410)	11,512 (9,559-12,700)	12,669 (10,861-13,898)	13,482 (11,450-14,867)	14,203 (11,790-15,529)
Meningitis	6,911 (4,964-8,354)	7,584 (6,096-8,943)	45,726 (39,032-52,634)	40,267 (32,128-48,750)	9,470 (6,928-11,905)	5,562 (4,260-7,270)	5,668 (4,341-7,619)	5,199 (4,125-6,864)	4,204 (3,362-5,620)	4,440 (3,636-6,100)	4,720 (3,850-6,474)	4,285 (3,523-6,095)
Pneumococcal meningitis	1,090 (775-1,433)	1,172 (890-1,514)	6,517 (4,903-8,271)	6,248 (4,441-8,263)	1,006 (689-1,398)	548 (384-803)	614 (432-890)	632 (455-884)	528 (389-743)	624 (469-868)	727 (540-994)	695 (515-991)
H influenzae type B meningitis	1,840 (1,144-2,484)	1,985 (1,490-2,628)	13,226 (10,406-16,598)	11,692 (8,607-15,489)	3,343 (2,342-4,407)	1,909 (1,455-2,635)	1,817 (1,401-2,524)	1,494 (1,147-2,042)	1,276 (1,009-1,724)	1,238 (1,016-1,779)	1,285 (1,035-1,906)	1,220 (990-1,856)
Meningococcal meningitis	1,049 (749-1,342)	1,204 (949-1,487)	6,671 (5,394-8,079)	5,611 (4,200-7,270)	1,072 (784-1,423)	542 (408-725)	498 (372-659)	429 (317-576)	345 (257-467)	373 (282-519)	388 (297-559)	342 (262-480)
Other meningitis	2,932 (2,088-3,628)	3,222 (2,568-3,926)	19,312 (15,965-23,018)	16,717 (12,996-21,113)	4,048 (2,887-5,026)	2,564 (1,920-3,288)	2,739 (2,062-3,626)	2,644 (1,995-3,432)	2,055 (1,580-2,652)	2,205 (1,710-3,028)	2,319 (1,754-3,104)	2,027 (1,590-2,762)
Encephalitis	339 (303-385)	540 (481-606)	6,641 (5,879-7,539)	6,429 (5,467-7,752)	3,106 (2,584-3,726)	2,436 (2,083-2,851)	1,901 (1,697-2,266)	1,621 (1,475-1,908)	1,384 (1,274-1,607)	1,621 (1,488-1,887)	1,701 (1,556-1,965)	1,575 (1,449-1,884)
Diphtheria	-	-	320 (186-530)	1,007 (528-1,829)	181 (107-295)	64 (40-101)	24 (16-36)	15 (10-23)	13 (8-18)	13 (9-20)	15 (10-22)	19 (13-26)
Whooping cough	-	-	23,547 (11,295-43,186)	21,227 (10,360-38,486)	2,020 (944-3,936)	405 (189-769)	183 (85-372)	122 (58-242)	65 (30-132)	54 (24-113)	38 (18-80)	54 (24-110)
Tetanus	7,625 (5,775-10,061)	7,209 (5,198-9,668)	2,807 (1,838-4,360)	1,513 (668-3,312)	962 (526-1,615)	557 (314-930)	945 (482-1,657)	852 (442-1,365)	920 (439-1,457)	855 (497-1,246)	1,002 (551-1,372)	1,294 (621-1,854)
Measles	-	-	24,309 (8,380-54,505)	52,594 (19,217-108,722)	8,209 (3,032-18,267)	2,010 (690-4,516)	759 (258-1,715)	333 (115-761)	136 (46-313)	92 (32-209)	58 (20-132)	48 (17-109)
Varicella and herpes zoster	68 (52-89)	186 (142-244)	1,619 (1,289-2,048)	1,855 (1,423-2,391)	730 (548-951)	233 (181-299)	127 (100-160)	134 (107-169)	165 (133-205)	212 (171-265)	186 (153-230)	123 (101-152)
Acute hepatitis	-	-	4,795 (2,347-5,982)	4,692 (2,342-6,253)	2,364 (1,262-3,107)	2,142 (1,183-2,630)	2,976 (1,622-3,593)	3,571 (2,176-4,163)	3,593 (2,136-4,205)	4,237 (2,451-4,944)	4,315 (2,602-4,951)	5,511 (3,347-6,399)
Acute hepatitis A	-	-	716 (265-1,278)	2,795 (1,409-4,011)	1,563 (832-2,111)	1,116 (614-1,499)	1,133 (627-1,567)	1,060 (583-1,536)	793 (409-1,215)	713 (315-1,244)	628 (259-1,112)	623 (274-1,221)
Acute hepatitis B	-	-	3,130 (1,409-4,329)	1,462 (653-2,518)	415 (174-772)	379 (165-658)	695 (335-1,183)	1,177 (617-1,902)	1,732 (968-2,571)	2,619 (1,409-3,625)	3,030 (1,732-3,942)	4,336 (2,531-5,405)
Acute hepatitis C	-	-	941 (414-1,666)	328 (140-619)	39 (14-87)	15 (6-33)	12 (4-26)	11 (3-24)	11 (3-26)	15 (4-39)	22 (5-60)	29 (8-75)
Acute hepatitis E	-	-	8 (3-17)	107 (42-216)	347 (151-583)	632 (296-992)	1,137 (561-1,593)	1,324 (639-1,942)	1,058 (502-1,610)	890 (335-1,597)	635 (231-1,197)	523 (182-1,093)
Other unspecified infectious diseases	2,879 (1,626-3,451)	2,633 (1,381-3,290)	6,221 (3,841-7,508)	4,837 (3,054-6,055)	1,611 (1,022-2,213)	1,109 (786-1,515)	1,634 (1,117-2,126)	1,236 (863-1,567)	1,033 (760-1,245)	1,144 (847-1,364)	1,446 (1,102-1,692)	1,295 (1,013-1,488)
Maternal and neonatal disorders	1,033,147 (987,452-1,077,357)	171,624 (161,349-183,041)	46,997 (39,603-52,860)	4,669 (3,622-5,355)	-	-	-	-	-	-	-	-
Maternal disorders	-	-	-	-	-	-	-	-	-	-	-	-
Maternal hemorrhage	-	-	-	-	-	-	-	-	-	-	-	-
Maternal sepsis and other maternal infections	-	-	-	-	-	-	-	-	-	-	-	-
Maternal hypertensive disorders	-	-	-	-	-	-	-	-	-	-	-	-
Maternal obstructed labor and uterine rupture	-	-	-	-	-	-	-	-	-	-	-	-
Maternal abortion and miscarriage	-	-	-	-	-	-	-	-	-	-	-	-
Ectopic pregnancy	-	-	-	-	-	-	-	-	-	-	-	-
Indirect maternal deaths	-	-	-	-	-	-	-	-	-	-	-	-
Late maternal deaths	-	-	-	-	-	-	-	-	-	-	-	-
Maternal deaths aggravated by HIV/AIDS	-	-	-	-	-	-	-	-	-	-	-	-
Other maternal disorders	-	-	-	-	-	-	-	-	-	-	-	-
Neonatal disorders	1,033,147 (987,452-1,077,357)	171,624 (161,349-183,041)	46,997 (39,603-52,860)	4,669 (3,622-5,355)	-	-	-	-	-	-	-	-
Neonatal preterm birth	402,830 (372,487-462,193)	49,220 (43,954-56,060)	16,927 (13,927-19,526)	1,216 (867-1,456)	-	-	-	-	-	-	-	-
Neonatal encephalopathy due to birth asphyxia and trauma	341,298 (300,284-370,853)	29,446 (22,995-34,276)	9,010 (6,780-10,754)	1,988 (1,471-2,316)	-	-	-	-	-	-	-	-
Neonatal sepsis and other neonatal infections	74,095 (61,903-95,009)	46,345 (39,421-57,436)	7,008 (5,766-9,023)	249 (155-440)	-	-	-	-	-	-	-	-
Hemolytic disease and other neonatal jaundice	26,026 (21,722-31,175)	11,686 (9,218-14,873)	2,245 (1,523-3,493)	135 (86-215)	-	-	-	-	-	-	-	-
Other neonatal disorders	188,898 (146,423-213,592)	34,927 (28,940-40,462)	11,806 (8,687-14,561)	1,081 (730-1,335)	-	-	-	-	-	-	-	-
Nutritional deficiencies	-	-	52,842 (47,193-59,095)	54,162 (45,141-63,246)	4,602 (3,952-5,300)	1,542 (1,364-1,728)	1,238 (1,124-1,363)	1,201 (1,106-1,326)	1,057 (983-1,190)	1,054 (982-1,166)	1,353 (1,275-1,468)	1,568 (1,476-1,695)
Protein-energy malnutrition	-	-	51,130 (45,657-57,266)	52,668 (43,838-61,683)	3,987 (3,394-4,606)	1,251 (1,095-1,423)	897 (800-1,011)	889 (801-1,016)	807 (731-947)	849 (779-968)	961 (886-1,079)	1,170 (1,078-1,295)
Other nutritional deficiencies	-	-	1,711 (1,397-2,048)	1,494 (1,075-1,906)	615 (466-791)	291 (240-355)	341 (290-409)	312 (273-380)	250 (220-303)	205 (176-262)	392 (347-472)	398 (350-483)
Non-communicable diseases	120,028 (109,220-137,727)	44,051 (40,558-49,038)	186,372 (166,516-210,154)	111,590 (99,716-125,743)	53,114 (47,861-57,705)	48,607 (44,771-52,005)	82,030 (77,150-86,042)	115,686 (111,458-119,850)	150,872 (146,136-156,035)	219,291 (213,647-225,760)	334,926 (327,307-344,334)	523,431 (512,863-535,645)
Neoplasms	843 (683-935)	530 (459-587)	5,159 (4,643-5,809)	21,495 (18,878-24,875)	19,741 (17,616-21,546)	17,313 (15,890-18,678)	21,521 (19,831-22,616)	23,918 (22,644-24,836)	27,921 (26,544-28,906)	41,314 (39,874-42,648)	69,487 (67,656-71,501)	123,018 (120,198-126,022)
Lip and oral cavity cancer	-	-	-	-	-	-	344 (310-388)	475 (439-519)	761 (696-837)	1,374 (1,281-1,495)	2,837 (2,632-3,071)	5,242 (4,902-5,667)
Nasopharynx cancer	-	-	-	-	132 (117-147)	209 (188-231)	273 (255-291)	321 (299-341)	409 (387-432)	719 (669-768)	1,499 (1,402-1,598)	2,840 (2,696-2,992)
Other pharynx cancer	-	-	-	-	-	-	141 (93-163)	198 (113-227)	253 (154-287)	625 (397-699)	1,367 (945-1,517)	2,977 (2,349-3,249)
Esophageal cancer	-	-	-	-	-	-	147 (134-161)	266 (249-283)	381 (362-402)	885 (847-924)	2,231 (2,144-2,334)	5,897 (5,700-6,131)
Stomach cancer	-	-	-	-	-	-	418 (395-445)	934 (889-978)	1,625 (1,565-1,700)	3,370 (3,252-3,498)	6,355 (6,149-6,585)	12,598 (12,228-13,024)
Colon and rectum cancer	-	-	-	-	-	-	523 (420-577)	1,073 (900-1,148)	1,752 (1,527-1,850)	3,238 (2,994-3,398)	5,161 (4,895-5,379)	8,584 (8,246-8,896)
Liver cancer	-	-	-	-	436 (379-500)	506 (449-598)	666 (609-804)	1,341 (1,190-1,604)	2,555 (2,361-2,998)	5,920 (5,467-6,469)	13,056 (12,197-14,062)	24,340 (23,196-25,854)
Liver cancer due to hepatitis B	-	-	-	-	313 (271-363)	402 (354-485)	515 (464-631)	1,033 (906-1,249)	1,860 (1,680-2,201)	4,144 (3,739-4,564)	8,785 (7,999-9,570)	15,912 (14,618-17,238)
Liver cancer due to hepatitis C	-	-	-	-	8 (6-10)	10 (8-13)	12 (9-15)	40 (32-51)	132 (101-172)	397 (326-478)	1,066 (853-1,301)	2,390 (2,005-2,827)
Liver cancer due to alcohol use	-	-	-	-	-	-	4 (2-6)	41 (32-55)	167 (124-228)	548 (433-690)	1,563 (1,111-2,071)	3,158 (2,381-4,041)
Liver cancer due to NASH	-	-	-	-	-	-	51 (41-65)	77 (61-98)	127 (96-162)	248 (195-314)	487 (385-603)	897 (722-1,114)
Liver cancer due to other causes	-	-	-	-	115 (96-136)	93 (76-112)	85 (68-107)	149 (122-184)	269 (213-343)	583 (456-717)	1,154 (912-1,430)	1,984 (1,582-2,484)
Gallbladder and biliary tract cancer	-	-	-	-	-	-	46 (38-52)	70 (57-83)	116 (93-138)	290 (229-353)	572 (456-678)	1,106 (873-1,290)
Pancreatic cancer	-	-	-	-	-	-	87 (82-92)	172 (163-181)	319 (308-331)	848 (811-887)	1,870 (1,797-1,947)	3,847 (3,735-3,971)
Larynx cancer	-	-	-	-	-	-	76 (71-83)	120 (112-128)	149 (141-157)	421 (394-449)	1,142 (1,031-1,240)	2,845 (2,662-3,044)
Tracheal, bronchus, and lung cancer	-	-	-	-	-	-	556 (522-596)	984 (922-1,055)	1,612 (1,533-1,704)	3,296 (3,167-3,429)	7,553 (7,286-7,848)	17,935 (17,439-18,487)
Malignant skin melanoma	-	-	-	-	-	-	129 (100-171)	232 (182-308)	377 (285-495)	644 (488-861)	982 (722-1,242)	1,468 (1,066-1,858)
Non-melanoma skin cancer	-	-	-	-	-	-	102 (96-108)	149 (141-158)	190 (182-199)	353 (335-374)	489 (467-520)	829 (798-860)
Non-melanoma skin cancer (squamous-cell carcinoma)	-	-	-	-	-	-	102 (96-108)	149 (141-158)	190 (182-199)	353 (335-374)	489 (467-520)	829 (798-860)
Breast cancer	-	-	-	-	-	-	34 (33-36)	35 (34-37)	39 (37-41)	72 (68-77)	192 (178-204)	311 (292-330)
Cervical cancer	-	-	-	-	-	-	-	-	-	-	-	-
Uterine cancer	-	-	-	-	-	-	-	-	-	-	-	-
Ovarian cancer	-	-	-	-	-	-	-	-	-	-	-	-
Prostate cancer	-	-	-	-	-	-	71 (61-86)	123 (102-144)	167 (143-203)	208 (172-243)	284 (233-327)	566 (477-659)
Testicular cancer	-	-	-	-	-	-	373 (346-409)	876 (817-948)	1,138 (1,071-1,223)	1,045 (990-1,117)	829 (778-886)	616 (581-658)
Kidney cancer	26 (20-30)	14 (11-16)	173 (148-196)	837 (685-1,013)	332 (297-363)	131 (117-140)	118 (106-124)	172 (153-181)	262 (233-275)	405 (363-426)	820 (736-857)	1,578 (1,428-1,635)
Bladder cancer	-	-	-	-	-	-	51 (46-58)	88 (78-102)	119 (105-134)	260 (230-291)	510 (460-571)	1,114 (1,015-1,209)
Brain and nervous system cancer	88 (64-101)	65 (49-76)	865 (669-1,053)	3,564 (2,749-4,557)	4,099 (3,476-4,969)	3,340 (2,708-4,012)	2,373 (1,938-2,736)	2,401 (1,921-2,697)	2,995 (2,494-3,427)	3,921 (3,232-4,480)	5,049 (4,169-5,862)	6,649 (5,669-7,784)
Thyroid cancer	-	-	-	-	-	80 (71-91)	110 (100-120)	169 (155-183)	303 (277-334)	237 (221-254)	323 (299-348)	484 (457-513)
Mesothelioma	-	-	-	-	-	-	45 (41-51)	64 (59-71)	94 (88-104)	128 (120-142)	228 (212-254)	408 (384-447)
Hodgkin lymphoma	9 (6-12)	2 (1-2)	50 (36-69)	499 (321-676)	956 (667-1,317)	906 (664-1,192)	1,017 (762-1,345)	1,087 (854-1,410)	1,244 (1,008-1,598)	1,213 (973-1,573)	1,233 (986-1,583)	1,187 (935-1,565)
Non-Hodgkin lymphoma	41 (36-47)	31 (27-35)	372 (323-442)	2,602 (2,151-3,172)	2,383 (2,045-2,754)	1,766 (1,569-1,978)	1,891 (1,750-2,036)	2,281 (2,140-2,450)	2,265 (2,149-2,401)	2,626 (2,506-2,769)	3,445 (3,263-3,692)	4,549 (4,354-4,795)

Cause	45 to 49	50 to 54	54 to 59	60 to 64	65 to 69	70 to 74	75 to 79	80 to 84	85 to 89	90 to 94	95 plus
All causes	1,244,887 (1,230,751-1,259,578)	1,490,465 (1,475,790-1,507,801)	1,830,932 (1,813,997-1,848,995)	2,202,225 (2,182,472-2,222,798)	2,367,986 (2,348,163-2,388,455)	2,862,204 (2,839,893-2,885,191)	3,002,663 (2,980,602-3,024,910)	2,780,256 (2,763,220-2,797,330)	1,733,612 (1,725,218-1,742,455)	726,100 (722,735-729,384)	240,932 (240,308-241,553)
Communicable, maternal, neonatal, and nutritional diseases	232,686 (222,597-247,754)	214,360 (204,245-232,830)	202,875 (191,225-223,405)	216,055 (198,303-244,539)	228,277 (206,637-267,186)	261,849 (231,761-312,806)	258,720 (226,864-314,589)	245,704 (213,110-299,871)	161,966 (142,718-196,389)	71,237 (63,639-84,098)	25,342 (23,481-28,588)
HIV/AIDS and sexually transmitted infections	90,318 (84,672-97,673)	54,588 (51,431-58,086)	28,253 (26,127-30,502)	16,207 (14,782-17,829)	10,036 (9,090-11,138)	6,037 (5,382-6,749)	4,012 (3,606-4,499)	709 (636-795)	237 (207-272)	97 (85-110)	22 (19-25)
HIV/AIDS	90,254 (84,612-97,609)	54,509 (51,354-58,013)	28,164 (26016-30,391)	16,128 (14,712-17,758)	9,945 (9,004-11,046)	5,954 (5,304-6,665)	3,918 (3,518-4,410)	653 (584-735)	195 (169-226)	78 (68-89)	13 (12-15)
HIV/AIDS - Drug-susceptible Tuberculosis	19,838 (13,264-26,594)	13,886 (9,907-17,328)	7,447 (5,812-8,937)	4,303 (3,408-5,110)	2,724 (2,171-3,240)	1,716 (1,364-2,058)	1,081 (850-1,292)	164 (128-199)	47 (37-58)	20 (16-24)	3 (2-3)
HIV/AIDS - Multidrug-resistant Tuberculosis without extensive drug resistance	2,235 (1,284-3,360)	1,564 (942-2,281)	847 (538-1,197)	485 (316-684)	298 (198-421)	187 (122-265)	121 (79-174)	19 (12-28)	6 (3-8)	2 (1-3)	0 (0-0)
HIV/AIDS - Extensively drug-resistant Tuberculosis	99 (60-148)	61 (37-88)	34 (21-48)	18 (12-26)	10 (6-14)	6 (4-8)	5 (3-7)	1 (1-1)	0 (0-0)	0 (0-0)	0 (0-0)
HIV/AIDS resulting in other diseases	68,081 (58,900-78,093)	38,999 (34,414-44,680)	19,837 (17,564-22,693)	11,322 (9,924-13,025)	6,913 (5,938-8,172)	4,045 (3,397-4,794)	2,712 (2,299-3,211)	469 (402-556)	142 (119-174)	56 (47-68)	10 (9-12)
Sexually transmitted infections excluding HIV	63 (40-99)	79 (48-137)	89 (57-153)	79 (49-141)	91 (56-160)	82 (53-139)	93 (66-142)	56 (40-84)	42 (27-59)	19 (12-26)	9 (6-11)
Syphilis	56 (35-87)	71 (43-122)	80 (52-139)	74 (46-133)	85 (53-150)	77 (49-129)	85 (60-130)	51 (36-76)	36 (24-52)	16 (10-21)	7 (5-9)
Chlamydial infection	0 (0-0)	0 (0-0)	0 (0-0)	0 (0-0)	0 (0-0)	0 (0-0)	0 (0-0)	0 (0-0)	0 (0-0)	0 (0-0)	0 (0-0)
Gonococcal infection	4 (3-6)	4 (2-6)	4 (2-7)	2 (1-4)	3 (2-5)	3 (2-5)	4 (3-5)	3 (2-4)	2 (1-3)	1 (1-1)	0 (0-1)
Other sexually transmitted infections	3 (2-5)	4 (3-8)	4 (3-8)	2 (1-4)	3 (2-5)	3 (2-5)	5 (3-7)	3 (2-4)	3 (2-5)	2 (1-3)	2 (1-2)
Respiratory infections and tuberculosis	96,236 (91,883-101,486)	111,147 (105,623-116,816)	120,883 (115,309-126,416)	135,399 (127,141-142,218)	143,859 (133,957-151,149)	163,329 (151,329-172,451)	164,282 (152,022-174,845)	157,474 (147,330-170,206)	107,836 (102,196-115,551)	49,373 (46,983-52,405)	18,658 (18,066-19,610)
Tuberculosis	68,509 (65,038-72,060)	74,409 (70,636-78,696)	75,908 (72,031-80,007)	75,568 (71,530-79,670)	74,120 (69,495-78,733)	72,337 (67,980-76,628)	57,491 (53,973-60,864)	42,060 (38,944-45,141)	19,722 (18,187-21,292)	5,908 (5,444-6,393)	1,169 (1,080-1,253)
Drug-susceptible tuberculosis	59,945 (55,320-64,243)	65,120 (60,411-69,825)	66,847 (62,168-71,530)	66,803 (61,647-71,418)	66,014 (61,156-71,049)	64,489 (59,762-69,291)	51,292 (47,311-54,910)	37,658 (34,528-40,737)	17,750 (16,179-19,244)	5,350 (4,874-5,815)	1,062 (978-1,143)
Multidrug-resistant tuberculosis without extensive drug resistance	7,670 (5,450-10,627)	8,313 (5,969-11,349)	8,185 (5,804-11,321)	8,054 (5,379-11,557)	7,541 (5,012-10,859)	7,275 (4,918-10,229)	5,752 (3,887-8,184)	4,078 (2,778-5,698)	1,827 (1,294-2,488)	518 (378-692)	98 (74-127)
Extensively drug-resistant tuberculosis	894 (709-1,107)	976 (772-1,211)	875 (698-1,090)	711 (548-920)	565 (426-762)	572 (436-758)	447 (332-604)	325 (246-433)	144 (109-191)	41 (31-54)	9 (7-11)
Lower respiratory infections	27,507 (24,186-29,969)	36,511 (31,994-39,230)	44,790 (39,143-48,208)	59,621 (51,625-63,852)	69,502 (59,715-74,275)	90,722 (79,065-98,177)	106,465 (94,928-116,944)	114,996 (105,711-126,268)	87,830 (82,076-95,764)	43,318 (41,130-46,239)	17,445 (16,894-18,372)
Upper respiratory infections	141 (89-200)	142 (92-201)	158 (110-212)	174 (119-239)	189 (133-279)	226 (165-348)	291 (210-469)	388 (288-644)	269 (200-435)	143 (109-240)	43 (35-67)
Otitis media	79 (31-188)	84 (36-180)	27 (17-54)	36 (22-76)	49 (28-101)	45 (27-94)	35 (22-72)	29 (20-60)	15 (11-28)	4 (3-8)	1 (1-2)
Enteric infections	17,145 (8,865-34,118)	19,562 (9,588-39,797)	23,828 (11,684-49,004)	33,280 (16,182-67,091)	44,456 (20,573-90,941)	59,982 (28,027-119,400)	64,395 (30,728-126,851)	67,000 (31,453-130,037)	41,736 (20,445-79,027)	16,670 (8,404-31,313)	4,671 (2,579-8,331)
Diarrheal diseases	15,063 (6,635-32,238)	17,826 (8,008-38,055)	22,269 (10,103-47,885)	31,882 (14,661-65,915)	43,258 (19,382-89,840)	59,073 (27,246-118,493)	63,768 (29,885-126,334)	66,596 (30,969-129,799)	41,553 (20,249-78,871)	16,618 (8,339-31,275)	4,658 (2,560-8,305)
Typhoid and paratyphoid	1,280 (681-2,146)	942 (487-1,621)	743 (357-1,377)	614 (301-1,117)	520 (294-852)	374 (224-602)	234 (139-361)	123 (73-188)	51 (32-77)	15 (10-23)	4 (3-6)
Typhoid fever	1,062 (566-1,803)	783 (404-1,358)	620 (297-1,160)	513 (251-944)	437 (243-716)	317 (185-510)	200 (119-313)	105 (62-162)	44 (28-66)	13 (9-20)	4 (3-5)
Paratyphoid fever	219 (91-444)	159 (62-332)	123 (47-267)	101 (41-211)	83 (37-165)	57 (27-108)	35 (17-64)	17 (8-33)	7 (3-13)	2 (1-4)	1 (0-1)
Invasive Non-typhoidal Salmonella (iNTS)	785 (377-1,493)	780 (354-1,504)	805 (383-1,413)	773 (357-1,393)	664 (309-1,152)	525 (247-932)	385 (179-708)	270 (113-509)	121 (48-225)	35 (10-76)	8 (0-23)
Other intestinal infectious diseases	16 (2-41)	13 (1-34)	11 (1-28)	12 (1-32)	14 (2-36)	10 (1-25)	7 (1-20)	11 (1-29)	10 (1-28)	2 (0-6)	1 (0-3)
Neglected tropical diseases and malaria	11,505 (8,398-15,529)	12,059 (8,670-16,546)	12,045 (8,799-16,708)	12,624 (9,163-17,662)	11,398 (8,182-15,953)	12,590 (8,887-17,970)	6,171 (4,555-8,625)	3,445 (2,735-4,730)	1,328 (1,091-1,838)	410 (329-587)	116 (94-193)
Malaria	8,506 (5,534-12,656)	9,313 (6,081-13,670)	9,053 (5,924-13,462)	9,748 (6,332-14,646)	8,775 (5,567-13,435)	10,051 (6,349-15,309)	4,123 (2,595-6,390)	1,879 (1,257-2,906)	517 (370-797)	130 (76-238)	30 (17-75)
Chagas disease	229 (212-247)	331 (310-354)	423 (388-462)	605 (564-656)	649 (605-699)	715 (658-782)	437 (400-477)	341 (318-364)	204 (192-218)	80 (73-87)	22 (20-25)
Leishmaniasis	366 (5-1,276)	321 (13-1,053)	295 (18-947)	273 (15-826)	238 (14-701)	177 (14-504)	106 (9-301)	65 (7-183)	26 (2-74)	6 (0-19)	1 (0-4)
Visceral leishmaniasis	366 (5-1,276)	321 (13-1,053)	295 (18-947)	273 (15-826)	238 (14-701)	177 (14-504)	106 (9-301)	65 (7-183)	26 (2-74)	6 (0-19)	1 (0-4)
African trypanosomiasis	173 (33-706)	130 (24-472)	118 (24-436)	63 (13-214)	48 (8-187)	29 (6-98)	19 (4-67)	9 (2-31)	3 (1-11)	1 (0-3)	0 (0-1)
Schistosomiasis	362 (284-452)	422 (331-535)	482 (383-598)	480 (382-594)	344 (271-426)	445 (358-550)	357 (286-441)	204 (161-257)	85 (65-108)	22 (16-30)	4 (3-7)
Cysticercosis	31 (4-84)	28 (3-75)	24 (2-68)	18 (1-54)	14 (0-42)	3 (0-11)	8 (0-23)	5 (0-14)	2 (0-7)	1 (0-2)	0 (0-1)
Cystic echinococcosis	45 (3-114)	49 (4-122)	51 (6-128)	51 (5-131)	48 (5-122)	47 (4-122)	36 (3-100)	26 (1-74)	15 (0-46)	5 (0-16)	1 (0-5)
Dengue	592 (123-786)	557 (136-752)	633 (116-868)	561 (142-782)	620 (150-880)	536 (207-784)	570 (224-983)	652 (235-994)	341 (140-562)	122 (57-181)	40 (23-67)
Yellow fever	140 (26-411)	116 (22-364)	93 (19-267)	67 (13-216)	48 (9-147)	29 (6-88)	16 (3-52)	6 (1-19)	10 (2-29)	1 (0-2)	0 (0-1)
Rabies	808 (642-1,169)	555 (439-734)	614 (509-776)	485 (406-635)	366 (301-456)	279 (231-344)	257 (209-322)	88 (71-112)	34 (27-43)	9 (7-11)	3 (2-4)
Intestinal nematode infections	13 (9-17)	12 (8-17)	11 (8-15)	23 (17-29)	11 (7-15)	17 (12-23)	14 (10-19)	9 (6-13)	4 (2-5)	2 (1-3)	1 (0-1)
Ascariasis	13 (9-17)	12 (8-17)	11 (8-15)	23 (17-29)	11 (7-15)	17 (12-23)	14 (10-19)	9 (6-13)	4 (2-5)	2 (1-3)	1 (0-1)
Ebola	0 (0-0)	0 (0-0)	0 (0-0)	0 (0-0)	0 (0-0)	0 (0-0)	0 (0-0)	0 (0-0)	0 (0-0)	0 (0-0)	0 (0-0)
Zika virus	0 (0-0)	0 (0-0)	0 (0-0)	0 (0-0)	0 (0-0)	0 (0-0)	0 (0-0)	0 (0-0)	0 (0-0)	0 (0-0)	0 (0-0)
Other neglected tropical diseases	239 (138-431)	225 (138-442)	248 (154-463)	251 (156-434)	235 (143-444)	260 (165-487)	228 (144-441)	162 (110-308)	87 (59-171)	33 (23-67)	13 (10-27)
Other infectious diseases	15,402 (12,811-16,828)	14,624 (12,406-16,429)	14,557 (12,457-15,946)	14,730 (12,740-16,354)	13,742 (12,134-15,863)	13,467 (11,915-15,304)	12,564 (11,084-13,922)	9,944 (8,623-10,866)	5,316 (4,693-5,868)	1,777 (1,548-1,931)	553 (474-607)
Meningitis	4,615 (3,904-6,430)	4,295 (3,743-6,024)	4,108 (3,525-5,888)	4,171 (3,584-6,189)	3,316 (2,866-5,171)	3,354 (2,916-5,190)	3,144 (2,704-5,041)	2,227 (1,902-3,299)	1,375 (1,167-2,009)	395 (335-545)	110 (93-145)
Pneumococcal meningitis	809 (589-1,176)	782 (588-1,081)	728 (555-1,023)	744 (556-1,094)	562 (422-842)	595 (450-900)	543 (406-843)	374 (281-548)	229 (177-320)	58 (44-79)	16 (12-23)
H influenzae type B meningitis	1,323 (1,081-1,954)	1,179 (945-1,785)	1,151 (933-1,806)	1,225 (979-1,980)	1,082 (857-1,853)	1,061 (839-1,786)	1,051 (837-1,785)	763 (609-1,228)	461 (373-701)	148 (120-210)	40 (32-56)
Meningococcal meningitis	360 (277-507)	360 (274-506)	353 (267-489)	358 (265-529)	274 (206-429)	279 (211-416)	262 (196-421)	185 (139-283)	108 (83-157)	29 (21-40)	8 (6-12)
Other meningitis	2,123 (1,717-2,907)	1,973 (1,624-2,786)	1,875 (1,534-2,647)	1,845 (1,466-2,635)	1,397 (1,119-2,136)	1,420 (1,147-2,179)	1,288 (1,044-2,000)	904 (722-1,308)	577 (457-815)	159 (126-218)	45 (36-58)
Encephalitis	1,504 (1,385-1,920)	1,487 (1,380-1,820)	1,634 (1,515-2,080)	1,945 (1,793-2,332)	2,170 (1,945-2,693)	2,506 (2,248-3,058)	2,669 (2,377-3,096)	2,485 (2,128-2,879)	1,069 (912-1,234)	289 (251-334)	75 (67-86)
Diphtheria	30 (22-43)	23 (17-33)	15 (10-21)	-	-	-	-	-	-	-	-
Whooping cough	42 (19-84)	32 (15-65)	43 (20-86)	-	-	-	-	-	-	-	-
Tetanus	1,645 (759-2,235)	1,404 (701-1,955)	1,514 (672-2,130)	1,104 (557-1,550)	1,076 (581-1,743)	855 (483-1,219)	626 (338-926)	398 (212-634)	181 (95-300)	58 (33-90)	17 (8-24)
Measles	37 (13-83)	27 (9-61)	29 (10-66)	-	-	-	-	-	-	-	-
Varicella and herpes zoster	118 (96-143)	110 (89-135)	123 (101-152)	147 (119-185)	259 (211-322)	259 (212-317)	377 (309-464)	555 (457-685)	508 (411-637)	262 (212-332)	125 (97-161)
Acute hepatitis	5,830 (3,678-6,784)	5,600 (3,555-6,425)	5,369 (3,418-6,068)	5,139 (3,242-5,855)	4,632 (2,950-5,299)	4,053 (2,640-4,689)	3,486 (2,200-3,971)	2,334 (1,445-2,718)	981 (654-1,124)	256 (176-294)	54 (39-62)
Acute hepatitis A	492 (217-954)	317 (154-587)	182 (94-312)	100 (49-174)	51 (27-90)	26 (11-47)	11 (3-23)	3 (1-7)	0 (0-1)	0 (0-0)	0 (0-0)
Acute hepatitis B	4,893 (3,139-6,015)	4,968 (3,098-5,864)	4,949 (3,085-5,685)	4,800 (3,011-5,535)	4,316 (2,766-5,011)	3,722 (2,469-4,347)	3,112 (1,900-3,675)	1,966 (1,220-2,409)	768 (484-951)	181 (107-234)	33 (19-47)
Acute hepatitis C	40 (11-108)	44 (12-110)	47 (13-126)	59 (15-145)	68 (17-184)	70 (20-172)	71 (20-188)	62 (16-182)	36 (11-90)	12 (3-29)	3 (1-9)
Acute hepatitis E	405 (148-936)	271 (97-618)	191 (71-376)	180 (65-364)	196 (73-437)	236 (87-484)	293 (107-605)	303 (115-635)	177 (73-345)	63 (25-112)	18 (8-31)
Other unspecified infectious diseases	1,581 (1,239-1,818)	1,645 (1,321-1,851)	1,722 (1,419-1,946)	2,224 (1,670-2,516)	2,289 (1,752-2,556)	2,440 (1,850-2,768)	2,262 (1,675-2,506)	1,946 (1,445-2,175)	1,201 (886-1,358)	517 (370-585)	171 (124-194)
Maternal and neonatal disorders	-	-	-	-	-	-	-	-	-	-	-
Maternal disorders	-	-	-	-	-	-	-	-	-	-	-
Maternal hemorrhage	-	-	-	-	-	-	-	-	-	-	-
Maternal sepsis and other maternal infections	-	-	-	-	-	-	-	-	-	-	-
Maternal hypertensive disorders	-	-	-	-	-	-	-	-	-	-	-
Maternal obstructed labor and uterine rupture	-	-	-	-	-	-	-	-	-	-	-
Maternal abortion and miscarriage	-	-	-	-	-	-	-	-	-	-	-
Ectopic pregnancy	-	-	-	-	-	-	-	-	-	-	-
Indirect maternal deaths	-	-	-	-	-	-	-	-	-	-	-
Late maternal deaths	-	-	-	-	-	-	-	-	-	-	-
Maternal deaths aggravated by HIV/AIDS	-	-	-	-	-	-	-	-	-	-	-
Other maternal disorders	-	-	-	-	-	-	-	-	-	-	-
Neonatal disorders	-	-	-	-	-	-	-	-	-	-	-
Neonatal preterm birth	-	-	-	-	-	-	-	-	-	-	-
Neonatal encephalopathy due to birth asphyxia and trauma	-	-	-	-	-	-	-	-	-	-	-
Neonatal sepsis and other neonatal infections	-	-	-	-	-	-	-	-	-	-	-
Hemolytic disease and other neonatal jaundice	-	-	-	-	-	-	-	-	-	-	-
Other neonatal disorders	-	-	-	-	-	-	-	-	-	-	-
Nutritional deficiencies	2,081 (1,965-2,239)	2,381 (2,239-2,599)	3,309 (3,103-3,551)	3,814 (3,603-4,081)	4,788 (4,496-5,102)	6,445 (6,030-6,903)	7,296 (6,860-7,731)	7,131 (6,752-7,545)	5,513 (5,248-5,825)	2,909 (2,778-3,066)	1,321 (1,271-1,379)
Protein-energy malnutrition	1,520 (1,398-1,697)	1,785 (1,630-2,015)	2,420 (2,239-2,687)	2,672 (2,478-2,945)	3,360 (3,130-3,658)	4,665 (4,290-5,100)	5,309 (4,933-5,734)	5,490 (5,090-5,919)	4,355 (4,007-4,683)	2,314 (2,012-2,487)	1,086 (935-1,164)
Other nutritional deficiencies	561 (483-677)	596 (520-740)	890 (777-1,052)	1,142 (987-1,359)	1,428 (1,242-1,660)	1,780 (1,556-2,116)	1,987 (1,716-2,344)	1,641 (1,402-2,097)	1,159 (971-1,577)	595 (484-869)	236 (177-399)
Non-communicable diseases	780,139 (765,703-793,408)	1,072,668 (1,055,476-1,086,452)	1,444,643 (1,422,180-1,462,204)	1,833,556 (1,802,345-1,856,119)	2,011,866 (1,972,195-2,039,254)	2,477,179 (2,428,257-2,512,264)	2,631,407 (2,577,882-2,665,748)	2,442,329 (2,385,930-2,477,045)	1,514,761 (1,480,583-1,534,409)	631,685 (618,686-639,540)	207,888 (204,723-209,851)
Neoplasms	205,864 (201,240-209,698)	303,352 (297,342-308,806)	451,426 (443,586-458,018)	565,928 (556,139-573,587)	589,862 (579,217-598,352)	665,918 (653,290-675,061)	631,858 (617,197-641,507)	496,217 (481,517-504,059)	249,835 (239,312-254,722)	79,913 (75,517-81,493)	21,183 (19,430-21,643)
Lip and oral cavity cancer	8,564 (8,112-9,088)	11,884 (11,368-12,488)	14,070 (13,557-14,602)	15,177 (14,551-15,856)	12,601 (12,068-13,228)	11,628 (11,156-12,090)	8,840 (8,477-9,152)	6,454 (6,164-6,691)	3,064 (2,935-3,181)	1,061 (1,007-1,104)	277 (266-289)
Nasopharynx cancer	4,228 (4,034-4,429)	4,977 (4,751-5,228)	6,218 (5,988-6,468)	5,928 (5,727-6,133)	4,925 (4,741-5,108)	4,495 (4,326-4,653)	3,199 (3,088-3,312)	2,173 (2,097-2,256)	947 (914-982)	296 (286-305)	74 (71-77)
Other pharynx cancer	5,518 (4,485-5,934)	8,535 (7,263-9,178)	10,073 (8,698-10,822)	10,968 (9,359-11,731)	9,189 (7,685-9,879)	7,647 (6,243-8,246)	5,124 (4,122-5,487)	3,437 (2,736-3,718)	1,276 (1,001-1,370)	333 (271-355)	62 (55-66)
Esophageal cancer	12,570 (12,197-12,985)	20,807 (20,242-21,413)	33,027 (32,139-33,970)	41,058 (40,034-42,196)	40,628 (39,602-41,789)	41,524 (40,427-42,667)	36,595 (35,651-37,568)	25,391 (24,734-26,032)	10,834 (10,575-11,094)	2,905 (2,838-2,979)	585 (568-602)
Stomach cancer	22,013 (21,387-22,636)	31,029 (30,266-31,916)	49,455 (48,347-50,683)	65,404 (63,905-66,937)	68,685 (67,282-70,241)	79,781 (78,023-81,388)	75,853 (74,386-77,340)	56,329 (55,271-57,399)	26,062 (25,585-26,536)	7,897 (7,747-8,042)	2,000 (1,961-2,039)
Colon and rectum cancer	14,844 (14,404-15,290)	22,094 (21,619-22,659)	34,110 (33,385-34,873)	45,998 (44,997-46,930)	50,364 (49,434-51,447)	61,003 (59,735-62,235)	61,489 (60,325-62,624)	52,538 (51,300-53,567)	28,446 (27,805-29,042)	9,234 (9,050-9,426)	2,758 (2,699-2,830)
Liver cancer	36,100 (34,221-38,103)	44,324 (42,149-47,845)	60,319 (57,855-63,693)	63,893 (61,410-67,285)	57,732 (55,631-61,391)	57,234 (55,315-60,410)	49,831 (48,322-51,691)	32,634 (31,545-33,720)	13,443 (13,018-13,914)	3,493 (3,379-3,608)	738 (716-761)
Liver cancer due to hepatitis B	22,509 (20,561-24,288)	24,666 (22,187-27,232)	30,722 (27,802-33,966)	29,087 (26,169-32,334)	23,928 (21,770-26,526)	21,260 (19,086-23,700)	16,005 (14,485-17,423)	8,617 (7,670-9,581)	2,973 (2,686-3,259)	664 (588-745)	107 (86-128)
Liver cancer due to hepatitis C	4,483 (3,646-5,434)	7,600 (6,395-8,939)	13,285 (11,437-15,244)	16,373 (14,194-18,665)	14,996 (13,421-16,760)	15,889 (14,316-17,662)	15,907 (14,359-17,531)	12,163 (11,058-13,219)	5,718 (5,250-6,122)	1,657 (1,524-1,781)	395 (359-429)
Liver cancer due to alcohol use	4,959 (3,675-6,837)	6,860 (5,148-9,355)	8,916 (6,654-11,653)	10,528 (8,274-13,389)	11,873 (10,082-13,906)	12,867 (11,123-15,115)	10,693 (9,440-12,187)	6,729 (5,834-7,643)	2,641 (2,318-2,972)	618 (530-711)	123 (98-148)
Liver cancer due to NASH	1,451 (1,139-1,814)	2,131 (1,671-2,639)	3,353 (2,616-4,193)	4,002 (3,230-4,888)	3,875 (3,099-4,799)	4,340 (3,492-5,402)	4,554 (3,731-5,505)	3,374 (2,749-4,094)	1,432 (1,204-1,671)	384 (316-462)	81 (62-102)
Liver cancer due to other causes	2,698 (2,170-3,303)	3,066 (2,453-3,834)	4,043 (3,194-4,879)	3,902 (3,116-4,740)	3,060 (2,472-3,720)	2,878 (2,303-3,522)	2,671 (2,190-3,193)	1,751 (1,420-2,115)	680 (575-801)	170 (140-204)	33 (25-41)
Gallbladder and biliary tract cancer	1,944 (1,549-2,193)	3,299 (2,658-3,699)	4,826 (3,934-5,439)	6,829 (5,610-7,526)	7,910 (6,611-8,775)	9,549 (8,099-10,429)	9,969 (8,516-10,792)	8,292 (7,056-8,859)	4,538 (3,790-4,827)	1,551 (1,299-1,652)	402 (326-420)
Pancreatic cancer	7,274 (7,115-7,444)	11,952 (11,738-12,179)	18,528 (18,211-18,867)	24,144 (23,754-24,613)	25,807 (25,342-26,311)	28,470 (27,991-28,967)	25,806 (25,378-26,255)	19,483 (19,111-19,847)	9,688 (9,527-9,870)	2,913 (2,861-2,965)	713 (695-731)
Larynx cancer	5,759 (5,426-6,098)	9,742 (9,369-10,188)	12,777 (12,315-13,219)	14,661 (14,093-15,211)	13,462 (12,883-14,039)	12,458 (11,991-12,898)	9,253 (8,955-9,563)	6,556 (6,317-6,787)	2,665 (2,583-2,743)	732 (711-753)	163 (158-168)
Tracheal, bronchus, and lung cancer	39,438 (38,486-40,379)	69,031 (67,527-70,721)	114,977 (112,997-117,055)	151,631 (149,207-153,952)	160,568 (157,904-163,449)	181,525 (178,550-184,462)	163,851 (161,220-166,446)	114,984 (112,834-117,077)	49,522 (48,697-50,369)	12,993 (12,798-13,210)	2,777 (2,726-2,830)
Malignant skin melanoma	1,854 (1,284-2,445)	2,457 (1,690-3,225)	3,073 (2,032-3,890)	3,040 (1,836-3,800)	2,993 (1,712-3,448)	3,230 (1,802-3,671)	3,089 (1,694-3,562)	2,723 (1,468-3,118)	1,594 (870-1,803)	565 (307-647)	161 (87-184)
Non-melanoma skin cancer	1,145 (1,109-1,184)	1,764 (1,671-1,840)	2,380 (2,291-2,459)	3,930 (3,773-4,061)	4,228 (4,053-4,376)	4,980 (4,747-5,147)	4,886 (4,676-5,049)	4,655 (4,467-4,810)	3,076 (2,979-3,157)	1,471 (1,416-1,513)	595 (579-610)
Non-melanoma skin cancer (squamous-cell carcinoma)	1,145 (1,109-1,184)	1,764 (1,671-1,840)	2,380 (2,291-2,459)	3,930 (3,773-4,061)	4,228 (4,053-4,376)	4,980 (4,747-5,147)	4,886 (4,676-5,049)	4,655 (4,467-4,810)	3,076 (2,979-3,157)	1,471 (1,416-1,513)	595 (579-610)
Breast cancer	601 (567-635)	753 (720-785)	1,022 (986-1,060)	1,270 (1,226-1,313)	1,185 (1,139-1,227)	1,326 (1,278-1,373)	830 (802-856)	722 (695-748)	445 (430-462)	146 (140-152)	48 (45-50)
Cervical cancer	-	-	-	-	-	-	-	-	-	-	-
Uterine cancer	-	-	-	-	-	-	-	-	-	-	-
Ovarian cancer	-	-	-	-	-	-	-	-	-	-	-
Prostate cancer	1,645 (1,370-1,931)	4,414 (3,608-5,144)	10,863 (8,829-12,372)	22,173 (18,016-25,613)	34,868 (28,614-40,410)	53,709 (45,575-62,655)	68,479 (57,940-83,313)	72,013 (59,261-84,351)	45,384 (36,421-53,086)	18,231 (14,223-20,662)	5,431 (3,785-5,855)
Testicular cancer	476 (453-501)	355 (342-370)	289 (279-301)	285 (274-297)	243 (233-253)	277 (267-287)	266 (256-277)	265 (254-275)	146 (140-152)	50 (48-53)	20 (19-21)
Kidney cancer	3,292 (3,011-3,399)	5,503 (5,121-5,661)	8,041 (7,460-8,241)	9,281 (8,847-9,502)	9,147 (8,645-9,368)	10,249 (9,772-10,483)	9,975 (9,323-10,211)	8,220 (7,788-8,429)	4,380 (4,233-4,493)	1,379 (1,338-1,419)	352 (340-365)
Bladder cancer	2,210 (2,051-2,370)	4,108 (3,849-4,295)	6,998 (6,683-7,446)	11,028 (10,635-11,846)	13,743 (13,294-14,650)	18,624 (18,119-20,029)	21,046 (20,428-22,932)	21,194 (20,590-23,012)	13,214 (12,936-14,003)	4,870 (4,754-5,108)	1,478 (1,441-1,526)
Brain and nervous system cancer	8,101 (6,935-9,323)	9,484 (8,293-10,892)	11,756 (10,145-13,471)	12,265 (10,530-13,769)	10,911 (9,154-12,195)	10,111 (8,433-11,011)	8,179 (6,471-8,801)	5,059 (3,833-5,502)	2,017 (1,443-2,214)	567 (419-619)	99 (69-109)
Thyroid cancer	789 (751-831)	1,139 (1,096-1,186)	1,543 (1,487-1,606)	1,497 (1,442-1,556)	1,510 (1,449-1,578)	1,622 (1,565-1,679)	2,101 (2,030-2,172)	1,547 (1,495-1,605)	710 (685-737)	210 (203-217)	51 (49-53)
Mesothelioma	748 (710-813)	1,145 (1,094-1,222)	1,789 (1,724-1,897)	1,986 (1,917-2,095)	2,433 (2,352-2,552)	2,843 (2,753-2,952)	2,920 (2,841-3,007)	2,285 (2,221-2,347)	1,207 (1,174-1,240)	329 (319-340)	74 (72-77)
Hodgkin lymphoma	1,280 (1,040-1,619)	1,405 (1,136-1,813)	1,658 (1,335-2,187)	1,668 (1,335-2,154)	1,693 (1,341-2,134)	1,731 (1,392-2,213)	1,015 (773-1,266)	718 (550-845)	329 (248-386)	60 (45-71)	11 (9-15)
Non-Hodgkin lymphoma	6,213 (5,986-6,502)	8,041 (7,749-8,317)	10,518 (10,245-10,842)	12,311 (11,928-12,728)	13,007 (12,573-13,395)	14,232 (13,827-14,554)	13,328 (12,999-13,619)	10,960 (10,710-11,182)	5,990 (5,874-6,107)	1,822 (1,787-1,856)	425 (414-439)

SR Table 6: Global deaths by cause and age group for males (with 95% confidence intervals), 2010

Cause	Early Neonatal	Late Neonatal	Post Neonatal	1 to 4	5 to 9	10 to 14	15 to 19	20 to 24	25 to 29	30 to 34	35 to 39	40 to 44
Multiple myeloma	-	-	-	-	-	-	98 (78-111)	125 (104-155)	141 (116-164)	299 (255-352)	533 (454-630)	1,054 (908-1,292)
Leukemia	175 (132-197)	148 (117-166)	1,994 (1,623-2,366)	7,647 (6,398-9,244)	7,946 (6,745-9,069)	6,419 (5,473-7,347)	6,559 (5,571-7,282)	5,654 (4,700-6,139)	4,928 (4,091-5,325)	4,890 (4,125-5,226)	5,591 (4,731-6,101)	6,537 (5,627-7,141)
Acute lymphoid leukemia	38 (28-50)	32 (24-42)	554 (439-727)	2,672 (2,086-3,541)	3,698 (3,106-4,453)	2,682 (2,267-3,174)	2,301 (1,952-2,642)	1,704 (1,449-1,919)	1,263 (1,062-1,401)	1,095 (928-1,208)	1,182 (995-1,341)	1,175 (991-1,335)
Chronic lymphoid leukemia	-	-	-	-	-	-	90 (71-104)	96 (78-119)	82 (66-95)	94 (76-111)	127 (107-153)	216 (186-250)
Acute myeloid leukemia	44 (31-56)	39 (29-49)	401 (310-522)	1,818 (1,318-2,601)	1,181 (927-1,501)	1,146 (928-1,362)	1,346 (1,109-1,539)	1,278 (1,046-1,436)	1,263 (1,044-1,406)	1,366 (1,150-1,536)	1,534 (1,305-1,731)	1,824 (1,529-2,047)
Chronic myeloid leukemia	-	-	-	-	-	-	174 (141-203)	264 (220-305)	429 (365-502)	477 (403-533)	549 (456-613)	585 (503-660)
Other leukemia	93 (68-107)	76 (58-89)	1,039 (811-1,273)	3,158 (2,302-4,090)	3,067 (2,376-3,650)	2,591 (2,102-3,065)	2,648 (2,149-3,051)	2,311 (1,779-2,588)	1,892 (1,428-2,079)	1,859 (1,432-2,049)	2,200 (1,717-2,461)	2,737 (2,193-3,095)
Other malignant neoplasms	469 (359-545)	249 (201-285)	1,436 (1,214-1,732)	5,757 (4,605-7,375)	2,951 (2,557-3,361)	3,537 (3,075-3,938)	4,844 (4,215-5,278)	4,056 (3,638-4,334)	3,224 (2,874-3,413)	3,351 (3,036-3,564)	4,476 (4,015-4,714)	6,285 (5,590-6,606)
Other neoplasms	36 (25-48)	22 (16-29)	268 (202-356)	588 (400-823)	507 (359-657)	418 (313-558)	430 (323-550)	451 (337-595)	501 (363-653)	676 (499-893)	859 (634-1,137)	1,171 (881-1,567)
Myelodysplastic, myeloproliferative, and other hematopoietic neoplasms	35 (24-47)	19 (14-25)	229 (173-304)	469 (312-682)	350 (241-475)	278 (199-378)	285 (209-371)	315 (228-425)	376 (275-501)	537 (395-728)	735 (542-986)	1,059 (802-1,422)
Other benign and in situ neoplasms	1 (0-1)	3 (2-4)	40 (26-54)	120 (73-160)	157 (103-205)	141 (96-184)	145 (102-188)	136 (94-179)	125 (81-168)	139 (88-187)	124 (79-172)	111 (68-163)
Cardiovascular diseases	2,063 (1,885-2,254)	1,241 (1,154-1,336)	10,294 (9,665-10,973)	7,700 (6,984-8,364)	4,264 (3,928-4,591)	5,350 (4,986-5,697)	17,055 (16,219-18,007)	31,049 (29,821-32,531)	45,968 (44,424-47,803)	77,241 (75,026-80,294)	127,436 (124,018-131,254)	212,425 (207,749-218,187)
Rheumatic heart disease	-	-	-	1,902 (1,615-2,313)	1,113 (996-1,264)	1,820 (1,640-2,026)	3,090 (2,760-3,544)	3,621 (3,251-4,222)	3,389 (3,019-3,984)	3,797 (3,378-4,579)	4,722 (4,195-5,597)	5,802 (5,138-6,934)
Ischemic heart disease	-	-	-	-	-	-	5,543 (5,202-5,917)	13,094 (12,504-13,866)	22,015 (21,159-23,291)	41,115 (39,569-43,514)	69,905 (67,538-72,960)	117,226 (114,028-121,846)
Stroke	714 (597-832)	396 (350-449)	3,019 (2,613-3,584)	2,212 (1,861-2,628)	1,531 (1,404-1,664)	1,850 (1,713-1,979)	4,606 (4,346-4,876)	8,261 (7,878-8,687)	10,903 (10,407-11,511)	18,394 (17,658-19,345)	32,876 (31,776-34,394)	60,298 (58,478-62,664)
Ischemic stroke	81 (58-117)	56 (44-76)	259 (201-372)	278 (183-477)	147 (127-172)	159 (136-189)	533 (462-627)	980 (867-1,138)	1,378 (1,228-1,600)	2,342 (2,085-2,676)	4,159 (3,739-4,730)	7,968 (7,290-8,934)
Intracerebral hemorrhage	375 (265-495)	190 (148-245)	1,601 (1,241-2,270)	808 (565-1,262)	807 (681-934)	956 (823-1,072)	2,871 (2,608-3,110)	5,188 (4,805-5,571)	6,801 (6,316-7,343)	12,234 (11,432-13,056)	22,571 (21,400-23,787)	42,307 (40,044-44,439)
Subarachnoid hemorrhage	257 (175-325)	150 (105-189)	1,159 (843-1,445)	1,125 (778-1,428)	577 (445-701)	735 (592-855)	1,202 (974-1,470)	2,093 (1,650-2,497)	2,724 (2,213-3,261)	3,818 (3,140-4,663)	6,146 (5,118-7,375)	10,024 (8,480-12,168)
Hypertensive heart disease	-	-	-	-	-	-	322 (228-364)	594 (424-682)	971 (648-1,107)	1,752 (1,230-2,011)	3,258 (2,304-3,741)	6,164 (4,412-6,969)
Non-rheumatic valvular heart disease	-	-	-	-	-	-	365 (312-423)	452 (389-524)	515 (445-598)	542 (477-626)	802 (696-933)	1,148 (1,004-1,330)
Non-rheumatic calcific aortic valve disease	-	-	-	-	-	-	125 (96-150)	159 (124-196)	208 (167-248)	239 (197-283)	391 (322-458)	594 (490-706)
Non-rheumatic degenerative mitral valve disease	-	-	-	-	-	-	162 (128-198)	206 (166-251)	217 (173-260)	242 (195-298)	325 (257-396)	437 (345-532)
Other non-rheumatic valve diseases	-	-	-	-	-	-	78 (49-112)	87 (52-124)	91 (55-132)	61 (37-95)	86 (50-124)	116 (64-181)
Cardiomyopathy and myocarditis	762 (667-846)	432 (387-470)	3,453 (3,055-3,828)	1,662 (1,427-1,936)	773 (704-844)	831 (773-891)	1,636 (1,463-1,809)	2,560 (2,276-2,815)	4,512 (3,853-4,925)	7,007 (5,528-7,595)	9,633 (7,589-10,361)	12,742 (9,851-13,865)
Myocarditis	83 (51-113)	94 (68-123)	942 (693-1,191)	496 (384-636)	253 (210-308)	253 (204-295)	503 (390-593)	592 (471-702)	738 (566-909)	627 (470-786)	815 (633-994)	1,107 (795-1,419)
Alcoholic cardiomyopathy	-	-	-	-	-	-	119 (99-145)	525 (429-583)	1,720 (1,338-1,937)	3,691 (2,635-4,121)	5,583 (3,941-6,070)	7,411 (5,137-7,957)
Other cardiomyopathy	679 (582-760)	337 (300-379)	2,511 (2,206-2,923)	1,165 (969-1,423)	520 (453-592)	578 (523-640)	1,015 (867-1,130)	1,444 (1,202-1,623)	2,055 (1,626-2,333)	2,689 (2,085-3,085)	3,235 (2,597-3,675)	4,224 (3,427-4,869)
Atrial fibrillation and flutter	-	-	-	-	-	-	-	-	-	38 (32-40)	61 (51-67)	279 (244-309)
Aortic aneurysm	-	-	-	-	-	-	174 (157-202)	285 (257-325)	486 (442-557)	714 (656-818)	1,230 (1,126-1,411)	1,880 (1,726-2,105)
Peripheral artery disease	-	-	-	-	-	-	-	-	-	-	-	138 (88-230)
Endocarditis	138 (111-184)	95 (74-126)	1,227 (995-1,562)	640 (398-875)	181 (160-205)	249 (222-282)	356 (326-405)	590 (542-662)	872 (795-991)	998 (914-1,133)	1,196 (1,093-1,346)	1,405 (1,290-1,604)
Other cardiovascular and circulatory diseases	449 (385-536)	318 (270-378)	2,596 (2,089-3,088)	1,284 (877-1,726)	666 (574-800)	600 (531-685)	965 (825-1,160)	1,592 (1,373-1,909)	2,305 (1,996-2,756)	2,884 (2,518-3,459)	3,753 (3,332-4,449)	5,343 (4,683-6,467)
Chronic respiratory diseases	0 (0-0)	0 (0-0)	0 (0-0)	6,702 (5,979-7,375)	2,119 (1,898-2,350)	2,311 (2,089-2,541)	4,142 (3,880-4,446)	6,076 (5,736-6,451)	7,319 (6,947-7,719)	9,353 (8,927-9,835)	14,350 (13,718-15,035)	22,515 (21,565-23,473)
Chronic obstructive pulmonary disease	-	-	0 (0-0)	604 (440-1,073)	257 (205-405)	217 (177-337)	1,325 (1,093-1,743)	2,001 (1,688-2,639)	2,574 (2,161-3,464)	3,827 (3,292-5,077)	6,229 (5,233-8,387)	11,397 (9,710-14,357)
Pneumoconiosis	-	-	-	-	-	-	16 (13-19)	28 (24-33)	56 (49-63)	99 (91-109)	272 (248-299)	488 (448-533)
Silicosis	-	-	-	-	-	-	4 (3-5)	9 (7-12)	22 (17-27)	56 (47-65)	177 (152-202)	332 (290-374)
Asbestosis	-	-	-	-	-	-	2 (1-3)	3 (2-5)	6 (3-9)	8 (4-12)	13 (6-20)	23 (12-35)
Coal workers pneumoconiosis	-	-	-	-	-	-	2 (1-3)	3 (2-6)	7 (5-9)	9 (7-12)	27 (19-37)	60 (44-78)
Other pneumoconiosis	-	-	-	-	-	-	7 (6-10)	12 (10-16)	21 (16-28)	26 (22-35)	55 (45-75)	73 (60-99)
Asthma	-	-	-	4,952 (4,252-5,764)	1,380 (1,168-1,595)	1,688 (1,471-1,900)	2,069 (1,512-2,491)	2,951 (2,024-3,564)	3,609 (2,418-4,315)	3,975 (2,432-4,836)	5,956 (3,527-7,367)	7,883 (4,349-10,119)
Interstitial lung disease and pulmonary sarcoidosis	-	-	-	179 (97-364)	65 (43-113)	58 (44-86)	103 (74-155)	170 (127-254)	249 (186-368)	348 (262-526)	576 (429-935)	913 (684-1,472)
Other chronic respiratory diseases	0 (0-0)	0 (0-0)	0 (0-0)	968 (606-1,382)	418 (325-521)	348 (279-441)	630 (490-803)	926 (718-1,208)	830 (631-1,086)	1,104 (852-1,444)	1,318 (994-1,719)	1,835 (1,409-2,428)
Digestive diseases	1,336 (1,155-1,559)	1,321 (1,155-1,501)	10,723 (9,636-12,393)	11,219 (9,551-13,767)	6,690 (5,650-7,906)	5,603 (4,905-6,657)	10,045 (8,889-11,562)	15,919 (14,597-18,290)	26,781 (25,045-30,535)	40,170 (37,819-44,904)	61,390 (58,108-69,268)	84,974 (80,220-95,403)
Cirrhosis and other chronic liver diseases	-	-	-	4,409 (3,624-5,560)	2,483 (2,033-3,081)	2,619 (2,238-3,192)	4,690 (4,029-5,392)	9,015 (8,121-10,359)	16,961 (15,595-19,490)	28,425 (26,593-31,902)	45,323 (42,671-51,057)	64,698 (60,888-72,332)
Cirrhosis and other chronic liver diseases due to hepatitis B	-	-	-	186 (134-263)	263 (198-350)	447 (340-584)	1,081 (817-1,414)	3,896 (3,251-4,692)	8,413 (7,018-10,022)	12,818 (11,308-14,882)	17,918 (15,220-21,279)	23,394 (20,137-27,684)
Cirrhosis and other chronic liver diseases due to hepatitis C	-	-	-	57 (39-83)	80 (58-111)	149 (108-203)	366 (264-487)	1,531 (1,186-1,935)	3,357 (2,573-4,215)	6,351 (5,297-7,526)	11,029 (8,711-13,626)	16,571 (13,567-19,714)
Cirrhosis and other chronic liver diseases due to alcohol use	-	-	-	-	-	-	36 (17-64)	789 (606-1,018)	2,358 (1,846-2,977)	5,915 (5,155-6,909)	11,928 (10,044-14,135)	18,339 (15,734-21,121)
Cirrhosis due to NASH	-	-	-	-	-	-	84 (64-111)	263 (211-331)	600 (470-759)	1,249 (1,068-1,458)	2,309 (1,831-2,822)	3,879 (3,259-4,562)
Cirrhosis and other chronic liver diseases due to other causes	-	-	-	4,165 (3,422-5,220)	2,140 (1,746-2,629)	2,023 (1,702-2,445)	3,124 (2,582-3,669)	2,537 (1,966-3,180)	2,233 (1,646-2,922)	2,093 (1,546-2,763)	2,139 (1,596-2,774)	2,515 (1,821-3,330)
Upper digestive system diseases	-	-	-	1,039 (752-1,642)	564 (465-678)	516 (439-647)	1,249 (1,086-1,521)	2,161 (1,919-2,603)	2,962 (2,688-3,610)	3,768 (3,437-4,471)	5,156 (4,718-6,158)	6,786 (6,268-8,165)
Peptic ulcer disease	-	-	-	625 (405-1,326)	438 (353-531)	417 (349-517)	958 (821-1,168)	1,717 (1,514-2,040)	2,280 (2,036-2,742)	2,819 (2,547-3,350)	4,063 (3,665-4,853)	5,569 (5,099-6,662)
Gastritis and duodenitis	-	-	-	413 (238-594)	126 (94-166)	99 (76-130)	292 (219-364)	444 (304-571)	682 (484-869)	949 (696-1,184)	1,093 (817-1,355)	1,218 (958-1,525)
Appendicitis	-	-	-	1,059 (637-1,448)	957 (769-1,199)	871 (732-1,093)	1,128 (934-1,454)	944 (797-1,216)	1,089 (936-1,412)	725 (634-864)	1,018 (874-1,310)	1,235 (1,070-1,576)
Paralytic ileus and intestinal obstruction	1,336 (1,155-1,559)	1,321 (1,155-1,501)	10,723 (9,636-12,393)	1,958 (1,312-2,506)	1,766 (1,208-2,190)	877 (638-1,072)	1,784 (1,286-2,207)	1,781 (1,198-2,187)	2,053 (1,506-2,582)	2,340 (1,651-2,909)	3,122 (2,236-3,886)	3,741 (2,725-4,809)
Inguinal, femoral, and abdominal hernia	-	-	-	447 (314-622)	190 (146-246)	135 (103-166)	252 (212-335)	292 (243-392)	370 (318-473)	362 (304-452)	501 (433-672)	610 (539-813)
Inflammatory bowel disease	-	-	-	532 (314-895)	108 (84-156)	68 (55-92)	94 (81-111)	176 (152-209)	256 (223-304)	337 (295-401)	394 (352-473)	543 (484-620)
Vascular intestinal disorders	-	-	-	316 (122-479)	66 (40-91)	30 (23-34)	48 (42-54)	102 (87-123)	160 (136-188)	159 (144-179)	301 (268-353)	528 (482-599)
Gallbladder and biliary diseases	-	-	-	308 (198-438)	117 (94-148)	151 (116-202)	161 (140-193)	254 (224-305)	326 (294-374)	422 (390-482)	641 (592-734)	940 (872-1,073)
Pancreatitis	-	-	-	76 (50-118)	78 (63-99)	91 (75-115)	287 (241-356)	898 (780-1,143)	2,124 (1,855-2,487)	2,983 (2,683-3,382)	4,059 (3,634-4,697)	4,815 (4,236-5,569)
Other digestive diseases	-	-	-	1,077 (686-1,696)	359 (274-493)	245 (198-332)	350 (262-545)	296 (249-413)	479 (396-676)	648 (546-886)	875 (752-1,142)	1,078 (977-1,300)
Neurological disorders	-	-	4,190 (3,593-5,105)	6,764 (4,763-9,177)	3,165 (2,677-3,835)	4,138 (3,586-4,836)	8,113 (7,018-9,512)	7,621 (6,708-8,666)	6,800 (5,968-7,930)	6,228 (5,433-7,214)	6,842 (6,031-8,032)	7,832 (7,062-8,785)
Alzheimer's disease and other dementias	-	-	-	-	-	-	-	-	-	-	-	406 (388-424)
Parkinson's disease	-	-	-	-	-	-	-	7 (5-7)	11 (9-12)	23 (21-26)	32 (29-36)	364 (342-424)
Epilepsy	-	-	2,572 (2,157-3,192)	5,408 (3,889-7,335)	2,574 (2,143-3,187)	3,232 (2,725-3,897)	6,215 (5,238-7,527)	6,068 (5,146-7,067)	5,775 (4,989-6,897)	5,205 (4,407-6,147)	5,441 (4,593-6,640)	5,042 (4,302-5,983)
Multiple sclerosis	-	-	-	-	-	-	-	56 (50-62)	112 (100-131)	226 (201-264)	377 (346-457)	615 (560-727)
Motor neuron disease	4 (3-4)	9 (7-10)	265 (195-332)	80 (53-103)	23 (20-25)	35 (30-45)	73 (61-84)	72 (60-83)	57 (52-64)	103 (93-121)	220 (207-265)	425 (401-484)
Other neurological disorders	-	-	1,353 (976-1,681)	1,277 (659-1,854)	568 (414-729)	871 (764-986)	1,824 (1,669-2,151)	1,419 (1,327-1,631)	846 (789-962)	671 (630-767)	771 (731-871)	980 (923-1,108)
Mental disorders	-	-	-	-	1 (0-1)	1 (0-1)	3 (2-4)	5 (4-6)	5 (3-6)	4 (3-5)	4 (4-5)	7 (6-8)
Eating disorders	-	-	-	-	1 (0-1)	1 (0-1)	3 (2-4)	5 (4-6)	5 (3-6)	4 (3-5)	4 (4-5)	7 (6-8)
Anorexia nervosa	-	-	-	-	0 (0-1)	0 (0-1)	2 (2-3)	3 (2-4)	2 (1-3)	2 (1-2)	3 (2-4)	5 (4-6)
Bulimia nervosa	-	-	-	-	0 (0-0)	0 (0-0)	1 (1-1)	2 (1-3)	3 (2-4)	2 (2-3)	1 (1-2)	2 (1-3)
Substance use disorders	0 (0-0)	0 (0-0)	0 (0-0)	13 (5-24)	31 (17-46)	33 (19-50)	3,064 (2,899-3,218)	9,324 (8,924-9,658)	14,902 (14,270-15,478)	20,183 (19,080-20,876)	22,880 (21,293-23,617)	25,979 (23,656-26,789)
Alcohol use disorders	-	-	-	-	-	-	841 (648-905)	2,614 (2,142-2,788)	5,448 (4,639-5,798)	9,880 (8,602-10,409)	13,415 (11,716-14,064)	17,283 (14,957-17,990)
Drug use disorders	0 (0-0)	0 (0-0)	0 (0-0)	13 (5-24)	31 (17-46)	33 (19-50)	2,223 (2,110-2,348)	6,710 (6,465-6,983)	9,454 (9,147-9,816)	10,303 (10,009-10,645)	9,465 (9,197-9,775)	8,697 (8,439-8,972)
Opioid use disorders	0 (0-0)	0 (0-0)	0 (0-0)	13 (5-24)	31 (17-46)	33 (19-50)	1,627 (1,488-1,765)	4,707 (4,391-5,037)	6,575 (6,218-6,994)	6,669 (6,321-7,097)	6,071 (5,729-6,493)	5,461 (5,149-5,804)
Cocaine use disorders	-	-	-	-	-	-	63 (52-78)	220 (188-259)	338 (299-382)	601 (536-676)	517 (448-598)	475 (406-557)
Amphetamine use disorders	-	-	-	-	-	-	71 (52-92)	217 (164-257)	319 (248-366)	409 (326-468)	375 (286-439)	349 (253-418)
Other drug use disorders	-	-	-	-	-	-	462 (397-568)	1,565 (1,398-1,826)	2,222 (2,033-2,478)	2,624 (2,418-2,911)	2,502 (2,270-2,826)	2,412 (2,196-2,728)
Diabetes and kidney diseases	22 (16-25)	61 (46-72)	4,377 (3,927-4,805)	4,258 (3,707-4,908)	2,893 (2,491-3,237)	3,041 (2,631-3,311)	7,114 (6,502-7,689)	10,553 (9,904-11,270)	11,501 (10,859-12,168)	15,538 (14,809-16,441)	21,823 (20,905-22,955)	33,968 (32,659-35,644)
Diabetes mellitus	22 (16-25)	61 (46-72)	652 (499-772)	285 (237-333)	410 (321-495)	455 (337-539)	1,179 (1,105-1,252)	2,224 (2,129-2,343)	2,780 (2,681-2,904)	4,979 (4,810-5,202)	7,346 (7,124-7,608)	13,966 (13,491-14,467)
Diabetes mellitus type 1	22 (16-25)	61 (46-72)	652 (499-772)	285 (237-333)	410 (321-495)	455 (337-539)	1,042 (976-1,112)	1,925 (1,832-2,036)	2,346 (2,252-2,461)	3,704 (3,504-3,922)	5,222 (4,970-5,508)	9,645 (9,107-10,248)
Diabetes mellitus type 2	-	-	-	-	-	-	137 (112-174)	299 (252-364)	434 (385-506)	1,274 (1,129-1,461)	2,124 (1,926-2,386)	4,321 (3,844-4,845)
Chronic kidney disease	-	-	3,615 (3,220-4,019)	3,861 (3,351-4,493)	2,387 (2,058-2,693)	2,511 (2,160-2,775)	5,838 (5,287-6,373)	8,200 (7,572-8,817)	8,595 (7,936-9,221)	10,396 (9,673-11,228)	14,240 (13,266-15,265)	19,715 (18,647-21,064)
Chronic kidney disease due to diabetes mellitus type 1	-	-	13 (7-21)	15 (8-28)	14 (7-25)	23 (13-39)	97 (50-158)	332 (172-543)	749 (449-1,159)	1,605 (1,028-2,314)	2,991 (1,965-4,273)	4,627 (3,019-6,482)
Chronic kidney disease due to diabetes mellitus type 2	-	-	10 (5-18)	11 (5-21)	9 (4-18)	13 (6-24)	46 (21-86)	117 (50-223)	206 (89-394)	419 (192-762)	950 (501-1,608)	2,185 (1,251-3,577)
Chronic kidney disease due to hypertension	-	-	18 (10-28)	30 (17-48)	30 (17-49)	54 (31-87)	231 (139-358)	553 (354-800)	807 (557-1,126)	1,170 (814-1,612)	1,814 (1,282-2,439)	2,806 (2,035-3,642)
Chronic kidney disease due to glomerulonephritis	-	-	830 (594-1,183)	1,183 (807-1,651)	760 (502-1,079)	843 (564-1,145)	2,280 (1,598-3,018)	3,262 (2,258-4,317)	3,206 (2,277-4,243)	3,253 (2,293-4,429)	3,760 (2,495-5,318)	4,542 (2,873-6,391)
Chronic kidney disease due to other and unspecified causes	-	-	2,744 (2,324-3,148)	2,622 (2,097-3,187)	1,574 (1,221-1,919)	1,578 (1,218-1,914)	3,184 (2,396-3,947)	3,937 (2,867-5,090)	3,627 (2,599-4,728)	3,948 (2,775-5,074)	4,724 (3,262-6,270)	5,556 (3,706-7,586)
Acute glomerulonephritis	-	-	110 (72-153)	112 (66-169)	96 (80-121)	75 (63-92)	97 (84-116)	129 (115-143)	126 (114-141)	164 (148-181)	237 (215-262)	287 (260-318)
Skin and subcutaneous diseases	-	-	1,482 (836-2,201)	906 (442-1,430)	241 (132-331)	295 (168-432)	577 (316-880)	492 (270-699)	453 (271-655)	909 (512-1,256)	929 (532-1,245)	1,150 (688-1,628)
Bacterial skin diseases	-	-	1,449 (811-2,155)	836 (406-1,337)	188 (97-256)	232 (127-323)	501 (266-754)	410 (221-566)	373 (219-539)	791 (424-1,064)	790 (438-1,063)	962 (558-1,341)
Cellulitis	-	-	114 (48-210)	156 (54-270)	61 (25-89)	87 (43-123)	115 (48-165)	96 (49-146)	95 (46-148)	172 (81-255)	212 (98-323)	259 (120-416)
Pyoderma	-	-	1,335 (745-1,988)	681 (343-1,097)	127 (68-177)	145 (76-210)	386 (211-598)	314 (154-425)	278 (157-415)	619 (312-864)	578 (303-802)	702 (384-988)
Decubitus ulcer	-	-	-	30 (16-61)	25 (15-44)	31 (19-58)	41 (22-69)	48 (25-80)	51 (26-83)	82 (45-153)	103 (55-181)	145 (79-268)
Other skin and subcutaneous diseases	-	-	33 (16-61)	40 (22-83)	28 (18-48)	32 (20-61)	34 (21-61)	35 (21-60)	29 (17-47)	36 (20-63)	35 (21-59)	43 (24-78)
Musculoskeletal disorders	-	-	-	-	238 (187-273)	335 (281-366)	469 (386-515)	529 (435-565)	612 (492-650)	696 (580-742)	898 (730-969)	1,143 (939-1,227)
Rheumatoid arthritis	-	-	-	-	32 (23-43)	37 (30-48)	46 (36-57)	48 (37-60)	52 (40-63)	62 (47-77)	101 (74-121)	181 (132-214)
Other musculoskeletal disorders	-	-	-	-	206 (163-237)	298 (250-325)	423 (350-465)	480 (392-513)	560 (458-597)	634 (527-679)	797 (648-864)	962 (800-1,038)
Other non-communicable diseases	115,760 (105,338-133,289)	40,889 (37,420-45,902)	150,146 (131,355-173,205)	52,533 (45,777-64,315)	13,733 (12,042-15,782)	10,187 (8,815-11,747)	9,927 (8,382-11,807)	10,201 (8,816-12,021)	8,609 (7,594-9,865)	7,654 (6,768-8,623)	8,887 (7,897-9,882)	10,419 (9,348-11,490)
Congenital birth defects	114,011 (103,886-131,396)	36,928 (33,991-41,515)	115,538 (107,073-133,482)	42,521 (37,331-55,010)	9,187 (8,295-10,393)	6,025 (5,570-6,602)	4,709 (4,431-5,166)	4,632 (4,379-5,074)	3,848 (3,639-4,243)	2,627 (2,477-2,905)	3,255 (3,052-3,527)	3,394 (3,184-3,712)
Neural tube defects	15,283 (11,121-21,961)	3,209 (2,314-4,665)	8,413 (5,311-13,937)	6,588 (3,486-13,700)	424 (310-594)	263 (213-348)	142 (118-186)	144 (122-183)	93 (81-117)	72 (63-91)	65 (58-75)	73 (64-88)
Congenital heart anomalies	37,502 (26,798-47,913)	17,244 (13,685-20,858)	60,943 (46,114-73,303)	18,282 (11,802-25,799)	5,384 (4,419-6,445)	3,767 (3,253-4,324)	2,934 (2,666-3,285)	2,864 (2,595-3,210)	2,679 (2,468-2,986)	1,731 (1,570-1,913)	2,241 (2,027-2,477)	2,179 (1,972-2,415)
Orofacial clefts	1,577 (482-4,171)	408 (140-1,022)	807 (275-2,232)	188 (32-718)	-	-	-	-	-	-	-	-
Down syndrome	2,233 (1,465-3,597)	874 (612-1,361)	4,544 (2,843-7,095)	2,434 (1,188-4,165)	350 (261-519)	213 (170-294)	276 (210-381)	291 (226-411)	248 (191-339)	240 (177-328)	283 (216-385)	382 (277-523)
Other chromosomal abnormalities	2,712 (1,518-5,005)	963 (592-1,651)	2,230 (1,254-4,052)	538 (269-1,125)	120 (68-191)	87 (46-126)	107 (67-149)	124 (78-172)	103 (67-139)	67 (39-84)	84 (52-111)	63 (35-87)
Congenital musculoskeletal and limb anomalies	2,415 (1,586-3,748)	539 (375-725)	1,460 (941-2,126)	519 (265-905)	191 (139-262)	131 (96-171)	165 (125-205)	133 (101-162)	80 (62-100)	53 (42-67)	57 (44-70)	49 (38-63)
Urogenital congenital anomalies	3,781 (2,207-5,219)	939 (600-1,251)	1,601 (941-2,315)	456 (216-805)	163 (111-230)	139 (101-181)	78 (61-101)	103 (82-129)	80 (65-103)	70 (56-88)	102 (77-130)	144 (110-184)
Digestive congenital anomalies	13,374 (8,905-19,265)	4,819 (3,564-6,543)	12,352 (8,436-18,971)	3,016 (1,460-7,113)	267 (197-358)	117 (93-141)	59 (51-70)	55 (47-65)	49 (41-60)	28 (25-36)	47 (37-58)	50 (39-61)
Other congenital birth defects	35,133 (22,998-45,896)	7,935 (5,400-10,395)	23,187 (13,946-32,179)	10,500 (5,013-15,982)	2,287 (1,686-3,033)	1,309 (1,032-1,667)	947 (826-1,153)	917 (817-1,107)	516 (453-631)	366 (324-465)	377 (316-472)	452 (389-550)
Urinary diseases and male infertility	454 (403-508)	314 (281-347)	3,279 (2,940-3,651)	1,829 (1,562-2,128)	866 (744-986)	636 (568-701)	1,234 (1,132-1,331)	1,703 (1,581-1,819)	1,541 (1,446-1,649)	1,871 (1,768-1,981)	2,273 (2,157-2,399)	3,169 (3,024-3,350)
Urinary tract infections	89 (55-145)	109 (79-151)	2,274 (1,796-2,783)	733 (502-1,059)	495 (392-612)	408 (347-486)	877 (771-1,026)	1,194 (1,060-1,405)	1,061 (944-1,200)	1,137 (1,013-1,333)	1,342 (1,186-1,580)	1,915 (1,672-2,213)
Urolithiasis	-	-	-	-	21 (11-30)	17 (10-23)	15 (11-22)	26 (20-35)	40 (31-52)	57 (44-74)	96 (75-127)	167 (128-220)
Other urinary diseases	365 (283-424)	204 (158-243)	1,005 (534-1,474)	1,096 (715-1,472)	350 (256-458)	211 (152-267)	341 (219-442)	482 (327-617)	440 (339-551)	677 (510-816)	835 (629-1,005)	1,087 (819-1,337)
Gynecological diseases	-	-	-	-	-	-	-	-	-	-	-	-
Uterine fibroids	-	-	-	-	-	-	-	-	-	-	-	-
Polycystic ovarian syndrome	-	-	-	-	-	-	-	-	-	-	-	-

Cause	45 to 49	50 to 54	54 to 59	60 to 64	65 to 69	70 to 74	75 to 79	80 to 84	85 to 89	90 to 94	95 plus
Multiple myeloma	1,610 (1,393-1,941)	2,668 (2,333-3,161)	4,080 (3,559-4,933)	5,306 (4,676-6,528)	5,904 (5,031-7,038)	6,813 (5,487-7,976)	6,545 (5,141-7,493)	5,338 (4,066-6,056)	2,967 (2,206-3,367)	821 (595-928)	217 (161-269)
Leukemia	7,714 (6,570-8,417)	9,196 (7,998-9,858)	11,978 (10,508-12,866)	14,489 (12,965-15,382)	15,752 (14,248-16,705)	18,269 (16,562-19,338)	17,875 (16,484-18,691)	14,888 (13,997-15,644)	8,342 (7,979-8,679)	2,709 (2,615-2,805)	714 (695-736)
Acute lymphoid leukemia	1,232 (1,023-1,398)	1,366 (1,162-1,487)	1,588 (1,358-1,764)	1,612 (1,379-1,749)	1,584 (1,352-1,737)	1,582 (1,374-1,736)	924 (802-1,038)	638 (568-705)	303 (278-329)	84 (78-90)	18 (17-19)
Chronic lymphoid leukemia	382 (329-432)	658 (587-704)	1,111 (1,016-1,187)	1,714 (1,575-1,812)	2,064 (1,891-2,165)	2,832 (2,627-2,972)	2,979 (2,784-3,109)	2,902 (2,766-3,021)	1,880 (1,812-1,947)	702 (679-727)	227 (216-237)
Acute myeloid leukemia	2,282 (1,936-2,499)	2,862 (2,454-3,106)	3,627 (3,165-3,983)	4,482 (3,969-4,742)	4,920 (4,440-5,212)	5,695 (5,183-5,987)	5,204 (4,770-5,428)	4,046 (3,777-4,226)	2,017 (1,907-2,104)	564 (533-589)	120 (115-126)
Chronic myeloid leukemia	684 (588-767)	786 (686-874)	938 (826-1,044)	1,026 (901-1,133)	1,202 (1,065-1,324)	1,372 (1,221-1,488)	1,661 (1,504-1,791)	1,471 (1,342-1,576)	809 (763-855)	256 (242-271)	63 (59-67)
Other leukemia	3,134 (2,511-3,586)	3,525 (2,887-3,928)	4,715 (3,911-5,228)	5,656 (4,709-6,270)	5,982 (5,170-6,694)	6,788 (5,934-7,490)	7,108 (6,339-7,792)	5,831 (5,347-6,398)	3,332 (3,109-3,627)	1,103 (1,043-1,176)	286 (273-301)
Other malignant neoplasms	8,440 (7,450-8,876)	11,312 (9,951-11,948)	14,472 (12,692-15,161)	16,442 (14,417-17,282)	16,595 (14,685-17,447)	17,857 (15,497-18,711)	16,095 (13,777-16,893)	12,196 (10,051-12,823)	5,905 (5,019-6,150)	1,928 (1,706-2,011)	511 (466-549)
Other neoplasms	1,493 (1,100-2,002)	1,934 (1,431-2,586)	2,586 (1,923-3,568)	3,265 (2,317-4,488)	3,779 (2,596-5,243)	4,730 (3,068-6,650)	5,418 (3,313-7,249)	5,166 (2,865-6,571)	3,643 (1,860-4,599)	1,347 (668-1,716)	446 (218-569)
Myelodysplastic, myeloproliferative, and other hematopoietic neoplasms	1,406 (1,054-1,893)	1,874 (1,392-2,516)	2,541 (1,895-3,495)	3,231 (2,295-4,446)	3,758 (2,582-5,215)	4,714 (3,059-6,635)	5,407 (3,307-7,234)	5,160 (2,863-6,566)	3,641 (1,859-4,596)	1,346 (668-1,715)	446 (218-569)
Other benign and in situ neoplasms	87 (53-131)	61 (39-94)	46 (28-68)	33 (18-46)	21 (10-29)	16 (7-23)	11 (5-17)	6 (3-10)	2 (1-4)	1 (0-1)	0 (0-0)
Cardiovascular diseases	328,960 (322,238-335,669)	464,640 (456,161-474,770)	618,157 (607,661-629,281)	790,613 (775,526-805,348)	871,583 (853,357-889,151)	1,116,656 (1,093,787-1,137,437)	1,192,360 (1,169,860-1,213,136)	1,144,889 (1,121,582-1,163,965)	721,095 (706,913-732,396)	306,542 (300,795-311,798)	104,638 (103,190-106,159)
Rheumatic heart disease	6,338 (5,742-7,396)	7,482 (6,712-8,764)	8,832 (8,092-10,135)	10,720 (9,800-12,449)	11,714 (10,687-13,665)	13,151 (12,111-14,932)	12,613 (11,775-14,021)	11,805 (11,106-13,034)	7,512 (7,140-8,166)	3,249 (3,101-3,497)	1,103 (1,059-1,176)
Ischemic heart disease	186,870 (182,484-193,187)	266,651 (260,792-275,319)	346,019 (339,390-356,218)	430,339 (422,104-440,908)	455,819 (446,109-467,284)	572,450 (559,032-586,409)	590,309 (578,271-605,063)	576,315 (564,265-592,238)	373,884 (366,205-385,440)	165,927 (162,781-171,592)	59,564 (58,467-61,877)
Stroke	95,208 (92,587-98,453)	137,210 (133,393-141,867)	196,562 (191,964-202,597)	271,290 (264,905-278,827)	319,588 (311,907-328,454)	421,741 (412,172-432,295)	462,372 (452,109-472,764)	421,838 (412,497-431,507)	242,423 (236,961-249,059)	91,352 (89,308-94,308)	27,238 (26,668-28,233)
Ischemic stroke	16,611 (15,309-18,333)	28,844 (26,629-31,954)	47,751 (44,696-52,041)	87,847 (81,555-95,702)	112,406 (104,489-122,390)	183,316 (174,031-193,677)	211,562 (201,926-221,816)	227,350 (219,377-235,174)	135,882 (131,313-140,720)	52,735 (50,854-54,817)	16,885 (16,416-17,647)
Intracerebral hemorrhage	67,011 (64,130-69,778)	92,759 (88,273-97,162)	128,841 (123,016-134,306)	164,225 (156,197-171,632)	186,681 (177,008-196,277)	217,257 (206,730-227,174)	229,184 (220,192-239,094)	177,621 (170,066-184,213)	96,633 (92,881-100,621)	34,684 (33,357-36,215)	9,154 (8,836-9,568)
Subarachnoid hemorrhage	11,586 (9,899-14,164)	15,606 (13,489-18,920)	19,969 (17,227-24,743)	19,218 (16,496-23,546)	20,501 (17,542-24,867)	21,168 (17,968-25,283)	21,626 (18,647-25,751)	16,867 (14,244-19,628)	9,908 (8,342-11,445)	3,933 (3,272-4,494)	1,200 (952-1,316)
Hypertensive heart disease	9,787 (6,888-10,994)	14,495 (10,403-16,096)	20,116 (14,997-22,142)	26,251 (19,430-28,884)	30,221 (22,775-33,371)	41,115 (30,577-44,586)	48,852 (37,172-52,650)	50,662 (37,711-54,294)	33,842 (23,544-36,213)	15,312 (10,399-16,401)	5,330 (3,214-5,722)
Non-rheumatic valvular heart disease	1,292 (1,174-1,487)	1,824 (1,666-2,126)	2,511 (2,278-2,864)	3,224 (2,882-3,514)	4,095 (3,631-4,554)	5,818 (5,011-6,195)	7,610 (6,367-8,051)	9,297 (7,103-9,789)	8,187 (5,627-8,625)	4,048 (2,768-4,282)	1,507 (967-1,600)
Non-rheumatic calcific aortic valve disease	726 (623-867)	1,065 (939-1,298)	1,486 (1,312-1,799)	1,978 (1,736-2,274)	2,592 (2,239-3,001)	3,745 (3,201-4,202)	5,124 (4,151-5,472)	6,702 (4,905-7,113)	6,442 (4,299-6,852)	3,302 (2,115-3,515)	1,269 (769-1,354)
Non-rheumatic degenerative mitral valve disease	508 (397-604)	671 (525-798)	901 (720-1,085)	1,077 (834-1,272)	1,269 (981-1,509)	1,695 (1,322-1,988)	1,995 (1,565-2,423)	2,081 (1,588-2,604)	1,450 (1,074-1,906)	632 (457-848)	208 (147-295)
Other non-rheumatic valve diseases	58 (30-81)	88 (49-123)	124 (64-162)	169 (81-226)	234 (116-298)	378 (174-476)	492 (220-622)	514 (250-651)	296 (156-367)	114 (63-152)	30 (19-40)
Cardiomyopathy and myocarditis	17,136 (13,094-18,487)	19,997 (14,681-21,626)	20,944 (15,991-22,563)	18,164 (14,412-19,499)	14,152 (12,201-15,326)	15,093 (12,471-16,256)	15,193 (12,505-16,248)	15,607 (12,037-16,706)	11,319 (8,430-12,098)	5,216 (3,837-5,579)	2,040 (1,368-2,190)
Myocarditis	955 (705-1,190)	898 (623-1,154)	1,107 (761-1,409)	968 (655-1,220)	957 (653-1,177)	1,302 (917-1,577)	1,551 (1,116-1,820)	1,854 (1,305-2,141)	1,557 (1,127-1,815)	934 (627-1,097)	432 (229-570)
Alcoholic cardiomyopathy	10,949 (7,380-11,704)	12,734 (8,222-13,726)	12,139 (8,018-13,005)	9,101 (5,983-9,816)	5,305 (3,894-5,741)	4,859 (3,150-5,353)	3,374 (2,332-3,894)	2,551 (1,664-3,303)	1,017 (732-1,422)	373 (289-528)	156 (108-229)
Other cardiomyopathy	5,232 (4,322-6,025)	6,365 (5,350-7,297)	7,698 (6,507-8,667)	8,094 (7,127-9,222)	7,891 (7,083-8,928)	8,932 (7,799-9,862)	10,268 (8,845-11,114)	11,203 (8,764-12,078)	8,744 (6,414-9,396)	3,909 (2,827-4,228)	1,453 (991-1,685)
Atrial fibrillation and flutter	473 (424-529)	897 (804-1,024)	1,733 (1,561-2,000)	2,998 (2,709-3,525)	4,426 (4,028-5,109)	8,142 (7,348-9,520)	12,335 (11,204-14,572)	19,240 (16,888-22,538)	17,363 (15,283-20,099)	10,613 (9,345-12,427)	4,051 (3,516-4,712)
Aortic aneurysm	2,859 (2,641-3,156)	3,985 (3,719-4,350)	6,073 (5,753-6,628)	8,610 (8,112-9,448)	10,474 (9,819-11,549)	13,104 (12,400-14,237)	14,428 (13,700-15,511)	13,430 (12,919-14,245)	8,769 (8,489-9,263)	3,328 (3,212-3,502)	938 (910-982)
Peripheral artery disease	309 (187-532)	697 (409-1,222)	1,296 (755-2,302)	1,999 (1,130-3,571)	2,466 (1,388-4,421)	3,689 (2,035-6,599)	4,116 (2,302-7,300)	4,457 (2,431-8,067)	3,437 (1,821-6,080)	1,772 (940-3,225)	881 (465-1,711)
Endocarditis	1,570 (1,460-1,768)	1,830 (1,705-2,080)	2,062 (1,947-2,289)	2,333 (2,201-2,597)	2,578 (2,428-2,900)	2,929 (2,790-3,250)	3,356 (3,216-3,645)	3,370 (3,240-3,566)	2,468 (2,385-2,589)	1,092 (1,053-1,143)	407 (393-429)
Other cardiovascular and circulatory diseases	7,119 (6,318-8,436)	9,571 (8,498-11,418)	12,010 (10,775-14,068)	14,685 (13,004-17,371)	16,050 (14,178-18,951)	19,424 (17,014-22,759)	21,177 (18,767-24,482)	18,867 (16,779-21,564)	11,891 (10,794-13,262)	4,632 (4,249-5,129)	1,580 (1,472-1,708)
Chronic respiratory diseases	36,567 (35,163-37,986)	60,332 (58,140-62,575)	95,569 (92,149-98,825)	163,912 (158,172-169,380)	225,003 (216,792-231,866)	315,151 (302,112-325,047)	365,833 (351,632-376,147)	350,895 (336,071-361,728)	207,196 (198,725-213,261)	77,983 (74,950-80,122)	21,902 (21,253-22,426)
Chronic obstructive pulmonary disease	21,305 (18,535-26,065)	38,945 (33,855-45,766)	67,159 (59,864-77,093)	126,648 (113,099-141,359)	180,298 (163,222-198,627)	263,850 (244,138-282,937)	314,129 (294,743-331,935)	307,848 (290,167-321,101)	184,400 (176,156-191,787)	69,994 (66,997-72,515)	19,781 (19,128-20,414)
Pneumoconiosis	677 (622-747)	793 (712-895)	1,095 (987-1,235)	1,450 (1,284-1,657)	1,765 (1,577-2,021)	2,572 (2,344-2,862)	3,190 (2,979-3,454)	2,910 (2,748-3,114)	1,680 (1,592-1,792)	500 (471-531)	124 (117-131)
Silicosis	452 (396-517)	494 (415-585)	677 (578-792)	825 (690-991)	962 (811-1,185)	1,354 (1,179-1,628)	1,752 (1,584-2,069)	1,597 (1,455-1,899)	871 (789-1,046)	218 (198-264)	51 (46-62)
Asbestosis	31 (16-45)	60 (35-86)	100 (62-141)	160 (102-225)	236 (148-311)	377 (236-474)	498 (273-601)	506 (254-588)	353 (185-406)	123 (71-139)	29 (16-33)
Coal workers pneumoconiosis	85 (61-118)	104 (75-143)	149 (109-199)	222 (164-298)	306 (229-386)	479 (395-593)	558 (480-699)	493 (432-610)	291 (257-360)	91 (79-115)	24 (21-30)
Other pneumoconiosis	108 (85-158)	136 (105-182)	169 (130-234)	242 (176-338)	262 (191-368)	361 (278-491)	382 (298-499)	314 (248-396)	164 (130-209)	68 (55-88)	20 (16-24)
Asthma	10,836 (5,609-14,435)	15,483 (7,543-21,241)	20,012 (9,879-28,014)	28,640 (13,002-41,523)	33,855 (15,096-51,645)	36,783 (17,487-54,679)	35,283 (18,402-52,578)	28,031 (15,693-41,988)	13,623 (8,145-20,110)	4,579 (2,948-6,641)	1,097 (793-1,574)
Interstitial lung disease and pulmonary sarcoidosis	1,421 (1,063-2,242)	2,292 (1,759-3,601)	3,522 (2,686-5,442)	5,305 (3,935-8,279)	7,123 (5,294-11,214)	9,504 (6,990-13,952)	11,072 (8,085-15,158)	9,909 (6,836-13,032)	6,065 (4,005-7,678)	2,211 (1,513-2,731)	658 (440-776)
Other chronic respiratory diseases	2,328 (1,815-2,999)	2,818 (2,186-3,800)	3,781 (2,995-4,990)	1,868 (1,484-2,411)	1,961 (1,568-2,459)	2,443 (2,067-3,057)	2,159 (1,853-2,612)	2,197 (1,890-2,698)	1,428 (1,254-1,686)	698 (613-815)	241 (219-270)
Digestive diseases	107,942 (103,639-118,794)	123,753 (117,311-135,224)	132,060 (126,676-145,842)	133,921 (127,060-149,379)	121,484 (115,855-133,447)	120,275 (112,045-130,206)	110,946 (105,607-123,255)	89,920 (84,903-99,069)	53,382 (50,626-58,372)	21,218 (20,236-22,595)	6,794 (6,537-7,086)
Cirrhosis and other chronic liver diseases	82,200 (78,532-89,895)	94,181 (88,862-101,841)	96,724 (92,371-106,650)	92,357 (87,531-102,121)	76,071 (71,752-82,864)	66,451 (61,542-72,320)	53,285 (49,630-58,953)	36,343 (33,444-39,659)	17,634 (16,121-19,201)	5,918 (5,424-6,421)	1,683 (1,571-1,798)
Cirrhosis and other chronic liver diseases due to hepatitis B	26,302 (22,313-30,997)	27,058 (22,536-32,101)	28,202 (23,506-34,264)	28,000 (23,235-33,794)	24,557 (20,785-29,644)	22,258 (18,305-26,554)	18,612 (15,642-21,799)	12,548 (10,465-14,895)	5,650 (4,821-6,554)	1,760 (1,451-2,133)	422 (321-538)
Cirrhosis and other chronic liver diseases due to hepatitis C	22,137 (18,287-26,482)	25,833 (21,162-30,776)	25,519 (21,840-29,643)	23,522 (19,464-27,958)	19,357 (16,577-22,473)	16,583 (14,211-19,260)	12,924 (10,806-15,522)	8,781 (7,307-10,628)	4,482 (3,780-5,210)	1,578 (1,312-1,884)	479 (378-592)
Cirrhosis and other chronic liver diseases due to alcohol use	24,435 (21,013-28,463)	29,213 (25,023-33,770)	29,741 (25,252-34,659)	27,560 (22,847-32,822)	20,806 (17,566-24,475)	17,248 (14,385-20,494)	13,071 (11,262-15,130)	8,617 (7,225-10,113)	4,061 (3,511-4,673)	1,308 (1,096-1,569)	380 (287-484)
Cirrhosis due to NASH	6,153 (5,117-7,274)	7,931 (6,584-9,428)	8,276 (7,234-9,410)	7,641 (6,545-8,766)	5,975 (5,234-6,838)	4,902 (4,279-5,667)	3,537 (2,987-4,158)	2,346 (1,970-2,762)	1,235 (1,059-1,428)	448 (366-551)	136 (103-178)
Cirrhosis and other chronic liver diseases due to other causes	3,172 (2,356-4,129)	4,146 (3,049-5,461)	4,985 (3,466-6,955)	5,634 (3,946-7,621)	5,376 (3,896-7,267)	5,459 (4,145-7,068)	5,141 (3,895-6,778)	4,051 (3,078-5,399)	2,206 (1,771-2,753)	825 (624-1,053)	267 (183-364)
Upper digestive system diseases	9,441 (8,834-11,036)	11,239 (10,546-13,018)	13,525 (12,686-15,740)	15,457 (14,485-17,994)	16,494 (15,352-19,061)	19,031 (17,582-21,476)	19,884 (18,614-22,856)	16,248 (15,093-18,640)	9,810 (9,122-11,257)	3,872 (3,668-4,326)	1,220 (1,166-1,336)
Peptic ulcer disease	7,791 (7,239-9,074)	9,450 (8,838-10,898)	11,320 (10,592-13,203)	13,187 (12,351-15,270)	14,040 (13,024-16,196)	16,177 (14,995-18,243)	16,704 (15,564-19,112)	13,686 (12,668-15,619)	8,243 (7,678-9,403)	3,222 (3,051-3,589)	1,012 (966-1,096)
Gastritis and duodenitis	1,650 (1,351-2,015)	1,789 (1,515-2,136)	2,205 (1,886-2,661)	2,271 (2,016-2,757)	2,455 (2,187-2,881)	2,854 (2,541-3,259)	3,180 (2,852-3,724)	2,562 (2,320-3,062)	1,566 (1,438-1,860)	650 (607-759)	208 (195-237)
Appendicitis	1,095 (962-1,335)	1,199 (1,048-1,441)	1,309 (1,154-1,567)	1,508 (1,333-1,846)	1,451 (1,287-1,741)	1,726 (1,506-2,021)	1,633 (1,437-1,930)	1,435 (1,247-1,669)	873 (756-1,027)	374 (329-441)	105 (94-119)
Paralytic ileus and intestinal obstruction	4,328 (3,082-5,417)	4,853 (3,672-6,197)	6,181 (4,591-7,810)	7,903 (6,026-9,863)	9,283 (6,941-11,681)	10,058 (7,988-12,426)	10,527 (8,547-13,393)	10,562 (8,511-13,038)	7,961 (6,600-9,436)	3,453 (2,781-4,116)	1,111 (882-1,325)
Inguinal, femoral, and abdominal hernia	769 (683-997)	1,080 (970-1,373)	1,363 (1,235-1,756)	1,759 (1,581-2,203)	2,198 (1,974-2,869)	2,603 (2,309-3,135)	3,163 (2,740-3,742)	3,340 (2,942-3,893)	2,148 (1,944-2,579)	978 (905-1,138)	328 (307-372)
Inflammatory bowel disease	695 (630-794)	827 (716-910)	1,040 (909-1,139)	1,213 (1,030-1,320)	1,353 (1,186-1,488)	1,690 (1,415-1,820)	1,942 (1,545-2,115)	2,013 (1,566-2,168)	1,465 (1,082-1,595)	687 (459-753)	246 (161-272)
Vascular intestinal disorders	905 (842-1,006)	1,434 (1,344-1,578)	2,228 (2,098-2,439)	2,950 (2,788-3,271)	3,854 (3,589-4,263)	5,518 (5,181-5,988)	5,992 (5,625-6,511)	5,811 (5,464-6,225)	3,644 (3,458-3,921)	1,488 (1,388-1,593)	477 (446-509)
Gallbladder and biliary diseases	1,191 (1,115-1,348)	1,613 (1,501-1,798)	2,033 (1,920-2,281)	2,720 (2,584-3,106)	3,441 (3,258-3,886)	4,767 (4,507-5,250)	5,961 (5,671-6,655)	6,340 (6,041-7,016)	4,631 (4,423-5,077)	2,184 (2,090-2,390)	814 (782-879)
Pancreatitis	5,750 (5,070-6,459)	5,335 (4,797-6,013)	5,380 (4,804-6,050)	4,801 (4,288-5,450)	4,202 (3,756-4,769)	4,397 (3,887-4,862)	4,300 (3,663-4,828)	3,306 (2,897-3,710)	1,890 (1,683-2,103)	665 (591-733)	196 (182-212)
Other digestive diseases	1,567 (1,405-1,935)	1,993 (1,767-2,495)	2,276 (2,087-2,659)	3,253 (2,808-4,273)	3,138 (2,832-3,817)	4,033 (3,600-4,920)	4,259 (3,931-4,894)	4,522 (4,182-5,080)	3,327 (3,160-3,583)	1,597 (1,512-1,713)	615 (587-656)
Neurological disorders	9,569 (8,728-10,568)	11,271 (10,554-11,896)	16,129 (15,487-16,724)	26,855 (25,985-27,699)	43,753 (42,212-45,081)	84,005 (81,112-86,073)	151,650 (146,907-154,591)	206,046 (199,487-209,643)	181,275 (176,272-184,480)	102,757 (100,152-104,202)	38,311 (37,652-38,785)
Alzheimer's disease and other dementias	1,030 (1,002-1,071)	2,617 (2,544-2,693)	5,874 (5,739-6,070)	14,181 (13,872-14,566)	26,626 (25,937-27,356)	55,275 (53,925-56,631)	107,747 (105,123-109,994)	158,911 (155,227-161,912)	152,586 (149,427-155,207)	93,641 (91,387-94,947)	36,174 (35,566-36,628)
Parkinson's disease	789 (744-916)	1,404 (1,321-1,645)	2,257 (2,145-2,606)	4,566 (4,303-5,257)	9,072 (8,546-10,399)	21,263 (19,496-23,726)	37,019 (33,652-39,836)	42,064 (37,561-44,352)	25,897 (22,251-27,797)	8,283 (7,294-9,000)	1,898 (1,731-2,099)
Epilepsy	4,749 (3,858-5,675)	3,625 (3,034-4,094)	3,449 (2,891-3,857)	3,197 (2,595-3,760)	3,188 (2,417-4,036)	2,628 (2,117-3,182)	2,490 (2,015-2,750)	1,926 (1,509-2,125)	1,153 (891-1,258)	373 (292-408)	114 (88-124)
Multiple sclerosis	830 (726-979)	917 (776-1,053)	998 (822-1,096)	930 (746-1,011)	729 (561-794)	595 (450-644)	439 (331-475)	278 (215-300)	127 (97-139)	33 (25-35)	8 (5-8)
Motor neuron disease	805 (762-1,005)	1,112 (1,060-1,218)	1,659 (1,594-1,890)	2,097 (2,012-2,301)	2,310 (2,234-2,528)	2,327 (2,253-2,455)	2,018 (1,956-2,091)	1,294 (1,255-1,338)	574 (557-594)	117 (113-122)	24 (24-25)
Other neurological disorders	1,366 (1,294-1,513)	1,595 (1,512-1,791)	1,893 (1,807-2,073)	1,883 (1,796-2,064)	1,828 (1,746-2,003)	1,918 (1,831-2,077)	1,938 (1,854-2,077)	1,573 (1,512-1,658)	938 (905-976)	309 (298-322)	93 (89-96)
Mental disorders	6 (6-7)	-	-	-	-	-	-	-	-	-	-
Eating disorders	6 (6-7)	-	-	-	-	-	-	-	-	-	-
Anorexia nervosa	5 (3-6)	-	-	-	-	-	-	-	-	-	-
Bulimia nervosa	2 (1-3)	-	-	-	-	-	-	-	-	-	-
Substance use disorders	30,009 (27,375-30,950)	26,293 (24,414-27,084)	23,205 (21,420-23,822)	17,401 (16,018-17,943)	11,227 (10,370-11,578)	8,761 (7,992-9,071)	6,472 (5,805-6,715)	3,913 (3,664-4,057)	2,038 (1,916-2,113)	742 (700-774)	233 (224-242)
Alcohol use disorders	22,364 (19,666-23,292)	20,213 (18,338-20,918)	18,360 (16,569-18,939)	14,010 (12,538-14,497)	8,552 (7,671-8,849)	6,195 (5,445-6,459)	4,027 (3,369-4,220)	1,921 (1,660-2,012)	903 (779-958)	274 (234-294)	86 (78-90)
Drug use disorders	7,645 (7,393-7,916)	6,080 (5,873-6,282)	4,844 (4,690-5,016)	3,391 (3,279-3,522)	2,675 (2,569-2,792)	2,566 (2,475-2,667)	2,446 (2,362-2,537)	1,993 (1,923-2,070)	1,135 (1,092-1,182)	468 (449-487)	148 (141-154)
Opioid use disorders	4,598 (4,328-4,849)	3,418 (3,214-3,598)	2,793 (2,598-2,968)	2,039 (1,896-2,168)	1,655 (1,483-1,798)	1,335 (1,213-1,471)	1,355 (1,242-1,461)	1,085 (1,005-1,170)	610 (568-656)	243 (224-262)	73 (68-78)
Cocaine use disorders	505 (442-590)	589 (531-670)	409 (364-480)	240 (208-295)	149 (117-206)	74 (58-99)	63 (47-86)	13 (11-18)	9 (7-11)	5 (4-6)	2 (1-2)
Amphetamine use disorders	335 (256-404)	280 (185-328)	203 (149-252)	121 (92-152)	78 (53-112)	26 (19-35)	23 (16-30)	7 (5-9)	4 (3-5)	2 (2-3)	1 (1-1)
Other drug use disorders	2,207 (2,024-2,469)	1,793 (1,656-1,991)	1,439 (1,296-1,662)	990 (890-1,156)	793 (674-1,013)	1,130 (1,021-1,273)	1,005 (912-1,126)	888 (802-972)	512 (472-557)	218 (201-240)	72 (67-78)
Diabetes and kidney diseases	48,066 (46,154-49,809)	67,875 (65,758-70,215)	90,270 (87,470-93,649)	112,889 (109,139-116,374)	124,241 (119,007-128,197)	137,367 (131,813-141,894)	141,323 (136,134-145,964)	119,745 (115,368-124,135)	77,761 (75,028-80,289)	31,968 (30,929-33,008)	10,709 (10,442-10,989)
Diabetes mellitus	23,101 (22,429-23,833)	34,925 (33,818-36,022)	48,672 (47,286-50,181)	62,425 (60,580-64,434)	68,281 (66,063-70,452)	74,516 (71,975-76,691)	73,944 (71,397-76,182)	58,629 (56,591-60,738)	34,098 (32,832-35,257)	12,418 (11,954-12,858)	3,504 (3,389-3,609)
Diabetes mellitus type 1	9,082 (7,991-10,431)	13,065 (11,574-15,109)	17,896 (15,470-20,659)	16,699 (14,228-20,196)	17,846 (15,356-21,222)	16,277 (13,370-19,876)	15,631 (12,599-19,095)	9,467 (7,503-11,822)	5,000 (4,021-6,114)	1,719 (1,386-2,127)	449 (372-529)
Diabetes mellitus type 2	14,019 (12,721-15,342)	21,860 (19,683-23,722)	30,776 (28,050-33,341)	45,727 (42,216-49,262)	50,435 (46,890-53,976)	58,239 (54,748-61,949)	58,314 (54,619-61,719)	49,162 (46,544-51,857)	29,098 (27,710-30,550)	10,699 (10,197-11,206)	3,055 (2,941-3,183)
Chronic kidney disease	24,599 (22,578-25,974)	32,612 (31,017-34,368)	41,121 (38,745-44,071)	49,975 (46,725-52,450)	55,441 (51,278-57,841)	62,240 (57,271-65,062)	66,740 (62,153-69,483)	60,586 (57,201-63,048)	43,363 (41,596-45,110)	19,430 (18,789-20,172)	7,162 (6,959-7,357)
Chronic kidney disease due to diabetes mellitus type 1	5,352 (3,469-7,556)	5,264 (3,169-7,664)	4,475 (2,489-7,038)	3,206 (1,607-5,476)	2,365 (1,196-4,250)	1,944 (945-3,470)	1,551 (733-2,899)	1,117 (554-1,989)	544 (285-942)	111 (55-202)	9 (4-19)
Chronic kidney disease due to diabetes mellitus type 2	4,421 (2,775-6,256)	8,166 (5,649-11,215)	12,445 (8,778-15,956)	17,364 (12,399-22,237)	19,999 (14,842-25,100)	21,467 (16,073-26,767)	21,285 (14,767-27,619)	17,780 (12,206-23,691)	10,999 (7,387-14,474)	4,387 (3,080-5,835)	1,476 (918-2,214)
Chronic kidney disease due to hypertension	3,871 (2,778-5,072)	6,000 (4,486-7,813)	8,646 (6,606-11,143)	11,767 (9,053-14,878)	14,731 (11,472-18,424)	18,551 (14,806-22,877)	22,517 (17,974-27,767)	22,676 (17,957-27,761)	18,613 (15,498-21,964)	9,342 (7,866-10,799)	3,676 (3,015-4,324)
Chronic kidney disease due to glomerulonephritis	5,040 (3,306-7,362)	5,976 (3,951-8,602)	6,475 (4,311-9,618)	6,615 (4,241-9,848)	6,965 (4,469-10,214)	8,082 (5,070-11,635)	8,543 (5,527-12,442)	7,527 (4,779-11,228)	4,938 (3,249-7,316)	2,035 (1,369-2,958)	736 (404-1,208)
Chronic kidney disease due to other and unspecified causes	5,916 (3,864-8,433)	7,205 (4,727-10,234)	9,080 (5,962-12,809)	11,024 (6,885-16,049)	11,380 (7,401-16,315)	12,196 (8,152-17,362)	12,845 (8,328-18,696)	11,485 (7,786-16,223)	8,270 (5,715-11,290)	3,554 (2,446-4,895)	1,265 (799-1,910)
Acute glomerulonephritis	365 (332-407)	338 (305-389)	477 (432-537)	489 (446-540)	519 (470-576)	611 (555-677)	638 (584-702)	530 (484-581)	299 (277-326)	121 (107-136)	43 (36-54)
Skin and subcutaneous diseases	1,660 (970-2,418)	1,889 (1,106-2,721)	2,386 (1,321-3,296)	2,918 (1,653-4,021)	3,071 (1,760-4,255)	3,858 (2,181-5,293)	4,556 (2,682-6,529)	4,103 (2,534-6,202)	3,162 (1,868-4,601)	1,559 (888-2,368)	599 (326-970)
Bacterial skin diseases	1,434 (834-2,080)	1,622 (898-2,319)	2,035 (1,083-2,732)	2,485 (1,298-3,358)	2,470 (1,364-3,322)	2,981 (1,632-4,128)	3,406 (1,875-4,780)	2,751 (1,563-4,078)	2,016 (1,058-2,882)	980 (532-1,477)	349 (175-561)
Cellulitis	391 (184-625)	474 (196-762)	571 (241-939)	668 (298-1,017)	565 (234-889)	701 (315-1,139)	696 (273-1,133)	665 (267-1,190)	481 (168-849)	242 (87-437)	95 (30-175)
Pyoderma	1,043 (594-1,522)	1,148 (605-1,646)	1,464 (756-2,039)	1,817 (944-2,512)	1,905 (994-2,652)	2,280 (1,195-3,183)	2,710 (1,432-3,846)	2,086 (1,184-3,185)	1,535 (808-2,225)	738 (403-1,127)	254 (128-412)
Decubitus ulcer	178 (97-343)	218 (116-408)	292 (154-530)	360 (188-700)	517 (263-893)	772 (416-1,392)	1,017 (532-1,920)	1,194 (644-2,152)	1,009 (506-1,736)	513 (252-980)	220 (97-417)
Other skin and subcutaneous diseases	48 (25-91)	49 (25-91)	59 (29-113)	72 (39-143)	84 (43-160)	105 (56-202)	134 (71-269)	158 (81-320)	138 (68-315)	66 (31-146)	30 (11-67)
Musculoskeletal disorders	1,345 (1,146-1,430)	1,252 (1,097-1,329)	1,838 (1,551-1,959)	3,060 (2,419-3,295)	3,631 (2,881-3,896)	4,577 (3,670-4,883)	5,032 (4,051-5,371)	4,349 (3,623-4,631)	2,916 (2,531-3,095)	1,180 (1,038-1,261)	387 (358-406)
Rheumatoid arthritis	262 (194-297)	409 (325-454)	747 (604-829)	1,366 (997-1,543)	1,748 (1,292-1,949)	2,297 (1,764-2,549)	2,549 (1,912-2,819)	1,894 (1,492-2,133)	1,131 (908-1,263)	369 (291-416)	102 (86-113)
Other musculoskeletal disorders	1,083 (927-1,161)	843 (739-908)	1,091 (933-1,196)	1,694 (1,360-1,898)	1,883 (1,492-2,121)	2,280 (1,809-2,581)	2,483 (1,980-2,827)	2,454 (2,045-2,757)	1,786 (1,540-1,963)	810 (718-885)	285 (265-304)
Other non-communicable diseases	10,151 (8,899-11,131)	12,011 (10,341-13,222)	13,605 (11,640-14,886)	16,060 (14,067-17,279)	18,010 (16,180-19,359)	20,611 (18,615-22,038)	21,376 (19,456-22,746)	22,252 (20,446-23,340)	16,101 (14,774-16,773)	7,824 (7,172-8,161)	3,131 (2,849-3,262)
Congenital birth defects	1,432 (1,264-1,618)	1,343 (1,134-1,495)	1,415 (1,171-1,540)	1,260 (1,034-1,366)	927 (805-1,058)	-	-	-	-	-	-
Neural tube defects	47 (41-56)	35 (31-43)	34 (29-41)	29 (25-39)	22 (19-28)	-	-	-	-	-	-
Congenital heart anomalies	659 (573-784)	534 (449-628)	524 (451-630)	437 (386-514)	345 (301-430)	-	-	-	-	-	-
Orofacial clefts	-	-	-	-	-	-	-	-	-	-	-
Down syndrome	355 (254-437)	407 (294-468)	456 (293-526)	390 (250-444)	173 (119-201)	-	-	-	-	-	-
Other chromosomal abnormalities	50 (30-61)	41 (22-53)	39 (21-48)	27 (13-35)	20 (10-27)	-	-	-	-	-	-
Congenital musculoskeletal and limb anomalies	29 (24-36)	28 (23-35)	22 (18-28)	26 (21-31)	21 (18-27)	-	-	-	-	-	-
Urogenital congenital anomalies	83 (70-103)	105 (84-126)	141 (111-163)	173 (136-199)	193 (156-222)	-	-	-	-	-	-
Digestive congenital anomalies	32 (26-40)	35 (28-42)	47 (37-56)	43 (35-52)	50 (41-58)	-	-	-	-	-	-
Other congenital birth defects	177 (148-209)	157 (130-183)	152 (125-176)	134 (111-157)	104 (84-120)	-	-	-	-	-	-
Urinary diseases and male infertility	4,095 (3,908-4,302)	4,975 (4,746-5,205)	5,739 (5,516-5,975)	7,858 (7,555-8,186)	9,923 (9,422-10,392)	12,679 (12,057-13,198)	13,203 (12,627-13,738)	14,768 (14,073-15,471)	10,484 (10,108-10,883)	5,233 (5,049-5,438)	2,120 (2,066-2,182)
Urinary tract infections	2,375 (2,081-2,723)	2,980 (2,625-3,426)	3,479 (3,105-3,969)	4,872 (4,333-5,505)	6,062 (5,395-6,946)	7,560 (6,698-8,595)	8,591 (7,759-9,593)	9,521 (8,708-10,517)	7,630 (7,074-8,173)	3,885 (3,644-4,141)	1,685 (1,553-1,764)
Urolithiasis	248 (197-319)	321 (264-405)	406 (332-520)	540 (432-684)	644 (501-830)	802 (658-1,025)	845 (698-1,106)	752 (626-970)	417 (357-540)	186 (158-244)	52 (45-71)
Other urinary diseases	1,472 (1,163-1,750)	1,675 (1,273-2,043)	1,854 (1,431-2,215)	2,445 (1,904-2,999)	3,216 (2,520-3,951)	4,317 (3,446-5,180)	3,768 (2,962-4,513)	4,495 (3,653-5,325)	2,436 (1,984-2,955)	1,163 (966-1,383)	383 (318-512)
Gynecological diseases	-	-	-	-	-	-	-	-	-	-	-
Uterine fibroids	-	-	-	-	-	-	-	-	-	-	-
Polycystic ovarian syndrome	-	-	-	-	-	-	-	-	-	-	-

SR Table 6: Global deaths by cause and age group for males (with 95% confidence intervals), 2010

Cause	Early Neonatal	Late Neonatal	Post Neonatal	1 to 4	5 to 9	10 to 14	15 to 19	20 to 24	25 to 29	30 to 34	35 to 39	40 to 44	45 to 49	50 to 54	54 to 59	60 to 64	65 to 69	70 to 74	75 to 79	80 to 84	85 to 89	90 to 94	95 plus
Endometriosis	-	-	-	-	-	-	-	-	-	-	-	-	-	-	-	-	-	-	-	-	-	-	-
Genital prolapse	-	-	-	-	-	-	-	-	-	-	-	-	-	-	-	-	-	-	-	-	-	-	-
Other gynecological diseases	-	-	-	-	-	-	-	-	-	-	-	-	-	-	-	-	-	-	-	-	-	-	-
Hemoglobinopathies and hemolytic anemias	578 (323-1,012)	391 (245-601)	3,123 (1,802-5,103)	5,852 (3,165-8,997)	2,691 (1,712-4,055)	2,661 (1,688-4,046)	3,159 (1,845-4,973)	2,893 (1,622-4,649)	2,063 (1,191-3,254)	1,605 (974-2,486)	1,548 (899-2,399)	1,641 (991-2,582)	1,651 (1,005-2,481)	1,969 (1,176-2,965)	2,214 (1,396-3,276)	2,347 (1,542-3,273)	2,536 (1,666-3,585)	2,921 (1,948-4,006)	3,087 (2,141-4,109)	2,760 (2,019-3,453)	1,960 (1,452-2,352)	918 (649-1,071)	336 (229-376)
Thalassemias	169 (121-204)	136 (99-169)	897 (685-1,211)	1,183 (871-1,517)	781 (549-1,105)	524 (364-693)	315 (225-389)	206 (133-269)	93 (62-122)	46 (33-59)	30 (23-38)	24 (19-30)	18 (14-23)	10 (8-12)	6 (5-7)	3 (2-3)	1 (1-1)	0 (0-1)	0 (0-0)	0 (0-0)	0 (0-0)	0 (0-0)	0 (0-0)
Sickle cell disorders	383 (169-797)	245 (123-452)	2,137 (961-4,096)	4,456 (1,941-7,487)	1,688 (943-2,948)	1,708 (903-2,987)	2,179 (1,029-3,904)	1,869 (842-3,524)	1,215 (494-2,360)	781 (290-1,568)	734 (250-1,491)	615 (196-1,307)	389 (116-883)	250 (87-596)	109 (51-222)	50 (33-78)	25 (17-35)	11 (7-15)	3 (2-5)	1 (1-1)	0 (0-1)	0 (0-0)	0 (0-0)
G6PD deficiency	11 (6-23)	8 (4-14)	73 (38-131)	192 (104-304)	194 (133-275)	348 (233-487)	526 (357-718)	614 (415-809)	574 (406-720)	602 (434-744)	600 (431-764)	725 (515-1,001)	900 (576-1,313)	1,108 (636-1,748)	1,147 (636-1,900)	1,007 (560-1,629)	795 (420-1,343)	587 (298-1,028)	359 (180-631)	154 (81-265)	56 (31-94)	12 (7-20)	2 (1-4)
Other hemoglobinopathies and hemolytic anemias	14 (9-21)	2 (2-3)	15 (10-22)	20 (14-28)	28 (21-37)	80 (57-110)	138 (92-192)	204 (141-277)	181 (130-233)	177 (124-229)	184 (133-232)	278 (201-349)	344 (250-450)	602 (412-811)	952 (676-1,293)	1,287 (893-1,726)	1,715 (1,203-2,316)	2,323 (1,617-3,137)	2,725 (1,930-3,559)	2,605 (1,923-3,238)	1,904 (1,415-2,276)	905 (643-1,052)	333 (227-373)
Endocrine, metabolic, blood, and immune disorders	717 (502-859)	658 (476-817)	4,183 (3,036-5,410)	2,331 (1,541-3,103)	989 (784-1,221)	865 (706-1,087)	825 (672-1,005)	973 (820-1,157)	1,157 (975-1,403)	1,551 (1,252-1,827)	1,810 (1,404-2,103)	2,215 (1,657-2,487)	2,974 (2,199-3,264)	3,724 (2,633-4,157)	4,237 (2,949-4,659)	4,596 (3,290-4,999)	4,625 (3,293-5,005)	5,012 (3,731-5,594)	5,085 (3,597-5,616)	4,724 (3,325-5,189)	3,657 (2,549-3,991)	1,672 (1,089-1,840)	675 (442-746)
Sudden infant death syndrome	-	2,598 (1,033-4,478)	24,023 (8,981-44,756)	-	-	-	-	-	-	-	-	-	-	-	-	-	-	-	-	-	-	-	-
Injuries	7,345 (6,337-8,022)	5,179 (4,620-5,540)	51,714 (47,704-55,437)	134,495 (120,410-146,982)	90,098 (83,150-95,858)	91,038 (85,595-95,500)	206,532 (199,769-212,639)	310,302 (302,713-317,043)	282,408 (274,529-287,780)	256,579 (248,632-261,178)	253,852 (244,322-258,916)	240,858 (230,355-245,708)	232,062 (222,653-236,535)	203,437 (195,934-207,348)	183,414 (174,837-187,298)	152,614 (143,829-156,106)	127,843 (118,174-131,744)	123,176 (113,893-126,850)	112,537 (102,879-116,389)	92,223 (83,823-95,957)	56,885 (51,811-59,044)	23,178 (21,313-23,968)	7,701 (7,319-7,922)
Transport injuries	806 (732-894)	653 (600-714)	7,316 (6,525-8,065)	29,194 (22,577-34,112)	27,632 (25,142-30,136)	25,344 (23,405-27,522)	78,411 (74,909-83,056)	120,193 (116,142-125,055)	104,365 (101,416-108,341)	90,707 (88,241-94,089)	92,400 (89,728-95,830)	88,212 (85,889-90,670)	80,876 (78,742-83,193)	67,727 (66,089-69,609)	60,534 (59,051-62,102)	48,723 (47,328-50,148)	39,543 (38,397-40,698)	33,761 (32,777-34,786)	27,454 (26,546-28,326)	18,659 (17,989-19,308)	8,892 (8,546-9,205)	2,777 (2,673-2,868)	720 (690-746)
Road injuries	697 (627-782)	572 (521-628)	6,758 (6,034-7,485)	27,420 (21,392-31,954)	25,997 (23,629-28,369)	23,065 (21,187-25,057)	72,122 (68,825-76,324)	112,189 (108,394-116,901)	96,240 (93,527-99,897)	84,440 (82,096-87,794)	85,517 (82,861-88,623)	81,770 (79,533-84,207)	74,726 (72,515-76,938)	62,060 (60,381-63,902)	55,682 (54,256-57,214)	45,307 (43,909-46,734)	36,748 (35,687-37,867)	31,472 (30,504-32,461)	25,566 (24,721-26,385)	17,461 (16,774-18,106)	8,353 (8,046-8,653)	2,599 (2,495-2,685)	664 (634-689)
Pedestrian road injuries	310 (260-395)	263 (219-330)	2,766 (2,391-3,464)	16,055 (12,701-20,172)	14,658 (13,186-16,684)	11,422 (10,109-13,246)	19,353 (17,281-23,131)	28,968 (26,673-33,633)	25,892 (23,951-30,184)	26,077 (24,439-29,644)	29,304 (27,443-33,071)	30,146 (28,474-33,996)	29,828 (27,997-33,475)	25,608 (24,016-28,639)	24,524 (23,129-27,457)	22,439 (21,177-24,765)	18,759 (17,760-20,634)	16,717 (15,834-18,304)	15,227 (14,462-16,534)	10,383 (9,802-11,248)	4,990 (4,717-5,368)	1,483 (1,405-1,595)	361 (342-388)
Cyclist road injuries	-	-	-	743 (544-1,009)	1,483 (1,190-1,895)	1,931 (1,564-2,442)	3,404 (2,731-4,158)	3,376 (2,659-3,932)	3,040 (2,375-3,549)	3,201 (2,555-3,724)	3,496 (2,668-3,969)	4,334 (3,365-4,943)	4,499 (3,513-5,135)	4,351 (3,359-5,034)	3,971 (3,028-4,440)	4,013 (3,052-4,549)	3,311 (2,563-3,756)	2,820 (2,235-3,181)	1,898 (1,507-2,112)	1,111 (914-1,226)	456 (382-511)	125 (105-146)	1 (1-1)
Motorcyclist road injuries	54 (39-65)	52 (38-63)	646 (497-765)	1,569 (1,116-2,012)	1,621 (1,328-1,904)	2,604 (2,073-2,974)	21,745 (18,906-23,972)	34,347 (30,061-36,995)	27,609 (23,936-29,551)	21,827 (18,973-23,508)	20,817 (17,177-22,459)	18,908 (15,595-20,154)	15,363 (13,019-16,390)	11,168 (9,385-12,060)	9,190 (7,457-9,932)	5,505 (4,443-5,947)	4,044 (3,207-4,441)	2,854 (2,203-3,125)	1,572 (1,218-1,710)	1,188 (936-1,316)	441 (341-494)	115 (87-130)	31 (24-34)
Motor vehicle road injuries	325 (256-390)	249 (193-292)	3,233 (2,671-3,805)	8,805 (6,288-11,342)	7,963 (6,851-8,954)	6,799 (5,923-7,582)	27,064 (24,942-30,684)	44,638 (41,594-49,517)	38,954 (36,292-43,613)	32,766 (30,676-36,466)	31,294 (28,877-34,620)	27,757 (25,703-31,101)	24,319 (21,899-27,008)	20,262 (18,467-21,656)	17,421 (15,626-18,939)	12,952 (11,659-13,904)	10,202 (9,110-11,127)	8,672 (7,801-9,479)	6,548 (5,864-7,087)	4,526 (4,092-4,827)	2,350 (2,109-2,492)	847 (768-896)	258 (238-274)
Other road injuries	8 (5-12)	9 (6-12)	114 (82-157)	248 (156-371)	273 (209-354)	308 (237-403)	556 (451-697)	859 (670-1,073)	745 (610-936)	571 (461-696)	606 (470-735)	626 (479-750)	717 (556-867)	672 (523-851)	577 (452-701)	399 (318-468)	433 (337-527)	409 (329-497)	321 (257-399)	253 (200-315)	117 (97-150)	29 (24-39)	13 (11-16)
Other transport injuries	109 (79-141)	81 (60-113)	557 (413-678)	1,774 (998-2,351)	1,635 (1,356-2,020)	2,279 (1,864-2,874)	6,289 (5,503-7,618)	8,004 (7,300-9,386)	8,125 (7,402-9,352)	6,266 (5,789-7,216)	6,882 (6,284-7,765)	6,441 (5,999-7,399)	6,150 (5,706-7,068)	5,667 (5,234-6,663)	4,852 (4,460-5,573)	3,416 (3,171-3,894)	2,795 (2,565-3,192)	2,290 (2,106-2,613)	1,888 (1,739-2,175)	1,199 (1,092-1,389)	539 (485-621)	178 (160-206)	56 (51-63)
Unintentional injuries	5,652 (4,794-6,209)	4,253 (3,714-4,581)	41,952 (38,270-45,252)	99,270 (90,342-108,745)	58,192 (53,455-62,199)	53,968 (50,616-56,668)	62,498 (59,709-64,501)	76,215 (72,283-78,417)	69,308 (65,910-71,268)	68,388 (64,241-70,255)	73,969 (68,204-76,291)	75,697 (69,481-78,105)	81,385 (74,792-83,924)	75,779 (70,622-77,930)	71,960 (65,841-74,374)	64,909 (58,858-67,131)	58,020 (51,153-60,769)	61,194 (53,328-64,111)	61,895 (53,792-64,818)	57,364 (50,111-60,215)	39,957 (35,133-41,850)	17,963 (16,157-18,700)	6,311 (5,925-6,512)
Falls	922 (619-1,104)	342 (219-410)	4,246 (3,016-4,954)	7,284 (5,125-9,184)	4,999 (3,794-5,877)	4,904 (3,838-5,622)	5,685 (4,648-6,199)	9,227 (7,406-9,938)	9,566 (7,910-10,336)	11,242 (9,044-12,087)	14,063 (11,003-15,080)	17,149 (13,214-18,301)	19,063 (15,128-20,214)	18,720 (15,577-19,745)	20,628 (16,938-21,956)	21,567 (17,677-22,962)	23,316 (18,900-24,937)	27,658 (22,011-29,687)	32,799 (26,411-35,006)	33,377 (27,161-35,751)	25,903 (21,438-27,495)	12,298 (10,571-12,965)	4,256 (3,864-4,442)
Drowning	303 (277-333)	153 (140-165)	4,449 (4,065-4,890)	47,177 (42,605-52,186)	26,056 (23,715-28,375)	19,880 (18,413-21,206)	17,665 (16,779-18,631)	16,742 (16,061-17,480)	12,117 (11,727-12,581)	11,336 (10,977-11,795)	11,280 (10,919-11,701)	10,879 (10,591-11,244)	10,389 (10,083-10,700)	9,189 (8,951-9,452)	7,888 (7,699-8,090)	7,127 (6,932-7,315)	5,886 (5,702-6,061)	5,834 (5,665-6,014)	4,690 (4,549-4,826)	3,905 (3,762-4,036)	1,957 (1,879-2,026)	599 (578-616)	150 (146-154)
Fire, heat, and hot substances	273 (173-330)	213 (133-261)	2,788 (1,795-3,334)	6,680 (4,047-8,431)	2,096 (1,524-2,501)	1,249 (919-1,473)	1,881 (1,360-2,210)	3,102 (2,365-3,534)	3,297 (2,585-3,818)	3,339 (2,765-3,803)	3,666 (3,035-4,163)	3,558 (2,993-3,964)	4,096 (3,463-4,480)	4,147 (3,579-4,503)	3,894 (3,398-4,300)	3,619 (3,102-3,942)	3,045 (2,563-3,410)	3,384 (2,844-3,759)	3,089 (2,596-3,401)	3,098 (2,612-3,404)	2,051 (1,786-2,218)	903 (808-960)	337 (314-351)
Poisonings	216 (118-255)	177 (117-206)	1,871 (1,231-2,177)	3,706 (2,475-4,480)	1,397 (913-1,596)	1,422 (898-1,650)	1,893 (1,306-2,099)	3,126 (2,192-3,393)	3,095 (2,344-3,351)	3,062 (2,254-3,289)	3,327 (2,471-3,581)	3,710 (2,685-4,026)	3,908 (2,850-4,239)	3,625 (2,756-3,924)	3,456 (2,570-3,738)	2,914 (2,126-3,149)	2,119 (1,449-2,331)	2,182 (1,535-2,377)	1,717 (1,134-1,881)	1,321 (865-1,451)	605 (417-662)	172 (125-186)	41 (32-44)
Poisoning by carbon monoxide	45 (25-55)	41 (25-49)	343 (196-421)	762 (504-946)	567 (361-650)	502 (317-570)	964 (662-1,086)	1,725 (1,235-1,882)	1,730 (1,362-1,857)	1,864 (1,448-1,992)	2,011 (1,579-2,152)	2,214 (1,713-2,380)	2,433 (1,938-2,606)	2,284 (1,889-2,447)	2,156 (1,725-2,314)	1,721 (1,343-1,844)	1,140 (822-1,257)	1,248 (908-1,361)	1,009 (669-1,116)	767 (498-858)	346 (233-386)	99 (69-108)	20 (15-22)
Poisoning by other means	171 (92-205)	136 (93-161)	1,528 (1,014-1,810)	2,944 (1,952-3,614)	830 (549-984)	920 (568-1,093)	929 (619-1,063)	1,401 (939-1,581)	1,365 (982-1,521)	1,198 (806-1,329)	1,316 (873-1,462)	1,495 (966-1,679)	1,475 (910-1,674)	1,341 (851-1,511)	1,300 (820-1,452)	1,193 (773-1,330)	979 (624-1,099)	934 (600-1,039)	708 (450-787)	555 (363-607)	259 (180-284)	73 (54-80)	21 (17-22)
Exposure to mechanical forces	767 (553-855)	704 (533-783)	3,402 (2,449-3,781)	5,749 (4,388-6,665)	3,163 (2,797-3,528)	2,684 (2,399-2,977)	6,072 (5,427-6,697)	9,432 (8,077-10,019)	8,886 (7,556-9,390)	8,695 (7,193-9,144)	10,248 (8,452-10,740)	9,603 (7,285-10,070)	9,828 (7,969-10,285)	8,106 (6,751-8,718)	6,988 (5,771-7,370)	4,895 (4,139-5,121)	3,706 (3,225-3,925)	3,259 (2,863-3,468)	2,765 (2,506-2,933)	1,886 (1,714-1,976)	1,134 (1,072-1,176)	490 (460-512)	182 (177-187)
Unintentional firearm injuries	83 (62-126)	65 (49-110)	522 (383-775)	804 (471-1,540)	390 (324-551)	475 (415-613)	1,500 (1,322-1,734)	2,124 (1,908-2,447)	2,190 (1,945-2,551)	1,549 (1,371-1,834)	1,370 (1,221-1,628)	1,085 (967-1,298)	1,179 (1,061-1,401)	969 (871-1,150)	736 (672-896)	612 (548-712)	506 (456-607)	477 (428-568)	433 (386-530)	330 (297-414)	185 (167-238)	84 (76-106)	26 (24-32)
Other exposure to mechanical forces	685 (478-771)	638 (473-716)	2,881 (1,999-3,211)	4,944 (3,787-5,799)	2,773 (2,389-3,098)	2,208 (1,927-2,453)	4,572 (3,941-5,096)	7,307 (6,036-7,851)	6,695 (5,526-7,105)	7,146 (5,810-7,555)	8,878 (7,029-9,353)	8,518 (6,162-8,969)	8,649 (6,680-9,093)	7,137 (5,735-7,718)	6,252 (4,961-6,630)	4,283 (3,489-4,489)	3,200 (2,715-3,400)	2,782 (2,345-2,967)	2,332 (2,021-2,474)	1,556 (1,346-1,631)	948 (852-988)	406 (371-423)	156 (149-161)
Adverse effects of medical treatment	596 (423-791)	352 (271-485)	3,620 (2,559-5,320)	2,975 (1,698-5,812)	1,136 (873-1,480)	946 (741-1,161)	1,356 (1,046-1,586)	2,134 (1,614-2,513)	1,926 (1,497-2,239)	2,048 (1,579-2,368)	2,308 (1,781-2,677)	2,580 (1,987-2,978)	2,780 (2,144-3,178)	2,499 (2,013-2,961)	2,963 (2,425-3,473)	3,904 (3,124-4,551)	4,553 (3,578-5,308)	4,400 (3,636-5,316)	4,520 (3,719-5,439)	3,843 (3,233-4,643)	2,429 (2,070-3,020)	1,003 (875-1,311)	336 (301-455)
Animal contact	208 (78-255)	197 (74-246)	1,267 (535-1,556)	3,469 (1,565-4,319)	3,406 (1,674-4,190)	3,455 (1,649-4,220)	2,490 (1,257-2,955)	2,417 (1,209-2,832)	2,430 (1,276-2,792)	2,405 (1,239-2,824)	2,431 (1,239-2,791)	2,612 (1,281-3,007)	3,267 (1,717-3,774)	2,336 (1,293-2,668)	2,708 (1,480-3,078)	2,569 (1,264-2,970)	2,648 (1,215-3,085)	2,245 (1,089-2,594)	1,967 (963-2,281)	1,251 (613-1,461)	548 (287-631)	161 (95-185)	42 (28-47)
Venomous animal contact	181 (62-228)	169 (58-217)	1,066 (404-1,359)	2,761 (1,083-3,686)	2,989 (1,402-3,745)	3,196 (1,467-3,936)	2,174 (1,059-2,622)	2,094 (984-2,512)	2,072 (1,009-2,427)	2,184 (1,072-2,617)	2,173 (1,045-2,537)	2,340 (1,079-2,738)	2,879 (1,405-3,376)	1,928 (1,005-2,245)	2,286 (1,174-2,652)	2,190 (1,004-2,597)	2,303 (1,008-2,733)	1,916 (867-2,261)	1,643 (759-1,965)	1,043 (479-1,252)	445 (218-533)	124 (68-146)	30 (18-35)
Non-venomous animal contact	26 (15-35)	28 (16-36)	201 (112-283)	708 (315-1,204)	417 (239-567)	259 (147-323)	316 (198-403)	322 (218-408)	358 (249-437)	221 (146-276)	258 (175-318)	272 (179-339)	388 (266-483)	407 (269-522)	422 (270-546)	379 (234-497)	345 (206-465)	329 (201-435)	325 (202-437)	207 (128-274)	104 (68-134)	37 (24-47)	13 (9-15)
Foreign body	1,816 (1,513-2,033)	1,626 (1,370-1,816)	15,809 (13,844-17,337)	6,975 (5,295-8,235)	1,883 (1,688-2,148)	1,522 (1,403-1,676)	1,229 (1,163-1,329)	2,184 (2,053-2,372)	2,151 (2,061-2,298)	2,277 (2,164-2,415)	2,514 (2,378-2,629)	2,783 (2,663-2,935)	3,127 (2,969-3,304)	3,431 (3,334-3,584)	3,512 (3,415-3,638)	3,165 (3,042-3,268)	2,745 (2,612-2,855)	3,006 (2,775-3,110)	3,313 (3,070-3,416)	3,661 (3,410-3,763)	2,954 (2,837-3,045)	1,489 (1,445-1,530)	688 (669-708)
Pulmonary aspiration and foreign body in airway	1,767 (1,475-1,993)	1,600 (1,352-1,781)	15,376 (13,546-16,827)	6,245 (4,945-7,624)	1,692 (1,506-1,934)	1,392 (1,288-1,537)	1,120 (1,057-1,235)	2,006 (1,891-2,210)	1,959 (1,873-2,126)	1,999 (1,899-2,140)	2,206 (2,074-2,315)	2,461 (2,333-2,563)	2,897 (2,773-3,067)	3,209 (3,100-3,341)	3,307 (3,214-3,443)	2,978 (2,841-3,068)	2,609 (2,486-2,703)	2,842 (2,592-2,938)	3,107 (2,863-3,202)	3,479 (3,237-3,579)	2,839 (2,707-2,920)	1,438 (1,392-1,478)	669 (651-688)
Foreign body in other body part	49 (23-73)	26 (16-42)	433 (260-746)	731 (298-1,511)	191 (134-269)	130 (95-180)	109 (73-133)	178 (124-217)	192 (135-238)	278 (223-346)	308 (251-399)	322 (263-427)	229 (184-293)	222 (173-281)	205 (162-258)	187 (154-266)	136 (112-198)	164 (133-237)	206 (173-315)	182 (153-277)	115 (99-179)	51 (44-75)	19 (16-27)
Environmental heat and cold exposure	179 (65-243)	113 (43-153)	1,133 (426-1,542)	950 (410-1,532)	412 (218-540)	380 (214-482)	1,001 (717-1,179)	1,956 (1,540-2,186)	2,660 (2,243-2,902)	3,847 (3,283-4,151)	5,025 (4,371-5,322)	6,573 (5,866-6,914)	9,647 (8,927-9,988)	11,886 (11,058-12,271)	9,840 (8,936-10,297)	7,318 (6,493-7,736)	4,292 (3,298-4,783)	4,599 (3,671-5,066)	3,575 (2,566-4,049)	2,607 (1,973-2,915)	1,361 (1,054-1,504)	535 (430-582)	186 (165-199)
Exposure to forces of nature	135 (55-272)	296 (128-596)	2,434 (1,111-4,797)	10,746 (4,399-21,468)	10,900 (4,936-21,458)	14,293 (6,260-28,775)	16,657 (7,511-33,029)	16,734 (7,106-35,467)	14,578 (6,729-29,051)	11,506 (5,057-22,630)	9,639 (4,368-19,210)	8,190 (3,694-16,600)	7,381 (3,554-14,405)	6,529 (3,090-13,058)	5,437 (2,712-10,027)	4,376 (2,067-8,756)	3,454 (1,651-6,860)	2,975 (1,417-5,644)	2,304 (1,101-4,428)	1,512 (734-2,958)	598 (300-1,109)	146 (72-289)	31 (17-53)
Other unintentional injuries	237 (204-273)	82 (72-92)	934 (842-1,026)	3,559 (3,072-4,055)	2,744 (2,431-3,045)	3,232 (2,905-3,535)	6,569 (6,125-7,069)	9,162 (8,680-9,701)	8,603 (8,202-9,048)	8,633 (8,240-9,118)	9,467 (9,023-9,945)	8,061 (7,727-8,389)	7,899 (7,576-8,279)	5,312 (5,096-5,553)	4,646 (4,465-4,852)	3,454 (3,304-3,616)	2,255 (2,137-2,377)	1,654 (1,569-1,734)	1,155 (1,100-1,213)	903 (841-955)	415 (390-437)	167 (157-175)	61 (58-64)
Self-harm and interpersonal violence	887 (662-997)	273 (219-305)	2,446 (1,993-2,794)	6,031 (4,932-6,729)	4,273 (3,663-4,680)	11,726 (10,402-12,530)	65,623 (60,529-68,141)	113,894 (106,411-117,341)	108,735 (101,226-111,680)	97,484 (90,104-100,328)	87,484 (80,887-90,258)	76,949 (70,687-79,167)	69,802 (64,881-71,685)	59,931 (56,309-61,591)	50,921 (47,030-52,416)	38,982 (35,126-40,118)	30,279 (26,682-31,299)	28,220 (25,027-29,091)	23,187 (19,978-24,013)	16,200 (13,771-16,868)	8,036 (7,213-8,323)	2,438 (2,262-2,524)	670 (647-698)
Self-harm	-	-	-	-	-	5,167 (4,449-5,717)	27,329 (24,633-28,988)	51,712 (46,990-54,388)	53,153 (47,794-55,535)	50,610 (44,964-52,602)	49,495 (44,309-51,429)	47,158 (41,836-48,957)	46,374 (42,203-47,818)	41,597 (38,135-42,860)	37,540 (33,942-38,712)	29,785 (26,066-30,697)	23,958 (20,674-24,839)	23,154 (19,862-23,998)	19,907 (16,716-20,689)	15,097 (12,661-15,755)	7,547 (6,713-7,819)	2,271 (2,091-2,352)	545 (523-571)
Self-harm by firearm	-	-	-	-	-	255 (187-419)	3,121 (2,348-4,491)	5,785 (4,336-8,012)	5,278 (3,989-7,185)	4,444 (3,367-5,978)	3,981 (3,157-5,409)	3,885 (3,070-5,125)	4,385 (3,616-5,703)	4,363 (3,569-5,572)	3,866 (3,147-4,893)	3,099 (2,535-3,845)	2,472 (1,995-3,012)	2,258 (1,809-2,820)	1,991 (1,602-2,478)	1,691 (1,401-2,105)	1,079 (900-1,392)	356 (292-464)	92 (75-126)
Self-harm by other specified means	-	-	-	-	-	4,911 (4,145-5,464)	24,209 (21,281-25,991)	45,927 (40,055-48,673)	47,875 (42,433-50,449)	46,165 (40,960-48,427)	45,514 (39,639-47,553)	43,272 (37,817-45,231)	41,989 (37,431-43,679)	37,235 (33,401-38,629)	33,674 (30,036-34,986)	26,686 (23,065-27,746)	21,486 (18,183-22,429)	20,896 (17,470-21,836)	17,917 (14,682-18,793)	13,406 (10,880-14,155)	6,468 (5,549-6,819)	1,916 (1,691-2,014)	453 (410-479)
Interpersonal violence	755 (530-865)	202 (148-234)	2,181 (1,737-2,544)	3,941 (2,846-4,642)	2,753 (2,141-3,160)	4,986 (3,958-5,596)	33,613 (28,866-35,719)	54,206 (47,541-58,170)	49,653 (44,125-52,475)	42,579 (37,649-45,743)	34,827 (30,519-37,912)	27,658 (24,039-29,838)	21,966 (19,431-24,143)	17,259 (15,184-18,901)	12,508 (11,036-13,819)	8,499 (7,288-9,295)	5,796 (4,832-6,359)	4,612 (3,886-5,099)	2,979 (2,543-3,333)	873 (756-987)	375 (339-434)	118 (108-133)	101 (95-107)
Physical violence by firearm	126 (67-165)	37 (21-51)	410 (245-519)	791 (451-1,140)	585 (400-717)	1,719 (1,332-1,954)	18,777 (15,947-20,276)	29,299 (25,172-31,903)	25,964 (22,031-28,117)	20,304 (17,007-22,220)	15,735 (12,765-17,726)	11,808 (9,700-13,320)	8,251 (6,777-9,292)	6,147 (4,907-6,983)	4,153 (3,385-4,856)	2,524 (2,014-2,961)	1,627 (1,237-1,887)	1,182 (880-1,443)	767 (567-934)	200 (148-242)	92 (71-109)	34 (28-40)	30 (21-33)
Physical violence by sharp object	74 (40-101)	22 (12-33)	195 (124-288)	551 (272-954)	365 (229-486)	736 (475-945)	6,888 (4,950-8,612)	11,920 (8,867-14,407)	11,539 (8,720-14,152)	10,239 (7,796-12,413)	8,682 (6,607-10,931)	7,245 (5,604-8,705)	6,037 (4,801-7,351)	4,695 (3,625-5,804)	3,185 (2,555-3,819)	2,364 (1,787-2,861)	1,566 (1,094-1,868)	1,176 (860-1,347)	741 (569-895)	215 (179-245)	89 (75-103)	29 (24-33)	24 (21-27)
Physical violence by other means	555 (403-661)	143 (109-176)	1,576 (1,283-2,009)	2,599 (1,892-3,216)	1,803 (1,412-2,201)	2,531 (1,949-3,190)	7,949 (6,411-10,323)	12,988 (10,785-16,516)	12,149 (10,112-15,148)	12,036 (10,257-14,978)	10,410 (8,729-13,059)	8,605 (7,334-10,474)	7,679 (6,659-9,141)	6,417 (5,515-7,590)	5,171 (4,435-6,186)	3,611 (3,086-4,332)	2,603 (2,196-3,209)	2,255 (1,922-2,765)	1,471 (1,247-1,761)	459 (395-567)	195 (172-243)	56 (51-68)	48 (45-56)
Conflict and terrorism	132 (118-151)	71 (64-82)	221 (199-256)	2,035 (1,820-2,349)	1,474 (1,314-1,718)	1,512 (1,335-1,751)	4,225 (3,757-4,932)	7,120 (6,295-8,360)	5,107 (4,505-5,923)	3,646 (3,188-4,263)	2,651 (2,322-3,096)	1,728 (1,503-2,042)	1,176 (1,008-1,420)	879 (724-1,109)	749 (600-965)	620 (488-809)	478 (381-630)	423 (327-570)	281 (225-364)	216 (173-279)	106 (86-136)	45 (37-56)	21 (18-25)
Executions and police conflict	-	-	44 (12-115)	54 (51-62)	46 (44-49)	61 (57-67)	456 (407-522)	855 (770-960)	821 (733-932)	649 (572-755)	511 (443-598)	405 (350-488)	285 (247-346)	196 (168-241)	124 (107-150)	77 (68-93)	48 (41-57)	31 (27-36)	19 (17-22)	14 (12-16)	7 (6-9)	3 (3-4)	3 (2-4)

SR Table 6: Global deaths by cause and age group for males (with 95% confidence intervals), 2017

Cause	Early Neonatal	Late Neonatal	Post Neonatal	1 to 4	5 to 9	10 to 14	15 to 19	20 to 24	25 to 29	30 to 34	35 to 39
All causes	1,069,764 (1,031,163-1,113,468)	272,690 (262,615-283,473)	845,068 (811,266-885,412)	728,674 (694,449-766,406)	221,045 (216,730-225,678)	182,066 (179,332-185,234)	311,138 (305,204-317,435)	449,756 (441,821-458,142)	523,975 (514,588-534,130)	633,628 (622,960-645,079)	730,485 (717,068-744,360)
Communicable, maternal, neonatal, and nutritional diseases	960,722 (922,683-1,001,328)	232,706 (223,373-242,712)	642,345 (609,756-677,284)	527,306 (495,232-563,335)	110,863 (102,543-121,290)	69,952 (64,183-77,444)	74,539 (67,664-83,408)	86,245 (79,428-95,606)	112,180 (103,843-122,537)	150,314 (141,452-161,056)	172,863 (162,841-185,130)
HIV/AIDS and sexually transmitted infections	-	10,953 (4,255-20,255)	49,165 (33,775-70,304)	15,862 (13,381-19,012)	12,808 (11,771-13,946)	12,433 (11,659-13,231)	18,348 (16,588-20,184)	18,418 (16,590-20,777)	41,033 (35,692-47,579)	65,522 (59,135-72,665)	75,189 (68,224-82,541)
HIV/AIDS	-	-	25,594 (22,668-28,730)	13,735 (11,954-16,283)	12,052 (11,147-13,008)	12,402 (11,629-13,200)	18,315 (16,554-20,148)	18,379 (16,560-20,737)	40,979 (35,656-47,526)	65,462 (59,083-72,603)	75,116 (68,157-82,473)
HIV/AIDS - Drug-susceptible Tuberculosis	-	-	6,804 (5,082-8,452)	3,466 (2,693-4,252)	3,649 (2,641-4,408)	3,638 (2,610-4,406)	5,096 (3,733-6,349)	4,769 (3,612-5,822)	8,595 (6,178-11,339)	12,176 (8,410-16,460)	13,061 (8,928-17,738)
HIV/AIDS - Multidrug-resistant Tuberculosis without extensive drug resistance	-	-	870 (505-1,389)	415 (246-650)	414 (242-638)	412 (244-621)	577 (344-871)	552 (343-812)	1,026 (607-1,564)	1,487 (871-2,302)	1,590 (936-2,476)
HIV/AIDS - Extensively drug-resistant Tuberculosis	-	-	24 (14-36)	13 (8-19)	10 (6-14)	9 (6-14)	15 (9-22)	24 (15-36)	68 (40-105)	126 (74-193)	139 (83-213)
HIV/AIDS resulting in other diseases	-	-	17,896 (15,094-21,202)	9,842 (8,149-12,387)	7,979 (6,962-9,333)	8,342 (7,320-9,626)	12,626 (10,839-14,737)	13,034 (11,216-15,637)	31,290 (25,480-38,103)	51,673 (44,064-59,929)	60,327 (51,991-69,641)
Sexually transmitted infections excluding HIV	31,905 (12,566-60,549)	10,953 (4,255-20,255)	23,571 (8,841-44,295)	2,127 (776-4,135)	756 (281-1,472)	31 (27-38)	33 (28-37)	39 (29-47)	54 (34-69)	60 (43-86)	73 (45-109)
Syphilis	31,905 (12,566-60,549)	10,953 (4,255-20,255)	23,571 (8,841-44,295)	2,127 (776-4,135)	756 (281-1,472)	26 (23-32)	27 (23-30)	32 (24-38)	45 (29-58)	46 (33-66)	60 (37-90)
Chlamydial infection	-	-	-	-	-	0 (0-0)	0 (0-0)	0 (0-0)	0 (0-0)	0 (0-0)	0 (0-0)
Gonococcal infection	-	-	-	-	-	2 (2-3)	4 (3-4)	4 (3-5)	5 (4-7)	11 (8-16)	3 (2-5)
Other sexually transmitted infections	-	-	-	-	-	2 (2-3)	2 (2-2)	3 (2-3)	3 (2-4)	2 (2-3)	9 (6-14)
Respiratory infections and tuberculosis	-	46,164 (41,451-51,156)	211,828 (191,307-234,501)	114,709 (97,180-132,559)	17,283 (14,519-19,930)	10,926 (9,581-12,293)	15,292 (14,003-17,097)	27,218 (25,290-29,834)	36,382 (33,969-39,519)	49,286 (46,374-52,788)	59,662 (55,988-63,969)
Tuberculosis	-	-	14,681 (13,136-16,373)	13,736 (11,693-15,781)	3,059 (2,623-3,526)	3,099 (2,714-3,536)	8,027 (7,064-9,079)	18,318 (16,610-20,408)	25,737 (23,612-28,187)	35,733 (33,076-38,783)	42,741 (39,585-46,301)
Drug-susceptible tuberculosis	-	-	13,099 (11,601-14,770)	12,357 (10,432-14,373)	2,741 (2,338-3,178)	2,771 (2,408-3,200)	7,184 (6,268-8,201)	16,320 (14,470-18,391)	22,717 (20,419-25,229)	31,291 (27,867-34,539)	37,313 (33,414-41,342)
Multidrug-resistant tuberculosis without extensive drug resistance	-	-	1,487 (925-2,205)	1,322 (840-2,000)	298 (195-427)	308 (198-457)	791 (510-1,165)	1,837 (1,196-2,735)	2,721 (1,748-4,033)	3,987 (2,390-6,117)	4,845 (2,838-7,591)
Extensively drug-resistant tuberculosis	-	-	94 (62-136)	57 (40-78)	20 (14-28)	21 (14-31)	52 (36-76)	161 (116-220)	300 (227-393)	455 (333-611)	583 (427-783)
Lower respiratory infections	73,714 (64,057-85,974)	46,111 (41,405-51,099)	196,134 (176,719-217,007)	99,857 (84,163-116,201)	14,098 (11,540-16,332)	7,741 (6,512-8,814)	7,210 (6,211-8,068)	8,850 (7,573-9,941)	10,585 (9,026-11,520)	13,482 (11,849-14,921)	16,821 (14,749-18,453)
Upper respiratory infections	20 (13-26)	47 (28-62)	965 (444-1,507)	1,082 (349-2,027)	106 (53-152)	67 (29-97)	42 (21-65)	39 (21-58)	50 (33-65)	63 (42-83)	87 (50-123)
Otitis media	7 (2-32)	7 (3-17)	48 (21-133)	33 (13-86)	19 (10-45)	19 (11-43)	14 (8-27)	12 (6-27)	9 (5-20)	8 (5-18)	13 (7-29)
Enteric infections	9,587 (7,941-11,551)	17,848 (15,012-21,060)	156,544 (133,598-183,042)	134,502 (110,331-161,736)	36,912 (26,517-51,547)	21,228 (14,660-30,423)	17,001 (11,185-26,812)	15,086 (9,506-24,175)	12,315 (7,648-20,614)	11,893 (7,074-21,255)	12,944 (7,270-24,304)
Diarrheal diseases	9,587 (7,941-11,551)	17,642 (14,825-20,877)	152,231 (129,543-177,809)	109,070 (87,894-134,471)	14,719 (6,943-29,344)	7,292 (3,421-15,203)	7,153 (3,214-14,583)	7,984 (3,492-16,593)	7,539 (3,227-15,536)	8,335 (3,765-18,257)	10,173 (4,549-21,528)
Typhoid and paratyphoid	-	0 (0-0)	162 (79-301)	13,017 (6,252-23,183)	18,282 (10,656-28,676)	12,360 (7,008-19,637)	8,905 (4,409-15,799)	6,233 (3,058-11,066)	4,004 (2,069-6,874)	2,792 (1,423-4,719)	2,014 (1,046-3,465)
Typhoid fever	-	-	143 (70-261)	11,339 (5,266-20,764)	15,824 (9,076-25,321)	10,656 (5,902-17,171)	7,639 (3,643-13,474)	5,332 (2,604-9,620)	3,419 (1,716-5,944)	2,378 (1,183-4,096)	1,712 (852-2,960)
Paratyphoid fever	-	-	19 (7-39)	1,678 (624-3,518)	2,458 (1,077-4,753)	1,703 (736-3,333)	1,267 (505-2,612)	902 (369-1,945)	585 (242-1,228)	414 (174-855)	302 (131-606)
Invasive Non-typhoidal Salmonella (iNTS)	-	205 (112-357)	3,894 (2,236-6,988)	12,278 (6,004-22,948)	3,890 (1,847-7,186)	1,562 (682-2,983)	927 (372-1,790)	849 (338-1,659)	760 (292-1,641)	748 (318-1,451)	740 (324-1,457)
Other intestinal infectious diseases	-	-	256 (102-665)	137 (46-404)	21 (4-55)	15 (2-41)	16 (2-42)	19 (3-50)	12 (1-33)	18 (2-47)	17 (2-43)
Neglected tropical diseases and malaria	247 (99-935)	8,825 (4,999-14,026)	65,012 (41,140-94,360)	124,498 (84,345-174,733)	22,304 (16,189-30,652)	13,385 (9,763-18,443)	12,345 (8,758-16,880)	13,689 (9,684-18,559)	11,795 (8,331-16,433)	10,907 (7,717-14,946)	11,540 (8,283-15,843)
Malaria	-	8,618 (4,839-13,831)	61,964 (37,561-91,536)	115,977 (75,423-164,965)	18,195 (12,522-26,697)	10,508 (7,161-15,346)	9,924 (6,502-14,295)	10,931 (7,221-16,004)	9,627 (6,279-14,167)	8,766 (5,805-12,982)	9,172 (6,051-13,498)
Chagas disease	-	-	1 (1-1)	0 (0-1)	1 (1-1)	2 (2-3)	5 (4-6)	11 (9-12)	17 (15-20)	42 (37-50)	89 (78-106)
Leishmaniasis	-	-	234 (0-905)	1,326 (1-6,345)	579 (0-3,780)	390 (0-2,464)	329 (0-1,804)	277 (0-1,379)	257 (0-1,176)	261 (0-1,128)	231 (0-922)
Visceral leishmaniasis	-	-	234 (0-905)	1,326 (1-6,345)	579 (0-3,780)	390 (0-2,464)	329 (0-1,804)	277 (0-1,379)	257 (0-1,176)	261 (0-1,128)	231 (0-922)
African trypanosomiasis	-	-	-	33 (2-171)	55 (6-244)	66 (7-318)	78 (7-391)	75 (7-366)	65 (6-324)	62 (6-335)	53 (5-250)
Schistosomiasis	-	-	0 (0-0)	220 (137-354)	103 (71-147)	149 (104-200)	173 (131-231)	203 (154-253)	196 (151-250)	207 (163-259)	248 (194-313)
Cysticercosis	0 (0-0)	0 (0-0)	0 (0-0)	41 (13-85)	20 (4-46)	15 (2-41)	18 (1-52)	28 (4-71)	33 (6-78)	34 (5-84)	31 (4-81)
Cystic echinococcosis	-	-	-	74 (28-131)	33 (5-76)	24 (1-61)	14 (0-42)	16 (0-49)	24 (0-67)	27 (0-80)	34 (1-98)
Dengue	-	-	1,466 (248-2,082)	2,351 (936-3,384)	1,325 (531-1,907)	748 (233-1,055)	809 (228-1,156)	1,148 (263-1,736)	913 (221-1,337)	820 (164-1,273)	1,000 (176-1,565)
Yellow fever	-	2 (0-5)	37 (7-115)	380 (72-1,184)	553 (110-1,623)	476 (95-1,383)	401 (75-1,201)	339 (66-965)	240 (48-727)	199 (40-616)	168 (32-545)
Rabies	-	-	91 (62-147)	890 (522-1,651)	905 (572-1,513)	796 (553-1,239)	468 (329-731)	521 (387-716)	259 (203-343)	315 (248-422)	306 (233-419)
Intestinal nematode infections	-	-	102 (71-145)	1,133 (752-1,694)	170 (123-235)	39 (28-51)	13 (9-18)	12 (8-16)	8 (6-12)	12 (8-15)	11 (8-16)
Ascariasis	-	-	102 (71-145)	1,133 (752-1,694)	170 (123-235)	39 (28-51)	13 (9-18)	12 (8-16)	8 (6-12)	12 (8-15)	11 (8-16)
Ebola	0 (0-0)	0 (0-0)	0 (0-0)	1 (1-1)	0 (0-0)	0 (0-0)	0 (0-0)	0 (0-0)	0 (0-0)	0 (0-0)	2 (2-3)
Zika virus	0 (0-0)	0 (0-0)	0 (0-0)	0 (0-2)	0 (0-2)	0 (0-3)	1 (0-3)	1 (0-5)	1 (0-4)	1 (0-4)	1 (0-5)
Other neglected tropical diseases	247 (99-935)	205 (92-762)	1,117 (533-4,007)	2,070 (654-9,464)	364 (175-1,114)	171 (93-488)	111 (74-272)	127 (82-315)	155 (105-345)	161 (111-318)	194 (119-387)
Other infectious diseases	11,761 (8,861-14,576)	12,420 (9,394-15,303)	87,953 (71,092-109,266)	98,418 (73,201-130,807)	18,819 (15,000-23,883)	10,929 (9,002-12,826)	10,699 (8,831-12,271)	10,927 (9,071-12,187)	9,822 (8,155-10,965)	11,841 (10,174-13,189)	12,449 (10,486-13,735)
Meningitis	5,535 (3,950-6,882)	5,600 (4,463-6,864)	37,688 (30,419-45,305)	33,753 (25,316-43,951)	7,137 (5,246-9,793)	4,682 (3,525-6,358)	4,790 (3,613-6,459)	4,679 (3,628-6,072)	3,747 (3,002-4,993)	4,241 (3,468-5,888)	4,485 (3,641-6,060)
Pneumococcal meningitis	882 (634-1,151)	874 (660-1,152)	5,786 (4,392-7,546)	5,592 (3,812-8,026)	840 (573-1,232)	504 (353-742)	563 (388-816)	603 (439-843)	494 (360-697)	616 (464-859)	722 (528-988)
H influenzae type B meningitis	1,150 (704-1,552)	1,160 (867-1,542)	8,782 (6,672-11,690)	7,125 (5,046-10,012)	2,077 (1,414-2,895)	1,358 (1,005-1,878)	1,282 (943-1,750)	1,158 (879-1,548)	1,027 (810-1,354)	1,106 (900-1,594)	1,107 (893-1,638)
Meningococcal meningitis	790 (555-1,040)	786 (601-1,012)	5,012 (3,816-6,396)	4,369 (3,024-6,034)	741 (503-1,094)	410 (286-591)	388 (270-543)	358 (249-489)	284 (205-390)	331 (243-471)	346 (253-504)
Other meningitis	2,712 (1,967-3,435)	2,780 (2,186-3,497)	18,109 (14,298-22,460)	16,666 (12,255-22,080)	3,479 (2,466-4,840)	2,410 (1,804-3,244)	2,557 (1,885-3,429)	2,560 (1,945-3,292)	1,942 (1,509-2,501)	2,188 (1,739-2,986)	2,311 (1,809-3,097)
Encephalitis	293 (258-329)	466 (378-541)	5,755 (4,903-6,542)	5,761 (4,948-6,693)	2,321 (1,760-2,935)	1,977 (1,591-2,379)	1,465 (1,204-1,761)	1,390 (1,198-1,726)	1,267 (1,117-1,567)	1,639 (1,442-1,999)	1,710 (1,522-2,113)
Diphtheria	-	-	321 (141-662)	1,173 (421-3,191)	176 (84-370)	62 (33-121)	21 (12-38)	14 (8-24)	12 (7-19)	13 (8-21)	14 (9-24)
Whooping cough	-	-	19,688 (9,845-35,662)	18,615 (9,455-33,806)	1,590 (795-2,958)	331 (165-616)	135 (70-244)	94 (49-169)	49 (23-91)	43 (20-81)	31 (15-57)
Tetanus	3,513 (2,327-5,773)	4,082 (2,486-6,338)	1,478 (908-2,691)	988 (341-2,473)	597 (282-1,196)	409 (212-724)	680 (326-1,266)	593 (292-1,062)	629 (282-1,132)	612 (345-981)	705 (359-1,069)
Measles	-	-	12,768 (4,537-28,848)	29,281 (10,476-62,540)	3,894 (1,390-8,635)	995 (359-2,208)	317 (112-703)	150 (53-340)	62 (22-139)	44 (16-96)	29 (11-64)
Varicella and herpes zoster	51 (38-67)	137 (103-176)	1,324 (1,016-1,695)	1,600 (1,197-2,063)	539 (408-721)	183 (139-238)	91 (71-118)	103 (82-130)	126 (102-158)	172 (141-215)	156 (126-195)
Acute hepatitis	-	-	3,417 (1,695-5,121)	2,934 (1,635-5,071)	1,260 (744-1,926)	1,318 (788-1,808)	1,814 (1,113-2,283)	2,761 (1,754-3,316)	2,904 (1,783-3,459)	3,884 (2,317-4,654)	3,846 (2,334-4,518)
Acute hepatitis A	-	-	563 (204-1,045)	1,769 (985-3,102)	846 (495-1,303)	687 (413-976)	685 (411-966)	790 (430-1,180)	624 (324-968)	639 (298-1,091)	550 (233-1,008)
Acute hepatitis B	-	-	2,131 (929-3,260)	864 (394-1,627)	214 (98-396)	244 (120-427)	464 (248-764)	959 (536-1,557)	1,431 (802-2,102)	2,416 (1,319-3,356)	2,703 (1,623-3,594)
Acute hepatitis C	-	-	717 (318-1,355)	235 (104-469)	24 (10-53)	10 (4-23)	8 (3-17)	9 (3-19)	9 (3-23)	15 (4-38)	20 (5-52)
Acute hepatitis E	-	-	6 (2-12)	66 (25-151)	177 (82-314)	376 (183-601)	657 (356-941)	1,003 (505-1,454)	840 (372-1,283)	814 (324-1,453)	573 (214-1,092)
Other unspecified infectious diseases	2,369 (1,430-2,848)	2,136 (1,190-2,624)	5,514 (3,535-6,616)	4,312 (2,835-5,358)	1,305 (812-1,788)	972 (681-1,332)	1,388 (952-1,862)	1,142 (811-1,454)	1,025 (800-1,221)	1,194 (929-1,427)	1,473 (1,176-1,717)
Maternal and neonatal disorders	833,480 (786,378-875,888)	136,496 (126,521-146,928)	40,103 (31,133-47,680)	4,281 (2,890-5,269)	-	-	-	-	-	-	-
Maternal disorders	-	-	-	-	-	-	-	-	-	-	-
Maternal hemorrhage	-	-	-	-	-	-	-	-	-	-	-
Maternal sepsis and other maternal infections	-	-	-	-	-	-	-	-	-	-	-
Maternal hypertensive disorders	-	-	-	-	-	-	-	-	-	-	-
Maternal obstructed labor and uterine rupture	-	-	-	-	-	-	-	-	-	-	-
Maternal abortion and miscarriage	-	-	-	-	-	-	-	-	-	-	-
Ectopic pregnancy	-	-	-	-	-	-	-	-	-	-	-
Indirect maternal deaths	-	-	-	-	-	-	-	-	-	-	-
Late maternal deaths	-	-	-	-	-	-	-	-	-	-	-
Maternal deaths aggravated by HIV/AIDS	-	-	-	-	-	-	-	-	-	-	-
Other maternal disorders	-	-	-	-	-	-	-	-	-	-	-
Neonatal disorders	833,480 (786,378-875,888)	136,496 (126,521-146,928)	40,103 (31,133-47,680)	4,281 (2,890-5,269)	-	-	-	-	-	-	-
Neonatal preterm birth	317,417 (290,636-366,648)	37,559 (33,221-42,717)	14,215 (10,571-16,852)	1,132 (684-1,453)	-	-	-	-	-	-	-
Neonatal encephalopathy due to birth asphyxia and trauma	278,312 (244,620-309,507)	23,097 (17,557-27,969)	7,474 (5,049-9,666)	1,740 (1,116-2,207)	-	-	-	-	-	-	-
Neonatal sepsis and other neonatal infections	65,706 (54,622-83,623)	40,150 (34,212-48,456)	6,196 (4,936-7,967)	311 (180-558)	-	-	-	-	-	-	-
Hemolytic disease and other neonatal jaundice	18,292 (15,242-21,592)	7,601 (6,036-9,540)	1,717 (1,132-2,693)	112 (68-181)	-	-	-	-	-	-	-
Other neonatal disorders	153,754 (119,959-175,814)	28,089 (23,756-32,840)	10,501 (7,518-13,414)	986 (618-1,254)	-	-	-	-	-	-	-
Nutritional deficiencies	-	-	31,740 (27,067-38,885)	35,036 (28,665-43,145)	2,736 (2,292-3,467)	1,050 (909-1,330)	853 (744-1,083)	906 (805-1,143)	833 (752-1,050)	864 (786-1,058)	1,078 (987-1,304)
Protein-energy malnutrition	-	-	30,766 (26,182-37,646)	34,187 (27,887-42,076)	2,475 (2,084-3,090)	908 (780-1,155)	683 (592-873)	715 (624-912)	660 (578-838)	702 (623-875)	802 (717-985)
Other nutritional deficiencies	-	-	974 (753-1,316)	848 (606-1,153)	261 (181-384)	142 (109-193)	170 (137-233)	191 (160-255)	173 (147-230)	162 (131-218)	277 (232-367)
Non-communicable diseases	103,117 (94,682-114,528)	36,216 (32,953-40,011)	161,585 (145,912-181,283)	101,921 (90,693-113,950)	46,655 (41,713-50,614)	44,619 (40,959-47,793)	71,259 (67,140-75,544)	103,311 (98,792-108,067)	149,962 (145,079-155,141)	232,624 (226,491-239,508)	328,747 (320,059-337,464)
Neoplasms	773 (637-866)	474 (421-524)	4,909 (4,346-5,461)	21,911 (19,035-24,701)	18,328 (16,025-20,165)	16,671 (15,158-18,061)	19,152 (17,833-20,358)	21,462 (20,381-22,408)	29,847 (28,533-31,075)	45,990 (44,350-47,602)	65,901 (63,835-68,303)
Lip and oral cavity cancer	-	-	-	-	-	-	297 (249-353)	458 (394-528)	759 (664-859)	1,518 (1,334-1,697)	2,977 (2,625-3,329)
Nasopharynx cancer	-	-	-	-	112 (98-130)	186 (166-210)	231 (213-253)	281 (261-306)	438 (410-474)	804 (747-875)	1,359 (1,271-1,465)
Other pharynx cancer	-	-	-	-	-	-	109 (75-131)	181 (105-215)	244 (151-288)	704 (453-816)	1,479 (1,054-1,702)
Esophageal cancer	-	-	-	-	-	-	120 (106-135)	226 (208-247)	384 (361-407)	947 (905-994)	2,090 (1,985-2,205)
Stomach cancer	-	-	-	-	-	-	303 (283-326)	702 (663-742)	1,576 (1,505-1,660)	3,328 (3,190-3,486)	5,178 (4,980-5,399)
Colon and rectum cancer	-	-	-	-	-	-	467 (388-522)	993 (841-1,076)	1,917 (1,729-2,040)	3,698 (3,438-3,901)	5,191 (4,884-5,475)
Liver cancer	-	-	-	-	414 (350-484)	491 (424-569)	585 (509-677)	1,127 (1,015-1,305)	3,186 (2,916-3,509)	6,943 (6,382-7,581)	10,822 (9,999-11,857)
Liver cancer due to hepatitis B	-	-	-	-	292 (245-344)	386 (333-450)	444 (379-520)	852 (761-996)	2,361 (2,112-2,615)	4,894 (4,416-5,428)	7,015 (6,339-7,872)
Liver cancer due to hepatitis C	-	-	-	-	8 (6-11)	10 (8-14)	11 (8-14)	35 (28-45)	153 (117-193)	449 (374-548)	913 (724-1,139)
Liver cancer due to alcohol use	-	-	-	-	-	-	3 (2-5)	40 (31-52)	183 (136-245)	612 (483-761)	1,503 (1,112-1,962)
Liver cancer due to NASH	-	-	-	-	-	-	50 (39-62)	74 (60-92)	156 (120-195)	301 (235-377)	449 (357-560)
Liver cancer due to other causes	-	-	-	-	114 (92-139)	94 (75-114)	77 (61-95)	125 (103-153)	332 (261-419)	687 (537-855)	942 (749-1,164)
Gallbladder and biliary tract cancer	-	-	-	-	-	-	40 (35-45)	61 (51-72)	114 (91-134)	306 (243-367)	547 (439-650)
Pancreatic cancer	-	-	-	-	-	-	80 (75-85)	159 (149-170)	356 (339-375)	992 (944-1,047)	1,907 (1,825-1,992)
Larynx cancer	-	-	-	-	-	-	65 (60-72)	103 (96-112)	144 (135-154)	427 (399-459)	1,087 (1,005-1,190)
Tracheal, bronchus, and lung cancer	-	-	-	-	-	-	459 (426-497)	807 (747-881)	1,749 (1,640-1,870)	3,562 (3,371-3,771)	6,565 (6,249-6,917)
Malignant skin melanoma	-	-	-	-	-	-	117 (92-157)	214 (172-295)	384 (299-513)	695 (542-943)	1,005 (760-1,304)
Non-melanoma skin cancer	-	-	-	-	-	-	94 (88-99)	139 (131-147)	202 (191-215)	395 (374-418)	490 (469-515)
Non-melanoma skin cancer (squamous-cell carcinoma)	-	-	-	-	-	-	94 (88-99)	139 (131-147)	202 (191-215)	395 (374-418)	490 (469-515)
Breast cancer	-	-	-	-	-	-	32 (30-34)	34 (32-36)	42 (40-44)	79 (74-85)	198 (184-215)
Cervical cancer	-	-	-	-	-	-	-	-	-	-	-
Uterine cancer	-	-	-	-	-	-	-	-	-	-	-
Ovarian cancer	-	-	-	-	-	-	-	-	-	-	-
Prostate cancer	-	-	-	-	-	-	68 (58-79)	118 (98-140)	180 (154-219)	238 (197-279)	310 (256-362)
Testicular cancer	-	-	-	-	-	-	315 (282-358)	797 (734-885)	1,055 (979-1,157)	1,046 (981-1,132)	817 (769-884)
Kidney cancer	23 (19-26)	12 (11-14)	162 (143-179)	872 (734-1,028)	323 (280-367)	138 (120-152)	113 (102-122)	166 (145-176)	299 (261-320)	477 (417-507)	868 (782-914)
Bladder cancer	-	-	-	-	-	-	49 (43-55)	86 (73-98)	126 (109-144)	301 (251-340)	547 (456-614)
Brain and nervous system cancer	81 (60-95)	59 (46-71)	835 (634-1,014)	3,600 (2,902-4,505)	4,023 (3,368-4,889)	3,405 (2,708-4,165)	2,299 (1,842-2,649)	2,305 (1,837-2,613)	3,436 (2,718-3,991)	4,721 (3,799-5,568)	5,493 (4,545-6,536)
Thyroid cancer	-	-	-	-	-	73 (65-83)	96 (85-109)	157 (142-176)	305 (276-341)	258 (241-279)	332 (309-358)
Mesothelioma	-	-	-	-	-	-	40 (37-44)	55 (50-60)	97 (91-103)	141 (134-150)	228 (216-241)
Hodgkin lymphoma	7 (5-9)	1 (1-2)	43 (33-58)	486 (329-701)	780 (533-1,130)	771 (569-1,025)	852 (647-1,142)	936 (749-1,226)	1,107 (897-1,441)	1,170 (939-1,528)	1,172 (948-1,530)
Non-Hodgkin lymphoma	36 (33-41)	27 (24-30)	355 (317-405)	2,748 (2,250-3,427)	2,339 (1,882-2,904)	1,783 (1,561-2,063)	1,750 (1,600-1,940)	2,157 (2,004-2,324)	2,426 (2,290-2,589)	2,963 (2,811-3,127)	3,612 (3,420-3,808)

Cause	40 to 44	45 to 49	50 to 54	54 to 59	60 to 64	65 to 69	70 to 74	75 to 79	80 to 84	85 to 89	90 to 94	95 plus
All causes	902,416 (887,234-918,440)	1,231,173 (1,209,046-1,253,098)	1,618,769 (1,590,980-1,648,060)	1,962,003 (1,932,839-1,990,389)	2,625,310 (2,585,373-2,665,290)	3,014,179 (2,970,129-3,058,702)	3,003,812 (2,960,935-3,045,088)	3,205,260 (3,161,356-3,249,121)	3,139,507 (3,101,760-3,176,990)	2,293,326 (2,271,127-2,315,722)	1,094,409 (1,085,859-1,103,020)	329,102 (327,262-330,988)
Communicable, maternal, neonatal, and nutritional diseases	184,725 (175,830-196,763)	193,229 (182,748-208,136)	193,034 (181,728-211,203)	204,064 (190,089-226,484)	220,653 (203,021-250,251)	239,364 (215,561-280,515)	257,663 (225,314-308,070)	254,731 (222,840-306,731)	259,367 (226,321-310,514)	199,299 (175,905-237,545)	97,633 (88,460-112,176)	33,305 (30,986-37,270)
HIV/AIDS and sexually transmitted infections	73,485 (68,935-78,025)	60,191 (55,326-66,759)	40,441 (37,815-43,561)	21,170 (19,390-23,045)	13,595 (12,385-15,152)	9,085 (8,220-10,282)	4,820 (4,304-5,568)	2,805 (2,517-3,181)	954 (850-1,081)	429 (372-492)	138 (116-161)	32 (25-41)
HIV/AIDS	73,414 (68,859-77,952)	60,128 (55,260-66,666)	40,360 (37,748-43,484)	21,084 (19,295-22,953)	13,509 (12,332-15,076)	8,992 (8,136-10,199)	4,741 (4,232-5,470)	2,716 (2,433-3,093)	889 (788-1,015)	364 (318-418)	107 (95-122)	19 (17-21)
HIV/AIDS - Drug-susceptible Tuberculosis	12,757 (8,797-17,204)	11,125 (7,692-14,859)	8,283 (5,807-10,894)	4,329 (3,087-5,538)	2,580 (1,891-3,263)	1,555 (1,125-2,000)	853 (626-1,095)	488 (348-632)	109 (75-148)	42 (28-59)	14 (10-19)	2 (2-3)
HIV/AIDS - Multidrug-resistant Tuberculosis without extensive drug resistance	1,521 (896-2,370)	1,298 (767-2,013)	952 (559-1,460)	497 (292-760)	293 (175-449)	171 (102-264)	93 (54-148)	54 (31-90)	12 (7-22)	5 (2-9)	2 (1-3)	0 (0-0)
HIV/AIDS - Extensively drug-resistant Tuberculosis	119 (72-181)	91 (55-136)	60 (37-91)	35 (21-52)	21 (13-33)	13 (8-22)	6 (3-11)	3 (2-7)	2 (1-3)	1 (0-1)	0 (0-0)	0 (0-0)
HIV/AIDS resulting in other diseases	59,018 (51,934-65,639)	47,613 (41,328-54,754)	31,065 (27,432-35,344)	16,223 (14,129-18,669)	10,614 (9,289-12,317)	7,252 (6,324-8,512)	3,789 (3,222-4,584)	2,171 (1,855-2,554)	766 (657-892)	317 (269-375)	92 (78-107)	16 (14-18)
Sexually transmitted infections excluding HIV	71 (43-115)	63 (38-96)	80 (44-137)	86 (55-151)	86 (49-156)	93 (57-165)	79 (48-133)	89 (59-139)	65 (37-96)	64 (29-101)	30 (12-49)	14 (6-22)
Syphilis	61 (37-98)	55 (34-85)	72 (39-123)	78 (50-137)	81 (46-147)	87 (53-155)	74 (44-123)	81 (54-126)	59 (33-87)	56 (25-88)	25 (10-41)	11 (5-17)
Chlamydial infection	0 ([illegible])	0 ([illegible])	0 ([illegible])	0 ([illegible])	0 ([illegible])	0 ([illegible])	0 ([illegible])	0 ([illegible])	0 ([illegible])	0 ([illegible])	0 ([illegible])	0 ([illegible])
Gonococcal infection	[illegible]	[illegible]	[illegible]	[illegible]	[illegible]	[illegible]	[illegible]	[illegible]	[illegible]	[illegible]	[illegible]	[illegible]
Other sexually transmitted infections	[illegible]	[illegible]	[illegible]	[illegible]	[illegible]	[illegible]	[illegible]	[illegible]	[illegible]	[illegible]	[illegible]	[illegible]
Respiratory infections and tuberculosis	[illegible]	[illegible]	[illegible]	[illegible]	[illegible]	[illegible]	[illegible]	[illegible]	[illegible]	[illegible]	[illegible]	[illegible]
Tuberculosis	[illegible]	[illegible]	[illegible]	[illegible]	[illegible]	[illegible]	[illegible]	[illegible]	[illegible]	[illegible]	[illegible]	[illegible]
Drug-susceptible tuberculosis	[illegible]	[illegible]	[illegible]	[illegible]	[illegible]	[illegible]	[illegible]	[illegible]	[illegible]	[illegible]	[illegible]	[illegible]
Multidrug-resistant tuberculosis without extensive drug resistance	[illegible]	[illegible]	[illegible]	[illegible]	[illegible]	[illegible]	[illegible]	[illegible]	[illegible]	[illegible]	[illegible]	[illegible]
Extensively drug-resistant tuberculosis	[illegible]	[illegible]	[illegible]	[illegible]	[illegible]	[illegible]	[illegible]	[illegible]	[illegible]	[illegible]	[illegible]	[illegible]
Lower respiratory infections	[illegible]	[illegible]	[illegible]	[illegible]	[illegible]	[illegible]	[illegible]	[illegible]	[illegible]	[illegible]	[illegible]	[illegible]
Upper respiratory infections	[illegible]	[illegible]	[illegible]	[illegible]	[illegible]	[illegible]	[illegible]	[illegible]	[illegible]	[illegible]	[illegible]	[illegible]
Otitis media	[illegible]	[illegible]	[illegible]	[illegible]	[illegible]	[illegible]	[illegible]	[illegible]	[illegible]	[illegible]	[illegible]	[illegible]
Enteric infections	[illegible]	[illegible]	[illegible]	[illegible]	[illegible]	[illegible]	[illegible]	[illegible]	[illegible]	[illegible]	[illegible]	[illegible]
Diarrheal diseases	[illegible]	[illegible]	[illegible]	[illegible]	[illegible]	[illegible]	[illegible]	[illegible]	[illegible]	[illegible]	[illegible]	[illegible]
Typhoid and paratyphoid	[illegible]	[illegible]	[illegible]	[illegible]	[illegible]	[illegible]	[illegible]	[illegible]	[illegible]	[illegible]	[illegible]	[illegible]
Typhoid fever	[illegible]	[illegible]	[illegible]	[illegible]	[illegible]	[illegible]	[illegible]	[illegible]	[illegible]	[illegible]	[illegible]	[illegible]
Paratyphoid fever	[illegible]	[illegible]	[illegible]	[illegible]	[illegible]	[illegible]	[illegible]	[illegible]	[illegible]	[illegible]	[illegible]	[illegible]
Invasive Non-typhoidal Salmonella (iNTS)	[illegible]	[illegible]	[illegible]	[illegible]	[illegible]	[illegible]	[illegible]	[illegible]	[illegible]	[illegible]	[illegible]	[illegible]
Other intestinal infectious diseases	[illegible]	[illegible]	[illegible]	[illegible]	[illegible]	[illegible]	[illegible]	[illegible]	[illegible]	[illegible]	[illegible]	[illegible]
Neglected tropical diseases and malaria	[illegible]	[illegible]	[illegible]	[illegible]	[illegible]	[illegible]	[illegible]	[illegible]	[illegible]	[illegible]	[illegible]	[illegible]
Malaria	[illegible]	[illegible]	[illegible]	[illegible]	[illegible]	[illegible]	[illegible]	[illegible]	[illegible]	[illegible]	[illegible]	[illegible]
Chagas disease	[illegible]	[illegible]	[illegible]	[illegible]	[illegible]	[illegible]	[illegible]	[illegible]	[illegible]	[illegible]	[illegible]	[illegible]
Leishmaniasis	[illegible]	[illegible]	[illegible]	[illegible]	[illegible]	[illegible]	[illegible]	[illegible]	[illegible]	[illegible]	[illegible]	[illegible]
Visceral leishmaniasis	[illegible]	[illegible]	[illegible]	[illegible]	[illegible]	[illegible]	[illegible]	[illegible]	[illegible]	[illegible]	[illegible]	[illegible]
African trypanosomiasis	[illegible]	[illegible]	[illegible]	[illegible]	[illegible]	[illegible]	[illegible]	[illegible]	[illegible]	[illegible]	[illegible]	[illegible]
Schistosomiasis	[illegible]	[illegible]	[illegible]	[illegible]	[illegible]	[illegible]	[illegible]	[illegible]	[illegible]	[illegible]	[illegible]	[illegible]
Cysticercosis	[illegible]	[illegible]	[illegible]	[illegible]	[illegible]	[illegible]	[illegible]	[illegible]	[illegible]	[illegible]	[illegible]	[illegible]
Cystic echinococcosis	[illegible]	[illegible]	[illegible]	[illegible]	[illegible]	[illegible]	[illegible]	[illegible]	[illegible]	[illegible]	[illegible]	[illegible]
Dengue	[illegible]	[illegible]	[illegible]	[illegible]	[illegible]	[illegible]	[illegible]	[illegible]	[illegible]	[illegible]	[illegible]	[illegible]
Yellow fever	[illegible]	[illegible]	[illegible]	[illegible]	[illegible]	[illegible]	[illegible]	[illegible]	[illegible]	[illegible]	[illegible]	[illegible]
Rabies	[illegible]	[illegible]	[illegible]	[illegible]	[illegible]	[illegible]	[illegible]	[illegible]	[illegible]	[illegible]	[illegible]	[illegible]
Intestinal nematode infections	[illegible]	[illegible]	[illegible]	[illegible]	[illegible]	[illegible]	[illegible]	[illegible]	[illegible]	[illegible]	[illegible]	[illegible]
Ascariasis	[illegible]	[illegible]	[illegible]	[illegible]	[illegible]	[illegible]	[illegible]	[illegible]	[illegible]	[illegible]	[illegible]	[illegible]
Ebola	[illegible]	[illegible]	[illegible]	[illegible]	[illegible]	[illegible]	[illegible]	[illegible]	[illegible]	[illegible]	[illegible]	[illegible]
Zika virus	[illegible]	[illegible]	[illegible]	[illegible]	[illegible]	[illegible]	[illegible]	[illegible]	[illegible]	[illegible]	[illegible]	[illegible]
Other neglected tropical diseases	[illegible]	[illegible]	[illegible]	[illegible]	[illegible]	[illegible]	[illegible]	[illegible]	[illegible]	[illegible]	[illegible]	[illegible]
Other infectious diseases	[illegible]	[illegible]	[illegible]	[illegible]	[illegible]	[illegible]	[illegible]	[illegible]	[illegible]	[illegible]	[illegible]	[illegible]
Meningitis	[illegible]	[illegible]	[illegible]	[illegible]	[illegible]	[illegible]	[illegible]	[illegible]	[illegible]	[illegible]	[illegible]	[illegible]
Pneumococcal meningitis	[illegible]	[illegible]	[illegible]	[illegible]	[illegible]	[illegible]	[illegible]	[illegible]	[illegible]	[illegible]	[illegible]	[illegible]
H influenzae type B meningitis	[illegible]	[illegible]	[illegible]	[illegible]	[illegible]	[illegible]	[illegible]	[illegible]	[illegible]	[illegible]	[illegible]	[illegible]
Meningococcal meningitis	[illegible]	[illegible]	[illegible]	[illegible]	[illegible]	[illegible]	[illegible]	[illegible]	[illegible]	[illegible]	[illegible]	[illegible]
Other meningitis	[illegible]	[illegible]	[illegible]	[illegible]	[illegible]	[illegible]	[illegible]	[illegible]	[illegible]	[illegible]	[illegible]	[illegible]
Encephalitis	[illegible]	[illegible]	[illegible]	[illegible]	[illegible]	[illegible]	[illegible]	[illegible]	[illegible]	[illegible]	[illegible]	[illegible]
Diphtheria	[illegible]	[illegible]	[illegible]	[illegible]	[illegible]	[illegible]	[illegible]	[illegible]	[illegible]	[illegible]	[illegible]	[illegible]
Whooping cough	[illegible]	[illegible]	[illegible]	[illegible]	[illegible]	[illegible]	[illegible]	[illegible]	[illegible]	[illegible]	[illegible]	[illegible]
Tetanus	[illegible]	[illegible]	[illegible]	[illegible]	[illegible]	[illegible]	[illegible]	[illegible]	[illegible]	[illegible]	[illegible]	[illegible]
Measles	[illegible]	[illegible]	[illegible]	[illegible]	[illegible]	[illegible]	[illegible]	[illegible]	[illegible]	[illegible]	[illegible]	[illegible]
Varicella and herpes zoster	[illegible]	[illegible]	[illegible]	[illegible]	[illegible]	[illegible]	[illegible]	[illegible]	[illegible]	[illegible]	[illegible]	[illegible]
Acute hepatitis	[illegible]	[illegible]	[illegible]	[illegible]	[illegible]	[illegible]	[illegible]	[illegible]	[illegible]	[illegible]	[illegible]	[illegible]
Acute hepatitis A	[illegible]	[illegible]	[illegible]	[illegible]	[illegible]	[illegible]	[illegible]	[illegible]	[illegible]	[illegible]	[illegible]	[illegible]
Acute hepatitis B	[illegible]	[illegible]	[illegible]	[illegible]	[illegible]	[illegible]	[illegible]	[illegible]	[illegible]	[illegible]	[illegible]	[illegible]
Acute hepatitis C	[illegible]	[illegible]	[illegible]	[illegible]	[illegible]	[illegible]	[illegible]	[illegible]	[illegible]	[illegible]	[illegible]	[illegible]
Acute hepatitis E	[illegible]	[illegible]	[illegible]	[illegible]	[illegible]	[illegible]	[illegible]	[illegible]	[illegible]	[illegible]	[illegible]	[illegible]
Other unspecified infectious diseases	[illegible]	[illegible]	[illegible]	[illegible]	[illegible]	[illegible]	[illegible]	[illegible]	[illegible]	[illegible]	[illegible]	[illegible]
Maternal and neonatal disorders	[illegible]	[illegible]	[illegible]	[illegible]	[illegible]	[illegible]	[illegible]	[illegible]	[illegible]	[illegible]	[illegible]	[illegible]
Maternal disorders	[illegible]	[illegible]	[illegible]	[illegible]	[illegible]	[illegible]	[illegible]	[illegible]	[illegible]	[illegible]	[illegible]	[illegible]
Maternal hemorrhage	[illegible]	[illegible]	[illegible]	[illegible]	[illegible]	[illegible]	[illegible]	[illegible]	[illegible]	[illegible]	[illegible]	[illegible]
Maternal sepsis and other maternal infections	[illegible]	[illegible]	[illegible]	[illegible]	[illegible]	[illegible]	[illegible]	[illegible]	[illegible]	[illegible]	[illegible]	[illegible]
Maternal hypertensive disorders	[illegible]	[illegible]	[illegible]	[illegible]	[illegible]	[illegible]	[illegible]	[illegible]	[illegible]	[illegible]	[illegible]	[illegible]
Maternal obstructed labor and uterine rupture	[illegible]	[illegible]	[illegible]	[illegible]	[illegible]	[illegible]	[illegible]	[illegible]	[illegible]	[illegible]	[illegible]	[illegible]
Maternal abortion and miscarriage	[illegible]	[illegible]	[illegible]	[illegible]	[illegible]	[illegible]	[illegible]	[illegible]	[illegible]	[illegible]	[illegible]	[illegible]
Ectopic pregnancy	[illegible]	[illegible]	[illegible]	[illegible]	[illegible]	[illegible]	[illegible]	[illegible]	[illegible]	[illegible]	[illegible]	[illegible]
Indirect maternal deaths	[illegible]	[illegible]	[illegible]	[illegible]	[illegible]	[illegible]	[illegible]	[illegible]	[illegible]	[illegible]	[illegible]	[illegible]
Late maternal deaths	[illegible]	[illegible]	[illegible]	[illegible]	[illegible]	[illegible]	[illegible]	[illegible]	[illegible]	[illegible]	[illegible]	[illegible]
Maternal deaths aggravated by HIV/AIDS	[illegible]	[illegible]	[illegible]	[illegible]	[illegible]	[illegible]	[illegible]	[illegible]	[illegible]	[illegible]	[illegible]	[illegible]
Other maternal disorders	[illegible]	[illegible]	[illegible]	[illegible]	[illegible]	[illegible]	[illegible]	[illegible]	[illegible]	[illegible]	[illegible]	[illegible]
Neonatal disorders	[illegible]	[illegible]	[illegible]	[illegible]	[illegible]	[illegible]	[illegible]	[illegible]	[illegible]	[illegible]	[illegible]	[illegible]
Neonatal preterm birth	[illegible]	[illegible]	[illegible]	[illegible]	[illegible]	[illegible]	[illegible]	[illegible]	[illegible]	[illegible]	[illegible]	[illegible]
Neonatal encephalopathy due to birth asphyxia and trauma	[illegible]	[illegible]	[illegible]	[illegible]	[illegible]	[illegible]	[illegible]	[illegible]	[illegible]	[illegible]	[illegible]	[illegible]
Neonatal sepsis and other neonatal infections	[illegible]	[illegible]	[illegible]	[illegible]	[illegible]	[illegible]	[illegible]	[illegible]	[illegible]	[illegible]	[illegible]	[illegible]
Hemolytic disease and other neonatal jaundice	[illegible]	[illegible]	[illegible]	[illegible]	[illegible]	[illegible]	[illegible]	[illegible]	[illegible]	[illegible]	[illegible]	[illegible]
Other neonatal disorders	[illegible]	[illegible]	[illegible]	[illegible]	[illegible]	[illegible]	[illegible]	[illegible]	[illegible]	[illegible]	[illegible]	[illegible]
Nutritional deficiencies	[illegible]	[illegible]	[illegible]	[illegible]	[illegible]	[illegible]	[illegible]	[illegible]	[illegible]	[illegible]	[illegible]	[illegible]
Protein-energy malnutrition	[illegible]	[illegible]	[illegible]	[illegible]	[illegible]	[illegible]	[illegible]	[illegible]	[illegible]	[illegible]	[illegible]	[illegible]
Other nutritional deficiencies	[illegible]	[illegible]	[illegible]	[illegible]	[illegible]	[illegible]	[illegible]	[illegible]	[illegible]	[illegible]	[illegible]	[illegible]
Non-communicable diseases	[illegible]	[illegible]	[illegible]	[illegible]	[illegible]	[illegible]	[illegible]	[illegible]	[illegible]	[illegible]	[illegible]	[illegible]
Neoplasms	[illegible]	[illegible]	[illegible]	[illegible]	[illegible]	[illegible]	[illegible]	[illegible]	[illegible]	[illegible]	[illegible]	[illegible]
Lip and oral cavity cancer	[illegible]	[illegible]	[illegible]	[illegible]	[illegible]	[illegible]	[illegible]	[illegible]	[illegible]	[illegible]	[illegible]	[illegible]
Nasopharynx cancer	[illegible]	[illegible]	[illegible]	[illegible]	[illegible]	[illegible]	[illegible]	[illegible]	[illegible]	[illegible]	[illegible]	[illegible]
Other pharynx cancer	[illegible]	[illegible]	[illegible]	[illegible]	[illegible]	[illegible]	[illegible]	[illegible]	[illegible]	[illegible]	[illegible]	[illegible]
Esophageal cancer	[illegible]	[illegible]	[illegible]	[illegible]	[illegible]	[illegible]	[illegible]	[illegible]	[illegible]	[illegible]	[illegible]	[illegible]
Stomach cancer	[illegible]	[illegible]	[illegible]	[illegible]	[illegible]	[illegible]	[illegible]	[illegible]	[illegible]	[illegible]	[illegible]	[illegible]
Colon and rectum cancer	[illegible]	[illegible]	[illegible]	[illegible]	[illegible]	[illegible]	[illegible]	[illegible]	[illegible]	[illegible]	[illegible]	[illegible]
Liver cancer	[illegible]	[illegible]	[illegible]	[illegible]	[illegible]	[illegible]	[illegible]	[illegible]	[illegible]	[illegible]	[illegible]	[illegible]
Liver cancer due to hepatitis B	[illegible]	[illegible]	[illegible]	[illegible]	[illegible]	[illegible]	[illegible]	[illegible]	[illegible]	[illegible]	[illegible]	[illegible]
Liver cancer due to hepatitis C	[illegible]	[illegible]	[illegible]	[illegible]	[illegible]	[illegible]	[illegible]	[illegible]	[illegible]	[illegible]	[illegible]	[illegible]
Liver cancer due to alcohol use	[illegible]	[illegible]	[illegible]	[illegible]	[illegible]	[illegible]	[illegible]	[illegible]	[illegible]	[illegible]	[illegible]	[illegible]
Liver cancer due to NASH	[illegible]	[illegible]	[illegible]	[illegible]	[illegible]	[illegible]	[illegible]	[illegible]	[illegible]	[illegible]	[illegible]	[illegible]
Liver cancer due to other causes	[illegible]	[illegible]	[illegible]	[illegible]	[illegible]	[illegible]	[illegible]	[illegible]	[illegible]	[illegible]	[illegible]	[illegible]
Gallbladder and biliary tract cancer	[illegible]	[illegible]	[illegible]	[illegible]	[illegible]	[illegible]	[illegible]	[illegible]	[illegible]	[illegible]	[illegible]	[illegible]
Pancreatic cancer	[illegible]	[illegible]	[illegible]	[illegible]	[illegible]	[illegible]	[illegible]	[illegible]	[illegible]	[illegible]	[illegible]	[illegible]
Larynx cancer	[illegible]	[illegible]	[illegible]	[illegible]	[illegible]	[illegible]	[illegible]	[illegible]	[illegible]	[illegible]	[illegible]	[illegible]
Tracheal, bronchus, and lung cancer	[illegible]	[illegible]	[illegible]	[illegible]	[illegible]	[illegible]	[illegible]	[illegible]	[illegible]	[illegible]	[illegible]	[illegible]
Malignant skin melanoma	[illegible]	[illegible]	[illegible]	[illegible]	[illegible]	[illegible]	[illegible]	[illegible]	[illegible]	[illegible]	[illegible]	[illegible]
Non-melanoma skin cancer	[illegible]	[illegible]	[illegible]	[illegible]	[illegible]	[illegible]	[illegible]	[illegible]	[illegible]	[illegible]	[illegible]	[illegible]
Non-melanoma skin cancer (squamous-cell carcinoma)	[illegible]	[illegible]	[illegible]	[illegible]	[illegible]	[illegible]	[illegible]	[illegible]	[illegible]	[illegible]	[illegible]	[illegible]
Breast cancer	[illegible]	[illegible]	[illegible]	[illegible]	[illegible]	[illegible]	[illegible]	[illegible]	[illegible]	[illegible]	[illegible]	[illegible]
Cervical cancer	[illegible]	[illegible]	[illegible]	[illegible]	[illegible]	[illegible]	[illegible]	[illegible]	[illegible]	[illegible]	[illegible]	[illegible]
Uterine cancer	[illegible]	[illegible]	[illegible]	[illegible]	[illegible]	[illegible]	[illegible]	[illegible]	[illegible]	[illegible]	[illegible]	[illegible]
Ovarian cancer	[illegible]	[illegible]	[illegible]	[illegible]	[illegible]	[illegible]	[illegible]	[illegible]	[illegible]	[illegible]	[illegible]	[illegible]
Prostate cancer	[illegible]	[illegible]	[illegible]	[illegible]	[illegible]	[illegible]	[illegible]	[illegible]	[illegible]	[illegible]	[illegible]	[illegible]
Testicular cancer	[illegible]	[illegible]	[illegible]	[illegible]	[illegible]	[illegible]	[illegible]	[illegible]	[illegible]	[illegible]	[illegible]	[illegible]
Kidney cancer	[illegible]	[illegible]	[illegible]	[illegible]	[illegible]	[illegible]	[illegible]	[illegible]	[illegible]	[illegible]	[illegible]	[illegible]
Bladder cancer	[illegible]	[illegible]	[illegible]	[illegible]	[illegible]	[illegible]	[illegible]	[illegible]	[illegible]	[illegible]	[illegible]	[illegible]
Brain and nervous system cancer	[illegible]	[illegible]	[illegible]	[illegible]	[illegible]	[illegible]	[illegible]	[illegible]	[illegible]	[illegible]	[illegible]	[illegible]
Thyroid cancer	[illegible]	[illegible]	[illegible]	[illegible]	[illegible]	[illegible]	[illegible]	[illegible]	[illegible]	[illegible]	[illegible]	[illegible]
Mesothelioma	[illegible]	[illegible]	[illegible]	[illegible]	[illegible]	[illegible]	[illegible]	[illegible]	[illegible]	[illegible]	[illegible]	[illegible]
Hodgkin lymphoma	[illegible]	[illegible]	[illegible]	[illegible]	[illegible]	[illegible]	[illegible]	[illegible]	[illegible]	[illegible]	[illegible]	[illegible]
Non-Hodgkin lymphoma	[illegible]	[illegible]	[illegible]	[illegible]	[illegible]	[illegible]	[illegible]	[illegible]	[illegible]	[illegible]	[illegible]	[illegible]

SR Table 6: Global deaths by cause and age group for males (with 95% confidence intervals), 2017

Cause	Early Neonatal	Late Neonatal	Post Neonatal	1 to 4	5 to 9	10 to 14	15 to 19	20 to 24	25 to 29	30 to 34	35 to 39	40 to 44	45 to 49	50 to 54	54 to 59	60 to 64	65 to 69	70 to 74	75 to 79	80 to 84	85 to 89	90 to 94	95 plus
Multiple myeloma	-	-	-	-	-	-	97 (74-110)	120 (99-142)	175 (130-195)	357 (298-418)	581 (494-692)	1,137 (976-1,384)	1,809 (1,568-2,165)	3,185 (2,754-3,799)	4,779 (4,185-5,845)	6,712 (5,868-8,140)	8,084 (6,876-9,669)	8,142 (6,601-9,637)	7,770 (6,045-9,079)	6,492 (5,027-7,394)	4,041 (2,990-4,531)	1,374 (961-1,511)	306 (226-386)
Leukemia	152 (114-171)	126 (100-142)	1,765 (1,395-2,039)	7,203 (6,021-8,392)	6,967 (5,800-8,027)	5,923 (5,029-6,770)	5,627 (4,893-6,390)	4,877 (4,144-5,457)	5,229 (4,293-5,675)	5,379 (4,483-5,780)	5,404 (4,661-5,988)	6,223 (5,505-7,008)	8,221 (6,972-9,115)	10,663 (9,129-11,743)	13,021 (11,445-14,276)	17,481 (15,283-18,888)	20,524 (18,303-22,285)	20,042 (18,053-21,366)	19,656 (18,175-20,901)	16,822 (15,698-17,926)	10,736 (10,242-11,412)	4,230 (4,063-4,513)	999 (951-1,055)
Acute lymphoid leukemia	33 (24-41)	27 (21-33)	502 (390-607)	2,430 (1,985-2,964)	3,236 (2,684-3,803)	2,523 (2,149-2,913)	2,013 (1,727-2,312)	1,561 (1,336-1,749)	1,375 (1,128-1,548)	1,249 (1,048-1,382)	1,188 (1,001-1,345)	1,168 (997-1,326)	1,385 (1,118-1,600)	1,630 (1,340-1,815)	1,769 (1,490-1,972)	2,055 (1,713-2,308)	2,194 (1,826-2,465)	1,770 (1,496-1,968)	1,104 (950-1,253)	797 (699-891)	425 (387-473)	132 (123-147)	27 (25-30)
Chronic lymphoid leukemia	-	-	-	-	-	-	81 (64-96)	89 (72-108)	120 (89-143)	122 (95-147)	120 (101-140)	215 (187-253)	419 (351-492)	730 (639-821)	1,187 (1,088-1,300)	2,001 (1,820-2,184)	2,613 (2,392-2,837)	2,934 (2,715-3,168)	3,226 (3,010-3,464)	3,161 (2,978-3,389)	2,366 (2,238-2,541)	1,091 (1,027-1,181)	310 (288-335)
Acute myeloid leukemia	43 (29-52)	37 (27-44)	411 (300-515)	2,125 (1,442-2,622)	1,185 (901-1,453)	1,146 (892-1,360)	1,273 (1,050-1,492)	1,252 (1,020-1,421)	1,335 (1,104-1,511)	1,534 (1,306-1,734)	1,688 (1,432-1,884)	1,979 (1,662-2,212)	2,540 (2,097-2,830)	3,277 (2,788-3,606)	4,337 (3,707-4,848)	5,449 (4,783-5,831)	6,455 (5,774-6,899)	6,632 (5,963-7,057)	6,088 (5,534-6,455)	4,823 (4,425-5,124)	2,719 (2,558-2,887)	903 (853-966)	172 (162-183)
Chronic myeloid leukemia	-	-	-	-	-	-	138 (113-164)	224 (189-260)	402 (343-479)	465 (403-528)	506 (428-575)	524 (458-603)	641 (547-728)	776 (672-877)	966 (846-1,075)	1,109 (963-1,232)	1,363 (1,207-1,512)	1,304 (1,162-1,433)	1,622 (1,467-1,770)	1,486 (1,342-1,620)	933 (868-1,009)	376 (351-407)	82 (75-89)
Other leukemia	76 (57-87)	61 (48-70)	851 (642-1,014)	2,648 (2,075-3,166)	2,546 (1,984-3,066)	2,254 (1,851-2,732)	2,123 (1,764-2,555)	1,752 (1,406-2,064)	1,997 (1,450-2,217)	2,008 (1,522-2,227)	1,902 (1,562-2,210)	2,337 (1,955-2,773)	3,236 (2,653-3,872)	4,250 (3,445-4,952)	4,763 (4,048-5,532)	6,867 (5,695-7,872)	7,899 (6,719-9,052)	7,402 (6,388-8,228)	7,616 (6,835-8,587)	6,556 (5,989-7,389)	4,292 (3,993-4,838)	1,730 (1,638-1,901)	409 (385-436)
Other malignant neoplasms	437 (340-503)	228 (195-261)	1,472 (1,253-1,741)	6,378 (5,223-7,920)	2,826 (2,349-3,281)	3,446 (2,978-3,877)	4,413 (3,882-4,937)	3,742 (3,371-4,048)	3,312 (2,921-3,510)	3,679 (3,317-3,907)	4,642 (4,191-4,975)	6,447 (5,761-6,859)	9,187 (8,074-9,723)	13,031 (11,393-13,809)	16,214 (14,430-17,123)	20,815 (17,857-22,003)	22,558 (19,460-23,876)	19,417 (17,200-20,399)	18,070 (15,777-18,999)	14,397 (12,186-15,131)	8,258 (6,897-8,687)	3,075 (2,619-3,239)	732 (630-783)
Other neoplasms	36 (25-49)	22 (16-29)	278 (214-380)	625 (442-879)	544 (386-717)	455 (334-615)	436 (324-577)	462 (336-622)	604 (452-802)	860 (645-1,179)	999 (731-1,384)	1,346 (1,001-1,878)	1,845 (1,372-2,537)	2,559 (1,978-3,488)	3,263 (2,444-4,590)	4,516 (3,325-6,228)	5,685 (4,137-7,903)	6,119 (3,963-8,675)	6,829 (4,240-9,356)	6,535 (3,723-8,742)	5,036 (2,675-6,516)	2,282 (1,144-2,992)	645 (313-834)
Myelodysplastic, myeloproliferative, and other hematopoietic neoplasms	36 (25-48)	19 (14-25)	231 (173-317)	479 (336-694)	366 (259-502)	295 (213-415)	283 (202-387)	312 (222-442)	449 (335-604)	673 (495-957)	842 (610-1,184)	1,207 (894-1,700)	1,730 (1,286-2,397)	2,471 (1,895-3,386)	3,201 (2,400-4,510)	4,466 (3,291-6,179)	5,652 (4,114-7,859)	6,098 (3,950-8,648)	6,814 (4,235-9,332)	6,526 (3,719-8,734)	5,032 (2,673-6,510)	2,281 (1,143-2,991)	644 (313-834)
Other benign and in situ neoplasms	1 (0-1)	3 (2-5)	48 (32-65)	146 (88-200)	178 (119-241)	160 (111-212)	153 (110-200)	150 (104-197)	155 (103-213)	187 (127-263)	157 (102-222)	139 (86-208)	115 (72-182)	88 (57-145)	61 (38-96)	50 (30-72)	33 (17-47)	21 (11-29)	15 (7-22)	9 (4-13)	4 (2-6)	1 (1-2)	0 (0-0)
Cardiovascular diseases	1,557 (1,428-1,728)	908 (832-1,008)	7,777 (7,220-8,431)	6,000 (5,412-6,630)	3,406 (3,101-3,763)	4,478 (4,167-4,829)	13,731 (12,875-14,643)	25,873 (24,613-27,368)	44,038 (42,353-45,892)	80,004 (77,457-82,926)	122,492 (118,780-126,282)	202,661 (196,833-208,370)	339,037 (330,076-347,768)	513,333 (501,387-526,247)	672,897 (657,356-687,760)	952,805 (932,776-972,338)	1,124,857 (1,098,827-1,148,794)	1,130,560 (1,104,899-1,155,893)	1,266,589 (1,241,765-1,292,482)	1,275,618 (1,250,128-1,302,188)	953,781 (935,653-971,338)	462,990 (455,515-470,101)	140,944 (138,803-142,709)
Rheumatic heart disease	-	-	-	1,290 (1,095-1,550)	750 (664-867)	1,320 (1,176-1,487)	1,994 (1,731-2,271)	2,557 (2,267-2,931)	2,563 (2,282-2,990)	3,243 (2,897-3,758)	3,781 (3,350-4,403)	4,725 (4,185-5,595)	5,731 (5,144-6,779)	7,279 (6,605-8,435)	8,399 (7,659-9,765)	10,771 (9,931-12,279)	12,453 (11,524-14,055)	12,346 (11,420-13,898)	12,159 (11,364-13,455)	12,385 (11,632-13,549)	9,161 (8,673-10,022)	4,471 (4,269-4,806)	1,430 (1,369-1,523)
Ischemic heart disease	-	-	-	-	-	-	4,541 (4,157-4,905)	11,003 (10,211-11,829)	21,235 (20,107-22,405)	43,449 (41,716-45,639)	68,909 (66,372-71,778)	114,521 (110,752-118,739)	194,946 (189,274-201,174)	289,487 (282,019-297,942)	386,855 (377,995-396,541)	514,947 (502,963-527,266)	580,310 (566,489-594,331)	573,031 (558,714-588,378)	631,548 (616,424-648,885)	640,438 (625,114-659,976)	496,621 (486,220-511,379)	251,886 (246,715-260,194)	80,231 (78,560-82,926)
Stroke	497 (418-574)	265 (238-295)	2,048 (1,811-2,423)	1,649 (1,423-1,943)	1,171 (1,059-1,292)	1,534 (1,410-1,675)	3,693 (3,433-3,964)	6,841 (6,426-7,316)	10,988 (10,453-11,643)	19,165 (18,289-20,137)	29,857 (28,538-31,387)	54,532 (52,205-57,137)	98,249 (94,379-102,184)	160,633 (154,989-167,077)	203,561 (196,620-210,272)	329,024 (319,347-338,929)	416,341 (403,293-429,003)	422,184 (408,973-435,559)	475,379 (461,116-490,320)	457,272 (443,090-473,006)	311,673 (302,738-322,808)	132,360 (128,954-137,228)	35,270 (34,408-36,550)
Ischemic stroke	52 (37-71)	35 (28-43)	175 (135-244)	203 (141-344)	114 (95-137)	130 (109-156)	430 (372-510)	821 (717-971)	1,335 (1,191-1,544)	2,409 (2,156-2,779)	3,888 (3,510-4,463)	7,412 (6,747-8,375)	17,406 (16,096-19,232)	33,959 (31,612-37,040)	50,562 (47,025-55,977)	111,325 (104,315-119,961)	155,478 (146,399-166,764)	184,807 (175,461-196,987)	225,384 (215,547-236,755)	252,939 (242,998-262,630)	180,451 (174,193-187,328)	79,163 (76,602-82,334)	22,311 (21,701-23,290)
Intracerebral hemorrhage	267 (187-349)	129 (104-157)	1,070 (841-1,487)	511 (362-813)	596 (512-670)	778 (691-878)	2,279 (2,071-2,497)	4,209 (3,829-4,575)	6,963 (6,541-7,477)	12,809 (12,033-13,663)	20,059 (18,909-21,283)	37,426 (35,219-39,697)	68,515 (65,314-72,155)	108,500 (103,771-113,722)	130,633 (124,277-136,951)	193,540 (184,946-201,972)	233,010 (222,460-244,278)	214,405 (204,482-225,621)	225,590 (216,100-237,404)	184,226 (176,355-194,060)	117,538 (112,741-124,124)	47,148 (45,477-49,767)	11,323 (10,890-11,907)
Subarachnoid hemorrhage	178 (123-233)	101 (76-133)	803 (610-1,008)	935 (695-1,150)	461 (370-575)	626 (527-750)	984 (845-1,190)	1,812 (1,483-2,203)	2,690 (2,301-3,149)	3,947 (3,411-4,705)	5,909 (5,143-7,046)	9,695 (8,537-11,701)	12,328 (10,383-14,776)	18,175 (15,565-21,505)	22,366 (19,396-27,101)	24,159 (20,549-29,363)	27,853 (23,300-32,603)	22,972 (19,007-27,425)	24,405 (20,192-28,293)	20,107 (16,549-23,156)	13,684 (11,369-15,869)	6,049 (5,056-6,874)	1,636 (1,280-1,808)
Hypertensive heart disease	-	-	-	-	-	-	286 (199-322)	535 (368-613)	1,048 (657-1,194)	1,968 (1,272-2,260)	3,366 (2,226-3,866)	6,249 (4,148-7,141)	10,961 (7,111-12,457)	17,915 (11,952-19,895)	23,114 (16,090-25,874)	35,131 (24,400-38,701)	43,784 (30,508-47,982)	48,045 (33,357-52,472)	58,699 (40,401-64,018)	66,323 (45,081-72,006)	50,837 (32,424-55,049)	25,672 (16,147-27,691)	7,813 (4,604-8,440)
Non-rheumatic valvular heart disease	-	-	-	-	-	-	333 (284-383)	422 (365-489)	518 (454-605)	581 (517-682)	842 (738-961)	1,205 (1,051-1,403)	1,394 (1,259-1,628)	2,080 (1,900-2,409)	2,900 (2,636-3,411)	3,924 (3,539-4,370)	5,228 (4,751-5,980)	6,321 (5,749-7,034)	8,213 (7,177-8,966)	10,528 (8,295-11,400)	10,387 (7,311-11,178)	6,425 (4,259-6,909)	2,227 (1,358-2,403)
Non-rheumatic calcific aortic valve disease	-	-	-	-	-	-	123 (95-149)	158 (125-195)	225 (181-275)	272 (228-323)	434 (359-498)	649 (519-754)	812 (698-944)	1,263 (1,100-1,509)	1,780 (1,552-2,096)	2,509 (2,210-2,868)	3,485 (3,056-4,090)	4,309 (3,666-4,865)	5,834 (4,847-6,372)	7,872 (5,770-8,608)	8,303 (5,535-9,024)	5,367 (3,329-5,772)	1,907 (1,099-2,059)
Non-rheumatic degenerative mitral valve disease	-	-	-	-	-	-	136 (106-167)	180 (145-219)	201 (160-243)	244 (194-301)	320 (251-388)	436 (344-529)	523 (405-620)	713 (560-876)	1,007 (784-1,246)	1,226 (962-1,420)	1,474 (1,118-1,813)	1,691 (1,286-2,036)	1,974 (1,496-2,454)	2,180 (1,694-2,814)	1,754 (1,361-2,284)	917 (682-1,273)	285 (196-421)
Other non-rheumatic valve diseases	-	-	-	-	-	-	74 (47-107)	84 (51-122)	92 (56-135)	66 (40-110)	88 (55-136)	120 (69-197)	59 (33-93)	104 (60-159)	113 (65-172)	189 (101-291)	269 (153-408)	320 (178-470)	406 (241-605)	475 (298-738)	330 (214-511)	142 (94-223)	35 (25-52)
Cardiomyopathy and myocarditis	593 (524-651)	325 (291-367)	2,610 (2,364-2,917)	1,414 (1,257-1,604)	728 (659-797)	810 (741-878)	1,513 (1,348-1,707)	2,189 (1,939-2,506)	4,003 (3,645-4,444)	6,598 (5,896-7,239)	9,170 (7,991-10,022)	12,022 (10,301-13,291)	14,669 (12,680-16,147)	17,205 (14,582-18,930)	21,297 (18,187-23,478)	21,401 (18,682-23,292)	19,251 (17,369-20,961)	16,013 (14,168-17,601)	17,360 (14,854-18,737)	17,750 (14,731-19,199)	15,078 (11,925-16,171)	8,185 (6,322-8,714)	2,747 (2,023-2,947)
Myocarditis	61 (42-79)	72 (56-93)	669 (540-867)	389 (323-499)	233 (196-280)	247 (200-290)	454 (360-562)	547 (422-705)	811 (637-1,017)	724 (551-942)	861 (663-1,136)	1,176 (847-1,625)	1,123 (826-1,476)	1,118 (777-1,476)	1,280 (884-1,762)	1,305 (903-1,712)	1,371 (978-1,713)	1,449 (1,034-1,840)	1,748 (1,318-2,119)	2,149 (1,678-2,563)	2,168 (1,677-2,685)	1,447 (1,040-1,782)	529 (339-642)
Alcoholic cardiomyopathy	-	-	-	-	-	-	90 (75-121)	274 (240-341)	1,042 (932-1,261)	2,685 (2,411-3,143)	4,404 (3,925-5,062)	5,881 (4,975-6,557)	7,511 (6,389-8,248)	8,624 (6,799-9,519)	10,253 (8,200-11,218)	9,304 (7,096-10,167)	6,778 (5,461-7,431)	3,996 (2,898-4,516)	3,540 (2,652-4,190)	2,353 (1,745-3,037)	1,163 (941-1,615)	516 (420-755)	175 (138-268)
Other cardiomyopathy	532 (469-589)	253 (226-291)	1,940 (1,726-2,226)	1,024 (883-1,210)	495 (431-557)	563 (504-625)	969 (822-1,083)	1,368 (1,121-1,570)	2,150 (1,769-2,421)	3,188 (2,464-3,678)	3,906 (2,994-4,508)	4,965 (3,862-5,767)	6,035 (4,950-7,008)	7,463 (6,221-8,585)	9,764 (8,209-11,177)	10,793 (9,405-12,248)	11,102 (9,731-12,653)	10,569 (9,244-11,757)	12,072 (10,487-13,203)	13,248 (10,809-14,435)	11,748 (8,996-12,742)	6,222 (4,663-6,854)	2,043 (1,489-2,321)
Atrial fibrillation and flutter	-	-	-	-	-	-	-	-	-	43 (37-46)	67 (55-73)	300 (256-335)	529 (467-587)	1,065 (946-1,200)	2,078 (1,818-2,350)	3,815 (3,397-4,441)	6,058 (5,449-6,969)	9,580 (8,385-10,959)	14,813 (13,217-17,428)	24,103 (21,257-27,991)	24,683 (21,714-28,339)	16,989 (14,883-19,757)	5,848 (5,047-6,696)
Aortic aneurysm	-	-	-	-	-	-	158 (141-179)	256 (225-289)	509 (454-572)	786 (698-879)	1,269 (1,133-1,416)	1,906 (1,707-2,116)	3,005 (2,707-3,306)	4,415 (4,013-4,815)	6,803 (6,235-7,458)	10,235 (9,501-11,179)	13,624 (12,697-14,793)	14,269 (13,157-15,461)	16,021 (14,901-17,229)	15,307 (14,377-16,309)	11,382 (10,810-12,070)	5,111 (4,901-5,399)	1,289 (1,245-1,353)
Peripheral artery disease	-	-	-	-	-	-	-	-	-	-	-	158 (99-268)	333 (200-581)	749 (440-1,324)	1,566 (902-2,875)	2,709 (1,536-4,893)	3,810 (2,199-6,974)	4,145 (2,339-7,615)	5,373 (2,936-9,603)	5,637 (3,166-10,429)	4,925 (2,751-8,980)	3,001 (1,668-5,506)	1,379 (752-2,660)
Endocarditis	119 (94-161)	81 (63-105)	1,096 (896-1,409)	589 (384-808)	167 (146-189)	252 (224-286)	351 (320-390)	605 (551-674)	947 (874-1,043)	1,185 (1,092-1,312)	1,395 (1,272-1,538)	1,596 (1,462-1,798)	1,821 (1,685-2,008)	2,229 (2,062-2,478)	2,613 (2,434-2,879)	3,176 (2,984-3,420)	3,660 (3,432-3,928)	3,648 (3,416-3,942)	4,028 (3,772-4,274)	4,157 (3,897-4,398)	3,412 (3,201-3,627)	1,833 (1,715-1,972)	610 (572-663)
Other cardiovascular and circulatory diseases	348 (298-420)	237 (203-286)	2,022 (1,674-2,424)	1,058 (759-1,455)	590 (513-720)	562 (495-645)	863 (745-1,036)	1,465 (1,257-1,759)	2,228 (1,945-2,623)	2,985 (2,633-3,521)	3,836 (3,406-4,495)	5,446 (4,739-6,520)	7,398 (6,512-8,722)	10,275 (9,067-12,076)	13,711 (12,239-15,995)	17,672 (15,832-20,283)	20,339 (18,045-23,101)	20,978 (18,381-24,169)	22,997 (20,437-26,095)	21,719 (19,402-24,406)	15,622 (14,175-17,138)	7,056 (6,582-7,653)	2,099 (1,973-2,251)
Chronic respiratory diseases	0 (0-0)	0 (0-0)	0 (0-0)	4,814 (4,306-5,368)	1,543 (1,364-1,737)	1,857 (1,674-2,044)	3,336 (3,066-3,639)	5,115 (4,780-5,491)	6,325 (5,939-6,743)	8,734 (8,270-9,265)	12,712 (12,028-13,508)	20,180 (19,116-21,256)	36,487 (34,605-38,416)	64,146 (61,252-67,227)	105,329 (100,336-110,332)	180,773 (173,500-188,256)	256,267 (245,657-265,785)	320,093 (306,387-331,850)	365,616 (351,102-378,000)	375,766 (360,902-388,187)	258,643 (248,471-267,443)	108,336 (104,053-111,541)	28,361 (27,492-29,055)
Chronic obstructive pulmonary disease	-	-	0 (0-0)	441 (345-671)	200 (167-266)	181 (148-242)	1,140 (946-1,366)	1,705 (1,427-2,076)	2,350 (2,020-2,814)	3,731 (3,223-4,520)	5,639 (4,798-7,099)	10,288 (8,761-12,528)	21,663 (18,777-25,406)	42,952 (37,691-48,377)	74,014 (65,169-83,405)	143,043 (130,126-154,443)	210,921 (193,772-226,012)	270,056 (249,581-286,812)	316,214 (296,411-331,908)	330,949 (313,434-346,357)	231,033 (220,244-240,879)	97,616 (93,728-101,130)	25,647 (24,801-26,484)
Pneumoconiosis	-	-	-	-	-	-	13 (11-15)	23 (19-27)	58 (52-65)	94 (84-104)	176 (161-193)	364 (330-401)	809 (740-891)	1,188 (1,086-1,303)	1,145 (1,031-1,279)	1,847 (1,685-2,028)	2,164 (1,997-2,373)	2,423 (2,237-2,634)	2,999 (2,809-3,222)	2,962 (2,786-3,154)	2,041 (1,916-2,164)	747 (703-794)	160 (150-169)
Silicosis	-	-	-	-	-	-	3 (2-4)	7 (5-10)	23 (16-27)	50 (41-58)	102 (85-117)	238 (200-273)	559 (452-634)	825 (679-928)	707 (576-814)	1,135 (956-1,291)	1,254 (1,078-1,438)	1,246 (1,099-1,428)	1,534 (1,380-1,761)	1,552 (1,401-1,820)	1,035 (932-1,219)	336 (300-397)	61 (54-72)
Asbestosis	-	-	-	-	-	-	2 (1-3)	3 (1-4)	6 (3-9)	9 (4-13)	12 (6-17)	21 (11-32)	33 (18-50)	68 (39-99)	110 (70-153)	180 (116-255)	280 (190-361)	417 (277-515)	533 (312-632)	554 (289-656)	453 (240-533)	195 (111-226)	42 (22-48)
Coal workers pneumoconiosis	-	-	-	-	-	-	2 (1-3)	2 (2-4)	7 (5-9)	8 (6-11)	17 (13-22)	42 (31-56)	92 (72-145)	128 (99-184)	139 (110-191)	239 (196-317)	318 (265-409)	407 (347-490)	548 (476-678)	523 (462-644)	342 (305-424)	116 (102-149)	29 (25-36)
Other pneumoconiosis	-	-	-	-	-	-	6 (5-8)	10 (8-13)	22 (18-28)	27 (23-35)	45 (37-59)	63 (51-90)	125 (102-193)	167 (137-249)	189 (147-261)	292 (229-397)	311 (247-418)	353 (276-479)	383 (309-523)	333 (272-438)	211 (175-282)	99 (82-128)	28 (24-35)
Asthma	-	-	-	3,369 (2,903-3,872)	908 (766-1,057)	1,275 (1,100-1,442)	1,513 (1,144-1,784)	2,317 (1,664-2,741)	2,828 (2,043-3,319)	3,335 (2,278-4,032)	4,922 (3,133-6,082)	6,635 (3,982-8,563)	9,781 (5,588-13,117)	14,059 (7,914-19,731)	20,816 (11,281-30,117)	26,794 (14,970-38,933)	31,059 (17,352-46,842)	32,955 (18,108-49,720)	30,516 (17,825-44,711)	26,703 (16,680-38,778)	15,004 (10,095-21,489)	5,371 (3,989-7,360)	1,263 (991-1,656)
Interstitial lung disease and pulmonary sarcoidosis	-	-	-	160 (100-282)	60 (44-91)	60 (48-79)	98 (73-133)	169 (130-228)	266 (207-349)	405 (318-553)	644 (506-921)	1,024 (788-1,509)	1,675 (1,286-2,480)	2,791 (2,190-3,978)	4,720 (3,603-7,011)	6,849 (5,250-9,716)	9,671 (7,433-13,385)	11,932 (8,793-16,448)	13,540 (10,196-17,512)	12,623 (8,855-16,169)	8,705 (5,891-10,833)	3,624 (2,448-4,408)	970 (638-1,149)
Other chronic respiratory diseases	0 (0-0)	0 (0-0)	0 (0-0)	844 (564-1,131)	374 (308-453)	342 (276-416)	573 (453-720)	901 (711-1,149)	823 (653-1,038)	1,170 (967-1,468)	1,329 (1,059-1,695)	1,869 (1,472-2,410)	2,559 (2,067-3,248)	3,156 (2,587-4,022)	4,635 (3,799-5,914)	2,241 (1,891-2,779)	2,452 (2,086-2,958)	2,726 (2,349-3,321)	2,347 (2,054-2,767)	2,529 (2,198-3,006)	1,861 (1,669-2,140)	978 (871-1,106)	322 (294-354)
Digestive diseases	1,143 (945-1,343)	1,104 (892-1,295)	9,390 (7,844-11,117)	10,795 (8,652-13,423)	5,761 (4,511-6,884)	5,059 (4,222-5,951)	8,537 (7,333-9,905)	14,003 (12,592-16,031)	24,228 (22,330-27,460)	40,053 (37,374-44,810)	60,654 (56,477-66,862)	83,591 (77,496-91,920)	111,798 (105,088-122,930)	134,428 (126,559-149,453)	146,625 (139,396-163,073)	160,205 (152,127-180,953)	151,671 (144,023-169,310)	127,873 (119,835-141,679)	120,951 (114,714-135,027)	103,353 (97,261-115,593)	70,175 (66,599-77,482)	31,224 (30,021-34,134)	9,132 (8,791-9,737)
Cirrhosis and other chronic liver diseases	-	-	-	4,187 (3,279-5,401)	2,169 (1,631-2,700)	2,309 (1,911-2,739)	4,032 (3,379-4,683)	7,846 (6,924-8,995)	15,073 (13,641-17,239)	28,207 (26,359-31,836)	44,678 (41,649-49,185)	63,449 (58,739-69,710)	85,188 (80,225-93,648)	102,796 (96,884-113,407)	107,473 (102,062-119,673)	111,641 (105,667-126,577)	95,615 (89,864-107,158)	71,626 (66,420-79,842)	59,024 (54,922-65,808)	42,517 (39,321-47,259)	23,780 (21,994-26,495)	8,737 (8,172-9,696)	2,325 (2,169-2,546)
Cirrhosis and other chronic liver diseases due to hepatitis B	-	-	-	166 (114-239)	214 (148-289)	376 (278-488)	880 (662-1,137)	3,246 (2,652-3,949)	7,279 (6,018-8,771)	12,364 (10,861-14,370)	16,748 (13,960-19,955)	21,760 (18,442-25,575)	26,731 (22,541-31,806)	29,670 (24,475-35,253)	29,496 (24,180-36,289)	32,661 (26,724-40,292)	29,674 (24,928-36,522)	22,976 (18,891-28,086)	19,506 (16,287-23,103)	14,018 (11,612-16,833)	7,396 (6,274-8,726)	2,447 (2,037-2,979)	558 (422-719)
Cirrhosis and other chronic liver diseases due to hepatitis C	-	-	-	50 (33-70)	69 (48-96)	133 (95-182)	332 (235-445)	1,401 (1,075-1,787)	3,113 (2,378-3,950)	6,439 (5,382-7,558)	11,167 (8,777-13,902)	16,617 (13,577-20,056)	23,085 (19,082-27,908)	28,271 (23,129-34,150)	28,493 (24,286-33,423)	28,802 (23,623-34,998)	24,599 (21,026-28,614)	18,154 (15,418-21,258)	14,529 (12,038-17,547)	10,377 (8,496-12,589)	6,051 (5,122-7,125)	2,333 (1,919-2,810)	651 (509-812)
Cirrhosis and other chronic liver diseases due to alcohol use	-	-	-	-	-	-	30 (13-53)	675 (511-884)	2,095 (1,627-2,631)	5,948 (5,147-6,958)	12,074 (10,112-14,460)	18,377 (15,726-21,229)	25,349 (21,503-29,680)	31,251 (26,577-36,507)	33,986 (28,751-40,060)	33,378 (27,655-40,044)	26,397 (22,426-30,967)	18,618 (15,467-22,416)	14,794 (12,744-17,237)	10,301 (8,642-12,190)	5,569 (4,755-6,471)	2,002 (1,645-2,444)	531 (392-687)
Cirrhosis due to NASH	-	-	-	-	-	-	76 (56-100)	242 (193-307)	568 (432-717)	1,329 (1,116-1,568)	2,475 (1,966-3,015)	4,108 (3,459-4,822)	6,728 (5,587-7,955)	9,188 (7,644-10,961)	9,713 (8,464-11,189)	9,955 (8,522-11,484)	8,147 (7,046-9,381)	5,720 (4,970-6,618)	4,263 (3,618-5,056)	2,951 (2,503-3,475)	1,761 (1,511-2,071)	690 (566-855)	198 (149-262)
Cirrhosis and other chronic liver diseases due to other causes	-	-	-	3,972 (3,096-5,132)	1,886 (1,417-2,358)	1,801 (1,474-2,143)	2,716 (2,171-3,235)	2,281 (1,718-2,878)	2,018 (1,485-2,629)	2,127 (1,547-2,830)	2,214 (1,650-2,874)	2,587 (1,886-3,416)	3,295 (2,430-4,288)	4,416 (3,260-5,813)	5,785 (4,042-8,044)	6,845 (4,854-9,247)	6,798 (4,974-9,007)	6,158 (4,747-7,962)	5,932 (4,492-7,724)	4,869 (3,725-6,428)	3,003 (2,435-3,722)	1,264 (971-1,607)	387 (271-522)
Upper digestive system diseases	-	-	-	939 (654-1,558)	403 (307-488)	418 (343-498)	971 (820-1,158)	1,833 (1,597-2,140)	2,551 (2,241-3,000)	3,426 (3,031-3,912)	4,380 (3,863-5,025)	5,832 (5,191-6,781)	8,846 (8,002-10,203)	11,396 (10,309-13,201)	13,252 (12,133-15,537)	16,483 (15,328-19,355)	18,807 (17,463-21,980)	17,859 (16,376-20,412)	19,509 (18,029-22,490)	17,178 (16,047-20,190)	11,932 (11,079-13,741)	5,161 (4,880-5,912)	1,495 (1,421-1,671)
Peptic ulcer disease	-	-	-	523 (331-1,151)	314 (239-383)	336 (273-405)	707 (591-850)	1,408 (1,218-1,662)	1,883 (1,642-2,211)	2,461 (2,175-2,826)	3,328 (2,962-3,834)	4,665 (4,189-5,409)	7,068 (6,401-8,179)	9,230 (8,361-10,586)	11,054 (10,142-13,011)	13,876 (12,867-16,161)	15,696 (14,561-18,256)	14,918 (13,732-17,045)	16,126 (14,916-18,581)	14,175 (13,247-16,636)	9,878 (9,211-11,384)	4,298 (4,058-4,886)	1,241 (1,177-1,375)
Gastritis and duodenitis	-	-	-	416 (277-623)	89 (67-114)	82 (66-102)	264 (205-334)	425 (327-534)	668 (527-857)	965 (790-1,195)	1,052 (855-1,299)	1,167 (964-1,434)	1,779 (1,514-2,124)	2,165 (1,863-2,593)	2,198 (1,919-2,616)	2,606 (2,322-3,175)	3,112 (2,774-3,708)	2,941 (2,614-3,400)	3,383 (3,010-3,924)	3,002 (2,745-3,582)	2,054 (1,860-2,418)	863 (799-1,040)	254 (237-298)
Appendicitis	-	-	-	889 (625-1,206)	711 (538-862)	724 (583-869)	842 (703-1,049)	787 (674-964)	930 (809-1,154)	703 (611-830)	955 (821-1,173)	1,168 (994-1,436)	1,115 (974-1,339)	1,269 (1,086-1,517)	1,521 (1,320-1,822)	1,666 (1,460-1,986)	1,621 (1,450-1,928)	1,803 (1,578-2,133)	1,679 (1,477-1,979)	1,563 (1,374-1,823)	1,082 (952-1,245)	501 (453-574)	138 (126-155)
Paralytic ileus and intestinal obstruction	1,143 (945-1,343)	1,104 (892-1,295)	9,390 (7,844-11,117)	2,191 (1,349-2,849)	1,619 (1,080-2,026)	882 (604-1,092)	1,572 (1,109-1,964)	1,705 (1,129-2,109)	2,036 (1,449-2,550)	2,524 (1,749-3,116)	3,516 (2,413-4,298)	4,152 (2,903-5,347)	5,039 (3,441-6,311)	5,588 (4,007-7,199)	7,777 (5,457-9,869)	9,716 (7,079-12,399)	11,679 (8,533-14,765)	11,504 (8,957-14,455)	11,918 (9,817-15,296)	12,555 (10,172-15,524)	10,677 (8,880-12,878)	5,141 (4,101-6,162)	1,490 (1,170-1,775)
Inguinal, femoral, and abdominal hernia	-	-	-	383 (268-521)	150 (102-189)	115 (79-139)	191 (158-234)	258 (217-320)	330 (274-397)	333 (279-400)	485 (412-594)	604 (524-754)	801 (701-979)	1,150 (1,013-1,404)	1,630 (1,456-2,022)	2,036 (1,815-2,463)	2,649 (2,340-3,290)	2,860 (2,480-3,386)	3,459 (2,941-4,027)	3,773 (3,314-4,392)	2,749 (2,483-3,338)	1,391 (1,285-1,633)	432 (401-496)
Inflammatory bowel disease	-	-	-	447 (283-704)	92 (74-116)	63 (53-76)	85 (68-101)	162 (137-187)	260 (227-298)	361 (317-411)	412 (356-466)	567 (476-647)	747 (652-834)	935 (783-1,041)	1,202 (1,012-1,341)	1,485 (1,236-1,639)	1,788 (1,498-1,958)	1,875 (1,525-2,061)	2,216 (1,696-2,475)	2,342 (1,805-2,588)	1,898 (1,395-2,086)	1,035 (696-1,154)	332 (216-375)
Vascular intestinal disorders	-	-	-	333 (159-556)	62 (38-88)	26 (21-30)	42 (35-47)	99 (84-119)	163 (138-193)	166 (149-183)	310 (270-345)	536 (466-590)	925 (833-1,013)	1,485 (1,342-1,623)	2,497 (2,273-2,735)	3,505 (3,248-3,821)	5,000 (4,616-5,438)	5,606 (5,011-6,107)	6,630 (5,998-7,161)	6,532 (5,956-7,049)	4,685 (4,346-5,030)	2,238 (2,099-2,424)	621 (585-672)
Gallbladder and biliary diseases	-	-	-	308 (210-472)	109 (80-140)	169 (123-227)	153 (126-183)	244 (213-290)	343 (306-403)	454 (414-516)	664 (593-756)	963 (856-1,102)	1,299 (1,194-1,492)	1,918 (1,739-2,197)	2,322 (2,159-2,641)	3,432 (3,198-3,973)	4,608 (4,310-5,315)	5,299 (4,936-6,002)	6,749 (6,324-7,593)	7,612 (7,168-8,480)	6,393 (6,031-7,032)	3,568 (3,373-3,963)	1,179 (1,115-1,284)
Pancreatitis	-	-	-	69 (51-95)	65 (50-79)	80 (64-98)	251 (202-302)	774 (651-960)	2,033 (1,717-2,385)	3,158 (2,735-3,522)	4,293 (3,670-4,896)	5,162 (4,311-5,951)	6,119 (5,151-7,042)	5,592 (4,825-6,327)	6,286 (5,476-7,134)	6,066 (5,328-7,015)	5,723 (5,052-6,695)	4,815 (4,224-5,628)	4,962 (4,240-5,653)	3,953 (3,417-4,594)	2,624 (2,324-3,007)	1,052 (950-1,186)	276 (257-305)
Other digestive diseases	-	-	-	1,049 (753-1,522)	380 (280-529)	272 (206-373)	397 (277-623)	296 (244-395)	509 (411-692)	721 (589-958)	961 (778-1,252)	1,157 (982-1,376)	1,719 (1,460-2,132)	2,299 (1,951-2,822)	2,665 (2,363-3,077)	4,175 (3,467-5,203)	4,180 (3,695-4,793)	4,627 (4,012-5,501)	4,806 (4,316-5,449)	5,329 (4,814-5,966)	4,356 (4,035-4,702)	2,401 (2,241-2,633)	845 (788-929)
Neurological disorders	-	-	3,688 (3,199-4,409)	6,279 (5,144-8,303)	2,616 (2,210-3,346)	3,569 (3,111-4,342)	7,030 (6,097-8,783)	6,843 (6,059-8,333)	6,843 (6,073-8,068)	6,586 (5,773-7,868)	6,873 (6,150-8,418)	7,903 (7,148-9,374)	10,345 (9,377-11,803)	13,229 (12,391-14,132)	18,650 (17,768-19,679)	34,094 (32,872-35,541)	59,085 (56,945-61,359)	96,556 (92,087-100,595)	175,366 (168,302-181,880)	252,293 (242,401-260,365)	256,216 (248,808-261,800)	163,662 (160,347-166,585)	54,641 (53,771-55,727)
Alzheimer's disease and other dementias	-	-	-	-	-	-	-	-	-	-	-	403 (375-443)	1,102 (1,062-1,145)	3,207 (3,113-3,299)	6,614 (6,389-6,872)	18,163 (17,665-18,652)	36,210 (35,145-37,221)	63,274 (60,952-65,707)	124,561 (120,005-129,307)	195,212 (188,412-201,423)	216,493 (210,758-220,905)	149,145 (146,213-151,598)	51,566 (50,720-52,627)
Parkinson's disease	-	-	-	-	-	-	-	6 (5-6)	11 (9-12)	26 (23-29)	32 (29-35)	375 (349-433)	891 (840-1,016)	1,725 (1,632-2,000)	2,597 (2,452-2,993)	5,870 (5,517-6,546)	12,267 (11,620-14,034)	24,363 (21,844-27,145)	42,643 (39,089-45,987)	50,869 (45,222-54,258)	35,874 (31,250-38,768)	13,149 (11,692-14,475)	2,731 (2,480-2,990)
Epilepsy	-	-	2,186 (1,840-2,801)	5,015 (4,045-6,777)	2,042 (1,682-2,796)	2,661 (2,232-3,434)	5,250 (4,441-6,848)	5,360 (4,646-6,739)	5,707 (5,009-6,899)	5,405 (4,595-6,664)	5,369 (4,585-6,881)	5,016 (4,294-6,411)	5,086 (4,116-6,477)	4,085 (3,407-4,779)	4,039 (3,363-4,694)	3,857 (3,169-4,633)	3,889 (2,990-5,063)	2,954 (2,360-3,714)	2,811 (2,307-3,158)	2,319 (1,844-2,592)	1,599 (1,243-1,759)	597 (459-649)	159 (124-174)
Multiple sclerosis	-	-	-	-	-	-	-	58 (47-63)	125 (106-142)	263 (231-309)	402 (369-495)	633 (575-764)	877 (775-1,058)	1,017 (862-1,185)	1,146 (936-1,281)	1,129 (905-1,231)	978 (737-1,050)	718 (510-778)	527 (386-572)	338 (248-364)	177 (131-192)	54 (39-58)	11 (7-13)
Motor neuron disease	3 (3-4)	8 (6-9)	241 (176-283)	70 (48-85)	22 (19-24)	37 (31-41)	69 (55-74)	65 (57-71)	70 (60-76)	122 (107-137)	241 (225-277)	442 (412-490)	874 (817-1,092)	1,296 (1,206-1,399)	1,977 (1,873-2,300)	2,623 (2,467-2,919)	3,164 (2,984-3,464)	2,859 (2,707-3,015)	2,402 (2,268-2,533)	1,534 (1,438-1,621)	751 (701-798)	193 (178-205)	37 (34-39)
Other neurological disorders	-	-	1,261 (984-1,557)	1,195 (710-1,678)	552 (430-694)	870 (802-976)	1,711 (1,550-1,980)	1,355 (1,230-1,539)	930 (861-1,025)	772 (715-861)	828 (763-933)	1,035 (946-1,179)	1,515 (1,403-1,679)	1,899 (1,766-2,106)	2,277 (2,117-2,511)	2,452 (2,299-2,653)	2,577 (2,423-2,773)	2,388 (2,233-2,596)	2,421 (2,248-2,603)	2,019 (1,896-2,157)	1,322 (1,246-1,397)	524 (499-549)	137 (131-143)
Mental disorders	-	-	-	-	1 (0-1)	1 (0-1)	3 (2-4)	5 (4-6)	6 (4-8)	6 (5-7)	5 (4-7)	9 (7-10)	10 (8-12)	-	-	-	-	-	-	-	-	-	-
Eating disorders	-	-	-	-	1 (0-1)	1 (0-1)	3 (2-4)	5 (4-6)	6 (4-8)	6 (5-7)	5 (4-7)	9 (7-10)	10 (8-12)	-	-	-	-	-	-	-	-	-	-
Anorexia nervosa	-	-	-	-	0 (0-1)	0 (0-1)	2 (2-3)	3 (2-4)	2 (1-4)	3 (2-4)	3 (2-5)	7 (5-8)	8 (5-10)	-	-	-	-	-	-	-	-	-	-
Bulimia nervosa	-	-	-	-	0 (0-0)	0 (0-0)	1 (1-1)	2 (1-3)	4 (3-5)	3 (2-4)	2 (1-3)	2 (2-4)	2 (1-4)	-	-	-	-	-	-	-	-	-	-
Substance use disorders	0 (0-0)	0 (0-0)	0 (0-0)	0 (0-0)	0 (0-0)	0 (0-0)	3,234 (3,037-3,403)	9,986 (9,445-10,429)	17,422 (16,564-18,090)	24,661 (23,108-25,722)	26,691 (25,178-27,798)	28,640 (26,308-29,967)	32,706 (29,468-34,467)	29,829 (27,454-31,140)	28,023 (25,988-29,156)	22,970 (21,055-23,942)	16,341 (14,895-17,078)	10,298 (9,336-10,782)	8,279 (7,502-8,690)	5,285 (4,903-5,536)	3,227 (3,023-3,381)	1,352 (1,277-1,417)	383 (368-398)
Alcohol use disorders	-	-	-	-	-	-	721 (546-792)	2,188 (1,764-2,405)	5,272 (4,447-5,695)	10,297 (8,805-11,031)	13,414 (11,880-14,241)	17,161 (14,801-18,300)	22,158 (18,849-23,732)	20,315 (17,864-21,500)	20,007 (18,047-21,027)	16,984 (15,095-17,880)	11,521 (10,065-12,206)	6,690 (5,731-7,115)	4,796 (3,995-5,117)	2,378 (1,996-2,551)	1,282 (1,088-1,383)	438 (372-480)	125 (114-134)
Drug use disorders	0 (0-0)	0 (0-0)	0 (0-0)	0 (0-0)	0 (0-0)	0 (0-0)	2,513 (2,381-2,662)	7,798 (7,418-8,153)	12,150 (11,698-12,643)	14,364 (13,892-14,890)	13,277 (12,848-13,770)	11,479 (11,074-11,937)	10,549 (10,081-11,041)	9,514 (9,126-9,957)	8,015 (7,661-8,420)	5,987 (5,744-6,238)	4,819 (4,608-5,007)	3,608 (3,445-3,773)	3,483 (3,329-3,613)	2,907 (2,771-3,040)	1,945 (1,865-2,031)	914 (877-953)	258 (247-269)
Opioid use disorders	0 (0-0)	0 (0-0)	0 (0-0)	0 (0-0)	0 (0-0)	0 (0-0)	1,948 (1,826-2,088)	5,857 (5,504-6,165)	9,009 (8,607-9,468)	10,021 (9,599-10,488)	9,308 (8,920-9,753)	7,959 (7,559-8,417)	7,117 (6,724-7,601)	6,131 (5,822-6,537)	5,150 (4,848-5,458)	3,970 (3,767-4,188)	3,297 (3,096-3,498)	2,051 (1,899-2,200)	2,134 (2,017-2,255)	1,755 (1,632-1,859)	1,159 (1,093-1,225)	515 (487-544)	141 (133-148)
Cocaine use disorders	-	-	-	-	-	-	67 (54-80)	231 (201-266)	382 (342-429)	764 (688-846)	676 (606-749)	553 (463-621)	562 (470-645)	716 (625-803)	622 (545-712)	395 (339-450)	254 (206-309)	103 (83-124)	91 (71-112)	19 (15-24)	14 (12-17)	8 (7-10)	3 (2-3)
Amphetamine use disorders	-	-	-	-	-	-	65 (47-79)	198 (141-228)	342 (260-385)	471 (336-525)	429 (289-483)	377 (253-435)	366 (269-429)	333 (187-387)	290 (175-352)	181 (114-213)	115 (85-146)	33 (23-40)	29 (20-36)	9 (6-11)	6 (4-7)	4 (3-5)	1 (1-2)
Other drug use disorders	-	-	-	-	-	-	433 (382-505)	1,513 (1,353-1,724)	2,416 (2,215-2,672)	3,108 (2,890-3,382)	2,865 (2,653-3,117)	2,590 (2,373-2,860)	2,503 (2,265-2,760)	2,333 (2,146-2,523)	1,954 (1,765-2,234)	1,441 (1,316-1,607)	1,154 (1,026-1,342)	1,422 (1,296-1,563)	1,229 (1,130-1,345)	1,124 (1,044-1,223)	766 (708-840)	386 (359-421)	113 (105-122)
Diabetes and kidney diseases	19 (14-23)	52 (41-62)	3,735 (3,328-4,234)	3,774 (3,223-4,439)	2,456 (2,134-2,767)	2,644 (2,367-2,909)	6,187 (5,673-6,817)	9,568 (8,896-10,279)	11,363 (10,720-12,132)	16,407 (15,651-17,492)	22,094 (21,006-23,243)	35,267 (33,670-37,027)	54,145 (51,705-56,235)	81,927 (78,813-85,129)	111,779 (107,789-116,034)	147,027 (141,671-151,733)	165,444 (158,619-170,624)	161,644 (155,553-167,262)	163,262 (157,395-168,539)	146,534 (140,728-151,361)	106,196 (102,188-109,613)	49,387 (47,722-50,818)	15,432 (14,952-15,842)
Diabetes mellitus	19 (14-23)	52 (41-62)	658 (505-804)	267 (225-321)	367 (297-446)	420 (324-488)	1,085 (1,007-1,174)	2,098 (1,976-2,235)	2,843 (2,717-2,999)	5,380 (5,173-5,629)	7,725 (7,425-8,071)	15,047 (14,482-15,684)	26,370 (25,403-27,312)	42,319 (40,930-43,810)	61,249 (59,111-63,385)	82,219 (79,498-84,875)	91,264 (88,176-94,277)	88,702 (85,690-91,904)	86,115 (83,334-89,222)	72,964 (70,447-75,875)	47,391 (45,630-49,133)	19,163 (18,454-19,775)	5,079 (4,903-5,223)
Diabetes mellitus type 1	19 (14-23)	52 (41-62)	658 (505-804)	267 (225-321)	367 (297-446)	420 (324-488)	948 (885-1,025)	1,798 (1,693-1,909)	2,338 (2,233-2,459)	3,861 (3,678-4,086)	5,307 (5,038-5,599)	10,025 (9,437-10,631)	9,608 (8,478-11,015)	14,613 (12,979-16,574)	20,778 (17,813-23,976)	19,972 (17,025-23,365)	21,704 (18,766-25,187)	17,626 (14,375-21,093)	16,438 (13,374-19,596)	10,709 (8,495-12,924)	6,459 (5,128-7,731)	2,417 (1,950-2,823)	578 (485-658)
Diabetes mellitus type 2	-	-	-	-	-	-	137 (113-172)	300 (246-373)	504 (462-586)	1,519 (1,370-1,770)	2,417 (2,209-2,731)	5,022 (4,515-5,644)	16,762 (15,281-18,421)	27,706 (25,249-29,813)	40,471 (36,888-43,776)	62,247 (57,987-66,336)	69,560 (65,478-74,085)	71,077 (66,753-75,337)	69,677 (65,528-73,649)	62,256 (58,960-65,375)	40,932 (38,964-43,145)	16,746 (16,130-17,413)	4,501 (4,355-4,651)
Chronic kidney disease	-	-	2,992 (2,621-3,407)	3,429 (2,900-4,066)	2,021 (1,756-2,309)	2,163 (1,917-2,413)	5,039 (4,567-5,589)	7,394 (6,831-8,004)	8,391 (7,768-9,093)	10,874 (10,091-11,737)	14,206 (13,150-15,130)	20,004 (18,695-21,324)	27,393 (24,909-28,795)	39,155 (36,723-41,019)	50,098 (46,894-53,170)	64,211 (58,283-66,896)	73,455 (66,422-76,589)	72,308 (65,840-75,299)	76,428 (70,493-79,440)	72,908 (67,331-75,692)	58,373 (55,536-60,804)	30,043 (28,964-31,062)	10,294 (9,901-10,665)
Chronic kidney disease due to diabetes mellitus type 1	-	-	10 (6-17)	13 (7-24)	12 (6-21)	20 (11-35)	85 (44-141)	300 (158-492)	755 (455-1,171)	1,693 (1,081-2,424)	2,947 (1,923-4,162)	4,634 (3,020-6,570)	5,970 (3,888-8,495)	6,311 (3,803-9,195)	5,500 (3,095-8,731)	4,074 (2,007-7,033)	3,107 (1,559-5,622)	2,237 (1,072-3,977)	1,766 (847-3,344)	1,326 (660-2,347)	715 (365-1,260)	179 (87-323)	13 (6-27)
Chronic kidney disease due to diabetes mellitus type 2	-	-	8 (4-15)	9 (4-18)	8 (4-15)	11 (5-21)	40 (18-75)	106 (45-200)	208 (91-399)	443 (206-793)	935 (479-1,574)	2,187 (1,267-3,578)	4,939 (3,126-6,983)	9,819 (6,816-13,419)	15,367 (10,965-19,740)	22,238 (15,949-28,199)	26,369 (19,594-33,227)	25,125 (18,832-31,336)	24,450 (16,919-31,459)	21,594 (14,888-28,714)	15,018 (10,075-19,752)	6,768 (4,745-8,980)	2,142 (1,330-3,210)
Chronic kidney disease due to hypertension	-	-	15 (9-24)	28 (15-45)	26 (15-43)	48 (27-77)	200 (120-304)	497 (319-716)	781 (538-1,086)	1,235 (865-1,716)	1,846 (1,307-2,492)	2,886 (2,097-3,743)	4,351 (3,141-5,712)	7,296 (5,440-9,475)	10,543 (8,061-13,641)	15,355 (11,755-19,560)	19,803 (15,549-24,823)	21,878 (17,455-26,938)	25,892 (20,745-31,968)	27,321 (21,592-33,487)	24,769 (20,272-29,360)	14,253 (11,984-16,439)	5,249 (4,286-6,216)
Chronic kidney disease due to glomerulonephritis	-	-	710 (507-1,005)	1,106 (765-1,550)	678 (460-956)	782 (533-1,042)	2,105 (1,480-2,774)	3,041 (2,144-3,979)	3,172 (2,246-4,164)	3,394 (2,409-4,642)	3,805 (2,518-5,384)	4,670 (2,958-6,565)	5,530 (3,582-8,185)	7,073 (4,702-10,249)	7,698 (5,098-11,499)	8,444 (5,403-12,579)	9,191 (5,917-13,576)	9,165 (5,765-13,192)	9,783 (6,293-14,304)	8,976 (5,740-13,391)	6,657 (4,387-9,823)	3,207 (2,106-4,658)	1,073 (590-1,757)
Chronic kidney disease due to other and unspecified causes	-	-	2,247 (1,905-2,641)	2,272 (1,792-2,799)	1,298 (1,009-1,596)	1,302 (1,005-1,602)	2,609 (1,954-3,267)	3,451 (2,510-4,485)	3,475 (2,505-4,523)	4,109 (2,884-5,327)	4,673 (3,227-6,189)	5,625 (3,747-7,679)	6,604 (4,290-9,507)	8,655 (5,613-12,350)	10,990 (7,145-15,654)	14,101 (8,889-20,719)	14,985 (9,632-21,405)	13,902 (9,146-19,788)	14,538 (9,471-21,305)	13,692 (9,253-19,551)	11,214 (7,841-15,282)	5,636 (3,867-7,667)	1,816 (1,167-2,741)
Acute glomerulonephritis	-	-	85 (57-119)	78 (50-115)	68 (54-93)	61 (50-80)	63 (53-81)	76 (65-93)	129 (112-150)	153 (132-178)	163 (143-195)	216 (190-253)	381 (334-441)	453 (398-524)	432 (379-504)	597 (524-695)	725 (634-830)	634 (557-734)	719 (625-832)	661 (584-756)	432 (385-496)	182 (161-208)	59 (49-73)
Skin and subcutaneous diseases	-	-	1,541 (914-2,446)	966 (521-1,639)	206 (131-321)	266 (163-420)	590 (350-977)	493 (281-710)	477 (286-719)	1,027 (588-1,517)	1,052 (606-1,464)	1,300 (759-1,875)	1,991 (1,179-2,872)	2,293 (1,320-3,389)	3,192 (1,711-4,387)	3,927 (2,159-5,489)	4,303 (2,452-6,123)	4,897 (2,789-6,902)	5,662 (3,407-8,402)	5,269 (3,296-8,183)	4,666 (2,710-6,865)	2,547 (1,415-3,912)	902 (488-1,443)
Bacterial skin diseases	-	-	1,512 (896-2,412)	894 (477-1,511)	155 (92-240)	204 (122-312)	519 (301-864)	412 (234-590)	390 (230-591)	893 (494-1,285)	907 (511-1,245)	1,106 (625-1,549)	1,737 (1,027-2,491)	1,965 (1,099-2,821)	2,770 (1,436-3,725)	3,355 (1,769-4,583)	3,491 (1,886-4,776)	3,844 (2,171-5,314)	4,342 (2,439-6,327)	3,684 (2,102-5,500)	3,182 (1,660-4,464)	1,672 (870-2,429)	563 (265-863)
Cellulitis	-	-	126 (53-245)	172 (63-310)	51 (25-75)	80 (43-117)	117 (54-168)	97 (49-160)	98 (49-166)	209 (98-321)	267 (122-405)	320 (147-505)	481 (220-758)	583 (257-951)	801 (339-1,229)	999 (434-1,502)	916 (369-1,443)	990 (444-1,633)	1,002 (409-1,657)	947 (384-1,622)	764 (279-1,349)	428 (157-759)	158 (50-288)
Pyoderma	-	-	1,386 (810-2,203)	722 (403-1,267)	105 (63-166)	124 (74-201)	402 (238-698)	315 (165-458)	292 (168-444)	684 (356-985)	640 (335-927)	786 (431-1,143)	1,256 (709-1,848)	1,382 (747-2,053)	1,969 (1,004-2,623)	2,357 (1,210-3,374)	2,576 (1,360-3,630)	2,854 (1,503-4,078)	3,340 (1,834-4,864)	2,737 (1,550-4,275)	2,418 (1,221-3,342)	1,244 (625-1,802)	404 (194-612)
Decubitus ulcer	-	-	-	32 (18-64)	24 (15-44)	31 (18-57)	38 (20-65)	47 (25-79)	55 (27-91)	92 (50-165)	106 (57-192)	147 (77-271)	199 (106-375)	267 (139-498)	352 (173-658)	478 (246-902)	696 (348-1,235)	924 (490-1,681)	1,160 (611-2,267)	1,388 (774-2,623)	1,289 (664-2,285)	765 (371-1,506)	298 (138-601)
Other skin and subcutaneous diseases	-	-	28 (13-52)	40 (23-81)	27 (18-47)	32 (20-60)	32 (19-59)	34 (21-60)	32 (18-53)	42 (25-78)	40 (24-67)	47 (26-86)	55 (29-104)	61 (32-111)	70 (34-133)	94 (49-181)	116 (58-228)	128 (69-247)	160 (84-319)	198 (105-397)	195 (100-431)	111 (52-241)	41 (16-91)
Musculoskeletal disorders	-	-	-	-	215 (177-241)	313 (266-345)	414 (346-461)	480 (405-517)	623 (523-663)	748 (629-800)	914 (751-1,000)	1,153 (954-1,263)	1,456 (1,232-1,562)	1,469 (1,279-1,563)	2,119 (1,786-2,262)	3,742 (2,994-4,014)	4,663 (3,822-4,981)	5,370 (4,318-5,730)	5,660 (4,700-6,044)	5,273 (4,404-5,628)	4,018 (3,487-4,272)	1,823 (1,611-1,951)	529 (494-563)
Rheumatoid arthritis	-	-	-	-	28 (22-37)	33 (27-43)	38 (30-48)	38 (30-47)	48 (36-60)	58 (44-73)	84 (63-102)	152 (114-180)	260 (186-299)	463 (352-522)	767 (618-862)	1,531 (1,126-1,732)	2,060 (1,532-2,293)	2,487 (1,895-2,784)	2,651 (2,029-2,942)	2,158 (1,675-2,402)	1,492 (1,188-1,664)	522 (407-589)	133 (111-148)
Other musculoskeletal disorders	-	-	-	-	187 (153-209)	280 (236-309)	376 (314-420)	441 (371-476)	575 (478-612)	690 (580-740)	829 (688-909)	1,002 (833-1,099)	1,196 (1,021-1,292)	1,006 (883-1,099)	1,352 (1,145-1,497)	2,211 (1,798-2,461)	2,603 (2,137-2,898)	2,883 (2,352-3,246)	3,009 (2,461-3,426)	3,115 (2,607-3,439)	2,527 (2,215-2,782)	1,301 (1,170-1,417)	396 (370-428)
Other non-communicable diseases	99,621 (91,401-110,923)	33,671 (30,540-37,407)	130,546 (115,727-149,546)	47,381 (41,184-55,946)	12,124 (10,554-13,856)	9,761 (8,389-11,376)	9,044 (7,518-10,961)	9,484 (8,134-11,219)	8,790 (7,793-10,012)	8,409 (7,548-9,410)	9,359 (8,394-10,378)	10,934 (9,827-12,102)	11,071 (9,754-12,167)	13,910 (12,055-15,264)	16,341 (14,115-17,738)	20,456 (18,161-21,924)	23,752 (21,431-25,291)	24,234 (22,095-25,760)	25,209 (23,048-26,678)	27,789 (25,672-29,226)	22,468 (20,745-23,398)	12,507 (11,462-13,011)	4,663 (4,241-4,845)
Congenital birth defects	98,112 (89,955-109,192)	30,429 (27,880-33,478)	99,544 (91,691-110,481)	37,444 (32,722-44,877)	7,955 (7,199-8,898)	5,723 (5,281-6,207)	4,118 (3,834-4,597)	4,156 (3,910-4,585)	3,973 (3,751-4,448)	2,970 (2,810-3,271)	3,434 (3,190-3,737)	3,576 (3,325-3,927)	1,538 (1,381-1,719)	1,590 (1,366-1,732)	1,647 (1,346-1,764)	1,597 (1,320-1,704)	1,252 (1,071-1,400)	-	-	-	-	-	-
Neural tube defects	13,022 (9,636-18,186)	2,590 (1,928-3,641)	7,738 (5,177-11,703)	6,527 (3,899-11,811)	382 (293-528)	252 (204-340)	136 (108-178)	148 (117-184)	104 (87-130)	87 (74-105)	76 (67-92)	86 (73-106)	51 (43-63)	41 (34-51)	39 (33-49)	37 (31-48)	29 (25-35)	-	-	-	-	-	-
Congenital heart anomalies	31,275 (23,217-40,005)	13,959 (11,740-17,045)	50,668 (39,952-61,442)	15,026 (10,512-21,179)	4,539 (3,956-5,304)	3,525 (3,178-3,927)	2,475 (2,252-2,796)	2,442 (2,229-2,745)	2,692 (2,492-3,040)	1,899 (1,744-2,103)	2,297 (2,097-2,543)	2,235 (2,023-2,495)	674 (589-805)	589 (505-696)	565 (486-678)	520 (458-607)	437 (380-543)	-	-	-	-	-	-
Orofacial clefts	1,162 (314-3,135)	270 (81-711)	574 (165-1,752)	126 (22-475)	-	-	-	-	-	-	-	-	-	-	-	-	-	-	-	-	-	-	-
Down syndrome	2,063 (1,436-3,260)	796 (579-1,231)	4,457 (2,997-6,939)	2,387 (1,225-4,103)	348 (265-486)	228 (186-304)	274 (220-366)	299 (243-402)	299 (232-404)	300 (226-405)	314 (245-411)	409 (304-551)	411 (289-508)	540 (375-635)	572 (362-658)	530 (329-611)	269 (176-315)	-	-	-	-	-	-
Other chromosomal abnormalities	2,755 (1,598-4,722)	954 (617-1,513)	2,291 (1,317-4,017)	540 (272-1,113)	127 (76-194)	97 (56-137)	113 (73-150)	134 (90-176)	128 (90-162)	89 (55-109)	106 (70-135)	76 (47-98)	55 (33-66)	45 (24-55)	47 (26-57)	31 (15-40)	28 (14-35)	-	-	-	-	-	-
Congenital musculoskeletal and limb anomalies	2,182 (1,472-3,209)	473 (355-609)	1,369 (948-1,910)	480 (267-792)	171 (122-223)	129 (96-161)	151 (114-191)	123 (94-151)	80 (63-99)	60 (49-76)	62 (50-77)	54 (42-67)	31 (26-38)	32 (27-40)	25 (20-31)	31 (26-38)	26 (22-34)	-	-	-	-	-	-
Urogenital congenital anomalies	3,520 (2,053-4,730)	846 (554-1,100)	1,511 (922-2,125)	425 (208-711)	153 (110-207)	138 (106-178)	73 (57-94)	103 (81-131)	87 (70-109)	82 (66-101)	113 (89-143)	161 (124-207)	94 (78-116)	127 (102-150)	170 (135-196)	229 (186-263)	262 (215-297)	-	-	-	-	-	-
Digestive congenital anomalies	11,373 (7,548-15,816)	3,899 (2,964-4,810)	10,629 (7,567-14,615)	2,596 (1,336-5,442)	229 (176-276)	111 (91-131)	53 (45-64)	52 (44-61)	52 (44-63)	32 (28-40)	50 (41-61)	54 (44-66)	32 (26-41)	39 (32-48)	51 (42-62)	52 (43-63)	66 (54-78)	-	-	-	-	-	-
Other congenital birth defects	30,760 (21,408-39,516)	6,641 (5,005-8,677)	20,307 (13,268-27,253)	9,337 (4,976-13,877)	2,006 (1,617-2,538)	1,242 (1,036-1,551)	844 (727-1,038)	854 (747-1,078)	531 (454-657)	421 (362-529)	415 (349-511)	502 (424-609)	190 (161-222)	178 (150-204)	177 (148-211)	166 (140-195)	134 (107-155)	-	-	-	-	-	-
Urinary diseases and male infertility	395 (353-442)	244 (218-279)	2,925 (2,552-3,390)	1,628 (1,341-1,935)	666 (571-774)	557 (499-621)	1,088 (980-1,201)	1,635 (1,507-1,782)	1,481 (1,370-1,613)	2,015 (1,903-2,173)	2,386 (2,246-2,552)	3,371 (3,184-3,619)	4,592 (4,333-4,914)	5,801 (5,538-6,112)	7,268 (6,945-7,645)	10,130 (9,670-10,703)	12,964 (12,375-13,686)	14,789 (14,056-15,625)	15,652 (14,968-16,539)	18,601 (17,736-19,652)	14,835 (14,226-15,551)	8,430 (8,136-8,797)	3,188 (3,088-3,309)
Urinary tract infections	84 (55-137)	96 (75-135)	2,163 (1,753-2,705)	633 (463-955)	405 (336-504)	369 (322-445)	764 (638-927)	1,165 (1,011-1,393)	1,037 (921-1,192)	1,269 (1,139-1,502)	1,468 (1,310-1,732)	2,121 (1,863-2,453)	2,786 (2,480-3,189)	3,595 (3,221-4,116)	4,629 (4,178-5,267)	6,596 (5,973-7,447)	8,419 (7,690-9,502)	9,277 (8,294-10,508)	10,661 (9,803-11,913)	12,357 (11,421-13,742)	10,991 (10,203-11,813)	6,386 (5,980-6,805)	2,551 (2,363-2,693)
Urolithiasis	-	-	-	-	15 (9-22)	14 (9-19)	13 (9-19)	21 (16-28)	39 (31-53)	59 (47-78)	91 (72-125)	160 (124-213)	261 (206-357)	391 (315-516)	443 (359-597)	681 (560-891)	869 (715-1,147)	840 (683-1,134)	956 (784-1,302)	890 (739-1,207)	583 (487-791)	308 (256-421)	79 (66-112)
Other urinary diseases	311 (246-361)	148 (122-180)	762 (447-1,115)	996 (672-1,307)	246 (187-311)	174 (135-212)	311 (214-387)	449 (316-560)	405 (331-499)	687 (535-812)	827 (663-981)	1,090 (845-1,316)	1,545 (1,256-1,834)	1,815 (1,424-2,141)	2,196 (1,738-2,649)	2,853 (2,291-3,467)	3,676 (3,006-4,404)	4,673 (3,862-5,752)	4,035 (3,249-4,869)	5,354 (4,389-6,537)	3,261 (2,721-4,001)	1,737 (1,467-2,081)	557 (457-754)
Gynecological diseases	-	-	-	-	-	-	-	-	-	-	-	-	-	-	-	-	-	-	-	-	-	-	-
Uterine fibroids	-	-	-	-	-	-	-	-	-	-	-	-	-	-	-	-	-	-	-	-	-	-	-
Polycystic ovarian syndrome	-	-	-	-	-	-	-	-	-	-	-	-	-	-	-	-	-	-	-	-	-	-	-

SR Table 6: Global deaths by cause and age group for males (with 95% confidence intervals), 2017

Cause	Early Neonatal	Late Neonatal	Post Neonatal	1 to 4	5 to 9	10 to 14
Endometriosis	-	-	-	-	-	-
Genital prolapse	-	-	-	-	-	-
Other gynecological diseases	-	-	-	-	-	-
Hemoglobinopathies and hemolytic anemias	498 (261-901)	330 (196-530)	3,003 (1,681-5,219)	6,187 (3,453-9,961)	2,576 (1,513-4,026)	2,639 (1,524-4,164)
Thalassemias	129 (92-153)	104 (79-131)	728 (549-985)	981 (726-1,259)	674 (462-964)	449 (319-611)
Sickle cell disorders	350 (149-736)	218 (106-408)	2,197 (995-4,298)	5,003 (2,375-8,564)	1,702 (859-3,058)	1,806 (846-3,187)
G6PD deficiency	10 (4-20)	6 (3-12)	66 (33-130)	188 (102-312)	178 (117-263)	325 (212-465)
Other hemoglobinopathies and hemolytic anemias	10 (7-16)	2 (1-2)	13 (8-20)	15 (11-22)	22 (16-29)	59 (41-82)
Endocrine, metabolic, blood, and immune disorders	616 (446-733)	566 (429-706)	3,836 (2,849-4,916)	2,122 (1,519-2,684)	928 (736-1,137)	843 (671-1,043)
Sudden infant death syndrome	-	2,102 (918-3,527)	21,238 (8,459-41,220)	-	-	-
Injuries	5,925 (5,150-6,516)	3,767 (3,371-4,081)	41,138 (37,596-44,663)	99,447 (90,779-108,149)	63,528 (58,214-68,326)	67,496 (63,372-71,329)
Transport injuries	558 (502-624)	415 (373-467)	5,813 (5,227-6,506)	22,869 (19,972-26,285)	21,541 (19,428-23,828)	21,268 (19,685-22,930)
Road injuries	487 (436-549)	363 (324-413)	5,354 (4,803-6,025)	21,377 (18,674-24,525)	20,227 (18,302-22,495)	19,396 (17,949-20,975)
Pedestrian road injuries	194 (163-251)	153 (128-193)	2,026 (1,718-2,582)	11,591 (9,820-14,358)	10,535 (9,354-12,147)	8,962 (7,858-10,569)
Cyclist road injuries	-	-	-	655 (511-861)	1,256 (968-1,704)	1,725 (1,397-2,222)
Motorcyclist road injuries	39 (29-46)	33 (26-40)	544 (421-655)	1,333 (1,014-1,687)	1,356 (1,083-1,670)	2,330 (1,746-2,709)
Motor vehicle road injuries	249 (193-294)	171 (138-204)	2,696 (2,263-3,143)	7,602 (5,914-9,437)	6,873 (5,896-8,033)	6,140 (5,379-6,817)
Other road injuries	5 (4-7)	5 (4-8)	88 (69-123)	197 (144-280)	207 (159-291)	239 (192-310)
Other transport injuries	71 (53-91)	52 (40-71)	459 (359-567)	1,491 (885-1,982)	1,313 (1,054-1,671)	1,872 (1,501-2,403)
Unintentional injuries	4,462 (3,810-4,966)	3,023 (2,672-3,282)	32,900 (29,707-35,835)	68,019 (61,688-74,521)	35,652 (32,089-38,805)	32,869 (30,356-35,335)
Falls	812 (553-983)	287 (196-339)	3,808 (2,864-4,732)	6,498 (5,262-7,572)	4,370 (3,513-5,215)	4,630 (3,705-5,458)
Drowning	238 (216-265)	113 (103-125)	3,249 (2,911-3,682)	32,059 (28,892-35,466)	18,265 (16,483-20,110)	15,994 (14,936-17,094)
Fire, heat, and hot substances	227 (146-276)	172 (115-212)	2,359 (1,622-2,893)	5,582 (3,899-6,729)	1,731 (1,277-2,143)	1,067 (800-1,300)
Poisonings	168 (101-214)	139 (96-182)	1,637 (1,107-2,060)	3,016 (2,256-3,674)	1,146 (765-1,401)	1,167 (767-1,408)
Poisoning by carbon monoxide	38 (23-50)	34 (21-46)	303 (189-418)	632 (449-786)	494 (306-621)	457 (291-548)
Poisoning by other means	130 (76-170)	105 (74-138)	1,334 (922-1,689)	2,384 (1,786-2,968)	652 (457-801)	710 (476-880)
Exposure to mechanical forces	587 (428-665)	499 (397-577)	2,606 (1,976-2,968)	4,780 (3,790-5,686)	2,469 (2,165-2,820)	2,191 (1,971-2,442)
Unintentional firearm injuries	66 (49-99)	51 (37-80)	517 (380-755)	793 (489-1,460)	348 (284-485)	420 (366-540)
Other exposure to mechanical forces	521 (365-597)	448 (349-517)	2,090 (1,522-2,398)	3,987 (3,140-4,817)	2,121 (1,819-2,415)	1,771 (1,554-1,990)
Adverse effects of medical treatment	504 (376-672)	292 (241-404)	3,505 (2,630-5,193)	3,051 (1,883-5,639)	1,041 (847-1,383)	888 (729-1,117)
Animal contact	164 (62-214)	147 (58-191)	1,053 (491-1,400)	2,874 (1,553-3,707)	2,414 (1,365-3,267)	2,530 (1,335-3,348)
Venomous animal contact	142 (48-188)	124 (45-165)	864 (362-1,200)	2,185 (1,062-3,019)	2,039 (1,104-2,812)	2,287 (1,162-3,042)
Non-venomous animal contact	22 (13-29)	23 (13-30)	189 (102-250)	689 (300-1,073)	375 (205-509)	244 (134-317)
Foreign body	1,446 (1,237-1,664)	1,223 (1,082-1,403)	12,997 (11,563-14,758)	6,297 (5,158-7,504)	1,749 (1,542-2,005)	1,450 (1,319-1,610)
Pulmonary aspiration and foreign body in airway	1,407 (1,206-1,622)	1,205 (1,064-1,384)	12,670 (11,301-14,355)	5,718 (4,653-7,028)	1,593 (1,411-1,834)	1,345 (1,227-1,504)
Foreign body in other body part	40 (18-59)	19 (12-28)	327 (191-520)	579 (242-1,187)	156 (107-211)	105 (77-146)
Environmental heat and cold exposure	129 (41-180)	81 (28-115)	879 (297-1,286)	799 (315-1,261)	322 (160-434)	281 (138-374)
Exposure to forces of nature	3 (3-4)	7 (6-9)	55 (48-73)	266 (225-375)	299 (248-468)	410 (338-622)
Other unintentional injuries	183 (160-214)	62 (54-72)	752 (671-847)	2,797 (2,409-3,224)	1,846 (1,645-2,059)	2,261 (2,057-2,491)
Self-harm and interpersonal violence	904 (736-1,011)	329 (289-361)	2,425 (2,011-2,775)	8,559 (7,636-9,198)	6,335 (5,871-6,771)	13,358 (12,330-14,173)
Self-harm	-	-	-	-	-	4,562 (3,926-5,187)
Self-harm by firearm	-	-	-	-	-	239 (188-364)
Self-harm by other specified means	-	-	-	-	-	4,323 (3,687-4,881)
Interpersonal violence	600 (431-706)	164 (124-196)	1,860 (1,456-2,200)	3,496 (2,573-4,134)	2,289 (1,822-2,723)	4,303 (3,490-4,934)
Physical violence by firearm	104 (53-140)	31 (17-42)	355 (215-455)	742 (395-1,060)	505 (343-623)	1,500 (1,153-1,715)
Physical violence by sharp object	60 (30-83)	18 (10-26)	163 (105-240)	529 (261-894)	299 (191-397)	633 (409-834)
Physical violence by other means	435 (329-534)	115 (90-147)	1,342 (1,087-1,691)	2,225 (1,707-2,804)	1,485 (1,200-1,837)	2,170 (1,725-2,762)
Conflict and terrorism	303 (221-423)	164 (120-224)	511 (376-721)	4,868 (3,538-6,995)	3,907 (2,765-5,617)	4,288 (2,965-6,593)
Executions and police conflict	-	-	54 (16-131)	195 (191-203)	139 (135-144)	206 (201-212)

Cause	15 to 19	20 to 24	25 to 29	30 to 34	35 to 39	40 to 44
Endometriosis	-	-	-	-	-	-
Genital prolapse	-	-	-	-	-	-
Other gynecological diseases	-	-	-	-	-	-
Hemoglobinopathies and hemolytic anemias	3,066 (1,651-4,944)	2,759 (1,531-4,460)	2,083 (1,191-3,294)	1,680 (1,008-2,690)	1,583 (893-2,561)	1,679 (969-2,733)
Thalassemias	268 (193-340)	172 (110-225)	96 (62-127)	47 (33-62)	24 (18-31)	21 (16-27)
Sickle cell disorders	2,193 (950-3,961)	1,862 (807-3,450)	1,262 (511-2,382)	849 (316-1,739)	823 (277-1,675)	725 (229-1,566)
G6PD deficiency	497 (323-719)	551 (360-759)	575 (405-716)	623 (444-776)	567 (400-744)	678 (473-971)
Other hemoglobinopathies and hemolytic anemias	107 (69-162)	175 (115-250)	151 (106-201)	161 (112-215)	170 (121-218)	255 (185-333)
Endocrine, metabolic, blood, and immune disorders	771 (628-926)	934 (776-1,118)	1,253 (1,044-1,505)	1,744 (1,421-2,054)	1,955 (1,502-2,310)	2,308 (1,704-2,614)
Sudden infant death syndrome	-	-	-	-	-	-
Injuries	165,341 (158,890-172,543)	260,200 (252,405-268,109)	261,834 (254,276-268,899)	250,690 (242,434-256,604)	228,875 (220,431-235,045)	211,776 (202,779-217,300)
Transport injuries	63,563 (60,167-68,203)	96,506 (92,430-101,315)	97,868 (94,494-101,944)	89,592 (86,322-92,990)	81,157 (76,576-84,789)	75,620 (71,848-78,411)
Road injuries	58,554 (55,509-63,045)	89,743 (85,726-94,233)	90,470 (87,321-94,366)	83,313 (80,016-86,512)	74,693 (70,022-78,320)	69,693 (65,650-72,490)
Pedestrian road injuries	14,700 (12,788-17,984)	20,686 (18,317-24,719)	24,102 (22,258-27,534)	24,521 (22,786-27,718)	22,725 (20,779-26,149)	22,933 (21,094-25,680)
Cyclist road injuries	2,865 (2,214-3,833)	2,841 (2,223-3,543)	3,204 (2,465-3,859)	3,440 (2,731-4,055)	3,197 (2,515-3,789)	3,941 (3,072-4,670)
Motorcyclist road injuries	17,440 (15,006-20,117)	27,734 (24,500-30,393)	25,628 (22,351-28,231)	21,690 (18,471-23,389)	18,493 (15,511-20,225)	16,326 (13,655-17,827)
Motor vehicle road injuries	23,109 (21,289-25,516)	37,802 (35,207-41,150)	36,876 (34,605-40,567)	33,149 (30,866-36,485)	29,780 (27,250-32,664)	25,975 (23,897-28,508)
Other road injuries	441 (360-570)	680 (542-855)	661 (538-851)	512 (411-626)	499 (392-632)	517 (406-637)
Other transport injuries	5,009 (4,145-6,713)	6,763 (5,890-8,440)	7,397 (6,458-8,850)	6,280 (5,743-7,424)	6,464 (5,751-7,760)	5,927 (5,368-7,090)
Unintentional injuries	35,393 (33,196-37,511)	46,256 (43,230-48,557)	50,521 (46,875-52,997)	53,825 (50,116-56,327)	54,453 (50,648-57,148)	56,374 (51,090-59,079)
Falls	4,773 (4,002-5,528)	7,630 (6,357-8,542)	9,726 (7,743-10,701)	11,700 (9,525-12,996)	12,844 (10,604-14,090)	15,917 (12,916-17,379)
Drowning	13,514 (12,717-14,385)	13,041 (12,380-13,776)	11,477 (11,010-12,031)	11,088 (10,651-11,635)	10,207 (9,789-10,676)	9,886 (9,515-10,314)
Fire, heat, and hot substances	1,499 (1,070-1,857)	2,485 (1,881-2,987)	2,870 (2,231-3,442)	3,129 (2,552-3,685)	3,317 (2,711-3,833)	3,152 (2,640-3,587)
Poisonings	1,464 (991-1,716)	2,288 (1,645-2,652)	2,954 (2,107-3,337)	2,938 (2,065-3,300)	2,806 (2,092-3,104)	3,097 (2,236-3,455)
Poisoning by carbon monoxide	743 (501-874)	1,198 (866-1,362)	1,591 (1,133-1,779)	1,741 (1,276-1,926)	1,679 (1,331-1,815)	1,779 (1,365-1,951)
Poisoning by other means	721 (480-878)	1,090 (756-1,314)	1,363 (937-1,588)	1,197 (771-1,394)	1,128 (755-1,306)	1,317 (850-1,536)
Exposure to mechanical forces	4,619 (4,181-5,029)	7,080 (6,377-7,563)	8,173 (6,991-8,621)	8,063 (6,817-8,494)	8,055 (7,150-8,490)	7,290 (6,076-7,660)
Unintentional firearm injuries	1,341 (1,182-1,548)	1,916 (1,721-2,194)	2,067 (1,808-2,379)	1,505 (1,322-1,775)	1,318 (1,172-1,544)	1,030 (912-1,211)
Other exposure to mechanical forces	3,278 (2,839-3,631)	5,164 (4,511-5,616)	6,106 (5,096-6,502)	6,558 (5,450-6,984)	6,737 (5,821-7,144)	6,260 (5,062-6,608)
Adverse effects of medical treatment	1,218 (959-1,449)	1,970 (1,522-2,364)	1,879 (1,473-2,257)	2,173 (1,690-2,587)	2,459 (1,936-2,877)	2,729 (2,141-3,192)
Animal contact	1,890 (980-2,517)	2,030 (1,073-2,554)	2,162 (1,132-2,714)	2,381 (1,240-2,912)	2,338 (1,178-2,840)	2,561 (1,250-3,063)
Venomous animal contact	1,614 (783-2,201)	1,747 (873-2,240)	1,820 (885-2,327)	2,162 (1,074-2,670)	2,100 (1,015-2,562)	2,308 (1,079-2,808)
Non-venomous animal contact	276 (174-364)	283 (187-366)	342 (238-427)	219 (149-276)	238 (160-302)	253 (162-326)
Foreign body	1,109 (1,025-1,200)	2,038 (1,890-2,246)	2,149 (2,010-2,310)	2,368 (2,240-2,509)	2,507 (2,349-2,629)	2,700 (2,561-2,834)
Pulmonary aspiration and foreign body in airway	1,014 (934-1,111)	1,878 (1,741-2,088)	1,962 (1,847-2,144)	2,076 (1,964-2,221)	2,202 (2,052-2,315)	2,385 (2,232-2,489)
Foreign body in other body part	95 (62-117)	160 (106-193)	187 (130-237)	292 (227-361)	305 (246-391)	315 (252-412)
Environmental heat and cold exposure	550 (311-707)	868 (509-1,076)	1,175 (764-1,397)	1,820 (1,277-2,083)	2,214 (1,603-2,495)	2,573 (1,897-2,915)
Exposure to forces of nature	487 (388-808)	495 (390-850)	487 (383-869)	432 (341-705)	394 (305-722)	362 (274-665)
Other unintentional injuries	4,270 (3,924-4,650)	6,330 (5,902-6,908)	7,469 (7,111-7,944)	7,732 (7,314-8,206)	7,312 (6,890-7,816)	6,108 (5,817-6,463)
Self-harm and interpersonal violence	66,385 (62,298-70,107)	117,439 (110,529-122,085)	113,446 (106,831-118,307)	107,273 (99,892-111,080)	93,265 (86,771-96,763)	79,782 (73,762-83,002)
Self-harm	22,149 (19,960-24,796)	44,213 (40,011-47,536)	50,504 (45,717-54,217)	52,685 (46,809-55,573)	48,953 (43,423-51,329)	45,688 (40,382-47,989)
Self-harm by firearm	2,650 (2,069-3,600)	5,266 (4,077-7,117)	5,102 (3,831-6,723)	4,633 (3,490-6,131)	4,099 (3,212-5,326)	3,815 (3,054-5,021)
Self-harm by other specified means	19,500 (17,317-21,526)	38,947 (34,272-41,737)	45,402 (40,668-48,464)	48,052 (42,761-50,976)	44,853 (39,005-47,199)	41,873 (36,493-44,397)
Interpersonal violence	30,957 (27,026-33,255)	51,981 (45,643-55,480)	48,448 (43,224-51,836)	43,284 (38,710-46,668)	35,569 (31,575-38,841)	28,104 (24,891-30,588)
Physical violence by firearm	17,964 (15,256-19,382)	29,251 (24,840-31,789)	26,006 (22,119-28,292)	21,161 (17,727-23,279)	17,100 (14,320-18,865)	12,762 (10,470-14,437)
Physical violence by sharp object	5,495 (3,979-6,921)	10,020 (7,308-12,672)	10,598 (8,032-13,393)	10,060 (7,773-12,593)	8,306 (6,526-10,569)	6,811 (5,341-8,376)
Physical violence by other means	7,498 (6,283-9,352)	12,710 (10,722-15,843)	11,844 (9,967-14,642)	12,063 (10,153-14,939)	10,164 (8,631-12,834)	8,531 (7,339-10,501)
Conflict and terrorism	11,786 (8,123-17,115)	18,912 (13,047-27,400)	12,415 (8,841-18,001)	9,611 (6,887-13,626)	7,395 (5,076-11,197)	4,962 (3,410-7,341)
Executions and police conflict	1,493 (1,425-1,588)	2,333 (2,229-2,475)	2,078 (1,982-2,201)	1,694 (1,614-1,780)	1,348 (1,292-1,418)	1,028 (980-1,086)

Cause	45 to 49	50 to 54	54 to 59	60 to 64	65 to 69	70 to 74
Endometriosis	-	-	-	-	-	-
Genital prolapse	-	-	-	-	-	-
Other gynecological diseases	-	-	-	-	-	-
Hemoglobinopathies and hemolytic anemias	1,758 (1,054-2,704)	2,235 (1,346-3,396)	2,417 (1,507-3,640)	2,854 (1,865-4,098)	3,083 (2,102-4,247)	3,117 (2,086-4,265)
Thalassemias	20 (16-27)	16 (12-21)	7 (6-9)	4 (3-5)	2 (1-2)	1 (1-1)
Sickle cell disorders	476 (140-1,089)	330 (111-774)	141 (63-299)	68 (43-111)	33 (23-46)	14 (10-20)
G6PD deficiency	936 (599-1,388)	1,296 (757-2,058)	1,306 (703-2,197)	1,278 (681-2,163)	1,029 (546-1,720)	742 (373-1,291)
Other hemoglobinopathies and hemolytic anemias	326 (240-428)	594 (418-807)	963 (690-1,319)	1,505 (1,071-2,041)	2,020 (1,469-2,641)	2,360 (1,680-3,130)
Endocrine, metabolic, blood, and immune disorders	3,183 (2,381-3,514)	4,283 (3,052-4,710)	5,010 (3,433-5,473)	5,874 (4,171-6,360)	6,452 (4,494-6,886)	6,328 (4,592-6,853)
Sudden infant death syndrome	-	-	-	-	-	-
Injuries	220,204 (210,513-226,834)	206,803 (197,265-212,827)	180,653 (172,492-186,880)	171,400 (160,910-176,962)	155,626 (144,280-161,348)	126,957 (116,968-132,284)
Transport injuries	80,279 (76,064-83,590)	75,656 (71,562-78,701)	61,379 (56,619-64,078)	57,482 (54,150-59,822)	49,337 (46,325-51,363)	35,829 (33,926-37,315)
Road injuries	74,144 (69,490-77,530)	69,616 (65,122-72,564)	55,981 (50,814-58,549)	53,496 (49,870-55,795)	45,947 (42,860-47,896)	33,415 (31,442-34,865)
Pedestrian road injuries	28,173 (25,980-31,624)	28,302 (25,802-31,642)	22,274 (20,092-25,170)	25,248 (23,041-28,233)	22,751 (20,847-25,365)	16,921 (15,534-18,721)
Cyclist road injuries	4,902 (3,822-5,660)	5,242 (4,166-6,074)	3,964 (3,233-4,528)	4,979 (3,967-5,661)	4,525 (3,457-5,104)	3,296 (2,525-3,751)
Motorcyclist road injuries	15,916 (13,270-17,329)	13,510 (11,070-14,736)	10,379 (8,220-11,440)	7,356 (5,406-8,095)	5,674 (4,023-6,311)	3,369 (2,450-3,731)
Motor vehicle road injuries	24,489 (21,996-27,070)	21,878 (19,730-23,644)	18,787 (16,650-20,344)	15,465 (13,693-16,882)	12,512 (11,027-13,611)	9,422 (8,411-10,307)
Other road injuries	664 (525-805)	685 (543-851)	577 (456-697)	448 (355-524)	486 (383-596)	407 (332-501)
Other transport injuries	6,135 (5,540-7,229)	6,040 (5,453-7,147)	5,398 (4,854-6,338)	3,986 (3,608-4,600)	3,390 (3,048-3,921)	2,415 (2,188-2,813)
Unintentional injuries	67,319 (60,375-70,645)	66,608 (59,698-70,148)	63,161 (57,739-66,653)	67,260 (59,718-70,700)	67,184 (58,504-71,223)	61,868 (53,133-66,056)
Falls	20,426 (15,913-22,374)	22,375 (17,262-24,497)	22,558 (18,652-25,024)	27,173 (21,469-29,692)	30,440 (23,946-33,524)	32,042 (25,159-35,551)
Drowning	10,265 (9,867-10,707)	9,857 (9,486-10,267)	8,206 (7,916-8,509)	8,387 (8,094-8,672)	7,552 (7,277-7,842)	6,247 (5,996-6,487)
Fire, heat, and hot substances	3,572 (2,934-3,993)	3,707 (3,095-4,101)	3,712 (3,187-4,138)	3,881 (3,267-4,201)	3,591 (2,990-4,017)	3,225 (2,660-3,658)
Poisonings	3,522 (2,367-3,967)	3,591 (2,443-4,021)	3,079 (2,246-3,375)	3,431 (2,315-3,837)	2,804 (1,822-3,186)	2,106 (1,330-2,424)
Poisoning by carbon monoxide	2,005 (1,459-2,234)	2,015 (1,475-2,229)	1,864 (1,473-2,029)	1,976 (1,419-2,198)	1,546 (1,036-1,748)	1,121 (702-1,304)
Poisoning by other means	1,517 (888-1,773)	1,576 (961-1,820)	1,215 (755-1,382)	1,455 (882-1,673)	1,259 (774-1,467)	984 (611-1,146)
Exposure to mechanical forces	9,337 (7,744-9,875)	8,965 (7,204-9,480)	6,649 (5,759-6,950)	5,594 (4,655-5,874)	4,533 (3,812-4,765)	3,334 (2,863-3,484)
Unintentional firearm injuries	1,133 (1,006-1,366)	931 (832-1,128)	766 (695-928)	673 (607-787)	573 (519-685)	470 (422-556)
Other exposure to mechanical forces	8,205 (6,534-8,699)	8,034 (6,251-8,533)	5,883 (4,847-6,182)	4,921 (3,960-5,193)	3,960 (3,218-4,184)	2,863 (2,362-3,013)
Adverse effects of medical treatment	3,052 (2,369-3,494)	2,823 (2,301-3,404)	3,588 (2,925-4,268)	4,738 (3,837-5,623)	5,724 (4,561-6,820)	5,106 (4,193-6,226)
Animal contact	3,497 (1,776-4,179)	2,543 (1,401-2,982)	3,145 (1,634-3,828)	2,823 (1,443-3,336)	2,932 (1,379-3,480)	2,415 (1,164-2,869)
Venomous animal contact	3,100 (1,474-3,796)	2,107 (1,076-2,509)	2,702 (1,353-3,405)	2,387 (1,143-2,891)	2,521 (1,138-3,016)	2,076 (931-2,506)
Non-venomous animal contact	396 (269-498)	436 (293-549)	443 (287-561)	435 (282-561)	411 (256-522)	339 (214-434)
Foreign body	2,957 (2,741-3,113)	3,284 (3,109-3,409)	3,570 (3,429-3,720)	3,692 (3,503-3,851)	3,742 (3,495-3,908)	3,314 (3,040-3,473)
Pulmonary aspiration and foreign body in airway	2,736 (2,554-2,874)	3,055 (2,908-3,183)	3,344 (3,225-3,510)	3,457 (3,274-3,580)	3,560 (3,340-3,691)	3,133 (2,853-3,269)
Foreign body in other body part	222 (169-298)	229 (173-308)	226 (174-279)	236 (193-339)	182 (150-276)	181 (146-262)
Environmental heat and cold exposure	3,017 (2,277-3,369)	3,477 (2,615-3,855)	3,910 (2,932-4,415)	3,426 (2,548-3,847)	2,996 (1,900-3,544)	2,279 (1,275-2,808)
Exposure to forces of nature	350 (262-636)	337 (242-657)	285 (197-582)	282 (189-618)	237 (149-592)	211 (125-553)
Other unintentional injuries	7,324 (6,907-7,777)	5,649 (5,354-5,993)	4,460 (4,233-4,716)	3,834 (3,640-4,048)	2,633 (2,499-2,789)	1,591 (1,506-1,679)
Self-harm and interpersonal violence	72,606 (67,706-75,992)	64,539 (60,788-67,489)	56,113 (52,277-58,464)	46,657 (42,651-48,581)	39,104 (35,499-40,911)	29,260 (26,511-30,712)
Self-harm	46,403 (41,793-48,872)	43,835 (39,984-46,009)	40,047 (36,354-42,049)	34,964 (30,921-36,633)	30,675 (27,289-32,265)	23,703 (20,929-25,025)
Self-harm by firearm	4,116 (3,342-5,288)	4,333 (3,572-5,577)	4,423 (3,728-5,607)	3,732 (3,120-4,676)	3,216 (2,610-3,944)	2,729 (2,244-3,335)
Self-harm by other specified means	42,287 (37,346-44,790)	39,502 (35,498-41,646)	35,623 (31,631-37,744)	31,232 (27,232-33,075)	27,459 (23,884-29,199)	20,973 (17,984-22,369)
Interpersonal violence	22,115 (19,666-24,680)	17,854 (15,797-20,111)	13,805 (12,153-15,368)	10,029 (8,772-11,030)	7,238 (6,103-7,994)	4,718 (3,938-5,346)
Physical violence by firearm	8,859 (7,244-10,035)	6,966 (5,617-7,794)	5,017 (4,016-5,801)	3,171 (2,569-3,701)	2,088 (1,615-2,448)	1,392 (1,051-1,677)
Physical violence by sharp object	5,717 (4,547-7,166)	4,591 (3,532-5,904)	3,325 (2,681-4,124)	2,693 (2,022-3,318)	1,930 (1,390-2,282)	1,177 (842-1,386)
Physical violence by other means	7,539 (6,516-9,215)	6,297 (5,412-7,679)	5,463 (4,711-6,647)	4,166 (3,645-5,130)	3,220 (2,764-4,022)	2,149 (1,835-2,764)
Conflict and terrorism	3,318 (2,211-4,917)	2,298 (1,524-3,482)	1,823 (1,219-2,845)	1,383 (933-2,096)	1,029 (686-1,574)	765 (519-1,169)
Executions and police conflict	770 (735-815)	551 (528-581)	439 (424-458)	281 (272-293)	162 (156-170)	74 (71-79)

Cause	75 to 79	80 to 84	85 to 89	90 to 94	95 plus
Endometriosis	-	-	-	-	-
Genital prolapse	-	-	-	-	-
Other gynecological diseases	-	-	-	-	-
Hemoglobinopathies and hemolytic anemias	3,260 (2,281-4,296)	3,169 (2,267-3,991)	2,527 (1,849-3,052)	1,332 (974-1,522)	443 (293-498)
Thalassemias	0 (0-0)	0 (0-0)	0 (0-0)	0 (0-0)	0 (0-0)
Sickle cell disorders	4 (2-6)	1 (1-2)	0 (0-1)	0 (0-0)	0 (0-0)
G6PD deficiency	429 (213-750)	202 (102-345)	81 (44-133)	19 (11-29)	3 (2-5)
Other hemoglobinopathies and hemolytic anemias	2,827 (2,051-3,659)	2,966 (2,142-3,688)	2,446 (1,793-2,925)	1,313 (959-1,496)	440 (291-494)
Endocrine, metabolic, blood, and immune disorders	6,297 (4,298-6,815)	6,020 (4,045-6,480)	5,106 (3,418-5,449)	2,745 (1,708-2,953)	1,032 (637-1,123)
Sudden infant death syndrome	-	-	-	-	-
Injuries	119,407 (109,082-124,685)	104,814 (94,999-110,533)	75,187 (68,578-79,652)	35,645 (32,689-37,624)	10,815 (10,197-11,258)
Transport injuries	28,569 (27,210-29,588)	20,781 (19,649-21,604)	11,301 (10,508-11,809)	3,952 (3,636-4,121)	945 (848-991)
Road injuries	26,552 (25,136-27,515)	19,400 (18,227-20,213)	10,577 (9,777-11,058)	3,693 (3,374-3,850)	869 (774-911)
Pedestrian road injuries	15,117 (13,987-16,382)	11,165 (10,282-12,110)	6,185 (5,593-6,686)	2,077 (1,883-2,231)	473 (427-510)
Cyclist road injuries	2,205 (1,734-2,496)	1,368 (1,128-1,524)	660 (548-742)	198 (166-222)	2 (1-2)
Motorcyclist road injuries	1,797 (1,351-1,972)	1,491 (1,142-1,665)	644 (481-728)	172 (131-194)	44 (34-50)
Motor vehicle road injuries	7,106 (6,297-7,643)	5,101 (4,553-5,472)	2,939 (2,633-3,145)	1,208 (1,084-1,294)	334 (289-362)
Other road injuries	327 (262-404)	275 (223-349)	148 (124-196)	38 (32-49)	16 (14-20)
Other transport injuries	2,017 (1,820-2,359)	1,380 (1,231-1,594)	724 (635-830)	259 (229-289)	77 (68-88)
Unintentional injuries	65,866 (56,637-70,299)	65,879 (56,871-70,698)	53,332 (46,633-57,326)	27,834 (24,851-29,692)	8,908 (8,287-9,320)
Falls	38,282 (30,489-41,872)	41,243 (33,139-45,496)	36,075 (29,485-39,767)	19,674 (16,651-21,455)	6,219 (5,573-6,613)
Drowning	5,031 (4,839-5,214)	4,559 (4,356-4,746)	2,686 (2,574-2,789)	899 (866-930)	200 (193-206)
Fire, heat, and hot substances	3,165 (2,688-3,504)	3,317 (2,775-3,686)	2,517 (2,192-2,770)	1,292 (1,167-1,386)	430 (400-451)
Poisonings	1,804 (1,154-2,081)	1,469 (918-1,678)	797 (550-897)	248 (183-272)	53 (42-57)
Poisoning by carbon monoxide	1,042 (671-1,201)	842 (518-972)	462 (307-526)	144 (102-159)	26 (20-28)
Poisoning by other means	763 (470-888)	627 (401-718)	335 (238-379)	104 (79-116)	27 (22-29)
Exposure to mechanical forces	2,826 (2,534-2,960)	2,044 (1,828-2,131)	1,402 (1,322-1,453)	699 (651-723)	236 (229-243)
Unintentional firearm injuries	444 (397-542)	357 (321-445)	229 (207-297)	116 (105-147)	33 (30-42)
Other exposure to mechanical forces	2,382 (2,031-2,507)	1,686 (1,431-1,767)	1,173 (1,049-1,225)	582 (528-607)	203 (191-209)
Adverse effects of medical treatment	5,115 (4,254-6,320)	4,567 (3,838-5,646)	3,269 (2,767-4,129)	1,526 (1,347-2,060)	457 (410-629)
Animal contact	2,111 (1,054-2,527)	1,452 (723-1,707)	734 (383-869)	229 (129-269)	59 (39-68)
Venomous animal contact	1,772 (835-2,126)	1,224 (555-1,479)	604 (286-741)	179 (93-217)	42 (24-51)
Non-venomous animal contact	339 (217-444)	228 (147-290)	129 (90-158)	50 (35-60)	17 (13-20)
Foreign body	3,786 (3,549-3,956)	4,315 (4,028-4,501)	3,936 (3,737-4,105)	2,400 (2,315-2,506)	987 (955-1,028)
Pulmonary aspiration and foreign body in airway	3,550 (3,312-3,692)	4,089 (3,799-4,263)	3,772 (3,580-3,929)	2,319 (2,235-2,412)	959 (929-997)
Foreign body in other body part	235 (197-356)	226 (187-337)	164 (139-257)	81 (70-120)	28 (24-42)
Environmental heat and cold exposure	2,442 (1,378-2,966)	1,771 (1,043-2,138)	1,246 (813-1,472)	578 (418-668)	167 (136-185)
Exposure to forces of nature	172 (99-429)	138 (76-360)	74 (38-223)	28 (12-87)	12 (4-46)
Other unintentional injuries	1,133 (1,076-1,191)	1,004 (935-1,082)	596 (548-648)	261 (240-284)	89 (82-96)
Self-harm and interpersonal violence	24,972 (22,661-26,157)	18,154 (16,277-19,108)	10,555 (9,921-11,196)	3,859 (3,651-4,060)	961 (920-1,019)
Self-harm	21,068 (18,774-22,212)	16,690 (14,798-17,631)	9,772 (9,125-10,369)	3,556 (3,350-3,758)	760 (719-815)
Self-harm by firearm	2,272 (1,876-2,817)	1,888 (1,570-2,368)	1,299 (1,061-1,695)	538 (442-725)	130 (107-176)
Self-harm by other specified means	18,796 (16,505-19,949)	14,802 (12,723-15,833)	8,473 (7,742-9,052)	3,018 (2,759-3,203)	630 (585-676)
Interpersonal violence	3,277 (2,834-3,708)	977 (853-1,134)	494 (447-584)	171 (156-194)	120 (109-129)
Physical violence by firearm	896 (659-1,078)	234 (174-283)	115 (87-140)	46 (37-54)	34 (26-39)
Physical violence by sharp object	796 (607-979)	224 (186-261)	111 (93-131)	41 (34-47)	29 (24-33)
Physical violence by other means	1,585 (1,372-1,947)	518 (449-657)	267 (237-338)	83 (75-101)	57 (53-68)
Conflict and terrorism	583 (379-895)	457 (300-695)	273 (173-433)	126 (82-195)	75 (45-123)
Executions and police conflict	43 (41-46)	30 (29-32)	16 (15-18)	7 (6-8)	6 (5-7)

SR Table 7: Global deaths by cause and age group for both sexes combined (with 95% confidence intervals), 1990

Cause	Early Neonatal	Late Neonatal	Post Neonatal	1 to 4	5 to 9	10 to 14
All causes	3,037,044 (2,966,409-3,104,943)	1,263,098 (1,220,453-1,308,864)	3,854,632 (3,771,297-3,942,560)	3,615,809 (3,519,278-3,715,515)	853,941 (844,956-862,845)	466,727 (462,844-470,900)
Communicable, maternal, neonatal, and nutritional diseases	2,783,954 (2,694,383-2,857,464)	1,115,904 (1,065,548-1,165,296)	3,197,902 (3,092,999-3,309,909)	2,840,909 (2,711,057-3,005,868)	475,533 (442,408-516,429)	202,900 (185,966-226,463)
HIV/AIDS and sexually transmitted infections	-	23,359 (8,579-43,653)	75,146 (51,851-107,045)	31,800 (27,955-35,912)	3,399 (2,286-4,915)	616 (516-788)
HIV/AIDS	-	-	38,975 (35,106-43,043)	28,475 (25,681-31,748)	1,950 (1,384-2,488)	483 (392-655)
HIV/AIDS - Drug-susceptible Tuberculosis	-	-	15,473 (12,562-17,802)	11,569 (9,641-13,228)	691 (453-914)	109 (66-162)
HIV/AIDS - Multidrug-resistant Tuberculosis without extensive drug resistance	-	-	95 (52-171)	83 (47-141)	4 (2-7)	1 (1-2)
HIV/AIDS - Extensively drug-resistant Tuberculosis	-	-	0 (0-0)	0 (0-0)	0 (0-0)	0 (0-0)
HIV/AIDS resulting in other diseases	-	-	23,407 (20,400-27,407)	16,823 (14,836-19,567)	1,256 (913-1,712)	373 (306-520)
Sexually transmitted infections excluding HIV	66,278 (25,348-123,702)	23,359 (8,579-43,653)	36,171 (13,786-68,965)	3,325 (1,203-6,400)	1,448 (514-2,783)	133 (99-167)
Syphilis	66,278 (25,348-123,702)	23,359 (8,579-43,653)	36,171 (13,786-68,965)	3,325 (1,203-6,400)	1,448 (514-2,783)	78 (57-103)
Chlamydial infection	-	-	-	-	-	13 (7-17)
Gonococcal infection	-	-	-	-	-	26 (17-33)
Other sexually transmitted infections	-	-	-	-	-	16 (11-20)
Respiratory infections and tuberculosis	-	296,595 (251,769-331,543)	1,241,837 (1,138,596-1,326,024)	733,013 (618,231-829,999)	88,806 (73,583-101,534)	38,838 (33,624-43,013)
Tuberculosis	-	-	84,227 (76,497-91,140)	88,181 (72,835-101,820)	21,345 (17,649-25,024)	15,214 (12,950-17,379)
Drug-susceptible tuberculosis	-	-	83,147 (75,336-90,125)	87,487 (72,344-101,186)	21,147 (17,456-24,859)	15,060 (12,802-17,239)
Multidrug-resistant tuberculosis without extensive drug resistance	-	-	1,080 (499-2,167)	694 (379-1,254)	198 (95-385)	153 (75-295)
Extensively drug-resistant tuberculosis	-	-	0 (0-0)	0 (0-0)	0 (0-0)	0 (0-0)
Lower respiratory infections	253,461 (227,675-282,478)	296,060 (251,225-330,989)	1,149,686 (1,054,703-1,228,976)	638,331 (537,460-725,303)	66,314 (54,075-75,684)	23,094 (19,388-25,574)
Upper respiratory infections	229 (127-293)	443 (248-568)	7,373 (3,626-10,462)	6,008 (2,094-11,348)	877 (409-1,231)	333 (181-437)
Otitis media	107 (16-489)	93 (49-187)	551 (345-885)	493 (183-1,017)	270 (151-465)	197 (126-328)
Enteric infections	44,705 (37,901-55,247)	101,229 (84,888-120,790)	833,465 (726,264-941,556)	749,733 (605,143-885,902)	143,539 (99,370-192,402)	67,677 (47,717-90,911)
Diarrheal diseases	44,705 (37,901-55,247)	100,848 (84,595-120,369)	825,129 (717,749-931,743)	691,270 (549,176-825,630)	62,310 (33,186-95,541)	22,896 (13,052-35,929)
Typhoid and paratyphoid	-	0 (0-0)	555 (364-913)	42,090 (19,045-79,313)	76,162 (42,871-124,745)	42,527 (24,719-67,299)
Typhoid fever	-	-	507 (331-830)	37,391 (17,039-71,555)	67,016 (37,485-110,043)	37,340 (21,232-59,531)
Paratyphoid fever	-	-	47 (25-85)	4,699 (1,732-10,071)	9,146 (3,856-17,875)	5,187 (2,259-10,373)
Invasive Non-typhoidal Salmonella (iNTS)	-	381 (209-642)	5,380 (2,976-9,258)	14,968 (7,491-28,267)	4,869 (2,363-8,887)	2,167 (962-4,144)
Other intestinal infectious diseases	-	-	2,401 (1,400-4,472)	1,404 (708-3,001)	198 (83-443)	87 (33-192)
Neglected tropical diseases and malaria	478 (280-1,331)	24,214 (15,002-36,950)	186,682 (116,333-282,738)	395,594 (236,129-658,268)	87,271 (51,471-172,991)	42,539 (25,669-83,804)
Malaria	-	23,708 (14,538-36,419)	152,266 (98,928-222,592)	283,387 (179,983-419,305)	46,945 (29,093-67,190)	21,348 (13,398-30,162)
[illegible]	[illegible]	[illegible]	[illegible]	[illegible]	[illegible]	[illegible]

Cause	15 to 19	20 to 24	25 to 29	30 to 34	35 to 39	40 to 44	45 to 49	50 to 54	54 to 59
All causes	674,033 (666,858-681,883)	883,892 (874,035-894,880)	915,948 (904,038-929,703)	949,207 (937,387-962,431)	1,055,373 (1,042,126-1,070,054)	1,169,841 (1,156,552-1,184,653)	1,350,268 (1,335,582-1,366,935)	1,826,812 (1,808,731-1,845,693)	2,296,854 (2,275,929-2,319,147)
Communicable, maternal, neonatal, and nutritional diseases	214,900 (198,037-242,804)	273,554 (257,297-299,543)	293,238 (276,689-316,878)	290,118 (274,160-311,231)	285,845 (269,299-307,547)	261,883 (245,612-282,658)	257,441 (241,013-278,285)	281,238 (262,522-305,333)	308,760 (288,125-336,032)
HIV/AIDS and sexually transmitted infections	4,423 (3,739-5,351)	23,820 (20,282-28,242)	49,988 (41,858-59,024)	53,025 (45,251-61,618)	45,772 (38,506-54,037)	34,602 (29,784-39,455)	25,615 (21,856-30,231)	16,580 (14,306-18,998)	12,061 (10,417-13,937)
HIV/AIDS	3,977 (3,320-4,879)	23,151 (19,744-27,506)	49,043 (40,993-58,012)	52,156 (44,370-60,829)	44,962 (37,682-53,203)	33,713 (28,914-38,649)	24,905 (21,122-29,594)	15,954 (13,680-18,409)	11,312 (9,723-13,157)
HIV/AIDS - Drug-susceptible Tuberculosis	1,388 (1,108-1,722)	7,438 (5,521-9,353)	12,538 (9,007-16,362)	12,494 (8,913-16,252)	10,948 (7,859-14,158)	8,655 (6,254-11,166)	7,146 (5,251-9,093)	5,103 (3,783-6,452)	3,774 (2,876-4,696)
HIV/AIDS - Multidrug-resistant Tuberculosis without extensive drug resistance	11 (6-20)	54 (29-105)	101 (54-183)	109 (59-194)	100 (55-173)	79 (44-136)	62 (35-106)	43 (24-73)	33 (19-57)
HIV/AIDS - Extensively drug-resistant Tuberculosis	0 (0-0)	0 (0-0)	0 (0-0)	0 (0-0)	0 (0-0)	0 (0-0)	0 (0-0)	0 (0-0)	0 (0-0)
HIV/AIDS resulting in other diseases	2,578 (2,069-3,377)	15,658 (12,238-20,174)	36,405 (28,052-46,025)	39,552 (31,655-48,132)	33,914 (27,074-42,828)	24,979 (20,125-30,259)	17,697 (13,939-22,549)	10,808 (8,817-13,198)	7,505 (6,175-9,302)
Sexually transmitted infections excluding HIV	445 (289-558)	669 (400-801)	945 (526-1,156)	870 (521-1,056)	810 (525-957)	889 (564-1,058)	710 (454-826)	626 (422-747)	749 (470-932)
Syphilis	107 (66-149)	124 (78-158)	192 (110-268)	191 (110-271)	181 (110-263)	214 (109-331)	157 (89-232)	208 (139-297)	296 (182-414)
Chlamydial infection	55 (34-69)	148 (85-178)	147 (79-179)	80 (46-95)	106 (65-123)	132 (81-153)	101 (64-116)	76 (47-90)	86 (51-104)
Gonococcal infection	149 (94-184)	236 (137-284)	406 (224-494)	382 (224-456)	304 (188-354)	349 (213-403)	285 (181-326)	226 (143-267)	237 (143-286)
Other sexually transmitted infections	134 (84-166)	161 (93-194)	200 (111-244)	217 (126-258)	218 (139-255)	193 (118-223)	166 (106-190)	116 (75-137)	130 (79-156)
Respiratory infections and tuberculosis	51,557 (46,353-55,721)	74,345 (68,409-79,661)	88,700 (81,684-94,606)	98,353 (91,717-104,966)	110,639 (103,183-117,654)	118,341 (111,404-125,085)	131,981 (124,827-140,419)	163,473 (155,566-173,213)	183,994 (174,696-195,267)
Tuberculosis	33,192 (29,426-36,545)	55,415 (50,395-60,155)	68,736 (62,600-74,539)	78,049 (72,101-84,246)	87,510 (81,045-94,157)	93,332 (87,054-99,706)	103,256 (96,527-110,954)	123,768 (116,208-132,400)	133,802 (125,310-143,997)
Drug-susceptible tuberculosis	32,905 (29,081-36,278)	54,861 (49,917-59,513)	68,052 (61,902-73,810)	77,267 (71,374-83,443)	86,646 (80,247-93,278)	92,387 (86,369-98,486)	102,293 (95,639-109,661)	122,611 (114,987-131,270)	132,452 (124,014-142,542)
Multidrug-resistant tuberculosis without extensive drug resistance	287 (143-546)	553 (282-1,044)	684 (359-1,251)	782 (406-1,441)	864 (450-1,589)	945 (485-1,774)	963 (507-1,785)	1,158 (611-2,086)	1,350 (687-2,502)
Extensively drug-resistant tuberculosis	0 (0-0)	0 (0-0)	0 (0-0)	0 (0-0)	0 (0-0)	0 (0-0)	0 (0-0)	0 (0-0)	0 (0-0)
Lower respiratory infections	18,021 (15,686-19,490)	18,569 (16,570-19,910)	19,586 (17,242-20,890)	19,945 (18,077-21,192)	22,726 (20,586-24,332)	24,575 (22,328-26,030)	28,185 (25,493-29,814)	39,107 (35,304-41,775)	49,584 (44,980-52,617)
Upper respiratory infections	223 (123-301)	281 (147-358)	312 (178-392)	299 (176-386)	327 (187-429)	355 (205-472)	368 (211-497)	406 (242-549)	498 (298-667)
Otitis media	121 (87-171)	81 (58-121)	66 (46-106)	60 (43-87)	76 (55-116)	78 (57-114)	172 (92-377)	191 (113-402)	111 (85-149)
Enteric infections	47,697 (32,875-69,504)	35,156 (23,474-50,488)	29,158 (19,507-41,877)	26,906 (18,006-39,281)	27,105 (17,965-40,981)	26,498 (17,526-40,965)	31,198 (20,390-48,992)	38,584 (24,282-61,411)	50,150 (31,485-80,622)
Diarrheal diseases	22,701 (12,597-36,755)	22,194 (12,395-36,243)	19,534 (10,820-31,838)	19,379 (10,858-31,527)	21,029 (12,047-34,538)	21,786 (12,383-36,109)	27,292 (15,967-45,276)	35,254 (20,690-58,034)	47,152 (28,547-77,375)
Typhoid and paratyphoid	23,345 (11,638-41,625)	11,526 (5,946-20,005)	8,333 (4,410-14,128)	6,298 (3,376-10,472)	4,821 (2,586-7,789)	3,576 (1,909-5,868)	2,826 (1,550-4,693)	2,217 (1,157-3,822)	1,831 (901-3,379)
Typhoid fever	20,422 (10,178-36,820)	10,111 (5,168-17,721)	7,236 (3,802-12,436)	5,407 (2,852-9,121)	4,084 (2,126-6,728)	3,001 (1,572-4,962)	2,348 (1,279-3,917)	1,845 (974-3,200)	1,537 (762-2,840)
Paratyphoid fever	2,923 (1,153-6,114)	1,415 (577-3,087)	1,097 (465-2,322)	891 (388-1,826)	737 (332-1,467)	575 (257-1,161)	478 (203-969)	372 (146-769)	294 (117-608)
Invasive Non-typhoidal Salmonella (iNTS)	1,572 (656-2,970)	1,355 (561-2,610)	1,245 (493-2,583)	1,169 (488-2,244)	1,201 (517-2,361)	1,096 (516-2,100)	1,042 (488-2,008)	1,082 (486-2,100)	1,140 (526-2,063)
Other intestinal infectious diseases	79 (27-174)	81 (30-175)	46 (14-104)	60 (19-132)	55 (18-119)	40 (11-82)	38 (10-87)	32 (8-69)	28 (7-58)
Neglected tropical diseases and malaria	33,632 (17,331-77,235)	33,342 (17,236-71,758)	29,074 (14,094-62,999)	27,008 (13,037-57,896)	27,791 (13,388-58,790)	26,456 (12,920-54,005)	25,945 (12,863-50,165)	27,996 (14,518-51,288)	27,482 (14,903-49,484)
Malaria	12,801 (8,507-18,131)	13,252 (8,747-18,787)	10,837 (7,132-15,434)	9,663 (6,346-13,655)	9,994 (6,648-14,385)	9,461 (6,248-13,739)	8,891 (5,914-12,816)	11,251 (7,554-16,176)	11,861 (7,963-17,332)
[illegible]	[illegible]	[illegible]	[illegible]	[illegible]	[illegible]	[illegible]	[illegible]	[illegible]	[illegible]

Cause	60 to 64	65 to 69	70 to 74	75 to 79	80 to 84	85 to 89	90 to 94	95 plus
All causes	3,152,401 (3,127,381-3,179,328)	3,558,979 (3,532,204-3,586,138)	3,807,202 (3,778,702-3,836,159)	4,142,826 (4,117,037-4,167,923)	3,775,380 (3,757,544-3,792,388)	2,484,332 (2,475,759-2,492,302)	1,039,808 (1,037,169-1,042,367)	303,906 (303,400-304,373)
Communicable, maternal, neonatal, and nutritional diseases	376,560 (349,301-418,425)	391,539 (361,480-440,064)	423,914 (388,867-478,916)	393,370 (358,348-445,597)	365,367 (330,716-412,703)	236,334 (215,042-262,911)	93,014 (85,141-102,797)	28,025 (26,260-30,219)
HIV/AIDS and sexually transmitted infections	8,800 (7,609-10,276)	6,510 (5,484-7,659)	3,769 (3,063-4,553)	2,531 (2,073-3,033)	692 (513-827)	333 (233-410)	124 (91-148)	33 (26-39)
HIV/AIDS	8,117 (6,941-9,613)	5,887 (4,914-7,008)	3,249 (2,576-4,004)	1,922 (1,515-2,392)	266 (216-328)	71 (58-90)	25 (21-31)	4 (4-5)
HIV/AIDS - Drug-susceptible Tuberculosis	2,781 (2,163-3,476)	2,067 (1,602-2,532)	1,240 (948-1,579)	735 (566-920)	92 (71-116)	21 (16-28)	8 (6-10)	1 (1-1)
HIV/AIDS - Multidrug-resistant Tuberculosis without extensive drug resistance	25 (14-42)	18 (10-31)	9 (5-16)	6 (3-12)	1 (1-2)	0 (0-1)	0 (0-0)	0 (0-0)
HIV/AIDS - Extensively drug-resistant Tuberculosis	0 (0-0)	0 (0-0)	0 (0-0)	0 (0-0)	0 (0-0)	0 (0-0)	0 (0-0)	0 (0-0)
HIV/AIDS resulting in other diseases	5,312 (4,441-6,721)	3,802 (3,092-4,772)	2,000 (1,525-2,639)	1,181 (904-1,597)	174 (137-226)	50 (40-64)	17 (14-22)	3 (3-4)
Sexually transmitted infections excluding HIV	683 (392-861)	623 (408-803)	520 (339-671)	608 (373-814)	426 (254-544)	262 (161-335)	99 (66-123)	29 (21-35)
Syphilis	303 (170-457)	299 (179-461)	278 (157-413)	334 (172-515)	206 (105-298)	128 (61-190)	40 (22-58)	12 (8-17)
Chlamydial infection	74 (43-89)	40 (26-47)	41 (29-47)	44 (29-52)	49 (33-59)	29 (21-34)	12 (9-15)	3 (3-4)
Gonococcal infection	188 (108-226)	213 (138-252)	145 (102-166)	172 (114-202)	129 (85-153)	77 (55-89)	34 (25-39)	9 (7-10)
Other sexually transmitted infections	118 (68-142)	71 (47-84)	56 (39-64)	59 (39-72)	42 (27-50)	29 (19-36)	13 (9-16)	5 (3-6)
Respiratory infections and tuberculosis	225,409 (214,511-238,667)	230,476 (219,805-244,343)	247,277 (235,905-261,359)	233,635 (223,853-246,962)	216,938 (207,646-229,280)	147,187 (142,726-155,103)	60,396 (58,781-63,502)	19,164 (18,761-19,938)
Tuberculosis	148,814 (138,849-161,120)	138,209 (128,515-150,746)	130,486 (121,342-142,546)	97,362 (90,113-106,023)	67,773 (62,430-74,653)	29,777 (27,348-32,952)	7,782 (7,090-8,632)	1,480 (1,348-1,643)
Drug-susceptible tuberculosis	147,172 (137,427-159,435)	136,482 (126,575-149,055)	128,748 (119,423-140,561)	96,000 (88,717-104,362)	66,888 (61,522-73,647)	29,392 (26,973-32,501)	7,691 (7,002-8,527)	1,463 (1,330-1,626)
Multidrug-resistant tuberculosis without extensive drug resistance	1,642 (795-3,097)	1,727 (825-3,325)	1,737 (821-3,430)	1,362 (664-2,626)	886 (444-1,660)	385 (205-687)	90 (54-145)	17 (11-26)
Extensively drug-resistant tuberculosis	0 (0-0)	0 (0-0)	0 (0-0)	0 (0-0)	0 (0-0)	0 (0-0)	0 (0-0)	0 (0-0)
Lower respiratory infections	75,815 (68,593-80,340)	91,238 (82,781-96,913)	115,366 (106,333-121,879)	134,544 (127,090-142,750)	146,761 (139,662-155,550)	115,601 (111,615-122,114)	51,917 (50,597-54,797)	17,454 (17,092-18,199)
Upper respiratory infections	666 (375-894)	883 (460-1,157)	1,310 (610-1,709)	1,650 (738-2,167)	2,340 (965-3,127)	1,772 (735-2,408)	684 (307-891)	225 (98-288)
Otitis media	113 (87-153)	147 (113-199)	116 (89-148)	79 (62-99)	64 (51-83)	37 (30-46)	13 (11-16)	5 (4-6)
Enteric infections	75,050 (45,457-120,061)	91,248 (56,791-145,506)	111,269 (69,908-171,156)	111,234 (70,261-172,209)	114,828 (72,119-169,894)	68,875 (44,590-98,965)	24,845 (15,610-35,941)	6,187 (4,101-8,507)
Diarrheal diseases	72,351 (42,402-118,121)	89,039 (54,846-143,238)	109,660 (68,238-169,536)	110,161 (69,215-171,187)	114,148 (71,427-168,945)	68,548 (44,177-98,678)	24,746 (15,547-35,797)	6,161 (4,062-8,481)
Typhoid and paratyphoid	1,506 (760-2,663)	1,144 (669-1,823)	805 (499-1,250)	495 (317-742)	296 (195-430)	137 (97-198)	44 (31-63)	12 (8-18)
Typhoid fever	1,271 (636-2,313)	977 (564-1,577)	694 (428-1,071)	426 (267-652)	256 (169-379)	120 (84-177)	38 (28-58)	10 (7-17)
Paratyphoid fever	234 (97-477)	167 (77-327)	111 (54-210)	69 (35-123)	39 (20-68)	17 (9-28)	6 (2-10)	2 (1-4)
Invasive Non-typhoidal Salmonella (iNTS)	1,162 (542-2,068)	1,028 (481-1,765)	780 (369-1,370)	559 (268-1,022)	358 (155-669)	165 (66-304)	50 (14-106)	12 (0-33)
Other intestinal infectious diseases	32 (7-73)	37 (9-80)	24 (6-52)	18 (2-42)	26 (4-58)	26 (4-62)	5 (0-13)	2 (0-6)
Neglected tropical diseases and malaria	29,306 (16,361-50,506)	25,162 (13,970-43,052)	23,252 (14,163-37,148)	11,782 (7,212-19,650)	5,657 (3,553-9,430)	2,092 (1,396-3,504)	548 (375-898)	133 (96-191)
Malaria	13,819 (9,305-19,918)	12,151 (7,957-18,134)	12,879 (8,385-19,346)	5,172 (3,425-7,591)	2,139 (1,513-3,021)	540 (386-721)	129 (81-168)	45 (20-62)
[illegible]	[illegible]	[illegible]	[illegible]	[illegible]	[illegible]	[illegible]	[illegible]	[illegible]

SR Table 7: Global deaths by cause and age group for both sexes combined (with 95% confidence intervals), 1990

Cause	Early Neonatal	Late Neonatal	Post Neonatal	1 to 4	5 to 9	10 to 14	15 to 19	20 to 24	25 to 29	30 to 34	35 to 39	40 to 44	45 to 49	50 to 54	54 to 59	60 to 64	65 to 69	70 to 74	75 to 79	80 to 84	85 to 89	90 to 94	95 plus
Thyroid cancer	-	-	-	-	-	171 (134-204)	332 (258-395)	409 (334-474)	695 (554-809)	480 (397-544)	581 (499-655)	756 (672-838)	950 (872-1,034)	1,488 (1,373-1,623)	1,952 (1,828-2,123)	2,549 (2,389-2,824)	2,761 (2,605-3,075)	2,629 (2,480-2,963)	2,737 (2,634-2,988)	2,142 (2,071-2,320)	1,058 (1,025-1,141)	311 (301-329)	64 (61-67)
Mesothelioma	-	-	-	-	-	-	82 (55-121)	127 (80-194)	188 (122-273)	220 (151-312)	348 (256-484)	545 (418-731)	644 (526-821)	1,068 (906-1,317)	1,748 (1,469-2,106)	2,226 (1,824-2,654)	2,645 (2,148-3,120)	2,417 (2,013-2,826)	2,522 (2,157-2,897)	1,658 (1,440-1,879)	728 (647-818)	202 (187-223)	37 (35-41)
Hodgkin lymphoma	22 (11-27)	10 (4-14)	224 (93-296)	1,152 (471-1,737)	1,835 (970-2,380)	1,585 (1,015-1,928)	2,136 (1,559-2,478)	2,421 (1,826-2,769)	2,603 (1,945-2,921)	2,360 (1,761-2,653)	2,144 (1,629-2,424)	1,861 (1,381-2,100)	1,767 (1,329-2,031)	2,073 (1,571-2,340)	2,436 (1,821-2,816)	2,751 (2,042-3,107)	2,810 (2,175-3,194)	2,396 (1,870-2,787)	1,687 (1,265-1,929)	1,108 (850-1,244)	462 (365-528)	92 (71-104)	13 (10-15)
Non-Hodgkin lymphoma	82 (74-96)	84 (68-110)	1,167 (928-1,419)	5,049 (3,302-7,231)	3,787 (3,119-4,652)	2,562 (2,234-2,968)	2,683 (2,440-2,905)	3,112 (2,853-3,373)	3,307 (3,049-3,557)	3,581 (3,347-3,826)	4,315 (4,051-4,618)	5,025 (4,770-5,374)	5,926 (5,660-6,282)	8,055 (7,721-8,533)	10,440 (10,040-10,915)	13,976 (13,483-14,548)	15,626 (15,109-16,091)	14,731 (14,315-15,068)	13,884 (13,524-14,167)	10,286 (10,046-10,489)	5,177 (5,059-5,275)	1,542 (1,510-1,573)	290 (284-297)
Multiple myeloma	-	-	-	-	-	-	234 (163-274)	278 (191-325)	288 (217-332)	394 (339-453)	672 (599-768)	1,166 (1,043-1,321)	1,700 (1,561-1,913)	3,017 (2,759-3,420)	4,663 (4,216-5,322)	6,768 (6,196-7,845)	7,995 (7,482-9,373)	7,656 (7,043-9,185)	7,769 (7,221-9,354)	5,781 (5,449-7,004)	2,904 (2,693-3,597)	846 (790-1,050)	145 (134-179)
Leukemia	431 (305-511)	451 (297-604)	7,049 (4,438-9,435)	19,370 (12,305-31,174)	19,134 (14,101-24,934)	13,945 (11,224-16,253)	13,061 (10,933-14,305)	11,361 (9,150-12,635)	10,123 (8,054-11,295)	9,203 (7,270-10,294)	9,603 (7,902-10,551)	9,595 (8,058-10,338)	9,907 (8,572-10,658)	12,207 (10,914-13,036)	14,987 (13,694-16,253)	18,992 (17,807-20,588)	20,386 (19,326-22,073)	19,117 (18,160-20,671)	19,371 (18,701-20,654)	14,941 (14,425-15,966)	8,159 (7,883-8,658)	2,849 (2,770-2,981)	644 (627-665)
Acute lymphoid leukemia	76 (59-100)	72 (49-112)	1,187 (823-1,980)	4,855 (3,148-9,184)	6,224 (4,714-9,410)	4,080 (3,403-5,320)	3,172 (2,785-3,876)	2,160 (1,891-2,668)	1,700 (1,459-2,161)	1,291 (1,118-1,608)	1,307 (1,138-1,597)	1,147 (1,005-1,366)	1,044 (944-1,231)	1,244 (1,132-1,422)	1,443 (1,316-1,668)	1,608 (1,483-1,892)	1,513 (1,401-1,742)	1,246 (1,153-1,453)	903 (844-1,020)	605 (565-690)	285 (268-319)	85 (80-93)	16 (16-17)
Chronic lymphoid leukemia	-	-	-	-	-	-	189 (143-230)	207 (150-262)	197 (142-255)	190 (137-238)	206 (164-244)	270 (228-304)	381 (336-415)	751 (685-797)	1,231 (1,144-1,299)	2,145 (2,027-2,257)	2,667 (2,543-2,800)	2,813 (2,661-2,929)	3,491 (3,349-3,636)	3,035 (2,923-3,160)	1,892 (1,819-1,963)	730 (699-756)	187 (179-194)
Acute myeloid leukemia	61 (44-86)	63 (40-111)	725 (426-1,431)	2,866 (1,446-6,806)	1,966 (1,259-3,423)	1,852 (1,415-2,645)	2,069 (1,728-2,651)	1,898 (1,611-2,469)	1,869 (1,590-2,466)	1,859 (1,608-2,356)	2,000 (1,758-2,453)	2,142 (1,913-2,532)	2,426 (2,200-2,791)	2,952 (2,726-3,288)	3,626 (3,347-3,995)	4,615 (4,340-5,029)	4,925 (4,666-5,406)	4,565 (4,321-4,977)	4,329 (4,147-4,681)	3,016 (2,876-3,348)	1,423 (1,358-1,609)	433 (413-507)	87 (82-102)
Chronic myeloid leukemia	-	-	-	-	-	-	486 (401-574)	695 (558-828)	953 (778-1,108)	1,081 (902-1,227)	1,197 (1,033-1,341)	1,259 (1,119-1,392)	1,313 (1,184-1,442)	1,684 (1,527-1,857)	1,962 (1,814-2,173)	2,359 (2,218-2,592)	2,545 (2,399-2,790)	2,259 (2,125-2,465)	2,656 (2,494-2,832)	2,138 (2,017-2,294)	1,117 (1,053-1,189)	401 (379-428)	93 (88-99)
Other leukemia	293 (189-357)	316 (190-428)	5,137 (2,872-7,054)	11,649 (6,140-19,899)	10,944 (6,743-14,488)	8,013 (5,660-9,337)	7,145 (5,394-8,051)	6,402 (4,655-7,333)	5,404 (3,860-6,344)	4,784 (3,377-5,613)	4,893 (3,641-5,580)	4,778 (3,619-5,396)	4,743 (3,772-5,304)	5,576 (4,703-6,125)	6,726 (5,821-7,350)	8,265 (7,390-9,119)	8,736 (7,980-9,721)	8,234 (7,587-9,077)	7,991 (7,510-8,806)	6,148 (5,787-6,767)	3,443 (3,248-3,748)	1,200 (1,140-1,284)	261 (249-273)
Other malignant neoplasms	999 (749-1,131)	636 (437-854)	3,878 (2,582-5,090)	11,684 (7,365-18,840)	6,078 (4,821-7,578)	6,068 (5,211-6,825)	7,337 (6,634-8,003)	6,116 (5,543-6,650)	5,463 (4,913-5,887)	5,460 (4,857-5,885)	6,681 (6,049-7,064)	8,187 (7,358-8,600)	9,798 (8,823-10,300)	14,271 (12,927-14,969)	17,951 (16,112-18,923)	22,333 (20,675-23,449)	23,013 (21,207-24,393)	20,171 (18,995-21,682)	18,979 (17,935-20,659)	13,877 (13,014-15,073)	7,499 (6,753-7,953)	2,562 (2,280-2,694)	593 (533-631)
Other neoplasms	54 (39-63)	38 (26-46)	499 (306-652)	1,222 (667-1,744)	1,004 (681-1,239)	686 (521-805)	610 (460-722)	630 (477-731)	728 (556-831)	890 (674-1,020)	1,081 (800-1,277)	1,238 (915-1,460)	1,424 (1,022-1,690)	1,990 (1,390-2,375)	2,564 (1,774-3,032)	3,432 (2,463-4,169)	4,079 (2,946-5,066)	4,171 (3,126-5,481)	4,917 (3,738-6,620)	4,106 (3,233-5,767)	2,517 (1,965-3,706)	940 (710-1,471)	240 (175-390)
Myelodysplastic, myeloproliferative, and other hematopoietic neoplasms	53 (38-62)	33 (22-41)	446 (270-599)	1,012 (550-1,528)	711 (493-926)	469 (357-558)	420 (320-489)	450 (348-527)	556 (431-653)	708 (538-819)	915 (693-1,056)	1,114 (833-1,296)	1,331 (962-1,571)	1,908 (1,337-2,264)	2,500 (1,730-2,945)	3,383 (2,428-4,120)	4,047 (2,926-5,043)	4,150 (3,113-5,467)	4,904 (3,727-6,611)	4,097 (3,227-5,752)	2,513 (1,960-3,703)	939 (709-1,469)	240 (175-390)
Other benign and in situ neoplasms	2 (1-3)	5 (3-8)	53 (31-74)	210 (110-368)	294 (183-392)	217 (154-275)	190 (132-249)	180 (120-238)	172 (111-229)	182 (117-245)	166 (105-228)	123 (77-171)	93 (57-132)	82 (48-118)	64 (36-101)	49 (26-81)	32 (16-53)	21 (10-37)	14 (6-23)	9 (4-18)	4 (2-7)	1 (1-2)	0 (0-0)
Cardiovascular diseases	4,892 (4,496-5,436)	4,554 (3,929-5,098)	35,008 (32,042-37,859)	28,577 (24,272-32,321)	14,664 (12,972-16,233)	13,524 (12,180-14,604)	36,715 (34,140-38,977)	56,954 (53,974-59,548)	76,226 (72,832-79,072)	109,176 (104,912-113,409)	164,300 (158,474-169,287)	244,077 (236,283-250,718)	342,169 (332,925-351,050)	542,653 (531,336-554,719)	751,236 (736,888-766,866)	1,127,351 (1,109,065-1,150,720)	1,362,987 (1,341,155-1,392,410)	1,533,030 (1,507,154-1,572,042)	1,843,092 (1,821,044-1,883,768)	1,759,946 (1,738,682-1,802,409)	1,211,284 (1,197,283-1,237,918)	520,430 (515,505-528,855)	158,687 (157,434-160,543)
Rheumatic heart disease	-	-	-	9,206 (7,237-11,679)	5,252 (4,534-6,051)	5,707 (5,065-6,309)	8,529 (7,583-9,524)	10,740 (9,532-11,962)	10,974 (9,871-12,216)	11,938 (10,644-13,333)	14,286 (12,814-15,947)	16,770 (15,155-18,611)	17,660 (16,061-19,625)	22,655 (20,934-25,065)	26,144 (24,295-28,970)	32,104 (29,946-35,957)	33,635 (31,622-37,564)	34,081 (31,789-38,539)	31,077 (29,135-35,171)	25,171 (23,524-28,290)	13,689 (12,856-15,288)	4,972 (4,686-5,451)	1,287 (1,228-1,386)
Ischemic heart disease	-	-	-	-	-	-	10,828 (9,978-11,571)	19,511 (18,400-20,650)	29,366 (27,904-30,774)	48,506 (46,220-50,685)	77,167 (73,918-80,162)	118,072 (113,902-122,031)	171,480 (165,990-177,061)	271,942 (264,789-279,447)	378,497 (369,894-388,289)	565,284 (553,906-579,556)	668,304 (655,374-685,722)	724,451 (710,217-746,906)	901,813 (888,239-925,288)	877,652 (864,239-900,955)	625,796 (615,972-641,925)	284,097 (280,438-289,830)	92,449 (91,413-93,995)
Stroke	2,183 (1,863-2,569)	2,020 (1,535-2,601)	14,522 (11,336-18,526)	8,576 (6,601-10,906)	5,543 (4,840-6,322)	4,602 (4,149-4,974)	10,487 (9,785-11,067)	16,894 (15,993-17,725)	22,673 (21,575-23,727)	32,509 (31,111-33,811)	50,949 (48,899-52,903)	79,886 (76,900-82,723)	115,771 (111,818-119,879)	191,953 (186,186-198,105)	269,326 (262,759-277,793)	419,071 (408,603-432,988)	521,342 (508,851-541,480)	608,882 (593,687-638,550)	708,890 (692,940-738,672)	652,502 (637,927-681,014)	419,387 (409,651-436,754)	160,665 (157,315-165,934)	43,865 (43,071-45,039)
Ischemic stroke	308 (242-416)	335 (217-551)	1,347 (920-2,069)	1,033 (674-1,720)	556 (468-699)	414 (356-479)	1,418 (1,253-1,592)	2,321 (2,057-2,589)	3,114 (2,824-3,414)	4,466 (4,070-4,894)	6,821 (6,266-7,466)	11,279 (10,369-12,304)	19,573 (17,998-21,431)	38,658 (35,762-42,161)	62,256 (57,760-67,441)	138,038 (129,608-147,838)	186,812 (176,582-197,637)	259,763 (246,986-273,509)	342,141 (329,566-356,167)	385,964 (374,662-399,570)	264,080 (256,472-273,686)	105,130 (102,569-108,099)	29,907 (29,266-30,648)
Intracerebral hemorrhage	972 (808-1,229)	833 (606-1,244)	6,989 (5,260-10,377)	3,390 (2,386-5,307)	2,746 (2,312-3,435)	2,332 (2,052-2,736)	6,160 (5,622-6,711)	9,936 (9,207-10,723)	13,515 (12,647-14,724)	19,930 (18,642-21,326)	31,754 (29,991-33,905)	50,116 (47,379-53,528)	75,861 (71,742-80,890)	122,939 (116,096-131,104)	166,690 (157,524-178,146)	235,630 (220,006-253,592)	280,793 (263,146-304,943)	296,283 (277,066-326,709)	314,084 (297,604-342,724)	230,161 (219,444-252,695)	135,218 (129,354-147,675)	48,897 (47,025-52,256)	12,338 (11,932-12,911)
Subarachnoid hemorrhage	903 (704-1,046)	852 (616-1,061)	6,186 (4,501-7,652)	4,153 (3,043-5,168)	2,241 (1,776-2,546)	1,856 (1,584-2,036)	2,909 (2,520-3,271)	4,637 (4,026-5,195)	6,044 (5,310-6,678)	8,113 (7,170-9,038)	12,374 (11,008-13,767)	18,491 (16,076-20,824)	20,338 (17,281-23,121)	30,357 (25,251-34,911)	40,380 (32,499-46,900)	45,404 (35,037-53,282)	53,737 (40,451-62,894)	52,836 (36,866-62,166)	52,665 (38,340-60,465)	36,378 (25,743-41,696)	20,089 (15,378-22,603)	6,637 (5,499-7,343)	1,619 (1,454-1,760)
Hypertensive heart disease	-	-	-	-	-	-	933 (667-1,105)	1,600 (1,134-1,864)	2,337 (1,688-2,737)	3,581 (2,689-4,135)	5,805 (4,481-6,575)	9,141 (7,131-10,189)	13,609 (10,348-15,123)	22,072 (16,865-24,433)	31,748 (24,874-35,116)	47,628 (37,243-52,392)	63,454 (46,927-69,432)	81,136 (60,601-88,855)	89,150 (68,549-97,354)	83,783 (67,551-91,499)	54,510 (44,191-59,852)	22,919 (18,241-25,209)	6,466 (5,282-7,367)
Non-rheumatic valvular heart disease	-	-	-	-	-	-	664 (521-856)	773 (607-1,005)	873 (702-1,112)	818 (683-1,006)	1,074 (933-1,296)	1,298 (1,154-1,532)	1,451 (1,302-1,688)	2,158 (1,969-2,449)	3,135 (2,886-3,521)	4,503 (4,075-4,960)	6,203 (5,571-6,758)	7,471 (6,693-8,311)	10,918 (9,641-12,239)	11,943 (10,699-13,691)	9,199 (8,489-11,224)	4,567 (4,192-5,812)	1,398 (1,290-1,830)
Non-rheumatic calcific aortic valve disease	-	-	-	-	-	-	192 (137-295)	222 (160-355)	286 (214-425)	308 (242-423)	442 (360-586)	557 (468-701)	650 (539-804)	1,013 (874-1,221)	1,506 (1,275-1,781)	2,346 (2,039-2,698)	3,430 (2,993-3,928)	4,288 (3,768-4,926)	6,612 (5,715-7,588)	7,657 (6,754-9,056)	6,234 (5,605-7,829)	3,225 (2,879-4,314)	1,004 (905-1,353)
Non-rheumatic degenerative mitral valve disease	-	-	-	-	-	-	349 (258-488)	423 (313-596)	450 (342-630)	423 (338-564)	525 (423-664)	619 (511-751)	716 (600-849)	1,019 (850-1,193)	1,463 (1,240-1,669)	1,953 (1,578-2,238)	2,509 (2,061-2,882)	2,879 (2,323-3,388)	3,817 (3,127-4,534)	3,781 (3,136-4,644)	2,647 (2,130-3,330)	1,221 (951-1,547)	360 (262-485)
Other non-rheumatic valve diseases	-	-	-	-	-	-	124 (89-162)	129 (91-169)	137 (99-185)	87 (63-120)	108 (72-138)	122 (81-163)	85 (58-116)	126 (84-162)	166 (111-216)	203 (133-255)	265 (168-340)	305 (190-384)	489 (313-608)	504 (320-638)	318 (208-398)	121 (81-156)	34 (23-44)
Cardiomyopathy and myocarditis	1,505 (1,276-1,713)	1,306 (1,052-1,596)	9,130 (7,456-10,803)	5,001 (3,398-6,177)	1,876 (1,509-2,241)	1,562 (1,367-1,791)	2,521 (2,184-2,951)	3,263 (2,807-3,820)	4,406 (3,806-5,072)	5,354 (4,707-6,045)	7,143 (6,262-8,105)	8,687 (7,573-9,797)	9,653 (8,392-10,960)	13,408 (11,728-15,207)	16,035 (14,092-17,924)	18,350 (16,346-20,278)	18,199 (15,613-19,876)	17,401 (14,464-18,980)	23,878 (19,922-25,561)	27,749 (23,087-29,553)	26,274 (21,622-28,507)	12,089 (10,270-13,056)	3,785 (3,320-4,052)
Myocarditis	200 (130-259)	271 (180-350)	2,731 (1,976-3,339)	1,727 (1,207-2,195)	730 (578-929)	508 (419-612)	892 (711-1,096)	1,112 (866-1,395)	1,168 (917-1,447)	908 (729-1,123)	1,046 (820-1,288)	1,167 (906-1,444)	881 (673-1,091)	941 (699-1,155)	1,191 (892-1,465)	1,078 (799-1,305)	1,204 (918-1,445)	1,297 (985-1,527)	1,830 (1,337-2,183)	2,258 (1,632-2,804)	2,382 (1,721-3,228)	1,194 (860-1,688)	401 (297-573)
Alcoholic cardiomyopathy	-	-	-	-	-	-	278 (205-340)	495 (403-581)	1,079 (927-1,239)	1,915 (1,625-2,221)	3,050 (2,618-3,556)	3,778 (3,208-4,358)	4,517 (3,835-5,118)	6,665 (5,701-8,021)	6,850 (5,910-7,993)	6,930 (6,066-8,540)	5,340 (4,695-6,252)	3,777 (3,345-4,506)	4,206 (3,654-5,399)	3,393 (2,840-4,604)	2,210 (1,781-3,423)	939 (753-1,583)	276 (233-462)
Other cardiomyopathy	1,305 (1,101-1,484)	1,035 (806-1,257)	6,399 (5,058-7,659)	3,274 (2,142-4,202)	1,146 (884-1,353)	1,054 (901-1,194)	1,352 (1,133-1,585)	1,656 (1,384-1,950)	2,159 (1,804-2,550)	2,530 (2,147-2,948)	3,047 (2,598-3,599)	3,742 (3,151-4,386)	4,255 (3,551-5,068)	5,802 (4,893-6,825)	7,994 (6,483-9,388)	10,343 (8,474-11,964)	11,656 (9,141-13,158)	12,327 (9,464-13,785)	17,841 (13,516-19,598)	22,097 (16,303-24,039)	21,682 (15,525-23,906)	9,956 (7,334-10,961)	3,107 (2,354-3,386)
Atrial fibrillation and flutter	-	-	-	-	-	-	-	-	-	55 (49-58)	72 (66-76)	316 (297-359)	501 (474-562)	976 (923-1,090)	2,007 (1,896-2,228)	3,923 (3,722-4,393)	6,308 (6,015-7,052)	10,358 (9,869-11,517)	17,524 (16,690-19,173)	26,854 (25,191-28,642)	24,682 (22,912-25,919)	15,103 (14,023-15,617)	4,710 (4,384-4,855)
Aortic aneurysm	-	-	-	-	-	-	270 (228-326)	433 (357-530)	670 (547-807)	902 (750-1,083)	1,331 (1,163-1,551)	1,766 (1,594-2,019)	2,408 (2,216-2,686)	3,824 (3,598-4,186)	6,083 (5,743-6,629)	9,925 (9,397-10,818)	13,849 (13,230-14,795)	15,689 (15,037-16,631)	18,351 (17,677-19,196)	15,430 (14,843-16,053)	9,453 (9,150-9,780)	3,571 (3,460-3,680)	851 (825-873)
Peripheral artery disease	-	-	-	-	-	-	-	-	-	-	-	118 (79-157)	183 (117-264)	415 (237-620)	753 (429-1,155)	1,471 (839-2,270)	1,929 (1,079-3,074)	2,251 (1,285-3,548)	3,499 (1,856-5,607)	3,841 (1,964-6,486)	3,109 (1,501-5,521)	1,711 (796-3,131)	698 (317-1,360)
Endocarditis	263 (214-336)	290 (221-393)	3,824 (3,022-4,933)	2,193 (1,546-3,079)	519 (429-656)	506 (425-623)	677 (582-822)	1,005 (867-1,247)	1,329 (1,126-1,601)	1,246 (1,073-1,438)	1,379 (1,196-1,596)	1,496 (1,312-1,752)	1,339 (1,190-1,585)	1,668 (1,472-1,951)	1,957 (1,741-2,362)	2,688 (2,382-3,176)	3,240 (2,841-3,895)	3,431 (2,942-4,136)	4,128 (3,383-5,073)	3,961 (3,127-5,091)	3,014 (2,264-3,874)	1,495 (1,044-2,001)	480 (341-666)
Other cardiovascular and circulatory diseases	941 (779-1,198)	939 (720-1,216)	7,533 (5,217-10,481)	3,600 (1,999-6,064)	1,474 (1,060-2,143)	1,148 (934-1,515)	1,805 (1,520-2,272)	2,736 (2,301-3,438)	3,598 (3,113-4,488)	4,268 (3,753-5,078)	5,094 (4,551-6,052)	6,526 (5,865-7,872)	8,113 (7,278-9,867)	11,582 (10,503-14,014)	15,551 (14,162-18,630)	22,404 (20,558-26,497)	26,523 (24,543-30,554)	27,878 (25,480-32,160)	33,863 (31,364-38,055)	31,062 (28,609-34,629)	22,172 (20,553-24,237)	9,240 (8,648-9,934)	2,699 (2,544-2,865)
Chronic respiratory diseases	0 (0-0)	0 (0-0)	0 (0-0)	38,577 (31,096-44,800)	9,840 (8,363-11,198)	6,855 (5,930-7,706)	10,901 (9,901-11,758)	14,420 (13,320-15,417)	17,708 (16,324-18,956)	21,744 (19,991-23,037)	30,891 (28,256-32,595)	43,675 (40,516-45,765)	67,015 (61,197-70,072)	119,313 (107,080-124,657)	189,663 (169,941-197,534)	334,921 (300,518-347,594)	431,938 (387,463-448,250)	547,541 (491,696-567,136)	559,927 (506,871-580,623)	490,264 (443,995-507,664)	274,507 (248,983-285,139)	87,975 (80,993-91,441)	19,530 (18,273-20,239)
Chronic obstructive pulmonary disease	-	-	0 (0-0)	4,390 (2,550-8,002)	1,424 (953-2,446)	807 (590-1,282)	3,399 (2,663-4,757)	5,120 (4,085-6,796)	6,870 (5,798-8,827)	9,552 (8,113-11,927)	13,861 (11,610-17,857)	22,384 (18,879-28,468)	37,583 (31,330-47,488)	75,146 (62,749-92,263)	128,339 (108,169-152,862)	252,626 (216,992-289,123)	343,810 (297,138-383,032)	458,251 (398,738-495,195)	476,487 (421,153-511,222)	424,504 (379,394-452,320)	241,027 (216,083-254,984)	77,412 (70,351-81,774)	17,151 (15,879-17,989)
Pneumoconiosis	-	-	-	-	-	-	55 (43-67)	80 (62-97)	162 (124-195)	292 (224-355)	536 (414-654)	671 (514-831)	725 (557-891)	1,062 (819-1,318)	1,481 (1,207-1,827)	2,344 (1,936-2,830)	3,010 (2,518-3,596)	3,206 (2,694-3,821)	3,196 (2,813-3,760)	2,233 (1,984-2,652)	1,006 (895-1,198)	267 (241-304)	52 (48-59)
Silicosis	-	-	-	-	-	-	16 (11-24)	28 (20-39)	76 (54-107)	184 (136-242)	367 (270-481)	451 (318-607)	459 (313-619)	648 (474-867)	868 (656-1,147)	1,341 (1,025-1,727)	1,726 (1,350-2,177)	1,780 (1,374-2,260)	1,747 (1,414-2,153)	1,168 (950-1,433)	495 (399-610)	116 (98-139)	21 (18-25)
Asbestosis	-	-	-	-	-	-	5 (2-8)	8 (4-12)	11 (5-16)	14 (6-24)	19 (9-29)	23 (11-39)	28 (14-44)	60 (34-90)	105 (59-156)	162 (94-245)	201 (121-286)	244 (162-346)	246 (162-325)	182 (125-237)	99 (69-129)	33 (25-44)	7 (5-8)
Coal workers pneumoconiosis	-	-	-	-	-	-	8 (5-14)	11 (6-17)	31 (15-45)	35 (18-54)	65 (29-110)	107 (49-190)	125 (60-231)	179 (99-303)	277 (178-421)	507 (333-741)	743 (526-1,054)	823 (560-1,187)	856 (652-1,153)	625 (487-857)	289 (231-386)	75 (64-94)	14 (12-17)
Other pneumoconiosis	-	-	-	-	-	-	25 (17-35)	33 (20-46)	44 (26-63)	59 (35-89)	85 (50-127)	90 (56-133)	113 (68-162)	175 (111-246)	231 (147-327)	334 (217-486)	340 (228-482)	359 (243-519)	347 (243-477)	258 (194-351)	124 (98-165)	42 (34-56)	10 (8-13)
Asthma	-	-	-	29,810 (22,675-36,432)	7,131 (5,789-8,415)	5,323 (4,461-6,213)	6,382 (4,736-7,492)	7,858 (5,329-9,209)	9,198 (6,167-10,721)	10,062 (6,315-11,800)	14,075 (8,628-16,531)	17,452 (10,020-20,909)	24,718 (13,251-30,895)	36,627 (19,069-47,268)	50,332 (24,839-66,017)	71,382 (34,673-94,823)	75,213 (36,310-102,284)	75,246 (38,514-101,607)	68,410 (38,229-91,831)	53,589 (31,695-73,500)	26,497 (16,939-35,807)	8,183 (5,635-10,974)	1,790 (1,349-2,276)
Interstitial lung disease and pulmonary sarcoidosis	-	-	-	557 (278-1,341)	199 (115-447)	114 (79-195)	164 (113-280)	239 (168-411)	353 (255-563)	491 (350-752)	762 (546-1,173)	1,013 (720-1,513)	1,480 (1,007-2,241)	2,585 (1,721-3,858)	3,949 (2,591-5,947)	6,170 (4,103-9,096)	7,408 (5,332-10,559)	8,199 (6,113-11,215)	9,211 (7,094-11,674)	7,462 (5,824-9,185)	4,296 (3,389-5,182)	1,425 (1,180-1,723)	354 (300-451)
Other chronic respiratory diseases	0 (0-0)	0 (0-0)	0 (0-0)	3,821 (1,677-6,535)	1,087 (683-1,585)	611 (435-817)	900 (623-1,214)	1,124 (773-1,551)	1,124 (759-1,523)	1,347 (930-1,816)	1,658 (1,111-2,212)	2,155 (1,455-2,883)	2,509 (1,716-3,367)	3,893 (2,835-4,976)	5,563 (4,078-7,084)	2,399 (1,808-3,036)	2,498 (1,939-3,158)	2,639 (2,138-3,246)	2,623 (2,204-3,068)	2,476 (2,091-2,940)	1,680 (1,472-1,938)	688 (605-792)	183 (163-207)
Digestive diseases	3,086 (2,732-3,396)	3,947 (3,357-5,125)	29,139 (23,459-41,775)	33,942 (24,615-51,693)	17,635 (14,235-22,354)	12,174 (10,260-14,243)	20,068 (17,512-22,296)	27,462 (24,530-29,855)	38,121 (34,041-41,137)	51,298 (47,295-54,657)	73,250 (67,018-77,949)	92,941 (84,913-98,177)	108,593 (100,069-114,695)	133,271 (122,859-139,702)	151,693 (140,853-158,842)	177,827 (162,288-185,901)	175,018 (159,264-182,694)	159,608 (143,956-167,038)	155,302 (141,462-161,661)	128,768 (118,103-134,191)	80,612 (74,538-83,835)	32,383 (30,236-33,498)	8,842 (8,341-9,122)
Cirrhosis and other chronic liver diseases	-	-	-	13,654 (8,541-24,848)	6,775 (5,102-9,723)	5,358 (4,373-6,592)	8,676 (7,558-9,775)	13,621 (11,971-14,944)	21,272 (18,685-23,091)	32,804 (29,976-35,293)	49,207 (44,682-52,292)	64,713 (58,842-68,657)	75,022 (68,772-79,509)	90,333 (82,267-95,411)	98,733 (90,192-104,498)	107,669 (97,599-114,036)	96,419 (86,499-102,880)	76,428 (67,416-82,424)	63,523 (55,914-68,493)	43,311 (37,309-47,124)	22,074 (18,774-24,058)	7,489 (6,360-8,130)	1,912 (1,630-2,066)
Cirrhosis and other chronic liver diseases due to hepatitis B	-	-	-	441 (252-846)	587 (434-840)	785 (610-1,016)	1,745 (1,326-2,210)	5,626 (4,646-6,631)	10,580 (8,797-12,240)	15,041 (13,149-16,966)	19,607 (16,700-22,456)	23,428 (19,898-26,789)	24,215 (20,497-28,067)	27,090 (22,472-32,087)	29,219 (24,029-34,809)	32,263 (26,090-38,371)	30,011 (24,413-35,884)	24,990 (19,784-29,744)	19,976 (15,890-23,885)	13,110 (10,053-15,949)	6,082 (4,650-7,328)	1,819 (1,344-2,318)	397 (268-543)
Cirrhosis and other chronic liver diseases due to hepatitis C	-	-	-	145 (76-293)	185 (122-284)	273 (196-369)	591 (430-781)	2,310 (1,783-2,878)	4,426 (3,378-5,645)	7,774 (6,479-9,158)	12,661 (10,169-15,574)	17,297 (14,395-20,578)	20,702 (17,156-24,736)	25,090 (20,366-29,795)	26,533 (22,740-30,757)	28,183 (23,518-33,792)	25,142 (21,489-29,273)	19,649 (16,520-23,042)	15,605 (12,691-19,095)	10,520 (8,283-13,073)	5,653 (4,554-6,745)	2,008 (1,586-2,475)	519 (388-667)
Cirrhosis and other chronic liver diseases due to alcohol use	-	-	-	-	-	-	57 (26-102)	1,003 (760-1,289)	2,453 (1,908-3,135)	5,826 (5,034-6,771)	11,685 (9,827-13,783)	17,035 (14,712-19,576)	21,037 (17,989-24,604)	26,053 (22,192-30,232)	28,496 (24,030-33,261)	29,981 (24,730-35,488)	24,453 (20,481-28,543)	17,242 (14,147-20,384)	14,353 (12,078-16,681)	9,305 (7,714-11,142)	4,444 (3,779-5,120)	1,416 (1,143-1,734)	350 (256-462)
Cirrhosis due to NASH	-	-	-	-	-	-	145 (113-188)	395 (314-486)	725 (563-913)	1,407 (1,175-1,646)	2,510 (1,974-3,083)	3,882 (3,234-4,575)	5,479 (4,493-6,509)	7,341 (6,089-8,707)	8,142 (7,022-9,328)	8,863 (7,489-10,287)	7,632 (6,522-8,766)	5,741 (4,870-6,671)	4,320 (3,521-5,246)	2,903 (2,288-3,575)	1,633 (1,303-1,953)	599 (459-759)	163 (116-221)
Cirrhosis and other chronic liver diseases due to other causes	-	-	-	13,068 (8,193-23,815)	6,004 (4,491-8,597)	4,300 (3,484-5,360)	6,138 (5,202-7,099)	4,286 (3,357-5,239)	3,087 (2,285-4,021)	2,756 (2,003-3,576)	2,744 (2,050-3,515)	3,071 (2,282-4,034)	3,590 (2,662-4,650)	4,759 (3,500-6,281)	6,343 (4,391-8,851)	8,378 (5,906-11,310)	9,181 (6,663-12,243)	8,806 (6,744-11,182)	9,268 (6,976-12,091)	7,473 (5,611-9,665)	4,262 (3,409-5,198)	1,646 (1,261-2,028)	483 (337-629)
Upper digestive system diseases	-	-	-	3,025 (2,071-4,320)	2,202 (1,715-2,854)	1,608 (1,328-1,933)	3,194 (2,700-3,740)	5,481 (4,736-6,308)	6,570 (5,671-7,579)	7,838 (6,938-8,808)	10,406 (9,274-11,721)	12,821 (11,428-14,157)	16,082 (14,551-17,687)	21,578 (19,602-23,534)	25,052 (22,693-27,153)	32,647 (29,361-34,840)	35,033 (31,748-37,385)	35,449 (31,564-37,746)	35,598 (32,623-37,895)	30,478 (27,971-32,479)	19,665 (18,323-20,797)	7,706 (7,204-8,105)	2,111 (1,964-2,218)
Peptic ulcer disease	-	-	-	2,298 (1,478-3,594)	1,685 (1,299-2,257)	1,323 (1,094-1,621)	2,718 (2,324-3,229)	4,715 (4,088-5,558)	5,542 (4,865-6,426)	6,561 (5,831-7,447)	8,941 (8,013-10,192)	11,286 (10,160-12,510)	14,199 (12,914-15,788)	19,194 (17,489-21,136)	22,244 (20,371-24,255)	29,291 (26,718-31,510)	31,388 (28,763-33,860)	31,678 (28,733-33,963)	31,864 (29,601-34,012)	27,266 (25,258-29,088)	17,604 (16,481-18,654)	6,869 (6,423-7,224)	1,863 (1,736-1,957)
Gastritis and duodenitis	-	-	-	727 (349-1,181)	516 (276-782)	285 (178-407)	476 (331-634)	765 (496-1,051)	1,028 (691-1,362)	1,277 (906-1,612)	1,465 (1,076-1,833)	1,535 (1,169-1,853)	1,884 (1,455-2,270)	2,384 (1,816-2,918)	2,809 (2,189-3,453)	3,356 (2,497-4,127)	3,644 (2,683-4,493)	3,770 (2,739-4,566)	3,735 (2,708-4,438)	3,213 (2,328-3,814)	2,061 (1,558-2,377)	837 (671-934)	248 (209-271)
Appendicitis	-	-	-	3,723 (1,958-6,174)	2,701 (1,994-3,837)	1,952 (1,572-2,469)	3,073 (2,531-3,835)	2,259 (1,918-2,736)	2,351 (1,983-2,901)	1,423 (1,230-1,696)	1,744 (1,515-2,087)	1,771 (1,514-2,084)	1,737 (1,517-2,023)	1,763 (1,558-1,992)	2,381 (2,119-2,733)	2,714 (2,418-3,014)	2,562 (2,297-2,825)	2,635 (2,283-3,001)	2,611 (2,286-2,975)	2,137 (1,861-2,475)	1,389 (1,200-1,653)	567 (483-689)	135 (115-164)
Paralytic ileus and intestinal obstruction	3,086 (2,732-3,396)	3,947 (3,357-5,125)	29,139 (23,459-41,775)	3,476 (2,548-4,766)	3,081 (1,895-4,070)	1,632 (1,069-1,991)	2,976 (2,031-3,605)	2,735 (1,871-3,276)	3,010 (2,100-3,520)	2,879 (1,972-3,390)	3,686 (2,587-4,322)	4,308 (3,108-5,017)	4,896 (3,451-5,662)	5,647 (4,252-6,426)	7,758 (5,900-8,782)	9,914 (7,871-11,281)	11,419 (9,127-12,994)	11,693 (9,732-13,225)	13,126 (11,182-15,152)	13,088 (11,333-15,174)	10,050 (8,485-11,675)	4,611 (3,909-5,344)	1,296 (1,075-1,610)
Inguinal, femoral, and abdominal hernia	-	-	-	1,137 (743-1,960)	485 (376-672)	255 (197-321)	374 (296-455)	382 (305-463)	449 (366-539)	501 (391-601)	683 (564-824)	738 (630-912)	953 (802-1,147)	1,473 (1,274-1,703)	2,108 (1,741-2,383)	2,930 (2,320-3,283)	3,481 (2,682-3,920)	3,709 (2,889-4,151)	4,423 (3,373-4,920)	4,763 (3,614-5,292)	3,260 (2,385-3,603)	1,416 (1,014-1,560)	383 (268-424)
Inflammatory bowel disease	-	-	-	1,751 (785-3,903)	331 (187-626)	177 (120-272)	230 (170-324)	393 (292-548)	493 (378-673)	527 (423-683)	609 (501-758)	686 (583-810)	827 (700-965)	1,010 (879-1,147)	1,278 (1,118-1,482)	1,649 (1,448-1,876)	1,950 (1,724-2,233)	2,249 (2,003-2,536)	2,783 (2,527-3,095)	2,892 (2,473-3,220)	2,082 (1,744-2,298)	930 (752-1,026)	282 (232-316)
Vascular intestinal disorders	-	-	-	538 (188-809)	107 (57-139)	50 (37-60)	78 (61-90)	161 (123-189)	225 (177-258)	257 (211-290)	420 (349-471)	653 (556-731)	980 (846-1,090)	1,621 (1,457-1,776)	2,531 (2,291-2,740)	4,210 (3,829-4,514)	5,858 (5,375-6,213)	7,190 (6,482-7,653)	9,771 (9,113-10,257)	9,577 (8,951-10,084)	6,504 (5,876-6,820)	2,631 (2,374-2,781)	650 (583-690)
Gallbladder and biliary diseases	-	-	-	1,135 (765-1,593)	436 (342-548)	300 (246-348)	390 (334-434)	633 (534-691)	801 (706-876)	1,065 (927-1,167)	1,410 (1,219-1,523)	1,704 (1,455-1,830)	2,031 (1,779-2,176)	3,088 (2,677-3,299)	4,119 (3,532-4,390)	6,327 (5,293-6,715)	8,010 (6,732-8,497)	9,181 (7,838-9,722)	11,400 (9,824-12,065)	11,133 (9,765-11,835)	7,469 (6,772-7,902)	3,477 (3,193-3,687)	1,015 (919-1,071)
Pancreatitis	-	-	-	181 (102-363)	190 (130-310)	175 (137-241)	465 (391-594)	1,024 (856-1,320)	1,973 (1,688-2,388)	2,840 (2,518-3,280)	3,714 (3,343-4,303)	4,040 (3,618-4,514)	4,238 (3,738-4,710)	4,440 (4,055-5,005)	4,859 (4,331-5,365)	5,326 (4,762-5,848)	5,288 (4,670-5,835)	4,939 (4,163-5,398)	5,149 (4,368-5,561)	4,132 (3,518-4,511)	2,498 (2,100-2,720)	918 (751-1,011)	232 (194-259)
Other digestive diseases	-	-	-	5,322 (3,954-7,926)	1,328 (1,074-1,784)	667 (565-836)	611 (507-764)	773 (665-899)	978 (832-1,159)	1,164 (1,017-1,370)	1,371 (1,182-1,668)	1,507 (1,330-1,762)	1,827 (1,613-2,201)	2,317 (2,057-2,759)	2,874 (2,604-3,302)	4,441 (3,947-5,404)	4,998 (4,597-5,693)	6,137 (5,621-6,920)	6,917 (6,468-7,512)	7,257 (6,750-7,749)	5,622 (5,273-5,902)	2,638 (2,473-2,758)	825 (765-863)
Neurological disorders	-	-	12,733 (8,108-15,635)	17,742 (8,828-23,960)	8,650 (6,267-10,496)	8,472 (6,792-9,712)	13,462 (11,556-14,854)	12,738 (10,855-13,754)	11,034 (9,519-11,947)	10,221 (8,818-10,990)	9,885 (8,661-10,594)	10,047 (9,123-10,631)	10,521 (9,856-11,050)	14,378 (13,821-15,038)	20,476 (19,857-21,373)	37,733 (36,663-39,774)	64,714 (62,919-68,193)	108,810 (105,887-114,849)	208,777 (204,087-217,466)	288,497 (282,369-297,366)	272,497 (267,469-276,335)	160,136 (158,041-162,101)	52,545 (51,846-53,034)
Alzheimer's disease and other dementias	-	-	-	-	-	-	-	-	-	-	-	574 (535-623)	1,408 (1,332-1,525)	4,119 (3,909-4,463)	8,959 (8,557-9,596)	22,616 (21,674-24,071)	43,961 (42,487-46,261)	79,849 (77,513-83,771)	165,540 (162,225-170,369)	245,028 (240,743-249,608)	246,795 (242,491-249,831)	152,141 (150,335-153,764)	51,018 (50,356-51,498)
Parkinson's disease	-	-	-	-	-	-	-	15 (11-17)	20 (16-23)	41 (33-46)	47 (40-53)	435 (393-488)	851 (784-971)	1,636 (1,527-1,841)	2,553 (2,395-2,919)	5,384 (5,062-6,222)	10,741 (10,101-12,854)	20,785 (19,569-24,177)	35,970 (34,035-41,214)	38,133 (36,076-43,706)	22,910 (21,785-25,669)	7,145 (6,822-8,179)	1,319 (1,263-1,473)
Epilepsy	-	-	8,415 (5,150-10,347)	13,980 (6,756-19,623)	7,218 (5,221-8,780)	6,864 (5,456-7,968)	11,021 (9,096-12,299)	10,807 (8,951-11,723)	9,526 (8,060-10,386)	8,571 (7,220-9,297)	7,717 (6,599-8,348)	6,319 (5,495-6,884)	4,938 (4,240-5,432)	4,539 (3,971-4,899)	4,015 (3,541-4,324)	3,967 (3,351-4,362)	3,845 (3,269-4,460)	3,130 (2,673-3,483)	2,508 (2,182-2,802)	1,865 (1,601-2,044)	1,104 (945-1,316)	339 (295-399)	85 (76-97)
Multiple sclerosis	-	-	-	-	-	-	-	142 (103-215)	295 (208-410)	547 (405-721)	888 (670-1,129)	1,186 (927-1,464)	1,295 (1,080-1,556)	1,477 (1,287-1,762)	1,501 (1,318-1,848)	1,479 (1,348-1,840)	1,274 (1,157-1,564)	864 (789-1,093)	691 (631-871)	403 (367-506)	189 (158-214)	48 (40-55)	9 (8-11)
Motor neuron disease	14 (5-21)	30 (13-49)	774 (575-1,248)	255 (152-389)	50 (42-72)	59 (51-90)	93 (84-135)	80 (71-121)	73 (66-100)	120 (110-155)	224 (210-287)	388 (365-465)	632 (589-787)	978 (914-1,121)	1,525 (1,427-1,770)	2,097 (1,984-2,343)	2,569 (2,442-2,798)	2,082 (1,993-2,189)	1,862 (1,777-1,958)	1,019 (967-1,065)	404 (383-426)	84 (80-89)	14 (13-15)
Other neurological disorders	-	-	3,543 (1,783-5,087)	3,508 (1,336-5,397)	1,382 (803-1,988)	1,549 (1,103-2,011)	2,348 (2,029-2,848)	1,695 (1,445-2,043)	1,120 (932-1,349)	943 (783-1,128)	1,009 (890-1,221)	1,146 (1,051-1,380)	1,396 (1,279-1,689)	1,630 (1,500-1,932)	1,923 (1,773-2,202)	2,191 (2,047-2,486)	2,323 (2,188-2,581)	2,100 (1,983-2,303)	2,205 (2,098-2,362)	1,744 (1,604-1,844)	1,094 (890-1,166)	378 (314-401)	101 (89-107)
Mental disorders	-	-	-	-	0 (0-1)	3 (3-4)	18 (15-23)	28 (24-36)	30 (26-38)	29 (25-34)	26 (23-30)	25 (23-29)	21 (19-25)	-	-	-	-	-	-	-	-	-	-
Eating disorders	-	-	-	-	0 (0-1)	3 (3-4)	18 (15-23)	28 (24-36)	30 (26-38)	29 (25-34)	26 (23-30)	25 (23-29)	21 (19-25)	-	-	-	-	-	-	-	-	-	-
Anorexia nervosa	-	-	-	-	0 (0-0)	3 (3-4)	16 (13-21)	18 (14-23)	18 (12-23)	19 (13-23)	19 (14-23)	19 (14-22)	17 (14-20)	-	-	-	-	-	-	-	-	-	-
[illegible]	[illegible]	[illegible]	[illegible]	[illegible]	[illegible]	[illegible]	[illegible]	[illegible]	[illegible]	[illegible]	[illegible]	[illegible]	[illegible]	[illegible]	[illegible]	[illegible]	[illegible]	[illegible]	[illegible]	[illegible]	[illegible]	[illegible]	[illegible]

SR Table 7: Global deaths by cause and age group for both sexes combined (with 95% confidence intervals), 1990

Cause	Early Neonatal	Late Neonatal	Post Neonatal	1 to 4	5 to 9	10 to 14	15 to 19	20 to 24	25 to 29	30 to 34	35 to 39	40 to 44	45 to 49	50 to 54	54 to 59	60 to 64	65 to 69	70 to 74	75 to 79	80 to 84	85 to 89	90 to 94	95 plus
Urolithiasis	-	-	-	-	114 (52-177)	73 (36-107)	71 (34-102)	139 (67-203)	192 (96-275)	248 (142-341)	285 (172-376)	357 (226-443)	446 (304-544)	656 (512-779)	785 (623-923)	1,187 (946-1,418)	1,401 (1,114-1,670)	1,350 (1,020-1,568)	1,475 (1,196-1,754)	1,088 (848-1,252)	568 (424-651)	221 (172-254)	53 (42-60)
Other urinary diseases	747 (578-896)	713 (475-907)	2,755 (1,475-3,954)	3,671 (2,228-5,073)	1,050 (724-1,438)	574 (431-743)	628 (417-841)	831 (571-1,100)	707 (518-953)	913 (699-1,162)	997 (743-1,248)	1,180 (891-1,472)	1,563 (1,211-1,925)	1,856 (1,389-2,297)	2,160 (1,659-2,656)	3,028 (2,330-3,756)	3,418 (2,628-4,201)	3,836 (2,917-4,687)	3,136 (2,381-3,804)	3,045 (2,361-3,676)	1,626 (1,282-1,950)	711 (547-850)	246 (177-291)
Gynecological diseases	-	-	-	-	-	-	468 (359-607)	498 (369-635)	480 (363-608)	514 (387-623)	557 (439-639)	564 (454-631)	636 (521-741)	499 (409-583)	336 (280-390)	276 (240-320)	321 (274-379)	201 (177-231)	177 (161-195)	286 (239-341)	185 (157-225)	69 (60-82)	23 (20-27)
Uterine fibroids	-	-	-	-	-	-	78 (49-104)	101 (60-155)	123 (75-185)	125 (79-187)	145 (97-199)	170 (115-214)	200 (153-259)	178 (141-255)	79 (64-119)	70 (56-94)	70 (55-103)	45 (35-71)	36 (29-51)	72 (49-114)	52 (33-80)	23 (16-33)	8 (5-11)
Polycystic ovarian syndrome	-	-	-	-	-	-	1 (0-4)	2 (1-6)	2 (0-4)	1 (0-3)	1 (0-2)	1 (0-2)	1 (0-2)	1 (0-2)	-	-	-	-	-	-	-	-	-
Endometriosis	-	-	-	-	-	-	9 (4-14)	11 (5-17)	11 (5-18)	16 (8-26)	15 (7-25)	18 (8-30)	23 (9-41)	16 (8-29)	-	-	-	-	-	-	-	-	-
Genital prolapse	-	-	-	-	-	-	18 (10-29)	13 (7-20)	15 (6-24)	16 (7-25)	13 (5-22)	24 (7-43)	30 (7-54)	61 (17-102)	49 (17-86)	53 (26-86)	87 (41-135)	66 (37-94)	57 (31-76)	76 (43-120)	45 (28-69)	17 (10-23)	6 (4-8)
Other gynecological diseases	-	-	-	-	-	-	362 (267-515)	371 (259-536)	330 (229-482)	357 (255-467)	383 (284-516)	350 (262-459)	382 (292-479)	243 (180-327)	208 (163-266)	153 (121-206)	164 (123-229)	89 (67-122)	84 (69-113)	138 (107-181)	88 (70-114)	30 (25-39)	9 (8-12)
Hemoglobinopathies and hemolytic anemias	1,226 (930-1,568)	1,026 (767-1,302)	7,931 (4,896-11,571)	18,438 (9,036-28,669)	6,036 (4,282-7,641)	4,694 (3,655-5,853)	5,685 (4,110-7,269)	5,230 (3,643-6,648)	3,909 (2,816-4,820)	3,333 (2,384-4,063)	2,721 (1,953-3,432)	2,619 (1,929-3,298)	2,359 (1,721-3,030)	2,911 (2,075-3,816)	3,305 (2,374-4,323)	3,832 (2,811-4,913)	4,394 (3,284-5,709)	4,725 (3,543-6,050)	4,978 (3,760-6,135)	4,691 (3,523-5,565)	3,566 (2,591-4,205)	1,662 (1,204-1,955)	509 (370-590)
Thalassemias	541 (421-649)	463 (325-681)	3,525 (2,335-5,261)	5,043 (2,884-7,713)	2,394 (1,731-3,197)	1,312 (1,031-1,604)	972 (735-1,159)	729 (507-886)	417 (289-519)	211 (147-273)	94 (70-116)	53 (43-65)	32 (27-40)	19 (16-23)	10 (9-13)	5 (4-6)	2 (2-3)	1 (1-1)	0 (0-0)	0 (0-0)	0 (0-0)	0 (0-0)	0 (0-0)
Sickle cell disorders	598 (387-948)	533 (369-709)	4,173 (2,352-5,987)	12,809 (5,627-20,117)	3,090 (2,029-4,175)	2,516 (1,680-3,535)	3,422 (2,216-4,821)	2,922 (1,795-4,249)	1,937 (1,199-2,803)	1,369 (828-2,062)	973 (529-1,597)	750 (408-1,294)	490 (257-896)	343 (188-649)	189 (110-363)	73 (47-119)	34 (23-50)	12 (7-17)	4 (2-6)	1 (1-2)	1 (0-1)	0 (0-0)	0 (0-0)
G6PD deficiency	16 (10-26)	14 (9-21)	125 (70-197)	382 (193-650)	337 (228-488)	506 (355-675)	728 (535-940)	837 (634-1,042)	844 (649-1,046)	921 (704-1,119)	815 (617-995)	814 (604-1,031)	815 (565-1,110)	952 (571-1,442)	873 (497-1,425)	736 (415-1,195)	552 (299-936)	347 (182-600)	196 (106-333)	84 (48-133)	33 (20-51)	8 (5-12)	1 (1-2)
Other hemoglobinopathies and hemolytic anemias	71 (54-93)	17 (12-22)	107 (70-160)	204 (118-311)	215 (165-275)	360 (300-437)	562 (443-682)	743 (598-872)	711 (577-827)	833 (650-965)	839 (669-971)	1,002 (818-1,157)	1,022 (814-1,202)	1,598 (1,239-1,926)	2,232 (1,729-2,756)	3,018 (2,317-3,737)	3,805 (2,901-4,824)	4,365 (3,315-5,524)	4,777 (3,635-5,857)	4,606 (3,461-5,453)	3,532 (2,568-4,163)	1,653 (1,199-1,943)	508 (369-589)
Endocrine, metabolic, blood, and immune disorders	1,913 (1,213-2,224)	1,972 (1,148-2,551)	10,740 (6,172-14,647)	6,108 (3,201-9,354)	2,316 (1,650-2,982)	1,639 (1,353-1,963)	1,668 (1,410-1,988)	1,981 (1,616-2,278)	2,244 (1,747-2,584)	2,507 (2,054-2,913)	2,615 (2,175-3,054)	2,614 (2,247-3,126)	2,951 (2,555-3,535)	3,716 (3,175-4,355)	3,992 (3,467-4,789)	5,257 (4,579-6,241)	5,869 (5,039-6,954)	5,617 (4,827-6,714)	6,026 (4,999-6,998)	5,359 (4,273-6,321)	3,944 (3,102-4,620)	1,714 (1,387-2,103)	544 (445-700)
Sudden infant death syndrome	-	6,982 (2,598-14,167)	57,662 (23,370-124,017)	-	-	-	-	-	-	-	-	-	-	-	-	-	-	-	-	-	-	-	-
Injuries	19,970 (17,116-22,332)	19,514 (16,196-22,427)	157,632 (136,023-175,776)	437,922 (348,740-498,983)	239,658 (214,162-258,843)	161,331 (147,684-171,029)	300,823 (284,565-312,332)	403,155 (387,544-414,450)	363,925 (351,798-374,018)	313,791 (303,021-322,939)	280,288 (271,034-288,994)	230,286 (221,893-237,774)	194,961 (186,880-201,689)	191,583 (183,074-198,833)	170,776 (162,582-177,278)	165,382 (156,292-171,196)	146,117 (137,287-151,983)	129,327 (120,709-134,713)	124,971 (117,716-130,233)	104,922 (98,866-109,490)	69,032 (64,913-71,926)	27,630 (26,211-28,755)	7,499 (7,183-7,742)
Transport injuries	2,624 (2,311-3,192)	3,517 (2,762-4,150)	24,190 (18,480-27,112)	90,906 (59,342-107,011)	71,804 (62,826-79,419)	44,839 (40,172-48,991)	104,415 (97,176-111,107)	137,965 (129,813-145,840)	122,756 (116,722-129,420)	102,643 (97,620-108,034)	92,983 (88,536-98,070)	76,279 (72,942-80,766)	62,495 (59,755-66,212)	60,705 (58,168-64,859)	53,221 (51,022-56,983)	48,283 (46,322-51,638)	40,417 (38,746-43,442)	32,900 (31,573-35,328)	26,751 (25,750-28,488)	17,298 (16,705-18,447)	8,070 (7,782-8,597)	2,413 (2,331-2,566)	572 (554-598)
Road injuries	2,206 (1,935-2,703)	3,110 (2,414-3,712)	22,329 (16,900-25,038)	85,452 (55,142-101,214)	68,515 (59,987-75,862)	41,964 (37,511-46,003)	97,564 (90,801-103,814)	130,048 (122,012-137,599)	114,986 (109,096-121,356)	96,403 (91,486-101,500)	86,875 (82,541-91,787)	70,970 (67,676-75,065)	57,806 (55,066-61,193)	56,201 (53,698-59,828)	49,609 (47,467-52,923)	45,213 (43,316-48,123)	38,045 (36,425-40,758)	31,178 (29,892-33,313)	25,328 (24,343-26,930)	16,410 (15,827-17,467)	7,674 (7,397-8,153)	2,294 (2,215-2,436)	538 (521-562)
Pedestrian road injuries	1,222 (1,000-1,665)	1,662 (1,233-2,263)	10,596 (8,481-12,943)	54,831 (39,000-68,024)	44,681 (39,148-50,392)	22,999 (20,230-25,838)	29,320 (25,799-33,600)	37,988 (32,928-42,935)	35,202 (30,776-39,693)	33,704 (29,814-37,417)	32,359 (27,810-36,149)	27,679 (24,122-30,863)	24,491 (21,701-27,266)	25,570 (22,751-28,677)	23,505 (21,163-26,184)	24,223 (22,278-26,659)	20,642 (18,908-22,775)	17,581 (16,300-19,530)	15,260 (14,330-16,754)	10,059 (9,499-11,029)	4,692 (4,414-5,159)	1,298 (1,228-1,421)	287 (274-310)
Cyclist road injuries	-	-	-	1,607 (809-2,509)	2,467 (1,798-3,318)	2,460 (2,047-3,031)	3,387 (2,863-4,020)	2,997 (2,516-3,595)	2,766 (2,314-3,352)	2,836 (2,355-3,440)	2,707 (2,204-3,265)	2,766 (2,239-3,316)	2,576 (2,156-3,050)	2,666 (2,282-3,102)	2,304 (1,997-2,666)	2,345 (2,001-2,733)	2,083 (1,809-2,382)	1,653 (1,452-1,894)	1,211 (1,079-1,355)	686 (621-766)	254 (230-284)	62 (54-71)	1 (0-1)
Motorcyclist road injuries	155 (126-224)	191 (120-286)	2,021 (1,488-2,833)	4,106 (2,365-6,617)	3,432 (2,713-4,692)	3,989 (3,265-5,145)	22,578 (19,846-27,384)	30,442 (26,961-36,726)	25,280 (22,542-30,640)	18,803 (16,611-22,503)	16,016 (13,933-19,312)	12,590 (11,117-15,331)	9,010 (7,841-11,050)	7,483 (6,485-9,287)	5,911 (5,142-7,503)	3,631 (3,100-4,640)	2,789 (2,392-3,600)	1,934 (1,651-2,462)	1,061 (909-1,370)	627 (532-815)	224 (186-304)	56 (46-74)	14 (12-18)
Motor vehicle road injuries	794 (606-982)	1,219 (730-1,642)	9,376 (6,000-11,399)	24,204 (11,790-32,448)	17,360 (13,560-21,373)	12,075 (10,133-13,827)	41,517 (37,503-45,321)	57,599 (52,659-62,670)	50,834 (47,059-55,556)	40,333 (37,128-44,459)	35,109 (31,947-38,774)	27,358 (25,373-30,460)	21,091 (19,240-23,859)	19,834 (18,187-22,550)	17,343 (15,968-19,831)	14,679 (13,541-16,790)	12,197 (11,199-13,943)	9,740 (8,916-11,136)	7,530 (7,005-8,526)	4,861 (4,535-5,495)	2,419 (2,259-2,744)	856 (803-953)	229 (216-245)
Other road injuries	35 (19-57)	37 (15-65)	337 (150-550)	704 (231-1,295)	575 (299-806)	442 (292-578)	761 (521-1,087)	1,022 (690-1,419)	904 (609-1,251)	726 (511-967)	685 (483-911)	576 (401-763)	637 (451-831)	648 (463-851)	546 (396-721)	334 (240-448)	333 (234-461)	270 (188-384)	265 (197-352)	176 (130-251)	86 (63-123)	22 (17-32)	7 (6-9)
Other transport injuries	418 (302-593)	407 (256-621)	1,862 (1,494-2,488)	5,453 (4,063-7,969)	3,289 (2,711-3,928)	2,874 (2,451-3,261)	6,852 (6,098-7,653)	7,917 (7,211-8,687)	7,770 (7,149-8,354)	6,240 (5,759-6,737)	6,109 (5,617-6,651)	5,309 (4,920-5,893)	4,689 (4,337-5,144)	4,504 (4,153-5,070)	3,612 (3,327-4,083)	3,070 (2,847-3,450)	2,372 (2,201-2,693)	1,722 (1,603-1,970)	1,423 (1,324-1,633)	888 (820-1,000)	396 (361-448)	119 (108-135)	34 (32-38)
Unintentional injuries	13,788 (11,480-15,721)	14,566 (11,862-16,868)	123,883 (107,608-139,663)	321,910 (263,174-371,799)	151,646 (134,830-165,313)	88,935 (81,035-95,001)	84,416 (78,665-88,925)	93,347 (87,984-97,721)	83,523 (78,784-87,078)	78,705 (74,438-81,850)	76,249 (72,248-79,149)	67,298 (63,336-69,966)	63,773 (59,901-66,171)	66,415 (62,286-68,812)	64,278 (59,621-66,697)	69,394 (63,785-72,433)	66,366 (60,614-69,667)	63,766 (57,970-67,233)	70,507 (64,472-74,351)	69,197 (63,931-73,002)	52,090 (48,524-54,593)	22,915 (21,567-24,035)	6,476 (6,177-6,713)
Falls	1,872 (1,420-2,214)	963 (687-1,332)	11,425 (8,840-14,618)	19,676 (13,534-28,783)	10,189 (7,877-13,973)	6,934 (5,633-8,502)	8,206 (7,128-9,749)	11,381 (9,792-13,270)	11,770 (10,091-13,927)	12,561 (11,015-14,550)	13,776 (12,170-15,736)	13,907 (12,095-16,125)	14,360 (12,876-16,565)	16,222 (14,656-18,549)	18,637 (16,595-20,749)	23,579 (21,252-26,264)	26,341 (23,486-28,743)	29,271 (25,814-31,864)	37,943 (34,336-40,646)	41,463 (37,485-44,431)	33,423 (30,670-35,511)	15,400 (14,376-16,350)	4,312 (4,074-4,520)
Drowning	753 (675-831)	672 (563-744)	17,298 (15,042-18,999)	190,366 (157,770-217,515)	90,442 (80,842-99,481)	45,550 (41,711-49,239)	31,909 (29,412-34,284)	27,398 (25,486-29,291)	20,134 (18,816-21,360)	17,145 (16,152-18,088)	14,919 (14,118-15,699)	12,326 (11,711-12,936)	10,228 (9,718-10,694)	10,424 (9,933-10,864)	8,605 (8,210-8,956)	8,649 (8,204-9,008)	7,295 (6,930-7,612)	6,315 (5,980-6,634)	5,152 (4,860-5,464)	3,911 (3,688-4,177)	1,890 (1,776-2,016)	486 (460-517)	91 (86-96)
Fire, heat, and hot substances	572 (410-694)	784 (418-1,021)	10,576 (6,217-13,140)	28,193 (14,637-37,437)	7,927 (5,539-9,299)	4,402 (3,283-5,136)	7,031 (4,910-8,450)	9,200 (6,585-11,029)	8,311 (6,313-9,731)	7,884 (6,216-8,998)	6,850 (5,603-7,746)	5,474 (4,629-6,056)	5,128 (4,365-5,697)	5,311 (4,616-5,863)	4,925 (4,264-5,489)	5,350 (4,627-5,928)	5,405 (4,628-6,040)	5,296 (4,515-5,971)	5,800 (5,061-6,383)	5,720 (5,065-6,282)	4,143 (3,748-4,490)	1,693 (1,567-1,815)	509 (481-536)
Poisonings	411 (271-484)	467 (296-604)	5,307 (3,522-6,944)	12,930 (8,066-17,799)	4,093 (2,943-5,331)	3,266 (2,523-4,251)	3,895 (3,262-5,042)	5,231 (4,281-6,811)	5,000 (4,201-6,360)	4,537 (3,885-5,660)	4,603 (4,066-5,416)	4,356 (3,868-5,099)	3,629 (3,253-4,291)	4,212 (3,900-4,850)	3,502 (3,225-4,154)	3,404 (3,083-4,034)	2,543 (2,292-3,189)	2,129 (1,893-2,747)	1,787 (1,635-2,221)	1,335 (1,230-1,652)	673 (626-806)	191 (180-217)	41 (38-44)
Poisoning by carbon monoxide	97 (62-114)	117 (75-145)	1,275 (902-1,588)	3,054 (2,397-3,788)	1,893 (1,384-2,606)	1,395 (1,094-1,900)	2,133 (1,773-2,883)	2,909 (2,364-3,869)	2,758 (2,303-3,627)	2,694 (2,328-3,410)	2,809 (2,525-3,362)	2,709 (2,455-3,173)	2,245 (2,057-2,651)	2,747 (2,581-3,132)	2,181 (2,034-2,565)	2,089 (1,922-2,485)	1,436 (1,283-1,850)	1,178 (1,030-1,577)	1,128 (1,017-1,451)	819 (737-1,042)	413 (378-513)	107 (99-128)	18 (17-21)
Poisoning by other means	315 (207-376)	350 (223-461)	4,032 (2,558-5,449)	9,876 (5,586-14,185)	2,200 (1,528-2,853)	1,871 (1,420-2,356)	1,762 (1,452-2,200)	2,322 (1,893-2,961)	2,242 (1,853-2,762)	1,843 (1,526-2,279)	1,794 (1,515-2,129)	1,647 (1,377-1,947)	1,384 (1,163-1,672)	1,465 (1,255-1,770)	1,321 (1,159-1,615)	1,314 (1,129-1,593)	1,106 (978-1,368)	951 (854-1,218)	659 (596-829)	515 (467-624)	260 (233-303)	84 (75-91)	22 (21-24)
Exposure to mechanical forces	1,296 (1,075-1,489)	1,768 (1,297-2,073)	7,889 (6,750-8,928)	13,795 (10,339-16,192)	8,044 (6,953-9,058)	5,254 (4,696-5,803)	8,853 (8,012-9,994)	11,882 (10,870-13,600)	11,341 (10,491-12,934)	10,574 (9,879-11,690)	10,445 (9,702-11,694)	8,255 (7,716-9,339)	7,897 (7,356-8,798)	7,427 (6,972-8,190)	6,401 (5,990-7,124)	5,237 (4,888-5,761)	4,195 (3,918-4,643)	3,335 (3,077-3,715)	2,812 (2,596-3,107)	2,179 (2,022-2,380)	1,498 (1,415-1,651)	659 (623-716)	203 (193-219)
Unintentional firearm injuries	155 (111-198)	157 (104-260)	1,202 (819-1,594)	1,865 (1,196-3,314)	1,253 (979-1,457)	1,234 (1,077-1,371)	2,427 (2,109-2,731)	3,318 (2,760-3,791)	2,969 (2,454-3,412)	2,322 (1,927-2,649)	1,947 (1,621-2,223)	1,506 (1,243-1,725)	1,507 (1,233-1,726)	1,357 (1,105-1,576)	1,005 (864-1,160)	852 (738-991)	731 (635-852)	625 (535-721)	642 (562-737)	503 (447-588)	329 (293-407)	138 (122-176)	41 (36-52)
Other exposure to mechanical forces	1,141 (931-1,317)	1,611 (1,159-1,914)	6,687 (5,602-7,596)	11,930 (8,754-14,180)	6,791 (5,761-7,705)	4,020 (3,557-4,481)	6,426 (5,723-7,369)	8,563 (7,676-10,241)	8,372 (7,613-9,989)	8,252 (7,630-9,523)	8,497 (7,807-9,877)	6,749 (6,249-7,935)	6,390 (5,917-7,161)	6,071 (5,634-6,756)	5,396 (5,001-6,010)	4,385 (4,060-4,788)	3,464 (3,194-3,833)	2,710 (2,471-3,024)	2,170 (1,987-2,408)	1,676 (1,549-1,834)	1,169 (1,105-1,272)	521 (493-559)	162 (154-174)
Adverse effects of medical treatment	1,751 (1,134-2,119)	1,436 (831-1,945)	10,660 (5,914-15,123)	7,731 (3,268-13,887)	2,867 (1,773-3,656)	1,893 (1,365-2,232)	2,363 (1,746-2,759)	3,746 (2,771-4,297)	3,598 (2,650-4,084)	3,279 (2,468-3,710)	3,467 (2,657-3,938)	3,587 (2,760-4,050)	3,621 (2,843-4,084)	3,466 (2,732-3,864)	4,254 (3,349-4,758)	6,054 (4,740-6,819)	6,487 (5,154-7,262)	5,954 (4,725-6,658)	6,327 (5,135-6,980)	5,755 (4,692-6,338)	4,458 (3,637-4,872)	1,955 (1,656-2,117)	568 (489-610)
Animal contact	474 (174-625)	584 (185-881)	3,917 (1,613-5,353)	10,753 (4,168-18,378)	10,009 (4,410-14,670)	6,480 (3,130-8,522)	4,516 (2,192-5,766)	4,242 (2,003-5,288)	3,932 (1,894-4,837)	3,955 (1,895-4,887)	3,653 (1,772-4,472)	4,093 (1,878-5,038)	4,607 (2,170-5,630)	4,105 (2,084-5,001)	3,978 (1,965-4,786)	3,957 (1,943-4,789)	3,648 (1,706-4,542)	2,917 (1,452-3,641)	2,232 (1,100-2,838)	1,276 (666-1,615)	544 (319-675)	151 (95-186)	32 (24-38)
Venomous animal contact	387 (122-536)	465 (123-716)	3,076 (1,220-4,153)	8,604 (3,237-13,721)	8,662 (3,705-12,545)	5,883 (2,773-7,790)	3,975 (1,866-5,052)	3,614 (1,632-4,567)	3,283 (1,514-4,082)	3,499 (1,652-4,378)	3,209 (1,530-3,971)	3,675 (1,673-4,539)	4,102 (1,872-5,036)	3,568 (1,766-4,392)	3,436 (1,659-4,131)	3,466 (1,662-4,200)	3,181 (1,469-3,928)	2,525 (1,232-3,138)	1,900 (903-2,398)	1,058 (533-1,343)	428 (245-541)	112 (68-142)	22 (16-27)
Non-venomous animal contact	86 (44-142)	119 (54-205)	841 (356-1,814)	2,149 (697-6,111)	1,347 (591-3,160)	596 (328-1,155)	540 (313-988)	628 (340-1,044)	649 (369-906)	456 (231-649)	445 (229-603)	417 (206-583)	505 (273-698)	538 (301-807)	543 (302-772)	492 (260-684)	467 (243-660)	392 (204-584)	332 (179-494)	217 (124-311)	115 (70-161)	38 (25-51)	10 (7-13)
Foreign body	5,590 (4,473-6,536)	7,164 (5,607-8,422)	49,075 (41,480-55,352)	19,959 (14,504-23,223)	4,949 (4,491-5,565)	3,166 (2,934-3,431)	2,030 (1,924-2,206)	2,740 (2,605-2,994)	2,862 (2,743-3,138)	2,916 (2,809-3,140)	3,077 (2,974-3,264)	3,011 (2,924-3,197)	2,806 (2,725-2,996)	3,410 (3,304-3,555)	3,299 (3,202-3,421)	3,440 (3,332-3,559)	3,230 (3,121-3,344)	3,177 (3,052-3,297)	3,713 (3,595-3,840)	3,801 (3,682-3,941)	3,017 (2,914-3,130)	1,403 (1,351-1,457)	453 (438-468)
Pulmonary aspiration and foreign body in airway	5,373 (4,304-6,311)	6,896 (5,447-8,127)	44,879 (37,528-51,897)	16,737 (12,199-20,408)	4,170 (3,733-4,770)	2,788 (2,585-3,045)	1,690 (1,585-1,876)	2,299 (2,163-2,565)	2,411 (2,290-2,682)	2,439 (2,340-2,653)	2,578 (2,479-2,766)	2,565 (2,479-2,731)	2,520 (2,438-2,682)	3,098 (2,994-3,228)	3,018 (2,921-3,137)	3,148 (3,046-3,254)	2,994 (2,889-3,109)	2,911 (2,792-3,045)	3,422 (3,305-3,561)	3,527 (3,410-3,677)	2,832 (2,725-2,952)	1,329 (1,276-1,385)	431 (416-447)
Foreign body in other body part	217 (109-285)	268 (94-388)	4,196 (1,356-5,651)	3,222 (1,436-4,770)	780 (494-962)	379 (264-438)	340 (253-386)	441 (327-508)	451 (337-511)	476 (388-539)	499 (404-557)	446 (366-500)	286 (230-320)	312 (254-361)	281 (226-323)	291 (232-335)	236 (177-272)	266 (195-303)	292 (214-327)	274 (199-307)	185 (134-210)	74 (56-83)	22 (18-27)
Environmental heat and cold exposure	487 (200-655)	358 (127-591)	3,791 (1,371-5,419)	2,159 (823-3,816)	1,072 (582-1,370)	666 (432-794)	1,503 (1,063-1,754)	2,121 (1,479-2,435)	2,544 (1,909-2,881)	3,429 (2,666-3,845)	3,975 (3,146-4,464)	4,057 (3,167-4,609)	3,898 (3,058-4,508)	5,212 (4,318-5,918)	4,633 (3,650-5,211)	4,546 (3,524-5,155)	3,333 (2,371-3,812)	2,787 (1,892-3,202)	2,936 (2,022-3,371)	2,464 (1,726-2,784)	1,684 (1,308-1,869)	678 (558-739)	185 (164-200)
Exposure to forces of nature	43 (26-69)	118 (70-193)	968 (604-1,529)	5,067 (3,057-8,113)	5,495 (3,326-8,679)	5,839 (3,566-9,574)	5,222 (3,158-8,568)	4,755 (2,814-8,104)	4,095 (2,535-6,632)	3,245 (1,955-5,136)	2,621 (1,575-4,264)	2,144 (1,389-3,503)	1,729 (1,072-2,712)	1,784 (1,130-2,847)	1,801 (1,102-2,896)	1,692 (1,063-2,726)	1,367 (850-2,271)	1,088 (690-1,734)	547 (376-798)	310 (224-452)	216 (143-340)	82 (51-131)	14 (9-21)
Other unintentional injuries	538 (467-627)	252 (205-294)	2,977 (2,602-3,319)	11,282 (9,136-13,090)	6,559 (5,525-7,594)	5,483 (4,728-6,151)	8,888 (7,925-9,733)	10,653 (9,705-11,443)	9,936 (9,196-10,592)	9,180 (8,526-9,761)	8,863 (8,287-9,436)	6,088 (5,727-6,419)	5,871 (5,444-6,217)	4,841 (4,557-5,117)	4,244 (4,013-4,474)	3,486 (3,278-3,681)	2,521 (2,384-2,672)	1,496 (1,409-1,577)	1,259 (1,196-1,339)	984 (932-1,063)	544 (514-588)	216 (204-230)	68 (64-72)
Self-harm and interpersonal violence	3,557 (2,948-3,891)	1,430 (1,200-1,543)	9,558 (7,875-10,585)	25,106 (21,957-27,689)	16,208 (14,363-17,723)	27,558 (24,990-29,532)	111,992 (104,456-117,500)	171,843 (159,600-179,084)	157,645 (146,669-164,700)	132,443 (122,054-138,522)	111,056 (101,768-116,221)	86,709 (79,230-90,727)	68,693 (62,339-72,207)	64,463 (59,264-67,537)	53,277 (48,458-55,680)	47,705 (43,047-50,015)	39,334 (35,208-41,495)	32,660 (29,338-34,354)	27,713 (25,680-29,056)	18,427 (17,059-19,259)	8,872 (8,356-9,249)	2,302 (2,196-2,376)	451 (434-464)
Self-harm	-	-	-	-	-	12,737 (10,567-14,203)	65,284 (59,306-69,655)	99,383 (89,283-105,003)	93,670 (84,021-99,199)	78,910 (70,165-83,670)	69,585 (62,079-73,839)	56,847 (50,529-60,161)	47,541 (42,299-50,575)	46,744 (41,890-49,399)	40,136 (35,966-42,402)	37,164 (32,939-39,197)	31,821 (28,184-33,769)	27,366 (24,137-28,918)	23,738 (21,695-25,009)	16,949 (15,601-17,758)	8,168 (7,639-8,528)	2,117 (2,010-2,188)	360 (345-371)
Self-harm by firearm	-	-	-	-	-	547 (419-765)	4,868 (4,009-6,118)	7,563 (6,249-9,658)	6,634 (5,484-8,300)	5,861 (4,844-7,514)	5,075 (4,205-6,433)	4,393 (3,644-5,528)	3,738 (3,040-4,647)	3,525 (2,862-4,382)	3,221 (2,634-3,968)	3,084 (2,567-3,812)	2,841 (2,407-3,460)	2,455 (2,156-2,977)	2,347 (2,076-2,907)	1,721 (1,501-2,219)	877 (745-1,168)	262 (228-342)	51 (46-62)
Self-harm by other specified means	-	-	-	-	-	12,190 (9,988-13,700)	60,416 (54,858-64,857)	91,820 (82,574-97,645)	87,036 (77,508-92,395)	73,048 (64,463-77,648)	64,510 (56,706-68,674)	52,453 (46,332-55,724)	43,803 (38,736-46,749)	43,220 (38,561-45,865)	36,915 (32,709-39,205)	34,080 (29,936-36,201)	28,979 (25,483-30,886)	24,911 (21,804-26,530)	21,391 (19,317-22,760)	15,228 (13,729-16,152)	7,291 (6,653-7,722)	1,855 (1,720-1,946)	309 (291-321)
Interpersonal violence	2,433 (1,823-2,767)	823 (593-937)	7,568 (5,891-8,588)	12,669 (9,519-15,235)	8,798 (6,955-10,317)	8,294 (7,069-9,519)	33,563 (29,680-36,653)	53,471 (47,420-58,456)	50,793 (45,469-54,260)	44,023 (39,516-46,817)	34,818 (30,773-37,245)	25,542 (22,808-27,319)	18,215 (15,998-19,657)	15,512 (13,700-16,628)	11,365 (9,895-12,410)	9,111 (7,991-9,787)	6,381 (5,532-6,961)	4,473 (3,943-4,960)	3,439 (3,112-3,775)	1,129 (1,052-1,205)	529 (501-567)	132 (126-142)	55 (50-58)
Physical violence by firearm	195 (128-260)	81 (53-106)	861 (653-1,165)	1,381 (945-2,081)	1,030 (855-1,361)	1,870 (1,644-2,217)	13,997 (12,326-15,282)	22,669 (19,618-24,717)	20,790 (17,896-22,448)	16,568 (13,954-17,942)	12,838 (10,514-13,927)	8,851 (7,225-9,765)	5,848 (4,767-6,400)	4,498 (3,625-5,025)	3,239 (2,589-3,717)	2,254 (1,844-2,534)	1,481 (1,210-1,689)	1,007 (804-1,174)	664 (538-768)	165 (138-187)	72 (63-83)	20 (17-23)	12 (9-13)
Physical violence by sharp object	162 (115-202)	84 (55-112)	725 (473-1,037)	1,605 (897-2,669)	1,406 (882-1,989)	1,521 (1,083-1,889)	8,226 (6,267-9,905)	14,225 (11,319-16,820)	14,585 (11,662-17,332)	12,985 (10,526-15,338)	10,092 (8,228-12,186)	7,463 (6,071-8,701)	5,225 (4,183-6,247)	4,358 (3,536-5,092)	2,952 (2,418-3,441)	2,558 (2,099-3,012)	1,659 (1,320-1,917)	1,188 (965-1,394)	927 (802-1,060)	272 (248-310)	118 (108-135)	30 (28-34)	14 (12-16)
Physical violence by other means	2,076 (1,528-2,383)	658 (458-749)	5,982 (4,549-6,855)	9,684 (7,221-11,924)	6,361 (4,953-7,605)	4,904 (3,950-5,847)	11,341 (9,452-13,576)	16,577 (14,174-19,858)	15,417 (13,126-18,227)	14,470 (12,396-17,013)	11,889 (10,346-13,817)	9,228 (8,004-10,816)	7,142 (6,027-8,378)	6,655 (5,749-7,697)	5,173 (4,448-5,923)	4,300 (3,758-4,931)	3,241 (2,779-3,707)	2,278 (1,961-2,664)	1,847 (1,633-2,087)	692 (641-754)	338 (315-364)	82 (78-90)	29 (27-32)
Conflict and terrorism	1,125 (1,105-1,150)	606 (595-620)	1,935 (1,900-1,978)	12,361 (12,144-12,638)	7,323 (7,187-7,478)	6,447 (6,327-6,579)	12,798 (12,534-13,110)	17,781 (17,419-18,263)	11,753 (11,508-12,048)	8,382 (8,195-8,587)	5,788 (5,661-5,942)	3,816 (3,735-3,907)	2,623 (2,570-2,684)	2,007 (1,966-2,056)	1,661 (1,624-1,698)	1,343 (1,316-1,375)	1,075 (1,051-1,100)	783 (766-800)	511 (498-526)	323 (314-334)	161 (156-168)	48 (46-50)	35 (33-36)
Executions and police conflict	-	-	55 (28-72)	76 (51-94)	87 (68-102)	81 (68-92)	347 (218-438)	1,209 (595-1,546)	1,429 (828-1,809)	1,128 (559-1,382)	864 (447-1,074)	504 (276-615)	314 (163-385)	200 (135-242)	115 (77-142)	87 (63-103)	58 (37-69)	38 (29-46)	26 (20-31)	26 (22-31)	14 (11-17)	5 (4-6)	2 (1-2)

SR Table 7: Global deaths by cause and age group for both sexes combined (with 95% confidence intervals), 1995

Cause	Early Neonatal	Late Neonatal	Post Neonatal	1 to 4	5 to 9	10 to 14	15 to 19	20 to 24	25 to 29	30 to 34	35 to 39
All causes	2,901,923 (2,835,004-2,971,017)	1,072,883 (1,039,173-1,111,065)	3,476,737 (3,410,452-3,550,653)	3,307,355 (3,215,104-3,400,091)	834,537 (826,006-842,992)	469,641 (466,119-473,428)	647,362 (640,985-654,603)	926,070 (914,851-937,626)	1,083,981 (1,067,644-1,101,703)	1,176,353 (1,161,080-1,190,738)	1,210,324 (1,193,621-1,226,871)
Communicable, maternal, neonatal, and nutritional diseases	2,656,178 (2,576,113-2,722,620)	945,795 (909,461-983,080)	2,883,239 (2,787,057-2,982,859)	2,625,583 (2,509,685-2,763,082)	445,288 (416,548-477,556)	198,270 (184,019-216,092)	203,028 (189,606-221,449)	291,044 (277,113-308,394)	367,079 (348,802-387,462)	391,648 (375,915-409,687)	366,532 (349,821-385,122)
HIV/AIDS and sexually transmitted infections	-	23,123 (8,745-43,142)	130,877 (106,807-164,163)	68,913 (63,713-74,327)	10,913 (9,601-12,516)	2,069 (1,573-2,534)	7,946 (6,867-9,448)	47,951 (42,150-55,278)	120,645 (107,166-136,147)	146,118 (134,807-158,197)	128,211 (116,337-140,889)
HIV/AIDS	-	-	93,841 (87,720-100,368)	65,466 (61,102-70,080)	9,424 (8,603-10,321)	1,938 (1,433-2,412)	7,554 (6,480-9,062)	47,299 (41,478-54,542)	119,663 (106,206-135,073)	145,176 (133,737-157,186)	127,385 (115,747-140,049)
HIV/AIDS - Drug-susceptible Tuberculosis	-	-	34,655 (26,357-39,477)	24,974 (19,832-28,049)	3,326 (2,532-3,848)	611 (396-813)	2,440 (1,893-2,975)	13,337 (9,500-16,765)	26,058 (17,820-34,767)	29,087 (19,741-39,107)	26,686 (18,178-35,642)
HIV/AIDS - Multidrug-resistant Tuberculosis without extensive drug resistance	-	-	1,455 (862-2,360)	1,068 (649-1,722)	135 (78-222)	24 (13-45)	108 (67-168)	578 (339-931)	1,166 (691-1,854)	1,351 (796-2,134)	1,256 (741-1,974)
HIV/AIDS - Extensively drug-resistant Tuberculosis	-	-	5 (3-8)	5 (3-7)	1 (1-1)	0 (0-0)	1 (1-1)	4 (2-6)	9 (6-14)	13 (9-19)	14 (9-21)
HIV/AIDS resulting in other diseases	-	-	57,725 (51,552-66,496)	39,419 (35,515-45,250)	5,961 (5,227-6,918)	1,302 (987-1,678)	5,005 (4,155-6,376)	33,380 (27,239-41,735)	92,430 (76,377-109,752)	114,725 (99,076-129,247)	99,430 (84,235-114,535)
Sexually transmitted infections excluding HIV	68,162 (26,109-127,657)	23,123 (8,745-43,142)	37,037 (14,043-70,311)	3,447 (1,295-6,729)	1,489 (547-2,858)	131 (100-157)	392 (276-464)	652 (406-774)	982 (565-1,187)	942 (573-1,118)	826 (563-948)
Syphilis	68,162 (26,109-127,657)	23,123 (8,745-43,142)	37,037 (14,043-70,311)	3,447 (1,295-6,729)	1,489 (547-2,858)	76 (59-95)	91 (61-121)	121 (76-155)	204 (114-274)	225 (122-311)	203 (128-276)
Chlamydial infection	-	-	-	-	-	13 (7-16)	49 (33-59)	144 (88-173)	152 (86-184)	83 (49-97)	104 (69-118)
Gonococcal infection	-	-	-	-	-	26 (17-32)	132 (90-157)	230 (141-274)	419 (239-507)	405 (243-475)	301 (199-341)
Other sexually transmitted infections	-	-	-	-	-	16 (11-19)	119 (80-141)	157 (96-187)	207 (118-251)	229 (137-267)	218 (146-246)
Respiratory infections and tuberculosis	-	243,301 (212,537-269,239)	1,066,799 (974,376-1,137,494)	634,426 (533,726-715,867)	85,627 (72,056-96,564)	38,890 (34,198-42,429)	48,890 (45,648-52,135)	75,346 (71,163-79,776)	94,703 (89,834-99,579)	107,697 (102,795-113,269)	115,197 (109,776-121,561)
Tuberculosis	-	-	74,684 (67,364-80,511)	79,974 (66,253-91,637)	19,676 (16,600-22,547)	15,036 (13,104-16,775)	30,984 (28,442-33,696)	55,537 (51,836-59,568)	72,409 (67,942-77,024)	84,363 (79,674-89,612)	89,157 (84,136-94,863)
Drug-susceptible tuberculosis	-	-	70,576 (63,413-76,532)	76,626 (63,407-88,196)	18,634 (15,643-21,539)	14,271 (12,432-15,975)	29,550 (27,037-32,255)	52,563 (48,568-56,754)	68,334 (63,630-73,014)	79,555 (74,557-84,895)	84,388 (78,936-90,198)
Multidrug-resistant tuberculosis without extensive drug resistance	-	-	4,040 (2,773-6,020)	3,311 (2,054-5,461)	1,024 (636-1,760)	752 (475-1,305)	1,412 (862-2,581)	2,916 (1,911-4,758)	3,994 (2,693-6,320)	4,709 (3,170-7,554)	4,664 (3,127-7,667)
Extensively drug-resistant tuberculosis	-	-	69 (47-98)	38 (24-58)	18 (12-26)	13 (8-18)	23 (15-34)	57 (39-80)	81 (57-111)	99 (72-133)	104 (77-140)
Lower respiratory infections	240,206 (215,313-268,852)	242,887 (212,225-268,776)	985,869 (901,905-1,052,965)	548,728 (462,199-622,182)	64,779 (52,532-73,016)	23,317 (19,755-25,552)	17,616 (15,622-18,897)	19,481 (17,503-20,744)	21,891 (19,384-23,108)	22,942 (20,868-24,174)	25,664 (23,687-27,497)
Upper respiratory infections	179 (94-227)	342 (188-436)	5,848 (2,743-8,369)	5,260 (1,760-9,967)	922 (383-1,250)	340 (172-453)	189 (102-257)	260 (133-324)	343 (179-415)	338 (184-418)	311 (177-405)
Otitis media	79 (11-394)	72 (35-157)	398 (191-749)	465 (158-1,028)	251 (134-466)	198 (119-334)	101 (69-153)	68 (45-108)	60 (38-99)	54 (37-83)	65 (45-102)
Enteric infections	40,166 (34,323-48,090)	82,949 (70,904-96,219)	705,240 (617,065-790,215)	674,661 (550,851-801,173)	130,508 (93,114-174,630)	65,405 (46,749-88,093)	45,441 (31,961-64,689)	35,973 (24,894-51,319)	30,702 (21,314-44,634)	28,499 (19,014-41,952)	27,609 (18,708-42,182)
Diarrheal diseases	40,166 (34,323-48,090)	82,494 (70,465-95,776)	696,509 (609,822-781,037)	617,583 (496,369-739,567)	56,734 (31,376-87,944)	23,153 (13,968-36,878)	22,067 (12,895-36,118)	22,656 (12,939-36,888)	20,777 (11,956-33,743)	20,898 (11,873-33,730)	21,823 (13,038-35,825)
Typhoid and paratyphoid	-	0 (0-0)	440 (262-759)	38,626 (17,602-72,661)	68,048 (39,172-109,898)	39,599 (22,796-62,757)	21,509 (10,659-38,238)	11,692 (5,999-20,228)	8,510 (4,486-14,552)	6,277 (3,288-10,522)	4,463 (2,351-7,372)
Typhoid fever	-	-	396 (235-672)	34,081 (15,720-64,410)	59,618 (33,533-97,173)	34,603 (19,541-54,674)	18,774 (9,139-33,861)	10,207 (5,117-17,958)	7,343 (3,786-12,675)	5,357 (2,733-9,123)	3,771 (1,943-6,416)
Paratyphoid fever	-	-	44 (22-82)	4,545 (1,669-10,034)	8,430 (3,582-16,782)	4,996 (2,186-9,662)	2,735 (1,077-5,717)	1,484 (599-3,178)	1,167 (498-2,442)	920 (402-1,895)	692 (305-1,401)
Invasive Non-typhoidal Salmonella (iNTS)	-	455 (258-744)	6,247 (3,501-10,596)	17,245 (8,657-31,565)	5,548 (2,677-10,215)	2,572 (1,141-4,871)	1,794 (743-3,367)	1,548 (639-2,954)	1,368 (538-2,839)	1,262 (530-2,427)	1,271 (551-2,462)
Other intestinal infectious diseases	-	-	2,045 (1,130-3,931)	1,206 (612-2,790)	178 (74-429)	82 (29-188)	71 (23-160)	78 (27-160)	47 (17-96)	62 (21-130)	51 (14-107)
Neglected tropical diseases and malaria	492 (262-1,468)	25,818 (16,558-39,398)	182,566 (127,107-260,361)	368,947 (249,183-532,536)	76,642 (51,564-123,504)	38,342 (26,579-63,813)	27,996 (18,459-51,400)	28,295 (18,766-49,405)	23,944 (15,010-42,833)	22,238 (14,405-38,418)	21,996 (14,434-35,937)
Malaria	-	25,340 (15,977-38,921)	169,408 (110,948-248,213)	318,266 (210,011-462,413)	51,861 (34,219-72,359)	24,145 (16,169-33,061)	15,357 (10,519-21,466)	15,919 (10,710-22,596)	12,976 (8,753-18,501)	11,834 (8,026-17,179)	12,160 (8,221-17,314)
Chagas disease	-	-	5 (4-5)	3 (2-3)	4 (3-4)	9 (8-10)	22 (20-25)	54 (49-60)	102 (94-111)	200 (187-213)	322 (301-346)
Leishmaniasis	-	-	6,601 (4-29,569)	25,682 (11-128,116)	9,665 (3-61,118)	5,511 (2-33,429)	5,777 (2-30,492)	5,294 (2-26,674)	5,214 (3-24,593)	4,701 (3-21,010)	4,258 (3-17,726)
Visceral leishmaniasis	-	-	6,601 (4-29,569)	25,682 (11-128,116)	9,665 (3-61,118)	5,511 (2-33,429)	5,777 (2-30,492)	5,294 (2-26,674)	5,214 (3-24,593)	4,701 (3-21,010)	4,258 (3-17,726)
African trypanosomiasis	-	-	-	1,081 (271-3,231)	1,733 (533-4,000)	1,881 (683-3,905)	2,461 (973-4,897)	2,615 (943-5,284)	2,219 (749-4,612)	1,998 (671-4,495)	1,780 (574-4,106)
Schistosomiasis	-	-	0 (0-0)	1,048 (720-1,461)	359 (266-474)	446 (343-570)	463 (371-574)	512 (422-616)	539 (448-650)	561 (463-659)	645 (540-760)
Cysticercosis	0 (0-0)	0 (0-0)	0 (0-0)	215 (104-355)	91 (29-175)	63 (16-130)	56 (9-124)	84 (21-166)	116 (38-220)	116 (36-220)	94 (25-197)
Cystic echinococcosis	-	-	-	630 (397-893)	256 (145-395)	135 (57-237)	67 (10-149)	83 (16-173)	109 (32-209)	108 (28-211)	124 (32-240)
Dengue	-	-	2,662 (868-5,110)	4,802 (1,900-7,993)	2,006 (844-3,053)	786 (351-1,238)	670 (305-1,002)	721 (352-1,102)	583 (304-919)	440 (211-696)	416 (196-664)
Yellow fever	-	6 (1-18)	93 (20-295)	892 (183-2,860)	1,296 (257-3,818)	1,188 (251-3,299)	1,119 (223-3,244)	970 (200-2,788)	715 (149-2,052)	603 (130-1,756)	528 (110-1,623)
Rabies	-	-	890 (592-1,334)	4,849 (2,793-7,497)	7,472 (5,281-10,090)	3,694 (2,744-5,050)	1,725 (1,290-2,514)	1,747 (1,328-2,476)	1,035 (782-1,526)	1,354 (1,008-1,972)	1,305 (977-1,834)
Intestinal nematode infections	-	-	672 (523-845)	7,104 (5,401-9,306)	1,216 (968-1,558)	201 (161-250)	57 (45-70)	49 (39-61)	38 (30-47)	48 (38-60)	42 (33-52)
Ascariasis	-	-	672 (523-845)	7,104 (5,401-9,306)	1,216 (968-1,558)	201 (161-250)	57 (45-70)	49 (39-61)	38 (30-47)	48 (38-60)	42 (33-52)
Ebola	0 (0-0)	0 (0-0)	13 (11-16)	20 (17-24)	16 (13-19)	7 (6-8)	14 (11-17)	34 (27-42)	56 (45-67)	44 (35-53)	74 (60-89)
Zika virus	0 (0-0)	0 (0-0)	0 (0-0)	0 (0-0)	0 (0-0)	0 (0-0)	0 (0-0)	0 (0-0)	0 (0-0)	0 (0-0)	0 (0-0)
Other neglected tropical diseases	491 (262-1,468)	471 (247-1,416)	2,222 (1,172-7,537)	4,356 (1,544-18,657)	668 (427-1,670)	276 (189-648)	209 (149-459)	213 (148-442)	242 (173-491)	231 (169-413)	249 (182-492)
Other infectious diseases	72,215 (62,409-83,423)	106,600 (91,190-122,386)	457,946 (338,130-656,024)	661,240 (439,024-1,007,950)	121,651 (88,797-176,232)	47,077 (37,377-63,343)	33,836 (28,924-41,151)	30,124 (26,112-34,526)	26,194 (22,811-29,002)	26,040 (23,036-28,397)	24,433 (21,095-26,497)
Meningitis	13,185 (10,222-15,875)	22,235 (17,855-25,445)	129,967 (111,911-144,848)	127,434 (99,699-149,566)	28,098 (20,812-34,492)	14,687 (11,498-17,717)	11,880 (9,625-14,383)	10,453 (8,390-12,955)	9,041 (7,336-11,210)	8,467 (7,016-10,634)	8,072 (6,793-10,210)
Pneumococcal meningitis	1,931 (1,441-2,481)	3,124 (2,362-3,814)	16,864 (13,714-20,692)	16,161 (11,681-20,842)	2,426 (1,648-3,247)	1,291 (938-1,758)	1,262 (922-1,705)	1,265 (962-1,688)	1,140 (860-1,553)	1,122 (859-1,472)	1,207 (932-1,629)
H influenzae type B meningitis	3,748 (2,803-4,708)	6,421 (5,076-7,754)	41,887 (34,001-49,480)	45,224 (34,986-55,592)	11,690 (8,376-14,400)	5,791 (4,380-7,051)	4,237 (3,253-5,192)	3,425 (2,701-4,179)	2,963 (2,333-3,576)	2,646 (2,164-3,288)	2,333 (1,919-2,978)
Meningococcal meningitis	2,007 (1,539-2,477)	3,364 (2,642-4,047)	18,363 (15,138-21,877)	16,617 (12,497-21,074)	2,991 (2,230-3,906)	1,392 (1,086-1,770)	1,088 (848-1,357)	882 (681-1,112)	778 (613-1,002)	755 (597-975)	726 (580-965)
Other meningitis	5,500 (4,242-6,747)	9,327 (7,274-11,192)	52,853 (44,444-61,905)	49,432 (37,720-60,615)	10,991 (8,081-13,780)	6,213 (4,734-7,716)	5,293 (4,175-6,683)	4,881 (3,835-6,105)	4,161 (3,309-5,356)	3,946 (3,169-5,123)	3,805 (3,108-4,934)
Encephalitis	829 (599-1,003)	1,528 (1,091-2,105)	16,344 (11,320-23,436)	18,409 (12,377-31,899)	9,461 (7,354-13,040)	5,306 (4,448-6,515)	3,272 (2,934-3,722)	3,078 (2,770-3,594)	2,644 (2,392-3,083)	2,845 (2,548-3,296)	2,608 (2,377-2,941)
Diphtheria	-	-	2,470 (1,649-3,582)	8,500 (5,387-13,074)	1,558 (1,007-2,334)	435 (298-623)	152 (106-216)	90 (63-127)	76 (55-106)	84 (61-115)	98 (75-128)
Whooping cough	-	-	75,989 (35,453-147,008)	70,791 (31,856-137,447)	7,639 (3,155-15,979)	1,373 (576-2,832)	532 (224-1,096)	409 (175-846)	252 (104-540)	185 (75-394)	130 (54-276)
Tetanus	51,189 (43,021-61,222)	76,463 (63,671-91,104)	20,669 (16,000-28,332)	14,372 (5,941-19,740)	4,618 (2,991-6,066)	2,882 (1,881-3,869)	3,975 (2,414-5,462)	3,778 (2,427-5,176)	3,583 (2,182-4,834)	3,644 (2,474-4,859)	3,450 (2,144-4,449)
Measles	-	-	177,865 (64,247-382,022)	386,451 (150,325-779,854)	55,700 (20,946-116,593)	14,298 (5,050-31,681)	5,188 (1,756-11,961)	2,600 (877-6,062)	1,209 (407-2,806)	706 (243-1,646)	425 (147-985)
Varicella and herpes zoster	216 (178-259)	616 (499-751)	4,578 (3,814-5,425)	5,078 (4,064-6,112)	2,027 (1,594-2,491)	627 (511-751)	315 (264-380)	346 (292-411)	454 (388-539)	553 (472-643)	457 (390-532)
Acute hepatitis	-	-	15,184 (9,782-17,686)	18,815 (12,845-23,528)	8,724 (6,100-10,499)	5,393 (3,760-6,497)	6,029 (4,283-6,988)	7,274 (5,590-8,204)	7,030 (5,089-7,819)	7,488 (5,480-8,259)	7,102 (5,200-7,961)
Acute hepatitis A	-	-	2,469 (1,220-3,959)	11,683 (7,636-15,659)	5,769 (3,720-7,329)	2,727 (1,788-3,653)	2,227 (1,434-2,963)	2,127 (1,356-2,917)	1,536 (898-2,269)	1,282 (685-2,036)	1,078 (552-1,801)
Acute hepatitis B	-	-	9,425 (5,635-12,486)	5,312 (2,845-8,100)	1,463 (740-2,394)	947 (489-1,589)	1,415 (796-2,252)	2,430 (1,428-3,747)	3,404 (2,195-4,758)	4,662 (3,100-6,027)	4,968 (3,347-6,285)
Acute hepatitis C	-	-	3,266 (1,670-5,584)	1,420 (654-2,620)	172 (72-353)	52 (20-113)	33 (12-70)	30 (10-64)	29 (10-70)	36 (10-90)	49 (14-118)
Acute hepatitis E	-	-	24 (11-42)	400 (197-687)	1,320 (729-2,039)	1,666 (994-2,467)	2,354 (1,495-3,176)	2,687 (1,721-3,627)	2,061 (1,218-3,050)	1,508 (734-2,529)	1,007 (454-1,758)
Other unspecified infectious diseases	6,796 (3,270-8,462)	5,757 (2,918-6,881)	14,881 (8,778-18,940)	11,389 (6,752-17,236)	3,825 (2,732-4,657)	2,076 (1,546-2,543)	2,494 (1,829-3,052)	2,096 (1,562-2,438)	1,905 (1,429-2,184)	2,067 (1,524-2,422)	2,091 (1,593-2,442)
Maternal and neonatal disorders	2,234,679 (2,140,791-2,317,865)	464,004 (420,362-529,429)	115,553 (98,692-146,571)	10,155 (7,709-14,663)	-	1,174 (978-1,352)	35,268 (32,369-37,986)	69,386 (65,053-73,613)	66,732 (62,856-70,589)	56,947 (53,754-60,689)	44,855 (41,936-47,829)
Maternal disorders	-	-	-	-	-	1,174 (978-1,352)	35,268 (32,369-37,986)	69,386 (65,053-73,613)	66,732 (62,856-70,589)	56,947 (53,754-60,689)	44,855 (41,936-47,829)
Maternal hemorrhage	-	-	-	-	-	392 (308-486)	10,174 (6,477-15,987)	25,139 (16,540-37,176)	26,278 (19,001-36,156)	24,377 (18,609-31,926)	19,276 (15,512-23,663)
Maternal sepsis and other maternal infections	-	-	-	-	-	106 (73-149)	5,355 (3,031-9,850)	8,177 (4,629-15,077)	6,898 (4,186-12,778)	5,247 (3,246-8,866)	4,153 (2,867-6,433)
Maternal hypertensive disorders	-	-	-	-	-	127 (96-165)	5,513 (3,651-9,229)	9,108 (6,061-14,848)	8,104 (5,581-13,401)	5,853 (4,099-9,129)	4,260 (3,008-6,540)
Maternal obstructed labor and uterine rupture	-	-	-	-	-	119 (81-172)	2,103 (1,030-4,630)	3,897 (1,743-10,007)	3,886 (1,948-9,026)	3,731 (2,097-7,304)	3,388 (2,136-5,831)
Maternal abortion and miscarriage	-	-	-	-	-	119 (87-167)	3,369 (2,073-5,697)	4,677 (2,847-8,059)	4,285 (2,770-7,617)	4,012 (2,557-7,016)	3,461 (2,341-5,400)
Ectopic pregnancy	-	-	-	-	-	48 (29-90)	1,898 (577-5,428)	3,228 (1,084-8,624)	2,887 (1,106-8,704)	2,394 (966-6,403)	1,590 (675-4,150)
Indirect maternal deaths	-	-	-	-	-	140 (107-180)	3,776 (2,295-7,214)	8,133 (4,902-15,269)	7,731 (4,984-13,549)	6,045 (4,047-10,292)	4,682 (3,356-7,325)
Late maternal deaths	-	-	-	-	-	14 (9-20)	385 (285-524)	742 (562-1,018)	738 (556-1,000)	629 (461-871)	468 (351-636)
Maternal deaths aggravated by HIV/AIDS	-	-	-	-	-	0 (0-0)	100 (55-148)	299 (181-404)	373 (237-489)	334 (212-444)	251 (156-339)
Other maternal disorders	-	-	-	-	-	109 (81-145)	2,596 (1,322-5,388)	5,986 (2,912-12,632)	5,553 (2,988-10,812)	4,325 (2,576-8,040)	3,326 (2,163-5,545)
Neonatal disorders	2,234,679 (2,140,791-2,317,865)	464,004 (420,362-529,429)	115,553 (98,692-146,571)	10,155 (7,709-14,663)	-	-	-	-	-	-	-
Neonatal preterm birth	965,443 (901,267-1,051,305)	159,907 (142,192-190,129)	50,695 (43,010-63,388)	3,006 (2,254-4,419)	-	-	-	-	-	-	-
Neonatal encephalopathy due to birth asphyxia and trauma	680,307 (619,201-741,740)	77,334 (66,865-92,429)	20,377 (16,499-29,455)	3,830 (2,815-6,320)	-	-	-	-	-	-	-
Neonatal sepsis and other neonatal infections	130,378 (108,345-175,771)	92,435 (74,556-130,770)	10,818 (8,587-16,938)	446 (303-798)	-	-	-	-	-	-	-
Hemolytic disease and other neonatal jaundice	61,835 (52,543-72,696)	42,807 (33,765-51,529)	8,097 (6,067-10,619)	566 (367-843)	-	-	-	-	-	-	-
Other neonatal disorders	396,716 (336,729-447,063)	91,521 (75,924-120,074)	25,566 (20,361-35,408)	2,307 (1,709-3,593)	-	-	-	-	-	-	-
Nutritional deficiencies	-	-	224,258 (199,526-245,386)	207,242 (171,502-240,718)	19,947 (16,569-23,440)	5,312 (4,595-5,990)	3,650 (3,211-4,031)	3,969 (3,582-4,308)	4,159 (3,865-4,447)	4,108 (3,853-4,315)	4,230 (3,909-4,479)
Protein-energy malnutrition	-	-	216,228 (192,633-236,662)	196,834 (163,516-229,978)	16,689 (14,059-19,419)	4,217 (3,694-4,724)	2,639 (2,357-2,891)	2,805 (2,543-3,053)	3,017 (2,793-3,250)	3,139 (2,923-3,352)	3,207 (2,997-3,401)
Other nutritional deficiencies	-	-	8,030 (6,008-10,191)	10,408 (7,225-13,549)	3,258 (2,315-4,244)	1,095 (840-1,355)	1,011 (813-1,225)	1,165 (985-1,390)	1,142 (976-1,340)	970 (834-1,142)	1,023 (842-1,265)
Non-communicable diseases	226,926 (205,143-272,360)	110,780 (98,620-129,522)	455,684 (403,755-517,912)	310,103 (257,412-358,738)	146,018 (132,304-161,647)	109,793 (101,455-116,752)	155,272 (147,141-161,400)	219,614 (212,449-226,310)	302,550 (294,833-310,793)	414,290 (405,575-423,411)	533,269 (524,448-542,467)
Neoplasms	1,782 (1,412-1,995)	1,307 (1,011-1,595)	14,246 (10,369-17,641)	47,560 (35,863-65,715)	48,583 (40,644-57,524)	35,916 (32,058-39,624)	38,739 (36,453-40,704)	48,811 (46,375-51,560)	71,818 (68,450-75,876)	110,507 (106,131-115,204)	149,740 (145,867-153,852)
Lip and oral cavity cancer	-	-	-	-	-	-	533 (479-590)	791 (725-876)	1,148 (1,063-1,241)	1,895 (1,785-2,013)	3,054 (2,893-3,241)
Nasopharynx cancer	-	-	-	-	492 (421-555)	496 (442-542)	626 (581-669)	901 (815-975)	1,368 (1,224-1,506)	2,142 (1,943-2,357)	2,469 (2,302-2,650)
Other pharynx cancer	-	-	-	-	-	-	286 (239-325)	352 (290-393)	506 (419-561)	846 (665-919)	1,489 (1,234-1,606)
Esophageal cancer	-	-	-	-	-	-	305 (282-330)	655 (616-693)	1,121 (1,060-1,185)	2,237 (2,128-2,354)	3,807 (3,622-4,012)
Stomach cancer	-	-	-	-	-	-	1,053 (993-1,119)	2,922 (2,785-3,086)	6,428 (6,124-6,820)	12,256 (11,736-12,921)	16,227 (15,698-16,848)
Colon and rectum cancer	-	-	-	-	-	-	880 (780-1,001)	2,103 (1,924-2,407)	3,993 (3,626-4,516)	7,283 (6,764-8,092)	9,176 (8,692-9,949)
Liver cancer	-	-	-	-	1,589 (1,316-1,803)	1,217 (1,065-1,347)	1,326 (1,215-1,478)	3,101 (2,670-3,399)	7,224 (6,102-8,218)	14,499 (12,696-16,077)	19,244 (17,731-20,493)
Liver cancer due to hepatitis B	-	-	-	-	1,077 (894-1,225)	930 (811-1,034)	986 (892-1,110)	2,375 (2,047-2,633)	5,451 (4,525-6,266)	10,531 (9,128-11,804)	13,091 (11,790-14,190)
Liver cancer due to hepatitis C	-	-	-	-	26 (19-34)	24 (19-30)	26 (20-32)	95 (77-113)	341 (261-429)	933 (756-1,115)	1,613 (1,315-1,957)
Liver cancer due to alcohol use	-	-	-	-	-	-	5 (3-8)	58 (44-76)	255 (178-353)	853 (645-1,096)	1,928 (1,352-2,567)
Liver cancer due to NASH	-	-	-	-	-	-	93 (74-116)	151 (119-185)	291 (220-371)	538 (407-683)	698 (555-859)
Liver cancer due to other causes	-	-	-	-	485 (384-574)	262 (214-308)	215 (174-257)	422 (341-503)	886 (683-1,108)	1,644 (1,264-2,035)	1,914 (1,504-2,349)
Gallbladder and biliary tract cancer	-	-	-	-	-	-	97 (87-111)	184 (157-214)	343 (286-403)	772 (651-890)	1,238 (1,101-1,413)
Pancreatic cancer	-	-	-	-	-	-	153 (144-163)	319 (298-340)	699 (636-765)	1,540 (1,421-1,649)	2,694 (2,575-2,804)
Larynx cancer	-	-	-	-	-	-	164 (152-178)	300 (274-331)	451 (421-486)	795 (758-835)	1,685 (1,611-1,764)
Tracheal, bronchus, and lung cancer	-	-	-	-	-	-	938 (875-1,022)	2,031 (1,868-2,232)	4,592 (4,226-5,022)	8,780 (8,199-9,496)	14,277 (13,679-15,000)
Malignant skin melanoma	-	-	-	-	-	-	238 (192-315)	454 (358-588)	781 (607-967)	1,312 (1,017-1,619)	1,869 (1,464-2,277)
Non-melanoma skin cancer	-	-	-	-	-	-	151 (139-164)	234 (213-256)	354 (322-393)	529 (490-571)	652 (613-700)
Non-melanoma skin cancer (squamous-cell carcinoma)	-	-	-	-	-	-	151 (139-164)	234 (213-256)	354 (322-393)	529 (490-571)	652 (613-700)
Breast cancer	-	-	-	-	-	-	293 (197-367)	888 (620-1,098)	3,131 (2,441-3,750)	9,247 (7,603-10,819)	17,902 (15,669-20,229)
Cervical cancer	-	-	-	-	-	-	297 (257-326)	1,330 (1,151-1,497)	3,766 (3,291-4,266)	7,825 (6,957-8,939)	12,349 (11,291-13,755)
Uterine cancer	-	-	-	-	-	-	84 (75-92)	252 (214-282)	545 (470-606)	922 (800-1,022)	1,239 (1,131-1,330)
Ovarian cancer	-	-	-	-	-	-	483 (435-553)	755 (677-883)	1,062 (960-1,242)	1,705 (1,565-1,974)	2,636 (2,464-2,961)
Prostate cancer	-	-	-	-	-	-	71 (54-79)	116 (88-128)	161 (126-186)	195 (152-218)	242 (194-272)
Testicular cancer	-	-	-	-	-	-	366 (339-396)	856 (802-919)	1,170 (1,091-1,254)	1,136 (1,065-1,210)	842 (796-892)
Kidney cancer	47 (38-55)	28 (22-35)	372 (286-461)	1,666 (1,137-2,247)	806 (679-928)	294 (263-327)	198 (183-213)	275 (250-297)	425 (385-464)	606 (560-653)	1,065 (1,007-1,119)
Bladder cancer	-	-	-	-	-	-	102 (92-111)	174 (152-190)	306 (271-331)	545 (470-587)	858 (744-917)
Brain and nervous system cancer	207 (131-258)	188 (113-243)	2,510 (1,297-3,456)	9,112 (4,832-13,699)	11,204 (7,042-15,419)	6,663 (4,990-8,237)	4,110 (3,341-4,930)	4,325 (3,232-5,370)	5,746 (4,213-7,133)	7,175 (5,426-8,449)	7,527 (6,230-8,466)

Cause	40 to 44	45 to 49	50 to 54	54 to 59	60 to 64	65 to 69	70 to 74	75 to 79	80 to 84	85 to 89	90 to 94	95 plus
All causes	1,472,233 (1,457,702-1,487,086)	1,612,208 (1,598,120-1,627,306)	1,822,019 (1,806,309-1,838,616)	2,445,261 (2,427,243-2,464,894)	3,286,043 (3,263,487-3,309,227)	4,007,304 (3,982,742-4,032,639)	4,428,927 (4,402,334-4,455,658)	4,086,774 (4,063,468-4,110,168)	4,101,330 (4,085,016-4,117,895)	2,944,084 (2,934,798-2,953,191)	1,367,821 (1,364,308-1,371,048)	402,333 (401,547-403,132)
Communicable, maternal, neonatal, and nutritional diseases	346,212 (332,767-361,848)	305,431 (292,516-322,439)	297,454 (283,785-317,948)	322,833 (306,697-348,314)	388,208 (364,799-428,804)	418,960 (390,747-471,617)	450,786 (416,033-509,352)	406,945 (373,741-465,191)	384,131 (350,619-433,315)	277,480 (254,936-309,673)	122,082 (113,185-134,263)	38,450 (36,330-41,173)
HIV/AIDS and sexually transmitted infections	103,480 (96,229-110,854)	71,828 (66,252-78,126)	44,945 (42,060-48,136)	29,764 (27,476-32,161)	21,195 (19,457-23,169)	15,087 (13,747-16,550)	8,703 (7,778-9,720)	5,048 (4,446-5,638)	1,182 (994-1,341)	504 (392-594)	208 (160-244)	49 (38-58)
HIV/AIDS	102,505 (95,231-109,884)	71,039 (65,471-77,360)	44,350 (41,469-47,530)	28,985 (26,721-31,325)	20,531 (18,769-22,504)	14,412 (13,088-15,885)	8,159 (7,243-9,136)	4,492 (3,957-5,051)	772 (663-891)	237 (197-281)	86 (73-98)	13 (12-15)
HIV/AIDS - Drug-susceptible Tuberculosis	23,346 (16,026-30,942)	18,001 (12,392-23,523)	13,172 (9,264-16,126)	9,137 (6,785-10,983)	6,740 (5,108-8,011)	4,841 (3,781-5,734)	2,923 (2,289-3,461)	1,632 (1,287-1,920)	252 (203-299)	69 (54-85)	26 (20-31)	3 (2-4)
HIV/AIDS - Multidrug-resistant Tuberculosis without extensive drug resistance	1,116 (662-1,769)	854 (503-1,338)	593 (347-944)	425 (257-662)	309 (190-479)	221 (136-346)	128 (78-200)	71 (43-113)	17 (11-25)	6 (4-8)	2 (1-2)	0 (0-0)
HIV/AIDS - Extensively drug-resistant Tuberculosis	13 (8-19)	9 (6-14)	5 (3-7)	4 (3-6)	3 (2-4)	2 (1-3)	1 (1-1)	0 (0-1)	0 (0-0)	0 (0-0)	0 (0-0)	0 (0-0)
HIV/AIDS resulting in other diseases	78,030 (67,784-88,070)	52,175 (44,483-60,339)	30,581 (26,689-35,411)	19,419 (17,057-22,648)	13,478 (11,787-15,665)	9,347 (8,097-11,041)	5,108 (4,356-5,998)	2,789 (2,375-3,309)	502 (413-613)	162 (130-206)	58 (48-70)	10 (9-12)
Sexually transmitted infections excluding HIV	974 (631-1,154)	789 (516-910)	595 (411-701)	779 (512-936)	664 (408-836)	675 (448-852)	544 (369-675)	556 (349-737)	409 (256-513)	267 (171-341)	122 (78-155)	36 (25-44)
Syphilis	257 (142-385)	207 (110-297)	218 (142-308)	347 (211-480)	322 (176-480)	347 (211-508)	297 (176-422)	317 (154-482)	203 (101-295)	132 (60-196)	53 (25-80)	16 (9-23)
Chlamydial infection	139 (92-158)	105 (71-119)	68 (46-77)	81 (53-93)	66 (43-76)	40 (28-45)	41 (32-46)	38 (28-43)	46 (34-53)	29 (23-32)	14 (11-16)	4 (3-4)
Gonococcal infection	372 (246-420)	300 (203-339)	203 (140-230)	226 (151-260)	169 (111-194)	215 (148-241)	148 (112-164)	149 (111-169)	121 (88-140)	77 (60-86)	39 (30-45)	10 (9-11)
Other sexually transmitted infections	206 (137-234)	176 (120-198)	106 (72-120)	126 (84-144)	107 (70-123)	73 (50-83)	57 (43-64)	52 (36-62)	40 (27-47)	29 (20-35)	16 (10-20)	6 (4-7)
Respiratory infections and tuberculosis	133,083 (127,635-139,562)	139,741 (133,847-147,329)	160,766 (154,072-168,536)	186,604 (178,686-196,040)	229,111 (219,171-240,520)	245,748 (235,660-259,895)	264,708 (253,359-278,814)	240,856 (231,104-252,807)	234,995 (225,838-246,213)	174,744 (169,149-182,655)	80,375 (78,453-83,630)	26,249 (25,740-27,151)
Tuberculosis	101,980 (96,734-108,102)	105,227 (99,576-112,217)	118,970 (112,822-126,531)	131,265 (123,372-140,409)	146,393 (137,953-157,417)	141,921 (132,620-154,067)	132,484 (123,505-144,404)	98,032 (91,215-106,544)	68,514 (63,546-75,283)	33,199 (30,831-36,504)	9,232 (8,520-10,107)	1,758 (1,614-1,943)
Drug-susceptible tuberculosis	95,984 (89,924-102,279)	99,292 (92,774-106,650)	112,935 (105,578-120,785)	124,035 (115,194-133,464)	138,156 (128,698-149,329)	133,590 (123,549-145,730)	124,493 (115,091-135,986)	92,166 (84,572-100,298)	64,510 (59,262-71,006)	31,293 (28,677-34,578)	8,733 (7,977-9,597)	1,670 (1,530-1,851)
Multidrug-resistant tuberculosis without extensive drug resistance	5,858 (3,985-9,562)	5,802 (3,971-9,587)	5,914 (3,834-10,298)	7,071 (4,654-11,924)	8,069 (5,290-13,293)	8,159 (5,503-13,230)	7,833 (5,278-12,129)	5,751 (3,887-8,761)	3,924 (2,660-5,956)	1,866 (1,278-2,736)	488 (347-708)	86 (62-125)
Extensively drug-resistant tuberculosis	138 (104-182)	133 (99-178)	121 (88-169)	159 (116-217)	168 (121-231)	172 (126-237)	158 (112-217)	115 (83-158)	80 (57-110)	39 (28-53)	10 (7-14)	2 (1-2)
Lower respiratory infections	30,623 (28,259-32,380)	33,931 (31,240-35,877)	41,258 (37,359-44,211)	54,776 (50,201-57,805)	81,969 (74,089-86,472)	102,815 (93,400-108,679)	130,788 (120,827-138,092)	141,190 (132,106-150,040)	164,059 (155,270-171,868)	139,573 (134,651-146,480)	70,282 (68,658-73,433)	24,229 (23,779-25,146)
Upper respiratory infections	409 (226-528)	405 (223-534)	351 (197-477)	469 (279-610)	643 (339-848)	867 (431-1,120)	1,327 (582-1,702)	1,562 (646-2,032)	2,364 (898-3,075)	1,939 (767-2,532)	849 (375-1,082)	258 (121-321)
Otitis media	71 (49-112)	178 (90-416)	188 (104-398)	95 (71-139)	106 (78-159)	145 (107-215)	109 (83-153)	71 (56-98)	57 (46-77)	33 (28-43)	12 (10-15)	4 (4-5)
Enteric infections	28,103 (18,391-43,987)	30,886 (20,237-49,258)	36,596 (23,464-59,213)	48,748 (31,083-77,897)	74,151 (45,620-120,403)	95,000 (60,180-154,071)	113,574 (72,575-178,386)	112,848 (72,788-178,254)	111,124 (71,246-166,252)	76,833 (50,626-113,498)	29,242 (18,850-42,573)	7,363 (4,905-10,346)
Diarrheal diseases	23,430 (13,673-39,360)	27,204 (16,053-45,267)	33,496 (19,993-56,126)	45,889 (28,027-75,457)	71,480 (42,980-117,483)	92,725 (58,178-152,164)	111,926 (70,827-177,042)	111,765 (71,730-177,291)	110,429 (70,619-165,429)	76,491 (50,218-113,247)	29,137 (18,787-42,484)	7,338 (4,889-10,325)
Typhoid and paratyphoid	3,418 (1,788-5,719)	2,545 (1,360-4,267)	1,933 (995-3,332)	1,600 (782-2,961)	1,383 (690-2,480)	1,107 (630-1,798)	743 (450-1,177)	445 (273-683)	244 (153-365)	119 (80-177)	39 (27-60)	9 (6-13)
Typhoid fever	2,849 (1,435-4,742)	2,096 (1,115-3,525)	1,591 (817-2,760)	1,323 (637-2,474)	1,149 (560-2,098)	931 (527-1,531)	628 (373-996)	377 (229-586)	209 (131-326)	102 (68-158)	33 (23-54)	7 (5-12)
Paratyphoid fever	569 (255-1,122)	449 (190-903)	343 (135-709)	277 (111-577)	234 (96-475)	177 (80-341)	115 (55-216)	67 (34-122)	34 (18-61)	17 (9-28)	6 (3-9)	2 (1-3)
Invasive Non-typhoidal Salmonella (iNTS)	1,213 (587-2,303)	1,099 (512-2,117)	1,138 (516-2,202)	1,233 (582-2,209)	1,259 (580-2,218)	1,131 (538-1,955)	882 (417-1,549)	621 (294-1,134)	426 (184-796)	195 (81-355)	60 (17-124)	14 (1-39)
Other intestinal infectious diseases	42 (12-90)	37 (10-78)	29 (7-66)	26 (5-58)	30 (6-65)	36 (8-77)	24 (5-52)	17 (3-40)	26 (3-58)	28 (4-64)	6 (0-15)	2 (0-7)
Neglected tropical diseases and malaria	21,687 (14,671-34,145)	19,552 (13,298-29,169)	21,324 (14,795-30,397)	21,991 (15,392-30,957)	24,124 (17,172-33,655)	21,266 (14,945-30,091)	21,501 (15,353-29,850)	10,534 (7,800-14,387)	5,143 (3,988-6,866)	2,007 (1,655-2,671)	606 (500-794)	152 (121-196)
Malaria	11,828 (7,953-17,361)	10,663 (7,077-15,590)	12,714 (8,549-18,469)	13,860 (9,194-20,393)	16,068 (10,835-23,476)	14,346 (9,441-21,595)	15,001 (9,636-22,595)	6,321 (4,185-9,320)	2,619 (1,861-3,744)	698 (516-928)	193 (120-246)	57 (26-76)
Chagas disease	474 (449-500)	640 (608-674)	799 (756-844)	933 (878-992)	1,120 (1,060-1,187)	1,169 (1,108-1,231)	1,078 (1,013-1,150)	724 (678-772)	532 (503-562)	348 (326-372)	131 (118-145)	23 (21-25)
Leishmaniasis	3,852 (3-15,470)	3,368 (6-12,380)	2,991 (12-10,847)	2,610 (8-9,497)	2,483 (15-8,708)	2,059 (12-7,180)	1,447 (14-4,999)	857 (10-3,080)	441 (13-1,589)	180 (19-615)	53 (6-171)	9 (0-29)
Visceral leishmaniasis	3,852 (3-15,470)	3,368 (6-12,380)	2,991 (12-10,847)	2,610 (8-9,497)	2,483 (15-8,708)	2,059 (12-7,180)	1,447 (14-4,999)	857 (10-3,080)	441 (13-1,589)	180 (19-615)	53 (6-171)	9 (0-29)
African trypanosomiasis	1,652 (518-3,995)	1,228 (397-3,118)	1,164 (323-3,025)	966 (279-2,757)	849 (240-2,542)	655 (178-1,726)	496 (138-1,514)	277 (77-786)	139 (37-398)	38 (9-112)	10 (3-30)	1 (0-4)
Schistosomiasis	758 (623-892)	796 (666-947)	939 (789-1,109)	1,084 (917-1,288)	1,217 (1,020-1,430)	922 (772-1,083)	1,195 (1,002-1,406)	926 (781-1,107)	513 (423-618)	212 (171-260)	52 (40-67)	9 (6-12)
Cysticercosis	90 (19-185)	76 (15-163)	67 (11-152)	60 (10-137)	54 (10-126)	44 (6-99)	10 (0-26)	21 (1-56)	14 (0-37)	7 (0-19)	2 (0-7)	1 (0-2)
Cystic echinococcosis	125 (33-244)	122 (29-243)	134 (37-263)	151 (39-302)	156 (42-298)	147 (37-293)	125 (29-257)	84 (12-188)	64 (6-140)	41 (3-100)	14 (0-40)	4 (0-12)
Dengue	361 (165-544)	350 (160-529)	398 (166-616)	439 (193-677)	413 (195-644)	399 (205-625)	503 (287-916)	384 (228-717)	430 (257-919)	261 (150-563)	84 (46-191)	22 (12-52)
Yellow fever	442 (93-1,336)	342 (71-1,026)	294 (57-892)	235 (46-721)	178 (33-535)	124 (25-362)	565 (365-789)	40 (8-125)	15 (3-44)	5 (1-16)	1 (0-4)	0 (0-1)
Rabies	1,750 (1,282-2,413)	1,627 (1,255-2,484)	1,504 (1,136-2,270)	1,321 (1,032-1,914)	1,185 (914-1,702)	1,050 (775-1,644)	731 (554-1,132)	609 (456-892)	176 (127-270)	107 (71-173)	22 (15-37)	9 (6-14)
Intestinal nematode infections	33 (26-41)	32 (25-39)	31 (24-38)	30 (24-37)	68 (55-84)	32 (25-41)	52 (42-63)	39 (31-49)	26 (21-33)	12 (9-15)	7 (5-9)	2 (1-3)
Ascariasis	33 (26-41)	32 (25-39)	31 (24-38)	30 (24-37)	68 (55-84)	32 (25-41)	52 (42-63)	39 (31-49)	26 (21-33)	12 (9-15)	7 (5-9)	2 (1-3)
Ebola	56 (46-67)	57 (47-70)	44 (36-54)	34 (28-42)	32 (26-39)	12 (10-14)	5 (4-7)	1 (1-1)	0 (0-1)	0 (0-0)	0 (0-0)	0 (0-0)
Zika virus	0 (0-0)	0 (0-0)	0 (0-0)	0 (0-0)	0 (0-0)	0 (0-0)	0 (0-0)	0 (0-0)	0 (0-0)	0 (0-0)	0 (0-0)	0 (0-0)
Other neglected tropical diseases	267 (199-487)	252 (183-464)	245 (181-458)	268 (200-506)	300 (226-579)	308 (230-600)	294 (223-545)	249 (185-492)	174 (130-347)	100 (75-194)	35 (27-67)	15 (11-29)
Other infectious diseases	26,116 (22,247-28,232)	25,858 (21,926-28,043)	25,577 (21,956-28,110)	26,782 (23,211-29,254)	28,503 (25,305-31,077)	28,408 (25,257-31,278)	26,102 (23,141-28,595)	20,873 (18,258-22,792)	15,822 (13,857-17,005)	9,444 (8,317-10,158)	3,246 (2,831-3,525)	974 (845-1,084)
Meningitis	7,574 (6,313-9,543)	7,634 (6,410-9,469)	7,419 (6,262-9,345)	7,673 (6,483-9,651)	8,166 (7,023-10,477)	7,493 (6,373-9,463)	7,515 (6,321-9,489)	5,648 (4,679-7,149)	4,186 (3,447-5,116)	2,586 (2,119-3,133)	758 (613-883)	218 (174-256)
Pneumococcal meningitis	1,167 (869-1,562)	1,285 (972-1,709)	1,271 (994-1,658)	1,249 (972-1,637)	1,357 (1,043-1,763)	1,232 (966-1,608)	1,341 (1,022-1,779)	999 (782-1,320)	726 (568-922)	429 (336-537)	120 (95-146)	35 (26-46)
H influenzae type B meningitis	2,383 (1,961-2,999)	2,403 (1,974-3,049)	2,311 (1,854-2,948)	2,482 (2,046-3,251)	2,676 (2,184-3,491)	2,536 (2,081-3,260)	2,438 (1,948-3,146)	1,896 (1,505-2,458)	1,440 (1,137-1,799)	910 (723-1,115)	290 (222-342)	80 (59-97)
Meningococcal meningitis	656 (523-828)	647 (521-822)	654 (525-862)	692 (546-884)	724 (559-954)	663 (529-852)	652 (512-828)	501 (390-661)	370 (291-480)	210 (170-260)	55 (43-69)	16 (12-22)
Other meningitis	3,368 (2,794-4,374)	3,298 (2,763-4,269)	3,183 (2,578-4,179)	3,251 (2,702-4,201)	3,409 (2,804-4,423)	3,061 (2,555-3,944)	3,083 (2,520-3,890)	2,252 (1,839-2,948)	1,650 (1,331-2,045)	1,036 (842-1,283)	292 (236-362)	87 (68-108)
Encephalitis	2,643 (2,419-3,020)	2,468 (2,262-2,804)	2,377 (2,176-2,758)	3,017 (2,760-3,437)	3,868 (3,543-4,449)	4,253 (3,860-4,821)	4,390 (3,891-4,958)	3,870 (3,325-4,366)	3,117 (2,600-3,498)	1,639 (1,398-1,815)	426 (379-468)	102 (90-111)
Diphtheria	149 (123-186)	231 (192-287)	155 (122-203)	100 (79-131)	-	-	-	-	-	-	-	-
Whooping cough	149 (61-318)	116 (47-250)	98 (41-208)	122 (51-261)	-	-	-	-	-	-	-	-
Tetanus	4,224 (2,526-5,782)	4,904 (2,691-6,536)	4,983 (2,806-6,845)	4,945 (2,669-7,169)	4,031 (2,503-5,492)	4,081 (2,603-6,224)	3,021 (2,061-4,386)	2,179 (1,403-3,457)	1,109 (757-1,698)	583 (379-893)	164 (111-259)	41 (25-62)
Measles	300 (105-689)	199 (70-460)	150 (52-349)	148 (52-341)	-	-	-	-	-	-	-	-
Varicella and herpes zoster	294 (253-344)	267 (232-310)	247 (211-289)	297 (255-347)	366 (317-425)	684 (589-797)	622 (535-725)	853 (740-1,004)	1,249 (1,063-1,499)	1,297 (1,105-1,562)	712 (586-875)	315 (246-420)
Acute hepatitis	8,754 (6,402-9,783)	7,914 (5,880-8,872)	7,869 (5,800-8,907)	7,988 (6,046-8,927)	8,745 (6,481-9,778)	8,117 (6,168-9,067)	6,762 (5,315-7,519)	5,095 (3,906-5,707)	3,344 (2,550-3,773)	1,517 (1,196-1,708)	413 (321-454)	74 (57-83)
Acute hepatitis A	1,119 (578-1,913)	792 (436-1,466)	486 (281-776)	328 (196-494)	264 (140-426)	203 (105-332)	112 (49-193)	39 (12-85)	10 (2-25)	1 (0-4)	0 (0-0)	0 (0-0)
Acute hepatitis B	6,757 (4,565-8,172)	6,519 (4,582-7,785)	6,939 (5,061-8,106)	7,296 (5,491-8,275)	8,060 (5,907-9,090)	7,413 (5,609-8,402)	6,092 (4,703-6,935)	4,447 (3,321-5,106)	2,743 (2,013-3,259)	1,150 (828-1,387)	279 (191-352)	43 (25-61)
Acute hepatitis C	60 (17-146)	65 (19-159)	71 (24-158)	81 (28-191)	118 (33-287)	144 (40-351)	144 (48-362)	133 (40-335)	114 (35-286)	70 (23-167)	25 (7-62)	6 (1-14)
Acute hepatitis E	818 (345-1,519)	538 (235-1,155)	372 (166-734)	282 (134-522)	303 (131-605)	357 (159-688)	414 (212-760)	475 (214-882)	476 (233-855)	296 (150-511)	110 (59-176)	26 (13-42)
Other unspecified infectious diseases	2,029 (1,601-2,340)	2,126 (1,674-2,434)	2,279 (1,848-2,561)	2,491 (1,993-2,816)	3,328 (2,705-3,780)	3,781 (3,021-4,236)	3,793 (2,952-4,424)	3,228 (2,497-3,618)	2,818 (2,145-3,236)	1,822 (1,373-2,124)	773 (576-901)	224 (168-257)
Maternal and neonatal disorders	28,767 (26,849-30,880)	12,245 (11,340-13,212)	1,846 (1,657-2,033)	-	-	-	-	-	-	-	-	-
Maternal disorders	28,767 (26,849-30,880)	12,245 (11,340-13,212)	1,846 (1,657-2,033)	-	-	-	-	-	-	-	-	-
Maternal hemorrhage	10,867 (8,669-13,352)	3,323 (2,564-4,182)	551 (433-691)	-	-	-	-	-	-	-	-	-
Maternal sepsis and other maternal infections	3,211 (2,393-4,507)	2,573 (2,011-3,157)	249 (189-329)	-	-	-	-	-	-	-	-	-
Maternal hypertensive disorders	2,485 (1,819-3,601)	609 (463-821)	88 (64-125)	-	-	-	-	-	-	-	-	-
Maternal obstructed labor and uterine rupture	2,498 (1,752-3,888)	1,476 (1,111-1,989)	124 (80-200)	-	-	-	-	-	-	-	-	-
Maternal abortion and miscarriage	2,780 (1,970-3,939)	2,059 (1,625-2,586)	207 (157-278)	-	-	-	-	-	-	-	-	-
Ectopic pregnancy	1,025 (427-2,433)	423 (198-923)	167 (86-287)	-	-	-	-	-	-	-	-	-
Indirect maternal deaths	3,029 (2,233-4,525)	700 (554-936)	222 (176-282)	-	-	-	-	-	-	-	-	-
Late maternal deaths	269 (205-367)	103 (87-123)	14 (12-17)	-	-	-	-	-	-	-	-	-
Maternal deaths aggravated by HIV/AIDS	148 (90-203)	100 (63-136)	11 (7-16)	-	-	-	-	-	-	-	-	-
Other maternal disorders	2,456 (1,685-3,738)	878 (679-1,146)	213 (164-284)	-	-	-	-	-	-	-	-	-
Neonatal disorders	-	-	-	-	-	-	-	-	-	-	-	-
Neonatal preterm birth	-	-	-	-	-	-	-	-	-	-	-	-
Neonatal encephalopathy due to birth asphyxia and trauma	-	-	-	-	-	-	-	-	-	-	-	-
Neonatal sepsis and other neonatal infections	-	-	-	-	-	-	-	-	-	-	-	-
Hemolytic disease and other neonatal jaundice	-	-	-	-	-	-	-	-	-	-	-	-
Other neonatal disorders	-	-	-	-	-	-	-	-	-	-	-	-
Nutritional deficiencies	4,977 (4,618-5,266)	5,321 (4,862-5,647)	6,401 (5,892-6,807)	8,943 (8,126-9,585)	11,124 (10,090-11,971)	13,450 (12,116-14,494)	16,198 (14,624-17,565)	16,785 (15,179-18,152)	15,864 (14,694-17,049)	13,948 (13,080-14,901)	8,405 (7,713-9,235)	3,664 (3,238-4,268)
Protein-energy malnutrition	3,682 (3,424-3,927)	4,085 (3,796-4,330)	4,880 (4,548-5,173)	6,753 (6,280-7,245)	8,419 (7,850-8,973)	10,262 (9,495-11,013)	12,869 (11,868-13,854)	13,479 (12,393-14,525)	13,254 (12,368-14,198)	11,763 (10,821-12,564)	7,267 (6,434-8,142)	3,224 (2,716-3,835)
Other nutritional deficiencies	1,295 (1,097-1,561)	1,235 (992-1,506)	1,521 (1,248-1,840)	2,190 (1,722-2,630)	2,704 (2,119-3,263)	3,188 (2,492-3,824)	3,329 (2,539-4,146)	3,306 (2,555-4,166)	2,610 (2,083-3,448)	2,184 (1,725-3,145)	1,138 (864-1,868)	441 (300-823)
Non-communicable diseases	834,650 (823,245-847,404)	1,066,234 (1,052,635-1,079,146)	1,331,362 (1,313,895-1,347,009)	1,928,476 (1,904,523-1,948,631)	2,717,679 (2,678,685-2,745,566)	3,416,279 (3,367,745-3,449,696)	3,824,637 (3,767,844-3,863,839)	3,551,731 (3,497,466-3,589,347)	3,602,225 (3,557,331-3,635,789)	2,585,388 (2,555,305-2,607,656)	1,210,199 (1,199,182-1,219,427)	353,889 (351,384-356,018)
Neoplasms	257,361 (251,406-264,178)	340,559 (334,193-348,345)	417,784 (410,545-427,348)	593,699 (585,123-606,149)	783,035 (771,929-801,977)	905,709 (895,616-923,040)	885,458 (875,233-907,366)	673,217 (664,229-688,360)	548,645 (540,229-558,947)	299,109 (292,790-303,910)	104,976 (102,105-106,513)	22,810 (22,183-23,179)
Lip and oral cavity cancer	5,805 (5,540-6,114)	8,399 (8,053-8,772)	10,856 (10,354-11,412)	13,950 (13,406-14,589)	16,257 (15,518-17,086)	14,775 (14,195-15,449)	11,757 (11,260-12,306)	8,334 (7,974-8,670)	6,857 (6,581-7,111)	3,910 (3,777-4,042)	1,467 (1,418-1,511)	329 (320-338)
Nasopharynx cancer	4,664 (4,377-5,025)	5,150 (4,882-5,463)	5,429 (5,151-5,693)	6,823 (6,496-7,134)	6,772 (6,439-7,071)	6,004 (5,729-6,269)	4,989 (4,771-5,224)	2,911 (2,776-3,061)	2,144 (2,050-2,260)	991 (945-1,040)	300 (289-314)	51 (49-53)
Other pharynx cancer	3,172 (2,726-3,412)	5,477 (4,809-5,837)	7,536 (6,659-8,037)	9,437 (8,504-10,041)	10,438 (9,339-11,352)	9,176 (8,169-9,853)	6,773 (6,066-7,269)	3,688 (3,238-3,982)	2,588 (2,255-2,779)	1,161 (1,014-1,236)	321 (294-337)	58 (55-60)
Esophageal cancer	11,393 (10,895-12,011)	18,278 (17,550-19,134)	24,748 (23,894-25,678)	36,120 (35,070-37,259)	53,103 (51,597-54,778)	58,823 (57,103-60,644)	57,690 (55,904-59,968)	40,175 (38,770-41,913)	26,484 (25,546-27,665)	12,544 (12,118-13,073)	3,597 (3,477-3,740)	593 (576-612)
Stomach cancer	30,640 (29,657-31,830)	39,409 (38,245-40,800)	47,321 (46,179-48,741)	72,479 (70,854-74,609)	104,742 (102,387-107,493)	121,921 (119,531-125,056)	121,063 (118,547-124,463)	90,605 (88,797-93,521)	71,008 (69,727-73,343)	37,707 (37,059-38,640)	12,322 (12,128-12,588)	2,522 (2,481-2,568)
Colon and rectum cancer	15,009 (14,208-16,135)	21,633 (20,686-23,070)	28,488 (27,246-30,233)	43,810 (42,197-45,962)	61,747 (59,480-64,748)	79,233 (77,129-82,277)	85,505 (83,103-88,935)	72,692 (71,193-75,349)	69,972 (68,670-71,821)	43,042 (42,255-43,978)	17,049 (16,765-17,372)	4,071 (4,003-4,148)
Liver cancer	36,383 (32,259-38,697)	42,107 (39,208-44,495)	43,249 (40,879-45,901)	55,253 (52,652-58,208)	70,599 (67,424-73,468)	72,843 (70,221-75,888)	64,900 (62,438-67,588)	44,283 (42,568-46,512)	29,573 (28,596-30,687)	13,829 (13,427-14,317)	3,823 (3,727-3,930)	674 (656-692)
Liver cancer due to hepatitis B	24,164 (21,100-26,373)	26,272 (23,690-28,423)	23,884 (21,437-26,106)	26,150 (23,467-28,793)	29,381 (26,049-32,723)	27,111 (24,567-29,915)	21,893 (19,509-24,655)	12,336 (11,139-13,629)	6,284 (5,538-7,120)	2,371 (2,130-2,635)	512 (446-580)	67 (53-82)
Liver cancer due to hepatitis C	3,656 (3,083-4,284)	5,583 (4,594-6,609)	8,173 (6,946-9,489)	14,158 (12,303-16,021)	21,131 (18,662-23,759)	22,579 (20,703-24,811)	21,168 (19,318-23,051)	16,504 (14,991-18,126)	12,833 (11,728-13,904)	6,776 (6,317-7,197)	2,074 (1,932-2,204)	401 (371-433)
Liver cancer due to alcohol use	3,870 (2,883-5,013)	4,881 (3,638-6,771)	5,572 (4,166-7,568)	7,401 (5,655-9,569)	10,356 (8,193-13,115)	13,339 (11,340-15,660)	12,523 (10,744-14,774)	7,974 (7,030-9,194)	5,195 (4,508-5,979)	2,205 (1,921-2,515)	548 (464-632)	84 (66-101)
Liver cancer due to NASH	1,303 (1,054-1,592)	1,664 (1,302-2,075)	2,031 (1,617-2,520)	3,100 (2,445-3,873)	4,424 (3,561-5,402)	4,938 (3,974-6,028)	5,038 (4,081-6,197)	4,274 (3,494-5,154)	3,180 (2,596-3,833)	1,547 (1,318-1,798)	446 (370-534)	81 (62-103)
Liver cancer due to other causes	3,389 (2,657-4,207)	3,706 (2,947-4,530)	3,589 (2,910-4,403)	4,444 (3,562-5,286)	5,308 (4,288-6,358)	4,876 (3,980-5,907)	4,279 (3,437-5,192)	3,194 (2,665-3,832)	2,081 (1,710-2,506)	931 (792-1,088)	243 (202-291)	40 (31-51)
Gallbladder and biliary tract cancer	2,243 (2,049-2,546)	3,770 (3,473-4,261)	5,789 (5,415-6,646)	8,780 (8,262-10,197)	13,227 (12,463-15,455)	16,578 (15,747-19,242)	17,937 (17,123-20,271)	15,684 (15,112-17,792)	14,331 (13,793-15,978)	8,531 (8,190-9,210)	3,117 (2,967-3,285)	690 (667-716)
Pancreatic cancer	5,295 (5,042-5,516)	8,517 (8,216-8,792)	12,277 (11,933-12,598)	19,958 (19,428-20,450)	27,897 (27,147-28,583)	35,048 (34,342-35,758)	36,159 (35,445-37,005)	29,117 (28,713-29,570)	25,208 (24,785-25,608)	14,338 (14,110-14,586)	5,283 (5,205-5,362)	1,106 (1,087-1,127)
Larynx cancer	4,097 (3,928-4,271)	7,029 (6,746-7,295)	10,173 (9,740-10,625)	14,407 (13,926-14,895)	16,907 (16,309-17,447)	16,903 (16,368-17,417)	12,925 (12,496-13,328)	7,586 (7,324-7,867)	5,439 (5,250-5,627)	2,367 (2,295-2,435)	652 (636-672)	107 (104-109)
Tracheal, bronchus, and lung cancer	32,820 (31,731-34,175)	52,677 (51,299-54,545)	74,754 (73,062-76,534)	122,793 (120,880-125,237)	173,913 (170,843-177,609)	208,606 (205,623-212,512)	193,579 (190,583-197,193)	132,915 (130,848-135,612)	90,291 (88,883-92,083)	40,662 (40,046-41,380)	11,640 (11,481-11,833)	2,152 (2,122-2,186)
Malignant skin melanoma	2,541 (2,005-3,154)	2,754 (2,125-3,392)	2,961 (2,367-3,648)	3,703 (2,983-4,621)	3,840 (3,169-4,883)	4,507 (3,671-5,664)	4,458 (3,610-5,633)	3,625 (2,950-4,511)	3,420 (2,824-4,375)	2,048 (1,646-2,559)	818 (648-997)	219 (164-272)
Non-melanoma skin cancer	1,030 (978-1,094)	1,265 (1,204-1,341)	1,617 (1,541-1,715)	2,281 (2,187-2,405)	3,580 (3,446-3,755)	4,316 (4,144-4,515)	4,475 (4,309-4,667)	3,847 (3,656-4,016)	4,045 (3,874-4,187)	3,154 (3,067-3,249)	1,672 (1,628-1,720)	595 (581-610)
Non-melanoma skin cancer (squamous-cell carcinoma)	1,030 (978-1,094)	1,265 (1,204-1,341)	1,617 (1,541-1,715)	2,281 (2,187-2,405)	3,580 (3,446-3,755)	4,316 (4,144-4,515)	4,475 (4,309-4,667)	3,847 (3,656-4,016)	4,045 (3,874-4,187)	3,154 (3,067-3,249)	1,672 (1,628-1,720)	595 (581-610)
Breast cancer	28,858 (26,429-32,128)	36,733 (34,370-40,093)	40,295 (38,014-44,080)	45,342 (43,333-48,968)	43,977 (42,070-47,710)	43,968 (42,524-47,460)	40,910 (39,687-43,925)	30,579 (29,758-32,893)	28,395 (27,813-29,837)	17,966 (17,602-18,667)	7,573 (7,393-7,813)	2,168 (2,116-2,225)
Cervical cancer	18,165 (16,594-20,765)	20,305 (18,748-22,934)	20,989 (19,111-23,763)	22,506 (20,901-24,999)	22,181 (20,550-24,610)	21,051 (19,657-23,084)	18,572 (17,439-20,357)	12,704 (12,072-14,106)	9,760 (9,375-10,375)	5,055 (4,885-5,394)	1,773 (1,726-1,841)	444 (431-460)
Uterine cancer	2,140 (1,979-2,292)	3,329 (3,106-3,531)	4,105 (3,864-4,325)	6,222 (5,898-6,540)	8,421 (7,995-8,866)	9,819 (9,440-10,268)	9,327 (8,995-9,688)	6,980 (6,771-7,237)	5,917 (5,763-6,083)	3,036 (2,970-3,113)	1,149 (1,121-1,176)	273 (264-283)
Ovarian cancer	4,822 (4,554-5,368)	7,468 (7,097-8,200)	9,510 (9,050-10,415)	12,012 (11,530-12,903)	13,515 (13,015-14,514)	14,801 (14,307-15,582)	14,091 (13,662-14,768)	10,075 (9,846-10,448)	8,418 (8,211-8,654)	4,644 (4,526-4,771)	1,723 (1,679-1,763)	361 (350-372)
Prostate cancer	479 (390-538)	1,247 (1,022-1,473)	3,056 (2,482-3,542)	7,906 (6,442-9,027)	17,311 (13,924-20,011)	31,014 (24,929-35,679)	42,961 (34,449-47,958)	46,781 (36,598-52,180)	48,224 (36,148-52,613)	28,997 (20,860-31,155)	10,870 (7,857-11,559)	2,097 (1,431-2,237)
Testicular cancer	620 (585-658)	417 (395-441)	277 (261-294)	263 (247-279)	283 (266-301)	281 (266-297)	253 (239-267)	191 (181-202)	183 (173-195)	97 (92-101)	30 (29-32)	8 (7-8)
Kidney cancer	2,053 (1,941-2,154)	3,542 (3,340-3,686)	5,043 (4,704-5,228)	8,026 (7,533-8,329)	9,912 (9,299-10,223)	12,174 (11,211-12,507)	11,492 (10,576-11,806)	8,755 (8,067-8,969)	7,453 (6,878-7,623)	3,903 (3,569-4,012)	1,362 (1,186-1,406)	267 (223-278)
Bladder cancer	1,728 (1,548-1,821)	2,725 (2,486-2,841)	4,206 (3,897-4,367)	7,362 (6,909-7,611)	12,394 (11,823-12,781)	18,024 (17,277-18,534)	21,261 (20,525-21,853)	19,804 (18,848-20,356)	20,369 (19,504-20,925)	12,680 (12,334-12,941)	4,798 (4,685-4,896)	1,055 (1,034-1,076)
Brain and nervous system cancer	9,512 (7,999-10,552)	10,344 (8,755-11,462)	10,725 (9,232-11,862)	13,694 (12,034-14,959)	15,286 (13,380-17,058)	15,236 (13,627-17,176)	12,474 (11,216-14,036)	8,081 (7,242-9,031)	4,949 (4,415-5,478)	2,115 (1,839-2,338)	616 (534-673)	102 (88-111)

SR Table 7: Global deaths by cause and age group for both sexes combined (with 95% confidence intervals), 1995

Cause	Early Neonatal	Late Neonatal	Post Neonatal	1 to 4	5 to 9	10 to 14	15 to 19	20 to 24	25 to 29	30 to 34	35 to 39
Thyroid cancer	-	-	-	-	-	194 (158-227)	346 (273-407)	455 (371-524)	814 (658-943)	573 (482-644)	638 (561-711)
Mesothelioma	-	-	-	-	-	-	84 (59-117)	136 (88-198)	225 (146-310)	267 (185-361)	398 (305-522)
Hodgkin lymphoma	18 (10-22)	7 (3-9)	171 (79-220)	1,058 (467-1,555)	1,819 (1,028-2,301)	1,655 (1,098-1,984)	2,104 (1,559-2,441)	2,451 (1,892-2,791)	2,711 (2,032-3,046)	2,502 (1,902-2,802)	2,193 (1,743-2,479)
Non-Hodgkin lymphoma	80 (73-92)	75 (64-93)	1,061 (861-1,266)	4,967 (3,454-6,795)	4,228 (3,604-4,989)	2,881 (2,585-3,219)	2,770 (2,586-2,966)	3,439 (3,162-3,727)	3,833 (3,517-4,123)	4,306 (4,018-4,576)	4,914 (4,661-5,188)
Multiple myeloma	-	-	-	-	-	-	222 (159-251)	301 (204-364)	363 (263-440)	495 (410-574)	758 (670-859)
Leukemia	397 (290-470)	389 (276-499)	6,124 (3,871-7,970)	18,066 (12,053-27,371)	20,769 (15,025-26,622)	15,054 (12,055-17,322)	12,384 (10,451-13,524)	11,553 (9,275-13,161)	11,595 (9,017-13,438)	10,813 (8,558-12,407)	9,940 (8,396-10,895)
Acute lymphoid leukemia	75 (57-97)	66 (47-100)	1,103 (781-1,770)	4,729 (3,192-8,365)	6,473 (4,988-9,554)	4,452 (3,770-5,675)	3,216 (2,819-3,820)	2,279 (2,019-2,820)	1,931 (1,669-2,530)	1,518 (1,307-1,927)	1,454 (1,287-1,720)
Chronic lymphoid leukemia	-	-	-	-	-	-	172 (129-206)	217 (150-279)	249 (171-337)	245 (170-319)	215 (176-248)
Acute myeloid leukemia	61 (46-86)	61 (43-100)	696 (438-1,280)	2,881 (1,612-6,141)	2,091 (1,400-3,491)	2,063 (1,636-2,862)	2,142 (1,837-2,661)	2,030 (1,718-2,649)	2,059 (1,708-2,773)	2,122 (1,804-2,750)	2,238 (1,990-2,683)
Chronic myeloid leukemia	-	-	-	-	-	-	475 (389-556)	711 (568-849)	1,028 (823-1,175)	1,171 (968-1,324)	1,238 (1,075-1,377)
Other leukemia	261 (174-316)	262 (170-340)	4,325 (2,438-5,826)	10,455 (5,895-16,548)	12,206 (7,473-15,795)	8,538 (5,979-9,912)	6,378 (4,854-7,195)	6,315 (4,453-7,544)	6,328 (4,202-7,821)	5,757 (3,883-6,980)	4,794 (3,631-5,437)
Other malignant neoplasms	976 (737-1,105)	581 (424-736)	3,505 (2,395-4,565)	11,449 (7,384-17,741)	6,516 (5,142-7,980)	6,671 (5,872-7,485)	7,427 (6,876-8,130)	6,428 (5,875-7,096)	6,063 (5,391-6,692)	6,188 (5,507-6,778)	7,083 (6,586-7,519)
Other neoplasms	57 (41-66)	38 (27-46)	503 (311-670)	1,243 (727-1,721)	1,161 (794-1,455)	791 (611-926)	650 (502-753)	730 (551-839)	895 (662-1,039)	1,117 (839-1,271)	1,277 (970-1,465)
Myelodysplastic, myeloproliferative, and other hematopoietic neoplasms	55 (40-65)	33 (23-41)	448 (274-614)	1,021 (594-1,490)	825 (571-1,083)	539 (415-651)	440 (339-520)	522 (401-628)	687 (518-834)	888 (666-1,041)	1,077 (825-1,227)
Other benign and in situ neoplasms	2 (1-3)	6 (3-8)	55 (34-76)	222 (130-368)	336 (221-433)	252 (186-307)	209 (153-260)	208 (146-265)	207 (138-268)	228 (150-299)	199 (131-262)
Cardiovascular diseases	4,499 (4,149-4,926)	3,712 (3,300-4,057)	29,894 (27,545-31,848)	24,737 (21,315-27,523)	14,954 (13,653-16,363)	13,978 (12,962-14,878)	34,544 (32,857-36,279)	58,553 (56,406-60,704)	88,517 (85,717-91,919)	129,689 (126,095-134,036)	179,686 (175,661-184,009)
Rheumatic heart disease	-	-	-	7,426 (5,937-9,382)	5,039 (4,450-5,731)	5,541 (5,049-6,036)	7,520 (6,857-8,293)	9,907 (9,010-10,826)	11,680 (10,727-12,758)	12,937 (11,903-14,119)	13,067 (11,914-14,362)
Ischemic heart disease	-	-	-	-	-	-	10,372 (9,802-10,965)	20,810 (19,871-21,819)	35,033 (33,567-36,719)	58,561 (56,623-60,966)	86,865 (84,370-89,366)
Stroke	1,855 (1,581-2,148)	1,501 (1,193-1,828)	11,431 (9,043-14,608)	7,146 (5,634-8,881)	5,660 (5,049-6,469)	4,788 (4,387-5,195)	9,610 (9,166-10,107)	17,185 (16,408-17,990)	26,466 (25,293-28,039)	38,611 (36,967-40,540)	53,748 (51,991-55,598)
Ischemic stroke	271 (212-354)	252 (175-378)	1,078 (740-1,610)	846 (554-1,397)	534 (456-660)	425 (371-491)	1,338 (1,195-1,496)	2,332 (2,105-2,573)	3,486 (3,203-3,784)	5,168 (4,778-5,593)	7,258 (6,754-7,809)
Intracerebral hemorrhage	841 (678-1,058)	621 (464-879)	5,517 (4,167-8,193)	2,774 (1,955-4,266)	2,805 (2,416-3,431)	2,425 (2,152-2,822)	5,623 (5,241-6,095)	10,144 (9,460-11,017)	16,050 (15,019-17,517)	23,987 (22,518-26,005)	33,562 (31,844-35,520)
Subarachnoid hemorrhage	744 (587-847)	628 (488-745)	4,836 (3,580-5,938)	3,525 (2,658-4,385)	2,321 (1,842-2,584)	1,938 (1,658-2,111)	2,649 (2,319-2,960)	4,709 (4,083-5,184)	6,930 (5,959-7,485)	9,456 (8,213-10,304)	12,928 (11,533-14,279)
Hypertensive heart disease	-	-	-	-	-	-	823 (603-982)	1,525 (1,119-1,804)	2,625 (1,942-3,068)	4,059 (3,113-4,648)	5,876 (4,668-6,672)
Non-rheumatic valvular heart disease	-	-	-	-	-	-	686 (554-864)	829 (662-1,065)	989 (793-1,261)	943 (790-1,152)	1,185 (1,053-1,402)
Non-rheumatic calcific aortic valve disease	-	-	-	-	-	-	205 (148-303)	242 (175-386)	328 (244-491)	361 (288-499)	504 (420-652)
Non-rheumatic degenerative mitral valve disease	-	-	-	-	-	-	352 (267-489)	446 (335-633)	503 (377-715)	478 (382-636)	562 (459-701)
Other non-rheumatic valve diseases	-	-	-	-	-	-	129 (93-170)	141 (99-184)	158 (112-218)	104 (74-144)	120 (80-154)
Cardiomyopathy and myocarditis	1,519 (1,261-1,705)	1,175 (966-1,370)	8,296 (6,885-9,662)	4,673 (3,394-5,601)	2,135 (1,783-2,464)	1,801 (1,615-2,018)	2,652 (2,354-3,033)	3,755 (3,348-4,274)	5,324 (4,829-5,932)	7,020 (6,427-7,668)	9,898 (8,987-10,768)
Myocarditis	197 (127-252)	241 (167-302)	2,360 (1,733-2,838)	1,561 (1,153-1,955)	878 (709-1,078)	595 (495-703)	902 (728-1,085)	1,229 (973-1,506)	1,426 (1,145-1,707)	1,135 (921-1,366)	1,169 (943-1,389)
Alcoholic cardiomyopathy	-	-	-	-	-	-	298 (230-359)	651 (548-768)	1,412 (1,216-1,588)	2,857 (2,520-3,134)	5,052 (4,426-5,555)
Other cardiomyopathy	1,322 (1,108-1,475)	934 (744-1,091)	5,936 (4,768-6,963)	3,112 (2,177-3,858)	1,257 (991-1,469)	1,206 (1,050-1,356)	1,453 (1,234-1,676)	1,875 (1,603-2,164)	2,486 (2,132-2,866)	3,028 (2,621-3,469)	3,677 (3,188-4,230)
Atrial fibrillation and flutter	-	-	-	-	-	-	-	-	-	63 (56-67)	80 (73-85)
Aortic aneurysm	-	-	-	-	-	-	280 (241-331)	471 (387-569)	788 (623-977)	1,083 (884-1,317)	1,527 (1,345-1,751)
Peripheral artery disease	-	-	-	-	-	-	-	-	-	-	-
Endocarditis	277 (230-346)	268 (209-350)	3,649 (2,930-4,557)	2,181 (1,564-2,992)	608 (525-739)	596 (520-724)	740 (655-897)	1,157 (1,025-1,406)	1,595 (1,406-1,868)	1,541 (1,364-1,741)	1,671 (1,487-1,904)
Other cardiovascular and circulatory diseases	848 (702-1,125)	768 (608-1,016)	6,517 (4,572-9,091)	3,313 (1,894-5,567)	1,513 (1,105-2,192)	1,252 (1,034-1,642)	1,860 (1,581-2,337)	2,914 (2,453-3,709)	4,016 (3,466-5,097)	4,872 (4,280-5,917)	5,770 (5,140-6,799)
Chronic respiratory diseases	0 (0-0)	0 (0-0)	0 (0-0)	33,705 (27,515-38,624)	9,149 (7,953-10,274)	6,789 (6,056-7,491)	10,152 (9,374-10,884)	14,514 (13,638-15,415)	19,629 (18,353-20,682)	24,662 (22,929-25,868)	30,446 (28,536-31,768)
Chronic obstructive pulmonary disease	-	-	0 (0-0)	3,726 (2,190-6,804)	1,407 (982-2,246)	793 (598-1,246)	3,032 (2,425-4,170)	5,219 (4,271-6,866)	8,117 (7,009-10,039)	11,463 (10,003-13,932)	13,237 (11,204-17,218)
Pneumoconiosis	-	-	-	-	-	-	45 (35-54)	81 (63-94)	208 (173-244)	375 (314-441)	479 (401-557)
Silicosis	-	-	-	-	-	-	12 (8-17)	27 (20-36)	100 (70-138)	240 (187-305)	316 (248-393)
Asbestosis	-	-	-	-	-	-	5 (2-7)	9 (4-13)	14 (7-21)	19 (8-32)	21 (11-29)
Coal workers pneumoconiosis	-	-	-	-	-	-	7 (4-11)	11 (6-16)	39 (22-57)	42 (25-62)	57 (31-90)
Other pneumoconiosis	-	-	-	-	-	-	21 (15-29)	34 (22-47)	55 (35-78)	74 (49-108)	85 (59-115)
Asthma	-	-	-	25,733 (20,051-31,191)	6,377 (5,192-7,510)	5,193 (4,440-5,971)	5,957 (4,433-6,949)	7,710 (5,373-8,981)	9,586 (6,685-11,088)	10,651 (6,922-12,385)	13,934 (8,756-16,413)
Interstitial lung disease and pulmonary sarcoidosis	-	-	-	543 (282-1,217)	212 (129-412)	128 (91-209)	177 (124-279)	271 (195-432)	411 (300-634)	586 (423-854)	903 (649-1,298)
Other chronic respiratory diseases	0 (0-0)	0 (0-0)	0 (0-0)	3,703 (1,771-6,032)	1,154 (780-1,587)	675 (502-871)	941 (675-1,281)	1,233 (883-1,678)	1,307 (935-1,722)	1,586 (1,150-2,091)	1,894 (1,335-2,487)
Digestive diseases	2,878 (2,607-3,215)	3,368 (2,952-4,173)	25,205 (20,616-36,076)	30,336 (22,618-44,478)	17,294 (14,485-21,446)	12,627 (11,032-14,365)	19,761 (17,818-21,559)	28,500 (26,036-30,527)	42,288 (39,077-45,658)	58,973 (55,221-62,960)	79,896 (74,861-84,378)
Cirrhosis and other chronic liver diseases	-	-	-	12,518 (8,341-21,537)	6,738 (5,319-9,534)	5,686 (4,845-6,905)	8,798 (7,928-9,875)	14,457 (12,993-15,783)	23,954 (21,718-26,079)	38,217 (35,551-41,141)	54,356 (50,353-57,685)
Cirrhosis and other chronic liver diseases due to hepatitis B	-	-	-	398 (242-722)	614 (471-864)	831 (654-1,074)	1,708 (1,310-2,163)	5,904 (4,956-6,915)	12,199 (10,370-14,181)	17,921 (15,966-20,306)	21,167 (18,084-24,437)
Cirrhosis and other chronic liver diseases due to hepatitis C	-	-	-	128 (72-247)	189 (133-281)	290 (216-390)	603 (444-800)	2,444 (1,903-3,072)	4,827 (3,732-6,135)	8,803 (7,270-10,388)	13,911 (11,166-17,062)
Cirrhosis and other chronic liver diseases due to alcohol use	-	-	-	-	-	-	57 (26-100)	1,064 (812-1,359)	2,740 (2,145-3,416)	6,753 (5,916-7,794)	13,332 (11,266-15,708)
Cirrhosis due to NASH	-	-	-	-	-	-	149 (116-194)	428 (341-527)	824 (644-1,027)	1,658 (1,394-1,940)	2,887 (2,289-3,521)
Cirrhosis and other chronic liver diseases due to other causes	-	-	-	11,992 (8,026-20,673)	5,935 (4,654-8,359)	4,565 (3,847-5,565)	6,281 (5,437-7,180)	4,618 (3,694-5,667)	3,364 (2,503-4,325)	3,081 (2,254-3,994)	3,059 (2,282-3,914)
Upper digestive system diseases	-	-	-	2,596 (1,785-3,636)	2,037 (1,641-2,564)	1,561 (1,342-1,847)	2,877 (2,502-3,325)	5,385 (4,804-6,147)	6,927 (6,215-7,860)	8,556 (7,826-9,458)	10,349 (9,547-11,557)
Peptic ulcer disease	-	-	-	1,942 (1,255-3,035)	1,548 (1,222-2,058)	1,274 (1,085-1,535)	2,419 (2,100-2,843)	4,576 (4,069-5,358)	5,770 (5,192-6,648)	7,079 (6,475-7,948)	8,813 (8,127-9,886)
Gastritis and duodenitis	-	-	-	655 (343-1,017)	489 (285-710)	287 (196-385)	458 (336-579)	810 (547-1,067)	1,157 (828-1,484)	1,477 (1,110-1,824)	1,537 (1,160-1,838)
Appendicitis	-	-	-	3,365 (1,812-5,414)	2,492 (1,926-3,403)	1,947 (1,648-2,400)	2,936 (2,509-3,575)	2,280 (1,973-2,757)	2,547 (2,214-3,104)	1,550 (1,378-1,819)	1,798 (1,592-2,073)
Paralytic ileus and intestinal obstruction	2,878 (2,607-3,215)	3,368 (2,952-4,173)	25,205 (20,616-36,076)	3,458 (2,497-4,605)	3,112 (1,956-4,012)	1,796 (1,189-2,160)	3,085 (2,137-3,637)	2,922 (1,984-3,424)	3,373 (2,361-3,867)	3,366 (2,271-3,892)	4,242 (2,932-4,938)
Inguinal, femoral, and abdominal hernia	-	-	-	916 (609-1,483)	438 (346-591)	250 (192-306)	362 (288-430)	397 (317-469)	491 (414-597)	566 (454-666)	757 (627-888)
Inflammatory bowel disease	-	-	-	1,551 (714-3,255)	354 (200-654)	180 (125-279)	218 (166-306)	393 (293-569)	528 (404-758)	589 (477-781)	648 (554-793)
Vascular intestinal disorders	-	-	-	517 (191-745)	108 (62-136)	52 (40-61)	79 (64-91)	170 (134-198)	243 (194-279)	279 (233-320)	461 (396-521)
Gallbladder and biliary diseases	-	-	-	1,000 (682-1,365)	427 (344-520)	306 (256-355)	348 (303-386)	615 (531-677)	857 (758-959)	1,162 (1,040-1,296)	1,392 (1,234-1,497)
Pancreatitis	-	-	-	158 (92-309)	184 (130-294)	182 (148-251)	463 (400-590)	1,111 (951-1,463)	2,252 (1,931-2,849)	3,336 (2,965-3,982)	4,446 (4,114-5,143)
Other digestive diseases	-	-	-	4,256 (3,201-6,141)	1,404 (1,176-1,830)	666 (584-816)	595 (494-784)	769 (683-897)	1,115 (968-1,333)	1,352 (1,194-1,576)	1,447 (1,275-1,704)
Neurological disorders	-	-	11,757 (7,894-14,152)	16,914 (9,048-22,487)	8,935 (6,757-10,612)	9,142 (7,581-10,204)	13,481 (11,700-14,613)	13,504 (11,480-14,398)	12,851 (11,078-13,797)	12,165 (10,436-12,936)	10,819 (9,499-11,550)
Alzheimer's disease and other dementias	-	-	-	-	-	-	-	-	-	-	-
Parkinson's disease	-	-	-	-	-	-	-	15 (12-18)	23 (18-26)	49 (40-54)	50 (42-56)
Epilepsy	-	-	7,868 (5,196-9,448)	13,365 (7,104-18,341)	7,412 (5,605-8,914)	7,379 (5,869-8,326)	11,051 (9,277-12,125)	11,449 (9,386-12,299)	11,105 (9,328-12,006)	10,219 (8,485-11,009)	8,381 (7,097-9,145)
Multiple sclerosis	-	-	-	-	-	-	-	156 (109-234)	336 (222-471)	619 (445-818)	991 (749-1,243)
Motor neuron disease	13 (4-19)	27 (12-40)	664 (474-992)	237 (139-356)	54 (44-76)	66 (55-94)	91 (84-117)	85 (74-120)	86 (72-116)	144 (129-184)	271 (254-331)
Other neurological disorders	-	-	3,225 (1,705-4,533)	3,312 (1,315-5,073)	1,468 (864-2,031)	1,697 (1,231-2,147)	2,340 (2,049-2,818)	1,798 (1,528-2,148)	1,301 (1,046-1,557)	1,133 (924-1,323)	1,127 (1,017-1,330)
Mental disorders	-	-	-	-	1 (0-1)	4 (3-5)	20 (18-26)	33 (29-43)	36 (31-46)	37 (33-44)	34 (30-38)
Eating disorders	-	-	-	-	1 (0-1)	4 (3-5)	20 (18-26)	33 (29-43)	36 (31-46)	37 (33-44)	34 (30-38)
Anorexia nervosa	-	-	-	-	0 (0-1)	4 (3-5)	18 (16-23)	23 (18-28)	23 (17-29)	26 (19-31)	25 (19-29)
Bulimia nervosa	-	-	-	-	0 (0-0)	0 (0-0)	2 (1-3)	10 (8-18)	13 (10-23)	12 (9-20)	9 (6-16)
Substance use disorders	0 (0-0)	0 (0-0)	0 (0-0)	2 (0-8)	5 (1-13)	5 (1-12)	5,396 (5,174-5,635)	13,378 (12,833-13,965)	21,298 (20,460-22,354)	27,562 (26,639-28,626)	29,716 (28,652-30,596)
Alcohol use disorders	-	-	-	-	-	-	1,449 (1,278-1,572)	3,606 (3,233-3,856)	6,712 (6,153-7,198)	12,675 (11,736-13,439)	18,680 (17,504-19,494)
Drug use disorders	0 (0-0)	0 (0-0)	0 (0-0)	2 (0-8)	5 (1-13)	5 (1-12)	3,947 (3,767-4,170)	9,772 (9,271-10,319)	14,586 (13,870-15,497)	14,887 (14,332-15,685)	11,035 (10,676-11,483)
Opioid use disorders	0 (0-0)	0 (0-0)	0 (0-0)	2 (0-8)	5 (1-13)	5 (1-12)	2,364 (2,057-2,720)	5,803 (4,830-6,895)	8,719 (7,267-10,448)	8,961 (7,631-10,532)	6,757 (6,069-7,433)
Cocaine use disorders	-	-	-	-	-	-	97 (75-124)	241 (169-361)	349 (242-503)	529 (411-734)	480 (406-609)
Amphetamine use disorders	-	-	-	-	-	-	217 (117-378)	589 (242-1,111)	856 (383-1,584)	622 (317-1,093)	445 (271-683)
Other drug use disorders	-	-	-	-	-	-	1,268 (942-1,625)	3,138 (2,189-4,306)	4,661 (3,253-6,413)	4,775 (3,592-6,328)	3,354 (2,852-4,159)
Diabetes and kidney diseases	36 (26-45)	116 (87-140)	11,668 (10,501-12,586)	14,523 (12,357-16,425)	8,062 (7,061-8,928)	7,459 (6,665-8,130)	12,125 (11,371-12,932)	19,647 (18,647-20,545)	24,880 (23,640-26,144)	30,693 (29,364-32,143)	34,642 (33,455-36,031)
Diabetes mellitus	36 (26-45)	116 (87-140)	1,368 (1,050-1,618)	920 (710-1,141)	1,003 (819-1,191)	1,360 (1,033-1,677)	2,532 (2,398-2,668)	4,271 (4,075-4,466)	6,092 (5,852-6,407)	8,708 (8,391-9,117)	11,580 (11,167-12,147)
Diabetes mellitus type 1	36 (26-45)	116 (87-140)	1,368 (1,050-1,618)	920 (710-1,141)	1,003 (819-1,191)	1,360 (1,033-1,677)	2,266 (2,111-2,411)	3,753 (3,537-3,986)	5,320 (5,073-5,586)	6,951 (6,600-7,300)	9,080 (8,677-9,549)
Diabetes mellitus type 2	-	-	-	-	-	-	266 (196-388)	518 (383-745)	771 (584-1,139)	1,757 (1,460-2,301)	2,500 (2,141-3,031)
Chronic kidney disease	-	-	9,885 (8,769-10,749)	13,002 (10,964-14,754)	6,481 (5,592-7,210)	5,748 (5,172-6,232)	9,267 (8,567-9,983)	14,834 (13,937-15,654)	18,074 (16,869-19,169)	21,235 (19,957-22,344)	22,434 (21,382-23,589)
Chronic kidney disease due to diabetes mellitus type 1	-	-	38 (21-63)	57 (29-104)	43 (21-78)	63 (33-105)	180 (94-293)	699 (370-1,142)	1,870 (1,138-2,881)	3,793 (2,457-5,411)	4,973 (3,304-6,969)
Chronic kidney disease due to diabetes mellitus type 2	-	-	28 (15-49)	37 (18-72)	26 (12-49)	32 (14-60)	81 (36-150)	234 (100-444)	494 (216-938)	944 (438-1,694)	1,479 (772-2,481)
Chronic kidney disease due to hypertension	-	-	49 (28-75)	96 (53-152)	79 (44-130)	122 (69-192)	348 (210-533)	879 (565-1,270)	1,450 (981-2,053)	2,069 (1,430-2,909)	2,573 (1,784-3,506)
Chronic kidney disease due to glomerulonephritis	-	-	2,331 (1,658-3,181)	3,939 (2,707-5,539)	2,007 (1,335-2,838)	1,986 (1,337-2,713)	3,701 (2,597-4,901)	5,910 (4,147-7,872)	6,475 (4,562-8,606)	6,445 (4,525-8,686)	6,092 (4,083-8,477)
Chronic kidney disease due to other and unspecified causes	-	-	7,438 (6,368-8,367)	8,872 (6,970-10,639)	4,326 (3,381-5,232)	3,546 (2,792-4,319)	4,956 (3,736-6,112)	7,112 (5,141-9,073)	7,785 (5,648-9,959)	7,983 (5,597-10,298)	7,318 (5,031-9,742)
Acute glomerulonephritis	-	-	415 (276-526)	602 (365-816)	578 (488-681)	351 (305-402)	326 (291-364)	542 (490-603)	715 (636-805)	750 (686-827)	627 (570-685)
Skin and subcutaneous diseases	-	-	4,258 (1,856-5,705)	2,496 (903-4,015)	680 (306-909)	584 (305-743)	792 (453-1,005)	777 (440-939)	784 (443-932)	1,216 (683-1,463)	1,225 (764-1,468)
Bacterial skin diseases	-	-	4,165 (1,780-5,612)	2,328 (786-3,847)	567 (211-804)	464 (214-620)	647 (333-847)	625 (322-780)	615 (322-755)	1,007 (540-1,242)	1,019 (614-1,222)
Cellulitis	-	-	385 (85-776)	437 (95-881)	182 (59-278)	163 (65-229)	169 (72-236)	155 (74-196)	152 (75-195)	222 (122-284)	236 (139-327)
Pyoderma	-	-	3,780 (1,644-4,959)	1,891 (680-2,968)	385 (148-543)	301 (142-401)	478 (253-623)	470 (234-594)	462 (235-578)	785 (386-994)	783 (426-973)
Decubitus ulcer	-	-	-	68 (38-142)	52 (38-90)	58 (40-90)	80 (52-119)	78 (49-116)	107 (66-161)	139 (88-217)	140 (89-219)
Other skin and subcutaneous diseases	-	-	93 (49-205)	100 (59-223)	61 (43-105)	62 (43-93)	65 (43-98)	74 (48-108)	63 (41-87)	70 (44-104)	65 (41-98)
Musculoskeletal disorders	-	-	-	-	783 (604-950)	1,072 (912-1,225)	1,645 (1,461-1,981)	2,108 (1,863-2,707)	2,360 (2,084-3,026)	2,535 (2,251-3,114)	2,406 (2,227-2,685)
Rheumatoid arthritis	-	-	-	-	145 (98-214)	116 (89-150)	126 (99-164)	167 (130-217)	216 (171-285)	299 (233-382)	315 (261-375)
Other musculoskeletal disorders	-	-	-	-	638 (484-785)	956 (816-1,097)	1,519 (1,326-1,832)	1,941 (1,695-2,523)	2,144 (1,877-2,831)	2,236 (1,972-2,792)	2,091 (1,924-2,326)
Other non-communicable diseases	217,718 (196,332-262,919)	102,249 (90,442-121,245)	358,656 (311,628-416,936)	139,830 (116,298-172,241)	37,573 (33,963-41,652)	22,217 (20,304-24,414)	18,617 (16,831-20,460)	19,790 (18,106-21,620)	18,090 (16,610-19,355)	16,250 (14,833-17,270)	14,658 (13,431-15,655)
Congenital birth defects	213,789 (192,572-258,722)	92,537 (81,779-112,032)	282,496 (257,488-331,208)	110,799 (93,506-142,249)	26,556 (24,079-29,759)	14,015 (12,973-15,476)	8,348 (7,893-9,174)	8,314 (7,891-9,060)	7,593 (7,197-8,204)	5,175 (4,879-5,606)	4,387 (4,107-4,853)
Neural tube defects	32,998 (23,898-45,090)	10,245 (6,948-14,512)	27,701 (16,556-41,642)	14,686 (6,812-27,465)	1,653 (1,173-2,108)	846 (661-1,028)	363 (312-432)	340 (282-410)	278 (228-328)	202 (174-240)	132 (117-148)
Congenital heart anomalies	73,819 (51,617-95,347)	43,187 (30,135-54,794)	151,257 (103,281-192,539)	56,716 (31,251-78,967)	16,880 (12,700-20,408)	9,251 (7,860-10,685)	5,365 (4,887-6,098)	5,373 (4,807-6,069)	5,270 (4,725-5,925)	3,291 (2,991-3,699)	2,951 (2,706-3,349)
Orofacial clefts	4,658 (1,889-10,039)	1,954 (753-4,300)	3,767 (1,442-8,641)	789 (206-2,410)	-	-	-	-	-	-	-
Down syndrome	4,145 (2,393-6,267)	2,009 (1,076-3,038)	9,732 (4,444-15,742)	5,132 (1,876-9,888)	762 (415-1,179)	416 (290-541)	474 (347-625)	586 (408-775)	672 (445-975)	599 (407-836)	410 (319-491)
Other chromosomal abnormalities	4,378 (2,504-7,824)	2,301 (1,225-4,506)	4,986 (2,803-9,058)	1,185 (561-2,826)	254 (150-454)	148 (98-221)	134 (90-221)	134 (86-210)	113 (78-168)	80 (56-124)	78 (55-109)
Congenital musculoskeletal and limb anomalies	4,456 (3,126-6,506)	1,338 (946-1,984)	3,556 (2,428-5,747)	1,357 (867-2,674)	477 (325-711)	313 (227-396)	273 (194-342)	218 (149-286)	142 (101-179)	117 (85-145)	93 (70-115)
Urogenital congenital anomalies	5,488 (3,822-7,399)	1,816 (1,154-2,490)	3,152 (1,736-4,933)	989 (391-1,867)	366 (231-576)	261 (193-348)	118 (100-150)	141 (117-176)	117 (91-152)	111 (87-135)	123 (99-149)
Digestive congenital anomalies	22,520 (15,412-38,158)	11,052 (7,291-19,143)	26,908 (16,756-48,569)	6,505 (3,045-16,447)	597 (373-971)	219 (158-303)	101 (79-130)	91 (68-130)	70 (54-89)	53 (42-68)	53 (44-64)
Other congenital birth defects	61,326 (39,495-82,131)	18,636 (9,962-26,874)	51,437 (23,987-77,943)	23,440 (9,056-40,777)	5,566 (3,308-8,139)	2,562 (1,835-3,364)	1,522 (1,252-1,947)	1,433 (1,109-1,839)	931 (692-1,207)	722 (572-918)	547 (460-676)
Urinary diseases and male infertility	942 (798-1,103)	844 (689-986)	7,081 (6,370-7,792)	5,802 (4,824-6,737)	2,218 (1,923-2,549)	1,414 (1,268-1,567)	2,277 (2,099-2,464)	3,279 (3,079-3,509)	3,093 (2,941-3,286)	3,729 (3,554-3,954)	3,815 (3,637-4,018)
Urinary tract infections	171 (116-234)	240 (151-331)	4,480 (3,474-5,431)	2,203 (1,222-3,291)	1,070 (776-1,411)	754 (609-908)	1,575 (1,364-1,820)	2,229 (1,947-2,583)	2,033 (1,720-2,336)	2,326 (1,976-2,675)	2,431 (2,125-2,737)

Cause	40 to 44	45 to 49	50 to 54	54 to 59	60 to 64	65 to 69	70 to 74	75 to 79	80 to 84	85 to 89	90 to 94	95 plus
Thyroid cancer	919 (822-1,004)	1,134 (1,046-1,224)	1,509 (1,390-1,646)	2,118 (1,983-2,303)	2,704 (2,518-2,988)	3,181 (3,001-3,529)	3,250 (3,082-3,633)	2,745 (2,641-3,023)	2,438 (2,363-2,624)	1,314 (1,276-1,412)	428 (415-453)	92 (89-96)
Mesothelioma	670 (532-857)	789 (666-953)	1,120 (986-1,322)	1,873 (1,648-2,163)	2,267 (1,952-2,602)	2,816 (2,428-3,178)	2,973 (2,588-3,333)	2,410 (2,182-2,646)	1,861 (1,688-2,026)	899 (814-978)	271 (255-290)	50 (48-53)
Hodgkin lymphoma	2,040 (1,570-2,300)	1,885 (1,478-2,187)	1,876 (1,483-2,156)	2,350 (1,843-2,785)	2,602 (2,021-2,979)	2,881 (2,322-3,299)	2,610 (2,095-3,059)	1,539 (1,192-1,782)	1,135 (890-1,278)	515 (417-582)	111 (88-127)	16 (12-18)
Non-Hodgkin lymphoma	6,193 (5,884-6,540)	7,247 (6,963-7,583)	8,700 (8,416-9,098)	11,605 (11,281-12,033)	15,357 (14,898-15,932)	18,391 (17,980-18,878)	19,262 (18,858-19,691)	15,375 (15,098-15,629)	13,184 (12,968-13,391)	7,197 (7,074-7,318)	2,327 (2,289-2,369)	445 (436-453)
Multiple myeloma	1,447 (1,282-1,629)	2,111 (1,930-2,349)	3,206 (2,901-3,586)	5,191 (4,689-5,873)	7,360 (6,708-8,510)	9,323 (8,572-10,665)	9,892 (8,914-11,468)	8,256 (7,465-9,403)	7,007 (6,379-8,104)	3,857 (3,460-4,590)	1,224 (1,107-1,403)	213 (195-256)
Leukemia	11,351 (9,569-12,356)	11,559 (10,095-12,402)	12,025 (10,819-12,995)	15,708 (14,496-17,095)	19,678 (18,465-21,322)	23,104 (22,056-24,997)	23,777 (22,840-25,555)	19,307 (18,654-20,689)	16,963 (16,489-18,042)	10,089 (9,859-10,656)	3,817 (3,733-3,986)	874 (855-898)
Acute lymphoid leukemia	1,398 (1,242-1,662)	1,258 (1,147-1,471)	1,256 (1,150-1,457)	1,576 (1,469-1,815)	1,687 (1,565-1,988)	1,796 (1,682-2,080)	1,563 (1,464-1,801)	900 (850-1,039)	685 (645-789)	346 (327-389)	111 (106-122)	22 (21-23)
Chronic lymphoid leukemia	335 (289-378)	470 (429-513)	735 (688-792)	1,364 (1,290-1,458)	2,194 (2,094-2,325)	3,082 (2,950-3,261)	3,653 (3,516-3,824)	3,342 (3,238-3,466)	3,509 (3,391-3,643)	2,421 (2,334-2,498)	1,017 (980-1,050)	267 (257-276)
Acute myeloid leukemia	2,537 (2,281-3,006)	2,905 (2,663-3,329)	3,079 (2,854-3,432)	3,988 (3,723-4,406)	4,961 (4,652-5,456)	5,800 (5,524-6,334)	5,977 (5,707-6,454)	4,599 (4,443-4,940)	3,622 (3,502-3,919)	1,830 (1,765-1,983)	588 (566-665)	118 (113-138)
Chronic myeloid leukemia	1,383 (1,225-1,517)	1,449 (1,312-1,569)	1,606 (1,451-1,773)	1,990 (1,847-2,192)	2,328 (2,187-2,552)	2,763 (2,606-3,004)	2,754 (2,611-2,953)	2,427 (2,307-2,605)	2,329 (2,225-2,469)	1,364 (1,302-1,440)	523 (498-547)	118 (112-125)
Other leukemia	5,698 (4,360-6,402)	5,478 (4,419-6,139)	5,350 (4,541-5,853)	6,789 (5,952-7,404)	8,508 (7,623-9,447)	9,663 (8,823-10,756)	9,831 (9,120-10,806)	8,039 (7,559-8,913)	6,818 (6,486-7,507)	4,128 (3,941-4,486)	1,578 (1,516-1,693)	349 (337-365)
Other malignant neoplasms	9,668 (8,942-10,169)	11,388 (10,562-11,854)	13,757 (12,820-14,387)	18,762 (17,703-19,939)	22,861 (21,538-24,149)	25,910 (24,515-27,600)	24,339 (23,177-26,038)	18,797 (17,916-20,334)	15,709 (14,579-16,939)	8,964 (7,990-9,584)	3,410 (3,003-3,579)	800 (711-843)
Other neoplasms	1,604 (1,231-1,844)	1,871 (1,400-2,176)	2,189 (1,588-2,571)	2,966 (2,142-3,477)	3,902 (2,867-4,652)	5,004 (3,688-6,129)	5,805 (4,351-7,573)	5,377 (3,980-6,937)	5,319 (4,035-6,904)	3,499 (2,662-4,812)	1,433 (1,079-2,066)	381 (289-577)
Myelodysplastic, myeloproliferative, and other hematopoietic neoplasms	1,441 (1,115-1,644)	1,748 (1,308-2,042)	2,101 (1,528-2,455)	2,891 (2,086-3,405)	3,845 (2,833-4,608)	4,963 (3,653-6,095)	5,776 (4,325-7,548)	5,361 (3,972-6,913)	5,307 (4,024-6,895)	3,493 (2,656-4,808)	1,430 (1,078-2,064)	380 (289-576)
Other benign and in situ neoplasms	163 (106-216)	123 (77-164)	88 (55-123)	75 (43-111)	57 (30-90)	41 (21-64)	29 (14-48)	16 (7-26)	12 (5-22)	6 (2-9)	2 (1-4)	0 (0-1)
Cardiovascular diseases	300,688 (294,579-307,612)	409,324 (401,979-417,777)	532,709 (523,822-541,882)	810,241 (797,139-823,050)	1,171,846 (1,151,870-1,192,343)	1,553,147 (1,530,551-1,582,099)	1,795,638 (1,769,533-1,830,240)	1,731,205 (1,709,552-1,772,612)	1,879,366 (1,859,816-1,921,449)	1,404,030 (1,390,109-1,430,066)	667,065 (661,477-676,784)	203,535 (202,175-205,603)
Rheumatic heart disease	17,515 (16,214-19,046)	17,988 (16,730-19,531)	18,849 (17,435-20,781)	23,966 (22,402-26,216)	29,829 (28,106-32,886)	32,742 (31,044-36,189)	34,049 (32,274-37,809)	28,127 (26,646-31,339)	24,719 (23,501-27,363)	15,542 (14,843-17,013)	6,399 (6,125-6,867)	1,688 (1,627-1,783)
Ischemic heart disease	146,554 (142,918-150,410)	207,472 (202,809-212,382)	272,210 (266,421-278,226)	417,310 (409,752-426,075)	583,374 (572,570-595,297)	769,017 (756,949-783,543)	859,025 (845,090-875,761)	824,695 (813,417-845,388)	931,659 (920,752-953,071)	719,413 (711,260-733,937)	358,967 (355,318-366,019)	116,765 (115,620-118,518)
Stroke	98,797 (95,976-102,018)	137,629 (134,026-141,687)	185,496 (181,374-191,070)	285,582 (279,231-293,092)	444,488 (434,371-457,592)	600,336 (587,932-619,991)	713,351 (699,298-741,742)	684,474 (670,808-714,323)	703,478 (690,111-731,890)	492,108 (483,637-510,534)	209,263 (206,013-215,808)	56,625 (55,773-57,991)
Ischemic stroke	14,034 (13,200-14,974)	23,726 (22,451-25,250)	36,523 (34,041-39,399)	68,223 (64,618-72,386)	144,049 (136,664-152,482)	221,371 (211,832-231,517)	308,613 (297,367-322,147)	316,923 (305,786-330,239)	412,367 (401,995-427,065)	306,443 (299,975-315,999)	134,995 (132,462-138,580)	38,351 (37,664-39,183)
Intracerebral hemorrhage	62,360 (59,341-66,278)	89,584 (85,462-94,915)	119,574 (113,962-127,426)	175,320 (166,377-186,588)	251,712 (236,597-269,867)	319,086 (301,833-343,400)	343,508 (325,131-374,827)	312,861 (298,929-343,081)	249,375 (239,578-274,239)	160,780 (154,968-174,712)	64,972 (62,948-69,279)	16,076 (15,627-16,787)
Subarachnoid hemorrhage	22,403 (19,053-24,735)	24,318 (20,150-27,065)	29,399 (24,194-33,516)	42,039 (34,077-47,869)	48,727 (36,371-56,277)	59,879 (44,451-68,508)	61,231 (42,016-69,837)	54,690 (37,891-62,377)	41,736 (28,113-47,007)	24,885 (18,049-27,388)	9,296 (7,388-10,050)	2,197 (1,925-2,331)
Hypertensive heart disease	10,586 (8,422-11,789)	15,053 (11,721-16,738)	20,778 (16,317-22,898)	31,077 (25,149-34,192)	47,478 (37,742-52,050)	64,114 (49,512-69,533)	85,533 (66,373-92,203)	85,557 (67,394-92,207)	85,833 (71,292-92,649)	61,798 (51,293-66,561)	29,529 (23,845-32,482)	8,560 (6,833-9,647)
Non-rheumatic valvular heart disease	1,534 (1,368-1,789)	1,691 (1,531-1,941)	2,210 (2,035-2,486)	3,249 (3,001-3,618)	4,609 (4,193-5,072)	6,735 (6,149-7,331)	9,173 (8,154-10,055)	10,869 (9,480-11,959)	14,125 (12,113-15,362)	12,054 (10,463-13,520)	6,662 (5,561-7,632)	2,164 (1,867-2,562)
Non-rheumatic calcific aortic valve disease	682 (579-853)	794 (674-971)	1,082 (934-1,277)	1,617 (1,386-1,886)	2,467 (2,166-2,826)	3,856 (3,369-4,376)	5,544 (4,836-6,184)	6,752 (5,769-7,556)	9,530 (7,942-10,451)	8,633 (7,144-9,870)	4,971 (4,048-5,903)	1,669 (1,352-1,953)
Non-rheumatic degenerative mitral valve disease	705 (584-857)	794 (670-944)	1,001 (841-1,179)	1,459 (1,236-1,685)	1,924 (1,576-2,232)	2,581 (2,138-3,002)	3,269 (2,623-3,882)	3,579 (2,951-4,224)	4,006 (3,285-4,915)	3,025 (2,424-3,852)	1,525 (1,232-1,966)	454 (368-619)
Other non-rheumatic valve diseases	147 (98-200)	103 (70-140)	127 (84-167)	173 (112-224)	218 (145-280)	298 (190-385)	361 (226-458)	538 (344-672)	588 (377-735)	396 (262-490)	166 (111-215)	42 (29-55)
Cardiomyopathy and myocarditis	13,183 (11,694-14,412)	14,317 (12,586-15,461)	14,147 (12,468-15,360)	20,332 (17,883-21,838)	19,934 (17,594-21,516)	21,808 (18,810-23,264)	21,940 (18,193-23,425)	21,675 (18,123-23,129)	28,753 (24,193-30,261)	28,754 (24,317-30,498)	14,934 (12,984-15,871)	4,898 (4,435-5,171)
Myocarditis	1,446 (1,144-1,720)	1,076 (848-1,272)	968 (742-1,153)	1,282 (971-1,535)	1,205 (913-1,422)	1,401 (1,092-1,642)	1,613 (1,228-1,873)	1,722 (1,340-1,984)	2,454 (1,825-2,977)	2,780 (2,069-3,693)	1,526 (1,161-2,166)	516 (400-772)
Alcoholic cardiomyopathy	6,952 (5,932-7,751)	7,866 (6,797-8,775)	6,861 (5,887-7,586)	10,130 (9,150-11,286)	7,640 (7,036-8,728)	7,209 (6,630-8,295)	5,018 (4,496-6,181)	3,810 (3,442-4,782)	3,765 (3,286-5,018)	2,715 (2,262-3,996)	1,334 (1,109-2,120)	404 (345-642)
Other cardiomyopathy	4,785 (4,094-5,485)	5,375 (4,619-6,175)	6,318 (5,320-7,243)	8,920 (7,336-10,171)	11,090 (9,248-12,571)	13,198 (10,656-14,638)	15,309 (11,798-16,788)	16,143 (12,615-17,598)	22,534 (17,019-24,204)	23,260 (17,076-25,201)	12,074 (9,111-13,085)	3,978 (3,090-4,278)
Atrial fibrillation and flutter	381 (360-432)	600 (570-682)	1,012 (956-1,126)	2,156 (2,039-2,425)	4,131 (3,926-4,633)	7,171 (6,840-8,031)	12,715 (12,200-14,302)	17,530 (16,735-18,980)	30,530 (28,844-32,424)	29,860 (28,317-31,837)	20,382 (19,238-21,344)	6,375 (6,021-6,605)
Aortic aneurysm	2,240 (2,011-2,576)	3,062 (2,821-3,411)	3,994 (3,758-4,329)	6,839 (6,503-7,393)	10,381 (9,831-11,182)	15,776 (15,106-16,720)	19,215 (18,533-20,277)	18,722 (18,014-19,507)	17,936 (17,380-18,567)	11,772 (11,434-12,176)	4,780 (4,661-4,910)	1,159 (1,134-1,184)
Peripheral artery disease	165 (109-220)	271 (168-388)	454 (268-659)	1,038 (599-1,539)	1,698 (1,005-2,547)	2,801 (1,574-4,224)	3,362 (1,914-5,272)	3,681 (2,029-5,609)	5,095 (2,647-8,522)	4,423 (2,229-7,617)	2,690 (1,286-5,099)	1,118 (516-2,219)
Endocarditis	1,922 (1,721-2,214)	1,696 (1,547-1,957)	1,854 (1,664-2,173)	2,228 (2,013-2,637)	3,024 (2,719-3,514)	3,825 (3,397-4,543)	4,391 (3,729-5,176)	4,488 (3,730-5,324)	4,867 (3,817-6,072)	3,981 (2,961-4,960)	2,157 (1,498-2,775)	730 (505-995)
Other cardiovascular and circulatory diseases	7,812 (7,006-9,268)	9,546 (8,616-11,248)	11,706 (10,553-14,086)	16,465 (14,963-19,489)	22,901 (20,947-26,777)	28,822 (26,611-33,037)	32,884 (30,492-37,076)	31,385 (28,720-35,424)	32,372 (29,863-35,791)	24,325 (22,636-26,337)	11,303 (10,640-12,070)	3,453 (3,272-3,648)
Chronic respiratory diseases	48,712 (45,553-50,632)	70,442 (64,534-72,916)	109,769 (98,686-114,060)	183,338 (165,041-189,564)	336,123 (301,734-347,469)	461,988 (417,450-477,857)	601,114 (545,038-619,903)	573,483 (516,309-593,101)	527,711 (477,315-545,936)	332,538 (301,622-344,089)	122,337 (112,272-126,357)	26,648 (24,866-27,472)
Chronic obstructive pulmonary disease	25,755 (22,250-31,756)	40,460 (34,302-49,554)	68,445 (57,074-84,574)	123,763 (104,626-147,610)	256,152 (218,641-291,800)	368,969 (322,055-409,188)	506,359 (445,335-548,846)	490,170 (430,696-525,029)	460,204 (410,071-488,313)	293,965 (263,819-308,145)	108,909 (98,674-113,441)	23,507 (21,796-24,401)
Pneumoconiosis	804 (662-942)	771 (634-909)	899 (738-1,101)	1,355 (1,148-1,642)	2,190 (1,874-2,599)	3,078 (2,676-3,588)	3,736 (3,314-4,281)	2,981 (2,666-3,551)	2,430 (2,218-2,846)	1,260 (1,151-1,455)	357 (330-403)	70 (64-78)
Silicosis	540 (426-677)	479 (364-615)	515 (383-669)	717 (546-927)	1,209 (952-1,538)	1,741 (1,418-2,144)	2,070 (1,703-2,523)	1,605 (1,321-1,959)	1,273 (1,083-1,526)	624 (527-746)	155 (134-184)	27 (24-31)
Asbestosis	31 (15-48)	35 (19-52)	62 (34-92)	124 (66-180)	176 (100-267)	245 (144-336)	313 (207-428)	278 (183-362)	235 (158-296)	140 (95-175)	49 (36-63)	10 (7-12)
Coal workers pneumoconiosis	122 (70-186)	125 (68-202)	150 (93-238)	258 (182-365)	430 (284-629)	678 (485-931)	909 (683-1,213)	719 (546-993)	618 (492-822)	334 (274-428)	93 (81-113)	18 (16-21)
Other pneumoconiosis	111 (76-153)	132 (88-178)	173 (118-234)	256 (162-349)	374 (261-513)	414 (302-560)	444 (325-604)	380 (282-517)	305 (231-396)	162 (130-208)	60 (50-76)	15 (12-18)
Asthma	18,316 (11,027-21,987)	24,531 (14,075-30,397)	34,124 (17,901-43,850)	47,982 (25,113-62,675)	68,925 (34,805-91,730)	78,093 (39,596-104,057)	77,795 (41,315-106,149)	68,041 (38,720-92,267)	53,542 (33,066-72,988)	29,728 (19,528-40,004)	10,055 (7,073-13,059)	2,278 (1,757-2,838)
Interstitial lung disease and pulmonary sarcoidosis	1,268 (916-1,784)	1,803 (1,256-2,538)	2,662 (1,824-3,889)	4,367 (2,922-6,249)	6,514 (4,462-9,317)	9,079 (6,441-12,653)	10,290 (7,880-13,571)	9,809 (7,677-12,480)	8,850 (7,014-10,679)	5,540 (4,395-6,630)	2,058 (1,696-2,428)	523 (440-649)
Other chronic respiratory diseases	2,568 (1,814-3,362)	2,876 (2,112-3,775)	3,639 (2,641-4,750)	5,871 (4,464-7,307)	2,341 (1,752-2,953)	2,769 (2,196-3,444)	2,935 (2,390-3,595)	2,482 (2,083-2,958)	2,685 (2,307-3,127)	2,044 (1,809-2,312)	958 (859-1,079)	270 (246-298)
Digestive diseases	111,122 (103,113-116,746)	123,430 (116,139-129,017)	132,942 (124,176-138,475)	159,759 (150,626-166,123)	184,065 (170,172-191,164)	192,647 (177,249-199,076)	181,081 (167,064-187,650)	153,981 (141,149-160,155)	137,905 (128,173-142,707)	93,694 (88,160-96,612)	41,582 (39,569-42,647)	11,719 (11,230-11,999)
Cirrhosis and other chronic liver diseases	78,868 (73,011-83,220)	86,952 (81,079-91,433)	92,705 (85,398-97,435)	106,456 (98,918-112,030)	114,494 (105,474-120,377)	108,527 (99,293-114,229)	89,798 (81,768-94,737)	65,394 (58,298-69,936)	47,892 (42,479-51,299)	26,120 (23,007-27,805)	9,629 (8,511-10,179)	2,544 (2,263-2,683)
Cirrhosis and other chronic liver diseases due to hepatitis B	28,510 (24,485-32,524)	27,874 (23,874-32,277)	27,421 (22,768-32,367)	30,951 (25,554-36,714)	34,612 (28,346-41,223)	33,794 (28,150-40,090)	28,750 (23,272-33,918)	21,510 (17,463-25,345)	14,777 (11,625-17,660)	7,258 (5,849-8,503)	2,313 (1,810-2,830)	514 (369-688)
Cirrhosis and other chronic liver diseases due to hepatitis C	20,802 (17,399-24,698)	23,669 (19,601-28,184)	25,583 (20,825-30,718)	28,051 (24,070-32,402)	29,582 (24,838-35,385)	27,989 (24,067-32,216)	22,769 (19,584-26,377)	15,963 (13,152-19,365)	11,607 (9,380-14,186)	6,561 (5,433-7,725)	2,519 (2,058-3,058)	688 (527-866)
Cirrhosis and other chronic liver diseases due to alcohol use	21,018 (18,282-23,962)	24,685 (21,328-28,523)	26,923 (23,034-31,151)	31,407 (26,546-36,389)	31,921 (26,455-37,721)	27,903 (23,509-32,354)	21,108 (17,450-24,838)	14,153 (11,955-16,386)	10,156 (8,494-12,050)	5,338 (4,611-6,102)	1,895 (1,550-2,287)	475 (350-625)
Cirrhosis due to NASH	4,885 (4,104-5,754)	6,618 (5,480-7,812)	7,869 (6,572-9,310)	9,201 (8,031-10,519)	9,711 (8,231-11,201)	8,911 (7,752-10,112)	6,997 (6,022-8,030)	4,650 (3,841-5,525)	3,346 (2,719-4,070)	2,001 (1,651-2,356)	805 (629-1,001)	225 (166-303)
Cirrhosis and other chronic liver diseases due to other causes	3,653 (2,702-4,755)	4,106 (3,094-5,312)	4,909 (3,669-6,456)	6,845 (4,739-9,502)	8,668 (6,106-11,750)	9,929 (7,253-13,108)	10,174 (7,837-12,927)	9,118 (6,957-11,660)	8,006 (6,108-10,396)	4,961 (4,029-6,047)	2,097 (1,632-2,599)	642 (453-825)
Upper digestive system diseases	13,909 (12,823-15,177)	16,587 (15,399-18,066)	19,508 (17,981-21,235)	24,157 (22,507-25,909)	31,624 (28,803-33,619)	36,289 (33,156-38,614)	36,780 (33,386-38,855)	33,977 (31,010-36,109)	31,019 (28,864-32,792)	22,039 (20,791-23,084)	9,607 (9,095-10,036)	2,669 (2,505-2,783)
Peptic ulcer disease	12,170 (11,278-13,356)	14,567 (13,579-16,055)	17,227 (15,950-18,950)	21,401 (20,013-23,204)	28,180 (25,964-30,197)	32,427 (29,933-34,918)	32,742 (30,105-34,858)	30,074 (27,750-32,076)	27,507 (25,574-29,180)	19,533 (18,440-20,658)	8,486 (8,028-8,887)	2,345 (2,204-2,459)
Gastritis and duodenitis	1,739 (1,355-2,070)	2,020 (1,585-2,392)	2,282 (1,773-2,757)	2,756 (2,176-3,291)	3,444 (2,665-4,211)	3,862 (2,923-4,647)	4,038 (3,035-4,858)	3,903 (2,844-4,581)	3,512 (2,653-4,074)	2,506 (1,938-2,843)	1,121 (906-1,235)	325 (279-352)
Appendicitis	1,933 (1,692-2,234)	1,777 (1,579-2,027)	1,663 (1,484-1,873)	2,368 (2,124-2,687)	2,664 (2,380-2,964)	2,671 (2,408-2,938)	2,776 (2,425-3,138)	2,523 (2,185-2,917)	2,141 (1,871-2,490)	1,514 (1,315-1,791)	664 (577-793)	165 (143-196)
Paralytic ileus and intestinal obstruction	5,052 (3,645-5,777)	5,392 (3,797-6,123)	5,680 (4,289-6,409)	8,184 (6,301-9,165)	10,361 (8,228-11,690)	12,708 (10,166-14,369)	13,332 (11,263-15,017)	13,420 (11,428-15,544)	14,401 (12,542-16,762)	11,795 (9,903-13,675)	6,014 (5,076-6,948)	1,770 (1,434-2,168)
Inguinal, femoral, and abdominal hernia	834 (722-1,005)	1,028 (888-1,219)	1,419 (1,235-1,652)	2,128 (1,768-2,367)	2,929 (2,345-3,233)	3,740 (2,927-4,162)	4,151 (3,290-4,550)	4,184 (3,290-4,666)	4,783 (3,730-5,285)	3,477 (2,662-3,844)	1,648 (1,278-1,841)	453 (332-531)
Inflammatory bowel disease	801 (702-953)	939 (826-1,113)	960 (850-1,103)	1,341 (1,191-1,576)	1,663 (1,482-1,905)	2,177 (1,950-2,438)	2,648 (2,402-2,979)	2,819 (2,488-3,121)	3,249 (2,746-3,620)	2,565 (2,136-2,835)	1,279 (1,017-1,406)	400 (315-437)
Vascular intestinal disorders	783 (682-878)	1,155 (1,019-1,294)	1,542 (1,394-1,700)	2,746 (2,488-3,018)	4,196 (3,822-4,583)	6,803 (6,279-7,289)	8,786 (8,168-9,354)	9,255 (8,536-9,721)	10,672 (9,997-11,289)	7,956 (7,021-8,493)	3,624 (3,188-3,830)	925 (798-987)
Gallbladder and biliary diseases	1,894 (1,697-2,037)	2,172 (1,994-2,329)	2,741 (2,469-2,911)	3,903 (3,518-4,143)	5,927 (5,291-6,271)	8,128 (7,288-8,598)	9,766 (8,763-10,281)	10,196 (9,144-10,820)	11,065 (10,254-11,708)	8,285 (7,819-8,822)	4,295 (4,082-4,605)	1,312 (1,225-1,400)
Pancreatitis	5,272 (4,823-5,956)	5,340 (4,803-5,992)	4,392 (4,049-4,987)	5,527 (5,037-6,158)	5,525 (4,989-6,144)	6,141 (5,539-6,801)	6,023 (5,162-6,564)	5,103 (4,289-5,537)	4,613 (3,932-5,009)	3,053 (2,545-3,292)	1,250 (1,018-1,355)	329 (266-359)
Other digestive diseases	1,776 (1,625-2,043)	2,088 (1,886-2,454)	2,332 (2,079-2,808)	2,950 (2,723-3,349)	4,683 (4,205-5,638)	5,463 (5,081-6,203)	7,020 (6,532-7,835)	7,109 (6,661-7,720)	8,071 (7,567-8,576)	6,889 (6,551-7,219)	3,572 (3,403-3,747)	1,151 (1,091-1,204)
Neurological disorders	12,250 (11,131-12,898)	12,566 (11,743-13,114)	14,692 (14,169-15,281)	21,632 (21,073-22,567)	40,085 (38,908-42,383)	73,095 (71,230-77,080)	131,160 (127,557-138,589)	213,332 (208,771-221,440)	327,349 (321,724-337,079)	330,026 (325,676-338,340)	216,766 (214,680-219,968)	71,661 (71,035-72,387)
Alzheimer's disease and other dementias	694 (651-752)	1,656 (1,574-1,786)	4,056 (3,881-4,364)	9,238 (8,879-9,858)	23,890 (22,845-25,554)	49,461 (47,898-51,964)	95,428 (92,422-100,233)	168,518 (165,315-173,193)	277,601 (273,412-282,721)	298,060 (294,292-304,285)	205,754 (203,840-208,466)	69,556 (68,935-70,272)
Parkinson's disease	529 (479-581)	1,005 (938-1,129)	1,643 (1,552-1,852)	2,696 (2,560-3,093)	5,791 (5,466-6,683)	12,335 (11,749-14,500)	25,482 (24,318-29,400)	37,140 (35,400-41,883)	43,845 (41,961-49,110)	28,437 (27,361-31,620)	9,832 (9,496-11,053)	1,810 (1,755-1,993)
Epilepsy	7,662 (6,699-8,316)	5,718 (4,893-6,314)	4,620 (3,990-4,985)	4,246 (3,716-4,509)	4,270 (3,565-4,653)	4,376 (3,692-5,102)	3,630 (3,108-4,030)	2,696 (2,316-3,007)	2,103 (1,787-2,286)	1,395 (1,177-1,625)	466 (403-531)	115 (104-129)
Multiple sclerosis	1,459 (1,123-1,785)	1,635 (1,321-1,936)	1,537 (1,315-1,790)	1,647 (1,440-1,954)	1,524 (1,390-1,821)	1,396 (1,258-1,652)	1,129 (980-1,332)	706 (620-817)	486 (417-555)	244 (193-269)	68 (56-74)	13 (11-15)
Motor neuron disease	489 (461-579)	825 (787-989)	1,105 (1,055-1,250)	1,717 (1,641-1,962)	2,278 (2,194-2,534)	2,931 (2,828-3,210)	2,821 (2,744-2,962)	2,012 (1,953-2,076)	1,290 (1,243-1,326)	552 (531-567)	125 (120-130)	21 (21-22)
Other neurological disorders	1,417 (1,297-1,645)	1,727 (1,601-2,025)	1,730 (1,633-2,020)	2,086 (1,973-2,355)	2,332 (2,228-2,631)	2,597 (2,487-2,856)	2,669 (2,558-2,864)	2,259 (2,164-2,418)	2,024 (1,876-2,127)	1,338 (1,119-1,404)	520 (436-545)	145 (127-152)
Mental disorders	32 (30-36)	31 (28-35)	-	-	-	-	-	-	-	-	-	-
Eating disorders	32 (30-36)	31 (28-35)	-	-	-	-	-	-	-	-	-	-
Anorexia nervosa	24 (18-28)	25 (21-29)	-	-	-	-	-	-	-	-	-	-
Bulimia nervosa	9 (6-14)	6 (4-11)	-	-	-	-	-	-	-	-	-	-
Substance use disorders	33,626 (32,301-34,821)	30,102 (28,621-31,385)	22,897 (21,994-23,676)	24,971 (23,412-26,048)	18,205 (17,540-18,936)	13,732 (13,108-14,211)	8,289 (7,996-8,581)	5,673 (5,416-5,941)	3,611 (3,461-3,750)	2,244 (2,103-2,335)	846 (795-878)	206 (192-213)
Alcohol use disorders	23,390 (21,995-24,550)	23,529 (22,034-24,810)	18,437 (17,572-19,198)	20,516 (18,860-21,563)	14,191 (13,424-14,904)	9,911 (9,205-10,338)	5,003 (4,609-5,260)	3,002 (2,650-3,241)	1,601 (1,347-1,691)	920 (708-982)	298 (229-319)	74 (56-78)
Drug use disorders	10,236 (9,854-10,702)	6,573 (6,329-6,839)	4,460 (4,313-4,648)	4,455 (4,302-4,647)	4,014 (3,881-4,176)	3,821 (3,694-4,001)	3,286 (3,158-3,480)	2,671 (2,567-2,851)	2,010 (1,929-2,145)	1,325 (1,264-1,412)	548 (527-578)	132 (128-138)
Opioid use disorders	6,077 (5,347-6,876)	3,655 (3,179-4,037)	2,314 (1,978-2,614)	2,379 (2,038-2,641)	2,096 (1,709-2,433)	2,033 (1,662-2,365)	1,379 (1,068-1,723)	1,130 (894-1,411)	824 (660-1,014)	587 (474-694)	248 (207-281)	56 (48-62)
Cocaine use disorders	432 (358-579)	301 (249-403)	207 (172-283)	199 (164-281)	142 (111-201)	129 (101-191)	46 (35-70)	35 (26-52)	11 (9-14)	7 (6-9)	4 (3-5)	1 (1-1)
Amphetamine use disorders	431 (248-681)	300 (170-488)	130 (82-204)	130 (81-201)	98 (59-155)	87 (50-142)	21 (13-31)	16 (10-24)	6 (4-8)	4 (3-5)	2 (1-2)	1 (0-1)
Other drug use disorders	3,295 (2,805-4,167)	2,317 (2,008-2,895)	1,808 (1,592-2,189)	1,748 (1,513-2,144)	1,678 (1,441-2,092)	1,572 (1,338-1,988)	1,840 (1,631-2,153)	1,490 (1,326-1,706)	1,170 (1,056-1,306)	727 (656-815)	295 (269-331)	75 (70-81)
Diabetes and kidney diseases	49,991 (48,344-51,980)	60,346 (58,381-62,676)	80,067 (77,516-83,043)	109,841 (106,783-114,704)	151,785 (147,899-157,376)	175,909 (171,446-182,210)	180,274 (175,647-187,020)	161,691 (157,810-167,271)	138,595 (135,676-142,844)	92,865 (91,051-95,293)	41,052 (40,271-41,940)	11,836 (11,642-12,032)
Diabetes mellitus	19,851 (19,167-20,686)	28,192 (27,245-29,266)	41,776 (40,427-43,307)	61,070 (59,345-63,022)	89,134 (86,560-91,836)	105,045 (102,010-108,061)	106,345 (103,451-109,110)	90,814 (88,397-93,235)	73,927 (72,136-75,926)	44,228 (43,160-45,320)	18,259 (17,850-18,658)	4,891 (4,792-4,994)
Diabetes mellitus type 1	15,206 (14,454-16,077)	14,143 (13,009-15,532)	19,994 (18,270-22,124)	28,725 (26,335-31,946)	31,479 (28,388-36,019)	36,010 (32,456-40,928)	30,183 (26,548-34,599)	24,939 (21,616-28,826)	15,008 (12,514-17,695)	8,533 (6,925-9,951)	3,356 (2,660-3,878)	854 (653-969)
Diabetes mellitus type 2	4,645 (3,986-5,522)	14,049 (12,807-15,539)	21,782 (19,812-23,886)	32,345 (29,739-35,016)	57,655 (53,945-61,627)	69,035 (65,171-73,161)	76,162 (72,590-80,358)	65,876 (62,397-69,686)	58,919 (56,454-62,103)	35,696 (34,290-37,715)	14,903 (14,403-15,714)	4,037 (3,920-4,261)
Chronic kidney disease	29,378 (28,141-30,895)	31,366 (30,083-33,003)	37,656 (36,146-39,962)	47,913 (45,961-51,890)	61,685 (59,368-66,507)	69,777 (67,087-74,623)	72,817 (70,375-78,145)	69,973 (67,796-74,043)	63,968 (62,230-66,925)	48,224 (47,062-50,026)	22,625 (22,113-23,215)	6,889 (6,751-7,021)
Chronic kidney disease due to diabetes mellitus type 1	6,912 (4,564-9,614)	6,747 (4,497-9,438)	6,104 (3,680-8,897)	5,318 (2,991-8,357)	4,043 (2,018-6,922)	3,033 (1,555-5,369)	2,320 (1,117-4,140)	1,739 (830-3,213)	1,397 (694-2,440)	693 (360-1,229)	139 (66-251)	9 (4-18)
Chronic kidney disease due to diabetes mellitus type 2	3,053 (1,730-4,983)	5,182 (3,213-7,410)	8,877 (6,031-12,447)	13,748 (9,640-17,784)	20,660 (14,791-26,405)	24,018 (17,802-30,392)	24,290 (18,135-30,386)	21,959 (15,323-28,385)	18,569 (12,864-24,839)	12,667 (8,612-16,517)	5,368 (3,803-7,051)	1,518 (957-2,238)
Chronic kidney disease due to hypertension	3,847 (2,768-5,061)	4,714 (3,362-6,277)	6,739 (5,012-8,875)	9,776 (7,445-12,698)	14,275 (10,770-18,233)	18,405 (14,327-23,170)	21,970 (17,483-27,074)	23,876 (19,157-29,347)	23,842 (19,033-28,990)	20,481 (17,036-24,095)	10,694 (9,000-12,304)	3,490 (2,860-4,092)
Chronic kidney disease due to glomerulonephritis	7,263 (4,774-10,071)	7,040 (4,691-10,092)	7,328 (4,790-10,412)	8,250 (5,567-11,938)	8,758 (5,614-12,894)	9,678 (6,281-14,147)	10,058 (6,319-14,338)	9,460 (6,144-13,711)	8,579 (5,541-12,751)	5,969 (3,961-8,765)	2,655 (1,770-3,893)	796 (441-1,286)
Chronic kidney disease due to other and unspecified causes	8,302 (5,576-11,288)	7,682 (5,106-10,919)	8,609 (5,651-12,154)	10,820 (7,112-15,260)	13,951 (8,617-20,312)	14,644 (9,360-20,590)	14,179 (9,290-19,878)	12,938 (8,504-19,118)	11,581 (7,805-16,357)	8,414 (5,786-11,590)	3,769 (2,587-5,214)	1,075 (686-1,652)
Acute glomerulonephritis	762 (703-830)	788 (719-880)	635 (570-719)	858 (778-967)	965 (887-1,052)	1,086 (994-1,186)	1,112 (1,013-1,231)	904 (814-1,017)	699 (631-780)	413 (375-457)	168 (149-193)	57 (46-70)
Skin and subcutaneous diseases	1,502 (927-1,804)	1,783 (1,115-2,193)	2,063 (1,288-2,562)	2,727 (1,719-3,411)	3,423 (2,138-4,369)	4,065 (2,631-5,165)	4,734 (3,076-6,024)	5,233 (3,348-6,954)	5,334 (3,414-7,248)	4,556 (2,735-5,921)	2,492 (1,418-3,473)	936 (516-1,390)
Bacterial skin diseases	1,231 (730-1,461)	1,498 (878-1,795)	1,747 (1,070-2,117)	2,251 (1,381-2,712)	2,848 (1,741-3,500)	3,118 (1,965-3,846)	3,349 (2,127-4,169)	3,554 (2,334-4,502)	3,019 (1,995-4,157)	2,293 (1,495-3,155)	1,193 (754-1,729)	413 (264-653)
Cellulitis	286 (159-408)	330 (189-462)	379 (220-566)	509 (304-749)	603 (352-909)	650 (376-1,003)	655 (403-1,066)	620 (337-1,026)	638 (376-1,094)	459 (270-813)	266 (149-485)	103 (55-193)
Pyoderma	945 (517-1,159)	1,168 (638-1,431)	1,368 (763-1,663)	1,741 (985-2,125)	2,245 (1,263-2,803)	2,468 (1,408-3,004)	2,695 (1,612-3,290)	2,934 (1,819-3,686)	2,382 (1,486-3,187)	1,834 (1,150-2,450)	927 (582-1,305)	310 (196-472)
Decubitus ulcer	193 (118-297)	212 (128-324)	244 (149-369)	394 (245-596)	466 (290-715)	803 (459-1,163)	1,218 (726-1,700)	1,484 (851-2,126)	2,067 (1,160-2,908)	2,032 (1,044-2,651)	1,169 (569-1,511)	479 (227-672)
Other skin and subcutaneous diseases	78 (49-129)	73 (44-119)	71 (44-122)	82 (49-144)	110 (67-191)	145 (84-246)	167 (103-299)	195 (108-334)	248 (140-444)	231 (123-408)	130 (62-239)	44 (19-85)
Musculoskeletal disorders	2,894 (2,611-3,293)	3,018 (2,763-3,306)	2,767 (2,545-3,025)	3,821 (3,523-4,123)	6,081 (5,394-6,567)	7,774 (6,991-8,380)	8,657 (7,879-9,319)	8,116 (7,347-8,640)	7,248 (6,666-7,750)	5,807 (5,437-6,123)	2,591 (2,449-2,715)	802 (768-834)
Rheumatoid arthritis	530 (434-629)	785 (647-949)	984 (849-1,123)	1,773 (1,588-2,013)	3,216 (2,813-3,639)	4,408 (3,895-4,951)	5,026 (4,488-5,676)	4,683 (4,184-5,232)	3,727 (3,352-4,193)	2,520 (2,279-2,832)	902 (818-1,004)	205 (186-232)
Other musculoskeletal disorders	2,364 (2,116-2,663)	2,233 (2,034-2,422)	1,783 (1,630-1,972)	2,048 (1,870-2,268)	2,866 (2,507-3,276)	3,366 (2,877-3,895)	3,631 (3,228-4,129)	3,433 (2,976-3,787)	3,520 (3,110-3,864)	3,287 (2,981-3,514)	1,689 (1,511-1,785)	597 (556-623)
Other non-communicable diseases	16,471 (15,263-17,586)	14,633 (13,566-15,521)	15,671 (14,360-16,828)	18,447 (17,086-19,676)	23,030 (21,386-24,496)	28,213 (26,195-29,903)	28,231 (26,215-29,966)	25,801 (23,938-27,415)	26,461 (24,512-27,730)	20,519 (18,952-21,384)	10,492 (9,663-10,924)	3,736 (3,486-3,881)
Congenital birth defects	4,570 (4,287-5,068)	2,038 (1,875-2,321)	1,623 (1,494-1,897)	1,677 (1,561-2,016)	1,585 (1,469-1,899)	1,461 (1,354-1,741)	-	-	-	-	-	-
Neural tube defects	131 (114-152)	82 (73-96)	55 (48-64)	56 (49-64)	46 (40-54)	39 (34-46)	-	-	-	-	-	-
Congenital heart anomalies	3,074 (2,838-3,494)	1,078 (996-1,261)	763 (706-915)	771 (713-954)	714 (660-874)	705 (649-860)	-	-	-	-	-	-
Orofacial clefts	-	-	-	-	-	-	-	-	-	-	-	-
Down syndrome	517 (395-654)	393 (316-465)	382 (325-480)	375 (321-512)	307 (260-441)	154 (125-233)	-	-	-	-	-	-
Other chromosomal abnormalities	59 (42-87)	41 (33-66)	36 (29-61)	38 (30-62)	32 (24-52)	26 (21-45)	-	-	-	-	-	-
Congenital musculoskeletal and limb anomalies	73 (55-90)	51 (41-63)	43 (32-50)	45 (35-54)	54 (41-65)	60 (39-73)	-	-	-	-	-	-
Urogenital congenital anomalies	149 (121-180)	102 (85-120)	110 (91-132)	155 (125-187)	203 (168-248)	252 (211-305)	-	-	-	-	-	-
Digestive congenital anomalies	53 (43-64)	40 (34-50)	40 (34-47)	50 (44-63)	55 (47-67)	70 (60-85)	-	-	-	-	-	-
Other congenital birth defects	515 (441-630)	250 (216-298)	195 (171-231)	188 (168-231)	174 (157-210)	155 (139-191)	-	-	-	-	-	-
Urinary diseases and male infertility	4,960 (4,758-5,223)	5,641 (5,396-5,968)	6,605 (6,326-7,011)	8,634 (8,306-9,086)	11,484 (11,026-12,032)	15,080 (14,424-15,700)	15,854 (15,195-16,499)	14,648 (14,102-15,174)	15,169 (14,674-15,649)	11,629 (11,312-11,966)	6,075 (5,908-6,223)	2,305 (2,260-2,352)
Urinary tract infections	3,155 (2,754-3,518)	3,368 (2,968-3,736)	4,156 (3,742-4,623)	5,552 (5,034-6,102)	7,196 (6,508-8,098)	9,794 (8,965-10,747)	10,092 (9,294-11,226)	10,106 (9,394-11,125)	10,778 (10,208-11,665)	9,054 (8,678-9,603)	4,866 (4,694-5,106)	1,949 (1,891-2,027)

SR Table 7: Global deaths by cause and age group for both sexes combined (with 95% confidence intervals), 1995

Cause	Early Neonatal	Late Neonatal	Post Neonatal	1 to 4	5 to 9	10 to 14	15 to 19	20 to 24
Urolithiasis	-	-	-	-	111 (58-195)	68 (38-107)	56 (32-88)	134 (71-219)
Other urinary diseases	770 (593-921)	604 (427-752)	2,601 (1,437-3,758)	3,599 (2,166-4,946)	1,037 (741-1,397)	592 (454-738)	646 (432-826)	915 (636-1,154)
Gynecological diseases	-	-	-	-	-	-	434 (347-566)	492 (380-635)
Uterine fibroids	-	-	-	-	-	-	70 (43-95)	94 (53-153)
Polycystic ovarian syndrome	-	-	-	-	-	-	1 (0-4)	3 (1-7)
Endometriosis	-	-	-	-	-	-	9 (4-13)	11 (6-17)
Genital prolapse	-	-	-	-	-	-	15 (8-24)	13 (6-19)
Other gynecological diseases	-	-	-	-	-	-	339 (258-490)	372 (268-546)
Hemoglobinopathies and hemolytic anemias	1,172 (876-1,521)	915 (720-1,102)	7,323 (4,622-10,540)	17,573 (9,063-26,592)	6,315 (4,592-7,943)	5,025 (3,946-6,137)	5,901 (4,222-7,522)	5,587 (3,919-7,102)
Thalassemias	481 (376-577)	389 (284-528)	2,957 (2,021-4,357)	4,189 (2,639-6,111)	2,581 (1,915-3,394)	1,355 (1,087-1,622)	904 (690-1,071)	741 (533-887)
Sickle cell disorders	616 (398-973)	501 (347-676)	4,169 (2,395-5,881)	12,867 (6,056-19,909)	3,196 (2,131-4,318)	2,773 (1,896-3,856)	3,720 (2,394-5,255)	3,299 (2,050-4,792)
G6PD deficiency	16 (10-26)	13 (8-19)	117 (67-180)	364 (192-621)	354 (251-502)	551 (392-733)	748 (552-967)	889 (676-1,112)
Other hemoglobinopathies and hemolytic anemias	59 (45-77)	13 (10-17)	81 (54-121)	154 (94-229)	183 (142-233)	346 (290-414)	529 (420-639)	658 (529-783)
Endocrine, metabolic, blood, and immune disorders	1,815 (1,164-2,097)	1,768 (1,098-2,191)	9,640 (5,897-13,241)	5,657 (3,137-8,407)	2,484 (1,766-3,203)	1,762 (1,442-2,111)	1,657 (1,402-1,951)	2,118 (1,674-2,412)
Sudden infant death syndrome	-	6,185 (2,323-12,094)	52,116 (19,489-114,320)	-	-	-	-	-
Injuries	18,820 (15,955-20,779)	16,309 (13,662-18,299)	137,814 (120,816-152,683)	371,669 (303,185-420,370)	243,232 (223,451-259,448)	161,578 (151,092-170,110)	289,063 (277,553-297,761)	415,412 (403,585-423,793)
Transport injuries	2,768 (2,363-3,192)	2,857 (2,312-3,298)	21,436 (17,008-23,651)	80,168 (54,826-93,120)	75,312 (67,978-81,630)	47,627 (43,993-51,081)	103,533 (98,416-108,633)	144,817 (139,082-151,823)
Road injuries	2,343 (1,998-2,755)	2,552 (2,052-2,950)	19,854 (15,599-21,971)	75,269 (50,671-87,730)	71,767 (64,826-78,030)	44,465 (41,090-47,793)	96,516 (91,826-101,319)	136,394 (130,843-143,312)
Pedestrian road injuries	1,311 (1,020-1,645)	1,330 (1,015-1,779)	9,120 (7,567-11,068)	47,287 (34,816-58,496)	47,052 (42,213-51,587)	24,183 (21,962-26,293)	28,722 (26,152-32,870)	40,666 (37,112-46,030)
Cyclist road injuries	-	-	-	1,535 (831-2,291)	2,847 (2,014-3,714)	2,830 (2,323-3,385)	3,490 (3,036-4,039)	3,471 (2,907-4,068)
Motorcyclist road injuries	162 (122-226)	167 (108-237)	1,808 (1,341-2,450)	3,651 (2,101-5,748)	3,511 (2,714-4,690)	4,099 (3,447-5,059)	21,733 (19,412-25,676)	32,316 (28,943-37,964)
Motor vehicle road injuries	833 (598-1,064)	1,025 (656-1,352)	8,625 (5,986-10,229)	22,161 (11,847-28,981)	17,785 (14,673-21,462)	12,913 (11,323-14,509)	41,821 (38,201-45,118)	58,891 (53,674-63,966)
Other road injuries	36 (18-59)	30 (14-50)	300 (143-477)	636 (229-1,132)	572 (309-787)	439 (301-568)	751 (542-1,037)	1,051 (735-1,422)
Other transport injuries	425 (292-601)	305 (204-460)	1,582 (1,304-2,102)	4,899 (3,743-7,075)	3,545 (3,023-4,191)	3,162 (2,775-3,551)	7,017 (6,389-7,845)	8,423 (7,837-9,227)
Unintentional injuries	13,093 (10,820-14,590)	12,392 (10,159-13,997)	108,561 (95,399-121,520)	272,983 (226,570-312,643)	154,141 (140,943-164,996)	87,106 (80,688-92,305)	75,715 (70,938-79,245)	92,206 (87,269-95,440)
Falls	1,844 (1,437-2,148)	858 (647-1,126)	10,817 (8,667-13,327)	18,074 (12,866-25,420)	10,873 (8,454-14,298)	7,353 (6,050-8,942)	7,954 (7,111-9,153)	11,869 (10,221-13,838)
Drowning	682 (615-744)	524 (455-571)	14,484 (12,712-15,733)	159,032 (134,259-179,488)	97,490 (89,598-104,864)	47,359 (44,297-50,691)	28,622 (26,926-30,327)	27,938 (26,740-29,341)
Fire, heat, and hot substances	535 (377-637)	672 (376-849)	8,951 (5,344-11,014)	24,081 (12,626-31,539)	7,589 (5,197-8,829)	4,363 (3,265-5,024)	6,691 (4,783-7,897)	9,118 (6,673-10,668)
Poisonings	365 (241-423)	399 (265-491)	4,732 (3,271-6,005)	11,280 (7,326-15,193)	4,266 (3,083-5,632)	3,332 (2,615-4,243)	3,603 (3,135-4,441)	5,233 (4,387-6,749)
Poisoning by carbon monoxide	87 (56-100)	99 (65-120)	1,079 (753-1,309)	2,463 (1,879-3,059)	2,052 (1,477-2,835)	1,423 (1,136-1,929)	1,982 (1,737-2,500)	2,922 (2,473-3,853)
Poisoning by other means	278 (183-327)	300 (198-375)	3,653 (2,427-4,756)	8,817 (5,292-12,405)	2,215 (1,594-2,897)	1,908 (1,492-2,370)	1,621 (1,364-1,957)	2,310 (1,911-2,920)
Exposure to mechanical forces	1,285 (1,031-1,463)	1,600 (1,168-1,856)	7,239 (6,012-8,183)	12,072 (9,312-14,086)	7,710 (6,802-8,516)	5,099 (4,653-5,546)	8,360 (7,673-9,374)	12,049 (11,342-13,427)
Unintentional firearm injuries	141 (107-189)	129 (93-211)	968 (708-1,388)	1,416 (894-2,726)	1,035 (792-1,242)	1,056 (933-1,211)	2,288 (2,046-2,595)	3,221 (2,735-3,634)
Other exposure to mechanical forces	1,144 (900-1,310)	1,471 (1,042-1,727)	6,270 (5,026-7,173)	10,656 (8,144-12,579)	6,675 (5,797-7,431)	4,043 (3,622-4,436)	6,071 (5,498-6,891)	8,828 (8,177-9,943)
Adverse effects of medical treatment	1,643 (1,085-1,971)	1,244 (768-1,628)	9,701 (5,492-13,797)	7,417 (3,224-13,157)	2,936 (1,846-3,697)	1,983 (1,453-2,302)	2,409 (1,810-2,789)	3,983 (2,954-4,548)
Animal contact	443 (169-576)	497 (173-695)	3,776 (1,588-5,087)	10,386 (4,167-17,004)	9,898 (4,404-13,538)	6,794 (3,332-8,690)	4,328 (2,179-5,354)	4,227 (2,038-5,192)
Venomous animal contact	364 (118-494)	395 (116-565)	3,024 (1,216-3,970)	8,356 (3,142-12,686)	8,513 (3,740-11,527)	6,178 (2,956-7,947)	3,803 (1,856-4,757)	3,584 (1,656-4,442)
Non-venomous animal contact	80 (41-137)	101 (48-173)	752 (327-1,585)	2,030 (670-5,825)	1,385 (614-3,073)	615 (339-1,095)	526 (315-869)	642 (349-1,049)
Foreign body	5,349 (4,182-6,062)	6,062 (4,826-6,880)	42,837 (35,978-47,862)	17,369 (12,695-20,185)	4,919 (4,489-5,499)	3,128 (2,933-3,385)	2,063 (1,978-2,221)	3,010 (2,881-3,282)
Pulmonary aspiration and foreign body in airway	5,160 (4,039-5,873)	5,869 (4,706-6,685)	39,728 (33,538-45,353)	14,793 (10,936-18,002)	4,128 (3,725-4,729)	2,749 (2,578-3,010)	1,732 (1,645-1,897)	2,542 (2,414-2,815)
Foreign body in other body part	189 (102-247)	193 (78-264)	3,110 (1,122-4,089)	2,575 (1,222-3,827)	790 (482-969)	379 (264-441)	331 (258-372)	469 (354-536)
Environmental heat and cold exposure	436 (178-584)	299 (111-464)	3,162 (1,143-4,537)	2,072 (775-3,617)	1,066 (563-1,338)	704 (453-841)	1,767 (1,348-2,004)	2,522 (1,895-2,802)
Exposure to forces of nature	11 (10-12)	30 (28-33)	266 (246-288)	1,263 (1,178-1,368)	1,341 (1,250-1,458)	1,551 (1,451-1,675)	1,513 (1,409-1,638)	1,620 (1,496-1,767)
Other unintentional injuries	500 (444-567)	208 (178-238)	2,597 (2,310-2,861)	9,936 (8,126-11,466)	6,055 (5,284-6,821)	5,440 (4,871-5,955)	8,406 (7,773-9,035)	10,638 (9,973-11,299)
Self-harm and interpersonal violence	2,959 (2,355-3,185)	1,059 (857-1,153)	7,817 (6,271-8,610)	18,518 (15,656-20,865)	13,778 (11,645-15,256)	26,846 (24,025-28,588)	109,815 (103,010-114,299)	178,389 (165,935-184,457)
Self-harm	-	-	-	-	-	13,624 (11,547-14,972)	62,430 (57,933-65,747)	103,446 (94,136-107,997)
Self-harm by firearm	-	-	-	-	-	583 (459-791)	4,929 (4,081-6,160)	7,731 (6,438-9,926)
Self-harm by other specified means	-	-	-	-	-	13,041 (10,909-14,403)	57,500 (53,219-60,988)	95,715 (86,848-100,810)
Interpersonal violence	2,348 (1,743-2,573)	730 (528-824)	6,754 (5,211-7,548)	11,828 (8,964-14,172)	9,511 (7,374-10,993)	9,338 (7,839-10,373)	39,392 (34,739-41,795)	62,541 (54,710-66,140)
Physical violence by firearm	216 (147-278)	77 (53-103)	833 (623-1,112)	1,439 (1,024-2,106)	1,112 (909-1,406)	2,238 (1,913-2,539)	17,632 (15,215-18,794)	26,779 (22,817-28,939)
Physical violence by sharp object	158 (114-196)	75 (51-100)	636 (418-907)	1,466 (850-2,425)	1,600 (977-2,354)	1,681 (1,200-2,112)	9,084 (7,042-10,799)	16,366 (12,933-19,109)
Physical violence by other means	1,974 (1,438-2,183)	578 (404-655)	5,285 (3,963-5,957)	8,923 (6,539-10,892)	6,798 (5,227-8,020)	5,419 (4,385-6,296)	12,675 (10,619-14,817)	19,396 (16,551-23,011)
Conflict and terrorism	611 (542-702)	329 (290-383)	1,023 (908-1,181)	6,628 (5,926-7,591)	4,175 (3,695-4,779)	3,803 (3,410-4,324)	7,659 (7,016-8,636)	11,103 (10,161-12,536)
Executions and police conflict	-	-	40 (23-50)	62 (47-71)	92 (74-105)	82 (74-90)	334 (272-388)	1,299 (734-1,547)

Cause	25 to 29	30 to 34	35 to 39	40 to 44	45 to 49	50 to 54	54 to 59	60 to 64
Urolithiasis	228 (122-362)	299 (178-449)	262 (184-354)	406 (285-528)	506 (388-636)	536 (427-649)	792 (653-960)	1,073 (864-1,284)
Other urinary diseases	831 (628-1,075)	1,104 (854-1,352)	1,122 (860-1,360)	1,398 (1,092-1,703)	1,767 (1,428-2,138)	1,913 (1,466-2,346)	2,290 (1,782-2,804)	3,215 (2,494-3,953)
Gynecological diseases	496 (384-638)	545 (432-669)	570 (464-644)	633 (528-709)	683 (583-772)	483 (409-567)	344 (297-390)	277 (244-323)
Uterine fibroids	125 (74-192)	134 (84-201)	148 (94-203)	196 (134-251)	223 (173-280)	163 (126-228)	80 (65-114)	67 (54-91)
Polycystic ovarian syndrome	2 (0-5)	1 (0-3)	1 (0-2)	1 (0-2)	1 (0-2)	1 (0-2)	-	-
Endometriosis	11 (5-18)	16 (8-25)	15 (7-25)	20 (9-33)	26 (11-45)	16 (7-29)	-	-
Genital prolapse	15 (6-23)	16 (6-25)	13 (4-22)	26 (7-45)	30 (7-53)	60 (17-98)	49 (17-86)	51 (24-84)
Other gynecological diseases	343 (249-503)	379 (287-496)	393 (305-512)	390 (299-497)	403 (314-491)	243 (183-319)	215 (174-271)	159 (127-209)
Hemoglobinopathies and hemolytic anemias	4,337 (3,157-5,263)	3,794 (2,746-4,583)	2,917 (2,073-3,729)	3,051 (2,223-3,858)	2,596 (1,901-3,266)	2,881 (2,074-3,800)	3,335 (2,440-4,361)	3,948 (2,951-5,045)
Thalassemias	467 (338-581)	244 (178-308)	98 (75-121)	65 (53-81)	41 (33-50)	21 (17-25)	12 (10-14)	6 (5-7)
Sickle cell disorders	2,235 (1,399-3,203)	1,592 (960-2,417)	1,117 (611-1,834)	919 (489-1,584)	554 (301-983)	380 (216-699)	223 (135-411)	90 (59-149)
G6PD deficiency	957 (753-1,174)	1,083 (826-1,297)	913 (689-1,118)	999 (755-1,253)	960 (677-1,283)	1,038 (620-1,546)	1,017 (589-1,626)	895 (508-1,436)
Other hemoglobinopathies and hemolytic anemias	677 (563-807)	875 (685-1,019)	790 (633-920)	1,068 (876-1,243)	1,041 (832-1,211)	1,442 (1,128-1,756)	2,083 (1,638-2,574)	2,957 (2,288-3,668)
Endocrine, metabolic, blood, and immune disorders	2,572 (1,915-2,929)	3,008 (2,352-3,437)	2,970 (2,446-3,389)	3,258 (2,726-3,800)	3,675 (3,121-4,305)	4,080 (3,446-4,678)	4,457 (3,787-5,153)	5,737 (4,937-6,644)
Sudden infant death syndrome	-	-	-	-	-	-	-	-
Injuries	414,352 (403,331-422,790)	370,416 (358,612-377,935)	310,523 (300,562-316,785)	291,371 (281,317-297,789)	240,542 (231,918-245,987)	193,203 (184,669-198,768)	193,952 (185,441-199,238)	180,156 (170,489-185,407)
Transport injuries	143,045 (137,655-149,903)	122,584 (118,441-127,841)	101,485 (98,414-105,428)	94,470 (91,795-97,905)	74,183 (72,043-77,044)	60,602 (58,609-63,587)	56,883 (55,101-59,521)	51,841 (50,267-54,041)
Road injuries	134,025 (128,897-140,629)	115,257 (111,334-120,189)	94,815 (91,835-98,665)	88,056 (85,442-91,421)	68,710 (66,707-71,208)	56,027 (54,186-58,547)	52,999 (51,337-55,342)	48,522 (47,023-50,525)
Pedestrian road injuries	43,777 (40,220-48,780)	42,174 (38,814-46,542)	34,997 (31,565-38,940)	35,002 (31,798-38,269)	29,315 (26,806-32,009)	24,753 (22,818-27,570)	24,918 (23,147-27,446)	25,784 (24,236-27,903)
Cyclist road injuries	3,776 (2,957-4,643)	3,960 (3,157-4,817)	3,208 (2,618-3,671)	3,829 (3,029-4,507)	3,404 (2,805-3,933)	2,951 (2,475-3,321)	2,642 (2,328-2,947)	2,804 (2,357-3,175)
Motorcyclist road injuries	31,381 (27,697-37,156)	23,756 (20,849-27,433)	17,557 (15,508-20,570)	15,771 (14,065-18,279)	10,554 (9,384-12,452)	7,629 (6,802-9,269)	6,239 (5,565-7,685)	3,989 (3,481-4,904)
Motor vehicle road injuries	54,097 (49,193-60,231)	44,554 (40,661-49,412)	38,323 (34,993-41,552)	32,778 (30,404-35,567)	24,734 (22,644-27,076)	20,060 (18,311-22,043)	18,625 (17,203-20,529)	15,596 (14,475-17,214)
Other road injuries	995 (671-1,340)	813 (572-1,059)	730 (546-929)	676 (495-851)	704 (534-888)	633 (473-806)	575 (450-717)	349 (270-451)
Other transport injuries	9,020 (8,404-9,638)	7,327 (6,873-7,781)	6,669 (6,239-7,224)	6,415 (6,039-6,890)	5,473 (5,130-5,929)	4,575 (4,269-5,139)	3,884 (3,619-4,352)	3,318 (3,120-3,663)
Unintentional injuries	91,327 (85,512-94,573)	89,646 (84,429-92,693)	83,522 (79,085-86,099)	84,493 (79,913-87,182)	77,487 (73,352-79,792)	65,840 (61,640-68,008)	73,339 (68,518-75,789)	74,990 (68,982-77,996)
Falls	13,823 (11,381-16,486)	15,020 (12,800-17,530)	15,215 (13,506-17,038)	17,521 (15,442-19,913)	17,285 (15,755-19,518)	16,644 (15,052-18,713)	20,593 (18,633-22,434)	25,880 (23,417-28,605)
Drowning	23,319 (22,325-24,326)	20,758 (20,056-21,514)	16,766 (16,228-17,365)	15,794 (15,327-16,273)	12,591 (12,169-12,962)	10,195 (9,838-10,547)	9,866 (9,555-10,171)	9,416 (9,065-9,716)
Fire, heat, and hot substances	8,778 (6,511-9,953)	8,663 (6,814-9,626)	7,610 (6,386-8,398)	7,032 (6,101-7,604)	6,510 (5,694-7,040)	5,470 (4,778-5,955)	6,077 (5,432-6,614)	5,878 (5,163-6,439)
Poisonings	5,613 (4,688-7,412)	5,304 (4,584-6,836)	5,417 (4,892-6,071)	6,060 (5,463-6,924)	5,066 (4,610-5,786)	3,977 (3,663-4,476)	4,544 (4,221-5,123)	3,668 (3,357-4,286)
Poisoning by carbon monoxide	3,133 (2,636-4,308)	3,208 (2,813-4,165)	3,521 (3,203-3,992)	4,062 (3,692-4,658)	3,466 (3,162-3,923)	2,555 (2,363-2,894)	3,140 (2,921-3,513)	2,297 (2,131-2,715)
Poisoning by other means	2,480 (2,013-3,157)	2,095 (1,731-2,712)	1,897 (1,612-2,135)	1,997 (1,690-2,317)	1,600 (1,382-1,886)	1,422 (1,216-1,665)	1,404 (1,247-1,667)	1,371 (1,187-1,632)
Exposure to mechanical forces	12,778 (12,031-14,447)	12,193 (11,528-13,439)	11,140 (10,585-12,066)	9,936 (9,391-10,986)	9,046 (8,591-9,785)	7,195 (6,787-7,879)	6,719 (6,319-7,362)	5,410 (5,032-5,840)
Unintentional firearm injuries	3,071 (2,423-3,456)	2,457 (1,986-2,711)	2,022 (1,707-2,258)	1,711 (1,398-1,895)	1,663 (1,366-1,874)	1,287 (1,041-1,488)	1,023 (902-1,174)	843 (729-965)
Other exposure to mechanical forces	9,707 (8,966-11,545)	9,736 (9,096-11,141)	9,118 (8,577-9,908)	8,225 (7,695-9,350)	7,383 (6,973-8,055)	5,908 (5,549-6,534)	5,695 (5,294-6,274)	4,567 (4,207-4,911)
Adverse effects of medical treatment	4,039 (2,912-4,603)	3,759 (2,764-4,244)	3,781 (2,908-4,301)	4,177 (3,217-4,687)	4,088 (3,225-4,574)	3,446 (2,744-3,808)	4,389 (3,520-4,864)	6,300 (5,031-7,045)
Animal contact	4,243 (2,052-5,096)	4,371 (2,114-5,318)	3,701 (1,857-4,459)	4,503 (2,119-5,431)	4,705 (2,314-5,628)	4,076 (2,083-4,918)	4,038 (2,028-4,806)	4,280 (2,072-5,171)
Venomous animal contact	3,491 (1,619-4,246)	3,818 (1,839-4,672)	3,250 (1,599-3,936)	3,996 (1,854-4,849)	4,125 (1,965-4,980)	3,540 (1,771-4,306)	3,468 (1,692-4,149)	3,750 (1,783-4,538)
Non-venomous animal contact	752 (408-1,014)	553 (264-732)	451 (251-592)	507 (259-666)	580 (329-770)	536 (307-771)	570 (331-782)	530 (279-714)
Foreign body	3,211 (3,097-3,581)	3,445 (3,337-3,732)	3,846 (3,737-4,061)	4,268 (4,158-4,480)	4,079 (3,965-4,274)	3,687 (3,585-3,822)	4,423 (4,292-4,570)	3,915 (3,795-4,037)
Pulmonary aspiration and foreign body in airway	2,698 (2,578-3,077)	2,904 (2,792-3,182)	3,303 (3,194-3,477)	3,722 (3,614-3,916)	3,730 (3,611-3,906)	3,389 (3,280-3,504)	4,106 (3,974-4,241)	3,604 (3,484-3,719)
Foreign body in other body part	513 (381-580)	541 (432-609)	543 (446-625)	546 (449-624)	349 (284-395)	298 (245-346)	317 (264-367)	311 (250-360)
Environmental heat and cold exposure	3,011 (2,247-3,326)	4,501 (3,556-4,914)	5,708 (4,831-6,165)	6,914 (5,882-7,457)	6,619 (5,656-7,156)	5,527 (4,658-5,988)	7,184 (6,186-7,761)	5,523 (4,453-6,016)
Exposure to forces of nature	1,439 (1,345-1,555)	1,254 (1,171-1,345)	1,114 (1,043-1,192)	1,095 (1,011-1,191)	1,004 (913-1,112)	1,013 (888-1,176)	1,067 (945-1,222)	1,108 (953-1,295)
Other unintentional injuries	11,073 (10,439-11,854)	10,376 (9,827-11,058)	9,225 (8,765-9,715)	7,194 (6,894-7,497)	6,496 (6,158-6,817)	4,610 (4,375-4,823)	4,439 (4,240-4,652)	3,612 (3,441-3,806)
Self-harm and interpersonal violence	179,980 (164,272-186,207)	158,186 (143,735-163,957)	125,516 (116,045-129,354)	112,408 (103,088-116,113)	88,872 (81,653-92,028)	66,761 (61,467-68,988)	63,729 (58,890-65,698)	53,326 (48,032-55,366)
Self-harm	110,536 (98,455-115,822)	96,548 (85,913-101,222)	76,189 (69,318-79,184)	72,898 (65,752-76,021)	60,959 (55,203-63,668)	47,565 (43,181-49,302)	46,581 (42,663-48,328)	40,963 (36,219-42,796)
Self-harm by firearm	6,800 (5,601-8,663)	6,253 (5,120-8,209)	5,681 (4,707-7,266)	5,259 (4,355-6,795)	4,605 (3,702-5,873)	3,736 (3,019-4,631)	3,461 (2,771-4,372)	3,019 (2,483-3,798)
Self-harm by other specified means	103,737 (91,387-109,467)	90,296 (79,890-95,261)	70,509 (63,427-73,680)	67,638 (60,503-70,892)	56,355 (50,878-59,213)	43,829 (39,649-45,605)	43,120 (39,087-44,910)	37,944 (33,402-39,828)
Interpersonal violence	59,674 (51,793-62,395)	54,005 (47,200-56,363)	44,049 (38,992-46,030)	35,828 (31,983-37,538)	25,503 (22,619-26,786)	17,452 (15,329-18,358)	15,487 (13,859-16,287)	10,913 (9,605-11,553)
Physical violence by firearm	23,786 (19,806-25,572)	19,618 (16,088-21,068)	15,666 (12,677-16,837)	11,217 (9,028-12,105)	7,267 (6,001-7,879)	5,115 (4,101-5,590)	3,743 (2,986-4,207)	2,521 (2,004-2,829)
Physical violence by sharp object	17,362 (13,495-20,338)	16,345 (12,843-18,926)	13,031 (10,806-15,148)	11,009 (9,106-12,319)	7,669 (6,381-8,707)	4,917 (4,002-5,681)	4,205 (3,588-4,698)	3,109 (2,542-3,557)
Physical violence by other means	18,525 (15,595-22,039)	18,042 (15,340-21,210)	15,351 (13,553-17,458)	13,602 (12,100-15,394)	10,567 (9,350-11,907)	7,420 (6,502-8,397)	7,539 (6,706-8,316)	5,283 (4,626-5,889)
Conflict and terrorism	7,790 (7,072-8,733)	6,040 (5,515-6,740)	4,399 (4,018-4,945)	3,029 (2,786-3,358)	2,027 (1,888-2,210)	1,542 (1,449-1,658)	1,539 (1,456-1,658)	1,359 (1,286-1,461)
Executions and police conflict	1,981 (1,085-2,398)	1,592 (801-1,858)	879 (540-1,030)	652 (409-766)	383 (231-470)	202 (156-245)	122 (95-154)	91 (75-110)

Cause	65 to 69	70 to 74	75 to 79	80 to 84	85 to 89	90 to 94	95 plus
Urolithiasis	1,464 (1,198-1,732)	1,462 (1,146-1,738)	1,222 (955-1,425)	1,041 (835-1,194)	614 (479-720)	270 (222-317)	63 (53-75)
Other urinary diseases	3,822 (2,978-4,660)	4,300 (3,317-5,216)	3,320 (2,555-4,048)	3,350 (2,621-3,980)	1,960 (1,553-2,332)	939 (746-1,108)	293 (230-342)
Gynecological diseases	340 (295-395)	214 (191-241)	159 (145-180)	287 (240-336)	205 (171-241)	84 (73-98)	28 (25-33)
Uterine fibroids	72 (56-104)	48 (37-72)	33 (26-48)	72 (50-111)	57 (37-85)	29 (21-41)	10 (7-14)
Polycystic ovarian syndrome	-	-	-	-	-	-	-
Endometriosis	-	-	-	-	-	-	-
Genital prolapse	89 (42-138)	69 (38-96)	49 (28-65)	74 (42-114)	46 (28-70)	18 (12-24)	6 (4-8)
Other gynecological diseases	178 (137-243)	97 (75-130)	78 (64-106)	142 (109-182)	102 (81-131)	37 (31-48)	12 (10-16)
Hemoglobinopathies and hemolytic anemias	4,688 (3,536-5,977)	5,254 (4,010-6,607)	4,881 (3,675-6,129)	5,020 (3,757-6,016)	4,039 (2,977-4,723)	2,071 (1,502-2,407)	659 (476-759)
Thalassemias	3 (2-4)	1 (1-1)	0 (0-0)	0 (0-0)	0 (0-0)	0 (0-0)	0 (0-0)
Sickle cell disorders	43 (30-64)	16 (10-23)	5 (3-8)	2 (1-3)	1 (0-1)	0 (0-1)	0 (0-0)
G6PD deficiency	694 (383-1,136)	445 (240-754)	238 (125-409)	106 (58-177)	40 (24-61)	10 (6-15)	2 (1-3)
Other hemoglobinopathies and hemolytic anemias	3,948 (3,050-4,922)	4,792 (3,693-5,945)	4,638 (3,525-5,758)	4,912 (3,683-5,855)	3,999 (2,950-4,676)	2,060 (1,495-2,392)	658 (475-756)
Endocrine, metabolic, blood, and immune disorders	6,644 (5,670-7,806)	6,909 (5,907-8,150)	6,112 (5,201-7,206)	5,985 (4,879-7,174)	4,646 (3,791-5,537)	2,262 (1,852-2,797)	744 (610-961)
Sudden infant death syndrome	-	-	-	-	-	-	-
Injuries	172,065 (162,322-177,716)	153,504 (143,993-158,210)	128,098 (120,234-133,267)	114,974 (108,721-119,012)	81,216 (76,787-84,023)	35,540 (33,954-36,670)	9,994 (9,613-10,274)
Transport injuries	46,399 (44,827-48,712)	38,830 (37,632-40,374)	27,729 (26,748-29,282)	19,259 (18,646-20,207)	9,740 (9,430-10,163)	3,158 (3,071-3,279)	749 (727-773)
Road injuries	43,619 (42,178-45,687)	36,787 (35,650-38,226)	26,226 (25,296-27,639)	18,277 (17,700-19,132)	9,260 (8,973-9,663)	3,000 (2,917-3,109)	705 (684-728)
Pedestrian road injuries	23,640 (22,133-25,861)	20,616 (19,528-22,276)	15,577 (14,764-17,059)	11,006 (10,477-11,906)	5,574 (5,321-5,990)	1,678 (1,611-1,795)	368 (352-391)
Cyclist road injuries	2,564 (2,263-2,853)	2,125 (1,888-2,353)	1,252 (1,113-1,401)	787 (723-871)	328 (299-363)	87 (78-98)	1 (1-1)
Motorcyclist road injuries	3,210 (2,810-3,962)	2,229 (1,950-2,700)	1,142 (982-1,432)	728 (627-900)	287 (244-367)	75 (64-93)	18 (16-21)
Motor vehicle road injuries	13,829 (12,792-15,325)	11,500 (10,637-12,592)	7,981 (7,376-8,902)	5,567 (5,234-6,103)	2,971 (2,808-3,243)	1,134 (1,079-1,214)	310 (293-325)
Other road injuries	376 (287-506)	316 (237-425)	275 (213-360)	190 (147-262)	100 (78-138)	27 (21-38)	9 (7-12)
Other transport injuries	2,779 (2,606-3,081)	2,044 (1,920-2,278)	1,502 (1,401-1,724)	981 (914-1,101)	480 (445-533)	157 (146-175)	44 (41-48)
Unintentional injuries	77,767 (71,372-81,152)	74,750 (68,436-78,164)	72,247 (65,801-76,194)	74,778 (69,294-78,371)	60,598 (56,673-63,205)	29,167 (27,663-30,234)	8,620 (8,257-8,902)
Falls	30,687 (27,424-33,118)	34,727 (30,806-37,282)	38,970 (35,164-41,685)	44,243 (40,153-46,987)	38,981 (35,778-41,117)	19,514 (18,364-20,417)	5,697 (5,422-5,938)
Drowning	8,563 (8,263-8,839)	7,357 (7,071-7,646)	5,528 (5,275-5,804)	4,502 (4,293-4,744)	2,387 (2,282-2,516)	705 (680-735)	129 (125-135)
Fire, heat, and hot substances	6,410 (5,630-7,009)	6,029 (5,293-6,591)	5,617 (4,905-6,156)	6,026 (5,416-6,516)	4,547 (4,150-4,847)	2,089 (1,942-2,202)	661 (629-689)
Poisonings	3,257 (3,015-3,849)	2,503 (2,281-3,154)	1,673 (1,545-2,125)	1,432 (1,337-1,757)	770 (724-927)	243 (230-275)	51 (48-55)
Poisoning by carbon monoxide	2,027 (1,879-2,449)	1,447 (1,306-1,869)	1,020 (923-1,347)	873 (799-1,114)	475 (441-591)	138 (129-165)	23 (21-26)
Poisoning by other means	1,230 (1,086-1,459)	1,055 (960-1,321)	653 (598-830)	559 (510-680)	296 (268-345)	105 (94-113)	28 (26-30)
Exposure to mechanical forces	4,729 (4,412-5,156)	3,784 (3,499-4,129)	2,844 (2,617-3,125)	2,329 (2,171-2,525)	1,667 (1,585-1,834)	801 (762-867)	254 (243-272)
Unintentional firearm injuries	795 (697-920)	668 (583-755)	632 (554-722)	532 (477-620)	369 (337-449)	175 (159-213)	50 (46-63)
Other exposure to mechanical forces	3,934 (3,636-4,298)	3,116 (2,848-3,403)	2,212 (2,007-2,440)	1,797 (1,658-1,954)	1,297 (1,229-1,404)	625 (595-668)	204 (193-215)
Adverse effects of medical treatment	7,116 (5,785-7,893)	6,880 (5,613-7,542)	6,348 (5,178-6,979)	6,179 (5,113-6,761)	4,972 (4,132-5,410)	2,433 (2,093-2,616)	741 (650-792)
Animal contact	4,138 (1,908-5,090)	3,284 (1,628-4,044)	2,471 (1,204-3,109)	1,369 (733-1,683)	667 (393-811)	196 (126-234)	42 (31-48)
Venomous animal contact	3,610 (1,648-4,430)	2,835 (1,358-3,491)	2,115 (995-2,639)	1,127 (582-1,408)	529 (304-655)	146 (90-179)	29 (21-34)
Non-venomous animal contact	528 (283-721)	449 (245-624)	356 (189-520)	243 (141-334)	139 (84-186)	50 (33-65)	13 (9-16)
Foreign body	3,987 (3,853-4,108)	3,938 (3,783-4,058)	3,808 (3,690-3,931)	4,349 (4,236-4,470)	3,741 (3,624-3,870)	1,940 (1,879-2,007)	682 (662-706)
Pulmonary aspiration and foreign body in airway	3,713 (3,582-3,826)	3,635 (3,509-3,771)	3,510 (3,395-3,637)	4,059 (3,952-4,214)	3,525 (3,410-3,654)	1,843 (1,782-1,915)	654 (633-677)
Foreign body in other body part	274 (211-312)	302 (224-339)	298 (217-334)	290 (213-323)	216 (157-243)	97 (73-108)	28 (23-34)
Environmental heat and cold exposure	4,863 (3,793-5,334)	3,521 (2,541-3,972)	2,984 (1,989-3,458)	2,611 (1,882-2,918)	1,900 (1,490-2,082)	867 (715-935)	249 (224-266)
Exposure to forces of nature	1,117 (946-1,347)	989 (808-1,221)	790 (634-989)	727 (576-916)	364 (290-462)	115 (89-149)	26 (22-32)
Other unintentional injuries	2,901 (2,760-3,067)	1,738 (1,654-1,817)	1,214 (1,156-1,286)	1,009 (959-1,078)	603 (572-644)	265 (252-280)	86 (82-91)
Self-harm and interpersonal violence	47,899 (43,363-49,547)	39,924 (35,795-41,402)	28,122 (25,786-29,187)	20,937 (19,465-21,547)	10,878 (10,217-11,189)	3,215 (3,060-3,292)	625 (601-637)
Self-harm	37,919 (34,041-39,408)	33,297 (29,452-34,668)	23,809 (21,538-24,784)	19,079 (17,625-19,687)	9,955 (9,312-10,262)	2,947 (2,798-3,023)	500 (479-511)
Self-harm by firearm	3,004 (2,488-3,746)	2,786 (2,383-3,437)	2,201 (1,917-2,652)	1,869 (1,649-2,377)	1,060 (914-1,400)	353 (307-458)	71 (62-86)
Self-harm by other specified means	34,915 (31,144-36,487)	30,510 (26,776-31,994)	21,608 (19,346-22,637)	17,210 (15,641-17,927)	8,895 (8,140-9,290)	2,594 (2,388-2,699)	429 (402-443)
Interpersonal violence	8,741 (7,705-9,304)	5,783 (5,072-6,203)	3,759 (3,346-4,046)	1,402 (1,315-1,476)	686 (650-724)	191 (182-202)	74 (68-77)
Physical violence by firearm	1,761 (1,415-1,987)	1,229 (981-1,412)	761 (608-867)	192 (159-218)	96 (82-107)	30 (26-33)	16 (12-18)
Physical violence by sharp object	2,346 (1,930-2,620)	1,554 (1,288-1,758)	1,022 (872-1,144)	351 (324-393)	160 (148-179)	44 (41-50)	18 (16-21)
Physical violence by other means	4,634 (4,051-5,123)	2,999 (2,604-3,383)	1,977 (1,729-2,209)	859 (795-917)	430 (402-457)	117 (111-125)	40 (37-43)
Conflict and terrorism	1,173 (1,111-1,256)	802 (759-853)	528 (499-564)	434 (414-458)	225 (216-235)	72 (69-75)	50 (48-52)
Executions and police conflict	67 (46-79)	42 (32-50)	25 (20-31)	23 (18-26)	11 (9-14)	5 (4-6)	2 (1-2)

SR Table 7: Global deaths by cause and age group for both sexes combined (with 95% confidence intervals), 2000

Cause	Early Neonatal	Late Neonatal	Post Neonatal	1 to 4	5 to 9	10 to 14	15 to 19
All causes	2,743,663 (2,683,053-2,807,354)	918,145 (887,698-954,872)	3,092,086 (3,030,247-3,163,815)	2,926,972 (2,853,818-3,007,898)	703,302 (696,132-711,149)	483,735 (479,703-487,837)	678,423 (670,973-686,282)
Communicable, maternal, neonatal, and nutritional diseases	2,502,246 (2,438,369-2,566,166)	804,573 (773,289-840,604)	2,547,750 (2,461,743-2,632,086)	2,334,551 (2,238,498-2,444,865)	392,871 (371,595-418,817)	201,557 (188,515-218,253)	210,218 (197,905-226,403)
HIV/AIDS and sexually transmitted infections	-	22,500 (8,615-43,138)	187,600 (162,531-222,035)	99,452 (92,594-106,266)	23,030 (21,280-24,906)	8,593 (7,871-9,441)	12,968 (11,078-15,165)
HIV/AIDS	-	-	149,783 (141,474-158,045)	95,976 (89,494-101,975)	21,555 (20,208-22,939)	8,478 (7,754-9,315)	12,615 (10,729-14,825)
HIV/AIDS - Drug-susceptible Tuberculosis	-	-	51,197 (38,333-59,559)	34,385 (26,797-39,283)	7,485 (5,794-8,664)	2,875 (2,238-3,362)	4,048 (3,159-4,947)
HIV/AIDS - Multidrug-resistant Tuberculosis without extensive drug resistance	-	-	4,467 (2,714-6,855)	2,957 (1,781-4,663)	602 (363-925)	234 (133-378)	370 (227-596)
HIV/AIDS - Extensively drug-resistant Tuberculosis	-	-	38 (25-55)	26 (17-37)	6 (4-8)	3 (2-5)	6 (4-9)
HIV/AIDS resulting in other diseases	-	-	94,081 (83,684-108,794)	58,608 (52,136-67,896)	13,463 (11,972-15,528)	5,366 (4,676-6,271)	8,190 (6,766-10,215)
Sexually transmitted infections excluding HIV	67,918 (26,572-128,864)	22,500 (8,615-43,138)	37,817 (14,085-73,431)	3,476 (1,291-6,906)	1,476 (539-2,865)	115 (94-135)	353 (263-405)
Syphilis	67,918 (26,572-128,864)	22,500 (8,615-43,138)	37,817 (14,085-73,431)	3,476 (1,291-6,906)	1,476 (539-2,865)	63 (54-77)	69 (57-80)
Chlamydial infection	-	-	-	-	-	12 (8-16)	47 (34-55)
Gonococcal infection	-	-	-	-	-	25 (17-30)	124 (90-144)
Other sexually transmitted infections	-	-	-	-	-	15 (11-18)	113 (81-131)
Respiratory infections and tuberculosis	-	197,221 (179,061-214,037)	886,232 (815,109-946,752)	520,202 (436,320-590,417)	68,133 (57,909-76,122)	38,734 (34,573-42,488)	50,627 (47,651-54,040)
Tuberculosis	-	-	64,409 (58,253-69,608)	67,813 (56,042-77,692)	16,252 (13,955-18,523)	15,219 (13,543-16,900)	32,682 (30,258-35,404)
Drug-susceptible tuberculosis	-	-	58,994 (53,120-64,340)	62,756 (51,680-72,057)	14,877 (12,635-17,055)	13,872 (12,191-15,494)	29,940 (27,269-32,957)
Multidrug-resistant tuberculosis without extensive drug resistance	-	-	5,246 (3,655-7,348)	4,954 (3,312-7,569)	1,333 (830-2,110)	1,303 (827-2,031)	2,658 (1,663-4,248)
Extensively drug-resistant tuberculosis	-	-	168 (121-230)	104 (72-149)	42 (28-60)	43 (29-62)	84 (58-122)
Lower respiratory infections	219,164 (198,953-241,424)	196,926 (178,840-213,724)	817,240 (751,913-874,173)	447,839 (375,714-510,438)	51,073 (42,681-57,161)	23,024 (19,642-25,332)	17,686 (16,014-18,951)
Upper respiratory infections	126 (66-161)	243 (139-312)	4,276 (2,019-6,224)	4,186 (1,323-7,988)	614 (272-839)	305 (150-404)	168 (90-238)
Otitis media	51 (8-272)	52 (25-119)	308 (142-617)	364 (135-770)	194 (102-357)	186 (104-324)	90 (59-148)
Enteric infections	34,468 (30,357-40,089)	66,704 (58,343-76,973)	571,759 (507,223-637,814)	571,351 (460,655-676,048)	114,969 (84,196-153,178)	64,342 (46,460-87,122)	46,860 (33,434-66,067)
Diarrheal diseases	34,468 (30,357-40,089)	66,152 (57,861-76,353)	561,836 (497,383-627,287)	514,344 (412,743-610,191)	49,106 (28,159-77,535)	23,492 (14,506-38,419)	23,141 (14,043-38,147)
Typhoid and paratyphoid	-	0 (0-0)	365 (194-673)	34,441 (15,830-64,074)	59,237 (34,619-95,476)	37,678 (21,508-60,467)	21,442 (10,589-38,490)
Typhoid fever	-	-	330 (173-598)	30,430 (14,090-56,987)	51,984 (29,712-84,133)	32,885 (18,333-52,815)	18,683 (9,154-33,015)
Paratyphoid fever	-	-	35 (14-72)	4,010 (1,493-8,667)	7,253 (3,100-14,229)	4,793 (2,092-9,431)	2,759 (1,083-5,833)
Invasive Non-typhoidal Salmonella (iNTS)	-	552 (309-938)	7,903 (4,433-13,992)	21,623 (11,056-39,444)	6,489 (3,159-11,430)	3,095 (1,389-5,939)	2,209 (919-4,152)
Other intestinal infectious diseases	-	-	1,654 (894-3,310)	944 (449-1,932)	136 (52-326)	77 (28-176)	68 (20-158)
Neglected tropical diseases and malaria	505 (258-1,590)	28,323 (17,937-42,541)	198,104 (136,764-277,087)	381,420 (262,540-531,265)	69,513 (50,422-96,590)	38,148 (28,140-52,962)	27,037 (19,554-40,193)
Malaria	-	27,870 (17,445-42,154)	189,507 (126,067-269,763)	350,470 (236,598-495,030)	52,490 (35,380-71,921)	27,652 (19,172-37,352)	18,000 (12,503-25,194)
Chagas disease	-	-	4 (3-4)	2 (2-2)	3 (2-3)	6 (5-7)	15 (14-17)
Leishmaniasis	-	-	2,278 (1-10,462)	9,320 (3-51,383)	4,277 (1-30,777)	2,578 (1-17,944)	2,569 (1-15,287)
Visceral leishmaniasis	-	-	2,278 (1-10,462)	9,320 (3-51,383)	4,277 (1-30,777)	2,578 (1-17,944)	2,569 (1-15,287)
African trypanosomiasis	-	-	-	900 (226-2,525)	1,489 (501-3,323)	1,673 (604-3,294)	2,129 (814-4,356)
Schistosomiasis	-	-	0 (0-0)	894 (616-1,275)	313 (232-416)	403 (310-515)	437 (351-532)
Cysticercosis	0 (0-0)	0 (0-0)	0 (0-0)	189 (91-316)	78 (21-159)	55 (10-120)	52 (6-124)
Cystic echinococcosis	-	-	-	463 (285-664)	193 (101-309)	116 (41-211)	59 (5-134)
Dengue	-	-	2,852 (1,009-4,698)	4,489 (2,148-6,951)	2,059 (862-2,923)	955 (435-1,371)	858 (373-1,200)
Yellow fever	-	5 (1-14)	77 (15-248)	724 (140-2,210)	1,070 (216-3,218)	957 (206-2,736)	945 (200-2,638)
Rabies	-	-	694 (497-1,012)	3,934 (2,495-5,824)	5,965 (4,259-8,313)	3,268 (2,542-4,441)	1,682 (1,282-2,566)
Intestinal nematode infections	-	-	570 (447-718)	5,960 (4,399-7,859)	952 (743-1,194)	190 (150-234)	54 (42-68)
Ascariasis	-	-	570 (447-718)	5,960 (4,399-7,859)	952 (743-1,194)	190 (150-234)	54 (42-68)
Ebola	2 (2-3)	3 (2-3)	42 (35-52)	112 (92-136)	42 (34-51)	21 (17-25)	14 (11-17)
Zika virus	0 (0-0)	0 (0-0)	0 (0-0)	0 (0-0)	0 (0-0)	0 (0-0)	0 (0-0)
Other neglected tropical diseases	503 (255-1,588)	446 (227-1,407)	2,082 (1,062-7,340)	3,963 (1,261-18,463)	581 (350-1,628)	274 (176-673)	223 (157-494)
Other infectious diseases	58,198 (50,465-66,674)	71,537 (61,364-82,974)	405,307 (299,240-561,189)	575,611 (380,294-863,444)	101,793 (74,566-146,938)	45,612 (37,267-59,644)	34,091 (29,743-40,815)
Meningitis	13,384 (10,479-15,937)	19,805 (16,370-22,692)	119,172 (102,447-133,881)	114,420 (90,191-135,003)	23,418 (18,158-29,201)	14,780 (11,922-18,488)	12,400 (10,194-15,207)
Pneumococcal meningitis	1,981 (1,474-2,513)	2,793 (2,139-3,433)	15,304 (12,313-18,946)	14,669 (10,739-19,000)	2,103 (1,448-2,846)	1,301 (953-1,777)	1,326 (965-1,788)
H influenzae type B meningitis	3,661 (2,726-4,647)	5,494 (4,442-6,732)	37,433 (30,517-44,547)	39,516 (30,074-48,890)	9,341 (7,064-11,957)	5,773 (4,583-7,320)	4,348 (3,484-5,408)
Meningococcal meningitis	2,110 (1,653-2,576)	3,213 (2,608-3,804)	17,908 (15,161-21,342)	15,596 (11,957-19,560)	2,687 (2,075-3,490)	1,445 (1,146-1,872)	1,158 (918-1,446)
Other meningitis	5,632 (4,401-6,869)	8,305 (6,682-9,882)	48,527 (40,709-57,169)	44,638 (34,355-55,675)	9,287 (6,988-11,808)	6,261 (4,863-7,798)	5,568 (4,527-7,016)
Encephalitis	761 (616-900)	1,380 (1,098-1,734)	15,881 (11,518-21,354)	17,289 (12,386-26,451)	8,228 (6,631-10,456)	5,885 (4,949-7,306)	3,680 (3,318-4,158)
Diphtheria	-	-	2,303 (1,536-3,335)	8,110 (4,894-13,138)	1,352 (876-2,068)	453 (299-665)	150 (98-224)
Whooping cough	-	-	71,546 (33,485-135,435)	66,502 (29,618-125,019)	6,580 (2,884-13,274)	1,330 (595-2,676)	524 (240-1,065)
Tetanus	37,064 (30,491-45,044)	44,169 (36,276-55,149)	14,316 (10,664-20,267)	9,785 (4,914-14,394)	3,432 (2,298-4,947)	2,477 (1,659-3,481)	3,617 (2,261-5,117)
Measles	-	-	150,443 (55,167-327,024)	328,285 (125,467-659,192)	46,465 (17,690-97,017)	12,445 (4,445-27,472)	4,566 (1,551-10,347)
Varicella and herpes zoster	195 (159-241)	548 (453-667)	4,273 (3,504-5,119)	4,703 (3,751-5,727)	1,826 (1,456-2,266)	621 (504-754)	316 (262-387)
Acute hepatitis	-	-	13,299 (8,829-15,505)	15,699 (10,658-19,194)	7,237 (5,176-8,729)	5,579 (3,964-6,527)	6,370 (4,513-7,363)
Acute hepatitis A	-	-	2,246 (1,081-3,481)	9,790 (6,340-12,832)	4,787 (3,275-6,124)	2,803 (1,839-3,691)	2,361 (1,530-3,080)
Acute hepatitis B	-	-	8,226 (4,960-10,803)	4,425 (2,525-6,674)	1,213 (613-1,991)	961 (472-1,637)	1,423 (797-2,214)
Acute hepatitis C	-	-	2,806 (1,407-4,882)	1,145 (538-2,083)	140 (59-289)	50 (19-110)	33 (12-71)
Acute hepatitis E	-	-	21 (10-38)	339 (172-602)	1,097 (621-1,695)	1,766 (1,048-2,504)	2,552 (1,670-3,340)
Other unspecified infectious diseases	6,794 (3,256-8,388)	5,635 (2,824-6,842)	14,073 (8,326-17,415)	10,817 (6,581-15,503)	3,255 (2,383-3,980)	2,042 (1,535-2,465)	2,469 (1,893-2,955)
Maternal and neonatal disorders	2,121,816 (2,042,002-2,196,430)	418,289 (389,484-458,755)	108,488 (96,136-127,450)	9,676 (7,934-12,368)	-	1,094 (936-1,240)	35,164 (32,542-37,709)
Maternal disorders	-	-	-	-	-	1,094 (936-1,240)	35,164 (32,542-37,709)
Maternal hemorrhage	-	-	-	-	-	354 (280-438)	10,352 (6,074-17,100)
Maternal sepsis and other maternal infections	-	-	-	-	-	102 (71-143)	5,412 (2,898-10,386)
Maternal hypertensive disorders	-	-	-	-	-	122 (96-152)	5,072 (3,323-8,876)
Maternal obstructed labor and uterine rupture	-	-	-	-	-	103 (72-148)	1,826 (982-3,876)
Maternal abortion and miscarriage	-	-	-	-	-	92 (70-126)	2,999 (1,776-5,705)
Ectopic pregnancy	-	-	-	-	-	49 (28-94)	2,060 (593-6,329)
Indirect maternal deaths	-	-	-	-	-	148 (115-192)	3,968 (2,430-7,558)
Late maternal deaths	-	-	-	-	-	13 (9-19)	409 (298-568)
Maternal deaths aggravated by HIV/AIDS	-	-	-	-	-	1 (0-1)	99 (54-150)
Other maternal disorders	-	-	-	-	-	110 (83-150)	2,965 (1,387-6,638)
Neonatal disorders	2,121,816 (2,042,002-2,196,430)	418,289 (389,484-458,755)	108,488 (96,136-127,450)	9,676 (7,934-12,368)	-	-	-
Neonatal preterm birth	874,808 (823,910-955,633)	135,113 (122,032-155,336)	43,218 (37,530-51,794)	2,756 (2,202-3,641)	-	-	-
Neonatal encephalopathy due to birth asphyxia and trauma	672,378 (615,942-722,843)	70,849 (59,785-79,273)	20,366 (17,230-24,422)	3,736 (3,007-5,126)	-	-	-
Neonatal sepsis and other neonatal infections	133,184 (112,728-182,231)	92,028 (77,210-125,428)	12,246 (10,122-16,873)	455 (317-784)	-	-	-
Hemolytic disease and other neonatal jaundice	56,151 (48,242-66,011)	34,490 (28,213-41,611)	7,156 (5,108-9,632)	475 (302-688)	-	-	-
Other neonatal disorders	385,295 (333,441-429,654)	85,808 (72,543-103,232)	25,501 (20,831-33,127)	2,255 (1,769-3,185)	-	-	-
Nutritional deficiencies	-	-	190,259 (170,462-208,758)	176,837 (146,311-205,404)	15,434 (13,076-17,750)	5,033 (4,424-5,624)	3,471 (3,132-3,785)
Protein-energy malnutrition	-	-	183,618 (164,483-202,235)	168,822 (139,107-196,320)	12,685 (10,816-14,622)	3,899 (3,481-4,334)	2,429 (2,217-2,622)
Other nutritional deficiencies	-	-	6,641 (5,122-8,452)	8,015 (5,714-10,205)	2,749 (2,084-3,472)	1,134 (911-1,377)	1,042 (873-1,252)
Non-communicable diseases	223,744 (204,979-260,103)	99,782 (91,639-113,419)	423,328 (377,994-479,410)	274,490 (232,749-315,640)	120,419 (109,297-131,682)	113,505 (106,130-120,425)	162,498 (155,812-168,237)
Neoplasms	1,696 (1,383-1,888)	1,165 (964-1,363)	12,247 (9,610-14,993)	43,844 (34,250-57,881)	40,522 (35,208-46,899)	37,373 (33,645-41,343)	40,756 (38,235-42,550)
Lip and oral cavity cancer	-	-	-	-	-	-	596 (536-667)
Nasopharynx cancer	-	-	-	-	328 (275-369)	462 (401-511)	576 (537-606)
Other pharynx cancer	-	-	-	-	-	-	325 (270-365)
Esophageal cancer	-	-	-	-	-	-	315 (295-339)
Stomach cancer	-	-	-	-	-	-	1,048 (992-1,114)
Colon and rectum cancer	-	-	-	-	-	-	886 (782-978)
Liver cancer	-	-	-	-	1,233 (1,026-1,401)	1,275 (1,099-1,411)	1,395 (1,297-1,503)
Liver cancer due to hepatitis B	-	-	-	-	818 (682-937)	976 (836-1,092)	1,036 (952-1,121)
Liver cancer due to hepatitis C	-	-	-	-	22 (17-29)	25 (20-31)	27 (21-33)
Liver cancer due to alcohol use	-	-	-	-	-	-	6 (4-8)
Liver cancer due to NASH	-	-	-	-	-	-	101 (82-125)
Liver cancer due to other causes	-	-	-	-	393 (319-464)	274 (225-323)	225 (186-265)
Gallbladder and biliary tract cancer	-	-	-	-	-	-	101 (91-116)
Pancreatic cancer	-	-	-	-	-	-	164 (155-172)
Larynx cancer	-	-	-	-	-	-	164 (152-177)
Tracheal, bronchus, and lung cancer	-	-	-	-	-	-	927 (878-994)
Malignant skin melanoma	-	-	-	-	-	-	249 (206-314)
Non-melanoma skin cancer	-	-	-	-	-	-	158 (148-169)
Non-melanoma skin cancer (squamous-cell carcinoma)	-	-	-	-	-	-	158 (148-169)
Breast cancer	-	-	-	-	-	-	329 (230-396)
Cervical cancer	-	-	-	-	-	-	290 (252-318)
Uterine cancer	-	-	-	-	-	-	75 (68-81)
Ovarian cancer	-	-	-	-	-	-	502 (455-566)
Prostate cancer	-	-	-	-	-	-	75 (59-83)
Testicular cancer	-	-	-	-	-	-	384 (358-414)
Kidney cancer	48 (39-56)	27 (22-32)	355 (281-431)	1,632 (1,177-2,117)	723 (618-816)	316 (279-353)	209 (194-225)
Bladder cancer	-	-	-	-	-	-	101 (92-110)
Brain and nervous system cancer	202 (135-244)	168 (112-208)	2,215 (1,303-2,875)	8,244 (4,951-11,524)	9,350 (6,549-12,139)	7,262 (5,444-8,752)	4,487 (3,690-5,126)

Cause	20 to 24	25 to 29	30 to 34	35 to 39	40 to 44	45 to 49	50 to 54
All causes	919,208 (905,953-933,595)	1,132,383 (1,109,523-1,156,380)	1,294,353 (1,273,826-1,314,873)	1,391,260 (1,368,221-1,414,170)	1,527,392 (1,511,297-1,544,467)	1,877,379 (1,859,618-1,895,759)	2,056,271 (2,038,043-2,073,692)
Communicable, maternal, neonatal, and nutritional diseases	305,159 (290,861-322,221)	434,599 (411,168-459,561)	492,214 (471,582-511,504)	477,010 (456,034-498,541)	426,632 (412,838-443,336)	390,407 (376,194-410,257)	335,369 (322,954-355,627)
HIV/AIDS and sexually transmitted infections	67,008 (59,036-77,439)	187,228 (167,034-208,846)	241,549 (225,058-259,749)	226,459 (207,846-246,640)	186,990 (177,188-196,997)	138,046 (129,719-147,346)	80,066 (76,270-84,070)
HIV/AIDS	66,406 (58,357-76,817)	186,327 (166,238-207,820)	240,703 (224,119-258,841)	225,662 (207,007-245,827)	186,141 (176,341-196,065)	137,292 (128,916-146,622)	79,533 (75,746-83,506)
HIV/AIDS - Drug-susceptible Tuberculosis	18,514 (13,115-23,702)	40,657 (26,101-55,677)	48,823 (30,900-69,410)	47,769 (30,502-66,667)	43,611 (27,715-59,000)	36,237 (23,815-46,967)	23,903 (16,880-29,045)
HIV/AIDS - Multidrug-resistant Tuberculosis without extensive drug resistance	1,598 (951-2,528)	3,435 (1,945-5,543)	4,139 (2,309-6,800)	4,048 (2,294-6,520)	3,684 (2,098-5,869)	3,070 (1,785-4,686)	2,012 (1,219-3,007)
HIV/AIDS - Extensively drug-resistant Tuberculosis	24 (15-36)	59 (37-88)	81 (50-121)	87 (55-128)	79 (50-117)	62 (39-90)	34 (22-48)
HIV/AIDS resulting in other diseases	46,270 (37,952-57,764)	142,177 (116,810-167,937)	187,660 (159,352-212,820)	173,758 (146,505-200,355)	138,767 (119,410-157,120)	97,923 (84,320-113,683)	53,584 (47,393-61,404)
Sexually transmitted infections excluding HIV	602 (402-703)	901 (557-1,060)	846 (568-978)	797 (564-894)	849 (601-968)	754 (540-848)	533 (401-618)
Syphilis	98 (68-119)	153 (100-188)	160 (110-207)	170 (114-222)	174 (115-252)	157 (106-215)	181 (131-247)
Chlamydial infection	138 (90-162)	147 (88-176)	82 (53-94)	106 (72-119)	133 (92-150)	109 (78-123)	64 (48-71)
Gonococcal infection	217 (142-254)	402 (244-479)	383 (250-441)	304 (209-341)	349 (245-391)	308 (221-345)	190 (143-211)
Other sexually transmitted infections	148 (97-174)	198 (120-236)	221 (143-255)	217 (152-242)	193 (135-216)	179 (129-201)	98 (73-109)
Respiratory infections and tuberculosis	76,048 (72,139-80,284)	98,725 (94,150-103,288)	113,409 (108,552-119,280)	125,248 (120,011-131,463)	133,930 (128,346-140,597)	155,049 (149,038-162,787)	165,456 (158,729-173,622)
Tuberculosis	56,974 (53,412-60,644)	76,518 (72,400-80,930)	89,340 (85,029-94,526)	97,064 (91,967-102,927)	101,633 (96,414-108,256)	115,350 (109,607-122,508)	118,076 (111,663-125,836)
Drug-susceptible tuberculosis	51,984 (47,988-55,873)	69,603 (64,681-74,579)	81,094 (75,706-86,885)	88,029 (82,140-94,131)	91,786 (85,017-98,858)	103,950 (96,822-111,579)	106,872 (99,199-115,024)
Multidrug-resistant tuberculosis without extensive drug resistance	4,786 (3,255-7,165)	6,615 (4,502-9,882)	7,888 (5,335-11,822)	8,605 (5,898-12,780)	9,328 (6,444-13,653)	10,796 (7,615-15,651)	10,661 (7,427-15,559)
Extensively drug-resistant tuberculosis	203 (151-274)	300 (227-393)	358 (270-465)	430 (332-554)	519 (406-657)	604 (472-758)	543 (420-690)
Lower respiratory infections	18,834 (17,349-20,298)	21,897 (19,967-23,310)	23,738 (21,798-25,291)	27,836 (25,645-29,622)	31,938 (29,645-33,816)	39,142 (35,997-41,448)	46,859 (42,823-50,001)
Upper respiratory infections	181 (99-238)	258 (149-323)	288 (159-361)	291 (162-379)	303 (177-414)	378 (214-493)	339 (191-455)
Otitis media	59 (35-99)	53 (32-92)	43 (27-74)	57 (35-104)	55 (34-96)	180 (88-435)	182 (98-419)
Enteric infections	36,995 (26,191-52,873)	31,611 (21,813-46,184)	29,301 (19,904-43,374)	28,644 (19,484-43,703)	27,701 (18,477-43,459)	32,188 (21,369-52,166)	35,210 (22,910-57,110)
Diarrheal diseases	23,191 (13,301-38,337)	21,440 (12,523-35,232)	21,524 (12,331-35,133)	22,679 (13,555-37,757)	23,073 (13,583-39,578)	28,325 (17,189-47,719)	32,139 (19,825-54,079)
Typhoid and paratyphoid	11,828 (6,051-20,527)	8,461 (4,435-14,455)	6,234 (3,236-10,489)	4,436 (2,315-7,416)	3,190 (1,656-5,228)	2,504 (1,344-4,243)	1,748 (895-3,012)
Typhoid fever	10,315 (5,089-18,140)	7,292 (3,747-12,550)	5,313 (2,694-9,076)	3,748 (1,908-6,417)	2,674 (1,348-4,539)	2,070 (1,097-3,492)	1,444 (735-2,493)
Paratyphoid fever	1,513 (609-3,230)	1,169 (501-2,485)	922 (391-1,921)	688 (298-1,381)	517 (222-1,021)	434 (180-880)	304 (121-629)
Invasive Non-typhoidal Salmonella (iNTS)	1,907 (811-3,606)	1,667 (658-3,416)	1,483 (617-2,829)	1,477 (657-2,812)	1,401 (663-2,630)	1,322 (625-2,518)	1,295 (585-2,468)
Other intestinal infectious diseases	69 (22-167)	43 (13-95)	59 (18-124)	51 (14-108)	36 (9-77)	37 (9-83)	27 (6-63)
Neglected tropical diseases and malaria	27,671 (20,290-38,395)	23,222 (16,325-33,603)	21,396 (15,300-30,521)	21,734 (15,609-30,283)	21,067 (15,445-28,796)	19,962 (14,783-27,125)	20,960 (15,330-28,360)
Malaria	18,890 (12,725-27,009)	15,532 (10,444-22,416)	14,113 (9,527-20,519)	14,736 (9,994-21,498)	14,308 (9,545-21,057)	13,107 (8,708-19,261)	14,902 (9,921-22,060)
Chagas disease	38 (35-42)	70 (65-77)	140 (130-151)	236 (223-252)	367 (347-387)	532 (502-563)	706 (674-742)
Leishmaniasis	2,133 (1-11,752)	2,291 (1-11,813)	2,021 (1-10,144)	1,761 (6-7,946)	1,399 (7-5,906)	1,266 (9-4,757)	1,040 (13-3,938)
Visceral leishmaniasis	2,133 (1-11,752)	2,291 (1-11,813)	2,021 (1-10,144)	1,761 (6-7,946)	1,399 (7-5,906)	1,266 (9-4,757)	1,040 (13-3,938)
African trypanosomiasis	2,364 (801-4,907)	2,005 (680-4,418)	1,759 (572-4,052)	1,592 (524-3,552)	1,428 (470-3,522)	1,401 (406-3,890)	895 (274-2,278)
Schistosomiasis	490 (399-586)	496 (409-607)	519 (436-617)	612 (512-731)	685 (574-804)	790 (661-930)	881 (741-1,026)
Cysticercosis	77 (15-160)	101 (27-204)	105 (28-216)	93 (20-198)	87 (17-186)	77 (13-171)	64 (9-150)
Cystic echinococcosis	70 (10-152)	98 (24-208)	98 (22-204)	113 (26-232)	106 (20-223)	112 (22-238)	115 (24-251)
Dengue	889 (430-1,298)	772 (382-1,149)	586 (277-862)	591 (259-919)	496 (234-757)	499 (234-743)	499 (216-759)
Yellow fever	835 (176-2,434)	589 (117-1,740)	467 (99-1,407)	401 (81-1,227)	345 (66-1,057)	283 (55-853)	222 (43-668)
Rabies	1,612 (1,221-2,355)	969 (759-1,369)	1,287 (968-1,765)	1,272 (985-1,776)	1,536 (1,178-2,108)	1,571 (1,231-2,387)	1,333 (1,029-1,921)
Intestinal nematode infections	44 (34-55)	34 (27-42)	44 (35-56)	41 (32-52)	29 (23-36)	32 (25-40)	29 (23-36)
Ascariasis	44 (34-55)	34 (27-42)	44 (35-56)	41 (32-52)	29 (23-36)	32 (25-40)	29 (23-36)
Ebola	11 (9-14)	10 (8-12)	9 (7-11)	9 (7-11)	8 (7-10)	8 (7-10)	8 (6-9)
Zika virus	0 (0-0)	0 (0-0)	0 (0-0)	0 (0-0)	0 (0-0)	0 (0-0)	0 (0-0)
Other neglected tropical diseases	217 (147-484)	255 (176-531)	246 (179-441)	276 (198-537)	273 (204-501)	282 (206-511)	266 (199-477)
Other infectious diseases	29,257 (25,823-33,222)	25,703 (22,691-28,716)	26,145 (23,403-28,594)	25,494 (22,125-27,411)	25,142 (21,696-27,213)	27,601 (23,687-29,709)	25,824 (22,502-27,981)
Meningitis	10,573 (8,597-13,137)	9,314 (7,692-11,785)	8,914 (7,373-11,332)	8,681 (7,242-11,047)	7,562 (6,365-9,583)	8,273 (7,034-10,432)	7,421 (6,318-9,483)
Pneumococcal meningitis	1,327 (1,008-1,782)	1,204 (910-1,638)	1,186 (913-1,544)	1,288 (984-1,732)	1,190 (888-1,588)	1,393 (1,046-1,873)	1,275 (998-1,673)
H influenzae type B meningitis	3,288 (2,656-4,130)	2,915 (2,380-3,622)	2,703 (2,248-3,485)	2,487 (2,037-3,259)	2,265 (1,869-2,940)	2,529 (2,102-3,337)	2,242 (1,837-2,955)
Meningococcal meningitis	918 (720-1,163)	801 (633-1,033)	790 (618-1,010)	764 (607-1,013)	645 (510-815)	691 (553-889)	659 (526-868)
Other meningitis	5,039 (3,986-6,322)	4,394 (3,511-5,687)	4,235 (3,379-5,517)	4,142 (3,332-5,319)	3,462 (2,871-4,416)	3,659 (3,044-4,710)	3,244 (2,648-4,261)
Encephalitis	3,243 (2,930-3,699)	2,815 (2,566-3,275)	3,070 (2,803-3,549)	2,900 (2,654-3,300)	2,746 (2,556-3,000)	2,722 (2,530-3,033)	2,463 (2,298-2,726)
Diphtheria	86 (57-128)	76 (53-109)	77 (53-111)	80 (55-115)	94 (67-128)	157 (116-214)	113 (83-155)
Whooping cough	378 (177-748)	236 (104-479)	177 (78-364)	127 (56-255)	133 (59-268)	114 (50-233)	92 (41-183)
Tetanus	3,332 (2,146-4,682)	3,128 (1,958-4,313)	3,112 (2,205-4,239)	2,986 (2,065-3,973)	3,502 (2,171-4,871)	4,474 (2,468-6,016)	4,217 (2,439-5,986)
Measles	2,246 (753-5,132)	1,068 (365-2,464)	622 (212-1,432)	372 (126-858)	251 (85-578)	175 (60-398)	125 (43-285)
Varicella and herpes zoster	336 (279-404)	446 (379-528)	550 (471-637)	465 (402-544)	283 (245-328)	275 (237-318)	242 (208-283)
Acute hepatitis	7,073 (5,511-8,045)	6,804 (5,051-7,608)	7,602 (5,592-8,388)	7,758 (5,679-8,696)	8,547 (6,305-9,664)	9,154 (6,735-10,205)	8,761 (6,468-9,986)
Acute hepatitis A	2,073 (1,344-2,879)	1,477 (894-2,184)	1,296 (697-2,118)	1,197 (607-2,029)	1,204 (650-2,031)	1,137 (609-1,925)	788 (457-1,194)
Acute hepatitis B	2,304 (1,316-3,491)	3,250 (1,962-4,559)	4,704 (2,980-6,270)	5,389 (3,478-6,853)	6,452 (4,486-7,876)	7,293 (5,117-8,687)	7,454 (5,391-8,671)
Acute hepatitis C	28 (9-61)	26 (9-66)	34 (10-85)	51 (14-135)	60 (18-144)	78 (23-188)	86 (28-199)
Acute hepatitis E	2,668 (1,699-3,673)	2,051 (1,194-2,977)	1,568 (728-2,542)	1,121 (494-2,018)	832 (365-1,565)	645 (277-1,329)	433 (203-843)
Other unspecified infectious diseases	1,991 (1,541-2,310)	1,817 (1,416-2,074)	2,020 (1,577-2,290)	2,125 (1,682-2,432)	2,024 (1,662-2,323)	2,257 (1,878-2,538)	2,390 (2,021-2,657)
Maternal and neonatal disorders	64,553 (60,344-68,416)	64,228 (60,532-67,836)	56,319 (53,030-59,690)	45,106 (42,276-47,981)	26,851 (25,047-28,708)	12,305 (11,351-13,272)	1,726 (1,570-1,883)
Maternal disorders	64,553 (60,344-68,416)	64,228 (60,532-67,836)	56,319 (53,030-59,690)	45,106 (42,276-47,981)	26,851 (25,047-28,708)	12,305 (11,351-13,272)	1,726 (1,570-1,883)
Maternal hemorrhage	23,210 (14,077-34,786)	24,199 (16,506-34,666)	23,325 (17,256-30,421)	18,997 (15,133-23,558)	9,342 (7,486-11,417)	3,259 (2,595-4,014)	503 (401-616)
Maternal sepsis and other maternal infections	7,455 (4,038-14,611)	6,616 (3,882-12,431)	5,073 (3,128-8,703)	4,059 (2,780-6,478)	2,994 (2,259-4,107)	2,542 (2,028-3,103)	220 (171-284)
Maternal hypertensive disorders	8,254 (5,469-14,513)	7,764 (5,273-12,761)	5,673 (3,955-9,274)	4,155 (2,998-6,333)	2,334 (1,807-3,193)	607 (466-801)	79 (60-106)
Maternal obstructed labor and uterine rupture	3,151 (1,407-8,398)	3,297 (1,620-7,446)	3,513 (2,010-6,819)	3,455 (2,220-5,753)	2,468 (1,742-3,574)	1,333 (978-1,768)	107 (72-163)
Maternal abortion and miscarriage	3,951 (2,345-6,849)	3,909 (2,367-7,508)	3,718 (2,287-6,712)	3,149 (2,090-5,072)	2,311 (1,686-3,364)	2,017 (1,619-2,507)	199 (152-259)
Ectopic pregnancy	3,230 (1,129-9,332)	3,036 (1,079-8,888)	2,626 (1,049-7,062)	1,669 (707-4,703)	875 (394-2,016)	380 (196-824)	133 (78-224)
Indirect maternal deaths	7,865 (4,828-14,688)	7,936 (5,109-13,966)	6,259 (4,268-10,650)	4,998 (3,660-7,541)	3,046 (2,339-4,267)	897 (740-1,115)	246 (198-300)
Late maternal deaths	744 (558-1,033)	769 (573-1,046)	667 (482-929)	499 (373-678)	262 (197-356)	106 (90-128)	15 (12-18)
Maternal deaths aggravated by HIV/AIDS	349 (214-473)	487 (315-631)	466 (300-605)	364 (232-481)	212 (132-288)	142 (90-194)	15 (9-20)
Other maternal disorders	6,345 (2,996-13,962)	6,216 (3,141-12,031)	4,999 (2,841-9,322)	3,761 (2,428-6,602)	3,005 (2,210-4,199)	1,022 (821-1,322)	210 (166-265)
Neonatal disorders	-	-	-	-	-	-	-
Neonatal preterm birth	-	-	-	-	-	-	-
Neonatal encephalopathy due to birth asphyxia and trauma	-	-	-	-	-	-	-
Neonatal sepsis and other neonatal infections	-	-	-	-	-	-	-
Hemolytic disease and other neonatal jaundice	-	-	-	-	-	-	-
Other neonatal disorders	-	-	-	-	-	-	-
Nutritional deficiencies	3,628 (3,334-3,890)	3,881 (3,635-4,127)	4,095 (3,893-4,271)	4,324 (4,080-4,513)	4,951 (4,679-5,182)	5,255 (4,900-5,528)	6,127 (5,737-6,409)
Protein-energy malnutrition	2,483 (2,278-2,673)	2,713 (2,530-2,911)	3,111 (2,944-3,286)	3,302 (3,127-3,462)	3,758 (3,557-3,963)	4,018 (3,750-4,244)	4,745 (4,477-5,012)
Other nutritional deficiencies	1,145 (985-1,360)	1,169 (1,017-1,351)	984 (863-1,157)	1,023 (878-1,213)	1,193 (1,039-1,415)	1,237 (1,043-1,477)	1,382 (1,178-1,662)
Non-communicable diseases	209,403 (203,111-214,939)	294,335 (287,623-301,530)	424,069 (416,105-432,625)	577,220 (567,976-587,004)	814,122 (802,455-824,644)	1,211,789 (1,196,543-1,226,639)	1,500,117 (1,482,662-1,515,982)
Neoplasms	45,464 (43,480-47,414)	68,670 (66,074-71,449)	113,881 (109,229-118,323)	164,474 (159,560-169,754)	243,643 (239,558-248,261)	391,823 (384,322-400,039)	487,640 (480,518-496,178)
Lip and oral cavity cancer	844 (768-943)	1,254 (1,153-1,376)	2,094 (1,957-2,238)	3,492 (3,298-3,702)	6,180 (5,881-6,519)	9,662 (9,260-10,110)	12,305 (11,847-12,856)
Nasopharynx cancer	683 (626-722)	1,120 (1,036-1,189)	2,044 (1,864-2,186)	2,661 (2,443-2,852)	3,945 (3,731-4,158)	5,829 (5,489-6,120)	6,268 (5,993-6,520)
Other pharynx cancer	390 (318-436)	589 (485-651)	981 (767-1,065)	1,736 (1,397-1,879)	3,326 (2,849-3,559)	6,351 (5,538-6,791)	8,525 (7,405-9,043)
Esophageal cancer	577 (548-606)	1,053 (1,002-1,106)	2,343 (2,239-2,454)	4,408 (4,226-4,604)	9,681 (9,317-10,066)	22,542 (21,731-23,382)	30,181 (29,326-31,175)
Stomach cancer	2,419 (2,306-2,545)	5,487 (5,222-5,851)	11,803 (11,264-12,681)	17,210 (16,582-17,954)	25,252 (24,627-26,039)	43,933 (42,571-45,507)	52,733 (51,536-54,125)
Colon and rectum cancer	1,831 (1,698-2,045)	3,533 (3,307-3,926)	7,126 (6,643-7,886)	9,853 (9,302-10,605)	14,362 (13,850-15,200)	24,416 (23,387-25,773)	32,979 (31,951-34,665)
Liver cancer	2,554 (2,294-2,742)	6,626 (5,843-7,182)	15,765 (13,657-17,385)	22,894 (20,562-24,877)	31,763 (28,877-33,953)	51,008 (46,621-54,137)	54,565 (52,228-57,226)
Liver cancer due to hepatitis B	1,913 (1,694-2,067)	4,951 (4,277-5,453)	11,443 (9,780-12,785)	15,697 (13,484-17,462)	20,544 (18,251-22,473)	32,013 (28,164-34,958)	30,575 (27,371-33,015)
Liver cancer due to hepatitis C	87 (71-104)	329 (256-404)	1,032 (833-1,230)	1,897 (1,514-2,334)	3,359 (2,798-3,940)	6,642 (5,423-7,884)	10,074 (8,622-11,583)
Liver cancer due to alcohol use	56 (44-73)	254 (181-346)	915 (687-1,180)	2,181 (1,504-2,931)	3,736 (2,840-4,753)	5,788 (4,243-8,109)	6,746 (4,972-9,316)
Liver cancer due to NASH	141 (114-170)	284 (218-357)	595 (453-752)	834 (659-1,036)	1,192 (954-1,460)	2,052 (1,593-2,546)	2,582 (2,041-3,186)
Liver cancer due to other causes	357 (296-419)	809 (633-1,000)	1,779 (1,357-2,194)	2,285 (1,809-2,824)	2,931 (2,336-3,644)	4,514 (3,575-5,568)	4,589 (3,709-5,676)
Gallbladder and biliary tract cancer	174 (154-205)	327 (278-390)	782 (674-911)	1,320 (1,163-1,518)	2,227 (1,992-2,488)	4,175 (3,752-4,637)	6,455 (5,947-7,244)
Pancreatic cancer	294 (276-309)	653 (606-698)	1,568 (1,451-1,680)	2,971 (2,795-3,117)	5,350 (5,213-5,499)	9,992 (9,643-10,306)	15,042 (14,710-15,361)
Larynx cancer	271 (245-304)	420 (393-454)	797 (760-839)	1,722 (1,634-1,799)	3,725 (3,576-3,894)	7,433 (7,111-7,735)	10,760 (10,371-11,143)
Tracheal, bronchus, and lung cancer	1,690 (1,588-1,815)	3,982 (3,751-4,268)	8,838 (8,228-9,520)	15,510 (14,726-16,196)	29,591 (28,677-30,574)	60,601 (58,644-62,653)	87,331 (85,638-89,563)
Malignant skin melanoma	447 (363-572)	769 (617-921)	1,264 (1,005-1,548)	1,889 (1,524-2,315)	2,605 (2,080-3,212)	3,057 (2,393-3,782)	3,555 (2,799-4,355)
Non-melanoma skin cancer	216 (198-235)	335 (308-364)	556 (514-600)	715 (674-761)	1,050 (1,001-1,116)	1,487 (1,428-1,568)	1,936 (1,861-2,037)
Non-melanoma skin cancer (squamous-cell carcinoma)	216 (198-235)	335 (308-364)	556 (514-600)	715 (674-761)	1,050 (1,001-1,116)	1,487 (1,428-1,568)	1,936 (1,861-2,037)
Breast cancer	927 (704-1,107)	3,303 (2,651-3,900)	9,753 (8,069-11,270)	19,324 (17,081-21,558)	29,723 (27,625-32,429)	41,567 (38,710-44,952)	46,848 (44,567-50,267)
Cervical cancer	1,288 (1,119-1,430)	3,805 (3,315-4,244)	8,103 (7,094-9,064)	13,279 (12,058-14,571)	18,887 (17,441-20,899)	23,341 (21,494-25,762)	23,008 (21,131-25,523)
Uterine cancer	197 (171-218)	452 (389-502)	876 (743-988)	1,333 (1,195-1,449)	2,076 (1,932-2,205)	3,819 (3,497-4,082)	4,912 (4,597-5,187)
Ovarian cancer	732 (662-846)	1,081 (975-1,255)	1,759 (1,589-2,049)	2,841 (2,646-3,195)	5,040 (4,773-5,516)	8,592 (8,156-9,376)	11,221 (10,755-12,081)
Prostate cancer	113 (88-127)	169 (135-195)	211 (165-236)	268 (212-298)	516 (420-582)	1,451 (1,166-1,672)	3,586 (2,880-4,111)
Testicular cancer	849 (794-914)	1,168 (1,083-1,252)	1,129 (1,037-1,203)	884 (825-939)	630 (598-670)	469 (441-493)	310 (294-327)
Kidney cancer	270 (249-288)	435 (400-465)	643 (592-692)	1,137 (1,071-1,206)	2,118 (2,022-2,196)	4,023 (3,834-4,176)	6,078 (5,745-6,249)
Bladder cancer	156 (138-170)	271 (244-292)	524 (468-566)	884 (798-944)	1,686 (1,522-1,778)	3,007 (2,797-3,148)	4,829 (4,518-5,001)
Brain and nervous system cancer	4,164 (3,344-4,830)	5,805 (4,575-6,738)	7,774 (5,926-8,854)	8,588 (6,920-9,481)	9,853 (8,399-10,714)	12,357 (10,322-13,442)	13,221 (11,280-14,295)

Cause	54 to 59	60 to 64	65 to 69	70 to 74	75 to 79	80 to 84	85 to 89	90 to 94	95 plus
All causes	2,313,278 (2,294,741-2,331,910)	3,274,926 (3,251,522-3,297,258)	4,037,002 (4,010,369-4,062,820)	4,875,469 (4,846,378-4,904,068)	4,761,462 (4,734,648-4,788,427)	4,081,576 (4,061,888-4,101,566)	3,257,317 (3,246,284-3,268,400)	1,655,268 (1,650,941-1,659,489)	541,132 (540,088-542,233)
Communicable, maternal, neonatal, and nutritional diseases	329,944 (315,346-355,718)	387,351 (366,527-429,614)	438,595 (408,657-497,092)	490,121 (452,932-556,410)	443,073 (409,078-506,304)	401,366 (365,449-457,770)	294,752 (272,575-328,471)	144,627 (134,980-159,542)	50,547 (48,183-54,036)
HIV/AIDS and sexually transmitted infections	47,252 (44,169-50,311)	32,578 (30,291-35,110)	23,320 (21,608-25,264)	14,171 (13,002-15,463)	8,043 (7,331-8,803)	1,531 (1,376-1,693)	600 (504-685)	271 (230-300)	66 (54-75)
HIV/AIDS	46,642 (43,543-49,683)	32,026 (29,765-34,560)	22,755 (21,014-24,687)	13,684 (12,509-14,966)	7,587 (6,878-8,327)	1,205 (1,074-1,353)	345 (298-398)	143 (126-164)	21 (19-25)
HIV/AIDS - Drug-susceptible Tuberculosis	14,970 (11,455-17,679)	10,555 (8,326-12,325)	7,694 (6,191-8,930)	4,762 (3,811-5,514)	2,665 (2,152-3,101)	398 (324-465)	105 (83-126)	44 (36-53)	6 (4-7)
HIV/AIDS - Multidrug-resistant Tuberculosis without extensive drug resistance	1,243 (777-1,821)	877 (553-1,297)	631 (399-929)	392 (249-575)	221 (140-328)	38 (25-54)	11 (7-16)	4 (3-6)	1 (0-1)
HIV/AIDS - Extensively drug-resistant Tuberculosis	18 (12-26)	14 (9-19)	9 (6-12)	5 (3-7)	3 (2-4)	1 (1-1)	0 (0-0)	0 (0-0)	0 (0-0)
HIV/AIDS resulting in other diseases	30,412 (27,010-34,915)	20,580 (18,276-23,575)	14,421 (12,752-16,600)	8,525 (7,475-9,831)	4,698 (4,113-5,479)	768 (661-912)	229 (190-281)	94 (80-114)	15 (13-18)
Sexually transmitted infections excluding HIV	610 (431-718)	552 (377-675)	566 (409-696)	487 (367-596)	456 (354-558)	326 (236-390)	256 (178-315)	128 (90-150)	45 (33-52)
Syphilis	231 (161-321)	245 (153-348)	254 (178-371)	242 (169-336)	227 (144-320)	137 (87-188)	109 (58-157)	46 (26-62)	17 (11-23)
Chlamydial infection	72 (50-81)	60 (43-68)	38 (28-43)	42 (33-46)	38 (31-42)	43 (33-49)	32 (26-36)	18 (14-20)	5 (5-6)
Gonococcal infection	199 (138-223)	152 (108-171)	205 (148-228)	147 (117-163)	145 (119-160)	111 (86-126)	85 (68-94)	47 (37-53)	15 (12-16)
Other sexually transmitted infections	108 (75-121)	96 (68-107)	68 (49-76)	56 (44-62)	47 (38-53)	35 (26-39)	30 (22-35)	17 (12-19)	7 (5-8)
Respiratory infections and tuberculosis	180,871 (173,284-189,782)	220,997 (211,358-232,769)	251,623 (241,605-264,740)	284,369 (271,773-298,631)	263,456 (253,153-277,036)	243,209 (233,582-255,614)	190,007 (184,234-198,530)	96,911 (94,542-100,661)	35,581 (34,970-36,733)
Tuberculosis	125,308 (117,760-133,478)	135,926 (128,208-146,253)	141,610 (133,314-152,485)	138,944 (130,400-150,072)	102,703 (96,096-110,552)	72,060 (67,177-78,609)	34,635 (32,266-37,827)	10,512 (9,751-11,470)	2,114 (1,968-2,309)
Drug-susceptible tuberculosis	114,070 (105,316-123,318)	123,171 (113,840-133,816)	128,481 (118,186-139,678)	126,045 (116,282-137,066)	93,283 (85,745-101,569)	65,617 (60,062-72,275)	31,565 (28,990-34,767)	9,616 (8,822-10,556)	1,941 (1,789-2,132)
Multidrug-resistant tuberculosis without extensive drug resistance	10,778 (7,210-16,386)	12,174 (8,419-18,209)	12,584 (8,425-18,664)	12,361 (8,481-18,009)	9,031 (6,192-13,041)	6,183 (4,253-8,924)	2,939 (2,047-4,139)	857 (609-1,198)	165 (119-228)
Extensively drug-resistant tuberculosis	460 (340-619)	580 (437-769)	544 (399-738)	539 (396-725)	389 (281-526)	260 (187-359)	132 (97-177)	39 (30-53)	8 (6-10)
Lower respiratory infections	55,141 (50,246-58,255)	84,475 (76,830-89,031)	109,173 (99,108-115,214)	144,230 (134,229-153,019)	159,314 (149,863-169,810)	169,040 (159,920-178,058)	153,474 (148,204-161,466)	85,483 (83,395-89,126)	33,159 (32,591-34,290)
Upper respiratory infections	346 (200-474)	502 (275-691)	703 (363-911)	1,089 (516-1,381)	1,370 (610-1,718)	2,058 (831-2,604)	1,868 (758-2,357)	905 (416-1,109)	305 (160-368)
Otitis media	78 (53-118)	93 (64-146)	137 (98-218)	105 (76-156)	68 (52-97)	52 (39-76)	29 (23-40)	11 (9-14)	4 (3-5)
Enteric infections	46,431 (29,751-75,351)	71,317 (45,408-117,460)	98,646 (63,040-162,322)	124,414 (79,950-197,327)	120,219 (77,390-193,151)	118,042 (77,398-181,542)	77,452 (51,320-116,630)	33,800 (22,403-49,956)	8,990 (6,192-12,922)
Diarrheal diseases	43,621 (27,603-72,556)	68,615 (42,537-114,774)	96,279 (60,878-160,479)	122,649 (78,143-195,536)	119,054 (76,043-191,623)	117,300 (76,711-180,711)	77,085 (50,772-116,311)	33,684 (22,265-49,801)	8,960 (6,142-12,890)
Typhoid and paratyphoid	1,401 (671-2,585)	1,244 (617-2,240)	1,057 (605-1,725)	742 (443-1,195)	426 (258-654)	224 (137-340)	104 (68-158)	35 (24-54)	9 (7-14)
Typhoid fever	1,163 (562-2,180)	1,037 (506-1,892)	888 (495-1,482)	627 (366-1,000)	361 (215-567)	192 (116-304)	90 (58-140)	31 (20-48)	8 (5-12)
Paratyphoid fever	238 (92-514)	207 (83-424)	169 (76-332)	115 (54-214)	65 (31-119)	32 (16-59)	14 (7-24)	5 (3-8)	1 (1-2)
Invasive Non-typhoidal Salmonella (iNTS)	1,386 (647-2,439)	1,432 (660-2,517)	1,275 (600-2,196)	999 (482-1,812)	722 (341-1,313)	493 (213-911)	235 (96-428)	74 (20-156)	18 (1-50)
Other intestinal infectious diseases	23 (4-52)	27 (5-58)	34 (7-76)	24 (5-52)	18 (3-37)	25 (4-54)	28 (4-65)	6 (0-18)	3 (0-9)
Neglected tropical diseases and malaria	21,432 (15,684-29,100)	24,108 (17,528-33,268)	22,440 (16,135-31,686)	22,859 (16,433-32,226)	11,436 (8,706-15,322)	5,706 (4,651-7,566)	2,234 (1,884-2,975)	753 (625-989)	220 (176-294)
Malaria	15,440 (10,118-22,902)	18,247 (12,138-27,194)	17,065 (11,136-25,799)	17,682 (11,379-26,717)	7,392 (4,909-10,964)	3,216 (2,273-4,644)	839 (641-1,130)	253 (159-330)	85 (39-114)
Chagas disease	829 (778-881)	1,032 (977-1,090)	1,085 (1,029-1,142)	1,121 (1,053-1,193)	787 (744-840)	571 (543-603)	375 (357-398)	161 (147-178)	33 (30-39)
Leishmaniasis	891 (18-3,350)	857 (21-3,051)	724 (27-2,517)	524 (19-1,808)	311 (11-1,095)	166 (5-576)	71 (6-231)	19 (1-65)	3 (0-12)
Visceral leishmaniasis	891 (18-3,350)	857 (21-3,051)	724 (27-2,517)	524 (19-1,808)	311 (11-1,095)	166 (5-576)	71 (6-231)	19 (1-65)	3 (0-12)
African trypanosomiasis	847 (216-2,496)	620 (164-1,683)	520 (131-1,492)	365 (91-1,125)	233 (61-664)	103 (29-286)	37 (10-108)	7 (2-21)	1 (0-3)
Schistosomiasis	976 (819-1,152)	1,096 (918-1,302)	848 (705-1,000)	1,101 (926-1,310)	867 (730-1,027)	512 (419-619)	221 (180-272)	55 (43-71)	10 (7-14)
Cysticercosis	56 (7-128)	48 (6-116)	41 (4-96)	10 (0-27)	22 (1-60)	14 (0-38)	7 (0-19)	3 (0-8)	1 (0-2)
Cystic echinococcosis	122 (24-259)	133 (29-276)	130 (27-277)	122 (23-261)	84 (10-184)	57 (5-129)	40 (3-99)	15 (0-42)	5 (0-14)
Dengue	566 (243-814)	532 (247-824)	556 (272-850)	764 (411-1,396)	574 (351-1,094)	672 (399-1,450)	422 (239-928)	160 (86-369)	50 (27-117)
Yellow fever	195 (38-630)	143 (30-444)	100 (20-320)	62 (12-190)	265 (110-420)	13 (2-40)	4 (1-13)	9 (2-30)	3 (0-9)
Rabies	1,205 (969-1,691)	1,029 (793-1,493)	1,005 (761-1,590)	722 (569-1,090)	579 (444-823)	164 (122-248)	95 (67-150)	21 (15-33)	9 (6-14)
Intestinal nematode infections	27 (21-34)	61 (49-75)	31 (25-38)	51 (40-63)	40 (32-48)	26 (20-33)	12 (9-15)	7 (5-10)	2 (2-3)
Ascariasis	27 (21-34)	61 (49-75)	31 (25-38)	51 (40-63)	40 (32-48)	26 (20-33)	12 (9-15)	7 (5-10)	2 (2-3)
Ebola	8 (7-10)	8 (7-10)	7 (6-9)	5 (4-7)	4 (3-4)	2 (2-3)	1 (1-1)	0 (0-0)	0 (0-0)
Zika virus	0 (0-0)	0 (0-0)	0 (0-0)	0 (0-0)	0 (0-0)	0 (0-0)	0 (0-0)	0 (0-0)	0 (0-0)
Other neglected tropical diseases	270 (203-513)	301 (230-574)	328 (248-640)	329 (253-596)	280 (212-542)	191 (144-378)	110 (83-216)	41 (31-77)	18 (13-37)
Other infectious diseases	25,671 (22,499-27,884)	27,276 (24,159-29,560)	29,046 (25,946-32,044)	27,789 (24,907-30,694)	22,557 (19,940-24,497)	16,543 (14,656-17,800)	9,893 (8,854-10,569)	3,758 (3,310-4,029)	1,209 (1,041-1,347)
Meningitis	7,335 (6,253-9,556)	7,812 (6,883-10,344)	7,515 (6,562-9,925)	7,846 (6,805-10,339)	5,975 (5,115-7,809)	4,304 (3,616-5,510)	2,665 (2,255-3,311)	847 (705-1,031)	254 (209-305)
Pneumococcal meningitis	1,215 (944-1,607)	1,304 (1,003-1,715)	1,229 (960-1,644)	1,369 (1,061-1,847)	1,042 (810-1,411)	725 (559-935)	435 (347-552)	129 (102-163)	40 (30-53)
H influenzae type B meningitis	2,304 (1,921-3,152)	2,478 (2,040-3,355)	2,529 (2,118-3,466)	2,563 (2,121-3,477)	2,008 (1,629-2,740)	1,484 (1,221-1,961)	935 (759-1,181)	332 (267-405)	94 (73-113)
Meningococcal meningitis	648 (506-847)	687 (529-906)	641 (501-870)	665 (523-866)	515 (402-699)	367 (286-486)	207 (164-264)	60 (47-77)	18 (13-25)
Other meningitis	3,168 (2,621-4,204)	3,343 (2,751-4,419)	3,116 (2,595-4,143)	3,249 (2,700-4,197)	2,411 (1,994-3,220)	1,728 (1,388-2,238)	1,088 (891-1,367)	326 (267-411)	101 (80-125)
Encephalitis	2,905 (2,685-3,267)	3,830 (3,566-4,215)	4,561 (4,199-5,045)	5,079 (4,604-5,665)	4,392 (3,950-4,933)	3,389 (3,004-3,777)	1,668 (1,493-1,843)	525 (476-574)	137 (124-148)
Diphtheria	72 (52-100)	-	-	-	-	-	-	-	-
Whooping cough	110 (48-219)	-	-	-	-	-	-	-	-
Tetanus	4,118 (2,307-5,944)	3,298 (2,067-4,568)	3,575 (2,376-5,584)	2,698 (1,943-3,947)	1,963 (1,284-3,127)	985 (671-1,510)	508 (323-804)	152 (102-236)	39 (25-56)
Measles	121 (42-276)	-	-	-	-	-	-	-	-
Varicella and herpes zoster	281 (242-328)	349 (297-407)	675 (581-782)	631 (544-735)	866 (747-1,011)	1,248 (1,077-1,465)	1,325 (1,146-1,565)	795 (649-971)	385 (296-509)
Acute hepatitis	8,281 (6,156-9,262)	8,671 (6,385-9,841)	8,857 (6,620-9,936)	7,459 (5,882-8,345)	5,770 (4,409-6,455)	3,631 (2,730-4,100)	1,712 (1,330-1,897)	511 (390-560)	101 (77-111)
Acute hepatitis A	452 (255-673)	315 (161-489)	221 (109-353)	113 (47-194)	52 (16-104)	10 (2-26)	2 (0-6)	0 (0-1)	0 (0-0)
Acute hepatitis B	7,426 (5,581-8,423)	7,913 (5,764-9,072)	8,065 (5,992-9,208)	6,715 (5,181-7,627)	5,011 (3,665-5,747)	2,986 (2,161-3,535)	1,290 (936-1,559)	339 (239-430)	58 (33-79)
Acute hepatitis C	91 (29-215)	129 (37-312)	171 (52-423)	171 (57-386)	160 (49-403)	126 (39-319)	82 (27-194)	31 (9-75)	8 (2-20)
Acute hepatitis E	312 (144-562)	314 (138-604)	400 (169-772)	461 (217-848)	547 (245-1,032)	508 (243-887)	338 (171-559)	140 (76-220)	35 (18-56)
Other unspecified infectious diseases	2,448 (2,031-2,726)	3,315 (2,716-3,743)	3,863 (3,166-4,293)	4,076 (3,313-4,596)	3,591 (2,869-3,911)	2,986 (2,368-3,311)	2,015 (1,584-2,247)	928 (717-1,021)	294 (227-322)
Maternal and neonatal disorders	-	-	-	-	-	-	-	-	-
Maternal disorders	-	-	-	-	-	-	-	-	-
Maternal hemorrhage	-	-	-	-	-	-	-	-	-
Maternal sepsis and other maternal infections	-	-	-	-	-	-	-	-	-
Maternal hypertensive disorders	-	-	-	-	-	-	-	-	-
Maternal obstructed labor and uterine rupture	-	-	-	-	-	-	-	-	-
Maternal abortion and miscarriage	-	-	-	-	-	-	-	-	-
Ectopic pregnancy	-	-	-	-	-	-	-	-	-
Indirect maternal deaths	-	-	-	-	-	-	-	-	-
Late maternal deaths	-	-	-	-	-	-	-	-	-
Maternal deaths aggravated by HIV/AIDS	-	-	-	-	-	-	-	-	-
Other maternal disorders	-	-	-	-	-	-	-	-	-
Neonatal disorders	-	-	-	-	-	-	-	-	-
Neonatal preterm birth	-	-	-	-	-	-	-	-	-
Neonatal encephalopathy due to birth asphyxia and trauma	-	-	-	-	-	-	-	-	-
Neonatal sepsis and other neonatal infections	-	-	-	-	-	-	-	-	-
Hemolytic disease and other neonatal jaundice	-	-	-	-	-	-	-	-	-
Other neonatal disorders	-	-	-	-	-	-	-	-	-
Nutritional deficiencies	8,286 (7,688-8,740)	11,076 (10,356-11,665)	13,520 (12,550-14,352)	16,518 (15,358-17,640)	17,363 (16,200-18,424)	16,335 (15,475-17,271)	14,567 (13,850-15,316)	9,135 (8,452-10,003)	4,480 (3,986-5,205)
Protein-energy malnutrition	6,322 (5,948-6,687)	8,674 (8,209-9,126)	10,364 (9,757-10,952)	13,081 (12,300-13,840)	13,913 (13,132-14,663)	13,757 (12,974-14,462)	12,415 (11,379-13,134)	7,859 (6,901-8,793)	3,942 (3,320-4,655)
Other nutritional deficiencies	1,965 (1,618-2,337)	2,402 (2,017-2,846)	3,156 (2,632-3,715)	3,438 (2,822-4,159)	3,450 (2,817-4,265)	2,578 (2,164-3,314)	2,152 (1,761-3,173)	1,276 (989-2,168)	538 (375-1,059)
Non-communicable diseases	1,804,797 (1,780,162-1,824,615)	2,702,631 (2,663,768-2,730,515)	3,421,141 (3,368,940-3,456,562)	4,212,340 (4,148,762-4,255,966)	4,167,925 (4,110,838-4,209,942)	3,562,821 (3,512,368-3,601,003)	2,873,112 (2,841,512-2,897,224)	1,467,515 (1,453,609-1,477,990)	477,124 (473,747-479,654)
Neoplasms	564,347 (557,439-572,732)	789,862 (780,680-802,590)	923,874 (913,517-937,997)	999,954 (988,819-1,017,084)	840,961 (829,782-852,850)	575,379 (565,332-583,045)	354,255 (346,097-358,332)	132,843 (128,685-134,404)	31,908 (30,863-32,292)
Lip and oral cavity cancer	13,971 (13,420-14,573)	17,117 (16,396-17,777)	15,582 (14,947-16,181)	13,748 (13,245-14,319)	10,080 (9,723-10,412)	7,191 (6,917-7,432)	4,481 (4,343-4,621)	1,864 (1,806-1,918)	462 (450-474)
Nasopharynx cancer	6,541 (6,242-6,818)	6,659 (6,401-6,905)	6,306 (6,058-6,536)	5,516 (5,306-5,714)	3,446 (3,311-3,585)	2,270 (2,174-2,372)	1,119 (1,076-1,164)	368 (355-381)	70 (69-73)
Other pharynx cancer	9,664 (8,451-10,364)	10,991 (9,799-11,760)	10,024 (8,836-10,749)	8,208 (7,242-8,758)	4,633 (4,041-4,947)	2,905 (2,476-3,118)	1,350 (1,180-1,434)	410 (370-431)	79 (74-82)
Esophageal cancer	35,098 (34,023-36,196)	51,119 (49,791-52,598)	60,777 (59,216-62,642)	64,235 (62,485-66,099)	49,262 (47,807-50,808)	30,745 (29,848-31,872)	15,119 (14,689-15,621)	4,599 (4,465-4,745)	842 (819-869)
Stomach cancer	61,031 (59,627-63,111)	96,081 (94,251-98,831)	117,493 (115,230-120,325)	130,518 (128,213-133,312)	105,452 (103,600-107,947)	70,897 (69,554-73,213)	41,936 (41,173-42,897)	14,821 (14,598-15,119)	3,456 (3,404-3,515)
Colon and rectum cancer	41,918 (40,882-43,605)	63,731 (62,314-65,852)	79,691 (77,951-82,165)	95,857 (93,955-98,770)	91,110 (89,472-93,668)	69,583 (68,447-71,250)	49,411 (48,689-50,314)	20,809 (20,503-21,157)	5,524 (5,439-5,614)
Liver cancer	56,268 (53,339-60,012)	72,455 (69,638-75,762)	81,722 (78,967-84,707)	78,643 (75,976-81,291)	58,717 (56,870-60,643)	35,680 (34,467-36,940)	18,076 (17,565-18,632)	5,412 (5,248-5,567)	1,076 (1,050-1,106)
Liver cancer due to hepatitis B	27,059 (23,975-30,332)	30,730 (27,360-34,143)	31,341 (28,555-34,454)	26,556 (23,726-29,623)	16,438 (14,885-18,023)	8,291 (7,336-9,322)	3,279 (2,954-3,622)	777 (680-879)	116 (92-141)
Liver cancer due to hepatitis C	14,166 (12,321-16,103)	21,112 (18,491-23,766)	24,859 (22,730-27,385)	25,786 (23,684-28,000)	21,785 (19,758-23,758)	15,111 (13,815-16,371)	8,795 (8,246-9,301)	2,912 (2,713-3,099)	639 (590-686)
Liver cancer due to alcohol use	7,296 (5,478-9,477)	10,563 (8,309-13,389)	14,422 (12,224-16,961)	14,990 (12,929-17,652)	10,693 (9,426-12,225)	5,736 (4,886-6,631)	2,714 (2,368-3,083)	736 (624-857)	126 (99-153)
Liver cancer due to NASH	3,232 (2,555-4,017)	4,670 (3,786-5,695)	5,645 (4,548-6,900)	6,217 (5,046-7,601)	5,702 (4,729-6,872)	3,944 (3,229-4,724)	2,049 (1,754-2,385)	636 (525-761)	130 (100-164)
Liver cancer due to other causes	4,515 (3,592-5,454)	5,379 (4,351-6,536)	5,454 (4,474-6,599)	5,094 (4,150-6,202)	4,099 (3,436-4,920)	2,598 (2,138-3,134)	1,239 (1,047-1,436)	351 (291-416)	66 (50-83)
Gallbladder and biliary tract cancer	8,693 (8,112-9,925)	13,580 (12,704-15,481)	17,003 (16,010-19,454)	19,664 (18,646-21,776)	18,783 (18,068-20,835)	13,941 (13,324-15,542)	9,768 (9,281-10,483)	3,881 (3,684-4,070)	969 (937-1,000)
Pancreatic cancer	19,983 (19,669-20,352)	30,061 (29,571-30,601)	36,888 (36,273-37,548)	42,050 (41,379-42,803)	37,777 (37,278-38,375)	26,203 (25,855-26,561)	17,289 (17,056-17,531)	6,815 (6,724-6,906)	1,555 (1,530-1,581)
Larynx cancer	12,547 (12,055-13,019)	16,175 (15,679-16,676)	16,351 (15,759-16,886)	14,391 (13,824-14,820)	8,965 (8,669-9,261)	5,459 (5,243-5,702)	2,552 (2,475-2,641)	750 (731-775)	136 (132-140)
Tracheal, bronchus, and lung cancer	110,876 (108,752-112,959)	172,241 (169,752-175,171)	209,464 (206,475-212,819)	224,291 (221,269-227,921)	172,572 (170,228-175,496)	101,265 (99,819-102,911)	51,165 (50,502-52,112)	15,695 (15,489-15,922)	3,227 (3,183-3,281)
Malignant skin melanoma	3,683 (2,916-4,429)	4,174 (3,327-5,020)	4,520 (3,575-5,472)	5,023 (3,963-6,050)	4,685 (3,599-5,603)	3,466 (2,769-4,224)	2,450 (1,898-2,969)	1,045 (799-1,250)	301 (226-378)
Non-melanoma skin cancer	2,297 (2,191-2,410)	3,864 (3,690-4,049)	4,667 (4,495-4,854)	5,364 (5,188-5,588)	4,912 (4,740-5,086)	4,259 (4,065-4,396)	3,697 (3,580-3,786)	2,156 (2,089-2,210)	867 (846-887)
Non-melanoma skin cancer (squamous-cell carcinoma)	2,297 (2,191-2,410)	3,864 (3,690-4,049)	4,667 (4,495-4,854)	5,364 (5,188-5,588)	4,912 (4,740-5,086)	4,259 (4,065-4,396)	3,697 (3,580-3,786)	2,156 (2,089-2,210)	867 (846-887)
Breast cancer	45,383 (43,683-48,411)	47,586 (46,106-51,167)	44,524 (43,132-47,878)	43,573 (42,294-46,509)	37,981 (37,098-40,240)	27,733 (27,138-29,229)	20,515 (20,123-21,364)	9,194 (8,999-9,471)	2,868 (2,807-2,939)
Cervical cancer	21,983 (20,402-23,954)	22,282 (20,936-24,417)	20,931 (19,525-22,901)	19,600 (18,445-21,644)	14,504 (13,812-15,897)	9,645 (9,249-10,251)	5,694 (5,512-5,984)	2,084 (2,020-2,158)	550 (535-568)
Uterine cancer	5,839 (5,595-6,065)	8,750 (8,411-9,099)	9,704 (9,351-10,096)	9,923 (9,594-10,286)	8,308 (8,087-8,534)	5,771 (5,617-5,941)	3,511 (3,433-3,590)	1,406 (1,376-1,438)	356 (346-367)
Ovarian cancer	11,805 (11,401-12,546)	14,632 (14,167-15,456)	15,051 (14,645-15,787)	15,345 (14,947-16,030)	12,960 (12,686-13,359)	8,413 (8,221-8,602)	5,511 (5,399-5,634)	2,159 (2,116-2,204)	488 (474-501)
Prostate cancer	8,082 (6,481-9,011)	18,451 (14,716-20,962)	31,973 (25,591-36,752)	47,859 (39,350-54,324)	56,191 (44,657-64,615)	49,434 (38,337-54,683)	33,755 (24,872-36,607)	13,548 (10,005-14,444)	2,945 (2,023-3,133)
Testicular cancer	231 (220-243)	280 (265-297)	264 (251-277)	280 (266-296)	226 (216-236)	171 (163-179)	104 (100-108)	36 (35-38)	11 (10-11)
Kidney cancer	7,549 (7,093-7,748)	10,595 (10,004-10,847)	12,203 (11,394-12,504)	13,483 (12,466-13,811)	11,628 (10,654-11,895)	7,756 (7,128-7,927)	5,015 (4,451-5,141)	1,846 (1,598-1,904)	394 (327-409)
Bladder cancer	6,976 (6,489-7,220)	12,593 (11,855-12,991)	17,695 (16,781-18,220)	23,539 (22,744-24,194)	24,173 (23,134-24,780)	19,934 (19,087-20,501)	14,500 (14,088-14,808)	5,948 (5,797-6,067)	1,458 (1,428-1,485)
Brain and nervous system cancer	13,983 (12,137-15,083)	16,406 (14,345-17,756)	16,675 (14,589-18,107)	14,775 (12,843-15,883)	10,957 (9,192-11,701)	5,875 (4,795-6,318)	2,910 (2,208-3,157)	940 (681-1,019)	180 (123-194)

SR Table 7: Global deaths by cause and age group for both sexes combined (with 95% confidence intervals), 2000

Cause	Early Neonatal	Late Neonatal	Post Neonatal	1 to 4	5 to 9	10 to 14	15 to 19	20 to 24	25 to 29	30 to 34	35 to 39	40 to 44
Thyroid cancer	-	-	-	-	-	212 (173-247)	396 (317-469)	491 (402-562)	896 (736-1,026)	616 (524-692)	709 (621-787)	944 (861-1,022)
Mesothelioma	-	-	-	-	-	-	89 (67-116)	130 (92-177)	222 (159-289)	281 (206-365)	429 (342-542)	691 (589-842)
Hodgkin lymphoma	16 (10-18)	5 (3-6)	120 (66-149)	892 (443-1,311)	1,479 (965-1,824)	1,618 (1,129-1,926)	2,113 (1,607-2,478)	2,336 (1,852-2,698)	2,627 (2,067-2,974)	2,393 (1,893-2,767)	2,168 (1,777-2,478)	1,926 (1,538-2,263)
Non-Hodgkin lymphoma	76 (68-86)	66 (57-78)	899 (729-1,078)	4,633 (3,368-6,185)	3,677 (3,157-4,309)	2,971 (2,634-3,342)	2,904 (2,723-3,097)	3,480 (3,243-3,718)	3,925 (3,648-4,182)	4,425 (4,105-4,720)	5,241 (4,940-5,573)	6,357 (6,104-6,628)
Multiple myeloma	-	-	-	-	-	-	219 (173-249)	256 (199-302)	328 (265-395)	521 (451-604)	857 (761-962)	1,517 (1,374-1,696)
Leukemia	361 (271-427)	334 (255-410)	5,061 (3,475-6,558)	16,432 (11,650-23,657)	17,071 (13,289-21,360)	15,437 (12,387-18,258)	13,041 (10,864-14,082)	10,561 (8,669-11,740)	11,014 (8,821-12,507)	11,319 (8,899-13,033)	11,140 (8,973-12,422)	11,195 (9,511-11,938)
Acute lymphoid leukemia	73 (57-94)	62 (47-87)	1,028 (779-1,553)	4,605 (3,318-7,504)	6,051 (4,876-8,399)	4,757 (4,051-6,074)	3,549 (3,093-4,100)	2,329 (2,045-2,746)	2,049 (1,764-2,558)	1,662 (1,421-2,074)	1,682 (1,444-1,993)	1,511 (1,323-1,697)
Chronic lymphoid leukemia	-	-	-	-	-	-	172 (135-200)	173 (129-207)	218 (164-269)	260 (186-326)	242 (191-288)	329 (284-361)
Acute myeloid leukemia	62 (48-86)	60 (45-90)	683 (477-1,148)	2,929 (1,821-5,758)	1,978 (1,443-3,058)	2,208 (1,766-2,975)	2,367 (2,040-2,834)	2,146 (1,848-2,620)	2,219 (1,913-2,806)	2,334 (1,996-2,961)	2,536 (2,243-3,004)	2,813 (2,514-3,169)
Chronic myeloid leukemia	-	-	-	-	-	-	478 (389-561)	666 (542-788)	983 (788-1,139)	1,151 (931-1,316)	1,254 (1,057-1,396)	1,311 (1,151-1,433)
Other leukemia	225 (160-274)	212 (151-264)	3,349 (2,063-4,484)	8,899 (5,330-13,508)	9,043 (5,961-11,609)	8,473 (6,006-10,306)	6,476 (4,868-7,202)	5,247 (3,856-6,136)	5,546 (3,955-6,632)	5,911 (4,034-7,184)	5,427 (3,881-6,375)	5,231 (4,064-5,766)
Other malignant neoplasms	935 (727-1,058)	527 (414-636)	3,103 (2,265-4,065)	10,790 (7,415-16,089)	5,600 (4,694-6,747)	6,942 (6,197-7,830)	7,913 (7,391-8,607)	6,372 (5,955-6,938)	6,056 (5,579-6,633)	6,326 (5,712-7,050)	7,509 (6,999-8,194)	9,648 (9,074-10,366)
Other neoplasms	60 (43-71)	38 (28-46)	494 (323-651)	1,223 (764-1,670)	1,062 (779-1,312)	878 (678-1,064)	725 (569-843)	751 (582-871)	964 (736-1,114)	1,269 (967-1,481)	1,501 (1,172-1,733)	1,781 (1,398-2,034)
Myelodysplastic, myeloproliferative, and other hematopoietic neoplasms	58 (42-69)	32 (24-40)	435 (283-592)	992 (616-1,448)	739 (537-953)	596 (460-755)	484 (381-576)	529 (419-643)	731 (575-879)	1,006 (771-1,220)	1,266 (996-1,484)	1,598 (1,260-1,837)
Other benign and in situ neoplasms	2 (1-3)	6 (4-8)	59 (38-77)	230 (144-359)	323 (227-395)	281 (212-335)	241 (179-291)	222 (162-274)	233 (158-293)	263 (177-334)	235 (161-298)	183 (128-229)
Cardiovascular diseases	4,136 (3,812-4,480)	3,085 (2,822-3,332)	25,720 (23,860-27,313)	20,723 (18,159-22,825)	11,548 (10,614-12,469)	14,116 (13,181-15,060)	35,450 (33,981-37,008)	54,824 (53,051-56,760)	85,494 (82,878-89,092)	133,529 (129,783-138,681)	193,962 (189,702-198,666)	294,994 (290,128-301,019)
Rheumatic heart disease	-	-	-	5,926 (4,825-7,307)	3,743 (3,315-4,186)	5,478 (5,038-5,961)	7,665 (7,066-8,397)	8,954 (8,133-9,821)	10,533 (9,701-11,569)	12,382 (11,391-13,449)	13,229 (12,103-14,416)	14,755 (13,415-16,197)
Ischemic heart disease	-	-	-	-	-	-	10,814 (10,273-11,360)	20,041 (19,254-20,960)	35,177 (33,789-36,982)	61,760 (59,722-64,521)	93,719 (91,183-96,710)	145,537 (142,351-149,399)
Stroke	1,575 (1,342-1,800)	1,137 (945-1,337)	8,805 (7,229-11,199)	5,764 (4,633-7,117)	4,131 (3,722-4,616)	4,750 (4,367-5,202)	9,521 (9,103-9,976)	15,149 (14,567-15,785)	23,852 (22,895-25,325)	39,013 (37,398-41,452)	58,783 (56,844-60,905)	94,331 (92,270-96,938)
Ischemic stroke	228 (170-298)	187 (136-264)	865 (608-1,277)	684 (451-1,103)	406 (348-488)	422 (369-491)	1,317 (1,166-1,492)	2,123 (1,884-2,375)	3,214 (2,918-3,532)	5,164 (4,713-5,616)	7,692 (7,164-8,334)	13,117 (12,216-14,141)
Intracerebral hemorrhage	770 (607-975)	504 (384-684)	4,497 (3,437-6,594)	2,295 (1,612-3,520)	2,124 (1,831-2,598)	2,489 (2,200-2,903)	5,679 (5,318-6,120)	9,104 (8,526-9,816)	14,668 (13,780-16,214)	24,911 (23,345-27,348)	37,977 (36,130-40,410)	61,046 (58,910-64,164)
Subarachnoid hemorrhage	576 (464-658)	445 (360-519)	3,443 (2,636-4,205)	2,785 (2,138-3,407)	1,601 (1,334-1,793)	1,839 (1,590-2,005)	2,525 (2,202-2,849)	3,922 (3,445-4,404)	5,969 (5,292-6,548)	8,939 (7,947-9,780)	13,114 (11,788-14,327)	20,168 (17,957-22,408)
Hypertensive heart disease	-	-	-	-	-	-	794 (575-951)	1,261 (936-1,523)	2,287 (1,752-2,691)	3,939 (3,033-4,587)	6,223 (4,895-7,110)	10,132 (8,119-11,298)
Non-rheumatic valvular heart disease	-	-	-	-	-	-	738 (613-905)	832 (690-1,035)	1,014 (850-1,250)	991 (848-1,190)	1,300 (1,162-1,525)	1,619 (1,478-1,845)
Non-rheumatic calcific aortic valve disease	-	-	-	-	-	-	222 (165-316)	245 (182-362)	334 (260-471)	378 (305-506)	557 (470-711)	734 (631-905)
Non-rheumatic degenerative mitral valve disease	-	-	-	-	-	-	376 (292-511)	443 (346-607)	512 (401-693)	499 (404-649)	610 (504-745)	727 (607-859)
Other non-rheumatic valve diseases	-	-	-	-	-	-	140 (104-186)	144 (104-188)	168 (121-226)	114 (81-159)	133 (89-177)	158 (107-218)
Cardiomyopathy and myocarditis	1,516 (1,255-1,683)	1,055 (892-1,189)	8,110 (6,953-9,203)	4,231 (3,268-4,944)	1,866 (1,611-2,112)	1,992 (1,808-2,195)	2,897 (2,593-3,293)	3,960 (3,498-4,496)	5,908 (5,292-6,579)	7,357 (6,600-8,104)	10,725 (9,502-11,630)	15,108 (13,028-16,312)
Myocarditis	188 (122-235)	215 (155-260)	2,236 (1,663-2,666)	1,396 (1,072-1,704)	727 (590-864)	683 (566-799)	989 (789-1,174)	1,202 (946-1,461)	1,513 (1,203-1,813)	1,300 (1,028-1,561)	1,368 (1,075-1,618)	1,579 (1,213-1,884)
Alcoholic cardiomyopathy	-	-	-	-	-	-	287 (224-345)	737 (598-866)	1,659 (1,372-1,841)	2,791 (2,362-2,996)	5,251 (4,395-5,611)	8,193 (6,619-8,741)
Other cardiomyopathy	1,328 (1,105-1,471)	839 (688-944)	5,874 (4,844-6,690)	2,835 (2,102-3,437)	1,139 (938-1,296)	1,309 (1,153-1,453)	1,621 (1,397-1,855)	2,020 (1,708-2,323)	2,736 (2,333-3,154)	3,266 (2,829-3,724)	4,107 (3,573-4,673)	5,337 (4,587-6,050)
Atrial fibrillation and flutter	-	-	-	-	-	-	-	-	-	70 (62-75)	93 (85-99)	412 (389-465)
Aortic aneurysm	-	-	-	-	-	-	285 (249-332)	447 (382-528)	766 (639-914)	1,101 (906-1,330)	1,640 (1,437-1,884)	2,295 (2,121-2,565)
Peripheral artery disease	-	-	-	-	-	-	-	-	-	-	-	196 (132-269)
Endocarditis	269 (221-337)	237 (189-305)	3,207 (2,590-3,997)	1,933 (1,414-2,594)	509 (443-613)	626 (555-751)	782 (702-945)	1,191 (1,069-1,426)	1,720 (1,551-2,025)	1,711 (1,559-1,959)	1,909 (1,737-2,149)	2,150 (1,958-2,468)
Other cardiovascular and circulatory diseases	776 (647-1,046)	656 (527-860)	5,598 (4,091-7,738)	2,869 (1,771-4,698)	1,298 (1,002-1,804)	1,269 (1,066-1,638)	1,955 (1,667-2,426)	2,988 (2,522-3,694)	4,237 (3,644-5,218)	5,205 (4,576-6,261)	6,340 (5,690-7,466)	8,459 (7,573-10,064)
Chronic respiratory diseases	0 (0-0)	0 (0-0)	0 (0-0)	25,438 (21,236-29,360)	6,932 (6,107-7,840)	6,461 (5,800-7,231)	9,983 (9,321-10,757)	13,344 (12,518-14,172)	18,420 (17,384-19,406)	24,229 (22,700-25,409)	32,151 (30,141-33,481)	43,171 (41,051-44,867)
Chronic obstructive pulmonary disease	-	-	0 (0-0)	2,707 (1,731-4,824)	895 (651-1,488)	696 (547-1,082)	2,859 (2,292-4,015)	4,270 (3,424-5,868)	6,831 (5,833-8,980)	10,525 (9,070-13,089)	13,740 (11,715-17,890)	20,967 (17,950-26,302)
Pneumoconiosis	-	-	-	-	-	-	41 (34-47)	59 (49-68)	162 (142-184)	356 (307-407)	524 (460-592)	575 (503-653)
Silicosis	-	-	-	-	-	-	10 (7-14)	18 (14-23)	71 (54-90)	222 (175-275)	346 (282-414)	359 (290-435)
Asbestosis	-	-	-	-	-	-	5 (2-7)	8 (4-11)	13 (7-19)	21 (9-32)	24 (12-34)	30 (16-44)
Coal workers pneumoconiosis	-	-	-	-	-	-	6 (4-8)	7 (5-10)	28 (19-38)	38 (24-54)	60 (38-80)	85 (54-125)
Other pneumoconiosis	-	-	-	-	-	-	20 (15-26)	26 (19-35)	49 (36-65)	76 (55-106)	94 (70-122)	101 (76-129)
Asthma	-	-	-	19,029 (15,136-23,208)	4,844 (4,007-5,794)	4,909 (4,222-5,716)	5,868 (4,365-6,953)	7,422 (5,237-8,697)	9,528 (6,572-11,079)	10,933 (7,227-12,701)	14,772 (9,334-17,511)	17,571 (10,837-21,232)
Interstitial lung disease and pulmonary sarcoidosis	-	-	-	473 (266-948)	181 (115-332)	137 (99-215)	199 (142-309)	296 (213-469)	459 (337-689)	652 (474-954)	1,029 (759-1,450)	1,416 (1,050-1,934)
Other chronic respiratory diseases	0 (0-0)	0 (0-0)	0 (0-0)	3,228 (1,695-4,981)	1,012 (711-1,346)	719 (539-916)	1,016 (739-1,360)	1,297 (928-1,768)	1,440 (1,045-1,854)	1,763 (1,293-2,261)	2,086 (1,506-2,727)	2,642 (1,872-3,523)
Digestive diseases	2,704 (2,448-3,004)	2,909 (2,592-3,475)	22,576 (19,016-30,387)	25,826 (19,602-37,298)	14,693 (12,511-17,545)	12,720 (11,251-14,525)	20,903 (19,103-22,784)	28,723 (26,486-30,756)	42,525 (39,331-46,174)	59,154 (55,595-64,570)	83,904 (79,699-91,129)	110,565 (102,520-115,943)
Cirrhosis and other chronic liver diseases	-	-	-	11,071 (7,849-17,998)	5,957 (4,862-8,139)	5,778 (4,998-7,120)	9,390 (8,525-10,607)	14,924 (13,451-16,243)	24,354 (22,276-26,611)	38,813 (36,193-42,944)	57,752 (54,285-63,237)	79,795 (74,077-84,369)
Cirrhosis and other chronic liver diseases due to hepatitis B	-	-	-	347 (224-597)	497 (378-687)	818 (650-1,068)	1,763 (1,371-2,235)	5,823 (4,840-6,866)	12,034 (10,197-14,053)	18,124 (16,033-20,613)	22,712 (19,493-26,521)	27,856 (23,869-32,021)
Cirrhosis and other chronic liver diseases due to hepatitis C	-	-	-	112 (67-203)	162 (114-235)	286 (213-381)	627 (465-837)	2,507 (1,965-3,141)	4,914 (3,798-6,269)	8,866 (7,385-10,491)	14,586 (11,615-17,866)	21,161 (17,573-25,087)
Cirrhosis and other chronic liver diseases due to alcohol use	-	-	-	-	-	-	60 (27-105)	1,112 (851-1,434)	2,858 (2,211-3,592)	6,855 (5,960-7,968)	14,061 (11,777-16,649)	21,708 (18,751-24,757)
Cirrhosis due to NASH	-	-	-	-	-	-	159 (124-207)	455 (359-563)	882 (688-1,107)	1,744 (1,456-2,042)	3,139 (2,478-3,861)	5,188 (4,341-6,113)
Cirrhosis and other chronic liver diseases due to other causes	-	-	-	10,611 (7,551-17,354)	5,298 (4,326-7,215)	4,673 (3,998-5,778)	6,780 (5,899-7,779)	5,027 (3,984-6,159)	3,666 (2,728-4,680)	3,225 (2,358-4,206)	3,255 (2,441-4,189)	3,882 (2,858-5,096)
Upper digestive system diseases	-	-	-	2,235 (1,574-3,166)	1,626 (1,335-1,977)	1,498 (1,282-1,789)	2,829 (2,461-3,300)	5,054 (4,522-5,757)	6,351 (5,723-7,190)	7,937 (7,273-8,818)	10,182 (9,374-11,590)	11,801 (10,934-12,869)
Peptic ulcer disease	-	-	-	1,623 (1,059-2,669)	1,225 (984-1,594)	1,208 (1,019-1,485)	2,347 (2,058-2,770)	4,214 (3,731-4,974)	5,135 (4,565-5,964)	6,345 (5,743-7,165)	8,454 (7,732-9,696)	10,144 (9,457-11,033)
Gastritis and duodenitis	-	-	-	612 (336-909)	401 (250-547)	290 (205-389)	481 (357-618)	840 (572-1,078)	1,216 (885-1,528)	1,592 (1,222-1,943)	1,728 (1,322-2,076)	1,657 (1,300-1,913)
Appendicitis	-	-	-	2,858 (1,499-4,496)	2,140 (1,709-2,822)	2,004 (1,697-2,482)	3,154 (2,687-3,812)	2,313 (2,013-2,745)	2,721 (2,372-3,311)	1,610 (1,424-1,884)	1,919 (1,699-2,234)	1,944 (1,690-2,232)
Paralytic ileus and intestinal obstruction	2,704 (2,448-3,004)	2,909 (2,592-3,475)	22,576 (19,016-30,387)	3,287 (2,434-4,286)	2,908 (1,845-3,619)	1,910 (1,286-2,305)	3,443 (2,448-4,015)	3,171 (2,206-3,674)	3,650 (2,624-4,182)	3,597 (2,496-4,132)	4,559 (3,240-5,239)	5,399 (3,931-6,195)
Inguinal, femoral, and abdominal hernia	-	-	-	699 (449-1,115)	344 (276-456)	239 (191-298)	389 (319-473)	396 (331-480)	492 (423-605)	564 (466-665)	776 (663-928)	818 (721-987)
Inflammatory bowel disease	-	-	-	1,283 (661-2,587)	267 (167-458)	175 (124-274)	223 (174-305)	373 (290-517)	529 (421-724)	608 (496-796)	699 (605-852)	849 (753-941)
Vascular intestinal disorders	-	-	-	463 (172-645)	94 (57-116)	52 (40-61)	81 (66-92)	172 (138-199)	254 (207-291)	285 (239-324)	489 (424-548)	830 (743-896)
Gallbladder and biliary diseases	-	-	-	921 (605-1,272)	358 (287-437)	324 (268-376)	350 (310-393)	578 (508-644)	810 (717-892)	1,129 (1,012-1,277)	1,458 (1,325-1,605)	1,768 (1,633-1,908)
Pancreatitis	-	-	-	130 (78-251)	156 (117-238)	188 (153-265)	482 (423-619)	1,131 (999-1,470)	2,385 (2,100-2,965)	3,367 (3,009-4,078)	4,611 (4,261-5,386)	5,657 (5,136-6,250)
Other digestive diseases	-	-	-	2,879 (1,997-4,449)	843 (676-1,135)	552 (467-703)	562 (459-804)	612 (535-743)	978 (839-1,181)	1,243 (1,080-1,493)	1,459 (1,296-1,745)	1,704 (1,550-2,031)
Neurological disorders	-	-	10,749 (7,800-12,585)	15,226 (8,847-19,622)	7,422 (5,965-8,680)	9,519 (8,023-10,597)	14,338 (12,341-15,458)	12,866 (11,052-13,766)	12,441 (10,892-13,262)	12,549 (10,902-13,352)	11,850 (10,468-12,690)	12,352 (11,324-13,195)
Alzheimer's disease and other dementias	-	-	-	-	-	-	-	-	-	-	-	666 (636-707)
Parkinson's disease	-	-	-	-	-	-	-	14 (11-16)	21 (17-24)	49 (41-54)	56 (46-62)	514 (475-562)
Epilepsy	-	-	7,250 (5,315-8,497)	12,044 (7,091-16,172)	6,147 (4,935-7,237)	7,691 (6,313-8,619)	11,786 (9,826-12,962)	10,899 (8,985-11,827)	10,711 (9,154-11,520)	10,540 (8,923-11,293)	9,216 (7,840-10,053)	7,630 (6,720-8,458)
Multiple sclerosis	-	-	-	-	-	-	-	154 (113-226)	325 (236-451)	601 (461-798)	991 (804-1,234)	1,478 (1,223-1,738)
Motor neuron disease	12 (5-18)	24 (12-32)	561 (404-798)	221 (124-319)	45 (37-55)	67 (55-93)	94 (86-115)	80 (72-101)	84 (74-104)	148 (133-182)	301 (284-364)	545 (523-618)
Other neurological disorders	-	-	2,938 (1,709-4,065)	2,962 (1,241-4,392)	1,231 (761-1,658)	1,761 (1,283-2,222)	2,458 (2,192-2,954)	1,719 (1,544-2,038)	1,300 (1,131-1,532)	1,211 (1,026-1,409)	1,286 (1,169-1,487)	1,519 (1,442-1,751)
Mental disorders	-	-	-	-	1 (0-1)	4 (3-5)	22 (20-27)	34 (30-43)	40 (36-49)	40 (36-48)	41 (37-46)	38 (35-42)
Eating disorders	-	-	-	-	1 (0-1)	4 (3-5)	22 (20-27)	34 (30-43)	40 (36-49)	40 (36-48)	41 (37-46)	38 (35-42)
Anorexia nervosa	-	-	-	-	0 (0-1)	4 (3-5)	19 (17-24)	24 (18-28)	27 (19-32)	29 (22-35)	31 (24-36)	28 (22-33)
Bulimia nervosa	-	-	-	-	0 (0-0)	0 (0-0)	3 (2-3)	10 (8-19)	14 (10-24)	12 (9-20)	10 (7-18)	10 (6-16)
Substance use disorders	0 (0-0)	0 (0-0)	0 (0-0)	7 (2-16)	16 (7-29)	17 (8-29)	5,471 (5,283-5,672)	12,593 (12,169-12,975)	20,013 (19,191-20,780)	26,206 (25,265-27,219)	30,306 (29,090-31,144)	34,331 (32,727-35,201)
Alcohol use disorders	-	-	-	-	-	-	1,352 (1,160-1,448)	3,572 (3,123-3,780)	7,089 (6,298-7,535)	11,656 (10,481-12,347)	17,530 (16,161-18,295)	23,567 (21,773-24,366)
Drug use disorders	0 (0-0)	0 (0-0)	0 (0-0)	7 (2-16)	16 (7-29)	17 (8-29)	4,119 (3,965-4,299)	9,021 (8,735-9,358)	12,924 (12,390-13,571)	14,549 (14,032-15,245)	12,776 (12,389-13,210)	10,764 (10,458-11,159)
Opioid use disorders	0 (0-0)	0 (0-0)	0 (0-0)	7 (2-16)	16 (7-29)	17 (8-29)	2,635 (2,359-2,909)	5,777 (5,238-6,307)	8,123 (7,271-9,122)	8,975 (7,975-10,132)	7,886 (7,192-8,665)	6,525 (6,029-7,039)
Cocaine use disorders	-	-	-	-	-	-	100 (84-122)	255 (217-315)	354 (283-440)	543 (434-690)	576 (494-696)	555 (499-650)
Amphetamine use disorders	-	-	-	-	-	-	184 (117-269)	397 (254-580)	630 (399-908)	571 (371-853)	494 (351-687)	415 (309-545)
Other drug use disorders	-	-	-	-	-	-	1,200 (971-1,481)	2,591 (2,169-3,170)	3,818 (3,036-4,660)	4,460 (3,544-5,521)	3,820 (3,242-4,567)	3,269 (2,975-3,773)
Diabetes and kidney diseases	34 (26-41)	104 (79-123)	10,295 (9,349-11,151)	12,593 (10,748-14,097)	6,641 (5,840-7,340)	7,693 (6,935-8,366)	13,027 (12,271-13,792)	19,063 (18,177-19,939)	24,919 (23,813-26,185)	32,865 (31,386-34,578)	39,652 (38,158-41,122)	52,827 (51,221-54,563)
Diabetes mellitus	34 (26-41)	104 (79-123)	1,245 (958-1,456)	844 (650-1,038)	909 (740-1,084)	1,439 (1,092-1,716)	2,879 (2,737-3,027)	4,453 (4,283-4,646)	6,461 (6,210-6,780)	9,549 (9,207-10,030)	13,330 (12,893-13,940)	22,048 (21,350-22,822)
Diabetes mellitus type 1	34 (26-41)	104 (79-123)	1,245 (958-1,456)	844 (650-1,038)	909 (740-1,084)	1,439 (1,092-1,716)	2,546 (2,375-2,705)	3,869 (3,662-4,093)	5,566 (5,308-5,851)	7,391 (7,035-7,799)	10,102 (9,649-10,585)	16,345 (15,569-17,252)
Diabetes mellitus type 2	-	-	-	-	-	-	333 (250-473)	584 (456-784)	895 (706-1,228)	2,158 (1,840-2,722)	3,228 (2,814-3,821)	5,703 (5,055-6,452)
Chronic kidney disease	-	-	8,728 (7,852-9,530)	11,315 (9,528-12,758)	5,355 (4,648-5,925)	5,931 (5,383-6,436)	9,866 (9,148-10,542)	14,248 (13,480-15,027)	17,919 (16,810-18,936)	22,644 (21,201-24,061)	25,720 (24,378-26,822)	30,217 (29,050-31,670)
Chronic kidney disease due to diabetes mellitus type 1	-	-	34 (19-54)	50 (25-90)	35 (17-64)	64 (35-108)	189 (99-307)	649 (344-1,046)	1,784 (1,085-2,724)	4,004 (2,609-5,690)	5,741 (3,790-8,080)	7,120 (4,717-9,932)
Chronic kidney disease due to diabetes mellitus type 2	-	-	24 (13-43)	32 (15-63)	21 (10-40)	33 (15-61)	85 (38-160)	217 (92-411)	471 (207-888)	1,003 (468-1,793)	1,730 (907-2,928)	3,165 (1,808-5,189)
Chronic kidney disease due to hypertension	-	-	43 (24-66)	84 (46-135)	65 (36-107)	124 (70-197)	371 (222-566)	855 (547-1,235)	1,462 (1,000-2,062)	2,241 (1,547-3,134)	2,978 (2,077-4,029)	4,006 (2,868-5,250)
Chronic kidney disease due to glomerulonephritis	-	-	2,078 (1,466-2,827)	3,487 (2,401-4,897)	1,697 (1,137-2,389)	2,039 (1,372-2,770)	3,896 (2,744-5,167)	5,718 (4,001-7,622)	6,450 (4,536-8,570)	6,779 (4,756-9,274)	6,766 (4,492-9,470)	7,395 (4,823-10,269)
Chronic kidney disease due to other and unspecified causes	-	-	6,548 (5,609-7,378)	7,661 (6,058-9,125)	3,538 (2,762-4,259)	3,671 (2,889-4,413)	5,326 (4,040-6,596)	6,809 (4,971-8,634)	7,753 (5,585-9,934)	8,617 (6,077-11,124)	8,505 (5,827-11,297)	8,532 (5,717-11,639)
Acute glomerulonephritis	-	-	323 (209-421)	435 (262-595)	377 (322-438)	323 (281-368)	282 (256-311)	362 (331-398)	538 (488-595)	672 (604-738)	602 (547-665)	562 (516-612)
Skin and subcutaneous diseases	-	-	4,160 (1,983-5,378)	2,378 (969-3,595)	605 (298-800)	653 (339-827)	897 (504-1,122)	808 (469-964)	818 (485-958)	1,346 (761-1,611)	1,417 (866-1,682)	1,672 (1,064-2,046)
Bacterial skin diseases	-	-	4,082 (1,917-5,297)	2,220 (859-3,428)	501 (210-701)	520 (239-692)	736 (379-952)	651 (353-797)	643 (360-766)	1,110 (601-1,342)	1,169 (688-1,383)	1,381 (842-1,647)
Cellulitis	-	-	354 (93-662)	400 (104-736)	161 (55-240)	186 (74-265)	194 (81-266)	163 (81-202)	160 (82-203)	237 (126-305)	271 (155-370)	339 (190-480)
Pyoderma	-	-	3,728 (1,802-4,772)	1,819 (732-2,746)	340 (149-469)	334 (157-442)	542 (285-699)	488 (253-604)	483 (264-584)	873 (449-1,087)	898 (488-1,096)	1,042 (600-1,260)
Decubitus ulcer	-	-	-	68 (40-136)	49 (36-82)	66 (44-101)	90 (57-131)	80 (49-115)	107 (66-152)	157 (97-237)	171 (103-251)	202 (126-309)
Other skin and subcutaneous diseases	-	-	78 (44-154)	91 (56-180)	55 (39-91)	67 (47-103)	71 (48-107)	77 (51-115)	68 (44-95)	80 (50-116)	77 (47-112)	88 (55-141)
Musculoskeletal disorders	-	-	-	-	665 (520-803)	1,145 (947-1,292)	1,783 (1,527-2,032)	2,145 (1,867-2,485)	2,545 (2,247-2,983)	2,832 (2,424-3,412)	2,833 (2,517-3,080)	3,150 (2,767-3,440)
Rheumatoid arthritis	-	-	-	-	113 (76-161)	109 (83-142)	122 (97-155)	145 (116-185)	204 (164-260)	301 (237-390)	352 (289-419)	512 (423-612)
Other musculoskeletal disorders	-	-	-	-	551 (426-668)	1,036 (863-1,171)	1,662 (1,414-1,885)	1,999 (1,730-2,343)	2,341 (2,055-2,768)	2,532 (2,137-3,066)	2,481 (2,188-2,705)	2,638 (2,311-2,896)
Other non-communicable diseases	215,161 (196,799-250,732)	92,495 (84,482-106,243)	337,581 (295,471-391,869)	128,455 (108,626-155,743)	31,374 (28,378-34,823)	23,803 (21,822-25,870)	19,868 (17,938-21,871)	19,540 (17,823-21,571)	18,450 (16,969-19,749)	17,437 (15,985-18,585)	16,631 (15,308-17,710)	17,378 (16,150-18,460)
Congenital birth defects	211,449 (193,278-247,251)	83,947 (76,824-97,378)	267,412 (245,636-306,850)	101,738 (86,647-127,224)	21,873 (19,943-24,477)	15,426 (14,370-16,637)	9,108 (8,663-9,818)	8,097 (7,718-8,742)	7,753 (7,383-8,293)	5,708 (5,392-6,070)	5,227 (4,922-5,645)	4,950 (4,669-5,369)
Neural tube defects	31,716 (23,601-42,834)	8,709 (6,174-12,133)	23,672 (14,492-35,683)	13,425 (6,239-25,044)	1,145 (839-1,480)	802 (637-984)	353 (312-419)	281 (246-344)	228 (196-271)	196 (171-236)	143 (127-163)	126 (112-146)
Congenital heart anomalies	72,356 (51,733-91,156)	39,016 (28,927-47,623)	144,472 (99,702-178,395)	51,465 (29,275-70,641)	13,659 (10,639-16,328)	10,251 (8,596-11,598)	5,792 (5,269-6,473)	5,090 (4,586-5,697)	5,341 (4,836-5,923)	3,577 (3,261-3,925)	3,473 (3,165-3,834)	3,318 (3,071-3,677)
Orofacial clefts	3,791 (1,711-8,143)	1,424 (659-3,016)	2,983 (1,322-6,709)	603 (180-1,816)	-	-	-	-	-	-	-	-
Down syndrome	4,244 (2,543-6,295)	1,885 (1,190-2,769)	9,398 (4,908-15,014)	4,853 (2,048-9,047)	680 (408-1,007)	479 (338-608)	546 (413-716)	591 (437-765)	688 (480-972)	708 (481-1,009)	535 (404-654)	551 (443-673)
Other chromosomal abnormalities	4,857 (2,937-8,251)	2,369 (1,361-4,245)	5,366 (3,117-9,237)	1,214 (600-2,734)	256 (152-421)	175 (118-244)	167 (117-247)	161 (110-240)	138 (95-192)	100 (68-144)	100 (69-130)	76 (52-100)
Congenital musculoskeletal and limb anomalies	4,462 (3,199-6,290)	1,248 (897-1,742)	3,370 (2,363-5,207)	1,260 (832-2,369)	414 (292-579)	332 (249-421)	296 (215-360)	223 (157-284)	146 (108-181)	129 (96-158)	107 (82-130)	77 (59-94)
Urogenital congenital anomalies	5,745 (3,965-7,734)	1,783 (1,193-2,383)	3,012 (1,756-4,595)	866 (393-1,618)	320 (212-474)	284 (211-383)	130 (112-165)	151 (127-187)	126 (101-156)	125 (99-151)	150 (119-182)	171 (141-206)
Digestive congenital anomalies	22,622 (15,899-35,921)	10,061 (7,001-16,083)	25,540 (16,645-43,382)	6,057 (3,040-14,457)	521 (362-811)	243 (179-332)	113 (91-144)	99 (76-139)	76 (61-94)	59 (46-74)	64 (53-76)	59 (50-69)
Other congenital birth defects	61,655 (41,087-80,152)	17,451 (10,114-23,918)	49,600 (24,912-73,627)	21,996 (9,155-37,031)	4,880 (3,055-6,894)	2,860 (2,013-3,815)	1,710 (1,416-2,164)	1,501 (1,200-1,880)	1,011 (776-1,304)	814 (646-1,027)	654 (542-793)	573 (503-680)
Urinary diseases and male infertility	931 (817-1,061)	712 (617-815)	6,642 (6,012-7,332)	5,218 (4,365-5,975)	1,934 (1,685-2,180)	1,540 (1,386-1,703)	2,522 (2,345-2,716)	3,447 (3,249-3,668)	3,316 (3,148-3,525)	4,094 (3,897-4,353)	4,340 (4,161-4,563)	5,103 (4,903-5,319)
Urinary tract infections	176 (117-248)	220 (148-293)	4,317 (3,372-5,239)	2,049 (1,191-2,984)	970 (728-1,240)	849 (686-1,011)	1,818 (1,596-2,069)	2,450 (2,177-2,792)	2,274 (1,960-2,567)	2,636 (2,251-2,963)	2,835 (2,487-3,159)	3,366 (3,035-3,718)

Cause	45 to 49	50 to 54	54 to 59	60 to 64	65 to 69	70 to 74	75 to 79	80 to 84	85 to 89	90 to 94	95 plus
Thyroid cancer	1,316 (1,214-1,425)	1,750 (1,625-1,876)	2,101 (1,983-2,246)	2,834 (2,688-3,082)	3,322 (3,146-3,653)	3,669 (3,490-4,030)	3,442 (3,323-3,708)	2,490 (2,411-2,680)	1,518 (1,478-1,611)	536 (520-567)	132 (128-137)
Mesothelioma	895 (791-1,041)	1,307 (1,204-1,470)	1,876 (1,754-2,072)	2,296 (2,144-2,508)	2,774 (2,576-3,020)	3,117 (2,907-3,356)	2,969 (2,815-3,152)	1,832 (1,766-1,923)	1,063 (1,021-1,110)	351 (340-364)	70 (68-72)
Hodgkin lymphoma	1,953 (1,575-2,262)	1,948 (1,582-2,284)	2,101 (1,702-2,557)	2,448 (1,972-2,883)	2,734 (2,239-3,225)	2,696 (2,207-3,191)	1,690 (1,343-1,941)	1,072 (849-1,211)	540 (446-616)	126 (101-145)	20 (16-23)
Non-Hodgkin lymphoma	8,059 (7,723-8,462)	10,018 (9,713-10,406)	11,977 (11,634-12,306)	16,247 (15,856-16,695)	19,553 (19,103-20,038)	21,790 (21,350-22,231)	19,731 (19,419-20,069)	14,341 (14,092-14,553)	9,148 (9,013-9,292)	3,166 (3,113-3,218)	657 (646-670)
Multiple myeloma	2,449 (2,262-2,737)	3,802 (3,512-4,289)	5,321 (4,837-6,018)	7,890 (7,211-8,974)	9,926 (9,110-11,189)	11,423 (10,082-12,709)	10,753 (9,529-12,003)	7,482 (6,636-8,233)	4,772 (4,190-5,440)	1,644 (1,462-1,812)	313 (276-358)
Leukemia	13,134 (11,184-14,210)	13,980 (12,325-14,753)	15,633 (14,120-16,637)	20,724 (19,098-22,030)	24,310 (22,673-25,913)	27,300 (25,629-28,691)	24,672 (23,614-25,863)	17,649 (16,880-18,470)	12,109 (11,727-12,517)	4,831 (4,678-4,974)	1,177 (1,151-1,207)
Acute lymphoid leukemia	1,493 (1,337-1,712)	1,528 (1,390-1,728)	1,618 (1,478-1,823)	1,881 (1,751-2,138)	1,942 (1,813-2,207)	1,895 (1,787-2,120)	1,143 (1,087-1,285)	720 (681-824)	408 (389-451)	137 (130-147)	29 (28-31)
Chronic lymphoid leukemia	526 (472-573)	848 (782-904)	1,253 (1,178-1,317)	2,312 (2,189-2,425)	3,030 (2,910-3,173)	4,101 (3,948-4,271)	4,289 (4,140-4,430)	3,441 (3,344-3,541)	2,870 (2,770-2,946)	1,319 (1,279-1,352)	362 (349-373)
Acute myeloid leukemia	3,395 (3,086-3,813)	3,719 (3,422-4,051)	4,292 (3,938-4,635)	5,625 (5,277-5,971)	6,550 (6,165-6,959)	7,331 (6,844-7,764)	6,374 (6,091-6,668)	4,206 (4,031-4,398)	2,458 (2,366-2,556)	817 (788-885)	173 (166-193)
Chronic myeloid leukemia	1,466 (1,304-1,582)	1,636 (1,467-1,798)	1,750 (1,603-1,940)	2,202 (2,051-2,418)	2,557 (2,389-2,790)	2,804 (2,642-3,001)	2,813 (2,678-2,990)	2,037 (1,918-2,158)	1,445 (1,382-1,509)	577 (553-602)	135 (129-141)
Other leukemia	6,254 (4,938-7,066)	6,249 (5,147-6,846)	6,720 (5,749-7,222)	8,704 (7,659-9,366)	10,230 (9,077-11,141)	11,170 (10,090-12,020)	10,053 (9,355-10,798)	7,245 (6,715-7,805)	4,928 (4,656-5,267)	1,980 (1,875-2,089)	478 (459-498)
Other malignant neoplasms	12,612 (11,812-13,456)	15,403 (14,540-16,426)	17,754 (16,834-18,967)	23,215 (22,195-25,013)	26,082 (25,061-28,157)	26,908 (25,953-29,029)	22,557 (21,610-24,264)	15,576 (14,767-16,821)	9,986 (9,219-10,647)	4,061 (3,696-4,285)	1,049 (960-1,130)
Other neoplasms	2,294 (1,798-2,691)	2,783 (2,117-3,239)	3,184 (2,355-3,755)	4,383 (3,263-5,282)	5,668 (4,177-6,715)	7,164 (5,223-8,838)	7,825 (5,490-9,346)	6,341 (4,289-7,422)	5,189 (3,395-6,141)	2,342 (1,447-2,784)	675 (419-834)
Myelodysplastic, myeloproliferative, and other hematopoietic neoplasms	2,141 (1,671-2,522)	2,671 (2,035-3,117)	3,104 (2,303-3,684)	4,318 (3,208-5,215)	5,621 (4,146-6,667)	7,128 (5,205-8,808)	7,803 (5,479-9,327)	6,328 (4,282-7,405)	5,182 (3,392-6,131)	2,339 (1,445-2,779)	674 (419-834)
Other benign and in situ neoplasms	153 (101-196)	112 (72-146)	80 (47-113)	66 (36-96)	46 (24-69)	36 (17-55)	21 (10-34)	13 (5-22)	8 (3-14)	3 (1-6)	1 (0-1)
Cardiovascular diseases	464,049 (455,951-472,627)	596,670 (587,744-605,581)	736,887 (726,010-750,632)	1,169,585 (1,152,636-1,190,272)	1,523,425 (1,500,864-1,552,650)	1,979,414 (1,951,451-2,009,286)	2,015,422 (1,989,711-2,048,934)	1,762,884 (1,741,795-1,802,730)	1,516,625 (1,503,396-1,542,791)	788,636 (782,657-798,378)	267,534 (265,798-270,169)
Rheumatic heart disease	18,043 (16,883-19,419)	18,727 (17,466-20,365)	19,899 (18,326-21,790)	26,360 (24,658-28,921)	31,085 (29,353-34,061)	34,489 (32,857-37,506)	30,124 (28,796-32,841)	24,689 (23,532-26,802)	17,506 (16,812-18,746)	8,100 (7,813-8,578)	2,388 (2,317-2,496)
Ischemic heart disease	232,989 (228,439-238,128)	301,620 (296,702-307,702)	379,562 (372,876-388,028)	588,746 (579,741-601,364)	738,580 (727,148-753,945)	946,493 (931,794-963,020)	958,310 (945,399-976,891)	836,862 (825,045-858,258)	755,493 (746,651-773,287)	415,336 (410,706-424,668)	150,542 (148,877-153,397)
Stroke	158,878 (154,758-163,607)	211,332 (206,769-216,319)	258,832 (253,616-266,554)	440,316 (432,427-452,838)	605,185 (594,063-622,281)	801,940 (787,947-821,734)	805,992 (792,885-828,883)	687,280 (674,732-712,195)	546,115 (537,942-562,580)	251,034 (247,420-257,610)	75,343 (74,268-77,098)
Ischemic stroke	26,929 (25,584-28,575)	41,071 (38,797-43,763)	57,608 (53,824-62,215)	143,231 (136,136-151,834)	213,247 (203,739-224,291)	348,002 (337,806-359,791)	370,627 (360,386-382,074)	377,411 (367,689-391,105)	328,087 (321,818-337,451)	157,173 (154,373-161,314)	50,034 (49,228-51,137)
Intracerebral hemorrhage	106,779 (102,607-111,945)	139,281 (133,964-146,176)	165,188 (158,218-174,771)	254,456 (241,910-269,480)	337,057 (322,030-356,063)	395,914 (378,774-418,269)	380,058 (367,012-402,931)	271,328 (262,276-291,043)	190,929 (185,217-203,332)	82,472 (80,363-86,688)	22,177 (21,641-23,020)
Subarachnoid hemorrhage	25,170 (21,754-27,556)	30,980 (26,369-34,790)	36,036 (30,586-41,078)	42,630 (34,591-48,846)	54,881 (43,312-62,196)	58,025 (44,451-65,113)	55,308 (42,525-61,460)	38,541 (27,807-43,121)	27,099 (20,488-29,502)	11,389 (9,164-12,207)	3,131 (2,695-3,295)
Hypertensive heart disease	16,657 (13,125-18,557)	22,570 (17,892-24,984)	29,025 (23,509-32,179)	44,564 (37,029-48,976)	62,282 (50,319-67,917)	84,597 (69,092-90,845)	90,588 (75,896-97,060)	84,950 (72,043-91,890)	68,933 (57,104-73,740)	36,764 (28,516-39,315)	12,204 (9,142-13,049)
Non-rheumatic valvular heart disease	1,918 (1,754-2,184)	2,490 (2,309-2,771)	3,221 (2,996-3,564)	4,568 (4,218-4,956)	6,700 (6,169-7,288)	9,638 (8,708-10,620)	13,227 (11,482-14,470)	14,317 (12,223-15,300)	14,989 (12,404-16,156)	9,050 (7,124-9,821)	3,179 (2,570-3,534)
Non-rheumatic calcific aortic valve disease	914 (790-1,111)	1,263 (1,118-1,490)	1,638 (1,436-1,898)	2,508 (2,252-2,879)	3,897 (3,481-4,449)	5,916 (5,270-6,666)	8,505 (7,293-9,482)	9,745 (8,079-10,534)	11,137 (8,859-12,038)	7,018 (5,301-7,670)	2,536 (1,900-2,761)
Non-rheumatic degenerative mitral valve disease	881 (739-1,026)	1,077 (911-1,245)	1,407 (1,207-1,604)	1,829 (1,542-2,082)	2,474 (2,093-2,817)	3,307 (2,714-3,826)	4,071 (3,310-4,785)	3,879 (3,152-4,659)	3,355 (2,657-4,239)	1,813 (1,460-2,374)	584 (464-797)
Other non-rheumatic valve diseases	123 (85-169)	149 (100-197)	176 (116-229)	230 (151-298)	329 (207-429)	415 (261-530)	651 (421-807)	693 (449-848)	497 (327-622)	219 (148-281)	59 (41-77)
Cardiomyopathy and myocarditis	17,982 (15,410-19,260)	18,080 (15,591-19,466)	18,315 (15,749-19,962)	21,893 (19,370-23,507)	21,459 (18,660-23,095)	23,627 (20,102-25,236)	26,013 (22,096-27,565)	24,679 (21,063-26,099)	28,526 (24,576-30,060)	16,876 (15,040-17,712)	6,300 (5,768-6,585)
Myocarditis	1,335 (1,025-1,564)	1,191 (893-1,422)	1,336 (977-1,631)	1,347 (961-1,631)	1,631 (1,212-1,921)	1,985 (1,447-2,330)	2,378 (1,740-2,770)	2,660 (1,940-3,056)	3,612 (2,518-4,193)	2,377 (1,603-2,776)	955 (630-1,114)
Alcoholic cardiomyopathy	10,305 (8,501-11,010)	9,648 (7,767-10,303)	8,142 (6,948-8,774)	9,610 (8,218-10,253)	6,983 (6,010-7,532)	5,821 (4,758-6,573)	4,394 (3,872-5,332)	2,767 (2,496-3,447)	2,299 (2,012-3,040)	1,219 (1,023-1,772)	419 (363-601)
Other cardiomyopathy	6,341 (5,542-7,184)	7,241 (6,342-8,132)	8,837 (7,481-10,029)	10,936 (9,683-12,247)	12,845 (11,084-14,130)	15,821 (13,302-17,171)	19,242 (15,877-20,616)	19,252 (15,795-20,499)	22,616 (18,222-24,105)	13,281 (10,917-14,238)	4,926 (4,191-5,305)
Atrial fibrillation and flutter	707 (672-795)	1,205 (1,146-1,347)	2,221 (2,098-2,448)	4,412 (4,191-4,896)	7,535 (7,192-8,297)	14,420 (13,809-16,082)	21,669 (20,812-23,695)	31,385 (29,481-32,949)	35,112 (33,452-37,485)	25,718 (24,584-27,146)	8,813 (8,421-9,250)
Aortic aneurysm	3,461 (3,187-3,810)	4,636 (4,399-4,969)	6,454 (6,151-6,941)	10,373 (9,884-11,092)	15,067 (14,336-16,061)	19,994 (19,293-21,073)	21,776 (21,116-22,676)	17,929 (17,363-18,578)	13,690 (13,348-14,094)	6,081 (5,933-6,235)	1,615 (1,580-1,654)
Peripheral artery disease	358 (226-510)	637 (384-926)	1,006 (607-1,497)	2,117 (1,226-3,198)	2,950 (1,711-4,529)	4,461 (2,540-6,945)	5,234 (2,922-8,244)	5,346 (2,946-8,941)	5,844 (2,982-10,171)	3,867 (1,908-7,649)	1,748 (840-3,619)
Endocarditis	2,070 (1,920-2,345)	2,248 (2,063-2,605)	2,378 (2,165-2,772)	3,258 (2,959-3,799)	4,152 (3,723-4,851)	5,006 (4,341-5,779)	5,700 (4,756-6,689)	5,262 (4,246-6,415)	4,921 (3,649-6,057)	2,936 (2,007-3,673)	1,085 (729-1,426)
Other cardiovascular and circulatory diseases	10,986 (9,981-12,914)	13,125 (11,910-15,579)	15,975 (14,433-19,146)	22,977 (20,921-27,060)	28,430 (26,043-33,052)	34,749 (32,020-39,452)	36,789 (33,931-41,161)	30,184 (27,437-33,786)	25,496 (23,600-27,533)	12,873 (12,082-13,764)	4,316 (4,077-4,563)
Chronic respiratory diseases	73,352 (67,771-75,681)	108,075 (99,048-111,569)	163,568 (149,115-169,127)	297,063 (272,084-307,084)	443,785 (405,864-457,794)	610,880 (565,816-628,142)	615,025 (568,611-632,141)	544,728 (498,785-561,033)	363,410 (334,528-373,441)	150,650 (141,031-154,647)	38,314 (36,322-39,247)
Chronic obstructive pulmonary disease	41,866 (35,765-50,888)	68,387 (58,895-82,479)	109,101 (92,229-130,480)	224,879 (194,492-256,322)	353,603 (312,572-392,754)	510,981 (460,068-549,264)	525,670 (474,365-559,588)	475,726 (430,630-501,577)	322,853 (295,962-335,233)	134,962 (125,535-139,533)	34,308 (32,531-35,371)
Pneumoconiosis	837 (736-943)	930 (806-1,082)	1,102 (952-1,311)	1,876 (1,634-2,190)	2,836 (2,517-3,237)	3,783 (3,420-4,225)	3,540 (3,250-3,966)	2,429 (2,249-2,770)	1,494 (1,391-1,656)	470 (440-515)	100 (93-108)
Silicosis	513 (422-629)	534 (426-663)	577 (442-747)	956 (768-1,210)	1,557 (1,300-1,907)	2,042 (1,732-2,443)	1,907 (1,648-2,265)	1,227 (1,055-1,477)	720 (625-854)	199 (175-234)	35 (31-41)
Asbestosis	43 (23-61)	72 (42-103)	112 (66-161)	182 (109-263)	268 (159-361)	403 (258-523)	419 (263-505)	318 (198-380)	222 (132-260)	80 (53-95)	18 (12-20)
Coal workers pneumoconiosis	132 (84-185)	141 (92-206)	184 (122-267)	348 (235-489)	553 (385-754)	783 (590-1,037)	724 (575-949)	530 (414-691)	342 (283-425)	107 (95-127)	24 (21-27)
Other pneumoconiosis	149 (110-194)	184 (134-239)	229 (164-308)	390 (281-514)	458 (350-595)	555 (426-720)	489 (374-630)	354 (266-447)	210 (167-255)	84 (71-103)	23 (20-27)
Asthma	25,489 (14,768-31,442)	32,032 (17,616-41,721)	44,047 (22,748-57,736)	61,356 (32,024-82,701)	75,165 (37,651-101,645)	80,632 (42,982-109,093)	70,369 (40,608-94,031)	53,948 (33,103-72,904)	29,829 (20,044-39,255)	11,197 (7,857-14,573)	2,729 (2,128-3,381)
Interstitial lung disease and pulmonary sarcoidosis	2,141 (1,546-2,957)	3,069 (2,205-4,259)	4,443 (3,135-6,279)	6,747 (4,864-9,245)	9,597 (7,105-13,135)	12,422 (9,539-16,140)	12,743 (9,931-15,833)	10,129 (7,847-12,293)	7,136 (5,489-8,241)	2,925 (2,274-3,303)	830 (650-962)
Other chronic respiratory diseases	3,020 (2,203-3,944)	3,657 (2,676-4,834)	4,874 (3,587-6,377)	2,205 (1,669-2,832)	2,585 (2,025-3,235)	3,062 (2,495-3,748)	2,704 (2,263-3,184)	2,496 (2,097-2,937)	2,097 (1,865-2,379)	1,095 (986-1,234)	348 (319-383)
Digestive diseases	136,103 (130,293-145,121)	144,873 (136,870-151,673)	153,127 (144,275-159,780)	184,773 (169,544-192,684)	194,809 (179,821-202,024)	197,067 (182,430-204,059)	175,500 (163,977-181,977)	138,802 (128,682-143,970)	103,547 (97,876-106,751)	50,016 (47,895-51,205)	15,638 (15,081-15,964)
Cirrhosis and other chronic liver diseases	97,643 (92,777-104,328)	103,187 (96,303-108,822)	104,207 (96,346-110,116)	117,667 (107,916-124,346)	111,795 (103,111-117,993)	99,312 (91,264-104,933)	75,859 (69,330-80,172)	49,441 (43,974-52,987)	29,097 (25,914-30,889)	11,347 (10,291-11,925)	3,293 (3,025-3,454)
Cirrhosis and other chronic liver diseases due to hepatitis B	30,949 (26,344-36,098)	30,037 (25,067-35,439)	30,276 (24,811-36,294)	35,029 (28,541-41,698)	35,138 (29,469-41,651)	31,785 (26,061-37,322)	24,315 (20,119-28,425)	15,872 (12,729-18,762)	8,267 (6,789-9,644)	2,773 (2,243-3,353)	669 (493-874)
Cirrhosis and other chronic liver diseases due to hepatitis C	26,515 (21,953-31,592)	28,538 (23,398-34,078)	27,608 (23,727-32,110)	30,372 (25,271-36,347)	28,789 (24,750-33,361)	25,172 (21,612-29,062)	18,471 (15,323-22,146)	11,945 (9,659-14,661)	7,320 (6,117-8,591)	2,933 (2,409-3,545)	892 (688-1,120)
Cirrhosis and other chronic liver diseases due to alcohol use	27,866 (24,090-32,578)	30,002 (25,628-34,800)	30,226 (25,537-35,374)	32,799 (26,912-38,877)	28,289 (23,821-32,868)	23,302 (19,328-27,459)	16,883 (14,421-19,496)	10,016 (8,368-11,827)	5,785 (5,000-6,631)	2,210 (1,804-2,678)	613 (451-802)
Cirrhosis due to NASH	7,660 (6,366-9,034)	9,088 (7,589-10,745)	9,321 (8,104-10,650)	10,432 (8,841-12,065)	9,457 (8,210-10,737)	8,036 (6,923-9,166)	5,630 (4,747-6,649)	3,625 (2,975-4,335)	2,300 (1,911-2,700)	973 (769-1,205)	299 (219-400)
Cirrhosis and other chronic liver diseases due to other causes	4,655 (3,483-6,031)	5,522 (4,136-7,208)	6,776 (4,742-9,296)	9,035 (6,378-12,245)	10,121 (7,462-13,350)	11,017 (8,493-14,015)	10,560 (8,077-13,623)	7,984 (6,120-10,271)	5,424 (4,393-6,584)	2,458 (1,919-3,042)	819 (589-1,059)
Upper digestive system diseases	16,083 (15,034-17,838)	18,495 (17,208-19,939)	20,607 (19,142-22,089)	27,910 (25,242-29,610)	34,276 (31,289-36,269)	37,654 (34,095-39,721)	35,442 (32,523-37,339)	29,615 (27,268-31,303)	22,279 (21,021-23,309)	10,595 (10,074-11,084)	3,228 (3,070-3,380)
Peptic ulcer disease	13,876 (12,932-15,473)	16,093 (15,005-17,543)	17,943 (16,746-19,430)	24,564 (22,295-26,078)	30,138 (27,633-32,353)	33,088 (30,200-35,336)	30,986 (28,808-32,996)	25,670 (23,776-27,283)	19,350 (18,202-20,460)	9,170 (8,705-9,736)	2,794 (2,654-2,946)
Gastritis and duodenitis	2,207 (1,787-2,606)	2,402 (1,925-2,849)	2,665 (2,121-3,069)	3,346 (2,634-3,877)	4,138 (3,188-4,848)	4,566 (3,535-5,349)	4,456 (3,402-5,101)	3,945 (2,923-4,550)	2,929 (2,253-3,315)	1,425 (1,159-1,579)	434 (372-476)
Appendicitis	1,936 (1,731-2,202)	1,730 (1,550-1,935)	2,349 (2,098-2,651)	2,691 (2,326-3,027)	2,784 (2,476-3,105)	3,027 (2,610-3,427)	2,826 (2,455-3,244)	2,256 (1,948-2,645)	1,640 (1,411-1,958)	771 (667-921)	206 (179-244)
Paralytic ileus and intestinal obstruction	6,041 (4,320-6,844)	6,340 (4,794-7,099)	8,472 (6,529-9,453)	11,309 (8,752-12,658)	13,897 (10,999-15,520)	15,284 (12,777-17,006)	16,046 (13,675-18,409)	15,304 (13,293-17,557)	13,866 (11,690-15,939)	7,656 (6,331-8,682)	2,498 (1,973-2,960)
Inguinal, femoral, and abdominal hernia	1,096 (954-1,290)	1,429 (1,256-1,675)	1,902 (1,642-2,165)	2,713 (2,219-3,005)	3,517 (2,903-3,917)	4,289 (3,555-4,707)	4,579 (3,772-5,068)	4,550 (3,706-5,018)	3,529 (2,842-4,064)	1,851 (1,494-2,166)	566 (434-688)
Inflammatory bowel disease	1,082 (968-1,245)	1,127 (1,018-1,230)	1,352 (1,208-1,488)	1,790 (1,602-1,944)	2,288 (2,046-2,480)	3,012 (2,689-3,237)	3,470 (2,953-3,760)	3,476 (2,784-3,834)	3,152 (2,488-3,455)	1,743 (1,308-1,905)	602 (439-664)
Vascular intestinal disorders	1,340 (1,209-1,473)	1,824 (1,674-1,954)	2,611 (2,408-2,774)	4,463 (4,112-4,786)	6,779 (6,260-7,136)	10,093 (9,317-10,662)	11,568 (10,741-12,237)	10,451 (9,644-10,974)	9,289 (8,190-9,896)	4,651 (3,991-4,962)	1,314 (1,108-1,422)
Gallbladder and biliary diseases	2,305 (2,141-2,535)	2,907 (2,715-3,127)	3,453 (3,170-3,709)	5,486 (5,038-5,806)	7,634 (6,973-8,089)	10,096 (9,318-10,711)	11,334 (10,606-12,120)	10,758 (10,050-11,524)	9,364 (8,934-10,079)	5,333 (5,099-5,736)	1,863 (1,740-2,016)
Pancreatitis	6,375 (5,777-7,116)	5,285 (4,863-5,865)	5,310 (4,711-5,847)	6,066 (5,393-6,594)	6,406 (5,707-6,967)	7,022 (6,083-7,548)	6,351 (5,413-6,832)	4,739 (4,003-5,093)	3,593 (2,972-3,828)	1,595 (1,281-1,717)	462 (371-499)
Other digestive diseases	2,202 (2,003-2,610)	2,549 (2,305-3,112)	2,864 (2,642-3,398)	4,679 (4,168-5,862)	5,433 (5,065-6,351)	7,278 (6,781-8,365)	8,025 (7,608-8,741)	8,212 (7,704-8,842)	7,737 (7,336-8,088)	4,474 (4,251-4,687)	1,607 (1,527-1,699)
Neurological disorders	14,515 (13,619-15,040)	17,123 (16,551-17,683)	22,006 (21,447-22,656)	41,745 (40,830-43,216)	78,070 (76,471-80,995)	149,181 (145,765-155,863)	259,591 (254,963-268,106)	337,135 (330,348-343,164)	381,012 (375,972-385,862)	269,520 (266,649-272,240)	98,555 (97,718-99,380)
Alzheimer's disease and other dementias	1,904 (1,803-2,056)	4,695 (4,498-5,050)	9,225 (8,932-9,727)	24,733 (24,045-25,930)	52,630 (51,246-54,976)	107,786 (104,830-112,653)	203,036 (199,425-208,995)	284,042 (278,830-287,915)	342,899 (338,523-346,827)	255,364 (252,774-257,875)	95,579 (94,760-96,418)
Parkinson's disease	1,168 (1,079-1,288)	1,931 (1,828-2,162)	2,715 (2,584-3,065)	6,038 (5,771-6,864)	13,287 (12,714-15,194)	29,694 (28,469-33,368)	46,636 (44,685-50,910)	46,683 (44,646-51,140)	33,894 (32,448-36,518)	12,625 (12,139-13,787)	2,567 (2,479-2,769)
Epilepsy	6,529 (5,566-7,125)	5,095 (4,400-5,519)	4,281 (3,737-4,554)	4,366 (3,649-4,878)	4,848 (4,052-5,620)	4,271 (3,576-4,732)	3,330 (2,847-3,651)	2,410 (2,015-2,601)	1,742 (1,439-1,997)	650 (551-730)	171 (151-187)
Multiple sclerosis	1,853 (1,530-2,145)	1,887 (1,591-2,128)	1,725 (1,472-1,938)	1,652 (1,477-1,866)	1,446 (1,267-1,636)	1,241 (1,053-1,388)	928 (765-1,010)	518 (420-560)	293 (225-320)	88 (69-95)	18 (14-20)
Motor neuron disease	973 (930-1,153)	1,371 (1,321-1,555)	1,853 (1,775-2,071)	2,475 (2,386-2,706)	3,149 (3,053-3,434)	3,304 (3,228-3,454)	2,895 (2,815-2,970)	1,531 (1,487-1,561)	746 (721-765)	181 (174-185)	32 (31-33)
Other neurological disorders	2,087 (1,943-2,392)	2,144 (2,044-2,417)	2,207 (2,104-2,468)	2,480 (2,381-2,765)	2,710 (2,608-2,987)	2,885 (2,785-3,102)	2,765 (2,672-2,932)	1,950 (1,877-2,060)	1,437 (1,310-1,499)	612 (550-639)	188 (177-197)
Mental disorders	36 (33-39)	-	-	-	-	-	-	-	-	-	-
Eating disorders	36 (33-39)	-	-	-	-	-	-	-	-	-	-
Anorexia nervosa	29 (24-33)	-	-	-	-	-	-	-	-	-	-
Bulimia nervosa	7 (4-12)	-	-	-	-	-	-	-	-	-	-
Substance use disorders	35,467 (33,551-36,640)	27,323 (25,769-28,117)	20,641 (19,488-21,264)	20,208 (19,105-20,846)	13,981 (13,277-14,369)	9,955 (9,409-10,218)	6,986 (6,552-7,255)	3,886 (3,695-4,012)	2,708 (2,530-2,797)	1,148 (1,063-1,183)	330 (303-339)
Alcohol use disorders	27,010 (25,027-28,200)	21,504 (19,902-22,259)	16,431 (15,172-17,034)	16,049 (14,830-16,663)	9,997 (9,203-10,355)	6,252 (5,625-6,501)	3,855 (3,343-4,121)	1,645 (1,383-1,719)	1,031 (796-1,091)	383 (283-407)	109 (79-115)
Drug use disorders	8,457 (8,192-8,743)	5,818 (5,655-5,992)	4,210 (4,077-4,375)	4,158 (4,042-4,313)	3,984 (3,859-4,147)	3,703 (3,589-3,849)	3,131 (3,034-3,262)	2,241 (2,162-2,351)	1,678 (1,615-1,757)	765 (740-793)	220 (214-227)
Opioid use disorders	4,829 (4,377-5,199)	3,150 (2,844-3,405)	2,273 (1,992-2,530)	2,338 (2,072-2,574)	2,248 (1,922-2,552)	1,679 (1,423-1,968)	1,422 (1,222-1,674)	981 (843-1,162)	769 (674-880)	350 (311-386)	93 (85-101)
Cocaine use disorders	459 (404-556)	339 (304-408)	209 (179-278)	180 (155-230)	143 (114-203)	66 (53-93)	49 (39-69)	13 (11-16)	11 (10-13)	6 (5-7)	2 (2-2)
Amphetamine use disorders	354 (255-490)	179 (139-238)	129 (88-188)	113 (81-158)	92 (58-142)	28 (20-39)	22 (15-30)	7 (5-9)	5 (4-6)	3 (2-3)	1 (1-1)
Other drug use disorders	2,815 (2,520-3,316)	2,149 (1,953-2,480)	1,600 (1,409-1,913)	1,528 (1,353-1,818)	1,501 (1,291-1,855)	1,929 (1,717-2,192)	1,638 (1,470-1,824)	1,240 (1,122-1,355)	893 (822-970)	407 (379-439)	124 (117-131)
Diabetes and kidney diseases	73,596 (71,246-76,159)	94,728 (92,407-97,456)	118,935 (115,954-123,588)	165,379 (161,653-170,891)	200,688 (196,028-206,830)	218,162 (212,734-224,362)	206,564 (201,603-211,773)	157,248 (153,729-162,188)	114,832 (112,647-117,585)	54,229 (53,260-55,361)	16,832 (16,581-17,116)
Diabetes mellitus	35,046 (33,870-36,290)	49,802 (48,399-51,392)	66,204 (64,442-68,102)	97,458 (95,003-100,062)	119,125 (115,963-122,296)	128,790 (125,434-131,992)	118,270 (115,189-121,029)	85,479 (83,266-87,745)	56,407 (55,062-57,796)	24,711 (24,168-25,276)	6,874 (6,734-7,020)
Diabetes mellitus type 1	16,693 (15,437-18,171)	22,634 (20,829-24,721)	29,275 (26,844-32,589)	32,146 (29,208-36,328)	38,302 (34,673-43,225)	34,280 (30,175-38,753)	30,308 (26,205-34,211)	16,545 (13,814-19,241)	10,330 (8,288-11,819)	4,269 (3,301-4,891)	1,133 (864-1,281)
Diabetes mellitus type 2	18,353 (16,991-19,937)	27,168 (25,204-29,130)	36,929 (34,310-39,586)	65,311 (61,733-68,911)	80,823 (76,754-85,214)	94,510 (90,505-98,922)	87,962 (84,128-93,381)	68,934 (65,834-72,436)	46,076 (44,279-48,838)	20,442 (19,779-21,601)	5,741 (5,571-6,047)
Chronic kidney disease	37,794 (36,053-39,481)	44,290 (42,883-46,534)	52,070 (50,204-55,756)	67,084 (65,005-71,506)	80,550 (77,688-84,539)	88,248 (85,556-93,184)	87,337 (84,666-91,213)	71,028 (69,155-73,836)	57,972 (56,576-59,963)	29,326 (28,739-30,021)	9,891 (9,708-10,104)
Chronic kidney disease due to diabetes mellitus type 1	8,216 (5,483-11,523)	7,220 (4,357-10,463)	5,807 (3,239-9,076)	4,408 (2,184-7,610)	3,531 (1,815-6,280)	2,863 (1,384-5,100)	2,215 (1,067-4,092)	1,494 (750-2,599)	852 (442-1,482)	190 (92-342)	13 (6-27)
Chronic kidney disease due to diabetes mellitus type 2	6,383 (3,984-9,174)	10,588 (7,306-14,735)	15,465 (10,935-19,882)	22,687 (16,135-28,871)	28,458 (21,212-35,852)	30,178 (22,777-37,553)	28,086 (19,749-35,857)	21,473 (14,992-28,345)	15,663 (10,705-20,545)	7,174 (5,105-9,466)	2,273 (1,437-3,327)
Chronic kidney disease due to hypertension	5,661 (3,993-7,543)	7,823 (5,825-10,367)	10,635 (8,050-13,792)	15,322 (11,567-19,500)	21,041 (16,288-26,453)	26,040 (20,652-32,161)	29,058 (23,289-35,797)	26,631 (21,189-32,485)	24,511 (20,346-28,884)	13,731 (11,550-15,897)	4,943 (4,031-5,805)
Chronic kidney disease due to glomerulonephritis	8,343 (5,468-12,080)	8,661 (5,714-12,276)	8,508 (5,625-12,500)	9,596 (6,175-14,118)	10,783 (6,988-15,847)	12,064 (7,590-17,300)	11,717 (7,579-17,048)	9,051 (5,827-13,464)	6,942 (4,635-10,205)	3,339 (2,234-4,855)	1,115 (620-1,831)
Chronic kidney disease due to other and unspecified causes	9,191 (6,112-13,056)	9,998 (6,583-14,165)	11,654 (7,769-16,424)	15,071 (9,313-22,014)	16,737 (10,672-23,603)	17,102 (11,237-24,059)	16,262 (10,694-23,858)	12,380 (8,405-17,543)	10,003 (6,877-13,755)	4,892 (3,367-6,756)	1,547 (988-2,357)
Acute glomerulonephritis	756 (695-821)	636 (580-698)	661 (595-740)	836 (769-909)	1,013 (927-1,098)	1,125 (1,035-1,225)	957 (873-1,044)	741 (678-817)	453 (415-498)	192 (172-216)	67 (56-81)
Skin and subcutaneous diseases	2,184 (1,374-2,649)	2,469 (1,547-3,012)	2,934 (1,814-3,626)	3,834 (2,378-4,828)	4,642 (2,871-5,734)	5,791 (3,590-7,183)	6,603 (4,180-8,647)	6,124 (3,792-8,114)	5,651 (3,258-7,377)	3,329 (1,841-4,492)	1,387 (741-1,982)
Bacterial skin diseases	1,833 (1,082-2,171)	2,084 (1,275-2,492)	2,424 (1,470-2,935)	3,211 (1,934-3,888)	3,603 (2,158-4,279)	4,199 (2,544-5,020)	4,482 (2,858-5,691)	3,602 (2,259-4,717)	2,951 (1,852-3,925)	1,659 (998-2,325)	633 (372-927)
Cellulitis	437 (236-599)	519 (286-748)	547 (305-798)	752 (416-1,080)	751 (405-1,078)	891 (510-1,327)	866 (443-1,326)	804 (441-1,272)	648 (344-1,074)	390 (205-676)	164 (81-280)
Pyoderma	1,396 (766-1,682)	1,566 (897-1,892)	1,877 (1,054-2,287)	2,459 (1,392-3,051)	2,851 (1,582-3,428)	3,308 (1,925-3,931)	3,615 (2,245-4,475)	2,798 (1,714-3,652)	2,303 (1,401-3,016)	1,269 (760-1,706)	469 (270-659)
Decubitus ulcer	260 (155-384)	296 (182-448)	421 (261-628)	501 (309-764)	882 (512-1,279)	1,397 (847-1,968)	1,875 (1,054-2,653)	2,256 (1,274-3,044)	2,414 (1,236-3,149)	1,499 (730-1,942)	691 (316-946)
Other skin and subcutaneous diseases	91 (55-143)	89 (54-153)	88 (51-151)	122 (71-205)	158 (89-269)	196 (120-337)	247 (137-428)	266 (151-464)	285 (155-522)	171 (80-323)	63 (27-124)
Musculoskeletal disorders	3,626 (3,270-3,928)	3,306 (2,986-3,560)	3,880 (3,534-4,143)	6,255 (5,547-6,641)	8,490 (7,476-9,030)	10,015 (8,932-10,668)	10,030 (9,009-10,532)	8,091 (7,262-8,570)	6,432 (5,983-6,725)	3,233 (3,020-3,380)	1,131 (1,082-1,169)
Rheumatoid arthritis	902 (747-1,061)	1,126 (976-1,270)	1,708 (1,503-1,901)	3,215 (2,761-3,583)	4,768 (4,069-5,280)	5,729 (4,991-6,330)	5,616 (5,021-6,159)	4,131 (3,664-4,564)	2,710 (2,440-3,045)	1,068 (962-1,236)	274 (250-320)
Other musculoskeletal disorders	2,724 (2,439-2,942)	2,180 (1,972-2,370)	2,172 (1,980-2,365)	3,039 (2,678-3,401)	3,721 (3,154-4,308)	4,286 (3,728-4,887)	4,413 (3,807-4,829)	3,961 (3,393-4,334)	3,722 (3,337-3,955)	2,165 (1,891-2,285)	857 (789-891)
Other non-communicable diseases	17,037 (15,774-18,082)	17,910 (16,386-19,056)	18,473 (17,047-19,751)	23,929 (22,172-25,411)	29,378 (27,243-31,138)	31,922 (29,767-33,678)	31,242 (29,171-32,860)	28,545 (26,743-29,835)	24,640 (22,902-25,576)	13,911 (12,839-14,449)	5,495 (5,076-5,688)
Congenital birth defects	2,473 (2,265-2,715)	2,032 (1,830-2,258)	1,817 (1,658-2,075)	1,731 (1,598-1,982)	1,575 (1,453-1,809)	-	-	-	-	-	-
Neural tube defects	91 (81-105)	62 (56-72)	55 (50-64)	46 (41-55)	40 (36-47)	-	-	-	-	-	-
Congenital heart anomalies	1,262 (1,146-1,435)	897 (813-1,044)	759 (695-903)	727 (677-856)	716 (655-847)	-	-	-	-	-	-
Orofacial clefts	-	-	-	-	-	-	-	-	-	-	-
Down syndrome	546 (431-646)	572 (476-651)	507 (421-588)	405 (344-492)	211 (175-262)	-	-	-	-	-	-
Other chromosomal abnormalities	55 (42-75)	48 (35-72)	41 (31-60)	34 (26-52)	29 (21-45)	-	-	-	-	-	-
Congenital musculoskeletal and limb anomalies	56 (46-70)	46 (36-57)	41 (32-51)	46 (38-59)	44 (35-58)	-	-	-	-	-	-
Urogenital congenital anomalies	124 (107-149)	132 (111-153)	167 (138-196)	226 (189-268)	293 (250-342)	-	-	-	-	-	-
Digestive congenital anomalies	48 (41-59)	49 (41-57)	51 (44-61)	59 (50-71)	74 (64-87)	-	-	-	-	-	-
Other congenital birth defects	290 (252-342)	226 (198-265)	195 (173-229)	187 (168-219)	169 (149-199)	-	-	-	-	-	-
Urinary diseases and male infertility	6,449 (6,189-6,760)	7,248 (6,986-7,563)	8,199 (7,880-8,563)	11,785 (11,340-12,228)	15,559 (14,940-16,177)	18,347 (17,625-18,962)	17,978 (17,362-18,538)	17,067 (16,520-17,622)	14,549 (14,218-14,928)	8,421 (8,240-8,620)	3,475 (3,412-3,540)
Urinary tract infections	4,014 (3,629-4,411)	4,728 (4,332-5,218)	5,379 (4,903-5,952)	7,726 (7,036-8,609)	10,400 (9,558-11,518)	12,117 (11,214-13,276)	12,911 (12,131-13,954)	12,461 (11,815-13,329)	11,732 (11,302-12,278)	6,958 (6,738-7,237)	3,007 (2,926-3,099)

SR Table 7: Global deaths by cause and age group for both sexes combined (with 95% confidence intervals), 2000

Cause	Early Neonatal	Late Neonatal	Post Neonatal	1 to 4	5 to 9	10 to 14	15 to 19
Urolithiasis	-	-	-	-	66 (41-110)	61 (39-97)	45 (32-69)
Other urinary diseases	755 (597-885)	492 (371-601)	2,325 (1,338-3,361)	3,168 (1,963-4,387)	898 (641-1,189)	630 (485-794)	660 (445-847)
Gynecological diseases	-	-	-	-	-	-	411 (346-554)
Uterine fibroids	-	-	-	-	-	-	59 (38-85)
Polycystic ovarian syndrome	-	-	-	-	-	-	1 (0-3)
Endometriosis	-	-	-	-	-	-	9 (4-14)
Genital prolapse	-	-	-	-	-	-	13 (7-21)
Other gynecological diseases	-	-	-	-	-	-	330 (263-482)
Hemoglobinopathies and hemolytic anemias	1,102 (817-1,492)	807 (636-981)	6,715 (4,530-9,189)	16,335 (8,892-24,162)	5,490 (4,129-6,821)	5,045 (3,888-6,300)	6,141 (4,399-8,107)
Thalassemias	408 (329-486)	315 (242-405)	2,410 (1,758-3,411)	3,420 (2,410-4,717)	1,962 (1,549-2,386)	1,304 (1,051-1,531)	846 (625-1,021)
Sickle cell disorders	634 (401-1,003)	471 (335-651)	4,141 (2,470-5,634)	12,474 (5,987-19,006)	3,096 (2,061-4,233)	2,895 (1,975-4,086)	4,026 (2,594-5,781)
G6PD deficiency	15 (9-26)	11 (7-18)	109 (64-162)	339 (193-552)	313 (228-428)	556 (400-726)	791 (577-1,040)
Other hemoglobinopathies and hemolytic anemias	44 (34-57)	9 (7-12)	55 (39-78)	102 (67-149)	119 (98-145)	289 (242-346)	479 (366-614)
Endocrine, metabolic, blood, and immune disorders	1,680 (1,153-1,926)	1,539 (1,059-1,876)	8,581 (5,742-11,876)	5,164 (3,094-7,498)	2,076 (1,630-2,640)	1,792 (1,514-2,189)	1,685 (1,483-2,046)
Sudden infant death syndrome	-	5,491 (2,105-10,158)	48,230 (17,805-103,062)	-	-	-	-
Injuries	17,673 (15,376-19,121)	13,791 (12,044-15,064)	121,008 (108,084-132,893)	317,931 (265,801-356,470)	190,012 (175,151-202,422)	168,673 (158,274-176,526)	305,707 (295,826-313,549)
Transport injuries	2,476 (2,117-2,814)	2,261 (1,976-2,506)	18,545 (15,617-20,278)	68,324 (48,661-78,359)	61,015 (55,797-65,770)	48,829 (45,457-51,871)	106,890 (102,328-111,588)
Road injuries	2,114 (1,801-2,449)	2,024 (1,765-2,251)	17,183 (14,283-18,893)	64,058 (45,118-73,641)	57,957 (52,909-62,645)	45,222 (42,096-48,124)	98,727 (94,643-103,316)
Pedestrian road injuries	1,152 (891-1,485)	1,005 (796-1,289)	7,547 (6,502-8,967)	38,866 (29,795-47,809)	36,217 (33,058-39,278)	24,416 (22,491-26,395)	28,995 (26,733-32,713)
Cyclist road injuries	-	-	-	1,435 (871-2,048)	2,489 (1,956-2,973)	3,268 (2,609-3,810)	4,035 (3,399-4,648)
Motorcyclist road injuries	165 (123-216)	154 (110-196)	1,643 (1,256-2,120)	3,286 (2,008-4,889)	2,954 (2,422-3,651)	4,187 (3,605-4,939)	23,009 (20,690-26,368)
Motor vehicle road injuries	764 (563-954)	840 (609-1,049)	7,720 (5,817-9,046)	19,903 (11,953-25,299)	15,781 (13,653-18,001)	12,898 (11,536-14,265)	41,914 (38,616-45,273)
Other road injuries	33 (18-50)	25 (14-38)	272 (146-402)	567 (239-965)	515 (323-656)	454 (331-563)	775 (593-1,017)
Other transport injuries	361 (251-518)	237 (167-346)	1,362 (1,131-1,834)	4,267 (3,298-5,920)	3,058 (2,677-3,497)	3,606 (3,223-4,019)	8,162 (7,485-9,175)
Unintentional injuries	11,740 (9,904-12,914)	10,189 (8,685-11,254)	94,524 (83,645-105,245)	224,573 (189,291-255,003)	112,702 (102,237-121,249)	88,571 (81,987-93,817)	76,260 (71,327-79,595)
Falls	1,778 (1,420-2,029)	760 (600-921)	10,088 (8,273-12,122)	15,886 (11,865-21,194)	8,851 (7,208-10,854)	7,648 (6,447-9,396)	8,215 (7,459-9,521)
Drowning	609 (551-666)	413 (368-445)	12,022 (10,728-13,126)	126,893 (106,999-141,905)	66,978 (61,064-72,280)	48,641 (45,652-51,469)	28,271 (26,865-29,800)
Fire, heat, and hot substances	492 (342-584)	568 (341-702)	7,501 (4,652-9,106)	19,847 (11,015-25,790)	5,867 (4,273-6,748)	4,184 (3,110-4,779)	6,857 (4,678-8,115)
Poisonings	340 (222-391)	356 (240-423)	4,285 (2,940-5,265)	9,726 (6,696-12,645)	3,213 (2,410-3,810)	3,477 (2,782-4,100)	3,643 (3,109-4,225)
Poisoning by carbon monoxide	82 (52-95)	92 (58-110)	974 (618-1,149)	2,116 (1,498-2,590)	1,462 (1,100-1,734)	1,488 (1,227-1,779)	2,045 (1,775-2,373)
Poisoning by other means	259 (170-301)	264 (181-316)	3,311 (2,281-4,173)	7,610 (4,904-10,266)	1,751 (1,310-2,122)	1,988 (1,571-2,343)	1,598 (1,325-1,855)
Exposure to mechanical forces	1,287 (965-1,460)	1,483 (1,038-1,717)	7,187 (5,307-8,241)	10,738 (8,614-12,422)	6,383 (5,569-7,094)	5,016 (4,559-5,412)	8,541 (7,885-9,480)
Unintentional firearm injuries	128 (100-181)	109 (84-178)	818 (644-1,239)	1,182 (762-2,353)	764 (656-1,000)	876 (786-1,042)	2,091 (1,875-2,372)
Other exposure to mechanical forces	1,159 (845-1,332)	1,374 (933-1,609)	6,369 (4,472-7,433)	9,556 (7,464-11,216)	5,619 (4,779-6,277)	4,141 (3,693-4,490)	6,450 (5,824-7,202)
Adverse effects of medical treatment	1,483 (1,027-1,747)	1,056 (703-1,312)	8,855 (5,327-12,383)	6,751 (3,143-11,872)	2,466 (1,643-3,043)	2,005 (1,483-2,307)	2,535 (1,917-2,934)
Animal contact	388 (155-490)	401 (160-516)	3,483 (1,529-4,510)	9,179 (3,943-14,260)	8,589 (4,143-11,274)	7,512 (3,536-9,427)	4,776 (2,376-5,960)
Venomous animal contact	318 (109-421)	319 (105-420)	2,862 (1,168-3,666)	7,385 (2,960-10,678)	7,555 (3,492-9,753)	6,913 (3,155-8,667)	4,249 (2,031-5,329)
Non-venomous animal contact	70 (39-116)	82 (44-135)	621 (293-1,358)	1,794 (662-4,843)	1,034 (530-2,277)	600 (347-1,144)	527 (329-920)
Foreign body	4,520 (3,689-5,044)	4,712 (3,968-5,215)	36,086 (30,438-40,017)	14,534 (10,622-16,885)	3,854 (3,497-4,322)	2,946 (2,756-3,208)	2,005 (1,924-2,150)
Pulmonary aspiration and foreign body in airway	4,385 (3,586-4,920)	4,599 (3,901-5,099)	34,308 (28,778-38,348)	12,729 (9,484-15,244)	3,315 (2,998-3,693)	2,593 (2,430-2,852)	1,682 (1,599-1,831)
Foreign body in other body part	135 (81-179)	113 (60-152)	1,778 (897-2,349)	1,805 (978-2,811)	539 (381-693)	354 (261-431)	323 (259-366)
Environmental heat and cold exposure	372 (151-481)	242 (95-341)	2,567 (967-3,600)	1,844 (679-3,101)	827 (439-1,033)	649 (390-779)	1,492 (1,027-1,758)
Exposure to forces of nature	8 (7-10)	17 (16-19)	147 (134-163)	653 (603-719)	670 (622-725)	770 (722-830)	759 (711-818)
Other unintentional injuries	463 (416-518)	181 (160-204)	2,305 (2,066-2,530)	8,523 (7,090-9,748)	5,006 (4,439-5,563)	5,722 (5,173-6,249)	9,165 (8,517-9,824)
Self-harm and interpersonal violence	3,457 (2,958-3,656)	1,340 (1,187-1,414)	7,939 (6,909-8,574)	25,033 (22,604-26,965)	16,295 (14,758-17,329)	31,273 (28,528-32,942)	122,558 (115,992-126,797)
Self-harm	-	-	-	-	-	13,723 (11,847-15,012)	63,032 (58,905-66,194)
Self-harm by firearm	-	-	-	-	-	542 (412-788)	4,775 (3,947-6,207)
Self-harm by other specified means	-	-	-	-	-	13,181 (11,321-14,463)	58,257 (54,120-61,463)
Interpersonal violence	2,126 (1,625-2,325)	618 (466-693)	5,618 (4,589-6,256)	10,467 (8,043-12,392)	7,558 (6,019-8,589)	9,706 (8,098-10,779)	42,763 (37,668-45,117)
Physical violence by firearm	218 (150-275)	73 (51-98)	757 (585-980)	1,390 (1,001-2,001)	1,072 (872-1,324)	2,225 (1,869-2,516)	19,282 (16,506-20,463)
Physical violence by sharp object	150 (110-184)	67 (47-88)	516 (364-705)	1,307 (786-2,094)	1,116 (740-1,527)	1,715 (1,221-2,183)	9,667 (7,367-11,449)
Physical violence by other means	1,758 (1,334-1,929)	479 (353-542)	4,344 (3,422-4,972)	7,769 (5,762-9,272)	5,370 (4,221-6,299)	5,765 (4,613-6,765)	13,814 (11,645-16,366)
Conflict and terrorism	1,331 (1,260-1,425)	722 (685-776)	2,291 (2,177-2,458)	14,484 (13,673-15,547)	8,654 (8,154-9,305)	7,751 (7,333-8,319)	16,313 (15,450-17,413)
Executions and police conflict	-	-	31 (21-48)	83 (76-88)	83 (77-90)	94 (88-100)	450 (401-508)

Cause	20 to 24	25 to 29	30 to 34	35 to 39	40 to 44	45 to 49	50 to 54	54 to 59
Urolithiasis	89 (58-138)	158 (107-243)	251 (168-382)	258 (189-359)	318 (246-409)	507 (404-644)	564 (464-683)	583 (473-709)
Other urinary diseases	908 (623-1,151)	884 (660-1,127)	1,207 (931-1,482)	1,246 (956-1,513)	1,419 (1,118-1,732)	1,928 (1,555-2,311)	1,956 (1,504-2,373)	2,237 (1,731-2,733)
Gynecological diseases	458 (373-611)	487 (390-638)	562 (454-678)	605 (509-669)	659 (553-728)	773 (667-872)	511 (448-585)	336 (292-379)
Uterine fibroids	74 (44-122)	107 (69-168)	128 (83-199)	153 (94-215)	200 (131-261)	255 (200-321)	181 (143-244)	73 (59-101)
Polycystic ovarian syndrome	2 (1-6)	2 (0-5)	1 (0-3)	1 (0-2)	1 (0-2)	1 (0-2)	1 (0-2)	-
Endometriosis	11 (5-16)	11 (5-18)	16 (8-27)	15 (7-26)	22 (9-37)	32 (12-55)	18 (8-31)	-
Genital prolapse	11 (5-17)	13 (5-20)	14 (5-22)	12 (4-20)	23 (7-40)	30 (7-52)	58 (17-94)	45 (15-81)
Other gynecological diseases	360 (275-541)	353 (264-522)	402 (311-519)	424 (334-548)	412 (317-514)	455 (349-550)	254 (188-324)	218 (181-269)
Hemoglobinopathies and hemolytic anemias	5,534 (3,873-7,335)	4,308 (3,129-5,459)	3,893 (2,847-4,807)	3,108 (2,235-4,018)	3,092 (2,269-4,001)	2,911 (2,148-3,713)	3,094 (2,291-3,958)	3,245 (2,399-4,225)
Thalassemias	614 (426-744)	409 (301-497)	251 (186-315)	109 (87-136)	64 (54-78)	51 (42-62)	29 (25-34)	14 (12-18)
Sickle cell disorders	3,482 (2,154-5,093)	2,387 (1,498-3,472)	1,743 (1,079-2,650)	1,213 (650-2,022)	989 (517-1,718)	649 (341-1,177)	412 (238-728)	246 (153-441)
G6PD deficiency	872 (649-1,099)	961 (751-1,170)	1,181 (904-1,403)	1,041 (792-1,258)	1,104 (827-1,395)	1,145 (820-1,514)	1,193 (743-1,732)	1,110 (665-1,742)
Other hemoglobinopathies and hemolytic anemias	566 (441-693)	551 (444-655)	718 (561-848)	744 (594-868)	934 (771-1,092)	1,066 (860-1,243)	1,459 (1,150-1,754)	1,875 (1,475-2,316)
Endocrine, metabolic, blood, and immune disorders	2,004 (1,690-2,345)	2,586 (2,055-2,997)	3,181 (2,505-3,737)	3,351 (2,803-3,916)	3,574 (3,037-4,134)	4,431 (3,742-5,088)	5,025 (4,181-5,653)	4,876 (4,079-5,566)
Sudden infant death syndrome	-	-	-	-	-	-	-	-
Injuries	404,647 (395,194-411,756)	403,450 (393,484-410,904)	378,071 (365,758-386,006)	337,030 (325,409-343,502)	286,639 (277,218-291,853)	275,184 (265,081-280,461)	220,785 (212,465-225,533)	178,537 (170,238-182,814)
Transport injuries	141,119 (136,872-146,562)	143,134 (136,173-148,966)	133,467 (127,501-138,099)	117,875 (114,600-121,339)	95,797 (93,204-98,684)	89,517 (87,324-91,841)	70,689 (68,766-73,197)	56,191 (54,441-58,334)
Road injuries	132,168 (128,100-137,455)	133,346 (126,314-139,186)	125,243 (119,332-129,926)	109,879 (106,658-113,228)	88,860 (86,365-91,664)	82,718 (80,491-85,045)	65,072 (63,306-67,338)	51,916 (50,355-53,807)
Pedestrian road injuries	37,579 (35,114-42,179)	42,229 (39,491-46,452)	45,805 (42,886-50,065)	40,833 (38,080-45,298)	33,495 (31,477-37,061)	35,166 (32,966-38,156)	28,702 (27,140-31,756)	23,437 (22,095-25,982)
Cyclist road injuries	3,651 (3,048-4,130)	4,089 (3,199-4,787)	4,947 (3,805-5,797)	4,364 (3,370-4,953)	4,227 (3,293-4,691)	4,761 (3,795-5,386)	4,008 (3,161-4,485)	2,961 (2,480-3,301)
Motorcyclist road injuries	32,456 (29,246-36,229)	32,412 (28,437-36,588)	27,883 (24,064-31,409)	22,336 (19,446-24,495)	16,449 (14,646-18,216)	13,360 (11,963-14,942)	9,084 (8,192-10,480)	6,558 (5,911-7,659)
Motor vehicle road injuries	57,448 (52,948-62,088)	53,635 (48,646-59,340)	45,732 (41,430-50,744)	41,525 (37,937-44,843)	33,981 (31,706-36,297)	28,603 (26,219-31,170)	22,572 (20,921-24,360)	18,388 (16,957-19,937)
Other road injuries	1,034 (785-1,321)	982 (715-1,259)	875 (649-1,103)	821 (633-1,005)	709 (569-865)	826 (655-1,005)	706 (553-876)	573 (462-692)
Other transport injuries	8,951 (8,401-10,083)	9,788 (9,175-10,612)	8,224 (7,754-8,774)	7,996 (7,528-8,529)	6,937 (6,592-7,729)	6,799 (6,438-7,314)	5,617 (5,290-6,325)	4,275 (4,017-4,836)
Unintentional injuries	84,124 (79,003-87,200)	86,254 (81,179-89,359)	88,157 (83,004-91,108)	86,370 (81,349-88,970)	80,414 (76,078-82,808)	85,908 (81,139-88,099)	72,638 (68,536-74,707)	64,920 (60,235-66,925)
Falls	10,908 (9,690-12,510)	12,847 (11,089-15,108)	14,930 (12,967-17,731)	16,340 (14,285-18,267)	17,107 (15,332-19,139)	19,890 (18,113-22,081)	18,805 (17,132-20,793)	19,998 (18,111-21,694)
Drowning	24,026 (23,030-25,107)	20,783 (19,992-21,574)	19,749 (19,033-20,436)	17,086 (16,552-17,636)	14,707 (14,271-15,146)	14,015 (13,611-14,391)	11,330 (11,023-11,632)	8,696 (8,440-8,965)
Fire, heat, and hot substances	8,904 (6,333-10,439)	8,649 (6,350-9,818)	8,479 (6,493-9,457)	7,707 (6,381-8,358)	6,960 (6,114-7,428)	7,361 (6,434-7,819)	6,205 (5,522-6,603)	5,230 (4,620-5,592)
Poisonings	4,862 (4,144-5,544)	5,375 (4,593-6,330)	5,250 (4,468-6,269)	5,385 (4,702-5,862)	5,690 (5,111-6,112)	5,618 (5,099-6,211)	4,569 (4,230-5,002)	3,410 (3,152-3,822)
Poisoning by carbon monoxide	2,789 (2,422-3,190)	3,069 (2,690-3,628)	3,186 (2,787-3,850)	3,439 (3,132-3,768)	3,872 (3,600-4,134)	3,921 (3,682-4,293)	3,087 (2,932-3,355)	2,241 (2,129-2,498)
Poisoning by other means	2,073 (1,695-2,419)	2,306 (1,883-2,671)	2,064 (1,659-2,494)	1,946 (1,562-2,159)	1,818 (1,501-1,990)	1,697 (1,402-1,940)	1,482 (1,253-1,667)	1,169 (1,012-1,358)
Exposure to mechanical forces	11,152 (10,423-12,275)	12,362 (11,580-13,222)	12,578 (11,795-13,236)	12,210 (11,571-12,781)	9,432 (8,920-10,096)	10,130 (9,628-10,631)	7,909 (7,462-8,540)	6,228 (5,802-6,823)
Unintentional firearm injuries	2,904 (2,548-3,275)	2,857 (2,420-3,248)	2,215 (1,865-2,521)	1,918 (1,644-2,182)	1,580 (1,373-1,756)	1,682 (1,442-1,893)	1,295 (1,085-1,477)	892 (792-1,050)
Other exposure to mechanical forces	8,247 (7,612-9,145)	9,505 (8,757-10,262)	10,363 (9,626-10,978)	10,292 (9,612-10,829)	7,852 (7,353-8,474)	8,448 (7,957-8,880)	6,614 (6,229-7,184)	5,336 (4,902-5,891)
Adverse effects of medical treatment	4,089 (3,066-4,705)	4,209 (3,084-4,781)	3,966 (2,909-4,500)	4,145 (3,137-4,688)	4,386 (3,389-4,948)	4,683 (3,712-5,217)	3,759 (3,055-4,145)	4,381 (3,566-4,863)
Animal contact	4,200 (2,088-5,213)	4,303 (2,131-5,310)	4,576 (2,250-5,692)	4,052 (2,012-4,855)	4,356 (2,131-5,218)	5,057 (2,601-5,937)	4,079 (2,139-4,843)	3,945 (2,008-4,626)
Venomous animal contact	3,641 (1,714-4,543)	3,643 (1,716-4,534)	4,072 (1,956-5,068)	3,596 (1,703-4,350)	3,926 (1,879-4,738)	4,435 (2,154-5,263)	3,509 (1,784-4,218)	3,409 (1,678-4,033)
Non-venomous animal contact	559 (345-1,036)	660 (410-1,011)	505 (273-736)	457 (270-634)	431 (258-586)	622 (384-837)	571 (350-786)	536 (313-740)
Foreign body	2,955 (2,822-3,173)	3,154 (3,040-3,450)	3,233 (3,116-3,499)	3,695 (3,587-3,916)	4,196 (4,107-4,417)	4,504 (4,387-4,724)	4,365 (4,265-4,513)	3,740 (3,648-3,890)
Pulmonary aspiration and foreign body in airway	2,520 (2,395-2,748)	2,669 (2,554-2,938)	2,712 (2,607-2,986)	3,131 (3,028-3,313)	3,658 (3,562-3,847)	4,106 (3,997-4,320)	4,020 (3,924-4,159)	3,454 (3,360-3,582)
Foreign body in other body part	436 (351-492)	485 (381-550)	521 (427-608)	564 (475-653)	538 (469-637)	398 (333-456)	345 (288-402)	286 (246-339)
Environmental heat and cold exposure	2,044 (1,490-2,314)	2,510 (1,886-2,793)	3,431 (2,539-3,823)	4,632 (3,718-5,066)	5,973 (5,087-6,456)	6,814 (5,850-7,359)	6,261 (5,377-6,724)	4,951 (3,950-5,457)
Exposure to forces of nature	680 (637-736)	646 (605-695)	584 (547-625)	526 (494-563)	461 (434-493)	417 (394-446)	348 (328-369)	285 (270-303)
Other unintentional injuries	10,303 (9,775-10,889)	11,417 (10,794-12,259)	11,380 (10,791-12,118)	10,592 (10,117-11,101)	7,146 (6,880-7,445)	7,418 (7,072-7,764)	5,007 (4,796-5,216)	4,055 (3,869-4,259)
Self-harm and interpersonal violence	179,403 (170,386-183,869)	174,061 (162,897-179,211)	156,447 (143,563-161,674)	132,786 (123,030-136,664)	110,428 (103,130-113,226)	99,759 (92,237-102,573)	77,458 (72,402-79,505)	57,426 (52,976-59,034)
Self-harm	90,374 (84,254-94,054)	96,746 (88,840-101,398)	90,134 (81,148-94,430)	78,572 (71,538-81,577)	67,843 (62,415-69,925)	66,696 (60,753-69,160)	54,063 (49,799-55,672)	41,556 (37,987-42,964)
Self-harm by firearm	7,262 (6,026-9,404)	6,478 (5,316-8,482)	5,700 (4,712-7,509)	5,453 (4,493-6,953)	5,294 (4,316-6,647)	4,951 (4,047-6,247)	4,097 (3,296-5,010)	3,255 (2,624-3,971)
Self-harm by other specified means	83,112 (77,159-87,288)	90,268 (82,423-95,257)	84,434 (75,646-89,118)	73,119 (65,413-76,443)	62,549 (57,118-64,712)	61,745 (55,557-64,395)	49,966 (45,721-51,620)	38,301 (34,766-39,667)
Interpersonal violence	65,901 (58,352-69,570)	61,813 (53,767-64,748)	54,850 (47,504-57,550)	46,182 (40,208-48,316)	37,278 (33,233-38,945)	29,378 (26,082-30,999)	20,846 (18,442-21,802)	13,859 (12,135-14,571)
Physical violence by firearm	29,827 (25,289-31,913)	25,794 (21,188-27,531)	21,023 (16,701-22,782)	17,279 (13,708-18,685)	12,768 (10,239-13,709)	8,671 (7,022-9,425)	6,028 (4,777-6,528)	4,031 (3,195-4,536)
Physical violence by sharp object	16,034 (12,800-18,835)	17,011 (13,350-20,153)	15,935 (12,672-19,041)	13,397 (10,844-15,893)	11,129 (9,259-12,583)	8,831 (7,495-10,099)	5,976 (5,020-6,963)	3,629 (3,018-4,178)
Physical violence by other means	20,040 (17,355-23,614)	19,008 (16,368-22,229)	17,891 (15,321-21,332)	15,506 (13,501-17,901)	13,381 (11,875-15,310)	11,877 (10,523-13,400)	8,841 (7,829-9,968)	6,199 (5,413-7,020)
Conflict and terrorism	22,082 (20,875-23,695)	14,094 (13,376-15,076)	10,069 (9,530-10,712)	7,100 (6,717-7,629)	4,701 (4,418-5,079)	3,289 (3,111-3,525)	2,321 (2,200-2,462)	1,884 (1,798-2,001)
Executions and police conflict	1,046 (887-1,173)	1,408 (1,168-1,621)	1,394 (961-1,619)	931 (758-1,080)	605 (465-723)	396 (313-492)	228 (196-277)	127 (107-157)

Cause	60 to 64	65 to 69	70 to 74	75 to 79	80 to 84	85 to 89	90 to 94	95 plus
Urolithiasis	935 (778-1,127)	1,194 (971-1,426)	1,403 (1,163-1,670)	1,284 (1,034-1,521)	932 (771-1,105)	648 (550-788)	313 (275-383)	83 (73-103)
Other urinary diseases	3,124 (2,442-3,826)	3,965 (3,105-4,850)	4,827 (3,780-5,820)	3,784 (2,998-4,537)	3,675 (2,955-4,355)	2,169 (1,760-2,547)	1,150 (933-1,348)	386 (316-451)
Gynecological diseases	284 (252-320)	367 (320-416)	238 (214-263)	182 (167-199)	310 (260-356)	227 (194-261)	102 (89-117)	38 (34-44)
Uterine fibroids	68 (55-89)	75 (58-107)	54 (43-77)	38 (31-51)	78 (57-115)	64 (45-93)	36 (26-49)	15 (10-19)
Polycystic ovarian syndrome	-	-	-	-	-	-	-	-
Endometriosis	-	-	-	-	-	-	-	-
Genital prolapse	51 (24-86)	91 (39-145)	75 (39-104)	55 (29-72)	78 (41-121)	51 (31-76)	21 (13-28)	8 (5-10)
Other gynecological diseases	166 (134-208)	201 (154-264)	110 (83-145)	89 (73-121)	154 (113-199)	111 (89-140)	45 (37-58)	16 (14-21)
Hemoglobinopathies and hemolytic anemias	3,904 (2,920-5,048)	4,761 (3,578-6,083)	5,511 (4,255-6,840)	5,417 (4,223-6,661)	4,782 (3,723-5,764)	4,253 (3,220-4,939)	2,422 (1,808-2,787)	852 (622-972)
Thalassemias	7 (6-9)	3 (3-4)	1 (1-2)	1 (0-1)	0 (0-0)	0 (0-0)	0 (0-0)	0 (0-0)
Sickle cell disorders	103 (69-165)	50 (34-74)	20 (13-29)	6 (4-9)	2 (1-3)	1 (0-1)	0 (0-0)	0 (0-0)
G6PD deficiency	1,000 (579-1,592)	823 (462-1,345)	567 (318-945)	308 (170-520)	127 (71-213)	51 (31-78)	14 (9-20)	2 (1-4)
Other hemoglobinopathies and hemolytic anemias	2,794 (2,177-3,491)	3,885 (3,017-4,849)	4,923 (3,887-6,018)	5,102 (4,011-6,210)	4,652 (3,641-5,573)	4,202 (3,186-4,872)	2,408 (1,800-2,769)	849 (620-969)
Endocrine, metabolic, blood, and immune disorders	6,224 (5,296-7,137)	7,115 (6,072-8,321)	7,825 (6,703-9,172)	7,666 (6,417-8,888)	6,385 (5,265-7,630)	5,612 (4,508-6,600)	2,966 (2,369-3,541)	1,130 (858-1,345)
Sudden infant death syndrome	-	-	-	-	-	-	-	-
Injuries	184,944 (175,809-189,691)	177,266 (167,391-181,845)	173,008 (163,157-177,744)	150,464 (142,194-155,137)	117,389 (110,673-121,522)	89,452 (85,405-91,855)	43,126 (41,547-44,286)	13,461 (13,096-13,760)
Transport injuries	53,964 (52,531-55,879)	49,955 (48,529-51,582)	44,929 (43,623-46,342)	33,682 (32,663-34,976)	20,930 (20,250-21,944)	11,481 (11,138-11,941)	4,006 (3,905-4,137)	1,031 (1,001-1,061)
Road injuries	50,158 (48,788-51,934)	46,609 (45,276-48,122)	42,250 (41,011-43,572)	31,661 (30,721-32,868)	19,751 (19,112-20,648)	10,875 (10,550-11,315)	3,793 (3,697-3,916)	968 (939-997)
Pedestrian road injuries	26,271 (25,083-28,487)	25,112 (23,936-27,074)	23,797 (22,899-25,602)	18,933 (18,230-20,407)	11,851 (11,360-12,822)	6,446 (6,187-6,931)	2,088 (2,015-2,232)	499 (480-530)
Cyclist road injuries	3,224 (2,660-3,573)	3,137 (2,667-3,448)	2,694 (2,294-2,939)	1,632 (1,415-1,796)	837 (747-937)	395 (356-438)	118 (106-133)	1 (1-1)
Motorcyclist road injuries	4,295 (3,815-5,066)	3,683 (3,237-4,285)	2,665 (2,387-3,098)	1,368 (1,152-1,611)	863 (730-1,032)	355 (299-429)	101 (86-122)	26 (22-30)
Motor vehicle road injuries	15,997 (14,924-17,390)	14,266 (13,162-15,418)	12,715 (11,803-13,619)	9,388 (8,750-10,151)	5,982 (5,608-6,510)	3,561 (3,347-3,817)	1,450 (1,371-1,521)	430 (410-446)
Other road injuries	371 (309-461)	412 (329-519)	379 (299-485)	340 (280-417)	218 (176-285)	118 (97-156)	36 (30-47)	13 (11-16)
Other transport injuries	3,806 (3,615-4,239)	3,346 (3,161-3,689)	2,679 (2,533-2,966)	2,021 (1,908-2,258)	1,179 (1,102-1,325)	606 (568-668)	213 (199-236)	62 (59-68)
Unintentional injuries	77,075 (70,898-80,140)	80,436 (73,694-83,644)	84,941 (77,931-88,472)	85,291 (78,494-89,038)	76,733 (70,911-80,018)	66,381 (62,608-68,609)	35,369 (33,819-36,441)	11,598 (11,245-11,893)
Falls	27,027 (24,610-29,260)	33,595 (30,294-36,113)	40,831 (36,530-43,644)	47,671 (43,707-50,147)	46,877 (42,516-49,338)	43,734 (40,665-45,597)	24,427 (23,135-25,369)	7,855 (7,546-8,120)
Drowning	9,626 (9,317-9,896)	8,812 (8,523-9,096)	8,504 (8,206-8,763)	6,680 (6,387-6,934)	5,118 (4,877-5,363)	2,907 (2,786-3,037)	930 (900-963)	194 (188-201)
Fire, heat, and hot substances	6,340 (5,644-6,837)	6,299 (5,484-6,778)	6,562 (5,817-7,079)	6,120 (5,453-6,559)	5,597 (5,043-6,044)	4,580 (4,210-4,834)	2,292 (2,158-2,393)	831 (796-857)
Poisonings	3,991 (3,691-4,403)	3,183 (2,933-3,634)	2,951 (2,729-3,445)	2,026 (1,868-2,397)	1,451 (1,342-1,700)	886 (829-1,018)	296 (277-323)	69 (65-74)
Poisoning by carbon monoxide	2,710 (2,565-2,986)	1,997 (1,871-2,271)	1,848 (1,721-2,142)	1,308 (1,206-1,553)	876 (808-1,059)	556 (522-647)	175 (165-198)	33 (31-37)
Poisoning by other means	1,281 (1,093-1,481)	1,187 (1,021-1,382)	1,103 (985-1,321)	717 (641-870)	576 (509-684)	330 (298-378)	121 (109-130)	36 (33-38)
Exposure to mechanical forces	5,439 (5,030-5,878)	4,917 (4,549-5,333)	4,294 (3,939-4,646)	3,361 (3,088-3,643)	2,433 (2,241-2,617)	1,839 (1,745-1,982)	954 (912-1,022)	333 (320-355)
Unintentional firearm injuries	834 (741-966)	781 (698-907)	729 (654-827)	705 (629-810)	553 (495-644)	424 (390-495)	220 (201-260)	70 (64-85)
Other exposure to mechanical forces	4,605 (4,210-4,977)	4,136 (3,761-4,498)	3,565 (3,240-3,874)	2,656 (2,387-2,885)	1,880 (1,697-2,030)	1,415 (1,329-1,517)	734 (699-781)	263 (251-277)
Adverse effects of medical treatment	6,448 (5,200-7,212)	7,568 (6,142-8,375)	7,509 (6,316-8,243)	7,233 (6,142-7,888)	5,835 (5,065-6,424)	4,853 (4,294-5,382)	2,530 (2,283-2,780)	845 (781-915)
Animal contact	4,210 (2,061-5,061)	4,515 (2,120-5,450)	3,759 (1,855-4,516)	2,842 (1,395-3,466)	1,563 (833-1,893)	739 (451-876)	249 (163-289)	58 (43-65)
Venomous animal contact	3,693 (1,772-4,437)	3,982 (1,797-4,820)	3,269 (1,555-3,972)	2,439 (1,153-2,999)	1,302 (666-1,587)	580 (337-699)	186 (115-222)	40 (28-46)
Non-venomous animal contact	516 (284-686)	533 (296-713)	490 (284-662)	403 (234-558)	261 (156-352)	159 (102-206)	63 (43-80)	18 (14-22)
Foreign body	4,235 (4,127-4,375)	3,984 (3,868-4,094)	4,383 (4,189-4,507)	4,581 (4,434-4,706)	4,422 (4,295-4,547)	4,373 (4,267-4,491)	2,479 (2,411-2,553)	1,031 (1,002-1,063)
Pulmonary aspiration and foreign body in airway	3,915 (3,808-4,030)	3,703 (3,589-3,821)	4,045 (3,902-4,189)	4,232 (4,107-4,382)	4,109 (4,004-4,263)	4,123 (4,017-4,258)	2,353 (2,283-2,437)	989 (959-1,021)
Foreign body in other body part	320 (275-388)	280 (221-324)	338 (269-386)	350 (268-400)	313 (235-353)	250 (191-279)	125 (94-138)	42 (33-48)
Environmental heat and cold exposure	5,903 (4,832-6,467)	4,363 (3,218-4,896)	3,966 (2,860-4,490)	3,219 (2,133-3,739)	2,326 (1,572-2,655)	1,776 (1,381-1,955)	896 (729-968)	276 (244-296)
Exposure to forces of nature	295 (279-313)	240 (227-255)	216 (204-230)	156 (147-166)	97 (92-103)	52 (49-56)	19 (17-21)	4 (3-4)
Other unintentional injuries	3,560 (3,398-3,725)	2,960 (2,815-3,133)	1,965 (1,870-2,055)	1,404 (1,342-1,480)	1,013 (961-1,084)	641 (612-680)	298 (284-314)	104 (100-110)
Self-harm and interpersonal violence	53,905 (49,474-55,390)	46,874 (42,460-48,229)	43,138 (39,091-44,407)	31,491 (28,581-32,515)	19,726 (18,094-20,430)	11,590 (10,867-11,912)	3,751 (3,562-3,840)	832 (802-846)
Self-harm	39,863 (36,128-41,121)	36,611 (33,070-37,796)	34,901 (31,186-36,018)	26,104 (23,449-27,036)	17,916 (16,294-18,591)	10,610 (9,878-10,925)	3,466 (3,269-3,550)	682 (655-695)
Self-harm by firearm	2,876 (2,337-3,551)	2,671 (2,161-3,257)	2,683 (2,244-3,298)	2,359 (2,015-2,868)	1,641 (1,425-1,973)	1,070 (938-1,362)	384 (338-493)	85 (75-103)
Self-harm by other specified means	36,987 (33,376-38,306)	33,940 (30,415-35,205)	32,218 (28,332-33,452)	23,745 (21,173-24,726)	16,275 (14,557-16,969)	9,540 (8,726-9,920)	3,083 (2,861-3,188)	597 (563-613)
Interpersonal violence	12,246 (10,958-12,959)	8,774 (7,674-9,271)	7,116 (6,202-7,525)	4,648 (4,138-4,920)	1,317 (1,206-1,393)	738 (699-776)	217 (206-231)	101 (94-105)
Physical violence by firearm	2,778 (2,242-3,085)	1,909 (1,508-2,127)	1,471 (1,159-1,673)	950 (754-1,064)	210 (172-239)	108 (91-119)	35 (29-38)	21 (16-23)
Physical violence by sharp object	3,498 (2,933-3,984)	2,355 (1,908-2,643)	1,922 (1,590-2,154)	1,273 (1,086-1,416)	330 (298-365)	178 (165-197)	51 (47-57)	24 (21-27)
Physical violence by other means	5,970 (5,346-6,609)	4,510 (3,891-5,050)	3,724 (3,280-4,149)	2,425 (2,135-2,675)	777 (706-842)	452 (424-483)	131 (124-141)	56 (53-60)
Conflict and terrorism	1,699 (1,622-1,799)	1,420 (1,352-1,507)	1,073 (1,023-1,129)	710 (678-749)	473 (454-495)	230 (221-242)	62 (59-65)	47 (44-50)
Executions and police conflict	98 (84-117)	70 (58-82)	48 (41-56)	29 (26-34)	20 (17-23)	12 (10-14)	6 (5-7)	3 (2-3)

SR Table 7: Global deaths by cause and age group for both sexes combined (with 95% confidence intervals), 2005

Cause	Early Neonatal	Late Neonatal	Post Neonatal	1 to 4	5 to 9	10 to 14	15 to 19	20 to 24	25 to 29	30 to 34	35 to 39	40 to 44
All causes	2,543,421 (2,486,970-2,605,029)	786,638 (758,302-815,455)	2,604,786 (2,545,401-2,666,651)	2,448,139 (2,380,351-2,517,572)	603,375 (596,614-610,286)	414,513 (410,345-418,669)	666,533 (658,477-675,764)	868,473 (855,235-884,250)	1,025,697 (1,000,354-1,052,780)	1,215,869 (1,192,486-1,239,504)	1,434,867 (1,408,527-1,459,209)	1,654,749 (1,637,361-1,673,329)
Communicable, maternal, neonatal, and nutritional diseases	2,308,551 (2,251,773-2,369,097)	685,302 (658,450-713,292)	2,115,490 (2,041,787-2,186,632)	1,934,463 (1,859,563-2,017,464)	350,274 (331,879-372,894)	184,878 (172,979-199,755)	204,876 (192,260-220,748)	285,849 (270,977-303,597)	431,751 (405,534-458,668)	522,858 (500,266-546,154)	538,850 (513,832-564,335)	495,123 (479,634-512,223)
HIV/AIDS and sexually transmitted infections	-	21,446 (8,276-40,255)	197,799 (172,150-232,342)	98,150 (91,236-105,059)	30,506 (28,900-32,350)	17,017 (15,967-18,156)	23,226 (20,296-26,323)	67,798 (58,954-78,155)	214,277 (191,725-239,271)	301,307 (280,857-322,359)	305,652 (282,770-328,922)	265,153 (253,851-275,548)
HIV/AIDS	-	-	159,318 (150,785-167,247)	94,625 (88,379-100,307)	29,017 (27,738-30,323)	16,924 (15,878-18,074)	22,922 (20,005-26,014)	67,302 (58,433-77,648)	213,571 (190,993-238,591)	300,656 (280,214-321,772)	304,985 (282,106-328,227)	264,425 (253,123-274,774)
HIV/AIDS - Drug-susceptible Tuberculosis	-	-	50,283 (37,267-59,850)	31,796 (24,699-36,983)	9,509 (7,137-11,128)	5,400 (3,995-6,411)	7,156 (5,360-8,781)	17,832 (13,038-22,602)	43,065 (28,036-59,126)	55,954 (35,043-78,591)	56,943 (35,654-80,773)	52,635 (33,181-73,669)
HIV/AIDS - Multidrug-resistant Tuberculosis without extensive drug resistance	-	-	5,184 (3,140-7,628)	3,212 (1,981-4,734)	900 (558-1,279)	493 (308-709)	686 (428-997)	2,018 (1,184-3,100)	4,681 (2,579-7,453)	6,053 (3,233-9,914)	6,086 (3,250-10,020)	5,581 (3,044-8,922)
HIV/AIDS - Extensively drug-resistant Tuberculosis	-	-	71 (45-102)	42 (27-59)	13 (8-18)	7 (5-10)	14 (9-19)	50 (32-72)	133 (83-196)	183 (113-272)	186 (115-276)	177 (110-263)
HIV/AIDS resulting in other diseases	-	-	103,780 (91,326-120,265)	59,575 (52,447-69,206)	18,595 (16,594-21,363)	11,024 (9,649-12,796)	15,066 (12,539-18,211)	47,402 (38,993-58,773)	165,692 (139,017-194,922)	238,466 (206,288-270,011)	241,769 (206,566-274,294)	206,032 (179,803-228,688)
Sexually transmitted infections excluding HIV	66,217 (25,820-124,722)	21,446 (8,276-40,255)	38,480 (14,731-73,898)	3,525 (1,325-6,962)	1,489 (548-2,873)	93 (78-108)	304 (244-340)	496 (367-560)	706 (485-816)	651 (491-729)	667 (503-740)	728 (563-810)
Syphilis	66,217 (25,820-124,722)	21,446 (8,276-40,255)	38,480 (14,731-73,898)	3,525 (1,325-6,962)	1,489 (548-2,873)	50 (44-61)	58 (51-64)	78 (60-92)	110 (79-134)	108 (81-138)	128 (85-168)	121 (87-172)
Chlamydial infection	-	-	-	-	-	10 (7-13)	41 (32-47)	115 (84-131)	118 (79-138)	65 (48-74)	92 (69-102)	121 (91-135)
Gonococcal infection	-	-	-	-	-	20 (15-24)	107 (85-122)	180 (133-205)	320 (215-373)	301 (224-339)	262 (198-292)	314 (238-350)
Other sexually transmitted infections	-	-	-	-	-	12 (9-14)	98 (77-111)	123 (90-140)	158 (106-184)	176 (130-198)	185 (139-204)	172 (131-192)
Respiratory infections and tuberculosis	-	159,328 (147,644-171,574)	713,842 (660,799-758,170)	415,027 (354,967-465,857)	56,045 (48,363-62,446)	31,795 (28,635-34,888)	46,268 (43,593-49,532)	69,226 (65,648-73,314)	85,789 (81,751-90,154)	100,454 (96,280-105,134)	116,063 (111,078-121,238)	128,511 (123,589-134,073)
Tuberculosis	-	-	51,668 (47,476-55,686)	52,830 (44,823-59,963)	13,077 (11,320-14,749)	12,252 (10,871-13,579)	29,213 (27,162-31,405)	50,886 (47,702-54,024)	65,165 (61,724-69,047)	77,055 (73,261-81,254)	87,711 (83,200-92,359)	94,675 (90,428-99,646)
Drug-susceptible tuberculosis	-	-	46,704 (42,633-50,701)	48,119 (40,774-54,756)	11,773 (10,170-13,379)	10,986 (9,678-12,240)	26,161 (24,119-28,337)	45,470 (42,310-48,606)	57,946 (54,407-61,837)	68,357 (64,452-72,551)	77,652 (73,146-82,436)	83,115 (78,432-87,873)
Multidrug-resistant tuberculosis without extensive drug resistance	-	-	4,757 (3,685-6,039)	4,579 (3,310-6,190)	1,253 (932-1,672)	1,214 (918-1,616)	2,915 (2,272-3,790)	5,087 (4,000-6,428)	6,709 (5,345-8,340)	8,078 (6,471-10,016)	9,312 (7,411-11,381)	10,582 (8,517-12,886)
Extensively drug-resistant tuberculosis	-	-	207 (162-261)	133 (101-173)	51 (39-68)	52 (39-68)	136 (108-175)	329 (266-405)	510 (412-621)	620 (502-749)	747 (604-901)	979 (784-1,184)
Lower respiratory infections	195,377 (177,743-215,607)	159,125 (147,441-171,350)	658,804 (610,453-700,144)	358,591 (306,794-403,069)	42,422 (36,156-47,551)	19,242 (16,800-21,193)	16,860 (15,397-18,216)	18,167 (16,754-19,753)	20,426 (18,749-21,741)	23,187 (21,296-24,660)	28,092 (25,913-29,941)	33,560 (31,372-35,687)
Upper respiratory infections	81 (48-107)	168 (97-226)	3,139 (1,415-4,528)	3,369 (955-6,309)	419 (189-653)	188 (104-296)	134 (79-194)	131 (81-183)	162 (103-212)	182 (122-232)	218 (138-290)	235 (159-313)
Otitis media	38 (6-219)	35 (17-70)	230 (103-464)	237 (96-499)	126 (70-226)	113 (64-193)	62 (41-108)	42 (26-79)	36 (22-62)	30 (18-55)	41 (25-74)	41 (26-73)
Enteric infections	29,345 (26,129-33,272)	54,560 (48,802-61,253)	464,255 (417,144-512,390)	472,514 (389,689-552,249)	106,034 (78,321-140,307)	58,684 (42,955-79,425)	45,157 (32,337-63,800)	36,362 (26,027-52,170)	29,774 (21,046-43,283)	27,156 (19,074-40,381)	27,623 (19,150-42,057)	26,881 (18,337-42,438)
Diarrheal diseases	29,345 (26,129-33,272)	53,830 (48,119-60,589)	452,117 (406,258-498,216)	412,882 (342,164-482,656)	43,973 (27,298-70,526)	21,042 (13,637-34,572)	21,541 (13,356-36,073)	21,852 (13,042-35,495)	19,533 (11,570-32,002)	19,335 (11,841-31,888)	21,419 (13,037-35,320)	21,973 (13,544-37,769)
Typhoid and paratyphoid	-	0 (0-0)	336 (172-627)	31,582 (14,339-58,597)	53,945 (31,392-86,409)	33,895 (19,522-54,118)	20,879 (10,225-37,391)	12,035 (6,116-21,011)	8,080 (4,183-13,803)	5,866 (3,045-9,949)	4,268 (2,213-7,152)	3,084 (1,587-5,188)
Typhoid fever	-	-	305 (153-559)	28,022 (12,938-52,709)	47,482 (26,927-77,708)	29,688 (16,773-47,537)	18,204 (8,929-32,154)	10,499 (5,128-18,552)	6,998 (3,573-12,096)	5,026 (2,543-8,651)	3,620 (1,851-6,205)	2,596 (1,298-4,398)
Paratyphoid fever	-	-	31 (12-66)	3,560 (1,338-7,656)	6,463 (2,772-12,514)	4,207 (1,840-8,334)	2,674 (1,056-5,672)	1,536 (615-3,304)	1,081 (456-2,255)	840 (357-1,781)	648 (283-1,299)	488 (214-983)
Invasive Non-typhoidal Salmonella (iNTS)	-	730 (408-1,234)	10,524 (5,906-18,066)	27,332 (14,566-47,014)	8,008 (4,067-13,603)	3,688 (1,670-6,691)	2,679 (1,099-5,027)	2,417 (1,015-4,596)	2,128 (828-4,428)	1,909 (807-3,683)	1,891 (822-3,689)	1,791 (838-3,362)
Other intestinal infectious diseases	-	-	1,279 (686-2,738)	718 (341-1,606)	107 (42-252)	59 (20-145)	58 (18-136)	58 (17-128)	33 (8-73)	44 (14-97)	46 (13-92)	33 (9-72)
Neglected tropical diseases and malaria	504 (248-1,635)	29,680 (19,334-43,939)	207,347 (146,558-287,579)	406,946 (290,159-547,529)	67,567 (49,555-90,389)	36,164 (26,808-48,793)	26,973 (19,647-37,420)	27,973 (20,261-38,052)	23,347 (16,645-32,149)	21,667 (15,547-29,580)	22,229 (16,087-30,424)	22,131 (15,782-30,182)
Malaria	-	29,264 (18,797-43,687)	199,998 (138,247-278,738)	382,451 (268,487-524,058)	53,873 (37,008-72,675)	28,050 (19,510-37,645)	19,789 (13,680-27,581)	21,126 (14,261-30,326)	17,692 (11,815-25,891)	16,232 (10,888-23,987)	16,697 (11,235-24,224)	16,625 (11,102-24,429)
Chagas disease	-	-	3 (2-3)	1 (1-2)	2 (2-3)	5 (4-5)	11 (10-12)	26 (24-29)	52 (48-57)	104 (97-112)	187 (176-199)	286 (270-304)
Leishmaniasis	-	-	1,399 (1-5,790)	6,294 (2-33,170)	3,376 (1-24,402)	1,956 (0-13,349)	1,928 (1-11,541)	1,550 (1-8,197)	1,467 (1-6,922)	1,336 (1-6,111)	1,278 (1-5,449)	1,042 (1-4,256)
Visceral leishmaniasis	-	-	1,399 (1-5,790)	6,294 (2-33,170)	3,376 (1-24,402)	1,956 (0-13,349)	1,928 (1-11,541)	1,550 (1-8,197)	1,467 (1-6,922)	1,336 (1-6,111)	1,278 (1-5,449)	1,042 (1-4,256)
African trypanosomiasis	-	-	-	534 (130-1,479)	890 (297-2,060)	1,002 (344-2,191)	1,272 (460-2,603)	1,356 (465-3,041)	1,150 (405-2,511)	1,006 (357-2,355)	881 (291-2,050)	788 (264-1,982)
Schistosomiasis	-	-	0 (0-0)	705 (512-977)	262 (201-346)	334 (261-429)	370 (296-463)	418 (337-513)	420 (346-512)	428 (353-513)	516 (429-607)	592 (496-692)
Cysticercosis	0 (0-0)	0 (0-0)	0 (0-0)	156 (74-272)	63 (16-127)	43 (7-97)	43 (5-101)	62 (11-138)	78 (16-163)	81 (19-172)	74 (15-160)	70 (13-153)
Cystic echinococcosis	-	-	-	344 (211-497)	152 (68-257)	91 (27-173)	47 (3-106)	55 (5-132)	78 (12-166)	81 (12-178)	96 (18-200)	91 (15-196)
Dengue	-	-	3,001 (993-4,417)	4,728 (2,022-6,374)	2,362 (843-3,124)	1,104 (439-1,445)	1,055 (393-1,443)	1,037 (476-1,396)	874 (406-1,134)	671 (314-894)	715 (289-954)	614 (283-790)
Yellow fever	-	4 (1-11)	64 (12-194)	620 (127-1,887)	929 (197-2,751)	820 (167-2,378)	760 (149-2,151)	681 (132-2,075)	487 (98-1,434)	384 (78-1,099)	320 (64-923)	272 (56-829)
Rabies	-	-	521 (377-748)	3,039 (2,181-4,670)	4,388 (3,205-6,325)	2,356 (1,801-3,368)	1,426 (1,091-2,292)	1,403 (1,077-2,120)	776 (608-1,119)	1,069 (828-1,479)	1,139 (880-1,553)	1,423 (1,125-1,911)
Intestinal nematode infections	-	-	422 (328-539)	4,499 (3,393-5,886)	711 (554-918)	138 (107-170)	42 (32-53)	33 (25-43)	24 (18-31)	31 (24-40)	31 (24-39)	23 (18-29)
Ascariasis	-	-	422 (328-539)	4,499 (3,393-5,886)	711 (554-918)	138 (107-170)	42 (32-53)	33 (25-43)	24 (18-31)	31 (24-40)	31 (24-39)	23 (18-29)
Ebola	0 (0-0)	0 (0-0)	0 (0-0)	0 (0-0)	0 (0-0)	0 (0-0)	0 (0-0)	0 (0-0)	4 (3-5)	4 (3-5)	4 (3-5)	4 (3-5)
Zika virus	0 (0-0)	0 (0-0)	0 (0-0)	0 (0-0)	0 (0-0)	0 (0-0)	0 (0-0)	0 (0-0)	0 (0-0)	0 (0-0)	0 (0-0)	0 (0-0)
Other neglected tropical diseases	504 (248-1,635)	413 (209-1,286)	1,940 (1,014-6,810)	3,575 (1,197-16,605)	560 (336-1,553)	266 (173-642)	231 (166-486)	224 (154-490)	246 (172-508)	239 (178-417)	290 (205-547)	300 (216-525)
Other infectious diseases	45,606 (39,326-52,889)	47,584 (40,666-54,765)	307,522 (235,965-413,167)	400,354 (276,839-585,541)	79,230 (59,597-110,929)	36,777 (30,759-46,509)	30,748 (26,794-35,491)	26,832 (23,421-29,906)	22,194 (19,300-24,455)	22,709 (20,192-24,738)	23,466 (20,321-25,439)	23,722 (20,279-25,676)
Meningitis	12,742 (9,840-14,966)	16,760 (14,039-19,238)	104,185 (91,017-116,536)	96,466 (77,512-112,513)	20,368 (16,272-25,568)	12,726 (10,629-16,153)	11,642 (9,641-14,401)	9,943 (8,068-12,288)	8,308 (6,864-10,397)	8,095 (6,716-10,190)	8,222 (6,924-10,517)	7,350 (6,218-9,431)
Pneumococcal meningitis	1,894 (1,413-2,421)	2,377 (1,870-2,911)	13,387 (10,657-16,327)	12,460 (9,208-16,080)	1,886 (1,341-2,552)	1,162 (848-1,590)	1,255 (923-1,691)	1,272 (960-1,716)	1,122 (853-1,511)	1,123 (861-1,428)	1,233 (935-1,654)	1,154 (863-1,551)
H influenzae type B meningitis	3,488 (2,536-4,447)	4,700 (3,814-5,796)	32,884 (26,878-39,067)	32,707 (24,991-40,112)	7,943 (6,098-10,265)	4,795 (3,911-6,265)	4,041 (3,271-5,143)	3,003 (2,429-3,824)	2,440 (2,020-3,076)	2,271 (1,893-2,980)	2,267 (1,882-3,090)	2,175 (1,798-2,926)
Meningococcal meningitis	1,893 (1,445-2,367)	2,512 (2,045-2,999)	14,355 (11,991-17,212)	12,407 (9,531-15,960)	2,147 (1,630-2,815)	1,140 (885-1,505)	968 (739-1,233)	789 (597-1,004)	666 (518-882)	676 (521-892)	674 (528-900)	577 (447-750)
Other meningitis	5,466 (4,244-6,609)	7,172 (5,883-8,409)	43,560 (36,705-51,087)	38,893 (30,906-47,491)	8,391 (6,494-10,483)	5,629 (4,505-7,068)	5,378 (4,364-6,783)	4,880 (3,817-6,026)	4,080 (3,231-5,141)	4,025 (3,218-5,179)	4,048 (3,261-5,129)	3,444 (2,821-4,360)
Encephalitis	692 (604-795)	1,202 (1,062-1,392)	14,565 (11,809-18,059)	15,234 (12,135-20,600)	7,282 (6,157-8,643)	5,247 (4,585-5,974)	3,607 (3,304-3,975)	3,144 (2,921-3,474)	2,507 (2,343-2,742)	2,798 (2,596-3,003)	2,784 (2,609-3,062)	2,693 (2,521-2,927)
Diphtheria	-	-	1,124 (760-1,640)	3,729 (2,221-6,017)	683 (434-1,049)	223 (152-327)	79 (55-115)	44 (30-64)	37 (26-53)	39 (27-56)	42 (30-60)	50 (37-69)
Whooping cough	-	-	60,752 (28,964-114,012)	57,284 (27,391-107,637)	5,631 (2,602-11,383)	1,092 (493-2,251)	444 (205-914)	322 (150-645)	181 (82-370)	136 (62-280)	106 (48-215)	111 (50-222)
Tetanus	26,095 (21,060-32,682)	24,353 (18,987-31,102)	9,658 (6,880-13,592)	5,091 (2,981-7,714)	2,196 (1,468-3,355)	1,541 (1,078-2,274)	2,351 (1,464-3,497)	2,197 (1,397-3,215)	2,076 (1,242-3,005)	2,058 (1,409-2,859)	2,053 (1,345-2,768)	2,478 (1,468-3,471)
Measles	-	-	88,945 (31,982-192,047)	194,366 (72,761-387,424)	31,175 (11,725-64,854)	8,145 (2,941-17,793)	2,999 (1,012-6,747)	1,491 (502-3,398)	686 (231-1,554)	391 (131-890)	242 (80-549)	163 (55-370)
Varicella and herpes zoster	162 (131-197)	448 (368-550)	3,725 (3,129-4,454)	4,209 (3,449-5,185)	1,641 (1,295-2,041)	542 (441-663)	280 (232-342)	300 (252-359)	375 (317-451)	466 (395-550)	416 (354-493)	258 (221-305)
Acute hepatitis	-	-	12,275 (7,828-14,406)	14,294 (8,987-17,703)	7,319 (4,648-8,977)	5,413 (3,490-6,482)	6,970 (4,599-8,137)	7,472 (5,332-8,774)	6,379 (4,556-7,293)	6,906 (4,940-7,865)	7,558 (5,387-8,669)	8,632 (6,035-9,786)
Acute hepatitis A	-	-	2,009 (998-3,136)	8,937 (5,515-11,564)	4,921 (3,108-6,282)	2,782 (1,737-3,643)	2,602 (1,531-3,507)	2,196 (1,317-3,058)	1,392 (796-2,039)	1,167 (620-1,886)	1,127 (537-1,907)	1,069 (555-1,835)
Acute hepatitis B	-	-	7,722 (4,390-10,082)	4,096 (2,202-6,443)	1,189 (548-2,021)	904 (431-1,569)	1,512 (806-2,471)	2,324 (1,276-3,684)	2,955 (1,814-4,273)	4,203 (2,649-5,577)	5,227 (3,375-6,771)	6,634 (4,496-8,121)
Acute hepatitis C	-	-	2,522 (1,223-4,396)	956 (462-1,773)	135 (55-278)	48 (18-105)	35 (12-76)	29 (10-63)	24 (8-62)	30 (8-76)	47 (13-121)	56 (17-140)
Acute hepatitis E	-	-	21 (9-38)	305 (148-538)	1,075 (578-1,641)	1,679 (966-2,499)	2,821 (1,696-3,792)	2,923 (1,825-4,032)	2,008 (1,187-2,942)	1,507 (687-2,576)	1,157 (479-2,015)	873 (354-1,667)
Other unspecified infectious diseases	5,915 (3,094-7,037)	4,820 (2,580-5,732)	12,292 (7,664-15,183)	9,681 (6,218-13,060)	2,936 (2,166-3,596)	1,848 (1,413-2,274)	2,375 (1,849-2,879)	1,918 (1,494-2,268)	1,645 (1,292-1,892)	1,819 (1,429-2,062)	2,042 (1,646-2,324)	1,988 (1,693-2,237)
Maternal and neonatal disorders	1,971,385 (1,897,298-2,042,324)	372,704 (350,948-396,571)	100,322 (87,863-112,370)	9,136 (7,883-10,515)	-	981 (845-1,124)	29,952 (27,714-32,220)	55,010 (51,665-58,224)	53,745 (50,698-56,616)	46,990 (44,124-49,468)	41,061 (38,429-43,636)	25,337 (23,589-27,172)
Maternal disorders	-	-	-	-	-	981 (845-1,124)	29,952 (27,714-32,220)	55,010 (51,665-58,224)	53,745 (50,698-56,616)	46,990 (44,124-49,468)	41,061 (38,429-43,636)	25,337 (23,589-27,172)
Maternal hemorrhage	-	-	-	-	-	289 (230-356)	8,094 (4,970-12,516)	18,004 (11,394-26,486)	18,498 (13,130-25,381)	17,686 (13,650-22,377)	15,888 (12,845-19,176)	7,910 (6,456-9,645)
Maternal sepsis and other maternal infections	-	-	-	-	-	92 (65-126)	4,591 (2,750-8,393)	6,349 (3,722-11,282)	5,599 (3,559-9,526)	4,235 (2,897-6,722)	3,732 (2,752-5,462)	3,011 (2,364-3,929)
Maternal hypertensive disorders	-	-	-	-	-	105 (83-130)	4,538 (3,144-7,383)	7,597 (5,283-12,163)	7,040 (5,008-10,999)	5,227 (3,864-7,528)	4,075 (3,100-5,739)	2,046 (1,624-2,728)
Maternal obstructed labor and uterine rupture	-	-	-	-	-	94 (65-133)	1,563 (871-3,241)	2,624 (1,321-6,212)	2,665 (1,365-5,456)	3,024 (1,796-5,262)	3,223 (2,102-4,989)	2,225 (1,573-3,169)
Maternal abortion and miscarriage	-	-	-	-	-	79 (59-106)	2,534 (1,511-4,620)	3,340 (2,046-6,022)	3,212 (2,044-5,681)	3,007 (1,956-4,826)	2,765 (1,870-4,234)	2,134 (1,569-2,985)
Ectopic pregnancy	-	-	-	-	-	42 (27-69)	1,649 (553-4,406)	2,766 (1,100-7,579)	2,578 (1,122-6,660)	2,274 (1,083-4,755)	1,500 (730-3,648)	766 (395-1,801)
Indirect maternal deaths	-	-	-	-	-	160 (125-200)	3,751 (2,501-6,398)	7,492 (5,211-12,576)	7,479 (5,273-11,725)	6,004 (4,508-8,409)	5,209 (4,099-7,160)	3,339 (2,676-4,355)
Late maternal deaths	-	-	-	-	-	13 (9-19)	385 (277-537)	729 (544-1,010)	744 (553-1,010)	634 (455-884)	494 (369-670)	262 (195-357)
Maternal deaths aggravated by HIV/AIDS	-	-	-	-	-	2 (1-4)	80 (43-119)	307 (184-423)	476 (310-621)	499 (336-641)	394 (255-518)	231 (141-317)
Other maternal disorders	-	-	-	-	-	105 (83-133)	2,768 (1,401-5,448)	5,802 (2,932-11,690)	5,453 (3,106-9,313)	4,401 (2,831-7,240)	3,780 (2,645-6,158)	3,413 (2,774-4,361)
Neonatal disorders	1,971,385 (1,897,298-2,042,324)	372,704 (350,948-396,571)	100,322 (87,863-112,370)	9,136 (7,883-10,515)	-	-	-	-	-	-	-	-
Neonatal preterm birth	772,276 (726,245-874,413)	112,242 (102,086-127,987)	36,218 (31,583-41,744)	2,453 (2,020-2,963)	-	-	-	-	-	-	-	-
Neonatal encephalopathy due to birth asphyxia and trauma	650,791 (583,517-696,692)	63,503 (51,550-70,830)	20,100 (15,772-23,384)	3,649 (3,081-4,326)	-	-	-	-	-	-	-	-
Neonatal sepsis and other neonatal infections	134,235 (115,742-186,888)	89,546 (77,623-116,472)	13,155 (10,980-16,792)	459 (332-758)	-	-	-	-	-	-	-	-
Hemolytic disease and other neonatal jaundice	49,594 (42,963-57,419)	28,290 (23,238-33,651)	6,028 (4,054-8,501)	361 (237-513)	-	-	-	-	-	-	-	-
Other neonatal disorders	364,489 (310,040-401,573)	79,124 (68,539-90,359)	24,822 (20,356-30,095)	2,214 (1,815-2,822)	-	-	-	-	-	-	-	-
Nutritional deficiencies	-	-	124,403 (111,787-136,465)	132,336 (111,182-152,370)	10,891 (9,329-12,333)	3,460 (3,063-3,837)	2,552 (2,347-2,774)	2,648 (2,461-2,857)	2,624 (2,469-2,821)	2,576 (2,438-2,718)	2,756 (2,619-2,926)	3,389 (3,224-3,567)
Protein-energy malnutrition	-	-	119,658 (107,195-131,505)	126,419 (106,242-145,942)	8,866 (7,586-10,162)	2,574 (2,265-2,857)	1,655 (1,509-1,804)	1,682 (1,536-1,828)	1,670 (1,525-1,831)	1,768 (1,639-1,918)	1,875 (1,736-2,022)	2,293 (2,121-2,479)
Other nutritional deficiencies	-	-	4,745 (3,920-5,916)	5,917 (4,457-7,573)	2,025 (1,642-2,475)	886 (734-1,034)	897 (801-1,015)	966 (868-1,076)	955 (845-1,068)	808 (720-909)	881 (801-994)	1,096 (992-1,232)
Non-communicable diseases	220,529 (203,322-248,109)	90,424 (84,544-99,371)	383,852 (345,927-431,951)	245,562 (216,602-278,032)	102,588 (92,857-111,727)	95,010 (88,141-100,907)	161,209 (154,512-166,843)	200,645 (194,227-206,623)	252,276 (246,370-257,993)	367,227 (360,411-374,575)	564,977 (555,652-574,907)	852,287 (840,902-864,028)
Neoplasms	1,629 (1,353-1,800)	1,055 (913-1,193)	10,580 (9,121-12,358)	40,394 (33,255-50,708)	34,958 (31,308-39,080)	31,457 (29,084-33,576)	41,367 (39,064-43,099)	43,732 (41,851-45,233)	55,910 (54,092-57,575)	91,728 (89,510-94,007)	158,610 (155,012-162,770)	250,821 (246,527-256,029)
Lip and oral cavity cancer	-	-	-	-	-	-	649 (577-726)	944 (849-1,068)	1,278 (1,171-1,406)	2,122 (1,979-2,306)	3,768 (3,561-4,000)	6,677 (6,340-7,069)
Nasopharynx cancer	-	-	-	-	233 (198-265)	341 (307-372)	501 (471-530)	529 (499-553)	677 (644-707)	1,228 (1,166-1,287)	2,383 (2,283-2,488)	3,844 (3,679-4,000)
Other pharynx cancer	-	-	-	-	-	-	345 (280-388)	403 (318-449)	556 (450-610)	935 (731-1,013)	1,766 (1,458-1,912)	3,473 (2,970-3,696)
Esophageal cancer	-	-	-	-	-	-	327 (306-350)	515 (490-545)	762 (725-803)	1,662 (1,594-1,744)	3,900 (3,755-4,055)	9,228 (8,929-9,542)
Stomach cancer	-	-	-	-	-	-	1,018 (967-1,077)	2,014 (1,932-2,110)	3,677 (3,539-3,848)	7,890 (7,626-8,212)	15,079 (14,580-15,657)	24,637 (24,047-25,414)
Colon and rectum cancer	-	-	-	-	-	-	887 (774-968)	1,757 (1,611-1,880)	2,856 (2,689-3,037)	5,898 (5,647-6,283)	9,594 (9,196-10,206)	15,222 (14,715-16,006)
Liver cancer	-	-	-	-	940 (810-1,078)	902 (816-1,064)	1,386 (1,278-1,562)	2,159 (2,001-2,512)	3,838 (3,572-4,407)	9,631 (9,056-10,567)	21,001 (19,798-22,534)	32,073 (30,563-34,316)
Liver cancer due to hepatitis B	-	-	-	-	598 (512-697)	674 (601-806)	1,021 (928-1,155)	1,578 (1,447-1,865)	2,706 (2,439-3,162)	6,723 (6,201-7,465)	14,216 (13,061-15,444)	20,620 (18,981-22,356)
Liver cancer due to hepatitis C	-	-	-	-	19 (14-25)	20 (16-26)	27 (22-34)	81 (66-100)	231 (176-297)	699 (584-839)	1,800 (1,461-2,179)	3,453 (2,920-4,069)
Liver cancer due to alcohol use	-	-	-	-	-	-	6 (4-9)	57 (45-74)	215 (160-293)	732 (579-919)	2,101 (1,484-2,776)	3,834 (2,915-4,901)
Liver cancer due to NASH	-	-	-	-	-	-	106 (85-130)	135 (109-166)	202 (155-255)	408 (321-514)	792 (633-976)	1,230 (989-1,524)
Liver cancer due to other causes	-	-	-	-	323 (267-379)	207 (174-244)	227 (185-270)	309 (259-368)	484 (393-603)	1,070 (866-1,308)	2,091 (1,678-2,552)	2,937 (2,342-3,621)
Gallbladder and biliary tract cancer	-	-	-	-	-	-	106 (94-120)	176 (154-200)	286 (247-334)	683 (595-788)	1,318 (1,149-1,497)	2,345 (2,050-2,584)
Pancreatic cancer	-	-	-	-	-	-	169 (159-177)	282 (271-293)	532 (516-549)	1,305 (1,264-1,349)	2,931 (2,831-3,045)	5,820 (5,642-5,991)
Larynx cancer	-	-	-	-	-	-	157 (146-171)	247 (223-280)	334 (307-365)	651 (617-689)	1,563 (1,485-1,641)	3,507 (3,359-3,673)
Tracheal, bronchus, and lung cancer	-	-	-	-	-	-	939 (889-1,003)	1,495 (1,424-1,576)	2,651 (2,536-2,780)	6,115 (5,910-6,361)	13,931 (13,487-14,541)	29,753 (29,069-30,531)
Malignant skin melanoma	-	-	-	-	-	-	254 (217-317)	455 (384-568)	740 (622-897)	1,205 (1,020-1,490)	1,843 (1,536-2,284)	2,687 (2,220-3,372)
Non-melanoma skin cancer	-	-	-	-	-	-	165 (155-175)	209 (197-223)	271 (257-288)	482 (459-513)	733 (694-779)	1,131 (1,084-1,189)
Non-melanoma skin cancer (squamous-cell carcinoma)	-	-	-	-	-	-	165 (155-175)	209 (197-223)	271 (257-288)	482 (459-513)	733 (694-779)	1,131 (1,084-1,189)
Breast cancer	-	-	-	-	-	-	361 (262-418)	966 (812-1,085)	3,195 (2,872-3,535)	9,155 (8,463-10,015)	19,735 (18,248-21,452)	31,481 (29,780-33,620)
Cervical cancer	-	-	-	-	-	-	280 (246-305)	1,188 (1,067-1,304)	3,404 (3,050-3,716)	7,365 (6,689-7,947)	13,248 (12,119-14,173)	19,611 (18,100-20,994)
Uterine cancer	-	-	-	-	-	-	71 (65-77)	160 (149-171)	322 (300-343)	651 (608-694)	1,265 (1,161-1,355)	2,145 (2,012-2,269)
Ovarian cancer	-	-	-	-	-	-	528 (480-590)	750 (687-827)	1,047 (971-1,139)	1,683 (1,564-1,851)	2,926 (2,740-3,236)	5,406 (5,145-5,847)
Prostate cancer	-	-	-	-	-	-	75 (62-87)	116 (96-135)	155 (131-186)	202 (166-233)	282 (230-323)	550 (454-637)
Testicular cancer	-	-	-	-	-	-	370 (342-400)	832 (774-898)	1,064 (994-1,142)	1,008 (944-1,072)	824 (781-875)	632 (600-672)
Kidney cancer	49 (40-56)	26 (22-30)	345 (290-404)	1,620 (1,266-2,006)	672 (593-752)	289 (264-314)	234 (214-250)	292 (266-308)	418 (389-440)	645 (597-679)	1,221 (1,150-1,277)	2,298 (2,165-2,378)
Bladder cancer	-	-	-	-	-	-	100 (91-108)	148 (133-161)	217 (196-234)	421 (387-450)	823 (766-883)	1,671 (1,568-1,760)
Brain and nervous system cancer	190 (136-225)	146 (110-180)	1,881 (1,324-2,347)	7,344 (5,176-9,981)	7,851 (6,448-9,811)	5,997 (5,113-6,875)	4,707 (3,943-5,220)	4,124 (3,449-4,612)	5,014 (4,257-5,530)	6,817 (5,715-7,485)	8,770 (7,467-9,659)	10,636 (9,305-11,710)

Cause	45 to 49	50 to 54	54 to 59	60 to 64	65 to 69	70 to 74	75 to 79	80 to 84	85 to 89	90 to 94	95 plus
All causes	1,884,948 (1,866,633-1,904,719)	2,367,858 (2,350,110-2,388,328)	2,640,834 (2,623,019-2,662,006)	3,115,925 (3,095,154-3,138,793)	4,086,587 (4,063,469-4,111,769)	4,866,336 (4,839,226-4,895,395)	5,330,887 (5,304,747-5,359,032)	4,898,006 (4,876,816-4,920,952)	3,361,601 (3,348,775-3,374,714)	1,910,817 (1,905,725-1,916,057)	692,405 (691,173-693,687)
Communicable, maternal, neonatal, and nutritional diseases	435,469 (419,383-455,949)	380,384 (367,416-402,232)	334,565 (319,858-359,234)	367,453 (347,686-406,568)	421,543 (392,868-479,455)	486,638 (447,258-555,635)	467,937 (431,186-538,376)	430,955 (394,215-492,208)	304,276 (280,084-343,332)	157,267 (147,080-173,737)	61,834 (58,993-66,170)
HIV/AIDS and sexually transmitted infections	197,072 (186,091-209,626)	120,483 (115,407-125,530)	63,549 (60,119-66,688)	39,985 (37,464-42,662)	27,711 (25,899-29,624)	17,362 (16,112-18,671)	9,949 (9,172-10,792)	1,763 (1,630-1,911)	658 (594-728)	305 (279-329)	78 (71-84)
HIV/AIDS	196,438 (185,466-209,016)	120,012 (114,894-125,032)	63,045 (59,616-66,218)	39,595 (37,089-42,237)	27,256 (25,453-29,212)	16,978 (15,714-18,286)	9,573 (8,815-10,424)	1,475 (1,346-1,619)	448 (394-508)	185 (166-207)	30 (27-33)
HIV/AIDS - Drug-susceptible Tuberculosis	44,268 (29,036-60,203)	32,320 (22,500-40,946)	18,740 (13,984-22,401)	12,479 (9,744-14,581)	8,788 (7,071-10,194)	5,651 (4,573-6,534)	3,175 (2,576-3,670)	468 (384-540)	134 (107-160)	56 (45-64)	8 (6-9)
HIV/AIDS - Multidrug-resistant Tuberculosis without extensive drug resistance	4,713 (2,654-7,301)	3,448 (2,026-5,236)	2,000 (1,213-2,958)	1,294 (800-1,909)	901 (561-1,330)	576 (362-865)	331 (210-504)	52 (33-80)	16 (9-25)	6 (4-10)	1 (1-1)
HIV/AIDS - Extensively drug-resistant Tuberculosis	140 (88-207)	87 (55-126)	47 (31-67)	26 (17-36)	18 (12-24)	10 (7-14)	7 (4-9)	1 (1-2)	0 (0-1)	0 (0-0)	0 (0-0)
HIV/AIDS resulting in other diseases	147,317 (127,557-167,530)	84,157 (73,662-95,625)	42,257 (37,384-48,103)	25,796 (22,926-29,271)	17,550 (15,631-19,908)	10,741 (9,508-12,249)	6,060 (5,342-6,935)	954 (845-1,096)	297 (256-351)	123 (107-143)	21 (19-24)
Sexually transmitted infections excluding HIV	634 (492-709)	471 (379-539)	505 (391-582)	390 (308-463)	456 (357-542)	384 (313-458)	376 (325-437)	288 (248-320)	211 (173-237)	120 (98-133)	48 (41-52)
Syphilis	101 (74-138)	137 (103-190)	160 (124-217)	136 (99-201)	175 (128-251)	160 (122-227)	155 (122-209)	99 (81-126)	72 (53-90)	36 (26-44)	16 (12-18)
Chlamydial infection	99 (75-111)	61 (49-66)	67 (49-74)	50 (39-56)	35 (27-39)	39 (32-44)	37 (32-41)	44 (38-48)	32 (27-35)	19 (17-20)	7 (6-7)
Gonococcal infection	275 (210-308)	181 (145-196)	181 (133-199)	126 (98-140)	185 (142-206)	135 (109-151)	141 (121-155)	112 (96-122)	82 (70-89)	49 (43-53)	18 (16-19)
Other sexually transmitted infections	159 (122-178)	92 (73-100)	97 (72-107)	78 (61-87)	60 (46-67)	50 (41-55)	43 (38-47)	33 (28-36)	26 (22-29)	16 (13-18)	7 (6-8)
Respiratory infections and tuberculosis	146,280 (140,764-152,782)	169,369 (162,951-177,257)	174,052 (167,477-182,144)	202,672 (194,676-211,883)	236,654 (227,206-247,285)	274,995 (262,997-288,811)	275,173 (264,677-290,286)	262,852 (252,828-278,586)	192,988 (187,213-204,204)	108,818 (106,308-113,463)	45,303 (44,510-46,986)
Tuberculosis	105,048 (100,008-110,920)	114,969 (109,418-121,742)	112,046 (106,123-118,329)	118,413 (111,972-125,608)	124,367 (117,901-132,378)	126,205 (118,996-134,464)	100,062 (94,244-106,500)	71,492 (67,416-76,711)	34,507 (32,356-37,295)	10,487 (9,842-11,312)	2,347 (2,208-2,525)
Drug-susceptible tuberculosis	92,093 (86,679-97,892)	101,074 (95,256-107,815)	99,190 (93,326-105,358)	105,611 (99,366-112,934)	110,699 (103,882-118,629)	112,719 (105,815-120,682)	89,371 (84,117-95,745)	64,051 (59,757-68,877)	31,003 (28,834-33,644)	9,462 (8,822-10,239)	2,126 (1,993-2,293)
Multidrug-resistant tuberculosis without extensive drug resistance	11,814 (9,534-14,492)	12,734 (10,346-15,552)	11,933 (9,556-14,641)	12,046 (9,581-14,945)	12,826 (10,265-15,944)	12,720 (10,139-15,817)	10,066 (8,088-12,539)	7,017 (5,588-8,766)	3,302 (2,647-4,136)	961 (757-1,213)	206 (163-260)
Extensively drug-resistant tuberculosis	1,141 (907-1,393)	1,161 (922-1,420)	923 (747-1,130)	756 (606-946)	842 (680-1,038)	766 (610-962)	625 (499-784)	424 (330-543)	202 (159-255)	63 (50-80)	14 (11-18)
Lower respiratory infections	40,869 (38,199-43,409)	53,986 (49,631-57,656)	61,649 (56,766-65,039)	83,849 (76,747-88,405)	111,732 (101,946-117,970)	148,043 (137,944-157,576)	174,169 (164,343-186,032)	190,050 (181,203-202,642)	157,231 (152,286-167,305)	97,655 (95,333-102,048)	42,691 (41,928-44,353)
Upper respiratory infections	249 (167-340)	286 (188-378)	298 (205-382)	333 (219-467)	446 (303-581)	656 (433-829)	876 (562-1,070)	1,259 (765-1,499)	1,222 (700-1,445)	666 (415-775)	261 (180-302)
Otitis media	114 (57-258)	128 (71-268)	59 (41-96)	76 (51-127)	109 (76-170)	91 (64-147)	66 (49-101)	50 (37-76)	28 (22-42)	11 (9-14)	4 (3-5)
Enteric infections	30,927 (20,855-51,087)	35,710 (23,760-58,419)	44,174 (28,791-72,196)	67,977 (43,931-110,692)	96,464 (62,454-161,201)	129,181 (84,115-206,430)	133,014 (86,803-214,484)	129,373 (86,357-200,329)	87,204 (59,182-130,905)	36,751 (24,945-54,102)	11,336 (8,043-15,916)
Diarrheal diseases	26,840 (16,513-46,061)	32,169 (20,064-55,431)	41,065 (25,812-69,167)	65,037 (40,621-107,997)	93,838 (60,303-157,814)	127,178 (81,925-205,191)	131,633 (85,563-213,090)	128,482 (85,319-199,897)	86,771 (58,722-130,403)	36,610 (24,772-54,000)	11,297 (8,020-15,895)
Typhoid and paratyphoid	2,357 (1,273-3,933)	1,772 (909-3,077)	1,346 (646-2,484)	1,169 (573-2,122)	1,017 (576-1,664)	750 (448-1,210)	456 (276-707)	230 (139-353)	105 (66-160)	34 (22-53)	9 (6-15)
Typhoid fever	1,966 (1,040-3,321)	1,474 (752-2,551)	1,126 (540-2,110)	980 (471-1,805)	858 (478-1,423)	637 (372-1,018)	389 (230-617)	198 (118-308)	91 (57-141)	30 (19-47)	8 (5-13)
Paratyphoid fever	391 (161-790)	298 (116-620)	221 (85-478)	189 (74-391)	159 (72-313)	113 (53-216)	67 (32-123)	32 (15-59)	14 (7-25)	4 (2-7)	1 (1-2)
Invasive Non-typhoidal Salmonella (iNTS)	1,698 (815-3,272)	1,744 (798-3,379)	1,742 (801-3,109)	1,748 (795-3,046)	1,579 (754-2,747)	1,232 (586-2,183)	907 (418-1,632)	636 (268-1,197)	301 (121-548)	100 (27-215)	26 (1-75)
Other intestinal infectious diseases	32 (7-70)	26 (6-62)	21 (4-48)	23 (5-49)	31 (7-65)	22 (5-51)	18 (4-40)	26 (6-57)	28 (5-65)	7 (1-18)	4 (0-11)
Neglected tropical diseases and malaria	20,932 (15,150-28,174)	23,264 (16,840-32,060)	22,902 (16,499-31,833)	24,874 (17,951-34,912)	24,256 (17,206-34,802)	25,805 (18,031-36,876)	12,562 (9,384-17,486)	6,452 (5,189-8,593)	2,507 (2,138-3,287)	835 (722-1,132)	328 (274-420)
Malaria	15,458 (10,185-22,884)	17,804 (11,701-26,401)	17,813 (11,582-26,624)	19,810 (13,070-29,828)	19,472 (12,608-29,727)	20,991 (13,450-31,689)	8,758 (5,801-13,164)	3,769 (2,670-5,493)	1,040 (787-1,414)	290 (204-396)	117 (56-161)
Chagas disease	422 (401-445)	604 (574-636)	755 (712-804)	936 (885-992)	1,042 (990-1,099)	1,097 (1,032-1,173)	862 (813-912)	628 (595-661)	401 (380-426)	191 (176-210)	50 (44-59)
Leishmaniasis	901 (3-3,185)	766 (7-2,605)	596 (5-1,982)	582 (5-1,829)	509 (13-1,547)	376 (21-1,097)	229 (14-676)	114 (6-340)	46 (2-140)	12 (0-40)	2 (0-8)
Visceral leishmaniasis	901 (3-3,185)	766 (7-2,605)	596 (5-1,982)	582 (5-1,829)	509 (13-1,547)	376 (21-1,097)	229 (14-676)	114 (6-340)	46 (2-140)	12 (0-40)	2 (0-8)
African trypanosomiasis	714 (208-1,716)	645 (196-1,746)	407 (128-1,095)	338 (90-902)	247 (67-657)	179 (51-472)	112 (35-314)	66 (20-195)	23 (6-67)	6 (1-20)	4 (1-11)
Schistosomiasis	662 (549-787)	819 (693-968)	871 (734-1,022)	928 (773-1,102)	722 (609-864)	950 (803-1,112)	772 (651-918)	461 (389-545)	212 (175-255)	58 (46-73)	11 (8-15)
Cysticercosis	63 (10-145)	59 (10-137)	48 (6-112)	39 (4-95)	34 (4-84)	8 (0-23)	19 (1-51)	12 (0-35)	6 (0-16)	2 (0-7)	1 (0-2)
Cystic echinococcosis	94 (14-208)	104 (18-225)	104 (19-220)	107 (18-222)	112 (19-238)	107 (18-235)	82 (8-189)	58 (6-139)	36 (2-93)	15 (0-39)	5 (0-14)
Dengue	628 (276-810)	621 (263-846)	688 (283-890)	667 (306-887)	700 (322-941)	940 (518-1,733)	749 (449-1,496)	842 (539-1,683)	503 (328-1,150)	183 (115-399)	62 (39-146)
Yellow fever	233 (46-715)	188 (36-568)	149 (29-462)	118 (25-368)	80 (16-240)	49 (10-155)	28 (6-86)	78 (22-162)	4 (1-12)	1 (0-3)	42 (29-48)
Rabies	1,432 (1,137-2,107)	1,306 (1,029-1,808)	1,138 (930-1,581)	985 (780-1,420)	961 (740-1,436)	700 (552-1,018)	587 (461-802)	173 (133-234)	94 (68-143)	20 (15-31)	9 (6-13)
Intestinal nematode infections	24 (18-31)	24 (19-31)	21 (16-26)	45 (35-55)	23 (17-29)	38 (30-48)	31 (24-40)	21 (16-26)	9 (6-12)	6 (4-8)	2 (1-3)
Ascariasis	24 (18-31)	24 (19-31)	21 (16-26)	45 (35-55)	23 (17-29)	38 (30-48)	31 (24-40)	21 (16-26)	9 (6-12)	6 (4-8)	2 (1-3)
Ebola	2 (2-3)	0 (0-0)	2 (2-3)	0 (0-0)	0 (0-0)	0 (0-0)	0 (0-0)	0 (0-0)	0 (0-0)	0 (0-0)	0 (0-0)
Zika virus	0 (0-0)	0 (0-0)	0 (0-0)	0 (0-0)	0 (0-0)	0 (0-0)	0 (0-0)	0 (0-0)	0 (0-0)	0 (0-0)	0 (0-0)
Other neglected tropical diseases	298 (215-519)	324 (225-519)	310 (232-532)	319 (237-581)	355 (261-649)	370 (273-632)	332 (246-589)	232 (177-417)	133 (104-247)	52 (41-89)	25 (20-46)
Other infectious diseases	24,830 (21,251-26,634)	25,490 (22,081-27,406)	24,270 (21,241-25,990)	24,950 (22,280-27,057)	27,024 (24,432-29,588)	27,405 (24,705-30,085)	23,734 (21,375-25,669)	17,943 (16,034-19,238)	10,240 (9,287-11,033)	3,908 (3,509-4,206)	1,387 (1,217-1,541)
Meningitis	7,805 (6,798-9,924)	7,448 (6,493-9,878)	6,887 (6,088-9,055)	7,074 (6,291-9,785)	6,831 (6,074-9,548)	7,431 (6,548-10,392)	5,999 (5,303-8,430)	4,413 (3,904-6,014)	2,727 (2,376-3,611)	866 (767-1,117)	288 (250-362)
Pneumococcal meningitis	1,338 (1,011-1,795)	1,289 (1,004-1,690)	1,157 (902-1,537)	1,207 (932-1,645)	1,126 (874-1,570)	1,298 (1,000-1,823)	1,035 (794-1,451)	746 (578-1,002)	437 (346-586)	133 (105-172)	46 (34-61)
H influenzae type B meningitis	2,303 (1,929-3,168)	2,186 (1,793-2,991)	2,097 (1,751-3,000)	2,186 (1,791-3,230)	2,255 (1,834-3,395)	2,409 (1,951-3,568)	2,025 (1,640-3,046)	1,511 (1,247-2,206)	949 (776-1,338)	334 (280-444)	105 (84-136)
Meningococcal meningitis	604 (475-790)	618 (488-838)	574 (442-766)	591 (449-811)	550 (422-792)	598 (455-820)	488 (377-705)	358 (275-497)	206 (162-276)	59 (46-79)	21 (15-29)
Other meningitis	3,560 (2,971-4,585)	3,355 (2,752-4,460)	3,059 (2,564-4,054)	3,090 (2,539-4,222)	2,899 (2,391-4,033)	3,127 (2,593-4,222)	2,451 (2,023-3,434)	1,799 (1,493-2,429)	1,135 (927-1,492)	340 (281-447)	117 (93-148)
Encephalitis	2,606 (2,442-3,027)	2,577 (2,428-2,988)	2,856 (2,671-3,348)	3,661 (3,406-4,214)	4,589 (4,242-5,077)	5,455 (4,997-6,015)	5,095 (4,625-5,594)	4,064 (3,606-4,495)	1,924 (1,726-2,119)	580 (529-637)	176 (162-189)
Diphtheria	80 (59-110)	63 (47-84)	38 (27-53)	-	-	-	-	-	-	-	-
Whooping cough	91 (41-189)	81 (37-165)	90 (42-185)	-	-	-	-	-	-	-	-
Tetanus	3,253 (1,699-4,329)	3,155 (1,789-4,423)	2,898 (1,617-4,097)	2,244 (1,458-3,102)	2,480 (1,624-3,831)	2,055 (1,456-2,846)	1,565 (1,029-2,286)	826 (546-1,223)	435 (267-657)	129 (83-191)	34 (21-47)
Measles	111 (37-249)	83 (28-188)	75 (25-169)	-	-	-	-	-	-	-	-
Varicella and herpes zoster	243 (210-281)	230 (197-272)	251 (217-292)	302 (260-352)	599 (516-699)	581 (496-689)	830 (719-979)	1,191 (1,022-1,412)	1,246 (1,052-1,487)	768 (629-958)	411 (308-556)
Acute hepatitis	8,379 (5,906-9,614)	9,270 (6,571-10,611)	8,553 (6,166-9,698)	8,349 (5,931-9,478)	8,622 (6,245-9,828)	7,708 (5,743-8,732)	6,315 (4,602-7,111)	4,079 (3,001-4,563)	1,765 (1,371-1,966)	530 (416-581)	115 (93-126)
Acute hepatitis A	835 (447-1,477)	665 (376-1,054)	392 (239-583)	220 (122-350)	142 (79-229)	73 (36-128)	37 (13-74)	11 (3-26)	1 (0-4)	0 (0-1)	0 (0-0)
Acute hepatitis B	6,859 (4,645-8,251)	8,067 (5,549-9,512)	7,756 (5,497-8,867)	7,713 (5,424-8,905)	7,942 (5,690-9,134)	7,008 (5,096-8,059)	5,550 (3,950-6,421)	3,375 (2,377-3,969)	1,362 (977-1,648)	360 (255-454)	68 (41-92)
Acute hepatitis C	67 (21-166)	83 (26-194)	88 (29-209)	114 (34-277)	154 (43-408)	166 (56-374)	166 (52-418)	140 (47-339)	80 (26-196)	32 (9-75)	9 (2-22)
Acute hepatitis E	617 (261-1,267)	455 (206-880)	318 (153-600)	303 (129-590)	384 (160-749)	460 (209-877)	562 (250-1,043)	554 (256-1,005)	321 (158-562)	138 (76-222)	38 (19-62)
Other unspecified infectious diseases	2,263 (1,881-2,535)	2,581 (2,227-2,854)	2,623 (2,219-2,870)	3,319 (2,720-3,708)	3,903 (3,182-4,269)	4,176 (3,446-4,632)	3,930 (3,153-4,253)	3,370 (2,700-3,693)	2,144 (1,746-2,374)	1,035 (816-1,132)	363 (282-391)
Maternal and neonatal disorders	11,743 (10,846-12,593)	1,749 (1,601-1,918)	-	-	-	-	-	-	-	-	-
Maternal disorders	11,743 (10,846-12,593)	1,749 (1,601-1,918)	-	-	-	-	-	-	-	-	-
Maternal hemorrhage	2,902 (2,331-3,484)	476 (379-585)	-	-	-	-	-	-	-	-	-
Maternal sepsis and other maternal infections	2,356 (1,938-2,811)	215 (168-277)	-	-	-	-	-	-	-	-	-
Maternal hypertensive disorders	593 (475-740)	81 (64-104)	-	-	-	-	-	-	-	-	-
Maternal obstructed labor and uterine rupture	793 (598-1,080)	111 (77-164)	-	-	-	-	-	-	-	-	-
Maternal abortion and miscarriage	2,059 (1,652-2,525)	203 (158-251)	-	-	-	-	-	-	-	-	-
Ectopic pregnancy	305 (172-651)	102 (69-166)	-	-	-	-	-	-	-	-	-
Indirect maternal deaths	1,169 (970-1,409)	304 (249-367)	-	-	-	-	-	-	-	-	-
Late maternal deaths	111 (92-134)	18 (14-22)	-	-	-	-	-	-	-	-	-
Maternal deaths aggravated by HIV/AIDS	145 (92-199)	16 (10-23)	-	-	-	-	-	-	-	-	-
Other maternal disorders	1,310 (1,036-1,651)	225 (185-268)	-	-	-	-	-	-	-	-	-
Neonatal disorders	-	-	-	-	-	-	-	-	-	-	-
Neonatal preterm birth	-	-	-	-	-	-	-	-	-	-	-
Neonatal encephalopathy due to birth asphyxia and trauma	-	-	-	-	-	-	-	-	-	-	-
Neonatal sepsis and other neonatal infections	-	-	-	-	-	-	-	-	-	-	-
Hemolytic disease and other neonatal jaundice	-	-	-	-	-	-	-	-	-	-	-
Other neonatal disorders	-	-	-	-	-	-	-	-	-	-	-
Nutritional deficiencies	3,685 (3,490-3,882)	4,320 (4,088-4,567)	5,617 (5,332-5,937)	6,996 (6,646-7,412)	9,433 (8,913-9,977)	11,889 (11,306-12,539)	13,505 (12,843-14,267)	12,572 (12,043-13,183)	10,678 (10,241-11,236)	6,649 (6,441-6,922)	3,401 (3,298-3,521)
Protein-energy malnutrition	2,581 (2,382-2,782)	3,013 (2,788-3,243)	3,963 (3,681-4,259)	4,886 (4,561-5,228)	6,637 (6,204-7,069)	8,688 (8,128-9,226)	9,969 (9,308-10,594)	9,753 (9,067-10,337)	8,425 (7,566-8,956)	5,277 (4,433-5,603)	2,745 (2,176-2,958)
Other nutritional deficiencies	1,104 (990-1,269)	1,307 (1,183-1,508)	1,654 (1,490-1,881)	2,110 (1,899-2,391)	2,796 (2,526-3,164)	3,201 (2,878-3,649)	3,536 (3,164-4,111)	2,818 (2,485-3,551)	2,253 (1,912-3,143)	1,372 (1,113-2,247)	656 (475-1,277)
Non-communicable diseases	1,173,897 (1,158,667-1,187,812)	1,722,990 (1,703,163-1,739,808)	2,088,667 (2,064,146-2,108,729)	2,565,038 (2,530,886-2,591,748)	3,472,474 (3,422,830-3,507,582)	4,191,855 (4,131,217-4,236,166)	4,681,754 (4,622,630-4,725,417)	4,315,209 (4,258,298-4,356,473)	2,955,620 (2,919,365-2,981,648)	1,699,799 (1,684,893-1,710,621)	611,906 (607,648-614,833)
Neoplasms	359,323 (354,672-364,874)	562,429 (555,531-570,888)	681,033 (673,617-689,837)	771,344 (762,335-781,214)	956,679 (945,913-967,516)	1,039,427 (1,025,759-1,050,841)	976,717 (962,094-987,419)	743,698 (729,595-751,995)	382,452 (373,614-386,696)	161,653 (156,630-163,519)	44,087 (42,568-44,628)
Lip and oral cavity cancer	10,324 (9,916-10,764)	14,415 (13,885-15,014)	16,150 (15,559-16,778)	17,707 (16,960-18,476)	17,201 (16,520-17,817)	15,254 (14,629-15,841)	12,286 (11,847-12,656)	9,050 (8,719-9,349)	4,952 (4,787-5,107)	2,254 (2,185-2,321)	646 (630-663)
Nasopharynx cancer	4,804 (4,605-4,984)	6,923 (6,676-7,170)	7,430 (7,158-7,674)	6,303 (6,093-6,496)	6,218 (5,992-6,403)	5,641 (5,447-5,813)	3,772 (3,645-3,890)	2,492 (2,418-2,567)	1,087 (1,053-1,121)	401 (390-413)	90 (88-93)
Other pharynx cancer	6,607 (5,742-7,001)	9,726 (8,506-10,200)	10,796 (9,611-11,373)	11,556 (10,192-12,264)	11,040 (9,751-11,654)	9,166 (8,026-9,722)	5,779 (4,950-6,125)	3,768 (3,205-4,009)	1,580 (1,361-1,676)	503 (450-526)	110 (103-114)
Esophageal cancer	16,894 (16,447-17,386)	33,673 (32,907-34,559)	42,974 (41,974-43,952)	48,970 (47,911-50,144)	58,274 (57,069-59,631)	64,453 (63,049-66,007)	54,834 (53,545-56,056)	37,474 (36,660-38,268)	17,386 (17,000-17,836)	5,614 (5,479-5,768)	1,149 (1,121-1,181)
Stomach cancer	33,952 (33,252-34,833)	57,636 (56,406-59,075)	71,927 (70,528-73,673)	85,258 (83,722-87,173)	112,715 (110,805-114,983)	127,148 (125,132-129,384)	118,212 (116,541-120,139)	86,193 (84,926-87,836)	43,679 (43,020-44,503)	17,424 (17,199-17,680)	4,701 (4,635-4,769)
Colon and rectum cancer	24,281 (23,643-25,121)	38,714 (37,656-40,194)	51,270 (49,936-53,062)	62,833 (61,582-64,759)	85,105 (83,552-87,250)	99,034 (97,404-101,270)	105,183 (103,536-107,352)	91,046 (89,606-92,931)	50,956 (50,297-51,864)	24,348 (24,005-24,731)	7,399 (7,280-7,513)
Liver cancer	40,616 (38,653-43,301)	65,596 (63,030-68,550)	71,628 (68,732-75,091)	71,802 (69,420-75,903)	81,697 (79,128-85,568)	85,530 (83,059-88,638)	71,473 (69,633-73,880)	47,042 (45,920-48,496)	21,390 (20,859-22,022)	7,038 (6,867-7,220)	1,609 (1,571-1,644)
Liver cancer due to hepatitis B	24,248 (22,011-26,411)	36,865 (33,407-39,758)	35,171 (31,801-38,660)	30,452 (27,297-33,989)	31,306 (28,431-34,453)	29,503 (26,402-32,674)	20,180 (18,374-22,084)	10,738 (9,583-12,010)	4,150 (3,757-4,562)	1,034 (909-1,166)	175 (142-212)
Liver cancer due to hepatitis C	5,732 (4,660-6,920)	11,892 (10,175-13,776)	17,508 (15,306-19,880)	20,752 (18,209-23,426)	24,372 (22,308-26,923)	27,532 (25,303-29,942)	26,434 (24,128-28,695)	19,947 (18,276-21,546)	10,224 (9,595-10,798)	3,795 (3,551-4,032)	961 (890-1,028)
Liver cancer due to alcohol use	5,401 (4,005-7,374)	8,146 (6,029-11,221)	9,003 (6,703-11,808)	10,425 (8,174-13,316)	14,654 (12,374-17,190)	16,019 (13,722-19,030)	12,857 (11,340-14,684)	7,725 (6,632-8,889)	3,003 (2,608-3,435)	913 (775-1,062)	180 (142-219)
Liver cancer due to NASH	1,756 (1,379-2,182)	3,164 (2,530-3,917)	4,116 (3,249-5,099)	4,788 (3,910-5,846)	5,876 (4,714-7,157)	6,910 (5,603-8,539)	7,045 (5,823-8,534)	5,260 (4,317-6,331)	2,494 (2,147-2,898)	835 (693-1,001)	194 (150-242)
Liver cancer due to other causes	3,479 (2,853-4,261)	5,528 (4,492-6,848)	5,830 (4,638-6,914)	5,385 (4,375-6,467)	5,488 (4,520-6,642)	5,566 (4,515-6,737)	4,957 (4,145-5,946)	3,372 (2,775-4,052)	1,519 (1,295-1,761)	461 (384-546)	98 (75-123)
Gallbladder and biliary tract cancer	4,204 (3,672-4,609)	7,369 (6,472-8,013)	9,908 (8,830-10,912)	13,660 (12,204-15,061)	17,852 (16,236-19,498)	20,862 (18,810-22,356)	21,368 (19,638-22,917)	17,596 (16,169-18,747)	10,147 (9,475-10,681)	4,717 (4,435-4,909)	1,403 (1,344-1,442)
Pancreatic cancer	10,364 (10,142-10,571)	18,196 (17,751-18,602)	25,444 (24,888-25,995)	31,849 (31,323-32,393)	41,820 (41,159-42,526)	46,483 (45,769-47,244)	45,611 (44,977-46,275)	35,633 (35,152-36,220)	19,042 (18,808-19,261)	8,529 (8,414-8,643)	2,210 (2,176-2,242)
Larynx cancer	6,745 (6,484-7,025)	11,296 (10,945-11,709)	13,359 (12,903-13,798)	14,623 (14,103-15,136)	15,970 (15,403-16,531)	14,007 (13,525-14,459)	10,121 (9,809-10,401)	6,502 (6,291-6,691)	2,650 (2,576-2,727)	847 (826-869)	174 (170-179)
Tracheal, bronchus, and lung cancer	54,418 (53,401-55,461)	102,576 (100,792-104,527)	137,829 (135,666-140,172)	166,423 (164,066-169,075)	215,960 (213,345-219,441)	236,645 (233,685-239,976)	209,560 (207,030-212,333)	138,300 (136,586-140,298)	61,116 (60,408-61,899)	20,777 (20,531-21,038)	4,861 (4,795-4,930)
Malignant skin melanoma	3,219 (2,587-4,044)	4,059 (3,280-5,054)	4,556 (3,564-5,448)	4,289 (3,292-4,940)	5,057 (3,848-5,830)	5,195 (4,002-5,982)	5,457 (4,036-6,278)	4,689 (3,500-5,395)	2,630 (1,975-3,043)	1,294 (953-1,498)	414 (302-506)
Non-melanoma skin cancer	1,503 (1,447-1,573)	2,315 (2,227-2,430)	2,778 (2,686-2,906)	3,973 (3,801-4,155)	5,143 (4,945-5,351)	5,901 (5,696-6,113)	6,019 (5,818-6,220)	5,682 (5,516-5,840)	4,082 (3,941-4,182)	2,679 (2,621-2,735)	1,217 (1,194-1,240)
Non-melanoma skin cancer (squamous-cell carcinoma)	1,503 (1,447-1,573)	2,315 (2,227-2,430)	2,778 (2,686-2,906)	3,973 (3,801-4,155)	5,143 (4,945-5,351)	5,901 (5,696-6,113)	6,019 (5,818-6,220)	5,682 (5,516-5,840)	4,082 (3,941-4,182)	2,679 (2,621-2,735)	1,217 (1,194-1,240)
Breast cancer	41,752 (40,045-44,183)	53,284 (51,217-56,131)	53,735 (51,948-56,430)	48,294 (46,925-51,381)	49,321 (48,110-51,890)	44,977 (43,906-47,814)	41,410 (40,618-43,341)	35,737 (35,043-36,971)	20,309 (19,938-21,208)	10,747 (10,541-11,014)	3,723 (3,643-3,819)
Cervical cancer	22,731 (21,222-24,057)	26,119 (24,138-27,590)	24,449 (22,921-25,673)	21,568 (20,206-23,119)	21,460 (20,248-22,613)	19,498 (18,430-21,021)	15,607 (14,929-16,587)	11,615 (11,136-12,124)	5,888 (5,649-6,132)	2,377 (2,304-2,448)	683 (662-702)
Uterine cancer	3,391 (3,240-3,538)	5,570 (5,266-5,849)	6,936 (6,591-7,237)	8,407 (8,089-8,751)	10,281 (9,947-10,634)	9,722 (9,431-9,999)	8,918 (8,715-9,128)	7,065 (6,893-7,220)	3,463 (3,393-3,535)	1,597 (1,564-1,630)	449 (437-461)
Ovarian cancer	9,027 (8,691-9,591)	13,340 (12,853-14,131)	14,444 (13,970-15,201)	14,978 (14,530-15,707)	16,816 (16,384-17,482)	15,942 (15,586-16,520)	14,201 (13,902-14,578)	10,943 (10,702-11,206)	5,574 (5,466-5,693)	2,558 (2,502-2,612)	654 (639-671)
Prostate cancer	1,547 (1,266-1,818)	4,160 (3,363-4,800)	9,605 (7,661-10,657)	18,891 (15,112-21,509)	34,259 (27,379-39,118)	50,023 (41,572-57,262)	63,909 (52,394-75,972)	61,071 (48,793-69,451)	35,117 (27,387-39,922)	16,069 (12,255-17,686)	4,008 (2,772-4,266)
Testicular cancer	466 (447-488)	349 (335-365)	261 (251-273)	245 (234-256)	258 (247-269)	265 (254-276)	255 (246-265)	209 (201-219)	107 (102-111)	43 (41-44)	14 (14-15)
Kidney cancer	4,356 (4,156-4,478)	7,255 (6,877-7,452)	9,681 (9,037-9,955)	10,642 (10,049-10,875)	13,497 (12,651-13,794)	14,347 (13,132-14,657)	14,350 (12,988-14,651)	11,248 (9,803-11,517)	5,627 (4,843-5,769)	2,452 (2,089-2,526)	606 (483-632)
Bladder cancer	2,946 (2,759-3,071)	5,390 (5,151-5,599)	8,096 (7,769-8,428)	11,814 (11,329-12,284)	17,942 (17,342-18,540)	23,117 (22,523-24,042)	27,129 (26,316-28,266)	25,188 (24,603-26,020)	14,778 (14,468-15,234)	7,093 (6,977-7,239)	2,014 (1,981-2,057)
Brain and nervous system cancer	12,200 (10,775-13,230)	15,644 (13,600-17,200)	17,229 (15,232-18,625)	16,812 (14,764-18,287)	18,031 (15,701-19,601)	16,305 (13,705-17,319)	13,299 (10,672-14,121)	8,060 (6,125-8,600)	3,403 (2,477-3,658)	1,216 (840-1,315)	259 (164-282)

SR Table 7: Global deaths by cause and age group for both sexes combined (with 95% confidence intervals), 2005

Cause	Early Neonatal	Late Neonatal	Post Neonatal	1 to 4	5 to 9	10 to 14	15 to 19	20 to 24	25 to 29	30 to 34	35 to 39
Thyroid cancer	-	-	-	-	-	189 (161-216)	409 (345-480)	502 (433-569)	854 (735-969)	558 (504-614)	725 (658-801)
Mesothelioma	-	-	-	-	-	-	93 (74-117)	126 (100-162)	181 (148-225)	240 (200-298)	419 (351-514)
Hodgkin lymphoma	12 (8-16)	3 (2-4)	81 (57-105)	728 (421-1,034)	1,282 (904-1,627)	1,405 (1,044-1,710)	1,986 (1,563-2,406)	2,203 (1,790-2,648)	2,372 (1,959-2,804)	2,126 (1,768-2,580)	1,996 (1,699-2,400)
Non-Hodgkin lymphoma	68 (61-78)	56 (49-64)	734 (606-867)	4,244 (3,239-5,399)	3,314 (2,917-3,788)	2,590 (2,374-2,849)	2,942 (2,755-3,122)	3,505 (3,306-3,701)	3,578 (3,396-3,765)	4,051 (3,876-4,250)	5,313 (5,064-5,580)
Multiple myeloma	-	-	-	-	-	-	204 (187-236)	219 (202-255)	248 (231-284)	445 (410-504)	842 (786-967)
Leukemia	330 (255-387)	290 (231-340)	4,208 (3,266-5,200)	14,808 (11,641-20,006)	14,435 (12,234-17,041)	12,453 (10,818-13,808)	12,960 (11,094-13,921)	9,968 (8,519-10,693)	8,790 (7,551-9,417)	9,321 (7,961-9,903)	10,975 (9,285-11,819)
Acute lymphoid leukemia	70 (55-89)	58 (46-78)	971 (792-1,345)	4,510 (3,479-6,655)	5,867 (4,910-7,523)	4,516 (3,963-5,341)	3,718 (3,310-4,248)	2,468 (2,210-2,801)	1,979 (1,745-2,271)	1,623 (1,444-1,823)	1,816 (1,575-2,049)
Chronic lymphoid leukemia	-	-	-	-	-	-	176 (142-202)	149 (124-171)	139 (119-156)	184 (154-204)	243 (204-280)
Acute myeloid leukemia	63 (48-83)	58 (45-81)	673 (518-1,010)	2,938 (2,035-5,008)	1,915 (1,504-2,661)	2,122 (1,799-2,551)	2,475 (2,170-2,867)	2,279 (1,986-2,552)	2,194 (1,952-2,478)	2,358 (2,063-2,670)	2,675 (2,363-3,044)
Chronic myeloid leukemia	-	-	-	-	-	-	433 (361-510)	590 (500-679)	814 (690-926)	946 (799-1,058)	1,094 (942-1,213)
Other leukemia	197 (144-236)	175 (132-208)	2,564 (1,829-3,277)	7,360 (5,027-10,507)	6,653 (5,132-7,938)	5,815 (4,634-6,428)	6,160 (4,828-6,796)	4,482 (3,522-4,849)	3,664 (2,893-3,976)	4,210 (3,308-4,598)	5,148 (4,008-5,750)
Other malignant neoplasms	919 (736-1,039)	496 (412-586)	2,858 (2,326-3,609)	10,427 (7,839-14,665)	5,242 (4,589-6,039)	6,489 (5,907-7,097)	8,338 (7,801-8,974)	6,656 (6,269-7,134)	5,701 (5,399-6,110)	5,990 (5,697-6,534)	7,801 (7,427-8,399)
Other neoplasms	61 (45-73)	37 (28-44)	473 (340-586)	1,222 (833-1,622)	992 (772-1,193)	800 (643-954)	807 (637-944)	793 (629-948)	895 (706-1,060)	1,241 (972-1,488)	1,634 (1,300-1,924)
Myelodysplastic, myeloproliferative, and other hematopoietic neoplasms	59 (43-71)	31 (24-39)	408 (293-521)	969 (660-1,364)	663 (528-824)	519 (409-634)	531 (421-639)	544 (437-659)	657 (521-809)	958 (763-1,166)	1,364 (1,092-1,627)
Other benign and in situ neoplasms	2 (1-3)	6 (4-8)	65 (44-83)	253 (165-364)	328 (239-392)	282 (219-334)	276 (207-328)	249 (187-301)	238 (169-290)	282 (199-346)	270 (190-332)
Cardiovascular diseases	3,851 (3,539-4,176)	2,597 (2,420-2,774)	21,987 (20,719-23,297)	17,638 (15,837-19,254)	9,135 (8,363-9,794)	11,378 (10,621-12,090)	34,786 (33,436-36,403)	51,737 (50,170-53,737)	72,696 (70,816-75,057)	118,348 (115,786-121,915)	193,311 (189,434-197,501)
Rheumatic heart disease	-	-	-	4,609 (3,869-5,648)	2,831 (2,535-3,149)	4,260 (3,881-4,641)	6,986 (6,408-7,711)	7,658 (6,909-8,480)	7,810 (6,977-8,619)	9,115 (8,184-10,086)	11,427 (10,362-12,599)
Ischemic heart disease	-	-	-	-	-	-	11,039 (10,494-11,661)	19,450 (18,662-20,486)	30,242 (29,162-31,704)	55,752 (54,102-58,336)	93,925 (91,511-97,233)
Stroke	1,378 (1,170-1,578)	877 (773-999)	6,731 (5,784-8,372)	4,754 (3,928-5,810)	3,080 (2,811-3,362)	3,687 (3,411-3,950)	9,183 (8,785-9,595)	13,690 (13,184-14,229)	18,299 (17,695-18,986)	31,051 (30,087-32,220)	56,262 (54,742-58,097)
Ischemic stroke	209 (157-270)	146 (114-188)	725 (544-1,021)	608 (421-941)	337 (292-391)	352 (306-404)	1,334 (1,177-1,517)	2,043 (1,794-2,331)	2,781 (2,500-3,097)	4,577 (4,176-5,073)	7,862 (7,290-8,546)
Intracerebral hemorrhage	702 (537-898)	410 (327-532)	3,573 (2,829-5,069)	1,960 (1,407-2,925)	1,612 (1,397-1,881)	1,941 (1,735-2,165)	5,555 (5,180-5,934)	8,240 (7,738-8,794)	10,991 (10,323-11,692)	19,563 (18,448-20,577)	36,789 (35,088-38,561)
Subarachnoid hemorrhage	467 (375-548)	320 (261-368)	2,433 (1,954-2,854)	2,186 (1,724-2,634)	1,132 (972-1,303)	1,395 (1,216-1,574)	2,294 (1,992-2,639)	3,406 (2,922-3,976)	4,527 (4,035-5,212)	6,911 (6,190-7,999)	11,610 (10,585-13,221)
Hypertensive heart disease	-	-	-	-	-	-	761 (562-902)	1,140 (865-1,356)	1,860 (1,429-2,181)	3,297 (2,597-3,817)	5,951 (4,728-6,802)
Non-rheumatic valvular heart disease	-	-	-	-	-	-	743 (628-900)	830 (709-1,000)	932 (812-1,107)	929 (832-1,082)	1,307 (1,187-1,516)
Non-rheumatic calcific aortic valve disease	-	-	-	-	-	-	228 (174-313)	251 (195-350)	313 (251-419)	361 (303-465)	572 (492-720)
Non-rheumatic degenerative mitral valve disease	-	-	-	-	-	-	374 (295-498)	431 (343-565)	458 (370-589)	455 (380-558)	593 (499-706)
Other non-rheumatic valve diseases	-	-	-	-	-	-	140 (105-189)	147 (109-199)	161 (121-215)	113 (84-159)	141 (99-188)
Cardiomyopathy and myocarditis	1,431 (1,224-1,590)	906 (799-994)	7,541 (6,671-8,343)	3,860 (3,115-4,398)	1,574 (1,384-1,772)	1,693 (1,538-1,873)	3,034 (2,737-3,422)	4,326 (3,788-4,847)	7,148 (5,933-7,815)	10,356 (8,274-11,203)	14,324 (11,412-15,400)
Myocarditis	169 (113-213)	184 (138-220)	2,092 (1,617-2,449)	1,231 (1,002-1,455)	548 (462-646)	513 (425-593)	1,022 (834-1,194)	1,130 (907-1,331)	1,266 (1,020-1,478)	1,151 (930-1,350)	1,460 (1,151-1,713)
Alcoholic cardiomyopathy	-	-	-	-	-	-	294 (241-343)	996 (780-1,115)	2,929 (2,147-3,242)	5,632 (4,030-6,055)	8,411 (5,944-8,984)
Other cardiomyopathy	1,262 (1,066-1,404)	722 (616-793)	5,449 (4,688-6,043)	2,628 (2,069-3,096)	1,026 (878-1,157)	1,180 (1,054-1,300)	1,718 (1,488-1,939)	2,200 (1,838-2,508)	2,954 (2,402-3,388)	3,572 (2,959-4,037)	4,453 (3,772-5,047)
Atrial fibrillation and flutter	-	-	-	-	-	-	-	-	-	71 (65-75)	100 (92-107)
Aortic aneurysm	-	-	-	-	-	-	277 (246-320)	428 (385-489)	664 (600-753)	983 (879-1,118)	1,625 (1,475-1,834)
Peripheral artery disease	-	-	-	-	-	-	-	-	-	-	-
Endocarditis	251 (206-312)	208 (167-260)	2,714 (2,232-3,381)	1,740 (1,307-2,277)	434 (374-528)	550 (487-652)	777 (702-922)	1,185 (1,073-1,390)	1,627 (1,481-1,861)	1,668 (1,534-1,876)	1,954 (1,794-2,183)
Other cardiovascular and circulatory diseases	791 (674-997)	606 (513-751)	5,001 (3,899-6,530)	2,676 (1,779-4,126)	1,216 (1,011-1,584)	1,188 (1,049-1,437)	1,986 (1,716-2,400)	3,030 (2,602-3,616)	4,113 (3,596-4,933)	5,126 (4,535-6,043)	6,436 (5,777-7,561)
Chronic respiratory diseases	0 (0-0)	0 (0-0)	0 (0-0)	17,675 (15,029-21,387)	4,991 (4,388-6,151)	4,840 (4,338-5,724)	8,557 (8,030-9,652)	11,301 (10,726-12,401)	13,745 (13,138-14,850)	18,312 (17,466-19,398)	27,328 (26,201-29,149)
Chronic obstructive pulmonary disease	-	-	0 (0-0)	1,877 (1,353-2,876)	596 (466-928)	464 (373-718)	2,547 (2,140-3,398)	3,510 (2,877-4,731)	4,598 (3,893-6,148)	7,136 (6,169-9,211)	11,440 (9,812-14,499)
Pneumoconiosis	-	-	-	-	-	-	35 (31-40)	45 (39-50)	79 (71-89)	175 (159-193)	422 (386-464)
Silicosis	-	-	-	-	-	-	8 (6-11)	12 (10-16)	28 (23-35)	94 (80-111)	268 (234-312)
Asbestosis	-	-	-	-	-	-	4 (2-6)	6 (4-8)	9 (5-12)	14 (7-19)	22 (12-29)
Coal workers pneumoconiosis	-	-	-	-	-	-	5 (3-7)	5 (4-8)	11 (8-14)	17 (13-23)	45 (31-57)
Other pneumoconiosis	-	-	-	-	-	-	18 (14-22)	21 (16-27)	32 (25-40)	49 (39-60)	87 (70-109)
Asthma	-	-	-	12,869 (10,456-16,322)	3,416 (2,845-4,509)	3,611 (3,103-4,478)	4,792 (3,708-5,983)	6,180 (4,458-7,501)	7,382 (5,253-8,785)	8,778 (6,015-10,497)	12,434 (8,220-15,158)
Interstitial lung disease and pulmonary sarcoidosis	-	-	-	377 (241-653)	148 (101-238)	118 (89-168)	207 (155-294)	302 (227-429)	441 (335-610)	639 (484-875)	1,030 (789-1,389)
Other chronic respiratory diseases	0 (0-0)	0 (0-0)	0 (0-0)	2,551 (1,443-3,785)	831 (628-1,079)	646 (502-804)	975 (757-1,239)	1,265 (956-1,639)	1,245 (927-1,586)	1,584 (1,205-1,981)	2,002 (1,501-2,540)
Digestive diseases	2,535 (2,274-2,796)	2,557 (2,336-2,915)	20,636 (18,298-25,416)	23,256 (18,704-30,895)	13,163 (11,340-15,323)	11,539 (10,302-12,956)	20,758 (19,140-22,481)	29,011 (26,480-31,185)	40,231 (37,756-42,919)	55,890 (53,036-59,821)	83,611 (79,508-90,288)
Cirrhosis and other chronic liver diseases	-	-	-	9,656 (7,382-14,876)	5,176 (4,266-7,032)	5,124 (4,489-6,260)	9,408 (8,534-10,715)	15,503 (13,854-16,957)	23,777 (21,950-25,991)	37,520 (35,292-40,878)	58,855 (55,645-64,428)
Cirrhosis and other chronic liver diseases due to hepatitis B	-	-	-	303 (205-489)	400 (307-549)	675 (530-875)	1,742 (1,365-2,238)	5,906 (4,838-7,065)	11,078 (9,199-13,172)	16,530 (14,491-19,061)	22,837 (19,482-26,691)
Cirrhosis and other chronic liver diseases due to hepatitis C	-	-	-	99 (64-164)	139 (99-202)	254 (189-342)	626 (465-838)	2,599 (2,010-3,254)	4,897 (3,742-6,150)	8,727 (7,276-10,304)	14,894 (11,875-18,302)
Cirrhosis and other chronic liver diseases due to alcohol use	-	-	-	-	-	-	59 (27-104)	1,153 (882-1,489)	2,945 (2,303-3,675)	7,020 (6,122-8,156)	14,443 (12,037-17,137)
Cirrhosis due to NASH	-	-	-	-	-	-	162 (124-211)	490 (391-609)	935 (729-1,179)	1,821 (1,536-2,115)	3,312 (2,622-4,061)
Cirrhosis and other chronic liver diseases due to other causes	-	-	-	9,255 (7,092-14,291)	4,637 (3,817-6,341)	4,196 (3,645-5,222)	6,819 (5,937-7,876)	5,355 (4,230-6,571)	3,923 (2,958-5,045)	3,422 (2,529-4,445)	3,370 (2,522-4,346)
Upper digestive system diseases	-	-	-	2,035 (1,496-2,886)	1,424 (1,187-1,693)	1,268 (1,109-1,476)	2,528 (2,243-2,959)	4,565 (4,072-5,089)	5,179 (4,712-5,807)	6,400 (5,838-7,029)	8,824 (8,190-9,939)
Peptic ulcer disease	-	-	-	1,433 (981-2,475)	1,054 (859-1,324)	1,006 (864-1,187)	2,057 (1,814-2,425)	3,725 (3,301-4,321)	4,054 (3,620-4,658)	4,920 (4,482-5,553)	7,105 (6,549-8,070)
Gastritis and duodenitis	-	-	-	602 (359-833)	370 (246-479)	261 (192-335)	471 (362-600)	840 (581-1,040)	1,124 (818-1,359)	1,480 (1,121-1,797)	1,719 (1,330-2,030)
Appendicitis	-	-	-	2,689 (1,496-3,852)	1,986 (1,639-2,515)	1,880 (1,635-2,257)	3,106 (2,632-3,764)	2,240 (1,950-2,565)	2,444 (2,167-2,847)	1,450 (1,284-1,651)	1,830 (1,630-2,096)
Paralytic ileus and intestinal obstruction	2,535 (2,274-2,796)	2,557 (2,336-2,915)	20,636 (18,298-25,416)	3,447 (2,500-4,437)	2,891 (1,881-3,543)	1,956 (1,302-2,337)	3,673 (2,584-4,275)	3,426 (2,356-4,013)	3,621 (2,593-4,160)	3,567 (2,530-4,128)	4,619 (3,367-5,276)
Inguinal, femoral, and abdominal hernia	-	-	-	633 (442-928)	312 (252-403)	217 (172-259)	357 (305-442)	371 (311-446)	425 (370-520)	508 (423-598)	698 (594-828)
Inflammatory bowel disease	-	-	-	1,155 (621-2,185)	216 (151-326)	145 (113-190)	222 (178-293)	365 (297-463)	476 (397-590)	567 (488-678)	695 (612-809)
Vascular intestinal disorders	-	-	-	410 (156-548)	84 (52-105)	48 (38-54)	83 (69-94)	174 (141-201)	245 (201-280)	277 (239-306)	483 (424-543)
Gallbladder and biliary diseases	-	-	-	850 (557-1,189)	313 (248-381)	286 (230-341)	338 (298-380)	550 (478-622)	707 (626-778)	953 (865-1,040)	1,385 (1,253-1,518)
Pancreatitis	-	-	-	129 (84-214)	152 (121-204)	186 (154-227)	526 (464-635)	1,278 (1,132-1,477)	2,553 (2,273-2,900)	3,614 (3,328-4,042)	4,896 (4,495-5,473)
Other digestive diseases	-	-	-	2,252 (1,580-3,320)	609 (499-766)	427 (369-518)	516 (407-723)	539 (468-666)	806 (684-1,025)	1,034 (891-1,266)	1,326 (1,167-1,598)
Neurological disorders	-	-	9,551 (7,599-10,981)	13,654 (8,707-17,351)	6,354 (5,375-7,333)	7,866 (6,886-8,673)	14,164 (12,372-15,456)	12,245 (10,743-13,548)	10,681 (9,654-11,664)	10,745 (9,632-11,688)	11,631 (10,422-12,808)
Alzheimer's disease and other dementias	-	-	-	-	-	-	-	-	-	-	-
Parkinson's disease	-	-	-	-	-	-	-	13 (10-14)	17 (15-18)	37 (35-40)	54 (48-59)
Epilepsy	-	-	6,406 (5,142-7,342)	10,753 (7,084-14,141)	5,226 (4,397-6,099)	6,280 (5,345-6,980)	11,524 (9,807-12,823)	10,270 (8,843-11,627)	9,134 (8,111-10,169)	8,928 (7,778-9,922)	8,987 (7,760-10,212)
Multiple sclerosis	-	-	-	-	-	-	-	155 (129-212)	286 (246-374)	541 (478-679)	944 (841-1,154)
Motor neuron disease	13 (5-19)	21 (13-26)	489 (387-605)	209 (122-284)	40 (36-44)	56 (52-67)	100 (92-119)	80 (76-93)	76 (72-88)	141 (134-161)	310 (295-362)
Other neurological disorders	-	-	2,656 (1,719-3,560)	2,692 (1,245-3,840)	1,088 (735-1,464)	1,531 (1,241-1,911)	2,540 (2,313-3,027)	1,727 (1,633-2,026)	1,169 (1,107-1,386)	1,099 (1,038-1,282)	1,337 (1,266-1,550)
Mental disorders	-	-	-	-	0 (0-1)	4 (3-4)	21 (19-25)	35 (31-43)	38 (34-44)	42 (38-49)	44 (40-50)
Eating disorders	-	-	-	-	0 (0-1)	4 (3-4)	21 (19-25)	35 (31-43)	38 (34-44)	42 (38-49)	44 (40-50)
Anorexia nervosa	-	-	-	-	0 (0-0)	3 (3-4)	18 (16-22)	24 (18-28)	24 (17-29)	30 (23-35)	34 (26-40)
Bulimia nervosa	-	-	-	-	0 (0-0)	0 (0-0)	3 (2-4)	11 (8-19)	14 (11-23)	12 (9-20)	10 (7-18)
Substance use disorders	0 (0-0)	0 (0-0)	0 (0-0)	4 (1-12)	14 (5-26)	18 (8-31)	5,122 (4,897-5,315)	12,173 (11,819-12,482)	18,524 (17,814-19,022)	24,590 (23,621-25,199)	29,533 (27,747-30,275)
Alcohol use disorders	-	-	-	-	-	-	1,281 (1,063-1,375)	3,413 (2,959-3,573)	7,260 (6,466-7,586)	12,541 (11,434-13,026)	17,779 (15,847-18,416)
Drug use disorders	0 (0-0)	0 (0-0)	0 (0-0)	4 (1-12)	14 (5-26)	18 (8-31)	3,841 (3,702-3,991)	8,760 (8,514-9,041)	11,264 (10,939-11,616)	12,049 (11,741-12,371)	11,754 (11,475-12,087)
Opioid use disorders	0 (0-0)	0 (0-0)	0 (0-0)	4 (1-12)	14 (5-26)	18 (8-31)	2,559 (2,321-2,762)	5,889 (5,504-6,199)	7,484 (7,063-7,807)	7,501 (7,046-7,874)	7,189 (6,728-7,604)
Cocaine use disorders	-	-	-	-	-	-	105 (90-125)	298 (267-342)	422 (382-479)	656 (597-737)	625 (566-703)
Amphetamine use disorders	-	-	-	-	-	-	155 (113-213)	329 (271-397)	433 (368-497)	485 (402-572)	465 (376-561)
Other drug use disorders	-	-	-	-	-	-	1,022 (854-1,264)	2,244 (2,042-2,605)	2,925 (2,706-3,280)	3,407 (3,154-3,828)	3,475 (3,193-3,913)
Diabetes and kidney diseases	31 (24-36)	91 (72-106)	9,153 (8,464-9,855)	11,083 (9,723-12,375)	5,850 (5,166-6,417)	6,715 (6,069-7,256)	13,481 (12,717-14,253)	18,548 (17,726-19,493)	21,327 (20,511-22,241)	28,227 (27,344-29,628)	39,855 (38,652-41,293)
Diabetes mellitus	31 (24-36)	91 (72-106)	1,102 (862-1,288)	772 (616-933)	856 (700-1,008)	1,341 (1,002-1,608)	3,088 (2,936-3,242)	4,561 (4,382-4,754)	6,053 (5,828-6,294)	9,067 (8,777-9,397)	14,008 (13,600-14,466)
Diabetes mellitus type 1	31 (24-36)	91 (72-106)	1,102 (862-1,288)	772 (616-933)	856 (700-1,008)	1,341 (1,002-1,608)	2,691 (2,514-2,864)	3,913 (3,704-4,134)	5,185 (4,927-5,458)	6,845 (6,512-7,234)	10,190 (9,757-10,674)
Diabetes mellitus type 2	-	-	-	-	-	-	396 (309-537)	649 (535-803)	868 (725-1,073)	2,222 (1,981-2,559)	3,818 (3,429-4,316)
Chronic kidney disease	-	-	7,793 (7,183-8,425)	9,996 (8,737-11,226)	4,761 (4,185-5,224)	5,194 (4,740-5,617)	10,146 (9,486-10,812)	13,740 (13,064-14,602)	15,019 (14,284-15,905)	18,795 (18,008-20,088)	25,350 (24,378-26,636)
Chronic kidney disease due to diabetes mellitus type 1	-	-	29 (16-48)	43 (22-78)	30 (15-54)	53 (29-89)	192 (100-314)	614 (326-999)	1,413 (860-2,152)	3,138 (2,070-4,452)	5,638 (3,747-7,892)
Chronic kidney disease due to diabetes mellitus type 2	-	-	21 (11-38)	28 (13-55)	18 (8-34)	27 (12-50)	87 (39-162)	206 (87-394)	370 (162-703)	781 (365-1,419)	1,710 (896-2,866)
Chronic kidney disease due to hypertension	-	-	38 (22-59)	74 (41-118)	57 (32-94)	108 (61-175)	389 (232-597)	887 (574-1,280)	1,334 (921-1,861)	1,994 (1,400-2,754)	3,049 (2,157-4,087)
[truncated: 2,110,835 more chars]
