# Supplementary material for: Global, regional, and national age-sex-specific mortality for 282 causes of death in 195 countries and territories, 1980–2017: a systematic analysis for the Global Burden of Disease Study 2017
Source: Lancet. 2018 Nov 10;392(10159):1736–88. doi: 10.1016/S0140-6736(18)32203-7 (PMC6227606; doi:10.1016/S0140-6736(18)32203-7)
Supplement: Supplementary appendix 1 [file mmc1.pdf]

THE LANCET

Supplementary appendix 1

This appendix formed part of the original submission and has been peer reviewed. We post it as supplied by the authors.

Supplement to: GBD 2017 Causes of Death Collaborators. Global, regional, and national age-sex-specific mortality for 282 causes of death in 195 countries and territories, 1980–2017: a systematic analysis for the Global Burden of Disease Study 2017. *Lancet* 2018; **392:** 1736–88.

Appendix to “Global, regional, and national age-sex specific mortality for 282 causes of death, 1980-2017: a systematic analysis for the Global Burden of Disease Study 2017”

This appendix provides further methodological detail, supplemental figures, and more detailed results for “Global, regional, and national age-sex specific mortality for 282 causes of death, 1980-2017: a systematic analysis for the Global Burden of Disease Study 2017.” This appendix is organized into sections that follow the structure of the main paper.

Preamble

This appendix provides further methodological detail and more detailed results for "Global, regional, and national age-sex specific mortality for 282 causes of death, 1980-2017: a systematic analysis for the Global Burden of Disease Study 2017." This study complies with the Guidelines for Accurate and Transparent Health Estimates Reporting (GATHER) recommendations. It includes detailed tables and information on data in an effort to maximize transparency in our estimation processes and provide a comprehensive description of analytical steps. We intend this appendix to be a living document, to be updated with each iteration of the Global Burden of Disease Study.

Table of Contents

Authors' Contributions i

Section 1: GBD Overview 7
Section 1.1: Locations of the Analysis 7
Section 1.2: Time Period of the Analysis 7
Section 1.3: Statement of GATHER Compliance 7
Section 1.4: List of abbreviations 7
Section 1.5: GBD results overview 12
Section 1.6: Data input sources overview 13
Section 1.7: Funding Sources 13

Section 2: GBD 2017 causes of death database 13
Section 2.1: Background 13
Section 2.2: CoD data identification 13
Section 2.3: Step 1 – Standardise input data 17
Section 2.4: Step 2 - Map to GBD cause list 20
Section 2.5: Step 3 – Age-sex splitting 20
Section 2.6: Step 4-- Correction for miscoding of Alzheimer's and other dementias, Parkinson's disease, and atrial fibrillation and flutter 22
Section 2.7: Step 5 - Redistribution 24
Section 2.8: Step 6 - HIV/AIDS misclassification correction 29
Section 2.9: Step 7 - Scale strata to province 29
Section 2.10: Step 8 - Restrictions post-redistribution 30
Section 2.11: Step 9 - Drop VR country years or mark as non-representative 30
Section 2.12: Step 10 - Cause aggregation 30
Section 2.13: Step 11 – Remove shocks and HIV/AIDS maternal adjustments 31
Section 2.14: Step 12 – Noise Reduction 35
Section 2.15: Step 13 – CoD database and outlier identification 36
Section 2.16: Causes of death data star rating calculation 36

Section 3: Causes of death modeling methods 38
Section 3.1: CODEm 38
Section 3.2: Causes modeled outside of CODEm 41
Section 3.3: CoD modeling descriptions 46

Section 4:Central computation 359
Section 4.1:Imported cases 359

Section 4.2:CodCorrect 359
Section 4.3:Years of life lost (YLLs) calculation 360
Section 5:SDI Analysis 361
Section 5.1:SDI definition 361
Section 5.2:Development of revised SDI indicator 361
Section 6:References 363
Section 7:Tables and Figures........ 366

List of Appendix Tables and Figures

Appendix Figures

Appendix Figure 1. Analytical flowchart for the development of the GBD 2017 causes of death database……366

Appendix Figure 2. GBD 2017 causes of death estimation flowchart……367

Appendix Figure 3. Vital Registration and Verbal Autopsy data availability by country, 1980-2017……368

Appendix Figure 4. Percent of vital registration deaths assigned to major garbage codes for all ages by country, 1980-2017……369

Appendix Figure 5A-B. Classification of national time series of vital registration and verbal autopsy data (A) 1980-2017 and (B) 2010-2017 on the basis of the fraction of deaths well-certified and assigned to a detailed GBD cause……371

Appendix Figure 6A-C. Recoding of Alzheimer's disease and other dementias, Parkinson's disease, and atrial fibrillation and flutter for Sweden and Mexico, 1990 to 2017……373

Appendix Figure 7. Dismod cascade……379

Appendix Tables

Appendix Table 1. GATHER checklist……380

Appendix Table 2. GBD 2017 location hierarchy with levels……383

Appendix Table 3. Total number of site years by cause and source type for 2017……397

Appendix Table 4. List of International Classification of Diseases (ICD) codes mapped to the Global Burden of Disease cause list for causes of death……400

Appendix Table 5. Restrictions on age and sex by cause for GBD 2017……408

Appendix Table 6. HIV/AIDS-related garbage code redistribution packages……413

Appendix Table 7. Data quality rating from 0 to 5 stars, maximum percent well-certified per 5-year interval and percent well-certified across time series for 195 countries, 1980–2017……415

Appendix Table 8. Underlying indicators for percent well-certified calculation, based on maximum percent well certified data source in each 5-year time interval for 195 countries, 1980-2017……418

Appendix Table 9. CODEm covariates used, level of covariate, and expected direction of covariate by cause, sex, and age……438

Appendix Table 10. Comparison of GBD 2016 and GBD 2017 covariates and level of covariates used in cause of death modeling……..551

Appendix Table 11. CODEm predictive validity results by cause, model type, sex, and age…………………583

Appendix Table 12. Modeling strategy for individual cause of death models in GBD 2017…………………..594

Appendix Table 13. Causes included in the 2017 Global Burden of Disease Study…………………………….600

Appendix Table 14. CoDCorrect cause hierarchy with levels…………………………………………………………….609

Appendix Table 15. Percent change after CoDCorrect by cause for all ages, both sexes, 2017……………615

Appendix Table 16. Theoretical minimum risk reference life table……………………………………………………624

Appendix Table 17. GBD world population age standard…………………………………………………………………625

Appendix Table 18. Socio-Demographic Index R-squared values with lags up to 10 years…………………..626

Appendix Table 19. Socio-Demographic Index groupings by location, based on 2017 values………………627

Appendix Table 20. Socio-Demographic Index values for all estimated GBD 2017 locations, 1980–2017……….641

Authors' Contributions

Managing the estimation process

Ashkan Afshin, Tahiya Alam, Molly H Biehl, Brigette F Blacker, Aaron J Cohen, Elizabeth A Cromwell, Lalit Dandona, Rakhi Dandona, Louisa Degenhardt, Samath D Dharmaratne, Daniel Dicker, Valery L Feigin, Christina Fitzmaurice, Nancy Fullman, Simon I Hay, Spencer James, Nicholas J Kassebaum, Hmwe H Kyu, Heidi J Larson, Stephen S Lim, Alan D Lopez, Rafael Lozano, Ashley Marks, Awoke T Misganaw, Ali H Mokdad, Christopher J L Murray, Mohsen Naghavi, Helen E Olsen, D M Pigott, Puja C Rao, Robert C Reiner, Gregory Roth, Joseph S Salama, Katya A Shackelford, Mari Smith, Jeffrey D Stanaway, Caitlyn Steiner, Stein Emil Vollset, Theo Vos, Harvey A Whiteford, Shelley Wilson.

Writing the first draft of the manuscript

Gregory Roth and Laurie B. Marczak

Providing data or critical feedback on data sources

Kalkidan Hassen Abate, Solomon Mequanente Abay, Ibrahim Abdollahpour, Rizwan Suliankatchi Abdulkader, Molla Abebe, Semaw Ferede Abera, Laith Abu-Raddad, Manfred Accrombessi, Oladimeji Adebayo, Rufus A. Adedoyin, Olatunji Adetokunboh, Mina Adib, Alireza Ahmadi, Mehdi Ahmadi, Muktar Ahmed, Ibtihel Aichour, Mohammad Esmaeil Akbari, Nadia Akseer, Fares Alahdab, Khurshid Alam, Kefyalew Addis Alene, Syed Mohamed Aljunid, Francois Alla, Peter Allebeck, Rajaa Al-Raddadi, Nelson Alvis-Guzman, Azmeraw T. Amare, Erfan Amini, Walid Ammar, Yaw Ampem Amoako, Catalina Liliana Andrei, Sofia Androudi, Hossein Ansari, Mustafa Ansha, Carl Abelardo Antonio, Olatunde Aremu, Johan Ärnlöv, Monika Arora, Al Artaman, Krishna K Aryal, Ephrem Tsegay Asfaw, Suleman Atique, Marcel Ausloos, Ashish Awasthi, Beatriz Paulina Ayala, Rakesh Ayer, Peter Azzopardi, Arefeh Babazadeh, Hamid Badali, Alaa Badawi, Maciej Banach, Kajori Banerjee, Joseph Banoub, Miguel A. Barboza, Till Bärnighausen, Lope Barrero, Sanjay Basu, Bernhard Baune, Habtamu Wondifraw Baynes, Ettore Beghi, Masoud Behzadifar, Meysam Behzadifar, Bayu Begashaw Bekele, Yihalem Abebe Belay, Aminu Bello, Derrick Bennett, Isabela Bensenor, Adam Berman, Eduardo Bernabe, Robert Bernstein, Mircea Beuran, Tina Beyranvand, Suraj Bhattarai, Soumyadeep Bhaumik, Burcu Kucuk Bicer, Boris Bikbov, Ver Bilano, Nigus Bililign, Muhammad Shahdaat Bin, Donal Bisanzio, Luisa Brant, Carol Brayne, Alexandra Brazinova, Gabrielle Britton, Traolach Brugha, Julio Cesar Campuzano, Mate Car, Giulia Carreras, Juan J Carrero, Carlos Castañeda-Orjuela, Franz Castro, Ferrán Catalá-López, Jung-Chen Chang, Fiona Charlson, Peggy Pei-Chia Chiang, Abdulaal Chitheer, Rajiv Chowdhury, Devasahayam Christopher, Sheng-Chia Chung, Flavia Cicuttini, Massimo Cirillo, Paolo Angelo Cortesi, Monica Cortinovis, Ewerton Cousin, Benjamin Cowie, Michael Criqui, John Crump, Matthew Cunningham, Alemneh Kabeta Daba, Abel Dadi, Lalit Dandona, Rakhi Dandona, Anh Kim Dang, Ahmad Daryani, Siddharth Das, José Das Neves, Adrian Davis, Kairat Davletov, Fernando De La, Barthelemy Kuate Defo, Louisa Degenhardt, Gebre Teklemariam Demoz, Kebede Deribe, Nikolaos Dervenis, Samath Dharmaratne, Eric L. Ding, M.Ashworth Dirac, Klara Dokova, David Teye Doku, Pratik Doshi, Kerrie Doyle, Manisha Dubey, Eyasu Ejeta Duken, Bruce Duncan, Hedyeh Ebrahimi, Soheil Ebrahimpour, Anne Elise Eggen, Ziad El-Khatib, Hajer Elkout, Christian Lycke Ellingsen, Matthias Endres, Aman Endries, Benjamin Er, Holly Erskine, Babak Eshrati, Sharareh Eskandarieh, Alireza Esteghamati, Mahdi Fakhar, Hamed Fakhim, Mohammad Fareed, Carla Farinha, Andre Faro, Farshad Farzadfar, Mohammad Hosein Farzaei, Valery Feigin, Andrea B. Feigl, Seyed-Mohammad Fereshtehnejad, Joao Fernandes, Irina Filip, Christina Fitzmaurice, Nataliya Foigt, Takeshi Fukumoto, Nancy Fullman, João M. Furtado, Neal Futran, M.A. Garcia-Gordillo, Tigist Gashaw, Tsegaye

Gebrehiwot, Amanuel Tesfay Gebremedhin, Bereket Gebremichael, Teklu Gebremichael, Tilayie Feto Gelano, Johanna Geleijnse, Ayele Geleto, Ricard Genova-Maleras, Peter Gething, Kebede Embaye Gezae, Mamata Ghimire, Katherine Gibney, Kidu Gidey, Ibrahim Ginawi, Maurice Giroud, Giorgia Giussani, Srinivas Goli, Hector Gomez-Dantes, Sameer Gopalani, Atsushi Goto, Alessandra C Goulart, Ayman Grada, Giuseppe Grosso, Andre Guimaraes, Prakash Gupta, Rahul Gupta, Rajat Das Gupta, Rajeev Gupta, Tanush Gupta, Reyna Alma Gutiérrez, Juanita Haagsma, Nima Hafezi-Nejad, Tekleberhan Beyene Hagos, Arvin Haj-Mirzaian, Arya Haj-Mirzaian, Randah Hamadeh, Hilda Harb, Sivadasanpillai Harikrishnan, Josep Maria Haro, Mehedi Hasan, Hadi Hassankhani, Hamid Y. Hassen, Rasmus Havmoeller, Mohamed Hegazy, Mohsen Heidari, Delia Hendrie, Claudiu Herteliu, Fatemeh Heydarpour, Pouria Heydarpour, Hans Hoek, Michael Hole, Praveen Hoogar, H Dean Hosgood, Mehdi Hosseinzadeh, Mihaela Hostiuc, Sorin Hostiuc, Damian Hoy, Guoqing Hu, Susan Hutfless, Bulat Idrisov, Olayinka Ilesanmi, Usman Iqbal, Farhad Islami, Kathryn H. Jacobsen, Mihajlo Jakovljevic, Spencer James, Don Des Jarlais, Mehdi Javanbakht, Achala Jayatilleke, Panniyammakal Jeemon, Vivekanand Jha, Catherine Johnson, Sarah Johnson, Jost B. Jonas, Bruno Sao Jose, Jacek Jozwiak, Mikk Jürisson, Zubair Kabir, Rajendra Kadel, Amaha Kahsay, Rizwan Kalani, Manoochehr Karami, Corine Karema, Hamidreza Karimi-Sari, Amir Kasaeian, Getachew Mullu Kassa, Nicholas J Kassebaum, Srinivasa Vittal Katikireddi, Anil Kaul, Dhruv Kazi, Peter Keiyoro, Andrei Keren, Chandrasekharan Nair Kesavachandran, Yousef Khader, Behzad Khafaie, Behzad Khafaie, Morteza Abdullatif Khafaie, Ibrahim Khalil, Young-Ho Khang, Mona Khater, Abdullah T. Khoja, Ardeshir Khosravi, Mohammad Hossein Khosravi, Jagdish Khubchandani, Aliasghar A Kiadaliri, Daniel Kim, Young-Eun Kim, Yun Jin Kim, Adnan Kisa, Mika Kivimaki, Ann Kristin Knudsen, Jonathan Kocarnik, Sonali Kochhar, Parvaiz Koul, Michael Kravchenko, Kewal Krishan, G Anil Kumar, Pushpendra Kumar, Michael Kutz, Ihor Kuzin, Deepesh P. Lad, Sheetal D. Lad, Alessandra Lafranconi, Dharmesh Lal, Van Lansingh, James Leigh, Yichong Li, Juan Liang, Lee-Ling Lim, Shiwei Liu, Alan Lopez, Stefan Lorkowski, Paulo Lotufo, Stefan Ma, Erlyn Rachelle Macarayan, Mark Mackay, Jennifer Maclachlan, Dhaval Maghavani, P A Mahesh, Marek Majdan, Reza Majdzadeh, Reza Malekzadeh, Deborah Carvalho Malta, Mohammedaman Mama, Mohammad Ali Mansournia, Joemer Maravilla, Guy Marks, Francisco Rogerlândio Martins-Melo, Melvin Marzan, Joseph Masci, Benjamin Massenburg, Manu Mathur, Richard Matzopoulos, Mohsen Mazidi, Colm Mcalinden, John Mcgrath, Man Mohan Mehndiratta, Varshil Mehta, Addisu Melese, Peter Memiah, Walter Mendoza, Getnet Mengistu, Zerihun Menlkalew, Atte Meretoja, Tuomo Meretoja, Tomislav Mestrovic, Haftay Berhane Mezgebe, Bartosz Miazgowski, Tomasz Miazgowski, Milena Santric Milicevic, Ted R Miller, Parvaneh Mirabi, Erkin Mirrakhimov, Babak Moazen, Karzan Mohammad, Moslem Mohammadi, Shafiu Mohammed, Viswanathan Mohan, Ali Mokdad, Lorenzo Monasta, Ghobad Moradi, Maziar Moradi-Lakeh, Mehdi Moradinazar, Lidia Morawska, Joana Morgado-Da-Costa, Marilita Moschos, Seyyed Meysam Mousavi, Kindie Fentahun Muchie, Ulrich Mueller, Kamarul Imran Musa, Ghulam Mustafa, Jean Nachega, Gabriele Nagel, Seyed Sina Naghibi, Sanjeev Nair, Luigi Naldi, Hae-Sung Nam, Bruno Nascimento, Ionut Negoi, Ruxandra Irina Negoi, Charles Newton, Frida Ngalesoni, Josephine Ngunjiri, Huong Nguyen, Long Hoang Nguyen, Trang Huyen Nguyen, Shuhei Nomura, Mehdi Noroozi, Bo Norrving, Jean Jacques Noubiap, Hamid Reza Nouri, Malihe Nourollahpour, Mohammad Reza Nowroozi, Christopher Odell, Felix Ogbo, Olanrewaju Oladimeji, Andrew T. Olagunju, Bolajoko Olusanya, Jacob Olusanya, Sokking Ong, Heather Orpana, Alberto Ortiz, Stanislav Otstavnov, Simon Øverland, Mayowa Owolabi, Raziye Özdemir, Smita Pakhale, Adrian Pana, Songhomitra Panda-Jonas, Andrea Parisi, Shanti Patel, Alexandre Pereira, David Pereira, Norberto Perico, Max Petzold, Frédéric Piel, David Pigott, Farhad Pishgar, Suzanne Polinder, Maarten Postma, Hossein Poustchi, Swayam Prakash, Ashwini Pujar, Mostafa Qorbani, D. Alex Quistberg, Anh Nguyen Quynh, Enayatollah Homaie Rad, Kirankumar Rade, Amir

Radfar, Anwar Rafay, Fakher Rahim, Afarin Rahimi-Movaghar, Mahfuzar Rahman, Rajesh Kumar Rai, Sasa Rajsic, Usha Ram, Chhabi Lal Ranabhat, PATURI RAO, David Rawaf, Salman Rawaf, Kolli Srinath Reddy, Giuseppe Remuzzi, Andre Renzaho, Shahab Rezaeian, Antonio Luiz Ribeiro, Aklilu Abrham Roba, Kedir Teji Roba, Leonardo Roever, Luca Ronfani, Gholamreza Roshandel, Ali Rostami, Gregory Roth, Dietrich Rothenbacher, Ambuj Roy, Enrico Rubagotti, Basema Saddik, Ehsan Sadeghi, Hosein Safari, Saeid Safiri, Mohammad Ali Sahraian, Nasir Salam, Payman Salamati, Yahya Salimi, Inbal Weiss Salz, Evanson Z Sambala, Abdallah M. Samy, Juan Sanabria, Diego Augusto Santos, João Vasco Santos, Muthupandian Saravanan, Abdur Razzaque Sarker, Rodrigo Sarmiento-Suárez, Nizal Sarrafzadegan, Benn Sartorius, Maheswar Satpathy, Monika Sawhney, Sonia Saxena, Mehdi Sayyah, Elke Schaeffner, David C Schwebel, Falk Schwendicke, Mario Šekerija, Sadaf Sepanlou, Edson Serván-Mori, Seyedmojtaba Seyedmousavi, Mehdi Shahbazi, Amira Shaheen, Masood Ali Shaikh, Mehran Shams-Beyranvand, Mehdi Sharif, Mahdi Sharif-Alhoseini, Sheikh Mohammed Shariful, Jun She, Aziz Sheikh, Peilin Shi, Ivy Shiue, Farhad Shokraneh, Si Si, Soraya Siabani, Dayane Silveira, Jasvinder Singh, Prashant Kumar Singh, Virendra Singh, Dhirendra N. Sinha, Mekonnen Sisay, Karen Sliwa, Soheila Sobhani, Eugene Sobngwi, Joan B Soriano, Luciano Sposato, Chandrashekhar T Sreeramareddy, Vasiliki Stathopoulou, Mark Stokes, Agus Sudaryanto, Muawiyyah Babale Sufiyan, Ipsita Sutradhar, Bryan L. Sykes, Dillon Sylte, Cassandra Szoeke, Rafael Tabarés-Seisdesdos, Mesfin Tadese, Nuno Taveira, Awoke Temesgen, Mohamad-Hani Temsah, Omar Temsah, Abdullah Terkawi, Belay Tessema, Nihal Thomas, Ha Nguyen Thu, Ruoyan Tobe-Gai, Marcello Tonelli, Miguel Tortajada-Girbés, Mathilde Touvier, Marcos Roberto Tovani-Palone, Bach Tran, Khanh Bao Tran, Christopher Troeger, Thomas Truelsen, Nu Thi Truong, Kingsley Nnanna Ukwaja, Irfan Ullah, Eduardo Undurraga, Mohammad Hifz Ur, Olalekan Uthman, Muthiah Vaduganathan, Afsane Vaezi, Gaurang Vaidya, Pascual Valdez, Tommi Vasankari, Ilais Moreno Velasquez, Narayanaswamy Venketasubramanian, Sergey Vladimirov, Vasiliy Vlassov, Stein Emil Vollset, Theo Vos, Fasil Wagnew, Yasir Waheed, Judd Walson, Yanping Wang, Yuan-Pang Wang, Elisabete Weiderpass, Robert Weintraub, Andrea Werdecker, Ronny Westerman, Harvey Whiteford, Justyna Widecka, Charles Shey Wiysonge, Charles Wolfe, Shouling Wu, Yun-Chun Wu, Grant Wyper, Denis Xavier, Gelin Xu, Ali Yadollahpour, Hossein Yahyazadeh, Bereket Yakob, Lijing Yan, Mehdi Yaseri, Yasin Jemal Yasin, Alex Yeshaneh, Alex Yeshanen, Manaye Yihune, Ebrahim M. Yimer, Biruck Desalegn Yirsaw, Naohiro Yonemoto, Marcel Yotebieng, Mustafa Younis, Vesna Zadnik, Zoubida Zaidi, Sojib Bin Zaman, Mohammad Zamani, Zohrah Zare, Kai Zhang, Sanjay Zodpey, and Liesl Zuhlke.

Developing methods or computational machinery

Ibrahim Abdollahpour, Rufus A. Adedoyin, Mehdi Ahmadi, Marlena Bannick, Donal Bisanzio, Fiona Charlson, Yilma Chisha, Devasahayam Christopher, Matthew Cunningham, Ahmad Daryani, Daniel Dicker, Hamed Fakhim, Farshad Farzadfar, Samuel Finegold, Christina Fitzmaurice, Kyle Foreman, Tahvi Frank, Segen G Meskel, Bereket Gebremichael, Ayele Geleto, Taren Gorman, Mehdi Hosseinzadeh, Kathy Jenkins, Catherine Johnson, Sarah Johnson, Nicholas J Kassebaum, Muhammad Shahzeb Khan, Adnan Kisa, Michael Kutz, Van Lansingh, Kathryn Lau, James Leigh, Tefera Chane Mekonnen, Zerihun Menlkalew, Grant Nguyen, Emma Nichols, Bolajoko Olusanya, Jacob Olusanya, Charles Parry, Chhabi Lal Ranabhat, Robert Reiner, Seyed Mohammad Riahi, Gregory Roth, Abdallah M. Samy, Shahabeddin Sarvi, David C Schwebel, Mehdi Sharif, Tariq Jamal Siddiqi, Virendra Singh, Dhirendra N. Sinha, Jeffrey Stanaway, Leo Stewart, Patrick Sur, Ipsita Sutradhar, Dillon Sylte, Andrew Theis, Christopher Troeger, Derrick Tsoi, Rachel L. Updike, Muhammad Shariq Usman, Theo Vos, Shadrach Wilson, Hossein Yahyazadeh, Lijing Yan, and Naohiro Yonemoto.

Applying analytical methods to produce estimates

Muhammed Magdy Abd, Haftom Abebe, Rufus A. Adedoyin, Kareha Agesa, Sutapa Agrawal, Mehdi Ahmadi, Azmeraw T. Amare, Olatunde Aremu, Amit Arora, Hamid Badali, Katherine Ballesteros, Marlena Bannick, Habtamu Wondifraw Baynes, Bayu Begashaw Bekele, Gregory Bertolacci, Donal Bisanzio, Charlton Callender, Chris Castle, Fiona Charlson, Elizabeth Cromwell, Matthew Cunningham, Anh Kim Dang, Ahmad Daryani, Barthelemy Kuate Defo, Getenet Dessie, Daniel Dicker, M.Ashworth Dirac, Maysaa El Sayed Zaki, Christian Lycke Ellingsen, Aman Endries, Holly Erskine, Mohammad Fareed, Samuel Finegold, Christina Fitzmaurice, Tahvi Frank, William Gardner, Teklu Gebremichael, Ayele Geleto, Gessessew Bugssa Hailu, Hamid Y. Hassen, Thomas Hsiao, Caleb Irvine, Spencer James, Catherine Johnson, Sarah Johnson, Manoochehr Karami, André Karch, Nicholas J Kassebaum, Behzad Khafaie, Ibrahim Khalil, Young-Eun Kim, Yun Jin Kim, Adnan Kisa, Jonathan Kocarnik, Michael Kutz, Hmwe Kyu, Van Lansingh, Kathryn Lau, Shai Linn, Helena Manguerra, Ira Martopullo, Melvin Marzan, Tefera Chane Mekonnen, Anoushka Millear, Shafiu Mohammed, Ali Mokdad, John Everett Mumford, Grant Nguyen, Long Hoang Nguyen, Emma Nichols, In-Hwan Oh, Bolajoko Olusanya, Jacob Olusanya, Katherine Paulson, David Pigott, Caroline Purcell, Chhabi Lal Ranabhat, Marissa Reitsma, Seyed Mohammad Riahi, Aklilu Abrham Roba, Nicholas Roberts, Gregory Roth, Sundeep Salvi, Inbal Weiss Salz, Abdallah M. Samy, Damian Santomauro, Seyedmojtaba Seyedmousavi, Masood Ali Shaikh, Mehdi Sharif, Virendra Singh, Mari Smith, Chandrashekhar T Sreeramareddy, Vinay Srinivasan, Jeffrey Stanaway, Leo Stewart, Patrick Sur, Dillon Sylte, Tamirat Tesfaye, Andrew Theis, Bach Tran, Khanh Bao Tran, Christopher Troeger, Nu Thi Truong, Derrick Tsoi, Irfan Ullah, Rachel L. Updike, Theo Vos, MItchell Wallin, Adhena A. Werkneh, Ronny Westerman, Lauren Wilner, Shadrach Wilson, Simon Yadgir, Paul Yip, Biruck Desalegn Yirsaw, Seok-Jun Yoon, Mustafa Younis, and Chuanhua Yu.

Providing critical feedback on methods or results

Degu Abate, Kalkidan Hassen Abate, Solomon Mequanente Abay, Cristiana Abbafati, Nooshin Abbasi, Hedayat Abbastabar, Foad Abd-Allah, Jemal Abdela, Ibrahim Abdollahpour, Rizwan Suliankatchi Abdulkader, Haftom Abebe, Molla Abebe, Zegeye Abebe, Semaw Ferede Abera, Haftom Abraha, Dilaram Acharya, Abdu Adamu, Oladimeji Adebayo, Rufus A. Adedoyin, Victor Adekanmbi, Olatunji Adetokunboh, Mina Adib, Ashkan Afshin, Gina Agarwal, Anurag Agrawal, Sutapa Agrawal, Mehdi Ahmadi, Muktar Ahmed, Sayem Ahmed, Amani Nidhal Aichour, Ibtihel Aichour, Mohammad Esmaeil Akbari, Nadia Akseer, Fares Alahdab, Ziyad Al-Aly, Khurshid Alam, Tahiya Alam, Animut Alebel, Kefyalew Addis Alene, Ayman Al-Eyadhy, Mehran Alijanzadeh, Reza Alizadeh-Navaei, Syed Mohamed Aljunid, Ala'a Alkerwi, Peter Allebeck, Jordi Alonso, Rajaa Al-Raddadi, Khalid Altirkawi, Nelson Alvis-Guzman, Azmeraw T. Amare, Leopold N. Aminde, Erfan Amini, Walid Ammar, Yaw Ampem Amoako, Catalina Liliana Andrei, Sofia Androudi, Dina Nur Anggraini, Mina Anjomshoa, Hossein Ansari, Mustafa Ansha, Carl Abelardo Antonio, Palwasha Anwari, Olatunde Aremu, Johan Ärnlöv, Amit Arora, Al Artaman, Krishna K Aryal, Hamid Asayesh, Ephrem Tsegay Asfaw, Zerihun Ataro, Suleman Atique, Sachin Atre, Marcel Ausloos, Euripide Avokpaho, Ashish Awasthi, Beatriz Paulina Ayala, Rakesh Ayer, Peter Azzopardi, Arefeh Babazadeh, Umar Bacha, Alaa Badawi, Katherine Ballesteros, Kajori Banerjee, Joseph Banoub, Miguel A. Barboza, Suzanne Barker-Collo, Till Bärnighausen, Simon Barquera, Lope Barrero, Quique Bassat, Sanjay Basu, Bernhard Baune, Habtamu Wondifraw Baynes, Shahrzad Bazargan-Hejazi, Neeraj Bedi, Ettore Beghi, Meysam Behzadifar, Yannick Béjot, Abate Bekele, Bayu Begashaw Bekele, Ezra Belay, Yihalem Abebe Belay, Michelle Bell, Aminu Bello, Derrick Bennett, Isabela Bensenor, Adam Berman, Eduardo Bernabe, Robert Bernstein, Mircea Beuran, Tina Beyranvand, Ashish Bhalla, Suraj Bhattarai, Soumyadeep Bhaumik, Zulfiqar Bhutta, Belete Biadgo, Burcu Kucuk Bicer, Ali Bijani, Boris Bikbov, Ver

Bilano, Nigus Bililign, Muhammad Shahdaat Bin, Tuhin Biswas, Brigette Blacker, Rohan Borschmann, Cristina Bosetti, Kayvan Bozorgmehr, Oliver Brady, Luisa Brant, Nicholas Breitborde, Hermann Brenner, Traolach Brugha, Reinhard Busse, Zahid Butt, Ismael Campos-Nonato, Jorge Cano, Lorainne Tudor Car, Mate Car, Rosario Cárdenas, Juan J Carrero, Felix Carvalho, Carlos Castañeda-Orjuela, Clara Castro, Franz Castro, Ferrán Catalá-López, Ester Cerin, Jung-Chen Chang, Fiona Charlson, Pankaj Chaturvedi, Peggy Pei-Chia Chiang, Odgerel Chimed-Ochir, Yilma Chisha, Vesper Chisumpa, Hanne Christensen, Devasahayam Christopher, Sheng-Chia Chung, Flavia Cicuttini, Liliana G Ciobanu, Aaron Cohen, Paolo Angelo Cortesi, Monica Cortinovis, Ewerton Cousin, Benjamin Cowie, Michael Criqui, Christopher Crowe, John Crump, Matthew Cunningham, Alemneh Kabeta Daba, Lalit Dandona, Rakhi Dandona, Anh Kim Dang, Paul I Dargan, Ahmad Daryani, José Das Neves, Adrian Davis, Dragos Davitoiu, Fernando De La, Megbaru Debalkie, Barthelemy Kuate Defo, Louisa Degenhardt, Tizta Degfie, Getiye Dejenu, Gebre Teklemariam Demoz, Balem Demtsu, Edgar Denova-Gutiérrez, Kebede Deribe, Nikolaos Dervenis, Subhojit Dey, Samath Dharmaratne, Daniel Dicker, Eric L. Ding, M.Ashworth Dirac, Shirin Djalalinia, Klara Dokova, David Teye Doku, Christl Donnelly, E. Ray Dorsey, Kerrie Doyle, Tim Driscoll, Manisha Dubey, Eleonora Dubljanin, Eyasu Ejeta Duken, Bruce Duncan, Hedyeh Ebrahimi, Soheil Ebrahimpour, Miloud Taki Eddine, Dumessa Edessa, David Edvardsson, Anne Elise Eggen, Maysaa El Sayed Zaki, Ziad El-Khatib, Christian Lycke Ellingsen, Matthias Endres, Aman Endries, Holly Erskine, Babak Eshrati, Sharareh Eskandarieh, Alireza Esteghamati, Hamed Fakhim, Khalil Ghasemi Falavarjani, Mahbobeh Faramarzi, Farzaneh Farhadi, Carla Farinha, Andre Faro, Farshad Farzadfar, Mohammad Hosein Farzaei, Valery Feigin, Andrea B. Feigl, Netsanet Fentahun, Seyed-Mohammad Fereshtehnejad, Eduarda Fernandes, Joao Fernandes, Garumma Tolu Feyissa, Irina Filip, Samuel Finegold, Florian Fischer, Nataliya Foigt, Kyle Foreman, Carla Fornari, Takeshi Fukumoto, Nancy Fullman, Thomas Fürst, João M. Furtado, Neal Futran, Segen G Meskel, Silvano Gallus, Alberto L. García-Basteiro, Tigist Gashaw, Abadi Kahsu Gebre, Tsegaye Gebrehiwot, Amanuel Tesfay Gebremedhin, Merhawi Gebremedhin, Afewerki Gebremeskel, Bereket Gebremichael, Tilayie Feto Gelano, Johanna Geleijnse, Ayele Geleto, Ricard Genova-Maleras, Kebede Embaye Gezae, Reza Ghadimi, Maryam Ghasemi-Kasman, Mamata Ghimire, Katherine Gibney, Kidu Gidey, Paramjit Gill, Tiffany Gill, Richard Gillum, Meaza Girma, Giorgia Giussani, Srinivas Goli, Philimon Gona, Sameer Gopalani, Taren Gorman, Alessandra C Goulart, Ayman Grada, Giuseppe Grosso, Harish Gugnani, Andre Guimaraes, Rahul Gupta, Rajat Das Gupta, Rajeev Gupta, Tanush Gupta, Reyna Alma Gutiérrez, Bishal Gyawali, Juanita Haagsma, Nima Hafezi-Nejad, Tekleberhan Beyene Hagos, Dessalegn Haile, Gessessew Bugssa Hailu, Arvin Haj-Mirzaian, Arya Haj-Mirzaian, Randah Hamadeh, Samer Hamidi, Alexis Handal, Graeme Hankey, Hilda Harb, Sivadasanpillai Harikrishnan, Josep Maria Haro, Mehedi Hasan, Hadi Hassankhani, Hamid Y. Hassen, Rasmus Havmoeller, Roderick Hay, Simon Hay, Akbar Hedayatizadeh-Omran, Mohamed Hegazy, Behzad Heibati, Mohsen Heidari, Delia Hendrie, Claudiu Herteliu, Fatemeh Heydarpour, Sousan Heydarpour, Desalegn Hibstu, Michael Hole, Praveen Hoogar, H Dean Hosgood, Mostafa Hosseini, Mihaela Hostiuc, Sorin Hostiuc, Peter Hotez, Guoqing Hu, Susan Hutfless, Olayinka Ilesanmi, Usman Iqbal, Nazrul Islam, Farhad Islami, Kathryn H. Jacobsen, Nader Jahanmehr, Sudhir Kumar Jain, Mihajlo Jakovljevic, Moti Jalu, Spencer James, Don Des Jarlais, Mehdi Javanbakht, Achala Jayatilleke, Panniyammakal Jeemon, Ravi Prakash Jha, Vivekanand Jha, Catherine Johnson, Sarah Johnson, Jost B. Jonas, Bruno Sao Jose, Ankur Joshi, Jacek Jozwiak, Suresh Jungari, Mikk Jürisson, Zubair Kabir, Rajendra Kadel, Amaha Kahsay, Rizwan Kalani, Manoochehr Karami, André Karch, Hamidreza Karimi-Sari, Ali Kazemi Karyani, Amir Kasaeian, Getachew Mullu Kassa, Tesfaye Kassa, Nicholas J Kassebaum, Srinivasa Vittal Katikireddi, Dhruv Kazi, Adane Kefale, Peter Keiyoro, Andre Pascal Kengne, Chandrasekharan Nair Kesavachandran, Yousef Khader, Behzad Khafaie, Morteza Abdullatif

Khafaie, Nauman Khalid, Ibrahim Khalil, Ejaz Khan, Muhammad Shahzeb Khan, Muhammad Ali Khan, Young-Ho Khang, Mona Khater, Abdullah T. Khoja, Mohammad Hossein Khosravi, Jagdish Khubchandani, Aliasghar A Kiadaliri, Daniel Kim, Young-Eun Kim, Yun Jin Kim, Ruth Kimokoti, Yohannes Kinfu, Adnan Kisa, Katarzyna Kissimova-Skarbek, Mika Kivimaki, Ann Kristin Knudsen, Jonathan Kocarnik, Sonali Kochhar, Yoshihiro Kokubo, Jacek Kopec, Parvaiz Koul, Ai Koyanagi, G Anil Kumar, Manasi Kumar, Pushpendra Kumar, Michael Kutz, Hmwe Kyu, Deepesh P. Lad, Sheetal D. Lad, Alessandra Lafranconi, Dharmesh Lal, Ratilal Lalloo, Tea Lallukka, Jennifer Lam, Faris Lami, Van Lansingh, Heidi Larson, Arman Latifi, Jeffrey Lazarus, Paul Lee, James Leigh, Cheru T Leshargie, Shanshan Li, Lee-Ling Lim, Miteku Limenih, Shai Linn, Shiwei Liu, Yang Liu, Rakesh Lodha, Chris Lonsdale, Alan Lopez, Stefan Lorkowski, Paulo Lotufo, Raimundas Lunevicius, Erlyn Rachelle Macarayan, Mark Mackay, Jennifer Maclachlan, Fabiana Madotto, Hassan Magdy Abd, P A Mahesh, Marek Majdan, Reza Majdzadeh, Azeem Majeed, Reza Malekzadeh, Deborah Carvalho Malta, Mohammedaman Mama, Ana Laura Manda, Luiz Garcia Mandarano-Filho, Mohammad Ali Mansournia, Christopher Chabila Mapoma, Dadi Marami, Joemer Maravilla, Wagner Marcenes, Guy Marks, Gabriel Martinez, Francisco Rogerlândio Martins-Melo, Ira Martopullo, Winfried März, Melvin Marzan, Benjamin Massenburg, Manu Mathur, Prashant Mathur, Richard Matzopoulos, Pallab K Maulik, Mohsen Mazidi, Colm Mcalinden, John Mcgrath, Martin Mckee, Brian Mcmahon, Suresh Mehata, Kala M. Mehta, Varshil Mehta, Tefera Chane Mekonnen, Addisu Melese, Mulugeta Melku, Peter Memiah, Ziad Memish, Walter Mendoza, Desalegn Tadese Mengistu, Getnet Mengistu, Zerihun Menlkalew, George Mensah, Beyene Meressa, Seid Tiku Mereta, Atte Meretoja, Tuomo Meretoja, Tomislav Mestrovic, Haftay Berhane Mezgebe, Bartosz Miazgowski, Tomasz Miazgowski, Milena Santric Milicevic, Ted R Miller, Molly Miller-Petrie, GK Mini, Mojde Mirarefin, Andreea Mirica, Erkin Mirrakhimov, Habtamu Mitiku, Babak Moazen, Moslem Mohammadi, Noushin Mohammadifard, Mohammed Mohammed, Shafiu Mohammed, Ali Mokdad, Mariam Molokhia, Lorenzo Monasta, Ghobad Moradi, Maziar Moradi-Lakeh, Mehdi Moradinazar, Paula Moraga, Lidia Morawska, Joana Morgado-Da-Costa, Shane Morrison, Marilita Moschos, Seyyed Meysam Mousavi, Kindie Fentahun Muchie, Ulrich Mueller, Satinath Mukhopadhyay, Jonah Musa, Kamarul Imran Musa, Ghulam Mustafa, Jean Nachega, Gabriele Nagel, Seyed Sina Naghibi, Aliya Naheed, Azin Nahvijou, Gurudatta Naik, Sanjeev Nair, Farid Najafi, Vinay Nangia, Jobert Richie Nansseu, Bruno Nascimento, Bruce Neal, Ayenew Negesse, Ionut Negoi, Ruxandra Irina Negoi, Subas Neupane, Jan-Walter De Neve, Frida Ngalesoni, Josephine Ngunjiri, Grant Nguyen, Long Hoang Nguyen, Trang Huyen Nguyen, Emma Nichols, Nomonde Nolutshungu, Ole Norheim, Mehdi Noroozi, Bo Norrving, Jean Jacques Noubiap, Hamid Reza Nouri, Malihe Nourollahpour, Peter Nyasulu, Richard Ofori-Asenso, Felix Ogbo, In-Hwan Oh, Olanrewaju Oladimeji, Andrew T. Olagunju, Pedro Olivares, Helen Olsen, Bolajoko Olusanya, Jacob Olusanya, Eyal Oren, Heather Orpana, Alberto Ortiz, Justin R Ortiz, Stanislav Otstavnov, Simon Øverland, Mayowa Owolabi, Rosana Pacella, Smita Pakhale, Abhijit Pakhare, Amir Pakpour, Adrian Pana, Songhomitra Panda-Jonas, Jeyaraj Pandian, Eun-Kee Park, Charles Parry, Hadi Parsian, Shanti Patel, Sanghamitra Pati, George Patton, Alexandre Pereira, David Pereira, Norberto Perico, Michael R. Phillips, Frédéric Piel, David Pigott, Julian Pillay, Meghdad Pirsaheb, Farhad Pishgar, Suzanne Polinder, Maarten Postma, Akram Pourshams, Hossein Poustchi, Swayam Prakash, Narayan Prasad, Ashwini Pujar, Mostafa Qorbani, D. Alex Quistberg, Enayatollah Homaie Rad, Amir Radfar, Anwar Rafay, Alireza Rafiei, Fakher Rahim, Kazem Rahimi, Afarin Rahimi-Movaghar, Mahfuzar Rahman, Muhammad Aziz Rahman, Rajesh Kumar Rai, Usha Ram, Chhabi Lal Ranabhat, Prabhat Ranjan, David Rawaf, Salman Rawaf, Christian Razo, Kolli Srinath Reddy, Robert Reiner, Marissa Reitsma, Giuseppe Remuzzi, Andre Renzaho, Serge Resnikoff, Shahab Rezaeian, Mohammad Sadegh Rezi, Seyed Mohammad Riahi, Antonio Luiz Ribeiro, Maria Jesus

Rios-Blancas, Jacqueline Castillo Rivas, Aklilu Abrham Roba, Nicholas Roberts, Stephen Robinson, Leonardo Roever, Luca Ronfani, Gholamreza Roshandel, Ali Rostami, Gregory Roth, Dietrich Rothenbacher, Ambuj Roy, Perminder Sachdev, Basema Saddik, Ehsan Sadeghi, Hosein Safari, Mahdi Safdarian, Saeid Safiri, Rajesh Sagar, Amirhossein Sahebkar, Mohammad Ali Sahraian, Nasir Salam, Joseph Salama, Raphael Saldanha, Zikria Saleem, Yahya Salimi, Inbal Weiss Salz, Evanson Z Sambala, Abdallah M. Samy, Juan Sanabria, Maria Dolores Sanchez-Niño, Diego Augusto Santos, Itamar Santos, João Vasco Santos, Muthupandian Saravanan, Abdur Razzaque Sarker, Rodrigo Sarmiento-Suárez, Benn Sartorius, Brijesh Sathian, Thirunavukkarasu Sathish, Maheswar Satpathy, Arundhati Sawant, Monika Sawhney, Sonia Saxena, Mehdi Sayyah, Elke Schaeffner, Ione Schneider, Ben Schöttker, Aletta Schutte, David C Schwebel, Falk Schwendicke, James Scott, Sadaf Sepanlou, Edson Serván-Mori, Seyedmojtaba Seyedmousavi, Hosein Shabaninejad, Azadeh Shafieesabet, Mehdi Shahbazi, Amira Shaheen, Masood Ali Shaikh, Mehran Shams-Beyranvand, Mohammad Bagher Shamsi, Kiomars Sharafi, Mehdi Sharif, Sheikh Mohammed Shariful, Rajesh Sharma, Jun She, Aziz Sheikh, Mika Shigematsu, Rahman Shiri, Ivy Shiue, Mark Shrime, Si Si, Soraya Siabani, Tariq Jamal Siddiqi, Inga Dora Sigfusdottir, Rannveig Sigurvinsdottir, Donald Silberberg, Dayane Silveira, Jasvinder Singh, Narinder Pal Singh, Prashant Kumar Singh, Virendra Singh, Mekonnen Sisay, Badr Sobaih, Soheila Sobhani, Eugene Sobngwi, Samir Soneji, Moslem Soofi, Reed Sorensen, Joan B Soriano, Ireneous Soyiri, Luciano Sposato, Chandrashekhar T Sreeramareddy, Jeffrey Stanaway, Dan J Stein, Mark Stokes, Muawiyyah Babale Sufiyan, Ipsita Sutradhar, Bryan L. Sykes, PN Sylaja, Dillon Sylte, Cassandra Szoeke, Rafael Tabarés-Seisdesdos, Takahiro TABUCHI, Santosh Tadakamadla, Mesfin Tadese, Ken Takahashi, Nikhil Tandon, Nuno Taveira, Arash Tehrani-Banihashemi, Zelalem Teklemariam, Awoke Temesgen, Habtamu Temesgen, Mohamad-Hani Temsah, Omar Temsah, Tewodros Tesfa, Tamirat Tesfaye, Belay Tessema, Gizachew Tessema, Kavumpurathu Thankappan, Nihal Thomas, Amanda Thrift, George Thurston, Binyam Tilahun, Quyen G. To, Ruoyan Tobe-Gai, Marcello Tonelli, Roman Topor-Madry, Natacha Torres Da, Miguel Tortajada-Girbés, Mathilde Touvier, Marcos Roberto Tovani-Palone, Bach Tran, Khanh Bao Tran, Christopher Troeger, Thomas Truelsen, Nu Thi Truong, Derrick Tsoi, E Murat Tuzcu, Stefanos Tyrovolas, Kingsley Nnanna Ukwaja, Irfan Ullah, Eduardo Undurraga, Muhammad Shariq Usman, Olalekan Uthman, Muthiah Vaduganathan, Afsane Vaezi, Pascual Valdez, Tommi Vasankari, Narayanaswamy Venketasubramanian, Santos Villafaina, Francesco S Violante, Sergey Vladimirov, Vasiliy Vlassov, Stein Emil Vollset, Theo Vos, Gregory Wagner, Fasil Wagnew, Yasir Waheed, Mitchell Wallin, Judd Walson, Yuan-Pang Wang, Molla Mesele Wassie, Robert Weintraub, Fitsum Weldegebreal, Andrea Werdecker, Adhena A. Werkneh, T. Eoin West, Ronny Westerman, Lauren Wilner, Andrea Winkler, Charles Shey Wiysonge, Charles Wolfe, Grant Wyper, Denis Xavier, Gelin Xu, Ali Yadollahpour, Bereket Yakob, Lijing Yan, Yuichiro Yano, Mehdi Yaseri, Yasin Jemal Yasin, Alex Yeshaneh, Alex Yeshanen, Manaye Yihune, Ebrahim M. Yimer, Biruck Desalegn Yirsaw, Engida Yisma, Naohiro Yonemoto, Gerald Yonga, Seok-Jun Yoon, Marcel Yotebieng, Mustafa Younis, Mahmoud Yousefifard, Chuanhua Yu, Vesna Zadnik, Zoubida Zaidi, Sojib Bin Zaman, Mohammad Zamani, Zohrah Zare, Ayalew Jejaw Zeleke, Olifan Zewdie, Anthony Zhang, And Liesl Zuhlke.

Drafting the work or revising is critically for important intellectual content

Nooshin Abbasi, Muhammed Magdy Abd, Haftom Abebe, Olatunji Adetokunboh, Mohammad Esmaeil Akbari, Fares Alahdab, Khurshid Alam, Tahiya Alam, Syed Mohamed Aljunid, Yaw Ampem Amoako, Nahla Anber, Amit Arora, Peter Azzopardi, Hamid Badali, Berrak Bora Basara, Shahrzad Bazargan-Hejazi, Yihalem Abebe Belay, Molly Biehl, Muhammad Shahdaat Bin, Oliver Brady, Hermann Brenner, Franz Castro, Devasahayam Christopher, Flavia Cicuttini, John Crump, Ahmad Daryani, Nicole Davis Weaver, Selina Deiparine, Kerrie Doyle, Andre Duraes, David Edvardsson, Maysaa El Sayed Zaki, Andre Faro,

Mohammad Hosein Farzaei, Seyed-Mohammad Fereshtehnejad, Joao Fernandes, Nataliya Foigt, Segen G Meskel, Ayele Geleto, Ricard Genova-Maleras, Kebede Embaye Gezae, Maryam Ghasemi-Kasman, Rajat Das Gupta, Arvin Haj-Mirzaian, Alexis Handal, Mehedi Hasan, Hadi Hassankhani, Rasmus Havmoeller, Behzad Heibati, John Huang, Farhad Islami, Spencer James, Mehdi Javanbakht, Ravi Prakash Jha, Jacek Jozwiak, Mikk Jürisson, Surya Kant, Manoochehr Karami, Nicholas J Kassebaum, Grant Kemp, Chandrasekharan Nair Kesavachandran, Morteza Abdullatif Khafaie, Alireza Khajavi, Mona Khater, Yun Jin Kim, Mika Kivimaki, Ann Kristin Knudsen, Manasi Kumar, Dharmesh Lal, Alan Lopez, Jennifer Maclachlan, Hassan Magdy Abd, Azeem Majeed, Joemer Maravilla, Francisco Rogerlândio Martins-Melo, Melvin Marzan, Benjamin Massenburg, Colm Mcalinden, Tuomo Meretoja, Milena Santric Milicevic, Karzan Mohammad, Ghobad Moradi, Mehdi Moradinazar, Nahid Neamati, Jan-Walter De Neve, Molly Nixon, Andrew T. Olagunju, Bolajoko Olusanya, Heather Orpana, Justin R Ortiz, Simon Øverland, Hadi Parsian, Konrad Pesudovs, Michael R. Phillips, Hedley Quintana, Enayatollah Homaie Rad, Muhammad Aziz Rahman, Seyed Mohammad Riahi, Jacqueline Castillo Rivas, Aklilu Abrham Roba, Leonardo Roever, Gregory Roth, Sare Safi, Saeid Safiri, Mohammad Ali Sahraian, Juan Sanabria, Damian Santomauro, Muthupandian Saravanan, Shahabeddin Sarvi, Maheswar Satpathy, Seyedmojtaba Seyedmousavi, Katya Shackelford, Mehdi Sharif, Mika Shigematsu, Inga Dora Sigfusdottir, Rannveig Sigurvinsdottir, Virendra Singh, Karen Sliwa, Joan B Soriano, Ipsita Sutradhar, Nuno Taveira, Nihal Thomas, Marcos Roberto Tovani-Palone, Thomas Truelsen, Irfan Ullah, Olalekan Uthman, Selen Begum Uzun, Yuan-Pang Wang, Robert Weintraub, Adhena A. Werkneh, Gokalp Kadri Yentur, And Manaye Yihune.

Extracting, cleaning, or cataloging data; designing or coding figures and tables

Hedayat Abbastabar, Ibrahim Abdollahpour, Rufus A. Adedoyin, Olatunji Adetokunboh, Tahiya Alam, Syed Mohamed Aljunid, Mustafa Ansha, Ashish Awasthi, Marlena Bannick, Habtamu Wondifraw Baynes, Neeraj Bedi, Bayu Begashaw Bekele, Gregory Bertolacci, Muhammad Shahdaat Bin, Donal Bisanzio, Alexandra Brazinova, Paul Briant, Julio Cesar Campuzano, Chris Castle, Devasahayam Christopher, MIchael Criqui, Matthew Cunningham, Anh Kim Dang, Ahmad Daryani, Barthelemy Kuate Defo, Getenet Dessie, Manisha Dubey, Aman Endries, Alireza Esteghamati, Hamed Fakhim, Takeshi Fukumoto, John Fuller, Nancy Fullman, Segen G Meskel, William Gardner, Ayele Geleto, Maurice Giroud, Taren Gorman, Nima Hafezi-Nejad, Mehedi Hasan, Roderick Hay, Yihua He, Behzad Heibati, Mostafa Hosseini, Mehdi Hosseinzadeh, Caleb Irvine, Moti Jalu, Vivekanand Jha, Catherine Johnson, Sarah Johnson, Manoochehr Karami, André Karch, Tesfaye Kassa, Nicholas J Kassebaum, Srinivasa Vittal Katikireddi, Grant Kemp, Andrei Keren, Behzad Khafaie, Behzad Khafaie, Mohammad Hossein Khosravi, Jagdish Khubchandani, Young-Eun Kim, Yun Jin Kim, Jonathan Kocarnik, Michael Kutz, Ihor Kuzin, Kathryn Lau, Shanshan Li, Shiwei Liu, Emilie Maddison, Dhaval Maghavani, Reza Malekzadeh, Mohammad Ali Mansournia, Wagner Marcenes, Winfried März, Mohsen Mazidi, Zerihun Menlkalew, Anoushka Millear, Lorenzo Monasta, Maziar Moradi-Lakeh, Hae-Sung Nam, Long Hoang Nguyen, Minh Nguyen, Mehdi Noroozi, Mohammad Reza Nowroozi, Jacob Olusanya, Mayowa Owolabi, Andrea Parisi, David Pereira, Max Petzold, David Pigott, Hossein Poustchi, Anwar Rafay, Seyed Mohammad Riahi, Luca Ronfani, Gregory Roth, Enrico Rubagotti, Hosein Safari, Saeid Safiri, Payman Salamati, Inbal Weiss Salz, Abdallah M. Samy, Damian Santomauro, Shahabeddin Sarvi, Monika Sawhney, Mehdi Sayyah, Sadaf Sepanlou, Seyedmojtaba Seyedmousavi, Mehdi Sharif, Peilin Shi, Farhad Shokraneh, Soraya Siabani, Virendra Singh, Jeffrey Stanaway, Michelle Subart, Patrick Sur, Dillon Sylte, Awoke Temesgen, Omar Temsah, Anna Torre, Bach Tran, Christopher Troeger, Nu Thi Truong, Kingsley Nnanna Ukwaja, Irfan Ullah, Rachel L. Updike, Gaurang Vaidya, Tommi Vasankari, Vasiliy Vlassov, Yasir Waheed, Shadrach Wilson, Shouling Wu, Simon

Yadgir, Ali Yadollahpour, Hossein Yahyazadeh, Naohiro Yonemoto, Mahmoud Yousefifard, and Sojib Bin Zama.

Managing the overall research enterprise

Ashkan Afshin, Tahiya Alam, Peter Allebeck, Zulfiqar Bhutta, Molly Biehl, Brigette Blacker, Aaron J Cohen, Elizabeth A Cromwell, Lalit Dandona, Rakhi Dandona, Louisa Degenhardt, Samath D Dharmaratne, Daniel Dicker, Valery L Feigin, Christina Fitzmaurice, Kyle Foreman, Nancy Fullman, Thomas Fürst, Tsegaye Gebrehiwot, Simon I Hay, Peter Hotez, Spencer James, Nicholas J Kassebaum, Hmwe Kyu, Heidi J Larson, Stephen S Lim, Alan D Lopez, Rafael Lozano, Deborah Carvalho Malta, Ashley Marks, George Mensah, Awoke T Misganaw, Ali H Mokdad, Christopher J L Murray, Kate Muller, Mohsen Naghavi, Molly R Nixon, Helen Olsen, D M Pigott, Puja C Rao, Robert C Reiner, Gregory Roth, Joseph Salama, Benn Sartorius, Katya A Shackelford, Mari Smith, Jeffrey D Stanaway, Caitlyn Steiner, Roman Topor-Madry, Stein Emil Vollset, Theo Vos, Harvey Whiteford, Shelley Wilson, Maigeng Zhou.

Did not provide contribution information

Yuming Guo and Maria Inês Schmidt.

Section 1: GBD Overview

Section 1.1: Locations of the Analysis

The locations included in GBD 2017 have been arranged into a set of hierarchical categories composed of seven super-regions and a further nested set of 21 regions containing 195 countries and territories (Appendix Table 2). Each year, the GBD Study undertakes subnational analysis for a small number of new countries, and continues to estimate subnationally any that were undertaken in previous cycles. Subnational estimation in GBD 2017 includes five new countries (Ethiopia, Iran, New Zealand, Norway, Russia), and all countries previously estimated at subnational levels (GBD 2013: China, Mexico, United Kingdom; GBD 2015: Brazil, India, Japan, Kenya, South Africa, Sweden, and the United States; GBD 2016: Indonesia, United Kingdom). All analyses are at the first level of administrative organization within each country with the exception of New Zealand separately by Maori ethnicity, Sweden which has been estimated for Stockholm and non-Stockholm, and the United Kingdom which has been estimated down to the local government authority level). All subnational estimates for these countries were incorporated into model development and evaluation as part of the GBD 2017 study. To meet data use requirements, in this publication we present only subnational estimates that have been already published elsewhere (Brazil, India, Japan, Kenya, Mexico, Sweden, United Kingdom, United States); given space constraints these results are presented in appendix tables and figures instead of the main text (see Supplementary Results). Subnational estimates for countries with populations larger than 200 million (measured using our most recent year of published estimates) that have not yet been published elsewhere are presented wherever estimates are illustrated with maps, but are not included in data tables.

Section 1.2: Time Period of the Analysis

A complete set of cause-specific mortality and years of life lost (YLL) numbers and rates were computed for the years 1980–2017.

All GBD 2017 results and online data visualisations will be made available upon paper acceptance.

Section 1.3: Statement of GATHER Compliance

This study complies with the Guidelines for Accurate and Transparent Health Estimates Reporting (GATHER) recommendations. We have documented the steps involved in our analytical procedures and detailed the data sources used. See Appendix Table 1 for the GATHER checklist.

The GATHER recommendations may be found here: http://gather-statement.org/

Section 1.4: List of abbreviations

5q0: probability of death from birth to age 5 years

45q15: probability of death from age 15 years to 60 years

Adol: adolescent

Air poll mort: mortality attributable to air pollution

ANC: antenatal care

ART: antiretroviral therapy

BMD: bone mineral density

BMI: body mass index

BTL: basic tabulation list

CBH: complete birth history

CD4: Cluster of differentiation 4+ T lymphocyte; an indicator of immune function

CDR: crude death rates

CKD: chronic kidney disease

CKD-DM: chronic kidney disease deaths attributable to diabetes

CoD: causes of death

CODEm: cause of death ensemble modelling

COPD: chronic obstructive pulmonary disease

CR: cancer registry

CRA: comparative risk assessment

CSA: childhood sexual abuse

CSMR: cause-specific mortality rate

CVD: cardiovascular disease

DAH: development assistance for health

DALYs: disability-adjusted life-years

DHS: Demographic and Health Survey

DPT: diphtheria-pertussis-tetanus

DRI: data representativeness index

DSP: Disease Surveillance Points

ELISA: enzyme-linked immunosorbent assay

EMR: excess mortality rate

EPEC: enteropathogenic *Escherichia coli*

EPP: Estimation and Projection Package

ETEC: enterotoxigenic *Escherichia coli*

FAO: Food and Agriculture Organization

GATHER: Guidelines for Accurate and Transparent Health Estimates Reporting

GBD: Global Burden of Diseases, Injuries, and Risk Factors Study

GEMS: Global Enteric Multicenter Study

GHDx: Global Health Data Exchange

GPRM: Global Price Reporting Mechanism

HAQ: Healthcare Access and Quality

HH air poll: household air pollution

Hib: *Haemophilus influenzae* type B

HIV CDR: Crude death rate due to HIV/AIDS

IAEG-SDGs: Inter-Agency and Expert Group on Sustainable Development Goal Indicators

IARC: International Agency for Research on Cancer

ICD: International Classification of Disease

IER: integrated exposure response

IHD: ischemic heart disease

IHR: International Health Regulations

ILO: International Labour Organization

IOTF: International Obesity Task Force

IPUMS: Integrated Public Use Microdata Series

IPV: intimate partner violence

ISIC: International Standard Industrial Classification

JMP: Joint Monitoring Programme

LDI: lag distributed income per capita

LMER: linear mixed effects regression

LRI: lower respiratory infection

MAP: Malaria Atlas Project

MCCD: Medical Certification of Causes of Death

MCEE: Maternal and Child Epidemiology Estimation group

MDG: Millennium Development Goal

MICS: Multiple Indicator Cluster Surveys

MIR: mortality/incidence ratio

MM: maternal mortality

MMR: maternal mortality ratio

MMS: Maternal Mortality Surveillance

Mort: mortality

NCD: non-communicable disease

NN mort: neonatal mortality

NTDs: neglected tropical diseases

Occ risk burden: burden attributable to occupational risks

ODA: Official development assistance

OECD: Organisation for Economic Co-operation and Development

PAF: population attributable fraction

PCV3: Three-dose pneumococcal conjugate vaccine

PM2.5: particulate matter <2.5μm in diameter

RCT: randomised controlled trial

RMSE: root mean square error

RSV: respiratory syncytial virus

SBA: skilled birth attendance

SBH: summary birth history

SBP: systolic blood pressure

SCD(R): Survey of Causes of Death (Rural)

SD: standard deviation

SDG: Sustainable Development Goal

SDI: Socio-demographic Index

SDSN: Sustainable Development Solutions Network

SEER: Surveillance, Epidemiology, and End Results Program

SEV: summary exposure value

SIR: smoking impact ratio

SRS: Sample Registration System

SSB: sugar-sweetened beverages

ST-GPR: spatiotemporal Gaussian process regression

TAC: TaqMan Array Card

TB: tuberculosis

TMREL: theoretical minimum-risk exposure level

TRIPS: Agreement on Trade-Related Aspects of Intellectual Property Rights

U5MR: under-5 mortality rate

UHC: universal health coverage

UI: uncertainty interval

UN: United Nations

UNICEF: United Nations Children's Fund

USSR: Union of Soviet Socialist Republics

VA: verbal autopsy

VR: vital registration

WaSH: water, sanitation, and hygiene

WFS: World Fertility Surveys

WHO: World Health Organization

YLDs: years lived with disability

YLLs: years of life lost

Section 1.5: GBD results overview

Results from the Global Burden of Disease Study (GBD 2017) are now measured in terabytes. Results are available in an interactive data downloading tool on the Global Health Data exchange (GHDx).

The current version of the data download tool is available in the GHDx and contains core summary results for the GBD 2017: http://ghdx.healthdata.org/gbd-results-tool.

The core summary results include deaths, YLLs, years lived with disability (YLDs), and disability-adjusted life-years (DALYs). The GHDx includes data for causes, risks, cause-risk attribution, aetiologies, and impairments.

In the GBD 2017 version, the GHDx tool also contains measures such as prevalence and incidence as well as rate of change data. Data above a certain size cannot be viewed online but can be downloaded. Depending on the size of the download, users may need to enter an email address; a download location will be sent to them when the files are prepared.

Section 1.6: Data input sources overview

GBD 2017 incorporated a large number and wide variety of input sources to estimate mortality, causes of death and illness, and risk factors for 195 countries and territories from 1990-2017. These input sources are accessible through an interactive citation tool available in IHME's GHDx.

Users can retrieve citations for a specific GBD component, cause or risk, and location by choosing from the available selection boxes. They can then view and access GHDx records for input sources and export a CSV file that includes the GHDx metadata, citations, and information about where the data were used in GBD. Additional metadata for each input source are available through the citation tool, as required by the GATHER statement.

The citation tool is accessible through the GHDx at http://ghdx.healthdata.org/gbd-2016/data-input-sources.

Section 1.7: Funding Sources

Research reported in this publication was supported by the Bill & Melinda Gates Foundation, the National Institute on Aging of the National Institutes of Health (award P30AG047845), and the National Institute of Mental Health of the National Institutes of Health (award R01MH110163). The content is solely the responsibility of the authors and does not necessarily represent the official views of the Bill & Melinda Gates Foundation or the National Institutes of Health.

Section 2: GBD 2017 causes of death database

Section 2.1: Background

Appendix Figures 1 and 2 show the high-level view of data inputs, analytical steps, and outputs of the CoD analysis frame. Section 2 of this appendix provides details on each step in the development of the CoD database as illustrated in Appendix Figure 1. The complexity of the overall process can be usefully divided into three broad phases: (1) data inputs on the event of death going into the CoD database that are analysed using CODEm; (2) data inputs on precursors to death that are modelled through a variety of strategies; and (3) the integration of these streams of analysis into a single set of CoD estimates by age, sex, year, and geography with uncertainty through the CodCorrect algorithm. A different process is used for cancer and HIV/AIDS estimations and is described in detail in Section 3.

Section 2.2: CoD data identification

Section 2.2.1: Overview of data types

The CoD database contains seven types of data sources (Appendix Table 3): vital registration (VR), verbal autopsy (VA), cancer registry (CR), police records, sibling history, surveillance, and survey/census. The highest-quality data have detailed demographic group characteristics and detailed CoD across the time series. Data from countries with complete VR systems are considered to be high-

quality. For countries with incomplete VR systems, vital statistics for causes of death may be supplemented with other data types to provide cause-specific estimates (Appendix Figure 3).

Section 2.2.2: ICD detail

A majority of the CoD data is VR data obtained from the World Health Organization (WHO) Mortality Database, a compilation of data submitted to the WHO by individual countries. VR is also obtained from country-specific mortality databases operated by official offices. VR data is considered to be the highest-quality data, as it is the most comprehensive. Each cause is coded directly to the most detailed CoD when possible, whereas cause codes in ICD-tabulated data are coded to aggregated cause groups. The CoD database contains 2,338 country-years of detailed data from 1980 to 2017, which includes underlying CoD coded with 3-5 digit codes, by country, year, sex, and age groups. Detailed causes are coded to one of the following International Classification of Disease (ICD) detail coding systems: ICD8, ICD9, or ICD10 (Appendix Table 4). Each coding system has a similar cause hierarchy and cause list that has continually developed over time. ICD10 is the current standard and the most exhaustive cause list. Within the cause lists, 5-digit codes are truncated to 4-digit codes to condense the lists. Updates to ICD detail occur biannually as WHO releases new versions or as country collaborators provide additional data. Updates to data from WHO increasingly include ICD10 CoD data, as it is the most current classification of CoD, while updates to ICD8 and ICD9 detailed lists are less common. In the case of overlapping data, preference is given to data from pre-determined country collaborations, which are updated annually.

Section 2.2.3: ICD tabulations list

The ICD tabulation lists include the ICD8 List A (ICD8A), ICD9 Basic Tabulation List (BTL), ICD10 Mortality Tabulation, Russia Tabulation list, and India Medical Certification of Cause of Death (MCCD). These data sources make up 1,183 country-years from 1980 to 2016 in the CoD database. All are condensed versions of the ICD9 and ICD10 detail lists, with some differences in the format of cause lists depending on the data source. ICD8A, ICD9 BTL, and ICD10 Mortality Tabulation CoD are assigned to subtotal groups (referred to as chapters) and cause groups respective to ICD detail groups. Additionally, ICD9 BTL includes ICD9 detail codes for some cancers and a custom tabulation scheme for the former Union of Soviet Socialist Republics (USSR) countries. The Russia Tabulation lists and India MCCD cause lists each have custom nomenclatures based on ICD detail cause codes.

Two of the drawbacks in using tabulation lists are discrepancies in the accuracy of death counts and lack of detail due to aggregated cause groups. There are instances where the sum of deaths in chapter subtotals are not equal to the sum of cause groups within the chapter. To account for any missing or duplicate deaths reported within the cause groupings, death counts are systematically adjusted by calculating the differences between subtotals and sub-causes within the cause groups. Any differences are assigned to a remainder cause group. To account for the lack of cause code detail, select cause groups are disaggregated (Step 1.1) to create a complete cause list. Updates to ICD Tabulation lists obtained from WHO occur less frequently compared to ICD detailed lists, as more countries are reporting deaths in ICD detail. In instances of overlapping data, preference is given first to detailed collaborator data, followed by detailed WHO data, then tabulated collaborator data, and finally tabulated WHO data.

China DSP/China CDC

The two primary sources of data for China are surveillance data from the China Disease Surveillance Points (DSP) system and VR data collected by the Chinese Center for Disease Control and Prevention (CDC). In the China DSP data, deaths were reported across 145 disease surveillance points used from 1991 to 2003, 161 points used from 2004 to 2007, and 605 disease surveillance points from 2008 to 2016. While China DSP with ICD10 coding is considered sample VR data, it provides national coverage and cause detail. Thus, it receives similar processing and treatment to the China CDC VR from 2008 to 2016. From 2008 to 2016, all of the deaths and CoD information from the Disease Surveillance Points system and other system points throughout China were collected and reported via the Mortality Registration and Reporting System, an online reporting system of the Chinese CDC. The deaths in these data are reported at the strata level, a metric that is specific to China. Counties are stratified by urban and rural classification, but definitions of urbanity vary across counties. In Step 7 we use a method developed to scale up deaths from strata level to the province level.

India MCCD

The India Medical Certification of Cause of Death (MCCD) has data for the urban parts of the majority of the states and union territories beginning in 1980. Deaths reported in this data source have been medically certified and are considered VR data. The CoD are reported in a tabulation list with a unique numbering scheme that conforms to ICD9 and ICD10 detail codes, which must be disaggregated. MCCD is state-split to fill in data gaps (Step 1.2 State Splitting) prior to age-sex splitting. Because the Sample Registration System (SRS) is widely considered a more credible assessment of CoD in India, we chose to use MCCD data only in certain cases for modelling with CODEm. We preserved MCCD data in the database for two primary reasons. First, where the three midpoint years of SRS data resulted in the loss of a clear time trend, as was the case for maternal mortality, we chose to preserve MCCD in addition to SRS. Second, MCCD has advantage over SRS in cases where VA is not a valid instrument for ascertaining CoD, like encephalitis, dengue fever, and peptic ulcer disease – in these cases, we kept MCCD over SRS.

Section 2.2.4: VA

VA coded to ICD10 and VA coded to other lists

In countries without VR systems, VA studies are a viable data source to inform CoD. Data are obtained by trained interviewers who use a standardised questionnaire to ask relatives about the signs, symptoms, and demographic characteristics of recently deceased family members. CoD is assigned based on the answers to the questionnaires.

VA data are highly heterogeneous: studies use different instruments, different cause lists (from single causes to full ICD cause lists), different methods for assigning CoD, different recall periods, and different age groups. Cultural differences may also affect the interpretation of specific questions. CoD validity must be considered when mapping to a GBD cause. VAs are likely accurate in assigning CoD to road injury or homicide, but less accurate for causes requiring medical certification, such as cardiovascular causes. Studies may also occur as stand-alone assessments or as part of an extended network, such as INDEPTH - a continuous surveillance source with several Demographic Surveillance Systems sites that collect data coded to ICD detail causes.

INTERVA-modelled VA

INTERVA, a set of computer models intended to facilitate interpreting VAs, was found to be non-credible by the Population Health Metrics Research Consortium (PHMRC). As a result, INTERVA-modelled VAs are typically excluded from our analysis due to low validations, with the exception of injuries and maternal causes where they are used to fill gaps and stabilise patterns.

India SCD

Deaths reported in VA studies in rural Indian states can be accounted for in the Survey of Causes of Death (SCD) from 1980 to 1994.[1] Data in the SCD were collected through a VA survey from a sample of villages. To expand our estimates to additional states and causes we used methods of state splitting post mapping to GBD causes (Step 1.2). For GBD 2017, the primary source of CoD data for India was India SRS, described below. With the exception of select causes for which SCD data were a critical source in CODEm models, SRS data were used in place of SCD data for causes included in both databases.

Sample registration system

Sample registration systems are expanding in several countries, and are key sources of data in Indonesia and India. The SRS is a dual-record system wherein a resident part-time enumerator continuously records births and deaths in each household within the sample unit every month. A full-time SRS supervisor thereafter independently collects the vital events along with other related details for each of the preceding six month periods during the calendar year.

Section 2.2.5: Other data types

Maternal mortality data

In locations with low-quality, or no, VR, maternal mortality metrics can be found in surveillance, surveys, census, and sibling history data sources. The best data have death counts due to maternal causes and the total number of deaths for women within the reproductive ages of 10 to 54 by year. If a data source is missing these components, it is necessary to create a complete cause list using live births and all-cause mortality deaths (Step 1.4).[2] Though death counts are the preferred metric, maternal mortality is often measured using the maternal mortality ratio (MMR), which is easily converted to deaths using live births. The China Maternal and Child Surveillance data is adjusted by scaling data from the strata to the province level (Step 7).

Surveys and censuses reporting fraction of deaths due to selected injuries

Surveys and censuses are often used in countries with less developed VR systems; in countries with adequate VR surveys and censuses are supplementary. Much like VAs, the CoD validity is a concern due to lack of medical certification at the time of death. For these data sources we only keep causes related to maternal mortality and injuries. The remaining causes are accounted for as a remainder of total deaths in the sample size.

Police records

In most countries, police and crime reports are an important source of information for some types of injury deaths, notably road injuries and interpersonal violence. Our police data come from reports on road traffic and crime trends. The police reports used in this analysis were obtained from published studies, national agencies, and institutional surveys such as the United Nations (UN) Crime Trends survey and the UN Office on Drugs and Crime Global study on Homicides. We assess whether police reports were likely to be complete and cover the entire country by comparing police trends with those seen in VR. Data are excluded in instances where police data for road traffic injuries are significantly lower than the VR. The threshold for exclusion is less than 80% of the cause fraction of the road traffic injuries in VR. Police data that meet our inclusion criteria and provide complete coverage are uploaded to the database for use in road injuries and interpersonal violence deaths estimation.

Section 2.2.6: Population-based cancer registries

Cancer registries with incidence

Data on cancer incidence were sought from individual population-based cancer registries as well as from databases that include multiple registries, including Cancer Incidence in Five Continents, NORDCAN, and EUREG. Cancer registries were identified through the membership list of the International Association of Cancer Registries, through the GBD collaborator network, or through the GHDx. Registries were excluded if they were not representative of the coverage population, if they did not contain incidence data tabulated by cancer site, if the data were limited to years prior to 1980, if the source did not provide details on the population covered, or if the list of cancer sites included was not comprehensive.

Cancer registries with incidence and high-quality mortality data

In addition to incidence, some high-quality cancer registries also report cancer mortality data. These data were also extracted and used as inputs to the mortality-to-incidence model.

Section 2.3: Step 1 – Standardise input data

The input data to the CoD database are received in various formats and must be standardised to run through central CoD machinery to then upload to the database. Raw data inputs come from data sources such as mortality databases, literature reviews, or reports. Usable data sources must have a clear sample size of the number of deaths in the population and exhaustive cause lists. The complexity of the data cleaning process varies drastically across data sources. For VR micro-data with the location, age, sex, year, and ICD-coded cause of every death, very little effort is necessary to standardise it into a consistent structure. Other sources may require weeks of careful review to accurately extract scans of hardcover CoD reports into spreadsheets that can be transformed and standardised.

At this point, data are assigned source identifiers so that they can be linked to the Global Health Data Exchange (GHDx) and cited appropriately. Any aggregate age and sex categories are flagged for age-sex splitting. The methods of cause-of-death assignment and data collection are reviewed to determine which source type to assign; for example, we distinguish sibling history data from surveys with a VA

module. Only data at the most detailed level of the Global Burden of Disease location hierarchy are used. Documentation from the source is reviewed to determine if the population is representative of the location or only a subset of the population in that location. Data sources representing a subset of the population are flagged as non-representative; this flag is used by CODEm to increase the variance associated with such data points.

Finally, diagnostics are reviewed at this stage to avoid sending cleaning errors downstream. We review cause-specific deaths for each demographic group to ensure the data are reasonable. For example, it is unlikely that male breast cancer deaths are higher than female breast cancer or deaths from neonatal causes occur in age groups over one year. All deaths totals are compared with the sum of cause-specific deaths to ensure the observed deaths are accounted for and sample size is complete.

Step 1.1 - Disaggregation

Causes of death in tabulated VR data are condensed into aggregated groups, some of which can be mapped directly to GBD causes while other aggregated cause groups are not informative and cannot be mapped to GBD causes. To correct for this, aggregated causes were mapped and split onto multiple ICD8, ICD9 and ICD10 detail causes, or targets, based on the ICD groupings within the aggregated causes. ICD8, ICD9 and ICD10 detail codes serve as targets because they are the highest-quality VR data and enable the calculation of proportions used to split the aggregated cause data into detailed causes. The proportions of deaths from nearby countries within the super-region were used to fill in data gaps as they were likely to have similar CoD trends.

We determined the targets based on detail causes missing from the tabulated cause list. For example, in ICD9 BTL, the tabulated cause list includes a viral diseases group. In the hierarchy of causes, this group consists of "measles", "yellow fever", "encephalitis", "hepatitis", "rabies", "other infectious diseases", "garbage code", and "remainder of viral diseases". We did not consider this list to be an exhaustive list of viral diseases based on the range of ICD detail codes given in the ICD9 BTL documentation. To make the cause list exhaustive and inclusive of other viral diseases, we split the remainder of the viral diseases group into "other meningitis", "other infectious diseases", "herpes", "dengue", "other neglected tropical diseases", and "garbage code". After a list of targets was determined, the aggregated deaths were disaggregated to the target causes using ICD8, ICD9, and ICD10 detail proportions generated at the super-region level for the corresponding sex and age groups across all years in the time series. For example, in ICD9 detail data, 54.8% of deaths in males in Latin America and the Caribbean within the target group for BTL Viral Diseases were designated "other meningitis", so 54.8% of deaths in the tabulated group "remainder of viral diseases", were assigned to "other meningitis" for any country within that particular super-region. For any cause and demographic group where we lacked ICD detail, global proportions were used.

Step 1.2 - State splitting

Two sources for CoD estimation in India are the Medical Certification of Causes of Death (MCCD) report, which reports medically certified deaths from health facilities in mostly urban areas[3], and the Survey of Causes of Death (SCD), which collects information via VA on about one-half of 1% of all rural deaths in India, based on populations living in about 1,300 primary health care centers spread throughout the country.[4] For both of these reports, missing data impedes estimation of trends at the

state level. We used a first-order, log-linear model of the four-way contingency table of deaths by sex, age, state, and year to estimate the missing state-years. We fit the model to all available data for MCCD and SCD separately for each cause, including state-specific all-age measurements and age-specific national measurements. From this, we produced estimates for each combination of sex, age, state, and year. We then used these estimates wherever the raw data did not include sex-, age-, and state-specific death counts.

For MCCD, the model was fit separately for ICD10- and ICD9-based reports using the tabulated cause list present in the data. In the SCD report, the model was fit for each GBD cause in the data. As data from the SCD reports were relatively sparse, the pooling of like causes together led to an improved model fit.

Step 1.3 - Calculate non-maternal deaths

In cases when maternal mortality metrics do not include both deaths due to maternal causes and deaths due to non-maternal causes for women of reproductive age, live births and all-cause mortality estimates can be used to calculate deaths. Many studies report maternal deaths as the maternal mortality ratio (MMR). MMR is the number of maternal deaths per 100,000 live births and can be used to calculate deaths when it has been derived from primary data and not estimated. Maternal deaths were calculated using MMR and live births; if live births were missing we substituted live birth estimates and used the following equation:

$$Maternal\ deaths\ = \frac{MMR}{100{,}000} \times Live\ births$$

If a study was non-representative, we extracted sample size and live births from that study. After maternal deaths were calculated, we used the difference from all-cause mortality estimates to determine non-maternal deaths.

A more accurate and data-inclusive method of calculating maternal and non-maternal deaths incorporates coverage and splits deaths for a range of years into individual years. If there were live births in the study, we adjusted the coverage.

$$Coverage = \frac{Live\ births}{GBD\ estimated\ live\ births}$$

After coverage was calculated, totals deaths were scaled to be more representative. This gives a more accurate death count since the envelope assumes representative coverage. Using all-cause mortality as an all-cause total, non-maternal deaths were subsequently calculated.

$$Maternal\ envelope\ with\ coverage\ = Maternal\ envelope \times Coverage$$

An additional adjustment can be applied to maternal data spanning over a range of consecutive years, which allows for more data inclusion. The years within specified year ranges are separated into individual years, and total deaths within the year range were split between each individual year using the fixed proportions of maternal deaths from VR in that particular country. We used only VR data

inform the proportions because it was both high-quality and representative.

Section 2.4: Step 2 - Map to GBD cause list

In GBD 2017 we used 529 maps to translate causes found in the input data to the GBD 2017 cause list. This included 33 maps for VR data, 289 for VA data sources, and 102 for other data types. The largest, and most universal, maps used were those for ICD9 and ICD10 detail VR data. The input data causes varied from 3-4 digit ICD codes to custom cause lists with cause names such as “cholera” or “hepatitis”. Our mapping process made it possible to compare these various data sources across demographic groups.

In GBD 2017, we developed additional maps to translate ICD codes found in the input data that are non-underlying causes to appropriate target codes based on the levels of the GBD cause list. These garbage codes were mapped to Levels 1-4 of the GBD cause list according to the following criteria:

1. **Level 1** includes all garbage codes for which a Level 1 GBD cause cannot be directly assigned. For example, the underlying causes of “sepsis” or “peritonitis”, if not specified in the data, could be an injury, a non-communicable disease, or a type of communicable disease. In these cases, deaths will be redistributed across all three of these Level 1 causes. In addition, deaths coded to impossible or ill-defined causes of death, including “senility” and “unspecified causes”, fall into this category, as they will be redistributed onto all causes.

2. **Level 2** includes all garbage codes that can be assigned to specific Level 1 causes in the GBD cause list. This would include deaths coded to “unspecified injuries” (X59), which are redistributed onto all injuries.

3. **Level 3** includes all garbage codes for which we know the Level 2 CoD, and can redistribute onto Level 3 causes. This includes deaths coded to causes such as “unspecified cardiovascular disease”, which falls within the Level 2 cause “cardiovascular diseases”, as well as those coded to “unspecified cancer site”, which falls within the Level 2 “neoplasms cause”.

4. **Level 4** includes all garbage codes for underlying causes of death that can be redistributed within a Level 3 cause. This includes garbage codes such as “unspecified stroke” or “unspecified road injuries.”

Section 2.5: Step 3 – Age-sex splitting

Different sources, particularly VA studies, report deaths for a wide range of age groups with varying intervals. For the analysis of causes of death, we mapped these different age intervals to the GBD standard set of age groups. The approach to undertake this mapping was the same as in the prior GBD studies (GBD 2016, GBD 2015, GBD 2013, and GBD 2010).

In the process of assembling a consolidated demographic database, perhaps the most impactful source of inconsistency is the aggregation of age groups. It is conventional to report such data in broad age groupings such as “0-4”, “5-14”, and “15-49,” or to report data with both sexes together. The issue of comparability between age-sex groups arose when assembling the GBD CoD database. The compiled database included 22 distinct tabulation formats for infants and 141 distinct tabulation formats for non-infants. We developed a tool, which we call age-sex splitting, that takes aggregated age groupings,

and likewise the “both sexes combined” grouping, and divides them into what their constituent age groups would likely have been using respective cause-specific and country-specific age distributions. The analytical framework for GBD includes three infant age categories: Early neonatal (0-6 days), late neonatal (7-27 days), and post-neonatal (28 days to 1 year), and 17 non-infant age categories starting with age 1-4 years, then proceeds in five-year age groups until the terminal age group of 95+. We treat unknown ages and sexes in the same manner we treated the “all ages combined” age category and “both sexes combined” sex group. Through this process, we were able to directly compare all data sources on even terms.

The approach to age splitting is based on the following formula. The key assumption underlying this formula is that the relative risk of death by age group compared to a reference age group is invariant across populations. While this assumption is likely violated in specific cases, there is a strong biologically based pattern of the relative risk of death for a cause by age that is observed for most causes. The basic formula is as follows:

$$D_a = R_a N_a \left(\frac{D_a^{a+x}}{\sum_a^{a+x}(R_a N_a)}\right)$$

Where:

D_a= the number of deaths from a cause in age group *a*

R_a= the relative risk of death in age group *a* compared to a reference group

N_a= the country-year-sex-specific population in age group *a*

D_a^{a+x}= the number of deaths in the age group *a* to *a+x*

With the assumption of invariant relative risks of death by age with respect to a reference age group, this equation can be used, along with population distribution by age, to split an aggregate number of deaths for the age groups *a* to *a+x* into specific deaths for each age group within the aggregate interval.

In some cases, deaths are reported for an aggregate age group for both sexes combined. The task in this case is more complicated, but the same principle can be applied. In this case we assumed that the relative risks of death by age and sex are constant.

$$D_{as} = R_{as} N_{as} \left(\frac{D_{as}^{a+x,s}}{\sum_a^{a+x}(R_{as} N_{as})}\right)$$

Where:

D_{as}= the number of deaths from a cause in age group *a,* sex *s*

R_{as}= the relative risk of death in age group *a* compared to a reference group for sex *s*

N_{as}= the country-year-sex-specific population in age group *a* for sex *s*

$D_a^{a+x,s}$= the number of deaths in the age group *a* to *a+x* for sex *s*

This equation can be used to split data aggregated over age and sex. The assumption, however, of invariant relative risks across age and sex is a stronger assumption. Fortunately, data pooled across sexes are less common in the published or unpublished CoD data.

The relative risk of death in a particular age group for a given sex is derived from the global distribution of cause-specific mortality rates found in available VR data. Location-years from the following code systems are used, provided they report the requisite age- and sex-detail: ICD7, ICD8, ICD9 BTL, ICD10 tabulated, ICD9, and ICD10. Upon compiling these data, we mapped them to GBD causes, and aggregated up to cause Level 3. This is the level at which a particular cause is split – that is, any daughter cause of a Level 3 parent is split using the age distribution of that parent (so, chronic kidney disease due to diabetes would be split using the age pattern of chronic kidney disease).

We next adjusted separately for estimated adult and child VR completeness. Location- year-age-sex-specific deaths and population were then aggregated across all location-years, in order to produce cause-specific mortality rates by age and sex. These were used to determine the risk of death at any age relative to any reference age group.

Section 2.5.1: Correct age-sex violations

Occasionally, data sources will include deaths by a cause for which there is medical consensus that death is impossible for the sex and age. For example, there may be some number of deaths due to cervical cancer in males, or deaths due to maternal causes in ages under 10. We have constructed a conservative list of age-sex restrictions. When deaths violate these restrictions, we redistribute them proportionally onto all causes. All restrictions are included in Appendix Table 5, Restrictions on age and sex by cause for GBD 2017.

Section 2.6: Step 4-- Correction for miscoding of Alzheimer’s and other dementias, Parkinson’s disease, and atrial fibrillation and flutter

Section 2.6.1: Objective

For certain causes of death, mortality rates reported in vital registration systems are impossible to reconcile with observed trends in disease prevalence and excess mortality. For dementia, Parkinson’s disease, and atrial fibrillation and flutter, these disparities can largely be attributed to death certification practices. We sought to address the known bias in cause of death data by first identifying the proportion of all deaths that should be assigned to these causes, and then determining the GBD causes and garbage groups to which these deaths are being incorrectly assigned.

In past GBD iterations, we estimated Alzheimer’s disease and other dementias, Parkinson’s disease, and atrial fibrillation and flutter on the basis of longitudinal prevalence and excess-mortality data in order to help account for changing patterns in death certification and corresponding implausible time trends in many VR sources. This method was first implemented for Alzheimer’s disease and other dementias in GBD 2013. We added atrial fibrillation and flutter and Parkinson’s disease to the causes modelled using

this strategy in GBD 2015 and GBD 2016, respectively. All of these causes were processed in CoDCorrect in a manner that was agnostic to the likely targets of misclassification, which inappropriately led to changes in mortality estimates for causes unrelated to these three in GBD 2015. For GBD 2016, we improved this process by completing a literature review to identify the causes of death most closely associated with Parkinson's and Alzheimer's diseases[5–8] and limiting the CoDCorrect adjustments to only include those causes. For GBD 2017, we have refined this approach further by using multiple CoD data to determine the GBD causes and garbage codes from which we move deaths, as well as the pattern of misclassification.

Section 2.6.2: Correction Process

We first estimated excess mortality from prevalence and CoD data in countries with the highest ratio of cause-specific mortality to prevalence, which represents the greatest willingness to code to an under-coded cause. Then, we derived estimates of cause-specific mortality rates from available prevalence surveys as well as the estimates of excess mortality rate, applied across all countries and over time, using DisMod-MR 2.1. We divide this value by the all-cause mortality rate to determine the fraction of overall mortality to attribute to each under-coded cause.

To ascertain the causes from which we would move deaths to under-coded causes, we leveraged multiple CoD data from the United States – by looking to the combinations of intermediate and immediate causes (i.e., chain causes) present on death certificates with an under-coded cause listed as underlying, and identifying other causes with similar or identical chain causes, we can determine the expected pattern of miscoded deaths.

The first stage in this process is to parse out years we believe coding practices in the United States to be relatively stable. For dementia, this "gold standard" dataset features 2010-2015, for Parkinson's 2005-2015, and for atrial fibrillation and flutter 2014-2015. We then collect all deaths in those years with the under-coded cause listed as underlying and remove any mention of the under-coded cause from the death certificate. Next, for each unique chain, we search the entire time series of data (1980-2015) to identify the distribution of underlying causes that share that chain. The premise here is that if the diagnosis of dementia, Parkinson's, or atrial fibrillation and flutter were missed, the other causes listed on the death certificate would have been the basis for certification. We then reallocate the under-coded deaths by chain based on that alternative underlying cause distribution.

Upon iterating through all unique chains, we are left with a dataset excluding under-coded causes of death, each remaining cause able to be subdivided into correctly coded deaths and deaths that have been recoded from an under-coded cause by the aforementioned process (though not all causes will necessarily be targeted by the recoding algorithm). The quantity of interest is the ratio of miscoded deaths to total deaths by cause, age, and sex in our counterfactual dataset.

We apply the ratios derived from the multiple cause data to all VR data to determine the local pattern of miscoding. In this way, the method is sensitive to the observed epidemiology of a given place and time. Then, we calculate the deficit in under-coded cause mortality for each location, year, age, and sex by taking the difference in the expected cause fraction based on prevalence and excess mortality compared to the proportion of deaths actually certified by the VR system. Lastly, we scale the cause-specific miscoded deaths to match the deficit and then move them accordingly. We assumed that

misclassification of actual dementia and Parkinson's deaths in past years only occurred for reported causes of death that might have plausibly been the direct result of dementia or resulted from misdiagnosis of other organic brain diseases, based on clinical expert judgement. A similar assumption is used for atrial fibrillation and flutter, for which only cardiovascular causes and ill-defined garbage are considered.

Because the deaths being reallocated vary by location-year, we need a mechanism to ensure plausible limits to how many deaths are extracted from each GBD cause and garbage code. To achieve this, we first run the above-mentioned algorithm on all 5-star VR data (see Section 2.16 of this appendix for an explanation of the star data quality rating system). Then, we determine the 95th percentile of the proportion of deaths moved for each GBD cause and garbage group by age and sex across location-years among these data. Those values are subsequently stored and applied as the limits for deaths moved by this process.

Section 2.7: Step 5 - Redistribution

A crucial aspect of enhancing the comparability of data for CoD is to deal with uninformative, so-called garbage codes. Garbage codes are codes to which deaths were assigned that cannot or should not be considered as the underlying CoD, for example: "heart failure", "ill-defined cancer site", "senility", "ill-defined external causes of injuries", and "septicaemia". The methods for redistributing these garbage-coded deaths were outlined in detail in Naghavi et al[9], and the underlying algorithm for redistributing deaths assigned to these codes has not changed since GBD 2013.

Step 5.1: Redistribute HIV-related garbage codes

Due to the disparate nature of HIV/AIDS mortality across space and time, dynamic redistribution of HIV/AIDS-related garbage codes was needed (Appendix Table 6). To inform this redistribution, we generated target proportions for each garbage group by age band (Under 1 month, 1-59 months, 5-19 years, 20-49 years, 50-59 years, 60-69 years, 70-79 years, and 80+ years), five-year time interval, and sex. The garbage groups either target HIV or a remainder target. The allotment of deaths to either of these is based on the regional increase in the mortality rate of all codes in the group relative to the rates seen in 1980–1984 – an increase greater than 5% is assumed to be HIV/AIDS-related, and the proportion of those deaths exceeding 5% are redistributed to HIV/AIDS. Any increase ≤ 5% is then assigned to the remainder target.

Step 5.2: Regress garbage codes versus non-garbage codes

As in GBD 2015, the statistical analysis used to determine proportions for garbage code redistribution for ill-defined cancer sites, ill-defined external causes of injury, unspecified stroke, heart failure, hypertension, and atherosclerosis was based on the approach outlined by Ahern et al.[10] For each redistribution package, we defined the "universe" of data as all deaths coded to either the package's garbage codes or the package's redistribution targets for each country, year, age, and sex. We then ran a regression based on the following equation, separately for each target group and sex:

$$TG_{crt} = \alpha + \beta_1 Gar_{crt} + \beta_2 Age_{crt} Gar_{crt} + \theta_r Gar_{crt} + \gamma_r + \varepsilon_{ct}$$

Where:

TG_{crt} = percentage of deaths within the given garbage code's universe which were coded to a given target group, by country

Gar_{crt} = percentage of deaths within the given garbage code's universe which were coded to a given set of garbage codes

α = constant

β_1 = slope coefficient describing the association between Gar_{crt} and TG_{crt}

β_2 = slope coefficient describing the association between the interaction $Age_{crt}Gar_{crt}$ and G_{crt}

γ_r = region-specific random intercept (or super-region if the random effect on region is not significant)

θ_r = region-specific random slope (or super-region if the random effect on region is not significant)

ε_{ct} = standard error, normally distributed and calculated by bootstrapping

This regression was adjusted from GBD 2013 to include fixed effects on the interaction of garbage and age to ensure smooth age patterns. We made this decision after investigating diagnostic visualisations that showed unlikely gaps between proportions assigned to different age groups.

Once proportions were produced for each country, sex, age, and target group, certain adjustments were made to conform our packages to the best medical evidence available. In some cases, we implemented restrictions on the proportions that the regressions could yield. For example, we did not allow any redistribution onto "Chagas disease" outside of Latin America and the Caribbean, or "suicide" under the age of 15. In other cases, we capped the proportion for some targets to the level that would be produced from proportional redistribution; for example, "haemoglobinopathy" and "haemolytic anaemia" were restricted to the level of proportional redistribution in the redistribution of "left heart failure". Occasionally, further adjustments were made on a case-by-case basis per country, age, sex, and target group to suppress the impact of outliers based on existing epidemiological evidence and expert judgment.

Development of an algorithm for redistribution of garbage codes based on multiple CoD data

Multiple CoD data are a form of individual record causes of death data which include an underlying CoD along with other causes in the death chain, including intermediate and immediate causes. By analysing this type of data, we can sometimes find the true underlying CoD in other CoD data where the underlying cause is a garbage code or a misassigned CoD.

For GBD 2017, this approach was implemented in redistribution for a few select causes. One example of this approach is seen in the correction of the misassignment of deaths due to drug overdoses to unintentional poisoning. More than 90% of these types of poisonings are due to exposure to narcotics, psychodysleptics, and other drugs, specified or unspecified. More than 97% of these poisonings by

substance or drug occurred in ages 15–65. It is clear that these are not cases of accidental ingestion of substances, but rather that the substance has been deliberately ingested, with unintentional poisoning occurring.

Using multiple CoD records for the United States, Mexico, Brazil, and Australia from 1980 to 2014, we selected all deaths with underlying causes coded to X40–X44 and X49. Table A below shows the combination of other potential causes that can be found in the multiple CoD data for these underlying causes. Based on this table, we proportionally redistributed misassigned unintentional poisoning deaths to one of these causes. The main assumption behind this algorithm is the dominancy of the fatality of some substances when considering a combination of drugs. Given the combination of different drugs and substances in these codes, opium is the main cause of fatality.[11,12] Other substances, like cocaine, methamphetamine, and alcohol in combination with cannabis are less likely to be dominant in fatality.[13]

Table A. Algorithm for the selection and assignment of a substance or drug use CoD for deaths coded to an underlying cause of unintentional poisoning using multiple CoD data

Selection Algorithm						
	Opioids	Cannabis	Cocaine	Amphetamines	alcohol	Psychoactive and psychedelic drug
Opioids	Opioids	Opioids	Opioids	Opioids	Opioids	Opioids
Cannabis	Opioids	Cannabis	Cocaine	Amphetamines	alcohol	Psychoactive and psychedelic drug
Cocaine	Opioids	Cocaine	Cocaine	Amphetamines + Cocaine	Cocaine + alcohol	Cocaine
Amphetamines	Opioids	Amphetamines	Amphetamines + Cocaine	Amphetamines	Amphetamines + alcohol	Amphetamines
alcohol	Opioids	alcohol	Cocaine + alcohol	Amphetamines + alcohol	alcohol	Psychoactive and psychedelic drug
Psychoactive and psychedelic drug	Opioids	Psychoactive and psychedelic drug	Cocaine	Amphetamines	Psychoactive and psychedelic drug	Psychoactive and psychedelic drug

For example, if the multiple CoD data show that 40% of deaths include opioid use disorders as an intermediate cause where the underlying cause is X40–X44, X49, the redistribution proportion for opioid use disorders will be exactly 40% due to the dominance of the fatality of opioid use disorders compared to other drugs in the above table. Similarly, because cannabis is not assumed to have dominance of fatality compared to any of the above drug use disorders, the proportion of X40–X44, X49 redistributed to cannabis use disorders was the percentage of deaths in the multiple CoD data that had only cannabis use disorders as an intermediate cause and none of the other above causes. If 40% of

deaths had cannabis use disorders listed in the intermediate cause list, but those 40% also had one or more of opioids, cocaine, amphetamines, alcohol, or psychoactive and psychedelic drugs, the redistribution proportion to cannabis use disorders would be 0%.

Multiple CoD data were only available to us for the United States, Brazil, Mexico, and Australia. Because of this limited sample, we applied the result from multiple CoD analysis from the United States to the United States and Canada, from Brazil and Mexico to Latin America and the Caribbean, and from Australia to Australia, New Zealand, and Western Europe. For other locations, we aggregated the results from the four countries and applied the aggregate pattern. We hope for increased availability of multiple causes of death data in future analyses in order to achieve a more precise distribution for more locations.

Step 5.3: VA anemia adjustment

To compensate for the over-representative cause fractions from anemia found in VA studies, we redistributed these deaths based on the causal attribution of severe anemia from the GBD 2013 study. The proportions were country-year-age-sex specific.

Step 5.4 Calculate redistribution uncertainty

We categorized garbage codes into four levels in order of increasing specificity (see Section 2.4 of this appendix). Some garbage codes are redistributed on all causes (e.g. unspecified causes of death) and others are only redistributed onto specific causes (e.g. unspecified cancer). Major garbage refers to garbage codes in levels one or two. Due to the variation in redistribution, estimating uncertainty due to garbage redistribution for CODEm modeling was an important goal for GBD 2017.

We assigned redistribution variance to each data point in the CoD database by calculating residual variance from a regression predicting the percent of garbage coded deaths redistributed to a cause, given the proportion of garbage we observed for that location, year, age, sex, cause and the age standardized relative rate of major garbage across all causes. If there is a cause that has greater residual variance, we assume greater redistribution uncertainty.

The two model inputs are the observed percent level 1, 2, 3 garbage (by cause, age, sex, location, and year) in redistributed CoD data and the percent garbage in the raw data (calculated as the age standardized mortality rate ratio of major garbage deaths to all deaths in the raw data by location, year, sex). Level four garbage was excluded from the model to avoid over estimating uncertainty in low major garbage countries. Additionally, the classification of level 4 garbage codes is not stable between successive GBD rounds – for example, “unspecified diabetes” was not a garbage code in GBD2016, and in GBD2017 was re-classified as a level 4 garbage code to permit estimation of diabetes by type. These deaths are still taken into account later in the uncertainty estimation process. The model predicts the percent of garbage coded deaths redistributed to a cause, given the proportion of garbage we observed for that location, year, age, sex, cause and the age standardized relative rate of major garbage across all causes. From this model we calculate residual variance. It is important to note that the variance here is a measurement of uncertainty of redistribution, not of the level of miscoding in the raw cause of death data for a given demographic.

In order to calculate variance, a dataset was generated containing percent garbage by location, year, age, sex and cause, where percent garbage is:

$$pct_{garbage} = \frac{deaths_{redistributed} - deaths_{raw}}{deaths_{redistributed}}$$

A mixed-effect linear regression model was then fit to predict the logit percent of deaths from redistribution by age-standardized relative rate of major garbage.

$$logit\left(pct_{garbage_{ij}}\right) = \beta_0 + \beta_1 * \log\left(ASR_{majorgarbage_{ij}}\right) + \beta_2 * 15yearage_{ij} + \gamma_{1j} * \log\left(ASR_{majorgarbage_{ij}}\right) + u_j + e_{ij},\ \ \theta_{\{i\}} \sim N(0, \sigma^2)$$

Where:

i indexes dataset-location-year-age-sex-cause data points nested within *j* groups by GBD region

ASR major garbage: age standardized relative rate of major garbage

Residual variance, as estimated by the MAD, was calculated for each cause, sex, and age.

The next step was to use the residual variance to calculate uncertainty around each data point in the CoD database. First, we calculated the percent garbage of each data point, treating all deaths that could not be directly mapped to a GBD cause as garbage, including level four garbage. Percent garbage was calculated as:

$$pct_{garbage} = \frac{deaths_{redistributed} - deaths_{corrected}}{deaths_{corrected}}$$

Where:

death corrected: deaths post misdiagnosis correction (Appendix Section 2.6)

deaths redistributed: deaths post redistribution (Appendix Section 2.7)

Residual variance was matched to each data point and one hundred draws were sampled from a normal distribution using the cause, age, sex, specific residual variance and mean of zero. The logit transformed percent garbage was added to each value in the distribution. Each draw was then transformed out of logit space and the post-redistribution deaths were calculated as:

$$deaths = \frac{deaths_{corrected}}{1 - pct\_garbage}$$

Draws of deaths were processed through noise reduction before calculating the final redistribution variance passed to CODEm, which was added to the total data variance. The mean of the draws was not used as the final estimate, because it was found that the logit transformation biases the distribution of cause fractions higher than if only point estimates are used.

Section 2.8: Step 6 - HIV/AIDS misclassification correction

In many location-years, certain causes of death known to be comorbid with HIV/AIDS (eg, tuberculosis, other infectious diseases) are seen to have age patterns that diverge from those observed in location-years without widespread HIV epidemics and are in fact more reflective of HIV mortality trends. In order to identify these instances, a global relative age pattern is generated using all VR deaths in countries with observed HIV prevalence less than 1% using the following:

$$RR_{asc} = \frac{R_{asc}}{\bar{x}(R_{65sc}, R_{70sc}, R_{75sc})}$$

Where RR_{asc} is the relative death rate for age group *a*, sex *s*, and cause *c*; R_{asc} is the rate for that age group; and $\bar{x}(R_{65sc}, R_{70sc}, R_{75sc})$ is the mean of the rates in ages 65–69, 60–74, and 75–79 for that sex and cause. This is preferable to comparing mortality rates because we are able to isolate divergence in age pattern while accounting for varying levels of overall mortality by fixing death rates to age groups that are unlikely to be confounded by the presence of HIV. Expected deaths for an identified cause were then determined to be:

$$ED_{lyasc} = \bar{x}\left(R_{ly65sc}, R_{ly70sc}, R_{ly75sc}\right) \times p_{lasc} \times RR_{asc}$$

Where ED_{lasc} are deaths for location *l*, year *y*, age group *a*, sex *s*, and cause *c*; $\bar{x}(R_{l65sc}, R_{l70sc}, R_{l75sc})$ is the mean of the rates for ages 65–69, 60–74, and 75–79 for that location-year-sex-cause; p_{lasc} is the population for that location-year-age-sex-cause; and RR_{asc} is the global standard relative rate determined in the previous step for that age-sex-cause. The expected deaths remain attributed to that particular cause, while the difference between observed and expected are reallocated to HIV/AIDS.

Section 2.9: Step 7 - Scale strata to province

Over time, a higher proportion of deaths have been registered in China through the expansion of the DSP system and provincial/county efforts to increase CoD registration. With the expansion of coverage, it is possible that province aggregates do not accurately represent the population distribution between urban and rural areas in each year. For this reason, we stratified the data preparation by urban and rural status for each county within each province. Stratification was based on the median level of urbanisation across counties within each province as recorded in the 2010 China census. In the provinces of Tibet and Hainan, all counties were placed into one strata based on largely homogeneous urbanisation levels within each province. This yielded a total of 62 analytical province-strata. Macao and Hong Kong were not included in this stratification system as the VR systems there are independent from that on the mainland; no weighting scheme needs to be carried out in these complete VR systems with quality data on causes of death.

Within each province-strata, a larger proportion of deaths in-hospital might be reported than that of deaths outside of hospital because of the internet hospital reporting system. To avoid bias, we reweighted in-hospital and out-of-hospital deaths based on the age-sex-province-specific fraction of deaths in and out of hospital in the DSP system. DSP data have been used to establish these percentages because, in these communities, there is a concerted effort to identify all out-of-hospital

deaths. Province-strata death rates are combined to produce overall province death rates by weighting each strata by population in each age-sex-year group. Province death rates are rescaled so that all-cause mortality equals the estimated death rate in each age-sex-year estimated in the life-table analysis. The Bayesian noise reduction algorithm was used to deal with zero counts and small number issues for rare causes.[14]

Section 2.10: Step 8 - Restrictions post-redistribution

Some causes of death can only be reliably assigned through an autopsy by a trained physician. For example, it is unlikely that a VA would reliably distinguish between ischaemic and haemorrhagic stroke.

This step ensures that the detail of the cause list at this point in the data prep process is reasonable given the detail of the original data source and the methods by which the CoD was assigned. Two primary corrections are applied. First, any cause which is purely an artifact of the redistribution machinery targeting too detailed a cause is aggregated up to the parent cause. Second, a "bridge map" is applied over a certain set of sources to ensure that these sources do not contain causes which could not reliably be determined by the methodology. These two corrections are applied to ICD9-BTL, ICD10-tabulated, USSR tabulated ICD9, India MCCD reports, China-DSP-tabulated-ICD9, India SCD reports, India SRS, and all VA sources.

Section 2.11: Step 9 - Drop VR country years or mark as non-representative

Lozano and colleagues[15] describe the negative impact that low-completeness VR data could have on CoD modelling for GBD 2010. In particular, in settings where a data source does not capture all deaths in a population, the cause composition of deaths captured might be different from those that are not. However, a completeness sensitivity test found that low- completeness VR data had little impact on the cause-specific mortality trends at the global level.

For GBD 2017, we investigated the impact of these data at the country and subnational levels using the more thorough diagnostic visualisations available to us. It was determined that these data produced unlikely trends in the models affected. Despite the minimal impact on global trends, better models were produced by eliminating or marking as non-representative data with extremely low completeness. VR completeness was estimated as the number of deaths registered divided by the number of deaths estimated in the GBD mortality envelope.

For this round, VR location-years with completeness below 50% were dropped, while location-years with completeness between 50% and 69% were marked as non-representative.

In addition, any country-year with a number of deaths registered to major garbage codes greater than 50% of the deaths registered was dropped.

Section 2.12: Step 10 - Cause aggregation

The cause list is organised in a top-down hierarchical format containing four levels. The first group, or Level 1, sums all causes. Following all cause-mortality are Level 2 causes, which include three broad groupings of causes of deaths: "communicable, maternal, neonatal, and nutritional diseases"; "non-

communicable diseases"; and "injuries". Within those Level 2 groupings are finer levels used for modelling. Level 3, or parent causes, are aggregated; the mortality estimate for a parent cause in the hierarchy represents the sum of the causes under that rubric. Sub-causes within Level 3 causes – Level 4 – are more detailed. For example, the parent cause "intestinal infectious diseases" contains the three sub-causes: "typhoid fever", "paratyphoid fever", and "other intestinal infectious diseases". Included in the parent cause estimate are deaths mapped directly to the parent and any Level 4 sub-causes. In data where there was not enough information to assign a Level 4 cause, we aggregated to the Level 3 parent cause. Exceptions to aggregating the Level 4 sub-causes to the parent are instances when certain sub-causes are not present. The United Nations Crime Trends police data only identify homicides, and aggregating homicides to injuries would not accurately represent all injuries.

Section 2.13: Step 11 – Remove shocks and HIV/AIDS maternal adjustments

For GBD 2017, CODEm models use an HIV/AIDS- and shock-free envelope. In order to be comparable, cause fractions must also be HIV/AIDS- and shock-free. Cause fractions were uploaded to the CoD database as the number of deaths due to the cause over an adjusted sample in which the number of deaths due to "HIV/AIDS", "conflict and terrorism", "executions and police conflict", and "exposure to forces of nature" were removed.

Step 11.1 Remove HIV/AIDS, shocks from denominator where cause list includes HIV/AIDS

The first step to generate HIV- and shock-free cause fractions was to remove any deaths from the sample which were directly coded to "HIV/AIDS", "collective violence and legal intervention", or "exposure to forces of nature". The resulting equation for a cause fraction uploaded to the database is simple:

$$CF_{l,t,a,x,c} = \frac{D_{l,t,a,x,c}}{D_{l,t,a,x} - D_{l,t,a,x,hiv} - D_{l,t,a,x,war} - D_{l,t,a,x,disaster}}$$

Where:

$CF_{l,t,a,x,c}$ is the cause fraction for a location (l), year (t), age (a), sex (x), and cause (c)

$D_{l,t,a,x,c}$ is the number of deaths observed in the sample for the same

$D_{l,t,a,x}$ is the total number of deaths observed in the sample in the location, year, age and sex

$D_{l,t,a,x,hiv}$, $D_{l,t,a,x,war}$, and $D_{l,t,a,x,disaster}$ are the number of deaths observed in the sample for "HIV/AIDS", "collective violence and legal intervention", and "exposure to forces of nature", respectively

Cause fractions for HIV/AIDS and shock causes were also uploaded to the database for use in separate estimation processes described by Wang et al.[12] In this case, cause fractions followed the standard equation, with variables following the same explanation as above:

$$CF_{l,t,a,x,c} = \frac{D_{l,t,a,x,c}}{D_{l,t,a,x}}$$

Step 11.2 Remove HIV/AIDS deaths from maternal mortality sources

HIV-free cause fractions were also uploaded for sources on mortality due to maternal causes. In these cases, the sample of all deaths observed in the study is likely to contain some amount of deaths due to HIV/AIDS and shocks, but the sample only includes cause information on maternal deaths. To account for the presence of HIV/AIDS and shocks in the entire sample, we assumed the same proportion of total deaths due to HIV/AIDS by location, age, sex, and year as provided from the estimation of HIV/AIDS and all-cause mortality described by Wang et al.[2]

Maternal mortality studies were only corrected for HIV/AIDS if the sample of total deaths was provided in the data source. Where sources only provided the Maternal Mortality Rate (MMR), we applied the rate to the HIV- and shock-free envelope produced by the analysis described in Wang et al.[2] and thus did not need to adjust cause fractions at this point in the process.

Where a correction was applied, we used the following equation:

$$CF_{l,t,a,x,mat} = D_{l,t,a,x,mat} \times \frac{E[D_{l,t,a,x,hiv\_shock\_free}]}{E[D_{l,t,a,x}]}$$

Where:

$CF_{l,t,a,x,mat}$ is the resulting cause fraction due to maternal causes for the location (l), year (t), age (a), sex (x);

$D_{l,t,a,x,mat}$ is the number of observed deaths in the sample due to maternal causes

$E[D_{l,t,a,x}]$ is the GBD estimate of all-cause mortality in the location, year, age, and sex

$E[D_{l,t,a,x,hiv\_shock\_free}$ is the GBD estimate of HIV- and shock-free mortality in the location, year, age, and sex

Step 11.3 HIV/AIDS correction of sibling history, census, and survey data

As described in our analysis from GBD 2013, many studies have failed to find increased mortality in HIV+ pregnant mothers, but those who have advanced HIV are known to have increased baseline mortality. Prior to GBD 2013, we did not distinguish between deaths in HIV+ women that were caused by pregnancy and those for which the pregnancy was incidental to their death. In order to more explicitly quantify the contribution of pregnancy to death in HIV+ women, and therefore more accurately estimate the maternal death count, we completed two additional analyses for GBD 2013 and all subsequent GBD analyses. First, we determined the population attributable fraction (PAF) of HIV/AIDS to pregnancy-related death. Second, we determined the proportion of pregnancy-related deaths in HIV-positive persons that are aggravated by pregnancy and are therefore by definition

maternal deaths.

$$PAF = \frac{P(RR-1)}{1+P(RR-1)}$$

Where PAF is the population attributable fraction, P denotes the prevalence of HIV in pregnancy, and RR is relative risk of mortality in HIV+ vs HIV- pregnant females.

To recap our analysis for GBD 2013, we used the paper published by Calvert and Ronsmans to identify sources[16] that could inform Step 1 of our HIV-correction analysis. We independently reviewed each of the component studies in Calvert and Ronsmans' review and extracted data directly, not from the systematic review paper. We identified only one additional study that was not used in Calvert and Ronsmans' analysis. We have, however, not used all the studies included in that review. Specific details are as follows:

1) Figueroa-Damian, et al. was excluded for not including any postpartum deaths at all. [17]

2) In the case of Ryder, et al. and Zvandasara, et al. we excluded those deaths > 12 months after delivery.[18,19]

3) We excluded the results from Chilongozi, et al. from the site that did not include any HIV-negative patients.[20]

4) Leroy, et al. was not in the bibliography. We could not locate it for review so it was excluded.[21]

5) Kourtis, et al. was extracted with adjustment of the denominator based on the average number of hospitalisations per delivery in each group.[22]

6) Ticconi, et al. was excluded for being both non- representative and including subgroup data from mothers with malaria infection.[23]

A total of 21 sources were included in our analysis of the increased mortality risk of HIV+ versus HIV- women in pregnancy.[13] We performed DerSimonian-Laird random effects meta-analysis to derive a pooled estimate of RR of death during pregnancy given HIV positivity.[24] The pooled effect size was 6·40 (95% uncertainty interval [UI] 3·98−10·29), which was then used to calculate an HIV PAF for each country, age group, and year. In order to determine the proportion of those HIV-related deaths that were attributable to maternal causes, we performed a second systematic literature review. This time we sought evidence for the excess mortality risk of pregnancy in those women who are already HIV+. Most studies have failed to find such an effect, but most also did not stratify their study population by stage of HIV or ART status. Only two studies did this stratification, with a pooled effect size of 1·13 (95% UI 0·73−1·77).

An updated literature review to inform the relative risk of mortality in pregnancy in HIV-positive versus HIV-negative women had 14 no usable sources. We completed this search on July 20, 2017, using the following search strings:

((HIV[Title/Abstract] OR "Acquired Immunodeficiency Syndrome"[Title/Abstract] OR AIDS[Title/Abstract]) AND (“pregnant”[Title/Abstract] OR “pregnancy”[Title/Abstract] OR “postpartum”[Title/Abstract] OR "post partum"[Title/Abstract]) AND (“mortality”[Title/Abstract] OR “death”[Title/Abstract]) NOT "case report" NOT (animals[MeSH] NOT humans[MeSH])

AND (2016/08/15[PDat] : 2017/12/31[PDat]))

Prevalence of HIV in pregnant women was calculated using UNAIDS’ Spectrum model, a compartmental HIV progression model used to generate age-specific incidence, prevalence, and death rates from pre-calculated incidence curves and assumptions about intervention scale-up and local variation in epidemiology. For each location, we used UNAIDS’ age-specific ratios of fertility in women living with HIV to fertility in women not living with HIV. In most locations, this ratio is assumed to be greater than one in women aged 15–24 and less than one and decreasing as age increases beyond 24. Since Spectrum assumes fertile ages of 15–49, we used the ratio of HIV prevalence in pregnant women to HIV prevalence in the general population at either end of that range to extend estimates to age bands 10–14 and 50–54.

Unlike GBD 2013, when we applied the PAF correction to the envelope of maternal deaths predicted by CODEm, we instead applied country-year-age-group-specific PAF to maternal mortality input data prior to modelling in CODEm. This ensured that both the numerator and denominator of all CF data were internally consistent in their exclusion of background HIV/AIDS mortality. The cause fractions for maternal deaths in sibling history, survey, and census data were therefore adjusted as follows:

$$CF_{l,t,a,x,mat_{adj}} = CF_{l,t,a,x,mat} \times \left(1 - ProP_{hiv_{l,t,a,x}}\right)$$

$$ProP_{hiv_{l,t,a,x}} = PAF_{l,t,a,x,hivpos} \times (1 - rr_{mat})$$

$$CF_{l,t,a,x,mat_{hiv}} = CF_{l,t,a,x,mat} \times ProP_{maternalhiv_{l,t,a,x}}$$

$$ProP_{maternalhiv_{l,t,a,x}} = PAF_{l,t,a,x,hivpos} \times rr_{mat}$$

Where:

$CF_{l,t,a,x,mat}$ = The proportion of deaths due to all maternal causes before HIV/AIDS correction for the location, year, age, and sex.

$CF_{l,t,a,x,mat_{adj}}$= The proportion of deaths due to maternal causes after the adjustment for the location, year, age, and sex.

$CF_{l,t,a,x,mat_{hiv}}$ = The proportion of deaths due to maternal deaths aggravated by HIV/AIDS after the adjustment for the location, year, age, and sex.

$PAF_{l,t,a,x,hivpos}$ = The population-attributable fraction (PAF) that describes the percentage of all maternal deaths that were HIV-related for the location (l), year (t), age (a), and sex (x=Female))

$ProP_{hiv_{l,t,a,x}}$ = The proportion of deaths in pregnancy for the location, year, age, and sex that are estimated to be incidental deaths due to HIV/AIDS, and therefore not a maternal CoD.

$ProP_{maternalhiv_{l,t,a,x}}$ =The proportion of deaths in pregnancy for the location, year, age, and sex that are estimated to be HIV-positive and maternal deaths which are aggravated by HIV/AIDS.

rr_{mat} = .13/1.13 = The proportion of HIV/AIDS deaths during pregnancy that were exacerbated by the pregnancy.

Step 11.4 HIV/AIDS correction of other maternal mortality data

Although there are a specific subset of codes in ICD10 that correspond to HIV/AIDS deaths aggravated by pregnancy, these codes are sparsely used and unreliable. We therefore adapted the method above to also correct VR and VA sources for the systematic exclusion of HIV-related maternal deaths. This correction was calculated in the same manner, using the same input data as above, with the only difference that HIV correction of VR and VA sources resulted in a net increase in maternal CF. Maternal deaths aggravated by HIV/AIDS are calculated as the following (where all symbols are the same as described above):

$$CF_{l,t,a,x,mat_{hiv}} = CF_{l,t,a,x,mat} \times ProP_{maternalhiv_{l,t,a,x}}$$

$$ProP_{maternalhiv_{l,t,a,x}} = \frac{PAF_{l,t,a,x,hivpos} \times rr_{mat}}{1 - PAF_{l,t,a,x,hivpos} \times rr_{mat}}$$

Section 2.14: Step 12 – Noise Reduction

To deal with problems of zero counts in VR, VA, cancer registries, or sibling histories for a given age group in a given year, we use a Bayesian noise-reduction algorithm. For this algorithm, we assume a normal prior and a normal data likelihood. We estimate the normal prior for a given country-series of data by estimating a negative binomial for the fraction of deaths in each age group due to each respective cause with dummy variables for age and year. With two notable exceptions (detailed below), these regressions are country-specific, so borrowing strength over age is only within a data type in a country. The variance of the prior, τ^2, is estimated from the negative binomial regression, taking into account the variance-covariance matrix of the regression coefficients. For the data variance, we use the Wilson approximation which provides an estimate of σ^2 even in cases with a zero count of cause-specific deaths. The posterior estimate for each data point is:

$$Mean = \left(\frac{\tau^2}{\tau^2 + \sigma^2} X + \frac{\sigma^2}{\tau^2 + \sigma^2} \mu\right)$$

$$Variance = \left(\frac{\tau^2 \sigma^2}{\tau^2 + \sigma^2}\right)$$

Where *X* is the mean of the data and μ is the mean of the prior. This approach to noise reduction avoids the problem that zero counts in an *ln* rates model or a logit cause fraction model will be dropped from the regression and lead to upward bias in the estimates. This is particularly important in two settings: high-income countries with small numbers of cause-specific deaths, and in the analysis of sibling history

data where for any given age group in any given year the number of deaths reported in the survey that are pregnancy-related or the number of deaths from all causes in that age group may be small.

Regarding the exceptions to the regression, the first is that country-years with populations under 1 million are pooled with the region data in order to prevent overdispersion and provide a stronger signal. Additionally, VA data diverge from the above description in two ways. First, all data for a given super-region are pooled together and a study dummy variable is added, allowing for different studies and surveillance sites to borrow strength from one another within a super-region. Second, unless the data are part of a time series (eg, Matlab HDSS), there is no year component to the regression.

Section 2.15: Step 13 – CoD database and outlier identification

Death rates for different causes of death generally have a stable age pattern. In large populations, these patterns will not change very rapidly over time. We can assume a relatively stable pattern in death rates for all causes except for some epidemic diseases and specific types of injuries. Rare causes in large populations and prevalent causes in small populations usually have stochastic patterns. To correct for these stochastic patterns, we implement a noise-reduction process, explained in Step 12.

In VR data, we infrequently find one or more data points for specific geography/age/sex/years that lie very far from the stable pattern of death rates. In these situations, the model will usually ignore the data point(s). If the model fails to ignore these data, dramatic jumps or drops can occur in the death rates. When there is no logical explanation for variation in the death rates to this degree, we outlier the data point(s). The selection of data points to outlier occurs after data have been prepped for modelling, as well as during preliminary reviews of the models.

In non-VR sources, data-collection methods and data quality can vary widely from source to source. Where data points in each age-sex-geography-year are very sparse, extreme data points can have a bad effect on regional estimation. In these situations, we investigate the study's methods and outlier lower-quality data points.

Identifying outliers in the CoD data occurs prior to finalisation of models for each cause. We do not automate the selection of outliers, but investigate the source of the offending data as well as reviewing other data sources for the same cause, geography, and year. Ultimately, outliers are identified based on the judgement of the modeller and senior faculty. Outlier decisions are reversible and may be revisited.

Section 2.16: Causes of death data star rating calculation

GBD estimates are most accurate when computed with a full time series of complete VR with a low percentage of garbage codes. For GBD 2016, we developed a simple star-rating system from 0 to 5 to give a picture of the quality of data available in a given country over the full time series used in GBD estimates. Countries improve in the star rating as they increase availability, completeness, and detail of their mortality data and reduce the percentage of deaths coded to ill-defined garbage codes or highly aggregated causes (Appendix Figure 5).

To assign stars, we measure the proportion of deaths registered to a well-defined cause from 1980 to 2016. We call this proportion "percent well-certified". We measure this proportion for each location-

year of VR and each VA study separately, and then combine the yearly measurements into a percent well-certified for the full time series.

For each year of VR, percent well-certified is:

$$pct_{wellcertified} = completeness \times (1 - pct_{majgarbage})$$

Where:

$$completeness = \frac{registered\ deaths}{GBD\ mortality\ envelope}$$

$$pct_{majgarbage} = \frac{deaths\ coded\ to\ level\ 1\ or\ 2\ garbage\ or\ highly\ aggregated\ cause}{registered\ deaths}$$

Simplifying this equation, one can see that in this case "percent well-certified" is simply the number of deaths that are registered to a well-defined cause (those codes which are not Level 1 or 2 garbage or highly aggregated) divided by the GBD mortality envelope.

ICD10 and ICD9 codes assigned to Level 1 or 2 garbage can be found in Appendix Table 4.

For each VA data source, percent well-certified is:

$$pct_{wellcertified} = VerbalAutopsyAdjustment \times (1 - pct_{majgarbage})$$

Where:

$$VerbalAutopsyAdjustment = SubAdj \times RegAdj \times AgeSexCoverage$$

And:

SubAdj:

10% for subnationally representative studies, 100% for nationally representative studies. This adjustment, while arbitrary in its specific value, reflects the bias that can be associated with studies that only cover a potentially non-representative sample of a country's population.

RegAdj:

64% for all VA data sources. This accounts for the inaccuracy of VA in assigning CoD compared to medically verified VR. The specific multiplier 0·64 is based on the chance-corrected concordance of Physician Certified Verbal Autopsy (PCVA) versus medical certification by the Population Health Metrics Research Consortium.[25]

Age-Sex Coverage:

The number of deaths estimated in the GBD mortality envelope for the ages and sexes in the study for the country and year divided by the number of deaths estimated in the

GBD mortality envelope for the country and year. Studies that only cover children under 5 or maternal mortality, for example, will be highly discounted by this multiplier.

In the case of VA, all garbage codes are considered ill-defined, as redistribution for VA is highly imprecise. Causes such as “Injuries” or “Cancer” will also be included in major garbage percentage, as this percentage includes use of highly aggregated causes.

Once percent well-certified is calculated for each location-year of VR and each VA study-year, we then combine these into one measurement for each five-year time interval and the full time series 1980–2017. For each five-year time interval, we take the maximum percent well-certified. Then for 1980–2017, we take the average of the maximum percentages well-certified for the seven five-year time intervals, including any five-year time interval where no data were available as a zero.

Once these values are calculated, we assign stars as follows:

5 stars: 85%–100% well-certified

4 stars: 65%–84% well-certified

3 stars: 35%–64% well-certified

2 stars: 10%–34% well-certified

1 star: >0%–9% well-certified

0 stars: No VR or VA data available from 1980–2017

While stars are calculated for each five-year time interval, as well as the full time series from 1980 to 2017, stars in the main text are presented for the full time series only.

Appendix Table 7 shows the percent well-certified, stars, data source, and underlying values for percent well certified used for each country and time interval. Underlying indicators for the percent well-certified calculation are listed in Appendix Table 8.

Section 3: Causes of death modeling methods

Section 3.1: CODEm

Section 3.1.1: Overview of methods

CoD ensemble modelling (CODEm) is the framework used to model most cause-specific death rates in the GBD.[26] It relies on four key components:

First, all available data are identified and gathered to be used in the modelling process. Though the data may vary in quality, they all contain some signal of the true epidemiological process.

Second, a diverse set of plausible models are developed to capture well-documented associations in the estimates. Using a wide variety of individual models to create an ensemble predictive model has

been shown to outperform techniques using only a single model both in CoD estimation[26] and in more general prediction applications.[27,28]

Third, the out-of-sample predictive validity is assessed for all individual models, which are then ranked for use in the ensemble modelling stage.

Finally, differently weighted combinations of individual models are evaluated to select the ensemble model with the highest out-of-sample predictive validity.

For some causes (see, for example, “lower respiratory infections”), there is evidence that the relationship between covariates and death rates might differ between children and adults. Separate models are therefore run for different age ranges, when applicable. Additionally, separate models are developed for countries with extensive, complete, and representative VR for every cause to ensure that uncertainty can better reflect the more complete data in these locations.

Section 3.1.2: Model pool development

Because many factors may covary with any given CoD, a range of plausible statistical models are developed for each cause. In the CODEm framework, four families of statistical models are used: linear mixed effects regression (LMER) models of the natural log of the cause-specific death rate, LMER models of the logit of the cause fraction, spatiotemporal Gaussian process regression (ST-GPR) models of the natural logarithm of the cause-specific death rate, and ST-GPR models of the logit of the cause fraction (see the 2x2 table in Foreman et al).[26] For each family of models, all plausible relationships between covariates and the response variable are identified. Because all possible combinations of selected covariates are considered for each family of models, multicollinearity between covariates may produce implausible signs on coefficients or unstable coefficients. Each combination is therefore tested for statistical significance (covariate coefficients must have a coefficient with p-value $< 0{\cdot}05$) and plausibility (the coefficients must have the directions expected based on the literature). Only covariate combinations meeting these criteria are retained. This selection process is run for both cause fractions and death rates, then ST-GPR and LMER-only models are created for each set of covariates. For a detailed explanation of the covariate selection algorithm, see Foreman et al 2012.[26]

Section 3.1.3: Data Variance Estimation

The families of models that go through ST-GPR described in Section 3.1.2 incorporate information about data variance. The main inputs for a Gaussian process regression (GPR) are a mean function, a covariance function, and data variance for each data point. These inputs are described in detail in Foreman et al 2012.[26] For GBD 2017, we have updated this calculation to incorporate garbage code redistribution uncertainty.

There are now three components of data variance used in CODEm: sampling variance, non-sampling variance, and garbage code redistribution variance. The computation of sampling variance and non-sampling variance have not changed since previous iterations of the GBD and are also described in Foreman et al 2012.[26] Garbage code redistribution variance is computed in the cause of death database process described in Section 2.7 of this appendix. Since variance is additive, we calculate

total data variance as the sum of sampling variance, non-sampling variance, and redistribution variance. Increased data variance in GPR results in the GPR draws not following the data point as closely.

Section 3.1.4: Testing model pool on 15% sample

The performance of all models (individual and ensemble) is evaluated using out-of-sample predictive validity tests. Thirty percent of the data are excluded from the initial model fits. These individual model fits are evaluated and ranked using half of the excluded data (15% of the total), then used to construct the ensembles based on their performance. Data are held out from the analysis based on the cause-specific missingness patterns for ages and years across locations. Out-of-sample predictive validity testing is repeated 20 times for each model, which has been shown to produce stable results.[26] These performance tests include the root mean square error (RMSE) for the log of the cause-specific death rate, the direction of the predicted versus actual trend in the data, and the coverage of the predicted 95% UI.

Section 3.1.5: Ensemble development and testing

The component models are weighted based on their predictive validity rank in order to determine their contribution to the ensemble estimate. The relative weights are determined both by the model ranks and by a parameter ψ, whose value determines how quickly the weights taper off as rank decreases. The distribution of ψ is described in more detail in Foreman et al 2012.[26] A set of ensemble models is then created using the weights constructed from the combinations of ranks and ψ values. These ensembles are tested using the predictive validity metrics described in Section 3.1.3 on the remaining 15% of the data, and the ensemble with the best performance in out-of-sample trend and RMSE is chosen as the final model.

Section 3.1.6: Final Estimation

Once a weighting scheme has been chosen, 1,000 draws are created for the final ensemble, with the number of draws contributed by each model proportional to its weight. The mean of the draws is used as the final estimate for the CODEm process, and a 95% uncertainty interval (UI) is created from the 0·025 and 0·975 quantiles of the draws. The validity of the UI can be checked via its coverage of the out-of-sample data; ideally, the 95% UI would capture 95% of these data. Higher coverage suggests that the UIs are too large, and lower coverage suggests overfitting.

Section 3.1.7: Selection of causes for which CODEm is used

CODEm is used to model 193 causes, described in detail below. However, it is unsuitable for use in modelling certain causes, including those with very low death counts, those where cause-specific death record availability is inadequate, or those for which there are marked biases or variability for CoD certification over time that cannot be fully accounted for with the current garbage code redistribution algorithms. Criteria for causes where CODEm is not used are discussed in further detail in Section 3.2.

Section 3.1.8: Model-specific covariates

A table of CODEm covariates used, level of the covariate, and expected direction of the covariate by cause, sex, age, and location can be found in Appendix Table 9. Modelers select covariates to be used in CODEm, but those covariates may not be significant or in the direction specified during the covariate selection step of CODEm, and will therefore not be used in the model. These covariates are listed with a '—' for number of draws. Additionally, covariates may be selected by CODEm, but only exist in submodels that perform poorly, and may end up with zero draws included in the final ensemble. Finally, all other covariates are listed with the number of draws in the final ensemble from submodels that had the covariate. A comparison of GBD 2016 and GBD 2017 covariates using CoD modeling is provided in Appendix Table 10.

Section 3.1.9: Fit statistics for CODEm models

A table of CODEm predictive validity results by cause, sex, and, and location can be found in Appendix Table 11.

Section 3.2: Causes modeled outside of CODEm

Section 3.2.1: Overview

A number of causes required alternative modelling strategies to those used for CODEm, as they were not compatible with CODEm estimation infrastructure and processes. Such unsuitability included having very low death counts; inadequate availability of cause-specific death records; and marked biases or variability for CoD certification over time which could not be fully accounted for with current garbage code redistribution algorithms. The inclusion of these causes in CODEm often renders its out-of-sample predictive validity testing, a key advantage of using CODEm for CoD estimation, unstable, or CODEm simply fails to generate plausible mortality rates in the absence of enough VR or VA data. Due to increased data availability and redistribution algorithm refinements, we were able to incorporate several new causes, which were modelled separately for GBD 2013, into CODEm for this iteration of the GBD study; with each annual update of GBD, we aim to add more causes within the CODEm estimation space. For GBD 2017, we used alternative modelling approaches for these causes, including negative binomial models, natural history models, sub-cause proportion models, and prevalence-based model (Appendix Table 12) A full list of causes in GBD 2017 can be found in Appendix Table 13.

Section 3.2.2: Negative binomial models

For eight rare causes of death, there were too few observed deaths in the CoD database to produce stable estimates. For these causes, we ran negative binomial regression models with either a constant or constant multiplied by the mean assumption for the dispersion parameter, using reverse step-wise model building. We selected between the two model dispersion assumptions on the basis of best fit to the data, using the same method as GBD 2013. For GBD 2015 we also tested zero-inflated Poisson models for these rare causes of death, but rejected them after finding that they did not substantially affect the mean predictions but produced unrealistically large UIs. Descriptions of the modelling process for each of these causes follow.

Section 3.2.3: DisMod-MR 2.1

Until GBD 2010, non-fatal estimates were based on a single data source on prevalence, incidence, remission, or a mortality risk selected by the researcher as most relevant to a particular location and time. For GBD 2010, we set a more ambitious goal: to evaluate all available information on a disease that passes a minimum quality standard. That required a different analytical tool that would be able to pool disparate information presented in varying age groupings and from data sources using different methods. The DisMod-MR 1.0 tool used in GBD 2010 evaluated and pooled all available data, adjusted data for systematic bias associated with methods that varied from the reference and produced estimates by world regions with uncertainty intervals. For GBD 2013, the improved DisMod-MR 2.0 had increased computational speed, allowing computations that were consistent between all disease parameters at the country rather than region level. The hundred-fold increase in speed of DisMod-MR 2.0 was partly due to a more efficient rewrite of the code in C++ but also by changing to a model specification using log rates rather than a negative binomial model used in DisMod-MR 1.0. In cross-validation tests, the log rates specification worked as well or better than the negative binomial specification.[29] For GBD 2015, the computational engine (DisMod-MR 2.1) remained substantively unchanged but we re-wrote the "wrapper" code that organised the flow of data and settings at each level of the analytical cascade. The sequence of estimation occurred at five levels: global, super-region, region, country, and, where applicable, subnational locations (see flow diagram of DisMod-MR 2.1 cascade, below). The super-region priors were generated at the global level with mixed-effects, non-linear regression using all available data; the super-region fit, in turn, informed the region fit, and so on down the cascade. The wrapper gave analysts the choice to branch the cascade in terms of time and sex at different levels depending on data density. The default used in most models was to branch by sex after the global fit but to retain all years of data until the lowest level in the cascade. For GBD 2015, we generated fits for the years 1990, 1995, 2000, 2005, 2010, and 2015.

In updating the "wrapper," we consolidated the code base into a single language, Python, to make the code more transparent and efficient and to better deal with subnational estimation. The computational engine is limited to three levels of random effects; we differentiated estimates at the super-region, region, and country levels. In GBD 2013, the subnational units of China, Mexico, and the UK were treated as "countries" such that a random effect was estimated for every location with contributing data. However, the lack of a hierarchy between country and subnational units meant that the fit to country data contributed as much to the estimation of a subnational unit as the fits for all other countries in the region. We found inconsistency between the country fit and the aggregation of subnational estimates when the country's epidemiology varied from the average of the region. Adding an additional level of random effects required a prohibitively comprehensive rewrite of the underlying DisMod-MR engine. Instead, we added a fifth layer to the cascade, with subnational estimation informed by the country fit and country covariates, plus an adjustment based on the average of the residuals between the subnational unit's available data and its prior. This mimicked the impact of a random effect on estimates between subnationals.

For GBD 2015 we improved how country covariates differentiate non-fatal estimates for diseases with sparse data. The coefficients for country covariates were re-estimated at each level of the cascade. For a given location, country coefficients were calculated using both data and prior information available for that location. In the absence of data, the coefficient of its parent location was chosen in order to utilise the predictive power of our covariates in data sparse situations.

For GBD 2017, the DisMod-MR 2.1 tool was used. Updates included estimation of new age groups through the GBD 2017 terminal age group of 95+, in addition to the new locations added for the GBD 2017 cycle. Please see Appendix Figure 7 for details of the GBD 2017 DisMod-MR 2.1 analytical cascade.

DisMod-MR 2.1 likelihood estimation

Analysts have the choice of using a Gaussian, log-Guassian, Laplace, or log-Laplace likelihood function in DisMod-MR 2.1. The default log-Gaussian equation for the data likelihood is:

$$-log\left[p\left(y_j\middle|\Phi\right)\right] = \log\left(\sqrt{2\pi}\right) + \log\left(\delta_j + s_j\right) + \frac{1}{2}\left(\frac{\log\left(a_j + \eta_j\right) - \log\left(m_j + \eta_j\right)}{\delta_j + s_j}\right)^2$$

Where:

y_j is a "measurement value" (ie, data point)

Φ denotes all model random variables

η_j is the offset value, eta, for a particular "integrand" (prevalence, incidence, remission, excess mortality rate, with-condition mortality rate, cause-specific mortality rate, relative risk, or standardised mortality ratio)

a_j is the adjusted measurement for data point j, defined by:

$$a_j = e^{(-u_j - c_j)} y_j$$

Where:

u_j is the total "area effect" (ie, the sum of the random effects at three levels of the cascade: super-region, region, and country)

c_j is the total covariate effect (ie, the mean combined fixed effects for sex, study-level, and country-level covariates), defined by:

$$c_j = \sum_{k=0}^{K[I(j)]-1} \beta_{I(j),k} \hat{X}_{k,j}$$

with standard deviation

$$s_j = \sum_{l=0}^{L[I(j)]-1} \zeta_{I(j),l} \hat{Z}_{k,j}$$

Where:

k denotes the mean value of each data point in relation to a covariate (also called x-covariate)

$I(j)$ denotes a data point for a particular integrand, j

$\beta_{I(j),k}$ is the multiplier of the kth x-covariate for the ith integrand

$\hat{X}_{k,j}$ is the covariate value corresponding to the data point j for covariate k

l denotes the standard deviation of each data point in relation to a covariate (also called z-covariate)

$\zeta_{I(j),k}$ is the multiplier of the lth z-covariate for the ith integrand

δ_j is the standard deviation for adjusted measurement j, defined by:

$$\delta_j = log\left[y_j + e^{(-u_j - c_j)}\eta_j + c_j\right] - log\left[y_j + e^{(-u_j - c_j)}\eta_j\right]$$

Where mj denotes the model for the jth measurement, not counting effects or measurement noise and defined by:

$$m_j = \frac{1}{B(j) - A(j)} \int_{A(j)}^{B(j)} I_j(\mathrm{a})\, \mathrm{da}$$

Where:

$A(j)$ is the lower bound of the age range for a data point j

$B(j)$ is the upper bound of the age range for a data point j

$I(j)$ denotes the function of age corresponding to the integrand for data point j

The source code for DisMod-MR 2.1 as well as the wrapper code are available at http://ihmeuw.org/dismod-ode.

Section 3.2.4: Natural history models

For some causes where CoD data may be systematically biased due to either misclassification or because the disease exists in focal communities without VR or VA studies, we have developed natural history models. In natural history models incidence and case-fatality rates are modelled separately and then combined to produce estimates of cause-specific mortality.

Section 3.2.5: Prevalence-based models

The modelling strategies for atrial fibrillation and flutter are distinct from those used for other causes modelled as natural history models. These models use prevalence estimates and excess mortality rates (EMR) generated through DisMod-MR 2.1, rather than incidence and case-fatality rates.

Section 3.2.6: Sub-cause proportion models

For certain sub-causes for which accurate diagnoses are known to be very difficult, we first modelled the parent cause in the GBD hierarchy with CODEm and then allocated deaths to specific causes using proportions of the parent cause for each age-sex-location-year for each sub-cause. For these causes, we identified no significant predictors in negative binomial regressions. This approach was taken because the available data on these specific causes may come from sources other than VR, such as end-stage renal disease registries, or come from too few places to model the death rates directly. Details for each cluster of causes analysed in this way are below.

Section 3.3: CoD modeling descriptions

HIV/AIDS

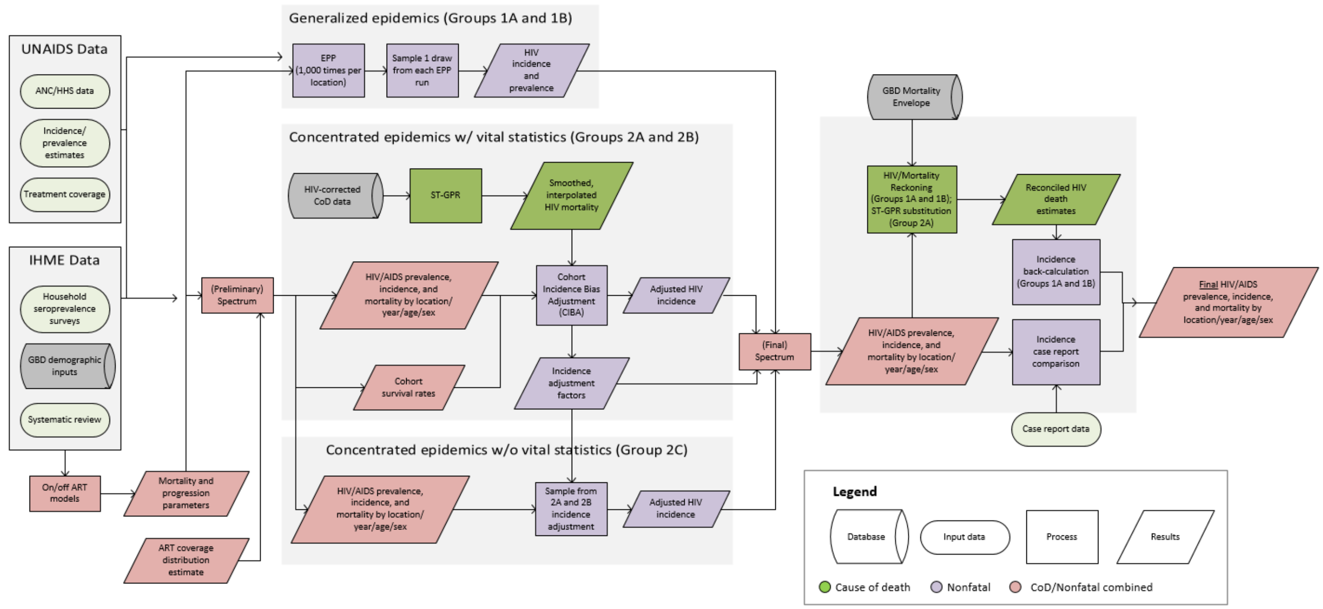

Case definition

Infection with the human immunodeficiency virus (HIV) causes influenza-like symptoms during the acute period following infection and can lead to acquired immunodeficiency syndrome (AIDS) if untreated. HIV attacks the immune system of its host, leaving infected individuals more susceptible to opportunistic infections like tuberculosis. Although there are two different subtypes of HIV, HIV-1 and HIV-2, no distinction is made in our estimation process or presentation of results. For HIV, ICD 10 codes are B20-B24, C46-C469, D84.9; ICD 9 codes are 042-044, 112-118 (after 1980), 130 (after 1980), 136.3-136.8 (after 1980), 176.0-176.9 (after 1980), 279 (after 1980); and ICD9 BTL codes are B184-B185.

Input data

Household seroprevalence surveys
Geographically representative HIV seroprevalence survey results were used as inputs to the model for countries with generalised HIV epidemics where available.

GBD demographic inputs
Location-specific population, fertility, and HIV-free survival rates from GBD 2017 and migration data from UNAIDS were used as inputs in modelling all locations.

UNAIDS data
The files compiled by UNAIDS for their HIV/AIDS estimation process were our main source of data for producing estimates of HIV burden. These files are typically country-specific and contain both demographic data (population, fertility, migration, and HIV-free survival rates) and HIV-specific information. In all cases except migration, we substituted in our own, internally consistent demographic estimates. The HIV-specific information includes what is needed to run both the Spectrum and

Estimation and Projection Package (EPP) models. Spectrum requires the following input data: AIDS mortality among people living with HIV with and without ART, CD4 progression among people living with HIV not on ART, ART coverage among adults and children, Cotrimoxazole coverage among children, coverage of breastfeeding among women living with HIV, prevention of mother-to-child transmission coverage, and CD4 thresholds for treatment eligibility. EPP uses many of the same assumptions as Spectrum but fits a simpler model to HIV prevalence data from surveillance sites and representative surveys. Antenatal care (ANC), incidence, prevalence, and treatment coverage data from UNAIDS were used in modelling for all locations. We extracted all of these data from the proprietary format used by UNAIDS.

For GBD 2017, we received updated national-level files for 97 countries and updated subnational-level files for eight countries. For many of the GBD locations not covered by these files, we had UNAIDS files from an earlier year of estimation, which we used again. After combining, we were left with a set of 35 countries for which we have never received a UNAIDS file, many of them countries with small populations and/or low HIV prevalence. In those places, we generated regional averages of all needed inputs. This enabled us to run Spectrum for every GBD location.

In several cases, we have modified the structure or data in the UNAIDS files. In South Africa, Russia, Iran, New Zealand, Great Britain, Japan, Indonesia, Mexico, United States, Norway, Brazil, Ukraine, and China, which we estimate at the subnational level, we split the national-level UNAIDS file into subnational datasets using assumptions from GBD 2017 demographics and GBD 2016 HIV prevalence. We also estimate at the subnational level in Ethiopia, Kenya, and India, but have subnational-level UNAIDS files for these locations; however, in Kenya and India, we must split larger subnationals to more granular locations. The subnational locations in Ethiopia, Kenya, India, and South Africa are fit as separate subpopulations in EPP, so we extracted the prevalence data for the individual subnationals. In Benin, Côte d'Ivoire, Haiti, Moldova, Mozambique, Nigeria, Togo, Zambia, and Zimbabwe, the country files that we received from UNAIDS contained only subnational data without national-level aggregates. In these cases, we aggregated the UNAIDS files and ran EPP and Spectrum at the national level.

Vital registration data

We used all available sources of vital registration and sample registration data from the GBD Causes of Death database after garbage code redistribution and HIV/AIDS mis-coding correction, except in Group 1A countries as described below.[1, 2] There are two different cause of death data sources for HIV/AIDS in China: the Disease Surveillance Point (DSP) system and the Notifiable Infectious Disease Reporting (NIDR) system. Both systems are administered by the Chinese Center for Disease Control and Prevention, but the reported number of deaths due to HIV is significantly lower in DSP. Therefore, we have used the provincial-level ratio of deaths due to HIV/AIDS from NIDR to those from DSP, choosing the larger ratio between years 2013 and 2014, and scaled the reported deaths in the DSP system, which is in turn used in the spatiotemporal Gaussian process regression (ST-GPR).

On-ART literature data

Data were identified by using search terms “HIV,” “mortality,” and “antiretroviral therapy” in PubMed searches across the literature. To be included, studies must include only HIV-positive people who receive antiretroviral therapy (ART) but who were ART-naïve prior to the study. In addition, studies must report either a duration-specific mortality proportion or a hazard ratio across age or sex, and must not include children.

For duration-specific survival data, studies must report uncertainty on mortality estimates or provide stratum-specific sample sizes and must include duration-specific data to allow for calculation of 0-6, 7-

12, or 13-24 month conditional mortality. In addition, studies must either report separate mortality and loss-to-follow-up (LTFU) curves, be corrected for LTFU using vital registration data or double sampling, or be conducted in a high-income setting. Finally, studies must report the percent of participants who are male and the median age of participants.

Hazard ratio data for ages or sexes can only be used if the hazard ratios are controlled for other variables of interest (age, sex, and CD4 category). In GBD 2013, we identified 102 papers for extraction. For GBD 2015, we included 13 additional studies informing the duration-specific mortality estimation process and 26 studies informing the age and sex hazard ratio estimation process (some studies were used and counted in both). We also added one study to our LTFU analysis. For GBD 2016, we included 12 additional studies informing the duration-specific mortality estimation process and 11 studies informing the age and sex hazard ratio estimation process (some studies were used and counted in both). For GBD 2017, we included 17 additional studies informing the duration-specific mortality estimation process and 13 studies informing the age and sex hazard ratio estimation process (some studies were used and counted in both). We also included two new studies in our LTFU analysis.

Off-ART literature data
In GBD 2013, to characterise uncertainty in the progression and death rates, we systematically reviewed the literature on mortality without ART. We searched terms related to pre-ART or ART-naive survival since seroconversion.[3] After screening, we identified 13 cohort studies that included the cohorts used by UNAIDS, from which we extracted survival at each one-year point after infection. Screening for additional, recently published studies in GBD 2015, GBD 2016 and GBD 2017 identified no new cohort studies for inclusion in this analysis.

Severity splits and disability weights
The basis of the GBD disability weight survey assessments are lay descriptions of sequelae highlighting major functional consequences and symptoms. The lay descriptions and disability weights for HIV/AIDS severity levels are shown below.

**Severity level**	**Lay description**	**DW (95% CI)**
Symptomatic HIV	Has weight loss, fatigue, and frequent infections.	0.274 (0.184–0.377)
AIDS with antiretroviral treatment	Has occasional fevers and infections. The person takes daily medication that sometimes causes diarrhoea.	0.078 (0.052–0.111)
AIDS without antiretroviral treatment	Has severe weight loss, weakness, fatigue, cough and fever, and frequent infections, skin rashes, and diarrhoea.	0.582 (0.406–0.743)

The proportion of people living with HIV/AIDS who are being treated with antiretroviral therapy is an output of Spectrum, the compartmental model used to make consistent incidence, prevalence, and mortality estimates described below.

Modelling strategy

In GBD 2017, our general modelling strategy for estimating HIV incidence, prevalence, and mortality is very similar to the strategy used in GBD 2016. We continue to use the Spectrum program rewritten in Python for GBD 2013 to facilitate faster and more flexible execution necessary for our more intensive computational needs. We made several changes to the modelling strategy in Spectrum comparing to the Spectrum software used by UNAIDS. We also, again, ran EPP using an open-source computer program in R written by Jeffrey Eaton.[4] We ran EPP for all Group 1 countries in order to produce incidence and prevalence estimates that were consistent with the demographic and epidemiological assumptions used in GBD 2017.

On-ART

First, we corrected reported probabilities of death for loss to follow-up using an update of the approach developed by Verguet and colleagues.[5] Verguet and colleagues used tracing and follow-up studies to empirically estimate the relationship between death in LTFU and the rate of LTFU.
To create estimates of age-specific hazard ratios, we synthesised hazard ratio data in five broad age groups: 15-25, 25-35, 35-45, 45-55, 55-100, and modelled the data using DisMod-MR 2.1.

To create estimates of sex-specific hazard ratios, we use the *metan* function in Stata to create estimates of relative risks separately by region, using female age groups as the reference group.
The age and sex hazard ratios were applied to the study-level mortality rates, accounting for the distribution of ages and sexes in the mortality data. We then subtracted HIV-free mortality from the model life table process to calculate study-level age-sex HIV-specific mortality.

We used DisMod-MR 2.1 to synthesise the age-sex-split study-level data into estimates of conditional probability of death over initial CD4 count.[3] We modelled the data separately by duration, age, sex, and region and added a fixed effect on whether the study was conducted prior to 2002. Finally, we replaced our on-ART mortality rates with those estimated off treatment if they were higher.

Off-ART

Following UNAIDS assumptions, no-ART mortality is modelled as shown in the figure below.[3]

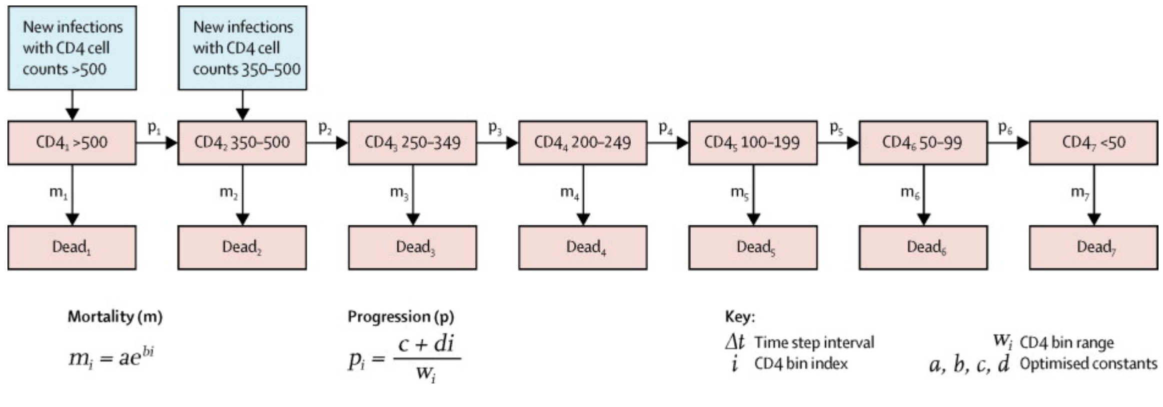

The death and progression rates between CD4 categories vary by age according to four age groups: 15–24 years, 25–34 years, 35–44 years, and 45 years or older. We modelled the logit of the conditional probability of death between years in these studies using the following formula:

$$logit\ (m_{ijk}) = \beta_0 + \sum_{i=1}^{4} \beta_{1i}\, a_i + \sum_{j=1}^{12} \beta_{2j} t_j + u_k + \varepsilon_{ijk}$$

In the formula, m is conditional probability of death from year t_j to t_{j+1}, a_i is an indicator variable for age group at seroconversion (15–24 years, 25–34 years, 35–44 years, and 45 years or older), t_j is an indicator variable of year since seroconversion, and u_κ is a study-level random effect.

By sampling the variance-covariance matrix of the regression coefficients and the study-level random effect, we generated 1,000 survival curves for each age group that capture the systematic variation in survival across the available studies. For each of the 1,000 survival curves, we used a framework modelled after the UNAIDS optimisation framework in which we find a set of progression and death rates that minimises the sum of the squared errors for the fit to the survival curve.[8, 9]

Changes for GBD 2017

In GBD 2017, we chose to estimate mortality for each region in its own DisMod model, whereas previous GBD iterations estimated all regions together with fixed effects. This change was driven by new results from the IeDEA cohort collaboration, which provided enough data to estimate mortality trends by CD4 in each region separately.[6] We also added a year covariate to our LTFU model reflecting evidence from a large meta-analysis by Zurcher and colleagues, which showed that mortality among the LTFU has declined in recent years.[7] Finally we replaced our estimated on-ART mortality rates by rates off ART, accounting for progression to lower CD4 categories, if the on-ART rates were higher. This was done to ensure individuals would not experience higher mortality when they entered treatment in Spectrum.

Burden estimation overview

UNAIDS uses two key analytical components in their epidemiological estimation. EPP is used to estimate incidence and prevalence trajectories that are consistent with prevalence surveys and other prevalence measurements such as antenatal clinic serosurveillance. Spectrum is a compartmental HIV progression model used to generate age-specific incidence, prevalence, and death rates from the EPP incidence and prevalence curves and assumptions about intervention scale-up and local variation in epidemiology.

Due to the substantial differences in the quality and types of data available across different countries, we used three different methodologies to produce year-, age-, and sex-specific estimates of HIV incidence, prevalence, and mortality. All three methodologies incorporate Spectrum to produce final estimates of disease burden and mortality.

Spectrum

For GBD 2013, we created an exact replica of Spectrum in Python. This enabled us to run thousands of iterations of the model at once on our computing cluster and allowed for more flexible input data structures. Additionally, in order to generate estimates with more realistic ranges of uncertainty than those in UNAIDS 2012, we adjusted all input data by uniformly sampled factors between 0.9 and 1.1. These changes, along with our new estimation of with- and without-ART mortality and CD4 progression parameters, persist into GBD 2017.

Changes for GBD 2017

For GBD 2017, we implemented a new approach to address selection bias resulting from temporal and geographic variation in ANC reporting, which has the potential to skew unadjusted estimates, especially

early in the epidemic when there are no nationally representative prevalence surveys to anchor estimates.[14] To address this issue, the specification of the likelihood of observed ANC clinic data within EPP includes random intercepts for each clinic. While this approach largely accounts for differences in level between clinics, it does not impact the estimated shape of the epidemic. In order to leverage available information from nearby geographies, we developed a model for data imputation which establishes an epidemic peak from a first-stage model fit to ANC clinic data from a location and its nearest neighbours. The model included random effects for country, clinic, and time. The year of the largest random effect was used as t_{max}, the location of a single knot in an imputation model which predicted the logit of prevalence in each year for a clinic as a linear spline. We can write this method mathematically in the following way:

$$logit(\rho_i(t)) = \beta_0 + S(t) + \beta_3 X_i + \epsilon$$

$$S(t) = \beta_1 S_1(t) + \beta_2 S_2(t)$$

$$S_1(t) = \begin{cases} t_{max} - t & t \leq t_{max} \\ 0 & t > t_{max} \end{cases}$$

$$S_2(t) = \begin{cases} t - t_{max} & t \geq t_{max} \\ 0 & t < t_{max} \end{cases}$$

One thousand draws of imputed clinic prevalence, accounting for covariance between predictors, were generated for clinic-years where at least one clinic had an observation in a given year. These draws were used for each of the 1,000 EPP runs we ran for each location.

Additionally, for GBD 2017 we improved our sex-specific modelling strategy in Spectrum by sex-splitting incidence based on a model fit to the sex ratio of prevalence observed in countries with representative surveys. We also updated the Spectrum pediatric module to reflect changes made by UNAIDS.[10] Our child module was revised to include CD4 progression and CD4-specific mortality rates taken from a model fit to survival data from IeDEA. We also updated child initiation of ART to include data on ART distribution from IeDEA.

ART coverage distribution

In UNAIDS' implementation of Spectrum, initiation of ART is constrained by eligibility criteria and distributed across CD4 count groups according to both the expected number of deaths and the number of people in each untreated CD4 count group – groups with a large proportion of PLHIV and high numbers of expected deaths initiated the most individuals into treatment. Three surveys were available at the time of publication that contained questions which can help inform the CD4 count distribution of ART coverage, Uganda AIS 2011 and Kenya AIS 2007 and 2012. Both of these surveys conducted CD4 count measurements and include a question regarding the amount of time that an individual receiving ART had been enrolled in treatment. Survey data provide cross-sectional CD4 count information; however, the Spectrum modelling framework tracks individuals by categorical CD4 count at the initiation of treatment. In order to cross-walk the cross-sectional survey data into estimates of CD4 count at treatment initiation, we built a model using relevant cohort data which tracked changes in CD4 count after initiation of treatment to translate an individual's current CD4 count and duration on treatment into CD4 count at initiation of treatment. The functional form for changes in CD4 count as a function of duration on treatment was a natural spline on duration with knots at 3, 12, 24 and 36 months, and an interaction between initial CD4 count and duration.

After cross-walking, we predicted the probability of being on treatment as a function of individual income (measured through an asset-based index), stratified by CD4 count, age, and sex. The results of this prediction were translated into country-specific age-sex-year-CD4 count probabilities of coverage using a conversion factor between individual income and LDI. We used stochastic frontier analysis to constrain the maximum possible coverage for a given degree of income and CD4 count. Predicted probabilities of coverage were input to Spectrum to inform the distribution, and not the overall level, of ART treatment by CD4 count. Spectrum converted counts of expected individuals on treatment in each CD4 count group and scaled the distribution across CD4 count groups to match the input data on the number of people on ART coming from UNAIDS country files. In cases where the predicted number of individuals initiating treatment exceeds the total number of untreated individuals in a CD4 count group, we reallocate treatment evenly to other CD4 count groups.

Countries with seroprevalence surveys and antenatal clinic data (Groups 1A and 1B)
We identified 50 countries – as well as subnational locations in India, Kenya, Ethiopia, and South Africa – with at least 0.5% adult HIV prevalence and at least one geographically representative HIV seroprevalence survey or available antenatal care clinic (ANC) data. In order to ensure that our estimates of incidence and prevalence in these places were consistent with our estimates of HIV progression, we used a version of EPP written in R and C++ by Jeffrey Eaton to create new fits to the available prevalence data. The version of EPP used in GBD 2017 was updated in 2017 by Jeffrey Eaton. In this new version, an ANC prevalence adjustment was included and incorporated with the 2016 lookup database for the relative risk between pregnant women and the whole adult population, and an additional parameter to estimate ANC variance inflation was included as well.

For adjusting ANC data to align with the national 15–49 both sexes population, we extracted data on HIV prevalence among pregnant women who gave birth within the last year and attended an ANC clinic, from available DHS surveys. A simple model with regional random effects was run to generate location-specific prior distributions for the ANC bias adjustment where surveys were available, and regional priors for locations without a survey. The adjustment using a time-series of relative risk between pregnant women and the adult population was removed, and the ANC bias parameter was changed to account for all of the biases observed between these two populations.

In the new version of EPP, in addition to the equilibrium prior assumption of the force of infection in projection, a random walk approach is available as an alternative method. There were two locations, Madagascar and Papua New Guinea, which had no prevalence surveys and an increasing trend in ANC data. We chose the random walk approach to project the force of infection for these locations. We assumed the change of the log-scaled force of infection was following a normal distribution with mean equal to the median of the change of the modelled force of infection among the years having ART implemented or prevalence data, and the SD was equal to the default setting as the mean SD of the change of the modelled force of infections among the years having prevalence data. The projection year was chosen from the most recent year between the year with the lowest model force of infection and the year of the second latest survey data.

In the new EPP code, an optimisation step was added into IMIS function to speed up the parameter sampling step based on Raftery and Bao.[11] Two optimisation methods have been introduced. The main algorithm is Broyden–Fletcher–Goldfarb–Shanno (BFGS) optimization. If BFGS fails, Nelder-Mead optimum is used instead. In our 2016 EPP model, by substituting in our own assumptions about HIV progression rates and on/off ART mortality, we were able to ensure that the implied relationship between incidence and mortality/prevalence in EPP is similar to that in Spectrum.

To incorporate uncertainty in our mortality and progression parameters, we run EPP with separate draws of each of these parameters. This process produced 1,000 sets of EPP output for each of the locations that make up the 48 countries in the group. Every set of EPP outputs contains 500 consistent draws of HIV incidence and prevalence in adults aged 15-49.

For every location in the group, we sampled one of the 500 incidence/prevalence draws from each of the sets of EPP results. By sampling one draw from each set, we ensured that the distribution of progression parameters dictating the relationship between incidence and prevalence was exactly the same as the distribution of the sorted parameters generated in the previous step. At the end of this process, for every location in the set of 48 countries, we were left with 1,000 linked draws of adult incidence and prevalence and the exact mortality and progression parameters that generated those draws. We then ran these results, along with the previously described demographic and HIV-specific inputs, through Spectrum to produce location-, year-, age-, and sex-specific estimates of HIV incidence, prevalence, and mortality.

The HIV/mortality reckoning process is intended as a method of reconciling separate estimates of HIV mortality (and its resulting effect on estimates of HIV-free and all-cause mortality) in Group 1 countries by averaging estimates of HIV mortality from the model life table process and EPP-Spectrum. Additional details on the reckoning can be found in the GBD 2017 mortality manuscript.[12]

Since Spectrum produces HIV incidence, prevalence, and deaths that are consistent with one another over time, the reckoning process results in death numbers that are no longer consistent with the incidence and prevalence produced in Spectrum. In order to recreate this consistency, we recalculated incidence for all Group 1 locations using the difference between reckoned deaths and deaths produced by Spectrum. The updated incidence is calculated by aggregating counts of new infections, HIV deaths from Spectrum, and HIV deaths after reckoning at the year-sex level. The difference between reckoned HIV deaths and HIV deaths from Spectrum is added to Spectrum incidence, and we calculate the ratio between updated incidence and Spectrum incidence. Age-specific counts of new infections are then scaled by their corresponding sex-year ratios.

Countries with vital registration data (Group 2A and 2B)
Vital registration is one of the highest-quality sources of data on HIV burden in many countries, so generating estimates that are consistent with these data with necessary adjustment to account for any potential underreporting is critical. We identified 122 countries – as well as 578 subnational locations from China, Japan, Indonesia, India, Mexico, Sweden, the United Kingdom, Ukraine, Russia, New Zealand, Iran, Norway and the United States – with usable points of vital registration data, verbal autopsy (VA) data, or sample registration system (SRS) data. In India, Vietnam and Indonesia, we used SRS and VA data, respectively, as input mortality for CIBA. For India we extracted the resulting age-sex distribution of incidence but scaled the level to match the adult incidence rate estimated from EPP for each state.

We imputed missing years of data to generate a complete time series for HIV from the estimated start year of the epidemic using ST-GPR. We analysed mortality trends using ST-GPR starting in 1981, the year that HIV was first identified in the United States.[13] For ST-GPR, we adjusted the lambda (time weight) and GPR scale according to the completeness of vital registration data, with 4- and 5-star quality VR using parameters designed to follow the data more closely. We produced separate splines by country/age group, up to the peak year of death rate. We then ran a linear regression with fixed effects on region, age, and sex. Following this, we ran space-time residual smoothing, in which time, age, and space weights are used to inform smoothing of the residuals between data points and the linear

regression estimate. From this process, we generated space-time estimates with the applied weights, along with the median absolute deviation (MAD) of the space-time estimates from the data. The MAD was calculated at various levels of the geographic hierarchy (eg, subnational and national), and was added into the data variance term. The data variance and space-time estimates were then analysed using Gaussian process regression to return a final estimate of mortality along with uncertainty.

Although Spectrum produces HIV mortality estimates that are within the realm of possibility in most countries using the incidence curves provided in the UNAIDS country files, it is a deterministic model that has not yet been integrated into an optimisable framework. Therefore, in order to "fit" it to vital registration data, we need to adjust input incidence.

To improve the fit of this process, in GBD 2015, we restructured Spectrum to track cohorts by year of HIV infection. With this version of Spectrum we can output, among many other metrics, HIV deaths by year, age, sex, and infection cohort. This enables us to adjust incidence to fit to death much more precisely and without making any rigid assumptions about the time from HIV infection to HIV death.

We have incorporated these improvements into a cohort incidence bias adjustment (CIBA) process. First, we ran Spectrum normally to produce 1,000 draws of incidence, prevalence, and mortality. Then, by year, age, and sex, we took the ratio of VR deaths to Spectrum deaths to quantify the amount of bias in Spectrum. Using draw-level duration data from the new version of Spectrum, for every year-, age-, and sex-specific infection cohort, we calculated the share of all HIV deaths observed over the course of the projection period in that cohort that would occur in each year after the year of infection. For example, projecting from 1970 through 2017, we identified the cohort of men infected in 1992 at the age of 16, calculated the total number of HIV deaths in that cohort in all subsequent years through the end of 2017, and divided the annual number of deaths by that total. This showed us the distribution of deaths among that cohort over the projection period. In the most extreme case (infections in 2016), we could only produce one point of that distribution (2017), so that single value is exactly 1·0; 100% of the deaths observed in that cohort occurred in 2017.

We then used these distributions of death to weigh the ratio of VR deaths to Spectrum deaths, meaning that ratios in the years where we expect the largest share of deaths were weighed most heavily. We then multiplied the initial size of that cohort from the normal run of Spectrum by the sum of the combined ratios to get a new estimate of new cases in that year/age/sex combination. We can write this method mathematically in the following way:

$$r_t = \frac{VR_t}{D_t}$$

$$\rho_t^{t-i} = \frac{d_t^{t-i}}{\sum_{k=t-i+1}^{n} d_k^{t-i}}$$

$$\alpha^{t-i} = \sum_{k=t-i+1}^{n} r_k * \rho_k^{t-i}$$

$$n_{\text{adjusted}}^{t-i} = \alpha^{t-i} * n^{t-i}$$

VR_t is the number of HIV/AIDS deaths in year t from ST-GPR, and D_t is the number of HIV/AIDS deaths from the first run of Spectrum. In the second equation, d_t^{t-i} is the number of HIV/AIDS deaths among members of infection cohort $t-i$ in year t, with $i \geq 1$, from the new, duration-tracking version of Spectrum, and n is final year of the projection. Therefore, ρ_t^{t-i} is the share of observed deaths in cohort $t-i$ that we expect to occur in year t. It follows that α^{t-i} is the weighted adjustment ratio described

above, which we multiply by the estimated initial size of infection cohort $t - i$ as calculated in the first-stage Spectrum run to get the adjusted number of new cases, $n_{\text{adjusted}}^{t-i}$. This process is run separately for every sex, single-age, and draw.

CIBA allows ratios in each year after a given infection year to influence the final adjustment to incidence. The size of that influence is determined by the relative importance of that year in the cohort-year's distribution of deaths over time. The result is a new set of 1,000 draws of incidence and a set of 1,000 ratios of post-adjustment incidence to pre-adjustment incidence. We perform this adjustment using mean durations from the new version of Spectrum in order to try to shift the mean of the regular distribution of deaths.

Finally, to produce location-, year-, age-, and sex-specific estimates of HIV incidence, prevalence, and mortality, we ran the new estimates of incidence and all previously input data through Spectrum.

Countries without survey data and vital registration data (Group 2C)
The remaining 30 countries – as well as Macau Special Administrative Region of China – had neither geographically representative seroprevalence surveys nor reliable vital registration systems. To produce estimates of HIV burden in these countries, we assumed that Spectrum is similarly biased as in other Group 2 countries within the same super-region. This involved running Spectrum, adjusting incidence using 1,000 adjustment ratios randomly sampled from CIBA results from the same super-region, and rerunning Spectrum using the new draws of adjusted incidence. As above, the estimates of incidence, prevalence, and mortality were incorporated into the rest of the machinery via the reckoning process.

References

1. Global, regional, and national age–sex specific all-cause and cause-specific mortality for 240 causes of death, 1990–2013: a systematic analysis for the Global Burden of Disease Study 2013. *The Lancet* 2015; **385**: 117–71.

2. Birnbaum JK, Murray CJ, Lozano R. Exposing misclassified HIV/AIDS deaths in South Africa. *Bull World Health Organ* 2011; **89**: 278–85.

3. Murray CJL, Ortblad KF, Guinovart C, *et al.* Global, regional, and national incidence and mortality for HIV, tuberculosis, and malaria during 1990–2013: a systematic analysis for the Global Burden of Disease Study 2013. *The Lancet* 2014; **384**: 1005–70.

4. jeffeaton/epp. GitHub. https://github.com/jeffeaton/epp (accessed April 21, 2016).

5. Verguet S, Lim SS, Murray CJL, Gakidou E, Salomon JA. Incorporating Loss to Follow-up in Estimates of Survival Among HIV-Infected Individuals in Sub-Saharan Africa Enrolled in Antiretroviral Therapy Programs. *J Infect Dis* 2013; **207**: 72–9.

6. Anderegg N, Johnson LF, Zaniewski E, *et al*. All-cause mortality in HIV-positive adults starting combination antiretroviral therapy: correcting for loss to follow-up. *AIDS* 2017; **31 Suppl 1:** S31-40.

7. Zürcher K, Mooser A, Anderegg N, *et* al. Outcomes of HIV-positive patients lost to follow-up in African treatment programmes. *Trop Med Int Health* 2017; **22**: 375-387.

8. Ghys PD, Zaba B, Prins M. Survival and mortality of people infected with HIV in low and middle income countries: results from the extended ALPHA network. *AIDS Lond Engl* 2007; **21 Suppl 6**: S1–4.

9. Hallett TB, Zaba B, Todd J, *et al.*, ALPHA Network. Estimating incidence from prevalence in generalised HIV epidemics: methods and validation. *PLoS Med* 2008; **5**: e80.

10. Mahy M, Penazzato M, Ciaranello A, et al. Improving estimates of children living with HIV from the Spectrum AIDS Impact Model. Aids 2017;31: S13–S22

11. Raftery AE, Bao L. Estimating and Projecting Trends in HIV/AIDS Generalized Epidemics Using Incremental Mixture Importance Sampling. *Biometrics* 2010; **66**: 1162–73.

12. Wang H, Murray CJL, Carter A, He F. Global, regional, and national under-5 mortality, adult mortality, age-specific mortality, and life expectancy, 1970-2016: a systematic analysis for the Global Burden of Disease Study 2016. *The Lancet* 2017; **390**: 1151-1210.

13. CDC. Pneumocystis Pneumonia --- Los Angeles. MMWR Wkly. 1981; published online June 5. http://www.cdc.gov/mmwr/preview/mmwrhtml/june_5.htm (accessed April 21, 2016).

14. Ng M, Gakidou E, Murray CJL, Lim S. A comparison of missing data procedures for addressing selection bias in HIV sentinel surveillance data. *Population Health Metrics* 2013; **11:** 12.

HIV/AIDS – Multidrug-resistant tuberculosis without extensive drug resistance, HIV/AIDS – Extensively drug-resistant tuberculosis, and HIV/AIDS – Drug-susceptible tuberculosis

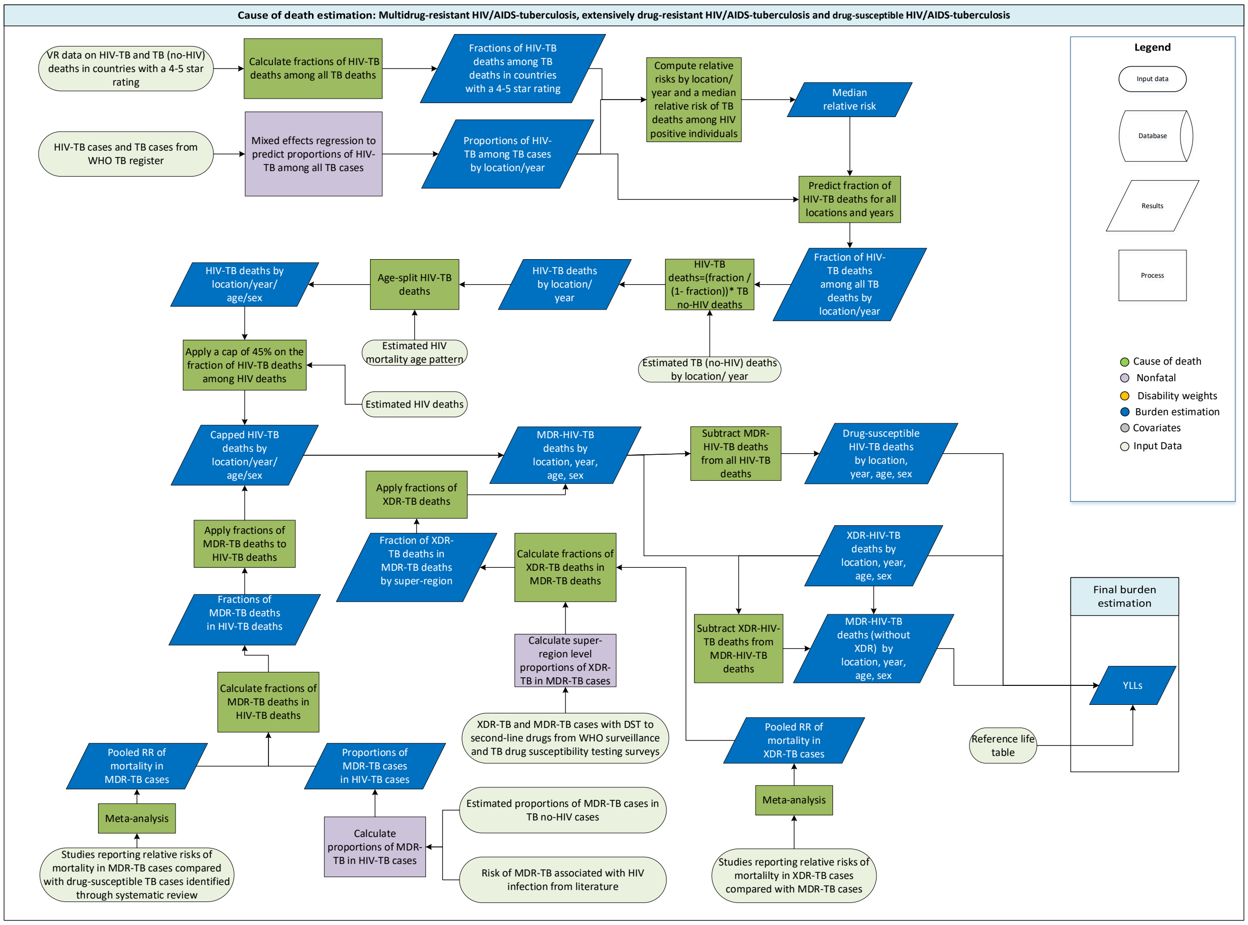

Input data

Input data for HIV/AIDS-tuberculosis (HIV-TB) mortality estimation include (i) 438 site-years of vital registration data from countries with a four- or five-star rating where cause of death data for directly coded HIV-TB and tuberculosis (TB) were available, and (ii) the number of TB cases (new and re-treatment) recorded as HIV-positive and the number of TB cases (new and re-treatment) with an HIV test result recorded in the TB register from the World Health Organization (WHO). We excluded data from countries with ten HIV-TB deaths or less. We also excluded data that were largely conflicting with the majority of data for other years from the same country.

Input data for estimation of multidrug-resistant and extensively drug-resistant HIV-TB include: (i) the number of drug-resistant cases by type (multidrug-resistant tuberculosis [MDR-TB], extensively drug-resistant tuberculosis [XDR-TB], all TB cases with a drug sensitivity testing [DST] result for isoniazid and rifampicin, and MDR-TB cases with DST for second-line drugs) from routine surveillance and surveys reported to the World Health Organization. Additional input data include relative risks of mortality in MDR-TB cases compared with drug-susceptible TB cases, and relative risks of mortality in XDR-TB cases

compared with MDR-TB cases reported by studies identified through our systematic review, and the risk of MDR-TB associated with HIV infection from the literature.[1]

Modelling strategy

To determine TB deaths in HIV-positive individuals, we first computed the fraction of HIV-TB deaths among all TB deaths using vital registration data from countries with a four- or five-star rating. We also calculated the proportion of TB cases that are HIV-positive (ie, number of TB cases recorded as HIV-positive/number of TB cases with an HIV test result recorded in the WHO TB register). We used these proportions as input data for a mixed effects regression to predict the proportions of HIV-TB cases among all TB cases for all locations and years using an adult HIV death rate covariate. We estimated the fraction of HIV-TB deaths among all TB deaths in each location and year $(D_{c,y})$, defined by

$$D_{c,y} = \frac{P_{c,y}RR}{P_{c,y}RR + 1 - P_{c,y}}$$

where $P_{c,y}$ is the proportion of HIV-TB cases among all TB cases and *RR* is the relative risk of TB deaths in HIV positive individuals, defined by:

$$RR = \frac{D_{c,y}P_{c,y} - D_{c,y}}{D_{c,y}P_{c,y} - P_{c,y}}$$

We took the median relative risk (RR) from each calculation. We then applied the median RR and the predicted proportions of HIV-TB cases among all TB cases to get the fractions of HIV-TB deaths among all TB deaths for all locations and years. Location-year-specific HIV-TB deaths were then calculated using the following equation:

$$Deaths_{HIV-TB} = \frac{D_{c,y}}{1 - D_{c,y}} Deaths_{TB}$$

where $Deaths_{TB}$ is location-year specific deaths from the CODEm TB no-HIV model. Finally, we applied the age-sex pattern of the HIV mortality estimates to these HIV-TB deaths to generate location-year-age-sex-specific HIV-TB deaths. As the HIV-TB deaths were estimated based on the fraction of HIV-TB deaths among all TB deaths, the total number of HIV-TB deaths could exceed the total number of HIV deaths in some locations. To avoid this, we applied a cap of 45% on the fraction of HIV-TB deaths among HIV deaths, based on a review by Cox and colleagues, 2010,[2] and a systematic review and meta-analysis by Ford and colleagues, 2016.[3]

To split HIV-TB into HIV-MDR-TB and HIV-drug-susceptible-TB, we first calculated the proportion of HIV-MDR-TB among all HIV-TB cases ($P_{MDR-HIV c,y,a,s}$) for each location, year, age, and sex using the following formula:

$$P_{MDR-HIVc,y,a,s} = P_{MDRnoHIVc,y,a,s} RR_{HIV}$$

where $P_{MDRnoHIVc,y,a,s}$ is the estimated proportion of MDR-TB among HIV-negative TB cases for each location, year, age, and sex (see MDR-TB modelling strategy for the detail) and RR_{HIV} is the relative risk of MDR-TB associated with HIV infection.

We then computed the fraction of HIV-MDR-TB deaths among all HIV-TB deaths ($D_{MDR-HIVc,y,a,s}$) using the following formula:

$$D_{MDR-HIVc,y,a,s} = \frac{P_{MDR-HIVc,y,a,s} RR_{MDR}}{P_{MDR-HIVc,y,a,s} RR_{MDR} + 1 - P_{MDR-HIVc,y,a,s}}$$

where RR_{MDR} is the pooled relative risk of mortality in MDR-TB cases compared with drug-susceptible TB cases. We then applied the predicted HIV-MDR-TB death fractions to all HIV-TB death estimates to generate HIV-MDR-TB deaths by location, year, age, and sex. Next, we subtracted HIV-MDR-TB deaths from all HIV-TB deaths at the 1,000 draw level to generate drug-susceptible HIV-TB deaths by location, year, age, and sex.

To separate out HIV-XDR-TB from HIV-MDR-TB, we aggregated the XDR-TB cases and MDR-TB cases (with DST for second-line drugs) up to the super-region level and calculated the super-region-level proportions of XDR-TB among MDR-TB cases. Next, we computed the super-region-specific fraction of XDR-TB deaths among all MDR-TB deaths (D_{XDRsr}) using the following formula:

$$D_{XDRsr} = \frac{P_{XDRsr} RR_{XDR}}{P_{XDRsr} RR_{XDR} + 1 - P_{XDRsr}}$$

where P_{XDRsr} is the proportion of XDR-TB among MDR-TB cases for each super-region, and RR_{XDR} is the pooled relative risk of mortality in XDR-TB cases compared with MDR-TB cases. These fractions were then applied to MDR-TB deaths in corresponding countries within the super-regions to produce XDR-TB deaths by location, age, and sex for the most recent year of estimation. We linearly extrapolated XDR-TB mortality rates back, assuming the mortality rates were zero in 1992, one year before 1993 when XDR-TB was first recorded in USA surveillance data.[4] Finally, we subtracted HIV-XDR-TB deaths from HIV-MDR-TB deaths to generate HIV-MDR-TB (without extensive drug resistance) deaths by location, year, age, and sex.

References

1 Mesfin YM, Hailemariam D, Biadglign S, Kibret KT. Association between HIV/AIDS and multi-drug resistance tuberculosis: a systematic review and meta-analysis. PLoS One. 2014 Jan 8;9(1):e82235.

2 Cox JA, Lukande RL, Lucas S, Nelson AM, Van Marck E, Colebunders R. Autopsy causes of death in HIV-positive individuals in sub-Saharan Africa and correlation with clinical diagnoses. *AIDS Rev* 2010; **12**: 183–94.

3 Ford N, Matteelli A, Shubber Z, *et al.* TB as a cause of hospitalization and in-hospital mortality among people living with HIV worldwide: a systematic review and meta-analysis. *J Int AIDS Soc* 2016; **19**: 20714.

4 Centers for Disease Control and Prevention (CDC). Extensively Drug-Resistant Tuberculosis --- United States, 1993—2006. MMWR. 2007; 56(11);250-253

Sexually Transmitted Infections Excluding HIV

Total sexually transmitted diseases (STI), chlamydia, gonorrhea, syphilis, and other STI.

Input data

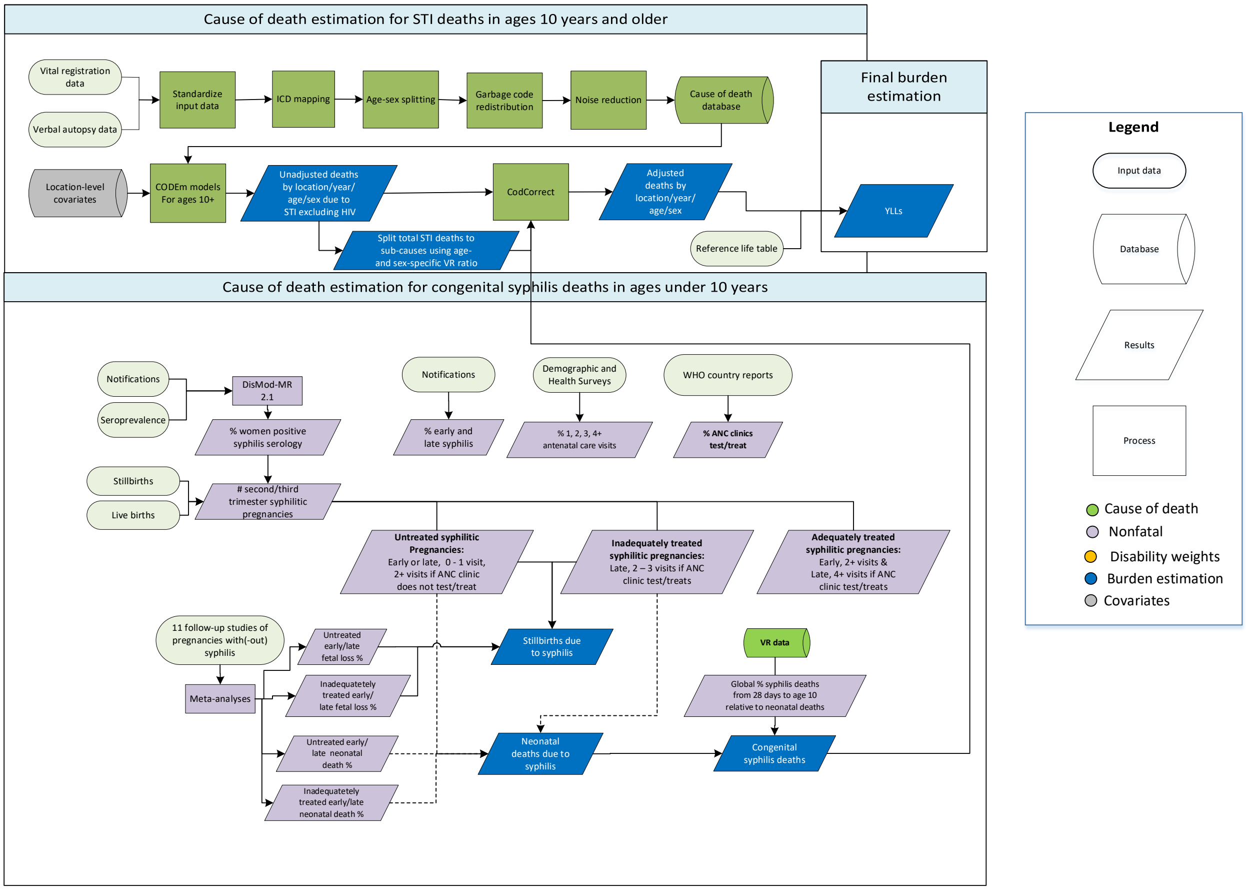

For GBD 2017, STI cause of death models included syphilis, chlamydial infection, gonococcal infection, and other STIs. CODEm models for males and females 10 years and older were informed from centrally processed data from the cause of death (COD) database. All data from all geographies were reviewed. Outliers were identified as those data where age patterns or temporal patterns were inconsistent with neighbouring age groups or locations or where sparse data were predicting implausible overall temporal or age patterns for a given location.

Four different types of data were used for the natural history model of congenital syphilis. First, we used notification and survey data to estimate the prevalence of a positive syphilis serology test in women of reproductive age in DisMod-MR 2.1. Second, we used estimates of the number of antenatal care (ANC) visits from Demographic and Health Surveys and live birth estimates from our demographic analyses. Third, we used published data from the Global Health Observatory on the proportion of ANC clinics that test for syphilis and the proportion of women testing positive who receive treatment. Fourth, we used

the results of meta-analyses on the increased risk of fetal loss and neonatal death in syphilitic women to inform the risk of fetal loss and neonatal mortality from pregnancies in women with syphilis.

Modelling strategy

We completed data-rich (DR) and global CODEm models for ages 10 years and over for males and females separately. Ten covariates were used in each CODEm model, including 1) prevalence of positive syphilis serology in pregnancy from the DisMod-MR 2.1 analysis; 2) coverage of one antenatal care (ANC) visit; 3) coverage of four or more ANC visits; 4) age-specific fertility rate; 5) total fertility rate; 6) health system access, a principal components analysis of ANC, in-facility delivery, skilled birth attendance, and vaccine coverage; 7) heath care access and quality index (HAQI), a measure of mortality amenable to heath care, 8) national income per capita (LDI); 9) years of education per capita; and 10) abortion legality, an index that includes a categorical rating of abortion laws that range from 1 (always illegal) to 7 (always legal on demand).

Covariates are shown in the following table.

Level	Covariate	Direction
1	Syphilis prevalence	+
2	Abortion legality	-
	Age-specific fertility rate	+
	Education (years per capita)	-
	Total fertility rate	+
	Health system access, capped	-
	Health care access and quality index	-
3	Antenatal care coverage, 1+ visits	-
	Antenatal care coverage, 4+ visits	-
	Lag-distributed income	-

The overall CODEm model for STI was split into the sub-causes using vital registration (VR) data from the COD database. Trichomoniasis and HSV-2 were assumed not to cause mortality. Chlamydia was further assumed not to cause death in males. Cause-specific mortality rate VR data for each age group, sex, and year were summed and scaled to match the total STI cause-specific mortality rate predicted by CODEm. This global VR pattern was applied to all locations.

Our model for congenital syphilis deaths began with estimation of the prevalence of a positive syphilis serology in adults in DisMod-MR 2.1, restricting incidence to zero before the age of 10 and assuming no excess mortality. A positive non-treponemal test (RPR or VDRL) with confirmation by a treponemal test was chosen as the reference. Incidence data, primarily in the form of notifications, were ignored due to varying degrees of under-reporting that lead to irreconcilable differences between incidence and prevalence data. Study-level covariates for non-reference categories (pregnant, blood donors) and non-reference testing modalities (eg, treponemal test only, non-treponemal test only) were used to crosswalk to the reference category.

To account for differing access to testing and treatment across geographies in the seroprevalence model, consistent duration ranges were calculated using a sum of the duration of untreated and treated syphilis, weighted by the percent of individuals that are symptomatic and the probability of receiving treatment if symptomatic with the formula below. These estimates were taken from published numbers

$$\mathbf{Duration} = (\%\,Symptomatic)(Prob_{Rx})(Duration_{Rx}) + (1 - \%\,Symptomatic)(Duration_{not\,Rx}) + (\%\,Symptomatic)(1 - Prob_{Rx})(Duration_{not\,Rx})$$

in GBD 2000 and WHO 2005, which were largely expert-driven. The probability of treatment if symptomatic was scaled using the Healthcare Access and Quality index (HAQI) to compute this probability for each location and year. Durations per stage (primary, secondary, latent, and tertiary) were calculated individually and summed along with the average seroreversion by stage, weighing by the proportion of cases remaining at each stage and including the time it would take to serorevert after adequate treatment.

Seroprevalence data in women of reproductive age from this DisMod model were paired with age-specific live birth estimates from our demographic analyses to generate the number of pregnancies in women with syphilis. To adjust for fetal loss, the following steps were taken: 1) we ran ST-GPR on the ANC syphilis testing and treatment data from the Global Health Observatory (GHO) using SDI as a covariate; 2) pregnancies were categorized by the proportion of pregnant women with 0–1, 2+, and 4+ ANC visits; 3) we used notification data on the proportions with early and late syphilis and multiplied these with the data on ANC visits. Women with 0–1 ANC visits or any number of visits to clinics that did not test and treat were considered untreated. Those with late syphilis and 2–3 ANC visits to clinics that tested and treated were classified as inadequately treated, and those with early syphilis and 2+ ANC visits to clinics that tested and treated or late syphilis and 4+ visits were categorised as adequately treated.

From 11 follow-up studies of pregnant women with syphilis we derived excess fetal loss proportions and excess neonatal death proportions relative to non-syphilitic women by meta-analyses. Only one study (Ingraham 1950) provided additional information on fetal loss and neonatal mortality for women with early and late syphilis. Japanese notification data detailed by syphilis stage and patient age were used to determine the proportion with early/late syphilis by 5-year age groups. As the time periods these studies were conducted in varies greatly, we took into account the higher risk of an adverse pregnancy outcome in the past by subtracting rates of healthy mothers from syphilitic mothers from the same study. The ratio of mothers with early/late syphilis and the mortality rates by stage from this study were used to split the overall fetal loss and neonatal mortality rates for both untreated and inadequately treated syphilis into early and late syphilis rates for each category.

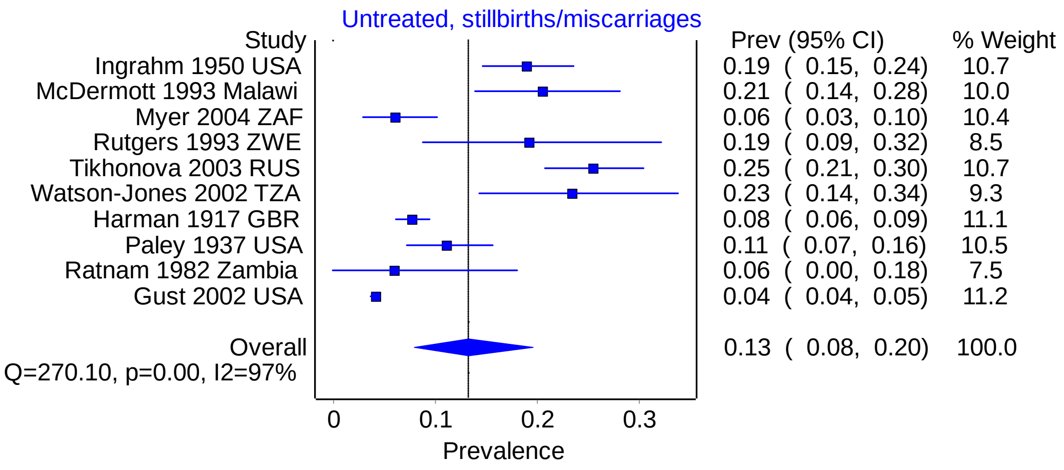
Untreated, stillbirths/miscarriages
Study
Prev (95% CI)
% Weight
Ingrahm 1950 USA 0.19 (0.15, 0.24) 10.7
McDermott 1993 Malawi 0.21 (0.14, 0.28) 10.0
Myer 2004 ZAF 0.06 (0.03, 0.10) 10.4
Rutgers 1993 ZWE 0.19 (0.09, 0.32) 8.5
Tikhonova 2003 RUS 0.25 (0.21, 0.30) 10.7
Watson-Jones 2002 TZA 0.23 (0.14, 0.34) 9.3
Harman 1917 GBR 0.08 (0.06, 0.09) 11.1
Paley 1937 USA 0.11 (0.07, 0.16) 10.5
Ratnam 1982 Zambia 0.06 (0.00, 0.18) 7.5
Gust 2002 USA 0.04 (0.04, 0.05) 11.2
Overall 0.13 (0.08, 0.20) 100.0
Q=270.10, p=0.00, I2=97%
0
0.1
0.2
0.3
Prevalence

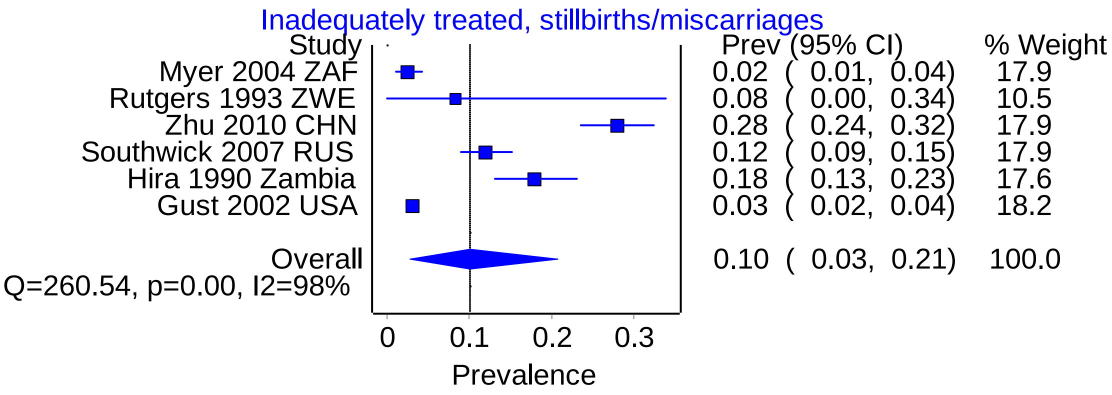
Inadequately treated, stillbirths/miscarriages
Study
Prev (95% CI)
% Weight
Myer 2004 ZAF 0.02 (0.01, 0.04) 17.9
Rutgers 1993 ZWE 0.08 (0.00, 0.34) 10.5
Zhu 2010 CHN 0.28 (0.24, 0.32) 17.9
Southwick 2007 RUS 0.12 (0.09, 0.15) 17.9
Hira 1990 Zambia 0.18 (0.13, 0.23) 17.6
Gust 2002 USA 0.03 (0.02, 0.04) 18.2
Overall 0.10 (0.03, 0.21) 100.0
Q=260.54, p=0.00, I2=98%
0
0.1
0.2
0.3
Prevalence

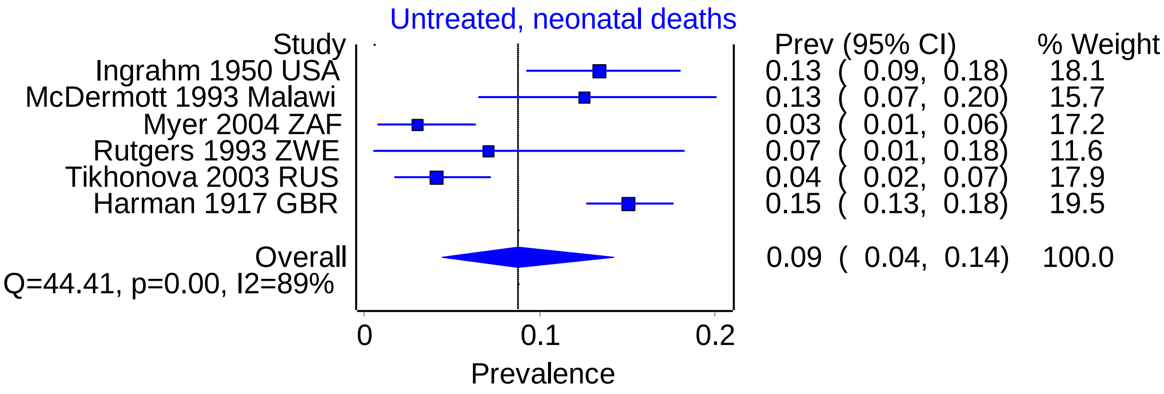
Untreated, neonatal deaths
Study
Prev (95% CI)
% Weight
Ingrahm 1950 USA 0.13 (0.09, 0.18) 18.1
McDermott 1993 Malawi 0.13 (0.07, 0.20) 15.7
Myer 2004 ZAF 0.03 (0.01, 0.06) 17.2
Rutgers 1993 ZWE 0.07 (0.01, 0.18) 11.6
Tikhonova 2003 RUS 0.04 (0.02, 0.07) 17.9
Harman 1917 GBR 0.15 (0.13, 0.18) 19.5
Overall 0.09 (0.04, 0.14) 100.0
Q=44.41, p=0.00, I2=89%
0
0.1
0.2
Prevalence

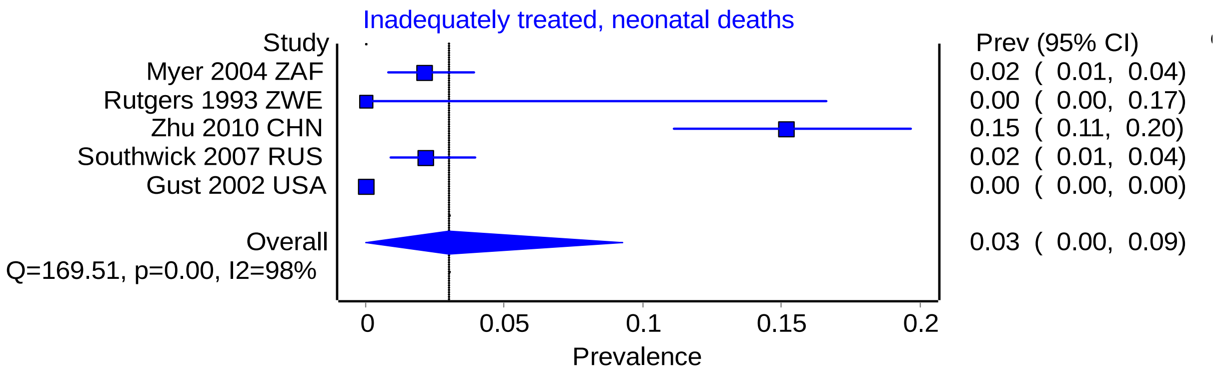

No excess mortality or fetal loss was assumed for adequately treated cases of maternal syphilis. A comparison of the neonatal mortality rates between adequately treated women and uninfected women showed a smaller proportion of babies from adequately treated women died than babies from uninfected women.

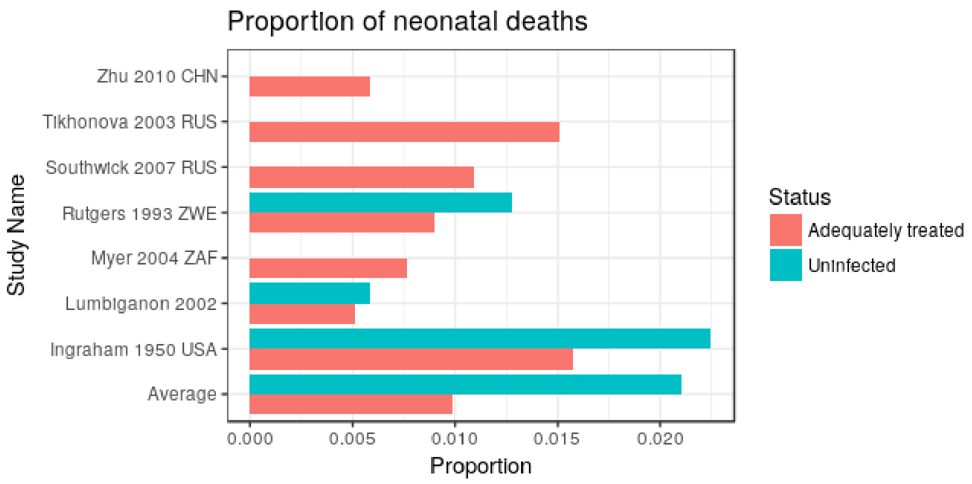

We first adjusted syphilitic pregnancies for the excess risk of stillbirth to estimate the number of stillbirths attributable to congenital syphilis. We then multiplied the live births in syphilitic mothers by the proportions of ANC visit categories, the probability of attending a clinic that tests and treats, and the proportions of early and late syphilis in pregnant women. We applied the appropriate neonatal excess mortality rates derived from our meta-analyses. This total number of neonatal syphilis deaths was split between the 0–6 days and 7–27 days age groups using sex- and age-specific VR data from the COD database. Congenital syphilis deaths beyond the neonatal period were likewise estimated using sex- and age-specific VR data. This VR pattern was applied globally to all locations.

The primary limitation of our estimation of STI deaths in those over 10 years old is data availability, especially from countries where VR systems are not available. Even in countries with VR, there may be some variation in practices for coding deaths to STI as the underlying cause, especially given the potentially variable presentation of many of the conditions in this category. Such variation is more likely to lead to underestimation of STI deaths than overestimation. The estimation of STI-specific deaths is

similarly limited by data availability in those locations without VR data, and our estimates are thus based on the overall pattern of deaths in generally higher-income geographies.

The primary implication of this limitation is that it decreases the resolution with which we can decompose the relationship between mortality from HIV and other STI. Our model for congenital syphilis continues to improve but still is limited by data availability issues and assumptions backed by sparse evidence, especially on the coverage and effectiveness of ANC interventions to prevent congenital syphilis. We do not have information on the proportion of women who tested positive who may have received treatment elsewhere, or information on the coverage of treatment for neonates, infants, and children born with congenital syphilis.

Tuberculosis

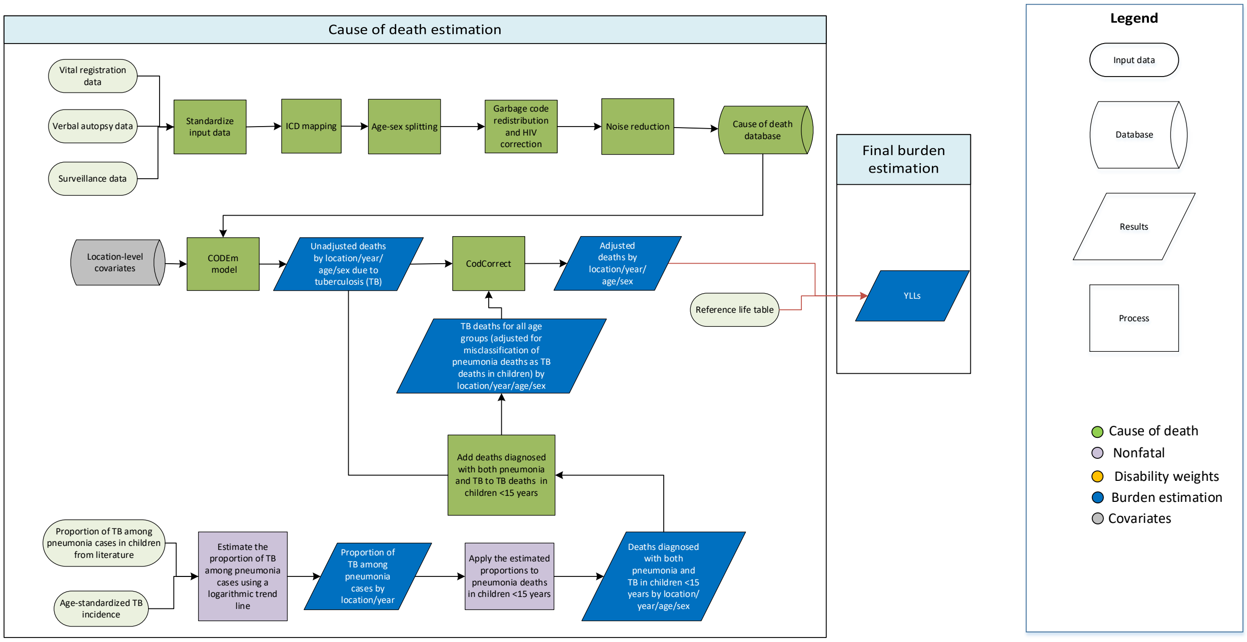

Input data

Input data for modelling tuberculosis (TB) mortality among HIV-negative individuals include vital registration, verbal autopsy, and surveillance data. Vital registration data were adjusted for garbage coding (including ill-defined codes and the use of intermediate causes) following GBD algorithms and misclassified HIV deaths (ie, HIV deaths being assigned to other underlying causes of death such as tuberculosis or diarrhea because of stigma or misdiagnosis).

Verbal autopsy data in countries with age-standardised HIV prevalence greater than 5% were removed because of a high probability of misclassification, as verbal autopsy studies have poor validity in distinguishing HIV deaths from HIV-TB deaths.

Modelling strategy

A general CODEm modelling strategy was used. We added a new covariate, namely the TB strain prevalence-weighted transmission risk. We also included the cigarettes per capita covariate. Other location-level covariates included in the CODEm model were the same as in GBD 2016: adult underweight proportion, alcohol (litres per capita), diabetes (fasting plasma glucose mmol/L), education (years per capita), Healthcare Access and Quality Index, lag-distributed income, indoor air pollution, outdoor air pollution, population density, prevalence of active tuberculosis, prevalence of latent tuberculosis infection, smoking prevalence, Socio-demographic Index, and a summary exposure variable reflecting the average exposure to all of the risk factors.

Covariate table

	Covariate	Direction
Level 1	TB prevalence<br>Latent TB infection prevalence<br>SEV scalar<br>Alcohol per capita<br>Smoking prevalence<br>Cigarettes per capita<br>Fasting plasma glucose<br>TB strain prevalence-weighted transmission risk	+<br>+<br>+<br>+<br>+<br>+<br>+<br>+
Level 2	HAQ Index<br>Adult underweight proportion<br>Indoor air pollution<br>Outdoor air pollution<br>Population density	-<br>+<br>+<br>+<br>+
Level 3	Log LDI<br>Education (years per capita)<br>Socio-demographic Index (SDI)	-<br>-<br>-

Correcting for a potential misclassification of tuberculosis deaths as pneumonia deaths in children

In locations with high TB burden, TB deaths may be misclassified as pneumonia deaths in children,[1] and we addressed this potential misclassification in GBD 2017. First, we estimated the proportion of tuberculosis among pneumonia cases as a function of age-standardised TB incidence using data from eight clinical studies[2,3,4,5,6,7,8,9] reporting the proportion of pneumonia cases that had tuberculosis (or the data to calculate them) and the age-standardised TB incidence estimates. We used a logarithmic trend line to fit these data. Next, we applied the estimated proportions to pneumonia deaths (estimated for GBD 2017) among children younger than 15 years to compute the number of deaths diagnosed with both pneumonia and TB, which were then added to child TB deaths from the CODEm model. Finally, these estimates were adjusted using CoDCorrect, which ensures that the number of deaths from each cause add up to all-cause mortality deaths for a given year.

References

1. Graham SM, Sismanidis C, Menzies HJ, Marais BJ, Detjen AK, Black RE. Importance of tuberculosis control to address child survival. *Lancet* 2014; **383**(9928): 1605-7.
2. Adegbola RA, Falade AG, Sam BE, et al. The etiology of pneumonia in malnourished and well-nourished Gambian children. Pediatr Infect Dis J 1994; 13: 975–82.
3. Chisti MJ, Graham SM, Duke T, et al. A prospective study of the prevalence of tuberculosis and bacteraemia in Bangladeshi children with severe malnutrition and pneumonia including an evaluation of Xpert MTB/RIF assay. PloS One 2014; 9: e93776.

4. Madhi SA, Petersen K, Madhi A, Khoosal M, Klugman KP. Increased disease burden and antibiotic resistance of bacteria causing severe community-acquired lower respiratory tract infections in human immunodeficiency virus type 1-infected children. Clin Infect Dis 2000; 31: 170–76.
5. McNally LM, Jeena PM, Gajee K, et al. Effect of age, polymicrobial disease, and maternal HIV status on treatment response and cause of severe pneumonia in South African children: a prospective descriptive study. Lancet 2007; 369: 1440–51.
6. Moore DP, Klugman KP, Madhi SA. Role of Streptococcus pneumoniae in hospitalisation for acute community-acquired pneumonia associated with culture-confirmed Mycobacterium tuberculosis in children: a pneumococcal conjugate vaccine probe study. Pediatr Infect Dis J 2010; 29: 1099–104.
7. Nantongo JM, Wobudeya E, Mupere E, et al. High incidence of pulmonary tuberculosis in children admitted with severe pneumonia in Uganda. BMC Pediatr 2013; 13: 16.
8. Zar HJ, Hanslo D, Tannenbaum E, et al. Aetiology and outcome of pneumonia in human immunodeficiency virus-infected children hospitalized in South Africa. Acta Paediatr 2001; 90: 119–25.
9. Moore DP, Higdon MM, Hammitt LL, Prosperi C, DeLuca AN, Da Silva P, Baillie VL, Adrian PV, Mudau A, Deloria Knoll M, Feikin DR. The incremental value of repeated induced sputum and gastric aspirate samples for the diagnosis of pulmonary tuberculosis in young children with acute community-acquired pneumonia. Clinical Infectious Diseases. 2017 May 27;64(suppl_3):S309-16.

TB strain prevalence-weighted transmission risk covariate

We added a new TB covariate added in this round of GBD that incorporated data on the global distribution of TB strains and the relative risk of transmission associated with those strains. For this covariate, we defined TB strains according to the seven phylogenetic lineages of the *Mycobacterium tuberculosis* complex (MTBC) identified by S. Gagneaux and colleagues.[1] We determined the global distribution of these strains using a systematic review of human TB molecular epidemiology studies from 1990 to 2017 in PubMed and Scopus, as described in greater detail elsewhere.[2] All studies that used population-based sampling methods or collected isolates from all culture-positive TB cases in a given location and time period were included. All genotypes that could be converted to phylogenetic lineages were extracted, including genotypes determined by spoligotyping, MIRU-VNTR typing, and PCR or whole-genome sequencing. Studies of sub-populations, such as prison populations or drug-resistant cases only, were excluded. In total, 206 studies representing 85 countries and over 200,000 bacterial isolates were included. A map of these strains highlighted the widespread global distribution of Euro-American Lineage 4 strains and East Asian Lineage 2 strains, and the geographic restriction of Lineage 5 and 6 strains to West Africa. Thirty of these studies also reported transmission chains associated with bacterial genotypes, as defined by genetic clustering.[3]

We used spatiotemporal Gaussian process regression (ST-GPR) to model the distribution of each strain in each GBD location across all ages and sexes, as described in greater detail elsewhere.[4] The covariates tested in each model included HIV age-standardised prevalence, population density, and a custom-made human movement covariate. The human movement covariate took into account (1) immigration and emigration patterns[5] and (2) airplane passenger flow[6] to and from each country. In the ST-GPR models we assumed strong correlation and smoothing over both space and time. We then used

a random-effects meta-analysis to determine the relative risk (RR) of transmission associated with each strain, as defined by genetic clustering. We used the most widespread strains, Euro-American Lineage 4 strains, as the reference group. We found that East Asian Lineage 2 strains were associated with increased risk of transmission overall (Relative Risk [95% CI] = 1.24 [1.07, 1.45]), while West African Lineage 5 and 6 strains were associated with reduced transmission (Relative Risk [95% CI] = 0.61 [0.43, 0.86]). We used the following formula to calculate a TB strain prevalence-weighted risk of transmission based on these estimates:

$$\sum_{i=1}^{n} Pr_i RR_i$$

i=TB strain; Pr=proportion; RR=relative risk

References

1. Comas I, Coscolla M, Luo T, *et al.* Out-of-Africa migration and Neolithic coexpansion of *Mycobacterium tuberculosis* with modern humans. *Nat Genet* 2013; **45**: 1176–82.
2. Wiens KE, Woyczynski LP, Ledesma JR, *et al*. Global variation in bacterial strains that cause tuberculosis disease: a systematic review and meta-analysis. *BMC Medicine*; In Review.
3. Dheda K, Gumbo T, Maartens G, *et al.* The epidemiology, pathogenesis, transmission, diagnosis, and management of multidrug-resistant, extensively drug-resistant, and incurable tuberculosis. *Lancet Respir Med* 2017; **5**: 291–360.
4. Manuscript in preparation.
5. United Nations Population Division. United Nations Trends in International Migrant Stock: The 2015 Revision. New York City, United States: United Nations Population Division, 2015.
6. Huang Z, Wu X, Garcia AJ, *et al*. An open-access modeled passenger flow matrix for the global air network in 2010. *PLoS ONE* **8(5)**: e64317.

Multidrug-resistant tuberculosis, extensively drug-resistant tuberculosis, and drug-susceptible tuberculosis

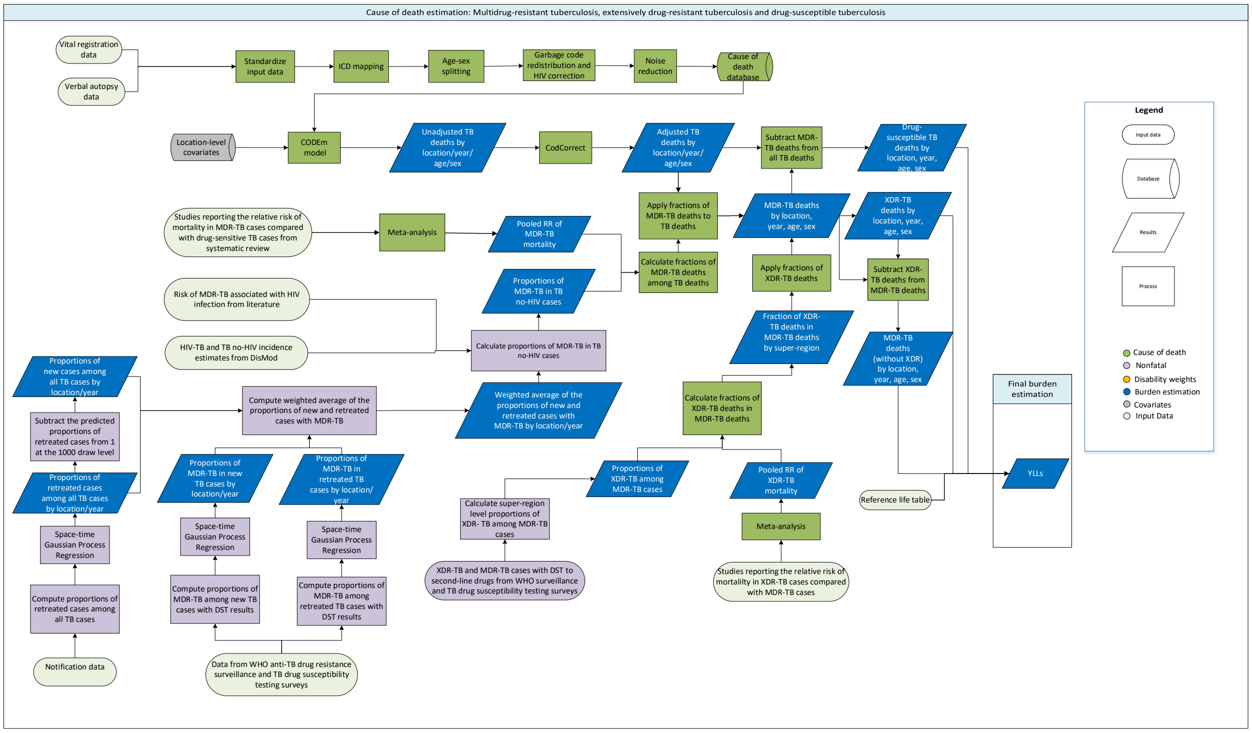

Input data

Input data include: (i) the number of drug-resistant cases by type (multidrug-resistant tuberculosis [MDR-TB], extensively drug-resistant tuberculosis [XDR-TB], all TB cases with a drug-susceptible testing [DST] result for isoniazid and rifampicin, and MDR-TB cases with DST for second-line drugs) from routine surveillance and surveys reported to the World Health Organization, (ii) data from studies (identified through our systematic review) reporting on the relative risk of death in MDR-TB cases compared with non-MDR TB (drug-susceptible TB) cases, and the relative risk of death in XDR-TB cases compared with MDR-TB cases, and (iii) the risk of MDR-TB associated with HIV infection from the literature.[1]

Modelling strategy

We conducted a systematic review and meta-analysis of studies reporting the relative risk of death in MDR-TB cases compared with drug-susceptible TB cases. We ran spatiotemporal Gaussian process regressions to predict the proportions of new TB cases with MDR-TB, proportions of retreated TB cases with MDR-TB, and proportions of retreated cases among all TB cases for all locations and years. We also calculated the proportions of new TB cases among all TB cases. We then computed the weighted average of the proportions of new and retreated cases with MDR-TB at the 1,000-draw level. We then used the weighted average proportions of MDR-TB, along with the HIV-TB and TB no-HIV incidence estimates (from our modeling of non-fatal TB), and the relative risk of MDR-TB associated with HIV

infection from the literature[1] to compute the proportions of MDR-TB cases among HIV-negative TB cases $\left(P_{MDRnoHIVc,y,a,s}\right)$ by location, year, age, and sex using the following formula:

$$P_{MDRnoHIVc,y,a,s} = \frac{MDR_{c,y}}{\left(1 + \left(RR_{HIV} \frac{HIVTB_{c,y,a,s}}{TBnoHIV_{c,y,a,s}}\right)\right) TBnoHIV_{c,y,a,s}}$$

where $MDR_{c,y}$ is the number of all MDR-TB cases among HIV-positive and HIV-negative individuals by location and year, RR_{HIV} is the relative risk of MDR-TB associated with HIV infection, $HIVTB_{c,y,a,s}$ is the number of HIV-TB incident cases by location, year, age, and sex, and $TBnoHIV_{c,y,a,s}$ is the number of TB no-HIV incident cases by location, year, age, and sex.

We then computed the fraction of MDR-TB deaths among all HIV-negative TB deaths ($D_{MDRnoHIVc,y,a,s}$) using the following formula:

$$D_{MDRnoHIVc,y,a,s} = \frac{P_{MDRnoHIVc,y,a,s}RR_{MDR}}{P_{MDRnoHIVc,y,a,s}RR_{MDR} + 1 - P_{MDRnoHIVc,y,a,s}}$$

where RR_{MDR} is the relative risk of death in MDR-TB cases compared with drug-susceptible TB cases. We then applied the predicted fractions of MDR-TB deaths among HIV-negative TB deaths to our CODEm TB death estimates to generate MDR-TB deaths by location, year, age, and sex. Next, we subtracted MDR-TB deaths from all TB deaths to generate drug-susceptible TB deaths by location, year, age, and sex.

To separate out XDR-TB from MDR-TB, we aggregated the XDR-TB cases and MDR-TB cases (with DST for second-line drugs) up to the super-region level and calculated the super-region-level proportions of XDR-TB among MDR-TB cases. Next, we computed the super-region-specific fractions of XDR-TB deaths among all MDR-TB deaths (D_{XDRsr}) using the following formula:

$$D_{XDRsr} = \frac{P_{XDRsr}RR_{XDR}}{P_{XDRsr}RR_{XDR} + 1 - P_{XDRsr}}$$

where P_{XDRsr} is the proportion of XDR-TB among MDR-TB cases for each super-region, and RR_{XDR} is the pooled relative risk of mortality in XDR-TB cases compared with MDR-TB cases. These fractions were then applied to MDR-TB deaths in corresponding countries within the super-regions to produce XDR-TB deaths by location, age, and sex for the most recent year of estimation. We linearly extrapolated XDR-TB mortality rates back, assuming the mortality rates were zero in 1992, one year before 1993 when XDR-TB was first recorded in USA surveillance data.[2] Finally, we subtracted XDR-TB deaths from MDR-TB deaths to generate MDR-TB (without extensive drug resistance) deaths by location, year, age, and sex.

References

1. Mesfin YM, Hailemariam D, Biadglign S, Kibret KT. Association between HIV/AIDS and multi-drug resistance tuberculosis: a systematic review and meta-analysis. PLoS One. 2014;9(1):e82235.

2. Centers for Disease Control and Prevention (CDC). Extensively Drug-Resistant Tuberculosis --- United States, 1993—2006. MMWR. 2007; 56(11);250-253

Lower Respiratory Infections

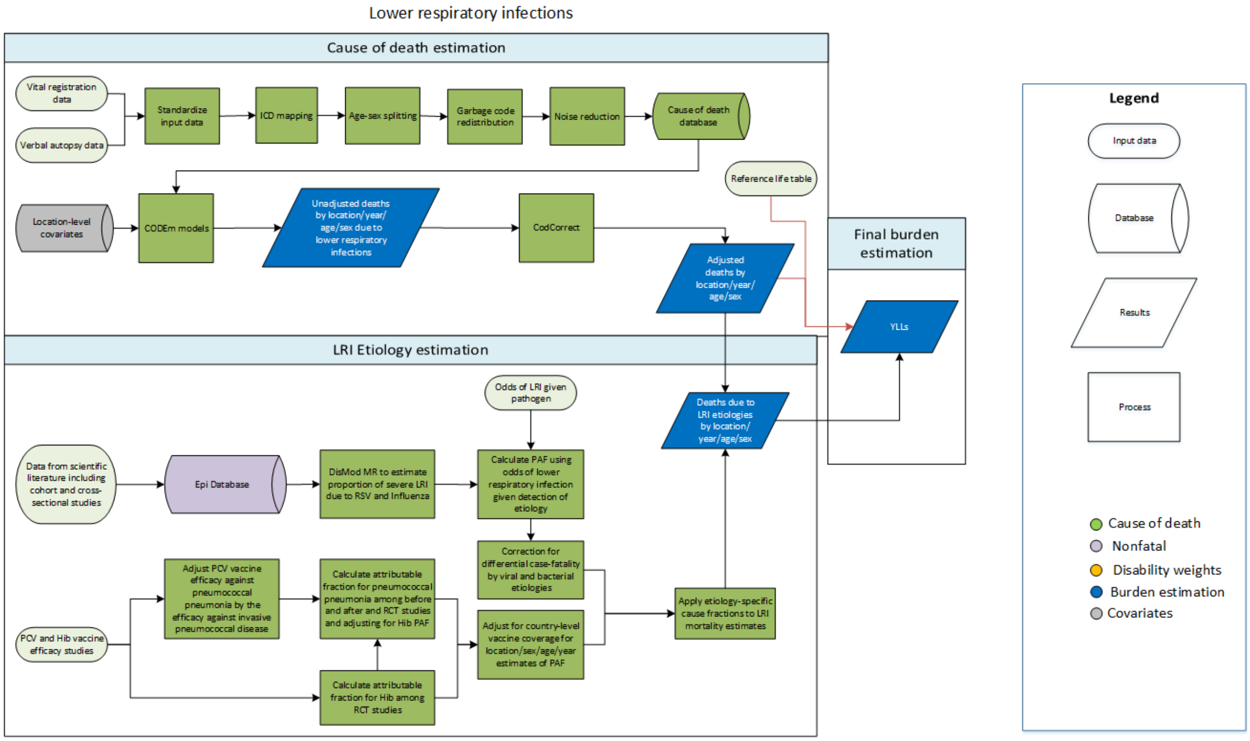

Input data

Cause of death

Lower respiratory infection (LRI) mortality was estimated in CODEm. We estimated LRI mortality separately for males and females and for children under 5 years and older than 5 years. We used all available data from vital registration systems, surveillance systems, and verbal autopsy (**Table 1**). We checked for and excluded outliers from our data by country or region. We also excluded ICD9-coded mortality data in Sri Lanka (1982, 1987–1992), ICD9-coded neonatal mortality data in Guatemala (1980, 1981, 1984, 2000–2004), and medically coded cause of death data (MCCD) and Civil Registration System data in many Indian states (1986–2013).

Table 1. Summary of cause-specific mortality modelling input data.

Input data	GBD 2017
**Total data sources**	**19,827 geography-years**
Vital registration data	17,374 geography-years
Sample registration data	740 geography-years
Verbal autopsy data	1,153 geography-years
Surveillance	560 geography-years

Figure 1. The number of geography-years of LRI mortality data by GBD geography is shown

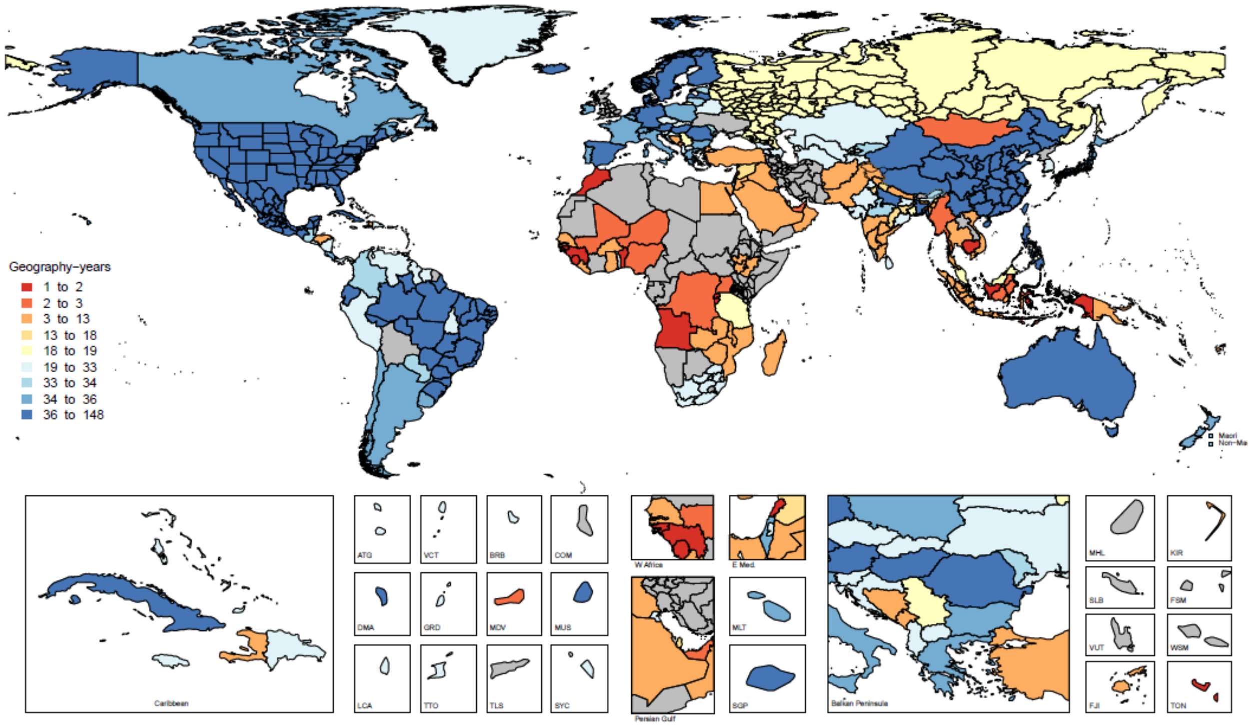

Aetiologies

We updated our systematic review of scientific literature for the proportion of LRI that tested positive for influenza and respiratory syncytial virus (RSV) to include all data from GBD 2016 and from studies published between January 1, 2016 and May 26, 2017. We performed the search using PubMed and the following search string:

(“lower respiratory”[title/abstract] OR pneumonia[title/abstract]) AND (2016/01/01[PDat] : 2017/12/31[PDat]) AND (incidence OR prevalence OR epidemiology OR etiolog*[title/abstract] OR influenza[title/abstract] OR “respiratory syncytial virus”[title/abstract]) AND Humans[MeSH Terms] NOT(autoimmune[title/abstract] OR COPD [title/abstract] OR “cystic fibrosis”[title/abstract] OR Review[ptyp])

Inclusion criteria were studies that had a sample size of at least 100, studies that were at least one year in duration, and studies describing lower respiratory infections, pneumonia, or bronchiolitis as the case definition. During our literature review we identified 595 studies, of which 75 met our inclusion criteria and were extracted. We excluded studies that described pandemic H1N1 influenza solely and studies that used influenza-like illness as the case definition. We assigned an age range based on the prevalence-weighted mean age of LRI in the appropriate year/sex/location if the ages of the study participants were not reported.

We also conducted a systematic literature review of studies on the Hib vaccine and PCV effectiveness studies against X-ray-confirmed pneumonia and against pneumococcal and Hib disease until May 2017. For PCV studies, we extracted, if available, the distribution of pneumococcal pneumonia serotypes and the serotypes included in the PCV used in the study. No new studies were identified for GBD 2017. We excluded observational and case-control studies due to implausibly high vaccine efficacy estimates. Hib trial data were exclusively from children <5 years, so we did not include the effect of Hib on ages over 5 years of age. PCV trial data are also frequently limited to younger populations. To understand the contribution of pneumococcal pneumonia in older populations, we also included PCV efficacy studies that used before-after approaches.

Modelling strategy

Cause of death. We used country-level covariates to inform our CODEm models (**Table 2**). We evaluated our LRI cause of death models using in and out of sample predictive performance.

Like all models of mortality in GBD, LRI mortality models are single-cause, requiring in effect that the sum of all mortality models must be equal to the all-cause mortality envelope. We correct LRI mortality estimates, and other causes of mortality, by rescaling them according to the uncertainty around the cause-specific mortality rate. This process is called CoDCorrect and is essential to ensure internal consistency among causes of death.

Table 2. Covariates used in LRI mortality modelling. Table 2A is for children under 5 and Table 2B shows the covariates used for ages 5-95+. The *Level* is the associated strength of relationship between the covariate and LRI mortality, ranked from 1 (proximally related) to 3 (distally related). The direction is the forced direction of the association between the covariate and LRI mortality.

Table 2A. Covariates used in under 5 years model

Level	Covariate	Direction
1	Childhood stunting SEV	+
	Childhood underweight SEV	+
	Childhood wasted SEV	+
	Indoor air pollution	+
	Short gestation SEV	+
	Low weight gestation	+
	LRI summary exposure variable	+
	Second-hand smoking prevalence	+
	Antibiotics for LRI	-
	Hib vaccine coverage	-
	Pneumococcal conjugate vaccine coverage	-
2	Discontinued breastfeeding SEV	+
	Vitamin A deficiency	+
	Zinc deficiency	+
	DTP3 vaccine coverage	-
	Healthcare access and quality index	-
3	Outdoor air pollution ($PM_{2.5}$)	+
	Population density > 1000/km^2	+
	Sanitation SEV	+
	Handwashing	-
	LDI per capita	-
	Maternal education	-
	Socio-demographic Index	-

Table 2B. Covariates used in 5-95+ years model

Level	Covariate	Direction
1	Indoor air pollution	+
	LRI summary exposure variable	+
	Outdoor air pollution	+
	Secondhand smoking prevalence	+
	Smoking prevalence	+
2	DTP3 vaccine coverage	-
	Healthcare access and quality index	-
	Mean BMI	-
	Pneumococcal conjugate vaccine coverage	-
	Handwashing	+
3	Education years per capita	-
	LDI per capita	-
	Socio-demographic Index	-
	Alcohol consumption	+
	Sanitation summary exposure variable	+

Aetiologies

We estimated LRI aetiologies separately from overall LRI mortality using two distinct counterfactual modelling strategies to estimate population attributable fractions (PAFs), described in detail below. The PAF represents the relative reduction in LRI mortality if there was no exposure to a given aetiology. As LRIs can be caused by multiple pathogens and the pathogens may co-infect, PAFs can overlap and add up to more than 100%. Separate strategies were used for viral – influenza and respiratory syncytial virus (RSV) – and bacterial – *Streptococcus pneumoniae* and *Haemophilus influenzae* type B – aetiologies. We did not attribute aetiologies to neonatal pneumonia deaths due to a dearth of reliable data in this age group. We calculated uncertainty of our PAF estimates from 1,000 draws of each parameter using normal distributions in log space.

Influenza and RSV. We calculated the population attributable fraction (PAF) from the proportion of severe LRI cases positive for influenza and RSV. We assumed that hospitalised LRI cases are a proxy of severe cases. We used the following formula to estimate PAF:[1]

$$PAF = Proportion * (1\text{-}1/OR)$$

Where *Proportion* is the proportion of LRI cases that test positive for influenza or RSV and *OR* is the odds ratio of LRI given the presence of the pathogen. We used an odds ratio of 5.1 (3.19–8.14) for influenza and 9.79 (4.98–19.27) for RSV from a recently published meta-analysis.[2]

We modelled the proportion data using the meta-regression tool DisMod-MR to estimate the proportion of LRI cases that are positive for influenza and RSV, separately, by location/year/age/sex. We accounted for study-level covariates in our models such as PCR as the diagnostic technique, studies that investigated RSV or influenza exclusively, and studies from inpatient populations.

As the case-fatality of viral causes of pneumonia is lower than for bacterial causes, we adjusted for differential case-fatality by determining the aetiological fractions for mortality attributable to RSV and influenza (**Table 3**). We measured the aetiologic fractions by applying a relative case-fatality adjustment based on in-hospital case-fatality, which we coded to specific pneumonia aetiologies. Hospital admissions data of this type were limited to data from Austria, Brazil, Chile, China, Ecuador, Italy, Kenya, Mexico, New Zealand, the Philippines, Portugal, and the United States. We generated the pooled estimate of the case-fatality differential between bacterial (pneumococcus, Hib) and viral aetiologies (RSV, influenza) using DisMod-MR to determine an age pattern for this ratio.

Pneumococcal pneumonia and Hib. For *Streptococcus pneumoniae* (pneumococcal pneumonia) and *Haemophilus influenzae* type B (Hib), we calculated the population attributable fraction using a vaccine probe design.[3,4] The ratio of vaccine effectiveness against nonspecific pneumonia to pathogen-specific disease represents the fraction of pneumonia cases attributable to each pathogen.

To estimate the PAF for Hib and pneumococcal pneumonia, we calculated the ratio of vaccine effectiveness against nonspecific pneumonia to pathogen-specific pneumonia (Equations 1 and 3). We estimated a study-level estimate of PAF from a meta-analysis of these ratios. To estimate the PAF for Hib, we only used randomised controlled trials because of implausibly high values of vaccine efficacy in case-control studies. To estimate the PAF for pneumococcal pneumonia, we included RCTs and before and after vaccine introduction longitudinal studies.

We adjusted the study-level PAF estimate by vaccine coverage and expected vaccine performance to estimate country- and year-specific PAF values. For pneumococcal pneumonia, we adjusted the PAF by the final Hib PAF estimate and by vaccine serotype coverage. Finally, we used an age distribution of PAF modelled in DisMod to determine the PAF by age. Because of an absence of data describing vaccine efficacy against Hib in children older than 2 years, we did not attribute Hib to episodes of LRI in ages 5 years and older.

We used a vaccine probe design to estimate the PAF for pneumococcal pneumonia and (Hib) by first calculating the ratio of vaccine effectiveness against nonspecific pneumonia to pathogen-specific pneumonia at the study level (Equations 1 and 2).[3–5] We then adjusted this estimate by vaccine coverage and expected vaccine performance to estimate country- and year-specific PAF values (Equations 3 and 4).

$$1)\quad HibPAF_{Base} = 1 - \frac{VE_{Pneumonia}}{VE_{Hib}}$$

$$2)\quad PneumoPAF_{Base} = 1 - \frac{VE_{Pneumonia} * (1 - PAF_{Hib} * VE_{Hib\ Optimal})}{VE_{Streptococcus} * Cov_{Serotype}}$$

$$3)\quad PAF_{Hib} = PAF_{Base} * \frac{(1 - Cov_{Hib} * VE_{Hib\ Optimal})}{(1 - PAF_{Base} * Cov_{Hib} * VE_{Hib\ Optimal})}$$

$$4)\quad PAF_{Pneumo} = \frac{PAF_{Base} * (1 - Cov_{PCV} * VE_{PCV\ Optimal})}{(1 - PAF_{Hib} * Cov_{Hib} * VE_{Hib\ Optimal}) * \left(1 - \frac{PAF_{Base} Cov_{PCV} * VE_{PCV\ Optimal}}{(1 - PAF_{Hib} * Cov_{Hib} * VE_{Hib\ Optimal})}\right)}$$

Where $VE_{Pneumonia}$ is the vaccine efficacy against nonspecific pneumonia, VE_{Hib} is the vaccine efficacy against invasive Hib disease, $VE_{Streptococcus}$ is the vaccine efficacy against serotype-specific pneumococcal pneumonia, $Cov_{serotype}$ is the serotype-specific vaccine coverage for PCV,[6] $VE_{Hib\ Optimal}$ is the Hib effectiveness in the community (0.8)[7], PAF_{Hib} is the final PAF for Hib, Cov_{PCV} is the PCV coverage, Cov_{Hib} is the Hib coverage by country, and $VE_{PCV\ Optimal}$ is the vaccine effectiveness in the community (0.8).[8]

For Hib, we assumed that the vaccine efficacy against invasive Hib disease is the same against Hib pneumonia. For pneumococcal pneumonia, a recent study in adults[9] found that the vaccine efficacy against invasive pneumococcal disease may be significantly higher than against pneumococcal pneumonia. We used this ratio to adjust estimates of vaccine efficacy against invasive pneumococcal disease from other studies. However, recognising that the study is unique in that it uses a urine antigen test among adults, we added uncertainty around our adjustment using a wide uniform distribution (median 0.65, 0.3–1.0). This has increased the estimates of pneumococcal pneumonia mortality in a meaningful way.

The only substantive changes to the cause of death estimation for LRI in GBD 2017 were the addition of new cause of death data and inclusion of several additional covariates. The ratio of mortality in bacterial

to viral aaetiologies was updated for GBD 2017 and the new results substantively increase the attribution of influenza and RSV to LRI deaths.

Table 3: **The median values for the ratio of viral to bacterial pneumonia case fatality ratio by age is shown.** These estimates are modelled using hospital-based, ICD-coded admissions and mortality for aetiology-specified pneumonia. Values in parentheses represent 95% uncertainty interval.

**Age Group**	**Ratio**
Early Neonatal	0.59 (0.36–0.84)
Late Neonatal	0.58 (0.37–0.84)
Post Neonatal	0.58 (0.41–0.77)
1 to 4	0.69 (0.64–0.74)
5 to 9	0.85 (0.77–0.93)
10 to 14	0.84 (0.79–0.89)
15 to 19	0.83 (0.78–0.87)
20 to 24	0.82 (0.77–0.87)
25 to 29	0.82 (0.78–0.86)
30 to 34	0.82 (0.79–0.85)
35 to 39	0.82 (0.8–0.85)
40 to 44	0.82 (0.8-0.85)
45 to 49	0.82 (0.8–0.85)
50 to 54	0.82 (0.79–0.85)
55 to 59	0.82 (0.79–0.86)
60 to 64	0.82 (0.79–0.86)
65 to 69	0.82 (0.8–0.85)
70 to 74	0.82 (0.79–0.85)
75 to 79	0.82 (0.78–0.85)
80 to 84	0.83 (0.8–0.87)
85 to 89	0.86 (0.83–0.89)
90 to 94	0.89 (0.85–0.93)
95 to 99	0.92 (0.86–0.97)

References

1 Miettinen OS. Proportion of disease caused or prevented by a given exposure, trait or intervention. *Am J Epidemiol* 1974; **99**: 325–32.

2 Shi T, McLean K, Campbell H, Nair H. Aetiological role of common respiratory viruses in acute lower respiratory infections in children under five years: A systematic review and meta-analysis. *J Glob Health* 2015; **5**: 10408.

3 Feikin DR, Scott JAG, Gessner BD. Use of vaccines as probes to define disease burden. *Lancet Lond Engl* 2014; **383**: 1762–70.

4 O'Brien KL, Wolfson LJ, Watt JP, *et al.* Burden of disease caused by Streptococcus pneumoniae in children younger than 5 years: global estimates. *Lancet Lond Engl* 2009; **374**: 893–902.

5 Watt JP, Wolfson LJ, O'Brien KL, *et al.* Burden of disease caused by Haemophilus influenzae type b in children younger than 5 years: global estimates. *Lancet Lond Engl* 2009; **374**: 903–11.

6 Johnson HL, Deloria-Knoll M, Levine OS, *et al.* Systematic evaluation of serotypes causing invasive pneumococcal disease among children under five: the pneumococcal global serotype project. *PLoS Med* 2010; **7**. DOI:10.1371/journal.pmed.1000348.

7 Swingler G, Fransman D, Hussey G. Conjugate vaccines for preventing Haemophilus influenzae type B infections. *Cochrane Database Syst Rev* 2007; : CD001729.

8 Lucero MG, Dulalia VE, Nillos LT, *et al.* Pneumococcal conjugate vaccines for preventing vaccine-type invasive pneumococcal disease and X-ray defined pneumonia in children less than two years of age. *Cochrane Database Syst Rev* 2009; : CD004977.

9 Bonten MJM, Huijts SM, Bolkenbaas M, *et al.* Polysaccharide conjugate vaccine against pneumococcal pneumonia in adults. *N Engl J Med* 2015; **372**: 1114–25.

Upper Respiratory Infections

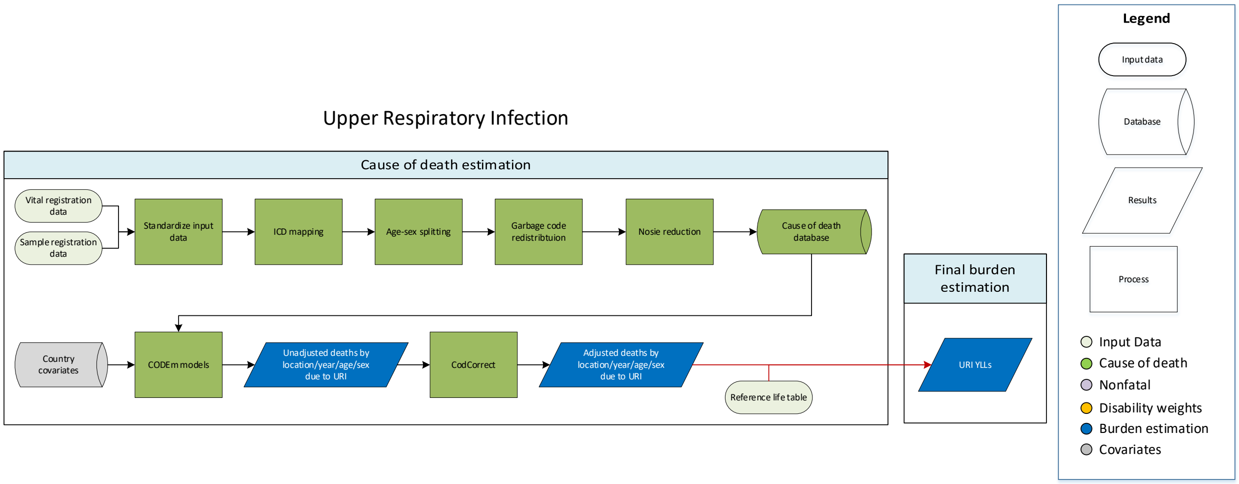

Input Data and Methodological Summary for Upper Respiratory Infections

Input data

Vital registration and surveillance data from the cause of death database were used. Outliers were identified by systematic examination of data points. Data points that violated well-established age or time trends, were inconsistent with other country- or region-specific points, or that resulted in extremely high or low mortality rates were determined to be outliers.

Modelling strategy

A generic CODEm approach was used to estimate mortality due to upper respiratory infections (URI) in GBD 2017. In GBD 2016, mortality from URI was modelled using a negative binomial regression. It was determined that a negative binomial regression was an appropriate approach for estimating URI due to a small number of deaths due to URI in the cause of death database. However, due to changes in how we redistribute cause of death codes, more deaths were attributed to URI in the COD database and thus it was determined that a generic CODEm approach is feasible for estimating URI mortality in GBD 2017. The covariates used are displayed below.

Level	Covariate	Direction
1	Smoking prevalence	+
2	Indoor pollution	+
	Outdoor pollution ($PM_{2.5}$)	+
	Healthcare Access and Quality Index	+
3	SDI	-
	LDI	-
	Education (years per capita)	-

Otitis Media

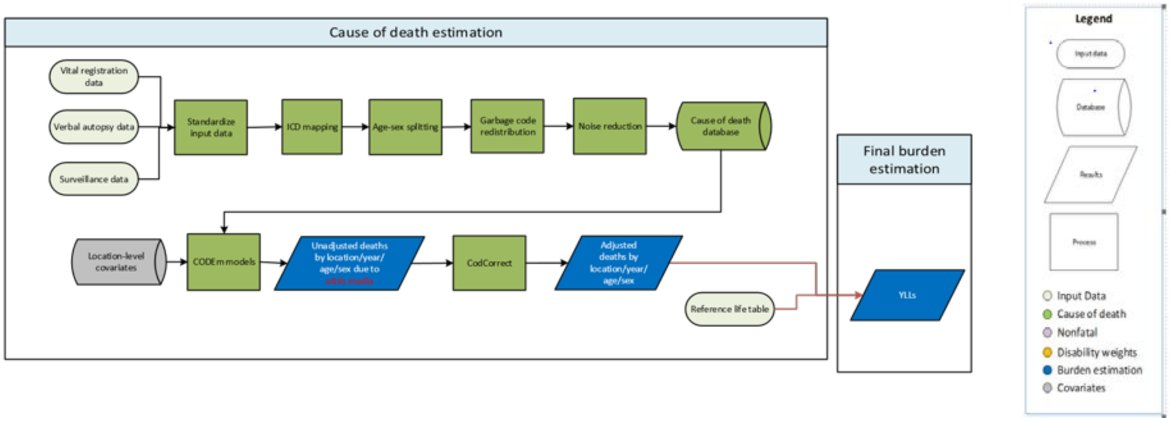

Input Data and Methodological Summary for Otitis Media

Input data

Vital registration, verbal autopsy, and surveillance data were used. Outliers were identified by systematic examination of data points. Data points that violated well-established age or time trends, were inconsistent with other country- or region-specific points, or that resulted in extremely high or low mortality rates were determined to be outliers.

Modeling strategy

A general CODEm modelling strategy was used. There were no substantive changes from GBD 2016 in terms of modelling strategy. The covariates used are displayed below.

Level	Covariate	Direction
1	Otitis SEV	+
	Smoking prevalence	+
2	Indoor pollution	+
	Healthcare Access and Quality Index	-
	Outdoor pollution ($PM_{2.5}$)	+
3	SDI	-
	LDI	-
	Education (years per capita)	-

Diarrhoeal Diseases

Diarrheal diseases

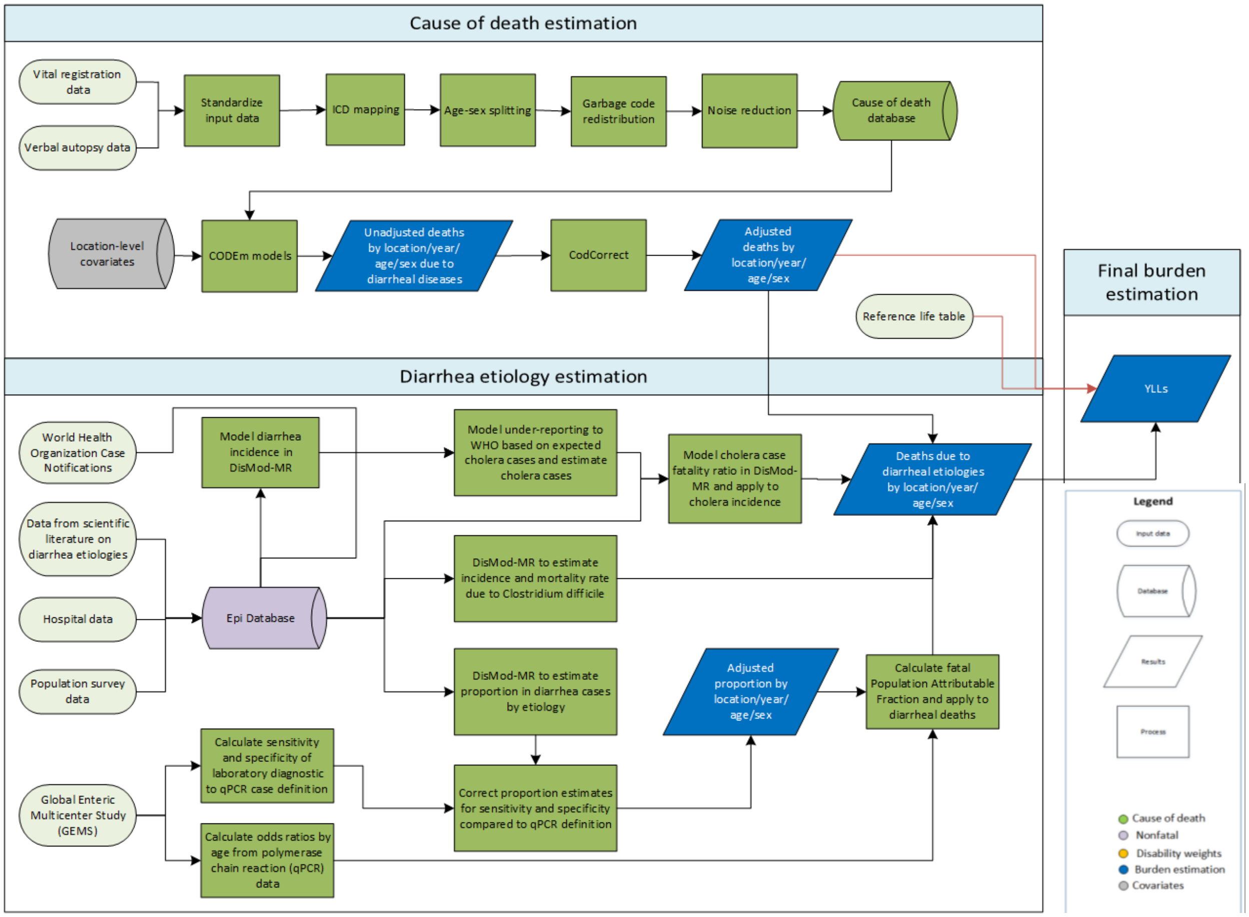

Input data

Cause of death. We used all available data from vital registration systems, surveillance systems and verbal autopsy (**Table 1 and Figure 1**). We checked for and excluded outliers from our data by country or region. We also excluded early neonatal mortality data in the Philippines (1994–1998) and India Civil Registration System data and medically certified cause of death (MCCD) data in all states (1986–2013).

Aetiologies. We conducted a systematic literature review for the proportion of diarrhoea cases that tested positive for each aetiology. We updated our review of literature to include studies published between January 2016 and May 2017. The search was performed in PubMed using the following search string:

(diarrhoea[title/abstract] OR diarrhea[title/abstract]) AND (2016/01/01:2017/12/31[PDat]) AND Humans[MeSH Terms] AND (incidence[title/abstract] OR prevalence[title/abstract] OR epidemiology[title/abstract] OR salmonella[title/abstract] OR aeromonad*[title/abstract] OR

shigell*[title/abstract] OR enteropathogenic[title/abstract] OR enterotoxigenic[title/abstract] OR campylobacter[title/abstract] OR amoebiasis[title/abstract] OR entamoeb*[title/abstract] OR cryptosporid*[title/abstract] OR rotavirus[title/abstract] OR norovirus[title/abstract] OR adenovirus[title/abstract] OR etiology[title/abstract]) NOT (appendicitis[title/abstract] OR esophag*[title/abstract] OR surger*[title/abstract] OR gastritis[title/abstract] OR liver[title/abstract] OR case report[title] OR case-report[title] OR therapy[title] OR treatment[title] Crohn[title/abstract] OR "inflammatory bowel"[title/abstract] OR irritable[title/abstract] OR travel*[title] OR Outbreak[title] OR Review[ptyp] OR vomiting[title/abstract)

Inclusion criteria included diarrhoea as the case definition, studies with a sample size of at least 100, and studies with at least one year of follow-up. We excluded studies that reported on diarrhoeal outbreaks exclusively and those that used acute gastroenteritis with or without diarrhoea. We identified 225 studies, of which 51 met our criteria of inclusion and were included. We extracted data points for location, sex, year, and age. We assigned an age range based on the prevalence-weighted mean age of diarrhoea in the appropriate year/sex/location if the age of the study participants was not reported.

We used the Global Enteric Multicenter Study (GEMS), a seven-site, case-control study of moderate-to-severe diarrhoea in children under 5 years,[1] to calculate odds ratios for the diarrhoeal pathogens based on a molecular diagnostic case definition.

For GBD 2017, we added an additional 40,000 stool samples analyzed using quantitative polymerase chain reaction (qPCR) from The Etiology, Risk Factors, and Interactions of Enteric Infections and Malnutrition and the Consequences for Child Health and Development Project (MAL-ED) study to the roughly 10,000 samples from the GEMS reanalysis.[2–6]

Modelling strategy

Cause of death. Diarrhoeal disease mortality was estimated in the Cause of Death Ensemble modelling platform (CODEm). We estimated diarrhoea mortality separately for males and females and for children under 5 years and older than 5 years. We used country-level covariates to inform our CODEm models (**Table 2**). We evaluated our diarrhoeal disease cause of death models using in and out of sample predictive performance.

Aetiologies. We estimated diarrhoeal disease aetiologies independently from overall diarrhoea mortality using a counterfactual strategy for enteric adenovirus, *Aeromonas*, *Entamoeba histolytica* (amoebiasis), *Campylobacter*, *Cryptosporidium*, typical enteropathogenic *Escherichia coli* (t-EPEC), enterotoxigenic *Escherichia coli* (ETEC), norovirus, non-typhoidal salmonella infections, rotavirus, and *Shigella*. *Vibrio cholerae* and *Clostridium difficile* were modelled separately.

Diarrhoeal aetiologies are attributed to diarrhoeal deaths using a counterfactual approach. We calculated a population attributable fraction (PAF) from the proportion of severe diarrhoea cases that are positive for each aetiology. The PAF represents the relative reduction in

diarrhoea mortality if there was no exposure to a given aetiology. As diarrhoea can be caused by multiple pathogens and the pathogens may co-infect, PAFs can overlap and add up to more than 100%. We calculated the PAF from the proportion of severe diarrhoea cases that are positive for each aetiology. We assumed that hospitalised diarrhoea cases are a proxy of severe and fatal cases. We used the following formula to estimate PAF:[7]

$$PAF = Proportion * (1 - \frac{1}{OR})$$

Where *Proportion* is the proportion of diarrhoea cases positive for an aetiology and *OR* is the odds ratio of diarrhoea given the presence of the pathogen.

We dichotomised the continuous qPCR test result using the value of the cycle threshold (Ct) that most accurately discriminated between cases and controls. The Ct values range from 0 to 35 cycles representing the relative concentration of the target gene in the stool sample. A low value indicates a higher concentration of the pathogen while a value of 35 indicates the absence of the target in the sample. We used the lower Ct value when we had multiple Ct values for the cutpoint. The case definition for each pathogen is a Ct value that is below the established cutoff point.

We used a mixed effects conditional logistic regression model to calculate the odds ratio for under 1 year and 1-4 years old for each of our pathogens. The stool samples from cases and controls in GEMS were used exclusively to calculate these odds ratios as we assumed that the association between pathogens and moderate-to-severe diarrhoea is a proxy for fatal outcomes. The odds ratio for 1-4 years was applied to all GBD age groups over 5 years. There were three pathogen-age odds ratios that were not statistically significant: Aeromonas and Amoebiasis in under 1 year and Campylobacter in 1-4 years. The mean value of the odds ratio was above 1 in all three cases so we transformed the odds ratios for these three exceptions only in log-space such that exponentiated values could not be below 1. The transformation was:

Odds ratio = exp(log(OR) – 1)) + 1

We modelled the proportion data using the meta-regression tool DisMod-MR to estimate the proportion of positive diarrhoea cases for each separate aetiology by location/year/age/sex and to adjust for the covariates.

We used the estimated sensitivity and specificity of the original laboratory diagnostic test results from the pooled GEMS and MALED qPCR stool samples compared to the qPCR test result to adjust our proportion before we computed the PAF:[8]

$$Proportion_{True} = \frac{(Proportion_{Observed} + Specificity - 1)}{(Sensitivity + Specificity - 1)}$$

We used this correction to account for the fact that the proportions we used are based on a new test that is not consistent with the laboratory-based case definition (qPCR versus GEMS conventional laboratory testing for pathogens).[9] Because differences in the type of PCR used in

the original (non-reference qPCR diagnostic) between GEMS and MALED in detecting norovirus, we combined the sensitivity and specificity results for norovirus such that 50% of the draws were coming from GEMS test results exclusively and 50% of the draws were coming from MALED test results exclusively. Additionally, because the original laboratory diagnostic technique used for *Campylobacter* in MALED was one not commonly used, we only used GEMS to determine the sensitivity and specificity of bacterial culture compared to qPCR in detecting *Campylobacter.*[10]

Our literature review extracted the proportion of any enteropathogenic *Escherichia coli* (EPEC) without differentiating between typical (tEPEC) and atypical (aEPEC). In order to be consistent with the odds ratios that we obtained, we adjusted our proportion estimates of any EPEC to typical EPEC only. This adjustment was informed by a subset of our literature review that reported both atypical and typical EPEC. We estimated a ratio by super-region of tEPEC to any EPEC and adjusted our proportion estimates accordingly. We found that the majority of EPEC diarrhoea cases were positive for atypical EPEC, consistent with other published work.[11]

For *Vibrio cholerae* (cholera), we used the literature review to estimate expected number of cholera cases for each country-year using the incidence of diarrhoea, estimated using DisMod-MR, and the proportion of diarrhoea cases that are positive for cholera. We assigned cholera PAF using odds ratios from the qPCR results to estimate a number of cholera-attributable cases. We compared this expected number of cholera cases to the number reported to the World Health Organization at the country-year level.[12] We modelled the underreporting fraction to correct the cholera case notification data for all countries using health system access and the diarrhoea SEV scalar to predict total cholera cases. We used the age-specific proportion of positive cholera samples in DisMod and our incidence estimates to predict the number of cholera cases for each age/sex/year/location. Finally, we modelled the case fatality ratio of cholera using DisMod-MR and to estimate the number of cholera deaths.

For *Clostridium difficile*, we modelled incidence and mortality in DisMod-MR for each age, sex, year, location. DisMod-MR is a Bayesian meta-regression tool that uses spatiotemporal information as priors to estimate prevalence, incidence, remission, and mortality for *Clostridium difficile* infection. DisMod-MR uses a compartmental model to relate prevalence, incidence, remission, and mortality. We set remission in our model to 1 month.

Table 1. Cause-specific mortality input data.

**Input data**	**GBD 2017**
**Total data sources**	**19,665 geography-years**
Vital registration data	17,734 geography-years
Sample registration data	740 geography-years
Verbal autopsy data	1,042 geography-years
Surveillance data	509 geography-years

Figure 1. Number of geography-years of mortality data used in diarrhoea mortality modelling

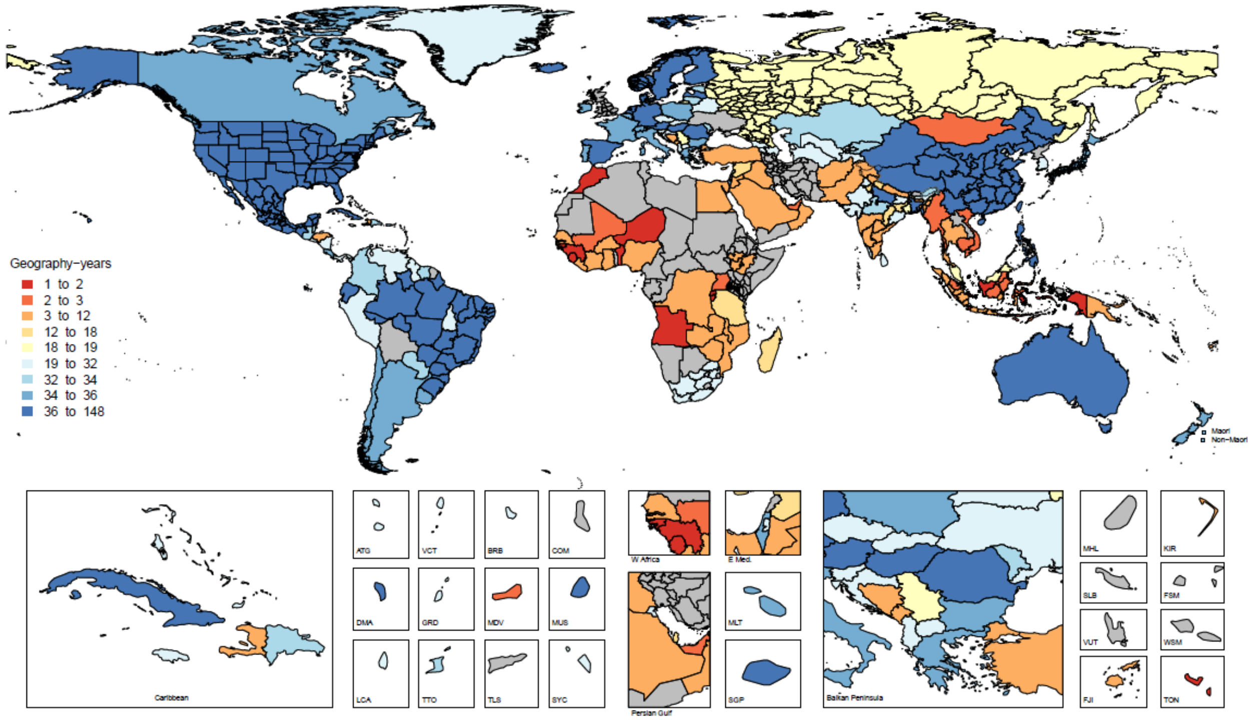

Table 2. The covariates used in diarrhoea mortality modelling. Table 2A shows the covariates used in the 0-4 years model and Table 2B shows the covariates used in the 5-95+ years model. The *Level* represents the strength of the association between the covariate and diarrhoea mortality from 1 (proximally related) to 3 (distally related). The *Direction* indicates the positive or negative association between the covariate and diarrhoea mortality.

Table 2A. The covariates used in the 0-4 years model

Level	Covariate	Direction
1	Diarrhoea SEV	+
	Childhood stunting SEV	+
	Sanitation SEV	+
	Water SEV	+
	Childhood underweight SEV	+
	Childhood wasting SEV	+
	Short gestation SEV	+
	Low weight gestation SEV	+
	Oral rehydration solution treatment	-
	Safe sanitation	-
	Safe water	-
2	Vitamin A deficiency	+
	Zinc deficiency	+
	Healthcare access and quality index	-
	Rotavirus vaccine	-
	Zinc treatment for diarrhoea	-
3	Breastfeeding SEV	+
	Handwashing	-
	LDI per capita	-
	Maternal education years	-
	Socio-demographic Index	-
	Population density < 150/km2	0
	Population density > 1000/km2	0

Table 2B. The covariates used in the 5-95+ years model.

Level	Covariate	Direction
1	Diarrhoea summary exposure variable	+
	Safe sanitation summary exposure variable	+
	Safe water summary exposure variable	+
	Improved sanitation	-
	Improved water	-
2	Healthcare access and quality index	-
	Rotavirus vaccine coverage	-
3	Education years per capita	-
	LDI per capita	-
	Mean BMI	-
	Socio-demographic Index	-
	Population density greater than 1000/km^2	0

References

1 Kotloff KL, Nataro JP, Blackwelder WC, *et al.* Burden and aetiology of diarrhoeal disease in infants and young children in developing countries (the Global Enteric Multicenter Study, GEMS): a prospective, case-control study. *Lancet Lond Engl* 2013; **382**: 209–22.

2 Platts-Mills JA, Babji S, Bodhidatta L, *et al.* Pathogen-specific burdens of community diarrhoea in developing countries: a multisite birth cohort study (MAL-ED). *Lancet Glob Health* 2015; **3**: e564-575.

3 MAL-ED Network Investigators. The MAL-ED study: a multinational and multidisciplinary approach to understand the relationship between enteric pathogens, malnutrition, gut physiology, physical growth, cognitive development, and immune responses in infants and children up to 2 years of age in resource-poor environments. *Clin Infect Dis Off Publ Infect Dis Soc Am* 2014; **59 Suppl 4**: S193-206.

4 Kotloff KL, Nataro JP, Blackwelder WC, *et al.* Burden and aetiology of diarrhoeal disease in infants and young children in developing countries (the Global Enteric Multicenter Study, GEMS): a prospective, case-control study. *Lancet Lond Engl* 2013; **382**: 209–22.

5 Liu J, Platts-Mills JA, Juma J, *et al.* Use of quantitative molecular diagnostic methods to identify causes of diarrhoea in children: a reanalysis of the GEMS case-control study. *Lancet Lond Engl* 2016; **388**: 1291–301.

6 Platts-Mills J, Liu J, Rogawski E. Aetiology, burden and clinical characteristics of diarrhoea in children in low-resource settings using quantitative molecular diagnostics: results from the MAL-ED cohort study. *Lancet Glob Health* 2018; Accepted.

7 Miettinen OS. Proportion of disease caused or prevented by a given exposure, trait or intervention. *Am J Epidemiol* 1974; **99**: 325–32.

8 Reiczigel J, Földi J, Ozsvári L. Exact confidence limits for prevalence of a disease with an imperfect diagnostic test. *Epidemiol Infect* 2010; **138**: 1674–8.

9 Platts-Mills JA, Operario DJ, Houpt ER. Molecular diagnosis of diarrhea: current status and future potential. *Curr Infect Dis Rep* 2012; **14**: 41–6.

10 Platts-Mills JA, Liu J, Gratz J, *et al.* Detection of Campylobacter in stool and determination of significance by culture, enzyme immunoassay, and PCR in developing countries. *J Clin Microbiol* 2014; **52**: 1074–80.

11 Ochoa TJ, Barletta F, Contreras C, Mercado E. New insights into the epidemiology of enteropathogenic Escherichia coli infection. *Trans R Soc Trop Med Hyg* 2008; **102**: 852–6.

12 World Health Organization. Global Health Observatory data repository: Cholera. 2016. http://apps.who.int/gho/data/node.main.174?lang=en (accessed Aug 25, 2016).

Typhoid fever

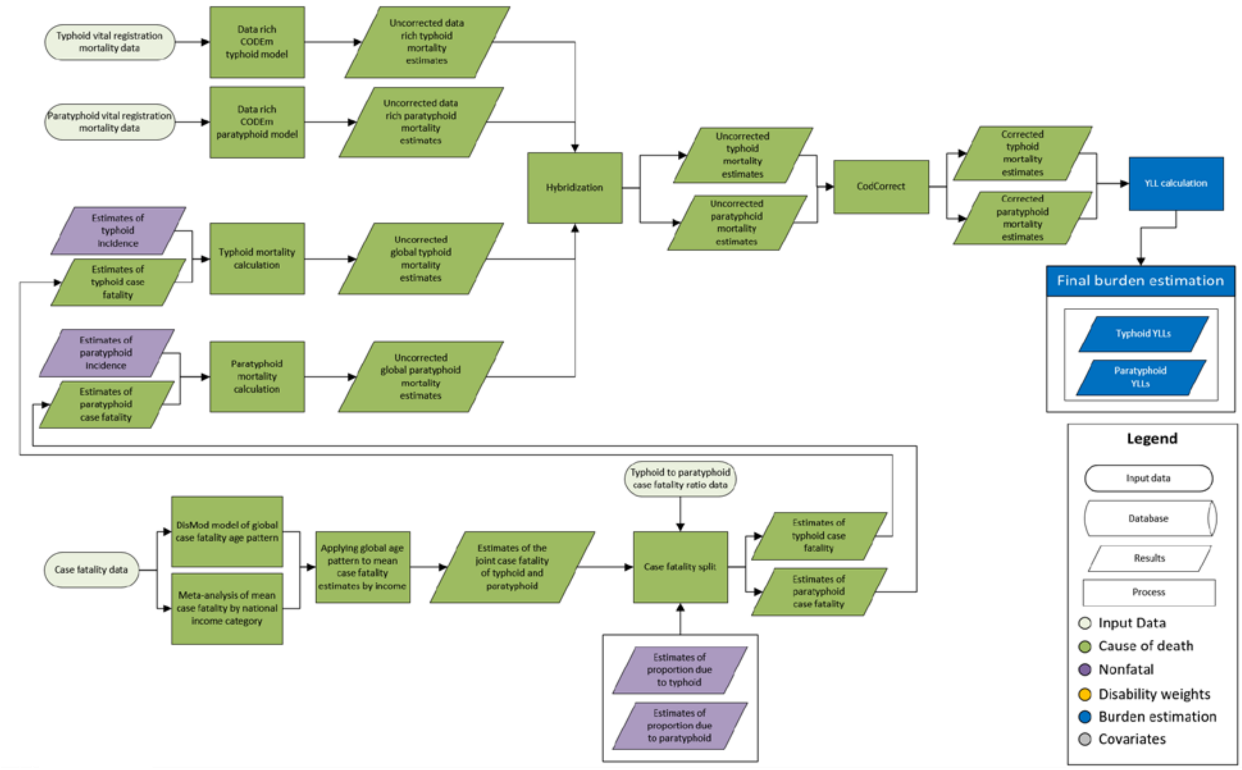

Input data

Our CODEm model used all available data in the cause of death database from data-rich countries. No data were outliered for this cause. For the natural history model, our incidence dataset included a combination of data from prospective cohort studies and national surveillance systems. Similarly, data on proportions due to typhoid and paratyphoid included a combination of prospective cohort studies and national surveillance systems. Case fatality data were from national surveillance systems and hospital databases.

Modelling strategy

We model typhoid deaths using a hybrid modelling strategy with two components: 1) for data-rich locations we estimate typhoid mortality using a CODEm model of CoD data; and 2) in all other locations (i., not data-rich) we use a natural history model in which we derive deaths as the product of cases and case fatality.

The CODEm model included six covariates:

Level	Covariate	Direction
1	Sanitation (proportion with access)	-
	Improved water source (proportion of the population with access)	-
	Proportion of the population living in the Indian Ocean monsoon belt	+
	SEV unsafe water	+

	SEV unsafe sanitation	+
2	Healthcare access and quality index	-

For the natural history model, we first model total incidence of typhoid and paratyphoid combined. Second, we model the proportion of this total due to typhoid and the proportion due to paratyphoid. Third, we estimate case fatality by age and national income category for typhoid and paratyphoid combined. Fourth, we use data on the relative fatality of typhoid and paratyphoid to split the joint case fatality estimates into typhoid- and paratyphoid-specific case fatality estimates. Finally, we estimate cause-specific mortality rates as the product of incidence and case fatality.

Total incidence was modelled using DisMod-MR 2.1, using the proportion of the population with access to clean water, and the proportion of the population living in the Indian Ocean monsoon belt as covariates. We performed a crosswalk using a study-level covariate indicating sources that were based on passive versus active surveillance, with active surveillance as the reference. This adjusts for incomplete case capture by passive surveillance. Incidence data were inflated to account for poor diagnostic sensitivity, based on a meta-analysis of the sensitivity of blood culture, the most common diagnostic used for typhoid. Similarly, we used two DisMod models to estimate aetiologic proportions: one for the proportion of total incidence due to typhoid, and one for the proportion due to paratyphoid.

Case fatality data were too limited to allow for a complete DisMod model, or to allow for varying estimates by time and space. We had sufficient data, however, to estimate case fatality by age and by three categories of national income. We used DisMod to extract a global age-pattern in case fatality, and meta-regression to estimate the mean case fatality by income category. Finally, we estimated the relative risk of death from typhoid relative to paratyphoid based on data from Chinese surveillance and used that relative risk to estimate case fatality separately for typhoid and paratyphoid, by age and income.

Finally, we estimated typhoid mortality as the product of total incidence, the proportion of the total due to typhoid, and case fatality for typhoid. We propagated uncertainty through every step of the modelling process by pulling 1,000 draws from the distribution of each model component (eg, incidence, proportion due to typhoid, overall case fatality, case fatality age pattern, relative fatalness of typhoid versus paratyphoid), and performing all calculations at the draw level.

For GBD 2016 we estimated typhoid mortality in all locations using a natural history model, and the introduction of the data-rich CODEm estimates is a new improvement for GBD 2017. We have made no substantive changes to our natural history modelling strategy between GBD 2016 and 2017.

Paratyphoid fever

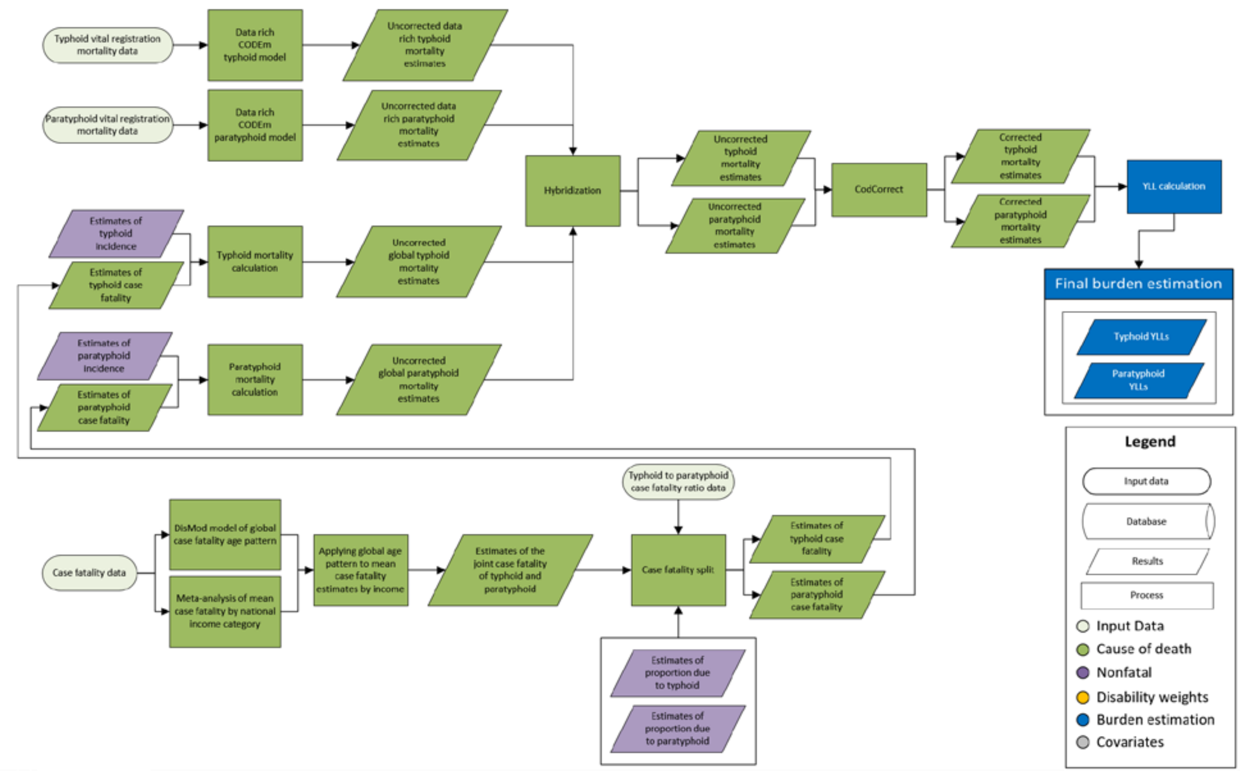

Input data

Our CODEm model used all available data in the cause of death database from data-rich countries. No data were outliered for this cause. For the natural history model, our incidence dataset included a combination of data from prospective cohort studies and national surveillance systems. Similarly, data on proportions due to typhoid and paratyphoid included a combination of prospective cohort studies and national surveillance systems. Case fatality data were from national surveillance systems and hospital databases.

Modelling strategy

We model paratyphoid deaths using a hybrid modelling strategy with two components: 1) for data-rich locations we estimate paratyphoid mortality using a CODEm model of CoD data; and 2) in all other locations (ie, not data-rich) we use a natural history model in which we derive deaths as the product of cases and case fatality.

The CODEm model included six covariates:

Level	Covariate	Direction
1	Sanitation (proportion with access)	-
	Improved water source (proportion of the population with access)	-
	Proportion of the population living in the Indian Ocean monsoon belt	+
	SEV unsafe water	+

	SEV unsafe sanitation	+
2	Healthcare access and quality index	-

For the natural history model, we first model total incidence of typhoid and paratyphoid combined. Second, we model the proportion of this total due to typhoid and the proportion due to paratyphoid. Third, we estimate case fatality by age and national income category for typhoid and paratyphoid combined. Fourth, we use data on the relative fatality of typhoid and paratyphoid to split the joint case fatality estimates into typhoid- and paratyphoid-specific case fatality estimates. Finally, we estimate cause-specific mortality rates as the product of incidence and case fatality.

Total incidence was modelled using DisMod-MR 2.1, using the proportion of the population with access to clean water, and the proportion of the population living in the Indian Ocean monsoon belt as covariates. We performed a crosswalk using a study-level covariate indicating sources that were based on passive versus active surveillance, with active surveillance as the reference. This adjusts for incomplete case capture by passive surveillance. Incidence data were inflated to account for poor diagnostic sensitivity, based on a meta-analysis of the sensitivity of blood culture, the most common diagnostic used for typhoid. Similarly, we used two DisMod models to estimate aetiologic proportions: one for the proportion of total incidence due to typhoid, and one for the proportion due to paratyphoid.

Case fatality data were too limited to allow for a complete DisMod model, or to allow for varying estimates by time and space. We had sufficient data, however, to estimate case fatality by age and by three categories of national income. We used DisMod to extract a global age-pattern in case fatality, and meta-regression to estimate the mean case fatality by income category. Finally, we estimated the relative risk of death from typhoid relative to paratyphoid based on data from Chinese surveillance and used that relative risk to estimate case fatality separately for typhoid and paratyphoid, by age and income.

Finally, we estimated paratyphoid mortality as the product of total incidence, the proportion of the total due to paratyphoid, and case fatality for paratyphoid. We propagated uncertainty through every step of the modelling process by pulling 1,000 draws from the distribution of each model component (eg, incidence, proportion due to typhoid, overall case fatality, case fatality age pattern, relative fatalness of typhoid versus paratyphoid), and performing all calculations at the draw level.

For GBD 2016 we estimated paratyphoid mortality in all locations using a natural history model, and the introduction of the data-rich CODEm estimates is a new improvement for GBD 2017. We have made no substantive changes to our natural history modelling strategy between GBD 2016 and 2017.

Invasive non-typhoidal salmonella (iNTS)

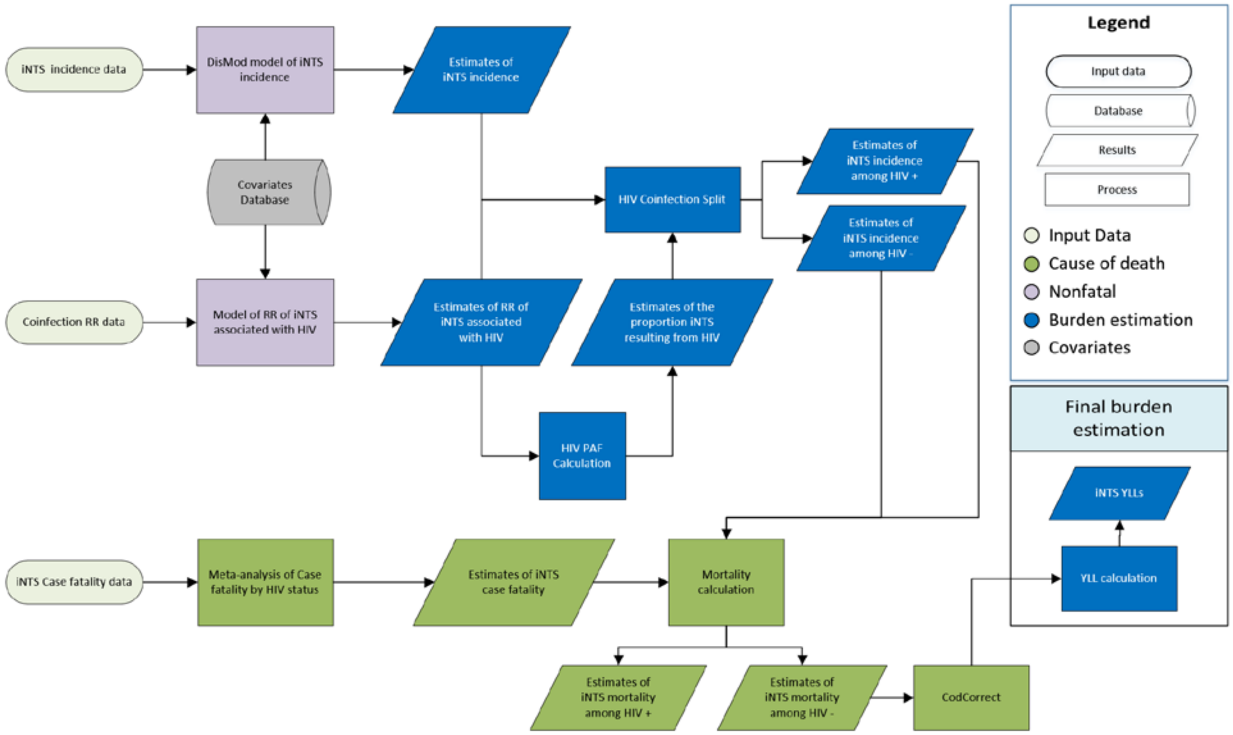

Input data

Incidence estimates for the natural history model are modelled using an incidence dataset based principally on prospective cohort studies and facility-based surveillance. Similarly, data on case fatality and co-infection come from prospective cohort studies and facility-based surveillance.

Modelling strategy

We model iNTS deaths using a natural history model in which we derive deaths as the product of cases and case fatality. Incidence was modelled using DisMod-MR 2.1, using the HIV mortality rate, malaria incidence adjusted for antimalarial coverage and drug effectiveness, and the summary exposure value (SEV), unsafe water, as covariates. We estimated the relative risk of iNTS comparing people with HIV to those without using a negative binomial model with log-age and log of the summary exposure value (SEV) for water as predictors. We used the resulting relative risk estimates and HIV prevalence estimates to calculate the proportion of iNTS that was attributable to HIV in each location, year, age and sex. Using these proportions, we divided iNTS cases into those that were attributable to HIV and those that were not. We modelled case fatality by age and Socio-demographic Index (SDI) separately for those with and without HIV using a generalised additive model, parameterising age with P-splines, and estimated mortality as the product of incidence and case fatality. Where iNTS occurs among those with HIV, we assume that iNTS is an opportunistic infection and that HIV is therefore the underlying cause of death. We therefore estimate deaths with iNTS as the underlying cause as total iNTS deaths times the proportion of cases not attributable to HIV. iNTS is a new cause for GBD 2017.

Other Intestinal Infectious Diseases

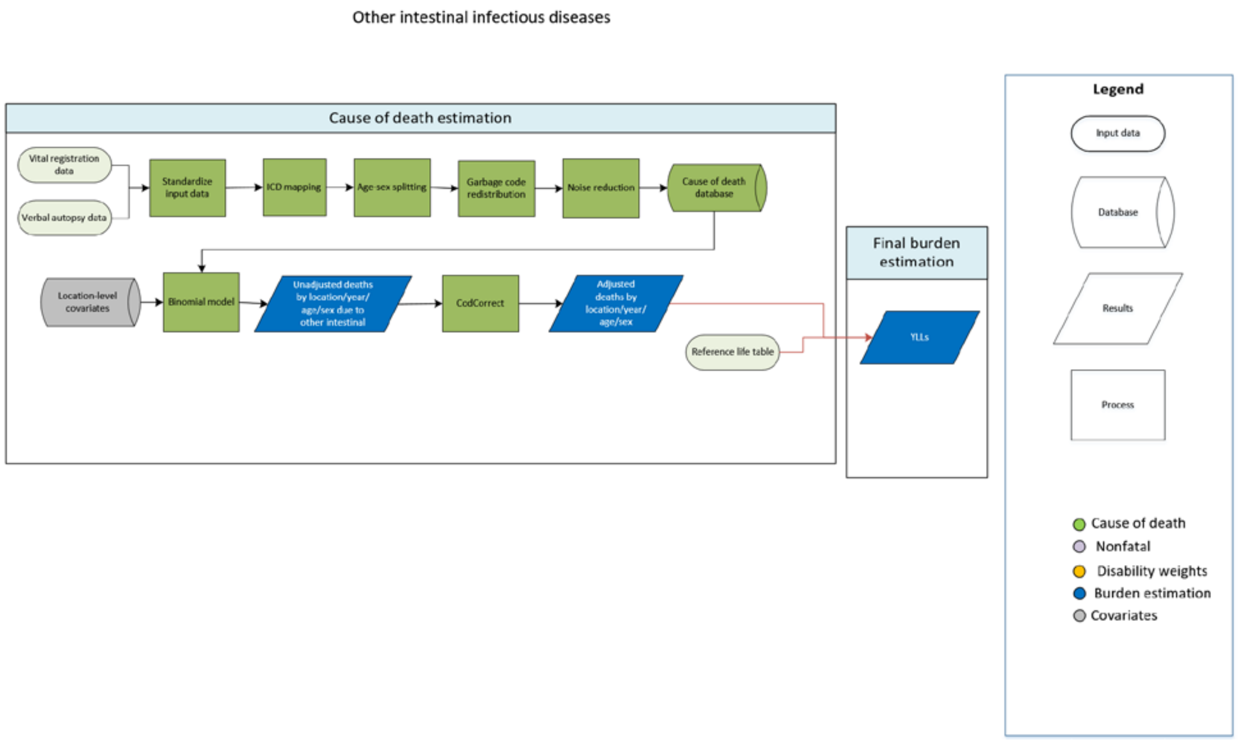

Input data

We modelled other intestinal infectious disease mortality using all available data in the cause of death database. Data points were outliered if they reported an improbable number of deaths or if their inclusion in the model yielded distorted trends. In some cases, multiple data sources for the same location differed dramatically both in their quality and reported other intestinal infectious disease mortality (eg, a verbal autopsy and vital registration source). In these cases the lower-quality data source was outliered.

Modelling strategy

We modelled other intestinal infectious disease mortality using a custom binomial model of all data in the CoD database. We used the number of cause-specific deaths as the outcome, with the all-cause mortality envelope as the exposure term. We included the square root of Socio-demographic Index, age group, and sex as covariates, and included a random effect on region

We have made no substantive changes to the modelling strategy in 2017.

Malaria

Malaria cause of death estimation in Sub-Saharan Africa

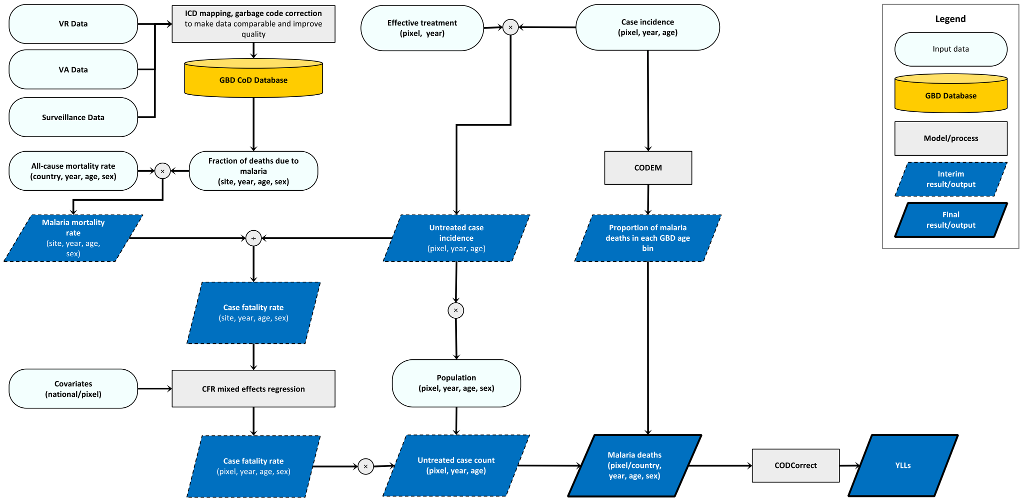

Malaria cause of death estimation outside Sub-Saharan Africa

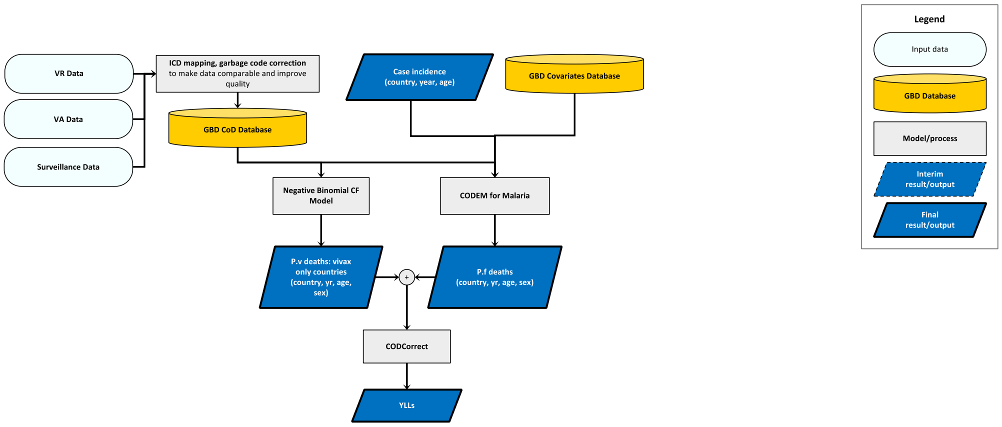

Input data and methodological summary for malaria

Overview

Variability in the type and abundance of CoD and related data meant that three distinct approaches were developed to estimate malaria mortality due to (i) *Plasmodium falciparum* inside sub-Saharan Africa; (ii) *P. falciparum* outside sub-Saharan Africa; and (iii) *P. vivax* in countries without Falciparum malaria.

Input data

For the Outside of sub-Saharan Africa and *P. vivax* models, data included vital registration, verbal autopsy, and surveillance data from the cause of death (CoD) database. Unlike other causes of death, we did not redistribute deaths to malaria. For the Africa models, we only used CoD data (mostly verbal autopsy) where we have been able to successfully geo-reference the site (ie, find associated geographic coordinates). Systematic literature reviews for malaria were not conducted.

Our outlier criteria excluded data points that (i) were implausibly high or low relative to global or regional patterns, (ii) substantially conflicted with established age or temporal patterns, or (iii) significantly conflicted with other data sources conducted from the same locations or locations with similar characteristics (ie, local Socio-demographic Index).

Modelling strategy

The malaria modelling strategy was carried out in three parts, and the covariates are:

Level	Covariate	Direction
1	*Pf*-only incidence	1
1	Effective antimalarial treatment ratio	-1
1	Effective antimalarial treatment	-1

***P. falciparum*: sub-Saharan Africa**

For most GBD causes, epidemiologic measures may be used as covariates in a traditional CODEm approach, if at all. To estimate the fatal burden of *P. falciparum* malaria in Africa, we used epidemiological measures in our estimation process directly. The Malaria Atlas Project (MAP) at the University of Oxford has generated updated spatiotemporal "cubes" estimating clinical incidence (rates and case counts) for each 5x5 km pixel across Africa, by year, from 1980 to 2017, specified by three broad age bins (0–5, 5–14 and 15+). MAP has also generated an equivalent spatiotemporal prediction of access to effective antimalarial drugs (combining access to care, the fraction of malaria cases receiving different classes of antimalarial, and the estimated country-year-specific efficacy of each antimalarial class though time). This estimated treatment rate was combined with the incidence rate cube to derive a third cube estimating the incidence of untreated cases at the pixel level.

For each site-year for which CoD malaria cause fraction data were available we (i) estimated a site-year-specific malaria mortality rate as the product of malaria cause fraction and all-cause mortality rate (with the latter drawn from national-level values); (ii) divided the malaria mortality rate by the site-year-specific estimate of untreated malaria incidence rate (drawn from the MAP cube) to estimate a site-year-specific case fatality rate (CFR) among untreated malaria cases. These derived site-year-specific CFR values were then used in a mixed-effects regression model to estimate pixel-year CFR for each 5x5 km

grid cell. The models used the following as covariates: the log of country-year all-cause mortality, pixel-year night-time lights, accessibility, fractional land-cover classes, and study-specific age and sex, with the location of each study site as a national-level random effect. Data were weighted by sample size (ie, the number of all-cause deaths observed in each study site-year).

Pixel-year predictions of CFR were then multiplied by the corresponding untreated incidence rate from the MAP cube to yield a pixel-year mortality rate estimate, which was then multiplied by pixel-year population to compute pixel-year malaria death counts. These were then aggregated to yield the required GBD national or subnational death estimates.

To disaggregate into GBD age bins, we separately ran a traditional national-level CODEm model with covariates: *Pf* incidence rate and access to effective antimalarial drugs. The resulting predicted age-patterns were used to split the country-year mortality estimates proportionally.

***P. falciparum:* outside of sub-Saharan Africa**

In locations outside of sub-Saharan Africa, we continued to use a traditional CODEm approach, mirroring closely what was used in GBD 2016. The approach was also applied to some countries on the continent of Africa with either very low incidence or relatively robust routine surveillance systems, including Algeria, Egypt, Morocco, Comoros, Mauritius, Cape Verde, Sao Tome, Principe, Botswana, Namibia, Eritrea, Djibouti, and South Africa. The model included the following covariates: *Pf* incidence rate, access to effective antimalarial drugs.

***P. vivax*: countries without *P. falciparum* transmission**

For countries where the main/exclusive strain of malaria was *P. vivax*, deaths were estimated using a zero-inflated negative binomial mixed model where the outcome is study deaths. The model included as fixed effect the logarithm of mortality rate, age, and sex. Locations were included as random effects.

The results from the *P. vivax*, Outside of Africa, and Africa models were collated, uploaded in CODEm and marked as best model in order to incorporate the estimation in the CodCorrect algorithm.

References

1 Bhatt S, Weiss DJ, Cameron E, *et al.* The effect of malaria control on Plasmodium falciparum in Africa between 2000 and 2015. *Nature* 2015; **526**: 207–11.

Chagas Disease

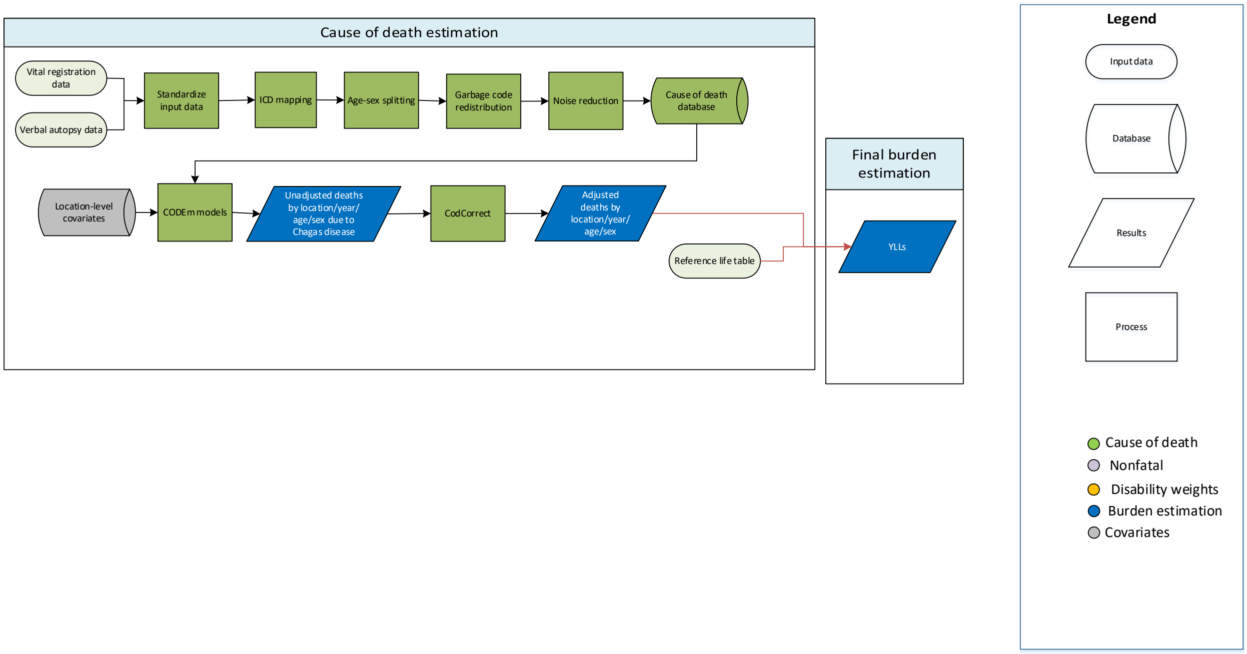

Input data

We modelled Chagas mortality using all available data in the cause of death database. No data were outliered for this cause.

Modelling strategy

We modelled Chagas mortality using a two-model hybrid approach: 1) a CODEm model of all Chagas-endemic countries of Latin America using all data in the CoD database; and 2) estimates of mortality from imported cases in non-endemic, data-rich countries. Where Chagas deaths were reported in non-endemic data-rich countries, we produced non-zero estimates by drawing from a beta distribution defined based on number of reported deaths and the underlying sample size. Estimates of Chagas mortality in endemic countries were drawn from the CODEm model.

The CODEm models included three covariates:

Level	Covariate	Direction
1	Chagas prevalence	+
2	Healthcare access and quality index	-
	Socio-demographic Index	-

We have made no substantive changes in the modelling strategy from GBD 2016 to GBD 2017 for Chagas-endemic countries.

Visceral Leishmaniasis

Visceral leishmaniasis – GBD2017

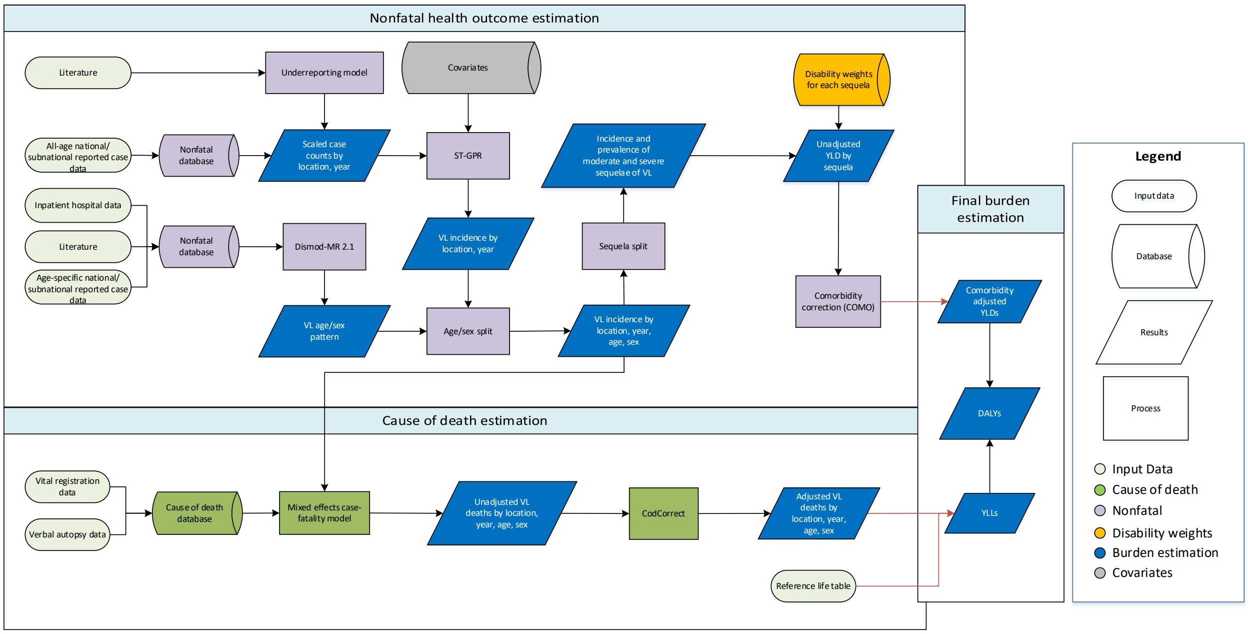

Visceral leishmaniasis (VL) is the most serious manifestation of disease caused by the *Leishmania* parasite, transmitted through the bite of phlebotomine sandflies. Those infected typically present with fever, weight loss, anaemia, leukopenia, thrombocytopenia, and enlargement of the spleen and liver. If left untreated, it can be fatal. Transmission varies by geographic region, with a variety of reservoir hosts implicated, and different vector species associated, maintaining both zoonotic and anthroponotic transmission cycles. The ICD9 code related to visceral leishmaniasis is 085.0, and the ICD10 code is B55.0.

Description of general methodology

The fatal estimation process for visceral leishmaniasis is built from incident case notification data representative of the GBD geographic location, which is adjusted for underreporting. The upscaled all-age, both-sex case counts are modelled using spatiotemporal Gaussian process regression (ST-GPR) in order to impute for missing location-year combinations as well as to account for further biases and inaccuracies in reporting. Datasets that disaggregate VL cases by age and sex are modelled using DisMod to produce a global age-sex split which is applied to the all-age, both-sex envelope estimates resulting from ST-GPR. The mean incidence estimates are compared with estimated death counts to generate a case-fatality rate model that is subsequently used to estimate deaths for each age, sex, location, year.

Input Data – Case Notification time series

Current estimation for the all-age, both-sex incidence envelope is based upon location-representative information rather than site-specific epidemiological measures due to the absence of global foci maps allowing for upscaling of geographically precise information. The primary data resource therefore is the case notification time-series reported by National Control Programs and Ministries of Health to the World Health Organization. This is supplemented by systematic literature review (last updated for GBD 2015) to identify alternate sources of data for years missing information. For countries with subnational

estimates, in-country collaborators have compiled information for respective programs, or identified key resources.

Input Data – Underreporting assessments

It is recognised that case notification series record only a subset of the true cases present. A review was undertaken to identify articles that compared reported cases with alternate measures to estimate the degree of underreporting. The following search strings were used: 'leish* AND under*'; 'active passive leish*'. Inclusion criteria were broad to maximise spatiotemporal coverage in potential estimates – any report that compared reported statistics with some notion of "truth" (whether capture-recapture, active surveillance, etc.) were extracted. Values for both cutaneous and visceral leishmaniasis were included. For GBD 2017, 12 articles were included, summarised in Table 1.

Input Data – Mortality

Deaths were extracted from a variety of sources, ranging from vital registration (VR) records, to verbal autopsy (VA) assessments. Deaths assigned to visceral leishmaniasis were processed following central cause of death processing, outlined elsewhere.

Citation	GBD location	Time period	Pathogen	Method synopsis	Proportion of "true" cases reported
Copeland *et al.*, 1990 "Comparison of active and passive case detection of cutaneous leishmaniasis in Guatemala" (Copeland, Arana, and Navin 1990)	Guatemala	1990	CL	Comparison of Ministry of Health data with cross-sectional population-based survey to inform estimated number of cases	64/2574
Yadon *et al.* 2001 "Assessment of Leishmaniasis notification system in Santiago del Estero, Argentina, 1990-1993" (Yadón et al. 2001)	Argentina	1990–1993	CL	Capture-recapture methods were used to evaluate four reporting sources.	94/210
Sesma *et al.* 1997 "Leishmaniasis in Navarra: a review of activities" (Sesma and Barricarte 1997)	Spain	1990–1997	CL, VL	Comparison of active searching within the region with reporting via Epidemiological Surveillance System	8/21
Maia-Elkhoury *et al.* 2007 "Analysis of visceral leishmaniasis reports by the capture-recapture method" (Maia-Elkhoury et al. 2007)	Brazil	2002–2003	VL	Comparison of three notification systems for completeness	5896/10691
Singh *et al.* 2006 "Serious underreporting of visceral leishmaniasis through passive case reporting in Bihar, India" (S. P. Singh et al. 2006)	Bihar, India	2003	VL	Comparison of actively detected cases (identified via household surveys) and governmental health system records. Estimate is among study population	8/65
Singh *et al.* 2006 "Serious underreporting of visceral leishmaniasis through passive case reporting in Bihar, India" (S. P. Singh et al. 2006)	Bihar, India	2003	VL	Comparison of actively detected cases (identified via household surveys) and governmental health system records.	109/876
Gkolfinopoulou *et al.* 2013 "Epidemiology of human leishmaniasis in Greece, 1981-2011" (Gkolfinopoulou et al. 2013)	Greece	2004–2009	VL	Comparing number of cases identified at national reference laboratory with mandatory notification system.	260/361

Singh *et al.* 2010 "Estimation of under-reporting of Visceral Leishmaniasis cases in Bihar India" (V. P. Singh et al. 2010)	Bihar, India	2006	VL	Comparison of actual reported number of cases with estimates age-sex stratified incidence proportions for a cohort of 31,324 persons	34/177
Hirve *et al.* 2010 "Effectiveness and feasibility of active and passive case detection in the Visceral Leishmaniasis Elimination Initiative in India, Bangladesh, and Nepal" (Hirve et al. 2010)	Bihar, India<br>Nepal<br>Bangladesh	2008	VL	Comparing active case detection evaluations (conducting via house-to-house screening) with passive case detection systems	111/130<br>119/127<br>18/25<br>20/32
Faraj *et al.* 2016 "Effectiveness and cost of insecticide-treated bed nets and indoor residual spraying for the control of cutaneous leishmaniasis: A cluster-randomized control trial in Morocco" (Faraj et al. 2016)	Morocco	2008–2013	CL	Comparison of incidence of new CL cases by both active and passive case detection	409/670
Das *et al.* 2014 "Active and passive case detection strategies for the control of leishmaniasis in Bangladesh" (Das et al. 2014)	Bangladesh	2010–2011	VL	Comparing two districts' estimates [identified in the paper as being directly comparable] of cases, one via active case detection, the other via passive case detection. Active case detection was via community education and outreach workers targeting households	756/1087
Rahman *et al.* 2015 "Performance of Kala-azar surveillance in Gaffargaon subdistrict of Mymensingh, Bangladesh" (Rahman et al. 2015)	Bangladesh	2010–2011	VL	Comparison of cases reported to the local health complex versus active search for kala-azar cases	29/58
Eid *et al.* 2017 "Assessment of a Leishmaniasis reporting system in tropical Bolivia using the capture-recapture method" (Eid et al. 2017)	Bolivia	2013–2014	CL	Active surveillance during medical campaigns were compared to registered cases reported by the National Program of Leishmaniasis Control	23/86.4

Table 1: Metadata for underreporting scalars used in GBD 2017. For each record, a citation, GBD location of relevance, year, pathogen, brief summary of methods, and output values used in modelling are listed.

Input data – age/sex-split data

Where possible, information disaggregating location-level statistics by age and sex were extracted.

Method – geographic restrictions

There are strong climatic and biogeographic constraints on the geographic distribution of VL resulting in a focal rather than cosmopolitan global distribution. As a result, it is necessary to identify locations burdened by the disease through space and time as distinct from countries were VL is absent. Tags were assigned to each location-year based upon the outcome of a search of IHME databases, as well as location-specific searches of PubMed. Each location-year is tagged as follows:

- Present – where a specific citation of either an autochthonous laboratory-confirmed case (ie, a case with PCR, serological, or parasitological diagnosis), reported case (ie, a case noted as VL, but with no supporting diagnostic), or supporting evidence (ie, confirmed infection in animal reservoirs or sandfly vectors)
- Protocol Present – for a given location-year, where no specific citation is used, but is present for another year in the same location, it is assumed that VL is present given that eradication of the pathogen has not been achieved
- Absent – where PubMed location-specific searches returned zero relevant results, in locations scoring -25 or lower as evaluated by Pigott *et al.* (2014) [the threshold for "absence" in that study (Pigott et al. 2014)], locations were tagged as Absent
- Protocol Absent – as with Absent, locations with zero relevant PubMed results, but with greater than -25 as evaluated by Pigott *et al.* (2014), were tagged as Protocol Absent (Pigott et al. 2014)

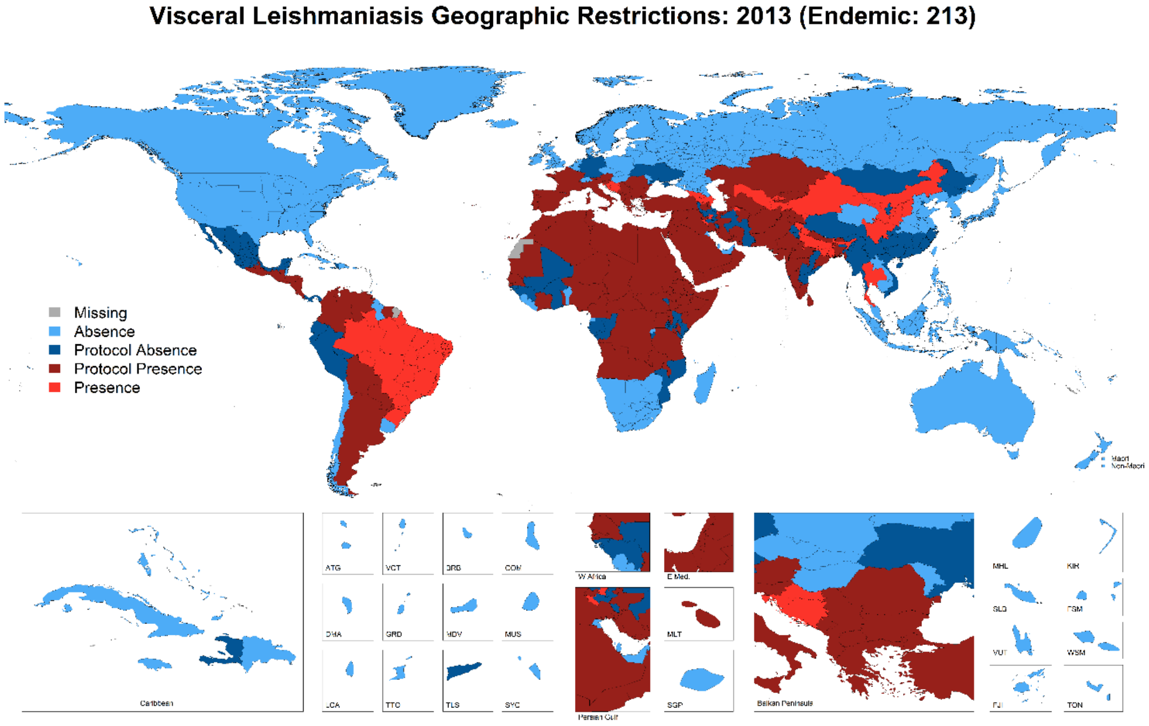

Figure 1: Visceral Leishmaniasis geographic restrictions for the year 2013. GBD locations tagged as present are coloured in red, yellow represents protocol presence, dark blue represents protocol absence, and absence is represented by light blue. Locations missing tags are presented in grey.

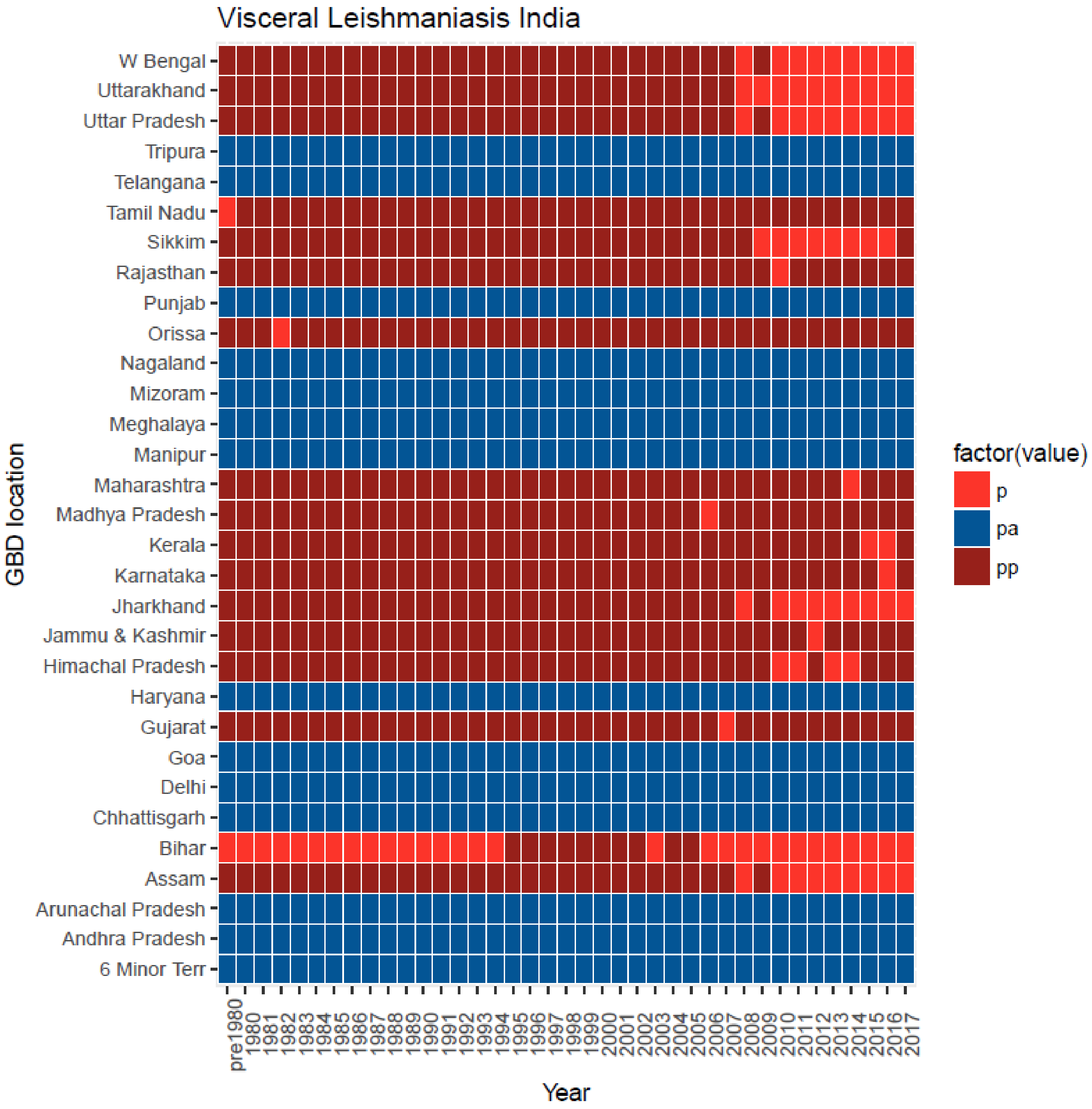

Figure 2: Visceral Leishmaniasis geographic restrictions for Indian subnationals. Locations tagged as present are coloured in red, yellow represents protocol presence, and dark blue represents protocol absence.

Full time series of maps and tables, with relevant GHDx NIDs, are available upon request from gbdsec@uw.edu.

Method – underreporting modelling and scaled case counts

Underreporting scalars were modelled as a generalised linear model estimating the proportion of true cases captured by reporting systems: a value of 1 therefore represents all actual cases of leishmaniasis being reported through notification systems. The specific models is as follows:

$$\frac{reported\ cases}{\text{"true" cases}} = Pathogen + Year + Sociodemographic\ Index$$

To account for potential biases inherently present based upon differing survey methods or location-specific confounders, 1,000 models were run, with each model randomly dropping all data from a specific location, and then one additional data point from the remaining dataset. Similarly, for estimates that spanned multiple years, for each model one of the years within the range of possible years was randomly assigned.
To generate scaled case counts, for each of the 1,000 models a random number was generated, using a normal distribution with mean being that of the mean estimated scalar bounded by the upper and lower confidence interval. With these 1,000 scalars, 1,000 scaled case counts were calculated and summarised for modelling within ST-GPR.

Method – ST-GPR

Using existing IHME tools, the summarised values were modelled using ST-GPR to produce a complete time series of estimates for each location-year tagged "Present" or "Protocol Present". In short, ST-GPR attempts to model non-linear trends utilising a Gaussian process to fit a trend, rather than a definitive functional form. The following model specifications were used:

$$\begin{aligned} Incidence = \ & Health\ Access\ and\ Quality\ Index + Sociodemographic\ Index + (1|level\ 1) \\ & + (1|level\ 2) + (1|level\ 3) \end{aligned}$$

where levels 1, 2, and 3, referring to GBD location hierarchies, treated as random effects. The following hyperparameters were used: st-lambda = 0.4, st-omega = 1, st-zeta = 0.01, gpr-scale = 10.

Method – DisMod

DisMod was used to generate an age-sex curve to disaggregate all-age, both-sex incidence data. DisMod is an integrated metaregression framework that allows for multiple datasets to be integrated into a singular analysis regardless of age-binning, sources, and geographies. As a consequence, a variety of differently aggregated information can be evaluated to generate a consensus output. From this model, the global fit was used.

Method – YLL estimation

Deaths were modelled using a mixed effect model parameterising case-fatality rate, with data derived from taking attributed-death data and dividing it by the mean predicted incident cases.

$$Case\ Fatality\ Rate \sim Age + Sex + (Age|Super\ Region\ /\ Region) + (Sex|Super\ Region)$$

Only data from countries defined as present or protocol present were used, as these represent locations that are generalisable to all endemic regions for VL. The deaths in non-endemic countries, while not used in the case-fatality rate model, are subsequently added back into the death envelope as-is by central computation.

Changes from GBD 2016

A number of changes to the methodology were implemented for GBD 2017:

Geographic restrictions – to improve transparency and tractability of geographic restrictions, maps of restricted locations and years are available, with clear designation of data (or assumptions) used to inform a GBD location-year's status. As a result of updating, the status of some GBD locations

has changed in the light of new evidence (eg, Angola). While we explore how best to host this information, it is currently available upon request to gbdsec@uw.edu.

All-age, both-sex incidence envelope – new data were acquired and an ST-GPR methodology implemented consistently across the globe. Relevant covariates were updated from GBD 2016. One important change was the removal of the "High endemicity" covariate, which constrained predictions, particularly in low SDI countries in Africa, since its construction and subsequent use in models are not independent of each other.

Age-sex breakdown – age-sex curves were taken from a DisMod model using an updated dataset of age-sex specific information

Underreporting model – considerable changes were undertaken from GBD 2016 for underreporting. Rather than using a single scalar, taken from expert opinion (Alvar et al. 2012), applied across the entire time series, a model was developed, parameterised by real data, allowing for spatiotemporal variation in estimates. These variable scalars were then applied to their relevant location-year case count values.

Results specific to visceral leishmaniasis model

The aim here is to provide insights in some of the sub-models that are involved in the VL estimation process that are not published as part of the GBD capstones or readily available via the supplemental materials. For further questions, please direct toward gbdsec@uw.edu.

Underreporting

Coefficients
Pathogen: 0.6371 (-0.0456 – 1.5868) (where pathogen order is CL, VL)
Year: 0.1350 (0.0714 – 0.2058)
SDI: 4.6230 (2.0290 – 9.3287)

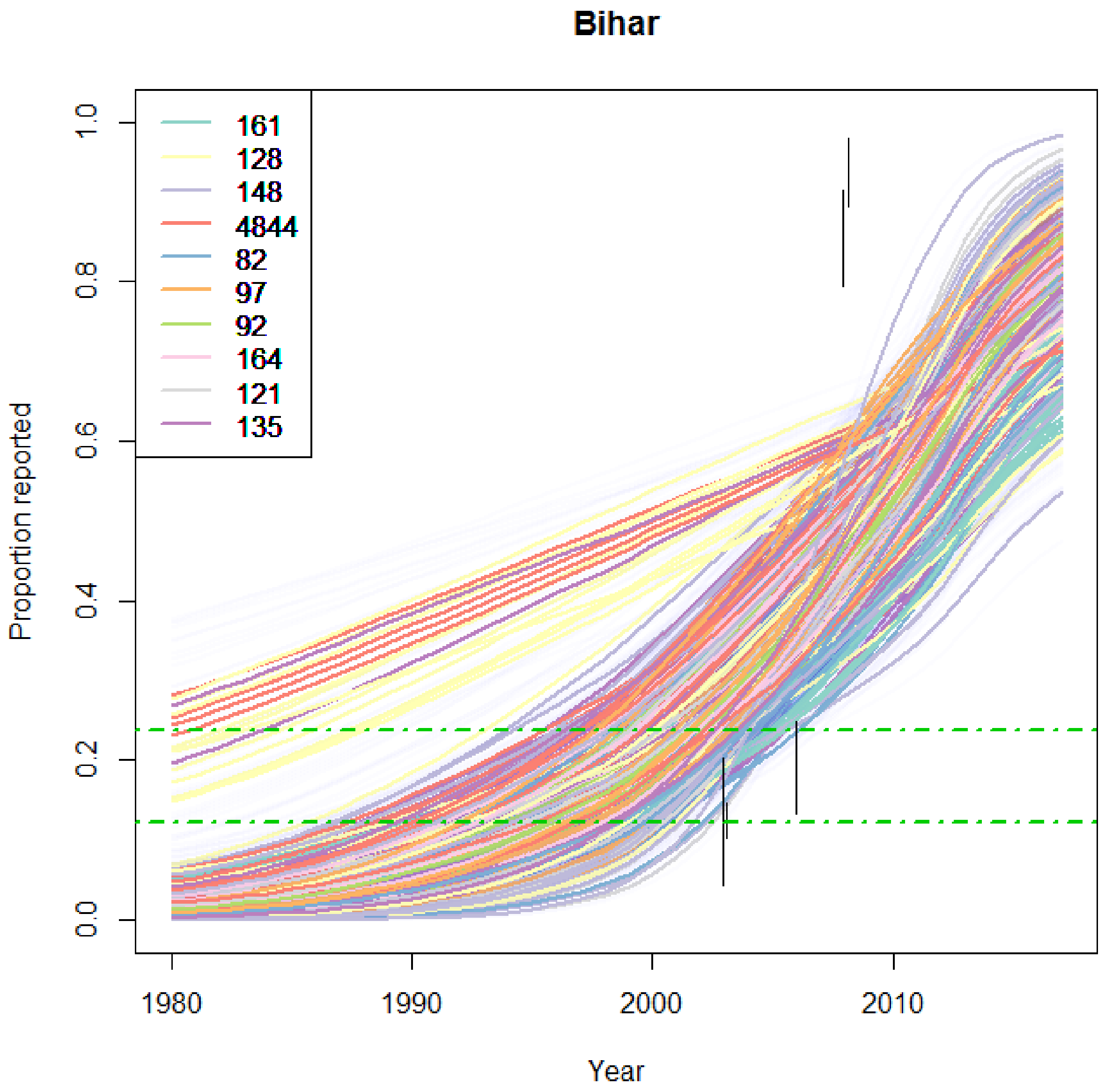

Figure 3: Example of VL underreporting model for Bihar, India. Plot showing each of the 1,000 iterations of the underreporting model ran, coloured by the location that has been held out [colours coded by their GBD location id: 161 = Bangladesh, 128 = Guatemala, 148 = Morocco, 4844 = Bihar, India, 82 = Greece, 97 = Argentina, 92 = Spain, 164 = Nepal, 121 = Bolivia, 135 = Brazil]. The black vertical lines represent data points (with standard errors) for Bihar as listed in Table 1, and the green dashed line is the upper and lower bound of the underreporting factor recorded by Alvar *et al.* (2012), which was applied across all time in GBD 2016.

<u>Age- and sex-specific trends in incidence rate</u>

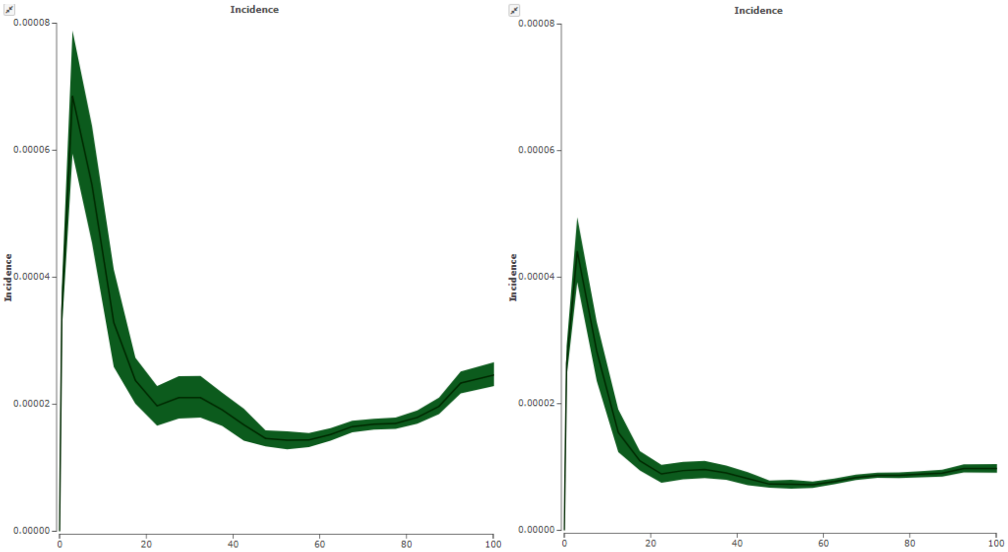

Figure 4: Global age-specific incidence estimates for males (left) and females (right) for the year 2010. Incidence is on the Y axis (rate per total population), and age in years on the X axis. Screenshot from EpiViz.

Figure 4 shows the age-specific variation in incidence rates, differentiated by sex. When considered as a global aggregate, we see that reported male incident rates are approximately double those of females, with highest rates observed in younger age groupings. In adults, levels are comparatively flat, but there is an uptick in older age groups.

<u>ST-GPR</u>

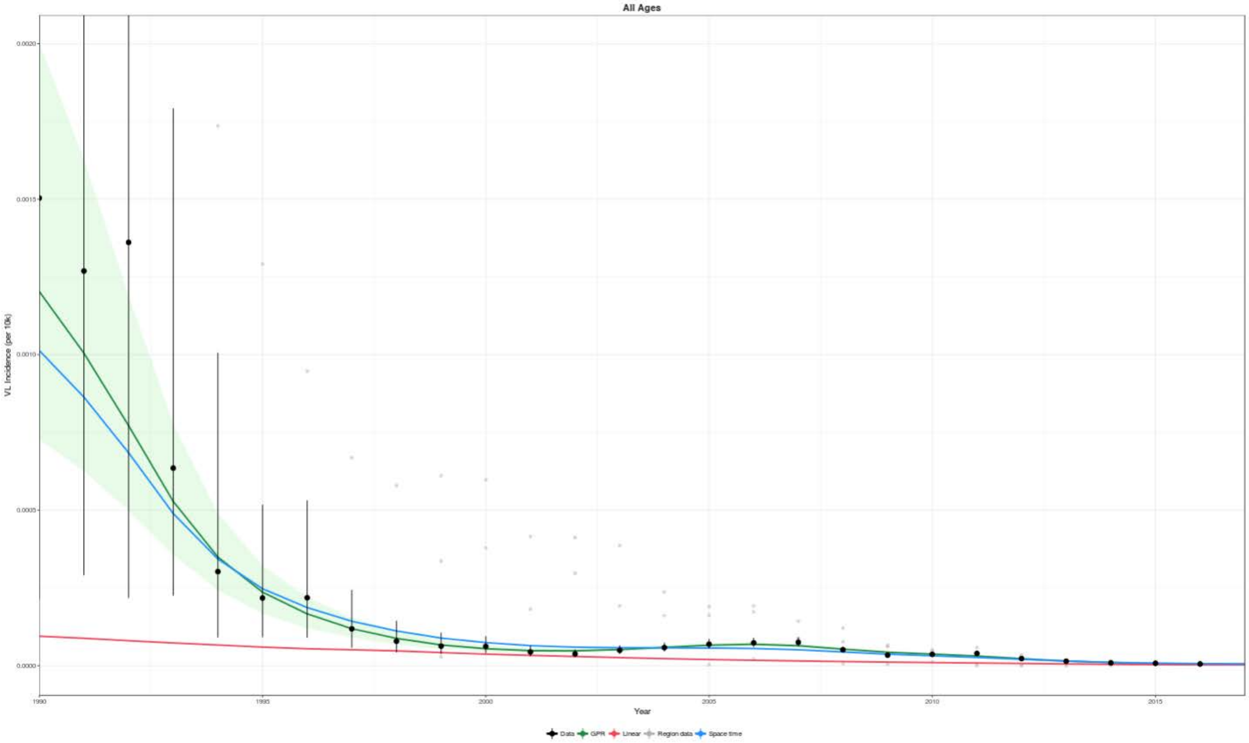

Figure 5: ST-GPR estimates for India (all-age, both sex) for years 1990–2017. Black dots represent input data points (post processing for underreporting) with the black lines indicating variance. The green line

represents the mean GPR estimated value, with uncertainty shown by the green polygon. The blue line indicates the space-time component of the ST-GPR; the red indicates the linear regression component derived off of global data. Transparent black dots represent data from other locations in the GBD region CFR.

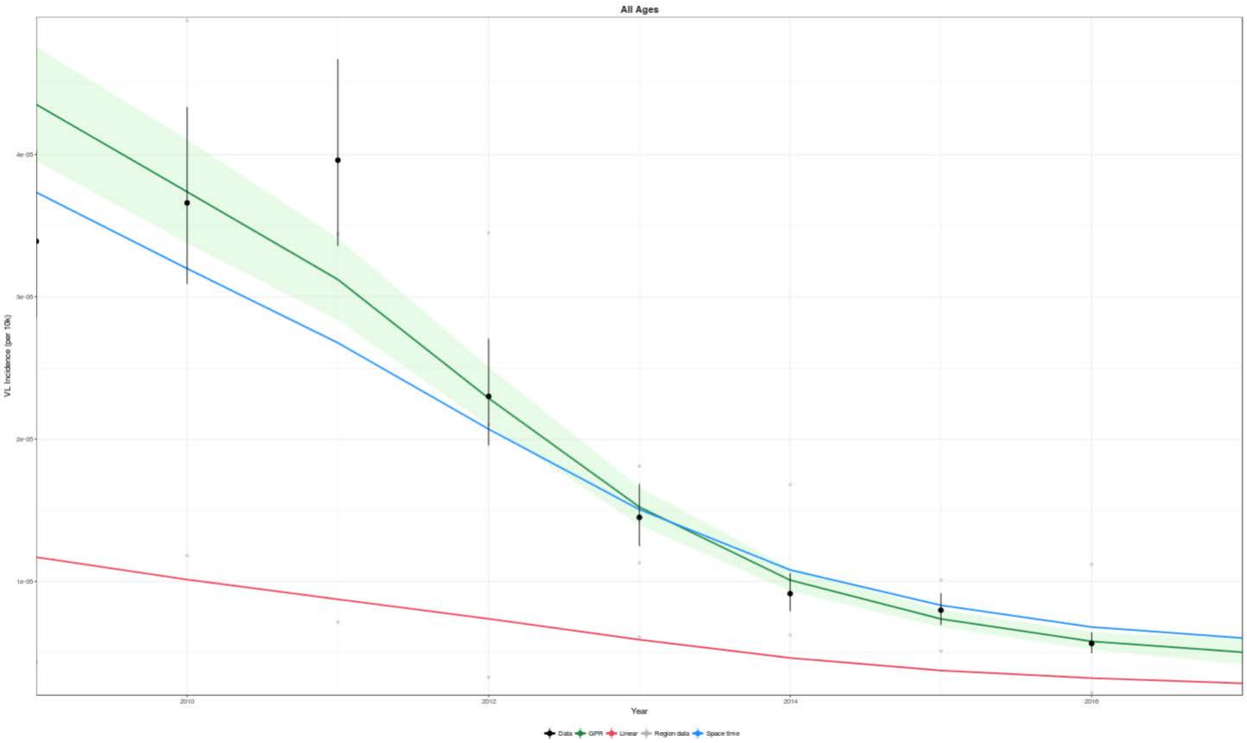

Figure 6: ST-GPR estimates for India (all-age, both sex) for years 2009–2017. Colouration and symbols are as stated in caption for Figure 5.

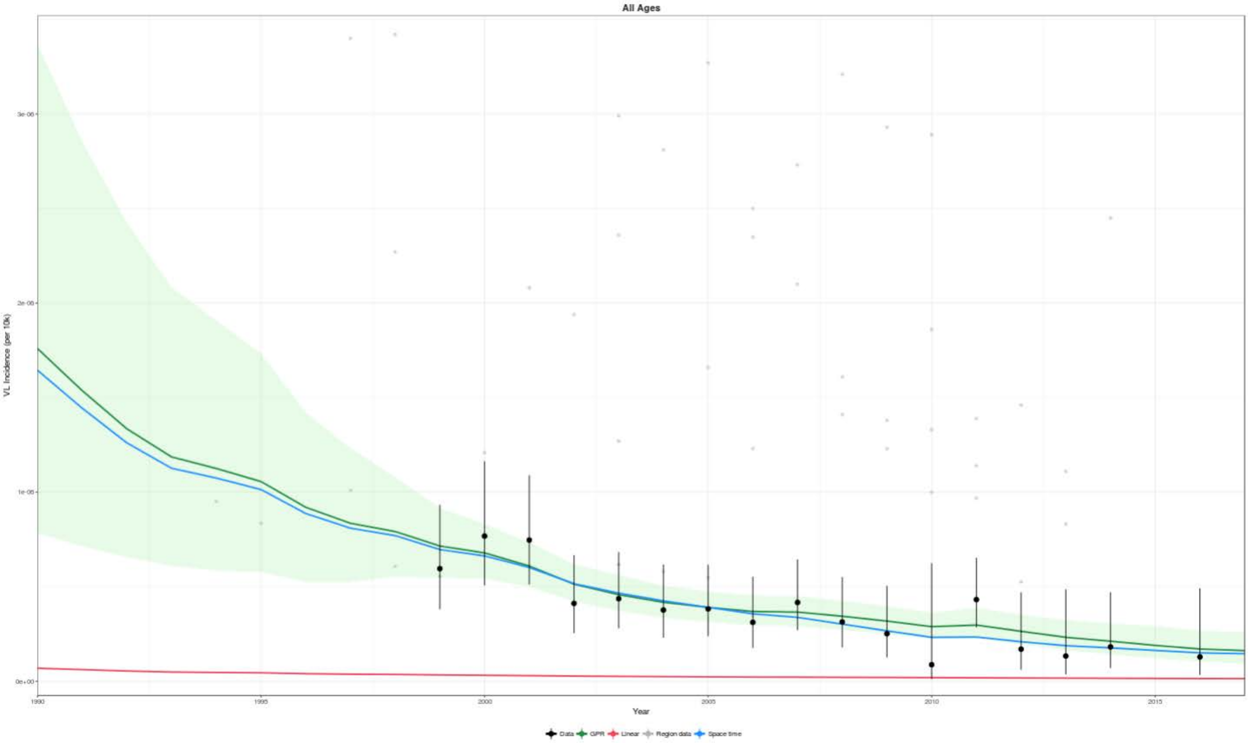

Figure 7: ST-GPR estimates for France (all-age, both sex) for years 1990–2017. Colouration and symbols are as stated in caption for Figure 5.

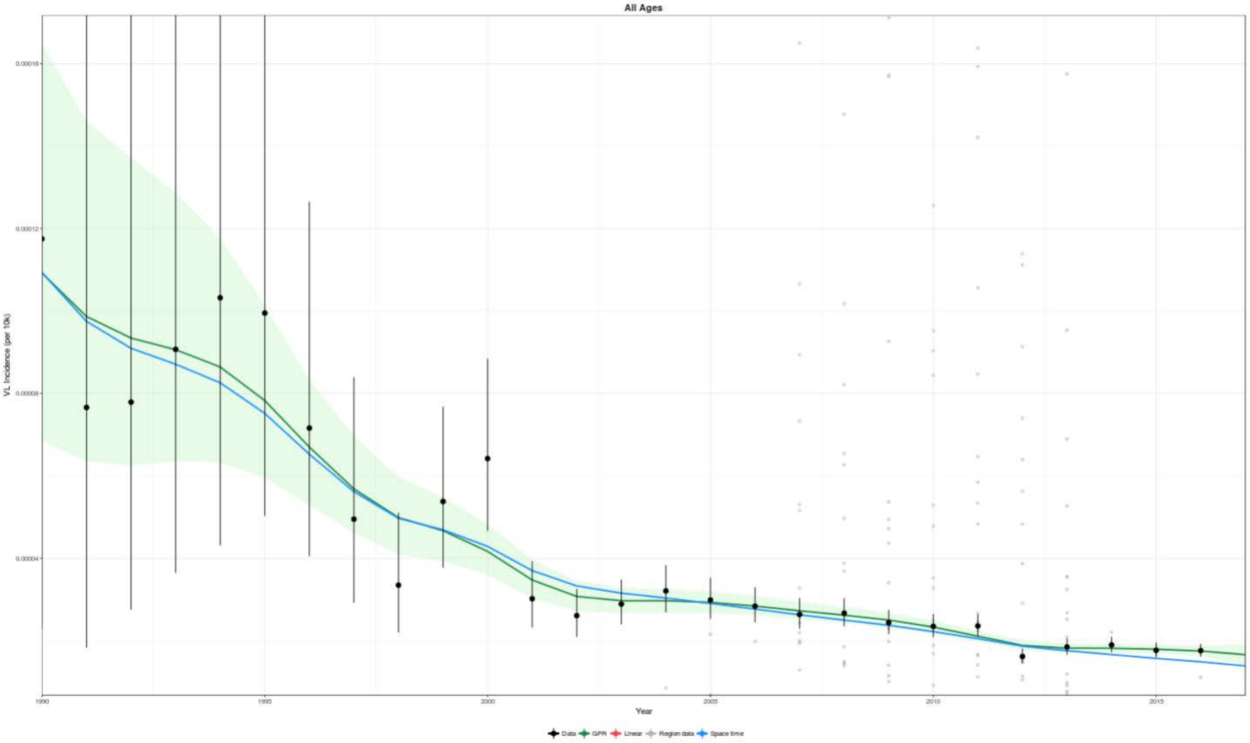

Figure 8: ST-GPR estimates for Brazil (all-age, both sex) for years 1990–2017. Colouration and symbols are as stated in caption for Figure 5.

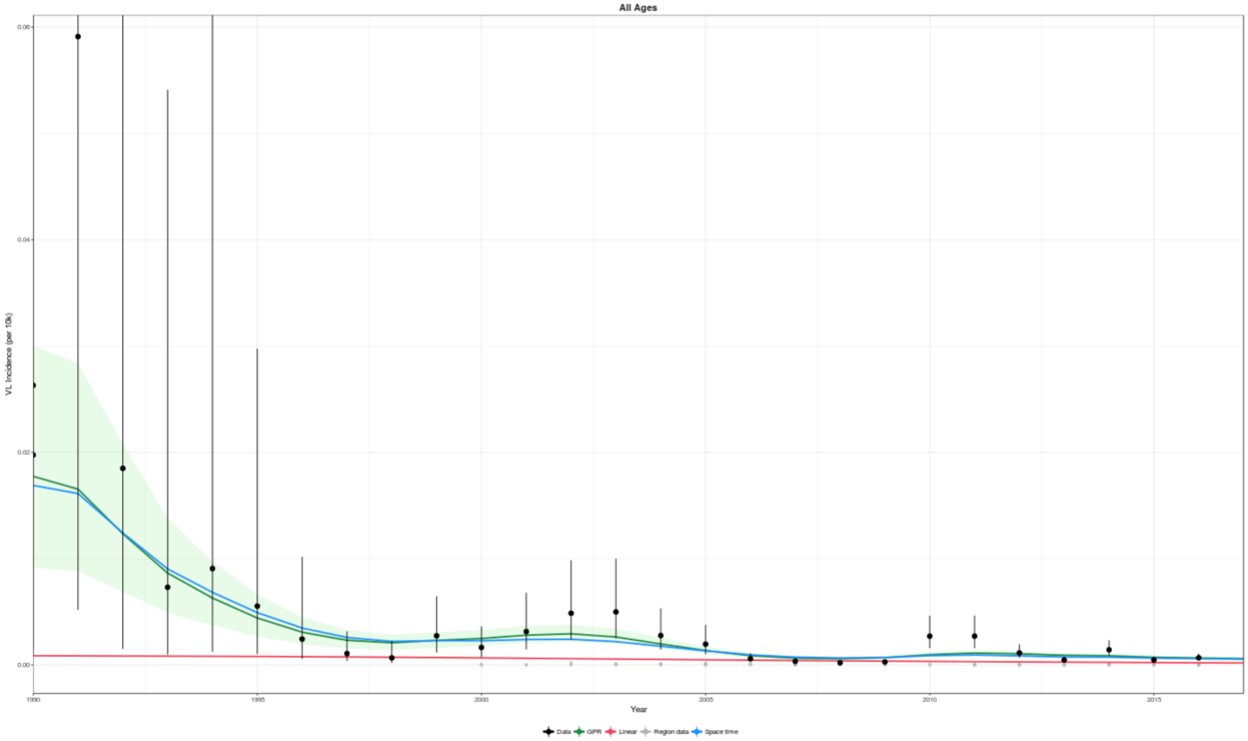

Figure 9: ST-GPR estimates for South Sudan (all-age, both sex) for years 1990–2017. Colouration and symbols are as stated in caption for Figure 5.

Case-fatality rate estimates

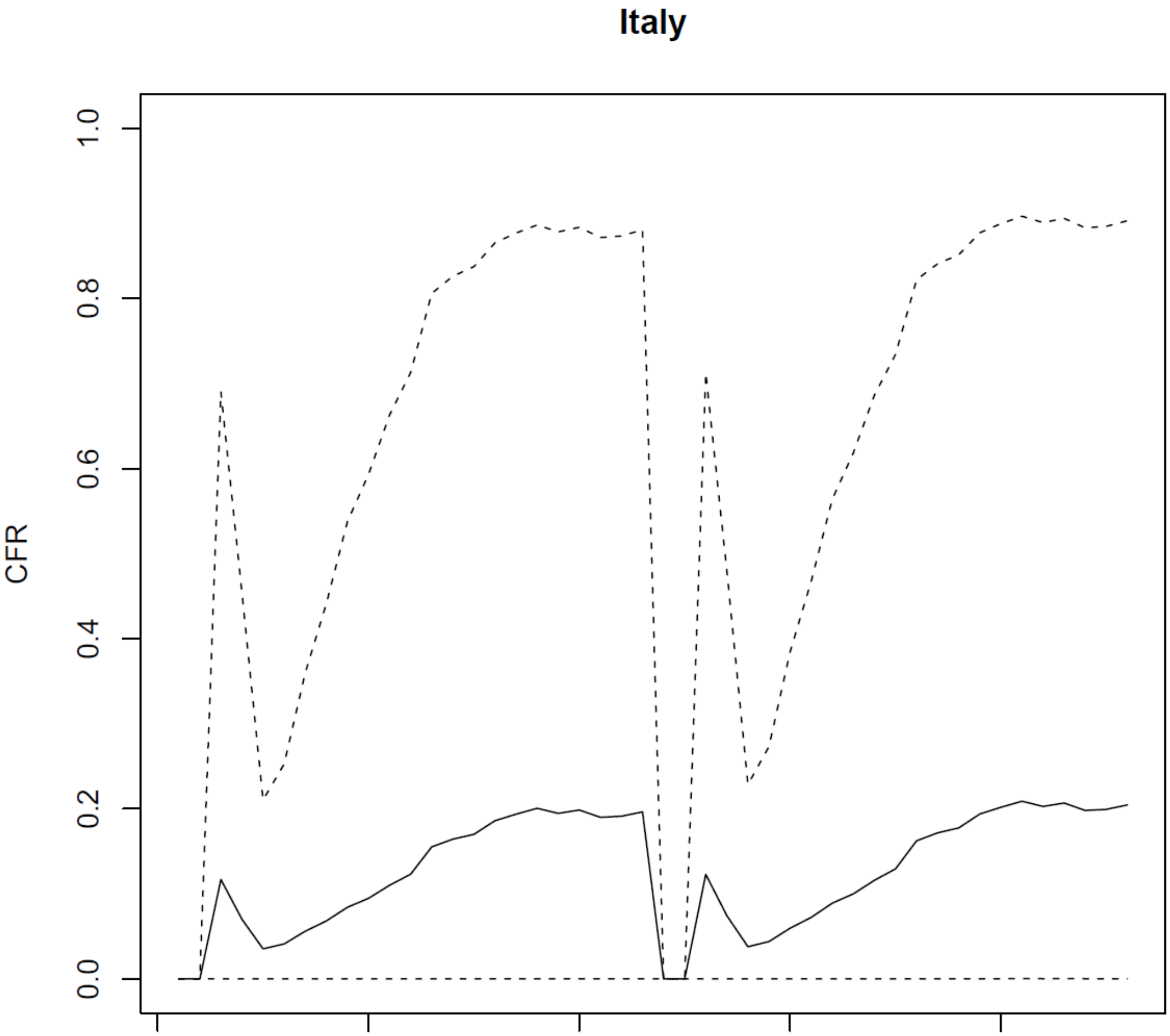

Figure 10: Case fatality rate values for Italy plotted sequentially for GBD age groups, female then male. The solid line represents the mean value of the 1,000 draws; the dashed lines represent the upper and lower bounds (95%).

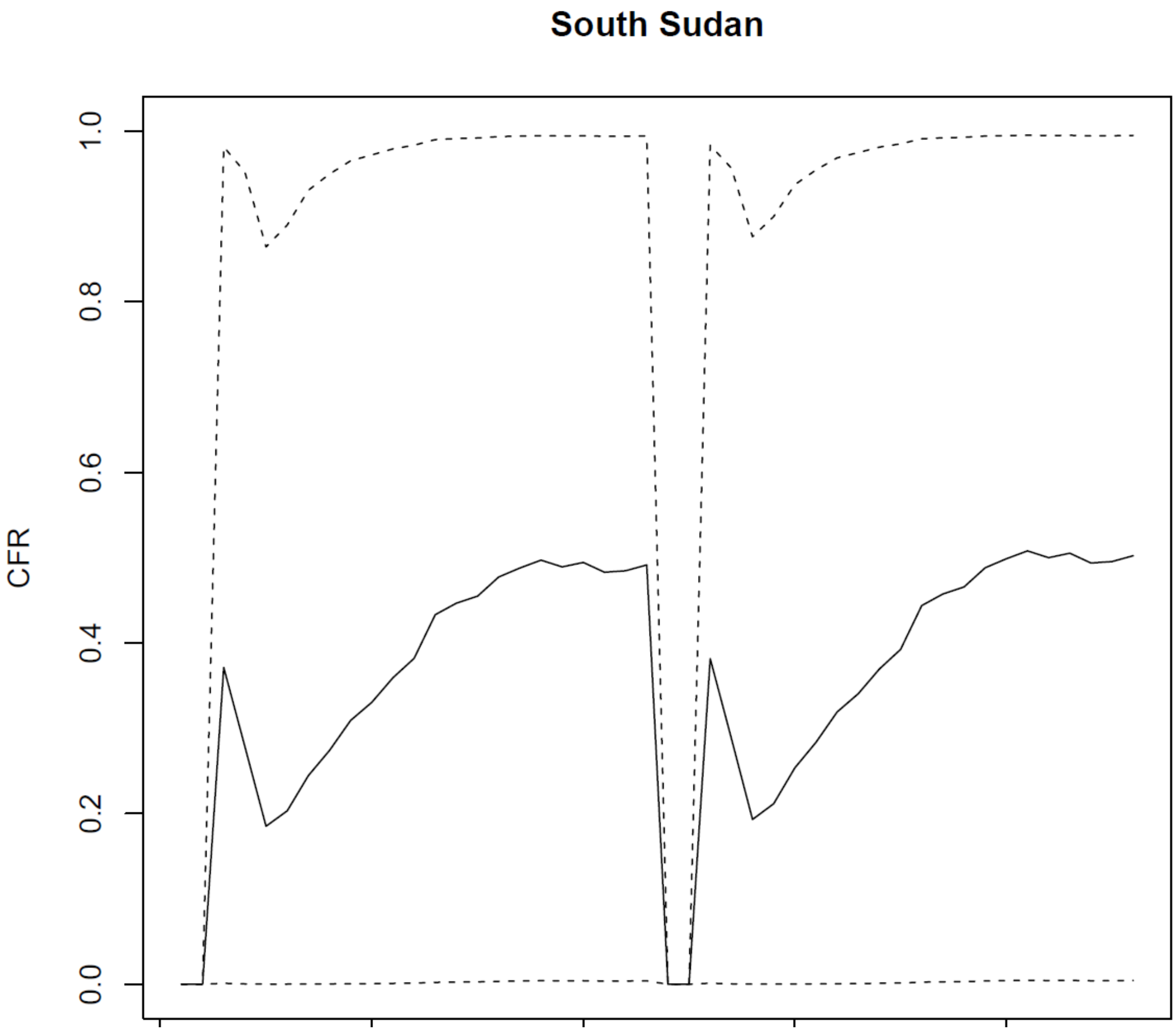

Figure 11: Case fatality rate values for South Sudan plotted sequentially for GBD age groups, male then female. The solid line represents the mean value of the 1,000 draws; the dashed lines represent the upper and lower bounds (95%).

Case fatality rate estimates had high uncertainty in some geographies. In general, female mean case fatality rates were higher than male case fatality rates. Typically an all-age estimate of 10% case-fatality rate is discussed when looking at visceral leishmaniasis (Alvar et al. 2012).

Limitations

As with any modelling process, a number of limitations are known, which will be the focus of additional effort in upcoming GBD cycles, and engagement with collaborators. Given the focus on location-representative estimates, the existing model is based upon national case counts. This excludes a large resource of published literature and grey literature focused on site-specific surveillance or surveys. While some pathogens have integrated subnational approaches as a building block for national estimates (eg, schistosomiasis) this is yet to be implemented for visceral leishmaniasis. Regardless of contribution to the global incidence model, these data can be used to inform age-sex splits, as well as a

variety of other key parameters, particularly duration parameters, which are currently lacking uncertainty and support from a full literature review.

The removal of the "high endemicity" dataset in the ST-GPR framework led to some sub-Saharan African nations having considerably higher burden estimates than prior cycles. For many nations, this is reflective of the sporadic reporting of cases in these countries (*eg,* in Angola and the Democratic Republic of Congo), and a consensus on pathogen presence is highly uncertain. It was important to remove this covariate, however, as it was a prior imposed on the model, a model designed to evaluate this status that leveraged the same data that was in the model itself. This high degree of dependence we aimed to eliminate. In the next cycle of GBD, there is a need to identify an independent resource to aid in quantifying the population-at-risk, which the high endemicity layer was designed to approximate, as well as engaging with self-identified pathogen-specific and country-specific collaborators to re-evaluate the presence/protocol presence status assigned to these nations.

Similarly, existing death data are limited in geographic distribution (due to primarily coming from countries with robust vital registration systems), and could lack in external validity when extrapolated to other nations. While region-level random effects help account for some of this (for instance, mitigating some of the higher case fatality rates in immunocompromised individuals in the high-income region in GBD), this doesn't eliminate all possible confounding, and furthermore, does not negate the fact that for most of the high incidence countries do not report a full time series of deaths. Further cycles should explicitly consider the reported case fatality rates in the literature, many of which come from those VR data-poor regions.

Age-sex patterns are highly reflective of the countries from which data is obtained. Importantly, there is a large skew in information coming from Brazil. This information has potential biases due to the nature of the data inputs (notification and hospital data) and the corresponding age-sex variation in health-seeking behaviours. Yet again, consulting some of the detailed household surveys that do exist will increase geographic coverage of these estimates, and provide an important independent comparator to determine whether these disparities are genuine, or an artefact of the reporting systems consulted in this current model.

References

Alvar, Jorge, Iván D Vélez, Caryn Bern, Mercé Herrero, Philippe Desjeux, Jorge Cano, Jean Jannin, and Margriet den Boer. 2012. "Leishmaniasis Worldwide and Global Estimates of Its Incidence." *PLoS One* 7 (5): e35671.

Copeland, H W, B A Arana, and T R Navin. 1990. "Comparison of Active and Passive Case Detection of Cutaneous Leishmaniasis in Guatemala." *Am. J. Trop. Med. Hyg.* 43 (3): 257–259.

Das, A K, A D Harries, S G Hinderaker, R Zachariah, B Ahmed, G N Shah, M A Khogali, G I Das, E M Ahmed, and K Ritmeijer. 2014. "Active and Passive Case Detection Strategies for the Control of Leishmaniasis in Bangladesh." *Public Health Action* 4 (1): 15–21.

Eid, Daniel, Miguel Guzman-Rivero, Ernesto Rojas, Isabel Goicolea, Anna-Karin Hurtig, Daniel Illanes, and Miguel San Sebastian. 2017. "Assessment of a Leishmaniasis Reporting System in Tropical Bolivia Using the Capture-Recapture Method," October, tpmd170308.

Faraj, Chafika, Joshua Yukich, El Bachir Adlaoui, Rachid Wahabi, Abraham Peter Mnzava, Mustapha Kaddaf, Abderrahmane Laamrani El Idrissi, Btissam Ameur, and Immo Kleinschmidt. 2016. "Effectiveness and Cost of Insecticide-Treated Bed Nets and Indoor Residual Spraying for the Control of Cutaneous Leishmaniasis: A Cluster-Randomized Control Trial in Morocco." *Am. J. Trop. Med. Hyg.* 94 (3): 679–685.

Gkolfinopoulou, K, N Bitsolas, S Patrinos, L Veneti, A Marka, G Dougas, D Pervanidou, et al. 2013. "Epidemiology of Human Leishmaniasis in Greece, 1981-2011." *Euro Surveill.* 18 (29): 20532.

Hirve, S, S P Singh, N Kumar, M R Banjara, P Das, S Sundar, S Rijal, et al. 2010. "Effectiveness and Feasibility of Active and Passive Case Detection in the Visceral Leishmaniasis Elimination Initiative in India, Bangladesh, and Nepal." *Am. J. Trop. Med. Hyg.* 83 (3): 507–511.

Maia-Elkhoury, Ana Nilce Silveira, Eduardo Hage Carmo, Marcia Leite Sousa-Gomes, and Eduardo Mota. 2007. "[Analysis of visceral leishmaniasis reports by the capture-recapture method]." *Rev. Saude Publica* 41 (6): 931–937.

Pigott, David M, Samir Bhatt, Nick Golding, Kirsten A Duda, Katherine E Battle, Oliver J Brady, Jane P Messina, et al. 2014. "Global Distribution Maps of the Leishmaniases." *Elife* 3 (January): e02851.

Rahman, Kazi Mizanur, Indira V M Samarawickrema, David Harley, Anna Olsen, Colin D Butler, Shariful Amin Sumon, Subrata Kumar Biswas, Stephen P Luby, and Adrian C Sleigh. 2015. "Performance of Kala-Azar Surveillance in Gaffargaon Subdistrict of Mymensingh, Bangladesh." Edited by Carlos Franco-Paredes. *PLoS Negl. Trop. Dis.* 9 (4): e0003531.

Sesma, B, and A Barricarte. 1997. "[Leishmaniasis in Navarra: review of activities]." *An. Sist. Sanit. Navar.* 20 (2): 209–216.

Singh, S P, D C S Reddy, M Rai, and S Sundar. 2006. "Serious Underreporting of Visceral Leishmaniasis through Passive Case Reporting in Bihar, India." *Trop. Med. Int. Health* 11 (6): 899–905.

Singh, V P, A Ranjan, R K Topno, R B Verma, N A Siddique, V N Ravidas, N Kumar, K Pandey, and P Das. 2010. "Estimation of Under-Reporting of Visceral Leishmaniasis Cases in Bihar, India." *Am. J. Trop. Med. Hyg.* 82 (1): 9–11.

Yadón, Z E, M A Quigley, C R Davies, L C Rodrigues, and E L Segura. 2001. "Assessment of Leishmaniasis Notification System in Santiago Del Estero, Argentina, 1990-1993." *Am. J. Trop. Med. Hyg.* 65 (1): 27–30.

Human African Trypanosomiasis (HAT)

Flowchart

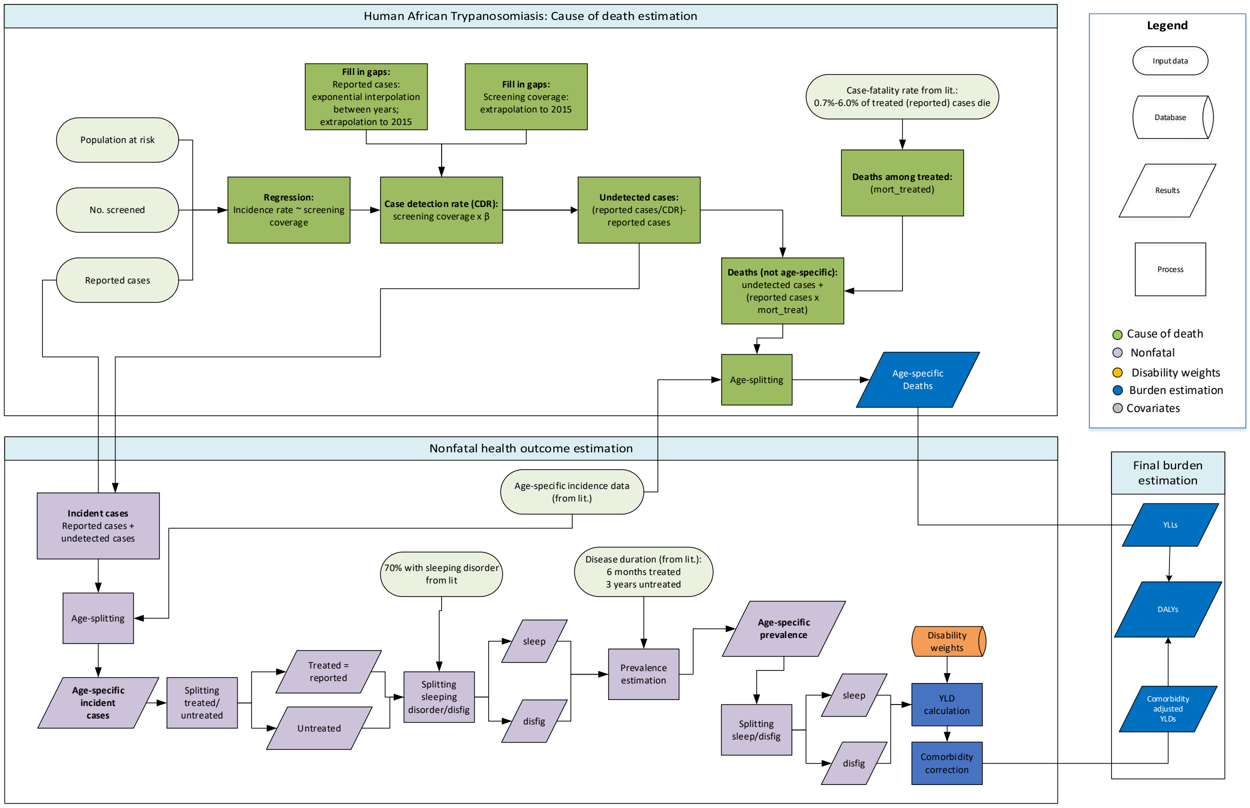

Input Data & Methodological Summary

Case Definition

Human African trypanosomiasis (HAT), also known as sleeping sickness, is a vector-borne disease which is transmitted by the bite of the tsetse fly. It is caused by the parasite *Trypanosoma brucei* with two subspecies, namely *T.b. rhodesience* (makes up less than 5% of total HAT cases) and *T.b. gambiense*. Cases are diagnosed through laboratory methods which rest on finding the parasite in body fluid or tissue by microscopy. In highly endemic or epidemic areas where the likelihood of false positives in serological tests is deemed lower, a seropositive individual is considered affected even in the absence of parasitological confirmation. The ICD-10 codes for HAT are B56.0, B56.1 and B56.9.

Input data

Model inputs

Data sources for GBD 2017:

1) Annual case totals 1980–2016: National-level annual case totals from 1990–2016 were obtained from WHO's publicly dataset, available here: http://apps.who.int/gho/data/node.main.A1635?lang=en

   Subnational data:

   Kenya: Deaths due to HAT were attributed to Busia county. Identification of subnational locations for Kenyan case data were obtained via studies published in the peer-reviewed literature[1] and review of maps published from via the WHO HAT Atlas: http://www.who.int/entity/trypanosomiasis_african/country/Kenya_whole_0014.jpg?ua=1

2) Age/sex data: Data on the age and sex distribution of HAT cases were extracted from the peer-reviewed literature via a systematic review of sources identified in PubMed using the following search string:

   ((African trypanosomiasis[Title/Abstract] AND (incidence[Title/Abstract] OR burden[Title/Abstract] OR prevalence[Title/Abstract] OR community[Title/Abstract])) AND ("1990"[Date – Publication] : "2017"[Date – Publication]))

   This yielded 219 studies of which only three met the inclusion criteria and were extracted. The inclusion criteria were:

   1. Studies representative of the national population
   2. Population-based studies
   3. Studies with primary data on incidence
   4. Studies of human African trypanosomiasis (excluded studies on animal African trypanosomiasis)

3) Population at risk estimates 1980-2015: population at risk estimates from GBD 2010 ArcGIS analysis using geocoded case notifications for 2000 to 2009[2] and population Count Grid estimates from Gridded Population of the World 3.

4) Screening coverage: Data on active vs. passive screening coverage were obtained from a Weekly Epidemiological Report[3] identifying the population screened from 1997 to 2004 at the national level.

5) Geographic restrictions: Data file of all GBD locations, defining location as either endemic or non-endemic for HAT. Estimates are not produced for non-endemic countries, nor are they generated for countries with a history of HAT transmission but no data reported by WHO from 1990–2016.

Modelling strategy

Geographic restrictions

For countries historically considered endemic for HAT, but which have no reported case data or estimate of the population at risk, estimates are not produced. These countries include Botswana, Ethiopia, Guinea-Bissau, and Rwanda.

Among countries where population at-risk data are available, if no cases were reported to WHO, we assume the incidence of HAT is zero for those years and generate model estimates accordingly.

Modelling steps

The cause of death model for HAT is implemented as follows:

1. The incidence of reported HAT cases among the population at-risk was calculated as the total number of reported cases divided by the population at-risk estimates generated by the GBD working group for the period 1980–2015. Population at-risk estimates for 2016–2017 were generated by assuming an annual 2% rate of population growth.

2. To estimate the number of cases that were likely undetected by country and year, a multilevel mixed-effects linear regression of log-transformed incidence rate (ratio of reported HAT cases to population at risk) on log-transformed screening coverage[3] (ratio of number screened for HAT to population at risk), with country random effects, was performed. Gaps were then filled using interpolation between years and extrapolation from 2016 to 2017 for reported cases. This model generates a beta-coefficient which is used to estimate the case detection rate (see step 4).

   For country-years in which no screening coverage data were reported:
   - Among countries with data reported, 1997–2004, the proportion of the at-risk population screened from 1997 was used retrospectively for the period 1980–1996 and the screening coverage from 2004 was carried forward from 2005 to 2017.
   - For countries with no screening data reported, the mean screening coverage for the region was used to impute a value over time.

3. To construct an estimate of total deaths, we first assume that all detected cases receive treatment, and that mortality among the treated occurs for a small proportion of cases. Deaths among detected cases are estimated by generating 1,000 draws of mortality among treated cases, assuming that between 0.7% and 6.0% of all reported (and therefore assumed to have received treatment) cases die.[4-6]

4. We then assume that all undetected cases experience mortality. This is estimated via generation of 1,000 draws of the case detection rate (CDR), given the expected screening coverage from the regression (in Step 2). Undetected deaths were then estimated as the difference between the ratio of reported cases to CDR and reported cases (reported cases/CDR – reported cases).

5. Estimates of death were obtained by adding the deaths among treated cases to the total number of undetected cases. Without information on sex-specific incidence or deaths, death rates between both sexes were equal.

6. Finally, an age-pattern was applied to the mortality estimates using the incidence studies from Sudan[7], DRC[8], and Uganda[9]. The age-pattern in GBD 2017 employed a cubic spline to account for the higher risk of infection among working age adults.

References

1. Rutto JJ, Osano O, Thuranira EG, Kurgat RK, Odenyo VA. Socio-economic and cultural determinants of human african trypanosomiasis at the Kenya - Uganda transboundary. *PLoS Negl Trop Dis* 2013; **7**(4): e2186.
2. Simarro PP, Cecchi G, Paone M, et al. The Atlas of human African trypanosomiasis: a contribution to global mapping of neglected tropical diseases. *Int J Health Geogr* 2010; **9**: 57.
3. World Health O. Human African trypanosomiasis (sleeping sickness): epidemiological update. *Weekly epidemiological record* 2006; **February 24**(8): 69-80.
4. Kato CD, Nanteza A, Mugasa C, Edyelu A, Matovu E, Alibu VP. Clinical profiles, disease outcome and co-morbidities among T. b. rhodesiense sleeping sickness patients in Uganda. *PLoS One* 2015; **10**(2): e0118370.
5. Balasegaram M, Harris S, Checchi F, Hamel C, Karunakara U. Treatment outcomes and risk factors for relapse in patients with early-stage human African trypanosomiasis (HAT) in the Republic of the Congo. *Bull World Health Organ* 2006; **84**(10): 777-82.
6. Odiit M, Kansiime F, Enyaru JC. Duration of symptoms and case fatality of sleeping sickness caused by Trypanosoma brucei rhodesiense in Tororo, Uganda. *East Afr Med J* 1997; **74**(12): 792-5.
7. Moore A, Richer M, Enrile M, Losio E, Roberts J, Levy D. Resurgence of sleeping sickness in Tambura County, Sudan. *Am J Trop Med Hyg* 1999; **61**(2): 315-8.
8. Lutumba P, Makieya E, Shaw A, Meheus F, Boelaert M. Human African trypanosomiasis in a rural community, Democratic Republic of Congo. *Emerg Infect Dis* 2007; **13**(2): 248-54.
9. Fevre EM, Odiit M, Coleman PG, Woolhouse ME, Welburn SC. Estimating the burden of rhodesiense sleeping sickness during an outbreak in Serere, eastern Uganda. *BMC Public Health* 2008; **8**: 96.

Schistosomiasis

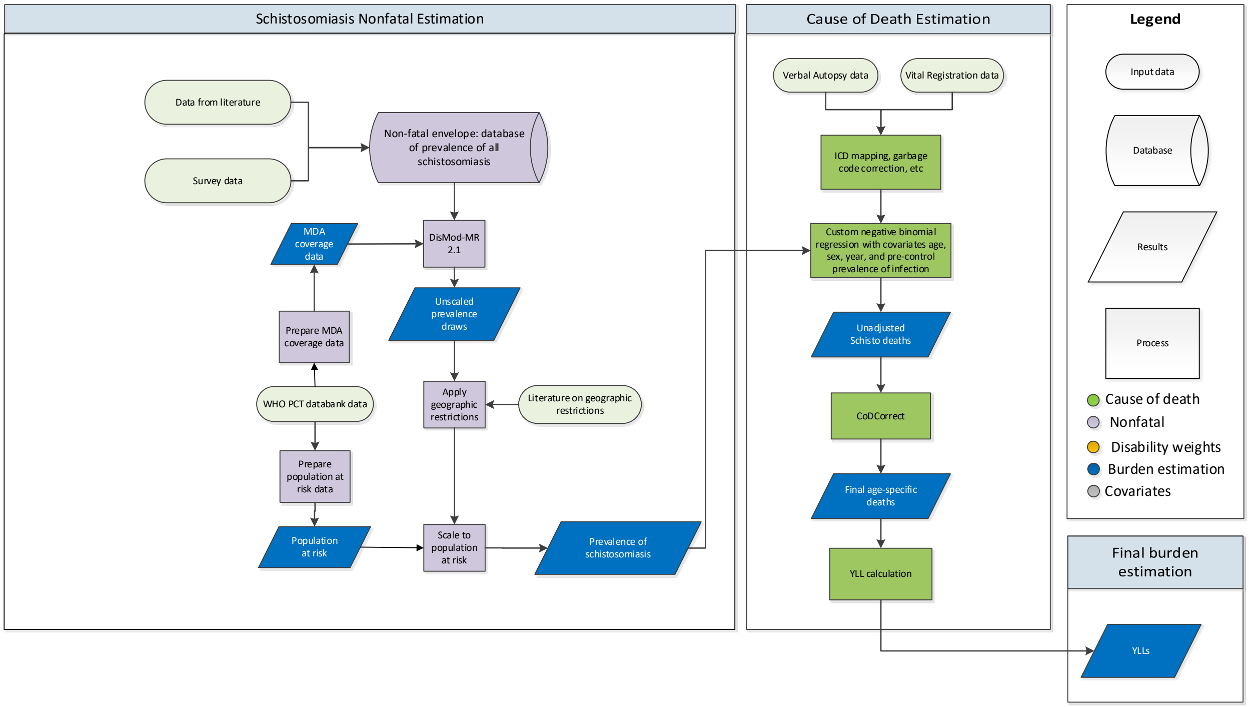

Input data

To estimate mortality due to schistosomiasis, data on deaths and prevalence of infection were used. The prevalence data were prepared this year for GBD 2017, and further information on prevalence data is available in the nonfatal write-up for this cause. Country-year-age-sex-specific verbal autopsy and vital registration data were used in the mortality model.

Geographic restrictions

We conducted a literature review to determine the geographic extent of the disease and classify locations based on whether the disease is absent or present in each year. Locations that were geographically restricted in any given year did not have estimates made for them but could have imported cases attributed to them at a later stage. Of note, we did not attempt a complete systematic review, since a single high-quality source could offer sufficient evidence of presence. Evidence of absence or presence was not available for every location for each year and so assumptions were made for missing years by taking into consideration the epidemiological characteristics of the disease. If evidence indicated disease presence for two non-consecutive years, we assumed presence for all years between the two. If evidence indicated disease absence for two non-consecutive years, we assumed absence for all years between the two. If evidence indicated a change in status (ie, from absent to present, or present to absent) between two non-consecutive years, than we conducted targeted searches to ascertain the relevant year of introduction or elimination for that location. In the cases where presence or absence information was missing for the start or end years of our study interval (1990–2017) without evidence of any introduction or elimination events within the interval, we applied the status of the first and last presence/absence observations respectively to all years between the

interval bound and the observation year. For schistosomiasis, we used a combination of Chitsulo et al's *The global status of schistosomiasis and its control* (1) and WHO's *Preventative chemotherapy in human helminthiasis* (2) report as a baseline. Where country-level endemicity statuses conflicted between the two sources, we searched Pubmed and Google Scholar for country- and subnational-specific endemicity status. Our search yielded 22 sources that were used to develop our annual geographic restriction map for schistosomiasis.

Modelling strategy

To estimate deaths due to schistosomiasis, a negative binomial regression model of country-year-age-sex-specific deaths on natural log-transformed prevalence of total schistosomiasis infection with a 15-year lag was used. The negative binomial regression was selected due to its suitability for modelling count data. In addition, there are relatively low number of deaths attributable to schistosomiasis. Covariates for endemic Brazil subnationals and South Africa subnationals were used to allow the model to follow data in those areas. A multivariate normal distribution using the mean and variance-covariance matrix from the model was used to generate 1,000 draws of deaths due to schistosomiasis.

Models were evaluated by assessing the AIC and plotting the predicted deaths against time, age, and sex. In addition, the Cause of Death visualisation tool was used to evaluate time trends across locations, age, and sex. A map of the global distribution of schistosomiasis across age groups was also used to assess the changes in death rates over time. The final model was selected based on how well the estimated numbers fit the input data and how plausible the predicted distribution of disease was over time and with age.

References

(1) Chitsulo, L., Engels, D., Montresor, A., & Savioli, L. (2000). The global status of schistosomiasis and its control. Acta Tropica, 77(1), 41-51. doi:10.1016/s0001-706x(00)00122-4
(2) World Health Organization (2006). Preventive chemotherapy in human helminthiasis: coordinated use of anthelminthic drugs in control interventions : a manual for health professionals and programme managers.

Cysticercosis

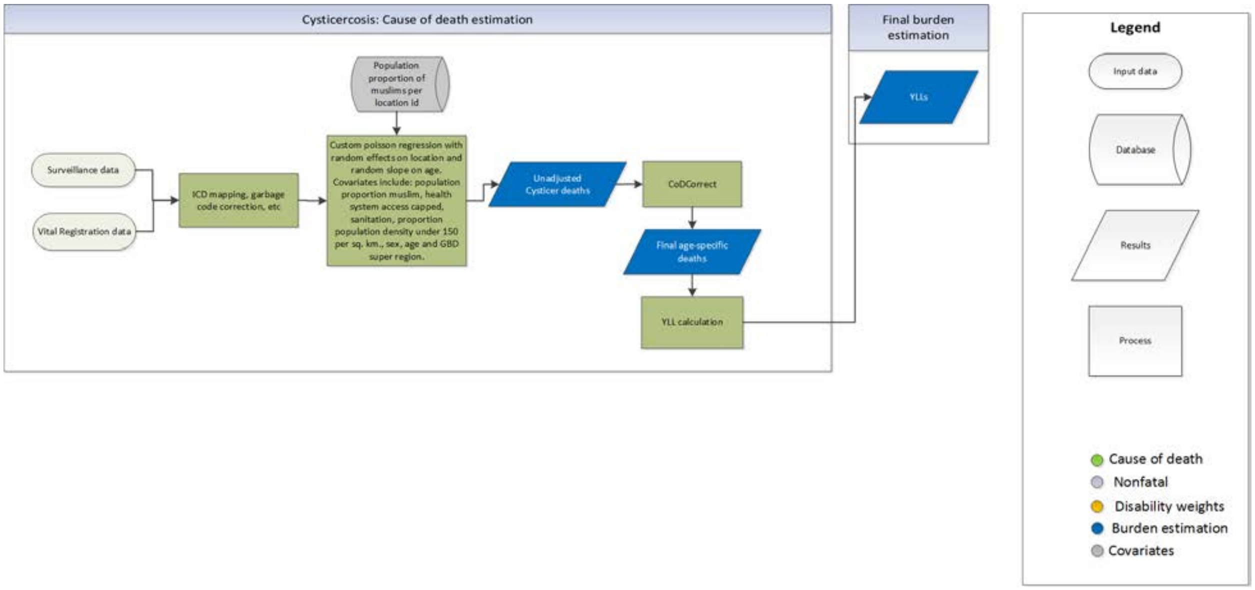

Input data

The model for mortality due to cysticercosis relied on vital registration and surveillance data from endemic countries. In addition, we used data from the Pew Research Center on percentage of population that is Muslim by country. The primary covariates adjusted for in the model were proportion of the population that is Muslim, health system access capped, proportion of the population with access to sanitation, proportion of the country with population density under 150 people per square kilometer, sex, age, and GBD super-region.

Geographic restrictions

We conducted a literature review to determine the geographic extent of the disease and classify locations based on whether the disease is absent or present in each year. Locations that were geographically restricted in any given year did not have estimates made for them but could have imported cases attributed to them at a later stage. Of note, we did not attempt a complete systematic review, since a single high-quality source could offer sufficient evidence of presence. Evidence of absence or presence was not available for every location for each year, and so assumptions were made for missing years by taking into consideration the epidemiological characteristics of the disease. If evidence indicated disease presence for two non-consecutive years, we assumed presence for all years between the two. If evidence indicated disease absence for two non-consecutive years, we assumed absence for all years between the two. If evidence indicated a change in status (ie, from absent to present, or present to absent) between two non-consecutive years than we conducted targeted searches to ascertain the relevant year of introduction or elimination for that location. In the cases where presence or absence information was missing for the start or end years of our study interval (1990–2016) without evidence of any introduction or elimination events within the interval, we applied the status of the first and last presence/absence observations respectively to all years between the interval bound and the observation year. For cysticercosis, we performed targeted searches to classify

location-years in PubMed and Google Scholar. Our map was populated by 21 peer-reviewed articles and meta-analyses and WHO reports.

Modelling strategy

Globally, deaths due to cysticercosis are relatively low. Therefore, a Poisson model was used to model cysticercosis deaths due to its suitability for count data. This model choice was validated by tests for overdispersion. Random effects were used on location with random slopes on age by location. A multivariate normal distribution using the mean and variance-covariance matrix from the model was used to generate 1,000 draws of deaths due to cysticercosis.

Estimates for new subnational locations were also added in GBD 2016. Since the Pew Research Center only has data on proportion of Muslims by country, we applied the national proportions to subnational locations. We understand that this does not account for sometimes large expected differences in proportions of Muslims within a country but were limited by data availability.

Cystic Echinococcosis

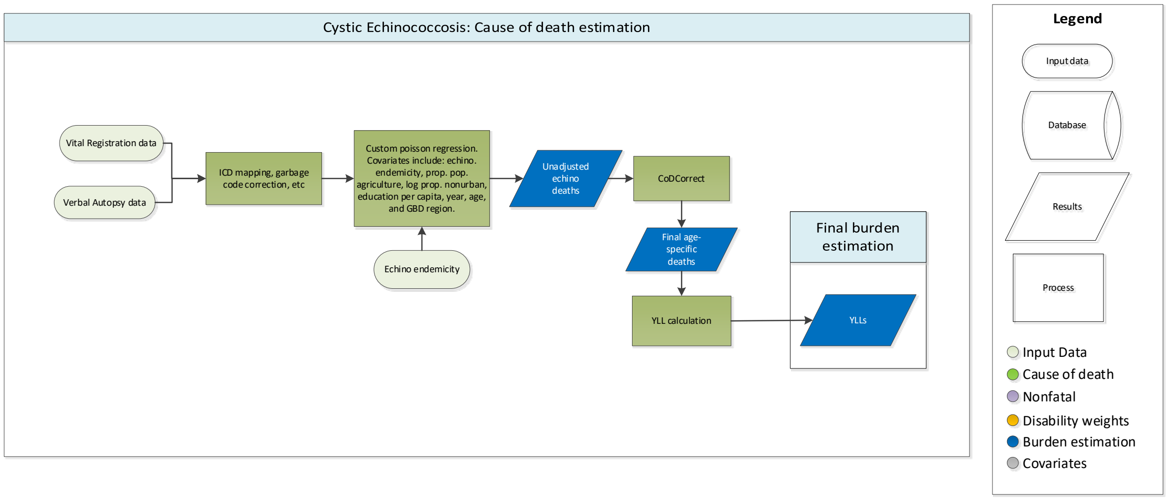

Input Data

Geographic restrictions

We conducted a literature review to determine the geographic extent of the disease and classify locations based on whether the disease is absent or present in each year. Locations that were geographically restricted in any given year did not have estimates made for them but could have imported cases attributed to them at a later stage. Of note, we did not attempt a complete systematic review, since a single high-quality source could offer sufficient evidence of presence. Evidence of absence or presence was not available for every location for each year, and so assumptions were made for missing years by taking into consideration the epidemiological characteristics of the disease. If evidence indicated disease presence at a given point in time, we assumed presence for all years. If evidence indicated disease absence, we assumed absence for all years. If evidence indicated a change in status (ie, from absent to present, or present to absent) between two non-consecutive years than we conducted targeted searches to ascertain the relevant year of introduction or elimination for that location. In the cases where presence or absence information was missing from the start or end years of our study interval (1990–2017) without evidence of any introduction or elimination events within the interval, we applied the status of the first and last presence/absence observations respectively to all years between the interval bound and the observation year. For cystic echinococcosis, we reviewed all references pertaining to CE in Global Distribution of Alveolar and Cystic Echinococcosis by Deplazes et al. and supplemented with targeted searches to classify location-years in PubMed and the GHDx. Below is a map of geographic restrictions for the year 2009 as an example.

Figure 1: Geographic restrictions for 2009

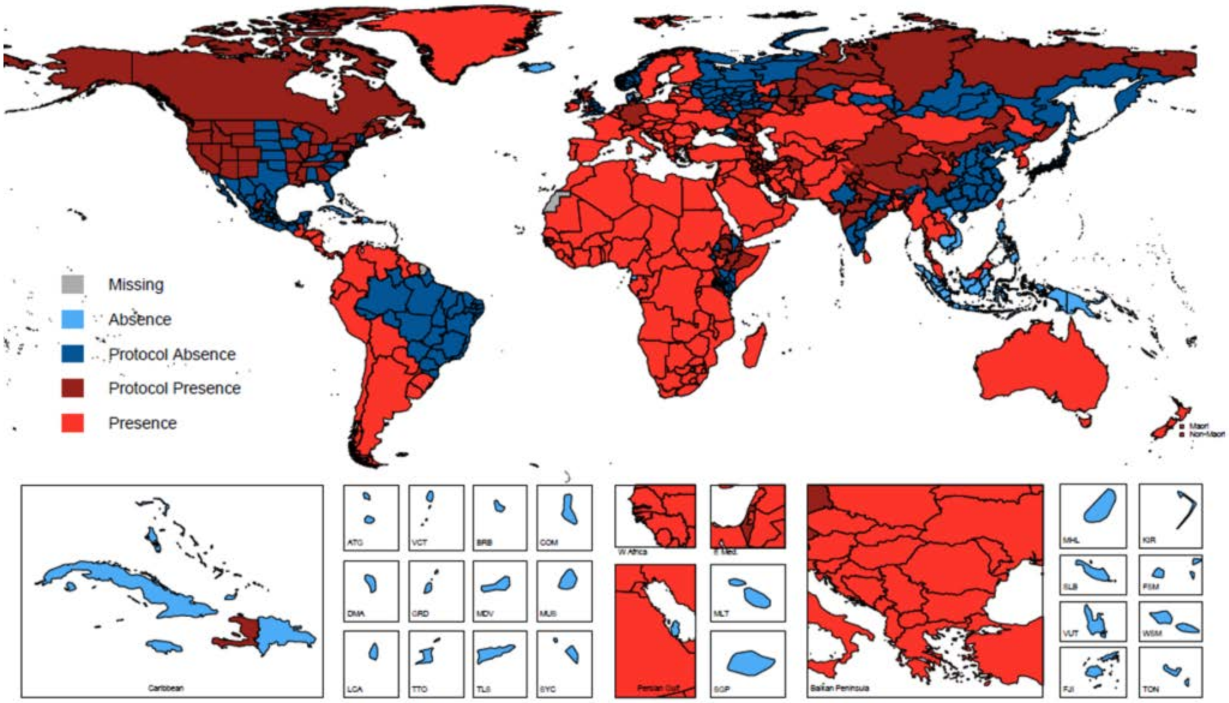

Data sources

Mortality due to cystic echinococcosis was modelled using vital registration data and covariates. The Mortality and Cause of Death team provided country-year-age-sex-specific vital registration. Of note, the ICD codes mapped to cystic echinococcosis are:

Table 1: ICD-9 codes mapped to CE

ICD Code	ICD Name
**122**	Echinococcosis
**122.0**	*Echinococcus granulosus* infection of liver
**122.1**	*Echinococcus granulosus* infection of lung
**122.2**	*Echinococcus granulosus* infection of thyroid
**122.3**	*Echinococcus granulosus* infection, other
**122.4**	*Echinococcus granulosus* infection, unspecified
**122.8**	Echinococcosis unspecified, of liver
**122.9**	Echinococcosis other and unspecified

Table 2: ICD-10 codes mapped to CE

ICD Code	ICD Name
**B67.0**	*Echinococcus granulosus* infection of liver
**B67.1**	*Echinococcus granulosus* infection of lung
**B67.2**	*Echinococcus granulosus* infection of bone
**B67.3**	*Echinococcus granulosus* infection, other and multiple sites
**B67.31**	*Echinococcus granulosus* infection, thyroid gland
**B67.32**	*Echinococcus granulosus* infection, multiple sites

**B67.39**	*Echinococcus granulosus* infection, other sites
**B67.4**	*Echinococcus granulosus* infection, unspecified
**B67.8**	Echinococcosis, unspecified, of liver
**B67.9**	Echinococcosis, other and unspecified
**B67.90**	Echinococcosis, unspecified
**B67.99**	Other echinococcosis

Due to the scarcity of hospital data, especially in endemic areas we incorporated covariates to drive global distribution of deaths in the model.

We created a categorical cystic echinococcosis endemicity covariate based on expert opinion and an endemicity map published by WHO [1]. We assigned GBD locations to one of four categories: probable absence, rare and/or sporadic transmission, suspected and/or confirmed transmission, and high endemic areas.

We based further selection of covariates on a meta-analysis of potential risk factors associated with cystic echinococcosis [2]. According to the meta-analysis, statistically significant potential risk factors include living in rural endemic areas, slaughtering, feeding dogs with viscera, and low income. Hence, we also included two other covariates: the proportion of the population participating in agricultural activities and the log of proportion non-urban.

Modelling strategy

We implemented a Poisson regression model to estimate deaths due to cystic echinococcosis. The Poisson regression was selected due to its suitability for modelling count data that are not over-dispersed. Since there is a relatively low number of deaths attributable to cystic echinococcosis, a Cause of Death Ensemble Model, which currently does not support count space models, could not be implemented. Covariates for the model, including echinococcosis endemicity, log of proportion non-urban, proportion of the population participating in agricultural activities, and education (years per capita), were incorporated into the model to influence the global trend due to paucity of data. Random effects were used on location with random slopes on age by location. A multivariate normal distribution using the mean and variance-covariance matrix from the model was used to generate 1,000 draws of deaths due to cystic echinococcosis. The final model was selected based on how well the estimated numbers fit the input data and how plausible the predicted distribution of disease was over time and with age.

References

1. World Health Organization (2010). Global Echinococcosis Granulosus and Cystic Echinococcosis (hydatisosis) Worldwide 2009.

2. Possenti A, Manzano-Román R, Sánchez-Ovejero C, et al. Potential Risk Factors Associated with Human Cystic Echinococcosis: Systematic Review and Meta-analysis. Flisser A, ed. *PLoS Neglected Tropical Diseases*. 2016;10(11):e0005114. doi:10.1371/journal.pntd.0005114.

Dengue

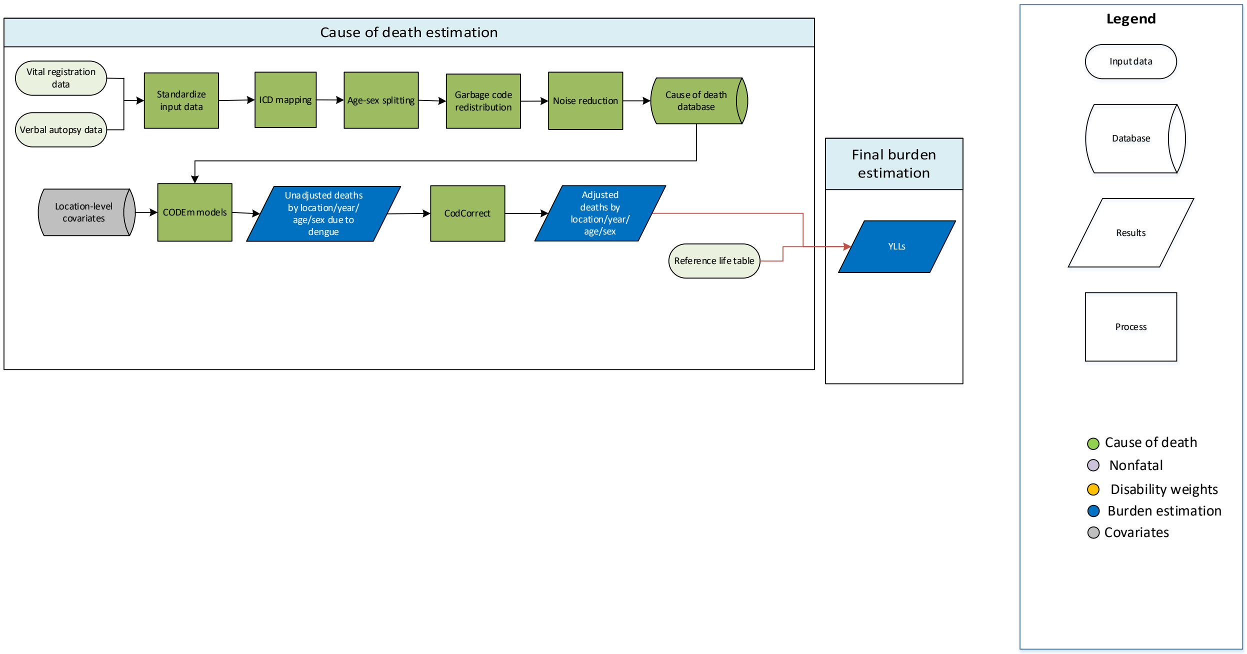

Input data

We modelled dengue mortality using all available data in the cause of death database. Data points were outliered if they reported an improbably low number of dengue deaths (eg, zero dengue deaths in a hyper-endemic country) or an improbably high number of dengue deaths.

Modelling strategy

We modelled dengue mortality using three-model hybrid approach: 1) a global CODEm model of all locations, using all data in the CoD database; 2) a CODEm model restricted to data-rich countries; and 3) estimates of mortality from imported cases in non-endemic, data-rich countries. Where dengue deaths were reported in non-endemic data-rich countries, we produced non-zero estimates by drawing from a beta distribution based on number of reported deaths and the underlying sample size. Estimates of dengue mortality in endemic data-rich countries were drawn from the data-rich CODEm model. Finally, estimates in other endemic countries were drawn from the global CODEm model.

We use county-level covarites to inform our model. The *Level* is the associated strength of relationship between the covariate and LRI mortality, ranked from 1 (proximally related) to 3 (distally related). The direction is the forced direction of the association between the covariate and dengue mortality

Level	Covariate	Direction
1	Population density (over 1000 ppl/sqkm, proportion)	+
	Population weighted probability of dengue transmission	+
2	Health system access	-
	Latitude under 15 (proportion)	+
	Elevation under 100m (proportion)	+

	Rainfall quintile 4 (proportion)	+
	Rainfall quintile 5 (proportion)	+
	Dengue outbreaks (binary)	+
3	Education (years per capita)	-
	LDI (1$ per capita)	-

While we've made no substantive changes to the modelling strategy in 2017, we have updated the geographic restrictions that determine whether a location is considered non-endemic (and, therefore, will have estimates based on the imported case model) in a given year. As for GBD 2016, we derived our geographic restrictions for 2010 from Brady and colleagues(1). We have also refreshed our literature review to determine locations and years in which dengue was introduced or eliminated, to allow for time-varying geographic restrictions.

References

1. Brady OJ, Gething PW, Bhatt S, Messina JP, Brownstein JS, Hoen AG, et al. Refining the Global Spatial Limits of Dengue Virus Transmission by Evidence-Based Consensus. PLoS Negl Trop Dis. 2012 Aug 7;6(8):e1760.

2. Al ST et. Autochthonous Dengue Fever, Tokyo, Japan, 2014 - Volume 21, Number 3—March 2015 - Emerging Infectious Disease journal - CDC. [cited 2017 Apr 28]; Available from: https://wwwnc.cdc.gov/eid/article/21/3/14-1662_article

3. Guzman MG, Kouri G. Dengue and dengue hemorrhagic fever in the Americas: lessons and challenges. J Clin Virol. 2003 May;27(1):1–13.

4. Boshell J, Groot H, Gacharna M, Márquez G, González M, Gaitán MO, et al. Dengue en Colombia. Biomédica. 1986;6(3–4):101–6.

5. Effler PV, Pang L, Kitsutani P, Vorndam V, Nakata M, Ayers T, et al. Dengue Fever, Hawaii, 2001–2002. Emerg Infect Dis. 2005 May;11(5):742–9.

6. McBride WJH. Dengue fever: is it endemic in Australia? Intern Med J. 2010 Apr 1;40(4):247–9.

7. Kay BH, Barker-Hudson P, Stallman ND, Wiemers MA, Marks EN, Holt PJ, et al. Dengue fever. Reappearance in northern Queensland after 26 years. Med J Aust. 1984 Mar 3;140(5):264–8.

8. Al GA et. Dengue Reemergence in Argentina - Volume 5, Number 4—August 1999 - Emerging Infectious Disease journal - CDC. [cited 2017 Apr 28]; Available from: https://wwwnc.cdc.gov/eid/article/5/4/99-0424_article

9. Ramos MM, Mohammed H, Zielinski-Gutierrez E, Hayden MH, Lopez JLR, Fournier M, et al. Epidemic Dengue and Dengue Hemorrhagic Fever at the Texas–Mexico Border: Results of a Household-based Seroepidemiologic Survey, December 2005. Am J Trop Med Hyg. 2008 Mar 1;78(3):364–9.

10. Luo L, Liang H, Hu Y, Liu W, Wang Y, Jing Q, et al. Epidemiological, virological, and entomological characteristics of dengue from 1978 to 2009 in Guangzhou, China. J Vector Ecol. 2012 Jun 1;37(1):230–40.

11. Murray KO, Rodriguez LF, Herrington E, Kharat V, Vasilakis N, Walker C, et al. Identification of Dengue Fever Cases in Houston, Texas, with Evidence of Autochthonous Transmission Between 2003 and 2005. Vector Borne Zoonotic Dis. 2013 Dec 1;13(12):835–45.

12. Locally Acquired Dengue --- Key West, Florida, 2009--2010 [Internet]. [cited 2017 Apr 28]. Available from: https://www.cdc.gov/mmwr/preview/mmwrhtml/mm5919a1.htm

13. Bouri N, Sell TK, Franco C, Adalja AA, Henderson DA, Hynes NA. Return of Epidemic Dengue in the United States: Implications for the Public Health Practitioner. Public Health Rep. 2012;127(3):259–66.

14. Wilder-Smith A, Quam M, Sessions O, Rocklov J, Liu-Helmersson J, Franco L, et al. The 2012 dengue outbreak in Madeira: exploring the origins. 2014 [cited 2017 Apr 28]; Available from: https://dr.ntu.edu.sg/handle/10220/19685

15. Brathwaite Dick O, San Martín JL, Montoya RH, del Diego J, Zambrano B, Dayan GH. The History of Dengue Outbreaks in the Americas. Am J Trop Med Hyg. 2012 Oct 3;87(4):584–93.

Yellow fever

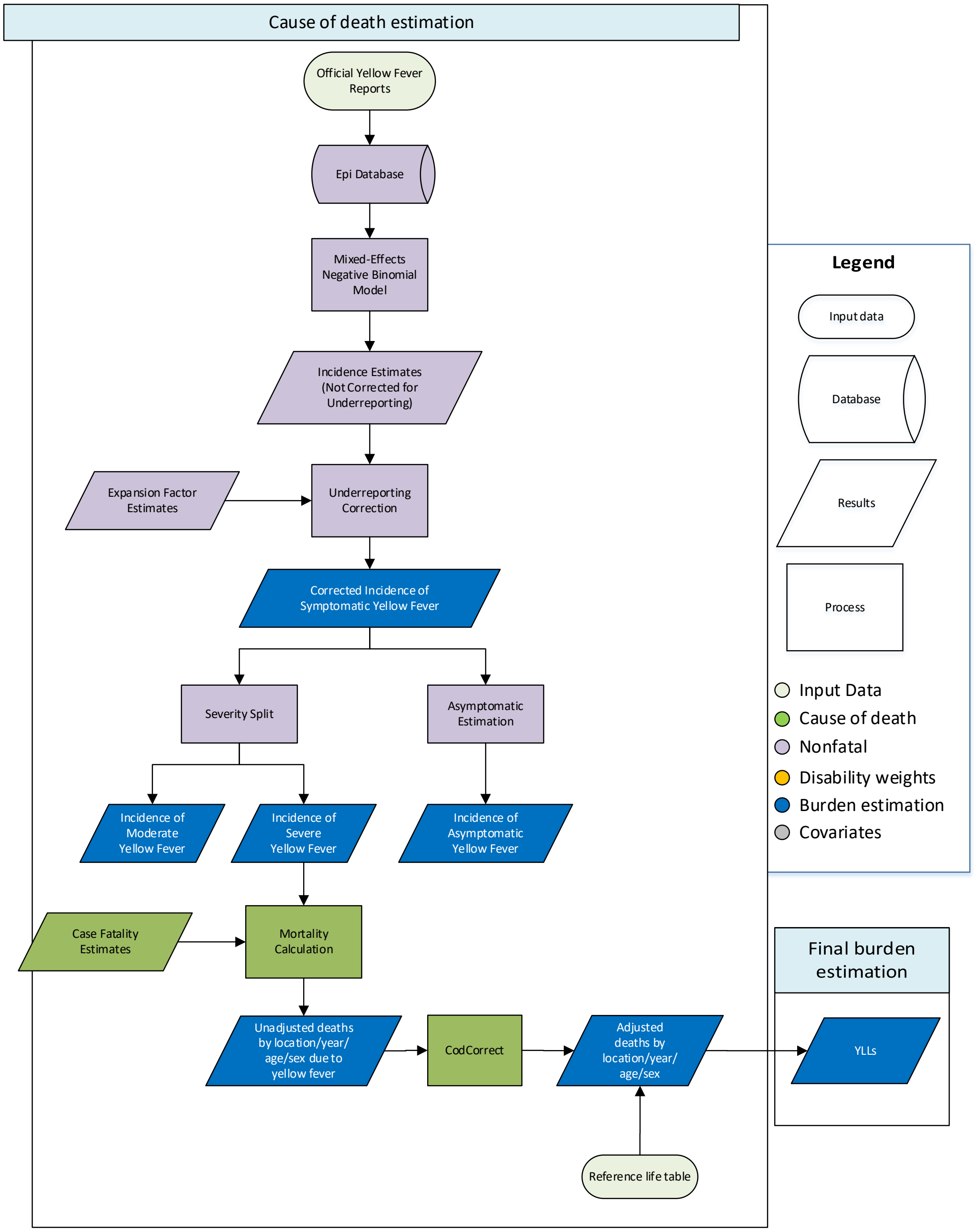

Input data

Case data come from official case reports filed with the World Health Organization. Data on case fatality come from published studies of yellow fever fatality. Data on deaths in non-endemic countries are restricted to only vital registration data.

Modelling strategy

We model yellow fever deaths using a hybrid approach. For countries in which yellow fever is endemic, we use a natural history approach in which we estimate deaths as the product of cases and case fatality. For non-endemic countries, we allow for deaths among imported cases where we have vital registration data indicating yellow fever deaths. That is, we assume no yellow fever deaths in non-endemic countries; however, where yellow fever deaths are reported in vital registration data, we accept those as true imported yellow fever deaths.

We model reported cases using a mixed-effects negative binomial model, with fixed effects for year and random effects for super-region, region, and country. We assume that yellow fever cases are underreported and that this underreporting mirrors that of dengue (a disease for which we have better data on underreporting). With that, we estimate symptomatic cases as the product of our base case estimates and dengue expansion factors (ie, the factor by which you must multiply reported cases to derive true cases). Based on published estimates, we assume that 27% of symptomatic cases will be severe.[1]

We performed a meta-analyses of case fatality using data from published studies of yellow fever fatality. Studies tend to report deaths among those with severe infection (eg, hospitalized cases), rather than among all cases. We assume that no deaths occur with asymptomatic infection or among those with only moderate symptoms. With that, we estimate deaths as the product of severe cases and case fatality.

We have improved our method for correcting for underreporting of yellow fever. In estimating yellow fever deaths for GBD 2013, our model assumed that all severe cases were reported and that reported cases reflected only severe cases. With that, we adjusted our base estimates upward to account for non-severe cases, and based our mortality estimates off these adjusted numbers. Based on feedback from collaborators, we believe that this adjustment was inadequate to fully account for underreporting. Accordingly, we have adopted the expansion factor-based method described above for GBD 2015.

Moreover, we have adopted the hybrid approach for GBD 2015. Whereas we previously allowed no yellow fever deaths in non-endemic countries, we now accept deaths reported in vital registration data as true imported deaths.

We have made no substantive changes to the modelling strategy for GBD 2017.

Reference

1 Johansson MA, Vasconcelos PFC, Staples JE. The whole iceberg: estimating the incidence of yellow fever virus infection from the number of severe cases. *Trans R Soc Trop Med Hyg* 2014; 108: 482–7.

Rabies

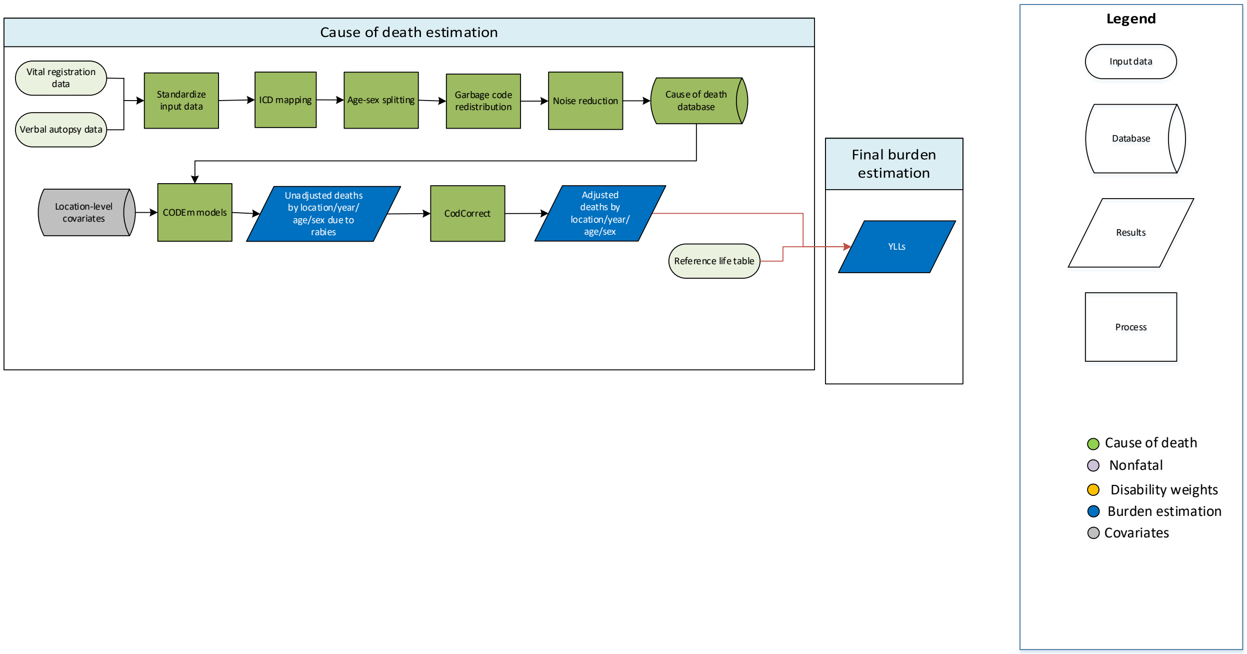

Input data

We modelled rabies mortality using all available data in the cause of death database. Data points were outliered if they reported an improbable number of rabies deaths (eg, zero rabies deaths in a hyper-endemic country) or if their inclusion in the model yielded distorted trends. In some cases, multiple data sources for the same location differed dramatically both in their quality and reported rabies mortality (eg, a verbal autopsy and vital registration source). In these cases the lower-quality data source was outliered.

Modelling strategy

We modelled rabies mortality using a two-model hybrid approach: 1) a global CODEm model of all locations, using all data in the CoD database; and 2) a CODEm model restricted to data-rich countries. The CODEm models included nine covariates:

Level	Covariate	Direction
1	Antenatal care coverage (4 visits)	-
	Health system access	-
	In-facility delivery coverage	-
2	Healthcare access and quality index	-
	Skilled birth attendance coverage	-
	Health system access (capped)	-
3	Population density, 500-1000 per km^2	0
	Population density, <150 per km^2	0
	Socio-demographic Index	-

We have made no substantive changes to the modelling strategy in GBD 2017.

Ascariasis

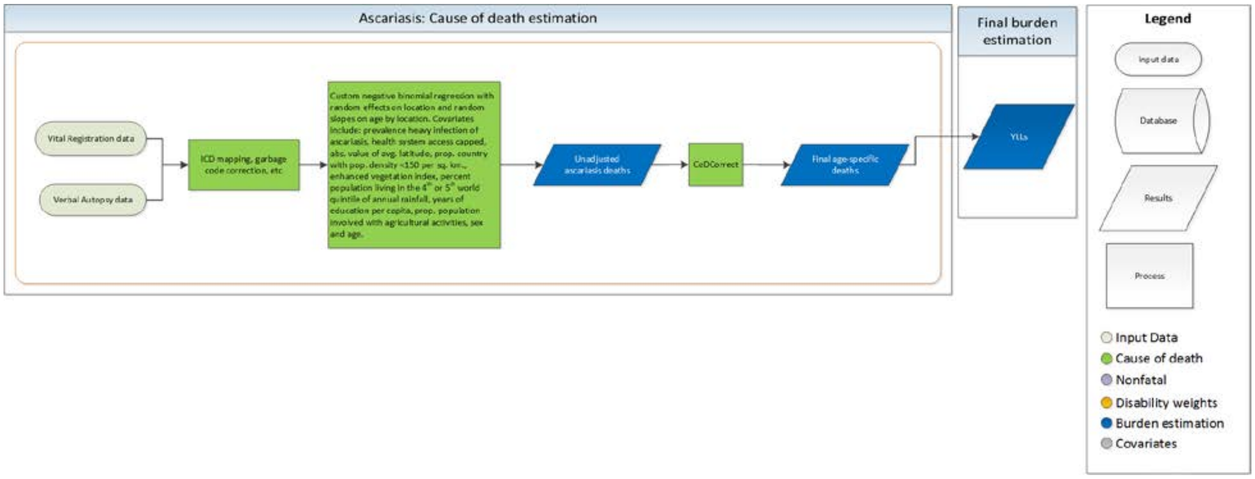

Input data

To estimate mortality due to ascariasis, country-year-age-sex-specific verbal autopsy and vital registration data were used. Covariates used include prevalence of heavy infection of ascariasis, health system access capped by the minimum OECD value, the absolute value of average latitude, the proportion of the country with population density under 150 people per square kilometer, enhanced vegetation index, percent of the population living in the fourth or fifth world quintile of annual rainfall, number of years of education per capita, proportion of the population involved with agricultural activities, sex, and age.

Geographic restrictions

We conducted a literature review to determine the geographic extent of the disease and classify locations based on whether the disease is absent or present in each year. Locations that were geographically restricted in any given year did not have estimates made for them but could have imported cases attributed to them at a later stage. Of note, we did not attempt a complete systematic review, since a single high-quality source could offer sufficient evidence of presence. Evidence of absence or presence was not available for every location for each year and so assumptions were made for missing years by taking into consideration the epidemiological characteristics of the disease. If evidence indicated disease presence for two non-consecutive years, we assumed presence for all years between the two. If evidence indicated disease absence for two non-consecutive years, we assumed absence for all years between the two. If evidence indicated a change in status (ie, from absent to present, or present to absent) between two non-consecutive years than we conducted targeted searches to ascertain the relevant year of introduction or elimination for that location. In the cases where presence or absence information was missing for the start or end years of our study interval (1990–2016) without evidence of any introduction or elimination events within the interval, we applied the status of the first and last presence/absence observations respectively to all years between the interval bound and the observation year. Our search was done in conjunction with the title/abstract screening portion of a systematic literature review for prevalence data. The search strings and yield can be viewed in the table below for each of the databases queried.

Database	Search string	Yield
PubMed	(Ascariasis[Title/Abstract] OR Ascaris[Title/Abstract] OR "A. lumbricoides"[Title/Abstract] OR Ascaris[MeSH] OR Trichuris[Title/Abstract] OR Trichuriasis[Title/Abstract] OR "Whip Worm"[Title/Abstract] OR "T. trichura"[Title/Abstract] OR Trichuris[MeSH] OR Hookworm[Title/Abstract] OR "A. duodenale"[Title/Abstract] OR "Ancylostoma duodenale"[Title/Abstract] OR ancylostomiasis[Title/Abstract] OR "N. americanus"[Title/Abstract] OR "Necator americanus"[Title/Abstract] OR necatoriasis[Title/Abstract] OR Ancylostoma [MeSH] OR Necator[MeSH]) AND (prevalence[Title/Abstract] OR incidence[Title/Abstract] OR epidemiology[Title/Abstract] OR surveillance[Title/Abstract]) NOT(Animals[MeSH] NOT Humans[MeSH])	2376
Web of Science	(Ascariasis OR Ascaris OR A. lumbricoides OR Trichuris OR Trichuriasis OR Whip Worm OR T. trichura OR Hookworm OR A. duodenale OR Ancylostoma duodenale OR anclyostomiasis OR N. americanus OR Necator americanus OR necatoriasis) AND TOPIC:(prevalence OR incidence OR epidemiology OR surveillance) NOTTOPIC: ((Animals NOT Humans))<br>Timespan: 1980-2016. Indexes: SCI-EXPANDED, SSCI, A&HCI, ESCI.	2266
SCOPUS	TITLE-ABS_KEY (ascariasis OR ascaris OR a. lumbricoides OR trichuris OR trichuriasis OR whip worm OR t. trichura OR hookworm OR a. duodenale OR ancylostoma duodenale OR anclyostomiasis OR n. americanus OR necator americanus OR necatoriasis) AND PUBYEAR>1979	29

These papers were used to classify location-years for all locations and years present in the literature. Additionally, systematic literature reviews, meta-analyses, national health statistics publications, and collaborator input were used to classify location-years not present in the literature review wherever possible.

Modelling strategy

A negative binomial model was used to estimate deaths from ascariasis with random intercepts for locations and random slopes for age groups by location. A multivariate normal distribution using the mean and variance-covariance matrix from the model was used to generate 1,000 draws of deaths due to ascariasis. The final model was selected based on how well the estimated number fit the input data and how plausible the predicted distribution of disease was over time and with age.

Ebola Virus Disease

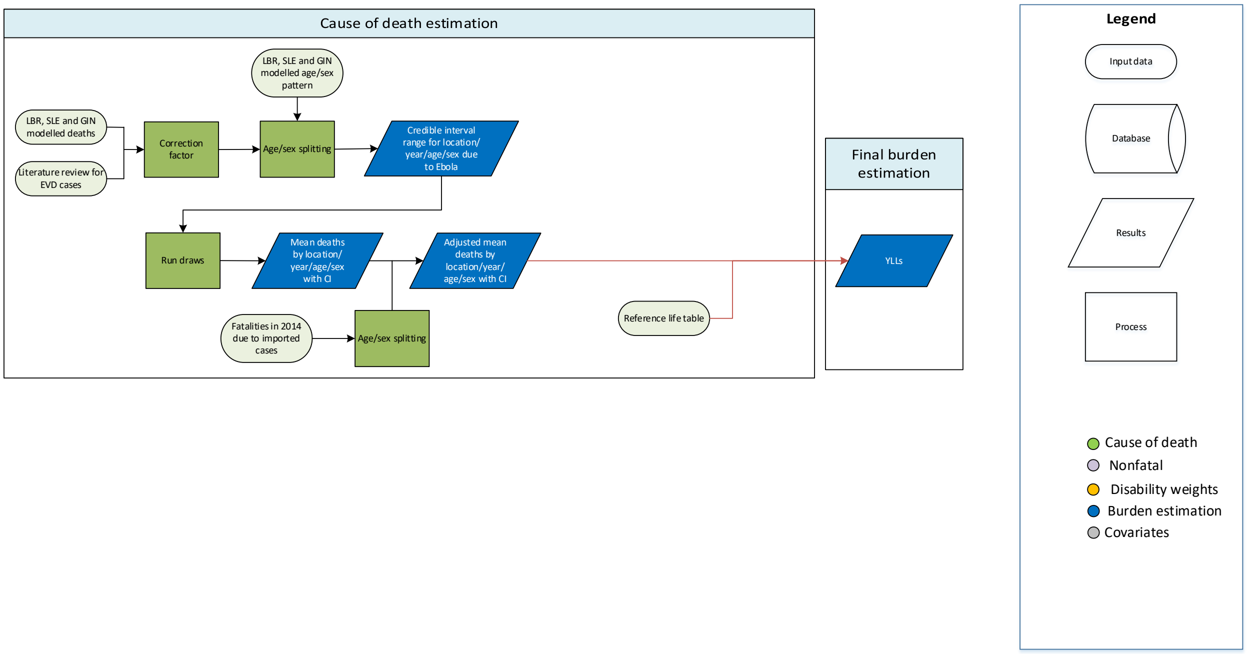

Input data

The input data for deaths due to Ebola virus disease (EVD) came in two forms: (i) total case count tallies and modelled estimates for the West African outbreak from 2013 to 2016 provided by the World Health Organization (WHO) focused specifically on the three worst-affected countries (Liberia, Guinea, and Sierra Leone) and (ii) literature searches for reported deaths due to EVD not captured by the West African dataset. This is further explained below:

i. WHO estimates for Liberia, Guinea, and Sierra Leone, 2014–2016

   1. The final tallies as reported by WHO and CDC were used for each country. Researchers from Imperial College London (UK), as part of the WHO Ebola response team, provided modelled estimates for the number of fatalities that result from a given number of reported cases (provided by line lists from WHO). This method was used in a variety of papers to generate baseline estimates of case fatality rates and other key epidemiological measures while correcting for the lag period between initially reporting a case and the final outcome of that case (whether it be death or survival). The full data cleaning and methodology are reported elsewhere.[1,2] Bespoke estimates were provided for GBD for Liberia, Sierra Leone, and Guinea and were stratified by age, sex, and year. Death data from Guinea ranged from February 18, 2014, until September 27, 2015, with data from Liberia ranging from March 20, 2014, to May 4, 2015, and data from Sierra Leone ranging from May 21 until

September 28, 2015. These models were used to disaggregate total case numbers by age and sex to generate the total case counts.

2. Reported clusters of cases in 2016 were identified by consulting WHO situation reports from the year 2016.

ii. Literature searches for reported deaths due to EVD outside of Liberia, Guinea, and Sierra Leone

1. In order to capture the small number of fatalities that occurred in countries outside of the core three mentioned above, WHO Situation Reports were consulted. Fatalities were reported in the US (specifically Texas), Mali, and Nigeria.[3] All deaths occurred in 2014. Additional age and sex information could only be obtained for the death that occurred in the US.

Using a previous review of historical outbreaks,[4,5] original articles describing the progression of historical outbreaks were consulted, which was supplemented by additional searches. This resulted in datasets describing each outbreak with variable degrees of detail – some fully describing the age and sex breakdown of all deaths [eg, Rosello and colleagues[7]] and others simply providing the final total. Only confirmed or probable deaths were included; suspected EVD deaths were omitted. Outbreaks that spanned multiple years, in the absence of sufficient data providing an accurate breakdown, were apportioned between the years by evenly assigning a uniform number of deaths to each month of the outbreak's duration.

A full tabulation of death metadata availability is found in Table 1.

Outbreak	Number of deaths	Sex metadata	Age metadata	Year metadata
Côte d'Ivoire 1994	No deaths	N/A	N/A	N/A
Gabon 1994/1995	Georges 1999	Imputed	Imputed	Georges 1999
Democratic Republic of the Congo 1995	Rosello 2015	Rosello 2015	Rosello 2015 [94.5% coverage]	Rosello 2015
Gabon 1996	Milleliri 2004	Imputed	Imputed	Milleliri 2004
Gabon 1996/1997	Milleliri 2004	Imputed	Imputed	Imputed
Uganda 2000/2001	Okware 2002	Imputed	Imputed	Imputed
Congo 2002/2003	Kuhn 2008	Imputed	Imputed	Imputed
Congo 2003	Boumandouki 2005	Imputed	Imputed	Boumandouki 2005
South Sudan 2004	WHO 2004	WHO 2004	WHO 2004 [42.86% coverage]	WHO 2004
Congo 2005	Nkoghe 2011	Nkoghe 2011	Nkoghe 2011	Nkoghe 2011
Democratic Republic of the Congo 2007	Rosello 2015	Rosello 2015	Rosello 2015	Rosello 2015
Uganda 2007	Wamala 2010	Wamala 2010	Imputed	Wamala 2010
Democratic Republic of the Congo 2008	Rosello 2015	Rosello 2015	Rosello 2015	Rosello 2015

Uganda 2011	Shoemaker 2012	Shoemaker 2012	Shoemaker 2012	Shoemaker 2012
Democratic Republic of the Congo 2012	Rosello 2015	Rosello 2015	Rosello 2015	Rosello 2015
Uganda 2012	Albarino 2013	Imputed	Imputed	Albarino 2013
Uganda 2012/2013	Albarino 2013	Imputed	Imputed	Imputed
West Africa 2013/2015	WHO/CDC	WHO/Imperial [incomplete]	WHO/Imperial [incomplete]	WHO/CDC
Democratic Republic of the Congo 2014	Rosello 2015	Rosello 2015	Rosello 2015	Rosello 2015

Modelling strategy

Data on deaths resulting from imported cases from 2014 were used as specific count data as it was assumed to be an accurate representation of the cases and outbreaks in these countries, all of which were on high alert for importation of cases.[8,9]

The other input data were processed prior to inclusion in GBD to account for any potential underreporting of deaths. A meta-analysis of existing underreporting studies from the literature was performed, using a random effects model with a DerSimonian-Laird estimator. A variety of sources were included, capturing a number of different estimation processes, all identified by literature review. The figure below shows the different effect sizes of the different studies,[10–15] as well as the resulting GBD 2016 correction factor, with the GBD 2015 correction factor for reference. The correction factor ranged from 1.4580 to 2.5475, with a mean of 2.0027. For GBD 2017 the GBD 2016 factor was used.

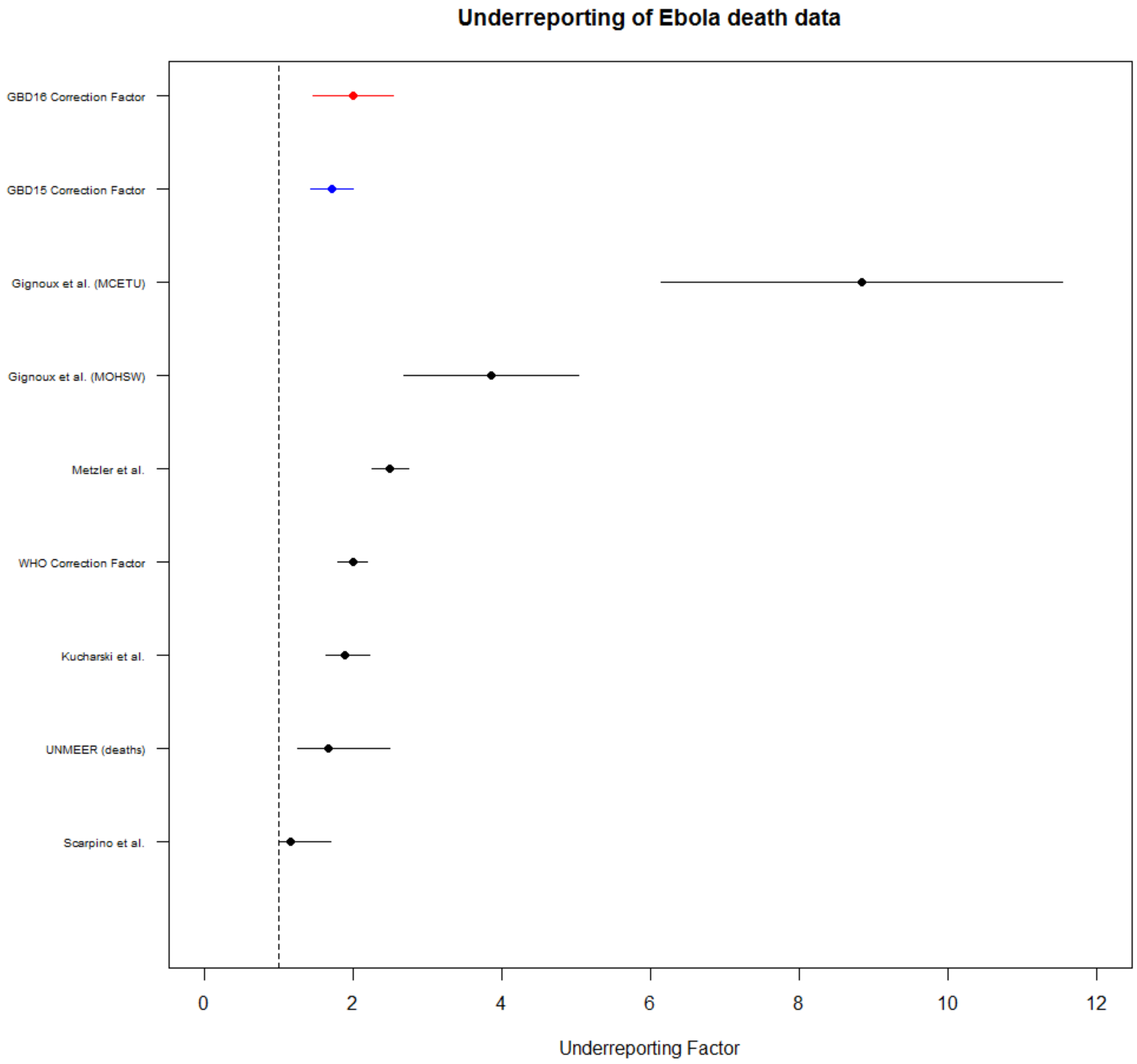

In order to capture this potential variation, all input data were multiplied by the lower and upper limit of this estimated correction factor; these numbers then provided the lower and upper bounds from which draw values were taken. For outbreaks where no data were supplied for age and/or sex, the pattern observed in the West African outbreak (for which there were the most comprehensive data) was used to apportion these total values.

One thousand draws were taken from a normal distribution fitted between these lower and upper bound values, which generated mean estimates stratified by age, sex, location, and year along with credible intervals for these numbers. These estimates were then adjusted by including the count data for imported cases from 2014.

Data on Ebola outbreaks prior to 2014 are sparse, and as a result many values derived from the West African outbreak were assumed to be valid for historical outbreaks as well. This may mask significant differences that exist between these outbreaks, some of which were caused by different species of

Ebolavirus. In order to minimize this problem, we chose to implement a data-driven approach – for those outbreaks where sufficiently detailed historical data could be obtained, these were used in preference to any assumed age/sex breakdown.

References

1 Agua-Agum J, Ariyarajah A, Aylward B, *et al.* West African Ebola Epidemic after One Year — Slowing but Not Yet under Control. *N Engl J Med* 2015; **372**: 584–7.

2 Ebola Virus Disease in West Africa - The First 9 Months of the Epidemic and Forward Projections. *N Engl J Med* 2014; **371**: 1481–95.

3 World Health Organization. Ebola Situation Reports. 2016. Interview (accessed March 14, 2016).

4 Pigott DM, Golding N, Mylne A, *et al.* Mapping the zoonotic niche of Ebola virus disease in Africa. *Elife* 2014; **3**: e04395.

5 Mylne A, Brady OJ, Huang Z, *et al.* A comprehensive database of the geographic spread of past human Ebola outbreaks. *Sci Data* 2014; **1**: 140042.

6 Maganga GD, Kapetshi J, Berthet N, *et al.* Ebola virus disease in the Democratic Republic of Congo. *N Engl J Med* 2014; **371**: 2083–91.

7 Rosello A, Mossoko M, Flasche S, *et al.* Ebola virus disease in the Democratic Republic of the Congo, 1976-2014. *Elife* 2015; **4**. DOI:10.7554/eLife.09015.

8 Fasina FO, Shittu A, Lazarus D, *et al.* Transmission dynamics and control of Ebola virus disease outbreak in Nigeria, July to September 2014. *Euro Surveill* 2014; **19**: 20920.

9 Althaus CL, Low N, Musa EO, Shuaib F, Gsteiger S. Ebola virus disease outbreak in Nigeria: Transmission dynamics and rapid control. *Epidemics* 2015; **11**: 80–4.

10 Gignoux E, Idowu R, Bawo L, *et al.* Use of Capture-Recapture to Estimate Underreporting of Ebola Virus Disease, Montserrado County, Liberia. *Emerg Infect Dis* 2015; **21**: 2265–7.

11 Meltzer MI, Atkins CY, Santibanez S, *et al.* Estimating the future number of cases in the Ebola epidemic--Liberia and Sierra Leone, 2014-2015. *MMWR Suppl* 2014; **63**: 1–14.

12 Scarpino S V, Iamarino A, Wells C, *et al.* Epidemiological and viral genomic sequence analysis of the 2014 ebola outbreak reveals clustered transmission. *Clin Infect Dis* 2015; **60**: 1079–82.

13 Kucharski AJ, Camacho A, Flasche S, Glover RE, Edmunds WJ, Funk S. Measuring the impact of Ebola control measures in Sierra Leone. *Proc Natl Acad Sci U S A* 2015; **112**: 14366–71.

14 UNMEER. Sierra Leone: Ebola emergency Weekly Situation Report No. 7. 2014 https://www.humanitarianresponse.info/system/files/documents/files/UNMEER_NERC_SitRep_07Dec.pdf.

15 Enserink M. How many Ebola cases are there really? | Science | AAAS. 2014. http://www.sciencemag.org/news/2014/10/how-many-ebola-cases-are-there-really (accessed Jan 28, 2017).

Zika Virus Disease

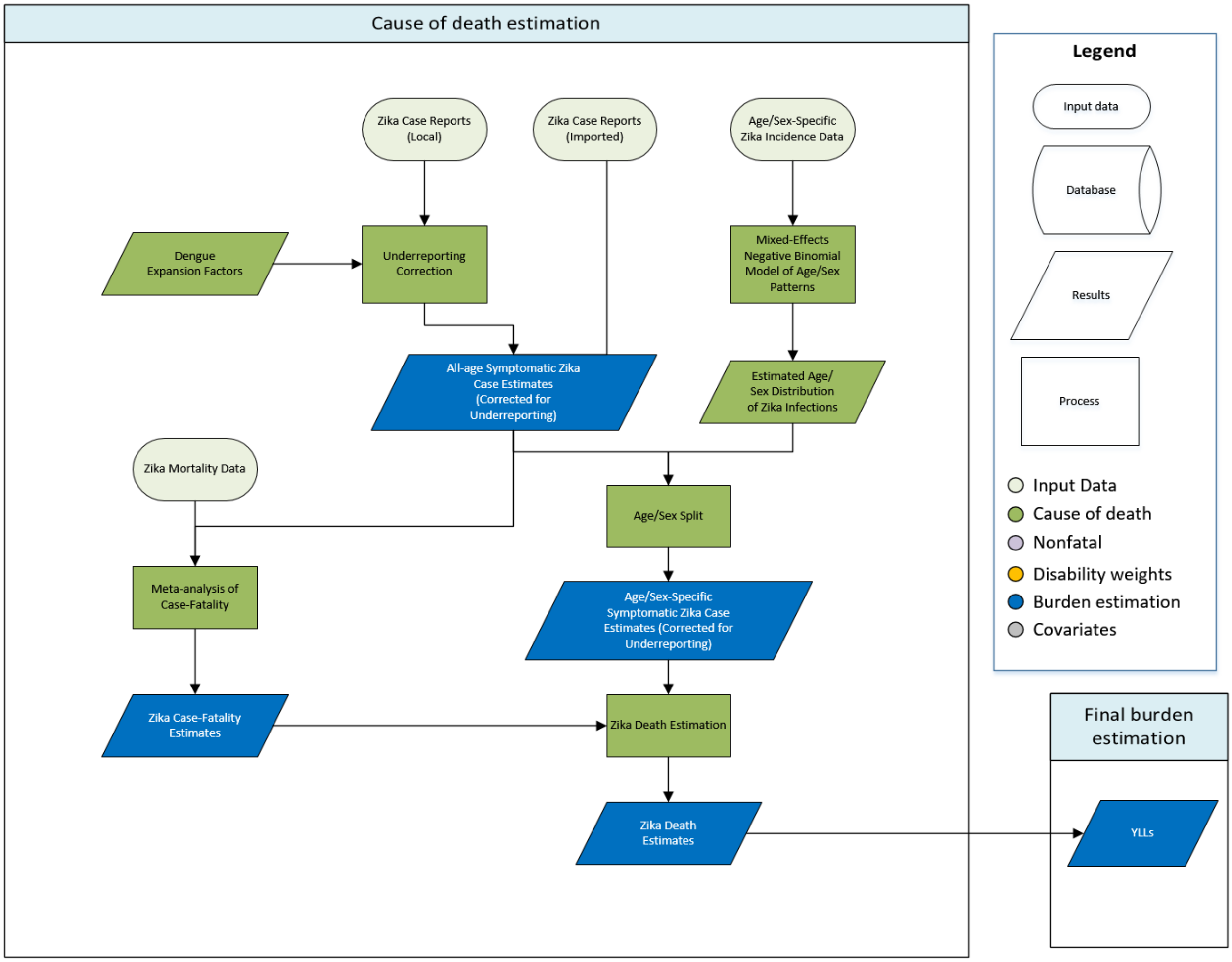

Input data

Case data and death data come from official reports, primarily from PAHO.

Modelling strategy

We model Zika deaths using a natural history approach in which we estimate deaths as the product of cases and case fatality. We estimate the number of true symptomatic cases as the product of reported cases and country-specific expansion factors that adjust for underreporting. Those expansion factors are derived from our dengue model and the methods used for their estimation are detailed in the dengue model documentation and by Stanaway and colleagues[1].

We then use an intercept only, mixed-effects Poisson regression model, with random effects on location, to estimate case fatality. Here, our outcome variable is reported Zika deaths and our exposure variable is estimated number of symptomatic Zika cases. For location-years with reported Zika deaths, we estimate deaths from the fixed effects (ie, intercept and offset) and random effects, including

uncertainty from both effects. For location-years with no Zika death reports, but with reported Zika cases, we estimate deaths from the fixed effects and sample from the distribution of all random effects.

For GBD 2017, we have both refreshed the data as well as conducted two systematic literature reviews to more completely incorporate the growing literature on Zika.

Reference

1 Stanaway JD, Shepard DS, Undurraga EA, Halasa YA, Coffeng LE, Brady OJ, et al. The global burden of dengue: an analysis from the Global Burden of Disease Study 2013. The Lancet Infectious Diseases [Internet]. 2016 Feb

Other neglected tropical diseases (NTDs)

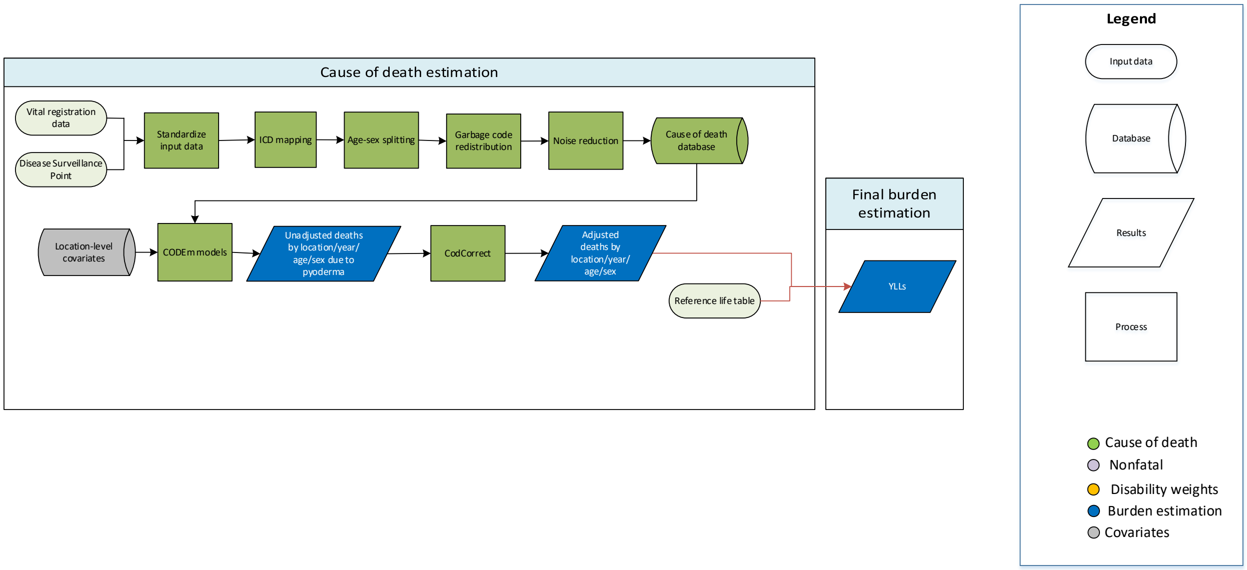

Input data

We modelled other neglected tropical disease mortality using all available data in the cause of death database. Data points were outliered if they reported an improbable number of deaths or if their inclusion in the model yielded distorted trends.

Modelling strategy

We modelled other neglected tropical disease mortality using a two-model hybrid approach: 1) a global CODEm model of all locations, using all data in the CoD database; and 2) a CODEm model restricted to data-rich countries.

We have made no substantive changes in the modelling strategy for other neglected tropical diseases from GBD 2016.

Level	Covariate	Direction
1	Healthcare access and quality index	–
	Proportion of the population living between 0 and 15 degrees latitude	+
2	Proportion of the population living in the 5th quintile of rainfall	+
	Sanitation	–
3	Education (years per capita)	–
	Lag-distributed income (per capita)	–
	Socio-demographic Index	–

Meningitis

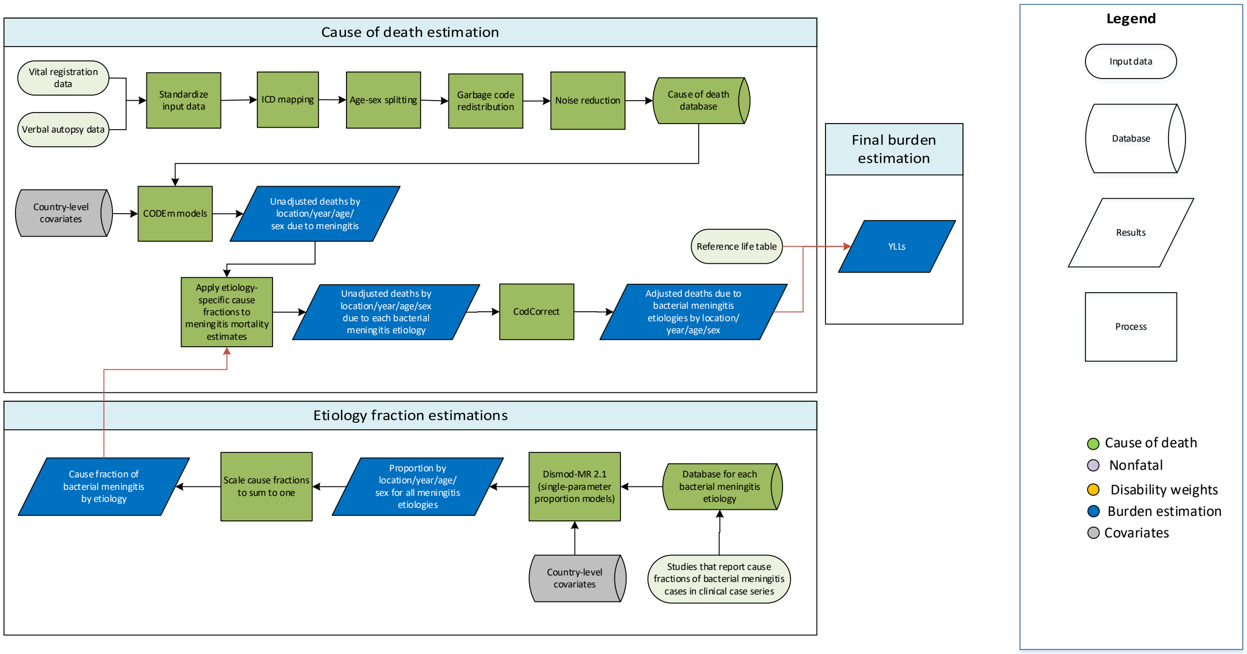

Input data

Input data for the overall meningitis model came from the cause of death database, which includes vital registration (VR) and verbal autopsy (VA) data. We outliered data in instances where garbage code redistribution and noise reduction, in combination with small sample sizes, resulted in unreasonable cause fractions when compared to regional, super-regional, and global rates, and data that violated well-established time or age trends. Outliering methods were consistent across both VR and VA data.

Etiology splits are derived from cause-specific VR mortality data. Viral meningitis mortality is rare except in the youngest age groups and is included with "other meningitis" etiology.

Modelling strategy

We modeled deaths due to all bacterial meningitis with two CODEm models, separately for each sex and two age categories – under 5 and 5 years and above. The mortality trends differ substantially between children and adults, and there are a significant number of data sources that only have data for children under 5. The two models used the same covariates and otherwise standard CODEm parameters. The final sex-specific models for deaths due to all bacterial meningitis were a hybridised model of separate global and data-rich models for males and females.

To obtain estimates for each of the four etiologies of bacterial meningitis – meningococcal, pneumococcal, *H. influenzae* type B, and other bacterial – we ran separate proportion models in DisMod-MR 2.1 using cause-specific VR data. The meningococcal meningitis proportion model used two country-level covariates to inform the model – proportion of the population living within the meningitis belt[1], and proportion of the population covered by the meningococcal meningitis type A vaccine (an initiative called Menafrivac). The pneumococcal meningitis model was informed by PCV3 vaccine

coverage, and the H influenza type B meningitis model was informed by HiB3 vaccine coverage. The other meningitis proportion model did not use any country-level covariates.

Since DisMod-MR 2.1 estimates in 5-year intervals, the etiological proportions for years between the intervals were interpolated at the draw level. The four proportion models were scaled to 1 at the draw level for each location, year, sex, and age combination. We applied these proportions to the total meningitis cause of death models to produce estimates for each of the four etiologies. In GBD 2017 we added the same vaccine-specific covariates used in the mortality proportion models to the parent meningitis mortality model. Covariates were weighted and selected in the ensemble modelling process. The covariate inputs for the final published CODEm parent models, and the coefficients for the VR etiology proportion models are listed below.

Parent Meningitis CODEm model covariate table (0-4 years, 5-95+ years)

Covariate Name	Level	Direction
Meningitis belt (proportion of population in belt)	1	1
Menafravac coverage	1	-1
H influenzae B proportion covered	1	-1
Underweight proportion	2	1
Water (proportion with access)	2	-1
Health system access	2	-1
Healthcare access and quality index	2	-1
DTP3 Coverage	3	-1
LDI	3	-1
Sanitation (proportion with access)	3	-1
Maternal education (years per capita)	3	-1
Socio-demographic Index	3	-1

Vital registration etiology proportion DisMod model covariate tables

Meningococcal mortality proportion

Measure	Covariate	Type	beta	exp beta
Proportion	meningitis belt (proportion)	Country	0.029 (0.00052 — 0.14)	1.03 (1.00 — 1.15)
Proportion	Proportion of total population covered by menafrivac initiative (meningitis meningococcal type A vaccine)	Country	-1 (-2 — -0.0053)	0.37 (0.14 — 0.99)

H influenzae B mortality proportion

Measure	Covariate	Type	beta	exp beta
Proportion	Hib3 Vaccine Coverage (proportion)	Country	-0.04 (-0.098 — -0.0022)	0.96 (0.91 — 1.00)

Pneumococcal mortality proportion

Measure	Covariate	Type	beta	exp beta
Proportion	PCV3 Coverage (proportion)	Country	-0.0017 (-0.0048 — -0.00026)	1.00 (1.00 — 1.00)

References

(1) Centers for Disease Control (CDC). CDC health information for international travel 2016: the yellow book. New York City, United States: Oxford University Press, USA, 2016.

Encephalitis

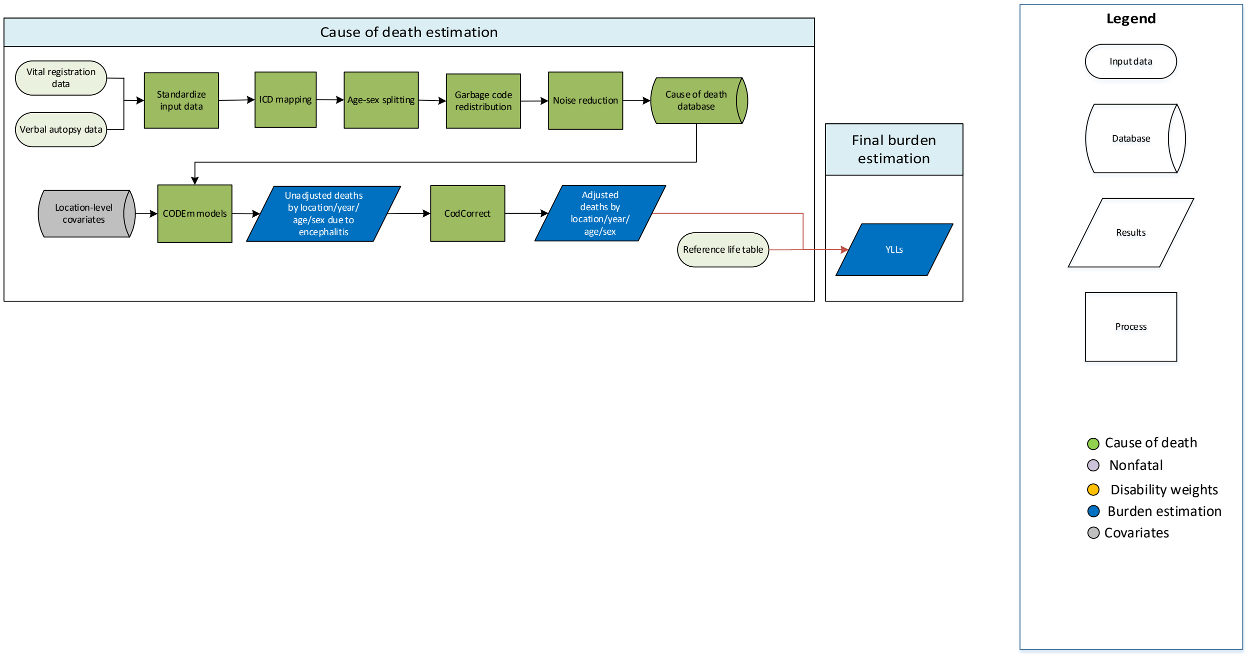

Input data

For GBD 2017, vital registration and verbal autopsy data were used to model this cause. We outliered data in instances where garbage code redistribution and noise reduction, in combination with small sample sizes, resulted in unreasonable cause fractions when compared to regional, super-regional, and global rates, and data that violated well-established time or age trends. Outliering methods were consistent across both vital registration and verbal autopsy data.

Modelling strategy

We modelled deaths due to encephalitis with a standard CODEm model using the cause of death database and location-level covariates as inputs. We hybridised separate global and data-rich models to acquire unadjusted results, which were adjusted using CodCorrect to reach final years of life lost (YLLs) due to encephalitis.

We previously used two separate age models for encephalitis, 0-5 years and 5-95. Starting in GBD 2015, we modelled encephalitis using the full age range in one model. Another significant change was the addition of the Japanese encephalitis covariate, which is a binary covariate indicating if the location is known to be endemic for Japanese encephalitis. The covariate was modelled according to data from the Centers for Disease Control and Prevention.[1] For GBD 2017, we updated the Japanese encephalitis covariate to include regions of Russia that are included as endemic regions in the CDC report. We also added the DTP3 coverage covariate to the model. A full list of covariate inputs in the published model can be found below. Covariates were weighted and selected based on the ensemble model process.

Covariate Name	Level	Direction
Japanese encephalitis binary	1	1
Underweight proportion	1	1
LDI	2	-1
Health system access	2	-1
Healthcare access and quality index	2	-1
DTP3 coverage	3	-1
Proportion of in-facility deliveries	3	-1
Sanitation (proportion with access)	3	-1
Water (proportion with access)	3	-1
Maternal education (years per capita)	3	-1
Socio-demographic Index	3	-1

References

(1) Centers for Disease Control (CDC). CDC health information for international travel 2016: the yellow book. New York City, United States: Oxford University Press, USA, 2016.

Diphtheria

Model flowchart

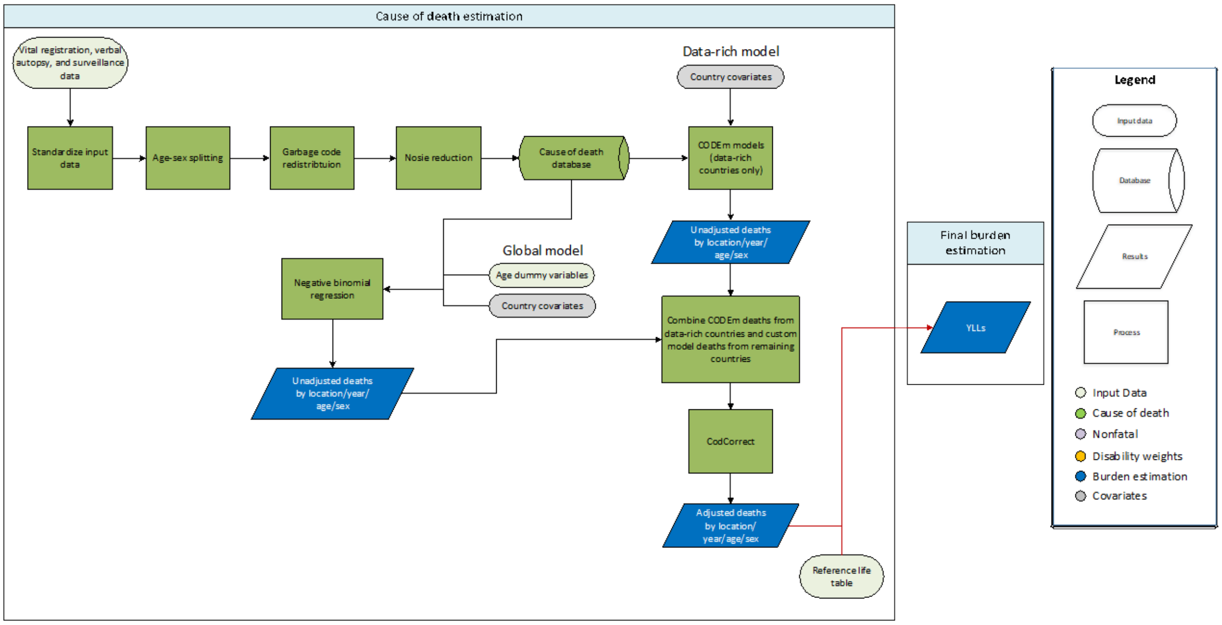

Modelling strategy overview

For this round of the GBD, we implemented two separate methods for modelling diphtheria mortality based on the quality of available vital registration data. For countries with well-defined vital registration (ie, “data-rich” countries), we used a cause of death ensemble model (CODEm) approach. For the remaining countries, we used a custom count model approach.

Data-rich countries

For data-rich countries, we used a CODEm strategy in count space to model vital registration (VR) data through time using the following country covariates:

Level	Covariate	Direction
1	Diphtheria-tetanus-pertussis third-dose vaccination coverage (DTP3)	-
	Health systems access (capped)	-
	Healthcare access and quality (HAQ) index	-
	Wasting (proportion under 2SD)	+
3	Lagged-distributed income (LDI)	-
	Socio-demographic Index (SDI)	-
	Mean years of education per capita	-

Models in count space had lower out-of-sample root mean squared error (RMSE) than rate-space models and were thus chosen as final models for these data-rich countries.

Custom count model

To inform the custom model of diphtheria mortality, vital registration and surveillance data from the cause of death database were used. Data with very high cause fractions (those greater than the 99th percentile values) were excluded.

Due to the small number of recorded deaths, diphtheria mortality was modelled using a negative binomial regression. Cause fractions representing number of deaths as a proportion of the all-cause mortality envelope were regressed on the diphtheria-pertussis-tetanus third-dose (DPT3) vaccine coverage covariate with dummy variables for each GBD age group, with death counts as the dependent variable and the offset as the total number of deaths:

$$Y_{ij} = \beta_0 + \beta_1\, DTP3_{ij} + age_{a\,ij} + e_{ij}\,,$$

where Y_{ij} is the log-transformed cause fraction, β_0 is the fixed-effect intercept, β_1 is the fixed-effects slope on vaccine coverage, $age_{a\,ij}$ is the dummy variable for each GBD age group in the estimation, e_{ij} is the residual, i is the year, and j is the location. The negative binomial model was used here over a Poisson count model because it more appropriately accounts for large variance (over-dispersion) in the data. Uncertainty was estimated by taking 1,000 iterations of the predictions based on the variance-covariance matrix and a random sample of the dispersion parameter from a gamma distribution.

Pertussis (whooping cough)

Model flowchart

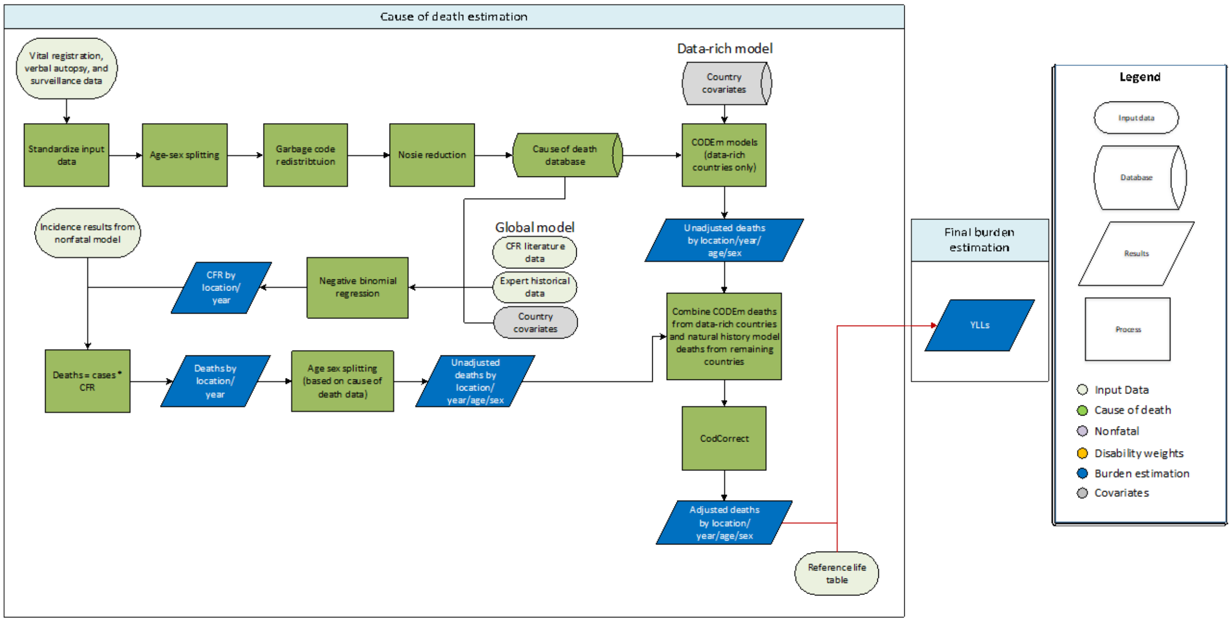

Modelling strategy overview

We implemented two separate methods for modeling pertussis mortality based on the quality of available vital registration data. For countries with well-defined vital registration (ie, "data-rich" countries), we used a cause of death ensemble model (CODEm) approach. For the remaining countries, we used a natural history model approach. For all countries, we estimated for the age range post-neonatal to 59 years.

Data-rich countries

For data-rich countries (ie, countries with vital registration more than 65% complete for all GBD estimation years, 1980–2017), we used a strategy in count space to model vital registration (VR) data through time using the following country covariates:

Level	Covariate	Direction
1	Diphtheria-tetanus-pertussis third-dose vaccination coverage (DTP3)	-
	Health system access (capped)	-
	Malnutrition	+
	Healthcare access and quality (HAQ) index	-
3	Lagged-distributed income (LDI)	-
	Socio-demographic Index (SDI)	-
	Mean years of education per capita	-

Models in count space (as opposed to rate space) had lower out-of-sample root mean squared error (RMSE) and were thus chosen as final models for these countries.

Natural history model

To inform the natural history model that was used as the modelling approach for countries without well-defined vital registration, we used data from the following sources: World Health Organization (WHO) case notifications; historical case notifications for the United Kingdom back to 1940; vital registration (VR) data in countries defined as "data-rich"; case fatality data identified by collaborators; and case fatality data identified through systematic literature reviews. The PubMed search query was: (whooping cough [Title/Abstract]) OR (pertussis [Title/Abstract]) AND (case fatality [Title/Abstract]). Studies were included if they reported case fatality rate, number of deaths, and number of cases. Studies were excluded if they included non-representative samples only.

Pertussis mortality in these remaining countries was modelled using a natural history-based model because CODEm does not predict well for countries without data.

First, we modelled log-transformed incidence from whooping cough case notifications reported to WHO (1985–2017) with diphtheria-tetanus-pertussis dose three (DTP3) vaccination coverage using the following equation:

$$Y_{ij} = \beta_0 + \beta_1\, DTP3_{ij} + u_j + e_{ij},$$

where Y_{ij} is the log-transformed incidence rate (in cases per 100,000 persons using WHO case notifications and GBD populations), β_0 is the fixed effect intercept, β_1 is the fixed effects slope on the log-transformed proportion of unvaccinated individuals, u_j is the country random effect, e_{ij} is the residual, i is the year, and j is the location. Historical data of UK pertussis cases and UK DTP3 coverage rates (both back to 1940) were also used to inform the incidence model. The random effect by country allowed for registration completeness to vary by country.

The results of this model were then used to predict incidence as a function of vaccine coverage. To correct for underreporting in case notifications, we used a value of the random effect that matched the highest random effect in a high-income region – Switzerland (which has a pertussis monitoring system which captures a high percentage of cases) – to get an implied attack rate assumed to be the same for all unvaccinated populations. Uncertainty was estimated by taking 1,000 iterations of the predictions based on the variance-covariance matrix.

Second, we modelled the pertussis case fatality rate using a negative binomial model with the healthcare access and quality (HAQ) index as a covariate:

$$Y_{ij} = \beta_0 + \beta_1 HAQ_{ij} + u_j + e_{ij},$$

where Y_{ij} is the number of deaths (using pertussis cases as the offset term); β_0 is the fixed-effect intercept; β_1, *is* the fixed-effects slopes on the healthcare access and quality (HAQ) index; u_j is country-level random effects; e_{ij} is the residual; i is the year; and j is the location. Uncertainty was estimated by taking 1,000 iterations of the predictions based on the variance-covariance matrix and a random sample from a gamma distribution of the dispersion parameter.

Finally, whooping cough deaths were calculated at the 1,000-draw level as

$$deaths = incidence * CFR \ .$$

We estimated overall number of deaths and then assigned an age-sex distribution based on the age- and sex-specific patterns found in the cause of death data.

Tetanus

Model flowchart

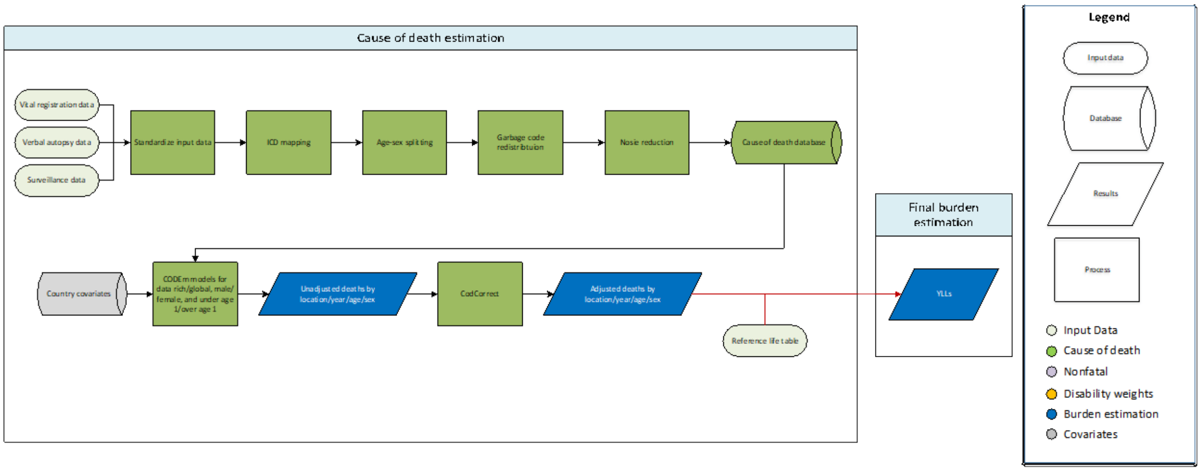

Input data

Mortality data from vital registration, verbal autopsy, and surveillance sources were used in tetanus cause of death models. Data were excluded if they largely conflicted with the majority of data from other studies conducted either in the same or different countries in the same region with similar sociodemographic characteristics.

Modelling strategy

A count-space cause of death ensemble modelling strategy (CODEm) was used. We ran separate models by age (under 1 year and 1 to 95+ years of age), sex (male and female), and data quality (data-rich and global). We used the following covariates for the under-1 models:

Level	Covariate	Direction
1	Diphtheria-tetanus-pertussis third-dose vaccination coverage (DTP3)	-
	Tetanus toxoid coverage	-
2	In-facility deliveries (proportion)	-
	Skilled birth attendance (proportion	-
	Health systems access (capped)	-
	Healthcare access and quality index (HAQ)	-
3	Lagged-distributed income (LDI)	-
	Socio-demographic Index (SDI)	-
	Mean years of education per capita	-

and the following covariates for the 1+ models:

Level	Covariate	Direction
1	Diphtheria-tetanus-pertussis third-dose vaccination coverage (DTP3)	-
2	Health systems access (capped)	-

	Healthcare access and quality index (HAQ)	-
3	Sanitation (proportion)	-
	Lagged-distributed income (LDI)	-
	Socio-demographic Index (SDI)	-
	Mean years of education per capita	-

Models in count space had lower out-of-sample root mean squared error (RMSE) than rate-space models and were thus chosen as final models for these data-rich countries.

Measles

Model flowchart

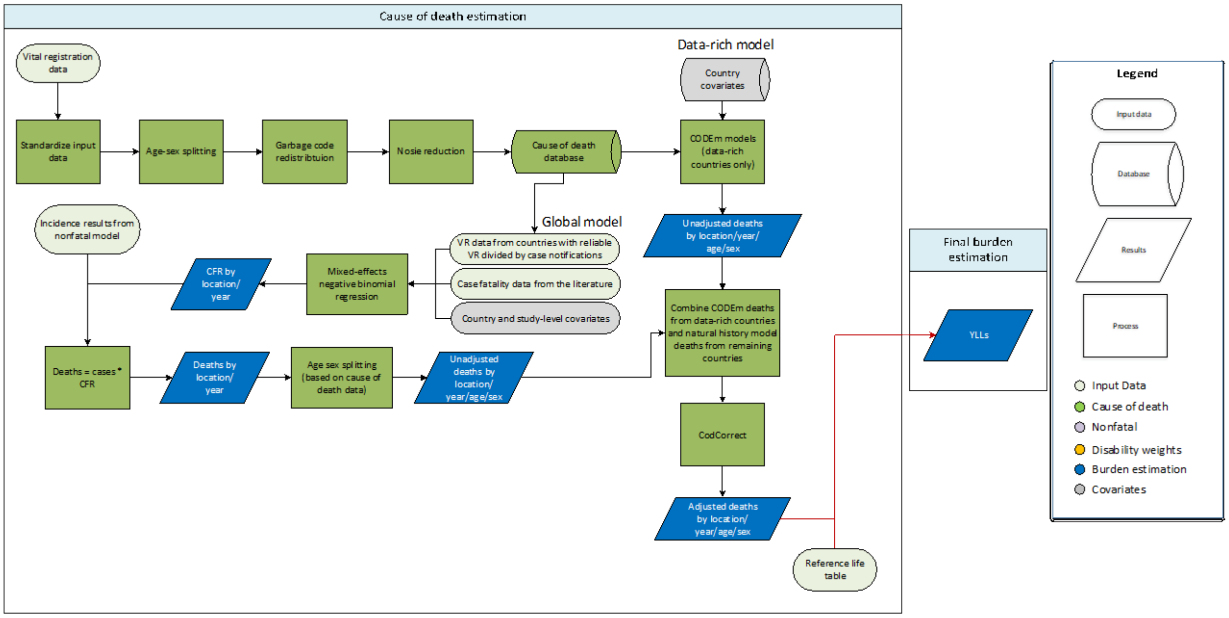

Modelling strategy overview

We implemented two separate methods for modelling measles mortality based on the quality of available vital registration data. For countries with well-defined vital registration (ie, “data-rich” countries), we used a cause of death ensemble model (CODEm) approach. For the remaining countries, we used a natural history model approach. For all countries, we estimated for the age range post-neonatal to 59 years.

Data-rich countries

For data-rich countries, we used a CODEm strategy in count space to model vital registration (VR) data through time using the following country covariates:

Level	Covariate	Direction
1	Measles-containing vaccination dose one (MCV1)	-
2	Healthcare access and quality (HAQ) index	-
3	Health systems access (capped)	-
	Socio-demographic Index (SDI)	-
	Mean years of education per capita	-

Covariates including measles-containing vaccination dose two (MCV2) coverage were excluded due to their collinearity with MCV1. Models in count space (as opposed to rate space) had lower out-of-sample root mean squared error (RMSE) and were thus chosen as final models for these countries.

Natural history model

To inform the natural history model, we used data from the following sources: World Health Organization (WHO) case notifications from 1995 to 2017 (most recently released in June 2018); additional case notification sources identified by collaborators (eg, Japan and USA subnational surveillance); vital registration (VR) data in countries with well-defined vital registration data; and case fatality data identified through systematic literature reviews. Studies were included in the literature review if they reported case fatality rate, number of deaths, and number of cases. Studies were excluded if they were conducted on non-representative samples.

Measles mortality in the non-data-rich countries was modelled using a natural-history-based model.

First, we modelled measles incidence with a mixed-effects linear regression of case notifications from WHO (1995–2017) on both doses of routine measles vaccination rates (MCV1 and MCV2) and supplementary immunization activity (SIA) coverage using the following equation:

$$Y_{ij} = \beta_0 + \beta_1 MCV1_{ij} + \beta_2 MCV2_{ij} + \beta_{a3} SIA_{aij} + u_j + e_{ij}\,,$$

where Y_{ij} is the log-transformed incidence rate (in cases per 100,000 persons using WHO case notifications and GBD populations); β_0 is the fixed-effect intercept; β_1, β_2, and β_{a3} are the fixed-effects slopes on the log-transformed proportion of population without the MCV1 vaccine, log-transformed proportion of population without the MCV2 vaccine, and supplementary vaccination coverage (administered doses over the target population of all under-15s) lagged by a=1-5 years, respectively; u_j is the super-region, region, and country-level random effects; e_{ij} is the residual; i is the year; and j is the location.

The results of this mixed effects regression model were then used to predict location-year-specific incidence as a function of routine vaccine coverage and SIAs. To correct for underreporting in case notifications, we added the effect of a 95% attack rate, assumed to be the same across all unvaccinated populations. Uncertainty was estimated by taking 1,000 iterations of the predictions based on the variance-covariance matrix. For locations in three super-regions – high-income, Central Europe/Eastern Europe/Central Asia and Latin America and Caribbean – we used reported measles cases as incident cases. More information on this part of the natural history model can be found in the non-fatal methods appendix for this round of the GBD.

Second, measles case fatality ratio was modelled using a mixed effects negative binomial regression using the Socio-demographic Index (SDI) as a country covariate and three indicators (hospital-based or not; outbreak or not; and rural or urban/mixed) as study-level covariates, with country random effects:

$$Y_{ij} = \beta_0 + \beta_1 SDI_{ij} + \beta_2 hospital_{ij} + \beta_3 outbreak_{ij} + \beta_3 rural_{ij} + u_j + e_{ij}\,,$$

where Y_{ij} is the number of deaths (using measles cases as the offset term); β_0 is the fixed-effect intercept; β_1, β_2, β_3, and β_4 are the fixed-effects slopes on the Socio-demographic Index (SDI) and hospital, outbreak, and rurality study-level covariates; u_j is country-level random effects; e_{ij} is the residual; i is the year; and j is the location. Uncertainty was estimated by taking 1,000 iterations of the predictions based on the variance-covariance matrix and uncertainty in country random effects. The fit of the model was evaluated using diagnostic plots of predicted versus observed values.

Finally, estimated deaths were calculated at the 1,000-draw level from the two sets of custom model predictions as:

$$deaths = incident\ cases * CFR\ .$$

We estimated overall number of deaths and then assigned an age-sex distribution based on the global-level age- and sex-specific patterns found in the cause of death data.

Varicella and Herpes Zoster

Model flowchart

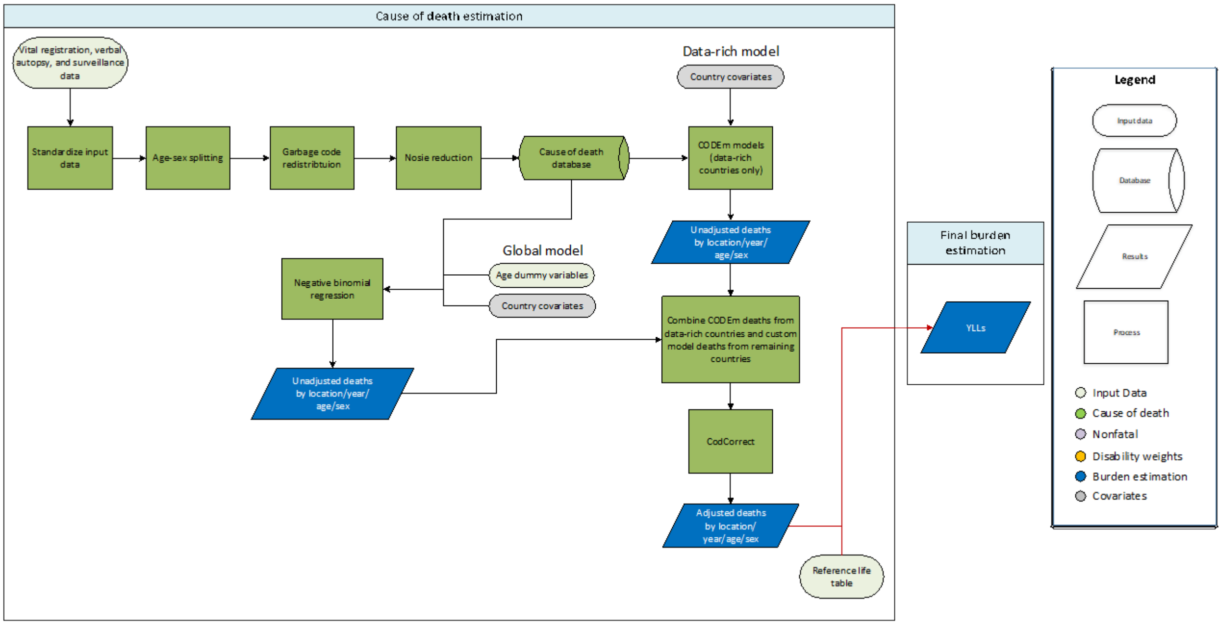

Modelling strategy overview

We implemented two separate methods for modelling varicella and herpes zoster mortality based on the quality of available vital registration data. For countries with well-defined vital registration (ie, "data-rich" countries), we used a cause of death ensemble model (CODEm) approach. For the remaining countries, we used a custom count model approach.

Data-rich countries

For data-rich countries, we used a CODEm strategy in count space to model vital registration (VR) data through time using the following country covariates:

Level	Covariate	Direction
1	Health systems access (capped)	0
	Healthcare access and quality (HAQ) index	0
	Malnutrition (proportion under 2SD)	+
3	Lagged-distributed income (LDI)	0
	Socio-demographic Index (SDI)	0

Models in count space had lower out-of-sample root mean squared error (RMSE) than rate-space models and were thus chosen as final models for these data-rich countries.

Custom count model

Since CODEm did not predict well in data-sparse areas, we used a custom model in count-space for countries without well-defined vital registration data. Vital registration, verbal autopsy, and surveillance

data from the cause of death database were used. Data with very high cause fractions (those greater than the 99th percentile values) were excluded in the negative binomial regression.

We used a negative binomial regression to model varicella mortality. Using the input data mentioned above, we modelled counts of deaths due to varicella using the healthcare access and quality (HAQ) index and age dummy variables with the offset set to the location- year- age- and sex-specific populations:

$$Y_{ij} = \beta_0 + \beta_1 \, HAQ_{ij} + age_{a\,ij} + e_{ij},$$

where Y_{ij} is the log-transformed cause fraction, β_0 is the fixed-effect intercept, β_1 is the fixed-effects slope on HAQ, $age_{a\,ij}$ is the dummy variable for each GBD age group in the estimation, e_{ij} is the residual, i is the year, and j is the location. Uncertainty was estimated by taking 1,000 iterations of the predictions based on the variance-covariance matrix and a random sample of the dispersion parameter from a gamma distribution.

Acute Hepatitis: Total and Virus-Specific

Overall estimation strategy:

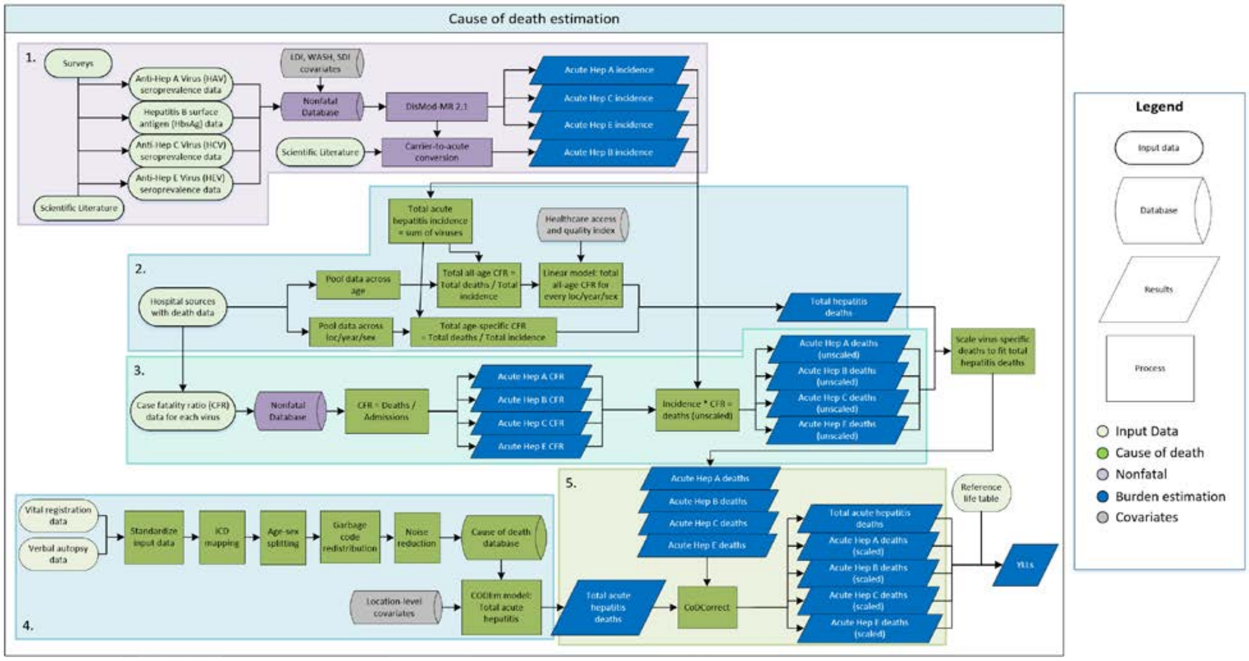

Virus-specific CoD data for acute hepatitis are inconsistently reported and of questionable accuracy. We therefore use a two-part modelling strategy for acute hepatitis A, B, C, and E. First, we develop a parent acute hepatitis mortality model using CODEm and all acute hepatitis mortality data within the CoD database (Part 4 above). Second, we develop four separate natural history models to estimate deaths from acute hepatitis A, B, C, and E (Parts 1–3). Finally, we rescale the virus-specific death estimates from the four natural history models to fit within the envelope defined by the parent acute hepatitis CoD model (Part 5).

Starting in GBD 2017, the virus-specific mortality modelling has changed in a few key ways. In GBD 2016, virus-specific case fatality ratio (CFR) was derived by calculating in-hospital case fatality ratio from Brazil hospital data and using published all-age population CFR from scientific literature to rescale the in-hospital CFR age pattern to a population CFR age pattern. Population CFR was multiplied by incidence to derive virus-specific deaths. The population CFR was specific to virus and age, but not location or year. In GBD 2017, we introduce a location- and year-specific CFR, which is then used to calculate virus-specific proportions of deaths as before.

Our new approach to calculating CFR is shown in parts two and three of the flowchart. We expanded the hospital data from Brazil to include 16 additional countries, and calculated virus-specific and age-specific CFR from this dataset by dividing deaths by admissions according to ICD code. Aggregating hospital deaths for all hepatitis ICD codes and dividing deaths by incidence from the non-fatal hepatitis models, we also modelled total hepatitis all-age CFR as a function of the healthcare access and quality (HAQ) index, thus deriving a location- and year-specific CFR. Aggregating hospital deaths across location, year, and sex in a separate step allowed us to calculate an age pattern for total hepatitis CFR, which was then

applied to the all-age CFR. The total hepatitis age-specific CFR was then multiplied by total incidence to get total deaths, and the virus-specific deaths were rescaled to fit within the total deaths envelope.

Total hepatitis

Input data

We modelled total hepatitis mortality using all available data in the cause of death database. Data points were outliered if they reported an improbable number of hepatitis deaths or if their inclusion in the model yielded distorted trends. In some cases, multiple data sources for the same location differed dramatically both in their quality and reported hepatitis mortality (eg, a verbal autopsy and vital registration source). In these cases, the lower-quality data source was outliered.

Modelling strategy

We modelled hepatitis mortality using a two-model hybrid approach: 1) a global CODEm model of all locations, using all data in the CoD database; and 2) a CODEm model restricted to data-rich countries. This model is represented as part four in the diagram above. Some covariates were changed in GBD 2017. We introduced a new covariate of hepatitis B vaccination, switched from an older measure of health system access to the healthcare access and quality index, and switched from proportion of unsafe water and unsafe sanitation to summary exposure values (SEV) of unsafe water and unsafe sanitation. The full covariate list is included below:

Level	Covariate	Direction	Transformation
1	SEV scalar (hepatitis)	+	
	Seroprevalence (HBsAg)	+	
	Seroprevalence (anti-HCV)	+	
	Seroprevalence (anti-HAV)	+	
	Seroprevalence (anti-HEV)	+	
2	HAQ index	-	
	SEV unsafe sanitation	+	
	SEV unsafe water	+	
	Socio-demographic Index	-	
	Hep B vaccine coverage (1 and 3 doses) proportion aged cohort	-	
3	Education (years per capita)	-	
	LDI	-	LN

Acute hepatitis A

Input data

We use anti-HAV seroprevalence data from population-based studies and surveys for the incidence model.

Modelling strategy

As outlined above, the hepatitis A model produces a proportion which is applied to the total hepatitis deaths. We estimate acute hepatitis A deaths as the product of our non-fatal incidence estimates and the case fatality rate:

$$Acute\ deaths = Acute\ incidence * \beta$$

Where:
$\beta = Case\ fatality\ rate$

Case fatality rate is derived by dividing the number of deaths due to acute hepatitis A by number of admissions (based on hospital data). This approach to calculating case fatality rate is a change from GBD 2016 (described in more detail in the introductory section on the overall modelling strategy). In GBD 2016, we used published estimates of acute hepatitis A case fatality [Rein et al, 2007[1]], sampling from a beta distribution to propagate uncertainty.

We model the prevalence of hepatitis A using a full DisMod model of anti-HAV seroprevalence. Given its reasonably stable force of infection among susceptible people across age groups, we derive incidence from the prevalence estimates using the following formula:

$$incid = \frac{-\ln(1 - prev)}{\mathrm{age_{mid}}} * (1 - prev)$$

This approach is a modification from GBD 2016, where we used a binomial generalised linear model with a complementary log-log link, and an offset term for log-age. That previous model used a predictive covariate derived from principal components analysis of lag-distributed income (LDI) and the proportion of the population with access to improved water.

Acute hepatitis B

Input data

We use hepatitis B surface antigen (HBsAg) seroprevalence data from population-based studies and surveys for the incidence model.

Modelling strategy

As outlined above, the hepatitis B model produces a proportion which is applied to the total hepatitis deaths. We estimate acute hepatitis B deaths as the product of our non-fatal incidence estimates and the case fatality rate:

$$Acute\ deaths = Acute\ incidence * \beta$$

Where:

$$\beta = Case\ fatality\ rate$$

Case fatality rate is derived by dividing the number of deaths due to acute hepatitis B by number of admissions (based on hospital data). This approach to calculating case fatality rate is a change from GBD 2016 (described in more detail in the introductory section on the overall modelling strategy). In GBD

2016, we used published estimates of acute hepatitis B case fatality [Bianco et al 2003[2]], sampling from a beta distribution to propagate uncertainty.

We model the incidence of chronic HBsAg carriage using a full DisMod model of HBsAg seroprevalence. We then convert incidence of chronic carriage to total incidence of hepatitis B infection by dividing age-specific estimates of the incidence of chronic carriage by age-specific estimates of the probability of infection resulting in carriage from Edmunds et al 1993[3]. We then multiply incident infection estimates by estimates of probability of acute incidence, derived from cases in McMahon et al 1997[4] and sampling from a beta distribution to propagate uncertainty.

$$Incident\ infection = \frac{Chronic\ carriage}{\beta_1}$$
$$Incident\ acute\ infection = Incidence\ infection\ *\ \beta_2$$

Where:
$\beta_1 = Probability\ of\ chronic\ infection$
$\beta_2 = Probability\ of\ acute\ infection$

Acute hepatitis C

Input data

We use anti-HCV seroprevalence data from population-based studies and surveys for the incidence model.

Modelling strategy

As outlined above, the hepatitis C model produces a proportion which is applied to the total hepatitis deaths. We estimate acute hepatitis C deaths as the product of our non-fatal incidence estimates and the case fatality rate:

$$Acute\ deaths = Acute\ incidence\ *\ \beta$$

Where:
$\beta = Case\ fatality\ rate$

Case fatality rate is derived by dividing the number of deaths due to acute hepatitis C by number of admissions (based on hospital data). This approach to calculating case fatality rate is a change from GBD 2016 (described in more detail in the introductory section on the overall modelling strategy). In GBD 2016, we used published estimates of acute hepatitis C case fatality [Stroffolini et al 1997[5]], sampling from a beta distribution to propagate uncertainty.

We model the incidence of hepatitis C using a full DisMod model of anti-HCV seroprevalence.

Acute hepatitis E

Input data

We use anti-HEV seroprevalence data from population-based studies and surveys for the incidence model.

Modelling strategy

As outlined above, the hepatitis E model produces a proportion which is applied to the total hepatitis deaths. We estimate acute hepatitis E deaths as the product of our non-fatal incidence estimates and the case fatality rate:

$$Acute\ deaths = Acute\ incidence * \beta$$

Where:

$\beta = Case\ fatality\ rate$

Case fatality rate is derived by dividing the number of deaths due to acute hepatitis E by number of admissions (based on hospital data). This approach to calculating case fatality rate is a change from GBD 2016 (described in more detail in the introductory section on the overall modelling strategy). In GBD 2016, we used published estimates of acute hepatitis E case fatality [Rein et al, 2012[6]], sampling from a beta distribution to propagate uncertainty.

We model the incidence of hepatitis E using a full DisMod model of anti-HEV seroprevalence.

Reference

1. Rein DB, Hicks KA, Wirth KE, Billah K, Finelli L, Fiore AE, et al. Cost-effectiveness of routine childhood vaccination for hepatitis a in the United States. Pediatrics. 2007;119:e12–21.
2. Bianco, E., et al. Case fatality rate of acute viral hepatitis in Italy: 1995–2000. An update. *Digestive and liver disease* 35.6 (2003): 404-408.
3. Stroffolini, T., et al. Prevalence, risk factors, and genotype distribution of hepatitis C virus infection in the general population: a community-based survey in southern Italy. *Hepatology* 26.4 (1997): 1006-1011.
4. Edmunds, W. J., et al. The influence of age on the development of the hepatitis B carrier state. *Proceedings of the Royal Society of London B: Biological Sciences* 253.1337 (1993): 197-201.
5. McMahon, Brian J. Epidemiology and natural history of hepatitis B. *Seminars in liver disease*. Vol. 25. No. S 1. Published in 2005 by Thieme Medical Publishers, Inc., 333 Seventh Avenue, New York, NY 10001, USA., 2005.
6. Rein DB, Stevens GA, Theaker J, Wittenborn JS, Wiersma ST. The global burden of hepatitis E virus genotypes 1 and 2 in 2005. *Hepatology* 2012; 55: 988–97.

Other Unspecified Infectious Diseases

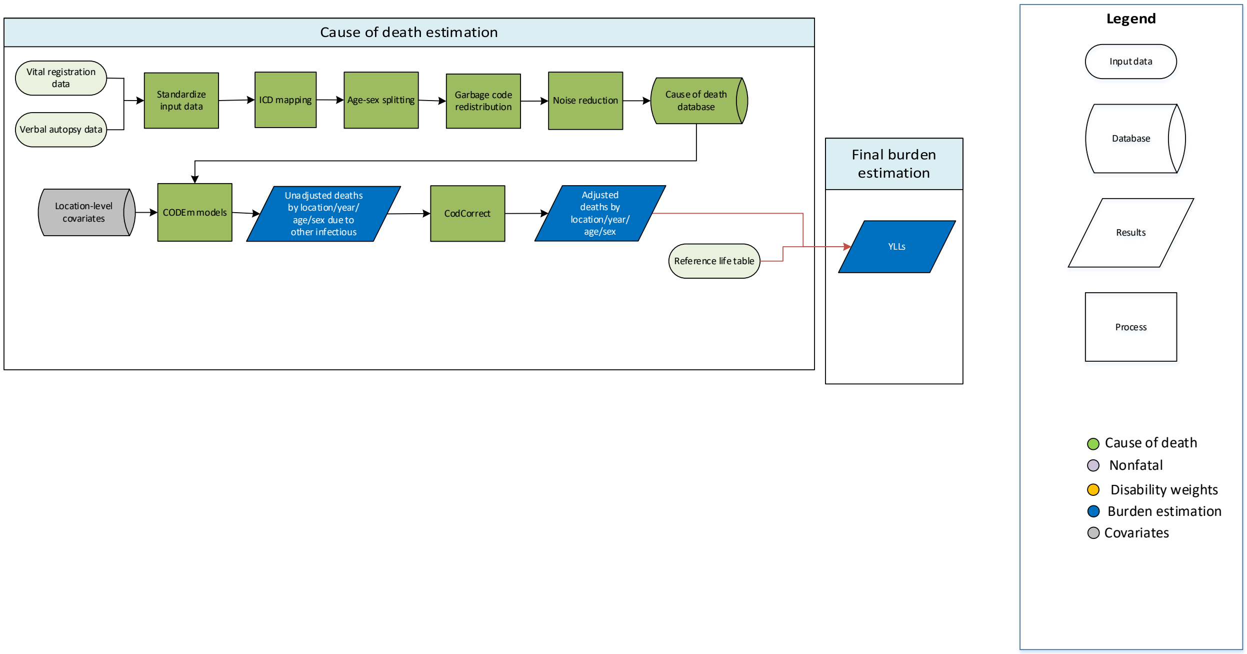

Input data

We modelled other infectious disease mortality using all available data in the cause of death database. Data points were outliered if they reported an improbable number of deaths or if their inclusion in the model yielded distorted trends. In some cases multiple data sources for the same location differed dramatically both in their quality and reported other infectious diseases mortality (eg, a verbal autopsy and vital registration source). In these cases the lower-quality data source was outliered.

Modelling strategy

We modeled other unspecified infectious disease mortality using a two-model hybrid approach: 1) a global CODEm model of all locations, using all data in the CoD database; and 2) a CODEm model restricted to data-rich countries.

The covariates used in the model include:

Covariate name	Level	Direction
ANC proportion	2	-
DPT3 coverage	1	-
Education	3	-
Health system access	1	-
Underweight	2	+
Measles vaccine coverage	1	-
Sanitation proportion	2	-
Clear water proportion	2	-

Socio-demographic Index	3	-

We have made no substantive changes to the modelling strategy since GBD 2016.

Maternal disorders

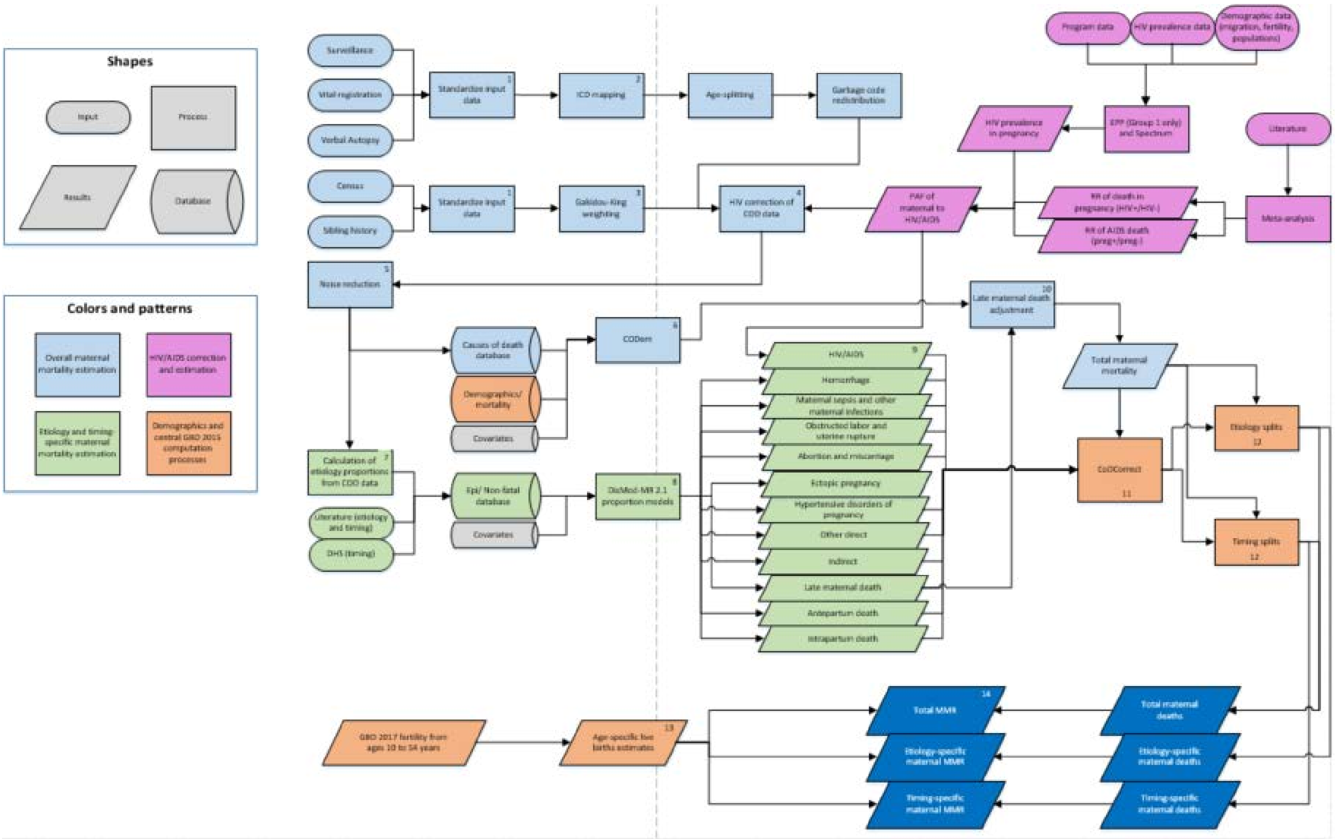

Input data

CODEm models were informed by centrally prepped data stored in the cause of death (COD) database. All data were corrected for incidental HIV deaths. Spectrum outputs of HIV prevalence in pregnancy were combined with relative risk of mortality during pregnancy (HIV-positive versus HIV-negative) to calculate population attributable fractions (PAFs). A proportion of these deaths are incidental and a proportion are maternal. PAFs were applied to all sibling history and census data to remove incidental HIV deaths. We performed an updated literature review to search for new scientific articles reporting data on maternal mortality, morbidity, and relative risk of mortality in pregnancy in HIV-positive versus HIV-negative women. We completed this search on July 20, 2017, using the following search string:

(((((("Postpartum Hemorrhage" OR "Uterine Hemorrhage") OR (maternal[Title/Abstract] OR pregnant[Title/Abstract] OR pregnancy[Title/Abstract] OR mothers) AND (haemorrhag*[Title/Abstract] OR hemorrhag*[Title/Abstract]) NOT "case report"[All fields]) OR (("induced abortion" OR "Therapeutic abortion" OR "legal Abortion" OR "medical abortion" OR "miscarriage" OR "Abortion, Induced"[Mesh] OR "Abortion, Therapeutic"[Mesh] OR "Abortion, Legal"[Mesh] OR "ectopic Pregnancy") NOT ("case report"[Title/Abstract] OR "birth defect"[Title/Abstract] OR congenital[Title/Abstract])) OR ("obstructed labour" OR "obstructed labor" OR "labour dystocia" OR "labor dystocia" OR dystocia OR "cephalopelvic disproportion" OR "cephalo-pelvic disproportion") OR (("obstetric fistula" OR "vesicovaginal fistula") OR "rectovaginal fistula") OR (("Puerperal Infection"[Mesh] OR "Puerperal Infection" OR ((maternal[Title/Abstract] OR pregnant[Title/Abstract] OR pregnancy[Title/Abstract]) AND (Sepsis OR infection[Title/Abstract]))) NOT "case report") OR ((Pre-Eclampsia[Title/Abstract] OR

preeclampsia[Title/Abstract] OR Eclampsia[Title/Abstract] OR Pre-Eclampsia[Mesh] OR Eclampsia[Mesh] OR "Hypertension, Pregnancy-Induced"[Mesh] OR "pregnancy induced hypertension"[Title/Abstract] OR "gestational hypertension"[Title/Abstract] OR "Hypertensive disorders of pregnancy"[Title/Abstract]) NOT ("case report" OR "kidney don*"[Title/Abstract] OR polymorphism*[Title/Abstract] OR endotheli*[Title/Abstract]))) AND (2016/08/01[PDat] : 2017/12/31[PDat]) NOT (animals[MeSH] NOT humans[MeSH]))) OR (((((("maternal mortality"[Title/Abstract] OR "maternal death"[Title/Abstract] OR "MM"[Title/Abstract] OR "confidential enquiry"[Title/Abstract] OR ((obstetric[Title/Abstract] OR pregnancy[Title/Abstract]) AND (etiology[Title/Abstract] OR cause[Title/Abstract] or pattern[Title/Abstract]) AND (death[Title/Abstract] OR mortality[Title/Abstract]))) NOT (fetal[Title/Abstract] OR newborns[Title/Abstract] OR newborn[Title/Abstract] OR neonatal[Title/Abstract] OR “case report”[Title/Abstract] OR “case study”[Title/Abstract] OR pathogenesis[Title/Abstract] OR thromboprophylaxis[Title/Abstract])) NOT (animals[MeSH] NOT humans[MeSH]) OR (((“maternal mortality”[Title/Abstract] OR “maternal death*”[Title/Abstract] OR “MMR”[Title/Abstract]) AND (“Afghanistan”[Title/Abstract] OR “Albania”[Title/Abstract] OR “Algeria”[Title/Abstract] OR “Andorra”[Title/Abstract] OR “Angola”[Title/Abstract] OR “Antigua and Barbuda”[Title/Abstract] OR “Argentina”[Title/Abstract] OR “Armenia”[Title/Abstract] OR “Azerbaijan”[Title/Abstract] OR “Bahrain”[Title/Abstract] OR “Bangladesh”[Title/Abstract] OR “Barbados”[Title/Abstract] OR “Belarus”[Title/Abstract] OR “Belize”[Title/Abstract] OR “Benin”[Title/Abstract] OR “Bhutan”[Title/Abstract] OR “Bolivia”[Title/Abstract] OR “Bosnia and Herzegovina”[Title/Abstract] OR “Botswana”[Title/Abstract] OR “Brazil”[Title/Abstract] OR “Brunei”[Title/Abstract] OR “Bulgaria”[Title/Abstract] OR “Burkina Faso”[Title/Abstract] OR “Burundi”[Title/Abstract] OR “Cambodia”[Title/Abstract] OR “Cameroon”[Title/Abstract] OR “Cape Verde”[Title/Abstract] OR “Central African Republic”[Title/Abstract] OR “Chad”[Title/Abstract] OR “China”[Title/Abstract] OR “Colombia”[Title/Abstract] OR “Comoros”[Title/Abstract] OR “Congo”[Title/Abstract] OR “Costa Rica”[Title/Abstract] OR “Croatia”[Title/Abstract] OR “Cuba”[Title/Abstract] OR “Cyprus”[Title/Abstract] OR “Côte d’Ivoire”[Title/Abstract] OR “Democratic Republic of the Congo”[Title/Abstract] OR “Djibouti”[Title/Abstract] OR “Dominica”[Title/Abstract] OR “Dominican Republic”[Title/Abstract] OR “Ecuador”[Title/Abstract] OR “Egypt”[Title/Abstract] OR “El Salvador”[Title/Abstract] OR “Equatorial Guinea”[Title/Abstract] OR “Eritrea”[Title/Abstract] OR “Ethiopia”[Title/Abstract] OR “Federated States of Micronesia”[Title/Abstract] OR “Fiji”[Title/Abstract] OR “Gabon”[Title/Abstract] OR “Georgia”[Title/Abstract] OR “Ghana”[Title/Abstract] OR “Grenada”[Title/Abstract] OR “Guatemala”[Title/Abstract] OR “Guinea”[Title/Abstract] OR “Guinea-Bissau”[Title/Abstract] OR “Guyana”[Title/Abstract] OR “Haiti”[Title/Abstract] OR “Honduras”[Title/Abstract] OR “India”[Title/Abstract] OR “Indonesia”[Title/Abstract] OR “Iran”[Title/Abstract] OR “Iraq”[Title/Abstract] OR “Jamaica”[Title/Abstract] OR “Jordan”[Title/Abstract] OR “Kazakhstan”[Title/Abstract] OR “Kenya”[Title/Abstract] OR “Kiribati”[Title/Abstract] OR “Kuwait”[Title/Abstract] OR “Kyrgyzstan”[Title/Abstract] OR “Laos”[Title/Abstract] OR “Latvia”[Title/Abstract] OR “Lebanon”[Title/Abstract] OR “Lesotho”[Title/Abstract] OR “Liberia”[Title/Abstract] OR “Libya”[Title/Abstract] OR “Lithuania”[Title/Abstract] OR “Macedonia”[Title/Abstract] OR “Madagascar”[Title/Abstract] OR “Malawi”[Title/Abstract] OR “Malaysia”[Title/Abstract] OR “Maldives”[Title/Abstract] OR “Mali”[Title/Abstract] OR “Malta”[Title/Abstract] OR “Marshall Islands”[Title/Abstract] OR “Mauritania”[Title/Abstract] OR “Mauritius”[Title/Abstract] OR “Moldova”[Title/Abstract] OR “Mongolia”[Title/Abstract] OR “Montenegro”[Title/Abstract] OR “Morocco”[Title/Abstract] OR “Mozambique”[Title/Abstract] OR

“Myanmar”[Title/Abstract] OR “Namibia”[Title/Abstract] OR “Nepal”[Title/Abstract] OR “Nicaragua”[Title/Abstract] OR “Niger”[Title/Abstract] OR “Nigeria”[Title/Abstract] OR “North Korea”[Title/Abstract] OR “Oman”[Title/Abstract] OR “Pakistan”[Title/Abstract] OR “Palestine”[Title/Abstract] OR “Panama”[Title/Abstract] OR “Papua New Guinea”[Title/Abstract] OR “Paraguay”[Title/Abstract] OR “Peru”[Title/Abstract] OR “Philippines”[Title/Abstract] OR “Qatar”[Title/Abstract] OR “Romania”[Title/Abstract] OR “Russia”[Title/Abstract] OR “Rwanda”[Title/Abstract] OR “Saint Lucia”[Title/Abstract] OR “Saint Vincent and the Grenadines”[Title/Abstract] OR “Samoa”[Title/Abstract] OR “Saudi Arabia”[Title/Abstract] OR “Senegal”[Title/Abstract] OR “Serbia”[Title/Abstract] OR “Seychelles”[Title/Abstract] OR “Sierra Leone”[Title/Abstract] OR “Singapore”[Title/Abstract] OR “Solomon Islands”[Title/Abstract] OR “Somalia”[Title/Abstract] OR “South Africa”[Title/Abstract] OR "South Sudan"[Title/Abstract] OR “Sri Lanka”[Title/Abstract] OR “Sudan”[Title/Abstract] OR “Suriname”[Title/Abstract] OR “Swaziland”[Title/Abstract] OR “Syria”[Title/Abstract] OR “São Tomé and Príncipe”[Title/Abstract] OR “Taiwan”[Title/Abstract] OR “Tajikistan”[Title/Abstract] OR “Tanzania”[Title/Abstract] OR “Thailand”[Title/Abstract] OR “The Bahamas”[Title/Abstract] OR “The Gambia”[Title/Abstract] OR “Timor-Leste”[Title/Abstract] OR “Togo”[Title/Abstract] OR “Tonga”[Title/Abstract] OR “Trinidad and Tobago”[Title/Abstract] OR “Tunisia”[Title/Abstract] OR “Turkmenistan”[Title/Abstract] OR “Uganda”[Title/Abstract] OR “Ukraine”[Title/Abstract] OR “United Arab Emirates”[Title/Abstract] OR “Uruguay”[Title/Abstract] OR “Uzbekistan”[Title/Abstract] OR “Vanuatu”[Title/Abstract] OR “Venezuela”[Title/Abstract] OR “Vietnam”[Title/Abstract] OR “Yemen”[Title/Abstract] OR “Zambia”[Title/Abstract] OR “Zimbabwe”[Title/Abstract]) NOT (animals[MeSH] NOT humans[MeSH]) NOT (“demographic and health survey*”[Title/Abstract] OR DHS[Title/Abstract] OR “reproductive health survey*”[Title/Abstract] OR RHS[Title/Abstract]))) AND (2016/08/15[PDat] : 2017/12/31[PDat]))))) OR ((HIV[Title/Abstract] OR "Acquired Immunodeficiency Syndrome"[Title/Abstract] OR AIDS[Title/Abstract]) AND (“pregnant”[Title/Abstract] OR “pregnancy”[Title/Abstract] OR “postpartum”[Title/Abstract] OR "post partum"[Title/Abstract]) AND (“mortality”[Title/Abstract] OR “death”[Title/Abstract]) NOT "case report" NOT (animals[MeSH] NOT humans[MeSH]) AND (2016/08/15[PDat] : 2017/12/31[PDat]))

All data from all geographies were reviewed in CODEm models. Outliers were identified as those data where age patterns or temporal patterns were inconsistent with neighbouring age groups or locations or where sparse data were predicting implausible overall temporal or age patterns for a given location.

We used scientific literature data identified through the search string above to inform DisMod-MR 2.1 aetiology proportion models as well as data from the COD database.

A total of 6,554 literature sources were reviewed for their title and abstract. Of those selected for full text review, 41 of them were extracted to inform maternal disorder models (fatal and non-fatal). All cause-specific maternal mortality data were prepped as “proportion” of total maternal deaths due to that cause. Because many sources do not include the entire cause list, a series of study covariates were used to facilitate crosswalking back to the reference definition. The reference definition ***includes*** “other” direct obstetric complications, indirect maternal deaths, and late maternal death. All aetiology-specific COD data were processed to be “proportion” data by calculating the cause-specific deaths divided by the total maternal deaths for the matching data source, year, age, and location. Late maternal death

data were only included for the subset of locations where they were reliably coded in raw VR. All data were uploaded to the non-fatal database.

Modelling strategy

Overall maternal mortality was estimated with CODEm. Covariates included in this model and their level and directionality are show in the table below:

Level	Covariate	Direction
Level 1	Age-specific fertility rate	+
	Total fertility rate (log-transformed)	+
	Maternal education (years per capita)	–
	In-facility delivery (proportion)	–
	Skilled birth attendance (proportion)	–
	Neonatal mortality ratio (log-transformed)	+
	Age-specific HIV mortality in females 10-54 (log-transformed)	+
Level 2	Antenatal care 1-visit coverage (proportion)	–
	Antenatal care 4-visits coverage (proportion)	–
	Age-standardised wasting (weight-for-height) summary exposure value	+
	Age-standardised stunting (height-for-age) summary exposure value	+
	Healthcare access and quality index	-
	Prevalence of obesity	+
Level 3		+
	Socio-demographic Index	–
	Mortality shock (cumulative rate in last 10 years)	+
	LDI (log-transformed)	–
	Hospital beds (per 1,000 population)	–

We used STGPR to estimate MMRs for each of the eight maternal subcauses. This modeling strategy requires data to be in standard GBD age groups. To achieve this, we used the age pattern of the COD data for each cause and applied it to the literature data that were not in the standard GBD age groups.

STGPR also requires variance for each data point. In order to compute variance we ran a Lowess regression on the data by year and used the variance of the residuals resulting from the difference between the data and the predicted values.

Country covariates were specific for each model and included abortion legality (for abortion and miscarriage as well as ectopic pregnancy), log-transformed lag-distributed income (other maternal deaths, and indirect maternal deaths), unsafe sanitation summary exposure value (for maternal sepsis and other maternal infections), Socio-demographic Index (for late maternal deaths), logit-transformed in-facility delivery proportion (for haemorrhage), mean systolic blood pressure (for hypertensive disorders of pregnancy), and age-specific underweight women (for obstructed labour).

Aetiology-specific estimates were derived by scaling the results from the STGPR subcause-specific models scaled in relation to each other to equal one and then multiplying them by the total maternal deaths, corrected for late maternal deaths, for that age group, location, and year. HIV-related maternal deaths were estimated for all locations using the PAF approach described above for mortality data processing. Incidental HIV deaths during pregnancy were by definition excluded.

Neonatal Disorders

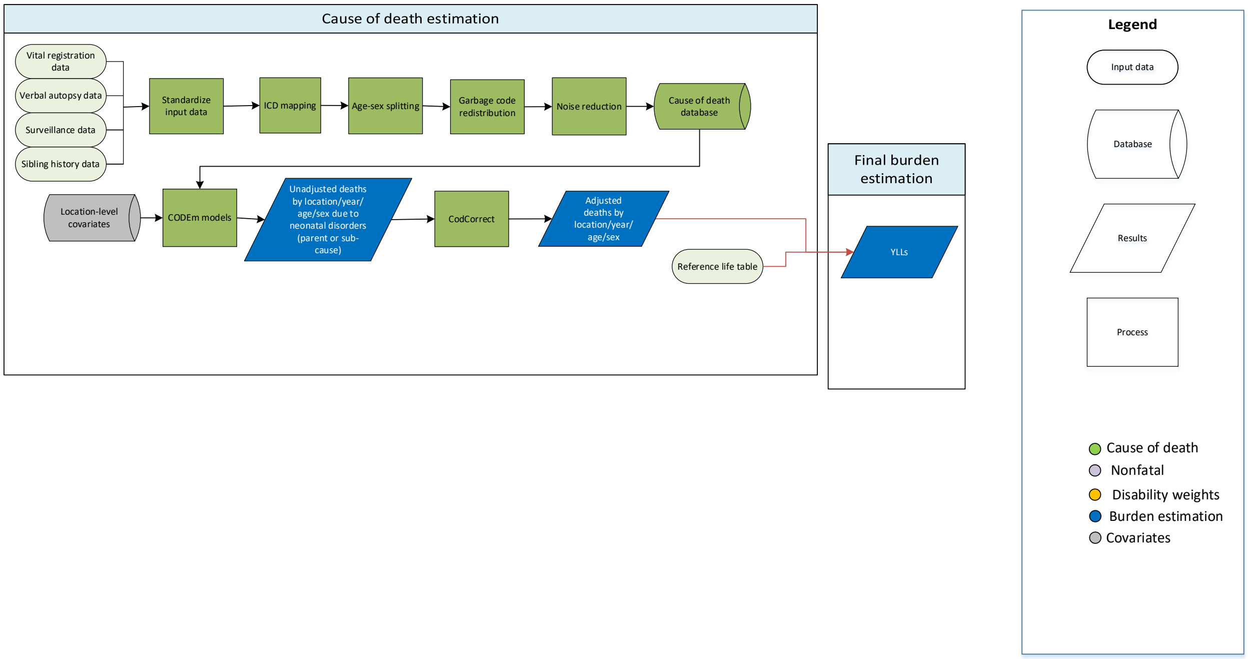

Input data

Mortality for five causes are modeled within "neonatal disorders": preterm birth complications, neonatal encephalopathy and birth trauma, neonatal sepsis and other infections, hemolytic disease and neonatal jaundice, and other neonatal disorders. An overall neonatal disorders "parent" envelope is also estimated, to which all neonatal causes are squeezed.

For the neonatal disorders envelope, preterm birth complications, neonatal encephalopathy and birth trauma, neonatal sepsis and other infections, hemolytic disease and neonatal jaundice, and other neonatal disorders, vital registration and surveillance were the majority of data sources used for GBD 2017 to estimate number of deaths from each condition. In Indian states, only verbal autopsy was used to inform estimates. Only deaths among males and females under age 5 were modelled, in four separate age groups: early neonatal period, late neonatal period, post-neonatal period, and 1-4 years. Data points were selected as outliers if they were implausibly high, low, or significantly conflicted with established age or temporal patterns.

Modelling strategy

For GBD 2017, the standard CODEm modelling approach was used to model each of the neonatal conditions. This same method was employed in GBD 2013, 2015, and 2016.

Varying levels of data quality and coding issues may still have affected our results. Validation studies suggest that verbal autopsy methods tend to be less accurate for cause of death ascertainment in the neonatal age groups.[1–4] Thus, for GBD 2017, except for the Indian states, the majority of verbal autopsy data were excluded.

All neonatal causes used the following pool of covariates in covariate selection:

Level	Covariate	Direction
1	Indoor air pollution (all cooking fuels)	+
	Smoking prevalence (reproductive age-standardized)	+
2	Antenatal care (4 visits) coverage (proportion)	-
	In-facility delivery (proportion)	-
	Live births 35+ (proportion)	+
	Skilled birth attendance (proportion)	-
	Health system access (capped)	-
	Healthcare access and quality index	-
	Age-standardised underweight (weight-for-age) SEV	+
3	Education (years per capita)	-
	Lag distributed income per capita (I$)	-
	Total fertility rate	+
	Socio-demographic Index	-

References

1 Anker M, Black RE, Coldham C, *et al.* A Standard Verbal Autopsy Method for Investigating Causes of Death in Infants and Children. Geneva, Switzerland: World Health Organization Department of Communicable Disease Surveillance and Response; The Johns Hopkins School of Hygiene and Public Health; The London School of Hygiene and Tropical Medicine, 1999.

2 Kalter HD, Gray RH, Black RE, Gultiano SA. Validation of postmortem interviews to ascertain selected causes of death in children. *Int J Epidemiol* 1990; **19**: 380–6.

3 Quigley MA, Armstrong Schellenberg JR, Snow RW. Algorithms for verbal autopsies: a validation study in Kenyan children. *Bull World Health Organ* 1996; **74**: 147–54.

4 Snow RW, Armstrong JR, Forster D, *et al.* Childhood deaths in Africa: uses and limitations of verbal autopsies. *The Lancet* 1992; **340**: 351–5.

Nutritional deficiencies: *Parent nutritional deficiencies, protein-energy malnutrition, and other nutritional deficiencies*

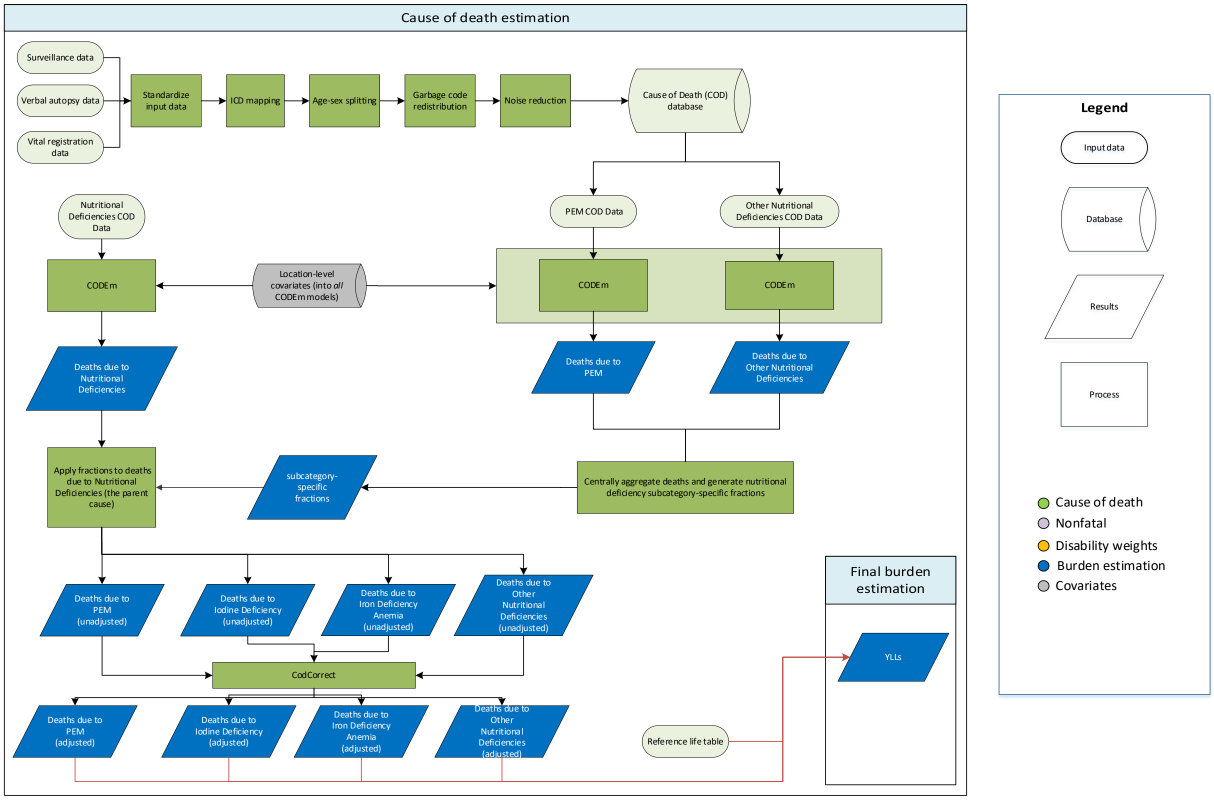

Input data and case definitions

For GBD 2017, vital registration, verbal autopsy, and surveillance data were used to model deaths due to nutritional deficiencies. As described in other sections, the volume of new data was significant. Notable additions include Sample Registration System (SRS) from states of India and provinces of Indonesia. ICD codes, which can be interpreted as case definitions, for each of the nutritional deficiencies is listed in the table below.

GBD cause	ICD-10 code
Protein-energy malnutrition	E40-E46.9 (Kwashiorkor, marasmus, specified and unspecified protein-calorie malnutrition)
Other nutritional deficiencies	D51-D52.0 (vitamin B12 deficiency anaemia and folate deficiency anaemia)
Other nutritional deficiencies	D52.8-D53.9 (other nutritional anaemias)
Other nutritional deficiencies	D64.3 (other sideroblastic anaemias)
Other nutritional deficiencies	E51-E61.9 (thiamine, niacin, other B group vitamins, ascorbic acid, vitamin D, other vitamin, dietary calcium, dietary selenium, dietary zinc, and other nutrient element deficiencies)

Other nutritional deficiencies	E63-E64.0 (other nutritional deficiencies and sequelae of protein-calorie malnutrition)
Other nutritional deficiencies	E64.2-E64.9 (sequelae of vitamin C deficiency, rickets, other nutritional deficiencies, and unspecified nutritional deficiencies)
Other nutritional deficiencies	M12.1-M12.19 (Kashin-Beck disease)
Garbage code	D50, D50.0 and D50.9 (unspecified anaemia)

Modelling strategy

Other than data and covariate updates, we did not make any modelling strategy changes for GBD 2017. Additionally, iodine deficiency and iron-deficiency anaemia, which were estimated as causes of death in previous GBDs, were changed to non-fatal-only causes for GBD 2017.

We estimated mortality for each of the nutritional deficiencies in two steps. CODEm was first used to generate mortality estimates for total nutritional deficiencies. The sub-categories of nutritional deficiencies, namely protein-energy malnutrition and other nutritional deficiencies, were modelled separately. We assumed zero mortality due to vitamin A deficiency, instead analysing it as a cause of non-fatal disease burden and a risk factor for mortality due to other causes. We outliered data that were largely conflicting with the majority of data from other studies conducted either in the same or different countries (with similar socio-demographic characteristics) in the same region.

CODEm was used to model all sub-categories except for iodine deficiency. The CODEm covariates (including level and direction) used for each of the models are listed in the table below.

**Nutritional deficiencies (overall)**		
**Level**	**Covariate**	**Direction**
1	Age-standardised prevalence of severe anaemia	+
	Malnutrition shock mortality rate	+
	Proportion of children 0-5 with weight-for-age z-score < -2	+
	Proportion of children 0-5 with weight-for-height z-score < -2	+
	Proportion of households using iodised salt	–
	Total kcal per person per day availability	–
	Proportion underweight (<20 yrs: <-2 BMI z-scores; >20 yrs: <17 BMI)	+
2	Population living in the 1st world quintile (least) of annual rainfall	+/–
	Population living in the 2nd world quintile (2nd least) of annual rainfall	+/–
	Unsafe sanitation SEV	+
	Mortality rate due to war shocks	+
	Unsafe water SEV	+
	Health Systems Access Capped	–
	Healthcare access and quality index	+
	Log-transformed SEV scalar: Diarrhoea	+
	Alcohol SEV (age, sex specific)	+
3	Education (years per capita)	–
	Lag distributed income per capita	–
	Socio-demographic Index	–
	Maternal education (years per capita)	–

Protein-energy malnutrition		
**Level**	**Covariate**	**Direction**
1	Age-standardised prevalence of severe anaemia	+
	Malnutrition shock mortality rate	+
	Proportion of children 0-5 with weight-for-height z-score < -2	+
	Total kcal per person per day availability	–
	Proportion underweight (<20 yrs: <-2 BMI z-scores; >20 yrs: <17 BMI)	+
2	Population living in the 1st world quintile (least) of annual rainfall	+/–
	Population living in the 2nd world quintile (2nd least) of annual rainfall	+/–
	Unsafe sanitation SEV	+
	Mortality rate due to war shocks	+
	Unsafe water SEV	+
	Health Systems Access Capped	–
	Healthcare access and quality index	+
	Log-transformed SEV scalar: Diarrhoea	+
	Alcohol SEV (age, sex specific)	+
3	Education (years per capita)	–
	Lag distributed income per capita	–
	Socio-demographic Index	–
**Other nutritional deficiencies**		
**Level**	**Covariate**	**Direction**
1	Age-standardised prevalence of severe anaemia	+
	Malnutrition shock mortality rate	+
	Proportion of children 0-5 with weight-for-age z-score < -2	+
	Total kcal per person per day availability	–
	Proportion underweight (<20 yrs: <-2 BMI z-scores; >20 yrs: <17 BMI)	+
2	Population living in the 1st world quintile (least) of annual rainfall	+/–
	Population living in the 2nd world quintile (2nd least) of annual rainfall	+/–
	Unsafe sanitation SEV	+
	Mortality rate due to war shocks	+
	Unsafe water SEV	+
	Health Systems Access Capped	–
	Healthcare access and quality index	+
	Log-transformed SEV scalar: Diarrhoea	+
	Alcohol SEV (age, sex specific)	+
3	Lag distributed income per capita	–
	Socio-demographic Index	–
	Maternal education (years per capita)	–

Estimates from the two nutritional sub-categories were then scaled at the 1,000 draw level in CODCorrect to match that for total nutritional deficiencies.

Cancers

Input data and methodological summary for all cancers except for non-melanoma skin cancer

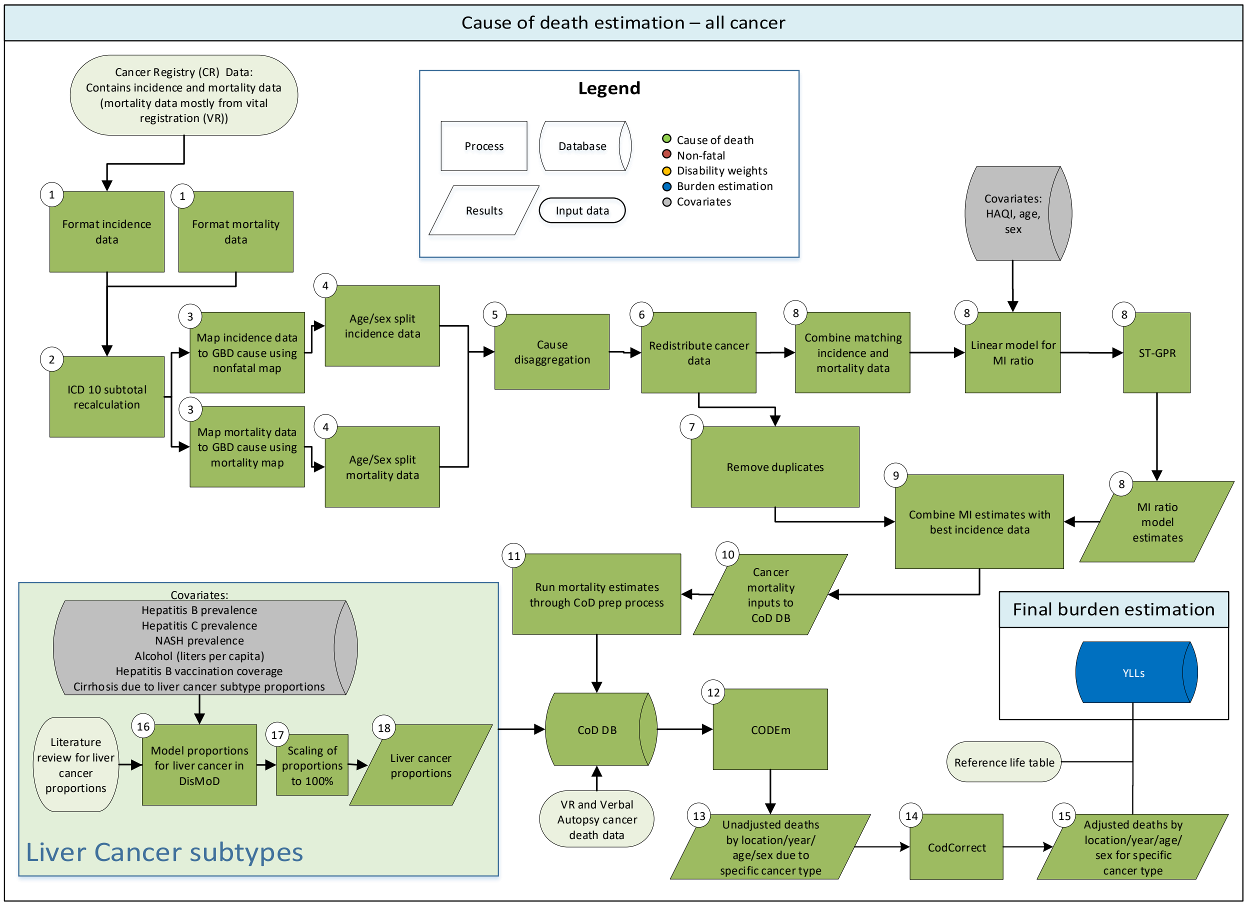

Abbreviations: ICD: International classification of diseases; DB: database, ST-GPR: Space-time smoothing, Gaussian process regression, COD: Causes of death

Data

The cause of death (COD) database contains multiple sources of cancer mortality data. These sources include vital registration, verbal autopsy, and cancer registry data. The cancer registry mortality estimates that are uploaded into the COD database stem from cancer registry incidence data that have been transformed to mortality estimates through the use of mortality-to-incidence ratios (MIR).

Data-seeking processes

Cancer mortality data in the cause of death database other than cancer registry data

Sources for cancer mortality data other than cancer registry data are described in the COD database description (Appendix Section 2).

Cancer registry data

Cancer registry data were used from publicly available sources or provided by collaborators. We used all data from GBD 2016 and added registry data from Russia, Iran, Ethiopia, Norway, as well as the newly released CI5 XI (Cancer Registry in Five Continents).

Inclusion and exclusion criteria

Only population-based cancer registries were included, and only those that included all cancers (no specialty registries), data for all age groups, and data for both sexes. Pathology-based cancer registries were included if they had a defined population. Hospital-based cancer registries were excluded. Cancer registry data were excluded from either the final incidence data input or the MI model input if a more detailed source (eg, providing more detailed age or diagnostic groups) was available for the same population. Preference was given to registries with national coverage over those with only local coverage, except those from countries where the GBD study provides subnational estimates. Data were excluded if the coverage population was unknown.

Bias of categories of input data

Cancer registry data can be biased in multiple ways. A high proportion of ill-defined cancer cases in the registry data requires redistribution of these cases to other cancers, which introduces a potential for bias. Changes between coding systems can lead to artificial differences in disease estimates; however, we adjust for this bias by mapping the different coding systems to the GBD causes. Underreporting of cancers that require advanced diagnostic techniques (eg, leukaemia and brain, pancreatic, and liver cancer) can be an issue in cancer registries from low-income countries. On the other hand, misclassification of metastatic sites as primary cancer can lead to overestimation of cancer sites that are common sites for metastases, like brain or liver. Since many cancer registries are located in urban areas, the representativeness of the registry for the general population can also be problematic. The accuracy of mortality data reported in cancer registries usually depends on the quality of the vital registration system. If the vital registration system is incomplete or of poor quality, the mortality-to-incidence ratio can be biased to lower ratios.

Data for liver cancer aetiology splits

For GBD 2017, the aetiologies for liver cancer were expanded to include a separate aetiology of liver cancer due to non-alcoholic steatohepatitis (NASH). To find the proportion of liver cancer cases due to the five aetiology groups included in GBD (1. Liver cancer due to hepatitis B, 2. Liver cancer due to hepatitis C, 3. Liver cancer due to alcohol, 4. Liver cancer due to NASH, 5. Liver cancer due to other causes), a systematic literature search was performed in PubMed on 10/24/2016 using the following search string: “("liver neoplasms"[All Fields] OR "HCC"[All Fields] OR "liver cancer"[All Fields] OR "Carcinoma, Hepatocellular"[Mesh]) AND (("hepatitis B"[All Fields] OR "Hepatitis B"[Mesh] OR "Hepatitis B virus"[Mesh] OR "Hepatitis B Antibodies"[Mesh] OR "Hepatitis B Antigens"[Mesh]) OR ("hepatitis C"[All Fields] OR "Hepatitis C"[Mesh] OR "hepatitis C antibodies"[MESH] OR "Hepatitis C Antigens"[Mesh] OR "Hepacivirus"[Mesh]) OR ("alcohol"[All Fields] OR "Alcohol Drinking"[Mesh] OR "Alcohol-Related Disorders"[Mesh] OR "Alcoholism"[Mesh] OR "Alcohol-Induced Disorders"[Mesh])) NOT (animals[MeSH] NOT humans[MeSH])”. Also, studies not found through this search but included in the meta-analysis by de Martel and colleagues were included.[10] We also included the study by Hong and colleagues after the authors provided us with additional data on the overlap in risk factors.[11]

Studies were included if the study population was representative of liver cancer population for the respective location. For each study, the proportions of liver cancer due to the five specific risk factors were calculated. Cases were considered to be due to NASH when the manuscript explicitly listed the aetiology to be NASH or non-alcoholic fatty liver disease (NAFLD). Cases where the aetiology was listed as “cryptogenic”, “idiopathic”, or “unknown” were included within the “other causes” category. In

manuscripts where the aetiology for a case was not known but major categories could not be ruled out (for example, the study tested for hepatitis B and C but did not assess alcohol use), these cases were excluded from the numerator of the study (in other words, did not contribute a proportion to any aetiology). Remaining risk factors were included under a combined "other" group (for example, haemochromatosis, autoimmune hepatitis, Wilson's disease, etc.). If multiple risk factors were reported for an individual patient, these were apportioned proportionally to the individual risk factors.

Methods

Steps of analysis and data transformation processes

Cancer registry data went through multiple processing steps before integration with the COD database. First, the original data were transformed into standardised files, which included standardisation of format, categorisation, and registry names (#1 in flowchart).

Second, some cancer registries report individual codes as well as aggregated totals (eg, C18, C19, and C20 are reported individually but the aggregated group of C18-C20 [colorectal cancer] is also reported in the registry data). The data processing step "subtotal recalculation" (#2 in flowchart) verifies these totals and subtracts the values of any individual codes from the aggregates.

In the third step (#3 in the flowchart), cancer registry incidence data and cancer registry mortality data are mapped to GBD causes. A different map is used for incidence and for mortality data because of the assumption that there are no deaths for certain cancers. One example is basal cell carcinoma of the skin. In the cancer registry incidence data, basal cell carcinoma is mapped to non-melanoma skin cancer (basal cell carcinoma). However, if basal cell skin cancer is recorded in the cancer registry mortality data, the deaths are instead mapped to non-melanoma skin cancer (squamous cell carcinoma) under the assumption that they were indeed misclassified squamous cell skin cancers. Other examples are benign or in situ neoplasms. Benign or in situ neoplasms found in the cancer registry incidence dataset were simply dropped from that dataset. The same neoplasms reported in a cancer registry mortality dataset were mapped to the respective invasive cancer (eg, melanoma in situ in the cancer registry incidence dataset was dropped from the dataset; melanoma in situ in the cancer registry mortality dataset was mapped to melanoma).

In the fourth data processing step (#4 in the flowchart) cancer registry data were standardised to the GBD age groups. Age-specific incidence rates were generated using all datasets that include microdata, and datasets that report age groups up to 95+ years of age, while age-specific mortality rates were generated from the CoD data through a method described in Appendix section 2.5. Age-specific proportions were then generated by applying the age-specific rates to a given registry population that required age-splitting to produce the expected number of cases/deaths for that registry by age. The expected number of cases/deaths for each sex, age, and cancer were then normalised to 1, creating final, age-specific proportions. These proportions were then applied to the total number of cases/deaths by sex and cancer to get the age-specific number of cases/deaths.

In the rare case that the cancer registry only contained data for both sexes combined, the now-age-specific cases/deaths were split and reassigned to separate sexes using the same weights that are used for the age-splitting process. Starting from the expected number of deaths, proportions were generated

by sex for each age (eg, if for ages 15 to 19 years old there are six expected deaths for males and four expected deaths for females, then 60% of the combined-sex deaths for ages 15-19 years would be assigned to males and the remaining 40% would be assigned to females).

In the fifth step (#5 in the flowchart) data for cause entries that are aggregates of GBD causes were redistributed. Examples of these aggregated causes include some registries reporting ICD10 codes C00-C14 together as "lip, oral cavity, and pharyngeal cancer." These groups were broken down into sub-causes that could be mapped to single GBD causes. In this example, those include lip and oral cavity cancer (C00-C08), nasopharyngeal cancer (C11), cancer of other parts of the pharynx (C09-C10, C12-C13), and "Malignant neoplasm of other and ill-defined sites in the lip, oral cavity, and pharynx" (C14). To redistribute the data, weights were created using the same "rate-applied-to-population" method employed in age-sex splitting (see step four above). For the undefined code (C14 in the example) an "average all cancer" weight was used, which was generated by adding all cases from SEER/NORDCAN/CI5 and dividing the total by the combined population. Then, proportions were generated by sub-cause for each aggregate cause as in the sex-splitting example above (see step four). The total number of cases from the aggregated group (C00-C14) was then recalculated for each subgroup and the undefined code (C14). C14 was then redistributed as a "garbage code" in step six. Distinct proportions were used for C44 (non-melanoma skin cancer) and C46 (Kaposi's sarcoma). Non-melanoma skin cancer processing is described under section "Input data and methodological summary for non-melanoma skin cancer (squamous-cell carcinoma)." C46 entries were redistributed as "other cancer," and HIV using proportions described in Appendix Section 2.
In the sixth step (#6 in the flowchart) unspecified codes ("garbage codes") were redistributed. Redistribution of cancer registry incidence and mortality data mirrored the process of the redistribution used in the cause of death database (Appendix Section 2.7).

In the seventh step (#7 in the flowchart) duplicate or redundant sources were removed from the processed cancer registry dataset. Duplicate sources were present if, for example, the cancer registry was part of the CI5 database but we also had data from the registry directly. Redundancies occurred and were removed as described in "Inclusion and Exclusion Criteria," where more detailed data were available, or when national registry data could replace regionally representative data. From here, two parallel selection processes were run to generate input data for the MI models and to generate incidence for final mortality estimation. Higher priority was given to registry data from the most standardised source when creating the final incidence input, whereas for the MI model input only sources that reported incidence and mortality were used.

In the eighth step (#8 in the flowchart) the processed incidence and mortality data from cancer registries were matched by cancer, age, sex, year, and location to generate MI ratios. These MI ratios were used as input for a three-step modelling approach using the updated GBD 2017 ST-GPR approach. with the Healthcare Access and Quality (HAQ) Index as a covariate in the linear step mixed effects model using a logit link function. This is different from GBD 2016, where we used Socio-demographic Index (SDI) as a predictive covariate. Predictions were made without the random effects. The ST-GPR model has three main hyper-parameters that control for smoothing across time, age, and geography. The time adjustment parameter (λ) was set to 2, which aims to borrow strength from neighbouring time points (ie, the exposure in this year is highly correlated with exposure in the previous year but less so further back in time). The age adjustment parameter ω was set to 0.5, which borrows strength from data in

neighbouring age groups. The space adjustment parameter ξ was set to 0.95 in locations with data and to 0.5 in locations without data (the higher ξ was applied when at least one age-sex group in the country of estimation had at least five unique data points. The lower ξ was applied when estimating data-scarce countries). Zeta aims to borrow strength across the hierarchy of geographical locations.[12] For the amplitude parameter in the Gaussian process regression we used 2, and for the scale we used a value of 15.

For GBD 2017 we slightly changed the data cleaning process and used HAQ rather than SDI to exclude data. For each cancer, MI ratios from locations in HAQ quintiles 1-4 were dropped if they were below the median of MI ratios from locations in HAQ quintile 5. We also dropped MI ratios from locations in HAQ quintiles 1-4 if the MI ratios were above the third quartile + 1.5 * IQR (inter-quartile range). We dropped all MIR that were based on less than 25 cases to avoid noise due to small numbers except for mesothelioma and acute lymphoid leukaemia, where we dropped MIR that were based on fewer than ten cases because of lower data availability for these two cancers. We also aggregated incidence and mortality to the youngest five-year age bin where we had at least 50 data points to avoid MIR predictions in young age groups that were based on few data points. The MIR in the age-bin that was used to aggregate MIR was used to backfill the MIR for younger age groups.

Since MI ratios can be above 1, especially in older age groups and cancers with low cure rates, we used the 95th percentile of the cleaned dataset that only included MIR that were based on 50 or more cases, to cap the MIR input data. This “upper cap” was used to allow MIR over 1 but to constrain the MIR to a maximum level. To run the logit model, the input data were divided by the upper caps and model predictions after ST-GPR was rescaled by multiplying them by the upper caps. To constrain the model at the lower end, we used the fifth percentile of the cancer-specific cleaned MIR input data to replace all model predictions with this lower cap.

Final MI ratios were matched with the cancer registry incidence dataset in the ninth step (#9 in the flowchart) to generate mortality estimates (Incidence * Mortality/Incidence = Mortality) (#10 in the flowchart). The final mortality estimates were then uploaded into the COD database (#11 in the flowchart). Cancer-specific mortality modelling then followed the general CODEm process.

Liver cancer aetiology split models

The proportion data found through the systematic literature review were used as input for five separate DisMod-MR 2.1 models to determine the proportion of liver cancers due to the five subgroups for all locations, both sexes, and all age groups (step #16 in the flowchart). A study covariate was used for publications that only assessed liver cancer in a cirrhotic population. The reference, or “gold standard”, that was used for crosswalking was the compilation of all studies that assessed the aetiology of liver cancer in a general population. For liver cancer due to hepatitis C and hepatitis B, a prior value of 0 was set between age 0 and 0.01. For liver cancer due to alcohol, a prior value of 0 was set for ages 0 to 5 years. For liver cancer due to hepatitis C, hepatitis C (IgG) seroprevalence was used as a covariate as well as a covariate for alcohol (litres per capita), hepatitis B prevalence (HBsAg seroprevalence), and NASH/NAFLD prevalence, forcing a negative relationship between the alcohol, hepatitis B, hepatitis C, and NASH/NAFLD covariates and the outcome of liver cancer due to alcohol proportion. For liver cancer due to hepatitis B, seroprevalence of HBsAg was used as a covariate as well as a covariate for alcohol, hepatitis C IgG seroprevalence, NASH/NAFLD prevalence, and the population coverage of three-dose

hepatitis B vaccination, forcing a negative relationship between these covariates and the outcome of liver cancer due to hepatitis B proportion. For liver cancer due to alcohol, alcohol (litres per capita) was used as a covariate as well as a covariate for proportion of alcohol abstainers, hepatitis B and hepatitis C seroprevalence, and NASH/NAFLD prevalence, forcing a negative relationship between the proportion of alcohol abstainers, NASH/NAFLD, and hepatitis B and hepatitis C covariates and the outcome of liver cancer due to alcohol proportion. For liver cancer due to NASH, NASH/NAFLD prevalence was used as a covariate as well as a covariate for obesity prevalence and mean body-mass index (BMI), forcing a positive relationship between these covariates and the outcome of liver cancer due to NASH proportion. All covariates used were modelled independently. To ensure consistency between cirrhosis and liver cancer estimates and to take advantage of the data for the respective other related cause (eg, liver cancer due to hepatitis C and the related cause cirrhosis due to hepatitis C), we generated covariates from the liver cancer proportion models that we used in the cirrhosis aetiology proportion models. We then created covariates from the cirrhosis aetiology proportion models and used those in the liver cancer aetiology models.

Since the proportion models are run independently of each other, the final proportion models were scaled to sum to 100% within each age, sex, year, and location, by dividing each proportion by the sum of the five (step # 17). For the liver cancer subtype mortality estimates, we multiplied the parent cause "liver cancer" by the corresponding scaled proportions (step # 18). Single-cause estimates were adjusted to fit into the separately modelled all-cause mortality in the process CoDCorrect.

Results

Interpretation of results

Cancer mortality estimates for GBD 2017 can differ from the GBD 2016 results for multiple reasons. Updated cancer mortality data were added from vital registration system data, verbal autopsy studies, as well as cancer registry incidence data. A new cause "Myelodysplastic, myeloproliferative, and other haematopoietic neoplasms" was added. In GBD 2016 all deaths due to this new cause were counted in the category "other neoplasms". The mortality-to-incidence ratio estimation has been updated compared to GBD 2016, using HAQ rather than SDI in the data cleaning and modelling process, and the ST-GPR approach was also updated. Covariate inputs for the CODEm models were changed based on recommendations from collaborators. Covariates used in CODEm models were updated for GBD 2017.

The other group producing country-level cancer mortality estimates is the International Agency for Research on Cancer (IARC) with their GLOBOCAN database. Significantly different methods between the GBD study and GLOBOCAN can lead to differences in results. Whereas estimates in GLOBOCAN are based on the assumption that there are "In theory, […] as many methods as countries,"[13] the cancer estimation process for the GBD study follows a coherent, well-documented method for all cancers, which allows cross-validation of models as well as determination of uncertainty. Another major difference is the ability in the GBD study to adjust single-cause estimates to the all-cause mortality, which is being determined independently. This also allows us to adjust individual causes of death to the all-cause mortality envelope, which permits us to correct for the underdiagnosis of cancer in countries with inadequate diagnostic resources. Redistribution of a fraction of undefined causes of death to certain cancers is another methodological advantage the GBD study has over GLOBOCAN, and estimates for cancer mortality can therefore differ substantially in countries with a large proportion of undefined

causes of deaths in their vital registration data or a large proportion of undefined cancer cases in their cancer registry data.

Limitations

There are certain limitations to consider when interpreting the GBD cancer mortality estimates. First, even though every effort is made to include the most recently available data for each country, data-seeking resources are not limitless and new data cannot always be accessed as soon as they are made available. It is therefore possible that the GBD study does not include all available data sources for cancer incidence or cancer mortality. Second, different redistribution methods can potentially change the cancer estimates substantially if the data sources used for the estimated location contain a large number of undefined causes; however, neglecting to account for these undefined deaths would likely introduce an even greater bias in the disease estimates. Third, using mortality-to-incidence ratios to transform cancer registry incidence data to mortality estimates requires accurate MIR. For GBD 2017 we have made further changes to the MIR estimation, but the method remains sensitive to underdiagnosis of cancer cases or underascertainment of cancer deaths. However, given that the majority of data used for the cancer mortality estimation come from vital registration data and not cancer registry data, this is not a major limitation.

Non-melanoma skin cancer (squamous cell carcinoma)

Data

Data seeking processes

Since squamous cell carcinomas are only very infrequently recorded by cancer registries, only vital registration system data were used as input for the squamous cell carcinoma mortality modelling.

Inclusion and exclusion criteria

Inclusion and exclusion criteria followed the same methods as described for the vital registration data sources (Appendix Section 2).

Bias of categories of input data

The potential biases of the input data are the same as for other cancers (see above).

Methods

Overall methodological process

Vital registration system data were used as input to model deaths due to squamous cell skin cancer.

Steps of analysis and data transformation processes

Since mortality estimates for non-melanoma skin cancer are only produced for squamous cell carcinoma under the assumption that basal cell carcinoma causes almost no deaths, all mortalities reported as “C44” or “173” were mapped to the “squamous cell carcinoma” GBD cause.

Model selection

The modelling strategy for non-melanoma skin cancer (squamous cell carcinoma) followed the general CODEm process.

Model performance and sensitivity

The modelling performance and sensitivity for non-melanoma skin cancer (squamous cell carcinoma) mirrored that of the general CODEm process.

Uncertainty intervals

Uncertainty was determined using standard CODEm methodology.

Results

Interpretation of results

Non-melanoma skin cancer mortality estimates are not available from other sources. GLOBOCAN, for example, does not report deaths due to non-melanoma skin cancer. Even though the data availability for non-melanoma skin cancer is poor, the fact that it is the most common incident cancer, with rates expected to rise, makes it a necessity to include the disease in the GBD framework.

Limitations

Cancer registry data for non-melanoma skin cancer incidence have to be interpreted with caution due to a substantial amount of underreporting or rules that only the first non-melanoma skin cancer has to be registered. Many cancer registries therefore do not include non-melanoma skin cancers at all. However, information on whether registries capture NMSC or not is not consistently available. Therefore, no cancer registry data were used to estimate deaths due to squamous cell carcinoma of the skin. For vital registration data, we make the assumption that there are no deaths due to basal cell non-melanoma skin cancer; therefore, all deaths attributed to basal cell carcinoma were included instead as squamous cell carcinoma.

Covariates by cancer:

Lip and oral cavity cancer

Level	Covariate	Direction
1	*Alcohol (litres per capita)*	+
	Cumulative cigarettes (5 years)	+
	Cumulative cigarettes (10 years)	+
	Cumulative cigarettes (15 years)	+
	Cumulative cigarettes (20 years)	+
	Smoking prevalence	+
	Tobacco (cigarettes per capita)	+
	Log-transformed SEV scalar: Mouth C	+
	Vegetables adjusted (g)	–
2	*Red meats adjusted (g)*	+
	Fruits adjusted (g)	–
	*Health system access 2 (unitless)**	–
	Healthcare access and quality index	–
3	*Education (years per capita)*	–
	LDI (I$ per capita)	0
	Socio-demographic Index	0

Nasopharynx cancer

Level	Covariate	Direction
1	*Alcohol (litres per capita)*	+
	Cumulative cigarettes (5 years)	+
	Cumulative cigarettes (10 years)	+
	Cumulative cigarettes (15 years)	+
	Cumulative cigarettes (20 years)	+
	Smoking prevalence	+
	Tobacco (cigarettes per capita)	+
	Log-transformed SEV scalar: Nasoph C	+
	Vegetables adjusted (g)	–
2	*Red meats adjusted (g)*	+
	Fruits adjusted (g)	–
	*Health system access 2 (unitless)**	–
	Healthcare access and quality index	–
3	*Education (years per capita)*	–
	LDI (I$ per capita)	0
	Socio-demographic Index	0

Oesophageal cancer

Level	Covariate	Direction
1	*Alcohol (litres per capita)*	+
	Log-transformed SEV scalar: Esophag C	+
	Log-transformed age-standardized SEV scalar: Esophag C	+
	Mean BMI	+
	Smoking prevalence	+
	Tobacco (cigarettes per capita)	+
	Fruits adjusted (g)	-
2	*Indoor air pollution (all cooking fuels)*	+
	Sanitation (proportion with access)	–
	Vegetables adjusted (g)	–
	Improved water source (proportion with access)	–
	Healthcare access and quality index	–
3	*Education (years per capita)*	–
	LDI (I$ per capita)	0
	Socio-demographic Index	0

Other pharynx cancer

Level	Covariate	Direction
1	*Alcohol (litres per capita)*	+
	Smoking prevalence	+
	Log-transformed SEV scalar: Oth Phar C	+
	Vegetables adjusted (g)	–
2	*Cumulative cigarettes (5 years)*	+
	Fruits adjusted (g)	–
	Vegetables adjusted (g)	–
	Population density (over 1000 ppl/sqkm, proportion)	+
	Population density (under 150 ppl/sqkm, proportion)	+
	Healthcare access and quality index	–
3	*Education (years per capita)*	–
	LDI (I$ per capita)	0
	Socio-demographic Index	0

Stomach cancer

Level	Covariate	Direction
1	*Diet high in sodium*	+
	Cumulative cigarettes (5 years)	+
	Cumulative cigarettes (10 years)	+
	Cumulative cigarettes (15 years)	+
	Cumulative cigarettes (20 years)	+
	Smoking prevalence	+
	Tobacco (cigarettes per capita)	+
	Log-transformed SEV scalar: Stomach C	+
	SEV unsafe water	+
	SEV unsafe sanitation	+
2	*Vegetables adjusted (g)*	–
	Fruits adjusted (g)	–
	Mean BMI	+
	Sanitation (proportion with access)	–
	Improved water source (proportion with access)	–
	Healthcare access and quality index	–
3	*Education (years per capita)*	–
	LDI (I$ per capita)	0
	Socio-demographic Index	0

Colon and rectum cancer

Level	Covariate	Direction
1	*Alcohol (litres per capita)*	+
	Mean BMI	+
	Smoking prevalence	+
	Tobacco (cigarettes per capita)	+
	Log-transformed SEV scalar: Colorect C	+
	Red meats adjusted (g)	+
2	*Milk adjusted (g)*	–
	Fruits adjusted (g)	–
	Nuts seeds adjusted (g)	–
	PUFA adjusted (percent)	–
	Vegetables adjusted (g)	–
	Cumulative cigarettes (5 years)	+
	Cumulative cigarettes (10 years)	+
	Cumulative cigarettes (15 years)	+
	Cumulative cigarettes (20 years)	+
	Diabetes age-specific prevalence (proportion)	+
	Healthcare access and quality index	–
3	*Education (years per capita)*	–
	LDI (I$ per capita)	0
	Socio-demographic Index	0

Liver cancer

Level	Covariate	Direction
1	*Alcohol (litres per capita)*	+
	HIV age-standardised prevalence	+
	Hepatitis B (HBsAg) seroprevalence	+
	Hepatitis C (IgG) seroprevalence	+
	Log-transformed SEV scalar: Liver C	+
2	*Hepatitis B 3-dose coverage (proportion)*	–
	Hepatitis B 3-dose coverage (proportion), lagged 5 years	–
	Hepatitis B 3-dose coverage (proportion), lagged 10 years	–
	Hepatitis B vaccine coverage (proportion), aged through time	–
	Intravenous drug use (age-standardised proportion)	+
	Cumulative cigarettes (5 years)	+
	Cumulative cigarettes (10 years)	+
	Cumulative cigarettes (15 years)	+
	Cumulative cigarettes (20 years)	+
	Diabetes age-specific prevalence (proportion)	+
	Diabetes fasting plasma glucose (mmol/L)	+

Liver cancer, continued

Level	Covariate	Direction
2	*Mean BMI*	+
	Tobacco (cigarettes per capita)	+
	Red meats adjusted (g)	+
	Healthcare access and quality index	–
3	*Education (years per capita)*	–
	LDI (I$ per capita)	0
	Socio-demographic Index	0

Gallbladder and biliary tract cancer

Level	Covariate	Direction
1	*Log-transformed SEV scalar: Gallblad C*	+
	Mean BMI	+
2	*Alcohol (litres per capita)*	+
	Cumulative cigarettes (5 years)	+
	Cumulative cigarettes (10 years)	+
	Smoking prevalence	+
	Tobacco (cigarettes per capita)	+
	Fruits adjusted (g)	–
	Vegetables adjusted (g)	–
	Diabetes age-standardised prevalence (proportion)	+
	Healthcare access and quality index	–
3	*Education (years per capita)*	–
	LDI (I$ per capita)	0
	Socio-demographic Index	0

Pancreatic cancer

Level	Covariate	Direction
1	Alcohol (litres per capita)	+
	Cumulative cigarettes (5 years)	+
	Cumulative cigarettes (10 years)	+
	Cumulative cigarettes (15 years)	+
	Cumulative cigarettes (20 years)	+
	Smoking prevalence	+
	Tobacco (cigarettes per capita)	+
	Log-transformed SEV scalar: Pancreas C	+
	Mean BMI	+
2	Red meats adjusted (g)	+
	Fruits adjusted (g)	–
	Vegetables adjusted (g)	–
	Energy unadjusted (kcal)	+
	Diabetes fasting plasma glucose (mmol/L)	+
	Diabetes age-standardised prevalence (proportion)	+
	Healthcare access and quality index	–
3	Education (years per capita)	–
	LDI (I$ per capita)	0
	Socio-demographic Index	0

Larynx cancer

Level	Covariate	Direction
1	Alcohol (litres per capita)	+
	Log-transformed SEV scalar: Larynx C	+
2	Smoking prevalence	+
	Tobacco (cigarettes per capita)	+
	Asbestos consumption (metric tons per year per capita)	+
	Fruits adjusted (g)	–
	Vegetables adjusted (g)	–
	Cumulative cigarettes (5 years)	+
	Cumulative cigarettes (10 years)	+
	Cumulative cigarettes (15 years)	+
	Cumulative cigarettes (20 years)	+
	Population density (over 1000 ppl/sqkm, proportion)	+
	Population density (under 150 ppl/sqkm, proportion)	+
	Healthcare access and quality index	–
3	Education (years per capita)	–
	LDI (I$ per capita)	0
	Socio-demographic Index	0

Tracheal, bronchus, and lung cancer

Level	Covariate	Direction
1	Alcohol (litres per capita)	+
	Cumulative cigarettes (5 yYears)	+
	Cumulative cigarettes (10 years)	+
	Cumulative cigarettes (15 years)	+
	Cumulative cigarettes (20 years)	+
	Smoking prevalence	+
	Tobacco (cigarettes per capita)	+
	Secondhand smoke	+
	Log-transformed SEV scalar: Lung C	+
	Log-transformed age-standardised SEV scalar: Lung C	+
2	Indoor air pollution (all cooking fuels)	+
	Outdoor air pollution ($PM_{2.5}$)	+
	Residential radon	+
	Diabetes fasting plasma glucose (mmol/L)	+
	Healthcare access and quality index	–
3	Education (years per capita)	–
	LDI (I$ per capita)	0
	Socio-demographic Index	0

Malignant skin melanoma

Level	Covariate	Direction
1	Alcohol (litres per capita)	+
2	Fruits adjusted (g)	–
	Vegetables adjusted (g)	–
	Latitude under 15 (proportion)	–
	Latitude 15 to 30 (proportion)	0
	Latitude 30 to 45 (proportion)	–
	Latitude over 45 (proportion)	–
	Healthcare access and quality index	–
3	Education (years per capita)	–
	LDI (I$ per capita)	0
	Socio-demographic Index	0

Non-melanoma skin cancer

Level	Covariate	Direction
1	Cumulative cigarettes (5 years)	+
	Cumulative cigarettes (10 years)	+
	Cumulative cigarettes (15 years)	+
	Smoking prevalence	+
2	Average latitude	0
	Healthcare access and quality index	–
3	Education (years per capita)	0
	LDI (I$ per capita)	0
	Socio-demographic Index	0

Breast cancer

Level	Covariate	Direction
1	Alcohol (litres per capita)	+
	Mean BMI	+
	Log-transformed SEV scalar: Breast C	+
2	Age-specific fertility rate	–
	Total fertility rate	–
	Fruits adjusted (g)	–
	Vegetables adjusted (g)	–
	Cumulative cigarettes (5 years)	+
	Cumulative cigarettes (10 years)	+
	Cumulative cigarettes (15 years)	+
	Cumulative cigarettes (20 years)	+
	Smoking prevalence	+
	Secondhand smoke	+
	Diabetes fasting plasma glucose (mmol/L)	+
	Healthcare access and quality index	–
3	Education (years per capita)	–
	LDI (I$ per capita)	0
	Socio-demographic Index	0

Cervical cancer

Level	Covariate	Direction
1	*Cumulative cigarettes (5 years)*	+
	Cumulative cigarettes (10 years)	+
	Cumulative cigarettes (15 years)	+
	HIV age-standardised prevalence	+
2	*Age-specific fertility rate*	+
	Total fertility rate	+
	Smoking prevalence	+
	Fruits adjusted (g)	–
	Vegetables adjusted (g)	–
	Healthcare access and quality index	–
3	*Education (years per capita)*	–
	LDI (I$ per capita)	0
	Socio-demographic Index	0

Uterine cancer

Level	Covariate	Direction
1	*Log-transformed SEV scalar: Uterus C*	+
	Mean BMI	+
2	*Cumulative cigarettes (5 years)*	+
	Cumulative cigarettes (10 years)	+
	Smoking prevalence	+
	Tobacco (cigarettes per capita)	+
	Diabetes age-standardized prevalence (proportion)	+
	Total fertility rate	0
	Fruits adjusted (g)	–
	Vegetables adjusted (g)	–
	Healthcare access and quality index	–
3	*Education (years per capita)*	–
	LDI (I$ per capita)	0
	Socio-demographic Index	0

Prostate cancer

Level	Covariate	Direction
1	*Log-transformed SEV scalar: Prostate C*	+
2	*Smoking prevalence*	0
	Healthcare access and quality index	–
3	*Education (years per capita)*	0
	LDI (I$ per capita)	0
	Socio-demographic Index	0

Ovarian cancer

Level	*Covariate*	*Direction*
1	*Alcohol (litres per capita)*	+
	Cumulative cigarettes (5 years)	+
	Cumulative cigarettes (10 years)	+
	Cumulative cigarettes (15 years)	+
	Cumulative cigarettes (20 years)	+
	Tobacco (cigarettes per capita)	+
	Contraception (modern) prevalence (proportion)	–
	Log-transformed SEV scalar: Ovary C	+
2	*Asbestos consumption (metric tons per year per capita)*	+
	Smoking prevalence	+
	Total fertility rate	0
	Energy unadjusted (kcal)	+
	Fruits adjusted (g)	–
	Vegetables adjusted (g)	–
	Mean BMI	+
	Diabetes age-standardised prevalence (proportion)	+
	Diabetes fasting plasma glucose (mmol/L)	+
	Healthcare access and quality index	–

Ovarian cancer, continued

3	*Education (years per capita)*	–
	LDI (I$ per capita)	0
	Socio-demographic Index	0

Testicular cancer

Level	*Covariate*	*Direction*
2	*Cumulative cigarettes (5 years)*	+
	Cumulative cigarettes (10 years)	+
	Cumulative cigarettes (15 years)	+
	Cumulative cigarettes (20 years)	+
	Tobacco (cigarettes per capita)	+
	Smoking prevalence	+
	Fruits adjusted (g)	–
	Vegetables adjusted (g)	–
	Healthcare access and quality index	–
3	*Education (years per capita)*	–
	LDI (I$ per capita)	0
	Socio-demographic Index	0

Kidney cancer

Level	Covariate	Direction
1	*Cumulative cigarettes (5 years)*	+
	Cumulative cigarettes (10 years)	+
	Cumulative cigarettes (15 years)	+
	Mean BMI	+
	Log-transformed SEV scalar: Kidney C	+
2	*Alcohol (litres per capita)*	+
	Diabetes age-standardised prevalence (proportion)	+
	Systolic blood pressure (mmHg)	+
	Smoking prevalence	+
	Healthcare access and quality index	–
3	*Education (years per capita)*	–
	LDI (I$ per capita)	0
	Socio-demographic Index	0

Bladder cancer

Level	Covariate	Direction
1	*Schistosomiasis prevalence (proportion)*	+
	Cumulative cigarettes (5 years)	+
	Cumulative cigarettes (10 years)	+
	Cumulative cigarettes (15 years)	+
	Smoking prevalence	+
	Log-transformed SEV scalar: Bladder C	+
2	*Alcohol (litres per capita)*	+
	Diabetes gasting plasma glucose (mmol/L)	+
	Vegetables adjusted (g)	–
	Fruits adjusted (g)	–
	Healthcare access and quality index	–
3	*Education (years per capita)*	–
	LDI (I$ per capita)	0
	Socio-demographic Index	0

Brain and nervous system cancer

Level	Covariate	Direction
1	*Alcohol (litres per capita)*	+
	Cumulative cigarettes (10 years)	+
	Cumulative cigarettes (15 years)	+
	Smoking prevalence	+
2	*Cholesterol (total, mean per capita)*	+
	Systolic blood pressure (mmHg)	+
	Red meats adjusted (g)	+
	Vegetables adjusted (g)	–
	Fruits adjusted (g)	–
	Healthcare access and quality index	–
3	*Education (years per capita)*	–
	LDI (I$ per capita)	0
	Socio-demographic Index	0

Thyroid cancer

Level	Covariate	Direction
1	*Alcohol (litres per capita)*	+
	Smoking prevalence	+
	Log-transformed SEV scalar: Thyroid C	+
2	*Vegetables adjusted (g)*	–
	Fruits adjusted (g)	–
	Red meats adjusted (g)	+
	Tobacco (cigarettes per capita)	+
	Mean BMI	+
	Smoking prevalence	+
	Sanitation (proportion with access)	–
	Improved water source (proportion with access)	–
	Healthcare access and quality index	–
3	*Education (years per capita)*	–
	LDI (I$ per capita)	0
	Socio-demographic Index	0

Mesothelioma

Level	Covariate	Direction
1	*Asbestos consumption (metric tons per year per capita)*	+
	Cumulative cigarettes (5 years)	+
	Asbestos production (binary)	+
	Smoking prevalence	+
	Indoor air pollution (all cooking fuels)	+
	Log-transformed SEV scalar: Mesothel	+
	Log-transformed age-standardised SEV scalar: Mesothel	+
2	*Asbestos production (kg) per capita*	+
	Gold production (binary)	+
	Gold production (kg) per capita	+
	Population density (over 1000 ppl/sqkm, proportion)	+
	Healthcare access and quality index	–
3	*Education (years per capita)*	–
	LDI (I$ per capita)	0
	Socio-demographic Index	0

Hodgkin's lymphoma

Level	Covariate	Direction
2	*Healthcare access and quality index*	–
3	*Education (years per capita)*	–
	LDI (I$ per capita)	0
	Socio-demographic Index	0

Non-Hodgkin lymphoma

Level	Covariate	Direction
2	*Cumulative cigarettes (5 years)*	+
	Cumulative cigarettes (10 years)	+
	Cumulative cigarettes (15 years)	+
	Cumulative cigarettes (20 years)	+
	Alcohol (litres per capita)	+
	Smoking prevalence	+
	Mean BMI	+
	Healthcare access and quality index	–
3	*Total fertility rate*	0
	LDI (I$ per capita)	0
	Socio-demographic Index	0

Multiple myeloma

Level	Covariate	Direction
1	*Alcohol (litres per capita)*	+
	Smoking prevalence	+
	Tobacco (cigarettes per capita)	+
2	*Vegetables adjusted (g)*	–
	Fruits adjusted (g)	–
	Red meats adjusted (g)	+
	Tobacco (cigarettes per capita)	+
	Mean BMI	+
	Sanitation (proportion with access)	–
	Improved water source (proportion with access)	–
	Healthcare access and quality index	–
3	*Education (years per capita)*	–
	LDI (I$ per capita)	0
	Socio-demographic Index	0

Leukaemia

Level	Covariate	Direction
1	*Log-transformed age-standardised SEV scalar: Leukaemia*	+
	Log-transformed SEV scalar: Leukaemia	+
2	*Alcohol (litres per capita)*	+
	Mean BMI	+
	Cumulative cigarettes (5 years)	+
	Cumulative cigarettes (10 years)	+
	Cumulative cigarettes (15 years)	+
	Cumulative cigarettes (20 years)	+
	Smoking prevalence	+
	Tobacco (cigarettes per capita)	+
	Healthcare access and quality index	–
3	*Education (years per capita)*	–
	LDI (I$ per capita)	0
	Socio-demographic Index	0

Myelodysplastic, myeloproliferative, other haematopoietic neoplasms

Level	Covariate	Direction
1	*Log-transformed age-standardised SEV scalar: Leukaemia*	+
	Log-transformed SEV scalar: Leukaemia	+
2	*Alcohol (litres per capita)*	+
	Mean BMI	+
	Cumulative cigarettes (5 years)	+
	Cumulative cigarettes (10 years)	+
	Cumulative cigarettes (15 years)	+
	Cumulative cigarettes (20 years)	+
	Smoking prevalence	+
	Tobacco (cigarettes per capita)	+
	Healthcare access and quality index	–
3	*Education (years per capita)*	–
	LDI (I$ per capita)	0
	Socio-demographic Index	0

Other malignant cancers

Level	Covariate	Direction
1	*Smoking prevalence*	+
	Tobacco (cigarettes per capita)	+
2	*Vegetables adjusted (g)*	–
	Fruits adjusted (g)	–
	Nuts seeds adjusted (g)	–
	PUFA adjusted (percent)	–
	Mean BMI	–
	Healthcare access and quality index	–
3	*Education (years per capita)*	–
	LDI (I$ per capita)	0
	Socio-demographic Index	0

Other neoplasms

Level	Covariate	Direction
2	*Healthcare access and quality index*	–
3	*Education (years per capita)*	-
	LDI (I$ per capita)	0
	Socio-demographic Index	0

References

1 Waterhouse J, Muir C, Shanmugaratnam K, Powell J. Cancer Incidence in Five Continents IV. Lyon: IARC, 1982.

2 Curado M, Edwards B, Shin H, *et al.* Cancer Incidence in Five Continents IX. Lyon: IARC, 2007 http://www.iarc.fr/en/publications/pdfs-online/epi/sp160/CI5vol9-A.pdf.

3 Muir C, Mack T, Powell J, Whelan S. Cancer Incidence in Five Continents V. Lyon: IARC, 1987.

4 Parkin D, Muir C, Whelan S, Gao Y, Ferlay J, Powell J. Cancer Incidence in Five Continents VI. Lyon: IARC, 1992.

5 Parkin D, Whelan S, Ferlay J, Raymond L, Young J. Cancer Incidence in Five Continents VII. Lyon: IARC, 1997.

6 Parkin D, Whelan S, Ferlay J, Teppo L, Thomas D. Cancer Incidence in Five Continents VIII. Lyon: IARC, 2002.

7 Forman D, Bray F, Brewster D, *et al.* Cancer Incidence in Five Continents X. 2013. http://ci5.iarc.fr.

8 Engholm G, Ferlay J, Christensen N, *et al.* NORDCAN: Cancer Incidence, Mortality, Prevalence and Survival in the Nordic Countries, Version 7.3 Association of the Nordic Cancer Registries. Danish Cancer Society. 2016; published online Aug 7. http://www.ancr.nu.

9 Steliarova-Foucher E, O'Callaghan M, Ferlay J, Masuyer E, Forman D, Comber H, Bray F. European Cancer Observatory: Cancer Incidence, Mortality, Prevalence and Survival in Europe. Version 1.0 European Network of Cancer Registries, International Agency for Research on Cancer. 2012; published online Sept. http://eco.iarc.fr.

10 de Martel C, Maucort-Boulch D, Plummer M, Franceschi S. World-wide relative contribution of hepatitis B and C viruses in hepatocellular carcinoma. *Hepatology* 2015; **62**: 1190–200.

11 Hong TP, Gow P, Fink M, *et al.* Novel population-based study finding higher than reported hepatocellular carcinoma incidence suggests an updated approach is needed. *Hepatology* 2016; **63**: 1205–12.

12 GBD 2015 Risk Factors Collaborators. Global, regional, and national comparative risk assessment of 79 behavioural, environmental and occupational, and metabolic risks or clusters of risks, 1990–2015: a systematic analysis for the Global Burden of Disease Study 2015. *The Lancet* 2016; **388**: 1659–724.

13 International Agency for Research on Cancer, World Health Organization. GLOBOCAN estimated cancer incidence, mortality, and prevalence worldwide in 2012. Lyon, France: IARC, 2014 http://globocan.iarc.fr/Default.aspx (accessed April 19, 2016).

14 Karagas MR, Greenberg ER, Spencer SK, Stukel TA, Mott LA. Increase in incidence rates of basal cell and squamous cell skin cancer in New Hampshire, USA. New Hampshire Skin Cancer Study Group. *Int J Cancer* 1999; **81**: 555–9.

Cardiovascular Diseases

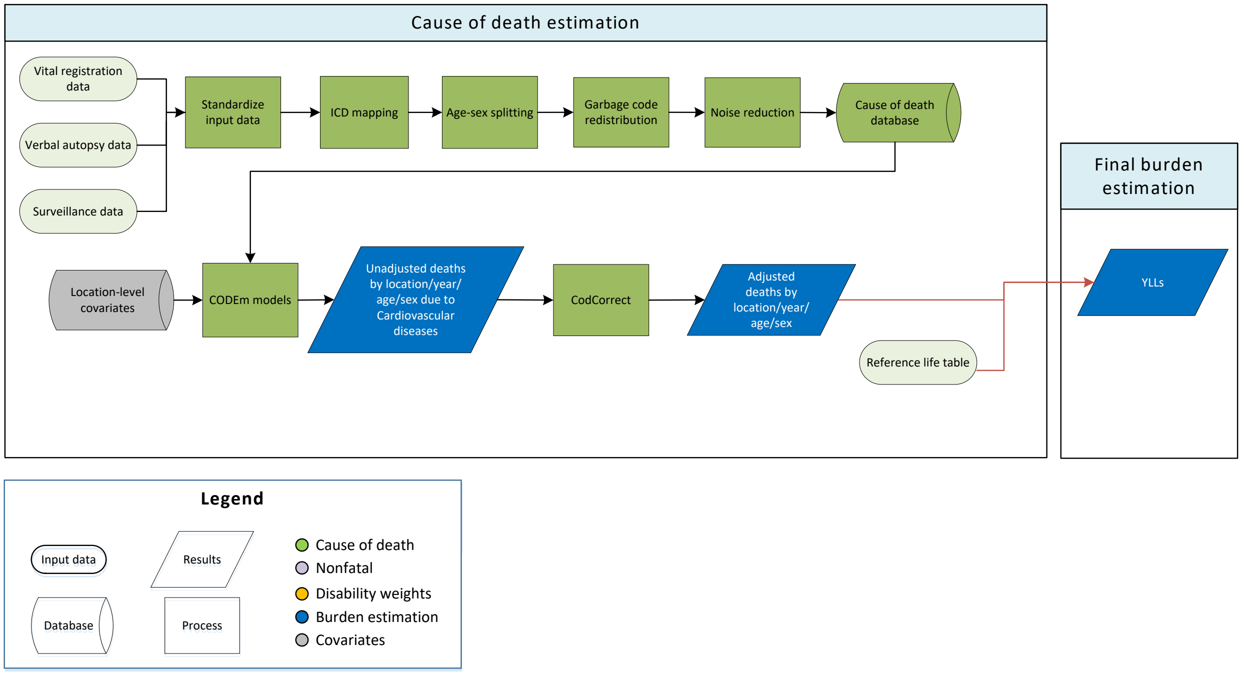

Input data

Vital registration, verbal autopsy, and surveillance data were used to model the parent cardiovascular envelope. We outliered non-representative subnational verbal autopsies from a number of Indian states. We also outliered verbal autopsy data sources that were implausibly low in all age groups and ICD8 and ICD9 BTL data points that were inconsistent with the rest of the data and created implausible time trends.

Modelling strategy

We used a standard CODEm approach to model deaths from cardiovascular diseases. The covariates included in the ensemble modelling process are listed in the table below. There have been no substantive changes from the approach used in GBD 2016.

Table: Selected covariates for CODEm models, cardiovascular diseases

Covariate	Transformation	Level	Direction
Summary exposure variable, cardiovascular disease	None	1	1
Cholesterol (total, mean per capita)	None	1	1
Smoking prevalence	None	1	1
Systolic blood pressure (mmHg)	None	1	1
Trans fatty acid	None	1	1
Mean BMI	None	2	1
Elevation over 1500m (proportion)	None	2	-1
Fasting plasma glucose (mmol/L)	None	2	1
Outdoor pollution ($PM_{2.5}$)	None	2	1
Indoor air pollution (all fuel types)	None	2	1
Healthcare access and quality index	None	2	-1
Lag distributed income per capita (I$)	Log	3	-1
Socio-demographic Index	None	3	0
Omega-3 (kcal/capita, adjusted)	Log	3	-1
Fruits (kcal/capita, adjusted)	None	3	-1
Vegetables (kcal/capita, adjusted)	None	3	-1
Nuts and seeds (kcal/capita, adjusted)	None	3	-1
Whole grains (kcal/capita, adjusted)	None	3	-1
Pulses/legumes (kcal/capita, adjusted)	None	3	-1
PUFA adjusted (percent)	None	3	-1
Alcohol (litres per capita)	None	3	0

Rheumatic Heart Disease

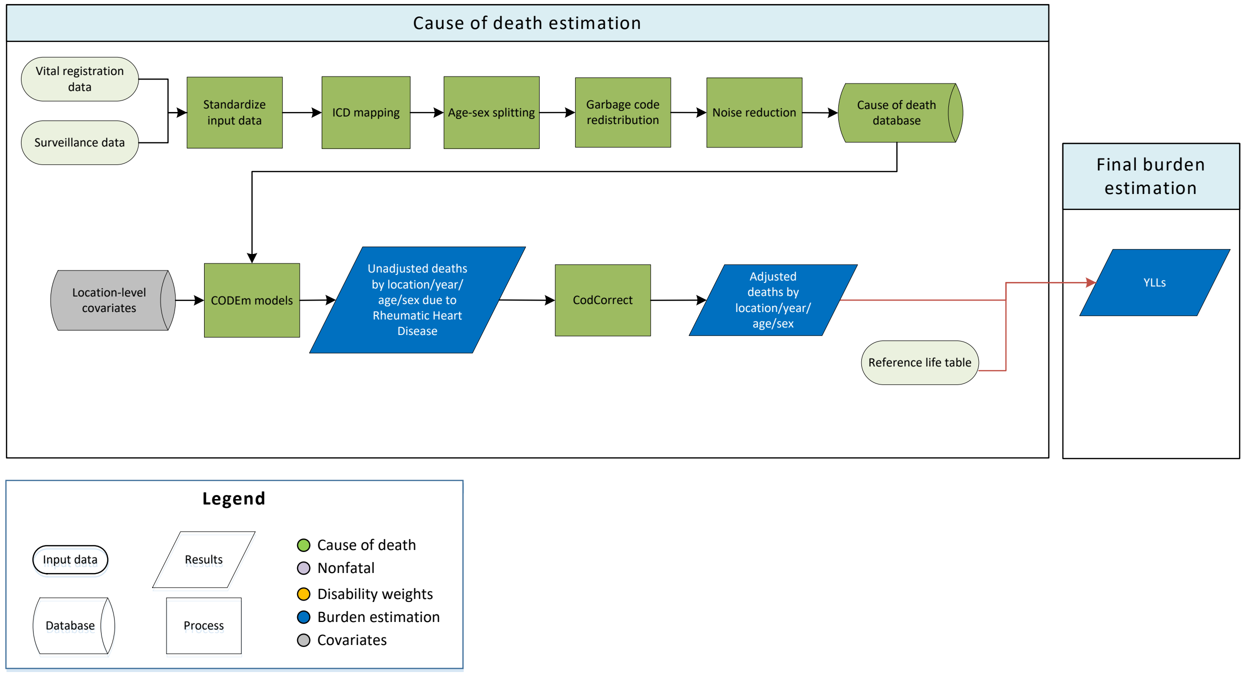

Input data

Vital registration and surveillance data were used to model rheumatic heart disease. We outliered ICD8 and ICD9 BTL data points which were inconsistent with the rest of the data and created implausible time trends. We also outliered data points which were too high after the redistribution process in a number of age groups.

Modelling strategy

We used a standard CODEm approach to model deaths from rheumatic heart disease. We have made no updates to the covariates used in the modelling process since GBD 2016. There have been no other substantive changes from the approach used in GBD 2016.

Table 1: Selected covariates for CODEm models, rheumatic heart disease

Covariate	Transformation	Level	Direction
Rheumatic heat disease SEV scalar	None	1	1
Improved water (proportion)	None	1	-1
Malnutrition	None	1	1
Sanitation (proportion with access)	None	1	-1
Healthcare access and quality index	None	2	-1
LDI	Log	3	-1
SDI	None	3	-1
Education (years per capita)	None	3	-1

Ischaemic Heart Disease

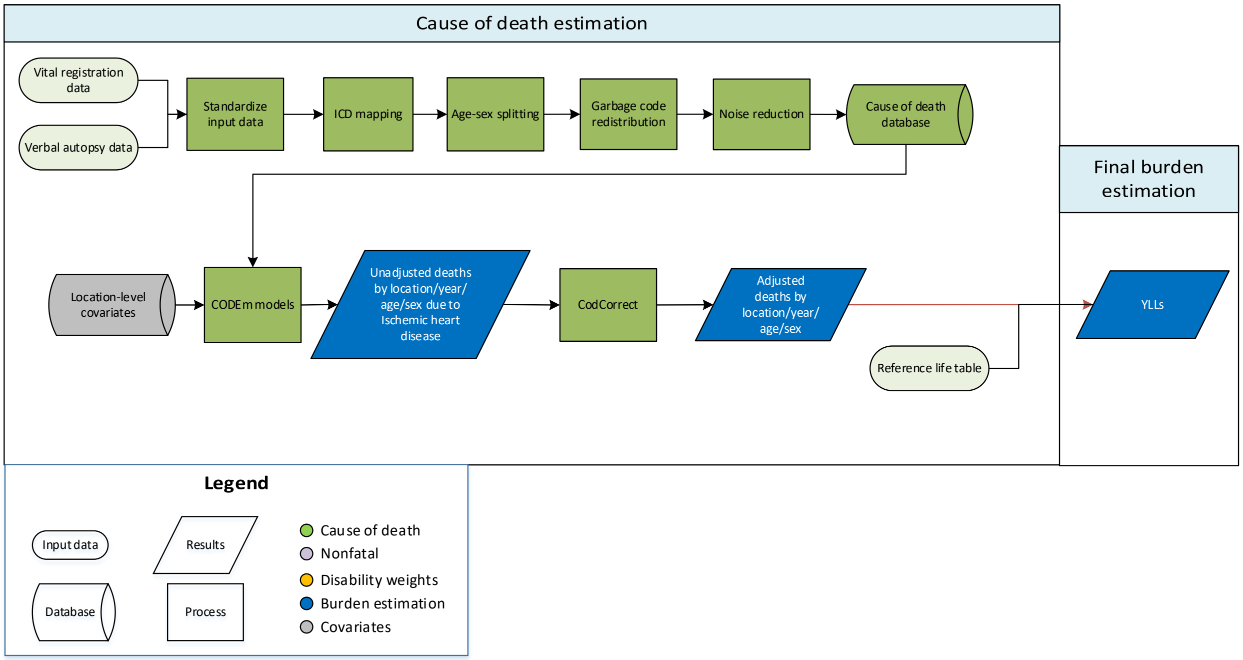

Input data

Vital registration, verbal autopsy data were used to model ischaemic heart disease. We outliered verbal autopsy data in countries and subnational locations where high-quality vital registration data were also available. We also outliered non-representative subnational verbal autopsy data points, ICD8 and ICD9 BTL data points which were inconsistent with the rest of the data and created implausible time trends, and data in a number of Indian states identified by experts as poor-quality.

Modelling strategy

We used a standard CODEm approach to model deaths from ischemic heart disease. The covariates chosen for inclusion in the ensemble modelling process are listed in the table below. There have been no substantive changes from the approach used in GBD 2016.

Table: Selected covariates for CODEm models, ischaemic heart disease

Covariate	Transformation	Level	Direction
Summary exposure variable	None	1	1
Cholesterol (total, mean per capita)	None	1	1
Smoking prevalence	None	1	1
Systolic blood pressure (mmHg)	None	1	1
Trans fatty acid	None	1	1
Mean BMI	None	2	1
Elevation over 1500m (proportion)	None	2	-1
Fasting plasma glucose	None	2	1
Outdoor pollution ($PM_{2.5}$)	None	2	1
Indoor air pollution	None	2	1
Healthcare access and quality index	None	2	-1
Lag distributed income per capita (I$)	Log	3	-1
Socio-demographic Index	None	3	0
Omega-3 (kcal/capita, adjusted)	Log	3	-1
Fruits (kcal/capita, adjusted)	None	3	-1
Vegetables (kcal/capita, adjusted)	None	3	-1
Nuts and seeds (kcal/capita, adjusted)	None	3	-1
Whole grains (kcal/capita, adjusted)	None	3	-1
Pulses/legumes (kcal/capita, adjusted)	None	3	-1
PUFA (percent, adjusted)	None	3	-1
Alcohol (litres per capita)	None	3	0

Stroke

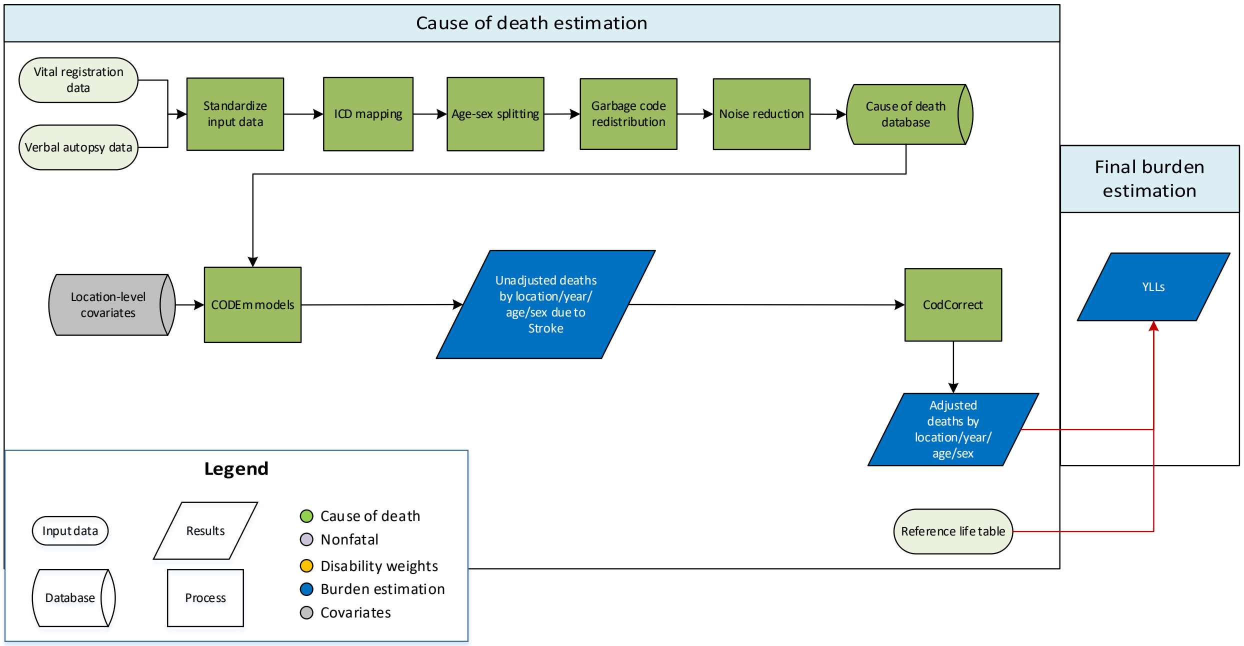

Input data

Verbal autopsy and vital registration data were used to model cerebrovascular disease. We reassigned deaths from verbal autopsy reports for cerebrovascular disease to the parent cardiovascular disease for both sexes for those under 20 years of age. We outliered non-representative subnational verbal autopsy data points. We also outliered ICD8, ICD9 BTL, and ICD10 Tabulated data points which were inconsistent with the rest of the data and created implausible time trends. Data points from sources which were implausibly low in all age groups and data points that were causing the regional estimates to be improbably high were outliered.

Modelling strategy

We used a standard CODEm approach to model deaths from stroke. The covariates included in the ensemble modelling process are listed in the table below. Apart from the updated strategy to reassign mis-coded deaths which should have been attributed to Alzheimer’s and other dementias, Parkinson’s disease, or atrial fibrillation and flutter, there have been no substantive changes from the approach used in GBD 2016.

Table: Selected covariates for CODEm models, stroke

Covariate	Transformation	Level	Direction
Summary exposure variable, stroke	None	1	1
Cholesterol (total, mean per capita)	None	1	1
Smoking prevalence	None	1	1
Systolic blood pressure (mmHg)	None	1	1
Trans fatty acid	None	1	1
Mean BMI	None	2	1
Elevation over 1500m (proportion)	None	2	-1
Fasting plasma glucose	None	2	1
Outdoor pollution ($PM_{2.5}$)	None	2	1
Indoor air pollution	None	2	1
Healthcare access and quality index	None	2	-1
Lag distributed income per capita (I$)	Log	3	-1
Socio-demographic Index	None	3	0
Omega-3 (kcal/capita, adjusted)	Log	3	-1
Fruits (kcal/capita, adjusted)	None	3	-1
Vegetables (kcal/capita, adjusted)	None	3	-1
Nuts and seeds (kcal/capita, adjusted)	None	3	-1
Whole grains (kcal/capita, adjusted)	None	3	-1
Pulses/legumes (kcal/capita, adjusted)	None	3	-1
PUFA adjusted (percent)	None	3	-1
Alcohol (litres per capita)	None	3	0

Ischaemic Stroke

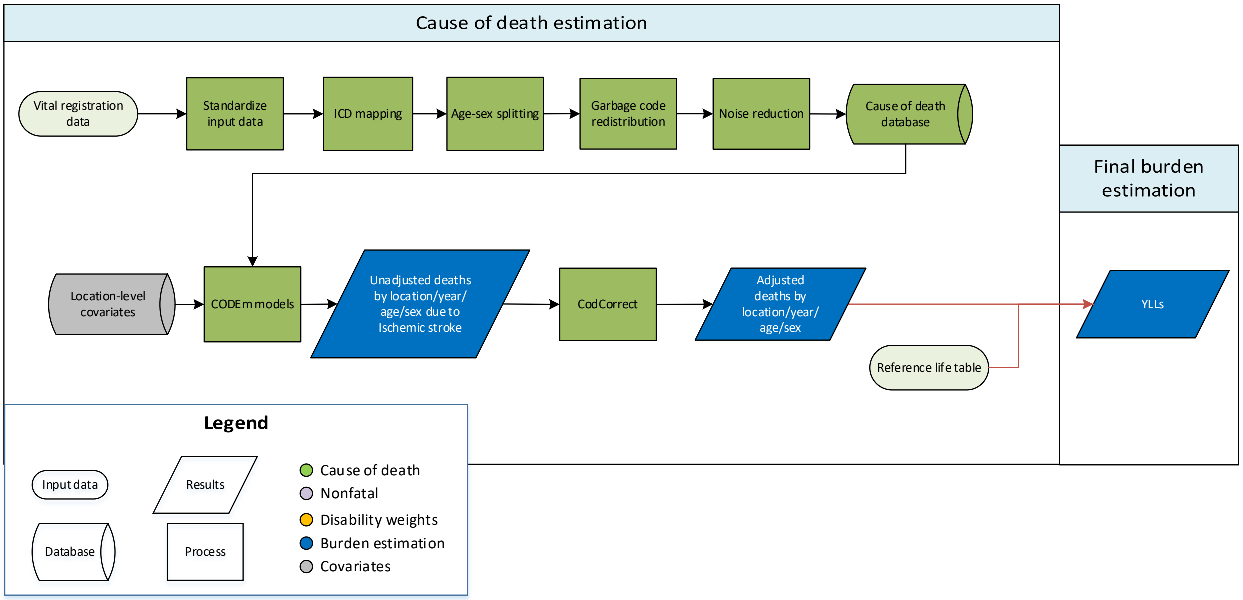

Input data

Vital registration data were used to model ischaemic stroke. We outliered ICD8 data points which were inconsistent with the rest of the data and created implausible time trends.

Modelling strategy

We used a standard CODEm approach to model deaths from ischemic stroke. In locations with limited data on ischaemic stroke, the subtype-specific deaths were estimated by squeezing both ischaemic and haemorrhagic stroke to the overall cerebrovascular envelope. The covariates selected for inclusion in the ensemble modelling process are listed in the table below. Apart from the updated strategy to reassign mis-coded deaths which should have been attributed to Alzheimer's and other dementias, Parkinson's disease, or atrial fibrillation and flutter, there have been no substantive changes from the approach used in GBD 2016.

Table: Selected covariates for CODEm models, ischaemic stroke

Covariate	Transformation	Level	Direction
Summary exposure variable, ischaemic stroke	None	1	1
Cholesterol (total, mean per capita)	None	1	1
Smoking prevalence	None	1	1
Systolic blood pressure (mmHg)	None	1	1
Trans fatty acid	None	1	1
Mean BMI	None	2	1
Elevation over 1500m (proportion)	None	2	-1
Fasting plasma glucose	None	2	1
Outdoor pollution ($PM_{2.5}$)	None	2	1
Indoor air pollution	None	2	1
Healthcare access and quality index	None	2	-1
Lag distributed income per capita (I$)	Log	3	-1
Socio-demographic Index	None	3	0
Omega-3 (kcal/capita, adjusted)	Log	3	-1
Fruits (kcal/capita, adjusted)	None	3	-1
Vegetables (kcal/capita, adjusted)	None	3	-1
Nuts and seeds (kcal/capita, adjusted)	None	3	-1
Whole grains (kcal/capita, adjusted)	None	3	-1
Pulses/legumes (kcal/capita, adjusted)	None	3	-1
PUFA adjusted (percent)	None	3	-1
Alcohol (litres per capita)	None	3	0

Intracerebral Haemorrhage

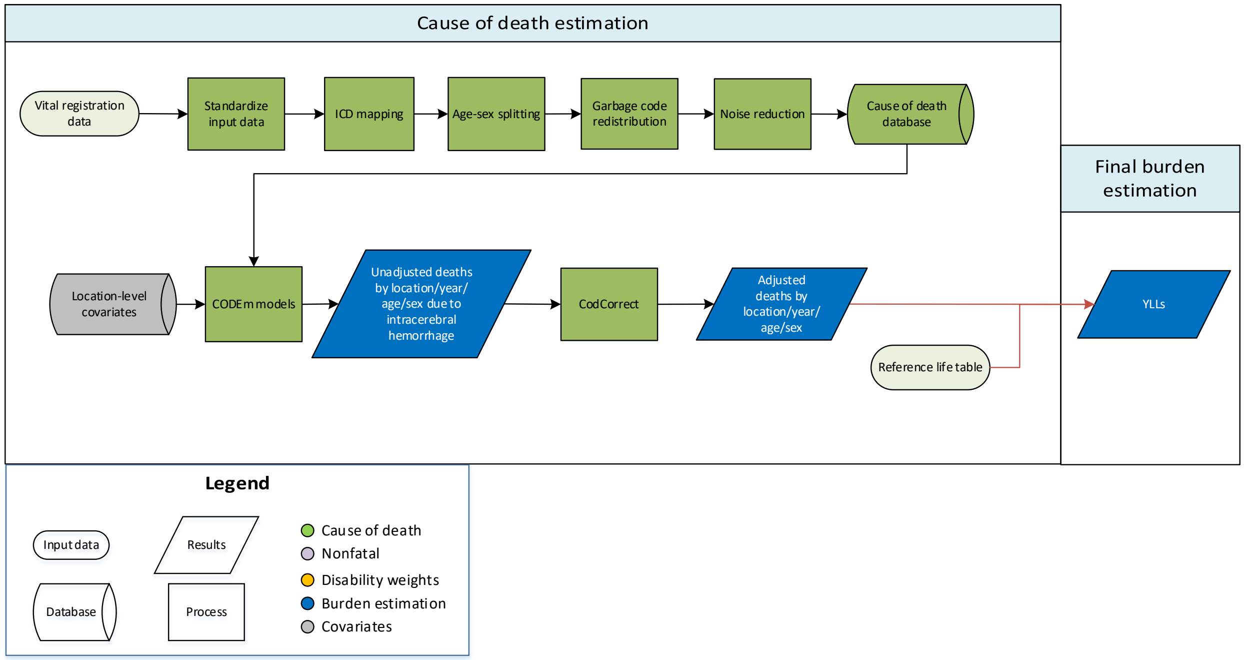

Input data

Vital registration data were used to model intracerebral haemorrhage. We outliered ICD8 data points which were inconsistent with the rest of the data and created implausible time trends.

Modelling strategy

We used a standard CODEm approach to model deaths from intracerebral haemorrhage. The covariates chosen for inclusion in the ensemble modelling process are listed in the table below. In previous iterations of GBD, we modelled intracerebral haemorrhage and subarachnoid haemorrhage together as haemorrhagic stroke. Other that this change, there have been no substantive changes from the modelling approach used in GBD 2016.

Table: Selected covariates for CODEm models, intracerebral haemorrhage

Covariate	Transformation	Level	Direction
Summary exposure variable, ICH	None	1	1
Cholesterol (total, mean per capita)	None	1	0
Smoking prevalence	None	1	1
Systolic blood pressure (mmHg)	None	1	1
Trans fatty acid	None	1	1
Mean BMI	None	2	1
Elevation over 1500m (proportion)	None	2	-1
Fasting plasma glucose	None	2	1
Outdoor pollution ($PM_{2.5}$)	None	2	1
Indoor air pollution	None	2	1
Healthcare access and quality index	None	2	-1
Lag distributed income per capita (I$)	Log	3	-1
Socio-demographic Index	None	3	0
Omega-3 (kcal/capita, adjusted)	Log	3	-1
Fruits (kcal/capita, adjusted)	None	3	-1
Vegetables (kcal/capita, adjusted)	None	3	-1
Nuts and seeds (kcal/capita, adjusted)	None	3	-1
Whole grains (kcal/capita, adjusted)	None	3	-1
Pulses/legumes (kcal/capita, adjusted)	None	3	-1
PUFA adjusted (percent)	None	3	-1
Alcohol (litres per capita)	None	3	0

Subarachnoid Haemorrhage

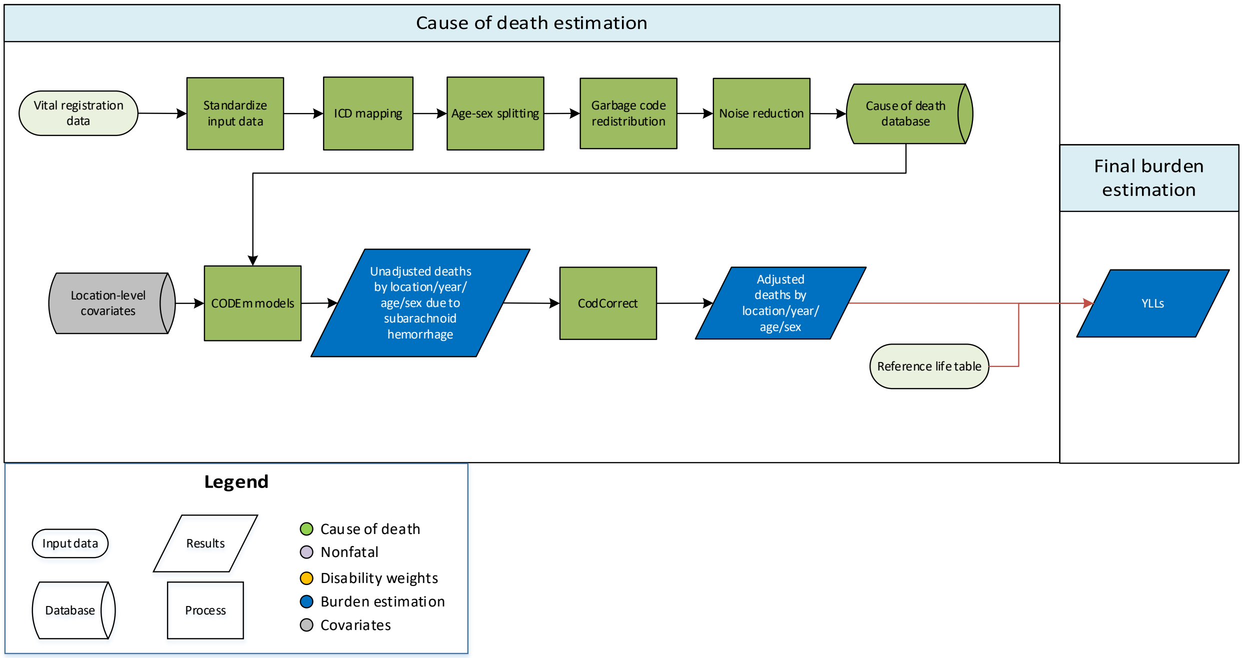

Input data

Vital registration data were used to model subarachnoid haemorrhage. We outliered ICD8 data points which were inconsistent with the rest of the data and created implausible time trends.

Modelling strategy

We used a standard CODEm approach to model deaths from subarachnoid haemorrhage. The covariates chosen for inclusion in the ensemble modelling process are listed in the table below. In previous iterations of GBD, we modelled intracerebral haemorrhage and subarachnoid haemorrhage together as haemorrhagic stroke. GBD 2017 is the first iteration where we have produced estimates specific to subarachnoid haemorrhage.

Table: Selected covariates for CODEm models, subarachnoid haemorrhage

Covariate	Transformation	Level	Direction
Smoking prevalence	None	1	1
Systolic blood pressure (mmHg)	None	1	1
Healthcare access and quality index	None	2	-1
Lag distributed income per capita (I$)	Log	3	-1
Socio-demographic Index	None	3	0
Alcohol (litres per capita)	None	3	0

Hypertensive Heart Disease

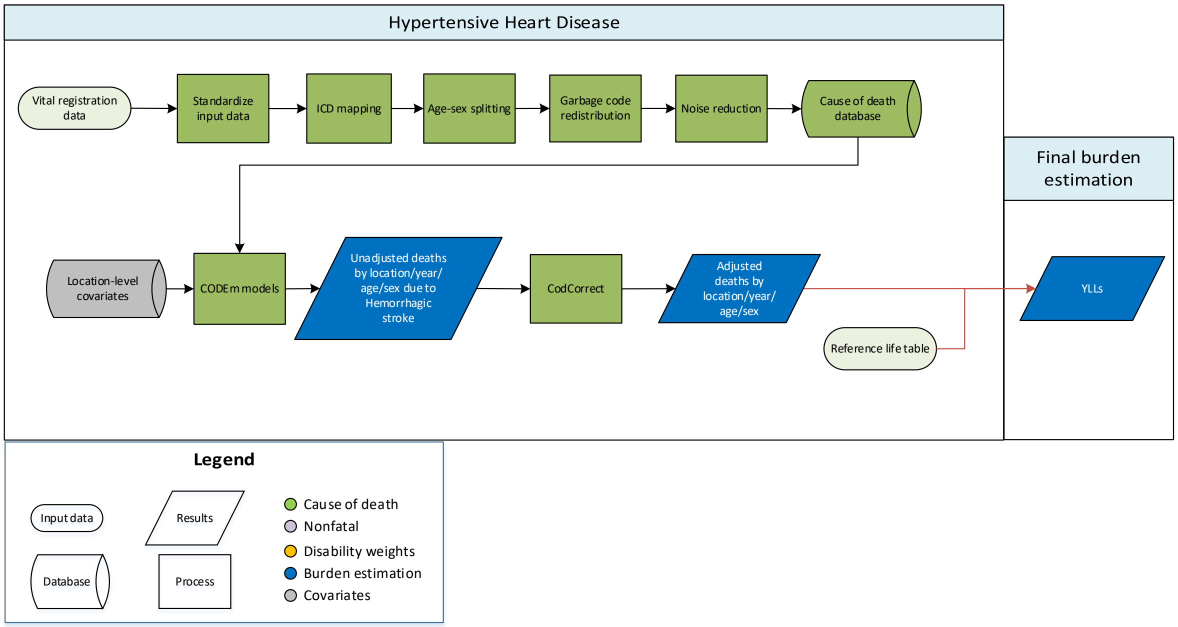

Input data

Vital registration data were used to model hypertensive heart disease. We outliered ICD9 BTL data points, which were inconsistent with the rest of the data and created implausible time trends.

Modelling strategy

We used a standard CODEm approach to model deaths from hypertensive heart disease. We have updated the covariates included in the ensemble modelling process (see Table). Otherwise, there have been no substantive changes from the approach used in GBD 2016.

Table: Selected covariates for CODEm models, hypertensive heart disease

Covariate	Transformation	Level	Direction
Systolic blood pressure (mmHg)	None	1	1
Cholesterol (total, mean per capita)	None	2	1
Smoking prevalence	None	2	1
Mean BMI	None	2	1
Healthcare access and quality index	None	2	-1
Lag distributed income per capita (I$)	Log	3	-1
Socio-demographic Index	None	3	0
Alcohol (litres per capita)	None	3	0
Omega-3 (kcal/capita, adjusted)	Log	3	-1
Fruits (kcal/capita, adjusted)	None	3	-1
Nuts and seeds (kcal/capita, adjusted)	None	3	-1
PUFA (percent, adjusted)	None	3	-1
Vegetables (kcal/capita, adjusted)	None	3	-1
Trans fatty acid (percent)	None	3	1

Non-rheumatic valvular heart disease, non-rheumatic calcific aortic valve disease, non-rheumatic degenerative mitral valve disease, and other non-rheumatic valvular heart diseases

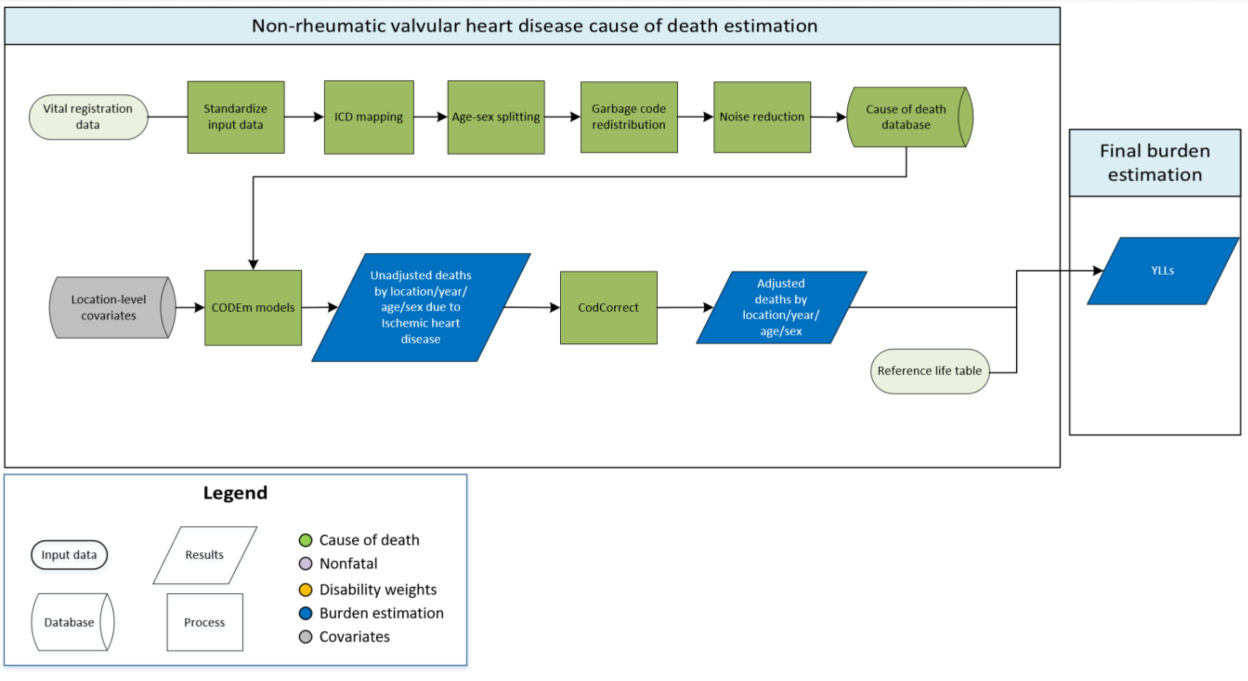

Input data

Vital registration data were used to model non-rheumatic valvular heart disease, non-rheumatic calcific valve disease, non-rheumatic degenerative mitral valve disease, and other non-rheumatic valve diseases. ICD codes used for these causes are listed above. We also outliered ICD8, ICD9 BTL, and ICD10 tabulated data points which were inconsistent with the rest of the data and created implausible time trends. Data points from sources which were implausibly low in all age groups and data points that were causing the regional estimates to be improbably high were outliered.

Modelling strategy

We used a standard CODEm approach to model deaths from non-rheumatic valvular heart disease and its three subcauses. The covariates used along with their transformations, importance levels, and imposed directions are reported by cause in the tables below.

Table 1: Selected covariates for CODEm models, non-rheumatic valvular heart disease

Covariate	Transformation	Level	Direction
Smoking prevalence	None	1	1
Systolic blood pressure (mmHg)	None	1	1
Cholesterol (total, mean per capita)	None	2	1
Mean BMI	None	2	1
Fasting plasma glucose	None	2	1
Healthcare access and quality index	None	2	-1
Lag distributed income per capita (I$)	Log	3	-1
Socio-demographic Index	None	3	0
Alcohol (litres per capita)	None	3	0

Table 2: Selected covariates for CODEm models, non-rheumatic calcific aortic valve disease

Covariate	Transformation	Level	Direction
Smoking prevalence	None	1	1
Systolic blood pressure (mmHg)	None	1	1
Cholesterol (total, mean per capita)	None	2	1
Mean BMI	None	2	1
Fasting plasma glucose	None	2	1
Healthcare access and quality index	None	2	-1
Lag distributed income per capita (I$)	Log	3	-1
Socio-demographic Index	None	3	0
Alcohol (litres per capita)	None	3	0

Table 3: Selected covariates for CODEm models, non-rheumatic degenerative mitral valve disease

Covariate	Transformation	Level	Direction
Healthcare access and quality index	None	1	-1
Lag distributed income per capita (I$)	Log	1	-1
Socio-demographic Index	None	1	0

Table 4: Selected covariates for CODEm models, other non-rheumatic valvular heart diseases

Covariate	Transformation	Level	Direction
Cardiovascular diseases SEV scalar	None	1	0

Cardiomyopathy and Myocarditis

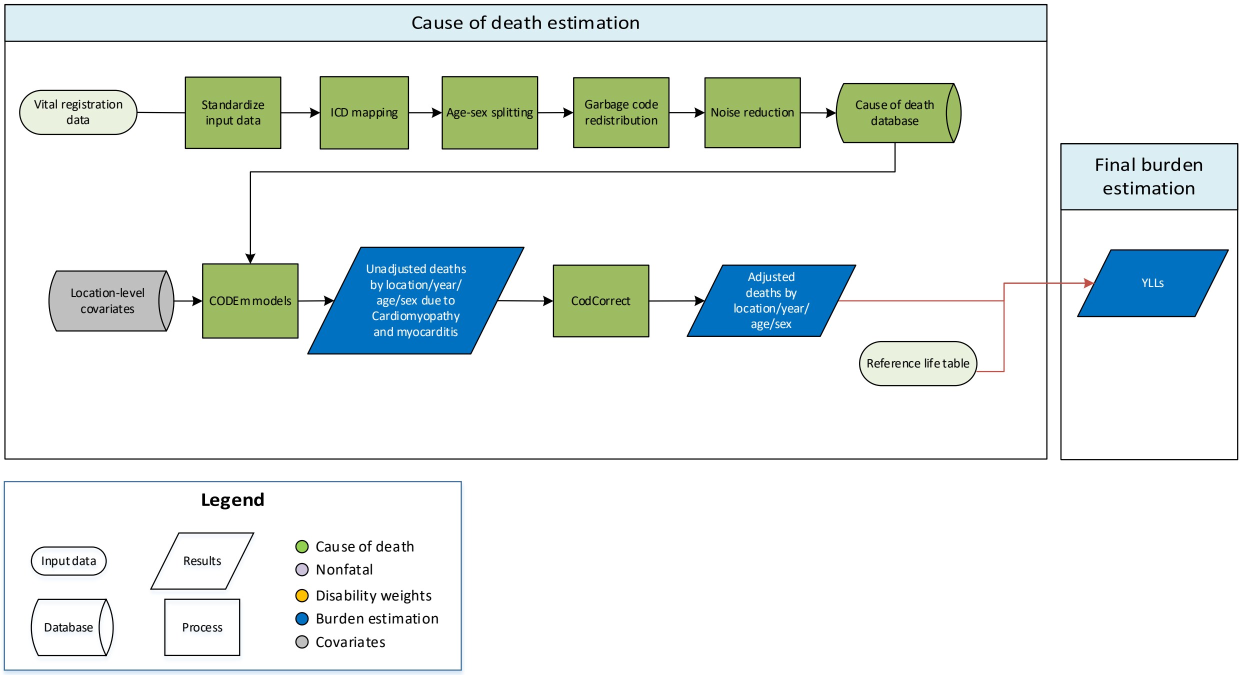

Input data

Vital registration data were used to model deaths due to cardiomyopathy and myocarditis. We outliered data points in Central Asia, Central Europe, and Eastern Europe due to implausibly high values which we attributed to variation in local coding practices. We also outliered ICD8 data points in countries where they were discontinuous with other data in the time series.

Modelling strategy

We used a standard CODEm approach to model deaths from cardiomyopathy and myocarditis. The covariates selected for inclusion in the CODEm modelling process can be found in the table below. There have been no substantive changes to the modelling strategy since GBD 2016.

Table: Selected covariates for CODEm models, cardiomyopathy and myocarditis

Covariate	Transformation	Level	Direction
Summary exposure variable, CMP	none	1	1
Mean systolic blood pressure (mmHg)	none	1	1
Smoking prevalence	none	1	1
Mean BMI (kg/m^2)	None	2	1
Healthcare access and quality index	none	2	-1
Lag distributed income per capita (I$)	log	3	0
Socio-demographic Index	none	3	0
Alcohol (litres per capita)	None	3	0

Myocarditis

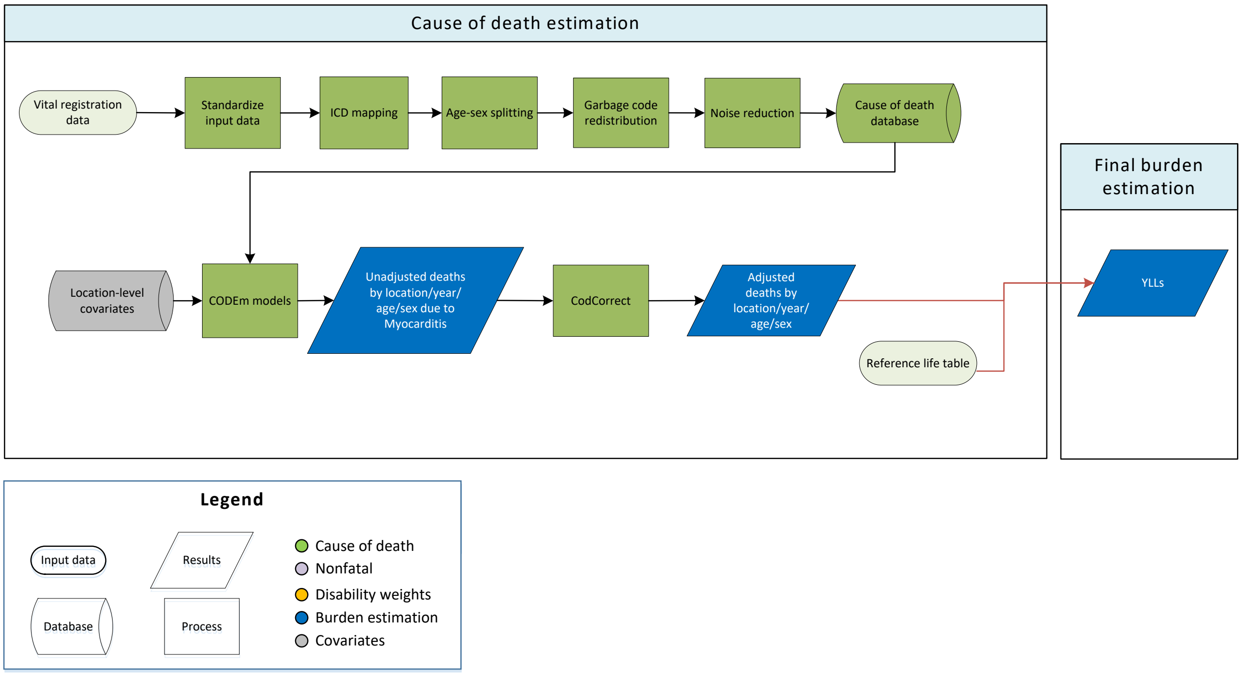

Input data
Vital registration data were used to model deaths due to myocarditis.

Modelling strategy
We used a standard CODEm approach to model deaths from myocarditis. The covariates selected for evaluation in the CODEm ensemble modelling process can be found in the table below. There have been no substantive changes to the modelling strategy since GBD 2016.

Table: Selected covariates for CODEm models, myocarditis

Covariate	Transformation	Level	Direction
Summary exposure variable, CMP	none	1	1
Systolic blood pressure (mm Hg)	none	1	1
Healthcare access and quality index	none	2	-1
Lag distributed income per capita (I$)	log	3	0
Socio-demographic Index	none	3	0

Alcoholic Cardiomyopathy

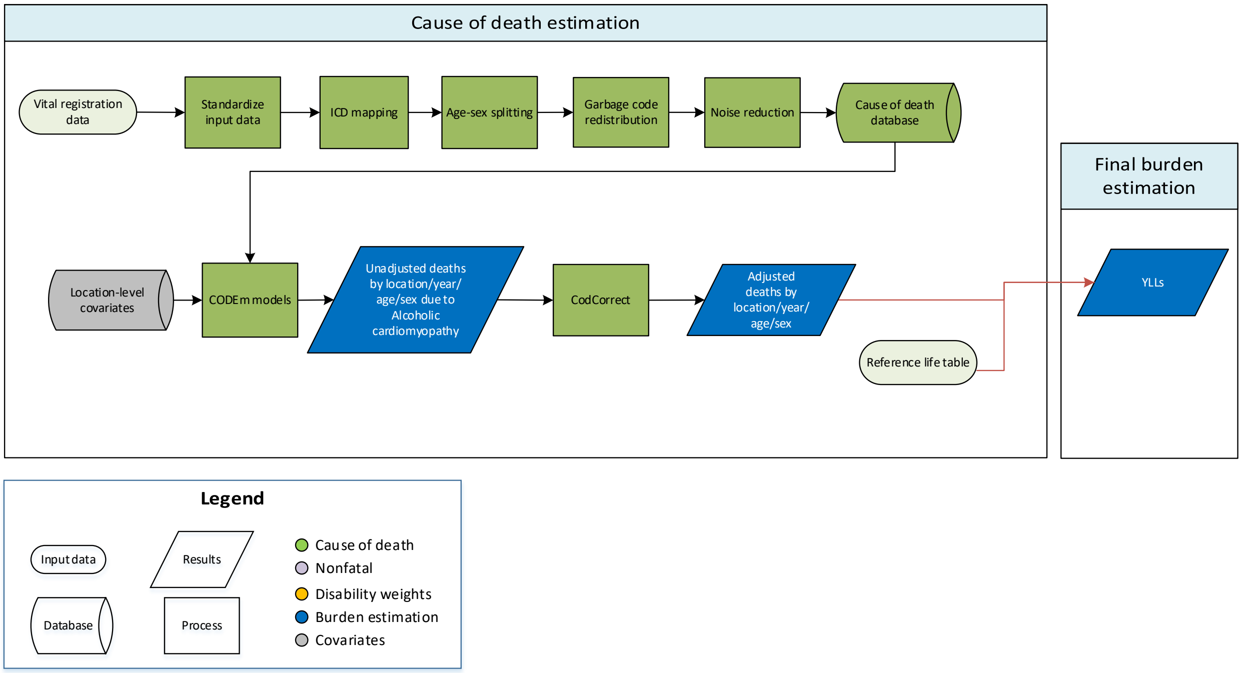

Input data

Vital registration and verbal autopsy data were used to model deaths due to alcoholic cardiomyopathy. We outliered ICD9 data points in Cyprus that were implausibly high and discontinuous with the rest of the time series. We also dropped ICD9BTL data points in locations in Central and Eastern Europe where we were unable to disaggregate them appropriately.

Modelling strategy

We used a standard CODEm approach to model deaths from alcoholic cardiomyopathy. The covariates selected for inclusion in the CODEm modelling process can be found in the table below. There have been no substantive changes from the approach used in GBD 2016.

Table: Selected covariates for CODEm models, alcoholic cardiomyopathy

Covariate	Transformation	Level	Direction
Summary exposure variable, CMP	none	1	1
Smoking prevalence	none	1	1
Alcohol (litres per capita)	none	1	1
Healthcare access and quality index	none	2	-1
Lag distributed income per capita (I$)	log	3	0
Socio-demographic Index	none	3	0

Other Cardiomyopathy

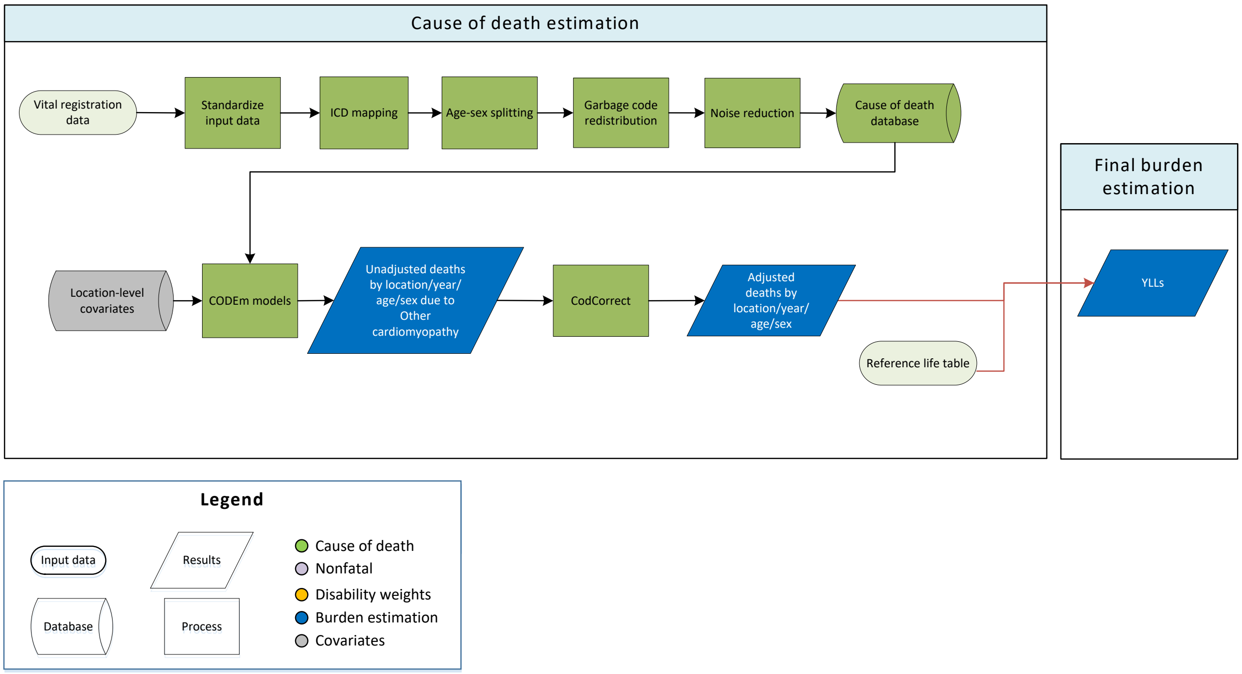

Input data

Vital registration data were used to model deaths due to other cardiomyopathy. We outliered data points in Central Asia and Central and Eastern Europe due to implausibly high values which we attributed to variation in local coding practices after review with experts.

Modelling strategy

We used a standard CODEm approach to model deaths from other cardiomyopathy. The covariates selected for inclusion in the CODEm modelling process can be found in the table below. There have been no substantive changes to the modelling process since GBD 2016.

Table: Selected covariates for CODEm models, other cardiomyopathy

Covariate	Transformation	Level	Direction
Summary exposure variable, CMP	none	1	1
Systolic blood pressure (mmHg)	none	1	1
Smoking prevalence	none	1	1
Body mass index (kg/m^2)	none	2	1
Healthcare access and quality index	none	2	-1
Lag distributed income per capita (I$)	log	3	0
Socio-demographic Index	none	3	0

Atrial Fibrillation and Flutter

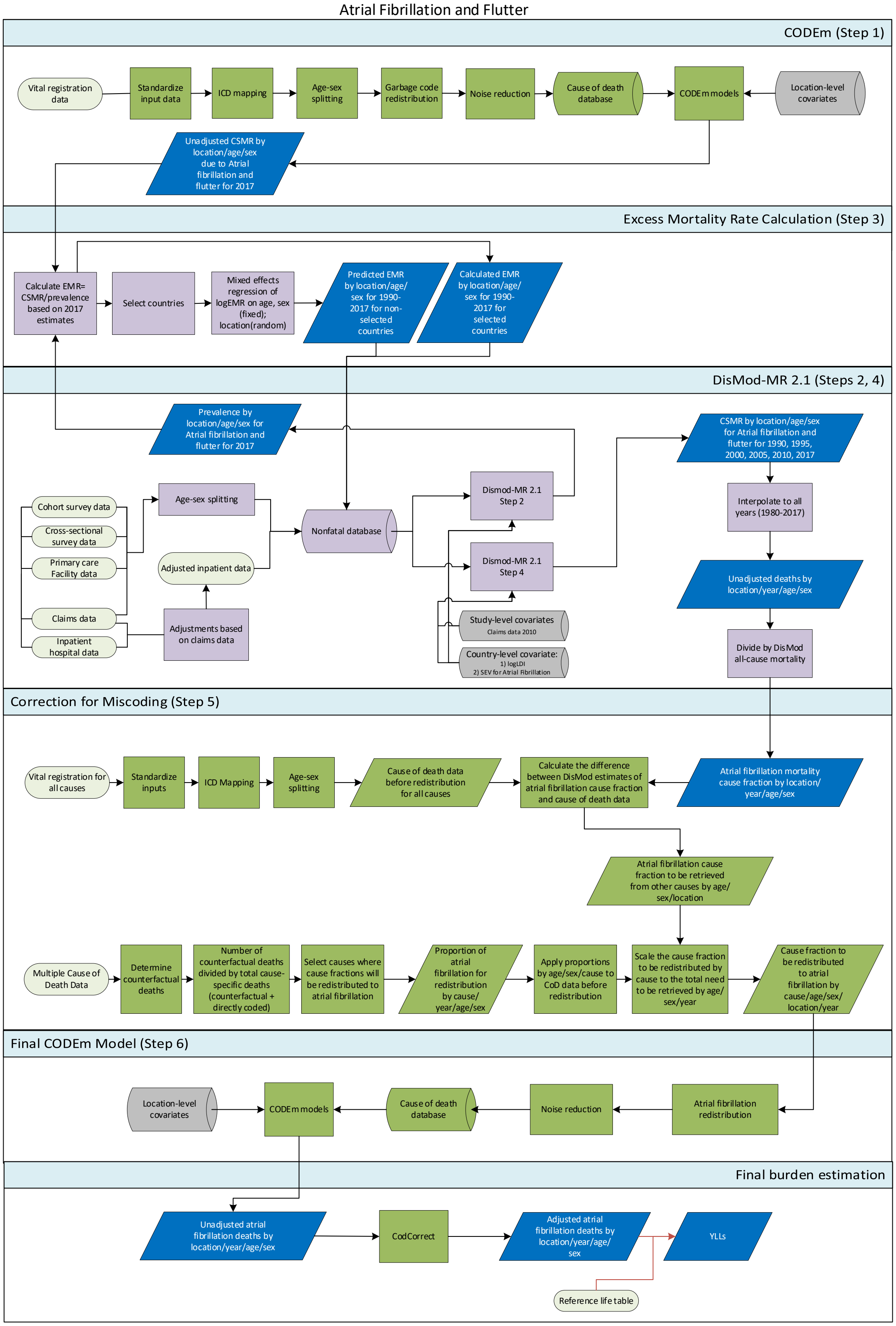

Input data

Vital registration (VR) data: We outliered ICD8 and ICD9 data points that were discontinuous from other data in the time series and created an unlikely time trend. We also outliered data points that were implausibly low in multiple age groups.

Modelling strategy

In order to address changes in coding practices for atrial fibrillation, we used an integrated approach that combined DisMod-MR and CODEm models to estimate deaths from atrial fibrillation and flutter. This approach allowed us to adjust estimates to more accurately reflect the number of deaths for which atrial fibrillation was the true underlying cause of death.

The modelling steps are illustrated in the above flowchart. Covariates included in both the DisMod-MR 2.1 and CODEm models can be found in the table below. In Step 1, we estimated deaths for atrial fibrillation using a standard CODEm approach. In Step 2, we estimated prevalence rates in DisMod-MR 2.1 using data from published reports of cross-sectional and cohort surveys, as well as primary care facility data. We also used claims data covering inpatient and outpatient visits for the United States along with inpatient hospital data from 163 locations in 15 countries. For GBD 2017, inpatient hospital data were adjusted using age- and sex-specific information from USA claims data for: 1) readmission within one year; 2) primary diagnosis code to secondary codes; and, 3) the ratio of inpatient to outpatient visits. We set priors of no remission and no excess mortality prior to age 30.

In Step 3, we calculated the excess mortality rate (EMR) for 2017 (defined as the cause-specific mortality rate (CSMR) estimated from CODEm divided by the prevalence rate from DisMod-MR 2.1). We then selected 17 countries based on four conditions: 1) ranking of 4 or 5 stars on the newly developed system for assessing the quality of VR data; 2) prevalence data available from the literature were included in the DisMod-MR 2.1 estimation; 3) prevalence rate ≥ 0.005; and, 4) CSMR ≥ 0.00002. Using information from these countries as input data, we ran a linear mixed-effects regression of logEMR on sex, age, and location. Sex and age were treated as fixed effects for the regression, while location was considered a random effect. We then predicted age- and sex-specific EMR using the results of this regression for all non-selected countries. Countries included in the regression were assigned their directly calculated values. These EMR data points were assigned to the time period 1990–2017 and uploaded into the nonfatal database in order to be used in modelling.

In Step 4, we reran DisMod-MR 2.1 including the EMR estimated in Step 3 as input data using the same priors as in Step 2 to obtain CSMR estimates from DisMod-MR 2.1 that are consistent with the available data for incidence and prevalence. As DisMod-MR 2.1 only generates estimates for six years (1990, 1995, 2000, 2005, 2010, 2017), we interpolated using a log-linear approach for 1990–2017. Estimates for 1980–1990 were generated via regression on the entire time series, using Socio-demographic Index as a predictor.

In Step 5, the CSMR estimates were divided by the all-cause mortality estimates used in DisMod-MR 2.1 to calculate the cause fraction for atrial fibrillation and flutter. We then calculated the difference between the cause fraction estimated by DisMod-MR 2.1 and the cause fraction in the VR data

generated by the Cause of Death data preparation process. This yielded the cause fraction that would need to be retrieved from other causes via the process described in Section 2.8: Correction for miscoding of Alzheimer's and other dementias and Parkinson's disease. After this correction process, the cause fraction data are processed through the standard redistribution and noise reduction processes.

In Step 6, these data are then used as inputs for a final CODEm model, using the same covariates as the model in Step 1. The results from the CODEm model are processed through CoDCorrect; these post-CoDCorrected results are the final estimates for cause-specific mortality for atrial fibrillation and flutter.

CODEm Covariates, atrial fibrillation and flutter

Covariate	Transformation	Level	Direction
Summary exposure variable, atrial fibrillation	None	1	1
Cholesterol (total, mean per capita)	None	1	1
Smoking prevalence	None	1	1
Systolic blood pressure (mmHg)	None	1	1
Mean BMI	None	2	1
Elevation over 1500m (proportion)	None	2	-1
Fasting plasma glucose	None	2	1
Outdoor pollution ($PM_{2.5}$)	None	2	1
Indoor air pollution	None	2	1
Healthcare Access and Quality Index	None	2	-1
Lag distributed income per capita (I$)	Log	3	-1
Socio-demographic Index	None	3	0
Omega-3 (kcal/capita, adjusted)	Log	3	-1
Fruits (kcal/capita, adjusted)	None	3	-1
Vegetables (kcal/capita, adjusted)	None	3	-1
Nuts and seeds (kcal/capita, adjusted)	None	3	-1
Whole grains (kcal/capita, adjusted)	None	3	-1
Pulses/legumes (kcal/capita, adjusted)	None	3	-1
PUFA adjusted (percent)	None	3	-1
Alcohol (litres per capita)	None	3	0
Trans fatty acid	None	1	1

DisMod Covariates – Step 2

Covariate	Parameter	Beta	Exponentiated beta
All MarketScan, year 2010	Prevalence	-0.077 (-0.099 to -0.051)	0.93 (0.91 to 0.95)
SEV scalar: Atrial fibrillation	Prevalence	0.75 (0.75 to 0.75)	2.12 (2.12 to 2.12)
Healthcare access and quality index	Excess mortality rate	-0.11 (-0.13 to -0.088)	0.90 (0.88 to 0.92)

DisMod Covariates – Step 4

Covariate	Parameter	Beta	Exponentiated beta
All MarketScan, year 2010	Prevalence	0.017 (-0.013 to 0.040)	1.02 (0.99 to 1.04)
SEV scalar: Atrial fibrillation	Prevalence	0.75 (0.75 to 0.75)	2.12 (2.12 to 2.12)
LDI (I$ per capita)	Excess mortality rate	-0.1 (-0.1 to -0.1)	0.90 (0.90 to 0.90)

Aortic Aneurysm

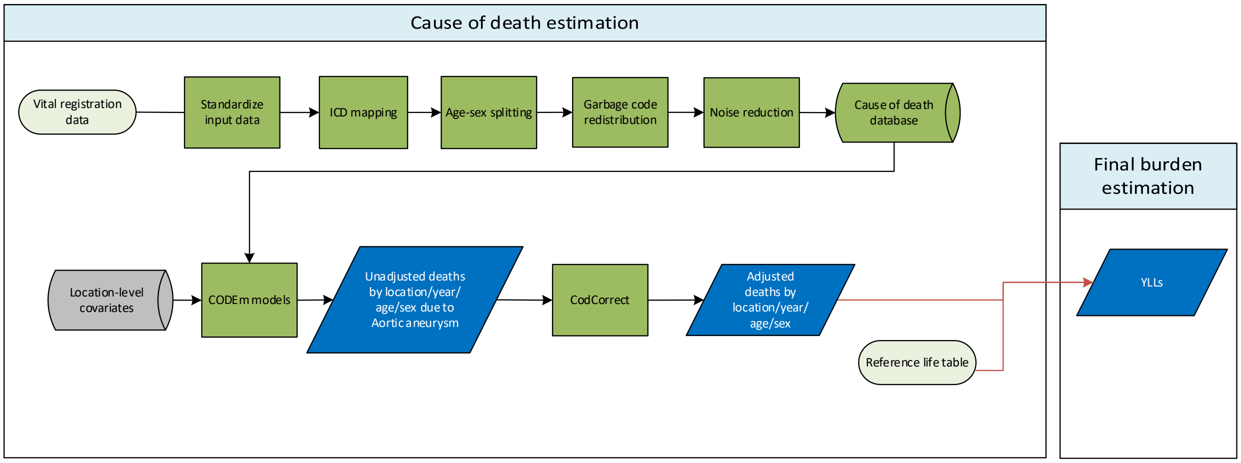

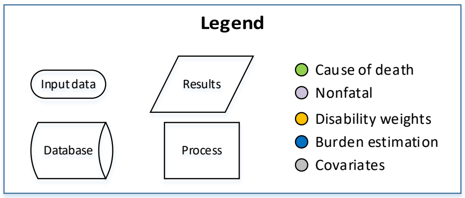

Input data

Vital registration data were used to model this cause. We outliered data in Oman as they were improbably high in comparison with the rest of the region. We also outliered ICD8 data that were discontinuous with the rest of the time series and created implausible time trends.

Modelling strategy

We used a standard CODEm approach to model deaths from aortic aneurysm. Please see the following table for a list of covariates selected for evaluation in the ensemble modelling process. There have been no substantive changes from the approach used in GBD 2016.

Table: Selected covariates for CODEm models, aortic aneurysm

Covariate	Transformation	Level	Direction
Summary exposure variable, aortic aneurysm	None	1	1
Cholesterol (total, mean per capita)	None	1	1
Cumulative cigarettes (10 yrs)	None	1	1
Systolic blood pressure (mmHg)	None	1	1
Mean BMI	None	2	1
Healthcare access and quality index	None	2	-1
Lag distributed income per capita (I$)	Log	3	-1
Socio-demographic Index	None	3	0
Omega-3 (kcal/capita, adjusted)	Log	3	-1
Fruits (kcal/capita, adjusted)	None	3	-1
Vegetables (kcal/capita, adjusted)	None	3	-1
Nuts and seeds (kcal/capita, adjusted)	None	3	-1
Pulses/legumes (kcal/capita, adjusted)	None	3	-1
PUFA adjusted (percent)	None	3	-1
Alcohol (litres per capita)	None	3	0

Peripheral Artery Disease

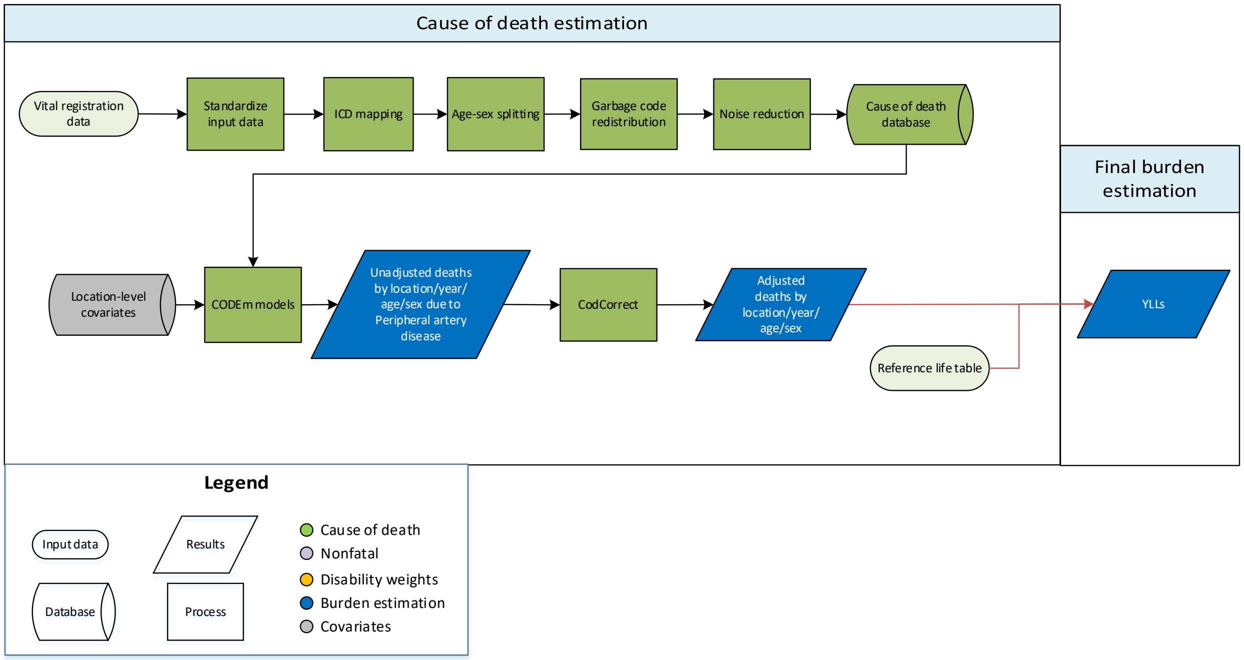

Input data

Vital registration data were used to model peripheral artery disease. We outliered all data points with <1 death in Egypt per expert review.

Modelling strategy

We used a standard CODEm approach to model deaths from peripheral artery disease. Covariates selected for inclusion in the ensemble modelling process are listed in the table below. There have been no substantive changes from the approach used in GBD 2016.

Table: Selected covariates for CODEm models, peripheral artery disease

Covariate	Transformation	Level	Direction
Summary exposure variable, PAD	None	1	1
Systolic blood pressure (mmHg)	None	1	1
Cholesterol (total, mean per capita)	None	1	1
Smoking prevalence	None	1	1
Mean body mass index (kg/m^2)	None	2	1
Healthcare access and quality index	None	2	-1
Diabetes fasting plasma glucose (mmol/L)	None	2	1
Lag distributed income per capita (I$)	Log	3	-1
Socio-demographic Index	None	3	0
Omega-3 (kcal/capita, adjusted)	Log	3	-1
Fruits (kcal/capita, adjusted)	None	3	-1
Vegetables (kcal/capita, adjusted)	None	3	-1
Nuts and seeds (kcal/capita, adjusted)	None	3	-1
Whole grains (kcal/capita, adjusted)	None	3	-1
Pulses/legumes (kcal/capita, adjusted)	None	3	-1
PUFA adjusted (percent)	None	3	-1
Trans fatty acid (percent)	None	3	1
Alcohol (litres per capita)	None	3	0

Endocarditis

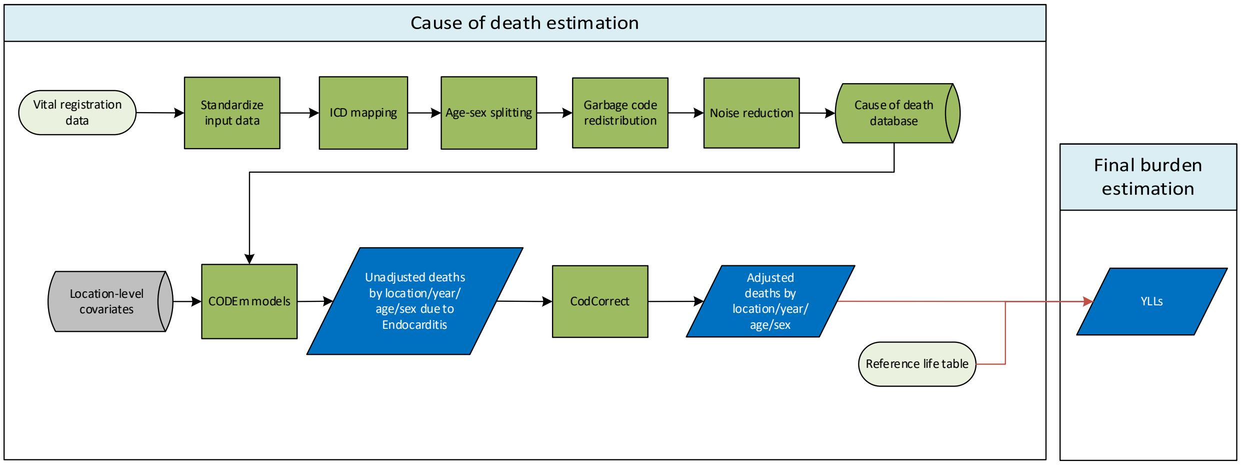

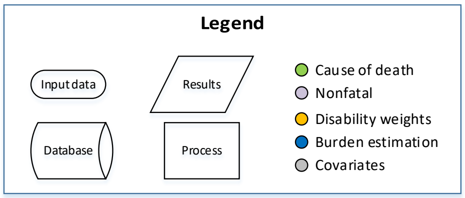

Input data

Vital registration and surveillance data were used to model endocarditis. We outliered vital registration data in Mozambique as these were non-representative for sub-Saharan Africa and were causing regional estimates to be implausibly low. We also outliered ICD8 data that were discontinuous from the rest of the data series and created an implausible time trend.

Modelling strategy

We used a standard CODEm approach to model deaths from endocarditis. Covariates selected for inclusion in the CODEm ensemble modelling process are listed in the table below. There have been no substantive changes from the approach used in GBD 2016.

Table: Selected covariates for CODEm models, endocarditis

Covariate	Transformation	Level	Direction
Summary exposure variable, endocarditis	None	1	1
Improved water (proportion)	None	1	-1
Sanitation (proportion with access)	None	1	-1
Healthcare access and quality index	None	1	-1
Lag distributed income per capita (I$)	Log	3	-1
Socio-demographic Index	None	3	0

Other Cardiovascular and Circulatory Diseases

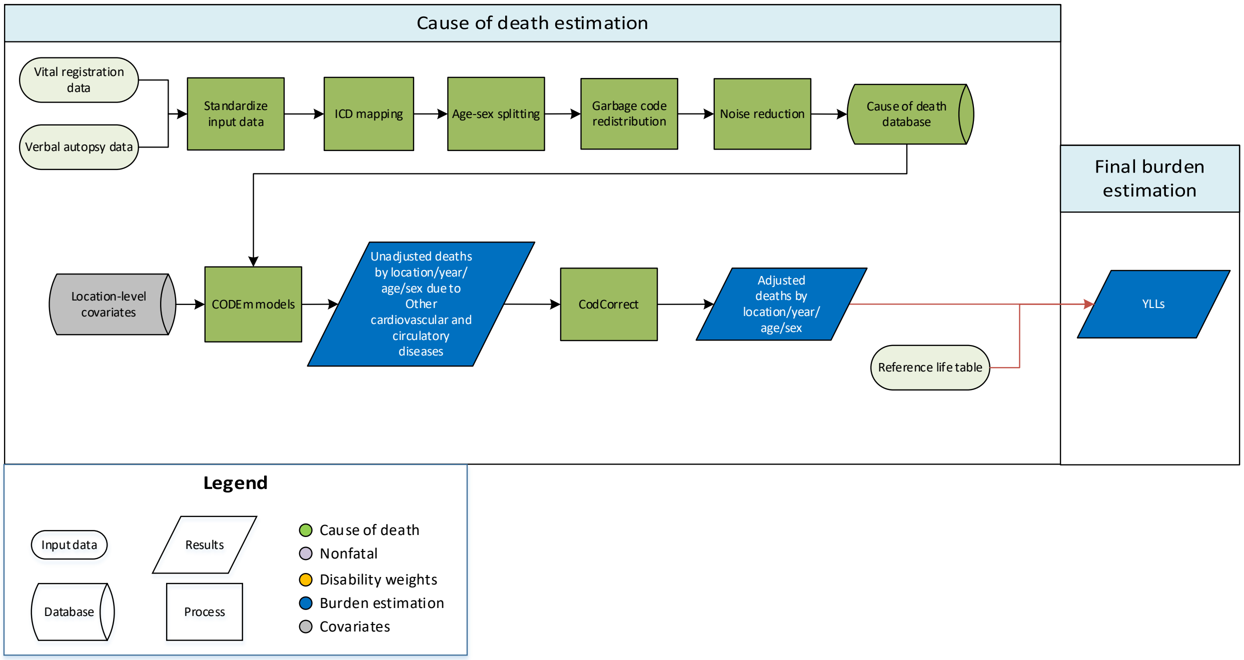

Input data

Vital registration and verbal autopsy data were used to model other cardiovascular and circulatory diseases. We outliered ICD8 and ICD9 BTL data points that were inconsistent with the rest of the data and created implausible time trends. We also outliered ICD8 data points which were not nationally representative.

Modelling strategy

We used a standard CODEm approach to model deaths from other circulatory and cardiovascular diseases. Covariates in the selected for inclusion in the ensemble model are listed in the table below. For GBD 2017, we have generated separate estimates for non-rheumatic valvular diseases, which were previously estimated as part of the other cardiovascular disease. A description of the modelling process for these causes can be found elsewhere in this appendix. Other than this, there have been no substantive changes from the approach used in GBD 2016.

Table: Selected covariates for CODEm models, cardiovascular diseases

Covariate	Transformation	Level	Direction
Summary exposure variable, Other CVD	None	1	1
Cholesterol (total, mean per capita)	None	1	1
Smoking prevalence	None	1	1
Systolic blood pressure (mmHg)	None	1	1
Trans fatty acid (percent)	None	1	1
Mean BMI	None	2	1
Elevation over 1500m (proportion)	None	2	-1
Fasting plasma glucose (mmol/L)	None	2	1
Indoor air pollution (all fuel types)	None	2	1
Outdoor air pollution ($PM_{2.5}$)	None	2	1
Healthcare access and quality index	None	2	-1
Lag distributed income per capita (I$)	Log	3	-1
Socio-demographic Index	None	3	0
Omega-3 (kcal/capita, adjusted)	Log	3	-1
Fruits (kcal/capita, adjusted)	None	3	-1
Vegetables (kcal/capita, adjusted)	None	3	-1
Nuts and seeds (kcal/capita, adjusted)	None	3	-1
Whole grains (kcal/capita, adjusted)	None	3	-1
Pulses/legumes (kcal/capita, adjusted)	None	3	-1
PUFA adjusted (percent)	None	3	-1
Alcohol (litres per capita)	None	3	0

Chronic Respiratory Diseases

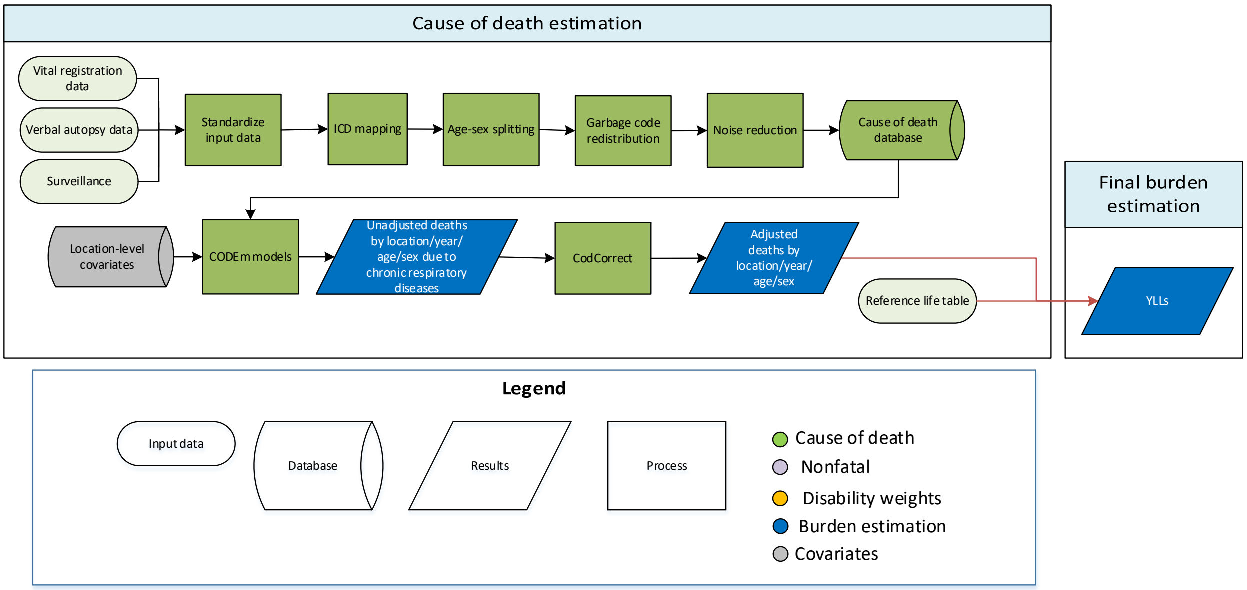

Input data

Sources used to estimate chronic respiratory disease mortality included vital registration, verbal autopsy, and surveillance data from China. Our outlier criteria excluded data points that (1) were implausibly high or low, (2) substantially conflicted with established age or temporal patterns, or (3) significantly conflicted with other data sources conducted from the same locations or locations with similar characteristics (ie, Socio-demographic Index).

Modelling strategy

The standard CODEm modelling approach was applied to estimate deaths due to chronic respiratory diseases. Chronic respiratory diseases served as the parent cause to chronic obstructive pulmonary disease, pneumoconiosis (including silicosis, asbestosis, coal worker's pneumoconiosis, other pneumoconiosis), asthma, interstitial lung disease and pulmonary sarcoidosis, and other chronic respiratory diseases. Functionally, this means the death estimates for chronic respiratory diseases serve as a "parent" envelope into which the "child" causes are squeezed by the CodCorrect algorithm. This approach allows us to use a broader range of data – specifically verbal autopsy data – which cannot be accurately mapped to specific respiratory diseases.

Separate models were conducted for male and female mortality, and the age range for both models was 1 to 95+ years. The same covariates from GBD 2016 were used.

Level	Covariate	Direction
1	log-transformed SEV scalar: chronic respiratory diseases	+
	cumulative cigarettes (10 years)	+

	cumulative cigarettes (5 years)	+
	healthcare quality and access index	-
2	smoking prevalence	+
	indoor air pollution (all cooking fuels)	+
	outdoor air pollution ($PM_{2.5}$)	+
	population above 1500m elevation (proportion)	+
3	log LDI (I$ per capita)	-
	education (years per capita)	-
	Socio-demographic Index	-
	population between 500 and 1,500m elevation (proportion)	+
	population density over 1,000 people/kilometer^2 (proportion)	+

Chronic Obstructive Pulmonary Disease

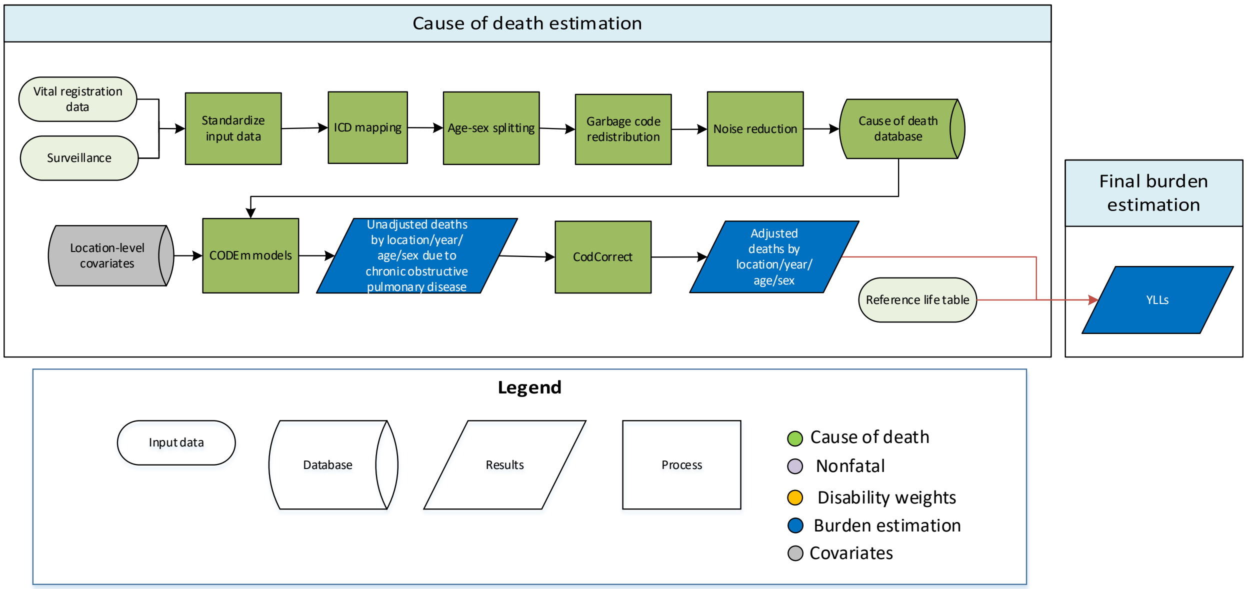

Input data

Data used to estimate chronic obstructive pulmonary disease (COPD) mortality included vital registration and surveillance data from the cause of death (COD) database. Our outlier criteria excluded data points that (1) were implausibly high or low, (2) substantially conflicted with established age or temporal patterns, or (3) significantly conflicted with other data sources conducted from the same locations or locations with similar characteristics (ie, Socio-demographic Index).

Modelling strategy

The standard CODEm modelling approach was applied to estimate deaths due to COPD. Separate models were conducted for male and female mortality, and the age range for both models was 1-95+ years. The mortality estimates from the COPD models were ultimately fit into the chronic respiratory diseases envelope.

The same covariates from GBD 2016 were used, but outdoor air pollution was moved to level 1.

**Level**	**Covariate**	**Direction**
1	log-transformed SEV scalar: COPD	+
	cumulative cigarettes (10 years)	+
	cumulative cigarettes (5 years)	+
	elevation over 1,500m (proportion)	+
	outdoor air pollution ($PM_{2.5}$)	+
2	smoking prevalence	+

	indoor air pollution (all cooking fuels)	+
	healthcare access and quality index	-
3	Socio-demographic Index	-
	log LDI (I$ per capita)	-
	education (years per capita)	-

Pneumoconiosis Diseases: Silicosis, Asbestosis, Coal Worker's Pneumoconiosis, and Other Pneumoconiosis

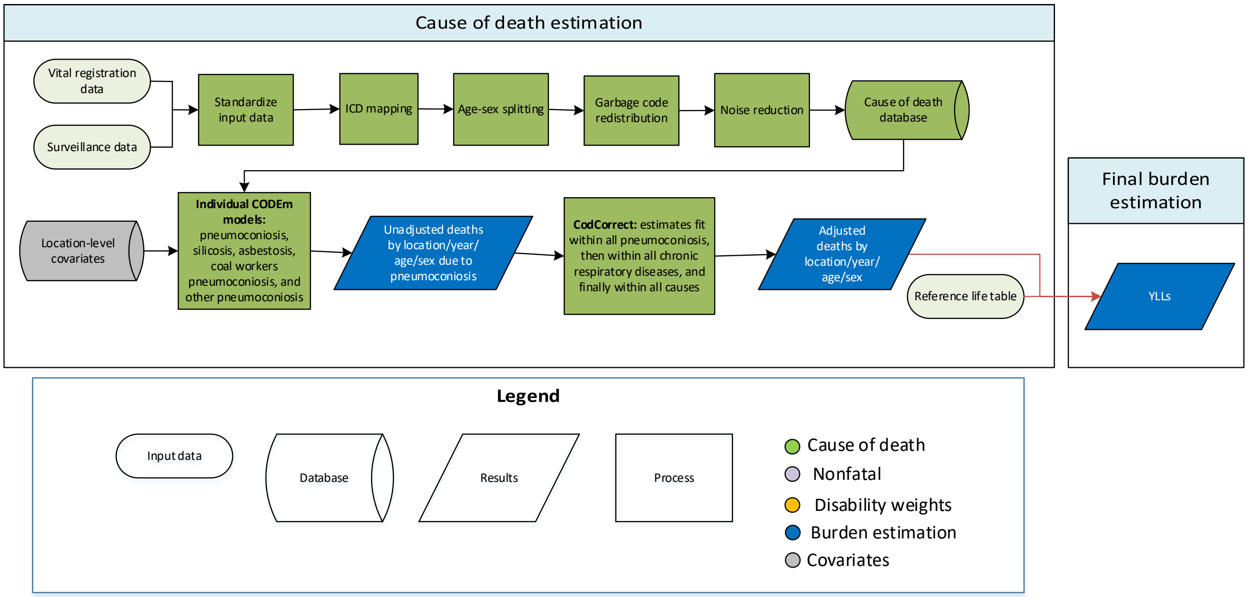

Input data

Data used to estimate pneumoconiosis diseases mortality included vital registration and China mortality surveillance data from the cause of death (COD) database. Our outlier criteria excluded data points that (1) were implausibly high or low, (2) substantially conflicted with established age or temporal patterns, or (3) significantly conflicted with other data sources conducted from the same locations or locations with similar characteristics (ie, socio-demographic index).

Modelling strategy

The standard CODEm modelling approach was applied to estimate deaths due to pneumoconiosis diseases. Separate models were conducted for male and female mortality, and the age range for both models was 15–95+ years. The mortality estimates from pneumoconiosis disease models were ultimately fit into the chronic respiratory envelope, which is the parent cause for pneumoconiosis disease. The pneumoconiosis model serves as an envelope for silicosis, asbestosis, coal worker's pneumoconiosis, and other pneumoconiosis. In CoDCorrect, estimates are first fit within all pneumoconiosis, then within all chronic respiratory disease, before being fit to the all-cause mortality envelope.

For the most part, the same covariates from GBD 2016 were used. The log-transformed SEV scalars were dropped, however, because the associated risk factors for GBD are occupational silica, asbestos, and particulate exposure, which each have a population attributable fraction (PAF) of 1 for pneumoconiosis. When PAF is equal to one, SEV=1/(1-PAF) is undefined. Subnational adjustments were also made to the coal, asbestos, and gold covariates.

The following table indicates covariates used in the pneumoconiosis models, their level, and direction:

Level	Covariate	Direction
1	asbestos consumption per capita*	+
	coal production per capita*	+
	gold production per capita*	+
2	smoking prevalence	+
	indoor air pollution (all cooking fuels)	+
	cumulative cigarettes (5 years)	+
	elevation over 1,500m (proportion)	+
	elevation 500 to 1,500m (proportion)	+
	healthcare access and quality index	-
3	log LDI (I$ per capita)	-
	education (years per capita)	-
	Socio-demographic Index	-

* asbestos, coal, and gold covariates are each only used in a subset of the pneumoconiosis models, as follows: all three are included in the parent all pneumoconiosis model, asbestos consumption is included in the asbestosis model, coal production is included in the coal worker's pneumoconiosis model, and gold production is included in the silicosis model.

Asthma

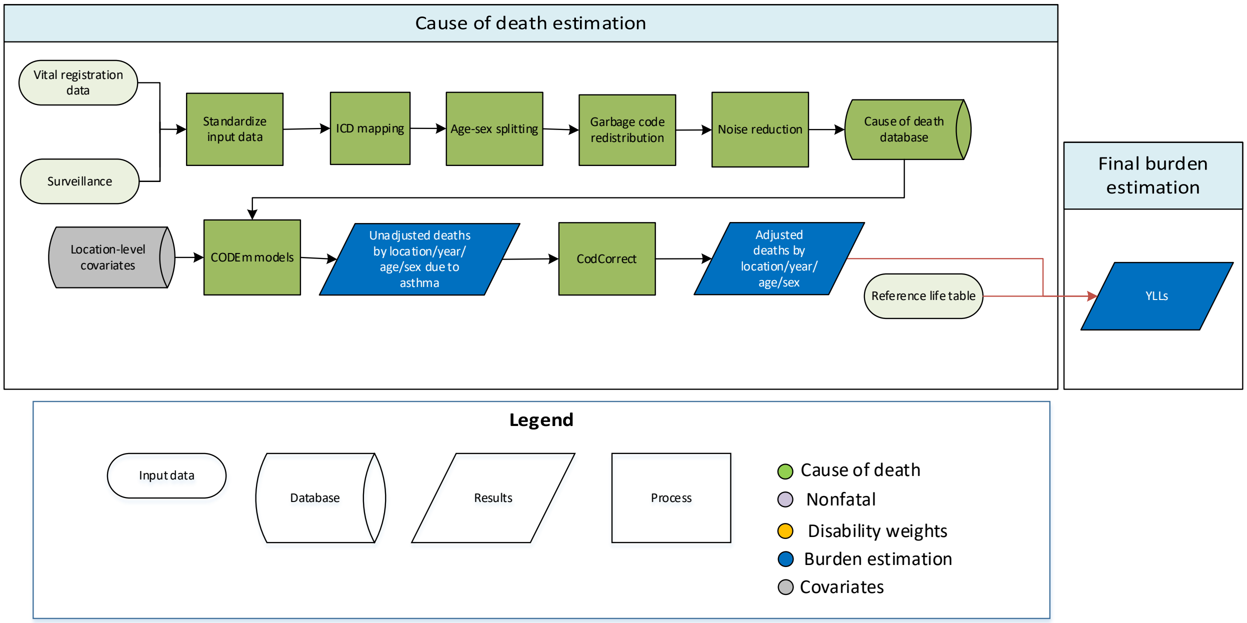

Input data

Data used to estimate asthma mortality included vital registration and surveillance data from the cause of death (COD) database. Verbal autopsy data were not included and were instead mapped to the parent model (chronic respiratory diseases). Our outlier criteria excluded data points that (1) were implausibly high or low relative to global or regional patterns, (2) substantially conflicted with established age or temporal patterns, or (3) significantly conflicted with other data sources conducted from the same locations or locations with similar characteristics (ie, Socio-demographic Index).

Modelling strategy

The standard CODEm modelling approach was applied to estimate deaths due to asthma. Separate models were conducted for male and female mortality, and the age range for both models was 1–95+ years. The mortality estimates from the asthma models were ultimately fit into the chronic respiratory diseases envelope.

The same covariates from GBD 2016 were used.

Level	Covariate	Direction
1	log-transformed SEV scalar: asthma	+
	cumulative cigarettes (10 years)	+
	cumulative cigarettes (5 years)	+
	healthcare access and quality index	-

2	smoking prevalence	+
	indoor air pollution (all cooking fuels)	+
	outdoor air pollution ($PM_{2.5}$)	+
3	log LDI (I$ per capita)	-
	education (years per capita)	-
	Socio-demographic Index	-

Interstitial Lung Disease and Pulmonary Sarcoidosis

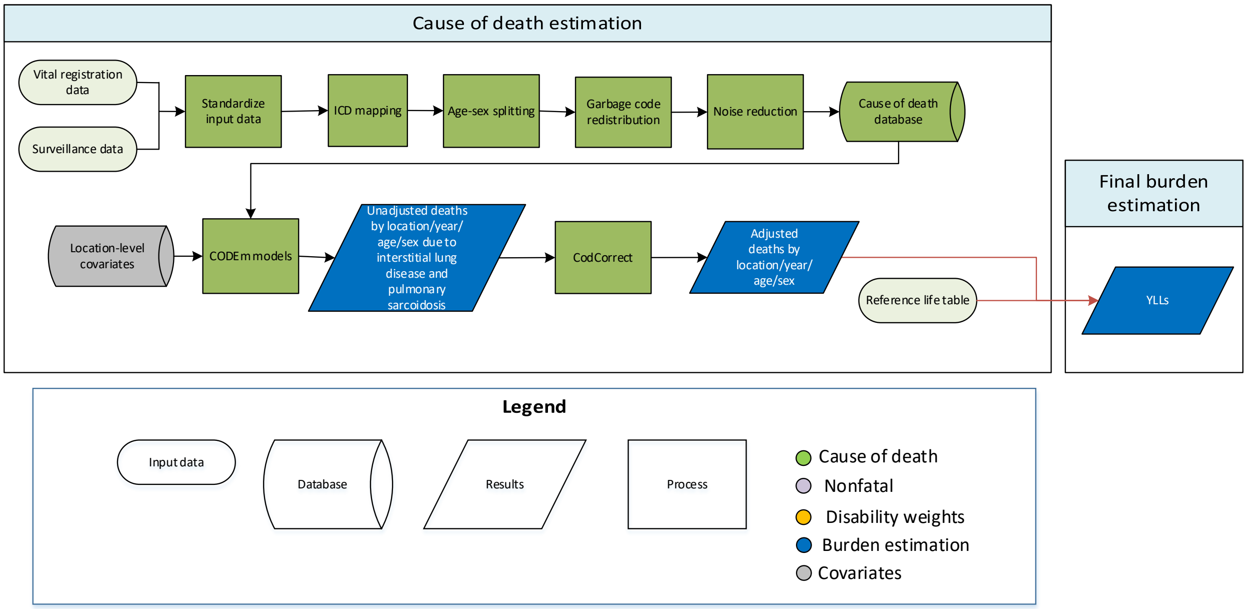

Input data

Data used to estimate interstitial lung disease and pulmonary sarcoidosis mortality included vital registration and surveillance data from the cause of death (COD) database. Our outlier criteria excluded data points that (1) were implausibly high or low, (2) substantially conflicted with established age or temporal patterns, or (3) significantly conflicted with other data sources conducted from the same locations or locations with similar characteristics (ie, Socio-demographic Index).

Modelling strategy

The standard CODEm modelling approach was applied to estimate deaths due to interstitial lung disease and pulmonary sarcoidosis. Separate models were conducted for male and female mortality, and the age range for both models was 1–95+ years. The mortality estimates from the interstitial lung disease and pulmonary sarcoidosis models were ultimately fit into the chronic respiratory envelope.

The same covariates from GBD 2016 were used.

**Level**	**Covariate**	**Direction**
1	log-transformed SEV scalar: interstitial lung disease	+
	smoking prevalence	+
	cumulative cigarettes (5 years)	+
2	elevation over 1,500m (proportion)	+
	elevation between 500 and 1,500m (proportion)	+

	population density over 1,000 ppl/km^2 (proportion)	+
	indoor air pollution (all cooking fuels)	+
	outdoor air pollution ($PM_{2.5}$)	+
	healthcare access and quality index	-
3	log LDI (I$ per capita)	-
	education (years per capita)	-
	Socio-demographic Index	-

Other Chronic Respiratory Diseases

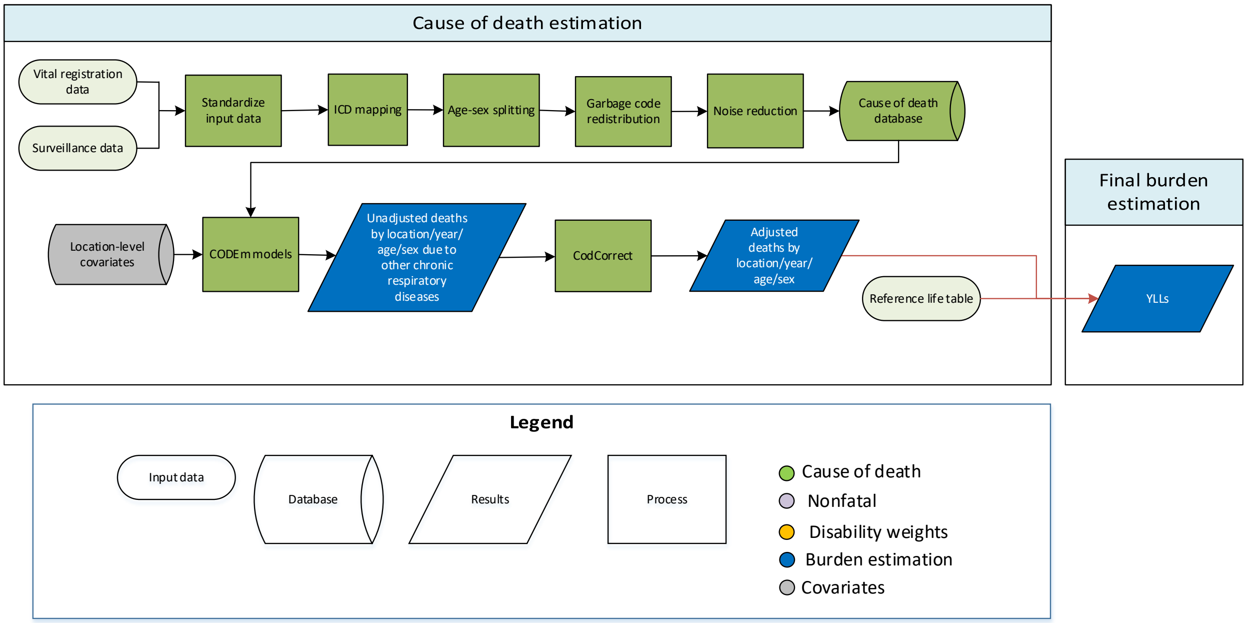

Input data

Data used to estimate other chronic respiratory diseases included vital registration and surveillance data from the cause of death (COD) database. Our outlier criteria excluded data points that (1) were implausibly high or low, (2) substantially conflicted with established age or temporal patterns, or (3) significantly conflicted with other data sources conducted from the same locations or locations with similar characteristics (ie, Socio-demographic Index).

Modelling strategy

The standard CODEm modelling approach was applied to estimate deaths due to other chronic respiratory diseases. Separate models were conducted for male and female mortality, and the age range for both models was 1 year to 95+ years. Like other respiratory causes, the mortality estimates from other chronic respiratory diseases were ultimately fit into the chronic respiratory envelope.

The same covariates from GBD 2016 were used.

Level	Covariate	Direction
1	log-transformed SEV scalar: other chronic respiratory diseases	+
	smoking prevalence	+
	cumulative cigarettes (5 years)	+
	indoor air pollution (all cooking fuels)	+
	outdoor air pollution ($PM_{2.5}$)	+

2	elevation over 1,500m (proportion)	+
	elevation between 500 and 1,500m (proportion)	+
	population density over 1,000 ppl/km^2 (proportion)	+
	healthcare access and quality index	-
3	log LDI (I$ per capita)	-
	education (years per capita)	-
	Socio-demographic Index	-

Digestive Diseases

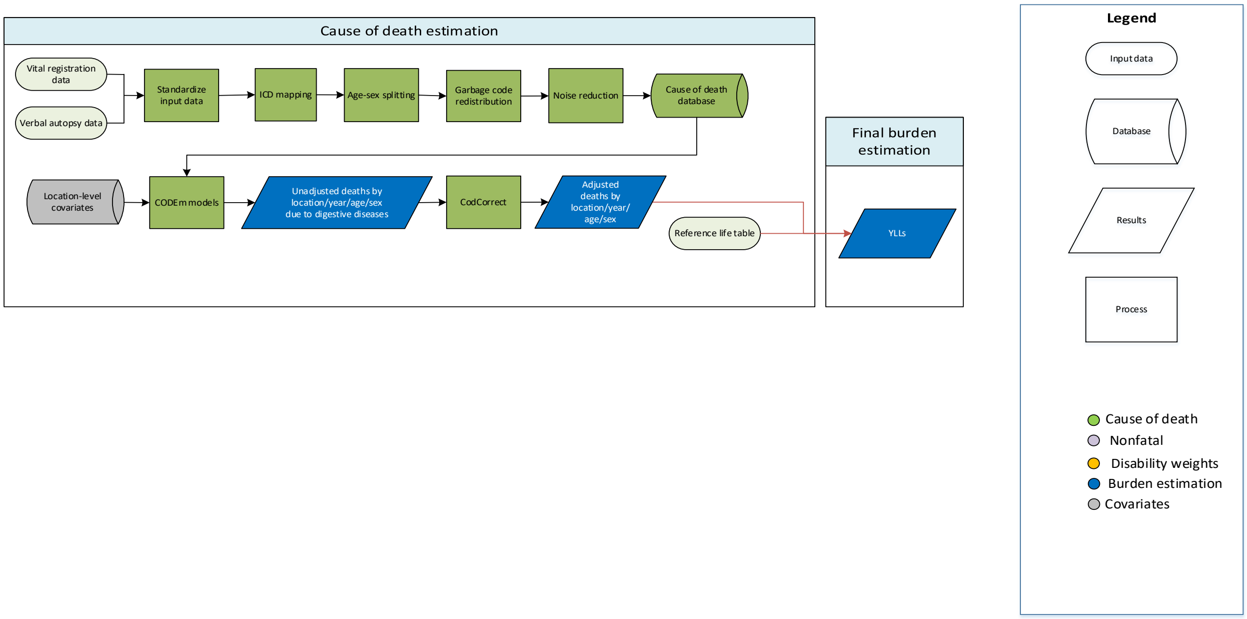

Input data

Data used to estimate mortality of digestive diseases consisted of vital registration data and verbal autopsy data from the cause of death (COD) database. The data in digestive diseases consist of aggregated data from all other specific digestive diseases (peptic ulcer disease, gastritis and duodenitis, gallbladder and biliary diseases, pancreatitis, cirrhosis and other chronic liver diseases, inguinal, femoral and abdominal hernias, inflammatory bowel disease, vascular intestinal disorders, paralytic ileus and intestinal obstruction), as well as unique data points from deaths reported with a set of non-specific digestive disease codes. In GBD 2017, in contrast to previous rounds, cirrhosis and other chronic liver diseases were included in the model of total deaths due to digestive diseases. We marked data as outliers in instances where garbage code redistribution and noise reduction, in combination with small sample sizes, resulted in unreasonable cause fractions. We also marked as outliers those data that violated well-established time or age trends. Methods for selecting outliers were consistent across both vital registration and verbal autopsy data.

Modelling strategy

We modelled deaths due to all digestive diseases with a standard CODEm model using the cause of death database and location-level covariates as inputs. The model followed standard parameters. We hybridised separate global and data-rich models to acquire unadjusted results, which we finalised and adjusted using CoDCorrect to reach final years of life lost (YLLs) due to digestive diseases. Compared to GBD 2016, in 2017 we removed the underweight covariate, and cigarette consumption was quantified as 5-year cumulative rather than 10-year cumulative.

Covariate	Level	Direction
Alcohol (litres per capita)	1	1
Cumulative cigarettes (5 years)	1	1
Education (years per capita)	3	-1
Lag distributed income (per capita)	3	-1

Sanitation (proportion with access)	1	-1
Socio-demographic Index	3	-1
Fruits (grams adjusted)	2	-1
Red meats (grams adjusted)	2	1
Healthcare access and quality index	2	-1

Cirrhosis

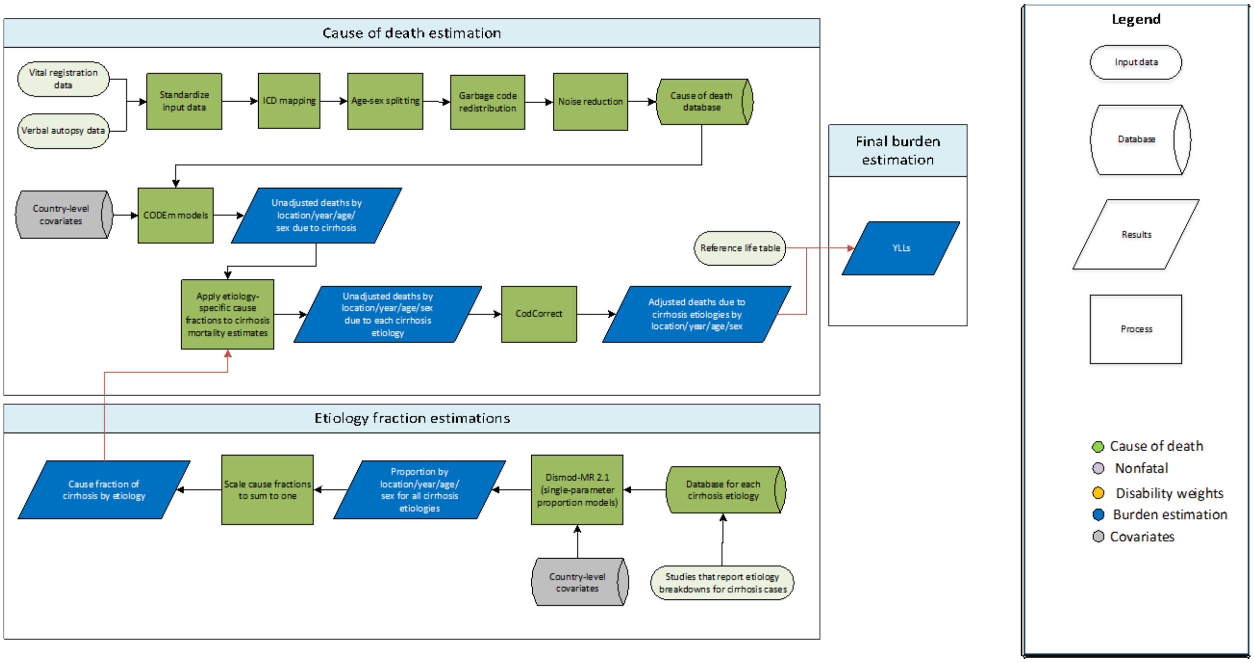

Input data

We modelled cirrhosis mortality using all available data in the cause of death database. Data points were outliered based on country data completeness, and for implausible values informing the models.

Modelling strategy

We modelled cirrhosis mortality using a two-model hybrid approach: 1) a global CODEm model of all locations, using all data in the CoD database; and 2) a CODEm model restricted to data-rich countries, both from ages 1 to 95+. We modelled cirrhosis deaths encompassing all aetiologies in a parent CODEm model. We then applied proportion splits to generate cirrhosis deaths for each cirrhosis aetiology.

To estimate mortality from cirrhosis due to alcohol, cirrhosis due to hepatitis B, cirrhosis due to hepatitis C, cirrhosis due to other causes, and cirrhosis due to NASH, we developed aetiological proportion models using DisMod MR to split the parent cirrhosis mortality estimates. In GBD 2017 we incorporated cirrhosis due to non-alcoholic steatohepatitis (NASH) as a fifth etiology for cirrhosis. Cases of cirrhosis due to NASH were previously nestled within the "other cirrhosis" aetiology and are occasionally listed as cryptogenic cirrhosis in literature.

Given the similar aetiologies for liver cancer and cirrhosis, we integrated the aetiology models for these two causes. We used liver cancer aetiology proportion models as covariates for our cirrhosis aetiology proportion models. The aetiology proportion models included as covariates such as alcohol consumption (litres per capita), hepatitis B surface antigen (HBsAg) seroprevalence, hepatitis C (anti-HCV IgG) seroprevalence, and obesity. Proportions from the five aetiology models were then rescaled to sum to one at the draw level and used to split the parent cirrhosis mortality estimates. Covariates inputs for

published models for the CODEm cirrhosis parent model and coefficient tables for GBD 2017 for the five DisMod proportion models are listed below.

Parent cirrhosis CODEm model covariate table

**Covariate name**	**Level**	**Direction**
Alcohol (litres per capita)	1	1
Schistosomiasis prevalence	1	1
Hepatitis B prevalence	1	1
Hepatitis C prevalence	1	1
Hepatitis B3 vaccine (proportion covered)	1	-1
Diabetes prevalence	2	1
BMI (mean)	2	1
Healthcare access and quality index	2	-1
Education (years per capita)	3	-1
Health system access	3	-1
LDI (per capita)	3	-1
Socio-demographic Index	3	-1

Etiology proportion DisMod model covariate tables

Proportion of cirrhosis due to Hep B covariate table

Measure	Covariate	Type	beta	exp beta
Proportion	Hepatitis B (HBsAg) seroprevalence	Country	0.50 (0.029 to 0.97)	1.65 (1.03 to 2.63)
Proportion	Proportion of liver cancer due to hepatitis B (age-standardised)	Country	0.62 (0.067 to 0.99)	1.86 (1.07 to 2.68)
Proportion	Hepatitis B 3-dose coverage (proportion), lagged 10 years	Country	-0.21 (-0.74 to -0.0077)	0.81 (0.48 to 0.99)

Proportion of cirrhosis due to Hep C covariate table

Measure	Covariate	Type	beta	exp beta
Proportion	Hepatitis C (IgG) seroprevalence	Country	0.49 (0.022 – 0.97)	1.63 (1.02 – 2.65)
Proportion	Proportion of liver cancer due to hepatitis C (age-standardised)	Country	0.54 (0.041 – 0.98)	1.72 (1.04 – 2.66)

Proportion of cirrhosis due to alcohol covariate table

Measure	Covariate	Type	beta	exp beta
Proportion	Alcohol (litres per capita)	Country	0.028 (0.0013 – 0.075)	1.03 (1.00 – 1.08)
Proportion	Proportion of liver cancer due to alcohol (age-standardised)	Country	0.53 (0.032 – 0.98)	1.70 (1.03 – 2.66)
Proportion	Alcohol abstainer proportion, age-standardised	Country	-0.55 (-0.99 to -0.035)	0.58 (0.37 – 0.97)

Proportion of cirrhosis due to NASH covariate table

Measure	Covariate	Type	beta	exp beta
Proportion	Mean BMI	Country	0.054 (0.00085 – 0.18)	1.06 (1.00 – 1.20)
Proportion	Prevalence of obesity	Country	0.81 (0.029 – 1.92)	2.24 (1.03 – 6.79)
Proportion	Proportion of liver cancer due to NASH (age-standardised)	Country	0.98 (0.040 – 1.95)	2.67 (1.04 – 7.02)
Proportion	NAFLD/NASH prevalence	Country	0.85 (0.026 – 1.93)	2.34 (1.03 – 6.87)

Proportion of cirrhosis due to other causes covariate table

Measure	Covariate	Type	beta	exp beta
Proportion	Proportion of liver cancer due to other causes (age-standardised)	Country	0.54 (0.037 – 0.98)	1.72 (1.04 – 2.66)

Upper Digestive Diseases

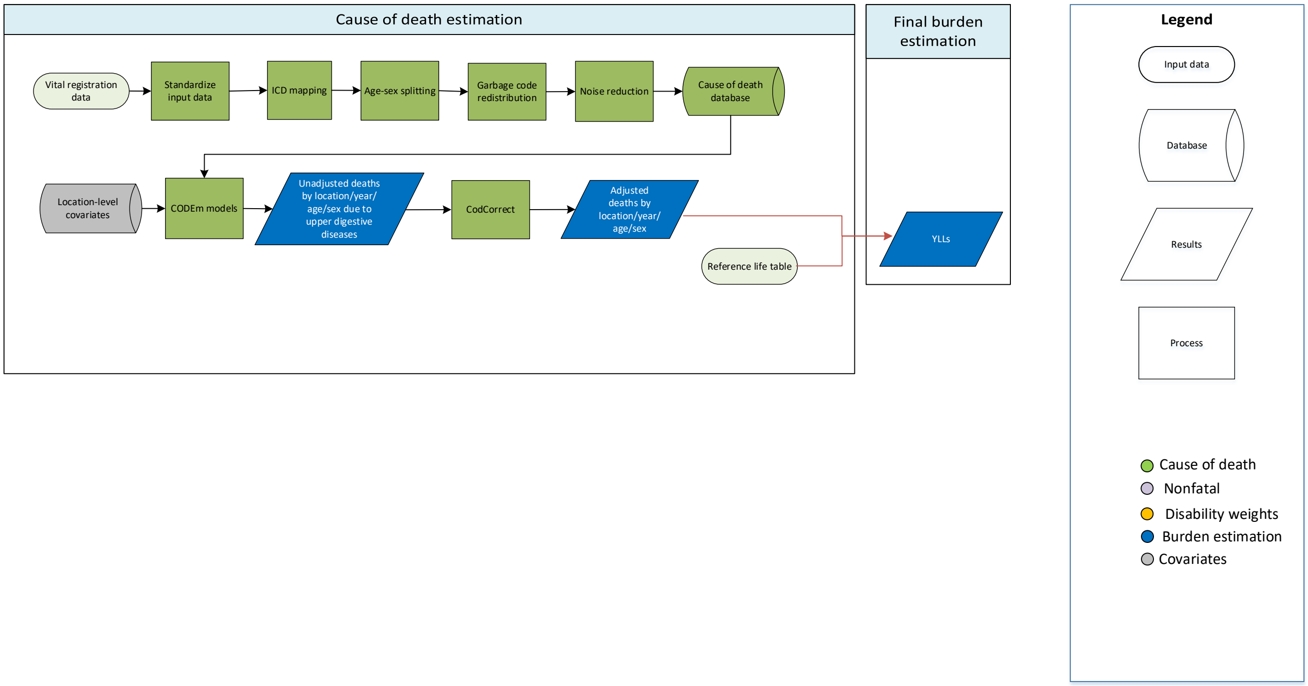

Input data

Data used to estimate mortality due to upper digestive diseases consisted of vital registration data from the cause of death (COD) database. Upper digestive disease data aggregate deaths due to peptic ulcer disease and gastritis and duodenitis, which are also modelled separately. For sources of data that were considered too low-quality to definitively assign peptic ulcer or gastritis deaths to one of these two causes, data were included only in the upper digestive disease dataset. We marked data as outliers in instances where garbage code redistribution and noise reduction, in combination with small sample sizes, resulted in unreasonable cause fractions; and data that violated well-established time or age trends.

Modelling strategy

We modelled deaths due to upper digestive diseases with a standard CODEm model using the cause of death database and location-level covariates as inputs. The model followed standard parameters, with the exception that the start age of the model was 1 year. We hybridised separate global and data-rich models to acquire unadjusted results, which we finalised and adjusted using CoDCorrect to reach final YLLs due to upper digestive diseases. This was the first year upper digestive diseases, in aggregate, were modelled as a cause of death. The following covariates were entered into CODEm for the selection process.

Covariate	Level	Direction
Alcohol (litres per capita)	1	1
Cumulative cigarettes (5 years)	1	1
Cumulative cigarettes (10 years)	1	1
Log LDI (I$ per capita)	3	-1
Sanitation (proportion with access)	2	-1

Smoking prevalence	1	1
Maternal education (years per capita)	3	-1
Unsafe water (summary exposure variable)	2	1
Socio-demographic Index	3	-1
Vegetables adjusted (g)	2	0
Healthcare access and quality index	2	-1

Peptic Ulcer Disease

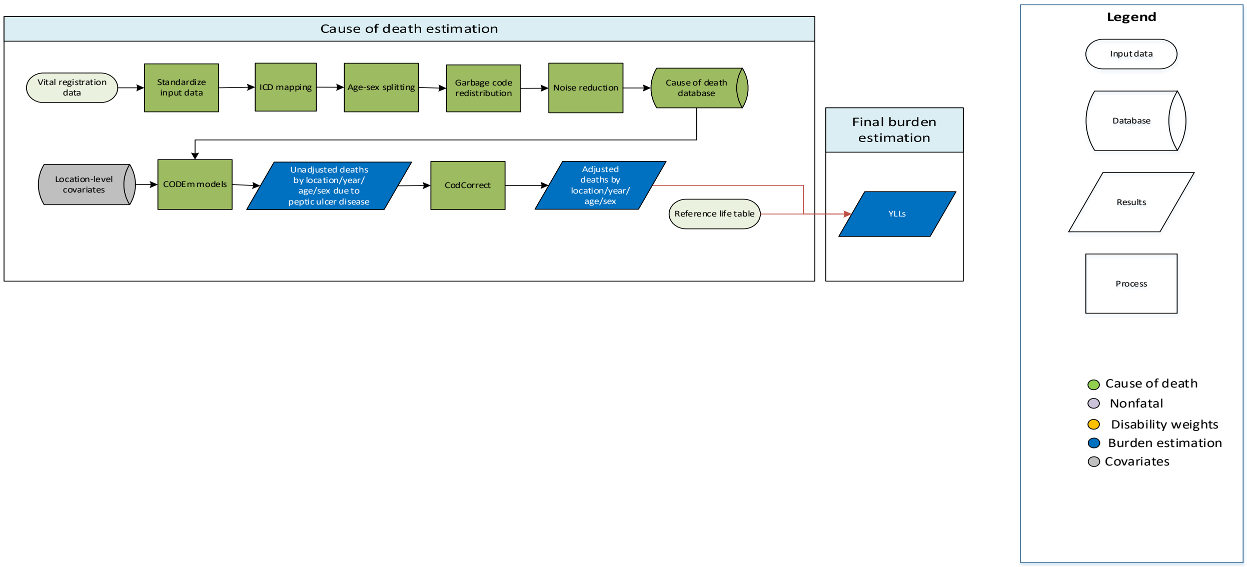

Input data

Data used to estimate mortality of peptic ulcer disease consisted of vital registration data from the cause of death (COD) database. We marked data as outliers in instances where garbage code redistribution and noise reduction, in combination with small sample sizes, resulted in unreasonable cause fractions, and data that violated well-established time or age trends.

Modelling strategy

We modelled deaths due to peptic ulcer disease with a standard CODEm model using the cause of death database and location-level covariates as inputs. The model followed standard parameters, with the exception that the start age of the model was 1 year instead of 0 and the linear floor rate was lowered to 0.0001 in order to better capture low data. We hybridised separate global and data-rich models to acquire unadjusted results, which we finalised and adjusted using CoDCorrect to reach final years of life lost (YLLs) due to peptic ulcer disease. The covariates tried and their expected strengths and directions are unchanged in GBD 2017 compared to GBD 2016.

Covariate	Level	Direction
Alcohol (litres per capita)	1	1
Cumulative cigarettes (10 years)	1	1
Cumulative cigarettes (5 years)	1	1
Lag distributed income (per capita)	3	-1
Sanitation (proportion with access)	2	-1
Smoking (prevalence)	1	1
Maternal education (years per capita)	3	-1
Improved water source (proportion with access)	2	1
Socio-demographic Index	3	-1
Vegetables (grams adjusted)	2	0
Healthcare access and quality index	2	-1

Gastritis and Duodenitis

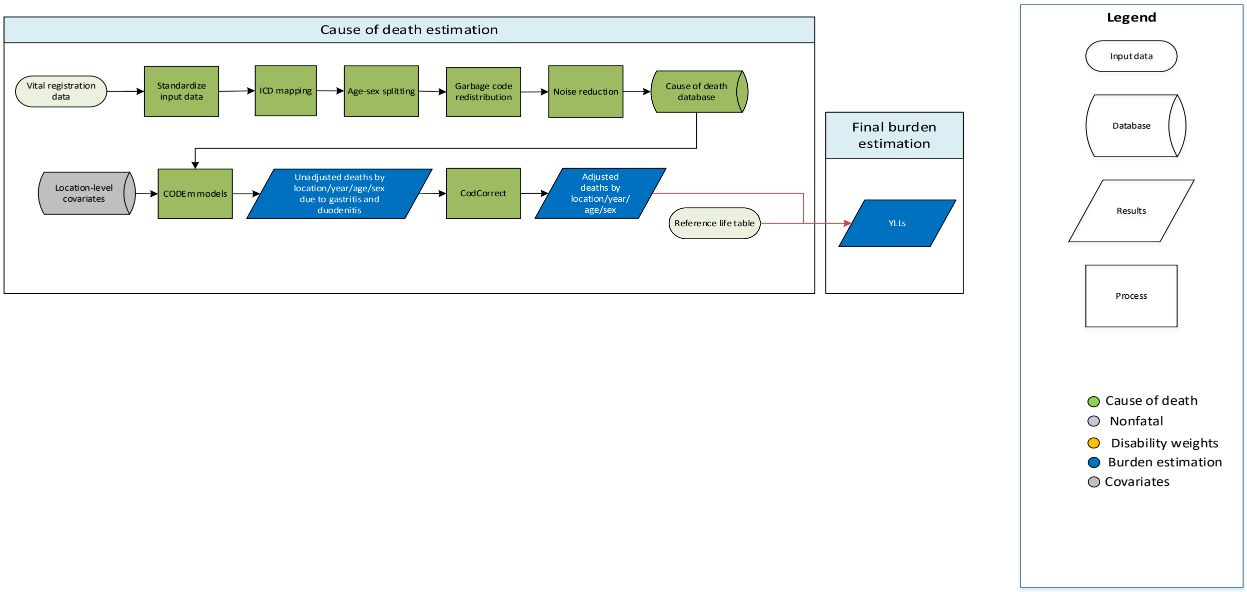

Input data

Vital registration data were used to model this cause. We marked data as outliers in instances where garbage code redistribution and noise reduction, in combination with small sample sizes, resulted in unreasonable cause fractions, and data that violated well-established time or age trends.

Modelling strategy

We modelled deaths due to gastritis and duodenitis with a standard CODEm model using the cause of death database and location-level covariates as inputs. The model followed standard parameters, with the exception that the start age of the model was 1 year instead of 0 and the linear floor rate was lowered to 0.001 in order to better capture low data. We hybridised separate global and data-rich models to acquire unadjusted results, which we finalised and adjusted using CoDCorrect to reach final years of life lost (YLLs) due to gastritis and duodenitis. Covariates tried and their expected strengths and directions in GBD 2017 were not changed compared to GBD 2016.

**Covariate**	**Level**	**Direction**
Alcohol (litres per capita)	1	1
Cumulative cigarettes (10 years)	1	1
Cumulative cigarettes (5 years)	1	1
Education (years per capita)	3	-1
Lag distributed income (per capita)	3	-1
Sanitation (proportion with access)	2	-1
Smoking prevalence	1	1
Unsafe water (summary exposure variable)	2	1
Socio-demographic Index	3	-1
Vegetables (grams adjusted)	2	0
Healthcare access and quality index	2	-1

Appendicitis

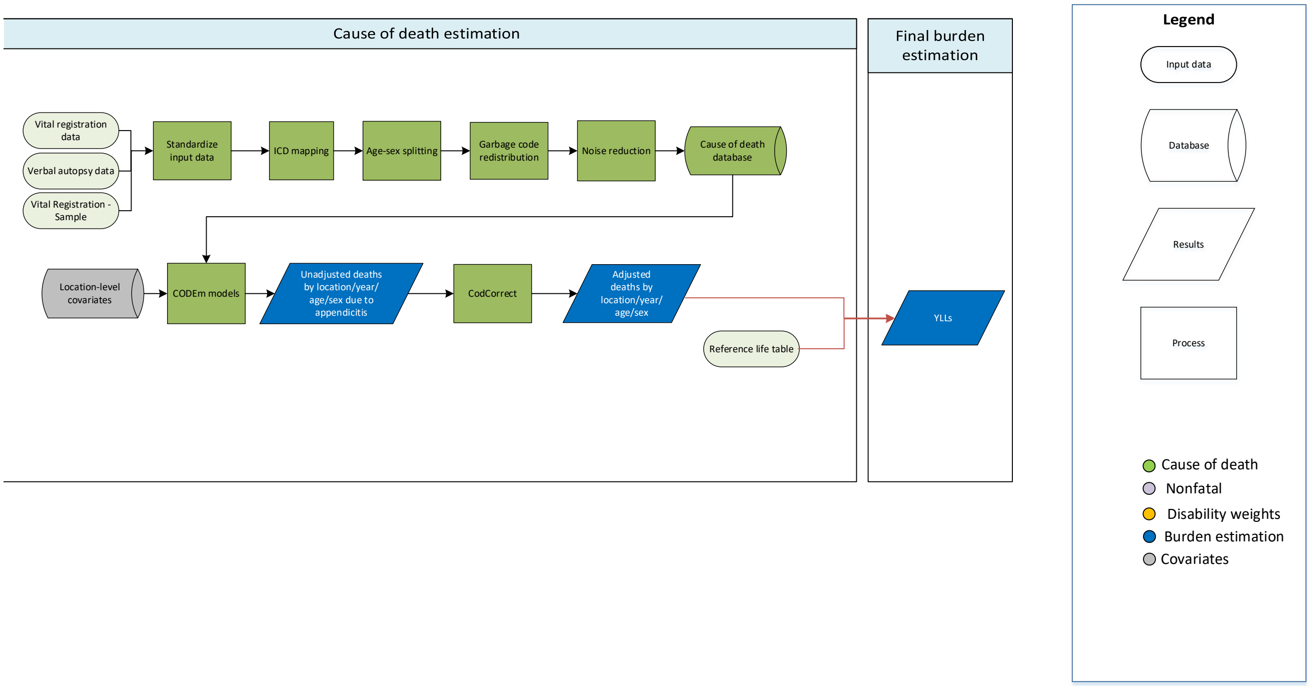

Input data

Data used to estimate appendicitis mortality consisted of vital registration and verbal autopsy data from the cause of death (COD) database. We outliered data in instances where garbage code redistribution and noise reduction, in combination with small sample sizes, resulted in unreasonable cause fractions; and data that violated well-established time or age trends.

Modelling strategy

We modelled deaths due to appendicitis with a standard CODEm model using the cause of death database and location-level covariates as inputs. The model followed standard parameters, with the exception that the start age of the model was 1 year old instead of 0 and the linear floor rate was lowered to 0.0001 in order to better capture low data. We hybridized separate global and data-rich models to acquire unadjusted results, which we finalised and adjusted using CodCorrect to reach final YLLs due to appendicitis.

There were no significant changes in the modelling process between GBD 2016 and GBD 2017.

Level	Covariate	Direction
2	Healthcare access and quality index	-
	Fruits adjusted (g)	-
	Vegetables adjusted (g)	-
3	Education (years per capita)	-
	Log LDI (I$ per capita)	-
	Socio-demographic Index	-
	Health system access (capped)	-

Paralytic Ileus and Intestinal Obstruction

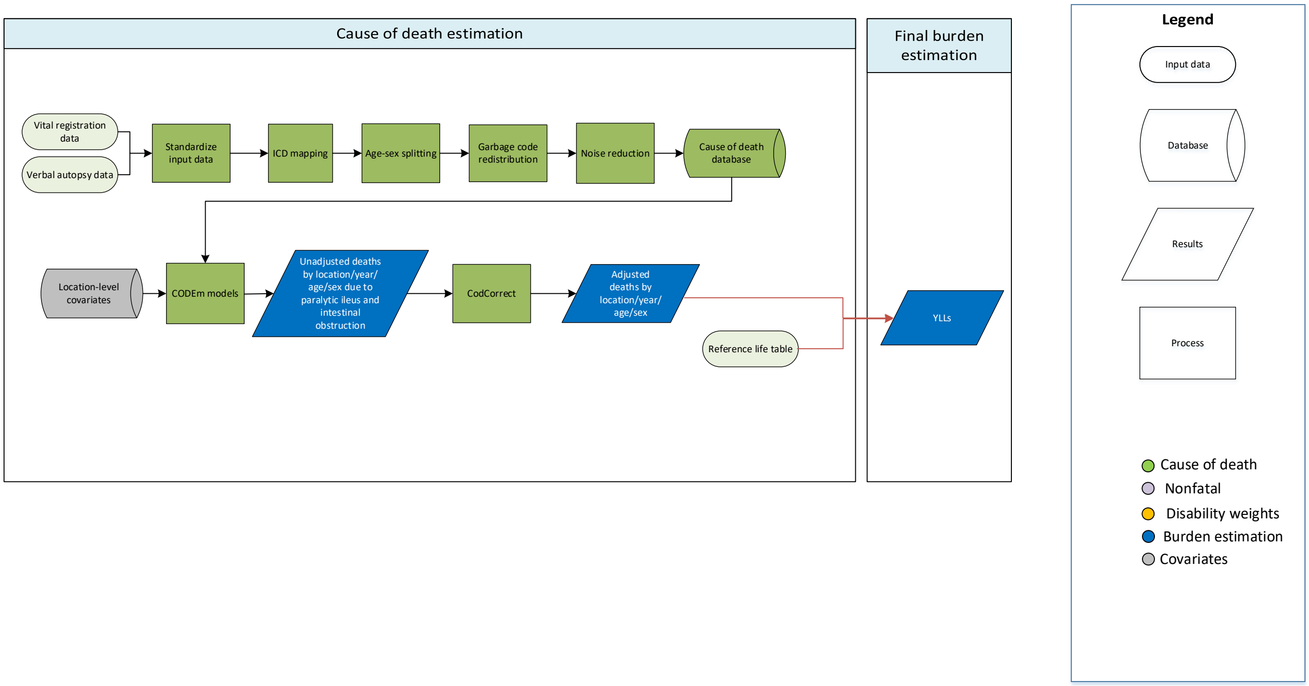

Input data

Data used to estimate paralytic ileus and intestinal obstruction mortality consisted of vital registration and verbal autopsy data from the cause of death (COD) database. We outliered all VA data in children under the age of 1 because it is not possible to accurately diagnose paralytic ileus or intestinal obstruction in this age group; and data that violated well-established time or age trends; and data in instances where garbage code redistribution and noise reduction, in combination with small sample sizes, resulted in unreasonable cause fractions.

Modelling strategy

We modelled deaths due to paralytic ileus and intestinal obstruction with a standard CODEm model using the cause of death database and location-level covariates as inputs. The model followed standard parameters, with the exception that the linear floor rate was lowered to 0.0001 in order to better capture low data. We hybridised separate global and data-rich models to acquire unadjusted results, which we finalised and adjusted using CodCorrect to reach final YLLs due to paralytic ileus and intestinal obstruction.

There were no significant changes in the modelling process between GBD 2016 and GBD 2017.

Level	Covariate	Direction
2	Healthcare access and quality index	-
	Fruits adjusted (g)	-
	Vegetables adjusted (g)	-
3	Education (years per capita)	-
	Log LDI (I$ per capita)	-
	Socio-demographic Index	-
	Health system access (capped)	-

Inguinal, Femoral, and Abdominal Hernias

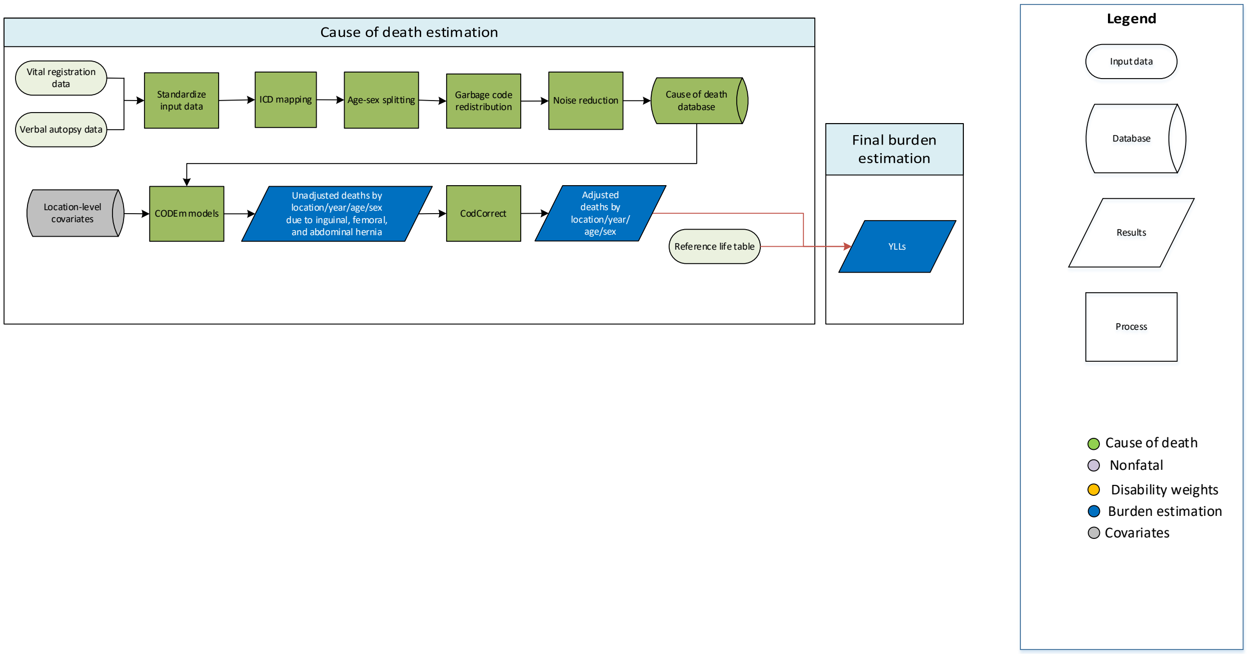

Input data

Vital registration and verbal autopsy data were used to model this cause. We marked data as outliers in instances where garbage code redistribution and noise reduction, in combination with small sample sizes, resulted in unreasonable cause fractions. We also marked as outliers those data that violated well-established time or age trends. Methods for assigning outlier status were consistent across both vital registration and verbal autopsy data.

Modelling strategy

We modelled deaths due to inguinal, femoral, and abdominal hernias with a standard CODEm model using the cause of death database and location-level covariates as inputs. The model followed standard parameters, with the exception that the start age of the model was 1 year instead of 0 and the linear floor rate was lowered to 0.0001 in order to better capture low data. We hybridised separate global and data-rich models to acquire unadjusted results, which we finalised and adjusted using CoDCorrect to reach final years of life lost (YLLs) due to inguinal, femoral, and abdominal hernias. In contrast to GBD 2016, covariates for smoking and BMI were included for possible selection during CODEm modelling in GBD 2017.

Covariate	Level	Direction
Education (years per capita)	3	-1
Lag distributed income (per capita)	3	-1
Socio-demographic Index	3	0
Healthcare access and quality index	2	-1
Cumulative cigarettes (10 years)	1	1
Cumulative cigarettes (5 years)	1	1

Smoking prevalence	1	1
Body mass index (mean)	1	-1

Inflammatory Bowel Disease

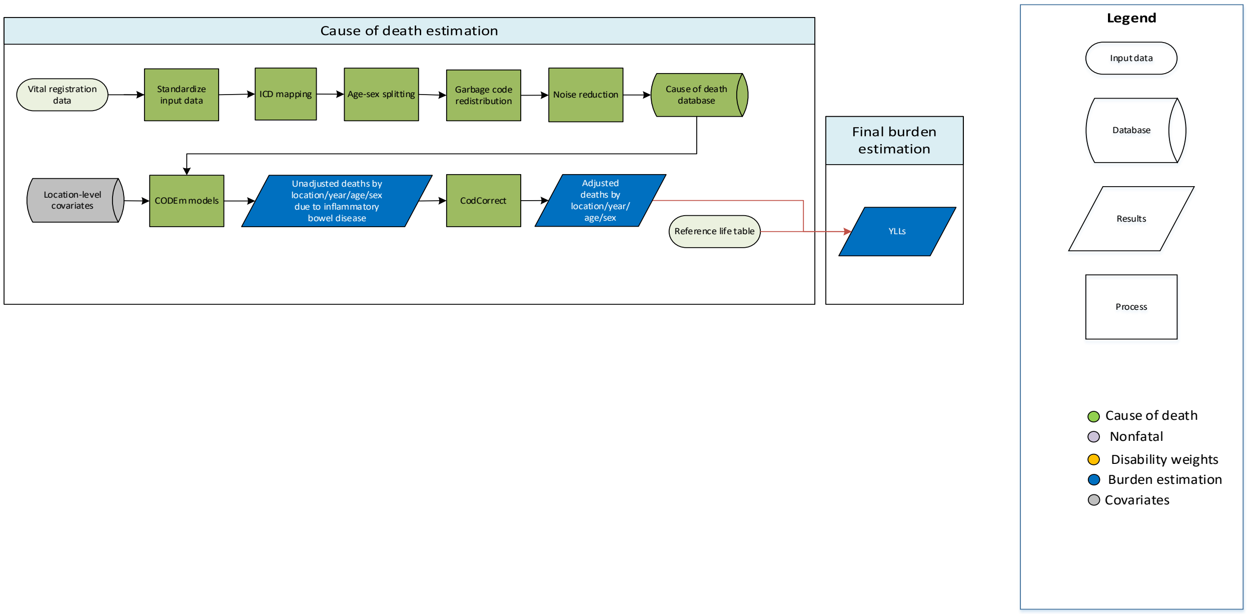

Input data

Vital registration data were used to model this cause. We marked data as outliers in instances where garbage code redistribution and noise reduction, in combination with small sample sizes, resulted in unreasonable cause fractions; and data that violated well-established time or age trends.

Modelling strategy

We modelled deaths due to inflammatory bowel disease with a standard CODEm model using the cause of death database and location-level covariates as inputs. The model followed standard parameters, with the exception that the start age of the model was 1 year instead of 0 and the linear floor rate was lowered to 0.0001 in order to better capture low data. We hybridised separate global and data-rich models to acquire unadjusted results, which we finalised and adjusted using CoDCorrect to reach final years of life lost (YLLs) due to inflammatory bowel disease. In GBD 2017 there were no changes in covariates tried or their expected strengths or directions, as compared to GBD 2016.

Covariate	Level	Direction
Education (years per capita)	3	-1
Lag distributed income (per capita)	3	0
Latitude 15 to 30 (proportion)	2	-1
Latitude 30 to 45 (proportion)	2	1
Latitude 45 plus (proportion)	2	1
Socio-demographic Index	3	0
Fruits (grams adjusted)	1	-1
Red meats (grams adjusted)	1	1
Saturated fats (adjusted percent)	1	1
Vegetables (grams adjusted)	1	-1
Healthcare access and quality index	2	-1

Vascular Intestinal Disorders

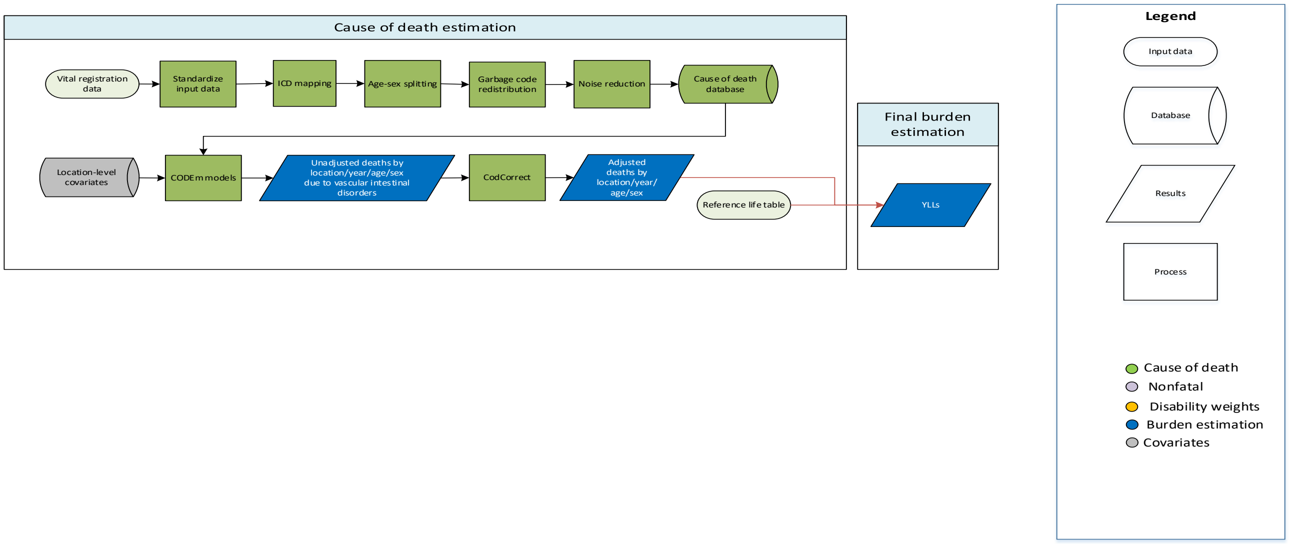

Input data

Vital registration data were used to model this cause. We marked data as outliers in instances where garbage code redistribution and noise reduction, in combination with small sample sizes, resulted in unreasonable cause fractions; and data that violated well-established time or age trends.

Modelling strategy

We modelled deaths due to vascular intestinal disorders with a standard CODEm model using the cause of death database and location-level covariates as inputs. The model followed standard parameters, with the exception that the start age of the model was 1 year instead of 0 and the linear floor rate was lowered to 0.0001 in order to better capture low data. We hybridised separate global and data-rich models to acquire unadjusted results, which we finalised and adjusted using CoDCorrect to reach final years of life lost (YLLs) due to vascular intestinal disorders. In GBD 2017, compared to GBD 2016, there were no changes in covariates or their expected strength and direction.

Covariate	Level	Direction
Alcohol (litres per capita)	2	1
Diabetes fasting plasma glucose (mmol/L)	1	1
Diabetes age-specific prevalence (proportion)	1	1
Education (years per capita)	3	-1
Lag distributed income (per capita)	3	-1
Cholesterol (mean)	1	1
Systolic blood pressure (mean)	1	1
Latitude over 45 (proportion)	3	1
Socio-demographic Index	3	0
Fruits (grams adjusted)	2	-1
Saturated fats (adjusted percent)	1	1
Vegetables (grams adjusted)	2	-1
Healthcare access and quality index	2	-1

Gallbladder and Biliary Diseases

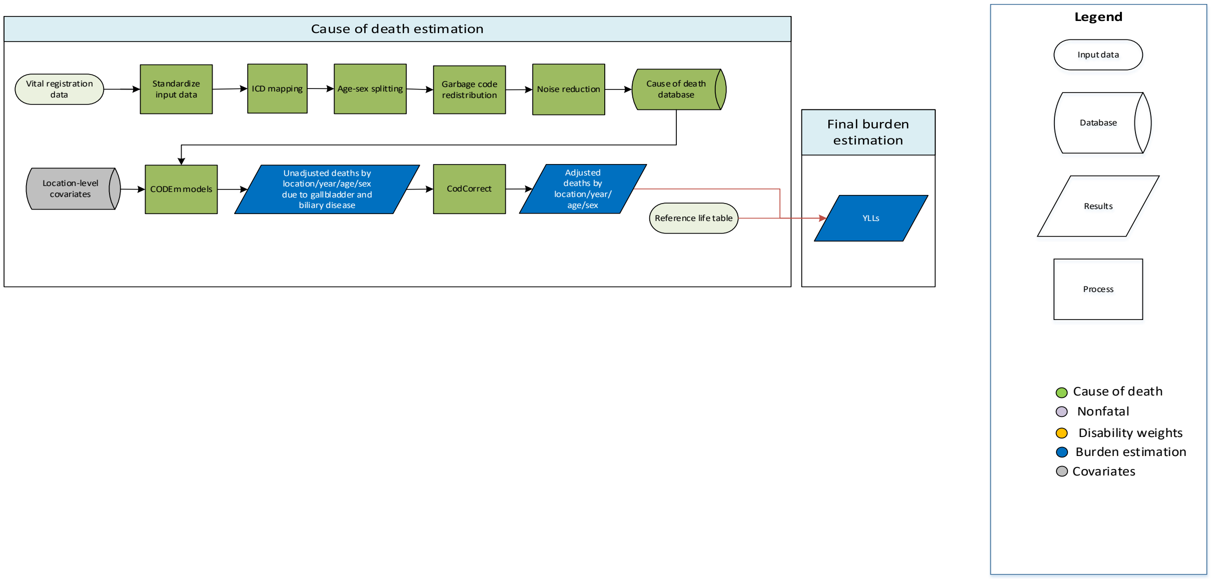

Input data

Data used to estimate mortality of gallbladder and biliary diseases consisted of vital registration data from the cause of death (COD) database. We outliered data in instances where garbage code redistribution and noise reduction, in combination with small sample sizes, resulted in unreasonable cause fractions; and data that violated well-established time or age trends.

Modelling strategy

We modelled deaths due to gallbladder and biliary diseases with a standard CODEm model using the cause of death database and location-level covariates as inputs. The model followed standard parameters, with the exception that the start age of the model was 1 year old instead of 0 and the linear floor rate was lowered to 0.0001 in order to better capture low data. We hybridised separate global and data-rich models to acquire unadjusted results, which we finalised and adjusted using CodCorrect to reach final years of life lost (YLLs) due to gallbladder and biliary diseases. In GBD 2016 we added the Healthcare Access and Quality (HAQ) index covariate and replaced the animal fats (kcal per capita) covariate with an updated saturated fats covariate (adjusted percent). There were no significant changes in the modelling process between GBD 2016 and GBD 2017.

Level	Covariate	Direction
1	Body-mass index (mean)	+
	Saturated fats (adjusted percent)	+
2	Alcohol (litres per capita)	+
	Healthcare access and quality index	-
	Red meats (grams adjusted)	+
	Population over 65 (proportion)	+
3	Socio-demographic Index	0
	Lag distributed income (per capita)	0
	Education (years per capita)	0

Pancreatitis

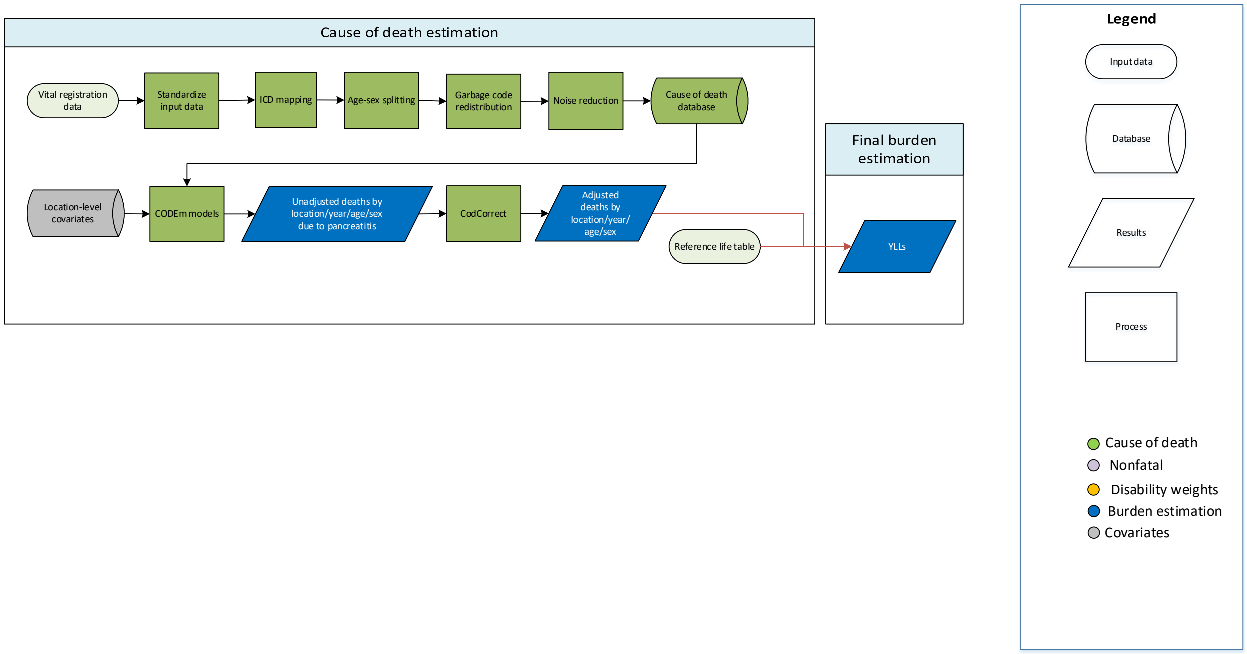

Input data

Vital registration data were used to model this cause. We marked data as outliers in instances where garbage code redistribution and noise reduction, in combination with small sample sizes, resulted in unreasonable cause fractions, and data that violated well-established time or age trends.

Modelling strategy

We modelled deaths due to pancreatitis with a standard CODEm model using the cause of death database and location-level covariates as inputs. The model followed standard parameters, with the exception that the start age of the model was 1 year instead of 0 and the linear floor rate was lowered to 0.0001 in order to better capture low data. We hybridised separate global and data-rich models to acquire unadjusted results, which we finalised and adjusted using CoDCorrect to reach final YLLs due to pancreatitis. In GBD 2017, we did not change covariates tried, or their expected strengths and directions, as compared to GBD 2016.

Covariate	Level	Direction
Alcohol (litres per capita)	1	1
Education (years per capita)	3	-1
Lag distributed income (per capita)	3	0
Body mass index (mean)	2	1
Pancreatitis scalar (summary exposure variable)	1	1
Socio-demographic Index	3	0
Healthcare access and quality index	2	-1

Other Digestive Diseases

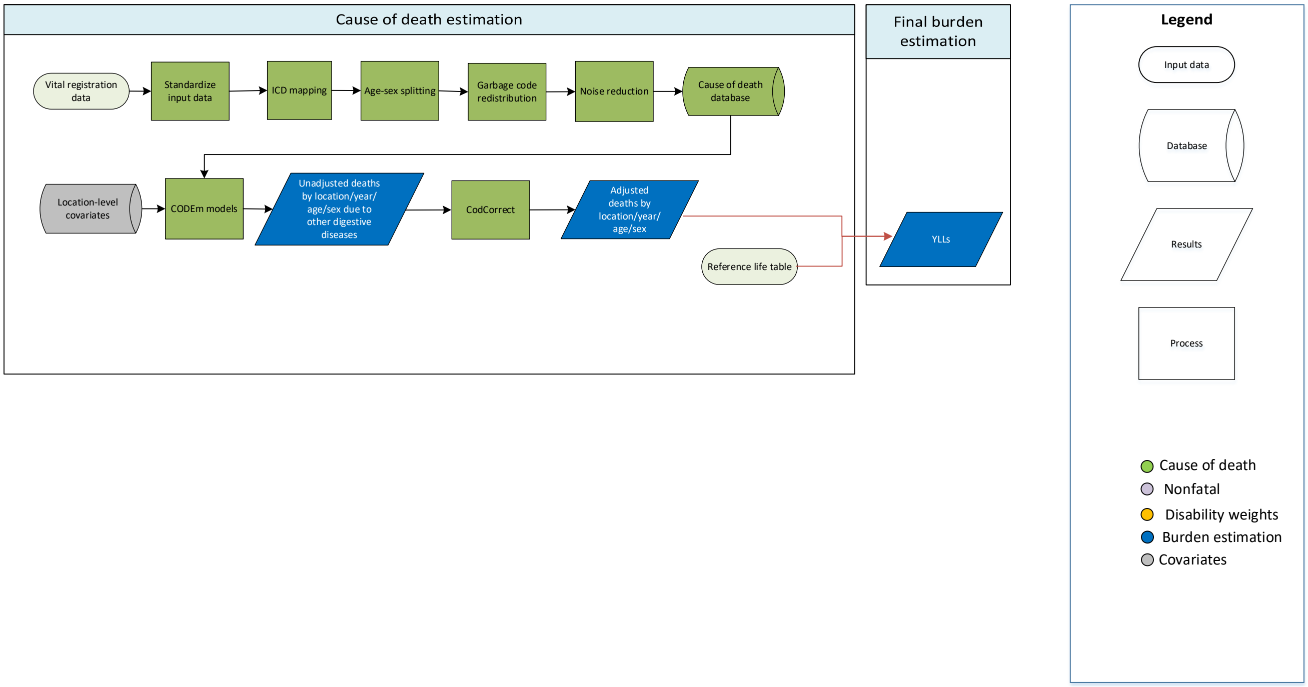

Input data

Data used to estimate mortality due to other digestive diseases consisted of vital registration data from the cause of death (COD) database. We outliered data in instances where garbage code redistribution and noise reduction, in combination with small sample sizes, resulted in unreasonable cause fractions; and data that violated well-established time or age trends.

Modelling strategy

We modelled deaths due to other digestive diseases with a standard CODEm model using the cause of death database and location-level covariates as inputs. The model followed standard parameters, with the exception that the start age of the model was 1 year old instead of 0. We hybridised separate global and data-rich models to acquire unadjusted results, which we finalised and adjusted using CodCorrect to reach final YLLs due to other digestive diseases.

There were no significant changes in the modelling process between GBD 2016 and GBD 2017.

Level	Covariate	Direction
1	Alcohol (litres per capita)	+
	Cumulative cigarettes (5 years)	+
	Cumulative cigarettes (10 years)	+
	Smoking prevalence	+
2	Diabetes age-standardised prevalence (proportion)	+
	Mean BMI	+
	Sanitation (proportion with access)	-
	Improved water source (proportion with access)	-
	Fruits adjusted (g)	-
	Red meats adjusted (g)	+
	Saturated fats adjusted (percentage)	+
	Healthcare access and quality index	-
	Vegetables adjusted (g)	0
3	Education (years per capita)	-
	Log LDI (I$ per capita)	-
	Socio-demographic Index	0
	Health system access 2	-

Alzheimer's Disease and Other Dementias

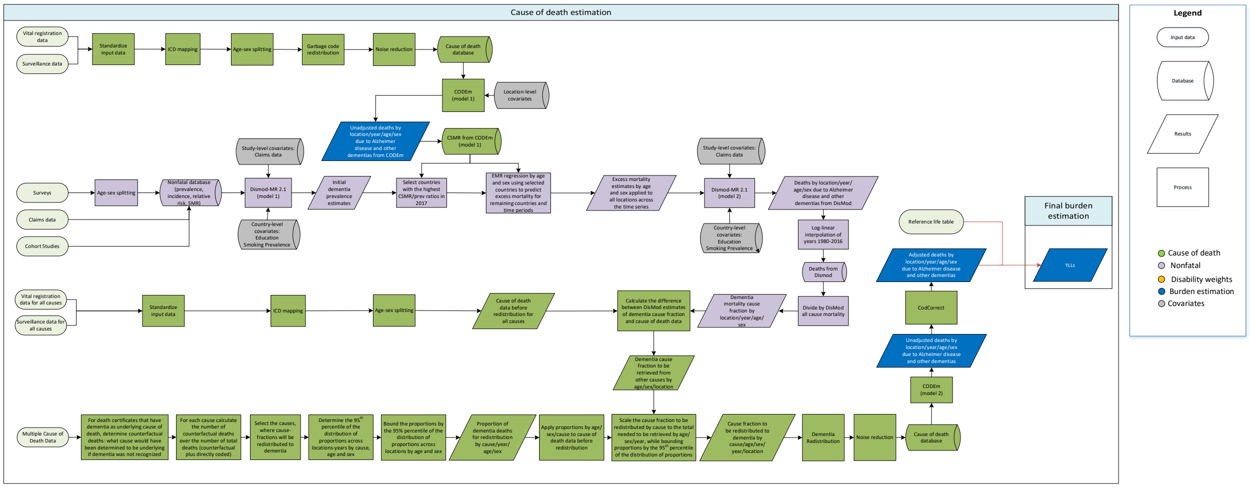

Input Data

In GBD 2017, data used to estimate deaths due to Alzheimer's disease and other dementias (dementias hereafter) included mortality data from vital registration systems and prevalence data from surveys and medical claims sources.

An updated systematic review was conducted from September 2016 to August 2017, and search terms[1] were set to capture studies for all dementia, including its sub-types. The search yielded 970 initial hits and 27 were marked for extraction. Inclusion criteria comprised studies that reported prevalence, incidence, remission rate, excess mortality rate, relative risk of mortality, standardised mortality ratio, or with-condition mortality rate. Studies with non-representative samples or no clearly defined sample were excluded. A flow chart documenting this review is displayed below. We also added US claims data for 2011 and 2012–2015.

[1] ((dementia[Title/Abstract]) AND (incidence[Title/Abstract] OR prevalence[Title/Abstract] OR epidemiology[Title/Abstract])) AND ("2016/09/01"[Date - Publication] : "2017/08/29"[Date - Publication])

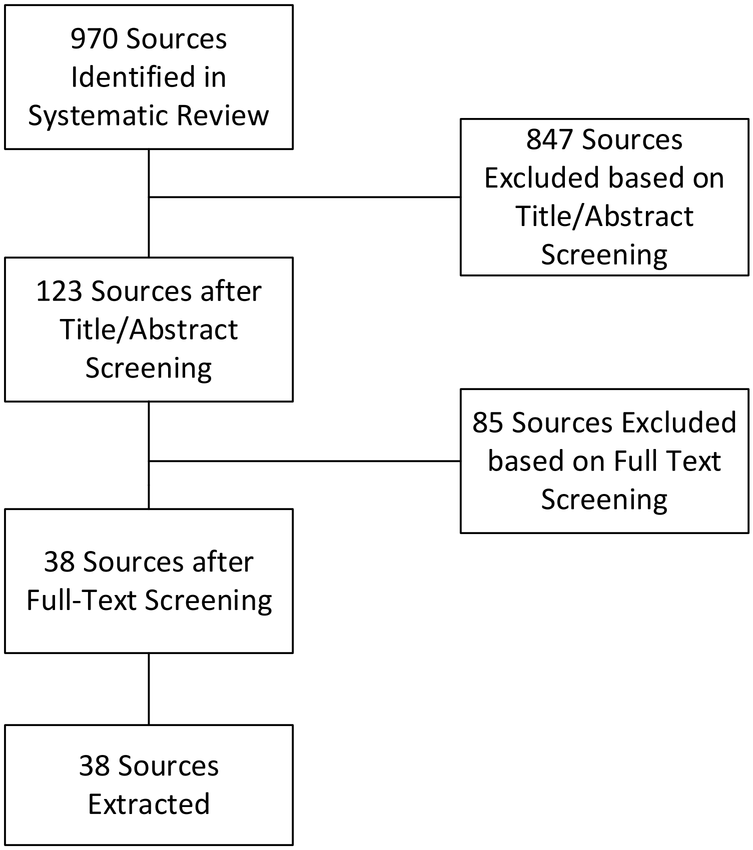

Modelling Strategy

Overview

Dementia mortality rates have increased more than five-fold since 1980 in high-quality vital registration systems such as in the US and Scandinavia. We have not seen an equivalent increase in prevalence and incidence data sources. If at all, there has been a modest decline in incidence and prevalence of dementia in studies in the UK and the US.[2,3] Also, the greater than 20-fold variation in mortality rates of dementia between countries is much greater than the four-fold difference in prevalence and incidence between countries. As it is unlikely that case fatality from dementia has dramatically increased over the time period and that it would differ by a very large margin between countries, the hypothesis is that certifying and coding practices have changed over time and at a different pace between countries. To avoid spurious large trends over time in the fatal component of the burden of dementia, we decided for GBD 2013 to make dementia mortality rates consistent with the most recent rates relative to prevalence of countries that are most likely to certify or code dementia as an underlying cause of death. This approach was applied again for GBD 2017 with some modifications, described further below.

[2] Akushevich I, Kravchenko J, Ukraintseva S, Arbeev K, Yashin AI. Time trends of incidence of age-associated diseases in the US elderly population: Medicare-based analysis. *Age and ageing*. 2013 Jul 1;42(4):494-500.

[3] Matthews FE, Arthur A, Barnes LE, Bond J, Jagger C, Robinson L, Brayne C, Medical Research Council Cognitive Function and Ageing Collaboration. A two-decade comparison of prevalence of dementia in individuals aged 65 years and older from three geographical areas of England: results of the Cognitive Function and Ageing Study I and II. *The Lancet*. 2013 Nov 1;382(9902):1405-12.

Modelling steps

First, we ran a CODEm model for dementia and extracted the mortality rates by age, sex, and geography for 2017. The covariates used in this model are displayed below.

Level	Covariate	Direction
1	Diabetes age-specific prevalence	+
	Mean BMI	+
	Cholesterol (total, mean per capita)	+
	Systolic blood pressure (mmHg)	+
	Smoking prevalence	+
	Education (years per capita)	-
2	Outdoor air pollution ($PM_{2.5}$)	+
	Physical activity (MET-mins per week)	-
	Latitude over 45 (proportion)	+
	Red meat consumption adjusted (g)	+
	Healthcare access and quality index	-
3	LDI per capita(I$ per capita)	0
	Sanitation (proportion with access)	-
	Improved water source (proportion with access)	-

Second, we ran a DisMod-MR 2.1 model with all data on incidence, prevalence, and mortality risk (RR, SMR, or with-condition mortality rates) and a setting of zero remission and extracted 2017 prevalence by age, sex, and geography. To account for potential systematic differences between medical claims and survey data, we crosswalked the US claims data and added a covariate for European GP data. We also used a linear regression to adjust data collected using the 10/66 diagnostic algorithm, which includes more cases of mild cognitive impairment in comparison to the reference DSM definition.[4] Additionally, to account for systematic differences in different methods of case ascertainment between different studies we used study-level covariates on whether or not studies used a doctor-given diagnosis, whether or not they used clinical records to ascertain a diagnosis, and whether or not a study used an algorithmic diagnosis.

Third, we selected countries where the sum of cause-specific mortality rate to prevalence ratio for males and females exceeded 2.5 (excluding small island nations, those without vital registration, and countries without data on prevalence). This resulted in choosing the Finland, Scotland, Sweden, the United States, England, the Netherlands, and Canada. The choice to pick fewer countries for this regression compared to GBD 2015, which used 30 countries in the EMR regression, was motivated by a desire to reduce the spread in EMR values, as some countries used in the regression can retain their original EMR values. We decided to exclude Finland from the regression, as it is a high outlier compared to the other countries included in the regression. However, we still allowed Finland to retain its original EMR values.

[4] Paddick S-M, Longdon AR, Kisoli A, *et al.* Dementia prevalence estimates in sub-Saharan Africa: comparison of two diagnostic criteria. *Glob Health Action* 2013; **6**. DOI:10.3402/gha.v6i0.19646 PMCID: PMC3617645.

Fourth, we used a linear effects regression with dummies on age group and sex to predict excess mortality (ie, the ratio of cause-specific mortality rate and prevalence) by age and sex, the results of which are found in the tables below.

Table: Fixed effect coefficients of EMR regression. Outcome: ln(EMR)

Independent variables	Coef	Std. error	P value	95% Confidence Interval	
Male	0.004	0.035	0.910	-0.064	0.072
Age 40-59	-2.897	0.074	0.000	-3.042	-2.752
Age 60-64	-2.267	0.074	0.000	-2.412	-2.122
Age 65-69	-2.103	0.074	0.000	-2.248	-1.958
Age 70-74	-1.845	0.074	0.000	-1.99	-1.7
Age 75- 80	-1.524	0.074	0.000	-1.669	-1.379
Age 80-84	-1.217	0.074	0.000	-1.362	-1.072
Age 85-89	-0.865	0.074	0.000	-1.01	-0.72
Age 90-94	-0.406	0.074	0.000	-0.551	-0.261
Constant	-1.748	0.055	0.000	-1.856	-1.64

Table: Predicted EMR values by age and sex (95% CI)

	Male	Female
Age 40-59	0.01 (0.009 - 0.011)	0.01 (0.009 - 0.011)
Age 60-64	0.018 (0.016 - 0.02)	0.018 (0.016 - 0.02)
Age 65-69	0.021 (0.019 - 0.024)	0.021 (0.019 - 0.024)
Age 70-74	0.028 (0.025 - 0.031)	0.028 (0.025 - 0.031)
Age 75- 80	0.038 (0.034 - 0.042)	0.038 (0.034 - 0.042)
Age 80-84	0.052 (0.046 - 0.057)	0.052 (0.047 - 0.057)
Age 85-89	0.074 (0.067 - 0.082)	0.074 (0.066 - 0.082)
Age 90-94	0.116 (0.105 - 0.13)	0.116 (0.104 - 0.129)
Age 95+	0.175 (0.157 - 0.196)	0.175 (0.157 - 0.194)

Fifth, these estimates were added to a second DisMod-MR 2.1 model as pertaining to the full 1990–2017 estimation period. For Finland and the countries included in the regression, we allowed them to retain their original EMR values when the age-standardised EMR for a country was higher than the age-standardised EMR prediction generated from the regression. These countries retained their age- and sex-specific ratios and entered those also as pertaining to the full 1990–2017 estimation period. We excluded data for standardised mortality ratio, with-condition mortality rate, and relative risk as we wanted to estimate cause-specific mortality rates that were consistent with the level of excess mortality from the four chosen countries in 2017. No random effects were used in the model in order to prevent spurious inflation of regional differences due to differences in measurement and measurement error. Smoking prevalence and age-standardised years of education were used as country-level covariates. We did a meta-analysis of the relative risk of dementia per additional year of education and used the results to set bounds on the covariate in the model. The result of that meta-analysis is shown below.

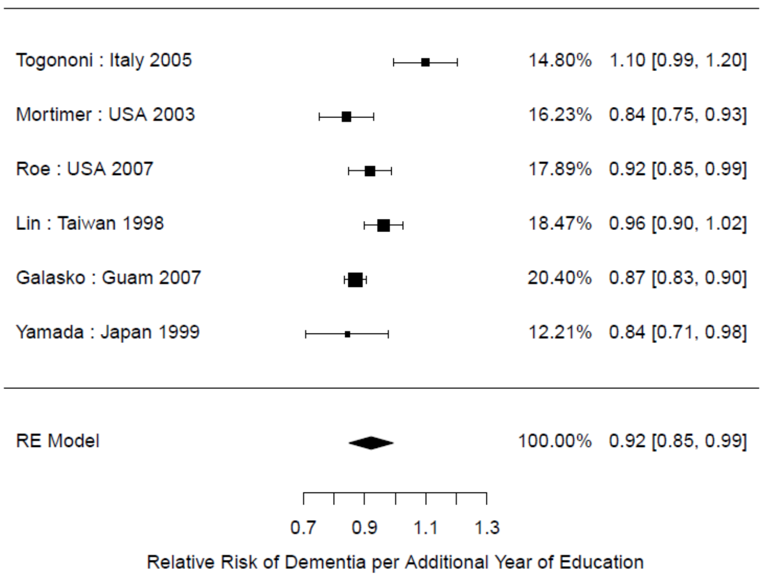

Sixth, we took the predictions of cause-specific mortality by age, sex, geography, and year that DisMod-MR 2.1 calculated as being consistent with the data on incidence and prevalence, and the priors on excess mortality from step five. Because DisMod-MR 2.1 produces estimates in five-year intervals only, we expanded the time series by log-linear interpolation; values for 1980–1990 were generated using a regression on the entire time series with Socio-demographic Index included as a predictor. We divided this cause-specific mortality by the all-cause mortality used in DisMod to calculate the dementia cause-fraction based on prevalence data and the excess mortality derived from countries most likely to code to dementia as a cause of death.

Seventh, we calculated the difference between this cause-fraction derived from DisMod and the cause-fraction derived from the cause of death data prep process before redistribution in order to get the amount of cause fraction that needed to be retrieved from other causes through the dementia redistribution process.

Eighth, in order to calculate where these dementia deaths should be retrieved from, we analysed multiple cause of death (MCOD) data. We only used data from the USA and asserted that the data from 2010–2015, during which the increases in coding to dementia as a cause of death leveled off, are the reference data.

Ninth, for deaths where dementia is the underlying cause of death in the years 2010–2015, we calculated what the underlying cause of death would have been in the counterfactual scenario in which dementia had not been recognised. In order to calculate this counterfactual, we examined the causes listed in part one of the chain of the death certificate. For each death certificate chain, we looked across the entire dataset from 1980 to 2015 and determined what the distribution of underlying causes of death was in individuals with that particular death certificate chain. Then, we assigned the counterfactual deaths proportionally to the causes that are listed as underlying in these death certificates. If, over the time period, there were less than 1,000 death certificates that had exactly the same death certificate chain, then we included all death certificate chains that had those same causes, but which could additionally include other causes in the chain as well. To assign counterfactual deaths for these chains, we further subsetted the data to death certificate chains where any of the causes in

the original death certificate chain were listed as underlying, determined the distribution of underlying causes of death among just this subset, and then assigned counterfactual deaths proportionally in the same manner.

Tenth, once we determined the counterfactual causes of death stemming from all dementia deaths from 2010–2015, we calculated the proportion of deaths by cause that should be dementia deaths according to the reference data by taking the counterfactual deaths for each cause and dividing by the sum of the counterfactual deaths for that cause plus the directly coded deaths for that cause.

Eleventh, we applied the proportions to cause of death data in cause fraction space and scaled the cause fractions to the total mortality cause fraction to be retrieved based on the DisMod model. We set caps on the percent of deaths that were moved by age, sex, and cause. The caps were determined by finding the 95th percentile of the percentages of deaths moved in each age-sex-cause category across all five-star VR locations. The COD data are then processed using general redistribution strategies and noise reduction.

Finally, the data derived from this process are used in a final CODEm model, using the same covariates as the original CODEm model. These results are then adjusted through CodCorrect and become the final cause of death estimates for dementia.

Parkinson’s Disease

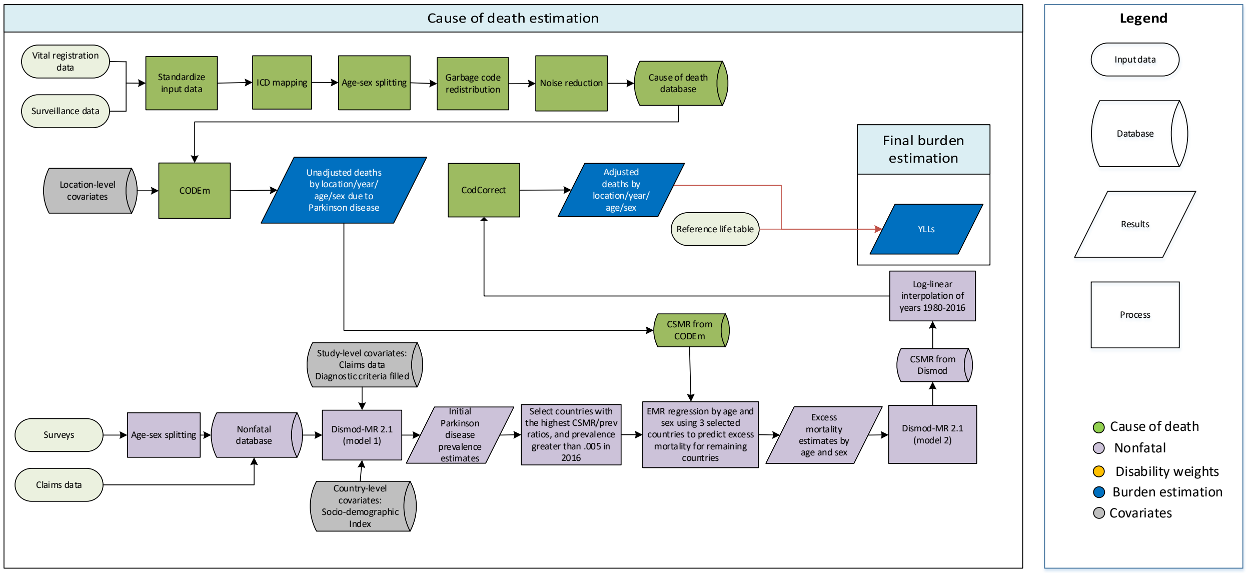

Input Data

In GBD 2017, data used to estimate deaths due to Parkinson’s disease included mortality data from vital registration systems and prevalence data from surveys and claims sources.

An updated systematic review was conducted from September 2015 to August 2017, and search terms[1] were set to capture studies for Parkinson’s disease. The search yielded 660 initial hits and 20 were marked for extraction. Inclusion criteria comprised studies that reported prevalence, incidence, remission rate, excess mortality rate, relative risk of mortality, standardised mortality ratio, or with-condition mortality rate. Studies with no clearly defined sample or that drew from specific clinic/patient organizations were excluded. We also added US claims data for 2011 and 2012–2015. A flow chart documenting this review is displayed below.

[1] (Parkinson disease[Title/Abstract] OR Parkinson's disease[Title/Abstract]) AND (epidemiology[Title/Abstract] OR prevalence[Title/Abstract] OR incidence[Title/Abstract]) AND ("2015/09/31"[PDAT] : "2017/08/23"[PDAT])

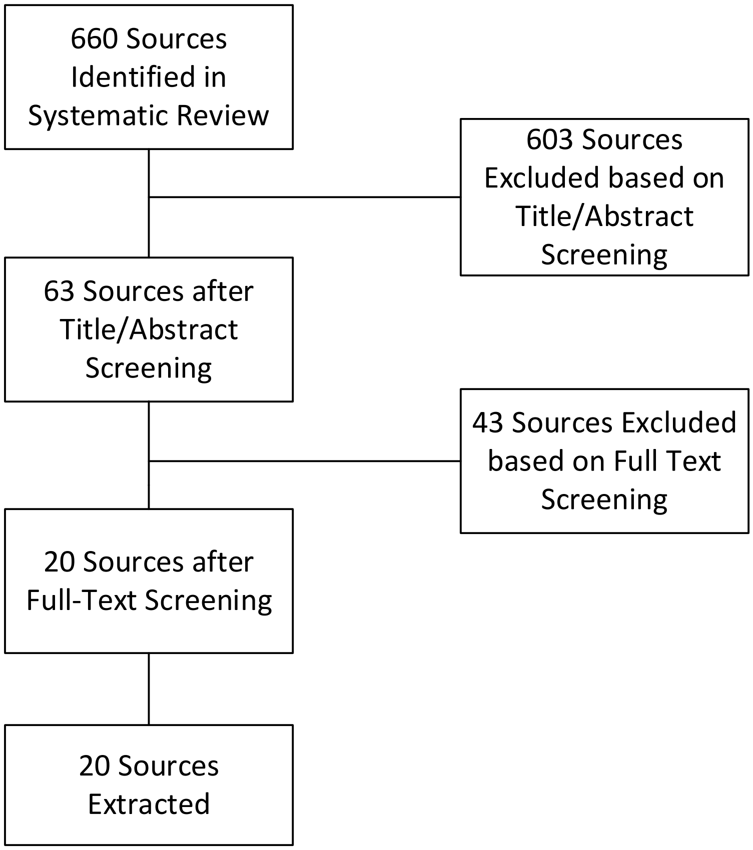

Modelling Strategy

Overview

Parkinson's disease mortality rates have more than doubled since 1980 in high-quality vital registration systems such as in the USA, Canada, Australia, France, Germany, the United Kingdom, and Finland, while other European countries like the Netherlands, Sweden, and Norway have not seen such increases over time. We have not seen an equivalent increase in prevalence and incidence data sources. Additionally, the greater than 15-fold variation in mortality rates of Parkinson's disease between countries is much greater the three-fold difference in prevalence and incidence between high-income countries. As it is unlikely that case fatality from Parkinson's disease has dramatically increased over the time period and that it would differ by a very large margin between countries, the hypothesis is that certifying and coding practices have changed over time and at a different pace between countries. For GBD 2016, we decided to employ a modelling strategy which we have previously used to model mortality from Alzheimer's disease and other dementias, which avoids spurious large trends over time in the fatal component of the burden of Parkinson's disease by making Parkinson's mortality rates consistent with the rates observed in 2016, relative to prevalence in countries that are most likely to certify or code Parkinson's disease as an underlying cause of death. For GBD 2017, we again employed this strategy.

Modelling steps

First, we ran a CODEm model for Parkinson's disease and extracted the mortality rates by age, sex, and geography for 2017. The covariates used in this model are displayed below.

Level	Covariate	Direction
1	Cumulative cigarette consumption (10 years)	-
2	Absolute latitude	+
	Cholesterol (total, mean per capita)	+
	Sanitation (proportion with access)	0
	Improved water source (proportion with access)	0
	Fruit consumption adjusted (g)	-
	Healthcare access and quality index	-
3	Education (years per capita)	-
	Socio-demographic Index	+

Second, we ran a DisMod-MR 2.1 model with all data on incidence, prevalence, and mortality risk (RR, SMR, or with-condition mortality rates) and a setting of zero remission and extracted prevalence by age, sex, and geography. Studies where the case definition of two of the four cardinal symptoms of Parkinson's disease was not filled were crosswalked to studies using the reference case definition. No random effects were used in the model in order to prevent spurious inflation of regional differences due to differences in measurement and measurement error.

Third, we selected the seven countries (France, England, the United States, the Netherlands, Finland, Scotland, and Wales) with the highest cause-specific mortality rate (from step 1) to prevalence (from step 2) ratio in 2017, which also had an age-standardised prevalence rate greater than 0.0005 and a population greater than 1 million.

Fourth, we used a linear effects regression with dummies on age group and sex to predict excess mortality (ie, the ratio of cause-specific mortality rate and prevalence) by age and sex, the results of which are found in the tables below.

Table: Fixed effect coefficients of EMR regression. Outcome: ln(EMR)

Independent variables	Coef	Std. error	P value	95% Confidence Interval	
Male	0.288	0.036	0.000	0.218	0.358
Age 40-59	-3.25	0.076	0.000	-3.399	-3.101
Age 60-64	-2.557	0.076	0.000	-2.706	-2.407
Age 65-69	-2.021	0.076	0.000	-2.17	-1.871
Age 70-74	-1.42	0.076	0.000	-1.57	-1.271
Age 75- 80	-0.898	0.076	0.000	-1.047	-0.749
Age 80-84	-0.502	0.076	0.000	-0.651	-0.352
Age 85-89	-0.248	0.076	0.001	-0.397	-0.099
Age 90-94	-0.047	0.076	0.537	-0.196	0.102
Constant	-2.357	0.057	0.000	-2.469	-2.246

Table: Predicted EMR values by age and sex (95% CI)

	Male	Female
Age 40-59	0.005 (0.004 - 0.005)	0.004 (0.003 - 0.004)
Age 60-64	0.01 (0.009 - 0.011)	0.007 (0.007 - 0.008)
Age 65-69	0.017 (0.015 - 0.019)	0.013 (0.011 - 0.014)

Age 70-74	0.031 (0.027 - 0.034)	0.023 (0.02 - 0.025)
Age 75- 80	0.051 (0.046 - 0.057)	0.039 (0.035 - 0.043)
Age 80-84	0.076 (0.068 - 0.085)	0.058 (0.052 - 0.064)
Age 85-89	0.099 (0.089 - 0.111)	0.074 (0.066 - 0.083)
Age 90-94	0.12 (0.108 - 0.135)	0.09 (0.081 - 0.1)
Age 95+	0.126 (0.113 - 0.142)	0.095 (0.085 - 0.106)

Fifth, these estimates were added to a second DisMod-MR 2.1 model as pertaining to the full 1990–2017 estimation period. For the countries included in the regression, we allowed them to retain their original EMR values when the age-standardised EMR for a country was higher than the age-standardised EMR prediction generated from the regression. These countries retained their age- and sex-specific ratios and entered those also as pertaining to the full 1990–2017 estimation period. Smoking prevalence was used as a country-level covariate. We excluded data for standardised mortality ratio, with-condition mortality rate, and relative risk as we wanted to estimate cause-specific mortality rates that were consistent with the level of excess mortality from the seven chosen countries in 2017.

Sixth, we took the predictions of cause-specific mortality by age, sex, geography, and year that DisMod-MR 2.1 calculated as being consistent with the data on incidence and prevalence, and the priors on excess mortality from step five. Because DisMod-MR 2.1 produces estimates in five-year intervals only, we expanded the time series by log-linear interpolation; values for 1980–1990 were generated using a regression on the entire time series with Socio-demographic Index included as a predictor. We divided this cause-specific mortality by the all-cause mortality used in DisMod to calculate the Parkinson's disease cause-fraction based on prevalence data and the excess mortality derived from countries most likely to code to Parkinson's disease as a cause of death.

Seventh, we calculated the difference between this cause-fraction derived from DisMod and the cause-fraction derived from the cause of death data prep process before redistribution in order to get the amount of cause fraction that needed to be retrieved from other causes through the Parkinson's disease redistribution process.

Eighth, in order to calculate where these Parkinson's disease deaths should be retrieved from, we analysed multiple cause of death (MCOD) data. We only used data from the USA and asserted that the data from 2010–2015, during which the increases in coding to Parkinson's disease as a cause of death leveled off, are the reference data.

Ninth, for deaths where Parkinson's disease is the underlying cause of death in the years 2010–2015, we calculated what the underlying cause of death would have been in the counterfactual scenario in which Parkinson's disease had not been recognised. In order to calculate this counterfactual, we examined the causes listed in part one of the chain of the death certificate. For each death certificate chain, we looked across the entire dataset from 1980–2015 and determined what the distribution of underlying causes of death was in individuals with that particular death certificate chain. Then, we assigned the counterfactual deaths proportionally to the causes that are listed as underlying in these death certificates. If, over the time period, there were less than 1,000 death certificates that had exactly the same death certificate chain, then we included all death certificate chains that had those same causes, but which could additionally include other causes in the chain as well. To assign counterfactual deaths for these chains, we further subsetted the data to death certificate chains where any of the causes in the original death certificate chain were listed as underlying, determined the distribution of underlying

causes of death among just this subset, and then assigned counterfactual deaths proportionally in the same manner.

Tenth, once we determined the counterfactual causes of death stemming from all Parkinson's disease deaths from 2010–2015, we calculated the proportion of deaths by cause that should be Parkinson's disease deaths according to the reference data by taking the counterfactual deaths for each cause and dividing by the sum of the counterfactual deaths for that cause plus the directly coded deaths for that cause.

Eleventh, we applied the proportions to cause of death data in cause fraction space and scaled the cause fractions to the total mortality cause fraction to be retrieved based on the DisMod model. We set caps on the percent of deaths that were moved by age, sex and cause. The caps were determined by finding the 95th percentile of the percentages of deaths moved in each age-sex-cause category across all five-star VR locations. The COD data are then processed using general redistribution strategies and noise reduction.

Finally, the data derived from this process are used in a final CODEm model, using the same covariates as the original CODEm model. These results are then adjusted through CodCorrect and become the final cause of death estimates for Parkinson's disease.

Epilepsy

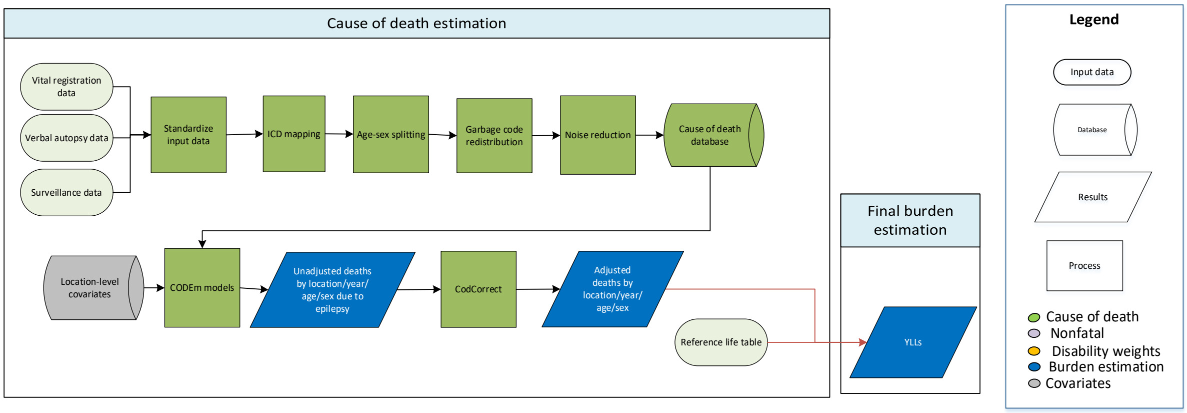

Input data

Data used to estimate epilepsy mortality included vital registration (VR), verbal autopsy, and China mortality surveillance data from the cause of death (COD) database. Our outlier criteria were to exclude data points that (1) were implausibly high or low relative to global or regional patterns, (2) substantially conflicted with established age or temporal patterns, or (3) significantly conflicted with other data sources based from the same locations or locations with similar characteristics (ie, Socio-demographic Index).

Based on these criteria, we excluded ICD-9 BTL data for Sri Lanka, Fiji, and Kiribati as the estimates varied from year to year between zero and high values. We also excluded the Survey of Causes of Death Data and Medical Certification of Cause of Death Data for India, as these data types were not consistent with the Sample Registration System Data and would have led to discontinuities in our estimates over time.

Modelling strategy

The standard CODEm modelling approach was applied to estimate deaths due to epilepsy. Separate models were conducted for male and female mortality, and the age range for both models was 28 days–95+ years. There were no substantial changes for GBD 2017. The covariates used are displayed below.

Level	Covariate	Direction
1	pig meat consumption (kcal per capita)	+
	pigs (per capita)	+
	SEV scalar: epilepsy	+
	mean systolic blood pressure (mmHg)	+

2	healthcare access and quality index	-
	mean body-mass index	+
	mean serum total cholesterol (mmol/L)	+
3	cumulative cigarettes (10 years)	+
	cumulative cigarettes (5 years)	+
	education (years per capita)	-
	log LDI (per capita)	-
	Socio-demographic Index	-

Multiple Sclerosis

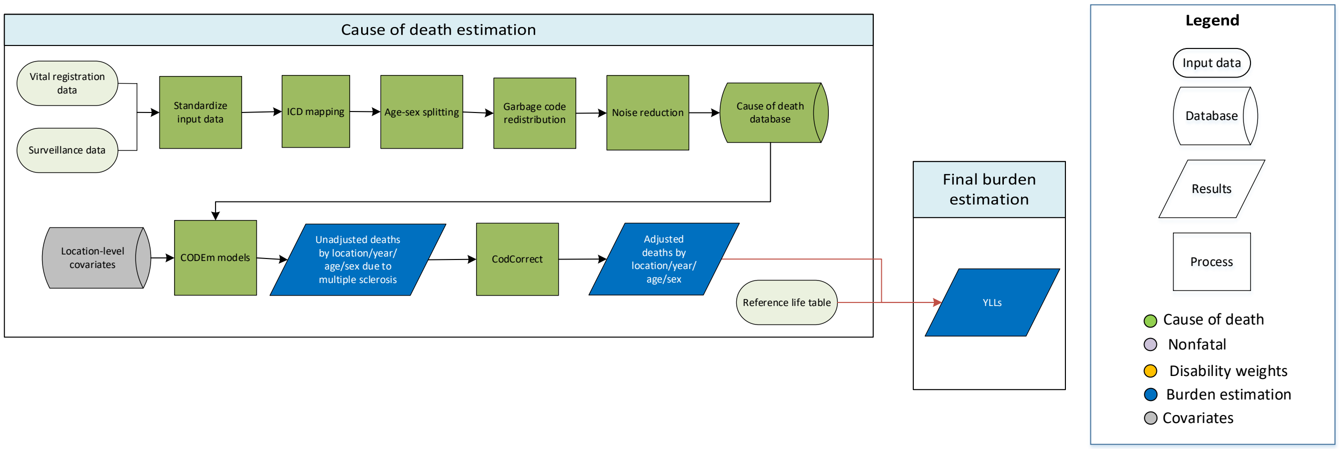

Input data

Data used to estimate multiple sclerosis included vital registration and surveillance data from the cause of death (COD) database. Our outlier criteria were to exclude data points that (1) were implausibly high or low, (2) substantially conflicted with established age or temporal patterns, or (3) significantly conflicted with other data sources conducted from the same locations or locations with similar characteristics (ie, Socio-demographic Index).

Modelling strategy

The standard CODEm modelling approach was used to estimate deaths due to multiple sclerosis. Separate models were conducted for male and female mortality, and the age range for both models was 20–95+ years. There were no substantial changes from GBD 2016. The covariates used are displayed below.

Level	Covariate	Direction
1	absolute value of average latitude	+
2	mean serum total cholesterol (mmol/L)	+
	healthcare access and quality index	-
3	cumulative cigarettes (10 years)	+
	cumulative cigarettes (5 years)	+
	education (years per capita)	-
	log-transformed LDI (per capita)	-
	smoking prevalence	+
	Socio-demographic Index	+

Motor Neuron Disease

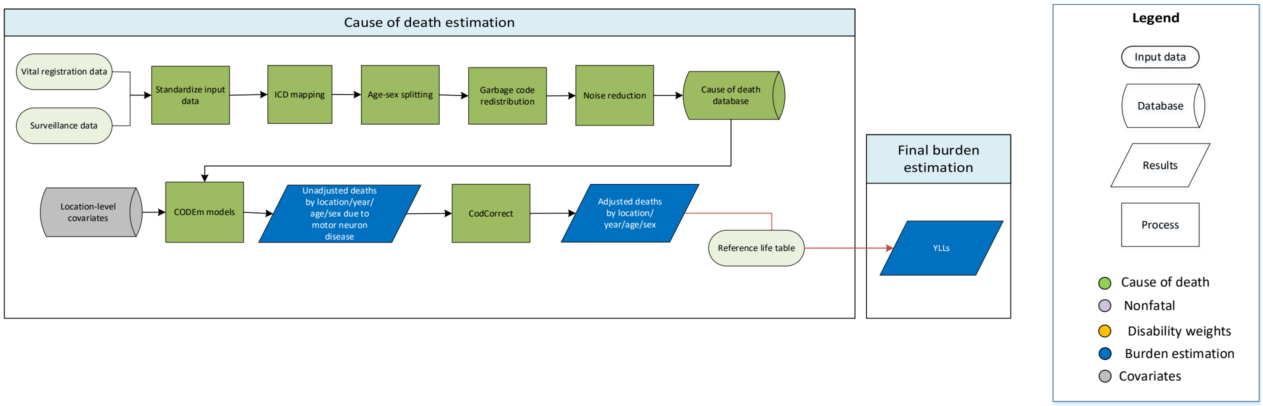

Input data

Data used to estimate motor neuron disease mortality included vital registration and surveillance data from the cause of death (COD) database. Our outlier criteria excluded data points that (1) were implausibly high or low, (2) substantially conflicted with established age or temporal patterns, or (3) significantly conflicted with other data sources conducted from the same locations or locations with similar characteristics (ie, Socio-demographic Index).

Modelling strategy

The standard CODEm modelling approach was used to estimate deaths due to motor neuron disease. Separate models were conducted for male and female mortality, and the age range for both models was 20–95+ years. For GBD 2017, we added covariates for BMI, diabetes, and temperature, all of which have been linked to motor neuron disease[i]. There were no other substantial changes from the GBD 2016 modelling strategy. The covariates used are displayed below.

Level	Covariate	Direction
1	absolute value of average latitude	+
	mean serum total cholesterol (mmol/L)	0
	fruit consumption (grams per day adjusted)	0
	mean diabetes fasting plasma glucose (mmol/L)	+
	mean body mass index (kg/m^2)	-
	Socio-demographic Index	0
2	population-weighted mean temperature	0
	sanitation (proportion with access)	0
	improved water source (proportion with access)	0
	healthcare access and quality index	-
3	education (years per capita)	0
	log-transformed LDI (per capita)	0

[i] Ingre C, Roos PM, Piehl F, Kamel F, Fang F. Risk factors for amyotrophic lateral sclerosis. *Clin Epidemiol* 2015; **7**: 181–93. PMCID: PMC4334292.

Other Neurological Disorders

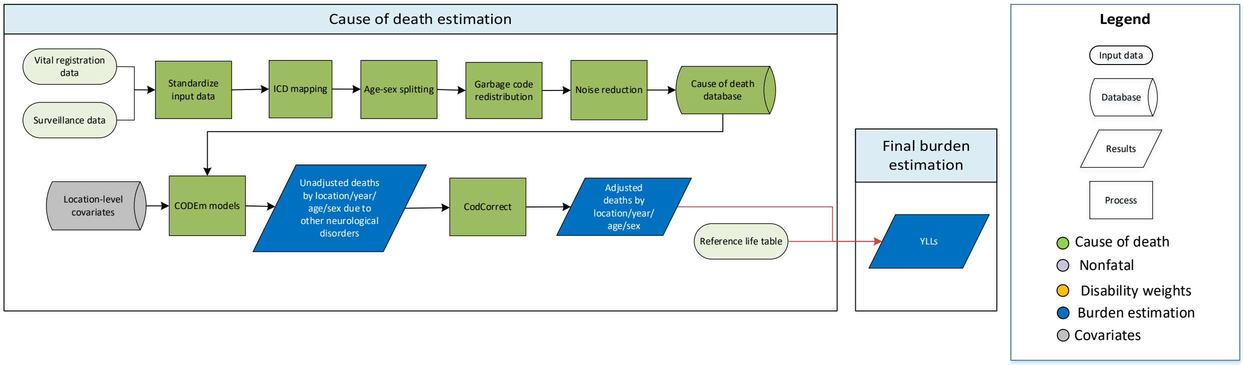

Input data

Data used to estimate other neurological disorders included vital registration and surveillance data from the cause of death (COD) database. Our outlier criteria were to exclude data points that (1) were implausibly high or low, (2) substantially conflicted with established age or temporal patterns, or (3) significantly conflicted with other data sources conducted from the same locations or locations with similar characteristics (ie, Socio-demographic Index).

Modelling strategy

The standard CODEm modelling approach was applied to estimate deaths due to other neurological disorders. Male and female CODEm models were run for deaths occurring between ages 28 days to 95+ years. There were no significant changes from the GBD 2016 modelling strategy. The covariates used are displayed below.

Level	Covariate	Direction
1	underweight proportion under 2 standard deviations	+
	mean body-mass index	+
	mean cholesterol	+
	mean systolic blood pressure	+
	pig meat consumption (kcal per capita)	+
	red meat consumption adjusted	+
2	alcohol (litres per capita)	+
	healthcare access and quality index	-
	fruit consumption adjusted	-
	population density over 1,000 per square kilometer pct	+
3	cumulative cigarette consumption (10 years)	+
	cumulative cigarette consumption (5 years)	+
	education (years per captia)	-
	log-transformed LDI (per capita)	-
	smoking prevalence	+
	Socio-demographic Index	0

Eating Disorders

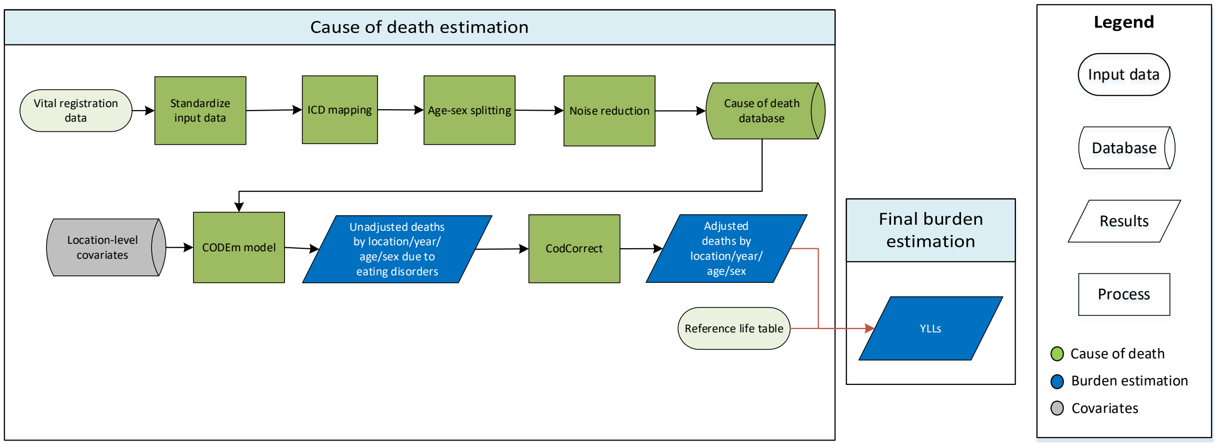

Input data

Data used to estimate eating disorders mortality included vital registration data from the cause of death (COD) database. No garbage codes were redistributed to eating disorders given previous issues with dehydration deaths in low- and middle-income countries causing unfeasible results.

Modelling strategy

Eating disorders were modelled using standard CODEm modelling approach and encompassing the two child models of anorexia nervosa and bulimia nervosa. Age was restricted to deaths occurring between 5 and 49 years of age based on expert advice and patterns of prevalence seen in the non-fatal models of anorexia nervosa and bulimia nervosa. Several covariates were applied to this model and are listed in the table below, along with the direction in which they were applied.

Level	Covariate	Direction
1	education (years per capita)	+
	log LDI (I$ per capita)	+
	underweight (proportion <2SD weight for age, <5 years)	-
	sanitation (proportion with access)	+
	maternal education (years per capita)	+
2	healthcare access and quality index	-
3	Socio-demographic Index	+

In GBD 2013, eating disorders were modelled as a negative binomial model using a custom approach. This approach was changed in GBD 2015, with eating disorders being modelled as a standard CODEm model, as no obvious benefit was seen from using the custom modelling approach. GBD 2016 utilised the same approach as GBD 2015 with the only difference being the inclusion of covariates. For GBD

2017, garbage codes were no longer redistributed to eating disorders given the impact of these codes on the feasibility of the geographical distribution. For example, while only a relatively small proportion of dehydration garbage code deaths were redistributed to eating disorders, this added a comparatively large number of deaths to eating disorders, particularly in regions with higher rates of infectious diseases, and they were redistributed equally between males and females despite the prevalence of eating disorders known to be up to ten times higher in females. As such, a decision was made to no longer redistribute garbage codes to eating disorders.

Anorexia Nervosa

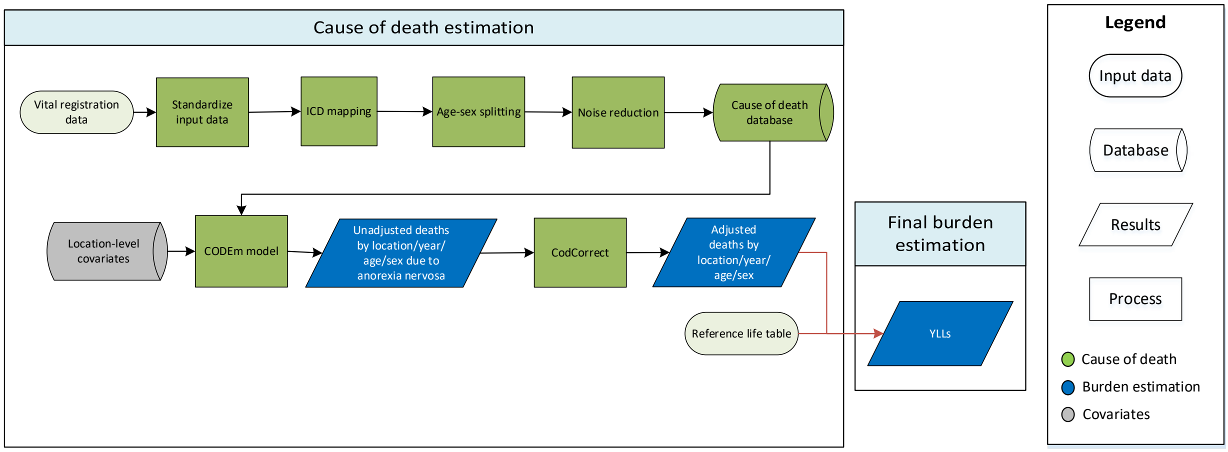

Input data

Data used to estimate anorexia nervosa mortality included centrally prepped vital registration data from the cause of death (COD) database. No garbage codes were redistributed to anorexia nervosa given previous issues with dehydration deaths in low- and middle-income countries causing unfeasible results.

Modelling strategy

Anorexia nervosa was modelled using the standard CODEm approach and came under the eating disorders parent model. Age was restricted to deaths occurring between 5 and 49 years based on expert advice and patterns of prevalence seen in the non-fatal model. Several covariates were applied to this model and are listed in the table below, along with the direction in which they were applied.

**Level**	**Covariate**	**Direction**
1	education (years per capita)	+
	log LDI (I$ per capita)	+
	underweight (proportion <2SD weight for age, <5 years)	-
	sanitation (proportion with access)	+
	maternal education (years per capita)	+
2	healthcare access and quality index	-
3	Socio-demographic Index	+

In GBD 2013, anorexia nervosa deaths were extrapolated from the eating disorders model, which was modelled through a negative binomial approach. This approach was changed in GBD 2015, with anorexia nervosa deaths being modelled through a standard CODEm approach under the overarching eating disorders model, as there was no benefit observed from applying the custom approach. GBD 2016 utilised the same approach as GBD 2015 with the only difference being the inclusion of covariates. For

GBD 2017, garbage codes were no longer redistributed to anorexia nervosa given the impact of these codes on the feasibility of the geographical distribution. For example, while only a relatively small proportion of dehydration garbage code deaths were redistributed to anorexia nervosa, this added a comparatively large number of deaths to anorexia nervosa, particularly in regions with higher rates of infectious diseases, and were redistributed equally between males and females despite the prevalence of anorexia nervosa known to be up to ten times higher in females. As such, a decision was made to no longer redistribute garbage codes to anorexia nervosa.

Bulimia Nervosa

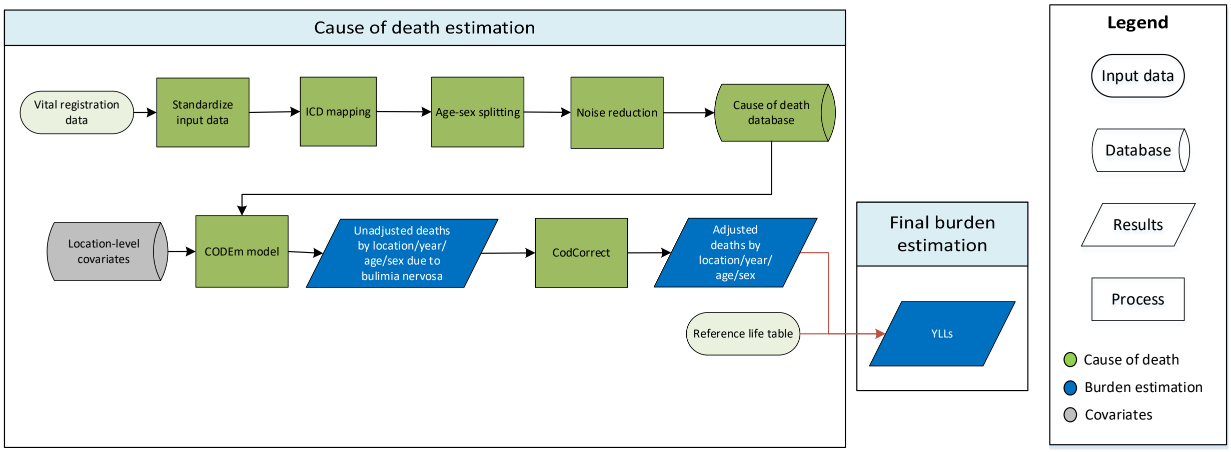

Input data

Data used to estimate bulimia nervosa mortality included centrally prepped vital registration data from the cause of death (COD) database. No garbage codes were redistributed to bulimia nervosa given previous issues with deaths in low- and middle-income countries causing unfeasible results.

Modelling strategy

Bulimia nervosa was modelled using the standard CODEm approach and comes under the eating disorders parent model. Age was restricted to deaths occurring between 5 and 49 years based on expert advice and patterns of prevalence seen in the non-fatal model. Several covariates were applied to this model and are listed in the table below, along with the direction in which they were applied.

Level	Covariate	Direction
1	education (years per capita)	+
	log LDI (I$ per capita)	+
	underweight (proportion <2SD weight for age, <5 years)	-
	sanitation (proportion with access)	+
	maternal education (years per capita)	+
2	healthcare access and quality index	-
3	Socio-demographic Index	+

In GBD 2013, bulimia nervosa was not modelled as a distinct cause of death. Any deaths due to bulimia nervosa were attributed to the eating disorders model. We changed this approach in GBD 2015, recognising bulimia nervosa as an individual cause of death, and therefore modelled it as a standard CODEm model under the overarching eating disorders model. This decision was based on observing

deaths due to bulimia nervosa in high-quality vital registration data, such as data from the USA. These data also include eating disorders not otherwise specified. GBD 2016 utilised the same approach as GBD 2015 with the only difference being the inclusion of covariates. For GBD 2017, garbage codes were no longer redistributed to bulimia nervosa given the impact of these codes on the feasibility of the geographical distribution.

Alcohol Use Disorders

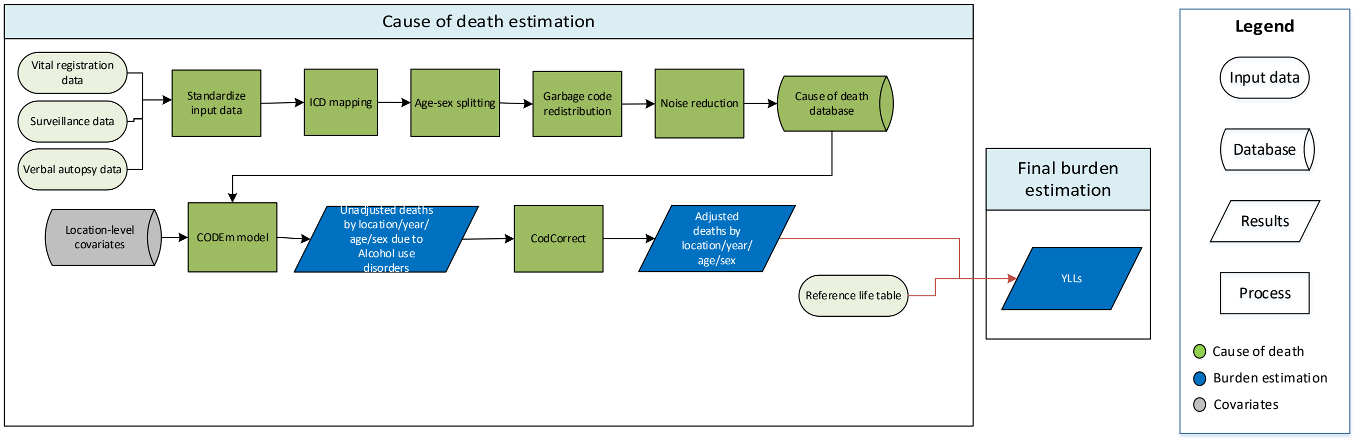

Input data

All data were from vital registration, China surveillance, and verbal autopsy sources. Some data were outliered from countries with sparse yet heterogeneous data if they created implausible fluctuations in deaths and regional patterns. As an example, Medical Certification of Cause of Death data from India were excluded for alcohol use disorders due to the extremely low estimates.

Modelling strategy

Cause of death modelling for alcohol use disorders followed the general CODEm strategy. There were no substantial changes from GBD 2017. Model covariate inclusion was based on empirical evidence and expert feedback, which resulted in a set of model covariates that reflected alcohol consumption, smoking, education, health system access, percent of population Muslim, domestic income, and Socio-demographic Index (SDI).

Table: covariates used in alcohol use disorders CODEm model

Level	Covariate	Direction
1	alcohol consumption (litres per capita)	+
	alcohol binge drinking	+
2	Muslim religion	-
	cumulative cigarettes (10 years)	0
	smoking prevalence	0
	healthcare access and quality index	-
3	log LDI (I$ per capita)	-
	education (years per capita)	-
	Socio-demographic Index	0

In GBD 2017, ICD codes for accidental poisoning (X40-44) were redistributed to the underlying GBD cause (substance use disorder) using an algorithm devised from analysing national registry data from several countries and expert feedback.

Drug Use Disorders

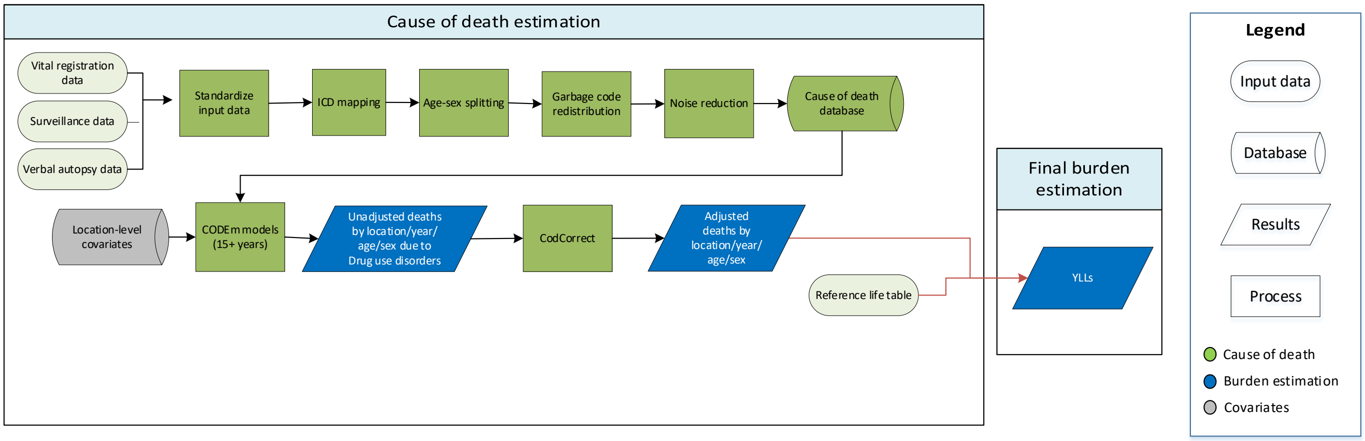

Input data

All data were from vital registration, verbal autopsy, and surveillance sources. Data from countries with sparse yet heterogeneous data were excluded as the data exaggerated fluctuations in deaths and gave implausible regional patterns. Excluded data were typically from low-income countries. Notably, a considerable amount of Medical Certification of Cause of Death (MCCD) data from India were excluded for drug use disorders. Specifically, it was decided to remove the MCCD ICD-9 data, as a specific garbage redistribution package was not available for that time series. Additionally, it was decided to remove MCCD-ICD10 data from the Northeastern states of Meghalaya, Mizoram, Nagaland, and Manipur (where the much lower values in MCCD compared to SRS removed the expected higher death rates there) and also from the four states of Punjab, Uttarakhand, Jharkand, and Karnataka (where the raw data showed almost no deaths from drug use disorders).

Modelling strategy

A few key changes were made to the modelling strategy in GBD 2017. Cause of death modelling for drug use disorders still followed the general CODEm strategy; however, in GBD 2017 we specified CODEm to model only space-time (rather than mixed effects) submodels. This was to address unsatisfactory model fits in some locations, particularly in the USA, where the fit from space-time models was better able to follow the large increase in deaths in later years. Additionally, we introduced new covariates: intravenous drug use prevalence, and opioid consumption per million inhabitants per day. The latter covariate is derived from data from the International Narcotics Control Board (INCB), which measures *"defined daily doses for statistical purposes" (S-DDD),* which is considered an approximate measure to rank consumption in different countries.

Another key change in the modelling strategy decision was to no longer run a model for the 0-28 day age group due to the extremely small number of deaths being recorded. Instead, for locations with data in infants, the data were directly applied during post-processing steps, rather than being included in a model.

Table: covariates used in drug use disorders CODEm model

Level	Covariate	Direction
1	Intravenous drug use age-standardised	+
	Intravenous drug use age-specific	+
	Opioid standard doses per million per day	+
	Opioid standard doses per million per day (5-year lag)	+
	Opioid standard doses per million per day (10-year lag)	+
2	alcohol use (litres per capita)	+
	cumulative cigarettes (10 years)	+
	cumulative cigarettes (5 years)	+
	opium cultivation bin	+
	smoking prevalence	+
	healthcare access and quality index	-
3	log LDI (I$ per capita)	0
	education (years per capita)	0
	Socio-demographic Index	0

The drug use model is the parent model of all other drug use causes (ie, amphetamine, cocaine, opioid, and other drug). It forms an envelope into which all four individual drug use models are scaled during the CoDCorrect process.

Opioid Use Disorders

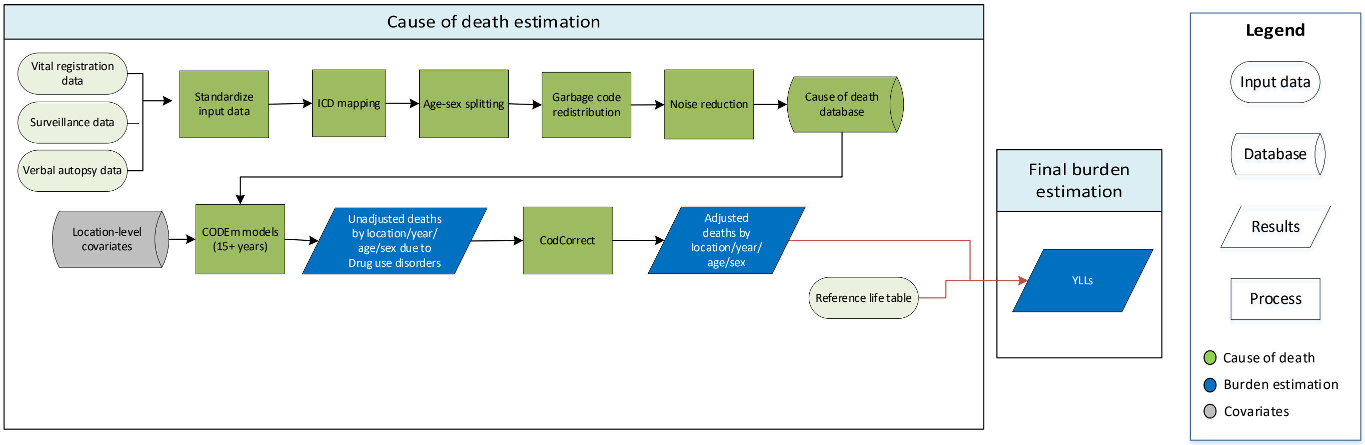

Input data

All input data were from vital registration and surveillance sources. The locations for which there was the most data included North America, Australia, Western Europe, and parts of Latin America. Mortality data were largely absent from sub-Saharan Africa and South Asia.

Modelling strategy

A few key changes were made to the modelling strategy in GBD 2017. Cause of death modelling for drug use disorders still followed the general CODEm strategy; however, in GBD 2017 we specified CODEm to model only space-time (rather than mixed effects) models. This was to address unsatisfactory model fits in some locations, particularly in the USA, where the fit from space-time models was better able to follow the large increase in deaths in later years. Additionally, we introduced new covariates: intravenous drug use prevalence from the model used to estimate exposure for the drug use as a risk analyses, and opioid consumption per million inhabitants per day. The latter covariate was derived from data from the International Narcotics Control Board (INCB) which measures *"defined daily doses for statistical purposes" (S-DDD),* which translates all different opioids of different types and dosages into comparable units to quantify consumption in different countries.

Another key change in the modelling strategy decision was to no longer run a model for the 0-28 day age group due to the extremely small number of deaths being recorded. Instead, for locations with data in infants, the data were directly applied during post-processing steps, rather than being included in a model.

Table 1: Covariates used in opioid use CODEm model

Level	Covariate	Direction
1	Intravenous drug use age-standardised	+
	Intravenous drug use age-specific	+
	Opioid standard doses per million per day	+
	Opioid standard doses per million per day (5-year lag)	+
	Opioid standard doses per million per day (10-year lag)	+
2	alcohol use (litres per capita)	+
	cumulative cigarettes (10 years)	+
	cumulative cigarettes (5 years)	+
	opium cultivation bin	+
	smoking prevalence	+
	healthcare access and quality index	-
3	log LDI (I$ per capita)	0
	education (years per capita)	0
	Socio-demographic Index	0

A significant limitation to previous drug use models was assumptions surrounding the redistribution of garbage codes. In GBD 2016, and continued in GBD 2017, ICD codes for accidental poisoning (X40-44 and X49) were redistributed to the underlying GBD cause (substance use disorder) using an algorithm devised from analysing multiple cause of death records for the United States, Mexico, Brazil, and Australia from 1980 to 2014. The main assumption behind this algorithm is the dominancy of the fatality of some substances when considering a combination of drugs (Table 2).

Table 2. Algorithm for the selection and assignment of a substance or drug use cause of death for deaths coded to an underlying cause of unintentional poisoning using multiple cause of death data

Selection Algorithm						
	Opioids	Cannabis	Cocaine	Amphetamines	alcohol	Psychoactive and psychedelic drug
Opioids	Opioids	Opioids	Opioids	Opioids	Opioids	Opioids
Cannabis	Opioids	Cannabis	Cocaine	Amphetamines	alcohol	Psychoactive and psychedelic drug
Cocaine	Opioids	Cocaine	Cocaine	Amphetamines + Cocaine	Cocaine + alcohol	Cocaine
Amphetamines	Opioids	Amphetamines	Amphetamines + Cocaine	Amphetamines	Amphetamines + alcohol	Amphetamines
alcohol	Opioids	alcohol	Cocaine + alcohol	Amphetamines + alcohol	alcohol	Psychoactive and psychedelic drug
Psychoactive and psychedelic drug	Opioids	Psychoactive and psychedelic drug	Cocaine	Amphetamines	Psychoactive and psychedelic drug	Psychoactive and psychedelic drug

Given that multiple cause of death data were only available to us for the United States, Brazil, Mexico, and Australia, we applied the result from multiple cause of death analysis from the United States to the United States and Canada, from Brazil and Mexico to Latin America and the Caribbean, and from Australia to Australia, New Zealand, Southeast Asia, and Western Europe. For other locations, we aggregated the results from the four countries and applied the aggregate pattern. This is an improvement on the opioid use model from previous rounds of GBD. We hope for increased availability of multiple causes of death data in future analyses in order to achieve a more precise distribution for more locations.

Cocaine Use Disorders

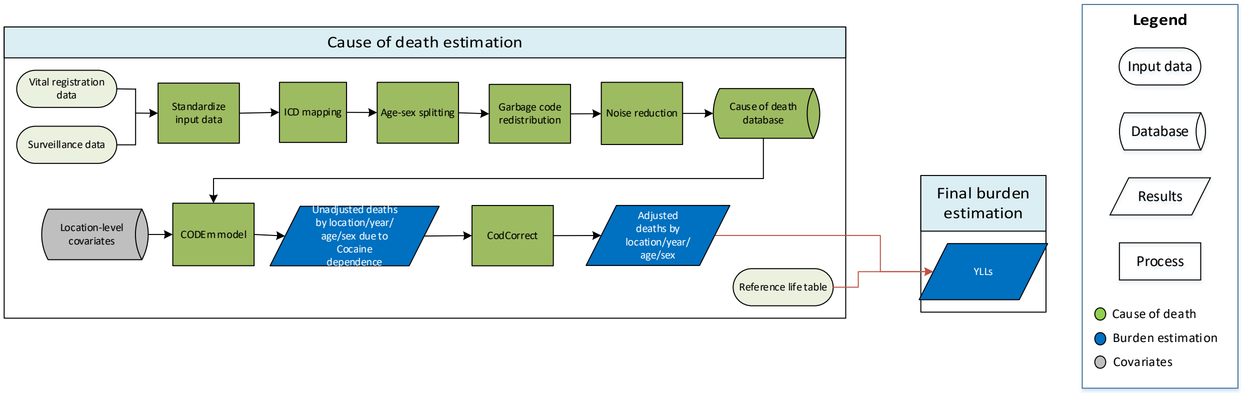

Input data

All data were from vital registration and surveillance sources. Data from countries with sparse yet heterogeneous data were excluded as the data exaggerated fluctuations in deaths and gave implausible regional patterns. Excluded data were typically from low- and middle-income countries.

Modelling strategy

Cause of death modelling for cocaine use followed the general CODEm strategy. There were no substantial changes from GBD 2016. Model covariate inclusion was based on empirical evidence and expert feedback, which resulted in a set of model covariates that reflected alcohol consumption, smoking, education, health system access, income per capita, and Socio-demographic Index (SDI) (Table 1).

Table 1: Covariates used in cocaine use CODEm model

Level	Covariate	Direction
1	alcohol use (litres per capita)	+
	cumulative cigarettes (10 years)	+
	cumulative cigarettes (5 years)	+
	smoking prevalence	+
2	healthcare access and quality index	-
3	log LDI (I$ per capita)	0
	education (years per capita)	0
	Socio-demographic Index	+

A significant limitation to previous drug use models was assumptions surrounding the redistribution of garbage codes. In GBD 2016, and continued in GBD 2017, ICD codes for accidental poisoning (X40-44 and X49) were redistributed to the underlying GBD cause (substance use disorder) using an algorithm devised from analysing multiple cause of death records for the United States, Mexico, Brazil, and

Australia from 1980 to 2014. The main assumption behind this algorithm is the dominancy of the fatality of some substances when considering a combination of drugs (Table 2).

Table 2. Algorithm for the selection and assignment of a substance or drug use cause of death for deaths coded to an underlying cause of unintentional poisoning using multiple cause of death data

Selection Algorithm						
	Opioids	Cannabis	Cocaine	Amphetamines	alcohol	Psychoactive and psychedelic drug
Opioids	Opioids	Opioids	Opioids	Opioids	Opioids	Opioids
Cannabis	Opioids	Cannabis	Cocaine	Amphetamines	alcohol	Psychoactive and psychedelic drug
Cocaine	Opioids	Cocaine	Cocaine	Amphetamines + Cocaine	Cocaine + alcohol	Cocaine
Amphetamines	Opioids	Amphetamines	Amphetamines + Cocaine	Amphetamines	Amphetamines + alcohol	Amphetamines
alcohol	Opioids	alcohol	Cocaine + alcohol	Amphetamines + alcohol	alcohol	Psychoactive and psychedelic drug
Psychoactive and psychedelic drug	Opioids	Psychoactive and psychedelic drug	Cocaine	Amphetamines	Psychoactive and psychedelic drug	Psychoactive and psychedelic drug

Given that multiple cause of death data were only available to us for the United States, Brazil, Mexico, and Australia, we applied the result from multiple cause of death analysis from the United States to the United States and Canada, from Brazil and Mexico to Latin America and the Caribbean, and from Australia to Australia, New Zealand, Southeast Asia, and Western Europe. For other locations, we aggregated the results from the four countries and applied the aggregate pattern. This is an improvement on the cocaine use model from previous rounds of GBD. We hope for increased availability of multiple causes of death data in future analyses in order to achieve a more precise distribution for more locations.

Amphetamine Use Disorders

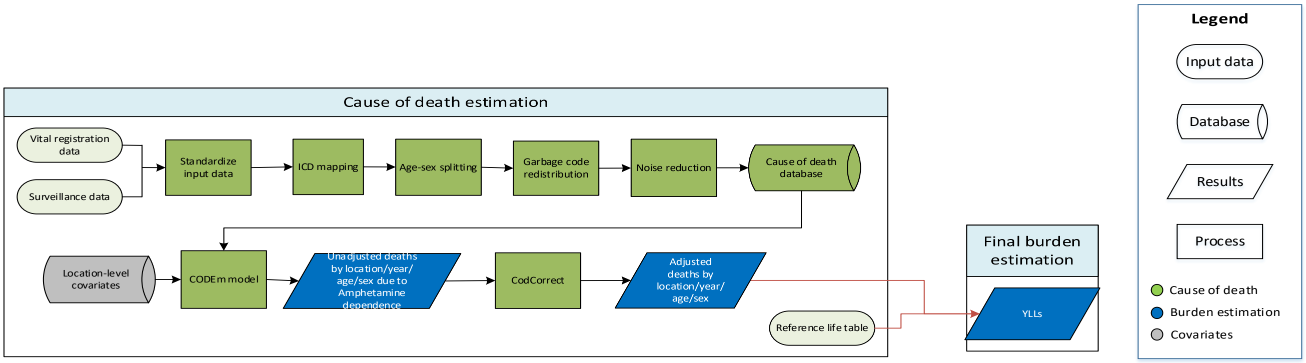

Input data

All data were from vital registration and surveillance sources. Data from countries with sparse yet heterogeneous data were excluded as the data exaggerated fluctuations in deaths and gave implausible regional patterns. Excluded data were typically from lower-income countries.

Modelling strategy

Cause of death modelling for amphetamine use followed the general CODEm strategy. There were no substantial changes from GBD 2016. Model covariate inclusion was based on empirical evidence and expert feedback, which resulted in a set of model covariates that reflected alcohol consumption, smoking, education, health system access, domestic income, and Socio-demographic Index (SDI) (Table 1).

Table 1: Covariates used in amphetamine use CODEm model

Level	Covariate	Direction
1	alcohol use (litres per capita)	+
	cumulative cigarettes (10 years)	+
	cumulative cigarettes (5 years)	+
	smoking prevalence	+
2	healthcare access and quality index	-
3	log LDI (I$ per capita)	0
	education (years per capita)	0
	Socio-demographic Index	+

A significant limitation to previous drug use models was assumptions surrounding the redistribution of garbage codes. In GBD 2016, and continued in GBD 2017, ICD codes for accidental poisoning (X40-44 and X49) were redistributed to the underlying GBD cause (substance use disorder) using an algorithm devised from analysing multiple cause of death records for the United States, Mexico, Brazil, and Australia from 1980 to 2014. The main assumption behind this algorithm is the dominancy of the fatality of some substances when considering a combination of drugs (Table 2).

Table 2. Algorithm for the selection and assignment of a substance or drug use cause of death for deaths coded to an underlying cause of unintentional poisoning using multiple cause of death data

Selection Algorithm						
	Opioids	Cannabis	Cocaine	Amphetamines	alcohol	Psychoactive and psychedelic drug
Opioids	Opioids	Opioids	Opioids	Opioids	Opioids	Opioids
Cannabis	Opioids	Cannabis	Cocaine	Amphetamines	alcohol	Psychoactive and psychedelic drug
Cocaine	Opioids	Cocaine	Cocaine	Amphetamines + Cocaine	Cocaine + alcohol	Cocaine
Amphetamines	Opioids	Amphetamines	Amphetamines + Cocaine	Amphetamines	Amphetamines + alcohol	Amphetamines
alcohol	Opioids	alcohol	Cocaine + alcohol	Amphetamines + alcohol	alcohol	Psychoactive and psychedelic drug
Psychoactive and psychedelic drug	Opioids	Psychoactive and psychedelic drug	Cocaine	Amphetamines	Psychoactive and psychedelic drug	Psychoactive and psychedelic drug

Given that multiple cause of death data were only available to us for the United States, Brazil, Mexico, and Australia, we applied the result from multiple cause of death analysis from the United States to the United States and Canada, from Brazil and Mexico to Latin America and the Caribbean, and from Australia to Australia, New Zealand, Southeast Asia, and Western Europe. For other locations, we aggregated the results from the four countries and applied the aggregate pattern. This is an improvement on the amphetamine use model from previous rounds of GBD. We hope for increased availability of multiple causes of death data in future analyses in order to achieve a more precise distribution for more locations.

Other Drug Use Disorders

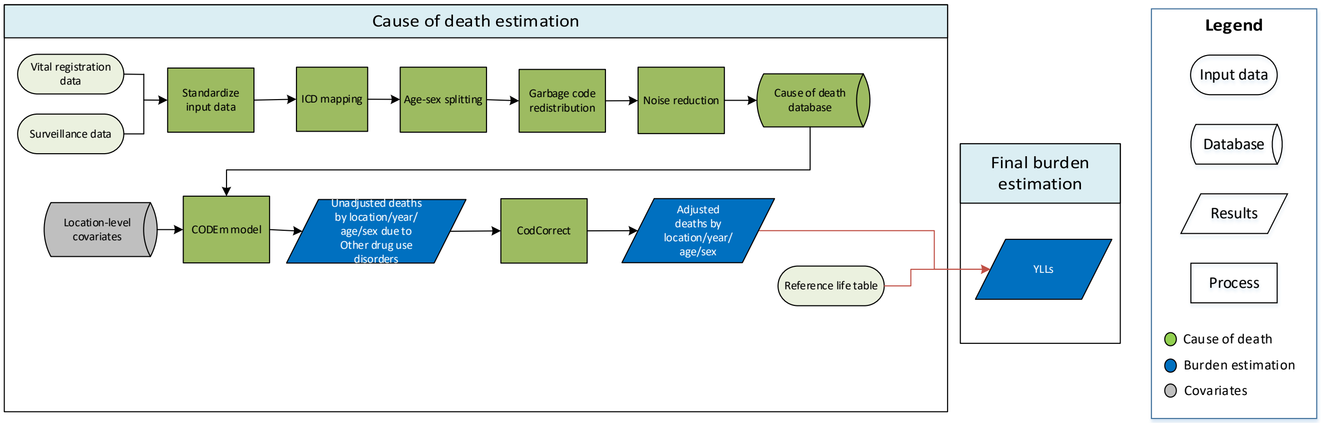

Input data

All data were from vital registration and China surveillance sources. Data from countries with sparse yet heterogeneous data were excluded as the data exaggerated fluctuations in deaths and gave implausible regional patterns. Excluded data were typically from lower-income countries.

Modelling strategy

Cause of death modelling for other drug use followed the general CODEm strategy. There were no substantial changes from GBD 2016. Model covariate inclusion was based on empirical evidence and expert feedback, which resulted in a set of model covariates that reflected alcohol consumption, smoking, education, health system access, domestic income, and Socio-demographic Index (SDI) (Table 1).

Table 1: Covariates used in other drug use CODEm model

Level	Covariate	Direction
1	alcohol (litres per capita)	+
	cumulative cigarettes (10 years)	+
	cumulative cigarettes (5 years)	+
	smoking prevalence	+
2	healthcare access and quality index	-
3	log LDI (I$ per capita)	0
	education (years per capita)	0
	Socio-demographic Index	0

A significant limitation to previous drug use models was assumptions surrounding the redistribution of garbage codes. In GBD 2016, and continued in GBD 2017, ICD codes for accidental poisoning (X40-44 and X49) were redistributed to the underlying GBD cause (substance use disorder) using an algorithm devised from analysing multiple cause of death records for the United States, Mexico, Brazil, and Australia from 1980 to 2014. The main assumption behind this algorithm is the dominancy of the fatality of some substances when considering a combination of drugs (Table 2).

Table 2. Algorithm for the selection and assignment of a substance or drug use cause of death for deaths coded to an underlying cause of unintentional poisoning using multiple cause of death data

Selection Algorithm						
	Opioids	Cannabis	Cocaine	Amphetamines	alcohol	Psychoactive and psychedelic drug
Opioids	Opioids	Opioids	Opioids	Opioids	Opioids	Opioids
Cannabis	Opioids	Cannabis	Cocaine	Amphetamines	alcohol	Psychoactive and psychedelic drug
Cocaine	Opioids	Cocaine	Cocaine	Amphetamines + Cocaine	Cocaine + alcohol	Cocaine
Amphetamines	Opioids	Amphetamines	Amphetamines + Cocaine	Amphetamines	Amphetamines + alcohol	Amphetamines
alcohol	Opioids	alcohol	Cocaine + alcohol	Amphetamines + alcohol	alcohol	Psychoactive and psychedelic drug
Psychoactive and psychedelic drug	Opioids	Psychoactive and psychedelic drug	Cocaine	Amphetamines	Psychoactive and psychedelic drug	Psychoactive and psychedelic drug

Given that multiple cause of death data were only available to us for the United States, Brazil, Mexico, and Australia, we applied the result from multiple cause of death analysis from the United States to the United States and Canada, from Brazil and Mexico to Latin America and the Caribbean, and from Australia to Australia, New Zealand, Southeast Asia, and Western Europe. For other locations, we aggregated the results from the four countries and applied the aggregate pattern. This is an improvement on the other drug use model from previous rounds of GBD. We hope for increased availability of multiple causes of death data in future analyses in order to achieve a more precise distribution for more locations.

Diabetes Mellitus

Diabetes mellitus mortality was estimated for overall diabetes mellitus, diabetes mellitus type 1, and diabetes mellitus type 2 in GBD 2017. We included neonatal diabetes with type 1 diabetes and gestational diabetes with type 2 diabetes. For full accounting of associated ICD 9 and ICD 10 codes, please refer to Appendix Table 4.

Overall Diabetes Mellitus

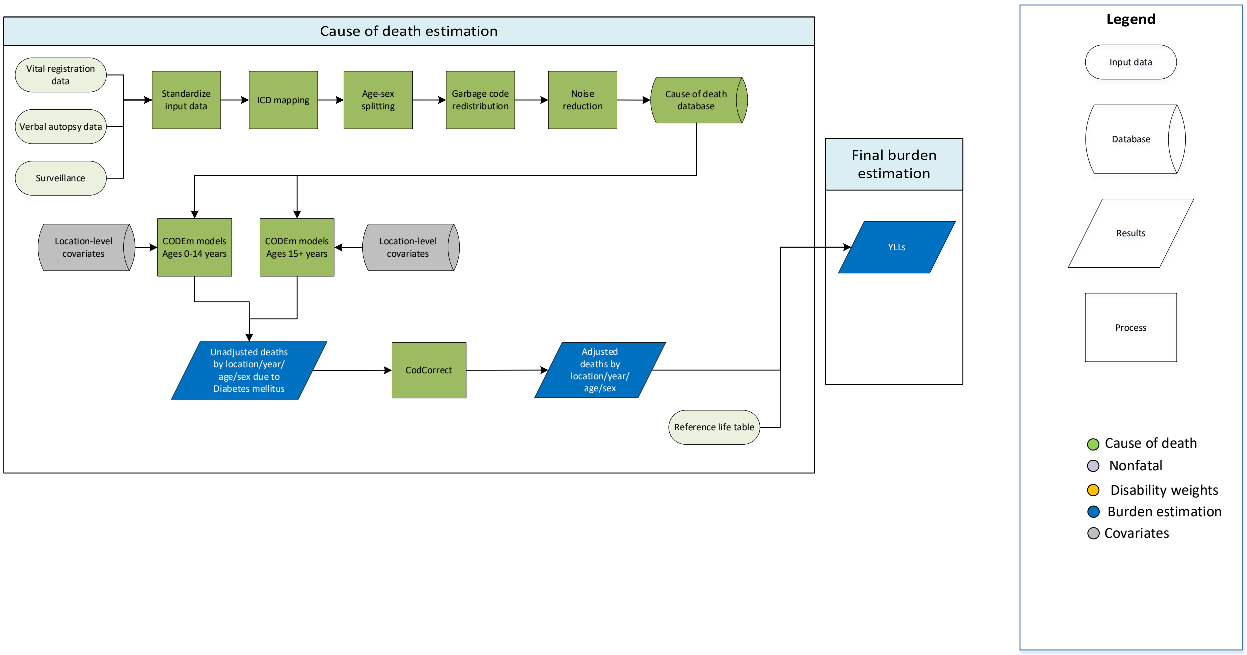

Input data

Overall diabetes mellitus mortality was estimated using deaths directly attributed to diabetes mellitus. We used verbal autopsy and vital registration data as inputs into the model.

Verbal autopsy data: We outliered data points from sources where there were zero deaths estimated in an age group as this was not realistic for deaths due to diabetes and we determined that these data sources were unreliable.

Vital registration data: We outliered all data from the India Medical Certification of Cause of Death report since the source of the data was unreliable according to expert opinion. We also outliered ICD9BTL data points that were inconsistent with the rest of the data series and created unlikely time trends.

Modelling strategy

The Cause of Death Ensemble model (CODEm) was used for deaths due to diabetes mellitus estimation.

In the overall diabetes mellitus model, we used two models to estimate overall diabetes deaths with different age restrictions. This is because deaths in younger age groups are almost exclusively due to type 1 diabetes, while deaths in older ages are primarily due to type 2 diabetes. This allowed us to select predictive covariates that are specific to the pathophysiology of diabetes type 1 and type 2. We set the younger age model from 0-14 years and the older age model from 15-95+ years. We determined the age threshold based on evidence that the onset age of diabetes type 2 is occurring at younger ages.

Covariate selection

The following are the covariates included in the model. We were able to set an expected direction on each covariate. This requires that the covariate selected for the model must have the directional relationship with diabetes mellitus deaths.

Model	Level	Covariate	Direction
0-14 years	1	Healthcare access and quality index	-
	3	Education years per capita	-
	2	Age-standardised fertility rate	+
	2	Latitude	+
	2	Age-standardised underweight (weight-for-age) summary exposure variable	-
	2	Percentage of births occurring in women >35 years old	+
	2	Percentage of births occurring in women >40 years old	+
	3	Socio-demographic Index	-
	2	Age-standardised stunting (height-for-age) summary exposure variable	-
	2	Mean birth weight	-
15 + model	1	Age-standardised mean fasting plasma glucose (mmol/L)	+
	1	Age-standardised prevalence of diabetes	+
	3	Education years per capita	+/-
	3	Lag-distributed income per capita	+/-
	1	Mean BMI	+
	2	Mean cholesterol	+/-
	2	Mean systolic blood pressure	+/-
	1	Prevalence of obesity	+
	2	Energy-adjusted grams of fruits	+/-
	2	Energy-adjusted grams of sugar	+
	2	Energy-adjusted grams of vegetables	+/-
	3	Healthcare access and quality index	+/-

Diabetes mellitus Type 1 and Type 2

Diabetes mellitus Type 1

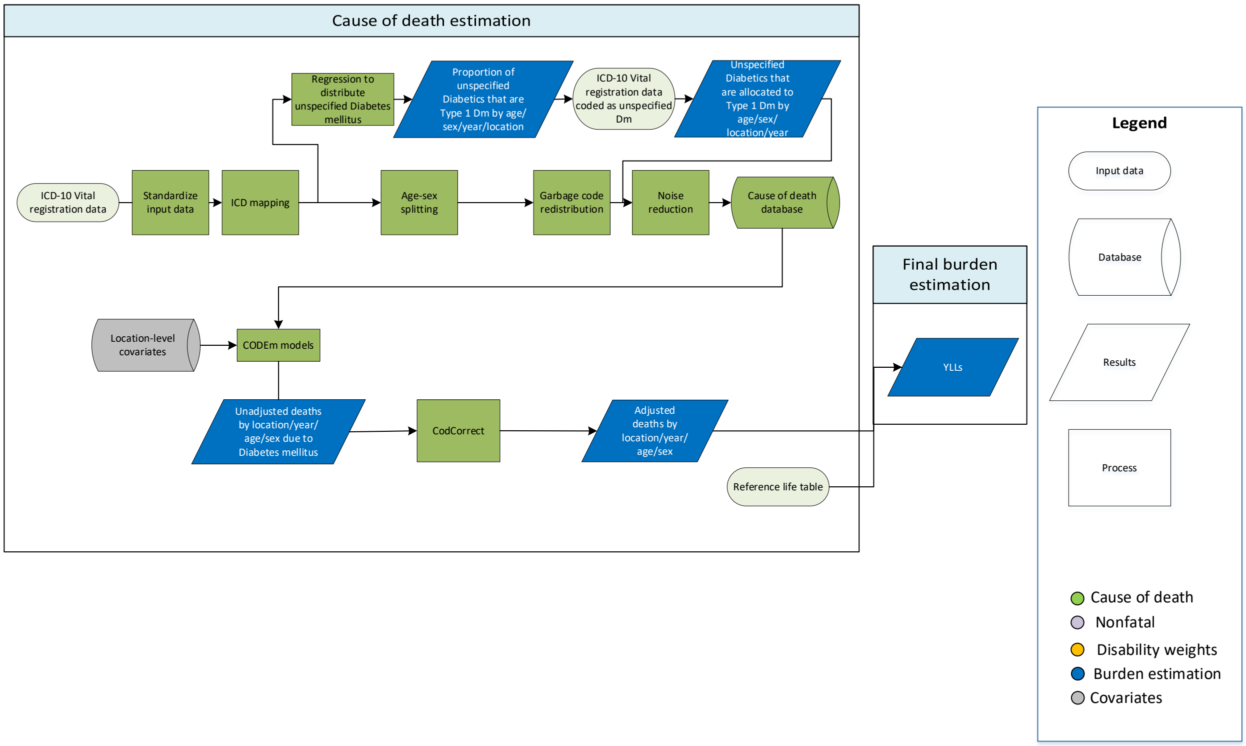

Diabetes mellitus Type 2

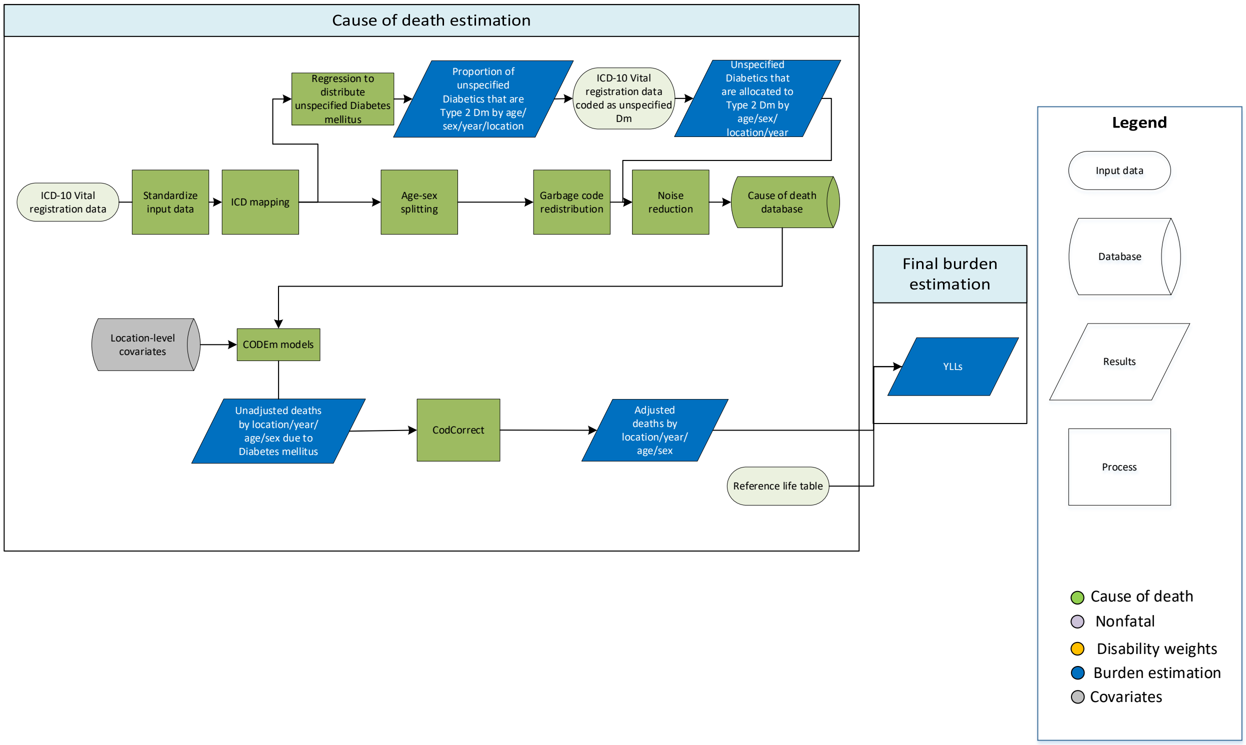

Input data

Type-specific diabetes mellitus mortality was estimated using deaths from vital registration sources in ICD-10 codes only. Diabetes type-specific information was not available in ICD-9 codes or deaths determined by verbal autopsy.

Modelling strategy

The Cause of Death Ensemble model (CODEm) was used for deaths due to diabetes mellitus estimation.

Deaths in younger age groups are almost exclusively due to type 1 diabetes, while deaths in older ages are primarily due to type 2 diabetes. To account for this age pattern, we set the age range of the diabetes type 1 model to 0-95+ years and the age range of the diabetes type 2 model to 15-95+ years. We used the same covariates in the diabetes type 1 model and diabetes type 2 model as the 0-14 year and 15-95+ year in the overall diabetes models, respectively.

There were two unique data manipulation steps that occurred in order to prepare the data as part of the modelling process.

1. We assumed that all deaths <15 years were due to type 1 regardless of the ICD-10 code assigned to the death. We imposed 100% attribution of diabetes mellitus deaths in <15 years to type 1 diabetes mellitus.
2. ICD-10 diabetes data were reported as type 1, type 2, or unspecified. We developed a regression to estimate the fraction of unspecified diabetes mellitus that was type 1 and type 2. Since there is a separate regression to estimate the proportion of type 1 diabetes mellitus and type 2 diabetes mellitus, we scaled the predicted proportions to one. These scaled proportions were then applied to number of deaths coded to unspecified diabetes in each location, year, sex where ICD-10 data was reported.

<u>Regression equation</u>

Type 1:

$$logit\left(\frac{number\ type\ 1\ DM}{number\ total\ DM}\right) \sim logit\left(\frac{number\ unspecified\ DM}{number\ total\ DM}\right) + \beta_1 age\ group \\ + \beta_2 age\text{-}st\ prev\ obesity * age\ group + age\text{-}st\ prev\ obesity$$

Type 2:

$$logit\left(\frac{number\ type\ 2\ DM}{number\ total\ DM}\right) \sim logit\left(\frac{number\ unspecified\ DM}{number\ total\ DM}\right) + \beta_1 age\ group \\ + \beta_2 age\text{-}st\ prev\ obesity * age\ group + age\text{-}st\ prev\ obesit$$

Covariate selection

The following are the covariates included in the model. We were able to set an expected direction on each covariate. This requires that the covariate selected for the model must have the directional relationship with diabetes mellitus deaths.

Model	Level	Covariate	Direction
Type 1	1	Healthcare access and quality index	-
	3	Education years per capita	-
	2	Age-standardised fertility rate	+
	2	Latitude	+
	2	Age-standardised underweight (weight-for-age) summary exposure variable	-
	2	Percentage of births occurring in women >35 years old	+
	2	Percentage of births occurring in women >40 years old	+
	3	Socio-demographic Index	-

	2	Age-standardised stunting (height-for-age) summary exposure variable	-
	2	Mean birth weight	-
Type 2	1	Age-standardised mean fasting plasma glucose (mmol/L)	+
	1	Age-standardised prevalence of diabetes	+
	3	Education years per capita	+/-
	3	Lag-distributed income per capita	+/-
	1	Mean BMI	+
	2	Mean cholesterol	+/-
	2	Mean systolic blood pressure	+/-
	1	Prevalence of obesity	+
	2	Energy-adjusted grams of fruits	+/-
	2	Energy-adjusted grams of sugar	+
	2	Energy-adjusted grams of vegetables	+/-
	3	Healthcare access and quality index	+/-

Chronic Kidney Disease

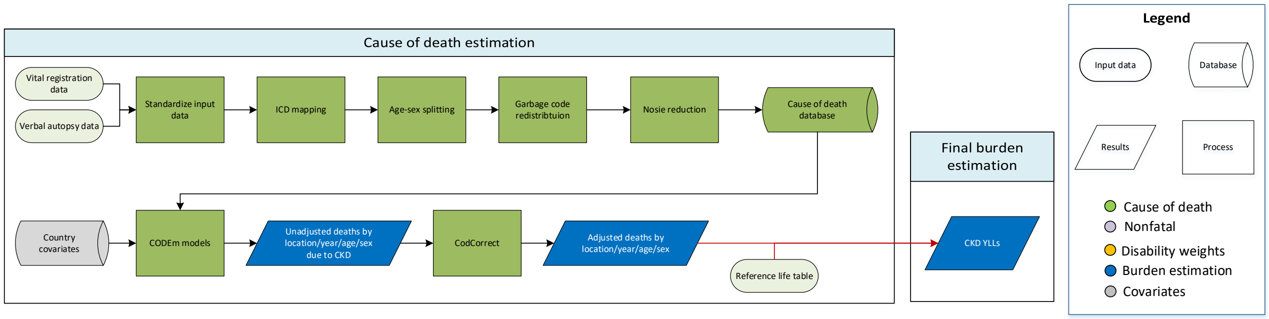

Input data

Vital registration and verbal autopsy data were used to model mortality due to chronic kidney disease. Outliers were identified by systematic examination of data points for all location-years. Data were standardised and mapped according to the GBD causes of death ICD mapping method. These data were then age-sex split, and appropriate redistribution of garbage code data was performed. Data points that violated well-established age or time trends or that resulted in extremely high or low cause fractions were determined to be outliers. Deaths due to congenital kidney anomalies (cystic kidney disease and reflux hydronephrosis) were attributed to chronic kidney disease, marking a change from GBD 2015, when these deaths were assigned to congenital anomalies.

Modelling strategy

The estimation strategy used for fatal chronic kidney disease is largely similar to methods used in GBD 2016. A standard CODEm model with location-level covariates was used to model deaths due to chronic kidney disease. Iterations of models were assessed at the location/year/age group/sex level to determine whether data points merited exclusion via outliering. Unadjusted death estimates were adjusted using CoDCorrect to produce final estimates of YLLs. The covariates used are displayed below.

Level	Covariate	Direction
1	Diabetes fasting plasma glucose (mmol/L)	+
	Diabetes age-standardised prevalence (proportion)	+
	Mean systolic blood pressure (mmHg)	+
	Mean BMI	+
	Healthcare access and quality index	–
2	Mean cholesterol	+
	Total calories (kcal per capita)	–
	Red meat (kcal per capita)	0
	Whole grains (kcal per capita)	0
	Animal fat (kcal per capita)	0
3	Socio-demographic Index	0
	Education (years per capita)	–
	Log LDI ($I per capita)	–

Chronic Kidney Disease subtypes

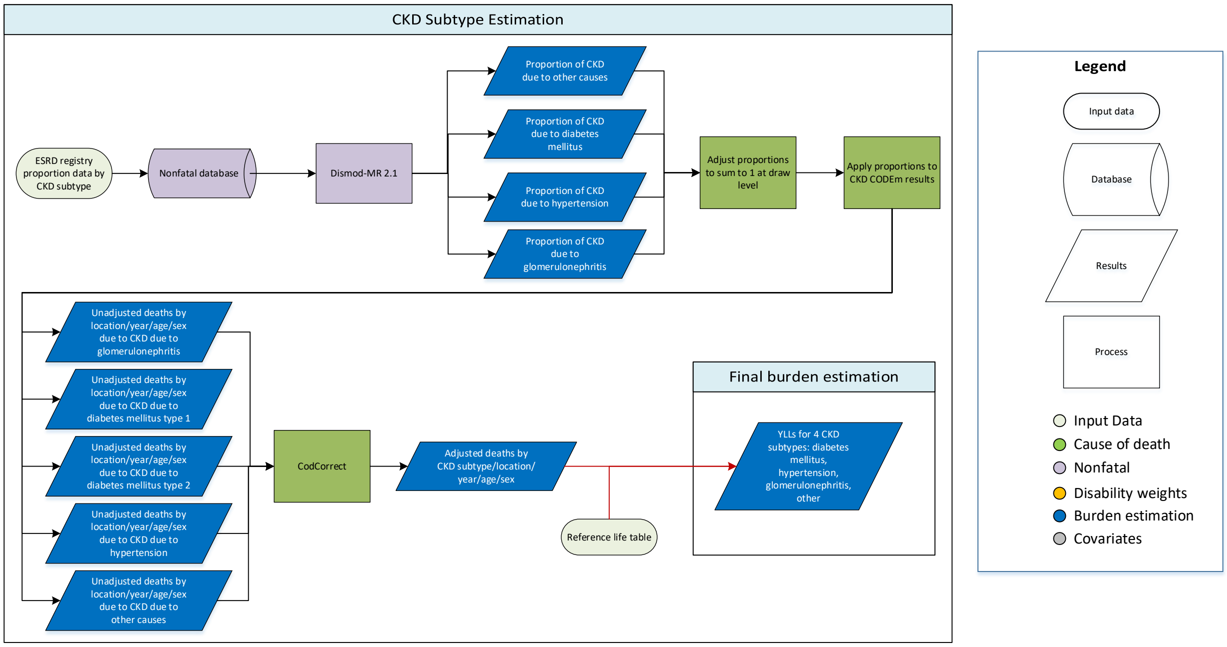

Input data

We estimated deaths due to five subtypes of chronic kidney disease: diabetes mellitus (DM) type 1, diabetes mellitus (DM) type 2, hypertension, glomerulonephritis, and other causes. Data from end-stage renal disease registries were used to inform estimates of proportion of CKD mortality attributable to each CKD subtype. Age-specific data on the proportion of ESRD by subtype was available from the United States, Australia, New Zealand, Nigeria, and Russia. Given the geographic spread in availability of age-specific proportion data, input data were not age-split, marking a change from GBD 2016.

Vital registration (VR) data were excluded from estimates, as aetiology coding in VR sources was considered highly variable and inconsistent between countries.

Modelling strategy

We ran DisMod-MR 2.1 models including diabetes prevalence and mean systolic blood pressure as country-level covariates to obtain estimates of proportions for each subtype by location, year, age, and sex. Data for CKD due to overall DM were more widely available than data by type of DM. In order to make use of all available data, we modelled the proportion of CKD due to overall DM, DM type 1, and DM type 2. Proportion of CKD due to DM type 1 and DM type 2 were then scaled to sum to the proportion of overall DM at the gender, age, and country-matched level. The results from all subtype-specific models were adjusted so that estimates across the subtypes equaled 1 at each of 1,000 draws. These adjusted proportions were applied to the parent CKD CODEm model.

Model	Covariate	Value	Exponentiated
CKD proportion due to diabetes mellitus	Diabetes age-standardised prevalence	0.49 (0.36–0.61)	1.63 (1.44–1.84)
CKD proportion due to hypertension	Mean systolic blood pressure	0.30 (0.010–1.05)	1.35 (1.01–2.86)

Acute Glomerulonephritis

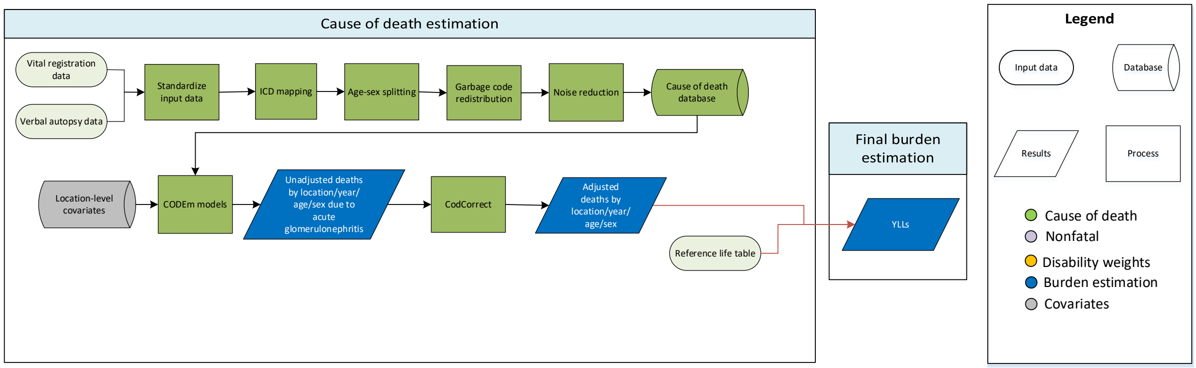

Input data

Vital registration data were used to model mortality due to acute glomerulonephritis. Vital registration data were standardised and mapped according to the GBD causes of death ICD mapping method. These data were then age-sex split, and appropriate redistribution of garbage code data was performed. After applying noise reduction, these data were uploaded to the COD database. Outliers were identified by systematic examination of data points for all location-years. Data points that violated well-established age or time trends or that resulted in extremely high or low cause fractions were determined to be outliers.

Modelling strategy

The estimation strategy used for fatal acute glomerulonephritis is largely similar to methods used in GBD 2016. A standard CODEm model with location-level covariates was used to model deaths due to acute glomerulonephritis. The linear floor is a model setting which indicates the lowest death rate (per 100,000) to be used in the linear prediction step of the cause of death modelling process. For GBD 2017, the linear floor was reduced from a value of 0.01 used in GBD 2016 to a value of 0.0001 to allow the model to better fit input data when the cause fraction is extremely low. This change has resulted in lower estimates of mortality rate due to acute glomerulonephritis in many geographies. Age-restrictions for death estimations secondary to acute glomerulonephritis include 28 days for lower bound and 95+ for upper bound. Iterations of models were assessed at the location/year/age group/sex level to determine whether data points merited exclusion via outliering. Unadjusted death estimates were adjusted using CoDCorrect to produce final estimates of YLLs. Estimates are limited by a paucity of data for regions such as Eastern and Central sub-Saharan Africa. The covariates used are displayed below.

Level	Covariate	Direction
2	Diabetes age-standardised prevalence	+
	Mean systolic blood pressure (mmHg)	+
	Sanitation (proportion with access)	−
	Improved water sources (proportion with access)	−
	Healthcare access and quality index	−
3	Socio-demographic Index	−
	Education (years per capita)	−
	Log LDI ($I per capita)	−

Skin and Subcutaneous Diseases

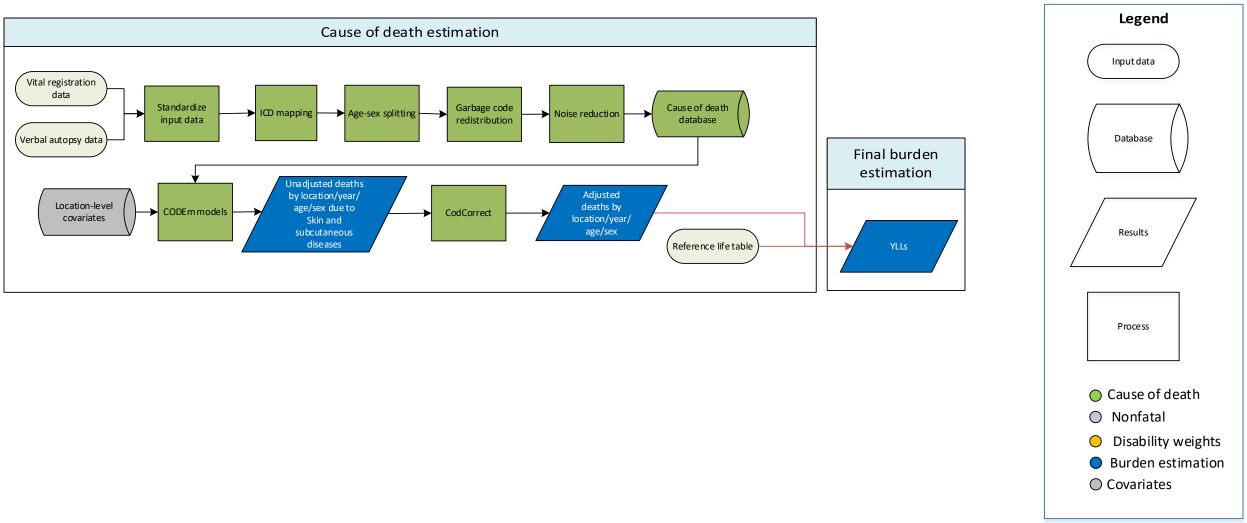

Input data

Data used to estimate mortality of skin and subcutaneous diseases consisted of vital registration data and verbal autopsy data from the cause of death (COD) database. We marked data as outliers in instances where garbage code redistribution and noise reduction, in combination with small sample sizes, resulted in unreasonable cause fractions; and data that violated well-established time or age trends. The data in skin and subcutaneous diseases consists of aggregated data from all other specific skin diseases (cellulitis, pyoderma, decubitus ulcer) as well as unique data points from unspecified codes of skin and subcutaneous disease.

Modelling strategy

We modelled deaths due to skin and subcutaneous diseases with a standard CODEm model using the cause of death database and location-level covariates as inputs. The model followed standard parameters, with the exception that the start age of the model was 28 days instead of 0. We hybridised separate global and data-rich models to acquire unadjusted results, which we finalised and adjusted using CoDCorrect to reach final years of life lost (YLLs) due to skin and subcutaneous diseases. In GBD 2017, we did not change covariates tried, or their expected strengths or directions, as compared to GBD 2016.

Covariate	Level	Direction
Alcohol (litres per capita)	2	1
Cumulative cigarettes (10 years)	2	1
Cumulative cigarettes (5 years)	2	1
Education (years per capita)	3	-1
Lag distributed income (per capita)	3	-1
Smoking prevalence	2	1
Improved water source (proportion with access)	1	-1
Unsafe sanitation (summary exposure variable)	1	1
Socio-demographic Index	3	0
Healthcare access and quality index	2	-1

Bacterial Skin Diseases

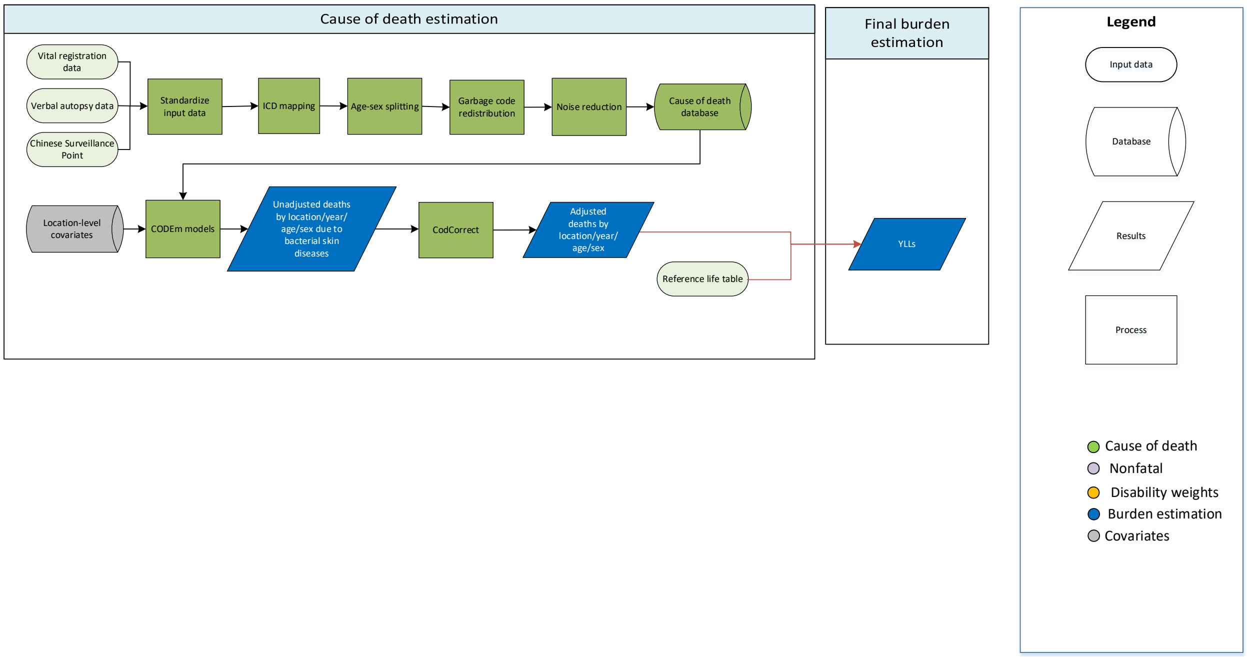

Input Data

Data used to estimate bacterial diseases consisted of vital registration, verbal autopsy, and Chinese disease surveillance point (DSP) data from the cause of death (COD) database. Outlier criteria excluded data points that were implausibly high or low relative to global or regional patterns and data from countries with small populations.

Modelling Strategy

This is a parent model of pyoderma and cellulitis. The standard CODEm modelling approach was used to estimate deaths due to bacterial skin diseases. CODEm parameters were a combination of those from pyoderma and cellulitis.

Level	Covariate	Direction
1	Summary exposure variable (SEV) scalar for unsafe sanitation	+
	Improved water source (proportion with access)	-
2	Alcohol (litres per capita)	+
	Cumulative cigarettes (5 years)	+
	Cumulative cigarettes (10 years)	+
	Healthcare access and quality index	-
	Smoking prevalence	+
3	Education (years per capita)	-
	Log LDI (I$ per capita)	-
	Socio-demographic Index	0

Cellulitis

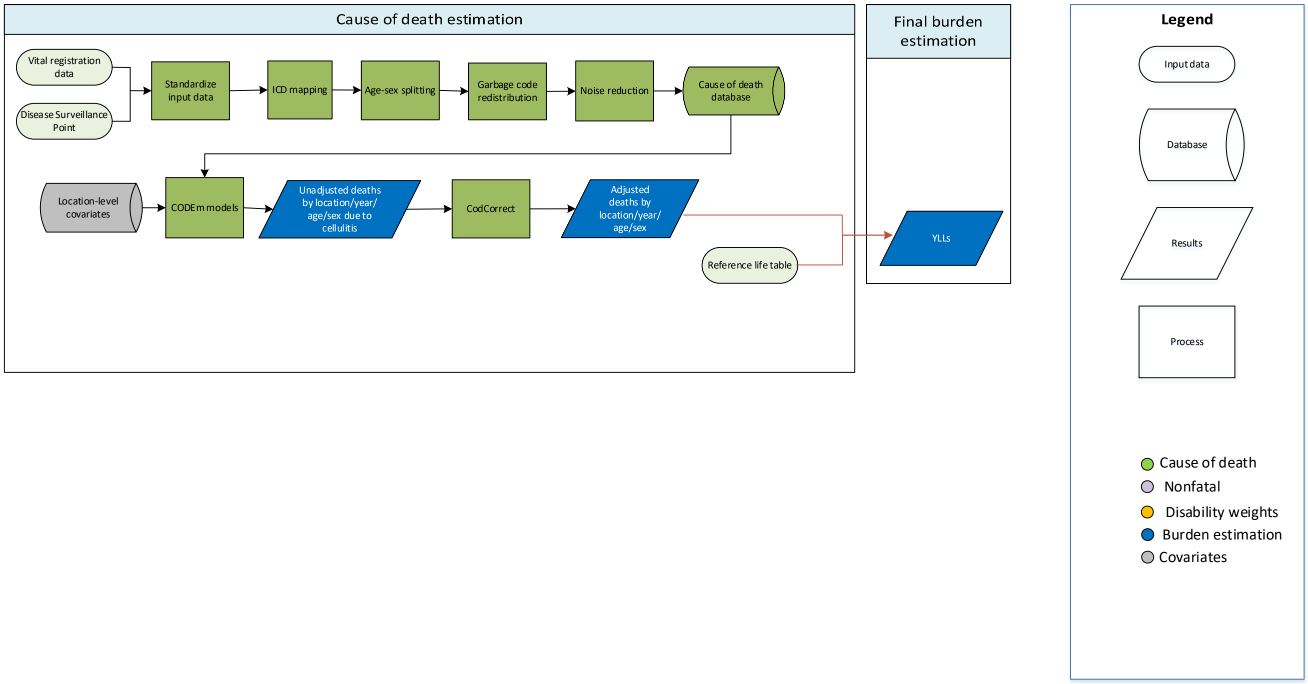

Input data

Data used to estimate cellulitis mortality consisted of vital registration and Chinese disease surveillance point (DSP) data from the cause of death (COD) database. Outlier criteria excluded data points that were implausibly high or low relative to global or regional patterns and data from countries with small populations.

Modelling strategy

The standard CODEm modelling approach was used to estimate deaths due to cellulitis. CODEm parameters were centrally defined. COD models were evaluated by comparing age-standardised death rates per 100,000 people to the GBD 2016 best model for 1990 and 2016 – individually for males and females. We also compared the age-standardised annualised rate of change for death rates per 100,000 persons to GBD 2016.

There were no significant changes in the modelling process between GBD 2016 and GBD 2017.

Level	Covariate	Direction
2	Healthcare access and quality index	-
3	Education (years per capita)	0
	Log LDI (I$ per capita)	0

Pyoderma

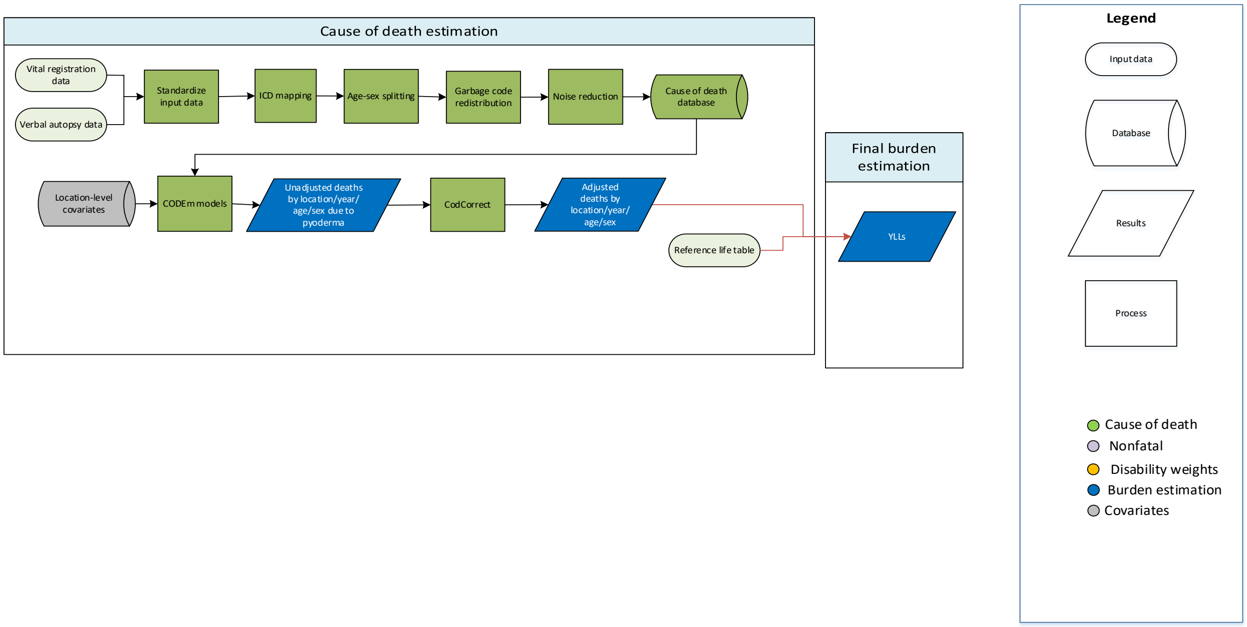

Input data

Data used to estimate pyoderma mortality included centrally prepped vital registration and verbal autopsy data from the cause of death (COD) database. Outlier criteria excluded data points that were implausibly high or low relative to global or regional patterns and data from countries with small populations.

Modelling strategy

We modelled deaths due to pyoderma with a standard CODEm model using the cause of death database and location-level covariates as inputs. The model followed standard parameters. We hybridised separate global and data-rich models to acquire unadjusted results, which we finalised and adjusted using CodCorrect to reach final years of life lost (YLLs) due to pyoderma. In GBD 2016 we added the healthcare access and quality (HAQ) index covariate to the model.
There were no significant changes in the modelling process between GBD 2016 and GBD 2017.

Level	Covariate	Direction
1	Improved water source (proportion with access)	-
	Unsafe sanitation (summary exposure variable)	+
2	Alcohol (litres per capita)	+
	Cumulative cigarettes (10 years)	+
	Cumulative cigarettes (5 years)	+
	Smoking prevalence	+
	Healthcare access and quality index	-
3	Lag distributed income (per capita)	-
	Education (years per capita)	-
	Socio-demographic Index	0

Decubitus Ulcer

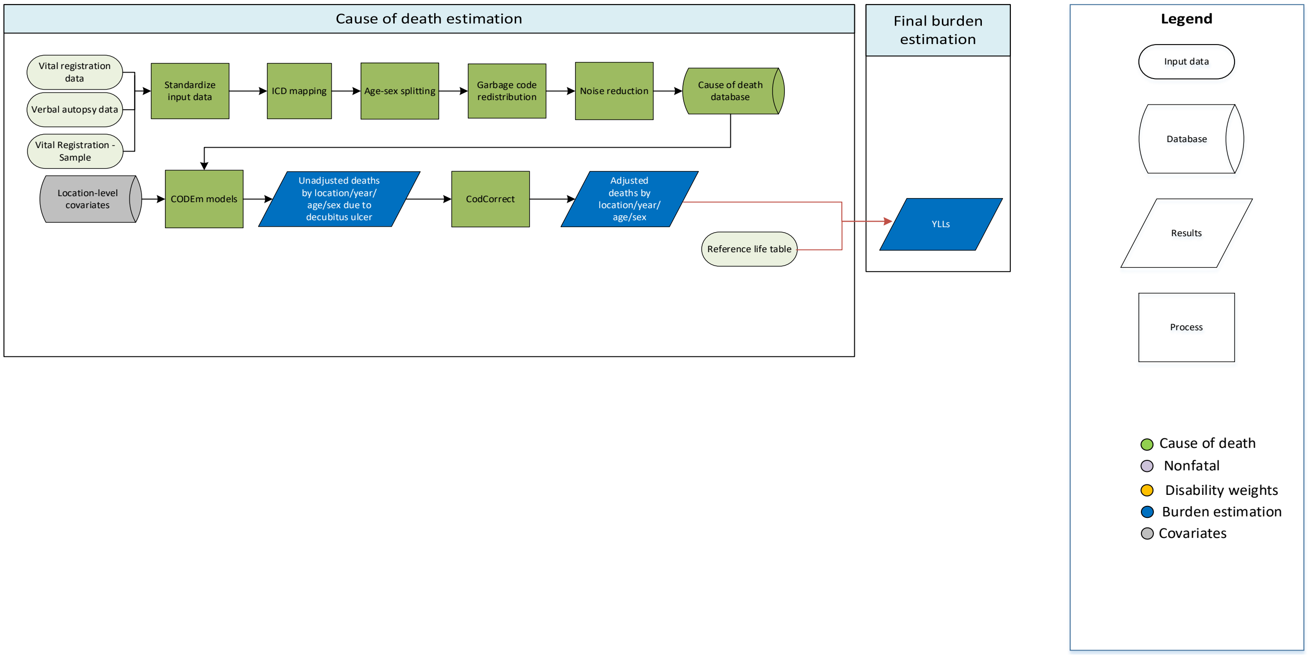

Input data

Data used to estimate decubitus ulcer mortality consisted of vital registration sources and verbal autopsy sources from the cause of death (COD) database. Outlier criteria excluded data points that were implausibly high or low relative to global or regional patterns and data from countries with small populations.

Modelling strategy

The standard CODEm modelling approach was used to estimate deaths due to decubitus ulcer. CODEm parameters were centrally defined. COD models were evaluated by comparing age-standardised death rates per 100,000 people to the GBD 2016 best model for 1990 and 2016 – individually for males and females. We also compared the age-standardised annualised rate of change for death rates per 100,000 persons to GBD 2016.

There were no significant changes in the modelling process between GBD 2016 and GBD 2017.

Level	Covariate	Direction
1	Summary exposure variable (SEV) scalar for unsafe sanitation	+
	Improved water source (proportion with access)	-
2	Alcohol (litres per capita)	+
	Healthcare access and quality index	–
	Cumulative cigarettes (5 years)	+
	Cumulative cigarettes (10 years)	+
	Smoking prevalence	+
3	Education (years per capita)	-
	Health system access 2	-
	Log LDI (I$ per capita)	-

Other Skin and Subcutaneous Diseases

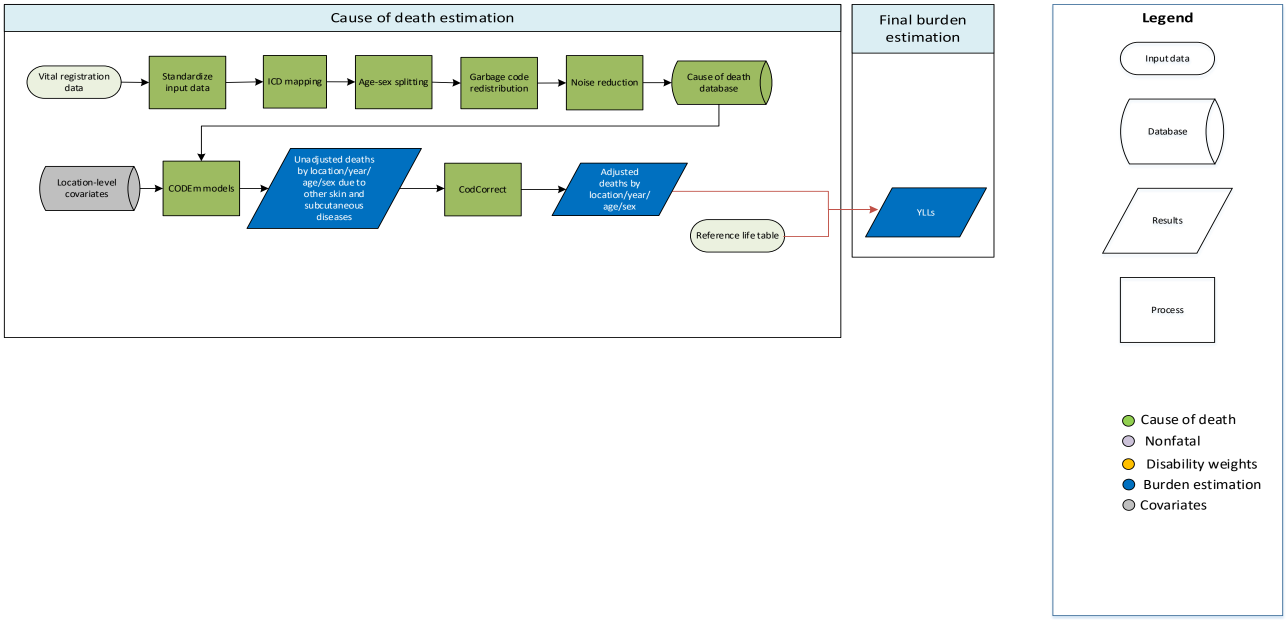

Input data

Data used to estimate mortality due to other skin and subcutaneous diseases consisted of vital registration data from the cause of death (COD) database. We outliered data in instances where garbage code redistribution and noise reduction, in combination with small sample sizes, resulted in unreasonable cause fractions; and data that violated well-established time or age trends.

Modelling strategy

The standard CODEm modelling approach was used to estimate deaths due to other skin and subcutaneous diseases. CODEm parameters were centrally defined. COD models were evaluated by comparing age-standardised death rates per 100,000 people to the GBD 2016 best model for 1990 and 2016 – individually for males and females. We also compared the age-standardised annualised rate of change for death rates per 100,000 people to GBD 2016.

There were no significant changes in the modelling process between GBD 2016 and GBD 2017.

Level	Covariate	Direction
1	Underweight (proportion <2SD weight for age, <5 years)	+
	Improved water source (proportion with access)	-
	Summary exposure variable (SEV) scalar for unsafe sanitation	+
2	Healthcare access and quality index	-
	Smoking prevalence	+
	Alcohol (litres per capita)	+
	Cumulative cigarettes (5 years)	+
	Cumulative cigarettes (10 years)	+
3	Education (years per capita)	-
	Health system access 2	-
	Log LDI (I$ per capita)	-
	Socio-demographic Index	0

Musculoskeletal Disorders

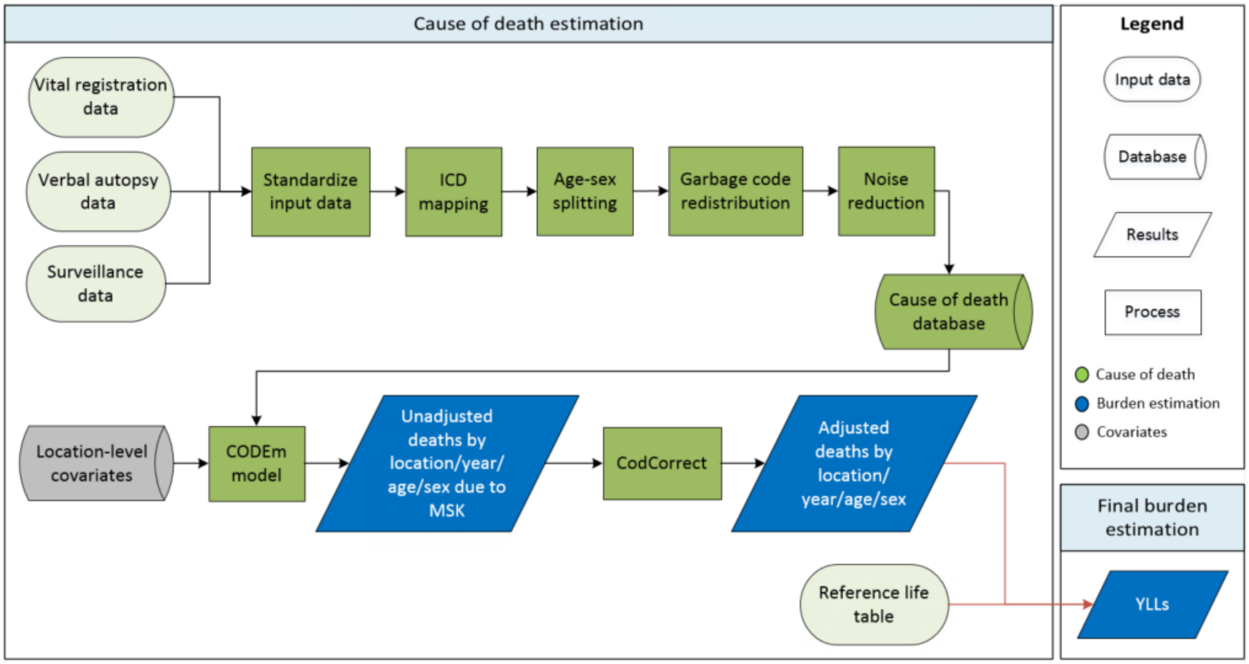

Input data

Data used to estimate mortality from musculoskeletal disorders (MSK) included vital registration (VR), verbal autopsy (VA), and China disease surveillance point data from the cause of death (COD) database. Our outlier criteria excluded data points that were implausibly high or low relative to global or regional patterns, substantially conflicted with established age or temporal patterns, or significantly conflicted with other data sources based from the same locations or locations with similar characteristics (ie, Socio-demographic Index).

Based on these criteria, in GBD 2017 we excluded VA data from Bangladesh, Vietnam, South Africa, Burkina Faso, Ghana, and all countries in Eastern sub-Saharan Africa, including Ethiopia, Kenya, Tanzania, Mozambique, and Zambia, as VA tools have poor validity in identifying MSK deaths. In India, the number of deaths from new Sample Registration System (SRS) data in urban parts of states was substantially higher than the number of deaths from Medical Certification of Cause of Death (MCCD) data. In rural India, the SRS data are the only source. We have outliered the MCCD data to make the models follow the SRS data. This does lead to higher estimates in India compared to other parts of the world. For Indonesia, we excluded mortality surveillance data from a few states with high estimates based on small numbers, ie, Kalimantan Selatan and Kalimantan Timur in males, and Maluku in females. Recent years of data from Kazakhstan (2013–2015) were outliered as they presented a discontinuity with previous years, which has been ascribed to the country's attempt to reduce deaths due to CVD leading to an increase of deaths. ICD9-BTL data from Latin American countries (Ecuador, Costa Rica, El Salvador, Guatemala, Honduras, Nicaragua, Panama, Venezuela, Antigua and Barbuda, the Bahamas,

Barbados, Belize, Bermuda, Cuba, Dominica, Grenada, Guyana, Jamaica, Saint Lucia, Saint Vincent and Grenadines, Suriname, and Trinidad and Tobago) were outliered. The data from these countries provided in ICD9-detail or ICD10 were kept in the analysis.

Modelling strategy

The standard CODEm modelling approach was applied to estimate deaths due to musculoskeletal disorders. We applied mostly the same covariates used in GBD 2016, with a few changes. Otherwise, there were no changes from the GBD 2016 modelling strategy.

Covariates are shown in the following table.

Level	Covariate	Direction
1	BMI (mean per capita)	+
2	Alcohol consumption (litres per capita)	+
	Cumulative cigarettes (10 years)	+
	Cumulative cigarettes (5 years)	+
	Smoking prevalence	+
	Cholesterol (mean per capita)	+
	Vegetable consumption (g per capita)	0
	Education (years per capita)	0
	Log-transformed LDI: lag-distributed income ($ per capita)	0
	Healthcare access and quality index	-
	Bone mineral density	+
	Age-standardised bone mineral density	+
3	SDI: Socio-demographic Index	0

Rheumatoid Arthritis

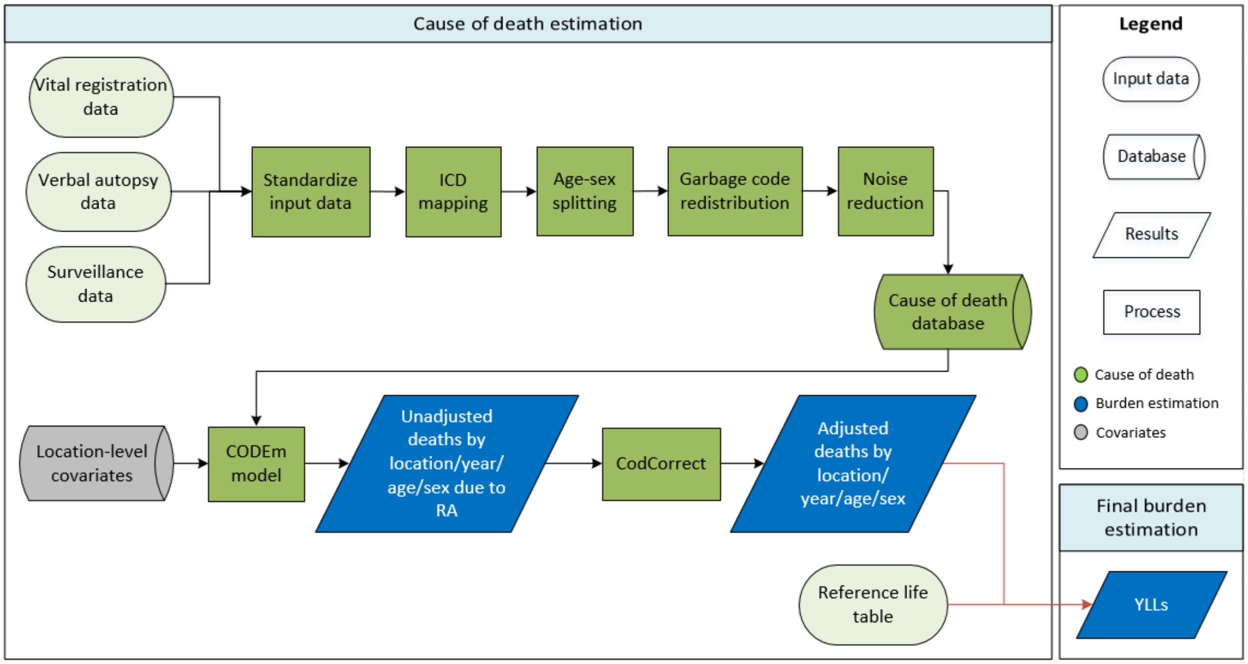

Input data

Data used to estimate rheumatoid arthritis mortality included vital registration, verbal autopsy, and China disease surveillance data from the cause of death database. Our outlier criteria were to exclude data points that were (1) implausibly high or low relative to global or regional patterns, (2) substantially conflicted with established age or temporal patterns, or (3) significantly conflicted with other data sources based from the same locations or locations with similar characteristics (ie, Socio-demographic Index).

Based on these criteria, we excluded a few data points from China. For males, we outliered data points from all sources in Tibet and data points from China disease surveillance in 1991 in all states, as these led to disproportionately high estimates. For females, we outliered Tibet data points from all sources up to 2007 and China disease surveillance data points in several southern states, ie, Guangxi, Hainan, and Yunnan. In addition, as the vital registration data in Limpopo for both males and females in 2003 and before are implausibly higher than the other provinces in South Africa, we outliered this data source and kept the data for 2004–2014 in the analysis. Also, as the vital registration data of mid-age males in Greenland are unrealistically high and much higher than, eg, in Canada and Denmark, the data for males age 45 and above were outliered. Recent years of data from Kazakhstan (2013–2015) were outliered as they presented a discontinuity with previous years, which has been ascribed to the country's attempt to reduce deaths due to CVD leading to an increase of deaths from all other causes including rheumatoid arthritis. Lastly, we outliered ICD9-BTL data from Latin American countries (Ecuador, Costa Rica, El Salvador, Guatemala, Honduras, Nicaragua, Panama, Venezuela, Antigua and Barbuda, the Bahamas, Barbados, Belize, Bermuda, Cuba, Dominica, Grenada, Guyana, Jamaica, Saint Lucia, Saint Vincent and

Grenadines, Suriname, and Trinidad and Tobago). The data from these countries in the years that used ICD10 were kept in the analysis.

Modelling strategy

The standard CODEm modelling approach was applied to estimate deaths due to rheumatoid arthritis. We mostly applied the same covariates used in GBD 2016, with a few changes. Otherwise, there were no changes from the GBD 2016 modelling strategy. All the covariates are shown in the following table.

Level	Covariate	Direction
1	Alcohol consumption (litres per capita)	+
	Cumulative cigarettes (10 years)	+
	Cumulative cigarettes (5 years)	+
	Smoking prevalence	+
	Healthcare access and quality index	-
2	Cholesterol (mean per capita)	+
	Vegetable consumption (g per capita)	0
3	BMI (mean per capita)	+
	SDI: Socio-demographic Index	0
	Log-transformed LDI: lag-distributed income ($ per capita)	-
	Education (years per capita)	-
	Milk (g), adjusted	0

Other Musculoskeletal Disorders

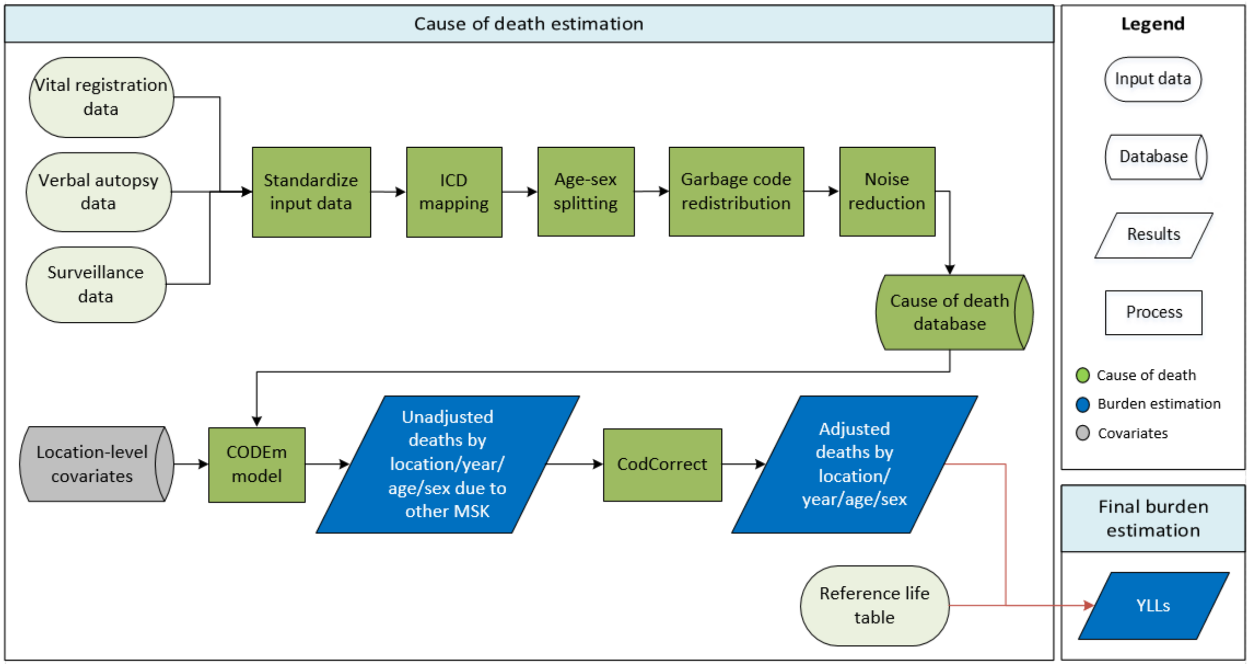

Input data

Data used to estimate mortality of other musculoskeletal disorders (MSK) included vital registration, verbal autopsy (VA), and China disease surveillance point data from the cause of death database. Our outlier criteria excluded data points that were implausibly high or low relative to global or regional patterns, substantially conflicted with established age or temporal patterns, or significantly conflicted with other data sources based from the same locations or locations with similar characteristics (ie, socio-demographic index).

Based on these criteria, we excluded VA studies from Eastern and Western sub-Saharan Africa, as VA studies cannot distinguish other MSK deaths and estimates for the regions were disproportionately high. Recent years of data from Kazakhstan (2013–2015) were outliered as they presented a discontinuity with previous years, which has been ascribed to the country's attempt to reduce deaths from CVD leading to an increase of deaths from all other causes, including other MSK. We also outliered all data in Latin American countries (Ecuador, Costa Rica, El Salvador, Guatemala, Honduras, Nicaragua, Panama, Venezuela, Antigua and Barbuda, the Bahamas, Barbados, Belize, Bermuda, Cuba, Dominica, Grenada, Guyana, Jamaica, Saint Lucia, Saint Vincent and Grenadines, Suriname, and Trinidad and Tobago). The data from these countries in the years that used ICD9-detail or ICD10 were kept in the analysis.

Modelling strategy

The standard CODEm modelling approach was applied to estimate deaths due to other musculoskeletal disorders. We applied the same covariates used in GBD 2016 and there were no major changes from the GBD 2016 modelling strategy. Covariates are shown in the following table.

Level	Covariate	Direction

1	BMI (mean per capita)	+
2	Alcohol consumption (litres per capita)	+
	Cumulative cigarettes (10 years)	+
	Cumulative cigarettes (5 years)	+
	Smoking prevalence	+
	Cholesterol (mean per capita)	+
	Vegetable consumption (g per capita)	0
	Education (years per capita)	0
	Log-transformed LDI: lag-distributed income ($ per capita)	0
	Healthcare access and quality index	-
3	SDI: Socio-demographic Index	0

Congenital Birth Defects: *Neural tube defects, congenital heart anomalies, orofacial clefts, Down syndrome, Turner syndrome, Klinefelter syndrome, other chromosomal disorders, congenital musculoskeletal anomalies, urogenital congenital anomalies, digestive congenital anomalies, and other congenital birth defects.*

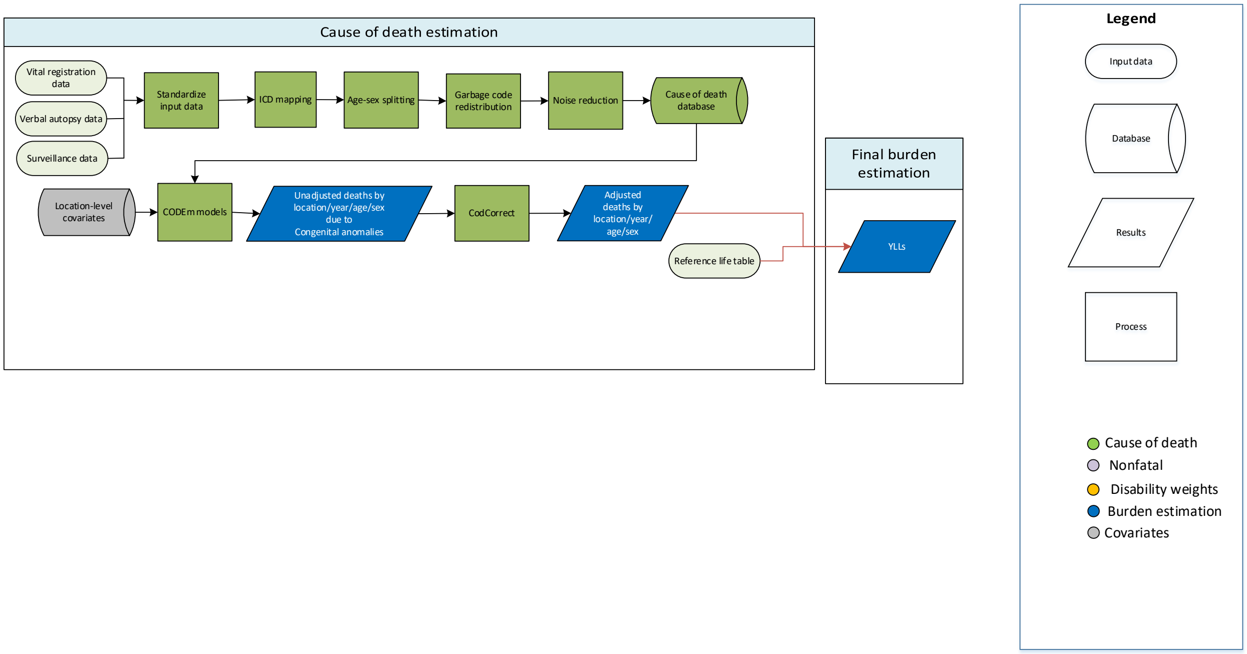

Input data

For GBD 2017, input data for estimating mortality due to congenital anomalies was centrally extracted, processed, and stored in the causes of death (CoD) database. Vital registration (VR) was the dominant data type, followed by verbal autopsy (VA) and surveillance. Those CoD data sources that specified the sub-cause of birth defect were included in estimation of both the parent congenital anomalies model as well as in sub-type-specific models.

For GBD 2017, data exclusions were limited. The majority of VA data were outliered in those over 5 years old as the age patterns were unreliable and led to poor model performance in the under-5 age groups. We also excluded some data sources from the parent model where only a subset of sub-causes were specified (eg, congenital heart disease, neural tube defects, and other congenital anomalies) and the sum of the sub-causes clearly represented systematic underreporting of one of the sub-causes. Systematic underreporting was suspected when sex- and age-specific rates were more than an order of magnitude lower than neighboring or comparable locations. Data sources for those locations were still included by default for sub-cause-specific models because under-reporting of the total was not assumed to necessarily be associated with under-reporting of all of the component conditions.

Modelling strategy

All types of congenital anomalies were estimated using cause of death ensemble modeling (CODEm) for GBD 2017, as was done for previous iterations of the GBD study. Specific causes included neural tube defects, congenital heart anomalies, orofacial clefts, Down syndrome, other chromosomal anomalies, congenital musculoskeletal anomalies, urogenital congenital anomalies, digestive congenital anomalies, and other congenital birth defects. We assumed no mortality from either Klinefelter syndrome or Turner syndrome, for which we model non-fatal outcomes only. For GBD 2017, we modelled congenital anomalies as a cause of death for ages 0-69 years only, assuming that all mortality from congenital conditions occurs before age 70 years of age.

For GBD 2016, we added three new causes to the congenital anomalies: congenital musculoskeletal and limb anomalies; urogenital congenital anomalies; and digestive congenital anomalies. We made no additions to the causes of congenital anomalies for GBD 2017.

Covariates selected for CODEm model of overall congenital birth defects

Covariate	Transformation	Level	Direction
Maternal alcohol consumption during pregnancy (proportion)	None	1	Positive
In-facility delivery (proportion)	None	1	Negative
Live births 35+ (proportion)	None	1	Positive
Folic acid unadjusted (ug)	None	1	Negative
Legality of abortion	None	2	Negative
Antenatal care (1 visit) coverage (proportion)	None	2	Not specified
Smoking prevalence (reproductive-age-standardised)	None	2	Positive
Antenatal care (4 visits) coverage (proportion)	None	2	Negative
Healthcare access and quality index	None	2	Negative
Education (years per capita)	None	2	Negative
Alcohol (litres per capita)	None	3	Positive
Fruits unadjusted (g)	None	3	Positive
Outdoor air pollution ($PM_{2.5}$)	None	3	Positive
Indoor air pollution (all cooking fuels)	None	3	Positive
Socio-demographic Index	None	3	Negative
Vegetables unadjusted (g)	None	3	Positive

Covariates selected for CODEm model of neural tube defects

Covariate	Transformation	Level	Direction
Health system access (capped)	None	1	Negative
Fruits adjusted (g)	None	2	Negative
Vegetables adjusted (g)	None	2	Negative
Healthcare access and quality index	None	2	Negative

Education (years per capita)	None	3	Negative
LDI (I$ per capita)	Log	3	Negative
Socio-demographic Index	None	3	Negative

Covariates selected for CODEm model of congenital heart anomalies

Covariate	Transformation	Level	Direction
Maternal alcohol consumption during pregnancy (proportion)	None	1	Positive
Socio-demographic Index	Log	2	Negative
Smoking prevalence (reproductive-age-standardised)	None	2	Positive
Diabetes age-standardised prevalence (proportion)	None	2	Positive
Healthcare access and quality index	None	2	Negative
Legality of abortion	None	2	Negative
Antenatal care (1 visit) coverage (proportion)	None	2	Negative
In-facility delivery (proportion)	None	2	Negative
Education (years per capita)	None	2	Negative
Alcohol (litres per capita)	None	3	Positive
Antenatal care (4 visits) coverage (proportion)	None	3	Negative
Skilled birth attendance (proportion)	None	3	Negative
Live births 35+ (proportion)	None	3	Positive

Covariates selected for CODEm model of cleft lip and cleft palate

Covariate	Transformation	Level	Direction
Indoor air pollution (all cooking fuels)	None	1	Positive
Diabetes age-standardised prevalence (proportion)	None	2	Positive
Maternal alcohol consumption during pregnancy (proportion)	None	2	Positive
Healthcare access and quality index	None	2	Negative
Outdoor air pollution ($PM_{2.5}$)	None	2	Positive
Legality of abortion	None	2	Negative
Skilled birth attendance (proportion)	None	2	Negative
Smoking prevalence (reproductive-age-standardised)	None	2	Positive
Vegetables unadjusted (g)	None	3	Not specified
Alcohol (litres per capita)	None	3	Positive
Antenatal care (4 visits) coverage (proportion)	None	3	Negative
Education (years per capita)	None	3	Negative
Fruits unadjusted (g)	None	3	Not specified
Antenatal care (1 visit) coverage (proportion)	None	3	Negative

Covariates selected for CODEm model of Down syndrome

Covariate	Transformation	Level	Direction
Live births 35+ (proportion)	None	1	Positive
Legality of abortion	None	1	Negative
Live births 40+ (proportion)	None	1	Positive
Socio-demographic Index	None	2	Negative
LDI (I$ per capita)	Log	2	Negative
In-facility delivery (proportion)	None	2	Negative
Healthcare access and quality index	None	2	Negative
Maternal alcohol consumption during pregnancy (proportion)	None	3	Positive
Antenatal care (1 visit) coverage (proportion)	None	3	Negative
Education (years per capita)	None	3	Negative
Indoor air pollution (all cooking fuels)	None	3	Positive
Antenatal care (4 visits) coverage (proportion)	None	3	Negative
Vegetables unadjusted (g)	None	3	Negative
Smoking prevalence (reproductive age-standardised)	None	3	Positive

Covariates selected for CODEm model of other chromosomal abnormalities

Covariate	Transformation	Level	Direction
Live births 35+ (proportion)	None	1	Positive
Live births 40+ (proportion)	None	1	Positive
Legality of abortion	None	1	Negative
LDI (I$ per capita)	Log	2	Negative
Healthcare access and quality index	None	2	Negative
Antenatal care (4 visits) coverage (proportion)	None	2	Negative
Antenatal care (1 visit) coverage (proportion)	None	2	Negative
In-facility delivery (proportion)	None	2	Negative
Maternal alcohol consumption during pregnancy (proportion)	None	2	Positive
Socio-demographic Index	None	3	Not specified
Alcohol (litres per capita)	None	3	Positive
Smoking prevalence (reproductive age-standardised)	None	3	Positive
Education (years per capita)	None	3	Negative
Skilled birth attendance (proportion)	None	3	Negative

Covariates selected for CODEm model of congenital musculoskeletal and limb anomalies

Covariate	Transformation	Level	Direction
Maternal alcohol consumption during pregnancy (proportion)	None	1	Positive
Legality of abortion	None	1	Negative
In-facility delivery (proportion)	None	2	Negative
Diabetes age-standardised prevalence (proportion)	None	2	Positive

Socio-demographic Index	None	2	Negative
Healthcare access and quality index	None	2	Negative
Indoor air pollution (all cooking fuels)	None	2	Positive
Smoking prevalence (reproductive age standardised)	None	2	Positive
Antenatal care (4 visits) coverage (proportion)	None	3	Negative
Alcohol (litres per capita)	None	3	Positive
Vegetables unadjusted (g)	None	3	Not specified
Fruits unadjusted (g)	None	3	Not specified
Education (years per capita)	None	3	Negative
Antenatal care (1 visit) coverage (proportion)	None	3	Negative

Covariates selected for CODEm model of urogenital congenital anomalies

Covariate	Transformation	Level	Direction
Smoking prevalence (reproductive age-standardised)	None	1	Positive
Maternal alcohol consumption during pregnancy (proportion)	None	1	Positive
Healthcare access and quality index	None	2	Negative
Diabetes age-standardised prevalence (proportion)	None	2	Positive
Socio-demographic Index	None	2	Negative
Outdoor air pollution ($PM_{2.5}$)	None	2	Positive
In-facility delivery (proportion)	None	2	Negative
Indoor air pollution (all cooking fuels)	None	2	Positive
Antenatal care (1 visit) coverage (proportion)	None	3	Negative
Alcohol (litres per capita)	None	3	Positive
Education (years per capita)	None	3	Negative
LDI (I$ per capita)	Log	3	Negative
Antenatal care (4 visits) coverage (proportion)	None	3	Negative

Covariates selected for CODEm model of digestive congenital anomalies

Covariate	Transformation	Level	Direction
Maternal alcohol consumption during pregnancy (proportion)	None	1	Positive
Smoking prevalence (reproductive age-standardised)	None	1	Positive
Indoor air pollution (all cooking fuels)	None	2	Positive
Diabetes age-standardised prevalence (proportion)	None	2	Positive
Socio-demographic Index	None	2	Negative
Prevalence of obesity (age-standardised)	None	2	Positive
In-facility delivery (proportion)	None	2	Negative
Healthcare access and quality index	None	2	Negative
Alcohol (liters per capita)	None	3	Positive
Health system access (capped)	None	3	Negative
Education (years per capita)	None	3	Negative
Vegetables unadjusted (g)	None	3	Not specified
Antenatal care (1 visit) coverage (proportion)	None	3	Negative
Antenatal care (4 visits) coverage (proportion)	None	3	Negative

Fruits unadjusted (g)	None	3	Not specified
LDI (I$ per capita)	Log	3	Negative

Covariates selected for CODEm model of other congenital birth defects

**Covariate**	**Transformation**	**Level**	**Direction**
Maternal alcohol consumption during pregnancy (proportion)	None	1	Positive
Live births 35+ (proportion)	None	1	Positive
Education (years per capita)	None	2	Negative
Smoking prevalence (reproductive age-standardised)	None	2	Positive
Legality of abortion	None	2	Negative
In-facility delivery (proportion)	None	2	Negative
Indoor air pollution (all cooking fuels)	None	2	Positive
Healthcare access and quality index	None	2	Negative
Antenatal care (1 visit) coverage (proportion)	None	3	Negative
Diabetes age-standardised prevalence (proportion)	None	3	Positive
LDI (I$ per capita)	Log	3	Negative
Socio-demographic Index	None	3	Negative
Antenatal care (4 visits) coverage (proportion)	None	3	Negative
Alcohol (litres per capita)	None	3	Positive

Urinary Diseases and Male Infertility

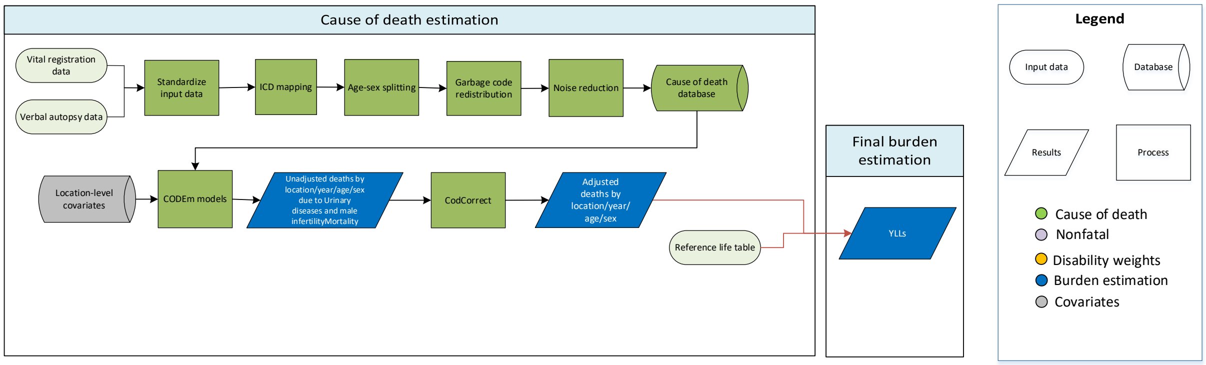

Input data

Vital registration data were used to model mortality due to urinary diseases and male infertility. Vital registration data were standardised and mapped according to the GBD causes of death ICD mapping method. These data were then age-sex split, and appropriate redistribution of garbage code data was performed. After applying noise reduction, these data were uploaded to the COD database. Outliers were identified by systematic examination of data points for all location-years. Data points that violated well-established age or time trends or that resulted in extremely high or low cause fractions were determined to be outliers.

Modelling strategy

The estimation strategy used for fatal urinary diseases and male infertility is largely similar to methods used in GBD 2016. A standard CODEm model with location-level covariates was used to model deaths due to urinary diseases and male infertility. Iterations of models were assessed at the location/year/age group/sex level to determine whether data points merited exclusion via outliering. The estimates are limited by a paucity of data for regions such as Eastern and Central sub-Saharan Africa. The results of this disease differ by gender as no “male infertility” estimates were performed among women. The covariates used are displayed below.

Level	Covariate	Direction
2	Mean BMI	+
	Healthcare access and quality index	–
	Latitude under 15 (proportion)	0
	Latitude 15 to 30 (proportion)	0
	Latitude 30 to 45 (proportion)	0
	Latitude over 45 (proportion)	0
3	Education (years per capita)	–
	Log LDI ($I per capita)	–
	Socio-demographic Index	0

Urinary Tract Infections

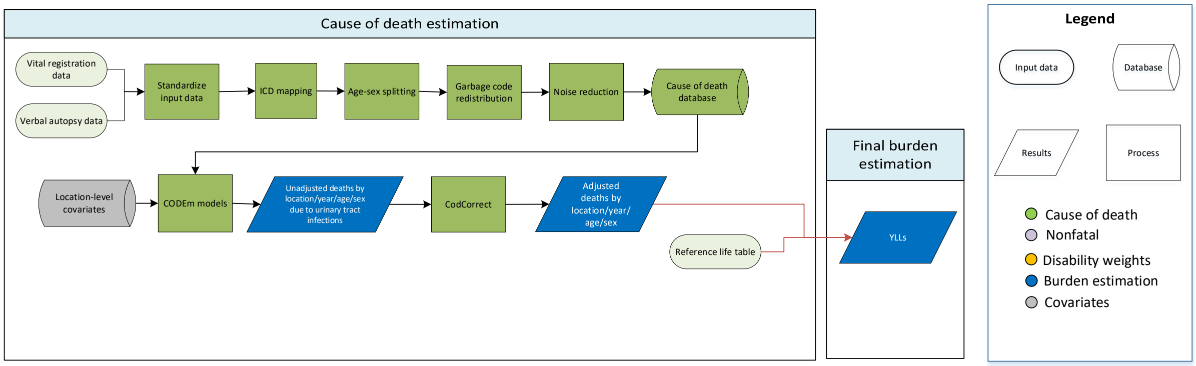

Input data

Vital registration and verbal autopsy data were used to model mortality due to urinary tract infections. Data were standardised and mapped according to the GBD causes of death ICD mapping method. These data were then age-sex split, and appropriate redistribution of garbage code data was performed. After applying noise reduction, these data were uploaded to the COD database. Outliers were identified by systematic examination of data points for all location-years. Data points that violated well-established age or time trends or that resulted in extremely high or low cause fractions were determined to be outliers.

Modelling strategy

The estimation strategy used for fatal urinary tract infections is largely similar to methods used in GBD 2016. A standard CODEm model with location-level covariates was used to model deaths due to interstitial nephritis. Age-restrictions for death estimations secondary to urinary tract infections include 0 days for lower bound, 95+ for upper bound. Iterations of models were assessed at the location/year/age group/sex level to determine whether data points merited exclusion via outliering. Unadjusted death estimates were adjusted using CoDCorrect to produce final estimates of YLLs. The estimates are limited by a paucity of data for regions such as Eastern and Central sub-Saharan Africa. The covariates used are displayed below.

Level	Covariate	Direction
1	Sanitation (proportion with access)	–
2	Education (years per capita)	–
	Log LDI ($I per capita)	–
	Healthcare access and quality index	–
3	Socio-demographic Index	n/a

Urolithiasis

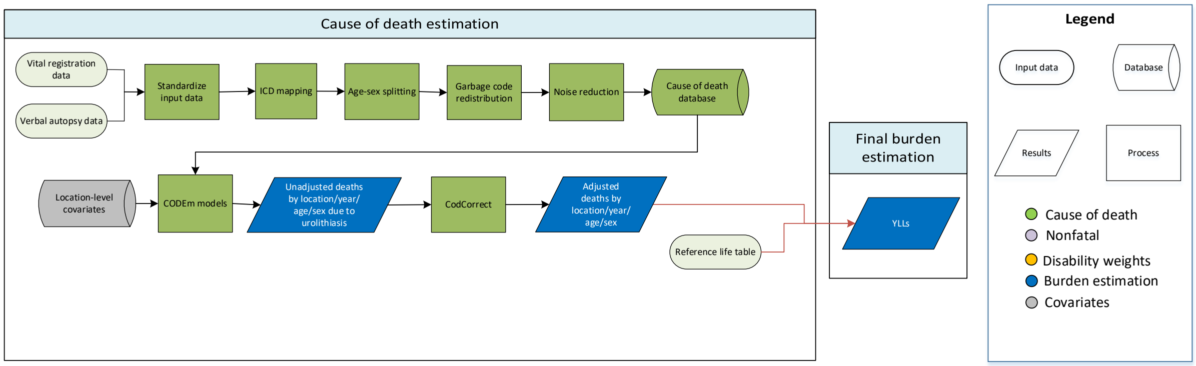

Input data

Vital registration and verbal autopsy data were used to model mortality due to urolithiasis. Data were standardised and mapped according to the GBD causes of death ICD mapping method. These data were then age-sex split, and appropriate redistribution of garbage code data was performed. After applying noise reduction, these data were uploaded to the COD database. Outliers were identified by systematic examination of data points for all location-years. Data points that violated well-established age or time trends or that resulted in extremely high or low cause fractions were determined to be outliers.

Modelling strategy

The estimation strategy used for fatal urolithiasis is largely similar to methods used in GBD 2016. A standard CODEm model including location-level covariates was used to model deaths due to urolithiasis. Age-restrictions for death estimations secondary to urolithiasis include 5 years for lower bound, 95+ for upper bound. Iterations of models were assessed at the location/year/age group/sex level to determine whether data points merited exclusion via outliering. Unadjusted death estimates were adjusted using CoDCorrect to produce final estimates of YLLs. The estimates are limited by a paucity of data for regions such as Eastern and Central sub-Saharan Africa. The covariates used are displayed below.

Level	Covariate	Direction
1	Temperature (90th percentile)	+
2	Animal fat (kcal per capita)	+
	Fruits (kcal per capita)	−
	Vegetables (kcal per capita)	−
	Red meat (kcal per capita)	+
	Healthcare access and quality index	−
3	Socio-demographic Index	0
	Log LDI ($I per capita)	−

Other Urinary Diseases

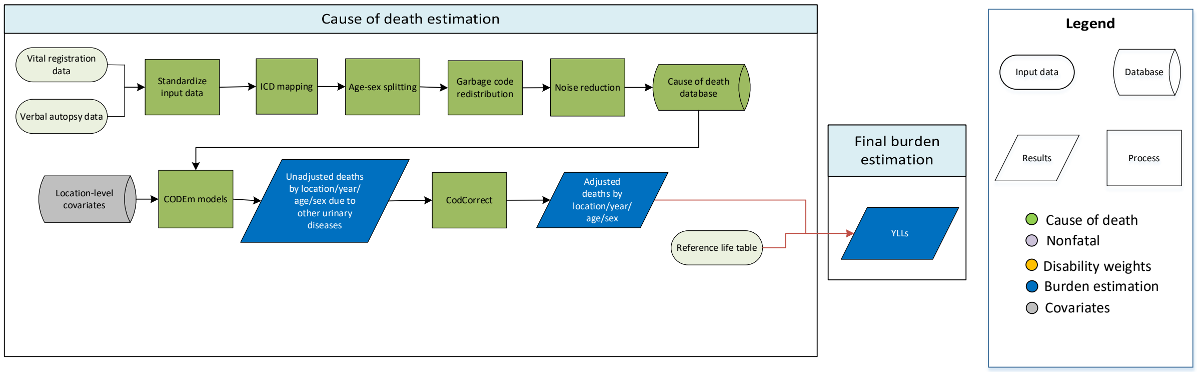

Input data

Vital registration and verbal autopsy data were used to model mortality due to other urinary diseases. Data were standardised and mapped according to the GBD causes of death ICD mapping method. These data were then age-sex split, and appropriate redistribution of garbage code data was performed. After applying noise reduction, these data were uploaded to the COD database. Outliers were identified by systematic examination of data points for all location-years. Data points that violated well-established age or time trends or that resulted in extremely high or low cause fractions were determined to be outliers.

Modelling strategy

The estimation strategy used for other urinary diseases is largely similar to methods used in GBD 2016. A standard CODEm model with location-level covariates was used to model deaths due to other urinary diseases. Age-restrictions for death estimations secondary to urinary diseases and male infertility include 0 days for lower bound, 95+ for upper bound. Iterations of models were assessed at the location/year/age group/sex level to determine whether data points merited exclusion via outliering. Unadjusted death estimates were adjusted using CoDCorrect to produce final estimates of YLLs. The estimates are limited by a paucity of data for regions such as Eastern and Central sub-Saharan Africa. The covariates used are displayed below.

Level	Covariate	Direction
1	Mean BMI	+
2	Education (years per capita)	−
	Log LDI ($I per capita)	−
	Healthcare access and quality index	−
3	Socio-demographic Index	0

Gynaecological Conditions

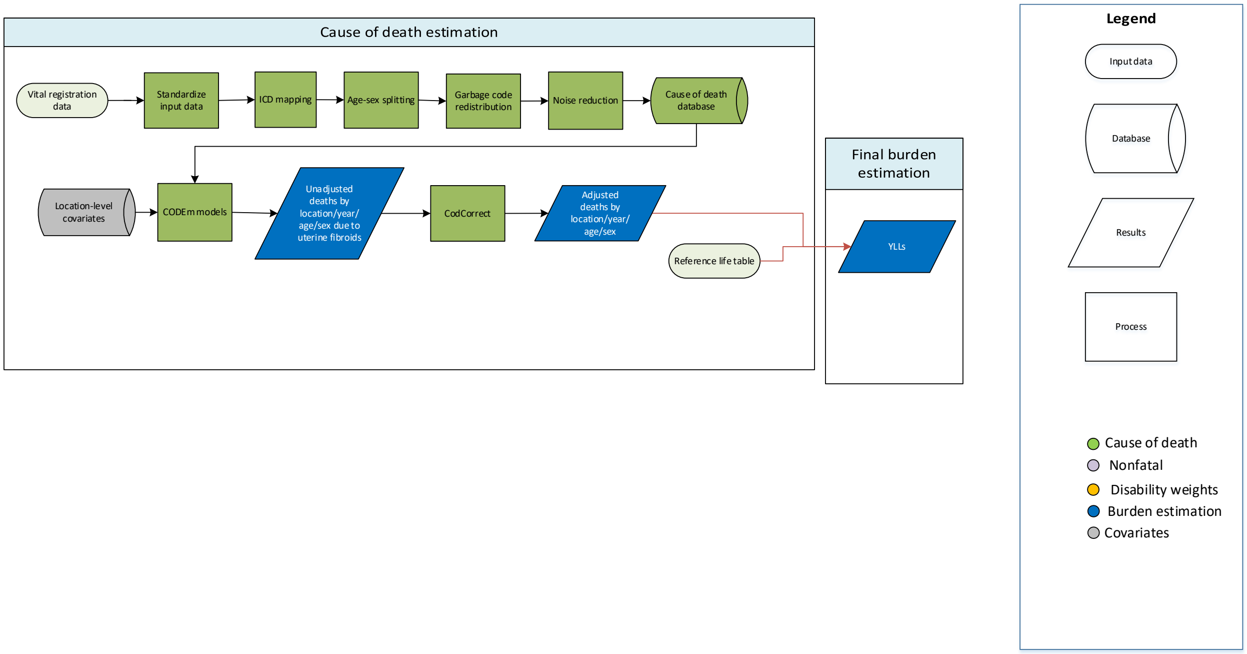

Input data

For GBD 2017, vital registration data were used to estimate deaths for each of the five fatal gynaecological conditions, which include uterine fibroids, polycystic ovarian syndrome (PCOS), endometriosis, genital prolapse, and other gynaecological conditions. These causes are sex-specific to women and we only model deaths among women. ICD9 and ICD10 codes for each are listed below. Data points were selected as outliers if they were implausibly high, low, or significantly conflicted with established age or temporal patterns.

Modelling strategy

For GBD 2017, we estimated mortality due to the total of all gynaecological diseases as well as each of the sub-categories using CODEm. We assumed no deaths from premenstrual syndrome and primary infertility, which we model for non-fatal outcomes.

Continuing in GBD 2017, we have reassigned deaths due to leiomyomas and other benign uterine tumors to uterine fibroids.

Covariates included in the CODEm models for each gynaecological disorder by level and direction:

Gynaecological disorders

Covariate name	Level	Direction
Education, years per capita	3	-1
LDI per capita	3	-1
Percentage births in over 35s	2	1
Skilled birth attendance proportion	2	-1
Smoking prevalence	1	0

TFR	2	1
Health system access capped	2	-1
Socio-demographic Index	3	-1
Healthcare access and quality index	2	-1

Uterine Fibroids

Covariate Name	Level	Direction
Education, years per capita	3	-1
LDI per capita	3	-1
percentage births in over 35s	2	1
Skilled birth attendance proportion	2	-1
Smoking prevalence	1	0
TFR	2	1
health system access capped	2	-1
Socio-demographic Index	3	-1
Healthcare access and quality index	2	-1

PCOS

Covariate Name	Level	Direction
Education, years per capita	3	-1
LDI per capita	3	-1
percentage births in over 35s	2	1
Skilled birth attendance proportion	2	-1
Smoking prevalence	1	0
TFR	2	1
health system access capped	2	-1
Socio-demographic Index	3	-1
Healthcare access and quality index	2	-1

Endometriosis

Covariate Name	Level	Direction
Education, years per capita	3	-1
LDI per capita	3	-1
percentage births in over 35s	2	1
Skilled birth attendance proportion	2	-1
Smoking prevalence	1	0
TFR	2	1
health system access capped	2	-1
Socio-demographic Index	3	-1
Healthcare access and quality index	2	-1

Genital Prolapse

Covariate Name	Level	Direction
Education, years per capita	3	-1
LDI per capita	3	-1
Percentage births in over 35s	2	1
Skilled birth attendance proportion	2	-1
Smoking prevalence	1	0
TFR	2	1
health system access capped	2	-1
Socio-demographic Index	3	-1
Healthcare access and quality index	2	-1

Other gynecological disorders

Covariate Name	Level	Direction
Education, years per capita	3	-1
LDI per capita	3	-1
Percentage births in over 35s	2	1
Skilled birth attendance proportion	2	-1
Smoking prevalence	1	0
TFR	2	1
health system access capped	2	-1
Socio-demographic Index	3	-1
Healthcare access and quality index	2	-1

ICD10 and ICD9 codes used for gynecological disorders

Model	ICD10 code	ICD9 code
Uterine fibroids	D25-D26.9, D28.2	218-219.9, 236.0
Endometriosis	N80-N80.9	617-617.9
Genital prolapse	N81-N81.9	618-618.9
Polycystic ovarian syndrome	E28.2	256.4
Other gynaecological disorders	B37.3-B37.49, N61 - N64.9, N72, N75 – N77.8, N83 – N86, N88 – N91.5, N94 – N95.9	112.1-112.2, 611-619, 620-629.81

Haemoglobinopathies and Haemolytic Anaemias

This write-up covers the following sub-causes: Sickle cell disorders, thalassaemias, glucose-6-phosphate dehydrogenase (G6PD) deficiency, and other haemoglobinopathies and haemolytic anaemias

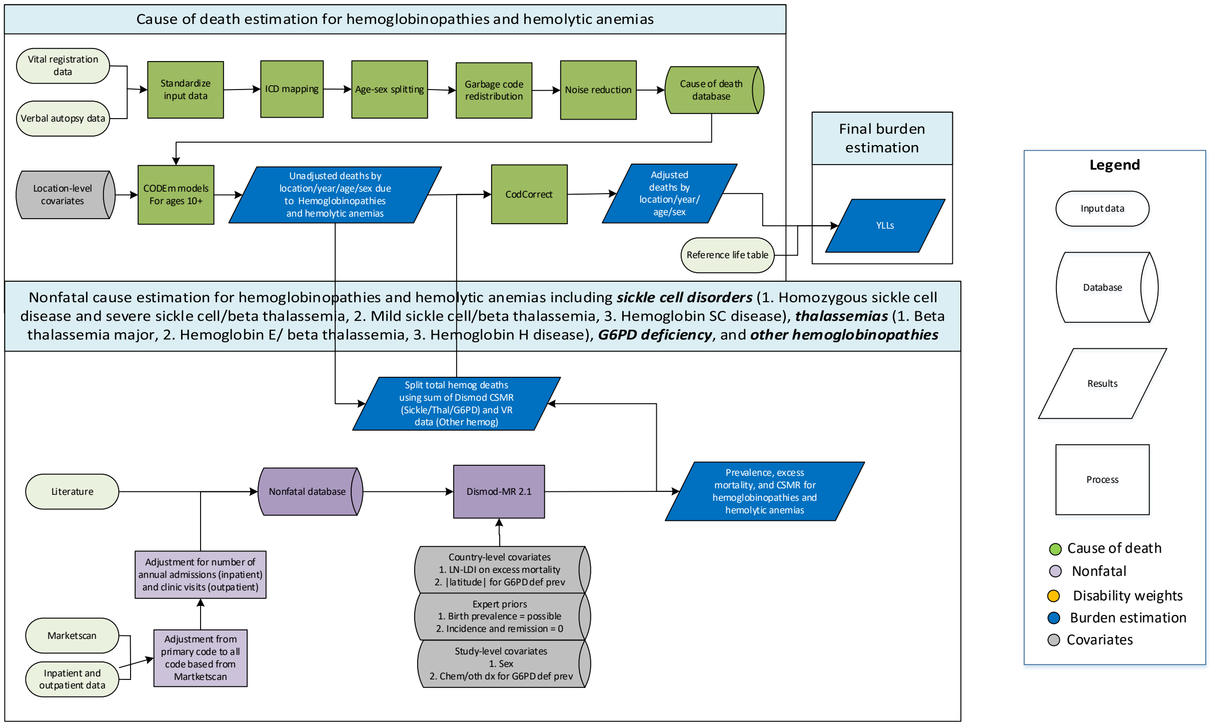

Input data

For GBD 2017, the overall CODEm model for haemoglobinopathies and haemolytic anemias was informed by centrally prepped data stored in the cause of death (COD) database. All data from all geographies were reviewed. Outliers were identified as those data where age patterns or temporal patterns were inconsistent with neighbouring age groups or locations or where sparse data were predicting implausible overall temporal or age patterns for a given location.

DisMod-MR 2.1 was used to estimate sickle cell disorders, thalassaemias, and G6PD deficiency age- and sex-specific prevalence and mortality for each location and year in the GBD. Three sources of data were used for DisMod-MR 2.1 models: literature, Marketscan data, and ICD-9 & ICD-10 hospital data. Each datum for sickle cell disease models was used for one of three mutually exclusive conditions: 1) homozygous sickle cell disease and severe sickle cell/beta thalassaemia, 2) mild sickle cell/beta thalassaemia, or 3) Hemoglobin SC disease. We similarly extracted data for thalassaemias using three mutually exclusive disease states: 1) beta thalassaemia major, 2) haemoglobin E/beta thalassaemia, and 3) haemoglobin H disease. G6PD deficiency was estimated as a single model. Cause-specific mortality rates for other haemoglobinopathies and haemolytic anaemias, lacking more specific data, was assumed to be geographically uniform, but did vary by age and sex; the levels and trends were informed by analysis of VR data from the COD database.

We added data from select geographies identified by GBD collaborators for GBD 2017. Our last comprehensive literature review was completed in GBD 2015, where we identified data on prevalence, excess mortality rate, or with-condition mortality rate. Age-specific survival probabilities from cohort studies were converted to corresponding with-condition mortality rates. G6PD deficiency is an X-linked recessive genetic disease, and genetic homozygosity served as the reference definition for our DisMod-MR 2.1 models in 2015. This was a change from GBD 2013, when we quantified G6PD deficiency on reagent tests as the reference category. Second, we extracted ICD-9-coded Marketscan data from the United States, correcting for multiple admissions, primary versus non-primary coding, and outpatient versus inpatient visits as determined from patient linkage analysis. Third, we used ICD-9 and ICD-10 inpatient and outpatient hospital data from all those locations where it was available, applying correction factors from analysis of claims data. These included correction factors for multiple admissions, age- and sex-specific ratio of prevalence that would be derived from only using the primary discharge ICD code versus that derived from using any of the discharge diagnosis codes, also accounting for differences in geography-specific overall hospitalisation rate when calculating the ratio. We applied this as a correction factor for those sources where only a single ICD code is given for each discharge. Of note, there were no hospital data available for haemoglobin E/beta-thalassaemia, haemoglobin H disease, or G6PD deficiency. All prevalence data from Marketscan, hospital sources, and literature were uploaded to the non-fatal database.

Modelling strategy

We completed seven separate DisMod-MR 2.1 models, as listed above. Several used log-transformed lag-distributed income as a country-level covariate on excess mortality, which had the effect of predicting higher excess mortality in those locations with lower national income. The only study covariate used for most models was for sex. Genetic G6PD deficiency is far more common in males, but for all others the male to female ratio is nearly equivalent. Our G6PD deficiency model included additional study covariates to crosswalk from non-genetic diagnostic tests (eg, chemical reagent testing) back to the reference definition. Incidence and remission were both set to be zero.

We completed data-rich (DR) and global CODEm models for males and females separately. The sum of CSMR from all seven DisMod-MR 2.1 models was used as a predictive covariate for CODEm model development. CODEm results were then split between sickle cell disorders, thalassaemias, G6PD deficiency, and other haemoglobinopathies and haemolytic anaemias using summed and scaled CSMR outputs from the same models. Other haemoglobinopathies and haemolytic anaemias did not have a separate DisMod-MR 2.1 model, but was instead informed by location-specific VR data.

The primary limitation of our estimation is data availability. We elected a hybrid approach of CODEm and DisMod-MR 2.1 to improve the quality of estimates in data-poor locations, but in most of these locations data are still relatively sparse for non-fatal models, which leads to relatively large uncertainty. Further adding to the uncertainty is the fact that haemoglobinopathies dramatically increase the risk of mortality due to infectious agents such as malaria, lower respiratory infections, and diarrhoea, as well as increasing the risk of maternal mortality. In locations with poor diagnostic capabilities and high infectious burden, it is thus very plausible that mortality due to haemoglobinopathies may be even higher. Secondly, our specification of seven distinct entities for DisMod-MR 2.1 models does not align perfectly with the cause categories in the central COD prep, which limits the extent to which CSMR data from the COD database can inform non-fatal models. We will continue to work to expand our dataset and consolidate the GBD analysis of haemoglobinopathies going forward.

Covariates in parent CODEm model

Covariate	Transformation	Level*	Direction*
Education	None	3	-1
LDI	Log	3	-1
Haemoglobinopathies (sum of prevalence * excess-mortality from all DisMod models)	None	1	1
Sickle Cell & Thal (sum of prevalence * excess-mortality from all DisMod models – excluding G6PD deficiency)	None	1	1
Latitude under 15 (proportion)	None	3	1
Latitude 15 to 30 (proportion)	None	3	1
Latitude 30 to 45 (proportion)	None	3	0
Latitude over 45 (proportion)	None	3	-1
Socio-demographic Index	None	3	-1
Healthcare access and quality index	None	2	-1
Health System Access, Capped	None	2	-1
Malaria Lysenko PFPR 1 (Holoendemic)	None	3	1

*Level refers to the likelihood of a relationship between the covariate and mortality (1 = more likely; 3 = less likely); direction refers to the direction of the relationship (1 = positive correlation; -1 = negative correlation).

Endocrine, Metabolic, Blood, and Immune Disorders

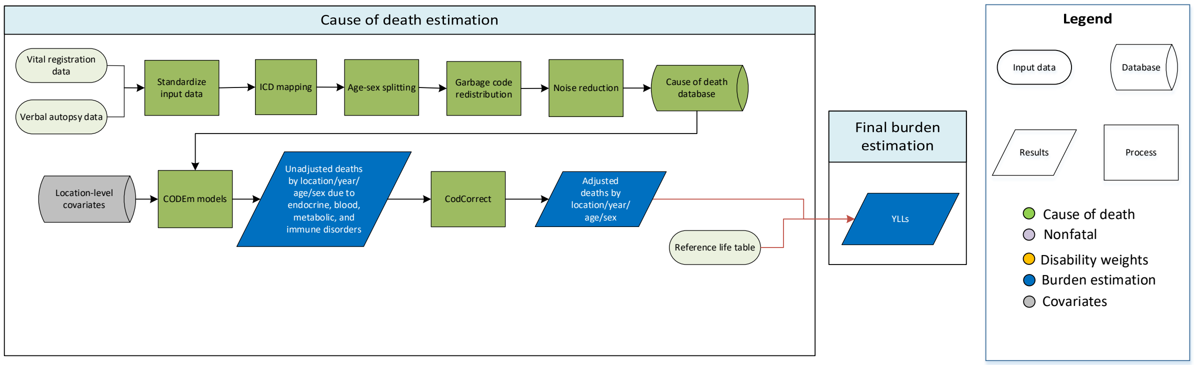

Input data

Vital registration and verbal autopsy data were used to model mortality due to endocrine, metabolic, blood, and immune disorders. Data were standardised and mapped according to the GBD causes of death ICD mapping method. These data were then age-sex split, and appropriate redistribution of garbage code data was performed. After applying noise reduction, these data were uploaded to the COD database. Outliers were identified by systematic examination of data points for all location-years. Data points that violated well-established age or time trends or that resulted in extremely high or low cause fractions were determined to be outliers.

Modelling strategy

The estimation strategy used for fatal endocrine, blood, metabolic, and immune disorders is largely similar to methods used in GBD 2016. A standard CODEm model with location-level covariates was used to model deaths due to endocrine, blood, metabolic, and immune disorders. Iterations of models were assessed at the location/year/age group/sex level to determine whether data points merited exclusion via outliering. Unadjusted death estimates were adjusted using CoDCorrect to produce final estimates of YLLs. The covariates used are displayed below.

Level	Covariate	Direction
1	Mean BMI	+
2	Animal fat (kcal per capita)	+
	Alcohol (litres per capita)	+
	Total calories (kcal per capita)	+
	Mean cholesterol	+
	Healthcare access and quality index	–
3	Socio-demographic Index	0
	Log LDI ($I per capita)	–
	Education (years per capita)	–

Sudden Infant Death Syndrome (SIDS)

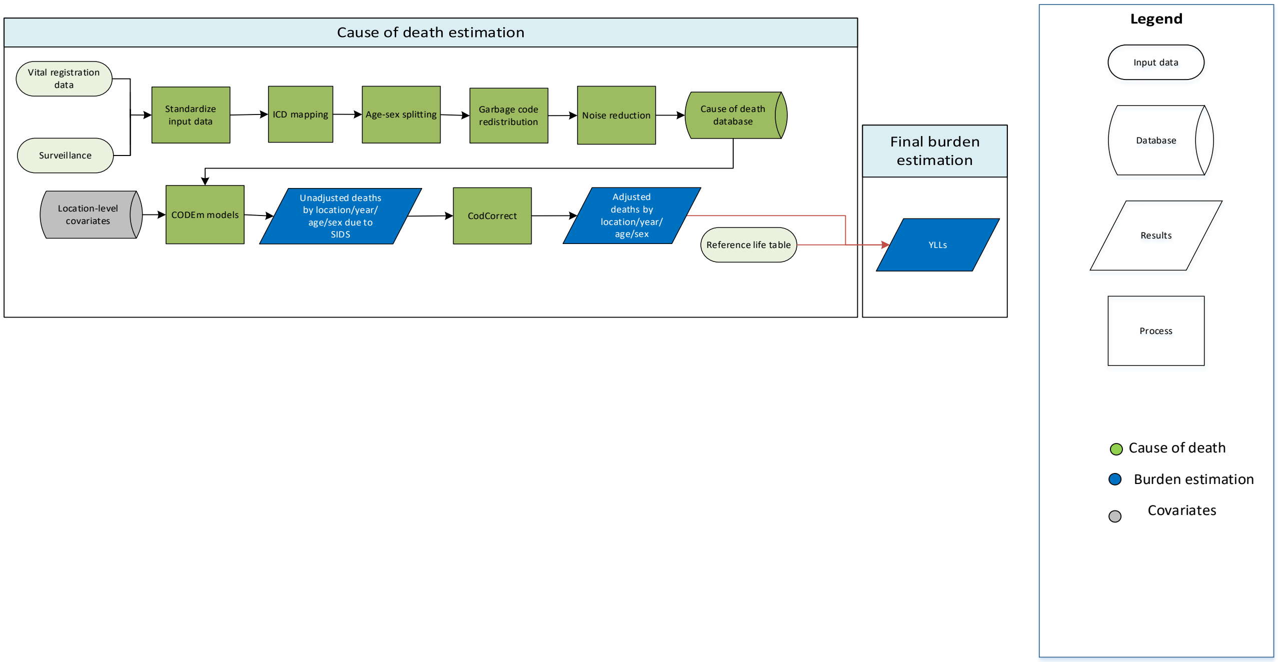

Input data

Vital registration data were used to estimate deaths due to sudden infant death syndrome (SIDS). Data points were selected as outliers if they met the following criteria: (1) implausibly high values relative to country time trends or global or regional patterns, based on the assumption that there are not "outbreaks" of SIDS, or (2) substantially conflicting with established age or temporal patterns. In addition, for GBD 2017, all deaths assigned to SIDS outside of 4- and 5-star countries were reassigned to neonatal disorders. SIDS can only be ascertained as a cause of death by autopsy, which is unlikely to have been used outside of 4- and 5-star countries.

Modelling strategy

The standard CODEm modelling approach was applied to estimate deaths due to SIDS. We ran CODEm models for ages 7–27 days and 28–364 days because we believe that deaths assigned to SIDS in other age groups are mis-assigned and these are therefore treated as garbage codes. Surveillance data and verbal autopsy data were not used as inputs to this model because these sources do not use data collection methods that can accurately diagnose deaths due to SIDS.

Notable differences between the GBD 2013 and GBD 2015 strategy included updates across the board to smoking-related covariates, total fertility rate, and Socio-demographic Index covariates. The addition of American Samoa to the Oceania region was also of note, as well as the shift to including more ICD detail codes in the input data for some countries that previously reported only aggregated codes. There were no significant changes in strategy from GBD 2015 to GBD 2017.

Covariates are shown in the following table.

Level	Covariate	Direction
1	Tobacco (cigarettes per capita)	+
	In-facility delivery (proportion)	-
2	Underweight (proportion <2SD weight for age, <5 years)	+
	Skilled birth attendance (proportion)	-
3	LDI (I$ per capita)	0
	Education (years per capita)	-
	Total fertility rate	+
	Socio-demographic Index	0

Injuries

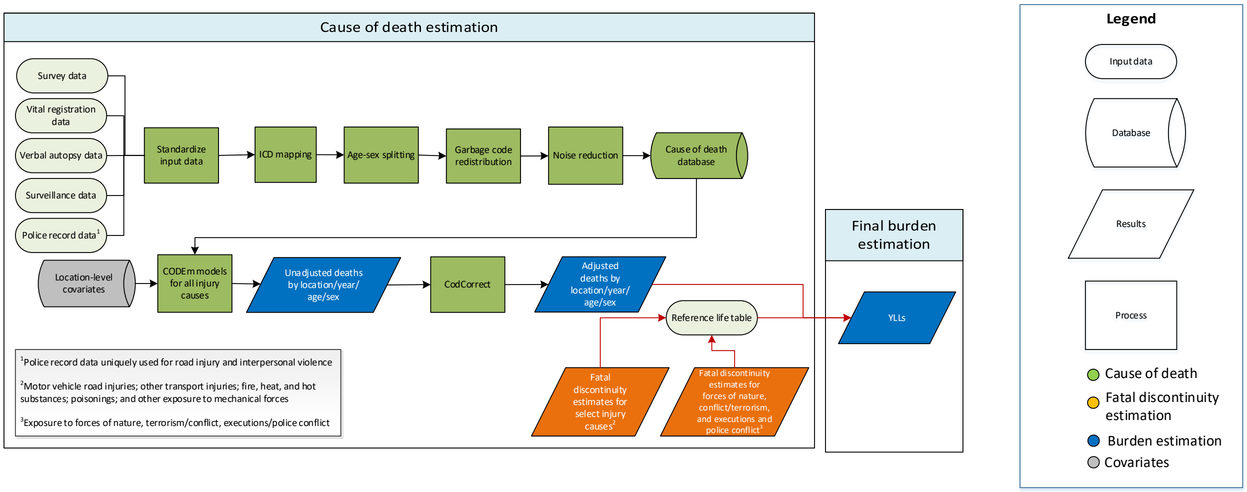

Input data

In GBD 2017, we estimated injury mortality from vital registration, verbal autopsy, mortality surveillance, censuses, surveys, and police record data. Police and crime reports were data sources uniquely used for the estimation of deaths from road traffic injury and interpersonal violence. The police data were collected from published studies, national agencies, and institutional surveys such as the United Nations Crime Trends Survey and the WHO Global Status Report on Road Safety Survey. For countries with vital registration data we did not use police records, except if the recorded number of road injury and interpersonal violence deaths from police records exceeded that in the vital registration.

Infrequently, data points were marked as outliers. Outlier criteria excluded data points that (1) were implausibly high or low relative to global or regional patterns, (2) substantially conflicted with established age or temporal patterns, or (3) significantly conflicted with other data sources conducted from the same locations or locations with similar characteristics (ie, Socio-demographic Index).

Modelling strategy

Overview

In GBD 2017, the standard CODEm modelling approach was applied to estimate deaths due to all causes of injury, excluding “Exposure to forces of nature,” and “Conflict and terrorism”. These causes were modelled solely outside of the CODEm process as fatal discontinuities estimation; this process is detailed further in the section on fatal discontinuities estimation in the appendix.

Fatal discontinuity was estimated for ten injury causes also modelled in CODEm. These causes included “Road injuries”, “Motor vehicle road injuries”, “Other transport injuries”, “Fire, heat, and hot substances”, “Poisonings”, “Environmental exposure to heat and cold “, “Other unintentional injuries”, “Interpersonal violence”, “Other exposure to mechanical forces”, and “Executions and police conflict”. Final fatal discontinuity estimations for these causes were merged with CODEm results post-CoDCorrect to produce final cause of death results.

Refer to the table at the end of this section for a complete list of the cause-of-injury categories, modelling strategies, and covariate changes from GBD 2016.

GBD injury codes and categories

The International Classification of Diseases (ICD) was used to classify injuries. In GBD, injury incidence and death are defined as ICD-9 codes E000-E999 and ICD-10 chapters V to Y. There is one exception: deaths and cases of alcohol poisoning and drug overdoses are classified under drug and alcohol use disorders. In GBD 2017, injury causes were organized into 28 mutually exclusive and collectively exhaustive external cause-of-injury categories. For GBD 2017, "Poisoning" was differentiated into "Poisoning by carbon monoxide," and "Poisoning by other", and "Unintentional suffocation" was removed as its ICD codes were added to the "Pulmonary aspiration and foreign body in airway" cause.

Preparation of data

The preparation of cause of death data includes age splitting, age-sex splitting, smoothing, and outlier detection. These steps are described in detail by Naghavi and colleagues and Lozano and colleagues.[1,2,3] The concept of "garbage codes" and redistribution of these codes was proposed in GBD 1990.[4] Garbage codes are causes of death that should not be identified as specific underlying causes of death but have been entered as the underlying cause of death on death certificates. A classic example of these types of codes in injuries chapters are "Exposure to unspecified factor" (X59 in ICD-10 and E887 in ICD-9) and all undetermined intent codes (Y10-Y34 in ICD-10 and E980-E988 in ICD-9). Other examples of garbage codes in injuries are the coding of an injury death to intermediate codes like septicaemia or peritonitis or as an ill-defined and unknown cause of mortality (R99). Approximately 2% of total deaths in countries with vital registration data are assigned to these three injury garbage code categories.

Splitting into sublevel causes

In countries with non-detail ICD code data, cause-of-injury categories were proportionally split into sublevel cause-of-injury categories. The sublevel cause-of-injury causes were created in the CoDCorrect process. One of the countries with non-detail ICD code data is South Africa, and in GBD 2013 the proportions of sublevel cause-of-injury were based on vital registration data. For GBD iterations of 2015, 2016, and 2017, the proportions were based on post-mortem investigation of injury deaths as described in the paper by Matzopoulos and colleagues 2015.[5]

Limitations and model assumptions

We added police data for road injuries and interpersonal violence to help predict level and age patterns in countries with sparse or absent cause of death data even though we know from countries with near-complete vital registration data that police records tend to underestimate the true level of deaths. However, we applied police data estimates in instances where reported deaths were higher than vital registration numbers.

Covariates

The following covariates were included.

**Transport injuries**		
Level	Covariate	Direction
1	Alcohol (litres per capita)	+
1	Vehicles – 2 wheels fraction (proportion)	+

1	Vehicles – 2+4 wheels (per capita)	+
2	Healthcare access and quality index	-
2	LDI (I$ per capita)	0
2	Population density (300-500 ppl/sqkm, proportion)	0
2	Population density (500-1000 ppl/sqkm, proportion)	0
3	Education (years per capita)	-
3	Rainfall quintile 5 (proportion)	+
3	Socio-demographic Index	-
**Road injuries**		
Level	Covariate	Direction
1	Alcohol (litres per capita)	+
1	Log-transformed SEV scalar: Road Inj	+
1	Vehicles – 2 wheels (per capita)	+
1	Vehicles – 2 wheels fraction (proportion)	+
1	Vehicles – 2+4 wheels (per capita)	+
1	Vehicles – 4 wheels (per capita)	+
2	Healthcare access and quality index	-
2	Population 15 to 30 (proportion)	+
2	Population density (300-500 ppl/sqkm, proportion)	0
2	Population density (500-1000 ppl/sqkm, proportion)	0
3	Education (years per capita)	-
3	LDI (I$ per capita)	0
3	Socio-demographic Index	-
**Pedestrian road injuries**		
Level	Covariate	Direction
1	Alcohol (litres per capita)	+
1	Log-transformed SEV scalar: Pedest	+
1	Vehicles – 2 wheels fraction (proportion)	+
1	Vehicles – 2+4 wheels (per capita)	+
2	Healthcare access and quality index	-
2	LDI (I$ per capita)	0
2	Population density (300-500 ppl/sqkm, proportion)	0
2	Population density (500-1000 ppl/sqkm, proportion)	0
3	Education (years per capita)	-
3	Rainfall quintile 5 (proportion)	+
3	Socio-demographic Index	-

**Cyclist road injuries**		
Level	Covariate	Direction
1	Alcohol (litres per capita)	+
1	Log-transformed SEV scalar: Cyclist	+
1	Vehicles – 2 wheels fraction (proportion)	+
1	Vehicles – 2+4 wheels (per capita)	+
2	Healthcare access and quality index	-
2	LDI (I$ per capita)	0
2	Population density (300-500 ppl/sqkm, proportion)	0
2	Population density (500-1000 ppl/sqkm, proportion)	0
3	Education (years per capita)	-
3	Socio-demographic Index	0
**Motorcyclist road injuries**		
Level	Covariate	Direction
1	Alcohol (litres per capita)	+
1	Log-transformed SEV scalar: Mot Cyc	+
1	Vehicles – 2 wheels (per capita)	+
2	Healthcare access and quality index	-
2	LDI (I$ per capita)	0
2	Population density (300-500 ppl/sqkm, proportion)	0
2	Population density (500-1000 ppl/sqkm, proportion)	0
3	Education (years per capita)	-
3	Rainfall quintile 5 (proportion)	+
3	Socio-demographic Index	0
**Motor vehicle road injuries**		
Level	Covariate	Direction
1	Alcohol (litres per capita)	+
1	Log-transformed SEV scalar: Mot Veh	+
1	Vehicles – 4 wheels (per capita)	+
2	Healthcare access and quality index	-
2	Population density (300-500 ppl/sqkm, proportion)	0
2	Population density (500-1000 ppl/sqkm, proportion)	0
3	Education (years per capita)	0
3	LDI (I$ per capita)	0
3	Rainfall quintile 5 (proportion)	+
3	Socio-demographic Index	0

**Other road injuries**		
Level	Covariate	Direction
1	Alcohol (litres per capita)	+
1	Log-transformed SEV scalar: Oth Road	+
1	Vehicles – 2 wheels fraction (proportion)	+
1	Vehicles – 2+4 wheels (per capita)	+
2	Healthcare access and quality index	-
2	LDI (I$ per capita)	0
3	Rainfall quintile 5 (proportion)	+
3	Socio-demographic Index	-
**Other transport injuries**		
Level	Covariate	Direction
1	Alcohol (litres per capita)	+
1	Log-transformed SEV scalar: Oth Trans	+
1	Vehicles – 2 wheels fraction (proportion)	+
1	Vehicles – 2+4 wheels (per capita)	+
2	Healthcare access and quality index	-
2	Population density (300-500 ppl/sqkm, proportion)	0
2	Population density (500-1000 ppl/sqkm, proportion)	0
3	Education (years per capita)	0
3	LDI (I$ per capita)	0
3	LDI (I$ per capita)	+
3	Rainfall quintile 5 (proportion)	+
3	Socio-demographic Index	0
**Unintentional injuries**		
Level	Covariate	Direction
1	Cumulative cigarettes (5 Years)	+
1	Diabetes fasting plasma glucose (mmol/L)	+
1	Health system access 2 (unitless)	-
1	Indoor air pollution (all cooking fuels)	+
1	Smoking prevalence	+
1	Underweight (proportion <2SD weight for age, <5 years)	+
2	Alcohol (litres per capita)	+
2	Population density (500-1000 ppl/sqkm, proportion)	+
2	Population density (over 1000 ppl/sqkm, proportion)	+
3	Education (years per capita)	-
3	LDI (I$ per capita)	-

**Falls**		
Level	Covariate	Direction
1	Alcohol (litres per capita)	+
1	Log-transformed SEV scalar: Falls	+
2	Healthcare access and quality index	-
2	Milk adjusted (g)	-
3	Elevation over 1500m (proportion)	+
3	LDI (I$ per capita)	0
3	Socio-demographic Index	0
**Drowning**		
Level	Covariate	Direction
1	Alcohol (litres per capita)	+
1	Coastal population within 10km (proportion)	+
1	Landlocked nation (binary)	-
1	Log-transformed SEV scalar: Drown	+
1	Rainfall quintile 1 (proportion)	-
1	Rainfall quintile 5 (proportion)	+
2	Elevation under 100m (proportion)	+
3	Education (years per capita)	-
3	LDI (I$ per capita)	0
3	Socio-demographic Index	-
**Fire, heat, and hot substances**		
Level	Covariate	Direction
1	Log-transformed SEV scalar: Fire	+
2	Alcohol (litres per capita)	+
2	Healthcare access and quality index	-
2	Indoor air pollution (all cooking fuels)	+
2	Population density (over 1000 ppl/sqkm, proportion)	0
2	Tobacco (cigarettes per capita)	+
3	Education (years per capita)	-
3	LDI (I$ per capita)	0
3	Socio-demographic Index	-
**Poisonings**		
Level	Covariate	Direction
1	Log-transformed SEV scalar: Poison	+

1	Opium cultivation (binary)	+
2	Healthcare access and quality index	-
2	Population density (over 1000 ppl/sqkm, proportion)	0
2	Population density (under 150 ppl/sqkm, proportion)	0
3	Education (years per capita)	-
3	LDI (I$ per capita)	0
3	Socio-demographic Index	-
**Poisoning by carbon monoxide**		
Level	Covariate	Direction
3	Education (years per capita)	-
3	Healthcare access and quality index	-
3	LDI (I$ per capita)	0
3	Socio-demographic Index	0
**Poisoning by other means**		
Level	Covariate	Direction
3	Education (years per capita)	-
3	Healthcare access and quality index	-
3	LDI (I$ per capita)	0
3	Socio-demographic Index	0
**Exposure to mechanical forces**		
Level	Covariate	Direction
2	Alcohol (litres per capita)	+
2	Healthcare access and quality index	-
2	Population density (over 1000 ppl/sqkm, proportion)	0
2	Population density (under 150 ppl/sqkm, proportion)	0
3	Education (years per capita)	-
3	LDI (I$ per capita)	0
3	Socio-demographic Index	-
**Unintentional firearm injuries**		
Level	Covariate	Direction
1	Log-transformed SEV scalar: Mech Gun	+
2	Alcohol (litres per capita)	+
2	Health system access (unitless)	-
2	Healthcare access and quality index	-
3	Education (years per capita)	-

3	LDI (I$ per capita)	0
3	Population density (over 1000 ppl/sqkm, proportion)	0
3	Population density (under 150 ppl/sqkm, proportion)	0
3	Socio-demographic Index	-
**Other exposure to mechanical forces**		
Level	Covariate	Direction
1	Log-transformed SEV scalar: Oth Mech	+
2	Alcohol (litres per capita)	+
2	Health system access (unitless)	-
2	Healthcare access and quality index	-
2	Population density (over 1000 ppl/sqkm, proportion)	0
2	Population density (under 150 ppl/sqkm, proportion)	0
3	Education (years per capita)	-
3	LDI (I$ per capita)	0
3	Socio-demographic Index	-
**Adverse effects of medical treatment**		
Level	Covariate	Direction
2	Healthcare access and quality index	0
3	LDI (I$ per capita)	0
3	Socio-demographic Index	0
**Animal contact**		
Level	Covariate	Direction
1	Alcohol (litres per capita)	+
1	Log-transformed SEV scalar: Animal	+
2	Healthcare access and quality index	-
2	Population 15 to 30 (proportion)	+
3	Education (years per capita)	-
3	Elevation over 1500m (proportion)	0
3	Elevation under 100m (proportion)	0
3	LDI (I$ per capita)	0
3	Population density (over 1000 ppl/sqkm, proportion)	0
3	Population density (under 150 ppl/sqkm, proportion)	0
3	Socio-demographic Index	-
**Venomous animal contact**		
Level	Covariate	Direction

1	Alcohol (litres per capita)	+
1	Log-transformed SEV scalar: Venom	+
2	Healthcare access and quality index	-
3	Education (years per capita)	-
3	Elevation over 1500m (proportion)	0
3	Elevation under 100m (proportion)	0
3	LDI (I$ per capita)	0
3	Population density (over 1000 ppl/sqkm, proportion)	0
3	Population density (under 150 ppl/sqkm, proportion)	0
3	Socio-demographic Index	-
**Non-venomous animal contact**		
Level	Covariate	Direction
1	Alcohol (litres per capita)	+
1	Log-transformed SEV scalar: Non Ven	+
2	Healthcare access and quality index	-
3	Alcohol (litres per capita)	+
3	Education (years per capita)	-
3	Elevation over 1500m (proportion)	0
3	Elevation under 100m (proportion)	0
3	Healthcare access and quality index	-
3	LDI (I$ per capita)	0
3	Population density (over 1000 ppl/sqkm, proportion)	0
3	Population density (under 150 ppl/sqkm, proportion)	0
3	Socio-demographic Index	-
**Foreign body**		
Level	Covariate	Direction
1	Education (years per capita)	+
1	Indoor air pollution (all cooking fuels)	+
1	LDI (I$ per capita)	+
1	Population density (over 1000 ppl/sqkm, proportion)	+
1	Population over 65 (proportion)	+
2	Healthcare access and quality index	-
3	Socio-demographic Index	0
**Pulmonary aspiration and foreign body in airway**		
Level	Covariate	Direction
1	Log-transformed SEV scalar: F Body Asp	+

2	Alcohol (litres per capita)	+
2	Alcohol binge drinker proportion, age-standardised	+
2	Healthcare access and quality index	-
2	Mean BMI	+
3	Education (years per capita)	-
3	LDI (I$ per capita)	0
3	Socio-demographic Index	0
**Foreign body in other body part**		
Level	Covariate	Direction
1	Alcohol (litres per capita)	+
1	Log-transformed SEV scalar: Oth F Body	+
2	Healthcare access and quality index	-
3	Education (years per capita)	-
3	Elevation Over 1500m (proportion)	0
3	Elevation Under 100m (proportion)	0
3	LDI (I$ per capita)	0
3	Population density (over 1000 ppl/sqkm, proportion)	0
3	Population density (under 150 ppl/sqkm, proportion)	0
3	Socio-demographic Index	-
**Other unintentional injuries**		
Level	Covariate	Direction
1	Alcohol (liters per capita)	+
1	Log-transformed SEV scalar: Oth Unint	+
1	Vehicles – 2 wheels (per capita)	+
1	Vehicles – 4 wheels (per capita)	0
2	Healthcare access and quality index	-
3	Education (years per capita)	-
3	Elevation over 1500m (proportion)	0
3	Elevation under 100m (proportion)	0
3	LDI (I$ per capita)	0
3	Population density (over 1000 ppl/sqkm, proportion)	0
3	Population density (under 150 ppl/sqkm, proportion)	0
3	Socio-demographic Index	0
**Self-harm and interpersonal violence**		
Level	Covariate	Direction
1	Alcohol (litres per capita)	+

1	Healthcare access and quality index	+
1	Log-transformed SEV scalar: Oth Unint	+
3	Education (years per capita)	-
3	Elevation over 1500m (proportion)	0
3	Elevation under 100m (proportion)	0
3	LDI (I$ per capita)	0
3	Population density (over 1000 ppl/sqkm, proportion)	0
3	Population density (under 150 ppl/sqkm, proportion)	0
**Self-harm**		
Level	Covariate	Direction
1	Alcohol (litres per capita)	+
1	Log-transformed SEV scalar: Self Harm	+
1	Major depressive disorder	+
1	Non-partner lifetime prevalence of sexual violence (female-only)	+
1	Risk of self-harm due to major depressive disorder	+
2	Healthcare access and quality index	-
2	Muslim religion (proportion of population)	-
2	Population density (150-300 ppl/sqkm, proportion)	0
2	Population density (300-500 ppl/sqkm, proportion)	0
2	Population density (500-1000 ppl/sqkm, proportion)	0
2	Population density (over 1000 ppl/sqkm, proportion)	0
2	Population density (under 150 ppl/sqkm, proportion)	0
2	Religion (binary, >50% Muslim)	-
3	Education (years per capita)	0
3	LDI (I$ per capita)	0
3	Socio-demographic Index	0
**Self-harm by firearm**		
Level	Covariate	Direction
1	Alcohol (litres per capita)	+
1	Log-transformed SEV scalar: Self Harm	+
1	Major depressive disorder	+
2	Healthcare access and quality index	-
2	Population density (150-300 ppl/sqkm, proportion)	0
2	Population density (300-500 ppl/sqkm, proportion)	0
2	Population density (500-1000 ppl/sqkm, proportion)	0
2	Population density (over 1000 ppl/sqkm, proportion)	0
2	Population density (under 150 ppl/sqkm, proportion)	0

2	Religion (binary, >50% Muslim)	-
3	Education (years per capita)	0
3	LDI (I$ per capita)	0
3	Socio-demographic Index	0
**Self-harm by other specified means**		
Level	Covariate	Direction
1	Alcohol (litres per capita)	+
1	Log-transformed SEV scalar: Self Harm	+
1	Major depressive disorder	+
2	Healthcare access and quality index	-
2	Population density (150-300 ppl/sqkm, proportion)	0
2	Population density (300-500 ppl/sqkm, proportion)	0
2	Population density (500-1000 ppl/sqkm, proportion)	0
2	Population density (over 1000 ppl/sqkm, proportion)	0
2	Population density (under 150 ppl/sqkm, proportion)	0
2	Religion (binary, >50% Muslim)	-
3	Education (years per capita)	0
3	LDI (I$ per capita)	0
3	Socio-demographic Index	0
**Interpersonal violence**		
Level	Covariate	Direction
1	Alcohol (liters per capita)	+
1	Log-transformed SEV scalar: Violence	+
2	Healthcare access and quality index	-
2	Opium cultivation (binary)	+
2	Population density (over 1000 ppl/sqkm, proportion)	+
3	Education (years per capita)	0
3	LDI (I$ per capita)	0
3	Socio-demographic Index	0
**Assault by firearm**		
Level	Covariate	Direction
1	Alcohol (liters per capita)	+
1	Log-transformed SEV scalar: Viol Gun	+
2	Healthcare access and quality index	-
2	Opium cultivation (binary)	+
2	Population density (over 1000 ppl/sqkm, proportion)	+

3	Education (years per capita)	0
3	LDI (I$ per capita)	0
3	Socio-demographic Index	0
**Assault by sharp object**		
Level	Covariate	Direction
1	Alcohol (litres per capita)	+
1	Log-transformed SEV scalar: Viol Knife	+
2	Healthcare access and quality index	-
2	Opium cultivation (binary)	+
2	Population density (over 1000 ppl/sqkm, proportion)	+
3	Education (years per capita)	0
3	LDI (I$ per capita)	0
3	Socio-demographic Index	0
**Assault by other means**		
Level	Covariate	Direction
1	Alcohol (litres per capita)	+
1	Log-transformed SEV scalar: Oth Viol	+
2	Healthcare access and quality index	-
2	Opium cultivation (binary)	+
2	Population density (over 1000 ppl/sqkm, proportion)	+
3	Education (years per capita)	0
3	LDI (I$ per capita)	0
3	Socio-demographic Index	0
**Environmental heat and cold exposure**		
Level	Covariate	Direction
2	Healthcare access and quality index	-
3	90th percentile climatic temperature in the given country-year	0
3	Education (years per capita)	-
3	Elevation 500 to 1500m (proportion)	0
3	Elevation over 1500m (proportion)	0
3	LDI (I$ per capita)	0
3	Population fensity (150-300 ppl/sqkm, proportion)	0
3	Population-weighted mean temperature	0
3	Rainfall (quintiles 4-5)	0
3	Sanitation (proportion with access)	0
3	Socio-demographic Index	-

Table – Injury cause list			
**ID**	**Cause**	**Modelling strategy**	**Covariate changes from GBD 2016**
1	Transport injuries	CODEm	
1.1	Road injuries	CODEm and fatal discontinuity estimation	
1.1.1	Pedestrian road injuries	CODEm	
1.1.2	Cyclist road injuries	CODEm	
1.1.3	Motorcyclist road injuries	CODEm	
1.1.4	Motor vehicle road injuries	CODEm and fatal discontinuity estimation	
1.1.5	Other road injuries	CODEm	
1.2	Other transport injuries	CODEm and fatal discontinuity estimation	
2	Unintentional injuries	CODEm	
2.1	Falls	CODEm	
2.2	Drowning	CODEm	
2.3	Fire, heat, and hot substances	CODEm and fatal discontinuity estimation	
2.4	Poisonings	CODEm and fatal discontinuity estimation	
2.4.1	Poisoning by carbon monoxide	CODEm	Same covariates used as Poisoning in GBD 2016
2.4.2	Poisoning by other means	CODEm	Same covariates used as Poisoning in GBD 2016
2.5	Exposure to mechanical forces	CODEm	
2.5.1	Unintentional firearm injuries	CODEm	
2.5.2	Other exposure to mechanical forces	CODEm and fatal discontinuity estimation	
2.6	Adverse effects of medical treatment	CODEm	
2.7	Animal contact	CODEm	
2.7.1	Venomous animal contact	CODEm	
2.7.2	Non-venomous animal contact	CODEm	
2.8	Foreign body	CODEm	
2.8.1	Pulmonary aspiration and foreign body in airway	CODEm	
2.8.2	Foreign body in other body part	CODEm	

Table – Injury cause list			
**ID**	**Cause**	**Modelling strategy**	**Covariate changes from GBD 2016**
2.9	Environmental exposure to heat and cold	CODEm and fatal discontinuity estimation	
2.10	Exposure to forces of nature	Fatal discontinuity estimation	
2.11	Other unintentional injuries	CODEm and fatal discontinuity estimation	
3	Self-harm and interpersonal violence	CODEm	
3.1	Self-harm	CODEm	
3.1.1	Self-harm by firearm	CODEm	
3.1.2	Self-harm by other specified means	CODEm	
3.2	Interpersonal violence	CODEm and fatal discontinuity estimation	
3.2.1	Physical violence by firearm	CODEm	
3.2.2	Physical violence by sharp object	CODEm	
3.2.3	Physical violence by other means	CODEm	
3.3	Conflict and terrorism	Fatal discontinuity estimation	
3.4	Executions and police conflict	CODEm and fatal discontinuity estimation	

References

1 Lozano R, Naghavi M, Foreman K, *et al.* Global and regional mortality from 235 causes of death for 20 age groups in 1990 and 2010: a systematic analysis for the Global Burden of Disease Study 2010. *The Lancet* 2012; **380**: 2095–128.

2 Global, regional, and national age–sex specific all-cause and cause-specific mortality for 240 causes of death, 1990–2013: a systematic analysis for the Global Burden of Disease Study 2013. *The Lancet* 2015; **385**: 117–71.

3 Global, regional, and national life expectancy, all-cause mortality, and cause-specific mortality for 249 causes of death, 1980-2015: a systematic analysis for the Global Burden of Disease Study 2015. *The Lancet* 2016; **388**: 1459-1544.

4 Murray CJL, Lopez AD, Harvard School of Public Health, World Health Organization, World Bank. The global burden of disease: a comprehensive assessment of mortality and disability from diseases, injuries, and risk factors in 1990 and projected to 2020. Cambridge, MA: Published by the Harvard School of Public Health on behalf of the World Health Organization and the World Bank : Distributed by Harvard University Press, 1996.

5 Matzopoulos R, Prinsloo M, Wyk VP, Gwebushe N, Mathews S, et al. Injury-related mortality in South Africa: a retrospective descriptive study of postmortem investigations. *Bull World Health Organ* 2015; **93**: 303–13.

Fatal Discontinuities

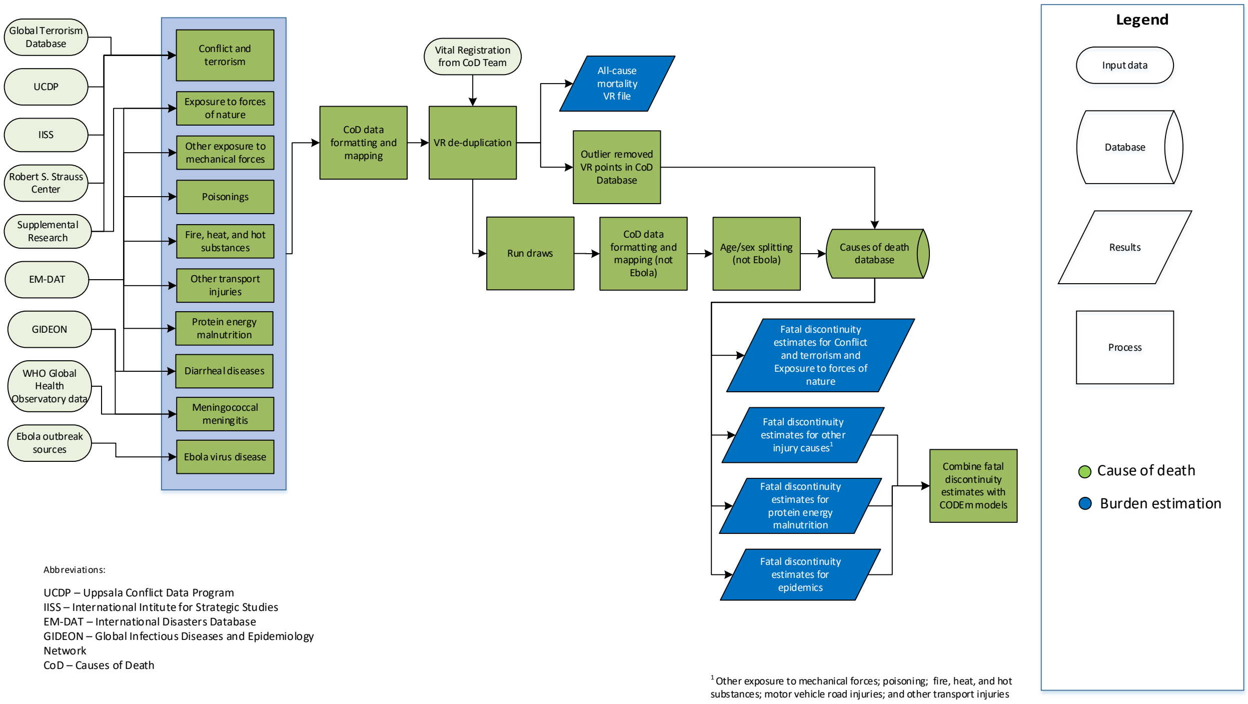

Input data

Overall

Input data for fatal discontinuities are compiled a range of sources, including country vital registration (VR) data; international databases that capture several cause-specific fatal discontinuities; and supplemental data in the presence of known issues with data quality or representativeness, or time lags in reporting. A systematic literature review was not used to identify input data for fatal discontinuities, though some literature sources were identified through online supplemental research. Below we provide more detail on the different input data sources by sub-causes of fatal discontinuities.

Subnational locations and population splitting

In locations where we produced estimates at the subnational level for GBD 2017, deaths due to all fatal discontinuity causes were assigned to the relevant subnational location(s) when that information could be obtained either through country data sources (eg, VR) or through additional online research. In the rare case that no subnational location could be found, the deaths were split proportionally by population across all subnational locations.

In locations that have experienced boundary changes or split from other locations that we currently estimate (eg, the former Yugoslavia, Czechoslovakia, the Soviet Union, Sudan and South Sudan), we split deaths due to events that occurred prior to boundary changes proportionally based on the populations residing within the boundaries of present-day locations unless we found documentation that clearly indicated whether the event and corresponding deaths occurred in one of the present-day GBD 2017 locations.

Choosing between multiple sources for same event

Where multiple sources reported shock deaths for the same location-year-cause, a cause-specific prioritisation scheme was followed that reflected the available detail in the cause-specific datasets. For example, the Generalized Event Dataset from UCDP was prioritised above all other non-VR sources because it included detail on how deaths were distributed between multiple actors and locations in each conflict event. In most cases, VR from 4- or 5-star locations was used where available. In some cases, VR from 4- or 5-star locations was not chosen if there were well-known data-quality issues or discrepancies in the cause of death data reporting related to a particular event (eg, supplemental death data for Louisiana was used for Hurricane Katrina because of established data reporting issues). The process for prioritidation among various sources for location-year fatal discontinuities is described more in the Modelling strategy below.

Major data sources other than country vital registration for each fatal discontinuity cause follow.

Conflict and terrorism. In GBD 2016, data for conflict and terrorism came from the Uppsala Conflict Data Program (UCDP), International Institute for Strategic Studies, and Robert S. Strauss Center for International Security and Law. For GBD 2017, data from the Global Terrorism Database (GTD), the University of Chicago Suicide Attack Database, and the RAND Database of Worldwide Terrorism Incidents were used in addition to those used in GBD 2016. The table below provides details about the various datasets we utilised from these sources, the dates they were last accessed, and the years for which we used the data provided. Where these data sources reported deaths due to gang violence, the cause was re-mapped to physical violence by other means. Where these data sources reported deaths due to legal intervention, the cause was re-mapped to executions and police conflict.

Data source name	Date accessed	Years of data downloaded	Type of data included
**Uppsala Conflict Data Program[1]**			
Georeferenced Event Dataset, Version 17.1	1/16/2018	1989-2015	UCDP battles, non-state, and one-sided conflict deaths with the most disaggregated location information available
PRIO Battles Deaths Dataset, Version 3.1	1/16/2018	1970-1988	Armed conflict (civil wars, etc.)
**International Institute for Strategic Studies**			
Armed Conflict Dataset	11/17/2016	1997-2016	Insurgency, Inter-state, Intra-state conflict deaths
**Robert S. Strauss Center For International Security And Law**			
Armed Conflict Location and Event Dataset (ACLED)	1/16/2018	1997-2016	Actions of opposition groups, governments, and militias in selected locations in Africa, Asia, and the Middle East specifying the exact location and date of battle events, transfers of military control, headquarter establishment, civilian violence, and rioting
Social Conflict Analysis Database (SCAD)	1/16/2018	1990-2016	Protests, riots, strikes, inter-communal conflict, government violence against civilians, and other forms of social conflict (covers Africa, Latin America, and Asia)
**University of Maryland, Global Terrorism Database**			
Global Terrorism Database (GTD)	1/16/2018	1970-2016	Attacks aimed at attaining political, economic, religious, or social goal, includes evidence of intention to coerce, action was outside precepts of International Humanitarian Law.
**University of Chicago, Chicago Project on Security and Threats**			
Suicide Attack Database (CPOST SAD)	8/5/2017	1974-2016	Attacks in which an attacker kills him/herself in a deliberate attempt to kill others, includes only attacks perpetrated by non-state actors
**RAND National Security Research Division**			
RAND Database of Worldwide Terrorism Incidents	9/8/2017	1968-2009	Terrorism, defined by the nature of the act, not by the identity of the perpetrators or the nature of the cause; including violence, calculated to create fear/alarm, intended to coerce certain actions, motive is political, group, or individual

Supplemental online research was conducted for recent conflicts where the databases above were not up to date. In addition, deaths due to conflict and terrorism in Iraq from 2003 to present were estimated using a combination of supplemental sources. The source found with the lowest number of deaths, Iraq Body Count[2], was used as the lower bound of the uncertainty interval from 2003 to 2016. Estimates from the Iraq Mortality Study by Hagopian et al[3] from 2003 to 2006, the deadliest years of the war, were used to scale deaths to generate the upper uncertainty interval limits using the following formula:

$$deaths_{GBD\ 2017,\ high} = deaths_{IBC} \cdot \overline{\left[\frac{deaths_{IMS}}{deaths_{IBC}}\right]}_{2003-2006}$$

We used the average ratio between IMS and IBC reported deaths between 2003 and 2006, multiplied by the number of deaths reported by the IBC. This high estimate was carried forward through 2017 under the assumption that the Iraq Body Count similarly undercounts the number of deaths due to the ongoing civil war in Iraq. The final, best estimate for conflict and terrorism deaths in Iraq from 2003 to 2016 is the midpoint of the high and low estimates given above.

We identified four major conflicts that were not represented in these databases: 1997 civil conflict in Albania[4]; 1971 genocide in Bangladesh[5]; 1972 genocide in Burundi[6]; and 1993 genocide in Burundi[6]. In these cases, we used literature sources in order to account for these fatal discontinuities.

For country-years where multiple sources provided estimates, we prioritised sources in the following order: (1) country VR data, if death estimates were highest of all sources; (2) UCDP; (3) IISS; (4) country VR if death estimates were not the highest of all sources; (5) Robert Strauss Center; (6) Global Terrorism DB; (7) CPOST Suicide Attack Database; (8) online supplemental research.

Exposure to forces of nature, other injury causes, and protein-energy malnutrition. The Centre for Research on the Epidemiology of Disasters' International Disaster Database (EM-DAT) served as the primary non-VR source of fatal discontinuities due to exposure to forces of nature (ie, natural disasters); other transport injuries (eg, plane, train, and boat accidents); poisonings; fire, heat, and hot substances; other exposure to mechanical forces (eg, building collapse); and protein-energy malnutrition (ie, famine or severe drought). Data from EM-DAT were last accessed February 14, 2018. Supplemental online research was conducted for events where EM-DAT was not up to date.

For country-years where multiple sources provided estimates, we prioritised sources in the following order: (1) country VR data, if data quality rating is 4 or 5 stars; (2) country VR data if data quality rating is less than 4 stars and death estimates were highest of all sources; (3) EM-DAT; (4) online supplemental research. Exceptions were made where it was clear that VR systems had been compromised by the event being measured.

Meningococcal meningitis and diarrhoeal diseases. For GBD 2017, we included fatal discontinuities due to a subset of infectious diseases: meningococcal meningitis (or meningococcal infection) and diarrhoeal disease caused by cholera. These two infectious diseases were first included on the fatal discontinuity cause list for GBD 2016 because (1) their current modelling strategies with the Cause of Death Ensemble model (CODEm) do not optimally capture the potentially highly variable – or epidemic – mortality levels and trends characteristic of these two causes; and (2) they can contribute to significant total fatalities in a given location-year. Other infectious diseases for which the latter is true – high death rates in the presence of an outbreak or epidemic – are currently modelled with alternative cause of death methods (eg, natural history models for measles and yellow fever), which allow for greater variation year-over-year if or when outbreaks occur. In future iterations of the GBD, we plan to revisit the inclusion criteria for infectious diseases as fatal discontinuities and develop more of an ensemble approach to modelling causes that can be both endemic (and thus result in more uniform levels and trends over time) and epidemic (and subsequently lead to rapid increases – and decreases – in deaths for a given location-year).

The Global Infectious Diseases and Epidemiology Network (GIDEON) served as the primary data source for collating cholera and meningococcal meningitis or meningococcal infection death reports.[7,8] For any year in which cholera or meningococcal meningitis deaths were recorded in a country or territory

covered by the GBD, we directly extracted reported deaths from 1970 to 2016. When there were reporting gaps in cholera or meningococcal meningitis deaths over this period of time and the World Health Organization (WHO) annual cholera or meningitis reports had death reports for those years, we used the WHO reports. The primary exception were two major cholera outbreaks in Bangladesh – 1982 to 1983 and 1991 – which were not captured by either GIDEON or WHO. As result, we used the EM-DAT records for the 1982–1983 outbreak and literature for the 1991 outbreak.[9] For the Yemen Cholera outbreak in 2016 and 2017, we used estimates from local collaborators in the absence of other data sources.

Ebola. Since GBD 2015, outbreaks due to Ebola virus disease have been estimated using the data and methods described in the Ebola write-up of this appendix and included in GBD death estimates in the same way as other fatal discontinuity causes.

Modelling strategy

All input data for fatal discontinuity causes were run through the causes of death data formatting and mapping process.

VR de-duplication

For injury causes that also have continuous background mortality and a CODEm model, a process was established to avoid duplication of fatal discontinuity deaths in the two models. First, location-years with fatal discontinuities data from non-VR sources were identified. If these location-cause-years also had VR death estimates that were greater than 40% higher than the immediately surrounding years and could be linked to a specific fatal discontinuity event, these years were marked as outliers in the VR data and the difference between the outlier year and the average of the surrounding years was included in the relevant cause in the fatal discontinuities database. The deaths from the identified events were subtracted from the all-cause VR estimates used in the all-cause mortality estimation process.

Uncertainty analysis for input and draw-level input to age-sex splitting

Uncertainty intervals for deaths due to conflict and terrorism were generated using UCDP high and low death estimates, except in the case of Iraq 2003–2016, as explained above. In cases where low and high estimates were not included in the available data, the regional average uncertainty interval was applied to the available death estimate across all fatal discontinuity causes.

We assumed a log-normal distribution using mean death rates and standard error based on high and low estimates. In the case that standard error was less than 10e-8, the draws were set equal to the mean rate. 1,000 draws were sampled from this log-normal distribution. These 1,000 draws were then converted back to count space and used for final calculations of means and uncertainty intervals.

Age-sex splitting

All compiled data were run through the causes of death age-sex splitting process, except for where we had strong supplemental information on the age distribution of specific, large events, such as United States mortality in the Vietnam War and Iranian mortality from the Iran-Iraq conflict in the early 1980s.

Changes from GBD 2016

GBD 2017 saw an effort to systematise the collection of up-to-date fatal discontinuity data through supplemental online research. New tools included use of Twitter to identify events not covered by other sources, most notably in identifying events that occurred recently (2016, 2017). This process resulted in a more comprehensive set of conflict and terrorism data for 2017, as well as large natural disasters not contained in EM-DAT or VR.

For GBD 2017, efforts were also made to improve location tagging in raw data to the GBD location hierarchy using several approaches. Identifying the correct GBD location for each event is difficult, as reports of fatal discontinuities come in many formats, often with limited metadata. The approaches used for improving the location tagging included a) utilising the collaborator network to more accurately tag events to subnational locations when information in the data was scarce, b) automated matching with GBD location names, c) overlaying a spatial file of the most-detailed GBD geographies, d) geo-coding using precise place names, and e) for events spanning multiple GBD locations, but without detail in the raw data, deaths were split using population.

We completed a detailed review of the fatal discontinuity cause mappings for conflict and terrorism, police conflict and executions, using the text descriptions of each event when provided in the data. This exercise resulted in updating the GBD cause assigned for a number of events present in the GBD 2016 analysis, which is one contributor in the differences seen in the GBD 2016 and GBD 2017 fatal discontinuity estimates.

References

1 UCDP/PRIO Armed Conflict Dataset Codebook. Uppsala Conflict Data Program (UCDP); Centre for the Study of Civil Wars, International Peace Research Institute, Oslo (PRIO), 2013.

2 Iraq Body Count. https://www.iraqbodycount.org/database/ (accessed May 8, 2017).

3 Hagopian A, Flaxman AD, Takaro TK, *et al.* Mortality in Iraq Associated with the 2003–2011 War and Occupation: Findings from a National Cluster Sample Survey by the University Collaborative Iraq Mortality Study. *PLOS Medicine* 2013; **10**: e1001533.

4 Jarvis C. The Rise and Fall of Albania's Pyramid Schemes. *F&D* 2000; **37**. http://www.imf.org/external/pubs/ft/fandd/2000/03/jarvis.htm.

5 Obermeyer Z, Murray CJL, Gakidou E. Fifty years of violent war deaths from Vietnam to Bosnia: analysis of data from the world health survey programme. *BMJ* 2008; **336**: 1482–6.

6 Milton L. Rwanda, 1994: International incompetence produces genocide. 1994 https://ezproxy.uwc.edu/login?url=http://search.proquest.com/docview/234405747?accountid=42411.

7 Inc GI, Berger DS. Cholera: Global Status: 2017 edition. GIDEON Informatics Inc, 2017.

8 Inc GI, Berger DS. Bacterial Meningitis: Global Status: 2017 edition. GIDEON Informatics Inc, 2017.

9 Siddique A, Zaman K, Baqui A, *et al.* Cholera Epidemics in Bangladesh: 1985-1991. *Journal of Diarrhoeal Diseases Research* 1992; **10**: 79–86.

Section 4: Central computation

Section 4.1: Imported cases

Imported cases are fatalities that occur in a geographic area where a particular CoD is known to be eradicated in a specific time period or where infection cannot occur. We apply space-time restrictions to these causes in the modeling strategy for that location and time period. However, in some rare cases, there are deaths from these causes outside of restricted locations and time periods. These deaths are referred to as Imported Cases.

Illustrating this concept, Chagas Disease is transmitted by insect vectors that only exist in the Americas. For this reason, Chagas Disease is restricted in the models for countries such as Russia. However, it is possible that someone traveling in Latin America could contract Chagas Disease and then die after returning home to Russia. Imported cases accounts for these kinds of deaths.

To calculate these Imported cases, we find all cases from the VRs of data-rich countries for any CoD that is otherwise geographically or temporally restricted. We then create a beta distribution from that data point, using the sample size of the VR for that data point, and upload these draws as a custom CoD model. This model is then used as an input to CoDCorrect.

Section 4.2: CodCorrect

Section 4.2.1: Objective of CodCorrect

As mentioned in the main text, the CoD models are cause-specific. As such, there is no guarantee that the sum of these models will equal the results of the all-cause mortality estimates or that model results of child causes add up to the parent model results. The CoDCorrect process is used to make the CoD and all-cause mortality estimates internally consistent using a very simple algorithm.

Section 4.2.2: Algorithm and levels

The core algorithm remains the same as it did in GBD 2013. The equation can be written as follows:

$$CD_{lyasjd} = D_{lyasjd}\left(\frac{PD_{lyasjd}}{\sum_{j=1}^{j=k} D_{lyasjd}}\right)$$

Where:

CD_{lyasjd} is the corrected number of deaths for a location l, year y, age a, sex s, cause j, and draw d

PD_{lyasjd} is the parent cause deaths for a location l, year y, age a, sex s, cause j, and draw d

D_{lyasjd} is the uncorrected number of deaths estimated from a cause-specific model for a l, year y, age a, sex s, cause j, and draw d

The CoDCorrect process starts by rescaling the Level 1 causes to match the all-cause mortality estimates (which is used for PD_{lyasjd} in the above equation). Level 2 causes are then rescaled to their corrected parent causes. This continues until all levels of the hierarchy have been rescaled. Causes and their levels within the CoDCorrect hierarchy can be found in Appendix Table 14.

Unlike in GBD 2013, HIV is not included in the CoDCorrect process for GBD 2017. To account for this change, Level 1 CoDCorrect causes are rescaled to HIV-deleted mortality estimates which are produced as part of the mortality and HIV estimation process. Results from the GBD version of Spectrum are added to the post-CoDCorrect death estimates, along with fatal discontinuities and imported cases, to generate the full set of death estimates.

Section 4.2.3: Diagnostic results of CodCorrect by cause and location

For more detail on diagnostic results of CodCorrect by cause see Appendix Table 15.

Section 4.3: Years of life lost (YLLs) calculation

Years of life lost due to premature mortality (YLLs) were computed for 918 locations and 37 years. First, we used the lowest observed age-specific mortality rates by location and sex across all estimation years from locations with total populations greater than 5 million in 2016 to establish a theoretical minimum risk reference life table. The values can be found in Appendix Table 16.

The YLL is a metric that is computed by multiplying the number of estimated deaths by the standard life expectancy at age of death. The metric therefore highlights premature deaths by applying a larger weight to deaths that occur at younger age groups. We propagated uncertainty from CoDCorrected deaths for all demographics. The core equation can be written as follows:

$$YLL = \sum_{c=1,a=0,s=1}^{\infty} d_{cas} e_a$$

Section 4.3.1: GBD world population age standard

Age-standardized populations in the GBD were calculated using the GBD world population age standard. For GBD 2013, GBD 2015 and GBD 2016, the age-specific proportional distributions of all national locations from the UNPOP World Population Prospects 2012 revision for all years from 2010 to 2035 were used to generate a standard population age structure using the non-weighted mean across all the aforementioned country-years. For GBD 2017 we have used the non-weighted mean of 2017 age-specific proportional distributions from the GBD 2017 population estimates for all national locations with a population greater than 5 million people in 2017 to generate an updated standard population age structure.[30] The values used for the age standard are found in Appendix Table 17.

Section 5: SDI Analysis

Section 5.1: SDI definition

The Socio-demographic Index (SDI) is a composite indicator of development status strongly correlated with health outcomes. In short, it is the geometric mean of 0 to 1 indices of total fertility rate under the age of 25 (TFU25), mean education for those aged 15 and older (EDU15+), and lag distributed income (LDI) per capita.

Section 5.2: Development of revised SDI indicator

SDI was originally constructed for GBD 2015 using the Human Development Index (HDI) methodology, wherein a 0 to 1 index value was determined for each of the original three covariate inputs (TFR in ages 15 to 49, EDU15+, and LDI per capita) using the observed minima and maxima over the estimation period to set the scales.[31]

In response to feedback from collaborators and the evolution of the GBD, we have refined the indicator with each GBD cycle. For GBD 2017, in conjunction with our expanded estimation of age-specific fertility, we replaced TFR with TFU25 as one of the three component indices. The TFU25 provides a better measure of women's status in society, as it focuses on ages where childbearing disrupts the pursuit of education and entrance into the workforce. In addition, we observe that in highly developed countries the TFU25 has tended to decline consistently over time, even amidst rebounds in TFR driven by increasing fertility in older ages. The concordance correlation coefficient between SDI using the GBD 2016 method and the updated method for GBD 2017 was 0.981.

During GBD 2016 we moved from using relative index scales to absolute scales to enhance the stability of SDI's interpretation over time, as we noticed that the measure was highly sensitive to the addition of subnational units that tended to stretch the empirical minima and maxima.[32] We selected the minima and maxima of the scales by examining the relationships each of the inputs had with life expectancy at birth and under-5 mortality and identifying points of limiting returns at both high and low values, if they occurred prior to theoretical limits (e.g., a TFU25 of 0).

Thus, an index score of 0 represents the minimum level of each covariate input past which selected health outcomes can get no worse, while an index score of 1 represents the maximum level of each covariate input past which selected health outcomes cease to improve. As a composite, a location with an SDI of 0 would have a theoretical minimum level of development relevant to these health outcomes, while a location with an SDI of 1 would have a theoretical maximum level of development relevant to these health outcomes.

We summarize the final scales for GBD 2017 in the table below.

Input	Lower Bound	Upper Bound
TFU25	0	3
LDI per capita	250 USD (5.52 log USD)[b]	60,000 USD (11.00 log USD)

EDU15+	0 years	17 years

[b] The minimum for the LDI scale was originally set at the theoretical limit of 0 USD, as we did not observe an asymptotic relationship between log(LDI) and E_0 or 5q0 at lower values of log(LDI). Empirically, however, we also did not observe an LDI below 350 USD (5.86 log USD) for the estimation period 1970-2016. In log-space, this meant that approximately half of our scale was not being utilized, compressing the observed variation in LDI and diminishing its meaningful contribution to SDI. Accordingly, we set the lower limit on LDI to 250 USD (5.52 log USD) to ensure we were fully utilizing the range of the scale to capture its variation across space and time, as is the case with the other two inputs.

Using scales described above, we computed the index scores underlying SDI as follows:

$$I_{Cly} = \frac{(C_{ly} - C_{low})}{(C_{high} - C_{low})}$$

Where I_{cly} – the index for covariate *C*, location *l*, and year *y* – is equal to the difference between the value of that covariate in that location-year and the lower bound of the covariate divided by the difference between the upper and lower bounds for that covariate. If the values of input covariates fell outside the upper or lower bounds (e.g. LDI per capita greater than 60,000 USD), they were mapped to the respective upper or lower bounds. We also note that the index value for TFU25 was computed as $1 - I_{TFU25ly}$, as lower TFU25s correspond to higher levels of development, and thus higher index scores. For GBD 2017 we expanded the computation of SDI to 890 national and subnational locations spanning the time period 1950-2017.

The composite Socio-demographic Index is the geometric mean of these three indices for a given location-year. The cutoff values used to determine quintiles for analysis were then computed using country-level estimates of SDI for the year 2017, excluding countries with populations less than 1 million. SDI groupings by geography are provided in Appendix Table 19, SDI values by location are provided in Appendix Table 20, and SDI R-squared values with lags up to 10 years are provided in Appendix Table 18.

Example Calculation

Below we present the calculation of SDI for a hypothetical country in the year 2010:

$$TFU25 = 1.09;\ Mean\ educ\ yrs\ pc = 8.23;\ lnLDI = 9.60$$

$$I_{TFU25} = 1 - \frac{1.09 - 0}{3 - 0} = .637$$

$$I_{Educ} = \frac{8.23 - 0}{17 - 0} = .484$$

$$I_{lnLDI} = \frac{9.60 - 5.52}{11.00 - 5.52} = .744$$

$$SDI = \sqrt[3]{I_{TFU25} * I_{Educ} * I_{lnLDI}} = \sqrt[3]{.637 * .484 * .744} = .611$$

$$I_{lnLDI} = \frac{9.58 - 5.52}{11.00 - 5.52} = .741$$

$$SDI = \sqrt[3]{I_{TFR} * I_{Educ} * I_{lnLDI}} = \sqrt[3]{.855 * .543 * .741} = .701$$

Section 6: References

1 Office of the Registrar General and Census Commissioner. India Special Survey of Deaths. India, 2004.

2 GBD 2016 Mortality Collaborators. Global, regional, and national under-5 mortality, adult mortality, age-specific mortality, and life expectancy, 1970-2016: a systematic analysis for the Global Burden of Disease Study 2016. *Lancet Rev* 2017.

3 Office of the Registrar General and Census Commissioner. India Medical Certification of Cause of Death Reports 1990-2010. India, 2014.

4 Office of the Registrar General and Census Commissioner. India Medical Certification of Cause of Death Reports 1980-1986. India.

5 Todd S, Barr S, Passmore AP. Cause of death in Alzheimer's disease: a cohort study. *QJM Mon J Assoc Physicians* 2013; **106**: 747–53.

6 Brunnström HR, Englund EM. Cause of death in patients with dementia disorders. *Eur J Neurol* 2009; **16**: 488–92.

7 Keene J, Hope T, Fairburn CG, Jacoby R. Death and dementia. *Int J Geriatr Psychiatry* 2001; **16**: 969–74.

8 Thomas BM, Starr JM, Whalley LJ. Death certification in treated cases of presenile Alzheimer's disease and vascular dementia in Scotland. *Age Ageing* 1997; **26**: 401–6.

9 Naghavi M, Makela S, Foreman K, O'Brien J, Pourmalek F, Lozano R. Algorithms for enhancing public health utility of national causes-of-death data. *Popul Health Metr* 2010; **8**: 9.

10 Ahern RM, Lozano R, Naghavi M, Foreman K, Gakidou E, Murray CJ. Improving the public health utility of global cardiovascular mortality data: the rise of ischemic heart disease. *Popul Health Metr* 2011; **9**: 8.

11 Barker B, Degenhardt L, National Drug and Alcohol Research Centre (Australia). Accidental drug-induced deaths in Australia 1997-2001. Sydney, Australia: National Drug and Alcohol Research Centre, University of New South Wales, 2003.

12 Roxburgh A, Burns L. Accidental drug-induced deaths due to opioids in Australia, 2011. Sydney, Australia: National Drug and Alcohol Research Centre, University of New South Wales, 2015.

13 Roxburgh A, Burns L. Cocaine and methamphetamine related drug-induced deaths in Australia, 2011. Sydney, Australia: National Drug and Alcohol Research Centre, University of New South Wales, 2015.

14 Naghavi M, Wang H, Lozano R, *et al.* Global, regional, and national age-sex specific all-cause and cause-specific mortality for 240 causes of death, 1990–2013: a systematic analysis for the Global Burden of Disease Study 2013. *The Lancet* 2015; **385**: 117–71.

15 Lozano R, Naghavi M, Foreman K, *et al.* Global and regional mortality from 235 causes of death for 20 age groups in 1990 and 2010: a systematic analysis for the Global Burden of Disease Study 2010. *The Lancet* 2012; **380**: 2095–128.

16 Calvert C, Ronsmans C. The contribution of HIV to pregnancy-related mortality: a systematic review and meta-analysis. *AIDS Lond Engl* 2013; **27**: 1631–9.

17 Figueroa-Damián R. [Pregnancy outcome in women infected with the human immunodeficiency virus]. *Salud Publica Mex* 1999; **41**: 362–7.

18 Ryder RW, Nsuami M, Nsa W, *et al.* Mortality in HIV-1-seropositive women, their spouses and their newly born children during 36 months of follow-up in Kinshasa, Zaïre. *AIDS Lond Engl* 1994; **8**: 667–72.

19 Zvandasara P, Saungweme G, Mlambo JT, Moyo J. Post Caesarean section infective morbidity in HIV-positive women at a tertiary training hospital in Zimbabwe. *Cent Afr J Med* 2007; **53**: 43–7.

20 Chilongozi D, Wang L, Brown L, *et al.* Morbidity and mortality among a cohort of human immunodeficiency virus type 1-infected and uninfected pregnant women and their infants from Malawi, Zambia, and Tanzania. *Pediatr Infect Dis J* 2008; **27**: 808–14.

21 Leroy V, Ladner J, Nyiraziraje M, *et al.* Effect of HIV-1 infection on pregnancy outcome in women in Kigali, Rwanda, 1992-1994. Pregnancy and HIV Study Group. *AIDS Lond Engl* 1998; **12**: 643–50.

22 Kourtis AP, Bansil P, McPheeters M, Meikle SF, Posner SF, Jamieson DJ. Hospitalizations of pregnant HIV-infected women in the USA prior to and during the era of HAART, 1994-2003. *AIDS Lond Engl* 2006; **20**: 1823–31.

23 Ticconi C, Mapfumo M, Dorrucci M, *et al.* Effect of maternal HIV and malaria infection on pregnancy and perinatal outcome in Zimbabwe. *J Acquir Immune Defic Syndr 1999* 2003; **34**: 289–94.

24 DerSimonian R, Laird N. Meta-analysis in clinical trials. *Control Clin Trials* 1986; **7**: 177–88.

25 Lozano R, Lopez AD, Atkinson C, Naghavi M, Flaxman AD, Murray CJ. Performance of physician-certified verbal autopsies: multisite validation study using clinical diagnostic gold standards. *Popul Health Metr* 2011; **9**: 32.

26 Foreman KJ, Lozano R, Lopez AD, Murray CJ. Modeling causes of death: an integrated approach using CODEm. *Popul Health Metr* 2012; **10**: 1.

27 Bell RM, Koren Y. Lessons from the Netflix Prize Challenge. *SIGKDD Explor Newsl* 2007; **9**: 75–79.

28 Bell RM, Koren Y, Volinsky C. All together now: A perspective on the NETFLIX PRIZE. *CHANCE* 2010; **23**: 24–24.

29 Flaxman AD, Vos T, Murray CJL, editors. An Integrative Metaregression Framework for Descriptive Epidemiology, 1 edition. Seattle: University of Washington Press, 2015.

30 GBD 2017 Population and Fertility collaborators. Population and fertility by age and sex for 195 countries and territories 1950-2017: a systematic analysis for the Global Burden of Disease 2017. *The Lancet* in review.

31 Wang H, Naghavi M, Allen C, *et al.* Global, regional, and national life expectancy, all-cause mortality, and cause-specific mortality for 249 causes of death, 1980–2015: a systematic analysis for the Global Burden of Disease Study 2015. *The Lancet* 2016; **388**: 1459–544.

32 Wang H, Abajobir AA, Abate KH, *et al.* Global, regional, and national under-5 mortality, adult mortality, age-specific mortality, and life expectancy, 1970–2016: a systematic analysis for the Global Burden of Disease Study 2016. *The Lancet* 2017; **390**: 1084–150.

Appendix Figure 1. Analytical flowchart for the development of the GBD 2017 cause of death database (A) and different strategies used to model different causes (B) and ultimately combine them into a consistent set of cause-specific deaths for each location, age, sex, and year.

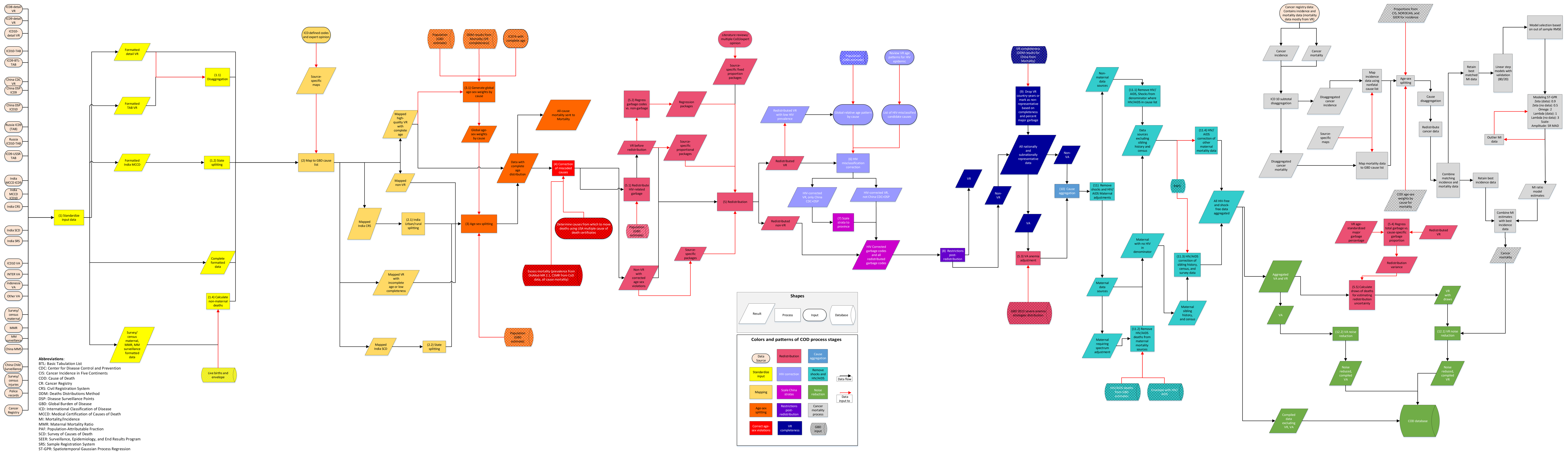

Abbreviations:
BTL: Basic Tabulation List
CDC: Center for Disease Control and Prevention
CI5: Cancer Incidence in Five Continents
COD: Cause of Death
CR: Cancer Registry
CRS: Civil Registration System
DDM: Deaths Distributions Method
DSP: Disease Surveillance Points
GBD: Global Burden of Disease
ICD: International Classification of Disease
MCCD: Medical Certification of Causes of Death
MI: Mortality/Incidence
MMR: Maternal Mortality Ratio
PAF: Population-Attributable Fraction
SCD: Survey of Causes of Death
SEER: Surveillance, Epidemiology, and End Results Program
SRS: Sample Registration System
ST-GPR: Spatiotemporal Gaussian Process Regression
TAB: Tabulation List
VA: Verbal Autopsy
VR: Vital Registration

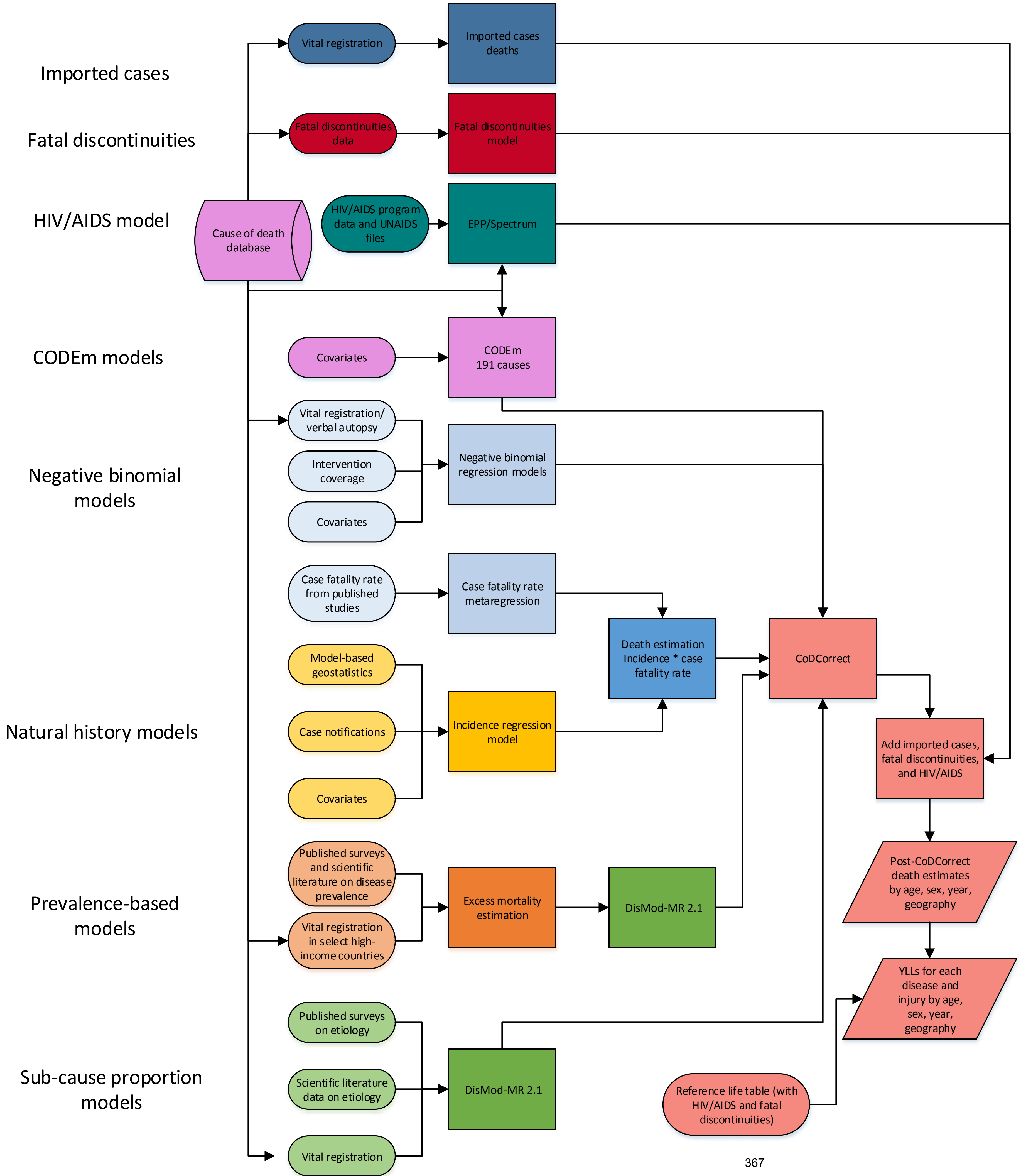

Appendix Figure 2. GBD 2017 Causes of death estimation flowchart by modeling group

Abbreviations:
CODEm: Cause of death ensemble model
GBD: Global Burden of Disease
YLL: years of life lost

Appendix Figure 3. Vital Registration and Verbal Autopsy data availability by country, 1980−2017

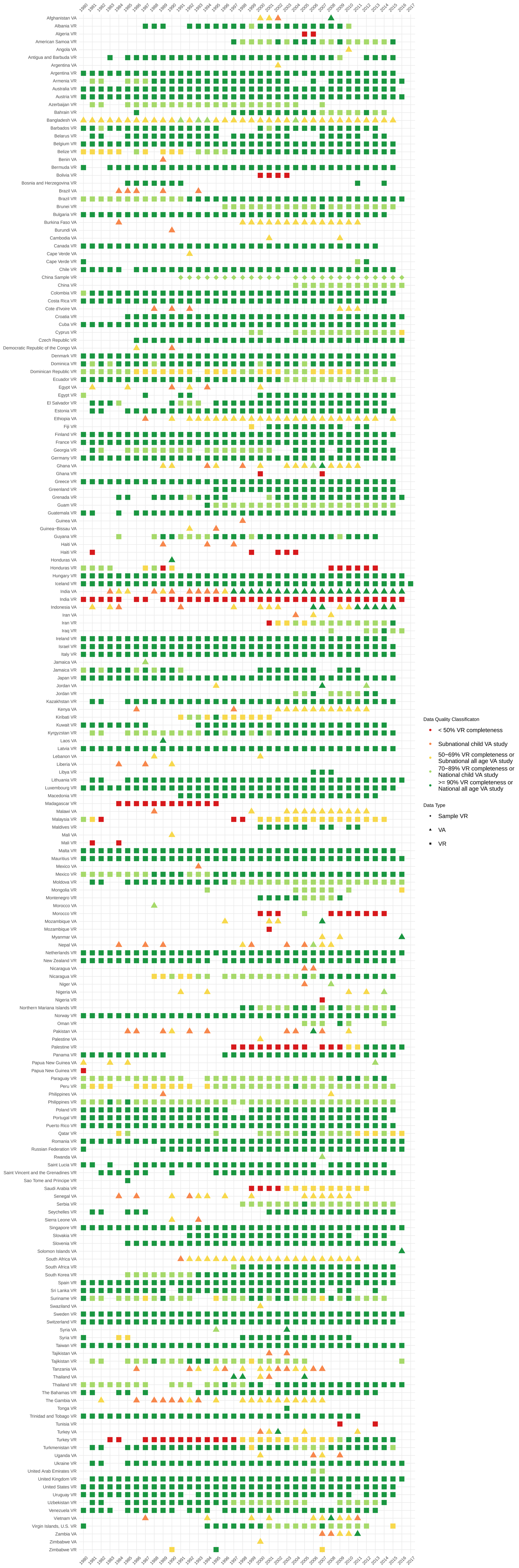

Appendix Figure 4: Percent of vital registration deaths assigned to major garbage codes for all ages by country, 1980-2017

Country	'80	'81	'82	'83	'84	'85	'86	'87	'88	'89	'90	'91	'92	'93	'94	'95	'96	'97	'98	'99	'00	'01	'02	'03	'04	'05	'06	'07	'08	'09	'10	'11	'12	'13	'14	'15	'16	'17
Albania								36	35	34			35	31	34	30	30	25	28	26	28	29	28	29	28	36	35	28	27	32	24							
Algeria																										44	44											
American Samoa																		22	21	18	16	15	19	14	21	17	26	26	14	17	17	22	24	19	17	15		
Antigua and Barbuda				33		38	38	37	33	28	26	25	28	27	25	31	21	28	22	21	21	26	23	22	21	30	30	20	21	20			28	29	25	27		
Argentina	23	22	27	29	30	30	29	29	29	29	30	30	31	32	31	32	32	33	33	33	33	34	35	34	33	33	34	35	35	35	35	35	34	34	32	29		
Armenia		13	14			14	12	13	51	14	13	12	13	17	20	15	14	12	12	11	11	11	12	13			7.6		7.1	7.8	8.1	8.7	9.4	9.2	8.8	8.3	8.6	
Australia	7.1	6.7	6.8	6.8	6.9	7	6.7	6.4	6.8	7.2	7.1	7	7.2	7.2	7.2	8.5	7.5	8.5	8.6	9	8.7	8.4	8.9	9	9.1	9.2	8.7	9.7	9.5	9.5	9.8	9.8	10	9.2	9.7	9.8		
Austria	10	10	11	17	9.7	9.4	9.3	9.1	10	9.8	10	11	12	12	12	11	11	12	12	13	13	13	9.3	8.1	8.2	9.2	9.3	9.5	9.2	11	11	11	11	11	11	12	12	
Azerbaijan		14	13			13	12	12	13	15	13	13	12	12	16	14	15	14	14	15	15	7.3	7.1	7.5	9.7			43										
Bahrain							22											32	44	43	35	39	40	38	42	39	41	40	42	45	48	46	42	39	36			
Barbados	25	27	27	26	27	26	25	24	25	25	25	26	22	25	25	23					24	25	26	28	31	29	21	18	23	19	19	18	19	20				
Belarus		14	14			12	11	11	12	13	20	23	22	22	21	21		20	18	17	17	17	15	15				16	17	16	15	18		19	16			
Belgium	23	22	22	23	23	24	23	22	23	24	23	23	21	19	19	18	16	17	16	17	17	17	17	18	17	17	17	17	17	18	20	20	21	20	20	20		
Belize	33	24	34	39	32		27	23		32	39	37		35	37	32	37	30	23	23	28	23	22	23	23	21	20	22	19	13	13	11	12	12	12	12		
Bermuda	11			18	9.6	11	21	8.6	15	14	12	9.8	13	14	17	21	6.5	6.6	9.1	12	11	14	8.9	11	13	14	17	15	15	16	17	15	13	11	9.7	13		
Bolivia																					74	72	71	68														
Bosnia and Herzegovina						38	38	34	33	34	33	33																				31			28			
Brazil	35	35	34	35	36	35	34	34	33	32	31	31	31	32	31	29	28	27	28	28	27	26	26	25	24	22	20	20	19	19	19	18	18	18	17	18	19	
Brunei																	25	17	14	18	17	17	17	17	17	17	17	18	18	23	21	15	12	15	16	15		
Bulgaria	19	20	20	20	18	18	18	20	20	19	20	20	20	20	21	23	26	28	28	28	29	29	28	29	30	26	28	28	28	32	34	35	30	32	31			
Canada	11	10	11	11	11	12	10	10	10	10	11	11	11	11	11	11	12	13	12	12	11	10	11	11	10	11	11	10	10	9.9	10	10	10	9.4				
Cape Verde	41																															31	29					
Chile	27	26	26	24	26		25	25	25	24	21	21	20	20	17	16	15	16	15	15	13	12	10	9.1	9.2	9.2	8.9	9.8	9.9	10	11	11	10	11	11	11		
China												22	21	17	13	15	15	16	14	14	16	26	24		5.3	5.1	9.2	8	14	12	12	12	11	10	8	7.3	7.3	
Colombia	24	25	23	21	21	21	21	20	20	20	19	19	18	16	16	17	17	17	12	11	10	10	9.6	10	11	11	11	11	11	11	11	12	11	10	9.7	9.4		
Costa Rica	22	23	20	18	18	17	17	17	17	17	21	20	18	17	18	18	18	11	11	8.9	8.5	8.9	9.6	7.7	8.8	9	11	10	11	11	9.6	10	9.7	10	9.5			
Croatia						18	18	18	17	16	15	15	17	19	19	20	19	20	18	17	16	16	17	16	14	13	13	14	13	12	9.5	9.8	9	8	7.7	7.9	7.9	
Cuba	15	15	15	15	14	15	14	15	14	14	15	15	16	16	17	17	17	11	11	11	12	11	10	9.8	9.9	10	9.3	9	9.3	9	9	8.7	8.5	8.7	8.7	8.5		
Cyprus																				65	64				31	28	25	23	23	24	24	20	23	18	18	19	18	
Czech Republic							9.3	11	11	11	10	11	12	12	13	15	17	20	18	16	15	15	16	16	17	16	15	14	15	16	16	15	15	12	13	14	15	
Denmark	15	15	16	16	16	17	18	18	19	20	21	22	23	23	16	16	20	16	15	13	15	15	17	17	17	15	16	17	17	17	16	17	15	16	16	16		
Dominica	33	30	30	31	28	42	37	38	33	37	36	40	38	37	42	38	42	39	38	34	33	36	37	34	31	14	14	18	21	20	19	16	23	17	29	23		
Dominican Republic	44	42	38	34	33	31	30	30	32	33	32	33	32		32	32	31	29	27	27	25	25	25	24	24	21	18	19	17	15	17	18	21	18				
Ecuador	28	27	27	28	28	28	28	28	28	28	28	27	27	27	32	32	28	33	34	34	34	33	35	33	31	29	27	26	28	25	26	26	26	23	21	18		
Egypt	57							50				50	50								55	58	57	57	58	57	58	58	59	59	58	54	52	57	52	55		
El Salvador		27	31	40	39						33	32	33	33		30	28	33	33	29	29	30	31	32	30	29	29	31	32	32	33	32	33	37	37			
Estonia		7.8	7.9			7.8	7.3	7.5	7.4	8	9.7	11	12	12	6.2	6.8	6.9	7.3	7.1	7.5	7.5	7.6	7.7	8	7.9	8.7	8.8	9.8	7	5.8	6.1	7	7.7	8.5	7.8	8.5		
Fiji																				43		53	44	42	41	39	39	37	36	36		30	30					
Finland	16	17	17	17	17	18	18	8.3	8.8	9.2	9.3	9.2	8.4	8.3	8.3	8.2	4.7	4.7	4.1	4.2	4.2	4.4	4.4	4.4	4.5	4.6	4.9	5.1	5	4.9	4.5	4.6	4.6	4.3	4.7	4.1		
France	25	25	25	25	24	23	22	22	22	22	22	21	21	21	21	22	22	22	21	21	22	21	21	22	20	21	20	21	21	21	22	22	23	23	22			
Georgia		10	11			11	11	11	8.4	7.6	7.1	8.2	8.8		9.3	8.9	11	13	13	14	12	11			23	37	41	42		48	71	53	48	51	41	43		
Germany	22	22	22	22	22	21	22	21	21	20	20	17	16	16	16	16	16	16	16	16	17	17	17	17	16	16	16	17	17	17	17	16	16	16	16	16		
Ghana																					33							33										
Greece	22	22	20	20	19	18	23	30	30	29	30	30	29	29	28	28	28	28	30	29	29	27	28	28	28	28	28	22	29	22	24	25	26	25	26	26		
Greenland																13	14	14	12	9.8	12	10	11	13	11	10	16	13	13	12	12	20	19	20	28	34		
Grenada					29	43			37	44	39	45	42	38	36	37	32					27	23	28	27	22	25	24	28	20	17	15	16	15	20	19	14	
Guam															6.3	7.2	8.7	7.3	7.3	7.9	10	8.8	9.5	9.9	8	9.6	7.1	7.3	7.5	8.2	10	9.4	11	7.7	12	9.2		
Guatemala	20	27			22		26	25	26	27	26	30	32	32	30	29	29	28	27	28	29	29	27	27	28	29	29	29	28	25	24	23	22	22	22	24		
Guyana					34				21	23	24	24	23	22	21	21	23	26	30	24	13	18	16	13	16	13	15	16	16	19	18	17	19	18				
Haiti		54																		54			41	45	53													
Honduras	53	48	58	73				36	35	40	35																		12	12	11	11	8.8	9.6				
Hungary	9	9.1	9.8	10	11	11	11	10	10	11	11	11	10	10	10	9.4	9.3	9	9.2	9.6	9.1	8.8	9	7.9	7.4	7.7	6.8	7.1	6.9	6.7	6.7	6.4	6.8	6.3	6.2	6.3	7.7	
Iceland	13	8	6.5	7.4	7.1	6.6	7.5	8	6.2	6.9	7.3	5.8	6.9	5.4	6	7.3	6.6	6.4	6.7	5.9	7.4	7.8	7	8.4	6.5	7.1	8.9	8.7	8.9	8.8	8.6	9.3	10	11	9.7	12	11	11
India	25	29	32	31	29		24	32		37	35	36	36	35	35	37	37	37	37	38	38	37	36	35	36	38	39	34	40	42	41	35	34	45	38	42	60	
Iran																						38	35	31	25	21	19	18	18	17	18	17	16	19	18	20		
Iraq																													42				37	39	38	38	38	
Ireland	10	9.8	9.7	9.7	9.2	8.9	9.1	8.5	8.4	8.8	8.8	9	8.5	8.3	9.2	9.1	9.3	9	9.2	9.8	9.3	9.5	9.6	10	10	9.3	12	7.6	7.6	7.7	8.3	8	7.7	8.1	8			
Israel	20	20	20	19	20	19	18	18	19	17	18	18	20	17	17	17	17	19	18	19	19	18	18	18	20	20	20	20	20	20	19	21	22	22	22	23		
Italy	11	11	12	12	12	13	13	12	12	12	12	12	12	12	11	12	12	13	12	13	13	13	12	12	11	12	12	12	11	11	12	12	12	13	13	14		
Jamaica	35	35	31	28	30	28	29	27	26	28	32	30									23	29	32	23	17	20	13			12	14	11						
Japan	17	17	17	18	19	19	19	19	20	20	21	21	21	21	18	12	13	13	13	14	14	14	14	15	15	16	16	16	17	18	19	19	20	21	27	27		
Jordan																									21	20	17		17	16	17	18	19	20				
Kazakhstan		19	18			18	16	16	16	15	15	11	12	11	11	11	11	12	12	16	14	14	15	15	15	15	15	20	16	20	24	29	30	16	16	13		
Kiribati												39	36	33	35	33	34	33	31	43	46	42																
Kuwait	19	18	20	20	19	18	20	19						22	21	28	25	25	22	25	22	21	20	19	17	16	18	17	19	15	15	16	17	22	16			
Kyrgyzstan		17	17			16	14	14	13	15	17	19	19	20	22	22	21	19	19	18	11	8.8	8.2	8.7	7.8	7.9	6.9	7.8	7.1	6.2	6.9	6.8	7	6.9	6.8	8		
Latvia	8.3	8.8	8.4	7.6	7.5	7.3	7.6	8	7.9	8.5	11	11	13	14	14	16	10	9.1	8.1	8	8.9	9.8	11	11	12	12	13	11	12	11	11	9.6	8.4	6.2	6.2	5.7		
Libya																											87	91	89									
Lithuania		9.3	8.5			7.2	6.4	6.3	6	6.2	6.3	7.7	8.3	11	8.5	6.5	6.3	5.2	6.5	7.3	8.5	7	7.1	6.8	7.1	7.2	7.2	7.4	6.8	6.7	6.6	6.7	6.1	5.4	5.7	5.9	5.9	
Luxembourg	17	16	15	15	14	13	14	14	15	16	16	19	14	15	15	15	16	16	19	18	18	18	20	22	21	22	22	22	22	23	22	21	19	17	17	18		
Macedonia												19	20	19	18	19	19	17	16	17	16	15	17	15	28	29	19	20	20	18	20	22	21	22				
Madagascar					42	38	43	42	43	43	41	43	45	45	44	43																						
Malaysia	34	33	25															30	30		34	36	35	36	36	36	35	33	33	26	26	26	25	26	25			
Maldives																					71	58	56	60	57	83		55	52		47	40						
Mali		46			53																																	
Malta	20	22	19	20	19	21	27	27	21	12	13	13	11	11	13	9.6	10	10	11	13	11	8.9	11	11	8.4	9.4	8.6	8.8	7.2	7.1	8.5	10	13	9.5	7.7	8.5		
Mauritius	36	30	26	26	25	24	24	21	20	21	22	21	23	25	25	26	29	28	22	22	20	17	17	17	17	16	16	14	16	15	16	16	16	15	13	14	14	
Mexico	26	25	23	22	22	21	21	20	18	17	17	17	16	16	15	15	15	14	14	13	13	13	13	13	12	12	12	14	13	13	13	13	13	13	12	12	11	
Moldova		11	11			8.8	8.8	8.8	9.7	9.8	17	18	20	21	20	16	11	8.1	8.2	7.1	6.1	4.2	3.9	3.5	3.1	3.2	3.1	3.2	3	3.2	3.2	3.1	2.8	2.3	2.5	2.9	2.9	
Mongolia															21										76	77	78	76	73		6.3						6.5	
Montenegro																					30	32	31	32	29	24	27	27	27	30								
Morocco																					53	53	57			55			55	53	54	57	51	49	49			

Country	Year																																					
	'80	'81	'82	'83	'84	'85	'86	'87	'88	'89	'90	'91	'92	'93	'94	'95	'96	'97	'98	'99	'00	'01	'02	'03	'04	'05	'06	'07	'08	'09	'10	'11	'12	'13	'14	'15	'16	'17
Mozambique																						21																
Netherlands	12	11	14	15	14	14	14	13	13	14	15	15	15	17	16	17	16	16	17	17	18	18	18	18	17	17	17	17	17	17	17	16	17	17	17	17	17	
New Zealand	4.7	4.8	4.7	4.8	4.4	4.8	5.2	5.1	5.1	5.1	5.5	5.5	5.2	5.4	4.9		4.8	4.5	3.4	3.2	3.7	3.7	3.9	3.7	3.6	3.5	3.7	4	4.1	4.2	4.3	4.4	4.3	4.5	4.6	4.4		
Nicaragua									21	22	20	21	21	22	22		22	17	15	14	15	13	12	11	12	12	13	13	13	11	11	9.5	10	9.5	9.9	12		
Nigeria																												92										
Northern Mariana Islands																			25	34	26	14	25	24	21	25	19	17	25	20	25	25	24	15	12	12		
Norway	12	11	11	11	11	11	10	11	11	11	11	12	12	13	13	13	11	12	13	13	13	13	14	14	14	16	16	16	17	17	17	17	17	18	18	16		
Oman																										16	16	16		45	59				56			
Palestine																		46	40	38	38	41	44	43	33	33		33	35	31	30	29	29	30	26	38	38	
Panama	26	26	25	26	25	24	23	23	25	24							26	25	18	16	15	15	13	15	17	15	16	14	15	15	17	17	16	18	16	17		
Papua New Guinea	17																																					
Paraguay	35	33	36	37	38	37	34	35	35	33	32	31			26	27	26	32	31	33	32	30	32	33	28	27	24	26	25	24	23	21	22	21	21			
Peru	22	21	22	23			49	51	48	48	50	51	45		49	41	39	42	38	36	31	33	33	30	30	62	65	24	25	27	26	26	26	25	24	20		
Philippines	21	26	26	25	24	21	24	20	21	22	23	24	27	24	25	26	25	22	22	22	20	20	15	18	20	21	15	16	15	15	15	15	18	16	17	15		
Poland	39	38	38	40	40	41	41	41	40	41	41	41	40	40	40	40	40			28	26	26	26	26	26	26	26	27	28	29	29	27	29	30	31	31		
Portugal	29	29	29	30	23	23	24	24	24	24	24	24	23	24	24	24	25	25	25	25	25	23	21	22	25	24	26	23	23	22	22	21	22	21	18			
Puerto Rico	24	21	26	25	25	25	24	24	24	23	23	22	22	23	16	17	17	17	17	17	17	17	17	17	16	17	17	17	16	17	16	16	16	16	17	16		
Qatar					78	76										30					100	35	100	100	37	33	32	35	40	34	38	39	42	38	39	35	30	
Romania	24	24	25	25	25	24	23	23	23	22	22	23	24	17	17	17	18	16	16	15	15	14	15	15	14	14	14	14	14	15	15	15	15	16	16	16	16	
Russian Federation	7									7.8	9.3	10	11	12	13	13	13	13	12	12	12	12	12	12	12	12	11	11	11	11	11	12	12	13	15	15	15	
Saint Lucia	41	37		29			36	32	33	37	31	28	27	28	27	28	31	28	30	33	24	20	25	24	21	30	27		28	16	21	16	15	17	14			
Saint Vincent and the Grenadines			32	28	45	36	31	41			37					21	23	19	15	16	16	18	15	18	15	17	13	12	20	27	21	19	19	16	13	17		
Sao Tome and Principe						30																																
Saudi Arabia																				46	47	45	49	50	50	48	49	52	53	54	54	56	57					
Serbia																			21	21	22	21	21	20	22	17	17	18	17	17	17	20	19	20	22	22		
Seychelles		27	26			33	29	28														24	24	24	25	25	25	23	23	27	25	22	24	24	23	42		
Singapore	12	11	9.6	8.8	9.1	8	13	8.4	7.9	10	8.1	6.9	6.4	6.1	5.3	5.4	5.2	4.7	5.3	5	5.6	5	5.4	4.7	4.9	4.9	4.7	5.1	5.2	5.5	5.7	5.2	2.9	2.4	2.4	2.8	2.5	
Slovakia													19	18	17	19	19	19	19	17	15	16	16	15	15	17	25	19	9.6	10	11		7.1	7.9	8.5			
Slovenia						8.4	9.2	10	9.5	15	14	9	7.5	9.2	10	11	11	8.2	10	10	11	12	12	12	12	15	14	13	12	12	12	12	12	13	14	14		
South Africa																		39	37	32	31	30	30	29	28	28	29	29	29	29	29	30	30	29	29	30		
South Korea						33	31	32	31	29	27	28	24	22	23	24	25	26	25	25	21	19	15	16	17	19	19	18	19	19	18	19	18	18	18	18		
Spain	23	23	24	24	22	23	22	21	21	21	20	20	19	19	17	17	17	16	16	17	17	17	17	17	17	17	17	18	18	16	15	15	15	15	15	15		
Sri Lanka	48	49	55	49	50	50	50	53	52	52		55	54	52	54	50	48	45	45	45	40	39	39	37	36	40	38	33		36	35			37				
Suriname	45	41	33		32	30	30	28	31	30	29	29	29			29	31	29	28	28	25	21	21	20	22	23	17	18	19	17	18	19	21	21	23			
Sweden	12	13	13	14	14	14	14				12	12	12	12	13	13	13	13	14	14	14	14	14	14	14	14	14	15	15	15	17	16	16	15	15	16	15	
Switzerland	27	26	27	26	26	26	26	26	26	26	27	27	27	27	27	16	16	16	15	16	16	16	16	16	16	15	14	14	13	14	14	14	15	14	15	15		
Syria	70				78	80													52	46	45	48	47	41	32	40	36	32	34	25	40							
Taiwan (province of China)	15	16	16	17	17	18	18	18	18	19	20	20	21	21	21	19	18	18	18	16	16	16	16	16	16	15	16	16	19	19	19	19	17	16	16	17	17	
Tajikistan		16	17			22	25	26	23	20	20	19	23	31	27	29	32	33	35	33	35	34	31	31	32	33											33	
Thailand	63	62	63	64	64	65	66	65			61	60	60		59	59	57	59	60	57	55		51	48	53	54	54	54	54	54	52	49	47	43	41	39	38	
The Bahamas	25	24			21	24		17						23	17	18	23	16	16	10	12	13	24	16	15	16	14	15	14	13	14	14	15	14				
Tonga																								46														
Trinidad and Tobago	20	18	18	16	17	18	16	15	15	15	16	16	15	15	14	15	14	14	14	10	9.7	10	9.3	9.5	9.9	10	11	12	12	12	11	9.9						
Tunisia																														26				35				
Turkey				54	55			54	54	54	55	55	55	53	55	55	57	56	54	56	52	51	53	55	62	59	60	60	61	20	20	20	22	15	16	16		
Turkmenistan		12	12			12	12	15	14	17	19	17	18	17	19	19	21	25	27	14	12	11	12	11	11	13	15	16	16	16	17	18	18	20	20	23		
Ukraine	9.6	10	10	11	11	12	12	12	12	11	10	10	10	11	11	11	11	12	12	12	12	12	12	13	13	13	13	13	13	13	13	13	13	13	13	14		
United Arab Emirates		11	11			10	10	12	14	16	20	20	20	18	16	16	16	15	14	14	14	14	15	15	16	11	11	10	10	9.1	8.8	8.4	7.8	19	8.5	19	20	
United Kingdom																											51	49										
United States		6.9	6.6	6.3	5.8	5.9	6	6	6.1	6.2	6.1	5.9	5.9	7.3	7.5	8	8.2	8.6	8.9	9.1	8.9	8.6	8.7	8.7	8.6	8.7	8.6	8.6	8.8	8.5	8.6	7.9	7.9	8	7.6	7.6	7.7	
Uruguay	23	23	23	24	25	24	25	25	25	24	24	21		25	24	26	25	20	21	22	21	20	21	22	22	21	22	23	24	24	24		26	24	24	23		
Uzbekistan		15	15			14	14	14	15	14	14	15	15	16	17	17	19	19	19	19	18	19	22	23	12	11				9.1	9.8	9.8	10	11	13			
Venezuela	27	27	27	19		30	29	26	25	25	17		17	17	17		13	13	13	12	11	10	11	12	11	11	11	11	11	12	11	11	11	11				
Virgin Islands, U.S.	18														9.4	9.5	11	9.1	11	10	11	12	11	12	9.9	12	12	9.5	11	13	14	14	19			20		
Zimbabwe											24					26												18										

Color Scale: 0 | 10 | 20 | 30 | 40 | 50<

Countries without VR for 1980-2017 are not listed. VR=vital registration. Blank white cells indicate lack of VR for that year. Cells are colour coded by percent of data redistributed in a given country-year from garbage coding to a likely underlying cause of death. High percentages of vaguely-coded deaths highlight the importance of improvements in accurate reporting of mortality data to avoid redistribution of deaths to broad level causes.

Appendix Figure 5A: Classification of national time series of vital registration and verbal autopsy data 1980−2017

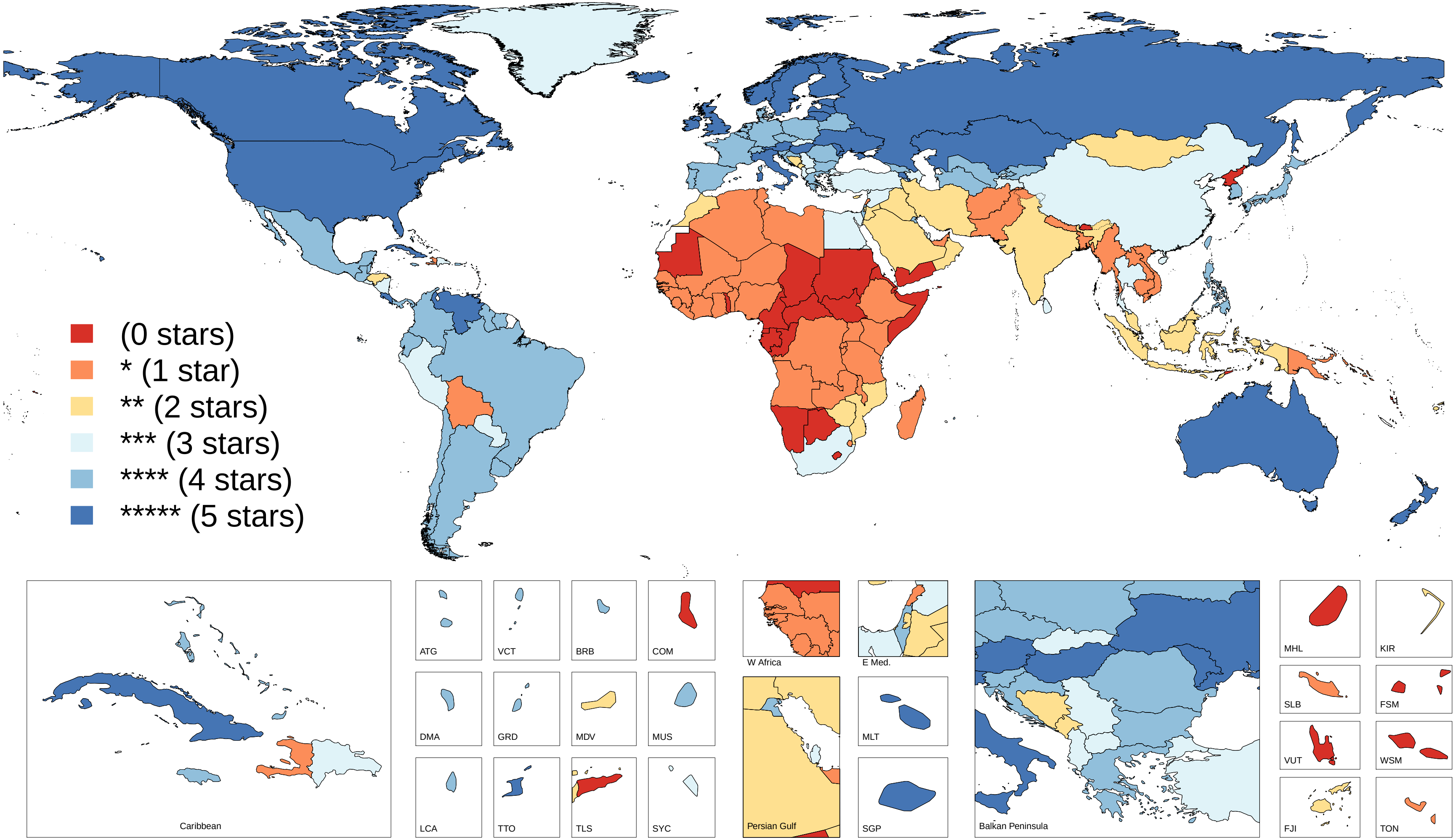

Appendix Figure 5B: Classification of national time series of vital registration and verbal autopsy data 2010−2017

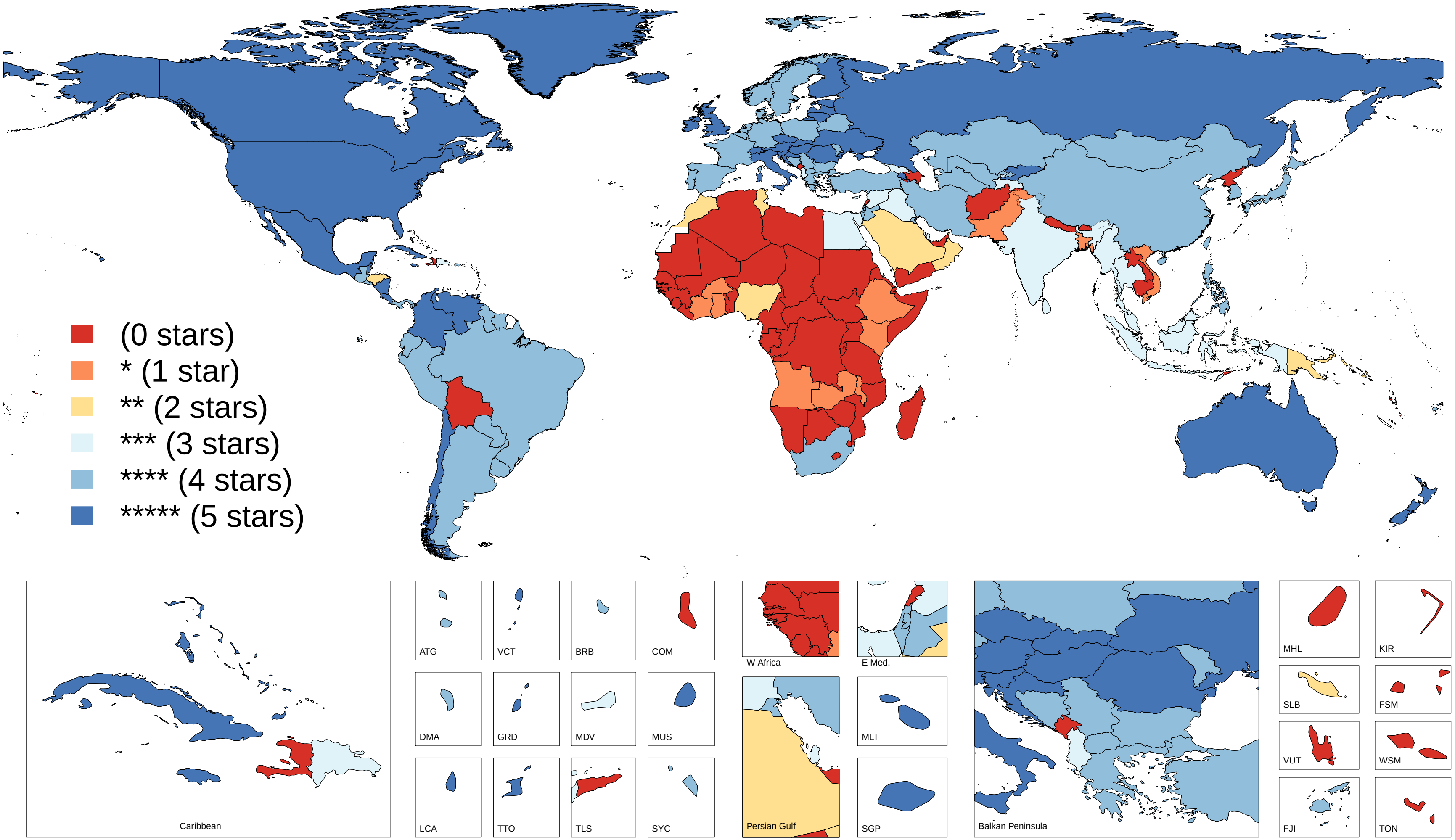

Appendix Figure 6a. Recoding of Alzheimer's disease and other dementias − Sweden

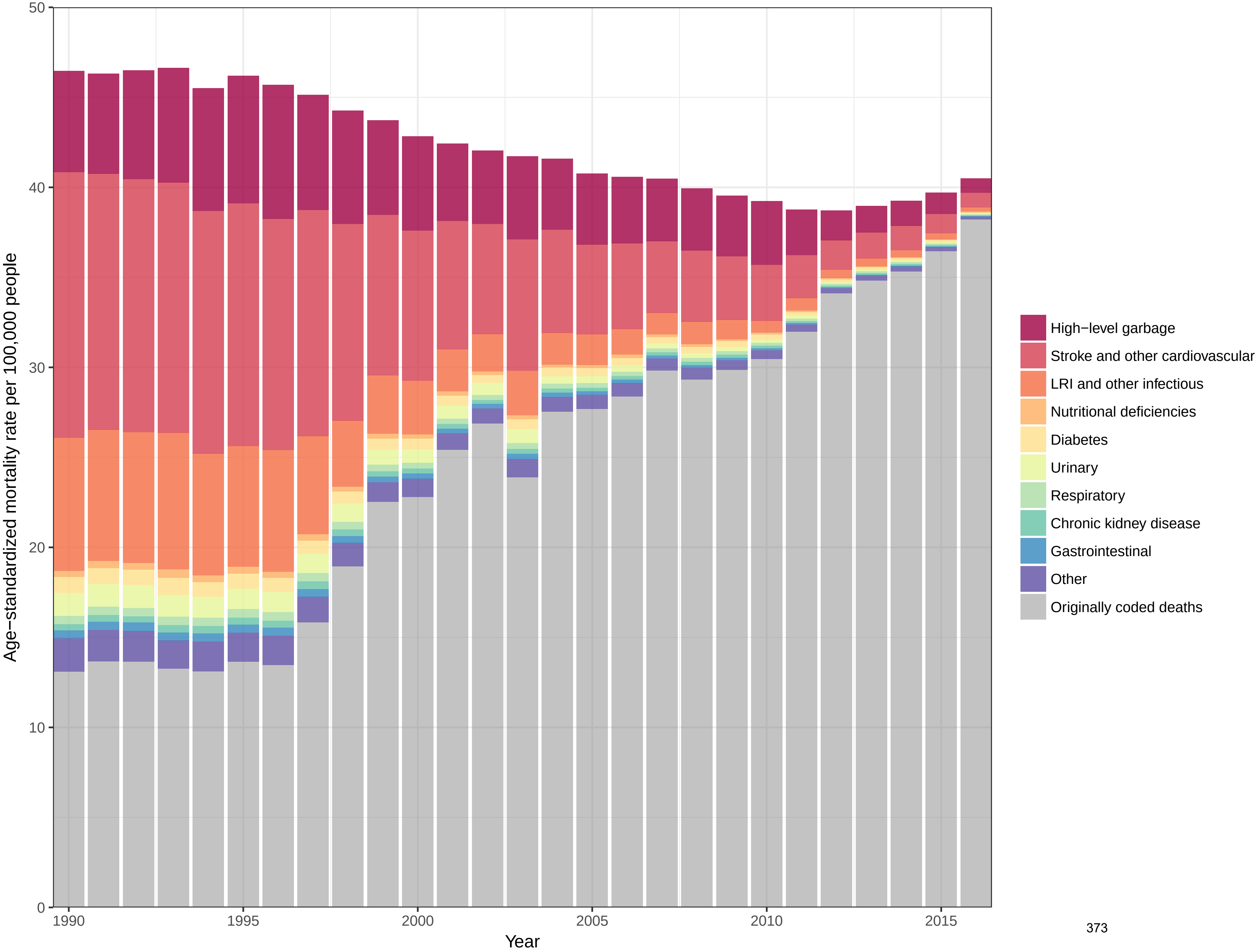

Appendix Figure 6a. Recoding of Alzheimer's disease and other dementias − Mexico

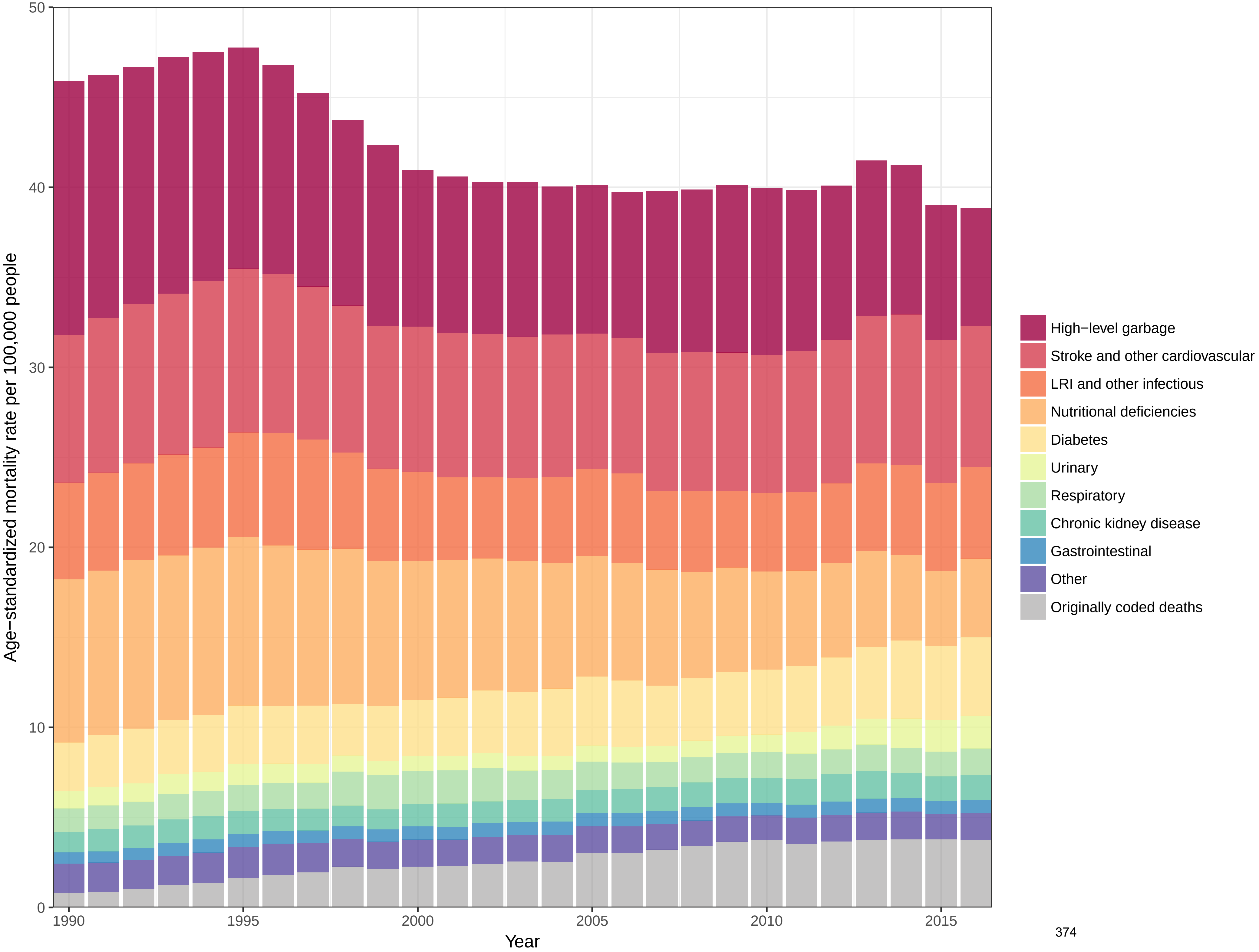

Appendix Figure 6b. Recoding of Parkinson's disease − Sweden

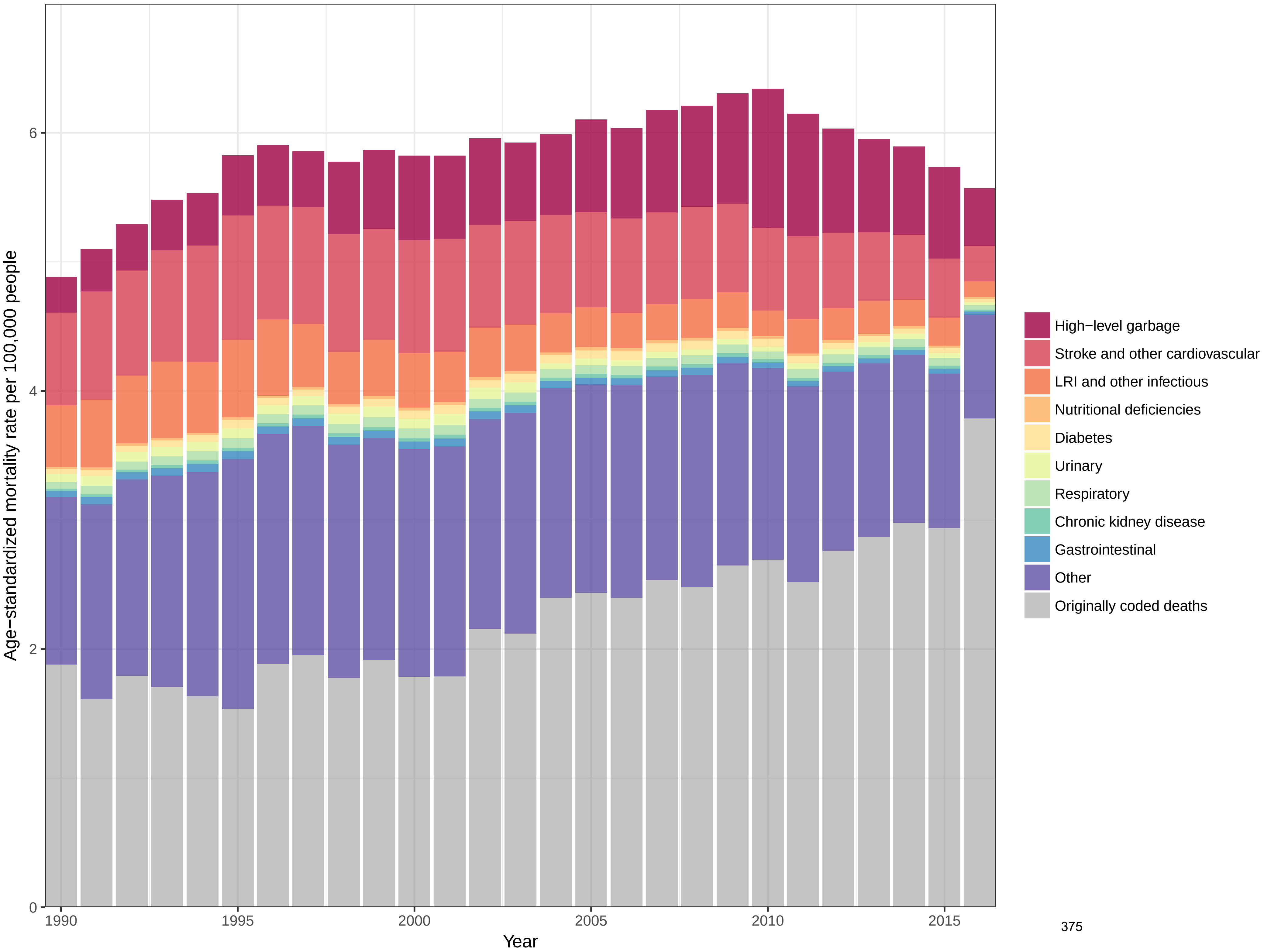

Appendix Figure 6b. Recoding of Parkinson's disease − Mexico

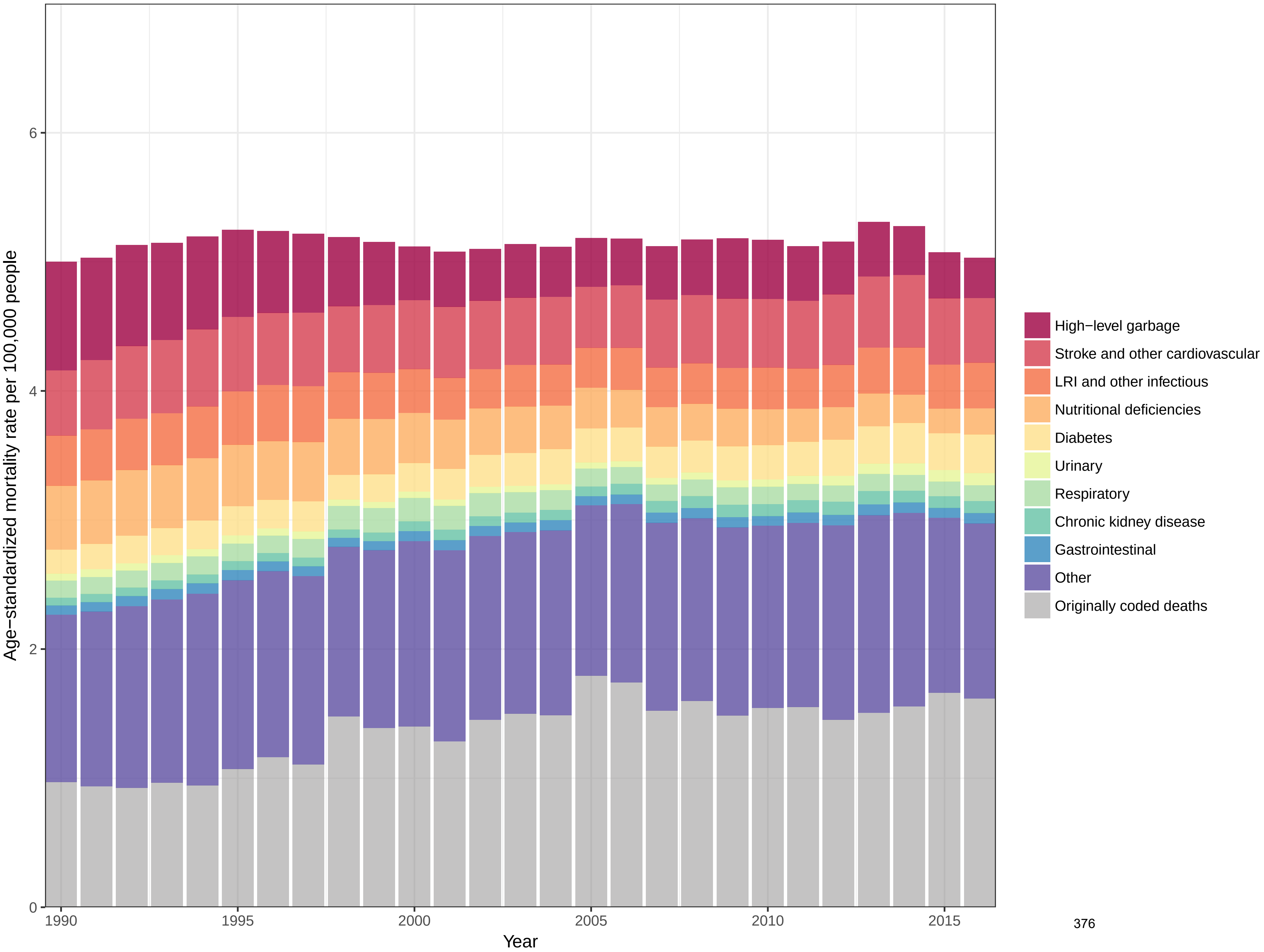

Appendix Figure 6c. Recoding of atrial fibrillation and flutter − Sweden

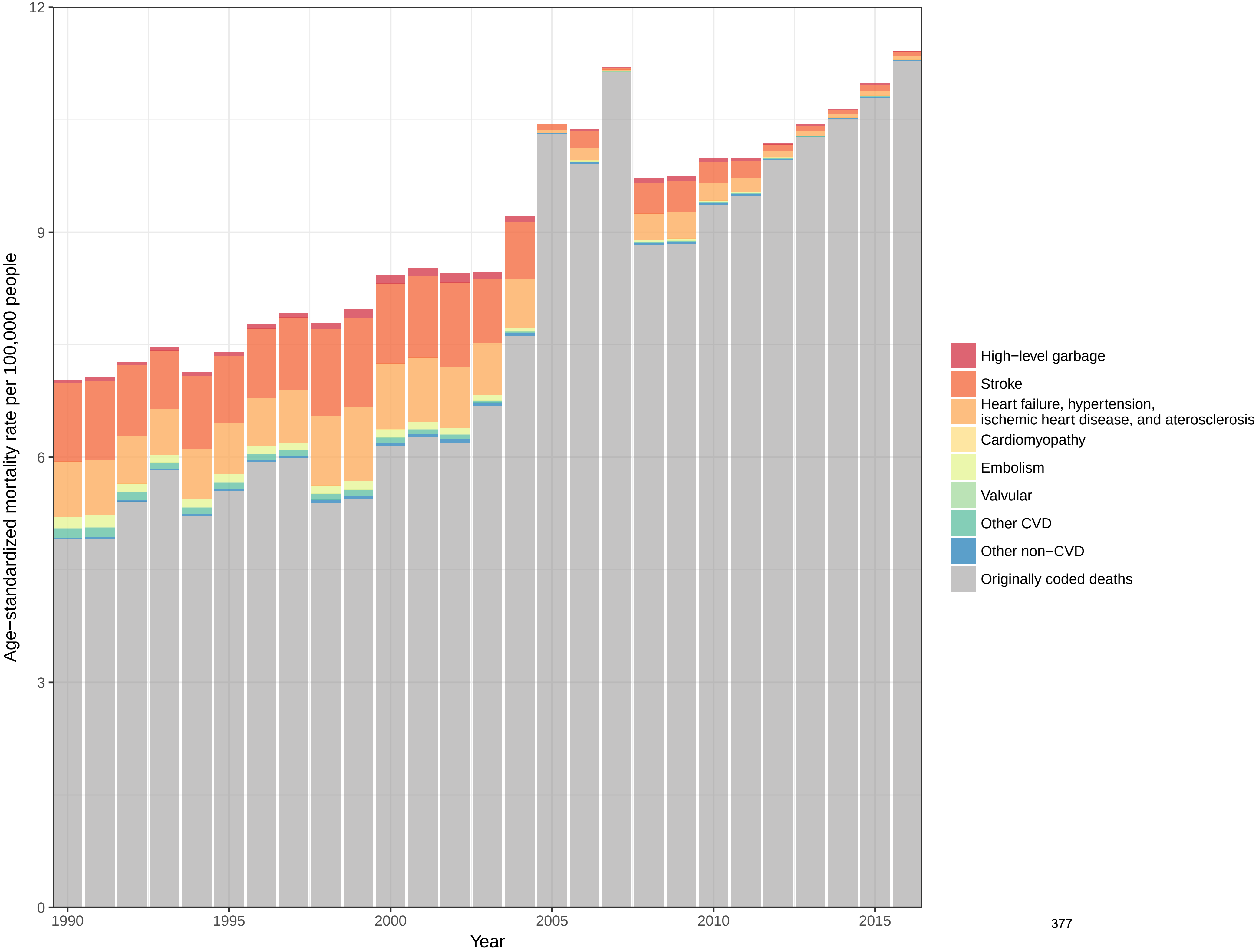

Appendix Figure 6c. Recoding of atrial fibrillation and flutter − Mexico

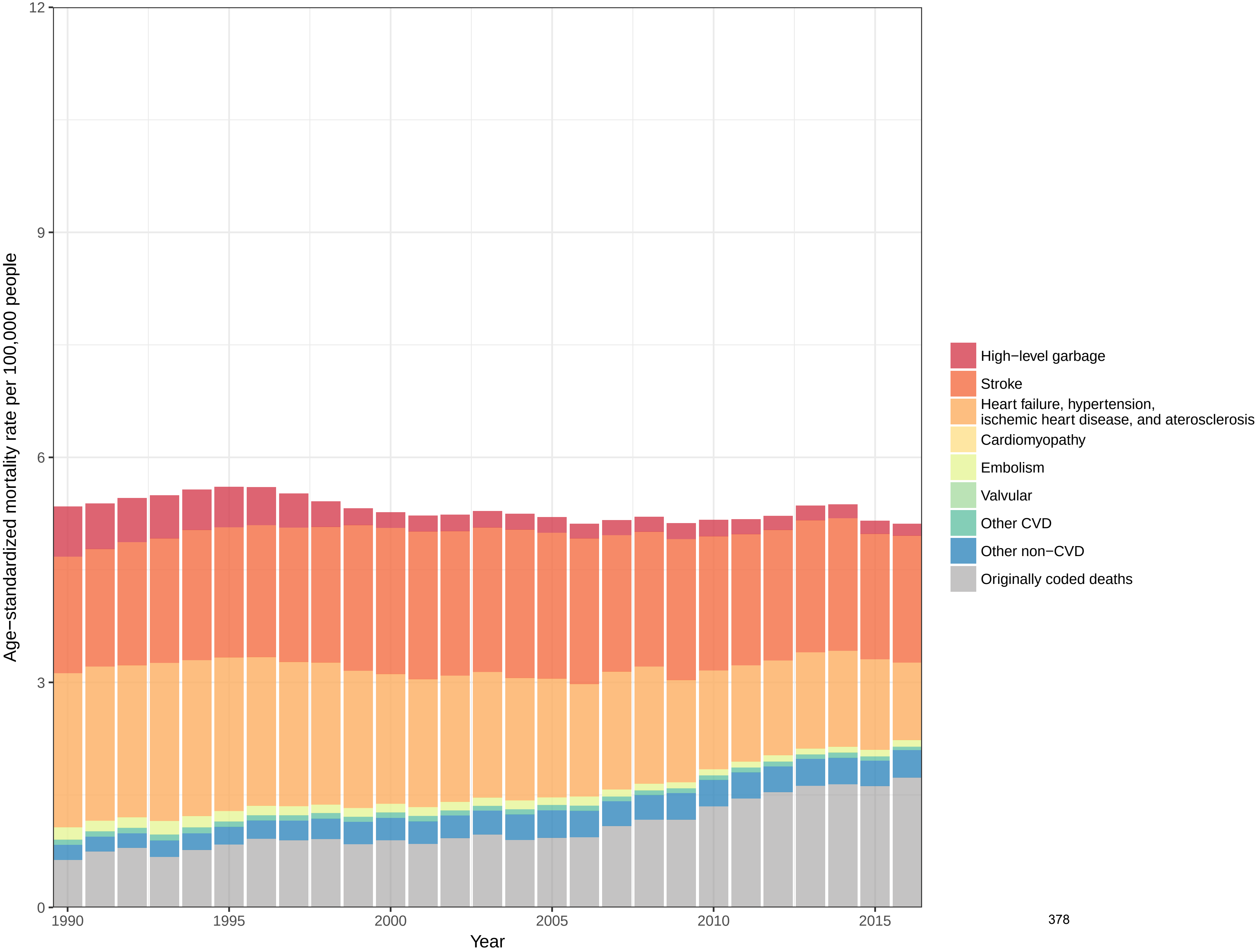

Appendix Figure 7. GBD 2017 DisMod-MR 2.1 analytical cascade

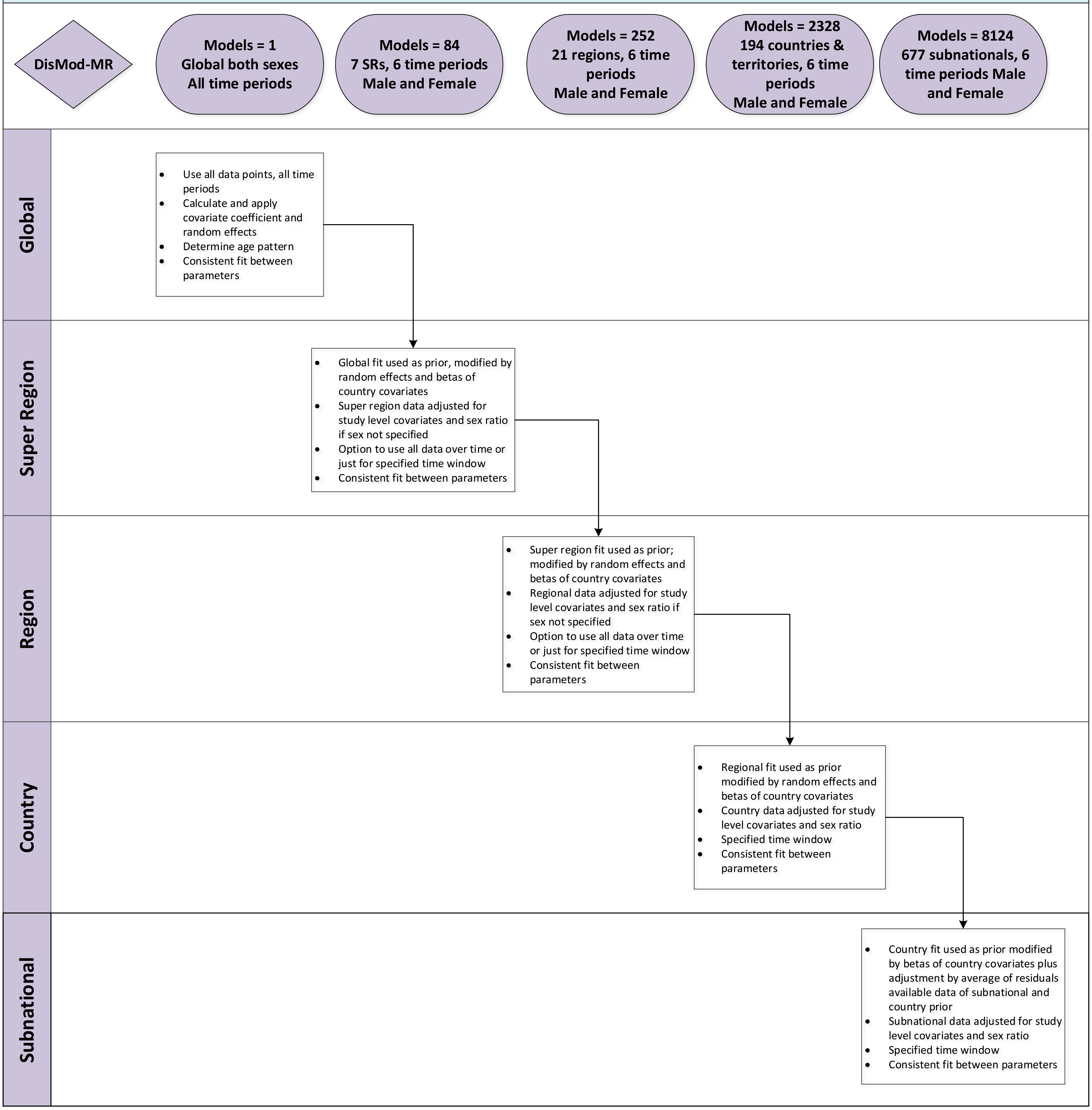

Appendix Table 1. GATHER checklist of information that should be included in reports of global health estimates, with description of compliance and location of information for GBD 2017 [Global, regional, and national age-sex specific mortality for 282 causes of death, 1980-2017: a systematic analysis for the Global Burden of Disease Study 2017]

#	GATHER checklist item	Description of compliance	Reference
**Objectives and funding**			
1	Define the indicators, populations, and time periods for which estimates were made.	Narrative provided in paper and methods appendix describing indicators, definitions, and populations	Main text (Methods—Overview, Geographic units and time periods) and methods appendix
2	List the funding sources for the work.	Funding sources listed in paper	Summary (Funding)
**Data Inputs**			
For all data inputs from multiple sources that are synthesized as part of the study:			
3	Describe how the data were identified and how the data were accessed.	Narrative description of data seeking methods provided	Main text (Methods) and methods appendix
4	Specify the inclusion and exclusion criteria. Identify all ad-hoc exclusions.	Narrative about inclusion and exclusion criteria by data type provided	Main text (Methods) and methods appendix
5	Provide information on all included data sources and their main characteristics. For each data source used, report reference information or contact name/institution, population represented, data collection method, year(s) of data collection, sex and age range, diagnostic criteria or measurement method, and sample size, as relevant.	An interactive, online data source tool that provides metadata for data sources by component, geography, cause, risk, or impairment has been developed	Online data citation tools
6	Identify and describe any categories of input data that have potentially important biases (e.g., based on characteristics listed in item 5).	Summary of known biases by cause included in methods appendix	Methods appendix
For data inputs that contribute to the analysis but were not synthesized as part of the study:			
7	Describe and give sources for any other data inputs.	Included in online data source tool, http://ghdx.healthdata.org/gbd-2017	Online data citation tools
For all data inputs:			
8	Provide all data inputs in a file format from which data can be efficiently extracted (e.g., a spreadsheet as opposed to a PDF), including all relevant meta-data listed in item 5. For any data inputs that cannot be shared due to ethical or legal	Downloads of input data available through online tools, including data visualization tools	Online data visualization tools, data query tools, and

	reasons, such as third-party ownership, provide a contact name or the name of the institution that retains the right to the data.	and data query tools, http://ghdx.healthdata.org/gbd-2017; input data not available in tools will be made available upon request	the Global Health Data Exchange, http://ghdx.healthdata.org
**Data analysis**			
9	Provide a conceptual overview of the data analysis method. A diagram may be helpful.	Flow diagrams of the overall methodological processes, as well as cause-specific modelling processes, have been provided	Main text (Methods) and methods appendix
10	Provide a detailed description of all steps of the analysis, including mathematical formulae. This description should cover, as relevant, data cleaning, data pre-processing, data adjustments and weighting of data sources, and mathematical or statistical model(s).	Flow diagrams and corresponding methodological write-ups for each cause, as well as the demographics and causes of death databases and modelling processes, have been provided	Main text (Methods) and methods appendix
11	Describe how candidate models were evaluated and how the final model(s) were selected.	Provided in the methodological write-ups	Methods appendix
12	Provide the results of an evaluation of model performance, if done, as well as the results of any relevant sensitivity analysis.	Provided in the methodological write-ups	Methods appendix
13	Describe methods for calculating uncertainty of the estimates. State which sources of uncertainty were, and were not, accounted for in the uncertainty analysis.	Provided in the methodological write-ups	Methods appendix
14	State how analytic or statistical source code used to generate estimates can be accessed.	Access statement provided	Code is provided in an online repository
**Results and Discussion**			
15	Provide published estimates in a file format from which data can be efficiently extracted.	Results are available through online data visualization tools, the Global Health Data Exchange, and the online data query tool (http://ghdx.healthdata.org/gbd-2017)	Main text, methods appendix, and online data tools (data visualization tools, data query tools, and the Global Health Data Exchange, http://ghdx.healthdata.org/gbd-2017)
16	Report a quantitative measure of the uncertainty of the estimates (e.g. uncertainty intervals).	Uncertainty intervals are provided with all results	Main text, methods appendix, and online data tools (data

			visualization tools, data query tools, and the Global Health Data Exchange, http://ghdx.healthdata.org/gbd-2017)
17	Interpret results in light of existing evidence. If updating a previous set of estimates, describe the reasons for changes in estimates.	Discussion of methodological changes between GBD rounds provided in the narrative of the Article and methods appendix	Main text (Methods and Discussion) and methods appendix
18	Discuss limitations of the estimates. Include a discussion of any modelling assumptions or data limitations that affect interpretation of the estimates.	Discussion of limitations provided in the narrative of the main paper, as well as in the methodological write-ups in the methods appendix	Main text (Limitations) and methods appendix

Appendix Table 2. GBD 2017 location hierarchy with levels	
**Geography**	**Level**
Global	0
Low SDI	1
Low-middle SDI	1
Middle SDI	1
High-middle SDI	1
High SDI	1
Central Europe, Eastern Europe, and Central Asia	1
Central Asia	2
Armenia	3
Azerbaijan	3
Georgia	3
Kazakhstan	3
Kyrgyzstan	3
Mongolia	3
Tajikistan	3
Turkmenistan	3
Uzbekistan	3
Central Europe	2
Albania	3
Bosnia and Herzegovina	3
Bulgaria	3
Croatia	3
Czech Republic	3
Hungary	3
Macedonia	3
Montenegro	3
Poland	3
Romania	3
Serbia	3
Slovakia	3
Slovenia	3
Eastern Europe	2
Belarus	3
Estonia	3
Latvia	3
Lithuania	3
Moldova	3
Russian Federation	3
Ukraine	3
High-income	1
Australasia	2
Australia	3
New Zealand	3
High-income Asia Pacific	2

Appendix Table 2. GBD 2017 location hierarchy with levels

Geography	Level
Brunei	3
Japan	3
Aichi	4
Akita	4
Aomori	4
Chiba	4
Ehime	4
Fukui	4
Fukuoka	4
Fukushima	4
Gifu	4
Gunma	4
Hiroshima	4
Hokkaidō	4
Hyōgo	4
Ibaraki	4
Ishikawa	4
Iwate	4
Kagawa	4
Kagoshima	4
Kanagawa	4
Kōchi	4
Kumamoto	4
Kyōto	4
Mie	4
Miyagi	4
Miyazaki	4
Nagano	4
Nagasaki	4
Nara	4
Niigata	4
Ōita	4
Okayama	4
Okinawa	4
Ōsaka	4
Saga	4
Saitama	4
Shiga	4
Shimane	4
Shizuoka	4
Tochigi	4
Tokushima	4
Tōkyō	4
Tottori	4
Toyama	4

Appendix Table 2. GBD 2017 location hierarchy with levels	
**Geography**	**Level**
Wakayama	4
Yamagata	4
Yamaguchi	4
Yamanashi	4
South Korea	3
Singapore	3
High-income North America	2
Canada	3
Greenland	3
United States	3
Alabama	4
Alaska	4
Arizona	4
Arkansas	4
California	4
Colorado	4
Connecticut	4
Delaware	4
District of Columbia	4
Florida	4
Georgia	4
Hawaii	4
Idaho	4
Illinois	4
Indiana	4
Iowa	4
Kansas	4
Kentucky	4
Louisiana	4
Maine	4
Maryland	4
Massachusetts	4
Michigan	4
Minnesota	4
Mississippi	4
Missouri	4
Montana	4
Nebraska	4
Nevada	4
New Hampshire	4
New Jersey	4
New Mexico	4
New York	4
North Carolina	4
North Dakota	4

Appendix Table 2. GBD 2017 location hierarchy with levels

Geography	Level
Ohio	4
Oklahoma	4
Oregon	4
Pennsylvania	4
Rhode Island	4
South Carolina	4
South Dakota	4
Tennessee	4
Texas	4
Utah	4
Vermont	4
Virginia	4
Washington	4
West Virginia	4
Wisconsin	4
Wyoming	4
Southern Latin America	2
Argentina	3
Chile	3
Uruguay	3
Western Europe	2
Andorra	3
Austria	3
Belgium	3
Cyprus	3
Denmark	3
Finland	3
France	3
Germany	3
Greece	3
Iceland	3
Ireland	3
Israel	3
Italy	3
Luxembourg	3
Malta	3
Netherlands	3
Norway	3
Portugal	3
Spain	3
Sweden	3
Stockholm	4
Sweden except Stockholm	4
Switzerland	3
United Kingdom	3

Appendix Table 2. GBD 2017 location hierarchy with levels	
**Geography**	**Level**
England	4
East Midlands	5
Derby	6
Derbyshire	6
Leicester	6
Leicestershire	6
Lincolnshire	6
Northamptonshire	6
Nottingham	6
Nottinghamshire	6
Rutland	6
East of England	5
Bedford	6
Cambridgeshire	6
Central Bedfordshire	6
Essex	6
Hertfordshire	6
Luton	6
Norfolk	6
Peterborough	6
Southend-on-Sea	6
Suffolk	6
Thurrock	6
Greater London	5
Barking and Dagenham	6
Barnet	6
Bexley	6
Brent	6
Bromley	6
Camden	6
Croydon	6
Ealing	6
Enfield	6
Greenwich	6
Hackney	6
Hammersmith and Fulham	6
Haringey	6
Harrow	6
Havering	6
Hillingdon	6
Hounslow	6
Islington	6
Kensington and Chelsea	6
Kingston upon Thames	6
Lambeth	6

Appendix Table 2. GBD 2017 location hierarchy with levels

Geography	Level
Lewisham	6
Merton	6
Newham	6
Redbridge	6
Richmond upon Thames	6
Southwark	6
Sutton	6
Tower Hamlets	6
Waltham Forest	6
Wandsworth	6
Westminster	6
North East England	5
County Durham	6
Darlington	6
Gateshead	6
Hartlepool	6
Middlesbrough	6
Newcastle upon Tyne	6
North Tyneside	6
Northumberland	6
Redcar and Cleveland	6
South Tyneside	6
Stockton-on-Tees	6
Sunderland	6
North West England	5
Blackburn with Darwen	6
Blackpool	6
Bolton	6
Bury	6
Cheshire East	6
Cheshire West and Chester	6
Cumbria	6
Halton	6
Knowsley	6
Lancashire	6
Liverpool	6
Manchester	6
Oldham	6
Rochdale	6
Salford	6
Sefton	6
St Helens	6
Stockport	6
Tameside	6
Trafford	6

Appendix Table 2. GBD 2017 location hierarchy with levels

Geography	Level
Warrington	6
Wigan	6
Wirral	6
South East England	5
Bracknell Forest	6
Brighton and Hove	6
Buckinghamshire	6
East Sussex	6
Hampshire	6
Isle of Wight	6
Kent	6
Medway	6
Milton Keynes	6
Oxfordshire	6
Portsmouth	6
Reading	6
Slough	6
Southampton	6
Surrey	6
West Berkshire	6
West Sussex	6
Windsor and Maidenhead	6
Wokingham	6
South West England	5
Bath and North East Somerset	6
Bournemouth	6
Bristol, City of	6
Cornwall	6
Devon	6
Dorset	6
Gloucestershire	6
North Somerset	6
Plymouth	6
Poole	6
Somerset	6
South Gloucestershire	6
Swindon	6
Torbay	6
Wiltshire	6
West Midlands	5
Birmingham	6
Coventry	6
Dudley	6
Herefordshire, County of	6
Sandwell	6

Appendix Table 2. GBD 2017 location hierarchy with levels	
Geography	Level
Shropshire	6
Solihull	6
Staffordshire	6
Stoke-on-Trent	6
Telford and Wrekin	6
Walsall	6
Warwickshire	6
Wolverhampton	6
Worcestershire	6
Yorkshire and the Humber	5
Barnsley	6
Bradford	6
Calderdale	6
Doncaster	6
East Riding of Yorkshire	6
Kingston upon Hull, City of	6
Kirklees	6
Leeds	6
North East Lincolnshire	6
North Lincolnshire	6
North Yorkshire	6
Rotherham	6
Sheffield	6
Wakefield	6
York	6
Northern Ireland	4
Scotland	4
Wales	4
Latin America and Caribbean	1
Andean Latin America	2
Bolivia	3
Ecuador	3
Peru	3
Caribbean	2
Antigua and Barbuda	3
The Bahamas	3
Barbados	3
Belize	3
Bermuda	3
Cuba	3
Dominica	3
Dominican Republic	3
Grenada	3
Guyana	3
Haiti	3

Appendix Table 2. GBD 2017 location hierarchy with levels	
**Geography**	**Level**
Jamaica	3
Puerto Rico	3
Saint Lucia	3
Saint Vincent and the Grenadines	3
Suriname	3
Trinidad and Tobago	3
Virgin Islands, U.S.	3
Central Latin America	2
Colombia	3
Costa Rica	3
El Salvador	3
Guatemala	3
Honduras	3
Mexico	3
Aguascalientes	4
Baja California	4
Baja California Sur	4
Campeche	4
Chiapas	4
Chihuahua	4
Coahuila	4
Colima	4
Mexico City	4
Durango	4
Guanajuato	4
Guerrero	4
Hidalgo	4
Jalisco	4
México	4
Michoacán de Ocampo	4
Morelos	4
Nayarit	4
Nuevo León	4
Oaxaca	4
Puebla	4
Querétaro	4
Quintana Roo	4
San Luis Potosí	4
Sinaloa	4
Sonora	4
Tabasco	4
Tamaulipas	4
Tlaxcala	4
Veracruz de Ignacio de la Llave	4
Yucatán	4

Appendix Table 2. GBD 2017 location hierarchy with levels	
**Geography**	**Level**
Zacatecas	4
Nicaragua	3
Panama	3
Venezuela	3
Tropical Latin America	2
Brazil	3
Acre	4
Alagoas	4
Amapá	4
Amazonas	4
Bahia	4
Ceará	4
Distrito Federal	4
Espírito Santo	4
Goiás	4
Maranhão	4
Mato Grosso	4
Mato Grosso do Sul	4
Minas Gerais	4
Pará	4
Paraíba	4
Paraná	4
Pernambuco	4
Piaui	4
Rio de Janeiro	4
Rio Grande do Norte	4
Rio Grande do Sul	4
Rondônia	4
Roraima	4
Santa Catarina	4
São Paulo	4
Sergipe	4
Tocantins	4
Paraguay	3
North Africa and Middle East	1
North Africa and Middle East	2
Afghanistan	3
Algeria	3
Bahrain	3
Egypt	3
Iran	3
Iraq	3
Jordan	3
Kuwait	3
Lebanon	3

Appendix Table 2. GBD 2017 location hierarchy with levels

Geography	Level
Libya	3
Morocco	3
Palestine	3
Oman	3
Qatar	3
Saudi Arabia	3
Sudan	3
Syria	3
Tunisia	3
Turkey	3
United Arab Emirates	3
Yemen	3
South Asia	1
South Asia	2
Bangladesh	3
Bhutan	3
India	3
Andhra Pradesh	4
Arunachal Pradesh	4
Assam	4
Bihar	4
Chhattisgarh	4
Delhi	4
Goa	4
Gujarat	4
Haryana	4
Himachal Pradesh	4
Jammu and Kashmir	4
Jharkhand	4
Karnataka	4
Kerala	4
Madhya Pradesh	4
Maharashtra	4
Manipur	4
Meghalaya	4
Mizoram	4
Nagaland	4
Odisha	4
Punjab	4
Rajasthan	4
Sikkim	4
Tamil Nadu	4
Telangana	4
Tripura	4
Uttar Pradesh	4

Appendix Table 2. GBD 2017 location hierarchy with levels	
**Geography**	**Level**
Uttarakhand	4
West Bengal	4
Union Territories other than Delhi	4
Nepal	3
Pakistan	3
Southeast Asia, East Asia, and Oceania	1
East Asia	2
China	3
North Korea	3
Taiwan	3
Oceania	2
American Samoa	3
Federated States of Micronesia	3
Fiji	3
Guam	3
Kiribati	3
Marshall Islands	3
Northern Mariana Islands	3
Papua New Guinea	3
Samoa	3
Solomon Islands	3
Tonga	3
Vanuatu	3
Southeast Asia	2
Cambodia	3
Indonesia	3
Laos	3
Malaysia	3
Maldives	3
Mauritius	3
Myanmar	3
Philippines	3
Sri Lanka	3
Seychelles	3
Thailand	3
Timor-Leste	3
Vietnam	3
Sub-Saharan Africa	1
Central Sub-Saharan Africa	2
Angola	3
Central African Republic	3
Congo	3
Democratic Republic of the Congo	3
Equatorial Guinea	3
Gabon	3

Appendix Table 2. GBD 2017 location hierarchy with levels	
**Geography**	**Level**
Eastern Sub-Saharan Africa	2
Burundi	3
Comoros	3
Djibouti	3
Eritrea	3
Ethiopia	3
Kenya	3
Baringo	4
Bomet	4
Bungoma	4
Busia	4
Elgeyo-Marakwet	4
Embu	4
Garissa	4
HomaBay	4
Isiolo	4
Kajiado	4
Kakamega	4
Kericho	4
Kiambu	4
Kilifi	4
Kirinyaga	4
Kisii	4
Kisumu	4
Kitui	4
Kwale	4
Laikipia	4
Lamu	4
Machakos	4
Makueni	4
Mandera	4
Marsabit	4
Meru	4
Migori	4
Mombasa	4
Murang’a	4
Nairobi	4
Nakuru	4
Nandi	4
Narok	4
Nyamira	4
Nyandarua	4
Nyeri	4
Samburu	4
Siaya	4

Appendix Table 2. GBD 2017 location hierarchy with levels	
**Geography**	**Level**
TaitaTaveta	4
TanaRiver	4
TharakaNithi	4
TransNzoia	4
Turkana	4
UasinGishu	4
Vihiga	4
Wajir	4
WestPokot	4
Madagascar	3
Malawi	3
Mozambique	3
Rwanda	3
Somalia	3
South Sudan	3
Tanzania	3
Uganda	3
Zambia	3
Southern Sub-Saharan Africa	2
Botswana	3
Lesotho	3
Namibia	3
South Africa	3
Swaziland	3
Zimbabwe	3
Western Sub-Saharan Africa	2
Benin	3
Burkina Faso	3
Cameroon	3
Cape Verde	3
Chad	3
Cote d'Ivoire	3
The Gambia	3
Ghana	3
Guinea	3
Guinea-Bissau	3
Liberia	3
Mali	3
Mauritania	3
Niger	3
Nigeria	3
Sao Tome and Principe	3
Senegal	3
Sierra Leone	3
Togo	3

Appendix Table 3. Total number of site years by cause and source type for GBD 2017

Cause	Level	Vital Registration	Vital Registration - Sample	Verbal Autopsy	Surveillance	Survey/Census	Sibling History	Cancer Registry	Police Records
All causes	0	19377	793	3095	2261	1144	4746	5545	1567
Communicable, maternal, neonatal, and nutritional diseases	1	19377	793	2777	2261	1078	4746		
HIV/AIDS and sexually transmitted infections	2	19331	793	527	411				
HIV/AIDS	3	19290	793	477	411				
HIV-AIDS - Drug-susceptible Tuberculosis	4	9706	416		11				
HIV/AIDS resulting in other diseases	4	16206	416		58				
Sexually transmitted diseases excluding HIV	3	19325	793	405	239				
Syphilis	4	18852	793		239				
Chlamydial infection	4	17624	416						
Gonococcal infection	4	18551	793						
Other sexually transmitted diseases	4	18840	416						
Respiratory infections and tuberculosis	2	19343	793	1790	560				
Tuberculosis	3	19343	793	1543	360				
Drug-susceptible tuberculosis	4	16189	416						
Multidrug-resistant tuberculosis without extensive drug resistance	4	9646	416						
Lower respiratory infections	3	19343	793	1748	560				
Influenza	4	16471	416						
Pneumococcal pneumonia	4	16331	416						
H influenzae type B pneumonia	4	16331	416						
Respiratory syncytial virus pneumonia	4	16189	416						
Other lower respiratory infections	4	16331	416						
Upper respiratory infections	3	18434	793						
Otitis media	3	17865	793	150					
Enteric infections	2	19343	793	1744	509				
Diarrheal diseases	3	19343	793	1735	509				
Cholera	4	16508	416						
Other salmonella infections	4	16351	416						
Shigellosis	4	16333	416						
Enteropathogenic E coli infection	4	9649	416						
Enterotoxigenic E coli infection	4	9649	416						
Campylobacter enteritis	4	9649	416						
Amoebiasis	4	16330	416						
Cryptosporidiosis	4	16189	416						
Rotaviral enteritis	4	9649	416						
Aeromonas	4	6540							
Clostridium difficile	4	16331	416						
Norovirus	4	9649	416						
Adenovirus	4	9649	416						
Other bacterial foodborne diarrhea	4	16353	416						
Other diarrheal diseases	4	16471	416						
Typhoid and paratyphoid	3	19324	793	420					
Typhoid fever	4	17673	416						
Paratyphoid fever	4	17043	416						
Invasive Non-typhoidal Salmonella (iNTS)	3	17239	416						
Poliomyelitis	3	18262	416	146					
Other intestinal infectious diseases	3	18262	416	146					
Neglected tropical diseases and malaria	2	19347	793	1525	289				
Malaria	3	19289	793	1388	1				
Chagas disease	3	6494	325						
Leishmaniasis	3	18689	793	353					
Visceral leishmaniasis	4	18134	793	353					
African trypanosomiasis	3	16957	416	148					
Schistosomiasis	3	17143	416						
Cysticercosis	3	18110	793						
Cystic echinococcosis	3	18256	793						
Dengue	3	18434	416	345	1				
Yellow fever	3	17145	416	160					
Rabies	3	18997	793	970	278				
Intestinal nematode infections	3	18841	793	287					
Ascariasis	4	16956	416						
Ebola	3	268	52		9				
Zika virus	3	10274	416						
Other neglected tropical diseases	3	17698	793						
Other infectious diseases	2	19343	793	1668	807				
Meningitis	3	19331	793	1470	546				
Pneumococcal meningitis	4	16753	416						
H influenzae type B meningitis	4	16760	416						
Meningococcal meningitis	4	17864	416						
Other meningitis	4	16760	416						
Encephalitis	3	19028	793	395					
Diphtheria	3	19046	793						
Whooping cough	3	19337	793	589					
Tetanus	3	19198	793	1295	393				
Measles	3	19332	793	1151	524				
Varicella and herpes zoster	3	18227	793	377					
Acute hepatitis	3	19336	793	1189					
Acute hepatitis A	4	16618	416						
Acute hepatitis B	4	16615	416						
Acute hepatitis C	4	16608	416						
Acute hepatitis E	4	10068	416						
Other unspecified infectious diseases	3	19053	793	1089	804				
Maternal and neonatal disorders	2	19370	793	2526	1903	1078	4746		
Maternal disorders	3	19367	793	2120	1345	1078	4746		
Maternal hemorrhage	4	19139	793	1097	848	9	1		
Maternal sepsis and other maternal infections	4	18839	793	804	544	7	1		
Maternal hypertensive disorders	4	19144	793	895	823	9	1		
Maternal obstructed labor and uterine rupture	4	18843	793	800	615	8	1		
Maternal abortion and miscarriage	4	19161	793	333	400	7	1		
Ectopic pregnancy	4	18239	793	70	515		1		
Indirect maternal deaths	4	18822	793	798	923	9	1		
Late maternal deaths	4	10922	416		152				
Maternal deaths aggravated by HIV/AIDS	4	19367	793	2120	1345	1078	4746		
Other maternal disorders	4	18856	793	290	771	4	1		
Neonatal disorders	3	19345	791	1597	558				
Neonatal preterm birth	4	18225	791	511	558				
Neonatal encephalopathy due to birth asphyxia and trauma	4	18218	791	497	558				
Neonatal sepsis and other neonatal infections	4	18175	791	165	546				
Hemolytic disease and other neonatal jaundice	4	18204	791	347					
Other neonatal disorders	4	18205	791	173	552				
Nutritional deficiencies	2	19343	793	1414	523				
Protein-energy malnutrition	3	19002	793	1366					
Dietary iron deficiency	3			741					
Other nutritional deficiencies	3	18223	793						
Non-communicable diseases	1	19353	793	1814	632			5545	64
Neoplasms	2	19327	793	1460	624			5545	
Lip and oral cavity cancer	3	18987	793	405				4627	
Nasopharynx cancer	3	18985	793					5490	
Other pharynx cancer	3	18985	793	187				5478	
Esophageal cancer	3	19323	793	419				5497	
Stomach cancer	3	19327	793	187				5518	
Colon and rectum cancer	3	19327	793	432				5518	
Liver cancer	3	18999	793	187				5508	
Gallbladder and biliary tract cancer	3	18392	793					5465	
Pancreatic cancer	3	18986	793					5516	
Larynx cancer	3	19323	793	187				5503	
Tracheal, bronchus, and lung cancer	3	19327	793	424				5503	
Malignant skin melanoma	3	18987	793					5395	
Non-melanoma skin cancer	3	18994	793						
Non-melanoma skin cancer (squamous-cell carcinoma)	4	18393	793						
Breast cancer	3	19327	793	445	1			5507	
Cervical cancer	3	19327	793	183				5334	
Uterine cancer	3	19313	793	185				5382	
Ovarian cancer	3	18983	793					5390	
Prostate cancer	3	19324	793					5376	
Testicular cancer	3	18262	793	132				5352	
Kidney cancer	3	18983	793					5322	
Bladder cancer	3	18985	793					5369	

Appendix Table 3. Total number of site years by cause and source type for GBD 2017

Cause	Level	Vital Registration	Vital Registration - Sample	Verbal Autopsy	Surveillance	Survey/Census	Sibling History	Cancer Registry	Police Records
Brain and nervous system cancer	3	18986	793	404				5493	
Thyroid cancer	3	18984	793					5501	
Mesothelioma	3	10942	416						
Hodgkin lymphoma	3	18984	793					5437	
Non-Hodgkin lymphoma	3	18986	793					5472	
Multiple myeloma	3	18983	793					5464	
Leukemia	3	19326	793	187	551			5511	
Acute lymphoid leukemia	4	16294	416					2649	
Chronic lymphoid leukemia	4	16214	403					2625	
Acute myeloid leukemia	4	16288	416					3452	
Chronic myeloid leukemia	4	16248	416					2637	
Other leukemia	4	16748	416					4436	
Other malignant neoplasms	3	19041	793	403	72			5524	
Other neoplasms	3	16750	416						
Myelodysplastic, myeloproliferative, and other hematopoietic neoplasms	4	16608	416						
Other benign and in situ neoplasms	4	16608	416						
Cardiovascular diseases	2	19353	793	1656	2				
Rheumatic heart disease	3	19329	793	443					
Ischemic heart disease	3	19341	793	1514					
Stroke	3	19341	793	1369	1				
Ischemic stroke	4	18259	793						
Intracerebral hemorrhage	4	18252	793						
Subarachnoid hemorrhage	4	18252	793						
Hypertensive heart disease	3	18275	793						
Non-rheumatic valvular heart disease	3	17754	793						
Non-rheumatic calcific aortic valve disease	4	17199	416						
Non-rheumatic degenerative mitral valve disease	4	17199	416						
Other non-rheumatic valve diseases	4	17199	416						
Cardiomyopathy and myocarditis	3	18394	793						
Myocarditis	4	17193	416						
Alcoholic cardiomyopathy	4	17193	416						
Other cardiomyopathy	4	17193	416						
Atrial fibrillation and flutter	3	16753	416						
Aortic aneurysm	3	17634	416						
Peripheral artery disease	3	16750	416						
Endocarditis	3	17625	793						
Other cardiovascular and circulatory diseases	3	18275	793	405					
Chronic respiratory diseases	2	19343	793	1568	517				
Chronic obstructive pulmonary disease	3	17631	793						
Pneumoconiosis	3	17631	416						
Silicosis	4	17624	416						
Asbestosis	4	17624	416						
Coal workers pneumoconiosis	4	17482	416						
Other pneumoconiosis	4	17624	416						
Asthma	3	17621	416						
Interstitial lung disease and pulmonary sarcoidosis	3	17624	416						
Other chronic respiratory diseases	3	17631	793						
Digestive diseases	2	19329	793	1406	546				
Cirrhosis and other chronic liver diseases	3	19329	793	1263					
Upper digestive system diseases	3	19315	793	347					
Peptic ulcer disease	4	17939	416						
Gastritis and duodenitis	4	17924	416						
Appendicitis	3	19325	793	187					
Paralytic ileus and intestinal obstruction	3	18984	793	378					
Inguinal, femoral, and abdominal hernia	3	18854	416	679					
Inflammatory bowel disease	3	17628	416						
Vascular intestinal disorders	3	17621	416						
Gallbladder and biliary diseases	3	19149	793						
Pancreatitis	3	17933	416						
Other digestive diseases	3	18850	793						
Neurological disorders	2	19284	793	920					
Alzheimer disease and other dementias	3	18986	793	32					
Parkinson disease	3	18119	793						
Epilepsy	3	19148	793	918					
Multiple sclerosis	3	18382	744						
Motor neuron disease	3	16750	416						
Other neurological disorders	3	17628	416						
Mental disorders	2	17057	793						
Eating disorders	3	14044	351						
Anorexia nervosa	4	12755	351						
Bulimia nervosa	4	2177	65						
Substance use disorders	2	19261	793	317					64
Alcohol use disorders	3	19261	793	315					
Drug use disorders	3	19260	793	302					64
Opioid use disorders	4	17202	416						
Cocaine use disorders	4	17202	416						
Amphetamine use disorders	4	17202	416						
Other drug use disorders	4	17202	416						
Diabetes and kidney diseases	2	19329	793	1494					
Diabetes mellitus	3	19329	793	1261					
Diabetes mellitus type 1	4	17482	416						
Diabetes mellitus type 2	4	17479	416						
Chronic kidney disease	3	19329	793	1402					
Chronic kidney disease due to diabetes mellitus type 1	4	16611	416						
Chronic kidney disease due to diabetes mellitus type 2	4	16611	416						
Chronic kidney disease due to hypertension	4	16331	416						
Chronic kidney disease due to glomerulonephritis	4	16331	416						
Chronic kidney disease due to other causes	4	16331	416						
Acute glomerulonephritis	3	18748	793						
Skin and subcutaneous diseases	2	19302	793	421					
Bacterial skin diseases	3	18977	793	389					
Cellulitis	4	16753	416						
Pyoderma	4	16755	416						
Decubitus ulcer	3	17621	416	221					
Other skin and subcutaneous diseases	3	16750	416						
Musculoskeletal disorders	2	18983	793	404					
Rheumatoid arthritis	3	17621	793						
Other musculoskeletal disorders	3	17632	793						
Other non-communicable diseases	2	19353	793	1433	559				
Congenital birth defects	3	19353	793	1342	559				
Neural tube defects	4	19176	793	104	548				
Congenital heart anomalies	4	19173	793	424	557				
Orofacial clefts	4	17277	416						
Down syndrome	4	17628	416		540				
Other chromosomal abnormalities	4	17621	416						
Congenital musculoskeletal and limb anomalies	4	16608	416						
Urogenital congenital anomalies	4	16753	416		481				
Digestive congenital anomalies	4	16750	416						
Other congenital birth defects	4	17621	793	370					
Urinary diseases and male infertility	3	19323	793	405					
Urinary tract infections	4	18694	793	183					
Urolithiasis	4	17639	793	170					
Other urinary diseases	4	16755	793						
Gynecological diseases	3	19129	793	671					
Uterine fibroids	4	16753	416	101					
Polycystic ovarian syndrome	4	16567	416						
Endometriosis	4	16585	416						
Genital prolapse	4	16750	416						
Other gynecological diseases	4	16611	416						
Hemoglobinopathies and hemolytic anemias	3	19307	793	1059					
Thalassemias	4	17931	793	214					
Sickle cell disorders	4	17631	793	416					
G6PD deficiency	4	6390	351						
Other hemoglobinopathies and hemolytic anemias	4	17628	793						
Endocrine, metabolic, blood, and immune disorders	3	19311	793	776					
Sudden infant death syndrome	3	14934	39						
Injuries	1	19329	793	1852	562	292			1567

Appendix Table 3. Total number of site years by cause and source type for GBD 2017

Cause	Level	Vital Registration	Vital Registration - Sample	Verbal Autopsy	Surveillance	Survey/Census	Sibling History	Cancer Registry	Police Records
Transport injuries	2	19329	793	1464	557	265			75
Road injuries	3	18563	793	188		13			75
Pedestrian road injuries	4	16753	416	188					70
Cyclist road injuries	4	16753	416	188					45
Motorcyclist road injuries	4	16753	416	187		2			52
Motor vehicle road injuries	4	16753	416	188		9			63
Other road injuries	4	16753	416	188					31
Other transport injuries	3	17392	416	188					2
Unintentional injuries	2	19327	793	1588	558	13			223
Falls	3	19327	793	1192	554	5			
Drowning	3	19315	793	1357	553	2			223
Fire, heat, and hot substances	3	19326	793	1344		1			191
Poisonings	3	19286	793	456	523				188
Poisoning by carbon monoxide	4	18487	793	129					188
Poisoning by other means	4	16563	793						
Exposure to mechanical forces	3	19038	793	380	558	8			
Unintentional firearm injuries	4	17973	793	358		1			
Other exposure to mechanical forces	4	17381	793	373	558	4			
Adverse effects of medical treatment	3	19026	793	187					
Animal contact	3	18694	793	1151		1			
Venomous animal contact	4	17060	793	443					
Non-venomous animal contact	4	16753	793						
Foreign body	3	18395	793	410					
Pulmonary aspiration and foreign body in airway	4	16753	793	181					
Foreign body in other body part	4	16753	793						
Environmental heat and cold exposure	3	18685	793	338					
Exposure to forces of nature	3	18998	793	328		6			
Other unintentional injuries	3	18459	793	411		1			
Self-harm and interpersonal violence	2	19329	793	1582	1	14			1269
Self-harm	3	19329	793	1522		3			
Self-harm by firearm	4	16563	416						
Self-harm by other specified means	4	16563	416						
Interpersonal violence	3	19329	793	1331		9			1269
Physical violence by firearm	4	16753	416	352		5			
Physical violence by sharp object	4	16753	416	360		1			
Physical violence by other means	4	16753	416	359					
Conflict and terrorism	3	18839	793	341		3			
Executions and police conflict	3	18200	793	156					

Appendix Table 4. List of International Classification of Diseases (ICD) codes mapped to the Global Burden of Disease cause list for causes of death

Cause	ICD10	ICD9
Communicable, maternal, neonatal, and nutritional diseases	A00-A00.9, A01.0-A14, A15-A28.9, A32-A39.9, A48.1-A48.2, A48.4-A48.5, A50-A58, A60-A60.9, A63-A63.8, A65-A65.0, A68-A70, A74, A74.8-A75.9, A77-A96.9, A98-A98.8, B00-B06.9, B10-B10.8, B15-B17.9, B19-B27.9, B29.4, B33-B33.1, B33.3-B33.8, B47-B48.8, B50-B53.8, B55.0, B56-B57.5, B60-B60.8, B63, B65-B67.9, B69-B72.0, B74.3-B75, B77-B77.9, B83-B83.8, B90-B91, B94.1-B94.2, B95-B95.5, B97.4-B97.6, D50.1-D50.8, D51-D52.0, D52.8-D53.9, E00-E02, E40-E46.9, E51-E61.9, E63-E64.0, E64.2-E64.9, F07.1, G00.0-G00.8, G03-G03.8, G04-G05.8, G14-G14.6, G21.3, H70-H70.9, I00, I02, I02.9, I98.0-I98.1, J00-J02.8, J03-J03.8, J04-J04.2, J05-J05.1, J06.0-J06.8, J09-J15.8, J16-J16.9, J20-J21.9, J36-J36.0, K67.0-K67.8, K75.3, K76.3, K77.0, K93.0-K93.1, M03.1, M12.1, M49.0-M49.1, M73.0-M73.1, M89.6, N74.1, N96, N98-N98.9, O00-O07.9, O09-O16.9, O20-O26.9, O28-O36.9, O40-O48.1, O60-O77.9, O80-O92.7, O96-O98.6, O98.8-P04.2, P04.5-P05.9, P07-P15.9, P19-P22.9, P23.0-P23.4, P24-P29.9, P35-P37.2, P37.5-P39.9, P50-P61.9, P70-P70.1, P70.3-P72.9, P74-P78.9, P80-P81.9, P83-P84, P90-P94.9, P96, P96.3-P96.4, P96.8, R19.7, U04-U04.9, U06-U06.9, U82-U89, Z16-Z16.3	001-001.9, 002.0-029, 032-034.9, 036-036.3, 036.5-037.9, 040, 040.1-041.0, 042-066.9, 070-075.9, 078.3-078.7, 079-079.7, 080-083.9, 084.0-084.5, 084.7-084.9, 085.0, 086-088, 088.8-088.9, 090-101.6, 104-104.9, 120-124.9, 125.4-125.9, 127-127.1, 128-129.0, 136-136.2, 137-139.0, 244.2, 260-263.9, 265-269.9, 281.0-281.9, 320.0-320.8, 321-323.9, 381-383.9, 390-390.9, 392, 392.9, 425.6, 460-464.4, 464.8-464.9, 465.0-465.8, 466-469, 470.0, 475-475.9, 476.9, 480-482.8, 483.0-483.9, 484.0-484.7, 487-489, 630-636.9, 638-638.9, 640-679.1, 716.0, 730.4-730.6, 760-760.6, 760.8-768, 768.2-770, 770.1-775.0, 775.4-779.3, 779.6-779.8, V09-V09.9
HIV/AIDS and sexually transmitted infections	A50-A58, A60-A60.9, A63-A63.8, B20-B24.9, B63, I98.0, K67.0-K67.2, M03.1, M73.0-M73.1	042-044.9, 054.1, 090-099.9
HIV/AIDS	B20-B24.9	042-044.9
HIV-AIDS – Drug-susceptible Tuberculosis	B20.0	
HIV/AIDS - Multidrug-resistant Tuberculosis without extensive drug resistance		
HIV/AIDS - Extensively drug-resistant Tuberculosis		
HIV/AIDS resulting in other diseases	B20.1-B23.9, B24.0	042-044.9
Sexually transmitted diseases excluding HIV	A50-A58, A60-A60.9, A63-A63.8, B63, I98.0, K67.0-K67.2, M03.1, M73.0-M73.1	054.1, 090-099.9
Syphilis	A50-A53.9, I98.0, K67.2, M03.1, M73.1	090-097.9
Chlamydial infection	A55-A56.8, K67.0	099
Gonococcal infection	A54-A54.9, K67.1, M73.0	098-098.9
Other sexually transmitted diseases	A57-A58, A63-A63.8, B63	099.0-099.9
Respiratory infections and tuberculosis	A10-A14, A15-A19.9, A48.1, A70, B90-B90.9, B97.4-B97.6, H70-H70.9, J00-J02.8, J03-J03.8, J04-J04.2, J05-J05.1, J06.0-J06.8, J09-J15.8, J16-J16.9, J20-J21.9, J36-J36.0, K67.3, K93.0, M49.0, N74.1, P23.0-P23.4, P37.0, U04-U04.9, U84.3	010-019.9, 034.0, 079.6, 137-137.9, 138.0-138.9, 381-383.9, 460-464.4, 464.8-464.9, 465.0-465.8, 466-469, 470.0, 475-475.9, 476.9, 480-482.8, 483.0-483.9, 484.1-484.2, 484.6-484.7, 487-489, 730.4-730.6
Tuberculosis	A15-A19.9, B90-B90.9, K67.3, K93.0, M49.0, N74.1, P37.0, U84.3	010-019.9, 137-137.9, 138.0-138.9, 730.4-730.6
Drug-susceptible tuberculosis	A15-A19.9, B90-B90.9, K67.3, K93.0, M49.0, N74.1, P37.0	010-019.9, 137-137.9, 138.0-138.9, 730.4-730.6
Multidrug-resistant tuberculosis without extensive drug resistance	U84.3	
Extensively drug-resistant tuberculosis		
Lower respiratory infections	A48.1, A70, B97.4-B97.6, J09-J15.8, J16-J16.9, J20-J21.9, P23.0-P23.4, U04-U04.9	079.6, 466-469, 470.0, 480-482.8, 483.0-483.9, 484.1-484.2, 484.6-484.7, 487-489
Influenza	J09-J11.8, U04-U04.9	079.6, 487-489
Pneumococcal pneumonia	J13-J13.9, J15.3-J15.4, J15.6	481-481.9
H influenzae type B pneumonia	J14-J14.0	482.2
Respiratory syncytial virus pneumonia	B97.4-B97.6, J12.1	480.1
Other lower respiratory infections	A48.1, A70, J12-J12.0, J12.2-J12.9, J15-J15.2, J15.5, J15.7-J15.8, J16-J16.9, J20-J21.9, P23.0-P23.4	466-469, 470.0, 480-480.0, 480.2-480.9, 482-482.1, 482.3-482.8, 483.0-483.9, 484.1-484.2, 484.6-484.7
Upper respiratory infections	J00-J02.8, J03-J03.8, J04-J04.2, J05-J05.1, J06.0-J06.8, J36-J36.0	034.0, 460-464.4, 464.8-464.9, 465.0-465.8, 475-475.9, 476.9
Otitis media	H70-H70.9	381-383.9
Enteric infections	A00-A00.9, A01.0-A09.9, A80-A80.9, R19.7	001-001.9, 002.0-009.9, 045-045.9, 138
Diarrheal diseases	A00-A00.9, A02-A02.0, A02.8-A07, A07.2-A07.4, A08-A09.9, R19.7	001-001.9, 003.8-006.9, 007.4-007.8, 008.2-009.9
Cholera	A00-A00.9	001-001.9
Other salmonella infections	A02-A02.0, A02.8-A02.9	003.8-003.9
Shigellosis	A03-A03.9	004-004.9
Enteropathogenic E coli infection	A04.0	
Enterotoxigenic E coli infection	A04.1-A04.4	
Campylobacter enteritis	A04.5	
Amoebiasis	A06-A06.9	006-006.9
Cryptosporidiosis	A07.2	007.4-007.7
Rotaviral enteritis	A08.0	
Aeromonas		008.2
Clostridium difficile	A04.7	
Norovirus	A08.1	
Adenovirus	A08.2	
Other bacterial foodborne diarrhea	A05-A05.9	005-005.9
Other diarrheal diseases	A04, A04.6, A04.8-A04.9, A07, A07.3-A07.4, A08, A08.3-A09.9, R19.7	007.8, 008.3-009.9
Typhoid and paratyphoid	A01.0-A01.4	002.0-002.9
Typhoid fever	A01.0	002.0

Cause	ICD10	ICD9
Paratyphoid fever	A01.1-A01.4	002.1-002.9
Invasive Non-typhoidal Salmonella (iNTS)	A02.1-A02.2	003-003.7
Poliomyelitis	A80-A80.9	045-045.9, 138
Other intestinal infectious diseases	A07.0-A07.1, A07.8-A07.9	007-007.3, 007.9-008.1
Neglected tropical diseases and malaria	A68-A68.9, A69.2-A69.9, A75-A75.9, A77-A79.9, A82-A82.9, A90-A96.9, A98-A98.8, B33.0-B33.1, B50-B53.8, B55.0, B56-B57.5, B60-B60.8, B65-B67.9, B69-B72.0, B74.3-B75, B77-B77.9, B83-B83.8, K93.1, P37.1, U06-U06.9	060-061.8, 065-066.9, 071-071.9, 080-083.9, 084.0-084.5, 084.7-084.9, 085.0, 086-088, 088.8-088.9, 120-124.9, 125.4-125.9, 127-127.1, 128-129.0, 425.6
Malaria	B50-B53.8	084.0-084.5, 084.7-084.9
Leprosy	A30-A30.9	030-030.9
Chagas disease	B57-B57.5, K93.1	086-086.2, 086.9, 425.6
Leishmaniasis	B55.0	085.0
Visceral leishmaniasis	B55.0	085.0
African trypanosomiasis	B56-B56.9	086.3-086.5
Schistosomiasis	B65-B65.9	120-120.9
Cysticercosis	B69-B69.9	123.1
Cystic echinococcosis	B67-B67.4, B67.8-B67.9	122-122.4, 122.8-122.9
Dengue	A90-A91.9	061-061.8
Yellow fever	A95-A95.9	060-060.9
Rabies	A82-A82.9	071-071.9
Intestinal nematode infections	B77-B77.9	127.0
Ascariasis	B77-B77.9	127.0
Ebola	A98.4	
Zika virus	U06-U06.9	
Other neglected tropical diseases	A68-A68.9, A69.2-A69.9, A75-A75.9, A77-A79.9, A92-A94.0, A96-A96.9, A98-A98.3, A98.5-A98.8, B33.0-B33.1, B60-B60.8, B67.5-B67.7, B70-B71.9, B74.3-B75, B83-B83.8, P37.1	065-066.9, 080-083.9, 087-088, 088.8-088.9, 122.5-122.7, 123-123.0, 123.2-124.9, 125.4-125.6, 125.9, 127, 127.1, 128-129.0
Other infectious diseases	A20-A28.9, A32-A39.9, A48.2, A48.4-A48.5, A65-A65.0, A69-A69.1, A74, A74.8-A74.9, A81-A81.9, A83-A89.9, B00-B06.9, B10-B10.8, B15-B17.9, B19-B19.9, B25-B27.9, B29.4, B33, B33.3-B33.8, B47-B48.8, B91, B94.1-B94.2, B95-B95.5, F07.1, G00.0-G00.8, G03-G03.8, G04-G05.8, G14-G14.6, G21.3, I00, I02, I02.9, I98.1, K67.8, K75.3, K76.3, K77.0, M49.1, M89.6, P35-P35.9, P37, P37.2, P37.5-P37.9, U82-U84, U85-U89, Z16-Z16.3	020-029, 032-034, 034.1-034.9, 036-036.3, 036.5-037.9, 040, 040.1-041.0, 046-054.0, 054.2-059.9, 062-064.9, 070-070.9, 072-075.9, 078.3-078.7, 079-079.5, 079.7, 100-101.6, 104-104.9, 136-136.2, 139-139.0, 320.0-320.8, 321-323.9, 390-390.9, 392, 392.9, 484.0, 484.3-484.5, 771.0-771.3, V09-V09.9
Meningitis	A39-A39.9, A87-A87.9, G00.0-G00.8, G03-G03.8	036-036.3, 036.5-036.9, 047-049.9, 320.0-320.8, 321-322.9
Pneumococcal meningitis	G00.1	320.1
H influenzae type B meningitis	G00.0	320.0
Meningococcal meningitis	A39-A39.9	036-036.3, 036.5-036.9
Other meningitis	A87-A87.9, G00.2-G00.8, G03-G03.8	047-049.9, 320.2-320.8, 321-322.9
Encephalitis	A83-A86.4, B94.1, F07.1, G04-G05.8, G21.3	062-064.9, 139.0, 323, 323.4-323.9
Diphtheria	A36-A36.9	032-032.9
Whooping cough	A37-A37.9	033-033.9, 484.3
Tetanus	A33-A35.0	037-037.9, 771.3
Measles	B05-B05.9	055-055.9, 484.0
Varicella and herpes zoster	B01-B02.9, P35.8	052-053.9
Acute hepatitis	B15-B17.9, B19-B19.9, B94.2, P35.3	070-070.9
Acute hepatitis A	B15-B15.9	070.0-070.1
Acute hepatitis B	B16-B16.9, B17.0, B19.1, P35.3	070.2-070.3
Acute hepatitis C	B17.1, B19.2	070.7
Acute hepatitis E	B17.2	
Other unspecified infectious diseases	A20-A28.9, A32-A32.9, A38-A38.9, A48.2, A48.4-A48.5, A65-A65.0, A69-A69.1, A74, A74.8-A74.9, A81-A81.9, A88-A89.9, B00-B00.9, B03-B04, B06-B06.9, B10-B10.8, B25-B27.9, B29.4, B33, B33.3-B33.8, B47-B48.8, B91, B95-B95.5, G14-G14.6, I00, I02, I02.9, I98.1, K67.8, K75.3, K76.3, K77.0, M49.1, M89.6, P35-P35.2, P35.9, P37, P37.2, P37.5-P37.9, U82-U84, U85-U89, Z16-Z16.3	020-029, 034, 034.1-034.9, 040, 040.1-041.0, 046-046.9, 050-051.9, 054-054.0, 054.2-054.9, 056-059.9, 072-075.9, 078.3-078.7, 079-079.5, 079.7, 100-101.6, 104-104.9, 136-136.2, 139, 323.0-323.3, 390-390.9, 392, 392.9, 484.4-484.5, 771.0-771.2, V09-V09.9
Maternal and neonatal disorders	N96, N98-N98.9, O00-O07.9, O09-O16.9, O20-O26.9, O28-O36.9, O40-O48.1, O60-O77.9, O80-O92.7, O96-O98.6, O98.8-P04.2, P04.5-P05.9, P07-P15.9, P19-P22.9, P24-P29.9, P36-P36.9, P38-P39.9, P50-P61.9, P70-P70.1, P70.3-P72.9, P74-P78.9, P80-P81.9, P83-P84, P90-P94.9, P96, P96.3-P96.4, P96.8	630-636.9, 638-638.9, 640-679.1, 760-760.6, 760.8-768, 768.2-770, 770.1-771, 771.4-775.0, 775.4-779.3, 779.6-779.8
Maternal disorders	N96, N98-N98.9, O00-O07.9, O09-O16.9, O20-O26.9, O28-O36.9, O40-O48.1, O60-O77.9, O80-O92.7, O96-O98.6, O98.8-O99.9	630-636.9, 638-638.9, 640-679.1
Maternal hemorrhage	O20-O20.9, O43.2, O44-O46.9, O62-O62.9, O67-O67.9, O70, O72-O72.3	640-641.9, 661-661.9, 665, 666-666.9
Maternal sepsis and other maternal infections	O23-O23.9, O85-O86.8, O91-O91.2	659.3, 670-670.9
Maternal hypertensive disorders	O10-O16.9	642-642.9
Maternal obstructed labor and uterine rupture	O32-O33.9, O64-O66.9, O71-O71.9	652-653.9, 660-660.9, 665.0-665.3
Maternal abortion and miscarriage	N96, O01-O07.9	630-632.9, 634-636.9, 638-638.9, 646.3
Ectopic pregnancy	O00-O00.9	633-633.9

Cause	ICD10	ICD9
Indirect maternal deaths	O24-O25.3, O98-O98.6, O98.8-O99.9	646-646.2, 646.4-649.9
Late maternal deaths	O96-O97.9	
Maternal deaths aggravated by HIV/AIDS		
Other maternal disorders	N98-N98.9, O09-O09.9, O21-O22.9, O26-O26.9, O28-O31.8, O34-O36.9, O40-O43.1, O43.8-O43.9, O47-O48.1, O60-O61.9, O63-O63.9, O68-O69.9, O70.0-O70.9, O73-O77.9, O80-O84.9, O87-O90.9, O92-O92.7	643-645.2, 650-651.9, 654-659.2, 659.4-659.9, 662-664.9, 665.4-665.9, 667-669.9, 671-679.1
Neonatal disorders	P00-P04.2, P04.5-P05.9, P07-P15.9, P19-P22.9, P24-P29.9, P36-P36.9, P38-P39.9, P50-P61.9, P70-P70.1, P70.3-P72.9, P74-P78.9, P80-P81.9, P83-P84, P90-P94.9, P96, P96.3-P96.4, P96.8	760-760.6, 760.8-768, 768.2-770, 770.1-771, 771.4-775.0, 775.4-779.3, 779.6-779.8
Neonatal preterm birth	P01.0-P01.1, P07-P07.3, P22-P22.9, P25-P28.9, P61.2, P77-P77.9	761.0-761.1, 765-765.9, 769-769.9, 770.2-770.9, 776.6, 777.5-777.6
Neonatal encephalopathy due to birth asphyxia and trauma	P01.7, P02-P03.9, P10-P15.9, P20-P21.9, P24-P24.9, P90-P91.9	761.7-763.9, 767-768, 768.2-768.9, 770.1, 772.1-772.9, 779.0-779.2
Neonatal sepsis and other neonatal infections	P36-P36.9, P38-P39.9	771.4-771.9
Hemolytic disease and other neonatal jaundice	P55-P59.9	773-774.9
Other neonatal disorders	P00-P01, P01.2-P01.6, P01.8-P01.9, P04-P04.2, P04.5-P05.9, P08-P09, P19-P19.9, P29-P29.9, P50-P54.9, P60-P61.1, P61.3-P61.9, P70-P70.1, P70.3-P72.9, P74-P76.9, P78-P78.9, P80-P81.9, P83-P84, P92-P94.9, P96, P96.3-P96.4, P96.8	760-760.6, 760.8-761, 761.2-761.6, 764-764.9, 766-766.9, 770, 771, 772-772.0, 775-775.0, 775.4-776.5, 776.7-777.4, 777.7-779, 779.3, 779.6-779.8
Nutritional deficiencies	D50.1-D50.8, D51-D52.0, D52.8-D53.9, E00-E02, E40-E46.9, E51-E61.9, E63-E64.0, E64.2-E64.9, M12.1	244.2, 260-263.9, 265-269.9, 281.0-281.9, 716.0
Protein-energy malnutrition	E40-E46.9, E64.0	260-263.9
Iodine deficiency		
Iron-deficiency anemia	D50.1-D50.8	
Other nutritional deficiencies	D51-D52.0, D52.8-D53.9, E00-E02, E51-E61.9, E63-E64, E64.2-E64.9, M12.1	244.2, 265-269.9, 281.0-281.9, 716.0
Non-communicable diseases	A46-A46.0, A66-A67.9, B18-B18.9, B33.2, B86, C00-C13.9, C15-C25.9, C30-C34.9, C37-C38.8, C40-C41.9, C43-C45.9, C47-C54.9, C56-C57.8, C58-C58.0, C60-C63.8, C64-C67.9, C68.0-C68.8, C69-C75.8, C81-C86.6, C88-C96.9, D00.1-D00.2, D01.0-D01.3, D02.0-D02.3, D03-D06.9, D07.0-D07.2, D07.4-D07.5, D09.0, D09.2-D09.3, D09.8, D10.0-D10.7, D11-D12.9, D13.0-D13.7, D14.0-D14.3, D15-D16.9, D22-D27.9, D28.0-D28.7, D29.0-D29.8, D30.0-D30.8, D31-D36, D36.1-D36.7, D37.1-D37.5, D38.0-D38.5, D39.1-D39.2, D39.8, D40.0-D40.8, D41.0-D41.8, D42-D43.9, D44.0-D44.8, D45-D47.9, D48.0-D48.6, D49.2-D49.4, D49.6, D52.1, D55-D58.9, D59.0-D59.3, D59.5-D59.6, D60-D61.9, D63.1, D64.0, D66-D67, D68.0-D69.8, D70-D75.8, D76-D78.8, D86-D86.9, D89-D89.3, E03-E07.1, E09-E11.9, E15.0, E16.0-E16.9, E20-E34.8, E36-E36.8, E65-E68, E70-E85.2, E88-E89.9, F00-F03.9, F10-F16.9, F18-F19.9, F24, F50.0-F50.5, G10-G13.8, G20-G20.9, G23-G26.0, G30-G31.9, G35-G37.9, G40-G41.9, G45-G46.8, G47.3, G61-G61.9, G70-G73.7, G90-G90.9, G93.7, G95-G95.9, G97-G97.9, H05.0-H05.1, I01-I01.9, I02.0, I05-I09.9, I11-I13.9, I20-I25.9, I27.1, I28-I28.8, I30-I31.1, I31.8-I37.8, I38-I41.9, I42.1-I42.8, I43-I43.9, I47-I48.9, I51.0-I51.4, I60-I63.9, I65-I66.9, I67.0-I67.3, I67.5-I67.7, I68.0-I68.2, I69.0-I69.3, I70.2-I70.8, I71-I73.9, I77-I89.9, I95.2-I95.3, I97-I98, I98.2, I98.9, J30-J35.9, J37-J39.9, J41-J46.9, J60-J63.8, J65-J68.9, J70-J70.9, J82, J84-J84.9, J91-J92.9, J95-J95.9, K20-K29.9, K31-K31.8, K35-K38.9, K40-K46.9, K50-K52.9, K55-K62.9, K63.5, K64-K64.9, K66.8, K67, K68-K68.9, K70-K70.3, K71.7, K74-K74.9, K75.1-K75.2, K75.4-K76.2, K76.4-K77, K77.8, K80-K83.9, K85-K86.9, K90-K91.9, K92.8, K93.8-K95.8, L00-L05.9, L08-L08.9, L10-L14.0, L51-L51.9, L88-L89.9, L93-L93.2, L97-L98.4, M00-M03.0, M03.2-M03.6, M05-M09.8, M30-M36.8, M40-M43.1, M65-M65.0, M71.0-M71.1, M72.5-M72.6, M80-M82.8, M86.3-M86.4, M87-M87.1, M88-M89.0, M89.5, M89.7-M89.9, N00-N08.8, N10-N12.9, N14-N16.8, N18-N18.9, N20-N23.0, N25-N28.1, N29-N32.0, N32.3-N32.4, N34-N34.3, N36-N36.9, N39-N39.2, N41-N41.9, N44-N44.0, N45-N45.9, N49-N49.9, N60-N60.9, N65-N65.1, N72-N72.0, N75-N77.8, N80-N81.9, N83-N83.9, N84.0-N84.1, N87-N87.9, N99-N99.9, P04.3-P04.4, P70.2, P96.0-P96.2, P96.5, Q00-Q07.9, Q10.4-Q18.9, Q20-Q28.9, Q30-Q36, Q37-Q45.9, Q50-Q87.8, Q89-Q89.8, Q90-Q93.9, Q95-Q99.8, R50.2, R78.0-R78.5, R95-R95.9, X45-X45.9, X65-X65.9, Y15-Y15.9	035-035.9, 036.4, 102-103.9, 133-133.6, 135-135.9, 136.6, 140-148.9, 150-158.9, 160-164.9, 170-175.9, 180-183.8, 184.0-184.4, 184.8, 185-186.9, 187.1-187.8, 188-188.9, 189.0-189.8, 190-194.8, 200-208.9, 209.0-209.1, 209.4-209.5, 210.0-210.9, 211.0-211.8, 212.0-212.8, 213-213.9, 217-220.9, 221.0-221.8, 222.0-222.8, 223.0-223.8, 224-228.9, 229.0, 229.8, 230.1-230.8, 231.0-231.2, 232-232.9, 233.0-233.2, 233.4-233.5, 233.7, 234.0-234.8, 235.0, 235.4, 235.6-235.8, 236.0-236.2, 236.4-236.5, 236.7, 237-237.3, 237.5-237.9, 238.0-238.9, 239.2-239.4, 239.6, 240-243.9, 244.0-244.1, 244.3-244.8, 245-246.9, 251-259.9, 270-273.9, 275-276, 277-277.2, 277.4-277.9, 278.0-278.8, 282-284.9, 286-286.5, 286.7-289.7, 290-292.9, 294.1-294.9, 303-303.9, 304.0-304.8, 305-305.9, 307.1, 327.2-327.8, 330-331.2, 331.5-332.0, 333-337.9, 340-341.9, 345-345.9, 349, 349.2-349.8, 353.6-353.9, 356-356.9, 357.0-357.1, 357.3-357.7, 358-359.9, 376.0-376.1, 391-391.9, 392.0, 393-398.9, 402-404.9, 410-414.9, 416.1, 417-417.9, 420-423, 423.1-423.9, 424.0-424.9, 425.0-425.3, 425.5, 425.7-425.8, 427-427.3, 427.6-427.8, 429.0, 430-435.9, 437.0-437.2, 437.4-437.8, 440.2, 440.4, 441-443.9, 446-457, 457.1-457.9, 459, 459.1-459.3, 470, 470.9-474.9, 476-476.1, 477-479, 491-493.9, 495-504.9, 506-506.9, 508-509, 515, 516-517.8, 518.6-518.7, 518.9, 519.0-519.4, 530-536.1, 536.4, 537-537.6, 537.8, 538-543.9, 550-553.6, 555-558.9, 560-560.3, 560.8-560.9, 562-562.1, 564-564.7, 565-566.9, 569.0-569.7, 571-571.9, 572.2-573.0, 573.4-577.9, 579-583.9, 585-585.9, 588-590.9, 592-593.8, 594-599.6, 599.8, 601-602.9, 604-604.9, 608.2, 610-610.9, 617-618.9, 620-620.9, 621.4-621.9, 622.1-622.7, 629-629.8, 680-689, 694-695.5, 707-707.9, 710-711.9, 714-714.3, 714.8-714.9, 730.1, 732-732.9, 733.0-733.1, 740-749.0, 749.2-758.9, 759.0-759.8, 760.7, 775.1-775.3, 779.4-779.5, 787.1, 788.0, 790.3, 798-798.0, E850, E860
Neoplasms	C00-C13.9, C15-C25.9, C30-C34.9, C37-C38.8, C40-C41.9, C43-C45.9, C47-C54.9, C56-C57.8, C58-C58.0, C60-C63.8, C64-C67.9, C68.0-C68.8, C69-C75.8, C81-C86.6, C88-C96.9, D00.1-D00.2, D01.0-D01.3, D02.0-D02.3, D03-D06.9, D07.0-D07.2, D07.4-D07.5, D09.0, D09.2-D09.3, D09.8, D10.0-D10.7, D11-D12.9, D13.0-D13.7, D14.0-D14.3, D15-D16.9, D22-D24.9, D26.0-D27.9, D28.0-D28.1, D28.7, D29.0-D29.8, D30.0-D30.8, D31-D36, D36.1-D36.7, D37.1-D37.5, D38.0-D38.5, D39.1-D39.2, D39.8, D40.0-D40.8, D41.0-D41.8, D42-D43.9, D44.0-D44.8, D45-D47.9, D48.0-D48.6, D49.2-D49.4, D49.6, K62.0-K62.1, K63.5, N60-N60.9, N84.0-N84.1, N87-N87.9	140-148.9, 150-158.9, 160-164.9, 170-175.9, 180-183.8, 184.0-184.4, 184.8, 185-186.9, 187.1-187.8, 188-188.9, 189.0-189.8, 190-194.8, 200-208.9, 209.0-209.1, 209.4-209.5, 210.0-210.9, 211.0-211.8, 212.0-212.8, 213-213.9, 217-217.8, 219.0, 220-220.9, 221.0-221.8, 222.0-222.8, 223.0-223.8, 224-228.9, 229.0, 229.8, 230.1-230.8, 231.0-231.2, 232-232.9, 233.0-233.2, 233.4-233.5, 233.7, 234.0-234.8, 235.0, 235.4, 235.6-235.8, 236.1-236.2, 236.4-236.5, 236.7, 237-237.3, 237.5-237.9, 238.0-238.9, 239.2-239.4, 239.6, 569.0, 610-610.9, 622.1-622.2, 622.7
Lip and oral cavity cancer	C00-C08.9, D10.0-D10.5, D11-D11.9	140-145.9, 210.0-210.6, 235.0
Nasopharynx cancer	C11-C11.9, D10.6	147-147.9, 210.7-210.9
Other pharynx cancer	C09-C10.9, C12-C13.9, D10.7	146-146.9, 148-148.9
Esophageal cancer	C15-C15.9, D00.1, D13.0	150-150.9, 211.0, 230.1
Stomach cancer	C16-C16.9, D00.2, D13.1, D37.1	151-151.9, 211.1, 230.2
Colon and rectum cancer	C18-C21.9, D01.0-D01.3, D12-D12.9, D37.3-D37.5	153-154.9, 209.1, 209.5, 211.3-211.4, 230.3-230.6, 569.0

Cause	ICD10	ICD9
Liver cancer	C22-C22.9, D13.4	155-155.9, 211.5
Liver cancer due to hepatitis B		
Liver cancer due to hepatitis C		
Liver cancer due to alcohol use		
Liver cancer due to NASH		
Liver cancer due to other causes		
Gallbladder and biliary tract cancer	C23-C24.9, D13.5	156-156.9
Pancreatic cancer	C25-C25.9, D13.6-D13.7	157-157.9, 211.6-211.7
Larynx cancer	C32-C32.9, D02.0, D14.1, D38.0	161-161.9, 212.1, 231.0, 235.6
Tracheal, bronchus, and lung cancer	C33-C34.9, D02.1-D02.3, D14.2-D14.3, D38.1	162-162.9, 212.2-212.3, 231.1-231.2, 235.7
Malignant skin melanoma	C43-C43.9, D03-D03.9, D22-D23.9, D48.5	172-172.9
Non-melanoma skin cancer	C44-C44.9, D04-D04.9, D49.2	173-173.9, 222.4, 232-232.9, 238.2
Non-melanoma skin cancer (squamous-cell carcinoma)	C44-C44.9, D04-D04.9, D49.2	173-173.9, 222.4, 232-232.9, 238.2
Breast cancer	C50-C50.9, D05-D05.9, D24-D24.9, D48.6, D49.3	174-175.9, 217-217.8, 233.0, 238.3, 239.3, 610-610.9
Cervical cancer	C53-C53.9, D06-D06.9, D26.0	180-180.9, 219.0, 233.1, 622.1-622.2, 622.7
Uterine cancer	C54-C54.9, D07.0-D07.2, D26.1-D26.9	182-182.9, 233.2
Ovarian cancer	C56-C56.9, D27-D27.9, D39.1	183-183.0, 220-220.9, 236.2
Prostate cancer	C61-C61.9, D07.5, D29.1, D40.0	185-185.9, 222.2, 236.5
Testicular cancer	C62-C62.9, D29.2-D29.8, D40.1-D40.8	186-186.9, 222.0, 222.3, 236.4
Kidney cancer	C64-C65.9, D30.0-D30.1, D41.0-D41.1	189.0-189.1, 189.5-189.6, 223.0-223.1
Bladder cancer	C67-C67.9, D09.0, D30.3, D41.4-D41.8, D49.4	188-188.9, 223.3, 233.7, 236.7, 239.4
Brain and nervous system cancer	C70-C72.9	191-192.9
Thyroid cancer	C73-C73.9, D09.3, D09.8, D34-D34.9, D44.0	193-193.9, 226-226.9
Mesothelioma	C45-C45.9	
Hodgkin lymphoma	C81-C81.9	201-201.9
Non-Hodgkin lymphoma	C82-C86.6, C96-C96.9	200-200.9, 202-202.9
Multiple myeloma	C88-C90.9	203-203.9
Leukemia	C91-C95.9	204-208.9
Acute lymphoid leukemia	C91.0	204.0
Chronic lymphoid leukemia	C91.1	204.1
Acute myeloid leukemia	C92.0, C92.3-C92.6, C93.0, C94.0, C94.2, C94.4-C94.5	205.0, 205.3, 206.0, 207.0
Chronic myeloid leukemia	C92.1	205.1, 206.1, 207.1
Other leukemia	C91.2-C91.9, C92.2, C92.7-C92.9, C93.1-C93.9, C94.1, C94.3, C94.6-C95.9	204.2-204.9, 205.2, 205.8-205.9, 206.2-207, 207.2-208.9
Other malignant cancers	C17-C17.9, C30-C31.9, C37-C38.8, C40-C41.9, C47-C4A, C51-C52.9, C57-C57.8, C58-C58.0, C60-C60.9, C63-C63.8, C66-C66.9, C68.0-C68.8, C69-C69.9, C74-C75.8, D07.4, D09.2, D13.2-D13.3, D14.0, D15-D16.9, D28.0-D28.1, D28.7, D29.0, D30.2, D30.4-D30.8, D31-D31.9, D35-D35.2, D35.5-D36, D36.1-D36.7, D37.2, D38.2-D38.5, D39.2, D39.8, D41.2-D41.3, D44.1-D44.8, D48.0-D48.4	152-152.9, 158-158.9, 160-160.9, 163-164.9, 170-171.9, 181-181.9, 183.2-183.8, 184.0-184.4, 184.8, 187.1-187.8, 189.2-189.4, 189.8, 190-190.9, 194-194.8, 209.0, 209.4, 211.2, 211.8, 212.0, 212.4-212.8, 213-213.9, 221.0-221.8, 222.1, 222.8, 223.2, 223.8, 224-224.9, 227-228.9, 229.0, 229.8, 230.7-230.8, 233.4-233.5, 234.0-234.8, 235.4, 235.8, 236.1, 238.0-238.1, 239.2
Other neoplasms	D32-D33.9, D35.3-D35.4, D42-D43.9, D45-D47.9, D49.6, K62.0-K62.1, K63.5, N60-N60.9, N84.0-N84.1, N87-N87.9	225-225.9, 237-237.3, 237.5-237.9, 238.4-238.9, 239.6
Myelodysplastic, myeloproliferative, and other hematopoietic neoplasms	D45-D47.9	238.4-238.9
Benign and in situ intestinal neoplasms	K62.0-K62.1, K63.5	
Benign and in situ cervical and uterine neoplasms	N84.0-N84.1, N87-N87.9	
Other benign and in situ neoplasms	N60-N60.9	
Cardiovascular diseases	B33.2, G45-G46.8, I01-I01.9, I02.0, I05-I09.9, I11-I11.9, I20-I25.9, I28-I28.8, I30-I31.1, I31.8-I37.8, I38-I41.9, I42.1-I42.8, I43-I43.9, I47-I48.9, I51.0-I51.4, I60-I63.9, I65-I66.9, I67.0-I67.3, I67.5-I67.6, I68.0-I68.2, I69.0-I69.3, I70.2-I70.8, I71-I73.9, I77-I83.9, I86-I89.0, I89.9, I98, K75.1	036.4, 391-391.9, 392.0, 393-398.9, 402-402.9, 410-414.9, 417-417.9, 420-423, 423.1-423.9, 424.0-424.9, 425.0-425.3, 425.5, 425.7-425.8, 427-427.3, 427.6-427.8, 429.0, 430-435.9, 437.0-437.2, 437.5-437.8, 440.2, 440.4, 441-443.9, 447-454.9, 456, 456.3-457, 457.1, 457.8-457.9, 459, 459.1-459.3
Rheumatic heart disease	I01-I01.9, I02.0, I05-I09.9	391-391.9, 392.0, 393-398.9
Ischemic heart disease	I20-I25.9	410-414.9
Stroke	G45-G46.8, I60-I63.9, I65-I66.9, I67.0-I67.3, I67.5-I67.6, I68.1-I68.2, I69.0-I69.3	430-435.9, 437.0-437.2, 437.5-437.8
Ischemic stroke	G45-G46.8, I63-I63.9, I65-I66.9, I67.2-I67.3, I67.5-I67.6, I69.3	433-435.9, 437.0-437.1, 437.5-437.8
Intracerebral hemorrhage	I61-I62, I62.1-I62.9, I68.1-I68.2, I69.1-I69.2	431-432.9, 437.2
Subarachnoid hemmorrhage	I60-I60.9, I62.0, I67.0-I67.1, I69.0	430-430.9
Hypertensive heart disease	I11-I11.9	402-402.9
Non-rheumatic valvular heart disease	I34-I37.8	424.0-424.3, 424.8
Non-rheumatic calcific aortic valve disease	I35-I35.9	424.1
Non-rheumatic degenerative mitral valve disease	I34-I34.9	424.0
Other non-rheumatic valve diseases	I36-I37.8	424.2-424.3, 424.8
Cardiomyopathy and myocarditis	B33.2, I40-I41.9, I42.1-I42.8, I43-I43.9, I51.4	422-422.9, 425.0-425.3, 425.5, 425.7-425.8, 429.0
Myocarditis	B33.2, I40-I41.9, I51.4	422-422.9
Alcoholic cardiomyopathy	I42.6	425.5

Cause	ICD10	ICD9
Other cardiomyopathy	I42.1-I42.5, I42.7-I42.8, I43-I43.9	425.0-425.3, 425.7-425.8, 429.0
Atrial fibrillation and flutter	I48-I48.9	427.3
Aortic aneurysm	I71-I71.9	441-441.9
Peripheral artery disease	I70.2-I70.8, I73-I73.9	440.2, 440.4, 443.0-443.9
Endocarditis	I33-I33.9, I38-I39.9	421-421.9, 424.4-424.5, 424.9
Other cardiovascular and circulatory diseases	I28-I28.8, I30-I31.1, I31.8-I32.8, I47-I47.9, I51.0-I51.3, I68.0, I72-I72.9, I77-I83.9, I86-I89.0, I89.9, I98, K75.1	036.4, 417-417.9, 420-420.9, 423, 423.1-423.9, 427-427.2, 427.6-427.8, 442-443, 447-454.9, 456, 456.3-457, 457.1, 457.8-457.9, 459, 459.1-459.3
Chronic respiratory diseases	D86-D86.2, D86.9, G47.3, J30-J35.9, J37-J39.9, J41-J46.9, J60-J63.8, J65-J68.9, J70, J70.8-J70.9, J82, J84-J84.9, J91-J92.9	135-135.9, 136.6, 327.2-327.8, 470, 470.9-474.9, 476-476.1, 477-479, 491-493.9, 495-504.9, 506-506.9, 508-509, 515, 516-517.8, 518.6, 518.9, 519.1-519.4
Chronic obstructive pulmonary disease	J41-J44.9	491-492.9, 496-499
Pneumoconiosis	J60-J63.8, J65-J65.0, J92.0	500-504.9
Silicosis	J62-J62.9	502-502.9, 503.0, 503.9
Asbestosis	J61-J61.0, J92.0	501
Coal workers pneumoconiosis	J60-J60.0	500-500.9, 501.0-501.9
Other pneumoconiosis	J63-J63.8, J65-J65.0	503, 503.1, 504-504.9
Asthma	J45-J46.9	493-493.9
Interstitial lung disease and pulmonary sarcoidosis	D86-D86.2, D86.9, J84-J84.9	135-135.9, 136.6, 515, 516-516.9
Other chronic respiratory diseases	G47.3, J30-J35.9, J37-J39.9, J66-J68.9, J70, J70.8-J70.9, J82, J91-J92, J92.9	327.2-327.8, 470, 470.9-474.9, 476-476.1, 477-479, 495-495.9, 506-506.9, 508-509, 517-517.8, 518.6, 518.9, 519.1-519.4
Digestive diseases	B18-B18.9, I84-I85.9, I98.2, K20-K29.9, K31-K31.8, K35-K38.9, K40-K42.9, K44-K46.9, K50-K52.9, K55-K62, K62.2-K62.6, K62.8-K62.9, K64-K64.9, K66.8, K67, K68-K68.9, K70-K70.3, K71.7, K74-K74.9, K75.2, K75.4-K76.2, K76.4-K77, K77.8, K80-K83.9, K85-K86.9, K90-K90.9, K92.8, K93.8, M09.1	455-455.9, 456.0-456.2, 530-536.1, 537-537.6, 537.8, 538, 540-543.9, 550-551.1, 551.3-552.1, 552.3-553.6, 555-558.9, 560-560.3, 560.8-560.9, 562-562.1, 564-564.1, 564.5-564.7, 565-566.9, 569.1-569.5, 569.7, 571-571.9, 572.2-573.0, 573.4-577.9, 579-579.2, 579.4-579.9, 787.1
Cirrhosis and other chronic liver diseases	B18-B18.9, I85-I85.9, I98.2, K70-K70.3, K71.7, K74-K74.9, K75.2, K75.4-K76.2, K76.4-K76.9, K77.8	456.0-456.2, 571-571.9, 572.2-573.0, 573.4-573.9
Cirrhosis and other chronic liver diseases due to hepatitis B		
Cirrhosis and other chronic liver diseases due to hepatitis C		
Cirrhosis and other chronic liver diseases due to alcohol use		
Non-alcoholic fatty liver disease/Non-alcoholic steatohepatitis (NFLD/NASH)		
Cirrhosis and other chronic liver diseases due to other causes		
Upper digestive system diseases	K21-K21.9, K22.7, K25-K29.9, K31, K31.1-K31.6, K31.8	530.1, 530.8, 531-535.9, 787.1
Peptic ulcer disease	K25-K28.9, K31, K31.1-K31.6, K31.8	531-534.9
Gastritis and duodenitis	K29-K29.9	535-535.9
Appendicitis	K35-K37.9, K38.3-K38.9	540-542.9
Paralytic ileus and intestinal obstruction	K56-K56.9	560-560.3, 560.8-560.9
Inguinal, femoral, and abdominal hernia	K40-K42.9, K44-K46.9	550-551.1, 551.3-552.1, 552.3-553.0, 553.6
Inflammatory bowel disease	K50-K52.9, M09.1	555-556.9, 558-558.9, 569.5
Vascular intestinal disorders	K55-K55.9	557-557.9
Gallbladder and biliary diseases	K80-K83.9	574-576.9
Pancreatitis	K85-K86.9	577-577.9, 579.4
Other digestive diseases	I84-I84.9, K20-K20.9, K22-K22.6, K22.8-K24, K31.0, K31.7, K38-K38.2, K57-K62, K62.2-K62.6, K62.8-K62.9, K64-K64.9, K66.8, K67, K68-K68.9, K77, K90-K90.9, K92.8, K93.8	455-455.9, 530-530.0, 530.2-530.7, 530.9, 536-536.1, 537-537.6, 537.8, 538, 543-543.9, 553.1-553.3, 562-562.1, 564-564.1, 564.5-564.7, 565-566.9, 569.1-569.4, 569.7, 579-579.2, 579.8-579.9
Neurological disorders	F00-F03.9, G10-G13.8, G20-G20.9, G23-G24, G24.1-G25.0, G25.2-G25.3, G25.5, G25.8-G26.0, G30-G31.1, G31.8-G31.9, G35-G37.9, G40-G41.9, G61-G61.9, G70-G72, G72.2-G73.7, G90-G90.9, G95-G95.9, M33-M33.9	290-290.9, 294.1-294.9, 330-331.2, 331.5-332.0, 333-337.9, 340-341.9, 345-345.9, 349, 349.2-349.8, 353.6-353.9, 356-356.9, 357.0-357.1, 357.3-357.4, 357.7, 358-359.9, 775.2
Alzheimer disease and other dementias	F00-F03.9, G30-G31.1, G31.8-G31.9	290-290.9, 294.1-294.9, 331-331.2
Parkinson disease	G20-G20.9	332-332.0
Epilepsy	G40-G41.9	345-345.9
Multiple sclerosis	G35-G35.9	340-340.9
Motor neuron disease	G12.2-G12.9	335-335.2, 335.8-335.9
Other neurological disorders	G10-G12.1, G13-G13.8, G23-G24, G24.1-G25.0, G25.2-G25.3, G25.5, G25.8-G26.0, G36-G37.9, G61-G61.9, G70-G72, G72.2-G73.7, G90-G90.9, G95-G95.9, M33-M33.9	330-330.9, 331.5-331.9, 333-334.9, 335.3, 336-337.9, 341-341.9, 349, 349.2-349.8, 353.6-353.9, 356-356.9, 357.0-357.1, 357.3-357.4, 357.7, 358-359.9, 775.2
Mental disorders	F24, F50.0-F50.5	307.1
Eating disorders	F50.0-F50.5	307.1
Anorexia nervosa	F50.0-F50.1	307.1
Bulimia nervosa	F50.2-F50.5	
Substance use disorders	F10-F16.9, F18-F19.9, G31.2, G72.1, P04.3-P04.4, P96.1, Q86.0, R78.0-R78.5, X45-X45.9, X65-X65.9, Y15-Y15.9	291-292.9, 303-303.9, 304.0-304.8, 305-305.9, 357.5, 760.7, 790.3, E850, E860
Alcohol use disorders	F10-F10.9, G31.2, G72.1, P04.3, Q86.0, R78.0, X45-X45.9, X65-X65.9, Y15-Y15.9	291-291.9, 303-303.9, 305.0, 357.5, 790.3, E860
Drug use disorders	F11-F16.9, F18-F19.9, P04.4, P96.1, R78.1-R78.5	292-292.9, 304.0-304.8, 305, 305.1-305.9, 760.7, E850
Opioid use disorders	F11-F11.9, P96.1, R78.1	304.0, 305.5
Cocaine use disorders	F14-F14.9, R78.2	304.2, 305.6
Amphetamine use disorders	F15-F15.9	304.4, 305.7

Cause	ICD10	ICD9
Other drug use disorders	F13-F13.9, F16-F16.9, F18-F19.9, P04.4, R78.3-R78.5	292-292.9, 304.1, 304.5-304.8, 305, 305.1, 305.3-305.4, 305.8-305.9, 760.7
Diabetes and kidney diseases	D63.1, E10-E11.9, I12-I13.9, N00-N08.8, N15.0, N18-N18.9, P70.2, Q61-Q62.8	403-404.9, 580-583.9, 585-585.9, 589-589.9, 753-753.3, 775.1
Diabetes mellitus	E10-E10.1, E10.3-E11.1, E11.3-E11.9, P70.2	775.1
Diabetes mellitus type 1	E10-E10.1, E10.3-E10.9, P70.2	775.1
Diabetes mellitus type 2	E11-E11.1, E11.3-E11.9	
Chronic kidney disease	D63.1, E10.2, E11.2, I12-I13.9, N02-N08.8, N15.0, N18-N18.9, Q61-Q62.8	403-404.9, 581-583.9, 585-585.9, 589-589.9, 753-753.3
Chronic kidney disease due to diabetes mellitus type 1	E10.2	
Chronic kidney disease due to diabetes mellitus type 2	E11.2	
Chronic kidney disease due to hypertension	I12-I13.9	403-404.9
Chronic kidney disease due to glomerulonephritis	N03-N06.9	581-583.9
Chronic kidney disease due to other causes	N02-N02.9, N07-N08.8, N15.0, Q61-Q62.8	589-589.9, 753-753.3
Acute glomerulonephritis	N00-N01.9	580-580.9
Skin and subcutaneous diseases	A46-A46.0, A66-A67.9, B86, D86.3, I89.1-I89.8, L00-L05.9, L08-L08.9, L10-L14.0, L51-L51.9, L88-L89.9, L97-L98.4, M72.5-M72.6	035-035.9, 102-103.9, 133-133.6, 457.2-457.3, 680-689, 694-695.3, 707-707.9
Bacterial skin diseases	A46-A46.0, A66-A67.9, I89.1-I89.8, L00-L05.9, L08-L08.9, L88, L97-L98.4, M72.5-M72.6	035-035.9, 102-103.9, 457.2-457.3, 680-689
Cellulitis	L03-L03.9, M72.5-M72.6	681-682.9
Pyoderma	A46-A46.0, A66-A67.9, I89.1-I89.8, L00-L02.9, L04-L05.9, L08-L08.9, L88, L97-L98.4	035-035.9, 102-103.9, 457.2-457.3, 680-680.9, 683-689
Decubitus ulcer	L89-L89.9	707-707.9
Other skin and subcutaneous diseases	D86.3, L10-L14.0, L51-L51.9	694-695.3
Musculoskeletal disorders	I27.1, I67.7, L93-L93.2, M00-M03.0, M03.2-M03.6, M05-M09.0, M09.2-M09.8, M30-M32.9, M34-M36.8, M40-M43.1, M65-M65.0, M71.0-M71.1, M80-M82.8, M86.3-M86.4, M87-M87.0, M88-M89.0, M89.5, M89.7-M89.9	416.1, 437.4, 446-446.9, 695.4-695.5, 710-711.9, 714-714.3, 714.8-714.9, 730.1, 732-732.9, 733.0-733.1
Rheumatoid arthritis	M05-M06.9, M08.0-M08.8	714-714.3, 714.8-714.9
Other musculoskeletal disorders	I27.1, I67.7, L93-L93.2, M00-M03.0, M03.2-M03.6, M07-M08, M08.9-M09.0, M09.2-M09.8, M30-M32.9, M34-M36.8, M40-M43.1, M65-M65.0, M71.0-M71.1, M80-M82.8, M86.3-M86.4, M87-M87.0, M88-M89.0, M89.5, M89.7-M89.9	416.1, 437.4, 446-446.9, 695.4-695.5, 710-711.9, 730.1, 732-732.9, 733.0-733.1
Other non-communicable diseases	D25-D26, D28.2, D52.1, D55-D58.9, D59.0-D59.3, D59.5-D59.6, D60-D61.9, D64.0, D66-D67, D68.0-D69.8, D70-D75.8, D76-D78.8, D86.8, D89-D89.3, E03-E07.1, E09-E09.9, E15.0, E16.0-E16.9, E20-E34.8, E36-E36.8, E65-E68, E70-E85.2, E88-E89.9, G24.0, G25.1, G25.4, G25.6-G25.7, G72.0, G93.7, G97-G97.9, I95.2-I95.3, I97-I97.9, I98.9, J70.0-J70.5, J95-J95.9, K43-K43.9, K62.7, K91-K91.9, K94-K95.8, M87.1, N10-N12.9, N14-N15, N15.1-N16.8, N20-N23.0, N25-N28.1, N29-N32.0, N32.3-N32.4, N34-N34.3, N36-N36.9, N39-N39.2, N41-N41.9, N44-N44.0, N45-N45.9, N49-N49.9, N65-N65.1, N72-N72.0, N75-N77.8, N80-N81.9, N83-N83.9, N99-N99.9, P96.0, P96.2, P96.5, Q00-Q07.9, Q10.4-Q18.9, Q20-Q28.9, Q30-Q36, Q37-Q45.9, Q50-Q60.6, Q63-Q86, Q86.1-Q87.8, Q89-Q89.8, Q90-Q93.9, Q95-Q99.8, R50.2, R95-R95.9	218-219, 219.1-219.9, 236.0, 240-243.9, 244.0-244.1, 244.3-244.8, 245-246.9, 251-259.9, 270-273.9, 275-276, 277-277.2, 277.4-277.9, 278.0-278.8, 282-284.9, 286-286.5, 286.7-289.7, 357.6, 518.7, 519.0, 536.4, 539-539.9, 551.2, 552.2, 564.2-564.4, 569.6, 579.3, 588-588.9, 590-590.9, 592-593.8, 594-599.6, 599.8, 601-602.9, 604-604.9, 608.2, 617-618.9, 620-620.9, 621.4-621.9, 622.3-622.6, 629-629.8, 740-749.0, 749.2-752.9, 753.4-758.9, 759.0-759.8, 775.3, 779.4-779.5, 788.0, 798-798.0
Congenital birth defects	P96.0, Q00-Q07.9, Q10.4-Q18.9, Q20-Q28.9, Q30-Q36, Q37-Q45.9, Q50-Q60.6, Q63-Q86, Q86.1-Q87.8, Q89-Q89.8, Q90-Q93.9, Q95-Q99.8	740-749.0, 749.2-752.9, 753.4-758.9, 759.0-759.8
Neural tube defects	Q00-Q01.9, Q05-Q05.9	740-741.9, 742.0
Congenital heart anomalies	Q20-Q28.9	745-747.9
Orofacial clefts	Q35-Q36, Q37-Q37.9	749-749.0, 749.2-749.9
Down syndrome	Q90-Q90.9	758.0
Other chromosomal abnormalities	Q87-Q87.8, Q91-Q93.9, Q95-Q95.9, Q97-Q97.9, Q99-Q99.8	758, 758.1-758.6, 758.8-758.9
Congenital musculoskeletal and limb anomalies	Q65-Q79, Q79.6-Q79.9	742.5, 754-756.5, 756.8-756.9
Urogenital congenital anomalies	P96.0, Q50-Q56.4, Q60-Q60.6, Q63-Q64.9	752-752.9, 753.4-753.9
Digestive congenital anomalies	Q38-Q45.9, Q79.0-Q79.5	750-751.9, 756.6-756.7
Other congenital birth defects	Q02-Q04.9, Q06-Q07.9, Q10.4-Q18.9, Q30-Q34.9, Q57, Q80-Q86, Q86.1-Q86.8, Q89-Q89.8	742, 742.1-742.4, 742.8-744.9, 748-748.9, 757-757.9, 759.0-759.8
Urinary diseases and male infertility	N10-N12.9, N15, N15.1-N16.8, N20-N23.0, N25-N28.1, N29-N32.0, N32.3-N32.4, N34-N34.3, N36-N36.9, N39-N39.2, N41-N41.9, N44-N44.0, N45-N45.9, N49-N49.9	588-588.9, 590-590.9, 592-593.8, 594-598.1, 598.8-599.6, 599.8, 601-602.9, 604-604.9, 608.2, 788.0
Urinary tract infections	N10-N12.9, N15, N15.1-N16.8, N30-N30.9, N34-N34.3, N39.0-N39.2	590-590.9, 595-595.9, 597-597.9, 599.0
Urolithiasis	N20-N23.0	592-592.9, 594-594.9, 788.0
Other urinary diseases	N25-N28.1, N29-N29.8, N31-N32.0, N32.3-N32.4, N36-N36.9, N39, N41-N41.9, N44-N44.0, N45-N45.9, N49-N49.9	588-588.9, 593-593.8, 596-596.9, 598-598.1, 598.8-599, 599.1-599.6, 599.8, 601-602.9, 604-604.9, 608.2
Gynecological diseases	D25-D26, D28.2, E28.2, N72-N72.0, N75-N77.8, N80-N81.9, N83-N83.9	218-219, 219.1-219.9, 236.0, 256.4, 617-618.9, 620-620.9, 621.4-621.9, 622.3-622.6, 629-629.8
Uterine fibroids	D25-D26, D28.2	218-219, 219.1-219.9, 236.0
Polycystic ovarian syndrome	E28.2	256.4
Endometriosis	N80-N80.9	617-617.9
Genital prolapse	N81-N81.9	618-618.9
Other gynecological diseases	N72-N72.0, N75-N77.8, N83-N83.9	620-620.9, 621.4-621.9, 622.3-622.6, 629-629.8
Hemoglobinopathies and hemolytic anemias	D55-D58.9, D59.1, D59.3, D59.5, D60-D61.9, D64.0	282-284.9
Thalassemias	D56-D56.9	282.4-282.5

Cause	ICD10	ICD9
Sickle cell disorders	D57-D57.8	282.6
G6PD deficiency	D55-D55.2	282.2-282.3
Other hemoglobinopathies and hemolytic anemias	D55.3-D55.9, D58-D58.9, D59.1, D59.3, D59.5, D60-D61.9, D64.0	282-282.1, 282.7-284.9
Endocrine, metabolic, blood, and immune disorders	D52.1, D59.0, D59.2, D59.6, D66-D67, D68.0-D69.8, D70-D75.8, D76-D78.8, D86.8, D89-D89.3, E03-E07.1, E09-E09.9, E15.0, E16.0-E16.9, E20-E28.1, E28.3-E34.8, E36-E36.8, E65-E68, E70-E85.2, E88-E89.9, G24.0, G25.1, G25.4, G25.6-G25.7, G72.0, G93.7, G97-G97.9, I95.2-I95.3, I97-I97.9, I98.9, J70.0-J70.5, J95-J95.9, K43-K43.9, K62.7, K91-K91.9, K94-K95.8, M87.1, N14-N14.4, N65-N65.1, N99-N99.9, P96.2, P96.5, R50.2	240-243.9, 244.0-244.1, 244.3-244.8, 245-246.9, 251-256.3, 256.8-259.9, 270-273.9, 275-276, 277-277.2, 277.4-277.9, 278.0-278.8, 286-286.5, 286.7-289.7, 357.6, 518.7, 519.0, 536.4, 539-539.9, 551.2, 552.2, 564.2-564.4, 569.6, 579.3, 598.2, 775.3, 779.4-779.5
Sudden infant death syndrome	R95-R95.9	798-798.0
Injuries	L55-L55.9, L56.3, L56.8-L56.9, L58-L58.9, U00-U03, V00-V86.9, V87.2-V87.3, V88.2-V88.3, V90-V98.8, W00-W46.2, W49-W62.9, W64-W70.9, W73-W75.9, W77-W81.9, W83-W94.9, W97.9, W99-X06.9, X08-X39.9, X46-X48.9, X50-X54.9, X57-X58.9, X60-X64.9, X66-Y08.9, Y35-Y84.9, Y87.0-Y87.1, Y88-Y88.3, Y89.0-Y89.1	349.0-349.1, 457.0, E800-E807, E830-E838, E840-E849, E856-E857, E861-E865, E867-E869, E870-E876, E878-E879, E880-E886, E888-E928, E930-E979, E990-E999
Transport injuries	V00-V86.9, V87.2-V87.3, V88.2-V88.3, V90-V98.8	E800-E807, E830-E838, E840-E849
Road injuries	V01-V04.9, V06-V80.9, V82-V82.9, V87.2-V87.3	
Pedestrian road injuries	V01-V04.9, V06-V09.9	
Cyclist road injuries	V10-V19.9	
Motorcyclist road injuries	V20-V29.9	
Motor vehicle road injuries	V30-V79.9, V87.2-V87.3	
Other road injuries	V80-V80.9, V82-V82.9	
Other transport injuries	V00-V00.8, V05-V05.9, V81-V81.9, V83-V86.9, V88.2-V88.3, V90-V98.8	E800-E807, E830-E838, E840-E849
Unintentional injuries	L55-L55.9, L56.3, L56.8-L56.9, L58-L58.9, W00-W46.2, W49-W62.9, W64-W70.9, W73-W75.9, W77-W81.9, W83-W94.9, W97.9, W99-X06.9, X08-X39.9, X46-X48.9, X50-X54.9, X57-X58.9, Y40-Y84.9, Y88-Y88.3	349.0-349.1, 457.0, E856-E857, E861-E865, E867-E869, E870-E876, E878-E879, E880-E886, E888-E928, E930-E949
Falls	W00-W19.9	E880-E886, E888
Drowning	W65-W70.9, W73-W74.9	E910
Fire, heat, and hot substances	X00-X06.9, X08-X19.9	E890-E899, E924
Poisonings	X46-X48.9	E856-E857, E861-E865, E867-E869
Poisoning by carbon monoxide	X47-X47.9	E862, E868-E869
Poisoning by other means	X46-X46.9, X48-X48.9	E856-E857, E861, E863-E865, E867
Exposure to mechanical forces	W20-W38.9, W40-W43.9, W45.0-W45.2, W46-W46.2, W49-W52	E916-E922
Unintentional firearm injuries	W32-W34.9	E922
Other exposure to mechanical forces	W20-W31.9, W35-W38.9, W40-W43.9, W45.0-W45.2, W46-W46.2, W49-W52	E916-E921
Adverse effects of medical treatment	Y40-Y84.9, Y88-Y88.3	349.0-349.1, 457.0, E870-E876, E878-E879, E930-E949
Animal contact	W52.0-W62.9, W64-W64.9, X20-X29.9	E905-E906
Venomous animal contact	X20-X29.9	E905
Non-venomous animal contact	W52.0-W62.9, W64-W64.9	E906
Foreign body	W44-W45, W45.3-W45.9, W75-W75.9, W78-W80.9, W83-W84.9	E911-E915
Pulmonary aspiration and foreign body in airway	W75-W75.9, W78-W80.9, W83-W84.9	E911-E913
Foreign body in other body part	W44-W45, W45.3-W45.9	E914-E915
Environmental heat and cold exposure	L55-L55.9, L56.3, L56.8-L56.9, L58-L58.9, W88-W94.9, W97.9, W99-W99.9, X30-X32.9, X39-X39.9	E900-E902, E926
Exposure to forces of nature	X33-X38.9	E907-E909
Other unintentional injuries	W39-W39.9, W77-W77.9, W81-W81.9, W85-W87.9, X50-X54.9, X57-X58.9	E903-E904, E923, E925, E927-E928
Self-harm and interpersonal violence	U00-U03, X60-X64.9, X66-Y08.9, Y35-Y38.9, Y87.0-Y87.1, Y89.0-Y89.1	E950-E979, E990-E999
Self-harm	X60-X64.9, X66-X84.9, Y87.0	E950-E959
Self-harm by firearm	X72-X74.9	E955
Self-harm by other specified means	X60-X64.9, X66-X71.9, X75-X84.9, Y87.0	E950-E954, E956-E959
Interpersonal violence	X85-Y08.9, Y87.1	E960-E969
Physical violence by firearm	X93-X95.9	E965
Physical violence by sharp object	X99-X99.9	E966
Physical violence by other means	X85-X92.9, X96-X98.9, Y00-Y04.9, Y06-Y08.9, Y87.1	E961-E964, E967-E969
Conflict and terrorism	U00-U03, Y36-Y38.9, Y89.1	E979, E990-E999
Executions and police conflict	Y35-Y35.9, Y89.0	E970-E978
None		
Still Born	P95-P95.9	768.0-768.1

Cause	ICD10	ICD9
Garbage Code (GBD Level 1)	A31-A31.9, A40-A44.9, A48.0, A48.3, A49.0-A49.1, A59-A59.9, A71-A71.9, A74.0, B07-B07.9, B30-B30.9, B35-B46.9, B49-B49.9, B55.1-B55.2, B58-B59.9, B85-B85.4, B87-B88.9, B94.0, C46-C46.9, D50-D50.0, D50.9, D62-D63.0, D63.8-D64, D64.1-D65.9, D68, D69.9, D80-D84.9, D89.8-D89.9, E12-E15, E16, E50-E50.9, E64.1, E85.3-E87.6, E87.8-E87.9, F06.2-F06.4, F07.2, F09-F09.9, F17-F17.9, F20-F23.9, F25-F49, F51-F99.0, G06-G08.0, G32-G32.8, G43-G44.2, G44.4-G44.8, G47-G47.2, G47.4-G47.9, G50-G60.9, G62-G65.2, G80-G83.9, G89-G89.4, G91-G91.2, G91.4-G92.9, G93.1-G93.2, G93.4-G93.6, G98, G99-H05, H05.2-H69.9, H71-H99, I26-I26.9, I31.2-I31.4, I37.9, I46-I46.9, I50-I50.9, I51.7, I67.4, I76, I95-I95.1, I95.8-I95.9, J40-J40.9, J47-J47.9, J69-J69.9, J80-J80.9, J85-J85.3, J86-J86.9, J93-J93.1, J93.8-J93.9, J94.2, J96-J96.9, J98.1-J98.3, K00-K19, K30, K65-K66.1, K66.9, K70.4-K71.6, K71.8-K73.9, K75.0, L20-L30.9, L40-L50.9, L52-L54.8, L56-L56.2, L56.4-L56.5, L57-L57.9, L59-L68.9, L70-L76.8, L80-L87.9, L90-L92.9, L94-L96, L98.5-L99.8, M04, M10-M12.0, M12.2-M29, M37-M39, M43.2-M49, M49.2-M64, M65.1-M71, M71.2-M72.4, M72.8-M73, M73.8-M79.9, M83-M86.2, M86.5-M86.9, M87.2-M87.9, M89.1-M89.4, M90-M99.9, N17-N17.9, N19-N19.9, N32.1-N32.2, N32.8-N33.8, N35-N35.9, N37-N37.8, N39.3-N39.8, N42-N43.4, N44.1-N44.8, N46-N48.9, N50-N53.9, N61-N64.9, N82-N82.9, N91-N91.5, N95, N95.1-N95.9, N97-N97.9, R02-R02.9, R03.1, R07.0, R08-R09, R09.3, R11-R12.0, R14-R15.9, R19-R19.6, R19.8-R23, R23.1-R30.9, R32-R50.1, R50.8-R57.9, R58.0-R78, R78.6-R94.8, R96-R99.9, U05, U07-U81, U89.9-U99, X40-X44.9, X49-X49.9, Y10-Y14.9, Y16-Y19.9, Z00-Z15.8, Z17-unsp.	031-031.9, 038-039.9, 040.0, 041.1, 076-078.2, 085.1-085.5, 088.0-088.7, 110-118.9, 125-125.3, 126-126.9, 127.2-127.9, 130-132.9, 133.8-134.9, 136.3-136.5, 139.1, 139.9, 176-176.9, 247-248, 250-250.9, 264-264.9, 274-274.9, 276.0-276.5, 276.7-276.9, 277.3, 279-281, 285-285.9, 286.6, 293, 294-294.0, 295-302.9, 306-307.0, 307.2-307.4, 307.6-319.9, 324-327.1, 328-329, 338-339.1, 339.3-339.8, 342-344.9, 346-347.9, 350-353.5, 354-355.9, 357.2, 360-376, 376.2-380.9, 384-389.9, 415-415.9, 423.0, 424, 427.5, 427.9-428.9, 429.3, 437.3, 458-458.9, 459.0, 490-490.9, 494-494.9, 507-507.9, 510-510.9, 512-513.9, 518.0-518.2, 518.5, 520-529.9, 536.3, 536.8-536.9, 537.7, 537.9, 564.8-564.9, 567-568.9, 570-570.9, 572-572.1, 573.1-573.3, 584-584.9, 586-587.9, 603-603.9, 605-608.1, 608.3-609, 611-612.1, 615-616.9, 619-619.9, 621-621.3, 622-622.0, 622.8-623.6, 623.8-624.5, 624.8-628.9, 629.9, 690-693.9, 695.8-706.9, 708-709.9, 712-713.8, 715-716, 716.2-721.6, 721.8-730.0, 730.2-730.3, 730.7-731.9, 733, 733.2-734.2, 737-738, 738.2-739.9, 780-782.3, 782.6-784.6, 784.9, 785.4-786, 786.6, 786.8, 787, 787.3-788, 788.3-789, 790-790.2, 790.4-796.1, 796.3-797.9, 798.1-799, 799.2-799.9, 999.0-999.9, E851-E855, E858, E866, E980-E982, V01-V08, V10-uns
Garbage Code (GBD Level 2)	A14.9, A29-A30.9, A45-A45.9, A47-A48, A48.8-A49, A49.3-A49.9, A61-A62, A72-A73, A76, A97, B08-B09, B11-B14, B28-B29, B31-B32.4, B34-B34.9, B61-B62, B68-B68.9, B73-B74.2, B76-B76.9, B78-B81.8, B84, B92-B94, B94.8-B94.9, B95.6-B97.3, B97.7-B99.9, D59, D59.4, D59.8-D59.9, G44.3, G91.3, G93.0, G93.3, I10-I10.9, I15-I15.9, I27-I27.0, I27.2-I27.9, I28.9, I70-I70.1, I70.9, I74-I75.8, J81-J81.1, J90-J90.0, J94-J94.1, J94.8-J94.9, K92.0-K92.2, N70-N71.9, N73-N74.0, N74.2-N74.8, R03-R03.0, R04-R06.9, R09.0-R09.2, R09.8-R10.9, R13-R13.9, R16-R18.9, R23.0, R58, S00-T98.3, W47-W48, W63, W71-W72, W76-W76.9, W82, W95-W97, W98, X07, X55-X56, X59-X59.9, Y20-Y34.9, Y86-Y87, Y87.2, Y89, Y89.9-Y99.9	000-000.9, 030-030.9, 041.2-041.9, 067-069, 078.8-078.9, 079.8-079.9, 089-089.9, 105-109.9, 119, 136.8-136.9, 139.8, 304, 304.9, 339.2, 401-401.9, 405-405.9, 416-416.0, 416.2-416.9, 440-440.1, 440.3, 440.8-440.9, 444-445.8, 511-511.9, 514-514.9, 515.0-515.9, 518, 518.3-518.4, 518.8, 536.2, 578-578.9, 599.7, 613-614.9, 714.4, 716.1, 721.7, 735-736.9, 738.0-738.1, 782.4, 784.7-784.8, 786.3, 787.0, 787.2, 789.0-789.9, 796.2, 799.0-799.1, 800-999, E000-E80, E83, E839, E85, E859, E87, E877, E88, E887, E929, E983-E985, E988-E989
Garbage Code (GBD Level 3)	A01, A49.2, A64-A64.0, A99-A99.0, B89, C14-C14.9, C26-C29, C35-C36, C39-C39.9, C42, C55-C55.9, C57.9, C59, C63.9, C68, C68.9, C75.9-C80.9, C87, C97-D00.0, D01, D01.4-D02, D02.4-D02.9, D07, D07.3, D07.6-D09, D09.1, D09.7, D09.9-D10, D10.9, D13, D13.9-D14, D14.4, D17-D21.9, D28, D28.9-D29, D29.9-D30, D30.9, D36.0, D36.9-D37.0, D37.6-D38, D38.6-D39.0, D39.7, D39.9-D40, D40.9-D41, D41.9, D44, D44.9, D48, D48.7-D49.1, D49.5, D49.7-D49.9, D54, D75.9, D79, D85, D87-D88, D90-D99, E07.8-E08.9, E17-E19, E34.9-E35.8, E37-E39, E47-E49., E62, E69, E87.7, E90-E998, F04-F06.1, F06.5-F07.0, F07.8-F08, F50, F50.8-F50.9, G09-G09.9, G15-G19, G21-G21.2, G21.4-G22.0, G27-G29, G33-G34, G38-G39., G42, G48-G49, G66-G69, G74-G79, G84-G88, G93, G93.8-G94.8, G96-G96.9, G98.0-G98.9, I00.0, I03-I04., I14-I14., I16-I19, I29-I29.9, I44-I45.9, I49-I49.9, I51, I51.6, I51.8-I59, I90-I94, I96-I96.9, I98.4-I98.8, I99-ID5.9, J02.9, J03.9, J04.3, J06, J06.9, J48-J59, J71-J79, J81.9, J83, J85.9, J87-J89, J90.9, J93.6, J97-J98.0, J98.4-J99.8, K31.9-K34, K39, K47-K49, K53-K54, K63-K63.4, K63.8-K63.9, K69, K75, K78-K79, K84, K87-K89, K92, K92.9-K93, K96-K99, L06-L07, L09, L15-L19, L31-L39, L69, L77-L79, N09, N13-N13.9, N24, N28.8-N28.9, N38, N39.9-N40.9, N54-N59, N66-N69, N78-N79, N84, N84.2-N86, N88-N90.9, N92-N94.9, N95.0, O08-O08.9, O17-O19, O27, O37-O39, O49-O59, O78-O79, O93-O95.9, P06, P16-P18, P30-P34.2, P40-P49, P62-P69, P73, P79, P82, P85-P89, P96.9-P99.9, Q08-Q10.3, Q19, Q29-Q29., Q36.0-Q36.9, Q46-Q49, Q88, Q89.9, Q94, Q99.9-R01.2, R07, R07.1-R07.9, R31-R31.9	002, 085, 085.9, 149-149.9, 159-159.9, 165-169, 177-179.9, 183.9-184, 184.5, 184.9, 187, 187.9, 189, 189.9, 194.9-199.9, 209, 209.2-209.3, 209.6-210, 211, 211.9-212, 212.9, 214-216.9, 221, 221.9-222, 222.9-223, 223.9, 229, 229.1, 229.9-230.0, 230.9-231, 231.8-231.9, 233, 233.3, 233.6, 233.9-234, 234.9-235, 235.1-235.3, 235.5, 235.9-236, 236.3, 236.6, 236.9, 237.4, 239-239.1, 239.5, 239.7-239.9, 249-249.9, 276.6, 278, 293.0-293.9, 331.3-331.4, 332.1-332.9, 348-348.9, 349.9, 357, 357.8-357.9, 399-400.0, 406-409.4, 418-419.9, 426-426.9, 427.4, 429, 429.2, 429.4-429.9, 459.5-459.9, 464.5, 465, 465.9, 505-505.9, 519, 519.8-519.9, 544-549, 553.8-553.9, 559-559.0, 560.4-560.7, 561, 562.2-563, 569, 569.8-569.9, 591-591.9, 593.9, 599.9-600.9, 623.7, 624.6, 637-637.9, 639-639.9, 749.1, 759, 759.9, 779.9, 782.5, 785-785.3, 786.0-786.2, 786.4-786.5, 786.7, 786.9, 788.1-788.2, E986-E987
Garbage Code (GBD Level 4)	B54-B55, B55.9, B64, B82-B82.9, B83.9, G00, G00.9-G02.8, G03.9, I42-I42.0, I42.9, I51.5, I64-I64.9, I67, I67.8-I68, I68.8-I69, I69.4-I69.9, J07-J08, J15.9, J17-J19.6, J22-J29, J64-J64.9, P23, P23.5-P23.9, P37.3-P37.4, V87-V87.1, V87.4-V88.1, V88.4-V89.9, V99-V99.0, Y09-Y09.9, Y85-Y85.9	084, 084.6, 238, 244, 244.9, 289.8-289.9, 307.5, 320, 320.9, 425, 425.4, 425.9, 429.1, 436-437, 437.9-439.6, 482.9-483, 484, 484.8-486.9, 770.0, E808-E829

Appendix Table 5. Restrictions on age and sex by cause for GBD 2017			
**Cause**	**Minimum Age**	**Maximum Age**	**Sex Restrictions**
HIV/AIDS and sexually transmitted infections	7 days		
HIV/AIDS	28 days		
HIV/AIDS - Drug-susceptible Tuberculosis	28 days		
HIV/AIDS - Multidrug-resistant Tuberculosis without extensive drug resistance	28 days		
HIV/AIDS - Extensively drug-resistant Tuberculosis	28 days		
HIV/AIDS resulting in other diseases	28 days		
Sexually transmitted infections excluding HIV			
Syphilis			
Chlamydial infection	10		
Gonococcal infection	10		
Other sexually transmitted infections	10		
Respiratory infections and tuberculosis	7 days		
Tuberculosis	28 days		
Drug-susceptible tuberculosis	28 days		
Multidrug-resistant tuberculosis without extensive drug resistance	28 days		
Extensively drug-resistant tuberculosis	28 days		
Lower respiratory infections			
Upper respiratory infections			
Otitis media			
Enteric infections			
Diarrhoeal diseases			
Typhoid and paratyphoid	7 days		
Typhoid fever	28 days		
Paratyphoid fever	28 days		
iNTS	7 days		
Other intestinal infectious diseases	28 days		
Neglected tropical diseases and malaria			
Malaria	7 days		
Chagas disease	28 days		
Leishmaniasis	28 days		
Visceral leishmaniasis	28 days		
African trypanosomiasis	1		
Schistosomiasis	28 days		
Cysticercosis			
Cystic echinococcosis	1		
Dengue	28 days		
Yellow fever	7 days		
Rabies	28 days		
Intestinal nematode infections	28 days		
Ascariasis	28 days		
Ebola virus disease			
Zika virus disease			
Other neglected tropical diseases			
Other infectious diseases			
Meningitis			
Pneumococcal meningitis			
H influenzae type B meningitis			
Meningococcal infection			
Other meningitis			
Encephalitis			
Diphtheria	28 days	59	
Whooping cough	28 days	59	
Tetanus			
Measles	28 days	59	
Varicella and herpes zoster			
Acute hepatitis	28 days		

Appendix Table 5. Restrictions on age and sex by cause for GBD 2017			
Cause	Minimum Age	Maximum Age	Sex Restrictions
Acute hepatitis A	28 days		
Acute hepatitis B	28 days		
Acute hepatitis C	28 days		
Acute hepatitis E	28 days		
Other unspecified infectious diseases			
Maternal and neonatal disorders		54	
Maternal disorders	10	54	Females Only
Maternal haemorrhage	10	54	Females Only
Maternal sepsis and other pregnancy related infections	10	54	Females Only
Maternal hypertensive disorders	10	54	Females Only
Maternal obstructed labour and uterine rupture	10	54	Females Only
Maternal abortive outcome	10	54	Females Only
Ectopic pregnancy	10	54	Females Only
Indirect maternal deaths	10	54	Females Only
Late maternal deaths	10	54	Females Only
Maternal deaths aggravated by HIV/AIDS	10	54	Females Only
Other maternal disorders	10	54	Females Only
Neonatal disorders		4	
Neonatal preterm birth		4	
Neonatal encephalopathy due to birth asphyxia and trauma		4	
Neonatal sepsis and other neonatal infections		4	
Hemolytic disease and other neonatal jaundice		4	
Other neonatal disorders		4	
Nutritional deficiencies	28 days		
Protein-energy malnutrition	28 days		
Other nutritional deficiencies	28 days		
Neoplasms			
Lip and oral cavity cancer	15		
Nasopharynx cancer	5		
Other pharynx cancer	15		
Oesophageal cancer	15		
Stomach cancer	15		
Colon and rectum cancer	15		
Liver cancer	5		
Liver cancer due to hepatitis B	5		
Liver cancer due to hepatitis C	5		
Liver cancer due to alcohol use	15		
Liver cancer due to NASH	15		
Liver cancer due to other causes	5		
Gallbladder and biliary tract cancer	15		
Pancreatic cancer	15		
Larynx cancer	15		
Tracheal, bronchus, and lung cancer	15		
Malignant skin melanoma	15		
Non-melanoma skin cancer	15		
Non-melanoma skin cancer (squamous-cell carcinoma)	15		
Breast cancer	15		
Cervical cancer	15		Females Only
Uterine cancer	15		Females Only
Ovarian cancer	15		Females Only
Prostate cancer	15		Males Only
Testicular cancer	15		Males Only
Kidney cancer			
Bladder cancer	15		
Brain and nervous system cancer			
Thyroid cancer	10		
Mesothelioma	15		

Appendix Table 5. Restrictions on age and sex by cause for GBD 2017			
Cause	Minimum Age	Maximum Age	Sex Restrictions
Hodgkin lymphoma			
Non-Hodgkin's lymphoma			
Multiple myeloma	15		
Leukaemia			
Acute lymphoid leukaemia			
Chronic lymphoid leukaemia	15		
Acute myeloid leukaemia			
Chronic myeloid leukaemia	15		
Other leukaemia			
Other malignant cancers			
Other neoplasms			
Myelodysplastic, myeloproliferative, and other hematopoietic neoplasms			
Other benign and in situ neoplasms			
Cardiovascular diseases			
Rheumatic heart disease	1		
Ischaemic heart disease	15		
Stroke			
Ischaemic stroke			
Intracerebral hemorrhage			
Subarachnoid hemorrhage			
Hypertensive heart disease	15		
Non-rheumatic valvular heart disease	15		
Non-rheumatic calcific aortic valve disease	15		
Non-rheumatic degenerative mitral valve disease	15		
Other non-rheumatic valve diseases	15		
Cardiomyopathy and myocarditis			
Myocarditis			
Alcoholic cardiomyopathy	15		
Other cardiomyopathy			
Atrial fibrillation and flutter	30		
Aortic aneurysm	15		
Peripheral vascular disease	40		
Endocarditis			
Other cardiovascular and circulatory diseases			
Chronic respiratory diseases			
Chronic obstructive pulmonary disease	28 days		
Pneumoconiosis	15		
Silicosis	15		
Asbestosis	15		
Coal workers pneumoconiosis	15		
Other pneumoconiosis	15		
Asthma	1		
Interstitial lung disease and pulmonary sarcoidosis	1		
Other chronic respiratory diseases			
Digestive diseases			
Cirrhosis and other chronic liver diseases	1		
Cirrhosis and other chronic liver diseases due to hepatitis B	1		
Cirrhosis and other chronic liver diseases due to hepatitis C	1		
Cirrhosis and other chronic liver diseases due to alcohol use	15		
Cirrhosis due to NASH	15		
Cirrhosis and other chronic liver diseases due to other causes	1		
Upper digestive system diseases	1		
Peptic ulcer disease	1		
Gastritis and duodenitis	1		
Appendicitis	1		
Paralytic ileus and intestinal obstruction			

Appendix Table 5. Restrictions on age and sex by cause for GBD 2017			
Cause	Minimum Age	Maximum Age	Sex Restrictions
Inguinal, femoral, and abdominal hernia	1		
Inflammatory bowel disease	1		
Vascular intestinal disorders	1		
Gallbladder and biliary diseases	1		
Pancreatitis	1		
Other digestive diseases	1		
Neurological disorders	28 days		
Alzheimer's disease and other dementias	40		
Parkinson's disease	20		
Epilepsy	28 days		
Multiple sclerosis	20		
Motor neuron disease			
Other neurological disorders	28 days		
Mental disorders			
Eating disorders	5	49	
Anorexia nervosa	5	49	
Bulimia nervosa	5	49	
Substance use disorders			
Alcohol use disorders	15		
Drug use disorders			
Opioid use disorders			
Cocaine use disorders	15		
Amphetamine use disorders	15		
Other drug use disorders	15		
Diabetes and kidney diseases			
Diabetes mellitus			
Diabetes mellitus type 1			
Diabetes mellitus type 2	15		
Chronic kidney disease	28 days		
Chronic kidney disease due to diabetes mellitus type 1	28 days		
Chronic kidney disease due to diabetes mellitus type 2	28 days		
Chronic kidney disease due to hypertension	28 days		
Chronic kidney disease due to glomerulonephritis	28 days		
Chronic kidney disease due to other and unspecified causes	28 days		
Acute glomerulonephritis	28 days		
Skin and subcutaneous diseases	28 days		
Bacterial skin diseases	7 days		
Cellulitis	28 days		
Pyoderma	28 days		
Decubitus ulcer	1		
Other skin and subcutaneous diseases	28 days		
Musculoskeletal disorders	5		
Rheumatoid arthritis	5		
Other musculoskeletal disorders	5		
Other non-communicable diseases			
Congenital anomalies		69	
Neural tube defects		69	
Congenital heart anomalies		69	
Orofacial clefts		4	
Down's syndrome		69	
Other chromosomal abnormalities		69	
Congenital musculoskeletal and limb anomalies		69	
Urogenital congenital anomalies		69	
Digestive congenital anomalies		69	
Other congenital anomalies		69	
Urinary diseases and male infertility			
Urinary tract infections			

Appendix Table 5. Restrictions on age and sex by cause for GBD 2017			
**Cause**	**Minimum Age**	**Maximum Age**	**Sex Restrictions**
Urolithiasis	5		
Other urinary diseases			
Gynecological diseases	15		Females Only
Uterine fibroids	15		Females Only
Polycystic ovarian syndrome	15	54	Females Only
Endometriosis	15	54	Females Only
Genital prolapse	15		Females Only
Other gynecological diseases	15		Females Only
Hemoglobinopathies and hemolytic anaemias			
Thalassemias			
Sickle cell disorders			
G6PD deficiency			
Other hemoglobinopathies and hemolytic anaemias			
Endocrine, metabolic, blood, and immune disorders			
Sudden infant death syndrome	7 days	364 days	
Transport injuries			
Road injuries			
Pedestrian road injuries			
Cyclist road injuries	1		
Motorcyclist road injuries			
Motor vehicle road injuries			
Other road injuries			
Other transport injuries			
Unintentional injuries			
Falls			
Drowning			
Fire, heat, and hot substances			
Poisonings			
Poisoning by carbon monoxide			
Poisoning by other means			
Exposure to mechanical forces			
Unintentional firearm injuries			
Other exposure to mechanical forces			
Adverse effects of medical treatment			
Animal contact			
Venomous animal contact			
Non-venomous animal contact			
Foreign body			
Pulmonary aspiration and foreign body in airway			
Foreign body in other body part			
Environmental heat and cold exposure			
Exposure to forces of nature			
Other unintentional injuries			
Self-harm and interpersonal violence			
Self-harm	10		
Self-harm by firearm	10		
Self-harm by other specified means	10		
Interpersonal violence			
Assault by firearm			
Assault by sharp object			
Assault by other means			
Conflict and terrorism			
Executions and police conflict	28 days		

Appendix Table 6. HIV/AIDS-related garbage code redistribution packages

Package name	ICD9 codes	ICD10 codes
Actinomycosis	39, 39.0, 39.1, 39.2, 39.3, 39.4, 39.6, 39.8, 39.9, 113, 113.2, 113.4, 113.5, 113.6	A42, A42.0, A42.1, A42.2, A42.7, A42.8, A42.81, A42.82, A42.89, A42.9
Bartonellosis	88.0, 88.2, 88.3, 88.5, 88.7	A44, A44.0, A44.1, A44.8, A44.9
Urogenital Candidiasis	112.1, 112.2	B37.3, B37.4, B37.41, B37.42, B37.49
Candidiasis	112, 112.0, 112.3, 112.4, 112.5, 112.6, 112.8, 112.81, 112.82, 112.83, 112.84, 112.85, 112.89, 112.9	B37, B37.0, B37.1, B37.2, B37.5, B37.6, B37.7, B37.8, B37.81, B37.82, B37.83, B37.84, B37.89, B37.9
Coccidioidomycosis	114, 114.0, 114.1, 114.2, 114.3, 114.4, 114.5, 114.6, 114.9	B38, B38.0, B38.1, B38.2, B38.3, B38.4, B38.7, B38.8, B38.81, B38.89, B38.9
Histoplasmosis	115, 115.0, 115.00, 115.01, 115.02, 115.03, 115.04, 115.05, 115.09, 115.1, 115.10, 115.11, 115.12, 115.13, 115.14, 115.15, 115.19, 115.2, 115.3, 115.4, 115.5, 115.9, 115.90, 115.91, 115.92, 115.93, 115.94, 115.95, 115.99	B39, B39.0, B39.1, B39.2, B39.3, B39.4, B39.5, B39.9
Blastomycosis	116, 116.0, 116.2, 116.3, 116.4, 116.5, 116.6, 116.9	B40, B40.0, B40.1, B40.2, B40.3, B40.7, B40.8, B40.81, B40.89, B40.9
Paracoccidioidomycosis	116.1	B41, B41.0, B41.7, B41.8, B41.9
Sporotrichosis & Chromomycosis	117.1	B42, B42.0, B42.1, B42.7, B42.8, B42.81, B42.82, B42.89, B42.9, B43, B43.0, B43.1, B43.2, B43.8, B43.9
Aspergillosis	117.3	B44, B44.0, B44.1, B44.2, B44.7, B44.8, B44.81, B44.89, B44.9
Zygomycosis	117.7	B46, B46.0, B46.1, B46.2, B46.3, B46.4, B46.5, B46.8, B46.9
Toxoplasmosis	130, 130.0, 130.1, 130.2, 130.3, 130.4, 130.5, 130.6, 130.7, 130.8, 130.9	B58, B58.0, B58.00, B58.01, B58.09, B58.1, B58.2, B58.3, B58.8, B58.81, B58.82, B58.83, B58.89, B58.9
Pneumocystosis, Psorospermiasis, Sarcosporidiosis	136.3, 136.4, 136.5	B59, B59.0, B59.9
Cryptococcosis	117.5	B45, B45.0, B45.1, B45.2, B45.3, B45.7, B45.8, B45.9
Chromoblastomycosis / Nocardiosis	117.2	A43, A43.0, A43.1, A43.8, A43.9
Mycoses / Unspecified mycosis	117, 117.0, 117.4, 117.6, 117.8, 117.9, 118, 118.0, 118.1, 118.2, 118.3, 118.4, 118.5, 118.6, 118.9	B49, B49.5, B49.9
Cutaneous leishmaniasis	85.1, 85.2, 85.3, 85.4, 85.5	B55.1, B55.2
Other Mycobacterial infection	31, 31.0, 31.2, 31.8, 31.9	A31, A31.0, A31.8, A31.9
Mycobacterial skin infection	31.1	A31.1, A31.2
Immunodeficiency - antibody	279.0, 279.00, 279.01, 279.02, 279.03, 279.04, 279.05, 279.06, 279.09, 279.1	D80, D80.0, D80.1, D80.2, D80.3, D80.4, D80.5, D80.6, D80.7, D80.8, D80.9
Immunodeficiency - WBC	279.10, 279.11, 279.12, 279.13, 279.19, 279.2, 279.3, 279.4, 279.41, 279.49	D81, D81.0, D81.1, D81.2, D81.3, D81.4, D81.5, D81.6, D81.7, D81.8, D81.81, D81.810, D81.818, D81.819, D81.89, D81.9, D82, D82.0, D82.1, D82.2, D82.3, D82.4, D82.8, D82.9
Immunodeficiency - other	279, 279.5, 279.50, 279.51, 279.52, 279.53, 279.6, 279.8, 279.9	D83, D83.0, D83.1, D83.2, D83.8, D83.9, D84, D84.0, D84.1, D84.8, D84.9, D89.8, D89.81, D89.810, D89.811, D89.812, D89.813, D89.82, D89.89, D89.9

Appendix Table 6 HIV/AIDS-related garbage code redistribution packages

Package name	ICD9 codes	ICD10 codes
Kaposi's sarcoma	176, 176.0, 176.1, 176.2, 176.3, 176.4, 176.5, 176.8, 176.9	C46, C46.0, C46.1, C46.2, C46.3, C46.4, C46.5, C46.50, C46.51, C46.52, C46.6, C46.7, C46.8, C46.9

Appendix Table 7. Data quality rating from 0 to 5 stars, maximum percent well certified per 5-year interval and percent well certified across time series for 195 countries, 1980-2017								
**Country**	**Data Quality Rating**	**1980-1984**	**1985-1989**	**1990-1994**	**1995-1999**	**2000-2004**	**2005-2009**	**2010-2017**	**1980-2017**
Afghanistan	1	0.0	0.0	0.0	0.0	4.6	38.2	0.0	6.1
Albania	3	0.0	66.1	68.6	70.7	71.8	72.5	54.7	57.8
Algeria	1	0.0	0.0	0.0	0.0	0.0	17.3	0.0	2.5
American Samoa	3	0.0	0.0	0.0	73.7	78.2	83.4	79.4	45.0
Andorra	0	0.0	0.0	0.0	0.0	0.0	0.0	0.0	0.0
Angola	1	0.0	0.0	0.0	0.0	0.0	0.0	4.4	0.6
Antigua and Barbuda	4	60.9	72.0	75.1	79.5	79.3	80.0	74.7	74.5
Argentina	4	77.4	70.9	69.7	68.5	66.7	66.6	69.9	70.0
Armenia	5	74.2	78.1	85.0	88.8	88.9	92.7	91.9	85.6
Australia	5	93.2	93.6	92.9	92.5	91.1	90.5	90.0	91.9
Austria	5	89.9	90.9	90.0	88.6	91.9	90.8	89.2	90.2
Azerbaijan	3	75.5	75.4	78.1	72.0	68.2	41.8	0.0	58.7
Bahrain	3	0.0	78.1	0.0	61.6	64.6	55.9	56.8	45.3
Bangladesh	1	2.8	4.5	25.7	4.4	12.4	6.3	6.3	8.9
Barbados	4	75.0	75.8	77.6	77.4	75.6	82.1	82.0	77.9
Belarus	4	84.0	88.9	80.1	82.9	85.4	84.5	85.0	84.4
Belgium	4	77.8	77.6	81.3	84.1	82.8	82.7	80.4	81.0
Belize	4	47.5	52.4	49.8	76.6	78.3	87.1	89.1	68.7
Benin	1	0.0	0.6	0.0	0.0	0.0	0.0	0.0	0.1
Bermuda	5	90.4	88.7	90.2	93.5	91.1	86.4	90.3	90.1
Bhutan	0	0.0	0.0	0.0	0.0	0.0	0.0	0.0	0.0
Bolivia	1	0.0	0.0	0.0	0.0	14.7	0.0	0.0	2.1
Bosnia and Herzegovina	2	0.0	65.8	67.1	0.0	0.0	0.0	71.4	29.2
Botswana	0	0.0	0.0	0.0	0.0	0.0	0.0	0.0	0.0
Brazil	4	54.0	58.9	65.2	68.9	74.6	80.7	82.5	69.3
Brunei	3	0.0	0.0	0.0	66.7	73.2	76.1	74.5	41.5
Bulgaria	4	81.7	82.3	79.6	76.5	71.8	73.5	70.2	76.5
Burkina Faso	1	0.2	0.0	0.0	4.6	6.3	4.6	0.3	2.3
Burundi	1	0.0	0.0	2.2	0.0	0.0	0.0	0.0	0.3
Cambodia	1	0.0	0.0	0.0	0.0	1.6	4.4	0.0	0.9
Cameroon	0	0.0	0.0	0.0	0.0	0.0	0.0	0.0	0.0
Canada	5	89.4	90.0	89.1	88.6	89.3	89.4	90.1	89.4
Cape Verde	2	56.1	0.0	0.1	0.0	0.0	0.0	67.7	17.7
Central African Republic	0	0.0	0.0	0.0	0.0	0.0	0.0	0.0	0.0
Chad	0	0.0	0.0	0.0	0.0	0.0	0.0	0.0	0.0
Chile	4	75.7	75.5	81.7	84.7	90.8	90.8	89.8	84.1
China	3	0.0	0.9	73.8	75.1	70.3	73.0	73.5	52.4
Colombia	4	72.4	74.4	77.3	88.0	88.6	88.9	88.5	82.6
Comoros	0	0.0	0.0	0.0	0.0	0.0	0.0	0.0	0.0
Congo (Brazzaville)	0	0.0	0.0	0.0	0.0	0.0	0.0	0.0	0.0
Costa Rica	5	81.6	83.4	82.5	91.1	92.3	90.6	90.5	87.4
Cote d'Ivoire	1	0.0	1.2	1.2	0.0	0.0	0.2	0.2	0.4
Croatia	4	0.0	84.4	85.3	82.6	86.1	88.3	92.3	74.1
Cuba	5	84.7	85.1	84.9	88.9	90.2	91.0	91.5	88.0
Cyprus	2	0.0	0.0	0.0	26.8	53.8	59.5	60.9	28.7
Czech Republic	4	0.0	90.7	89.6	84.8	84.7	85.8	87.8	74.8
DR Congo	1	0.0	2.3	3.0	0.0	0.0	0.0	0.0	0.8
Denmark	4	84.5	82.9	83.1	85.8	85.4	84.5	84.6	84.4
Djibouti	0	0.0	0.0	0.0	0.0	0.0	0.0	0.0	0.0
Dominica	4	71.6	63.1	62.1	65.8	68.6	81.7	83.9	71.0
Dominican Republic	3	53.6	53.5	44.5	53.8	57.2	56.8	59.9	54.2
Ecuador	4	73.0	72.4	72.9	69.8	66.4	62.9	70.4	69.7
Egypt	3	35.0	48.2	46.8	0.0	43.9	42.7	48.2	37.8
El Salvador	3	73.3	0.0	60.4	67.2	69.4	70.8	67.4	58.4
Equatorial Guinea	0	0.0	0.0	0.0	0.0	0.0	0.0	0.0	0.0
Eritrea	0	0.0	0.0	0.0	0.0	0.0	0.0	0.0	0.0
Estonia	5	92.1	92.7	93.8	93.1	92.5	94.1	93.9	93.2
Ethiopia	1	0.0	1.2	2.5	0.6	5.0	4.6	4.6	2.6
Federated States of Micronesia	0	0.0	0.0	0.0	0.0	0.0	0.0	0.0	0.0
Fiji	2	0.0	0.0	0.0	34.8	58.0	63.9	70.0	32.4
Finland	5	83.7	91.7	91.7	95.8	95.6	94.9	95.9	92.8
France	4	76.1	78.4	79.1	79.0	79.1	79.4	77.4	78.3
Gabon	0	0.0	0.0	0.0	0.0	0.0	0.0	0.0	0.0
Georgia	4	83.4	80.7	81.3	77.7	78.7	62.2	58.9	74.7
Germany	4	78.4	80.5	84.1	83.9	83.5	83.8	84.0	82.6
Ghana	1	0.0	0.1	1.5	0.8	7.2	16.7	0.5	3.8
Greece	4	80.2	81.6	71.7	72.0	72.6	77.9	76.2	76.0
Greenland	3	0.0	0.0	0.0	90.2	89.7	89.6	87.6	51.0
Grenada	4	67.7	63.4	64.0	63.2	77.0	79.9	85.9	71.6
Guam	3	0.0	0.0	85.9	80.7	76.7	79.9	79.2	57.5
Guatemala	4	80.1	75.0	73.9	72.6	73.3	75.2	78.0	75.4
Guinea	1	0.0	0.0	0.0	3.2	0.0	0.0	0.0	0.5
Guinea-Bissau	1	0.0	0.0	0.1	1.2	0.0	0.0	0.0	0.2
Guyana	4	55.5	76.2	69.8	78.3	84.3	85.5	78.6	75.5

Appendix Table 7. Data quality rating from 0 to 5 stars, maximum percent well certified per 5-year interval and percent well certified across time series for 195 countries, 1980-2017								
Country	Data Quality Rating	1980-1984	1985-1989	1990-1994	1995-1999	2000-2004	2005-2009	2010-2017	1980-2017
Haiti	1	22.3	1.3	1.0	13.9	6.1	0.0	0.0	6.4
Honduras	2	41.2	46.0	43.2	0.0	0.0	17.5	19.1	23.8
Hungary	5	91.0	89.8	89.9	91.0	92.1	92.6	93.8	91.5
Iceland	5	92.6	93.8	94.2	94.1	93.5	92.9	91.4	93.2
India	2	2.8	3.0	2.9	5.2	6.3	52.7	49.0	17.4
Indonesia	2	1.4	0.0	1.3	0.4	0.3	53.7	62.3	17.1
Iran	2	0.0	0.0	0.0	0.0	52.8	63.7	80.3	28.1
Iraq	2	0.0	0.0	0.0	0.0	0.0	45.8	56.7	14.6
Ireland	5	90.3	91.6	91.7	90.9	90.2	92.4	92.3	91.3
Israel	4	81.2	82.6	83.2	82.7	81.8	80.1	80.7	81.8
Italy	5	88.8	88.4	88.5	87.8	88.1	88.7	88.3	88.4
Jamaica	4	70.5	72.8	64.6	0.0	83.0	87.5	89.1	66.8
Japan	4	82.6	81.0	81.5	87.6	85.7	84.4	81.3	83.4
Jordan	2	0.0	0.0	0.0	1.9	70.1	76.5	74.1	31.8
Kazakhstan	5	80.0	84.9	89.1	88.6	85.0	84.2	84.1	85.1
Kenya	1	0.0	2.7	0.0	0.4	4.9	5.4	0.8	2.0
Kiribati	2	0.0	0.0	47.9	66.6	35.6	0.0	0.0	21.4
Kuwait	4	82.1	81.9	76.4	77.5	83.1	84.9	84.6	81.5
Kyrgyzstan	4	67.5	75.9	72.5	74.4	87.4	92.9	93.2	80.6
Laos	1	0.0	1.3	0.0	0.0	0.0	0.0	0.0	0.2
Latvia	5	92.5	92.7	89.3	92.0	91.1	88.7	94.3	91.5
Lebanon	1	0.0	3.5	0.0	0.0	0.0	0.0	0.0	0.5
Lesotho	0	0.0	0.0	0.0	0.0	0.0	0.0	0.0	0.0
Liberia	1	2.1	2.1	3.2	0.0	0.0	0.0	0.0	1.1
Libya	1	0.0	0.0	0.0	0.0	0.0	12.6	0.0	1.8
Lithuania	5	90.1	94.0	93.7	94.8	92.9	93.3	94.6	93.3
Luxembourg	4	86.4	85.8	85.5	84.5	80.6	77.9	82.5	83.3
Macedonia	3	0.0	0.0	82.5	83.8	84.5	81.7	79.6	58.9
Madagascar	1	2.8	3.5	2.4	2.4	0.0	0.0	0.0	1.6
Malawi	1	0.0	2.5	0.0	0.6	2.5	4.5	0.6	1.5
Malaysia	2	46.9	0.0	0.0	32.1	37.3	42.0	40.2	28.4
Maldives	2	0.0	0.0	0.0	0.0	44.0	46.6	60.1	21.5
Mali	1	4.0	0.0	0.1	0.0	0.0	0.0	0.0	0.6
Malta	5	81.2	86.3	88.7	90.0	90.3	92.8	89.5	88.4
Marshall Islands	0	0.0	0.0	0.0	0.0	0.0	0.0	0.0	0.0
Mauritania	0	0.0	0.0	0.0	0.0	0.0	0.0	0.0	0.0
Mauritius	4	75.2	79.4	78.7	78.2	83.2	85.1	86.3	80.9
Mexico	4	68.3	77.7	78.5	79.7	82.4	84.1	88.1	79.8
Moldova	5	88.8	91.2	81.9	87.8	85.0	83.5	83.7	86.0
Mongolia	2	0.0	0.0	61.2	0.0	19.6	21.4	83.4	26.5
Montenegro	2	0.0	0.0	0.0	0.0	69.2	69.2	0.0	19.8
Morocco	2	0.0	15.4	0.0	0.0	10.2	33.3	13.7	10.4
Mozambique	2	0.0	0.0	0.0	0.1	16.2	63.4	0.0	11.4
Myanmar	1	0.0	0.0	0.0	0.0	0.0	2.9	49.5	7.5
Namibia	0	0.0	0.0	0.0	0.0	0.0	0.0	0.0	0.0
Nepal	1	2.8	2.6	0.0	0.6	0.6	9.2	0.0	2.3
Netherlands	4	88.5	86.7	85.2	83.9	82.3	83.2	83.3	84.7
New Zealand	5	95.3	95.2	95.1	96.8	96.4	96.3	95.7	95.8
Nicaragua	3	0.0	54.3	56.9	69.0	77.2	84.9	90.5	61.8
Niger	1	0.0	0.0	0.0	0.0	0.0	35.7	0.0	5.1
Nigeria	1	0.0	0.0	3.3	0.0	0.0	0.1	32.3	5.1
North Korea	0	0.0	0.0	0.0	0.0	0.0	0.0	0.0	0.0
Northern Mariana Islands	3	0.0	0.0	0.0	75.3	71.4	80.3	82.5	44.2
Norway	5	88.6	89.4	88.6	88.5	86.3	84.2	84.1	87.1
Oman	2	0.0	0.0	0.0	0.0	0.0	73.2	34.4	15.4
Pakistan	1	0.0	2.8	1.6	0.0	1.5	17.8	1.9	3.7
Palestine	2	0.0	0.0	0.0	29.7	32.2	34.2	74.1	24.3
Panama	4	72.3	75.3	0.0	83.5	86.6	86.3	84.0	69.7
Papua New Guinea	1	9.4	3.4	0.0	0.0	0.0	0.0	28.8	6.0
Paraguay	3	51.3	51.2	57.2	62.7	63.6	68.8	74.0	61.3
Peru	3	54.9	36.1	37.2	51.1	63.3	65.4	65.9	53.4
Philippines	4	68.2	71.1	65.9	68.6	74.3	74.1	74.8	71.0
Poland	4	61.8	59.5	59.9	71.6	73.7	73.5	72.6	67.5
Portugal	4	76.9	77.1	76.3	76.2	79.1	77.7	80.1	77.6
Puerto Rico	4	78.6	76.7	83.9	83.2	83.6	83.6	84.4	82.0
Qatar	3	14.7	18.9	0.0	55.1	56.3	64.9	46.1	36.6
Romania	4	76.0	78.0	83.3	84.8	85.9	86.2	85.4	82.8
Russian Federation	5	93.0	87.3	90.7	87.6	88.0	89.3	88.8	89.3
Rwanda	1	0.0	0.0	0.0	0.0	0.0	22.6	0.0	3.2
Saint Lucia	4	66.8	68.2	73.2	72.1	80.0	79.0	85.6	75.0
Saint Vincent and the Grenadines	4	72.3	63.4	61.2	85.3	85.1	87.6	87.3	77.5
Samoa	0	0.0	0.0	0.0	0.0	0.0	0.0	0.0	0.0
Sao Tome and Principe	1	0.0	69.6	0.0	0.0	0.0	0.0	0.0	9.9
Saudi Arabia	2	0.0	0.0	0.0	23.1	27.5	29.4	28.8	15.5

Appendix Table 7. Data quality rating from 0 to 5 stars, maximum percent well certified per 5-year interval and percent well certified across time series for 195 countries, 1980-2017

Country	Data Quality Rating	1980-1984	1985-1989	1990-1994	1995-1999	2000-2004	2005-2009	2010-2017	1980-2017
Senegal	1	2.0	2.5	2.6	2.5	0.0	0.0	0.0	1.4
Serbia	3	0.0	0.0	0.0	66.7	70.8	74.9	69.6	40.3
Seychelles	3	74.4	71.5	0.0	0.0	75.9	76.9	78.1	53.8
Sierra Leone	1	0.0	0.0	3.4	0.0	0.0	0.0	0.0	0.5
Singapore	5	91.2	92.0	94.6	95.1	95.3	94.9	95.7	94.1
Slovakia	3	0.0	0.0	82.7	81.9	84.9	90.2	92.9	61.8
Slovenia	4	0.0	91.6	92.5	91.8	89.3	88.3	88.5	77.4
Solomon Islands	1	0.0	0.0	0.0	0.0	0.0	0.0	34.6	4.9
Somalia	0	0.0	0.0	0.0	0.0	0.0	0.0	0.0	0.0
South Africa	3	0.0	0.0	1.0	67.8	72.0	72.3	70.7	40.5
South Korea	4	0.0	57.8	74.2	75.4	84.5	82.2	81.9	65.1
South Sudan	0	0.0	0.0	0.0	0.0	0.0	0.0	0.0	0.0
Spain	4	77.3	79.4	82.6	83.8	83.1	84.0	84.8	82.1
Sri Lanka	3	52.0	50.2	47.7	55.4	63.7	67.4	64.8	57.3
Sudan	0	0.0	0.0	0.0	0.0	0.0	0.0	0.0	0.0
Suriname	4	60.0	63.7	61.2	66.4	74.3	73.3	73.9	67.5
Swaziland	1	0.0	0.0	0.0	0.0	0.0	0.0	0.0	0.0
Sweden	5	87.4	88.6	88.2	87.1	85.7	85.9	84.8	86.8
Switzerland	4	73.8	74.2	73.4	84.6	84.4	86.7	86.1	80.5
Syria	3	29.5	13.2	0.0	54.3	66.7	74.4	59.8	42.5
Taiwan (Province of China)	4	84.3	82.4	79.2	83.4	84.4	84.8	83.7	83.2
Tajikistan	3	74.9	71.4	71.6	58.5	51.4	50.6	50.7	61.3
Tanzania	1	0.0	2.8	1.8	2.1	6.3	2.5	0.0	2.2
Thailand	3	29.9	28.3	35.9	62.6	52.0	61.6	62.3	47.5
The Bahamas	4	76.2	82.6	83.0	89.8	87.7	86.7	86.3	84.6
The Gambia	1	2.8	2.2	2.1	0.9	0.7	1.0	0.0	1.4
Timor-Leste	0	0.0	0.0	0.0	0.0	0.0	0.0	0.0	0.0
Togo	0	0.0	0.0	0.0	0.0	0.0	0.0	0.0	0.0
Tonga	1	0.0	0.0	0.0	0.0	53.6	0.0	0.0	7.7
Trinidad and Tobago	5	79.2	83.4	85.5	89.5	90.7	89.6	90.1	86.9
Tunisia	1	0.0	0.0	0.0	0.0	0.0	28.9	24.8	7.7
Turkey	3	16.8	19.6	20.7	24.0	59.7	70.3	83.9	42.2
Turkmenistan	4	88.4	88.1	83.0	80.9	85.1	81.0	83.4	84.3
Uganda	1	0.0	0.0	0.0	0.0	0.0	2.7	0.0	0.4
Ukraine	5	87.4	90.0	83.5	86.0	86.4	90.9	92.2	88.1
United Arab Emirates	1	0.0	0.0	0.0	0.0	0.0	41.5	0.0	5.9
United Kingdom	5	94.2	94.1	94.1	92.0	91.4	91.5	92.4	92.8
USA	5	88.5	88.1	88.7	88.1	88.0	87.2	86.7	87.9
Uruguay	4	77.3	76.0	79.1	79.1	79.6	78.5	77.5	78.1
Uzbekistan	4	83.1	86.3	85.0	79.0	68.3	72.2	79.8	79.1
Vanuatu	0	0.0	0.0	0.0	0.0	0.0	0.0	0.0	0.0
Venezuela	5	81.3	75.2	83.3	87.8	89.8	89.5	88.9	85.1
Vietnam	1	0.0	0.7	0.2	1.3	0.9	62.0	4.8	10.0
Virgin Islands	4	82.0	0.0	90.6	90.5	84.1	81.9	74.0	71.8
Yemen	0	0.0	0.0	0.0	0.0	0.0	0.0	0.0	0.0
Zambia	1	0.0	0.0	0.0	0.0	0.0	6.3	9.5	2.3
Zimbabwe	2	0.0	0.0	45.3	74.2	0.0	45.3	0.0	23.5

Appendix Table 8. Underlying indicators for percent well-certified for data source with maximum percent well certified in each 5-year time interval for 195 countries, 1980-2017								
**Country**	**Time Window**	**Stars**	**Percent Well-Certified [PWC] (%)**	**Max PWC Data Year**	**Max PWC Data Source**	**Completeness (%)**	**Percent Major Garbage (%)**	**Verbal Autopsy Adjustment (None for VR) (%)**
Afghanistan	1980-1984	0						
Afghanistan	1985-1989	0						
Afghanistan	1990-1994	0						
Afghanistan	1995-1999	0						
Afghanistan	2000-2004	1	4.6	2001	Afghanistan - Badghis Nutrition and Health Survey 2002		27.8	6.4
Afghanistan	2005-2009	3	38.2	2008	Afghanistan Special Demographic and Health Survey 2010		40.3	64.0
Afghanistan	2010-2017	0						
Albania	1980-1984	0						
Albania	1985-1989	4	66.1	1989	Vital Registration	100.0	33.9	
Albania	1990-1994	4	68.6	1993	Vital Registration	100.0	31.4	
Albania	1995-1999	4	70.7	1998	Vital Registration	98.3	28.1	
Albania	2000-2004	4	71.8	2000	Vital Registration	100.0	28.2	
Albania	2005-2009	4	72.5	2008	Vital Registration	100.0	27.5	
Albania	2010-2017	3	54.7	2010	Vital Registration	72.2	24.2	
Algeria	1980-1984	0						
Algeria	1985-1989	0						
Algeria	1990-1994	0						
Algeria	1995-1999	0						
Algeria	2000-2004	0						
Algeria	2005-2009	2	17.3	2006	Vital Registration	30.7	43.7	
Algeria	2010-2017	0						
American Samoa	1980-1984	0						
American Samoa	1985-1989	0						
American Samoa	1990-1994	0						
American Samoa	1995-1999	4	73.7	1997	Vital Registration	94.3	21.8	
American Samoa	2000-2004	4	78.2	2002	Vital Registration	96.9	19.3	
American Samoa	2005-2009	4	83.4	2009	Vital Registration	100.0	16.6	
American Samoa	2010-2017	4	79.4	2015	Vital Registration	93.2	14.9	
Andorra	1980-1984	0						
Andorra	1985-1989	0						
Andorra	1990-1994	0						
Andorra	1995-1999	0						
Andorra	2000-2004	0						
Andorra	2005-2009	0						
Andorra	2010-2017	0						
Angola	1980-1984	0						
Angola	1985-1989	0						
Angola	1990-1994	0						
Angola	1995-1999	0						
Angola	2000-2004	0						
Angola	2005-2009	0						
Angola	2010-2017	1	4.4	2010	Angola - Dande Health and Demographic Surveillance System		30.9	6.4
Antigua and Barbuda	1980-1984	3	60.9	1983	Vital Registration	91.2	33.3	
Antigua and Barbuda	1985-1989	4	72.0	1989	Vital Registration	100.0	28.0	
Antigua and Barbuda	1990-1994	4	75.1	1991	Vital Registration	100.0	24.9	
Antigua and Barbuda	1995-1999	4	79.5	1996	Vital Registration	100.0	20.5	
Antigua and Barbuda	2000-2004	4	79.3	2004	Vital Registration	100.0	20.7	
Antigua and Barbuda	2005-2009	4	80.0	2007	Vital Registration	100.0	20.0	
Antigua and Barbuda	2010-2017	4	74.7	2014	Vital Registration	100.0	25.3	
Argentina	1980-1984	4	77.4	1981	Vital Registration	99.9	22.5	
Argentina	1985-1989	4	70.9	1987	Vital Registration	100.0	29.1	
Argentina	1990-1994	4	69.7	1990	Vital Registration	100.0	30.3	
Argentina	1995-1999	4	68.5	1996	Vital Registration	100.0	31.5	
Argentina	2000-2004	4	66.7	2000	Vital Registration	99.9	33.3	
Argentina	2005-2009	4	66.6	2005	Vital Registration	100.0	33.4	
Argentina	2010-2017	4	69.9	2015	Vital Registration	98.9	29.3	
Armenia	1980-1984	4	74.2	1982	Vital Registration	85.7	13.5	
Armenia	1985-1989	4	78.1	1989	Vital Registration	90.7	13.9	
Armenia	1990-1994	4	85.0	1992	Vital Registration	97.8	13.2	
Armenia	1995-1999	5	88.8	1999	Vital Registration	100.0	11.2	
Armenia	2000-2004	5	88.9	2000	Vital Registration	99.8	10.9	
Armenia	2005-2009	5	92.7	2008	Vital Registration	99.8	7.1	
Armenia	2010-2017	5	91.9	2010	Vital Registration	100.0	8.1	
Australia	1980-1984	5	93.2	1982	Vital Registration	100.0	6.8	
Australia	1985-1989	5	93.6	1987	Vital Registration	100.0	6.4	
Australia	1990-1994	5	92.9	1990	Vital Registration	100.0	7.1	
Australia	1995-1999	5	92.5	1996	Vital Registration	100.0	7.5	
Australia	2000-2004	5	91.1	2002	Vital Registration	100.0	8.9	
Australia	2005-2009	5	90.5	2008	Vital Registration	100.0	9.5	
Australia	2010-2017	5	90.0	2011	Vital Registration	99.8	9.8	
Austria	1980-1984	5	89.9	1980	Vital Registration	100.0	10.1	
Austria	1985-1989	5	90.9	1987	Vital Registration	100.0	9.1	
Austria	1990-1994	5	90.0	1990	Vital Registration	100.0	10.0	
Austria	1995-1999	5	88.6	1996	Vital Registration	99.9	11.2	

Appendix Table 8. Underlying indicators for percent well-certified for data source with maximum percent well certified in each 5-year time interval for 195 countries, 1980-2017								
**Country**	**Time Window**	**Stars**	**Percent Well-Certified [PWC] (%)**	**Max PWC Data Year**	**Max PWC Data Source**	**Completeness (%)**	**Percent Major Garbage (%)**	**Verbal Autopsy Adjustment (None for VR) (%)**
Austria	2000-2004	5	91.9	2003	Vital Registration	100.0	8.1	
Austria	2005-2009	5	90.8	2008	Vital Registration	100.0	9.2	
Austria	2010-2017	5	89.2	2013	Vital Registration	100.0	10.8	
Azerbaijan	1980-1984	4	75.5	1981	Vital Registration	87.4	13.6	
Azerbaijan	1985-1989	4	75.4	1988	Vital Registration	86.6	12.9	
Azerbaijan	1990-1994	4	78.1	1993	Vital Registration	88.4	11.6	
Azerbaijan	1995-1999	4	72.0	1995	Vital Registration	84.1	14.4	
Azerbaijan	2000-2004	4	68.2	2003	Vital Registration	73.7	7.5	
Azerbaijan	2005-2009	3	41.8	2007	Vital Registration	73.1	42.9	
Azerbaijan	2010-2017	0						
Bahrain	1980-1984	0						
Bahrain	1985-1989	4	78.1	1986	Vital Registration	100.0	21.9	
Bahrain	1990-1994	0						
Bahrain	1995-1999	3	61.6	1997	Vital Registration	90.7	32.1	
Bahrain	2000-2004	3	64.6	2000	Vital Registration	99.0	34.8	
Bahrain	2005-2009	3	55.9	2005	Vital Registration	91.8	39.1	
Bahrain	2010-2017	3	56.8	2014	Vital Registration	88.9	36.2	
Bangladesh	1980-1984	1	2.8	1984	Bangladesh - Matlab Health and Demographic Surveillance System		55.5	6.4
Bangladesh	1985-1989	1	4.5	1989	Bangladesh - Matlab Health and Demographic Surveillance System		29.3	6.4
Bangladesh	1990-1994	2	25.7	1991	Causes of childhood deaths in Bangladesh: an update		16.7	30.8
Bangladesh	1995-1999	1	4.4	1998	Bangladesh - Matlab Health and Demographic Surveillance System		31.9	6.4
Bangladesh	2000-2004	2	12.4	2004	Bangladesh Demographic and Health Survey 2004		40.9	20.9
Bangladesh	2005-2009	1	6.3	2006	Bangladesh - Matlab Health and Demographic Surveillance System		2.2	6.4
Bangladesh	2010-2017	1	6.3	2010	Bangladesh - Matlab Health and Demographic Surveillance System		1.6	6.4
Barbados	1980-1984	4	75.0	1980	Vital Registration	100.0	25.0	
Barbados	1985-1989	4	75.8	1987	Vital Registration	100.0	24.2	
Barbados	1990-1994	4	77.6	1992	Vital Registration	100.0	22.4	
Barbados	1995-1999	4	77.4	1995	Vital Registration	100.0	22.6	
Barbados	2000-2004	4	75.6	2000	Vital Registration	100.0	24.4	
Barbados	2005-2009	4	82.1	2007	Vital Registration	100.0	17.9	
Barbados	2010-2017	4	82.0	2011	Vital Registration	100.0	18.0	
Belarus	1980-1984	4	84.0	1982	Vital Registration	97.6	13.9	
Belarus	1985-1989	5	88.9	1987	Vital Registration	100.0	11.1	
Belarus	1990-1994	4	80.1	1990	Vital Registration	100.0	19.9	
Belarus	1995-1999	4	82.9	1999	Vital Registration	100.0	17.1	
Belarus	2000-2004	5	85.4	2002	Vital Registration	100.0	14.6	
Belarus	2005-2009	4	84.5	2007	Vital Registration	100.0	15.5	
Belarus	2010-2017	4	85.0	2010	Vital Registration	100.0	15.0	
Belgium	1980-1984	4	77.8	1981	Vital Registration	100.0	22.2	
Belgium	1985-1989	4	77.6	1987	Vital Registration	100.0	22.4	
Belgium	1990-1994	4	81.3	1993	Vital Registration	100.0	18.7	
Belgium	1995-1999	4	84.1	1998	Vital Registration	100.0	15.9	
Belgium	2000-2004	4	82.8	2000	Vital Registration	99.5	16.8	
Belgium	2005-2009	4	82.7	2008	Vital Registration	99.7	17.0	
Belgium	2010-2017	4	80.4	2010	Vital Registration	100.0	19.6	
Belize	1980-1984	3	47.5	1984	Vital Registration	69.5	31.7	
Belize	1985-1989	3	52.4	1987	Vital Registration	68.2	23.2	
Belize	1990-1994	3	49.8	1993	Vital Registration	76.1	34.6	
Belize	1995-1999	4	76.6	1998	Vital Registration	100.0	23.4	
Belize	2000-2004	4	78.3	2002	Vital Registration	100.0	21.7	
Belize	2005-2009	5	87.1	2009	Vital Registration	100.0	12.9	
Belize	2010-2017	5	89.1	2011	Vital Registration	100.0	10.9	
Benin	1980-1984	0						
Benin	1985-1989	1	0.6	1989	Incidence de décès de 0 à 1 an dans une cohorte de 802 enfants en milieu rural au sud du Bénir		72.0	2.0
Benin	1990-1994	0						
Benin	1995-1999	0						
Benin	2000-2004	0						
Benin	2005-2009	0						
Benin	2010-2017	0						
Bermuda	1980-1984	5	90.4	1984	Vital Registration	100.0	9.6	
Bermuda	1985-1989	5	88.7	1985	Vital Registration	100.0	11.3	
Bermuda	1990-1994	5	90.2	1991	Vital Registration	100.0	9.8	
Bermuda	1995-1999	5	93.5	1996	Vital Registration	100.0	6.5	
Bermuda	2000-2004	5	91.1	2002	Vital Registration	100.0	8.9	
Bermuda	2005-2009	5	86.4	2005	Vital Registration	100.0	13.6	
Bermuda	2010-2017	5	90.3	2014	Vital Registration	100.0	9.7	
Bhutan	1980-1984	0						
Bhutan	1985-1989	0						
Bhutan	1990-1994	0						
Bhutan	1995-1999	0						
Bhutan	2000-2004	0						
Bhutan	2005-2009	0						

Appendix Table 8. Underlying indicators for percent well-certified for data source with maximum percent well certified in each 5-year time interval for 195 countries, 1980-2017								
Country	Time Window	Stars	Percent Well-Certified [PWC] (%)	Max PWC Data Year	Max PWC Data Source	Completeness (%)	Percent Major Garbage (%)	Verbal Autopsy Adjustment (None for VR) (%)
Bhutan	2010-2017	0						
Bolivia	1980-1984	0						
Bolivia	1985-1989	0						
Bolivia	1990-1994	0						
Bolivia	1995-1999	0						
Bolivia	2000-2004	2	14.7	2003	Vital Registration	46.5	68.5	
Bolivia	2005-2009	0						
Bolivia	2010-2017	0						
Bosnia and Herzegovina	1980-1984	0						
Bosnia and Herzegovina	1985-1989	4	65.8	1988	Vital Registration	98.6	33.3	
Bosnia and Herzegovina	1990-1994	4	67.1	1990	Vital Registration	99.9	32.8	
Bosnia and Herzegovina	1995-1999	0						
Bosnia and Herzegovina	2000-2004	0						
Bosnia and Herzegovina	2005-2009	0						
Bosnia and Herzegovina	2010-2017	4	71.4	2014	Vital Registration	99.1	28.0	
Botswana	1980-1984	0						
Botswana	1985-1989	0						
Botswana	1990-1994	0						
Botswana	1995-1999	0						
Botswana	2000-2004	0						
Botswana	2005-2009	0						
Botswana	2010-2017	0						
Brazil	1980-1984	3	54.0	1984	Vital Registration	84.2	35.8	
Brazil	1985-1989	3	58.9	1989	Vital Registration	86.9	32.3	
Brazil	1990-1994	4	65.2	1994	Vital Registration	93.8	30.5	
Brazil	1995-1999	4	68.9	1999	Vital Registration	95.5	27.8	
Brazil	2000-2004	4	74.6	2004	Vital Registration	98.5	24.3	
Brazil	2005-2009	4	80.7	2009	Vital Registration	99.6	19.0	
Brazil	2010-2017	4	82.5	2014	Vital Registration	100.0	17.5	
Brunei	1980-1984	0						
Brunei	1985-1989	0						
Brunei	1990-1994	0						
Brunei	1995-1999	4	66.7	1998	Vital Registration	77.8	14.2	
Brunei	2000-2004	4	73.2	2002	Vital Registration	88.4	17.2	
Brunei	2005-2009	4	76.1	2007	Vital Registration	93.0	18.1	
Brunei	2010-2017	4	74.5	2015	Vital Registration	87.9	15.3	
Bulgaria	1980-1984	4	81.7	1984	Vital Registration	100.0	18.3	
Bulgaria	1985-1989	4	82.3	1986	Vital Registration	100.0	17.7	
Bulgaria	1990-1994	4	79.6	1991	Vital Registration	100.0	20.4	
Bulgaria	1995-1999	4	76.5	1995	Vital Registration	99.8	23.3	
Bulgaria	2000-2004	4	71.8	2002	Vital Registration	100.0	28.2	
Bulgaria	2005-2009	4	73.5	2005	Vital Registration	100.0	26.5	
Bulgaria	2010-2017	4	70.2	2012	Vital Registration	100.0	29.8	
Burkina Faso	1980-1984	1	0.2	1984	The burden of malaria mortality among African children in the year 2000		95.3	3.8
Burkina Faso	1985-1989	0						
Burkina Faso	1990-1994	0						
Burkina Faso	1995-1999	1	4.6	1998	Measuring the local burden of disease. A study of years of life lost in sub-Saharan Africa		27.4	6.4
Burkina Faso	2000-2004	1	6.3	2000	Burkina Faso - Nouna Health and Demographic Surveillance System		1.0	6.4
Burkina Faso	2005-2009	1	4.6	2009	An improved method for physician-certified verbal autopsy reduces the rate of discrepancy: experiences in the Nouna Health and Demographic Surveillance Site (NHDSS), Burkina Faso		28.8	6.4
Burkina Faso	2010-2017	1	0.3	2010	Africa, Asia, Oceania - INDEPTH Network Cause-Specific Mortality - Release 2014		95.4	6.4
Burundi	1980-1984	0						
Burundi	1985-1989	0						
Burundi	1990-1994	1	2.2	1990	Mortality and morbidity at young ages in a stable hyperendemic malaria region, community Nyanza-Lac, Imbo South, Burundi		23.1	2.8
Burundi	1995-1999	0						
Burundi	2000-2004	0						
Burundi	2005-2009	0						
Burundi	2010-2017	0						
Cambodia	1980-1984	0						
Cambodia	1985-1989	0						
Cambodia	1990-1994	0						
Cambodia	1995-1999	0						
Cambodia	2000-2004	1	1.6	2001	Community-based surveillance: a pilot study from rural Cambodia		74.8	6.4
Cambodia	2005-2009	1	4.4	2009	Mortality in Cambodia An 18-Month Prospective Community-based Surveillance of All-age Deaths Using Verbal Autopsies		31.4	6.4
Cambodia	2010-2017	0						
Cameroon	1980-1984	0						
Cameroon	1985-1989	0						
Cameroon	1990-1994	0						
Cameroon	1995-1999	0						
Cameroon	2000-2004	0						
Cameroon	2005-2009	0						

Appendix Table 8. Underlying indicators for percent well-certified for data source with maximum percent well certified in each 5-year time interval for 195 countries, 1980-2017								
**Country**	**Time Window**	**Stars**	**Percent Well-Certified [PWC] (%)**	**Max PWC Data Year**	**Max PWC Data Source**	**Completeness (%)**	**Percent Major Garbage (%)**	**Verbal Autopsy Adjustment (None for VR) (%)**
Cameroon	2010-2017	0						
Canada	1980-1984	5	89.4	1984	Vital Registration	100.0	10.6	
Canada	1985-1989	5	90.0	1986	Vital Registration	100.0	10.0	
Canada	1990-1994	5	89.1	1993	Vital Registration	100.0	10.9	
Canada	1995-1999	5	88.6	1995	Vital Registration	100.0	11.4	
Canada	2000-2004	5	89.3	2002	Vital Registration	100.0	10.7	
Canada	2005-2009	5	89.4	2007	Vital Registration	99.5	10.2	
Canada	2010-2017	5	90.1	2013	Vital Registration	99.4	9.4	
Cape Verde	1980-1984	3	56.1	1980	Vital Registration	95.5	41.2	
Cape Verde	1985-1989	0						
Cape Verde	1990-1994	1	0.1	1992	Deaths among women of reproductive age in Cape Verde: causes and avoidability		49.5	0.2
Cape Verde	1995-1999	0						
Cape Verde	2000-2004	0						
Cape Verde	2005-2009	0						
Cape Verde	2010-2017	4	67.7	2012	Vital Registration	95.9	29.3	
Central African Republic	1980-1984	0						
Central African Republic	1985-1989	0						
Central African Republic	1990-1994	0						
Central African Republic	1995-1999	0						
Central African Republic	2000-2004	0						
Central African Republic	2005-2009	0						
Central African Republic	2010-2017	0						
Chad	1980-1984	0						
Chad	1985-1989	0						
Chad	1990-1994	0						
Chad	1995-1999	0						
Chad	2000-2004	0						
Chad	2005-2009	0						
Chad	2010-2017	0						
Chile	1980-1984	4	75.7	1983	Vital Registration	100.0	24.3	
Chile	1985-1989	4	75.5	1988	Vital Registration	100.0	24.5	
Chile	1990-1994	4	81.7	1994	Vital Registration	98.8	17.3	
Chile	1995-1999	4	84.7	1999	Vital Registration	100.0	15.3	
Chile	2000-2004	5	90.8	2003	Vital Registration	99.8	9.1	
Chile	2005-2009	5	90.8	2005	Vital Registration	100.0	9.2	
Chile	2010-2017	5	89.8	2012	Vital Registration	100.0	10.2	
China	1980-1984	0						
China	1985-1989	1	0.9	1986	Infant mortality among various nationalities in the middle part of Guizhou, China		17.9	1.0
China	1990-1994	4	73.8	1994	Vital Registration - Sample	85.3	13.4	
China	1995-1999	4	75.1	1998	Vital Registration - Sample	87.4	14.0	
China	2000-2004	4	70.3	2004	Vital Registration	74.2	5.3	
China	2005-2009	4	73.0	2009	Vital Registration - Sample	77.7	6.0	
China	2010-2017	4	73.5	2012	Vital Registration - Sample	77.6	5.4	
Colombia	1980-1984	4	72.4	1983	Vital Registration	92.1	21.4	
Colombia	1985-1989	4	74.4	1985	Vital Registration	94.2	21.0	
Colombia	1990-1994	4	77.3	1994	Vital Registration	92.0	16.0	
Colombia	1995-1999	5	88.0	1999	Vital Registration	98.6	10.8	
Colombia	2000-2004	5	88.6	2001	Vital Registration	98.7	10.2	
Colombia	2005-2009	5	88.9	2008	Vital Registration	99.6	10.7	
Colombia	2010-2017	5	88.5	2013	Vital Registration	98.5	10.1	
Comoros	1980-1984	0						
Comoros	1985-1989	0						
Comoros	1990-1994	0						
Comoros	1995-1999	0						
Comoros	2000-2004	0						
Comoros	2005-2009	0						
Comoros	2010-2017	0						
Congo (Brazzaville)	1980-1984	0						
Congo (Brazzaville)	1985-1989	0						
Congo (Brazzaville)	1990-1994	0						
Congo (Brazzaville)	1995-1999	0						
Congo (Brazzaville)	2000-2004	0						
Congo (Brazzaville)	2005-2009	0						
Congo (Brazzaville)	2010-2017	0						
Costa Rica	1980-1984	4	81.6	1983	Vital Registration	100.0	18.4	
Costa Rica	1985-1989	4	83.4	1987	Vital Registration	100.0	16.6	
Costa Rica	1990-1994	4	82.5	1993	Vital Registration	100.0	17.5	
Costa Rica	1995-1999	5	91.1	1999	Vital Registration	100.0	8.9	
Costa Rica	2000-2004	5	92.3	2003	Vital Registration	100.0	7.7	
Costa Rica	2005-2009	5	90.6	2005	Vital Registration	99.5	9.0	
Costa Rica	2010-2017	5	90.5	2014	Vital Registration	100.0	9.5	
Cote d'Ivoire	1980-1984	0						

Appendix Table 8. Underlying indicators for percent well-certified for data source with maximum percent well certified in each 5-year time interval for 195 countries, 1980-2017								
Country	Time Window	Stars	Percent Well-Certified [PWC] (%)	Max PWC Data Year	Max PWC Data Source	Completeness (%)	Percent Major Garbage (%)	Verbal Autopsy Adjustment (None for VR) (%)
Cote d'Ivoire	1985-1989	1	1.2	1988	Effet de l'observance des d'approvisionnement en eau et de la therapie par voie orale sur les diarrhees chez les enfants de moins de 5 de la Cote d'Ivoire		68.0	3.7
Cote d'Ivoire	1990-1994	1	1.2	1992	Effet de l'observance des d'approvisionnement en eau et de la therapie par voie orale sur les diarrhees chez les enfants de moins de 5 de la Cote d'Ivoire		65.9	3.4
Cote d'Ivoire	1995-1999	0						
Cote d'Ivoire	2000-2004	0						
Cote d'Ivoire	2005-2009	1	0.2	2009	Africa, Asia, Oceania - INDEPTH Network Cause-Specific Mortality - Release 2014		97.1	6.4
Cote d'Ivoire	2010-2017	1	0.2	2011	Africa, Asia, Oceania - INDEPTH Network Cause-Specific Mortality - Release 2014		96.8	6.4
Croatia	1980-1984	0						
Croatia	1985-1989	4	84.4	1989	Vital Registration	100.0	15.6	
Croatia	1990-1994	5	85.3	1990	Vital Registration	100.0	14.7	
Croatia	1995-1999	4	82.6	1999	Vital Registration	100.0	17.4	
Croatia	2000-2004	5	86.1	2004	Vital Registration	100.0	13.9	
Croatia	2005-2009	5	88.3	2009	Vital Registration	100.0	11.7	
Croatia	2010-2017	5	92.3	2014	Vital Registration	100.0	7.7	
Cuba	1980-1984	4	84.7	1981	Vital Registration	99.6	15.0	
Cuba	1985-1989	5	85.1	1988	Vital Registration	98.9	13.9	
Cuba	1990-1994	4	84.9	1990	Vital Registration	99.4	14.5	
Cuba	1995-1999	5	88.9	1997	Vital Registration	100.0	11.1	
Cuba	2000-2004	5	90.2	2003	Vital Registration	100.0	9.8	
Cuba	2005-2009	5	91.0	2009	Vital Registration	100.0	9.0	
Cuba	2010-2017	5	91.5	2015	Vital Registration	100.0	8.5	
Cyprus	1980-1984	0						
Cyprus	1985-1989	0						
Cyprus	1990-1994	0						
Cyprus	1995-1999	2	26.8	1999	Vital Registration	76.8	65.0	
Cyprus	2000-2004	3	53.8	2004	Vital Registration	77.4	30.5	
Cyprus	2005-2009	3	59.5	2007	Vital Registration	77.1	22.8	
Cyprus	2010-2017	3	60.9	2015	Vital Registration	75.0	18.7	
Czech Republic	1980-1984	0						
Czech Republic	1985-1989	5	90.7	1986	Vital Registration	100.0	9.3	
Czech Republic	1990-1994	5	89.6	1990	Vital Registration	100.0	10.4	
Czech Republic	1995-1999	4	84.8	1995	Vital Registration	100.0	15.2	
Czech Republic	2000-2004	4	84.7	2000	Vital Registration	99.5	14.9	
Czech Republic	2005-2009	5	85.8	2007	Vital Registration	100.0	14.2	
Czech Republic	2010-2017	5	87.8	2013	Vital Registration	100.0	12.2	
DR Congo	1980-1984	0						
DR Congo	1985-1989	1	2.3	1986	Etude de la mortalité globale et de la mortalité liée au paludisme dans le Kivu montagneux, Zaïre		63.5	6.4
DR Congo	1990-1994	1	3.0	1990	Influence of nutritional status on child mortality in rural Zaire		14.2	3.5
DR Congo	1995-1999	0						
DR Congo	2000-2004	0						
DR Congo	2005-2009	0						
DR Congo	2010-2017	0						
Denmark	1980-1984	4	84.5	1981	Vital Registration	100.0	15.5	
Denmark	1985-1989	4	82.9	1985	Vital Registration	100.0	17.1	
Denmark	1990-1994	4	83.1	1994	Vital Registration	99.0	16.0	
Denmark	1995-1999	5	85.8	1999	Vital Registration	99.0	13.3	
Denmark	2000-2004	5	85.4	2000	Vital Registration	100.0	14.6	
Denmark	2005-2009	4	84.5	2005	Vital Registration	100.0	15.5	
Denmark	2010-2017	4	84.6	2012	Vital Registration	100.0	15.4	
Djibouti	1980-1984	0						
Djibouti	1985-1989	0						
Djibouti	1990-1994	0						
Djibouti	1995-1999	0						
Djibouti	2000-2004	0						
Djibouti	2005-2009	0						
Djibouti	2010-2017	0						
Dominica	1980-1984	4	71.6	1984	Vital Registration	100.0	28.4	
Dominica	1985-1989	3	63.1	1986	Vital Registration	100.0	36.9	
Dominica	1990-1994	3	62.1	1992	Vital Registration	99.4	37.6	
Dominica	1995-1999	4	65.8	1999	Vital Registration	100.0	34.2	
Dominica	2000-2004	4	68.6	2004	Vital Registration	98.7	30.5	
Dominica	2005-2009	4	81.7	2007	Vital Registration	100.0	18.3	
Dominica	2010-2017	4	83.9	2011	Vital Registration	100.0	16.1	
Dominican Republic	1980-1984	3	53.6	1984	Vital Registration	79.7	32.7	
Dominican Republic	1985-1989	3	53.5	1985	Vital Registration	77.9	31.3	
Dominican Republic	1990-1994	3	44.5	1990	Vital Registration	65.6	32.2	
Dominican Republic	1995-1999	3	53.8	1999	Vital Registration	73.9	27.3	
Dominican Republic	2000-2004	3	57.2	2004	Vital Registration	75.2	24.0	
Dominican Republic	2005-2009	3	56.8	2005	Vital Registration	72.2	21.3	
Dominican Republic	2010-2017	3	59.9	2013	Vital Registration	72.9	17.9	
Ecuador	1980-1984	4	73.0	1982	Vital Registration	100.0	27.0	
Ecuador	1985-1989	4	72.4	1988	Vital Registration	100.0	27.6	

Appendix Table 8. Underlying indicators for percent well-certified for data source with maximum percent well certified in each 5-year time interval for 195 countries, 1980-2017

Country	Time Window	Stars	Percent Well-Certified [PWC] (%)	Max PWC Data Year	Max PWC Data Source	Completeness (%)	Percent Major Garbage (%)	Verbal Autopsy Adjustment (None for VR) (%)
Ecuador	1990-1994	4	72.9	1992	Vital Registration	100.0	27.1	
Ecuador	1995-1999	4	69.8	1996	Vital Registration	97.1	28.1	
Ecuador	2000-2004	4	66.4	2001	Vital Registration	98.9	32.9	
Ecuador	2005-2009	3	62.9	2006	Vital Registration	86.5	27.3	
Ecuador	2010-2017	4	70.4	2015	Vital Registration	85.7	17.8	
Egypt	1980-1984	3	35.0	1980	Vital Registration	81.8	57.2	
Egypt	1985-1989	3	48.2	1987	Vital Registration	96.6	50.1	
Egypt	1990-1994	3	46.8	1991	Vital Registration	93.1	49.7	
Egypt	1995-1999	0						
Egypt	2000-2004	3	43.9	2000	Vital Registration	98.4	55.4	
Egypt	2005-2009	3	42.7	2005	Vital Registration	100.0	57.3	
Egypt	2010-2017	3	48.2	2014	Vital Registration	100.0	51.8	
El Salvador	1980-1984	4	73.3	1981	Vital Registration	100.0	26.7	
El Salvador	1985-1989	0						
El Salvador	1990-1994	3	60.4	1990	Vital Registration	90.4	33.2	
El Salvador	1995-1999	4	67.2	1996	Vital Registration	93.5	28.1	
El Salvador	2000-2004	4	69.4	2001	Vital Registration	99.0	29.8	
El Salvador	2005-2009	4	70.8	2006	Vital Registration	100.0	29.2	
El Salvador	2010-2017	4	67.4	2011	Vital Registration	99.2	32.0	
Equatorial Guinea	1980-1984	0						
Equatorial Guinea	1985-1989	0						
Equatorial Guinea	1990-1994	0						
Equatorial Guinea	1995-1999	0						
Equatorial Guinea	2000-2004	0						
Equatorial Guinea	2005-2009	0						
Equatorial Guinea	2010-2017	0						
Eritrea	1980-1984	0						
Eritrea	1985-1989	0						
Eritrea	1990-1994	0						
Eritrea	1995-1999	0						
Eritrea	2000-2004	0						
Eritrea	2005-2009	0						
Eritrea	2010-2017	0						
Estonia	1980-1984	5	92.1	1981	Vital Registration	99.9	7.8	
Estonia	1985-1989	5	92.7	1986	Vital Registration	100.0	7.3	
Estonia	1990-1994	5	93.8	1994	Vital Registration	100.0	6.2	
Estonia	1995-1999	5	93.1	1996	Vital Registration	100.0	6.9	
Estonia	2000-2004	5	92.5	2000	Vital Registration	100.0	7.5	
Estonia	2005-2009	5	94.1	2009	Vital Registration	100.0	5.8	
Estonia	2010-2017	5	93.9	2010	Vital Registration	100.0	6.1	
Ethiopia	1980-1984	0						
Ethiopia	1985-1989	1	1.2	1987	The Butajira rural health project in Ethiopia: mortality pattern of the under fives		62.5	3.1
Ethiopia	1990-1994	1	2.5	1992	Patterns of childhood mortality in three districts of north Gondar Administrative Zone. A community based study using the verbal autopsy method		19.5	3.1
Ethiopia	1995-1999	1	0.6	1995	Africa, Asia, Oceania - INDEPTH Network Cause-Specific Mortality - Release 2014		90.3	6.4
Ethiopia	2000-2004	1	5.0	2001	HIV/AIDS-Related Mortality In Addis Ababa City Administration		22.5	6.4
Ethiopia	2005-2009	1	4.6	2009	Ethiopia Demographic Surveillance Verbal Autopsy Data 2009-2013		27.6	6.4
Ethiopia	2010-2017	1	4.6	2010	Ethiopia Demographic Surveillance Verbal Autopsy Data 2009-2013		28.1	6.4
Federated States of Micronesia	1980-1984	0						
Federated States of Micronesia	1985-1989	0						
Federated States of Micronesia	1990-1994	0						
Federated States of Micronesia	1995-1999	0						
Federated States of Micronesia	2000-2004	0						
Federated States of Micronesia	2005-2009	0						
Federated States of Micronesia	2010-2017	0						
Fiji	1980-1984	0						
Fiji	1985-1989	0						
Fiji	1990-1994	0						
Fiji	1995-1999	2	34.8	1999	Vital Registration	61.0	42.9	
Fiji	2000-2004	3	58.0	2004	Vital Registration	97.9	40.8	
Fiji	2005-2009	3	63.9	2008	Vital Registration	100.0	36.1	
Fiji	2010-2017	4	70.0	2012	Vital Registration	99.4	29.6	
Finland	1980-1984	4	83.7	1980	Vital Registration	100.0	16.3	
Finland	1985-1989	5	91.7	1987	Vital Registration	100.0	8.3	
Finland	1990-1994	5	91.7	1993	Vital Registration	100.0	8.3	
Finland	1995-1999	5	95.8	1999	Vital Registration	100.0	4.2	
Finland	2000-2004	5	95.6	2003	Vital Registration	100.0	4.4	
Finland	2005-2009	5	94.9	2006	Vital Registration	99.8	4.9	
Finland	2010-2017	5	95.9	2015	Vital Registration	100.0	4.1	
France	1980-1984	4	76.1	1984	Vital Registration	99.6	23.6	
France	1985-1989	4	78.4	1988	Vital Registration	100.0	21.6	
France	1990-1994	4	79.1	1993	Vital Registration	100.0	20.9	

Appendix Table 8. Underlying indicators for percent well-certified for data source with maximum percent well certified in each 5-year time interval for 195 countries, 1980-2017								
Country	Time Window	Stars	Percent Well-Certified [PWC] (%)	Max PWC Data Year	Max PWC Data Source	Completeness (%)	Percent Major Garbage (%)	Verbal Autopsy Adjustment (None for VR) (%)
France	1995-1999	4	79.0	1998	Vital Registration	100.0	21.0	
France	2000-2004	4	79.1	2000	Vital Registration	100.0	20.9	
France	2005-2009	4	79.4	2005	Vital Registration	100.0	20.6	
France	2010-2017	4	77.4	2010	Vital Registration	99.6	22.3	
Gabon	1980-1984	0						
Gabon	1985-1989	0						
Gabon	1990-1994	0						
Gabon	1995-1999	0						
Gabon	2000-2004	0						
Gabon	2005-2009	0						
Gabon	2010-2017	0						
Georgia	1980-1984	4	83.4	1981	Vital Registration	92.7	10.0	
Georgia	1985-1989	4	80.7	1988	Vital Registration	88.1	8.4	
Georgia	1990-1994	4	81.3	1992	Vital Registration	89.1	8.8	
Georgia	1995-1999	4	77.7	1995	Vital Registration	85.4	8.9	
Georgia	2000-2004	4	78.7	2000	Vital Registration	88.9	11.5	
Georgia	2005-2009	3	62.2	2005	Vital Registration	98.1	36.6	
Georgia	2010-2017	3	58.9	2014	Vital Registration	100.0	41.1	
Germany	1980-1984	4	78.4	1984	Vital Registration	100.0	21.6	
Germany	1985-1989	4	80.5	1989	Vital Registration	100.0	19.5	
Germany	1990-1994	4	84.1	1993	Vital Registration	100.0	15.9	
Germany	1995-1999	4	83.9	1998	Vital Registration	100.0	16.1	
Germany	2000-2004	4	83.5	2004	Vital Registration	100.0	16.5	
Germany	2005-2009	4	83.8	2006	Vital Registration	100.0	16.2	
Germany	2010-2017	4	84.0	2011	Vital Registration	99.9	15.9	
Ghana	1980-1984	0						
Ghana	1985-1989	1	0.1	1989	Maternal mortality among the Kassena-Nankana of northern Ghana		79.0	0.5
Ghana	1990-1994	1	1.5	1990	Vitamin A supplementation in northern Ghana: effects on clinic attendances, hospital admissions, and child mortalit		27.9	2.0
Ghana	1995-1999	1	0.8	1998	Trend and causes of neonatal mortality in the Kassena-Nankana district of northern Ghana, 1995-2002		6.0	0.8
Ghana	2000-2004	1	7.2	2000	Vital Registration	10.6	32.8	
Ghana	2005-2009	2	16.7	2006	Ghana Child Verbal Autopsy Study 2008		8.2	18.2
Ghana	2010-2017	1	0.5	2011	Africa, Asia, Oceania - INDEPTH Network Cause-Specific Mortality - Release 2014		92.7	6.4
Greece	1980-1984	4	80.2	1984	Vital Registration	99.0	19.0	
Greece	1985-1989	4	81.6	1985	Vital Registration	100.0	18.4	
Greece	1990-1994	4	71.7	1994	Vital Registration	100.0	28.3	
Greece	1995-1999	4	72.0	1995	Vital Registration	100.0	28.0	
Greece	2000-2004	4	72.6	2001	Vital Registration	100.0	27.4	
Greece	2005-2009	4	77.9	2009	Vital Registration	100.0	22.1	
Greece	2010-2017	4	76.2	2010	Vital Registration	100.0	23.8	
Greenland	1980-1984	0						
Greenland	1985-1989	0						
Greenland	1990-1994	0						
Greenland	1995-1999	5	90.2	1999	Vital Registration	100.0	9.8	
Greenland	2000-2004	5	89.7	2001	Vital Registration	100.0	10.3	
Greenland	2005-2009	5	89.6	2005	Vital Registration	100.0	10.4	
Greenland	2010-2017	5	87.6	2010	Vital Registration	100.0	12.4	
Grenada	1980-1984	4	67.7	1984	Vital Registration	95.4	29.0	
Grenada	1985-1989	3	63.4	1988	Vital Registration	100.0	36.6	
Grenada	1990-1994	3	64.0	1994	Vital Registration	100.0	36.0	
Grenada	1995-1999	3	63.2	1995	Vital Registration	100.0	36.8	
Grenada	2000-2004	4	77.0	2002	Vital Registration	100.0	23.0	
Grenada	2005-2009	4	79.9	2009	Vital Registration	100.0	20.1	
Grenada	2010-2017	5	85.9	2016	Vital Registration	100.0	14.1	
Guam	1980-1984	0						
Guam	1985-1989	0						
Guam	1990-1994	5	85.9	1994	Vital Registration	91.7	6.3	
Guam	1995-1999	4	80.7	1999	Vital Registration	87.6	7.9	
Guam	2000-2004	4	76.7	2001	Vital Registration	84.2	8.8	
Guam	2005-2009	4	79.9	2009	Vital Registration	87.0	8.2	
Guam	2010-2017	4	79.2	2010	Vital Registration	88.4	10.4	
Guatemala	1980-1984	4	80.1	1980	Vital Registration	100.0	19.9	
Guatemala	1985-1989	4	75.0	1987	Vital Registration	100.0	25.0	
Guatemala	1990-1994	4	73.9	1990	Vital Registration	100.0	26.1	
Guatemala	1995-1999	4	72.6	1998	Vital Registration	100.0	27.4	
Guatemala	2000-2004	4	73.3	2002	Vital Registration	100.0	26.7	
Guatemala	2005-2009	4	75.2	2009	Vital Registration	100.0	24.8	
Guatemala	2010-2017	4	78.0	2014	Vital Registration	99.7	21.7	
Guinea	1980-1984	0						
Guinea	1985-1989	0						
Guinea	1990-1994	0						
Guinea	1995-1999	1	3.2	1998	Guinea - Mandiana Mortality Study 1998-1999		5.5	3.4
Guinea	2000-2004	0						

Appendix Table 8. Underlying indicators for percent well-certified for data source with maximum percent well certified in each 5-year time interval for 195 countries, 1980-2017								
**Country**	**Time Window**	**Stars**	**Percent Well-Certified [PWC] (%)**	**Max PWC Data Year**	**Max PWC Data Source**	**Completeness (%)**	**Percent Major Garbage (%)**	**Verbal Autopsy Adjustment (None for VR) (%)**
Guinea	2005-2009	0						
Guinea	2010-2017	0						
Guinea-Bissau	1980-1984	0						
Guinea-Bissau	1985-1989	0						
Guinea-Bissau	1990-1994	1	0.1	1992	Maternal mortality in Guinea-Bissau: the use of verbal autopsy i a multi-ethnic populatior		80.9	0.4
Guinea-Bissau	1995-1999	1	1.2	1995	BCG vaccination scar associated with better childhood survival in Guinea-Bissau		45.6	2.2
Guinea-Bissau	2000-2004	0						
Guinea-Bissau	2005-2009	0						
Guinea-Bissau	2010-2017	0						
Guyana	1980-1984	3	55.5	1984	Vital Registration	84.2	34.1	
Guyana	1985-1989	4	76.2	1989	Vital Registration	99.1	23.1	
Guyana	1990-1994	4	69.8	1990	Vital Registration	91.8	24.0	
Guyana	1995-1999	4	78.3	1995	Vital Registration	99.0	20.9	
Guyana	2000-2004	4	84.3	2000	Vital Registration	96.8	12.9	
Guyana	2005-2009	5	85.5	2005	Vital Registration	98.6	13.2	
Guyana	2010-2017	4	78.6	2010	Vital Registration	95.7	17.9	
Haiti	1980-1984	2	22.3	1981	Vital Registration	48.1	53.7	
Haiti	1985-1989	1	1.3	1989	The utility of verbal autopsies for identifying HIV-1-related deaths in Haitian childrer		21.7	1.7
Haiti	1990-1994	1	1.0	1994	Survey on Infant mortality in Mirebalais, Haiti		36.5	1.5
Haiti	1995-1999	2	13.9	1999	Vital Registration	30.5	54.4	
Haiti	2000-2004	1	6.1	2002	Vital Registration	10.4	41.2	
Haiti	2005-2009	0						
Haiti	2010-2017	0						
Honduras	1980-1984	3	41.2	1981	Vital Registration	79.6	48.3	
Honduras	1985-1989	3	46.0	1988	Vital Registration	70.6	34.8	
Honduras	1990-1994	3	43.2	1990	Vital Registration	65.9	34.5	
Honduras	1995-1999	0						
Honduras	2000-2004	0						
Honduras	2005-2009	2	17.5	2008	Vital Registration	19.8	11.9	
Honduras	2010-2017	2	19.1	2013	Vital Registration	21.1	9.6	
Hungary	1980-1984	5	91.0	1980	Vital Registration	100.0	9.0	
Hungary	1985-1989	5	89.8	1988	Vital Registration	100.0	10.2	
Hungary	1990-1994	5	89.9	1994	Vital Registration	100.0	10.1	
Hungary	1995-1999	5	91.0	1997	Vital Registration	100.0	9.0	
Hungary	2000-2004	5	92.1	2003	Vital Registration	100.0	7.9	
Hungary	2005-2009	5	92.6	2008	Vital Registration	99.5	6.9	
Hungary	2010-2017	5	93.8	2014	Vital Registration	100.0	6.2	
Iceland	1980-1984	5	92.6	1983	Vital Registration	100.0	7.4	
Iceland	1985-1989	5	93.8	1988	Vital Registration	100.0	6.2	
Iceland	1990-1994	5	94.2	1991	Vital Registration	100.0	5.8	
Iceland	1995-1999	5	94.1	1999	Vital Registration	100.0	5.9	
Iceland	2000-2004	5	93.5	2004	Vital Registration	100.0	6.5	
Iceland	2005-2009	5	92.9	2005	Vital Registration	100.0	7.1	
Iceland	2010-2017	5	91.4	2010	Vital Registration	100.0	8.6	
India	1980-1984	1	2.8	1984	Vital Registration	3.9	28.7	
India	1985-1989	1	3.0	1986	Vital Registration	4.0	23.9	
India	1990-1994	1	2.9	1991	Vital Registration	4.6	35.6	
India	1995-1999	1	5.2	1999	Vital Registration	8.5	38.4	
India	2000-2004	1	6.3	2003	India Study on Causes of Death by Verbal Autopsy 2003		1.7	6.4
India	2005-2009	3	52.7	2005	Verbal Autopsy		17.7	64.0
India	2010-2017	3	49.0	2012	Verbal Autopsy		23.4	64.0
Indonesia	1980-1984	1	1.4	1983	The incidence of diarrhoeal diseases and diarrhoeal diseases related mortality in rural swampy low-landarea of south Sumatra Indonesia		77.9	6.4
Indonesia	1985-1989	0						
Indonesia	1990-1994	1	1.3	1991	Care-seeking for fatal illnesses in young children in Indramayu, West Java, Indonesia		23.4	1.7
Indonesia	1995-1999	1	0.4	1997	Age- and cause-specific childhood mortality in Lombok Indonesia, as a factor for determining the appropriateness of introducing Haemophilus influenzae type b and pneumococcal vaccines		34.1	0.6
Indonesia	2000-2004	1	0.3	2001	Determining the Cause of Death: Mortality Surveillance Using Verbal Autopsy in Indonesia		96.0	6.4
Indonesia	2005-2009	3	53.7	2007	Indonesia Basic Health Research 2007-2008		3.9	55.8
Indonesia	2010-2017	3	62.3	2013	Indonesia Sample Registration System - Deaths 2013		2.6	64.0
Iran	1980-1984	0						
Iran	1985-1989	0						
Iran	1990-1994	0						
Iran	1995-1999	0						
Iran	2000-2004	3	52.8	2004	Vital Registration	70.1	24.6	
Iran	2005-2009	3	63.7	2008	Vital Registration	77.3	17.6	
Iran	2010-2017	4	80.3	2015	Vital Registration	100.0	19.7	
Iraq	1980-1984	0						
Iraq	1985-1989	0						
Iraq	1990-1994	0						
Iraq	1995-1999	0						

Appendix Table 8. Underlying indicators for percent well-certified for data source with maximum percent well certified in each 5-year time interval for 195 countries, 1980-2017								
Country	Time Window	Stars	Percent Well-Certified [PWC] (%)	Max PWC Data Year	Max PWC Data Source	Completeness (%)	Percent Major Garbage (%)	Verbal Autopsy Adjustment (None for VR) (%)
Iraq	2000-2004	0						
Iraq	2005-2009	3	45.8	2008	Vital Registration	78.4	41.6	
Iraq	2010-2017	3	56.7	2014	Vital Registration	92.0	38.4	
Ireland	1980-1984	5	90.3	1983	Vital Registration	100.0	9.7	
Ireland	1985-1989	5	91.6	1988	Vital Registration	100.0	8.4	
Ireland	1990-1994	5	91.7	1993	Vital Registration	100.0	8.3	
Ireland	1995-1999	5	90.9	1995	Vital Registration	100.0	9.1	
Ireland	2000-2004	5	90.2	2002	Vital Registration	99.8	9.6	
Ireland	2005-2009	5	92.4	2007	Vital Registration	99.9	7.6	
Ireland	2010-2017	5	92.3	2012	Vital Registration	100.0	7.7	
Israel	1980-1984	4	81.2	1983	Vital Registration	99.9	18.7	
Israel	1985-1989	4	82.6	1989	Vital Registration	100.0	17.4	
Israel	1990-1994	4	83.2	1993	Vital Registration	100.0	16.8	
Israel	1995-1999	4	82.7	1995	Vital Registration	100.0	17.3	
Israel	2000-2004	4	81.8	2002	Vital Registration	100.0	18.2	
Israel	2005-2009	4	80.1	2005	Vital Registration	100.0	19.9	
Israel	2010-2017	4	80.7	2010	Vital Registration	100.0	19.3	
Italy	1980-1984	5	88.8	1980	Vital Registration	100.0	11.2	
Italy	1985-1989	5	88.4	1989	Vital Registration	100.0	11.6	
Italy	1990-1994	5	88.5	1994	Vital Registration	100.0	11.5	
Italy	1995-1999	5	87.8	1995	Vital Registration	100.0	12.2	
Italy	2000-2004	5	88.1	2003	Vital Registration	100.0	11.9	
Italy	2005-2009	5	88.7	2008	Vital Registration	100.0	11.3	
Italy	2010-2017	5	88.3	2010	Vital Registration	99.9	11.6	
Jamaica	1980-1984	4	70.5	1983	Vital Registration	97.9	28.0	
Jamaica	1985-1989	4	72.8	1987	Vital Registration	100.0	27.2	
Jamaica	1990-1994	3	64.6	1990	Vital Registration	94.4	31.6	
Jamaica	1995-1999	0						
Jamaica	2000-2004	4	83.0	2004	Vital Registration	100.0	17.0	
Jamaica	2005-2009	5	87.5	2009	Vital Registration	100.0	12.5	
Jamaica	2010-2017	5	89.1	2011	Vital Registration	100.0	10.9	
Japan	1980-1984	4	82.6	1980	Vital Registration	100.0	17.4	
Japan	1985-1989	4	81.0	1985	Vital Registration	100.0	19.0	
Japan	1990-1994	4	81.5	1994	Vital Registration	99.7	18.3	
Japan	1995-1999	5	87.6	1995	Vital Registration	100.0	12.4	
Japan	2000-2004	5	85.7	2000	Vital Registration	99.4	13.8	
Japan	2005-2009	4	84.4	2005	Vital Registration	100.0	15.6	
Japan	2010-2017	4	81.3	2010	Vital Registration	100.0	18.7	
Jordan	1980-1984	0						
Jordan	1985-1989	0						
Jordan	1990-1994	0						
Jordan	1995-1999	1	1.9	1995	Mortality and causes of death in Jordan 1995-96: assessment by verbal autopsy		69.6	6.4
Jordan	2000-2004	4	70.1	2004	Vital Registration	88.5	20.8	
Jordan	2005-2009	4	76.5	2006	Vital Registration	92.6	17.4	
Jordan	2010-2017	4	74.1	2012	Vital Registration	92.0	19.4	
Kazakhstan	1980-1984	4	80.0	1982	Vital Registration	97.5	18.0	
Kazakhstan	1985-1989	4	84.9	1989	Vital Registration	99.8	15.0	
Kazakhstan	1990-1994	5	89.1	1993	Vital Registration	100.0	10.9	
Kazakhstan	1995-1999	5	88.6	1995	Vital Registration	100.0	11.4	
Kazakhstan	2000-2004	5	85.0	2000	Vital Registration	98.9	14.0	
Kazakhstan	2005-2009	4	84.2	2005	Vital Registration	98.6	14.6	
Kazakhstan	2010-2017	4	84.1	2015	Vital Registration	96.3	12.7	
Kenya	1980-1984	0						
Kenya	1985-1989	1	2.7	1986	Mortality patterns in a rural Kenyan community		12.0	3.1
Kenya	1990-1994	0						
Kenya	1995-1999	1	0.4	1997	The burden of malaria mortality among African children in the year 2000		82.2	2.5
Kenya	2000-2004	1	4.9	2003	Kenya - Nairobi Urban Health and Demographic Surveillance System		19.8	6.1
Kenya	2005-2009	1	5.4	2006	Kenya - Nairobi Urban Health and Demographic Surveillance System		16.2	6.4
Kenya	2010-2017	1	0.8	2011	Africa, Asia, Oceania - INDEPTH Network Cause-Specific Mortality - Release 2014		87.7	6.4
Kiribati	1980-1984	0						
Kiribati	1985-1989	0						
Kiribati	1990-1994	3	47.9	1993	Vital Registration	71.6	33.1	
Kiribati	1995-1999	4	66.6	1995	Vital Registration	100.0	33.4	
Kiribati	2000-2004	3	35.6	2000	Vital Registration	65.4	45.5	
Kiribati	2005-2009	0						
Kiribati	2010-2017	0						
Kuwait	1980-1984	4	82.1	1981	Vital Registration	100.0	17.9	
Kuwait	1985-1989	4	81.9	1985	Vital Registration	100.0	18.1	
Kuwait	1990-1994	4	76.4	1994	Vital Registration	97.1	21.3	
Kuwait	1995-1999	4	77.5	1998	Vital Registration	99.4	22.0	
Kuwait	2000-2004	4	83.1	2004	Vital Registration	100.0	16.9	
Kuwait	2005-2009	4	84.9	2009	Vital Registration	100.0	15.1	

Appendix Table 8. Underlying indicators for percent well-certified for data source with maximum percent well certified in each 5-year time interval for 195 countries, 1980-2017								
**Country**	**Time Window**	**Stars**	**Percent Well-Certified [PWC] (%)**	**Max PWC Data Year**	**Max PWC Data Source**	**Completeness (%)**	**Percent Major Garbage (%)**	**Verbal Autopsy Adjustment (None for VR) (%)**
Kuwait	2010-2017	4	84.6	2010	Vital Registration	99.1	14.7	
Kyrgyzstan	1980-1984	4	67.5	1981	Vital Registration	81.7	17.3	
Kyrgyzstan	1985-1989	4	75.9	1988	Vital Registration	87.7	13.5	
Kyrgyzstan	1990-1994	4	72.5	1994	Vital Registration	93.1	22.1	
Kyrgyzstan	1995-1999	4	74.4	1998	Vital Registration	91.7	18.9	
Kyrgyzstan	2000-2004	5	87.4	2003	Vital Registration	95.7	8.7	
Kyrgyzstan	2005-2009	5	92.9	2008	Vital Registration	100.0	7.1	
Kyrgyzstan	2010-2017	5	93.2	2014	Vital Registration	100.0	6.8	
Laos	1980-1984	0						
Laos	1985-1989	1	1.3	1989	The Lao People's Democratic Republic: maternal mortality and female mortality: determining causes of death		68.7	4.2
Laos	1990-1994	0						
Laos	1995-1999	0						
Laos	2000-2004	0						
Laos	2005-2009	0						
Laos	2010-2017	0						
Latvia	1980-1984	5	92.5	1984	Vital Registration	100.0	7.5	
Latvia	1985-1989	5	92.7	1985	Vital Registration	100.0	7.3	
Latvia	1990-1994	5	89.3	1990	Vital Registration	100.0	10.7	
Latvia	1995-1999	5	92.0	1999	Vital Registration	100.0	8.0	
Latvia	2000-2004	5	91.1	2000	Vital Registration	100.0	8.9	
Latvia	2005-2009	5	88.7	2007	Vital Registration	99.4	10.8	
Latvia	2010-2017	5	94.3	2015	Vital Registration	100.0	5.7	
Lebanon	1980-1984	0						
Lebanon	1985-1989	1	3.5	1988	Non-communicable disease mortality rates using the verbal autopsy in a cohort of middle aged and older populations in Beirut during wartime, 1983-93		16.8	4.2
Lebanon	1990-1994	0						
Lebanon	1995-1999	0						
Lebanon	2000-2004	1	0.0	2000	Facility-based audit of maternal mortality in Lebanon: A feasibility study		94.6	0.4
Lebanon	2005-2009	0						
Lebanon	2010-2017	0						
Lesotho	1980-1984	0						
Lesotho	1985-1989	0						
Lesotho	1990-1994	0						
Lesotho	1995-1999	0						
Lesotho	2000-2004	0						
Lesotho	2005-2009	0						
Lesotho	2010-2017	0						
Liberia	1980-1984	1	2.1	1984	Infant and child mortality in two counties of Liberia: results of a survey in 1988 and trends since 1984		48.0	3.9
Liberia	1985-1989	1	2.1	1987	Infant and child mortality in two counties of Liberia: results of a survey in 1988 and trends since 1984		45.5	3.9
Liberia	1990-1994	1	3.2	1990	Application of the verbal autopsy during a clinical trial		29.2	4.6
Liberia	1995-1999	0						
Liberia	2000-2004	0						
Liberia	2005-2009	0						
Liberia	2010-2017	0						
Libya	1980-1984	0						
Libya	1985-1989	0						
Libya	1990-1994	0						
Libya	1995-1999	0						
Libya	2000-2004	0						
Libya	2005-2009	2	12.6	2006	Vital Registration	96.2	86.9	
Libya	2010-2017	0						
Lithuania	1980-1984	5	90.1	1982	Vital Registration	98.5	8.5	
Lithuania	1985-1989	5	94.0	1988	Vital Registration	100.0	6.0	
Lithuania	1990-1994	5	93.7	1990	Vital Registration	100.0	6.3	
Lithuania	1995-1999	5	94.8	1997	Vital Registration	100.0	5.2	
Lithuania	2000-2004	5	92.9	2002	Vital Registration	100.0	7.1	
Lithuania	2005-2009	5	93.3	2009	Vital Registration	100.0	6.7	
Lithuania	2010-2017	5	94.6	2013	Vital Registration	100.0	5.4	
Luxembourg	1980-1984	5	86.4	1984	Vital Registration	99.9	13.6	
Luxembourg	1985-1989	5	85.8	1985	Vital Registration	98.9	13.3	
Luxembourg	1990-1994	5	85.5	1992	Vital Registration	100.0	14.5	
Luxembourg	1995-1999	4	84.5	1997	Vital Registration	100.0	15.5	
Luxembourg	2000-2004	4	80.6	2000	Vital Registration	98.2	17.9	
Luxembourg	2005-2009	4	77.9	2007	Vital Registration	100.0	22.1	
Luxembourg	2010-2017	4	82.5	2015	Vital Registration	100.0	17.5	
Macedonia	1980-1984	0						
Macedonia	1985-1989	0						
Macedonia	1990-1994	4	82.5	1994	Vital Registration	100.0	17.5	
Macedonia	1995-1999	4	83.8	1998	Vital Registration	100.0	16.2	
Macedonia	2000-2004	4	84.5	2003	Vital Registration	99.5	15.1	
Macedonia	2005-2009	4	81.7	2009	Vital Registration	100.0	18.3	
Macedonia	2010-2017	4	79.6	2010	Vital Registration	99.2	19.7	

Appendix Table 8. Underlying indicators for percent well-certified for data source with maximum percent well certified in each 5-year time interval for 195 countries, 1980-2017								
**Country**	**Time Window**	**Stars**	**Percent Well-Certified [PWC] (%)**	**Max PWC Data Year**	**Max PWC Data Source**	**Completeness (%)**	**Percent Major Garbage (%)**	**Verbal Autopsy Adjustment (None for VR) (%)**
Madagascar	1980-1984	1	2.8	1984	Vital Registration	4.9	42.4	
Madagascar	1985-1989	1	3.5	1986	Vital Registration	6.0	42.7	
Madagascar	1990-1994	1	2.4	1990	Vital Registration	4.1	40.9	
Madagascar	1995-1999	1	2.4	1995	Vital Registration	4.3	43.0	
Madagascar	2000-2004	0						
Madagascar	2005-2009	0						
Madagascar	2010-2017	0						
Malawi	1980-1984	0						
Malawi	1985-1989	1	2.5	1988	Infant and second-year mortality in rural Malawi: causes and descriptive epidemiology		35.2	3.8
Malawi	1990-1994	0						
Malawi	1995-1999	1	0.6	1999	Estimation of AIDS adult mortality by verbal autopsy in rural Malawi		28.0	0.8
Malawi	2000-2004	1	2.5	2004	Adult mortality and probable cause of death in rural northern Malawi in the era of HIV treatmen		30.9	3.6
Malawi	2005-2009	1	4.5	2008	Rates and causes of death in Chiradzulu District, Malawi, 2008: a key informant study		30.0	6.4
Malawi	2010-2017	1	0.6	2011	Measuring causes of adult mortality in rural northern Malawi over a decade of change		84.2	4.0
Malaysia	1980-1984	3	46.9	1980	Vital Registration	70.6	33.6	
Malaysia	1985-1989	0						
Malaysia	1990-1994	0						
Malaysia	1995-1999	2	32.1	1997	Vital Registration	46.0	30.2	
Malaysia	2000-2004	3	37.3	2004	Vital Registration	58.4	36.2	
Malaysia	2005-2009	3	42.0	2008	Vital Registration	62.4	32.6	
Malaysia	2010-2017	3	40.2	2014	Vital Registration	53.9	25.4	
Maldives	1980-1984	0						
Maldives	1985-1989	0						
Maldives	1990-1994	0						
Maldives	1995-1999	0						
Maldives	2000-2004	3	44.0	2002	Vital Registration	100.0	56.0	
Maldives	2005-2009	3	46.6	2008	Vital Registration	97.0	52.0	
Maldives	2010-2017	3	60.1	2011	Vital Registration	100.0	39.9	
Mali	1980-1984	1	4.0	1981	Vital Registration	7.4	45.8	
Mali	1985-1989	0						
Mali	1990-1994	1	0.1	1990	Assessment of maternal mortality and late maternal mortality among a cohort of pregnant women in Bamako, Mal		79.0	0.3
Mali	1995-1999	0						
Mali	2000-2004	0						
Mali	2005-2009	0						
Mali	2010-2017	0						
Malta	1980-1984	4	81.2	1982	Vital Registration	100.0	18.8	
Malta	1985-1989	5	86.3	1989	Vital Registration	98.5	12.4	
Malta	1990-1994	5	88.7	1993	Vital Registration	100.0	11.3	
Malta	1995-1999	5	90.0	1997	Vital Registration	100.0	10.0	
Malta	2000-2004	5	90.3	2001	Vital Registration	99.1	8.9	
Malta	2005-2009	5	92.8	2008	Vital Registration	100.0	7.2	
Malta	2010-2017	5	89.5	2011	Vital Registration	99.6	10.1	
Marshall Islands	1980-1984	0						
Marshall Islands	1985-1989	0						
Marshall Islands	1990-1994	0						
Marshall Islands	1995-1999	0						
Marshall Islands	2000-2004	0						
Marshall Islands	2005-2009	0						
Marshall Islands	2010-2017	0						
Mauritania	1980-1984	0						
Mauritania	1985-1989	0						
Mauritania	1990-1994	0						
Mauritania	1995-1999	0						
Mauritania	2000-2004	0						
Mauritania	2005-2009	0						
Mauritania	2010-2017	0						
Mauritius	1980-1984	4	75.2	1984	Vital Registration	100.0	24.8	
Mauritius	1985-1989	4	79.4	1988	Vital Registration	99.6	20.3	
Mauritius	1990-1994	4	78.7	1991	Vital Registration	100.0	21.3	
Mauritius	1995-1999	4	78.2	1998	Vital Registration	100.0	21.8	
Mauritius	2000-2004	4	83.2	2002	Vital Registration	100.0	16.8	
Mauritius	2005-2009	5	85.1	2007	Vital Registration	98.9	13.9	
Mauritius	2010-2017	5	86.3	2014	Vital Registration	98.8	12.6	
Mexico	1980-1984	4	68.3	1984	Vital Registration	87.3	21.8	
Mexico	1985-1989	4	77.7	1989	Vital Registration	93.9	17.3	
Mexico	1990-1994	4	78.5	1990	Vital Registration	94.3	16.8	
Mexico	1995-1999	4	79.7	1999	Vital Registration	91.8	13.2	
Mexico	2000-2004	4	82.4	2004	Vital Registration	93.8	12.2	
Mexico	2005-2009	4	84.1	2007	Vital Registration	97.3	13.5	
Mexico	2010-2017	5	88.1	2014	Vital Registration	100.0	11.9	
Moldova	1980-1984	5	88.8	1981	Vital Registration	100.0	11.2	
Moldova	1985-1989	5	91.2	1985	Vital Registration	100.0	8.8	

Appendix Table 8. Underlying indicators for percent well-certified for data source with maximum percent well certified in each 5-year time interval for 195 countries, 1980-2017

Country	Time Window	Stars	Percent Well-Certified [PWC] (%)	Max PWC Data Year	Max PWC Data Source	Completeness (%)	Percent Major Garbage (%)	Verbal Autopsy Adjustment (None for VR) (%)
Moldova	1990-1994	4	81.9	1991	Vital Registration	100.0	18.1	
Moldova	1995-1999	5	87.8	1996	Vital Registration	98.4	10.8	
Moldova	2000-2004	5	85.0	2003	Vital Registration	88.1	3.5	
Moldova	2005-2009	4	83.5	2005	Vital Registration	86.2	3.2	
Moldova	2010-2017	4	83.7	2010	Vital Registration	86.5	3.2	
Mongolia	1980-1984	0						
Mongolia	1985-1989	0						
Mongolia	1990-1994	3	61.2	1994	Vital Registration	77.5	21.0	
Mongolia	1995-1999	0						
Mongolia	2000-2004	2	19.6	2004	Vital Registration	81.1	75.8	
Mongolia	2005-2009	2	21.4	2008	Vital Registration	80.6	73.4	
Mongolia	2010-2017	4	83.4	2010	Vital Registration	89.1	6.3	
Montenegro	1980-1984	0						
Montenegro	1985-1989	0						
Montenegro	1990-1994	0						
Montenegro	1995-1999	0						
Montenegro	2000-2004	4	69.2	2004	Vital Registration	98.0	29.4	
Montenegro	2005-2009	4	69.2	2009	Vital Registration	98.8	29.9	
Montenegro	2010-2017	0						
Morocco	1980-1984	0						
Morocco	1985-1989	2	15.4	1988	Morocco National Survey on Causes and Circumstances of Infar and Child Deaths 1988-1989		25.7	20.7
Morocco	1990-1994	0						
Morocco	1995-1999	0						
Morocco	2000-2004	2	10.2	2001	Vital Registration	21.7	53.0	
Morocco	2005-2009	2	33.3	2005	Vital Registration	73.5	54.7	
Morocco	2010-2017	2	13.7	2014	Vital Registration	26.8	49.0	
Mozambique	1980-1984	0						
Mozambique	1985-1989	0						
Mozambique	1990-1994	0						
Mozambique	1995-1999	1	0.1	1996	Quality of registration of maternal deaths in Mozambique: a community-based study in rural and urban area		81.3	0.4
Mozambique	2000-2004	2	16.2	2001	Vital Registration	20.5	21.0	
Mozambique	2005-2009	3	63.4	2007	Mozambique National Survey on the Causes of Death 2007-2008		0.9	64.0
Mozambique	2010-2017	0						
Myanmar	1980-1984	0						
Myanmar	1985-1989	0						
Myanmar	1990-1994	0						
Myanmar	1995-1999	0						
Myanmar	2000-2004	0						
Myanmar	2005-2009	1	2.9	2007	Cause of Death Verification Study in Myanmar		54.3	6.3
Myanmar	2010-2017	3	49.5	2016	Myanmar National Mortality Survey 2016		22.6	64.0
Namibia	1980-1984	0						
Namibia	1985-1989	0						
Namibia	1990-1994	0						
Namibia	1995-1999	0						
Namibia	2000-2004	0						
Namibia	2005-2009	0						
Namibia	2010-2017	0						
Nepal	1980-1984	1	2.8	1984	Impact of a pilot acute respiratory infection (ARI) control programme in a rural community of the hill region of Nepa		17.2	3.4
Nepal	1985-1989	1	2.6	1987	Reduction in total under-five mortality in western Nepal through community-based antimicrobial treatment of pneumoni		19.2	3.2
Nepal	1990-1994	0						
Nepal	1995-1999	1	0.6	1999	Evaluation of neonatal verbal autopsy using physician review versus algorithm-based cause-of-death assignment in rural Nepa		45.6	1.1
Nepal	2000-2004	1	0.6	2003	Effect of daily zinc supplementation on child mortality in southern Nepal: a community-based, cluster randomised, placebo controlled trial		31.8	0.9
Nepal	2005-2009	1	9.2	2006	Nepal Demographic and Health Survey 2006		40.3	15.4
Nepal	2010-2017	0						
Netherlands	1980-1984	5	88.5	1981	Vital Registration	100.0	11.5	
Netherlands	1985-1989	5	86.7	1988	Vital Registration	100.0	13.3	
Netherlands	1990-1994	5	85.2	1990	Vital Registration	100.0	14.8	
Netherlands	1995-1999	4	83.9	1996	Vital Registration	100.0	16.1	
Netherlands	2000-2004	4	82.3	2003	Vital Registration	100.0	17.7	
Netherlands	2005-2009	4	83.2	2006	Vital Registration	100.0	16.8	
Netherlands	2010-2017	4	83.3	2012	Vital Registration	100.0	16.7	
New Zealand	1980-1984	5	95.3	1980	Vital Registration	100.0	4.7	
New Zealand	1985-1989	5	95.2	1985	Vital Registration	100.0	4.8	
New Zealand	1990-1994	5	95.1	1994	Vital Registration	100.0	4.9	
New Zealand	1995-1999	5	96.8	1999	Vital Registration	100.0	3.2	
New Zealand	2000-2004	5	96.4	2004	Vital Registration	100.0	3.6	
New Zealand	2005-2009	5	96.3	2006	Vital Registration	100.0	3.7	
New Zealand	2010-2017	5	95.7	2012	Vital Registration	100.0	4.3	
Nicaragua	1980-1984	0						
Nicaragua	1985-1989	3	54.3	1988	Vital Registration	68.7	20.9	

Appendix Table 8. Underlying indicators for percent well-certified for data source with maximum percent well certified in each 5-year time interval for 195 countries, 1980-2017								
Country	Time Window	Stars	Percent Well-Certified [PWC] (%)	Max PWC Data Year	Max PWC Data Source	Completeness (%)	Percent Major Garbage (%)	Verbal Autopsy Adjustment (None for VR) (%)
Nicaragua	1990-1994	3	56.9	1990	Vital Registration	71.2	20.1	
Nicaragua	1995-1999	4	69.0	1998	Vital Registration	81.6	15.4	
Nicaragua	2000-2004	4	77.2	2004	Vital Registration	87.4	11.7	
Nicaragua	2005-2009	4	84.9	2009	Vital Registration	95.5	11.2	
Nicaragua	2010-2017	5	90.5	2013	Vital Registration	100.0	9.5	
Niger	1980-1984	0						
Niger	1985-1989	0						
Niger	1990-1994	0						
Niger	1995-1999	0						
Niger	2000-2004	0						
Niger	2005-2009	3	35.7	2008	Direct estimates of national neonatal and child cause-specific mortality proportions in Niger by expert algorithm and physician coded analysis of verbal autopsy interview		8.9	39.1
Niger	2010-2017	0						
Nigeria	1980-1984	0						
Nigeria	1985-1989	0						
Nigeria	1990-1994	1	3.3	1991	Community-based surveillance of paediatric deaths in Cross River State, Nigeria		9.7	3.7
Nigeria	1995-1999	0						
Nigeria	2000-2004	0						
Nigeria	2005-2009	1	0.1	2007	Vital Registration	1.7	91.6	
Nigeria	2010-2017	2	32.3	2014	Direct estimates of cause-specific mortality fractions and rates of under-five deaths in the northern and southern regions of Nigeria by verbal autopsy interview		5.5	34.2
North Korea	1980-1984	0						
North Korea	1985-1989	0						
North Korea	1990-1994	0						
North Korea	1995-1999	0						
North Korea	2000-2004	0						
North Korea	2005-2009	0						
North Korea	2010-2017	0						
Northern Mariana Islands	1980-1984	0						
Northern Mariana Islands	1985-1989	0						
Northern Mariana Islands	1990-1994	0						
Northern Mariana Islands	1995-1999	4	75.3	1998	Vital Registration	100.0	24.7	
Northern Mariana Islands	2000-2004	4	71.4	2004	Vital Registration	90.8	21.3	
Northern Mariana Islands	2005-2009	4	80.3	2009	Vital Registration	100.0	19.7	
Northern Mariana Islands	2010-2017	4	82.5	2015	Vital Registration	93.9	12.1	
Norway	1980-1984	5	88.6	1984	Vital Registration	99.3	10.8	
Norway	1985-1989	5	89.4	1985	Vital Registration	100.0	10.6	
Norway	1990-1994	5	88.6	1990	Vital Registration	100.0	11.4	
Norway	1995-1999	5	88.5	1997	Vital Registration	100.0	11.5	
Norway	2000-2004	5	86.3	2001	Vital Registration	99.8	13.5	
Norway	2005-2009	4	84.2	2006	Vital Registration	100.0	15.7	
Norway	2010-2017	4	84.1	2015	Vital Registration	100.0	15.9	
Oman	1980-1984	0						
Oman	1985-1989	0						
Oman	1990-1994	0						
Oman	1995-1999	0						
Oman	2000-2004	0						
Oman	2005-2009	4	73.2	2007	Vital Registration	87.6	16.5	
Oman	2010-2017	2	34.4	2014	Vital Registration	78.6	56.2	
Pakistan	1980-1984	0						
Pakistan	1985-1989	1	2.8	1986	Acute respiratory infections in children: a case management intervention in Abbottabad District, Pakistan		13.0	3.2
Pakistan	1990-1994	1	1.6	1994	Time to focus child survival programmes on the newborn: assessment of levels and causes of infant mortality in rural Pakistan		19.9	2.0
Pakistan	1995-1999	0						
Pakistan	2000-2004	1	1.5	2003	Impact of a community-based perinatal and newborn preventive care package on perinatal and neonatal mortality in a remote mountainous district in Northern Pakistan		0.0	1.5
Pakistan	2005-2009	2	17.8	2006	Pakistan Demographic and Health Survey 2006-2007		32.9	26.6
Pakistan	2010-2017	1	1.9	2010	Cause of Death in under 5 Children in a Demographic Surveillance Site in Pakistan		19.1	2.4
Palestine	1980-1984	0						
Palestine	1985-1989	0						
Palestine	1990-1994	0						
Palestine	1995-1999	2	29.7	1999	Vital Registration	48.2	38.3	
Palestine	2000-2004	2	32.2	2004	Vital Registration	48.1	33.1	
Palestine	2005-2009	2	34.2	2009	Vital Registration	49.5	30.9	
Palestine	2010-2017	4	74.1	2014	Vital Registration	100.0	25.9	
Panama	1980-1984	4	72.3	1983	Vital Registration	97.3	25.7	
Panama	1985-1989	4	75.3	1987	Vital Registration	97.3	22.6	
Panama	1990-1994	0						
Panama	1995-1999	4	83.5	1999	Vital Registration	100.0	16.5	
Panama	2000-2004	5	86.6	2002	Vital Registration	100.0	13.4	
Panama	2005-2009	5	86.3	2007	Vital Registration	100.0	13.7	
Panama	2010-2017	4	84.0	2014	Vital Registration	100.0	16.0	

Appendix Table 8. Underlying indicators for percent well-certified for data source with maximum percent well certified in each 5-year time interval for 195 countries, 1980-2017								
Country	Time Window	Stars	Percent Well-Certified [PWC] (%)	Max PWC Data Year	Max PWC Data Source	Completeness (%)	Percent Major Garbage (%)	Verbal Autopsy Adjustment (None for VR) (%)
Papua New Guinea	1980-1984	1	9.4	1980	Vital Registration	11.4	17.4	
Papua New Guinea	1985-1989	1	3.4	1985	Mortality rates and the utilization of health services during terminal illness in the Asaro Valley, Eastern Highlands Province Papua New Guinea		46.7	6.4
Papua New Guinea	1990-1994	0						
Papua New Guinea	1995-1999	0						
Papua New Guinea	2000-2004	0						
Papua New Guinea	2005-2009	0						
Papua New Guinea	2010-2017	2	28.8	2013	The epidemiological transition in Papua New Guinea: new evidence from verbal autopsy studie:		53.7	62.3
Paraguay	1980-1984	3	51.3	1980	Vital Registration	79.3	35.4	
Paraguay	1985-1989	3	51.2	1989	Vital Registration	76.3	32.9	
Paraguay	1990-1994	3	57.2	1994	Vital Registration	77.1	25.8	
Paraguay	1995-1999	3	62.7	1996	Vital Registration	84.9	26.1	
Paraguay	2000-2004	3	63.6	2004	Vital Registration	88.3	27.9	
Paraguay	2005-2009	4	68.8	2009	Vital Registration	90.9	24.3	
Paraguay	2010-2017	4	74.0	2013	Vital Registration	93.6	21.0	
Peru	1980-1984	3	54.9	1980	Vital Registration	70.4	21.9	
Peru	1985-1989	3	36.1	1989	Vital Registration	68.9	47.7	
Peru	1990-1994	3	37.2	1992	Vital Registration	68.0	45.3	
Peru	1995-1999	3	51.1	1999	Vital Registration	79.6	35.8	
Peru	2000-2004	3	63.3	2004	Vital Registration	90.4	29.9	
Peru	2005-2009	4	65.4	2007	Vital Registration	86.4	24.3	
Peru	2010-2017	4	65.9	2015	Vital Registration	82.8	20.4	
Philippines	1980-1984	4	68.2	1983	Vital Registration	91.0	25.1	
Philippines	1985-1989	4	71.1	1985	Vital Registration	90.2	21.2	
Philippines	1990-1994	4	65.9	1990	Vital Registration	85.5	22.9	
Philippines	1995-1999	4	68.6	1998	Vital Registration	87.9	21.9	
Philippines	2000-2004	4	74.3	2002	Vital Registration	87.1	14.7	
Philippines	2005-2009	4	74.1	2009	Vital Registration	87.3	15.1	
Philippines	2010-2017	4	74.8	2015	Vital Registration	87.9	14.9	
Poland	1980-1984	3	61.8	1981	Vital Registration	99.4	37.8	
Poland	1985-1989	3	59.5	1988	Vital Registration	100.0	40.5	
Poland	1990-1994	3	59.9	1994	Vital Registration	100.0	40.1	
Poland	1995-1999	4	71.6	1999	Vital Registration	99.9	28.4	
Poland	2000-2004	4	73.7	2002	Vital Registration	99.3	25.8	
Poland	2005-2009	4	73.5	2005	Vital Registration	99.9	26.4	
Poland	2010-2017	4	72.6	2011	Vital Registration	99.8	27.2	
Portugal	1980-1984	4	76.9	1984	Vital Registration	100.0	23.1	
Portugal	1985-1989	4	77.1	1985	Vital Registration	100.0	22.9	
Portugal	1990-1994	4	76.3	1991	Vital Registration	100.0	23.7	
Portugal	1995-1999	4	76.2	1995	Vital Registration	100.0	23.8	
Portugal	2000-2004	4	79.1	2002	Vital Registration	100.0	20.9	
Portugal	2005-2009	4	77.7	2009	Vital Registration	100.0	22.3	
Portugal	2010-2017	4	80.1	2014	Vital Registration	97.9	18.2	
Puerto Rico	1980-1984	4	78.6	1981	Vital Registration	100.0	21.4	
Puerto Rico	1985-1989	4	76.7	1989	Vital Registration	100.0	23.3	
Puerto Rico	1990-1994	4	83.9	1994	Vital Registration	100.0	16.1	
Puerto Rico	1995-1999	4	83.2	1996	Vital Registration	100.0	16.8	
Puerto Rico	2000-2004	4	83.6	2004	Vital Registration	100.0	16.4	
Puerto Rico	2005-2009	4	83.6	2008	Vital Registration	100.0	16.4	
Puerto Rico	2010-2017	4	84.4	2012	Vital Registration	100.0	15.6	
Qatar	1980-1984	2	14.7	1984	Vital Registration	65.6	77.6	
Qatar	1985-1989	2	18.9	1985	Vital Registration	77.9	75.7	
Qatar	1990-1994	0						
Qatar	1995-1999	3	55.1	1995	Vital Registration	78.9	30.1	
Qatar	2000-2004	3	56.3	2001	Vital Registration	87.0	35.3	
Qatar	2005-2009	3	64.9	2006	Vital Registration	95.1	31.8	
Qatar	2010-2017	3	46.1	2010	Vital Registration	74.2	37.8	
Romania	1980-1984	4	76.0	1980	Vital Registration	100.0	24.0	
Romania	1985-1989	4	78.0	1989	Vital Registration	100.0	22.0	
Romania	1990-1994	4	83.3	1994	Vital Registration	100.0	16.7	
Romania	1995-1999	4	84.8	1999	Vital Registration	100.0	15.2	
Romania	2000-2004	5	85.9	2004	Vital Registration	99.8	13.9	
Romania	2005-2009	5	86.2	2006	Vital Registration	100.0	13.8	
Romania	2010-2017	5	85.4	2010	Vital Registration	100.0	14.6	
Russian Federation	1980-1984	5	93.0	1980	Vital Registration	100.0	7.0	
Russian Federation	1985-1989	5	87.3	1989	Vital Registration	94.7	7.8	
Russian Federation	1990-1994	5	90.7	1990	Vital Registration	100.0	9.3	
Russian Federation	1995-1999	5	87.6	1998	Vital Registration	100.0	12.4	
Russian Federation	2000-2004	5	88.0	2004	Vital Registration	100.0	12.0	
Russian Federation	2005-2009	5	89.3	2009	Vital Registration	100.0	10.7	
Russian Federation	2010-2017	5	88.8	2010	Vital Registration	100.0	11.2	
Rwanda	1980-1984	0						
Rwanda	1985-1989	0						
Rwanda	1990-1994	0						

Country	Time Window	Stars	Percent Well-Certified [PWC] (%)	Max PWC Data Year	Max PWC Data Source	Completeness (%)	Percent Major Garbage (%)	Verbal Autopsy Adjustment (None for VR) (%)
Rwanda	1995-1999	0						
Rwanda	2000-2004	0						
Rwanda	2005-2009	2	22.6	2007	Rwanda Child Verbal Autopsy Study 2008		11.6	25.5
Rwanda	2010-2017	0						
Saint Lucia	1980-1984	4	66.8	1983	Vital Registration	94.7	29.5	
Saint Lucia	1985-1989	4	68.2	1987	Vital Registration	100.0	31.8	
Saint Lucia	1990-1994	4	73.2	1994	Vital Registration	100.0	26.8	
Saint Lucia	1995-1999	4	72.1	1997	Vital Registration	100.0	27.9	
Saint Lucia	2000-2004	4	80.0	2001	Vital Registration	100.0	20.0	
Saint Lucia	2005-2009	4	79.0	2009	Vital Registration	94.4	16.3	
Saint Lucia	2010-2017	5	85.6	2014	Vital Registration	100.0	14.4	
Saint Vincent and the Grenadines	1980-1984	4	72.3	1983	Vital Registration	100.0	27.7	
Saint Vincent and the Grenadines	1985-1989	3	63.4	1986	Vital Registration	92.2	31.2	
Saint Vincent and the Grenadines	1990-1994	3	61.2	1990	Vital Registration	96.7	36.7	
Saint Vincent and the Grenadines	1995-1999	5	85.3	1998	Vital Registration	100.0	14.7	
Saint Vincent and the Grenadines	2000-2004	5	85.1	2002	Vital Registration	100.0	14.9	
Saint Vincent and the Grenadines	2005-2009	5	87.6	2007	Vital Registration	99.2	11.7	
Saint Vincent and the Grenadines	2010-2017	5	87.3	2014	Vital Registration	100.0	12.7	
Samoa	1980-1984	0						
Samoa	1985-1989	0						
Samoa	1990-1994	0						
Samoa	1995-1999	0						
Samoa	2000-2004	0						
Samoa	2005-2009	0						
Samoa	2010-2017	0						
Sao Tome and Principe	1980-1984	0						
Sao Tome and Principe	1985-1989	4	69.6	1985	Vital Registration	100.0	30.4	
Sao Tome and Principe	1990-1994	0						
Sao Tome and Principe	1995-1999	0						
Sao Tome and Principe	2000-2004	0						
Sao Tome and Principe	2005-2009	0						
Sao Tome and Principe	2010-2017	0						
Saudi Arabia	1980-1984	0						
Saudi Arabia	1985-1989	0						
Saudi Arabia	1990-1994	0						
Saudi Arabia	1995-1999	2	23.1	1999	Vital Registration	42.3	45.5	
Saudi Arabia	2000-2004	2	27.5	2004	Vital Registration	55.1	50.0	
Saudi Arabia	2005-2009	2	29.4	2008	Vital Registration	63.1	53.5	
Saudi Arabia	2010-2017	2	28.8	2010	Vital Registration	63.2	54.5	
Senegal	1980-1984	1	2.0	1984	Senegal - Risk of Death Associated with Different Nutritional States in Children of Preschool age: Study Conducted in Niakhar (Senegal) 1983-1986		28.7	2.7
Senegal	1985-1989	1	2.5	1986	International differences in clinical patterns of diarrhoeal deaths: a comparison of children from Brazil, Senegal, Bangladesh, and India		30.0	3.6
Senegal	1990-1994	1	2.6	1993	Childhood mortality and probable causes of death using verbal autopsy in Niakhar, Senegal, 1989-2000		26.2	3.5
Senegal	1995-1999	1	2.5	1996	Childhood mortality and probable causes of death using verbal autopsy in Niakhar, Senegal, 1989-2000		25.1	3.4
Senegal	2000-2004	0						
Senegal	2005-2009	1	0.0	2008	Africa, Asia, Oceania - INDEPTH Network Cause-Specific Mortality - Release 2014		99.6	6.4
Senegal	2010-2017	0	0.0	2010	Africa, Asia, Oceania - INDEPTH Network Cause-Specific Mortality - Release 2014		100.0	6.4
Serbia	1980-1984	0						
Serbia	1985-1989	0						
Serbia	1990-1994	0						
Serbia	1995-1999	4	66.7	1999	Vital Registration	84.8	21.3	
Serbia	2000-2004	4	70.8	2003	Vital Registration	89.0	20.4	
Serbia	2005-2009	4	74.9	2005	Vital Registration	90.6	17.4	
Serbia	2010-2017	4	69.6	2010	Vital Registration	83.5	16.7	
Seychelles	1980-1984	4	74.4	1982	Vital Registration	100.0	25.6	
Seychelles	1985-1989	4	71.5	1987	Vital Registration	98.7	27.6	
Seychelles	1990-1994	0						
Seychelles	1995-1999	0						
Seychelles	2000-2004	4	75.9	2002	Vital Registration	100.0	24.1	
Seychelles	2005-2009	4	76.9	2008	Vital Registration	100.0	23.1	
Seychelles	2010-2017	4	78.1	2011	Vital Registration	100.0	21.9	
Sierra Leone	1980-1984	0						
Sierra Leone	1985-1989	0						
Sierra Leone	1990-1994	1	3.4	1990	Malaria in a rural area of Sierra Leone. I. Initial results		10.8	3.8
Sierra Leone	1995-1999	0						
Sierra Leone	2000-2004	0						
Sierra Leone	2005-2009	0						
Sierra Leone	2010-2017	0						
Singapore	1980-1984	5	91.2	1983	Vital Registration	100.0	8.8	
Singapore	1985-1989	5	92.0	1985	Vital Registration	100.0	8.0	
Singapore	1990-1994	5	94.6	1994	Vital Registration	99.9	5.3	

Table title: **Appendix Table 8. Underlying indicators for percent well-certified for data source with maximum percent well certified in each 5-year time interval for 195 countries, 1980-2017**

Appendix Table 8. Underlying indicators for percent well-certified for data source with maximum percent well certified in each 5-year time interval for 195 countries, 1980-2017								
Country	Time Window	Stars	Percent Well-Certified [PWC] (%)	Max PWC Data Year	Max PWC Data Source	Completeness (%)	Percent Major Garbage (%)	Verbal Autopsy Adjustment (None for VR) (%)
Singapore	1995-1999	5	95.1	1997	Vital Registration	99.7	4.7	
Singapore	2000-2004	5	95.3	2003	Vital Registration	100.0	4.7	
Singapore	2005-2009	5	94.9	2007	Vital Registration	100.0	5.1	
Singapore	2010-2017	5	95.7	2015	Vital Registration	98.5	2.8	
Slovakia	1980-1984	0						
Slovakia	1985-1989	0						
Slovakia	1990-1994	4	82.7	1994	Vital Registration	99.8	17.1	
Slovakia	1995-1999	4	81.9	1999	Vital Registration	99.1	17.4	
Slovakia	2000-2004	4	84.9	2003	Vital Registration	99.7	14.9	
Slovakia	2005-2009	5	90.2	2008	Vital Registration	99.7	9.6	
Slovakia	2010-2017	5	92.9	2012	Vital Registration	100.0	7.1	
Slovenia	1980-1984	0						
Slovenia	1985-1989	5	91.6	1985	Vital Registration	100.0	8.4	
Slovenia	1990-1994	5	92.5	1992	Vital Registration	100.0	7.5	
Slovenia	1995-1999	5	91.8	1997	Vital Registration	100.0	8.2	
Slovenia	2000-2004	5	89.3	2000	Vital Registration	100.0	10.7	
Slovenia	2005-2009	5	88.3	2009	Vital Registration	100.0	11.7	
Slovenia	2010-2017	5	88.5	2011	Vital Registration	100.0	11.5	
Solomon Islands	1980-1984	0						
Solomon Islands	1985-1989	0						
Solomon Islands	1990-1994	0						
Solomon Islands	1995-1999	0						
Solomon Islands	2000-2004	0						
Solomon Islands	2005-2009	0						
Solomon Islands	2010-2017	2	34.6	2016	Verbal Autopsy		37.4	55.3
Somalia	1980-1984	0						
Somalia	1985-1989	0						
Somalia	1990-1994	0						
Somalia	1995-1999	0						
Somalia	2000-2004	0						
Somalia	2005-2009	0						
Somalia	2010-2017	0						
South Africa	1980-1984	0						
South Africa	1985-1989	0						
South Africa	1990-1994	1	1.0	1991	'A bothersome death' -- narrative accounts of infant mortality in Cape Town, South Africa		19.0	1.2
South Africa	1995-1999	4	67.8	1999	Vital Registration	100.0	32.2	
South Africa	2000-2004	4	72.0	2004	Vital Registration	100.0	28.0	
South Africa	2005-2009	4	72.3	2005	Vital Registration	100.0	27.7	
South Africa	2010-2017	4	70.7	2013	Vital Registration	100.0	29.3	
South Korea	1980-1984	0						
South Korea	1985-1989	3	57.8	1985	Vital Registration	85.6	32.5	
South Korea	1990-1994	4	74.2	1994	Vital Registration	96.0	22.7	
South Korea	1995-1999	4	75.4	1995	Vital Registration	99.8	24.5	
South Korea	2000-2004	4	84.5	2002	Vital Registration	100.0	15.5	
South Korea	2005-2009	4	82.2	2007	Vital Registration	100.0	17.8	
South Korea	2010-2017	4	81.9	2014	Vital Registration	99.7	17.9	
South Sudan	1980-1984	0						
South Sudan	1985-1989	0						
South Sudan	1990-1994	0						
South Sudan	1995-1999	0						
South Sudan	2000-2004	0						
South Sudan	2005-2009	0						
South Sudan	2010-2017	0						
Spain	1980-1984	4	77.3	1984	Vital Registration	99.4	22.2	
Spain	1985-1989	4	79.4	1989	Vital Registration	100.0	20.6	
Spain	1990-1994	4	82.6	1994	Vital Registration	100.0	17.4	
Spain	1995-1999	4	83.8	1998	Vital Registration	100.0	16.2	
Spain	2000-2004	4	83.1	2002	Vital Registration	100.0	16.9	
Spain	2005-2009	4	84.0	2009	Vital Registration	100.0	16.0	
Spain	2010-2017	4	84.8	2015	Vital Registration	100.0	15.2	
Sri Lanka	1980-1984	3	52.0	1980	Vital Registration	100.0	48.0	
Sri Lanka	1985-1989	3	50.2	1986	Vital Registration	100.0	49.8	
Sri Lanka	1990-1994	3	47.7	1993	Vital Registration	100.0	52.3	
Sri Lanka	1995-1999	3	55.4	1999	Vital Registration	100.0	44.6	
Sri Lanka	2000-2004	3	63.7	2004	Vital Registration	100.0	36.3	
Sri Lanka	2005-2009	4	67.4	2007	Vital Registration	100.0	32.6	
Sri Lanka	2010-2017	3	64.8	2010	Vital Registration	100.0	35.2	
Sudan	1980-1984	0						
Sudan	1985-1989	0						
Sudan	1990-1994	0						
Sudan	1995-1999	0						
Sudan	2000-2004	0						
Sudan	2005-2009	0						
Sudan	2010-2017	0						

Appendix Table 8. Underlying indicators for percent well-certified for data source with maximum percent well certified in each 5-year time interval for 195 countries, 1980-2017								
Country	Time Window	Stars	Percent Well-Certified [PWC] (%)	Max PWC Data Year	Max PWC Data Source	Completeness (%)	Percent Major Garbage (%)	Verbal Autopsy Adjustment (None for VR) (%)
Suriname	1980-1984	3	60.0	1984	Vital Registration	87.6	31.5	
Suriname	1985-1989	3	63.7	1989	Vital Registration	91.3	30.2	
Suriname	1990-1994	3	61.2	1991	Vital Registration	86.2	29.0	
Suriname	1995-1999	4	66.4	1999	Vital Registration	92.4	28.2	
Suriname	2000-2004	4	74.3	2002	Vital Registration	93.5	20.5	
Suriname	2005-2009	4	73.3	2008	Vital Registration	91.1	19.5	
Suriname	2010-2017	4	73.9	2010	Vital Registration	90.3	18.2	
Swaziland	1980-1984	0						
Swaziland	1985-1989	0						
Swaziland	1990-1994	0						
Swaziland	1995-1999	0						
Swaziland	2000-2004	1	0.0	2000	Effect of HIV infection on pregnancy-related mortality in sul Saharan Africa: secondary analyses of pooled community-basec data from the network for Analysing Longitudinal Population-based HIV/AIDS data on Africa (ALPHA		94.9	0.4
Swaziland	2005-2009	0						
Swaziland	2010-2017	0						
Sweden	1980-1984	5	87.4	1980	Vital Registration	99.1	11.8	
Sweden	1985-1989	5	88.6	1988	Vital Registration	100.0	11.4	
Sweden	1990-1994	5	88.2	1990	Vital Registration	100.0	11.8	
Sweden	1995-1999	5	87.1	1995	Vital Registration	100.0	12.9	
Sweden	2000-2004	5	85.7	2002	Vital Registration	100.0	14.3	
Sweden	2005-2009	5	85.9	2005	Vital Registration	99.9	14.1	
Sweden	2010-2017	4	84.8	2013	Vital Registration	99.9	15.2	
Switzerland	1980-1984	4	73.8	1983	Vital Registration	100.0	26.2	
Switzerland	1985-1989	4	74.2	1986	Vital Registration	100.0	25.8	
Switzerland	1990-1994	4	73.4	1990	Vital Registration	100.0	26.6	
Switzerland	1995-1999	4	84.6	1998	Vital Registration	100.0	15.4	
Switzerland	2000-2004	4	84.4	2003	Vital Registration	100.0	15.6	
Switzerland	2005-2009	5	86.7	2008	Vital Registration	100.0	13.3	
Switzerland	2010-2017	5	86.1	2010	Vital Registration	100.0	13.9	
Syria	1980-1984	2	29.5	1980	Vital Registration	100.0	70.5	
Syria	1985-1989	2	13.2	1985	Vital Registration	66.4	80.1	
Syria	1990-1994	0						
Syria	1995-1999	3	54.3	1999	Vital Registration	100.0	45.7	
Syria	2000-2004	4	66.7	2004	Vital Registration	97.9	31.9	
Syria	2005-2009	4	74.4	2009	Vital Registration	99.7	25.4	
Syria	2010-2017	3	59.8	2010	Vital Registration	100.0	40.2	
Taiwan (Province of China)	1980-1984	4	84.3	1980	Vital Registration	99.2	15.0	
Taiwan (Province of China)	1985-1989	4	82.4	1986	Vital Registration	100.0	17.6	
Taiwan (Province of China)	1990-1994	4	79.2	1990	Vital Registration	99.3	20.2	
Taiwan (Province of China)	1995-1999	4	83.4	1999	Vital Registration	99.7	16.4	
Taiwan (Province of China)	2000-2004	4	84.4	2004	Vital Registration	100.0	15.6	
Taiwan (Province of China)	2005-2009	4	84.8	2005	Vital Registration	100.0	15.2	
Taiwan (Province of China)	2010-2017	4	83.7	2014	Vital Registration	100.0	16.3	
Tajikistan	1980-1984	4	74.9	1981	Vital Registration	89.2	16.0	
Tajikistan	1985-1989	4	71.4	1988	Vital Registration	92.9	23.1	
Tajikistan	1990-1994	4	71.6	1992	Vital Registration	92.8	22.9	
Tajikistan	1995-1999	3	58.5	1995	Vital Registration	82.9	29.4	
Tajikistan	2000-2004	3	51.4	2002	Vital Registration	74.0	30.5	
Tajikistan	2005-2009	3	50.6	2005	Vital Registration	75.4	32.9	
Tajikistan	2010-2017	3	50.7	2016	Vital Registration	75.6	33.0	
Tanzania	1980-1984	0						
Tanzania	1985-1989	1	2.8	1986	Risk factors for deaths in children under 5 years old in Bagamoy district, Tanzania		20.7	3.5
Tanzania	1990-1994	1	1.8	1993	Community based studies on childhood mortality in a malaria holoendemic area on the Tanzanian coas		46.6	3.3
Tanzania	1995-1999	1	2.1	1995	The Policy Implications of Tanzania's Mortality Burden		67.2	6.4
Tanzania	2000-2004	1	6.3	2000	Tanzania - Rufiji Health and Demographic Surveillance System		0.9	6.4
Tanzania	2005-2009	1	2.5	2005	The contribution of reduction in malaria as a cause of rapid decline of under-five mortality: evidence from the Rufiji Health and Demographic Surveillance System (HDSS) in rural Tanzania		6.7	2.7
Tanzania	2010-2017	0						
Thailand	1980-1984	2	29.9	1980	Vital Registration	80.3	62.8	
Thailand	1985-1989	2	28.3	1987	Vital Registration	80.3	64.8	
Thailand	1990-1994	3	35.9	1994	Vital Registration	87.6	59.0	
Thailand	1995-1999	3	62.6	1998	Thailand Burden of Disease and Injuries 1998-1999		2.2	64.0
Thailand	2000-2004	3	52.0	2003	Vital Registration	100.0	48.0	
Thailand	2005-2009	3	61.6	2005	Thailand Verbal Autopsy Study 2005		3.7	64.0
Thailand	2010-2017	3	62.3	2016	Vital Registration	100.0	37.7	
The Bahamas	1980-1984	4	76.2	1984	Vital Registration	95.8	20.5	
The Bahamas	1985-1989	4	82.6	1987	Vital Registration	100.0	17.4	
The Bahamas	1990-1994	4	83.0	1994	Vital Registration	100.0	17.0	
The Bahamas	1995-1999	5	89.8	1999	Vital Registration	100.0	10.2	
The Bahamas	2000-2004	5	87.7	2000	Vital Registration	100.0	12.3	
The Bahamas	2005-2009	5	86.7	2009	Vital Registration	100.0	13.3	
The Bahamas	2010-2017	5	86.3	2011	Vital Registration	100.0	13.7	

Appendix Table 8. Underlying indicators for percent well-certified for data source with maximum percent well certified in each 5-year time interval for 195 countries, 1980-2017								
Country	Time Window	Stars	Percent Well-Certified [PWC] (%)	Max PWC Data Year	Max PWC Data Source	Completeness (%)	Percent Major Garbage (%)	Verbal Autopsy Adjustment (None for VR) (%)
The Gambia	1980-1984	1	2.8	1982	Deaths in infancy and early childhood in a well-vaccinated, rural West African population		20.7	3.5
The Gambia	1985-1989	1	2.2	1989	Changes in the pattern of infant and childhood mortality in upper river division, The Gambia, from 1989 to 1993		29.4	3.1
The Gambia	1990-1994	1	2.1	1991	Changes in the pattern of infant and childhood mortality in upper river division, The Gambia, from 1989 to 1993		30.2	3.1
The Gambia	1995-1999	1	0.9	1999	Reaching millennium development goal 4 - the Gambia		65.1	2.7
The Gambia	2000-2004	1	0.7	2002	Reaching millennium development goal 4 - the Gambia		70.4	2.5
The Gambia	2005-2009	1	1.0	2006	Preventive measures in infancy to reduce under-five mortality: a case-control study in The Gambia		56.0	2.2
The Gambia	2010-2017	0						
Timor-Leste	1980-1984	0						
Timor-Leste	1985-1989	0						
Timor-Leste	1990-1994	0						
Timor-Leste	1995-1999	0						
Timor-Leste	2000-2004	0						
Timor-Leste	2005-2009	0						
Timor-Leste	2010-2017	0						
Togo	1980-1984	0						
Togo	1985-1989	0						
Togo	1990-1994	0						
Togo	1995-1999	0						
Togo	2000-2004	0						
Togo	2005-2009	0						
Togo	2010-2017	0						
Tonga	1980-1984	0						
Tonga	1985-1989	0						
Tonga	1990-1994	0						
Tonga	1995-1999	0						
Tonga	2000-2004	3	53.6	2003	Vital Registration	100.0	46.4	
Tonga	2005-2009	0						
Tonga	2010-2017	0						
Trinidad and Tobago	1980-1984	4	79.2	1983	Vital Registration	94.7	16.3	
Trinidad and Tobago	1985-1989	4	83.4	1987	Vital Registration	98.6	15.4	
Trinidad and Tobago	1990-1994	5	85.5	1994	Vital Registration	100.0	14.5	
Trinidad and Tobago	1995-1999	5	89.5	1999	Vital Registration	100.0	10.5	
Trinidad and Tobago	2000-2004	5	90.7	2002	Vital Registration	100.0	9.3	
Trinidad and Tobago	2005-2009	5	89.6	2005	Vital Registration	100.0	10.4	
Trinidad and Tobago	2010-2017	5	90.1	2011	Vital Registration	100.0	9.9	
Tunisia	1980-1984	0						
Tunisia	1985-1989	0						
Tunisia	1990-1994	0						
Tunisia	1995-1999	0						
Tunisia	2000-2004	0						
Tunisia	2005-2009	2	28.9	2009	Vital Registration	39.0	25.8	
Tunisia	2010-2017	2	24.8	2013	Vital Registration	38.2	35.2	
Turkey	1980-1984	2	16.8	1983	Vital Registration	36.2	53.6	
Turkey	1985-1989	2	19.6	1989	Vital Registration	42.2	53.5	
Turkey	1990-1994	2	20.7	1993	Vital Registration	44.5	53.4	
Turkey	1995-1999	2	24.0	1999	Vital Registration	54.8	56.2	
Turkey	2000-2004	3	59.7	2002	Turkey Verbal Autopsy Survey 2003		6.6	64.0
Turkey	2005-2009	4	70.3	2009	Vital Registration	88.1	20.1	
Turkey	2010-2017	4	83.9	2013	Vital Registration	99.3	15.5	
Turkmenistan	1980-1984	5	88.4	1981	Vital Registration	100.0	11.6	
Turkmenistan	1985-1989	5	88.1	1986	Vital Registration	100.0	11.9	
Turkmenistan	1990-1994	4	83.0	1991	Vital Registration	100.0	17.0	
Turkmenistan	1995-1999	4	80.9	1995	Vital Registration	100.0	19.1	
Turkmenistan	2000-2004	5	85.1	2002	Vital Registration	96.4	11.7	
Turkmenistan	2005-2009	4	81.0	2009	Vital Registration	96.9	16.4	
Turkmenistan	2010-2017	4	83.4	2010	Vital Registration	100.0	16.6	
USA	1980-1984	5	88.5	1980	Vital Registration	98.0	9.6	
USA	1985-1989	5	88.1	1989	Vital Registration	98.8	10.8	
USA	1990-1994	5	88.7	1993	Vital Registration	99.6	11.0	
USA	1995-1999	5	88.1	1999	Vital Registration	100.0	11.9	
USA	2000-2004	5	88.0	2000	Vital Registration	100.0	12.0	
USA	2005-2009	5	87.2	2005	Vital Registration	100.0	12.8	
USA	2010-2017	5	86.7	2010	Vital Registration	99.9	13.2	
Uganda	1980-1984	0						
Uganda	1985-1989	0						
Uganda	1990-1994	0						
Uganda	1995-1999	0						
Uganda	2000-2004	1	0.0	2000	Effect of HIV infection on pregnancy-related mortality in sub-Saharan Africa: secondary analyses of pooled community-based data from the network for Analysing Longitudinal Population-based HIV/AIDS data on Africa (ALPHA		95.7	0.4
Uganda	2005-2009	1	2.7	2006	Uganda Child Verbal Autopsy Study 2007		12.4	3.0
Uganda	2010-2017	0						
Ukraine	1980-1984	5	87.4	1982	Vital Registration	98.5	11.2	

Appendix Table 8. Underlying indicators for percent well-certified for data source with maximum percent well certified in each 5-year time interval for 195 countries, 1980-2017								
Country	Time Window	Stars	Percent Well-Certified [PWC] (%)	Max PWC Data Year	Max PWC Data Source	Completeness (%)	Percent Major Garbage (%)	Verbal Autopsy Adjustment (None for VR) (%)
Ukraine	1985-1989	5	90.0	1985	Vital Registration	100.0	10.0	
Ukraine	1990-1994	4	83.5	1994	Vital Registration	100.0	16.5	
Ukraine	1995-1999	5	86.0	1999	Vital Registration	100.0	14.0	
Ukraine	2000-2004	5	86.4	2000	Vital Registration	100.0	13.6	
Ukraine	2005-2009	5	90.9	2009	Vital Registration	100.0	9.1	
Ukraine	2010-2017	5	92.2	2012	Vital Registration	100.0	7.8	
United Arab Emirates	1980-1984	0						
United Arab Emirates	1985-1989	0						
United Arab Emirates	1990-1994	0						
United Arab Emirates	1995-1999	0						
United Arab Emirates	2000-2004	0						
United Arab Emirates	2005-2009	3	41.5	2007	Vital Registration	81.9	49.3	
United Arab Emirates	2010-2017	0						
United Kingdom	1980-1984	5	94.2	1984	Vital Registration	100.0	5.8	
United Kingdom	1985-1989	5	94.1	1985	Vital Registration	100.0	5.9	
United Kingdom	1990-1994	5	94.1	1992	Vital Registration	100.0	5.9	
United Kingdom	1995-1999	5	92.0	1995	Vital Registration	100.0	8.0	
United Kingdom	2000-2004	5	91.4	2001	Vital Registration	100.0	8.6	
United Kingdom	2005-2009	5	91.5	2009	Vital Registration	100.0	8.5	
United Kingdom	2010-2017	5	92.4	2014	Vital Registration	100.0	7.6	
Uruguay	1980-1984	4	77.3	1982	Vital Registration	100.0	22.7	
Uruguay	1985-1989	4	76.0	1985	Vital Registration	100.0	24.0	
Uruguay	1990-1994	4	79.1	1991	Vital Registration	100.0	20.9	
Uruguay	1995-1999	4	79.1	1998	Vital Registration	100.0	20.9	
Uruguay	2000-2004	4	79.6	2001	Vital Registration	100.0	20.4	
Uruguay	2005-2009	4	78.5	2005	Vital Registration	100.0	21.5	
Uruguay	2010-2017	4	77.5	2015	Vital Registration	100.0	22.5	
Uzbekistan	1980-1984	4	83.1	1982	Vital Registration	97.7	14.9	
Uzbekistan	1985-1989	5	86.3	1985	Vital Registration	100.0	13.7	
Uzbekistan	1990-1994	4	85.0	1992	Vital Registration	100.0	15.0	
Uzbekistan	1995-1999	4	79.0	1995	Vital Registration	94.9	16.8	
Uzbekistan	2000-2004	4	68.3	2000	Vital Registration	83.8	18.5	
Uzbekistan	2005-2009	4	72.2	2009	Vital Registration	79.4	9.1	
Uzbekistan	2010-2017	4	79.8	2012	Vital Registration	88.9	10.2	
Vanuatu	1980-1984	0						
Vanuatu	1985-1989	0						
Vanuatu	1990-1994	0						
Vanuatu	1995-1999	0						
Vanuatu	2000-2004	0						
Vanuatu	2005-2009	0						
Vanuatu	2010-2017	0						
Venezuela	1980-1984	4	81.3	1983	Vital Registration	99.8	18.6	
Venezuela	1985-1989	4	75.2	1988	Vital Registration	100.0	24.8	
Venezuela	1990-1994	4	83.3	1994	Vital Registration	99.8	16.5	
Venezuela	1995-1999	5	87.8	1999	Vital Registration	100.0	12.2	
Venezuela	2000-2004	5	89.8	2001	Vital Registration	100.0	10.2	
Venezuela	2005-2009	5	89.5	2005	Vital Registration	100.0	10.5	
Venezuela	2010-2017	5	88.9	2011	Vital Registration	100.0	11.1	
Vietnam	1980-1984	0						
Vietnam	1985-1989	1	0.7	1987	Are there social inequities in child morbidity and mortality in rural Vietnam		52.4	1.5
Vietnam	1990-1994	1	0.2	1994	Maternal mortality in Vietnam in 1994-95		77.6	1.0
Vietnam	1995-1999	1	1.3	1999	Applying verbal autopsy to determine cause of death in rural Vietnam		79.9	6.4
Vietnam	2000-2004	1	0.9	2001	Socio-economic status inequality and major causes of death in adults: a 5-year follow-up study in rural Vietnan		84.5	5.7
Vietnam	2005-2009	3	62.0	2008	Mortality measures from sample-based surveillance: evidence of the epidemiological transition in Viet Nam, Unpublished dat		3.1	64.0
Vietnam	2010-2017	1	4.8	2010	The causes of deaths in Chililab between 2008-2010 based on verbal autopsy methoc		24.3	6.4
Virgin Islands	1980-1984	4	82.0	1980	Vital Registration	100.0	18.0	
Virgin Islands	1985-1989	0						
Virgin Islands	1990-1994	5	90.6	1994	Vital Registration	100.0	9.4	
Virgin Islands	1995-1999	5	90.5	1995	Vital Registration	100.0	9.5	
Virgin Islands	2000-2004	4	84.1	2000	Vital Registration	94.5	11.0	
Virgin Islands	2005-2009	4	81.9	2007	Vital Registration	90.5	9.5	
Virgin Islands	2010-2017	4	74.0	2010	Vital Registration	86.6	14.5	
Yemen	1980-1984	0						
Yemen	1985-1989	0						
Yemen	1990-1994	0						
Yemen	1995-1999	0						
Yemen	2000-2004	0						
Yemen	2005-2009	0						
Yemen	2010-2017	0						
Zambia	1980-1984	0						
Zambia	1985-1989	0						
Zambia	1990-1994	0						

Appendix Table 8. Underlying indicators for percent well-certified for data source with maximum percent well certified in each 5-year time interval for 195 countries, 1980-2017								
**Country**	**Time Window**	**Stars**	**Percent Well-Certified [PWC] (%)**	**Max PWC Data Year**	**Max PWC Data Source**	**Completeness (%)**	**Percent Major Garbage (%)**	**Verbal Autopsy Adjustment (None for VR) (%)**
Zambia	1995-1999	0						
Zambia	2000-2004	0						
Zambia	2005-2009	1	6.3	2009	Zambia Sample Vital Registration with Verbal Autopsy (SAVVY) Data 2010		1.0	6.4
Zambia	2010-2017	1	9.5	2011	Adult Mortality in Sub-saharan Africa, Zambia: Where Do Adults Die?		42.7	16.6
Zimbabwe	1980-1984	0						
Zimbabwe	1985-1989	0						
Zimbabwe	1990-1994	3	45.3	1990	Vital Registration	59.8	24.2	
Zimbabwe	1995-1999	4	74.2	1995	Vital Registration	100.0	25.8	
Zimbabwe	2000-2004	1	0.0	2000	Effect of HIV infection on pregnancy-related mortality in sub Saharan Africa: secondary analyses of pooled community-based data from the network for Analysing Longitudinal Population-based HIV/AIDS data on Africa (ALPHA		96.6	0.5
Zimbabwe	2005-2009	3	45.3	2007	Vital Registration	55.0	17.7	
Zimbabwe	2010-2017	0						

Appendix Table 9: CODEm covariates used, level of covariate, and expected direction of covariate by cause, sex, and age								
Covariates that CODEm did not select during the covariate selection process have no draw counts listed.								
**Cause**	**Sex**	**Age Start**	**Age End**	**Model Type**	**Direction**	**Level**	**Covariate Name**	**Number of Draws**
Sexually transmitted infections excluding HIV	Female	10-14 years	95+ years	Data Rich	1	1	Syphilis prevalence (proportion)	--
Sexually transmitted infections excluding HIV	Female	10-14 years	95+ years	Data Rich	-1	2	Education (years per capita)	57
Sexually transmitted infections excluding HIV	Female	10-14 years	95+ years	Data Rich	-1	2	Health System Access (capped)	57
Sexually transmitted infections excluding HIV	Female	10-14 years	95+ years	Data Rich	-1	2	Healthcare access and quality index	112
Sexually transmitted infections excluding HIV	Female	10-14 years	95+ years	Data Rich	-1	2	Legality of Abortion	618
Sexually transmitted infections excluding HIV	Female	10-14 years	95+ years	Data Rich	1	2	Age-Specific Fertility Rate	--
Sexually transmitted infections excluding HIV	Female	10-14 years	95+ years	Data Rich	1	2	Total Fertility Rate	--
Sexually transmitted infections excluding HIV	Female	10-14 years	95+ years	Data Rich	-1	3	Antenatal Care (4 visits) Coverage (proportion)	138
Sexually transmitted infections excluding HIV	Female	10-14 years	95+ years	Data Rich	-1	3	Antenatal Care (1 visit) Coverage (proportion)	531
Sexually transmitted infections excluding HIV	Female	10-14 years	95+ years	Data Rich	-1	3	LDI (I$ per capita)	--
Sexually transmitted infections excluding HIV	Female	10-14 years	95+ years	Global	1	1	Syphilis prevalence (proportion)	--
Sexually transmitted infections excluding HIV	Female	10-14 years	95+ years	Global	-1	2	Education (years per capita)	59
Sexually transmitted infections excluding HIV	Female	10-14 years	95+ years	Global	-1	2	Health System Access (capped)	59
Sexually transmitted infections excluding HIV	Female	10-14 years	95+ years	Global	-1	2	Healthcare access and quality index	284
Sexually transmitted infections excluding HIV	Female	10-14 years	95+ years	Global	-1	2	Legality of Abortion	343
Sexually transmitted infections excluding HIV	Female	10-14 years	95+ years	Global	1	2	Age-Specific Fertility Rate	--
Sexually transmitted infections excluding HIV	Female	10-14 years	95+ years	Global	1	2	Total Fertility Rate	--
Sexually transmitted infections excluding HIV	Female	10-14 years	95+ years	Global	-1	3	Antenatal Care (1 visit) Coverage (proportion)	336
Sexually transmitted infections excluding HIV	Female	10-14 years	95+ years	Global	-1	3	Antenatal Care (4 visits) Coverage (proportion)	359
Sexually transmitted infections excluding HIV	Female	10-14 years	95+ years	Global	-1	3	LDI (I$ per capita)	--
Sexually transmitted infections excluding HIV	Male	10-14 years	95+ years	Data Rich	1	1	Syphilis prevalence (proportion)	--
Sexually transmitted infections excluding HIV	Male	10-14 years	95+ years	Data Rich	-1	2	Healthcare access and quality index	404
Sexually transmitted infections excluding HIV	Male	10-14 years	95+ years	Data Rich	-1	2	Education (years per capita)	449
Sexually transmitted infections excluding HIV	Male	10-14 years	95+ years	Data Rich	-1	2	Legality of Abortion	643
Sexually transmitted infections excluding HIV	Male	10-14 years	95+ years	Data Rich	-1	2	Health System Access (capped)	788
Sexually transmitted infections excluding HIV	Male	10-14 years	95+ years	Data Rich	1	2	Age-Specific Fertility Rate	--
Sexually transmitted infections excluding HIV	Male	10-14 years	95+ years	Data Rich	1	2	Total Fertility Rate	--
Sexually transmitted infections excluding HIV	Male	10-14 years	95+ years	Data Rich	-1	3	Antenatal Care (1 visit) Coverage (proportion)	175
Sexually transmitted infections excluding HIV	Male	10-14 years	95+ years	Data Rich	-1	3	Antenatal Care (4 visits) Coverage (proportion)	221
Sexually transmitted infections excluding HIV	Male	10-14 years	95+ years	Data Rich	-1	3	LDI (I$ per capita)	--
Sexually transmitted infections excluding HIV	Male	10-14 years	95+ years	Global	1	1	Syphilis prevalence (proportion)	--
Sexually transmitted infections excluding HIV	Male	10-14 years	95+ years	Global	-1	2	Education (years per capita)	385
Sexually transmitted infections excluding HIV	Male	10-14 years	95+ years	Global	-1	2	Healthcare access and quality index	385
Sexually transmitted infections excluding HIV	Male	10-14 years	95+ years	Global	-1	2	Legality of Abortion	385
Sexually transmitted infections excluding HIV	Male	10-14 years	95+ years	Global	-1	2	Health System Access (capped)	647
Sexually transmitted infections excluding HIV	Male	10-14 years	95+ years	Global	1	2	Age-Specific Fertility Rate	--
Sexually transmitted infections excluding HIV	Male	10-14 years	95+ years	Global	1	2	Total Fertility Rate	--
Sexually transmitted infections excluding HIV	Male	10-14 years	95+ years	Global	-1	3	Antenatal Care (4 visits) Coverage (proportion)	99
Sexually transmitted infections excluding HIV	Male	10-14 years	95+ years	Global	-1	3	Antenatal Care (1 visit) Coverage (proportion)	335
Sexually transmitted infections excluding HIV	Male	10-14 years	95+ years	Global	-1	3	LDI (I$ per capita)	--
Lower respiratory infections	Female	0-6 days	1-4 years	Data Rich	-1	1	Antibiotics for LRI	94
Lower respiratory infections	Female	0-6 days	1-4 years	Data Rich	-1	1	PCV3 Coverage (proportion)	401
Lower respiratory infections	Female	0-6 days	1-4 years	Data Rich	-1	1	Hib3 Vaccine Coverage (proportion)	506
Lower respiratory infections	Female	0-6 days	1-4 years	Data Rich	1	1	Age- and sex-specific wasting (weight-for-height) SEV	38
Lower respiratory infections	Female	0-6 days	1-4 years	Data Rich	1	1	Age- and sex-specific stunting (height-for-age) SEV	80
Lower respiratory infections	Female	0-6 days	1-4 years	Data Rich	1	1	Indoor Air Pollution (All Cooking Fuels)	208
Lower respiratory infections	Female	0-6 days	1-4 years	Data Rich	1	1	Age- and sex-specific underweight (weight-for-age) SEV	326
Lower respiratory infections	Female	0-6 days	1-4 years	Data Rich	1	1	Short gestation SEV (all ages, by sex)	328
Lower respiratory infections	Female	0-6 days	1-4 years	Data Rich	1	1	Low birth weight SEV (all ages, by sex)	384
Lower respiratory infections	Female	0-6 days	1-4 years	Data Rich	1	1	Log-transformed SEV scalar: LRI	918
Lower respiratory infections	Female	0-6 days	1-4 years	Data Rich	-1	2	Healthcare access and quality index	126
Lower respiratory infections	Female	0-6 days	1-4 years	Data Rich	-1	2	DTP3 Coverage (proportion)	407
Lower respiratory infections	Female	0-6 days	1-4 years	Data Rich	1	2	Zinc deficiency	0
Lower respiratory infections	Female	0-6 days	1-4 years	Data Rich	1	2	Vitamin A Deficiency Prevalence (age-standardized)	145
Lower respiratory infections	Female	0-6 days	1-4 years	Data Rich	1	2	Secondhand smoke	330
Lower respiratory infections	Female	0-6 days	1-4 years	Data Rich	1	2	Outdoor Air Pollution (PM2.5)	382
Lower respiratory infections	Female	0-6 days	1-4 years	Data Rich	1	2	Discontinued breastfeeding SEV	--
Lower respiratory infections	Female	0-6 days	1-4 years	Data Rich	-1	3	Maternal Education (years per capita)	0
Lower respiratory infections	Female	0-6 days	1-4 years	Data Rich	-1	3	Socio-demographic Index	90
Lower respiratory infections	Female	0-6 days	1-4 years	Data Rich	1	3	Population Density (over 1000 ppl/sqkm, proportion)	38
Lower respiratory infections	Female	0-6 days	1-4 years	Data Rich	1	3	No access to handwashing facility	404
Lower respiratory infections	Female	0-6 days	1-4 years	Data Rich	1	3	SEV unsafe sanitation	596
Lower respiratory infections	Female	0-6 days	1-4 years	Global	-1	1	Hib3 Vaccine Coverage (proportion)	259
Lower respiratory infections	Female	0-6 days	1-4 years	Global	-1	1	Antibiotics for LRI	389
Lower respiratory infections	Female	0-6 days	1-4 years	Global	-1	1	PCV3 Coverage (proportion)	428
Lower respiratory infections	Female	0-6 days	1-4 years	Global	1	1	Short gestation SEV (all ages, by sex)	143
Lower respiratory infections	Female	0-6 days	1-4 years	Global	1	1	Age- and sex-specific stunting (height-for-age) SEV	287
Lower respiratory infections	Female	0-6 days	1-4 years	Global	1	1	Indoor Air Pollution (All Cooking Fuels)	290
Lower respiratory infections	Female	0-6 days	1-4 years	Global	1	1	Age- and sex-specific underweight (weight-for-age) SEV	321
Lower respiratory infections	Female	0-6 days	1-4 years	Global	1	1	Age- and sex-specific wasting (weight-for-height) SEV	326
Lower respiratory infections	Female	0-6 days	1-4 years	Global	1	1	Low birth weight SEV (all ages, by sex)	382
Lower respiratory infections	Female	0-6 days	1-4 years	Global	1	1	Log-transformed SEV scalar: LRI	855
Lower respiratory infections	Female	0-6 days	1-4 years	Global	-1	2	Healthcare access and quality index	4
Lower respiratory infections	Female	0-6 days	1-4 years	Global	-1	2	DTP3 Coverage (proportion)	69
Lower respiratory infections	Female	0-6 days	1-4 years	Global	1	2	Vitamin A Deficiency Prevalence (age-standardized)	3
Lower respiratory infections	Female	0-6 days	1-4 years	Global	1	2	Secondhand smoke	34
Lower respiratory infections	Female	0-6 days	1-4 years	Global	1	2	Zinc deficiency	77
Lower respiratory infections	Female	0-6 days	1-4 years	Global	1	2	Discontinued breastfeeding SEV	--

**Appendix Table 9: CODEm covariates used, level of covariate, and expected direction of covariate by cause, sex, and age**<br>*Covariates that CODEm did not select during the covariate selection process have no draw counts listed.*								
**Cause**	**Sex**	**Age Start**	**Age End**	**Model Type**	**Direction**	**Level**	**Covariate Name**	**Number of Draws**
Lower respiratory infections	Female	0-6 days	1-4 years	Global	1	2	Outdoor Air Pollution (PM2.5)	--
Lower respiratory infections	Female	0-6 days	1-4 years	Global	-1	3	Socio-demographic Index	375
Lower respiratory infections	Female	0-6 days	1-4 years	Global	-1	3	Maternal Education (years per capita)	385
Lower respiratory infections	Female	0-6 days	1-4 years	Global	1	3	No access to handwashing facility	381
Lower respiratory infections	Female	0-6 days	1-4 years	Global	1	3	SEV unsafe sanitation	553
Lower respiratory infections	Female	0-6 days	1-4 years	Global	1	3	Population Density (over 1000 ppl/sqkm, proportion)	--
Lower respiratory infections	Female	5-9 years	95+ years	Data Rich	1	1	Secondhand smoke	3
Lower respiratory infections	Female	5-9 years	95+ years	Data Rich	1	1	Log-transformed SEV scalar: LRI	5
Lower respiratory infections	Female	5-9 years	95+ years	Data Rich	1	1	Outdoor Air Pollution (PM2.5)	19
Lower respiratory infections	Female	5-9 years	95+ years	Data Rich	1	1	Smoking Prevalence	562
Lower respiratory infections	Female	5-9 years	95+ years	Data Rich	1	1	Indoor Air Pollution (All Cooking Fuels)	828
Lower respiratory infections	Female	5-9 years	95+ years	Data Rich	-1	2	Mean BMI	1
Lower respiratory infections	Female	5-9 years	95+ years	Data Rich	-1	2	DTP3 Coverage (proportion)	51
Lower respiratory infections	Female	5-9 years	95+ years	Data Rich	-1	2	Healthcare access and quality index	52
Lower respiratory infections	Female	5-9 years	95+ years	Data Rich	-1	2	PCV3 Coverage (proportion)	--
Lower respiratory infections	Female	5-9 years	95+ years	Data Rich	1	2	No access to handwashing facility	346
Lower respiratory infections	Female	5-9 years	95+ years	Data Rich	-1	3	Socio-demographic Index	28
Lower respiratory infections	Female	5-9 years	95+ years	Data Rich	-1	3	Education (years per capita)	35
Lower respiratory infections	Female	5-9 years	95+ years	Data Rich	-1	3	LDI (I$ per capita)	--
Lower respiratory infections	Female	5-9 years	95+ years	Data Rich	1	3	SEV unsafe sanitation	45
Lower respiratory infections	Female	5-9 years	95+ years	Data Rich	1	3	Alcohol (liters per capita)	520
Lower respiratory infections	Female	5-9 years	95+ years	Global	1	1	Secondhand smoke	3
Lower respiratory infections	Female	5-9 years	95+ years	Global	1	1	Indoor Air Pollution (All Cooking Fuels)	7
Lower respiratory infections	Female	5-9 years	95+ years	Global	1	1	Log-transformed SEV scalar: LRI	116
Lower respiratory infections	Female	5-9 years	95+ years	Global	1	1	Smoking Prevalence	879
Lower respiratory infections	Female	5-9 years	95+ years	Global	1	1	Outdoor Air Pollution (PM2.5)	--
Lower respiratory infections	Female	5-9 years	95+ years	Global	-1	2	DTP3 Coverage (proportion)	0
Lower respiratory infections	Female	5-9 years	95+ years	Global	-1	2	PCV3 Coverage (proportion)	72
Lower respiratory infections	Female	5-9 years	95+ years	Global	-1	2	Mean BMI	191
Lower respiratory infections	Female	5-9 years	95+ years	Global	-1	2	Healthcare access and quality index	296
Lower respiratory infections	Female	5-9 years	95+ years	Global	1	2	No access to handwashing facility	254
Lower respiratory infections	Female	5-9 years	95+ years	Global	-1	3	Education (years per capita)	0
Lower respiratory infections	Female	5-9 years	95+ years	Global	-1	3	Socio-demographic Index	100
Lower respiratory infections	Female	5-9 years	95+ years	Global	-1	3	LDI (I$ per capita)	--
Lower respiratory infections	Female	5-9 years	95+ years	Global	1	3	SEV unsafe sanitation	287
Lower respiratory infections	Female	5-9 years	95+ years	Global	1	3	Alcohol (liters per capita)	655
Lower respiratory infections	Male	0-6 days	1-4 years	Data Rich	-1	1	Antibiotics for LRI	119
Lower respiratory infections	Male	0-6 days	1-4 years	Data Rich	-1	1	PCV3 Coverage (proportion)	268
Lower respiratory infections	Male	0-6 days	1-4 years	Data Rich	-1	1	Hib3 Vaccine Coverage (proportion)	421
Lower respiratory infections	Male	0-6 days	1-4 years	Data Rich	1	1	Indoor Air Pollution (All Cooking Fuels)	0
Lower respiratory infections	Male	0-6 days	1-4 years	Data Rich	1	1	Age- and sex-specific stunting (height-for-age) SEV	172
Lower respiratory infections	Male	0-6 days	1-4 years	Data Rich	1	1	Low birth weight SEV (all ages, by sex)	179
Lower respiratory infections	Male	0-6 days	1-4 years	Data Rich	1	1	Age- and sex-specific wasting (weight-for-height) SEV	259
Lower respiratory infections	Male	0-6 days	1-4 years	Data Rich	1	1	Short gestation SEV (all ages, by sex)	270
Lower respiratory infections	Male	0-6 days	1-4 years	Data Rich	1	1	Age- and sex-specific underweight (weight-for-age) SEV	292
Lower respiratory infections	Male	0-6 days	1-4 years	Data Rich	1	1	Log-transformed SEV scalar: LRI	997
Lower respiratory infections	Male	0-6 days	1-4 years	Data Rich	-1	2	DTP3 Coverage (proportion)	254
Lower respiratory infections	Male	0-6 days	1-4 years	Data Rich	-1	2	Healthcare access and quality index	338
Lower respiratory infections	Male	0-6 days	1-4 years	Data Rich	1	2	Zinc deficiency	0
Lower respiratory infections	Male	0-6 days	1-4 years	Data Rich	1	2	Vitamin A Deficiency Prevalence (age-standardized)	3
Lower respiratory infections	Male	0-6 days	1-4 years	Data Rich	1	2	Discontinued breastfeeding SEV	366
Lower respiratory infections	Male	0-6 days	1-4 years	Data Rich	1	2	Secondhand smoke	563
Lower respiratory infections	Male	0-6 days	1-4 years	Data Rich	1	2	Outdoor Air Pollution (PM2.5)	638
Lower respiratory infections	Male	0-6 days	1-4 years	Data Rich	-1	3	Maternal Education (years per capita)	66
Lower respiratory infections	Male	0-6 days	1-4 years	Data Rich	-1	3	Socio-demographic Index	232
Lower respiratory infections	Male	0-6 days	1-4 years	Data Rich	1	3	Population Density (over 1000 ppl/sqkm, proportion)	558
Lower respiratory infections	Male	0-6 days	1-4 years	Data Rich	1	3	No access to handwashing facility	679
Lower respiratory infections	Male	0-6 days	1-4 years	Data Rich	1	3	SEV unsafe sanitation	771
Lower respiratory infections	Male	0-6 days	1-4 years	Global	-1	1	Hib3 Vaccine Coverage (proportion)	269
Lower respiratory infections	Male	0-6 days	1-4 years	Global	-1	1	Antibiotics for LRI	408
Lower respiratory infections	Male	0-6 days	1-4 years	Global	-1	1	PCV3 Coverage (proportion)	754
Lower respiratory infections	Male	0-6 days	1-4 years	Global	1	1	Indoor Air Pollution (All Cooking Fuels)	159
Lower respiratory infections	Male	0-6 days	1-4 years	Global	1	1	Short gestation SEV (all ages, by sex)	185
Lower respiratory infections	Male	0-6 days	1-4 years	Global	1	1	Age- and sex-specific underweight (weight-for-age) SEV	203
Lower respiratory infections	Male	0-6 days	1-4 years	Global	1	1	Age- and sex-specific wasting (weight-for-height) SEV	354
Lower respiratory infections	Male	0-6 days	1-4 years	Global	1	1	Age- and sex-specific stunting (height-for-age) SEV	392
Lower respiratory infections	Male	0-6 days	1-4 years	Global	1	1	Low birth weight SEV (all ages, by sex)	423
Lower respiratory infections	Male	0-6 days	1-4 years	Global	1	1	Log-transformed SEV scalar: LRI	1000
Lower respiratory infections	Male	0-6 days	1-4 years	Global	-1	2	Healthcare access and quality index	0
Lower respiratory infections	Male	0-6 days	1-4 years	Global	-1	2	DTP3 Coverage (proportion)	3
Lower respiratory infections	Male	0-6 days	1-4 years	Global	1	2	Outdoor Air Pollution (PM2.5)	0
Lower respiratory infections	Male	0-6 days	1-4 years	Global	1	2	Secondhand smoke	0
Lower respiratory infections	Male	0-6 days	1-4 years	Global	1	2	Vitamin A Deficiency Prevalence (age-standardized)	0
Lower respiratory infections	Male	0-6 days	1-4 years	Global	1	2	Zinc deficiency	3
Lower respiratory infections	Male	0-6 days	1-4 years	Global	1	2	Discontinued breastfeeding SEV	--
Lower respiratory infections	Male	0-6 days	1-4 years	Global	-1	3	Socio-demographic Index	310
Lower respiratory infections	Male	0-6 days	1-4 years	Global	-1	3	Maternal Education (years per capita)	353
Lower respiratory infections	Male	0-6 days	1-4 years	Global	1	3	No access to handwashing facility	403

Appendix Table 9: CODEm covariates used, level of covariate, and expected direction of covariate by cause, sex, and age<br>*Covariates that CODEm did not select during the covariate selection process have no draw counts listed.*								
**Cause**	**Sex**	**Age Start**	**Age End**	**Model Type**	**Direction**	**Level**	**Covariate Name**	**Number of Draws**
Lower respiratory infections	Male	0-6 days	1-4 years	Global	1	3	SEV unsafe sanitation	812
Lower respiratory infections	Male	0-6 days	1-4 years	Global	1	3	Population Density (over 1000 ppl/sqkm, proportion)	--
Lower respiratory infections	Male	5-9 years	95+ years	Data Rich	1	1	Outdoor Air Pollution (PM2.5)	0
Lower respiratory infections	Male	5-9 years	95+ years	Data Rich	1	1	Log-transformed SEV scalar: LRI	61
Lower respiratory infections	Male	5-9 years	95+ years	Data Rich	1	1	Smoking Prevalence	326
Lower respiratory infections	Male	5-9 years	95+ years	Data Rich	1	1	Indoor Air Pollution (All Cooking Fuels)	699
Lower respiratory infections	Male	5-9 years	95+ years	Data Rich	1	1	Secondhand smoke	730
Lower respiratory infections	Male	5-9 years	95+ years	Data Rich	-1	2	Healthcare access and quality index	16
Lower respiratory infections	Male	5-9 years	95+ years	Data Rich	-1	2	DTP3 Coverage (proportion)	56
Lower respiratory infections	Male	5-9 years	95+ years	Data Rich	-1	2	PCV3 Coverage (proportion)	125
Lower respiratory infections	Male	5-9 years	95+ years	Data Rich	-1	2	Mean BMI	141
Lower respiratory infections	Male	5-9 years	95+ years	Data Rich	1	2	No access to handwashing facility	1
Lower respiratory infections	Male	5-9 years	95+ years	Data Rich	-1	3	Socio-demographic Index	35
Lower respiratory infections	Male	5-9 years	95+ years	Data Rich	-1	3	Education (years per capita)	95
Lower respiratory infections	Male	5-9 years	95+ years	Data Rich	-1	3	LDI (I$ per capita)	--
Lower respiratory infections	Male	5-9 years	95+ years	Data Rich	1	3	SEV unsafe sanitation	1
Lower respiratory infections	Male	5-9 years	95+ years	Data Rich	1	3	Alcohol (liters per capita)	491
Lower respiratory infections	Male	5-9 years	95+ years	Global	1	1	Smoking Prevalence	5
Lower respiratory infections	Male	5-9 years	95+ years	Global	1	1	Log-transformed SEV scalar: LRI	6
Lower respiratory infections	Male	5-9 years	95+ years	Global	1	1	Secondhand smoke	577
Lower respiratory infections	Male	5-9 years	95+ years	Global	1	1	Indoor Air Pollution (All Cooking Fuels)	647
Lower respiratory infections	Male	5-9 years	95+ years	Global	1	1	Outdoor Air Pollution (PM2.5)	--
Lower respiratory infections	Male	5-9 years	95+ years	Global	-1	2	DTP3 Coverage (proportion)	3
Lower respiratory infections	Male	5-9 years	95+ years	Global	-1	2	Mean BMI	8
Lower respiratory infections	Male	5-9 years	95+ years	Global	-1	2	Healthcare access and quality index	21
Lower respiratory infections	Male	5-9 years	95+ years	Global	-1	2	PCV3 Coverage (proportion)	331
Lower respiratory infections	Male	5-9 years	95+ years	Global	1	2	No access to handwashing facility	0
Lower respiratory infections	Male	5-9 years	95+ years	Global	-1	3	Socio-demographic Index	1
Lower respiratory infections	Male	5-9 years	95+ years	Global	-1	3	Education (years per capita)	2
Lower respiratory infections	Male	5-9 years	95+ years	Global	-1	3	LDI (I$ per capita)	--
Lower respiratory infections	Male	5-9 years	95+ years	Global	1	3	SEV unsafe sanitation	46
Lower respiratory infections	Male	5-9 years	95+ years	Global	1	3	Alcohol (liters per capita)	624
Upper respiratory infections	Female	0-6 days	95+ years	Data Rich	1	1	Smoking Prevalence	1000
Upper respiratory infections	Female	0-6 days	95+ years	Data Rich	-1	2	Healthcare access and quality index	--
Upper respiratory infections	Female	0-6 days	95+ years	Data Rich	1	2	Indoor Air Pollution (All Cooking Fuels)	488
Upper respiratory infections	Female	0-6 days	95+ years	Data Rich	1	2	Outdoor Air Pollution (PM2.5)	488
Upper respiratory infections	Female	0-6 days	95+ years	Data Rich	-1	3	Socio-demographic Index	145
Upper respiratory infections	Female	0-6 days	95+ years	Data Rich	-1	3	Education (years per capita)	--
Upper respiratory infections	Female	0-6 days	95+ years	Data Rich	-1	3	LDI (I$ per capita)	--
Upper respiratory infections	Female	0-6 days	95+ years	Global	1	1	Smoking Prevalence	1000
Upper respiratory infections	Female	0-6 days	95+ years	Global	-1	2	Healthcare access and quality index	--
Upper respiratory infections	Female	0-6 days	95+ years	Global	1	2	Indoor Air Pollution (All Cooking Fuels)	356
Upper respiratory infections	Female	0-6 days	95+ years	Global	1	2	Outdoor Air Pollution (PM2.5)	--
Upper respiratory infections	Female	0-6 days	95+ years	Global	-1	3	Socio-demographic Index	160
Upper respiratory infections	Female	0-6 days	95+ years	Global	-1	3	Education (years per capita)	--
Upper respiratory infections	Female	0-6 days	95+ years	Global	-1	3	LDI (I$ per capita)	--
Upper respiratory infections	Male	0-6 days	95+ years	Data Rich	1	1	Smoking Prevalence	1000
Upper respiratory infections	Male	0-6 days	95+ years	Data Rich	-1	2	Healthcare access and quality index	324
Upper respiratory infections	Male	0-6 days	95+ years	Data Rich	1	2	Indoor Air Pollution (All Cooking Fuels)	351
Upper respiratory infections	Male	0-6 days	95+ years	Data Rich	1	2	Outdoor Air Pollution (PM2.5)	--
Upper respiratory infections	Male	0-6 days	95+ years	Data Rich	-1	3	Socio-demographic Index	151
Upper respiratory infections	Male	0-6 days	95+ years	Data Rich	-1	3	Education (years per capita)	751
Upper respiratory infections	Male	0-6 days	95+ years	Data Rich	-1	3	LDI (I$ per capita)	--
Upper respiratory infections	Male	0-6 days	95+ years	Global	1	1	Smoking Prevalence	1000
Upper respiratory infections	Male	0-6 days	95+ years	Global	-1	2	Healthcare access and quality index	336
Upper respiratory infections	Male	0-6 days	95+ years	Global	1	2	Indoor Air Pollution (All Cooking Fuels)	336
Upper respiratory infections	Male	0-6 days	95+ years	Global	1	2	Outdoor Air Pollution (PM2.5)	--
Upper respiratory infections	Male	0-6 days	95+ years	Global	-1	3	Socio-demographic Index	208
Upper respiratory infections	Male	0-6 days	95+ years	Global	-1	3	Education (years per capita)	328
Upper respiratory infections	Male	0-6 days	95+ years	Global	-1	3	LDI (I$ per capita)	--
Otitis media	Female	0-6 days	95+ years	Data Rich	1	1	Log-transformed SEV scalar: Otitis	556
Otitis media	Female	0-6 days	95+ years	Data Rich	1	1	Smoking Prevalence	786
Otitis media	Female	0-6 days	95+ years	Data Rich	-1	2	Healthcare access and quality index	940
Otitis media	Female	0-6 days	95+ years	Data Rich	1	2	Indoor Air Pollution (All Cooking Fuels)	17
Otitis media	Female	0-6 days	95+ years	Data Rich	1	2	Outdoor Air Pollution (PM2.5)	209
Otitis media	Female	0-6 days	95+ years	Data Rich	-1	3	Socio-demographic Index	40
Otitis media	Female	0-6 days	95+ years	Data Rich	-1	3	Education (years per capita)	595
Otitis media	Female	0-6 days	95+ years	Data Rich	-1	3	LDI (I$ per capita)	--
Otitis media	Female	0-6 days	95+ years	Global	1	1	Log-transformed SEV scalar: Otitis	303
Otitis media	Female	0-6 days	95+ years	Global	1	1	Smoking Prevalence	889
Otitis media	Female	0-6 days	95+ years	Global	-1	2	Healthcare access and quality index	641
Otitis media	Female	0-6 days	95+ years	Global	1	2	Indoor Air Pollution (All Cooking Fuels)	175
Otitis media	Female	0-6 days	95+ years	Global	1	2	Outdoor Air Pollution (PM2.5)	361
Otitis media	Female	0-6 days	95+ years	Global	-1	3	Socio-demographic Index	278
Otitis media	Female	0-6 days	95+ years	Global	-1	3	Education (years per capita)	655
Otitis media	Female	0-6 days	95+ years	Global	-1	3	LDI (I$ per capita)	--
Otitis media	Male	0-6 days	95+ years	Data Rich	1	1	Log-transformed SEV scalar: Otitis	307
Otitis media	Male	0-6 days	95+ years	Data Rich	1	1	Smoking Prevalence	874

Appendix Table 9: CODEm covariates used, level of covariate, and expected direction of covariate by cause, sex, and age
Covariates that CODEm did not select during the covariate selection process have no draw counts listed.

Cause	Sex	Age Start	Age End	Model Type	Direction	Level	Covariate Name	Number of Draws
Otitis media	Male	0-6 days	95+ years	Data Rich	-1	2	Healthcare access and quality index	913
Otitis media	Male	0-6 days	95+ years	Data Rich	1	2	Indoor Air Pollution (All Cooking Fuels)	46
Otitis media	Male	0-6 days	95+ years	Data Rich	1	2	Outdoor Air Pollution (PM2.5)	812
Otitis media	Male	0-6 days	95+ years	Data Rich	-1	3	Socio-demographic Index	66
Otitis media	Male	0-6 days	95+ years	Data Rich	-1	3	Education (years per capita)	512
Otitis media	Male	0-6 days	95+ years	Data Rich	-1	3	LDI (I$ per capita)	--
Otitis media	Male	0-6 days	95+ years	Global	1	1	Log-transformed SEV scalar: Otitis	531
Otitis media	Male	0-6 days	95+ years	Global	1	1	Smoking Prevalence	673
Otitis media	Male	0-6 days	95+ years	Global	-1	2	Healthcare access and quality index	736
Otitis media	Male	0-6 days	95+ years	Global	1	2	Indoor Air Pollution (All Cooking Fuels)	116
Otitis media	Male	0-6 days	95+ years	Global	1	2	Outdoor Air Pollution (PM2.5)	669
Otitis media	Male	0-6 days	95+ years	Global	-1	3	Socio-demographic Index	170
Otitis media	Male	0-6 days	95+ years	Global	-1	3	Education (years per capita)	467
Otitis media	Male	0-6 days	95+ years	Global	-1	3	LDI (I$ per capita)	--
Diarrhoeal diseases	Female	0-6 days	1-4 years	Data Rich	-1	1	Improved Water Source (proportion with access)	0
Diarrhoeal diseases	Female	0-6 days	1-4 years	Data Rich	-1	1	Rotavirus coverage (proportion)	0
Diarrhoeal diseases	Female	0-6 days	1-4 years	Data Rich	-1	1	ORS (oral rehydration)	550
Diarrhoeal diseases	Female	0-6 days	1-4 years	Data Rich	-1	1	Sanitation (proportion with access)	553
Diarrhoeal diseases	Female	0-6 days	1-4 years	Data Rich	1	1	Short gestation SEV (all ages, by sex)	130
Diarrhoeal diseases	Female	0-6 days	1-4 years	Data Rich	1	1	SEV unsafe sanitation	190
Diarrhoeal diseases	Female	0-6 days	1-4 years	Data Rich	1	1	Age- and sex-specific stunting (height-for-age) SEV	332
Diarrhoeal diseases	Female	0-6 days	1-4 years	Data Rich	1	1	Age- and sex-specific underweight (weight-for-age) SEV	398
Diarrhoeal diseases	Female	0-6 days	1-4 years	Data Rich	1	1	Age- and sex-specific wasting (weight-for-height) SEV	424
Diarrhoeal diseases	Female	0-6 days	1-4 years	Data Rich	1	1	Low birth weight SEV (all ages, by sex)	615
Diarrhoeal diseases	Female	0-6 days	1-4 years	Data Rich	1	1	SEV unsafe water	810
Diarrhoeal diseases	Female	0-6 days	1-4 years	Data Rich	1	1	Log-transformed SEV scalar: Diarrhea	910
Diarrhoeal diseases	Female	0-6 days	1-4 years	Data Rich	-1	2	Healthcare access and quality index	0
Diarrhoeal diseases	Female	0-6 days	1-4 years	Data Rich	-1	2	Zinc treatment for diarrhea	74
Diarrhoeal diseases	Female	0-6 days	1-4 years	Data Rich	1	2	Vitamin A Deficiency Prevalence (age-standardized)	13
Diarrhoeal diseases	Female	0-6 days	1-4 years	Data Rich	1	2	Discontinued breastfeeding SEV	17
Diarrhoeal diseases	Female	0-6 days	1-4 years	Data Rich	1	2	Zinc deficiency	416
Diarrhoeal diseases	Female	0-6 days	1-4 years	Data Rich	-1	3	Maternal Education (years per capita)	0
Diarrhoeal diseases	Female	0-6 days	1-4 years	Data Rich	-1	3	Socio-demographic Index	0
Diarrhoeal diseases	Female	0-6 days	1-4 years	Data Rich	-1	3	LDI (I$ per capita)	--
Diarrhoeal diseases	Female	0-6 days	1-4 years	Data Rich	1	3	No access to handwashing facility	661
Diarrhoeal diseases	Female	0-6 days	1-4 years	Global	-1	1	Improved Water Source (proportion with access)	0
Diarrhoeal diseases	Female	0-6 days	1-4 years	Global	-1	1	Rotavirus coverage (proportion)	12
Diarrhoeal diseases	Female	0-6 days	1-4 years	Global	-1	1	ORS (oral rehydration)	541
Diarrhoeal diseases	Female	0-6 days	1-4 years	Global	-1	1	Sanitation (proportion with access)	708
Diarrhoeal diseases	Female	0-6 days	1-4 years	Global	1	1	Age- and sex-specific wasting (weight-for-height) SEV	151
Diarrhoeal diseases	Female	0-6 days	1-4 years	Global	1	1	Low birth weight SEV (all ages, by sex)	154
Diarrhoeal diseases	Female	0-6 days	1-4 years	Global	1	1	Short gestation SEV (all ages, by sex)	262
Diarrhoeal diseases	Female	0-6 days	1-4 years	Global	1	1	Age- and sex-specific stunting (height-for-age) SEV	404
Diarrhoeal diseases	Female	0-6 days	1-4 years	Global	1	1	SEV unsafe water	441
Diarrhoeal diseases	Female	0-6 days	1-4 years	Global	1	1	SEV unsafe sanitation	514
Diarrhoeal diseases	Female	0-6 days	1-4 years	Global	1	1	Age- and sex-specific underweight (weight-for-age) SEV	523
Diarrhoeal diseases	Female	0-6 days	1-4 years	Global	1	1	Log-transformed SEV scalar: Diarrhea	553
Diarrhoeal diseases	Female	0-6 days	1-4 years	Global	-1	2	Healthcare access and quality index	0
Diarrhoeal diseases	Female	0-6 days	1-4 years	Global	-1	2	Zinc treatment for diarrhea	--
Diarrhoeal diseases	Female	0-6 days	1-4 years	Global	1	2	Zinc deficiency	44
Diarrhoeal diseases	Female	0-6 days	1-4 years	Global	1	2	Vitamin A Deficiency Prevalence (age-standardized)	131
Diarrhoeal diseases	Female	0-6 days	1-4 years	Global	1	2	Discontinued breastfeeding SEV	659
Diarrhoeal diseases	Female	0-6 days	1-4 years	Global	-1	3	Maternal Education (years per capita)	0
Diarrhoeal diseases	Female	0-6 days	1-4 years	Global	-1	3	Socio-demographic Index	0
Diarrhoeal diseases	Female	0-6 days	1-4 years	Global	-1	3	LDI (I$ per capita)	--
Diarrhoeal diseases	Female	0-6 days	1-4 years	Global	1	3	No access to handwashing facility	421
Diarrhoeal diseases	Female	5-9 years	95+ years	Data Rich	-1	1	Improved Water Source (proportion with access)	0
Diarrhoeal diseases	Female	5-9 years	95+ years	Data Rich	-1	1	Sanitation (proportion with access)	558
Diarrhoeal diseases	Female	5-9 years	95+ years	Data Rich	1	1	Log-transformed SEV scalar: Diarrhea	286
Diarrhoeal diseases	Female	5-9 years	95+ years	Data Rich	1	1	SEV unsafe sanitation	369
Diarrhoeal diseases	Female	5-9 years	95+ years	Data Rich	1	1	SEV unsafe water	525
Diarrhoeal diseases	Female	5-9 years	95+ years	Data Rich	-1	2	Healthcare access and quality index	--
Diarrhoeal diseases	Female	5-9 years	95+ years	Data Rich	-1	2	Rotavirus coverage (proportion)	--
Diarrhoeal diseases	Female	5-9 years	95+ years	Data Rich	-1	3	Education (years per capita)	--
Diarrhoeal diseases	Female	5-9 years	95+ years	Data Rich	-1	3	LDI (I$ per capita)	--
Diarrhoeal diseases	Female	5-9 years	95+ years	Data Rich	-1	3	Mean BMI	--
Diarrhoeal diseases	Female	5-9 years	95+ years	Data Rich	-1	3	Socio-demographic Index	--
Diarrhoeal diseases	Female	5-9 years	95+ years	Data Rich	0	3	Population Density (over 1000 ppl/sqkm, proportion)	446
Diarrhoeal diseases	Female	5-9 years	95+ years	Global	-1	1	Sanitation (proportion with access)	508
Diarrhoeal diseases	Female	5-9 years	95+ years	Global	-1	1	Improved Water Source (proportion with access)	--
Diarrhoeal diseases	Female	5-9 years	95+ years	Global	1	1	Log-transformed SEV scalar: Diarrhea	398
Diarrhoeal diseases	Female	5-9 years	95+ years	Global	1	1	SEV unsafe sanitation	455
Diarrhoeal diseases	Female	5-9 years	95+ years	Global	1	1	SEV unsafe water	602
Diarrhoeal diseases	Female	5-9 years	95+ years	Global	-1	2	Healthcare access and quality index	--
Diarrhoeal diseases	Female	5-9 years	95+ years	Global	-1	2	Rotavirus coverage (proportion)	--
Diarrhoeal diseases	Female	5-9 years	95+ years	Global	-1	3	Education (years per capita)	--
Diarrhoeal diseases	Female	5-9 years	95+ years	Global	-1	3	LDI (I$ per capita)	--
Diarrhoeal diseases	Female	5-9 years	95+ years	Global	-1	3	Mean BMI	--

Appendix Table 9: CODEm covariates used, level of covariate, and expected direction of covariate by cause, sex, and age
Covariates that CODEm did not select during the covariate selection process have no draw counts listed.

Cause	Sex	Age Start	Age End	Model Type	Direction	Level	Covariate Name	Number of Draws
Diarrhoeal diseases	Female	5-9 years	95+ years	Global	-1	3	Socio-demographic Index	--
Diarrhoeal diseases	Female	5-9 years	95+ years	Global	0	3	Population Density (over 1000 ppl/sqkm, proportion)	348
Diarrhoeal diseases	Male	0-6 days	1-4 years	Data Rich	-1	1	Improved Water Source (proportion with access)	0
Diarrhoeal diseases	Male	0-6 days	1-4 years	Data Rich	-1	1	Rotavirus coverage (proportion)	0
Diarrhoeal diseases	Male	0-6 days	1-4 years	Data Rich	-1	1	ORS (oral rehydration)	301
Diarrhoeal diseases	Male	0-6 days	1-4 years	Data Rich	-1	1	Sanitation (proportion with access)	558
Diarrhoeal diseases	Male	0-6 days	1-4 years	Data Rich	1	1	Short gestation SEV (all ages, by sex)	0
Diarrhoeal diseases	Male	0-6 days	1-4 years	Data Rich	1	1	Low birth weight SEV (all ages, by sex)	1
Diarrhoeal diseases	Male	0-6 days	1-4 years	Data Rich	1	1	Age- and sex-specific wasting (weight-for-height) SEV	222
Diarrhoeal diseases	Male	0-6 days	1-4 years	Data Rich	1	1	SEV unsafe sanitation	260
Diarrhoeal diseases	Male	0-6 days	1-4 years	Data Rich	1	1	Age- and sex-specific stunting (height-for-age) SEV	287
Diarrhoeal diseases	Male	0-6 days	1-4 years	Data Rich	1	1	Log-transformed SEV scalar: Diarrhea	349
Diarrhoeal diseases	Male	0-6 days	1-4 years	Data Rich	1	1	Age- and sex-specific underweight (weight-for-age) SEV	671
Diarrhoeal diseases	Male	0-6 days	1-4 years	Data Rich	1	1	SEV unsafe water	878
Diarrhoeal diseases	Male	0-6 days	1-4 years	Data Rich	-1	2	Healthcare access and quality index	0
Diarrhoeal diseases	Male	0-6 days	1-4 years	Data Rich	-1	2	Zinc treatment for diarrhea	35
Diarrhoeal diseases	Male	0-6 days	1-4 years	Data Rich	1	2	Zinc deficiency	34
Diarrhoeal diseases	Male	0-6 days	1-4 years	Data Rich	1	2	Vitamin A Deficiency Prevalence (age-standardized)	67
Diarrhoeal diseases	Male	0-6 days	1-4 years	Data Rich	1	2	Discontinued breastfeeding SEV	76
Diarrhoeal diseases	Male	0-6 days	1-4 years	Data Rich	-1	3	Maternal Education (years per capita)	0
Diarrhoeal diseases	Male	0-6 days	1-4 years	Data Rich	-1	3	Socio-demographic Index	0
Diarrhoeal diseases	Male	0-6 days	1-4 years	Data Rich	-1	3	LDI (I$ per capita)	--
Diarrhoeal diseases	Male	0-6 days	1-4 years	Data Rich	1	3	No access to handwashing facility	378
Diarrhoeal diseases	Male	0-6 days	1-4 years	Global	-1	1	Improved Water Source (proportion with access)	0
Diarrhoeal diseases	Male	0-6 days	1-4 years	Global	-1	1	Rotavirus coverage (proportion)	56
Diarrhoeal diseases	Male	0-6 days	1-4 years	Global	-1	1	ORS (oral rehydration)	562
Diarrhoeal diseases	Male	0-6 days	1-4 years	Global	-1	1	Sanitation (proportion with access)	841
Diarrhoeal diseases	Male	0-6 days	1-4 years	Global	1	1	Age- and sex-specific wasting (weight-for-height) SEV	35
Diarrhoeal diseases	Male	0-6 days	1-4 years	Global	1	1	SEV unsafe sanitation	153
Diarrhoeal diseases	Male	0-6 days	1-4 years	Global	1	1	Short gestation SEV (all ages, by sex)	207
Diarrhoeal diseases	Male	0-6 days	1-4 years	Global	1	1	Age- and sex-specific underweight (weight-for-age) SEV	276
Diarrhoeal diseases	Male	0-6 days	1-4 years	Global	1	1	Low birth weight SEV (all ages, by sex)	425
Diarrhoeal diseases	Male	0-6 days	1-4 years	Global	1	1	Log-transformed SEV scalar: Diarrhea	648
Diarrhoeal diseases	Male	0-6 days	1-4 years	Global	1	1	SEV unsafe water	776
Diarrhoeal diseases	Male	0-6 days	1-4 years	Global	1	1	Age- and sex-specific stunting (height-for-age) SEV	854
Diarrhoeal diseases	Male	0-6 days	1-4 years	Global	-1	2	Healthcare access and quality index	33
Diarrhoeal diseases	Male	0-6 days	1-4 years	Global	-1	2	Zinc treatment for diarrhea	--
Diarrhoeal diseases	Male	0-6 days	1-4 years	Global	1	2	Zinc deficiency	1
Diarrhoeal diseases	Male	0-6 days	1-4 years	Global	1	2	Vitamin A Deficiency Prevalence (age-standardized)	123
Diarrhoeal diseases	Male	0-6 days	1-4 years	Global	1	2	Discontinued breastfeeding SEV	363
Diarrhoeal diseases	Male	0-6 days	1-4 years	Global	-1	3	Maternal Education (years per capita)	77
Diarrhoeal diseases	Male	0-6 days	1-4 years	Global	-1	3	Socio-demographic Index	92
Diarrhoeal diseases	Male	0-6 days	1-4 years	Global	-1	3	LDI (I$ per capita)	--
Diarrhoeal diseases	Male	0-6 days	1-4 years	Global	1	3	No access to handwashing facility	149
Diarrhoeal diseases	Male	5-9 years	95+ years	Data Rich	-1	1	Sanitation (proportion with access)	395
Diarrhoeal diseases	Male	5-9 years	95+ years	Data Rich	-1	1	Improved Water Source (proportion with access)	--
Diarrhoeal diseases	Male	5-9 years	95+ years	Data Rich	1	1	SEV unsafe water	142
Diarrhoeal diseases	Male	5-9 years	95+ years	Data Rich	1	1	SEV unsafe sanitation	562
Diarrhoeal diseases	Male	5-9 years	95+ years	Data Rich	1	1	Log-transformed SEV scalar: Diarrhea	778
Diarrhoeal diseases	Male	5-9 years	95+ years	Data Rich	-1	2	Healthcare access and quality index	--
Diarrhoeal diseases	Male	5-9 years	95+ years	Data Rich	-1	2	Rotavirus coverage (proportion)	--
Diarrhoeal diseases	Male	5-9 years	95+ years	Data Rich	-1	3	Education (years per capita)	--
Diarrhoeal diseases	Male	5-9 years	95+ years	Data Rich	-1	3	LDI (I$ per capita)	--
Diarrhoeal diseases	Male	5-9 years	95+ years	Data Rich	-1	3	Mean BMI	--
Diarrhoeal diseases	Male	5-9 years	95+ years	Data Rich	-1	3	Socio-demographic Index	--
Diarrhoeal diseases	Male	5-9 years	95+ years	Data Rich	0	3	Population Density (over 1000 ppl/sqkm, proportion)	443
Diarrhoeal diseases	Male	5-9 years	95+ years	Global	-1	1	Improved Water Source (proportion with access)	0
Diarrhoeal diseases	Male	5-9 years	95+ years	Global	-1	1	Sanitation (proportion with access)	466
Diarrhoeal diseases	Male	5-9 years	95+ years	Global	1	1	Log-transformed SEV scalar: Diarrhea	368
Diarrhoeal diseases	Male	5-9 years	95+ years	Global	1	1	SEV unsafe water	544
Diarrhoeal diseases	Male	5-9 years	95+ years	Global	1	1	SEV unsafe sanitation	564
Diarrhoeal diseases	Male	5-9 years	95+ years	Global	-1	2	Healthcare access and quality index	0
Diarrhoeal diseases	Male	5-9 years	95+ years	Global	-1	2	Rotavirus coverage (proportion)	--
Diarrhoeal diseases	Male	5-9 years	95+ years	Global	-1	3	Education (years per capita)	--
Diarrhoeal diseases	Male	5-9 years	95+ years	Global	-1	3	LDI (I$ per capita)	--
Diarrhoeal diseases	Male	5-9 years	95+ years	Global	-1	3	Mean BMI	--
Diarrhoeal diseases	Male	5-9 years	95+ years	Global	-1	3	Socio-demographic Index	--
Diarrhoeal diseases	Male	5-9 years	95+ years	Global	0	3	Population Density (over 1000 ppl/sqkm, proportion)	471
Dengue	Female	28-364 days	95+ years	Data Rich	1	1	Population weighted probability of dengue transmission	720
Dengue	Female	28-364 days	95+ years	Data Rich	1	1	Population Density (over 1000 ppl/sqkm, proportion)	--
Dengue	Female	28-364 days	95+ years	Data Rich	0	2	Health System Access (unitless)	740
Dengue	Female	28-364 days	95+ years	Data Rich	1	2	Elevation Under 100m (proportion)	259
Dengue	Female	28-364 days	95+ years	Data Rich	1	2	Latitude Under 15 (proportion)	528
Dengue	Female	28-364 days	95+ years	Data Rich	1	2	Dengue outbreaks (binary)	787
Dengue	Female	28-364 days	95+ years	Data Rich	1	2	Rainfall Quintile 4 (proportion)	--
Dengue	Female	28-364 days	95+ years	Data Rich	1	2	Rainfall Quintile 5 (proportion)	--
Dengue	Female	28-364 days	95+ years	Data Rich	0	3	Education (years per capita)	299
Dengue	Female	28-364 days	95+ years	Data Rich	0	3	LDI (I$ per capita)	--

Appendix Table 9: CODEm covariates used, level of covariate, and expected direction of covariate by cause, sex, and age

Covariates that CODEm did not select during the covariate selection process have no draw counts listed.

Cause	Sex	Age Start	Age End	Model Type	Direction	Level	Covariate Name	Number of Draws
Dengue	Female	28-364 days	95+ years	Global	1	1	Population weighted probability of dengue transmission	1000
Dengue	Female	28-364 days	95+ years	Global	1	1	Population Density (over 1000 ppl/sqkm, proportion)	--
Dengue	Female	28-364 days	95+ years	Global	0	2	Health System Access (unitless)	647
Dengue	Female	28-364 days	95+ years	Global	1	2	Elevation Under 100m (proportion)	129
Dengue	Female	28-364 days	95+ years	Global	1	2	Dengue outbreaks (binary)	725
Dengue	Female	28-364 days	95+ years	Global	1	2	Latitude Under 15 (proportion)	725
Dengue	Female	28-364 days	95+ years	Global	1	2	Rainfall Quintile 4 (proportion)	--
Dengue	Female	28-364 days	95+ years	Global	1	2	Rainfall Quintile 5 (proportion)	--
Dengue	Female	28-364 days	95+ years	Global	0	3	Education (years per capita)	--
Dengue	Female	28-364 days	95+ years	Global	0	3	LDI (I$ per capita)	--
Dengue	Male	28-364 days	95+ years	Data Rich	1	1	Population weighted probability of dengue transmission	668
Dengue	Male	28-364 days	95+ years	Data Rich	1	1	Population Density (over 1000 ppl/sqkm, proportion)	--
Dengue	Male	28-364 days	95+ years	Data Rich	0	2	Health System Access (unitless)	672
Dengue	Male	28-364 days	95+ years	Data Rich	1	2	Elevation Under 100m (proportion)	244
Dengue	Male	28-364 days	95+ years	Data Rich	1	2	Rainfall Quintile 5 (proportion)	244
Dengue	Male	28-364 days	95+ years	Data Rich	1	2	Dengue outbreaks (binary)	428
Dengue	Male	28-364 days	95+ years	Data Rich	1	2	Latitude Under 15 (proportion)	672
Dengue	Male	28-364 days	95+ years	Data Rich	1	2	Rainfall Quintile 4 (proportion)	--
Dengue	Male	28-364 days	95+ years	Data Rich	0	3	Education (years per capita)	600
Dengue	Male	28-364 days	95+ years	Data Rich	0	3	LDI (I$ per capita)	--
Dengue	Male	28-364 days	95+ years	Global	1	1	Population Density (over 1000 ppl/sqkm, proportion)	101
Dengue	Male	28-364 days	95+ years	Global	1	1	Population weighted probability of dengue transmission	899
Dengue	Male	28-364 days	95+ years	Global	0	2	Health System Access (unitless)	483
Dengue	Male	28-364 days	95+ years	Global	1	2	Elevation Under 100m (proportion)	87
Dengue	Male	28-364 days	95+ years	Global	1	2	Rainfall Quintile 4 (proportion)	570
Dengue	Male	28-364 days	95+ years	Global	1	2	Dengue outbreaks (binary)	594
Dengue	Male	28-364 days	95+ years	Global	1	2	Latitude Under 15 (proportion)	612
Dengue	Male	28-364 days	95+ years	Global	1	2	Rainfall Quintile 5 (proportion)	--
Dengue	Male	28-364 days	95+ years	Global	0	3	Education (years per capita)	381
Dengue	Male	28-364 days	95+ years	Global	0	3	LDI (I$ per capita)	--
Rabies	Female	28-364 days	95+ years	Data Rich	-1	1	Antenatal Care (4 visits) Coverage (proportion)	206
Rabies	Female	28-364 days	95+ years	Data Rich	-1	1	In-Facility Delivery (proportion)	384
Rabies	Female	28-364 days	95+ years	Data Rich	-1	1	Health System Access (unitless)	700
Rabies	Female	28-364 days	95+ years	Data Rich	-1	2	Health System Access (capped)	1
Rabies	Female	28-364 days	95+ years	Data Rich	-1	2	Healthcare access and quality index	82
Rabies	Female	28-364 days	95+ years	Data Rich	-1	2	Skilled Birth Attendance (proportion)	952
Rabies	Female	28-364 days	95+ years	Data Rich	-1	3	Socio-demographic Index	44
Rabies	Female	28-364 days	95+ years	Data Rich	0	3	Population Density (500-1000 ppl/sqkm, proportion)	--
Rabies	Female	28-364 days	95+ years	Data Rich	0	3	Population Density (under 150 ppl/sqkm, proportion)	--
Rabies	Female	28-364 days	95+ years	Global	-1	1	Antenatal Care (4 visits) Coverage (proportion)	352
Rabies	Female	28-364 days	95+ years	Global	-1	1	In-Facility Delivery (proportion)	919
Rabies	Female	28-364 days	95+ years	Global	-1	2	Skilled Birth Attendance (proportion)	246
Rabies	Female	28-364 days	95+ years	Global	-1	2	Health System Access (unitless)	342
Rabies	Female	28-364 days	95+ years	Global	1	2	Healthcare access and quality index	--
Rabies	Female	28-364 days	95+ years	Global	-1	3	Socio-demographic Index	329
Rabies	Female	28-364 days	95+ years	Global	0	3	Population Density (500-1000 ppl/sqkm, proportion)	--
Rabies	Female	28-364 days	95+ years	Global	0	3	Population Density (under 150 ppl/sqkm, proportion)	--
Rabies	Male	28-364 days	95+ years	Data Rich	-1	1	Health System Access (unitless)	288
Rabies	Male	28-364 days	95+ years	Data Rich	-1	1	Antenatal Care (4 visits) Coverage (proportion)	444
Rabies	Male	28-364 days	95+ years	Data Rich	-1	1	In-Facility Delivery (proportion)	793
Rabies	Male	28-364 days	95+ years	Data Rich	-1	2	Health System Access (capped)	25
Rabies	Male	28-364 days	95+ years	Data Rich	-1	2	Healthcare access and quality index	353
Rabies	Male	28-364 days	95+ years	Data Rich	-1	2	Skilled Birth Attendance (proportion)	959
Rabies	Male	28-364 days	95+ years	Data Rich	-1	3	Socio-demographic Index	57
Rabies	Male	28-364 days	95+ years	Data Rich	0	3	Population Density (500-1000 ppl/sqkm, proportion)	--
Rabies	Male	28-364 days	95+ years	Data Rich	0	3	Population Density (under 150 ppl/sqkm, proportion)	--
Rabies	Male	28-364 days	95+ years	Global	-1	1	In-Facility Delivery (proportion)	697
Rabies	Male	28-364 days	95+ years	Global	-1	1	Antenatal Care (4 visits) Coverage (proportion)	747
Rabies	Male	28-364 days	95+ years	Global	-1	2	Skilled Birth Attendance (proportion)	38
Rabies	Male	28-364 days	95+ years	Global	-1	2	Health System Access (unitless)	130
Rabies	Male	28-364 days	95+ years	Global	1	2	Healthcare access and quality index	231
Rabies	Male	28-364 days	95+ years	Global	-1	3	Socio-demographic Index	735
Rabies	Male	28-364 days	95+ years	Global	0	3	Population Density (500-1000 ppl/sqkm, proportion)	--
Rabies	Male	28-364 days	95+ years	Global	0	3	Population Density (under 150 ppl/sqkm, proportion)	--
Other neglected tropical diseases	Female	0-6 days	95+ years	Data Rich	-1	1	Healthcare access and quality index	521
Other neglected tropical diseases	Female	0-6 days	95+ years	Data Rich	1	1	Latitude Under 15 (proportion)	728
Other neglected tropical diseases	Female	0-6 days	95+ years	Data Rich	-1	2	Sanitation (proportion with access)	155
Other neglected tropical diseases	Female	0-6 days	95+ years	Data Rich	1	2	Rainfall Quintile 5 (proportion)	377
Other neglected tropical diseases	Female	0-6 days	95+ years	Data Rich	-1	3	Socio-demographic Index	173
Other neglected tropical diseases	Female	0-6 days	95+ years	Data Rich	-1	3	Education (years per capita)	--
Other neglected tropical diseases	Female	0-6 days	95+ years	Data Rich	-1	3	LDI (I$ per capita)	--
Other neglected tropical diseases	Female	0-6 days	95+ years	Global	-1	1	Healthcare access and quality index	674
Other neglected tropical diseases	Female	0-6 days	95+ years	Global	1	1	Latitude Under 15 (proportion)	252
Other neglected tropical diseases	Female	0-6 days	95+ years	Global	-1	2	Sanitation (proportion with access)	237
Other neglected tropical diseases	Female	0-6 days	95+ years	Global	1	2	Rainfall Quintile 5 (proportion)	563
Other neglected tropical diseases	Female	0-6 days	95+ years	Global	-1	3	Education (years per capita)	--
Other neglected tropical diseases	Female	0-6 days	95+ years	Global	-1	3	LDI (I$ per capita)	--
Other neglected tropical diseases	Female	0-6 days	95+ years	Global	-1	3	Socio-demographic Index	--

Appendix Table 9: CODEm covariates used, level of covariate, and expected direction of covariate by cause, sex, and age<br>*Covariates that CODEm did not select during the covariate selection process have no draw counts listed.*								
**Cause**	**Sex**	**Age Start**	**Age End**	**Model Type**	**Direction**	**Level**	**Covariate Name**	**Number of Draws**
Other neglected tropical diseases	Male	0-6 days	95+ years	Data Rich	-1	1	Healthcare access and quality index	487
Other neglected tropical diseases	Male	0-6 days	95+ years	Data Rich	1	1	Latitude Under 15 (proportion)	646
Other neglected tropical diseases	Male	0-6 days	95+ years	Data Rich	-1	2	Sanitation (proportion with access)	475
Other neglected tropical diseases	Male	0-6 days	95+ years	Data Rich	1	2	Rainfall Quintile 5 (proportion)	475
Other neglected tropical diseases	Male	0-6 days	95+ years	Data Rich	-1	3	Education (years per capita)	--
Other neglected tropical diseases	Male	0-6 days	95+ years	Data Rich	-1	3	LDI (I$ per capita)	--
Other neglected tropical diseases	Male	0-6 days	95+ years	Data Rich	-1	3	Socio-demographic Index	--
Other neglected tropical diseases	Male	0-6 days	95+ years	Global	-1	1	Healthcare access and quality index	537
Other neglected tropical diseases	Male	0-6 days	95+ years	Global	1	1	Latitude Under 15 (proportion)	629
Other neglected tropical diseases	Male	0-6 days	95+ years	Global	-1	2	Sanitation (proportion with access)	406
Other neglected tropical diseases	Male	0-6 days	95+ years	Global	1	2	Rainfall Quintile 5 (proportion)	100
Other neglected tropical diseases	Male	0-6 days	95+ years	Global	-1	3	Education (years per capita)	--
Other neglected tropical diseases	Male	0-6 days	95+ years	Global	-1	3	LDI (I$ per capita)	--
Other neglected tropical diseases	Male	0-6 days	95+ years	Global	-1	3	Socio-demographic Index	--
Meningitis	Female	0-6 days	1-4 years	Data Rich	-1	1	Hib3 Vaccine Coverage (proportion)	1000
Meningitis	Female	0-6 days	1-4 years	Data Rich	-1	1	Proportion of total population covered by menafrivac initiative (meningitis meningococcal type A vaccine)	--
Meningitis	Female	0-6 days	1-4 years	Data Rich	1	1	meningitis belt (proportion)	--
Meningitis	Female	0-6 days	1-4 years	Data Rich	-1	2	Health System Access (capped)	125
Meningitis	Female	0-6 days	1-4 years	Data Rich	-1	2	Healthcare access and quality index	233
Meningitis	Female	0-6 days	1-4 years	Data Rich	-1	2	Improved Water Source (proportion with access)	--
Meningitis	Female	0-6 days	1-4 years	Data Rich	1	2	Underweight (proportion <2SD weight for age, <5 years)	502
Meningitis	Female	0-6 days	1-4 years	Data Rich	-1	3	DTP3 Coverage (proportion)	65
Meningitis	Female	0-6 days	1-4 years	Data Rich	-1	3	Sanitation (proportion with access)	192
Meningitis	Female	0-6 days	1-4 years	Data Rich	-1	3	Maternal Education (years per capita)	229
Meningitis	Female	0-6 days	1-4 years	Data Rich	-1	3	Socio-demographic Index	274
Meningitis	Female	0-6 days	1-4 years	Data Rich	-1	3	LDI (I$ per capita)	--
Meningitis	Female	0-6 days	1-4 years	Global	-1	1	Proportion of total population covered by menafrivac initiative (meningitis meningococcal type A vaccine)	3
Meningitis	Female	0-6 days	1-4 years	Global	-1	1	Hib3 Vaccine Coverage (proportion)	819
Meningitis	Female	0-6 days	1-4 years	Global	1	1	meningitis belt (proportion)	421
Meningitis	Female	0-6 days	1-4 years	Global	-1	2	Health System Access (capped)	47
Meningitis	Female	0-6 days	1-4 years	Global	-1	2	Healthcare access and quality index	252
Meningitis	Female	0-6 days	1-4 years	Global	-1	2	Improved Water Source (proportion with access)	--
Meningitis	Female	0-6 days	1-4 years	Global	1	2	Underweight (proportion <2SD weight for age, <5 years)	193
Meningitis	Female	0-6 days	1-4 years	Global	-1	3	Maternal Education (years per capita)	65
Meningitis	Female	0-6 days	1-4 years	Global	-1	3	Socio-demographic Index	73
Meningitis	Female	0-6 days	1-4 years	Global	-1	3	Sanitation (proportion with access)	254
Meningitis	Female	0-6 days	1-4 years	Global	-1	3	DTP3 Coverage (proportion)	283
Meningitis	Female	0-6 days	1-4 years	Global	-1	3	LDI (I$ per capita)	--
Meningitis	Female	5-9 years	95+ years	Data Rich	-1	1	Hib3 Vaccine Coverage (proportion)	1000
Meningitis	Female	5-9 years	95+ years	Data Rich	-1	1	Proportion of total population covered by menafrivac initiative (meningitis meningococcal type A vaccine)	--
Meningitis	Female	5-9 years	95+ years	Data Rich	1	1	meningitis belt (proportion)	--
Meningitis	Female	5-9 years	95+ years	Data Rich	-1	2	Healthcare access and quality index	40
Meningitis	Female	5-9 years	95+ years	Data Rich	-1	2	Health System Access (capped)	--
Meningitis	Female	5-9 years	95+ years	Data Rich	-1	2	Improved Water Source (proportion with access)	--
Meningitis	Female	5-9 years	95+ years	Data Rich	1	2	Underweight (proportion <2SD weight for age, <5 years)	542
Meningitis	Female	5-9 years	95+ years	Data Rich	-1	3	DTP3 Coverage (proportion)	13
Meningitis	Female	5-9 years	95+ years	Data Rich	-1	3	Maternal Education (years per capita)	176
Meningitis	Female	5-9 years	95+ years	Data Rich	-1	3	Sanitation (proportion with access)	408
Meningitis	Female	5-9 years	95+ years	Data Rich	-1	3	Socio-demographic Index	414
Meningitis	Female	5-9 years	95+ years	Data Rich	-1	3	LDI (I$ per capita)	--
Meningitis	Female	5-9 years	95+ years	Global	-1	1	Hib3 Vaccine Coverage (proportion)	992
Meningitis	Female	5-9 years	95+ years	Global	-1	1	Proportion of total population covered by menafrivac initiative (meningitis meningococcal type A vaccine)	--
Meningitis	Female	5-9 years	95+ years	Global	1	1	meningitis belt (proportion)	8
Meningitis	Female	5-9 years	95+ years	Global	-1	2	Healthcare access and quality index	90
Meningitis	Female	5-9 years	95+ years	Global	-1	2	Health System Access (capped)	--
Meningitis	Female	5-9 years	95+ years	Global	-1	2	Improved Water Source (proportion with access)	--
Meningitis	Female	5-9 years	95+ years	Global	1	2	Underweight (proportion <2SD weight for age, <5 years)	525
Meningitis	Female	5-9 years	95+ years	Global	-1	3	DTP3 Coverage (proportion)	26
Meningitis	Female	5-9 years	95+ years	Global	-1	3	Maternal Education (years per capita)	70
Meningitis	Female	5-9 years	95+ years	Global	-1	3	Socio-demographic Index	302
Meningitis	Female	5-9 years	95+ years	Global	-1	3	Sanitation (proportion with access)	507
Meningitis	Female	5-9 years	95+ years	Global	-1	3	LDI (I$ per capita)	--
Meningitis	Male	0-6 days	1-4 years	Data Rich	-1	1	Hib3 Vaccine Coverage (proportion)	1000
Meningitis	Male	0-6 days	1-4 years	Data Rich	-1	1	Proportion of total population covered by menafrivac initiative (meningitis meningococcal type A vaccine)	--
Meningitis	Male	0-6 days	1-4 years	Data Rich	1	1	meningitis belt (proportion)	--
Meningitis	Male	0-6 days	1-4 years	Data Rich	-1	2	Healthcare access and quality index	305
Meningitis	Male	0-6 days	1-4 years	Data Rich	-1	2	Health System Access (capped)	333
Meningitis	Male	0-6 days	1-4 years	Data Rich	-1	2	Improved Water Source (proportion with access)	--
Meningitis	Male	0-6 days	1-4 years	Data Rich	1	2	Underweight (proportion <2SD weight for age, <5 years)	531
Meningitis	Male	0-6 days	1-4 years	Data Rich	-1	3	DTP3 Coverage (proportion)	108
Meningitis	Male	0-6 days	1-4 years	Data Rich	-1	3	Sanitation (proportion with access)	279
Meningitis	Male	0-6 days	1-4 years	Data Rich	-1	3	Maternal Education (years per capita)	306
Meningitis	Male	0-6 days	1-4 years	Data Rich	-1	3	Socio-demographic Index	375
Meningitis	Male	0-6 days	1-4 years	Data Rich	-1	3	LDI (I$ per capita)	--
Meningitis	Male	0-6 days	1-4 years	Global	-1	1	Hib3 Vaccine Coverage (proportion)	635

Appendix Table 9: CODEm covariates used, level of covariate, and expected direction of covariate by cause, sex, and age

Covariates that CODEm did not select during the covariate selection process have no draw counts listed.

Cause	Sex	Age Start	Age End	Model Type	Direction	Level	Covariate Name	Number of Draws
Meningitis	Male	0-6 days	1-4 years	Global	-1	1	Proportion of total population covered by menafrivac initiative (meningitis meningococcal type A vaccine)	--
Meningitis	Male	0-6 days	1-4 years	Global	1	1	meningitis belt (proportion)	606
Meningitis	Male	0-6 days	1-4 years	Global	-1	2	Health System Access (capped)	225
Meningitis	Male	0-6 days	1-4 years	Global	-1	2	Healthcare access and quality index	372
Meningitis	Male	0-6 days	1-4 years	Global	-1	2	Improved Water Source (proportion with access)	--
Meningitis	Male	0-6 days	1-4 years	Global	1	2	Underweight (proportion <2SD weight for age, <5 years)	145
Meningitis	Male	0-6 days	1-4 years	Global	-1	3	DTP3 Coverage (proportion)	80
Meningitis	Male	0-6 days	1-4 years	Global	-1	3	Maternal Education (years per capita)	113
Meningitis	Male	0-6 days	1-4 years	Global	-1	3	Socio-demographic Index	126
Meningitis	Male	0-6 days	1-4 years	Global	-1	3	Sanitation (proportion with access)	525
Meningitis	Male	0-6 days	1-4 years	Global	-1	3	LDI (I$ per capita)	--
Meningitis	Male	5-9 years	95+ years	Data Rich	-1	1	Hib3 Vaccine Coverage (proportion)	1000
Meningitis	Male	5-9 years	95+ years	Data Rich	-1	1	Proportion of total population covered by menafrivac initiative (meningitis meningococcal type A vaccine)	--
Meningitis	Male	5-9 years	95+ years	Data Rich	1	1	meningitis belt (proportion)	--
Meningitis	Male	5-9 years	95+ years	Data Rich	-1	2	Healthcare access and quality index	73
Meningitis	Male	5-9 years	95+ years	Data Rich	-1	2	Health System Access (capped)	--
Meningitis	Male	5-9 years	95+ years	Data Rich	-1	2	Improved Water Source (proportion with access)	--
Meningitis	Male	5-9 years	95+ years	Data Rich	1	2	Underweight (proportion <2SD weight for age, <5 years)	153
Meningitis	Male	5-9 years	95+ years	Data Rich	-1	3	Maternal Education (years per capita)	202
Meningitis	Male	5-9 years	95+ years	Data Rich	-1	3	Socio-demographic Index	241
Meningitis	Male	5-9 years	95+ years	Data Rich	-1	3	Sanitation (proportion with access)	358
Meningitis	Male	5-9 years	95+ years	Data Rich	-1	3	DTP3 Coverage (proportion)	--
Meningitis	Male	5-9 years	95+ years	Data Rich	-1	3	LDI (I$ per capita)	--
Meningitis	Male	5-9 years	95+ years	Global	-1	1	Hib3 Vaccine Coverage (proportion)	--
Meningitis	Male	5-9 years	95+ years	Global	-1	1	Proportion of total population covered by menafrivac initiative (meningitis meningococcal type A vaccine)	--
Meningitis	Male	5-9 years	95+ years	Global	1	1	meningitis belt (proportion)	24
Meningitis	Male	5-9 years	95+ years	Global	-1	2	Healthcare access and quality index	420
Meningitis	Male	5-9 years	95+ years	Global	-1	2	Health System Access (capped)	--
Meningitis	Male	5-9 years	95+ years	Global	-1	2	Improved Water Source (proportion with access)	--
Meningitis	Male	5-9 years	95+ years	Global	1	2	Underweight (proportion <2SD weight for age, <5 years)	325
Meningitis	Male	5-9 years	95+ years	Global	-1	3	DTP3 Coverage (proportion)	182
Meningitis	Male	5-9 years	95+ years	Global	-1	3	Socio-demographic Index	285
Meningitis	Male	5-9 years	95+ years	Global	-1	3	Sanitation (proportion with access)	381
Meningitis	Male	5-9 years	95+ years	Global	-1	3	LDI (I$ per capita)	--
Meningitis	Male	5-9 years	95+ years	Global	-1	3	Maternal Education (years per capita)	--
Encephalitis	Female	0-6 days	95+ years	Data Rich	1	1	Japanese encelphalitis endemic area (binary)	611
Encephalitis	Female	0-6 days	95+ years	Data Rich	1	1	Underweight (proportion <2SD weight for age, <5 years)	698
Encephalitis	Female	0-6 days	95+ years	Data Rich	-1	2	Health System Access (capped)	--
Encephalitis	Female	0-6 days	95+ years	Data Rich	-1	2	Healthcare access and quality index	--
Encephalitis	Female	0-6 days	95+ years	Data Rich	-1	2	LDI (I$ per capita)	--
Encephalitis	Female	0-6 days	95+ years	Data Rich	-1	3	Improved Water Source (proportion with access)	69
Encephalitis	Female	0-6 days	95+ years	Data Rich	-1	3	Sanitation (proportion with access)	658
Encephalitis	Female	0-6 days	95+ years	Data Rich	-1	3	DTP3 Coverage (proportion)	--
Encephalitis	Female	0-6 days	95+ years	Data Rich	-1	3	In-Facility Delivery (proportion)	--
Encephalitis	Female	0-6 days	95+ years	Data Rich	-1	3	Maternal Education (years per capita)	--
Encephalitis	Female	0-6 days	95+ years	Data Rich	-1	3	Socio-demographic Index	--
Encephalitis	Female	0-6 days	95+ years	Global	1	1	Japanese encelphalitis endemic area (binary)	800
Encephalitis	Female	0-6 days	95+ years	Global	1	1	Underweight (proportion <2SD weight for age, <5 years)	--
Encephalitis	Female	0-6 days	95+ years	Global	-1	2	Healthcare access and quality index	322
Encephalitis	Female	0-6 days	95+ years	Global	-1	2	Health System Access (capped)	--
Encephalitis	Female	0-6 days	95+ years	Global	-1	2	LDI (I$ per capita)	--
Encephalitis	Female	0-6 days	95+ years	Global	-1	3	Socio-demographic Index	19
Encephalitis	Female	0-6 days	95+ years	Global	-1	3	DTP3 Coverage (proportion)	--
Encephalitis	Female	0-6 days	95+ years	Global	-1	3	Improved Water Source (proportion with access)	--
Encephalitis	Female	0-6 days	95+ years	Global	-1	3	In-Facility Delivery (proportion)	--
Encephalitis	Female	0-6 days	95+ years	Global	-1	3	Maternal Education (years per capita)	--
Encephalitis	Female	0-6 days	95+ years	Global	-1	3	Sanitation (proportion with access)	--
Encephalitis	Male	0-6 days	95+ years	Data Rich	1	1	Underweight (proportion <2SD weight for age, <5 years)	546
Encephalitis	Male	0-6 days	95+ years	Data Rich	1	1	Japanese encelphalitis endemic area (binary)	695
Encephalitis	Male	0-6 days	95+ years	Data Rich	-1	2	Healthcare access and quality index	51
Encephalitis	Male	0-6 days	95+ years	Data Rich	-1	2	Health System Access (capped)	--
Encephalitis	Male	0-6 days	95+ years	Data Rich	-1	2	LDI (I$ per capita)	--
Encephalitis	Male	0-6 days	95+ years	Data Rich	-1	3	Improved Water Source (proportion with access)	38
Encephalitis	Male	0-6 days	95+ years	Data Rich	-1	3	Sanitation (proportion with access)	418
Encephalitis	Male	0-6 days	95+ years	Data Rich	-1	3	DTP3 Coverage (proportion)	--
Encephalitis	Male	0-6 days	95+ years	Data Rich	-1	3	In-Facility Delivery (proportion)	--
Encephalitis	Male	0-6 days	95+ years	Data Rich	-1	3	Maternal Education (years per capita)	--
Encephalitis	Male	0-6 days	95+ years	Data Rich	-1	3	Socio-demographic Index	--
Encephalitis	Male	0-6 days	95+ years	Global	1	1	Japanese encelphalitis endemic area (binary)	548
Encephalitis	Male	0-6 days	95+ years	Global	1	1	Underweight (proportion <2SD weight for age, <5 years)	--
Encephalitis	Male	0-6 days	95+ years	Global	-1	2	Healthcare access and quality index	276
Encephalitis	Male	0-6 days	95+ years	Global	-1	2	Health System Access (capped)	--
Encephalitis	Male	0-6 days	95+ years	Global	-1	2	LDI (I$ per capita)	--
Encephalitis	Male	0-6 days	95+ years	Global	-1	3	Socio-demographic Index	33
Encephalitis	Male	0-6 days	95+ years	Global	-1	3	DTP3 Coverage (proportion)	--
Encephalitis	Male	0-6 days	95+ years	Global	-1	3	Improved Water Source (proportion with access)	--

Appendix Table 9: CODEm covariates used, level of covariate, and expected direction of covariate by cause, sex, and age
Covariates that CODEm did not select during the covariate selection process have no draw counts listed.

Cause	Sex	Age Start	Age End	Model Type	Direction	Level	Covariate Name	Number of Draws
Encephalitis	Male	0-6 days	95+ years	Global	-1	3	In-Facility Delivery (proportion)	--
Encephalitis	Male	0-6 days	95+ years	Global	-1	3	Maternal Education (years per capita)	--
Encephalitis	Male	0-6 days	95+ years	Global	-1	3	Sanitation (proportion with access)	--
Tetanus	Female	0-6 days	28-364 days	Data Rich	-1	1	DTP3 Coverage (proportion)	13
Tetanus	Female	0-6 days	28-364 days	Data Rich	-1	1	Tetanus Toxoid Coverage Smooth (proportion)	1000
Tetanus	Female	0-6 days	28-364 days	Data Rich	-1	2	In-Facility Delivery (proportion)	115
Tetanus	Female	0-6 days	28-364 days	Data Rich	-1	2	Skilled Birth Attendance (proportion)	133
Tetanus	Female	0-6 days	28-364 days	Data Rich	-1	2	Healthcare access and quality index	768
Tetanus	Female	0-6 days	28-364 days	Data Rich	-1	2	Health System Access (capped)	791
Tetanus	Female	0-6 days	28-364 days	Data Rich	-1	3	Education (years per capita)	238
Tetanus	Female	0-6 days	28-364 days	Data Rich	-1	3	Socio-demographic Index	479
Tetanus	Female	0-6 days	28-364 days	Data Rich	-1	3	LDI (I$ per capita)	--
Tetanus	Female	0-6 days	28-364 days	Global	-1	1	DTP3 Coverage (proportion)	613
Tetanus	Female	0-6 days	28-364 days	Global	-1	1	Tetanus Toxoid Coverage Smooth (proportion)	670
Tetanus	Female	0-6 days	28-364 days	Global	-1	2	In-Facility Delivery (proportion)	339
Tetanus	Female	0-6 days	28-364 days	Global	-1	2	Healthcare access and quality index	496
Tetanus	Female	0-6 days	28-364 days	Global	-1	2	Skilled Birth Attendance (proportion)	629
Tetanus	Female	0-6 days	28-364 days	Global	-1	2	Health System Access (capped)	652
Tetanus	Female	0-6 days	28-364 days	Global	-1	3	Socio-demographic Index	353
Tetanus	Female	0-6 days	28-364 days	Global	-1	3	Education (years per capita)	454
Tetanus	Female	0-6 days	28-364 days	Global	-1	3	LDI (I$ per capita)	--
Tetanus	Female	1-4 years	95+ years	Data Rich	-1	1	DTP3 Coverage (proportion)	1000
Tetanus	Female	1-4 years	95+ years	Data Rich	-1	2	Healthcare access and quality index	828
Tetanus	Female	1-4 years	95+ years	Data Rich	-1	2	Health System Access (capped)	--
Tetanus	Female	1-4 years	95+ years	Data Rich	-1	3	Socio-demographic Index	537
Tetanus	Female	1-4 years	95+ years	Data Rich	-1	3	Education (years per capita)	625
Tetanus	Female	1-4 years	95+ years	Data Rich	-1	3	Sanitation (proportion with access)	745
Tetanus	Female	1-4 years	95+ years	Data Rich	-1	3	LDI (I$ per capita)	--
Tetanus	Female	1-4 years	95+ years	Global	-1	1	DTP3 Coverage (proportion)	1000
Tetanus	Female	1-4 years	95+ years	Global	-1	2	Health System Access (capped)	188
Tetanus	Female	1-4 years	95+ years	Global	-1	2	Healthcare access and quality index	540
Tetanus	Female	1-4 years	95+ years	Global	-1	3	Socio-demographic Index	402
Tetanus	Female	1-4 years	95+ years	Global	-1	3	Education (years per capita)	496
Tetanus	Female	1-4 years	95+ years	Global	-1	3	Sanitation (proportion with access)	569
Tetanus	Female	1-4 years	95+ years	Global	-1	3	LDI (I$ per capita)	--
Tetanus	Male	0-6 days	28-364 days	Data Rich	-1	1	DTP3 Coverage (proportion)	428
Tetanus	Male	0-6 days	28-364 days	Data Rich	-1	1	Tetanus Toxoid Coverage Smooth (proportion)	819
Tetanus	Male	0-6 days	28-364 days	Data Rich	-1	2	Skilled Birth Attendance (proportion)	124
Tetanus	Male	0-6 days	28-364 days	Data Rich	-1	2	In-Facility Delivery (proportion)	199
Tetanus	Male	0-6 days	28-364 days	Data Rich	-1	2	Healthcare access and quality index	216
Tetanus	Male	0-6 days	28-364 days	Data Rich	-1	2	Health System Access (capped)	278
Tetanus	Male	0-6 days	28-364 days	Data Rich	-1	3	Socio-demographic Index	550
Tetanus	Male	0-6 days	28-364 days	Data Rich	-1	3	Education (years per capita)	728
Tetanus	Male	0-6 days	28-364 days	Data Rich	-1	3	LDI (I$ per capita)	--
Tetanus	Male	0-6 days	28-364 days	Global	-1	1	Tetanus Toxoid Coverage Smooth (proportion)	615
Tetanus	Male	0-6 days	28-364 days	Global	-1	1	DTP3 Coverage (proportion)	688
Tetanus	Male	0-6 days	28-364 days	Global	-1	2	Health System Access (capped)	524
Tetanus	Male	0-6 days	28-364 days	Global	-1	2	Skilled Birth Attendance (proportion)	714
Tetanus	Male	0-6 days	28-364 days	Global	-1	2	In-Facility Delivery (proportion)	744
Tetanus	Male	0-6 days	28-364 days	Global	-1	2	Healthcare access and quality index	766
Tetanus	Male	0-6 days	28-364 days	Global	-1	3	Education (years per capita)	238
Tetanus	Male	0-6 days	28-364 days	Global	-1	3	Socio-demographic Index	327
Tetanus	Male	0-6 days	28-364 days	Global	-1	3	LDI (I$ per capita)	--
Tetanus	Male	1-4 years	95+ years	Data Rich	-1	1	DTP3 Coverage (proportion)	1000
Tetanus	Male	1-4 years	95+ years	Data Rich	-1	2	Health System Access (capped)	514
Tetanus	Male	1-4 years	95+ years	Data Rich	-1	2	Healthcare access and quality index	--
Tetanus	Male	1-4 years	95+ years	Data Rich	-1	3	Socio-demographic Index	548
Tetanus	Male	1-4 years	95+ years	Data Rich	-1	3	Education (years per capita)	--
Tetanus	Male	1-4 years	95+ years	Data Rich	-1	3	LDI (I$ per capita)	--
Tetanus	Male	1-4 years	95+ years	Data Rich	-1	3	Sanitation (proportion with access)	--
Tetanus	Male	1-4 years	95+ years	Global	-1	1	DTP3 Coverage (proportion)	665
Tetanus	Male	1-4 years	95+ years	Global	-1	2	Health System Access (capped)	351
Tetanus	Male	1-4 years	95+ years	Global	-1	2	Healthcare access and quality index	576
Tetanus	Male	1-4 years	95+ years	Global	-1	3	Socio-demographic Index	660
Tetanus	Male	1-4 years	95+ years	Global	-1	3	Education (years per capita)	786
Tetanus	Male	1-4 years	95+ years	Global	-1	3	Sanitation (proportion with access)	786
Tetanus	Male	1-4 years	95+ years	Global	-1	3	LDI (I$ per capita)	--
Acute hepatitis	Female	28-364 days	95+ years	Data Rich	1	1	Seroprevalence of anti-HAV (IgG)	474
Acute hepatitis	Female	28-364 days	95+ years	Data Rich	1	1	Hepatitis B (HBsAg) Seroprevalence	770
Acute hepatitis	Female	28-364 days	95+ years	Data Rich	1	1	Hepatitis C (IgG) Seroprevalence	--
Acute hepatitis	Female	28-364 days	95+ years	Data Rich	1	1	Log-transformed SEV scalar: Hep	--
Acute hepatitis	Female	28-364 days	95+ years	Data Rich	1	1	Seroprevalence of anti-HEV (IgG)	--
Acute hepatitis	Female	28-364 days	95+ years	Data Rich	-1	2	Socio-demographic Index	170
Acute hepatitis	Female	28-364 days	95+ years	Data Rich	-1	2	Healthcare access and quality index	194
Acute hepatitis	Female	28-364 days	95+ years	Data Rich	-1	2	Hepatitis B vaccine coverage (proportion), aged through time	282
Acute hepatitis	Female	28-364 days	95+ years	Data Rich	1	2	SEV unsafe sanitation	122
Acute hepatitis	Female	28-364 days	95+ years	Data Rich	1	2	SEV unsafe water	304
Acute hepatitis	Female	28-364 days	95+ years	Data Rich	-1	3	Education (years per capita)	24

Appendix Table 9: CODEm covariates used, level of covariate, and expected direction of covariate by cause, sex, and age<br>*Covariates that CODEm did not select during the covariate selection process have no draw counts listed.*								
**Cause**	**Sex**	**Age Start**	**Age End**	**Model Type**	**Direction**	**Level**	**Covariate Name**	**Number of Draws**
Acute hepatitis	Female	28-364 days	95+ years	Data Rich	-1	3	LDI (I$ per capita)	--
Acute hepatitis	Female	28-364 days	95+ years	Global	1	1	Hepatitis B (HBsAg) Seroprevalence	369
Acute hepatitis	Female	28-364 days	95+ years	Global	1	1	Seroprevalence of anti-HAV (IgG)	725
Acute hepatitis	Female	28-364 days	95+ years	Global	1	1	Hepatitis C (IgG) Seroprevalence	--
Acute hepatitis	Female	28-364 days	95+ years	Global	1	1	Log-transformed SEV scalar: Hep	--
Acute hepatitis	Female	28-364 days	95+ years	Global	1	1	Seroprevalence of anti-HEV (IgG)	--
Acute hepatitis	Female	28-364 days	95+ years	Global	-1	2	Healthcare access and quality index	221
Acute hepatitis	Female	28-364 days	95+ years	Global	-1	2	Hepatitis B vaccine coverage (proportion), aged through time	313
Acute hepatitis	Female	28-364 days	95+ years	Global	-1	2	Socio-demographic Index	323
Acute hepatitis	Female	28-364 days	95+ years	Global	1	2	SEV unsafe sanitation	396
Acute hepatitis	Female	28-364 days	95+ years	Global	1	2	SEV unsafe water	621
Acute hepatitis	Female	28-364 days	95+ years	Global	-1	3	Education (years per capita)	295
Acute hepatitis	Female	28-364 days	95+ years	Global	-1	3	LDI (I$ per capita)	--
Acute hepatitis	Male	28-364 days	95+ years	Data Rich	1	1	Seroprevalence of anti-HAV (IgG)	172
Acute hepatitis	Male	28-364 days	95+ years	Data Rich	1	1	Hepatitis B (HBsAg) Seroprevalence	865
Acute hepatitis	Male	28-364 days	95+ years	Data Rich	1	1	Hepatitis C (IgG) Seroprevalence	--
Acute hepatitis	Male	28-364 days	95+ years	Data Rich	1	1	Seroprevalence of anti-HEV (IgG)	--
Acute hepatitis	Male	28-364 days	95+ years	Data Rich	-1	2	Healthcare access and quality index	20
Acute hepatitis	Male	28-364 days	95+ years	Data Rich	-1	2	Hepatitis B vaccine coverage (proportion), aged through time	453
Acute hepatitis	Male	28-364 days	95+ years	Data Rich	1	2	Log-transformed SEV scalar: Hep	59
Acute hepatitis	Male	28-364 days	95+ years	Data Rich	1	2	SEV unsafe sanitation	231
Acute hepatitis	Male	28-364 days	95+ years	Data Rich	1	2	SEV unsafe water	233
Acute hepatitis	Male	28-364 days	95+ years	Data Rich	-1	3	Education (years per capita)	43
Acute hepatitis	Male	28-364 days	95+ years	Data Rich	-1	3	Socio-demographic Index	133
Acute hepatitis	Male	28-364 days	95+ years	Data Rich	-1	3	LDI (I$ per capita)	--
Acute hepatitis	Male	28-364 days	95+ years	Global	1	1	Log-transformed SEV scalar: Hep	230
Acute hepatitis	Male	28-364 days	95+ years	Global	1	1	Hepatitis B (HBsAg) Seroprevalence	523
Acute hepatitis	Male	28-364 days	95+ years	Global	1	1	Seroprevalence of anti-HAV (IgG)	603
Acute hepatitis	Male	28-364 days	95+ years	Global	1	1	Hepatitis C (IgG) Seroprevalence	--
Acute hepatitis	Male	28-364 days	95+ years	Global	1	1	Seroprevalence of anti-HEV (IgG)	--
Acute hepatitis	Male	28-364 days	95+ years	Global	-1	2	Socio-demographic Index	31
Acute hepatitis	Male	28-364 days	95+ years	Global	-1	2	Healthcare access and quality index	238
Acute hepatitis	Male	28-364 days	95+ years	Global	-1	2	Hepatitis B vaccine coverage (proportion), aged through time	453
Acute hepatitis	Male	28-364 days	95+ years	Global	1	2	SEV unsafe sanitation	227
Acute hepatitis	Male	28-364 days	95+ years	Global	1	2	SEV unsafe water	299
Acute hepatitis	Male	28-364 days	95+ years	Global	-1	3	Education (years per capita)	36
Acute hepatitis	Male	28-364 days	95+ years	Global	-1	3	LDI (I$ per capita)	--
Other unspecified infectious diseases	Female	0-6 days	95+ years	Data Rich	-1	1	DTP3 Coverage (proportion)	525
Other unspecified infectious diseases	Female	0-6 days	95+ years	Data Rich	-1	1	Health System Access (unitless)	--
Other unspecified infectious diseases	Female	0-6 days	95+ years	Data Rich	-1	1	Measles Vaccine Coverage (proportion)	--
Other unspecified infectious diseases	Female	0-6 days	95+ years	Data Rich	-1	2	Antenatal Care (1 visit) Coverage (proportion)	--
Other unspecified infectious diseases	Female	0-6 days	95+ years	Data Rich	-1	2	Health System Access (capped)	--
Other unspecified infectious diseases	Female	0-6 days	95+ years	Data Rich	-1	2	Healthcare access and quality index	--
Other unspecified infectious diseases	Female	0-6 days	95+ years	Data Rich	-1	2	Improved Water Source (proportion with access)	--
Other unspecified infectious diseases	Female	0-6 days	95+ years	Data Rich	-1	2	Latitude Over 45 (proportion)	--
Other unspecified infectious diseases	Female	0-6 days	95+ years	Data Rich	-1	2	Rainfall Quintile 1 (proportion)	--
Other unspecified infectious diseases	Female	0-6 days	95+ years	Data Rich	-1	2	Sanitation (proportion with access)	--
Other unspecified infectious diseases	Female	0-6 days	95+ years	Data Rich	0	2	Rainfall Quintile 2 (proportion)	236
Other unspecified infectious diseases	Female	0-6 days	95+ years	Data Rich	0	2	Rainfall Quintile 4 (proportion)	236
Other unspecified infectious diseases	Female	0-6 days	95+ years	Data Rich	0	2	Latitude 30 to 45 (proportion)	246
Other unspecified infectious diseases	Female	0-6 days	95+ years	Data Rich	0	2	Latitude 15 to 30 (proportion)	452
Other unspecified infectious diseases	Female	0-6 days	95+ years	Data Rich	0	2	Rainfall Quintile 3 (proportion)	620
Other unspecified infectious diseases	Female	0-6 days	95+ years	Data Rich	1	2	Latitude Under 15 (proportion)	431
Other unspecified infectious diseases	Female	0-6 days	95+ years	Data Rich	1	2	Rainfall Quintile 5 (proportion)	--
Other unspecified infectious diseases	Female	0-6 days	95+ years	Data Rich	1	2	Underweight (proportion <2SD weight for age, <5 years)	--
Other unspecified infectious diseases	Female	0-6 days	95+ years	Data Rich	-1	3	Education (years per capita)	--
Other unspecified infectious diseases	Female	0-6 days	95+ years	Data Rich	-1	3	LDI (I$ per capita)	--
Other unspecified infectious diseases	Female	0-6 days	95+ years	Data Rich	-1	3	Socio-demographic Index	--
Other unspecified infectious diseases	Female	0-6 days	95+ years	Global	-1	1	Antenatal Care (1 visit) Coverage (proportion)	--
Other unspecified infectious diseases	Female	0-6 days	95+ years	Global	1	1	Underweight (proportion <2SD weight for age, <5 years)	1000
Other unspecified infectious diseases	Female	0-6 days	95+ years	Global	-1	2	Sanitation (proportion with access)	459
Other unspecified infectious diseases	Female	0-6 days	95+ years	Global	-1	2	Latitude Over 45 (proportion)	662
Other unspecified infectious diseases	Female	0-6 days	95+ years	Global	-1	2	Healthcare access and quality index	--
Other unspecified infectious diseases	Female	0-6 days	95+ years	Global	-1	2	Improved Water Source (proportion with access)	--
Other unspecified infectious diseases	Female	0-6 days	95+ years	Global	-1	3	DTP3 Coverage (proportion)	--
Other unspecified infectious diseases	Female	0-6 days	95+ years	Global	-1	3	Socio-demographic Index	--
Other unspecified infectious diseases	Female	0-6 days	95+ years	Global	0	3	Latitude 30 to 45 (proportion)	557
Other unspecified infectious diseases	Female	0-6 days	95+ years	Global	0	3	Latitude 15 to 30 (proportion)	582
Other unspecified infectious diseases	Female	0-6 days	95+ years	Global	1	3	Latitude Under 15 (proportion)	573
Other unspecified infectious diseases	Male	0-6 days	95+ years	Data Rich	-1	1	Measles Vaccine Coverage (proportion)	892
Other unspecified infectious diseases	Male	0-6 days	95+ years	Data Rich	-1	1	DTP3 Coverage (proportion)	--
Other unspecified infectious diseases	Male	0-6 days	95+ years	Data Rich	-1	1	Health System Access (unitless)	--
Other unspecified infectious diseases	Male	0-6 days	95+ years	Data Rich	-1	2	Antenatal Care (1 visit) Coverage (proportion)	--
Other unspecified infectious diseases	Male	0-6 days	95+ years	Data Rich	-1	2	Improved Water Source (proportion with access)	--
Other unspecified infectious diseases	Male	0-6 days	95+ years	Data Rich	-1	2	Latitude Over 45 (proportion)	--
Other unspecified infectious diseases	Male	0-6 days	95+ years	Data Rich	-1	2	Rainfall Quintile 1 (proportion)	--
Other unspecified infectious diseases	Male	0-6 days	95+ years	Data Rich	-1	2	Sanitation (proportion with access)	--
Other unspecified infectious diseases	Male	0-6 days	95+ years	Data Rich	0	2	Latitude 15 to 30 (proportion)	108

Appendix Table 9: CODEm covariates used, level of covariate, and expected direction of covariate by cause, sex, and age
Covariates that CODEm did not select during the covariate selection process have no draw counts listed.

Cause	Sex	Age Start	Age End	Model Type	Direction	Level	Covariate Name	Number of Draws
Other unspecified infectious diseases	Male	0-6 days	95+ years	Data Rich	0	2	Latitude 30 to 45 (proportion)	108
Other unspecified infectious diseases	Male	0-6 days	95+ years	Data Rich	0	2	Rainfall Quintile 4 (proportion)	162
Other unspecified infectious diseases	Male	0-6 days	95+ years	Data Rich	0	2	Rainfall Quintile 3 (proportion)	434
Other unspecified infectious diseases	Male	0-6 days	95+ years	Data Rich	0	2	Rainfall Quintile 2 (proportion)	492
Other unspecified infectious diseases	Male	0-6 days	95+ years	Data Rich	1	2	Rainfall Quintile 5 (proportion)	374
Other unspecified infectious diseases	Male	0-6 days	95+ years	Data Rich	1	2	Latitude Under 15 (proportion)	--
Other unspecified infectious diseases	Male	0-6 days	95+ years	Data Rich	1	2	Underweight (proportion <2SD weight for age, <5 years)	--
Other unspecified infectious diseases	Male	0-6 days	95+ years	Data Rich	-1	3	Education (years per capita)	--
Other unspecified infectious diseases	Male	0-6 days	95+ years	Data Rich	-1	3	LDI (I$ per capita)	--
Other unspecified infectious diseases	Male	0-6 days	95+ years	Data Rich	-1	3	Socio-demographic Index	--
Other unspecified infectious diseases	Male	0-6 days	95+ years	Global	-1	1	Antenatal Care (1 visit) Coverage (proportion)	381
Other unspecified infectious diseases	Male	0-6 days	95+ years	Global	1	1	Underweight (proportion <2SD weight for age, <5 years)	884
Other unspecified infectious diseases	Male	0-6 days	95+ years	Global	-1	2	Improved Water Source (proportion with access)	358
Other unspecified infectious diseases	Male	0-6 days	95+ years	Global	-1	2	Sanitation (proportion with access)	358
Other unspecified infectious diseases	Male	0-6 days	95+ years	Global	-1	2	Latitude Over 45 (proportion)	532
Other unspecified infectious diseases	Male	0-6 days	95+ years	Global	-1	3	DTP3 Coverage (proportion)	--
Other unspecified infectious diseases	Male	0-6 days	95+ years	Global	-1	3	Socio-demographic Index	--
Other unspecified infectious diseases	Male	0-6 days	95+ years	Global	0	3	Latitude 30 to 45 (proportion)	545
Other unspecified infectious diseases	Male	0-6 days	95+ years	Global	0	3	Latitude 15 to 30 (proportion)	576
Other unspecified infectious diseases	Male	0-6 days	95+ years	Global	1	3	Latitude Under 15 (proportion)	165
Neonatal disorders	Female	0-6 days	1-4 years	Data Rich	1	1	Indoor Air Pollution (All Cooking Fuels)	177
Neonatal disorders	Female	0-6 days	1-4 years	Data Rich	1	1	Smoking Prevalence (Reproductive Age Standardized)	743
Neonatal disorders	Female	0-6 days	1-4 years	Data Rich	-1	2	In-Facility Delivery (proportion)	61
Neonatal disorders	Female	0-6 days	1-4 years	Data Rich	-1	2	Antenatal Care (4 visits) Coverage (proportion)	103
Neonatal disorders	Female	0-6 days	1-4 years	Data Rich	-1	2	Skilled Birth Attendance (proportion)	150
Neonatal disorders	Female	0-6 days	1-4 years	Data Rich	-1	2	Healthcare access and quality index	253
Neonatal disorders	Female	0-6 days	1-4 years	Data Rich	-1	2	Health System Access (capped)	497
Neonatal disorders	Female	0-6 days	1-4 years	Data Rich	1	2	Age-standardized underweight (weight-for-age) SEV	80
Neonatal disorders	Female	0-6 days	1-4 years	Data Rich	1	2	Live Births 35+ (proportion)	187
Neonatal disorders	Female	0-6 days	1-4 years	Data Rich	-1	3	Socio-demographic Index	80
Neonatal disorders	Female	0-6 days	1-4 years	Data Rich	-1	3	Education (years per capita)	126
Neonatal disorders	Female	0-6 days	1-4 years	Data Rich	-1	3	LDI (I$ per capita)	--
Neonatal disorders	Female	0-6 days	1-4 years	Data Rich	1	3	Total Fertility Rate	--
Neonatal disorders	Female	0-6 days	1-4 years	Global	1	1	Indoor Air Pollution (All Cooking Fuels)	169
Neonatal disorders	Female	0-6 days	1-4 years	Global	1	1	Smoking Prevalence (Reproductive Age Standardized)	581
Neonatal disorders	Female	0-6 days	1-4 years	Global	-1	2	Health System Access (capped)	47
Neonatal disorders	Female	0-6 days	1-4 years	Global	-1	2	Antenatal Care (4 visits) Coverage (proportion)	48
Neonatal disorders	Female	0-6 days	1-4 years	Global	-1	2	In-Facility Delivery (proportion)	89
Neonatal disorders	Female	0-6 days	1-4 years	Global	-1	2	Skilled Birth Attendance (proportion)	89
Neonatal disorders	Female	0-6 days	1-4 years	Global	-1	2	Healthcare access and quality index	356
Neonatal disorders	Female	0-6 days	1-4 years	Global	1	2	Age-standardized underweight (weight-for-age) SEV	137
Neonatal disorders	Female	0-6 days	1-4 years	Global	1	2	Live Births 35+ (proportion)	323
Neonatal disorders	Female	0-6 days	1-4 years	Global	-1	3	Education (years per capita)	125
Neonatal disorders	Female	0-6 days	1-4 years	Global	-1	3	Socio-demographic Index	150
Neonatal disorders	Female	0-6 days	1-4 years	Global	-1	3	LDI (I$ per capita)	--
Neonatal disorders	Female	0-6 days	1-4 years	Global	1	3	Total Fertility Rate	--
Neonatal disorders	Male	0-6 days	1-4 years	Data Rich	1	1	Smoking Prevalence (Reproductive Age Standardized)	316
Neonatal disorders	Male	0-6 days	1-4 years	Data Rich	1	1	Indoor Air Pollution (All Cooking Fuels)	498
Neonatal disorders	Male	0-6 days	1-4 years	Data Rich	-1	2	Antenatal Care (4 visits) Coverage (proportion)	8
Neonatal disorders	Male	0-6 days	1-4 years	Data Rich	-1	2	Skilled Birth Attendance (proportion)	166
Neonatal disorders	Male	0-6 days	1-4 years	Data Rich	-1	2	In-Facility Delivery (proportion)	240
Neonatal disorders	Male	0-6 days	1-4 years	Data Rich	-1	2	Health System Access (capped)	--
Neonatal disorders	Male	0-6 days	1-4 years	Data Rich	-1	2	Healthcare access and quality index	--
Neonatal disorders	Male	0-6 days	1-4 years	Data Rich	1	2	Age-standardized underweight (weight-for-age) SEV	210
Neonatal disorders	Male	0-6 days	1-4 years	Data Rich	1	2	Live Births 35+ (proportion)	250
Neonatal disorders	Male	0-6 days	1-4 years	Data Rich	-1	3	Socio-demographic Index	61
Neonatal disorders	Male	0-6 days	1-4 years	Data Rich	-1	3	Education (years per capita)	70
Neonatal disorders	Male	0-6 days	1-4 years	Data Rich	-1	3	LDI (I$ per capita)	--
Neonatal disorders	Male	0-6 days	1-4 years	Data Rich	1	3	Total Fertility Rate	--
Neonatal disorders	Male	0-6 days	1-4 years	Global	1	1	Indoor Air Pollution (All Cooking Fuels)	387
Neonatal disorders	Male	0-6 days	1-4 years	Global	1	1	Smoking Prevalence (Reproductive Age Standardized)	394
Neonatal disorders	Male	0-6 days	1-4 years	Global	-1	2	Antenatal Care (4 visits) Coverage (proportion)	57
Neonatal disorders	Male	0-6 days	1-4 years	Global	-1	2	In-Facility Delivery (proportion)	92
Neonatal disorders	Male	0-6 days	1-4 years	Global	-1	2	Skilled Birth Attendance (proportion)	210
Neonatal disorders	Male	0-6 days	1-4 years	Global	-1	2	Health System Access (capped)	217
Neonatal disorders	Male	0-6 days	1-4 years	Global	-1	2	Healthcare access and quality index	--
Neonatal disorders	Male	0-6 days	1-4 years	Global	1	2	Age-standardized underweight (weight-for-age) SEV	248
Neonatal disorders	Male	0-6 days	1-4 years	Global	1	2	Live Births 35+ (proportion)	323
Neonatal disorders	Male	0-6 days	1-4 years	Global	-1	3	Socio-demographic Index	85
Neonatal disorders	Male	0-6 days	1-4 years	Global	-1	3	Education (years per capita)	156
Neonatal disorders	Male	0-6 days	1-4 years	Global	-1	3	LDI (I$ per capita)	--
Neonatal disorders	Male	0-6 days	1-4 years	Global	1	3	Total Fertility Rate	--
Neonatal preterm birth	Female	0-6 days	1-4 years	Data Rich	-1	1	Health System Access (capped)	700
Neonatal preterm birth	Female	0-6 days	1-4 years	Data Rich	1	1	Indoor Air Pollution (All Cooking Fuels)	12
Neonatal preterm birth	Female	0-6 days	1-4 years	Data Rich	1	1	Smoking Prevalence (Reproductive Age Standardized)	396
Neonatal preterm birth	Female	0-6 days	1-4 years	Data Rich	-1	2	In-Facility Delivery (proportion)	1
Neonatal preterm birth	Female	0-6 days	1-4 years	Data Rich	-1	2	Skilled Birth Attendance (proportion)	53
Neonatal preterm birth	Female	0-6 days	1-4 years	Data Rich	-1	2	Antenatal Care (4 visits) Coverage (proportion)	106

Appendix Table 9: CODEm covariates used, level of covariate, and expected direction of covariate by cause, sex, and age								
Covariates that CODEm did not select during the covariate selection process have no draw counts listed.								
**Cause**	**Sex**	**Age Start**	**Age End**	**Model Type**	**Direction**	**Level**	**Covariate Name**	**Number of Draws**
Neonatal preterm birth	Female	0-6 days	1-4 years	Data Rich	-1	2	Healthcare access and quality index	483
Neonatal preterm birth	Female	0-6 days	1-4 years	Data Rich	1	2	Age-standardized underweight (weight-for-age) SEV	54
Neonatal preterm birth	Female	0-6 days	1-4 years	Data Rich	1	2	Live Births 35+ (proportion)	191
Neonatal preterm birth	Female	0-6 days	1-4 years	Data Rich	-1	3	Education (years per capita)	27
Neonatal preterm birth	Female	0-6 days	1-4 years	Data Rich	-1	3	Socio-demographic Index	197
Neonatal preterm birth	Female	0-6 days	1-4 years	Data Rich	-1	3	LDI (I$ per capita)	--
Neonatal preterm birth	Female	0-6 days	1-4 years	Data Rich	1	3	Total Fertility Rate	--
Neonatal preterm birth	Female	0-6 days	1-4 years	Global	-1	1	Health System Access (capped)	610
Neonatal preterm birth	Female	0-6 days	1-4 years	Global	1	1	Indoor Air Pollution (All Cooking Fuels)	79
Neonatal preterm birth	Female	0-6 days	1-4 years	Global	1	1	Smoking Prevalence (Reproductive Age Standardized)	199
Neonatal preterm birth	Female	0-6 days	1-4 years	Global	-1	2	Antenatal Care (4 visits) Coverage (proportion)	18
Neonatal preterm birth	Female	0-6 days	1-4 years	Global	-1	2	In-Facility Delivery (proportion)	106
Neonatal preterm birth	Female	0-6 days	1-4 years	Global	-1	2	Skilled Birth Attendance (proportion)	114
Neonatal preterm birth	Female	0-6 days	1-4 years	Global	-1	2	Healthcare access and quality index	412
Neonatal preterm birth	Female	0-6 days	1-4 years	Global	1	2	Age-standardized underweight (weight-for-age) SEV	248
Neonatal preterm birth	Female	0-6 days	1-4 years	Global	1	2	Live Births 35+ (proportion)	324
Neonatal preterm birth	Female	0-6 days	1-4 years	Global	-1	3	Education (years per capita)	53
Neonatal preterm birth	Female	0-6 days	1-4 years	Global	-1	3	Socio-demographic Index	131
Neonatal preterm birth	Female	0-6 days	1-4 years	Global	-1	3	LDI (I$ per capita)	--
Neonatal preterm birth	Female	0-6 days	1-4 years	Global	1	3	Total Fertility Rate	--
Neonatal preterm birth	Male	0-6 days	1-4 years	Data Rich	-1	1	Health System Access (capped)	759
Neonatal preterm birth	Male	0-6 days	1-4 years	Data Rich	1	1	Indoor Air Pollution (All Cooking Fuels)	19
Neonatal preterm birth	Male	0-6 days	1-4 years	Data Rich	1	1	Smoking Prevalence (Reproductive Age Standardized)	32
Neonatal preterm birth	Male	0-6 days	1-4 years	Data Rich	-1	2	Antenatal Care (4 visits) Coverage (proportion)	42
Neonatal preterm birth	Male	0-6 days	1-4 years	Data Rich	-1	2	Healthcare access and quality index	122
Neonatal preterm birth	Male	0-6 days	1-4 years	Data Rich	-1	2	In-Facility Delivery (proportion)	122
Neonatal preterm birth	Male	0-6 days	1-4 years	Data Rich	-1	2	Skilled Birth Attendance (proportion)	187
Neonatal preterm birth	Male	0-6 days	1-4 years	Data Rich	1	2	Live Births 35+ (proportion)	193
Neonatal preterm birth	Male	0-6 days	1-4 years	Data Rich	1	2	Age-standardized underweight (weight-for-age) SEV	231
Neonatal preterm birth	Male	0-6 days	1-4 years	Data Rich	-1	3	Education (years per capita)	44
Neonatal preterm birth	Male	0-6 days	1-4 years	Data Rich	-1	3	Socio-demographic Index	172
Neonatal preterm birth	Male	0-6 days	1-4 years	Data Rich	-1	3	LDI (I$ per capita)	--
Neonatal preterm birth	Male	0-6 days	1-4 years	Data Rich	1	3	Total Fertility Rate	--
Neonatal preterm birth	Male	0-6 days	1-4 years	Global	-1	1	Health System Access (capped)	640
Neonatal preterm birth	Male	0-6 days	1-4 years	Global	1	1	Smoking Prevalence (Reproductive Age Standardized)	33
Neonatal preterm birth	Male	0-6 days	1-4 years	Global	1	1	Indoor Air Pollution (All Cooking Fuels)	66
Neonatal preterm birth	Male	0-6 days	1-4 years	Global	-1	2	Antenatal Care (4 visits) Coverage (proportion)	45
Neonatal preterm birth	Male	0-6 days	1-4 years	Global	-1	2	In-Facility Delivery (proportion)	58
Neonatal preterm birth	Male	0-6 days	1-4 years	Global	-1	2	Healthcare access and quality index	121
Neonatal preterm birth	Male	0-6 days	1-4 years	Global	-1	2	Skilled Birth Attendance (proportion)	160
Neonatal preterm birth	Male	0-6 days	1-4 years	Global	1	2	Age-standardized underweight (weight-for-age) SEV	254
Neonatal preterm birth	Male	0-6 days	1-4 years	Global	1	2	Live Births 35+ (proportion)	324
Neonatal preterm birth	Male	0-6 days	1-4 years	Global	-1	3	Education (years per capita)	67
Neonatal preterm birth	Male	0-6 days	1-4 years	Global	-1	3	Socio-demographic Index	102
Neonatal preterm birth	Male	0-6 days	1-4 years	Global	-1	3	LDI (I$ per capita)	--
Neonatal preterm birth	Male	0-6 days	1-4 years	Global	1	3	Total Fertility Rate	--
Neonatal encephalopathy due to birth asphyxia and trauma	Female	0-6 days	1-4 years	Data Rich	1	1	Indoor Air Pollution (All Cooking Fuels)	326
Neonatal encephalopathy due to birth asphyxia and trauma	Female	0-6 days	1-4 years	Data Rich	1	1	Smoking Prevalence (Reproductive Age Standardized)	854
Neonatal encephalopathy due to birth asphyxia and trauma	Female	0-6 days	1-4 years	Data Rich	-1	2	Antenatal Care (4 visits) Coverage (proportion)	37
Neonatal encephalopathy due to birth asphyxia and trauma	Female	0-6 days	1-4 years	Data Rich	-1	2	In-Facility Delivery (proportion)	78
Neonatal encephalopathy due to birth asphyxia and trauma	Female	0-6 days	1-4 years	Data Rich	-1	2	Health System Access (capped)	117
Neonatal encephalopathy due to birth asphyxia and trauma	Female	0-6 days	1-4 years	Data Rich	-1	2	Healthcare access and quality index	239
Neonatal encephalopathy due to birth asphyxia and trauma	Female	0-6 days	1-4 years	Data Rich	-1	2	Skilled Birth Attendance (proportion)	256
Neonatal encephalopathy due to birth asphyxia and trauma	Female	0-6 days	1-4 years	Data Rich	1	2	Age-standardized underweight (weight-for-age) SEV	368
Neonatal encephalopathy due to birth asphyxia and trauma	Female	0-6 days	1-4 years	Data Rich	1	2	Live Births 35+ (proportion)	--
Neonatal encephalopathy due to birth asphyxia and trauma	Female	0-6 days	1-4 years	Data Rich	-1	3	Socio-demographic Index	284
Neonatal encephalopathy due to birth asphyxia and trauma	Female	0-6 days	1-4 years	Data Rich	-1	3	Education (years per capita)	--
Neonatal encephalopathy due to birth asphyxia and trauma	Female	0-6 days	1-4 years	Data Rich	-1	3	LDI (I$ per capita)	--
Neonatal encephalopathy due to birth asphyxia and trauma	Female	0-6 days	1-4 years	Data Rich	1	3	Total Fertility Rate	--
Neonatal encephalopathy due to birth asphyxia and trauma	Female	0-6 days	1-4 years	Global	1	1	Smoking Prevalence (Reproductive Age Standardized)	384
Neonatal encephalopathy due to birth asphyxia and trauma	Female	0-6 days	1-4 years	Global	1	1	Indoor Air Pollution (All Cooking Fuels)	412
Neonatal encephalopathy due to birth asphyxia and trauma	Female	0-6 days	1-4 years	Global	-1	2	Skilled Birth Attendance (proportion)	106
Neonatal encephalopathy due to birth asphyxia and trauma	Female	0-6 days	1-4 years	Global	-1	2	Antenatal Care (4 visits) Coverage (proportion)	112
Neonatal encephalopathy due to birth asphyxia and trauma	Female	0-6 days	1-4 years	Global	-1	2	In-Facility Delivery (proportion)	160
Neonatal encephalopathy due to birth asphyxia and trauma	Female	0-6 days	1-4 years	Global	-1	2	Health System Access (capped)	242
Neonatal encephalopathy due to birth asphyxia and trauma	Female	0-6 days	1-4 years	Global	-1	2	Healthcare access and quality index	--
Neonatal encephalopathy due to birth asphyxia and trauma	Female	0-6 days	1-4 years	Global	1	2	Age-standardized underweight (weight-for-age) SEV	262
Neonatal encephalopathy due to birth asphyxia and trauma	Female	0-6 days	1-4 years	Global	1	2	Live Births 35+ (proportion)	324
Neonatal encephalopathy due to birth asphyxia and trauma	Female	0-6 days	1-4 years	Global	-1	3	Socio-demographic Index	115
Neonatal encephalopathy due to birth asphyxia and trauma	Female	0-6 days	1-4 years	Global	-1	3	Education (years per capita)	--
Neonatal encephalopathy due to birth asphyxia and trauma	Female	0-6 days	1-4 years	Global	-1	3	LDI (I$ per capita)	--
Neonatal encephalopathy due to birth asphyxia and trauma	Female	0-6 days	1-4 years	Global	1	3	Total Fertility Rate	--
Neonatal encephalopathy due to birth asphyxia and trauma	Male	0-6 days	1-4 years	Data Rich	1	1	Indoor Air Pollution (All Cooking Fuels)	364
Neonatal encephalopathy due to birth asphyxia and trauma	Male	0-6 days	1-4 years	Data Rich	1	1	Smoking Prevalence (Reproductive Age Standardized)	550
Neonatal encephalopathy due to birth asphyxia and trauma	Male	0-6 days	1-4 years	Data Rich	-1	2	Antenatal Care (4 visits) Coverage (proportion)	75
Neonatal encephalopathy due to birth asphyxia and trauma	Male	0-6 days	1-4 years	Data Rich	-1	2	Skilled Birth Attendance (proportion)	103
Neonatal encephalopathy due to birth asphyxia and trauma	Male	0-6 days	1-4 years	Data Rich	-1	2	In-Facility Delivery (proportion)	152
Neonatal encephalopathy due to birth asphyxia and trauma	Male	0-6 days	1-4 years	Data Rich	-1	2	Health System Access (capped)	325

Appendix Table 9: CODEm covariates used, level of covariate, and expected direction of covariate by cause, sex, and age

Covariates that CODEm did not select during the covariate selection process have no draw counts listed.

Cause	Sex	Age Start	Age End	Model Type	Direction	Level	Covariate Name	Number of Draws
Neonatal encephalopathy due to birth asphyxia and trauma	Male	0-6 days	1-4 years	Data Rich	-1	2	Healthcare access and quality index	--
Neonatal encephalopathy due to birth asphyxia and trauma	Male	0-6 days	1-4 years	Data Rich	1	2	Age-standardized underweight (weight-for-age) SEV	265
Neonatal encephalopathy due to birth asphyxia and trauma	Male	0-6 days	1-4 years	Data Rich	1	2	Live Births 35+ (proportion)	--
Neonatal encephalopathy due to birth asphyxia and trauma	Male	0-6 days	1-4 years	Data Rich	-1	3	Socio-demographic Index	140
Neonatal encephalopathy due to birth asphyxia and trauma	Male	0-6 days	1-4 years	Data Rich	-1	3	Education (years per capita)	207
Neonatal encephalopathy due to birth asphyxia and trauma	Male	0-6 days	1-4 years	Data Rich	-1	3	LDI (I$ per capita)	--
Neonatal encephalopathy due to birth asphyxia and trauma	Male	0-6 days	1-4 years	Data Rich	1	3	Total Fertility Rate	--
Neonatal encephalopathy due to birth asphyxia and trauma	Male	0-6 days	1-4 years	Global	1	1	Indoor Air Pollution (All Cooking Fuels)	226
Neonatal encephalopathy due to birth asphyxia and trauma	Male	0-6 days	1-4 years	Global	1	1	Smoking Prevalence (Reproductive Age Standardized)	558
Neonatal encephalopathy due to birth asphyxia and trauma	Male	0-6 days	1-4 years	Global	-1	2	Antenatal Care (4 visits) Coverage (proportion)	12
Neonatal encephalopathy due to birth asphyxia and trauma	Male	0-6 days	1-4 years	Global	-1	2	Skilled Birth Attendance (proportion)	74
Neonatal encephalopathy due to birth asphyxia and trauma	Male	0-6 days	1-4 years	Global	-1	2	In-Facility Delivery (proportion)	172
Neonatal encephalopathy due to birth asphyxia and trauma	Male	0-6 days	1-4 years	Global	-1	2	Healthcare access and quality index	213
Neonatal encephalopathy due to birth asphyxia and trauma	Male	0-6 days	1-4 years	Global	-1	2	Health System Access (capped)	404
Neonatal encephalopathy due to birth asphyxia and trauma	Male	0-6 days	1-4 years	Global	1	2	Age-standardized underweight (weight-for-age) SEV	178
Neonatal encephalopathy due to birth asphyxia and trauma	Male	0-6 days	1-4 years	Global	1	2	Live Births 35+ (proportion)	--
Neonatal encephalopathy due to birth asphyxia and trauma	Male	0-6 days	1-4 years	Global	-1	3	Socio-demographic Index	164
Neonatal encephalopathy due to birth asphyxia and trauma	Male	0-6 days	1-4 years	Global	-1	3	Education (years per capita)	--
Neonatal encephalopathy due to birth asphyxia and trauma	Male	0-6 days	1-4 years	Global	-1	3	LDI (I$ per capita)	--
Neonatal encephalopathy due to birth asphyxia and trauma	Male	0-6 days	1-4 years	Global	1	3	Total Fertility Rate	--
Neonatal sepsis and other neonatal infections	Female	0-6 days	1-4 years	Data Rich	1	1	Smoking Prevalence (Reproductive Age Standardized)	953
Neonatal sepsis and other neonatal infections	Female	0-6 days	1-4 years	Data Rich	1	1	Indoor Air Pollution (All Cooking Fuels)	--
Neonatal sepsis and other neonatal infections	Female	0-6 days	1-4 years	Data Rich	-1	2	Healthcare access and quality index	127
Neonatal sepsis and other neonatal infections	Female	0-6 days	1-4 years	Data Rich	-1	2	Antenatal Care (4 visits) Coverage (proportion)	187
Neonatal sepsis and other neonatal infections	Female	0-6 days	1-4 years	Data Rich	-1	2	Health System Access (capped)	187
Neonatal sepsis and other neonatal infections	Female	0-6 days	1-4 years	Data Rich	-1	2	In-Facility Delivery (proportion)	--
Neonatal sepsis and other neonatal infections	Female	0-6 days	1-4 years	Data Rich	-1	2	Skilled Birth Attendance (proportion)	--
Neonatal sepsis and other neonatal infections	Female	0-6 days	1-4 years	Data Rich	1	2	Live Births 35+ (proportion)	47
Neonatal sepsis and other neonatal infections	Female	0-6 days	1-4 years	Data Rich	1	2	Age-standardized underweight (weight-for-age) SEV	--
Neonatal sepsis and other neonatal infections	Female	0-6 days	1-4 years	Data Rich	-1	3	Education (years per capita)	111
Neonatal sepsis and other neonatal infections	Female	0-6 days	1-4 years	Data Rich	-1	3	Socio-demographic Index	204
Neonatal sepsis and other neonatal infections	Female	0-6 days	1-4 years	Data Rich	-1	3	LDI (I$ per capita)	--
Neonatal sepsis and other neonatal infections	Female	0-6 days	1-4 years	Data Rich	1	3	Total Fertility Rate	--
Neonatal sepsis and other neonatal infections	Female	0-6 days	1-4 years	Global	1	1	Smoking Prevalence (Reproductive Age Standardized)	920
Neonatal sepsis and other neonatal infections	Female	0-6 days	1-4 years	Global	1	1	Indoor Air Pollution (All Cooking Fuels)	--
Neonatal sepsis and other neonatal infections	Female	0-6 days	1-4 years	Global	-1	2	Antenatal Care (4 visits) Coverage (proportion)	38
Neonatal sepsis and other neonatal infections	Female	0-6 days	1-4 years	Global	-1	2	Health System Access (capped)	148
Neonatal sepsis and other neonatal infections	Female	0-6 days	1-4 years	Global	-1	2	Healthcare access and quality index	269
Neonatal sepsis and other neonatal infections	Female	0-6 days	1-4 years	Global	-1	2	In-Facility Delivery (proportion)	--
Neonatal sepsis and other neonatal infections	Female	0-6 days	1-4 years	Global	-1	2	Skilled Birth Attendance (proportion)	--
Neonatal sepsis and other neonatal infections	Female	0-6 days	1-4 years	Global	1	2	Live Births 35+ (proportion)	80
Neonatal sepsis and other neonatal infections	Female	0-6 days	1-4 years	Global	1	2	Age-standardized underweight (weight-for-age) SEV	--
Neonatal sepsis and other neonatal infections	Female	0-6 days	1-4 years	Global	-1	3	Education (years per capita)	129
Neonatal sepsis and other neonatal infections	Female	0-6 days	1-4 years	Global	-1	3	Socio-demographic Index	129
Neonatal sepsis and other neonatal infections	Female	0-6 days	1-4 years	Global	-1	3	LDI (I$ per capita)	--
Neonatal sepsis and other neonatal infections	Female	0-6 days	1-4 years	Global	1	3	Total Fertility Rate	--
Neonatal sepsis and other neonatal infections	Male	0-6 days	1-4 years	Data Rich	1	1	Indoor Air Pollution (All Cooking Fuels)	89
Neonatal sepsis and other neonatal infections	Male	0-6 days	1-4 years	Data Rich	1	1	Age-standardized underweight (weight-for-age) SEV	209
Neonatal sepsis and other neonatal infections	Male	0-6 days	1-4 years	Data Rich	1	1	Smoking Prevalence (Reproductive Age Standardized)	765
Neonatal sepsis and other neonatal infections	Male	0-6 days	1-4 years	Data Rich	-1	2	Skilled Birth Attendance (proportion)	24
Neonatal sepsis and other neonatal infections	Male	0-6 days	1-4 years	Data Rich	-1	2	Antenatal Care (4 visits) Coverage (proportion)	107
Neonatal sepsis and other neonatal infections	Male	0-6 days	1-4 years	Data Rich	-1	2	Healthcare access and quality index	324
Neonatal sepsis and other neonatal infections	Male	0-6 days	1-4 years	Data Rich	-1	2	Health System Access (capped)	431
Neonatal sepsis and other neonatal infections	Male	0-6 days	1-4 years	Data Rich	-1	2	In-Facility Delivery (proportion)	--
Neonatal sepsis and other neonatal infections	Male	0-6 days	1-4 years	Data Rich	1	2	Live Births 35+ (proportion)	33
Neonatal sepsis and other neonatal infections	Male	0-6 days	1-4 years	Data Rich	-1	3	Socio-demographic Index	118
Neonatal sepsis and other neonatal infections	Male	0-6 days	1-4 years	Data Rich	-1	3	Education (years per capita)	202
Neonatal sepsis and other neonatal infections	Male	0-6 days	1-4 years	Data Rich	-1	3	LDI (I$ per capita)	--
Neonatal sepsis and other neonatal infections	Male	0-6 days	1-4 years	Data Rich	1	3	Total Fertility Rate	--
Neonatal sepsis and other neonatal infections	Male	0-6 days	1-4 years	Global	1	1	Indoor Air Pollution (All Cooking Fuels)	62
Neonatal sepsis and other neonatal infections	Male	0-6 days	1-4 years	Global	1	1	Smoking Prevalence (Reproductive Age Standardized)	772
Neonatal sepsis and other neonatal infections	Male	0-6 days	1-4 years	Global	-1	2	Skilled Birth Attendance (proportion)	26
Neonatal sepsis and other neonatal infections	Male	0-6 days	1-4 years	Global	-1	2	In-Facility Delivery (proportion)	32
Neonatal sepsis and other neonatal infections	Male	0-6 days	1-4 years	Global	-1	2	Antenatal Care (4 visits) Coverage (proportion)	39
Neonatal sepsis and other neonatal infections	Male	0-6 days	1-4 years	Global	-1	2	Healthcare access and quality index	440
Neonatal sepsis and other neonatal infections	Male	0-6 days	1-4 years	Global	-1	2	Health System Access (capped)	479
Neonatal sepsis and other neonatal infections	Male	0-6 days	1-4 years	Global	1	2	Age-standardized underweight (weight-for-age) SEV	55
Neonatal sepsis and other neonatal infections	Male	0-6 days	1-4 years	Global	1	2	Live Births 35+ (proportion)	190
Neonatal sepsis and other neonatal infections	Male	0-6 days	1-4 years	Global	-1	3	Socio-demographic Index	249
Neonatal sepsis and other neonatal infections	Male	0-6 days	1-4 years	Global	-1	3	Education (years per capita)	281
Neonatal sepsis and other neonatal infections	Male	0-6 days	1-4 years	Global	-1	3	LDI (I$ per capita)	--
Neonatal sepsis and other neonatal infections	Male	0-6 days	1-4 years	Global	1	3	Total Fertility Rate	--
Hemolytic disease and other neonatal jaundice	Female	0-6 days	1-4 years	Data Rich	1	1	Indoor Air Pollution (All Cooking Fuels)	332
Hemolytic disease and other neonatal jaundice	Female	0-6 days	1-4 years	Data Rich	1	1	Smoking Prevalence (Reproductive Age Standardized)	737
Hemolytic disease and other neonatal jaundice	Female	0-6 days	1-4 years	Data Rich	-1	2	Health System Access (capped)	0
Hemolytic disease and other neonatal jaundice	Female	0-6 days	1-4 years	Data Rich	-1	2	Antenatal Care (4 visits) Coverage (proportion)	84
Hemolytic disease and other neonatal jaundice	Female	0-6 days	1-4 years	Data Rich	-1	2	Healthcare access and quality index	141
Hemolytic disease and other neonatal jaundice	Female	0-6 days	1-4 years	Data Rich	-1	2	Skilled Birth Attendance (proportion)	174

Appendix Table 9: CODEm covariates used, level of covariate, and expected direction of covariate by cause, sex, and age
Covariates that CODEm did not select during the covariate selection process have no draw counts listed.

Cause	Sex	Age Start	Age End	Model Type	Direction	Level	Covariate Name	Number of Draws
Hemolytic disease and other neonatal jaundice	Female	0-6 days	1-4 years	Data Rich	-1	2	In-Facility Delivery (proportion)	446
Hemolytic disease and other neonatal jaundice	Female	0-6 days	1-4 years	Data Rich	1	2	Age-standardized underweight (weight-for-age) SEV	5
Hemolytic disease and other neonatal jaundice	Female	0-6 days	1-4 years	Data Rich	1	2	Live Births 35+ (proportion)	--
Hemolytic disease and other neonatal jaundice	Female	0-6 days	1-4 years	Data Rich	-1	3	Education (years per capita)	187
Hemolytic disease and other neonatal jaundice	Female	0-6 days	1-4 years	Data Rich	-1	3	Socio-demographic Index	338
Hemolytic disease and other neonatal jaundice	Female	0-6 days	1-4 years	Data Rich	-1	3	LDI (I$ per capita)	--
Hemolytic disease and other neonatal jaundice	Female	0-6 days	1-4 years	Data Rich	1	3	Total Fertility Rate	--
Hemolytic disease and other neonatal jaundice	Female	0-6 days	1-4 years	Global	1	1	Age-standardized underweight (weight-for-age) SEV	16
Hemolytic disease and other neonatal jaundice	Female	0-6 days	1-4 years	Global	1	1	Indoor Air Pollution (All Cooking Fuels)	269
Hemolytic disease and other neonatal jaundice	Female	0-6 days	1-4 years	Global	1	1	Smoking Prevalence (Reproductive Age Standardized)	911
Hemolytic disease and other neonatal jaundice	Female	0-6 days	1-4 years	Global	-1	2	Health System Access (capped)	0
Hemolytic disease and other neonatal jaundice	Female	0-6 days	1-4 years	Global	-1	2	Antenatal Care (4 visits) Coverage (proportion)	39
Hemolytic disease and other neonatal jaundice	Female	0-6 days	1-4 years	Global	-1	2	Skilled Birth Attendance (proportion)	68
Hemolytic disease and other neonatal jaundice	Female	0-6 days	1-4 years	Global	-1	2	Healthcare access and quality index	115
Hemolytic disease and other neonatal jaundice	Female	0-6 days	1-4 years	Global	-1	2	In-Facility Delivery (proportion)	741
Hemolytic disease and other neonatal jaundice	Female	0-6 days	1-4 years	Global	1	2	Live Births 35+ (proportion)	--
Hemolytic disease and other neonatal jaundice	Female	0-6 days	1-4 years	Global	-1	3	Education (years per capita)	5
Hemolytic disease and other neonatal jaundice	Female	0-6 days	1-4 years	Global	-1	3	Socio-demographic Index	279
Hemolytic disease and other neonatal jaundice	Female	0-6 days	1-4 years	Global	-1	3	LDI (I$ per capita)	--
Hemolytic disease and other neonatal jaundice	Female	0-6 days	1-4 years	Global	1	3	Total Fertility Rate	--
Hemolytic disease and other neonatal jaundice	Male	0-6 days	1-4 years	Data Rich	1	1	Indoor Air Pollution (All Cooking Fuels)	354
Hemolytic disease and other neonatal jaundice	Male	0-6 days	1-4 years	Data Rich	1	1	Smoking Prevalence (Reproductive Age Standardized)	854
Hemolytic disease and other neonatal jaundice	Male	0-6 days	1-4 years	Data Rich	-1	2	Skilled Birth Attendance (proportion)	5
Hemolytic disease and other neonatal jaundice	Male	0-6 days	1-4 years	Data Rich	-1	2	Antenatal Care (4 visits) Coverage (proportion)	89
Hemolytic disease and other neonatal jaundice	Male	0-6 days	1-4 years	Data Rich	-1	2	In-Facility Delivery (proportion)	156
Hemolytic disease and other neonatal jaundice	Male	0-6 days	1-4 years	Data Rich	-1	2	Healthcare access and quality index	242
Hemolytic disease and other neonatal jaundice	Male	0-6 days	1-4 years	Data Rich	-1	2	Health System Access (capped)	278
Hemolytic disease and other neonatal jaundice	Male	0-6 days	1-4 years	Data Rich	1	2	Age-standardized underweight (weight-for-age) SEV	172
Hemolytic disease and other neonatal jaundice	Male	0-6 days	1-4 years	Data Rich	1	2	Live Births 35+ (proportion)	--
Hemolytic disease and other neonatal jaundice	Male	0-6 days	1-4 years	Data Rich	-1	3	Education (years per capita)	218
Hemolytic disease and other neonatal jaundice	Male	0-6 days	1-4 years	Data Rich	-1	3	Socio-demographic Index	350
Hemolytic disease and other neonatal jaundice	Male	0-6 days	1-4 years	Data Rich	-1	3	LDI (I$ per capita)	--
Hemolytic disease and other neonatal jaundice	Male	0-6 days	1-4 years	Data Rich	1	3	Total Fertility Rate	--
Hemolytic disease and other neonatal jaundice	Male	0-6 days	1-4 years	Global	1	1	Indoor Air Pollution (All Cooking Fuels)	419
Hemolytic disease and other neonatal jaundice	Male	0-6 days	1-4 years	Global	1	1	Smoking Prevalence (Reproductive Age Standardized)	791
Hemolytic disease and other neonatal jaundice	Male	0-6 days	1-4 years	Global	-1	2	In-Facility Delivery (proportion)	82
Hemolytic disease and other neonatal jaundice	Male	0-6 days	1-4 years	Global	-1	2	Healthcare access and quality index	121
Hemolytic disease and other neonatal jaundice	Male	0-6 days	1-4 years	Global	-1	2	Skilled Birth Attendance (proportion)	143
Hemolytic disease and other neonatal jaundice	Male	0-6 days	1-4 years	Global	-1	2	Antenatal Care (4 visits) Coverage (proportion)	241
Hemolytic disease and other neonatal jaundice	Male	0-6 days	1-4 years	Global	-1	2	Health System Access (capped)	412
Hemolytic disease and other neonatal jaundice	Male	0-6 days	1-4 years	Global	1	2	Age-standardized underweight (weight-for-age) SEV	25
Hemolytic disease and other neonatal jaundice	Male	0-6 days	1-4 years	Global	1	2	Live Births 35+ (proportion)	--
Hemolytic disease and other neonatal jaundice	Male	0-6 days	1-4 years	Global	-1	3	Socio-demographic Index	142
Hemolytic disease and other neonatal jaundice	Male	0-6 days	1-4 years	Global	-1	3	Education (years per capita)	194
Hemolytic disease and other neonatal jaundice	Male	0-6 days	1-4 years	Global	-1	3	LDI (I$ per capita)	--
Hemolytic disease and other neonatal jaundice	Male	0-6 days	1-4 years	Global	1	3	Total Fertility Rate	--
Other neonatal disorders	Female	0-6 days	1-4 years	Data Rich	1	1	Indoor Air Pollution (All Cooking Fuels)	484
Other neonatal disorders	Female	0-6 days	1-4 years	Data Rich	1	1	Smoking Prevalence (Reproductive Age Standardized)	679
Other neonatal disorders	Female	0-6 days	1-4 years	Data Rich	-1	2	Skilled Birth Attendance (proportion)	49
Other neonatal disorders	Female	0-6 days	1-4 years	Data Rich	-1	2	In-Facility Delivery (proportion)	50
Other neonatal disorders	Female	0-6 days	1-4 years	Data Rich	-1	2	Health System Access (capped)	127
Other neonatal disorders	Female	0-6 days	1-4 years	Data Rich	-1	2	Antenatal Care (4 visits) Coverage (proportion)	268
Other neonatal disorders	Female	0-6 days	1-4 years	Data Rich	-1	2	Healthcare access and quality index	550
Other neonatal disorders	Female	0-6 days	1-4 years	Data Rich	1	2	Age-standardized underweight (weight-for-age) SEV	1
Other neonatal disorders	Female	0-6 days	1-4 years	Data Rich	1	2	Live Births 35+ (proportion)	186
Other neonatal disorders	Female	0-6 days	1-4 years	Data Rich	-1	3	Socio-demographic Index	67
Other neonatal disorders	Female	0-6 days	1-4 years	Data Rich	-1	3	Education (years per capita)	164
Other neonatal disorders	Female	0-6 days	1-4 years	Data Rich	-1	3	LDI (I$ per capita)	--
Other neonatal disorders	Female	0-6 days	1-4 years	Data Rich	1	3	Total Fertility Rate	--
Other neonatal disorders	Female	0-6 days	1-4 years	Global	1	1	Indoor Air Pollution (All Cooking Fuels)	320
Other neonatal disorders	Female	0-6 days	1-4 years	Global	1	1	Smoking Prevalence (Reproductive Age Standardized)	490
Other neonatal disorders	Female	0-6 days	1-4 years	Global	-1	2	Skilled Birth Attendance (proportion)	165
Other neonatal disorders	Female	0-6 days	1-4 years	Global	-1	2	In-Facility Delivery (proportion)	376
Other neonatal disorders	Female	0-6 days	1-4 years	Global	-1	2	Antenatal Care (4 visits) Coverage (proportion)	464
Other neonatal disorders	Female	0-6 days	1-4 years	Global	-1	2	Health System Access (capped)	680
Other neonatal disorders	Female	0-6 days	1-4 years	Global	-1	2	Healthcare access and quality index	--
Other neonatal disorders	Female	0-6 days	1-4 years	Global	1	2	Live Births 35+ (proportion)	190
Other neonatal disorders	Female	0-6 days	1-4 years	Global	1	2	Age-standardized underweight (weight-for-age) SEV	355
Other neonatal disorders	Female	0-6 days	1-4 years	Global	-1	3	Education (years per capita)	74
Other neonatal disorders	Female	0-6 days	1-4 years	Global	-1	3	Socio-demographic Index	79
Other neonatal disorders	Female	0-6 days	1-4 years	Global	-1	3	LDI (I$ per capita)	--
Other neonatal disorders	Female	0-6 days	1-4 years	Global	1	3	Total Fertility Rate	--
Other neonatal disorders	Male	0-6 days	1-4 years	Data Rich	1	1	Indoor Air Pollution (All Cooking Fuels)	451
Other neonatal disorders	Male	0-6 days	1-4 years	Data Rich	1	1	Smoking Prevalence (Reproductive Age Standardized)	719
Other neonatal disorders	Male	0-6 days	1-4 years	Data Rich	-1	2	Antenatal Care (4 visits) Coverage (proportion)	51
Other neonatal disorders	Male	0-6 days	1-4 years	Data Rich	-1	2	Health System Access (capped)	313
Other neonatal disorders	Male	0-6 days	1-4 years	Data Rich	-1	2	In-Facility Delivery (proportion)	321
Other neonatal disorders	Male	0-6 days	1-4 years	Data Rich	-1	2	Skilled Birth Attendance (proportion)	359

Appendix Table 9: CODEm covariates used, level of covariate, and expected direction of covariate by cause, sex, and age

Covariates that CODEm did not select during the covariate selection process have no draw counts listed.

Cause	Sex	Age Start	Age End	Model Type	Direction	Level	Covariate Name	Number of Draws
Other neonatal disorders	Male	0-6 days	1-4 years	Data Rich	-1	2	Healthcare access and quality index	--
Other neonatal disorders	Male	0-6 days	1-4 years	Data Rich	1	2	Live Births 35+ (proportion)	188
Other neonatal disorders	Male	0-6 days	1-4 years	Data Rich	1	2	Age-standardized underweight (weight-for-age) SEV	225
Other neonatal disorders	Male	0-6 days	1-4 years	Data Rich	-1	3	Education (years per capita)	--
Other neonatal disorders	Male	0-6 days	1-4 years	Data Rich	-1	3	LDI (I$ per capita)	--
Other neonatal disorders	Male	0-6 days	1-4 years	Data Rich	-1	3	Socio-demographic Index	--
Other neonatal disorders	Male	0-6 days	1-4 years	Data Rich	1	3	Total Fertility Rate	--
Other neonatal disorders	Male	0-6 days	1-4 years	Global	1	1	Smoking Prevalence (Reproductive Age Standardized)	234
Other neonatal disorders	Male	0-6 days	1-4 years	Global	1	1	Indoor Air Pollution (All Cooking Fuels)	807
Other neonatal disorders	Male	0-6 days	1-4 years	Global	-1	2	Antenatal Care (4 visits) Coverage (proportion)	173
Other neonatal disorders	Male	0-6 days	1-4 years	Global	-1	2	Skilled Birth Attendance (proportion)	228
Other neonatal disorders	Male	0-6 days	1-4 years	Global	-1	2	In-Facility Delivery (proportion)	230
Other neonatal disorders	Male	0-6 days	1-4 years	Global	-1	2	Health System Access (capped)	346
Other neonatal disorders	Male	0-6 days	1-4 years	Global	-1	2	Healthcare access and quality index	--
Other neonatal disorders	Male	0-6 days	1-4 years	Global	1	2	Age-standardized underweight (weight-for-age) SEV	182
Other neonatal disorders	Male	0-6 days	1-4 years	Global	1	2	Live Births 35+ (proportion)	193
Other neonatal disorders	Male	0-6 days	1-4 years	Global	-1	3	Education (years per capita)	--
Other neonatal disorders	Male	0-6 days	1-4 years	Global	-1	3	LDI (I$ per capita)	--
Other neonatal disorders	Male	0-6 days	1-4 years	Global	-1	3	Socio-demographic Index	--
Other neonatal disorders	Male	0-6 days	1-4 years	Global	1	3	Total Fertility Rate	--
Nutritional deficiencies	Female	28-364 days	95+ years	Data Rich	-1	1	Proportion of households using iodized salt (adjusted)	0
Nutritional deficiencies	Female	28-364 days	95+ years	Data Rich	-1	1	energy unadjusted(kcal)	0
Nutritional deficiencies	Female	28-364 days	95+ years	Data Rich	1	1	Age-Standardize Prevalence of Severe Anemia	0
Nutritional deficiencies	Female	28-364 days	95+ years	Data Rich	1	1	Age-standardized wasting (weight-for-height) SEV	205
Nutritional deficiencies	Female	28-364 days	95+ years	Data Rich	1	1	Underweight, age and sex specific	358
Nutritional deficiencies	Female	28-364 days	95+ years	Data Rich	1	1	Age-standardized underweight (weight-for-age) SEV	795
Nutritional deficiencies	Female	28-364 days	95+ years	Data Rich	-1	2	Health System Access (capped)	0
Nutritional deficiencies	Female	28-364 days	95+ years	Data Rich	-1	2	Healthcare access and quality index	0
Nutritional deficiencies	Female	28-364 days	95+ years	Data Rich	0	2	Rainfall Quintile 2 (proportion)	49
Nutritional deficiencies	Female	28-364 days	95+ years	Data Rich	0	2	Rainfall Quintile 1 (proportion)	74
Nutritional deficiencies	Female	28-364 days	95+ years	Data Rich	1	2	Log-transformed SEV scalar: Diarrhea	0
Nutritional deficiencies	Female	28-364 days	95+ years	Data Rich	1	2	SEV unsafe water	17
Nutritional deficiencies	Female	28-364 days	95+ years	Data Rich	1	2	SEV unsafe sanitation	27
Nutritional deficiencies	Female	28-364 days	95+ years	Data Rich	1	2	Alcohol SEV, age and sex specific	79
Nutritional deficiencies	Female	28-364 days	95+ years	Data Rich	1	2	Mortality Rate Due to War Shocks (per 1 person)	--
Nutritional deficiencies	Female	28-364 days	95+ years	Data Rich	-1	3	Maternal Education (years per capita)	0
Nutritional deficiencies	Female	28-364 days	95+ years	Data Rich	-1	3	Education (years per capita)	167
Nutritional deficiencies	Female	28-364 days	95+ years	Data Rich	-1	3	Socio-demographic Index	269
Nutritional deficiencies	Female	28-364 days	95+ years	Data Rich	-1	3	LDI (I$ per capita)	--
Nutritional deficiencies	Female	28-364 days	95+ years	Global	-1	1	energy unadjusted(kcal)	73
Nutritional deficiencies	Female	28-364 days	95+ years	Global	-1	1	Proportion of households using iodized salt (adjusted)	511
Nutritional deficiencies	Female	28-364 days	95+ years	Global	1	1	Age-standardized underweight (weight-for-age) SEV	15
Nutritional deficiencies	Female	28-364 days	95+ years	Global	1	1	Age-Standardize Prevalence of Severe Anemia	60
Nutritional deficiencies	Female	28-364 days	95+ years	Global	1	1	Age-standardized wasting (weight-for-height) SEV	322
Nutritional deficiencies	Female	28-364 days	95+ years	Global	1	1	Underweight, age and sex specific	949
Nutritional deficiencies	Female	28-364 days	95+ years	Global	-1	2	Health System Access (capped)	0
Nutritional deficiencies	Female	28-364 days	95+ years	Global	-1	2	Healthcare access and quality index	7
Nutritional deficiencies	Female	28-364 days	95+ years	Global	0	2	Rainfall Quintile 1 (proportion)	70
Nutritional deficiencies	Female	28-364 days	95+ years	Global	0	2	Rainfall Quintile 2 (proportion)	399
Nutritional deficiencies	Female	28-364 days	95+ years	Global	1	2	Log-transformed SEV scalar: Diarrhea	6
Nutritional deficiencies	Female	28-364 days	95+ years	Global	1	2	SEV unsafe sanitation	388
Nutritional deficiencies	Female	28-364 days	95+ years	Global	1	2	SEV unsafe water	388
Nutritional deficiencies	Female	28-364 days	95+ years	Global	1	2	Alcohol SEV, age and sex specific	598
Nutritional deficiencies	Female	28-364 days	95+ years	Global	1	2	Mortality Rate Due to War Shocks (per 1 person)	--
Nutritional deficiencies	Female	28-364 days	95+ years	Global	-1	3	Socio-demographic Index	2
Nutritional deficiencies	Female	28-364 days	95+ years	Global	-1	3	Education (years per capita)	158
Nutritional deficiencies	Female	28-364 days	95+ years	Global	-1	3	LDI (I$ per capita)	--
Nutritional deficiencies	Male	28-364 days	95+ years	Data Rich	-1	1	Proportion of households using iodized salt (adjusted)	3
Nutritional deficiencies	Male	28-364 days	95+ years	Data Rich	-1	1	energy unadjusted(kcal)	138
Nutritional deficiencies	Male	28-364 days	95+ years	Data Rich	1	1	Age-standardized wasting (weight-for-height) SEV	197
Nutritional deficiencies	Male	28-364 days	95+ years	Data Rich	1	1	Age-Standardize Prevalence of Severe Anemia	350
Nutritional deficiencies	Male	28-364 days	95+ years	Data Rich	1	1	Age-standardized underweight (weight-for-age) SEV	724
Nutritional deficiencies	Male	28-364 days	95+ years	Data Rich	1	1	Underweight, age and sex specific	920
Nutritional deficiencies	Male	28-364 days	95+ years	Data Rich	-1	2	Healthcare access and quality index	0
Nutritional deficiencies	Male	28-364 days	95+ years	Data Rich	-1	2	Sanitation (proportion with access)	0
Nutritional deficiencies	Male	28-364 days	95+ years	Data Rich	-1	2	Health System Access (capped)	11
Nutritional deficiencies	Male	28-364 days	95+ years	Data Rich	-1	2	Improved Water Source (proportion with access)	12
Nutritional deficiencies	Male	28-364 days	95+ years	Data Rich	0	2	Rainfall Quintile 2 (proportion)	34
Nutritional deficiencies	Male	28-364 days	95+ years	Data Rich	0	2	Rainfall Quintile 1 (proportion)	285
Nutritional deficiencies	Male	28-364 days	95+ years	Data Rich	1	2	Alcohol SEV, age and sex specific	0
Nutritional deficiencies	Male	28-364 days	95+ years	Data Rich	1	2	Mortality Rate Due to War Shocks (per 1 person)	--
Nutritional deficiencies	Male	28-364 days	95+ years	Data Rich	-1	3	Education (years per capita)	0
Nutritional deficiencies	Male	28-364 days	95+ years	Data Rich	-1	3	Socio-demographic Index	4
Nutritional deficiencies	Male	28-364 days	95+ years	Data Rich	-1	3	Maternal Education (years per capita)	27
Nutritional deficiencies	Male	28-364 days	95+ years	Data Rich	-1	3	LDI (I$ per capita)	--
Nutritional deficiencies	Male	28-364 days	95+ years	Global	-1	1	Proportion of households using iodized salt (adjusted)	0
Nutritional deficiencies	Male	28-364 days	95+ years	Global	-1	1	energy unadjusted(kcal)	16
Nutritional deficiencies	Male	28-364 days	95+ years	Global	1	1	Age-standardized wasting (weight-for-height) SEV	105

**Appendix Table 9: CODEm covariates used, level of covariate, and expected direction of covariate by cause, sex, and age** *Covariates that CODEm did not select during the covariate selection process have no draw counts listed.*								
Cause	Sex	Age Start	Age End	Model Type	Direction	Level	Covariate Name	Number of Draws
Nutritional deficiencies	Male	28-364 days	95+ years	Global	1	1	Age-Standardize Prevalence of Severe Anemia	212
Nutritional deficiencies	Male	28-364 days	95+ years	Global	1	1	Underweight, age and sex specific	467
Nutritional deficiencies	Male	28-364 days	95+ years	Global	1	1	Age-standardized underweight (weight-for-age) SEV	828
Nutritional deficiencies	Male	28-364 days	95+ years	Global	-1	2	Health System Access (capped)	0
Nutritional deficiencies	Male	28-364 days	95+ years	Global	-1	2	Healthcare access and quality index	174
Nutritional deficiencies	Male	28-364 days	95+ years	Global	0	2	Rainfall Quintile 1 (proportion)	177
Nutritional deficiencies	Male	28-364 days	95+ years	Global	0	2	Rainfall Quintile 2 (proportion)	215
Nutritional deficiencies	Male	28-364 days	95+ years	Global	1	2	Alcohol SEV, age and sex specific	0
Nutritional deficiencies	Male	28-364 days	95+ years	Global	1	2	Log-transformed SEV scalar: Diarrhea	14
Nutritional deficiencies	Male	28-364 days	95+ years	Global	1	2	SEV unsafe water	101
Nutritional deficiencies	Male	28-364 days	95+ years	Global	1	2	SEV unsafe sanitation	189
Nutritional deficiencies	Male	28-364 days	95+ years	Global	1	2	Mortality Rate Due to War Shocks (per 1 person)	--
Nutritional deficiencies	Male	28-364 days	95+ years	Global	-1	3	Education (years per capita)	3
Nutritional deficiencies	Male	28-364 days	95+ years	Global	-1	3	Socio-demographic Index	114
Nutritional deficiencies	Male	28-364 days	95+ years	Global	-1	3	Maternal Education (years per capita)	147
Nutritional deficiencies	Male	28-364 days	95+ years	Global	-1	3	LDI (I$ per capita)	--
Protein-energy malnutrition	Female	28-364 days	1-4 years	Data Rich	-1	1	energy unadjusted(kcal)	--
Protein-energy malnutrition	Female	28-364 days	1-4 years	Data Rich	1	1	Age-Standardize Prevalence of Severe Anemia	342
Protein-energy malnutrition	Female	28-364 days	1-4 years	Data Rich	1	1	Underweight, age and sex specific	502
Protein-energy malnutrition	Female	28-364 days	1-4 years	Data Rich	1	1	Age- and sex-specific wasting (weight-for-height) SEV	534
Protein-energy malnutrition	Female	28-364 days	1-4 years	Data Rich	1	1	Malnutrition Shock mortality rate	--
Protein-energy malnutrition	Female	28-364 days	1-4 years	Data Rich	-1	2	Health System Access (capped)	0
Protein-energy malnutrition	Female	28-364 days	1-4 years	Data Rich	-1	2	Healthcare access and quality index	115
Protein-energy malnutrition	Female	28-364 days	1-4 years	Data Rich	0	2	Rainfall Quintile 1 (proportion)	0
Protein-energy malnutrition	Female	28-364 days	1-4 years	Data Rich	0	2	Rainfall Quintile 2 (proportion)	365
Protein-energy malnutrition	Female	28-364 days	1-4 years	Data Rich	1	2	Log-transformed SEV scalar: Diarrhea	115
Protein-energy malnutrition	Female	28-364 days	1-4 years	Data Rich	1	2	SEV unsafe sanitation	115
Protein-energy malnutrition	Female	28-364 days	1-4 years	Data Rich	1	2	SEV unsafe water	115
Protein-energy malnutrition	Female	28-364 days	1-4 years	Data Rich	1	2	Alcohol SEV, age and sex specific	--
Protein-energy malnutrition	Female	28-364 days	1-4 years	Data Rich	1	2	Mortality Rate Due to War Shocks (per 1 person)	--
Protein-energy malnutrition	Female	28-364 days	1-4 years	Data Rich	-1	3	Antenatal Care (4 visits) Coverage (proportion)	76
Protein-energy malnutrition	Female	28-364 days	1-4 years	Data Rich	-1	3	Socio-demographic Index	179
Protein-energy malnutrition	Female	28-364 days	1-4 years	Data Rich	-1	3	Education (years per capita)	212
Protein-energy malnutrition	Female	28-364 days	1-4 years	Data Rich	-1	3	LDI (I$ per capita)	--
Protein-energy malnutrition	Female	28-364 days	1-4 years	Global	-1	1	energy unadjusted(kcal)	--
Protein-energy malnutrition	Female	28-364 days	1-4 years	Global	1	1	Age-Standardize Prevalence of Severe Anemia	320
Protein-energy malnutrition	Female	28-364 days	1-4 years	Global	1	1	Age- and sex-specific wasting (weight-for-height) SEV	482
Protein-energy malnutrition	Female	28-364 days	1-4 years	Global	1	1	Underweight, age and sex specific	576
Protein-energy malnutrition	Female	28-364 days	1-4 years	Global	1	1	Malnutrition Shock mortality rate	--
Protein-energy malnutrition	Female	28-364 days	1-4 years	Global	-1	2	Health System Access (capped)	0
Protein-energy malnutrition	Female	28-364 days	1-4 years	Global	-1	2	Healthcare access and quality index	0
Protein-energy malnutrition	Female	28-364 days	1-4 years	Global	0	2	Rainfall Quintile 1 (proportion)	178
Protein-energy malnutrition	Female	28-364 days	1-4 years	Global	0	2	Rainfall Quintile 2 (proportion)	179
Protein-energy malnutrition	Female	28-364 days	1-4 years	Global	1	2	Log-transformed SEV scalar: Diarrhea	0
Protein-energy malnutrition	Female	28-364 days	1-4 years	Global	1	2	SEV unsafe water	34
Protein-energy malnutrition	Female	28-364 days	1-4 years	Global	1	2	SEV unsafe sanitation	125
Protein-energy malnutrition	Female	28-364 days	1-4 years	Global	1	2	Alcohol SEV, age and sex specific	--
Protein-energy malnutrition	Female	28-364 days	1-4 years	Global	1	2	Mortality Rate Due to War Shocks (per 1 person)	--
Protein-energy malnutrition	Female	28-364 days	1-4 years	Global	-1	3	Education (years per capita)	0
Protein-energy malnutrition	Female	28-364 days	1-4 years	Global	-1	3	Socio-demographic Index	0
Protein-energy malnutrition	Female	28-364 days	1-4 years	Global	-1	3	Antenatal Care (4 visits) Coverage (proportion)	504
Protein-energy malnutrition	Female	28-364 days	1-4 years	Global	-1	3	LDI (I$ per capita)	--
Protein-energy malnutrition	Female	5-9 years	95+ years	Data Rich	-1	1	energy unadjusted(kcal)	--
Protein-energy malnutrition	Female	5-9 years	95+ years	Data Rich	1	1	Age-Standardize Prevalence of Severe Anemia	--
Protein-energy malnutrition	Female	5-9 years	95+ years	Data Rich	1	1	Age-standardized wasting (weight-for-height) SEV	--
Protein-energy malnutrition	Female	5-9 years	95+ years	Data Rich	1	1	Malnutrition Shock mortality rate	--
Protein-energy malnutrition	Female	5-9 years	95+ years	Data Rich	1	1	Underweight, age and sex specific	--
Protein-energy malnutrition	Female	5-9 years	95+ years	Data Rich	-1	2	Health System Access (capped)	--
Protein-energy malnutrition	Female	5-9 years	95+ years	Data Rich	-1	2	Healthcare access and quality index	--
Protein-energy malnutrition	Female	5-9 years	95+ years	Data Rich	0	2	Rainfall Quintile 2 (proportion)	1000
Protein-energy malnutrition	Female	5-9 years	95+ years	Data Rich	0	2	Rainfall Quintile 1 (proportion)	--
Protein-energy malnutrition	Female	5-9 years	95+ years	Data Rich	1	2	Alcohol SEV, age and sex specific	--
Protein-energy malnutrition	Female	5-9 years	95+ years	Data Rich	1	2	Log-transformed SEV scalar: Diarrhea	--
Protein-energy malnutrition	Female	5-9 years	95+ years	Data Rich	1	2	Mortality Rate Due to War Shocks (per 1 person)	--
Protein-energy malnutrition	Female	5-9 years	95+ years	Data Rich	1	2	SEV unsafe sanitation	--
Protein-energy malnutrition	Female	5-9 years	95+ years	Data Rich	1	2	SEV unsafe water	--
Protein-energy malnutrition	Female	5-9 years	95+ years	Data Rich	-1	3	Antenatal Care (4 visits) Coverage (proportion)	--
Protein-energy malnutrition	Female	5-9 years	95+ years	Data Rich	-1	3	Education (years per capita)	--
Protein-energy malnutrition	Female	5-9 years	95+ years	Data Rich	-1	3	LDI (I$ per capita)	--
Protein-energy malnutrition	Female	5-9 years	95+ years	Data Rich	-1	3	Socio-demographic Index	--
Protein-energy malnutrition	Female	5-9 years	95+ years	Global	-1	1	energy unadjusted(kcal)	--
Protein-energy malnutrition	Female	5-9 years	95+ years	Global	1	1	Age-Standardize Prevalence of Severe Anemia	197
Protein-energy malnutrition	Female	5-9 years	95+ years	Global	1	1	Age-standardized wasting (weight-for-height) SEV	628
Protein-energy malnutrition	Female	5-9 years	95+ years	Global	1	1	Underweight, age and sex specific	783
Protein-energy malnutrition	Female	5-9 years	95+ years	Global	1	1	Malnutrition Shock mortality rate	--
Protein-energy malnutrition	Female	5-9 years	95+ years	Global	-1	2	Health System Access (capped)	--
Protein-energy malnutrition	Female	5-9 years	95+ years	Global	-1	2	Healthcare access and quality index	--
Protein-energy malnutrition	Female	5-9 years	95+ years	Global	0	2	Rainfall Quintile 1 (proportion)	255

Appendix Table 9: CODEm covariates used, level of covariate, and expected direction of covariate by cause, sex, and age

Covariates that CODEm did not select during the covariate selection process have no draw counts listed.

Cause	Sex	Age Start	Age End	Model Type	Direction	Level	Covariate Name	Number of Draws
Protein-energy malnutrition	Female	5-9 years	95+ years	Global	0	2	Rainfall Quintile 2 (proportion)	597
Protein-energy malnutrition	Female	5-9 years	95+ years	Global	1	2	Alcohol SEV, age and sex specific	7
Protein-energy malnutrition	Female	5-9 years	95+ years	Global	1	2	Log-transformed SEV scalar: Diarrhea	62
Protein-energy malnutrition	Female	5-9 years	95+ years	Global	1	2	SEV unsafe sanitation	84
Protein-energy malnutrition	Female	5-9 years	95+ years	Global	1	2	SEV unsafe water	279
Protein-energy malnutrition	Female	5-9 years	95+ years	Global	1	2	Mortality Rate Due to War Shocks (per 1 person)	--
Protein-energy malnutrition	Female	5-9 years	95+ years	Global	-1	3	Socio-demographic Index	2
Protein-energy malnutrition	Female	5-9 years	95+ years	Global	-1	3	Education (years per capita)	5
Protein-energy malnutrition	Female	5-9 years	95+ years	Global	-1	3	Antenatal Care (4 visits) Coverage (proportion)	426
Protein-energy malnutrition	Female	5-9 years	95+ years	Global	-1	3	LDI (I$ per capita)	--
Protein-energy malnutrition	Male	28-364 days	1-4 years	Data Rich	-1	1	energy unadjusted(kcal)	--
Protein-energy malnutrition	Male	28-364 days	1-4 years	Data Rich	1	1	Underweight, age and sex specific	337
Protein-energy malnutrition	Male	28-364 days	1-4 years	Data Rich	1	1	Age-Standardize Prevalence of Severe Anemia	388
Protein-energy malnutrition	Male	28-364 days	1-4 years	Data Rich	1	1	Age- and sex-specific wasting (weight-for-height) SEV	672
Protein-energy malnutrition	Male	28-364 days	1-4 years	Data Rich	1	1	Malnutrition Shock mortality rate	--
Protein-energy malnutrition	Male	28-364 days	1-4 years	Data Rich	-1	2	Healthcare access and quality index	9
Protein-energy malnutrition	Male	28-364 days	1-4 years	Data Rich	-1	2	Health System Access (capped)	11
Protein-energy malnutrition	Male	28-364 days	1-4 years	Data Rich	0	2	Rainfall Quintile 1 (proportion)	294
Protein-energy malnutrition	Male	28-364 days	1-4 years	Data Rich	0	2	Rainfall Quintile 2 (proportion)	359
Protein-energy malnutrition	Male	28-364 days	1-4 years	Data Rich	1	2	Log-transformed SEV scalar: Diarrhea	11
Protein-energy malnutrition	Male	28-364 days	1-4 years	Data Rich	1	2	SEV unsafe water	11
Protein-energy malnutrition	Male	28-364 days	1-4 years	Data Rich	1	2	SEV unsafe sanitation	52
Protein-energy malnutrition	Male	28-364 days	1-4 years	Data Rich	1	2	Alcohol SEV, age and sex specific	--
Protein-energy malnutrition	Male	28-364 days	1-4 years	Data Rich	1	2	Mortality Rate Due to War Shocks (per 1 person)	--
Protein-energy malnutrition	Male	28-364 days	1-4 years	Data Rich	-1	3	Education (years per capita)	17
Protein-energy malnutrition	Male	28-364 days	1-4 years	Data Rich	-1	3	Socio-demographic Index	18
Protein-energy malnutrition	Male	28-364 days	1-4 years	Data Rich	-1	3	LDI (I$ per capita)	--
Protein-energy malnutrition	Male	28-364 days	1-4 years	Global	-1	1	energy unadjusted(kcal)	--
Protein-energy malnutrition	Male	28-364 days	1-4 years	Global	1	1	Age-Standardize Prevalence of Severe Anemia	190
Protein-energy malnutrition	Male	28-364 days	1-4 years	Global	1	1	Age- and sex-specific wasting (weight-for-height) SEV	641
Protein-energy malnutrition	Male	28-364 days	1-4 years	Global	1	1	Underweight, age and sex specific	759
Protein-energy malnutrition	Male	28-364 days	1-4 years	Global	1	1	Malnutrition Shock mortality rate	--
Protein-energy malnutrition	Male	28-364 days	1-4 years	Global	-1	2	Health System Access (capped)	0
Protein-energy malnutrition	Male	28-364 days	1-4 years	Global	-1	2	Healthcare access and quality index	0
Protein-energy malnutrition	Male	28-364 days	1-4 years	Global	0	2	Rainfall Quintile 1 (proportion)	755
Protein-energy malnutrition	Male	28-364 days	1-4 years	Global	0	2	Rainfall Quintile 2 (proportion)	803
Protein-energy malnutrition	Male	28-364 days	1-4 years	Global	1	2	Log-transformed SEV scalar: Diarrhea	0
Protein-energy malnutrition	Male	28-364 days	1-4 years	Global	1	2	SEV unsafe water	15
Protein-energy malnutrition	Male	28-364 days	1-4 years	Global	1	2	SEV unsafe sanitation	716
Protein-energy malnutrition	Male	28-364 days	1-4 years	Global	1	2	Alcohol SEV, age and sex specific	--
Protein-energy malnutrition	Male	28-364 days	1-4 years	Global	1	2	Mortality Rate Due to War Shocks (per 1 person)	--
Protein-energy malnutrition	Male	28-364 days	1-4 years	Global	-1	3	Education (years per capita)	0
Protein-energy malnutrition	Male	28-364 days	1-4 years	Global	-1	3	Socio-demographic Index	0
Protein-energy malnutrition	Male	28-364 days	1-4 years	Global	-1	3	LDI (I$ per capita)	--
Protein-energy malnutrition	Male	5-9 years	95+ years	Data Rich	-1	1	energy unadjusted(kcal)	--
Protein-energy malnutrition	Male	5-9 years	95+ years	Data Rich	1	1	Age-Standardize Prevalence of Severe Anemia	254
Protein-energy malnutrition	Male	5-9 years	95+ years	Data Rich	1	1	Underweight, age and sex specific	254
Protein-energy malnutrition	Male	5-9 years	95+ years	Data Rich	1	1	Malnutrition Shock mortality rate	--
Protein-energy malnutrition	Male	5-9 years	95+ years	Data Rich	-1	2	Health System Access (capped)	--
Protein-energy malnutrition	Male	5-9 years	95+ years	Data Rich	-1	2	Healthcare access and quality index	--
Protein-energy malnutrition	Male	5-9 years	95+ years	Data Rich	0	2	Rainfall Quintile 2 (proportion)	294
Protein-energy malnutrition	Male	5-9 years	95+ years	Data Rich	0	2	Rainfall Quintile 1 (proportion)	--
Protein-energy malnutrition	Male	5-9 years	95+ years	Data Rich	1	2	SEV unsafe sanitation	185
Protein-energy malnutrition	Male	5-9 years	95+ years	Data Rich	1	2	Alcohol SEV, age and sex specific	--
Protein-energy malnutrition	Male	5-9 years	95+ years	Data Rich	1	2	Log-transformed SEV scalar: Diarrhea	--
Protein-energy malnutrition	Male	5-9 years	95+ years	Data Rich	1	2	Mortality Rate Due to War Shocks (per 1 person)	--
Protein-energy malnutrition	Male	5-9 years	95+ years	Data Rich	1	2	SEV unsafe water	--
Protein-energy malnutrition	Male	5-9 years	95+ years	Data Rich	-1	3	Education (years per capita)	--
Protein-energy malnutrition	Male	5-9 years	95+ years	Data Rich	-1	3	LDI (I$ per capita)	--
Protein-energy malnutrition	Male	5-9 years	95+ years	Data Rich	-1	3	Socio-demographic Index	--
Protein-energy malnutrition	Male	5-9 years	95+ years	Global	-1	1	energy unadjusted(kcal)	--
Protein-energy malnutrition	Male	5-9 years	95+ years	Global	1	1	Age-Standardize Prevalence of Severe Anemia	25
Protein-energy malnutrition	Male	5-9 years	95+ years	Global	1	1	Underweight, age and sex specific	535
Protein-energy malnutrition	Male	5-9 years	95+ years	Global	1	1	Malnutrition Shock mortality rate	--
Protein-energy malnutrition	Male	5-9 years	95+ years	Global	-1	2	Health System Access (capped)	--
Protein-energy malnutrition	Male	5-9 years	95+ years	Global	-1	2	Healthcare access and quality index	--
Protein-energy malnutrition	Male	5-9 years	95+ years	Global	0	2	Rainfall Quintile 2 (proportion)	5
Protein-energy malnutrition	Male	5-9 years	95+ years	Global	0	2	Rainfall Quintile 1 (proportion)	13
Protein-energy malnutrition	Male	5-9 years	95+ years	Global	1	2	SEV unsafe sanitation	56
Protein-energy malnutrition	Male	5-9 years	95+ years	Global	1	2	Log-transformed SEV scalar: Diarrhea	185
Protein-energy malnutrition	Male	5-9 years	95+ years	Global	1	2	SEV unsafe water	410
Protein-energy malnutrition	Male	5-9 years	95+ years	Global	1	2	Alcohol SEV, age and sex specific	--
Protein-energy malnutrition	Male	5-9 years	95+ years	Global	1	2	Mortality Rate Due to War Shocks (per 1 person)	--
Protein-energy malnutrition	Male	5-9 years	95+ years	Global	-1	3	Education (years per capita)	10
Protein-energy malnutrition	Male	5-9 years	95+ years	Global	-1	3	Socio-demographic Index	94
Protein-energy malnutrition	Male	5-9 years	95+ years	Global	-1	3	Maternal Education (years per capita)	155
Protein-energy malnutrition	Male	5-9 years	95+ years	Global	-1	3	LDI (I$ per capita)	--
Other nutritional deficiencies	Female	28-364 days	95+ years	Data Rich	1	1	Age-Standardize Prevalence of Severe Anemia	191

Appendix Table 9: CODEm covariates used, level of covariate, and expected direction of covariate by cause, sex, and age								
Covariates that CODEm did not select during the covariate selection process have no draw counts listed.								
**Cause**	**Sex**	**Age Start**	**Age End**	**Model Type**	**Direction**	**Level**	**Covariate Name**	**Number of Draws**
Other nutritional deficiencies	Female	28-364 days	95+ years	Data Rich	1	1	Age-standardized underweight (weight-for-age) SEV	224
Other nutritional deficiencies	Female	28-364 days	95+ years	Data Rich	1	1	Underweight, age and sex specific	879
Other nutritional deficiencies	Female	28-364 days	95+ years	Data Rich	1	1	Malnutrition Shock mortality rate	--
Other nutritional deficiencies	Female	28-364 days	95+ years	Data Rich	-1	2	energy unadjusted(kcal)	22
Other nutritional deficiencies	Female	28-364 days	95+ years	Data Rich	-1	2	Healthcare access and quality index	459
Other nutritional deficiencies	Female	28-364 days	95+ years	Data Rich	-1	2	Health System Access (capped)	486
Other nutritional deficiencies	Female	28-364 days	95+ years	Data Rich	0	2	Rainfall Quintile 2 (proportion)	92
Other nutritional deficiencies	Female	28-364 days	95+ years	Data Rich	0	2	Rainfall Quintile 1 (proportion)	496
Other nutritional deficiencies	Female	28-364 days	95+ years	Data Rich	1	2	SEV unsafe sanitation	13
Other nutritional deficiencies	Female	28-364 days	95+ years	Data Rich	1	2	Log-transformed SEV scalar: Diarrhea	27
Other nutritional deficiencies	Female	28-364 days	95+ years	Data Rich	1	2	SEV unsafe water	28
Other nutritional deficiencies	Female	28-364 days	95+ years	Data Rich	1	2	Alcohol SEV, age and sex specific	--
Other nutritional deficiencies	Female	28-364 days	95+ years	Data Rich	1	2	Mortality Rate Due to War Shocks (per 1 person)	--
Other nutritional deficiencies	Female	28-364 days	95+ years	Data Rich	-1	3	Socio-demographic Index	453
Other nutritional deficiencies	Female	28-364 days	95+ years	Data Rich	-1	3	Education (years per capita)	522
Other nutritional deficiencies	Female	28-364 days	95+ years	Data Rich	-1	3	LDI (I$ per capita)	--
Other nutritional deficiencies	Female	28-364 days	95+ years	Global	1	1	Age-standardized underweight (weight-for-age) SEV	151
Other nutritional deficiencies	Female	28-364 days	95+ years	Global	1	1	Age-Standardize Prevalence of Severe Anemia	323
Other nutritional deficiencies	Female	28-364 days	95+ years	Global	1	1	Underweight, age and sex specific	744
Other nutritional deficiencies	Female	28-364 days	95+ years	Global	1	1	Malnutrition Shock mortality rate	--
Other nutritional deficiencies	Female	28-364 days	95+ years	Global	-1	2	energy unadjusted(kcal)	32
Other nutritional deficiencies	Female	28-364 days	95+ years	Global	-1	2	Healthcare access and quality index	535
Other nutritional deficiencies	Female	28-364 days	95+ years	Global	-1	2	Health System Access (capped)	547
Other nutritional deficiencies	Female	28-364 days	95+ years	Global	0	2	Rainfall Quintile 1 (proportion)	86
Other nutritional deficiencies	Female	28-364 days	95+ years	Global	0	2	Rainfall Quintile 2 (proportion)	582
Other nutritional deficiencies	Female	28-364 days	95+ years	Global	1	2	SEV unsafe sanitation	4
Other nutritional deficiencies	Female	28-364 days	95+ years	Global	1	2	SEV unsafe water	40
Other nutritional deficiencies	Female	28-364 days	95+ years	Global	1	2	Log-transformed SEV scalar: Diarrhea	85
Other nutritional deficiencies	Female	28-364 days	95+ years	Global	1	2	Alcohol SEV, age and sex specific	--
Other nutritional deficiencies	Female	28-364 days	95+ years	Global	1	2	Mortality Rate Due to War Shocks (per 1 person)	--
Other nutritional deficiencies	Female	28-364 days	95+ years	Global	-1	3	Socio-demographic Index	170
Other nutritional deficiencies	Female	28-364 days	95+ years	Global	-1	3	Education (years per capita)	471
Other nutritional deficiencies	Female	28-364 days	95+ years	Global	-1	3	LDI (I$ per capita)	--
Other nutritional deficiencies	Male	28-364 days	95+ years	Data Rich	1	1	Age-standardized underweight (weight-for-age) SEV	104
Other nutritional deficiencies	Male	28-364 days	95+ years	Data Rich	1	1	Age-Standardize Prevalence of Severe Anemia	191
Other nutritional deficiencies	Male	28-364 days	95+ years	Data Rich	1	1	Underweight, age and sex specific	899
Other nutritional deficiencies	Male	28-364 days	95+ years	Data Rich	1	1	Malnutrition Shock mortality rate	--
Other nutritional deficiencies	Male	28-364 days	95+ years	Data Rich	-1	2	Healthcare access and quality index	7
Other nutritional deficiencies	Male	28-364 days	95+ years	Data Rich	-1	2	Health System Access (capped)	245
Other nutritional deficiencies	Male	28-364 days	95+ years	Data Rich	-1	2	energy unadjusted(kcal)	299
Other nutritional deficiencies	Male	28-364 days	95+ years	Data Rich	0	2	Rainfall Quintile 2 (proportion)	411
Other nutritional deficiencies	Male	28-364 days	95+ years	Data Rich	0	2	Rainfall Quintile 1 (proportion)	500
Other nutritional deficiencies	Male	28-364 days	95+ years	Data Rich	1	2	SEV unsafe sanitation	22
Other nutritional deficiencies	Male	28-364 days	95+ years	Data Rich	1	2	SEV unsafe water	324
Other nutritional deficiencies	Male	28-364 days	95+ years	Data Rich	1	2	Log-transformed SEV scalar: Diarrhea	356
Other nutritional deficiencies	Male	28-364 days	95+ years	Data Rich	1	2	Alcohol SEV, age and sex specific	--
Other nutritional deficiencies	Male	28-364 days	95+ years	Data Rich	1	2	Mortality Rate Due to War Shocks (per 1 person)	--
Other nutritional deficiencies	Male	28-364 days	95+ years	Data Rich	-1	3	Socio-demographic Index	249
Other nutritional deficiencies	Male	28-364 days	95+ years	Data Rich	-1	3	Education (years per capita)	--
Other nutritional deficiencies	Male	28-364 days	95+ years	Data Rich	-1	3	LDI (I$ per capita)	--
Other nutritional deficiencies	Male	28-364 days	95+ years	Global	1	1	Age-Standardize Prevalence of Severe Anemia	412
Other nutritional deficiencies	Male	28-364 days	95+ years	Global	1	1	Age-standardized underweight (weight-for-age) SEV	935
Other nutritional deficiencies	Male	28-364 days	95+ years	Global	1	1	Underweight, age and sex specific	949
Other nutritional deficiencies	Male	28-364 days	95+ years	Global	1	1	Malnutrition Shock mortality rate	--
Other nutritional deficiencies	Male	28-364 days	95+ years	Global	-1	2	Healthcare access and quality index	15
Other nutritional deficiencies	Male	28-364 days	95+ years	Global	-1	2	energy unadjusted(kcal)	170
Other nutritional deficiencies	Male	28-364 days	95+ years	Global	-1	2	Health System Access (capped)	237
Other nutritional deficiencies	Male	28-364 days	95+ years	Global	0	2	Rainfall Quintile 1 (proportion)	120
Other nutritional deficiencies	Male	28-364 days	95+ years	Global	0	2	Rainfall Quintile 2 (proportion)	383
Other nutritional deficiencies	Male	28-364 days	95+ years	Global	1	2	SEV unsafe sanitation	1
Other nutritional deficiencies	Male	28-364 days	95+ years	Global	1	2	SEV unsafe water	24
Other nutritional deficiencies	Male	28-364 days	95+ years	Global	1	2	Log-transformed SEV scalar: Diarrhea	150
Other nutritional deficiencies	Male	28-364 days	95+ years	Global	1	2	Alcohol SEV, age and sex specific	--
Other nutritional deficiencies	Male	28-364 days	95+ years	Global	1	2	Mortality Rate Due to War Shocks (per 1 person)	--
Other nutritional deficiencies	Male	28-364 days	95+ years	Global	-1	3	Education (years per capita)	202
Other nutritional deficiencies	Male	28-364 days	95+ years	Global	-1	3	LDI (I$ per capita)	--
Other nutritional deficiencies	Male	28-364 days	95+ years	Global	-1	3	Socio-demographic Index	--
Lip and oral cavity cancer	Female	15-19 years	95+ years	Data Rich	-1	1	vegetables adjusted(g)	434
Lip and oral cavity cancer	Female	15-19 years	95+ years	Data Rich	1	1	Cumulative Cigarettes (10 Years)	0
Lip and oral cavity cancer	Female	15-19 years	95+ years	Data Rich	1	1	Cumulative Cigarettes (5 Years)	0
Lip and oral cavity cancer	Female	15-19 years	95+ years	Data Rich	1	1	Cumulative Cigarettes (15 Years)	16
Lip and oral cavity cancer	Female	15-19 years	95+ years	Data Rich	1	1	Smoking Prevalence	35
Lip and oral cavity cancer	Female	15-19 years	95+ years	Data Rich	1	1	Log-transformed SEV scalar: Mouth C	121
Lip and oral cavity cancer	Female	15-19 years	95+ years	Data Rich	1	1	Alcohol (liters per capita)	201
Lip and oral cavity cancer	Female	15-19 years	95+ years	Data Rich	1	1	Cumulative Cigarettes (20 Years)	278
Lip and oral cavity cancer	Female	15-19 years	95+ years	Data Rich	1	1	Tobacco (cigarettes per capita)	--
Lip and oral cavity cancer	Female	15-19 years	95+ years	Data Rich	-1	2	Healthcare access and quality index	22
Lip and oral cavity cancer	Female	15-19 years	95+ years	Data Rich	-1	2	Health System Access 2 (unitless)	366

**Appendix Table 9: CODEm covariates used, level of covariate, and expected direction of covariate by cause, sex, and age** *Covariates that CODEm did not select during the covariate selection process have no draw counts listed.*								
**Cause**	**Sex**	**Age Start**	**Age End**	**Model Type**	**Direction**	**Level**	**Covariate Name**	**Number of Draws**
Lip and oral cavity cancer	Female	15-19 years	95+ years	Data Rich	-1	2	fruits adjusted(g)	--
Lip and oral cavity cancer	Female	15-19 years	95+ years	Data Rich	1	2	red meats adjusted(g)	479
Lip and oral cavity cancer	Female	15-19 years	95+ years	Data Rich	-1	3	Education (years per capita)	--
Lip and oral cavity cancer	Female	15-19 years	95+ years	Data Rich	0	3	Socio-demographic Index	810
Lip and oral cavity cancer	Female	15-19 years	95+ years	Data Rich	0	3	LDI (I$ per capita)	--
Lip and oral cavity cancer	Female	15-19 years	95+ years	Global	-1	1	vegetables adjusted(g)	1000
Lip and oral cavity cancer	Female	15-19 years	95+ years	Global	1	1	Cumulative Cigarettes (5 Years)	0
Lip and oral cavity cancer	Female	15-19 years	95+ years	Global	1	1	Tobacco (cigarettes per capita)	0
Lip and oral cavity cancer	Female	15-19 years	95+ years	Global	1	1	Cumulative Cigarettes (15 Years)	26
Lip and oral cavity cancer	Female	15-19 years	95+ years	Global	1	1	Log-transformed SEV scalar: Mouth C	74
Lip and oral cavity cancer	Female	15-19 years	95+ years	Global	1	1	Cumulative Cigarettes (10 Years)	102
Lip and oral cavity cancer	Female	15-19 years	95+ years	Global	1	1	Cumulative Cigarettes (20 Years)	304
Lip and oral cavity cancer	Female	15-19 years	95+ years	Global	1	1	Smoking Prevalence	566
Lip and oral cavity cancer	Female	15-19 years	95+ years	Global	1	1	Alcohol (liters per capita)	849
Lip and oral cavity cancer	Female	15-19 years	95+ years	Global	-1	2	fruits adjusted(g)	0
Lip and oral cavity cancer	Female	15-19 years	95+ years	Global	-1	2	Healthcare access and quality index	298
Lip and oral cavity cancer	Female	15-19 years	95+ years	Global	1	2	red meats adjusted(g)	99
Lip and oral cavity cancer	Female	15-19 years	95+ years	Global	-1	3	Education (years per capita)	0
Lip and oral cavity cancer	Female	15-19 years	95+ years	Global	0	3	Socio-demographic Index	684
Lip and oral cavity cancer	Female	15-19 years	95+ years	Global	0	3	LDI (I$ per capita)	--
Lip and oral cavity cancer	Male	15-19 years	95+ years	Data Rich	-1	1	vegetables adjusted(g)	60
Lip and oral cavity cancer	Male	15-19 years	95+ years	Data Rich	1	1	Tobacco (cigarettes per capita)	0
Lip and oral cavity cancer	Male	15-19 years	95+ years	Data Rich	1	1	Cumulative Cigarettes (10 Years)	6
Lip and oral cavity cancer	Male	15-19 years	95+ years	Data Rich	1	1	Cumulative Cigarettes (5 Years)	12
Lip and oral cavity cancer	Male	15-19 years	95+ years	Data Rich	1	1	Smoking Prevalence	13
Lip and oral cavity cancer	Male	15-19 years	95+ years	Data Rich	1	1	Cumulative Cigarettes (15 Years)	16
Lip and oral cavity cancer	Male	15-19 years	95+ years	Data Rich	1	1	Cumulative Cigarettes (20 Years)	30
Lip and oral cavity cancer	Male	15-19 years	95+ years	Data Rich	1	1	Alcohol (liters per capita)	458
Lip and oral cavity cancer	Male	15-19 years	95+ years	Data Rich	1	1	Log-transformed SEV scalar: Mouth C	745
Lip and oral cavity cancer	Male	15-19 years	95+ years	Data Rich	-1	2	Healthcare access and quality index	0
Lip and oral cavity cancer	Male	15-19 years	95+ years	Data Rich	-1	2	fruits adjusted(g)	--
Lip and oral cavity cancer	Male	15-19 years	95+ years	Data Rich	1	2	red meats adjusted(g)	604
Lip and oral cavity cancer	Male	15-19 years	95+ years	Data Rich	-1	3	Education (years per capita)	7
Lip and oral cavity cancer	Male	15-19 years	95+ years	Data Rich	0	3	Socio-demographic Index	623
Lip and oral cavity cancer	Male	15-19 years	95+ years	Data Rich	0	3	LDI (I$ per capita)	--
Lip and oral cavity cancer	Male	15-19 years	95+ years	Global	-1	1	vegetables adjusted(g)	293
Lip and oral cavity cancer	Male	15-19 years	95+ years	Global	1	1	Cumulative Cigarettes (5 Years)	0
Lip and oral cavity cancer	Male	15-19 years	95+ years	Global	1	1	Cumulative Cigarettes (10 Years)	1
Lip and oral cavity cancer	Male	15-19 years	95+ years	Global	1	1	Smoking Prevalence	4
Lip and oral cavity cancer	Male	15-19 years	95+ years	Global	1	1	Cumulative Cigarettes (15 Years)	41
Lip and oral cavity cancer	Male	15-19 years	95+ years	Global	1	1	Cumulative Cigarettes (20 Years)	233
Lip and oral cavity cancer	Male	15-19 years	95+ years	Global	1	1	Tobacco (cigarettes per capita)	254
Lip and oral cavity cancer	Male	15-19 years	95+ years	Global	1	1	Log-transformed SEV scalar: Mouth C	495
Lip and oral cavity cancer	Male	15-19 years	95+ years	Global	1	1	Alcohol (liters per capita)	568
Lip and oral cavity cancer	Male	15-19 years	95+ years	Global	-1	2	Healthcare access and quality index	329
Lip and oral cavity cancer	Male	15-19 years	95+ years	Global	-1	2	fruits adjusted(g)	--
Lip and oral cavity cancer	Male	15-19 years	95+ years	Global	1	2	red meats adjusted(g)	659
Lip and oral cavity cancer	Male	15-19 years	95+ years	Global	-1	3	Education (years per capita)	0
Lip and oral cavity cancer	Male	15-19 years	95+ years	Global	0	3	Socio-demographic Index	572
Lip and oral cavity cancer	Male	15-19 years	95+ years	Global	0	3	LDI (I$ per capita)	--
Nasopharynx cancer	Female	5-9 years	95+ years	Data Rich	1	1	Tobacco (cigarettes per capita)	0
Nasopharynx cancer	Female	5-9 years	95+ years	Data Rich	1	1	Cumulative Cigarettes (10 Years)	47
Nasopharynx cancer	Female	5-9 years	95+ years	Data Rich	1	1	Cumulative Cigarettes (15 Years)	163
Nasopharynx cancer	Female	5-9 years	95+ years	Data Rich	1	1	Log-transformed SEV scalar: Nasoph C	167
Nasopharynx cancer	Female	5-9 years	95+ years	Data Rich	1	1	Smoking Prevalence	286
Nasopharynx cancer	Female	5-9 years	95+ years	Data Rich	1	1	Cumulative Cigarettes (5 Years)	295
Nasopharynx cancer	Female	5-9 years	95+ years	Data Rich	1	1	Cumulative Cigarettes (20 Years)	738
Nasopharynx cancer	Female	5-9 years	95+ years	Data Rich	1	1	Alcohol (liters per capita)	--
Nasopharynx cancer	Female	5-9 years	95+ years	Data Rich	-1	2	Healthcare access and quality index	0
Nasopharynx cancer	Female	5-9 years	95+ years	Data Rich	-1	2	fruits adjusted(g)	0
Nasopharynx cancer	Female	5-9 years	95+ years	Data Rich	-1	2	vegetables adjusted(g)	0
Nasopharynx cancer	Female	5-9 years	95+ years	Data Rich	1	2	Population Density (over 1000 ppl/sqkm, proportion)	1
Nasopharynx cancer	Female	5-9 years	95+ years	Data Rich	1	2	Population Density (under 150 ppl/sqkm, proportion)	--
Nasopharynx cancer	Female	5-9 years	95+ years	Data Rich	-1	3	Education (years per capita)	0
Nasopharynx cancer	Female	5-9 years	95+ years	Data Rich	0	3	Socio-demographic Index	608
Nasopharynx cancer	Female	5-9 years	95+ years	Data Rich	0	3	LDI (I$ per capita)	--
Nasopharynx cancer	Female	5-9 years	95+ years	Global	1	1	Tobacco (cigarettes per capita)	39
Nasopharynx cancer	Female	5-9 years	95+ years	Global	1	1	Smoking Prevalence	57
Nasopharynx cancer	Female	5-9 years	95+ years	Global	1	1	Cumulative Cigarettes (10 Years)	200
Nasopharynx cancer	Female	5-9 years	95+ years	Global	1	1	Cumulative Cigarettes (20 Years)	212
Nasopharynx cancer	Female	5-9 years	95+ years	Global	1	1	Cumulative Cigarettes (5 Years)	237
Nasopharynx cancer	Female	5-9 years	95+ years	Global	1	1	Cumulative Cigarettes (15 Years)	311
Nasopharynx cancer	Female	5-9 years	95+ years	Global	1	1	Log-transformed SEV scalar: Nasoph C	394
Nasopharynx cancer	Female	5-9 years	95+ years	Global	1	1	Alcohol (liters per capita)	--
Nasopharynx cancer	Female	5-9 years	95+ years	Global	-1	2	fruits adjusted(g)	0
Nasopharynx cancer	Female	5-9 years	95+ years	Global	-1	2	Healthcare access and quality index	40
Nasopharynx cancer	Female	5-9 years	95+ years	Global	-1	2	vegetables adjusted(g)	449
Nasopharynx cancer	Female	5-9 years	95+ years	Global	1	2	Population Density (over 1000 ppl/sqkm, proportion)	496

Appendix Table 9: CODEm covariates used, level of covariate, and expected direction of covariate by cause, sex, and age
Covariates that CODEm did not select during the covariate selection process have no draw counts listed.

Cause	Sex	Age Start	Age End	Model Type	Direction	Level	Covariate Name	Number of Draws
Nasopharynx cancer	Female	5-9 years	95+ years	Global	1	2	Population Density (under 150 ppl/sqkm, proportion)	--
Nasopharynx cancer	Female	5-9 years	95+ years	Global	-1	3	Education (years per capita)	0
Nasopharynx cancer	Female	5-9 years	95+ years	Global	0	3	Socio-demographic Index	223
Nasopharynx cancer	Female	5-9 years	95+ years	Global	0	3	LDI (I$ per capita)	--
Nasopharynx cancer	Male	5-9 years	95+ years	Data Rich	1	1	Cumulative Cigarettes (15 Years)	4
Nasopharynx cancer	Male	5-9 years	95+ years	Data Rich	1	1	Smoking Prevalence	10
Nasopharynx cancer	Male	5-9 years	95+ years	Data Rich	1	1	Cumulative Cigarettes (5 Years)	12
Nasopharynx cancer	Male	5-9 years	95+ years	Data Rich	1	1	Cumulative Cigarettes (10 Years)	81
Nasopharynx cancer	Male	5-9 years	95+ years	Data Rich	1	1	Tobacco (cigarettes per capita)	110
Nasopharynx cancer	Male	5-9 years	95+ years	Data Rich	1	1	Cumulative Cigarettes (20 Years)	332
Nasopharynx cancer	Male	5-9 years	95+ years	Data Rich	1	1	Alcohol (liters per capita)	--
Nasopharynx cancer	Male	5-9 years	95+ years	Data Rich	1	1	Log-transformed SEV scalar: Nasoph C	--
Nasopharynx cancer	Male	5-9 years	95+ years	Data Rich	-1	2	Healthcare access and quality index	27
Nasopharynx cancer	Male	5-9 years	95+ years	Data Rich	-1	2	fruits adjusted(g)	193
Nasopharynx cancer	Male	5-9 years	95+ years	Data Rich	-1	2	vegetables adjusted(g)	257
Nasopharynx cancer	Male	5-9 years	95+ years	Data Rich	1	2	Population Density (over 1000 ppl/sqkm, proportion)	532
Nasopharynx cancer	Male	5-9 years	95+ years	Data Rich	1	2	Population Density (under 150 ppl/sqkm, proportion)	--
Nasopharynx cancer	Male	5-9 years	95+ years	Data Rich	-1	3	Education (years per capita)	27
Nasopharynx cancer	Male	5-9 years	95+ years	Data Rich	0	3	Socio-demographic Index	46
Nasopharynx cancer	Male	5-9 years	95+ years	Data Rich	0	3	LDI (I$ per capita)	--
Nasopharynx cancer	Male	5-9 years	95+ years	Global	1	1	Cumulative Cigarettes (5 Years)	0
Nasopharynx cancer	Male	5-9 years	95+ years	Global	1	1	Tobacco (cigarettes per capita)	5
Nasopharynx cancer	Male	5-9 years	95+ years	Global	1	1	Cumulative Cigarettes (20 Years)	84
Nasopharynx cancer	Male	5-9 years	95+ years	Global	1	1	Cumulative Cigarettes (10 Years)	87
Nasopharynx cancer	Male	5-9 years	95+ years	Global	1	1	Log-transformed SEV scalar: Nasoph C	138
Nasopharynx cancer	Male	5-9 years	95+ years	Global	1	1	Cumulative Cigarettes (15 Years)	189
Nasopharynx cancer	Male	5-9 years	95+ years	Global	1	1	Smoking Prevalence	241
Nasopharynx cancer	Male	5-9 years	95+ years	Global	1	1	Alcohol (liters per capita)	650
Nasopharynx cancer	Male	5-9 years	95+ years	Global	-1	2	fruits adjusted(g)	50
Nasopharynx cancer	Male	5-9 years	95+ years	Global	-1	2	Healthcare access and quality index	360
Nasopharynx cancer	Male	5-9 years	95+ years	Global	-1	2	vegetables adjusted(g)	602
Nasopharynx cancer	Male	5-9 years	95+ years	Global	1	2	Population Density (over 1000 ppl/sqkm, proportion)	311
Nasopharynx cancer	Male	5-9 years	95+ years	Global	1	2	Population Density (under 150 ppl/sqkm, proportion)	--
Nasopharynx cancer	Male	5-9 years	95+ years	Global	-1	3	Education (years per capita)	129
Nasopharynx cancer	Male	5-9 years	95+ years	Global	0	3	Socio-demographic Index	278
Nasopharynx cancer	Male	5-9 years	95+ years	Global	0	3	LDI (I$ per capita)	--
Other pharynx cancer	Female	15-19 years	95+ years	Data Rich	1	1	Log-transformed SEV scalar: Oth Phar C	94
Other pharynx cancer	Female	15-19 years	95+ years	Data Rich	1	1	Alcohol (liters per capita)	225
Other pharynx cancer	Female	15-19 years	95+ years	Data Rich	1	1	Smoking Prevalence	--
Other pharynx cancer	Female	15-19 years	95+ years	Data Rich	-1	2	Healthcare access and quality index	189
Other pharynx cancer	Female	15-19 years	95+ years	Data Rich	-1	2	vegetables adjusted(g)	236
Other pharynx cancer	Female	15-19 years	95+ years	Data Rich	-1	2	fruits adjusted(g)	--
Other pharynx cancer	Female	15-19 years	95+ years	Data Rich	1	2	Population Density (over 1000 ppl/sqkm, proportion)	309
Other pharynx cancer	Female	15-19 years	95+ years	Data Rich	1	2	Cumulative Cigarettes (5 Years)	--
Other pharynx cancer	Female	15-19 years	95+ years	Data Rich	1	2	Population Density (under 150 ppl/sqkm, proportion)	--
Other pharynx cancer	Female	15-19 years	95+ years	Data Rich	-1	3	Education (years per capita)	97
Other pharynx cancer	Female	15-19 years	95+ years	Data Rich	0	3	Socio-demographic Index	271
Other pharynx cancer	Female	15-19 years	95+ years	Data Rich	0	3	LDI (I$ per capita)	--
Other pharynx cancer	Female	15-19 years	95+ years	Global	1	1	Smoking Prevalence	170
Other pharynx cancer	Female	15-19 years	95+ years	Global	1	1	Log-transformed SEV scalar: Oth Phar C	271
Other pharynx cancer	Female	15-19 years	95+ years	Global	1	1	Alcohol (liters per capita)	729
Other pharynx cancer	Female	15-19 years	95+ years	Global	-1	2	fruits adjusted(g)	17
Other pharynx cancer	Female	15-19 years	95+ years	Global	-1	2	vegetables adjusted(g)	125
Other pharynx cancer	Female	15-19 years	95+ years	Global	-1	2	Healthcare access and quality index	205
Other pharynx cancer	Female	15-19 years	95+ years	Global	1	2	Cumulative Cigarettes (5 Years)	144
Other pharynx cancer	Female	15-19 years	95+ years	Global	1	2	Population Density (over 1000 ppl/sqkm, proportion)	636
Other pharynx cancer	Female	15-19 years	95+ years	Global	1	2	Population Density (under 150 ppl/sqkm, proportion)	--
Other pharynx cancer	Female	15-19 years	95+ years	Global	-1	3	Education (years per capita)	61
Other pharynx cancer	Female	15-19 years	95+ years	Global	0	3	Socio-demographic Index	315
Other pharynx cancer	Female	15-19 years	95+ years	Global	0	3	LDI (I$ per capita)	--
Other pharynx cancer	Male	15-19 years	95+ years	Data Rich	1	1	Log-transformed SEV scalar: Oth Phar C	335
Other pharynx cancer	Male	15-19 years	95+ years	Data Rich	1	1	Alcohol (liters per capita)	799
Other pharynx cancer	Male	15-19 years	95+ years	Data Rich	1	1	Smoking Prevalence	--
Other pharynx cancer	Male	15-19 years	95+ years	Data Rich	-1	2	Healthcare access and quality index	--
Other pharynx cancer	Male	15-19 years	95+ years	Data Rich	-1	2	fruits adjusted(g)	--
Other pharynx cancer	Male	15-19 years	95+ years	Data Rich	-1	2	vegetables adjusted(g)	--
Other pharynx cancer	Male	15-19 years	95+ years	Data Rich	1	2	Population Density (over 1000 ppl/sqkm, proportion)	423
Other pharynx cancer	Male	15-19 years	95+ years	Data Rich	1	2	Cumulative Cigarettes (5 Years)	--
Other pharynx cancer	Male	15-19 years	95+ years	Data Rich	1	2	Population Density (under 150 ppl/sqkm, proportion)	--
Other pharynx cancer	Male	15-19 years	95+ years	Data Rich	-1	3	Education (years per capita)	--
Other pharynx cancer	Male	15-19 years	95+ years	Data Rich	0	3	Socio-demographic Index	406
Other pharynx cancer	Male	15-19 years	95+ years	Data Rich	0	3	LDI (I$ per capita)	--
Other pharynx cancer	Male	15-19 years	95+ years	Global	1	1	Log-transformed SEV scalar: Oth Phar C	676
Other pharynx cancer	Male	15-19 years	95+ years	Global	1	1	Alcohol (liters per capita)	784
Other pharynx cancer	Male	15-19 years	95+ years	Global	1	1	Smoking Prevalence	--
Other pharynx cancer	Male	15-19 years	95+ years	Global	-1	2	Healthcare access and quality index	--
Other pharynx cancer	Male	15-19 years	95+ years	Global	-1	2	fruits adjusted(g)	--
Other pharynx cancer	Male	15-19 years	95+ years	Global	-1	2	vegetables adjusted(g)	--

Appendix Table 9: CODEm covariates used, level of covariate, and expected direction of covariate by cause, sex, and age
Covariates that CODEm did not select during the covariate selection process have no draw counts listed.

Cause	Sex	Age Start	Age End	Model Type	Direction	Level	Covariate Name	Number of Draws
Other pharynx cancer	Male	15-19 years	95+ years	Global	1	2	Population Density (over 1000 ppl/sqkm, proportion)	276
Other pharynx cancer	Male	15-19 years	95+ years	Global	1	2	Cumulative Cigarettes (5 Years)	--
Other pharynx cancer	Male	15-19 years	95+ years	Global	1	2	Population Density (under 150 ppl/sqkm, proportion)	--
Other pharynx cancer	Male	15-19 years	95+ years	Global	-1	3	Education (years per capita)	--
Other pharynx cancer	Male	15-19 years	95+ years	Global	0	3	Socio-demographic Index	198
Other pharynx cancer	Male	15-19 years	95+ years	Global	0	3	LDI (I$ per capita)	--
Oesophageal cancer	Female	15-19 years	95+ years	Data Rich	-1	1	fruits adjusted(g)	--
Oesophageal cancer	Female	15-19 years	95+ years	Data Rich	1	1	Smoking Prevalence	0
Oesophageal cancer	Female	15-19 years	95+ years	Data Rich	1	1	Tobacco (cigarettes per capita)	0
Oesophageal cancer	Female	15-19 years	95+ years	Data Rich	1	1	Mean BMI	62
Oesophageal cancer	Female	15-19 years	95+ years	Data Rich	1	1	Log-transformed age-standardized SEV scalar: Esophag C	300
Oesophageal cancer	Female	15-19 years	95+ years	Data Rich	1	1	Alcohol (liters per capita)	833
Oesophageal cancer	Female	15-19 years	95+ years	Data Rich	1	1	Log-transformed SEV scalar: Esophag C	--
Oesophageal cancer	Female	15-19 years	95+ years	Data Rich	-1	2	Healthcare access and quality index	0
Oesophageal cancer	Female	15-19 years	95+ years	Data Rich	-1	2	Improved Water Source (proportion with access)	0
Oesophageal cancer	Female	15-19 years	95+ years	Data Rich	-1	2	vegetables adjusted(g)	7
Oesophageal cancer	Female	15-19 years	95+ years	Data Rich	-1	2	Sanitation (proportion with access)	379
Oesophageal cancer	Female	15-19 years	95+ years	Data Rich	1	2	Indoor Air Pollution (All Cooking Fuels)	77
Oesophageal cancer	Female	15-19 years	95+ years	Data Rich	-1	3	Education (years per capita)	0
Oesophageal cancer	Female	15-19 years	95+ years	Data Rich	0	3	Socio-demographic Index	357
Oesophageal cancer	Female	15-19 years	95+ years	Data Rich	0	3	LDI (I$ per capita)	--
Oesophageal cancer	Female	15-19 years	95+ years	Global	-1	1	fruits adjusted(g)	313
Oesophageal cancer	Female	15-19 years	95+ years	Global	1	1	Smoking Prevalence	43
Oesophageal cancer	Female	15-19 years	95+ years	Global	1	1	Tobacco (cigarettes per capita)	53
Oesophageal cancer	Female	15-19 years	95+ years	Global	1	1	Log-transformed age-standardized SEV scalar: Esophag C	97
Oesophageal cancer	Female	15-19 years	95+ years	Global	1	1	Alcohol (liters per capita)	538
Oesophageal cancer	Female	15-19 years	95+ years	Global	1	1	Mean BMI	685
Oesophageal cancer	Female	15-19 years	95+ years	Global	1	1	Log-transformed SEV scalar: Esophag C	--
Oesophageal cancer	Female	15-19 years	95+ years	Global	-1	2	Improved Water Source (proportion with access)	0
Oesophageal cancer	Female	15-19 years	95+ years	Global	-1	2	Healthcare access and quality index	57
Oesophageal cancer	Female	15-19 years	95+ years	Global	-1	2	vegetables adjusted(g)	207
Oesophageal cancer	Female	15-19 years	95+ years	Global	-1	2	Sanitation (proportion with access)	442
Oesophageal cancer	Female	15-19 years	95+ years	Global	1	2	Indoor Air Pollution (All Cooking Fuels)	431
Oesophageal cancer	Female	15-19 years	95+ years	Global	-1	3	Education (years per capita)	170
Oesophageal cancer	Female	15-19 years	95+ years	Global	0	3	Socio-demographic Index	488
Oesophageal cancer	Female	15-19 years	95+ years	Global	0	3	LDI (I$ per capita)	--
Oesophageal cancer	Male	15-19 years	95+ years	Data Rich	-1	1	fruits adjusted(g)	--
Oesophageal cancer	Male	15-19 years	95+ years	Data Rich	1	1	Tobacco (cigarettes per capita)	3
Oesophageal cancer	Male	15-19 years	95+ years	Data Rich	1	1	Log-transformed SEV scalar: Esophag C	5
Oesophageal cancer	Male	15-19 years	95+ years	Data Rich	1	1	Smoking Prevalence	7
Oesophageal cancer	Male	15-19 years	95+ years	Data Rich	1	1	Log-transformed age-standardized SEV scalar: Esophag C	21
Oesophageal cancer	Male	15-19 years	95+ years	Data Rich	1	1	Alcohol (liters per capita)	147
Oesophageal cancer	Male	15-19 years	95+ years	Data Rich	1	1	Mean BMI	981
Oesophageal cancer	Male	15-19 years	95+ years	Data Rich	-1	2	Healthcare access and quality index	1
Oesophageal cancer	Male	15-19 years	95+ years	Data Rich	-1	2	vegetables adjusted(g)	288
Oesophageal cancer	Male	15-19 years	95+ years	Data Rich	-1	2	Sanitation (proportion with access)	460
Oesophageal cancer	Male	15-19 years	95+ years	Data Rich	-1	2	Improved Water Source (proportion with access)	--
Oesophageal cancer	Male	15-19 years	95+ years	Data Rich	1	2	Indoor Air Pollution (All Cooking Fuels)	305
Oesophageal cancer	Male	15-19 years	95+ years	Data Rich	-1	3	Education (years per capita)	0
Oesophageal cancer	Male	15-19 years	95+ years	Data Rich	0	3	Socio-demographic Index	547
Oesophageal cancer	Male	15-19 years	95+ years	Data Rich	0	3	LDI (I$ per capita)	--
Oesophageal cancer	Male	15-19 years	95+ years	Global	-1	1	fruits adjusted(g)	259
Oesophageal cancer	Male	15-19 years	95+ years	Global	1	1	Log-transformed SEV scalar: Esophag C	0
Oesophageal cancer	Male	15-19 years	95+ years	Global	1	1	Smoking Prevalence	0
Oesophageal cancer	Male	15-19 years	95+ years	Global	1	1	Tobacco (cigarettes per capita)	0
Oesophageal cancer	Male	15-19 years	95+ years	Global	1	1	Alcohol (liters per capita)	196
Oesophageal cancer	Male	15-19 years	95+ years	Global	1	1	Log-transformed age-standardized SEV scalar: Esophag C	274
Oesophageal cancer	Male	15-19 years	95+ years	Global	1	1	Mean BMI	999
Oesophageal cancer	Male	15-19 years	95+ years	Global	-1	2	Healthcare access and quality index	0
Oesophageal cancer	Male	15-19 years	95+ years	Global	-1	2	Improved Water Source (proportion with access)	0
Oesophageal cancer	Male	15-19 years	95+ years	Global	-1	2	vegetables adjusted(g)	344
Oesophageal cancer	Male	15-19 years	95+ years	Global	-1	2	Sanitation (proportion with access)	447
Oesophageal cancer	Male	15-19 years	95+ years	Global	1	2	Indoor Air Pollution (All Cooking Fuels)	379
Oesophageal cancer	Male	15-19 years	95+ years	Global	-1	3	Education (years per capita)	0
Oesophageal cancer	Male	15-19 years	95+ years	Global	0	3	Socio-demographic Index	583
Oesophageal cancer	Male	15-19 years	95+ years	Global	0	3	LDI (I$ per capita)	--
Stomach cancer	Female	15-19 years	95+ years	Data Rich	1	1	Smoking Prevalence	0
Stomach cancer	Female	15-19 years	95+ years	Data Rich	1	1	SEV unsafe water	6
Stomach cancer	Female	15-19 years	95+ years	Data Rich	1	1	Cumulative Cigarettes (5 Years)	15
Stomach cancer	Female	15-19 years	95+ years	Data Rich	1	1	Cumulative Cigarettes (15 Years)	54
Stomach cancer	Female	15-19 years	95+ years	Data Rich	1	1	SEV unsafe sanitation	178
Stomach cancer	Female	15-19 years	95+ years	Data Rich	1	1	Log-transformed SEV scalar: Stomach C	183
Stomach cancer	Female	15-19 years	95+ years	Data Rich	1	1	Cumulative Cigarettes (10 Years)	215
Stomach cancer	Female	15-19 years	95+ years	Data Rich	1	1	Tobacco (cigarettes per capita)	367
Stomach cancer	Female	15-19 years	95+ years	Data Rich	1	1	Cumulative Cigarettes (20 Years)	368
Stomach cancer	Female	15-19 years	95+ years	Data Rich	1	1	Diet high in sodium	--
Stomach cancer	Female	15-19 years	95+ years	Data Rich	-1	2	fruits adjusted(g)	20
Stomach cancer	Female	15-19 years	95+ years	Data Rich	-1	2	vegetables adjusted(g)	23

Appendix Table 9: CODEm covariates used, level of covariate, and expected direction of covariate by cause, sex, and age<br>*Covariates that CODEm did not select during the covariate selection process have no draw counts listed.*								
**Cause**	**Sex**	**Age Start**	**Age End**	**Model Type**	**Direction**	**Level**	**Covariate Name**	**Number of Draws**
Stomach cancer	Female	15-19 years	95+ years	Data Rich	-1	2	Healthcare access and quality index	27
Stomach cancer	Female	15-19 years	95+ years	Data Rich	-1	2	Sanitation (proportion with access)	152
Stomach cancer	Female	15-19 years	95+ years	Data Rich	-1	2	Improved Water Source (proportion with access)	153
Stomach cancer	Female	15-19 years	95+ years	Data Rich	1	2	Mean BMI	--
Stomach cancer	Female	15-19 years	95+ years	Data Rich	-1	3	Education (years per capita)	699
Stomach cancer	Female	15-19 years	95+ years	Data Rich	0	3	Socio-demographic Index	551
Stomach cancer	Female	15-19 years	95+ years	Data Rich	0	3	LDI (I$ per capita)	--
Stomach cancer	Female	15-19 years	95+ years	Global	1	1	Cumulative Cigarettes (5 Years)	0
Stomach cancer	Female	15-19 years	95+ years	Global	1	1	Smoking Prevalence	0
Stomach cancer	Female	15-19 years	95+ years	Global	1	1	Cumulative Cigarettes (10 Years)	2
Stomach cancer	Female	15-19 years	95+ years	Global	1	1	SEV unsafe sanitation	69
Stomach cancer	Female	15-19 years	95+ years	Global	1	1	SEV unsafe water	85
Stomach cancer	Female	15-19 years	95+ years	Global	1	1	Log-transformed SEV scalar: Stomach C	127
Stomach cancer	Female	15-19 years	95+ years	Global	1	1	Cumulative Cigarettes (15 Years)	281
Stomach cancer	Female	15-19 years	95+ years	Global	1	1	Cumulative Cigarettes (20 Years)	367
Stomach cancer	Female	15-19 years	95+ years	Global	1	1	Diet high in sodium	769
Stomach cancer	Female	15-19 years	95+ years	Global	1	1	Tobacco (cigarettes per capita)	946
Stomach cancer	Female	15-19 years	95+ years	Global	-1	2	fruits adjusted(g)	1
Stomach cancer	Female	15-19 years	95+ years	Global	-1	2	Sanitation (proportion with access)	12
Stomach cancer	Female	15-19 years	95+ years	Global	-1	2	Improved Water Source (proportion with access)	19
Stomach cancer	Female	15-19 years	95+ years	Global	-1	2	Healthcare access and quality index	28
Stomach cancer	Female	15-19 years	95+ years	Global	-1	2	vegetables adjusted(g)	129
Stomach cancer	Female	15-19 years	95+ years	Global	1	2	Mean BMI	--
Stomach cancer	Female	15-19 years	95+ years	Global	-1	3	Education (years per capita)	38
Stomach cancer	Female	15-19 years	95+ years	Global	0	3	Socio-demographic Index	234
Stomach cancer	Female	15-19 years	95+ years	Global	0	3	LDI (I$ per capita)	--
Stomach cancer	Male	15-19 years	95+ years	Data Rich	1	1	SEV unsafe water	0
Stomach cancer	Male	15-19 years	95+ years	Data Rich	1	1	Cumulative Cigarettes (20 Years)	22
Stomach cancer	Male	15-19 years	95+ years	Data Rich	1	1	SEV unsafe sanitation	33
Stomach cancer	Male	15-19 years	95+ years	Data Rich	1	1	Cumulative Cigarettes (15 Years)	99
Stomach cancer	Male	15-19 years	95+ years	Data Rich	1	1	Cumulative Cigarettes (10 Years)	105
Stomach cancer	Male	15-19 years	95+ years	Data Rich	1	1	Tobacco (cigarettes per capita)	185
Stomach cancer	Male	15-19 years	95+ years	Data Rich	1	1	Smoking Prevalence	297
Stomach cancer	Male	15-19 years	95+ years	Data Rich	1	1	Cumulative Cigarettes (5 Years)	452
Stomach cancer	Male	15-19 years	95+ years	Data Rich	1	1	Log-transformed SEV scalar: Stomach C	617
Stomach cancer	Male	15-19 years	95+ years	Data Rich	1	1	Diet high in sodium	--
Stomach cancer	Male	15-19 years	95+ years	Data Rich	-1	2	Healthcare access and quality index	14
Stomach cancer	Male	15-19 years	95+ years	Data Rich	-1	2	fruits adjusted(g)	14
Stomach cancer	Male	15-19 years	95+ years	Data Rich	-1	2	Sanitation (proportion with access)	138
Stomach cancer	Male	15-19 years	95+ years	Data Rich	-1	2	vegetables adjusted(g)	183
Stomach cancer	Male	15-19 years	95+ years	Data Rich	-1	2	Improved Water Source (proportion with access)	302
Stomach cancer	Male	15-19 years	95+ years	Data Rich	1	2	Mean BMI	--
Stomach cancer	Male	15-19 years	95+ years	Data Rich	-1	3	Education (years per capita)	751
Stomach cancer	Male	15-19 years	95+ years	Data Rich	0	3	Socio-demographic Index	868
Stomach cancer	Male	15-19 years	95+ years	Data Rich	0	3	LDI (I$ per capita)	--
Stomach cancer	Male	15-19 years	95+ years	Global	1	1	Cumulative Cigarettes (5 Years)	87
Stomach cancer	Male	15-19 years	95+ years	Global	1	1	Diet high in sodium	180
Stomach cancer	Male	15-19 years	95+ years	Global	1	1	Smoking Prevalence	210
Stomach cancer	Male	15-19 years	95+ years	Global	1	1	SEV unsafe sanitation	217
Stomach cancer	Male	15-19 years	95+ years	Global	1	1	SEV unsafe water	231
Stomach cancer	Male	15-19 years	95+ years	Global	1	1	Cumulative Cigarettes (10 Years)	235
Stomach cancer	Male	15-19 years	95+ years	Global	1	1	Cumulative Cigarettes (15 Years)	268
Stomach cancer	Male	15-19 years	95+ years	Global	1	1	Cumulative Cigarettes (20 Years)	385
Stomach cancer	Male	15-19 years	95+ years	Global	1	1	Log-transformed SEV scalar: Stomach C	450
Stomach cancer	Male	15-19 years	95+ years	Global	1	1	Tobacco (cigarettes per capita)	568
Stomach cancer	Male	15-19 years	95+ years	Global	-1	2	vegetables adjusted(g)	39
Stomach cancer	Male	15-19 years	95+ years	Global	-1	2	Sanitation (proportion with access)	80
Stomach cancer	Male	15-19 years	95+ years	Global	-1	2	fruits adjusted(g)	83
Stomach cancer	Male	15-19 years	95+ years	Global	-1	2	Healthcare access and quality index	221
Stomach cancer	Male	15-19 years	95+ years	Global	-1	2	Improved Water Source (proportion with access)	223
Stomach cancer	Male	15-19 years	95+ years	Global	1	2	Mean BMI	--
Stomach cancer	Male	15-19 years	95+ years	Global	-1	3	Education (years per capita)	73
Stomach cancer	Male	15-19 years	95+ years	Global	0	3	Socio-demographic Index	108
Stomach cancer	Male	15-19 years	95+ years	Global	0	3	LDI (I$ per capita)	--
Colon and rectum cancer	Female	15-19 years	95+ years	Data Rich	-1	1	Total Physical Activity (MET-min/week), Age-standardized	7
Colon and rectum cancer	Female	15-19 years	95+ years	Data Rich	-1	1	Total Physical Activity (MET-min/week), Age-specific	174
Colon and rectum cancer	Female	15-19 years	95+ years	Data Rich	1	1	Tobacco (cigarettes per capita)	0
Colon and rectum cancer	Female	15-19 years	95+ years	Data Rich	1	1	Log-transformed SEV scalar: Colorect C	79
Colon and rectum cancer	Female	15-19 years	95+ years	Data Rich	1	1	Smoking Prevalence	79
Colon and rectum cancer	Female	15-19 years	95+ years	Data Rich	1	1	Alcohol (liters per capita)	137
Colon and rectum cancer	Female	15-19 years	95+ years	Data Rich	1	1	Mean BMI	739
Colon and rectum cancer	Female	15-19 years	95+ years	Data Rich	1	1	red meats adjusted(g)	818
Colon and rectum cancer	Female	15-19 years	95+ years	Data Rich	-1	2	Healthcare access and quality index	0
Colon and rectum cancer	Female	15-19 years	95+ years	Data Rich	-1	2	fruits adjusted(g)	0
Colon and rectum cancer	Female	15-19 years	95+ years	Data Rich	-1	2	nuts seeds adjusted(g)	150
Colon and rectum cancer	Female	15-19 years	95+ years	Data Rich	-1	2	pufa adjusted(percent)	257
Colon and rectum cancer	Female	15-19 years	95+ years	Data Rich	-1	2	vegetables adjusted(g)	545
Colon and rectum cancer	Female	15-19 years	95+ years	Data Rich	-1	2	milk adjusted(g)	594

Appendix Table 9: CODEm covariates used, level of covariate, and expected direction of covariate by cause, sex, and age

Covariates that CODEm did not select during the covariate selection process have no draw counts listed.

Cause	Sex	Age Start	Age End	Model Type	Direction	Level	Covariate Name	Number of Draws
Colon and rectum cancer	Female	15-19 years	95+ years	Data Rich	-1	2	fiber adjusted(g)	815
Colon and rectum cancer	Female	15-19 years	95+ years	Data Rich	-1	2	calcium adjusted(g)	--
Colon and rectum cancer	Female	15-19 years	95+ years	Data Rich	1	2	Cumulative Cigarettes (10 Years)	21
Colon and rectum cancer	Female	15-19 years	95+ years	Data Rich	1	2	Diabetes Age-Specific Prevalence (proportion)	32
Colon and rectum cancer	Female	15-19 years	95+ years	Data Rich	1	2	Cumulative Cigarettes (5 Years)	156
Colon and rectum cancer	Female	15-19 years	95+ years	Data Rich	1	2	Cumulative Cigarettes (15 Years)	266
Colon and rectum cancer	Female	15-19 years	95+ years	Data Rich	1	2	Cumulative Cigarettes (20 Years)	410
Colon and rectum cancer	Female	15-19 years	95+ years	Data Rich	1	2	Diabetes Fasting Plasma Glucose (mmol/L)	592
Colon and rectum cancer	Female	15-19 years	95+ years	Data Rich	-1	3	Education (years per capita)	67
Colon and rectum cancer	Female	15-19 years	95+ years	Data Rich	0	3	Socio-demographic Index	0
Colon and rectum cancer	Female	15-19 years	95+ years	Data Rich	0	3	LDI (I$ per capita)	--
Colon and rectum cancer	Female	15-19 years	95+ years	Global	-1	1	Total Physical Activity (MET-min/week), Age-specific	163
Colon and rectum cancer	Female	15-19 years	95+ years	Global	-1	1	Total Physical Activity (MET-min/week), Age-standardized	370
Colon and rectum cancer	Female	15-19 years	95+ years	Global	1	1	Mean BMI	0
Colon and rectum cancer	Female	15-19 years	95+ years	Global	1	1	Tobacco (cigarettes per capita)	78
Colon and rectum cancer	Female	15-19 years	95+ years	Global	1	1	Smoking Prevalence	84
Colon and rectum cancer	Female	15-19 years	95+ years	Global	1	1	Alcohol (liters per capita)	242
Colon and rectum cancer	Female	15-19 years	95+ years	Global	1	1	red meats adjusted(g)	408
Colon and rectum cancer	Female	15-19 years	95+ years	Global	1	1	Log-transformed SEV scalar: Colorect C	451
Colon and rectum cancer	Female	15-19 years	95+ years	Global	-1	2	milk adjusted(g)	0
Colon and rectum cancer	Female	15-19 years	95+ years	Global	-1	2	fruits adjusted(g)	4
Colon and rectum cancer	Female	15-19 years	95+ years	Global	-1	2	vegetables adjusted(g)	17
Colon and rectum cancer	Female	15-19 years	95+ years	Global	-1	2	calcium adjusted(g)	22
Colon and rectum cancer	Female	15-19 years	95+ years	Global	-1	2	pufa adjusted(percent)	23
Colon and rectum cancer	Female	15-19 years	95+ years	Global	-1	2	Healthcare access and quality index	46
Colon and rectum cancer	Female	15-19 years	95+ years	Global	-1	2	nuts seeds adjusted(g)	612
Colon and rectum cancer	Female	15-19 years	95+ years	Global	-1	2	fiber adjusted(g)	961
Colon and rectum cancer	Female	15-19 years	95+ years	Global	1	2	Cumulative Cigarettes (5 Years)	1
Colon and rectum cancer	Female	15-19 years	95+ years	Global	1	2	Cumulative Cigarettes (15 Years)	215
Colon and rectum cancer	Female	15-19 years	95+ years	Global	1	2	Cumulative Cigarettes (20 Years)	216
Colon and rectum cancer	Female	15-19 years	95+ years	Global	1	2	Cumulative Cigarettes (10 Years)	244
Colon and rectum cancer	Female	15-19 years	95+ years	Global	1	2	Diabetes Fasting Plasma Glucose (mmol/L)	291
Colon and rectum cancer	Female	15-19 years	95+ years	Global	1	2	Diabetes Age-Specific Prevalence (proportion)	654
Colon and rectum cancer	Female	15-19 years	95+ years	Global	-1	3	Education (years per capita)	262
Colon and rectum cancer	Female	15-19 years	95+ years	Global	0	3	Socio-demographic Index	498
Colon and rectum cancer	Female	15-19 years	95+ years	Global	0	3	LDI (I$ per capita)	--
Colon and rectum cancer	Male	15-19 years	95+ years	Data Rich	-1	1	Total Physical Activity (MET-min/week), Age-specific	42
Colon and rectum cancer	Male	15-19 years	95+ years	Data Rich	-1	1	Total Physical Activity (MET-min/week), Age-standardized	199
Colon and rectum cancer	Male	15-19 years	95+ years	Data Rich	1	1	Alcohol (liters per capita)	0
Colon and rectum cancer	Male	15-19 years	95+ years	Data Rich	1	1	Tobacco (cigarettes per capita)	0
Colon and rectum cancer	Male	15-19 years	95+ years	Data Rich	1	1	Smoking Prevalence	79
Colon and rectum cancer	Male	15-19 years	95+ years	Data Rich	1	1	Mean BMI	397
Colon and rectum cancer	Male	15-19 years	95+ years	Data Rich	1	1	red meats adjusted(g)	401
Colon and rectum cancer	Male	15-19 years	95+ years	Data Rich	1	1	Log-transformed SEV scalar: Colorect C	463
Colon and rectum cancer	Male	15-19 years	95+ years	Data Rich	-1	2	Healthcare access and quality index	0
Colon and rectum cancer	Male	15-19 years	95+ years	Data Rich	-1	2	fruits adjusted(g)	4
Colon and rectum cancer	Male	15-19 years	95+ years	Data Rich	-1	2	pufa adjusted(percent)	96
Colon and rectum cancer	Male	15-19 years	95+ years	Data Rich	-1	2	milk adjusted(g)	138
Colon and rectum cancer	Male	15-19 years	95+ years	Data Rich	-1	2	vegetables adjusted(g)	246
Colon and rectum cancer	Male	15-19 years	95+ years	Data Rich	-1	2	nuts seeds adjusted(g)	270
Colon and rectum cancer	Male	15-19 years	95+ years	Data Rich	-1	2	fiber adjusted(g)	436
Colon and rectum cancer	Male	15-19 years	95+ years	Data Rich	-1	2	calcium adjusted(g)	--
Colon and rectum cancer	Male	15-19 years	95+ years	Data Rich	1	2	Cumulative Cigarettes (5 Years)	0
Colon and rectum cancer	Male	15-19 years	95+ years	Data Rich	1	2	Cumulative Cigarettes (15 Years)	4
Colon and rectum cancer	Male	15-19 years	95+ years	Data Rich	1	2	Cumulative Cigarettes (10 Years)	13
Colon and rectum cancer	Male	15-19 years	95+ years	Data Rich	1	2	Diabetes Age-Specific Prevalence (proportion)	24
Colon and rectum cancer	Male	15-19 years	95+ years	Data Rich	1	2	Cumulative Cigarettes (20 Years)	317
Colon and rectum cancer	Male	15-19 years	95+ years	Data Rich	1	2	Diabetes Fasting Plasma Glucose (mmol/L)	544
Colon and rectum cancer	Male	15-19 years	95+ years	Data Rich	-1	3	Education (years per capita)	0
Colon and rectum cancer	Male	15-19 years	95+ years	Data Rich	0	3	Socio-demographic Index	515
Colon and rectum cancer	Male	15-19 years	95+ years	Data Rich	0	3	LDI (I$ per capita)	--
Colon and rectum cancer	Male	15-19 years	95+ years	Global	-1	1	Total Physical Activity (MET-min/week), Age-standardized	84
Colon and rectum cancer	Male	15-19 years	95+ years	Global	-1	1	Total Physical Activity (MET-min/week), Age-specific	107
Colon and rectum cancer	Male	15-19 years	95+ years	Global	1	1	Alcohol (liters per capita)	0
Colon and rectum cancer	Male	15-19 years	95+ years	Global	1	1	Smoking Prevalence	57
Colon and rectum cancer	Male	15-19 years	95+ years	Global	1	1	Tobacco (cigarettes per capita)	77
Colon and rectum cancer	Male	15-19 years	95+ years	Global	1	1	red meats adjusted(g)	504
Colon and rectum cancer	Male	15-19 years	95+ years	Global	1	1	Log-transformed SEV scalar: Colorect C	642
Colon and rectum cancer	Male	15-19 years	95+ years	Global	1	1	Mean BMI	709
Colon and rectum cancer	Male	15-19 years	95+ years	Global	-1	2	Healthcare access and quality index	0
Colon and rectum cancer	Male	15-19 years	95+ years	Global	-1	2	fruits adjusted(g)	5
Colon and rectum cancer	Male	15-19 years	95+ years	Global	-1	2	nuts seeds adjusted(g)	157
Colon and rectum cancer	Male	15-19 years	95+ years	Global	-1	2	pufa adjusted(percent)	296
Colon and rectum cancer	Male	15-19 years	95+ years	Global	-1	2	milk adjusted(g)	364
Colon and rectum cancer	Male	15-19 years	95+ years	Global	-1	2	vegetables adjusted(g)	381
Colon and rectum cancer	Male	15-19 years	95+ years	Global	-1	2	fiber adjusted(g)	623
Colon and rectum cancer	Male	15-19 years	95+ years	Global	-1	2	calcium adjusted(g)	--
Colon and rectum cancer	Male	15-19 years	95+ years	Global	1	2	Cumulative Cigarettes (5 Years)	0

Appendix Table 9: CODEm covariates used, level of covariate, and expected direction of covariate by cause, sex, and age<br>*Covariates that CODEm did not select during the covariate selection process have no draw counts listed.*								
**Cause**	**Sex**	**Age Start**	**Age End**	**Model Type**	**Direction**	**Level**	**Covariate Name**	**Number of Draws**
Colon and rectum cancer	Male	15-19 years	95+ years	Global	1	2	Diabetes Age-Specific Prevalence (proportion)	175
Colon and rectum cancer	Male	15-19 years	95+ years	Global	1	2	Cumulative Cigarettes (10 Years)	190
Colon and rectum cancer	Male	15-19 years	95+ years	Global	1	2	Cumulative Cigarettes (20 Years)	255
Colon and rectum cancer	Male	15-19 years	95+ years	Global	1	2	Cumulative Cigarettes (15 Years)	294
Colon and rectum cancer	Male	15-19 years	95+ years	Global	1	2	Diabetes Fasting Plasma Glucose (mmol/L)	878
Colon and rectum cancer	Male	15-19 years	95+ years	Global	-1	3	Education (years per capita)	0
Colon and rectum cancer	Male	15-19 years	95+ years	Global	0	3	Socio-demographic Index	468
Colon and rectum cancer	Male	15-19 years	95+ years	Global	0	3	LDI (I$ per capita)	--
Liver cancer	Female	5-9 years	95+ years	Data Rich	1	1	Hepatitis B (HBsAg) Seroprevalence	10
Liver cancer	Female	5-9 years	95+ years	Data Rich	1	1	Alcohol (liters per capita)	196
Liver cancer	Female	5-9 years	95+ years	Data Rich	1	1	Log-transformed SEV scalar: Liver C	794
Liver cancer	Female	5-9 years	95+ years	Data Rich	1	1	HIV age-standardized prevalence	--
Liver cancer	Female	5-9 years	95+ years	Data Rich	1	1	Hepatitis C (IgG) Seroprevalence	--
Liver cancer	Female	5-9 years	95+ years	Data Rich	-1	2	Hepatitis B 3-dose coverage (proportion)	303
Liver cancer	Female	5-9 years	95+ years	Data Rich	-1	2	Hepatitis B 3-dose coverage (proportion), lagged 10 years	304
Liver cancer	Female	5-9 years	95+ years	Data Rich	-1	2	Hepatitis B vaccine coverage (proportion), aged through time	462
Liver cancer	Female	5-9 years	95+ years	Data Rich	-1	2	Healthcare access and quality index	--
Liver cancer	Female	5-9 years	95+ years	Data Rich	-1	2	Hepatitis B 3-dose coverage (proportion), lagged 5 years	--
Liver cancer	Female	5-9 years	95+ years	Data Rich	1	2	Diabetes Fasting Plasma Glucose (mmol/L)	94
Liver cancer	Female	5-9 years	95+ years	Data Rich	1	2	red meats adjusted(g)	252
Liver cancer	Female	5-9 years	95+ years	Data Rich	1	2	Intravenous drug use (age-standardized proportion)	333
Liver cancer	Female	5-9 years	95+ years	Data Rich	1	2	Diabetes Age-Standardized Prevalence (proportion)	502
Liver cancer	Female	5-9 years	95+ years	Data Rich	1	2	Mean BMI	671
Liver cancer	Female	5-9 years	95+ years	Data Rich	1	2	Cumulative Cigarettes (10 Years)	--
Liver cancer	Female	5-9 years	95+ years	Data Rich	1	2	Cumulative Cigarettes (15 Years)	--
Liver cancer	Female	5-9 years	95+ years	Data Rich	1	2	Cumulative Cigarettes (20 Years)	--
Liver cancer	Female	5-9 years	95+ years	Data Rich	1	2	Cumulative Cigarettes (5 Years)	--
Liver cancer	Female	5-9 years	95+ years	Data Rich	1	2	Tobacco (cigarettes per capita)	--
Liver cancer	Female	5-9 years	95+ years	Data Rich	-1	3	Education (years per capita)	--
Liver cancer	Female	5-9 years	95+ years	Data Rich	0	3	Socio-demographic Index	61
Liver cancer	Female	5-9 years	95+ years	Data Rich	0	3	LDI (I$ per capita)	--
Liver cancer	Female	5-9 years	95+ years	Global	1	1	Alcohol (liters per capita)	--
Liver cancer	Female	5-9 years	95+ years	Global	1	1	HIV age-standardized prevalence	--
Liver cancer	Female	5-9 years	95+ years	Global	1	1	Hepatitis B (HBsAg) Seroprevalence	--
Liver cancer	Female	5-9 years	95+ years	Global	1	1	Hepatitis C (IgG) Seroprevalence	--
Liver cancer	Female	5-9 years	95+ years	Global	1	1	Log-transformed SEV scalar: Liver C	--
Liver cancer	Female	5-9 years	95+ years	Global	-1	2	Healthcare access and quality index	--
Liver cancer	Female	5-9 years	95+ years	Global	-1	2	Hepatitis B 3-dose coverage (proportion)	--
Liver cancer	Female	5-9 years	95+ years	Global	-1	2	Hepatitis B 3-dose coverage (proportion), lagged 10 years	--
Liver cancer	Female	5-9 years	95+ years	Global	-1	2	Hepatitis B 3-dose coverage (proportion), lagged 5 years	--
Liver cancer	Female	5-9 years	95+ years	Global	-1	2	Hepatitis B vaccine coverage (proportion), aged through time	--
Liver cancer	Female	5-9 years	95+ years	Global	1	2	Cumulative Cigarettes (10 Years)	--
Liver cancer	Female	5-9 years	95+ years	Global	1	2	Cumulative Cigarettes (15 Years)	--
Liver cancer	Female	5-9 years	95+ years	Global	1	2	Cumulative Cigarettes (20 Years)	--
Liver cancer	Female	5-9 years	95+ years	Global	1	2	Cumulative Cigarettes (5 Years)	--
Liver cancer	Female	5-9 years	95+ years	Global	1	2	Diabetes Age-Standardized Prevalence (proportion)	--
Liver cancer	Female	5-9 years	95+ years	Global	1	2	Diabetes Fasting Plasma Glucose (mmol/L)	--
Liver cancer	Female	5-9 years	95+ years	Global	1	2	Intravenous drug use (age-standardized proportion)	--
Liver cancer	Female	5-9 years	95+ years	Global	1	2	Mean BMI	--
Liver cancer	Female	5-9 years	95+ years	Global	1	2	Tobacco (cigarettes per capita)	--
Liver cancer	Female	5-9 years	95+ years	Global	1	2	red meats adjusted(g)	--
Liver cancer	Female	5-9 years	95+ years	Global	-1	3	Education (years per capita)	--
Liver cancer	Female	5-9 years	95+ years	Global	0	3	LDI (I$ per capita)	--
Liver cancer	Female	5-9 years	95+ years	Global	0	3	Socio-demographic Index	--
Liver cancer	Male	5-9 years	95+ years	Data Rich	1	1	Alcohol (liters per capita)	579
Liver cancer	Male	5-9 years	95+ years	Data Rich	1	1	HIV age-standardized prevalence	--
Liver cancer	Male	5-9 years	95+ years	Data Rich	1	1	Hepatitis B (HBsAg) Seroprevalence	--
Liver cancer	Male	5-9 years	95+ years	Data Rich	1	1	Hepatitis C (IgG) Seroprevalence	--
Liver cancer	Male	5-9 years	95+ years	Data Rich	1	1	Log-transformed SEV scalar: Liver C	--
Liver cancer	Male	5-9 years	95+ years	Data Rich	-1	2	Hepatitis B vaccine coverage (proportion), aged through time	235
Liver cancer	Male	5-9 years	95+ years	Data Rich	-1	2	Hepatitis B 3-dose coverage (proportion), lagged 5 years	344
Liver cancer	Male	5-9 years	95+ years	Data Rich	-1	2	Hepatitis B 3-dose coverage (proportion), lagged 10 years	354
Liver cancer	Male	5-9 years	95+ years	Data Rich	-1	2	Hepatitis B 3-dose coverage (proportion)	413
Liver cancer	Male	5-9 years	95+ years	Data Rich	-1	2	Healthcare access and quality index	--
Liver cancer	Male	5-9 years	95+ years	Data Rich	1	2	Diabetes Fasting Plasma Glucose (mmol/L)	2
Liver cancer	Male	5-9 years	95+ years	Data Rich	1	2	red meats adjusted(g)	2
Liver cancer	Male	5-9 years	95+ years	Data Rich	1	2	Intravenous drug use (age-standardized proportion)	173
Liver cancer	Male	5-9 years	95+ years	Data Rich	1	2	Diabetes Age-Standardized Prevalence (proportion)	293
Liver cancer	Male	5-9 years	95+ years	Data Rich	1	2	Mean BMI	979
Liver cancer	Male	5-9 years	95+ years	Data Rich	1	2	Cumulative Cigarettes (10 Years)	--
Liver cancer	Male	5-9 years	95+ years	Data Rich	1	2	Cumulative Cigarettes (15 Years)	--
Liver cancer	Male	5-9 years	95+ years	Data Rich	1	2	Cumulative Cigarettes (20 Years)	--
Liver cancer	Male	5-9 years	95+ years	Data Rich	1	2	Cumulative Cigarettes (5 Years)	--
Liver cancer	Male	5-9 years	95+ years	Data Rich	1	2	Tobacco (cigarettes per capita)	--
Liver cancer	Male	5-9 years	95+ years	Data Rich	-1	3	Education (years per capita)	73
Liver cancer	Male	5-9 years	95+ years	Data Rich	0	3	Socio-demographic Index	252
Liver cancer	Male	5-9 years	95+ years	Data Rich	0	3	LDI (I$ per capita)	--
Liver cancer	Male	5-9 years	95+ years	Global	1	1	Alcohol (liters per capita)	--

Appendix Table 9: CODEm covariates used, level of covariate, and expected direction of covariate by cause, sex, and age<br>*Covariates that CODEm did not select during the covariate selection process have no draw counts listed.*								
**Cause**	**Sex**	**Age Start**	**Age End**	**Model Type**	**Direction**	**Level**	**Covariate Name**	**Number of Draws**
Liver cancer	Male	5-9 years	95+ years	Global	1	1	HIV age-standardized prevalence	--
Liver cancer	Male	5-9 years	95+ years	Global	1	1	Hepatitis B (HBsAg) Seroprevalence	--
Liver cancer	Male	5-9 years	95+ years	Global	1	1	Hepatitis C (IgG) Seroprevalence	--
Liver cancer	Male	5-9 years	95+ years	Global	1	1	Log-transformed SEV scalar: Liver C	--
Liver cancer	Male	5-9 years	95+ years	Global	-1	2	Healthcare access and quality index	--
Liver cancer	Male	5-9 years	95+ years	Global	-1	2	Hepatitis B 3-dose coverage (proportion)	--
Liver cancer	Male	5-9 years	95+ years	Global	-1	2	Hepatitis B 3-dose coverage (proportion), lagged 10 years	--
Liver cancer	Male	5-9 years	95+ years	Global	-1	2	Hepatitis B 3-dose coverage (proportion), lagged 5 years	--
Liver cancer	Male	5-9 years	95+ years	Global	-1	2	Hepatitis B vaccine coverage (proportion), aged through time	--
Liver cancer	Male	5-9 years	95+ years	Global	1	2	Cumulative Cigarettes (10 Years)	--
Liver cancer	Male	5-9 years	95+ years	Global	1	2	Cumulative Cigarettes (15 Years)	--
Liver cancer	Male	5-9 years	95+ years	Global	1	2	Cumulative Cigarettes (20 Years)	--
Liver cancer	Male	5-9 years	95+ years	Global	1	2	Cumulative Cigarettes (5 Years)	--
Liver cancer	Male	5-9 years	95+ years	Global	1	2	Diabetes Age-Standardized Prevalence (proportion)	--
Liver cancer	Male	5-9 years	95+ years	Global	1	2	Diabetes Fasting Plasma Glucose (mmol/L)	--
Liver cancer	Male	5-9 years	95+ years	Global	1	2	Intravenous drug use (age-standardized proportion)	--
Liver cancer	Male	5-9 years	95+ years	Global	1	2	Mean BMI	--
Liver cancer	Male	5-9 years	95+ years	Global	1	2	Tobacco (cigarettes per capita)	--
Liver cancer	Male	5-9 years	95+ years	Global	1	2	red meats adjusted(g)	--
Liver cancer	Male	5-9 years	95+ years	Global	-1	3	Education (years per capita)	--
Liver cancer	Male	5-9 years	95+ years	Global	0	3	LDI (I$ per capita)	--
Liver cancer	Male	5-9 years	95+ years	Global	0	3	Socio-demographic Index	--
Gallbladder and biliary tract cancer	Female	15-19 years	95+ years	Data Rich	1	1	Log-transformed SEV scalar: Gallblad C	--
Gallbladder and biliary tract cancer	Female	15-19 years	95+ years	Data Rich	1	1	Mean BMI	--
Gallbladder and biliary tract cancer	Female	15-19 years	95+ years	Data Rich	-1	2	fruits adjusted(g)	25
Gallbladder and biliary tract cancer	Female	15-19 years	95+ years	Data Rich	-1	2	Healthcare access and quality index	79
Gallbladder and biliary tract cancer	Female	15-19 years	95+ years	Data Rich	-1	2	vegetables adjusted(g)	125
Gallbladder and biliary tract cancer	Female	15-19 years	95+ years	Data Rich	1	2	Smoking Prevalence	354
Gallbladder and biliary tract cancer	Female	15-19 years	95+ years	Data Rich	1	2	Cumulative Cigarettes (10 Years)	502
Gallbladder and biliary tract cancer	Female	15-19 years	95+ years	Data Rich	1	2	Alcohol (liters per capita)	--
Gallbladder and biliary tract cancer	Female	15-19 years	95+ years	Data Rich	1	2	Cumulative Cigarettes (5 Years)	--
Gallbladder and biliary tract cancer	Female	15-19 years	95+ years	Data Rich	1	2	Diabetes Age-Standardized Prevalence (proportion)	--
Gallbladder and biliary tract cancer	Female	15-19 years	95+ years	Data Rich	1	2	Tobacco (cigarettes per capita)	--
Gallbladder and biliary tract cancer	Female	15-19 years	95+ years	Data Rich	-1	3	Education (years per capita)	113
Gallbladder and biliary tract cancer	Female	15-19 years	95+ years	Data Rich	0	3	Socio-demographic Index	77
Gallbladder and biliary tract cancer	Female	15-19 years	95+ years	Data Rich	0	3	LDI (I$ per capita)	--
Gallbladder and biliary tract cancer	Female	15-19 years	95+ years	Global	1	1	Log-transformed SEV scalar: Gallblad C	--
Gallbladder and biliary tract cancer	Female	15-19 years	95+ years	Global	1	1	Mean BMI	--
Gallbladder and biliary tract cancer	Female	15-19 years	95+ years	Global	-1	2	Healthcare access and quality index	532
Gallbladder and biliary tract cancer	Female	15-19 years	95+ years	Global	-1	2	fruits adjusted(g)	--
Gallbladder and biliary tract cancer	Female	15-19 years	95+ years	Global	-1	2	vegetables adjusted(g)	--
Gallbladder and biliary tract cancer	Female	15-19 years	95+ years	Global	1	2	Cumulative Cigarettes (10 Years)	262
Gallbladder and biliary tract cancer	Female	15-19 years	95+ years	Global	1	2	Smoking Prevalence	412
Gallbladder and biliary tract cancer	Female	15-19 years	95+ years	Global	1	2	Cumulative Cigarettes (5 Years)	420
Gallbladder and biliary tract cancer	Female	15-19 years	95+ years	Global	1	2	Alcohol (liters per capita)	682
Gallbladder and biliary tract cancer	Female	15-19 years	95+ years	Global	1	2	Diabetes Age-Standardized Prevalence (proportion)	--
Gallbladder and biliary tract cancer	Female	15-19 years	95+ years	Global	1	2	Tobacco (cigarettes per capita)	--
Gallbladder and biliary tract cancer	Female	15-19 years	95+ years	Global	-1	3	Education (years per capita)	282
Gallbladder and biliary tract cancer	Female	15-19 years	95+ years	Global	0	3	Socio-demographic Index	343
Gallbladder and biliary tract cancer	Female	15-19 years	95+ years	Global	0	3	LDI (I$ per capita)	--
Gallbladder and biliary tract cancer	Male	15-19 years	95+ years	Data Rich	1	1	Log-transformed SEV scalar: Gallblad C	--
Gallbladder and biliary tract cancer	Male	15-19 years	95+ years	Data Rich	1	1	Mean BMI	--
Gallbladder and biliary tract cancer	Male	15-19 years	95+ years	Data Rich	-1	2	fruits adjusted(g)	97
Gallbladder and biliary tract cancer	Male	15-19 years	95+ years	Data Rich	-1	2	vegetables adjusted(g)	497
Gallbladder and biliary tract cancer	Male	15-19 years	95+ years	Data Rich	-1	2	Healthcare access and quality index	--
Gallbladder and biliary tract cancer	Male	15-19 years	95+ years	Data Rich	1	2	Cumulative Cigarettes (5 Years)	155
Gallbladder and biliary tract cancer	Male	15-19 years	95+ years	Data Rich	1	2	Alcohol (liters per capita)	198
Gallbladder and biliary tract cancer	Male	15-19 years	95+ years	Data Rich	1	2	Cumulative Cigarettes (10 Years)	241
Gallbladder and biliary tract cancer	Male	15-19 years	95+ years	Data Rich	1	2	Smoking Prevalence	396
Gallbladder and biliary tract cancer	Male	15-19 years	95+ years	Data Rich	1	2	Tobacco (cigarettes per capita)	396
Gallbladder and biliary tract cancer	Male	15-19 years	95+ years	Data Rich	1	2	Diabetes Age-Standardized Prevalence (proportion)	--
Gallbladder and biliary tract cancer	Male	15-19 years	95+ years	Data Rich	-1	3	Education (years per capita)	324
Gallbladder and biliary tract cancer	Male	15-19 years	95+ years	Data Rich	0	3	Socio-demographic Index	207
Gallbladder and biliary tract cancer	Male	15-19 years	95+ years	Data Rich	0	3	LDI (I$ per capita)	--
Gallbladder and biliary tract cancer	Male	15-19 years	95+ years	Global	1	1	Log-transformed SEV scalar: Gallblad C	463
Gallbladder and biliary tract cancer	Male	15-19 years	95+ years	Global	1	1	Mean BMI	--
Gallbladder and biliary tract cancer	Male	15-19 years	95+ years	Global	-1	2	vegetables adjusted(g)	100
Gallbladder and biliary tract cancer	Male	15-19 years	95+ years	Global	-1	2	Healthcare access and quality index	--
Gallbladder and biliary tract cancer	Male	15-19 years	95+ years	Global	-1	2	fruits adjusted(g)	--
Gallbladder and biliary tract cancer	Male	15-19 years	95+ years	Global	1	2	Alcohol (liters per capita)	245
Gallbladder and biliary tract cancer	Male	15-19 years	95+ years	Global	1	2	Cumulative Cigarettes (10 Years)	562
Gallbladder and biliary tract cancer	Male	15-19 years	95+ years	Global	1	2	Cumulative Cigarettes (5 Years)	--
Gallbladder and biliary tract cancer	Male	15-19 years	95+ years	Global	1	2	Diabetes Age-Standardized Prevalence (proportion)	--
Gallbladder and biliary tract cancer	Male	15-19 years	95+ years	Global	1	2	Smoking Prevalence	--
Gallbladder and biliary tract cancer	Male	15-19 years	95+ years	Global	1	2	Tobacco (cigarettes per capita)	--
Gallbladder and biliary tract cancer	Male	15-19 years	95+ years	Global	-1	3	Education (years per capita)	293
Gallbladder and biliary tract cancer	Male	15-19 years	95+ years	Global	0	3	Socio-demographic Index	173
Gallbladder and biliary tract cancer	Male	15-19 years	95+ years	Global	0	3	LDI (I$ per capita)	--

Appendix Table 9: CODEm covariates used, level of covariate, and expected direction of covariate by cause, sex, and age<br>*Covariates that CODEm did not select during the covariate selection process have no draw counts listed.*								
Cause	Sex	Age Start	Age End	Model Type	Direction	Level	Covariate Name	Number of Draws
Pancreatic cancer	Female	15-19 years	95+ years	Data Rich	1	1	Alcohol (liters per capita)	0
Pancreatic cancer	Female	15-19 years	95+ years	Data Rich	1	1	Tobacco (cigarettes per capita)	5
Pancreatic cancer	Female	15-19 years	95+ years	Data Rich	1	1	Cumulative Cigarettes (5 Years)	105
Pancreatic cancer	Female	15-19 years	95+ years	Data Rich	1	1	Cumulative Cigarettes (10 Years)	177
Pancreatic cancer	Female	15-19 years	95+ years	Data Rich	1	1	Cumulative Cigarettes (15 Years)	230
Pancreatic cancer	Female	15-19 years	95+ years	Data Rich	1	1	Cumulative Cigarettes (20 Years)	279
Pancreatic cancer	Female	15-19 years	95+ years	Data Rich	1	1	Smoking Prevalence	446
Pancreatic cancer	Female	15-19 years	95+ years	Data Rich	1	1	Log-transformed SEV scalar: Pancreas C	700
Pancreatic cancer	Female	15-19 years	95+ years	Data Rich	1	1	Mean BMI	810
Pancreatic cancer	Female	15-19 years	95+ years	Data Rich	-1	2	Healthcare access and quality index	--
Pancreatic cancer	Female	15-19 years	95+ years	Data Rich	-1	2	fruits adjusted(g)	--
Pancreatic cancer	Female	15-19 years	95+ years	Data Rich	-1	2	vegetables adjusted(g)	--
Pancreatic cancer	Female	15-19 years	95+ years	Data Rich	1	2	red meats adjusted(g)	0
Pancreatic cancer	Female	15-19 years	95+ years	Data Rich	1	2	Diabetes Fasting Plasma Glucose (mmol/L)	75
Pancreatic cancer	Female	15-19 years	95+ years	Data Rich	1	2	energy unadjusted(kcal)	186
Pancreatic cancer	Female	15-19 years	95+ years	Data Rich	1	2	Diabetes Age-Standardized Prevalence (proportion)	192
Pancreatic cancer	Female	15-19 years	95+ years	Data Rich	-1	3	Education (years per capita)	--
Pancreatic cancer	Female	15-19 years	95+ years	Data Rich	0	3	Socio-demographic Index	980
Pancreatic cancer	Female	15-19 years	95+ years	Data Rich	0	3	LDI (I$ per capita)	--
Pancreatic cancer	Female	15-19 years	95+ years	Global	1	1	Alcohol (liters per capita)	0
Pancreatic cancer	Female	15-19 years	95+ years	Global	1	1	Cumulative Cigarettes (15 Years)	131
Pancreatic cancer	Female	15-19 years	95+ years	Global	1	1	Cumulative Cigarettes (5 Years)	133
Pancreatic cancer	Female	15-19 years	95+ years	Global	1	1	Cumulative Cigarettes (20 Years)	157
Pancreatic cancer	Female	15-19 years	95+ years	Global	1	1	Cumulative Cigarettes (10 Years)	179
Pancreatic cancer	Female	15-19 years	95+ years	Global	1	1	Tobacco (cigarettes per capita)	502
Pancreatic cancer	Female	15-19 years	95+ years	Global	1	1	Smoking Prevalence	542
Pancreatic cancer	Female	15-19 years	95+ years	Global	1	1	Log-transformed SEV scalar: Pancreas C	610
Pancreatic cancer	Female	15-19 years	95+ years	Global	1	1	Mean BMI	999
Pancreatic cancer	Female	15-19 years	95+ years	Global	-1	2	Healthcare access and quality index	--
Pancreatic cancer	Female	15-19 years	95+ years	Global	-1	2	fruits adjusted(g)	--
Pancreatic cancer	Female	15-19 years	95+ years	Global	-1	2	vegetables adjusted(g)	--
Pancreatic cancer	Female	15-19 years	95+ years	Global	1	2	energy unadjusted(kcal)	0
Pancreatic cancer	Female	15-19 years	95+ years	Global	1	2	Diabetes Age-Standardized Prevalence (proportion)	1
Pancreatic cancer	Female	15-19 years	95+ years	Global	1	2	Diabetes Fasting Plasma Glucose (mmol/L)	1
Pancreatic cancer	Female	15-19 years	95+ years	Global	1	2	red meats adjusted(g)	138
Pancreatic cancer	Female	15-19 years	95+ years	Global	-1	3	Education (years per capita)	--
Pancreatic cancer	Female	15-19 years	95+ years	Global	0	3	Socio-demographic Index	999
Pancreatic cancer	Female	15-19 years	95+ years	Global	0	3	LDI (I$ per capita)	--
Pancreatic cancer	Male	15-19 years	95+ years	Data Rich	1	1	Cumulative Cigarettes (5 Years)	12
Pancreatic cancer	Male	15-19 years	95+ years	Data Rich	1	1	Tobacco (cigarettes per capita)	46
Pancreatic cancer	Male	15-19 years	95+ years	Data Rich	1	1	Cumulative Cigarettes (10 Years)	93
Pancreatic cancer	Male	15-19 years	95+ years	Data Rich	1	1	Smoking Prevalence	165
Pancreatic cancer	Male	15-19 years	95+ years	Data Rich	1	1	Log-transformed SEV scalar: Pancreas C	180
Pancreatic cancer	Male	15-19 years	95+ years	Data Rich	1	1	Cumulative Cigarettes (20 Years)	205
Pancreatic cancer	Male	15-19 years	95+ years	Data Rich	1	1	Cumulative Cigarettes (15 Years)	702
Pancreatic cancer	Male	15-19 years	95+ years	Data Rich	1	1	Mean BMI	923
Pancreatic cancer	Male	15-19 years	95+ years	Data Rich	1	1	Alcohol (liters per capita)	--
Pancreatic cancer	Male	15-19 years	95+ years	Data Rich	-1	2	Healthcare access and quality index	--
Pancreatic cancer	Male	15-19 years	95+ years	Data Rich	-1	2	fruits adjusted(g)	--
Pancreatic cancer	Male	15-19 years	95+ years	Data Rich	-1	2	vegetables adjusted(g)	--
Pancreatic cancer	Male	15-19 years	95+ years	Data Rich	1	2	Diabetes Age-Standardized Prevalence (proportion)	46
Pancreatic cancer	Male	15-19 years	95+ years	Data Rich	1	2	energy unadjusted(kcal)	66
Pancreatic cancer	Male	15-19 years	95+ years	Data Rich	1	2	red meats adjusted(g)	84
Pancreatic cancer	Male	15-19 years	95+ years	Data Rich	1	2	Diabetes Fasting Plasma Glucose (mmol/L)	436
Pancreatic cancer	Male	15-19 years	95+ years	Data Rich	-1	3	Education (years per capita)	--
Pancreatic cancer	Male	15-19 years	95+ years	Data Rich	0	3	Socio-demographic Index	588
Pancreatic cancer	Male	15-19 years	95+ years	Data Rich	0	3	LDI (I$ per capita)	--
Pancreatic cancer	Male	15-19 years	95+ years	Global	1	1	Alcohol (liters per capita)	0
Pancreatic cancer	Male	15-19 years	95+ years	Global	1	1	Tobacco (cigarettes per capita)	38
Pancreatic cancer	Male	15-19 years	95+ years	Global	1	1	Cumulative Cigarettes (20 Years)	82
Pancreatic cancer	Male	15-19 years	95+ years	Global	1	1	Cumulative Cigarettes (10 Years)	85
Pancreatic cancer	Male	15-19 years	95+ years	Global	1	1	Smoking Prevalence	214
Pancreatic cancer	Male	15-19 years	95+ years	Global	1	1	Log-transformed SEV scalar: Pancreas C	288
Pancreatic cancer	Male	15-19 years	95+ years	Global	1	1	Cumulative Cigarettes (15 Years)	327
Pancreatic cancer	Male	15-19 years	95+ years	Global	1	1	Cumulative Cigarettes (5 Years)	532
Pancreatic cancer	Male	15-19 years	95+ years	Global	1	1	Mean BMI	935
Pancreatic cancer	Male	15-19 years	95+ years	Global	-1	2	Healthcare access and quality index	--
Pancreatic cancer	Male	15-19 years	95+ years	Global	-1	2	fruits adjusted(g)	--
Pancreatic cancer	Male	15-19 years	95+ years	Global	-1	2	vegetables adjusted(g)	--
Pancreatic cancer	Male	15-19 years	95+ years	Global	1	2	energy unadjusted(kcal)	67
Pancreatic cancer	Male	15-19 years	95+ years	Global	1	2	Diabetes Fasting Plasma Glucose (mmol/L)	122
Pancreatic cancer	Male	15-19 years	95+ years	Global	1	2	Diabetes Age-Standardized Prevalence (proportion)	254
Pancreatic cancer	Male	15-19 years	95+ years	Global	1	2	red meats adjusted(g)	337
Pancreatic cancer	Male	15-19 years	95+ years	Global	-1	3	Education (years per capita)	--
Pancreatic cancer	Male	15-19 years	95+ years	Global	0	3	Socio-demographic Index	679
Pancreatic cancer	Male	15-19 years	95+ years	Global	0	3	LDI (I$ per capita)	--
Larynx cancer	Female	15-19 years	95+ years	Data Rich	1	1	Alcohol (liters per capita)	180
Larynx cancer	Female	15-19 years	95+ years	Data Rich	1	1	Log-transformed SEV scalar: Larynx C	1000

Appendix Table 9: CODEm covariates used, level of covariate, and expected direction of covariate by cause, sex, and age
Covariates that CODEm did not select during the covariate selection process have no draw counts listed.

Cause	Sex	Age Start	Age End	Model Type	Direction	Level	Covariate Name	Number of Draws
Larynx cancer	Female	15-19 years	95+ years	Data Rich	-1	2	Healthcare access and quality index	0
Larynx cancer	Female	15-19 years	95+ years	Data Rich	-1	2	fruits adjusted(g)	0
Larynx cancer	Female	15-19 years	95+ years	Data Rich	-1	2	vegetables adjusted(g)	0
Larynx cancer	Female	15-19 years	95+ years	Data Rich	1	2	Tobacco (cigarettes per capita)	0
Larynx cancer	Female	15-19 years	95+ years	Data Rich	1	2	Smoking Prevalence	10
Larynx cancer	Female	15-19 years	95+ years	Data Rich	1	2	Cumulative Cigarettes (20 Years)	120
Larynx cancer	Female	15-19 years	95+ years	Data Rich	1	2	Cumulative Cigarettes (15 Years)	123
Larynx cancer	Female	15-19 years	95+ years	Data Rich	1	2	Cumulative Cigarettes (10 Years)	161
Larynx cancer	Female	15-19 years	95+ years	Data Rich	1	2	Cumulative Cigarettes (5 Years)	212
Larynx cancer	Female	15-19 years	95+ years	Data Rich	1	2	Population Density (over 1000 ppl/sqkm, proportion)	529
Larynx cancer	Female	15-19 years	95+ years	Data Rich	1	2	Asbestos consumption (metric tons per year per capita)	--
Larynx cancer	Female	15-19 years	95+ years	Data Rich	1	2	Population Density (under 150 ppl/sqkm, proportion)	--
Larynx cancer	Female	15-19 years	95+ years	Data Rich	-1	3	Education (years per capita)	8
Larynx cancer	Female	15-19 years	95+ years	Data Rich	0	3	Socio-demographic Index	375
Larynx cancer	Female	15-19 years	95+ years	Data Rich	0	3	LDI (I$ per capita)	--
Larynx cancer	Female	15-19 years	95+ years	Global	1	1	Alcohol (liters per capita)	253
Larynx cancer	Female	15-19 years	95+ years	Global	1	1	Log-transformed SEV scalar: Larynx C	875
Larynx cancer	Female	15-19 years	95+ years	Global	-1	2	vegetables adjusted(g)	0
Larynx cancer	Female	15-19 years	95+ years	Global	-1	2	fruits adjusted(g)	138
Larynx cancer	Female	15-19 years	95+ years	Global	-1	2	Healthcare access and quality index	372
Larynx cancer	Female	15-19 years	95+ years	Global	1	2	Tobacco (cigarettes per capita)	94
Larynx cancer	Female	15-19 years	95+ years	Global	1	2	Cumulative Cigarettes (20 Years)	152
Larynx cancer	Female	15-19 years	95+ years	Global	1	2	Cumulative Cigarettes (15 Years)	181
Larynx cancer	Female	15-19 years	95+ years	Global	1	2	Cumulative Cigarettes (10 Years)	233
Larynx cancer	Female	15-19 years	95+ years	Global	1	2	Cumulative Cigarettes (5 Years)	285
Larynx cancer	Female	15-19 years	95+ years	Global	1	2	Smoking Prevalence	413
Larynx cancer	Female	15-19 years	95+ years	Global	1	2	Population Density (over 1000 ppl/sqkm, proportion)	618
Larynx cancer	Female	15-19 years	95+ years	Global	1	2	Asbestos consumption (metric tons per year per capita)	--
Larynx cancer	Female	15-19 years	95+ years	Global	1	2	Population Density (under 150 ppl/sqkm, proportion)	--
Larynx cancer	Female	15-19 years	95+ years	Global	-1	3	Education (years per capita)	165
Larynx cancer	Female	15-19 years	95+ years	Global	0	3	Socio-demographic Index	39
Larynx cancer	Female	15-19 years	95+ years	Global	0	3	LDI (I$ per capita)	--
Larynx cancer	Male	15-19 years	95+ years	Data Rich	1	1	Log-transformed SEV scalar: Larynx C	59
Larynx cancer	Male	15-19 years	95+ years	Data Rich	1	1	Alcohol (liters per capita)	982
Larynx cancer	Male	15-19 years	95+ years	Data Rich	-1	2	fruits adjusted(g)	0
Larynx cancer	Male	15-19 years	95+ years	Data Rich	-1	2	Healthcare access and quality index	57
Larynx cancer	Male	15-19 years	95+ years	Data Rich	-1	2	vegetables adjusted(g)	732
Larynx cancer	Male	15-19 years	95+ years	Data Rich	1	2	Tobacco (cigarettes per capita)	97
Larynx cancer	Male	15-19 years	95+ years	Data Rich	1	2	Cumulative Cigarettes (10 Years)	143
Larynx cancer	Male	15-19 years	95+ years	Data Rich	1	2	Smoking Prevalence	347
Larynx cancer	Male	15-19 years	95+ years	Data Rich	1	2	Cumulative Cigarettes (15 Years)	400
Larynx cancer	Male	15-19 years	95+ years	Data Rich	1	2	Cumulative Cigarettes (5 Years)	467
Larynx cancer	Male	15-19 years	95+ years	Data Rich	1	2	Cumulative Cigarettes (20 Years)	468
Larynx cancer	Male	15-19 years	95+ years	Data Rich	1	2	Population Density (over 1000 ppl/sqkm, proportion)	789
Larynx cancer	Male	15-19 years	95+ years	Data Rich	1	2	Asbestos consumption (metric tons per year per capita)	--
Larynx cancer	Male	15-19 years	95+ years	Data Rich	1	2	Population Density (under 150 ppl/sqkm, proportion)	--
Larynx cancer	Male	15-19 years	95+ years	Data Rich	-1	3	Education (years per capita)	22
Larynx cancer	Male	15-19 years	95+ years	Data Rich	0	3	Socio-demographic Index	32
Larynx cancer	Male	15-19 years	95+ years	Data Rich	0	3	LDI (I$ per capita)	--
Larynx cancer	Male	15-19 years	95+ years	Global	1	1	Log-transformed SEV scalar: Larynx C	413
Larynx cancer	Male	15-19 years	95+ years	Global	1	1	Alcohol (liters per capita)	587
Larynx cancer	Male	15-19 years	95+ years	Global	-1	2	fruits adjusted(g)	0
Larynx cancer	Male	15-19 years	95+ years	Global	-1	2	Healthcare access and quality index	168
Larynx cancer	Male	15-19 years	95+ years	Global	-1	2	vegetables adjusted(g)	390
Larynx cancer	Male	15-19 years	95+ years	Global	1	2	Cumulative Cigarettes (20 Years)	79
Larynx cancer	Male	15-19 years	95+ years	Global	1	2	Cumulative Cigarettes (10 Years)	377
Larynx cancer	Male	15-19 years	95+ years	Global	1	2	Smoking Prevalence	466
Larynx cancer	Male	15-19 years	95+ years	Global	1	2	Population Density (over 1000 ppl/sqkm, proportion)	471
Larynx cancer	Male	15-19 years	95+ years	Global	1	2	Cumulative Cigarettes (5 Years)	484
Larynx cancer	Male	15-19 years	95+ years	Global	1	2	Tobacco (cigarettes per capita)	693
Larynx cancer	Male	15-19 years	95+ years	Global	1	2	Cumulative Cigarettes (15 Years)	727
Larynx cancer	Male	15-19 years	95+ years	Global	1	2	Asbestos consumption (metric tons per year per capita)	--
Larynx cancer	Male	15-19 years	95+ years	Global	1	2	Population Density (under 150 ppl/sqkm, proportion)	--
Larynx cancer	Male	15-19 years	95+ years	Global	-1	3	Education (years per capita)	155
Larynx cancer	Male	15-19 years	95+ years	Global	0	3	Socio-demographic Index	245
Larynx cancer	Male	15-19 years	95+ years	Global	0	3	LDI (I$ per capita)	--
Tracheal, bronchus, and lung cancer	Female	15-19 years	95+ years	Data Rich	1	1	Log-transformed SEV scalar: Lung C	12
Tracheal, bronchus, and lung cancer	Female	15-19 years	95+ years	Data Rich	1	1	Cumulative Cigarettes (10 Years)	15
Tracheal, bronchus, and lung cancer	Female	15-19 years	95+ years	Data Rich	1	1	Cumulative Cigarettes (15 Years)	89
Tracheal, bronchus, and lung cancer	Female	15-19 years	95+ years	Data Rich	1	1	Cumulative Cigarettes (20 Years)	120
Tracheal, bronchus, and lung cancer	Female	15-19 years	95+ years	Data Rich	1	1	Smoking Prevalence	167
Tracheal, bronchus, and lung cancer	Female	15-19 years	95+ years	Data Rich	1	1	Cumulative Cigarettes (5 Years)	179
Tracheal, bronchus, and lung cancer	Female	15-19 years	95+ years	Data Rich	1	1	Asbestos consumption (metric tons per year per capita)	429
Tracheal, bronchus, and lung cancer	Female	15-19 years	95+ years	Data Rich	1	1	Log-transformed age-standardized SEV scalar: Lung C	559
Tracheal, bronchus, and lung cancer	Female	15-19 years	95+ years	Data Rich	1	1	Secondhand smoke	--
Tracheal, bronchus, and lung cancer	Female	15-19 years	95+ years	Data Rich	1	1	Tobacco (cigarettes per capita)	--
Tracheal, bronchus, and lung cancer	Female	15-19 years	95+ years	Data Rich	-1	2	Healthcare access and quality index	--
Tracheal, bronchus, and lung cancer	Female	15-19 years	95+ years	Data Rich	1	2	Outdoor Air Pollution (PM2.5)	0

**Appendix Table 9: CODEm covariates used, level of covariate, and expected direction of covariate by cause, sex, and age** *Covariates that CODEm did not select during the covariate selection process have no draw counts listed.*								
**Cause**	**Sex**	**Age Start**	**Age End**	**Model Type**	**Direction**	**Level**	**Covariate Name**	**Number of Draws**
Tracheal, bronchus, and lung cancer	Female	15-19 years	95+ years	Data Rich	1	2	Diabetes Fasting Plasma Glucose (mmol/L)	605
Tracheal, bronchus, and lung cancer	Female	15-19 years	95+ years	Data Rich	1	2	Indoor Air Pollution (All Cooking Fuels)	--
Tracheal, bronchus, and lung cancer	Female	15-19 years	95+ years	Data Rich	1	2	Residential radon	--
Tracheal, bronchus, and lung cancer	Female	15-19 years	95+ years	Data Rich	-1	3	Education (years per capita)	--
Tracheal, bronchus, and lung cancer	Female	15-19 years	95+ years	Data Rich	0	3	Socio-demographic Index	571
Tracheal, bronchus, and lung cancer	Female	15-19 years	95+ years	Data Rich	0	3	LDI (I$ per capita)	--
Tracheal, bronchus, and lung cancer	Female	15-19 years	95+ years	Global	1	1	Asbestos consumption (metric tons per year per capita)	0
Tracheal, bronchus, and lung cancer	Female	15-19 years	95+ years	Global	1	1	Secondhand smoke	0
Tracheal, bronchus, and lung cancer	Female	15-19 years	95+ years	Global	1	1	Cumulative Cigarettes (10 Years)	27
Tracheal, bronchus, and lung cancer	Female	15-19 years	95+ years	Global	1	1	Cumulative Cigarettes (15 Years)	27
Tracheal, bronchus, and lung cancer	Female	15-19 years	95+ years	Global	1	1	Cumulative Cigarettes (20 Years)	71
Tracheal, bronchus, and lung cancer	Female	15-19 years	95+ years	Global	1	1	Cumulative Cigarettes (5 Years)	163
Tracheal, bronchus, and lung cancer	Female	15-19 years	95+ years	Global	1	1	Smoking Prevalence	404
Tracheal, bronchus, and lung cancer	Female	15-19 years	95+ years	Global	1	1	Log-transformed SEV scalar: Lung C	433
Tracheal, bronchus, and lung cancer	Female	15-19 years	95+ years	Global	1	1	Log-transformed age-standardized SEV scalar: Lung C	574
Tracheal, bronchus, and lung cancer	Female	15-19 years	95+ years	Global	1	1	Tobacco (cigarettes per capita)	--
Tracheal, bronchus, and lung cancer	Female	15-19 years	95+ years	Global	-1	2	Healthcare access and quality index	--
Tracheal, bronchus, and lung cancer	Female	15-19 years	95+ years	Global	1	2	Outdoor Air Pollution (PM2.5)	138
Tracheal, bronchus, and lung cancer	Female	15-19 years	95+ years	Global	1	2	Diabetes Fasting Plasma Glucose (mmol/L)	472
Tracheal, bronchus, and lung cancer	Female	15-19 years	95+ years	Global	1	2	Indoor Air Pollution (All Cooking Fuels)	--
Tracheal, bronchus, and lung cancer	Female	15-19 years	95+ years	Global	1	2	Residential radon	--
Tracheal, bronchus, and lung cancer	Female	15-19 years	95+ years	Global	-1	3	Education (years per capita)	--
Tracheal, bronchus, and lung cancer	Female	15-19 years	95+ years	Global	0	3	Socio-demographic Index	999
Tracheal, bronchus, and lung cancer	Female	15-19 years	95+ years	Global	0	3	LDI (I$ per capita)	--
Tracheal, bronchus, and lung cancer	Male	15-19 years	95+ years	Data Rich	1	1	Smoking Prevalence	0
Tracheal, bronchus, and lung cancer	Male	15-19 years	95+ years	Data Rich	1	1	Tobacco (cigarettes per capita)	0
Tracheal, bronchus, and lung cancer	Male	15-19 years	95+ years	Data Rich	1	1	Cumulative Cigarettes (5 Years)	27
Tracheal, bronchus, and lung cancer	Male	15-19 years	95+ years	Data Rich	1	1	Secondhand smoke	69
Tracheal, bronchus, and lung cancer	Male	15-19 years	95+ years	Data Rich	1	1	Cumulative Cigarettes (10 Years)	118
Tracheal, bronchus, and lung cancer	Male	15-19 years	95+ years	Data Rich	1	1	Cumulative Cigarettes (20 Years)	224
Tracheal, bronchus, and lung cancer	Male	15-19 years	95+ years	Data Rich	1	1	Log-transformed SEV scalar: Lung C	322
Tracheal, bronchus, and lung cancer	Male	15-19 years	95+ years	Data Rich	1	1	Cumulative Cigarettes (15 Years)	494
Tracheal, bronchus, and lung cancer	Male	15-19 years	95+ years	Data Rich	1	1	Asbestos consumption (metric tons per year per capita)	690
Tracheal, bronchus, and lung cancer	Male	15-19 years	95+ years	Data Rich	1	1	Log-transformed age-standardized SEV scalar: Lung C	765
Tracheal, bronchus, and lung cancer	Male	15-19 years	95+ years	Data Rich	-1	2	Healthcare access and quality index	0
Tracheal, bronchus, and lung cancer	Male	15-19 years	95+ years	Data Rich	1	2	Outdoor Air Pollution (PM2.5)	46
Tracheal, bronchus, and lung cancer	Male	15-19 years	95+ years	Data Rich	1	2	Diabetes Fasting Plasma Glucose (mmol/L)	599
Tracheal, bronchus, and lung cancer	Male	15-19 years	95+ years	Data Rich	1	2	Indoor Air Pollution (All Cooking Fuels)	--
Tracheal, bronchus, and lung cancer	Male	15-19 years	95+ years	Data Rich	1	2	Residential radon	--
Tracheal, bronchus, and lung cancer	Male	15-19 years	95+ years	Data Rich	-1	3	Education (years per capita)	547
Tracheal, bronchus, and lung cancer	Male	15-19 years	95+ years	Data Rich	0	3	Socio-demographic Index	673
Tracheal, bronchus, and lung cancer	Male	15-19 years	95+ years	Data Rich	0	3	LDI (I$ per capita)	--
Tracheal, bronchus, and lung cancer	Male	15-19 years	95+ years	Global	1	1	Tobacco (cigarettes per capita)	63
Tracheal, bronchus, and lung cancer	Male	15-19 years	95+ years	Global	1	1	Cumulative Cigarettes (15 Years)	68
Tracheal, bronchus, and lung cancer	Male	15-19 years	95+ years	Global	1	1	Smoking Prevalence	68
Tracheal, bronchus, and lung cancer	Male	15-19 years	95+ years	Global	1	1	Cumulative Cigarettes (10 Years)	231
Tracheal, bronchus, and lung cancer	Male	15-19 years	95+ years	Global	1	1	Log-transformed SEV scalar: Lung C	236
Tracheal, bronchus, and lung cancer	Male	15-19 years	95+ years	Global	1	1	Cumulative Cigarettes (5 Years)	239
Tracheal, bronchus, and lung cancer	Male	15-19 years	95+ years	Global	1	1	Cumulative Cigarettes (20 Years)	268
Tracheal, bronchus, and lung cancer	Male	15-19 years	95+ years	Global	1	1	Asbestos consumption (metric tons per year per capita)	361
Tracheal, bronchus, and lung cancer	Male	15-19 years	95+ years	Global	1	1	Secondhand smoke	365
Tracheal, bronchus, and lung cancer	Male	15-19 years	95+ years	Global	1	1	Log-transformed age-standardized SEV scalar: Lung C	369
Tracheal, bronchus, and lung cancer	Male	15-19 years	95+ years	Global	-1	2	Healthcare access and quality index	0
Tracheal, bronchus, and lung cancer	Male	15-19 years	95+ years	Global	1	2	Diabetes Fasting Plasma Glucose (mmol/L)	324
Tracheal, bronchus, and lung cancer	Male	15-19 years	95+ years	Global	1	2	Outdoor Air Pollution (PM2.5)	353
Tracheal, bronchus, and lung cancer	Male	15-19 years	95+ years	Global	1	2	Indoor Air Pollution (All Cooking Fuels)	--
Tracheal, bronchus, and lung cancer	Male	15-19 years	95+ years	Global	1	2	Residential radon	--
Tracheal, bronchus, and lung cancer	Male	15-19 years	95+ years	Global	-1	3	Education (years per capita)	9
Tracheal, bronchus, and lung cancer	Male	15-19 years	95+ years	Global	0	3	Socio-demographic Index	538
Tracheal, bronchus, and lung cancer	Male	15-19 years	95+ years	Global	0	3	LDI (I$ per capita)	--
Malignant skin melanoma	Female	15-19 years	95+ years	Data Rich	1	1	Alcohol (liters per capita)	--
Malignant skin melanoma	Female	15-19 years	95+ years	Data Rich	-1	2	Latitude 30 to 45 (proportion)	310
Malignant skin melanoma	Female	15-19 years	95+ years	Data Rich	-1	2	Healthcare access and quality index	--
Malignant skin melanoma	Female	15-19 years	95+ years	Data Rich	-1	2	Latitude Over 45 (proportion)	--
Malignant skin melanoma	Female	15-19 years	95+ years	Data Rich	-1	2	fruits adjusted(g)	--
Malignant skin melanoma	Female	15-19 years	95+ years	Data Rich	-1	2	vegetables adjusted(g)	--
Malignant skin melanoma	Female	15-19 years	95+ years	Data Rich	0	2	Latitude Under 15 (proportion)	367
Malignant skin melanoma	Female	15-19 years	95+ years	Data Rich	0	2	Latitude 15 to 30 (proportion)	--
Malignant skin melanoma	Female	15-19 years	95+ years	Data Rich	-1	3	Education (years per capita)	--
Malignant skin melanoma	Female	15-19 years	95+ years	Data Rich	0	3	Socio-demographic Index	566
Malignant skin melanoma	Female	15-19 years	95+ years	Data Rich	0	3	LDI (I$ per capita)	--
Malignant skin melanoma	Female	15-19 years	95+ years	Global	1	1	Alcohol (liters per capita)	181
Malignant skin melanoma	Female	15-19 years	95+ years	Global	-1	2	vegetables adjusted(g)	433
Malignant skin melanoma	Female	15-19 years	95+ years	Global	-1	2	Healthcare access and quality index	--
Malignant skin melanoma	Female	15-19 years	95+ years	Global	-1	2	Latitude 30 to 45 (proportion)	--
Malignant skin melanoma	Female	15-19 years	95+ years	Global	-1	2	Latitude Over 45 (proportion)	--
Malignant skin melanoma	Female	15-19 years	95+ years	Global	-1	2	fruits adjusted(g)	--
Malignant skin melanoma	Female	15-19 years	95+ years	Global	0	2	Latitude 15 to 30 (proportion)	472

Appendix Table 9: CODEm covariates used, level of covariate, and expected direction of covariate by cause, sex, and age<br>*Covariates that CODEm did not select during the covariate selection process have no draw counts listed.*								
**Cause**	**Sex**	**Age Start**	**Age End**	**Model Type**	**Direction**	**Level**	**Covariate Name**	**Number of Draws**
Malignant skin melanoma	Female	15-19 years	95+ years	Global	0	2	Latitude Under 15 (proportion)	--
Malignant skin melanoma	Female	15-19 years	95+ years	Global	-1	3	Education (years per capita)	--
Malignant skin melanoma	Female	15-19 years	95+ years	Global	0	3	Socio-demographic Index	331
Malignant skin melanoma	Female	15-19 years	95+ years	Global	0	3	LDI (I$ per capita)	--
Malignant skin melanoma	Male	15-19 years	95+ years	Data Rich	1	1	Alcohol (liters per capita)	224
Malignant skin melanoma	Male	15-19 years	95+ years	Data Rich	-1	2	Healthcare access and quality index	--
Malignant skin melanoma	Male	15-19 years	95+ years	Data Rich	-1	2	Latitude 30 to 45 (proportion)	--
Malignant skin melanoma	Male	15-19 years	95+ years	Data Rich	-1	2	Latitude Over 45 (proportion)	--
Malignant skin melanoma	Male	15-19 years	95+ years	Data Rich	-1	2	fruits adjusted(g)	--
Malignant skin melanoma	Male	15-19 years	95+ years	Data Rich	-1	2	vegetables adjusted(g)	--
Malignant skin melanoma	Male	15-19 years	95+ years	Data Rich	0	2	Latitude Under 15 (proportion)	520
Malignant skin melanoma	Male	15-19 years	95+ years	Data Rich	0	2	Latitude 15 to 30 (proportion)	--
Malignant skin melanoma	Male	15-19 years	95+ years	Data Rich	-1	3	Education (years per capita)	--
Malignant skin melanoma	Male	15-19 years	95+ years	Data Rich	0	3	Socio-demographic Index	362
Malignant skin melanoma	Male	15-19 years	95+ years	Data Rich	0	3	LDI (I$ per capita)	--
Malignant skin melanoma	Male	15-19 years	95+ years	Global	1	1	Alcohol (liters per capita)	1000
Malignant skin melanoma	Male	15-19 years	95+ years	Global	-1	2	Healthcare access and quality index	--
Malignant skin melanoma	Male	15-19 years	95+ years	Global	-1	2	Latitude 30 to 45 (proportion)	--
Malignant skin melanoma	Male	15-19 years	95+ years	Global	-1	2	Latitude Over 45 (proportion)	--
Malignant skin melanoma	Male	15-19 years	95+ years	Global	-1	2	fruits adjusted(g)	--
Malignant skin melanoma	Male	15-19 years	95+ years	Global	-1	2	vegetables adjusted(g)	--
Malignant skin melanoma	Male	15-19 years	95+ years	Global	0	2	Latitude Under 15 (proportion)	398
Malignant skin melanoma	Male	15-19 years	95+ years	Global	0	2	Latitude 15 to 30 (proportion)	--
Malignant skin melanoma	Male	15-19 years	95+ years	Global	-1	3	Education (years per capita)	--
Malignant skin melanoma	Male	15-19 years	95+ years	Global	0	3	LDI (I$ per capita)	--
Malignant skin melanoma	Male	15-19 years	95+ years	Global	0	3	Socio-demographic Index	--
Non-melanoma skin cancer	Female	15-19 years	95+ years	Data Rich	1	1	Cumulative Cigarettes (15 Years)	99
Non-melanoma skin cancer	Female	15-19 years	95+ years	Data Rich	1	1	Cumulative Cigarettes (10 Years)	182
Non-melanoma skin cancer	Female	15-19 years	95+ years	Data Rich	1	1	Smoking Prevalence	353
Non-melanoma skin cancer	Female	15-19 years	95+ years	Data Rich	1	1	Cumulative Cigarettes (5 Years)	519
Non-melanoma skin cancer	Female	15-19 years	95+ years	Data Rich	-1	2	Healthcare access and quality index	355
Non-melanoma skin cancer	Female	15-19 years	95+ years	Data Rich	0	2	Average latitude	215
Non-melanoma skin cancer	Female	15-19 years	95+ years	Data Rich	-1	3	Education (years per capita)	262
Non-melanoma skin cancer	Female	15-19 years	95+ years	Data Rich	0	3	Socio-demographic Index	957
Non-melanoma skin cancer	Female	15-19 years	95+ years	Data Rich	0	3	LDI (I$ per capita)	--
Non-melanoma skin cancer	Female	15-19 years	95+ years	Global	1	1	Smoking Prevalence	572
Non-melanoma skin cancer	Female	15-19 years	95+ years	Global	1	1	Cumulative Cigarettes (10 Years)	--
Non-melanoma skin cancer	Female	15-19 years	95+ years	Global	1	1	Cumulative Cigarettes (15 Years)	--
Non-melanoma skin cancer	Female	15-19 years	95+ years	Global	1	1	Cumulative Cigarettes (5 Years)	--
Non-melanoma skin cancer	Female	15-19 years	95+ years	Global	-1	2	Healthcare access and quality index	282
Non-melanoma skin cancer	Female	15-19 years	95+ years	Global	0	2	Average latitude	192
Non-melanoma skin cancer	Female	15-19 years	95+ years	Global	-1	3	Education (years per capita)	107
Non-melanoma skin cancer	Female	15-19 years	95+ years	Global	0	3	Socio-demographic Index	465
Non-melanoma skin cancer	Female	15-19 years	95+ years	Global	0	3	LDI (I$ per capita)	--
Non-melanoma skin cancer	Male	15-19 years	95+ years	Data Rich	1	1	Cumulative Cigarettes (5 Years)	73
Non-melanoma skin cancer	Male	15-19 years	95+ years	Data Rich	1	1	Cumulative Cigarettes (10 Years)	130
Non-melanoma skin cancer	Male	15-19 years	95+ years	Data Rich	1	1	Smoking Prevalence	153
Non-melanoma skin cancer	Male	15-19 years	95+ years	Data Rich	1	1	Cumulative Cigarettes (15 Years)	211
Non-melanoma skin cancer	Male	15-19 years	95+ years	Data Rich	-1	2	Healthcare access and quality index	392
Non-melanoma skin cancer	Male	15-19 years	95+ years	Data Rich	0	2	Average latitude	778
Non-melanoma skin cancer	Male	15-19 years	95+ years	Data Rich	-1	3	Education (years per capita)	2
Non-melanoma skin cancer	Male	15-19 years	95+ years	Data Rich	0	3	Socio-demographic Index	646
Non-melanoma skin cancer	Male	15-19 years	95+ years	Data Rich	0	3	LDI (I$ per capita)	--
Non-melanoma skin cancer	Male	15-19 years	95+ years	Global	1	1	Cumulative Cigarettes (5 Years)	70
Non-melanoma skin cancer	Male	15-19 years	95+ years	Global	1	1	Cumulative Cigarettes (10 Years)	151
Non-melanoma skin cancer	Male	15-19 years	95+ years	Global	1	1	Cumulative Cigarettes (15 Years)	237
Non-melanoma skin cancer	Male	15-19 years	95+ years	Global	1	1	Smoking Prevalence	473
Non-melanoma skin cancer	Male	15-19 years	95+ years	Global	-1	2	Healthcare access and quality index	250
Non-melanoma skin cancer	Male	15-19 years	95+ years	Global	0	2	Average latitude	562
Non-melanoma skin cancer	Male	15-19 years	95+ years	Global	-1	3	Education (years per capita)	4
Non-melanoma skin cancer	Male	15-19 years	95+ years	Global	0	3	Socio-demographic Index	605
Non-melanoma skin cancer	Male	15-19 years	95+ years	Global	0	3	LDI (I$ per capita)	--
Non-melanoma skin cancer (squamous-cell carcinoma)	Female	28-364 days	95+ years	Data Rich	1	1	Cumulative Cigarettes (5 Years)	195
Non-melanoma skin cancer (squamous-cell carcinoma)	Female	28-364 days	95+ years	Data Rich	1	1	Cumulative Cigarettes (10 Years)	217
Non-melanoma skin cancer (squamous-cell carcinoma)	Female	28-364 days	95+ years	Data Rich	1	1	Cumulative Cigarettes (15 Years)	260
Non-melanoma skin cancer (squamous-cell carcinoma)	Female	28-364 days	95+ years	Data Rich	1	1	Smoking Prevalence	264
Non-melanoma skin cancer (squamous-cell carcinoma)	Female	28-364 days	95+ years	Data Rich	-1	2	Healthcare access and quality index	516
Non-melanoma skin cancer (squamous-cell carcinoma)	Female	28-364 days	95+ years	Data Rich	0	2	Average latitude	307
Non-melanoma skin cancer (squamous-cell carcinoma)	Female	28-364 days	95+ years	Data Rich	-1	3	Education (years per capita)	30
Non-melanoma skin cancer (squamous-cell carcinoma)	Female	28-364 days	95+ years	Data Rich	0	3	Socio-demographic Index	501
Non-melanoma skin cancer (squamous-cell carcinoma)	Female	28-364 days	95+ years	Data Rich	0	3	LDI (I$ per capita)	621
Non-melanoma skin cancer (squamous-cell carcinoma)	Female	28-364 days	95+ years	Global	1	1	Smoking Prevalence	31
Non-melanoma skin cancer (squamous-cell carcinoma)	Female	28-364 days	95+ years	Global	1	1	Cumulative Cigarettes (10 Years)	150
Non-melanoma skin cancer (squamous-cell carcinoma)	Female	28-364 days	95+ years	Global	1	1	Cumulative Cigarettes (15 Years)	192
Non-melanoma skin cancer (squamous-cell carcinoma)	Female	28-364 days	95+ years	Global	1	1	Cumulative Cigarettes (5 Years)	198
Non-melanoma skin cancer (squamous-cell carcinoma)	Female	28-364 days	95+ years	Global	-1	2	Healthcare access and quality index	80
Non-melanoma skin cancer (squamous-cell carcinoma)	Female	28-364 days	95+ years	Global	0	2	Average latitude	669
Non-melanoma skin cancer (squamous-cell carcinoma)	Female	28-364 days	95+ years	Global	-1	3	Education (years per capita)	45

Appendix Table 9: CODEm covariates used, level of covariate, and expected direction of covariate by cause, sex, and age

Covariates that CODEm did not select during the covariate selection process have no draw counts listed.

Cause	Sex	Age Start	Age End	Model Type	Direction	Level	Covariate Name	Number of Draws
Non-melanoma skin cancer (squamous-cell carcinoma)	Female	28-364 days	95+ years	Global	0	3	Socio-demographic Index	375
Non-melanoma skin cancer (squamous-cell carcinoma)	Female	28-364 days	95+ years	Global	0	3	LDI (I$ per capita)	501
Non-melanoma skin cancer (squamous-cell carcinoma)	Male	28-364 days	95+ years	Data Rich	1	1	Cumulative Cigarettes (5 Years)	68
Non-melanoma skin cancer (squamous-cell carcinoma)	Male	28-364 days	95+ years	Data Rich	1	1	Cumulative Cigarettes (10 Years)	116
Non-melanoma skin cancer (squamous-cell carcinoma)	Male	28-364 days	95+ years	Data Rich	1	1	Smoking Prevalence	211
Non-melanoma skin cancer (squamous-cell carcinoma)	Male	28-364 days	95+ years	Data Rich	1	1	Cumulative Cigarettes (15 Years)	269
Non-melanoma skin cancer (squamous-cell carcinoma)	Male	28-364 days	95+ years	Data Rich	-1	2	Healthcare access and quality index	275
Non-melanoma skin cancer (squamous-cell carcinoma)	Male	28-364 days	95+ years	Data Rich	0	2	Average latitude	668
Non-melanoma skin cancer (squamous-cell carcinoma)	Male	28-364 days	95+ years	Data Rich	-1	3	Education (years per capita)	16
Non-melanoma skin cancer (squamous-cell carcinoma)	Male	28-364 days	95+ years	Data Rich	0	3	LDI (I$ per capita)	412
Non-melanoma skin cancer (squamous-cell carcinoma)	Male	28-364 days	95+ years	Data Rich	0	3	Socio-demographic Index	649
Non-melanoma skin cancer (squamous-cell carcinoma)	Male	28-364 days	95+ years	Global	1	1	Cumulative Cigarettes (10 Years)	141
Non-melanoma skin cancer (squamous-cell carcinoma)	Male	28-364 days	95+ years	Global	1	1	Cumulative Cigarettes (5 Years)	153
Non-melanoma skin cancer (squamous-cell carcinoma)	Male	28-364 days	95+ years	Global	1	1	Cumulative Cigarettes (15 Years)	473
Non-melanoma skin cancer (squamous-cell carcinoma)	Male	28-364 days	95+ years	Global	1	1	Smoking Prevalence	724
Non-melanoma skin cancer (squamous-cell carcinoma)	Male	28-364 days	95+ years	Global	-1	2	Healthcare access and quality index	292
Non-melanoma skin cancer (squamous-cell carcinoma)	Male	28-364 days	95+ years	Global	0	2	Average latitude	16
Non-melanoma skin cancer (squamous-cell carcinoma)	Male	28-364 days	95+ years	Global	-1	3	Education (years per capita)	15
Non-melanoma skin cancer (squamous-cell carcinoma)	Male	28-364 days	95+ years	Global	0	3	Socio-demographic Index	308
Non-melanoma skin cancer (squamous-cell carcinoma)	Male	28-364 days	95+ years	Global	0	3	LDI (I$ per capita)	395
Breast cancer	Female	15-19 years	95+ years	Data Rich	1	1	Mean BMI	388
Breast cancer	Female	15-19 years	95+ years	Data Rich	1	1	Log-transformed SEV scalar: Breast C	647
Breast cancer	Female	15-19 years	95+ years	Data Rich	1	1	Alcohol (liters per capita)	669
Breast cancer	Female	15-19 years	95+ years	Data Rich	-1	2	Healthcare access and quality index	0
Breast cancer	Female	15-19 years	95+ years	Data Rich	-1	2	vegetables adjusted(g)	0
Breast cancer	Female	15-19 years	95+ years	Data Rich	-1	2	Total Fertility Rate	626
Breast cancer	Female	15-19 years	95+ years	Data Rich	-1	2	Age-Specific Fertility Rate	811
Breast cancer	Female	15-19 years	95+ years	Data Rich	-1	2	fruits adjusted(g)	--
Breast cancer	Female	15-19 years	95+ years	Data Rich	1	2	Secondhand smoke	0
Breast cancer	Female	15-19 years	95+ years	Data Rich	1	2	Smoking Prevalence	0
Breast cancer	Female	15-19 years	95+ years	Data Rich	1	2	Cumulative Cigarettes (10 Years)	2
Breast cancer	Female	15-19 years	95+ years	Data Rich	1	2	Cumulative Cigarettes (5 Years)	40
Breast cancer	Female	15-19 years	95+ years	Data Rich	1	2	Cumulative Cigarettes (15 Years)	140
Breast cancer	Female	15-19 years	95+ years	Data Rich	1	2	Cumulative Cigarettes (20 Years)	350
Breast cancer	Female	15-19 years	95+ years	Data Rich	1	2	Diabetes Fasting Plasma Glucose (mmol/L)	814
Breast cancer	Female	15-19 years	95+ years	Data Rich	-1	3	Education (years per capita)	0
Breast cancer	Female	15-19 years	95+ years	Data Rich	0	3	Socio-demographic Index	0
Breast cancer	Female	15-19 years	95+ years	Data Rich	0	3	LDI (I$ per capita)	--
Breast cancer	Female	15-19 years	95+ years	Global	1	1	Log-transformed SEV scalar: Breast C	255
Breast cancer	Female	15-19 years	95+ years	Global	1	1	Mean BMI	289
Breast cancer	Female	15-19 years	95+ years	Global	1	1	Alcohol (liters per capita)	694
Breast cancer	Female	15-19 years	95+ years	Global	-1	2	Healthcare access and quality index	203
Breast cancer	Female	15-19 years	95+ years	Global	-1	2	Age-Specific Fertility Rate	382
Breast cancer	Female	15-19 years	95+ years	Global	-1	2	vegetables adjusted(g)	423
Breast cancer	Female	15-19 years	95+ years	Global	-1	2	Total Fertility Rate	718
Breast cancer	Female	15-19 years	95+ years	Global	-1	2	fruits adjusted(g)	--
Breast cancer	Female	15-19 years	95+ years	Global	1	2	Cumulative Cigarettes (5 Years)	155
Breast cancer	Female	15-19 years	95+ years	Global	1	2	Cumulative Cigarettes (20 Years)	202
Breast cancer	Female	15-19 years	95+ years	Global	1	2	Cumulative Cigarettes (10 Years)	219
Breast cancer	Female	15-19 years	95+ years	Global	1	2	Cumulative Cigarettes (15 Years)	253
Breast cancer	Female	15-19 years	95+ years	Global	1	2	Diabetes Fasting Plasma Glucose (mmol/L)	329
Breast cancer	Female	15-19 years	95+ years	Global	1	2	Smoking Prevalence	488
Breast cancer	Female	15-19 years	95+ years	Global	1	2	Secondhand smoke	--
Breast cancer	Female	15-19 years	95+ years	Global	-1	3	Education (years per capita)	8
Breast cancer	Female	15-19 years	95+ years	Global	0	3	Socio-demographic Index	269
Breast cancer	Female	15-19 years	95+ years	Global	0	3	LDI (I$ per capita)	--
Breast cancer	Male	15-19 years	95+ years	Data Rich	1	1	Log-transformed SEV scalar: Breast C	613
Breast cancer	Male	15-19 years	95+ years	Data Rich	1	1	Mean BMI	784
Breast cancer	Male	15-19 years	95+ years	Data Rich	1	1	Alcohol (liters per capita)	--
Breast cancer	Male	15-19 years	95+ years	Data Rich	-1	2	Healthcare access and quality index	21
Breast cancer	Male	15-19 years	95+ years	Data Rich	-1	2	fruits adjusted(g)	64
Breast cancer	Male	15-19 years	95+ years	Data Rich	-1	2	vegetables adjusted(g)	--
Breast cancer	Male	15-19 years	95+ years	Data Rich	1	2	Cumulative Cigarettes (15 Years)	10
Breast cancer	Male	15-19 years	95+ years	Data Rich	1	2	Cumulative Cigarettes (10 Years)	12
Breast cancer	Male	15-19 years	95+ years	Data Rich	1	2	Secondhand smoke	17
Breast cancer	Male	15-19 years	95+ years	Data Rich	1	2	Cumulative Cigarettes (5 Years)	24
Breast cancer	Male	15-19 years	95+ years	Data Rich	1	2	Smoking Prevalence	34
Breast cancer	Male	15-19 years	95+ years	Data Rich	1	2	Diabetes Fasting Plasma Glucose (mmol/L)	356
Breast cancer	Male	15-19 years	95+ years	Data Rich	1	2	Cumulative Cigarettes (20 Years)	--
Breast cancer	Male	15-19 years	95+ years	Data Rich	-1	3	Education (years per capita)	9
Breast cancer	Male	15-19 years	95+ years	Data Rich	0	3	Socio-demographic Index	94
Breast cancer	Male	15-19 years	95+ years	Data Rich	0	3	LDI (I$ per capita)	--
Breast cancer	Male	15-19 years	95+ years	Global	1	1	Mean BMI	541
Breast cancer	Male	15-19 years	95+ years	Global	1	1	Log-transformed SEV scalar: Breast C	689
Breast cancer	Male	15-19 years	95+ years	Global	1	1	Alcohol (liters per capita)	--
Breast cancer	Male	15-19 years	95+ years	Global	-1	2	Healthcare access and quality index	248
Breast cancer	Male	15-19 years	95+ years	Global	-1	2	fruits adjusted(g)	--
Breast cancer	Male	15-19 years	95+ years	Global	-1	2	vegetables adjusted(g)	--

Appendix Table 9: CODEm covariates used, level of covariate, and expected direction of covariate by cause, sex, and age

Covariates that CODEm did not select during the covariate selection process have no draw counts listed.

Cause	Sex	Age Start	Age End	Model Type	Direction	Level	Covariate Name	Number of Draws
Breast cancer	Male	15-19 years	95+ years	Global	1	2	Cumulative Cigarettes (5 Years)	10
Breast cancer	Male	15-19 years	95+ years	Global	1	2	Cumulative Cigarettes (10 Years)	25
Breast cancer	Male	15-19 years	95+ years	Global	1	2	Cumulative Cigarettes (15 Years)	96
Breast cancer	Male	15-19 years	95+ years	Global	1	2	Smoking Prevalence	112
Breast cancer	Male	15-19 years	95+ years	Global	1	2	Cumulative Cigarettes (20 Years)	177
Breast cancer	Male	15-19 years	95+ years	Global	1	2	Secondhand smoke	221
Breast cancer	Male	15-19 years	95+ years	Global	1	2	Diabetes Fasting Plasma Glucose (mmol/L)	603
Breast cancer	Male	15-19 years	95+ years	Global	-1	3	Education (years per capita)	153
Breast cancer	Male	15-19 years	95+ years	Global	0	3	Socio-demographic Index	159
Breast cancer	Male	15-19 years	95+ years	Global	0	3	LDI (I$ per capita)	--
Cervical cancer	Female	15-19 years	95+ years	Data Rich	1	1	Cumulative Cigarettes (5 Years)	348
Cervical cancer	Female	15-19 years	95+ years	Data Rich	1	1	Cumulative Cigarettes (10 Years)	633
Cervical cancer	Female	15-19 years	95+ years	Data Rich	1	1	Cumulative Cigarettes (15 Years)	--
Cervical cancer	Female	15-19 years	95+ years	Data Rich	1	1	HIV age-standardized prevalence	--
Cervical cancer	Female	15-19 years	95+ years	Data Rich	-1	2	fruits adjusted(g)	139
Cervical cancer	Female	15-19 years	95+ years	Data Rich	-1	2	vegetables adjusted(g)	216
Cervical cancer	Female	15-19 years	95+ years	Data Rich	-1	2	Healthcare access and quality index	465
Cervical cancer	Female	15-19 years	95+ years	Data Rich	1	2	Age-Specific Fertility Rate	4
Cervical cancer	Female	15-19 years	95+ years	Data Rich	1	2	Total Fertility Rate	243
Cervical cancer	Female	15-19 years	95+ years	Data Rich	1	2	Smoking Prevalence	300
Cervical cancer	Female	15-19 years	95+ years	Data Rich	-1	3	Education (years per capita)	474
Cervical cancer	Female	15-19 years	95+ years	Data Rich	0	3	Socio-demographic Index	295
Cervical cancer	Female	15-19 years	95+ years	Data Rich	0	3	LDI (I$ per capita)	--
Cervical cancer	Female	15-19 years	95+ years	Global	1	1	Cumulative Cigarettes (5 Years)	487
Cervical cancer	Female	15-19 years	95+ years	Global	1	1	Cumulative Cigarettes (15 Years)	513
Cervical cancer	Female	15-19 years	95+ years	Global	1	1	Cumulative Cigarettes (10 Years)	--
Cervical cancer	Female	15-19 years	95+ years	Global	1	1	HIV age-standardized prevalence	--
Cervical cancer	Female	15-19 years	95+ years	Global	-1	2	vegetables adjusted(g)	9
Cervical cancer	Female	15-19 years	95+ years	Global	-1	2	fruits adjusted(g)	15
Cervical cancer	Female	15-19 years	95+ years	Global	-1	2	Healthcare access and quality index	295
Cervical cancer	Female	15-19 years	95+ years	Global	1	2	Age-Specific Fertility Rate	15
Cervical cancer	Female	15-19 years	95+ years	Global	1	2	Total Fertility Rate	369
Cervical cancer	Female	15-19 years	95+ years	Global	1	2	Smoking Prevalence	--
Cervical cancer	Female	15-19 years	95+ years	Global	-1	3	Education (years per capita)	362
Cervical cancer	Female	15-19 years	95+ years	Global	0	3	Socio-demographic Index	302
Cervical cancer	Female	15-19 years	95+ years	Global	0	3	LDI (I$ per capita)	--
Uterine cancer	Female	15-19 years	95+ years	Data Rich	1	1	Mean BMI	369
Uterine cancer	Female	15-19 years	95+ years	Data Rich	1	1	Log-transformed SEV scalar: Uterus C	626
Uterine cancer	Female	15-19 years	95+ years	Data Rich	-1	2	fruits adjusted(g)	35
Uterine cancer	Female	15-19 years	95+ years	Data Rich	-1	2	Healthcare access and quality index	43
Uterine cancer	Female	15-19 years	95+ years	Data Rich	-1	2	vegetables adjusted(g)	655
Uterine cancer	Female	15-19 years	95+ years	Data Rich	0	2	Total Fertility Rate	674
Uterine cancer	Female	15-19 years	95+ years	Data Rich	1	2	Cumulative Cigarettes (10 Years)	2
Uterine cancer	Female	15-19 years	95+ years	Data Rich	1	2	Smoking Prevalence	2
Uterine cancer	Female	15-19 years	95+ years	Data Rich	1	2	Cumulative Cigarettes (5 Years)	5
Uterine cancer	Female	15-19 years	95+ years	Data Rich	1	2	Tobacco (cigarettes per capita)	52
Uterine cancer	Female	15-19 years	95+ years	Data Rich	1	2	Diabetes Age-Standardized Prevalence (proportion)	--
Uterine cancer	Female	15-19 years	95+ years	Data Rich	-1	3	Education (years per capita)	1
Uterine cancer	Female	15-19 years	95+ years	Data Rich	0	3	Socio-demographic Index	20
Uterine cancer	Female	15-19 years	95+ years	Data Rich	0	3	LDI (I$ per capita)	--
Uterine cancer	Female	15-19 years	95+ years	Global	1	1	Log-transformed SEV scalar: Uterus C	257
Uterine cancer	Female	15-19 years	95+ years	Global	1	1	Mean BMI	368
Uterine cancer	Female	15-19 years	95+ years	Global	-1	2	fruits adjusted(g)	265
Uterine cancer	Female	15-19 years	95+ years	Global	-1	2	Healthcare access and quality index	267
Uterine cancer	Female	15-19 years	95+ years	Global	-1	2	vegetables adjusted(g)	269
Uterine cancer	Female	15-19 years	95+ years	Global	0	2	Total Fertility Rate	340
Uterine cancer	Female	15-19 years	95+ years	Global	1	2	Smoking Prevalence	14
Uterine cancer	Female	15-19 years	95+ years	Global	1	2	Cumulative Cigarettes (10 Years)	17
Uterine cancer	Female	15-19 years	95+ years	Global	1	2	Cumulative Cigarettes (5 Years)	28
Uterine cancer	Female	15-19 years	95+ years	Global	1	2	Tobacco (cigarettes per capita)	294
Uterine cancer	Female	15-19 years	95+ years	Global	1	2	Diabetes Age-Standardized Prevalence (proportion)	--
Uterine cancer	Female	15-19 years	95+ years	Global	-1	3	Education (years per capita)	33
Uterine cancer	Female	15-19 years	95+ years	Global	0	3	Socio-demographic Index	144
Uterine cancer	Female	15-19 years	95+ years	Global	0	3	LDI (I$ per capita)	--
Ovarian cancer	Female	15-19 years	95+ years	Data Rich	-1	1	Contraception (Modern) Prevalence (proportion)	--
Ovarian cancer	Female	15-19 years	95+ years	Data Rich	1	1	Tobacco (cigarettes per capita)	0
Ovarian cancer	Female	15-19 years	95+ years	Data Rich	1	1	Cumulative Cigarettes (20 Years)	1
Ovarian cancer	Female	15-19 years	95+ years	Data Rich	1	1	Cumulative Cigarettes (15 Years)	8
Ovarian cancer	Female	15-19 years	95+ years	Data Rich	1	1	Cumulative Cigarettes (10 Years)	90
Ovarian cancer	Female	15-19 years	95+ years	Data Rich	1	1	Cumulative Cigarettes (5 Years)	230
Ovarian cancer	Female	15-19 years	95+ years	Data Rich	1	1	Log-transformed SEV scalar: Ovary C	827
Ovarian cancer	Female	15-19 years	95+ years	Data Rich	1	1	Alcohol (liters per capita)	--
Ovarian cancer	Female	15-19 years	95+ years	Data Rich	-1	2	fruits adjusted(g)	95
Ovarian cancer	Female	15-19 years	95+ years	Data Rich	-1	2	vegetables adjusted(g)	123
Ovarian cancer	Female	15-19 years	95+ years	Data Rich	-1	2	Healthcare access and quality index	--
Ovarian cancer	Female	15-19 years	95+ years	Data Rich	0	2	Total Fertility Rate	285
Ovarian cancer	Female	15-19 years	95+ years	Data Rich	1	2	energy unadjusted(kcal)	0
Ovarian cancer	Female	15-19 years	95+ years	Data Rich	1	2	Diabetes Fasting Plasma Glucose (mmol/L)	95

**Appendix Table 9: CODEm covariates used, level of covariate, and expected direction of covariate by cause, sex, and age**<br>*Covariates that CODEm did not select during the covariate selection process have no draw counts listed.*								
**Cause**	**Sex**	**Age Start**	**Age End**	**Model Type**	**Direction**	**Level**	**Covariate Name**	**Number of Draws**
Ovarian cancer	Female	15-19 years	95+ years	Data Rich	1	2	Asbestos consumption (metric tons per year per capita)	329
Ovarian cancer	Female	15-19 years	95+ years	Data Rich	1	2	Diabetes Age-Standardized Prevalence (proportion)	329
Ovarian cancer	Female	15-19 years	95+ years	Data Rich	1	2	Smoking Prevalence	329
Ovarian cancer	Female	15-19 years	95+ years	Data Rich	1	2	Mean BMI	371
Ovarian cancer	Female	15-19 years	95+ years	Data Rich	-1	3	Education (years per capita)	56
Ovarian cancer	Female	15-19 years	95+ years	Data Rich	0	3	Socio-demographic Index	429
Ovarian cancer	Female	15-19 years	95+ years	Data Rich	0	3	LDI (I$ per capita)	--
Ovarian cancer	Female	15-19 years	95+ years	Global	-1	1	Contraception (Modern) Prevalence (proportion)	--
Ovarian cancer	Female	15-19 years	95+ years	Global	1	1	Tobacco (cigarettes per capita)	6
Ovarian cancer	Female	15-19 years	95+ years	Global	1	1	Cumulative Cigarettes (5 Years)	52
Ovarian cancer	Female	15-19 years	95+ years	Global	1	1	Cumulative Cigarettes (10 Years)	66
Ovarian cancer	Female	15-19 years	95+ years	Global	1	1	Cumulative Cigarettes (15 Years)	223
Ovarian cancer	Female	15-19 years	95+ years	Global	1	1	Cumulative Cigarettes (20 Years)	325
Ovarian cancer	Female	15-19 years	95+ years	Global	1	1	Log-transformed SEV scalar: Ovary C	819
Ovarian cancer	Female	15-19 years	95+ years	Global	1	1	Alcohol (liters per capita)	--
Ovarian cancer	Female	15-19 years	95+ years	Global	-1	2	Healthcare access and quality index	--
Ovarian cancer	Female	15-19 years	95+ years	Global	-1	2	fruits adjusted(g)	--
Ovarian cancer	Female	15-19 years	95+ years	Global	-1	2	vegetables adjusted(g)	--
Ovarian cancer	Female	15-19 years	95+ years	Global	0	2	Total Fertility Rate	216
Ovarian cancer	Female	15-19 years	95+ years	Global	1	2	energy unadjusted(kcal)	12
Ovarian cancer	Female	15-19 years	95+ years	Global	1	2	Smoking Prevalence	42
Ovarian cancer	Female	15-19 years	95+ years	Global	1	2	Diabetes Fasting Plasma Glucose (mmol/L)	290
Ovarian cancer	Female	15-19 years	95+ years	Global	1	2	Diabetes Age-Standardized Prevalence (proportion)	300
Ovarian cancer	Female	15-19 years	95+ years	Global	1	2	Mean BMI	323
Ovarian cancer	Female	15-19 years	95+ years	Global	1	2	Asbestos consumption (metric tons per year per capita)	400
Ovarian cancer	Female	15-19 years	95+ years	Global	-1	3	Education (years per capita)	77
Ovarian cancer	Female	15-19 years	95+ years	Global	0	3	Socio-demographic Index	576
Ovarian cancer	Female	15-19 years	95+ years	Global	0	3	LDI (I$ per capita)	--
Prostate cancer	Male	15-19 years	95+ years	Data Rich	1	1	Log-transformed SEV scalar: Prostate C	810
Prostate cancer	Male	15-19 years	95+ years	Data Rich	-1	2	Healthcare access and quality index	--
Prostate cancer	Male	15-19 years	95+ years	Data Rich	1	2	Smoking Prevalence	--
Prostate cancer	Male	15-19 years	95+ years	Data Rich	-1	3	Education (years per capita)	--
Prostate cancer	Male	15-19 years	95+ years	Data Rich	0	3	Socio-demographic Index	692
Prostate cancer	Male	15-19 years	95+ years	Data Rich	0	3	LDI (I$ per capita)	--
Prostate cancer	Male	15-19 years	95+ years	Global	1	1	Log-transformed SEV scalar: Prostate C	924
Prostate cancer	Male	15-19 years	95+ years	Global	-1	2	Healthcare access and quality index	352
Prostate cancer	Male	15-19 years	95+ years	Global	1	2	Smoking Prevalence	218
Prostate cancer	Male	15-19 years	95+ years	Global	-1	3	Education (years per capita)	--
Prostate cancer	Male	15-19 years	95+ years	Global	0	3	Socio-demographic Index	504
Prostate cancer	Male	15-19 years	95+ years	Global	0	3	LDI (I$ per capita)	--
Testicular cancer	Male	15-19 years	95+ years	Data Rich	-1	2	Healthcare access and quality index	0
Testicular cancer	Male	15-19 years	95+ years	Data Rich	-1	2	vegetables adjusted(g)	440
Testicular cancer	Male	15-19 years	95+ years	Data Rich	-1	2	fruits adjusted(g)	--
Testicular cancer	Male	15-19 years	95+ years	Data Rich	1	2	Cumulative Cigarettes (5 Years)	4
Testicular cancer	Male	15-19 years	95+ years	Data Rich	1	2	Cumulative Cigarettes (15 Years)	11
Testicular cancer	Male	15-19 years	95+ years	Data Rich	1	2	Cumulative Cigarettes (20 Years)	18
Testicular cancer	Male	15-19 years	95+ years	Data Rich	1	2	Cumulative Cigarettes (10 Years)	40
Testicular cancer	Male	15-19 years	95+ years	Data Rich	1	2	Smoking Prevalence	99
Testicular cancer	Male	15-19 years	95+ years	Data Rich	1	2	Tobacco (cigarettes per capita)	893
Testicular cancer	Male	15-19 years	95+ years	Data Rich	-1	3	Education (years per capita)	280
Testicular cancer	Male	15-19 years	95+ years	Data Rich	0	3	Socio-demographic Index	695
Testicular cancer	Male	15-19 years	95+ years	Data Rich	0	3	LDI (I$ per capita)	--
Testicular cancer	Male	15-19 years	95+ years	Global	-1	2	Healthcare access and quality index	66
Testicular cancer	Male	15-19 years	95+ years	Global	-1	2	vegetables adjusted(g)	375
Testicular cancer	Male	15-19 years	95+ years	Global	-1	2	fruits adjusted(g)	--
Testicular cancer	Male	15-19 years	95+ years	Global	1	2	Cumulative Cigarettes (15 Years)	71
Testicular cancer	Male	15-19 years	95+ years	Global	1	2	Cumulative Cigarettes (5 Years)	152
Testicular cancer	Male	15-19 years	95+ years	Global	1	2	Cumulative Cigarettes (20 Years)	201
Testicular cancer	Male	15-19 years	95+ years	Global	1	2	Cumulative Cigarettes (10 Years)	217
Testicular cancer	Male	15-19 years	95+ years	Global	1	2	Tobacco (cigarettes per capita)	316
Testicular cancer	Male	15-19 years	95+ years	Global	1	2	Smoking Prevalence	322
Testicular cancer	Male	15-19 years	95+ years	Global	-1	3	Education (years per capita)	60
Testicular cancer	Male	15-19 years	95+ years	Global	0	3	Socio-demographic Index	221
Testicular cancer	Male	15-19 years	95+ years	Global	0	3	LDI (I$ per capita)	--
Kidney cancer	Female	0-6 days	95+ years	Data Rich	1	1	Mean BMI	433
Kidney cancer	Female	0-6 days	95+ years	Data Rich	1	1	Log-transformed SEV scalar: Kidney C	567
Kidney cancer	Female	0-6 days	95+ years	Data Rich	1	1	Cumulative Cigarettes (10 Years)	--
Kidney cancer	Female	0-6 days	95+ years	Data Rich	1	1	Cumulative Cigarettes (15 Years)	--
Kidney cancer	Female	0-6 days	95+ years	Data Rich	1	1	Cumulative Cigarettes (5 Years)	--
Kidney cancer	Female	0-6 days	95+ years	Data Rich	-1	2	Healthcare access and quality index	266
Kidney cancer	Female	0-6 days	95+ years	Data Rich	1	2	Alcohol (liters per capita)	313
Kidney cancer	Female	0-6 days	95+ years	Data Rich	1	2	Diabetes Age-Standardized Prevalence (proportion)	--
Kidney cancer	Female	0-6 days	95+ years	Data Rich	1	2	Smoking Prevalence	--
Kidney cancer	Female	0-6 days	95+ years	Data Rich	1	2	Systolic Blood Pressure (mmHg)	--
Kidney cancer	Female	0-6 days	95+ years	Data Rich	-1	3	Education (years per capita)	--
Kidney cancer	Female	0-6 days	95+ years	Data Rich	0	3	Socio-demographic Index	374
Kidney cancer	Female	0-6 days	95+ years	Data Rich	0	3	LDI (I$ per capita)	--
Kidney cancer	Female	0-6 days	95+ years	Global	1	1	Mean BMI	395

Appendix Table 9: CODEm covariates used, level of covariate, and expected direction of covariate by cause, sex, and age

Covariates that CODEm did not select during the covariate selection process have no draw counts listed.

Cause	Sex	Age Start	Age End	Model Type	Direction	Level	Covariate Name	Number of Draws
Kidney cancer	Female	0-6 days	95+ years	Global	1	1	Log-transformed SEV scalar: Kidney C	914
Kidney cancer	Female	0-6 days	95+ years	Global	1	1	Cumulative Cigarettes (10 Years)	--
Kidney cancer	Female	0-6 days	95+ years	Global	1	1	Cumulative Cigarettes (15 Years)	--
Kidney cancer	Female	0-6 days	95+ years	Global	1	1	Cumulative Cigarettes (5 Years)	--
Kidney cancer	Female	0-6 days	95+ years	Global	-1	2	Healthcare access and quality index	--
Kidney cancer	Female	0-6 days	95+ years	Global	1	2	Diabetes Age-Standardized Prevalence (proportion)	80
Kidney cancer	Female	0-6 days	95+ years	Global	1	2	Alcohol (liters per capita)	263
Kidney cancer	Female	0-6 days	95+ years	Global	1	2	Smoking Prevalence	--
Kidney cancer	Female	0-6 days	95+ years	Global	1	2	Systolic Blood Pressure (mmHg)	--
Kidney cancer	Female	0-6 days	95+ years	Global	-1	3	Education (years per capita)	--
Kidney cancer	Female	0-6 days	95+ years	Global	0	3	Socio-demographic Index	404
Kidney cancer	Female	0-6 days	95+ years	Global	0	3	LDI (I$ per capita)	--
Kidney cancer	Male	0-6 days	95+ years	Data Rich	1	1	Mean BMI	607
Kidney cancer	Male	0-6 days	95+ years	Data Rich	1	1	Log-transformed SEV scalar: Kidney C	937
Kidney cancer	Male	0-6 days	95+ years	Data Rich	1	1	Cumulative Cigarettes (10 Years)	--
Kidney cancer	Male	0-6 days	95+ years	Data Rich	1	1	Cumulative Cigarettes (15 Years)	--
Kidney cancer	Male	0-6 days	95+ years	Data Rich	1	1	Cumulative Cigarettes (5 Years)	--
Kidney cancer	Male	0-6 days	95+ years	Data Rich	-1	2	Healthcare access and quality index	--
Kidney cancer	Male	0-6 days	95+ years	Data Rich	1	2	Systolic Blood Pressure (mmHg)	22
Kidney cancer	Male	0-6 days	95+ years	Data Rich	1	2	Diabetes Age-Standardized Prevalence (proportion)	97
Kidney cancer	Male	0-6 days	95+ years	Data Rich	1	2	Alcohol (liters per capita)	--
Kidney cancer	Male	0-6 days	95+ years	Data Rich	1	2	Smoking Prevalence	--
Kidney cancer	Male	0-6 days	95+ years	Data Rich	-1	3	Education (years per capita)	--
Kidney cancer	Male	0-6 days	95+ years	Data Rich	0	3	Socio-demographic Index	720
Kidney cancer	Male	0-6 days	95+ years	Data Rich	0	3	LDI (I$ per capita)	--
Kidney cancer	Male	0-6 days	95+ years	Global	1	1	Log-transformed SEV scalar: Kidney C	513
Kidney cancer	Male	0-6 days	95+ years	Global	1	1	Mean BMI	619
Kidney cancer	Male	0-6 days	95+ years	Global	1	1	Cumulative Cigarettes (10 Years)	--
Kidney cancer	Male	0-6 days	95+ years	Global	1	1	Cumulative Cigarettes (15 Years)	--
Kidney cancer	Male	0-6 days	95+ years	Global	1	1	Cumulative Cigarettes (5 Years)	--
Kidney cancer	Male	0-6 days	95+ years	Global	-1	2	Healthcare access and quality index	--
Kidney cancer	Male	0-6 days	95+ years	Global	1	2	Diabetes Age-Standardized Prevalence (proportion)	57
Kidney cancer	Male	0-6 days	95+ years	Global	1	2	Systolic Blood Pressure (mmHg)	271
Kidney cancer	Male	0-6 days	95+ years	Global	1	2	Alcohol (liters per capita)	--
Kidney cancer	Male	0-6 days	95+ years	Global	1	2	Smoking Prevalence	--
Kidney cancer	Male	0-6 days	95+ years	Global	-1	3	Education (years per capita)	--
Kidney cancer	Male	0-6 days	95+ years	Global	0	3	Socio-demographic Index	481
Kidney cancer	Male	0-6 days	95+ years	Global	0	3	LDI (I$ per capita)	--
Bladder cancer	Female	15-19 years	95+ years	Data Rich	1	1	Cumulative Cigarettes (10 Years)	15
Bladder cancer	Female	15-19 years	95+ years	Data Rich	1	1	Cumulative Cigarettes (5 Years)	47
Bladder cancer	Female	15-19 years	95+ years	Data Rich	1	1	Log-transformed SEV scalar: Bladder C	48
Bladder cancer	Female	15-19 years	95+ years	Data Rich	1	1	Schistosomiasis Prevalence (proportion)	71
Bladder cancer	Female	15-19 years	95+ years	Data Rich	1	1	Cumulative Cigarettes (15 Years)	87
Bladder cancer	Female	15-19 years	95+ years	Data Rich	1	1	Smoking Prevalence	189
Bladder cancer	Female	15-19 years	95+ years	Data Rich	-1	2	fruits adjusted(g)	0
Bladder cancer	Female	15-19 years	95+ years	Data Rich	-1	2	Healthcare access and quality index	249
Bladder cancer	Female	15-19 years	95+ years	Data Rich	-1	2	vegetables adjusted(g)	249
Bladder cancer	Female	15-19 years	95+ years	Data Rich	1	2	Diabetes Fasting Plasma Glucose (mmol/L)	269
Bladder cancer	Female	15-19 years	95+ years	Data Rich	1	2	Alcohol (liters per capita)	509
Bladder cancer	Female	15-19 years	95+ years	Data Rich	-1	3	Education (years per capita)	180
Bladder cancer	Female	15-19 years	95+ years	Data Rich	0	3	LDI (I$ per capita)	341
Bladder cancer	Female	15-19 years	95+ years	Data Rich	0	3	Socio-demographic Index	678
Bladder cancer	Female	15-19 years	95+ years	Global	1	1	Cumulative Cigarettes (10 Years)	72
Bladder cancer	Female	15-19 years	95+ years	Global	1	1	Cumulative Cigarettes (5 Years)	100
Bladder cancer	Female	15-19 years	95+ years	Global	1	1	Cumulative Cigarettes (15 Years)	102
Bladder cancer	Female	15-19 years	95+ years	Global	1	1	Smoking Prevalence	235
Bladder cancer	Female	15-19 years	95+ years	Global	1	1	Schistosomiasis Prevalence (proportion)	251
Bladder cancer	Female	15-19 years	95+ years	Global	1	1	Log-transformed SEV scalar: Bladder C	843
Bladder cancer	Female	15-19 years	95+ years	Global	-1	2	fruits adjusted(g)	31
Bladder cancer	Female	15-19 years	95+ years	Global	-1	2	Healthcare access and quality index	387
Bladder cancer	Female	15-19 years	95+ years	Global	-1	2	vegetables adjusted(g)	413
Bladder cancer	Female	15-19 years	95+ years	Global	1	2	Diabetes Fasting Plasma Glucose (mmol/L)	301
Bladder cancer	Female	15-19 years	95+ years	Global	1	2	Alcohol (liters per capita)	526
Bladder cancer	Female	15-19 years	95+ years	Global	-1	3	Education (years per capita)	63
Bladder cancer	Female	15-19 years	95+ years	Global	0	3	LDI (I$ per capita)	533
Bladder cancer	Female	15-19 years	95+ years	Global	0	3	Socio-demographic Index	761
Bladder cancer	Male	15-19 years	95+ years	Data Rich	1	1	Cumulative Cigarettes (5 Years)	8
Bladder cancer	Male	15-19 years	95+ years	Data Rich	1	1	Smoking Prevalence	78
Bladder cancer	Male	15-19 years	95+ years	Data Rich	1	1	Cumulative Cigarettes (10 Years)	198
Bladder cancer	Male	15-19 years	95+ years	Data Rich	1	1	Cumulative Cigarettes (15 Years)	361
Bladder cancer	Male	15-19 years	95+ years	Data Rich	1	1	Log-transformed SEV scalar: Bladder C	560
Bladder cancer	Male	15-19 years	95+ years	Data Rich	1	1	Schistosomiasis Prevalence (proportion)	722
Bladder cancer	Male	15-19 years	95+ years	Data Rich	-1	2	fruits adjusted(g)	0
Bladder cancer	Male	15-19 years	95+ years	Data Rich	-1	2	Healthcare access and quality index	571
Bladder cancer	Male	15-19 years	95+ years	Data Rich	-1	2	vegetables adjusted(g)	571
Bladder cancer	Male	15-19 years	95+ years	Data Rich	1	2	Alcohol (liters per capita)	499
Bladder cancer	Male	15-19 years	95+ years	Data Rich	1	2	Diabetes Fasting Plasma Glucose (mmol/L)	571
Bladder cancer	Male	15-19 years	95+ years	Data Rich	-1	3	Education (years per capita)	0

Appendix Table 9: CODEm covariates used, level of covariate, and expected direction of covariate by cause, sex, and age
Covariates that CODEm did not select during the covariate selection process have no draw counts listed.

Cause	Sex	Age Start	Age End	Model Type	Direction	Level	Covariate Name	Number of Draws
Bladder cancer	Male	15-19 years	95+ years	Data Rich	0	3	Socio-demographic Index	282
Bladder cancer	Male	15-19 years	95+ years	Data Rich	0	3	LDI (I$ per capita)	315
Bladder cancer	Male	15-19 years	95+ years	Global	1	1	Schistosomiasis Prevalence (proportion)	0
Bladder cancer	Male	15-19 years	95+ years	Global	1	1	Cumulative Cigarettes (15 Years)	117
Bladder cancer	Male	15-19 years	95+ years	Global	1	1	Cumulative Cigarettes (10 Years)	175
Bladder cancer	Male	15-19 years	95+ years	Global	1	1	Cumulative Cigarettes (5 Years)	219
Bladder cancer	Male	15-19 years	95+ years	Global	1	1	Log-transformed SEV scalar: Bladder C	427
Bladder cancer	Male	15-19 years	95+ years	Global	1	1	Smoking Prevalence	639
Bladder cancer	Male	15-19 years	95+ years	Global	-1	2	fruits adjusted(g)	138
Bladder cancer	Male	15-19 years	95+ years	Global	-1	2	vegetables adjusted(g)	546
Bladder cancer	Male	15-19 years	95+ years	Global	-1	2	Healthcare access and quality index	658
Bladder cancer	Male	15-19 years	95+ years	Global	1	2	Alcohol (liters per capita)	923
Bladder cancer	Male	15-19 years	95+ years	Global	1	2	Diabetes Fasting Plasma Glucose (mmol/L)	995
Bladder cancer	Male	15-19 years	95+ years	Global	-1	3	Education (years per capita)	0
Bladder cancer	Male	15-19 years	95+ years	Global	0	3	LDI (I$ per capita)	819
Bladder cancer	Male	15-19 years	95+ years	Global	0	3	Socio-demographic Index	982
Brain and nervous system cancer	Female	0-6 days	95+ years	Data Rich	1	1	Alcohol (liters per capita)	1000
Brain and nervous system cancer	Female	0-6 days	95+ years	Data Rich	1	1	Cumulative Cigarettes (10 Years)	--
Brain and nervous system cancer	Female	0-6 days	95+ years	Data Rich	1	1	Cumulative Cigarettes (15 Years)	--
Brain and nervous system cancer	Female	0-6 days	95+ years	Data Rich	1	1	Smoking Prevalence	--
Brain and nervous system cancer	Female	0-6 days	95+ years	Data Rich	-1	2	Healthcare access and quality index	--
Brain and nervous system cancer	Female	0-6 days	95+ years	Data Rich	-1	2	fruits adjusted(g)	--
Brain and nervous system cancer	Female	0-6 days	95+ years	Data Rich	-1	2	vegetables adjusted(g)	--
Brain and nervous system cancer	Female	0-6 days	95+ years	Data Rich	1	2	Cholesterol (total, mean per capita)	471
Brain and nervous system cancer	Female	0-6 days	95+ years	Data Rich	1	2	red meats adjusted(g)	471
Brain and nervous system cancer	Female	0-6 days	95+ years	Data Rich	1	2	Systolic Blood Pressure (mmHg)	--
Brain and nervous system cancer	Female	0-6 days	95+ years	Data Rich	-1	3	Education (years per capita)	--
Brain and nervous system cancer	Female	0-6 days	95+ years	Data Rich	0	3	LDI (I$ per capita)	624
Brain and nervous system cancer	Female	0-6 days	95+ years	Data Rich	0	3	Socio-demographic Index	--
Brain and nervous system cancer	Female	0-6 days	95+ years	Global	1	1	Alcohol (liters per capita)	602
Brain and nervous system cancer	Female	0-6 days	95+ years	Global	1	1	Cumulative Cigarettes (10 Years)	--
Brain and nervous system cancer	Female	0-6 days	95+ years	Global	1	1	Cumulative Cigarettes (15 Years)	--
Brain and nervous system cancer	Female	0-6 days	95+ years	Global	1	1	Smoking Prevalence	--
Brain and nervous system cancer	Female	0-6 days	95+ years	Global	-1	2	Healthcare access and quality index	--
Brain and nervous system cancer	Female	0-6 days	95+ years	Global	-1	2	fruits adjusted(g)	--
Brain and nervous system cancer	Female	0-6 days	95+ years	Global	-1	2	vegetables adjusted(g)	--
Brain and nervous system cancer	Female	0-6 days	95+ years	Global	1	2	red meats adjusted(g)	328
Brain and nervous system cancer	Female	0-6 days	95+ years	Global	1	2	Cholesterol (total, mean per capita)	--
Brain and nervous system cancer	Female	0-6 days	95+ years	Global	1	2	Systolic Blood Pressure (mmHg)	--
Brain and nervous system cancer	Female	0-6 days	95+ years	Global	-1	3	Education (years per capita)	--
Brain and nervous system cancer	Female	0-6 days	95+ years	Global	0	3	LDI (I$ per capita)	398
Brain and nervous system cancer	Female	0-6 days	95+ years	Global	0	3	Socio-demographic Index	398
Brain and nervous system cancer	Male	0-6 days	95+ years	Data Rich	1	1	Alcohol (liters per capita)	--
Brain and nervous system cancer	Male	0-6 days	95+ years	Data Rich	1	1	Cumulative Cigarettes (10 Years)	--
Brain and nervous system cancer	Male	0-6 days	95+ years	Data Rich	1	1	Cumulative Cigarettes (15 Years)	--
Brain and nervous system cancer	Male	0-6 days	95+ years	Data Rich	1	1	Smoking Prevalence	--
Brain and nervous system cancer	Male	0-6 days	95+ years	Data Rich	-1	2	Healthcare access and quality index	--
Brain and nervous system cancer	Male	0-6 days	95+ years	Data Rich	-1	2	fruits adjusted(g)	--
Brain and nervous system cancer	Male	0-6 days	95+ years	Data Rich	-1	2	vegetables adjusted(g)	--
Brain and nervous system cancer	Male	0-6 days	95+ years	Data Rich	1	2	Cholesterol (total, mean per capita)	326
Brain and nervous system cancer	Male	0-6 days	95+ years	Data Rich	1	2	red meats adjusted(g)	434
Brain and nervous system cancer	Male	0-6 days	95+ years	Data Rich	1	2	Systolic Blood Pressure (mmHg)	--
Brain and nervous system cancer	Male	0-6 days	95+ years	Data Rich	-1	3	Education (years per capita)	--
Brain and nervous system cancer	Male	0-6 days	95+ years	Data Rich	0	3	Socio-demographic Index	566
Brain and nervous system cancer	Male	0-6 days	95+ years	Data Rich	0	3	LDI (I$ per capita)	742
Brain and nervous system cancer	Male	0-6 days	95+ years	Global	1	1	Alcohol (liters per capita)	454
Brain and nervous system cancer	Male	0-6 days	95+ years	Global	1	1	Cumulative Cigarettes (10 Years)	--
Brain and nervous system cancer	Male	0-6 days	95+ years	Global	1	1	Cumulative Cigarettes (15 Years)	--
Brain and nervous system cancer	Male	0-6 days	95+ years	Global	1	1	Smoking Prevalence	--
Brain and nervous system cancer	Male	0-6 days	95+ years	Global	-1	2	Healthcare access and quality index	--
Brain and nervous system cancer	Male	0-6 days	95+ years	Global	-1	2	fruits adjusted(g)	--
Brain and nervous system cancer	Male	0-6 days	95+ years	Global	-1	2	vegetables adjusted(g)	--
Brain and nervous system cancer	Male	0-6 days	95+ years	Global	1	2	red meats adjusted(g)	546
Brain and nervous system cancer	Male	0-6 days	95+ years	Global	1	2	Cholesterol (total, mean per capita)	--
Brain and nervous system cancer	Male	0-6 days	95+ years	Global	1	2	Systolic Blood Pressure (mmHg)	--
Brain and nervous system cancer	Male	0-6 days	95+ years	Global	-1	3	Education (years per capita)	--
Brain and nervous system cancer	Male	0-6 days	95+ years	Global	0	3	Socio-demographic Index	546
Brain and nervous system cancer	Male	0-6 days	95+ years	Global	0	3	LDI (I$ per capita)	--
Thyroid cancer	Female	10-14 years	95+ years	Data Rich	1	1	Alcohol (liters per capita)	665
Thyroid cancer	Female	10-14 years	95+ years	Data Rich	1	1	Log-transformed SEV scalar: Thyroid C	753
Thyroid cancer	Female	10-14 years	95+ years	Data Rich	-1	2	Sanitation (proportion with access)	5
Thyroid cancer	Female	10-14 years	95+ years	Data Rich	-1	2	Healthcare access and quality index	55
Thyroid cancer	Female	10-14 years	95+ years	Data Rich	-1	2	Improved Water Source (proportion with access)	55
Thyroid cancer	Female	10-14 years	95+ years	Data Rich	-1	2	fruits adjusted(g)	55
Thyroid cancer	Female	10-14 years	95+ years	Data Rich	-1	2	vegetables adjusted(g)	--
Thyroid cancer	Female	10-14 years	95+ years	Data Rich	1	2	Smoking Prevalence	27
Thyroid cancer	Female	10-14 years	95+ years	Data Rich	1	2	Tobacco (cigarettes per capita)	33
Thyroid cancer	Female	10-14 years	95+ years	Data Rich	1	2	red meats adjusted(g)	68

**Appendix Table 9: CODEm covariates used, level of covariate, and expected direction of covariate by cause, sex, and age**<br>*Covariates that CODEm did not select during the covariate selection process have no draw counts listed.*								
**Cause**	**Sex**	**Age Start**	**Age End**	**Model Type**	**Direction**	**Level**	**Covariate Name**	**Number of Draws**
Thyroid cancer	Female	10-14 years	95+ years	Data Rich	1	2	Mean BMI	192
Thyroid cancer	Female	10-14 years	95+ years	Data Rich	-1	3	Education (years per capita)	31
Thyroid cancer	Female	10-14 years	95+ years	Data Rich	0	3	Socio-demographic Index	279
Thyroid cancer	Female	10-14 years	95+ years	Data Rich	0	3	LDI (I$ per capita)	--
Thyroid cancer	Female	10-14 years	95+ years	Global	1	1	Log-transformed SEV scalar: Thyroid C	400
Thyroid cancer	Female	10-14 years	95+ years	Global	1	1	Alcohol (liters per capita)	796
Thyroid cancer	Female	10-14 years	95+ years	Global	-1	2	Sanitation (proportion with access)	72
Thyroid cancer	Female	10-14 years	95+ years	Global	-1	2	Healthcare access and quality index	134
Thyroid cancer	Female	10-14 years	95+ years	Global	-1	2	Improved Water Source (proportion with access)	134
Thyroid cancer	Female	10-14 years	95+ years	Global	-1	2	vegetables adjusted(g)	167
Thyroid cancer	Female	10-14 years	95+ years	Global	-1	2	fruits adjusted(g)	173
Thyroid cancer	Female	10-14 years	95+ years	Global	1	2	Tobacco (cigarettes per capita)	70
Thyroid cancer	Female	10-14 years	95+ years	Global	1	2	Smoking Prevalence	136
Thyroid cancer	Female	10-14 years	95+ years	Global	1	2	Mean BMI	234
Thyroid cancer	Female	10-14 years	95+ years	Global	1	2	red meats adjusted(g)	234
Thyroid cancer	Female	10-14 years	95+ years	Global	-1	3	Education (years per capita)	64
Thyroid cancer	Female	10-14 years	95+ years	Global	0	3	Socio-demographic Index	125
Thyroid cancer	Female	10-14 years	95+ years	Global	0	3	LDI (I$ per capita)	--
Thyroid cancer	Male	10-14 years	95+ years	Data Rich	1	1	Alcohol (liters per capita)	369
Thyroid cancer	Male	10-14 years	95+ years	Data Rich	1	1	Log-transformed SEV scalar: Thyroid C	770
Thyroid cancer	Male	10-14 years	95+ years	Data Rich	-1	2	Sanitation (proportion with access)	19
Thyroid cancer	Male	10-14 years	95+ years	Data Rich	-1	2	Improved Water Source (proportion with access)	43
Thyroid cancer	Male	10-14 years	95+ years	Data Rich	-1	2	Healthcare access and quality index	97
Thyroid cancer	Male	10-14 years	95+ years	Data Rich	-1	2	fruits adjusted(g)	384
Thyroid cancer	Male	10-14 years	95+ years	Data Rich	-1	2	vegetables adjusted(g)	421
Thyroid cancer	Male	10-14 years	95+ years	Data Rich	1	2	Tobacco (cigarettes per capita)	25
Thyroid cancer	Male	10-14 years	95+ years	Data Rich	1	2	Smoking Prevalence	43
Thyroid cancer	Male	10-14 years	95+ years	Data Rich	1	2	Mean BMI	663
Thyroid cancer	Male	10-14 years	95+ years	Data Rich	1	2	red meats adjusted(g)	--
Thyroid cancer	Male	10-14 years	95+ years	Data Rich	-1	3	Education (years per capita)	25
Thyroid cancer	Male	10-14 years	95+ years	Data Rich	0	3	Socio-demographic Index	149
Thyroid cancer	Male	10-14 years	95+ years	Data Rich	0	3	LDI (I$ per capita)	--
Thyroid cancer	Male	10-14 years	95+ years	Global	1	1	Alcohol (liters per capita)	483
Thyroid cancer	Male	10-14 years	95+ years	Global	1	1	Log-transformed SEV scalar: Thyroid C	614
Thyroid cancer	Male	10-14 years	95+ years	Global	2	1	Smoking Prevalence	--
Thyroid cancer	Male	10-14 years	95+ years	Global	-1	2	Improved Water Source (proportion with access)	17
Thyroid cancer	Male	10-14 years	95+ years	Global	-1	2	Sanitation (proportion with access)	19
Thyroid cancer	Male	10-14 years	95+ years	Global	-1	2	vegetables adjusted(g)	53
Thyroid cancer	Male	10-14 years	95+ years	Global	-1	2	Healthcare access and quality index	226
Thyroid cancer	Male	10-14 years	95+ years	Global	-1	2	fruits adjusted(g)	232
Thyroid cancer	Male	10-14 years	95+ years	Global	1	2	Tobacco (cigarettes per capita)	68
Thyroid cancer	Male	10-14 years	95+ years	Global	1	2	red meats adjusted(g)	116
Thyroid cancer	Male	10-14 years	95+ years	Global	1	2	Mean BMI	--
Thyroid cancer	Male	10-14 years	95+ years	Global	-1	3	Education (years per capita)	125
Thyroid cancer	Male	10-14 years	95+ years	Global	0	3	Socio-demographic Index	297
Thyroid cancer	Male	10-14 years	95+ years	Global	0	3	LDI (I$ per capita)	--
Mesothelioma	Female	15-19 years	95+ years	Data Rich	1	1	Asbestos consumption (metric tons per year per capita)	0
Mesothelioma	Female	15-19 years	95+ years	Data Rich	1	1	Cumulative Cigarettes (5 Years)	39
Mesothelioma	Female	15-19 years	95+ years	Data Rich	1	1	Smoking Prevalence	130
Mesothelioma	Female	15-19 years	95+ years	Data Rich	1	1	Log-transformed age-standardized SEV scalar: Mesothel	151
Mesothelioma	Female	15-19 years	95+ years	Data Rich	1	1	Log-transformed SEV scalar: Mesothel	878
Mesothelioma	Female	15-19 years	95+ years	Data Rich	1	1	Asbestos production (binary)	--
Mesothelioma	Female	15-19 years	95+ years	Data Rich	1	1	Indoor Air Pollution (All Cooking Fuels)	--
Mesothelioma	Female	15-19 years	95+ years	Data Rich	-1	2	Healthcare access and quality index	--
Mesothelioma	Female	15-19 years	95+ years	Data Rich	1	2	Gold production (kg) per capita	0
Mesothelioma	Female	15-19 years	95+ years	Data Rich	1	2	Gold production (binary)	498
Mesothelioma	Female	15-19 years	95+ years	Data Rich	1	2	Population Density (over 1000 ppl/sqkm, proportion)	498
Mesothelioma	Female	15-19 years	95+ years	Data Rich	1	2	Asbestos production (kg) per capita	--
Mesothelioma	Female	15-19 years	95+ years	Data Rich	-1	3	Education (years per capita)	--
Mesothelioma	Female	15-19 years	95+ years	Data Rich	0	3	Socio-demographic Index	648
Mesothelioma	Female	15-19 years	95+ years	Data Rich	0	3	LDI (I$ per capita)	--
Mesothelioma	Female	15-19 years	95+ years	Global	1	1	Asbestos consumption (metric tons per year per capita)	5
Mesothelioma	Female	15-19 years	95+ years	Global	1	1	Smoking Prevalence	86
Mesothelioma	Female	15-19 years	95+ years	Global	1	1	Cumulative Cigarettes (5 Years)	211
Mesothelioma	Female	15-19 years	95+ years	Global	1	1	Log-transformed age-standardized SEV scalar: Mesothel	652
Mesothelioma	Female	15-19 years	95+ years	Global	1	1	Log-transformed SEV scalar: Mesothel	798
Mesothelioma	Female	15-19 years	95+ years	Global	1	1	Asbestos production (binary)	--
Mesothelioma	Female	15-19 years	95+ years	Global	1	1	Indoor Air Pollution (All Cooking Fuels)	--
Mesothelioma	Female	15-19 years	95+ years	Global	-1	2	Healthcare access and quality index	--
Mesothelioma	Female	15-19 years	95+ years	Global	1	2	Gold production (binary)	113
Mesothelioma	Female	15-19 years	95+ years	Global	1	2	Gold production (kg) per capita	131
Mesothelioma	Female	15-19 years	95+ years	Global	1	2	Population Density (over 1000 ppl/sqkm, proportion)	463
Mesothelioma	Female	15-19 years	95+ years	Global	1	2	Asbestos production (kg) per capita	--
Mesothelioma	Female	15-19 years	95+ years	Global	-1	3	Education (years per capita)	--
Mesothelioma	Female	15-19 years	95+ years	Global	0	3	Socio-demographic Index	332
Mesothelioma	Female	15-19 years	95+ years	Global	0	3	LDI (I$ per capita)	--
Mesothelioma	Male	15-19 years	95+ years	Data Rich	1	1	Asbestos consumption (metric tons per year per capita)	0
Mesothelioma	Male	15-19 years	95+ years	Data Rich	1	1	Smoking Prevalence	0

**Appendix Table 9: CODEm covariates used, level of covariate, and expected direction of covariate by cause, sex, and age** *Covariates that CODEm did not select during the covariate selection process have no draw counts listed.*								
**Cause**	**Sex**	**Age Start**	**Age End**	**Model Type**	**Direction**	**Level**	**Covariate Name**	**Number of Draws**
Mesothelioma	Male	15-19 years	95+ years	Data Rich	1	1	Cumulative Cigarettes (5 Years)	375
Mesothelioma	Male	15-19 years	95+ years	Data Rich	1	1	Log-transformed SEV scalar: Mesothel	882
Mesothelioma	Male	15-19 years	95+ years	Data Rich	1	1	Log-transformed age-standardized SEV scalar: Mesothel	977
Mesothelioma	Male	15-19 years	95+ years	Data Rich	1	1	Asbestos production (binary)	--
Mesothelioma	Male	15-19 years	95+ years	Data Rich	1	1	Indoor Air Pollution (All Cooking Fuels)	--
Mesothelioma	Male	15-19 years	95+ years	Data Rich	-1	2	Healthcare access and quality index	--
Mesothelioma	Male	15-19 years	95+ years	Data Rich	1	2	Population Density (over 1000 ppl/sqkm, proportion)	6
Mesothelioma	Male	15-19 years	95+ years	Data Rich	1	2	Gold production (binary)	246
Mesothelioma	Male	15-19 years	95+ years	Data Rich	1	2	Asbestos production (kg) per capita	--
Mesothelioma	Male	15-19 years	95+ years	Data Rich	1	2	Gold production (kg) per capita	--
Mesothelioma	Male	15-19 years	95+ years	Data Rich	-1	3	Education (years per capita)	--
Mesothelioma	Male	15-19 years	95+ years	Data Rich	0	3	Socio-demographic Index	694
Mesothelioma	Male	15-19 years	95+ years	Data Rich	0	3	LDI (I$ per capita)	--
Mesothelioma	Male	15-19 years	95+ years	Global	1	1	Asbestos consumption (metric tons per year per capita)	0
Mesothelioma	Male	15-19 years	95+ years	Global	1	1	Smoking Prevalence	11
Mesothelioma	Male	15-19 years	95+ years	Global	1	1	Indoor Air Pollution (All Cooking Fuels)	167
Mesothelioma	Male	15-19 years	95+ years	Global	1	1	Cumulative Cigarettes (5 Years)	443
Mesothelioma	Male	15-19 years	95+ years	Global	1	1	Log-transformed SEV scalar: Mesothel	732
Mesothelioma	Male	15-19 years	95+ years	Global	1	1	Log-transformed age-standardized SEV scalar: Mesothel	968
Mesothelioma	Male	15-19 years	95+ years	Global	1	1	Asbestos production (binary)	--
Mesothelioma	Male	15-19 years	95+ years	Global	-1	2	Healthcare access and quality index	--
Mesothelioma	Male	15-19 years	95+ years	Global	1	2	Gold production (binary)	0
Mesothelioma	Male	15-19 years	95+ years	Global	1	2	Gold production (kg) per capita	0
Mesothelioma	Male	15-19 years	95+ years	Global	1	2	Population Density (over 1000 ppl/sqkm, proportion)	84
Mesothelioma	Male	15-19 years	95+ years	Global	1	2	Asbestos production (kg) per capita	--
Mesothelioma	Male	15-19 years	95+ years	Global	-1	3	Education (years per capita)	--
Mesothelioma	Male	15-19 years	95+ years	Global	0	3	Socio-demographic Index	630
Mesothelioma	Male	15-19 years	95+ years	Global	0	3	LDI (I$ per capita)	--
Hodgkin lymphoma	Female	0-6 days	95+ years	Data Rich	-1	2	Healthcare access and quality index	1000
Hodgkin lymphoma	Female	0-6 days	95+ years	Data Rich	-1	3	Education (years per capita)	308
Hodgkin lymphoma	Female	0-6 days	95+ years	Data Rich	0	3	Socio-demographic Index	308
Hodgkin lymphoma	Female	0-6 days	95+ years	Data Rich	0	3	LDI (I$ per capita)	--
Hodgkin lymphoma	Female	0-6 days	95+ years	Global	-1	2	Healthcare access and quality index	1000
Hodgkin lymphoma	Female	0-6 days	95+ years	Global	-1	3	Education (years per capita)	328
Hodgkin lymphoma	Female	0-6 days	95+ years	Global	0	3	Socio-demographic Index	328
Hodgkin lymphoma	Female	0-6 days	95+ years	Global	0	3	LDI (I$ per capita)	--
Hodgkin lymphoma	Male	0-6 days	95+ years	Data Rich	-1	2	Healthcare access and quality index	--
Hodgkin lymphoma	Male	0-6 days	95+ years	Data Rich	-1	3	Education (years per capita)	381
Hodgkin lymphoma	Male	0-6 days	95+ years	Data Rich	0	3	Socio-demographic Index	1000
Hodgkin lymphoma	Male	0-6 days	95+ years	Data Rich	0	3	LDI (I$ per capita)	--
Hodgkin lymphoma	Male	0-6 days	95+ years	Global	-1	2	Healthcare access and quality index	1000
Hodgkin lymphoma	Male	0-6 days	95+ years	Global	-1	3	Education (years per capita)	--
Hodgkin lymphoma	Male	0-6 days	95+ years	Global	0	3	Socio-demographic Index	452
Hodgkin lymphoma	Male	0-6 days	95+ years	Global	0	3	LDI (I$ per capita)	--
Non-Hodgkin's lymphoma	Female	0-6 days	95+ years	Data Rich	-1	2	Healthcare access and quality index	128
Non-Hodgkin's lymphoma	Female	0-6 days	95+ years	Data Rich	1	2	Cumulative Cigarettes (15 Years)	3
Non-Hodgkin's lymphoma	Female	0-6 days	95+ years	Data Rich	1	2	Cumulative Cigarettes (10 Years)	22
Non-Hodgkin's lymphoma	Female	0-6 days	95+ years	Data Rich	1	2	Smoking Prevalence	24
Non-Hodgkin's lymphoma	Female	0-6 days	95+ years	Data Rich	1	2	Cumulative Cigarettes (5 Years)	44
Non-Hodgkin's lymphoma	Female	0-6 days	95+ years	Data Rich	1	2	Cumulative Cigarettes (20 Years)	117
Non-Hodgkin's lymphoma	Female	0-6 days	95+ years	Data Rich	1	2	Mean BMI	407
Non-Hodgkin's lymphoma	Female	0-6 days	95+ years	Data Rich	1	2	Alcohol (liters per capita)	902
Non-Hodgkin's lymphoma	Female	0-6 days	95+ years	Data Rich	0	3	Total Fertility Rate	107
Non-Hodgkin's lymphoma	Female	0-6 days	95+ years	Data Rich	0	3	Socio-demographic Index	366
Non-Hodgkin's lymphoma	Female	0-6 days	95+ years	Data Rich	0	3	LDI (I$ per capita)	--
Non-Hodgkin's lymphoma	Female	0-6 days	95+ years	Global	-1	2	Healthcare access and quality index	247
Non-Hodgkin's lymphoma	Female	0-6 days	95+ years	Global	1	2	Cumulative Cigarettes (5 Years)	38
Non-Hodgkin's lymphoma	Female	0-6 days	95+ years	Global	1	2	Cumulative Cigarettes (10 Years)	86
Non-Hodgkin's lymphoma	Female	0-6 days	95+ years	Global	1	2	Smoking Prevalence	103
Non-Hodgkin's lymphoma	Female	0-6 days	95+ years	Global	1	2	Cumulative Cigarettes (20 Years)	161
Non-Hodgkin's lymphoma	Female	0-6 days	95+ years	Global	1	2	Cumulative Cigarettes (15 Years)	283
Non-Hodgkin's lymphoma	Female	0-6 days	95+ years	Global	1	2	Mean BMI	532
Non-Hodgkin's lymphoma	Female	0-6 days	95+ years	Global	1	2	Alcohol (liters per capita)	749
Non-Hodgkin's lymphoma	Female	0-6 days	95+ years	Global	0	3	Socio-demographic Index	361
Non-Hodgkin's lymphoma	Female	0-6 days	95+ years	Global	0	3	Total Fertility Rate	458
Non-Hodgkin's lymphoma	Female	0-6 days	95+ years	Global	0	3	LDI (I$ per capita)	--
Non-Hodgkin's lymphoma	Male	0-6 days	95+ years	Data Rich	-1	2	Healthcare access and quality index	233
Non-Hodgkin's lymphoma	Male	0-6 days	95+ years	Data Rich	1	2	Cumulative Cigarettes (10 Years)	13
Non-Hodgkin's lymphoma	Male	0-6 days	95+ years	Data Rich	1	2	Cumulative Cigarettes (20 Years)	17
Non-Hodgkin's lymphoma	Male	0-6 days	95+ years	Data Rich	1	2	Cumulative Cigarettes (15 Years)	23
Non-Hodgkin's lymphoma	Male	0-6 days	95+ years	Data Rich	1	2	Cumulative Cigarettes (5 Years)	169
Non-Hodgkin's lymphoma	Male	0-6 days	95+ years	Data Rich	1	2	Mean BMI	249
Non-Hodgkin's lymphoma	Male	0-6 days	95+ years	Data Rich	1	2	Alcohol (liters per capita)	568
Non-Hodgkin's lymphoma	Male	0-6 days	95+ years	Data Rich	1	2	Smoking Prevalence	--
Non-Hodgkin's lymphoma	Male	0-6 days	95+ years	Data Rich	0	3	Socio-demographic Index	344
Non-Hodgkin's lymphoma	Male	0-6 days	95+ years	Data Rich	0	3	LDI (I$ per capita)	--
Non-Hodgkin's lymphoma	Male	0-6 days	95+ years	Global	-1	2	Healthcare access and quality index	364
Non-Hodgkin's lymphoma	Male	0-6 days	95+ years	Global	1	2	Cumulative Cigarettes (10 Years)	50

Appendix Table 9: CODEm covariates used, level of covariate, and expected direction of covariate by cause, sex, and age
Covariates that CODEm did not select during the covariate selection process have no draw counts listed.

Cause	Sex	Age Start	Age End	Model Type	Direction	Level	Covariate Name	Number of Draws
Non-Hodgkin's lymphoma	Male	0-6 days	95+ years	Global	1	2	Cumulative Cigarettes (5 Years)	60
Non-Hodgkin's lymphoma	Male	0-6 days	95+ years	Global	1	2	Cumulative Cigarettes (20 Years)	73
Non-Hodgkin's lymphoma	Male	0-6 days	95+ years	Global	1	2	Cumulative Cigarettes (15 Years)	183
Non-Hodgkin's lymphoma	Male	0-6 days	95+ years	Global	1	2	Mean BMI	324
Non-Hodgkin's lymphoma	Male	0-6 days	95+ years	Global	1	2	Alcohol (liters per capita)	349
Non-Hodgkin's lymphoma	Male	0-6 days	95+ years	Global	1	2	Smoking Prevalence	--
Non-Hodgkin's lymphoma	Male	0-6 days	95+ years	Global	0	3	Socio-demographic Index	622
Non-Hodgkin's lymphoma	Male	0-6 days	95+ years	Global	0	3	LDI (I$ per capita)	--
Multiple myeloma	Female	15-19 years	95+ years	Data Rich	1	1	Smoking Prevalence	287
Multiple myeloma	Female	15-19 years	95+ years	Data Rich	1	1	Alcohol (liters per capita)	713
Multiple myeloma	Female	15-19 years	95+ years	Data Rich	1	1	Tobacco (cigarettes per capita)	--
Multiple myeloma	Female	15-19 years	95+ years	Data Rich	-1	2	Healthcare access and quality index	--
Multiple myeloma	Female	15-19 years	95+ years	Data Rich	-1	2	Improved Water Source (proportion with access)	--
Multiple myeloma	Female	15-19 years	95+ years	Data Rich	-1	2	Sanitation (proportion with access)	--
Multiple myeloma	Female	15-19 years	95+ years	Data Rich	-1	2	fruits adjusted(g)	--
Multiple myeloma	Female	15-19 years	95+ years	Data Rich	-1	2	vegetables adjusted(g)	--
Multiple myeloma	Female	15-19 years	95+ years	Data Rich	1	2	red meats adjusted(g)	191
Multiple myeloma	Female	15-19 years	95+ years	Data Rich	1	2	Mean BMI	379
Multiple myeloma	Female	15-19 years	95+ years	Data Rich	-1	3	Education (years per capita)	--
Multiple myeloma	Female	15-19 years	95+ years	Data Rich	0	3	Socio-demographic Index	370
Multiple myeloma	Female	15-19 years	95+ years	Data Rich	0	3	LDI (I$ per capita)	--
Multiple myeloma	Female	15-19 years	95+ years	Global	1	1	Smoking Prevalence	643
Multiple myeloma	Female	15-19 years	95+ years	Global	1	1	Alcohol (liters per capita)	796
Multiple myeloma	Female	15-19 years	95+ years	Global	1	1	Tobacco (cigarettes per capita)	--
Multiple myeloma	Female	15-19 years	95+ years	Global	-1	2	Healthcare access and quality index	--
Multiple myeloma	Female	15-19 years	95+ years	Global	-1	2	Improved Water Source (proportion with access)	--
Multiple myeloma	Female	15-19 years	95+ years	Global	-1	2	Sanitation (proportion with access)	--
Multiple myeloma	Female	15-19 years	95+ years	Global	-1	2	fruits adjusted(g)	--
Multiple myeloma	Female	15-19 years	95+ years	Global	-1	2	vegetables adjusted(g)	--
Multiple myeloma	Female	15-19 years	95+ years	Global	1	2	red meats adjusted(g)	430
Multiple myeloma	Female	15-19 years	95+ years	Global	1	2	Mean BMI	648
Multiple myeloma	Female	15-19 years	95+ years	Global	-1	3	Education (years per capita)	--
Multiple myeloma	Female	15-19 years	95+ years	Global	0	3	Socio-demographic Index	327
Multiple myeloma	Female	15-19 years	95+ years	Global	0	3	LDI (I$ per capita)	--
Multiple myeloma	Male	15-19 years	95+ years	Data Rich	1	1	Alcohol (liters per capita)	677
Multiple myeloma	Male	15-19 years	95+ years	Data Rich	1	1	Smoking Prevalence	--
Multiple myeloma	Male	15-19 years	95+ years	Data Rich	1	1	Tobacco (cigarettes per capita)	--
Multiple myeloma	Male	15-19 years	95+ years	Data Rich	-1	2	Healthcare access and quality index	--
Multiple myeloma	Male	15-19 years	95+ years	Data Rich	-1	2	Improved Water Source (proportion with access)	--
Multiple myeloma	Male	15-19 years	95+ years	Data Rich	-1	2	Sanitation (proportion with access)	--
Multiple myeloma	Male	15-19 years	95+ years	Data Rich	-1	2	fruits adjusted(g)	--
Multiple myeloma	Male	15-19 years	95+ years	Data Rich	-1	2	vegetables adjusted(g)	--
Multiple myeloma	Male	15-19 years	95+ years	Data Rich	1	2	red meats adjusted(g)	707
Multiple myeloma	Male	15-19 years	95+ years	Data Rich	1	2	Mean BMI	--
Multiple myeloma	Male	15-19 years	95+ years	Data Rich	-1	3	Education (years per capita)	--
Multiple myeloma	Male	15-19 years	95+ years	Data Rich	0	3	Socio-demographic Index	751
Multiple myeloma	Male	15-19 years	95+ years	Data Rich	0	3	LDI (I$ per capita)	--
Multiple myeloma	Male	15-19 years	95+ years	Global	1	1	Alcohol (liters per capita)	1000
Multiple myeloma	Male	15-19 years	95+ years	Global	1	1	Smoking Prevalence	--
Multiple myeloma	Male	15-19 years	95+ years	Global	1	1	Tobacco (cigarettes per capita)	--
Multiple myeloma	Male	15-19 years	95+ years	Global	-1	2	Healthcare access and quality index	--
Multiple myeloma	Male	15-19 years	95+ years	Global	-1	2	Improved Water Source (proportion with access)	--
Multiple myeloma	Male	15-19 years	95+ years	Global	-1	2	Sanitation (proportion with access)	--
Multiple myeloma	Male	15-19 years	95+ years	Global	-1	2	fruits adjusted(g)	--
Multiple myeloma	Male	15-19 years	95+ years	Global	-1	2	vegetables adjusted(g)	--
Multiple myeloma	Male	15-19 years	95+ years	Global	1	2	Mean BMI	535
Multiple myeloma	Male	15-19 years	95+ years	Global	1	2	red meats adjusted(g)	535
Multiple myeloma	Male	15-19 years	95+ years	Global	-1	3	Education (years per capita)	--
Multiple myeloma	Male	15-19 years	95+ years	Global	0	3	Socio-demographic Index	463
Multiple myeloma	Male	15-19 years	95+ years	Global	0	3	LDI (I$ per capita)	--
Leukaemia	Female	0-6 days	95+ years	Data Rich	1	1	Log-transformed SEV scalar: Leukemia	1000
Leukaemia	Female	0-6 days	95+ years	Data Rich	1	1	Log-transformed age-standardized SEV scalar: Leukemia	--
Leukaemia	Female	0-6 days	95+ years	Data Rich	-1	2	Healthcare access and quality index	351
Leukaemia	Female	0-6 days	95+ years	Data Rich	1	2	Tobacco (cigarettes per capita)	51
Leukaemia	Female	0-6 days	95+ years	Data Rich	1	2	Smoking Prevalence	184
Leukaemia	Female	0-6 days	95+ years	Data Rich	1	2	Cumulative Cigarettes (15 Years)	197
Leukaemia	Female	0-6 days	95+ years	Data Rich	1	2	Alcohol (liters per capita)	759
Leukaemia	Female	0-6 days	95+ years	Data Rich	1	2	Cumulative Cigarettes (10 Years)	--
Leukaemia	Female	0-6 days	95+ years	Data Rich	1	2	Cumulative Cigarettes (20 Years)	--
Leukaemia	Female	0-6 days	95+ years	Data Rich	1	2	Cumulative Cigarettes (5 Years)	--
Leukaemia	Female	0-6 days	95+ years	Data Rich	1	2	Mean BMI	--
Leukaemia	Female	0-6 days	95+ years	Data Rich	-1	3	Education (years per capita)	256
Leukaemia	Female	0-6 days	95+ years	Data Rich	0	3	Socio-demographic Index	191
Leukaemia	Female	0-6 days	95+ years	Data Rich	0	3	LDI (I$ per capita)	--
Leukaemia	Female	0-6 days	95+ years	Global	1	1	Log-transformed age-standardized SEV scalar: Leukemia	255
Leukaemia	Female	0-6 days	95+ years	Global	1	1	Log-transformed SEV scalar: Leukemia	--
Leukaemia	Female	0-6 days	95+ years	Global	-1	2	Healthcare access and quality index	525
Leukaemia	Female	0-6 days	95+ years	Global	1	2	Cumulative Cigarettes (15 Years)	144

Appendix Table 9: CODEm covariates used, level of covariate, and expected direction of covariate by cause, sex, and age
Covariates that CODEm did not select during the covariate selection process have no draw counts listed.

Cause	Sex	Age Start	Age End	Model Type	Direction	Level	Covariate Name	Number of Draws
Leukaemia	Female	0-6 days	95+ years	Global	1	2	Cumulative Cigarettes (20 Years)	381
Leukaemia	Female	0-6 days	95+ years	Global	1	2	Alcohol (liters per capita)	--
Leukaemia	Female	0-6 days	95+ years	Global	1	2	Cumulative Cigarettes (10 Years)	--
Leukaemia	Female	0-6 days	95+ years	Global	1	2	Cumulative Cigarettes (5 Years)	--
Leukaemia	Female	0-6 days	95+ years	Global	1	2	Mean BMI	--
Leukaemia	Female	0-6 days	95+ years	Global	1	2	Smoking Prevalence	--
Leukaemia	Female	0-6 days	95+ years	Global	1	2	Tobacco (cigarettes per capita)	--
Leukaemia	Female	0-6 days	95+ years	Global	-1	3	Education (years per capita)	328
Leukaemia	Female	0-6 days	95+ years	Global	0	3	Socio-demographic Index	220
Leukaemia	Female	0-6 days	95+ years	Global	0	3	LDI (I$ per capita)	--
Leukaemia	Male	0-6 days	95+ years	Data Rich	1	1	Log-transformed SEV scalar: Leukemia	465
Leukaemia	Male	0-6 days	95+ years	Data Rich	1	1	Log-transformed age-standardized SEV scalar: Leukemia	571
Leukaemia	Male	0-6 days	95+ years	Data Rich	-1	2	Healthcare access and quality index	329
Leukaemia	Male	0-6 days	95+ years	Data Rich	1	2	Smoking Prevalence	0
Leukaemia	Male	0-6 days	95+ years	Data Rich	1	2	Cumulative Cigarettes (10 Years)	55
Leukaemia	Male	0-6 days	95+ years	Data Rich	1	2	Cumulative Cigarettes (5 Years)	99
Leukaemia	Male	0-6 days	95+ years	Data Rich	1	2	Cumulative Cigarettes (20 Years)	103
Leukaemia	Male	0-6 days	95+ years	Data Rich	1	2	Cumulative Cigarettes (15 Years)	105
Leukaemia	Male	0-6 days	95+ years	Data Rich	1	2	Tobacco (cigarettes per capita)	296
Leukaemia	Male	0-6 days	95+ years	Data Rich	1	2	Mean BMI	429
Leukaemia	Male	0-6 days	95+ years	Data Rich	1	2	Alcohol (liters per capita)	--
Leukaemia	Male	0-6 days	95+ years	Data Rich	-1	3	Education (years per capita)	465
Leukaemia	Male	0-6 days	95+ years	Data Rich	0	3	Socio-demographic Index	636
Leukaemia	Male	0-6 days	95+ years	Data Rich	0	3	LDI (I$ per capita)	--
Leukaemia	Male	0-6 days	95+ years	Global	1	1	Log-transformed SEV scalar: Leukemia	--
Leukaemia	Male	0-6 days	95+ years	Global	1	1	Log-transformed age-standardized SEV scalar: Leukemia	--
Leukaemia	Male	0-6 days	95+ years	Global	-1	2	Healthcare access and quality index	416
Leukaemia	Male	0-6 days	95+ years	Global	1	2	Cumulative Cigarettes (5 Years)	198
Leukaemia	Male	0-6 days	95+ years	Global	1	2	Smoking Prevalence	230
Leukaemia	Male	0-6 days	95+ years	Global	1	2	Cumulative Cigarettes (10 Years)	256
Leukaemia	Male	0-6 days	95+ years	Global	1	2	Tobacco (cigarettes per capita)	324
Leukaemia	Male	0-6 days	95+ years	Global	1	2	Cumulative Cigarettes (20 Years)	514
Leukaemia	Male	0-6 days	95+ years	Global	1	2	Alcohol (liters per capita)	--
Leukaemia	Male	0-6 days	95+ years	Global	1	2	Cumulative Cigarettes (15 Years)	--
Leukaemia	Male	0-6 days	95+ years	Global	1	2	Mean BMI	--
Leukaemia	Male	0-6 days	95+ years	Global	-1	3	Education (years per capita)	32
Leukaemia	Male	0-6 days	95+ years	Global	0	3	Socio-demographic Index	457
Leukaemia	Male	0-6 days	95+ years	Global	0	3	LDI (I$ per capita)	--
Acute lymphoid leukaemia	Female	0-6 days	95+ years	Data Rich	1	1	Log-transformed age-standardized SEV scalar: Leukemia	523
Acute lymphoid leukaemia	Female	0-6 days	95+ years	Data Rich	1	1	Log-transformed SEV scalar: Leukemia	837
Acute lymphoid leukaemia	Female	0-6 days	95+ years	Data Rich	-1	2	Healthcare access and quality index	18
Acute lymphoid leukaemia	Female	0-6 days	95+ years	Data Rich	1	2	Alcohol (liters per capita)	18
Acute lymphoid leukaemia	Female	0-6 days	95+ years	Data Rich	1	2	Cumulative Cigarettes (20 Years)	22
Acute lymphoid leukaemia	Female	0-6 days	95+ years	Data Rich	1	2	Mean BMI	199
Acute lymphoid leukaemia	Female	0-6 days	95+ years	Data Rich	1	2	Tobacco (cigarettes per capita)	383
Acute lymphoid leukaemia	Female	0-6 days	95+ years	Data Rich	1	2	Cumulative Cigarettes (10 Years)	--
Acute lymphoid leukaemia	Female	0-6 days	95+ years	Data Rich	1	2	Cumulative Cigarettes (15 Years)	--
Acute lymphoid leukaemia	Female	0-6 days	95+ years	Data Rich	1	2	Cumulative Cigarettes (5 Years)	--
Acute lymphoid leukaemia	Female	0-6 days	95+ years	Data Rich	1	2	Smoking Prevalence	--
Acute lymphoid leukaemia	Female	0-6 days	95+ years	Data Rich	-1	3	Education (years per capita)	295
Acute lymphoid leukaemia	Female	0-6 days	95+ years	Data Rich	0	3	Socio-demographic Index	612
Acute lymphoid leukaemia	Female	0-6 days	95+ years	Data Rich	0	3	LDI (I$ per capita)	646
Acute lymphoid leukaemia	Female	0-6 days	95+ years	Global	1	1	Log-transformed age-standardized SEV scalar: Leukemia	649
Acute lymphoid leukaemia	Female	0-6 days	95+ years	Global	1	1	Log-transformed SEV scalar: Leukemia	685
Acute lymphoid leukaemia	Female	0-6 days	95+ years	Global	-1	2	Healthcare access and quality index	--
Acute lymphoid leukaemia	Female	0-6 days	95+ years	Global	1	2	Alcohol (liters per capita)	34
Acute lymphoid leukaemia	Female	0-6 days	95+ years	Global	1	2	Cumulative Cigarettes (20 Years)	109
Acute lymphoid leukaemia	Female	0-6 days	95+ years	Global	1	2	Mean BMI	229
Acute lymphoid leukaemia	Female	0-6 days	95+ years	Global	1	2	Tobacco (cigarettes per capita)	265
Acute lymphoid leukaemia	Female	0-6 days	95+ years	Global	1	2	Cumulative Cigarettes (10 Years)	--
Acute lymphoid leukaemia	Female	0-6 days	95+ years	Global	1	2	Cumulative Cigarettes (15 Years)	--
Acute lymphoid leukaemia	Female	0-6 days	95+ years	Global	1	2	Cumulative Cigarettes (5 Years)	--
Acute lymphoid leukaemia	Female	0-6 days	95+ years	Global	1	2	Smoking Prevalence	--
Acute lymphoid leukaemia	Female	0-6 days	95+ years	Global	-1	3	Education (years per capita)	463
Acute lymphoid leukaemia	Female	0-6 days	95+ years	Global	0	3	Socio-demographic Index	341
Acute lymphoid leukaemia	Female	0-6 days	95+ years	Global	0	3	LDI (I$ per capita)	462
Acute lymphoid leukaemia	Male	0-6 days	95+ years	Data Rich	1	1	Log-transformed age-standardized SEV scalar: Leukemia	440
Acute lymphoid leukaemia	Male	0-6 days	95+ years	Data Rich	1	1	Log-transformed SEV scalar: Leukemia	653
Acute lymphoid leukaemia	Male	0-6 days	95+ years	Data Rich	-1	2	Healthcare access and quality index	3
Acute lymphoid leukaemia	Male	0-6 days	95+ years	Data Rich	1	2	Tobacco (cigarettes per capita)	141
Acute lymphoid leukaemia	Male	0-6 days	95+ years	Data Rich	1	2	Mean BMI	508
Acute lymphoid leukaemia	Male	0-6 days	95+ years	Data Rich	1	2	Alcohol (liters per capita)	--
Acute lymphoid leukaemia	Male	0-6 days	95+ years	Data Rich	1	2	Cumulative Cigarettes (10 Years)	--
Acute lymphoid leukaemia	Male	0-6 days	95+ years	Data Rich	1	2	Cumulative Cigarettes (15 Years)	--
Acute lymphoid leukaemia	Male	0-6 days	95+ years	Data Rich	1	2	Cumulative Cigarettes (20 Years)	--
Acute lymphoid leukaemia	Male	0-6 days	95+ years	Data Rich	1	2	Cumulative Cigarettes (5 Years)	--
Acute lymphoid leukaemia	Male	0-6 days	95+ years	Data Rich	1	2	Smoking Prevalence	--
Acute lymphoid leukaemia	Male	0-6 days	95+ years	Data Rich	-1	3	Education (years per capita)	171

Appendix Table 9: CODEm covariates used, level of covariate, and expected direction of covariate by cause, sex, and age
Covariates that CODEm did not select during the covariate selection process have no draw counts listed.

Cause	Sex	Age Start	Age End	Model Type	Direction	Level	Covariate Name	Number of Draws
Acute lymphoid leukaemia	Male	0-6 days	95+ years	Data Rich	0	3	Socio-demographic Index	539
Acute lymphoid leukaemia	Male	0-6 days	95+ years	Data Rich	0	3	LDI (I$ per capita)	579
Acute lymphoid leukaemia	Male	0-6 days	95+ years	Global	1	1	Log-transformed SEV scalar: Leukemia	409
Acute lymphoid leukaemia	Male	0-6 days	95+ years	Global	1	1	Log-transformed age-standardized SEV scalar: Leukemia	722
Acute lymphoid leukaemia	Male	0-6 days	95+ years	Global	-1	2	Healthcare access and quality index	--
Acute lymphoid leukaemia	Male	0-6 days	95+ years	Global	1	2	Cumulative Cigarettes (20 Years)	12
Acute lymphoid leukaemia	Male	0-6 days	95+ years	Global	1	2	Smoking Prevalence	17
Acute lymphoid leukaemia	Male	0-6 days	95+ years	Global	1	2	Tobacco (cigarettes per capita)	131
Acute lymphoid leukaemia	Male	0-6 days	95+ years	Global	1	2	Mean BMI	289
Acute lymphoid leukaemia	Male	0-6 days	95+ years	Global	1	2	Alcohol (liters per capita)	--
Acute lymphoid leukaemia	Male	0-6 days	95+ years	Global	1	2	Cumulative Cigarettes (10 Years)	--
Acute lymphoid leukaemia	Male	0-6 days	95+ years	Global	1	2	Cumulative Cigarettes (15 Years)	--
Acute lymphoid leukaemia	Male	0-6 days	95+ years	Global	1	2	Cumulative Cigarettes (5 Years)	--
Acute lymphoid leukaemia	Male	0-6 days	95+ years	Global	-1	3	Education (years per capita)	103
Acute lymphoid leukaemia	Male	0-6 days	95+ years	Global	0	3	Socio-demographic Index	235
Acute lymphoid leukaemia	Male	0-6 days	95+ years	Global	0	3	LDI (I$ per capita)	536
Chronic lymphoid leukaemia	Female	15-19 years	95+ years	Data Rich	1	1	Log-transformed age-standardized SEV scalar: Leukemia	819
Chronic lymphoid leukaemia	Female	15-19 years	95+ years	Data Rich	1	1	Log-transformed SEV scalar: Leukemia	964
Chronic lymphoid leukaemia	Female	15-19 years	95+ years	Data Rich	-1	2	Healthcare access and quality index	102
Chronic lymphoid leukaemia	Female	15-19 years	95+ years	Data Rich	1	2	Cumulative Cigarettes (20 Years)	40
Chronic lymphoid leukaemia	Female	15-19 years	95+ years	Data Rich	1	2	Cumulative Cigarettes (5 Years)	53
Chronic lymphoid leukaemia	Female	15-19 years	95+ years	Data Rich	1	2	Cumulative Cigarettes (10 Years)	109
Chronic lymphoid leukaemia	Female	15-19 years	95+ years	Data Rich	1	2	Tobacco (cigarettes per capita)	180
Chronic lymphoid leukaemia	Female	15-19 years	95+ years	Data Rich	1	2	Smoking Prevalence	322
Chronic lymphoid leukaemia	Female	15-19 years	95+ years	Data Rich	1	2	Mean BMI	429
Chronic lymphoid leukaemia	Female	15-19 years	95+ years	Data Rich	1	2	Alcohol (liters per capita)	568
Chronic lymphoid leukaemia	Female	15-19 years	95+ years	Data Rich	1	2	Cumulative Cigarettes (15 Years)	594
Chronic lymphoid leukaemia	Female	15-19 years	95+ years	Data Rich	-1	3	Education (years per capita)	212
Chronic lymphoid leukaemia	Female	15-19 years	95+ years	Data Rich	0	3	Socio-demographic Index	46
Chronic lymphoid leukaemia	Female	15-19 years	95+ years	Data Rich	0	3	LDI (I$ per capita)	347
Chronic lymphoid leukaemia	Female	15-19 years	95+ years	Global	1	1	Log-transformed age-standardized SEV scalar: Leukemia	160
Chronic lymphoid leukaemia	Female	15-19 years	95+ years	Global	1	1	Log-transformed SEV scalar: Leukemia	600
Chronic lymphoid leukaemia	Female	15-19 years	95+ years	Global	1	1	Healthcare access and quality index	814
Chronic lymphoid leukaemia	Female	15-19 years	95+ years	Global	1	2	Mean BMI	0
Chronic lymphoid leukaemia	Female	15-19 years	95+ years	Global	1	2	Cumulative Cigarettes (5 Years)	111
Chronic lymphoid leukaemia	Female	15-19 years	95+ years	Global	1	2	Cumulative Cigarettes (15 Years)	206
Chronic lymphoid leukaemia	Female	15-19 years	95+ years	Global	1	2	Tobacco (cigarettes per capita)	239
Chronic lymphoid leukaemia	Female	15-19 years	95+ years	Global	1	2	Smoking Prevalence	338
Chronic lymphoid leukaemia	Female	15-19 years	95+ years	Global	1	2	Cumulative Cigarettes (10 Years)	552
Chronic lymphoid leukaemia	Female	15-19 years	95+ years	Global	1	2	Cumulative Cigarettes (20 Years)	600
Chronic lymphoid leukaemia	Female	15-19 years	95+ years	Global	1	2	Alcohol (liters per capita)	676
Chronic lymphoid leukaemia	Female	15-19 years	95+ years	Global	-1	3	Education (years per capita)	25
Chronic lymphoid leukaemia	Female	15-19 years	95+ years	Global	0	3	LDI (I$ per capita)	223
Chronic lymphoid leukaemia	Female	15-19 years	95+ years	Global	0	3	Socio-demographic Index	229
Chronic lymphoid leukaemia	Male	15-19 years	95+ years	Data Rich	1	1	Log-transformed SEV scalar: Leukemia	315
Chronic lymphoid leukaemia	Male	15-19 years	95+ years	Data Rich	1	1	Log-transformed age-standardized SEV scalar: Leukemia	1000
Chronic lymphoid leukaemia	Male	15-19 years	95+ years	Data Rich	-1	2	Healthcare access and quality index	226
Chronic lymphoid leukaemia	Male	15-19 years	95+ years	Data Rich	1	2	Tobacco (cigarettes per capita)	16
Chronic lymphoid leukaemia	Male	15-19 years	95+ years	Data Rich	1	2	Cumulative Cigarettes (10 Years)	42
Chronic lymphoid leukaemia	Male	15-19 years	95+ years	Data Rich	1	2	Cumulative Cigarettes (20 Years)	75
Chronic lymphoid leukaemia	Male	15-19 years	95+ years	Data Rich	1	2	Cumulative Cigarettes (15 Years)	107
Chronic lymphoid leukaemia	Male	15-19 years	95+ years	Data Rich	1	2	Mean BMI	578
Chronic lymphoid leukaemia	Male	15-19 years	95+ years	Data Rich	1	2	Alcohol (liters per capita)	--
Chronic lymphoid leukaemia	Male	15-19 years	95+ years	Data Rich	1	2	Cumulative Cigarettes (5 Years)	--
Chronic lymphoid leukaemia	Male	15-19 years	95+ years	Data Rich	1	2	Smoking Prevalence	--
Chronic lymphoid leukaemia	Male	15-19 years	95+ years	Data Rich	-1	3	Education (years per capita)	12
Chronic lymphoid leukaemia	Male	15-19 years	95+ years	Data Rich	0	3	LDI (I$ per capita)	253
Chronic lymphoid leukaemia	Male	15-19 years	95+ years	Data Rich	0	3	Socio-demographic Index	373
Chronic lymphoid leukaemia	Male	15-19 years	95+ years	Global	1	1	Log-transformed SEV scalar: Leukemia	134
Chronic lymphoid leukaemia	Male	15-19 years	95+ years	Global	1	1	Log-transformed age-standardized SEV scalar: Leukemia	866
Chronic lymphoid leukaemia	Male	15-19 years	95+ years	Global	-1	2	Healthcare access and quality index	250
Chronic lymphoid leukaemia	Male	15-19 years	95+ years	Global	1	2	Tobacco (cigarettes per capita)	22
Chronic lymphoid leukaemia	Male	15-19 years	95+ years	Global	1	2	Cumulative Cigarettes (15 Years)	27
Chronic lymphoid leukaemia	Male	15-19 years	95+ years	Global	1	2	Cumulative Cigarettes (10 Years)	137
Chronic lymphoid leukaemia	Male	15-19 years	95+ years	Global	1	2	Cumulative Cigarettes (20 Years)	137
Chronic lymphoid leukaemia	Male	15-19 years	95+ years	Global	1	2	Cumulative Cigarettes (5 Years)	146
Chronic lymphoid leukaemia	Male	15-19 years	95+ years	Global	1	2	Mean BMI	287
Chronic lymphoid leukaemia	Male	15-19 years	95+ years	Global	1	2	Alcohol (liters per capita)	--
Chronic lymphoid leukaemia	Male	15-19 years	95+ years	Global	1	2	Smoking Prevalence	--
Chronic lymphoid leukaemia	Male	15-19 years	95+ years	Global	-1	3	Education (years per capita)	55
Chronic lymphoid leukaemia	Male	15-19 years	95+ years	Global	0	3	Socio-demographic Index	345
Chronic lymphoid leukaemia	Male	15-19 years	95+ years	Global	0	3	LDI (I$ per capita)	469
Acute myeloid leukaemia	Female	0-6 days	95+ years	Data Rich	1	1	Log-transformed age-standardized SEV scalar: Leukemia	867
Acute myeloid leukaemia	Female	0-6 days	95+ years	Data Rich	1	1	Log-transformed SEV scalar: Leukemia	1000
Acute myeloid leukaemia	Female	0-6 days	95+ years	Data Rich	-1	2	Healthcare access and quality index	--
Acute myeloid leukaemia	Female	0-6 days	95+ years	Data Rich	1	2	Tobacco (cigarettes per capita)	147
Acute myeloid leukaemia	Female	0-6 days	95+ years	Data Rich	1	2	Alcohol (liters per capita)	356
Acute myeloid leukaemia	Female	0-6 days	95+ years	Data Rich	1	2	Cumulative Cigarettes (10 Years)	--

Appendix Table 9: CODEm covariates used, level of covariate, and expected direction of covariate by cause, sex, and age
Covariates that CODEm did not select during the covariate selection process have no draw counts listed.

Cause	Sex	Age Start	Age End	Model Type	Direction	Level	Covariate Name	Number of Draws
Acute myeloid leukaemia	Female	0-6 days	95+ years	Data Rich	1	2	Cumulative Cigarettes (15 Years)	--
Acute myeloid leukaemia	Female	0-6 days	95+ years	Data Rich	1	2	Cumulative Cigarettes (20 Years)	--
Acute myeloid leukaemia	Female	0-6 days	95+ years	Data Rich	1	2	Cumulative Cigarettes (5 Years)	--
Acute myeloid leukaemia	Female	0-6 days	95+ years	Data Rich	1	2	Mean BMI	--
Acute myeloid leukaemia	Female	0-6 days	95+ years	Data Rich	1	2	Smoking Prevalence	--
Acute myeloid leukaemia	Female	0-6 days	95+ years	Data Rich	-1	3	Education (years per capita)	--
Acute myeloid leukaemia	Female	0-6 days	95+ years	Data Rich	0	3	LDI (I$ per capita)	--
Acute myeloid leukaemia	Female	0-6 days	95+ years	Data Rich	0	3	Socio-demographic Index	--
Acute myeloid leukaemia	Female	0-6 days	95+ years	Global	1	1	Log-transformed age-standardized SEV scalar: Leukemia	577
Acute myeloid leukaemia	Female	0-6 days	95+ years	Global	1	1	Log-transformed SEV scalar: Leukemia	623
Acute myeloid leukaemia	Female	0-6 days	95+ years	Global	-1	2	Healthcare access and quality index	--
Acute myeloid leukaemia	Female	0-6 days	95+ years	Global	1	2	Tobacco (cigarettes per capita)	42
Acute myeloid leukaemia	Female	0-6 days	95+ years	Global	1	2	Cumulative Cigarettes (10 Years)	46
Acute myeloid leukaemia	Female	0-6 days	95+ years	Global	1	2	Cumulative Cigarettes (5 Years)	49
Acute myeloid leukaemia	Female	0-6 days	95+ years	Global	1	2	Alcohol (liters per capita)	485
Acute myeloid leukaemia	Female	0-6 days	95+ years	Global	1	2	Cumulative Cigarettes (15 Years)	--
Acute myeloid leukaemia	Female	0-6 days	95+ years	Global	1	2	Cumulative Cigarettes (20 Years)	--
Acute myeloid leukaemia	Female	0-6 days	95+ years	Global	1	2	Mean BMI	--
Acute myeloid leukaemia	Female	0-6 days	95+ years	Global	1	2	Smoking Prevalence	--
Acute myeloid leukaemia	Female	0-6 days	95+ years	Global	-1	3	Education (years per capita)	--
Acute myeloid leukaemia	Female	0-6 days	95+ years	Global	0	3	Socio-demographic Index	192
Acute myeloid leukaemia	Female	0-6 days	95+ years	Global	0	3	LDI (I$ per capita)	--
Acute myeloid leukaemia	Male	0-6 days	95+ years	Data Rich	1	1	Log-transformed SEV scalar: Leukemia	330
Acute myeloid leukaemia	Male	0-6 days	95+ years	Data Rich	1	1	Log-transformed age-standardized SEV scalar: Leukemia	779
Acute myeloid leukaemia	Male	0-6 days	95+ years	Data Rich	-1	2	Healthcare access and quality index	--
Acute myeloid leukaemia	Male	0-6 days	95+ years	Data Rich	1	2	Tobacco (cigarettes per capita)	105
Acute myeloid leukaemia	Male	0-6 days	95+ years	Data Rich	1	2	Alcohol (liters per capita)	271
Acute myeloid leukaemia	Male	0-6 days	95+ years	Data Rich	1	2	Cumulative Cigarettes (5 Years)	271
Acute myeloid leukaemia	Male	0-6 days	95+ years	Data Rich	1	2	Mean BMI	705
Acute myeloid leukaemia	Male	0-6 days	95+ years	Data Rich	1	2	Cumulative Cigarettes (10 Years)	--
Acute myeloid leukaemia	Male	0-6 days	95+ years	Data Rich	1	2	Cumulative Cigarettes (15 Years)	--
Acute myeloid leukaemia	Male	0-6 days	95+ years	Data Rich	1	2	Cumulative Cigarettes (20 Years)	--
Acute myeloid leukaemia	Male	0-6 days	95+ years	Data Rich	1	2	Smoking Prevalence	--
Acute myeloid leukaemia	Male	0-6 days	95+ years	Data Rich	-1	3	Education (years per capita)	12
Acute myeloid leukaemia	Male	0-6 days	95+ years	Data Rich	0	3	Socio-demographic Index	281
Acute myeloid leukaemia	Male	0-6 days	95+ years	Data Rich	0	3	LDI (I$ per capita)	--
Acute myeloid leukaemia	Male	0-6 days	95+ years	Global	1	1	Log-transformed age-standardized SEV scalar: Leukemia	243
Acute myeloid leukaemia	Male	0-6 days	95+ years	Global	1	1	Log-transformed SEV scalar: Leukemia	757
Acute myeloid leukaemia	Male	0-6 days	95+ years	Global	-1	2	Healthcare access and quality index	--
Acute myeloid leukaemia	Male	0-6 days	95+ years	Global	1	2	Cumulative Cigarettes (5 Years)	184
Acute myeloid leukaemia	Male	0-6 days	95+ years	Global	1	2	Mean BMI	189
Acute myeloid leukaemia	Male	0-6 days	95+ years	Global	1	2	Tobacco (cigarettes per capita)	259
Acute myeloid leukaemia	Male	0-6 days	95+ years	Global	1	2	Alcohol (liters per capita)	288
Acute myeloid leukaemia	Male	0-6 days	95+ years	Global	1	2	Cumulative Cigarettes (10 Years)	--
Acute myeloid leukaemia	Male	0-6 days	95+ years	Global	1	2	Cumulative Cigarettes (15 Years)	--
Acute myeloid leukaemia	Male	0-6 days	95+ years	Global	1	2	Cumulative Cigarettes (20 Years)	--
Acute myeloid leukaemia	Male	0-6 days	95+ years	Global	1	2	Smoking Prevalence	--
Acute myeloid leukaemia	Male	0-6 days	95+ years	Global	-1	3	Education (years per capita)	--
Acute myeloid leukaemia	Male	0-6 days	95+ years	Global	0	3	Socio-demographic Index	307
Acute myeloid leukaemia	Male	0-6 days	95+ years	Global	0	3	LDI (I$ per capita)	--
Chronic myeloid leukaemia	Female	28-364 days	95+ years	Data Rich	1	1	Log-transformed SEV scalar: Leukemia	--
Chronic myeloid leukaemia	Female	28-364 days	95+ years	Data Rich	1	1	Log-transformed age-standardized SEV scalar: Leukemia	--
Chronic myeloid leukaemia	Female	28-364 days	95+ years	Data Rich	-1	2	Healthcare access and quality index	702
Chronic myeloid leukaemia	Female	28-364 days	95+ years	Data Rich	1	2	Cumulative Cigarettes (5 Years)	50
Chronic myeloid leukaemia	Female	28-364 days	95+ years	Data Rich	1	2	Cumulative Cigarettes (10 Years)	118
Chronic myeloid leukaemia	Female	28-364 days	95+ years	Data Rich	1	2	Cumulative Cigarettes (20 Years)	239
Chronic myeloid leukaemia	Female	28-364 days	95+ years	Data Rich	1	2	Cumulative Cigarettes (15 Years)	317
Chronic myeloid leukaemia	Female	28-364 days	95+ years	Data Rich	1	2	Smoking Prevalence	327
Chronic myeloid leukaemia	Female	28-364 days	95+ years	Data Rich	1	2	Alcohol (liters per capita)	432
Chronic myeloid leukaemia	Female	28-364 days	95+ years	Data Rich	1	2	Tobacco (cigarettes per capita)	507
Chronic myeloid leukaemia	Female	28-364 days	95+ years	Data Rich	1	2	Mean BMI	--
Chronic myeloid leukaemia	Female	28-364 days	95+ years	Data Rich	-1	3	Education (years per capita)	418
Chronic myeloid leukaemia	Female	28-364 days	95+ years	Data Rich	0	3	Socio-demographic Index	673
Chronic myeloid leukaemia	Female	28-364 days	95+ years	Data Rich	0	3	LDI (I$ per capita)	--
Chronic myeloid leukaemia	Female	28-364 days	95+ years	Global	1	1	Log-transformed SEV scalar: Leukemia	--
Chronic myeloid leukaemia	Female	28-364 days	95+ years	Global	1	1	Log-transformed age-standardized SEV scalar: Leukemia	--
Chronic myeloid leukaemia	Female	28-364 days	95+ years	Global	-1	2	Healthcare access and quality index	939
Chronic myeloid leukaemia	Female	28-364 days	95+ years	Global	1	2	Cumulative Cigarettes (5 Years)	109
Chronic myeloid leukaemia	Female	28-364 days	95+ years	Global	1	2	Cumulative Cigarettes (15 Years)	148
Chronic myeloid leukaemia	Female	28-364 days	95+ years	Global	1	2	Cumulative Cigarettes (20 Years)	265
Chronic myeloid leukaemia	Female	28-364 days	95+ years	Global	1	2	Cumulative Cigarettes (10 Years)	417
Chronic myeloid leukaemia	Female	28-364 days	95+ years	Global	1	2	Smoking Prevalence	501
Chronic myeloid leukaemia	Female	28-364 days	95+ years	Global	1	2	Tobacco (cigarettes per capita)	669
Chronic myeloid leukaemia	Female	28-364 days	95+ years	Global	1	2	Alcohol (liters per capita)	778
Chronic myeloid leukaemia	Female	28-364 days	95+ years	Global	1	2	Mean BMI	--
Chronic myeloid leukaemia	Female	28-364 days	95+ years	Global	-1	3	Education (years per capita)	131
Chronic myeloid leukaemia	Female	28-364 days	95+ years	Global	0	3	Socio-demographic Index	217
Chronic myeloid leukaemia	Female	28-364 days	95+ years	Global	0	3	LDI (I$ per capita)	--

Appendix Table 9: CODEm covariates used, level of covariate, and expected direction of covariate by cause, sex, and age

Covariates that CODEm did not select during the covariate selection process have no draw counts listed.

Cause	Sex	Age Start	Age End	Model Type	Direction	Level	Covariate Name	Number of Draws
Chronic myeloid leukaemia	Male	28-364 days	95+ years	Data Rich	1	1	Log-transformed age-standardized SEV scalar: Leukemia	139
Chronic myeloid leukaemia	Male	28-364 days	95+ years	Data Rich	1	1	Log-transformed SEV scalar: Leukemia	888
Chronic myeloid leukaemia	Male	28-364 days	95+ years	Data Rich	-1	2	Healthcare access and quality index	274
Chronic myeloid leukaemia	Male	28-364 days	95+ years	Data Rich	1	2	Cumulative Cigarettes (20 Years)	37
Chronic myeloid leukaemia	Male	28-364 days	95+ years	Data Rich	1	2	Cumulative Cigarettes (15 Years)	47
Chronic myeloid leukaemia	Male	28-364 days	95+ years	Data Rich	1	2	Tobacco (cigarettes per capita)	54
Chronic myeloid leukaemia	Male	28-364 days	95+ years	Data Rich	1	2	Cumulative Cigarettes (10 Years)	79
Chronic myeloid leukaemia	Male	28-364 days	95+ years	Data Rich	1	2	Cumulative Cigarettes (5 Years)	111
Chronic myeloid leukaemia	Male	28-364 days	95+ years	Data Rich	1	2	Alcohol (liters per capita)	321
Chronic myeloid leukaemia	Male	28-364 days	95+ years	Data Rich	1	2	Smoking Prevalence	496
Chronic myeloid leukaemia	Male	28-364 days	95+ years	Data Rich	1	2	Mean BMI	--
Chronic myeloid leukaemia	Male	28-364 days	95+ years	Data Rich	-1	3	Education (years per capita)	274
Chronic myeloid leukaemia	Male	28-364 days	95+ years	Data Rich	0	3	Socio-demographic Index	286
Chronic myeloid leukaemia	Male	28-364 days	95+ years	Data Rich	0	3	LDI (I$ per capita)	--
Chronic myeloid leukaemia	Male	28-364 days	95+ years	Global	1	1	Log-transformed age-standardized SEV scalar: Leukemia	617
Chronic myeloid leukaemia	Male	28-364 days	95+ years	Global	1	1	Log-transformed SEV scalar: Leukemia	706
Chronic myeloid leukaemia	Male	28-364 days	95+ years	Global	-1	2	Healthcare access and quality index	224
Chronic myeloid leukaemia	Male	28-364 days	95+ years	Global	1	2	Alcohol (liters per capita)	55
Chronic myeloid leukaemia	Male	28-364 days	95+ years	Global	1	2	Cumulative Cigarettes (5 Years)	83
Chronic myeloid leukaemia	Male	28-364 days	95+ years	Global	1	2	Cumulative Cigarettes (10 Years)	187
Chronic myeloid leukaemia	Male	28-364 days	95+ years	Global	1	2	Cumulative Cigarettes (15 Years)	293
Chronic myeloid leukaemia	Male	28-364 days	95+ years	Global	1	2	Smoking Prevalence	320
Chronic myeloid leukaemia	Male	28-364 days	95+ years	Global	1	2	Cumulative Cigarettes (20 Years)	397
Chronic myeloid leukaemia	Male	28-364 days	95+ years	Global	1	2	Tobacco (cigarettes per capita)	790
Chronic myeloid leukaemia	Male	28-364 days	95+ years	Global	1	2	Mean BMI	--
Chronic myeloid leukaemia	Male	28-364 days	95+ years	Global	-1	3	Education (years per capita)	374
Chronic myeloid leukaemia	Male	28-364 days	95+ years	Global	0	3	Socio-demographic Index	302
Chronic myeloid leukaemia	Male	28-364 days	95+ years	Global	0	3	LDI (I$ per capita)	--
Other leukaemia	Female	0-6 days	95+ years	Data Rich	1	1	Log-transformed SEV scalar: Leukemia	--
Other leukaemia	Female	0-6 days	95+ years	Data Rich	1	1	Log-transformed age-standardized SEV scalar: Leukemia	--
Other leukaemia	Female	0-6 days	95+ years	Data Rich	-1	2	Healthcare access and quality index	380
Other leukaemia	Female	0-6 days	95+ years	Data Rich	1	2	Cumulative Cigarettes (15 Years)	206
Other leukaemia	Female	0-6 days	95+ years	Data Rich	1	2	Cumulative Cigarettes (20 Years)	245
Other leukaemia	Female	0-6 days	95+ years	Data Rich	1	2	Smoking Prevalence	267
Other leukaemia	Female	0-6 days	95+ years	Data Rich	1	2	Cumulative Cigarettes (5 Years)	279
Other leukaemia	Female	0-6 days	95+ years	Data Rich	1	2	Cumulative Cigarettes (10 Years)	459
Other leukaemia	Female	0-6 days	95+ years	Data Rich	1	2	Alcohol (liters per capita)	--
Other leukaemia	Female	0-6 days	95+ years	Data Rich	1	2	Mean BMI	--
Other leukaemia	Female	0-6 days	95+ years	Data Rich	1	2	Tobacco (cigarettes per capita)	--
Other leukaemia	Female	0-6 days	95+ years	Data Rich	-1	3	Education (years per capita)	18
Other leukaemia	Female	0-6 days	95+ years	Data Rich	0	3	LDI (I$ per capita)	327
Other leukaemia	Female	0-6 days	95+ years	Data Rich	0	3	Socio-demographic Index	357
Other leukaemia	Female	0-6 days	95+ years	Global	1	1	Log-transformed SEV scalar: Leukemia	--
Other leukaemia	Female	0-6 days	95+ years	Global	1	1	Log-transformed age-standardized SEV scalar: Leukemia	--
Other leukaemia	Female	0-6 days	95+ years	Global	-1	2	Healthcare access and quality index	--
Other leukaemia	Female	0-6 days	95+ years	Global	1	2	Smoking Prevalence	51
Other leukaemia	Female	0-6 days	95+ years	Global	1	2	Cumulative Cigarettes (10 Years)	97
Other leukaemia	Female	0-6 days	95+ years	Global	1	2	Cumulative Cigarettes (20 Years)	151
Other leukaemia	Female	0-6 days	95+ years	Global	1	2	Cumulative Cigarettes (15 Years)	174
Other leukaemia	Female	0-6 days	95+ years	Global	1	2	Cumulative Cigarettes (5 Years)	458
Other leukaemia	Female	0-6 days	95+ years	Global	1	2	Alcohol (liters per capita)	--
Other leukaemia	Female	0-6 days	95+ years	Global	1	2	Mean BMI	--
Other leukaemia	Female	0-6 days	95+ years	Global	1	2	Tobacco (cigarettes per capita)	--
Other leukaemia	Female	0-6 days	95+ years	Global	-1	3	Education (years per capita)	249
Other leukaemia	Female	0-6 days	95+ years	Global	0	3	LDI (I$ per capita)	401
Other leukaemia	Female	0-6 days	95+ years	Global	0	3	Socio-demographic Index	513
Other leukaemia	Male	0-6 days	95+ years	Data Rich	1	1	Log-transformed age-standardized SEV scalar: Leukemia	78
Other leukaemia	Male	0-6 days	95+ years	Data Rich	1	1	Log-transformed SEV scalar: Leukemia	119
Other leukaemia	Male	0-6 days	95+ years	Data Rich	-1	2	Healthcare access and quality index	70
Other leukaemia	Male	0-6 days	95+ years	Data Rich	1	2	Cumulative Cigarettes (10 Years)	38
Other leukaemia	Male	0-6 days	95+ years	Data Rich	1	2	Smoking Prevalence	39
Other leukaemia	Male	0-6 days	95+ years	Data Rich	1	2	Tobacco (cigarettes per capita)	103
Other leukaemia	Male	0-6 days	95+ years	Data Rich	1	2	Cumulative Cigarettes (20 Years)	125
Other leukaemia	Male	0-6 days	95+ years	Data Rich	1	2	Cumulative Cigarettes (15 Years)	182
Other leukaemia	Male	0-6 days	95+ years	Data Rich	1	2	Cumulative Cigarettes (5 Years)	493
Other leukaemia	Male	0-6 days	95+ years	Data Rich	1	2	Alcohol (liters per capita)	--
Other leukaemia	Male	0-6 days	95+ years	Data Rich	1	2	Mean BMI	--
Other leukaemia	Male	0-6 days	95+ years	Data Rich	-1	3	Education (years per capita)	96
Other leukaemia	Male	0-6 days	95+ years	Data Rich	0	3	Socio-demographic Index	382
Other leukaemia	Male	0-6 days	95+ years	Data Rich	0	3	LDI (I$ per capita)	439
Other leukaemia	Male	0-6 days	95+ years	Global	1	1	Log-transformed SEV scalar: Leukemia	158
Other leukaemia	Male	0-6 days	95+ years	Global	1	1	Log-transformed age-standardized SEV scalar: Leukemia	842
Other leukaemia	Male	0-6 days	95+ years	Global	-1	2	Healthcare access and quality index	122
Other leukaemia	Male	0-6 days	95+ years	Global	1	2	Mean BMI	38
Other leukaemia	Male	0-6 days	95+ years	Global	1	2	Cumulative Cigarettes (20 Years)	107
Other leukaemia	Male	0-6 days	95+ years	Global	1	2	Tobacco (cigarettes per capita)	122
Other leukaemia	Male	0-6 days	95+ years	Global	1	2	Cumulative Cigarettes (15 Years)	181
Other leukaemia	Male	0-6 days	95+ years	Global	1	2	Cumulative Cigarettes (10 Years)	361

Appendix Table 9: CODEm covariates used, level of covariate, and expected direction of covariate by cause, sex, and age

Covariates that CODEm did not select during the covariate selection process have no draw counts listed.

Cause	Sex	Age Start	Age End	Model Type	Direction	Level	Covariate Name	Number of Draws
Other leukaemia	Male	0-6 days	95+ years	Global	1	2	Cumulative Cigarettes (5 Years)	452
Other leukaemia	Male	0-6 days	95+ years	Global	1	2	Alcohol (liters per capita)	510
Other leukaemia	Male	0-6 days	95+ years	Global	1	2	Smoking Prevalence	--
Other leukaemia	Male	0-6 days	95+ years	Global	-1	3	Education (years per capita)	82
Other leukaemia	Male	0-6 days	95+ years	Global	0	3	Socio-demographic Index	82
Other leukaemia	Male	0-6 days	95+ years	Global	0	3	LDI (I$ per capita)	498
Other malignant cancers	Female	0-6 days	95+ years	Data Rich	1	1	Tobacco (cigarettes per capita)	764
Other malignant cancers	Female	0-6 days	95+ years	Data Rich	1	1	Smoking Prevalence	--
Other malignant cancers	Female	0-6 days	95+ years	Data Rich	-1	2	vegetables adjusted(g)	111
Other malignant cancers	Female	0-6 days	95+ years	Data Rich	-1	2	Healthcare access and quality index	352
Other malignant cancers	Female	0-6 days	95+ years	Data Rich	-1	2	fruits adjusted(g)	--
Other malignant cancers	Female	0-6 days	95+ years	Data Rich	-1	2	nuts seeds adjusted(g)	--
Other malignant cancers	Female	0-6 days	95+ years	Data Rich	-1	2	pufa adjusted(percent)	--
Other malignant cancers	Female	0-6 days	95+ years	Data Rich	-1	3	Education (years per capita)	--
Other malignant cancers	Female	0-6 days	95+ years	Data Rich	0	3	LDI (I$ per capita)	328
Other malignant cancers	Female	0-6 days	95+ years	Data Rich	0	3	Socio-demographic Index	529
Other malignant cancers	Female	0-6 days	95+ years	Global	1	1	Tobacco (cigarettes per capita)	669
Other malignant cancers	Female	0-6 days	95+ years	Global	1	1	Smoking Prevalence	--
Other malignant cancers	Female	0-6 days	95+ years	Global	-1	2	fruits adjusted(g)	51
Other malignant cancers	Female	0-6 days	95+ years	Global	-1	2	nuts seeds adjusted(g)	78
Other malignant cancers	Female	0-6 days	95+ years	Global	-1	2	Healthcare access and quality index	233
Other malignant cancers	Female	0-6 days	95+ years	Global	-1	2	pufa adjusted(percent)	233
Other malignant cancers	Female	0-6 days	95+ years	Global	-1	2	vegetables adjusted(g)	233
Other malignant cancers	Female	0-6 days	95+ years	Global	-1	3	Education (years per capita)	100
Other malignant cancers	Female	0-6 days	95+ years	Global	0	3	Socio-demographic Index	580
Other malignant cancers	Female	0-6 days	95+ years	Global	0	3	LDI (I$ per capita)	680
Other malignant cancers	Male	0-6 days	95+ years	Data Rich	1	1	Smoking Prevalence	392
Other malignant cancers	Male	0-6 days	95+ years	Data Rich	1	1	Tobacco (cigarettes per capita)	394
Other malignant cancers	Male	0-6 days	95+ years	Data Rich	-1	2	vegetables adjusted(g)	57
Other malignant cancers	Male	0-6 days	95+ years	Data Rich	-1	2	nuts seeds adjusted(g)	251
Other malignant cancers	Male	0-6 days	95+ years	Data Rich	-1	2	pufa adjusted(percent)	251
Other malignant cancers	Male	0-6 days	95+ years	Data Rich	-1	2	Healthcare access and quality index	--
Other malignant cancers	Male	0-6 days	95+ years	Data Rich	-1	2	fruits adjusted(g)	--
Other malignant cancers	Male	0-6 days	95+ years	Data Rich	-1	3	Education (years per capita)	388
Other malignant cancers	Male	0-6 days	95+ years	Data Rich	0	3	LDI (I$ per capita)	341
Other malignant cancers	Male	0-6 days	95+ years	Data Rich	0	3	Socio-demographic Index	580
Other malignant cancers	Male	0-6 days	95+ years	Global	1	1	Tobacco (cigarettes per capita)	745
Other malignant cancers	Male	0-6 days	95+ years	Global	1	1	Smoking Prevalence	--
Other malignant cancers	Male	0-6 days	95+ years	Global	-1	2	fruits adjusted(g)	105
Other malignant cancers	Male	0-6 days	95+ years	Global	-1	2	Healthcare access and quality index	333
Other malignant cancers	Male	0-6 days	95+ years	Global	-1	2	pufa adjusted(percent)	438
Other malignant cancers	Male	0-6 days	95+ years	Global	-1	2	nuts seeds adjusted(g)	--
Other malignant cancers	Male	0-6 days	95+ years	Global	-1	2	vegetables adjusted(g)	--
Other malignant cancers	Male	0-6 days	95+ years	Global	-1	3	Education (years per capita)	374
Other malignant cancers	Male	0-6 days	95+ years	Global	0	3	Socio-demographic Index	437
Other malignant cancers	Male	0-6 days	95+ years	Global	0	3	LDI (I$ per capita)	629
Other neoplasms	Female	0-6 days	95+ years	Data Rich	-1	2	Healthcare access and quality index	--
Other neoplasms	Female	0-6 days	95+ years	Data Rich	-1	3	Education (years per capita)	--
Other neoplasms	Female	0-6 days	95+ years	Data Rich	0	3	LDI (I$ per capita)	1000
Other neoplasms	Female	0-6 days	95+ years	Data Rich	0	3	Socio-demographic Index	1000
Other neoplasms	Female	0-6 days	95+ years	Global	-1	2	Healthcare access and quality index	--
Other neoplasms	Female	0-6 days	95+ years	Global	-1	3	Education (years per capita)	--
Other neoplasms	Female	0-6 days	95+ years	Global	0	3	Socio-demographic Index	546
Other neoplasms	Female	0-6 days	95+ years	Global	0	3	LDI (I$ per capita)	1000
Other neoplasms	Male	0-6 days	95+ years	Data Rich	-1	2	Healthcare access and quality index	--
Other neoplasms	Male	0-6 days	95+ years	Data Rich	-1	3	Education (years per capita)	--
Other neoplasms	Male	0-6 days	95+ years	Data Rich	0	3	Socio-demographic Index	591
Other neoplasms	Male	0-6 days	95+ years	Data Rich	0	3	LDI (I$ per capita)	1000
Other neoplasms	Male	0-6 days	95+ years	Global	-1	2	Healthcare access and quality index	--
Other neoplasms	Male	0-6 days	95+ years	Global	-1	3	Education (years per capita)	--
Other neoplasms	Male	0-6 days	95+ years	Global	0	3	LDI (I$ per capita)	1000
Other neoplasms	Male	0-6 days	95+ years	Global	0	3	Socio-demographic Index	1000
Myelodysplastic, myeloproliferative, and other hematopoietic neoplasms	Female	0-6 days	95+ years	Data Rich	1	1	Log-transformed SEV scalar: Leukemia	832
Myelodysplastic, myeloproliferative, and other hematopoietic neoplasms	Female	0-6 days	95+ years	Data Rich	1	1	Log-transformed age-standardized SEV scalar: Leukemia	934
Myelodysplastic, myeloproliferative, and other hematopoietic neoplasms	Female	0-6 days	95+ years	Data Rich	-1	2	Healthcare access and quality index	--
Myelodysplastic, myeloproliferative, and other hematopoietic neoplasms	Female	0-6 days	95+ years	Data Rich	1	2	Alcohol (liters per capita)	74
Myelodysplastic, myeloproliferative, and other hematopoietic neoplasms	Female	0-6 days	95+ years	Data Rich	1	2	Cumulative Cigarettes (10 Years)	186
Myelodysplastic, myeloproliferative, and other hematopoietic neoplasms	Female	0-6 days	95+ years	Data Rich	1	2	Cumulative Cigarettes (5 Years)	231
Myelodysplastic, myeloproliferative, and other hematopoietic neoplasms	Female	0-6 days	95+ years	Data Rich	1	2	Cumulative Cigarettes (15 Years)	--
Myelodysplastic, myeloproliferative, and other hematopoietic neoplasms	Female	0-6 days	95+ years	Data Rich	1	2	Cumulative Cigarettes (20 Years)	--
Myelodysplastic, myeloproliferative, and other hematopoietic neoplasms	Female	0-6 days	95+ years	Data Rich	1	2	Smoking Prevalence	--
Myelodysplastic, myeloproliferative, and other hematopoietic neoplasms	Female	0-6 days	95+ years	Data Rich	1	2	Tobacco (cigarettes per capita)	--

Appendix Table 9: CODEm covariates used, level of covariate, and expected direction of covariate by cause, sex, and age<br>*Covariates that CODEm did not select during the covariate selection process have no draw counts listed.*								
**Cause**	**Sex**	**Age Start**	**Age End**	**Model Type**	**Direction**	**Level**	**Covariate Name**	**Number of Draws**
Myelodysplastic, myeloproliferative, and other hematopoietic neoplasms	Female	0-6 days	95+ years	Data Rich	-1	3	Education (years per capita)	--
Myelodysplastic, myeloproliferative, and other hematopoietic neoplasms	Female	0-6 days	95+ years	Data Rich	0	3	Socio-demographic Index	449
Myelodysplastic, myeloproliferative, and other hematopoietic neoplasms	Female	0-6 days	95+ years	Data Rich	0	3	LDI (I$ per capita)	--
Myelodysplastic, myeloproliferative, and other hematopoietic neoplasms	Female	0-6 days	95+ years	Global	1	1	Log-transformed SEV scalar: Leukemia	395
Myelodysplastic, myeloproliferative, and other hematopoietic neoplasms	Female	0-6 days	95+ years	Global	1	1	Log-transformed age-standardized SEV scalar: Leukemia	822
Myelodysplastic, myeloproliferative, and other hematopoietic neoplasms	Female	0-6 days	95+ years	Global	-1	2	Healthcare access and quality index	--
Myelodysplastic, myeloproliferative, and other hematopoietic neoplasms	Female	0-6 days	95+ years	Global	1	2	Cumulative Cigarettes (5 Years)	3
Myelodysplastic, myeloproliferative, and other hematopoietic neoplasms	Female	0-6 days	95+ years	Global	1	2	Cumulative Cigarettes (10 Years)	19
Myelodysplastic, myeloproliferative, and other hematopoietic neoplasms	Female	0-6 days	95+ years	Global	1	2	Cumulative Cigarettes (15 Years)	33
Myelodysplastic, myeloproliferative, and other hematopoietic neoplasms	Female	0-6 days	95+ years	Global	1	2	Cumulative Cigarettes (20 Years)	92
Myelodysplastic, myeloproliferative, and other hematopoietic neoplasms	Female	0-6 days	95+ years	Global	1	2	Smoking Prevalence	139
Myelodysplastic, myeloproliferative, and other hematopoietic neoplasms	Female	0-6 days	95+ years	Global	1	2	Alcohol (liters per capita)	195
Myelodysplastic, myeloproliferative, and other hematopoietic neoplasms	Female	0-6 days	95+ years	Global	1	2	Tobacco (cigarettes per capita)	--
Myelodysplastic, myeloproliferative, and other hematopoietic neoplasms	Female	0-6 days	95+ years	Global	-1	3	Education (years per capita)	--
Myelodysplastic, myeloproliferative, and other hematopoietic neoplasms	Female	0-6 days	95+ years	Global	0	3	Socio-demographic Index	186
Myelodysplastic, myeloproliferative, and other hematopoietic neoplasms	Female	0-6 days	95+ years	Global	0	3	LDI (I$ per capita)	--
Myelodysplastic, myeloproliferative, and other hematopoietic neoplasms	Male	0-6 days	95+ years	Data Rich	1	1	Log-transformed age-standardized SEV scalar: Leukemia	374
Myelodysplastic, myeloproliferative, and other hematopoietic neoplasms	Male	0-6 days	95+ years	Data Rich	1	1	Log-transformed SEV scalar: Leukemia	516
Myelodysplastic, myeloproliferative, and other hematopoietic neoplasms	Male	0-6 days	95+ years	Data Rich	-1	2	Healthcare access and quality index	--
Myelodysplastic, myeloproliferative, and other hematopoietic neoplasms	Male	0-6 days	95+ years	Data Rich	1	2	Cumulative Cigarettes (20 Years)	88
Myelodysplastic, myeloproliferative, and other hematopoietic neoplasms	Male	0-6 days	95+ years	Data Rich	1	2	Cumulative Cigarettes (15 Years)	107
Myelodysplastic, myeloproliferative, and other hematopoietic neoplasms	Male	0-6 days	95+ years	Data Rich	1	2	Cumulative Cigarettes (10 Years)	128
Myelodysplastic, myeloproliferative, and other hematopoietic neoplasms	Male	0-6 days	95+ years	Data Rich	1	2	Alcohol (liters per capita)	--
Myelodysplastic, myeloproliferative, and other hematopoietic neoplasms	Male	0-6 days	95+ years	Data Rich	1	2	Cumulative Cigarettes (5 Years)	--
Myelodysplastic, myeloproliferative, and other hematopoietic neoplasms	Male	0-6 days	95+ years	Data Rich	1	2	Smoking Prevalence	--
Myelodysplastic, myeloproliferative, and other hematopoietic neoplasms	Male	0-6 days	95+ years	Data Rich	1	2	Tobacco (cigarettes per capita)	--
Myelodysplastic, myeloproliferative, and other hematopoietic neoplasms	Male	0-6 days	95+ years	Data Rich	-1	3	Education (years per capita)	--
Myelodysplastic, myeloproliferative, and other hematopoietic neoplasms	Male	0-6 days	95+ years	Data Rich	0	3	Socio-demographic Index	228
Myelodysplastic, myeloproliferative, and other hematopoietic neoplasms	Male	0-6 days	95+ years	Data Rich	0	3	LDI (I$ per capita)	--
Myelodysplastic, myeloproliferative, and other hematopoietic neoplasms	Male	0-6 days	95+ years	Global	1	1	Log-transformed SEV scalar: Leukemia	491
Myelodysplastic, myeloproliferative, and other hematopoietic neoplasms	Male	0-6 days	95+ years	Global	1	1	Log-transformed age-standardized SEV scalar: Leukemia	509
Myelodysplastic, myeloproliferative, and other hematopoietic neoplasms	Male	0-6 days	95+ years	Global	-1	2	Healthcare access and quality index	--
Myelodysplastic, myeloproliferative, and other hematopoietic neoplasms	Male	0-6 days	95+ years	Global	1	2	Alcohol (liters per capita)	--
Myelodysplastic, myeloproliferative, and other hematopoietic neoplasms	Male	0-6 days	95+ years	Global	1	2	Cumulative Cigarettes (10 Years)	--
Myelodysplastic, myeloproliferative, and other hematopoietic neoplasms	Male	0-6 days	95+ years	Global	1	2	Cumulative Cigarettes (15 Years)	--
Myelodysplastic, myeloproliferative, and other hematopoietic neoplasms	Male	0-6 days	95+ years	Global	1	2	Cumulative Cigarettes (20 Years)	--
Myelodysplastic, myeloproliferative, and other hematopoietic neoplasms	Male	0-6 days	95+ years	Global	1	2	Cumulative Cigarettes (5 Years)	--
Myelodysplastic, myeloproliferative, and other hematopoietic neoplasms	Male	0-6 days	95+ years	Global	1	2	Smoking Prevalence	--
Myelodysplastic, myeloproliferative, and other hematopoietic neoplasms	Male	0-6 days	95+ years	Global	1	2	Tobacco (cigarettes per capita)	--
Myelodysplastic, myeloproliferative, and other hematopoietic neoplasms	Male	0-6 days	95+ years	Global	-1	3	Education (years per capita)	--
Myelodysplastic, myeloproliferative, and other hematopoietic neoplasms	Male	0-6 days	95+ years	Global	0	3	LDI (I$ per capita)	--
Myelodysplastic, myeloproliferative, and other hematopoietic neoplasms	Male	0-6 days	95+ years	Global	0	3	Socio-demographic Index	--
Other benign and in situ neoplasms	Female	0-6 days	95+ years	Data Rich	-1	2	Healthcare access and quality index	--
Other benign and in situ neoplasms	Female	0-6 days	95+ years	Data Rich	-1	3	Education (years per capita)	--
Other benign and in situ neoplasms	Female	0-6 days	95+ years	Data Rich	0	3	LDI (I$ per capita)	1000
Other benign and in situ neoplasms	Female	0-6 days	95+ years	Data Rich	0	3	Socio-demographic Index	1000
Other benign and in situ neoplasms	Female	0-6 days	95+ years	Global	-1	2	Healthcare access and quality index	546
Other benign and in situ neoplasms	Female	0-6 days	95+ years	Global	-1	3	Education (years per capita)	--
Other benign and in situ neoplasms	Female	0-6 days	95+ years	Global	0	3	LDI (I$ per capita)	454
Other benign and in situ neoplasms	Female	0-6 days	95+ years	Global	0	3	Socio-demographic Index	454
Other benign and in situ neoplasms	Male	0-6 days	95+ years	Data Rich	-1	2	Healthcare access and quality index	--
Other benign and in situ neoplasms	Male	0-6 days	95+ years	Data Rich	-1	3	Education (years per capita)	--
Other benign and in situ neoplasms	Male	0-6 days	95+ years	Data Rich	0	3	Socio-demographic Index	546
Other benign and in situ neoplasms	Male	0-6 days	95+ years	Data Rich	0	3	LDI (I$ per capita)	1000
Other benign and in situ neoplasms	Male	0-6 days	95+ years	Global	-1	2	Healthcare access and quality index	--
Other benign and in situ neoplasms	Male	0-6 days	95+ years	Global	-1	3	Education (years per capita)	--
Other benign and in situ neoplasms	Male	0-6 days	95+ years	Global	0	3	LDI (I$ per capita)	1000

Appendix Table 9: CODEm covariates used, level of covariate, and expected direction of covariate by cause, sex, and age
Covariates that CODEm did not select during the covariate selection process have no draw counts listed.

Cause	Sex	Age Start	Age End	Model Type	Direction	Level	Covariate Name	Number of Draws
Other benign and in situ neoplasms	Male	0-6 days	95+ years	Global	0	3	Socio-demographic Index	1000
Cardiovascular diseases	Female	0-6 days	95+ years	Data Rich	1	1	Cholesterol (total, mean per capita)	195
Cardiovascular diseases	Female	0-6 days	95+ years	Data Rich	1	1	Systolic Blood Pressure (mmHg)	681
Cardiovascular diseases	Female	0-6 days	95+ years	Data Rich	1	1	Smoking Prevalence	997
Cardiovascular diseases	Female	0-6 days	95+ years	Data Rich	1	1	Log-transformed SEV scalar: CVD	1000
Cardiovascular diseases	Female	0-6 days	95+ years	Data Rich	-1	2	Healthcare access and quality index	0
Cardiovascular diseases	Female	0-6 days	95+ years	Data Rich	-1	2	Elevation Over 1500m (proportion)	256
Cardiovascular diseases	Female	0-6 days	95+ years	Data Rich	1	2	Indoor Air Pollution (All Cooking Fuels)	26
Cardiovascular diseases	Female	0-6 days	95+ years	Data Rich	1	2	Outdoor Air Pollution (PM2.5)	283
Cardiovascular diseases	Female	0-6 days	95+ years	Data Rich	1	2	Diabetes Fasting Plasma Glucose (mmol/L)	--
Cardiovascular diseases	Female	0-6 days	95+ years	Data Rich	1	2	Mean BMI	--
Cardiovascular diseases	Female	0-6 days	95+ years	Data Rich	-1	3	pulses legumes adjusted(g)	0
Cardiovascular diseases	Female	0-6 days	95+ years	Data Rich	-1	3	LDI (I$ per capita)	71
Cardiovascular diseases	Female	0-6 days	95+ years	Data Rich	-1	3	nuts seeds adjusted(g)	144
Cardiovascular diseases	Female	0-6 days	95+ years	Data Rich	-1	3	fruits adjusted(g)	172
Cardiovascular diseases	Female	0-6 days	95+ years	Data Rich	-1	3	pufa adjusted(percent)	211
Cardiovascular diseases	Female	0-6 days	95+ years	Data Rich	-1	3	omega 3 adjusted(g)	--
Cardiovascular diseases	Female	0-6 days	95+ years	Data Rich	-1	3	vegetables adjusted(g)	--
Cardiovascular diseases	Female	0-6 days	95+ years	Data Rich	0	3	Socio-demographic Index	0
Cardiovascular diseases	Female	0-6 days	95+ years	Data Rich	0	3	Alcohol (liters per capita)	168
Cardiovascular diseases	Female	0-6 days	95+ years	Data Rich	1	3	Diet high in trans fatty acids	15
Cardiovascular diseases	Female	0-6 days	95+ years	Global	1	1	Systolic Blood Pressure (mmHg)	45
Cardiovascular diseases	Female	0-6 days	95+ years	Global	1	1	Cholesterol (total, mean per capita)	139
Cardiovascular diseases	Female	0-6 days	95+ years	Global	1	1	Smoking Prevalence	652
Cardiovascular diseases	Female	0-6 days	95+ years	Global	1	1	Log-transformed SEV scalar: CVD	1000
Cardiovascular diseases	Female	0-6 days	95+ years	Global	-1	2	Elevation Over 1500m (proportion)	0
Cardiovascular diseases	Female	0-6 days	95+ years	Global	-1	2	Healthcare access and quality index	798
Cardiovascular diseases	Female	0-6 days	95+ years	Global	1	2	Outdoor Air Pollution (PM2.5)	25
Cardiovascular diseases	Female	0-6 days	95+ years	Global	1	2	Indoor Air Pollution (All Cooking Fuels)	47
Cardiovascular diseases	Female	0-6 days	95+ years	Global	1	2	Diabetes Fasting Plasma Glucose (mmol/L)	--
Cardiovascular diseases	Female	0-6 days	95+ years	Global	1	2	Mean BMI	--
Cardiovascular diseases	Female	0-6 days	95+ years	Global	-1	3	fruits adjusted(g)	0
Cardiovascular diseases	Female	0-6 days	95+ years	Global	-1	3	pulses legumes adjusted(g)	0
Cardiovascular diseases	Female	0-6 days	95+ years	Global	-1	3	nuts seeds adjusted(g)	140
Cardiovascular diseases	Female	0-6 days	95+ years	Global	-1	3	LDI (I$ per capita)	288
Cardiovascular diseases	Female	0-6 days	95+ years	Global	-1	3	pufa adjusted(percent)	360
Cardiovascular diseases	Female	0-6 days	95+ years	Global	-1	3	omega 3 adjusted(g)	--
Cardiovascular diseases	Female	0-6 days	95+ years	Global	-1	3	vegetables adjusted(g)	--
Cardiovascular diseases	Female	0-6 days	95+ years	Global	0	3	Socio-demographic Index	326
Cardiovascular diseases	Female	0-6 days	95+ years	Global	0	3	Alcohol (liters per capita)	476
Cardiovascular diseases	Female	0-6 days	95+ years	Global	1	3	Diet high in trans fatty acids	515
Cardiovascular diseases	Male	0-6 days	95+ years	Data Rich	1	1	Cholesterol (total, mean per capita)	265
Cardiovascular diseases	Male	0-6 days	95+ years	Data Rich	1	1	Systolic Blood Pressure (mmHg)	265
Cardiovascular diseases	Male	0-6 days	95+ years	Data Rich	1	1	Log-transformed SEV scalar: CVD	1000
Cardiovascular diseases	Male	0-6 days	95+ years	Data Rich	1	1	Smoking Prevalence	1000
Cardiovascular diseases	Male	0-6 days	95+ years	Data Rich	-1	2	Healthcare access and quality index	116
Cardiovascular diseases	Male	0-6 days	95+ years	Data Rich	-1	2	Elevation Over 1500m (proportion)	121
Cardiovascular diseases	Male	0-6 days	95+ years	Data Rich	1	2	Indoor Air Pollution (All Cooking Fuels)	84
Cardiovascular diseases	Male	0-6 days	95+ years	Data Rich	1	2	Outdoor Air Pollution (PM2.5)	121
Cardiovascular diseases	Male	0-6 days	95+ years	Data Rich	1	2	Diabetes Fasting Plasma Glucose (mmol/L)	--
Cardiovascular diseases	Male	0-6 days	95+ years	Data Rich	1	2	Mean BMI	--
Cardiovascular diseases	Male	0-6 days	95+ years	Data Rich	-1	3	fruits adjusted(g)	0
Cardiovascular diseases	Male	0-6 days	95+ years	Data Rich	-1	3	LDI (I$ per capita)	39
Cardiovascular diseases	Male	0-6 days	95+ years	Data Rich	-1	3	nuts seeds adjusted(g)	113
Cardiovascular diseases	Male	0-6 days	95+ years	Data Rich	-1	3	pufa adjusted(percent)	454
Cardiovascular diseases	Male	0-6 days	95+ years	Data Rich	-1	3	pulses legumes adjusted(g)	516
Cardiovascular diseases	Male	0-6 days	95+ years	Data Rich	-1	3	omega 3 adjusted(g)	--
Cardiovascular diseases	Male	0-6 days	95+ years	Data Rich	-1	3	vegetables adjusted(g)	--
Cardiovascular diseases	Male	0-6 days	95+ years	Data Rich	0	3	Socio-demographic Index	45
Cardiovascular diseases	Male	0-6 days	95+ years	Data Rich	0	3	Alcohol (liters per capita)	511
Cardiovascular diseases	Male	0-6 days	95+ years	Data Rich	1	3	Diet high in trans fatty acids	0
Cardiovascular diseases	Male	0-6 days	95+ years	Global	1	1	Systolic Blood Pressure (mmHg)	19
Cardiovascular diseases	Male	0-6 days	95+ years	Global	1	1	Cholesterol (total, mean per capita)	626
Cardiovascular diseases	Male	0-6 days	95+ years	Global	1	1	Smoking Prevalence	979
Cardiovascular diseases	Male	0-6 days	95+ years	Global	1	1	Log-transformed SEV scalar: CVD	1000
Cardiovascular diseases	Male	0-6 days	95+ years	Global	-1	2	Elevation Over 1500m (proportion)	285
Cardiovascular diseases	Male	0-6 days	95+ years	Global	-1	2	Healthcare access and quality index	411
Cardiovascular diseases	Male	0-6 days	95+ years	Global	1	2	Indoor Air Pollution (All Cooking Fuels)	264
Cardiovascular diseases	Male	0-6 days	95+ years	Global	1	2	Diabetes Fasting Plasma Glucose (mmol/L)	285
Cardiovascular diseases	Male	0-6 days	95+ years	Global	1	2	Outdoor Air Pollution (PM2.5)	285
Cardiovascular diseases	Male	0-6 days	95+ years	Global	1	2	Mean BMI	--
Cardiovascular diseases	Male	0-6 days	95+ years	Global	-1	3	fruits adjusted(g)	0
Cardiovascular diseases	Male	0-6 days	95+ years	Global	-1	3	LDI (I$ per capita)	216
Cardiovascular diseases	Male	0-6 days	95+ years	Global	-1	3	pufa adjusted(percent)	408
Cardiovascular diseases	Male	0-6 days	95+ years	Global	-1	3	nuts seeds adjusted(g)	494
Cardiovascular diseases	Male	0-6 days	95+ years	Global	-1	3	pulses legumes adjusted(g)	950
Cardiovascular diseases	Male	0-6 days	95+ years	Global	-1	3	omega 3 adjusted(g)	--
Cardiovascular diseases	Male	0-6 days	95+ years	Global	-1	3	vegetables adjusted(g)	--

Appendix Table 9: CODEm covariates used, level of covariate, and expected direction of covariate by cause, sex, and age
Covariates that CODEm did not select during the covariate selection process have no draw counts listed.

Cause	Sex	Age Start	Age End	Model Type	Direction	Level	Covariate Name	Number of Draws
Cardiovascular diseases	Male	0-6 days	95+ years	Global	0	3	Socio-demographic Index	351
Cardiovascular diseases	Male	0-6 days	95+ years	Global	0	3	Alcohol (liters per capita)	923
Cardiovascular diseases	Male	0-6 days	95+ years	Global	1	3	Diet high in trans fatty acids	0
Rheumatic heart disease	Female	1-4 years	95+ years	Data Rich	-1	1	Improved Water Source (proportion with access)	165
Rheumatic heart disease	Female	1-4 years	95+ years	Data Rich	-1	1	Sanitation (proportion with access)	606
Rheumatic heart disease	Female	1-4 years	95+ years	Data Rich	1	1	Underweight (proportion <2SD weight for age, <5 years)	281
Rheumatic heart disease	Female	1-4 years	95+ years	Data Rich	1	1	Log-transformed SEV scalar: RHD	860
Rheumatic heart disease	Female	1-4 years	95+ years	Data Rich	-1	2	Healthcare access and quality index	199
Rheumatic heart disease	Female	1-4 years	95+ years	Data Rich	-1	3	Socio-demographic Index	424
Rheumatic heart disease	Female	1-4 years	95+ years	Data Rich	-1	3	Education (years per capita)	782
Rheumatic heart disease	Female	1-4 years	95+ years	Data Rich	-1	3	LDI (I$ per capita)	--
Rheumatic heart disease	Female	1-4 years	95+ years	Global	-1	1	Improved Water Source (proportion with access)	162
Rheumatic heart disease	Female	1-4 years	95+ years	Global	-1	1	Sanitation (proportion with access)	395
Rheumatic heart disease	Female	1-4 years	95+ years	Global	1	1	Underweight (proportion <2SD weight for age, <5 years)	221
Rheumatic heart disease	Female	1-4 years	95+ years	Global	1	1	Log-transformed SEV scalar: RHD	880
Rheumatic heart disease	Female	1-4 years	95+ years	Global	-1	2	Healthcare access and quality index	282
Rheumatic heart disease	Female	1-4 years	95+ years	Global	-1	3	Socio-demographic Index	333
Rheumatic heart disease	Female	1-4 years	95+ years	Global	-1	3	Education (years per capita)	756
Rheumatic heart disease	Female	1-4 years	95+ years	Global	-1	3	LDI (I$ per capita)	--
Rheumatic heart disease	Male	1-4 years	95+ years	Data Rich	-1	1	Improved Water Source (proportion with access)	39
Rheumatic heart disease	Male	1-4 years	95+ years	Data Rich	-1	1	Sanitation (proportion with access)	772
Rheumatic heart disease	Male	1-4 years	95+ years	Data Rich	1	1	Underweight (proportion <2SD weight for age, <5 years)	478
Rheumatic heart disease	Male	1-4 years	95+ years	Data Rich	1	1	Log-transformed SEV scalar: RHD	690
Rheumatic heart disease	Male	1-4 years	95+ years	Data Rich	-1	2	Healthcare access and quality index	555
Rheumatic heart disease	Male	1-4 years	95+ years	Data Rich	-1	3	Education (years per capita)	336
Rheumatic heart disease	Male	1-4 years	95+ years	Data Rich	-1	3	Socio-demographic Index	363
Rheumatic heart disease	Male	1-4 years	95+ years	Data Rich	-1	3	LDI (I$ per capita)	--
Rheumatic heart disease	Male	1-4 years	95+ years	Global	-1	1	Improved Water Source (proportion with access)	23
Rheumatic heart disease	Male	1-4 years	95+ years	Global	-1	1	Sanitation (proportion with access)	546
Rheumatic heart disease	Male	1-4 years	95+ years	Global	1	1	Underweight (proportion <2SD weight for age, <5 years)	377
Rheumatic heart disease	Male	1-4 years	95+ years	Global	1	1	Log-transformed SEV scalar: RHD	746
Rheumatic heart disease	Male	1-4 years	95+ years	Global	-1	2	Healthcare access and quality index	681
Rheumatic heart disease	Male	1-4 years	95+ years	Global	-1	3	Socio-demographic Index	136
Rheumatic heart disease	Male	1-4 years	95+ years	Global	-1	3	Education (years per capita)	281
Rheumatic heart disease	Male	1-4 years	95+ years	Global	-1	3	LDI (I$ per capita)	--
Ischaemic heart disease	Female	15-19 years	95+ years	Data Rich	1	1	Cholesterol (total, mean per capita)	628
Ischaemic heart disease	Female	15-19 years	95+ years	Data Rich	1	1	Systolic Blood Pressure (mmHg)	723
Ischaemic heart disease	Female	15-19 years	95+ years	Data Rich	1	1	Smoking Prevalence	972
Ischaemic heart disease	Female	15-19 years	95+ years	Data Rich	1	1	Log-transformed SEV scalar: IHD	1000
Ischaemic heart disease	Female	15-19 years	95+ years	Data Rich	-1	2	Healthcare access and quality index	460
Ischaemic heart disease	Female	15-19 years	95+ years	Data Rich	-1	2	Elevation Over 1500m (proportion)	624
Ischaemic heart disease	Female	15-19 years	95+ years	Data Rich	1	2	Indoor Air Pollution (All Cooking Fuels)	374
Ischaemic heart disease	Female	15-19 years	95+ years	Data Rich	1	2	Outdoor Air Pollution (PM2.5)	648
Ischaemic heart disease	Female	15-19 years	95+ years	Data Rich	1	2	Diabetes Fasting Plasma Glucose (mmol/L)	--
Ischaemic heart disease	Female	15-19 years	95+ years	Data Rich	1	2	Mean BMI	--
Ischaemic heart disease	Female	15-19 years	95+ years	Data Rich	-1	3	nuts seeds adjusted(g)	12
Ischaemic heart disease	Female	15-19 years	95+ years	Data Rich	-1	3	fruits adjusted(g)	13
Ischaemic heart disease	Female	15-19 years	95+ years	Data Rich	-1	3	pufa adjusted(percent)	14
Ischaemic heart disease	Female	15-19 years	95+ years	Data Rich	-1	3	pulses legumes adjusted(g)	527
Ischaemic heart disease	Female	15-19 years	95+ years	Data Rich	-1	3	LDI (I$ per capita)	--
Ischaemic heart disease	Female	15-19 years	95+ years	Data Rich	-1	3	omega 3 adjusted(g)	--
Ischaemic heart disease	Female	15-19 years	95+ years	Data Rich	-1	3	vegetables adjusted(g)	--
Ischaemic heart disease	Female	15-19 years	95+ years	Data Rich	0	3	Socio-demographic Index	370
Ischaemic heart disease	Female	15-19 years	95+ years	Data Rich	0	3	Alcohol (liters per capita)	711
Ischaemic heart disease	Female	15-19 years	95+ years	Data Rich	1	3	Diet high in trans fatty acids	371
Ischaemic heart disease	Female	15-19 years	95+ years	Global	1	1	Cholesterol (total, mean per capita)	551
Ischaemic heart disease	Female	15-19 years	95+ years	Global	1	1	Smoking Prevalence	735
Ischaemic heart disease	Female	15-19 years	95+ years	Global	1	1	Systolic Blood Pressure (mmHg)	924
Ischaemic heart disease	Female	15-19 years	95+ years	Global	1	1	Log-transformed SEV scalar: IHD	961
Ischaemic heart disease	Female	15-19 years	95+ years	Global	-1	2	Healthcare access and quality index	71
Ischaemic heart disease	Female	15-19 years	95+ years	Global	-1	2	Elevation Over 1500m (proportion)	424
Ischaemic heart disease	Female	15-19 years	95+ years	Global	1	2	Indoor Air Pollution (All Cooking Fuels)	0
Ischaemic heart disease	Female	15-19 years	95+ years	Global	1	2	Outdoor Air Pollution (PM2.5)	210
Ischaemic heart disease	Female	15-19 years	95+ years	Global	1	2	Diabetes Fasting Plasma Glucose (mmol/L)	281
Ischaemic heart disease	Female	15-19 years	95+ years	Global	1	2	Mean BMI	--
Ischaemic heart disease	Female	15-19 years	95+ years	Global	-1	3	fruits adjusted(g)	0
Ischaemic heart disease	Female	15-19 years	95+ years	Global	-1	3	nuts seeds adjusted(g)	24
Ischaemic heart disease	Female	15-19 years	95+ years	Global	-1	3	pufa adjusted(percent)	32
Ischaemic heart disease	Female	15-19 years	95+ years	Global	-1	3	pulses legumes adjusted(g)	422
Ischaemic heart disease	Female	15-19 years	95+ years	Global	-1	3	LDI (I$ per capita)	--
Ischaemic heart disease	Female	15-19 years	95+ years	Global	-1	3	omega 3 adjusted(g)	--
Ischaemic heart disease	Female	15-19 years	95+ years	Global	-1	3	vegetables adjusted(g)	--
Ischaemic heart disease	Female	15-19 years	95+ years	Global	0	3	Alcohol (liters per capita)	182
Ischaemic heart disease	Female	15-19 years	95+ years	Global	0	3	Socio-demographic Index	275
Ischaemic heart disease	Female	15-19 years	95+ years	Global	1	3	Diet high in trans fatty acids	168
Ischaemic heart disease	Male	15-19 years	95+ years	Data Rich	1	1	Systolic Blood Pressure (mmHg)	81
Ischaemic heart disease	Male	15-19 years	95+ years	Data Rich	1	1	Cholesterol (total, mean per capita)	611
Ischaemic heart disease	Male	15-19 years	95+ years	Data Rich	1	1	Log-transformed SEV scalar: IHD	936

Appendix Table 9: CODEm covariates used, level of covariate, and expected direction of covariate by cause, sex, and age
Covariates that CODEm did not select during the covariate selection process have no draw counts listed.

Cause	Sex	Age Start	Age End	Model Type	Direction	Level	Covariate Name	Number of Draws
Ischaemic heart disease	Male	15-19 years	95+ years	Data Rich	1	1	Smoking Prevalence	1000
Ischaemic heart disease	Male	15-19 years	95+ years	Data Rich	-1	2	Elevation Over 1500m (proportion)	728
Ischaemic heart disease	Male	15-19 years	95+ years	Data Rich	-1	2	Healthcare access and quality index	757
Ischaemic heart disease	Male	15-19 years	95+ years	Data Rich	1	2	Indoor Air Pollution (All Cooking Fuels)	541
Ischaemic heart disease	Male	15-19 years	95+ years	Data Rich	1	2	Outdoor Air Pollution (PM2.5)	730
Ischaemic heart disease	Male	15-19 years	95+ years	Data Rich	1	2	Diabetes Fasting Plasma Glucose (mmol/L)	--
Ischaemic heart disease	Male	15-19 years	95+ years	Data Rich	1	2	Mean BMI	--
Ischaemic heart disease	Male	15-19 years	95+ years	Data Rich	-1	3	fruits adjusted(g)	1
Ischaemic heart disease	Male	15-19 years	95+ years	Data Rich	-1	3	pufa adjusted(percent)	180
Ischaemic heart disease	Male	15-19 years	95+ years	Data Rich	-1	3	nuts seeds adjusted(g)	620
Ischaemic heart disease	Male	15-19 years	95+ years	Data Rich	-1	3	pulses legumes adjusted(g)	896
Ischaemic heart disease	Male	15-19 years	95+ years	Data Rich	-1	3	LDI (I$ per capita)	--
Ischaemic heart disease	Male	15-19 years	95+ years	Data Rich	-1	3	omega 3 adjusted(g)	--
Ischaemic heart disease	Male	15-19 years	95+ years	Data Rich	-1	3	vegetables adjusted(g)	--
Ischaemic heart disease	Male	15-19 years	95+ years	Data Rich	0	3	Socio-demographic Index	234
Ischaemic heart disease	Male	15-19 years	95+ years	Data Rich	0	3	Alcohol (liters per capita)	843
Ischaemic heart disease	Male	15-19 years	95+ years	Data Rich	1	3	Diet high in trans fatty acids	783
Ischaemic heart disease	Male	15-19 years	95+ years	Global	1	1	Log-transformed SEV scalar: IHD	791
Ischaemic heart disease	Male	15-19 years	95+ years	Global	1	1	Cholesterol (total, mean per capita)	851
Ischaemic heart disease	Male	15-19 years	95+ years	Global	1	1	Smoking Prevalence	939
Ischaemic heart disease	Male	15-19 years	95+ years	Global	1	1	Systolic Blood Pressure (mmHg)	966
Ischaemic heart disease	Male	15-19 years	95+ years	Global	-1	2	Elevation Over 1500m (proportion)	667
Ischaemic heart disease	Male	15-19 years	95+ years	Global	-1	2	Healthcare access and quality index	674
Ischaemic heart disease	Male	15-19 years	95+ years	Global	1	2	Indoor Air Pollution (All Cooking Fuels)	233
Ischaemic heart disease	Male	15-19 years	95+ years	Global	1	2	Outdoor Air Pollution (PM2.5)	671
Ischaemic heart disease	Male	15-19 years	95+ years	Global	1	2	Diabetes Fasting Plasma Glucose (mmol/L)	786
Ischaemic heart disease	Male	15-19 years	95+ years	Global	1	2	Mean BMI	--
Ischaemic heart disease	Male	15-19 years	95+ years	Global	-1	3	fruits adjusted(g)	0
Ischaemic heart disease	Male	15-19 years	95+ years	Global	-1	3	nuts seeds adjusted(g)	298
Ischaemic heart disease	Male	15-19 years	95+ years	Global	-1	3	pufa adjusted(percent)	427
Ischaemic heart disease	Male	15-19 years	95+ years	Global	-1	3	pulses legumes adjusted(g)	553
Ischaemic heart disease	Male	15-19 years	95+ years	Global	-1	3	LDI (I$ per capita)	--
Ischaemic heart disease	Male	15-19 years	95+ years	Global	-1	3	omega 3 adjusted(g)	--
Ischaemic heart disease	Male	15-19 years	95+ years	Global	-1	3	vegetables adjusted(g)	--
Ischaemic heart disease	Male	15-19 years	95+ years	Global	0	3	Socio-demographic Index	65
Ischaemic heart disease	Male	15-19 years	95+ years	Global	0	3	Alcohol (liters per capita)	552
Ischaemic heart disease	Male	15-19 years	95+ years	Global	1	3	Diet high in trans fatty acids	33
Stroke	Female	0-6 days	95+ years	Data Rich	1	1	Cholesterol (total, mean per capita)	0
Stroke	Female	0-6 days	95+ years	Data Rich	1	1	Log-transformed SEV scalar: Stroke	829
Stroke	Female	0-6 days	95+ years	Data Rich	1	1	Smoking Prevalence	910
Stroke	Female	0-6 days	95+ years	Data Rich	1	1	Systolic Blood Pressure (mmHg)	1000
Stroke	Female	0-6 days	95+ years	Data Rich	-1	2	Elevation Over 1500m (proportion)	0
Stroke	Female	0-6 days	95+ years	Data Rich	-1	2	fruits adjusted(g)	386
Stroke	Female	0-6 days	95+ years	Data Rich	-1	2	Healthcare access and quality index	416
Stroke	Female	0-6 days	95+ years	Data Rich	-1	2	vegetables adjusted(g)	--
Stroke	Female	0-6 days	95+ years	Data Rich	1	2	Outdoor Air Pollution (PM2.5)	0
Stroke	Female	0-6 days	95+ years	Data Rich	1	2	Indoor Air Pollution (All Cooking Fuels)	416
Stroke	Female	0-6 days	95+ years	Data Rich	1	2	Diabetes Fasting Plasma Glucose (mmol/L)	--
Stroke	Female	0-6 days	95+ years	Data Rich	1	2	Mean BMI	--
Stroke	Female	0-6 days	95+ years	Data Rich	-1	3	pufa adjusted(percent)	61
Stroke	Female	0-6 days	95+ years	Data Rich	-1	3	pulses legumes adjusted(g)	160
Stroke	Female	0-6 days	95+ years	Data Rich	-1	3	nuts seeds adjusted(g)	316
Stroke	Female	0-6 days	95+ years	Data Rich	-1	3	LDI (I$ per capita)	--
Stroke	Female	0-6 days	95+ years	Data Rich	-1	3	omega 3 adjusted(g)	--
Stroke	Female	0-6 days	95+ years	Data Rich	0	3	Socio-demographic Index	488
Stroke	Female	0-6 days	95+ years	Data Rich	0	3	Alcohol (liters per capita)	849
Stroke	Female	0-6 days	95+ years	Data Rich	1	3	Diet high in trans fatty acids	285
Stroke	Female	0-6 days	95+ years	Global	1	1	Cholesterol (total, mean per capita)	28
Stroke	Female	0-6 days	95+ years	Global	1	1	Log-transformed SEV scalar: Stroke	478
Stroke	Female	0-6 days	95+ years	Global	1	1	Smoking Prevalence	738
Stroke	Female	0-6 days	95+ years	Global	1	1	Systolic Blood Pressure (mmHg)	940
Stroke	Female	0-6 days	95+ years	Global	-1	2	fruits adjusted(g)	245
Stroke	Female	0-6 days	95+ years	Global	-1	2	Healthcare access and quality index	817
Stroke	Female	0-6 days	95+ years	Global	-1	2	Elevation Over 1500m (proportion)	--
Stroke	Female	0-6 days	95+ years	Global	-1	2	vegetables adjusted(g)	--
Stroke	Female	0-6 days	95+ years	Global	1	2	Outdoor Air Pollution (PM2.5)	51
Stroke	Female	0-6 days	95+ years	Global	1	2	Indoor Air Pollution (All Cooking Fuels)	208
Stroke	Female	0-6 days	95+ years	Global	1	2	Diabetes Fasting Plasma Glucose (mmol/L)	--
Stroke	Female	0-6 days	95+ years	Global	1	2	Mean BMI	--
Stroke	Female	0-6 days	95+ years	Global	-1	3	pulses legumes adjusted(g)	180
Stroke	Female	0-6 days	95+ years	Global	-1	3	nuts seeds adjusted(g)	208
Stroke	Female	0-6 days	95+ years	Global	-1	3	pufa adjusted(percent)	317
Stroke	Female	0-6 days	95+ years	Global	-1	3	LDI (I$ per capita)	--
Stroke	Female	0-6 days	95+ years	Global	-1	3	omega 3 adjusted(g)	--
Stroke	Female	0-6 days	95+ years	Global	0	3	Socio-demographic Index	233
Stroke	Female	0-6 days	95+ years	Global	0	3	Alcohol (liters per capita)	432
Stroke	Female	0-6 days	95+ years	Global	1	3	Diet high in trans fatty acids	18
Stroke	Male	0-6 days	95+ years	Data Rich	1	1	Cholesterol (total, mean per capita)	18

Appendix Table 9: CODEm covariates used, level of covariate, and expected direction of covariate by cause, sex, and age

Covariates that CODEm did not select during the covariate selection process have no draw counts listed.

Cause	Sex	Age Start	Age End	Model Type	Direction	Level	Covariate Name	Number of Draws
Stroke	Male	0-6 days	95+ years	Data Rich	1	1	Systolic Blood Pressure (mmHg)	234
Stroke	Male	0-6 days	95+ years	Data Rich	1	1	Log-transformed SEV scalar: Stroke	311
Stroke	Male	0-6 days	95+ years	Data Rich	1	1	Smoking Prevalence	999
Stroke	Male	0-6 days	95+ years	Data Rich	-1	2	fruits adjusted(g)	148
Stroke	Male	0-6 days	95+ years	Data Rich	-1	2	Elevation Over 1500m (proportion)	170
Stroke	Male	0-6 days	95+ years	Data Rich	-1	2	Healthcare access and quality index	170
Stroke	Male	0-6 days	95+ years	Data Rich	-1	2	vegetables adjusted(g)	--
Stroke	Male	0-6 days	95+ years	Data Rich	1	2	Outdoor Air Pollution (PM2.5)	0
Stroke	Male	0-6 days	95+ years	Data Rich	1	2	Indoor Air Pollution (All Cooking Fuels)	33
Stroke	Male	0-6 days	95+ years	Data Rich	1	2	Diabetes Fasting Plasma Glucose (mmol/L)	--
Stroke	Male	0-6 days	95+ years	Data Rich	1	2	Mean BMI	--
Stroke	Male	0-6 days	95+ years	Data Rich	-1	3	nuts seeds adjusted(g)	157
Stroke	Male	0-6 days	95+ years	Data Rich	-1	3	pufa adjusted(percent)	310
Stroke	Male	0-6 days	95+ years	Data Rich	-1	3	pulses legumes adjusted(g)	612
Stroke	Male	0-6 days	95+ years	Data Rich	-1	3	LDI (I$ per capita)	--
Stroke	Male	0-6 days	95+ years	Data Rich	-1	3	omega 3 adjusted(g)	--
Stroke	Male	0-6 days	95+ years	Data Rich	0	3	Socio-demographic Index	304
Stroke	Male	0-6 days	95+ years	Data Rich	0	3	Alcohol (liters per capita)	915
Stroke	Male	0-6 days	95+ years	Data Rich	1	3	Diet high in trans fatty acids	255
Stroke	Male	0-6 days	95+ years	Global	1	1	Cholesterol (total, mean per capita)	107
Stroke	Male	0-6 days	95+ years	Global	1	1	Smoking Prevalence	467
Stroke	Male	0-6 days	95+ years	Global	1	1	Systolic Blood Pressure (mmHg)	565
Stroke	Male	0-6 days	95+ years	Global	1	1	Log-transformed SEV scalar: Stroke	708
Stroke	Male	0-6 days	95+ years	Global	-1	2	fruits adjusted(g)	102
Stroke	Male	0-6 days	95+ years	Global	-1	2	Elevation Over 1500m (proportion)	258
Stroke	Male	0-6 days	95+ years	Global	-1	2	Healthcare access and quality index	761
Stroke	Male	0-6 days	95+ years	Global	-1	2	vegetables adjusted(g)	--
Stroke	Male	0-6 days	95+ years	Global	1	2	Indoor Air Pollution (All Cooking Fuels)	3
Stroke	Male	0-6 days	95+ years	Global	1	2	Outdoor Air Pollution (PM2.5)	261
Stroke	Male	0-6 days	95+ years	Global	1	2	Diabetes Fasting Plasma Glucose (mmol/L)	--
Stroke	Male	0-6 days	95+ years	Global	1	2	Mean BMI	--
Stroke	Male	0-6 days	95+ years	Global	-1	3	pufa adjusted(percent)	179
Stroke	Male	0-6 days	95+ years	Global	-1	3	nuts seeds adjusted(g)	324
Stroke	Male	0-6 days	95+ years	Global	-1	3	pulses legumes adjusted(g)	483
Stroke	Male	0-6 days	95+ years	Global	-1	3	LDI (I$ per capita)	--
Stroke	Male	0-6 days	95+ years	Global	-1	3	omega 3 adjusted(g)	--
Stroke	Male	0-6 days	95+ years	Global	0	3	Alcohol (liters per capita)	261
Stroke	Male	0-6 days	95+ years	Global	0	3	Socio-demographic Index	371
Stroke	Male	0-6 days	95+ years	Global	1	3	Diet high in trans fatty acids	136
Ischaemic stroke	Female	0-6 days	95+ years	Data Rich	1	1	Cholesterol (total, mean per capita)	41
Ischaemic stroke	Female	0-6 days	95+ years	Data Rich	1	1	Log-transformed SEV scalar: Isch Stroke	107
Ischaemic stroke	Female	0-6 days	95+ years	Data Rich	1	1	Smoking Prevalence	239
Ischaemic stroke	Female	0-6 days	95+ years	Data Rich	1	1	Systolic Blood Pressure (mmHg)	736
Ischaemic stroke	Female	0-6 days	95+ years	Data Rich	-1	2	Elevation Over 1500m (proportion)	577
Ischaemic stroke	Female	0-6 days	95+ years	Data Rich	-1	2	Healthcare access and quality index	577
Ischaemic stroke	Female	0-6 days	95+ years	Data Rich	1	2	Outdoor Air Pollution (PM2.5)	0
Ischaemic stroke	Female	0-6 days	95+ years	Data Rich	1	2	Indoor Air Pollution (All Cooking Fuels)	604
Ischaemic stroke	Female	0-6 days	95+ years	Data Rich	1	2	Diabetes Fasting Plasma Glucose (mmol/L)	--
Ischaemic stroke	Female	0-6 days	95+ years	Data Rich	1	2	Mean BMI	--
Ischaemic stroke	Female	0-6 days	95+ years	Data Rich	-1	3	pulses legumes adjusted(g)	0
Ischaemic stroke	Female	0-6 days	95+ years	Data Rich	-1	3	pufa adjusted(percent)	219
Ischaemic stroke	Female	0-6 days	95+ years	Data Rich	-1	3	nuts seeds adjusted(g)	343
Ischaemic stroke	Female	0-6 days	95+ years	Data Rich	-1	3	LDI (I$ per capita)	--
Ischaemic stroke	Female	0-6 days	95+ years	Data Rich	-1	3	fruits adjusted(g)	--
Ischaemic stroke	Female	0-6 days	95+ years	Data Rich	-1	3	omega 3 adjusted(g)	--
Ischaemic stroke	Female	0-6 days	95+ years	Data Rich	-1	3	vegetables adjusted(g)	--
Ischaemic stroke	Female	0-6 days	95+ years	Data Rich	0	3	Alcohol (liters per capita)	92
Ischaemic stroke	Female	0-6 days	95+ years	Data Rich	0	3	Socio-demographic Index	443
Ischaemic stroke	Female	0-6 days	95+ years	Data Rich	1	3	Diet high in trans fatty acids	424
Ischaemic stroke	Female	0-6 days	95+ years	Global	1	1	Systolic Blood Pressure (mmHg)	184
Ischaemic stroke	Female	0-6 days	95+ years	Global	1	1	Log-transformed SEV scalar: Isch Stroke	355
Ischaemic stroke	Female	0-6 days	95+ years	Global	1	1	Cholesterol (total, mean per capita)	527
Ischaemic stroke	Female	0-6 days	95+ years	Global	1	1	Smoking Prevalence	553
Ischaemic stroke	Female	0-6 days	95+ years	Global	-1	2	Elevation Over 1500m (proportion)	728
Ischaemic stroke	Female	0-6 days	95+ years	Global	-1	2	Healthcare access and quality index	815
Ischaemic stroke	Female	0-6 days	95+ years	Global	1	2	Indoor Air Pollution (All Cooking Fuels)	138
Ischaemic stroke	Female	0-6 days	95+ years	Global	1	2	Outdoor Air Pollution (PM2.5)	457
Ischaemic stroke	Female	0-6 days	95+ years	Global	1	2	Diabetes Fasting Plasma Glucose (mmol/L)	--
Ischaemic stroke	Female	0-6 days	95+ years	Global	1	2	Mean BMI	--
Ischaemic stroke	Female	0-6 days	95+ years	Global	-1	3	nuts seeds adjusted(g)	324
Ischaemic stroke	Female	0-6 days	95+ years	Global	-1	3	pufa adjusted(percent)	483
Ischaemic stroke	Female	0-6 days	95+ years	Global	-1	3	LDI (I$ per capita)	--
Ischaemic stroke	Female	0-6 days	95+ years	Global	-1	3	fruits adjusted(g)	--
Ischaemic stroke	Female	0-6 days	95+ years	Global	-1	3	omega 3 adjusted(g)	--
Ischaemic stroke	Female	0-6 days	95+ years	Global	-1	3	pulses legumes adjusted(g)	--
Ischaemic stroke	Female	0-6 days	95+ years	Global	-1	3	vegetables adjusted(g)	--
Ischaemic stroke	Female	0-6 days	95+ years	Global	0	3	Socio-demographic Index	500
Ischaemic stroke	Female	0-6 days	95+ years	Global	0	3	Alcohol (liters per capita)	650

Appendix Table 9: CODEm covariates used, level of covariate, and expected direction of covariate by cause, sex, and age<br>*Covariates that CODEm did not select during the covariate selection process have no draw counts listed.*								
**Cause**	**Sex**	**Age Start**	**Age End**	**Model Type**	**Direction**	**Level**	**Covariate Name**	**Number of Draws**
Ischaemic stroke	Female	0-6 days	95+ years	Global	1	3	Diet high in trans fatty acids	509
Ischaemic stroke	Male	0-6 days	95+ years	Data Rich	1	1	Log-transformed SEV scalar: Isch Stroke	42
Ischaemic stroke	Male	0-6 days	95+ years	Data Rich	1	1	Cholesterol (total, mean per capita)	64
Ischaemic stroke	Male	0-6 days	95+ years	Data Rich	1	1	Systolic Blood Pressure (mmHg)	918
Ischaemic stroke	Male	0-6 days	95+ years	Data Rich	1	1	Smoking Prevalence	1000
Ischaemic stroke	Male	0-6 days	95+ years	Data Rich	-1	2	Elevation Over 1500m (proportion)	454
Ischaemic stroke	Male	0-6 days	95+ years	Data Rich	-1	2	Healthcare access and quality index	454
Ischaemic stroke	Male	0-6 days	95+ years	Data Rich	1	2	Outdoor Air Pollution (PM2.5)	0
Ischaemic stroke	Male	0-6 days	95+ years	Data Rich	1	2	Indoor Air Pollution (All Cooking Fuels)	454
Ischaemic stroke	Male	0-6 days	95+ years	Data Rich	1	2	Diabetes Fasting Plasma Glucose (mmol/L)	--
Ischaemic stroke	Male	0-6 days	95+ years	Data Rich	1	2	Mean BMI	--
Ischaemic stroke	Male	0-6 days	95+ years	Data Rich	-1	3	pulses legumes adjusted(g)	32
Ischaemic stroke	Male	0-6 days	95+ years	Data Rich	-1	3	nuts seeds adjusted(g)	219
Ischaemic stroke	Male	0-6 days	95+ years	Data Rich	-1	3	pufa adjusted(percent)	661
Ischaemic stroke	Male	0-6 days	95+ years	Data Rich	-1	3	LDI (I$ per capita)	--
Ischaemic stroke	Male	0-6 days	95+ years	Data Rich	-1	3	fruits adjusted(g)	--
Ischaemic stroke	Male	0-6 days	95+ years	Data Rich	-1	3	omega 3 adjusted(g)	--
Ischaemic stroke	Male	0-6 days	95+ years	Data Rich	-1	3	vegetables adjusted(g)	--
Ischaemic stroke	Male	0-6 days	95+ years	Data Rich	0	3	Alcohol (liters per capita)	764
Ischaemic stroke	Male	0-6 days	95+ years	Data Rich	0	3	Socio-demographic Index	825
Ischaemic stroke	Male	0-6 days	95+ years	Data Rich	1	3	Diet high in trans fatty acids	50
Ischaemic stroke	Male	0-6 days	95+ years	Global	1	1	Log-transformed SEV scalar: Isch Stroke	247
Ischaemic stroke	Male	0-6 days	95+ years	Global	1	1	Cholesterol (total, mean per capita)	384
Ischaemic stroke	Male	0-6 days	95+ years	Global	1	1	Systolic Blood Pressure (mmHg)	548
Ischaemic stroke	Male	0-6 days	95+ years	Global	1	1	Smoking Prevalence	952
Ischaemic stroke	Male	0-6 days	95+ years	Global	-1	2	Elevation Over 1500m (proportion)	408
Ischaemic stroke	Male	0-6 days	95+ years	Global	-1	2	Healthcare access and quality index	489
Ischaemic stroke	Male	0-6 days	95+ years	Global	1	2	Indoor Air Pollution (All Cooking Fuels)	250
Ischaemic stroke	Male	0-6 days	95+ years	Global	1	2	Outdoor Air Pollution (PM2.5)	557
Ischaemic stroke	Male	0-6 days	95+ years	Global	1	2	Diabetes Fasting Plasma Glucose (mmol/L)	574
Ischaemic stroke	Male	0-6 days	95+ years	Global	1	2	Mean BMI	--
Ischaemic stroke	Male	0-6 days	95+ years	Global	-1	3	pufa adjusted(percent)	90
Ischaemic stroke	Male	0-6 days	95+ years	Global	-1	3	nuts seeds adjusted(g)	274
Ischaemic stroke	Male	0-6 days	95+ years	Global	-1	3	LDI (I$ per capita)	--
Ischaemic stroke	Male	0-6 days	95+ years	Global	-1	3	fruits adjusted(g)	--
Ischaemic stroke	Male	0-6 days	95+ years	Global	-1	3	omega 3 adjusted(g)	--
Ischaemic stroke	Male	0-6 days	95+ years	Global	-1	3	pulses legumes adjusted(g)	--
Ischaemic stroke	Male	0-6 days	95+ years	Global	-1	3	vegetables adjusted(g)	--
Ischaemic stroke	Male	0-6 days	95+ years	Global	0	3	Socio-demographic Index	299
Ischaemic stroke	Male	0-6 days	95+ years	Global	0	3	Alcohol (liters per capita)	643
Ischaemic stroke	Male	0-6 days	95+ years	Global	1	3	Diet high in trans fatty acids	226
Intracerebral hemorrhage	Female	0-6 days	95+ years	Data Rich	0	1	Cholesterol (total, mean per capita)	821
Intracerebral hemorrhage	Female	0-6 days	95+ years	Data Rich	1	1	Log-transformed SEV scalar: Hem Stroke	92
Intracerebral hemorrhage	Female	0-6 days	95+ years	Data Rich	1	1	Smoking Prevalence	993
Intracerebral hemorrhage	Female	0-6 days	95+ years	Data Rich	1	1	Systolic Blood Pressure (mmHg)	1000
Intracerebral hemorrhage	Female	0-6 days	95+ years	Data Rich	-1	2	Elevation Over 1500m (proportion)	255
Intracerebral hemorrhage	Female	0-6 days	95+ years	Data Rich	-1	2	Healthcare access and quality index	402
Intracerebral hemorrhage	Female	0-6 days	95+ years	Data Rich	1	2	Outdoor Air Pollution (PM2.5)	441
Intracerebral hemorrhage	Female	0-6 days	95+ years	Data Rich	1	2	Indoor Air Pollution (All Cooking Fuels)	445
Intracerebral hemorrhage	Female	0-6 days	95+ years	Data Rich	1	2	Diabetes Fasting Plasma Glucose (mmol/L)	--
Intracerebral hemorrhage	Female	0-6 days	95+ years	Data Rich	1	2	Mean BMI	--
Intracerebral hemorrhage	Female	0-6 days	95+ years	Data Rich	-1	3	pulses legumes adjusted(g)	160
Intracerebral hemorrhage	Female	0-6 days	95+ years	Data Rich	-1	3	fruits adjusted(g)	299
Intracerebral hemorrhage	Female	0-6 days	95+ years	Data Rich	-1	3	nuts seeds adjusted(g)	422
Intracerebral hemorrhage	Female	0-6 days	95+ years	Data Rich	-1	3	LDI (I$ per capita)	--
Intracerebral hemorrhage	Female	0-6 days	95+ years	Data Rich	-1	3	omega 3 adjusted(g)	--
Intracerebral hemorrhage	Female	0-6 days	95+ years	Data Rich	-1	3	pufa adjusted(percent)	--
Intracerebral hemorrhage	Female	0-6 days	95+ years	Data Rich	-1	3	vegetables adjusted(g)	--
Intracerebral hemorrhage	Female	0-6 days	95+ years	Data Rich	0	3	Socio-demographic Index	145
Intracerebral hemorrhage	Female	0-6 days	95+ years	Data Rich	0	3	Alcohol (liters per capita)	755
Intracerebral hemorrhage	Female	0-6 days	95+ years	Data Rich	1	3	Diet high in trans fatty acids	0
Intracerebral hemorrhage	Female	0-6 days	95+ years	Global	0	1	Cholesterol (total, mean per capita)	960
Intracerebral hemorrhage	Female	0-6 days	95+ years	Global	1	1	Log-transformed SEV scalar: Hem Stroke	364
Intracerebral hemorrhage	Female	0-6 days	95+ years	Global	1	1	Smoking Prevalence	930
Intracerebral hemorrhage	Female	0-6 days	95+ years	Global	1	1	Systolic Blood Pressure (mmHg)	996
Intracerebral hemorrhage	Female	0-6 days	95+ years	Global	-1	2	Healthcare access and quality index	427
Intracerebral hemorrhage	Female	0-6 days	95+ years	Global	-1	2	Elevation Over 1500m (proportion)	--
Intracerebral hemorrhage	Female	0-6 days	95+ years	Global	1	2	Indoor Air Pollution (All Cooking Fuels)	36
Intracerebral hemorrhage	Female	0-6 days	95+ years	Global	1	2	Outdoor Air Pollution (PM2.5)	40
Intracerebral hemorrhage	Female	0-6 days	95+ years	Global	1	2	Diabetes Fasting Plasma Glucose (mmol/L)	--
Intracerebral hemorrhage	Female	0-6 days	95+ years	Global	1	2	Mean BMI	--
Intracerebral hemorrhage	Female	0-6 days	95+ years	Global	-1	3	fruits adjusted(g)	10
Intracerebral hemorrhage	Female	0-6 days	95+ years	Global	-1	3	nuts seeds adjusted(g)	364
Intracerebral hemorrhage	Female	0-6 days	95+ years	Global	-1	3	pulses legumes adjusted(g)	698
Intracerebral hemorrhage	Female	0-6 days	95+ years	Global	-1	3	LDI (I$ per capita)	--
Intracerebral hemorrhage	Female	0-6 days	95+ years	Global	-1	3	omega 3 adjusted(g)	--
Intracerebral hemorrhage	Female	0-6 days	95+ years	Global	-1	3	pufa adjusted(percent)	--
Intracerebral hemorrhage	Female	0-6 days	95+ years	Global	-1	3	vegetables adjusted(g)	--

Appendix Table 9: CODEm covariates used, level of covariate, and expected direction of covariate by cause, sex, and age<br>*Covariates that CODEm did not select during the covariate selection process have no draw counts listed.*								
**Cause**	**Sex**	**Age Start**	**Age End**	**Model Type**	**Direction**	**Level**	**Covariate Name**	**Number of Draws**
Intracerebral hemorrhage	Female	0-6 days	95+ years	Global	0	3	Alcohol (liters per capita)	230
Intracerebral hemorrhage	Female	0-6 days	95+ years	Global	0	3	Socio-demographic Index	481
Intracerebral hemorrhage	Female	0-6 days	95+ years	Global	1	3	Diet high in trans fatty acids	0
Intracerebral hemorrhage	Male	0-6 days	95+ years	Data Rich	0	1	Cholesterol (total, mean per capita)	811
Intracerebral hemorrhage	Male	0-6 days	95+ years	Data Rich	1	1	Log-transformed SEV scalar: Hem Stroke	12
Intracerebral hemorrhage	Male	0-6 days	95+ years	Data Rich	1	1	Systolic Blood Pressure (mmHg)	815
Intracerebral hemorrhage	Male	0-6 days	95+ years	Data Rich	1	1	Smoking Prevalence	1000
Intracerebral hemorrhage	Male	0-6 days	95+ years	Data Rich	-1	2	Healthcare access and quality index	805
Intracerebral hemorrhage	Male	0-6 days	95+ years	Data Rich	-1	2	Elevation Over 1500m (proportion)	833
Intracerebral hemorrhage	Male	0-6 days	95+ years	Data Rich	1	2	Indoor Air Pollution (All Cooking Fuels)	222
Intracerebral hemorrhage	Male	0-6 days	95+ years	Data Rich	1	2	Outdoor Air Pollution (PM2.5)	930
Intracerebral hemorrhage	Male	0-6 days	95+ years	Data Rich	1	2	Diabetes Fasting Plasma Glucose (mmol/L)	--
Intracerebral hemorrhage	Male	0-6 days	95+ years	Data Rich	1	2	Mean BMI	--
Intracerebral hemorrhage	Male	0-6 days	95+ years	Data Rich	-1	3	fruits adjusted(g)	46
Intracerebral hemorrhage	Male	0-6 days	95+ years	Data Rich	-1	3	nuts seeds adjusted(g)	89
Intracerebral hemorrhage	Male	0-6 days	95+ years	Data Rich	-1	3	pulses legumes adjusted(g)	681
Intracerebral hemorrhage	Male	0-6 days	95+ years	Data Rich	-1	3	LDI (I$ per capita)	--
Intracerebral hemorrhage	Male	0-6 days	95+ years	Data Rich	-1	3	omega 3 adjusted(g)	--
Intracerebral hemorrhage	Male	0-6 days	95+ years	Data Rich	-1	3	pufa adjusted(percent)	--
Intracerebral hemorrhage	Male	0-6 days	95+ years	Data Rich	-1	3	vegetables adjusted(g)	--
Intracerebral hemorrhage	Male	0-6 days	95+ years	Data Rich	0	3	Alcohol (liters per capita)	300
Intracerebral hemorrhage	Male	0-6 days	95+ years	Data Rich	0	3	Socio-demographic Index	585
Intracerebral hemorrhage	Male	0-6 days	95+ years	Data Rich	1	3	Diet high in trans fatty acids	0
Intracerebral hemorrhage	Male	0-6 days	95+ years	Global	0	1	Cholesterol (total, mean per capita)	509
Intracerebral hemorrhage	Male	0-6 days	95+ years	Global	1	1	Log-transformed SEV scalar: Hem Stroke	298
Intracerebral hemorrhage	Male	0-6 days	95+ years	Global	1	1	Systolic Blood Pressure (mmHg)	435
Intracerebral hemorrhage	Male	0-6 days	95+ years	Global	1	1	Smoking Prevalence	998
Intracerebral hemorrhage	Male	0-6 days	95+ years	Global	-1	2	Healthcare access and quality index	458
Intracerebral hemorrhage	Male	0-6 days	95+ years	Global	-1	2	Elevation Over 1500m (proportion)	--
Intracerebral hemorrhage	Male	0-6 days	95+ years	Global	1	2	Indoor Air Pollution (All Cooking Fuels)	478
Intracerebral hemorrhage	Male	0-6 days	95+ years	Global	1	2	Outdoor Air Pollution (PM2.5)	864
Intracerebral hemorrhage	Male	0-6 days	95+ years	Global	1	2	Diabetes Fasting Plasma Glucose (mmol/L)	--
Intracerebral hemorrhage	Male	0-6 days	95+ years	Global	1	2	Mean BMI	--
Intracerebral hemorrhage	Male	0-6 days	95+ years	Global	-1	3	fruits adjusted(g)	0
Intracerebral hemorrhage	Male	0-6 days	95+ years	Global	-1	3	nuts seeds adjusted(g)	247
Intracerebral hemorrhage	Male	0-6 days	95+ years	Global	-1	3	pulses legumes adjusted(g)	855
Intracerebral hemorrhage	Male	0-6 days	95+ years	Global	-1	3	LDI (I$ per capita)	--
Intracerebral hemorrhage	Male	0-6 days	95+ years	Global	-1	3	omega 3 adjusted(g)	--
Intracerebral hemorrhage	Male	0-6 days	95+ years	Global	-1	3	pufa adjusted(percent)	--
Intracerebral hemorrhage	Male	0-6 days	95+ years	Global	-1	3	vegetables adjusted(g)	--
Intracerebral hemorrhage	Male	0-6 days	95+ years	Global	0	3	Socio-demographic Index	492
Intracerebral hemorrhage	Male	0-6 days	95+ years	Global	0	3	Alcohol (liters per capita)	713
Intracerebral hemorrhage	Male	0-6 days	95+ years	Global	1	3	Diet high in trans fatty acids	242
Subarachnoid hemorrhage	Female	0-6 days	95+ years	Data Rich	1	1	Systolic Blood Pressure (mmHg)	468
Subarachnoid hemorrhage	Female	0-6 days	95+ years	Data Rich	1	1	Smoking Prevalence	866
Subarachnoid hemorrhage	Female	0-6 days	95+ years	Data Rich	-1	2	Healthcare access and quality index	308
Subarachnoid hemorrhage	Female	0-6 days	95+ years	Data Rich	-1	3	LDI (I$ per capita)	--
Subarachnoid hemorrhage	Female	0-6 days	95+ years	Data Rich	0	3	Socio-demographic Index	178
Subarachnoid hemorrhage	Female	0-6 days	95+ years	Data Rich	0	3	Alcohol (liters per capita)	237
Subarachnoid hemorrhage	Female	0-6 days	95+ years	Global	1	1	Systolic Blood Pressure (mmHg)	564
Subarachnoid hemorrhage	Female	0-6 days	95+ years	Global	1	1	Smoking Prevalence	936
Subarachnoid hemorrhage	Female	0-6 days	95+ years	Global	-1	2	Healthcare access and quality index	647
Subarachnoid hemorrhage	Female	0-6 days	95+ years	Global	-1	3	LDI (I$ per capita)	--
Subarachnoid hemorrhage	Female	0-6 days	95+ years	Global	0	3	Alcohol (liters per capita)	351
Subarachnoid hemorrhage	Female	0-6 days	95+ years	Global	0	3	Socio-demographic Index	446
Subarachnoid hemorrhage	Male	0-6 days	95+ years	Data Rich	1	1	Smoking Prevalence	330
Subarachnoid hemorrhage	Male	0-6 days	95+ years	Data Rich	1	1	Systolic Blood Pressure (mmHg)	1000
Subarachnoid hemorrhage	Male	0-6 days	95+ years	Data Rich	-1	2	Healthcare access and quality index	401
Subarachnoid hemorrhage	Male	0-6 days	95+ years	Data Rich	-1	3	LDI (I$ per capita)	--
Subarachnoid hemorrhage	Male	0-6 days	95+ years	Data Rich	0	3	Socio-demographic Index	476
Subarachnoid hemorrhage	Male	0-6 days	95+ years	Data Rich	0	3	Alcohol (liters per capita)	554
Subarachnoid hemorrhage	Male	0-6 days	95+ years	Global	1	1	Systolic Blood Pressure (mmHg)	66
Subarachnoid hemorrhage	Male	0-6 days	95+ years	Global	1	1	Smoking Prevalence	245
Subarachnoid hemorrhage	Male	0-6 days	95+ years	Global	-1	2	Healthcare access and quality index	592
Subarachnoid hemorrhage	Male	0-6 days	95+ years	Global	-1	3	LDI (I$ per capita)	--
Subarachnoid hemorrhage	Male	0-6 days	95+ years	Global	0	3	Alcohol (liters per capita)	228
Subarachnoid hemorrhage	Male	0-6 days	95+ years	Global	0	3	Socio-demographic Index	371
Hypertensive heart disease	Female	15-19 years	95+ years	Data Rich	1	1	Systolic Blood Pressure (mmHg)	615
Hypertensive heart disease	Female	15-19 years	95+ years	Data Rich	-1	2	Healthcare access and quality index	214
Hypertensive heart disease	Female	15-19 years	95+ years	Data Rich	1	2	Smoking Prevalence	141
Hypertensive heart disease	Female	15-19 years	95+ years	Data Rich	1	2	Cholesterol (total, mean per capita)	--
Hypertensive heart disease	Female	15-19 years	95+ years	Data Rich	1	2	Mean BMI	--
Hypertensive heart disease	Female	15-19 years	95+ years	Data Rich	-1	3	LDI (I$ per capita)	--
Hypertensive heart disease	Female	15-19 years	95+ years	Data Rich	-1	3	fruits adjusted(g)	--
Hypertensive heart disease	Female	15-19 years	95+ years	Data Rich	-1	3	nuts seeds adjusted(g)	--
Hypertensive heart disease	Female	15-19 years	95+ years	Data Rich	-1	3	omega 3 adjusted(g)	--
Hypertensive heart disease	Female	15-19 years	95+ years	Data Rich	-1	3	pufa adjusted(percent)	--
Hypertensive heart disease	Female	15-19 years	95+ years	Data Rich	-1	3	pulses legumes adjusted(g)	--

Appendix Table 9: CODEm covariates used, level of covariate, and expected direction of covariate by cause, sex, and age

Covariates that CODEm did not select during the covariate selection process have no draw counts listed.

Cause	Sex	Age Start	Age End	Model Type	Direction	Level	Covariate Name	Number of Draws
Hypertensive heart disease	Female	15-19 years	95+ years	Data Rich	-1	3	vegetables adjusted(g)	--
Hypertensive heart disease	Female	15-19 years	95+ years	Data Rich	0	3	Socio-demographic Index	433
Hypertensive heart disease	Female	15-19 years	95+ years	Data Rich	0	3	Alcohol (liters per capita)	448
Hypertensive heart disease	Female	15-19 years	95+ years	Data Rich	1	3	Diet high in trans fatty acids	--
Hypertensive heart disease	Female	15-19 years	95+ years	Global	1	1	Systolic Blood Pressure (mmHg)	480
Hypertensive heart disease	Female	15-19 years	95+ years	Global	-1	2	Healthcare access and quality index	389
Hypertensive heart disease	Female	15-19 years	95+ years	Global	1	2	Smoking Prevalence	277
Hypertensive heart disease	Female	15-19 years	95+ years	Global	1	2	Cholesterol (total, mean per capita)	--
Hypertensive heart disease	Female	15-19 years	95+ years	Global	1	2	Mean BMI	--
Hypertensive heart disease	Female	15-19 years	95+ years	Global	-1	3	nuts seeds adjusted(g)	14
Hypertensive heart disease	Female	15-19 years	95+ years	Global	-1	3	fruits adjusted(g)	47
Hypertensive heart disease	Female	15-19 years	95+ years	Global	-1	3	LDI (I$ per capita)	--
Hypertensive heart disease	Female	15-19 years	95+ years	Global	-1	3	omega 3 adjusted(g)	--
Hypertensive heart disease	Female	15-19 years	95+ years	Global	-1	3	pufa adjusted(percent)	--
Hypertensive heart disease	Female	15-19 years	95+ years	Global	-1	3	pulses legumes adjusted(g)	--
Hypertensive heart disease	Female	15-19 years	95+ years	Global	-1	3	vegetables adjusted(g)	--
Hypertensive heart disease	Female	15-19 years	95+ years	Global	0	3	Alcohol (liters per capita)	425
Hypertensive heart disease	Female	15-19 years	95+ years	Global	0	3	Socio-demographic Index	560
Hypertensive heart disease	Female	15-19 years	95+ years	Global	1	3	Diet high in trans fatty acids	--
Hypertensive heart disease	Male	15-19 years	95+ years	Data Rich	1	1	Systolic Blood Pressure (mmHg)	--
Hypertensive heart disease	Male	15-19 years	95+ years	Data Rich	-1	2	Healthcare access and quality index	285
Hypertensive heart disease	Male	15-19 years	95+ years	Data Rich	1	2	Smoking Prevalence	647
Hypertensive heart disease	Male	15-19 years	95+ years	Data Rich	1	2	Cholesterol (total, mean per capita)	--
Hypertensive heart disease	Male	15-19 years	95+ years	Data Rich	1	2	Mean BMI	--
Hypertensive heart disease	Male	15-19 years	95+ years	Data Rich	-1	3	pulses legumes adjusted(g)	145
Hypertensive heart disease	Male	15-19 years	95+ years	Data Rich	-1	3	LDI (I$ per capita)	--
Hypertensive heart disease	Male	15-19 years	95+ years	Data Rich	-1	3	fruits adjusted(g)	--
Hypertensive heart disease	Male	15-19 years	95+ years	Data Rich	-1	3	nuts seeds adjusted(g)	--
Hypertensive heart disease	Male	15-19 years	95+ years	Data Rich	-1	3	omega 3 adjusted(g)	--
Hypertensive heart disease	Male	15-19 years	95+ years	Data Rich	-1	3	pufa adjusted(percent)	--
Hypertensive heart disease	Male	15-19 years	95+ years	Data Rich	-1	3	vegetables adjusted(g)	--
Hypertensive heart disease	Male	15-19 years	95+ years	Data Rich	0	3	Alcohol (liters per capita)	248
Hypertensive heart disease	Male	15-19 years	95+ years	Data Rich	0	3	Socio-demographic Index	477
Hypertensive heart disease	Male	15-19 years	95+ years	Data Rich	1	3	Diet high in trans fatty acids	--
Hypertensive heart disease	Male	15-19 years	95+ years	Global	1	1	Systolic Blood Pressure (mmHg)	110
Hypertensive heart disease	Male	15-19 years	95+ years	Global	-1	2	Healthcare access and quality index	557
Hypertensive heart disease	Male	15-19 years	95+ years	Global	1	2	Mean BMI	430
Hypertensive heart disease	Male	15-19 years	95+ years	Global	1	2	Smoking Prevalence	547
Hypertensive heart disease	Male	15-19 years	95+ years	Global	1	2	Cholesterol (total, mean per capita)	--
Hypertensive heart disease	Male	15-19 years	95+ years	Global	-1	3	fruits adjusted(g)	31
Hypertensive heart disease	Male	15-19 years	95+ years	Global	-1	3	LDI (I$ per capita)	--
Hypertensive heart disease	Male	15-19 years	95+ years	Global	-1	3	nuts seeds adjusted(g)	--
Hypertensive heart disease	Male	15-19 years	95+ years	Global	-1	3	omega 3 adjusted(g)	--
Hypertensive heart disease	Male	15-19 years	95+ years	Global	-1	3	pufa adjusted(percent)	--
Hypertensive heart disease	Male	15-19 years	95+ years	Global	-1	3	pulses legumes adjusted(g)	--
Hypertensive heart disease	Male	15-19 years	95+ years	Global	-1	3	vegetables adjusted(g)	--
Hypertensive heart disease	Male	15-19 years	95+ years	Global	0	3	Socio-demographic Index	251
Hypertensive heart disease	Male	15-19 years	95+ years	Global	0	3	Alcohol (liters per capita)	429
Hypertensive heart disease	Male	15-19 years	95+ years	Global	1	3	Diet high in trans fatty acids	--
Non-rheumatic valvular heart disease	Female	15-19 years	95+ years	Data Rich	1	1	Systolic Blood Pressure (mmHg)	370
Non-rheumatic valvular heart disease	Female	15-19 years	95+ years	Data Rich	1	1	Smoking Prevalence	416
Non-rheumatic valvular heart disease	Female	15-19 years	95+ years	Data Rich	-1	2	Healthcare access and quality index	--
Non-rheumatic valvular heart disease	Female	15-19 years	95+ years	Data Rich	1	2	Mean BMI	276
Non-rheumatic valvular heart disease	Female	15-19 years	95+ years	Data Rich	1	2	Cholesterol (total, mean per capita)	--
Non-rheumatic valvular heart disease	Female	15-19 years	95+ years	Data Rich	-1	3	LDI (I$ per capita)	--
Non-rheumatic valvular heart disease	Female	15-19 years	95+ years	Data Rich	0	3	Socio-demographic Index	420
Non-rheumatic valvular heart disease	Female	15-19 years	95+ years	Data Rich	0	3	Alcohol (liters per capita)	431
Non-rheumatic valvular heart disease	Female	15-19 years	95+ years	Global	1	1	Systolic Blood Pressure (mmHg)	350
Non-rheumatic valvular heart disease	Female	15-19 years	95+ years	Global	1	1	Smoking Prevalence	414
Non-rheumatic valvular heart disease	Female	15-19 years	95+ years	Global	-1	2	Healthcare access and quality index	183
Non-rheumatic valvular heart disease	Female	15-19 years	95+ years	Global	1	2	Mean BMI	525
Non-rheumatic valvular heart disease	Female	15-19 years	95+ years	Global	1	2	Cholesterol (total, mean per capita)	--
Non-rheumatic valvular heart disease	Female	15-19 years	95+ years	Global	-1	3	LDI (I$ per capita)	--
Non-rheumatic valvular heart disease	Female	15-19 years	95+ years	Global	0	3	Socio-demographic Index	513
Non-rheumatic valvular heart disease	Female	15-19 years	95+ years	Global	0	3	Alcohol (liters per capita)	673
Non-rheumatic valvular heart disease	Male	15-19 years	95+ years	Data Rich	1	1	Systolic Blood Pressure (mmHg)	149
Non-rheumatic valvular heart disease	Male	15-19 years	95+ years	Data Rich	1	1	Smoking Prevalence	272
Non-rheumatic valvular heart disease	Male	15-19 years	95+ years	Data Rich	-1	2	Healthcare access and quality index	77
Non-rheumatic valvular heart disease	Male	15-19 years	95+ years	Data Rich	1	2	Mean BMI	599
Non-rheumatic valvular heart disease	Male	15-19 years	95+ years	Data Rich	1	2	Cholesterol (total, mean per capita)	--
Non-rheumatic valvular heart disease	Male	15-19 years	95+ years	Data Rich	-1	3	LDI (I$ per capita)	--
Non-rheumatic valvular heart disease	Male	15-19 years	95+ years	Data Rich	0	3	Alcohol (liters per capita)	280
Non-rheumatic valvular heart disease	Male	15-19 years	95+ years	Data Rich	0	3	Socio-demographic Index	401
Non-rheumatic valvular heart disease	Male	15-19 years	95+ years	Global	1	1	Smoking Prevalence	248
Non-rheumatic valvular heart disease	Male	15-19 years	95+ years	Global	1	1	Systolic Blood Pressure (mmHg)	395
Non-rheumatic valvular heart disease	Male	15-19 years	95+ years	Global	-1	2	Healthcare access and quality index	268
Non-rheumatic valvular heart disease	Male	15-19 years	95+ years	Global	1	2	Mean BMI	299
Non-rheumatic valvular heart disease	Male	15-19 years	95+ years	Global	1	2	Cholesterol (total, mean per capita)	--

**Appendix Table 9: CODEm covariates used, level of covariate, and expected direction of covariate by cause, sex, and age** *Covariates that CODEm did not select during the covariate selection process have no draw counts listed.*								
Cause	Sex	Age Start	Age End	Model Type	Direction	Level	Covariate Name	Number of Draws
Non-rheumatic valvular heart disease	Male	15-19 years	95+ years	Global	-1	3	LDI (I$ per capita)	--
Non-rheumatic valvular heart disease	Male	15-19 years	95+ years	Global	0	3	Alcohol (liters per capita)	402
Non-rheumatic valvular heart disease	Male	15-19 years	95+ years	Global	0	3	Socio-demographic Index	576
Non-rheumatic calcific aortic valve disease	Female	15-19 years	95+ years	Data Rich	1	1	Smoking Prevalence	277
Non-rheumatic calcific aortic valve disease	Female	15-19 years	95+ years	Data Rich	1	1	Mean BMI	1000
Non-rheumatic calcific aortic valve disease	Female	15-19 years	95+ years	Data Rich	1	1	Systolic Blood Pressure (mmHg)	--
Non-rheumatic calcific aortic valve disease	Female	15-19 years	95+ years	Data Rich	-1	2	Healthcare access and quality index	--
Non-rheumatic calcific aortic valve disease	Female	15-19 years	95+ years	Data Rich	1	2	Cholesterol (total, mean per capita)	--
Non-rheumatic calcific aortic valve disease	Female	15-19 years	95+ years	Data Rich	1	2	Diabetes Fasting Plasma Glucose (mmol/L)	--
Non-rheumatic calcific aortic valve disease	Female	15-19 years	95+ years	Data Rich	-1	3	LDI (I$ per capita)	--
Non-rheumatic calcific aortic valve disease	Female	15-19 years	95+ years	Data Rich	0	3	Socio-demographic Index	463
Non-rheumatic calcific aortic valve disease	Female	15-19 years	95+ years	Data Rich	0	3	Alcohol (liters per capita)	596
Non-rheumatic calcific aortic valve disease	Female	15-19 years	95+ years	Global	1	1	Mean BMI	513
Non-rheumatic calcific aortic valve disease	Female	15-19 years	95+ years	Global	1	1	Smoking Prevalence	513
Non-rheumatic calcific aortic valve disease	Female	15-19 years	95+ years	Global	1	1	Systolic Blood Pressure (mmHg)	--
Non-rheumatic calcific aortic valve disease	Female	15-19 years	95+ years	Global	-1	2	Healthcare access and quality index	--
Non-rheumatic calcific aortic valve disease	Female	15-19 years	95+ years	Global	1	2	Diabetes Fasting Plasma Glucose (mmol/L)	334
Non-rheumatic calcific aortic valve disease	Female	15-19 years	95+ years	Global	1	2	Cholesterol (total, mean per capita)	--
Non-rheumatic calcific aortic valve disease	Female	15-19 years	95+ years	Global	-1	3	LDI (I$ per capita)	--
Non-rheumatic calcific aortic valve disease	Female	15-19 years	95+ years	Global	0	3	Socio-demographic Index	282
Non-rheumatic calcific aortic valve disease	Female	15-19 years	95+ years	Global	0	3	Alcohol (liters per capita)	628
Non-rheumatic calcific aortic valve disease	Male	15-19 years	95+ years	Data Rich	1	1	Smoking Prevalence	278
Non-rheumatic calcific aortic valve disease	Male	15-19 years	95+ years	Data Rich	1	1	Mean BMI	1000
Non-rheumatic calcific aortic valve disease	Male	15-19 years	95+ years	Data Rich	1	1	Systolic Blood Pressure (mmHg)	--
Non-rheumatic calcific aortic valve disease	Male	15-19 years	95+ years	Data Rich	-1	2	Healthcare access and quality index	--
Non-rheumatic calcific aortic valve disease	Male	15-19 years	95+ years	Data Rich	1	2	Cholesterol (total, mean per capita)	--
Non-rheumatic calcific aortic valve disease	Male	15-19 years	95+ years	Data Rich	1	2	Diabetes Fasting Plasma Glucose (mmol/L)	--
Non-rheumatic calcific aortic valve disease	Male	15-19 years	95+ years	Data Rich	-1	3	LDI (I$ per capita)	--
Non-rheumatic calcific aortic valve disease	Male	15-19 years	95+ years	Data Rich	0	3	Socio-demographic Index	317
Non-rheumatic calcific aortic valve disease	Male	15-19 years	95+ years	Data Rich	0	3	Alcohol (liters per capita)	572
Non-rheumatic calcific aortic valve disease	Male	15-19 years	95+ years	Global	1	1	Smoking Prevalence	278
Non-rheumatic calcific aortic valve disease	Male	15-19 years	95+ years	Global	1	1	Mean BMI	1000
Non-rheumatic calcific aortic valve disease	Male	15-19 years	95+ years	Global	1	1	Systolic Blood Pressure (mmHg)	--
Non-rheumatic calcific aortic valve disease	Male	15-19 years	95+ years	Global	-1	2	Healthcare access and quality index	--
Non-rheumatic calcific aortic valve disease	Male	15-19 years	95+ years	Global	1	2	Cholesterol (total, mean per capita)	--
Non-rheumatic calcific aortic valve disease	Male	15-19 years	95+ years	Global	1	2	Diabetes Fasting Plasma Glucose (mmol/L)	--
Non-rheumatic calcific aortic valve disease	Male	15-19 years	95+ years	Global	-1	3	LDI (I$ per capita)	--
Non-rheumatic calcific aortic valve disease	Male	15-19 years	95+ years	Global	0	3	Alcohol (liters per capita)	572
Non-rheumatic calcific aortic valve disease	Male	15-19 years	95+ years	Global	0	3	Socio-demographic Index	572
Non-rheumatic degenerative mitral valve disease	Female	15-19 years	95+ years	Data Rich	-1	1	Healthcare access and quality index	557
Non-rheumatic degenerative mitral valve disease	Female	15-19 years	95+ years	Data Rich	0	1	Socio-demographic Index	619
Non-rheumatic degenerative mitral valve disease	Female	15-19 years	95+ years	Data Rich	0	1	LDI (I$ per capita)	--
Non-rheumatic degenerative mitral valve disease	Female	15-19 years	95+ years	Global	-1	1	Healthcare access and quality index	445
Non-rheumatic degenerative mitral valve disease	Female	15-19 years	95+ years	Global	0	1	Socio-demographic Index	533
Non-rheumatic degenerative mitral valve disease	Female	15-19 years	95+ years	Global	0	1	LDI (I$ per capita)	--
Non-rheumatic degenerative mitral valve disease	Male	15-19 years	95+ years	Data Rich	-1	1	Healthcare access and quality index	600
Non-rheumatic degenerative mitral valve disease	Male	15-19 years	95+ years	Data Rich	0	1	Socio-demographic Index	410
Non-rheumatic degenerative mitral valve disease	Male	15-19 years	95+ years	Data Rich	0	1	LDI (I$ per capita)	--
Non-rheumatic degenerative mitral valve disease	Male	15-19 years	95+ years	Global	-1	1	Healthcare access and quality index	443
Non-rheumatic degenerative mitral valve disease	Male	15-19 years	95+ years	Global	0	1	Socio-demographic Index	621
Non-rheumatic degenerative mitral valve disease	Male	15-19 years	95+ years	Global	0	1	LDI (I$ per capita)	--
Other non-rheumatic valve diseases	Female	15-19 years	95+ years	Data Rich	1	1	Log-transformed SEV scalar: CVD	1000
Other non-rheumatic valve diseases	Female	15-19 years	95+ years	Global	1	1	Log-transformed SEV scalar: CVD	1000
Other non-rheumatic valve diseases	Male	15-19 years	95+ years	Data Rich	1	1	Log-transformed SEV scalar: CVD	1000
Other non-rheumatic valve diseases	Male	15-19 years	95+ years	Global	1	1	Log-transformed SEV scalar: CVD	1000
Cardiomyopathy and myocarditis	Female	0-6 days	95+ years	Data Rich	1	1	Smoking Prevalence	193
Cardiomyopathy and myocarditis	Female	0-6 days	95+ years	Data Rich	1	1	Log-transformed SEV scalar: CMP	223
Cardiomyopathy and myocarditis	Female	0-6 days	95+ years	Data Rich	1	1	Systolic Blood Pressure (mmHg)	449
Cardiomyopathy and myocarditis	Female	0-6 days	95+ years	Data Rich	-1	2	Healthcare access and quality index	314
Cardiomyopathy and myocarditis	Female	0-6 days	95+ years	Data Rich	1	2	Mean BMI	422
Cardiomyopathy and myocarditis	Female	0-6 days	95+ years	Data Rich	0	3	Socio-demographic Index	438
Cardiomyopathy and myocarditis	Female	0-6 days	95+ years	Data Rich	0	3	Alcohol (liters per capita)	467
Cardiomyopathy and myocarditis	Female	0-6 days	95+ years	Data Rich	0	3	LDI (I$ per capita)	--
Cardiomyopathy and myocarditis	Female	0-6 days	95+ years	Global	0	1	Log-transformed SEV scalar: CMP	644
Cardiomyopathy and myocarditis	Female	0-6 days	95+ years	Global	0	1	Smoking Prevalence	755
Cardiomyopathy and myocarditis	Female	0-6 days	95+ years	Global	0	1	Systolic Blood Pressure (mmHg)	783
Cardiomyopathy and myocarditis	Female	0-6 days	95+ years	Global	-1	2	Healthcare access and quality index	49
Cardiomyopathy and myocarditis	Female	0-6 days	95+ years	Global	1	2	Mean BMI	554
Cardiomyopathy and myocarditis	Female	0-6 days	95+ years	Global	0	3	Socio-demographic Index	78
Cardiomyopathy and myocarditis	Female	0-6 days	95+ years	Global	0	3	Alcohol (liters per capita)	253
Cardiomyopathy and myocarditis	Female	0-6 days	95+ years	Global	0	3	LDI (I$ per capita)	--
Cardiomyopathy and myocarditis	Male	0-6 days	95+ years	Data Rich	1	1	Smoking Prevalence	208
Cardiomyopathy and myocarditis	Male	0-6 days	95+ years	Data Rich	1	1	Systolic Blood Pressure (mmHg)	298
Cardiomyopathy and myocarditis	Male	0-6 days	95+ years	Data Rich	1	1	Log-transformed SEV scalar: CMP	--
Cardiomyopathy and myocarditis	Male	0-6 days	95+ years	Data Rich	-1	2	Healthcare access and quality index	--
Cardiomyopathy and myocarditis	Male	0-6 days	95+ years	Data Rich	1	2	Mean BMI	600
Cardiomyopathy and myocarditis	Male	0-6 days	95+ years	Data Rich	0	3	Socio-demographic Index	527
Cardiomyopathy and myocarditis	Male	0-6 days	95+ years	Data Rich	0	3	Alcohol (liters per capita)	705

Appendix Table 9: CODEm covariates used, level of covariate, and expected direction of covariate by cause, sex, and age

Covariates that CODEm did not select during the covariate selection process have no draw counts listed.

Cause	Sex	Age Start	Age End	Model Type	Direction	Level	Covariate Name	Number of Draws
Cardiomyopathy and myocarditis	Male	0-6 days	95+ years	Data Rich	0	3	LDI (I$ per capita)	--
Cardiomyopathy and myocarditis	Male	0-6 days	95+ years	Global	1	1	Smoking Prevalence	187
Cardiomyopathy and myocarditis	Male	0-6 days	95+ years	Global	1	1	Systolic Blood Pressure (mmHg)	424
Cardiomyopathy and myocarditis	Male	0-6 days	95+ years	Global	1	1	Log-transformed SEV scalar: CMP	--
Cardiomyopathy and myocarditis	Male	0-6 days	95+ years	Global	-1	2	Healthcare access and quality index	273
Cardiomyopathy and myocarditis	Male	0-6 days	95+ years	Global	1	2	Mean BMI	265
Cardiomyopathy and myocarditis	Male	0-6 days	95+ years	Global	0	3	Alcohol (liters per capita)	487
Cardiomyopathy and myocarditis	Male	0-6 days	95+ years	Global	0	3	Socio-demographic Index	636
Cardiomyopathy and myocarditis	Male	0-6 days	95+ years	Global	0	3	LDI (I$ per capita)	--
Myocarditis	Female	0-6 days	95+ years	Data Rich	1	1	Log-transformed SEV scalar: CMP	--
Myocarditis	Female	0-6 days	95+ years	Data Rich	1	1	Systolic Blood Pressure (mmHg)	--
Myocarditis	Female	0-6 days	95+ years	Data Rich	-1	2	Healthcare access and quality index	489
Myocarditis	Female	0-6 days	95+ years	Data Rich	-1	3	LDI (I$ per capita)	--
Myocarditis	Female	0-6 days	95+ years	Data Rich	0	3	Socio-demographic Index	778
Myocarditis	Female	0-6 days	95+ years	Global	1	1	Log-transformed SEV scalar: CMP	--
Myocarditis	Female	0-6 days	95+ years	Global	1	1	Systolic Blood Pressure (mmHg)	--
Myocarditis	Female	0-6 days	95+ years	Global	-1	2	Healthcare access and quality index	424
Myocarditis	Female	0-6 days	95+ years	Global	-1	3	LDI (I$ per capita)	--
Myocarditis	Female	0-6 days	95+ years	Global	0	3	Socio-demographic Index	674
Myocarditis	Male	0-6 days	95+ years	Data Rich	1	1	Log-transformed SEV scalar: CMP	--
Myocarditis	Male	0-6 days	95+ years	Data Rich	1	1	Systolic Blood Pressure (mmHg)	--
Myocarditis	Male	0-6 days	95+ years	Data Rich	-1	2	Healthcare access and quality index	--
Myocarditis	Male	0-6 days	95+ years	Data Rich	-1	3	LDI (I$ per capita)	--
Myocarditis	Male	0-6 days	95+ years	Data Rich	0	3	Socio-demographic Index	1000
Myocarditis	Male	0-6 days	95+ years	Global	1	1	Log-transformed SEV scalar: CMP	--
Myocarditis	Male	0-6 days	95+ years	Global	1	1	Systolic Blood Pressure (mmHg)	--
Myocarditis	Male	0-6 days	95+ years	Global	-1	2	Healthcare access and quality index	489
Myocarditis	Male	0-6 days	95+ years	Global	-1	3	LDI (I$ per capita)	--
Myocarditis	Male	0-6 days	95+ years	Global	0	3	Socio-demographic Index	778
Alcoholic cardiomyopathy	Female	15-19 years	95+ years	Data Rich	1	1	Smoking Prevalence	182
Alcoholic cardiomyopathy	Female	15-19 years	95+ years	Data Rich	1	1	Alcohol (liters per capita)	899
Alcoholic cardiomyopathy	Female	15-19 years	95+ years	Data Rich	1	1	Log-transformed SEV scalar: CMP	927
Alcoholic cardiomyopathy	Female	15-19 years	95+ years	Data Rich	-1	2	Healthcare access and quality index	193
Alcoholic cardiomyopathy	Female	15-19 years	95+ years	Data Rich	-1	3	LDI (I$ per capita)	--
Alcoholic cardiomyopathy	Female	15-19 years	95+ years	Data Rich	0	3	Socio-demographic Index	168
Alcoholic cardiomyopathy	Female	15-19 years	95+ years	Global	1	1	Smoking Prevalence	453
Alcoholic cardiomyopathy	Female	15-19 years	95+ years	Global	1	1	Alcohol (liters per capita)	605
Alcoholic cardiomyopathy	Female	15-19 years	95+ years	Global	1	1	Log-transformed SEV scalar: CMP	997
Alcoholic cardiomyopathy	Female	15-19 years	95+ years	Global	-1	2	Healthcare access and quality index	169
Alcoholic cardiomyopathy	Female	15-19 years	95+ years	Global	-1	3	LDI (I$ per capita)	--
Alcoholic cardiomyopathy	Female	15-19 years	95+ years	Global	0	3	Socio-demographic Index	204
Alcoholic cardiomyopathy	Male	15-19 years	95+ years	Data Rich	1	1	Smoking Prevalence	84
Alcoholic cardiomyopathy	Male	15-19 years	95+ years	Data Rich	1	1	Alcohol (liters per capita)	648
Alcoholic cardiomyopathy	Male	15-19 years	95+ years	Data Rich	1	1	Log-transformed SEV scalar: CMP	750
Alcoholic cardiomyopathy	Male	15-19 years	95+ years	Data Rich	-1	2	Healthcare access and quality index	--
Alcoholic cardiomyopathy	Male	15-19 years	95+ years	Data Rich	-1	3	LDI (I$ per capita)	--
Alcoholic cardiomyopathy	Male	15-19 years	95+ years	Data Rich	0	3	Socio-demographic Index	714
Alcoholic cardiomyopathy	Male	15-19 years	95+ years	Global	1	1	Smoking Prevalence	186
Alcoholic cardiomyopathy	Male	15-19 years	95+ years	Global	1	1	Alcohol (liters per capita)	378
Alcoholic cardiomyopathy	Male	15-19 years	95+ years	Global	1	1	Log-transformed SEV scalar: CMP	931
Alcoholic cardiomyopathy	Male	15-19 years	95+ years	Global	-1	2	Healthcare access and quality index	50
Alcoholic cardiomyopathy	Male	15-19 years	95+ years	Global	-1	3	LDI (I$ per capita)	--
Alcoholic cardiomyopathy	Male	15-19 years	95+ years	Global	0	3	Socio-demographic Index	623
Other cardiomyopathy	Female	0-6 days	95+ years	Data Rich	1	1	Smoking Prevalence	316
Other cardiomyopathy	Female	0-6 days	95+ years	Data Rich	1	1	Systolic Blood Pressure (mmHg)	334
Other cardiomyopathy	Female	0-6 days	95+ years	Data Rich	1	1	Log-transformed SEV scalar: CMP	--
Other cardiomyopathy	Female	0-6 days	95+ years	Data Rich	-1	2	Healthcare access and quality index	--
Other cardiomyopathy	Female	0-6 days	95+ years	Data Rich	1	2	Mean BMI	426
Other cardiomyopathy	Female	0-6 days	95+ years	Data Rich	0	3	Socio-demographic Index	634
Other cardiomyopathy	Female	0-6 days	95+ years	Data Rich	0	3	LDI (I$ per capita)	--
Other cardiomyopathy	Female	0-6 days	95+ years	Global	1	1	Smoking Prevalence	718
Other cardiomyopathy	Female	0-6 days	95+ years	Global	1	1	Systolic Blood Pressure (mmHg)	718
Other cardiomyopathy	Female	0-6 days	95+ years	Global	1	1	Log-transformed SEV scalar: CMP	--
Other cardiomyopathy	Female	0-6 days	95+ years	Global	-1	2	Healthcare access and quality index	--
Other cardiomyopathy	Female	0-6 days	95+ years	Global	1	2	Mean BMI	364
Other cardiomyopathy	Female	0-6 days	95+ years	Global	0	3	Socio-demographic Index	585
Other cardiomyopathy	Female	0-6 days	95+ years	Global	0	3	LDI (I$ per capita)	--
Other cardiomyopathy	Male	0-6 days	95+ years	Data Rich	1	1	Systolic Blood Pressure (mmHg)	364
Other cardiomyopathy	Male	0-6 days	95+ years	Data Rich	1	1	Log-transformed SEV scalar: CMP	--
Other cardiomyopathy	Male	0-6 days	95+ years	Data Rich	1	1	Smoking Prevalence	--
Other cardiomyopathy	Male	0-6 days	95+ years	Data Rich	-1	2	Healthcare access and quality index	--
Other cardiomyopathy	Male	0-6 days	95+ years	Data Rich	1	2	Mean BMI	692
Other cardiomyopathy	Male	0-6 days	95+ years	Data Rich	0	3	Socio-demographic Index	463
Other cardiomyopathy	Male	0-6 days	95+ years	Data Rich	0	3	LDI (I$ per capita)	--
Other cardiomyopathy	Male	0-6 days	95+ years	Global	1	1	Systolic Blood Pressure (mmHg)	364
Other cardiomyopathy	Male	0-6 days	95+ years	Global	1	1	Log-transformed SEV scalar: CMP	--
Other cardiomyopathy	Male	0-6 days	95+ years	Global	1	1	Smoking Prevalence	--
Other cardiomyopathy	Male	0-6 days	95+ years	Global	-1	2	Healthcare access and quality index	--

Appendix Table 9: CODEm covariates used, level of covariate, and expected direction of covariate by cause, sex, and age
Covariates that CODEm did not select during the covariate selection process have no draw counts listed.

Cause	Sex	Age Start	Age End	Model Type	Direction	Level	Covariate Name	Number of Draws
Other cardiomyopathy	Male	0-6 days	95+ years	Global	1	2	Mean BMI	523
Other cardiomyopathy	Male	0-6 days	95+ years	Global	0	3	Socio-demographic Index	607
Other cardiomyopathy	Male	0-6 days	95+ years	Global	0	3	LDI (I$ per capita)	--
Atrial fibrillation and flutter	Female	30-34 years	95+ years	Data Rich	1	1	Smoking Prevalence	633
Atrial fibrillation and flutter	Female	30-34 years	95+ years	Data Rich	1	1	Log-transformed SEV scalar: A Fib	762
Atrial fibrillation and flutter	Female	30-34 years	95+ years	Data Rich	1	1	Systolic Blood Pressure (mmHg)	--
Atrial fibrillation and flutter	Female	30-34 years	95+ years	Data Rich	-1	2	Healthcare access and quality index	--
Atrial fibrillation and flutter	Female	30-34 years	95+ years	Data Rich	1	2	Mean BMI	334
Atrial fibrillation and flutter	Female	30-34 years	95+ years	Data Rich	1	2	Diabetes Fasting Plasma Glucose (mmol/L)	361
Atrial fibrillation and flutter	Female	30-34 years	95+ years	Data Rich	1	2	Cholesterol (total, mean per capita)	--
Atrial fibrillation and flutter	Female	30-34 years	95+ years	Data Rich	-1	3	vegetables adjusted(g)	7
Atrial fibrillation and flutter	Female	30-34 years	95+ years	Data Rich	-1	3	nuts seeds adjusted(g)	46
Atrial fibrillation and flutter	Female	30-34 years	95+ years	Data Rich	-1	3	pulses legumes adjusted(g)	194
Atrial fibrillation and flutter	Female	30-34 years	95+ years	Data Rich	-1	3	LDI (I$ per capita)	--
Atrial fibrillation and flutter	Female	30-34 years	95+ years	Data Rich	-1	3	fruits adjusted(g)	--
Atrial fibrillation and flutter	Female	30-34 years	95+ years	Data Rich	-1	3	omega 3 adjusted(g)	--
Atrial fibrillation and flutter	Female	30-34 years	95+ years	Data Rich	-1	3	pufa adjusted(percent)	--
Atrial fibrillation and flutter	Female	30-34 years	95+ years	Data Rich	0	3	Alcohol (liters per capita)	279
Atrial fibrillation and flutter	Female	30-34 years	95+ years	Data Rich	0	3	Socio-demographic Index	410
Atrial fibrillation and flutter	Female	30-34 years	95+ years	Data Rich	1	3	Diet high in trans fatty acids	96
Atrial fibrillation and flutter	Female	30-34 years	95+ years	Global	1	1	Smoking Prevalence	485
Atrial fibrillation and flutter	Female	30-34 years	95+ years	Global	1	1	Log-transformed SEV scalar: A Fib	583
Atrial fibrillation and flutter	Female	30-34 years	95+ years	Global	1	1	Systolic Blood Pressure (mmHg)	--
Atrial fibrillation and flutter	Female	30-34 years	95+ years	Global	-1	2	Healthcare access and quality index	378
Atrial fibrillation and flutter	Female	30-34 years	95+ years	Global	1	2	Mean BMI	250
Atrial fibrillation and flutter	Female	30-34 years	95+ years	Global	1	2	Diabetes Fasting Plasma Glucose (mmol/L)	378
Atrial fibrillation and flutter	Female	30-34 years	95+ years	Global	1	2	Cholesterol (total, mean per capita)	--
Atrial fibrillation and flutter	Female	30-34 years	95+ years	Global	-1	3	vegetables adjusted(g)	100
Atrial fibrillation and flutter	Female	30-34 years	95+ years	Global	-1	3	pulses legumes adjusted(g)	189
Atrial fibrillation and flutter	Female	30-34 years	95+ years	Global	-1	3	LDI (I$ per capita)	--
Atrial fibrillation and flutter	Female	30-34 years	95+ years	Global	-1	3	fruits adjusted(g)	--
Atrial fibrillation and flutter	Female	30-34 years	95+ years	Global	-1	3	nuts seeds adjusted(g)	--
Atrial fibrillation and flutter	Female	30-34 years	95+ years	Global	-1	3	omega 3 adjusted(g)	--
Atrial fibrillation and flutter	Female	30-34 years	95+ years	Global	-1	3	pufa adjusted(percent)	--
Atrial fibrillation and flutter	Female	30-34 years	95+ years	Global	0	3	Alcohol (liters per capita)	316
Atrial fibrillation and flutter	Female	30-34 years	95+ years	Global	0	3	Socio-demographic Index	487
Atrial fibrillation and flutter	Female	30-34 years	95+ years	Global	1	3	Diet high in trans fatty acids	67
Atrial fibrillation and flutter	Male	30-34 years	95+ years	Data Rich	1	1	Log-transformed SEV scalar: A Fib	--
Atrial fibrillation and flutter	Male	30-34 years	95+ years	Data Rich	1	1	Smoking Prevalence	--
Atrial fibrillation and flutter	Male	30-34 years	95+ years	Data Rich	1	1	Systolic Blood Pressure (mmHg)	--
Atrial fibrillation and flutter	Male	30-34 years	95+ years	Data Rich	-1	2	Healthcare access and quality index	--
Atrial fibrillation and flutter	Male	30-34 years	95+ years	Data Rich	1	2	Diabetes Fasting Plasma Glucose (mmol/L)	244
Atrial fibrillation and flutter	Male	30-34 years	95+ years	Data Rich	1	2	Mean BMI	428
Atrial fibrillation and flutter	Male	30-34 years	95+ years	Data Rich	1	2	Cholesterol (total, mean per capita)	--
Atrial fibrillation and flutter	Male	30-34 years	95+ years	Data Rich	-1	3	pulses legumes adjusted(g)	192
Atrial fibrillation and flutter	Male	30-34 years	95+ years	Data Rich	-1	3	LDI (I$ per capita)	--
Atrial fibrillation and flutter	Male	30-34 years	95+ years	Data Rich	-1	3	fruits adjusted(g)	--
Atrial fibrillation and flutter	Male	30-34 years	95+ years	Data Rich	-1	3	nuts seeds adjusted(g)	--
Atrial fibrillation and flutter	Male	30-34 years	95+ years	Data Rich	-1	3	omega 3 adjusted(g)	--
Atrial fibrillation and flutter	Male	30-34 years	95+ years	Data Rich	-1	3	pufa adjusted(percent)	--
Atrial fibrillation and flutter	Male	30-34 years	95+ years	Data Rich	-1	3	vegetables adjusted(g)	--
Atrial fibrillation and flutter	Male	30-34 years	95+ years	Data Rich	0	3	Socio-demographic Index	160
Atrial fibrillation and flutter	Male	30-34 years	95+ years	Data Rich	0	3	Alcohol (liters per capita)	680
Atrial fibrillation and flutter	Male	30-34 years	95+ years	Data Rich	1	3	Diet high in trans fatty acids	444
Atrial fibrillation and flutter	Male	30-34 years	95+ years	Global	1	1	Log-transformed SEV scalar: A Fib	--
Atrial fibrillation and flutter	Male	30-34 years	95+ years	Global	1	1	Smoking Prevalence	--
Atrial fibrillation and flutter	Male	30-34 years	95+ years	Global	1	1	Systolic Blood Pressure (mmHg)	--
Atrial fibrillation and flutter	Male	30-34 years	95+ years	Global	-1	2	Healthcare access and quality index	--
Atrial fibrillation and flutter	Male	30-34 years	95+ years	Global	1	2	Diabetes Fasting Plasma Glucose (mmol/L)	428
Atrial fibrillation and flutter	Male	30-34 years	95+ years	Global	1	2	Mean BMI	757
Atrial fibrillation and flutter	Male	30-34 years	95+ years	Global	1	2	Cholesterol (total, mean per capita)	--
Atrial fibrillation and flutter	Male	30-34 years	95+ years	Global	-1	3	pulses legumes adjusted(g)	36
Atrial fibrillation and flutter	Male	30-34 years	95+ years	Global	-1	3	LDI (I$ per capita)	--
Atrial fibrillation and flutter	Male	30-34 years	95+ years	Global	-1	3	fruits adjusted(g)	--
Atrial fibrillation and flutter	Male	30-34 years	95+ years	Global	-1	3	nuts seeds adjusted(g)	--
Atrial fibrillation and flutter	Male	30-34 years	95+ years	Global	-1	3	omega 3 adjusted(g)	--
Atrial fibrillation and flutter	Male	30-34 years	95+ years	Global	-1	3	pufa adjusted(percent)	--
Atrial fibrillation and flutter	Male	30-34 years	95+ years	Global	-1	3	vegetables adjusted(g)	--
Atrial fibrillation and flutter	Male	30-34 years	95+ years	Global	0	3	Socio-demographic Index	177
Atrial fibrillation and flutter	Male	30-34 years	95+ years	Global	0	3	Alcohol (liters per capita)	691
Atrial fibrillation and flutter	Male	30-34 years	95+ years	Global	1	3	Diet high in trans fatty acids	36
Aortic aneurysm	Female	15-19 years	95+ years	Data Rich	1	1	Systolic Blood Pressure (mmHg)	65
Aortic aneurysm	Female	15-19 years	95+ years	Data Rich	1	1	Cumulative Cigarettes (10 Years)	272
Aortic aneurysm	Female	15-19 years	95+ years	Data Rich	1	1	Cholesterol (total, mean per capita)	602
Aortic aneurysm	Female	15-19 years	95+ years	Data Rich	1	1	Log-transformed SEV scalar: Aort An	664
Aortic aneurysm	Female	15-19 years	95+ years	Data Rich	-1	2	Healthcare access and quality index	--
Aortic aneurysm	Female	15-19 years	95+ years	Data Rich	1	2	Mean BMI	187
Aortic aneurysm	Female	15-19 years	95+ years	Data Rich	-1	3	nuts seeds adjusted(g)	1

Appendix Table 9: CODEm covariates used, level of covariate, and expected direction of covariate by cause, sex, and age<br>*Covariates that CODEm did not select during the covariate selection process have no draw counts listed.*								
**Cause**	**Sex**	**Age Start**	**Age End**	**Model Type**	**Direction**	**Level**	**Covariate Name**	**Number of Draws**
Aortic aneurysm	Female	15-19 years	95+ years	Data Rich	-1	3	vegetables adjusted(g)	1
Aortic aneurysm	Female	15-19 years	95+ years	Data Rich	-1	3	pulses legumes adjusted(g)	66
Aortic aneurysm	Female	15-19 years	95+ years	Data Rich	-1	3	LDI (I$ per capita)	--
Aortic aneurysm	Female	15-19 years	95+ years	Data Rich	-1	3	fruits adjusted(g)	--
Aortic aneurysm	Female	15-19 years	95+ years	Data Rich	-1	3	omega 3 adjusted(g)	--
Aortic aneurysm	Female	15-19 years	95+ years	Data Rich	-1	3	pufa adjusted(percent)	--
Aortic aneurysm	Female	15-19 years	95+ years	Data Rich	0	3	Socio-demographic Index	685
Aortic aneurysm	Female	15-19 years	95+ years	Data Rich	0	3	Alcohol (liters per capita)	871
Aortic aneurysm	Female	15-19 years	95+ years	Global	1	1	Systolic Blood Pressure (mmHg)	113
Aortic aneurysm	Female	15-19 years	95+ years	Global	1	1	Cumulative Cigarettes (10 Years)	198
Aortic aneurysm	Female	15-19 years	95+ years	Global	1	1	Log-transformed SEV scalar: Aort An	213
Aortic aneurysm	Female	15-19 years	95+ years	Global	1	1	Cholesterol (total, mean per capita)	795
Aortic aneurysm	Female	15-19 years	95+ years	Global	-1	2	Healthcare access and quality index	39
Aortic aneurysm	Female	15-19 years	95+ years	Global	1	2	Mean BMI	253
Aortic aneurysm	Female	15-19 years	95+ years	Global	-1	3	nuts seeds adjusted(g)	33
Aortic aneurysm	Female	15-19 years	95+ years	Global	-1	3	pulses legumes adjusted(g)	96
Aortic aneurysm	Female	15-19 years	95+ years	Global	-1	3	vegetables adjusted(g)	337
Aortic aneurysm	Female	15-19 years	95+ years	Global	-1	3	LDI (I$ per capita)	--
Aortic aneurysm	Female	15-19 years	95+ years	Global	-1	3	fruits adjusted(g)	--
Aortic aneurysm	Female	15-19 years	95+ years	Global	-1	3	omega 3 adjusted(g)	--
Aortic aneurysm	Female	15-19 years	95+ years	Global	-1	3	pufa adjusted(percent)	--
Aortic aneurysm	Female	15-19 years	95+ years	Global	0	3	Socio-demographic Index	558
Aortic aneurysm	Female	15-19 years	95+ years	Global	0	3	Alcohol (liters per capita)	795
Aortic aneurysm	Male	15-19 years	95+ years	Data Rich	1	1	Systolic Blood Pressure (mmHg)	112
Aortic aneurysm	Male	15-19 years	95+ years	Data Rich	1	1	Cumulative Cigarettes (10 Years)	121
Aortic aneurysm	Male	15-19 years	95+ years	Data Rich	1	1	Log-transformed SEV scalar: Aort An	155
Aortic aneurysm	Male	15-19 years	95+ years	Data Rich	1	1	Cholesterol (total, mean per capita)	1000
Aortic aneurysm	Male	15-19 years	95+ years	Data Rich	-1	2	Healthcare access and quality index	122
Aortic aneurysm	Male	15-19 years	95+ years	Data Rich	1	2	Mean BMI	253
Aortic aneurysm	Male	15-19 years	95+ years	Data Rich	-1	3	nuts seeds adjusted(g)	76
Aortic aneurysm	Male	15-19 years	95+ years	Data Rich	-1	3	vegetables adjusted(g)	89
Aortic aneurysm	Male	15-19 years	95+ years	Data Rich	-1	3	LDI (I$ per capita)	--
Aortic aneurysm	Male	15-19 years	95+ years	Data Rich	-1	3	fruits adjusted(g)	--
Aortic aneurysm	Male	15-19 years	95+ years	Data Rich	-1	3	omega 3 adjusted(g)	--
Aortic aneurysm	Male	15-19 years	95+ years	Data Rich	-1	3	pufa adjusted(percent)	--
Aortic aneurysm	Male	15-19 years	95+ years	Data Rich	-1	3	pulses legumes adjusted(g)	--
Aortic aneurysm	Male	15-19 years	95+ years	Data Rich	0	3	Socio-demographic Index	504
Aortic aneurysm	Male	15-19 years	95+ years	Data Rich	0	3	Alcohol (liters per capita)	586
Aortic aneurysm	Male	15-19 years	95+ years	Global	1	1	Cumulative Cigarettes (10 Years)	8
Aortic aneurysm	Male	15-19 years	95+ years	Global	1	1	Systolic Blood Pressure (mmHg)	158
Aortic aneurysm	Male	15-19 years	95+ years	Global	1	1	Log-transformed SEV scalar: Aort An	197
Aortic aneurysm	Male	15-19 years	95+ years	Global	1	1	Cholesterol (total, mean per capita)	978
Aortic aneurysm	Male	15-19 years	95+ years	Global	-1	2	Healthcare access and quality index	99
Aortic aneurysm	Male	15-19 years	95+ years	Global	1	2	Mean BMI	374
Aortic aneurysm	Male	15-19 years	95+ years	Global	-1	3	nuts seeds adjusted(g)	127
Aortic aneurysm	Male	15-19 years	95+ years	Global	-1	3	vegetables adjusted(g)	248
Aortic aneurysm	Male	15-19 years	95+ years	Global	-1	3	LDI (I$ per capita)	--
Aortic aneurysm	Male	15-19 years	95+ years	Global	-1	3	fruits adjusted(g)	--
Aortic aneurysm	Male	15-19 years	95+ years	Global	-1	3	omega 3 adjusted(g)	--
Aortic aneurysm	Male	15-19 years	95+ years	Global	-1	3	pufa adjusted(percent)	--
Aortic aneurysm	Male	15-19 years	95+ years	Global	-1	3	pulses legumes adjusted(g)	--
Aortic aneurysm	Male	15-19 years	95+ years	Global	0	3	Socio-demographic Index	517
Aortic aneurysm	Male	15-19 years	95+ years	Global	0	3	Alcohol (liters per capita)	718
Peripheral vascular disease	Female	40-44 years	95+ years	Data Rich	1	1	Cholesterol (total, mean per capita)	--
Peripheral vascular disease	Female	40-44 years	95+ years	Data Rich	1	1	Log-transformed SEV scalar: PVD	--
Peripheral vascular disease	Female	40-44 years	95+ years	Data Rich	1	1	Smoking Prevalence	--
Peripheral vascular disease	Female	40-44 years	95+ years	Data Rich	1	1	Systolic Blood Pressure (mmHg)	--
Peripheral vascular disease	Female	40-44 years	95+ years	Data Rich	-1	2	Healthcare access and quality index	--
Peripheral vascular disease	Female	40-44 years	95+ years	Data Rich	1	2	Diabetes Fasting Plasma Glucose (mmol/L)	149
Peripheral vascular disease	Female	40-44 years	95+ years	Data Rich	1	2	Mean BMI	578
Peripheral vascular disease	Female	40-44 years	95+ years	Data Rich	-1	3	pulses legumes adjusted(g)	16
Peripheral vascular disease	Female	40-44 years	95+ years	Data Rich	-1	3	LDI (I$ per capita)	--
Peripheral vascular disease	Female	40-44 years	95+ years	Data Rich	-1	3	fruits adjusted(g)	--
Peripheral vascular disease	Female	40-44 years	95+ years	Data Rich	-1	3	nuts seeds adjusted(g)	--
Peripheral vascular disease	Female	40-44 years	95+ years	Data Rich	-1	3	omega 3 adjusted(g)	--
Peripheral vascular disease	Female	40-44 years	95+ years	Data Rich	-1	3	pufa adjusted(percent)	--
Peripheral vascular disease	Female	40-44 years	95+ years	Data Rich	-1	3	vegetables adjusted(g)	--
Peripheral vascular disease	Female	40-44 years	95+ years	Data Rich	0	3	Alcohol (liters per capita)	527
Peripheral vascular disease	Female	40-44 years	95+ years	Data Rich	0	3	Socio-demographic Index	666
Peripheral vascular disease	Female	40-44 years	95+ years	Global	1	1	Cholesterol (total, mean per capita)	--
Peripheral vascular disease	Female	40-44 years	95+ years	Global	1	1	Log-transformed SEV scalar: PVD	--
Peripheral vascular disease	Female	40-44 years	95+ years	Global	1	1	Smoking Prevalence	--
Peripheral vascular disease	Female	40-44 years	95+ years	Global	1	1	Systolic Blood Pressure (mmHg)	--
Peripheral vascular disease	Female	40-44 years	95+ years	Global	-1	2	Healthcare access and quality index	--
Peripheral vascular disease	Female	40-44 years	95+ years	Global	1	2	Diabetes Fasting Plasma Glucose (mmol/L)	142
Peripheral vascular disease	Female	40-44 years	95+ years	Global	1	2	Mean BMI	591
Peripheral vascular disease	Female	40-44 years	95+ years	Global	-1	3	pulses legumes adjusted(g)	12
Peripheral vascular disease	Female	40-44 years	95+ years	Global	-1	3	LDI (I$ per capita)	--

Appendix Table 9: CODEm covariates used, level of covariate, and expected direction of covariate by cause, sex, and age

Covariates that CODEm did not select during the covariate selection process have no draw counts listed.

Cause	Sex	Age Start	Age End	Model Type	Direction	Level	Covariate Name	Number of Draws
Peripheral vascular disease	Female	40-44 years	95+ years	Global	-1	3	fruits adjusted(g)	--
Peripheral vascular disease	Female	40-44 years	95+ years	Global	-1	3	nuts seeds adjusted(g)	--
Peripheral vascular disease	Female	40-44 years	95+ years	Global	-1	3	omega 3 adjusted(g)	--
Peripheral vascular disease	Female	40-44 years	95+ years	Global	-1	3	pufa adjusted(percent)	--
Peripheral vascular disease	Female	40-44 years	95+ years	Global	-1	3	vegetables adjusted(g)	--
Peripheral vascular disease	Female	40-44 years	95+ years	Global	0	3	Socio-demographic Index	664
Peripheral vascular disease	Female	40-44 years	95+ years	Global	0	3	Alcohol (liters per capita)	754
Peripheral vascular disease	Male	40-44 years	95+ years	Data Rich	1	1	Cholesterol (total, mean per capita)	--
Peripheral vascular disease	Male	40-44 years	95+ years	Data Rich	1	1	Log-transformed SEV scalar: PVD	--
Peripheral vascular disease	Male	40-44 years	95+ years	Data Rich	1	1	Smoking Prevalence	--
Peripheral vascular disease	Male	40-44 years	95+ years	Data Rich	1	1	Systolic Blood Pressure (mmHg)	--
Peripheral vascular disease	Male	40-44 years	95+ years	Data Rich	-1	2	Healthcare access and quality index	--
Peripheral vascular disease	Male	40-44 years	95+ years	Data Rich	1	2	Mean BMI	738
Peripheral vascular disease	Male	40-44 years	95+ years	Data Rich	1	2	Diabetes Fasting Plasma Glucose (mmol/L)	--
Peripheral vascular disease	Male	40-44 years	95+ years	Data Rich	-1	3	pulses legumes adjusted(g)	34
Peripheral vascular disease	Male	40-44 years	95+ years	Data Rich	-1	3	LDI (I$ per capita)	--
Peripheral vascular disease	Male	40-44 years	95+ years	Data Rich	-1	3	fruits adjusted(g)	--
Peripheral vascular disease	Male	40-44 years	95+ years	Data Rich	-1	3	nuts seeds adjusted(g)	--
Peripheral vascular disease	Male	40-44 years	95+ years	Data Rich	-1	3	omega 3 adjusted(g)	--
Peripheral vascular disease	Male	40-44 years	95+ years	Data Rich	-1	3	pufa adjusted(percent)	--
Peripheral vascular disease	Male	40-44 years	95+ years	Data Rich	-1	3	vegetables adjusted(g)	--
Peripheral vascular disease	Male	40-44 years	95+ years	Data Rich	0	3	Alcohol (liters per capita)	610
Peripheral vascular disease	Male	40-44 years	95+ years	Data Rich	0	3	Socio-demographic Index	648
Peripheral vascular disease	Male	40-44 years	95+ years	Global	1	1	Cholesterol (total, mean per capita)	--
Peripheral vascular disease	Male	40-44 years	95+ years	Global	1	1	Log-transformed SEV scalar: PVD	--
Peripheral vascular disease	Male	40-44 years	95+ years	Global	1	1	Smoking Prevalence	--
Peripheral vascular disease	Male	40-44 years	95+ years	Global	1	1	Systolic Blood Pressure (mmHg)	--
Peripheral vascular disease	Male	40-44 years	95+ years	Global	-1	2	Healthcare access and quality index	--
Peripheral vascular disease	Male	40-44 years	95+ years	Global	1	2	Mean BMI	511
Peripheral vascular disease	Male	40-44 years	95+ years	Global	1	2	Diabetes Fasting Plasma Glucose (mmol/L)	--
Peripheral vascular disease	Male	40-44 years	95+ years	Global	-1	3	LDI (I$ per capita)	--
Peripheral vascular disease	Male	40-44 years	95+ years	Global	-1	3	fruits adjusted(g)	--
Peripheral vascular disease	Male	40-44 years	95+ years	Global	-1	3	nuts seeds adjusted(g)	--
Peripheral vascular disease	Male	40-44 years	95+ years	Global	-1	3	omega 3 adjusted(g)	--
Peripheral vascular disease	Male	40-44 years	95+ years	Global	-1	3	pufa adjusted(percent)	--
Peripheral vascular disease	Male	40-44 years	95+ years	Global	-1	3	pulses legumes adjusted(g)	--
Peripheral vascular disease	Male	40-44 years	95+ years	Global	-1	3	vegetables adjusted(g)	--
Peripheral vascular disease	Male	40-44 years	95+ years	Global	0	3	Alcohol (liters per capita)	815
Peripheral vascular disease	Male	40-44 years	95+ years	Global	0	3	Socio-demographic Index	815
Endocarditis	Female	0-6 days	95+ years	Data Rich	-1	1	Improved Water Source (proportion with access)	602
Endocarditis	Female	0-6 days	95+ years	Data Rich	-1	1	Healthcare access and quality index	--
Endocarditis	Female	0-6 days	95+ years	Data Rich	-1	1	Sanitation (proportion with access)	--
Endocarditis	Female	0-6 days	95+ years	Data Rich	1	1	Log-transformed SEV scalar: Endocar	--
Endocarditis	Female	0-6 days	95+ years	Data Rich	-1	3	LDI (I$ per capita)	--
Endocarditis	Female	0-6 days	95+ years	Data Rich	0	3	Socio-demographic Index	726
Endocarditis	Female	0-6 days	95+ years	Global	0	1	Healthcare access and quality index	599
Endocarditis	Female	0-6 days	95+ years	Global	0	1	Improved Water Source (proportion with access)	648
Endocarditis	Female	0-6 days	95+ years	Global	0	1	Sanitation (proportion with access)	677
Endocarditis	Female	0-6 days	95+ years	Global	0	1	Log-transformed SEV scalar: Endocar	759
Endocarditis	Female	0-6 days	95+ years	Global	0	3	Socio-demographic Index	469
Endocarditis	Female	0-6 days	95+ years	Global	0	3	LDI (I$ per capita)	--
Endocarditis	Male	0-6 days	95+ years	Data Rich	-1	1	Healthcare access and quality index	--
Endocarditis	Male	0-6 days	95+ years	Data Rich	0	1	Sanitation (proportion with access)	689
Endocarditis	Male	0-6 days	95+ years	Data Rich	0	1	Improved Water Source (proportion with access)	763
Endocarditis	Male	0-6 days	95+ years	Data Rich	0	1	Log-transformed SEV scalar: Endocar	800
Endocarditis	Male	0-6 days	95+ years	Data Rich	-1	3	LDI (I$ per capita)	--
Endocarditis	Male	0-6 days	95+ years	Data Rich	0	3	Socio-demographic Index	700
Endocarditis	Male	0-6 days	95+ years	Global	0	1	Healthcare access and quality index	417
Endocarditis	Male	0-6 days	95+ years	Global	0	1	Improved Water Source (proportion with access)	545
Endocarditis	Male	0-6 days	95+ years	Global	0	1	Sanitation (proportion with access)	639
Endocarditis	Male	0-6 days	95+ years	Global	0	1	Log-transformed SEV scalar: Endocar	675
Endocarditis	Male	0-6 days	95+ years	Global	0	3	Socio-demographic Index	659
Endocarditis	Male	0-6 days	95+ years	Global	0	3	LDI (I$ per capita)	--
Other cardiovascular and circulatory diseases	Female	0-6 days	95+ years	Data Rich	1	1	Log-transformed SEV scalar: Oth Cardio	17
Other cardiovascular and circulatory diseases	Female	0-6 days	95+ years	Data Rich	1	1	Cholesterol (total, mean per capita)	177
Other cardiovascular and circulatory diseases	Female	0-6 days	95+ years	Data Rich	1	1	Systolic Blood Pressure (mmHg)	337
Other cardiovascular and circulatory diseases	Female	0-6 days	95+ years	Data Rich	1	1	Smoking Prevalence	359
Other cardiovascular and circulatory diseases	Female	0-6 days	95+ years	Data Rich	-1	2	Healthcare access and quality index	381
Other cardiovascular and circulatory diseases	Female	0-6 days	95+ years	Data Rich	-1	2	Elevation Over 1500m (proportion)	--
Other cardiovascular and circulatory diseases	Female	0-6 days	95+ years	Data Rich	1	2	Indoor Air Pollution (All Cooking Fuels)	126
Other cardiovascular and circulatory diseases	Female	0-6 days	95+ years	Data Rich	1	2	Diabetes Fasting Plasma Glucose (mmol/L)	301
Other cardiovascular and circulatory diseases	Female	0-6 days	95+ years	Data Rich	1	2	Outdoor Air Pollution (PM2.5)	507
Other cardiovascular and circulatory diseases	Female	0-6 days	95+ years	Data Rich	1	2	Mean BMI	--
Other cardiovascular and circulatory diseases	Female	0-6 days	95+ years	Data Rich	-1	3	nuts seeds adjusted(g)	10
Other cardiovascular and circulatory diseases	Female	0-6 days	95+ years	Data Rich	-1	3	vegetables adjusted(g)	16
Other cardiovascular and circulatory diseases	Female	0-6 days	95+ years	Data Rich	-1	3	LDI (I$ per capita)	--
Other cardiovascular and circulatory diseases	Female	0-6 days	95+ years	Data Rich	-1	3	fruits adjusted(g)	--
Other cardiovascular and circulatory diseases	Female	0-6 days	95+ years	Data Rich	-1	3	omega 3 adjusted(g)	--

**Appendix Table 9: CODEm covariates used, level of covariate, and expected direction of covariate by cause, sex, and age** *Covariates that CODEm did not select during the covariate selection process have no draw counts listed.*								
**Cause**	**Sex**	**Age Start**	**Age End**	**Model Type**	**Direction**	**Level**	**Covariate Name**	**Number of Draws**
Other cardiovascular and circulatory diseases	Female	0-6 days	95+ years	Data Rich	-1	3	pufa adjusted(percent)	--
Other cardiovascular and circulatory diseases	Female	0-6 days	95+ years	Data Rich	-1	3	pulses legumes adjusted(g)	--
Other cardiovascular and circulatory diseases	Female	0-6 days	95+ years	Data Rich	0	3	Socio-demographic Index	626
Other cardiovascular and circulatory diseases	Female	0-6 days	95+ years	Data Rich	0	3	Alcohol (liters per capita)	739
Other cardiovascular and circulatory diseases	Female	0-6 days	95+ years	Global	1	1	Log-transformed SEV scalar: Oth Cardio	27
Other cardiovascular and circulatory diseases	Female	0-6 days	95+ years	Global	1	1	Smoking Prevalence	327
Other cardiovascular and circulatory diseases	Female	0-6 days	95+ years	Global	1	1	Systolic Blood Pressure (mmHg)	649
Other cardiovascular and circulatory diseases	Female	0-6 days	95+ years	Global	1	1	Cholesterol (total, mean per capita)	--
Other cardiovascular and circulatory diseases	Female	0-6 days	95+ years	Global	-1	2	Healthcare access and quality index	359
Other cardiovascular and circulatory diseases	Female	0-6 days	95+ years	Global	-1	2	Elevation Over 1500m (proportion)	--
Other cardiovascular and circulatory diseases	Female	0-6 days	95+ years	Global	1	2	Indoor Air Pollution (All Cooking Fuels)	167
Other cardiovascular and circulatory diseases	Female	0-6 days	95+ years	Global	1	2	Diabetes Fasting Plasma Glucose (mmol/L)	--
Other cardiovascular and circulatory diseases	Female	0-6 days	95+ years	Global	1	2	Mean BMI	--
Other cardiovascular and circulatory diseases	Female	0-6 days	95+ years	Global	1	2	Outdoor Air Pollution (PM2.5)	--
Other cardiovascular and circulatory diseases	Female	0-6 days	95+ years	Global	-1	3	nuts seeds adjusted(g)	3
Other cardiovascular and circulatory diseases	Female	0-6 days	95+ years	Global	-1	3	vegetables adjusted(g)	23
Other cardiovascular and circulatory diseases	Female	0-6 days	95+ years	Global	-1	3	LDI (I$ per capita)	--
Other cardiovascular and circulatory diseases	Female	0-6 days	95+ years	Global	-1	3	fruits adjusted(g)	--
Other cardiovascular and circulatory diseases	Female	0-6 days	95+ years	Global	-1	3	omega 3 adjusted(g)	--
Other cardiovascular and circulatory diseases	Female	0-6 days	95+ years	Global	-1	3	pufa adjusted(percent)	--
Other cardiovascular and circulatory diseases	Female	0-6 days	95+ years	Global	-1	3	pulses legumes adjusted(g)	--
Other cardiovascular and circulatory diseases	Female	0-6 days	95+ years	Global	0	3	Socio-demographic Index	708
Other cardiovascular and circulatory diseases	Female	0-6 days	95+ years	Global	0	3	Alcohol (liters per capita)	719
Other cardiovascular and circulatory diseases	Male	0-6 days	95+ years	Data Rich	1	1	Log-transformed SEV scalar: Oth Cardio	28
Other cardiovascular and circulatory diseases	Male	0-6 days	95+ years	Data Rich	1	1	Smoking Prevalence	245
Other cardiovascular and circulatory diseases	Male	0-6 days	95+ years	Data Rich	1	1	Systolic Blood Pressure (mmHg)	429
Other cardiovascular and circulatory diseases	Male	0-6 days	95+ years	Data Rich	1	1	Cholesterol (total, mean per capita)	536
Other cardiovascular and circulatory diseases	Male	0-6 days	95+ years	Data Rich	-1	2	Healthcare access and quality index	176
Other cardiovascular and circulatory diseases	Male	0-6 days	95+ years	Data Rich	-1	2	Elevation Over 1500m (proportion)	--
Other cardiovascular and circulatory diseases	Male	0-6 days	95+ years	Data Rich	1	2	Indoor Air Pollution (All Cooking Fuels)	206
Other cardiovascular and circulatory diseases	Male	0-6 days	95+ years	Data Rich	1	2	Diabetes Fasting Plasma Glucose (mmol/L)	253
Other cardiovascular and circulatory diseases	Male	0-6 days	95+ years	Data Rich	1	2	Mean BMI	253
Other cardiovascular and circulatory diseases	Male	0-6 days	95+ years	Data Rich	1	2	Outdoor Air Pollution (PM2.5)	329
Other cardiovascular and circulatory diseases	Male	0-6 days	95+ years	Data Rich	-1	3	vegetables adjusted(g)	2
Other cardiovascular and circulatory diseases	Male	0-6 days	95+ years	Data Rich	-1	3	nuts seeds adjusted(g)	6
Other cardiovascular and circulatory diseases	Male	0-6 days	95+ years	Data Rich	-1	3	LDI (I$ per capita)	--
Other cardiovascular and circulatory diseases	Male	0-6 days	95+ years	Data Rich	-1	3	fruits adjusted(g)	--
Other cardiovascular and circulatory diseases	Male	0-6 days	95+ years	Data Rich	-1	3	omega 3 adjusted(g)	--
Other cardiovascular and circulatory diseases	Male	0-6 days	95+ years	Data Rich	-1	3	pufa adjusted(percent)	--
Other cardiovascular and circulatory diseases	Male	0-6 days	95+ years	Data Rich	-1	3	pulses legumes adjusted(g)	--
Other cardiovascular and circulatory diseases	Male	0-6 days	95+ years	Data Rich	0	3	Socio-demographic Index	301
Other cardiovascular and circulatory diseases	Male	0-6 days	95+ years	Data Rich	0	3	Alcohol (liters per capita)	725
Other cardiovascular and circulatory diseases	Male	0-6 days	95+ years	Global	1	1	Log-transformed SEV scalar: Oth Cardio	32
Other cardiovascular and circulatory diseases	Male	0-6 days	95+ years	Global	1	1	Smoking Prevalence	81
Other cardiovascular and circulatory diseases	Male	0-6 days	95+ years	Global	1	1	Cholesterol (total, mean per capita)	122
Other cardiovascular and circulatory diseases	Male	0-6 days	95+ years	Global	1	1	Systolic Blood Pressure (mmHg)	150
Other cardiovascular and circulatory diseases	Male	0-6 days	95+ years	Global	-1	2	Healthcare access and quality index	171
Other cardiovascular and circulatory diseases	Male	0-6 days	95+ years	Global	-1	2	Elevation Over 1500m (proportion)	--
Other cardiovascular and circulatory diseases	Male	0-6 days	95+ years	Global	1	2	Indoor Air Pollution (All Cooking Fuels)	121
Other cardiovascular and circulatory diseases	Male	0-6 days	95+ years	Global	1	2	Diabetes Fasting Plasma Glucose (mmol/L)	486
Other cardiovascular and circulatory diseases	Male	0-6 days	95+ years	Global	1	2	Mean BMI	486
Other cardiovascular and circulatory diseases	Male	0-6 days	95+ years	Global	1	2	Outdoor Air Pollution (PM2.5)	671
Other cardiovascular and circulatory diseases	Male	0-6 days	95+ years	Global	-1	3	nuts seeds adjusted(g)	0
Other cardiovascular and circulatory diseases	Male	0-6 days	95+ years	Global	-1	3	vegetables adjusted(g)	0
Other cardiovascular and circulatory diseases	Male	0-6 days	95+ years	Global	-1	3	LDI (I$ per capita)	--
Other cardiovascular and circulatory diseases	Male	0-6 days	95+ years	Global	-1	3	fruits adjusted(g)	--
Other cardiovascular and circulatory diseases	Male	0-6 days	95+ years	Global	-1	3	omega 3 adjusted(g)	--
Other cardiovascular and circulatory diseases	Male	0-6 days	95+ years	Global	-1	3	pufa adjusted(percent)	--
Other cardiovascular and circulatory diseases	Male	0-6 days	95+ years	Global	-1	3	pulses legumes adjusted(g)	--
Other cardiovascular and circulatory diseases	Male	0-6 days	95+ years	Global	0	3	Socio-demographic Index	394
Other cardiovascular and circulatory diseases	Male	0-6 days	95+ years	Global	0	3	Alcohol (liters per capita)	647
Chronic respiratory diseases	Female	1-4 years	95+ years	Data Rich	1	1	Cumulative Cigarettes (10 Years)	26
Chronic respiratory diseases	Female	1-4 years	95+ years	Data Rich	1	1	Cumulative Cigarettes (5 Years)	48
Chronic respiratory diseases	Female	1-4 years	95+ years	Data Rich	1	1	Log-transformed SEV scalar: Chr Resp	1000
Chronic respiratory diseases	Female	1-4 years	95+ years	Data Rich	1	1	Healthcare access and quality index	--
Chronic respiratory diseases	Female	1-4 years	95+ years	Data Rich	1	2	Smoking Prevalence	3
Chronic respiratory diseases	Female	1-4 years	95+ years	Data Rich	1	2	Indoor Air Pollution (All Cooking Fuels)	120
Chronic respiratory diseases	Female	1-4 years	95+ years	Data Rich	1	2	Outdoor Air Pollution (PM2.5)	242
Chronic respiratory diseases	Female	1-4 years	95+ years	Data Rich	1	2	Elevation Over 1500m (proportion)	365
Chronic respiratory diseases	Female	1-4 years	95+ years	Data Rich	-1	3	Education (years per capita)	142
Chronic respiratory diseases	Female	1-4 years	95+ years	Data Rich	-1	3	LDI (I$ per capita)	--
Chronic respiratory diseases	Female	1-4 years	95+ years	Data Rich	0	3	Socio-demographic Index	246
Chronic respiratory diseases	Female	1-4 years	95+ years	Data Rich	1	3	Population Density (over 1000 ppl/sqkm, proportion)	0
Chronic respiratory diseases	Female	1-4 years	95+ years	Data Rich	1	3	Elevation 500 to 1500m (proportion)	434
Chronic respiratory diseases	Female	1-4 years	95+ years	Global	1	1	Cumulative Cigarettes (10 Years)	22
Chronic respiratory diseases	Female	1-4 years	95+ years	Global	1	1	Cumulative Cigarettes (5 Years)	23
Chronic respiratory diseases	Female	1-4 years	95+ years	Global	1	1	Log-transformed SEV scalar: Chr Resp	963
Chronic respiratory diseases	Female	1-4 years	95+ years	Global	1	1	Healthcare access and quality index	--

Appendix Table 9: CODEm covariates used, level of covariate, and expected direction of covariate by cause, sex, and age
Covariates that CODEm did not select during the covariate selection process have no draw counts listed.

Cause	Sex	Age Start	Age End	Model Type	Direction	Level	Covariate Name	Number of Draws
Chronic respiratory diseases	Female	1-4 years	95+ years	Global	1	2	Smoking Prevalence	11
Chronic respiratory diseases	Female	1-4 years	95+ years	Global	1	2	Elevation Over 1500m (proportion)	622
Chronic respiratory diseases	Female	1-4 years	95+ years	Global	1	2	Indoor Air Pollution (All Cooking Fuels)	622
Chronic respiratory diseases	Female	1-4 years	95+ years	Global	1	2	Outdoor Air Pollution (PM2.5)	--
Chronic respiratory diseases	Female	1-4 years	95+ years	Global	-1	3	Education (years per capita)	400
Chronic respiratory diseases	Female	1-4 years	95+ years	Global	-1	3	LDI (I$ per capita)	--
Chronic respiratory diseases	Female	1-4 years	95+ years	Global	0	3	Socio-demographic Index	153
Chronic respiratory diseases	Female	1-4 years	95+ years	Global	1	3	Elevation 500 to 1500m (proportion)	684
Chronic respiratory diseases	Female	1-4 years	95+ years	Global	1	3	Population Density (over 1000 ppl/sqkm, proportion)	--
Chronic respiratory diseases	Male	1-4 years	95+ years	Data Rich	1	1	Cumulative Cigarettes (10 Years)	337
Chronic respiratory diseases	Male	1-4 years	95+ years	Data Rich	1	1	Cumulative Cigarettes (5 Years)	526
Chronic respiratory diseases	Male	1-4 years	95+ years	Data Rich	1	1	Log-transformed SEV scalar: Chr Resp	721
Chronic respiratory diseases	Male	1-4 years	95+ years	Data Rich	-1	2	Healthcare access and quality index	2
Chronic respiratory diseases	Male	1-4 years	95+ years	Data Rich	1	2	Indoor Air Pollution (All Cooking Fuels)	0
Chronic respiratory diseases	Male	1-4 years	95+ years	Data Rich	1	2	Smoking Prevalence	775
Chronic respiratory diseases	Male	1-4 years	95+ years	Data Rich	1	2	Elevation Over 1500m (proportion)	777
Chronic respiratory diseases	Male	1-4 years	95+ years	Data Rich	1	2	Outdoor Air Pollution (PM2.5)	777
Chronic respiratory diseases	Male	1-4 years	95+ years	Data Rich	-1	3	Education (years per capita)	140
Chronic respiratory diseases	Male	1-4 years	95+ years	Data Rich	-1	3	LDI (I$ per capita)	--
Chronic respiratory diseases	Male	1-4 years	95+ years	Data Rich	0	3	Socio-demographic Index	116
Chronic respiratory diseases	Male	1-4 years	95+ years	Data Rich	1	3	Population Density (over 1000 ppl/sqkm, proportion)	158
Chronic respiratory diseases	Male	1-4 years	95+ years	Data Rich	1	3	Elevation 500 to 1500m (proportion)	484
Chronic respiratory diseases	Male	1-4 years	95+ years	Global	1	1	Cumulative Cigarettes (10 Years)	75
Chronic respiratory diseases	Male	1-4 years	95+ years	Global	1	1	Indoor Air Pollution (All Cooking Fuels)	165
Chronic respiratory diseases	Male	1-4 years	95+ years	Global	1	1	Cumulative Cigarettes (5 Years)	234
Chronic respiratory diseases	Male	1-4 years	95+ years	Global	1	1	Smoking Prevalence	296
Chronic respiratory diseases	Male	1-4 years	95+ years	Global	1	1	Log-transformed SEV scalar: Chr Resp	858
Chronic respiratory diseases	Male	1-4 years	95+ years	Global	-1	2	Healthcare access and quality index	253
Chronic respiratory diseases	Male	1-4 years	95+ years	Global	1	2	Outdoor Air Pollution (PM2.5)	0
Chronic respiratory diseases	Male	1-4 years	95+ years	Global	1	2	Elevation Over 1500m (proportion)	548
Chronic respiratory diseases	Male	1-4 years	95+ years	Global	-1	3	Education (years per capita)	467
Chronic respiratory diseases	Male	1-4 years	95+ years	Global	-1	3	LDI (I$ per capita)	--
Chronic respiratory diseases	Male	1-4 years	95+ years	Global	0	3	Socio-demographic Index	262
Chronic respiratory diseases	Male	1-4 years	95+ years	Global	1	3	Population Density (over 1000 ppl/sqkm, proportion)	202
Chronic respiratory diseases	Male	1-4 years	95+ years	Global	1	3	Elevation 500 to 1500m (proportion)	846
Chronic obstructive pulmonary disease	Female	1-4 years	95+ years	Data Rich	1	1	Outdoor Air Pollution (PM2.5)	446
Chronic obstructive pulmonary disease	Female	1-4 years	95+ years	Data Rich	1	1	Log-transformed SEV scalar: COPD	581
Chronic obstructive pulmonary disease	Female	1-4 years	95+ years	Data Rich	1	1	Elevation Over 1500m (proportion)	678
Chronic obstructive pulmonary disease	Female	1-4 years	95+ years	Data Rich	1	1	Healthcare access and quality index	706
Chronic obstructive pulmonary disease	Female	1-4 years	95+ years	Data Rich	1	1	Cumulative Cigarettes (10 Years)	--
Chronic obstructive pulmonary disease	Female	1-4 years	95+ years	Data Rich	1	1	Cumulative Cigarettes (5 Years)	--
Chronic obstructive pulmonary disease	Female	1-4 years	95+ years	Data Rich	1	2	Indoor Air Pollution (All Cooking Fuels)	1
Chronic obstructive pulmonary disease	Female	1-4 years	95+ years	Data Rich	1	2	Smoking Prevalence	3
Chronic obstructive pulmonary disease	Female	1-4 years	95+ years	Data Rich	-1	3	Education (years per capita)	2
Chronic obstructive pulmonary disease	Female	1-4 years	95+ years	Data Rich	-1	3	LDI (I$ per capita)	--
Chronic obstructive pulmonary disease	Female	1-4 years	95+ years	Data Rich	0	3	Socio-demographic Index	782
Chronic obstructive pulmonary disease	Female	1-4 years	95+ years	Global	1	1	Healthcare access and quality index	588
Chronic obstructive pulmonary disease	Female	1-4 years	95+ years	Global	1	1	Log-transformed SEV scalar: COPD	680
Chronic obstructive pulmonary disease	Female	1-4 years	95+ years	Global	1	1	Elevation Over 1500m (proportion)	803
Chronic obstructive pulmonary disease	Female	1-4 years	95+ years	Global	1	1	Cumulative Cigarettes (10 Years)	--
Chronic obstructive pulmonary disease	Female	1-4 years	95+ years	Global	1	1	Cumulative Cigarettes (5 Years)	--
Chronic obstructive pulmonary disease	Female	1-4 years	95+ years	Global	1	1	Outdoor Air Pollution (PM2.5)	--
Chronic obstructive pulmonary disease	Female	1-4 years	95+ years	Global	1	2	Smoking Prevalence	3
Chronic obstructive pulmonary disease	Female	1-4 years	95+ years	Global	1	2	Indoor Air Pollution (All Cooking Fuels)	206
Chronic obstructive pulmonary disease	Female	1-4 years	95+ years	Global	-1	3	Education (years per capita)	35
Chronic obstructive pulmonary disease	Female	1-4 years	95+ years	Global	-1	3	LDI (I$ per capita)	--
Chronic obstructive pulmonary disease	Female	1-4 years	95+ years	Global	0	3	Socio-demographic Index	140
Chronic obstructive pulmonary disease	Male	1-4 years	95+ years	Data Rich	1	1	Cumulative Cigarettes (10 Years)	0
Chronic obstructive pulmonary disease	Male	1-4 years	95+ years	Data Rich	1	1	Cumulative Cigarettes (20 Years)	0
Chronic obstructive pulmonary disease	Male	1-4 years	95+ years	Data Rich	1	1	Cumulative Cigarettes (5 Years)	0
Chronic obstructive pulmonary disease	Male	1-4 years	95+ years	Data Rich	1	1	Elevation Over 1500m (proportion)	485
Chronic obstructive pulmonary disease	Male	1-4 years	95+ years	Data Rich	1	1	Outdoor Air Pollution (PM2.5)	551
Chronic obstructive pulmonary disease	Male	1-4 years	95+ years	Data Rich	1	1	Log-transformed SEV scalar: COPD	652
Chronic obstructive pulmonary disease	Male	1-4 years	95+ years	Data Rich	-1	2	Healthcare access and quality index	0
Chronic obstructive pulmonary disease	Male	1-4 years	95+ years	Data Rich	1	2	Indoor Air Pollution (All Cooking Fuels)	0
Chronic obstructive pulmonary disease	Male	1-4 years	95+ years	Data Rich	1	2	Smoking Prevalence	488
Chronic obstructive pulmonary disease	Male	1-4 years	95+ years	Data Rich	-1	3	Education (years per capita)	813
Chronic obstructive pulmonary disease	Male	1-4 years	95+ years	Data Rich	-1	3	LDI (I$ per capita)	--
Chronic obstructive pulmonary disease	Male	1-4 years	95+ years	Data Rich	0	3	Socio-demographic Index	940
Chronic obstructive pulmonary disease	Male	1-4 years	95+ years	Global	1	1	Cumulative Cigarettes (20 Years)	0
Chronic obstructive pulmonary disease	Male	1-4 years	95+ years	Global	1	1	Cumulative Cigarettes (10 Years)	4
Chronic obstructive pulmonary disease	Male	1-4 years	95+ years	Global	1	1	Cumulative Cigarettes (5 Years)	7
Chronic obstructive pulmonary disease	Male	1-4 years	95+ years	Global	1	1	Outdoor Air Pollution (PM2.5)	124
Chronic obstructive pulmonary disease	Male	1-4 years	95+ years	Global	1	1	Log-transformed SEV scalar: COPD	626
Chronic obstructive pulmonary disease	Male	1-4 years	95+ years	Global	1	1	Elevation Over 1500m (proportion)	939
Chronic obstructive pulmonary disease	Male	1-4 years	95+ years	Global	-1	2	Healthcare access and quality index	477
Chronic obstructive pulmonary disease	Male	1-4 years	95+ years	Global	1	2	Indoor Air Pollution (All Cooking Fuels)	71
Chronic obstructive pulmonary disease	Male	1-4 years	95+ years	Global	1	2	Smoking Prevalence	380

Appendix Table 9: CODEm covariates used, level of covariate, and expected direction of covariate by cause, sex, and age
Covariates that CODEm did not select during the covariate selection process have no draw counts listed.

Cause	Sex	Age Start	Age End	Model Type	Direction	Level	Covariate Name	Number of Draws
Chronic obstructive pulmonary disease	Male	1-4 years	95+ years	Global	-1	3	Education (years per capita)	437
Chronic obstructive pulmonary disease	Male	1-4 years	95+ years	Global	-1	3	LDI (I$ per capita)	--
Chronic obstructive pulmonary disease	Male	1-4 years	95+ years	Global	0	3	Socio-demographic Index	664
Pneumoconiosis	Female	15-19 years	95+ years	Data Rich	1	1	Asbestos consumption (metric tons per year per capita)	260
Pneumoconiosis	Female	15-19 years	95+ years	Data Rich	1	1	Coal Production (per capita)	--
Pneumoconiosis	Female	15-19 years	95+ years	Data Rich	1	1	Gold production (kg) per capita	--
Pneumoconiosis	Female	15-19 years	95+ years	Data Rich	-1	2	Healthcare access and quality index	43
Pneumoconiosis	Female	15-19 years	95+ years	Data Rich	1	2	Cumulative Cigarettes (5 Years)	38
Pneumoconiosis	Female	15-19 years	95+ years	Data Rich	1	2	Indoor Air Pollution (All Cooking Fuels)	38
Pneumoconiosis	Female	15-19 years	95+ years	Data Rich	1	2	Smoking Prevalence	404
Pneumoconiosis	Female	15-19 years	95+ years	Data Rich	-1	3	Education (years per capita)	106
Pneumoconiosis	Female	15-19 years	95+ years	Data Rich	-1	3	Socio-demographic Index	430
Pneumoconiosis	Female	15-19 years	95+ years	Data Rich	-1	3	LDI (I$ per capita)	--
Pneumoconiosis	Female	15-19 years	95+ years	Global	1	1	Asbestos consumption (metric tons per year per capita)	1000
Pneumoconiosis	Female	15-19 years	95+ years	Global	1	1	Coal Production (per capita)	--
Pneumoconiosis	Female	15-19 years	95+ years	Global	1	1	Gold production (kg) per capita	--
Pneumoconiosis	Female	15-19 years	95+ years	Global	-1	2	Healthcare access and quality index	190
Pneumoconiosis	Female	15-19 years	95+ years	Global	1	2	Cumulative Cigarettes (5 Years)	24
Pneumoconiosis	Female	15-19 years	95+ years	Global	1	2	Indoor Air Pollution (All Cooking Fuels)	24
Pneumoconiosis	Female	15-19 years	95+ years	Global	1	2	Smoking Prevalence	193
Pneumoconiosis	Female	15-19 years	95+ years	Global	-1	3	Socio-demographic Index	144
Pneumoconiosis	Female	15-19 years	95+ years	Global	-1	3	Education (years per capita)	266
Pneumoconiosis	Female	15-19 years	95+ years	Global	-1	3	LDI (I$ per capita)	--
Pneumoconiosis	Male	15-19 years	95+ years	Data Rich	1	1	Asbestos consumption (metric tons per year per capita)	365
Pneumoconiosis	Male	15-19 years	95+ years	Data Rich	1	1	Coal Production (per capita)	805
Pneumoconiosis	Male	15-19 years	95+ years	Data Rich	1	1	Gold production (kg) per capita	--
Pneumoconiosis	Male	15-19 years	95+ years	Data Rich	-1	2	Healthcare access and quality index	34
Pneumoconiosis	Male	15-19 years	95+ years	Data Rich	1	2	Indoor Air Pollution (All Cooking Fuels)	0
Pneumoconiosis	Male	15-19 years	95+ years	Data Rich	1	2	Smoking Prevalence	154
Pneumoconiosis	Male	15-19 years	95+ years	Data Rich	1	2	Cumulative Cigarettes (5 Years)	167
Pneumoconiosis	Male	15-19 years	95+ years	Data Rich	-1	3	Socio-demographic Index	29
Pneumoconiosis	Male	15-19 years	95+ years	Data Rich	-1	3	Education (years per capita)	323
Pneumoconiosis	Male	15-19 years	95+ years	Data Rich	-1	3	LDI (I$ per capita)	--
Pneumoconiosis	Male	15-19 years	95+ years	Global	1	1	Coal Production (per capita)	567
Pneumoconiosis	Male	15-19 years	95+ years	Global	1	1	Asbestos consumption (metric tons per year per capita)	750
Pneumoconiosis	Male	15-19 years	95+ years	Global	1	1	Gold production (kg) per capita	--
Pneumoconiosis	Male	15-19 years	95+ years	Global	-1	2	Healthcare access and quality index	160
Pneumoconiosis	Male	15-19 years	95+ years	Global	1	2	Indoor Air Pollution (All Cooking Fuels)	2
Pneumoconiosis	Male	15-19 years	95+ years	Global	1	2	Smoking Prevalence	126
Pneumoconiosis	Male	15-19 years	95+ years	Global	1	2	Cumulative Cigarettes (5 Years)	154
Pneumoconiosis	Male	15-19 years	95+ years	Global	-1	3	Education (years per capita)	299
Pneumoconiosis	Male	15-19 years	95+ years	Global	-1	3	Socio-demographic Index	413
Pneumoconiosis	Male	15-19 years	95+ years	Global	-1	3	LDI (I$ per capita)	--
Silicosis	Female	15-19 years	95+ years	Data Rich	1	1	Gold production (kg) per capita	--
Silicosis	Female	15-19 years	95+ years	Data Rich	-1	2	Healthcare access and quality index	568
Silicosis	Female	15-19 years	95+ years	Data Rich	1	2	Cumulative Cigarettes (5 Years)	--
Silicosis	Female	15-19 years	95+ years	Data Rich	1	2	Indoor Air Pollution (All Cooking Fuels)	--
Silicosis	Female	15-19 years	95+ years	Data Rich	1	2	Smoking Prevalence	--
Silicosis	Female	15-19 years	95+ years	Data Rich	-1	3	Socio-demographic Index	568
Silicosis	Female	15-19 years	95+ years	Data Rich	-1	3	Education (years per capita)	--
Silicosis	Female	15-19 years	95+ years	Data Rich	-1	3	LDI (I$ per capita)	--
Silicosis	Female	15-19 years	95+ years	Global	1	1	Gold production (kg) per capita	--
Silicosis	Female	15-19 years	95+ years	Global	-1	2	Healthcare access and quality index	546
Silicosis	Female	15-19 years	95+ years	Global	1	2	Cumulative Cigarettes (5 Years)	--
Silicosis	Female	15-19 years	95+ years	Global	1	2	Indoor Air Pollution (All Cooking Fuels)	--
Silicosis	Female	15-19 years	95+ years	Global	1	2	Smoking Prevalence	--
Silicosis	Female	15-19 years	95+ years	Global	-1	3	Socio-demographic Index	546
Silicosis	Female	15-19 years	95+ years	Global	-1	3	Education (years per capita)	--
Silicosis	Female	15-19 years	95+ years	Global	-1	3	LDI (I$ per capita)	--
Silicosis	Male	15-19 years	95+ years	Data Rich	1	1	Gold production (kg) per capita	823
Silicosis	Male	15-19 years	95+ years	Data Rich	-1	2	Healthcare access and quality index	110
Silicosis	Male	15-19 years	95+ years	Data Rich	1	2	Indoor Air Pollution (All Cooking Fuels)	10
Silicosis	Male	15-19 years	95+ years	Data Rich	1	2	Cumulative Cigarettes (5 Years)	70
Silicosis	Male	15-19 years	95+ years	Data Rich	1	2	Smoking Prevalence	504
Silicosis	Male	15-19 years	95+ years	Data Rich	-1	3	Education (years per capita)	277
Silicosis	Male	15-19 years	95+ years	Data Rich	-1	3	Socio-demographic Index	384
Silicosis	Male	15-19 years	95+ years	Data Rich	-1	3	LDI (I$ per capita)	--
Silicosis	Male	15-19 years	95+ years	Global	1	1	Gold production (kg) per capita	1000
Silicosis	Male	15-19 years	95+ years	Global	-1	2	Healthcare access and quality index	321
Silicosis	Male	15-19 years	95+ years	Global	1	2	Indoor Air Pollution (All Cooking Fuels)	121
Silicosis	Male	15-19 years	95+ years	Global	1	2	Cumulative Cigarettes (5 Years)	215
Silicosis	Male	15-19 years	95+ years	Global	1	2	Smoking Prevalence	--
Silicosis	Male	15-19 years	95+ years	Global	-1	3	Socio-demographic Index	192
Silicosis	Male	15-19 years	95+ years	Global	-1	3	Education (years per capita)	225
Silicosis	Male	15-19 years	95+ years	Global	-1	3	LDI (I$ per capita)	--
Asbestosis	Female	15-19 years	95+ years	Data Rich	1	1	Asbestos consumption (metric tons per year per capita)	--
Asbestosis	Female	15-19 years	95+ years	Data Rich	-1	2	Healthcare access and quality index	--
Asbestosis	Female	15-19 years	95+ years	Data Rich	1	2	Elevation Over 1500m (proportion)	744

Appendix Table 9: CODEm covariates used, level of covariate, and expected direction of covariate by cause, sex, and age

Covariates that CODEm did not select during the covariate selection process have no draw counts listed.

Cause	Sex	Age Start	Age End	Model Type	Direction	Level	Covariate Name	Number of Draws
Asbestosis	Female	15-19 years	95+ years	Data Rich	1	2	Cumulative Cigarettes (10 Years)	--
Asbestosis	Female	15-19 years	95+ years	Data Rich	1	2	Cumulative Cigarettes (5 Years)	--
Asbestosis	Female	15-19 years	95+ years	Data Rich	1	2	Elevation 500 to 1500m (proportion)	--
Asbestosis	Female	15-19 years	95+ years	Data Rich	1	2	Indoor Air Pollution (All Cooking Fuels)	--
Asbestosis	Female	15-19 years	95+ years	Data Rich	1	2	Smoking Prevalence	--
Asbestosis	Female	15-19 years	95+ years	Data Rich	-1	3	Education (years per capita)	--
Asbestosis	Female	15-19 years	95+ years	Data Rich	-1	3	LDI (I$ per capita)	--
Asbestosis	Female	15-19 years	95+ years	Data Rich	-1	3	Socio-demographic Index	--
Asbestosis	Female	15-19 years	95+ years	Global	1	1	Asbestos consumption (metric tons per year per capita)	--
Asbestosis	Female	15-19 years	95+ years	Global	-1	2	Healthcare access and quality index	546
Asbestosis	Female	15-19 years	95+ years	Global	1	2	Elevation Over 1500m (proportion)	546
Asbestosis	Female	15-19 years	95+ years	Global	1	2	Cumulative Cigarettes (10 Years)	--
Asbestosis	Female	15-19 years	95+ years	Global	1	2	Cumulative Cigarettes (5 Years)	--
Asbestosis	Female	15-19 years	95+ years	Global	1	2	Elevation 500 to 1500m (proportion)	--
Asbestosis	Female	15-19 years	95+ years	Global	1	2	Indoor Air Pollution (All Cooking Fuels)	--
Asbestosis	Female	15-19 years	95+ years	Global	1	2	Smoking Prevalence	--
Asbestosis	Female	15-19 years	95+ years	Global	-1	3	Education (years per capita)	--
Asbestosis	Female	15-19 years	95+ years	Global	-1	3	LDI (I$ per capita)	--
Asbestosis	Female	15-19 years	95+ years	Global	-1	3	Socio-demographic Index	--
Asbestosis	Male	15-19 years	95+ years	Data Rich	1	1	Asbestos consumption (metric tons per year per capita)	--
Asbestosis	Male	15-19 years	95+ years	Data Rich	1	1	Smoking Prevalence	--
Asbestosis	Male	15-19 years	95+ years	Data Rich	-1	2	Healthcare access and quality index	--
Asbestosis	Male	15-19 years	95+ years	Data Rich	1	2	Elevation 500 to 1500m (proportion)	516
Asbestosis	Male	15-19 years	95+ years	Data Rich	1	2	Elevation Over 1500m (proportion)	516
Asbestosis	Male	15-19 years	95+ years	Data Rich	1	2	Cumulative Cigarettes (5 Years)	--
Asbestosis	Male	15-19 years	95+ years	Data Rich	1	2	Indoor Air Pollution (All Cooking Fuels)	--
Asbestosis	Male	15-19 years	95+ years	Data Rich	-1	3	Education (years per capita)	--
Asbestosis	Male	15-19 years	95+ years	Data Rich	-1	3	LDI (I$ per capita)	--
Asbestosis	Male	15-19 years	95+ years	Data Rich	0	3	Socio-demographic Index	742
Asbestosis	Male	15-19 years	95+ years	Global	1	1	Asbestos consumption (metric tons per year per capita)	--
Asbestosis	Male	15-19 years	95+ years	Global	-1	2	Healthcare access and quality index	--
Asbestosis	Male	15-19 years	95+ years	Global	1	2	Elevation 500 to 1500m (proportion)	1000
Asbestosis	Male	15-19 years	95+ years	Global	1	2	Elevation Over 1500m (proportion)	1000
Asbestosis	Male	15-19 years	95+ years	Global	1	2	Cumulative Cigarettes (5 Years)	--
Asbestosis	Male	15-19 years	95+ years	Global	1	2	Indoor Air Pollution (All Cooking Fuels)	--
Asbestosis	Male	15-19 years	95+ years	Global	1	2	Smoking Prevalence	--
Asbestosis	Male	15-19 years	95+ years	Global	-1	3	Education (years per capita)	--
Asbestosis	Male	15-19 years	95+ years	Global	-1	3	LDI (I$ per capita)	--
Asbestosis	Male	15-19 years	95+ years	Global	-1	3	Socio-demographic Index	--
Coal workers pneumoconiosis	Female	15-19 years	95+ years	Data Rich	1	1	Coal Production (per capita)	--
Coal workers pneumoconiosis	Female	15-19 years	95+ years	Data Rich	-1	2	Healthcare access and quality index	325
Coal workers pneumoconiosis	Female	15-19 years	95+ years	Data Rich	1	2	Smoking Prevalence	325
Coal workers pneumoconiosis	Female	15-19 years	95+ years	Data Rich	1	2	Cumulative Cigarettes (5 Years)	--
Coal workers pneumoconiosis	Female	15-19 years	95+ years	Data Rich	1	2	Indoor Air Pollution (All Cooking Fuels)	--
Coal workers pneumoconiosis	Female	15-19 years	95+ years	Data Rich	-1	3	Education (years per capita)	--
Coal workers pneumoconiosis	Female	15-19 years	95+ years	Data Rich	-1	3	LDI (I$ per capita)	--
Coal workers pneumoconiosis	Female	15-19 years	95+ years	Data Rich	-1	3	Socio-demographic Index	--
Coal workers pneumoconiosis	Female	15-19 years	95+ years	Global	1	1	Coal Production (per capita)	--
Coal workers pneumoconiosis	Female	15-19 years	95+ years	Global	-1	2	Healthcare access and quality index	105
Coal workers pneumoconiosis	Female	15-19 years	95+ years	Global	1	2	Smoking Prevalence	354
Coal workers pneumoconiosis	Female	15-19 years	95+ years	Global	1	2	Cumulative Cigarettes (5 Years)	--
Coal workers pneumoconiosis	Female	15-19 years	95+ years	Global	1	2	Indoor Air Pollution (All Cooking Fuels)	--
Coal workers pneumoconiosis	Female	15-19 years	95+ years	Global	-1	3	Socio-demographic Index	281
Coal workers pneumoconiosis	Female	15-19 years	95+ years	Global	-1	3	Education (years per capita)	--
Coal workers pneumoconiosis	Female	15-19 years	95+ years	Global	-1	3	LDI (I$ per capita)	--
Coal workers pneumoconiosis	Male	15-19 years	95+ years	Data Rich	1	1	Coal Production (per capita)	1000
Coal workers pneumoconiosis	Male	15-19 years	95+ years	Data Rich	-1	2	Healthcare access and quality index	327
Coal workers pneumoconiosis	Male	15-19 years	95+ years	Data Rich	1	2	Indoor Air Pollution (All Cooking Fuels)	68
Coal workers pneumoconiosis	Male	15-19 years	95+ years	Data Rich	1	2	Cumulative Cigarettes (5 Years)	250
Coal workers pneumoconiosis	Male	15-19 years	95+ years	Data Rich	1	2	Smoking Prevalence	274
Coal workers pneumoconiosis	Male	15-19 years	95+ years	Data Rich	-1	3	Socio-demographic Index	195
Coal workers pneumoconiosis	Male	15-19 years	95+ years	Data Rich	-1	3	Education (years per capita)	464
Coal workers pneumoconiosis	Male	15-19 years	95+ years	Data Rich	-1	3	LDI (I$ per capita)	--
Coal workers pneumoconiosis	Male	15-19 years	95+ years	Global	1	1	Coal Production (per capita)	1000
Coal workers pneumoconiosis	Male	15-19 years	95+ years	Global	-1	2	Healthcare access and quality index	461
Coal workers pneumoconiosis	Male	15-19 years	95+ years	Global	1	2	Indoor Air Pollution (All Cooking Fuels)	64
Coal workers pneumoconiosis	Male	15-19 years	95+ years	Global	1	2	Cumulative Cigarettes (5 Years)	334
Coal workers pneumoconiosis	Male	15-19 years	95+ years	Global	1	2	Smoking Prevalence	339
Coal workers pneumoconiosis	Male	15-19 years	95+ years	Global	-1	3	Socio-demographic Index	191
Coal workers pneumoconiosis	Male	15-19 years	95+ years	Global	-1	3	Education (years per capita)	232
Coal workers pneumoconiosis	Male	15-19 years	95+ years	Global	-1	3	LDI (I$ per capita)	--
Other pneumoconiosis	Female	15-19 years	95+ years	Data Rich	-1	2	Healthcare access and quality index	89
Other pneumoconiosis	Female	15-19 years	95+ years	Data Rich	1	2	Indoor Air Pollution (All Cooking Fuels)	38
Other pneumoconiosis	Female	15-19 years	95+ years	Data Rich	1	2	Smoking Prevalence	408
Other pneumoconiosis	Female	15-19 years	95+ years	Data Rich	1	2	Cumulative Cigarettes (5 Years)	575
Other pneumoconiosis	Female	15-19 years	95+ years	Data Rich	-1	3	Socio-demographic Index	137
Other pneumoconiosis	Female	15-19 years	95+ years	Data Rich	-1	3	Education (years per capita)	--
Other pneumoconiosis	Female	15-19 years	95+ years	Data Rich	-1	3	LDI (I$ per capita)	--

Appendix Table 9: CODEm covariates used, level of covariate, and expected direction of covariate by cause, sex, and age

Covariates that CODEm did not select during the covariate selection process have no draw counts listed.

Cause	Sex	Age Start	Age End	Model Type	Direction	Level	Covariate Name	Number of Draws
Other pneumoconiosis	Female	15-19 years	95+ years	Global	-1	2	Healthcare access and quality index	38
Other pneumoconiosis	Female	15-19 years	95+ years	Global	1	2	Indoor Air Pollution (All Cooking Fuels)	63
Other pneumoconiosis	Female	15-19 years	95+ years	Global	1	2	Cumulative Cigarettes (5 Years)	431
Other pneumoconiosis	Female	15-19 years	95+ years	Global	1	2	Smoking Prevalence	625
Other pneumoconiosis	Female	15-19 years	95+ years	Global	-1	3	Education (years per capita)	22
Other pneumoconiosis	Female	15-19 years	95+ years	Global	-1	3	Socio-demographic Index	236
Other pneumoconiosis	Female	15-19 years	95+ years	Global	-1	3	LDI (I$ per capita)	--
Other pneumoconiosis	Male	15-19 years	95+ years	Data Rich	-1	2	Healthcare access and quality index	508
Other pneumoconiosis	Male	15-19 years	95+ years	Data Rich	1	2	Indoor Air Pollution (All Cooking Fuels)	169
Other pneumoconiosis	Male	15-19 years	95+ years	Data Rich	1	2	Cumulative Cigarettes (5 Years)	323
Other pneumoconiosis	Male	15-19 years	95+ years	Data Rich	1	2	Smoking Prevalence	323
Other pneumoconiosis	Male	15-19 years	95+ years	Data Rich	-1	3	Education (years per capita)	141
Other pneumoconiosis	Male	15-19 years	95+ years	Data Rich	-1	3	LDI (I$ per capita)	--
Other pneumoconiosis	Male	15-19 years	95+ years	Data Rich	-1	3	Socio-demographic Index	--
Other pneumoconiosis	Male	15-19 years	95+ years	Global	-1	2	Healthcare access and quality index	265
Other pneumoconiosis	Male	15-19 years	95+ years	Global	1	2	Indoor Air Pollution (All Cooking Fuels)	216
Other pneumoconiosis	Male	15-19 years	95+ years	Global	1	2	Cumulative Cigarettes (5 Years)	435
Other pneumoconiosis	Male	15-19 years	95+ years	Global	1	2	Smoking Prevalence	719
Other pneumoconiosis	Male	15-19 years	95+ years	Global	-1	3	Socio-demographic Index	52
Other pneumoconiosis	Male	15-19 years	95+ years	Global	-1	3	Education (years per capita)	201
Other pneumoconiosis	Male	15-19 years	95+ years	Global	-1	3	LDI (I$ per capita)	--
Asthma	Female	1-4 years	95+ years	Data Rich	-1	1	Healthcare access and quality index	566
Asthma	Female	1-4 years	95+ years	Data Rich	1	1	Cumulative Cigarettes (10 Years)	108
Asthma	Female	1-4 years	95+ years	Data Rich	1	1	Cumulative Cigarettes (5 Years)	199
Asthma	Female	1-4 years	95+ years	Data Rich	1	1	Log-transformed SEV scalar: Asthma	347
Asthma	Female	1-4 years	95+ years	Data Rich	1	2	Smoking Prevalence	5
Asthma	Female	1-4 years	95+ years	Data Rich	1	2	Indoor Air Pollution (All Cooking Fuels)	29
Asthma	Female	1-4 years	95+ years	Data Rich	1	2	Outdoor Air Pollution (PM2.5)	537
Asthma	Female	1-4 years	95+ years	Data Rich	-1	3	Socio-demographic Index	2
Asthma	Female	1-4 years	95+ years	Data Rich	-1	3	Education (years per capita)	180
Asthma	Female	1-4 years	95+ years	Data Rich	-1	3	LDI (I$ per capita)	--
Asthma	Female	1-4 years	95+ years	Global	-1	1	Healthcare access and quality index	593
Asthma	Female	1-4 years	95+ years	Global	1	1	Log-transformed SEV scalar: Asthma	106
Asthma	Female	1-4 years	95+ years	Global	1	1	Cumulative Cigarettes (5 Years)	145
Asthma	Female	1-4 years	95+ years	Global	1	1	Cumulative Cigarettes (10 Years)	160
Asthma	Female	1-4 years	95+ years	Global	1	2	Smoking Prevalence	2
Asthma	Female	1-4 years	95+ years	Global	1	2	Indoor Air Pollution (All Cooking Fuels)	91
Asthma	Female	1-4 years	95+ years	Global	1	2	Outdoor Air Pollution (PM2.5)	300
Asthma	Female	1-4 years	95+ years	Global	-1	3	Socio-demographic Index	12
Asthma	Female	1-4 years	95+ years	Global	-1	3	Education (years per capita)	273
Asthma	Female	1-4 years	95+ years	Global	-1	3	LDI (I$ per capita)	--
Asthma	Male	1-4 years	95+ years	Data Rich	-1	1	Healthcare access and quality index	159
Asthma	Male	1-4 years	95+ years	Data Rich	1	1	Cumulative Cigarettes (5 Years)	143
Asthma	Male	1-4 years	95+ years	Data Rich	1	1	Cumulative Cigarettes (10 Years)	283
Asthma	Male	1-4 years	95+ years	Data Rich	1	1	Log-transformed SEV scalar: Asthma	905
Asthma	Male	1-4 years	95+ years	Data Rich	1	2	Outdoor Air Pollution (PM2.5)	9
Asthma	Male	1-4 years	95+ years	Data Rich	1	2	Smoking Prevalence	27
Asthma	Male	1-4 years	95+ years	Data Rich	1	2	Indoor Air Pollution (All Cooking Fuels)	110
Asthma	Male	1-4 years	95+ years	Data Rich	-1	3	Socio-demographic Index	432
Asthma	Male	1-4 years	95+ years	Data Rich	-1	3	Education (years per capita)	571
Asthma	Male	1-4 years	95+ years	Data Rich	-1	3	LDI (I$ per capita)	--
Asthma	Male	1-4 years	95+ years	Global	-1	1	Healthcare access and quality index	788
Asthma	Male	1-4 years	95+ years	Global	1	1	Cumulative Cigarettes (5 Years)	268
Asthma	Male	1-4 years	95+ years	Global	1	1	Cumulative Cigarettes (10 Years)	427
Asthma	Male	1-4 years	95+ years	Global	1	1	Log-transformed SEV scalar: Asthma	457
Asthma	Male	1-4 years	95+ years	Global	1	2	Smoking Prevalence	63
Asthma	Male	1-4 years	95+ years	Global	1	2	Indoor Air Pollution (All Cooking Fuels)	70
Asthma	Male	1-4 years	95+ years	Global	1	2	Outdoor Air Pollution (PM2.5)	198
Asthma	Male	1-4 years	95+ years	Global	-1	3	Socio-demographic Index	76
Asthma	Male	1-4 years	95+ years	Global	-1	3	Education (years per capita)	549
Asthma	Male	1-4 years	95+ years	Global	-1	3	LDI (I$ per capita)	--
Interstitial lung disease and pulmonary sarcoidosis	Female	1-4 years	95+ years	Data Rich	1	1	Log-transformed SEV scalar: ILD	414
Interstitial lung disease and pulmonary sarcoidosis	Female	1-4 years	95+ years	Data Rich	1	1	Cumulative Cigarettes (10 Years)	--
Interstitial lung disease and pulmonary sarcoidosis	Female	1-4 years	95+ years	Data Rich	1	1	Cumulative Cigarettes (5 Years)	--
Interstitial lung disease and pulmonary sarcoidosis	Female	1-4 years	95+ years	Data Rich	1	1	Smoking Prevalence	--
Interstitial lung disease and pulmonary sarcoidosis	Female	1-4 years	95+ years	Data Rich	-1	2	Healthcare access and quality index	--
Interstitial lung disease and pulmonary sarcoidosis	Female	1-4 years	95+ years	Data Rich	1	2	Outdoor Air Pollution (PM2.5)	586
Interstitial lung disease and pulmonary sarcoidosis	Female	1-4 years	95+ years	Data Rich	1	2	Indoor Air Pollution (All Cooking Fuels)	--
Interstitial lung disease and pulmonary sarcoidosis	Female	1-4 years	95+ years	Data Rich	-1	3	Education (years per capita)	--
Interstitial lung disease and pulmonary sarcoidosis	Female	1-4 years	95+ years	Data Rich	-1	3	LDI (I$ per capita)	--
Interstitial lung disease and pulmonary sarcoidosis	Female	1-4 years	95+ years	Data Rich	0	3	Socio-demographic Index	618
Interstitial lung disease and pulmonary sarcoidosis	Female	1-4 years	95+ years	Global	1	1	Outdoor Air Pollution (PM2.5)	681
Interstitial lung disease and pulmonary sarcoidosis	Female	1-4 years	95+ years	Global	1	1	Log-transformed SEV scalar: ILD	739
Interstitial lung disease and pulmonary sarcoidosis	Female	1-4 years	95+ years	Global	1	1	Cumulative Cigarettes (10 Years)	--
Interstitial lung disease and pulmonary sarcoidosis	Female	1-4 years	95+ years	Global	1	1	Cumulative Cigarettes (5 Years)	--
Interstitial lung disease and pulmonary sarcoidosis	Female	1-4 years	95+ years	Global	1	1	Indoor Air Pollution (All Cooking Fuels)	--
Interstitial lung disease and pulmonary sarcoidosis	Female	1-4 years	95+ years	Global	1	1	Smoking Prevalence	--
Interstitial lung disease and pulmonary sarcoidosis	Female	1-4 years	95+ years	Global	-1	2	Healthcare access and quality index	--

Appendix Table 9: CODEm covariates used, level of covariate, and expected direction of covariate by cause, sex, and age<br>*Covariates that CODEm did not select during the covariate selection process have no draw counts listed.*								
**Cause**	**Sex**	**Age Start**	**Age End**	**Model Type**	**Direction**	**Level**	**Covariate Name**	**Number of Draws**
Interstitial lung disease and pulmonary sarcoidosis	Female	1-4 years	95+ years	Global	-1	3	Education (years per capita)	--
Interstitial lung disease and pulmonary sarcoidosis	Female	1-4 years	95+ years	Global	-1	3	LDI (I$ per capita)	--
Interstitial lung disease and pulmonary sarcoidosis	Female	1-4 years	95+ years	Global	0	3	Socio-demographic Index	693
Interstitial lung disease and pulmonary sarcoidosis	Male	1-4 years	95+ years	Data Rich	1	1	Cumulative Cigarettes (10 Years)	--
Interstitial lung disease and pulmonary sarcoidosis	Male	1-4 years	95+ years	Data Rich	1	1	Cumulative Cigarettes (5 Years)	--
Interstitial lung disease and pulmonary sarcoidosis	Male	1-4 years	95+ years	Data Rich	1	1	Log-transformed SEV scalar: ILD	--
Interstitial lung disease and pulmonary sarcoidosis	Male	1-4 years	95+ years	Data Rich	1	1	Smoking Prevalence	--
Interstitial lung disease and pulmonary sarcoidosis	Male	1-4 years	95+ years	Data Rich	-1	2	Healthcare access and quality index	--
Interstitial lung disease and pulmonary sarcoidosis	Male	1-4 years	95+ years	Data Rich	1	2	Outdoor Air Pollution (PM2.5)	1000
Interstitial lung disease and pulmonary sarcoidosis	Male	1-4 years	95+ years	Data Rich	1	2	Indoor Air Pollution (All Cooking Fuels)	--
Interstitial lung disease and pulmonary sarcoidosis	Male	1-4 years	95+ years	Data Rich	-1	3	Education (years per capita)	--
Interstitial lung disease and pulmonary sarcoidosis	Male	1-4 years	95+ years	Data Rich	-1	3	LDI (I$ per capita)	--
Interstitial lung disease and pulmonary sarcoidosis	Male	1-4 years	95+ years	Data Rich	0	3	Socio-demographic Index	1000
Interstitial lung disease and pulmonary sarcoidosis	Male	1-4 years	95+ years	Global	1	1	Cumulative Cigarettes (10 Years)	--
Interstitial lung disease and pulmonary sarcoidosis	Male	1-4 years	95+ years	Global	1	1	Cumulative Cigarettes (5 Years)	--
Interstitial lung disease and pulmonary sarcoidosis	Male	1-4 years	95+ years	Global	1	1	Log-transformed SEV scalar: ILD	--
Interstitial lung disease and pulmonary sarcoidosis	Male	1-4 years	95+ years	Global	1	1	Smoking Prevalence	--
Interstitial lung disease and pulmonary sarcoidosis	Male	1-4 years	95+ years	Global	-1	2	Healthcare access and quality index	--
Interstitial lung disease and pulmonary sarcoidosis	Male	1-4 years	95+ years	Global	1	2	Outdoor Air Pollution (PM2.5)	422
Interstitial lung disease and pulmonary sarcoidosis	Male	1-4 years	95+ years	Global	1	2	Indoor Air Pollution (All Cooking Fuels)	--
Interstitial lung disease and pulmonary sarcoidosis	Male	1-4 years	95+ years	Global	-1	3	Education (years per capita)	--
Interstitial lung disease and pulmonary sarcoidosis	Male	1-4 years	95+ years	Global	-1	3	LDI (I$ per capita)	--
Interstitial lung disease and pulmonary sarcoidosis	Male	1-4 years	95+ years	Global	0	3	Socio-demographic Index	815
Other chronic respiratory diseases	Female	1-4 years	95+ years	Data Rich	1	1	Indoor Air Pollution (All Cooking Fuels)	242
Other chronic respiratory diseases	Female	1-4 years	95+ years	Data Rich	1	1	Outdoor Air Pollution (PM2.5)	536
Other chronic respiratory diseases	Female	1-4 years	95+ years	Data Rich	1	1	Log-transformed SEV scalar: Oth Resp	772
Other chronic respiratory diseases	Female	1-4 years	95+ years	Data Rich	1	1	Cumulative Cigarettes (5 Years)	--
Other chronic respiratory diseases	Female	1-4 years	95+ years	Data Rich	1	1	Smoking Prevalence	--
Other chronic respiratory diseases	Female	1-4 years	95+ years	Data Rich	-1	2	Healthcare access and quality index	58
Other chronic respiratory diseases	Female	1-4 years	95+ years	Data Rich	1	2	Elevation Over 1500m (proportion)	12
Other chronic respiratory diseases	Female	1-4 years	95+ years	Data Rich	1	2	Elevation 500 to 1500m (proportion)	306
Other chronic respiratory diseases	Female	1-4 years	95+ years	Data Rich	1	2	Population Density (over 1000 ppl/sqkm, proportion)	--
Other chronic respiratory diseases	Female	1-4 years	95+ years	Data Rich	-1	3	Education (years per capita)	17
Other chronic respiratory diseases	Female	1-4 years	95+ years	Data Rich	-1	3	Socio-demographic Index	44
Other chronic respiratory diseases	Female	1-4 years	95+ years	Data Rich	-1	3	LDI (I$ per capita)	--
Other chronic respiratory diseases	Female	1-4 years	95+ years	Global	1	1	Indoor Air Pollution (All Cooking Fuels)	134
Other chronic respiratory diseases	Female	1-4 years	95+ years	Global	1	1	Log-transformed SEV scalar: Oth Resp	939
Other chronic respiratory diseases	Female	1-4 years	95+ years	Global	1	1	Cumulative Cigarettes (5 Years)	--
Other chronic respiratory diseases	Female	1-4 years	95+ years	Global	1	1	Outdoor Air Pollution (PM2.5)	--
Other chronic respiratory diseases	Female	1-4 years	95+ years	Global	1	1	Smoking Prevalence	--
Other chronic respiratory diseases	Female	1-4 years	95+ years	Global	-1	2	Healthcare access and quality index	215
Other chronic respiratory diseases	Female	1-4 years	95+ years	Global	1	2	Elevation 500 to 1500m (proportion)	337
Other chronic respiratory diseases	Female	1-4 years	95+ years	Global	1	2	Elevation Over 1500m (proportion)	405
Other chronic respiratory diseases	Female	1-4 years	95+ years	Global	1	2	Population Density (over 1000 ppl/sqkm, proportion)	--
Other chronic respiratory diseases	Female	1-4 years	95+ years	Global	-1	3	Socio-demographic Index	70
Other chronic respiratory diseases	Female	1-4 years	95+ years	Global	-1	3	Education (years per capita)	300
Other chronic respiratory diseases	Female	1-4 years	95+ years	Global	-1	3	LDI (I$ per capita)	--
Other chronic respiratory diseases	Male	1-4 years	95+ years	Data Rich	1	1	Smoking Prevalence	19
Other chronic respiratory diseases	Male	1-4 years	95+ years	Data Rich	1	1	Indoor Air Pollution (All Cooking Fuels)	230
Other chronic respiratory diseases	Male	1-4 years	95+ years	Data Rich	1	1	Outdoor Air Pollution (PM2.5)	253
Other chronic respiratory diseases	Male	1-4 years	95+ years	Data Rich	1	1	Cumulative Cigarettes (5 Years)	401
Other chronic respiratory diseases	Male	1-4 years	95+ years	Data Rich	1	1	Log-transformed SEV scalar: Oth Resp	997
Other chronic respiratory diseases	Male	1-4 years	95+ years	Data Rich	-1	2	Healthcare access and quality index	0
Other chronic respiratory diseases	Male	1-4 years	95+ years	Data Rich	1	2	Elevation Over 1500m (proportion)	60
Other chronic respiratory diseases	Male	1-4 years	95+ years	Data Rich	1	2	Elevation 500 to 1500m (proportion)	--
Other chronic respiratory diseases	Male	1-4 years	95+ years	Data Rich	1	2	Population Density (over 1000 ppl/sqkm, proportion)	--
Other chronic respiratory diseases	Male	1-4 years	95+ years	Data Rich	-1	3	Education (years per capita)	0
Other chronic respiratory diseases	Male	1-4 years	95+ years	Data Rich	-1	3	Socio-demographic Index	0
Other chronic respiratory diseases	Male	1-4 years	95+ years	Data Rich	-1	3	LDI (I$ per capita)	--
Other chronic respiratory diseases	Male	1-4 years	95+ years	Global	1	1	Cumulative Cigarettes (5 Years)	132
Other chronic respiratory diseases	Male	1-4 years	95+ years	Global	1	1	Smoking Prevalence	139
Other chronic respiratory diseases	Male	1-4 years	95+ years	Global	1	1	Indoor Air Pollution (All Cooking Fuels)	256
Other chronic respiratory diseases	Male	1-4 years	95+ years	Global	1	1	Log-transformed SEV scalar: Oth Resp	932
Other chronic respiratory diseases	Male	1-4 years	95+ years	Global	1	1	Outdoor Air Pollution (PM2.5)	--
Other chronic respiratory diseases	Male	1-4 years	95+ years	Global	-1	2	Healthcare access and quality index	182
Other chronic respiratory diseases	Male	1-4 years	95+ years	Global	1	2	Elevation Over 1500m (proportion)	390
Other chronic respiratory diseases	Male	1-4 years	95+ years	Global	1	2	Elevation 500 to 1500m (proportion)	410
Other chronic respiratory diseases	Male	1-4 years	95+ years	Global	1	2	Population Density (over 1000 ppl/sqkm, proportion)	--
Other chronic respiratory diseases	Male	1-4 years	95+ years	Global	-1	3	Socio-demographic Index	69
Other chronic respiratory diseases	Male	1-4 years	95+ years	Global	-1	3	Education (years per capita)	168
Other chronic respiratory diseases	Male	1-4 years	95+ years	Global	-1	3	LDI (I$ per capita)	--
Digestive diseases	Female	0-6 days	95+ years	Data Rich	-1	1	Sanitation (proportion with access)	417
Digestive diseases	Female	0-6 days	95+ years	Data Rich	1	1	Cumulative Cigarettes (5 Years)	596
Digestive diseases	Female	0-6 days	95+ years	Data Rich	1	1	Alcohol (liters per capita)	693
Digestive diseases	Female	0-6 days	95+ years	Data Rich	-1	2	fruits adjusted(g)	7
Digestive diseases	Female	0-6 days	95+ years	Data Rich	-1	2	Healthcare access and quality index	--
Digestive diseases	Female	0-6 days	95+ years	Data Rich	1	2	red meats adjusted(g)	--
Digestive diseases	Female	0-6 days	95+ years	Data Rich	-1	3	LDI (I$ per capita)	156

Appendix Table 9: CODEm covariates used, level of covariate, and expected direction of covariate by cause, sex, and age

Covariates that CODEm did not select during the covariate selection process have no draw counts listed.

Cause	Sex	Age Start	Age End	Model Type	Direction	Level	Covariate Name	Number of Draws
Digestive diseases	Female	0-6 days	95+ years	Data Rich	-1	3	Socio-demographic Index	317
Digestive diseases	Female	0-6 days	95+ years	Data Rich	-1	3	Education (years per capita)	472
Digestive diseases	Female	0-6 days	95+ years	Global	-1	1	Sanitation (proportion with access)	477
Digestive diseases	Female	0-6 days	95+ years	Global	1	1	Cumulative Cigarettes (5 Years)	150
Digestive diseases	Female	0-6 days	95+ years	Global	1	1	Alcohol (liters per capita)	814
Digestive diseases	Female	0-6 days	95+ years	Global	-1	2	fruits adjusted(g)	71
Digestive diseases	Female	0-6 days	95+ years	Global	-1	2	Healthcare access and quality index	211
Digestive diseases	Female	0-6 days	95+ years	Global	1	2	red meats adjusted(g)	--
Digestive diseases	Female	0-6 days	95+ years	Global	-1	3	LDI (I$ per capita)	137
Digestive diseases	Female	0-6 days	95+ years	Global	-1	3	Socio-demographic Index	168
Digestive diseases	Female	0-6 days	95+ years	Global	-1	3	Education (years per capita)	210
Digestive diseases	Male	0-6 days	95+ years	Data Rich	-1	1	Sanitation (proportion with access)	141
Digestive diseases	Male	0-6 days	95+ years	Data Rich	1	1	Cumulative Cigarettes (5 Years)	278
Digestive diseases	Male	0-6 days	95+ years	Data Rich	1	1	Alcohol (liters per capita)	905
Digestive diseases	Male	0-6 days	95+ years	Data Rich	-1	2	fruits adjusted(g)	85
Digestive diseases	Male	0-6 days	95+ years	Data Rich	-1	2	Healthcare access and quality index	183
Digestive diseases	Male	0-6 days	95+ years	Data Rich	1	2	red meats adjusted(g)	--
Digestive diseases	Male	0-6 days	95+ years	Data Rich	-1	3	LDI (I$ per capita)	27
Digestive diseases	Male	0-6 days	95+ years	Data Rich	-1	3	Education (years per capita)	142
Digestive diseases	Male	0-6 days	95+ years	Data Rich	-1	3	Socio-demographic Index	186
Digestive diseases	Male	0-6 days	95+ years	Global	-1	1	Sanitation (proportion with access)	414
Digestive diseases	Male	0-6 days	95+ years	Global	1	1	Cumulative Cigarettes (5 Years)	111
Digestive diseases	Male	0-6 days	95+ years	Global	1	1	Alcohol (liters per capita)	924
Digestive diseases	Male	0-6 days	95+ years	Global	-1	2	Healthcare access and quality index	116
Digestive diseases	Male	0-6 days	95+ years	Global	-1	2	fruits adjusted(g)	160
Digestive diseases	Male	0-6 days	95+ years	Global	1	2	red meats adjusted(g)	--
Digestive diseases	Male	0-6 days	95+ years	Global	-1	3	LDI (I$ per capita)	129
Digestive diseases	Male	0-6 days	95+ years	Global	-1	3	Socio-demographic Index	216
Digestive diseases	Male	0-6 days	95+ years	Global	-1	3	Education (years per capita)	314
Cirrhosis and other chronic liver diseases	Female	1-4 years	95+ years	Data Rich	-1	1	Hepatitis B 3-dose coverage (proportion)	--
Cirrhosis and other chronic liver diseases	Female	1-4 years	95+ years	Data Rich	1	1	Alcohol (liters per capita)	881
Cirrhosis and other chronic liver diseases	Female	1-4 years	95+ years	Data Rich	1	1	Schistosomiasis Prevalence (proportion)	931
Cirrhosis and other chronic liver diseases	Female	1-4 years	95+ years	Data Rich	1	1	Hepatitis B (HBsAg) Seroprevalence	--
Cirrhosis and other chronic liver diseases	Female	1-4 years	95+ years	Data Rich	1	1	Hepatitis C (IgG) Seroprevalence	--
Cirrhosis and other chronic liver diseases	Female	1-4 years	95+ years	Data Rich	-1	2	Healthcare access and quality index	187
Cirrhosis and other chronic liver diseases	Female	1-4 years	95+ years	Data Rich	1	2	Diabetes Age-Standardized Prevalence (proportion)	397
Cirrhosis and other chronic liver diseases	Female	1-4 years	95+ years	Data Rich	1	2	Mean BMI	--
Cirrhosis and other chronic liver diseases	Female	1-4 years	95+ years	Data Rich	-1	3	Health System Access 2 (unitless)	79
Cirrhosis and other chronic liver diseases	Female	1-4 years	95+ years	Data Rich	-1	3	Education (years per capita)	--
Cirrhosis and other chronic liver diseases	Female	1-4 years	95+ years	Data Rich	-1	3	LDI (I$ per capita)	--
Cirrhosis and other chronic liver diseases	Female	1-4 years	95+ years	Data Rich	0	3	Socio-demographic Index	363
Cirrhosis and other chronic liver diseases	Female	1-4 years	95+ years	Global	-1	1	Hepatitis B 3-dose coverage (proportion)	33
Cirrhosis and other chronic liver diseases	Female	1-4 years	95+ years	Global	1	1	Hepatitis B (HBsAg) Seroprevalence	22
Cirrhosis and other chronic liver diseases	Female	1-4 years	95+ years	Global	1	1	Schistosomiasis Prevalence (proportion)	247
Cirrhosis and other chronic liver diseases	Female	1-4 years	95+ years	Global	1	1	Alcohol (liters per capita)	937
Cirrhosis and other chronic liver diseases	Female	1-4 years	95+ years	Global	1	1	Hepatitis C (IgG) Seroprevalence	--
Cirrhosis and other chronic liver diseases	Female	1-4 years	95+ years	Global	-1	2	Healthcare access and quality index	54
Cirrhosis and other chronic liver diseases	Female	1-4 years	95+ years	Global	1	2	Mean BMI	365
Cirrhosis and other chronic liver diseases	Female	1-4 years	95+ years	Global	1	2	Diabetes Age-Standardized Prevalence (proportion)	--
Cirrhosis and other chronic liver diseases	Female	1-4 years	95+ years	Global	-1	3	Education (years per capita)	36
Cirrhosis and other chronic liver diseases	Female	1-4 years	95+ years	Global	-1	3	Health System Access 2 (unitless)	67
Cirrhosis and other chronic liver diseases	Female	1-4 years	95+ years	Global	-1	3	LDI (I$ per capita)	--
Cirrhosis and other chronic liver diseases	Female	1-4 years	95+ years	Global	0	3	Socio-demographic Index	579
Cirrhosis and other chronic liver diseases	Male	1-4 years	95+ years	Data Rich	-1	1	Hepatitis B 3-dose coverage (proportion)	42
Cirrhosis and other chronic liver diseases	Male	1-4 years	95+ years	Data Rich	1	1	Alcohol (liters per capita)	640
Cirrhosis and other chronic liver diseases	Male	1-4 years	95+ years	Data Rich	1	1	Schistosomiasis Prevalence (proportion)	683
Cirrhosis and other chronic liver diseases	Male	1-4 years	95+ years	Data Rich	1	1	Hepatitis B (HBsAg) Seroprevalence	--
Cirrhosis and other chronic liver diseases	Male	1-4 years	95+ years	Data Rich	1	1	Hepatitis C (IgG) Seroprevalence	--
Cirrhosis and other chronic liver diseases	Male	1-4 years	95+ years	Data Rich	-1	2	Healthcare access and quality index	50
Cirrhosis and other chronic liver diseases	Male	1-4 years	95+ years	Data Rich	1	2	Mean BMI	272
Cirrhosis and other chronic liver diseases	Male	1-4 years	95+ years	Data Rich	1	2	Diabetes Age-Standardized Prevalence (proportion)	--
Cirrhosis and other chronic liver diseases	Male	1-4 years	95+ years	Data Rich	-1	3	Health System Access 2 (unitless)	105
Cirrhosis and other chronic liver diseases	Male	1-4 years	95+ years	Data Rich	-1	3	Education (years per capita)	--
Cirrhosis and other chronic liver diseases	Male	1-4 years	95+ years	Data Rich	-1	3	LDI (I$ per capita)	--
Cirrhosis and other chronic liver diseases	Male	1-4 years	95+ years	Data Rich	0	3	Socio-demographic Index	739
Cirrhosis and other chronic liver diseases	Male	1-4 years	95+ years	Global	-1	1	Hepatitis B 3-dose coverage (proportion)	65
Cirrhosis and other chronic liver diseases	Male	1-4 years	95+ years	Global	1	1	Hepatitis B (HBsAg) Seroprevalence	63
Cirrhosis and other chronic liver diseases	Male	1-4 years	95+ years	Global	1	1	Schistosomiasis Prevalence (proportion)	415
Cirrhosis and other chronic liver diseases	Male	1-4 years	95+ years	Global	1	1	Alcohol (liters per capita)	837
Cirrhosis and other chronic liver diseases	Male	1-4 years	95+ years	Global	1	1	Hepatitis C (IgG) Seroprevalence	--
Cirrhosis and other chronic liver diseases	Male	1-4 years	95+ years	Global	-1	2	Healthcare access and quality index	60
Cirrhosis and other chronic liver diseases	Male	1-4 years	95+ years	Global	1	2	Mean BMI	535
Cirrhosis and other chronic liver diseases	Male	1-4 years	95+ years	Global	1	2	Diabetes Age-Standardized Prevalence (proportion)	--
Cirrhosis and other chronic liver diseases	Male	1-4 years	95+ years	Global	-1	3	Education (years per capita)	14
Cirrhosis and other chronic liver diseases	Male	1-4 years	95+ years	Global	-1	3	Health System Access 2 (unitless)	148
Cirrhosis and other chronic liver diseases	Male	1-4 years	95+ years	Global	-1	3	LDI (I$ per capita)	--
Cirrhosis and other chronic liver diseases	Male	1-4 years	95+ years	Global	0	3	Socio-demographic Index	517
Upper digestive system diseases	Female	1-4 years	95+ years	Data Rich	1	1	Cumulative Cigarettes (5 Years)	82

Appendix Table 9: CODEm covariates used, level of covariate, and expected direction of covariate by cause, sex, and age

Covariates that CODEm did not select during the covariate selection process have no draw counts listed.

Cause	Sex	Age Start	Age End	Model Type	Direction	Level	Covariate Name	Number of Draws
Upper digestive system diseases	Female	1-4 years	95+ years	Data Rich	1	1	Cumulative Cigarettes (10 Years)	288
Upper digestive system diseases	Female	1-4 years	95+ years	Data Rich	1	1	Smoking Prevalence	409
Upper digestive system diseases	Female	1-4 years	95+ years	Data Rich	1	1	Alcohol (liters per capita)	426
Upper digestive system diseases	Female	1-4 years	95+ years	Data Rich	-1	2	Sanitation (proportion with access)	18
Upper digestive system diseases	Female	1-4 years	95+ years	Data Rich	-1	2	Healthcare access and quality index	981
Upper digestive system diseases	Female	1-4 years	95+ years	Data Rich	0	2	vegetables adjusted(g)	999
Upper digestive system diseases	Female	1-4 years	95+ years	Data Rich	1	2	SEV unsafe water	807
Upper digestive system diseases	Female	1-4 years	95+ years	Data Rich	-1	3	Maternal Education (years per capita)	0
Upper digestive system diseases	Female	1-4 years	95+ years	Data Rich	-1	3	Socio-demographic Index	528
Upper digestive system diseases	Female	1-4 years	95+ years	Data Rich	-1	3	LDI (I$ per capita)	--
Upper digestive system diseases	Female	1-4 years	95+ years	Global	1	1	Smoking Prevalence	216
Upper digestive system diseases	Female	1-4 years	95+ years	Global	1	1	Cumulative Cigarettes (5 Years)	299
Upper digestive system diseases	Female	1-4 years	95+ years	Global	1	1	Cumulative Cigarettes (10 Years)	394
Upper digestive system diseases	Female	1-4 years	95+ years	Global	1	1	Alcohol (liters per capita)	640
Upper digestive system diseases	Female	1-4 years	95+ years	Global	-1	2	Sanitation (proportion with access)	69
Upper digestive system diseases	Female	1-4 years	95+ years	Global	-1	2	Healthcare access and quality index	654
Upper digestive system diseases	Female	1-4 years	95+ years	Global	0	2	vegetables adjusted(g)	699
Upper digestive system diseases	Female	1-4 years	95+ years	Global	1	2	SEV unsafe water	313
Upper digestive system diseases	Female	1-4 years	95+ years	Global	-1	3	Socio-demographic Index	286
Upper digestive system diseases	Female	1-4 years	95+ years	Global	-1	3	Maternal Education (years per capita)	344
Upper digestive system diseases	Female	1-4 years	95+ years	Global	-1	3	LDI (I$ per capita)	--
Upper digestive system diseases	Male	1-4 years	95+ years	Data Rich	1	1	Alcohol (liters per capita)	1
Upper digestive system diseases	Male	1-4 years	95+ years	Data Rich	1	1	Cumulative Cigarettes (10 Years)	362
Upper digestive system diseases	Male	1-4 years	95+ years	Data Rich	1	1	Smoking Prevalence	605
Upper digestive system diseases	Male	1-4 years	95+ years	Data Rich	1	1	Cumulative Cigarettes (5 Years)	637
Upper digestive system diseases	Male	1-4 years	95+ years	Data Rich	-1	2	Healthcare access and quality index	2
Upper digestive system diseases	Male	1-4 years	95+ years	Data Rich	-1	2	Sanitation (proportion with access)	690
Upper digestive system diseases	Male	1-4 years	95+ years	Data Rich	0	2	vegetables adjusted(g)	591
Upper digestive system diseases	Male	1-4 years	95+ years	Data Rich	1	2	SEV unsafe water	448
Upper digestive system diseases	Male	1-4 years	95+ years	Data Rich	-1	3	Maternal Education (years per capita)	153
Upper digestive system diseases	Male	1-4 years	95+ years	Data Rich	-1	3	Socio-demographic Index	337
Upper digestive system diseases	Male	1-4 years	95+ years	Data Rich	-1	3	LDI (I$ per capita)	--
Upper digestive system diseases	Male	1-4 years	95+ years	Global	1	1	Cumulative Cigarettes (5 Years)	242
Upper digestive system diseases	Male	1-4 years	95+ years	Global	1	1	Smoking Prevalence	286
Upper digestive system diseases	Male	1-4 years	95+ years	Global	1	1	Cumulative Cigarettes (10 Years)	406
Upper digestive system diseases	Male	1-4 years	95+ years	Global	1	1	Alcohol (liters per capita)	753
Upper digestive system diseases	Male	1-4 years	95+ years	Global	-1	2	Sanitation (proportion with access)	783
Upper digestive system diseases	Male	1-4 years	95+ years	Global	-1	2	Healthcare access and quality index	852
Upper digestive system diseases	Male	1-4 years	95+ years	Global	0	2	vegetables adjusted(g)	793
Upper digestive system diseases	Male	1-4 years	95+ years	Global	1	2	SEV unsafe water	100
Upper digestive system diseases	Male	1-4 years	95+ years	Global	-1	3	Maternal Education (years per capita)	85
Upper digestive system diseases	Male	1-4 years	95+ years	Global	-1	3	Socio-demographic Index	124
Upper digestive system diseases	Male	1-4 years	95+ years	Global	-1	3	LDI (I$ per capita)	--
Peptic ulcer disease	Female	1-4 years	95+ years	Data Rich	1	1	Cumulative Cigarettes (10 Years)	288
Peptic ulcer disease	Female	1-4 years	95+ years	Data Rich	1	1	Cumulative Cigarettes (5 Years)	296
Peptic ulcer disease	Female	1-4 years	95+ years	Data Rich	1	1	Alcohol (liters per capita)	478
Peptic ulcer disease	Female	1-4 years	95+ years	Data Rich	1	1	Smoking Prevalence	741
Peptic ulcer disease	Female	1-4 years	95+ years	Data Rich	-1	2	Sanitation (proportion with access)	0
Peptic ulcer disease	Female	1-4 years	95+ years	Data Rich	-1	2	Healthcare access and quality index	961
Peptic ulcer disease	Female	1-4 years	95+ years	Data Rich	0	2	vegetables adjusted(g)	956
Peptic ulcer disease	Female	1-4 years	95+ years	Data Rich	1	2	SEV unsafe water	965
Peptic ulcer disease	Female	1-4 years	95+ years	Data Rich	-1	3	Maternal Education (years per capita)	48
Peptic ulcer disease	Female	1-4 years	95+ years	Data Rich	-1	3	Socio-demographic Index	448
Peptic ulcer disease	Female	1-4 years	95+ years	Data Rich	-1	3	LDI (I$ per capita)	--
Peptic ulcer disease	Female	1-4 years	95+ years	Global	1	1	Cumulative Cigarettes (5 Years)	244
Peptic ulcer disease	Female	1-4 years	95+ years	Global	1	1	Cumulative Cigarettes (10 Years)	381
Peptic ulcer disease	Female	1-4 years	95+ years	Global	1	1	Smoking Prevalence	684
Peptic ulcer disease	Female	1-4 years	95+ years	Global	1	1	Alcohol (liters per capita)	966
Peptic ulcer disease	Female	1-4 years	95+ years	Global	-1	2	Sanitation (proportion with access)	15
Peptic ulcer disease	Female	1-4 years	95+ years	Global	-1	2	Healthcare access and quality index	930
Peptic ulcer disease	Female	1-4 years	95+ years	Global	0	2	vegetables adjusted(g)	933
Peptic ulcer disease	Female	1-4 years	95+ years	Global	1	2	SEV unsafe water	930
Peptic ulcer disease	Female	1-4 years	95+ years	Global	-1	3	Maternal Education (years per capita)	36
Peptic ulcer disease	Female	1-4 years	95+ years	Global	-1	3	Socio-demographic Index	368
Peptic ulcer disease	Female	1-4 years	95+ years	Global	-1	3	LDI (I$ per capita)	--
Peptic ulcer disease	Male	1-4 years	95+ years	Data Rich	1	1	Cumulative Cigarettes (10 Years)	101
Peptic ulcer disease	Male	1-4 years	95+ years	Data Rich	1	1	Smoking Prevalence	505
Peptic ulcer disease	Male	1-4 years	95+ years	Data Rich	1	1	Cumulative Cigarettes (5 Years)	580
Peptic ulcer disease	Male	1-4 years	95+ years	Data Rich	1	1	Alcohol (liters per capita)	775
Peptic ulcer disease	Male	1-4 years	95+ years	Data Rich	-1	2	Healthcare access and quality index	955
Peptic ulcer disease	Male	1-4 years	95+ years	Data Rich	-1	2	Sanitation (proportion with access)	979
Peptic ulcer disease	Male	1-4 years	95+ years	Data Rich	0	2	vegetables adjusted(g)	923
Peptic ulcer disease	Male	1-4 years	95+ years	Data Rich	1	2	SEV unsafe water	944
Peptic ulcer disease	Male	1-4 years	95+ years	Data Rich	-1	3	Maternal Education (years per capita)	75
Peptic ulcer disease	Male	1-4 years	95+ years	Data Rich	-1	3	Socio-demographic Index	129
Peptic ulcer disease	Male	1-4 years	95+ years	Data Rich	-1	3	LDI (I$ per capita)	--
Peptic ulcer disease	Male	1-4 years	95+ years	Global	1	1	Cumulative Cigarettes (5 Years)	346
Peptic ulcer disease	Male	1-4 years	95+ years	Global	1	1	Cumulative Cigarettes (10 Years)	409

Appendix Table 9: CODEm covariates used, level of covariate, and expected direction of covariate by cause, sex, and age<br>*Covariates that CODEm did not select during the covariate selection process have no draw counts listed.*								
**Cause**	**Sex**	**Age Start**	**Age End**	**Model Type**	**Direction**	**Level**	**Covariate Name**	**Number of Draws**
Peptic ulcer disease	Male	1-4 years	95+ years	Global	1	1	Smoking Prevalence	465
Peptic ulcer disease	Male	1-4 years	95+ years	Global	1	1	Alcohol (liters per capita)	719
Peptic ulcer disease	Male	1-4 years	95+ years	Global	-1	2	Sanitation (proportion with access)	789
Peptic ulcer disease	Male	1-4 years	95+ years	Global	-1	2	Healthcare access and quality index	852
Peptic ulcer disease	Male	1-4 years	95+ years	Global	0	2	vegetables adjusted(g)	855
Peptic ulcer disease	Male	1-4 years	95+ years	Global	1	2	SEV unsafe water	525
Peptic ulcer disease	Male	1-4 years	95+ years	Global	-1	3	Maternal Education (years per capita)	125
Peptic ulcer disease	Male	1-4 years	95+ years	Global	-1	3	Socio-demographic Index	135
Peptic ulcer disease	Male	1-4 years	95+ years	Global	-1	3	LDI (I$ per capita)	--
Gastritis and duodenitis	Female	1-4 years	95+ years	Data Rich	1	1	Cumulative Cigarettes (5 Years)	5
Gastritis and duodenitis	Female	1-4 years	95+ years	Data Rich	1	1	Cumulative Cigarettes (10 Years)	161
Gastritis and duodenitis	Female	1-4 years	95+ years	Data Rich	1	1	Alcohol (liters per capita)	907
Gastritis and duodenitis	Female	1-4 years	95+ years	Data Rich	1	1	Smoking Prevalence	--
Gastritis and duodenitis	Female	1-4 years	95+ years	Data Rich	-1	2	Sanitation (proportion with access)	142
Gastritis and duodenitis	Female	1-4 years	95+ years	Data Rich	-1	2	Healthcare access and quality index	185
Gastritis and duodenitis	Female	1-4 years	95+ years	Data Rich	0	2	vegetables adjusted(g)	81
Gastritis and duodenitis	Female	1-4 years	95+ years	Data Rich	1	2	SEV unsafe water	162
Gastritis and duodenitis	Female	1-4 years	95+ years	Data Rich	-1	3	Education (years per capita)	15
Gastritis and duodenitis	Female	1-4 years	95+ years	Data Rich	-1	3	Socio-demographic Index	62
Gastritis and duodenitis	Female	1-4 years	95+ years	Data Rich	-1	3	LDI (I$ per capita)	--
Gastritis and duodenitis	Female	1-4 years	95+ years	Global	1	1	Cumulative Cigarettes (10 Years)	100
Gastritis and duodenitis	Female	1-4 years	95+ years	Global	1	1	Alcohol (liters per capita)	--
Gastritis and duodenitis	Female	1-4 years	95+ years	Global	1	1	Cumulative Cigarettes (5 Years)	--
Gastritis and duodenitis	Female	1-4 years	95+ years	Global	1	1	Smoking Prevalence	--
Gastritis and duodenitis	Female	1-4 years	95+ years	Global	-1	2	Healthcare access and quality index	151
Gastritis and duodenitis	Female	1-4 years	95+ years	Global	-1	2	Sanitation (proportion with access)	463
Gastritis and duodenitis	Female	1-4 years	95+ years	Global	0	2	vegetables adjusted(g)	614
Gastritis and duodenitis	Female	1-4 years	95+ years	Global	1	2	SEV unsafe water	--
Gastritis and duodenitis	Female	1-4 years	95+ years	Global	-1	3	Socio-demographic Index	120
Gastritis and duodenitis	Female	1-4 years	95+ years	Global	-1	3	Education (years per capita)	--
Gastritis and duodenitis	Female	1-4 years	95+ years	Global	-1	3	LDI (I$ per capita)	--
Gastritis and duodenitis	Male	1-4 years	95+ years	Data Rich	1	1	Cumulative Cigarettes (5 Years)	28
Gastritis and duodenitis	Male	1-4 years	95+ years	Data Rich	1	1	Alcohol (liters per capita)	435
Gastritis and duodenitis	Male	1-4 years	95+ years	Data Rich	1	1	Smoking Prevalence	596
Gastritis and duodenitis	Male	1-4 years	95+ years	Data Rich	1	1	Cumulative Cigarettes (10 Years)	671
Gastritis and duodenitis	Male	1-4 years	95+ years	Data Rich	-1	2	Healthcare access and quality index	9
Gastritis and duodenitis	Male	1-4 years	95+ years	Data Rich	-1	2	Sanitation (proportion with access)	164
Gastritis and duodenitis	Male	1-4 years	95+ years	Data Rich	0	2	vegetables adjusted(g)	249
Gastritis and duodenitis	Male	1-4 years	95+ years	Data Rich	1	2	SEV unsafe water	16
Gastritis and duodenitis	Male	1-4 years	95+ years	Data Rich	-1	3	Education (years per capita)	5
Gastritis and duodenitis	Male	1-4 years	95+ years	Data Rich	-1	3	Socio-demographic Index	14
Gastritis and duodenitis	Male	1-4 years	95+ years	Data Rich	-1	3	LDI (I$ per capita)	--
Gastritis and duodenitis	Male	1-4 years	95+ years	Global	1	1	Cumulative Cigarettes (5 Years)	24
Gastritis and duodenitis	Male	1-4 years	95+ years	Global	1	1	Alcohol (liters per capita)	241
Gastritis and duodenitis	Male	1-4 years	95+ years	Global	1	1	Cumulative Cigarettes (10 Years)	465
Gastritis and duodenitis	Male	1-4 years	95+ years	Global	1	1	Smoking Prevalence	508
Gastritis and duodenitis	Male	1-4 years	95+ years	Global	-1	2	Healthcare access and quality index	83
Gastritis and duodenitis	Male	1-4 years	95+ years	Global	-1	2	Sanitation (proportion with access)	656
Gastritis and duodenitis	Male	1-4 years	95+ years	Global	0	2	vegetables adjusted(g)	484
Gastritis and duodenitis	Male	1-4 years	95+ years	Global	1	2	SEV unsafe water	3
Gastritis and duodenitis	Male	1-4 years	95+ years	Global	-1	3	Socio-demographic Index	8
Gastritis and duodenitis	Male	1-4 years	95+ years	Global	-1	3	Education (years per capita)	12
Gastritis and duodenitis	Male	1-4 years	95+ years	Global	-1	3	LDI (I$ per capita)	--
Appendicitis	Female	1-4 years	95+ years	Data Rich	-1	2	vegetables adjusted(g)	265
Appendicitis	Female	1-4 years	95+ years	Data Rich	-1	2	fruits adjusted(g)	425
Appendicitis	Female	1-4 years	95+ years	Data Rich	-1	2	Healthcare access and quality index	595
Appendicitis	Female	1-4 years	95+ years	Data Rich	-1	3	Education (years per capita)	12
Appendicitis	Female	1-4 years	95+ years	Data Rich	-1	3	Socio-demographic Index	48
Appendicitis	Female	1-4 years	95+ years	Data Rich	-1	3	Health System Access (capped)	460
Appendicitis	Female	1-4 years	95+ years	Data Rich	-1	3	LDI (I$ per capita)	--
Appendicitis	Female	1-4 years	95+ years	Global	-1	2	fruits adjusted(g)	225
Appendicitis	Female	1-4 years	95+ years	Global	-1	2	vegetables adjusted(g)	433
Appendicitis	Female	1-4 years	95+ years	Global	-1	2	Healthcare access and quality index	653
Appendicitis	Female	1-4 years	95+ years	Global	-1	3	Education (years per capita)	94
Appendicitis	Female	1-4 years	95+ years	Global	-1	3	Health System Access (capped)	136
Appendicitis	Female	1-4 years	95+ years	Global	-1	3	Socio-demographic Index	310
Appendicitis	Female	1-4 years	95+ years	Global	-1	3	LDI (I$ per capita)	--
Appendicitis	Male	1-4 years	95+ years	Data Rich	-1	2	fruits adjusted(g)	283
Appendicitis	Male	1-4 years	95+ years	Data Rich	-1	2	vegetables adjusted(g)	557
Appendicitis	Male	1-4 years	95+ years	Data Rich	-1	2	Healthcare access and quality index	654
Appendicitis	Male	1-4 years	95+ years	Data Rich	-1	3	Education (years per capita)	12
Appendicitis	Male	1-4 years	95+ years	Data Rich	-1	3	Health System Access (capped)	24
Appendicitis	Male	1-4 years	95+ years	Data Rich	-1	3	Socio-demographic Index	502
Appendicitis	Male	1-4 years	95+ years	Data Rich	-1	3	LDI (I$ per capita)	--
Appendicitis	Male	1-4 years	95+ years	Global	-1	2	vegetables adjusted(g)	217
Appendicitis	Male	1-4 years	95+ years	Global	-1	2	fruits adjusted(g)	394
Appendicitis	Male	1-4 years	95+ years	Global	-1	2	Healthcare access and quality index	728
Appendicitis	Male	1-4 years	95+ years	Global	-1	3	Education (years per capita)	58

**Appendix Table 9: CODEm covariates used, level of covariate, and expected direction of covariate by cause, sex, and age** *Covariates that CODEm did not select during the covariate selection process have no draw counts listed.*								
**Cause**	**Sex**	**Age Start**	**Age End**	**Model Type**	**Direction**	**Level**	**Covariate Name**	**Number of Draws**
Appendicitis	Male	1-4 years	95+ years	Global	-1	3	Health System Access (capped)	161
Appendicitis	Male	1-4 years	95+ years	Global	-1	3	Socio-demographic Index	257
Appendicitis	Male	1-4 years	95+ years	Global	-1	3	LDI (I$ per capita)	--
Paralytic ileus and intestinal obstruction	Female	0-6 days	95+ years	Data Rich	-1	2	Healthcare access and quality index	1000
Paralytic ileus and intestinal obstruction	Female	0-6 days	95+ years	Data Rich	-1	2	vegetables adjusted(g)	1000
Paralytic ileus and intestinal obstruction	Female	0-6 days	95+ years	Data Rich	-1	2	fruits adjusted(g)	--
Paralytic ileus and intestinal obstruction	Female	0-6 days	95+ years	Data Rich	-1	3	Education (years per capita)	--
Paralytic ileus and intestinal obstruction	Female	0-6 days	95+ years	Data Rich	-1	3	Health System Access (capped)	--
Paralytic ileus and intestinal obstruction	Female	0-6 days	95+ years	Data Rich	-1	3	LDI (I$ per capita)	--
Paralytic ileus and intestinal obstruction	Female	0-6 days	95+ years	Data Rich	-1	3	Socio-demographic Index	--
Paralytic ileus and intestinal obstruction	Female	0-6 days	95+ years	Global	-1	2	Healthcare access and quality index	463
Paralytic ileus and intestinal obstruction	Female	0-6 days	95+ years	Global	-1	2	fruits adjusted(g)	537
Paralytic ileus and intestinal obstruction	Female	0-6 days	95+ years	Global	-1	2	vegetables adjusted(g)	559
Paralytic ileus and intestinal obstruction	Female	0-6 days	95+ years	Global	-1	3	Socio-demographic Index	133
Paralytic ileus and intestinal obstruction	Female	0-6 days	95+ years	Global	-1	3	Education (years per capita)	166
Paralytic ileus and intestinal obstruction	Female	0-6 days	95+ years	Global	-1	3	Health System Access (capped)	166
Paralytic ileus and intestinal obstruction	Female	0-6 days	95+ years	Global	-1	3	LDI (I$ per capita)	--
Paralytic ileus and intestinal obstruction	Male	0-6 days	95+ years	Data Rich	-1	2	Healthcare access and quality index	326
Paralytic ileus and intestinal obstruction	Male	0-6 days	95+ years	Data Rich	-1	2	vegetables adjusted(g)	674
Paralytic ileus and intestinal obstruction	Male	0-6 days	95+ years	Data Rich	-1	2	fruits adjusted(g)	--
Paralytic ileus and intestinal obstruction	Male	0-6 days	95+ years	Data Rich	-1	3	Health System Access (capped)	222
Paralytic ileus and intestinal obstruction	Male	0-6 days	95+ years	Data Rich	-1	3	Education (years per capita)	267
Paralytic ileus and intestinal obstruction	Male	0-6 days	95+ years	Data Rich	-1	3	LDI (I$ per capita)	--
Paralytic ileus and intestinal obstruction	Male	0-6 days	95+ years	Data Rich	-1	3	Socio-demographic Index	--
Paralytic ileus and intestinal obstruction	Male	0-6 days	95+ years	Global	-1	2	vegetables adjusted(g)	514
Paralytic ileus and intestinal obstruction	Male	0-6 days	95+ years	Global	-1	2	Healthcare access and quality index	706
Paralytic ileus and intestinal obstruction	Male	0-6 days	95+ years	Global	-1	2	fruits adjusted(g)	796
Paralytic ileus and intestinal obstruction	Male	0-6 days	95+ years	Global	-1	3	Socio-demographic Index	161
Paralytic ileus and intestinal obstruction	Male	0-6 days	95+ years	Global	-1	3	Education (years per capita)	--
Paralytic ileus and intestinal obstruction	Male	0-6 days	95+ years	Global	-1	3	Health System Access (capped)	--
Paralytic ileus and intestinal obstruction	Male	0-6 days	95+ years	Global	-1	3	LDI (I$ per capita)	--
Inguinal, femoral, and abdominal hernia	Female	1-4 years	95+ years	Data Rich	-1	1	Mean BMI	751
Inguinal, femoral, and abdominal hernia	Female	1-4 years	95+ years	Data Rich	1	1	Smoking Prevalence	243
Inguinal, femoral, and abdominal hernia	Female	1-4 years	95+ years	Data Rich	1	1	Cumulative Cigarettes (10 Years)	249
Inguinal, femoral, and abdominal hernia	Female	1-4 years	95+ years	Data Rich	1	1	Cumulative Cigarettes (5 Years)	--
Inguinal, femoral, and abdominal hernia	Female	1-4 years	95+ years	Data Rich	-1	2	Healthcare access and quality index	--
Inguinal, femoral, and abdominal hernia	Female	1-4 years	95+ years	Data Rich	-1	3	Education (years per capita)	--
Inguinal, femoral, and abdominal hernia	Female	1-4 years	95+ years	Data Rich	-1	3	LDI (I$ per capita)	--
Inguinal, femoral, and abdominal hernia	Female	1-4 years	95+ years	Data Rich	0	3	Socio-demographic Index	--
Inguinal, femoral, and abdominal hernia	Female	1-4 years	95+ years	Global	-1	1	Mean BMI	816
Inguinal, femoral, and abdominal hernia	Female	1-4 years	95+ years	Global	1	1	Cumulative Cigarettes (10 Years)	409
Inguinal, femoral, and abdominal hernia	Female	1-4 years	95+ years	Global	1	1	Cumulative Cigarettes (5 Years)	485
Inguinal, femoral, and abdominal hernia	Female	1-4 years	95+ years	Global	1	1	Smoking Prevalence	--
Inguinal, femoral, and abdominal hernia	Female	1-4 years	95+ years	Global	-1	2	Healthcare access and quality index	--
Inguinal, femoral, and abdominal hernia	Female	1-4 years	95+ years	Global	-1	3	Education (years per capita)	330
Inguinal, femoral, and abdominal hernia	Female	1-4 years	95+ years	Global	-1	3	LDI (I$ per capita)	--
Inguinal, femoral, and abdominal hernia	Female	1-4 years	95+ years	Global	0	3	Socio-demographic Index	227
Inguinal, femoral, and abdominal hernia	Male	1-4 years	95+ years	Data Rich	-1	1	Mean BMI	185
Inguinal, femoral, and abdominal hernia	Male	1-4 years	95+ years	Data Rich	1	1	Smoking Prevalence	87
Inguinal, femoral, and abdominal hernia	Male	1-4 years	95+ years	Data Rich	1	1	Cumulative Cigarettes (10 Years)	175
Inguinal, femoral, and abdominal hernia	Male	1-4 years	95+ years	Data Rich	1	1	Cumulative Cigarettes (5 Years)	681
Inguinal, femoral, and abdominal hernia	Male	1-4 years	95+ years	Data Rich	-1	2	Healthcare access and quality index	430
Inguinal, femoral, and abdominal hernia	Male	1-4 years	95+ years	Data Rich	-1	3	Education (years per capita)	496
Inguinal, femoral, and abdominal hernia	Male	1-4 years	95+ years	Data Rich	-1	3	LDI (I$ per capita)	--
Inguinal, femoral, and abdominal hernia	Male	1-4 years	95+ years	Data Rich	0	3	Socio-demographic Index	340
Inguinal, femoral, and abdominal hernia	Male	1-4 years	95+ years	Global	-1	1	Mean BMI	635
Inguinal, femoral, and abdominal hernia	Male	1-4 years	95+ years	Global	1	1	Cumulative Cigarettes (5 Years)	73
Inguinal, femoral, and abdominal hernia	Male	1-4 years	95+ years	Global	1	1	Cumulative Cigarettes (10 Years)	380
Inguinal, femoral, and abdominal hernia	Male	1-4 years	95+ years	Global	1	1	Smoking Prevalence	560
Inguinal, femoral, and abdominal hernia	Male	1-4 years	95+ years	Global	-1	2	Healthcare access and quality index	67
Inguinal, femoral, and abdominal hernia	Male	1-4 years	95+ years	Global	-1	3	Education (years per capita)	191
Inguinal, femoral, and abdominal hernia	Male	1-4 years	95+ years	Global	-1	3	LDI (I$ per capita)	--
Inguinal, femoral, and abdominal hernia	Male	1-4 years	95+ years	Global	0	3	Socio-demographic Index	361
Inflammatory bowel disease	Female	1-4 years	95+ years	Data Rich	-1	1	fruits adjusted(g)	--
Inflammatory bowel disease	Female	1-4 years	95+ years	Data Rich	-1	1	vegetables adjusted(g)	--
Inflammatory bowel disease	Female	1-4 years	95+ years	Data Rich	1	1	saturated fats adjusted(percent)	455
Inflammatory bowel disease	Female	1-4 years	95+ years	Data Rich	1	1	red meats adjusted(g)	--
Inflammatory bowel disease	Female	1-4 years	95+ years	Data Rich	-1	2	Healthcare access and quality index	411
Inflammatory bowel disease	Female	1-4 years	95+ years	Data Rich	-1	2	Latitude 15 to 30 (proportion)	--
Inflammatory bowel disease	Female	1-4 years	95+ years	Data Rich	1	2	Latitude Over 45 (proportion)	623
Inflammatory bowel disease	Female	1-4 years	95+ years	Data Rich	1	2	Latitude 30 to 45 (proportion)	--
Inflammatory bowel disease	Female	1-4 years	95+ years	Data Rich	-1	3	Education (years per capita)	--
Inflammatory bowel disease	Female	1-4 years	95+ years	Data Rich	0	3	Socio-demographic Index	430
Inflammatory bowel disease	Female	1-4 years	95+ years	Data Rich	0	3	LDI (I$ per capita)	--
Inflammatory bowel disease	Female	1-4 years	95+ years	Global	-1	1	vegetables adjusted(g)	369
Inflammatory bowel disease	Female	1-4 years	95+ years	Global	-1	1	fruits adjusted(g)	428
Inflammatory bowel disease	Female	1-4 years	95+ years	Global	1	1	saturated fats adjusted(percent)	338
Inflammatory bowel disease	Female	1-4 years	95+ years	Global	1	1	red meats adjusted(g)	--

Appendix Table 9: CODEm covariates used, level of covariate, and expected direction of covariate by cause, sex, and age<br>*Covariates that CODEm did not select during the covariate selection process have no draw counts listed.*								
**Cause**	**Sex**	**Age Start**	**Age End**	**Model Type**	**Direction**	**Level**	**Covariate Name**	**Number of Draws**
Inflammatory bowel disease	Female	1-4 years	95+ years	Global	-1	2	Healthcare access and quality index	382
Inflammatory bowel disease	Female	1-4 years	95+ years	Global	-1	2	Latitude 15 to 30 (proportion)	--
Inflammatory bowel disease	Female	1-4 years	95+ years	Global	1	2	Latitude Over 45 (proportion)	501
Inflammatory bowel disease	Female	1-4 years	95+ years	Global	1	2	Latitude 30 to 45 (proportion)	--
Inflammatory bowel disease	Female	1-4 years	95+ years	Global	-1	3	Education (years per capita)	72
Inflammatory bowel disease	Female	1-4 years	95+ years	Global	0	3	Socio-demographic Index	281
Inflammatory bowel disease	Female	1-4 years	95+ years	Global	0	3	LDI (I$ per capita)	--
Inflammatory bowel disease	Male	1-4 years	95+ years	Data Rich	-1	1	fruits adjusted(g)	751
Inflammatory bowel disease	Male	1-4 years	95+ years	Data Rich	-1	1	vegetables adjusted(g)	--
Inflammatory bowel disease	Male	1-4 years	95+ years	Data Rich	1	1	saturated fats adjusted(percent)	249
Inflammatory bowel disease	Male	1-4 years	95+ years	Data Rich	1	1	red meats adjusted(g)	--
Inflammatory bowel disease	Male	1-4 years	95+ years	Data Rich	-1	2	Healthcare access and quality index	--
Inflammatory bowel disease	Male	1-4 years	95+ years	Data Rich	-1	2	Latitude 15 to 30 (proportion)	--
Inflammatory bowel disease	Male	1-4 years	95+ years	Data Rich	1	2	Latitude Over 45 (proportion)	485
Inflammatory bowel disease	Male	1-4 years	95+ years	Data Rich	1	2	Latitude 30 to 45 (proportion)	--
Inflammatory bowel disease	Male	1-4 years	95+ years	Data Rich	-1	3	Education (years per capita)	453
Inflammatory bowel disease	Male	1-4 years	95+ years	Data Rich	0	3	Socio-demographic Index	453
Inflammatory bowel disease	Male	1-4 years	95+ years	Data Rich	0	3	LDI (I$ per capita)	--
Inflammatory bowel disease	Male	1-4 years	95+ years	Global	-1	1	fruits adjusted(g)	337
Inflammatory bowel disease	Male	1-4 years	95+ years	Global	-1	1	vegetables adjusted(g)	432
Inflammatory bowel disease	Male	1-4 years	95+ years	Global	1	1	saturated fats adjusted(percent)	342
Inflammatory bowel disease	Male	1-4 years	95+ years	Global	1	1	red meats adjusted(g)	--
Inflammatory bowel disease	Male	1-4 years	95+ years	Global	-1	2	Healthcare access and quality index	387
Inflammatory bowel disease	Male	1-4 years	95+ years	Global	-1	2	Latitude 15 to 30 (proportion)	--
Inflammatory bowel disease	Male	1-4 years	95+ years	Global	1	2	Latitude Over 45 (proportion)	629
Inflammatory bowel disease	Male	1-4 years	95+ years	Global	1	2	Latitude 30 to 45 (proportion)	--
Inflammatory bowel disease	Male	1-4 years	95+ years	Global	-1	3	Education (years per capita)	66
Inflammatory bowel disease	Male	1-4 years	95+ years	Global	0	3	Socio-demographic Index	305
Inflammatory bowel disease	Male	1-4 years	95+ years	Global	0	3	LDI (I$ per capita)	--
Vascular intestinal disorders	Female	1-4 years	95+ years	Data Rich	1	1	saturated fats adjusted(percent)	87
Vascular intestinal disorders	Female	1-4 years	95+ years	Data Rich	1	1	Diabetes Fasting Plasma Glucose (mmol/L)	496
Vascular intestinal disorders	Female	1-4 years	95+ years	Data Rich	1	1	Diabetes Age-Standardized Prevalence (proportion)	504
Vascular intestinal disorders	Female	1-4 years	95+ years	Data Rich	1	1	Cholesterol (total, mean per capita)	--
Vascular intestinal disorders	Female	1-4 years	95+ years	Data Rich	1	1	Systolic Blood Pressure (mmHg)	--
Vascular intestinal disorders	Female	1-4 years	95+ years	Data Rich	-1	2	Healthcare access and quality index	--
Vascular intestinal disorders	Female	1-4 years	95+ years	Data Rich	-1	2	fruits adjusted(g)	--
Vascular intestinal disorders	Female	1-4 years	95+ years	Data Rich	-1	2	vegetables adjusted(g)	--
Vascular intestinal disorders	Female	1-4 years	95+ years	Data Rich	1	2	Alcohol (liters per capita)	--
Vascular intestinal disorders	Female	1-4 years	95+ years	Data Rich	-1	3	Education (years per capita)	--
Vascular intestinal disorders	Female	1-4 years	95+ years	Data Rich	-1	3	LDI (I$ per capita)	--
Vascular intestinal disorders	Female	1-4 years	95+ years	Data Rich	0	3	Socio-demographic Index	262
Vascular intestinal disorders	Female	1-4 years	95+ years	Data Rich	1	3	Latitude Over 45 (proportion)	535
Vascular intestinal disorders	Female	1-4 years	95+ years	Global	1	1	saturated fats adjusted(percent)	330
Vascular intestinal disorders	Female	1-4 years	95+ years	Global	1	1	Diabetes Age-Standardized Prevalence (proportion)	417
Vascular intestinal disorders	Female	1-4 years	95+ years	Global	1	1	Diabetes Fasting Plasma Glucose (mmol/L)	493
Vascular intestinal disorders	Female	1-4 years	95+ years	Global	1	1	Cholesterol (total, mean per capita)	--
Vascular intestinal disorders	Female	1-4 years	95+ years	Global	1	1	Systolic Blood Pressure (mmHg)	--
Vascular intestinal disorders	Female	1-4 years	95+ years	Global	-1	2	Healthcare access and quality index	--
Vascular intestinal disorders	Female	1-4 years	95+ years	Global	-1	2	fruits adjusted(g)	--
Vascular intestinal disorders	Female	1-4 years	95+ years	Global	-1	2	vegetables adjusted(g)	--
Vascular intestinal disorders	Female	1-4 years	95+ years	Global	1	2	Alcohol (liters per capita)	--
Vascular intestinal disorders	Female	1-4 years	95+ years	Global	-1	3	Education (years per capita)	--
Vascular intestinal disorders	Female	1-4 years	95+ years	Global	-1	3	LDI (I$ per capita)	--
Vascular intestinal disorders	Female	1-4 years	95+ years	Global	0	3	Socio-demographic Index	102
Vascular intestinal disorders	Female	1-4 years	95+ years	Global	1	3	Latitude Over 45 (proportion)	493
Vascular intestinal disorders	Male	1-4 years	95+ years	Data Rich	1	1	saturated fats adjusted(percent)	98
Vascular intestinal disorders	Male	1-4 years	95+ years	Data Rich	1	1	Diabetes Fasting Plasma Glucose (mmol/L)	167
Vascular intestinal disorders	Male	1-4 years	95+ years	Data Rich	1	1	Diabetes Age-Standardized Prevalence (proportion)	572
Vascular intestinal disorders	Male	1-4 years	95+ years	Data Rich	1	1	Cholesterol (total, mean per capita)	--
Vascular intestinal disorders	Male	1-4 years	95+ years	Data Rich	1	1	Systolic Blood Pressure (mmHg)	--
Vascular intestinal disorders	Male	1-4 years	95+ years	Data Rich	-1	2	Healthcare access and quality index	--
Vascular intestinal disorders	Male	1-4 years	95+ years	Data Rich	-1	2	fruits adjusted(g)	--
Vascular intestinal disorders	Male	1-4 years	95+ years	Data Rich	-1	2	vegetables adjusted(g)	--
Vascular intestinal disorders	Male	1-4 years	95+ years	Data Rich	1	2	Alcohol (liters per capita)	523
Vascular intestinal disorders	Male	1-4 years	95+ years	Data Rich	-1	3	Education (years per capita)	--
Vascular intestinal disorders	Male	1-4 years	95+ years	Data Rich	-1	3	LDI (I$ per capita)	--
Vascular intestinal disorders	Male	1-4 years	95+ years	Data Rich	0	3	Socio-demographic Index	180
Vascular intestinal disorders	Male	1-4 years	95+ years	Data Rich	1	3	Latitude Over 45 (proportion)	531
Vascular intestinal disorders	Male	1-4 years	95+ years	Global	1	1	saturated fats adjusted(percent)	331
Vascular intestinal disorders	Male	1-4 years	95+ years	Global	1	1	Diabetes Fasting Plasma Glucose (mmol/L)	498
Vascular intestinal disorders	Male	1-4 years	95+ years	Global	1	1	Diabetes Age-Standardized Prevalence (proportion)	645
Vascular intestinal disorders	Male	1-4 years	95+ years	Global	1	1	Cholesterol (total, mean per capita)	--
Vascular intestinal disorders	Male	1-4 years	95+ years	Global	1	1	Systolic Blood Pressure (mmHg)	--
Vascular intestinal disorders	Male	1-4 years	95+ years	Global	-1	2	vegetables adjusted(g)	24
Vascular intestinal disorders	Male	1-4 years	95+ years	Global	-1	2	Healthcare access and quality index	29
Vascular intestinal disorders	Male	1-4 years	95+ years	Global	-1	2	fruits adjusted(g)	--
Vascular intestinal disorders	Male	1-4 years	95+ years	Global	1	2	Alcohol (liters per capita)	622
Vascular intestinal disorders	Male	1-4 years	95+ years	Global	-1	3	Education (years per capita)	--

Appendix Table 9: CODEm covariates used, level of covariate, and expected direction of covariate by cause, sex, and age<br>*Covariates that CODEm did not select during the covariate selection process have no draw counts listed.*								
**Cause**	**Sex**	**Age Start**	**Age End**	**Model Type**	**Direction**	**Level**	**Covariate Name**	**Number of Draws**
Vascular intestinal disorders	Male	1-4 years	95+ years	Global	-1	3	LDI (I$ per capita)	--
Vascular intestinal disorders	Male	1-4 years	95+ years	Global	0	3	Socio-demographic Index	40
Vascular intestinal disorders	Male	1-4 years	95+ years	Global	1	3	Latitude Over 45 (proportion)	536
Gallbladder and biliary diseases	Female	1-4 years	95+ years	Data Rich	1	1	saturated fats adjusted(percent)	408
Gallbladder and biliary diseases	Female	1-4 years	95+ years	Data Rich	1	1	Mean BMI	473
Gallbladder and biliary diseases	Female	1-4 years	95+ years	Data Rich	-1	2	Healthcare access and quality index	365
Gallbladder and biliary diseases	Female	1-4 years	95+ years	Data Rich	1	2	red meats adjusted(g)	365
Gallbladder and biliary diseases	Female	1-4 years	95+ years	Data Rich	1	2	Alcohol (liters per capita)	--
Gallbladder and biliary diseases	Female	1-4 years	95+ years	Data Rich	1	2	Population Over 65 (proportion)	--
Gallbladder and biliary diseases	Female	1-4 years	95+ years	Data Rich	0	3	Socio-demographic Index	263
Gallbladder and biliary diseases	Female	1-4 years	95+ years	Data Rich	0	3	Education (years per capita)	304
Gallbladder and biliary diseases	Female	1-4 years	95+ years	Data Rich	0	3	LDI (I$ per capita)	--
Gallbladder and biliary diseases	Female	1-4 years	95+ years	Global	1	1	Mean BMI	276
Gallbladder and biliary diseases	Female	1-4 years	95+ years	Global	1	1	saturated fats adjusted(percent)	813
Gallbladder and biliary diseases	Female	1-4 years	95+ years	Global	-1	2	Healthcare access and quality index	650
Gallbladder and biliary diseases	Female	1-4 years	95+ years	Global	1	2	red meats adjusted(g)	83
Gallbladder and biliary diseases	Female	1-4 years	95+ years	Global	1	2	Alcohol (liters per capita)	--
Gallbladder and biliary diseases	Female	1-4 years	95+ years	Global	1	2	Population Over 65 (proportion)	--
Gallbladder and biliary diseases	Female	1-4 years	95+ years	Global	0	3	Socio-demographic Index	530
Gallbladder and biliary diseases	Female	1-4 years	95+ years	Global	0	3	Education (years per capita)	649
Gallbladder and biliary diseases	Female	1-4 years	95+ years	Global	0	3	LDI (I$ per capita)	--
Gallbladder and biliary diseases	Male	1-4 years	95+ years	Data Rich	1	1	Mean BMI	404
Gallbladder and biliary diseases	Male	1-4 years	95+ years	Data Rich	1	1	saturated fats adjusted(percent)	966
Gallbladder and biliary diseases	Male	1-4 years	95+ years	Data Rich	-1	2	Healthcare access and quality index	3
Gallbladder and biliary diseases	Male	1-4 years	95+ years	Data Rich	1	2	Alcohol (liters per capita)	3
Gallbladder and biliary diseases	Male	1-4 years	95+ years	Data Rich	1	2	red meats adjusted(g)	25
Gallbladder and biliary diseases	Male	1-4 years	95+ years	Data Rich	1	2	Population Over 65 (proportion)	492
Gallbladder and biliary diseases	Male	1-4 years	95+ years	Data Rich	0	3	Education (years per capita)	108
Gallbladder and biliary diseases	Male	1-4 years	95+ years	Data Rich	0	3	Socio-demographic Index	391
Gallbladder and biliary diseases	Male	1-4 years	95+ years	Data Rich	0	3	LDI (I$ per capita)	--
Gallbladder and biliary diseases	Male	1-4 years	95+ years	Global	1	1	Mean BMI	417
Gallbladder and biliary diseases	Male	1-4 years	95+ years	Global	1	1	saturated fats adjusted(percent)	713
Gallbladder and biliary diseases	Male	1-4 years	95+ years	Global	-1	2	Health System Access (capped)	13
Gallbladder and biliary diseases	Male	1-4 years	95+ years	Global	-1	2	Healthcare access and quality index	200
Gallbladder and biliary diseases	Male	1-4 years	95+ years	Global	1	2	red meats adjusted(g)	104
Gallbladder and biliary diseases	Male	1-4 years	95+ years	Global	1	2	Alcohol (liters per capita)	248
Gallbladder and biliary diseases	Male	1-4 years	95+ years	Global	1	2	Population Over 65 (proportion)	267
Gallbladder and biliary diseases	Male	1-4 years	95+ years	Global	0	3	Education (years per capita)	200
Gallbladder and biliary diseases	Male	1-4 years	95+ years	Global	0	3	Socio-demographic Index	233
Gallbladder and biliary diseases	Male	1-4 years	95+ years	Global	0	3	LDI (I$ per capita)	--
Pancreatitis	Female	1-4 years	95+ years	Data Rich	1	1	Log-transformed SEV scalar: Pancreatit	323
Pancreatitis	Female	1-4 years	95+ years	Data Rich	1	1	Alcohol (liters per capita)	--
Pancreatitis	Female	1-4 years	95+ years	Data Rich	-1	2	Healthcare access and quality index	428
Pancreatitis	Female	1-4 years	95+ years	Data Rich	1	2	Mean BMI	--
Pancreatitis	Female	1-4 years	95+ years	Data Rich	-1	3	Education (years per capita)	134
Pancreatitis	Female	1-4 years	95+ years	Data Rich	0	3	Socio-demographic Index	358
Pancreatitis	Female	1-4 years	95+ years	Data Rich	0	3	LDI (I$ per capita)	--
Pancreatitis	Female	1-4 years	95+ years	Global	1	1	Log-transformed SEV scalar: Pancreatit	282
Pancreatitis	Female	1-4 years	95+ years	Global	1	1	Alcohol (liters per capita)	--
Pancreatitis	Female	1-4 years	95+ years	Global	-1	2	Healthcare access and quality index	424
Pancreatitis	Female	1-4 years	95+ years	Global	1	2	Mean BMI	--
Pancreatitis	Female	1-4 years	95+ years	Global	-1	3	Education (years per capita)	133
Pancreatitis	Female	1-4 years	95+ years	Global	0	3	Socio-demographic Index	392
Pancreatitis	Female	1-4 years	95+ years	Global	0	3	LDI (I$ per capita)	--
Pancreatitis	Male	1-4 years	95+ years	Data Rich	1	1	Log-transformed SEV scalar: Pancreatit	453
Pancreatitis	Male	1-4 years	95+ years	Data Rich	1	1	Alcohol (liters per capita)	547
Pancreatitis	Male	1-4 years	95+ years	Data Rich	-1	2	Healthcare access and quality index	74
Pancreatitis	Male	1-4 years	95+ years	Data Rich	1	2	Mean BMI	706
Pancreatitis	Male	1-4 years	95+ years	Data Rich	-1	3	Education (years per capita)	5
Pancreatitis	Male	1-4 years	95+ years	Data Rich	0	3	Socio-demographic Index	626
Pancreatitis	Male	1-4 years	95+ years	Data Rich	0	3	LDI (I$ per capita)	--
Pancreatitis	Male	1-4 years	95+ years	Global	1	1	Alcohol (liters per capita)	114
Pancreatitis	Male	1-4 years	95+ years	Global	1	1	Log-transformed SEV scalar: Pancreatit	234
Pancreatitis	Male	1-4 years	95+ years	Global	-1	2	Health System Access (capped)	54
Pancreatitis	Male	1-4 years	95+ years	Global	-1	2	Healthcare access and quality index	207
Pancreatitis	Male	1-4 years	95+ years	Global	1	2	Mean BMI	513
Pancreatitis	Male	1-4 years	95+ years	Global	-1	3	Education (years per capita)	52
Pancreatitis	Male	1-4 years	95+ years	Global	0	3	Socio-demographic Index	456
Pancreatitis	Male	1-4 years	95+ years	Global	0	3	LDI (I$ per capita)	--
Other digestive diseases	Female	1-4 years	95+ years	Data Rich	1	1	Cumulative Cigarettes (5 Years)	19
Other digestive diseases	Female	1-4 years	95+ years	Data Rich	1	1	Cumulative Cigarettes (10 Years)	28
Other digestive diseases	Female	1-4 years	95+ years	Data Rich	1	1	Alcohol (liters per capita)	451
Other digestive diseases	Female	1-4 years	95+ years	Data Rich	1	1	Smoking Prevalence	906
Other digestive diseases	Female	1-4 years	95+ years	Data Rich	-1	2	Healthcare access and quality index	--
Other digestive diseases	Female	1-4 years	95+ years	Data Rich	-1	2	Improved Water Source (proportion with access)	--
Other digestive diseases	Female	1-4 years	95+ years	Data Rich	-1	2	Sanitation (proportion with access)	--
Other digestive diseases	Female	1-4 years	95+ years	Data Rich	-1	2	fruits adjusted(g)	--
Other digestive diseases	Female	1-4 years	95+ years	Data Rich	0	2	vegetables adjusted(g)	256

Appendix Table 9: CODEm covariates used, level of covariate, and expected direction of covariate by cause, sex, and age
Covariates that CODEm did not select during the covariate selection process have no draw counts listed.

Cause	Sex	Age Start	Age End	Model Type	Direction	Level	Covariate Name	Number of Draws
Other digestive diseases	Female	1-4 years	95+ years	Data Rich	1	2	Diabetes Age-Standardized Prevalence (proportion)	98
Other digestive diseases	Female	1-4 years	95+ years	Data Rich	1	2	Mean BMI	420
Other digestive diseases	Female	1-4 years	95+ years	Data Rich	1	2	red meats adjusted(g)	--
Other digestive diseases	Female	1-4 years	95+ years	Data Rich	1	2	saturated fats adjusted(percent)	--
Other digestive diseases	Female	1-4 years	95+ years	Data Rich	-1	3	Health System Access 2 (unitless)	192
Other digestive diseases	Female	1-4 years	95+ years	Data Rich	-1	3	Education (years per capita)	--
Other digestive diseases	Female	1-4 years	95+ years	Data Rich	-1	3	LDI (I$ per capita)	--
Other digestive diseases	Female	1-4 years	95+ years	Data Rich	0	3	Socio-demographic Index	547
Other digestive diseases	Female	1-4 years	95+ years	Global	1	1	Alcohol (liters per capita)	659
Other digestive diseases	Female	1-4 years	95+ years	Global	1	1	Smoking Prevalence	767
Other digestive diseases	Female	1-4 years	95+ years	Global	1	1	Cumulative Cigarettes (10 Years)	--
Other digestive diseases	Female	1-4 years	95+ years	Global	1	1	Cumulative Cigarettes (5 Years)	--
Other digestive diseases	Female	1-4 years	95+ years	Global	-1	2	Improved Water Source (proportion with access)	7
Other digestive diseases	Female	1-4 years	95+ years	Global	-1	2	Sanitation (proportion with access)	12
Other digestive diseases	Female	1-4 years	95+ years	Global	-1	2	fruits adjusted(g)	12
Other digestive diseases	Female	1-4 years	95+ years	Global	-1	2	Healthcare access and quality index	15
Other digestive diseases	Female	1-4 years	95+ years	Global	0	2	vegetables adjusted(g)	596
Other digestive diseases	Female	1-4 years	95+ years	Global	1	2	Mean BMI	720
Other digestive diseases	Female	1-4 years	95+ years	Global	1	2	Diabetes Age-Standardized Prevalence (proportion)	--
Other digestive diseases	Female	1-4 years	95+ years	Global	1	2	red meats adjusted(g)	--
Other digestive diseases	Female	1-4 years	95+ years	Global	1	2	saturated fats adjusted(percent)	--
Other digestive diseases	Female	1-4 years	95+ years	Global	-1	3	Education (years per capita)	10
Other digestive diseases	Female	1-4 years	95+ years	Global	-1	3	Health System Access 2 (unitless)	416
Other digestive diseases	Female	1-4 years	95+ years	Global	-1	3	LDI (I$ per capita)	--
Other digestive diseases	Female	1-4 years	95+ years	Global	0	3	Socio-demographic Index	610
Other digestive diseases	Male	1-4 years	95+ years	Data Rich	1	1	Cumulative Cigarettes (5 Years)	40
Other digestive diseases	Male	1-4 years	95+ years	Data Rich	1	1	Smoking Prevalence	288
Other digestive diseases	Male	1-4 years	95+ years	Data Rich	1	1	Alcohol (liters per capita)	924
Other digestive diseases	Male	1-4 years	95+ years	Data Rich	1	1	Cumulative Cigarettes (10 Years)	--
Other digestive diseases	Male	1-4 years	95+ years	Data Rich	-1	2	Healthcare access and quality index	--
Other digestive diseases	Male	1-4 years	95+ years	Data Rich	-1	2	Improved Water Source (proportion with access)	--
Other digestive diseases	Male	1-4 years	95+ years	Data Rich	-1	2	Sanitation (proportion with access)	--
Other digestive diseases	Male	1-4 years	95+ years	Data Rich	-1	2	fruits adjusted(g)	--
Other digestive diseases	Male	1-4 years	95+ years	Data Rich	0	2	vegetables adjusted(g)	263
Other digestive diseases	Male	1-4 years	95+ years	Data Rich	1	2	Diabetes Age-Standardized Prevalence (proportion)	409
Other digestive diseases	Male	1-4 years	95+ years	Data Rich	1	2	Mean BMI	592
Other digestive diseases	Male	1-4 years	95+ years	Data Rich	1	2	red meats adjusted(g)	--
Other digestive diseases	Male	1-4 years	95+ years	Data Rich	1	2	saturated fats adjusted(percent)	--
Other digestive diseases	Male	1-4 years	95+ years	Data Rich	-1	3	Health System Access 2 (unitless)	303
Other digestive diseases	Male	1-4 years	95+ years	Data Rich	-1	3	Education (years per capita)	--
Other digestive diseases	Male	1-4 years	95+ years	Data Rich	-1	3	LDI (I$ per capita)	--
Other digestive diseases	Male	1-4 years	95+ years	Data Rich	0	3	Socio-demographic Index	437
Other digestive diseases	Male	1-4 years	95+ years	Global	1	1	Smoking Prevalence	50
Other digestive diseases	Male	1-4 years	95+ years	Global	1	1	Cumulative Cigarettes (5 Years)	96
Other digestive diseases	Male	1-4 years	95+ years	Global	1	1	Cumulative Cigarettes (10 Years)	175
Other digestive diseases	Male	1-4 years	95+ years	Global	1	1	Alcohol (liters per capita)	854
Other digestive diseases	Male	1-4 years	95+ years	Global	-1	2	Healthcare access and quality index	67
Other digestive diseases	Male	1-4 years	95+ years	Global	-1	2	Improved Water Source (proportion with access)	82
Other digestive diseases	Male	1-4 years	95+ years	Global	-1	2	Sanitation (proportion with access)	88
Other digestive diseases	Male	1-4 years	95+ years	Global	-1	2	fruits adjusted(g)	93
Other digestive diseases	Male	1-4 years	95+ years	Global	0	2	vegetables adjusted(g)	470
Other digestive diseases	Male	1-4 years	95+ years	Global	1	2	Mean BMI	326
Other digestive diseases	Male	1-4 years	95+ years	Global	1	2	Diabetes Age-Standardized Prevalence (proportion)	--
Other digestive diseases	Male	1-4 years	95+ years	Global	1	2	red meats adjusted(g)	--
Other digestive diseases	Male	1-4 years	95+ years	Global	1	2	saturated fats adjusted(percent)	--
Other digestive diseases	Male	1-4 years	95+ years	Global	-1	3	Education (years per capita)	47
Other digestive diseases	Male	1-4 years	95+ years	Global	-1	3	Health System Access 2 (unitless)	233
Other digestive diseases	Male	1-4 years	95+ years	Global	-1	3	LDI (I$ per capita)	--
Other digestive diseases	Male	1-4 years	95+ years	Global	0	3	Socio-demographic Index	407
Alzheimer's disease and other dementias	Female	40-44 years	95+ years	Data Rich	-1	1	Education (years per capita)	125
Alzheimer's disease and other dementias	Female	40-44 years	95+ years	Data Rich	1	1	Systolic Blood Pressure (mmHg)	0
Alzheimer's disease and other dementias	Female	40-44 years	95+ years	Data Rich	1	1	Diabetes Age-Specific Prevalence (proportion)	214
Alzheimer's disease and other dementias	Female	40-44 years	95+ years	Data Rich	1	1	Smoking Prevalence	673
Alzheimer's disease and other dementias	Female	40-44 years	95+ years	Data Rich	1	1	Mean BMI	685
Alzheimer's disease and other dementias	Female	40-44 years	95+ years	Data Rich	1	1	Cholesterol (total, mean per capita)	--
Alzheimer's disease and other dementias	Female	40-44 years	95+ years	Data Rich	-1	2	Total Physical Activity (MET-min/week), Age-specific	178
Alzheimer's disease and other dementias	Female	40-44 years	95+ years	Data Rich	-1	2	Healthcare access and quality index	378
Alzheimer's disease and other dementias	Female	40-44 years	95+ years	Data Rich	1	2	red meats adjusted(g)	138
Alzheimer's disease and other dementias	Female	40-44 years	95+ years	Data Rich	1	2	Latitude Over 45 (proportion)	222
Alzheimer's disease and other dementias	Female	40-44 years	95+ years	Data Rich	1	2	Outdoor Air Pollution (PM2.5)	--
Alzheimer's disease and other dementias	Female	40-44 years	95+ years	Data Rich	-1	3	Sanitation (proportion with access)	54
Alzheimer's disease and other dementias	Female	40-44 years	95+ years	Data Rich	-1	3	Improved Water Source (proportion with access)	243
Alzheimer's disease and other dementias	Female	40-44 years	95+ years	Data Rich	0	3	LDI (I$ per capita)	--
Alzheimer's disease and other dementias	Female	40-44 years	95+ years	Global	-1	1	Education (years per capita)	314
Alzheimer's disease and other dementias	Female	40-44 years	95+ years	Global	1	1	Systolic Blood Pressure (mmHg)	7
Alzheimer's disease and other dementias	Female	40-44 years	95+ years	Global	1	1	Diabetes Age-Specific Prevalence (proportion)	104
Alzheimer's disease and other dementias	Female	40-44 years	95+ years	Global	1	1	Mean BMI	487
Alzheimer's disease and other dementias	Female	40-44 years	95+ years	Global	1	1	Smoking Prevalence	578

Appendix Table 9: CODEm covariates used, level of covariate, and expected direction of covariate by cause, sex, and age

Covariates that CODEm did not select during the covariate selection process have no draw counts listed.

Cause	Sex	Age Start	Age End	Model Type	Direction	Level	Covariate Name	Number of Draws
Alzheimer's disease and other dementias	Female	40-44 years	95+ years	Global	1	1	Cholesterol (total, mean per capita)	--
Alzheimer's disease and other dementias	Female	40-44 years	95+ years	Global	-1	2	Healthcare access and quality index	141
Alzheimer's disease and other dementias	Female	40-44 years	95+ years	Global	-1	2	Total Physical Activity (MET-min/week), Age-specific	294
Alzheimer's disease and other dementias	Female	40-44 years	95+ years	Global	1	2	red meats adjusted(g)	138
Alzheimer's disease and other dementias	Female	40-44 years	95+ years	Global	1	2	Latitude Over 45 (proportion)	246
Alzheimer's disease and other dementias	Female	40-44 years	95+ years	Global	1	2	Outdoor Air Pollution (PM2.5)	--
Alzheimer's disease and other dementias	Female	40-44 years	95+ years	Global	-1	3	Sanitation (proportion with access)	123
Alzheimer's disease and other dementias	Female	40-44 years	95+ years	Global	-1	3	Improved Water Source (proportion with access)	221
Alzheimer's disease and other dementias	Female	40-44 years	95+ years	Global	0	3	LDI (I$ per capita)	--
Alzheimer's disease and other dementias	Male	40-44 years	95+ years	Data Rich	-1	1	Education (years per capita)	87
Alzheimer's disease and other dementias	Male	40-44 years	95+ years	Data Rich	1	1	Diabetes Age-Specific Prevalence (proportion)	23
Alzheimer's disease and other dementias	Male	40-44 years	95+ years	Data Rich	1	1	Smoking Prevalence	278
Alzheimer's disease and other dementias	Male	40-44 years	95+ years	Data Rich	1	1	Mean BMI	687
Alzheimer's disease and other dementias	Male	40-44 years	95+ years	Data Rich	1	1	Cholesterol (total, mean per capita)	--
Alzheimer's disease and other dementias	Male	40-44 years	95+ years	Data Rich	1	1	Systolic Blood Pressure (mmHg)	--
Alzheimer's disease and other dementias	Male	40-44 years	95+ years	Data Rich	-1	2	Healthcare access and quality index	0
Alzheimer's disease and other dementias	Male	40-44 years	95+ years	Data Rich	-1	2	Total Physical Activity (MET-min/week), Age-specific	--
Alzheimer's disease and other dementias	Male	40-44 years	95+ years	Data Rich	1	2	red meats adjusted(g)	115
Alzheimer's disease and other dementias	Male	40-44 years	95+ years	Data Rich	1	2	Latitude Over 45 (proportion)	296
Alzheimer's disease and other dementias	Male	40-44 years	95+ years	Data Rich	1	2	Outdoor Air Pollution (PM2.5)	--
Alzheimer's disease and other dementias	Male	40-44 years	95+ years	Data Rich	-1	3	Sanitation (proportion with access)	138
Alzheimer's disease and other dementias	Male	40-44 years	95+ years	Data Rich	-1	3	Improved Water Source (proportion with access)	194
Alzheimer's disease and other dementias	Male	40-44 years	95+ years	Data Rich	0	3	LDI (I$ per capita)	--
Alzheimer's disease and other dementias	Male	40-44 years	95+ years	Global	-1	1	Education (years per capita)	273
Alzheimer's disease and other dementias	Male	40-44 years	95+ years	Global	1	1	Smoking Prevalence	54
Alzheimer's disease and other dementias	Male	40-44 years	95+ years	Global	1	1	Diabetes Age-Specific Prevalence (proportion)	524
Alzheimer's disease and other dementias	Male	40-44 years	95+ years	Global	1	1	Mean BMI	547
Alzheimer's disease and other dementias	Male	40-44 years	95+ years	Global	1	1	Cholesterol (total, mean per capita)	--
Alzheimer's disease and other dementias	Male	40-44 years	95+ years	Global	1	1	Systolic Blood Pressure (mmHg)	--
Alzheimer's disease and other dementias	Male	40-44 years	95+ years	Global	-1	2	Healthcare access and quality index	5
Alzheimer's disease and other dementias	Male	40-44 years	95+ years	Global	-1	2	Total Physical Activity (MET-min/week), Age-specific	--
Alzheimer's disease and other dementias	Male	40-44 years	95+ years	Global	1	2	red meats adjusted(g)	72
Alzheimer's disease and other dementias	Male	40-44 years	95+ years	Global	1	2	Latitude Over 45 (proportion)	670
Alzheimer's disease and other dementias	Male	40-44 years	95+ years	Global	1	2	Outdoor Air Pollution (PM2.5)	--
Alzheimer's disease and other dementias	Male	40-44 years	95+ years	Global	-1	3	Sanitation (proportion with access)	87
Alzheimer's disease and other dementias	Male	40-44 years	95+ years	Global	-1	3	Improved Water Source (proportion with access)	98
Alzheimer's disease and other dementias	Male	40-44 years	95+ years	Global	0	3	LDI (I$ per capita)	--
Parkinson's disease	Female	20-24 years	95+ years	Data Rich	-1	1	Cumulative Cigarettes (10 Years)	704
Parkinson's disease	Female	20-24 years	95+ years	Data Rich	-1	2	fruits adjusted(g)	17
Parkinson's disease	Female	20-24 years	95+ years	Data Rich	-1	2	Healthcare access and quality index	--
Parkinson's disease	Female	20-24 years	95+ years	Data Rich	0	2	Improved Water Source (proportion with access)	410
Parkinson's disease	Female	20-24 years	95+ years	Data Rich	0	2	Sanitation (proportion with access)	419
Parkinson's disease	Female	20-24 years	95+ years	Data Rich	1	2	Absolute value of average latitude	--
Parkinson's disease	Female	20-24 years	95+ years	Data Rich	1	2	Cholesterol (total, mean per capita)	--
Parkinson's disease	Female	20-24 years	95+ years	Data Rich	-1	3	Education (years per capita)	83
Parkinson's disease	Female	20-24 years	95+ years	Data Rich	0	3	LDI (I$ per capita)	--
Parkinson's disease	Female	20-24 years	95+ years	Data Rich	1	3	Socio-demographic Index	259
Parkinson's disease	Female	20-24 years	95+ years	Global	-1	1	Cumulative Cigarettes (10 Years)	680
Parkinson's disease	Female	20-24 years	95+ years	Global	-1	2	Healthcare access and quality index	188
Parkinson's disease	Female	20-24 years	95+ years	Global	-1	2	fruits adjusted(g)	--
Parkinson's disease	Female	20-24 years	95+ years	Global	0	2	Improved Water Source (proportion with access)	553
Parkinson's disease	Female	20-24 years	95+ years	Global	0	2	Sanitation (proportion with access)	553
Parkinson's disease	Female	20-24 years	95+ years	Global	1	2	Cholesterol (total, mean per capita)	77
Parkinson's disease	Female	20-24 years	95+ years	Global	1	2	Absolute value of average latitude	265
Parkinson's disease	Female	20-24 years	95+ years	Global	-1	3	Education (years per capita)	66
Parkinson's disease	Female	20-24 years	95+ years	Global	0	3	LDI (I$ per capita)	--
Parkinson's disease	Female	20-24 years	95+ years	Global	1	3	Socio-demographic Index	406
Parkinson's disease	Male	20-24 years	95+ years	Data Rich	-1	1	Cumulative Cigarettes (10 Years)	300
Parkinson's disease	Male	20-24 years	95+ years	Data Rich	-1	2	fruits adjusted(g)	119
Parkinson's disease	Male	20-24 years	95+ years	Data Rich	-1	2	Healthcare access and quality index	--
Parkinson's disease	Male	20-24 years	95+ years	Data Rich	0	2	Improved Water Source (proportion with access)	599
Parkinson's disease	Male	20-24 years	95+ years	Data Rich	0	2	Sanitation (proportion with access)	--
Parkinson's disease	Male	20-24 years	95+ years	Data Rich	1	2	Absolute value of average latitude	119
Parkinson's disease	Male	20-24 years	95+ years	Data Rich	1	2	Cholesterol (total, mean per capita)	--
Parkinson's disease	Male	20-24 years	95+ years	Data Rich	-1	3	Education (years per capita)	--
Parkinson's disease	Male	20-24 years	95+ years	Data Rich	0	3	LDI (I$ per capita)	--
Parkinson's disease	Male	20-24 years	95+ years	Data Rich	1	3	Socio-demographic Index	496
Parkinson's disease	Male	20-24 years	95+ years	Global	-1	1	Cumulative Cigarettes (10 Years)	1000
Parkinson's disease	Male	20-24 years	95+ years	Global	-1	2	Healthcare access and quality index	130
Parkinson's disease	Male	20-24 years	95+ years	Global	-1	2	fruits adjusted(g)	130
Parkinson's disease	Male	20-24 years	95+ years	Global	0	2	Sanitation (proportion with access)	312
Parkinson's disease	Male	20-24 years	95+ years	Global	0	2	Improved Water Source (proportion with access)	372
Parkinson's disease	Male	20-24 years	95+ years	Global	1	2	Absolute value of average latitude	130
Parkinson's disease	Male	20-24 years	95+ years	Global	1	2	Cholesterol (total, mean per capita)	--
Parkinson's disease	Male	20-24 years	95+ years	Global	-1	3	Education (years per capita)	--
Parkinson's disease	Male	20-24 years	95+ years	Global	0	3	LDI (I$ per capita)	--
Parkinson's disease	Male	20-24 years	95+ years	Global	1	3	Socio-demographic Index	398
Epilepsy	Female	28-364 days	95+ years	Data Rich	1	1	Log-transformed SEV scalar: Epilepsy	108

Appendix Table 9: CODEm covariates used, level of covariate, and expected direction of covariate by cause, sex, and age
Covariates that CODEm did not select during the covariate selection process have no draw counts listed.

Cause	Sex	Age Start	Age End	Model Type	Direction	Level	Covariate Name	Number of Draws
Epilepsy	Female	28-364 days	95+ years	Data Rich	1	1	Pig Meat (kg per capita)	630
Epilepsy	Female	28-364 days	95+ years	Data Rich	1	1	Pigs (per capita)	687
Epilepsy	Female	28-364 days	95+ years	Data Rich	1	1	Systolic Blood Pressure (mmHg)	--
Epilepsy	Female	28-364 days	95+ years	Data Rich	-1	2	Healthcare access and quality index	28
Epilepsy	Female	28-364 days	95+ years	Data Rich	1	2	Mean BMI	582
Epilepsy	Female	28-364 days	95+ years	Data Rich	1	2	Cholesterol (total, mean per capita)	--
Epilepsy	Female	28-364 days	95+ years	Data Rich	-1	3	Education (years per capita)	12
Epilepsy	Female	28-364 days	95+ years	Data Rich	-1	3	Socio-demographic Index	16
Epilepsy	Female	28-364 days	95+ years	Data Rich	-1	3	LDI (I$ per capita)	--
Epilepsy	Female	28-364 days	95+ years	Data Rich	1	3	Cumulative Cigarettes (10 Years)	--
Epilepsy	Female	28-364 days	95+ years	Data Rich	1	3	Cumulative Cigarettes (5 Years)	--
Epilepsy	Female	28-364 days	95+ years	Global	1	1	Log-transformed SEV scalar: Epilepsy	157
Epilepsy	Female	28-364 days	95+ years	Global	1	1	Pig Meat (kg per capita)	493
Epilepsy	Female	28-364 days	95+ years	Global	1	1	Pigs (per capita)	667
Epilepsy	Female	28-364 days	95+ years	Global	1	1	Systolic Blood Pressure (mmHg)	--
Epilepsy	Female	28-364 days	95+ years	Global	-1	2	Healthcare access and quality index	52
Epilepsy	Female	28-364 days	95+ years	Global	1	2	Mean BMI	429
Epilepsy	Female	28-364 days	95+ years	Global	1	2	Cholesterol (total, mean per capita)	--
Epilepsy	Female	28-364 days	95+ years	Global	-1	3	Education (years per capita)	39
Epilepsy	Female	28-364 days	95+ years	Global	-1	3	Socio-demographic Index	45
Epilepsy	Female	28-364 days	95+ years	Global	-1	3	LDI (I$ per capita)	--
Epilepsy	Female	28-364 days	95+ years	Global	1	3	Cumulative Cigarettes (10 Years)	--
Epilepsy	Female	28-364 days	95+ years	Global	1	3	Cumulative Cigarettes (5 Years)	--
Epilepsy	Male	28-364 days	95+ years	Data Rich	1	1	Log-transformed SEV scalar: Epilepsy	120
Epilepsy	Male	28-364 days	95+ years	Data Rich	1	1	Pig Meat (kg per capita)	580
Epilepsy	Male	28-364 days	95+ years	Data Rich	1	1	Pigs (per capita)	662
Epilepsy	Male	28-364 days	95+ years	Data Rich	1	1	Systolic Blood Pressure (mmHg)	--
Epilepsy	Male	28-364 days	95+ years	Data Rich	-1	2	Healthcare access and quality index	13
Epilepsy	Male	28-364 days	95+ years	Data Rich	1	2	Mean BMI	522
Epilepsy	Male	28-364 days	95+ years	Data Rich	1	2	Cholesterol (total, mean per capita)	--
Epilepsy	Male	28-364 days	95+ years	Data Rich	-1	3	Education (years per capita)	14
Epilepsy	Male	28-364 days	95+ years	Data Rich	-1	3	Socio-demographic Index	14
Epilepsy	Male	28-364 days	95+ years	Data Rich	-1	3	LDI (I$ per capita)	--
Epilepsy	Male	28-364 days	95+ years	Data Rich	1	3	Cumulative Cigarettes (10 Years)	26
Epilepsy	Male	28-364 days	95+ years	Data Rich	1	3	Cumulative Cigarettes (5 Years)	33
Epilepsy	Male	28-364 days	95+ years	Global	1	1	Log-transformed SEV scalar: Epilepsy	129
Epilepsy	Male	28-364 days	95+ years	Global	1	1	Pig Meat (kg per capita)	468
Epilepsy	Male	28-364 days	95+ years	Global	1	1	Pigs (per capita)	712
Epilepsy	Male	28-364 days	95+ years	Global	1	1	Systolic Blood Pressure (mmHg)	--
Epilepsy	Male	28-364 days	95+ years	Global	-1	2	Healthcare access and quality index	54
Epilepsy	Male	28-364 days	95+ years	Global	1	2	Mean BMI	394
Epilepsy	Male	28-364 days	95+ years	Global	1	2	Cholesterol (total, mean per capita)	--
Epilepsy	Male	28-364 days	95+ years	Global	-1	3	Socio-demographic Index	43
Epilepsy	Male	28-364 days	95+ years	Global	-1	3	Education (years per capita)	89
Epilepsy	Male	28-364 days	95+ years	Global	-1	3	LDI (I$ per capita)	--
Epilepsy	Male	28-364 days	95+ years	Global	1	3	Cumulative Cigarettes (5 Years)	33
Epilepsy	Male	28-364 days	95+ years	Global	1	3	Cumulative Cigarettes (10 Years)	--
Multiple sclerosis	Female	20-24 years	95+ years	Data Rich	1	1	Absolute value of average latitude	1000
Multiple sclerosis	Female	20-24 years	95+ years	Data Rich	-1	2	Healthcare access and quality index	--
Multiple sclerosis	Female	20-24 years	95+ years	Data Rich	1	2	Cholesterol (total, mean per capita)	--
Multiple sclerosis	Female	20-24 years	95+ years	Data Rich	-1	3	Education (years per capita)	--
Multiple sclerosis	Female	20-24 years	95+ years	Data Rich	-1	3	LDI (I$ per capita)	--
Multiple sclerosis	Female	20-24 years	95+ years	Data Rich	1	3	Socio-demographic Index	618
Multiple sclerosis	Female	20-24 years	95+ years	Data Rich	1	3	Cumulative Cigarettes (10 Years)	--
Multiple sclerosis	Female	20-24 years	95+ years	Data Rich	1	3	Cumulative Cigarettes (5 Years)	--
Multiple sclerosis	Female	20-24 years	95+ years	Data Rich	1	3	Smoking Prevalence	--
Multiple sclerosis	Female	20-24 years	95+ years	Global	1	1	Absolute value of average latitude	1000
Multiple sclerosis	Female	20-24 years	95+ years	Global	-1	2	Healthcare access and quality index	--
Multiple sclerosis	Female	20-24 years	95+ years	Global	1	2	Cholesterol (total, mean per capita)	--
Multiple sclerosis	Female	20-24 years	95+ years	Global	-1	3	Education (years per capita)	--
Multiple sclerosis	Female	20-24 years	95+ years	Global	-1	3	LDI (I$ per capita)	--
Multiple sclerosis	Female	20-24 years	95+ years	Global	1	3	Socio-demographic Index	569
Multiple sclerosis	Female	20-24 years	95+ years	Global	1	3	Cumulative Cigarettes (10 Years)	--
Multiple sclerosis	Female	20-24 years	95+ years	Global	1	3	Cumulative Cigarettes (5 Years)	--
Multiple sclerosis	Female	20-24 years	95+ years	Global	1	3	Smoking Prevalence	--
Multiple sclerosis	Male	20-24 years	95+ years	Data Rich	1	1	Absolute value of average latitude	1000
Multiple sclerosis	Male	20-24 years	95+ years	Data Rich	-1	2	Healthcare access and quality index	--
Multiple sclerosis	Male	20-24 years	95+ years	Data Rich	1	2	Cholesterol (total, mean per capita)	--
Multiple sclerosis	Male	20-24 years	95+ years	Data Rich	-1	3	Education (years per capita)	--
Multiple sclerosis	Male	20-24 years	95+ years	Data Rich	-1	3	LDI (I$ per capita)	--
Multiple sclerosis	Male	20-24 years	95+ years	Data Rich	1	3	Socio-demographic Index	324
Multiple sclerosis	Male	20-24 years	95+ years	Data Rich	1	3	Cumulative Cigarettes (10 Years)	--
Multiple sclerosis	Male	20-24 years	95+ years	Data Rich	1	3	Cumulative Cigarettes (5 Years)	--
Multiple sclerosis	Male	20-24 years	95+ years	Data Rich	1	3	Smoking Prevalence	--
Multiple sclerosis	Male	20-24 years	95+ years	Global	1	1	Absolute value of average latitude	1000
Multiple sclerosis	Male	20-24 years	95+ years	Global	-1	2	Healthcare access and quality index	--
Multiple sclerosis	Male	20-24 years	95+ years	Global	1	2	Cholesterol (total, mean per capita)	--
Multiple sclerosis	Male	20-24 years	95+ years	Global	-1	3	Education (years per capita)	--

Appendix Table 9: CODEm covariates used, level of covariate, and expected direction of covariate by cause, sex, and age
Covariates that CODEm did not select during the covariate selection process have no draw counts listed.

Cause	Sex	Age Start	Age End	Model Type	Direction	Level	Covariate Name	Number of Draws
Multiple sclerosis	Male	20-24 years	95+ years	Global	-1	3	LDI (I$ per capita)	--
Multiple sclerosis	Male	20-24 years	95+ years	Global	1	3	Socio-demographic Index	569
Multiple sclerosis	Male	20-24 years	95+ years	Global	1	3	Cumulative Cigarettes (10 Years)	--
Multiple sclerosis	Male	20-24 years	95+ years	Global	1	3	Cumulative Cigarettes (5 Years)	--
Multiple sclerosis	Male	20-24 years	95+ years	Global	1	3	Smoking Prevalence	--
Motor neuron disease	Female	0-6 days	95+ years	Data Rich	-1	1	Mean BMI	--
Motor neuron disease	Female	0-6 days	95+ years	Data Rich	0	1	fruits adjusted(g)	758
Motor neuron disease	Female	0-6 days	95+ years	Data Rich	0	1	Cholesterol (total, mean per capita)	761
Motor neuron disease	Female	0-6 days	95+ years	Data Rich	1	1	Diabetes Fasting Plasma Glucose (mmol/L)	286
Motor neuron disease	Female	0-6 days	95+ years	Data Rich	1	1	Absolute value of average latitude	312
Motor neuron disease	Female	0-6 days	95+ years	Data Rich	1	1	Socio-demographic Index	659
Motor neuron disease	Female	0-6 days	95+ years	Data Rich	-1	2	Healthcare access and quality index	--
Motor neuron disease	Female	0-6 days	95+ years	Data Rich	0	2	Population-weighted mean temperature	270
Motor neuron disease	Female	0-6 days	95+ years	Data Rich	0	2	Improved Water Source (proportion with access)	--
Motor neuron disease	Female	0-6 days	95+ years	Data Rich	0	2	Sanitation (proportion with access)	--
Motor neuron disease	Female	0-6 days	95+ years	Data Rich	0	3	Education (years per capita)	--
Motor neuron disease	Female	0-6 days	95+ years	Data Rich	0	3	LDI (I$ per capita)	--
Motor neuron disease	Female	0-6 days	95+ years	Global	0	1	fruits adjusted(g)	543
Motor neuron disease	Female	0-6 days	95+ years	Global	0	1	Cholesterol (total, mean per capita)	750
Motor neuron disease	Female	0-6 days	95+ years	Global	1	1	Absolute value of average latitude	240
Motor neuron disease	Female	0-6 days	95+ years	Global	1	1	Diabetes Fasting Plasma Glucose (mmol/L)	465
Motor neuron disease	Female	0-6 days	95+ years	Global	1	1	Socio-demographic Index	811
Motor neuron disease	Female	0-6 days	95+ years	Global	-1	2	Healthcare access and quality index	--
Motor neuron disease	Female	0-6 days	95+ years	Global	-1	2	Mean BMI	--
Motor neuron disease	Female	0-6 days	95+ years	Global	0	2	Improved Water Source (proportion with access)	40
Motor neuron disease	Female	0-6 days	95+ years	Global	0	2	Population-weighted mean temperature	50
Motor neuron disease	Female	0-6 days	95+ years	Global	0	2	Sanitation (proportion with access)	74
Motor neuron disease	Female	0-6 days	95+ years	Global	0	3	Education (years per capita)	194
Motor neuron disease	Female	0-6 days	95+ years	Global	0	3	LDI (I$ per capita)	--
Motor neuron disease	Male	0-6 days	95+ years	Data Rich	-1	1	Mean BMI	--
Motor neuron disease	Male	0-6 days	95+ years	Data Rich	0	1	Cholesterol (total, mean per capita)	134
Motor neuron disease	Male	0-6 days	95+ years	Data Rich	0	1	fruits adjusted(g)	743
Motor neuron disease	Male	0-6 days	95+ years	Data Rich	1	1	Diabetes Fasting Plasma Glucose (mmol/L)	165
Motor neuron disease	Male	0-6 days	95+ years	Data Rich	1	1	Absolute value of average latitude	473
Motor neuron disease	Male	0-6 days	95+ years	Data Rich	1	1	Socio-demographic Index	889
Motor neuron disease	Male	0-6 days	95+ years	Data Rich	-1	2	Healthcare access and quality index	--
Motor neuron disease	Male	0-6 days	95+ years	Data Rich	0	2	Sanitation (proportion with access)	4
Motor neuron disease	Male	0-6 days	95+ years	Data Rich	0	2	Improved Water Source (proportion with access)	20
Motor neuron disease	Male	0-6 days	95+ years	Data Rich	0	2	Population-weighted mean temperature	--
Motor neuron disease	Male	0-6 days	95+ years	Data Rich	0	3	Education (years per capita)	260
Motor neuron disease	Male	0-6 days	95+ years	Data Rich	0	3	LDI (I$ per capita)	--
Motor neuron disease	Male	0-6 days	95+ years	Global	-1	1	Mean BMI	--
Motor neuron disease	Male	0-6 days	95+ years	Global	0	1	Cholesterol (total, mean per capita)	420
Motor neuron disease	Male	0-6 days	95+ years	Global	0	1	fruits adjusted(g)	762
Motor neuron disease	Male	0-6 days	95+ years	Global	1	1	Diabetes Fasting Plasma Glucose (mmol/L)	37
Motor neuron disease	Male	0-6 days	95+ years	Global	1	1	Absolute value of average latitude	413
Motor neuron disease	Male	0-6 days	95+ years	Global	1	1	Socio-demographic Index	705
Motor neuron disease	Male	0-6 days	95+ years	Global	-1	2	Healthcare access and quality index	--
Motor neuron disease	Male	0-6 days	95+ years	Global	0	2	Sanitation (proportion with access)	164
Motor neuron disease	Male	0-6 days	95+ years	Global	0	2	Improved Water Source (proportion with access)	--
Motor neuron disease	Male	0-6 days	95+ years	Global	0	2	Population-weighted mean temperature	--
Motor neuron disease	Male	0-6 days	95+ years	Global	0	3	Education (years per capita)	--
Motor neuron disease	Male	0-6 days	95+ years	Global	0	3	LDI (I$ per capita)	--
Other neurological disorders	Female	28-364 days	95+ years	Data Rich	1	1	Pig Meat (kg per capita)	154
Other neurological disorders	Female	28-364 days	95+ years	Data Rich	1	1	Cholesterol (total, mean per capita)	395
Other neurological disorders	Female	28-364 days	95+ years	Data Rich	1	1	red meats adjusted(g)	398
Other neurological disorders	Female	28-364 days	95+ years	Data Rich	1	1	Mean BMI	960
Other neurological disorders	Female	28-364 days	95+ years	Data Rich	1	1	Systolic Blood Pressure (mmHg)	--
Other neurological disorders	Female	28-364 days	95+ years	Data Rich	1	1	Underweight (proportion <2SD weight for age, <5 years)	--
Other neurological disorders	Female	28-364 days	95+ years	Data Rich	-1	2	fruits adjusted(g)	119
Other neurological disorders	Female	28-364 days	95+ years	Data Rich	-1	2	Healthcare access and quality index	--
Other neurological disorders	Female	28-364 days	95+ years	Data Rich	1	2	Alcohol (liters per capita)	--
Other neurological disorders	Female	28-364 days	95+ years	Data Rich	1	2	Population Density (over 1000 ppl/sqkm, proportion)	--
Other neurological disorders	Female	28-364 days	95+ years	Data Rich	-1	3	Education (years per capita)	--
Other neurological disorders	Female	28-364 days	95+ years	Data Rich	-1	3	LDI (I$ per capita)	--
Other neurological disorders	Female	28-364 days	95+ years	Data Rich	0	3	Socio-demographic Index	179
Other neurological disorders	Female	28-364 days	95+ years	Data Rich	1	3	Smoking Prevalence	207
Other neurological disorders	Female	28-364 days	95+ years	Data Rich	1	3	Cumulative Cigarettes (10 Years)	--
Other neurological disorders	Female	28-364 days	95+ years	Data Rich	1	3	Cumulative Cigarettes (5 Years)	--
Other neurological disorders	Female	28-364 days	95+ years	Global	1	1	red meats adjusted(g)	176
Other neurological disorders	Female	28-364 days	95+ years	Global	1	1	Pig Meat (kg per capita)	222
Other neurological disorders	Female	28-364 days	95+ years	Global	1	1	Cholesterol (total, mean per capita)	544
Other neurological disorders	Female	28-364 days	95+ years	Global	1	1	Mean BMI	963
Other neurological disorders	Female	28-364 days	95+ years	Global	1	1	Systolic Blood Pressure (mmHg)	--
Other neurological disorders	Female	28-364 days	95+ years	Global	1	1	Underweight (proportion <2SD weight for age, <5 years)	--
Other neurological disorders	Female	28-364 days	95+ years	Global	-1	2	fruits adjusted(g)	62
Other neurological disorders	Female	28-364 days	95+ years	Global	-1	2	Healthcare access and quality index	--
Other neurological disorders	Female	28-364 days	95+ years	Global	1	2	Population Density (over 1000 ppl/sqkm, proportion)	10

Appendix Table 9: CODEm covariates used, level of covariate, and expected direction of covariate by cause, sex, and age<br>*Covariates that CODEm did not select during the covariate selection process have no draw counts listed.*								
**Cause**	**Sex**	**Age Start**	**Age End**	**Model Type**	**Direction**	**Level**	**Covariate Name**	**Number of Draws**
Other neurological disorders	Female	28-364 days	95+ years	Global	1	2	Alcohol (liters per capita)	--
Other neurological disorders	Female	28-364 days	95+ years	Global	-1	3	Education (years per capita)	--
Other neurological disorders	Female	28-364 days	95+ years	Global	-1	3	LDI (I$ per capita)	--
Other neurological disorders	Female	28-364 days	95+ years	Global	0	3	Socio-demographic Index	436
Other neurological disorders	Female	28-364 days	95+ years	Global	1	3	Smoking Prevalence	101
Other neurological disorders	Female	28-364 days	95+ years	Global	1	3	Cumulative Cigarettes (10 Years)	--
Other neurological disorders	Female	28-364 days	95+ years	Global	1	3	Cumulative Cigarettes (5 Years)	--
Other neurological disorders	Male	28-364 days	95+ years	Data Rich	1	1	Pig Meat (kg per capita)	7
Other neurological disorders	Male	28-364 days	95+ years	Data Rich	1	1	Cholesterol (total, mean per capita)	87
Other neurological disorders	Male	28-364 days	95+ years	Data Rich	1	1	Systolic Blood Pressure (mmHg)	468
Other neurological disorders	Male	28-364 days	95+ years	Data Rich	1	1	Mean BMI	715
Other neurological disorders	Male	28-364 days	95+ years	Data Rich	1	1	Underweight (proportion <2SD weight for age, <5 years)	--
Other neurological disorders	Male	28-364 days	95+ years	Data Rich	1	1	red meats adjusted(g)	--
Other neurological disorders	Male	28-364 days	95+ years	Data Rich	-1	2	Healthcare access and quality index	21
Other neurological disorders	Male	28-364 days	95+ years	Data Rich	-1	2	fruits adjusted(g)	21
Other neurological disorders	Male	28-364 days	95+ years	Data Rich	1	2	Alcohol (liters per capita)	293
Other neurological disorders	Male	28-364 days	95+ years	Data Rich	1	2	Population Density (over 1000 ppl/sqkm, proportion)	--
Other neurological disorders	Male	28-364 days	95+ years	Data Rich	-1	3	Education (years per capita)	--
Other neurological disorders	Male	28-364 days	95+ years	Data Rich	-1	3	LDI (I$ per capita)	--
Other neurological disorders	Male	28-364 days	95+ years	Data Rich	0	3	Socio-demographic Index	534
Other neurological disorders	Male	28-364 days	95+ years	Data Rich	1	3	Cumulative Cigarettes (10 Years)	--
Other neurological disorders	Male	28-364 days	95+ years	Data Rich	1	3	Cumulative Cigarettes (5 Years)	--
Other neurological disorders	Male	28-364 days	95+ years	Data Rich	1	3	Smoking Prevalence	--
Other neurological disorders	Male	28-364 days	95+ years	Global	1	1	Pig Meat (kg per capita)	102
Other neurological disorders	Male	28-364 days	95+ years	Global	1	1	Cholesterol (total, mean per capita)	245
Other neurological disorders	Male	28-364 days	95+ years	Global	1	1	Systolic Blood Pressure (mmHg)	260
Other neurological disorders	Male	28-364 days	95+ years	Global	1	1	Mean BMI	935
Other neurological disorders	Male	28-364 days	95+ years	Global	1	1	Underweight (proportion <2SD weight for age, <5 years)	--
Other neurological disorders	Male	28-364 days	95+ years	Global	1	1	red meats adjusted(g)	--
Other neurological disorders	Male	28-364 days	95+ years	Global	-1	2	Healthcare access and quality index	140
Other neurological disorders	Male	28-364 days	95+ years	Global	-1	2	fruits adjusted(g)	140
Other neurological disorders	Male	28-364 days	95+ years	Global	1	2	Population Density (over 1000 ppl/sqkm, proportion)	9
Other neurological disorders	Male	28-364 days	95+ years	Global	1	2	Alcohol (liters per capita)	140
Other neurological disorders	Male	28-364 days	95+ years	Global	-1	3	Education (years per capita)	--
Other neurological disorders	Male	28-364 days	95+ years	Global	-1	3	LDI (I$ per capita)	--
Other neurological disorders	Male	28-364 days	95+ years	Global	0	3	Socio-demographic Index	296
Other neurological disorders	Male	28-364 days	95+ years	Global	1	3	Cumulative Cigarettes (10 Years)	--
Other neurological disorders	Male	28-364 days	95+ years	Global	1	3	Cumulative Cigarettes (5 Years)	--
Other neurological disorders	Male	28-364 days	95+ years	Global	1	3	Smoking Prevalence	--
Eating disorders	Female	5-9 years	45-49 years	Data Rich	-1	1	Underweight (proportion <2SD weight for age, <5 years)	37
Eating disorders	Female	5-9 years	45-49 years	Data Rich	1	1	Maternal Education (years per capita)	568
Eating disorders	Female	5-9 years	45-49 years	Data Rich	1	1	Education (years per capita)	--
Eating disorders	Female	5-9 years	45-49 years	Data Rich	1	1	LDI (I$ per capita)	--
Eating disorders	Female	5-9 years	45-49 years	Data Rich	1	1	Sanitation (proportion with access)	--
Eating disorders	Female	5-9 years	45-49 years	Data Rich	-1	2	Healthcare access and quality index	138
Eating disorders	Female	5-9 years	45-49 years	Data Rich	1	3	Socio-demographic Index	161
Eating disorders	Female	5-9 years	45-49 years	Global	-1	1	Underweight (proportion <2SD weight for age, <5 years)	105
Eating disorders	Female	5-9 years	45-49 years	Global	1	1	Maternal Education (years per capita)	477
Eating disorders	Female	5-9 years	45-49 years	Global	1	1	Education (years per capita)	--
Eating disorders	Female	5-9 years	45-49 years	Global	1	1	LDI (I$ per capita)	--
Eating disorders	Female	5-9 years	45-49 years	Global	1	1	Sanitation (proportion with access)	--
Eating disorders	Female	5-9 years	45-49 years	Global	-1	2	Healthcare access and quality index	119
Eating disorders	Female	5-9 years	45-49 years	Global	1	3	Socio-demographic Index	211
Eating disorders	Male	5-9 years	45-49 years	Data Rich	-1	1	Underweight (proportion <2SD weight for age, <5 years)	--
Eating disorders	Male	5-9 years	45-49 years	Data Rich	1	1	Maternal Education (years per capita)	510
Eating disorders	Male	5-9 years	45-49 years	Data Rich	1	1	Education (years per capita)	--
Eating disorders	Male	5-9 years	45-49 years	Data Rich	1	1	LDI (I$ per capita)	--
Eating disorders	Male	5-9 years	45-49 years	Data Rich	1	1	Sanitation (proportion with access)	--
Eating disorders	Male	5-9 years	45-49 years	Data Rich	-1	2	Healthcare access and quality index	718
Eating disorders	Male	5-9 years	45-49 years	Data Rich	1	3	Socio-demographic Index	351
Eating disorders	Male	5-9 years	45-49 years	Global	-1	1	Underweight (proportion <2SD weight for age, <5 years)	15
Eating disorders	Male	5-9 years	45-49 years	Global	1	1	Maternal Education (years per capita)	542
Eating disorders	Male	5-9 years	45-49 years	Global	1	1	Education (years per capita)	--
Eating disorders	Male	5-9 years	45-49 years	Global	1	1	LDI (I$ per capita)	--
Eating disorders	Male	5-9 years	45-49 years	Global	1	1	Sanitation (proportion with access)	--
Eating disorders	Male	5-9 years	45-49 years	Global	-1	2	Healthcare access and quality index	162
Eating disorders	Male	5-9 years	45-49 years	Global	1	3	Socio-demographic Index	451
Anorexia nervosa	Female	5-9 years	45-49 years	Data Rich	-1	1	Underweight (proportion <2SD weight for age, <5 years)	27
Anorexia nervosa	Female	5-9 years	45-49 years	Data Rich	1	1	Maternal Education (years per capita)	751
Anorexia nervosa	Female	5-9 years	45-49 years	Data Rich	1	1	Education (years per capita)	--
Anorexia nervosa	Female	5-9 years	45-49 years	Data Rich	1	1	LDI (I$ per capita)	--
Anorexia nervosa	Female	5-9 years	45-49 years	Data Rich	1	1	Sanitation (proportion with access)	--
Anorexia nervosa	Female	5-9 years	45-49 years	Data Rich	-1	2	Healthcare access and quality index	386
Anorexia nervosa	Female	5-9 years	45-49 years	Data Rich	1	3	Socio-demographic Index	47
Anorexia nervosa	Female	5-9 years	45-49 years	Global	-1	1	Underweight (proportion <2SD weight for age, <5 years)	92
Anorexia nervosa	Female	5-9 years	45-49 years	Global	1	1	Maternal Education (years per capita)	565
Anorexia nervosa	Female	5-9 years	45-49 years	Global	1	1	Education (years per capita)	--
Anorexia nervosa	Female	5-9 years	45-49 years	Global	1	1	LDI (I$ per capita)	--

Appendix Table 9: CODEm covariates used, level of covariate, and expected direction of covariate by cause, sex, and age

Covariates that CODEm did not select during the covariate selection process have no draw counts listed.

Cause	Sex	Age Start	Age End	Model Type	Direction	Level	Covariate Name	Number of Draws
Anorexia nervosa	Female	5-9 years	45-49 years	Global	1	1	Sanitation (proportion with access)	--
Anorexia nervosa	Female	5-9 years	45-49 years	Global	-1	2	Healthcare access and quality index	185
Anorexia nervosa	Female	5-9 years	45-49 years	Global	1	3	Socio-demographic Index	174
Anorexia nervosa	Male	5-9 years	45-49 years	Data Rich	-1	1	Underweight (proportion <2SD weight for age, <5 years)	--
Anorexia nervosa	Male	5-9 years	45-49 years	Data Rich	1	1	Maternal Education (years per capita)	45
Anorexia nervosa	Male	5-9 years	45-49 years	Data Rich	1	1	Education (years per capita)	--
Anorexia nervosa	Male	5-9 years	45-49 years	Data Rich	1	1	LDI (I$ per capita)	--
Anorexia nervosa	Male	5-9 years	45-49 years	Data Rich	1	1	Sanitation (proportion with access)	--
Anorexia nervosa	Male	5-9 years	45-49 years	Data Rich	-1	2	Healthcare access and quality index	3
Anorexia nervosa	Male	5-9 years	45-49 years	Data Rich	1	3	Socio-demographic Index	71
Anorexia nervosa	Male	5-9 years	45-49 years	Global	-1	1	Underweight (proportion <2SD weight for age, <5 years)	47
Anorexia nervosa	Male	5-9 years	45-49 years	Global	1	1	Maternal Education (years per capita)	133
Anorexia nervosa	Male	5-9 years	45-49 years	Global	1	1	Education (years per capita)	--
Anorexia nervosa	Male	5-9 years	45-49 years	Global	1	1	LDI (I$ per capita)	--
Anorexia nervosa	Male	5-9 years	45-49 years	Global	1	1	Sanitation (proportion with access)	--
Anorexia nervosa	Male	5-9 years	45-49 years	Global	-1	2	Healthcare access and quality index	7
Anorexia nervosa	Male	5-9 years	45-49 years	Global	1	3	Socio-demographic Index	69
Bulimia nervosa	Female	5-9 years	45-49 years	Data Rich	-1	1	Underweight (proportion <2SD weight for age, <5 years)	432
Bulimia nervosa	Female	5-9 years	45-49 years	Data Rich	1	1	Maternal Education (years per capita)	1000
Bulimia nervosa	Female	5-9 years	45-49 years	Data Rich	1	1	Education (years per capita)	--
Bulimia nervosa	Female	5-9 years	45-49 years	Data Rich	1	1	LDI (I$ per capita)	--
Bulimia nervosa	Female	5-9 years	45-49 years	Data Rich	1	1	Sanitation (proportion with access)	--
Bulimia nervosa	Female	5-9 years	45-49 years	Data Rich	-1	2	Healthcare access and quality index	--
Bulimia nervosa	Female	5-9 years	45-49 years	Data Rich	1	3	Socio-demographic Index	--
Bulimia nervosa	Female	5-9 years	45-49 years	Global	-1	1	Underweight (proportion <2SD weight for age, <5 years)	231
Bulimia nervosa	Female	5-9 years	45-49 years	Global	1	1	Maternal Education (years per capita)	331
Bulimia nervosa	Female	5-9 years	45-49 years	Global	1	1	Education (years per capita)	--
Bulimia nervosa	Female	5-9 years	45-49 years	Global	1	1	LDI (I$ per capita)	--
Bulimia nervosa	Female	5-9 years	45-49 years	Global	1	1	Sanitation (proportion with access)	--
Bulimia nervosa	Female	5-9 years	45-49 years	Global	-1	2	Healthcare access and quality index	--
Bulimia nervosa	Female	5-9 years	45-49 years	Global	1	3	Socio-demographic Index	--
Bulimia nervosa	Male	5-9 years	45-49 years	Data Rich	-1	1	Underweight (proportion <2SD weight for age, <5 years)	795
Bulimia nervosa	Male	5-9 years	45-49 years	Data Rich	1	1	Maternal Education (years per capita)	144
Bulimia nervosa	Male	5-9 years	45-49 years	Data Rich	1	1	Education (years per capita)	--
Bulimia nervosa	Male	5-9 years	45-49 years	Data Rich	1	1	LDI (I$ per capita)	--
Bulimia nervosa	Male	5-9 years	45-49 years	Data Rich	1	1	Sanitation (proportion with access)	--
Bulimia nervosa	Male	5-9 years	45-49 years	Data Rich	-1	2	Healthcare access and quality index	--
Bulimia nervosa	Male	5-9 years	45-49 years	Data Rich	1	3	Socio-demographic Index	256
Bulimia nervosa	Male	5-9 years	45-49 years	Global	-1	1	Underweight (proportion <2SD weight for age, <5 years)	155
Bulimia nervosa	Male	5-9 years	45-49 years	Global	1	1	Maternal Education (years per capita)	311
Bulimia nervosa	Male	5-9 years	45-49 years	Global	1	1	Education (years per capita)	--
Bulimia nervosa	Male	5-9 years	45-49 years	Global	1	1	LDI (I$ per capita)	--
Bulimia nervosa	Male	5-9 years	45-49 years	Global	1	1	Sanitation (proportion with access)	--
Bulimia nervosa	Male	5-9 years	45-49 years	Global	-1	2	Healthcare access and quality index	73
Bulimia nervosa	Male	5-9 years	45-49 years	Global	1	3	Socio-demographic Index	266
Alcohol use disorders	Female	15-19 years	95+ years	Data Rich	1	1	Alcohol (liters per capita)	38
Alcohol use disorders	Female	15-19 years	95+ years	Data Rich	1	1	Alcohol binge drinker proportion, age-standardized	962
Alcohol use disorders	Female	15-19 years	95+ years	Data Rich	-1	2	Health System Access 2 (unitless)	--
Alcohol use disorders	Female	15-19 years	95+ years	Data Rich	-1	2	Healthcare access and quality index	--
Alcohol use disorders	Female	15-19 years	95+ years	Data Rich	-1	2	Religion (binary, >50% Muslim)	--
Alcohol use disorders	Female	15-19 years	95+ years	Data Rich	0	2	Smoking Prevalence	157
Alcohol use disorders	Female	15-19 years	95+ years	Data Rich	0	2	Cumulative Cigarettes (10 Years)	481
Alcohol use disorders	Female	15-19 years	95+ years	Data Rich	-1	3	Education (years per capita)	--
Alcohol use disorders	Female	15-19 years	95+ years	Data Rich	-1	3	LDI (I$ per capita)	--
Alcohol use disorders	Female	15-19 years	95+ years	Data Rich	0	3	Socio-demographic Index	691
Alcohol use disorders	Female	15-19 years	95+ years	Global	1	1	Alcohol (liters per capita)	290
Alcohol use disorders	Female	15-19 years	95+ years	Global	1	1	Alcohol binge drinker proportion, age-standardized	710
Alcohol use disorders	Female	15-19 years	95+ years	Global	-1	2	Healthcare access and quality index	--
Alcohol use disorders	Female	15-19 years	95+ years	Global	-1	2	Religion (binary, >50% Muslim)	--
Alcohol use disorders	Female	15-19 years	95+ years	Global	0	2	Smoking Prevalence	378
Alcohol use disorders	Female	15-19 years	95+ years	Global	0	2	Cumulative Cigarettes (10 Years)	670
Alcohol use disorders	Female	15-19 years	95+ years	Global	-1	3	Education (years per capita)	--
Alcohol use disorders	Female	15-19 years	95+ years	Global	-1	3	LDI (I$ per capita)	--
Alcohol use disorders	Female	15-19 years	95+ years	Global	0	3	Socio-demographic Index	521
Alcohol use disorders	Male	15-19 years	95+ years	Data Rich	1	1	Alcohol (liters per capita)	647
Alcohol use disorders	Male	15-19 years	95+ years	Data Rich	1	1	Alcohol binge drinker proportion, age-standardized	754
Alcohol use disorders	Male	15-19 years	95+ years	Data Rich	-1	2	Health System Access 2 (unitless)	--
Alcohol use disorders	Male	15-19 years	95+ years	Data Rich	-1	2	Healthcare access and quality index	--
Alcohol use disorders	Male	15-19 years	95+ years	Data Rich	-1	2	Religion (binary, >50% Muslim)	--
Alcohol use disorders	Male	15-19 years	95+ years	Data Rich	0	2	Cumulative Cigarettes (10 Years)	309
Alcohol use disorders	Male	15-19 years	95+ years	Data Rich	0	2	Smoking Prevalence	374
Alcohol use disorders	Male	15-19 years	95+ years	Data Rich	-1	3	Education (years per capita)	--
Alcohol use disorders	Male	15-19 years	95+ years	Data Rich	-1	3	LDI (I$ per capita)	--
Alcohol use disorders	Male	15-19 years	95+ years	Data Rich	0	3	Socio-demographic Index	780
Alcohol use disorders	Male	15-19 years	95+ years	Global	1	1	Alcohol binge drinker proportion, age-standardized	624
Alcohol use disorders	Male	15-19 years	95+ years	Global	1	1	Alcohol (liters per capita)	662
Alcohol use disorders	Male	15-19 years	95+ years	Global	-1	2	Religion (binary, >50% Muslim)	409
Alcohol use disorders	Male	15-19 years	95+ years	Global	-1	2	Healthcare access and quality index	--

Appendix Table 9: CODEm covariates used, level of covariate, and expected direction of covariate by cause, sex, and age

Covariates that CODEm did not select during the covariate selection process have no draw counts listed.

Cause	Sex	Age Start	Age End	Model Type	Direction	Level	Covariate Name	Number of Draws
Alcohol use disorders	Male	15-19 years	95+ years	Global	0	2	Cumulative Cigarettes (10 Years)	723
Alcohol use disorders	Male	15-19 years	95+ years	Global	0	2	Smoking Prevalence	723
Alcohol use disorders	Male	15-19 years	95+ years	Global	-1	3	Education (years per capita)	--
Alcohol use disorders	Male	15-19 years	95+ years	Global	-1	3	LDI (I$ per capita)	--
Alcohol use disorders	Male	15-19 years	95+ years	Global	0	3	Socio-demographic Index	583
Drug use disorders	Female	15-19 years	95+ years	Data Rich	1	1	Intravenous drug use (age-standardized proportion)	6
Drug use disorders	Female	15-19 years	95+ years	Data Rich	1	1	Intravenous drug use (proportion by age)	160
Drug use disorders	Female	15-19 years	95+ years	Data Rich	1	1	Opioids per million population per day (10 year lag)	1000
Drug use disorders	Female	15-19 years	95+ years	Data Rich	0	2	Healthcare access and quality index	962
Drug use disorders	Female	15-19 years	95+ years	Data Rich	1	2	Opium Cultivation (binary)	0
Drug use disorders	Female	15-19 years	95+ years	Data Rich	1	2	Cumulative Cigarettes (10 Years)	--
Drug use disorders	Female	15-19 years	95+ years	Data Rich	1	2	Cumulative Cigarettes (5 Years)	--
Drug use disorders	Female	15-19 years	95+ years	Data Rich	1	2	Smoking Prevalence	--
Drug use disorders	Female	15-19 years	95+ years	Data Rich	0	3	Education (years per capita)	1000
Drug use disorders	Female	15-19 years	95+ years	Data Rich	0	3	Socio-demographic Index	1000
Drug use disorders	Female	15-19 years	95+ years	Data Rich	0	3	LDI (I$ per capita)	--
Drug use disorders	Female	15-19 years	95+ years	Global	1	1	Intravenous drug use (age-standardized proportion)	220
Drug use disorders	Female	15-19 years	95+ years	Global	1	1	Intravenous drug use (proportion by age)	634
Drug use disorders	Female	15-19 years	95+ years	Global	1	1	Opioids per million population per day (10 year lag)	988
Drug use disorders	Female	15-19 years	95+ years	Global	0	2	Healthcare access and quality index	332
Drug use disorders	Female	15-19 years	95+ years	Global	1	2	Opium Cultivation (binary)	0
Drug use disorders	Female	15-19 years	95+ years	Global	1	2	Cumulative Cigarettes (10 Years)	--
Drug use disorders	Female	15-19 years	95+ years	Global	1	2	Cumulative Cigarettes (5 Years)	--
Drug use disorders	Female	15-19 years	95+ years	Global	1	2	Smoking Prevalence	--
Drug use disorders	Female	15-19 years	95+ years	Global	0	3	Socio-demographic Index	950
Drug use disorders	Female	15-19 years	95+ years	Global	0	3	Education (years per capita)	993
Drug use disorders	Female	15-19 years	95+ years	Global	0	3	LDI (I$ per capita)	--
Drug use disorders	Male	15-19 years	95+ years	Data Rich	1	1	Intravenous drug use (proportion by age)	199
Drug use disorders	Male	15-19 years	95+ years	Data Rich	1	1	Intravenous drug use (age-standardized proportion)	839
Drug use disorders	Male	15-19 years	95+ years	Data Rich	1	1	Opioids per million population per day (10 year lag)	999
Drug use disorders	Male	15-19 years	95+ years	Data Rich	0	2	Healthcare access and quality index	994
Drug use disorders	Male	15-19 years	95+ years	Data Rich	1	2	Opium Cultivation (binary)	0
Drug use disorders	Male	15-19 years	95+ years	Data Rich	1	2	Cumulative Cigarettes (10 Years)	--
Drug use disorders	Male	15-19 years	95+ years	Data Rich	1	2	Cumulative Cigarettes (5 Years)	--
Drug use disorders	Male	15-19 years	95+ years	Data Rich	1	2	Smoking Prevalence	--
Drug use disorders	Male	15-19 years	95+ years	Data Rich	0	3	Socio-demographic Index	968
Drug use disorders	Male	15-19 years	95+ years	Data Rich	0	3	Education (years per capita)	1000
Drug use disorders	Male	15-19 years	95+ years	Data Rich	0	3	LDI (I$ per capita)	--
Drug use disorders	Male	15-19 years	95+ years	Global	1	1	Intravenous drug use (proportion by age)	613
Drug use disorders	Male	15-19 years	95+ years	Global	1	1	Intravenous drug use (age-standardized proportion)	872
Drug use disorders	Male	15-19 years	95+ years	Global	1	1	Opioids per million population per day (10 year lag)	997
Drug use disorders	Male	15-19 years	95+ years	Global	0	2	Healthcare access and quality index	346
Drug use disorders	Male	15-19 years	95+ years	Global	1	2	Cumulative Cigarettes (10 Years)	--
Drug use disorders	Male	15-19 years	95+ years	Global	1	2	Cumulative Cigarettes (5 Years)	--
Drug use disorders	Male	15-19 years	95+ years	Global	1	2	Opium Cultivation (binary)	--
Drug use disorders	Male	15-19 years	95+ years	Global	1	2	Smoking Prevalence	--
Drug use disorders	Male	15-19 years	95+ years	Global	0	3	Socio-demographic Index	928
Drug use disorders	Male	15-19 years	95+ years	Global	0	3	Education (years per capita)	971
Drug use disorders	Male	15-19 years	95+ years	Global	0	3	LDI (I$ per capita)	--
Opioid use disorders	Female	15-19 years	95+ years	Data Rich	1	1	Opioids per million population per day (10 year lag)	190
Opioid use disorders	Female	15-19 years	95+ years	Data Rich	1	1	Opioids per million population per day	281
Opioid use disorders	Female	15-19 years	95+ years	Data Rich	1	1	Opioids per million population per day (5 year lag)	529
Opioid use disorders	Female	15-19 years	95+ years	Data Rich	0	2	Healthcare access and quality index	32
Opioid use disorders	Female	15-19 years	95+ years	Data Rich	1	2	Opium Cultivation (binary)	38
Opioid use disorders	Female	15-19 years	95+ years	Data Rich	1	2	Cumulative Cigarettes (10 Years)	--
Opioid use disorders	Female	15-19 years	95+ years	Data Rich	1	2	Cumulative Cigarettes (5 Years)	--
Opioid use disorders	Female	15-19 years	95+ years	Data Rich	1	2	Smoking Prevalence	--
Opioid use disorders	Female	15-19 years	95+ years	Data Rich	0	3	Socio-demographic Index	875
Opioid use disorders	Female	15-19 years	95+ years	Data Rich	0	3	Education (years per capita)	999
Opioid use disorders	Female	15-19 years	95+ years	Data Rich	0	3	LDI (I$ per capita)	--
Opioid use disorders	Female	15-19 years	95+ years	Global	1	1	Opioids per million population per day (10 year lag)	153
Opioid use disorders	Female	15-19 years	95+ years	Global	1	1	Opioids per million population per day (5 year lag)	332
Opioid use disorders	Female	15-19 years	95+ years	Global	1	1	Opioids per million population per day	515
Opioid use disorders	Female	15-19 years	95+ years	Global	0	2	Healthcare access and quality index	471
Opioid use disorders	Female	15-19 years	95+ years	Global	1	2	Opium Cultivation (binary)	25
Opioid use disorders	Female	15-19 years	95+ years	Global	1	2	Cumulative Cigarettes (10 Years)	--
Opioid use disorders	Female	15-19 years	95+ years	Global	1	2	Cumulative Cigarettes (5 Years)	--
Opioid use disorders	Female	15-19 years	95+ years	Global	1	2	Smoking Prevalence	--
Opioid use disorders	Female	15-19 years	95+ years	Global	0	3	Socio-demographic Index	700
Opioid use disorders	Female	15-19 years	95+ years	Global	0	3	Education (years per capita)	857
Opioid use disorders	Female	15-19 years	95+ years	Global	0	3	LDI (I$ per capita)	--
Opioid use disorders	Male	15-19 years	95+ years	Data Rich	1	1	Intravenous drug use (age-standardized proportion)	7
Opioid use disorders	Male	15-19 years	95+ years	Data Rich	1	1	Intravenous drug use (proportion by age)	833
Opioid use disorders	Male	15-19 years	95+ years	Data Rich	1	1	Opioids per million population per day (10 year lag)	1000
Opioid use disorders	Male	15-19 years	95+ years	Data Rich	0	2	Healthcare access and quality index	802
Opioid use disorders	Male	15-19 years	95+ years	Data Rich	1	2	Cumulative Cigarettes (10 Years)	--
Opioid use disorders	Male	15-19 years	95+ years	Data Rich	1	2	Cumulative Cigarettes (5 Years)	--
Opioid use disorders	Male	15-19 years	95+ years	Data Rich	1	2	Opium Cultivation (binary)	--

Appendix Table 9: CODEm covariates used, level of covariate, and expected direction of covariate by cause, sex, and age
Covariates that CODEm did not select during the covariate selection process have no draw counts listed.

Cause	Sex	Age Start	Age End	Model Type	Direction	Level	Covariate Name	Number of Draws
Opioid use disorders	Male	15-19 years	95+ years	Data Rich	1	2	Smoking Prevalence	--
Opioid use disorders	Male	15-19 years	95+ years	Data Rich	0	3	Socio-demographic Index	967
Opioid use disorders	Male	15-19 years	95+ years	Data Rich	0	3	Education (years per capita)	994
Opioid use disorders	Male	15-19 years	95+ years	Data Rich	0	3	LDI (I$ per capita)	--
Opioid use disorders	Male	15-19 years	95+ years	Global	1	1	Intravenous drug use (age-standardized proportion)	466
Opioid use disorders	Male	15-19 years	95+ years	Global	1	1	Intravenous drug use (proportion by age)	543
Opioid use disorders	Male	15-19 years	95+ years	Global	1	1	Opioids per million population per day (10 year lag)	997
Opioid use disorders	Male	15-19 years	95+ years	Global	0	2	Healthcare access and quality index	88
Opioid use disorders	Male	15-19 years	95+ years	Global	1	2	Opium Cultivation (binary)	0
Opioid use disorders	Male	15-19 years	95+ years	Global	1	2	Cumulative Cigarettes (10 Years)	--
Opioid use disorders	Male	15-19 years	95+ years	Global	1	2	Cumulative Cigarettes (5 Years)	--
Opioid use disorders	Male	15-19 years	95+ years	Global	1	2	Smoking Prevalence	--
Opioid use disorders	Male	15-19 years	95+ years	Global	0	3	Socio-demographic Index	571
Opioid use disorders	Male	15-19 years	95+ years	Global	0	3	Education (years per capita)	839
Opioid use disorders	Male	15-19 years	95+ years	Global	0	3	LDI (I$ per capita)	--
Cocaine use disorders	Female	15-19 years	95+ years	Data Rich	1	1	Alcohol (liters per capita)	1000
Cocaine use disorders	Female	15-19 years	95+ years	Data Rich	1	1	Cumulative Cigarettes (10 Years)	--
Cocaine use disorders	Female	15-19 years	95+ years	Data Rich	1	1	Cumulative Cigarettes (5 Years)	--
Cocaine use disorders	Female	15-19 years	95+ years	Data Rich	1	1	Smoking Prevalence	--
Cocaine use disorders	Female	15-19 years	95+ years	Data Rich	-1	2	Healthcare access and quality index	--
Cocaine use disorders	Female	15-19 years	95+ years	Data Rich	0	3	Education (years per capita)	926
Cocaine use disorders	Female	15-19 years	95+ years	Data Rich	0	3	LDI (I$ per capita)	--
Cocaine use disorders	Female	15-19 years	95+ years	Data Rich	1	3	Socio-demographic Index	--
Cocaine use disorders	Female	15-19 years	95+ years	Global	1	1	Alcohol (liters per capita)	1000
Cocaine use disorders	Female	15-19 years	95+ years	Global	1	1	Cumulative Cigarettes (10 Years)	--
Cocaine use disorders	Female	15-19 years	95+ years	Global	1	1	Cumulative Cigarettes (5 Years)	--
Cocaine use disorders	Female	15-19 years	95+ years	Global	1	1	Smoking Prevalence	--
Cocaine use disorders	Female	15-19 years	95+ years	Global	-1	2	Healthcare access and quality index	--
Cocaine use disorders	Female	15-19 years	95+ years	Global	0	3	Education (years per capita)	724
Cocaine use disorders	Female	15-19 years	95+ years	Global	0	3	LDI (I$ per capita)	--
Cocaine use disorders	Female	15-19 years	95+ years	Global	1	3	Socio-demographic Index	--
Cocaine use disorders	Male	15-19 years	95+ years	Data Rich	1	1	Alcohol (liters per capita)	193
Cocaine use disorders	Male	15-19 years	95+ years	Data Rich	1	1	Cumulative Cigarettes (10 Years)	--
Cocaine use disorders	Male	15-19 years	95+ years	Data Rich	1	1	Cumulative Cigarettes (5 Years)	--
Cocaine use disorders	Male	15-19 years	95+ years	Data Rich	1	1	Smoking Prevalence	--
Cocaine use disorders	Male	15-19 years	95+ years	Data Rich	-1	2	Healthcare access and quality index	--
Cocaine use disorders	Male	15-19 years	95+ years	Data Rich	0	3	Education (years per capita)	968
Cocaine use disorders	Male	15-19 years	95+ years	Data Rich	0	3	LDI (I$ per capita)	--
Cocaine use disorders	Male	15-19 years	95+ years	Data Rich	1	3	Socio-demographic Index	807
Cocaine use disorders	Male	15-19 years	95+ years	Global	1	1	Alcohol (liters per capita)	1000
Cocaine use disorders	Male	15-19 years	95+ years	Global	1	1	Cumulative Cigarettes (10 Years)	--
Cocaine use disorders	Male	15-19 years	95+ years	Global	1	1	Cumulative Cigarettes (5 Years)	--
Cocaine use disorders	Male	15-19 years	95+ years	Global	1	1	Smoking Prevalence	--
Cocaine use disorders	Male	15-19 years	95+ years	Global	-1	2	Healthcare access and quality index	--
Cocaine use disorders	Male	15-19 years	95+ years	Global	0	3	Education (years per capita)	724
Cocaine use disorders	Male	15-19 years	95+ years	Global	0	3	LDI (I$ per capita)	--
Cocaine use disorders	Male	15-19 years	95+ years	Global	1	3	Socio-demographic Index	449
Amphetamine use disorders	Female	15-19 years	95+ years	Data Rich	1	1	Alcohol (liters per capita)	166
Amphetamine use disorders	Female	15-19 years	95+ years	Data Rich	1	1	Cumulative Cigarettes (10 Years)	--
Amphetamine use disorders	Female	15-19 years	95+ years	Data Rich	1	1	Cumulative Cigarettes (5 Years)	--
Amphetamine use disorders	Female	15-19 years	95+ years	Data Rich	1	1	Smoking Prevalence	--
Amphetamine use disorders	Female	15-19 years	95+ years	Data Rich	-1	2	Healthcare access and quality index	--
Amphetamine use disorders	Female	15-19 years	95+ years	Data Rich	0	3	Education (years per capita)	834
Amphetamine use disorders	Female	15-19 years	95+ years	Data Rich	0	3	LDI (I$ per capita)	--
Amphetamine use disorders	Female	15-19 years	95+ years	Data Rich	1	3	Socio-demographic Index	834
Amphetamine use disorders	Female	15-19 years	95+ years	Global	1	1	Alcohol (liters per capita)	382
Amphetamine use disorders	Female	15-19 years	95+ years	Global	1	1	Cumulative Cigarettes (10 Years)	--
Amphetamine use disorders	Female	15-19 years	95+ years	Global	1	1	Cumulative Cigarettes (5 Years)	--
Amphetamine use disorders	Female	15-19 years	95+ years	Global	1	1	Smoking Prevalence	--
Amphetamine use disorders	Female	15-19 years	95+ years	Global	-1	2	Healthcare access and quality index	--
Amphetamine use disorders	Female	15-19 years	95+ years	Global	0	3	Education (years per capita)	618
Amphetamine use disorders	Female	15-19 years	95+ years	Global	0	3	LDI (I$ per capita)	--
Amphetamine use disorders	Female	15-19 years	95+ years	Global	1	3	Socio-demographic Index	618
Amphetamine use disorders	Male	15-19 years	95+ years	Data Rich	1	1	Alcohol (liters per capita)	1000
Amphetamine use disorders	Male	15-19 years	95+ years	Data Rich	1	1	Cumulative Cigarettes (10 Years)	--
Amphetamine use disorders	Male	15-19 years	95+ years	Data Rich	1	1	Cumulative Cigarettes (5 Years)	--
Amphetamine use disorders	Male	15-19 years	95+ years	Data Rich	1	1	Smoking Prevalence	--
Amphetamine use disorders	Male	15-19 years	95+ years	Data Rich	-1	2	Healthcare access and quality index	--
Amphetamine use disorders	Male	15-19 years	95+ years	Data Rich	0	3	Education (years per capita)	--
Amphetamine use disorders	Male	15-19 years	95+ years	Data Rich	0	3	LDI (I$ per capita)	--
Amphetamine use disorders	Male	15-19 years	95+ years	Data Rich	1	3	Socio-demographic Index	--
Amphetamine use disorders	Male	15-19 years	95+ years	Global	1	1	Alcohol (liters per capita)	1000
Amphetamine use disorders	Male	15-19 years	95+ years	Global	1	1	Cumulative Cigarettes (10 Years)	--
Amphetamine use disorders	Male	15-19 years	95+ years	Global	1	1	Cumulative Cigarettes (5 Years)	--
Amphetamine use disorders	Male	15-19 years	95+ years	Global	1	1	Smoking Prevalence	--
Amphetamine use disorders	Male	15-19 years	95+ years	Global	-1	2	Healthcare access and quality index	--
Amphetamine use disorders	Male	15-19 years	95+ years	Global	0	3	Education (years per capita)	500
Amphetamine use disorders	Male	15-19 years	95+ years	Global	0	3	LDI (I$ per capita)	--

Appendix Table 9: CODEm covariates used, level of covariate, and expected direction of covariate by cause, sex, and age

Covariates that CODEm did not select during the covariate selection process have no draw counts listed.

Cause	Sex	Age Start	Age End	Model Type	Direction	Level	Covariate Name	Number of Draws
Amphetamine use disorders	Male	15-19 years	95+ years	Global	1	3	Socio-demographic Index	--
Other drug use disorders	Female	15-19 years	95+ years	Data Rich	1	1	Alcohol (liters per capita)	1000
Other drug use disorders	Female	15-19 years	95+ years	Data Rich	1	1	Cumulative Cigarettes (10 Years)	--
Other drug use disorders	Female	15-19 years	95+ years	Data Rich	1	1	Cumulative Cigarettes (5 Years)	--
Other drug use disorders	Female	15-19 years	95+ years	Data Rich	1	1	Smoking Prevalence	--
Other drug use disorders	Female	15-19 years	95+ years	Data Rich	-1	2	Healthcare access and quality index	--
Other drug use disorders	Female	15-19 years	95+ years	Data Rich	0	3	Education (years per capita)	807
Other drug use disorders	Female	15-19 years	95+ years	Data Rich	0	3	LDI (I$ per capita)	--
Other drug use disorders	Female	15-19 years	95+ years	Data Rich	0	3	Socio-demographic Index	--
Other drug use disorders	Female	15-19 years	95+ years	Global	1	1	Alcohol (liters per capita)	1000
Other drug use disorders	Female	15-19 years	95+ years	Global	1	1	Cumulative Cigarettes (10 Years)	--
Other drug use disorders	Female	15-19 years	95+ years	Global	1	1	Cumulative Cigarettes (5 Years)	--
Other drug use disorders	Female	15-19 years	95+ years	Global	1	1	Smoking Prevalence	--
Other drug use disorders	Female	15-19 years	95+ years	Global	-1	2	Healthcare access and quality index	--
Other drug use disorders	Female	15-19 years	95+ years	Global	0	3	Education (years per capita)	724
Other drug use disorders	Female	15-19 years	95+ years	Global	0	3	LDI (I$ per capita)	--
Other drug use disorders	Female	15-19 years	95+ years	Global	0	3	Socio-demographic Index	--
Other drug use disorders	Male	15-19 years	95+ years	Data Rich	1	1	Alcohol (liters per capita)	1000
Other drug use disorders	Male	15-19 years	95+ years	Data Rich	1	1	Cumulative Cigarettes (10 Years)	--
Other drug use disorders	Male	15-19 years	95+ years	Data Rich	1	1	Cumulative Cigarettes (5 Years)	--
Other drug use disorders	Male	15-19 years	95+ years	Data Rich	1	1	Smoking Prevalence	--
Other drug use disorders	Male	15-19 years	95+ years	Data Rich	-1	2	Healthcare access and quality index	--
Other drug use disorders	Male	15-19 years	95+ years	Data Rich	0	3	Socio-demographic Index	802
Other drug use disorders	Male	15-19 years	95+ years	Data Rich	0	3	Education (years per capita)	962
Other drug use disorders	Male	15-19 years	95+ years	Data Rich	0	3	LDI (I$ per capita)	--
Other drug use disorders	Male	15-19 years	95+ years	Global	1	1	Alcohol (liters per capita)	1000
Other drug use disorders	Male	15-19 years	95+ years	Global	1	1	Cumulative Cigarettes (10 Years)	--
Other drug use disorders	Male	15-19 years	95+ years	Global	1	1	Cumulative Cigarettes (5 Years)	--
Other drug use disorders	Male	15-19 years	95+ years	Global	1	1	Smoking Prevalence	--
Other drug use disorders	Male	15-19 years	95+ years	Global	-1	2	Healthcare access and quality index	--
Other drug use disorders	Male	15-19 years	95+ years	Global	0	3	Education (years per capita)	724
Other drug use disorders	Male	15-19 years	95+ years	Global	0	3	LDI (I$ per capita)	--
Other drug use disorders	Male	15-19 years	95+ years	Global	0	3	Socio-demographic Index	--
Diabetes mellitus	Female	0-6 days	10-14 years	Data Rich	-1	1	Healthcare access and quality index	199
Diabetes mellitus	Female	0-6 days	10-14 years	Data Rich	-1	2	Mean birth weight	186
Diabetes mellitus	Female	0-6 days	10-14 years	Data Rich	-1	2	Age-standardized underweight (weight-for-age) SEV	368
Diabetes mellitus	Female	0-6 days	10-14 years	Data Rich	-1	2	Age-standardized stunting (height-for-age) SEV	470
Diabetes mellitus	Female	0-6 days	10-14 years	Data Rich	1	2	Age-Specific Fertility Rate	945
Diabetes mellitus	Female	0-6 days	10-14 years	Data Rich	1	2	Absolute value of average latitude	--
Diabetes mellitus	Female	0-6 days	10-14 years	Data Rich	1	2	Live Births 35+ (proportion)	--
Diabetes mellitus	Female	0-6 days	10-14 years	Data Rich	1	2	Live Births 40+ (proportion)	--
Diabetes mellitus	Female	0-6 days	10-14 years	Data Rich	-1	3	Education (years per capita)	198
Diabetes mellitus	Female	0-6 days	10-14 years	Data Rich	-1	3	Socio-demographic Index	252
Diabetes mellitus	Female	0-6 days	10-14 years	Global	-1	1	Healthcare access and quality index	632
Diabetes mellitus	Female	0-6 days	10-14 years	Global	-1	2	Age-standardized stunting (height-for-age) SEV	733
Diabetes mellitus	Female	0-6 days	10-14 years	Global	-1	2	Age-standardized underweight (weight-for-age) SEV	733
Diabetes mellitus	Female	0-6 days	10-14 years	Global	-1	2	Mean birth weight	--
Diabetes mellitus	Female	0-6 days	10-14 years	Global	1	2	Age-Specific Fertility Rate	733
Diabetes mellitus	Female	0-6 days	10-14 years	Global	1	2	Absolute value of average latitude	--
Diabetes mellitus	Female	0-6 days	10-14 years	Global	1	2	Live Births 35+ (proportion)	--
Diabetes mellitus	Female	0-6 days	10-14 years	Global	1	2	Live Births 40+ (proportion)	--
Diabetes mellitus	Female	0-6 days	10-14 years	Global	-1	3	Education (years per capita)	354
Diabetes mellitus	Female	0-6 days	10-14 years	Global	-1	3	Socio-demographic Index	354
Diabetes mellitus	Female	15-19 years	95+ years	Data Rich	1	1	Mean BMI	483
Diabetes mellitus	Female	15-19 years	95+ years	Data Rich	1	1	Diabetes Age-Standardized Prevalence (proportion)	517
Diabetes mellitus	Female	15-19 years	95+ years	Data Rich	1	1	Diabetes Fasting Plasma Glucose (mmol/L)	530
Diabetes mellitus	Female	15-19 years	95+ years	Data Rich	1	1	Prevalence of obesity	592
Diabetes mellitus	Female	15-19 years	95+ years	Data Rich	0	2	vegetables adjusted(g)	390
Diabetes mellitus	Female	15-19 years	95+ years	Data Rich	0	2	Cholesterol (total, mean per capita)	606
Diabetes mellitus	Female	15-19 years	95+ years	Data Rich	0	2	Systolic Blood Pressure (mmHg)	803
Diabetes mellitus	Female	15-19 years	95+ years	Data Rich	0	2	fruits adjusted(g)	--
Diabetes mellitus	Female	15-19 years	95+ years	Data Rich	1	2	sugar adjusted(g)	504
Diabetes mellitus	Female	15-19 years	95+ years	Data Rich	0	3	Education (years per capita)	284
Diabetes mellitus	Female	15-19 years	95+ years	Data Rich	0	3	Healthcare access and quality index	313
Diabetes mellitus	Female	15-19 years	95+ years	Data Rich	0	3	LDI (I$ per capita)	--
Diabetes mellitus	Female	15-19 years	95+ years	Global	1	1	Mean BMI	99
Diabetes mellitus	Female	15-19 years	95+ years	Global	1	1	Diabetes Fasting Plasma Glucose (mmol/L)	442
Diabetes mellitus	Female	15-19 years	95+ years	Global	1	1	Prevalence of obesity	620
Diabetes mellitus	Female	15-19 years	95+ years	Global	1	1	Diabetes Age-Standardized Prevalence (proportion)	985
Diabetes mellitus	Female	15-19 years	95+ years	Global	0	2	vegetables adjusted(g)	245
Diabetes mellitus	Female	15-19 years	95+ years	Global	0	2	Systolic Blood Pressure (mmHg)	915
Diabetes mellitus	Female	15-19 years	95+ years	Global	0	2	Cholesterol (total, mean per capita)	928
Diabetes mellitus	Female	15-19 years	95+ years	Global	0	2	fruits adjusted(g)	--
Diabetes mellitus	Female	15-19 years	95+ years	Global	1	2	sugar adjusted(g)	781
Diabetes mellitus	Female	15-19 years	95+ years	Global	0	3	Education (years per capita)	504
Diabetes mellitus	Female	15-19 years	95+ years	Global	0	3	Healthcare access and quality index	670
Diabetes mellitus	Female	15-19 years	95+ years	Global	0	3	LDI (I$ per capita)	--
Diabetes mellitus	Male	0-6 days	10-14 years	Data Rich	-1	1	Healthcare access and quality index	793

**Appendix Table 9: CODEm covariates used, level of covariate, and expected direction of covariate by cause, sex, and age** *Covariates that CODEm did not select during the covariate selection process have no draw counts listed.*								
Cause	Sex	Age Start	Age End	Model Type	Direction	Level	Covariate Name	Number of Draws
Diabetes mellitus	Male	0-6 days	10-14 years	Data Rich	-1	2	Age-standardized stunting (height-for-age) SEV	549
Diabetes mellitus	Male	0-6 days	10-14 years	Data Rich	-1	2	Age-standardized underweight (weight-for-age) SEV	584
Diabetes mellitus	Male	0-6 days	10-14 years	Data Rich	-1	2	Mean birth weight	718
Diabetes mellitus	Male	0-6 days	10-14 years	Data Rich	1	2	Live Births 35+ (proportion)	207
Diabetes mellitus	Male	0-6 days	10-14 years	Data Rich	1	2	Absolute value of average latitude	--
Diabetes mellitus	Male	0-6 days	10-14 years	Data Rich	1	2	Age-Specific Fertility Rate	--
Diabetes mellitus	Male	0-6 days	10-14 years	Data Rich	1	2	Live Births 40+ (proportion)	--
Diabetes mellitus	Male	0-6 days	10-14 years	Data Rich	-1	3	Education (years per capita)	265
Diabetes mellitus	Male	0-6 days	10-14 years	Data Rich	-1	3	Socio-demographic Index	347
Diabetes mellitus	Male	0-6 days	10-14 years	Global	-1	1	Healthcare access and quality index	718
Diabetes mellitus	Male	0-6 days	10-14 years	Global	-1	2	Age-standardized stunting (height-for-age) SEV	666
Diabetes mellitus	Male	0-6 days	10-14 years	Global	-1	2	Age-standardized underweight (weight-for-age) SEV	666
Diabetes mellitus	Male	0-6 days	10-14 years	Global	-1	2	Mean birth weight	666
Diabetes mellitus	Male	0-6 days	10-14 years	Global	1	2	Live Births 35+ (proportion)	282
Diabetes mellitus	Male	0-6 days	10-14 years	Global	1	2	Absolute value of average latitude	--
Diabetes mellitus	Male	0-6 days	10-14 years	Global	1	2	Age-Specific Fertility Rate	--
Diabetes mellitus	Male	0-6 days	10-14 years	Global	1	2	Live Births 40+ (proportion)	--
Diabetes mellitus	Male	0-6 days	10-14 years	Global	-1	3	Education (years per capita)	424
Diabetes mellitus	Male	0-6 days	10-14 years	Global	-1	3	Socio-demographic Index	424
Diabetes mellitus	Male	15-19 years	95+ years	Data Rich	1	1	Prevalence of obesity	76
Diabetes mellitus	Male	15-19 years	95+ years	Data Rich	1	1	Diabetes Age-Standardized Prevalence (proportion)	297
Diabetes mellitus	Male	15-19 years	95+ years	Data Rich	1	1	Diabetes Fasting Plasma Glucose (mmol/L)	417
Diabetes mellitus	Male	15-19 years	95+ years	Data Rich	1	1	Mean BMI	879
Diabetes mellitus	Male	15-19 years	95+ years	Data Rich	0	2	vegetables adjusted(g)	755
Diabetes mellitus	Male	15-19 years	95+ years	Data Rich	0	2	Cholesterol (total, mean per capita)	775
Diabetes mellitus	Male	15-19 years	95+ years	Data Rich	0	2	Systolic Blood Pressure (mmHg)	931
Diabetes mellitus	Male	15-19 years	95+ years	Data Rich	0	2	fruits adjusted(g)	--
Diabetes mellitus	Male	15-19 years	95+ years	Data Rich	1	2	sugar adjusted(g)	0
Diabetes mellitus	Male	15-19 years	95+ years	Data Rich	0	3	Education (years per capita)	387
Diabetes mellitus	Male	15-19 years	95+ years	Data Rich	0	3	Healthcare access and quality index	410
Diabetes mellitus	Male	15-19 years	95+ years	Data Rich	0	3	LDI (I$ per capita)	--
Diabetes mellitus	Male	15-19 years	95+ years	Global	1	1	Prevalence of obesity	208
Diabetes mellitus	Male	15-19 years	95+ years	Global	1	1	Mean BMI	660
Diabetes mellitus	Male	15-19 years	95+ years	Global	1	1	Diabetes Fasting Plasma Glucose (mmol/L)	670
Diabetes mellitus	Male	15-19 years	95+ years	Global	1	1	Diabetes Age-Standardized Prevalence (proportion)	1000
Diabetes mellitus	Male	15-19 years	95+ years	Global	0	2	Systolic Blood Pressure (mmHg)	295
Diabetes mellitus	Male	15-19 years	95+ years	Global	0	2	vegetables adjusted(g)	586
Diabetes mellitus	Male	15-19 years	95+ years	Global	0	2	Cholesterol (total, mean per capita)	615
Diabetes mellitus	Male	15-19 years	95+ years	Global	0	2	fruits adjusted(g)	--
Diabetes mellitus	Male	15-19 years	95+ years	Global	1	2	sugar adjusted(g)	--
Diabetes mellitus	Male	15-19 years	95+ years	Global	0	3	Education (years per capita)	529
Diabetes mellitus	Male	15-19 years	95+ years	Global	0	3	Healthcare access and quality index	593
Diabetes mellitus	Male	15-19 years	95+ years	Global	0	3	LDI (I$ per capita)	--
Diabetes mellitus type 1	Female	0-6 days	95+ years	Data Rich	-1	1	Healthcare access and quality index	1000
Diabetes mellitus type 1	Female	0-6 days	95+ years	Data Rich	-1	2	Age-standardized stunting (height-for-age) SEV	354
Diabetes mellitus type 1	Female	0-6 days	95+ years	Data Rich	-1	2	Age-standardized underweight (weight-for-age) SEV	--
Diabetes mellitus type 1	Female	0-6 days	95+ years	Data Rich	-1	2	Mean birth weight	--
Diabetes mellitus type 1	Female	0-6 days	95+ years	Data Rich	1	2	Absolute value of average latitude	158
Diabetes mellitus type 1	Female	0-6 days	95+ years	Data Rich	1	2	Age-Specific Fertility Rate	--
Diabetes mellitus type 1	Female	0-6 days	95+ years	Data Rich	1	2	Live Births 35+ (proportion)	--
Diabetes mellitus type 1	Female	0-6 days	95+ years	Data Rich	1	2	Live Births 40+ (proportion)	--
Diabetes mellitus type 1	Female	0-6 days	95+ years	Data Rich	-1	3	Socio-demographic Index	145
Diabetes mellitus type 1	Female	0-6 days	95+ years	Data Rich	-1	3	Education (years per capita)	491
Diabetes mellitus type 1	Female	0-6 days	95+ years	Global	-1	1	Healthcare access and quality index	1000
Diabetes mellitus type 1	Female	0-6 days	95+ years	Global	-1	2	Age-standardized underweight (weight-for-age) SEV	498
Diabetes mellitus type 1	Female	0-6 days	95+ years	Global	-1	2	Age-standardized stunting (height-for-age) SEV	555
Diabetes mellitus type 1	Female	0-6 days	95+ years	Global	-1	2	Mean birth weight	--
Diabetes mellitus type 1	Female	0-6 days	95+ years	Global	1	2	Absolute value of average latitude	269
Diabetes mellitus type 1	Female	0-6 days	95+ years	Global	1	2	Age-Specific Fertility Rate	--
Diabetes mellitus type 1	Female	0-6 days	95+ years	Global	1	2	Live Births 35+ (proportion)	--
Diabetes mellitus type 1	Female	0-6 days	95+ years	Global	1	2	Live Births 40+ (proportion)	--
Diabetes mellitus type 1	Female	0-6 days	95+ years	Global	-1	3	Education (years per capita)	450
Diabetes mellitus type 1	Female	0-6 days	95+ years	Global	-1	3	Socio-demographic Index	--
Diabetes mellitus type 1	Male	0-6 days	95+ years	Data Rich	-1	1	Healthcare access and quality index	1000
Diabetes mellitus type 1	Male	0-6 days	95+ years	Data Rich	-1	2	Mean birth weight	177
Diabetes mellitus type 1	Male	0-6 days	95+ years	Data Rich	-1	2	Age-standardized underweight (weight-for-age) SEV	430
Diabetes mellitus type 1	Male	0-6 days	95+ years	Data Rich	-1	2	Age-standardized stunting (height-for-age) SEV	437
Diabetes mellitus type 1	Male	0-6 days	95+ years	Data Rich	1	2	Absolute value of average latitude	195
Diabetes mellitus type 1	Male	0-6 days	95+ years	Data Rich	1	2	Age-Specific Fertility Rate	--
Diabetes mellitus type 1	Male	0-6 days	95+ years	Data Rich	1	2	Live Births 35+ (proportion)	--
Diabetes mellitus type 1	Male	0-6 days	95+ years	Data Rich	1	2	Live Births 40+ (proportion)	--
Diabetes mellitus type 1	Male	0-6 days	95+ years	Data Rich	-1	3	Socio-demographic Index	72
Diabetes mellitus type 1	Male	0-6 days	95+ years	Data Rich	-1	3	Education (years per capita)	492
Diabetes mellitus type 1	Male	0-6 days	95+ years	Global	-1	1	Healthcare access and quality index	1000
Diabetes mellitus type 1	Male	0-6 days	95+ years	Global	-1	2	Age-standardized underweight (weight-for-age) SEV	341
Diabetes mellitus type 1	Male	0-6 days	95+ years	Global	-1	2	Mean birth weight	440
Diabetes mellitus type 1	Male	0-6 days	95+ years	Global	-1	2	Age-standardized stunting (height-for-age) SEV	662
Diabetes mellitus type 1	Male	0-6 days	95+ years	Global	1	2	Absolute value of average latitude	119

Appendix Table 9: CODEm covariates used, level of covariate, and expected direction of covariate by cause, sex, and age
Covariates that CODEm did not select during the covariate selection process have no draw counts listed.

Cause	Sex	Age Start	Age End	Model Type	Direction	Level	Covariate Name	Number of Draws
Diabetes mellitus type 1	Male	0-6 days	95+ years	Global	1	2	Age-Specific Fertility Rate	--
Diabetes mellitus type 1	Male	0-6 days	95+ years	Global	1	2	Live Births 35+ (proportion)	--
Diabetes mellitus type 1	Male	0-6 days	95+ years	Global	1	2	Live Births 40+ (proportion)	--
Diabetes mellitus type 1	Male	0-6 days	95+ years	Global	-1	3	Education (years per capita)	414
Diabetes mellitus type 1	Male	0-6 days	95+ years	Global	-1	3	Socio-demographic Index	--
Diabetes mellitus type 2	Female	15-19 years	95+ years	Data Rich	1	1	Prevalence of obesity	487
Diabetes mellitus type 2	Female	15-19 years	95+ years	Data Rich	1	1	Mean BMI	489
Diabetes mellitus type 2	Female	15-19 years	95+ years	Data Rich	1	1	Diabetes Fasting Plasma Glucose (mmol/L)	742
Diabetes mellitus type 2	Female	15-19 years	95+ years	Data Rich	1	1	Diabetes Age-Standardized Prevalence (proportion)	942
Diabetes mellitus type 2	Female	15-19 years	95+ years	Data Rich	0	2	fruits adjusted(g)	654
Diabetes mellitus type 2	Female	15-19 years	95+ years	Data Rich	0	2	Alcohol SEV, age and sex specific	788
Diabetes mellitus type 2	Female	15-19 years	95+ years	Data Rich	0	2	Cholesterol (total, mean per capita)	869
Diabetes mellitus type 2	Female	15-19 years	95+ years	Data Rich	0	2	Systolic Blood Pressure (mmHg)	900
Diabetes mellitus type 2	Female	15-19 years	95+ years	Data Rich	0	2	vegetables adjusted(g)	951
Diabetes mellitus type 2	Female	15-19 years	95+ years	Data Rich	1	2	sugar adjusted(g)	1
Diabetes mellitus type 2	Female	15-19 years	95+ years	Data Rich	0	3	Healthcare access and quality index	333
Diabetes mellitus type 2	Female	15-19 years	95+ years	Data Rich	0	3	Education (years per capita)	334
Diabetes mellitus type 2	Female	15-19 years	95+ years	Data Rich	0	3	LDI (I$ per capita)	--
Diabetes mellitus type 2	Female	15-19 years	95+ years	Global	1	1	Prevalence of obesity	241
Diabetes mellitus type 2	Female	15-19 years	95+ years	Global	1	1	Mean BMI	537
Diabetes mellitus type 2	Female	15-19 years	95+ years	Global	1	1	Diabetes Fasting Plasma Glucose (mmol/L)	556
Diabetes mellitus type 2	Female	15-19 years	95+ years	Global	1	1	Diabetes Age-Standardized Prevalence (proportion)	837
Diabetes mellitus type 2	Female	15-19 years	95+ years	Global	0	2	Cholesterol (total, mean per capita)	219
Diabetes mellitus type 2	Female	15-19 years	95+ years	Global	0	2	vegetables adjusted(g)	253
Diabetes mellitus type 2	Female	15-19 years	95+ years	Global	0	2	Systolic Blood Pressure (mmHg)	256
Diabetes mellitus type 2	Female	15-19 years	95+ years	Global	0	2	fruits adjusted(g)	412
Diabetes mellitus type 2	Female	15-19 years	95+ years	Global	0	2	Alcohol SEV, age and sex specific	546
Diabetes mellitus type 2	Female	15-19 years	95+ years	Global	1	2	sugar adjusted(g)	252
Diabetes mellitus type 2	Female	15-19 years	95+ years	Global	0	3	Education (years per capita)	379
Diabetes mellitus type 2	Female	15-19 years	95+ years	Global	0	3	Healthcare access and quality index	443
Diabetes mellitus type 2	Female	15-19 years	95+ years	Global	0	3	LDI (I$ per capita)	--
Diabetes mellitus type 2	Male	15-19 years	95+ years	Data Rich	1	1	Diabetes Age-Standardized Prevalence (proportion)	83
Diabetes mellitus type 2	Male	15-19 years	95+ years	Data Rich	1	1	Prevalence of obesity	88
Diabetes mellitus type 2	Male	15-19 years	95+ years	Data Rich	1	1	Diabetes Fasting Plasma Glucose (mmol/L)	102
Diabetes mellitus type 2	Male	15-19 years	95+ years	Data Rich	1	1	Mean BMI	912
Diabetes mellitus type 2	Male	15-19 years	95+ years	Data Rich	0	2	Alcohol SEV, age and sex specific	583
Diabetes mellitus type 2	Male	15-19 years	95+ years	Data Rich	0	2	Cholesterol (total, mean per capita)	703
Diabetes mellitus type 2	Male	15-19 years	95+ years	Data Rich	0	2	fruits adjusted(g)	720
Diabetes mellitus type 2	Male	15-19 years	95+ years	Data Rich	0	2	vegetables adjusted(g)	879
Diabetes mellitus type 2	Male	15-19 years	95+ years	Data Rich	0	2	Systolic Blood Pressure (mmHg)	984
Diabetes mellitus type 2	Male	15-19 years	95+ years	Data Rich	1	2	sugar adjusted(g)	0
Diabetes mellitus type 2	Male	15-19 years	95+ years	Data Rich	0	3	Education (years per capita)	163
Diabetes mellitus type 2	Male	15-19 years	95+ years	Data Rich	0	3	Healthcare access and quality index	501
Diabetes mellitus type 2	Male	15-19 years	95+ years	Data Rich	0	3	LDI (I$ per capita)	--
Diabetes mellitus type 2	Male	15-19 years	95+ years	Global	1	1	Diabetes Fasting Plasma Glucose (mmol/L)	184
Diabetes mellitus type 2	Male	15-19 years	95+ years	Global	1	1	Prevalence of obesity	270
Diabetes mellitus type 2	Male	15-19 years	95+ years	Global	1	1	Mean BMI	418
Diabetes mellitus type 2	Male	15-19 years	95+ years	Global	1	1	Diabetes Age-Standardized Prevalence (proportion)	1000
Diabetes mellitus type 2	Male	15-19 years	95+ years	Global	0	2	Cholesterol (total, mean per capita)	361
Diabetes mellitus type 2	Male	15-19 years	95+ years	Global	0	2	Systolic Blood Pressure (mmHg)	542
Diabetes mellitus type 2	Male	15-19 years	95+ years	Global	0	2	Alcohol SEV, age and sex specific	587
Diabetes mellitus type 2	Male	15-19 years	95+ years	Global	0	2	fruits adjusted(g)	590
Diabetes mellitus type 2	Male	15-19 years	95+ years	Global	0	2	vegetables adjusted(g)	590
Diabetes mellitus type 2	Male	15-19 years	95+ years	Global	1	2	sugar adjusted(g)	48
Diabetes mellitus type 2	Male	15-19 years	95+ years	Global	0	3	Healthcare access and quality index	471
Diabetes mellitus type 2	Male	15-19 years	95+ years	Global	0	3	Education (years per capita)	507
Diabetes mellitus type 2	Male	15-19 years	95+ years	Global	0	3	LDI (I$ per capita)	--
Chronic kidney disease	Female	28-364 days	95+ years	Data Rich	-1	1	Healthcare access and quality index	9
Chronic kidney disease	Female	28-364 days	95+ years	Data Rich	1	1	Systolic Blood Pressure (mmHg)	1
Chronic kidney disease	Female	28-364 days	95+ years	Data Rich	1	1	Mean BMI	41
Chronic kidney disease	Female	28-364 days	95+ years	Data Rich	1	1	Diabetes Age-Standardized Prevalence (proportion)	248
Chronic kidney disease	Female	28-364 days	95+ years	Data Rich	1	1	Diabetes Fasting Plasma Glucose (mmol/L)	921
Chronic kidney disease	Female	28-364 days	95+ years	Data Rich	0	2	red meats adjusted(g)	138
Chronic kidney disease	Female	28-364 days	95+ years	Data Rich	1	2	Cholesterol (total, mean per capita)	0
Chronic kidney disease	Female	28-364 days	95+ years	Data Rich	1	2	energy unadjusted(kcal)	518
Chronic kidney disease	Female	28-364 days	95+ years	Data Rich	-1	3	Education (years per capita)	116
Chronic kidney disease	Female	28-364 days	95+ years	Data Rich	-1	3	LDI (I$ per capita)	--
Chronic kidney disease	Female	28-364 days	95+ years	Data Rich	0	3	Socio-demographic Index	163
Chronic kidney disease	Female	28-364 days	95+ years	Global	-1	1	Healthcare access and quality index	388
Chronic kidney disease	Female	28-364 days	95+ years	Global	1	1	Systolic Blood Pressure (mmHg)	19
Chronic kidney disease	Female	28-364 days	95+ years	Global	1	1	Diabetes Age-Standardized Prevalence (proportion)	59
Chronic kidney disease	Female	28-364 days	95+ years	Global	1	1	Diabetes Fasting Plasma Glucose (mmol/L)	954
Chronic kidney disease	Female	28-364 days	95+ years	Global	1	1	Mean BMI	1000
Chronic kidney disease	Female	28-364 days	95+ years	Global	0	2	red meats adjusted(g)	444
Chronic kidney disease	Female	28-364 days	95+ years	Global	1	2	Cholesterol (total, mean per capita)	0
Chronic kidney disease	Female	28-364 days	95+ years	Global	1	2	energy unadjusted(kcal)	0
Chronic kidney disease	Female	28-364 days	95+ years	Global	-1	3	Education (years per capita)	647
Chronic kidney disease	Female	28-364 days	95+ years	Global	-1	3	LDI (I$ per capita)	--

**Appendix Table 9: CODEm covariates used, level of covariate, and expected direction of covariate by cause, sex, and age** *Covariates that CODEm did not select during the covariate selection process have no draw counts listed.*								
Cause	Sex	Age Start	Age End	Model Type	Direction	Level	Covariate Name	Number of Draws
Chronic kidney disease	Female	28-364 days	95+ years	Global	0	3	Socio-demographic Index	489
Chronic kidney disease	Male	28-364 days	95+ years	Data Rich	-1	1	Healthcare access and quality index	48
Chronic kidney disease	Male	28-364 days	95+ years	Data Rich	1	1	Systolic Blood Pressure (mmHg)	1
Chronic kidney disease	Male	28-364 days	95+ years	Data Rich	1	1	Mean BMI	130
Chronic kidney disease	Male	28-364 days	95+ years	Data Rich	1	1	Diabetes Fasting Plasma Glucose (mmol/L)	427
Chronic kidney disease	Male	28-364 days	95+ years	Data Rich	1	1	Diabetes Age-Standardized Prevalence (proportion)	790
Chronic kidney disease	Male	28-364 days	95+ years	Data Rich	0	2	red meats adjusted(g)	273
Chronic kidney disease	Male	28-364 days	95+ years	Data Rich	1	2	Cholesterol (total, mean per capita)	3
Chronic kidney disease	Male	28-364 days	95+ years	Data Rich	1	2	energy unadjusted(kcal)	105
Chronic kidney disease	Male	28-364 days	95+ years	Data Rich	-1	3	Education (years per capita)	68
Chronic kidney disease	Male	28-364 days	95+ years	Data Rich	-1	3	LDI (I$ per capita)	--
Chronic kidney disease	Male	28-364 days	95+ years	Data Rich	0	3	Socio-demographic Index	114
Chronic kidney disease	Male	28-364 days	95+ years	Global	-1	1	Healthcare access and quality index	363
Chronic kidney disease	Male	28-364 days	95+ years	Global	1	1	Systolic Blood Pressure (mmHg)	0
Chronic kidney disease	Male	28-364 days	95+ years	Global	1	1	Diabetes Fasting Plasma Glucose (mmol/L)	452
Chronic kidney disease	Male	28-364 days	95+ years	Global	1	1	Mean BMI	828
Chronic kidney disease	Male	28-364 days	95+ years	Global	1	1	Diabetes Age-Standardized Prevalence (proportion)	860
Chronic kidney disease	Male	28-364 days	95+ years	Global	0	2	red meats adjusted(g)	319
Chronic kidney disease	Male	28-364 days	95+ years	Global	1	2	Cholesterol (total, mean per capita)	0
Chronic kidney disease	Male	28-364 days	95+ years	Global	1	2	energy unadjusted(kcal)	107
Chronic kidney disease	Male	28-364 days	95+ years	Global	-1	3	Education (years per capita)	215
Chronic kidney disease	Male	28-364 days	95+ years	Global	-1	3	LDI (I$ per capita)	--
Chronic kidney disease	Male	28-364 days	95+ years	Global	0	3	Socio-demographic Index	257
Acute glomerulonephritis	Female	28-364 days	95+ years	Data Rich	-1	2	Sanitation (proportion with access)	601
Acute glomerulonephritis	Female	28-364 days	95+ years	Data Rich	-1	2	Improved Water Source (proportion with access)	760
Acute glomerulonephritis	Female	28-364 days	95+ years	Data Rich	-1	2	Healthcare access and quality index	935
Acute glomerulonephritis	Female	28-364 days	95+ years	Data Rich	1	2	Systolic Blood Pressure (mmHg)	0
Acute glomerulonephritis	Female	28-364 days	95+ years	Data Rich	1	2	Diabetes Age-Standardized Prevalence (proportion)	--
Acute glomerulonephritis	Female	28-364 days	95+ years	Data Rich	-1	3	Education (years per capita)	16
Acute glomerulonephritis	Female	28-364 days	95+ years	Data Rich	-1	3	Socio-demographic Index	43
Acute glomerulonephritis	Female	28-364 days	95+ years	Data Rich	-1	3	LDI (I$ per capita)	--
Acute glomerulonephritis	Female	28-364 days	95+ years	Global	-1	2	Healthcare access and quality index	492
Acute glomerulonephritis	Female	28-364 days	95+ years	Global	-1	2	Sanitation (proportion with access)	558
Acute glomerulonephritis	Female	28-364 days	95+ years	Global	-1	2	Improved Water Source (proportion with access)	728
Acute glomerulonephritis	Female	28-364 days	95+ years	Global	1	2	Systolic Blood Pressure (mmHg)	15
Acute glomerulonephritis	Female	28-364 days	95+ years	Global	1	2	Diabetes Age-Standardized Prevalence (proportion)	--
Acute glomerulonephritis	Female	28-364 days	95+ years	Global	-1	3	Education (years per capita)	207
Acute glomerulonephritis	Female	28-364 days	95+ years	Global	-1	3	Socio-demographic Index	219
Acute glomerulonephritis	Female	28-364 days	95+ years	Global	-1	3	LDI (I$ per capita)	--
Acute glomerulonephritis	Male	28-364 days	95+ years	Data Rich	-1	2	Sanitation (proportion with access)	491
Acute glomerulonephritis	Male	28-364 days	95+ years	Data Rich	-1	2	Improved Water Source (proportion with access)	592
Acute glomerulonephritis	Male	28-364 days	95+ years	Data Rich	-1	2	Healthcare access and quality index	941
Acute glomerulonephritis	Male	28-364 days	95+ years	Data Rich	1	2	Systolic Blood Pressure (mmHg)	0
Acute glomerulonephritis	Male	28-364 days	95+ years	Data Rich	1	2	Diabetes Age-Standardized Prevalence (proportion)	242
Acute glomerulonephritis	Male	28-364 days	95+ years	Data Rich	-1	3	Socio-demographic Index	1
Acute glomerulonephritis	Male	28-364 days	95+ years	Data Rich	-1	3	Education (years per capita)	58
Acute glomerulonephritis	Male	28-364 days	95+ years	Data Rich	-1	3	LDI (I$ per capita)	--
Acute glomerulonephritis	Male	28-364 days	95+ years	Global	-1	2	Sanitation (proportion with access)	520
Acute glomerulonephritis	Male	28-364 days	95+ years	Global	-1	2	Improved Water Source (proportion with access)	538
Acute glomerulonephritis	Male	28-364 days	95+ years	Global	-1	2	Healthcare access and quality index	744
Acute glomerulonephritis	Male	28-364 days	95+ years	Global	1	2	Systolic Blood Pressure (mmHg)	154
Acute glomerulonephritis	Male	28-364 days	95+ years	Global	1	2	Diabetes Age-Standardized Prevalence (proportion)	--
Acute glomerulonephritis	Male	28-364 days	95+ years	Global	-1	3	Education (years per capita)	44
Acute glomerulonephritis	Male	28-364 days	95+ years	Global	-1	3	Socio-demographic Index	142
Acute glomerulonephritis	Male	28-364 days	95+ years	Global	-1	3	LDI (I$ per capita)	--
Skin and subcutaneous diseases	Female	28-364 days	95+ years	Data Rich	-1	1	Improved Water Source (proportion with access)	--
Skin and subcutaneous diseases	Female	28-364 days	95+ years	Data Rich	1	1	SEV unsafe sanitation	--
Skin and subcutaneous diseases	Female	28-364 days	95+ years	Data Rich	-1	2	Healthcare access and quality index	--
Skin and subcutaneous diseases	Female	28-364 days	95+ years	Data Rich	1	2	Alcohol (liters per capita)	1000
Skin and subcutaneous diseases	Female	28-364 days	95+ years	Data Rich	1	2	Cumulative Cigarettes (10 Years)	--
Skin and subcutaneous diseases	Female	28-364 days	95+ years	Data Rich	1	2	Cumulative Cigarettes (5 Years)	--
Skin and subcutaneous diseases	Female	28-364 days	95+ years	Data Rich	1	2	Smoking Prevalence	--
Skin and subcutaneous diseases	Female	28-364 days	95+ years	Data Rich	-1	3	Education (years per capita)	--
Skin and subcutaneous diseases	Female	28-364 days	95+ years	Data Rich	-1	3	LDI (I$ per capita)	--
Skin and subcutaneous diseases	Female	28-364 days	95+ years	Data Rich	0	3	Socio-demographic Index	568
Skin and subcutaneous diseases	Female	28-364 days	95+ years	Global	-1	1	Improved Water Source (proportion with access)	--
Skin and subcutaneous diseases	Female	28-364 days	95+ years	Global	1	1	SEV unsafe sanitation	--
Skin and subcutaneous diseases	Female	28-364 days	95+ years	Global	-1	2	Healthcare access and quality index	--
Skin and subcutaneous diseases	Female	28-364 days	95+ years	Global	1	2	Alcohol (liters per capita)	--
Skin and subcutaneous diseases	Female	28-364 days	95+ years	Global	1	2	Cumulative Cigarettes (10 Years)	--
Skin and subcutaneous diseases	Female	28-364 days	95+ years	Global	1	2	Cumulative Cigarettes (5 Years)	--
Skin and subcutaneous diseases	Female	28-364 days	95+ years	Global	1	2	Smoking Prevalence	--
Skin and subcutaneous diseases	Female	28-364 days	95+ years	Global	-1	3	Education (years per capita)	--
Skin and subcutaneous diseases	Female	28-364 days	95+ years	Global	-1	3	LDI (I$ per capita)	--
Skin and subcutaneous diseases	Female	28-364 days	95+ years	Global	0	3	Socio-demographic Index	1000
Skin and subcutaneous diseases	Male	28-364 days	95+ years	Data Rich	-1	1	Improved Water Source (proportion with access)	--
Skin and subcutaneous diseases	Male	28-364 days	95+ years	Data Rich	1	1	SEV unsafe sanitation	--
Skin and subcutaneous diseases	Male	28-364 days	95+ years	Data Rich	-1	2	Healthcare access and quality index	--

Appendix Table 9: CODEm covariates used, level of covariate, and expected direction of covariate by cause, sex, and age

Covariates that CODEm did not select during the covariate selection process have no draw counts listed.

Cause	Sex	Age Start	Age End	Model Type	Direction	Level	Covariate Name	Number of Draws
Skin and subcutaneous diseases	Male	28-364 days	95+ years	Data Rich	1	2	Alcohol (liters per capita)	1000
Skin and subcutaneous diseases	Male	28-364 days	95+ years	Data Rich	1	2	Cumulative Cigarettes (10 Years)	--
Skin and subcutaneous diseases	Male	28-364 days	95+ years	Data Rich	1	2	Cumulative Cigarettes (5 Years)	--
Skin and subcutaneous diseases	Male	28-364 days	95+ years	Data Rich	1	2	Smoking Prevalence	--
Skin and subcutaneous diseases	Male	28-364 days	95+ years	Data Rich	-1	3	Education (years per capita)	--
Skin and subcutaneous diseases	Male	28-364 days	95+ years	Data Rich	-1	3	LDI (I$ per capita)	--
Skin and subcutaneous diseases	Male	28-364 days	95+ years	Data Rich	0	3	Socio-demographic Index	568
Skin and subcutaneous diseases	Male	28-364 days	95+ years	Global	-1	1	Improved Water Source (proportion with access)	--
Skin and subcutaneous diseases	Male	28-364 days	95+ years	Global	1	1	SEV unsafe sanitation	674
Skin and subcutaneous diseases	Male	28-364 days	95+ years	Global	-1	2	Healthcare access and quality index	--
Skin and subcutaneous diseases	Male	28-364 days	95+ years	Global	1	2	Alcohol (liters per capita)	452
Skin and subcutaneous diseases	Male	28-364 days	95+ years	Global	1	2	Cumulative Cigarettes (10 Years)	--
Skin and subcutaneous diseases	Male	28-364 days	95+ years	Global	1	2	Cumulative Cigarettes (5 Years)	--
Skin and subcutaneous diseases	Male	28-364 days	95+ years	Global	1	2	Smoking Prevalence	--
Skin and subcutaneous diseases	Male	28-364 days	95+ years	Global	-1	3	Education (years per capita)	--
Skin and subcutaneous diseases	Male	28-364 days	95+ years	Global	-1	3	LDI (I$ per capita)	--
Skin and subcutaneous diseases	Male	28-364 days	95+ years	Global	0	3	Socio-demographic Index	593
Bacterial skin diseases	Female	0-6 days	95+ years	Data Rich	-1	1	Improved Water Source (proportion with access)	--
Bacterial skin diseases	Female	0-6 days	95+ years	Data Rich	1	1	SEV unsafe sanitation	--
Bacterial skin diseases	Female	0-6 days	95+ years	Data Rich	-1	2	Healthcare access and quality index	--
Bacterial skin diseases	Female	0-6 days	95+ years	Data Rich	1	2	Alcohol (liters per capita)	1000
Bacterial skin diseases	Female	0-6 days	95+ years	Data Rich	1	2	Cumulative Cigarettes (10 Years)	--
Bacterial skin diseases	Female	0-6 days	95+ years	Data Rich	1	2	Cumulative Cigarettes (5 Years)	--
Bacterial skin diseases	Female	0-6 days	95+ years	Data Rich	1	2	Smoking Prevalence	--
Bacterial skin diseases	Female	0-6 days	95+ years	Data Rich	-1	3	Education (years per capita)	--
Bacterial skin diseases	Female	0-6 days	95+ years	Data Rich	-1	3	LDI (I$ per capita)	--
Bacterial skin diseases	Female	0-6 days	95+ years	Data Rich	0	3	Socio-demographic Index	--
Bacterial skin diseases	Female	0-6 days	95+ years	Global	-1	1	Improved Water Source (proportion with access)	--
Bacterial skin diseases	Female	0-6 days	95+ years	Global	1	1	SEV unsafe sanitation	726
Bacterial skin diseases	Female	0-6 days	95+ years	Global	-1	2	Healthcare access and quality index	--
Bacterial skin diseases	Female	0-6 days	95+ years	Global	1	2	Alcohol (liters per capita)	274
Bacterial skin diseases	Female	0-6 days	95+ years	Global	1	2	Cumulative Cigarettes (10 Years)	--
Bacterial skin diseases	Female	0-6 days	95+ years	Global	1	2	Cumulative Cigarettes (5 Years)	--
Bacterial skin diseases	Female	0-6 days	95+ years	Global	1	2	Smoking Prevalence	--
Bacterial skin diseases	Female	0-6 days	95+ years	Global	-1	3	Education (years per capita)	--
Bacterial skin diseases	Female	0-6 days	95+ years	Global	-1	3	LDI (I$ per capita)	--
Bacterial skin diseases	Female	0-6 days	95+ years	Global	0	3	Socio-demographic Index	398
Bacterial skin diseases	Male	0-6 days	95+ years	Data Rich	-1	1	Improved Water Source (proportion with access)	--
Bacterial skin diseases	Male	0-6 days	95+ years	Data Rich	1	1	SEV unsafe sanitation	--
Bacterial skin diseases	Male	0-6 days	95+ years	Data Rich	-1	2	Healthcare access and quality index	--
Bacterial skin diseases	Male	0-6 days	95+ years	Data Rich	1	2	Alcohol (liters per capita)	691
Bacterial skin diseases	Male	0-6 days	95+ years	Data Rich	1	2	Cumulative Cigarettes (10 Years)	--
Bacterial skin diseases	Male	0-6 days	95+ years	Data Rich	1	2	Cumulative Cigarettes (5 Years)	--
Bacterial skin diseases	Male	0-6 days	95+ years	Data Rich	1	2	Smoking Prevalence	--
Bacterial skin diseases	Male	0-6 days	95+ years	Data Rich	-1	3	Education (years per capita)	--
Bacterial skin diseases	Male	0-6 days	95+ years	Data Rich	-1	3	LDI (I$ per capita)	--
Bacterial skin diseases	Male	0-6 days	95+ years	Data Rich	0	3	Socio-demographic Index	593
Bacterial skin diseases	Male	0-6 days	95+ years	Global	-1	1	Improved Water Source (proportion with access)	--
Bacterial skin diseases	Male	0-6 days	95+ years	Global	1	1	SEV unsafe sanitation	1000
Bacterial skin diseases	Male	0-6 days	95+ years	Global	-1	2	Healthcare access and quality index	--
Bacterial skin diseases	Male	0-6 days	95+ years	Global	1	2	Alcohol (liters per capita)	491
Bacterial skin diseases	Male	0-6 days	95+ years	Global	1	2	Cumulative Cigarettes (10 Years)	--
Bacterial skin diseases	Male	0-6 days	95+ years	Global	1	2	Cumulative Cigarettes (5 Years)	--
Bacterial skin diseases	Male	0-6 days	95+ years	Global	1	2	Smoking Prevalence	--
Bacterial skin diseases	Male	0-6 days	95+ years	Global	-1	3	Education (years per capita)	--
Bacterial skin diseases	Male	0-6 days	95+ years	Global	-1	3	LDI (I$ per capita)	--
Bacterial skin diseases	Male	0-6 days	95+ years	Global	0	3	Socio-demographic Index	--
Cellulitis	Female	28-364 days	95+ years	Data Rich	-1	2	Healthcare access and quality index	--
Cellulitis	Female	28-364 days	95+ years	Data Rich	0	3	Education (years per capita)	--
Cellulitis	Female	28-364 days	95+ years	Data Rich	0	3	LDI (I$ per capita)	--
Cellulitis	Female	28-364 days	95+ years	Global	-1	2	Healthcare access and quality index	--
Cellulitis	Female	28-364 days	95+ years	Global	0	3	Education (years per capita)	546
Cellulitis	Female	28-364 days	95+ years	Global	0	3	LDI (I$ per capita)	--
Cellulitis	Male	28-364 days	95+ years	Data Rich	-1	2	Healthcare access and quality index	--
Cellulitis	Male	28-364 days	95+ years	Data Rich	0	3	Education (years per capita)	546
Cellulitis	Male	28-364 days	95+ years	Data Rich	0	3	LDI (I$ per capita)	--
Cellulitis	Male	28-364 days	95+ years	Global	-1	2	Healthcare access and quality index	--
Cellulitis	Male	28-364 days	95+ years	Global	0	3	Education (years per capita)	454
Cellulitis	Male	28-364 days	95+ years	Global	0	3	LDI (I$ per capita)	--
Pyoderma	Female	0-6 days	95+ years	Data Rich	-1	1	Improved Water Source (proportion with access)	--
Pyoderma	Female	0-6 days	95+ years	Data Rich	1	1	SEV unsafe sanitation	1000
Pyoderma	Female	0-6 days	95+ years	Data Rich	-1	2	Healthcare access and quality index	--
Pyoderma	Female	0-6 days	95+ years	Data Rich	1	2	Alcohol (liters per capita)	--
Pyoderma	Female	0-6 days	95+ years	Data Rich	1	2	Cumulative Cigarettes (10 Years)	--
Pyoderma	Female	0-6 days	95+ years	Data Rich	1	2	Cumulative Cigarettes (5 Years)	--
Pyoderma	Female	0-6 days	95+ years	Data Rich	1	2	Smoking Prevalence	--
Pyoderma	Female	0-6 days	95+ years	Data Rich	-1	3	Education (years per capita)	--
Pyoderma	Female	0-6 days	95+ years	Data Rich	-1	3	LDI (I$ per capita)	--

Appendix Table 9: CODEm covariates used, level of covariate, and expected direction of covariate by cause, sex, and age<br>*Covariates that CODEm did not select during the covariate selection process have no draw counts listed.*								
**Cause**	**Sex**	**Age Start**	**Age End**	**Model Type**	**Direction**	**Level**	**Covariate Name**	**Number of Draws**
Pyoderma	Female	0-6 days	95+ years	Data Rich	0	3	Socio-demographic Index	546
Pyoderma	Female	0-6 days	95+ years	Global	-1	1	Improved Water Source (proportion with access)	--
Pyoderma	Female	0-6 days	95+ years	Global	1	1	SEV unsafe sanitation	1000
Pyoderma	Female	0-6 days	95+ years	Global	-1	2	Healthcare access and quality index	--
Pyoderma	Female	0-6 days	95+ years	Global	1	2	Alcohol (liters per capita)	--
Pyoderma	Female	0-6 days	95+ years	Global	1	2	Cumulative Cigarettes (10 Years)	--
Pyoderma	Female	0-6 days	95+ years	Global	1	2	Cumulative Cigarettes (5 Years)	--
Pyoderma	Female	0-6 days	95+ years	Global	1	2	Smoking Prevalence	--
Pyoderma	Female	0-6 days	95+ years	Global	-1	3	Education (years per capita)	--
Pyoderma	Female	0-6 days	95+ years	Global	-1	3	LDI (I$ per capita)	--
Pyoderma	Female	0-6 days	95+ years	Global	0	3	Socio-demographic Index	546
Pyoderma	Male	0-6 days	95+ years	Data Rich	-1	1	Improved Water Source (proportion with access)	--
Pyoderma	Male	0-6 days	95+ years	Data Rich	1	1	SEV unsafe sanitation	--
Pyoderma	Male	0-6 days	95+ years	Data Rich	-1	2	Healthcare access and quality index	--
Pyoderma	Male	0-6 days	95+ years	Data Rich	1	2	Alcohol (liters per capita)	--
Pyoderma	Male	0-6 days	95+ years	Data Rich	1	2	Cumulative Cigarettes (10 Years)	--
Pyoderma	Male	0-6 days	95+ years	Data Rich	1	2	Cumulative Cigarettes (5 Years)	--
Pyoderma	Male	0-6 days	95+ years	Data Rich	1	2	Smoking Prevalence	--
Pyoderma	Male	0-6 days	95+ years	Data Rich	-1	3	Education (years per capita)	--
Pyoderma	Male	0-6 days	95+ years	Data Rich	-1	3	LDI (I$ per capita)	--
Pyoderma	Male	0-6 days	95+ years	Data Rich	0	3	Socio-demographic Index	1000
Pyoderma	Male	0-6 days	95+ years	Global	-1	1	Improved Water Source (proportion with access)	--
Pyoderma	Male	0-6 days	95+ years	Global	1	1	SEV unsafe sanitation	1000
Pyoderma	Male	0-6 days	95+ years	Global	-1	2	Healthcare access and quality index	--
Pyoderma	Male	0-6 days	95+ years	Global	1	2	Alcohol (liters per capita)	--
Pyoderma	Male	0-6 days	95+ years	Global	1	2	Cumulative Cigarettes (10 Years)	--
Pyoderma	Male	0-6 days	95+ years	Global	1	2	Cumulative Cigarettes (5 Years)	--
Pyoderma	Male	0-6 days	95+ years	Global	1	2	Smoking Prevalence	--
Pyoderma	Male	0-6 days	95+ years	Global	-1	3	Education (years per capita)	--
Pyoderma	Male	0-6 days	95+ years	Global	-1	3	LDI (I$ per capita)	--
Pyoderma	Male	0-6 days	95+ years	Global	0	3	Socio-demographic Index	--
Decubitus ulcer	Female	1-4 years	95+ years	Data Rich	-1	1	Improved Water Source (proportion with access)	--
Decubitus ulcer	Female	1-4 years	95+ years	Data Rich	1	1	Diabetes Fasting Plasma Glucose (mmol/L)	--
Decubitus ulcer	Female	1-4 years	95+ years	Data Rich	1	1	Prevalence of obesity	--
Decubitus ulcer	Female	1-4 years	95+ years	Data Rich	-1	2	Healthcare access and quality index	--
Decubitus ulcer	Female	1-4 years	95+ years	Data Rich	1	2	Smoking Prevalence	105
Decubitus ulcer	Female	1-4 years	95+ years	Data Rich	1	2	Cumulative Cigarettes (10 Years)	128
Decubitus ulcer	Female	1-4 years	95+ years	Data Rich	1	2	Alcohol (liters per capita)	498
Decubitus ulcer	Female	1-4 years	95+ years	Data Rich	1	2	Cumulative Cigarettes (5 Years)	584
Decubitus ulcer	Female	1-4 years	95+ years	Data Rich	-1	3	Health System Access 2 (unitless)	113
Decubitus ulcer	Female	1-4 years	95+ years	Data Rich	-1	3	Education (years per capita)	334
Decubitus ulcer	Female	1-4 years	95+ years	Data Rich	-1	3	LDI (I$ per capita)	--
Decubitus ulcer	Female	1-4 years	95+ years	Data Rich	0	3	Socio-demographic Index	189
Decubitus ulcer	Female	1-4 years	95+ years	Data Rich	1	3	SEV unsafe sanitation	42
Decubitus ulcer	Female	1-4 years	95+ years	Global	-1	1	Improved Water Source (proportion with access)	194
Decubitus ulcer	Female	1-4 years	95+ years	Global	1	1	Diabetes Fasting Plasma Glucose (mmol/L)	194
Decubitus ulcer	Female	1-4 years	95+ years	Global	1	1	Prevalence of obesity	--
Decubitus ulcer	Female	1-4 years	95+ years	Global	-1	2	Healthcare access and quality index	--
Decubitus ulcer	Female	1-4 years	95+ years	Global	1	2	Cumulative Cigarettes (5 Years)	170
Decubitus ulcer	Female	1-4 years	95+ years	Global	1	2	Cumulative Cigarettes (10 Years)	557
Decubitus ulcer	Female	1-4 years	95+ years	Global	1	2	Alcohol (liters per capita)	727
Decubitus ulcer	Female	1-4 years	95+ years	Global	1	2	Smoking Prevalence	--
Decubitus ulcer	Female	1-4 years	95+ years	Global	-1	3	Education (years per capita)	279
Decubitus ulcer	Female	1-4 years	95+ years	Global	-1	3	Health System Access 2 (unitless)	361
Decubitus ulcer	Female	1-4 years	95+ years	Global	-1	3	LDI (I$ per capita)	--
Decubitus ulcer	Female	1-4 years	95+ years	Global	0	3	Socio-demographic Index	375
Decubitus ulcer	Female	1-4 years	95+ years	Global	1	3	SEV unsafe sanitation	104
Decubitus ulcer	Male	1-4 years	95+ years	Data Rich	-1	1	Improved Water Source (proportion with access)	572
Decubitus ulcer	Male	1-4 years	95+ years	Data Rich	1	1	Diabetes Fasting Plasma Glucose (mmol/L)	572
Decubitus ulcer	Male	1-4 years	95+ years	Data Rich	1	1	Prevalence of obesity	--
Decubitus ulcer	Male	1-4 years	95+ years	Data Rich	-1	2	Healthcare access and quality index	--
Decubitus ulcer	Male	1-4 years	95+ years	Data Rich	1	2	Cumulative Cigarettes (5 Years)	428
Decubitus ulcer	Male	1-4 years	95+ years	Data Rich	1	2	Alcohol (liters per capita)	--
Decubitus ulcer	Male	1-4 years	95+ years	Data Rich	1	2	Cumulative Cigarettes (10 Years)	--
Decubitus ulcer	Male	1-4 years	95+ years	Data Rich	1	2	Smoking Prevalence	--
Decubitus ulcer	Male	1-4 years	95+ years	Data Rich	-1	3	Health System Access 2 (unitless)	428
Decubitus ulcer	Male	1-4 years	95+ years	Data Rich	-1	3	Education (years per capita)	--
Decubitus ulcer	Male	1-4 years	95+ years	Data Rich	-1	3	LDI (I$ per capita)	--
Decubitus ulcer	Male	1-4 years	95+ years	Data Rich	0	3	Socio-demographic Index	--
Decubitus ulcer	Male	1-4 years	95+ years	Data Rich	1	3	SEV unsafe sanitation	325
Decubitus ulcer	Male	1-4 years	95+ years	Global	-1	1	Improved Water Source (proportion with access)	249
Decubitus ulcer	Male	1-4 years	95+ years	Global	1	1	Diabetes Fasting Plasma Glucose (mmol/L)	970
Decubitus ulcer	Male	1-4 years	95+ years	Global	1	1	Prevalence of obesity	--
Decubitus ulcer	Male	1-4 years	95+ years	Global	-1	2	Healthcare access and quality index	563
Decubitus ulcer	Male	1-4 years	95+ years	Global	1	2	Cumulative Cigarettes (10 Years)	121
Decubitus ulcer	Male	1-4 years	95+ years	Global	1	2	Smoking Prevalence	176
Decubitus ulcer	Male	1-4 years	95+ years	Global	1	2	Alcohol (liters per capita)	383
Decubitus ulcer	Male	1-4 years	95+ years	Global	1	2	Cumulative Cigarettes (5 Years)	529

**Appendix Table 9: CODEm covariates used, level of covariate, and expected direction of covariate by cause, sex, and age** *Covariates that CODEm did not select during the covariate selection process have no draw counts listed.*									
Cause	Sex	Age Start	Age End	Model Type	Direction	Level	Covariate Name	Number of Draws
Decubitus ulcer	Male	1-4 years	95+ years	Global	-1	3	Health System Access 2 (unitless)	32
Decubitus ulcer	Male	1-4 years	95+ years	Global	-1	3	Education (years per capita)	300
Decubitus ulcer	Male	1-4 years	95+ years	Global	-1	3	LDI (I$ per capita)	--
Decubitus ulcer	Male	1-4 years	95+ years	Global	0	3	Socio-demographic Index	177
Decubitus ulcer	Male	1-4 years	95+ years	Global	1	3	SEV unsafe sanitation	132
Other skin and subcutaneous diseases	Female	28-364 days	95+ years	Data Rich	-1	1	Improved Water Source (proportion with access)	--
Other skin and subcutaneous diseases	Female	28-364 days	95+ years	Data Rich	1	1	SEV unsafe sanitation	1000
Other skin and subcutaneous diseases	Female	28-364 days	95+ years	Data Rich	1	1	Age-standardized underweight (weight-for-age) SEV	--
Other skin and subcutaneous diseases	Female	28-364 days	95+ years	Data Rich	-1	2	Healthcare access and quality index	--
Other skin and subcutaneous diseases	Female	28-364 days	95+ years	Data Rich	1	2	Alcohol (liters per capita)	--
Other skin and subcutaneous diseases	Female	28-364 days	95+ years	Data Rich	1	2	Cumulative Cigarettes (10 Years)	--
Other skin and subcutaneous diseases	Female	28-364 days	95+ years	Data Rich	1	2	Cumulative Cigarettes (5 Years)	--
Other skin and subcutaneous diseases	Female	28-364 days	95+ years	Data Rich	1	2	Smoking Prevalence	--
Other skin and subcutaneous diseases	Female	28-364 days	95+ years	Data Rich	-1	3	Education (years per capita)	--
Other skin and subcutaneous diseases	Female	28-364 days	95+ years	Data Rich	-1	3	Health System Access 2 (unitless)	--
Other skin and subcutaneous diseases	Female	28-364 days	95+ years	Data Rich	-1	3	LDI (I$ per capita)	--
Other skin and subcutaneous diseases	Female	28-364 days	95+ years	Data Rich	0	3	Socio-demographic Index	--
Other skin and subcutaneous diseases	Female	28-364 days	95+ years	Global	-1	1	Improved Water Source (proportion with access)	1000
Other skin and subcutaneous diseases	Female	28-364 days	95+ years	Global	1	1	Age-standardized underweight (weight-for-age) SEV	--
Other skin and subcutaneous diseases	Female	28-364 days	95+ years	Global	1	1	SEV unsafe sanitation	--
Other skin and subcutaneous diseases	Female	28-364 days	95+ years	Global	-1	2	Healthcare access and quality index	--
Other skin and subcutaneous diseases	Female	28-364 days	95+ years	Global	1	2	Cumulative Cigarettes (5 Years)	407
Other skin and subcutaneous diseases	Female	28-364 days	95+ years	Global	1	2	Cumulative Cigarettes (10 Years)	414
Other skin and subcutaneous diseases	Female	28-364 days	95+ years	Global	1	2	Alcohol (liters per capita)	--
Other skin and subcutaneous diseases	Female	28-364 days	95+ years	Global	1	2	Smoking Prevalence	--
Other skin and subcutaneous diseases	Female	28-364 days	95+ years	Global	-1	3	Health System Access 2 (unitless)	275
Other skin and subcutaneous diseases	Female	28-364 days	95+ years	Global	-1	3	Education (years per capita)	--
Other skin and subcutaneous diseases	Female	28-364 days	95+ years	Global	-1	3	LDI (I$ per capita)	--
Other skin and subcutaneous diseases	Female	28-364 days	95+ years	Global	0	3	Socio-demographic Index	--
Other skin and subcutaneous diseases	Male	28-364 days	95+ years	Data Rich	-1	1	Improved Water Source (proportion with access)	1000
Other skin and subcutaneous diseases	Male	28-364 days	95+ years	Data Rich	1	1	Age-standardized underweight (weight-for-age) SEV	--
Other skin and subcutaneous diseases	Male	28-364 days	95+ years	Data Rich	1	1	SEV unsafe sanitation	--
Other skin and subcutaneous diseases	Male	28-364 days	95+ years	Data Rich	-1	2	Healthcare access and quality index	--
Other skin and subcutaneous diseases	Male	28-364 days	95+ years	Data Rich	1	2	Cumulative Cigarettes (10 Years)	275
Other skin and subcutaneous diseases	Male	28-364 days	95+ years	Data Rich	1	2	Alcohol (liters per capita)	--
Other skin and subcutaneous diseases	Male	28-364 days	95+ years	Data Rich	1	2	Cumulative Cigarettes (5 Years)	--
Other skin and subcutaneous diseases	Male	28-364 days	95+ years	Data Rich	1	2	Smoking Prevalence	--
Other skin and subcutaneous diseases	Male	28-364 days	95+ years	Data Rich	-1	3	Education (years per capita)	171
Other skin and subcutaneous diseases	Male	28-364 days	95+ years	Data Rich	-1	3	Health System Access 2 (unitless)	--
Other skin and subcutaneous diseases	Male	28-364 days	95+ years	Data Rich	-1	3	LDI (I$ per capita)	--
Other skin and subcutaneous diseases	Male	28-364 days	95+ years	Data Rich	0	3	Socio-demographic Index	361
Other skin and subcutaneous diseases	Male	28-364 days	95+ years	Global	-1	1	Improved Water Source (proportion with access)	570
Other skin and subcutaneous diseases	Male	28-364 days	95+ years	Global	1	1	SEV unsafe sanitation	430
Other skin and subcutaneous diseases	Male	28-364 days	95+ years	Global	1	1	Age-standardized underweight (weight-for-age) SEV	--
Other skin and subcutaneous diseases	Male	28-364 days	95+ years	Global	-1	2	Healthcare access and quality index	82
Other skin and subcutaneous diseases	Male	28-364 days	95+ years	Global	1	2	Cumulative Cigarettes (5 Years)	256
Other skin and subcutaneous diseases	Male	28-364 days	95+ years	Global	1	2	Smoking Prevalence	337
Other skin and subcutaneous diseases	Male	28-364 days	95+ years	Global	1	2	Alcohol (liters per capita)	--
Other skin and subcutaneous diseases	Male	28-364 days	95+ years	Global	1	2	Cumulative Cigarettes (10 Years)	--
Other skin and subcutaneous diseases	Male	28-364 days	95+ years	Global	-1	3	Education (years per capita)	28
Other skin and subcutaneous diseases	Male	28-364 days	95+ years	Global	-1	3	Health System Access 2 (unitless)	226
Other skin and subcutaneous diseases	Male	28-364 days	95+ years	Global	-1	3	LDI (I$ per capita)	--
Other skin and subcutaneous diseases	Male	28-364 days	95+ years	Global	0	3	Socio-demographic Index	46
Musculoskeletal disorders	Female	5-9 years	95+ years	Data Rich	1	1	Mean BMI	--
Musculoskeletal disorders	Female	5-9 years	95+ years	Data Rich	-1	2	Healthcare access and quality index	--
Musculoskeletal disorders	Female	5-9 years	95+ years	Data Rich	0	2	Education (years per capita)	--
Musculoskeletal disorders	Female	5-9 years	95+ years	Data Rich	0	2	LDI (I$ per capita)	--
Musculoskeletal disorders	Female	5-9 years	95+ years	Data Rich	0	2	vegetables adjusted(g)	--
Musculoskeletal disorders	Female	5-9 years	95+ years	Data Rich	1	2	Age-standardized bone mineral density among population age 60+ years	--
Musculoskeletal disorders	Female	5-9 years	95+ years	Data Rich	1	2	Alcohol (liters per capita)	--
Musculoskeletal disorders	Female	5-9 years	95+ years	Data Rich	1	2	Cholesterol (total, mean per capita)	--
Musculoskeletal disorders	Female	5-9 years	95+ years	Data Rich	1	2	Cumulative Cigarettes (10 Years)	--
Musculoskeletal disorders	Female	5-9 years	95+ years	Data Rich	1	2	Cumulative Cigarettes (5 Years)	--
Musculoskeletal disorders	Female	5-9 years	95+ years	Data Rich	1	2	Low bone mineral density	--
Musculoskeletal disorders	Female	5-9 years	95+ years	Data Rich	1	2	Smoking Prevalence	--
Musculoskeletal disorders	Female	5-9 years	95+ years	Data Rich	0	3	Socio-demographic Index	--
Musculoskeletal disorders	Female	5-9 years	95+ years	Global	1	1	Mean BMI	282
Musculoskeletal disorders	Female	5-9 years	95+ years	Global	-1	2	Healthcare access and quality index	--
Musculoskeletal disorders	Female	5-9 years	95+ years	Global	0	2	vegetables adjusted(g)	344
Musculoskeletal disorders	Female	5-9 years	95+ years	Global	0	2	Education (years per capita)	362
Musculoskeletal disorders	Female	5-9 years	95+ years	Global	0	2	LDI (I$ per capita)	--
Musculoskeletal disorders	Female	5-9 years	95+ years	Global	1	2	Smoking Prevalence	52
Musculoskeletal disorders	Female	5-9 years	95+ years	Global	1	2	Cumulative Cigarettes (5 Years)	66
Musculoskeletal disorders	Female	5-9 years	95+ years	Global	1	2	Cumulative Cigarettes (10 Years)	79
Musculoskeletal disorders	Female	5-9 years	95+ years	Global	1	2	Alcohol (liters per capita)	95
Musculoskeletal disorders	Female	5-9 years	95+ years	Global	1	2	Low bone mineral density	623
Musculoskeletal disorders	Female	5-9 years	95+ years	Global	1	2	Age-standardized bone mineral density among population age 60+ years	--

**Appendix Table 9: CODEm covariates used, level of covariate, and expected direction of covariate by cause, sex, and age**<br>*Covariates that CODEm did not select during the covariate selection process have no draw counts listed.*								
**Cause**	**Sex**	**Age Start**	**Age End**	**Model Type**	**Direction**	**Level**	**Covariate Name**	**Number of Draws**
Musculoskeletal disorders	Female	5-9 years	95+ years	Global	1	2	Cholesterol (total, mean per capita)	--
Musculoskeletal disorders	Female	5-9 years	95+ years	Global	0	3	Socio-demographic Index	246
Musculoskeletal disorders	Male	5-9 years	95+ years	Data Rich	1	1	Mean BMI	678
Musculoskeletal disorders	Male	5-9 years	95+ years	Data Rich	-1	2	Healthcare access and quality index	285
Musculoskeletal disorders	Male	5-9 years	95+ years	Data Rich	0	2	Education (years per capita)	545
Musculoskeletal disorders	Male	5-9 years	95+ years	Data Rich	0	2	vegetables adjusted(g)	593
Musculoskeletal disorders	Male	5-9 years	95+ years	Data Rich	0	2	LDI (I$ per capita)	--
Musculoskeletal disorders	Male	5-9 years	95+ years	Data Rich	1	2	Cumulative Cigarettes (10 Years)	9
Musculoskeletal disorders	Male	5-9 years	95+ years	Data Rich	1	2	Smoking Prevalence	9
Musculoskeletal disorders	Male	5-9 years	95+ years	Data Rich	1	2	Cumulative Cigarettes (5 Years)	12
Musculoskeletal disorders	Male	5-9 years	95+ years	Data Rich	1	2	Low bone mineral density	359
Musculoskeletal disorders	Male	5-9 years	95+ years	Data Rich	1	2	Alcohol (liters per capita)	613
Musculoskeletal disorders	Male	5-9 years	95+ years	Data Rich	1	2	Age-standardized bone mineral density among population age 60+ years	--
Musculoskeletal disorders	Male	5-9 years	95+ years	Data Rich	1	2	Cholesterol (total, mean per capita)	--
Musculoskeletal disorders	Male	5-9 years	95+ years	Data Rich	0	3	Socio-demographic Index	487
Musculoskeletal disorders	Male	5-9 years	95+ years	Global	1	1	Mean BMI	605
Musculoskeletal disorders	Male	5-9 years	95+ years	Global	-1	2	Healthcare access and quality index	144
Musculoskeletal disorders	Male	5-9 years	95+ years	Global	0	2	vegetables adjusted(g)	309
Musculoskeletal disorders	Male	5-9 years	95+ years	Global	0	2	Education (years per capita)	625
Musculoskeletal disorders	Male	5-9 years	95+ years	Global	0	2	LDI (I$ per capita)	--
Musculoskeletal disorders	Male	5-9 years	95+ years	Global	1	2	Smoking Prevalence	66
Musculoskeletal disorders	Male	5-9 years	95+ years	Global	1	2	Cumulative Cigarettes (10 Years)	99
Musculoskeletal disorders	Male	5-9 years	95+ years	Global	1	2	Alcohol (liters per capita)	309
Musculoskeletal disorders	Male	5-9 years	95+ years	Global	1	2	Cholesterol (total, mean per capita)	--
Musculoskeletal disorders	Male	5-9 years	95+ years	Global	1	2	Cumulative Cigarettes (5 Years)	--
Musculoskeletal disorders	Male	5-9 years	95+ years	Global	0	3	Socio-demographic Index	491
Rheumatoid arthritis	Female	5-9 years	95+ years	Data Rich	-1	1	Healthcare access and quality index	329
Rheumatoid arthritis	Female	5-9 years	95+ years	Data Rich	1	1	Smoking Prevalence	118
Rheumatoid arthritis	Female	5-9 years	95+ years	Data Rich	1	1	Alcohol (liters per capita)	137
Rheumatoid arthritis	Female	5-9 years	95+ years	Data Rich	1	1	Cumulative Cigarettes (10 Years)	149
Rheumatoid arthritis	Female	5-9 years	95+ years	Data Rich	1	1	Cumulative Cigarettes (5 Years)	839
Rheumatoid arthritis	Female	5-9 years	95+ years	Data Rich	1	2	Cholesterol (total, mean per capita)	12
Rheumatoid arthritis	Female	5-9 years	95+ years	Data Rich	1	2	Mean BMI	603
Rheumatoid arthritis	Female	5-9 years	95+ years	Data Rich	-1	3	Education (years per capita)	47
Rheumatoid arthritis	Female	5-9 years	95+ years	Data Rich	-1	3	LDI (I$ per capita)	--
Rheumatoid arthritis	Female	5-9 years	95+ years	Data Rich	0	3	milk adjusted(g)	61
Rheumatoid arthritis	Female	5-9 years	95+ years	Data Rich	0	3	Socio-demographic Index	361
Rheumatoid arthritis	Female	5-9 years	95+ years	Global	-1	1	Healthcare access and quality index	309
Rheumatoid arthritis	Female	5-9 years	95+ years	Global	1	1	Smoking Prevalence	36
Rheumatoid arthritis	Female	5-9 years	95+ years	Global	1	1	Cumulative Cigarettes (5 Years)	258
Rheumatoid arthritis	Female	5-9 years	95+ years	Global	1	1	Cumulative Cigarettes (10 Years)	563
Rheumatoid arthritis	Female	5-9 years	95+ years	Global	1	1	Alcohol (liters per capita)	--
Rheumatoid arthritis	Female	5-9 years	95+ years	Global	1	2	Cholesterol (total, mean per capita)	11
Rheumatoid arthritis	Female	5-9 years	95+ years	Global	1	2	Mean BMI	51
Rheumatoid arthritis	Female	5-9 years	95+ years	Global	-1	3	Education (years per capita)	13
Rheumatoid arthritis	Female	5-9 years	95+ years	Global	-1	3	LDI (I$ per capita)	--
Rheumatoid arthritis	Female	5-9 years	95+ years	Global	0	3	Socio-demographic Index	36
Rheumatoid arthritis	Female	5-9 years	95+ years	Global	0	3	milk adjusted(g)	205
Rheumatoid arthritis	Male	5-9 years	95+ years	Data Rich	-1	1	Healthcare access and quality index	260
Rheumatoid arthritis	Male	5-9 years	95+ years	Data Rich	1	1	Smoking Prevalence	36
Rheumatoid arthritis	Male	5-9 years	95+ years	Data Rich	1	1	Cumulative Cigarettes (10 Years)	149
Rheumatoid arthritis	Male	5-9 years	95+ years	Data Rich	1	1	Cumulative Cigarettes (5 Years)	704
Rheumatoid arthritis	Male	5-9 years	95+ years	Data Rich	1	1	Alcohol (liters per capita)	--
Rheumatoid arthritis	Male	5-9 years	95+ years	Data Rich	1	2	Mean BMI	642
Rheumatoid arthritis	Male	5-9 years	95+ years	Data Rich	1	2	Cholesterol (total, mean per capita)	--
Rheumatoid arthritis	Male	5-9 years	95+ years	Data Rich	-1	3	Education (years per capita)	--
Rheumatoid arthritis	Male	5-9 years	95+ years	Data Rich	-1	3	LDI (I$ per capita)	--
Rheumatoid arthritis	Male	5-9 years	95+ years	Data Rich	0	3	milk adjusted(g)	311
Rheumatoid arthritis	Male	5-9 years	95+ years	Data Rich	0	3	Socio-demographic Index	516
Rheumatoid arthritis	Male	5-9 years	95+ years	Global	-1	1	Healthcare access and quality index	444
Rheumatoid arthritis	Male	5-9 years	95+ years	Global	1	1	Smoking Prevalence	50
Rheumatoid arthritis	Male	5-9 years	95+ years	Global	1	1	Cumulative Cigarettes (5 Years)	379
Rheumatoid arthritis	Male	5-9 years	95+ years	Global	1	1	Cumulative Cigarettes (10 Years)	415
Rheumatoid arthritis	Male	5-9 years	95+ years	Global	1	1	Alcohol (liters per capita)	--
Rheumatoid arthritis	Male	5-9 years	95+ years	Global	1	2	Cholesterol (total, mean per capita)	--
Rheumatoid arthritis	Male	5-9 years	95+ years	Global	1	2	Mean BMI	--
Rheumatoid arthritis	Male	5-9 years	95+ years	Global	-1	3	Education (years per capita)	224
Rheumatoid arthritis	Male	5-9 years	95+ years	Global	-1	3	LDI (I$ per capita)	--
Rheumatoid arthritis	Male	5-9 years	95+ years	Global	0	3	Socio-demographic Index	13
Rheumatoid arthritis	Male	5-9 years	95+ years	Global	0	3	milk adjusted(g)	134
Other musculoskeletal disorders	Female	5-9 years	95+ years	Data Rich	1	1	Mean BMI	508
Other musculoskeletal disorders	Female	5-9 years	95+ years	Data Rich	-1	2	Healthcare access and quality index	--
Other musculoskeletal disorders	Female	5-9 years	95+ years	Data Rich	0	2	vegetables adjusted(g)	352
Other musculoskeletal disorders	Female	5-9 years	95+ years	Data Rich	0	2	Education (years per capita)	683
Other musculoskeletal disorders	Female	5-9 years	95+ years	Data Rich	0	2	LDI (I$ per capita)	--
Other musculoskeletal disorders	Female	5-9 years	95+ years	Data Rich	1	2	Alcohol (liters per capita)	95
Other musculoskeletal disorders	Female	5-9 years	95+ years	Data Rich	1	2	Cumulative Cigarettes (5 Years)	179

Appendix Table 9: CODEm covariates used, level of covariate, and expected direction of covariate by cause, sex, and age

Covariates that CODEm did not select during the covariate selection process have no draw counts listed.

Cause	Sex	Age Start	Age End	Model Type	Direction	Level	Covariate Name	Number of Draws
Other musculoskeletal disorders	Female	5-9 years	95+ years	Data Rich	1	2	Cumulative Cigarettes (10 Years)	239
Other musculoskeletal disorders	Female	5-9 years	95+ years	Data Rich	1	2	Cholesterol (total, mean per capita)	--
Other musculoskeletal disorders	Female	5-9 years	95+ years	Data Rich	1	2	Smoking Prevalence	--
Other musculoskeletal disorders	Female	5-9 years	95+ years	Data Rich	0	3	Socio-demographic Index	341
Other musculoskeletal disorders	Female	5-9 years	95+ years	Global	1	1	Mean BMI	359
Other musculoskeletal disorders	Female	5-9 years	95+ years	Global	-1	2	Healthcare access and quality index	--
Other musculoskeletal disorders	Female	5-9 years	95+ years	Global	0	2	vegetables adjusted(g)	253
Other musculoskeletal disorders	Female	5-9 years	95+ years	Global	0	2	Education (years per capita)	572
Other musculoskeletal disorders	Female	5-9 years	95+ years	Global	0	2	LDI (I$ per capita)	--
Other musculoskeletal disorders	Female	5-9 years	95+ years	Global	1	2	Alcohol (liters per capita)	47
Other musculoskeletal disorders	Female	5-9 years	95+ years	Global	1	2	Cumulative Cigarettes (5 Years)	85
Other musculoskeletal disorders	Female	5-9 years	95+ years	Global	1	2	Cumulative Cigarettes (10 Years)	102
Other musculoskeletal disorders	Female	5-9 years	95+ years	Global	1	2	Cholesterol (total, mean per capita)	--
Other musculoskeletal disorders	Female	5-9 years	95+ years	Global	1	2	Smoking Prevalence	--
Other musculoskeletal disorders	Female	5-9 years	95+ years	Global	0	3	Socio-demographic Index	470
Other musculoskeletal disorders	Male	5-9 years	95+ years	Data Rich	1	1	Mean BMI	141
Other musculoskeletal disorders	Male	5-9 years	95+ years	Data Rich	-1	2	Healthcare access and quality index	--
Other musculoskeletal disorders	Male	5-9 years	95+ years	Data Rich	0	2	vegetables adjusted(g)	378
Other musculoskeletal disorders	Male	5-9 years	95+ years	Data Rich	0	2	Education (years per capita)	496
Other musculoskeletal disorders	Male	5-9 years	95+ years	Data Rich	0	2	LDI (I$ per capita)	--
Other musculoskeletal disorders	Male	5-9 years	95+ years	Data Rich	1	2	Alcohol (liters per capita)	580
Other musculoskeletal disorders	Male	5-9 years	95+ years	Data Rich	1	2	Cholesterol (total, mean per capita)	--
Other musculoskeletal disorders	Male	5-9 years	95+ years	Data Rich	1	2	Cumulative Cigarettes (10 Years)	--
Other musculoskeletal disorders	Male	5-9 years	95+ years	Data Rich	1	2	Cumulative Cigarettes (5 Years)	--
Other musculoskeletal disorders	Male	5-9 years	95+ years	Data Rich	1	2	Smoking Prevalence	--
Other musculoskeletal disorders	Male	5-9 years	95+ years	Data Rich	0	3	Socio-demographic Index	618
Other musculoskeletal disorders	Male	5-9 years	95+ years	Global	1	1	Mean BMI	--
Other musculoskeletal disorders	Male	5-9 years	95+ years	Global	-1	2	Healthcare access and quality index	--
Other musculoskeletal disorders	Male	5-9 years	95+ years	Global	0	2	Education (years per capita)	264
Other musculoskeletal disorders	Male	5-9 years	95+ years	Global	0	2	vegetables adjusted(g)	381
Other musculoskeletal disorders	Male	5-9 years	95+ years	Global	0	2	LDI (I$ per capita)	--
Other musculoskeletal disorders	Male	5-9 years	95+ years	Global	1	2	Alcohol (liters per capita)	264
Other musculoskeletal disorders	Male	5-9 years	95+ years	Global	1	2	Cholesterol (total, mean per capita)	--
Other musculoskeletal disorders	Male	5-9 years	95+ years	Global	1	2	Cumulative Cigarettes (10 Years)	--
Other musculoskeletal disorders	Male	5-9 years	95+ years	Global	1	2	Cumulative Cigarettes (5 Years)	--
Other musculoskeletal disorders	Male	5-9 years	95+ years	Global	1	2	Smoking Prevalence	--
Other musculoskeletal disorders	Male	5-9 years	95+ years	Global	0	3	Socio-demographic Index	707
Congenital anomalies	Female	0-6 days	65-69 years	Data Rich	-1	1	Composite fortification standard and folic acid inclusion	86
Congenital anomalies	Female	0-6 days	65-69 years	Data Rich	-1	1	Folic acid unadjusted (ug)	743
Congenital anomalies	Female	0-6 days	65-69 years	Data Rich	-1	1	In-Facility Delivery (proportion)	--
Congenital anomalies	Female	0-6 days	65-69 years	Data Rich	1	1	Live Births 35+ (proportion)	460
Congenital anomalies	Female	0-6 days	65-69 years	Data Rich	1	1	Birth prevalence of congenital chromosomal anomalies	584
Congenital anomalies	Female	0-6 days	65-69 years	Data Rich	1	1	Birth prevalence of CHD	--
Congenital anomalies	Female	0-6 days	65-69 years	Data Rich	1	1	Maternal alcohol consumption during pregnancy (proportion)	--
Congenital anomalies	Female	0-6 days	65-69 years	Data Rich	-1	2	Antenatal Care (4 visits) Coverage (proportion)	68
Congenital anomalies	Female	0-6 days	65-69 years	Data Rich	-1	2	Healthcare access and quality index	--
Congenital anomalies	Female	0-6 days	65-69 years	Data Rich	-1	2	Legality of Abortion	--
Congenital anomalies	Female	0-6 days	65-69 years	Data Rich	0	2	Antenatal Care (1 visit) Coverage (proportion)	495
Congenital anomalies	Female	0-6 days	65-69 years	Data Rich	1	2	Smoking Prevalence (Reproductive Age Standardized)	10
Congenital anomalies	Female	0-6 days	65-69 years	Data Rich	-1	3	Socio-demographic Index	11
Congenital anomalies	Female	0-6 days	65-69 years	Data Rich	-1	3	Maternal Education (years per capita)	23
Congenital anomalies	Female	0-6 days	65-69 years	Data Rich	1	3	Outdoor Air Pollution (PM2.5)	21
Congenital anomalies	Female	0-6 days	65-69 years	Data Rich	1	3	Alcohol (liters per capita)	92
Congenital anomalies	Female	0-6 days	65-69 years	Data Rich	1	3	fruits unadjusted(g)	151
Congenital anomalies	Female	0-6 days	65-69 years	Data Rich	1	3	vegetables unadjusted(g)	319
Congenital anomalies	Female	0-6 days	65-69 years	Data Rich	1	3	Indoor Air Pollution (All Cooking Fuels)	--
Congenital anomalies	Female	0-6 days	65-69 years	Global	-1	1	Composite fortification standard and folic acid inclusion	0
Congenital anomalies	Female	0-6 days	65-69 years	Global	-1	1	In-Facility Delivery (proportion)	0
Congenital anomalies	Female	0-6 days	65-69 years	Global	-1	1	Folic acid unadjusted (ug)	855
Congenital anomalies	Female	0-6 days	65-69 years	Global	1	1	Birth prevalence of CHD	0
Congenital anomalies	Female	0-6 days	65-69 years	Global	1	1	Birth prevalence of congenital chromosomal anomalies	748
Congenital anomalies	Female	0-6 days	65-69 years	Global	1	1	Live Births 35+ (proportion)	758
Congenital anomalies	Female	0-6 days	65-69 years	Global	1	1	Maternal alcohol consumption during pregnancy (proportion)	--
Congenital anomalies	Female	0-6 days	65-69 years	Global	-1	2	Antenatal Care (4 visits) Coverage (proportion)	0
Congenital anomalies	Female	0-6 days	65-69 years	Global	-1	2	Healthcare access and quality index	0
Congenital anomalies	Female	0-6 days	65-69 years	Global	-1	2	Legality of Abortion	--
Congenital anomalies	Female	0-6 days	65-69 years	Global	0	2	Antenatal Care (1 visit) Coverage (proportion)	977
Congenital anomalies	Female	0-6 days	65-69 years	Global	1	2	Smoking Prevalence (Reproductive Age Standardized)	0
Congenital anomalies	Female	0-6 days	65-69 years	Global	-1	3	Maternal Education (years per capita)	0
Congenital anomalies	Female	0-6 days	65-69 years	Global	-1	3	Socio-demographic Index	0
Congenital anomalies	Female	0-6 days	65-69 years	Global	1	3	Alcohol (liters per capita)	0
Congenital anomalies	Female	0-6 days	65-69 years	Global	1	3	Indoor Air Pollution (All Cooking Fuels)	0
Congenital anomalies	Female	0-6 days	65-69 years	Global	1	3	Outdoor Air Pollution (PM2.5)	73
Congenital anomalies	Female	0-6 days	65-69 years	Global	1	3	vegetables unadjusted(g)	185
Congenital anomalies	Female	0-6 days	65-69 years	Global	1	3	fruits unadjusted(g)	268
Congenital anomalies	Male	0-6 days	65-69 years	Data Rich	-1	1	Composite fortification standard and folic acid inclusion	93
Congenital anomalies	Male	0-6 days	65-69 years	Data Rich	-1	1	Folic acid unadjusted (ug)	827
Congenital anomalies	Male	0-6 days	65-69 years	Data Rich	-1	1	In-Facility Delivery (proportion)	--

Appendix Table 9: CODEm covariates used, level of covariate, and expected direction of covariate by cause, sex, and age

Covariates that CODEm did not select during the covariate selection process have no draw counts listed.

Cause	Sex	Age Start	Age End	Model Type	Direction	Level	Covariate Name	Number of Draws
Congenital anomalies	Male	0-6 days	65-69 years	Data Rich	1	1	Birth prevalence of CHD	25
Congenital anomalies	Male	0-6 days	65-69 years	Data Rich	1	1	Birth prevalence of congenital chromosomal anomalies	402
Congenital anomalies	Male	0-6 days	65-69 years	Data Rich	1	1	Live Births 35+ (proportion)	644
Congenital anomalies	Male	0-6 days	65-69 years	Data Rich	1	1	Maternal alcohol consumption during pregnancy (proportion)	--
Congenital anomalies	Male	0-6 days	65-69 years	Data Rich	-1	2	Healthcare access and quality index	21
Congenital anomalies	Male	0-6 days	65-69 years	Data Rich	-1	2	Antenatal Care (4 visits) Coverage (proportion)	53
Congenital anomalies	Male	0-6 days	65-69 years	Data Rich	-1	2	Legality of Abortion	--
Congenital anomalies	Male	0-6 days	65-69 years	Data Rich	0	2	Antenatal Care (1 visit) Coverage (proportion)	624
Congenital anomalies	Male	0-6 days	65-69 years	Data Rich	1	2	Smoking Prevalence (Reproductive Age Standardized)	5
Congenital anomalies	Male	0-6 days	65-69 years	Data Rich	-1	3	Socio-demographic Index	23
Congenital anomalies	Male	0-6 days	65-69 years	Data Rich	-1	3	Maternal Education (years per capita)	25
Congenital anomalies	Male	0-6 days	65-69 years	Data Rich	1	3	Outdoor Air Pollution (PM2.5)	44
Congenital anomalies	Male	0-6 days	65-69 years	Data Rich	1	3	vegetables unadjusted(g)	236
Congenital anomalies	Male	0-6 days	65-69 years	Data Rich	1	3	fruits unadjusted(g)	286
Congenital anomalies	Male	0-6 days	65-69 years	Data Rich	1	3	Alcohol (liters per capita)	--
Congenital anomalies	Male	0-6 days	65-69 years	Data Rich	1	3	Indoor Air Pollution (All Cooking Fuels)	--
Congenital anomalies	Male	0-6 days	65-69 years	Global	-1	1	Composite fortification standard and folic acid inclusion	0
Congenital anomalies	Male	0-6 days	65-69 years	Global	-1	1	Folic acid unadjusted (ug)	524
Congenital anomalies	Male	0-6 days	65-69 years	Global	-1	1	In-Facility Delivery (proportion)	--
Congenital anomalies	Male	0-6 days	65-69 years	Global	1	1	Birth prevalence of CHD	0
Congenital anomalies	Male	0-6 days	65-69 years	Global	1	1	Birth prevalence of congenital chromosomal anomalies	491
Congenital anomalies	Male	0-6 days	65-69 years	Global	1	1	Live Births 35+ (proportion)	911
Congenital anomalies	Male	0-6 days	65-69 years	Global	1	1	Maternal alcohol consumption during pregnancy (proportion)	--
Congenital anomalies	Male	0-6 days	65-69 years	Global	-1	2	Antenatal Care (4 visits) Coverage (proportion)	0
Congenital anomalies	Male	0-6 days	65-69 years	Global	-1	2	Healthcare access and quality index	0
Congenital anomalies	Male	0-6 days	65-69 years	Global	-1	2	Legality of Abortion	--
Congenital anomalies	Male	0-6 days	65-69 years	Global	0	2	Antenatal Care (1 visit) Coverage (proportion)	894
Congenital anomalies	Male	0-6 days	65-69 years	Global	1	2	Smoking Prevalence (Reproductive Age Standardized)	0
Congenital anomalies	Male	0-6 days	65-69 years	Global	-1	3	Maternal Education (years per capita)	0
Congenital anomalies	Male	0-6 days	65-69 years	Global	-1	3	Socio-demographic Index	0
Congenital anomalies	Male	0-6 days	65-69 years	Global	1	3	Indoor Air Pollution (All Cooking Fuels)	0
Congenital anomalies	Male	0-6 days	65-69 years	Global	1	3	vegetables unadjusted(g)	251
Congenital anomalies	Male	0-6 days	65-69 years	Global	1	3	Outdoor Air Pollution (PM2.5)	317
Congenital anomalies	Male	0-6 days	65-69 years	Global	1	3	fruits unadjusted(g)	398
Congenital anomalies	Male	0-6 days	65-69 years	Global	1	3	Alcohol (liters per capita)	--
Neural tube defects	Female	0-6 days	65-69 years	Data Rich	-1	1	In-Facility Delivery (proportion)	29
Neural tube defects	Female	0-6 days	65-69 years	Data Rich	-1	1	Socio-demographic Index	186
Neural tube defects	Female	0-6 days	65-69 years	Data Rich	-1	1	Composite fortification standard and folic acid inclusion	573
Neural tube defects	Female	0-6 days	65-69 years	Data Rich	-1	1	Folic acid unadjusted (ug)	848
Neural tube defects	Female	0-6 days	65-69 years	Data Rich	-1	2	Antenatal Care (1 visit) Coverage (proportion)	10
Neural tube defects	Female	0-6 days	65-69 years	Data Rich	-1	2	Healthcare access and quality index	15
Neural tube defects	Female	0-6 days	65-69 years	Data Rich	-1	2	Antenatal Care (4 visits) Coverage (proportion)	18
Neural tube defects	Female	0-6 days	65-69 years	Data Rich	-1	2	vegetables unadjusted(g)	366
Neural tube defects	Female	0-6 days	65-69 years	Data Rich	-1	2	Legality of Abortion	389
Neural tube defects	Female	0-6 days	65-69 years	Data Rich	1	2	Smoking Prevalence (Reproductive Age Standardized)	215
Neural tube defects	Female	0-6 days	65-69 years	Data Rich	-1	3	Maternal Education (years per capita)	80
Neural tube defects	Female	0-6 days	65-69 years	Data Rich	0	3	fruits unadjusted(g)	263
Neural tube defects	Female	0-6 days	65-69 years	Data Rich	1	3	Indoor Air Pollution (All Cooking Fuels)	0
Neural tube defects	Female	0-6 days	65-69 years	Data Rich	1	3	Diabetes Age-Standardized Prevalence (proportion)	77
Neural tube defects	Female	0-6 days	65-69 years	Data Rich	1	3	Maternal alcohol consumption during pregnancy (proportion)	--
Neural tube defects	Female	0-6 days	65-69 years	Global	-1	1	Socio-demographic Index	250
Neural tube defects	Female	0-6 days	65-69 years	Global	-1	1	In-Facility Delivery (proportion)	287
Neural tube defects	Female	0-6 days	65-69 years	Global	-1	1	Folic acid unadjusted (ug)	479
Neural tube defects	Female	0-6 days	65-69 years	Global	-1	1	Composite fortification standard and folic acid inclusion	712
Neural tube defects	Female	0-6 days	65-69 years	Global	-1	2	Antenatal Care (1 visit) Coverage (proportion)	0
Neural tube defects	Female	0-6 days	65-69 years	Global	-1	2	Antenatal Care (4 visits) Coverage (proportion)	0
Neural tube defects	Female	0-6 days	65-69 years	Global	-1	2	Healthcare access and quality index	13
Neural tube defects	Female	0-6 days	65-69 years	Global	-1	2	Legality of Abortion	275
Neural tube defects	Female	0-6 days	65-69 years	Global	-1	2	vegetables unadjusted(g)	324
Neural tube defects	Female	0-6 days	65-69 years	Global	1	2	Smoking Prevalence (Reproductive Age Standardized)	764
Neural tube defects	Female	0-6 days	65-69 years	Global	-1	3	Maternal Education (years per capita)	12
Neural tube defects	Female	0-6 days	65-69 years	Global	0	3	fruits unadjusted(g)	115
Neural tube defects	Female	0-6 days	65-69 years	Global	1	3	Indoor Air Pollution (All Cooking Fuels)	11
Neural tube defects	Female	0-6 days	65-69 years	Global	1	3	Diabetes Age-Standardized Prevalence (proportion)	138
Neural tube defects	Female	0-6 days	65-69 years	Global	1	3	Maternal alcohol consumption during pregnancy (proportion)	--
Neural tube defects	Male	0-6 days	65-69 years	Data Rich	-1	1	In-Facility Delivery (proportion)	0
Neural tube defects	Male	0-6 days	65-69 years	Data Rich	-1	1	Socio-demographic Index	193
Neural tube defects	Male	0-6 days	65-69 years	Data Rich	-1	1	Composite fortification standard and folic acid inclusion	587
Neural tube defects	Male	0-6 days	65-69 years	Data Rich	-1	1	Folic acid unadjusted (ug)	687
Neural tube defects	Male	0-6 days	65-69 years	Data Rich	-1	2	Antenatal Care (1 visit) Coverage (proportion)	0
Neural tube defects	Male	0-6 days	65-69 years	Data Rich	-1	2	Healthcare access and quality index	23
Neural tube defects	Male	0-6 days	65-69 years	Data Rich	-1	2	Antenatal Care (4 visits) Coverage (proportion)	53
Neural tube defects	Male	0-6 days	65-69 years	Data Rich	-1	2	vegetables unadjusted(g)	65
Neural tube defects	Male	0-6 days	65-69 years	Data Rich	-1	2	Legality of Abortion	161
Neural tube defects	Male	0-6 days	65-69 years	Data Rich	1	2	Smoking Prevalence (Reproductive Age Standardized)	373
Neural tube defects	Male	0-6 days	65-69 years	Data Rich	-1	3	Maternal Education (years per capita)	165
Neural tube defects	Male	0-6 days	65-69 years	Data Rich	0	3	fruits unadjusted(g)	18
Neural tube defects	Male	0-6 days	65-69 years	Data Rich	1	3	Indoor Air Pollution (All Cooking Fuels)	0

Appendix Table 9: CODEm covariates used, level of covariate, and expected direction of covariate by cause, sex, and age

Covariates that CODEm did not select during the covariate selection process have no draw counts listed.

Cause	Sex	Age Start	Age End	Model Type	Direction	Level	Covariate Name	Number of Draws
Neural tube defects	Male	0-6 days	65-69 years	Data Rich	1	3	Maternal alcohol consumption during pregnancy (proportion)	41
Neural tube defects	Male	0-6 days	65-69 years	Data Rich	1	3	Diabetes Age-Standardized Prevalence (proportion)	82
Neural tube defects	Male	0-6 days	65-69 years	Global	-1	1	Folic acid unadjusted (ug)	18
Neural tube defects	Male	0-6 days	65-69 years	Global	-1	1	Composite fortification standard and folic acid inclusion	397
Neural tube defects	Male	0-6 days	65-69 years	Global	-1	1	In-Facility Delivery (proportion)	647
Neural tube defects	Male	0-6 days	65-69 years	Global	-1	1	Socio-demographic Index	797
Neural tube defects	Male	0-6 days	65-69 years	Global	-1	2	Antenatal Care (1 visit) Coverage (proportion)	4
Neural tube defects	Male	0-6 days	65-69 years	Global	-1	2	vegetables unadjusted(g)	23
Neural tube defects	Male	0-6 days	65-69 years	Global	-1	2	Antenatal Care (4 visits) Coverage (proportion)	43
Neural tube defects	Male	0-6 days	65-69 years	Global	-1	2	Healthcare access and quality index	157
Neural tube defects	Male	0-6 days	65-69 years	Global	-1	2	Legality of Abortion	187
Neural tube defects	Male	0-6 days	65-69 years	Global	1	2	Smoking Prevalence (Reproductive Age Standardized)	337
Neural tube defects	Male	0-6 days	65-69 years	Global	-1	3	Maternal Education (years per capita)	204
Neural tube defects	Male	0-6 days	65-69 years	Global	0	3	fruits unadjusted(g)	49
Neural tube defects	Male	0-6 days	65-69 years	Global	1	3	Diabetes Age-Standardized Prevalence (proportion)	10
Neural tube defects	Male	0-6 days	65-69 years	Global	1	3	Indoor Air Pollution (All Cooking Fuels)	491
Neural tube defects	Male	0-6 days	65-69 years	Global	1	3	Maternal alcohol consumption during pregnancy (proportion)	--
Congenital heart anomalies	Female	0-6 days	65-69 years	Data Rich	1	1	Birth prevalence of CHD	--
Congenital heart anomalies	Female	0-6 days	65-69 years	Data Rich	1	1	Maternal alcohol consumption during pregnancy (proportion)	--
Congenital heart anomalies	Female	0-6 days	65-69 years	Data Rich	-1	2	Healthcare access and quality index	235
Congenital heart anomalies	Female	0-6 days	65-69 years	Data Rich	-1	2	Socio-demographic Index	235
Congenital heart anomalies	Female	0-6 days	65-69 years	Data Rich	-1	2	Antenatal Care (1 visit) Coverage (proportion)	--
Congenital heart anomalies	Female	0-6 days	65-69 years	Data Rich	-1	2	In-Facility Delivery (proportion)	--
Congenital heart anomalies	Female	0-6 days	65-69 years	Data Rich	-1	2	Legality of Abortion	--
Congenital heart anomalies	Female	0-6 days	65-69 years	Data Rich	1	2	Smoking Prevalence (Reproductive Age Standardized)	367
Congenital heart anomalies	Female	0-6 days	65-69 years	Data Rich	1	2	Diabetes Age-Standardized Prevalence (proportion)	--
Congenital heart anomalies	Female	0-6 days	65-69 years	Data Rich	-1	3	Antenatal Care (4 visits) Coverage (proportion)	76
Congenital heart anomalies	Female	0-6 days	65-69 years	Data Rich	-1	3	Maternal Education (years per capita)	101
Congenital heart anomalies	Female	0-6 days	65-69 years	Data Rich	-1	3	Skilled Birth Attendance (proportion)	--
Congenital heart anomalies	Female	0-6 days	65-69 years	Data Rich	1	3	Alcohol (liters per capita)	516
Congenital heart anomalies	Female	0-6 days	65-69 years	Data Rich	1	3	Live Births 35+ (proportion)	--
Congenital heart anomalies	Female	0-6 days	65-69 years	Global	1	1	Birth prevalence of CHD	--
Congenital heart anomalies	Female	0-6 days	65-69 years	Global	1	1	Maternal alcohol consumption during pregnancy (proportion)	--
Congenital heart anomalies	Female	0-6 days	65-69 years	Global	-1	2	Antenatal Care (1 visit) Coverage (proportion)	25
Congenital heart anomalies	Female	0-6 days	65-69 years	Global	-1	2	In-Facility Delivery (proportion)	38
Congenital heart anomalies	Female	0-6 days	65-69 years	Global	-1	2	Healthcare access and quality index	168
Congenital heart anomalies	Female	0-6 days	65-69 years	Global	-1	2	Socio-demographic Index	168
Congenital heart anomalies	Female	0-6 days	65-69 years	Global	-1	2	Legality of Abortion	--
Congenital heart anomalies	Female	0-6 days	65-69 years	Global	1	2	Diabetes Age-Standardized Prevalence (proportion)	435
Congenital heart anomalies	Female	0-6 days	65-69 years	Global	1	2	Smoking Prevalence (Reproductive Age Standardized)	603
Congenital heart anomalies	Female	0-6 days	65-69 years	Global	-1	3	Antenatal Care (4 visits) Coverage (proportion)	32
Congenital heart anomalies	Female	0-6 days	65-69 years	Global	-1	3	Skilled Birth Attendance (proportion)	33
Congenital heart anomalies	Female	0-6 days	65-69 years	Global	-1	3	Maternal Education (years per capita)	101
Congenital heart anomalies	Female	0-6 days	65-69 years	Global	1	3	Alcohol (liters per capita)	494
Congenital heart anomalies	Female	0-6 days	65-69 years	Global	1	3	Live Births 35+ (proportion)	--
Congenital heart anomalies	Male	0-6 days	65-69 years	Data Rich	1	1	Birth prevalence of CHD	--
Congenital heart anomalies	Male	0-6 days	65-69 years	Data Rich	1	1	Maternal alcohol consumption during pregnancy (proportion)	--
Congenital heart anomalies	Male	0-6 days	65-69 years	Data Rich	-1	2	Healthcare access and quality index	329
Congenital heart anomalies	Male	0-6 days	65-69 years	Data Rich	-1	2	Socio-demographic Index	372
Congenital heart anomalies	Male	0-6 days	65-69 years	Data Rich	-1	2	Antenatal Care (1 visit) Coverage (proportion)	--
Congenital heart anomalies	Male	0-6 days	65-69 years	Data Rich	-1	2	In-Facility Delivery (proportion)	--
Congenital heart anomalies	Male	0-6 days	65-69 years	Data Rich	-1	2	Legality of Abortion	--
Congenital heart anomalies	Male	0-6 days	65-69 years	Data Rich	1	2	Smoking Prevalence (Reproductive Age Standardized)	782
Congenital heart anomalies	Male	0-6 days	65-69 years	Data Rich	1	2	Diabetes Age-Standardized Prevalence (proportion)	--
Congenital heart anomalies	Male	0-6 days	65-69 years	Data Rich	-1	3	Antenatal Care (4 visits) Coverage (proportion)	60
Congenital heart anomalies	Male	0-6 days	65-69 years	Data Rich	-1	3	Maternal Education (years per capita)	333
Congenital heart anomalies	Male	0-6 days	65-69 years	Data Rich	-1	3	Skilled Birth Attendance (proportion)	--
Congenital heart anomalies	Male	0-6 days	65-69 years	Data Rich	1	3	Alcohol (liters per capita)	--
Congenital heart anomalies	Male	0-6 days	65-69 years	Data Rich	1	3	Live Births 35+ (proportion)	--
Congenital heart anomalies	Male	0-6 days	65-69 years	Global	1	1	Birth prevalence of CHD	--
Congenital heart anomalies	Male	0-6 days	65-69 years	Global	1	1	Maternal alcohol consumption during pregnancy (proportion)	--
Congenital heart anomalies	Male	0-6 days	65-69 years	Global	-1	2	Healthcare access and quality index	237
Congenital heart anomalies	Male	0-6 days	65-69 years	Global	-1	2	Socio-demographic Index	237
Congenital heart anomalies	Male	0-6 days	65-69 years	Global	-1	2	Antenatal Care (1 visit) Coverage (proportion)	--
Congenital heart anomalies	Male	0-6 days	65-69 years	Global	-1	2	In-Facility Delivery (proportion)	--
Congenital heart anomalies	Male	0-6 days	65-69 years	Global	-1	2	Legality of Abortion	--
Congenital heart anomalies	Male	0-6 days	65-69 years	Global	1	2	Diabetes Age-Standardized Prevalence (proportion)	402
Congenital heart anomalies	Male	0-6 days	65-69 years	Global	1	2	Smoking Prevalence (Reproductive Age Standardized)	639
Congenital heart anomalies	Male	0-6 days	65-69 years	Global	-1	3	Antenatal Care (4 visits) Coverage (proportion)	164
Congenital heart anomalies	Male	0-6 days	65-69 years	Global	-1	3	Maternal Education (years per capita)	197
Congenital heart anomalies	Male	0-6 days	65-69 years	Global	-1	3	Skilled Birth Attendance (proportion)	--
Congenital heart anomalies	Male	0-6 days	65-69 years	Global	1	3	Alcohol (liters per capita)	--
Congenital heart anomalies	Male	0-6 days	65-69 years	Global	1	3	Live Births 35+ (proportion)	--
Orofacial clefts	Female	0-6 days	1-4 years	Data Rich	-1	1	Socio-demographic Index	574
Orofacial clefts	Female	0-6 days	1-4 years	Data Rich	-1	1	Composite fortification standard and folic acid inclusion	766
Orofacial clefts	Female	0-6 days	1-4 years	Data Rich	1	1	Indoor Air Pollution (All Cooking Fuels)	15
Orofacial clefts	Female	0-6 days	1-4 years	Data Rich	-1	2	Skilled Birth Attendance (proportion)	0
Orofacial clefts	Female	0-6 days	1-4 years	Data Rich	-1	2	Healthcare access and quality index	597

**Appendix Table 9: CODEm covariates used, level of covariate, and expected direction of covariate by cause, sex, and age** *Covariates that CODEm did not select during the covariate selection process have no draw counts listed.*								
**Cause**	**Sex**	**Age Start**	**Age End**	**Model Type**	**Direction**	**Level**	**Covariate Name**	**Number of Draws**
Orofacial clefts	Female	0-6 days	1-4 years	Data Rich	-1	2	Legality of Abortion	--
Orofacial clefts	Female	0-6 days	1-4 years	Data Rich	1	2	Smoking Prevalence (Reproductive Age Standardized)	619
Orofacial clefts	Female	0-6 days	1-4 years	Data Rich	1	2	Diabetes Age-Standardized Prevalence (proportion)	--
Orofacial clefts	Female	0-6 days	1-4 years	Data Rich	1	2	Maternal alcohol consumption during pregnancy (proportion)	--
Orofacial clefts	Female	0-6 days	1-4 years	Data Rich	-1	3	Antenatal Care (4 visits) Coverage (proportion)	10
Orofacial clefts	Female	0-6 days	1-4 years	Data Rich	-1	3	Maternal Education (years per capita)	450
Orofacial clefts	Female	0-6 days	1-4 years	Data Rich	-1	3	Antenatal Care (1 visit) Coverage (proportion)	--
Orofacial clefts	Female	0-6 days	1-4 years	Data Rich	0	3	vegetables unadjusted(g)	386
Orofacial clefts	Female	0-6 days	1-4 years	Data Rich	0	3	fruits unadjusted(g)	583
Orofacial clefts	Female	0-6 days	1-4 years	Data Rich	1	3	Alcohol (liters per capita)	--
Orofacial clefts	Female	0-6 days	1-4 years	Global	-1	1	Socio-demographic Index	533
Orofacial clefts	Female	0-6 days	1-4 years	Global	-1	1	Composite fortification standard and folic acid inclusion	818
Orofacial clefts	Female	0-6 days	1-4 years	Global	-1	2	Skilled Birth Attendance (proportion)	2
Orofacial clefts	Female	0-6 days	1-4 years	Global	-1	2	Healthcare access and quality index	569
Orofacial clefts	Female	0-6 days	1-4 years	Global	-1	2	Legality of Abortion	--
Orofacial clefts	Female	0-6 days	1-4 years	Global	1	2	Smoking Prevalence (Reproductive Age Standardized)	569
Orofacial clefts	Female	0-6 days	1-4 years	Global	1	2	Diabetes Age-Standardized Prevalence (proportion)	--
Orofacial clefts	Female	0-6 days	1-4 years	Global	1	2	Maternal alcohol consumption during pregnancy (proportion)	--
Orofacial clefts	Female	0-6 days	1-4 years	Global	-1	3	Antenatal Care (4 visits) Coverage (proportion)	2
Orofacial clefts	Female	0-6 days	1-4 years	Global	-1	3	Maternal Education (years per capita)	498
Orofacial clefts	Female	0-6 days	1-4 years	Global	-1	3	Antenatal Care (1 visit) Coverage (proportion)	--
Orofacial clefts	Female	0-6 days	1-4 years	Global	0	3	fruits unadjusted(g)	543
Orofacial clefts	Female	0-6 days	1-4 years	Global	0	3	vegetables unadjusted(g)	663
Orofacial clefts	Female	0-6 days	1-4 years	Global	1	3	Indoor Air Pollution (All Cooking Fuels)	2
Orofacial clefts	Female	0-6 days	1-4 years	Global	1	3	Alcohol (liters per capita)	--
Orofacial clefts	Male	0-6 days	1-4 years	Data Rich	-1	1	Composite fortification standard and folic acid inclusion	313
Orofacial clefts	Male	0-6 days	1-4 years	Data Rich	-1	1	Socio-demographic Index	535
Orofacial clefts	Male	0-6 days	1-4 years	Data Rich	-1	1	Folic acid unadjusted (ug)	950
Orofacial clefts	Male	0-6 days	1-4 years	Data Rich	-1	2	Legality of Abortion	0
Orofacial clefts	Male	0-6 days	1-4 years	Data Rich	-1	2	Skilled Birth Attendance (proportion)	0
Orofacial clefts	Male	0-6 days	1-4 years	Data Rich	-1	2	Healthcare access and quality index	79
Orofacial clefts	Male	0-6 days	1-4 years	Data Rich	1	2	Diabetes Age-Standardized Prevalence (proportion)	0
Orofacial clefts	Male	0-6 days	1-4 years	Data Rich	1	2	Smoking Prevalence (Reproductive Age Standardized)	215
Orofacial clefts	Male	0-6 days	1-4 years	Data Rich	1	2	Maternal alcohol consumption during pregnancy (proportion)	--
Orofacial clefts	Male	0-6 days	1-4 years	Data Rich	-1	3	Antenatal Care (1 visit) Coverage (proportion)	0
Orofacial clefts	Male	0-6 days	1-4 years	Data Rich	-1	3	Antenatal Care (4 visits) Coverage (proportion)	2
Orofacial clefts	Male	0-6 days	1-4 years	Data Rich	-1	3	Maternal Education (years per capita)	375
Orofacial clefts	Male	0-6 days	1-4 years	Data Rich	0	3	fruits unadjusted(g)	391
Orofacial clefts	Male	0-6 days	1-4 years	Data Rich	0	3	vegetables unadjusted(g)	627
Orofacial clefts	Male	0-6 days	1-4 years	Data Rich	1	3	Indoor Air Pollution (All Cooking Fuels)	2
Orofacial clefts	Male	0-6 days	1-4 years	Data Rich	1	3	Alcohol (liters per capita)	--
Orofacial clefts	Male	0-6 days	1-4 years	Global	-1	1	Composite fortification standard and folic acid inclusion	495
Orofacial clefts	Male	0-6 days	1-4 years	Global	-1	1	Socio-demographic Index	521
Orofacial clefts	Male	0-6 days	1-4 years	Global	-1	1	Folic acid unadjusted (ug)	604
Orofacial clefts	Male	0-6 days	1-4 years	Global	-1	2	Skilled Birth Attendance (proportion)	0
Orofacial clefts	Male	0-6 days	1-4 years	Global	-1	2	Healthcare access and quality index	511
Orofacial clefts	Male	0-6 days	1-4 years	Global	-1	2	Legality of Abortion	--
Orofacial clefts	Male	0-6 days	1-4 years	Global	1	2	Diabetes Age-Standardized Prevalence (proportion)	223
Orofacial clefts	Male	0-6 days	1-4 years	Global	1	2	Smoking Prevalence (Reproductive Age Standardized)	556
Orofacial clefts	Male	0-6 days	1-4 years	Global	1	2	Maternal alcohol consumption during pregnancy (proportion)	--
Orofacial clefts	Male	0-6 days	1-4 years	Global	-1	3	Antenatal Care (1 visit) Coverage (proportion)	0
Orofacial clefts	Male	0-6 days	1-4 years	Global	-1	3	Antenatal Care (4 visits) Coverage (proportion)	4
Orofacial clefts	Male	0-6 days	1-4 years	Global	-1	3	Maternal Education (years per capita)	64
Orofacial clefts	Male	0-6 days	1-4 years	Global	0	3	fruits unadjusted(g)	631
Orofacial clefts	Male	0-6 days	1-4 years	Global	0	3	vegetables unadjusted(g)	757
Orofacial clefts	Male	0-6 days	1-4 years	Global	1	3	Indoor Air Pollution (All Cooking Fuels)	0
Orofacial clefts	Male	0-6 days	1-4 years	Global	1	3	Alcohol (liters per capita)	--
Down's syndrome	Female	0-6 days	65-69 years	Data Rich	-1	1	Legality of Abortion	--
Down's syndrome	Female	0-6 days	65-69 years	Data Rich	1	1	Live Births 40+ (proportion)	269
Down's syndrome	Female	0-6 days	65-69 years	Data Rich	1	1	Live Births 35+ (proportion)	752
Down's syndrome	Female	0-6 days	65-69 years	Data Rich	1	1	Birth prevalence of congenital chromosomal anomalies	902
Down's syndrome	Female	0-6 days	65-69 years	Data Rich	-1	2	Healthcare access and quality index	--
Down's syndrome	Female	0-6 days	65-69 years	Data Rich	-1	2	In-Facility Delivery (proportion)	--
Down's syndrome	Female	0-6 days	65-69 years	Data Rich	-1	2	Socio-demographic Index	--
Down's syndrome	Female	0-6 days	65-69 years	Data Rich	-1	3	vegetables unadjusted(g)	249
Down's syndrome	Female	0-6 days	65-69 years	Data Rich	-1	3	Antenatal Care (1 visit) Coverage (proportion)	--
Down's syndrome	Female	0-6 days	65-69 years	Data Rich	-1	3	Antenatal Care (4 visits) Coverage (proportion)	--
Down's syndrome	Female	0-6 days	65-69 years	Data Rich	-1	3	Maternal Education (years per capita)	--
Down's syndrome	Female	0-6 days	65-69 years	Data Rich	1	3	Indoor Air Pollution (All Cooking Fuels)	--
Down's syndrome	Female	0-6 days	65-69 years	Data Rich	1	3	Maternal alcohol consumption during pregnancy (proportion)	--
Down's syndrome	Female	0-6 days	65-69 years	Data Rich	1	3	Smoking Prevalence (Reproductive Age Standardized)	--
Down's syndrome	Female	0-6 days	65-69 years	Global	-1	1	Legality of Abortion	--
Down's syndrome	Female	0-6 days	65-69 years	Global	1	1	Live Births 40+ (proportion)	332
Down's syndrome	Female	0-6 days	65-69 years	Global	1	1	Live Births 35+ (proportion)	758
Down's syndrome	Female	0-6 days	65-69 years	Global	1	1	Birth prevalence of congenital chromosomal anomalies	778
Down's syndrome	Female	0-6 days	65-69 years	Global	-1	2	Healthcare access and quality index	--
Down's syndrome	Female	0-6 days	65-69 years	Global	-1	2	In-Facility Delivery (proportion)	--
Down's syndrome	Female	0-6 days	65-69 years	Global	-1	2	Socio-demographic Index	--

Appendix Table 9: CODEm covariates used, level of covariate, and expected direction of covariate by cause, sex, and age
Covariates that CODEm did not select during the covariate selection process have no draw counts listed.

Cause	Sex	Age Start	Age End	Model Type	Direction	Level	Covariate Name	Number of Draws
Down's syndrome	Female	0-6 days	65-69 years	Global	-1	3	vegetables unadjusted(g)	227
Down's syndrome	Female	0-6 days	65-69 years	Global	-1	3	Antenatal Care (1 visit) Coverage (proportion)	--
Down's syndrome	Female	0-6 days	65-69 years	Global	-1	3	Antenatal Care (4 visits) Coverage (proportion)	--
Down's syndrome	Female	0-6 days	65-69 years	Global	-1	3	Maternal Education (years per capita)	--
Down's syndrome	Female	0-6 days	65-69 years	Global	1	3	Indoor Air Pollution (All Cooking Fuels)	--
Down's syndrome	Female	0-6 days	65-69 years	Global	1	3	Maternal alcohol consumption during pregnancy (proportion)	--
Down's syndrome	Female	0-6 days	65-69 years	Global	1	3	Smoking Prevalence (Reproductive Age Standardized)	--
Down's syndrome	Male	0-6 days	65-69 years	Data Rich	-1	1	Legality of Abortion	--
Down's syndrome	Male	0-6 days	65-69 years	Data Rich	1	1	Live Births 40+ (proportion)	432
Down's syndrome	Male	0-6 days	65-69 years	Data Rich	1	1	Birth prevalence of congenital chromosomal anomalies	756
Down's syndrome	Male	0-6 days	65-69 years	Data Rich	1	1	Live Births 35+ (proportion)	793
Down's syndrome	Male	0-6 days	65-69 years	Data Rich	-1	2	Healthcare access and quality index	--
Down's syndrome	Male	0-6 days	65-69 years	Data Rich	-1	2	In-Facility Delivery (proportion)	--
Down's syndrome	Male	0-6 days	65-69 years	Data Rich	-1	2	Socio-demographic Index	--
Down's syndrome	Male	0-6 days	65-69 years	Data Rich	-1	3	vegetables unadjusted(g)	292
Down's syndrome	Male	0-6 days	65-69 years	Data Rich	-1	3	Antenatal Care (1 visit) Coverage (proportion)	--
Down's syndrome	Male	0-6 days	65-69 years	Data Rich	-1	3	Antenatal Care (4 visits) Coverage (proportion)	--
Down's syndrome	Male	0-6 days	65-69 years	Data Rich	-1	3	Maternal Education (years per capita)	--
Down's syndrome	Male	0-6 days	65-69 years	Data Rich	1	3	Indoor Air Pollution (All Cooking Fuels)	--
Down's syndrome	Male	0-6 days	65-69 years	Data Rich	1	3	Maternal alcohol consumption during pregnancy (proportion)	--
Down's syndrome	Male	0-6 days	65-69 years	Data Rich	1	3	Smoking Prevalence (Reproductive Age Standardized)	--
Down's syndrome	Male	0-6 days	65-69 years	Global	-1	1	Legality of Abortion	--
Down's syndrome	Male	0-6 days	65-69 years	Global	1	1	Live Births 40+ (proportion)	216
Down's syndrome	Male	0-6 days	65-69 years	Global	1	1	Birth prevalence of congenital chromosomal anomalies	574
Down's syndrome	Male	0-6 days	65-69 years	Global	1	1	Live Births 35+ (proportion)	863
Down's syndrome	Male	0-6 days	65-69 years	Global	-1	2	Healthcare access and quality index	--
Down's syndrome	Male	0-6 days	65-69 years	Global	-1	2	In-Facility Delivery (proportion)	--
Down's syndrome	Male	0-6 days	65-69 years	Global	-1	2	Socio-demographic Index	--
Down's syndrome	Male	0-6 days	65-69 years	Global	-1	3	vegetables unadjusted(g)	270
Down's syndrome	Male	0-6 days	65-69 years	Global	-1	3	Antenatal Care (1 visit) Coverage (proportion)	--
Down's syndrome	Male	0-6 days	65-69 years	Global	-1	3	Antenatal Care (4 visits) Coverage (proportion)	--
Down's syndrome	Male	0-6 days	65-69 years	Global	-1	3	Maternal Education (years per capita)	--
Down's syndrome	Male	0-6 days	65-69 years	Global	1	3	Indoor Air Pollution (All Cooking Fuels)	--
Down's syndrome	Male	0-6 days	65-69 years	Global	1	3	Maternal alcohol consumption during pregnancy (proportion)	--
Down's syndrome	Male	0-6 days	65-69 years	Global	1	3	Smoking Prevalence (Reproductive Age Standardized)	--
Other chromosomal abnormalities	Female	0-6 days	65-69 years	Data Rich	-1	1	Legality of Abortion	--
Other chromosomal abnormalities	Female	0-6 days	65-69 years	Data Rich	1	1	Live Births 35+ (proportion)	1000
Other chromosomal abnormalities	Female	0-6 days	65-69 years	Data Rich	1	1	Live Births 40+ (proportion)	--
Other chromosomal abnormalities	Female	0-6 days	65-69 years	Data Rich	-1	2	Antenatal Care (1 visit) Coverage (proportion)	--
Other chromosomal abnormalities	Female	0-6 days	65-69 years	Data Rich	-1	2	Antenatal Care (4 visits) Coverage (proportion)	--
Other chromosomal abnormalities	Female	0-6 days	65-69 years	Data Rich	-1	2	Healthcare access and quality index	--
Other chromosomal abnormalities	Female	0-6 days	65-69 years	Data Rich	-1	2	In-Facility Delivery (proportion)	--
Other chromosomal abnormalities	Female	0-6 days	65-69 years	Data Rich	-1	2	LDI (I$ per capita)	--
Other chromosomal abnormalities	Female	0-6 days	65-69 years	Data Rich	1	2	Maternal alcohol consumption during pregnancy (proportion)	--
Other chromosomal abnormalities	Female	0-6 days	65-69 years	Data Rich	-1	3	Maternal Education (years per capita)	--
Other chromosomal abnormalities	Female	0-6 days	65-69 years	Data Rich	-1	3	Skilled Birth Attendance (proportion)	--
Other chromosomal abnormalities	Female	0-6 days	65-69 years	Data Rich	0	3	Socio-demographic Index	--
Other chromosomal abnormalities	Female	0-6 days	65-69 years	Data Rich	1	3	Alcohol (liters per capita)	481
Other chromosomal abnormalities	Female	0-6 days	65-69 years	Data Rich	1	3	Indoor Air Pollution (All Cooking Fuels)	--
Other chromosomal abnormalities	Female	0-6 days	65-69 years	Data Rich	1	3	Smoking Prevalence (Reproductive Age Standardized)	--
Other chromosomal abnormalities	Female	0-6 days	65-69 years	Global	-1	1	Legality of Abortion	--
Other chromosomal abnormalities	Female	0-6 days	65-69 years	Global	1	1	Live Births 35+ (proportion)	1000
Other chromosomal abnormalities	Female	0-6 days	65-69 years	Global	1	1	Live Births 40+ (proportion)	--
Other chromosomal abnormalities	Female	0-6 days	65-69 years	Global	-1	2	Antenatal Care (1 visit) Coverage (proportion)	--
Other chromosomal abnormalities	Female	0-6 days	65-69 years	Global	-1	2	Antenatal Care (4 visits) Coverage (proportion)	--
Other chromosomal abnormalities	Female	0-6 days	65-69 years	Global	-1	2	Healthcare access and quality index	--
Other chromosomal abnormalities	Female	0-6 days	65-69 years	Global	-1	2	In-Facility Delivery (proportion)	--
Other chromosomal abnormalities	Female	0-6 days	65-69 years	Global	-1	2	LDI (I$ per capita)	--
Other chromosomal abnormalities	Female	0-6 days	65-69 years	Global	1	2	Maternal alcohol consumption during pregnancy (proportion)	--
Other chromosomal abnormalities	Female	0-6 days	65-69 years	Global	-1	3	Maternal Education (years per capita)	--
Other chromosomal abnormalities	Female	0-6 days	65-69 years	Global	-1	3	Skilled Birth Attendance (proportion)	--
Other chromosomal abnormalities	Female	0-6 days	65-69 years	Global	0	3	Socio-demographic Index	326
Other chromosomal abnormalities	Female	0-6 days	65-69 years	Global	1	3	Alcohol (liters per capita)	593
Other chromosomal abnormalities	Female	0-6 days	65-69 years	Global	1	3	Indoor Air Pollution (All Cooking Fuels)	--
Other chromosomal abnormalities	Female	0-6 days	65-69 years	Global	1	3	Smoking Prevalence (Reproductive Age Standardized)	--
Other chromosomal abnormalities	Male	0-6 days	65-69 years	Data Rich	-1	1	Legality of Abortion	707
Other chromosomal abnormalities	Male	0-6 days	65-69 years	Data Rich	1	1	Live Births 35+ (proportion)	293
Other chromosomal abnormalities	Male	0-6 days	65-69 years	Data Rich	1	1	Live Births 40+ (proportion)	--
Other chromosomal abnormalities	Male	0-6 days	65-69 years	Data Rich	-1	2	Antenatal Care (1 visit) Coverage (proportion)	--
Other chromosomal abnormalities	Male	0-6 days	65-69 years	Data Rich	-1	2	Antenatal Care (4 visits) Coverage (proportion)	--
Other chromosomal abnormalities	Male	0-6 days	65-69 years	Data Rich	-1	2	Healthcare access and quality index	--
Other chromosomal abnormalities	Male	0-6 days	65-69 years	Data Rich	-1	2	In-Facility Delivery (proportion)	--
Other chromosomal abnormalities	Male	0-6 days	65-69 years	Data Rich	-1	2	LDI (I$ per capita)	--
Other chromosomal abnormalities	Male	0-6 days	65-69 years	Data Rich	1	2	Maternal alcohol consumption during pregnancy (proportion)	--
Other chromosomal abnormalities	Male	0-6 days	65-69 years	Data Rich	-1	3	Maternal Education (years per capita)	--
Other chromosomal abnormalities	Male	0-6 days	65-69 years	Data Rich	-1	3	Skilled Birth Attendance (proportion)	--
Other chromosomal abnormalities	Male	0-6 days	65-69 years	Data Rich	0	3	Socio-demographic Index	486
Other chromosomal abnormalities	Male	0-6 days	65-69 years	Data Rich	1	3	Alcohol (liters per capita)	--

Appendix Table 9: CODEm covariates used, level of covariate, and expected direction of covariate by cause, sex, and age
Covariates that CODEm did not select during the covariate selection process have no draw counts listed.

Cause	Sex	Age Start	Age End	Model Type	Direction	Level	Covariate Name	Number of Draws
Other chromosomal abnormalities	Male	0-6 days	65-69 years	Data Rich	1	3	Indoor Air Pollution (All Cooking Fuels)	--
Other chromosomal abnormalities	Male	0-6 days	65-69 years	Data Rich	1	3	Smoking Prevalence (Reproductive Age Standardized)	--
Other chromosomal abnormalities	Male	0-6 days	65-69 years	Global	-1	1	Legality of Abortion	328
Other chromosomal abnormalities	Male	0-6 days	65-69 years	Global	1	1	Live Births 35+ (proportion)	672
Other chromosomal abnormalities	Male	0-6 days	65-69 years	Global	1	1	Live Births 40+ (proportion)	--
Other chromosomal abnormalities	Male	0-6 days	65-69 years	Global	-1	2	Antenatal Care (1 visit) Coverage (proportion)	--
Other chromosomal abnormalities	Male	0-6 days	65-69 years	Global	-1	2	Antenatal Care (4 visits) Coverage (proportion)	--
Other chromosomal abnormalities	Male	0-6 days	65-69 years	Global	-1	2	Healthcare access and quality index	--
Other chromosomal abnormalities	Male	0-6 days	65-69 years	Global	-1	2	In-Facility Delivery (proportion)	--
Other chromosomal abnormalities	Male	0-6 days	65-69 years	Global	-1	2	LDI (I$ per capita)	--
Other chromosomal abnormalities	Male	0-6 days	65-69 years	Global	1	2	Maternal alcohol consumption during pregnancy (proportion)	--
Other chromosomal abnormalities	Male	0-6 days	65-69 years	Global	-1	3	Maternal Education (years per capita)	--
Other chromosomal abnormalities	Male	0-6 days	65-69 years	Global	-1	3	Skilled Birth Attendance (proportion)	--
Other chromosomal abnormalities	Male	0-6 days	65-69 years	Global	0	3	Socio-demographic Index	636
Other chromosomal abnormalities	Male	0-6 days	65-69 years	Global	1	3	Alcohol (liters per capita)	--
Other chromosomal abnormalities	Male	0-6 days	65-69 years	Global	1	3	Indoor Air Pollution (All Cooking Fuels)	--
Other chromosomal abnormalities	Male	0-6 days	65-69 years	Global	1	3	Smoking Prevalence (Reproductive Age Standardized)	--
Congenital musculoskeletal and limb anomalies	Female	0-6 days	65-69 years	Data Rich	-1	1	Legality of Abortion	674
Congenital musculoskeletal and limb anomalies	Female	0-6 days	65-69 years	Data Rich	1	1	Maternal alcohol consumption during pregnancy (proportion)	--
Congenital musculoskeletal and limb anomalies	Female	0-6 days	65-69 years	Data Rich	-1	2	Healthcare access and quality index	--
Congenital musculoskeletal and limb anomalies	Female	0-6 days	65-69 years	Data Rich	-1	2	In-Facility Delivery (proportion)	--
Congenital musculoskeletal and limb anomalies	Female	0-6 days	65-69 years	Data Rich	-1	2	Socio-demographic Index	--
Congenital musculoskeletal and limb anomalies	Female	0-6 days	65-69 years	Data Rich	1	2	Diabetes Age-Standardized Prevalence (proportion)	185
Congenital musculoskeletal and limb anomalies	Female	0-6 days	65-69 years	Data Rich	1	2	Indoor Air Pollution (All Cooking Fuels)	185
Congenital musculoskeletal and limb anomalies	Female	0-6 days	65-69 years	Data Rich	1	2	Smoking Prevalence (Reproductive Age Standardized)	--
Congenital musculoskeletal and limb anomalies	Female	0-6 days	65-69 years	Data Rich	-1	3	Antenatal Care (1 visit) Coverage (proportion)	--
Congenital musculoskeletal and limb anomalies	Female	0-6 days	65-69 years	Data Rich	-1	3	Antenatal Care (4 visits) Coverage (proportion)	--
Congenital musculoskeletal and limb anomalies	Female	0-6 days	65-69 years	Data Rich	-1	3	LDI (I$ per capita)	--
Congenital musculoskeletal and limb anomalies	Female	0-6 days	65-69 years	Data Rich	-1	3	Maternal Education (years per capita)	--
Congenital musculoskeletal and limb anomalies	Female	0-6 days	65-69 years	Data Rich	0	3	fruits unadjusted(g)	326
Congenital musculoskeletal and limb anomalies	Female	0-6 days	65-69 years	Data Rich	0	3	vegetables unadjusted(g)	--
Congenital musculoskeletal and limb anomalies	Female	0-6 days	65-69 years	Data Rich	1	3	Alcohol (liters per capita)	326
Congenital musculoskeletal and limb anomalies	Female	0-6 days	65-69 years	Global	-1	1	Legality of Abortion	254
Congenital musculoskeletal and limb anomalies	Female	0-6 days	65-69 years	Global	1	1	Maternal alcohol consumption during pregnancy (proportion)	--
Congenital musculoskeletal and limb anomalies	Female	0-6 days	65-69 years	Global	-1	2	Healthcare access and quality index	--
Congenital musculoskeletal and limb anomalies	Female	0-6 days	65-69 years	Global	-1	2	In-Facility Delivery (proportion)	--
Congenital musculoskeletal and limb anomalies	Female	0-6 days	65-69 years	Global	-1	2	Socio-demographic Index	--
Congenital musculoskeletal and limb anomalies	Female	0-6 days	65-69 years	Global	1	2	Diabetes Age-Standardized Prevalence (proportion)	541
Congenital musculoskeletal and limb anomalies	Female	0-6 days	65-69 years	Global	1	2	Indoor Air Pollution (All Cooking Fuels)	--
Congenital musculoskeletal and limb anomalies	Female	0-6 days	65-69 years	Global	1	2	Smoking Prevalence (Reproductive Age Standardized)	--
Congenital musculoskeletal and limb anomalies	Female	0-6 days	65-69 years	Global	-1	3	Antenatal Care (1 visit) Coverage (proportion)	--
Congenital musculoskeletal and limb anomalies	Female	0-6 days	65-69 years	Global	-1	3	Antenatal Care (4 visits) Coverage (proportion)	--
Congenital musculoskeletal and limb anomalies	Female	0-6 days	65-69 years	Global	-1	3	LDI (I$ per capita)	--
Congenital musculoskeletal and limb anomalies	Female	0-6 days	65-69 years	Global	-1	3	Maternal Education (years per capita)	--
Congenital musculoskeletal and limb anomalies	Female	0-6 days	65-69 years	Global	0	3	vegetables unadjusted(g)	197
Congenital musculoskeletal and limb anomalies	Female	0-6 days	65-69 years	Global	0	3	fruits unadjusted(g)	336
Congenital musculoskeletal and limb anomalies	Female	0-6 days	65-69 years	Global	1	3	Alcohol (liters per capita)	239
Congenital musculoskeletal and limb anomalies	Male	0-6 days	65-69 years	Data Rich	-1	1	Legality of Abortion	807
Congenital musculoskeletal and limb anomalies	Male	0-6 days	65-69 years	Data Rich	1	1	Maternal alcohol consumption during pregnancy (proportion)	--
Congenital musculoskeletal and limb anomalies	Male	0-6 days	65-69 years	Data Rich	-1	2	Healthcare access and quality index	44
Congenital musculoskeletal and limb anomalies	Male	0-6 days	65-69 years	Data Rich	-1	2	Socio-demographic Index	264
Congenital musculoskeletal and limb anomalies	Male	0-6 days	65-69 years	Data Rich	-1	2	In-Facility Delivery (proportion)	--
Congenital musculoskeletal and limb anomalies	Male	0-6 days	65-69 years	Data Rich	1	2	Indoor Air Pollution (All Cooking Fuels)	35
Congenital musculoskeletal and limb anomalies	Male	0-6 days	65-69 years	Data Rich	1	2	Smoking Prevalence (Reproductive Age Standardized)	79
Congenital musculoskeletal and limb anomalies	Male	0-6 days	65-69 years	Data Rich	1	2	Diabetes Age-Standardized Prevalence (proportion)	--
Congenital musculoskeletal and limb anomalies	Male	0-6 days	65-69 years	Data Rich	-1	3	Antenatal Care (1 visit) Coverage (proportion)	16
Congenital musculoskeletal and limb anomalies	Male	0-6 days	65-69 years	Data Rich	-1	3	Antenatal Care (4 visits) Coverage (proportion)	52
Congenital musculoskeletal and limb anomalies	Male	0-6 days	65-69 years	Data Rich	-1	3	Maternal Education (years per capita)	329
Congenital musculoskeletal and limb anomalies	Male	0-6 days	65-69 years	Data Rich	-1	3	LDI (I$ per capita)	--
Congenital musculoskeletal and limb anomalies	Male	0-6 days	65-69 years	Data Rich	0	3	fruits unadjusted(g)	240
Congenital musculoskeletal and limb anomalies	Male	0-6 days	65-69 years	Data Rich	0	3	vegetables unadjusted(g)	399
Congenital musculoskeletal and limb anomalies	Male	0-6 days	65-69 years	Data Rich	1	3	Alcohol (liters per capita)	--
Congenital musculoskeletal and limb anomalies	Male	0-6 days	65-69 years	Global	-1	1	Legality of Abortion	809
Congenital musculoskeletal and limb anomalies	Male	0-6 days	65-69 years	Global	1	1	Maternal alcohol consumption during pregnancy (proportion)	--
Congenital musculoskeletal and limb anomalies	Male	0-6 days	65-69 years	Global	-1	2	In-Facility Delivery (proportion)	58
Congenital musculoskeletal and limb anomalies	Male	0-6 days	65-69 years	Global	-1	2	Socio-demographic Index	143
Congenital musculoskeletal and limb anomalies	Male	0-6 days	65-69 years	Global	-1	2	Healthcare access and quality index	268
Congenital musculoskeletal and limb anomalies	Male	0-6 days	65-69 years	Global	1	2	Indoor Air Pollution (All Cooking Fuels)	140
Congenital musculoskeletal and limb anomalies	Male	0-6 days	65-69 years	Global	1	2	Diabetes Age-Standardized Prevalence (proportion)	191
Congenital musculoskeletal and limb anomalies	Male	0-6 days	65-69 years	Global	1	2	Smoking Prevalence (Reproductive Age Standardized)	350
Congenital musculoskeletal and limb anomalies	Male	0-6 days	65-69 years	Global	-1	3	Antenatal Care (4 visits) Coverage (proportion)	11
Congenital musculoskeletal and limb anomalies	Male	0-6 days	65-69 years	Global	-1	3	Antenatal Care (1 visit) Coverage (proportion)	84
Congenital musculoskeletal and limb anomalies	Male	0-6 days	65-69 years	Global	-1	3	Maternal Education (years per capita)	125
Congenital musculoskeletal and limb anomalies	Male	0-6 days	65-69 years	Global	-1	3	LDI (I$ per capita)	--
Congenital musculoskeletal and limb anomalies	Male	0-6 days	65-69 years	Global	0	3	fruits unadjusted(g)	86
Congenital musculoskeletal and limb anomalies	Male	0-6 days	65-69 years	Global	0	3	vegetables unadjusted(g)	482
Congenital musculoskeletal and limb anomalies	Male	0-6 days	65-69 years	Global	1	3	Alcohol (liters per capita)	314
Urogenital congenital anomalies	Female	0-6 days	65-69 years	Data Rich	1	1	Maternal alcohol consumption during pregnancy (proportion)	--

Appendix Table 9: CODEm covariates used, level of covariate, and expected direction of covariate by cause, sex, and age<br>*Covariates that CODEm did not select during the covariate selection process have no draw counts listed.*								
**Cause**	**Sex**	**Age Start**	**Age End**	**Model Type**	**Direction**	**Level**	**Covariate Name**	**Number of Draws**
Urogenital congenital anomalies	Female	0-6 days	65-69 years	Data Rich	1	1	Smoking Prevalence (Reproductive Age Standardized)	--
Urogenital congenital anomalies	Female	0-6 days	65-69 years	Data Rich	-1	2	Healthcare access and quality index	153
Urogenital congenital anomalies	Female	0-6 days	65-69 years	Data Rich	-1	2	Socio-demographic Index	480
Urogenital congenital anomalies	Female	0-6 days	65-69 years	Data Rich	-1	2	In-Facility Delivery (proportion)	--
Urogenital congenital anomalies	Female	0-6 days	65-69 years	Data Rich	1	2	Diabetes Age-Standardized Prevalence (proportion)	807
Urogenital congenital anomalies	Female	0-6 days	65-69 years	Data Rich	1	2	Indoor Air Pollution (All Cooking Fuels)	--
Urogenital congenital anomalies	Female	0-6 days	65-69 years	Data Rich	1	2	Outdoor Air Pollution (PM2.5)	--
Urogenital congenital anomalies	Female	0-6 days	65-69 years	Data Rich	-1	3	Maternal Education (years per capita)	193
Urogenital congenital anomalies	Female	0-6 days	65-69 years	Data Rich	-1	3	Antenatal Care (4 visits) Coverage (proportion)	211
Urogenital congenital anomalies	Female	0-6 days	65-69 years	Data Rich	-1	3	Antenatal Care (1 visit) Coverage (proportion)	--
Urogenital congenital anomalies	Female	0-6 days	65-69 years	Data Rich	-1	3	LDI (I$ per capita)	--
Urogenital congenital anomalies	Female	0-6 days	65-69 years	Data Rich	1	3	Alcohol (liters per capita)	--
Urogenital congenital anomalies	Female	0-6 days	65-69 years	Global	1	1	Maternal alcohol consumption during pregnancy (proportion)	--
Urogenital congenital anomalies	Female	0-6 days	65-69 years	Global	1	1	Smoking Prevalence (Reproductive Age Standardized)	--
Urogenital congenital anomalies	Female	0-6 days	65-69 years	Global	-1	2	In-Facility Delivery (proportion)	171
Urogenital congenital anomalies	Female	0-6 days	65-69 years	Global	-1	2	Healthcare access and quality index	180
Urogenital congenital anomalies	Female	0-6 days	65-69 years	Global	-1	2	Socio-demographic Index	364
Urogenital congenital anomalies	Female	0-6 days	65-69 years	Global	1	2	Indoor Air Pollution (All Cooking Fuels)	535
Urogenital congenital anomalies	Female	0-6 days	65-69 years	Global	1	2	Diabetes Age-Standardized Prevalence (proportion)	940
Urogenital congenital anomalies	Female	0-6 days	65-69 years	Global	1	2	Outdoor Air Pollution (PM2.5)	--
Urogenital congenital anomalies	Female	0-6 days	65-69 years	Global	-1	3	Antenatal Care (4 visits) Coverage (proportion)	172
Urogenital congenital anomalies	Female	0-6 days	65-69 years	Global	-1	3	Maternal Education (years per capita)	195
Urogenital congenital anomalies	Female	0-6 days	65-69 years	Global	-1	3	Antenatal Care (1 visit) Coverage (proportion)	--
Urogenital congenital anomalies	Female	0-6 days	65-69 years	Global	-1	3	LDI (I$ per capita)	--
Urogenital congenital anomalies	Female	0-6 days	65-69 years	Global	1	3	Alcohol (liters per capita)	--
Urogenital congenital anomalies	Male	0-6 days	65-69 years	Data Rich	1	1	Maternal alcohol consumption during pregnancy (proportion)	--
Urogenital congenital anomalies	Male	0-6 days	65-69 years	Data Rich	1	1	Smoking Prevalence (Reproductive Age Standardized)	--
Urogenital congenital anomalies	Male	0-6 days	65-69 years	Data Rich	-1	2	Healthcare access and quality index	546
Urogenital congenital anomalies	Male	0-6 days	65-69 years	Data Rich	-1	2	In-Facility Delivery (proportion)	--
Urogenital congenital anomalies	Male	0-6 days	65-69 years	Data Rich	-1	2	Socio-demographic Index	--
Urogenital congenital anomalies	Male	0-6 days	65-69 years	Data Rich	1	2	Outdoor Air Pollution (PM2.5)	546
Urogenital congenital anomalies	Male	0-6 days	65-69 years	Data Rich	1	2	Diabetes Age-Standardized Prevalence (proportion)	1000
Urogenital congenital anomalies	Male	0-6 days	65-69 years	Data Rich	1	2	Indoor Air Pollution (All Cooking Fuels)	--
Urogenital congenital anomalies	Male	0-6 days	65-69 years	Data Rich	-1	3	Antenatal Care (1 visit) Coverage (proportion)	--
Urogenital congenital anomalies	Male	0-6 days	65-69 years	Data Rich	-1	3	Antenatal Care (4 visits) Coverage (proportion)	--
Urogenital congenital anomalies	Male	0-6 days	65-69 years	Data Rich	-1	3	LDI (I$ per capita)	--
Urogenital congenital anomalies	Male	0-6 days	65-69 years	Data Rich	-1	3	Maternal Education (years per capita)	--
Urogenital congenital anomalies	Male	0-6 days	65-69 years	Data Rich	1	3	Alcohol (liters per capita)	--
Urogenital congenital anomalies	Male	0-6 days	65-69 years	Global	1	1	Maternal alcohol consumption during pregnancy (proportion)	--
Urogenital congenital anomalies	Male	0-6 days	65-69 years	Global	1	1	Smoking Prevalence (Reproductive Age Standardized)	--
Urogenital congenital anomalies	Male	0-6 days	65-69 years	Global	-1	2	Socio-demographic Index	131
Urogenital congenital anomalies	Male	0-6 days	65-69 years	Global	-1	2	Healthcare access and quality index	160
Urogenital congenital anomalies	Male	0-6 days	65-69 years	Global	-1	2	In-Facility Delivery (proportion)	385
Urogenital congenital anomalies	Male	0-6 days	65-69 years	Global	1	2	Outdoor Air Pollution (PM2.5)	214
Urogenital congenital anomalies	Male	0-6 days	65-69 years	Global	1	2	Indoor Air Pollution (All Cooking Fuels)	414
Urogenital congenital anomalies	Male	0-6 days	65-69 years	Global	1	2	Diabetes Age-Standardized Prevalence (proportion)	759
Urogenital congenital anomalies	Male	0-6 days	65-69 years	Global	-1	3	Maternal Education (years per capita)	49
Urogenital congenital anomalies	Male	0-6 days	65-69 years	Global	-1	3	Antenatal Care (4 visits) Coverage (proportion)	160
Urogenital congenital anomalies	Male	0-6 days	65-69 years	Global	-1	3	Antenatal Care (1 visit) Coverage (proportion)	--
Urogenital congenital anomalies	Male	0-6 days	65-69 years	Global	-1	3	LDI (I$ per capita)	--
Urogenital congenital anomalies	Male	0-6 days	65-69 years	Global	1	3	Alcohol (liters per capita)	--
Digestive congenital anomalies	Female	0-6 days	65-69 years	Data Rich	1	1	Smoking Prevalence (Reproductive Age Standardized)	532
Digestive congenital anomalies	Female	0-6 days	65-69 years	Data Rich	1	1	Maternal alcohol consumption during pregnancy (proportion)	--
Digestive congenital anomalies	Female	0-6 days	65-69 years	Data Rich	-1	2	Socio-demographic Index	114
Digestive congenital anomalies	Female	0-6 days	65-69 years	Data Rich	-1	2	Healthcare access and quality index	340
Digestive congenital anomalies	Female	0-6 days	65-69 years	Data Rich	-1	2	In-Facility Delivery (proportion)	--
Digestive congenital anomalies	Female	0-6 days	65-69 years	Data Rich	1	2	Diabetes Age-Standardized Prevalence (proportion)	187
Digestive congenital anomalies	Female	0-6 days	65-69 years	Data Rich	1	2	Prevalence of obesity (age-standardized)	768
Digestive congenital anomalies	Female	0-6 days	65-69 years	Data Rich	1	2	Indoor Air Pollution (All Cooking Fuels)	--
Digestive congenital anomalies	Female	0-6 days	65-69 years	Data Rich	-1	3	Maternal Education (years per capita)	20
Digestive congenital anomalies	Female	0-6 days	65-69 years	Data Rich	-1	3	Health System Access (capped)	267
Digestive congenital anomalies	Female	0-6 days	65-69 years	Data Rich	-1	3	Antenatal Care (1 visit) Coverage (proportion)	--
Digestive congenital anomalies	Female	0-6 days	65-69 years	Data Rich	-1	3	Antenatal Care (4 visits) Coverage (proportion)	--
Digestive congenital anomalies	Female	0-6 days	65-69 years	Data Rich	-1	3	LDI (I$ per capita)	--
Digestive congenital anomalies	Female	0-6 days	65-69 years	Data Rich	0	3	vegetables unadjusted(g)	285
Digestive congenital anomalies	Female	0-6 days	65-69 years	Data Rich	0	3	fruits unadjusted(g)	522
Digestive congenital anomalies	Female	0-6 days	65-69 years	Data Rich	1	3	Alcohol (liters per capita)	356
Digestive congenital anomalies	Female	0-6 days	65-69 years	Global	1	1	Smoking Prevalence (Reproductive Age Standardized)	282
Digestive congenital anomalies	Female	0-6 days	65-69 years	Global	1	1	Maternal alcohol consumption during pregnancy (proportion)	--
Digestive congenital anomalies	Female	0-6 days	65-69 years	Global	-1	2	Healthcare access and quality index	141
Digestive congenital anomalies	Female	0-6 days	65-69 years	Global	-1	2	In-Facility Delivery (proportion)	--
Digestive congenital anomalies	Female	0-6 days	65-69 years	Global	-1	2	Socio-demographic Index	--
Digestive congenital anomalies	Female	0-6 days	65-69 years	Global	1	2	Prevalence of obesity (age-standardized)	348
Digestive congenital anomalies	Female	0-6 days	65-69 years	Global	1	2	Diabetes Age-Standardized Prevalence (proportion)	397
Digestive congenital anomalies	Female	0-6 days	65-69 years	Global	1	2	Indoor Air Pollution (All Cooking Fuels)	--
Digestive congenital anomalies	Female	0-6 days	65-69 years	Global	-1	3	Health System Access (capped)	64
Digestive congenital anomalies	Female	0-6 days	65-69 years	Global	-1	3	Antenatal Care (1 visit) Coverage (proportion)	--
Digestive congenital anomalies	Female	0-6 days	65-69 years	Global	-1	3	Antenatal Care (4 visits) Coverage (proportion)	--

Appendix Table 9: CODEm covariates used, level of covariate, and expected direction of covariate by cause, sex, and age

Covariates that CODEm did not select during the covariate selection process have no draw counts listed.

Cause	Sex	Age Start	Age End	Model Type	Direction	Level	Covariate Name	Number of Draws
Digestive congenital anomalies	Female	0-6 days	65-69 years	Global	-1	3	LDI (I$ per capita)	--
Digestive congenital anomalies	Female	0-6 days	65-69 years	Global	-1	3	Maternal Education (years per capita)	--
Digestive congenital anomalies	Female	0-6 days	65-69 years	Global	0	3	fruits unadjusted(g)	414
Digestive congenital anomalies	Female	0-6 days	65-69 years	Global	0	3	vegetables unadjusted(g)	459
Digestive congenital anomalies	Female	0-6 days	65-69 years	Global	1	3	Alcohol (liters per capita)	472
Digestive congenital anomalies	Male	0-6 days	65-69 years	Data Rich	1	1	Smoking Prevalence (Reproductive Age Standardized)	333
Digestive congenital anomalies	Male	0-6 days	65-69 years	Data Rich	1	1	Maternal alcohol consumption during pregnancy (proportion)	--
Digestive congenital anomalies	Male	0-6 days	65-69 years	Data Rich	-1	2	Healthcare access and quality index	96
Digestive congenital anomalies	Male	0-6 days	65-69 years	Data Rich	-1	2	Socio-demographic Index	121
Digestive congenital anomalies	Male	0-6 days	65-69 years	Data Rich	-1	2	In-Facility Delivery (proportion)	--
Digestive congenital anomalies	Male	0-6 days	65-69 years	Data Rich	1	2	Diabetes Age-Standardized Prevalence (proportion)	307
Digestive congenital anomalies	Male	0-6 days	65-69 years	Data Rich	1	2	Prevalence of obesity (age-standardized)	347
Digestive congenital anomalies	Male	0-6 days	65-69 years	Data Rich	1	2	Indoor Air Pollution (All Cooking Fuels)	--
Digestive congenital anomalies	Male	0-6 days	65-69 years	Data Rich	-1	3	Maternal Education (years per capita)	24
Digestive congenital anomalies	Male	0-6 days	65-69 years	Data Rich	-1	3	Health System Access (capped)	184
Digestive congenital anomalies	Male	0-6 days	65-69 years	Data Rich	-1	3	Antenatal Care (1 visit) Coverage (proportion)	--
Digestive congenital anomalies	Male	0-6 days	65-69 years	Data Rich	-1	3	Antenatal Care (4 visits) Coverage (proportion)	--
Digestive congenital anomalies	Male	0-6 days	65-69 years	Data Rich	-1	3	LDI (I$ per capita)	--
Digestive congenital anomalies	Male	0-6 days	65-69 years	Data Rich	0	3	vegetables unadjusted(g)	406
Digestive congenital anomalies	Male	0-6 days	65-69 years	Data Rich	0	3	fruits unadjusted(g)	467
Digestive congenital anomalies	Male	0-6 days	65-69 years	Data Rich	1	3	Alcohol (liters per capita)	342
Digestive congenital anomalies	Male	0-6 days	65-69 years	Global	1	1	Maternal alcohol consumption during pregnancy (proportion)	260
Digestive congenital anomalies	Male	0-6 days	65-69 years	Global	1	1	Smoking Prevalence (Reproductive Age Standardized)	--
Digestive congenital anomalies	Male	0-6 days	65-69 years	Global	-1	2	Socio-demographic Index	88
Digestive congenital anomalies	Male	0-6 days	65-69 years	Global	-1	2	Healthcare access and quality index	118
Digestive congenital anomalies	Male	0-6 days	65-69 years	Global	-1	2	In-Facility Delivery (proportion)	--
Digestive congenital anomalies	Male	0-6 days	65-69 years	Global	1	2	Prevalence of obesity (age-standardized)	359
Digestive congenital anomalies	Male	0-6 days	65-69 years	Global	1	2	Diabetes Age-Standardized Prevalence (proportion)	487
Digestive congenital anomalies	Male	0-6 days	65-69 years	Global	1	2	Indoor Air Pollution (All Cooking Fuels)	--
Digestive congenital anomalies	Male	0-6 days	65-69 years	Global	-1	3	Maternal Education (years per capita)	39
Digestive congenital anomalies	Male	0-6 days	65-69 years	Global	-1	3	Health System Access (capped)	59
Digestive congenital anomalies	Male	0-6 days	65-69 years	Global	-1	3	Antenatal Care (1 visit) Coverage (proportion)	--
Digestive congenital anomalies	Male	0-6 days	65-69 years	Global	-1	3	Antenatal Care (4 visits) Coverage (proportion)	--
Digestive congenital anomalies	Male	0-6 days	65-69 years	Global	-1	3	LDI (I$ per capita)	--
Digestive congenital anomalies	Male	0-6 days	65-69 years	Global	0	3	vegetables unadjusted(g)	137
Digestive congenital anomalies	Male	0-6 days	65-69 years	Global	0	3	fruits unadjusted(g)	178
Digestive congenital anomalies	Male	0-6 days	65-69 years	Global	1	3	Alcohol (liters per capita)	461
Other congenital anomalies	Female	0-6 days	65-69 years	Data Rich	1	1	Live Births 35+ (proportion)	282
Other congenital anomalies	Female	0-6 days	65-69 years	Data Rich	1	1	Maternal alcohol consumption during pregnancy (proportion)	--
Other congenital anomalies	Female	0-6 days	65-69 years	Data Rich	-1	2	Healthcare access and quality index	354
Other congenital anomalies	Female	0-6 days	65-69 years	Data Rich	-1	2	In-Facility Delivery (proportion)	--
Other congenital anomalies	Female	0-6 days	65-69 years	Data Rich	-1	2	Legality of Abortion	--
Other congenital anomalies	Female	0-6 days	65-69 years	Data Rich	1	2	Smoking Prevalence (Reproductive Age Standardized)	354
Other congenital anomalies	Female	0-6 days	65-69 years	Data Rich	1	2	Indoor Air Pollution (All Cooking Fuels)	--
Other congenital anomalies	Female	0-6 days	65-69 years	Data Rich	-1	3	Maternal Education (years per capita)	153
Other congenital anomalies	Female	0-6 days	65-69 years	Data Rich	-1	3	Socio-demographic Index	211
Other congenital anomalies	Female	0-6 days	65-69 years	Data Rich	-1	3	Antenatal Care (1 visit) Coverage (proportion)	--
Other congenital anomalies	Female	0-6 days	65-69 years	Data Rich	-1	3	Antenatal Care (4 visits) Coverage (proportion)	--
Other congenital anomalies	Female	0-6 days	65-69 years	Data Rich	-1	3	LDI (I$ per capita)	--
Other congenital anomalies	Female	0-6 days	65-69 years	Data Rich	1	3	Diabetes Age-Standardized Prevalence (proportion)	415
Other congenital anomalies	Female	0-6 days	65-69 years	Data Rich	1	3	Alcohol (liters per capita)	--
Other congenital anomalies	Female	0-6 days	65-69 years	Global	1	1	Live Births 35+ (proportion)	405
Other congenital anomalies	Female	0-6 days	65-69 years	Global	1	1	Maternal alcohol consumption during pregnancy (proportion)	--
Other congenital anomalies	Female	0-6 days	65-69 years	Global	-1	2	Healthcare access and quality index	236
Other congenital anomalies	Female	0-6 days	65-69 years	Global	-1	2	In-Facility Delivery (proportion)	--
Other congenital anomalies	Female	0-6 days	65-69 years	Global	-1	2	Legality of Abortion	--
Other congenital anomalies	Female	0-6 days	65-69 years	Global	1	2	Smoking Prevalence (Reproductive Age Standardized)	236
Other congenital anomalies	Female	0-6 days	65-69 years	Global	1	2	Indoor Air Pollution (All Cooking Fuels)	--
Other congenital anomalies	Female	0-6 days	65-69 years	Global	-1	3	Antenatal Care (4 visits) Coverage (proportion)	133
Other congenital anomalies	Female	0-6 days	65-69 years	Global	-1	3	Socio-demographic Index	148
Other congenital anomalies	Female	0-6 days	65-69 years	Global	-1	3	Maternal Education (years per capita)	161
Other congenital anomalies	Female	0-6 days	65-69 years	Global	-1	3	Antenatal Care (1 visit) Coverage (proportion)	--
Other congenital anomalies	Female	0-6 days	65-69 years	Global	-1	3	LDI (I$ per capita)	--
Other congenital anomalies	Female	0-6 days	65-69 years	Global	1	3	Diabetes Age-Standardized Prevalence (proportion)	674
Other congenital anomalies	Female	0-6 days	65-69 years	Global	1	3	Alcohol (liters per capita)	--
Other congenital anomalies	Male	0-6 days	65-69 years	Data Rich	1	1	Live Births 35+ (proportion)	428
Other congenital anomalies	Male	0-6 days	65-69 years	Data Rich	1	1	Maternal alcohol consumption during pregnancy (proportion)	--
Other congenital anomalies	Male	0-6 days	65-69 years	Data Rich	-1	2	Healthcare access and quality index	196
Other congenital anomalies	Male	0-6 days	65-69 years	Data Rich	-1	2	In-Facility Delivery (proportion)	--
Other congenital anomalies	Male	0-6 days	65-69 years	Data Rich	-1	2	Legality of Abortion	--
Other congenital anomalies	Male	0-6 days	65-69 years	Data Rich	1	2	Smoking Prevalence (Reproductive Age Standardized)	196
Other congenital anomalies	Male	0-6 days	65-69 years	Data Rich	1	2	Indoor Air Pollution (All Cooking Fuels)	--
Other congenital anomalies	Male	0-6 days	65-69 years	Data Rich	-1	3	Socio-demographic Index	227
Other congenital anomalies	Male	0-6 days	65-69 years	Data Rich	-1	3	Maternal Education (years per capita)	256
Other congenital anomalies	Male	0-6 days	65-69 years	Data Rich	-1	3	Antenatal Care (1 visit) Coverage (proportion)	--
Other congenital anomalies	Male	0-6 days	65-69 years	Data Rich	-1	3	Antenatal Care (4 visits) Coverage (proportion)	--
Other congenital anomalies	Male	0-6 days	65-69 years	Data Rich	-1	3	LDI (I$ per capita)	--
Other congenital anomalies	Male	0-6 days	65-69 years	Data Rich	1	3	Diabetes Age-Standardized Prevalence (proportion)	719

Appendix Table 9: CODEm covariates used, level of covariate, and expected direction of covariate by cause, sex, and age								
Covariates that CODEm did not select during the covariate selection process have no draw counts listed.								
**Cause**	**Sex**	**Age Start**	**Age End**	**Model Type**	**Direction**	**Level**	**Covariate Name**	**Number of Draws**
Other congenital anomalies	Male	0-6 days	65-69 years	Data Rich	1	3	Alcohol (liters per capita)	--
Other congenital anomalies	Male	0-6 days	65-69 years	Global	1	1	Live Births 35+ (proportion)	405
Other congenital anomalies	Male	0-6 days	65-69 years	Global	1	1	Maternal alcohol consumption during pregnancy (proportion)	--
Other congenital anomalies	Male	0-6 days	65-69 years	Global	-1	2	Healthcare access and quality index	274
Other congenital anomalies	Male	0-6 days	65-69 years	Global	-1	2	In-Facility Delivery (proportion)	--
Other congenital anomalies	Male	0-6 days	65-69 years	Global	-1	2	Legality of Abortion	--
Other congenital anomalies	Male	0-6 days	65-69 years	Global	1	2	Smoking Prevalence (Reproductive Age Standardized)	274
Other congenital anomalies	Male	0-6 days	65-69 years	Global	1	2	Indoor Air Pollution (All Cooking Fuels)	--
Other congenital anomalies	Male	0-6 days	65-69 years	Global	-1	3	Antenatal Care (4 visits) Coverage (proportion)	65
Other congenital anomalies	Male	0-6 days	65-69 years	Global	-1	3	Maternal Education (years per capita)	161
Other congenital anomalies	Male	0-6 days	65-69 years	Global	-1	3	Socio-demographic Index	173
Other congenital anomalies	Male	0-6 days	65-69 years	Global	-1	3	Antenatal Care (1 visit) Coverage (proportion)	--
Other congenital anomalies	Male	0-6 days	65-69 years	Global	-1	3	LDI (I$ per capita)	--
Other congenital anomalies	Male	0-6 days	65-69 years	Global	1	3	Diabetes Age-Standardized Prevalence (proportion)	581
Other congenital anomalies	Male	0-6 days	65-69 years	Global	1	3	Alcohol (liters per capita)	--
Urinary diseases and male infertility	Female	0-6 days	95+ years	Data Rich	-1	2	Healthcare access and quality index	0
Urinary diseases and male infertility	Female	0-6 days	95+ years	Data Rich	0	2	Latitude Under 15 (proportion)	340
Urinary diseases and male infertility	Female	0-6 days	95+ years	Data Rich	0	2	Latitude 30 to 45 (proportion)	447
Urinary diseases and male infertility	Female	0-6 days	95+ years	Data Rich	0	2	Latitude Over 45 (proportion)	497
Urinary diseases and male infertility	Female	0-6 days	95+ years	Data Rich	0	2	Latitude 15 to 30 (proportion)	502
Urinary diseases and male infertility	Female	0-6 days	95+ years	Data Rich	1	2	Mean BMI	971
Urinary diseases and male infertility	Female	0-6 days	95+ years	Data Rich	-1	3	Education (years per capita)	--
Urinary diseases and male infertility	Female	0-6 days	95+ years	Data Rich	-1	3	LDI (I$ per capita)	--
Urinary diseases and male infertility	Female	0-6 days	95+ years	Data Rich	0	3	Socio-demographic Index	180
Urinary diseases and male infertility	Female	0-6 days	95+ years	Global	-1	2	Healthcare access and quality index	0
Urinary diseases and male infertility	Female	0-6 days	95+ years	Global	0	2	Latitude 15 to 30 (proportion)	305
Urinary diseases and male infertility	Female	0-6 days	95+ years	Global	0	2	Latitude Over 45 (proportion)	332
Urinary diseases and male infertility	Female	0-6 days	95+ years	Global	0	2	Latitude Under 15 (proportion)	508
Urinary diseases and male infertility	Female	0-6 days	95+ years	Global	0	2	Latitude 30 to 45 (proportion)	557
Urinary diseases and male infertility	Female	0-6 days	95+ years	Global	1	2	Mean BMI	996
Urinary diseases and male infertility	Female	0-6 days	95+ years	Global	-1	3	Education (years per capita)	0
Urinary diseases and male infertility	Female	0-6 days	95+ years	Global	-1	3	LDI (I$ per capita)	--
Urinary diseases and male infertility	Female	0-6 days	95+ years	Global	0	3	Socio-demographic Index	884
Urinary diseases and male infertility	Male	0-6 days	95+ years	Data Rich	-1	2	Healthcare access and quality index	0
Urinary diseases and male infertility	Male	0-6 days	95+ years	Data Rich	0	2	Latitude Under 15 (proportion)	258
Urinary diseases and male infertility	Male	0-6 days	95+ years	Data Rich	0	2	Latitude 15 to 30 (proportion)	330
Urinary diseases and male infertility	Male	0-6 days	95+ years	Data Rich	0	2	Latitude Over 45 (proportion)	344
Urinary diseases and male infertility	Male	0-6 days	95+ years	Data Rich	0	2	Latitude 30 to 45 (proportion)	618
Urinary diseases and male infertility	Male	0-6 days	95+ years	Data Rich	1	2	Mean BMI	952
Urinary diseases and male infertility	Male	0-6 days	95+ years	Data Rich	-1	3	Education (years per capita)	0
Urinary diseases and male infertility	Male	0-6 days	95+ years	Data Rich	-1	3	LDI (I$ per capita)	--
Urinary diseases and male infertility	Male	0-6 days	95+ years	Data Rich	0	3	Socio-demographic Index	86
Urinary diseases and male infertility	Male	0-6 days	95+ years	Global	-1	2	Healthcare access and quality index	507
Urinary diseases and male infertility	Male	0-6 days	95+ years	Global	0	2	Latitude 30 to 45 (proportion)	545
Urinary diseases and male infertility	Male	0-6 days	95+ years	Global	0	2	Latitude Over 45 (proportion)	549
Urinary diseases and male infertility	Male	0-6 days	95+ years	Global	0	2	Latitude Under 15 (proportion)	665
Urinary diseases and male infertility	Male	0-6 days	95+ years	Global	0	2	Latitude 15 to 30 (proportion)	673
Urinary diseases and male infertility	Male	0-6 days	95+ years	Global	1	2	Mean BMI	953
Urinary diseases and male infertility	Male	0-6 days	95+ years	Global	-1	3	Education (years per capita)	0
Urinary diseases and male infertility	Male	0-6 days	95+ years	Global	-1	3	LDI (I$ per capita)	--
Urinary diseases and male infertility	Male	0-6 days	95+ years	Global	0	3	Socio-demographic Index	548
Urinary tract infections	Female	0-6 days	95+ years	Data Rich	1	1	Sanitation (proportion with access)	1000
Urinary tract infections	Female	0-6 days	95+ years	Data Rich	-1	2	Education (years per capita)	--
Urinary tract infections	Female	0-6 days	95+ years	Data Rich	-1	2	Healthcare access and quality index	--
Urinary tract infections	Female	0-6 days	95+ years	Data Rich	-1	2	LDI (I$ per capita)	--
Urinary tract infections	Female	0-6 days	95+ years	Data Rich	0	3	Socio-demographic Index	--
Urinary tract infections	Female	0-6 days	95+ years	Global	1	1	Sanitation (proportion with access)	326
Urinary tract infections	Female	0-6 days	95+ years	Global	-1	2	Healthcare access and quality index	489
Urinary tract infections	Female	0-6 days	95+ years	Global	-1	2	Education (years per capita)	--
Urinary tract infections	Female	0-6 days	95+ years	Global	-1	2	LDI (I$ per capita)	--
Urinary tract infections	Female	0-6 days	95+ years	Global	0	3	Socio-demographic Index	452
Urinary tract infections	Male	0-6 days	95+ years	Data Rich	1	1	Sanitation (proportion with access)	1000
Urinary tract infections	Male	0-6 days	95+ years	Data Rich	-1	2	Education (years per capita)	--
Urinary tract infections	Male	0-6 days	95+ years	Data Rich	-1	2	Health System Access (capped)	--
Urinary tract infections	Male	0-6 days	95+ years	Data Rich	-1	2	Healthcare access and quality index	--
Urinary tract infections	Male	0-6 days	95+ years	Data Rich	-1	2	LDI (I$ per capita)	--
Urinary tract infections	Male	0-6 days	95+ years	Data Rich	0	3	Socio-demographic Index	--
Urinary tract infections	Male	0-6 days	95+ years	Global	1	1	Sanitation (proportion with access)	326
Urinary tract infections	Male	0-6 days	95+ years	Global	-1	2	Healthcare access and quality index	489
Urinary tract infections	Male	0-6 days	95+ years	Global	-1	2	Education (years per capita)	--
Urinary tract infections	Male	0-6 days	95+ years	Global	-1	2	LDI (I$ per capita)	--
Urinary tract infections	Male	0-6 days	95+ years	Global	0	3	Socio-demographic Index	452
Urolithiasis	Female	5-9 years	95+ years	Data Rich	1	1	Healthcare access and quality index	620
Urolithiasis	Female	5-9 years	95+ years	Data Rich	1	1	90th percentile climatic temperature in the given country-year.	683
Urolithiasis	Female	5-9 years	95+ years	Data Rich	-1	2	fruits adjusted(g)	369
Urolithiasis	Female	5-9 years	95+ years	Data Rich	-1	2	vegetables adjusted(g)	822
Urolithiasis	Female	5-9 years	95+ years	Data Rich	1	2	red meats adjusted(g)	903
Urolithiasis	Female	5-9 years	95+ years	Data Rich	-1	3	Education (years per capita)	5

Appendix Table 9: CODEm covariates used, level of covariate, and expected direction of covariate by cause, sex, and age
Covariates that CODEm did not select during the covariate selection process have no draw counts listed.

Cause	Sex	Age Start	Age End	Model Type	Direction	Level	Covariate Name	Number of Draws
Urolithiasis	Female	5-9 years	95+ years	Data Rich	-1	3	LDI (I$ per capita)	--
Urolithiasis	Female	5-9 years	95+ years	Data Rich	0	3	Socio-demographic Index	319
Urolithiasis	Female	5-9 years	95+ years	Global	1	1	Healthcare access and quality index	333
Urolithiasis	Female	5-9 years	95+ years	Global	1	1	90th percentile climatic temperature in the given country-year.	864
Urolithiasis	Female	5-9 years	95+ years	Global	-1	2	fruits adjusted(g)	466
Urolithiasis	Female	5-9 years	95+ years	Global	-1	2	vegetables adjusted(g)	466
Urolithiasis	Female	5-9 years	95+ years	Global	1	2	red meats adjusted(g)	466
Urolithiasis	Female	5-9 years	95+ years	Global	-1	3	Education (years per capita)	88
Urolithiasis	Female	5-9 years	95+ years	Global	-1	3	LDI (I$ per capita)	--
Urolithiasis	Female	5-9 years	95+ years	Global	0	3	Socio-demographic Index	267
Urolithiasis	Male	5-9 years	95+ years	Data Rich	-1	1	Healthcare access and quality index	267
Urolithiasis	Male	5-9 years	95+ years	Data Rich	1	1	90th percentile climatic temperature in the given country-year.	381
Urolithiasis	Male	5-9 years	95+ years	Data Rich	1	1	red meats adjusted(g)	935
Urolithiasis	Male	5-9 years	95+ years	Data Rich	-1	2	fruits adjusted(g)	18
Urolithiasis	Male	5-9 years	95+ years	Data Rich	-1	2	vegetables adjusted(g)	398
Urolithiasis	Male	5-9 years	95+ years	Data Rich	-1	3	Education (years per capita)	0
Urolithiasis	Male	5-9 years	95+ years	Data Rich	-1	3	LDI (I$ per capita)	--
Urolithiasis	Male	5-9 years	95+ years	Data Rich	0	3	Socio-demographic Index	397
Urolithiasis	Male	5-9 years	95+ years	Global	1	1	90th percentile climatic temperature in the given country-year.	1000
Urolithiasis	Male	5-9 years	95+ years	Global	-1	2	vegetables adjusted(g)	189
Urolithiasis	Male	5-9 years	95+ years	Global	-1	2	Healthcare access and quality index	414
Urolithiasis	Male	5-9 years	95+ years	Global	-1	2	fruits adjusted(g)	414
Urolithiasis	Male	5-9 years	95+ years	Global	1	2	red meats adjusted(g)	225
Urolithiasis	Male	5-9 years	95+ years	Global	-1	3	Education (years per capita)	222
Urolithiasis	Male	5-9 years	95+ years	Global	-1	3	LDI (I$ per capita)	--
Urolithiasis	Male	5-9 years	95+ years	Global	0	3	Socio-demographic Index	283
Other urinary diseases	Female	0-6 days	95+ years	Data Rich	1	1	Education (years per capita)	--
Other urinary diseases	Female	0-6 days	95+ years	Data Rich	1	1	LDI (I$ per capita)	--
Other urinary diseases	Female	0-6 days	95+ years	Data Rich	1	1	Mean BMI	--
Other urinary diseases	Female	0-6 days	95+ years	Data Rich	-1	2	Healthcare access and quality index	653
Other urinary diseases	Female	0-6 days	95+ years	Data Rich	0	3	Socio-demographic Index	614
Other urinary diseases	Female	0-6 days	95+ years	Global	1	1	Education (years per capita)	--
Other urinary diseases	Female	0-6 days	95+ years	Global	1	1	LDI (I$ per capita)	--
Other urinary diseases	Female	0-6 days	95+ years	Global	1	1	Mean BMI	--
Other urinary diseases	Female	0-6 days	95+ years	Global	-1	2	Healthcare access and quality index	807
Other urinary diseases	Female	0-6 days	95+ years	Global	0	3	Socio-demographic Index	557
Other urinary diseases	Male	0-6 days	95+ years	Data Rich	1	1	Mean BMI	--
Other urinary diseases	Male	0-6 days	95+ years	Data Rich	-1	2	LDI (I$ per capita)	89
Other urinary diseases	Male	0-6 days	95+ years	Data Rich	-1	2	Education (years per capita)	344
Other urinary diseases	Male	0-6 days	95+ years	Data Rich	-1	2	Healthcare access and quality index	453
Other urinary diseases	Male	0-6 days	95+ years	Data Rich	0	3	Socio-demographic Index	662
Other urinary diseases	Male	0-6 days	95+ years	Global	1	1	Mean BMI	--
Other urinary diseases	Male	0-6 days	95+ years	Global	-1	2	LDI (I$ per capita)	251
Other urinary diseases	Male	0-6 days	95+ years	Global	-1	2	Education (years per capita)	598
Other urinary diseases	Male	0-6 days	95+ years	Global	-1	2	Healthcare access and quality index	635
Other urinary diseases	Male	0-6 days	95+ years	Global	0	3	Socio-demographic Index	435
Gynecological diseases	Female	15-19 years	95+ years	Data Rich	0	1	Smoking Prevalence	486
Gynecological diseases	Female	15-19 years	95+ years	Data Rich	-1	2	Skilled Birth Attendance (proportion)	407
Gynecological diseases	Female	15-19 years	95+ years	Data Rich	-1	2	Healthcare access and quality index	514
Gynecological diseases	Female	15-19 years	95+ years	Data Rich	-1	2	Health System Access (capped)	662
Gynecological diseases	Female	15-19 years	95+ years	Data Rich	1	2	Total Fertility Rate	407
Gynecological diseases	Female	15-19 years	95+ years	Data Rich	1	2	Live Births 35+ (proportion)	--
Gynecological diseases	Female	15-19 years	95+ years	Data Rich	-1	3	Socio-demographic Index	274
Gynecological diseases	Female	15-19 years	95+ years	Data Rich	-1	3	Education (years per capita)	418
Gynecological diseases	Female	15-19 years	95+ years	Data Rich	-1	3	LDI (I$ per capita)	--
Gynecological diseases	Female	15-19 years	95+ years	Global	0	1	Smoking Prevalence	1000
Gynecological diseases	Female	15-19 years	95+ years	Global	-1	2	Skilled Birth Attendance (proportion)	145
Gynecological diseases	Female	15-19 years	95+ years	Global	-1	2	Healthcare access and quality index	307
Gynecological diseases	Female	15-19 years	95+ years	Global	-1	2	Health System Access (capped)	410
Gynecological diseases	Female	15-19 years	95+ years	Global	1	2	Total Fertility Rate	201
Gynecological diseases	Female	15-19 years	95+ years	Global	1	2	Live Births 35+ (proportion)	--
Gynecological diseases	Female	15-19 years	95+ years	Global	-1	3	Socio-demographic Index	171
Gynecological diseases	Female	15-19 years	95+ years	Global	-1	3	Education (years per capita)	379
Gynecological diseases	Female	15-19 years	95+ years	Global	-1	3	LDI (I$ per capita)	--
Uterine fibroids	Female	15-19 years	95+ years	Data Rich	0	1	Smoking Prevalence	--
Uterine fibroids	Female	15-19 years	95+ years	Data Rich	-1	2	Skilled Birth Attendance (proportion)	300
Uterine fibroids	Female	15-19 years	95+ years	Data Rich	-1	2	Health System Access (capped)	570
Uterine fibroids	Female	15-19 years	95+ years	Data Rich	-1	2	Healthcare access and quality index	--
Uterine fibroids	Female	15-19 years	95+ years	Data Rich	1	2	Live Births 35+ (proportion)	--
Uterine fibroids	Female	15-19 years	95+ years	Data Rich	1	2	Total Fertility Rate	--
Uterine fibroids	Female	15-19 years	95+ years	Data Rich	-1	3	Socio-demographic Index	221
Uterine fibroids	Female	15-19 years	95+ years	Data Rich	-1	3	Education (years per capita)	380
Uterine fibroids	Female	15-19 years	95+ years	Data Rich	-1	3	LDI (I$ per capita)	--
Uterine fibroids	Female	15-19 years	95+ years	Global	0	1	Smoking Prevalence	1000
Uterine fibroids	Female	15-19 years	95+ years	Global	-1	2	Skilled Birth Attendance (proportion)	168
Uterine fibroids	Female	15-19 years	95+ years	Global	-1	2	Healthcare access and quality index	430
Uterine fibroids	Female	15-19 years	95+ years	Global	-1	2	Health System Access (capped)	598
Uterine fibroids	Female	15-19 years	95+ years	Global	1	2	Total Fertility Rate	36

Appendix Table 9: CODEm covariates used, level of covariate, and expected direction of covariate by cause, sex, and age
Covariates that CODEm did not select during the covariate selection process have no draw counts listed.

Cause	Sex	Age Start	Age End	Model Type	Direction	Level	Covariate Name	Number of Draws
Uterine fibroids	Female	15-19 years	95+ years	Global	1	2	Live Births 35+ (proportion)	--
Uterine fibroids	Female	15-19 years	95+ years	Global	-1	3	LDI (I$ per capita)	31
Uterine fibroids	Female	15-19 years	95+ years	Global	-1	3	Socio-demographic Index	137
Uterine fibroids	Female	15-19 years	95+ years	Global	-1	3	Education (years per capita)	428
Polycystic ovarian syndrome	Female	15-19 years	50-54 years	Data Rich	0	1	Smoking Prevalence	454
Polycystic ovarian syndrome	Female	15-19 years	50-54 years	Data Rich	-1	2	Health System Access (capped)	--
Polycystic ovarian syndrome	Female	15-19 years	50-54 years	Data Rich	-1	2	Healthcare access and quality index	--
Polycystic ovarian syndrome	Female	15-19 years	50-54 years	Data Rich	-1	2	Skilled Birth Attendance (proportion)	--
Polycystic ovarian syndrome	Female	15-19 years	50-54 years	Data Rich	1	2	Live Births 35+ (proportion)	--
Polycystic ovarian syndrome	Female	15-19 years	50-54 years	Data Rich	1	2	Total Fertility Rate	--
Polycystic ovarian syndrome	Female	15-19 years	50-54 years	Data Rich	-1	3	LDI (I$ per capita)	546
Polycystic ovarian syndrome	Female	15-19 years	50-54 years	Data Rich	-1	3	Education (years per capita)	--
Polycystic ovarian syndrome	Female	15-19 years	50-54 years	Data Rich	-1	3	Socio-demographic Index	--
Polycystic ovarian syndrome	Female	15-19 years	50-54 years	Global	0	1	Smoking Prevalence	1000
Polycystic ovarian syndrome	Female	15-19 years	50-54 years	Global	-1	2	Health System Access (capped)	--
Polycystic ovarian syndrome	Female	15-19 years	50-54 years	Global	-1	2	Healthcare access and quality index	--
Polycystic ovarian syndrome	Female	15-19 years	50-54 years	Global	-1	2	Skilled Birth Attendance (proportion)	--
Polycystic ovarian syndrome	Female	15-19 years	50-54 years	Global	1	2	Live Births 35+ (proportion)	--
Polycystic ovarian syndrome	Female	15-19 years	50-54 years	Global	1	2	Total Fertility Rate	--
Polycystic ovarian syndrome	Female	15-19 years	50-54 years	Global	-1	3	Education (years per capita)	--
Polycystic ovarian syndrome	Female	15-19 years	50-54 years	Global	-1	3	LDI (I$ per capita)	--
Polycystic ovarian syndrome	Female	15-19 years	50-54 years	Global	-1	3	Socio-demographic Index	--
Endometriosis	Female	15-19 years	50-54 years	Data Rich	0	1	Smoking Prevalence	1000
Endometriosis	Female	15-19 years	50-54 years	Data Rich	-1	2	Skilled Birth Attendance (proportion)	76
Endometriosis	Female	15-19 years	50-54 years	Data Rich	-1	2	Healthcare access and quality index	559
Endometriosis	Female	15-19 years	50-54 years	Data Rich	-1	2	Health System Access (capped)	--
Endometriosis	Female	15-19 years	50-54 years	Data Rich	1	2	Total Fertility Rate	76
Endometriosis	Female	15-19 years	50-54 years	Data Rich	1	2	Live Births 35+ (proportion)	--
Endometriosis	Female	15-19 years	50-54 years	Data Rich	-1	3	Education (years per capita)	193
Endometriosis	Female	15-19 years	50-54 years	Data Rich	-1	3	Socio-demographic Index	312
Endometriosis	Female	15-19 years	50-54 years	Data Rich	-1	3	LDI (I$ per capita)	564
Endometriosis	Female	15-19 years	50-54 years	Global	0	1	Smoking Prevalence	1000
Endometriosis	Female	15-19 years	50-54 years	Global	-1	2	Health System Access (capped)	194
Endometriosis	Female	15-19 years	50-54 years	Global	-1	2	Skilled Birth Attendance (proportion)	210
Endometriosis	Female	15-19 years	50-54 years	Global	-1	2	Healthcare access and quality index	302
Endometriosis	Female	15-19 years	50-54 years	Global	1	2	Total Fertility Rate	192
Endometriosis	Female	15-19 years	50-54 years	Global	1	2	Live Births 35+ (proportion)	--
Endometriosis	Female	15-19 years	50-54 years	Global	-1	3	Education (years per capita)	122
Endometriosis	Female	15-19 years	50-54 years	Global	-1	3	Socio-demographic Index	218
Endometriosis	Female	15-19 years	50-54 years	Global	-1	3	LDI (I$ per capita)	311
Genital prolapse	Female	15-19 years	95+ years	Data Rich	0	1	Smoking Prevalence	1000
Genital prolapse	Female	15-19 years	95+ years	Data Rich	-1	2	Health System Access (capped)	161
Genital prolapse	Female	15-19 years	95+ years	Data Rich	-1	2	Healthcare access and quality index	397
Genital prolapse	Female	15-19 years	95+ years	Data Rich	-1	2	Skilled Birth Attendance (proportion)	405
Genital prolapse	Female	15-19 years	95+ years	Data Rich	1	2	Total Fertility Rate	35
Genital prolapse	Female	15-19 years	95+ years	Data Rich	1	2	Live Births 35+ (proportion)	--
Genital prolapse	Female	15-19 years	95+ years	Data Rich	-1	3	LDI (I$ per capita)	198
Genital prolapse	Female	15-19 years	95+ years	Data Rich	-1	3	Socio-demographic Index	384
Genital prolapse	Female	15-19 years	95+ years	Data Rich	-1	3	Education (years per capita)	713
Genital prolapse	Female	15-19 years	95+ years	Global	0	1	Smoking Prevalence	1000
Genital prolapse	Female	15-19 years	95+ years	Global	-1	2	Skilled Birth Attendance (proportion)	382
Genital prolapse	Female	15-19 years	95+ years	Global	-1	2	Healthcare access and quality index	438
Genital prolapse	Female	15-19 years	95+ years	Global	-1	2	Health System Access (capped)	548
Genital prolapse	Female	15-19 years	95+ years	Global	1	2	Total Fertility Rate	326
Genital prolapse	Female	15-19 years	95+ years	Global	1	2	Live Births 35+ (proportion)	--
Genital prolapse	Female	15-19 years	95+ years	Global	-1	3	Socio-demographic Index	168
Genital prolapse	Female	15-19 years	95+ years	Global	-1	3	Education (years per capita)	304
Genital prolapse	Female	15-19 years	95+ years	Global	-1	3	LDI (I$ per capita)	400
Other gynecological diseases	Female	15-19 years	95+ years	Data Rich	0	1	Smoking Prevalence	1000
Other gynecological diseases	Female	15-19 years	95+ years	Data Rich	-1	2	Health System Access (capped)	113
Other gynecological diseases	Female	15-19 years	95+ years	Data Rich	-1	2	Healthcare access and quality index	347
Other gynecological diseases	Female	15-19 years	95+ years	Data Rich	-1	2	Skilled Birth Attendance (proportion)	375
Other gynecological diseases	Female	15-19 years	95+ years	Data Rich	1	2	Live Births 35+ (proportion)	262
Other gynecological diseases	Female	15-19 years	95+ years	Data Rich	1	2	Total Fertility Rate	375
Other gynecological diseases	Female	15-19 years	95+ years	Data Rich	-1	3	LDI (I$ per capita)	78
Other gynecological diseases	Female	15-19 years	95+ years	Data Rich	-1	3	Education (years per capita)	151
Other gynecological diseases	Female	15-19 years	95+ years	Data Rich	-1	3	Socio-demographic Index	193
Other gynecological diseases	Female	15-19 years	95+ years	Global	0	1	Smoking Prevalence	1000
Other gynecological diseases	Female	15-19 years	95+ years	Global	-1	2	Healthcare access and quality index	86
Other gynecological diseases	Female	15-19 years	95+ years	Global	-1	2	Health System Access (capped)	118
Other gynecological diseases	Female	15-19 years	95+ years	Global	-1	2	Skilled Birth Attendance (proportion)	339
Other gynecological diseases	Female	15-19 years	95+ years	Global	1	2	Live Births 35+ (proportion)	339
Other gynecological diseases	Female	15-19 years	95+ years	Global	1	2	Total Fertility Rate	457
Other gynecological diseases	Female	15-19 years	95+ years	Global	-1	3	LDI (I$ per capita)	151
Other gynecological diseases	Female	15-19 years	95+ years	Global	-1	3	Education (years per capita)	188
Other gynecological diseases	Female	15-19 years	95+ years	Global	-1	3	Socio-demographic Index	204
Hemoglobinopathies and hemolytic anaemias	Female	0-6 days	95+ years	Data Rich	-1	2	Health System Access (capped)	--
Hemoglobinopathies and hemolytic anaemias	Female	0-6 days	95+ years	Data Rich	-1	2	Healthcare access and quality index	--

Appendix Table 9: CODEm covariates used, level of covariate, and expected direction of covariate by cause, sex, and age

Covariates that CODEm did not select during the covariate selection process have no draw counts listed.

Cause	Sex	Age Start	Age End	Model Type	Direction	Level	Covariate Name	Number of Draws
Hemoglobinopathies and hemolytic anaemias	Female	0-6 days	95+ years	Data Rich	-1	3	Education (years per capita)	1000
Hemoglobinopathies and hemolytic anaemias	Female	0-6 days	95+ years	Data Rich	-1	3	Latitude Over 45 (proportion)	1000
Hemoglobinopathies and hemolytic anaemias	Female	0-6 days	95+ years	Data Rich	-1	3	Socio-demographic Index	1000
Hemoglobinopathies and hemolytic anaemias	Female	0-6 days	95+ years	Data Rich	-1	3	LDI (I$ per capita)	--
Hemoglobinopathies and hemolytic anaemias	Female	0-6 days	95+ years	Data Rich	0	3	Latitude 30 to 45 (proportion)	1000
Hemoglobinopathies and hemolytic anaemias	Female	0-6 days	95+ years	Data Rich	1	3	Latitude 15 to 30 (proportion)	--
Hemoglobinopathies and hemolytic anaemias	Female	0-6 days	95+ years	Data Rich	1	3	Latitude Under 15 (proportion)	--
Hemoglobinopathies and hemolytic anaemias	Female	0-6 days	95+ years	Data Rich	1	3	Malaria Lysenko PFPR 1 (Holoendemic)	--
Hemoglobinopathies and hemolytic anaemias	Female	0-6 days	95+ years	Global	1	1	Hemoglobinopathies Prevalence x Excess Mortality	119
Hemoglobinopathies and hemolytic anaemias	Female	0-6 days	95+ years	Global	1	1	Hemoglobinopathies Prevalence x Excess Mortality (excluding G6PD deficiency)	201
Hemoglobinopathies and hemolytic anaemias	Female	0-6 days	95+ years	Global	-1	2	Healthcare access and quality index	257
Hemoglobinopathies and hemolytic anaemias	Female	0-6 days	95+ years	Global	-1	2	Health System Access (capped)	654
Hemoglobinopathies and hemolytic anaemias	Female	0-6 days	95+ years	Global	-1	3	Socio-demographic Index	221
Hemoglobinopathies and hemolytic anaemias	Female	0-6 days	95+ years	Global	-1	3	Education (years per capita)	305
Hemoglobinopathies and hemolytic anaemias	Female	0-6 days	95+ years	Global	-1	3	Latitude Over 45 (proportion)	374
Hemoglobinopathies and hemolytic anaemias	Female	0-6 days	95+ years	Global	-1	3	LDI (I$ per capita)	--
Hemoglobinopathies and hemolytic anaemias	Female	0-6 days	95+ years	Global	0	3	Latitude 30 to 45 (proportion)	633
Hemoglobinopathies and hemolytic anaemias	Female	0-6 days	95+ years	Global	1	3	Latitude 15 to 30 (proportion)	173
Hemoglobinopathies and hemolytic anaemias	Female	0-6 days	95+ years	Global	1	3	Malaria Lysenko PFPR 1 (Holoendemic)	247
Hemoglobinopathies and hemolytic anaemias	Female	0-6 days	95+ years	Global	1	3	Latitude Under 15 (proportion)	--
Hemoglobinopathies and hemolytic anaemias	Male	0-6 days	95+ years	Data Rich	-1	2	Health System Access (capped)	477
Hemoglobinopathies and hemolytic anaemias	Male	0-6 days	95+ years	Data Rich	-1	2	Healthcare access and quality index	613
Hemoglobinopathies and hemolytic anaemias	Male	0-6 days	95+ years	Data Rich	-1	3	Socio-demographic Index	50
Hemoglobinopathies and hemolytic anaemias	Male	0-6 days	95+ years	Data Rich	-1	3	Education (years per capita)	60
Hemoglobinopathies and hemolytic anaemias	Male	0-6 days	95+ years	Data Rich	-1	3	Latitude Over 45 (proportion)	445
Hemoglobinopathies and hemolytic anaemias	Male	0-6 days	95+ years	Data Rich	-1	3	LDI (I$ per capita)	--
Hemoglobinopathies and hemolytic anaemias	Male	0-6 days	95+ years	Data Rich	0	3	Latitude 30 to 45 (proportion)	477
Hemoglobinopathies and hemolytic anaemias	Male	0-6 days	95+ years	Data Rich	1	3	Latitude Under 15 (proportion)	202
Hemoglobinopathies and hemolytic anaemias	Male	0-6 days	95+ years	Data Rich	1	3	Latitude 15 to 30 (proportion)	312
Hemoglobinopathies and hemolytic anaemias	Male	0-6 days	95+ years	Data Rich	1	3	Malaria Lysenko PFPR 1 (Holoendemic)	--
Hemoglobinopathies and hemolytic anaemias	Male	0-6 days	95+ years	Global	1	1	Hemoglobinopathies Prevalence x Excess Mortality (excluding G6PD deficiency)	159
Hemoglobinopathies and hemolytic anaemias	Male	0-6 days	95+ years	Global	1	1	Hemoglobinopathies Prevalence x Excess Mortality	522
Hemoglobinopathies and hemolytic anaemias	Male	0-6 days	95+ years	Global	-1	2	Health System Access (capped)	386
Hemoglobinopathies and hemolytic anaemias	Male	0-6 days	95+ years	Global	-1	2	Healthcare access and quality index	386
Hemoglobinopathies and hemolytic anaemias	Male	0-6 days	95+ years	Global	-1	3	Socio-demographic Index	184
Hemoglobinopathies and hemolytic anaemias	Male	0-6 days	95+ years	Global	-1	3	Education (years per capita)	271
Hemoglobinopathies and hemolytic anaemias	Male	0-6 days	95+ years	Global	-1	3	Latitude Over 45 (proportion)	692
Hemoglobinopathies and hemolytic anaemias	Male	0-6 days	95+ years	Global	-1	3	LDI (I$ per capita)	--
Hemoglobinopathies and hemolytic anaemias	Male	0-6 days	95+ years	Global	0	3	Latitude 30 to 45 (proportion)	585
Hemoglobinopathies and hemolytic anaemias	Male	0-6 days	95+ years	Global	1	3	Malaria Lysenko PFPR 1 (Holoendemic)	462
Hemoglobinopathies and hemolytic anaemias	Male	0-6 days	95+ years	Global	1	3	Latitude 15 to 30 (proportion)	654
Hemoglobinopathies and hemolytic anaemias	Male	0-6 days	95+ years	Global	1	3	Latitude Under 15 (proportion)	--
Endocrine, metabolic, blood, and immune disorders	Female	0-6 days	95+ years	Data Rich	1	1	Mean BMI	--
Endocrine, metabolic, blood, and immune disorders	Female	0-6 days	95+ years	Data Rich	-1	2	Healthcare access and quality index	699
Endocrine, metabolic, blood, and immune disorders	Female	0-6 days	95+ years	Data Rich	1	2	Alcohol (liters per capita)	--
Endocrine, metabolic, blood, and immune disorders	Female	0-6 days	95+ years	Data Rich	1	2	Cholesterol (total, mean per capita)	--
Endocrine, metabolic, blood, and immune disorders	Female	0-6 days	95+ years	Data Rich	-1	3	Education (years per capita)	--
Endocrine, metabolic, blood, and immune disorders	Female	0-6 days	95+ years	Data Rich	-1	3	LDI (I$ per capita)	--
Endocrine, metabolic, blood, and immune disorders	Female	0-6 days	95+ years	Data Rich	0	3	Socio-demographic Index	572
Endocrine, metabolic, blood, and immune disorders	Female	0-6 days	95+ years	Global	1	1	Mean BMI	--
Endocrine, metabolic, blood, and immune disorders	Female	0-6 days	95+ years	Global	-1	2	Healthcare access and quality index	317
Endocrine, metabolic, blood, and immune disorders	Female	0-6 days	95+ years	Global	1	2	Alcohol (liters per capita)	--
Endocrine, metabolic, blood, and immune disorders	Female	0-6 days	95+ years	Global	1	2	Cholesterol (total, mean per capita)	--
Endocrine, metabolic, blood, and immune disorders	Female	0-6 days	95+ years	Global	-1	3	Education (years per capita)	271
Endocrine, metabolic, blood, and immune disorders	Female	0-6 days	95+ years	Global	-1	3	LDI (I$ per capita)	--
Endocrine, metabolic, blood, and immune disorders	Female	0-6 days	95+ years	Global	0	3	Socio-demographic Index	479
Endocrine, metabolic, blood, and immune disorders	Male	0-6 days	95+ years	Data Rich	1	1	Mean BMI	--
Endocrine, metabolic, blood, and immune disorders	Male	0-6 days	95+ years	Data Rich	-1	2	Healthcare access and quality index	137
Endocrine, metabolic, blood, and immune disorders	Male	0-6 days	95+ years	Data Rich	1	2	Cholesterol (total, mean per capita)	137
Endocrine, metabolic, blood, and immune disorders	Male	0-6 days	95+ years	Data Rich	1	2	Alcohol (liters per capita)	--
Endocrine, metabolic, blood, and immune disorders	Male	0-6 days	95+ years	Data Rich	-1	3	Education (years per capita)	78
Endocrine, metabolic, blood, and immune disorders	Male	0-6 days	95+ years	Data Rich	-1	3	LDI (I$ per capita)	--
Endocrine, metabolic, blood, and immune disorders	Male	0-6 days	95+ years	Data Rich	0	3	Socio-demographic Index	348
Endocrine, metabolic, blood, and immune disorders	Male	0-6 days	95+ years	Global	1	1	Mean BMI	--
Endocrine, metabolic, blood, and immune disorders	Male	0-6 days	95+ years	Global	-1	2	Healthcare access and quality index	317
Endocrine, metabolic, blood, and immune disorders	Male	0-6 days	95+ years	Global	1	2	Alcohol (liters per capita)	--
Endocrine, metabolic, blood, and immune disorders	Male	0-6 days	95+ years	Global	1	2	Cholesterol (total, mean per capita)	--
Endocrine, metabolic, blood, and immune disorders	Male	0-6 days	95+ years	Global	-1	3	Education (years per capita)	286
Endocrine, metabolic, blood, and immune disorders	Male	0-6 days	95+ years	Global	-1	3	LDI (I$ per capita)	--
Endocrine, metabolic, blood, and immune disorders	Male	0-6 days	95+ years	Global	0	3	Socio-demographic Index	531
Sudden infant death syndrome	Female	7-27 days	28-364 days	Data Rich	-1	1	In-Facility Delivery (proportion)	251
Sudden infant death syndrome	Female	7-27 days	28-364 days	Data Rich	1	1	Tobacco (cigarettes per capita)	900
Sudden infant death syndrome	Female	7-27 days	28-364 days	Data Rich	-1	2	Skilled Birth Attendance (proportion)	120
Sudden infant death syndrome	Female	7-27 days	28-364 days	Data Rich	1	2	Underweight (proportion <2SD weight for age, <5 years)	--
Sudden infant death syndrome	Female	7-27 days	28-364 days	Data Rich	-1	3	Education (years per capita)	208
Sudden infant death syndrome	Female	7-27 days	28-364 days	Data Rich	0	3	Socio-demographic Index	208
Sudden infant death syndrome	Female	7-27 days	28-364 days	Data Rich	0	3	LDI (I$ per capita)	--
Sudden infant death syndrome	Female	7-27 days	28-364 days	Data Rich	1	3	Total Fertility Rate	--

Appendix Table 9: CODEm covariates used, level of covariate, and expected direction of covariate by cause, sex, and age
Covariates that CODEm did not select during the covariate selection process have no draw counts listed.

Cause	Sex	Age Start	Age End	Model Type	Direction	Level	Covariate Name	Number of Draws
Sudden infant death syndrome	Female	7-27 days	28-364 days	Global	-1	1	In-Facility Delivery (proportion)	412
Sudden infant death syndrome	Female	7-27 days	28-364 days	Global	1	1	Tobacco (cigarettes per capita)	689
Sudden infant death syndrome	Female	7-27 days	28-364 days	Global	-1	2	Skilled Birth Attendance (proportion)	240
Sudden infant death syndrome	Female	7-27 days	28-364 days	Global	1	2	Underweight (proportion <2SD weight for age, <5 years)	--
Sudden infant death syndrome	Female	7-27 days	28-364 days	Global	-1	3	Education (years per capita)	146
Sudden infant death syndrome	Female	7-27 days	28-364 days	Global	0	3	Socio-demographic Index	146
Sudden infant death syndrome	Female	7-27 days	28-364 days	Global	0	3	LDI (I$ per capita)	--
Sudden infant death syndrome	Female	7-27 days	28-364 days	Global	1	3	Total Fertility Rate	--
Sudden infant death syndrome	Male	7-27 days	28-364 days	Data Rich	-1	1	In-Facility Delivery (proportion)	40
Sudden infant death syndrome	Male	7-27 days	28-364 days	Data Rich	1	1	Tobacco (cigarettes per capita)	960
Sudden infant death syndrome	Male	7-27 days	28-364 days	Data Rich	-1	2	Healthcare access and quality index	368
Sudden infant death syndrome	Male	7-27 days	28-364 days	Data Rich	-1	2	Health System Access (capped)	--
Sudden infant death syndrome	Male	7-27 days	28-364 days	Data Rich	-1	2	Skilled Birth Attendance (proportion)	--
Sudden infant death syndrome	Male	7-27 days	28-364 days	Data Rich	1	2	Underweight (proportion <2SD weight for age, <5 years)	--
Sudden infant death syndrome	Male	7-27 days	28-364 days	Data Rich	-1	3	Education (years per capita)	433
Sudden infant death syndrome	Male	7-27 days	28-364 days	Data Rich	0	3	Socio-demographic Index	242
Sudden infant death syndrome	Male	7-27 days	28-364 days	Data Rich	0	3	LDI (I$ per capita)	--
Sudden infant death syndrome	Male	7-27 days	28-364 days	Data Rich	1	3	Total Fertility Rate	--
Sudden infant death syndrome	Male	7-27 days	28-364 days	Global	-1	1	In-Facility Delivery (proportion)	120
Sudden infant death syndrome	Male	7-27 days	28-364 days	Global	1	1	Tobacco (cigarettes per capita)	880
Sudden infant death syndrome	Male	7-27 days	28-364 days	Global	-1	2	Health System Access (capped)	352
Sudden infant death syndrome	Male	7-27 days	28-364 days	Global	-1	2	Healthcare access and quality index	352
Sudden infant death syndrome	Male	7-27 days	28-364 days	Global	-1	2	Skilled Birth Attendance (proportion)	--
Sudden infant death syndrome	Male	7-27 days	28-364 days	Global	1	2	Underweight (proportion <2SD weight for age, <5 years)	--
Sudden infant death syndrome	Male	7-27 days	28-364 days	Global	-1	3	Education (years per capita)	212
Sudden infant death syndrome	Male	7-27 days	28-364 days	Global	0	3	Socio-demographic Index	116
Sudden infant death syndrome	Male	7-27 days	28-364 days	Global	0	3	LDI (I$ per capita)	--
Sudden infant death syndrome	Male	7-27 days	28-364 days	Global	1	3	Total Fertility Rate	--
Transport injuries	Female	0-6 days	95+ years	Data Rich	1	1	Alcohol (liters per capita)	410
Transport injuries	Female	0-6 days	95+ years	Data Rich	1	1	Vehicles - 2 wheels fraction (proportion)	694
Transport injuries	Female	0-6 days	95+ years	Data Rich	1	1	Vehicles - 2+4 wheels (per capita)	--
Transport injuries	Female	0-6 days	95+ years	Data Rich	-1	2	Healthcare access and quality index	462
Transport injuries	Female	0-6 days	95+ years	Data Rich	0	2	Population Density (300-500 ppl/sqkm, proportion)	462
Transport injuries	Female	0-6 days	95+ years	Data Rich	0	2	Population Density (500-1000 ppl/sqkm, proportion)	462
Transport injuries	Female	0-6 days	95+ years	Data Rich	0	2	LDI (I$ per capita)	--
Transport injuries	Female	0-6 days	95+ years	Data Rich	-1	3	Education (years per capita)	149
Transport injuries	Female	0-6 days	95+ years	Data Rich	-1	3	Socio-demographic Index	342
Transport injuries	Female	0-6 days	95+ years	Data Rich	1	3	Rainfall Quintile 5 (proportion)	--
Transport injuries	Female	0-6 days	95+ years	Global	1	1	Alcohol (liters per capita)	671
Transport injuries	Female	0-6 days	95+ years	Global	1	1	Vehicles - 2 wheels fraction (proportion)	812
Transport injuries	Female	0-6 days	95+ years	Global	1	1	Vehicles - 2+4 wheels (per capita)	--
Transport injuries	Female	0-6 days	95+ years	Global	-1	2	Healthcare access and quality index	323
Transport injuries	Female	0-6 days	95+ years	Global	0	2	Population Density (300-500 ppl/sqkm, proportion)	527
Transport injuries	Female	0-6 days	95+ years	Global	0	2	Population Density (500-1000 ppl/sqkm, proportion)	531
Transport injuries	Female	0-6 days	95+ years	Global	0	2	LDI (I$ per capita)	--
Transport injuries	Female	0-6 days	95+ years	Global	-1	3	Socio-demographic Index	294
Transport injuries	Female	0-6 days	95+ years	Global	-1	3	Education (years per capita)	344
Transport injuries	Female	0-6 days	95+ years	Global	1	3	Rainfall Quintile 5 (proportion)	--
Transport injuries	Male	0-6 days	95+ years	Data Rich	1	1	Alcohol (liters per capita)	1000
Transport injuries	Male	0-6 days	95+ years	Data Rich	1	1	Vehicles - 2 wheels fraction (proportion)	--
Transport injuries	Male	0-6 days	95+ years	Data Rich	1	1	Vehicles - 2+4 wheels (per capita)	--
Transport injuries	Male	0-6 days	95+ years	Data Rich	-1	2	Healthcare access and quality index	466
Transport injuries	Male	0-6 days	95+ years	Data Rich	0	2	Population Density (500-1000 ppl/sqkm, proportion)	373
Transport injuries	Male	0-6 days	95+ years	Data Rich	0	2	Population Density (300-500 ppl/sqkm, proportion)	424
Transport injuries	Male	0-6 days	95+ years	Data Rich	0	2	LDI (I$ per capita)	--
Transport injuries	Male	0-6 days	95+ years	Data Rich	-1	3	Education (years per capita)	385
Transport injuries	Male	0-6 days	95+ years	Data Rich	-1	3	Socio-demographic Index	437
Transport injuries	Male	0-6 days	95+ years	Data Rich	1	3	Rainfall Quintile 5 (proportion)	--
Transport injuries	Male	0-6 days	95+ years	Global	1	1	Alcohol (liters per capita)	470
Transport injuries	Male	0-6 days	95+ years	Global	1	1	Vehicles - 2 wheels fraction (proportion)	834
Transport injuries	Male	0-6 days	95+ years	Global	1	1	Vehicles - 2+4 wheels (per capita)	--
Transport injuries	Male	0-6 days	95+ years	Global	-1	2	Healthcare access and quality index	535
Transport injuries	Male	0-6 days	95+ years	Global	0	2	Population Density (300-500 ppl/sqkm, proportion)	352
Transport injuries	Male	0-6 days	95+ years	Global	0	2	Population Density (500-1000 ppl/sqkm, proportion)	481
Transport injuries	Male	0-6 days	95+ years	Global	0	2	LDI (I$ per capita)	--
Transport injuries	Male	0-6 days	95+ years	Global	-1	3	Socio-demographic Index	309
Transport injuries	Male	0-6 days	95+ years	Global	-1	3	Education (years per capita)	338
Transport injuries	Male	0-6 days	95+ years	Global	1	3	Rainfall Quintile 5 (proportion)	--
Road injuries	Female	0-6 days	95+ years	Data Rich	1	1	Alcohol (liters per capita)	3
Road injuries	Female	0-6 days	95+ years	Data Rich	1	1	Log-transformed SEV scalar: Road Inj	366
Road injuries	Female	0-6 days	95+ years	Data Rich	1	1	Vehicles - 2 wheels fraction (proportion)	961
Road injuries	Female	0-6 days	95+ years	Data Rich	1	1	Vehicles - 2 wheels (per capita)	--
Road injuries	Female	0-6 days	95+ years	Data Rich	1	1	Vehicles - 2+4 wheels (per capita)	--
Road injuries	Female	0-6 days	95+ years	Data Rich	1	1	Vehicles - 4 wheels (per capita)	--
Road injuries	Female	0-6 days	95+ years	Data Rich	-1	2	Healthcare access and quality index	240
Road injuries	Female	0-6 days	95+ years	Data Rich	0	2	Population Density (500-1000 ppl/sqkm, proportion)	115
Road injuries	Female	0-6 days	95+ years	Data Rich	0	2	Population Density (300-500 ppl/sqkm, proportion)	240
Road injuries	Female	0-6 days	95+ years	Data Rich	1	2	Population 15 to 30 (proportion)	240

Appendix Table 9: CODEm covariates used, level of covariate, and expected direction of covariate by cause, sex, and age<br>*Covariates that CODEm did not select during the covariate selection process have no draw counts listed.*								
**Cause**	**Sex**	**Age Start**	**Age End**	**Model Type**	**Direction**	**Level**	**Covariate Name**	**Number of Draws**
Road injuries	Female	0-6 days	95+ years	Data Rich	-1	3	Education (years per capita)	0
Road injuries	Female	0-6 days	95+ years	Data Rich	-1	3	Socio-demographic Index	0
Road injuries	Female	0-6 days	95+ years	Data Rich	0	3	LDI (I$ per capita)	--
Road injuries	Female	0-6 days	95+ years	Global	1	1	Alcohol (liters per capita)	338
Road injuries	Female	0-6 days	95+ years	Global	1	1	Vehicles - 2 wheels fraction (proportion)	908
Road injuries	Female	0-6 days	95+ years	Global	1	1	Log-transformed SEV scalar: Road Inj	--
Road injuries	Female	0-6 days	95+ years	Global	1	1	Vehicles - 2 wheels (per capita)	--
Road injuries	Female	0-6 days	95+ years	Global	1	1	Vehicles - 2+4 wheels (per capita)	--
Road injuries	Female	0-6 days	95+ years	Global	1	1	Vehicles - 4 wheels (per capita)	--
Road injuries	Female	0-6 days	95+ years	Global	-1	2	Healthcare access and quality index	500
Road injuries	Female	0-6 days	95+ years	Global	0	2	Population Density (300-500 ppl/sqkm, proportion)	534
Road injuries	Female	0-6 days	95+ years	Global	0	2	Population Density (500-1000 ppl/sqkm, proportion)	534
Road injuries	Female	0-6 days	95+ years	Global	1	2	Population 15 to 30 (proportion)	71
Road injuries	Female	0-6 days	95+ years	Global	-1	3	Socio-demographic Index	184
Road injuries	Female	0-6 days	95+ years	Global	-1	3	Education (years per capita)	242
Road injuries	Female	0-6 days	95+ years	Global	0	3	LDI (I$ per capita)	--
Road injuries	Male	0-6 days	95+ years	Data Rich	1	1	Alcohol (liters per capita)	519
Road injuries	Male	0-6 days	95+ years	Data Rich	1	1	Vehicles - 2 wheels fraction (proportion)	866
Road injuries	Male	0-6 days	95+ years	Data Rich	1	1	Log-transformed SEV scalar: Road Inj	--
Road injuries	Male	0-6 days	95+ years	Data Rich	1	1	Vehicles - 2 wheels (per capita)	--
Road injuries	Male	0-6 days	95+ years	Data Rich	1	1	Vehicles - 2+4 wheels (per capita)	--
Road injuries	Male	0-6 days	95+ years	Data Rich	1	1	Vehicles - 4 wheels (per capita)	--
Road injuries	Male	0-6 days	95+ years	Data Rich	-1	2	Healthcare access and quality index	726
Road injuries	Male	0-6 days	95+ years	Data Rich	0	2	Population Density (500-1000 ppl/sqkm, proportion)	68
Road injuries	Male	0-6 days	95+ years	Data Rich	0	2	Population Density (300-500 ppl/sqkm, proportion)	326
Road injuries	Male	0-6 days	95+ years	Data Rich	1	2	Population 15 to 30 (proportion)	382
Road injuries	Male	0-6 days	95+ years	Data Rich	-1	3	Socio-demographic Index	1
Road injuries	Male	0-6 days	95+ years	Data Rich	-1	3	Education (years per capita)	361
Road injuries	Male	0-6 days	95+ years	Data Rich	0	3	LDI (I$ per capita)	--
Road injuries	Male	0-6 days	95+ years	Global	1	1	Vehicles - 2 wheels fraction (proportion)	398
Road injuries	Male	0-6 days	95+ years	Global	1	1	Alcohol (liters per capita)	963
Road injuries	Male	0-6 days	95+ years	Global	1	1	Log-transformed SEV scalar: Road Inj	--
Road injuries	Male	0-6 days	95+ years	Global	1	1	Vehicles - 2 wheels (per capita)	--
Road injuries	Male	0-6 days	95+ years	Global	1	1	Vehicles - 2+4 wheels (per capita)	--
Road injuries	Male	0-6 days	95+ years	Global	1	1	Vehicles - 4 wheels (per capita)	--
Road injuries	Male	0-6 days	95+ years	Global	-1	2	Healthcare access and quality index	433
Road injuries	Male	0-6 days	95+ years	Global	0	2	Population Density (300-500 ppl/sqkm, proportion)	549
Road injuries	Male	0-6 days	95+ years	Global	0	2	Population Density (500-1000 ppl/sqkm, proportion)	615
Road injuries	Male	0-6 days	95+ years	Global	1	2	Population 15 to 30 (proportion)	397
Road injuries	Male	0-6 days	95+ years	Global	-1	3	Education (years per capita)	328
Road injuries	Male	0-6 days	95+ years	Global	-1	3	Socio-demographic Index	343
Road injuries	Male	0-6 days	95+ years	Global	0	3	LDI (I$ per capita)	--
Pedestrian road injuries	Female	0-6 days	95+ years	Data Rich	1	1	Vehicles - 2 wheels fraction (proportion)	246
Pedestrian road injuries	Female	0-6 days	95+ years	Data Rich	1	1	Alcohol (liters per capita)	298
Pedestrian road injuries	Female	0-6 days	95+ years	Data Rich	1	1	Log-transformed SEV scalar: Pedest	702
Pedestrian road injuries	Female	0-6 days	95+ years	Data Rich	1	1	Vehicles - 2+4 wheels (per capita)	--
Pedestrian road injuries	Female	0-6 days	95+ years	Data Rich	-1	2	Healthcare access and quality index	10
Pedestrian road injuries	Female	0-6 days	95+ years	Data Rich	0	2	Population Density (300-500 ppl/sqkm, proportion)	10
Pedestrian road injuries	Female	0-6 days	95+ years	Data Rich	0	2	Population Density (500-1000 ppl/sqkm, proportion)	10
Pedestrian road injuries	Female	0-6 days	95+ years	Data Rich	0	2	LDI (I$ per capita)	--
Pedestrian road injuries	Female	0-6 days	95+ years	Data Rich	-1	3	Socio-demographic Index	24
Pedestrian road injuries	Female	0-6 days	95+ years	Data Rich	-1	3	Education (years per capita)	976
Pedestrian road injuries	Female	0-6 days	95+ years	Data Rich	1	3	Rainfall Quintile 5 (proportion)	--
Pedestrian road injuries	Female	0-6 days	95+ years	Global	1	1	Log-transformed SEV scalar: Pedest	166
Pedestrian road injuries	Female	0-6 days	95+ years	Global	1	1	Vehicles - 2 wheels fraction (proportion)	479
Pedestrian road injuries	Female	0-6 days	95+ years	Global	1	1	Alcohol (liters per capita)	656
Pedestrian road injuries	Female	0-6 days	95+ years	Global	1	1	Vehicles - 2+4 wheels (per capita)	--
Pedestrian road injuries	Female	0-6 days	95+ years	Global	-1	2	Healthcare access and quality index	139
Pedestrian road injuries	Female	0-6 days	95+ years	Global	0	2	Population Density (300-500 ppl/sqkm, proportion)	298
Pedestrian road injuries	Female	0-6 days	95+ years	Global	0	2	Population Density (500-1000 ppl/sqkm, proportion)	440
Pedestrian road injuries	Female	0-6 days	95+ years	Global	0	2	LDI (I$ per capita)	--
Pedestrian road injuries	Female	0-6 days	95+ years	Global	-1	3	Socio-demographic Index	120
Pedestrian road injuries	Female	0-6 days	95+ years	Global	-1	3	Education (years per capita)	694
Pedestrian road injuries	Female	0-6 days	95+ years	Global	1	3	Rainfall Quintile 5 (proportion)	3
Pedestrian road injuries	Male	0-6 days	95+ years	Data Rich	1	1	Alcohol (liters per capita)	38
Pedestrian road injuries	Male	0-6 days	95+ years	Data Rich	1	1	Log-transformed SEV scalar: Pedest	962
Pedestrian road injuries	Male	0-6 days	95+ years	Data Rich	1	1	Vehicles - 2 wheels fraction (proportion)	--
Pedestrian road injuries	Male	0-6 days	95+ years	Data Rich	1	1	Vehicles - 2+4 wheels (per capita)	--
Pedestrian road injuries	Male	0-6 days	95+ years	Data Rich	-1	2	Healthcare access and quality index	7
Pedestrian road injuries	Male	0-6 days	95+ years	Data Rich	0	2	Population Density (500-1000 ppl/sqkm, proportion)	0
Pedestrian road injuries	Male	0-6 days	95+ years	Data Rich	0	2	Population Density (300-500 ppl/sqkm, proportion)	808
Pedestrian road injuries	Male	0-6 days	95+ years	Data Rich	0	2	LDI (I$ per capita)	--
Pedestrian road injuries	Male	0-6 days	95+ years	Data Rich	-1	3	Socio-demographic Index	834
Pedestrian road injuries	Male	0-6 days	95+ years	Data Rich	-1	3	Education (years per capita)	999
Pedestrian road injuries	Male	0-6 days	95+ years	Data Rich	1	3	Rainfall Quintile 5 (proportion)	--
Pedestrian road injuries	Male	0-6 days	95+ years	Global	1	1	Vehicles - 2 wheels fraction (proportion)	465
Pedestrian road injuries	Male	0-6 days	95+ years	Global	1	1	Alcohol (liters per capita)	928
Pedestrian road injuries	Male	0-6 days	95+ years	Global	1	1	Log-transformed SEV scalar: Pedest	--

Appendix Table 9: CODEm covariates used, level of covariate, and expected direction of covariate by cause, sex, and age

Covariates that CODEm did not select during the covariate selection process have no draw counts listed.

Cause	Sex	Age Start	Age End	Model Type	Direction	Level	Covariate Name	Number of Draws
Pedestrian road injuries	Male	0-6 days	95+ years	Global	1	1	Vehicles - 2+4 wheels (per capita)	--
Pedestrian road injuries	Male	0-6 days	95+ years	Global	-1	2	Healthcare access and quality index	578
Pedestrian road injuries	Male	0-6 days	95+ years	Global	0	2	Population Density (500-1000 ppl/sqkm, proportion)	425
Pedestrian road injuries	Male	0-6 days	95+ years	Global	0	2	Population Density (300-500 ppl/sqkm, proportion)	476
Pedestrian road injuries	Male	0-6 days	95+ years	Global	0	2	LDI (I$ per capita)	--
Pedestrian road injuries	Male	0-6 days	95+ years	Global	-1	3	Socio-demographic Index	384
Pedestrian road injuries	Male	0-6 days	95+ years	Global	-1	3	Education (years per capita)	525
Pedestrian road injuries	Male	0-6 days	95+ years	Global	1	3	Rainfall Quintile 5 (proportion)	--
Cyclist road injuries	Female	1-4 years	95+ years	Data Rich	1	1	Log-transformed SEV scalar: Cyclist	57
Cyclist road injuries	Female	1-4 years	95+ years	Data Rich	1	1	Vehicles - 2+4 wheels (per capita)	251
Cyclist road injuries	Female	1-4 years	95+ years	Data Rich	1	1	Vehicles - 2 wheels fraction (proportion)	385
Cyclist road injuries	Female	1-4 years	95+ years	Data Rich	1	1	Alcohol (liters per capita)	773
Cyclist road injuries	Female	1-4 years	95+ years	Data Rich	-1	2	Healthcare access and quality index	0
Cyclist road injuries	Female	1-4 years	95+ years	Data Rich	0	2	Population Density (300-500 ppl/sqkm, proportion)	179
Cyclist road injuries	Female	1-4 years	95+ years	Data Rich	0	2	Population Density (500-1000 ppl/sqkm, proportion)	179
Cyclist road injuries	Female	1-4 years	95+ years	Data Rich	0	2	LDI (I$ per capita)	--
Cyclist road injuries	Female	1-4 years	95+ years	Data Rich	-1	3	Education (years per capita)	0
Cyclist road injuries	Female	1-4 years	95+ years	Data Rich	0	3	Socio-demographic Index	714
Cyclist road injuries	Female	1-4 years	95+ years	Global	1	1	Vehicles - 2+4 wheels (per capita)	267
Cyclist road injuries	Female	1-4 years	95+ years	Global	1	1	Alcohol (liters per capita)	684
Cyclist road injuries	Female	1-4 years	95+ years	Global	1	1	Vehicles - 2 wheels fraction (proportion)	745
Cyclist road injuries	Female	1-4 years	95+ years	Global	1	1	Log-transformed SEV scalar: Cyclist	--
Cyclist road injuries	Female	1-4 years	95+ years	Global	-1	2	Healthcare access and quality index	74
Cyclist road injuries	Female	1-4 years	95+ years	Global	0	2	Population Density (500-1000 ppl/sqkm, proportion)	376
Cyclist road injuries	Female	1-4 years	95+ years	Global	0	2	Population Density (300-500 ppl/sqkm, proportion)	468
Cyclist road injuries	Female	1-4 years	95+ years	Global	0	2	LDI (I$ per capita)	--
Cyclist road injuries	Female	1-4 years	95+ years	Global	-1	3	Education (years per capita)	42
Cyclist road injuries	Female	1-4 years	95+ years	Global	0	3	Socio-demographic Index	475
Cyclist road injuries	Male	1-4 years	95+ years	Data Rich	1	1	Vehicles - 2+4 wheels (per capita)	0
Cyclist road injuries	Male	1-4 years	95+ years	Data Rich	1	1	Vehicles - 2 wheels fraction (proportion)	191
Cyclist road injuries	Male	1-4 years	95+ years	Data Rich	1	1	Alcohol (liters per capita)	1000
Cyclist road injuries	Male	1-4 years	95+ years	Data Rich	1	1	Log-transformed SEV scalar: Cyclist	--
Cyclist road injuries	Male	1-4 years	95+ years	Data Rich	-1	2	Healthcare access and quality index	0
Cyclist road injuries	Male	1-4 years	95+ years	Data Rich	0	2	Population Density (300-500 ppl/sqkm, proportion)	803
Cyclist road injuries	Male	1-4 years	95+ years	Data Rich	0	2	Population Density (500-1000 ppl/sqkm, proportion)	962
Cyclist road injuries	Male	1-4 years	95+ years	Data Rich	0	2	LDI (I$ per capita)	--
Cyclist road injuries	Male	1-4 years	95+ years	Data Rich	-1	3	Education (years per capita)	0
Cyclist road injuries	Male	1-4 years	95+ years	Data Rich	0	3	Socio-demographic Index	999
Cyclist road injuries	Male	1-4 years	95+ years	Global	1	1	Vehicles - 2+4 wheels (per capita)	246
Cyclist road injuries	Male	1-4 years	95+ years	Global	1	1	Vehicles - 2 wheels fraction (proportion)	575
Cyclist road injuries	Male	1-4 years	95+ years	Global	1	1	Alcohol (liters per capita)	682
Cyclist road injuries	Male	1-4 years	95+ years	Global	1	1	Log-transformed SEV scalar: Cyclist	--
Cyclist road injuries	Male	1-4 years	95+ years	Global	-1	2	Healthcare access and quality index	110
Cyclist road injuries	Male	1-4 years	95+ years	Global	0	2	Population Density (500-1000 ppl/sqkm, proportion)	524
Cyclist road injuries	Male	1-4 years	95+ years	Global	0	2	Population Density (300-500 ppl/sqkm, proportion)	559
Cyclist road injuries	Male	1-4 years	95+ years	Global	0	2	LDI (I$ per capita)	--
Cyclist road injuries	Male	1-4 years	95+ years	Global	-1	3	Education (years per capita)	32
Cyclist road injuries	Male	1-4 years	95+ years	Global	0	3	Socio-demographic Index	438
Motorcyclist road injuries	Female	0-6 days	95+ years	Data Rich	1	1	Alcohol (liters per capita)	1000
Motorcyclist road injuries	Female	0-6 days	95+ years	Data Rich	1	1	Log-transformed SEV scalar: Mot Cyc	--
Motorcyclist road injuries	Female	0-6 days	95+ years	Data Rich	1	1	Vehicles - 2 wheels (per capita)	--
Motorcyclist road injuries	Female	0-6 days	95+ years	Data Rich	-1	2	Healthcare access and quality index	8
Motorcyclist road injuries	Female	0-6 days	95+ years	Data Rich	0	2	Population Density (300-500 ppl/sqkm, proportion)	344
Motorcyclist road injuries	Female	0-6 days	95+ years	Data Rich	0	2	Population Density (500-1000 ppl/sqkm, proportion)	851
Motorcyclist road injuries	Female	0-6 days	95+ years	Data Rich	0	2	LDI (I$ per capita)	--
Motorcyclist road injuries	Female	0-6 days	95+ years	Data Rich	-1	3	Education (years per capita)	998
Motorcyclist road injuries	Female	0-6 days	95+ years	Data Rich	0	3	Socio-demographic Index	17
Motorcyclist road injuries	Female	0-6 days	95+ years	Data Rich	1	3	Rainfall Quintile 5 (proportion)	--
Motorcyclist road injuries	Female	0-6 days	95+ years	Global	1	1	Alcohol (liters per capita)	426
Motorcyclist road injuries	Female	0-6 days	95+ years	Global	1	1	Log-transformed SEV scalar: Mot Cyc	--
Motorcyclist road injuries	Female	0-6 days	95+ years	Global	1	1	Vehicles - 2 wheels (per capita)	--
Motorcyclist road injuries	Female	0-6 days	95+ years	Global	-1	2	Healthcare access and quality index	486
Motorcyclist road injuries	Female	0-6 days	95+ years	Global	0	2	Population Density (500-1000 ppl/sqkm, proportion)	288
Motorcyclist road injuries	Female	0-6 days	95+ years	Global	0	2	Population Density (300-500 ppl/sqkm, proportion)	450
Motorcyclist road injuries	Female	0-6 days	95+ years	Global	0	2	LDI (I$ per capita)	--
Motorcyclist road injuries	Female	0-6 days	95+ years	Global	-1	3	Education (years per capita)	596
Motorcyclist road injuries	Female	0-6 days	95+ years	Global	0	3	Socio-demographic Index	297
Motorcyclist road injuries	Female	0-6 days	95+ years	Global	1	3	Rainfall Quintile 5 (proportion)	--
Motorcyclist road injuries	Male	0-6 days	95+ years	Data Rich	1	1	Alcohol (liters per capita)	1000
Motorcyclist road injuries	Male	0-6 days	95+ years	Data Rich	1	1	Log-transformed SEV scalar: Mot Cyc	--
Motorcyclist road injuries	Male	0-6 days	95+ years	Data Rich	1	1	Vehicles - 2 wheels (per capita)	--
Motorcyclist road injuries	Male	0-6 days	95+ years	Data Rich	-1	2	Healthcare access and quality index	1
Motorcyclist road injuries	Male	0-6 days	95+ years	Data Rich	0	2	Population Density (300-500 ppl/sqkm, proportion)	0
Motorcyclist road injuries	Male	0-6 days	95+ years	Data Rich	0	2	Population Density (500-1000 ppl/sqkm, proportion)	161
Motorcyclist road injuries	Male	0-6 days	95+ years	Data Rich	0	2	LDI (I$ per capita)	--
Motorcyclist road injuries	Male	0-6 days	95+ years	Data Rich	-1	3	Education (years per capita)	32
Motorcyclist road injuries	Male	0-6 days	95+ years	Data Rich	0	3	Socio-demographic Index	801
Motorcyclist road injuries	Male	0-6 days	95+ years	Data Rich	1	3	Rainfall Quintile 5 (proportion)	--

Appendix Table 9: CODEm covariates used, level of covariate, and expected direction of covariate by cause, sex, and age
Covariates that CODEm did not select during the covariate selection process have no draw counts listed.

Cause	Sex	Age Start	Age End	Model Type	Direction	Level	Covariate Name	Number of Draws
Motorcyclist road injuries	Male	0-6 days	95+ years	Global	1	1	Alcohol (liters per capita)	1000
Motorcyclist road injuries	Male	0-6 days	95+ years	Global	1	1	Log-transformed SEV scalar: Mot Cyc	--
Motorcyclist road injuries	Male	0-6 days	95+ years	Global	1	1	Vehicles - 2 wheels (per capita)	--
Motorcyclist road injuries	Male	0-6 days	95+ years	Global	-1	2	Healthcare access and quality index	266
Motorcyclist road injuries	Male	0-6 days	95+ years	Global	0	2	Population Density (300-500 ppl/sqkm, proportion)	615
Motorcyclist road injuries	Male	0-6 days	95+ years	Global	0	2	Population Density (500-1000 ppl/sqkm, proportion)	615
Motorcyclist road injuries	Male	0-6 days	95+ years	Global	0	2	LDI (I$ per capita)	--
Motorcyclist road injuries	Male	0-6 days	95+ years	Global	-1	3	Education (years per capita)	328
Motorcyclist road injuries	Male	0-6 days	95+ years	Global	0	3	Socio-demographic Index	180
Motorcyclist road injuries	Male	0-6 days	95+ years	Global	1	3	Rainfall Quintile 5 (proportion)	--
Motor vehicle road injuries	Female	0-6 days	95+ years	Data Rich	1	1	Alcohol (liters per capita)	1000
Motor vehicle road injuries	Female	0-6 days	95+ years	Data Rich	1	1	Log-transformed SEV scalar: Mot Veh	--
Motor vehicle road injuries	Female	0-6 days	95+ years	Data Rich	1	1	Vehicles - 4 wheels (per capita)	--
Motor vehicle road injuries	Female	0-6 days	95+ years	Data Rich	-1	2	Healthcare access and quality index	57
Motor vehicle road injuries	Female	0-6 days	95+ years	Data Rich	0	2	Population Density (300-500 ppl/sqkm, proportion)	471
Motor vehicle road injuries	Female	0-6 days	95+ years	Data Rich	0	2	Population Density (500-1000 ppl/sqkm, proportion)	471
Motor vehicle road injuries	Female	0-6 days	95+ years	Data Rich	0	3	Education (years per capita)	609
Motor vehicle road injuries	Female	0-6 days	95+ years	Data Rich	0	3	Socio-demographic Index	762
Motor vehicle road injuries	Female	0-6 days	95+ years	Data Rich	0	3	LDI (I$ per capita)	--
Motor vehicle road injuries	Female	0-6 days	95+ years	Data Rich	1	3	Rainfall Quintile 5 (proportion)	--
Motor vehicle road injuries	Female	0-6 days	95+ years	Global	1	1	Alcohol (liters per capita)	1000
Motor vehicle road injuries	Female	0-6 days	95+ years	Global	1	1	Log-transformed SEV scalar: Mot Veh	--
Motor vehicle road injuries	Female	0-6 days	95+ years	Global	1	1	Vehicles - 4 wheels (per capita)	--
Motor vehicle road injuries	Female	0-6 days	95+ years	Global	-1	2	Healthcare access and quality index	199
Motor vehicle road injuries	Female	0-6 days	95+ years	Global	0	2	Population Density (300-500 ppl/sqkm, proportion)	665
Motor vehicle road injuries	Female	0-6 days	95+ years	Global	0	2	Population Density (500-1000 ppl/sqkm, proportion)	665
Motor vehicle road injuries	Female	0-6 days	95+ years	Global	0	3	Socio-demographic Index	399
Motor vehicle road injuries	Female	0-6 days	95+ years	Global	0	3	Education (years per capita)	477
Motor vehicle road injuries	Female	0-6 days	95+ years	Global	0	3	LDI (I$ per capita)	--
Motor vehicle road injuries	Female	0-6 days	95+ years	Global	1	3	Rainfall Quintile 5 (proportion)	--
Motor vehicle road injuries	Male	0-6 days	95+ years	Data Rich	1	1	Alcohol (liters per capita)	766
Motor vehicle road injuries	Male	0-6 days	95+ years	Data Rich	1	1	Log-transformed SEV scalar: Mot Veh	--
Motor vehicle road injuries	Male	0-6 days	95+ years	Data Rich	1	1	Vehicles - 4 wheels (per capita)	--
Motor vehicle road injuries	Male	0-6 days	95+ years	Data Rich	-1	2	Healthcare access and quality index	219
Motor vehicle road injuries	Male	0-6 days	95+ years	Data Rich	0	2	Population Density (300-500 ppl/sqkm, proportion)	--
Motor vehicle road injuries	Male	0-6 days	95+ years	Data Rich	0	2	Population Density (500-1000 ppl/sqkm, proportion)	--
Motor vehicle road injuries	Male	0-6 days	95+ years	Data Rich	0	3	Education (years per capita)	304
Motor vehicle road injuries	Male	0-6 days	95+ years	Data Rich	0	3	Socio-demographic Index	382
Motor vehicle road injuries	Male	0-6 days	95+ years	Data Rich	0	3	LDI (I$ per capita)	--
Motor vehicle road injuries	Male	0-6 days	95+ years	Data Rich	1	3	Rainfall Quintile 5 (proportion)	--
Motor vehicle road injuries	Male	0-6 days	95+ years	Global	1	1	Alcohol (liters per capita)	353
Motor vehicle road injuries	Male	0-6 days	95+ years	Global	1	1	Log-transformed SEV scalar: Mot Veh	--
Motor vehicle road injuries	Male	0-6 days	95+ years	Global	1	1	Vehicles - 4 wheels (per capita)	--
Motor vehicle road injuries	Male	0-6 days	95+ years	Global	-1	2	Healthcare access and quality index	242
Motor vehicle road injuries	Male	0-6 days	95+ years	Global	0	2	Population Density (300-500 ppl/sqkm, proportion)	770
Motor vehicle road injuries	Male	0-6 days	95+ years	Global	0	2	Population Density (500-1000 ppl/sqkm, proportion)	770
Motor vehicle road injuries	Male	0-6 days	95+ years	Global	0	3	Education (years per capita)	432
Motor vehicle road injuries	Male	0-6 days	95+ years	Global	0	3	Socio-demographic Index	571
Motor vehicle road injuries	Male	0-6 days	95+ years	Global	0	3	LDI (I$ per capita)	--
Motor vehicle road injuries	Male	0-6 days	95+ years	Global	1	3	Rainfall Quintile 5 (proportion)	--
Other road injuries	Female	0-6 days	95+ years	Data Rich	1	1	Vehicles - 2 wheels fraction (proportion)	1000
Other road injuries	Female	0-6 days	95+ years	Data Rich	1	1	Alcohol (liters per capita)	--
Other road injuries	Female	0-6 days	95+ years	Data Rich	1	1	Log-transformed SEV scalar: Oth Road	--
Other road injuries	Female	0-6 days	95+ years	Data Rich	1	1	Vehicles - 2+4 wheels (per capita)	--
Other road injuries	Female	0-6 days	95+ years	Data Rich	-1	2	Healthcare access and quality index	1
Other road injuries	Female	0-6 days	95+ years	Data Rich	0	2	LDI (I$ per capita)	--
Other road injuries	Female	0-6 days	95+ years	Data Rich	-1	3	Socio-demographic Index	166
Other road injuries	Female	0-6 days	95+ years	Data Rich	1	3	Rainfall Quintile 5 (proportion)	--
Other road injuries	Female	0-6 days	95+ years	Global	1	1	Vehicles - 2 wheels fraction (proportion)	1000
Other road injuries	Female	0-6 days	95+ years	Global	1	1	Alcohol (liters per capita)	--
Other road injuries	Female	0-6 days	95+ years	Global	1	1	Log-transformed SEV scalar: Oth Road	--
Other road injuries	Female	0-6 days	95+ years	Global	1	1	Vehicles - 2+4 wheels (per capita)	--
Other road injuries	Female	0-6 days	95+ years	Global	-1	2	Healthcare access and quality index	161
Other road injuries	Female	0-6 days	95+ years	Global	0	2	LDI (I$ per capita)	--
Other road injuries	Female	0-6 days	95+ years	Global	-1	3	Socio-demographic Index	193
Other road injuries	Female	0-6 days	95+ years	Global	1	3	Rainfall Quintile 5 (proportion)	--
Other road injuries	Male	0-6 days	95+ years	Data Rich	1	1	Vehicles - 2 wheels fraction (proportion)	6
Other road injuries	Male	0-6 days	95+ years	Data Rich	1	1	Alcohol (liters per capita)	--
Other road injuries	Male	0-6 days	95+ years	Data Rich	1	1	Log-transformed SEV scalar: Oth Road	--
Other road injuries	Male	0-6 days	95+ years	Data Rich	1	1	Vehicles - 2+4 wheels (per capita)	--
Other road injuries	Male	0-6 days	95+ years	Data Rich	-1	2	Healthcare access and quality index	1
Other road injuries	Male	0-6 days	95+ years	Data Rich	0	2	LDI (I$ per capita)	--
Other road injuries	Male	0-6 days	95+ years	Data Rich	-1	3	Socio-demographic Index	967
Other road injuries	Male	0-6 days	95+ years	Data Rich	1	3	Rainfall Quintile 5 (proportion)	--
Other road injuries	Male	0-6 days	95+ years	Global	1	1	Vehicles - 2 wheels fraction (proportion)	294
Other road injuries	Male	0-6 days	95+ years	Global	1	1	Alcohol (liters per capita)	--
Other road injuries	Male	0-6 days	95+ years	Global	1	1	Log-transformed SEV scalar: Oth Road	--
Other road injuries	Male	0-6 days	95+ years	Global	1	1	Vehicles - 2+4 wheels (per capita)	--

Appendix Table 9: CODEm covariates used, level of covariate, and expected direction of covariate by cause, sex, and age

Covariates that CODEm did not select during the covariate selection process have no draw counts listed.

Cause	Sex	Age Start	Age End	Model Type	Direction	Level	Covariate Name	Number of Draws
Other road injuries	Male	0-6 days	95+ years	Global	-1	2	Healthcare access and quality index	--
Other road injuries	Male	0-6 days	95+ years	Global	0	2	LDI (I$ per capita)	--
Other road injuries	Male	0-6 days	95+ years	Global	-1	3	Socio-demographic Index	674
Other road injuries	Male	0-6 days	95+ years	Global	1	3	Rainfall Quintile 5 (proportion)	--
Other transport injuries	Female	0-6 days	95+ years	Data Rich	1	1	Vehicles - 2 wheels fraction (proportion)	4
Other transport injuries	Female	0-6 days	95+ years	Data Rich	1	1	Alcohol (liters per capita)	702
Other transport injuries	Female	0-6 days	95+ years	Data Rich	1	1	Log-transformed SEV scalar: Oth Trans	766
Other transport injuries	Female	0-6 days	95+ years	Data Rich	1	1	Vehicles - 2+4 wheels (per capita)	--
Other transport injuries	Female	0-6 days	95+ years	Data Rich	-1	2	Healthcare access and quality index	4
Other transport injuries	Female	0-6 days	95+ years	Data Rich	0	2	Population Density (300-500 ppl/sqkm, proportion)	233
Other transport injuries	Female	0-6 days	95+ years	Data Rich	0	2	Population Density (500-1000 ppl/sqkm, proportion)	234
Other transport injuries	Female	0-6 days	95+ years	Data Rich	0	3	Education (years per capita)	36
Other transport injuries	Female	0-6 days	95+ years	Data Rich	0	3	Socio-demographic Index	62
Other transport injuries	Female	0-6 days	95+ years	Data Rich	0	3	LDI (I$ per capita)	--
Other transport injuries	Female	0-6 days	95+ years	Data Rich	1	3	Rainfall Quintile 5 (proportion)	--
Other transport injuries	Female	0-6 days	95+ years	Global	1	1	Log-transformed SEV scalar: Oth Trans	204
Other transport injuries	Female	0-6 days	95+ years	Global	1	1	Vehicles - 2 wheels fraction (proportion)	436
Other transport injuries	Female	0-6 days	95+ years	Global	1	1	Alcohol (liters per capita)	702
Other transport injuries	Female	0-6 days	95+ years	Global	1	1	Vehicles - 2+4 wheels (per capita)	--
Other transport injuries	Female	0-6 days	95+ years	Global	-1	2	Healthcare access and quality index	1
Other transport injuries	Female	0-6 days	95+ years	Global	0	2	Population Density (300-500 ppl/sqkm, proportion)	481
Other transport injuries	Female	0-6 days	95+ years	Global	0	2	Population Density (500-1000 ppl/sqkm, proportion)	481
Other transport injuries	Female	0-6 days	95+ years	Global	0	3	Education (years per capita)	136
Other transport injuries	Female	0-6 days	95+ years	Global	0	3	Socio-demographic Index	150
Other transport injuries	Female	0-6 days	95+ years	Global	0	3	LDI (I$ per capita)	--
Other transport injuries	Female	0-6 days	95+ years	Global	1	3	Rainfall Quintile 5 (proportion)	--
Other transport injuries	Male	0-6 days	95+ years	Data Rich	1	1	Log-transformed SEV scalar: Oth Trans	418
Other transport injuries	Male	0-6 days	95+ years	Data Rich	1	1	Alcohol (liters per capita)	564
Other transport injuries	Male	0-6 days	95+ years	Data Rich	1	1	Vehicles - 2 wheels fraction (proportion)	999
Other transport injuries	Male	0-6 days	95+ years	Data Rich	1	1	Vehicles - 2+4 wheels (per capita)	--
Other transport injuries	Male	0-6 days	95+ years	Data Rich	-1	2	Healthcare access and quality index	11
Other transport injuries	Male	0-6 days	95+ years	Data Rich	0	2	Population Density (300-500 ppl/sqkm, proportion)	414
Other transport injuries	Male	0-6 days	95+ years	Data Rich	0	2	Population Density (500-1000 ppl/sqkm, proportion)	586
Other transport injuries	Male	0-6 days	95+ years	Data Rich	0	3	Socio-demographic Index	70
Other transport injuries	Male	0-6 days	95+ years	Data Rich	0	3	Education (years per capita)	178
Other transport injuries	Male	0-6 days	95+ years	Data Rich	1	3	LDI (I$ per capita)	--
Other transport injuries	Male	0-6 days	95+ years	Data Rich	1	3	Rainfall Quintile 5 (proportion)	--
Other transport injuries	Male	0-6 days	95+ years	Global	1	1	Alcohol (liters per capita)	97
Other transport injuries	Male	0-6 days	95+ years	Global	1	1	Log-transformed SEV scalar: Oth Trans	507
Other transport injuries	Male	0-6 days	95+ years	Global	1	1	Vehicles - 2 wheels fraction (proportion)	938
Other transport injuries	Male	0-6 days	95+ years	Global	1	1	Vehicles - 2+4 wheels (per capita)	--
Other transport injuries	Male	0-6 days	95+ years	Global	-1	2	Healthcare access and quality index	103
Other transport injuries	Male	0-6 days	95+ years	Global	0	2	Population Density (300-500 ppl/sqkm, proportion)	507
Other transport injuries	Male	0-6 days	95+ years	Global	0	2	Population Density (500-1000 ppl/sqkm, proportion)	507
Other transport injuries	Male	0-6 days	95+ years	Global	0	3	Socio-demographic Index	376
Other transport injuries	Male	0-6 days	95+ years	Global	0	3	Education (years per capita)	486
Other transport injuries	Male	0-6 days	95+ years	Global	1	3	LDI (I$ per capita)	--
Other transport injuries	Male	0-6 days	95+ years	Global	1	3	Rainfall Quintile 5 (proportion)	--
Unintentional injuries	Female	28-364 days	95+ years	Data Rich	-1	1	Health System Access 2 (unitless)	650
Unintentional injuries	Female	28-364 days	95+ years	Data Rich	1	1	Cumulative Cigarettes (5 Years)	29
Unintentional injuries	Female	28-364 days	95+ years	Data Rich	1	1	Smoking Prevalence	162
Unintentional injuries	Female	28-364 days	95+ years	Data Rich	1	1	Indoor Air Pollution (All Cooking Fuels)	179
Unintentional injuries	Female	28-364 days	95+ years	Data Rich	1	1	Diabetes Fasting Plasma Glucose (mmol/L)	589
Unintentional injuries	Female	28-364 days	95+ years	Data Rich	1	1	Underweight (proportion <2SD weight for age, <5 years)	--
Unintentional injuries	Female	28-364 days	95+ years	Data Rich	1	2	Population Density (500-1000 ppl/sqkm, proportion)	569
Unintentional injuries	Female	28-364 days	95+ years	Data Rich	1	2	Alcohol (liters per capita)	583
Unintentional injuries	Female	28-364 days	95+ years	Data Rich	1	2	Population Density (over 1000 ppl/sqkm, proportion)	--
Unintentional injuries	Female	28-364 days	95+ years	Data Rich	-1	3	Education (years per capita)	51
Unintentional injuries	Female	28-364 days	95+ years	Data Rich	-1	3	LDI (I$ per capita)	--
Unintentional injuries	Female	28-364 days	95+ years	Global	-1	1	Health System Access 2 (unitless)	216
Unintentional injuries	Female	28-364 days	95+ years	Global	1	1	Smoking Prevalence	23
Unintentional injuries	Female	28-364 days	95+ years	Global	1	1	Cumulative Cigarettes (5 Years)	26
Unintentional injuries	Female	28-364 days	95+ years	Global	1	1	Indoor Air Pollution (All Cooking Fuels)	32
Unintentional injuries	Female	28-364 days	95+ years	Global	1	1	Diabetes Fasting Plasma Glucose (mmol/L)	746
Unintentional injuries	Female	28-364 days	95+ years	Global	1	1	Underweight (proportion <2SD weight for age, <5 years)	--
Unintentional injuries	Female	28-364 days	95+ years	Global	1	2	Alcohol (liters per capita)	509
Unintentional injuries	Female	28-364 days	95+ years	Global	1	2	Population Density (500-1000 ppl/sqkm, proportion)	565
Unintentional injuries	Female	28-364 days	95+ years	Global	1	2	Population Density (over 1000 ppl/sqkm, proportion)	--
Unintentional injuries	Female	28-364 days	95+ years	Global	-1	3	Education (years per capita)	57
Unintentional injuries	Female	28-364 days	95+ years	Global	-1	3	LDI (I$ per capita)	--
Unintentional injuries	Male	28-364 days	95+ years	Data Rich	-1	1	Health System Access 2 (unitless)	703
Unintentional injuries	Male	28-364 days	95+ years	Data Rich	1	1	Smoking Prevalence	3
Unintentional injuries	Male	28-364 days	95+ years	Data Rich	1	1	Indoor Air Pollution (All Cooking Fuels)	589
Unintentional injuries	Male	28-364 days	95+ years	Data Rich	1	1	Diabetes Fasting Plasma Glucose (mmol/L)	616
Unintentional injuries	Male	28-364 days	95+ years	Data Rich	1	1	Cumulative Cigarettes (5 Years)	897
Unintentional injuries	Male	28-364 days	95+ years	Data Rich	1	1	Underweight (proportion <2SD weight for age, <5 years)	--
Unintentional injuries	Male	28-364 days	95+ years	Data Rich	1	2	Population Density (500-1000 ppl/sqkm, proportion)	655
Unintentional injuries	Male	28-364 days	95+ years	Data Rich	1	2	Alcohol (liters per capita)	669

Appendix Table 9: CODEm covariates used, level of covariate, and expected direction of covariate by cause, sex, and age<br>*Covariates that CODEm did not select during the covariate selection process have no draw counts listed.*								
**Cause**	**Sex**	**Age Start**	**Age End**	**Model Type**	**Direction**	**Level**	**Covariate Name**	**Number of Draws**
Unintentional injuries	Male	28-364 days	95+ years	Data Rich	1	2	Population Density (over 1000 ppl/sqkm, proportion)	--
Unintentional injuries	Male	28-364 days	95+ years	Data Rich	-1	3	Education (years per capita)	5
Unintentional injuries	Male	28-364 days	95+ years	Data Rich	-1	3	LDI (I$ per capita)	--
Unintentional injuries	Male	28-364 days	95+ years	Global	-1	1	Health System Access 2 (unitless)	328
Unintentional injuries	Male	28-364 days	95+ years	Global	1	1	Smoking Prevalence	4
Unintentional injuries	Male	28-364 days	95+ years	Global	1	1	Indoor Air Pollution (All Cooking Fuels)	486
Unintentional injuries	Male	28-364 days	95+ years	Global	1	1	Diabetes Fasting Plasma Glucose (mmol/L)	798
Unintentional injuries	Male	28-364 days	95+ years	Global	1	1	Cumulative Cigarettes (5 Years)	905
Unintentional injuries	Male	28-364 days	95+ years	Global	1	1	Underweight (proportion <2SD weight for age, <5 years)	--
Unintentional injuries	Male	28-364 days	95+ years	Global	1	2	Population Density (500-1000 ppl/sqkm, proportion)	740
Unintentional injuries	Male	28-364 days	95+ years	Global	1	2	Alcohol (liters per capita)	787
Unintentional injuries	Male	28-364 days	95+ years	Global	1	2	Population Density (over 1000 ppl/sqkm, proportion)	--
Unintentional injuries	Male	28-364 days	95+ years	Global	-1	3	Education (years per capita)	0
Unintentional injuries	Male	28-364 days	95+ years	Global	-1	3	LDI (I$ per capita)	--
Falls	Female	0-6 days	95+ years	Data Rich	1	1	Log-transformed SEV scalar: Falls	214
Falls	Female	0-6 days	95+ years	Data Rich	1	1	Alcohol (liters per capita)	967
Falls	Female	0-6 days	95+ years	Data Rich	-1	2	Healthcare access and quality index	12
Falls	Female	0-6 days	95+ years	Data Rich	-1	2	milk adjusted(g)	432
Falls	Female	0-6 days	95+ years	Data Rich	0	3	Socio-demographic Index	21
Falls	Female	0-6 days	95+ years	Data Rich	0	3	LDI (I$ per capita)	--
Falls	Female	0-6 days	95+ years	Data Rich	1	3	Elevation Over 1500m (proportion)	276
Falls	Female	0-6 days	95+ years	Global	1	1	Log-transformed SEV scalar: Falls	387
Falls	Female	0-6 days	95+ years	Global	1	1	Alcohol (liters per capita)	836
Falls	Female	0-6 days	95+ years	Global	-1	2	milk adjusted(g)	45
Falls	Female	0-6 days	95+ years	Global	-1	2	Healthcare access and quality index	388
Falls	Female	0-6 days	95+ years	Global	0	3	Socio-demographic Index	658
Falls	Female	0-6 days	95+ years	Global	0	3	LDI (I$ per capita)	--
Falls	Female	0-6 days	95+ years	Global	1	3	Elevation Over 1500m (proportion)	494
Falls	Male	0-6 days	95+ years	Data Rich	1	1	Log-transformed SEV scalar: Falls	614
Falls	Male	0-6 days	95+ years	Data Rich	1	1	Alcohol (liters per capita)	806
Falls	Male	0-6 days	95+ years	Data Rich	-1	2	Healthcare access and quality index	48
Falls	Male	0-6 days	95+ years	Data Rich	-1	2	milk adjusted(g)	--
Falls	Male	0-6 days	95+ years	Data Rich	0	3	Socio-demographic Index	727
Falls	Male	0-6 days	95+ years	Data Rich	0	3	LDI (I$ per capita)	--
Falls	Male	0-6 days	95+ years	Data Rich	1	3	Elevation Over 1500m (proportion)	737
Falls	Male	0-6 days	95+ years	Global	1	1	Alcohol (liters per capita)	1000
Falls	Male	0-6 days	95+ years	Global	1	1	Log-transformed SEV scalar: Falls	--
Falls	Male	0-6 days	95+ years	Global	-1	2	Healthcare access and quality index	317
Falls	Male	0-6 days	95+ years	Global	-1	2	milk adjusted(g)	--
Falls	Male	0-6 days	95+ years	Global	0	3	Socio-demographic Index	561
Falls	Male	0-6 days	95+ years	Global	0	3	LDI (I$ per capita)	--
Falls	Male	0-6 days	95+ years	Global	1	3	Elevation Over 1500m (proportion)	561
Drowning	Female	0-6 days	95+ years	Data Rich	-1	1	Landlocked Nation (binary)	--
Drowning	Female	0-6 days	95+ years	Data Rich	-1	1	Rainfall Quintile 1 (proportion)	--
Drowning	Female	0-6 days	95+ years	Data Rich	1	1	Coastal Population within 10km (proportion)	945
Drowning	Female	0-6 days	95+ years	Data Rich	1	1	Alcohol (liters per capita)	1000
Drowning	Female	0-6 days	95+ years	Data Rich	1	1	Log-transformed SEV scalar: Drown	--
Drowning	Female	0-6 days	95+ years	Data Rich	1	1	Rainfall Quintile 5 (proportion)	--
Drowning	Female	0-6 days	95+ years	Data Rich	1	2	Elevation Under 100m (proportion)	--
Drowning	Female	0-6 days	95+ years	Data Rich	-1	3	Socio-demographic Index	38
Drowning	Female	0-6 days	95+ years	Data Rich	-1	3	Education (years per capita)	641
Drowning	Female	0-6 days	95+ years	Data Rich	0	3	LDI (I$ per capita)	--
Drowning	Female	0-6 days	95+ years	Global	-1	1	Landlocked Nation (binary)	--
Drowning	Female	0-6 days	95+ years	Global	-1	1	Rainfall Quintile 1 (proportion)	--
Drowning	Female	0-6 days	95+ years	Global	1	1	Alcohol (liters per capita)	393
Drowning	Female	0-6 days	95+ years	Global	1	1	Coastal Population within 10km (proportion)	754
Drowning	Female	0-6 days	95+ years	Global	1	1	Rainfall Quintile 5 (proportion)	767
Drowning	Female	0-6 days	95+ years	Global	1	1	Log-transformed SEV scalar: Drown	--
Drowning	Female	0-6 days	95+ years	Global	1	2	Elevation Under 100m (proportion)	22
Drowning	Female	0-6 days	95+ years	Global	-1	3	Socio-demographic Index	355
Drowning	Female	0-6 days	95+ years	Global	-1	3	Education (years per capita)	543
Drowning	Female	0-6 days	95+ years	Global	0	3	LDI (I$ per capita)	--
Drowning	Male	0-6 days	95+ years	Data Rich	-1	1	Landlocked Nation (binary)	--
Drowning	Male	0-6 days	95+ years	Data Rich	-1	1	Rainfall Quintile 1 (proportion)	--
Drowning	Male	0-6 days	95+ years	Data Rich	1	1	Coastal Population within 10km (proportion)	626
Drowning	Male	0-6 days	95+ years	Data Rich	1	1	Rainfall Quintile 5 (proportion)	858
Drowning	Male	0-6 days	95+ years	Data Rich	1	1	Alcohol (liters per capita)	1000
Drowning	Male	0-6 days	95+ years	Data Rich	1	1	Log-transformed SEV scalar: Drown	--
Drowning	Male	0-6 days	95+ years	Data Rich	1	2	Elevation Under 100m (proportion)	336
Drowning	Male	0-6 days	95+ years	Data Rich	-1	3	Education (years per capita)	17
Drowning	Male	0-6 days	95+ years	Data Rich	-1	3	Socio-demographic Index	983
Drowning	Male	0-6 days	95+ years	Data Rich	0	3	LDI (I$ per capita)	--
Drowning	Male	0-6 days	95+ years	Global	-1	1	Rainfall Quintile 1 (proportion)	546
Drowning	Male	0-6 days	95+ years	Global	-1	1	Landlocked Nation (binary)	--
Drowning	Male	0-6 days	95+ years	Global	1	1	Coastal Population within 10km (proportion)	259
Drowning	Male	0-6 days	95+ years	Global	1	1	Rainfall Quintile 5 (proportion)	339
Drowning	Male	0-6 days	95+ years	Global	1	1	Alcohol (liters per capita)	644
Drowning	Male	0-6 days	95+ years	Global	1	1	Log-transformed SEV scalar: Drown	--

**Appendix Table 9: CODEm covariates used, level of covariate, and expected direction of covariate by cause, sex, and age** *Covariates that CODEm did not select during the covariate selection process have no draw counts listed.*								
**Cause**	**Sex**	**Age Start**	**Age End**	**Model Type**	**Direction**	**Level**	**Covariate Name**	**Number of Draws**
Drowning	Male	0-6 days	95+ years	Global	1	2	Elevation Under 100m (proportion)	672
Drowning	Male	0-6 days	95+ years	Global	-1	3	Education (years per capita)	306
Drowning	Male	0-6 days	95+ years	Global	-1	3	Socio-demographic Index	500
Drowning	Male	0-6 days	95+ years	Global	0	3	LDI (I$ per capita)	--
Fire, heat, and hot substances	Female	0-6 days	95+ years	Data Rich	1	1	Log-transformed SEV scalar: Fire	--
Fire, heat, and hot substances	Female	0-6 days	95+ years	Data Rich	-1	2	Healthcare access and quality index	967
Fire, heat, and hot substances	Female	0-6 days	95+ years	Data Rich	0	2	Population Density (over 1000 ppl/sqkm, proportion)	967
Fire, heat, and hot substances	Female	0-6 days	95+ years	Data Rich	1	2	Alcohol (liters per capita)	967
Fire, heat, and hot substances	Female	0-6 days	95+ years	Data Rich	1	2	Indoor Air Pollution (All Cooking Fuels)	967
Fire, heat, and hot substances	Female	0-6 days	95+ years	Data Rich	1	2	Tobacco (cigarettes per capita)	967
Fire, heat, and hot substances	Female	0-6 days	95+ years	Data Rich	-1	3	Socio-demographic Index	33
Fire, heat, and hot substances	Female	0-6 days	95+ years	Data Rich	-1	3	Education (years per capita)	--
Fire, heat, and hot substances	Female	0-6 days	95+ years	Data Rich	0	3	LDI (I$ per capita)	--
Fire, heat, and hot substances	Female	0-6 days	95+ years	Global	1	1	Log-transformed SEV scalar: Fire	--
Fire, heat, and hot substances	Female	0-6 days	95+ years	Global	-1	2	Healthcare access and quality index	740
Fire, heat, and hot substances	Female	0-6 days	95+ years	Global	0	2	Population Density (over 1000 ppl/sqkm, proportion)	740
Fire, heat, and hot substances	Female	0-6 days	95+ years	Global	1	2	Alcohol (liters per capita)	740
Fire, heat, and hot substances	Female	0-6 days	95+ years	Global	1	2	Indoor Air Pollution (All Cooking Fuels)	740
Fire, heat, and hot substances	Female	0-6 days	95+ years	Global	1	2	Tobacco (cigarettes per capita)	740
Fire, heat, and hot substances	Female	0-6 days	95+ years	Global	-1	3	Education (years per capita)	260
Fire, heat, and hot substances	Female	0-6 days	95+ years	Global	-1	3	Socio-demographic Index	260
Fire, heat, and hot substances	Female	0-6 days	95+ years	Global	0	3	LDI (I$ per capita)	--
Fire, heat, and hot substances	Male	0-6 days	95+ years	Data Rich	1	1	Log-transformed SEV scalar: Fire	--
Fire, heat, and hot substances	Male	0-6 days	95+ years	Data Rich	-1	2	Healthcare access and quality index	366
Fire, heat, and hot substances	Male	0-6 days	95+ years	Data Rich	0	2	Population Density (over 1000 ppl/sqkm, proportion)	57
Fire, heat, and hot substances	Male	0-6 days	95+ years	Data Rich	1	2	Indoor Air Pollution (All Cooking Fuels)	3
Fire, heat, and hot substances	Male	0-6 days	95+ years	Data Rich	1	2	Tobacco (cigarettes per capita)	313
Fire, heat, and hot substances	Male	0-6 days	95+ years	Data Rich	1	2	Alcohol (liters per capita)	368
Fire, heat, and hot substances	Male	0-6 days	95+ years	Data Rich	-1	3	Education (years per capita)	140
Fire, heat, and hot substances	Male	0-6 days	95+ years	Data Rich	-1	3	Socio-demographic Index	509
Fire, heat, and hot substances	Male	0-6 days	95+ years	Data Rich	0	3	LDI (I$ per capita)	--
Fire, heat, and hot substances	Male	0-6 days	95+ years	Global	1	1	Log-transformed SEV scalar: Fire	--
Fire, heat, and hot substances	Male	0-6 days	95+ years	Global	-1	2	Healthcare access and quality index	303
Fire, heat, and hot substances	Male	0-6 days	95+ years	Global	0	2	Population Density (over 1000 ppl/sqkm, proportion)	303
Fire, heat, and hot substances	Male	0-6 days	95+ years	Global	1	2	Alcohol (liters per capita)	303
Fire, heat, and hot substances	Male	0-6 days	95+ years	Global	1	2	Indoor Air Pollution (All Cooking Fuels)	303
Fire, heat, and hot substances	Male	0-6 days	95+ years	Global	1	2	Tobacco (cigarettes per capita)	557
Fire, heat, and hot substances	Male	0-6 days	95+ years	Global	-1	3	Education (years per capita)	150
Fire, heat, and hot substances	Male	0-6 days	95+ years	Global	-1	3	Socio-demographic Index	593
Fire, heat, and hot substances	Male	0-6 days	95+ years	Global	0	3	LDI (I$ per capita)	--
Poisonings	Female	0-6 days	95+ years	Data Rich	1	1	Opium Cultivation (binary)	999
Poisonings	Female	0-6 days	95+ years	Data Rich	1	1	Log-transformed SEV scalar: Poison	--
Poisonings	Female	0-6 days	95+ years	Data Rich	-1	2	Healthcare access and quality index	962
Poisonings	Female	0-6 days	95+ years	Data Rich	0	2	Population Density (under 150 ppl/sqkm, proportion)	961
Poisonings	Female	0-6 days	95+ years	Data Rich	0	2	Population Density (over 1000 ppl/sqkm, proportion)	962
Poisonings	Female	0-6 days	95+ years	Data Rich	-1	3	Socio-demographic Index	32
Poisonings	Female	0-6 days	95+ years	Data Rich	-1	3	Education (years per capita)	160
Poisonings	Female	0-6 days	95+ years	Data Rich	0	3	LDI (I$ per capita)	--
Poisonings	Female	0-6 days	95+ years	Global	1	1	Opium Cultivation (binary)	1000
Poisonings	Female	0-6 days	95+ years	Global	1	1	Log-transformed SEV scalar: Poison	--
Poisonings	Female	0-6 days	95+ years	Global	-1	2	Healthcare access and quality index	218
Poisonings	Female	0-6 days	95+ years	Global	0	2	Population Density (over 1000 ppl/sqkm, proportion)	432
Poisonings	Female	0-6 days	95+ years	Global	0	2	Population Density (under 150 ppl/sqkm, proportion)	--
Poisonings	Female	0-6 days	95+ years	Global	-1	3	Socio-demographic Index	141
Poisonings	Female	0-6 days	95+ years	Global	-1	3	Education (years per capita)	264
Poisonings	Female	0-6 days	95+ years	Global	0	3	LDI (I$ per capita)	--
Poisonings	Male	0-6 days	95+ years	Data Rich	1	1	Log-transformed SEV scalar: Poison	--
Poisonings	Male	0-6 days	95+ years	Data Rich	1	1	Opium Cultivation (binary)	--
Poisonings	Male	0-6 days	95+ years	Data Rich	-1	2	Healthcare access and quality index	864
Poisonings	Male	0-6 days	95+ years	Data Rich	0	2	Population Density (over 1000 ppl/sqkm, proportion)	864
Poisonings	Male	0-6 days	95+ years	Data Rich	0	2	Population Density (under 150 ppl/sqkm, proportion)	864
Poisonings	Male	0-6 days	95+ years	Data Rich	-1	3	Education (years per capita)	25
Poisonings	Male	0-6 days	95+ years	Data Rich	-1	3	Socio-demographic Index	164
Poisonings	Male	0-6 days	95+ years	Data Rich	0	3	LDI (I$ per capita)	--
Poisonings	Male	0-6 days	95+ years	Global	1	1	Opium Cultivation (binary)	412
Poisonings	Male	0-6 days	95+ years	Global	1	1	Log-transformed SEV scalar: Poison	--
Poisonings	Male	0-6 days	95+ years	Global	-1	2	Healthcare access and quality index	352
Poisonings	Male	0-6 days	95+ years	Global	0	2	Population Density (over 1000 ppl/sqkm, proportion)	560
Poisonings	Male	0-6 days	95+ years	Global	0	2	Population Density (under 150 ppl/sqkm, proportion)	560
Poisonings	Male	0-6 days	95+ years	Global	-1	3	Education (years per capita)	236
Poisonings	Male	0-6 days	95+ years	Global	-1	3	Socio-demographic Index	236
Poisonings	Male	0-6 days	95+ years	Global	0	3	LDI (I$ per capita)	--
Poisoning by carbon monoxide	Female	0-6 days	95+ years	Data Rich	-1	3	Education (years per capita)	6
Poisoning by carbon monoxide	Female	0-6 days	95+ years	Data Rich	-1	3	Healthcare access and quality index	31
Poisoning by carbon monoxide	Female	0-6 days	95+ years	Data Rich	0	3	Socio-demographic Index	962
Poisoning by carbon monoxide	Female	0-6 days	95+ years	Data Rich	0	3	LDI (I$ per capita)	--
Poisoning by carbon monoxide	Female	0-6 days	95+ years	Global	-1	3	Education (years per capita)	444
Poisoning by carbon monoxide	Female	0-6 days	95+ years	Global	-1	3	Healthcare access and quality index	530

Appendix Table 9: CODEm covariates used, level of covariate, and expected direction of covariate by cause, sex, and age
Covariates that CODEm did not select during the covariate selection process have no draw counts listed.

Cause	Sex	Age Start	Age End	Model Type	Direction	Level	Covariate Name	Number of Draws
Poisoning by carbon monoxide	Female	0-6 days	95+ years	Global	0	3	Socio-demographic Index	395
Poisoning by carbon monoxide	Female	0-6 days	95+ years	Global	0	3	LDI (I$ per capita)	--
Poisoning by carbon monoxide	Male	0-6 days	95+ years	Data Rich	-1	3	Healthcare access and quality index	1
Poisoning by carbon monoxide	Male	0-6 days	95+ years	Data Rich	-1	3	Education (years per capita)	961
Poisoning by carbon monoxide	Male	0-6 days	95+ years	Data Rich	0	3	Socio-demographic Index	999
Poisoning by carbon monoxide	Male	0-6 days	95+ years	Data Rich	0	3	LDI (I$ per capita)	--
Poisoning by carbon monoxide	Male	0-6 days	95+ years	Global	-1	3	Healthcare access and quality index	274
Poisoning by carbon monoxide	Male	0-6 days	95+ years	Global	-1	3	Education (years per capita)	768
Poisoning by carbon monoxide	Male	0-6 days	95+ years	Global	0	3	Socio-demographic Index	410
Poisoning by carbon monoxide	Male	0-6 days	95+ years	Global	0	3	LDI (I$ per capita)	--
Poisoning by other means	Female	0-6 days	95+ years	Data Rich	-1	3	Education (years per capita)	161
Poisoning by other means	Female	0-6 days	95+ years	Data Rich	-1	3	Healthcare access and quality index	967
Poisoning by other means	Female	0-6 days	95+ years	Data Rich	0	3	Socio-demographic Index	193
Poisoning by other means	Female	0-6 days	95+ years	Data Rich	0	3	LDI (I$ per capita)	--
Poisoning by other means	Female	0-6 days	95+ years	Global	-1	3	Education (years per capita)	365
Poisoning by other means	Female	0-6 days	95+ years	Global	-1	3	Healthcare access and quality index	399
Poisoning by other means	Female	0-6 days	95+ years	Global	0	3	Socio-demographic Index	444
Poisoning by other means	Female	0-6 days	95+ years	Global	0	3	LDI (I$ per capita)	--
Poisoning by other means	Male	0-6 days	95+ years	Data Rich	-1	3	Education (years per capita)	967
Poisoning by other means	Male	0-6 days	95+ years	Data Rich	-1	3	Healthcare access and quality index	1000
Poisoning by other means	Male	0-6 days	95+ years	Data Rich	0	3	Socio-demographic Index	993
Poisoning by other means	Male	0-6 days	95+ years	Data Rich	0	3	LDI (I$ per capita)	--
Poisoning by other means	Male	0-6 days	95+ years	Global	-1	3	Education (years per capita)	501
Poisoning by other means	Male	0-6 days	95+ years	Global	-1	3	Healthcare access and quality index	536
Poisoning by other means	Male	0-6 days	95+ years	Global	0	3	Socio-demographic Index	524
Poisoning by other means	Male	0-6 days	95+ years	Global	0	3	LDI (I$ per capita)	--
Exposure to mechanical forces	Female	0-6 days	95+ years	Data Rich	-1	2	Healthcare access and quality index	0
Exposure to mechanical forces	Female	0-6 days	95+ years	Data Rich	0	2	Population Density (under 150 ppl/sqkm, proportion)	839
Exposure to mechanical forces	Female	0-6 days	95+ years	Data Rich	0	2	Population Density (over 1000 ppl/sqkm, proportion)	968
Exposure to mechanical forces	Female	0-6 days	95+ years	Data Rich	1	2	Alcohol (liters per capita)	7
Exposure to mechanical forces	Female	0-6 days	95+ years	Data Rich	-1	3	Education (years per capita)	0
Exposure to mechanical forces	Female	0-6 days	95+ years	Data Rich	-1	3	Socio-demographic Index	0
Exposure to mechanical forces	Female	0-6 days	95+ years	Data Rich	0	3	LDI (I$ per capita)	--
Exposure to mechanical forces	Female	0-6 days	95+ years	Global	-1	2	Healthcare access and quality index	847
Exposure to mechanical forces	Female	0-6 days	95+ years	Global	0	2	Population Density (under 150 ppl/sqkm, proportion)	436
Exposure to mechanical forces	Female	0-6 days	95+ years	Global	0	2	Population Density (over 1000 ppl/sqkm, proportion)	556
Exposure to mechanical forces	Female	0-6 days	95+ years	Global	1	2	Alcohol (liters per capita)	258
Exposure to mechanical forces	Female	0-6 days	95+ years	Global	-1	3	Education (years per capita)	51
Exposure to mechanical forces	Female	0-6 days	95+ years	Global	-1	3	Socio-demographic Index	168
Exposure to mechanical forces	Female	0-6 days	95+ years	Global	0	3	LDI (I$ per capita)	--
Exposure to mechanical forces	Male	0-6 days	95+ years	Data Rich	-1	2	Healthcare access and quality index	1000
Exposure to mechanical forces	Male	0-6 days	95+ years	Data Rich	0	2	Population Density (under 150 ppl/sqkm, proportion)	967
Exposure to mechanical forces	Male	0-6 days	95+ years	Data Rich	0	2	Population Density (over 1000 ppl/sqkm, proportion)	1000
Exposure to mechanical forces	Male	0-6 days	95+ years	Data Rich	1	2	Alcohol (liters per capita)	199
Exposure to mechanical forces	Male	0-6 days	95+ years	Data Rich	-1	3	Education (years per capita)	0
Exposure to mechanical forces	Male	0-6 days	95+ years	Data Rich	-1	3	Socio-demographic Index	161
Exposure to mechanical forces	Male	0-6 days	95+ years	Data Rich	0	3	LDI (I$ per capita)	--
Exposure to mechanical forces	Male	0-6 days	95+ years	Global	-1	2	Healthcare access and quality index	535
Exposure to mechanical forces	Male	0-6 days	95+ years	Global	0	2	Population Density (under 150 ppl/sqkm, proportion)	487
Exposure to mechanical forces	Male	0-6 days	95+ years	Global	0	2	Population Density (over 1000 ppl/sqkm, proportion)	694
Exposure to mechanical forces	Male	0-6 days	95+ years	Global	1	2	Alcohol (liters per capita)	482
Exposure to mechanical forces	Male	0-6 days	95+ years	Global	-1	3	Socio-demographic Index	154
Exposure to mechanical forces	Male	0-6 days	95+ years	Global	-1	3	Education (years per capita)	511
Exposure to mechanical forces	Male	0-6 days	95+ years	Global	0	3	LDI (I$ per capita)	--
Unintentional firearm injuries	Female	0-6 days	95+ years	Data Rich	1	1	Log-transformed SEV scalar: Mech Gun	--
Unintentional firearm injuries	Female	0-6 days	95+ years	Data Rich	-1	2	Healthcare access and quality index	689
Unintentional firearm injuries	Female	0-6 days	95+ years	Data Rich	-1	2	Health System Access (unitless)	--
Unintentional firearm injuries	Female	0-6 days	95+ years	Data Rich	1	2	Alcohol (liters per capita)	--
Unintentional firearm injuries	Female	0-6 days	95+ years	Data Rich	-1	3	Socio-demographic Index	150
Unintentional firearm injuries	Female	0-6 days	95+ years	Data Rich	-1	3	Education (years per capita)	--
Unintentional firearm injuries	Female	0-6 days	95+ years	Data Rich	0	3	Population Density (over 1000 ppl/sqkm, proportion)	235
Unintentional firearm injuries	Female	0-6 days	95+ years	Data Rich	0	3	Population Density (under 150 ppl/sqkm, proportion)	369
Unintentional firearm injuries	Female	0-6 days	95+ years	Data Rich	0	3	LDI (I$ per capita)	--
Unintentional firearm injuries	Female	0-6 days	95+ years	Global	1	1	Log-transformed SEV scalar: Mech Gun	--
Unintentional firearm injuries	Female	0-6 days	95+ years	Global	-1	2	Healthcare access and quality index	392
Unintentional firearm injuries	Female	0-6 days	95+ years	Global	-1	2	Health System Access (unitless)	--
Unintentional firearm injuries	Female	0-6 days	95+ years	Global	1	2	Alcohol (liters per capita)	--
Unintentional firearm injuries	Female	0-6 days	95+ years	Global	-1	3	Education (years per capita)	133
Unintentional firearm injuries	Female	0-6 days	95+ years	Global	-1	3	Socio-demographic Index	475
Unintentional firearm injuries	Female	0-6 days	95+ years	Global	0	3	Population Density (over 1000 ppl/sqkm, proportion)	513
Unintentional firearm injuries	Female	0-6 days	95+ years	Global	0	3	Population Density (under 150 ppl/sqkm, proportion)	839
Unintentional firearm injuries	Female	0-6 days	95+ years	Global	0	3	LDI (I$ per capita)	--
Unintentional firearm injuries	Male	0-6 days	95+ years	Data Rich	1	1	Log-transformed SEV scalar: Mech Gun	--
Unintentional firearm injuries	Male	0-6 days	95+ years	Data Rich	-1	2	Health System Access (unitless)	725
Unintentional firearm injuries	Male	0-6 days	95+ years	Data Rich	-1	2	Healthcare access and quality index	--
Unintentional firearm injuries	Male	0-6 days	95+ years	Data Rich	1	2	Alcohol (liters per capita)	224
Unintentional firearm injuries	Male	0-6 days	95+ years	Data Rich	-1	3	Socio-demographic Index	48
Unintentional firearm injuries	Male	0-6 days	95+ years	Data Rich	-1	3	Education (years per capita)	188

Appendix Table 9: CODEm covariates used, level of covariate, and expected direction of covariate by cause, sex, and age<br>*Covariates that CODEm did not select during the covariate selection process have no draw counts listed.*								
**Cause**	**Sex**	**Age Start**	**Age End**	**Model Type**	**Direction**	**Level**	**Covariate Name**	**Number of Draws**
Unintentional firearm injuries	Male	0-6 days	95+ years	Data Rich	0	3	Population Density (under 150 ppl/sqkm, proportion)	113
Unintentional firearm injuries	Male	0-6 days	95+ years	Data Rich	0	3	Population Density (over 1000 ppl/sqkm, proportion)	551
Unintentional firearm injuries	Male	0-6 days	95+ years	Data Rich	0	3	LDI (I$ per capita)	--
Unintentional firearm injuries	Male	0-6 days	95+ years	Global	1	1	Log-transformed SEV scalar: Mech Gun	--
Unintentional firearm injuries	Male	0-6 days	95+ years	Global	-1	2	Health System Access (unitless)	582
Unintentional firearm injuries	Male	0-6 days	95+ years	Global	-1	2	Healthcare access and quality index	692
Unintentional firearm injuries	Male	0-6 days	95+ years	Global	1	2	Alcohol (liters per capita)	504
Unintentional firearm injuries	Male	0-6 days	95+ years	Global	-1	3	Education (years per capita)	91
Unintentional firearm injuries	Male	0-6 days	95+ years	Global	-1	3	Socio-demographic Index	373
Unintentional firearm injuries	Male	0-6 days	95+ years	Global	0	3	Population Density (under 150 ppl/sqkm, proportion)	383
Unintentional firearm injuries	Male	0-6 days	95+ years	Global	0	3	Population Density (over 1000 ppl/sqkm, proportion)	609
Unintentional firearm injuries	Male	0-6 days	95+ years	Global	0	3	LDI (I$ per capita)	--
Other exposure to mechanical forces	Female	0-6 days	95+ years	Data Rich	1	1	Log-transformed SEV scalar: Oth Mech	1000
Other exposure to mechanical forces	Female	0-6 days	95+ years	Data Rich	-1	2	Health System Access (unitless)	132
Other exposure to mechanical forces	Female	0-6 days	95+ years	Data Rich	-1	2	Healthcare access and quality index	132
Other exposure to mechanical forces	Female	0-6 days	95+ years	Data Rich	0	2	Population Density (over 1000 ppl/sqkm, proportion)	397
Other exposure to mechanical forces	Female	0-6 days	95+ years	Data Rich	0	2	Population Density (under 150 ppl/sqkm, proportion)	397
Other exposure to mechanical forces	Female	0-6 days	95+ years	Data Rich	1	2	Alcohol (liters per capita)	265
Other exposure to mechanical forces	Female	0-6 days	95+ years	Data Rich	-1	3	Education (years per capita)	6
Other exposure to mechanical forces	Female	0-6 days	95+ years	Data Rich	-1	3	Socio-demographic Index	14
Other exposure to mechanical forces	Female	0-6 days	95+ years	Data Rich	0	3	LDI (I$ per capita)	--
Other exposure to mechanical forces	Female	0-6 days	95+ years	Global	1	1	Log-transformed SEV scalar: Oth Mech	--
Other exposure to mechanical forces	Female	0-6 days	95+ years	Global	-1	2	Health System Access (unitless)	354
Other exposure to mechanical forces	Female	0-6 days	95+ years	Global	-1	2	Healthcare access and quality index	354
Other exposure to mechanical forces	Female	0-6 days	95+ years	Global	0	2	Population Density (over 1000 ppl/sqkm, proportion)	614
Other exposure to mechanical forces	Female	0-6 days	95+ years	Global	0	2	Population Density (under 150 ppl/sqkm, proportion)	614
Other exposure to mechanical forces	Female	0-6 days	95+ years	Global	1	2	Alcohol (liters per capita)	614
Other exposure to mechanical forces	Female	0-6 days	95+ years	Global	-1	3	Education (years per capita)	104
Other exposure to mechanical forces	Female	0-6 days	95+ years	Global	-1	3	Socio-demographic Index	--
Other exposure to mechanical forces	Female	0-6 days	95+ years	Global	0	3	LDI (I$ per capita)	--
Other exposure to mechanical forces	Male	0-6 days	95+ years	Data Rich	1	1	Log-transformed SEV scalar: Oth Mech	1000
Other exposure to mechanical forces	Male	0-6 days	95+ years	Data Rich	-1	2	Healthcare access and quality index	804
Other exposure to mechanical forces	Male	0-6 days	95+ years	Data Rich	-1	2	Health System Access (unitless)	--
Other exposure to mechanical forces	Male	0-6 days	95+ years	Data Rich	0	2	Population Density (over 1000 ppl/sqkm, proportion)	812
Other exposure to mechanical forces	Male	0-6 days	95+ years	Data Rich	0	2	Population Density (under 150 ppl/sqkm, proportion)	812
Other exposure to mechanical forces	Male	0-6 days	95+ years	Data Rich	1	2	Alcohol (liters per capita)	8
Other exposure to mechanical forces	Male	0-6 days	95+ years	Data Rich	-1	3	Education (years per capita)	251
Other exposure to mechanical forces	Male	0-6 days	95+ years	Data Rich	-1	3	Socio-demographic Index	302
Other exposure to mechanical forces	Male	0-6 days	95+ years	Data Rich	0	3	LDI (I$ per capita)	--
Other exposure to mechanical forces	Male	0-6 days	95+ years	Global	1	1	Log-transformed SEV scalar: Oth Mech	773
Other exposure to mechanical forces	Male	0-6 days	95+ years	Global	-1	2	Health System Access (unitless)	449
Other exposure to mechanical forces	Male	0-6 days	95+ years	Global	-1	2	Healthcare access and quality index	449
Other exposure to mechanical forces	Male	0-6 days	95+ years	Global	0	2	Population Density (over 1000 ppl/sqkm, proportion)	449
Other exposure to mechanical forces	Male	0-6 days	95+ years	Global	0	2	Population Density (under 150 ppl/sqkm, proportion)	449
Other exposure to mechanical forces	Male	0-6 days	95+ years	Global	1	2	Alcohol (liters per capita)	449
Other exposure to mechanical forces	Male	0-6 days	95+ years	Global	-1	3	Education (years per capita)	218
Other exposure to mechanical forces	Male	0-6 days	95+ years	Global	-1	3	Socio-demographic Index	251
Other exposure to mechanical forces	Male	0-6 days	95+ years	Global	0	3	LDI (I$ per capita)	--
Adverse effects of medical treatment	Female	0-6 days	95+ years	Data Rich	0	2	Healthcare access and quality index	--
Adverse effects of medical treatment	Female	0-6 days	95+ years	Data Rich	0	3	Socio-demographic Index	166
Adverse effects of medical treatment	Female	0-6 days	95+ years	Data Rich	0	3	LDI (I$ per capita)	--
Adverse effects of medical treatment	Female	0-6 days	95+ years	Global	0	2	Healthcare access and quality index	602
Adverse effects of medical treatment	Female	0-6 days	95+ years	Global	0	3	Socio-demographic Index	692
Adverse effects of medical treatment	Female	0-6 days	95+ years	Global	0	3	LDI (I$ per capita)	--
Adverse effects of medical treatment	Male	0-6 days	95+ years	Data Rich	0	2	Healthcare access and quality index	--
Adverse effects of medical treatment	Male	0-6 days	95+ years	Data Rich	0	3	LDI (I$ per capita)	--
Adverse effects of medical treatment	Male	0-6 days	95+ years	Data Rich	0	3	Socio-demographic Index	--
Adverse effects of medical treatment	Male	0-6 days	95+ years	Global	0	2	Healthcare access and quality index	602
Adverse effects of medical treatment	Male	0-6 days	95+ years	Global	0	3	Socio-demographic Index	726
Adverse effects of medical treatment	Male	0-6 days	95+ years	Global	0	3	LDI (I$ per capita)	--
Animal contact	Female	0-6 days	95+ years	Data Rich	1	1	Alcohol (liters per capita)	--
Animal contact	Female	0-6 days	95+ years	Data Rich	1	1	Log-transformed SEV scalar: Animal	--
Animal contact	Female	0-6 days	95+ years	Data Rich	-1	2	Healthcare access and quality index	38
Animal contact	Female	0-6 days	95+ years	Data Rich	1	2	Population 15 to 30 (proportion)	38
Animal contact	Female	0-6 days	95+ years	Data Rich	-1	3	Socio-demographic Index	1
Animal contact	Female	0-6 days	95+ years	Data Rich	-1	3	Education (years per capita)	160
Animal contact	Female	0-6 days	95+ years	Data Rich	0	3	Elevation Over 1500m (proportion)	192
Animal contact	Female	0-6 days	95+ years	Data Rich	0	3	Population Density (over 1000 ppl/sqkm, proportion)	801
Animal contact	Female	0-6 days	95+ years	Data Rich	0	3	Population Density (under 150 ppl/sqkm, proportion)	801
Animal contact	Female	0-6 days	95+ years	Data Rich	0	3	Elevation Under 100m (proportion)	--
Animal contact	Female	0-6 days	95+ years	Data Rich	0	3	LDI (I$ per capita)	--
Animal contact	Female	0-6 days	95+ years	Global	1	1	Alcohol (liters per capita)	--
Animal contact	Female	0-6 days	95+ years	Global	1	1	Log-transformed SEV scalar: Animal	--
Animal contact	Female	0-6 days	95+ years	Global	-1	2	Healthcare access and quality index	424
Animal contact	Female	0-6 days	95+ years	Global	1	2	Population 15 to 30 (proportion)	424
Animal contact	Female	0-6 days	95+ years	Global	-1	3	Education (years per capita)	133
Animal contact	Female	0-6 days	95+ years	Global	-1	3	Socio-demographic Index	392
Animal contact	Female	0-6 days	95+ years	Global	0	3	Population Density (over 1000 ppl/sqkm, proportion)	282

**Appendix Table 9: CODEm covariates used, level of covariate, and expected direction of covariate by cause, sex, and age**<br>*Covariates that CODEm did not select during the covariate selection process have no draw counts listed.*								
**Cause**	**Sex**	**Age Start**	**Age End**	**Model Type**	**Direction**	**Level**	**Covariate Name**	**Number of Draws**
Animal contact	Female	0-6 days	95+ years	Global	0	3	Population Density (under 150 ppl/sqkm, proportion)	282
Animal contact	Female	0-6 days	95+ years	Global	0	3	Elevation Over 1500m (proportion)	513
Animal contact	Female	0-6 days	95+ years	Global	0	3	Elevation Under 100m (proportion)	807
Animal contact	Female	0-6 days	95+ years	Global	0	3	LDI (I$ per capita)	--
Animal contact	Male	0-6 days	95+ years	Data Rich	1	1	Alcohol (liters per capita)	969
Animal contact	Male	0-6 days	95+ years	Data Rich	1	1	Log-transformed SEV scalar: Animal	993
Animal contact	Male	0-6 days	95+ years	Data Rich	-1	2	Healthcare access and quality index	0
Animal contact	Male	0-6 days	95+ years	Data Rich	1	2	Population 15 to 30 (proportion)	0
Animal contact	Male	0-6 days	95+ years	Data Rich	-1	3	Education (years per capita)	0
Animal contact	Male	0-6 days	95+ years	Data Rich	-1	3	Socio-demographic Index	0
Animal contact	Male	0-6 days	95+ years	Data Rich	0	3	Population Density (under 150 ppl/sqkm, proportion)	1
Animal contact	Male	0-6 days	95+ years	Data Rich	0	3	Elevation Under 100m (proportion)	803
Animal contact	Male	0-6 days	95+ years	Data Rich	0	3	Population Density (over 1000 ppl/sqkm, proportion)	803
Animal contact	Male	0-6 days	95+ years	Data Rich	0	3	Elevation Over 1500m (proportion)	804
Animal contact	Male	0-6 days	95+ years	Data Rich	0	3	LDI (I$ per capita)	--
Animal contact	Male	0-6 days	95+ years	Global	1	1	Alcohol (liters per capita)	491
Animal contact	Male	0-6 days	95+ years	Global	1	1	Log-transformed SEV scalar: Animal	509
Animal contact	Male	0-6 days	95+ years	Global	-1	2	Healthcare access and quality index	170
Animal contact	Male	0-6 days	95+ years	Global	1	2	Population 15 to 30 (proportion)	161
Animal contact	Male	0-6 days	95+ years	Global	-1	3	Socio-demographic Index	195
Animal contact	Male	0-6 days	95+ years	Global	-1	3	Education (years per capita)	196
Animal contact	Male	0-6 days	95+ years	Global	0	3	Elevation Over 1500m (proportion)	114
Animal contact	Male	0-6 days	95+ years	Global	0	3	Elevation Under 100m (proportion)	207
Animal contact	Male	0-6 days	95+ years	Global	0	3	Population Density (under 150 ppl/sqkm, proportion)	352
Animal contact	Male	0-6 days	95+ years	Global	0	3	Population Density (over 1000 ppl/sqkm, proportion)	495
Animal contact	Male	0-6 days	95+ years	Global	0	3	LDI (I$ per capita)	--
Venomous animal contact	Female	0-6 days	95+ years	Data Rich	1	1	Alcohol (liters per capita)	--
Venomous animal contact	Female	0-6 days	95+ years	Data Rich	1	1	Log-transformed SEV scalar: Venom	--
Venomous animal contact	Female	0-6 days	95+ years	Data Rich	-1	2	Healthcare access and quality index	--
Venomous animal contact	Female	0-6 days	95+ years	Data Rich	-1	3	Education (years per capita)	181
Venomous animal contact	Female	0-6 days	95+ years	Data Rich	-1	3	Socio-demographic Index	181
Venomous animal contact	Female	0-6 days	95+ years	Data Rich	0	3	Elevation Over 1500m (proportion)	819
Venomous animal contact	Female	0-6 days	95+ years	Data Rich	0	3	Elevation Under 100m (proportion)	819
Venomous animal contact	Female	0-6 days	95+ years	Data Rich	0	3	Population Density (under 150 ppl/sqkm, proportion)	1000
Venomous animal contact	Female	0-6 days	95+ years	Data Rich	0	3	LDI (I$ per capita)	--
Venomous animal contact	Female	0-6 days	95+ years	Data Rich	0	3	Population Density (over 1000 ppl/sqkm, proportion)	--
Venomous animal contact	Female	0-6 days	95+ years	Global	1	1	Alcohol (liters per capita)	--
Venomous animal contact	Female	0-6 days	95+ years	Global	1	1	Log-transformed SEV scalar: Venom	--
Venomous animal contact	Female	0-6 days	95+ years	Global	-1	2	Healthcare access and quality index	--
Venomous animal contact	Female	0-6 days	95+ years	Global	-1	3	Education (years per capita)	546
Venomous animal contact	Female	0-6 days	95+ years	Global	-1	3	Socio-demographic Index	546
Venomous animal contact	Female	0-6 days	95+ years	Global	0	3	Elevation Under 100m (proportion)	454
Venomous animal contact	Female	0-6 days	95+ years	Global	0	3	Population Density (over 1000 ppl/sqkm, proportion)	454
Venomous animal contact	Female	0-6 days	95+ years	Global	0	3	Elevation Over 1500m (proportion)	--
Venomous animal contact	Female	0-6 days	95+ years	Global	0	3	LDI (I$ per capita)	--
Venomous animal contact	Female	0-6 days	95+ years	Global	0	3	Population Density (under 150 ppl/sqkm, proportion)	--
Venomous animal contact	Male	0-6 days	95+ years	Data Rich	1	1	Log-transformed SEV scalar: Venom	574
Venomous animal contact	Male	0-6 days	95+ years	Data Rich	1	1	Alcohol (liters per capita)	738
Venomous animal contact	Male	0-6 days	95+ years	Data Rich	-1	2	Healthcare access and quality index	241
Venomous animal contact	Male	0-6 days	95+ years	Data Rich	-1	3	Socio-demographic Index	21
Venomous animal contact	Male	0-6 days	95+ years	Data Rich	-1	3	Education (years per capita)	86
Venomous animal contact	Male	0-6 days	95+ years	Data Rich	0	3	Elevation Over 1500m (proportion)	16
Venomous animal contact	Male	0-6 days	95+ years	Data Rich	0	3	Elevation Under 100m (proportion)	111
Venomous animal contact	Male	0-6 days	95+ years	Data Rich	0	3	Population Density (under 150 ppl/sqkm, proportion)	399
Venomous animal contact	Male	0-6 days	95+ years	Data Rich	0	3	Population Density (over 1000 ppl/sqkm, proportion)	466
Venomous animal contact	Male	0-6 days	95+ years	Data Rich	0	3	LDI (I$ per capita)	--
Venomous animal contact	Male	0-6 days	95+ years	Global	1	1	Log-transformed SEV scalar: Venom	499
Venomous animal contact	Male	0-6 days	95+ years	Global	1	1	Alcohol (liters per capita)	661
Venomous animal contact	Male	0-6 days	95+ years	Global	-1	2	Healthcare access and quality index	566
Venomous animal contact	Male	0-6 days	95+ years	Global	-1	3	Education (years per capita)	125
Venomous animal contact	Male	0-6 days	95+ years	Global	-1	3	Socio-demographic Index	225
Venomous animal contact	Male	0-6 days	95+ years	Global	0	3	Population Density (under 150 ppl/sqkm, proportion)	575
Venomous animal contact	Male	0-6 days	95+ years	Global	0	3	Elevation Over 1500m (proportion)	579
Venomous animal contact	Male	0-6 days	95+ years	Global	0	3	Population Density (over 1000 ppl/sqkm, proportion)	755
Venomous animal contact	Male	0-6 days	95+ years	Global	0	3	Elevation Under 100m (proportion)	780
Venomous animal contact	Male	0-6 days	95+ years	Global	0	3	LDI (I$ per capita)	--
Non-venomous animal contact	Female	0-6 days	95+ years	Data Rich	1	1	Alcohol (liters per capita)	211
Non-venomous animal contact	Female	0-6 days	95+ years	Data Rich	1	1	Log-transformed SEV scalar: Non Ven	--
Non-venomous animal contact	Female	0-6 days	95+ years	Data Rich	-1	2	Healthcare access and quality index	--
Non-venomous animal contact	Female	0-6 days	95+ years	Data Rich	-1	3	Education (years per capita)	--
Non-venomous animal contact	Female	0-6 days	95+ years	Data Rich	-1	3	Socio-demographic Index	--
Non-venomous animal contact	Female	0-6 days	95+ years	Data Rich	0	3	Population Density (under 150 ppl/sqkm, proportion)	39
Non-venomous animal contact	Female	0-6 days	95+ years	Data Rich	0	3	Elevation Over 1500m (proportion)	828
Non-venomous animal contact	Female	0-6 days	95+ years	Data Rich	0	3	Elevation Under 100m (proportion)	828
Non-venomous animal contact	Female	0-6 days	95+ years	Data Rich	0	3	Population Density (over 1000 ppl/sqkm, proportion)	828
Non-venomous animal contact	Female	0-6 days	95+ years	Data Rich	0	3	LDI (I$ per capita)	--
Non-venomous animal contact	Female	0-6 days	95+ years	Global	1	1	Alcohol (liters per capita)	1000
Non-venomous animal contact	Female	0-6 days	95+ years	Global	1	1	Log-transformed SEV scalar: Non Ven	--

Appendix Table 9: CODEm covariates used, level of covariate, and expected direction of covariate by cause, sex, and age<br>*Covariates that CODEm did not select during the covariate selection process have no draw counts listed.*								
**Cause**	**Sex**	**Age Start**	**Age End**	**Model Type**	**Direction**	**Level**	**Covariate Name**	**Number of Draws**
Non-venomous animal contact	Female	0-6 days	95+ years	Global	-1	2	Healthcare access and quality index	--
Non-venomous animal contact	Female	0-6 days	95+ years	Global	-1	3	Education (years per capita)	161
Non-venomous animal contact	Female	0-6 days	95+ years	Global	-1	3	Socio-demographic Index	193
Non-venomous animal contact	Female	0-6 days	95+ years	Global	0	3	Elevation Over 1500m (proportion)	282
Non-venomous animal contact	Female	0-6 days	95+ years	Global	0	3	Population Density (over 1000 ppl/sqkm, proportion)	282
Non-venomous animal contact	Female	0-6 days	95+ years	Global	0	3	Elevation Under 100m (proportion)	636
Non-venomous animal contact	Female	0-6 days	95+ years	Global	0	3	Population Density (under 150 ppl/sqkm, proportion)	636
Non-venomous animal contact	Female	0-6 days	95+ years	Global	0	3	LDI (I$ per capita)	--
Non-venomous animal contact	Male	0-6 days	95+ years	Data Rich	1	1	Alcohol (liters per capita)	468
Non-venomous animal contact	Male	0-6 days	95+ years	Data Rich	1	1	Log-transformed SEV scalar: Non Ven	--
Non-venomous animal contact	Male	0-6 days	95+ years	Data Rich	-1	2	Healthcare access and quality index	277
Non-venomous animal contact	Male	0-6 days	95+ years	Data Rich	-1	3	Socio-demographic Index	255
Non-venomous animal contact	Male	0-6 days	95+ years	Data Rich	-1	3	Education (years per capita)	--
Non-venomous animal contact	Male	0-6 days	95+ years	Data Rich	0	3	Elevation Over 1500m (proportion)	374
Non-venomous animal contact	Male	0-6 days	95+ years	Data Rich	0	3	Elevation Under 100m (proportion)	643
Non-venomous animal contact	Male	0-6 days	95+ years	Data Rich	0	3	Population Density (over 1000 ppl/sqkm, proportion)	643
Non-venomous animal contact	Male	0-6 days	95+ years	Data Rich	0	3	Population Density (under 150 ppl/sqkm, proportion)	643
Non-venomous animal contact	Male	0-6 days	95+ years	Data Rich	0	3	LDI (I$ per capita)	--
Non-venomous animal contact	Male	0-6 days	95+ years	Global	1	1	Log-transformed SEV scalar: Non Ven	--
Non-venomous animal contact	Male	0-6 days	95+ years	Global	-1	3	Education (years per capita)	454
Non-venomous animal contact	Male	0-6 days	95+ years	Global	-1	3	Healthcare access and quality index	454
Non-venomous animal contact	Male	0-6 days	95+ years	Global	-1	3	Socio-demographic Index	454
Non-venomous animal contact	Male	0-6 days	95+ years	Global	0	3	LDI (I$ per capita)	--
Non-venomous animal contact	Male	0-6 days	95+ years	Global	1	3	Alcohol (liters per capita)	546
Foreign body	Female	0-6 days	95+ years	Data Rich	1	1	LDI (I$ per capita)	999
Foreign body	Female	0-6 days	95+ years	Data Rich	1	1	Education (years per capita)	1000
Foreign body	Female	0-6 days	95+ years	Data Rich	1	1	Indoor Air Pollution (All Cooking Fuels)	1000
Foreign body	Female	0-6 days	95+ years	Data Rich	1	1	Population Over 65 (proportion)	1000
Foreign body	Female	0-6 days	95+ years	Data Rich	1	1	Population Density (over 1000 ppl/sqkm, proportion)	--
Foreign body	Female	0-6 days	95+ years	Data Rich	-1	2	Healthcare access and quality index	165
Foreign body	Female	0-6 days	95+ years	Data Rich	0	3	Socio-demographic Index	963
Foreign body	Female	0-6 days	95+ years	Global	1	1	Indoor Air Pollution (All Cooking Fuels)	55
Foreign body	Female	0-6 days	95+ years	Global	1	1	Population Over 65 (proportion)	611
Foreign body	Female	0-6 days	95+ years	Global	1	1	LDI (I$ per capita)	614
Foreign body	Female	0-6 days	95+ years	Global	1	1	Education (years per capita)	748
Foreign body	Female	0-6 days	95+ years	Global	1	1	Population Density (over 1000 ppl/sqkm, proportion)	--
Foreign body	Female	0-6 days	95+ years	Global	-1	2	Healthcare access and quality index	34
Foreign body	Female	0-6 days	95+ years	Global	0	3	Socio-demographic Index	539
Foreign body	Male	0-6 days	95+ years	Data Rich	1	1	Education (years per capita)	395
Foreign body	Male	0-6 days	95+ years	Data Rich	1	1	Indoor Air Pollution (All Cooking Fuels)	992
Foreign body	Male	0-6 days	95+ years	Data Rich	1	1	LDI (I$ per capita)	1000
Foreign body	Male	0-6 days	95+ years	Data Rich	1	1	Population Over 65 (proportion)	1000
Foreign body	Male	0-6 days	95+ years	Data Rich	1	1	Population Density (over 1000 ppl/sqkm, proportion)	--
Foreign body	Male	0-6 days	95+ years	Data Rich	-1	2	Healthcare access and quality index	61
Foreign body	Male	0-6 days	95+ years	Data Rich	0	3	Socio-demographic Index	884
Foreign body	Male	0-6 days	95+ years	Global	1	1	Population Density (over 1000 ppl/sqkm, proportion)	21
Foreign body	Male	0-6 days	95+ years	Global	1	1	Indoor Air Pollution (All Cooking Fuels)	467
Foreign body	Male	0-6 days	95+ years	Global	1	1	Population Over 65 (proportion)	614
Foreign body	Male	0-6 days	95+ years	Global	1	1	LDI (I$ per capita)	648
Foreign body	Male	0-6 days	95+ years	Global	1	1	Education (years per capita)	835
Foreign body	Male	0-6 days	95+ years	Global	-1	2	Healthcare access and quality index	--
Foreign body	Male	0-6 days	95+ years	Global	0	3	Socio-demographic Index	475
Pulmonary aspiration and foreign body in airway	Female	0-6 days	95+ years	Data Rich	1	1	Log-transformed SEV scalar: F Body Asp	--
Pulmonary aspiration and foreign body in airway	Female	0-6 days	95+ years	Data Rich	-1	2	Healthcare access and quality index	32
Pulmonary aspiration and foreign body in airway	Female	0-6 days	95+ years	Data Rich	1	2	Mean BMI	32
Pulmonary aspiration and foreign body in airway	Female	0-6 days	95+ years	Data Rich	1	2	Alcohol (liters per capita)	--
Pulmonary aspiration and foreign body in airway	Female	0-6 days	95+ years	Data Rich	0	3	Socio-demographic Index	770
Pulmonary aspiration and foreign body in airway	Female	0-6 days	95+ years	Data Rich	0	3	LDI (I$ per capita)	--
Pulmonary aspiration and foreign body in airway	Female	0-6 days	95+ years	Global	1	1	Log-transformed SEV scalar: F Body Asp	--
Pulmonary aspiration and foreign body in airway	Female	0-6 days	95+ years	Global	-1	2	Healthcare access and quality index	264
Pulmonary aspiration and foreign body in airway	Female	0-6 days	95+ years	Global	1	2	Alcohol binge drinker proportion, age-standardized	428
Pulmonary aspiration and foreign body in airway	Female	0-6 days	95+ years	Global	1	2	Mean BMI	692
Pulmonary aspiration and foreign body in airway	Female	0-6 days	95+ years	Global	1	2	Alcohol (liters per capita)	--
Pulmonary aspiration and foreign body in airway	Female	0-6 days	95+ years	Global	-1	3	Education (years per capita)	100
Pulmonary aspiration and foreign body in airway	Female	0-6 days	95+ years	Global	0	3	Socio-demographic Index	352
Pulmonary aspiration and foreign body in airway	Female	0-6 days	95+ years	Global	0	3	LDI (I$ per capita)	--
Pulmonary aspiration and foreign body in airway	Male	0-6 days	95+ years	Data Rich	1	1	Log-transformed SEV scalar: F Body Asp	--
Pulmonary aspiration and foreign body in airway	Male	0-6 days	95+ years	Data Rich	-1	2	Healthcare access and quality index	250
Pulmonary aspiration and foreign body in airway	Male	0-6 days	95+ years	Data Rich	1	2	Mean BMI	750
Pulmonary aspiration and foreign body in airway	Male	0-6 days	95+ years	Data Rich	1	2	Alcohol (liters per capita)	1000
Pulmonary aspiration and foreign body in airway	Male	0-6 days	95+ years	Data Rich	0	3	Socio-demographic Index	1000
Pulmonary aspiration and foreign body in airway	Male	0-6 days	95+ years	Data Rich	0	3	LDI (I$ per capita)	--
Pulmonary aspiration and foreign body in airway	Male	0-6 days	95+ years	Global	1	1	Log-transformed SEV scalar: F Body Asp	--
Pulmonary aspiration and foreign body in airway	Male	0-6 days	95+ years	Global	-1	2	Healthcare access and quality index	585
Pulmonary aspiration and foreign body in airway	Male	0-6 days	95+ years	Global	1	2	Alcohol (liters per capita)	282
Pulmonary aspiration and foreign body in airway	Male	0-6 days	95+ years	Global	1	2	Mean BMI	--
Pulmonary aspiration and foreign body in airway	Male	0-6 days	95+ years	Global	0	3	Socio-demographic Index	475
Pulmonary aspiration and foreign body in airway	Male	0-6 days	95+ years	Global	0	3	LDI (I$ per capita)	--

Appendix Table 9: CODEm covariates used, level of covariate, and expected direction of covariate by cause, sex, and age
Covariates that CODEm did not select during the covariate selection process have no draw counts listed.

Cause	Sex	Age Start	Age End	Model Type	Direction	Level	Covariate Name	Number of Draws
Foreign body in other body part	Female	0-6 days	95+ years	Data Rich	1	1	Alcohol (liters per capita)	889
Foreign body in other body part	Female	0-6 days	95+ years	Data Rich	1	1	Log-transformed SEV scalar: Oth F Body	--
Foreign body in other body part	Female	0-6 days	95+ years	Data Rich	-1	2	Healthcare access and quality index	298
Foreign body in other body part	Female	0-6 days	95+ years	Data Rich	-1	3	Socio-demographic Index	239
Foreign body in other body part	Female	0-6 days	95+ years	Data Rich	-1	3	Education (years per capita)	--
Foreign body in other body part	Female	0-6 days	95+ years	Data Rich	0	3	Population Density (over 1000 ppl/sqkm, proportion)	290
Foreign body in other body part	Female	0-6 days	95+ years	Data Rich	0	3	Elevation Over 1500m (proportion)	350
Foreign body in other body part	Female	0-6 days	95+ years	Data Rich	0	3	Elevation Under 100m (proportion)	418
Foreign body in other body part	Female	0-6 days	95+ years	Data Rich	0	3	Population Density (under 150 ppl/sqkm, proportion)	529
Foreign body in other body part	Female	0-6 days	95+ years	Data Rich	0	3	LDI (I$ per capita)	--
Foreign body in other body part	Female	0-6 days	95+ years	Global	1	1	Alcohol (liters per capita)	1000
Foreign body in other body part	Female	0-6 days	95+ years	Global	1	1	Log-transformed SEV scalar: Oth F Body	--
Foreign body in other body part	Female	0-6 days	95+ years	Global	-1	2	Healthcare access and quality index	293
Foreign body in other body part	Female	0-6 days	95+ years	Global	-1	3	Education (years per capita)	92
Foreign body in other body part	Female	0-6 days	95+ years	Global	-1	3	Socio-demographic Index	111
Foreign body in other body part	Female	0-6 days	95+ years	Global	0	3	Elevation Over 1500m (proportion)	388
Foreign body in other body part	Female	0-6 days	95+ years	Global	0	3	Population Density (under 150 ppl/sqkm, proportion)	388
Foreign body in other body part	Female	0-6 days	95+ years	Global	0	3	Elevation Under 100m (proportion)	591
Foreign body in other body part	Female	0-6 days	95+ years	Global	0	3	Population Density (over 1000 ppl/sqkm, proportion)	591
Foreign body in other body part	Female	0-6 days	95+ years	Global	0	3	LDI (I$ per capita)	--
Foreign body in other body part	Male	0-6 days	95+ years	Data Rich	1	1	Alcohol (liters per capita)	968
Foreign body in other body part	Male	0-6 days	95+ years	Data Rich	1	1	Log-transformed SEV scalar: Oth F Body	--
Foreign body in other body part	Male	0-6 days	95+ years	Data Rich	-1	2	Healthcare access and quality index	153
Foreign body in other body part	Male	0-6 days	95+ years	Data Rich	-1	3	Socio-demographic Index	249
Foreign body in other body part	Male	0-6 days	95+ years	Data Rich	-1	3	Education (years per capita)	--
Foreign body in other body part	Male	0-6 days	95+ years	Data Rich	0	3	Elevation Under 100m (proportion)	46
Foreign body in other body part	Male	0-6 days	95+ years	Data Rich	0	3	Population Density (under 150 ppl/sqkm, proportion)	69
Foreign body in other body part	Male	0-6 days	95+ years	Data Rich	0	3	Population Density (over 1000 ppl/sqkm, proportion)	355
Foreign body in other body part	Male	0-6 days	95+ years	Data Rich	0	3	Elevation Over 1500m (proportion)	409
Foreign body in other body part	Male	0-6 days	95+ years	Data Rich	0	3	LDI (I$ per capita)	--
Foreign body in other body part	Male	0-6 days	95+ years	Global	1	1	Alcohol (liters per capita)	636
Foreign body in other body part	Male	0-6 days	95+ years	Global	1	1	Log-transformed SEV scalar: Oth F Body	--
Foreign body in other body part	Male	0-6 days	95+ years	Global	-1	2	Healthcare access and quality index	264
Foreign body in other body part	Male	0-6 days	95+ years	Global	-1	3	Socio-demographic Index	100
Foreign body in other body part	Male	0-6 days	95+ years	Global	-1	3	Education (years per capita)	--
Foreign body in other body part	Male	0-6 days	95+ years	Global	0	3	Population Density (under 150 ppl/sqkm, proportion)	426
Foreign body in other body part	Male	0-6 days	95+ years	Global	0	3	Elevation Over 1500m (proportion)	437
Foreign body in other body part	Male	0-6 days	95+ years	Global	0	3	Population Density (over 1000 ppl/sqkm, proportion)	499
Foreign body in other body part	Male	0-6 days	95+ years	Global	0	3	Elevation Under 100m (proportion)	581
Foreign body in other body part	Male	0-6 days	95+ years	Global	0	3	LDI (I$ per capita)	--
Environmental heat and cold exposure	Female	0-6 days	95+ years	Data Rich	-1	2	Healthcare access and quality index	1000
Environmental heat and cold exposure	Female	0-6 days	95+ years	Data Rich	-1	3	Education (years per capita)	6
Environmental heat and cold exposure	Female	0-6 days	95+ years	Data Rich	-1	3	Socio-demographic Index	--
Environmental heat and cold exposure	Female	0-6 days	95+ years	Data Rich	0	3	Elevation Over 1500m (proportion)	6
Environmental heat and cold exposure	Female	0-6 days	95+ years	Data Rich	0	3	LDI (I$ per capita)	6
Environmental heat and cold exposure	Female	0-6 days	95+ years	Data Rich	0	3	Population-weighted mean temperature	161
Environmental heat and cold exposure	Female	0-6 days	95+ years	Data Rich	0	3	Sanitation (proportion with access)	161
Environmental heat and cold exposure	Female	0-6 days	95+ years	Data Rich	0	3	90th percentile climatic temperature in the given country-year.	167
Environmental heat and cold exposure	Female	0-6 days	95+ years	Data Rich	0	3	Elevation 500 to 1500m (proportion)	167
Environmental heat and cold exposure	Female	0-6 days	95+ years	Data Rich	0	3	Population Density (150-300 ppl/sqkm, proportion)	167
Environmental heat and cold exposure	Female	0-6 days	95+ years	Data Rich	0	3	Rainfall (Quintiles 4-5)	167
Environmental heat and cold exposure	Female	0-6 days	95+ years	Global	-1	2	Healthcare access and quality index	1000
Environmental heat and cold exposure	Female	0-6 days	95+ years	Global	-1	3	Education (years per capita)	196
Environmental heat and cold exposure	Female	0-6 days	95+ years	Global	-1	3	Socio-demographic Index	231
Environmental heat and cold exposure	Female	0-6 days	95+ years	Global	0	3	Population-weighted mean temperature	107
Environmental heat and cold exposure	Female	0-6 days	95+ years	Global	0	3	LDI (I$ per capita)	200
Environmental heat and cold exposure	Female	0-6 days	95+ years	Global	0	3	Sanitation (proportion with access)	200
Environmental heat and cold exposure	Female	0-6 days	95+ years	Global	0	3	Elevation Over 1500m (proportion)	299
Environmental heat and cold exposure	Female	0-6 days	95+ years	Global	0	3	Population Density (150-300 ppl/sqkm, proportion)	320
Environmental heat and cold exposure	Female	0-6 days	95+ years	Global	0	3	Elevation 500 to 1500m (proportion)	324
Environmental heat and cold exposure	Female	0-6 days	95+ years	Global	0	3	90th percentile climatic temperature in the given country-year.	--
Environmental heat and cold exposure	Female	0-6 days	95+ years	Global	0	3	Rainfall (Quintiles 4-5)	--
Environmental heat and cold exposure	Male	0-6 days	95+ years	Data Rich	-1	2	Healthcare access and quality index	1000
Environmental heat and cold exposure	Male	0-6 days	95+ years	Data Rich	-1	3	Education (years per capita)	--
Environmental heat and cold exposure	Male	0-6 days	95+ years	Data Rich	-1	3	Socio-demographic Index	--
Environmental heat and cold exposure	Male	0-6 days	95+ years	Data Rich	0	3	Rainfall (Quintiles 4-5)	15
Environmental heat and cold exposure	Male	0-6 days	95+ years	Data Rich	0	3	LDI (I$ per capita)	62
Environmental heat and cold exposure	Male	0-6 days	95+ years	Data Rich	0	3	Population-weighted mean temperature	62
Environmental heat and cold exposure	Male	0-6 days	95+ years	Data Rich	0	3	Elevation 500 to 1500m (proportion)	132
Environmental heat and cold exposure	Male	0-6 days	95+ years	Data Rich	0	3	90th percentile climatic temperature in the given country-year.	253
Environmental heat and cold exposure	Male	0-6 days	95+ years	Data Rich	0	3	Population Density (150-300 ppl/sqkm, proportion)	385
Environmental heat and cold exposure	Male	0-6 days	95+ years	Data Rich	0	3	Elevation Over 1500m (proportion)	--
Environmental heat and cold exposure	Male	0-6 days	95+ years	Data Rich	0	3	Sanitation (proportion with access)	--
Environmental heat and cold exposure	Male	0-6 days	95+ years	Global	-1	2	Healthcare access and quality index	674
Environmental heat and cold exposure	Male	0-6 days	95+ years	Global	-1	3	Education (years per capita)	566
Environmental heat and cold exposure	Male	0-6 days	95+ years	Global	-1	3	Socio-demographic Index	--
Environmental heat and cold exposure	Male	0-6 days	95+ years	Global	0	3	Elevation 500 to 1500m (proportion)	326
Environmental heat and cold exposure	Male	0-6 days	95+ years	Global	0	3	Elevation Over 1500m (proportion)	326

**Appendix Table 9: CODEm covariates used, level of covariate, and expected direction of covariate by cause, sex, and age** *Covariates that CODEm did not select during the covariate selection process have no draw counts listed.*								
**Cause**	**Sex**	**Age Start**	**Age End**	**Model Type**	**Direction**	**Level**	**Covariate Name**	**Number of Draws**
Environmental heat and cold exposure	Male	0-6 days	95+ years	Global	0	3	LDI (I$ per capita)	326
Environmental heat and cold exposure	Male	0-6 days	95+ years	Global	0	3	Population Density (150-300 ppl/sqkm, proportion)	326
Environmental heat and cold exposure	Male	0-6 days	95+ years	Global	0	3	Sanitation (proportion with access)	452
Environmental heat and cold exposure	Male	0-6 days	95+ years	Global	0	3	90th percentile climatic temperature in the given country-year.	538
Environmental heat and cold exposure	Male	0-6 days	95+ years	Global	0	3	Population-weighted mean temperature	538
Environmental heat and cold exposure	Male	0-6 days	95+ years	Global	0	3	Rainfall (Quintiles 4-5)	--
Other unintentional injuries	Female	0-6 days	95+ years	Data Rich	0	1	Vehicles - 4 wheels (per capita)	1000
Other unintentional injuries	Female	0-6 days	95+ years	Data Rich	1	1	Alcohol (liters per capita)	0
Other unintentional injuries	Female	0-6 days	95+ years	Data Rich	1	1	Vehicles - 2 wheels (per capita)	1000
Other unintentional injuries	Female	0-6 days	95+ years	Data Rich	1	1	Log-transformed SEV scalar: Oth Unint	--
Other unintentional injuries	Female	0-6 days	95+ years	Data Rich	-1	2	Healthcare access and quality index	834
Other unintentional injuries	Female	0-6 days	95+ years	Data Rich	-1	3	Education (years per capita)	1000
Other unintentional injuries	Female	0-6 days	95+ years	Data Rich	0	3	Population Density (over 1000 ppl/sqkm, proportion)	166
Other unintentional injuries	Female	0-6 days	95+ years	Data Rich	0	3	Population Density (under 150 ppl/sqkm, proportion)	198
Other unintentional injuries	Female	0-6 days	95+ years	Data Rich	0	3	Socio-demographic Index	962
Other unintentional injuries	Female	0-6 days	95+ years	Data Rich	0	3	Elevation Under 100m (proportion)	999
Other unintentional injuries	Female	0-6 days	95+ years	Data Rich	0	3	Elevation Over 1500m (proportion)	1000
Other unintentional injuries	Female	0-6 days	95+ years	Data Rich	0	3	LDI (I$ per capita)	--
Other unintentional injuries	Female	0-6 days	95+ years	Global	0	1	Vehicles - 4 wheels (per capita)	668
Other unintentional injuries	Female	0-6 days	95+ years	Global	1	1	Alcohol (liters per capita)	509
Other unintentional injuries	Female	0-6 days	95+ years	Global	1	1	Vehicles - 2 wheels (per capita)	551
Other unintentional injuries	Female	0-6 days	95+ years	Global	1	1	Log-transformed SEV scalar: Oth Unint	--
Other unintentional injuries	Female	0-6 days	95+ years	Global	-1	2	Healthcare access and quality index	277
Other unintentional injuries	Female	0-6 days	95+ years	Global	-1	3	Education (years per capita)	717
Other unintentional injuries	Female	0-6 days	95+ years	Global	0	3	Elevation Under 100m (proportion)	463
Other unintentional injuries	Female	0-6 days	95+ years	Global	0	3	Population Density (over 1000 ppl/sqkm, proportion)	680
Other unintentional injuries	Female	0-6 days	95+ years	Global	0	3	Population Density (under 150 ppl/sqkm, proportion)	700
Other unintentional injuries	Female	0-6 days	95+ years	Global	0	3	Elevation Over 1500m (proportion)	797
Other unintentional injuries	Female	0-6 days	95+ years	Global	0	3	Socio-demographic Index	821
Other unintentional injuries	Female	0-6 days	95+ years	Global	0	3	LDI (I$ per capita)	--
Other unintentional injuries	Male	0-6 days	95+ years	Data Rich	0	1	Vehicles - 4 wheels (per capita)	1000
Other unintentional injuries	Male	0-6 days	95+ years	Data Rich	1	1	Alcohol (liters per capita)	189
Other unintentional injuries	Male	0-6 days	95+ years	Data Rich	1	1	Vehicles - 2 wheels (per capita)	1000
Other unintentional injuries	Male	0-6 days	95+ years	Data Rich	1	1	Log-transformed SEV scalar: Oth Unint	--
Other unintentional injuries	Male	0-6 days	95+ years	Data Rich	-1	2	Healthcare access and quality index	765
Other unintentional injuries	Male	0-6 days	95+ years	Data Rich	-1	3	Education (years per capita)	765
Other unintentional injuries	Male	0-6 days	95+ years	Data Rich	0	3	Population Density (over 1000 ppl/sqkm, proportion)	233
Other unintentional injuries	Male	0-6 days	95+ years	Data Rich	0	3	Elevation Under 100m (proportion)	811
Other unintentional injuries	Male	0-6 days	95+ years	Data Rich	0	3	Elevation Over 1500m (proportion)	989
Other unintentional injuries	Male	0-6 days	95+ years	Data Rich	0	3	Population Density (under 150 ppl/sqkm, proportion)	1000
Other unintentional injuries	Male	0-6 days	95+ years	Data Rich	0	3	Socio-demographic Index	1000
Other unintentional injuries	Male	0-6 days	95+ years	Data Rich	0	3	LDI (I$ per capita)	--
Other unintentional injuries	Male	0-6 days	95+ years	Global	0	1	Vehicles - 4 wheels (per capita)	1000
Other unintentional injuries	Male	0-6 days	95+ years	Global	1	1	Alcohol (liters per capita)	401
Other unintentional injuries	Male	0-6 days	95+ years	Global	1	1	Vehicles - 2 wheels (per capita)	922
Other unintentional injuries	Male	0-6 days	95+ years	Global	1	1	Log-transformed SEV scalar: Oth Unint	--
Other unintentional injuries	Male	0-6 days	95+ years	Global	-1	2	Healthcare access and quality index	156
Other unintentional injuries	Male	0-6 days	95+ years	Global	-1	3	Education (years per capita)	724
Other unintentional injuries	Male	0-6 days	95+ years	Global	0	3	Population Density (over 1000 ppl/sqkm, proportion)	116
Other unintentional injuries	Male	0-6 days	95+ years	Global	0	3	Elevation Under 100m (proportion)	603
Other unintentional injuries	Male	0-6 days	95+ years	Global	0	3	Socio-demographic Index	605
Other unintentional injuries	Male	0-6 days	95+ years	Global	0	3	Population Density (under 150 ppl/sqkm, proportion)	735
Other unintentional injuries	Male	0-6 days	95+ years	Global	0	3	Elevation Over 1500m (proportion)	890
Other unintentional injuries	Male	0-6 days	95+ years	Global	0	3	LDI (I$ per capita)	--
Self-harm and interpersonal violence	Female	0-6 days	95+ years	Data Rich	1	1	Healthcare access and quality index	940
Self-harm and interpersonal violence	Female	0-6 days	95+ years	Data Rich	1	1	Alcohol (liters per capita)	--
Self-harm and interpersonal violence	Female	0-6 days	95+ years	Data Rich	1	1	Log-transformed SEV scalar: Oth Unint	--
Self-harm and interpersonal violence	Female	0-6 days	95+ years	Data Rich	-1	3	Education (years per capita)	641
Self-harm and interpersonal violence	Female	0-6 days	95+ years	Data Rich	0	3	Population Density (under 150 ppl/sqkm, proportion)	323
Self-harm and interpersonal violence	Female	0-6 days	95+ years	Data Rich	0	3	Population Density (over 1000 ppl/sqkm, proportion)	407
Self-harm and interpersonal violence	Female	0-6 days	95+ years	Data Rich	0	3	Elevation Under 100m (proportion)	692
Self-harm and interpersonal violence	Female	0-6 days	95+ years	Data Rich	0	3	Elevation Over 1500m (proportion)	859
Self-harm and interpersonal violence	Female	0-6 days	95+ years	Data Rich	0	3	LDI (I$ per capita)	--
Self-harm and interpersonal violence	Female	0-6 days	95+ years	Global	1	1	Healthcare access and quality index	403
Self-harm and interpersonal violence	Female	0-6 days	95+ years	Global	1	1	Alcohol (liters per capita)	883
Self-harm and interpersonal violence	Female	0-6 days	95+ years	Global	1	1	Log-transformed SEV scalar: Oth Unint	--
Self-harm and interpersonal violence	Female	0-6 days	95+ years	Global	-1	3	Education (years per capita)	117
Self-harm and interpersonal violence	Female	0-6 days	95+ years	Global	0	3	Population Density (over 1000 ppl/sqkm, proportion)	366
Self-harm and interpersonal violence	Female	0-6 days	95+ years	Global	0	3	Elevation Under 100m (proportion)	442
Self-harm and interpersonal violence	Female	0-6 days	95+ years	Global	0	3	Elevation Over 1500m (proportion)	559
Self-harm and interpersonal violence	Female	0-6 days	95+ years	Global	0	3	Population Density (under 150 ppl/sqkm, proportion)	559
Self-harm and interpersonal violence	Female	0-6 days	95+ years	Global	0	3	LDI (I$ per capita)	--
Self-harm and interpersonal violence	Male	0-6 days	95+ years	Data Rich	1	1	Healthcare access and quality index	726
Self-harm and interpersonal violence	Male	0-6 days	95+ years	Data Rich	1	1	Alcohol (liters per capita)	--
Self-harm and interpersonal violence	Male	0-6 days	95+ years	Data Rich	1	1	Log-transformed SEV scalar: Oth Unint	--
Self-harm and interpersonal violence	Male	0-6 days	95+ years	Data Rich	-1	3	Education (years per capita)	274
Self-harm and interpersonal violence	Male	0-6 days	95+ years	Data Rich	0	3	Elevation Under 100m (proportion)	274
Self-harm and interpersonal violence	Male	0-6 days	95+ years	Data Rich	0	3	Elevation Over 1500m (proportion)	627

Appendix Table 9: CODEm covariates used, level of covariate, and expected direction of covariate by cause, sex, and age

Covariates that CODEm did not select during the covariate selection process have no draw counts listed.

Cause	Sex	Age Start	Age End	Model Type	Direction	Level	Covariate Name	Number of Draws
Self-harm and interpersonal violence	Male	0-6 days	95+ years	Data Rich	0	3	Population Density (over 1000 ppl/sqkm, proportion)	627
Self-harm and interpersonal violence	Male	0-6 days	95+ years	Data Rich	0	3	Population Density (under 150 ppl/sqkm, proportion)	627
Self-harm and interpersonal violence	Male	0-6 days	95+ years	Data Rich	0	3	LDI (I$ per capita)	--
Self-harm and interpersonal violence	Male	0-6 days	95+ years	Global	1	1	Healthcare access and quality index	780
Self-harm and interpersonal violence	Male	0-6 days	95+ years	Global	1	1	Alcohol (liters per capita)	--
Self-harm and interpersonal violence	Male	0-6 days	95+ years	Global	1	1	Log-transformed SEV scalar: Oth Unint	--
Self-harm and interpersonal violence	Male	0-6 days	95+ years	Global	-1	3	Education (years per capita)	--
Self-harm and interpersonal violence	Male	0-6 days	95+ years	Global	0	3	Elevation Over 1500m (proportion)	353
Self-harm and interpersonal violence	Male	0-6 days	95+ years	Global	0	3	Population Density (under 150 ppl/sqkm, proportion)	353
Self-harm and interpersonal violence	Male	0-6 days	95+ years	Global	0	3	Elevation Under 100m (proportion)	573
Self-harm and interpersonal violence	Male	0-6 days	95+ years	Global	0	3	Population Density (over 1000 ppl/sqkm, proportion)	573
Self-harm and interpersonal violence	Male	0-6 days	95+ years	Global	0	3	LDI (I$ per capita)	--
Self-harm	Female	10-14 years	95+ years	Data Rich	1	1	Log-transformed SEV scalar: Self Harm	0
Self-harm	Female	10-14 years	95+ years	Data Rich	1	1	Alcohol (liters per capita)	38
Self-harm	Female	10-14 years	95+ years	Data Rich	1	1	Major depressive disorder	1000
Self-harm	Female	10-14 years	95+ years	Data Rich	-1	2	Healthcare access and quality index	0
Self-harm	Female	10-14 years	95+ years	Data Rich	-1	2	Religion (binary, >50% Muslim)	--
Self-harm	Female	10-14 years	95+ years	Data Rich	0	2	Population Density (300-500 ppl/sqkm, proportion)	0
Self-harm	Female	10-14 years	95+ years	Data Rich	0	2	Population Density (500-1000 ppl/sqkm, proportion)	31
Self-harm	Female	10-14 years	95+ years	Data Rich	0	2	Population Density (under 150 ppl/sqkm, proportion)	31
Self-harm	Female	10-14 years	95+ years	Data Rich	0	2	Population Density (over 1000 ppl/sqkm, proportion)	803
Self-harm	Female	10-14 years	95+ years	Data Rich	0	2	Population Density (150-300 ppl/sqkm, proportion)	804
Self-harm	Female	10-14 years	95+ years	Data Rich	0	3	Education (years per capita)	0
Self-harm	Female	10-14 years	95+ years	Data Rich	0	3	Socio-demographic Index	0
Self-harm	Female	10-14 years	95+ years	Data Rich	0	3	LDI (I$ per capita)	--
Self-harm	Female	10-14 years	95+ years	Global	1	1	Risk of selfharm due to major depressive disorder	0
Self-harm	Female	10-14 years	95+ years	Global	1	1	Log-transformed SEV scalar: Self Harm	57
Self-harm	Female	10-14 years	95+ years	Global	1	1	Non-partner lifetime prevalence of sexual violence (female-only)	371
Self-harm	Female	10-14 years	95+ years	Global	1	1	Alcohol (liters per capita)	404
Self-harm	Female	10-14 years	95+ years	Global	1	1	Major depressive disorder	998
Self-harm	Female	10-14 years	95+ years	Global	-1	2	Healthcare access and quality index	0
Self-harm	Female	10-14 years	95+ years	Global	-1	2	Religion (binary, >50% Muslim)	417
Self-harm	Female	10-14 years	95+ years	Global	0	2	Population Density (500-1000 ppl/sqkm, proportion)	136
Self-harm	Female	10-14 years	95+ years	Global	0	2	Population Density (300-500 ppl/sqkm, proportion)	291
Self-harm	Female	10-14 years	95+ years	Global	0	2	Population Density (over 1000 ppl/sqkm, proportion)	354
Self-harm	Female	10-14 years	95+ years	Global	0	2	Population Density (under 150 ppl/sqkm, proportion)	369
Self-harm	Female	10-14 years	95+ years	Global	0	2	Population Density (150-300 ppl/sqkm, proportion)	417
Self-harm	Female	10-14 years	95+ years	Global	0	3	Socio-demographic Index	678
Self-harm	Female	10-14 years	95+ years	Global	0	3	Education (years per capita)	754
Self-harm	Female	10-14 years	95+ years	Global	0	3	LDI (I$ per capita)	--
Self-harm	Male	10-14 years	95+ years	Data Rich	1	1	Alcohol (liters per capita)	--
Self-harm	Male	10-14 years	95+ years	Data Rich	-1	2	Religion (binary, >50% Muslim)	15
Self-harm	Male	10-14 years	95+ years	Data Rich	-1	2	Healthcare access and quality index	--
Self-harm	Male	10-14 years	95+ years	Data Rich	0	2	Population Density (500-1000 ppl/sqkm, proportion)	84
Self-harm	Male	10-14 years	95+ years	Data Rich	0	2	Population Density (under 150 ppl/sqkm, proportion)	703
Self-harm	Male	10-14 years	95+ years	Data Rich	0	2	Population Density (300-500 ppl/sqkm, proportion)	718
Self-harm	Male	10-14 years	95+ years	Data Rich	0	2	Population Density (150-300 ppl/sqkm, proportion)	--
Self-harm	Male	10-14 years	95+ years	Data Rich	0	2	Population Density (over 1000 ppl/sqkm, proportion)	--
Self-harm	Male	10-14 years	95+ years	Data Rich	0	3	Education (years per capita)	760
Self-harm	Male	10-14 years	95+ years	Data Rich	0	3	Socio-demographic Index	791
Self-harm	Male	10-14 years	95+ years	Data Rich	0	3	LDI (I$ per capita)	--
Self-harm	Male	10-14 years	95+ years	Global	1	1	Alcohol (liters per capita)	--
Self-harm	Male	10-14 years	95+ years	Global	-1	2	Muslim Religion (proportion of population)	668
Self-harm	Male	10-14 years	95+ years	Global	-1	2	Healthcare access and quality index	--
Self-harm	Male	10-14 years	95+ years	Global	0	2	Population Density (under 150 ppl/sqkm, proportion)	436
Self-harm	Male	10-14 years	95+ years	Global	0	2	Population Density (500-1000 ppl/sqkm, proportion)	512
Self-harm	Male	10-14 years	95+ years	Global	0	2	Population Density (300-500 ppl/sqkm, proportion)	572
Self-harm	Male	10-14 years	95+ years	Global	0	2	Population Density (150-300 ppl/sqkm, proportion)	760
Self-harm	Male	10-14 years	95+ years	Global	0	2	Population Density (over 1000 ppl/sqkm, proportion)	760
Self-harm	Male	10-14 years	95+ years	Global	0	3	Education (years per capita)	677
Self-harm	Male	10-14 years	95+ years	Global	0	3	Socio-demographic Index	722
Self-harm	Male	10-14 years	95+ years	Global	0	3	LDI (I$ per capita)	--
Self-harm by firearm	Female	10-14 years	95+ years	Data Rich	1	1	Major depressive disorder	1000
Self-harm by firearm	Female	10-14 years	95+ years	Data Rich	1	1	Alcohol (liters per capita)	--
Self-harm by firearm	Female	10-14 years	95+ years	Data Rich	1	1	Log-transformed SEV scalar: Self Harm	--
Self-harm by firearm	Female	10-14 years	95+ years	Data Rich	-1	2	Healthcare access and quality index	14
Self-harm by firearm	Female	10-14 years	95+ years	Data Rich	-1	2	Religion (binary, >50% Muslim)	--
Self-harm by firearm	Female	10-14 years	95+ years	Data Rich	0	2	Population Density (over 1000 ppl/sqkm, proportion)	14
Self-harm by firearm	Female	10-14 years	95+ years	Data Rich	0	2	Population Density (under 150 ppl/sqkm, proportion)	319
Self-harm by firearm	Female	10-14 years	95+ years	Data Rich	0	2	Population Density (150-300 ppl/sqkm, proportion)	588
Self-harm by firearm	Female	10-14 years	95+ years	Data Rich	0	2	Population Density (300-500 ppl/sqkm, proportion)	641
Self-harm by firearm	Female	10-14 years	95+ years	Data Rich	0	2	Population Density (500-1000 ppl/sqkm, proportion)	698
Self-harm by firearm	Female	10-14 years	95+ years	Data Rich	0	3	Education (years per capita)	872
Self-harm by firearm	Female	10-14 years	95+ years	Data Rich	0	3	Socio-demographic Index	938
Self-harm by firearm	Female	10-14 years	95+ years	Data Rich	0	3	LDI (I$ per capita)	--
Self-harm by firearm	Female	10-14 years	95+ years	Global	1	1	Major depressive disorder	1000
Self-harm by firearm	Female	10-14 years	95+ years	Global	1	1	Alcohol (liters per capita)	--

Appendix Table 9: CODEm covariates used, level of covariate, and expected direction of covariate by cause, sex, and age<br>*Covariates that CODEm did not select during the covariate selection process have no draw counts listed.*								
**Cause**	**Sex**	**Age Start**	**Age End**	**Model Type**	**Direction**	**Level**	**Covariate Name**	**Number of Draws**
Self-harm by firearm	Female	10-14 years	95+ years	Global	1	1	Log-transformed SEV scalar: Self Harm	--
Self-harm by firearm	Female	10-14 years	95+ years	Global	-1	2	Healthcare access and quality index	200
Self-harm by firearm	Female	10-14 years	95+ years	Global	-1	2	Religion (binary, >50% Muslim)	--
Self-harm by firearm	Female	10-14 years	95+ years	Global	0	2	Population Density (150-300 ppl/sqkm, proportion)	149
Self-harm by firearm	Female	10-14 years	95+ years	Global	0	2	Population Density (300-500 ppl/sqkm, proportion)	687
Self-harm by firearm	Female	10-14 years	95+ years	Global	0	2	Population Density (500-1000 ppl/sqkm, proportion)	700
Self-harm by firearm	Female	10-14 years	95+ years	Global	0	2	Population Density (over 1000 ppl/sqkm, proportion)	711
Self-harm by firearm	Female	10-14 years	95+ years	Global	0	2	Population Density (under 150 ppl/sqkm, proportion)	749
Self-harm by firearm	Female	10-14 years	95+ years	Global	0	3	Socio-demographic Index	390
Self-harm by firearm	Female	10-14 years	95+ years	Global	0	3	Education (years per capita)	517
Self-harm by firearm	Female	10-14 years	95+ years	Global	0	3	LDI (I$ per capita)	--
Self-harm by firearm	Male	10-14 years	95+ years	Data Rich	1	1	Major depressive disorder	1000
Self-harm by firearm	Male	10-14 years	95+ years	Data Rich	1	1	Alcohol (liters per capita)	--
Self-harm by firearm	Male	10-14 years	95+ years	Data Rich	1	1	Log-transformed SEV scalar: Self Harm	--
Self-harm by firearm	Male	10-14 years	95+ years	Data Rich	-1	2	Healthcare access and quality index	--
Self-harm by firearm	Male	10-14 years	95+ years	Data Rich	-1	2	Religion (binary, >50% Muslim)	--
Self-harm by firearm	Male	10-14 years	95+ years	Data Rich	0	2	Population Density (under 150 ppl/sqkm, proportion)	47
Self-harm by firearm	Male	10-14 years	95+ years	Data Rich	0	2	Population Density (300-500 ppl/sqkm, proportion)	181
Self-harm by firearm	Male	10-14 years	95+ years	Data Rich	0	2	Population Density (150-300 ppl/sqkm, proportion)	211
Self-harm by firearm	Male	10-14 years	95+ years	Data Rich	0	2	Population Density (over 1000 ppl/sqkm, proportion)	218
Self-harm by firearm	Male	10-14 years	95+ years	Data Rich	0	2	Population Density (500-1000 ppl/sqkm, proportion)	219
Self-harm by firearm	Male	10-14 years	95+ years	Data Rich	0	3	Socio-demographic Index	210
Self-harm by firearm	Male	10-14 years	95+ years	Data Rich	0	3	Education (years per capita)	991
Self-harm by firearm	Male	10-14 years	95+ years	Data Rich	0	3	LDI (I$ per capita)	--
Self-harm by firearm	Male	10-14 years	95+ years	Global	1	1	Alcohol (liters per capita)	--
Self-harm by firearm	Male	10-14 years	95+ years	Global	1	1	Log-transformed SEV scalar: Self Harm	--
Self-harm by firearm	Male	10-14 years	95+ years	Global	1	1	Major depressive disorder	--
Self-harm by firearm	Male	10-14 years	95+ years	Global	-1	2	Healthcare access and quality index	96
Self-harm by firearm	Male	10-14 years	95+ years	Global	-1	2	Religion (binary, >50% Muslim)	--
Self-harm by firearm	Male	10-14 years	95+ years	Global	0	2	Population Density (over 1000 ppl/sqkm, proportion)	85
Self-harm by firearm	Male	10-14 years	95+ years	Global	0	2	Population Density (500-1000 ppl/sqkm, proportion)	98
Self-harm by firearm	Male	10-14 years	95+ years	Global	0	2	Population Density (150-300 ppl/sqkm, proportion)	546
Self-harm by firearm	Male	10-14 years	95+ years	Global	0	2	Population Density (300-500 ppl/sqkm, proportion)	--
Self-harm by firearm	Male	10-14 years	95+ years	Global	0	2	Population Density (under 150 ppl/sqkm, proportion)	--
Self-harm by firearm	Male	10-14 years	95+ years	Global	0	3	Education (years per capita)	177
Self-harm by firearm	Male	10-14 years	95+ years	Global	0	3	Socio-demographic Index	295
Self-harm by firearm	Male	10-14 years	95+ years	Global	0	3	LDI (I$ per capita)	--
Self-harm by other specified means	Female	10-14 years	95+ years	Data Rich	1	1	Alcohol (liters per capita)	0
Self-harm by other specified means	Female	10-14 years	95+ years	Data Rich	1	1	Log-transformed SEV scalar: Self Harm	0
Self-harm by other specified means	Female	10-14 years	95+ years	Data Rich	1	1	Major depressive disorder	1000
Self-harm by other specified means	Female	10-14 years	95+ years	Data Rich	-1	2	Healthcare access and quality index	0
Self-harm by other specified means	Female	10-14 years	95+ years	Data Rich	-1	2	Religion (binary, >50% Muslim)	--
Self-harm by other specified means	Female	10-14 years	95+ years	Data Rich	0	2	Population Density (over 1000 ppl/sqkm, proportion)	32
Self-harm by other specified means	Female	10-14 years	95+ years	Data Rich	0	2	Population Density (150-300 ppl/sqkm, proportion)	198
Self-harm by other specified means	Female	10-14 years	95+ years	Data Rich	0	2	Population Density (300-500 ppl/sqkm, proportion)	839
Self-harm by other specified means	Female	10-14 years	95+ years	Data Rich	0	2	Population Density (500-1000 ppl/sqkm, proportion)	967
Self-harm by other specified means	Female	10-14 years	95+ years	Data Rich	0	2	Population Density (under 150 ppl/sqkm, proportion)	993
Self-harm by other specified means	Female	10-14 years	95+ years	Data Rich	0	3	Education (years per capita)	0
Self-harm by other specified means	Female	10-14 years	95+ years	Data Rich	0	3	Socio-demographic Index	0
Self-harm by other specified means	Female	10-14 years	95+ years	Data Rich	0	3	LDI (I$ per capita)	--
Self-harm by other specified means	Female	10-14 years	95+ years	Global	1	1	Alcohol (liters per capita)	200
Self-harm by other specified means	Female	10-14 years	95+ years	Global	1	1	Log-transformed SEV scalar: Self Harm	720
Self-harm by other specified means	Female	10-14 years	95+ years	Global	1	1	Major depressive disorder	910
Self-harm by other specified means	Female	10-14 years	95+ years	Global	-1	2	Healthcare access and quality index	7
Self-harm by other specified means	Female	10-14 years	95+ years	Global	-1	2	Religion (binary, >50% Muslim)	673
Self-harm by other specified means	Female	10-14 years	95+ years	Global	0	2	Population Density (300-500 ppl/sqkm, proportion)	41
Self-harm by other specified means	Female	10-14 years	95+ years	Global	0	2	Population Density (150-300 ppl/sqkm, proportion)	438
Self-harm by other specified means	Female	10-14 years	95+ years	Global	0	2	Population Density (500-1000 ppl/sqkm, proportion)	444
Self-harm by other specified means	Female	10-14 years	95+ years	Global	0	2	Population Density (under 150 ppl/sqkm, proportion)	462
Self-harm by other specified means	Female	10-14 years	95+ years	Global	0	2	Population Density (over 1000 ppl/sqkm, proportion)	523
Self-harm by other specified means	Female	10-14 years	95+ years	Global	0	3	Education (years per capita)	268
[truncated: 752,381 more chars]
